
JOURNAL INFORMATION
==============================
NLM Title Abbreviation: Insights Imaging
Journal ID: Insights Imaging
NLM Journal ID: 101532453

Insights into Imaging

EISSN: 1869-4101
Publisher: Springer

ARTICLE INFORMATION
==============================
PMCID: PMC6396437
PMID: 30806851
DOI: 10.1186/s13244-019-0713-y
Article ID: 713
Article version: 1
Subjects: Meeting Abstracts

ECR 2019: Book of Abstracts

Vienna, Austria. February 27 - March 3, 2019

Electronic publication date: 2019 Feb 26
Publication date: 2019 Feb
Volume: 10
Issue: Suppl 1
Issue sponsor: The European Congress of Radiology (ECR) is the annual meeting of the European Society of Radiology (ESR). Publication costs were not supported by sponsorship.
Electronic Location ID: 22
Copyright: © The Author(s). 2019
License: Open AccessThis article is distributed under the terms of the Creative Commons Attribution 4.0 International License (http://creativecommons.org/licenses/by/4.0/), which permits unrestricted use, distribution, and reproduction in any medium, provided you give appropriate credit to the original author(s) and the source, provide a link to the Creative Commons license, and indicate if changes were made.
License URL: https://creativecommons.org/licenses/by/4.0/

Conference name: European Congress of Radiology 2019
Conference Acronym: ECR 2019
Conference location: Vienna, Austria
Conference date: February 27 - March 3, 2019
issue-copyright-statement: © The Author(s) 2019

==============================
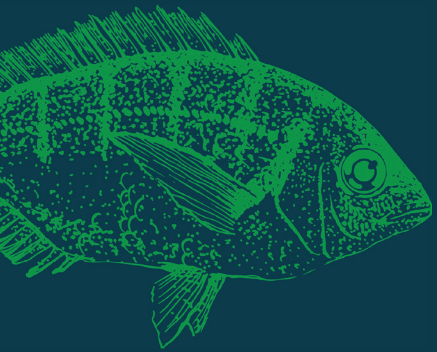

### ECR2019 the bigger picture

### BOOK OF ABSTRACTS

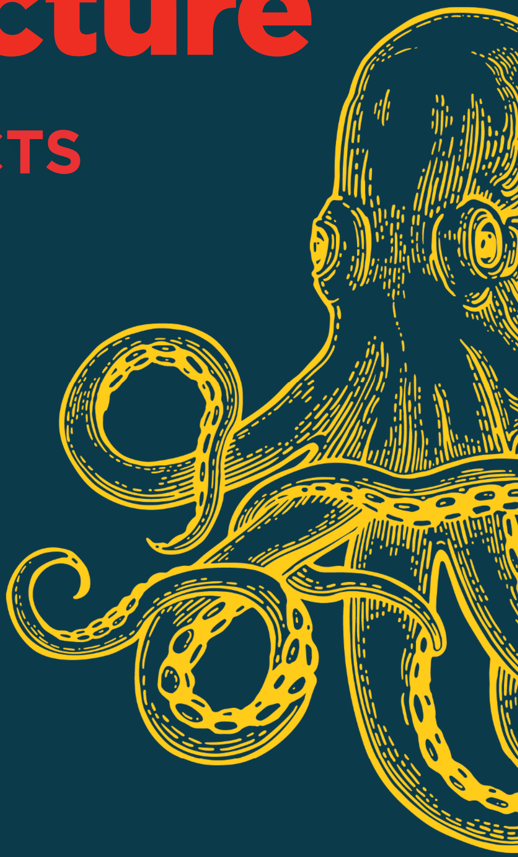

### CONTENTS

| | |
|---|---|
| Postgraduate Educational Programme (A) | S3 - 184 |
| Scientific Sessions (SS), My Thesis in 3 Minutes (MY), Clinical Trials in Radiology (CT) (B) | S185 - 585 |
| Scientific and Educational Exhibits (C) | S586 |
| Satellite Symposia (D) | S587 - 596 |
| Author's Index (E) | S597 - 683 |
| List of Authors Co-Authors (F) | S684 - 717 |
| List of Moderators (G) | S718 - 720 |

*Insights Imaging* 2019, **10**(Suppl 1):S1-S720
https://doi.org/10.1186/s13244-019-0713-y

**Disclaimer**

The ECR 2019 Book of Abstracts is published by the European Society of Radiology (ESR) and summarises the presentations accepted to be held at the European Congress of Radiology 2019 (Vienna, Austria, February 27 - March 3, 2019). Abstracts were submitted by the authors warranting that good scientific practice, copyrights and data privacy regulations have been observed and relevant conflicts of interest declared.

Abstracts reflect the authors' opinions and knowledge. The ESR does not give any warranty about the accuracy or completeness of medical procedures, diagnostic procedures or treatments contained in the material included in this publication. The views and opinions presented in ECR abstracts and presentations, including scientific, educational and professional matters, do not necessarily reflect the views and opinions of the ESR.

In no event will the ESR be liable for any direct or indirect, special, incidental, consequential, punitive or exemplary damages arising from the use of these abstracts.

The Book of Abstracts and all of its component elements are for general educational purposes for health care professionals only and must not take the place of professional medical advice. Those seeking medical advice should always consult their physician or other medical professional.

In preparing this publication, every effort has been made to provide the most current, accurate, and clearly expressed information possible. Nevertheless, inadvertent errors in information can occur. The ESR is not responsible for typographical errors, accuracy, completeness or timeliness of the information contained in this publication.

The ECR 2019 Book of Abstracts is a supplement to Insights into Imaging (1869-4101) and published under the Creative Commons Attribution License 4.0 (CC BY 4.0).

Published: 26 February 2019

### A

### Postgraduate Educational Programme

EFOMP Workshop (EF)
ESR/EFRS/ISRRT meets Sessions (EM)
European Excellence in Education ($E^3$)
Headline Sessions (HL)
Joint Sessions
Multidisciplinary Sessions (MS)
New Horizons Sessions (NH)
Professional Challenges Sessions (PC)
Pros & Cons Session (PS)
Refresher Courses (RC)
Special Focus Sessions (SF)
State of the Art Symposia (SA)
Transatlantic Courses (TC)

**Wednesday, February 27 ......... 4**
**Thursday, February 28 .......... 37**
**Friday, March 1 ..................... 80**
**Saturday, March 2 ............... 121**
**Sunday, March 3 ................. 166**

### Wednesday, February 27

**08:30 - 10:00** **Room B**

### Abdominal Viscera

### RC 101

### Imaging of benign liver lesions: still difficult?

**A-0001** 08:30
**Chairperson's introduction**
P. Ricci; *Rome/IT (paolo.ricci@uniroma1.it)*

Focal liver lesions are routinely encountered by clinical radiologists and represent a wide spectrum of pathology. Majority of these lesions are likely to be benign in nature, especially in the absence of chronic liver disease or primary cancer. A radiologist must be aware of common and uncommon imaging features of benign lesions across the various imaging modalities. Benign focal liver lesions can origin from all kind of liver cells: hepatocytes, mesenchymal and cholangiocellular line. Their features at imaging may sometimes pose difficulties in differential diagnosis with malignant primary and secondary lesions. In particular, the use of MDCT and MRI with extracellular and hepatobiliary contrast agents may non invasively help in correct interpretation and definition of hepatocellular or mesenchymal and inflammatory nature, allowing to choose the best treatment option. The correct differential diagnosis is based on the knowledge of the main diagnostic features. There are some malignant lesions which may mimic benign liver conditions. The most challenging lesions should be cystic lesions, hemangiomas, focal nodular hyperplasia and adenoma, whose features are not always so clear and unequivocal.
***Session Objectives:***
1. To understand the role of different imaging techniques in the characterisation of focal liver lesions.
2. To be aware of malignant lesions which may mimic benign liver conditions.
3. To underline current guidelines for the characterisation of equivocal liver lesions.

**A-0002** 08:35
**A. Hepatic cysts: always simple?**
I. Santiago; *Lisbon/PT (ines_agp_santiago@hotmail.com)*

The differential diagnosis of cystic liver lesions is as broad as their clinical significance, which ranges from benign to malignant and/or potentially lethal conditions. These lesions include foregut and ductal plate malformations, infectious conditions, primary and secondary neoplasms and traumatic/iatrogenic fluid collections. The number of lesions, morphology, fluid content characteristics, presence/absence of septae and/or solid components are the key imaging features for the diagnostic approach, and different imaging modalities pose different advantages and disadvantages for their characterisation. Given imaging characteristics may overlap between distinct conditions, patient history and laboratory data should be integrated to allow a more definitive diagnosis.
***Learning Objectives:***
1. To learn about different types of cystic lesions in the liver, including giant biliary hamartomas or foregut cysts.
2. To understand the diagnostic approach to differentiate simple and complex cysts/cystic lesions.
3. To appreciate advantages and limitations of imaging for differentiating simple cysts from other cystic lesions.

**A-0003** 08:58
**B. Liver haemangiomas and mimickers**
F. Caseiro Alves; *Coimbra/PT (caseiroalves@gmail.com)*

Haemangiomas are common focal liver lesions, generally detected in the workup of asymptomatic patients and do not require further workup, follow-up, or treatment. From the morphologic point of view, they can be classified as small (capillary) or large, with cavernous vascular spaces that may prone to show thrombosis, calcifications and hyalinisation. The polymorphic imaging appearance of haemangiomas depends on their histological features and flow pattern. The widespread use of cross-sectional imaging has allowed a better characterisation of this benign vascular tumour, and for this lecture, illustrative cases will be displayed especially using CT and multiparametric MRI including hepato-biliary contrast agents. Haemangiomas imaging findings may range from the commonly known aspects especially after extra-cellular Gd-chelates administration to atypical patterns where its recognition and positive diagnosis may not be so straightforward. The scope of the present lecture to present and discuss the patterns of those vascular liver lesions, describing normal findings, pitfalls, potential differentials, confounders and complications.
***Learning Objectives:***
1. To learn about typical imaging findings of liver haemangioma.
2. To understand the atypical imaging findings of liver haemangiomas.
3. To appreciate the role of multiparametric and liver-specific contrast MRI in differentiation between haemangiomas and malignant lesions mimicking haemangiomas.
*Author Disclosure:*
**F. Caseiro Alves:** Speaker; Bayer.

**A-0004** 09:21
**C. FNH or adenoma?**
A. Ba-Ssalamah; *Vienna/AT (ahmed.ba-ssalamah@meduniwien.ac.at)*

FNH and hepatocellular adenoma (HCA) is liver lesions of hepatocellular origin. Whereas FNH commonly occurs, HCA is very rare. Distinguishing FNH from HCA is of great importance clinically, as FNH is considered a benign lesion and needs no further management. In contrast, HCA, according to its subtype, can be considered a borderline tumour due to the risk of haemorrhage, growth, and even malignant transformation, and therefore requires individualised management. The genotype/phenotype classification of HCA is an evolving issue, and there is now a recent HCA molecular classification by which to stratify patients according to the risk of complications. On imaging, HCA is no longer a distinct entity, and imaging features reflect the tumour subtypes. Contrast-enhanced MRI is much more accurate because of its ability to visualise the textural composition of the HCA. However, even after the administration of MRI hepatobiliary contrast agents, a small proportion of HCA subtypes still show either inhomogeneous uptake or inhomogeneous washout in the hepatobiliary phase (HBP); making them difficult to differentiate from FNH on the basis of imaging presentation alone. This may pose a diagnostic dilemma as both FNH and HCA tend to occur in women of child-bearing age. Clinical presentation and risk factors, male gender, patient age, the presence of storage disease, obesity, metabolic or inflammatory syndrome, hepatitis, alcohol abuse and tumour over five cms, as well as significant growth have been identified as factors associated with an HCA-subtype of higher malignant potential. In difficult cases, histology remains the gold standard.
***Learning Objectives:***
1. To learn the imaging features associated with FNH and adenoma on contrast-enhanced CT and MRI.
2. To understand how the imaging characteristics are related to the underlying pathological findings.
3. To appreciate the optimal use of liver-specific contrast media for distinguishing between FNH and adenoma, and current classification and pathological characteristics and its impact on the management.
*Author Disclosure:*
**A. Ba-Ssalamah:** Consultant; Bayer. Speaker; Bayer, Siemens.

09:44
**Panel discussion: What imaging strategy for my routine clinical practice?**

**08:30 - 10:00** **Room C**

### General Radiography (Radiographers)

### RC 114

### Contemporary breast imaging

**A-0005** 08:30
**Chairperson's introduction (Part 1)**
E. Metsala; *Helsinki/FI (eija.metsala@metropolia.fi)*

This session covers many very up to date issues related to breast imaging. The final aim of breast imaging is to find breast cancer. For that, we need optimal screening policies and protocols for finding breast cancer from various types of breast. What is good for most women, might not be optimal for all of us. However, we want to produce optimal diagnostic services for all types of women. In addition to breast ultrasound, tomosynthesis and breast MRI seem to be the most used imaging modalities for finding breast cancer at the moment. It is important to discuss the options we may have for screening purposes. However, there is no use for excellent technical and diagnostic devices and processes in case the client/patient does now show up for examinations. To ensure that we must carefully pay attention to the information we deliver about breast cancer and breast examinations, patient guidance and make the imaging and diagnostic process as patient-centred as possible. To achieve this, we need interprofessional co-operation between various health care professionals: radiologists, radiographers, physicists, biomedical laboratory scientists and pathologists to mention a few.

***Session Objectives:***
1. To discuss the relevance and application of tomosynthesis in breast imaging.
2. To understand the indications and use of breast MRI in pre-menopausal women.
3. To appreciate the current debate regarding the appropriateness of breast screening programmes.

**A-0006** 08:33
**Chairperson's introduction (Part 2)**
G. Ivanac; *Zagreb/HR (gordana.augustan@gmail.com)*

Tomosynthesis is a relatively new modality in digital mammography that increases conspicuity if lesions that are hidden by superposition of breast parenchyma. Also, MRI is established diagnostic modality for numerous indications: for high-risk screening, detection of residual lesions or recurrence after surgery, for the detection of breast cancer when axillary lymph nodes are positive and mammography and ultrasound negative, and for monitoring of neoadjuvant treatment, and in some cases for preoperative evaluation of the extent of cancer. Breast cancer screening is a matter of many controversies, in the light of overdiagnosis and overtreatment, but it is generally accepted that biannual mammographic screening should be offered to healthy women of the age group of 50-69. Supplemental screening with ultrasound and tomosynthesis is indicated in many cases of dense breasts, and a special regimen should be applied to women with a high risk of acquiring breast cancer.
***Session Objectives:***
1. To discuss the relevance and application of tomosynthesis in breast imaging.
2. To understand the indications and use of breast MRI in pre-menopausal women.
3. To appreciate the current debate regarding the appropriateness of breast screening programmes.

**A-0007** 08:35
**A. Tomosynthesis**
H. Yule; *Cardiff/UK*

The aim of the presentation is to provide a basic introduction to digital breast tomosynthesis (DBT) and the principles that underpin this technique. An overview in general of the current evidence to support the use of DBT in clinical practice. A discussion of the impact of DBT in terms of mammographic practice to include technique and positioning.
***Learning Objectives:***
1. To review the technology underpinning tomosynthesis.
2. To understand the current evidence base for use of tomosynthesis.
3. To appreciate results from the UK TOMMY trial and others.

**A-0008** 08:58
**B. Breast imaging in young females: the role of MRI**
M.M. Ribeiro; *Lisbon/PT (margarida.ribeiro@estesl.ipl.pt)*

Nowadays, Magnetic Resonance Imaging (MRI) is used routinely to provide anatomic details and contrast while, allows the evaluation of the functionality of the organ, the quantification of the lesions and histological information. Although breast cancer in young women is relatively uncommon, affecting about 4-6% of women under the age of 40 and less than 4% are diagnosed under the age of 35, there has been an increase in the number of breast cancers diagnosed in the premenopausal women. The first indication to Breast MRI from EUSOBI is the screening of women at high risk of breast cancer; however, diagnosing breast cancer in women under 40 years old is more difficult as their breast tissue is, generally, denser. There have been significant advances in technical developments and clinical applications with the goal to improve image acquisition, early cancer diagnosis, treatment monitoring, the prognostic indication and predictive biomarkers. The discussion is centred on the new acquisition techniques to improve cancer detection specificity in premenopausal women and the comparatively to mammography and ultrasound. The limitations of the technique and, in the other hand, the technology improvements like technical developments on advanced quantitative MRI, the breast MRI in clinical trials, the fast techniques, updates on DWI, perfusion, novel contrasts, spectroscopy and the impact of artificial intelligence on breast MRI research and clinic, will be addressed. We cannot forget the strategies to increase the early prevention, regarding the appliance of best practices around the world and incorporating the psychosocial concerns of the disease.
***Learning Objectives:***
1. To understand current sensitivity of MRI when imaging the breast.
2. To discuss the varying sensitivities of mammography and ultrasound according to age.
3. To appreciate the potential use of abbreviated MRI sequences.

**A-0009** 09:21
**C. Breast screening programmes: current evidence**
S. Hofvind; *Oslo/NO*

"no abstract submitted"
***Learning Objectives:***
1. To identify advantages and disadvantages of breast screening regarding false positives.
2. To appreciate current debate both for and against organised screening programmes.
3. To consider alternatives to breast screening.

09:44
**Panel discussion: Breast imaging: a true multidisciplinary team effort?**

**08:30 - 10:00 Room X**

### Imaging Informatics

### RC 105

### Mobile devices in radiology

**A-0010** 08:30
**Chairperson's introduction**
O. Ratib; *Geneva/CH*

Mobile devices such as tablets and high-resolution smartphones are becoming widely available providing convenient mobile solutions for physicians and healthcare providers to access imaging data. This is particularly attractive in medicine where “nomad” physicians who need to be able to access relevant patient data and images anywhere-anytime in their daily practice where they are rarely a single location. While they may not always be adequate for routine diagnostic tasks, they provide a convenient mobile solution for on-call and remote consultations. There are different types of software architecture that can be implemented for such tasks. Two major different design are: (1) online web-based applications where the device serves as a “thin-client” to display images rendered and manipulated on a remote computer and (2) local applications that reside on the mobile device and can run independently after images have been downloaded on the device. The first solution requires the user to be constantly connected to the network, while the second solution can continue to function after disconnecting from the network. Most vendors are starting to provide web access to their imaging solutions that can be accessed from mobile devices. Web access can, however, be slow and dependent on reliable access to wireless network.
***Session Objectives:***
1. To give an overview of tools available on mobile devices for education and exam reporting.
2. To underline the impact of mobile devices in routine clinical activity.
3. To learn about the legislative backbone and potential drawbacks of mobile technology.

**A-0011** 08:35
**A. Security and confidentiality aspects of mobile computing**
E.R. Ranschaert; *Tilburg/NL (ranschaert@telenet.be)*

Medical specialists and radiologists are using mobile devices to share and exchange medical information and images with other healthcare professionals. Usually, they need advice regarding a diagnosis or treatment, sometimes in an acute setting. Mobile devices with viewing apps and messaging services such as WhatsApp are frequently used for such purpose. Transmission of patient data by viewing apps or messaging services, however, does have several risks and limitations, mostly related to the security and privacy of patients, but also from an ethical and legal point of view. Some questions need to be answered: is this type of communication accurate for diagnosis, is it secure and legally allowed? What regulations or guidelines are available and what legislation is applicable? What secure options are available? In this scientific focus session these issues will be discussed in more depth.
***Learning Objectives:***
1. To provide an overview of technical solutions for patients' image and data mobility.
2. To provide a risk assessment analysis (data loss, privacy, etc.) of mobile technology.
3. To give an overview of European legislation in relation to patient image and data mobility.
*Author Disclosure:*
**E.R. Ranschaert:** Advisory Board; MedicalPHIT. Board Member; EuSoMII. Shareholder; Diagnose.me.

**A-0012** 08:58
**B. Added value of mobile devices in education**
E. Kotter; *Freiburg/DE (elmar.kotter@uniklinik-freiburg.de)*

E-learning has been used in radiology for more than 30 years. The lecture will give an introduction to and an overview of e-learning systems for radiology with emphasis on e-learning on mobile devices. Advantages and limitations of mobile e-learning will be discussed. An outlook to the future development of e-learning will be given.
***Learning Objectives:***
1. To give an overview of tools available for e-learning.
2. To explore the potential impact of e-learning in the daily radiological practice.
3. To explore future developments and limits of e-learning.
*Author Disclosure:*
**E. Kotter:** Advisory Board; Agfa.

**A-0013** 09:21
**C. Recommendations for reporting on mobile devices**
N.H. Strickland; *London/UK*

"no abstract submitted"
***Learning Objectives:***
1. To give an overview of available DICOM viewers and software for reporting imaging studies.
2. To discuss technical requirements of mobile devices for use in imaging interpretation.
3. To provide insight on future developments of imaging viewing technology.

09:44
**Panel discussion: Can mobile technology supplement stationary technology in radiology?**
*Discussion will address main controversies of the use of mobile technology in a clinical environment such as image quality, data mobility, safety and legal issues, etc.*

**08:30 - 10:00** **Room N**

### EuroSafe Imaging Session

### EU 1

### Cardiovascular effects after radiotherapy for breast cancer: the European MEDIRAD Project

**A-0014** 08:30
**Chairpersons' introduction (part 1)**
A. Crijns; *Groningen/NL (a.p.g.crijns@umcg.nl)*

Radiation-induced major cardiac events (MCEs) are becoming increasingly relevant for breast cancer (BC) patients, affecting the quality of life and increasing morbidity and mortality. Information regarding the relationship between radiation dose to cardiac substructures and MCEs and their early subclinical precursor cardiovascular effects is needed to develop preventive strategies for radiation-induced MCEs. In this session, current knowledge of dose-effect relationships will be discussed as well as which important information is still lacking. The European MEDIRAD project comprises a multicenter retrospective cohort study aiming at the development and external validation of prediction models for MCEs. In addition, a prospective multicenter study for identification of early subclinical cardiovascular effects with three different cardiac imaging modalities; echocardiography, cardiac MRI and cardiac CT is ongoing. We will present how the European MEDIRAD project provides the data needed for the development of individualised preventive strategies for radiation-induced MCEs. Finally, the technical issues of cardiac CT and MRI in detecting early subclinical morphological and/or functional cardiovascular effects will be discussed.
***Session Objectives:***
1. To learn about major cardiovascular events after radiotherapy for breast cancer.
2. To understand the importance of assessing their relationship with radiation dose to cardiac substructures.
3. To appreciate the potential of imaging biomarkers for their early detection and their role in prevention strategies.
*Author Disclosure:*
**A. Crijns:** Research/Grant Support; supported by the European Community's Horizon 2020 Programme.

**A-0015** 08:33
**Chairpersons' introduction (part 2)**
G. Frija; *Paris/FR (guy.frija@aphp.fr)*

MEDIRAD (Implications of Medical Low Dose Radiation Exposure) is a multidisciplinary, cross-cutting project funded under the Euratom research and training programme 2014-2018 under grant agreement No. 662287. The four-year MEDIRAD project kicked off in June 2017 and is led by the European Institute for Biomedical Imaging Research (EIBIR). The consortium brings together a wide range of expertise, with 33 partners from 14 European countries, and involves research groups that focus on radiology, nuclear medicine, radiotherapy, dosimetry, epidemiology, biology, bioinformatics, modelling, radiation protection, and public health. MEDIRAD has three major operational objectives. Improving organ dose estimation and registration; evaluating and understanding the mechanisms of the effects of medical radiation exposure, focusing on two outcomes of public health relevance (cardiovascular effects of radiotherapy in breast cancer treatment and cancer risk following CT scanning of children and adolescents); and, developing science-based policy recommendations for the effective protection of patients, workers, and the general public. This session will focus on cardiovascular effects after radiotherapy for breast cancer and introduce the Early-Heart Study developed under work package 4 of the MEDIRAD project.
***Session Objectives:***
1. To learn about major cardiovascular events after radiotherapy for breast cancer.
2. To understand the importance of assessing their relationship with radiation dose to cardiac substructures.
3. To appreciate the potential of imaging biomarkers for their early detection and their role in prevention strategies.

**A-0016** 08:36
**Overview of dose-effect relationships and their use in strategies for prevention of cardiovascular effects after radiotherapy for breast cancer**
A. Crijns; *Groningen/NL (a.p.g.crijns@umcg.nl)*

Breast cancer (BC) radiotherapy leads to incidental cardiac irradiation, resulting in an increased risk of various major cardiac events (MCEs). The risk of a MCEs increases linearly by 7.4% per Gray of the mean heart dose. Recent studies have shown that MCEs may already appear within 5-10 years of radiotherapy. Due to an increased incidence of BC in Europe and a continuously improving survival rate, the prevalence of BC survivors at risk of MCEs is also growing. Information regarding the relationship between radiation dose to cardiac substructures and MCEs is scarce. Knowledge about early subclinical cardiovascular effects (ESCEs) induced by radiotherapy that eventually develop into MCEs is also largely lacking. We need this information to optimise radiation dose distributions as a primary preventive strategy. In addition, we need tools to identify BC patients -already treated with radiotherapy- who have a high risk of future treatment-related MCEs. These patients may benefit from secondary preventive strategies. Finally, information on potential targets for developing strategies to prevent or delay progression into clinically apparent is not currently available. The European MEDIRAD Project; Modern imaging techniques such as echocardiography, cardiac MRI and cardiac CT can be used to identify morphological and functional ESCEs, considered risk factors for developing MCEs. This information will provide better insight into the biological mechanisms of radiation-induced MCE and may provide potential targets for secondary prevention. In addition, by relating imaging-derived ESCEs and/or MCEs to local radiation dose, prediction models can be developed to optimise radiation planning techniques.
***Learning Objectives:***
1. To learn about the increased risk of various major cardiac events after incidental exposure of the heart during breast radiotherapy.
2. To understand that multivariable prediction models are needed describing the relationship between radiation dose to cardiac substructures and (early) cardiovascular effects.
3. To appreciate the role of modern cardiac imaging techniques in providing detailed information regarding early cardiovascular effects after radiotherapy.
*Author Disclosure:*
**A. Crijns:** Research/Grant Support; supported by the European Community's Horizon 2020 Programme.

08:52
**Discussion**

**A-0017** 08:57
**Optimisation of multivariable prediction models for major cardiac events after radiotherapy for breast cancer**
D.S. Spoor; *Groningen/NL (d.s.spoor@umcg.nl)*

The relationship between radiation dose to the heart in breast cancer radiotherapy (RT) and various major cardiac events (MCEs) can be described with normal tissue complication probability (NTCP) models. Previous studies showed the predictive value of the mean heart dose (MHD) for the risk of acute coronary events (ACE). Recent studies suggest that the dose delivered to cardiac substructures, like the left ventricle, might be better predictors of MCEs. Furthermore, preliminary results of ongoing work suggest that radiation dose to coronary arteries, particularly the left anterior descending artery, may have superior predictive value. More accurate risk estimations for MCEs requires improved NTCP-models, which requires large retrospective cohort studies with sufficient length of follow-up to capture sufficient numbers of cardiac events.MEDIRAD-BRACE is an international multicenter retrospective cohort study. The study will include 7000 female breast cancer patients treated with primary surgery and postoperative RT treated from 2005 to 2014 and who were aged 40-75 years at a time of RT start. A test cohort of 5000 patients will be used to develop NTCP-models for MCEs, which will be validated using a validation cohort consisting of 2000 patients. The primary endpoint of the study is an ACE after completion of treatment, and secondary endpoint comprises other MCEs. The resulting models can be used to identify patients at increased risk for MCE, which allows for the development of primary and secondary preventive measures. Moreover, NTCP-models may be further improved by incorporating imaging-derived subclinical cardiac effects that precede MCE. This is studied in MEDIRAD-EARLY HEART.

***Learning Objectives:***
1. To appreciate how the MEDIRAD-BRACE study will provide optimised multivariable prediction models for major cardiac events.
2. To learn that optimal prevention of radiation-induced major cardiac events requires early detection/imaging biomarkers.

09:13
**Discussion**

**A-0018** 09:18
**The MEDIRAD Early-Heart study**
S. Jacob; *Fontenay aux Roses/FR (sophie.jacob@irsn.fr)*

Radiotherapy (RT) plays a major role in breast cancer (BC) treatment. However, BC RT can lead to incidental irradiation of the heart, resulting in cardiac complications with an increased risk of various heart diseases arising many years after RT. Long before the onset of clinically significant late cardiac complications, subclinical cardiac changes may occur over months or years after RT that can be detected using anatomical and functional cardiac imaging. Therefore, detecting early signs of cardiotoxicity and determining the relationship between radiation dose to specific cardiac structures and subclinical cardiac changes is crucial for primary/secondary prevention. In the frame of the MEDIRAD European project, the EARLY-HEART study was launched in 2017. This five European centres prospective cohort study will include approximately 250 unilateral BC women aged 40-75 years treated with RT without chemotherapy and followed for two years. Baseline (before RT) and follow-up data (6 and 24 months after RT) include cardiac imaging measurements: myocardial deformation is evaluated with myocardial strain imaging (2D-speckle-tracking echocardiography); cardiac magnetic resonance imaging is performed to detect subtle changes in cardiac function and structure; cardiac computed tomography is performed to evaluate coronary artery lesions. Radiation exposure to cardiac structures based on 3D-dosimetry is precisely evaluated for all patients. Dose-response relationship will allow modelling radiation-induced occurrence and evolution of subclinical cardiac lesions to develop early cardiotoxicity prediction models. Based on emerging research in advanced imaging modalities, this study will allow enhanced detection and prediction of early radiotherapy-induced cardiotoxicity and patients' care.

***Learning Objectives:***
1. To appreciate that there is already some information from cardiac imaging studies regarding early cardiovascular effects after breast cancer radiotherapy.
2. To learn how the MEDIRAD Early-Heart study will provide information regarding imaging biomarkers of early cardiovascular effects after radiotherapy for breast cancer.
3. To understand that cardiac imaging can provide clues for underlying mechanisms of radiation-induced major cardiac events.

09:34
**Discussion**

**A-0019** 09:39
**Imaging biomarkers of cardiovascular effects of incidental cardiac radiation**
E. Mousseaux; *Paris/FR (elie.mousseaux@egp.ap-hop-paris.fr)*

Since Radiotherapy (RT) has been linked to cardiac diseases, detection of early signs of radiotherapy-induced cardiotoxicity (RTIC) is crucial and challenging for prevention. To identify and validate cardiac imaging biomarkers of RTIC arising within two years after breast cancer RT. CT and MRI will be done at baseline, and at RT+6 months (MRI), and RT+24 months (CT+MRI) during follow-up. By using MRI, in association to conventional ventricular function analysis of both ventricles, myocardial strain (deformation) will be quantified by studying cavity contours length changes ($\Delta l/l°$) during the cardiac cycle in each of four cardiac chambers. In addition, after myocardial T1 estimation of the left ventricle before and after gadolinium injection, derived indices of intra- and extra-cellular lesions will be calculated since RT is likely to modify components of these two compartments (cell destruction, inflammation and/or appearance of interstitial fibrosis). Cardiac CT will also be performed to evaluate coronary artery, valves and pericardium lesions by estimating local and global calcium score with a non-enhanced acquisition, and by subjectively analysed all these structures with enhanced acquisitions before and after RT. All analysis will be done in a core lab without knowledge of any possible clinical events that may occur and of information regarding the site of a tumour and RT procedures. Based on emerging results in CT and MRI to analyse sensitive biomarkers of cardiac lesions, hypotheses of the study are that early signs of RTIC could be detected to predict irreversible cardiotoxicity better and to improve patients' care.

***Learning Objectives:***
1. To appreciate how radiation-induced coronary artery lesions can be studied by cardiac CT.
2. To understand how MRI can be used to evaluate myocardial tissues abnormalities induced by incidental cardiac radiation, including morphology, function and tissue abnormalities.

09:55
**Discussion**

**08:30 - 10:00** **Room O**

### Vascular

### RC 115

### Visceral arteries

**A-0020** 08:30
**Chairperson's introduction**
J.A. Reekers; *Amsterdam/NL*

The visceral vessels are the superior and inferior mesenteric artery, coeliac trunc, hepatic artery and splenic artery and the renal arteries. There is a wide variety of pathology including both stenotic disease and dilating vessel disease. There is also a wide variety of interventional treatment options to treat these problems.

***Session Objectives:***
1. To learn about incidence and aetiology of visceral arteries diseases.
2. To become familiar with clinical symptoms and evaluation in visceral arteries diseases.
3. To indicate the role of radiology in diagnosis and treatment of the visceral arteries.

**A-0021** 08:35
**A. Diagnosis of vascular compression syndromes**
B.E. Cil; *Istanbul/TR (bcil@kuh.ku.edu.tr)*

Vascular compression syndromes are clinical entities caused by the entrapment of veins or arteries between rigid or semirigid anatomic structures or compression of hollow viscera by vascular structures. Although they occur infrequently (less than 1% of the general population are affected), they may cause significant disease, including median arcuate ligament syndrome, May-Thurner syndrome, nutcracker syndrome, superior mesenteric artery syndrome, and ureteropelvic junction obstruction. These syndromes are usually seen in otherwise healthy young patients, among whom underdiagnosis is common. Most occurrences of vascular compression are associated with an underlying anatomic abnormality. The diagnosis must be based on both clinical and radiologic findings. Digital subtraction angiography, venography or Doppler ultrasonography can provide hemodynamic information in cases of vascular compression. However, computed tomographic angiography and magnetic resonance angiography are particularly useful in that it allows comprehensive, fast evaluation of the anatomy and resultant morphologic changes. Symptomatic patients may require treatment, which is generally surgical due to the need for the relief of external compression. However, endovascular techniques are also increasingly being used to treat venous compressions.

***Learning Objectives:***
1. To become familiar with abdominal arterial and venous compression syndromes.
2. To learn about functional imaging techniques in assessment of vascular compression syndromes.
3. To become familiar with the typical imaging findings in abdominal compression syndromes and their clinical relevance.

**A-0022** 08:58
**B. Acute and chronic mesenteric ischaemia**
M. Zins; *Paris/FR (mzins@hpsj.fr)*

Acute mesenteric ischemia (AMI) corresponds to the inadequate blood supply to the gastrointestinal tract resulting in an ischemic and inflammatory injury that may progress to necrosis of the bowel wall. Prognosis is poor with a mortality rate superior to 95 % without treatment, dropping to around 70% when surgical treatment is performed. Contrast-enhanced computed tomography (CT) has become the cornerstone of the diagnosis by showing features of vascular disorder (occlusion and/or low blood flow) and features of intestinal ischemic injury. Imaging-based patient management is required, and multimodal and multidisciplinary management should be introduced. The treatment involves gastroenterologists, vascular and digestive surgeons, cardiologists, intensivists, and diagnostic and interventional radiologists. This lecture will give an overview of pathophysiology, diagnosis proves, and treatment of Acute and Chronic Mesenteric Ischemia. The goal is to improve the understanding and management of this life-threatening condition.
***Learning Objectives:***
1. To become familiar with occlusive and non-occlusive, mesenteric ischaemia.
2. To understand the differences between acute and chronic ischaemia.
3. To learn about the importance of fast and correct diagnosis in acute mesenteric ischaemia.

**A-0023** 09:21
**C. Endovascular treatment of mesenteric ischaemia**
R. Morgan; *London/UK (robert.morgan@stgeorges.nhs.uk)*

Mesenteric ischaemia is divided into acute and chronic mesenteric ischaemia. The main imaging modalities of mesenteric ischaemia are CT angiography and duplex ultrasound. Acute mesenteric ischaemia (AMI) is an abdominal emergency and is usually due to acute thrombotic or embolic occlusion of one or more of the arteries supplying the gastrointestinal tract. In practice, this is often the superior mesenteric artery (SMA). In some patients, acute mesenteric ischaemia is caused by acute mesenteric venous thrombosis, although such cases are unusual. Standard therapy is by surgical thrombectomy, embolectomy or bypass. In cases where there are no signs of irreversible intestinal ischaemia, patients may be suitable for endovascular treatment, either transcatheter thrombolysis or thrombectomy. Chronic mesenteric ischaemia (CMI) causes postprandial abdominal pain, “food-fear” and weight loss. CMI is usually due to severe stenosis or occlusion of two or three of the coeliac trunk, SMA or inferior mesenteric artery. Endovascular treatment is the primary first method used to treat CMI. Stenting is usually performed in preference to angioplasty. There are insufficient data to decide which endovascular method is best and there are no data on the use of drug-eluting technology.
***Learning Objectives:***
1. To review indications for endovascular treatment of mesenteric ischaemia.
2. To become familiar with the technical possibilities of endovascular repair of visceral arteries.
3. To learn about risks and complications of endovascular treatment of mesenteric ischaemia.

09:44
**Panel discussion: Radiologists as the best case managers in acute and chronic mesenteric ischaemia**

**08:30 - 10:00** **Room E1**

### E³ - ECR Master Class (Neuro)

### E³ 126a

### How to implement MRI neuro advanced techniques at home

*Moderator:*
P.C. Maly Sundgren; Lund/SE

**A-0024** 08:30
**A. Practical approach to cerebral perfusion techniques**
H.R. Jäger; *London/UK*

"no abstract submitted"
***Learning Objectives:***
1. To learn about the essentials in MRI perfusion techniques.
2. To understand how to interpret the data in brain perfusion MRI.
3. To know the limitations in perfusion MRI studies.

**A-0025** 09:00
**B. How to read susceptibility-weighted imaging (SWI)**
S. Haller; *Carouge/CH (sven.haller@me.com)*

Susceptibility weighted imaging (SWI) is increasingly used in clinical routine and is useful notably for the detection of hemosiderin (in a variety of hemorrhagic conditions) as well as iron deposition (in a variety of neurodegenerative diseases). The first part will discuss the essential technical aspects of SWI, and how imaging parameters will influence imaging contrast and consequently image analysis and results. The second part will assess imaging, interpretation and implications and notably the differential diagnosis of cerebral microbleeds, which occur in a variety of conditions including arterial hypertension, cerebral amyloid angiopathy (CAA), CADASIL (Cerebral Autosomal Dominant Arteriopathy with Subcortical Infarcts and Leukoencephalopathy), trauma (hemorrhagic diffuse axonal injury DAI) and brain irradiation. The third part will discuss imaging of the nigrosome 1 of the substantia nigra, also known as swallowtail sign, as an imaging marker of Parkinson Disease and Dementia with Lewy bodies. The final fourth part will discuss applications of SWI in a variety of vascular and neurodegenerative disorders including NBAI (neurodegeneration with brain iron accumulation), ASL (amyotrophic lateral sclerosis), clot imaging in acute stroke, and iron deposition during ageing.
***Learning Objectives:***
1. To be familiar with the practical basics of MRI SWI sequences.
2. To learn what information we can get from the SWI sequence.
3. To understand the importance of applying the SWI technique in individual patients.

**A-0026** 09:30
**C. How to read diffusion tensor imaging (DTI)**
R. Gasparotti; *Brescia/IT (gasparo@med.unibs.it)*

Diffusion Tensor Imaging provides in vivo visualisation of white matter tracts by voxel x voxel mapping of the anisotropy and local direction of fibres. Conventional MR imaging is limited to detecting macroscopic brain changes, while DTI quantifies diffusion characteristics within microscopic nerve fibre bundles and is thought to represent axon density, diameter, and continuity, myelin and interstitial water content. Due to recent advances in the technical design of sequences a DTI study of the brain with 64 directions and 1.8 mm voxel size can be obtained in 5 minutes with a 3T MR unit. Therefore DTI can be easily included in routine clinical MR protocols. There are no standardised approaches in DTI pre and postprocessing despite the availability of proprietary software and several freely distributed software packages providing good quality tractography of the bran white matter bundles. The easiest implementation of DTI in clinical practice is based on deterministic tractography, which provides an acceptable reproducibility in the identification and quantitative analysis of the brain white matter bundles through a rather standardised approach. Common clinical applications of DTI in single subjects are represented by brain neoplasms, neurodegenerative disorders and traumatic brain injuries. DTI-based tractography is widely used for presurgical planning and is a powerful tool in the evaluation of major WM fibre bundles; it has also a positive impact on neurosurgical resection, disease prognosis, and preservation of brain function.
***Learning Objectives:***
1. To learn the principals of DTI imaging in neuroradiology.
2. To appreciate the practical value of DTI in different neurological disorders.
3. To be familiar with pitfalls of DWI and DTI imaging - common language with neurosurgeons.

**08:30 - 10:00** **Room E2**

### Special Focus Session

### SF 1

### Screening with imaging: a new era?

**A-0027** 08:30
**Chairperson's introduction**
A. Devaraj; *London/UK*

This session will review common aspects of screening for lung cancer, colorectal cancer and breast cancer: namely the evidence for the particular screening programme, the additional challenges of implementing screening in the real world compared to a research setting, and future developments in each area. Common areas of debate such as overdiagnosis will also be addressed.

***Session Objectives:***
1. To understand how screening is currently implemented in Europe.
2. To understand the challenges familiar to all screening programmes when aiming to continually enhance quality and minimise harms.
3. To understand the emerging technologies in screening of lung, breast and colorectal cancer.

**A-0028** 08:35
**Screening of lung cancer with low dose CT**
M. Prokop; *Nijmegen/NL*

"no abstract submitted"
***Learning Objectives:***
1. To understand the current situation with lung cancer screening in Europe.
2. To understand the challenges of implementing a new screening programme for lung cancer.
3. To discuss areas for quality assurance and standardisation in lung cancer screening.

**A-0029** 08:58
**Screening of colorectal cancer with CT colonography**
A. Laghi; *Latina/IT (andrea.laghi@uniroma1.it)*

Colo-rectal cancer (CRC) is currently the second leading cause of cancer-related deaths in males and females. However, it could be easily prevented because of the favourable natural history. Radiologists can now fully support CRC screening, thanks to the availability of Computed Tomography Colonography (CTC). Three randomised clinical trials, investigating the performances of CTC in asymptomatic average-risk individuals, demonstrated good CTC patient acceptance, lower than FOBT, but higher than FS and CC, and good CTC adenoma detection rate, slightly lower than CC, but much higher than FS and FOBT.CTC in the setting of CRC screening has different roles, depending if an organised CRC screening program is available or not. If it is not available, CTC might be used as an opportunistic screening test together with FOBT/ FIT, FS and CC. In this case, individuals should be informed about the benefits and possible drawbacks of CTC in comparison with other tests. In many European countries, where established screening programs based on FOBT/FIT are available, CTC can be used as a back-up for an incomplete CC, performed after a positive FOBT/FIT. Alternatively, CTC might be offered as an alternative test in those patients (they are not few) tested positive at FOBT/FIT, but who refuse CC. The next goal would be to introduce CTC as a population screening test, replacing FOBT/FIT. However, the demonstration of higher cost-effectiveness compared with the available screening test is still missing and the major barrier preventing the endorsement by policymakers.
***Learning Objectives:***
1. To understand the rationale for a colorectal cancer screening programme.
2. To be updated about current experiences on screening CT colonography.
3. To learn about advantages and limitations of CT colonography in comparison with other colorectal cancer screening tests.
*Author Disclosure:*
**A. Laghi:** Speaker; Bracco, General Electric Healthcare, Bayer, Merck, Bristol-Myers Squibb.

**A-0030** 09:21
**Screening of breast cancer with abbreviated MRI**
C.K. Kuhl; *Aachen/DE (ckuhl@ukaachen.de)*

Early diagnosis improves the survival of women with breast cancer. Mammographic screening improves early diagnosis of breast cancer. And yet, there appears to be room for improvement. Major shortcomings of mammographic screening are overdiagnosis of prognostically unimportant cancer, as well as underdiagnosis of cancers that are indeed relevant. Failure to detect biologically relevant breast cancer with mammographic screening is driven by host-related factors, i.e. breast tissue density, but also tumour-related factors: Biologically relevant cancers may exhibit imaging features that renders them indistinguishable from normal or benign breast tissue on mammography. These cancers will then progress to become the advanced-stage interval cancers observed in women undergoing mammographic screening. Since breast cancer continues to represent a major cause of cancer death in women, the search for improved breast cancer screening method continues. Abbreviated breast MRI has been proposed for this purpose because it will greatly reduce the cost associated with this method, due to a greatly reduced magnet time (down to 3 minutes), but especially also due to a greatly abridged image interpretation time, i.e. radiologist reading time. This lecture will review the current evidence and presents the EA1141 trial designed to investigate the utility of abbreviated breast MRI for screening average-risk women with dense breast tissue.
***Learning Objectives:***
1. To list the cancer detection rates of radiographic, ultrasound and MR imaging for screening of breast cancer.
2. To describe the respective diagnostic accuracies published for breast MRI with abbreviated vs full diagnostic protocols.
3. To list rates and causes of over- and underdiagnosis of breast cancer in current breast cancer screening programmes.

09:44
**Panel discussion: Optimising screening and taking it to the next level**

**08:30 - 10:00** **Room F1**

### E³ - Rising Stars Programme: Basic Session

### BS 1
### Musculoskeletal: bones and soft tissues

*Moderator:*
A.H. Karantanas; Iraklion/GR

**A-0031** 08:30
**Bone marrow diseases**
K. Verstraete; *Ghent/BE*

Bone marrow consists of trabecular bone, a stroma of connective tissue, hematopoietic cells (red marrow) and fat (yellow marrow). Distribution of red and yellow marrow is age-dependent, with gradual conversion of red to yellow marrow in the limbs during childhood, and patchy heterogeneity in the spine in the elderly patient. There are many causes of reconversion from yellow to red bone marrow, like smoking, long distance running, obesity, anaemia, erythropoietin, etc. Depletion may occur in aplastic anaemia and after radiation therapy. Gelatinous transformation is seen in anorexia nervosa, cachexia, HIV and after successful therapy in multiple myeloma. Bone infarction and avascular necrosis are well-delineated areas of dead bone marrow. The value of different imaging techniques, including plain radiography, (dual-energy)-CT, bone scintigraphy, PET and the most sensitive technique, MRI (conventional T1, T2, fat suppression techniques, in-phase, out-phase and diffusion imaging), will be explained. The imaging characteristics of many diseases will be reviewed (diffuse bone marrow replacement in hematologic diseases, multiple myeloma, metastases; treatment-related changes of bone marrow, primary bone tumors, and multiple causes of bone marrow edema, like bone contusion, stress fracture, insufficiency fracture, Modic changes, spondylodiscitis, osteomyelitis, abscess, arthritis and specific bone tumors).
***Learning Objectives:***
1. To describe the typical features of normal bone marrow.
2. To determine origin of bone marrow changes.
3. To present the imaging characteristics of a bone marrow disease of the different types.

**A-0032** 09:00
**Soft tissue tumours**
V.N. Cassar-Pullicino; *Oswestry/UK (Victor.Pullicino@nhs.net)*

All imaging modalities can play a role in the diagnosis and management of soft tissue tumours and pseudotumours with a variable contributory performance to both sensitivity and specificity. MRI steals the show with an unparalleled role in soft tissue assessment ranging from detection, localisation, characterisation, identifying multiple lesions, other syndrome stigmata, the probability of benignity/malignancy, local staging and recurrence identification. Sonography does have a supporting role especially in the initial assessment of the likelihood of cystic/benign/abnormal malignant Colour Doppler flow patterns. This presentation aims to provide a distillation of the knowledge regarding soft tissue tumour imaging which can be applied in practice using a stepwise analytical approach. Despite an overwhelming spectrum of potential histological diagnosis, the radiologist needs to remember that eight benign and six malignant lesions account for 80% of all soft tissue tumours.
***Learning Objectives:***
1. To determine the origin of a soft tissue tumour (e.g. fat, neural, vascular, etc.).
2. To present current imaging techniques for evaluation of soft tissue tumours.
3. To discuss the imaging findings which are important for the diagnosis of soft tissue tumours.

**A-0033** 09:30
**Bone tumours**
J.L. Bloem; *Leiden/NL (j.l.bloem@lumc.nl)*

Bone sarcomas are rare (0.2% of all neoplasms, annual incidence in Europe is 0.8 per 100.000 population), in contrast to benign bone tumours and the so-called tumour-like lesions. The incidence of these benign entities is relatively high, but not known exactly as these are often asymptomatic. The WHO (version 2013) classified benign and malignant bone tumours in 13 main categories; chondrogenic, osteogenic, fibrogenic, fibrohistiocytic, hematopoietic, osteoclastic giant cell rich, notochordal, vascular, myogenic, lipogenic, undefined neoplastic nature and miscellaneous tumours. Each category is further subdivided into 1-14 tumour types. Imaging plays an important role in diagnosis, monitoring therapy, staging, and detecting recurrent disease. Diagnosis is mainly based on conventional radiography using morphologic appearance in combination with location, and age. Advanced imaging techniques are used for local staging (MR), detection of metastases (chest CT), monitoring therapy (MR, ultra-sound, PET-CT), detecting recurrence (MR, ultra-sound, PET-CT). Typical imaging features based on the WHO classification system will be presented with a focus on conventional radiography, common tumours, and relevance.
***Learning Objectives:***
1. To review the classification of bone tumours.
2. To present current imaging techniques for evaluation of bone tumours.
3. To describe the typical features of common bone tumours.

**08:30 - 10:00** **Room F2**

### New Horizons Session

### NH 1

### The role of imaging in the era of liquid biopsy

**A-0034** 08:30
**Chairperson's introduction**
D. Regge; *Turin/IT (daniele.regge@ircc.it)*

Tumours shed DNA fragments in the bloodstream when undergoing apoptosis. Technology is now available that allows genotyping of circulating tumour DNA (ctDNA) to detect somatic alterations found in tumours by sampling blood, a test commonly described as a liquid biopsy. This technique has shown promise in the detection of cancer in its early stages, in the identification of cancer recurrence following surgery and to monitor antineoplastic treatment longitudinally. Detecting the evolving polyclonal mechanisms of drug resistance hints at what personalised treatment could look like in the future. If liquid biopsy proves up to expectations, the role of imaging in the assessment of cancer will have to be revised. This session will explore the potential impact of liquid biopsy on diagnostic imaging from the perspective of the molecular biologist and of the imaging doctor with the aim of drawing-up a shared view.
***Session Objectives:***
1. To learn about the role of liquid biopsy in cancer detection and tumour surveillance.
2. To become familiar with advances of imaging in cancer detection and characterisation.
3. To understand the revised role of imaging in monitoring of cancer therapy.
4. To explore how combining molecular and imaging metrics could improve clinical decision in cancer patients.
*Author Disclosure:*
**D. Regge:** Author; Springer. Consultant; im3D. Research/Grant Support; Sanitas. Speaker; GE Healthcare, GE Medical Systems.

**A-0035** 08:35
**Liquid biopsy in perspective: colorectal cancer as a model system**
A. Bardelli; *Turin/IT (alberto.bardelli@ircc.it)*

When metastatic colorectal cancers are challenged with targeted agents almost invariably a subset of cells insensitive to the drug emerges. As a result, in most instances, targeted therapies are only transiently effective in patients. Strategies to prevent or overcome resistance are therefore essential to design the next generation of clinical trials. How can we overcome the near-certainty of disease recurrence following treatment with targeted agents? To address this question a deeper understanding of the evolutive nature of cancer cells is necessary. We used colorectal cancer (CRC) as a model system to test the hypothesis that by understanding tumour's evolution the emergence of drug resistance can be controlled. We find that clonal dynamics can be monitored in real time in the blood of patients, and liquid biopsies can be used to intercept the emergence of resistant clones before relapses are clinically manifest. We discovered that a multistep clonal evolution process driven by progressive increases in drug fitness underlies the development of resistance in cells and patient avatars. To have long-term efficacy, the use of targeted therapies must take into account the continuous evolution of cancer cells, that is to say, therapies must adapt to tumour evolution. One possibility is to anticipate the changes the tumours will make. For example, we propose to use liquid biopsies to know early the mechanisms of resistance to EGFR blockade in individual patients, and devised further rounds of therapy accordingly.
***Learning Objectives:***
1. To explain the principles of liquid biopsy.
2. To review the role of liquid biopsy as a diagnostic tool for early diagnosis.
3. To learn about the role of liquid biopsy as a predictor of cancer recurrence.
4. To explore how liquid biopsy could complement imaging, from a molecular biologist's perspective.
*Author Disclosure:*
**A. Bardelli:** Advisory Board; Horizon Discovery, Biocartis, Neophore. Consultant; Roche, Illumina, Novartis, Guardant Health, Merck. Shareholder; Neophore, Phoremost.

**A-0036** 08:53
**New imaging tools for cancer detection and characterisation**
H.-P. Schlemmer; *Heidelberg/DE (h.schlemmer@dkfz.de)*

Prognostic and predictive imaging biomarkers are essential for personalised oncology, regarding both, research as well as clinical practice. With the backing of computer assistance, an increased amount and complexity of multiparametric and multimodal imaging data enable to precisely detect cancer including its origin, local infiltration pattern and distant spreading, to gain important functional/biological information about its individual aggressiveness and to monitor or even predict morphologic and functional tumour changes during therapy. Sophisticated image postprocessing tools serve to extract information in a quantitative, objective and reproducible way. Recent research on radiomics, deep learning and artificial intelligence even envisage gaining information, which is otherwise inaccessible to conventional visual image analyses by radiologists. But the potential of imaging is inevitably limited for intrinsic reasons, why in many clinical situations microscopic/molecular tissue analyses are still imperative. The collection and molecular analysis of circulating tumour cells, extracellular vesicles and/or cell-free nucleid acids from fluids, especially blood, is currently object of intensive research. It is even hoped that in certain cases non-invasive/ minimal-invasive, the so-called liquid biopsy may replace tissue biopsy. The integration of imaging with liquid biopsy accordingly opens the door to new diagnostic opportunities. Personalised oncology may significantly benefit from the integration of spatial/functional information from imaging and molecular information from a liquid biopsy. But various scientific and methodological issues have still to be addressed before this concept will become a valuable tool for clinical practice.
***Learning Objectives:***
1. To review the modern approach to early diagnosis with imaging.
2. To become familiar with the new imaging biomarkers for tumour characterisation.
3. To envisage how liquid biopsy and imaging could complement each other in cancer diagnostics.

**A-0037** 09:11
**Is there still a role for imaging surveillance? And when?**
V.J. Goh; *London/UK (vicky.goh@kcl.ac.uk)*

Cancer surveillance aims to detect disease recurrence at an early enough stage for further definitive treatment to be a success. Accurate quantification of disease burden & comprehensive localisation of disease sites is required to improve patient stratification for further therapy - definitive or otherwise. This ensures that progression-free survival is improved particularly for patients undergoing definitive therapy. Imaging may be utilised either as the primary surveillance tool or to localise disease sites once other techniques have detected recurrence. A liquid biopsy is a highly sensitive test, and one of the challenges for imaging is to be able to localise small burden disease if further treatment is an option. Ultimately, the choice of imaging modality and strategy for active surveillance has to balance sensitivity with cost-effectiveness. This lecture will explore current surveillance protocols for common cancers and the future role of imaging in the era of liquid biopsy.
***Learning Objectives:***
1. To explain the rationale of cancer surveillance.
2. To review current cancer surveillance imaging strategies.
3. To become familiar with new imaging tools for surveillance of patients with cancer.
4. To explore how liquid biopsy and imaging could improve detection of minimal residual disease.
*Author Disclosure:*
**V.J. Goh:** Research/Grant Support; Siemens Healthcare.

**A-0038** 09:29
**Combining molecular and imaging metrics in cancer (radiogenomics)**
K. Pinker-Domenig; *Vienna/AT (pinkerdk@mskcc.org)*

With the genomic revolution in the early 1990s, medical research has been driven to study the basis of human disease on a genomic level and to devise precise cancer therapies tailored to the specific genetic makeup of a tumour. To match novel therapeutic concepts conceived in the era of precision medicine, diagnostic tests must be equally sufficient, multilayered and complex to identify the relevant genetic alterations that render cancers susceptible to treatment. With significant advances in training and medical imaging techniques, image analysis and the development of high-throughput methods to extract and correlate multiple imaging parameters with genomic data, a new direction in medical research has emerged. This novel approach has been termed radiogenomics. Radiogenomics aims to correlate imaging characteristics (i.e., the imaging phenotype) from different imaging modalities with gene expression patterns, gene mutations, and other genome-related characteristics and is designed to facilitate a deeper understanding of tumour biology and capture the intrinsic tumour heterogeneity. Ultimately, the goal of radiogenomics is to develop imaging biomarkers for an outcome that incorporate both phenotypic and genotypic metrics. Due to the non-invasive nature of medical imaging and its ubiquitous use in clinical practice, the field of radiogenomics is rapidly evolving, and initial results are encouraging. In this article, we will briefly discuss the background and then summarise the current role and the potential of radiogenomics in oncology.
***Learning Objectives:***
1. To explain the basic principles of radiogenomics.
2. To summarise the current clinical applications of radiogenomics.
3. To explore how radiogenomics could guide clinical decisions in the future.
*Author Disclosure:*
**K. Pinker-Domenig:** Research/Grant Support; 2020 - Research and Innovation Framework Programme PHC-11-2015 # 667211-2, NIH/NCI Cancer Center Support Grant (P30 CA008748, 828978 — CANCER SCAN — H2020-FETOPEN-2018-2020/H2020-FETOPEN-2018-2019-2020-01.

09:47
**Panel discussion: Will liquid biopsy be a game changer for radiologists?**

**08:30 - 10:00 Room Y**

### Joint Session of the ESR Ultrasound Subcommittee with EFSUMB

### ESR US SC/EFSUMB

### Ultrasound simulation models in training and education: where are we going?

**A-0039/A-0040** 08:30
**Chairpersons' introduction**
D.A. Clevert; *Munich/DE (Dirk.Clevert@med.uni-muenchen.de),*
P.S. Sidhu; *London/UK (paulsidhu@btinternet.com)*

This session deals with the ever advancing practice of using simulators for education and training of ultrasound practitioners. Many new developments of technology related to the machines and simulators make this an attractive proposal. Experts in the field will detail experience and discuss the future directions. New simulators will be presented, and data will be discussed.
***Session Objectives:***
1. To learn about which technical procedures are proposed in a simulation-based curriculum in radiology.
2. To learn about advantages and disadvantages of the different types of ultrasound simulators available.
3. To learn about the possibility of using virtual reality and gamification for training.
4. To learn about the experience from different parts of Europe in using ultrasound simulation training.
*Author Disclosure:*
**D.A. Clevert:** Speaker; Bracco, Siemens, Philips, Samsung. **P.S. Sidhu:** Advisory Board; Samsung Medison. Consultant; Itreas Inc. Speaker; Bracco SpA, Philips Healthcare, GE Healthcare, Siemens Healthineers, Hitachi Inc.

**A-0041** 08:40
**Needs assessment of simulations-based training in radiology**
L. Nayahangan; *Copenhagen/DK (leizl.joy.nayahangan@regionh.dk)*

Radiology is rapidly evolving with the advent of sophisticated, state-of-the-art imaging modalities. The implementation of these advanced technologies requires the need for alternative training methods such as simulation to supplement the traditional apprenticeship approach. Simulation-based education has been adapted in radiology to provide trainees with the opportunities to practice different technical and diagnostic skills, including interpretative and non-interpretative skills. Many simulation-based training programs exist; however, they are most often developed based on the availability of simulation equipment, local interests or other practical considerations. The development of training programs should follow a systematic approach by starting with a general needs assessment to ensure that these are aligned with current trainee needs. The needs assessment follows a structured three-round Delphi method to identify technical procedures that are suitable for simulation-based training. This iterative approach involves a panel of key opinion leaders to gather information and achieve consensus regarding procedures for simulation training. Round 1 is a brainstorming phase to gather procedures that a newly qualified radiologist should be able to perform. Round 2 is rating the procedures using a needs assessment formula to explore frequency, the number of doctors, impact on patients and feasibility for simulation-based training. Round 3 involves the elimination and final prioritisation of the procedures. The needs assessment process using the Delphi method identifies and prioritises a list of technical procedures for simulation-based training. This list provides an important foundation for the planning and development of simulation-based training programs in Radiology.
***Learning Objectives:***
1. To learn about how to perform a needs assessment using the Delphi method.
2. To learn about how a needs assessment identified and prioritised technical procedures to be included in a simulation-based curriculum.
3. To learn about how the list of procedures may be used as guide for development of training programmes.

**A-0042** 09:00
**Review of the available market of ultrasound simulators**
M. Bachmann Nielsen; *Copenhagen/DK (mbn@dadlnet.dk)*

A number of simulators are available for training abdominal ultrasound examinations. A screen with limited number of knobs aims to resemble a clinical ultrasound machine. Most simulators will use a dummy torso and a look-a-like transducer and others will use a joystick or a mouse. To train the coordination of the probe positioning and the screen a simple cartoonish sonogram may suffice. The presentation will aim to present a number of simulators available as per February 2019 including simple simulators, high-end simulators comprised of 3D patient examinations, virtual reality simulators and simulators aimed for biopsy training. Each simulator will have its strengths and weaknesses. It is not clear which simulator to use for specific needs.
***Learning Objectives:***
1. To learn about different types of simulators on the market.
2. To learn about which are suitable for beginners or advanced levels, and which can be used for training in interventional ultrasound.
3. To learn about advantages and disadvantages of different types of ultrasound simulators.

**A-0043** 09:20
**Gamification and virtual reality in medical simulation**
L. Konge; *Copenhagen/DK (lars.konge@regionh.dk)*

The old “see-one, do-one, teach-one” approach to medical education is outdated. Simulation-based training provides evidence for better efficacy, less stress, better patient outcomes, and improved return-on-investment. The question is no longer if we should use simulators to train our future doctors but how. There is a huge implementation gap regarding simulation in medical education. Despite the solid evidence, simulation is still severely under-utilised as a teaching modality, and initial training on patients are still very common. Virtual reality simulators and gamification allow new trainees to measure their own progress and compete against peers - this is highly motivating and could be a driver for implementation.
***Learning Objectives:***
1. To learn about gamification as a tool in medical education.
2. To learn about virtual reality in medical education.
3. To learn which possible ways virtual reality and gamification could be implemented in ultrasound.

09:40
**Panel discussion: Will there be an option to include these technics in our daily work and how could we improve the outcome?**

**08:30 - 10:00** **Room D**

### E³ - ECR Academies: Interactive Teaching Session for Young (and not so Young) Radiologists

### E³ 121

### Emergency radiology I

**A-0044** 08:30
**A. Acute aortic syndrome**
H. Alkadhi; *Zurich/CH (hatem.alkadhi@usz.ch)*

Computed tomography (CT) angiography represents the major imaging modality for the diagnosis of acute aortic syndromes. This lecture will review the various underlying diseases of the acute aortic syndrome, demonstrate typical imaging features enabling the diagnosis and discuss management options depending on the type, extent, and location of the disease. Clinical examples will be shown in this presentation.
***Learning Objectives:***
1. To understand the different types of acute aortic syndrome.
2. To learn about imaging findings and management options.

**A-0045** 09:15
**B. Abdominal trauma**
R. Basilico; *Chieti/IT (rbasilic@unich.it)*

Abdominal traumas can be classified into two categories: penetrating and blunt traumas. Abdominal injuries are more often observed in the setting of polytrauma: in fact, they are present in about 10% of patients admitted to level 1 trauma. However, only 11% of patients with abdominal trauma require laparotomy when a correct imaging-guide approach is performed. In fact, because the management of trauma patients mainly depends on the mechanism and severity of the trauma, it is crucial to choose the correct imaging modality and/or technique when evaluating a trauma patient on the basis of these two parameters. For example, an ultrasound examination, possibily integrated by contrast-enhanced ultrasonography, may be adequate to image a minor blunt abdominal trauma. This modality, however, is not appropriate when evaluating a severe blunt or penetrating abdominal trauma or even a polytrauma patient with a minor mechanism of injury. Multidetector CT is actually the modality of choice for evaluating severe trauma patients, accompanied by an appropriate CT protocol to image these patients so as to avoid missed injuries and to correctly detect abdominal solid organ injuries, mesenteric and intestinal injuries and abdominal vascular traumatic lesions. Moreover, due to the fact that during the past decades there has been a major change from operative to increasingly conservative management of abdominal traumatic injuries, even in patients with higher grades of injuries or those with older age, imaging features together with hemodynamic considerations play an essential role in the treatment choice: surgery, conservative management, endovascular treatment.
***Learning Objectives:***
1. To identify the signs of trauma.
2. To provide an indication of their clinical significance.

**08:30 - 10:00** **Room G**

### Physics in Medical Imaging

### RC 113

### Patient-specific dosimetry

**A-0046** 08:30
**Chairperson's introduction**
E. Samara; *Sion/CH (elina.samara@hopitalvs.ch)*

The use of ionising radiation in medical imaging offers substantial benefits for the diagnosis and treatment of numerous medical conditions in children and adults. However, exposure to ionising radiation may be associated with harmful risks. Justification and optimisation as principles of radiation protection make radiation dosimetry fundamental. Physicians need to know the levels of exposure, and hence the risks from imaging examinations that they have to justify and operators of X-ray cases such as examinations of children and pregnant patients or screening examinations require specific organ dose estimations. Moreover, to prevent tissue reactions patient skin dose needs to be estimated during radiological interventional procedures. Patient characteristics, such as age, sex and size, should be taken into consideration for specific patient dose assessments. Each modality (radiography, fluoroscopy, mammography, computed tomography, etc.) has its specificities and demands different methods to calculate the dose. Modern imaging modalities usually display conventional dosimetry metrics, such as dose-area product, incident air-kerma or computed tomography dose index that do not represent individual patient dose. Thus, numerous sophisticated concepts and methods have been proposed to estimate patient radiation dose that includes either physical measurements with dosimeters and anthropomorphic phantoms or computational measurements using Monte Carlo simulations. All these methods progressively allow to more accurate estimations of individual patient doses.
***Session Objectives:***
1. To understand the needs for personalised dosimetry.
2. To learn about existing and new methodologies used for patient dosimetry.
3. To understand the challenges for the implementation of patient-specific dosimetry.

**A-0047** 08:35
**A. Breast imaging dosimetry**
I. Sechopoulos; *Nijmegen/NL (ioannis.sechopoulos@radboudumc.nl)*

Mammographic dosimetry is a subject of intense interest due to the use of this imaging modality for population-based screening. However, established breast dosimetry methods do not result in patient-specific dose estimates. Rather, our current methods provide estimates of dose to a model breast, even if the actual technique used for a specific acquisition is taken into account. New insights into breast anatomy have provided us, for the first time, with estimates of how current dose predictions can differ from actual patient-specific doses. During this talk, the current method and model for breast dosimetry in mammography and digital breast tomosynthesis will be reviewed, and its capabilities and limitations discussed. How patient-specific breast dosimetry could be achieved and the current progress towards this goal will be presented, and what its potential applications could be will be discussed.
***Learning Objectives:***
1. To understand the current method to estimate organ dose in mammography and its limitations.
2. To understand breast dosimetry in emerging modalities.
3. To learn about upcoming approaches in breast dosimetry.
*Author Disclosure:*
**I. Sechopoulos:** Advisory Board; Fischer Imaging. Research/Grant Support; Siemens Healthcare, Canon Medical Systems. Speaker; Siemens Healthcare.

**A-0048** 08:58
**B. Patient dosimetry in CT and CBCT**
S. Edyvean; *London/UK (sue.edyvean@phe.gov.uk)*

Conventional CT is a complex imaging device which can utilise narrow or wide beams, helical scanning, varying tube current, varying kV, and operating with various and dynamic collimations - all of which can affect patient dose. Cone beam CT (or flat panel CT) uses different technology, referring to cross-sectional imaging with digital x-ray systems used in dental, digital radiography or radiotherapy. Many of the challenges are similar for both conventional CT and cone-beam CT; however, there has been substantially more research, and literature available, for the former. The dose distribution for both can be complex, due to the geometry of irradiation. When considering patient-specific dosimetry, the technology, exposure settings, and the patient, all need to be considered. As with other modalities, the scope to directly measure doses using physical dosimeters is limited. Therefore, doses need to be estimated using regular-shaped, or anthropomorphic, phantoms, or calculated using mathematical modelling techniques. The appropriate application, and the limitations, of standard dose indices (such as the computed tomography dose index, the size-specific dose index, the cone beam dose index), together with their associated methodologies and phantoms, will be addressed. Monte Carlo calculations from a modelled scanner and patient characteristics give more precise organ dose information, and there are a number of applied software packages developed to utilise these data. The advantages and limitations of these will be discussed.
***Learning Objectives:***
1. To understand what is estimated.
2. To learn how to measure it.

**A-0049** 09:21
**C. Patient dose in fluoroscopy and interventional**
A. Trianni; *Udine/IT (annalisa.trianni@asuiud.sanita.fvg.it)*

Biological effects induced by exposure to radiation made evident the need for accurate dosimetry from the early days of X-ray use in medicine. Later the scientific interest turned also to the estimation of risks of radiation-induced cancer and genetic effects. Various dosimetric quantities have been used in diagnostic radiology and sometimes confusion is caused because the same name is used for different quantities. The question arises: what is patient dose and what is not? It's common practice to indicate the dose received by a patient in radiology using the dosimetric indexes defined for the different imaging modalities. However, dosimetric indexes are a useful tool for practices optimisation and comparison, but certainly, they don't describe patient dose. To accurately estimate patient dose there is the need to collect a lot of information concerning patient characteristics and irradiation events in order to obtain organ or tissue dose. Nowadays, thanks to the digital technology used in medical imaging, various ways of collecting these data are available. The information is communicated using specific standards (i.e. DICOM), such as the non-image information object definitions. These objects are carrying information about equipment output and other dose-related information which allow the user to provide a more accurate estimate of patient dose. This talk will review the basic concepts of patient dosimetry as well as the existing DICOM objects and IHE profiles used to monitor patient exposure, highlighting the pros and cons and introducing to future developments.

***Learning Objectives:***
1. To review the fundamental patient dosimetry quantities.
2. To learn about calculation of patient dose for interventional procedures.
3. To learn about real time patient dose monitoring strategies.

09:44
**Panel discussion: The future of patient-specific dosimetry**

**08:30 - 10:00** **Room K**

### Multidisciplinary Session

### MS 1

### Lung cancer team

**A-0050** 08:30
**Chairperson's introduction**
A.R. Larici; *Rome/IT (annarita.larici@unicatt.it)*

Lung cancer is the leading cause of cancer-related mortality worldwide. Non-small cell lung cancer (NSCLC), the most common subtype, has an overall 5-year survival rate of 16%, which has not improved significantly for several decades. The poor prognosis could be attributed to the diagnosis of lung cancer at an advanced stage and the lack of a cure. Radiological imaging plays a crucial role in the diagnostic workup of lung cancer, from the early identification to the accurate staging. The new staging TNM system introduced relevant changes in order to better reflects different patient prognosis, even though with some limitations. It is mandatory when lung cancer is suspected, to obtain a tissue diagnosis to ascertain the tumour type. Imaging modalities represent effective tools to guide further investigative procedures for tissue sampling and, therefore, to adequately guide patient management and treatment planning. In the past decade, the therapeutic arsenal for NSCLCs has diversified significantly, with the emergence of targeted therapies and, more recently, immunotherapies. Personalised treatment has grown with the integration of predictive biomarkers, giving the potential to identify patients who may experience the lowest toxicity and/or derive the greatest benefit from these new treatments based on individual tumour profile. Lung cancer treatment planning is a complex process that involves multiple specialities. In the past two decades, multidisciplinary care has emerged as the standard of care in lung cancer management. This session will be focused on discussing the main aspects of lung cancer diagnosis and management.

***Session Objectives:***
1. To understand how to carry out an accurate diagnosis of lung cancer.
2. To learn about the actual therapeutic approach to lung cancer in the era of "personalised treatment".
3. To appreciate how a multidisciplinary team can make the difference in effectively managing lung cancer.

**A-0051** 08:35
**Imaging and staging**
M. Silva; *Parma/IT (mariosilvamed@gmail.com)*

Lung cancer (LC) staging is mandatory to formulate effective treatment strategies and optimise patient outcomes. The staging has traditionally relied on the TNM system, for which the International Association for the Study of Lung Cancer (IASLC), which is now in his eighth edition (TNM-8). The TNM-8 is based on detailed analysis of a new large international database of lung cancer cases assembled by the IASLC. Fundamental cornerstones of TNM-8 include the modifications to the T classification on the basis of 1-cm increments in tumour size, the grouping of lung cancers that result in partial or complete lung atelectasis or pneumonitis, and grouping of tumours with involvement of a main bronchus irrespective of the distance from the carina. Furthermore, reassignment of diaphragmatic invasion was included in terms of T classification, whilst the mediastinal pleural invasion was removed from the T classification, and the M classification was divided into different descriptors on the basis of the number and site of extrathoracic metastases. In response to these revisions, established stage groups have been modified, and others have been created. In addition, recommendations for classifying patterns of disease that result in multiple sites of pulmonary involvement, including multiple primary lung cancers, lung cancers with separate tumour nodules, multiple ground-glass/lepidic lesions, and consolidation, as well as recommendations for lesion measurement, are addressed. Understanding the key revisions introduced in TNM-8 allows radiologists to accurately stage patients with lung cancer and optimise therapy.

***Learning Objectives:***
1. To learn how to optimise the diagnostic algorithm of lung cancer.
2. To understand the evolving meaning of TNM classification.
3. To become familiar with key imaging criteria to manage lung cancer.

**A-0052** 08:53
**Role of the pathologist: making the most of the sample**
G. Rossi; *Ravenna/IT (giurossi68@gmail.com)*

The advent of effective targeted therapies in lung cancer has significantly changed the standard of care and contemporarily stressed the need for even more tumour tissue finalised at predictive biomarker determinations. Lung cancer presentation, patients characteristics, tumor location and stage profoundly impact on the better approach to maximize the amount of neoplastic material using conventional invasive methods, while liquid biopsy does represent a new source of tumour cells, particularly helpful in monitoring disease progression and in the comprehension of the mechanisms of drug resistance when using specific inhibitors in oncogenic driven malignancies. Actually, radiologists and bronchoscopists are both involved in the correct choice to sample lung cancer, but no universally perfect procedures do exist. The significant increase of adenocarcinoma histology, representing about 60% of all lung malignancies, led to a huge number of peripheral tumours with more frequent mediastinal lymph node involvement. Then, computed-tomography (CT)-guided and transbronchial (with/without endobronchial ultrasound guidance, EBUS) fine-needle aspiration (FNA) are the most common adopted techniques, equally allowing a fair-to-optimal tumour specimen. First line treatment initially requires a precise histological definition of lung cancer. Indeed, about 30% of non-small-cell carcinoma (NSCLC) are poorly-differentiated requiring immunostains with TTF-1 (quite specific for adenocarcinoma) and p40 (indicating squamous cell differentiation). Advanced/metastatic adenocarcinoma, squamous cell carcinoma arising in non-smokers and NSCLC not otherwise specified require a prompt determination of EGFR mutations, ALK and ROS1 rearrangements and PD-L1 expression, whereas conventional squamous cell carcinoma needs only PD-L1 investigation.

***Learning Objectives:***
1. To learn about the best way to obtain tumour tissue using different approaches.
2. To optimise tumour tissue management increasing the diagnostic yield in histologic subtyping and predictive molecular determinations.
3. To understand the correct information to relate to the oncologist as the first line of treatment.

**A-0053** 09:11
**Role of the oncologist: personalising the treatment**
S. Novello, M.L. Reale; *Orbassano/IT (silvia.novello@unito.it)*

Lung cancer treatment has become a paradigmatic example of personalised medicine. Therapeutic management takes into consideration histology, molecular pathology, staging, patients' characteristics. The identification of oncogenic driver alterations (EGFR activating mutations, ALK/ROS1 translocations) allowed the development of targeted therapies based on molecular profiling, enriching the previous 'histology-directed treatment' paradigm. These drugs, counteracting the deregulated pathways, improved outcomes substantially, quality of life and toxicity in molecular selected populations and represent today the standard of care in the oncogene-addicted advanced disease. Nevertheless, acquired resistance arises. Newer agents, overcoming cancer escape, showed remarkable outcomes. Unfortunately, the best sequence of the available treatments, in most cases, is not defined. The landscape is evolving, and other oncogenes are emerging as potential targets implementing personalised medicine algorithms. In the case of non-oncogene addiction and permissive comorbidities, immunotherapy demonstrated successful results in different settings. PDL-1 is the current biomarker to identify patients who may benefit from frontline immune checkpoint inhibitors. However, because of its high variability and dynamic expression, complementary predictive factors are exploring to maximise patients' selection. In this scenario, tissue availability, after histologic definition, is necessary; non-invasive methods are emerging to overcome the potential limits of biopsies or aspirates. Lung cancer is an increasingly complex and heterogeneous disease. Understanding how to best sequence and combine therapies could represent another important step in treatment personalisation. Cooperation and multidisciplinary approaches are the keys for the shared vision of a precision medicine aimed to offer the best treatment to every single lung cancer patient.

***Learning Objectives:***
1. To learn about the meaning of personalised treatment and the evolution of lung cancer therapies in the last 10 years.
2. To understand the role of the newer targeted therapies.
3. To become familiar with the needs of the oncologist to optimise lung cancer treatment.

*Author Disclosure:*
**S. Novello:** Speaker; Bureau for Roche, Boheringer Ingelheim, Eli Lilly, Astra Zeneca, MSD, BMS, Takeda.

**A-0054** 09:29
**What the surgeon needs to know**
U. Pastorino; *Milan/IT (ugo.pastorino@istitutotumori.mi.it)*

The optimal management of non-small cell lung cancer (NSCLC) requires multidisciplinary collaboration. The incidence of stage I and II NSCLC is likely to increase with the ageing population and the introduction of lung cancer screening for high-risk individuals. The surgical approach is to be tailored upon each individual case, and options are lobectomy, sublobar resections (SLR), sleeve resections, and minimally invasive techniques such as video-assisted thoracic surgery (VATS) and robot-assisted thoracic surgery (RATS). Furthermore, radiation therapy (especially stereotactic body radiation therapy - SBRT), is a valid alternative in compromised patients who are high-risk candidates for surgery. Minimally invasive techniques are suitable for a subset of patients who can be selected on the basis of a pre-surgical investigation by imaging and bronchoscopic sampling by ultrasonography. Stage III NSCLC is also treated surgically with curative purpose when specific conditions are fulfilled. A variable combination with neoadjuvant chemotherapy in specific cases is discussed by the lung cancer team before surgical resection, to improve long-term outcome. The synergistic collaboration of the lung cancer team is based on diagnostic cornerstones, which are demanded by the surgeon for the optimal planning of lung cancer resection (either minimally invasive or enlarged) with or without pre-surgical neo-adjuvant therapy, or for the definition of those subjects at high risk for surgical procedures (alternative treatment such as SBRT).

***Learning Objectives:***
1. To learn about the advances in the surgical approach for lung cancer treatment.
2. To understand how the clinical staging may affect surgical results in lung cancer.
3. To become familiar with the needs of the surgeon to define the appropriate surgical approach.

**A-0055** 09:47
**Multidisciplinary case presentation and discussion**
A.R. Larici; *Rome/IT (annarita.larici@unicatt.it)*

Lung cancer management and treatment planning are complex processes involving multiple specialities. In the past two decades, multidisciplinary care has emerged as the standard of care for lung cancer patients. Multidisciplinary care facilitates discussions between the different specialists involved in the diagnostic process to ensure that the site with the highest chance of obtaining tissue from is targeted first, thereby minimising the risk to the patient of recurrent procedures. There is evidence that access to the most accurate staging investigations is improved by multidisciplinary care, helping to limit unnecessary surgery which does not appear to occur at the expense of patient undertreatment. The impact of multidisciplinary care on measures of quality lung cancer treatment includes staging accuracy, access to diagnostic investigations, improvements in clinical decision making, better utilisation of radiotherapy and palliative care services, and improved quality of life for patients. Multidisciplinary care reduces variation in care, overcomes barriers to treatment, promotes standardised treatment through adherence to guidelines, and allows the audit of clinical services and for these reasons is more likely to provide quality care for lung cancer patients. This presentation is a case-based review demonstrating the added role of the multidisciplinary discussion on lung cancer patient management.

**08:30 - 10:00 Room M 1**

### Joint Session of the ESR and EIBALL

### ESR/EIBALL

### Imaging biomarkers and their combinations in the era of artificial intelligence

**A-0056** 08:30
**Chairpersons' introduction (part 1)**
O. Clément; *Paris/FR (olivier.clement@aphp.fr)*

Imaging biomarkers have raised a major interest over the last ten years by a radiologist to quantify and monitor diseases. The rapid spread of artificial intelligence in our discipline might trigger a new era where complex information will be more easily integrated. This session focuses on techniques, examples, opportunities and pitfalls in the use if AI for biomarkers development.

***Session Objectives:***
1. To introduce the potential of AI to accelerate the introduction of imaging biomarkers.
2. To introduce the challenges of managing biomarkers with AI systems.
3. To introduce the speakers.

*Author Disclosure:*
**O. Clément:** Advisory Board; Bayer. Speaker; Bracco, Guerbet.

**A-0057** 08:33
**Chairpersons' introduction (part 2)**
N.M. deSouza; *Sutton/UK*

This session will focus on the use of artificial intelligence for selecting and managing imaging biomarkers. Clarification around the use of terms such as 'artificial intelligence' and 'biomarkers' will be discussed. The strengths and limitations of the technologies used will be considered. The development of researcher-driven science and technology networks through the EU COST (Co-operation in Science and Technology) action initiative will be showcased using renal biomarkers as an exemplar. Finally, the selection of combinations of biomarkers from hybrid imaging technologies using AI will be addressed.

***Session Objectives:***
1. To introduce the potential of AI to accelerate the introduction of imaging biomarkers.
2. To introduce the challenges of managing biomarkers with AI systems.
3. To introduce the speakers.

*Author Disclosure:*
**N.M. deSouza:** Grant Recipient; Cancer Research UK, EU Framework 7.

**A-0058** 08:35
**Building and discovering biomarkers with AI**
B. Rance; *Paris/FR (bastien.rance@aphp.fr)*

In recent years, Deep Neural Networks (DNN) have achieved unprecedented performances in many domains, especially with the analysis of images. Major results have been announced for several applications, including skin lesions, pneumonia, pathology and so forth. Several algorithms have even been approved by regulatory agencies, e.g. for the diagnosis of diabetic retinopathy in specific circumstances. It now makes no doubt that artificial intelligence algorithms will be part of the medical experts' toolboxes. In this presentation, we will explore the basic principles behind artificial intelligence and neural networks: supervised and unsupervised algorithms, neurons, activation functions, and the overall architectures of networks. We will discuss more specific classes of DNN used today for the exploration of images, namely the convolutional network and the autoencoder-denoiser, and how they can be used to identify new biomarkers. We will emphasise the crucial role played by expert annotations on images, and explore how experts annotations are used to build models. Finally, we will discuss the implications of the use of deep neural networks in medicine and radiology, and briefly explore new risks (for example adversarial attacks) linked with the use of such technologies.

***Learning Objectives:***
1. To learn about available supervised vs unsupervised machine learning techniques.
2. To learn about deep-learning methods to discover biomarkers.
3. To understand the strength, but also limits and pitfalls, of machine learning methods.

**A-0059** 09:00
**COST action initiatives as a platform for image biomarker selection**
A. Caroli; *Ranica/IT (acaroli@marionegri.it)*

COST actions are EU-funded, open, and growing pan-European networking tools aimed at building, bridging and expanding interdisciplinary research communities. A successful example is the COST action PARENCHYMA (Magnetic Resonance Imaging Biomarkers for Chronic Kidney Disease - www.renalmri.org), which coordinates the research of leading European groups working on renal MRI. The rising prevalence of Chronic Kidney Disease (CKD) poses a major public health challenge, and an alarming number of negative clinical trials on CKD progression point out the urgent need for better biomarkers to identify patients that are at risk of progression or are likely to respond to candidate therapeutics. MRI biomarkers have shown a high potential to help fill this gap, as they are non-invasive and sensitive to CKD pathophysiology. Based on this rationale, PARENCHYMA is trying to unlock renal MRI biomarker potential by improving their standardisation and availability, and by generating strong multicentre clinical evidence of biological validity and clinical utility. As different imaging biomarkers provide complementary information on pathophysiology, they need to be combined to reach the highest sensitivity. Artificial intelligence could help to identify best disease-specific combinations.
***Learning Objectives:***
1. To learn about how COST actions work and the outputs generated.
2. To appreciate examples of a successful COST action for selecting imaging biomarkers.
3. To understand difficulties in selecting biomarkers for specific indications.

**A-0060** 09:25
**Role of AI in the introduction of imaging biomarkers: accelerator or obstacle?**
J.C. Waterton; *Manchester/UK (John.Waterton@manchester.ac.uk)*

According to the FDA/NIH BEST resource, a biomarker is a "defined characteristic that is measured as an indicator of normal biological processes, pathogenic processes, or responses to exposure or intervention, including therapeutic interventions; ... radiographic characteristics are types of biomarkers". Imaging biomarkers include scores from scoring systems, such as objective tumour response; extensive variables, such as LVEF; and intensive variables, such as the CT Hounsfield Unit. Biomarkers are essential for evidence-based based medicine, for regulatory approvals, and for prescribing information. However biomarkers must be reproducible over time and space: the measured imaging biomarker value must not drift when measured in a different clinic, or when better scanners are introduced. Radiologic examinations often provide imaging biomarkers which guide patient care. However much of the information in single images, or from multi-modal imaging examinations, is not captured in currently available imaging biomarkers. In the hands of an expert radiologist, this additional information further improves patient care: AI offers the hope of creating new biomarkers by quantifying this additional information. AI-derived imaging biomarkers are, however, not exempt from the need to follow established scientific and regulatory validation pathways and roadmaps. They should show which aspect of the underlying pathology is captured by the new biomarker and must demonstrate reproducibility over time and space (including strategies to maintain validity with future scanners yet to be designed). Otherwise, AI-derived imaging biomarkers will not translate and will remain academic curiosities.
***Learning Objectives:***
1. To learn how AI can improve imaging biomarkers from multiple imaging modalities.
2. To appreciate the challenges "big data" pose to regulators, whether from imaging biomarkers or from the more conventional biospecimen (genomic, proteomic) biomarkers.
3. To understand how best to utilise AI for imaging biomarkers and to avoid the pitfalls.
*Author Disclosure:*
**J.C. Waterton:** Consultant; Bioxydyn.

09:50
**Panel discussion: What infrastructure do we need to exploit AI for selecting, validating and managing imaging biomarkers?**

**08:30 - 10:00** **Room M 2**

### Paediatric

### RC 112

### Interventional radiology (IR) in children: what a non-interventional radiologist needs to know

*Moderator:*
E. Alexopoulou; Athens/GR

**A-0061** 08:30
**A. Common IR procedures in children: state-of-the-art**
A. Barnacle; *London/UK (Alex.Barnacle@gosh.nhs.uk)*

Interventional radiology (IR) procedures are a vital part of paediatric care for most complex diseases, providing reliable intravenous access and feeding tube support during intensive therapy such as chemotherapy and delivering innovative minimally-invasive therapies for other conditions to avoid surgery and often save lives. Central venous access in children can be technically challenging but is quicker and less invasive than open surgical techniques, and there is evidence to suggest that the central veins are preserved for longer, which is key in children with chronic disease. Angiography in children is usually straightforward, with just a few important technical points to remember in small children. Looking beyond central venous access, paediatric IR delivers therapies such as sclerotherapy for vascular malformations, percutaneous nephrolithotomy (PCNL) for renal stone extraction, oesophageal dilatations to manage oesophageal strictures and biopsy to safely and accurately stage childhood tumours. As with all of IR, the key to safe and successful practice is an understanding of the different pathology processes in children and the underlying decision making processes involved in delivering best practice.
***Learning Objectives:***
1. To learn about the most common causes for image-guided intervention in children.
2. To understand how the practical and technical approach to intervention in a child differs from that in adults.
3. To appreciate tips, tricks and pitfalls in paediatric intervention procedures.

**A-0062** 09:00
**B. Vascular malformations: diagnosis and interventions**
M. Beeres; *Frankfurt a. Main/DE (beeres@gmx.net)*

Diagnosis and treatment of vascular malformations in children is a combination of different diagnostic tools adapted to the given clinical setting. It always has to be an interdisciplinary team approach mostly based in dedicated centres to first find the correct diagnosis and then decide whether or not treatment is necessary. From the clinical background, most vascular malformations don't need invasive treatment because they do not cause any symptoms. However, the clinical decision has to be based on a reliable diagnosis, and different imaging pathways have to be considered. Imaging is predominantly performed by Ultrasound and MRI. Valuable information is added by clinical examination and history as well as sometimes histopathologic specimens. Different MRI examination approaches exist that help to find the correct diagnosis in the end. In imaging, it is important to distinguish between high-flow lesions e.g. arteriovenous malformations (AVM) or low-flow lesions e.g. venous malformations. Ultrasound and MRI can help to look beyond the focal vascular malformation because other body-regions might be affected as well. The correct diagnostic workup will help to define the right clinical pathway in a multidisciplinary team discussion. The treatment decision should then include non-interventional procedures such as physiotherapy or compression hosiery as well as interventional procedures such as sclerotherapy, embolization and surgery. It is essential to talk to and support the children and their parents, so they understand the different pathways and options. When all this comes together, they can decide well-informed leading to a hopefully satisfied patient in the end.
***Learning Objectives:***
1. To learn about the classification of paediatric vascular malformations.
2. To understand the diagnostic work-up and the indications for percutaneous treatment of paediatric vascular malformations.
3. To appreciate the treatment options for different vascular malformations.
*Author Disclosure:*
**M. Beeres:** Research/Grant Support; Travel Grant: Bayer Vital.

**A-0063** 09:30
**C. Osteoid osteoma: diagnosis and treatment**
D. Filippiadis; *Athens/GR (dfilippiadis@yahoo.gr)*

Osteoid osteoma is a benign inflammatory bone tumour encompassing 2-3% of all bone tumours and 10% of benign bone tumours; it is most common in males < 25 years of age with patients typically complaining of pain that worsens at night and is promptly relieved by salicylates. A tumour was first reported by Jaffe in 1953; osteoid osteoma is composed of the nidus which is bone at various maturity stages surrounded by highly vascular connective tissue stroma. Depending on the location and axial imaging findings, osteoid osteoma can be classified into subperiosteal, intracortical, endosteal or intramedullary and intra-articular with the latter being the least common type and refers to lesions located within or near a joint. The application of radiofrequency ablation (RFA) was introduced in clinical practice by Rosenthal in 1992 performing a percutaneous approach for the treatment of osteoid osteoma. Nowadays thermal ablation of osteoid osteoma constitutes a first-line therapy. Numerous studies upon all ablation techniques, others with lesser and others with higher numbers of patients report high pain reduction rates (up to 96%) and low recurrence rates (~7% at two years). Comparing percutaneous ablation to the traditional surgical techniques for osteoid osteoma (wide excision removing a bone block, marginal resection of the entire nidus, curettage or high-speed burr techniques) favours a percutaneous approach in terms of minimum trauma, minimum functional restriction and significantly lower cost.

***Learning Objectives:***
1. To learn how to diagnose, and how to treat, osteoid osteomas.
2. To understand the techniques for percutaneous treatment of osteoid osteoma: preparations, procedure and follow-up.
3. To appreciate advantages and potential complications in percutaneous treatment of osteoid osteoma in children.

*Author Disclosure:*
**D. Filippiadis:** Advisory Board; BTG/Galil Medical, Medtronic.

**08:30 - 10:00** **Room M 3**

### Oncologic Imaging

### RC 116

### Imaging tumour response to immunotherapy

**A-0064** 08:30
**Chairperson's introduction**
O.L. Sedlaczek; *Heidelberg/DE (sedlaczek@web.de)*

Cancer Immunotherapies include a broad variety of interactions between applied substances and the immune system to treat cancer. Passive mechanisms include the delivery of compounds that may use the immune system. The more recently extensively used drugs lead to an active priming of the immune system via disinhibition of immune checkpoints either at the level of the lymphnode (CTLA 4 - interaction) or at/near the malignancy itself (PD(L) -1 - interaction). Checkpoint-inhibitors are a breakthrough in the treatment of a variety of human malignancies including lung, renal, bladder cancer, and of course melanoma. They led to significant improvements in response and survival rates. However, the disinhibition of mechanisms normally protecting from autoimmunity and prolonged immunoreactions can lead to both unusual tumour response patterns and atypical toxicities. Concerning the response patterns the continued application of an immune treatment after the first observation of a classical RECIST - progression (pseudoprogression) is the common difference in all the response criteria specifically suggested to be used in immunotherapies (irRC, irRECIST, iRECIST). Although there are data for several substances and entities showing a beneficial effect of an ongoing treatment with checkpoint inhibitors even beyond a confirmed progression, there is a relevant role for imaging in the early prediction of treatment success particularly due the enormous cost of the treatments and the relevant toxicities associated.

***Session Objectives:***
1. To discuss the mechanism of action of immunotherapies.
2. To understand immunotherapy response and immune-related adverse events.
3. To become familiar with immune related response criteria (irRC), immune-related RECIST (irRECIST) and immune RECIST (iRECIST).
4. To discuss future directions for advanced imaging of immunotherapy.

**A-0065** 08:35
**A. Imaging the immune system in cancer**
C. Dromain; *Lausanne/CH (Clarisse.Dromain@chuv.ch)*

A wide range of cancer immunotherapy approaches has been developed including non-specific immune-stimulant such as cytokines (Interferon, IL2), cancer vaccines (peptide or dendritic-cell-based vaccines), adoptive T-cell therapy (TILs, CAR, TRC) and immune checkpoint inhibitors (anti-CTLA-4, anti PD1 and anti PDL1). The most commonly used are the immune checkpoint inhibitors (ICIs). Their mechanism of action signifies a true shift in oncology where instead of targeting the tumour cells, ICIs target the immune system to break the cancer tolerance and stimulate the anti-tumour immune response. These new drugs have, since 2011, received marketing authorisation for melanoma, lung, bladder, renal, and head and neck cancer with remarkable and long-lasting treatment response. The novel mechanism of action of these drugs, with immune and T-cell activation, lead to unusual patterns of response with presence pseudo-progression more pronounced and more frequent than previously described responses. Pseudo-progression that has been described in about 3-10% of patients treated using ICIs corresponds to increase of tumour burden and/or appearance of new lesions due to Infiltration of a tumour by activated T-cells before the disease responds to treatment. To overcome the limitation of RECIST criteria to assess this specific changes in tumour burden, new criteria so-called irRC and then irRECIST were proposed. The major modification involved the inclusion of the measurements of new target lesions into disease assessments and the need for a 4-week CT re-assessment to confirm progression. More recently (2017) a consensus guideline iRECIST was developed by the RECIST working group.

***Learning Objectives:***
1. To discuss the concept of immunotherapy treatment in cancer.
2. To review what the radiologist needs to know when assessing immunotherapy treatment.
3. To understand the challenges of assessing immunotherapy response using imaging.

**A-0066** 08:53
**B. Monitoring immune response with CT**
F. Gentili; *Siena/IT (francescogentili@gmail.com)*

Computed tomography (CT) is the most widely available imaging technique for evaluating tumour response to chemotherapy. In the last years' cancer immunotherapy is changing response evaluation criteria to treatment since new patterns of treatment response have been observed employing immunomodulating agents; such therapies indeed can be associated with a significantly delayed decrease in tumour size, and new or enlarging lesions observed soon after completion of treatment may not indicate disease progression. For this reason, in this scenario, the traditional response criteria, such as WHO and RECIST 1.1, cannot be applied and therefore, during these years, several response criteria (irRC, irRECIST, iRECIST and imRECIST) were proposed and applied in clinical trials on immunotherapy. Moreover, changes of intratumoral vascularisation, objectively assessed by Perfusion-CT and Dual-energy CT, may reflect the effects of treatment and therefore be incorporated in response criteria. Finally, CT radiomics is a promising method, applied to conventional images, that seems to be able to detect subtle differences in CT values which cannot be recognised by human eyes, providing quantitative data on tumour microenvironment by analysing the distribution and relationship of pixel intensities.

***Learning Objectives:***
1. To discuss different types of immunotherapies and to review their mode of action as imaged with CT.
2. To understand the limitations of RECIST and become aware of immune response criteria.
3. To discuss the limitations of CT for assessment of immunotherapies.

**A-0067** 09:11
**C. Monitoring immune response with PET**
C.C. Cyran; *Munich/DE (clemens.cyran@med.uni-muenchen.de)*

In recent years a range of novel immunomodulatory cancer therapeutics were introduced into clinical use aiming to boost anti-tumour immune response in cancer patients. Among these immunotherapies, neutralising antibodies targeting the immune checkpoints T-lymphocyte-associated protein 4 (CTLA-4) and programmed cell death protein 1 (PD-1) have shown effectiveness in the treatment of different tumour entities. Unlike cytototoxic radio- and chemotherapy, which directly interferes with tumour cell growth and survival, immunotherapies target the tumour indirectly stimulating the physiological anti-tumour immune response. Established methods of assessing cytotoxic tumour therapies (e.g. RECIST) have demonstrated limitations for monitoring the early therapeutic effects of immunotherapy. This has led to the development of immune-specific related response criteria (e.g. irRC, irRECIST,iRECIST) that allow continued treatment beyond progression defined by RECIST, however with also limited applicability. The complementary acquisition of functional and molecular information using PET in line with hybrid imaging techniques may allow for a higher diagnostic accuracy in monitoring immunotherapy and the timely differentiation of responders from non-responders. The applicability of 18F-FDG PET for monitoring treatment with immune checkpoint inhibitors has been questioned because the infiltration of the tumour by immune cells may cause a transient increase in metabolic activity. Dedicated tracers for immune PET mostly aim to visualise the presence and abundance of various subsets of immune cells with different targets including PD-L1, PD-1, CTLA-4, CD3 and IFNγ. Dedicated immune PET with specific tracers may provide valuable in vivo insights into the pathophysiology of tumours under immunotherapy and new opportunities to evaluate antitumor immune response.

***Learning Objectives:***
1. To discuss the framework for the use of FDG PET/CT for immunotherapy assessment.
2. To become familiar with the best response criteria for assessment of immunotherapy using PET/CT.
3. To discuss the limitations of PET/CT for assessing of immunotherapies.

**A-0068** 09:29

**D. Monitoring immune response with MRI**

D.-M. Koh; *Sutton/UK (dowmukoh@icr.ac.uk)*

Drugs that modulate the body immune responses are increasingly used to treat cancers, either alone in combination, such as in malignant melanoma, non-small cell lung cancer, hepatocellular carcinomas. In patients receiving immunotherapies, CT is still the most widely used imaging technique to assess the treatment response of tumours and to identify drug-related side-effects. However, anatomical MR imaging is also effective in evaluating tumour regression by size measurement criteria (e.g. iRECIST). Like CT, an increase in tumour size may be observed on MRI in pseudo-progression, which can confound response assessment. Nonetheless, the superior soft tissue contrast of MRI helps to depict subtle disease (e.g. intracranial) and specific complications (e.g. hypophysitis) that are difficult to visualise on CT. There is great interest in applying quantitative MR imaging, including whole-body MRI techniques, to study functional changes in tumour cellularity (using diffusion-weighted MRI), vascularity (using contrast-enhanced MRI) and macromolecules (magnetisation transfer). These are areas of on-going research, including the use of texture analysis and radiomics analysis. Whole body MRI can provide information about inter-tumoural heterogeneity, thus allowing insights into tumour evolution and differential response to treatment. Active research in molecular probes is being undertaken to develop PET imaging tracers that can identify and predict treatment response, which can be explored alongside multi-parametric MRI measurements on an MRI/PET hybrid system as imaging biomarkers.

***Learning Objectives:***
1. To learn the advantages and limitations of MRI for assessment of immunotherapies.
2. To describe the potential role of whole-body MRI and quantitative MRI techniques for patient follow-up.
3. To consider the potential of integrated PET/MRI for the assessment of immunotherapies.

09:47

**Panel discussion: What is the best way to monitor immunotherapy response?**

**08:30 - 10:00** **Room M 4**

### E³ - ECR Academies: Hot Topics in GU Cancer

### E³ 119

### Early detection of ovarian cancer

**A-0069** 08:30

**Chairperson's introduction**

R. Forstner; *Salzburg/AT (r.forstner@salk.at)*

Ovarian cancer is still among the deadliest of cancers in females. Its prognosis depends on different factors including histologic subtype, stage at diagnosis, tumour burden after cytoreductive surgery and sensitivity to chemotherapy. However, it is early stage and detection of precursor lesions of ovarian cancer that are of utmost prognostic impact. New insights in biology and genetics in ovarian cancer hold promise in earlier detection and are pivotal in personalised treatment. Blood biomarkers and/or imaging have been widely used in screening programs of ovarian cancer. Currently, a screening test is widely recommended in patients at risk, e. g in familial history, BRCA1and 2 mutation carriers or HPCN families, which are estimated to account for approximately 5-15% of ovarian cancers. Imaging using US and MRI plays a central role in identifying early ovarian cancer and precursors of invasive cancer, e.g. cystadenomas or Borderline tumours. The "O RADS" working group has developed a lexicon for both US and MRI. Such standardised terminology will allow an unequivocal and generally applicable description of ovarian masses. It will also provide an essential tool for risk stratification of ovarian lesions.

**A-0070/A-0071** 08:36

**A. Current guidance on screening and familial ovarian cancer**

A.G. Rockall; *London/UK (a.rockall@imperial.ac.uk)*
A. George; *London/UK (angela.george@rmh.nhs.uk)*

Germline BRCA1/2 (gBRCA) mutations underlie 15-20% of epithelial ovarian cancer diagnoses and are associated with biologically distinct tumours. Those with a gBRCA mutation typically have a younger age of diagnosis, higher rates of visceral and nodal metastases and an improved prognosis compared to those with gBRCA wildtype tumours. Mutation carriers also have increased rates of synchronous/metachronous tumours, particularly breast and pancreatic cancers. As ovarian cancer is typically diagnosed at a late stage, with poor long-term survival, there has been interest in screening, to try and improve outcomes. A number of screening studies have investigated the role of transvaginal ultrasound +/- CA-125 testing in both those with a family history of ovarian cancer; and those at population risk. To date, no improvement in overall survival has been observed with screening, although studies assessing relative CA-125 levels are ongoing. The methods and results of the largest screening trials will be presented with a discussion of findings.

***Learning Objectives:***
1. To be aware of the implications of results of recent screening trials.
2. To be aware of BRCA gene testing and treatment implications.
3. To know the guidance on screening high risk groups.

*Author Disclosure:*
**A.G. Rockall:** Speaker; Guerbet.

**A-0072** 09:04

**B. ORADS: ultrasound and International Ovarian Tumor Analysis (IOTA) group models**

R.F. Andreotti; *Nashville, TN/US (rochelle.f.andreotti@vanderbilt.edu)*

"O-RADS" is an acronym for an Ovarian-Adnexal Imaging-Reporting-Data System which is being developed by an international committee that is sponsored by the American College of Radiology (ACR) and will function as a quality assurance tool for the standardised ultrasound (US) and magnetic resonance imaging (MRI) description of ovarian/adnexal pathology. The O-RADS Committee has published a lexicon for describing US imaging characteristics of ovarian/adnexal masses. The terminology has been applied to a risk stratification classification for consistent follow up and management in clinical practice. The use of these internationally agreed upon standardised descriptors should result in consistent interpretations and decrease or eliminate ambiguity in reports resulting in a higher probability of accuracy in assigning the risk of malignancy and, subsequently, optimal patient management. The US lexicon is based upon supporting evidence for the performance of terms with regards to classification of the mass as benign or malignant and common usage of terms. A large part of the lexicon is based upon terms in use by the International Ovarian Tumor Analysis Group (IOTA). These terms demonstrate consistency regarding performance in the evaluation of malignancy risk and have been supplemented with other modifying, non-IOTA descriptors. Risk categories were developed using the most predictive descriptors in the lexicon that, in combination, include all possible lesions. The risk table is based on statistics collected by IOTA/ADNEX models. Cases that illustrate the O-RADS-US Lexicon and Risk Stratification System will be discussed.

***Learning Objectives:***
1. To understand a new lexicon for ovarian/adnexal mass evaluation on ultrasound.
2. To understand the use of the lexicon in the development of a risk stratification and management system.
3. To apply O-RADS-US to case management.

**A-0073** 09:32

**C. ORADS: MRI**

I. Thomassin-Naggara; *Paris/FR (isabelle.thomassin@tnn.aphp.fr)*

The O-RADS classification has been developed by the American College of Radiology in collaboration with ESR members. This classification helps the radiologist to evaluate the risk of malignancy of an adnexal mass and was built as a five categories steps. O-RADS needs the analysis of morphological and functional criteria including DCE MR images and DWI. This lecture will present the new lexicon.

***Learning Objectives:***
1. To understand a new lexicon for ovarian/adnexal mass evaluation on MRI.
2. To understand the use of the lexicon in the development of a risk stratification and management system.
3. To apply O-RADS-MRI to case management.

*Author Disclosure:*
**I. Thomassin-Naggara:** Advisory Board; SIEMENS. Speaker; GE, Samsung, Canon, Guerbet.

**08:30 - 10:00** **Room M 5**

### E³ - ECR Master Class (Head and Neck)

### E³ 126b

### Functional imaging in head and neck oncology

**A-0074** 08:30
**Chairperson's introduction**
F. Bidault; *Villejuif/FR (francois.bidault@gustaveroussy.fr)*

Squamous cell carcinoma of the head and neck is one of the most common cancers worldwide. Advanced tumours are associated with high morbidity and mortality rate. Risk factors are tobacco, alcohol, wood dust (professional disease), and viral infections (EBV, HPV). A multidisciplinary team approach is required to undertake effective personalised care planning. Decision process requires understanding patient's wish, conducting a physical examination, making imaging examination, panendoscopy, tumour biopsy with a pathological result, checking the patient's suitability for surgery. Imaging is the extension of physical examination and definitely is of tremendous importance for decision making. Speakers of this master session are leading experts in the field of imaging for locoregional tumour assessment with M.G. Mack, from Munich, incidence and prognosis of synchronous cancer or distant metastases from head and neck tumours with A.D. King, from Hong Kong, and question of the need for functional imaging to detect distant metastases with R. Maroldi, from Brescia.
***Session Objectives:***
1. To discuss the role of functional imaging in tumour staging.
2. To learn about the presentation and prognosis of head and neck cancer patients.
3. To understand the clinical impact of imaging findings.

**A-0075** 08:35
**A. Functional imaging for locoregional tumour assessment**
M.G. Mack; *Munich/DE (m.mack@radiologie-muenchen.de)*

Head and neck cancer is one of the most common cancers worldwide. MRI based diffusion and perfusion techniques enable the non-invasive assessment of tumour biology and physiology and add additional information to standard structural MRI scans. Head and neck squamous cell carcinoma may cause a decreased apparent diffusion coefficient (ADC) on diffusion-weighted magnetic resonance imaging (DW MRI) and an increased standardised uptake value (SUV) on fluorodeoxyglucose (FDG) positron emission tomography (PET/CT). These techniques can improve the initial staging and can help to monitor treatment response. In addition, these techniques can improve patient selection for therapeutic strategies and provide evidence for a change of therapy regime. This presentation summarizes recent literature and provides an overview of the various studies in which diffusion or perfusion-based MRI studies are applied to head and neck cancer and will provide an overview of commonly used acquisition protocols and postprocessing methods, followed by advanced data analysis, imaging findings regarding tumour characterization and differentiation, tumour risk stratification and staging, monitoring and prediction of treatment response. Limitations will be highlighted followed by a conclusion with recommendations for the future.
***Learning Objectives:***
1. To learn about functional imaging (perfusion, DWI, PET) in head and neck cancer patients.
2. To understand the value of functional imaging for tumour assessment.
3. To discuss nodal assessment in head and neck cancer.

**A-0076** 08:58
**B. Incidence and prognosis of synchronous cancer or distant metastases from head and neck tumours**
A.D. King; *Hong Kong/CN*

Second primary tumours Head & neck cancer has one of the highest associations with second primary tumours (SPT). SCC is associated with smoking and high alcohol intake, and so patients are also at risk of SPTs in other sites of the head & neck, lung and oesophagus. Less commonly SPTs arise in the colon, pancreas and bladder. SPTs are usually metachronous with synchronous tumours being diagnosed in only ~ 4% of patients. SPTs are a leading cause of death in patients with early-stage SCC, but SPTs detected early are potentially curable. Distant metastases With advances in locoregional control distant metastases (DM) now are emerging as a major determinant of survival. Therefore, a more active approach is being taken to detect and manage distant site disease, including the use of a new generation of immunotherapy agents. The incidence, risk factors, sites, prognosis, and management of DM vary with histology and will be discussed in three main groups comprising carcinomas of the aerodigestive tract, thyroid and salivary glands. Most DM manifest after initial treatment, the most common sites being lung or bone, followed by the liver. Prognosis tends to be poor with most patients succumbing to disease within a year. However, the prognosis is dependent on many factors which include histology, site and number of DM. Notably, some patients survive, and there is a subgroup of patients with oligometastases who have a potentially curable disease.
***Learning Objectives:***
1. To become familiar with the incidence of the synchronous tumours in the head and neck population.
2. To become familiar with the incidence of the distant metastases in the patients with newly diagnosed head and neck cancer.
3. To understand the consequences in a prognosis.

**A-0077** 09:21
**C. Is functional imaging necessary to detect distant metastases in head and neck cancers?**
R. Maroldi; *Brescia/IT (roberto.maroldi@unibs.it)*

Head and neck squamous cell carcinoma (HNSCC) is a predominantly locoregional disease. The reported incidence of distant metastasis (DM) varies in a wide range, between 3% and 50%, reflecting heterogeneity in population characteristics disease stages included, timing of diagnosis of DM, etc. DM factors do not only influence prognosis, but it is a major determinant of the choice of treatment. Patients with known DM can possibly be spared the toxicities of aggressive and often unnecessary locoregional therapy. FdG-PET-CT is the most used imaging techniques for detecting DM in HNSCC. Because the sensitivity for lesions with size less than 10mm is poor, the negative predictive value is considered insufficient for a low-volume disease. In fact, if the follow-up window increases from 12 to 30 months, the sensitivity of Fdg-PET-CT decreases from 83-97% to 60-82%, probably because of metastases too small for the technique. False positive findings are an additional troublesome factor, as the imply increased costs and delay of treatment. Therefore, FdG-PET-CT has a diagnostic yield low in early-stage disease. The highest clinical value is obtained in the pretreatment evaluation of advanced-stage (III/IV) and recurrent HNSCC. A new oncologic perspective has been recently explored: the concept of treating oligometastases (metastasectomy or stereotactic body radiotherapy). If the locoregional disease is controlled/resected, and the distant sites ablated, a prolonged disease-free interval and possible cure may be achieved. In addition to Fdg-PET-CT, a growing body of evidence has been published in the recent literature, supporting Whole-Body MRas screening for DM.
***Learning Objectives:***
1. To review the advanced imaging for detection of distant metastases of head and neck cancer.
2. To understand the advantages and disadvantages of functional imaging modalities for M staging.
3. To discuss when to image for distant metastases.

09:44
**Panel discussion: How to stage head and neck tumours correctly**

**10:30 - 12:00** **Room F1**

### E³ - Rising Stars Programme: Basic Session

### BS 2

### Thoracic emergencies

*Moderator:*
A.P. Parkar; Bergen/NO

**A-0086** 10:30
**Acute aortic syndrome**
T. Jargiello; *Lublin/PL (tojarg@interia.pl)*

Acute aortic syndrome (AAS) describes symptoms relevant to severe chest or back pain caused by several aortic pathologies which can be potentially life-threatening. The most common aetiology compromises the majority of AAS is aortic dissection. Other causes of AAS with similar presentation include intramural haematoma, penetrating aortic ulcer (atherosclerotic), aneurysm formation (rupture, enlargement) and traumatic transection (pseudoaneurysm). Imaging plays a vital role in diagnosing AAS. The role of the plain chest radiograph is nowadays historic. Echocardiography (plain or transoesophageal) is an effective portable tool but does not visualise the whole thoracic aorta. It may only suggest a certain AAS aetiology. Contrast-enhanced

CT is now considered as the diagnostic method of choice. It provides an accurate aortic assessment as well as a detailed evaluation of other thoracic structures. CT permits 3D reconstructions, essential for intervention planning and it is widely available. Its sensitivity for aortic disorders reaches 100%. MRI is another imaging modality to diagnose AAS. Although it provides greater anatomical detail than CT, it is seriously limited. The limitation involves procedure time, lesser availability, and the expense.
***Learning Objectives:***
1. To become familiar with the most common aetiologies of acute aortic diseases.
2. To present current imaging techniques for evaluation of acute aortic diseases.
3. To demonstrate the most important imaging findings.

**A-0087** 11:00
**Pulmonary embolism**
C. Loewe; *Vienna/AT (christian.loewe@meduniwien.ac.at)*

Pulmonary embolism (PE) represent a daily clinical challenge at the emergency department: out of the patients admitted to an emergency room because of the very unspecific clinical symptom of chest pain and/or dyspnea, the early identification of patients with a high clinical likelihood for PE is a difficult task. The D-Dimer test is very sensitive but unspecific for pulmonary embolism, and by using this test without clinical assessment, a high number of patients will be diagnosed as "false positives". On the other hand, PE is potentially life-threatening; and early and safe diagnosis of this relevant disease is important to improve patient's outcome. CT angiography of the pulmonary arteries has been established as the first method of choice to accurately detect PE with a high PPV and PPV. This presentation will provide an overview of the diagnostic challenges in the chest pain unit and will explain the need for accurate clinical assessment to select the right patient for the appropriate diagnostic test. Furthermore, the risks due to overdiagnosis or false-positive diagnosis will be exemplified. In the second part, this presentation will Focus on the Radiological view on this picture by explaining an optimised Imaging technique and possible pitfalls and problems. Challenging situations in the daily clinical practice will be discussed by addressing the role of imaging in suspected PE in pregnancy and the oncologic patients. Finally, typical findings in acute and chronic pulmonary embolism will be reviewed.
***Learning Objectives:***
1. To review the most common pathologies leading to pulmonary embolism.
2. To present current imaging techniques for evaluation of pulmonary embolism.
3. To become familiar with the typical findings in acute and chronic pulmonary embolism.
*Author Disclosure:*
**C. Loewe:** Speaker; Siemens, Bracco, GE Healthcare.

**A-0088** 11:30
**Acute coronary syndrome**
R.M.M. Hinzpeter; *Zurich/CH (Ricarda.Hinzpeter@usz.ch)*

Normal anatomic conditions of the coronary artery tree consist of two main coronary arteries, a left coronary artery and a right coronary artery arising from the coronary sinuses in the proximal ascending aorta immediately distal of the aortic valve. The right coronary artery descends in the right atrioventricular groove to the inferior surface of the heart, continuing as the posterior descending artery representing a right dominant circulation. After a short common stem, the left coronary artery bifurcates into the left anterior descending, coursing in the anterior interventricular groove and the left circumflex artery coursing in the left atrioventricular groove. After the initial workup of an acute coronary syndrome in the emergency department, including laboratory values and ECG changes, coronary CT angiography (CCTA) as a non-invasive examination plays an important role besides invasive techniques. Especially in patients with low to intermediate risk CCTA is a quick and reliable method for ruling out coronary artery disease with a very high negative predictive value in the absence of coronary artery calcifications. Besides the detection of significant coronary artery stenosis, the presence, amount and composition of non-calcified plaques and the degree of coronary remodelling is substantial. In addition, information about structural changes of the myocardium and myocardial perfusion can be obtained by CCTA.
***Learning Objectives:***
1. To become familiar with segmental coronary anatomy.
2. To present different techniques for assessment of acute coronary syndrome.
3. To become familiar with the typical findings of acute coronary syndrome.

**10:30 - 12:00 Room D**

### E³ - ECR Academies: Interactive Teaching Session for Young (and not so Young) Radiologists

### E³ 221

### Musculoskeletal radiology: inflammation

**A-0089** 10:30
**A. Inflammatory and infections in the soft tissues**
S. Martin; *Palma de Mallorca/ES (silvia.m.martin@gmail.com)*

The diagnosis of infections is based on the presence of clinical symptoms like erythema, swelling and pain. Also, the diagnosis is based on the presence of clinical signs such as fever, tachycardia and shock and laboratory test such as leukocytosis and C protein reactive. However, the clinical symptoms and signs of infection may not be specific, especially in the early stages of the disease. In these cases, Imaging tests play a fundamental role in the early diagnosis of infections and the differential diagnosis. The most important radiological findings for inflammatory and infections soft tissue are: 1/ Intramuscular fluid collections. 2/ Soft tissue air. 3/ Fascial fluid collections and 4/Muscle oedema. Potential causes of these radiological findings are diverse, including, infectious, autoimmune, inflammatory, neoplastic, neurologic, traumatic and iatrogenic conditions. Some of these conditions require prompt medical or surgical management, whereas others do not benefit from medical intervention. Necrotising fasciitis is a rare, life-threatening soft-tissue infection and a medical and surgical emergency that radiologist must know. The presence of gas within the necrotised fascia is characteristic, but may be lacking. The main finding is thickening of the deep fascia due to fluid accumulation and reactive hyperemia. All these findings may be seen in other different conditions. The ability to accurately diagnose these conditions is therefore necessary. Clues to the correct diagnosis and whether a biopsy is necessary or appropriate are often present on the images techniques, especially when they are correlated with clinical features.
***Learning Objectives:***
1. To learn the key signs for differential diagnosis.
2. To learn about imaging findings and management options.

**A-0090** 11:15
**B. Arthropathies**
U. Aydingoz; *Ankara/TR (uaydingo@hacettepe.edu.tr)*

Arthropathies are one of the most common health problems and the leading cause of disability in adults. Imaging plays an essential role in their diagnosis and follow-up. Plain films remain the first line imaging tool in the diagnosis and management of arthropathies, whereas MR imaging is essential to ascertain the presence of active inflammation and disruption of intra- and periarticular structures. This interactive presentation focuses on radiological features of common (and several less common) arthropathies and how they help in narrowing the differential diagnostic considerations.
***Learning Objectives:***
1. To explain the key points in the differential diagnosis of common arthropathies.
2. To describe the imaging findings of common arthropathies as they relate to pathophysiology.

**10:30 - 12:00 Room M 4**

### E³ - ECR Academies: Hot Topics in GU Cancer

### E³ 219

### Early detection of prostate cancer

**A-0091** 10:30
**Chairperson's introduction**
G.M. Villeirs; *Ghent/BE*

Prostate cancer is traditionally diagnosed on the basis of the combination of elevated PSA, abnormal digital rectal examination and transrectal biopsy. New biomarkers, including imaging, are currently challenging this gold standard diagnostic test. They are equally useful in the choice between immediate versus deferred treatment. Both aspects will be covered in detail during this session.

**A-0092** 10:36
**A. Screening for prostate cancer: where are we now? (part 1)**
A. George; *London/UK (angela.george@rmh.nhs.uk)*

Screening for prostate cancer with prostate-specific antigen (PSA) has been widely investigated. Several large screening studies, e.g. prostate, lung, colorectal and ovarian (PLCO), European randomised study of screening for prostate cancer (ERSPC) and cluster randomised trial of PSA testing (CAP), have shown PSA screening may lead to increased prostate cancer diagnoses, and at best may result in a small benefit in disease-specific mortality over 10 years but does not improve overall mortality. These benefits need to be considered against possible harms of PSA screening, including complications from a biopsy and subsequent treatments and the risk of overdiagnosis and treatment. Recent studies suggest incorporating MRI in the investigation of those with positive PSA test reduces the false positive rate and unnecessary biopsies and also increases the accuracy of biopsies in those with clinically significant cancer. Trials screening for prostate cancer with MRI is being proposed. Incorporating the patient's genetic mutation status into risk algorithms may allow development of targeted screening programs for early cancer detection and treatment, and may improve survival. Those with germline mutations such as BRCA2 have an increased risk from the age of 40yrs, and often more aggressive disease. There is also ongoing work stratifying prostate cancer risk at a population level with the use of single nucleotide polymorphism (SNP) panels. This use of targeted PSA and MRI screening in men with DNA repair mutations, adverse SNP profile and Afro-Caribbean ethnicity may result in improved outcomes and management algorithms based on biological disease behaviour.
***Learning Objectives:***
1. To be aware of important prostate cancer genetics and familial cancer.
2. To learn about the difference in low risk vs high risk genetic groups.
3. To be aware of the use of imaging and other biomarkers.

*Author Disclosure:*
**A. George:** Advisory Board; Astra Zeneca, Pfizer, Tesaro.

**A-0093** 10:50
**A. Screening for prostate cancer: where are we now? (part 2)**
A. Sohaib; *London/UK*

Screening for prostate cancer with prostate-specific antigen (PSA) has been widely investigated. Several large screening studies, eg prostate, lung, colorectal and ovarian (PLCO), European randomised study of screening for prostate cancer (ERSPC) and cluster randomised trial of PSA testing (CAP), have shown PSA screening may lead to increased diagnoses of prostate cancer and at best may result in a small benefit in disease-specific mortality over 10 years but does not improve overall mortality. These benefits need to be considered against the possible harms of PSA screening, including complications from a biopsy and subsequent treatments and the risk of overdiagnosis and treatment. Recent studies suggest incorporating MRI in the investigation of those with positive PSA test reduces false positive rate and patients undergoing unnecessary biopsies and also increases the accuracy of biopsies in those with clinically significant cancer. Trials screening for prostate cancer with MRI is being proposed. Incorporating the patient's genetic mutation status into risk algorithms may allow development of targeted screening programs for early cancer detection and treatment, and may improve survival. Those with germline mutations such as BRCA2 have an increased risk from the age of 40yrs, and often more aggressive disease. There is also ongoing work stratifying prostate cancer risk at a population level with the use of single nucleotide polymorphism (SNP) panels. This use of targeted PSA and MRI screening in men with DNA repair mutations, adverse SNP profile and those of Afro-Caribbean ethnicity may result in improved outcomes and management algorithms based on biological disease behaviour.
***Learning Objectives:***
1. To be aware of important prostate cancer genetics and familial cancer.
2. To learn about the difference in low risk vs high risk genetic groups.
3. To be aware of the use of imaging and other biomarkers.

**A-0094** 11:04
**B. Pre-biopsy detection and new techniques for detection in prostate cancer**
S. Punwani; *London/UK*

"no abstract submitted"
***Learning Objectives:***
1. To understand the role of mpMRI in tumour detection.
2. To be aware of texture features of prostate cancer.
3. To learn how texture analysis differentiate benign from malignancy prostate lesions.

**A-0095** 11:32
**C. Active surveillance: best practice**
J.J. Fütterer; *Nijmegen/NL (jurgen.futterer@radboudumc.nl)*

In order to avoid unnecessary radical treatment, active surveillance (AS) is becoming a viable treatment alternative in low-risk prostate cancer. Because most low-risk prostate tumours have an indolent course and the slow growth rate allows ample time during follow-up to detect tumours that begin more aggressive while remaining in a window of definitive curability. Patients are carefully observed every three or four months for changes in PSA, digital rectal examination or changes upon performed transrectal ultrasound (TRUS) guided biopsy. MR imaging is an appealing imaging technique to select and to surveil patients who choose for active surveillance. The addition of prostate MR imaging to the biopsy strategy or, in select patients, using MR imaging as a substitute for a repeat biopsy improves prostate cancer detection.
***Learning Objectives:***
1. To be familiar with case selection for active surveillance.
2. To know the frequency of imaging.
3. To understand when treatment will be commenced.

**12:30 - 13:30** **Room C**

### E³ - The Beauty of Basic Knowledge: Pancreas

### E³ 24A

### Acute pancreatitis

*Moderator:*
R. Manfredi; Rome/IT

**A-0096** 12:30
**Atlanta Classification of acute pancreatitis**
T. Bollen; *Nijmegen/NL (tlbollen@hotmail.com)*

The 2012 Revised Atlanta Classification (RAC) distinguishes between clinical severity of acute pancreatitis (AP) based on clinical parameters and morphologic severity based on CT parameters. The RAC defines three grades of clinical severity with increasing morbidity and mortality rates: Mild AP: defined as the absence of systemic or local complications. Moderately severe AP: defined as the presence of transient organ failure, deteriorating pre-existing co-morbid disease and/or presence of local complications requiring prolonged stay or intervention. Severe AP: defined as persistent organ failure (>48 h). Morphologically, interstitial and necrotising pancreatitis are discriminated, depending on the absence or presence of tissue necrosis, respectively. In general, patients with interstitial pancreatitis have clinically mild disease, and conversely, most patients with necrotising pancreatitis will sustain clinically severe AP. CT findings, however, are not absolutely predictive of outcome for an individual patient. Approximately 5% of patients with minimal changes on CT will have significant complications with mortality of around 1-3%. An even larger number of patients, up to 30%, will have a relatively benign clinical course despite the presence of pancreatic necrosis. Therefore, ultimate severity of disease is based on clinical parameters, primarily organ failure. CT is regarded as the frontline imaging technique for full evaluation of AP severity, especially in those who are predicted severe by clinical assessment. Follow-up studies are dictated by clinical findings that include sudden-onset or increase of abdominal pain and organ failure, signs of sepsis or other clinical signs of local complications or when invasive treatment is contemplated.
***Learning Objectives:***
1. To understand grading of acute pancreatitis: Atlanta Classification.
2. To learn about the clinical impact of Atlanta Classification.
3. To understand the follow up of acute pancreatitis.

**A-0097** 13:00
**Role of imaging**
C. Triantopoulou; *Athens/GR (ctriantopoulou@gmail.com)*

Imaging is frequently recommended in patients with acute pancreatitis (AP) to confirm the clinical diagnosis, ascertain the cause, grade the extent and severity of the disease, evaluate severe complications and indicate interventional procedures. The revised 2012 Atlanta criteria for classification of the severity of AP are widely accepted. The challenge for imaging remains to recognise patients suffering from severe or moderately severe AP. But, a direct correlation between clinical severity and morphology may not exist. Imaging is of utmost importance in the 2nd phase of the disease evolution (usually >2 weeks after onset) where AP resolves or evolves secondary to the presence of necrosis and infection, thus morphologic criteria are needed as defined by imaging techniques. It is important to evaluate the extent of necrosis (intrapancreatic, extrapancreatic or both) and also to define if this is sterile or infected. Contrast-enhanced CT is the best technique; however the staging of severity and detection of complications depend on the timing of CT scanning. In the first 24-48 hours, the CT findings of necrosis may be equivocal. In severe AP, unless the patient is critically ill and in need of emergency intervention, the initial CT scan should ideally be obtained at least 72 hours following symptoms onset. As 50% of AP cases are gallstone-related, transabdominal ultrasound is the most common initial radiologic investigation of choice. MRI can better differentiate complex fluid collections from mature pseudocysts and hemorrhagic collections, while MRCP is the best technique to identify pancreatic duct disconnection.
***Learning Objectives:***
1. To learn about diagnosis of acute pancreatitis.
2. To understand how to apply Atlanta Classification to imaging.
3. To learn about new trends in diagnosis of acute pancreatitis.

**12:30 - 13:30 Room D**

### E³ - The Beauty of Basic Knowledge: A Survival Guide to Musculoskeletal Imaging

### E³ 25A

### Infection: bone and soft tissue

*Moderator:*
V.N. Cassar-Pullicino; Oswestry/UK

**A-0106** 12:30
**Infection: bone and soft tissue**
J.L. Bloem; *Leiden/NL (j.l.bloem@lumc.nl)*

In this lecture, we'll focus on imaging osteomyelitis of the appendicular and axial skeleton, and their differential diagnosis using radiographs, US, and especially MRI and PET-CT. Understanding the interaction between invading organisms and intact or compromised host response is essential in the interpretation of imaging features. Major routes of infection include hematogeneous or contiguous routes occurring in respectively pediatric-geriatric, or immunocompromised and diabetic, or post-procedure infections. Location in the metaphysis and vertebral body are hallmarks of hematogenous spread. Location in bones close to skin defects, pressure points, and soft tissue infection are hallmarks of contiguous spread, especially in diabetic foot, intensive care patients, and following surgical or image guided procedures. Knowledge of age and the comorbidity-related relationship between vasculature on one-hand and growth plates and discs, on the other hand, is essential in the diagnosis of infection. Also, imaging features of host response depend on the pressure within anatomical compartments. The most relevant differential diagnostic issues including posttraumatic sequelae, degenerative disease, Charcot foot and spine, sterile inflammatory disease (CRMO, SAPHO), tumours, and also the differentiation between mild and life-threatening infection like necrotising fasciitis will be discussed. The impact of imaging on clinical outcome as it depends on treatment options, cost-effectiveness, predictive values of various imaging studies relative to clinical and laboratory tests, is addressed.
***Learning Objectives:***
1. To become familiar with pathophysiology of infectious diseases pertinent to imaging features.
2. To understand imaging features of infectious diseases and their differential diagnosis.
3. To appreciate the clinical perspective of imaging infectious diseases.

**14:00 - 15:30 Room B**

### Abdominal Viscera

### RC 301

### Tumour response assessment in abdominal imaging

*Moderator:*
S.M. Ertürk; Istanbul/TR

**A-0107** 14:00
**A. Colorectal liver metastases**
S.K. Venkatesh; *Rochester, MN/US (venkatesh.sudhakar@mayo.edu)*

Colorectal liver metastases (CRLM) occur in nearly half of the patients with colorectal cancer. Neoadjuvant chemotherapy (NAC) is the treatment of choice. Surgical resection and locoregional control have evolved as treatment options in the patients with single or oligometastatic disease and in those who show excellent response to NAC. The number,location and response of CRLM are critical in determining their surgical resection. Assessment of treatment response of CRLM to NAC and locoregional treatment is therefore very important to provide guidance for their management. Standard methods of assessment (RECIST) use size (one-dimensional, bi-dimensional, volume) and a reduction in the size is considered a response. However there are pitfalls to these methods and they will highlighted during the presentation. Variable responses of CRLM and to different chemotherapy regimens add another dimension to complexity of assessment of the response. The most important information is a viable or a residual tumour which tumour size alone cannot predict but can be assessed with additional information from dynamic contrast-enhanced MRI (DCE-MRI), diffusion-weighted imaging (DWI), positron emission tomography (PET). NAC and some locoregional treatment also cause liver toxicity including sinusoidal obstruction syndrome (SOS), chemotherapy-associated steatohepatitis (CASH) or simple fatty change that can influence the assessment of response. In this presentation, an overview of response of CRLM to treatment will be presented and this will be followed by review of different methods for response assessment including their limitations and advantages.
***Learning Objectives:***
1. To learn about different methods to assess the tumour response in the colorectal metastases.
2. To understand usefulness and limitation of techniques and the role of different contrast media.
3. To appreciate the variable response pattern of colorectal liver metastases.

**A-0108** 14:30
**B. Rectal carcinoma**
M.J. Lahaye; *Amsterdam/NL (MJ.Lahaye@gmail.com)*

MRI is the modality of choice for restaging rectal cancer. The high soft-tissue contrast of MRI can accurately assess the extramural tumour spread and relation to surrounding structures after chemoradiation. This lecture will have a practical approach to determining the role of MRI in the restaging of rectal cancer. The relevant anatomy, MRI techniques, the rationale for neoadjuvant treatment, and post-chemoradiation therapy imaging (including detection of patients with a complete response) will be discussed with special attention to how to apply recent advances in knowledge to daily clinical practice.
***Learning Objectives:***
1. To learn about the rationale for neoadjuvant treatment in rectal cancer and the impact on subsequent surgery.
2. To understand why imaging is needed to assess the response to neoadjuvant therapy, what to look for when judging response and where the challenges lie.
3. To appreciate when surgery can be deferred or avoided and how to best follow-up on these patients.

**A-0109** 15:00
**C. Pancreatic adenocarcinoma**
M. Zins; *Paris/FR (mzins@hpsj.fr)*

Pancreatic ductal adenocarcinoma (PDA) remains among the most challenging malignancies to treat. At diagnosis, the tumour often already extends beyond the confines of the pancreas, spreading to an extent such that primary surgery with curative intent is very rarely feasible. Considerable momentum is now being given to a treatment strategy involving neoadjuvant chemotherapy or chemoradiotherapy in patients with nonmetastatic PDA. The main advantage of this strategy is a better selection of patients likely to benefit from curative-intent surgery via the achievement of negative resection margins. Patients with a rapidly progressive disease are identified and spared ineffective surgery with its attendant morbidity. Neoadjuvant therapy can convert tumours classified as locally advanced by initial imaging studies to resectable tumours. However, the imaging study evaluation of the response to neoadjuvant therapy is extremely complex. Thus, the diagnostic performance of imaging studies is not sufficient to ensure the accurate selection of patients in whom negative-margin resection is likely to be achieved. More specifically, standard criteria for predicting vascular invasion, based on the amount of tumour-vessel contact, are not valid after neoadjuvant therapy.
***Learning Objectives:***
1. To understand the rationale for neoadjuvant treatment in pancreatic adenocarcinoma.
2. To learn the limitations of CT in assessing treatment response.
3. To learn how to accurately select patients for curative-intent surgery after neoadjuvant therapy.

**14:00 - 15:30 Room C**

### General Radiography (Radiographers)

### RC 314

### Hybrid imaging: the best of both worlds

*Moderators:*
N.N.
K. Riklund; Umea/SE

***Session Objectives:***
1. To review the principles of hybrid medical imaging.
2. To understand the indications, limitations and comparative merits of hybrid medical imaging across a wide range of cardiac, vascular and oncological conditions.
3. To appreciate the need for optimisation and dose management within hybrid imaging.

### A-0110 14:00
**A. PET/CT**
M. Zeilinger; *Wiener Neustadt/AT (markus.zeilinger@fhwn.ac.at)*

The establishment of highly sensitive molecular imaging techniques, in combination with the synthesis of novel radiolabelled bioactive molecules for the visualisation and quantification of numerous specific (patho)physiological processes and biochemical targets, merged the fields of molecular imaging and personalised medicine. In this context, PET belongs the most sensitive imaging modality with an excellent molecular sensitivity, capable of providing high temporal resolution for kinetic analysis of biochemical phenomena and molecular processes. The combination of a dedicated PET with a CT modality, as a morphological imaging technique, has the potential to provide essential, comprehensive information and to increase the scope of application for modern molecular imaging.
***Learning Objectives:***
1. To review the technology underpinning PET/CT.
2. To understand important staff and patient safety issues.
3. To discuss the current and potential clinical applications of PET/CT and optimal imaging protocols.

### A-0111 14:30
**B. Clinical impact of hybrid interventional imaging**
S. Robinson; *Manchester/UK (sharon.robinson@mft.nhs.uk)*

With many interventions no longer only open surgery but a combination of open and endovascular or purely endovascular, a hybrid endovascular theatre is recommended. Hybrid theatres combine access for safe open surgery with the ability to perform high quality rotational fluoroscopic imaging. As lead Interventional Radiographer at a busy tertiary vascular referral centre, the author has knowledge and experience in the implementation and use of the Hybrid theatre setting and will discuss the benefits of patient care and advanced imaging techniques provided by Hybrid imaging.
***Learning Objectives:***
1. To review clinical indications for hybrid interventional procedures.
2. To understand the radiographer's role in patient care and management in the hybrid suite.
3. To discuss image acquisition and processing techniques required for hybrid examinations.

### A-0112 15:00
**C. Optimisation/dose management in the hybrid interventional suite**
D. Maccagni; *Milan/IT (maccagni.davide@gmail.com)*

The hybrid room is a high-complexity operating room, fully equipped for minimally invasive procedures and provided with imaging techniques such as fluoroscopy/angiography, ultrasound, computed tomography, or magnetic resonance imaging. In this environment, the radiographer works with a team of health professionals including cardiologists, cardiac surgeons, vascular surgeons, urologists, anesthesiologists, sonographers, radiologists, engineers and nurses, sharing the knowledge and skills necessary to assist these specific hybrid procedures. Managing radiation exposure in the hybrid room is of utmost importance. The risk related to radiation exposure is represented by tissue reactions, (e.g., skin burns), and stochastic effects, where the risk of an outcome is proportional to the dose received, (e.g., cancer). This issue can affect patients but can also have harmful consequences for the operators that are present in the room, during the entire procedure. Nowadays, with the possibility offered by technology in a hybrid room, there are different options for dose optimisation. Modern fluoroscopy machines reduce the dose delivered while maintaining high-level image quality and some tools have been introduced to limit operators' x-ray exposure. This has indeed allowed an improvement in terms of safety, but it should not be forgotten that the continuous application of the basic rules of radiological protection remains of fundamental importance. The proper application of radiological protection is one of the most prominent responsibilities of the radiographer's duty. A proper application of radiation protection, with continuous and updated training, can minimise the radiation issues, increasing the procedure safety, without compromising the effectiveness of the procedures.
***Learning Objectives:***
1. To review current options for optimisation in the hybrid interventional suite.
2. To understand the radiographer's role in optimisation and dose management in the hybrid suite.
3. To discuss occupational radiation protection issues in the hybrid suite.

**14:00 - 15:30 Room F1**

### E³ - Rising Stars Programme: Joint Session with ESOR

### BE 3

### Radiologic anatomy: chest

*Moderator:*
J. Vilar; Valencia/ES

### A-0122 14:00
**Mediastinal**
M. Occhipinti; *Florence/IT (mariaelena.occhipinti@gmail.com)*

Several pathological conditions can arise from such a complex anatomical structure as the mediastinum, including large airways, large and small vessels, lymph nodes, adipose tissue, nerves, oesophagus, heart, and pericardium. The wide spectrum of mediastinal diseases makes their differential diagnosis very challenging at times. To simplify the clinical approach to the mediastinal lesions many classifications of the mediastinum based either on either chest radiography or CT have been proposed. Knowledge of the most appropriate technique to use in each clinical context as well as knowledge of specific imaging signs and features are needed to narrow the spectrum of differential diagnosis and to address most questions arisen when a mediastinal mass is either seen or suspected at chest radiography. Old and new classifications will be compared and their value discussed as well as signs useful in localising and characterising mediastinal lesions will be examined.
***Learning Objectives:***
1. To review the mediastinal anatomy according to old and new classifications.
2. To recap signs useful in localising and characterising mediastinal lesions.
3. To know how to choose the most appropriate imaging modality to explore the mediastinum according to the diagnostic question.
*Author Disclosure:*
**M. Occhipinti:** Consultant; Imbio LLC. Grant Recipient; Menarini Foundation.

### A-0123 14:30
**Lungs**
C.M. Schaefer-Prokop; *Amersfoort/NL*

"no abstract submitted"
***Learning Objectives:***
1. To know how to interpret mediastinal lines and contours in chest radiography.
2. To get familiar with "signs" in chest radiography helpful for diagnosis.
3. To know the normal appearance of lung parenchyma on CT and to use the anatomy of the secondary lobule for analysis of chest diseases.

### A-0124 15:00
**Vasculature**
M. Francone; *Rome/IT (marco.francone@uniroma1.it)*

Present lecture aims to review the normal anatomy of chest vasculature including caval venous system, great vessels and coronary arteries. Common and less common variants and anomalies will also be displayed and classified, analysing strength and weakness of different imaging methods. Some of these anomalies are found in children having impact, but they are more commonly discovered later in adulthood. Many of these anomalies are asymptomatic or 'leave alone' lesions, but some of these anomalies are symptomatic and need to be treated. Not only cardiovascular imaging dedicated physicians but all general radiologist we have to be familiar with normal vascular anatomy as well as common and less common anomalies.
***Learning Objectives:***
1. To learn peculiarities of coronary arteries anatomy with specific referral to its implications on myocardial physiology.
2. To review anatomic features of pulmonary arteries and thoracic aorta.
3. To examine patterns and variants of the venous drainage of both the pulmonary and caval system.

**14:00 - 15:30** **Room K**

### New Horizons Session

### NH 3

### Artificial intelligence (AI): driven by radiologists

**A-0125** 14:00
**Chairperson's introduction**
E. Kotter; *Freiburg/DE (elmar.kotter@uniklinik-freiburg.de)*

The development of Artificial Intelligence in Radiology has been surpassing expectations over the last years. Radiologists have both great expectations and many questions regarding the introduction of these disrupting technologies in clinical routine. Many questions are raised concerning the integration of AI in the clinical workflow, and continuous clinical validation of results. Additionally, the future role of the radiologist is questioned, and there are many discussions on the ethics of AI applications in clinical routine. This session will address these questions with the support of internationally renowned speakers.
***Session Objectives:***
1. To learn what can be expected from AI in radiology and to know the limitations of the technology.
2. To learn how AI technology can be integrated in the radiology workflow.
3. To learn how to make radiology knowledge available for AI training.
4. To learn how the introduction of AI in radiology will change the doctor-patient and radiologist-clinician relationship.
*Author Disclosure:*
**E. Kotter:** Advisory Board; Agfa.

**A-0126** 14:05
**The impact of AI technologies in patient care: advantages and limitations**
E.L. Siegel; *Baltimore, MD/US (esiegel@umaryland.edu)*

In their recent editorial in Academic Radiology, Cohan and Davenport refer to radiologist “burnout” and having reached a “tipping point”. They suggest that despite improvements in PACS and EMR's, “Radiologists are still being told to work faster as the screws continue to tighten; more images, greater case volume, increasing complexity and less time to do the work”. Radiologists are increasingly asked to perform quantitative analysis on complex dynamic studies such as prostate and breast MRI, analyse multi-parametric imaging from MRI, PET, CT, and to follow new guidelines for lung cancer and other screening studies. Deep learning represents a fundamentally different approach to the development of algorithms for image acquisition, quantitative analysis, and interpretation based on learning by example from large image sets. It offers numerous advantages over more “traditional” Computer Aided Design approaches including decreased time, and less specialised medical imaging expertise required for development as well as the potential for continuous and personalised refinement of algorithms. In fact, Deep Learning may actually have its greatest initial success in solving non-image related challenges such as image quality, workflow efficiency, improved communication and patient safety. This technology, however, is also fraught with limitations including the requirement for large amounts of annotated data, regulatory, medicolegal, and relative brittleness with regard to lack of generalizability from a few to a multitude of different scanners. Overall, despite the challenges, Deep Learning will undoubtedly have a major impact in the next several years on positively resetting radiology’s current “tipping point”.
***Learning Objectives:***
1. To know the challenges of knowledge management in radiology.
2. To know the advantages of machine learning technologies compared to traditional approaches.
3. To know the technical limitations of machine learning.

**A-0127** 14:28
**How to integrate AI technology in radiology today**
W.J. Niessen; *Rotterdam/NL (w.niessen@erasmusmc.nl)*

The combination of big data and artificial intelligence are dramatically increasing the possibilities for prevention, cure and care, and are changing the landscape of the healthcare system. Biomedical imaging data will play a central role in this revolution. In this presentation, I will show examples of possible large benefits of big data analytics of imaging, genetic and clinical data in dementia and oncology. Both conventional machine learning techniques, such as radiomics for tumour characterisation, and deep learning techniques that directly learn from the imaging data will be addressed. Also, the concept of deep imaging, full integration of medical imaging and machine learning, will be discussed. Finally, I will address the challenges of how to successfully integrate these technologies into the daily clinical workflow.
***Learning Objectives:***
1. To know the challenge: multitude of AI engines to be integrated in one workflow.
2. To learn how to collect and annotate radiology data for machine learning.
3. To learn how to organise radiologist's workflow when using AI.
4. To learn to manage radiology data to be used for machine learning.
*Author Disclosure:*
**W.J. Niessen:** Consultant; I am Scientific Lead at Quantib BV. Shareholder; I am shareholder of Quantib BV.

**A-0128** 14:51
**How will the introduction of AI change the role of the radiologist?**
H. Harvey; *London/UK (hugh@kheironmed.com)*

An overview of how machine learning technologies may play a role in the workflow and task automation of radiologists, from appropriateness criteria to image acquisition, to image perception tasks and report generation, this talk will look at the entire ecosystem of diagnostic radiology and AI in the coming years.
***Learning Objectives:***
1. To learn how radiologists will use machine learning in clinical routine.
2. To learn how machine learning will change the role of the radiologist (doctors-patient relationship, relationship between radiologists and referring physicians).
3. To learn how radiologists can prepare for machine learning.

15:14
**Panel discussion: The radiologist's work after integrating AI**
*Is there a future for radiology without the usage of AI?*
*Discuss the main challenges from radiologist's perspective*
*Clinical validation of AI: it looks like it is lacking*
*Ethical considerations when using AI in radiology*

**14:00 - 15:30** **Room M 4**

### E³ - ECR Academies: Hot Topics in GU Cancer

### E³ 319

### Whole-body imaging in gynaecological malignancy

**A-0129** 14:00
**Chairperson's introduction**
L.S. Fournier; *Paris/FR*

**A-0130** 14:06
**A. WB MRI for staging and treatment planning in ovarian cancer**
V. Vandecaveye; *Leuven/BE (vincent.vandecaveye@uzleuven.be)*

By their ability to evaluate distant nodal or visceral metastatic disease spread, computed tomography (CT), Fluorodeoxyglucose positron emission tomography/CT (FDG-PET/CT) and magnetic resonance imaging (MRI) have an increasingly important role in the management of gynaecological cancers. MRI has established its clinical importance by its superior contrast resolution and ability to extract morphological and functional information, beneficial for tumour detection, characterisation, staging and response assessment. The single most important prognostic factor in ovarian cancer is complete tumour resection at debulking surgery. In patients suspected for ovarian cancer, imaging workup has a major role in depicting metastases in order to assess resectability. CT tends to underestimate peritoneal disease extent; a problem that is not adequately solved by FDG-PET/CT. Recent technological progress has enabled time efficient whole-body MRI (WB-MRI) integrating diffusion-weighted imaging (DWI); the latter allows for the depiction of millimetric tumour deposits. In recent studies, WB-MRI/DWI has shown greater accuracy for a comprehensive presurgical depiction of peritoneal metastases over CT or FDG-PET/CT, while WB-MRI/DWI has shown similar accuracy to FDG-PET/CT for depicting distant metastases and lymphadenopathies. Important for surgical planning, MRI has shown better sensitivity over conventional imaging for detecting involvement of surgically critical tumour sites including mesenteric root infiltration small bowel and colon carcinomatosis and unresectable distant metastases. This presentation will discuss sequence optimisation, DWI anatomy, physiological signal and artefacts, the appearance of tumoral lesions in relation to their size and correlation of WB-MRI/DWI findings to surgical criteria that determine complete resection.

***Learning Objectives:***
1. To learn the MRI technique for imaging the peritoneum in advanced ovarian cancer.
2. To learn the appearances of WB-MRI in metastatic ovarian cancer.
3. To be aware of the pitfalls to interpretation.

**A-0131** 14:34
**B. PET/CT and PET/MRI in cervix and endometrial cancer: current status**
L. Umutlu; *Essen/DE*

"no abstract submitted"
***Learning Objectives:***
1. To learn the indications for use of hybrid imaging in cervix and endometrial cancer.
2. To know the strengths and weaknesses of the technique.
3. To be familiar with the role of hybrid imaging in patient prognosis.

**A-0132** 15:02
**C. Advanced imaging techniques in metastatic gynaecological cancer**
E. Sala; *Cambridge/UK (es220@cam.ac.uk)*

Tumour heterogeneity in metastatic gynaecological cancer and especially advanced ovarian cancer has been reported at the histological and genetic levels and found to be associated with adverse clinical outcomes. Classic tumour evaluation using standard CT or MRI techniques does not account for the intra- or inter-tumoural heterogeneity in advanced ovarian cancer with peritoneal carcinomatosis. As such, computational approaches in assessing tumour heterogeneity have been proposed using radiomics and radiogenomics to capitalise the whole tumour heterogeneity as opposed to single biopsy sampling. As part of radiomics, texture analysis which includes the extraction of multiple data from the images has been proposed recently to evaluate advanced ovarian tumour heterogeneity. The preliminary data suggests that it can unravel tumour heterogeneity and predict response to treatment both conventional and immunotherapy.
***Learning Objectives:***
1. To learn about the concept and technique of texture analysis.
2. To be familiar with the key associations of biology and texture features.
3. To be familiar with the potential added value of texture analysis in image interpretation.

**14:00 - 15:30** **Room M 5**

### E³ - ECR Academies: Functional Imaging for Disease Management: Research to Medical Practice

### E³ 320

### Functional imaging of the brain

**A-0133** 14:00
**Chairperson's introduction**
T.A. Yousry; *London/UK (t.yousry@ucl.ac.uk)*

Functional imaging has come a long way from being a demanding research tool to becoming an integral part of clinical MRI protocols essential for the decision-making process and therefore management of various CNS diseases. In this exciting session, we will explore the spectrum of its use from research to clinical practice in 3 distinct diseases: stroke, brain tumours, and small vessel diseases.

**A-0134** 14:06
**A. Stroke**
T. Tourdias; *Bordeaux/FR*

Brain imaging is an emergency when an acute stroke is clinically suspected. Such acute imaging is crucial (i) to establish the diagnosis of an ischemic or hemorrhagic stroke while ruling out stroke mimics, (ii) to provide etiological and prognosis clues and mainly (iii) to guide the acute therapeutic strategies. Either computer tomography (CT) or magnetic resonance imaging (MRI) can be used depending on their availability. The protocol is typically multimodal including for CT: non-contrast acquisition, angio-CT of head and neck vessels and brain perfusion CT; and for MRI: FLAIR, diffusion, T2*, angio-MR of head and neck vessels and brain perfusion MR. In ischemic stroke, the functional information provided by diffusion and/or perfusion can now be used to select the appropriate candidates for mechanical recanalisation within an extended time window (up to 24h after stroke onset) based on the concept of penumbra.

***Learning Objectives:***
1. To be aware of relevant functional studies in the emergency room.
2. To learn about the most useful functional parameter to be quantified.
3. To learn about the impact of functional data for patient management.

**A-0135** 14:34
**B. Brain tumours**
A. Majos; *Lodz/PL (agata.majos@umed.lodz.pl)*

There are quite many advanced MR imaging techniques which are currently available in the majority of MRI centres and can be used in daily routine. They offer different kinds of functional data resulting from their physical basis which can be helpful in differentiation between brain tumours and other pathological items as well as in the determination of tumour types and grades. They are also the source of vital information for clinicians to provide the most effective and safe ways of treatment. The essentials of main functional methods will be introduced including diffusion and perfusion technics, functional MRI and just a touch of susceptibility weighted imaging. The selection of technics will be discussed, and some diagnostic algorithms will be proposed. The examples of clinical cases will be presented to illustrate the validity of multiparametric imaging in practice. Lastly, future perspectives will be mentioned, e.g. -randomised and use of machine learning developments.
***Learning Objectives:***
1. To learn about how functional data may help in characterising brain tumours.
2. To understand significance of quantitative data patient management.
3. To become familiar with limitations and pitfalls of functional technics.

**A-0136** 15:02
**C. Cerebral small vessel diseases**
M.A. Van Buchem; *Leiden/NL*

"no abstract submitted"
***Learning Objectives:***
1. To know about main imaging criteria for characterisation of diseases.
2. To learn about the role of functional techniques for grading diseases.
3. To understand how functional imaging allows following diseases process.

**16:00 - 17:30** **Room X**

### ESR Audit and Standards Session

### ESR Audit

### Audit across Europe: directive and perspective

**A-0137** 16:00
**Chairperson's introduction**
D.C. Howlett; *Eastbourne/UK (david.howlett@nhs.net)*

This session on clinical audit aims to further promote knowledge and understanding of clinical audit and its application in the departmental radiology setting. Topics include radiographic and industry perspectives, giving insight and guidance on local implementation of clinical audit programmes. An important update on inspection is also provided. The ESR Guide to Clinical Audit, Esperanto, is designed to support radiology departments in developing audit programmes, with an emphasis on compulsory, regulatory, radiation protection type audit and a brief update on the latest version of this guide will be provided.
***Session Objectives:***
1. To understand the principles of clinical audit.
2. To update on the Esperanto audit project.
3. To evaluate individual experience on implementation of a clinical audit programme.

**A-0138** 16:10
**Clinical audit and inspection: HERCA update**
S. Ebdon-Jackson; *Didcot/UK (steve.ebdon-jackson@phe.gov.uk)*

Carrying out clinical audits in accordance with national procedures is a requirement of the Basic Safety Standards Directive (BSSD) 2013/59/Euratom Article 58(e). The Heads of the European Radiation protection Competent Authorities (HERCA) intends to publish a position paper on the differences between inspection and audit and the expectations of radiation protection competent authorities regarding the requirements. The contents of this paper will be discussed.
***Learning Objectives:***
1. To understand the differences between inspection and clinical audit.
2. To update on Heads of the European Radiological Protection Competent Authorities (HERCA) and departmental inspection.

**A-0139** 16:20
**Implementing a clinical audit programme: the Finland experience**
T. Autti; *Helsinki/FI (taina.autti@hus.fi)*

The purpose of clinical audits is to improve the quality and outcome of patient care by reviewing imaging practices against agreed standards for good medical practices and evidence-based medicine. The national advisory committee for the clinical audit (NACA) was founded to support this goal in 2004. The members of the NACA are clinical experts independent of the auditing organisations and public officers from radiation and nuclear safety authority (STUK) in Finland and the ministry of social affairs and health. So far NACA has given 14 recommendations for the clinical implementation of the clinical audit process. These recommendations will be presented and discussed in this presentation. In a clinical audit, the auditors should be independent of the audited organisations. Their role is to produce an independent assessment and report the findings and recommendations to the audited unit. The interval of clinical audits for Finnish medical units using radiation has been five years. Thus, we have experience of three completed audit rounds. Interestingly, the largest number of auditor recommendations were given in the second audit round. Based on our experience, the coordinating and advisory role of the NACA has turned out to be highly important in ensuring good quality of the clinical audit process. In addition, the recommendations of NACA help to harmonise the clinical audit process. Finally, consistent guidelines for auditors given by the recommendations seem to be necessary to produce comparable audit results.
***Learning Objectives:***
1. To learn about Finnish clinical audit rounds performed between 2002 and 2016.
2. To introduce the recommendations issued by the Finnish advisory committee for clinical audit and evaluate the effectiveness of these recommendations.
3. To evaluate the future of clinical audits in Finland.

**A-0140** 16:40
**Implementing a clinical audit programme: the radiographic perspective**
M.-L. Ryan; *Dublin/IE (marielouise.ryan@ucd.ie)*

Clinical audit is arguably the most important part of a quality programme. It allows the radiology team to measure performance against specified standards- local, national and international. This session will help the audience understand something that is often feared in clinical practice and make clinical audit something that is useable, practical and achievable. This session will bring the audience through suggested clinical audits in their department and show real like examples- namely patient identification, workflow optimisation and radiation dose optimisation and justification. The presenter will show examples of where real-life audits have fed into quality improvement in the radiology department around patient and staff safety, financial savings and optimising processes. Different challenges and how to overcome them will be discussed including staff involvement, time management and creating a culture of quality improvement.
***Learning Objectives:***
1. To learn how important clinical audit is in day-to-day practice.
2. To appreciate the opportunities and challenges of clinical audit in practice.
3. To understand the impact on quality improvement of clinical audit in a radiographer department, using real life examples.

**A-0141** 17:00
**Quality improvement and change management: audit in industry**
S. Lee; *Guildford/UK (steven.lee_1@philips.com)*

A clinical audit might be likened to the process of ‘continuous improvement’ of quality in the industry. The later focusing on improving competitive performance by raising the quality (for customers) and reducing the cost of products and services. While the former focuses on improving the quality of patient care by ensuring clinical practice is delivered in line with defined standards. Both face the challenges of engaging people, developing supportive cultures and of using systematic methods for sustaining the improvement process. While the industry has been deploying quality improvement approaches for many decades, the practice of clinical audit in many healthcare organisations is by comparison relatively immature. The talk considers the evolution of different industrial management 'paradigms' concerned with quality improvement from the middle of the last century. The growing emphasis on the ‘people dimension’ of quality improvement is highlighted, as is the significance of using an agreed and systematic process (e.g. PDSA: Plan, Do, Study, Act) for effecting quality improvement. The central importance of quality improvement for achieving competitiveness in an industrial setting is described, as are the major ‘trends’ deployed in realising such improvement. Potential areas of symbiosis between clinical audit and management paradigms (e.g. Lean, Six Sigma etc.) are explored, particularly Lean principles, people change management and alignment of structured quality improvement processes. Working to align the intrinsic motivation of staff with the practice of audit and quality improvement, by highlighting the benefits offered to patients (and internal customers) is emphasised as the key to securing staff engagement.
***Learning Objectives:***
1. To learn about the genesis of audit.
2. To appreciate the importance of audit in industry.
3. To understand the importance of clinical audit in successful management.
*Author Disclosure:*
**S. Lee:** Author; The author is an employee of Philips. Speaker; The speaker is an employee of Philips.

17:20
**Panel discussion: What are the benefits of good clinical audit?**

**16:00 - 17:30** **Room N**

### Imaging Informatics

### RC 405

### Everything you need to know about 3D post-processing

**A-0142** 16:00
**Chairperson's introduction**
E. Sorantin; *Graz/AT (erich.sorantin@medunigraz.at)*

Progress in imaging technology equipment enables to scan patients in high geometrical and temporal resolution as well as in multidimensional space (e.g. 4D). The amount of resulting data cannot be read any more in 2D as done in the last millennium. Furthermore advances in computational power enables to use sophisticated processing algorithms in real time. Thus reading in 2D, as done in the previous millennium, will be gradually replaced by volumetric reading as well as extracting diagnostic information from parametric images. Moreover, for personalised medicine Radiology has to deliver more detailed information, especially to measure tumour volumes or characterise contrast uptake on perfusion imaging. In order to get familiar with those now really emerging techniques, three well-known speakers will cover essential subtopics and provide a roadmap of how to migrate from reading style in the last millennium to current millennium.
***Session Objectives:***
1. To learn about the state-of-the-art information regarding 3D post-processing.
2. To understand how 3D post-processing can most optimally be used in daily clinical practice.
3. To appreciate how automated 3D post-processing and quantification will lead to increased use of 3D visualisations for diagnostics and therapy planning, over 2D viewing.
*Author Disclosure:*
**E. Sorantin:** Board Member; Austrian Society of Radiology and Nuclear Medicine. Consultant; Ulrich Medical GmbH Germany. Employee; Medical University Graz / Austria.

**A-0143** 16:05
**A. 3D post-processing in 2019**
A. Alberich-Bayarri; *Valencia/ES (alberich_ang@gva.es)*

There is a need for the incorporation of advanced tools to assist the specialist in the study evaluation. Automated segmentation of structures based on convolutional neural networks (CNN) has already been established as a powerful tool to detect organs and regions within the body. However, there is a computational limit when working with 3D CNN. Current technology allows for the automated detection of the organs' location and identification of most of the tissue using bounding boxes. These applications may be used today in clinics for the automated assessment of tissue properties. These zero-click algorithms can run seamlessly in the back-end between image acquisition and radiological reporting, with no humans involved, and will provide a paradigm shift in 3D post-processing for radiologists, having the results of the 3D assessment already generated in their PACS even before starting a review of the study. The results of these algorithms though must be verified to provide expected results by a quality check process before getting integrated with the electronic health records.
***Learning Objectives:***
1. To learn about recent advances in 3D post-processing techniques.
2. To understand how these techniques can now be used in clinical practice.
3. To learn new tips and tricks to use in your daily practice.
*Author Disclosure:*
**A. Alberich-Bayarri:** CEO; QUIBIM. Founder; QUIBIM.

**A-0144** 16:28
**B. Making better use of your 3D package: tips and tricks**
P.M.A. van Ooijen; *Groningen/NL (p.m.a.van.ooijen@umcg.nl)*

Advanced visualisation, simulation and planning software is increasingly used in clinical practice providing a shift from 2D to 3D visualisation, processing and interpretation. With this ongoing trend, the radiological profession should not only focus on the diagnosis to be made but also on the utilisation of our imaging data in patient simulation, planning, and treatment. Current functionality moves in this direction with providing extensive possibilities for support of surgical interventions and treatment planning in 3D including the advent of Virtual and Augmented Reality. With this 3D is also moving into the operating theatre. Although these new possibilities are interesting and exciting one should be very aware of the pitfalls that come with 3D visualisation and processing of data. This not only includes the technical but also the procedural pitfalls where image acquisition optimal for diagnosis is not always optimised for the intended use by the referring physician. To adequately use the new techniques and to provide optimal support from radiology to the referring physicians, training is required, and dedicated staff should be involved in this process.
***Learning Objectives:***
1. To learn about the functionality of state-of-the-art 3D packages.
2. To understand the pitfalls in use of 3D post-processing.
3. To appreciate the need for training in 3D post-processing techniques.

**A-0145** 16:51
**C. Interpretation of 3D processing results: from image to volume reading**
T. Frauenfelder; *Zurich/CH (thomas.frauenfelder@usz.ch)*

The widespread introduction of multidetector computed tomography has revolutionised the field of computed tomography (CT). This revolution can be attributed to three primary properties of MDCT: its ability to produce a vast quantity of volumetric data in a reduced amount of time, the high resolution, and the ability to create isotropic voxel data and, consequently, reliable multiplanar and three-dimensional reconstructions. Specialised 3D reconstruction techniques permit the visualisation of anatomical details, which would be difficult to evaluate using axial reconstructions alone. The architecture of the IT systems has undergone dramatic changes. From thick-client systems to thin-client systems and continue to cloud-based architectures. The current trend is to merge the routine diagnostic console and 3D-reconstruction workstation. Whereas there was only a single post-processing station for a whole department, a variety of functions are now available at every single workstation. Although many of us believe that the use of 3D-reconstructions greatly increases total exam evaluation time, there are reports show how using 3D reconstruction techniques for examining volumetric data are effective and improve the speed of interpretation, recognition, and description of specific clinical conditions. But this lean “infrastructure” has not only advantages. Whereas in the old days the radiologist could decide by himself about the systems, radiology is now embedded in a hospital-wide network asking for standards. This may enable the use of new software. Nevertheless, AI and other features ask for a rapid adaptation of IT-software and increase the need for storage.
***Learning Objectives:***
1. To learn about different developments in creating 3D anatomical and functional models for diagnostic and therapy planning purposes.
2. To understand the pros and cons of such technologies.
3. To appreciate that automated 3D image analysis will lead to new ways in which diagnosis and therapy planning will be performed.

17:14
**Panel discussion: Will we still look at 2D images in 10 years' time?**

**16:00 - 17:30** **Room O**

### Cardiac

### RC 403

### From diagnosis to prognosis: how does cardiac imaging affect patient outcome?

*Moderator:*
A. Kallifatidis; Thessaloniki/GR

**A-0146** 16:00
**A. In myocarditis**
M. Francone; *Rome/IT (marco.francone@uniroma1.it)*

Natural history of acute and chronic myocarditis is often unpredictable being independent on the clinical onset of disease and even poorly correlated with more traditional clinical predictors like end-diastolic volume or ejection fraction. In most cases, patients typically improve within weeks to months, but an unfavourable clinical evolution may be observed in a minority of individuals consisting with the development of chronic ventricular dysfunction progressing leading to transplantation or death in 25% of cases.CMR has emerged as a central prognostic indicator in inflammatory cardiomyopathies, mainly because of its ability to detect tissue fibrosis in vivo. This has been demonstrated with a growing and consistent scientific evidence, derived from late enhancement imaging and, more recently from native T1 mapping and ECV. Present lecture will aim to illustrate CMR capabilities as a prognostic indicator in acute and chronic myocarditis, providing an overview of the most important published studies and ongoing clinical trials. Particular emphasis will be given to the importance of T1 and T2 mapping techniques, being a new standard of reference for CMR diagnosis and the patient’s prognostic stratification.
***Learning Objectives:***
1. To learn about the correlation between clinical presentation and imaging findings.
2. To understand the current state-of-art MRI methods for the evaluation of patients with suspected myocarditis.
3. To learn how MRI could be used to inform prognosis and management decisions in myocarditis.
*Author Disclosure:*
**M. Francone:** Speaker; Bracco Medical Imaging, General Electric Speaker Bureau.

**A-0147** 16:30
**B. In non-ischaemic cardiomyopathy**
B.K. Velthuis; *Utrecht/NL (b.k.velthuis@umcutrecht.nl)*

Imaging has established a diagnostic and prognostic value for non-ischaemic cardiomyopathies (CMPs). Early recognition of non-ischaemic CMPs is important for timely management and risk stratification. Cardiac MRI (CMR) provides both the most multiparametric information of all imaging modalities as well as different powerful imaging biomarkers for diagnosis and risk stratification. CMR should help differentiate early CMP from normal variation to prevent over-diagnosis and unnecessary anxiety. Ventricular volumes and function, as well as biventricular involvement, are important for all cardiomyopathies. Atrial size and function are underused but easily obtainable with CMR. Late gadolinium enhancement (LGE) can range from a specific hinge-point (insertion) fibrosis in all types of cardiomyopathies to more specific or diffuse LGE. Location, extent and biventricular involvement of LGE are important diagnostic and prognostic factors that are underutilised in current clinical risk stratification calculators. Promising tools are T1 mapping and feature tracking (FT-CMR). T1 mapping without (native) and with contrast - to calculate the extracellular volume (ECV) - are easily obtainable and have additional value to ventricular volume, function and LGE. Feature tracking (FT-CMR) is a post-processing technique performed on the standard cine imaging and is used to assess global and regional myocardial function. It is comparable to echocardiographic strain analysis and has potential as a new CMR biomarker. The presentation will provide information how to recognise early and typical manifestations of arrhythmogenic (ACM), hypertrophic (HCM) and dilating (DCM) cardiomyopathies on CMR, as well as signs that indicate a risk of progression or adverse prognosis.
***Learning Objectives:***
1. To recognise typical and early signs of ARVC, HCM and dilated cardiomyopathy that indicate progression and prognosis.
2. To understand what findings should be reported.
3. To appreciate the role of MRI in early diagnosis and prognosis assessment in these cardiomyopathies.
*Author Disclosure:*
**B.K. Velthuis:** Board Member; ESCR.

**A-0148** 17:00
**C. In coronary artery disease**
M. Kantarci; *Erzurum/TR (akkanrad@hotmail.com)*

In most patients with coronary artery disease, the decision to perform revascularisation procedures should be based not only on coronary anatomy but also on the functional severity of the lesion. If such a lesion can be clearly demonstrated by myocardial ischemia, exercise, or pharmacological stress testing, revascularisation is appropriate when medical therapy cannot control symptoms. Noninvasive tests, however, are inadequate in some patients. The technology estimates the reference standard fractional flow reserve (FFR) measurement in catheter angiography to determine lesion-specific ischemia. Myocardial FFR is a new index of functional severity of coronary stenosis calculated from pressure measurements during coronary arteriography. In 2015, FDA approved a computational flow dynamics (CFD) method for the diagnosis of hemodynamically significant coronary artery disease by noninvasive coronary CT angiography. FFR is the reference standard in catheter angiography to determine if the lesion is causing ischemia. The clinical significance of moderate coronary artery stenosis may be difficult to determine. FFR = Distal Pressure/Proximal Pressure. If Causes 0.8 ischemia or> 0.8 if it does not cause ischaemia The understanding of the functional evaluation of coronary arteries is explosive in the last five years and will be increasingly encountered in clinical practice. However, calculation of FFR from pressure measurements is limited to small vessel disease, diffuse coronary artery disease and left ventricular hypertrophy. A precise understanding of FFR by the radiologist is important in terms of causing new discoveries for new or improved diagnostic tools related to the pathophysiology of vascular diseases.
***Learning Objectives:***
1. To appreciate the role of anatomic assessment of the coronary arteries versus functional imaging in different patient groups.
2. To learn to report on anatomic and functional imaging in patients with suspected coronary artery diseases.
3. To appreciate the role of non-invasive imaging in prognosis assessment.

**16:00 - 17:30** **Room E1**

### E³ - ECR Master Class (Breast)

### E³ 426a

### The high-risk patient enigma

*Moderator:*
F. Kilburn-Toppin; Cambridge/UK

**A-0149** 16:00
**A. Lesions with an elevated risk for breast cancer**
G. Forrai; *Budapest/HU (forrai.gabor@t-online.hu)*

The histological, clinical presentation, together with the mammography and US appearance of the high-risk lesions would be demonstrated. They have very different probability figures for developing cancer. This would be detailed for each type of lesion. The concept and the global problem of the management of these lesions will be explained, together with the conflict of over/under diagnosis/treatment.
***Learning Objectives:***
1. To learn about different types of high-risk lesions.
2. To become familiar with the risk of developing cancer.
3. To appreciate the different imaging modalities for diagnosis.

**A-0150** 16:30
**B. Value of breast MRI. Rate of underestimation and impact on treatment decision: is breast MRI increasing the number of high-risk lesions?**
R.M. Mann; *Nijmegen/NL (r.mann@rad.umcn.nl)*

The evaluation of high-risk lesions on biopsy with MRI is commonly performed. One of the major ideas is to ascertain that there is not more disease present than seen with other techniques. As many high-risk lesions are, in fact, accidental findings on needle biopsy, the absence of enhancement within the breast reduces the likelihood of more extensive disease. Nevertheless, the underestimation rate with MRI is dependent on the type of high-risk lesion initially found and is still substantial for lesions like atypical ductal hyperplasia and lobular neoplasia. Moreover, several high-risk lesions may, in fact, enhance strongly in breast MRI, but are not upgraded at subsequent pathological assessment. In this lecture, MRI features of common and less common high-risk lesions will be discussed. In general, MRI cannot preclude the need for extensive tissue sampling, but it may be used for guiding of the biopsy and may obviate complete surgical excision.
***Learning Objectives:***
1. To learn about the evidence on MRI for evaluating high-risk lesions.
2. To become familiar with various imaging appearances of high-risk lesions.
3. To appreciate the added value for diagnosis and treatment decision.
*Author Disclosure:*
**R.M. Mann:** Grant Recipient; European Union (FP7, EUROSTARS, EFRO), Dutch Research Council, Dutch Cancer Society. Research/Grant Support; Siemens Healthineers; Bayer Healthcare; Medtronic; Seno Medical, Identification Solutions, Screenpoint medical, Volpara. Shareholder; Transonic Imaging.

**A-0151** 17:00
**C. Can surgery be avoided?**
S.J. Vinnicombe; *Dundee/UK (s.vinnicombe@dundee.ac.uk)*

Within the UK National Health Service Breast Screening Programme (NHSBSP), a median of 7% of screen-detected lesions subjected to needle core biopsy are categorised as B3, of uncertain malignant potential. These lesions comprise a diverse group of pathological entities, with or without epithelial atypia, mostly manifest as mammographic microcalcification. B3 lesions tend to be heterogeneous (such that the area sampled by needle core biopsy may not be representative) and are associated with a variable rate of upgrade to malignancy, either in-situ or invasive, at surgical excision biopsy, which has been the treatment of choice until recently. However, the approach to B3 lesions is changing. With increasing concerns about overdiagnosis (and subsequent overtreatment) in breast screening and the widespread availability of large bore needle biopsy devices, minimally invasive modes of treatment are increasingly used for these lesions. Various techniques are available, including large bore vacuum-assisted devices and devices which remove the lesion in its entirety with a single pass. These approaches to management of B3 lesions are cost-effective and well tolerated, but oncological safety must be the key consideration. In this respect the literature is challenging to synthesise, since there is wide variability in the rates of surgical excision in differing series, introducing a source of bias. Nonetheless, many countries are gradually adopting minimally invasive approaches, and indeed, the UK NHS Breast Screening programme now recommends that no more than 25% of B3 lesions should undergo surgical excision. These developments will be discussed during this presentation.
***Learning Objectives:***
1. To learn about the different image guided minimally-invasive modalities to excise high-risk lesions.
2. To become familiar with the risk of avoiding surgery.
3. To appreciate the standard protocols in different countries.
*Author Disclosure:*
**S.J. Vinnicombe:** Consultant; Siemens Healthineers. Grant Recipient; Breast Cancer Now, CRUK, CSO Scotland.

**16:00 - 17:30** **Room E2**

### Special Focus Session

### SF 4

### My three top tips in neuroimaging (not only for neuroradiologists)

**A-0152** 16:00
**Chairperson's introduction**
M. Stajgis; *Poznan/PL (stajgis@gmail.com)*

I do hope that this new format of education will gain a lot of interest among radiologists. Nine hot topics in neuroradiology were deliberately chosen to be presented as short 8 minutes mini-lectures. All presenters will focus on fundamental knowledge of different pathological entities. The role of different diagnostic imaging methods and techniques as well as typical imaging findings will be overviewed. Every lecture will end with ‘3 top tips’ based on lecturer’s practical experience.
***Session Objectives:***
1. To highlight the importance of 'key point' knowledge of the pathological entities listed below.
2. To be familiar with most common and typical imaging features in these conditions.
3. To present crucial tips and tricks that allow you to make confident diagnoses.

**A-0153** 16:05
**MRI T2 brain white lesions**
A. Krainik; *Grenoble/FR (akrainik@chu-grenoble.fr)*

T2 hyperintensities are a very common finding on brain MRI, especially in elderly. Indeed, white matter hyperintensities (WMH) might be detected in up to 50% in healthy subjects before 50yo. Beside non-specific WMH of the centrum semiovale and the brainstem suggesting microangiopathy, numerous diseases harbour brain T2 hyperintensities. To further discuss their aetiology and improve MR protocol, three tips are given on their location, their appearance on other weighted images, and patient’s history.
***Learning Objectives:***
1. To be able to identify physiological T2 white matter hyperintensities.
2. To be aware of age-related most common T2 white matter lesions.
3. To give practical clues in quick differentiation of T2 white matter lesions based on their location and appearance.

**A-0154** 16:13
**Multiple sclerosis**
K. Katulska; *Poznan/PL (katarzyna_katulska@op.pl)*

Multiple sclerosis (MS) is an inflammatory demyelinating disease of the central nervous system that is characterised by inflammation, demyelination and degenerative changes. The clinical use of Magnetic Resonance Imaging (MRI) in patients with MS has advanced markedly over the past few years. The benefits of the 2010 McDonald MRI criteria included the focus on lesion location; the presence of gadolinium-enhancing and gadolinium-non-enhancing lesions allows very early diagnosis in some patients who undergo a single MRI examination at any time after symptom onset. Be presented the basic symptoms differentiating demyelinating changes from other hyperintensive lesions in T2 images dependent in the brain and spinal cord. To assure how to be confident with the correct diagnosis of MS lesions present.
***Learning Objectives:***
1. To give a short overview of McDonald criteria in multiple sclerosis.
2. To present the typical findings in cerebral and spinal cord multiple sclerosis.
3. To assure you how confident one can feel with correct diagnosis of the multiple sclerosis lesions presence.

**A-0155** 16:21
**Acute disseminated encephalomyelitis (ADEM)**
A. Rovira-Cañellas; *Barcelona/ES (alex.rovira@idi.gencat.cat)*

Acute disseminated encephalomyelitis (ADEM) is a severe, immune-mediated inflammatory-demyelinating disorder of the central nervous system that predominantly affects the white matter of the brain and spinal cord characterised clinically by new-onset polyfocal neurologic symptoms including encephalopathy, coupled with neuroimaging evidence of multifocal white matter inflammatory-demyelinating lesions. The disorder is mainly a condition of the pediatric age group, but on rare occasions, adults and elderly patients can also be affected. In the absence of specific biologic markers, the diagnosis of ADEM is based on clinical and radiologic features. This presentation includes special consideration of the value of neuroimaging in the diagnoses of ADEM and the distinction with and other immune-mediated inflammatory demyelinating diseases, in particular, multiple sclerosis.
***Learning Objectives:***
1. To describe the basics of ADEM entity.
2. To be familiar with morphology, distribution and other characteristic radiographic features in patients with ADEM.
3. To highlight my point of view in the most correct diagnosis of ADEM.

**A-0156** 16:29
**Encephalitis**
H.R. Jäger; *London/UK*

"no abstract submitted"
***Learning Objectives:***
1. To be aware of different etiology of encephalitis.
2. To learn the typical imaging picture of brain structural abnormalities in patients with encephalitis.
3. To present my tips in evaluation of encephalitis-suspected brain lesions.

**A-0157** 16:37
**Meningitis**
M.M. Thurnher; *Vienna/AT (majda.thurnher@meduniwien.ac.at)*

Imaging findings in meningitis are unspecific. Computed tomography (CT) is the initial method to exclude other pathologies. Magnetic resonance imaging (MRI) findings include several indirect features suggesting meningeal pathology. Most important MR techniques to detect meningitis are FLAIR with and without gadolinium, diffusion-weighted MR imaging (DWI), postcontrast-T1WI. This lecture will teach you how to interpret MR imaging findings on different MR techniques to make a correct diagnosis of meningitis.
***Learning Objectives:***
1. To be familiar with different causes of meningitis.
2. To describe the most common MRI findings in acute and chronic meningitis.
3. To present my advice: 'does thickened and enhancing dura always mean meningitis?'

**A-0158** 16:45
**Transient ischemic attack**
J. Hodel; *Créteil/FR*

TIA represents a major public health issue. It is crucial to identify the cause of TIA to avoid an acute stroke. The radiologist plays an important role in the management of patients with TIA. Indeed, the objective of imaging is to detect parenchymal and/or vascular abnormalities that can explain the transient clinical symptoms. Such abnormalities are frequently subtle, and the imaging protocol can be thus optimised to further improve the diagnostic performance. MRI or CT can be used, with the advantage for MRI of better visualisation of parenchymal lesions. Imaging of the supra-aortic trunks is an important step of the diagnostic work-up. Using MRI, optimised diffusion, perfusion (including arterial spin labelling) and post-contrast 3D TSE sequences can be also very useful.
***Learning Objectives:***
1. To understand the importance of imaging in patients with transient ischemic attack (TIA).
2. To highlight the most important imaging features.
3. To present my tips in evaluation of patients with TIA.

**A-0159** 16:53
**Venous sinus thrombosis**
P. Vilela; *Almada/PT (ferrovilela@sapo.pt)*

Intracranial venous sinus thrombosis is an important cause of stroke, especially in young patients and children, and only imaging can make the diagnosis. Imaging is essential to establish the venous thrombosis diagnosis but also to evaluate and monitor associated brain lesions. Obtaining orthogonal imaging planes improves the venous thrombosis diagnosis accuracy. CT multiplanar reconstructions (MPR) views improve the CT accuracy for depicting venous thrombosis, especially cortical venous thrombosis. On MRI, the use of T2*WI and T1WI on two orthogonal planes, such as axial and coronal, are highly accurate to depict vessel thrombosis. An important pitfall is that a low T2 signal inside of a venous structure does not exclude thrombosis since a subacute thrombus is hypointense on T2WI (and isointense on T1WI) due to the presence of deoxyhaemoglobin. Computed Tomography Venogram (CTV) or Contrast-Enhanced Magnetic Resonance Venogram (CE-MRV) prove the presence of venous thrombosis by exhibiting complete or partial filling defects inside the dural sinus and/or veins. Venous thrombosis is well-known clinical and imaging mimicker with different forms of presentation. It is extremely important to think on venous thrombosis in the differential diagnosis of a brain lesion, since in a significant number of cases is not suspected by the clinician, and choose the correct imaging protocol.
***Learning Objectives:***
1. To be familiar with basics of pathology in cortical venous and venous sinus thrombosis.
2. To learn the most common radiologic presentation in venous sinus thrombosis.
3. To provide the hints how to avoid the confusion with sinus thrombosis mimics.

**A-0160** 17:01
**Hydrocephalus**
A. Ramos; *Madrid/ES (ramosana3@yahoo.es)*

Imaging plays a central role in the diagnosis of hydrocephalus, CT is the first imaging test in emergency patients, but MRI is the first-line imaging modality. Hydrocephalus can be divided into communicating and non-communicating and obstructive and non-obstructive, depending if there is an obstruction in the ventricular system or the subarachnoid space. Communicating hydrocephalus can be further subdivided in: a) cases with no obstruction to CSF absorption, normal pressure hydrocephalus (NPH) is the most important cause in this category. b) cases with obstruction to CSF absorption due to damage to arachnoid granulations, as occurs in subarachnoid haemorrhage and meningitis. Non-communicating hydrocephalus implies a mechanical problem affecting the passage of CSF through the ventricular system (masses or stenosis in the ventricular system). MRI is useful in detecting the cause and location of the obstruction, imaging protocol should always include sagittal high-resolution T2WI, and especially rewarding are balanced steady-state gradient echo sequences that appear as highly T2 weighted sequences without fluid artefact. When an inflammatory aetiology is suspected, imaging with contrast agent administration is necessary. However, the diagnosis of NPH is difficult because of commonly associated diseases, such as Alzheimer's disease and microangiopathy. With MRI we try to predict which patients are going to respond to a ventricular shunt and include the following parameters: ventriculomegaly with frontal and temporal horns of the lateral ventricles most affected, dilated sylvian fissures, tight high convexity, acute callosal angle, and focal sulcal dilation.
***Learning Objectives:***
1. To gain a short knowledge of different types of hydrocephalus.
2. To learn how to differentiate normal pressure and obstructive hydrocephalus.
3. To present my 'hot points' in evaluation of patient with hydrocephalus.

**A-0161** 17:09
**Microbleeds**
L. van den Hauwe; *Antwerp (Edegem)/BE (lucvdhauwe@mac.com)*

Cerebral microbleeds (CMBs) are detected with increasing frequency since MRI has become more widely available over the last decade. Especially with the introduction of high magnetic field strength (3T) and newer dedicated MR imaging sequences such as susceptibility weighted imaging (SWI), CMBs are increasingly recognized. CMBs are detected on SWI as small hypointense foci with a maximum size of 5-10 mm. They represent focal accumulations of hemosiderin-containing macrophages with paramagnetic properties causing signal loss due to susceptibility effects. CMBs may be observed as an incidental finding in the normal aging population, as well as in various disorders such as Alzheimer dementia, cerebral amyloid angiopathy, stroke, and trauma. Rare causes include endocarditis, cerebral autosomal dominant arteriopathy with subcortical infarcts and leukoencephalopathy (CADASIL), and radiation therapy. Small (so-called type IV) cavernous malformations may be indistinguishable from CMBs. Not every black dot on SWI constitutes a CMB; phase information obtained with SWI allows to discriminate CMBs from calcifications. Clinical information and previous medical history of the patient, spatial distribution of the lesions (superficial or lobar versus deep, supratentorial versus infratentorial), morphology, and associated imaging findings such as convexity subarachnoid hemorrhage, cortical superficial siderosis, prior lobar intracerebral hemorrhage, white matter abnormalities, are important clues to obtain the correct diagnosis.
***Learning Objectives:***
1. To understand the impact of presence and number of cerebral microbleeds.
2. To learn the distribution of cerebral microbleeds in different pathological conditions.
3. To give you advice how not to overdetect cerebral microbleeds and how to differentiate them from their mimics.
*Author Disclosure:*
**L. van den Hauwe:** Consultant; icometrix.

17:17
**Panel discussion: 'You can ask me for additional tips' - questions from the audience, answers from experts**

**16:00 - 17:30** **Room F1**

### E³ - Rising Stars Programme: Joint Session with ESOR

### BE 4

### Radiologic anatomy: abdomen

*Moderator:*
A. Palkó; Szeged/HU

**A-0162** 16:00
**Small bowel**
S.A. Taylor; *London/UK (csytaylor@yahoo.co.uk)*

Cross-sectional imaging is increasingly replacing fluoroscopic techniques for imaging the small bowel, but both still have a role. It is crucial to recognise the normal appearances of the bowel on all imaging modalities to diagnose abnormality and recognise normal variants. This talk will review the embryological development of the small bowel of importance to radiologists. The aetiology of common congenital malformations such as malrotations and Meckel's diverticulum will be outlined. The appearance of the normal small bowel on the various imaging modalities will be reviewed, along with pitfalls in interpterion.
***Learning Objectives:***
1. To appreciate the embryological development of the small bowel.
2. To learn about the anatomy of the small bowel relevant to radiological imaging.
3. To understand the pitfalls of the normal small bowel appearance during imaging interpretation.
*Author Disclosure:*
**S.A. Taylor:** Consultant; Robarts.

**A-0163** 16:30
**Anorectal**
S. Gourtsoyianni; *Athens/GR*

"no abstract submitted"
***Learning Objectives:***
1. To review the normal anatomy of the anorectum.
2. To learn how well depicted the different anatomical landmarks of the anorectum are with different available imaging methods.
3. To discuss the clinical scenarios for which it is necessary to be aware of the anatomy of the anorectum.

**A-0164** 17:00
**Peritoneum and mesentery**
P.K. Prassopoulos; *Thessaloniki/GR (pprasopo@med.duth.gr)*

Peritoneal anatomy plays a key role in the dissemination process of intraabdominal inflammatory fluid collections or malignant diseases. The peritoneum, a serous membrane, is reflected over abdominal organs to form a series of folds known as ligaments, mesenteries and omenta. They may act as conduits or barriers in the spread of intra- or extra-peritoneal diseases and they influence the peritoneal fluid circulation- responsible for malignant intraperitoneal seeding - along with bowel peristalsis, gravity and hydrostatic pressure gradients. Although not directly visible on imaging, the twelve major peritoneal ligaments and mesenteries can be identified by their typical position, organ relationships, fatty composition and anatomic landmarks provided by their constituent vessels. Peritoneal folds involved by oedema, inflammation or neoplastic infiltration alter their composition, become thickened, and they are directly recognised on imaging. Multiplanar CT reconstructions and specific MRI sequences are useful in demonstrating ligaments and mesenteries. Peritoneal reflections subdivide the peritoneal cavity into multiple compartments and recesses that are visualised when distended by the fluid. These subdivisions provide the anatomic basis for localisation of ascites, abscesses, seeded metastases and traumatic effusions. Familiarity with the normal anatomy is essential in the clinical radiologic analysis and differential diagnosis of peritoneal and mesenteric pathology.
***Learning Objectives:***
1. To review the radiologic anatomy of peritoneal ligaments, mesenteries and omenta on cross-sectional imaging.
2. To describe the most clinically important peritoneal compartments and fluid collections.
3. To discuss the key role of anatomy in the dissemination of malignancies, or the restriction of inflammatory processes in the abdomen.

**16:00 - 17:30** **Room F2**

### Emergency Imaging

### RC 417

### Imaging of 'foreign bodies'

**A-0165** 16:00
**Chairperson's introduction**
U. Linsenmaier; *Munich/DE (Ulrich.Linsenmaier@helios-kliniken.de)*

This integrated refresher course (iRC) will provide a thorough overview on the role of diagnostic imaging in the assessment of (I) implanted surgical and orthopaedic devices, (II) diagnosis of sharp foreign bodies as well as(III) the role of interventional radiology in the management of foreign bodies. The speakers will explain the current role of CR, CT and US in the assessment and diagnosis of implanted devices as well as ingested foreign bodies. The correct assessment of surgical and orthopaedic devices is a daily routine in many radiological institutions. The speaker will explain the appearance of established and newly introduced materials and discuss their proper positioning as well as early signs of postoperative complications. Many foreign bodies pass through the gastrointestinal tract without problems. However sharp foreign bodies may cause perforation, haemorrhage or bowel obstruction an can be complicated by abscess formation and septicemia. The speaker will discuss characteristics of commonly ingested foreign bodies as well as signs of early complications. Interventional treatment for foreign bodies is relatively rare in many radiological institutions. It comprises not only endovascular removal techniques but also techniques the treatment of complications resulting from inserted or ingested foreign bodies.
***Session Objectives:***
1. To become familiar with commonly used surgical and orthopaedic devices and materials.
2. To learn their proper positioning and early signs of postoperative complications.
3. To understand the imaging pathway in management of ingested foreign bodies.

**A-0166** 16:05
**A. Surgical and orthopaedic devices: are they really properly positioned?**
E. Dick; *London/UK (elizabeth.dick@imperial.nhs.uk)*

This session will address the common surgical and orthopaedic devices that we encounter in everyday Radiology practice. What do the clinicians consider to be the optimal placement of each device? How can we assess if positioning is suboptimal? What kind of language should we use to describe devices? It will cover lines and tubes, how to evaluate if an endotracheal tube, chest and abdominal drains are correctly placed, including assessing the position of ETT beyond vocal cords, satisfactory inflation of ETT balloon Surgical packing used in damage control surgery. How to evaluate the post-operative trauma/emergency abdomen when radiopaque and non-radiopaque packing is present. Neurosurgical devices: cervical and thoracolumbar spinal fixation: principles of stabilisation, how to evaluate if correctly positioned, what language to use, complications including malplacement and neurovascular injury. Orthopaedic devices: Rib and Pelvic fixation devices: how to assess stability, considerations of neurovascular compromise.
***Learning Objectives:***
1. To become familiar with different types of commonly used surgical, neurosurgical and orthopaedic devices in clinical practice.
2. To understand how to evaluate their proper positioning.
3. To be familiar with imaging signs of incorrect implementation of neurosurgical and orthopaedic devices.

**A-0167** 16:30
**B. Did I swallow that? US and CT of sharp foreign bodies penetrating stomach and bowel**
J.B.C.M. Puylaert; *The Hague/NL (dr.jbcmpuylaert@wxs.nl)*

If a sharp foreign body gets stuck in the oesophagus, the patient usually will notice its presence and will seek medical help rapidly. However, when it reaches stomach or bowel and penetrates the wall there, the ensuing symptoms are atypical and treacherous and serious delay may ensue. Perforating foreign bodies in stomach and bowel are increasingly recognised by the use of US and spiral CT with multiplanar reconstructions, and may be of help in guiding minimal invasive treatment. The most frequently encountered penetrating sharp foreign bodies are chicken bones, fish bones and wooden sticks as toothpicks. Most patients do not recall having swallowed such an object. Over two-thirds has a history of a previous operation with kinking of bowel loops caused by adhesions, which inhibit the foreign body to "take the corner". Once a sharp object penetrates the wall of the stomach or bowel, omentum and mesentery will try to wall-off the ensuing perforation. Often an abscess develops in the peritoneal cavity, in the liver or the abdominal wall. Eventually, significant migration of the object may be the result. If the sharp foreign body is beyond the reach of the endoscope, patients usually undergo laparotomy for removal of the foreign body and drainage of the abscess. US and CT are important in the diagnosis and the minimal invasive treatment of sharp foreign bodies.
***Learning Objectives:***
1. To be familiar with characteristics of commonly ingested foreign bodies.
2. To understand which imaging modalities are best suited for detection of foreign bodies in different clinical scenarios.
3. To recognise the first signs of early complications resulting from ingested foreign bodies.

**A-0168** 16:55
**C. The role of interventional radiology in the management of foreign bodies and following complications**
H. Leonhardt; *Gothenburg/SE (henrik.leonhardt@vgregion.se)*

The main purpose of the presentation is to give the audience an insight into how the interventional radiologist can contribute to the management of foreign bodies. Some minimally invasive methods and tools to remove foreign bodies will be presented. Strategies for high-risk situations of heavy bleeding after removal of the item by the use of occlusion balloon technique in a multidisciplinary approach within an integrated angiographic - operating room (hybrid OR) will be discussed.
***Learning Objectives:***
1. To recognise the role of interventional radiology in the treatment of complications resulting from the presence of foreign bodies.
2. To learn when to call the interventional radiologist.
3. To learn about techniques of removal of foreign bodies with an endovascular approach.

17:20
**Panel discussion: Common language with clinicians: how to report 'foreign bodies' presence and indicate the optimal management**

**16:00 - 17:30** **Room Y**

### ESR Ultrasound Subcommittee Session

### US 1

### How to perform ultrasound image fusion

*Moderator:*
P.S. Sidhu; London/UK

**A-0169** 16:00
**Liver**
E.M. Jung; *Regensburg/DE (ernst-michael.jung@ukr.de)*

Ultrasound image Fusion means a combination of real-time imaging of ultrasound B-Mode or colour-coded Doppler sonography (CCDS), Power Doppler (PD) or contrast-enhanced ultrasound (CEUS) with computed tomography (CT) or magnetic resonance tomography (MRI). These techniques of image fusion are integrated into high-end ultrasound machines and need the experienced examiners. Beginning with the imaging registration step by step would be shown for a successful imaging fusion. For interventional procedures fusion could be combined with volume navigation with GPS markers. This technique could be used for difficult punctures, biopsies, drainages or also interventional procedures of ablation therapies like radiofrequency ablation (RFA) or irreversible electroporation (IRE). The results from literature would be discussed about interventional procedures using US fusion techniques. For better detection and characterisation of tumours fusion could be used by a combination of CEUS with contrast-enhanced CT or MRI. Fusion could be used for differentiation of benign from malignant lesions, for detection and characterisation of microvascular changes, and for the planning of interventional procedures. Actually, fusion by CEUS with CT or MRI could be used to realise interventional procedures like operative procedures, intra-arterial chemoembolization (TACE) or radionuclide application (SIRT) in cases of hepatocellular carcinomas or diffuse malignant tumour lesions. There are more and more reports about post-interventional control and follow up after interventional procedures like RFA, TACE or surgery. But also for the follow up after chemotherapies fusion could be used.
***Learning Objectives:***
1. To learn about the advanced liver imaging fusion technique.
2. To learn how to optimise scanning protocols by using the image fusion tool.
3. To learn about common benign and malignant lesions and differential diagnosis by using image fusion.
4. To understand how to use fusion imaging of US, CT and MRI for treatment of liver tumour.
*Author Disclosure:*
**E.M. Jung:** Speaker; BRACCO.

**A-0170** 16:20
**Kidney**
J. Rübenthaler; *Munich/DE*

Ultrasound is a widely accepted imaging technique for the diagnosis and monitoring of cystic renal lesions. The widely used Bosniak classification (I-IV) categorises renal cystic lesions in five distinctive groups by the help of ultrasound and CT image criteria. In unclear cases, Contrast-enhanced ultrasound (CEUS) and MRI/CT-ultrasound image fusion are able to detect and characterise difficult pathologies. In contrast to CT or MRI, ultrasound image fusion is a real-time imaging technique that can be used in combination with other cross-sectional imaging techniques. This talk explains the latest possibilities of image fusion with CEUS to detect and characterise unclear renal pathologies.
***Learning Objectives:***
1. To understand the imaging features of kidney lesions.
2. To become familiar with unclear cystic lesions by using the Bosniak classification.
3. To learn about the differential diagnoses of complex cystic renal masses and compare the modalities by using the image fusion tool.
4. To understand the potential pitfalls in image fusion.

**A-0171** 16:40
**Prostate**
D.A. Clevert; *Munich/DE (Dirk.Clevert@med.uni-muenchen.de)*

Prostate cancer (PCa) is one of the most common malignancies in men. The diagnostic standard to confirm prostate cancer is the transrectal ultrasound-guided biopsy. During that procedure 10-12 biopsy cores are taken. However, the standard transrectal biopsy of the prostate is associated with the underdetection of clinically significant prostate cancer and therefore needs to be improved. In the last years, MRI fusion based targeted biopsy gained importance as a consequence. Several studies already confirmed that MRI guided biopsy of the prostate improves the accuracy in detecting and localising clinical significant cancer.
***Learning Objectives:***
1. To explain the PIRADS system in prostate imaging.
2. To become familiar with the technical requirements for performing image fusion of the prostate.
3. To understand how to detect and biopsy guided suspicious MR-lesions using the ultrasound image fusion technique.
4. To understand the potential pitfalls and discuss the evidence for the use of these technologies in routine clinical practice.
*Author Disclosure:*
**D.A. Clevert:** Speaker; Bracco, Siemens, Philips, Samsung.

**A-0172** 17:00
**Minimise the risk of infection transmission and effective decontamination protocols in the setting of ultrasound fusion**
C. Nyhsen; *Strasbourg/FR (nyhsenc@doctors.org.uk)*

Ultrasound is generally considered one of the safest diagnostic modalities available. Causing significant harm to imaged patients by performing ultrasound is neither anticipated by professionals nor patients. However, risks of infection transmission through ultrasound have been highlighted, and public awareness is increasing. A previous survey by the ESR ultrasound working group showed a wide range of decontamination protocols throughout Europe. This led to the publication of best practice recommendations on decontamination of equipment as well as the choice of US gel and transducer covers. Ultrasound fusion is a well-established technique for diagnostic but also interventional procedures, necessitating the use of extra equipment, which may be installed as needed in different settings. Strict hygiene protocols are essential to ensure that transmission of pathogens is avoided and patient safety is ensured at all times.

***Learning Objectives:***

1. To understand the contamination risks in the setting of ultrasound fusion.
2. To learn how cross infection may occur, in particular when fusion is used for invasive procedures.
3. To appreciate the different steps of an effective decontamination, including fusion equipment.

17:20
**Panel discussion: How to start ultrasound image fusion**

**16:00 - 17:30 Room D**

### Musculoskeletal

### RC 410

### Bone, joint and soft tissue infection

*Moderator:*
V. Njagulj; Novi Sad/RS

**A-0178** 16:00
**A. Osteomyelitis**
K. Verstraete; *Ghent/BE*

Osteomyelitis (OM) is an infection of the bone and can present clinically in an acute, subacute or chronic form. Acute OM affects mostly children, just like subacute OM which predominantly presents as a Brodie's abscess. Chronic OM is more prevalent in adults (vascular insufficiency, diabetes mellitus). Routes of contamination are hematogenous (predominantly in children), spread from a contiguous source (cutaneous, dental), or direct implantation (bites, open fractures, postoperative). One to 2 weeks after onset, when >50 % of trabecular bone is destroyed, radiography can detect OM as ill-defined radiolucencies; later findings include extensive osteolysis, periosteal reaction, sequestra, involucrum, sinus (cloaca) through the cortex, and permeative bone destruction, which may resemble a malignant bone tumour. Bone scintigraphy is highly sensitive for OM, and within 48 hours a focus of increased activity in the affected bone can be seen. Specificity is low, however, as tumours, trauma, and neuropathic osteoarthropathy can result in hot spots. CT has a limited role in acute OM but is useful to evaluate complex findings in chronic OM (e.g. sequestra). MRI is the modality of choice to detect acute OM in an early stage: it is highly sensitive and specific demonstrating bone marrow oedema, periosteal reaction, soft-tissue oedema, abscess formation and sequestra. Gadolinium administration demonstrates enhancement of granulation tissue in the abscess wall, along with sinus tracts and around sequestra. Special manifestations are Garré's sclerosing OM, tuberculous OM, diabetic foot (may resemble neuropathic osteoarthropathy), spondylodiscitis, CRMO (chronic recurrent multifocal OM) and SAPHO.

***Learning Objectives:***

1. To explain the pathophysiology and disease spectrum of osteomyelitis.
2. To describe the imaging criteria for diagnosis of acute and chronic osteomyelitis in children and adults.

**A-0179** 16:30
**B. Septic arthritis**
M. Zanetti; *Zurich/CH (marco.zanetti@hirslanden.ch)*

Septic arthritis is often a clinical diagnosis based on physical examination and prompt arthrocentesis. Sampling at arthrocentesis can usually be achieved with needle aspiration imaging-guided, such as fluoroscopy, ultrasound, MRI, or CT. Plain radiography and ultrasound have been suggested to be the preferred initial imaging method. If further imaging is required, MRI is the most accurate technique. FDG-PET is also used less commonly. Staphylococcus aureus infection is most commonly seen in acute bacterial arthritis in adults and children older than two years. Most septic joints develop hematogenously. Rarely, acute septic arthritis may also occur as a result of joint aspiration or local corticosteroid joint injection. In addition, bacterial arthritis may arise secondary to trauma even without an obvious break in the skin. Bacterial arthritis, in association with arthroplasties, is commonly seen in our areas. In infants, small capillaries cross the epiphyseal growth plate and permit extension of infection into the epiphysis and joint space. In children older than one year, osteomyelitis infection presumably starts in the metaphyseal sinusoidal veins and is usually contained by the growth plate. The joint is spared unless the metaphysis is intracapsular. In adults, the growth plate has resorbed, and the infection may again extend to the joint spaces. Based on these considerations the basic imaging criteria for the diagnosis of septic arthritis in adults will be shown.

***Learning Objectives:***

1. To explain the pathophysiology and disease spectrum of septic arthritis.
2. To describe the imaging criteria for diagnosis of septic arthritis in children and adults.

**A-0180** 17:00
**C. Pyomyositis and other soft tissue infections**
D.J. Wilson; *Oxford/UK (davidwilson.stlukes@btconnect.com)*

Soft tissue infection can range from a minor wound infection through cellulitis to pyomyositis and necrotising fasciitis. At one end of the spectrum, the treatment may be limited to anti-sepsis and oral antibiotics whilst at the other many months of intravenous antibiotics, and radical surgery may be necessary. Severe infection may be rapidly fatal. Imaging plays a crucial role in the detection of the extent of infection, the detection of abscesses and the planning of surgical procedures. It is also very useful in determining response to therapy. Ultrasound and MRI are the principal techniques. The strength of ultrasound has its rapid availability and discrimination of free fluid from oedematous solid tissue. It is also critically helpful in detecting foreign material which could be a source and irritant causing persisting infection. MRI gives us a better overview showing bone involvement and the extent of tissue damage. There is a role for both techniques and their often complementary. The first lesson is to recognise the risk of infection especially for patients who are immunosuppressed or diabetic. Imaging may be used to guide aspiration of cavities and biopsy of tissue to identify organisms. On rare occasions the primary treatment of abscesses may be via sump drainage catheter is placed using ultrasound guidance. Normally these patients are treated with surgery to include excision of necrotic tissue, but there are occasions when patients are unfit for anaesthetic, and ultrasound-guided techniques assist.

***Learning Objectives:***

1. To explain the pathophysiology and disease spectrum of pyomyositis and other soft-tissue infections.
2. To describe the imaging criteria for diagnosis of pyomyositis and other soft-tissue infections.

*Author Disclosure:*
**D.J. Wilson:** Consultant; St Luke's Radiology Oxford Ltd.

**16:00 - 17:30 Room G**

### Physics in Medical Imaging

### RC 413

### Striking the balance: image quality assessment in radiological optimisation

**A-0181** 16:00
**Chairperson's introduction: The big picture, can we be objective about image quality?**
N. Marshall; *Leuven/BE (nicholas.marshall@kuleuven.be)*

Current generations of medical imaging devices come with a great deal of flexibility "designed-in", where for example, the number, energy and the temporal rate of the X-ray photons used to generate the images can be varied. Where applicable, reconstruction algorithms add further flexibility. The medical physicist is faced with the challenge of ensuring the optimal setup of imaging systems for the tasks relevant to the local clinical site. This optimisation cannot be performed without a relevant definition of image quality, and this session covers methods of assessing imaging quality in projection radiography, interventional radiology and CT. At the equipment level, we can use Fourier transforms to make technical, physics-based assessments of x-ray component performance such as the sharpness and noise. These parameters are reproducible and relatively quick to implement, but are not task-weighted and come with potential limitations in terms of task description and validity in anatomical backgrounds. An alternative, classic approach is to assess image quality using the Receiver Operating Curve (ROC) or related methods, giving a task-based figure of merit (FOM) for use in optimisation. These methods require observers, either human or algorithmic and a dataset of tasks/cases, which can be time-consuming to assemble but deliver a quantitative measurement of image quality for the imaging system and task in question. This session surveys the available methods for image quality evaluation in radiological imaging and how they are applied, thus allowing an informed choice for optimisation studies.

***Session Objectives:***

1. To appreciate the reasons why image quality is important in radiological optimisation.
2. To understand the main methods of image quality assessment and optimisation.
3. To learn how image quality assessment is applied in clinical practice.

**A-0182** 16:05
**A. Between a ROC(k) and a hard place: classical and pragmatic methods of determining clinical image quality**
O.J. O'Connor; *Cork/IE (oj.oconnor@ucc.ie)*

Image quality assessment is an important yet challenging aspect of radiation protection and integral to the ALARA principle. Radiological image quality may pertain to the fidelity of the radiological image relative to the imaged subject, the information that may be gained from the image and the radiation cost required. Assessment methods may, therefore, measure physical or subjective/psychophysical parameters. Physical parameters include spatial resolution, signal to noise ratio and contrast to noise ratio. Spatial resolution is a measure of how close two anatomical can be in an image and still be distinguished in space. Signal to noise ratio is the mean Hounsfield Unit value within a region of interest divided by the standard deviation. Contrast to noise ratio reflects the ability to distinguish differences in intensity and radiation dose. Subjective assessment remains vital as X-ray images are visual representations of clinical information and human judgement is required. Parameters such as lesion detection can be quantified using receiver operating characteristic curves, which relate observer findings to the image contents and which reduce bias by compensating of varying reader confidence. The area under the curve serves as a figure of merit of image quality and can be used to compare systems or assess new techniques. This method is more suited to clinical trials, however, and in practice, subjective assessment of image quality entails observers reviewing anatomy on appropriate clinical images in 'normal' patients. Ultimately we aim to standardise imaging performance to provide image quality tailored to clinical indications.
***Learning Objectives:***
1. To learn about traditional and practical methods of determining image quality.
2. To understand how the methods are applied to projection radiography, interventional radiology and CT.
3. To identify limitations of current techniques.

**A-0183** 16:28
**B. Bridging the gap between physical and clinical image quality**
E. Samei; *Durham, NC/US (samei@duke.edu)*

Clinical image quality is the cornerstone of imaging optimisation, as images are as good as the value that they offer clinically. Over years various have been developed and used to measure image quality in terms of first-order physics-based metrics of contrast, resolution, and noise. While foundational, these attributes fall short of fully capturing the quality of images for clinical tasks. The first-order metrics need to be extended to second- and third-order metrology of task transfer function (TTF), noise power spectrum (NPS), detectability and estimability indices, and noise heterogeneity. Recent work has shown that these metrics can offer better reflections of clinical quality and integrate them in the optimisation process. This presentation offers a description of the traditional and new CT image quality metrology, outlines the new methods of their assessment, and highlights their utility for improved patient care.
***Learning Objectives:***
1. To learn about physics-based methods of determining image quality.
2. To understand how they are applied to projection radiography, interventional radiology and CT.
3. To identify how to bridge the gap between the physics methods and the clinical image quality assessment.
*Author Disclosure:*
**E. Samei:** Other; The author lists relationships with the following entities unrelated to the present publication: GE, Siemens, Bracco, Imalogix, 12Sigma, Gammex, and Metis Health Analytics..

**A-0184** 16:51
**C. Sharing the message: image quality optimisation for multiple scanners and clinics**
J. Sjöberg; *Stockholm/SE (johan.a.sjoberg@sll.se)*

The management of quality of radiological examinations in any department with multiple CT scanners is a non-trivial task. More so in larger multi-departmental university hospital settings, as both the specialisation of the examinations and the complexity of the organisation multiplies. The situation is also exacerbated by the increasingly sophisticated and diverse imaging and reconstruction technology. A systematic approach to assuring a high-quality practice can be greatly benefited through the adoption of a quality management system, in which two key components are: 1. All quality improvement processes are documented, measured and regularly revised and 2. All quality improvement efforts are directed by the lead radiologist and thereafter initiated and executed by a multi-disciplinary working group consisting, as a minimum, of the section lead radiologist, section lead radiographer and a medical physicist. In this manner, all aspects of quality are properly managed by a representation of the relevant speciality such as consistency and balance of clinical image quality, radiation dose and patient management. Any decent quality management system in a complex high technology environment with limited resources must also increasingly rely on the fundamental principles of automation and quantification. Efforts should be invested in automating basic and repetitive tasks, such as aggregation of data and computation of key performance indicators of image quality and equipment performance.
***Learning Objectives:***
1. To learn about how to apply image quality assessment techniques across multiple scanners and clinics.
2. To learn how to apply image quality assessment methods to partner clinically-indicated DRLs.
3. To understand how to ensure consistency using standardised protocols.

17:14
**Panel discussion: Can we balance image quality and dose needs in an objective manner?**

**16:00 - 17:30** **Room M 1**

### ESHI$^{MT}$ Session

### ESHI(MT) 1

### Clinical and methodological pitfalls in hybrid imaging: problems and solutions

*Moderators:*
C.C. Cyran; Munich/DE
F.A. Gallagher; Cambridge/UK

**A-0185** 16:00
**Artefacts in hybrid imaging**
T. Beyer; *Vienna/AT (thomas.beyer@meduniwien.ac.at)*

A proposal to combine PET with CT was made in the early 1990s by Townsend, Nutt et al. In addition to the intrinsic alignment of complementary images, the anticipated benefit of PET/CT was to use the CT-images to derive the mandatory attenuation maps for the PET data. In short, CT-based attenuation correction (CT-AC) is based on the assumption that a CT image can be segmented into bone and non-bone tissues; voxels in each tissue class are then scaled with corresponding scale-factors.CT-AC is prone to several errors arising from the methodological shortcomings of the segmentation-scaling method in light of CT-transmission measurements in clinical conditions. These include truncation artefacts, artefacts from high-density implants and positive contrast agents and others. More frequently, errors from patient motion during the examination propagate through CT-AC into the final emission images and lead to distortions/bias of the reconstructed data. In addition, artefacts and biases may occur from involuntary mistakes made during the set-up/conduct of the imaging procedure. During this presentation, we will rehearse the principles of CT-AC in PET/CT and point to the source of artefacts arising from the methodology of CT-AC and from specific imaging workflow scenarios not optimised for routine PET/CT. Following this presentation, the audience will understand image distortions, artefacts and bias from methodological pitfalls in PET/CT imaging, and appreciate and understand solutions to frequent image distortions in PET/CT, also, understand the methodological limitations of PET/CT.
***Learning Objectives:***
1. To understand methodological pitfalls in hybrid imaging.
2. To highlight frequent image distortions.
3. To review solutions to limit clinical misinterpretation from image artefacts.

**A-0186** 16:30
**Clinical pitfalls in FDG-PET/CT and PET/MR**
C. Pfannenberg; *Tübingen/DE (christina.pfannenberg@med.uni-tuebingen.de)*

Besides potential artefactual uptake patterns, the use of hybrid imaging is also prone to some potential clinical pitfalls and interpretative challenges you should be aware of when reading the images. A variety of benign conditions like physiological variants of normal tracer uptake increased FDG uptake due to inflammatory disorders, treatment-related effects (e.g.recent surgery, chemoradiation) and hypermetabolic benign tumours are sources of potential false positive interpretations of FDG-PET scans. Less frequent, lesions may be missed on FDG-PET/CT due to low FDG uptake of some tumours, small lesion size or high neighbourhood activity. To avoid misinterpretations in hybrid imaging, it is therefore very important to have a profound knowledge of normal tracer distribution and its physiological variants to reduce false positive interpretations and overdiagnosis of benign conditions. Each tracer uptake in PET should be precisely correlated with CT or MRI and interpreted in the context of all available clinical and other imaging data. Furthermore, to minimise potential artefacts and pitfalls the adequate timing of PET/CT, a careful patient preparation (e.g.diabetic patients, BAT) and an optimised scanning protocol concerning contrast agents, breathing pattern and positioning are important factors. In the lecture, we will discuss characteristic pitfalls in PET/CT and PET/MRI and how to avoid misdiagnosis.
***Learning Objectives:***
1. To learn about the key imaging features of the physiological distribution of 18F-FDG and its frequently encountered normal variants.
2. To become familiar with the most common clinical pitfalls caused by non-physiological FDG uptake due to
benign conditions like inflammation, medication and benign tumours.
3. To discuss the principles of adequate patient preparation and scanning protocols to avoid artefacts and misinterpretation in PET/CT and PET/MRI.

**A-0187** 17:00
**Clinical and technical pitfalls in SPECT/CT**
J. Howard; *Manchester/UK (james.howard@mft.nhs.uk)*

Molecular and anatomical imaging with SPECT/CT provides accurate localisation and specificity of disease. As with all imaging studies clinical and technical pitfalls are encountered, and in hybrid imaging the combination of modalities provides a new challenge. In this lecture, we will discuss how to recognise the pitfalls and limitations that can be encountered with SPECT/CT and give some recommendations to help overcome these issues.
***Learning Objectives:***
1. To discuss common clinical and technical pitfalls that can be encountered in SPECT/CT imaging.
2. To recognise possible clinical and technical limitations/pitfalls and correctly interpret these pitfalls.
3. To learn about solutions and recommendations to deal with clinical and technical pitfalls in routine clinical practice.

**16:00 - 17:30 Room M 2**

### Paediatric

### RC 412

### Imaging in abdominal emergencies: an (evidence-based) update

*Moderator:*
C.E. de Lange; Oslo/NO

**A-0188** 16:00
**A. The acute abdomen in neonates**
A. Coma; *Barcelona/ES (acoma@vhebron.net)*

The acute abdomen in neonates is a frequent emergency room challenge. Neonatal abdominal emergencies comprise a wide variety of congenital and acquired conditions. This review discusses the most common ones in which radiologic evaluation contributes substantially to the diagnosis. Hepatobiliary, genitourinary, gastrointestinal, intraperitoneal, retroperitoneal, and iatrogenic emergencies are discussed. Establishing the diagnosis of acute abdominal conditions in neonates requires a high index of suspicion clinically and detection of subtle findings on imaging. Several imaging techniques are available for this purpose. A general radiologic approach to these conditions is outlined, and individual investigations are subsequently analysed in each case. Although the diagnosis can sometimes be made on prenatal imaging, patients present more commonly after birth and require a prompt diagnosis to facilitate early treatment. Plain radiography, conventional contrast studies and ultrasonography have major roles in evaluating the acute abdomen in neonates. CT and MRI contribute to the diagnosis in certain situations. Particular emphasis is placed on the radiologic clues found in typical neonatal abdominal emergencies. A practical approach to the acute abdomen in neonates is discussed and illustrated. Because the findings can be subtle, use of an appropriate imaging modality and a correct differential diagnosis are essential. To provide optimal patient care radiologists should be aware of the clinical presentation and imaging tips related to the acute abdomen in neonates.
***Learning Objectives:***
1. To learn about typical neonatal abdominal emergencies.
2. To understand the choice of modalities in acute abdomen in neonates.
3. To appreciate typical findings and 'red flag' features.

**A-0189** 16:30
**B. The acute abdomen in young children**
A.D. Calder; *London/UK*

Common causes of the acute abdomen in children beyond the neonatal period are reviewed, with a focus on three conditions with major roles for imaging: hypertrophic pyloric stenosis, intussusception and acute appendicitis. The evidence base underlying the roles imaging plays is evaluated for each condition. The role of ultrasound in pyloric stenosis is well established and is generally highly sensitive and specific. The pyloric length and muscle thickness parameters remain robust measures for correct diagnosis but may need to be adjusted for age and size. Additional signs which may be useful, such as pyloric shape and colour Doppler findings are discussed. Surgical management remains the mainstay of treatment, despite evidence of the effectiveness of medical therapy in some children. The role of ultrasound in the diagnosis of intussusception is similarly well supported and is highly accurate even in relatively inexperienced hands. Radiologists also typically play the leading role in initial attempts to reduce intussusception and are mostly successful. Various techniques for reduction are available, each with their own advocates and evidence base. Acute appendicitis is the most common abdominal emergency requiring surgery. Pre-operative imaging diagnosis is now the norm. Ultrasound is recommended as the primary imaging tool in children but can lack sensitivity, and in most centres, equivocal sonography is followed by cross-sectional imaging. CT has a strong evidence base for accurate diagnosis of appendicitis but confers significant radiation burden. Low dose protocol CT and MRI offer alternatives with potentially similar levels of accuracy.
***Learning Objectives:***
1. To learn about the causes of acute abdominal pain in children.
2. To understand the choice of imaging techniques and their limitations.
3. To appreciate typical radiological features of abdominal emergencies.

**A-0190** 17:00
**C. Polytrauma: differences between adult and paediatric protocols**
M. Raissaki; *Iraklion/GR (mraissaki@yahoo.gr)*

Trauma is a frequent cause of mortality and disability in childhood and adolescence. Emergency physicians have to thoroughly assess injured children following high-energy trauma. Traumatised children differ from adults: haemodynamically stable children may be actively bleeding. Conversely, children have smaller calibre vessels, stronger vasoconstriction, stronger solid organ capsules; bleedings may stop spontaneously, organ rupture is more difficult and delayed rupture rare. Consequently, children are imaged aggressively although few will undergo surgery or trans-arterial embolisation. Imaging is mandatory for diagnosis and management, especially during the primary survey. Its goal is to exclude life-threatening injuries, identify children that may rapidly deteriorate because of clinically silent active bleeding, increase the surgeon’s confidence level by clarifying injuries that need to be treated and ultimately determine short and long-term management. Radiographs and ultrasonography play a basic role in haemodynamically unstable patients. Contrast-enhanced ultrasound is an emerging diagnostic tool in low-energy trauma and suspected isolated abdominal injuries. In stabilised patients, CT scanning has been considered a sensitive, specific, and accurate test for identification and grading of multi-organ injuries. Due to children’ increased radiosensitivity, CT scans should be indicated based on appropriate early clinical evaluation of the closely monitored child and assessment of risk factors for CNS, chest, MSK and abdominal injuries. ALARA includes avoiding non-contrast scans, avoiding multiple phases, applying age/weight-dependent exposure parameters and radiation-saving reconstruction algorithms. CT should not be performed for follow-up unless there is clinical deterioration. Objective documentation of injuries is extremely important in suspected child abuse.
***Learning Objectives:***
1. To learn how paediatric trauma differs from adult trauma.
2. To understand how examination techniques and protocols must be tailored accordingly.
3. To appreciate the importance of multidisciplinary team collaboration in planning and conducting radiological investigations in a trauma setting.

**16:00 - 17:30 Room M 3**

### E³ - ECR Master Class (Oncologic Imaging)

### E³ 426b

### Novelties in oncologic imaging

*Moderator:*
P. Brader; Graz/AT

**A-0191** 16:00
**A. Imaging-guided liver interventions in oncology**
B. Gebauer; *Berlin/DE (bernhard.gebauer@charite.de)*

Interventional techniques in primary and secondary liver tumours play a crucial role in most therapy algorithms, especially in hepatocellular cancer (HCC). Local ablation using thermal ablation (e.g. RFA or MWA) are widely used in liver tumours smaller 35 mm, in larger tumours combined therapies together with transhepatic (chemo-) embolisation might be possible. TACE is mostly performed in HCC, but also in other entities like colorectal or neuroendocrine liver metastasis. In the follow-up imaging after TACE, a precise knowledge about the used TACE intervention and material is necessary to interpret the post-TACE imaging correctly. Another transarterial treatment option is radioembolisation (RE, SIRT). In RE new multicenter studies like SARAH, SIRVENIB, SORAMIC and FOXFIRE are recently published and influenced the use and indications for RE. Localised therapy has a systemic effect on micrometastasis at other sides. This effect and the combination of local ablation and systemic treatments including immunotherapeutic agents will be the next scientific challenges in hepatic interventional radiology.

***Learning Objectives:***
1. To learn about newly established treatment options in interventional oncology.
2. To understand the role of pre- and post-treatment imaging for improving clinical outcome.
3. To appreciate potential future applications of interventional oncology.

*Author Disclosure:*
**B. Gebauer:** Advisory Board; BAYER. Author; Pfizer. Research/Grant Support; AngioDynamics, Boston. Speaker; ROCHE, Sirtex. Other; PAREXEL, ICON.

**A-0192** 16:30
**B. Radiomics: the role of imaging**
S. Rizzo; *Milan/IT (stefania.rizzo@ieo.it)*

Radiomics, is an emerging translational field of research, aiming to extract mineable data from clinical images. The concept underlying radiomics is that digital images, commonly acquired for staging or follow-up of patients, contain much more information than those commonly used for reporting. The digital data may indeed reflect the pathophysiology of the tumoral tissue. The extracted information may be associated with clinical data and can be used to assess prognosis and to support a clinical decision. Specific software allows the extraction of radiomic features from digital images (CT, MR, PET), and convert them into high-dimensional data for hypothesis generation, testing, or both. The steps necessary for a radiomic study include: acquisition of the images; identification and segmentation of volumes of interest that may contain prognostic value; extraction of radiomic features; clustering of the extracted features; creation of a database; inclusion of the extracted data to develop models, possibly in combination with demographic, clinical and genomic data. Imaging is used in routine practice for oncological patients worldwide, at many stages of diagnosis and treatment. In the current era of targeted therapies, radiomics offers the possibility to quantify and monitor phenotypic changes many times during treatment. The power of a predictive classifier model is dependent on the amount of data; hence, it is desirable that the radiomic studies will consider sharing of data among different centres, with the creation of databases including radiomics data and covariates, such as genomic profiles, histology, serum markers, patient histories, and biomarkers.

***Learning Objectives:***
1. To learn about the concept of radiomics and personalised medicine.
2. To learn how radiomics can be extracted from standard clinical examinations.
3. To appreciate the consequences of radiomics for radiologists in the future.

**A-0193** 17:00
**C. Is PET/MRI better than PET/CT plus MRI?**
H.-P. Schlemmer; *Heidelberg/DE (h.schlemmer@dkfz.de)*

PET/CT has emerged as an important imaging tool to detect, characterise, stage, monitor and follow-up various cancers in clinical routine. MRI, on the other hand, is known to provide superior soft tissue contrast and functional information about, e.g. tissue perfusion and diffusion. Accordingly, sequential MR and PET/CT imaging is performed in certain clinical situations. Since 2010 fully integrated whole-body PET/MRI systems are commercially available from different vendors. Temporal and spatial coregistration of PET and MR data has been proven beneficial concerning image coregistration. Simultaneous imaging furthermore enables to improve PET imaging of moving organs by taking advantage of MR-gating. The X-ray exposure of the patients is furthermore reduced, which is important particularly for pediatric patients and patients who need multiple follow-up examinations during treatment and follow-up. Research on simultaneous PET/MR imaging has proven advantages for the diagnosis and therapy monitoring of different tumour entities, e.g. lung cancer, breast cancer, prostate cancer, rectal carcinoma, lymphoma, melanoma, etc. But until an improvement in diagnostic accuracy, influence on therapeutic management and economic reasonability has been evidenced by clinical studies, the crossfire of criticism is still justified from a practical perspective. The presentation will discuss what unmet clinical needs can be addressed by the PET/MR imaging technology. The added value of simultaneous PET/MR imaging will be discussed in comparison to sequential PET/CT and MR imaging.

***Learning Objectives:***
1. To learn in which clinical scenarios PET/MRI is considered to be the most helpful.
2. To understand the difference between the use of PET/MRI and PET/CT plus MRI in the patient pathway.
3. To discuss impediments for future adoption of PET/MRI.

**16:00 - 17:30** **Room M 4**

### E³ - ECR Academies: Hot Topics in GU Cancer

### E³ 419

### Whole-body imaging in metastatic urinary tract and prostate cancer

**A-0194** 16:00
**Chairperson's introduction**
A.R. Padhani; *London/UK*

Current imaging tools lead to poor confidence for assigning cancer clinical states & assessing therapy benefits, particularly for bone disease. There are three major issues with profound clinical consequences. We are unable to accurately predict metastatic disease presence and extent, we have a limited ability to depict heterogeneity of the expressed biologic phenotype, and we are unable to identify who is not benefiting early after starting treatment. This leads to 3 important clinical consequences: the late detection & volume underestimation of metastatic disease extent which misdirects the therapy approach, non-detection of heterogeneity of response which predicts for poor patient outcomes, and failure to rapidly detect primary & secondary resistance; impedes patient progress to alternative therapies.

**A-0195** 16:06
**A. WB-MRI: technique and reporting system Met Rads P**
F.E. Lecouvet; *Brussels/BE*

The presentation first highlights the different time points in the course of prostate cancer (PCa) where imaging is needed, i.e. new diagnosis, biochemical recurrence and metastatic castration-resistant PCa. For each of these stages, the current standard of care and needs for modern imaging approaches are discussed. Whole body MRI technique is presented, with emphasis on the evolution of protocols and current minimal and optimal requirements. Examples are provided illustrating findings in the most frequently affected organs: bones and nodes. Anatomic and functional (DWI) sequences are introduced, and their respective roles and limitations are highlighted, by the time of lesion detection and for the assessment of response to treatment. A practical presentation of the Met Rads P system is provided, with emphasis on standardisation of acquisitions, reporting, quantitative evaluation of disease burden, and response assessment.

***Learning Objectives:***
1. To review patient selection for WB-MRI in prostate cancer.
2. To learn state of the art technical tips.
3. To learn a systematic reporting system.

**A-0196** 16:34
**B. WB-MRI and response assessment**
N. Tunariu; *London/UK (nina.tunariu@icr.ac.uk)*

The availability of multiple life-prolonging agents for Advanced Prostate Cancer (APC), in conjunction with the inter- and intra-patient molecular heterogeneity of this disease, present major challenges for therapy selection and treatment switch decisions. In addition, the standard imaging in APC (CT and Bone Scans), are unable to provide objective criteria for assessing treatment response of bone metastases, the dominant site of metastatic disease in APC and the only site of involvement in 40-60% of APC patients. Earlier identification of treatment failure would avoid potential toxicity, reduce the costs of ineffective treatments and decrease the time to initiation of a next-line, potentially effective treatment. WBMRI has an improved performance compared to standard imaging in the detection of active bone metastases extent, it can identify early response to therapy without the confounder of FLARE, and it has the ability to identify mixed response to treatment, reflecting clonal disease evolution. WBMR-DWI strengths come from its wide availability, lack of radiation and the opportunity of performing a “one-stop shop examination” which can stage and assess response in local disease and in the bone, nodal and visceral metastases without routine contrast administration. Like any other new technique, there is ongoing development towards optimisation and standardisation both in terms of acquisition and data interpretation.

***Learning Objectives:***
1. To recognise active disease.
2. To learn pitfalls of interpretation.
3. To recognise the appearance of treatment response.

*Author Disclosure:*
**N. Tunariu:** Advisory Board; Janssen, Astellas. Speaker; Janssen, Sanofi, Bayer, Siemens.

**A-0197** 17:02
**C. PET and PET/MRI in prostate cancer**
I.A. Burger; *Zurich/CH (irene.burger@usz.ch)*

The introduction of the new PET Tracers targeting the prostate specific membrane antigen (PSMA) changed the approach in patients with biochemical recurrence after radical prostatectomy dramatically. With promising detection rates of 58% and 76% for PSA ranges of 0.2-1 ng/ml and 1-2 ng/ml, PSMA-PET surpasses all other imaging modalities and enables an early localisation of recurrent disease. For restaging prostate cancer PSMA-PET/CT is usually performed, with the CT component used for attenuation correction, localisation of the tracer activity (e.g. ureter vs lymph node) and characterisation of the lesions (e.g. sclerotic vs lytic bone lesions). Given the high sensitivity for metastasis in the recurrence situation, there is an increasing interest for PSMA-PET to stage primary high-risk cancers. With PSMA-PET/MRI not only the detection of metastasis but also the utility of PSMA-PET to improve the detection of primary prostate cancer is under investigation. First results showed an improved detection rate for significant prostate cancer with PSMA-PET/MRI compared to mpMRI. However, despite these promising results it is important to keep in mind that around 10% of the prostate cancers (low grade and high-grade tumours) are PSMA negative and therefore will not be detected with PSMA-PET. Furthermore, the term "prostate specific" is misleading. It is important to know that there are physiological structures (e.g. duodenum, kidneys) as well as neovasculature with high PSMA expression, leading to potential false positive lesions on PSMA-PET such as secondary primaries (e.g. lung cancer, renal cancer), hemangiomas or Padgett's disease.
***Learning Objectives:***
1. To learn about the role of PET in prostate cancer.
2. To understand the advantages and limitations of PET and PET/MRI.
3. To become familiar with potential pitfalls through case review.

*Author Disclosure:*
**I.A. Burger:** Research/Grant Support; GE Healthcare, Swiss Life, Iten-Kohaut foundation. Speaker; GE Healthcare, Bayer Healthcare, Astellas Pharma AG.

**16:00 - 17:30** **Room M 5**

### E³ - ECR Academies: Functional Imaging for Disease Management: Research to Medical Practice

### E³ 420
### Functional imaging of the heart

**A-0198** 16:00
**Chairperson's introduction**
M. Gutberlet; *Leipzig/DE*

**A-0199** 16:06
**A. Functional techniques for clinical cardiac MR**
R. Manka; *Zurich/CH (robert.manka@usz.ch)*

Over the past decade, cardiovascular magnetic resonance (CMR) has become an established non-invasive imaging modality in cardiovascular medicine. CMR provides clinicians and researchers with an unmatched flexibility of diagnostic options such as cardiac morphology, function, myocardial texture, perfusion and flow. CMR is safe, free of ionising radiation and proved higher diagnostic accuracy.
***Learning Objectives:***
1. To learn about main functional techniques useful in clinical cardiac MR imaging.
2. To become familiar with pharmacological testing approaches.
3. To learn about some more advanced techniques.

**A-0200** 16:34
**B. Ischaemic diseases**
J. Bogaert; *Leuven/BE (Jan.Bogaert@uz.kuleuven.ac.be)*

Ischemic heart disease (IHD) remains the most frequent cause of mortality. Cessation of blood flow, due to temporary or persistent occlusion of the CA lumen, causes loss of function in the myocardium downstream the occluded vessel. The impact on the pump capacity of the heart directly depends on the loss in contractile power. Therefore, functional imaging is obligatory. Amongst cardiac imaging modalities, CMR is the most comprehensive technique to study IHD, including functional imaging. The latter is achieved using bright-blood cine steady-state free-precession (SSFP) sequences. Typically, the heart is imaged along the different cardiac axes. These images allow calculating ventricular volumes, ejection fraction and myocardial mass. Moreover, the regional function can be assessed by visual analysis or by using dedicated software - e.g. feature tracking - providing data with regard to myocardial strain (in the different directions). Cine imaging is combined with myocardial tissue characterisation and/or first-pass perfusion imaging. In this way, functional impairment can be directly related to the presence and severity of myocardial damage and/or impaired myocardial perfusion. This approach allows to describe different presentations and link them to clinical scenarios. Moreover, stress functional imaging is of valuable interest to depict hemodynamically significant stenoses and to determine myocardial viability in patients with chronic myocardial ischemia. This lecture will provide a depth view of how to use functional imaging by CMR in different clinical scenarios of IHD, i.e. acute chest paint (diagnosis/differential diagnosis) - angor pectoris - chronic IHD.
***Learning Objectives:***
1. To learn about the impact of functional parameters for therapeutic orientation in emergency.
2. To learn about the validated biomarkers of prognosis.
3. To learn about how to follow the patients.

**A-0201** 17:02
**C. Non-ischaemic cardiopathies**
L. Natale; *Rome/IT (luigi.natale@unicatt.it)*

Non-ischemic cardiopathies potentially represent a huge amount of cardiac diseases; nevertheless, the term is frequently used for myocardial diseases. Multi-modality non-invasive imaging (Echo, MR, CT) has taken the lead in diagnosis, choice of treatment and treatment assessment. Particularly, MR has unique capabilities in morpho-functional phenotyping and tissue characterisation, but more recently CT showed new tools (i.e. spectral imaging) and potential role in this field. Inflammatory diseases of the myocardium represent an important application of multimodality imaging, particularly MRI, because of its multiparametric approach, that allows the assessment of oedema, capillary leak and necrosis/scar. Furthermore, imaging techniques can provide important data about the outcome, both with functional approach (Echo, MRI) or tissue characterisation approach (MRI). Tissue characterisation is mainly aimed to discover fibrosis: replacement fibrosis is typically assessed by late Gadolinium enhancement, while interstitial and plexiform fibrosis are recognised and quantified by parametric CMR imaging (T1 mapping, ECV). A huge amount of data have been published about fibrosis and prognosis, demonstrating that fibrosis is a stronger independent predictor or events than ejection fraction. Finally, morphology, function and tissue characterisation are extremely helpful in the assessment of response to treatment. Again, MR plays a major role in this field.
***Learning Objectives:***
1. To learn about diagnostic criteria of non-ischaemic cardiopathies.
2. To understand the impact of functional imaging for outcome prediction.
3. To learn about their impact in treatment response.

### Thursday, February 28

08:30 - 10:00 Room A

### $E^3$ - ECR Academies: Interactive Teaching Session for Young (and not so Young) Radiologists

### $E^3$ 521

### Emergency radiology II

**A-0202** 08:30
**A. Urinary system trauma**
V. Logager; *Copenhagen/DK (vibeke.loegager@regionh.dk)*

According to the American Association of Surgeons in Trauma (AAST), approximately 10% of all trauma admissions have kidney injuries. Blunt traumas can be graded in a 5-point Renal Injury Scale. On the basis of the patient's clinical findings, an imaging algorithm is set. In general, patients that are normotensive with microscopic haematuria have less than 0.2% risk of serious kidney damage and imaging is unnecessary, whereas patients with either: (A) gross haematuria, (B) microscopic haematuria and blood pressure less than 90 mmHg or occasionally, (C) microscopic haematuria, will require imaging. Contrast-enhanced CT is the way to go. Imaging should be in 3 phases (cortico-medullary, delayed 3-5 min and late phase (more than 10 min). Image reading should be by the multiplane approach. Most of the findings do not require surgical intervention. Some can be treated with minimal invasive therapy. Only a few cases need surgical intervention. On the basis of case presentations, findings will be analysed, discussed and correlated to the patient's clinical status and treatment possibilities, including where and which signs to look for. Which modality could be used to solve the diagnostic problem when the clinical picture does not fit with the radiological picture. Relevant questions will be asked during this session for the participants to vote and the results will be discussed.
***Learning Objectives:***
1. To identify the signs of trauma.
2. To outline the clinical impact of these findings.

**A-0203** 09:15
**B. Non-traumatic urinary tract emergencies**
G. Masselli; *Rome/IT (gabriele.masselli@uniroma1.it)*

Urinary tract (UT) obstruction secondary to urolithiasis is the most common urologic emergency in patients presenting with abdominal pain. Serious complications of acute obstruction include ureteral rupture, pyelonephrosis or abscesses. Radiologists need to define the extent of obstruction, its likely duration and whether an intervention is required, aware that ultrasound (US) is usually normal in case of acute onset. In such cases, computerised tomography (CT) is the gold standard diagnostic tool. UT infection (UTI) is another common emergency, and it may vary in severity, from ureteral to focal renal infection, emphysematous pyelonephritis or pyonephrosis. UTI diagnosis is usually clinical, but in the case of uncertainty, CT provides early diagnosis, outlining the extent and severity of the disease. Magnetic resonance diffusion-weighted imaging (DWI) is of particular value when differentiating pyonephrosis from simple hydronephrosis. DWI of the kidneys is highly sensitive for the detection of focal or diffuse infections, a reason why is gaining more and more popularity. T2-weighted (static fluid) urography is performed in pregnant women to outline the ureters in their entirety. A challenge of MR urography is the differentiation between physiologic hydronephrosis and pathologic obstruction. Vascular UT emergencies include renal infarcts (commonly of thromboembolic origin), renal vein thrombosis (typical of hypercoagulable states and neoplastic patients), and spontaneous haemorrhage (due to angiomyolipoma rupture). These are among the most common non-traumatic UT emergencies, and it is fundamental for every radiologist to be fully confident in their diagnosis. The role of each imaging modality will be interactively discussed in the different clinical scenarios.
***Learning Objectives:***
1. To understand the imaging technique.
2. To become familiar with the differential diagnosis.

08:30 - 10:00 Room B

### GI Tract

### RC 501

### GI bleeding: how can the problem be solved?

**A-0204** 08:30
**Chairperson's introduction**
D. Negru; *Iasi/RO (draneg@gmail.com)*

Gastrointestinal (GI) bleeding is common in the daily practice and needs rapid diagnosis and treatment. It might be acute, overt and chronic. GI bleeding has many causes and can appear anywhere throughout the GI tract. Hematemesis, melena or hematochezia are forms of acute or overt GI bleeding. Chronic GI bleeding presents as iron deficiency or positive faecal occult blood. Computed tomography (CT) is the method of choice in the diagnosis of acute massive GI bleeding. The CT examinations used are CT angiography and CT enterography depending on the clinical context. Angiography is used in well-selected cases for the treatment of GI bleeding. CT also can complement scintigraphy in chronic bleeding.
***Session Objectives:***
1. To define acute, overt and occult GI bleeding.
2. To learn about different imaging modalities that can be utilised in the work-up of GI bleeding.
3. To define the role of the interventional radiologist in the management of the GI bleeding.

**A-0205** 08:35
**A. Acute GI bleeding**
G.H. Mostbeck; *Vienna/AT (gerhard.mostbeck@wienkav.at)*

Acute gastrointestinal (GI) bleeding has high morbidity and mortality. The clinical presentation of acute GI bleeding varies with the location of the bleeding site, the cause, the amount of blood loss and the presence of comorbidities. The ligament of Treitz is the border between the upper (mouth to Treitz) and lower GI bleeding. However, it is not always possible to differentiate between upper and lower GI bleeding clinically, although the clinical presentation is different, as is the aetiology. In decreasing order, erosions and ulcer, variceal bleeding, Mallory-Weiss-tears, vascular lesions and neoplasms are responsible for upper GI bleeding. In contrast, lower GI bleeding occurs in the elderly population, with diverticular disease, angiodysplasia, neoplasms, colitis and benign anorectal lesions being the major etiologies. The main diagnostic objective is the identification of the aetiology and site of bleeding. Endoscopy is the initial diagnostic step in upper GI bleeding, but limited in lower GI bleeding due to difficulties in colonic cleansing in an emergency situation. Accordingly, CT is the imaging method of choice (technique: no positive oral contrast, high dose /iodine content/flow of contrast material (e.g.100-150ml, 350 mg/ml, 4-6ml/sec), plain, arterial and parenchymal phases, dual-energy CT with iodine maps if available, multiplanar reformation, high anatomic resolution). We search for high-attenuation (> 80HU) luminal or wall lesions not seen on unenhanced CT data. Detection rates vary, and an amount of bleeding > 0.35 to 0.5ml/min is required. As bleeding might be intermittent, CT should be performed when active bleeding is present.
***Learning Objectives:***
1. To learn about the common causes of the acute upper and lower GI bleeding.
2. To understand the rationale for different investigative pathways depending on the likely site of bleeding.
3. To appreciate how to best optimise imaging protocols to identify the site and cause of bleeding, and assist with treatment planning.

**A-0206** 08:58
**B. Occult and overt GI bleeding: the role of radiology**
C. Stroszczynski; *Regensburg/DE (Christian.Stroszczynski@klinik.uni-regensburg.de)*

Acute gastrointestinal bleeding often presents a challenging clinical situation. The impact on radiological imaging depends on the clinical setting and the presence or absence of sufficient endoscopic procedures. Thus, diagnostic pathways are strongly varying from hospital to hospital. Causes of GI bleeding strongly depend on the topography of the bleeding source (upper or lower GI bleeding) and age of the patient. Most common causes of upper and lower GI bleeding will be discussed in this presentation. In addition, differences between obscure, occult and overt GI bleeding in interaction with gastroenterologist and surgeons will be focused on. The clinical value of radiologic methods for imaging the GI tract such as MS-CT, MRI, scintigraphy and digital subtraction angiography will be demonstrated as well as common diagnostic pathways proposed by interdisciplinary societies such as ESGE.

***Learning Objectives:***
1. To learn about the differences between obscure, occult and overt GI bleeding, and the most common causes of each.
2. To understand which tests to perform when imaging is indicated, and the most important diagnoses to look for.
3. To appreciate the interaction between endoscopic and radiologic investigations in managing patients with obscure GI bleeding.

**A-0207** 09:21
**C. When is the interventional radiologist needed?**
D.K. Tsetis; *Iraklion/GR (tsetis@med.uoc.gr)*

Gastrointestinal (non-variceal) bleeding is a medical emergency with an associated mortality of approximately 10%. Acute significant gastrointestinal bleeding is defined as bleeding requiring transfusion of at least four units of blood within 24 hours or showing signs of hemodynamic instability. Most cases are resolved endoscopically, pharmacologically, or by correction of coagulation parameters. Due to its minimally invasive nature, endovascular treatment is the method of choice, after the failure of the previous methods. Standard access for embolisation is via common femoral, brachial or radial artery. After reaching the appropriate visceral artery through diagnostic 4 or 5F catheter, and verification of the source of bleeding, microcatheres are introduced coaxially. Embolic materials include microcoils, PVA microspheres, gelatin foam and occasionally tissue glue. The upper gastrointestinal tract is characterised by a rich network of collateral supply with a lower risk of ischemia. In the risk of rebleeding via collaterals, it is necessary to perform embolisation proximally and distally from the site of bleeding (so-called sandwich method). In the lower gastrointestinal tract, ischemia risk is higher due to the higher portion of terminal branches, and embolisation should be as selective as possible. In the case of too proximal embolisation, there is a higher risk of bowel ischemia.

***Learning Objectives:***
1. To learn about the role of interventional radiology in the management of acute and chronic GI bleeding.
2. To learn about the variety of techniques available to the interventional radiologist to evaluate obscure GI bleeding and control acute GI bleeding.
3. To understand when interventional radiology is clearly indicated, when it should be considered and when it should be avoided, if possible.

09:44
**Panel discussion: Guidelines for management of GI bleeding and real life: why are they different?**

**08:30 - 10:00** **Room C**

### General Radiography (Radiographers)

### RC 514

### Radiography audit and quality management

*Moderators:*
F. Zarb; Msida/MT
E.J. Adam; London/UK

***Session Objectives:***
1. To understand the importance of Radiography Quality Management.
2. To appreciate the role of PACS in driving quality improvements.
3. To promote discussion on practical concerns in a Radiology department.

**A-0208** 08:30
**A. Understanding PACS to support radiography quality management processes**
M. O'Connor; *Dublin/IE (michelle.oconnor@ucd.ie)*

Quality management in the field of radiography plays an important role in the improvement of the performance of and processes related to diagnostic procedures, including the selection of imaging and procedural services, the quality and safety of healthcare delivered and the effectiveness and management of all imaging services. Many factors have contributed to the growing importance of evaluating quality in radiography practice such as the rapid evolution of technology, legislative requirements, increased reliance on diagnostic imaging in healthcare, role development and patient safety factors particularly related to radiation risks. One of the key tools for evaluating services is clinical audit. Through clinical audit, healthcare staff may identify and measure areas of risk within their service. The Medical Exposure Directive 97/43/EURATOM defines clinical audit as ‘a systematic examination or review of medical radiological procedures. It seeks to improve the quality and the outcome of patient care through structured review whereby radiological practices, procedures, and results are examined against agreed standards for good medical radiological procedures’. Modifications of the practices are implemented where indicated and new standards applied if necessary. Picture archive and communications system (PACS) is commonly used to support radiography service evaluation as it contains a large database of radiology data which is easily filtered thus provides extensive audit opportunities, e.g. identifying workflow inefficiencies, image reject analysis, assessing compliance with protocols and policies etc. Practical tips to enhance audit readiness have been developed.

***Learning Objectives:***
1. To highlight the need for evaluating radiographic quality within clinical practice.
2. To understand the ability of PACS to support radiography service evaluation.
3. To identify practical tips to enhance audit readiness.

**A-0209** 09:00
**B. An expert in radiography quality management**
D. Catania; *Milan/IT (cataniadiego@hotmail.com)*

The presentation will relay current literature on the topic of the audit with respect to patient referrals, waiting times and turnaround times. The focus will be predominantly related to radiography and radiography professional activity. The discussion will include guidelines from professional agencies such as the IAEA, Radiology professional Societies and Radiography Societies. Radiography research published on the topics above and key current/future considerations for our professional societies will be discussed. To broaden the discussion on quality management, the many aspects of radiography activity which require quality management will be proposed, and as part of the presentation, the role of a designated radiographer to manage these radiography quality needs will be discussed. Current structures which support such roles will be presented, and the presentation will explore the current evidence for developing additional roles within the profession of radiography, to support the broad spectrum of possible radiography quality needs. Consideration will be given to how professional staffing structures vary across Europe and the potential impact on effective radiography quality management. Specific examples from the clinical environment will be provided to illustrate "real world" examples of radiography quality management. These examples will cover a broad spectrum of medical imaging activity. The discussion will include how other professionals interact within the quality processes undertaken by radiographers and the need for interdisciplinary cooperation will be emphasised. Barriers to successful quality management activity performed by radiographers will be included.

***Learning Objectives:***
1. To understand the importance of auditing the status of referrals, waiting times and turnaround times.
2. To introduce the role of a designated radiographer to manage the radiography quality process.
3. To provide "real world" examples of radiography quality management.

**A-0210** 09:30
**C. Back to basics: auditing radiographic technique**
L. Harding; *Warrington/UK*

"no abstract submitted"

***Learning Objectives:***
1. To discuss the importance of regular auditing of radiographic practice.
2. To highlight the importance of Radiology Information System (RIS) dashboards in promoting high quality imaging.
3. To understand how a departmental quality improvement team, using audit, can maintain and raise imaging standards.

**08:30 - 10:00** **Room X**

### Joint Session of the ESR and UEMS

### ESR/UEMS

### Imaging professionals in the EU: radiologists without borders

**A-0211/A-0212** 08:30
**Chairpersons' introduction**
M. Adriaensen; *Heerlen/NL (miraude@gmail.com)*
L. Bonomo; *Rome/IT*

The UEMS (European Union of Medical Specialists) is the oldest European medical organisation representing the interests of more than 50 different medical specialities and involving more than 1.6 million healthcare professionals. The ESR (European Society of Radiology) is the world’s biggest radiological society, encompassing more than 82,000 members from 161 different countries. It was founded in 2005 by merging the European Congress of Radiology and the European Association of Radiology, aiming at establishing a single, powerful and unified voice for European radiologists. Through the section of radiology of the UEMS, UEMS and ESR share the same ambitious objective of promoting the highest quality of care and medical practice in radiology, by serving the needs of patients and the general public, harmonising radiological training and defending free movement and professional interests of European radiologists. This Joint Session will focus on the concept and importance of advocacy at the level of the European Union, with specific emphasis on the value of qualifications across borders and the consequences of a Brexit. With regard to harmonisation of radiological training, the revised European Training Curriculum will be discussed. Furthermore, you will be informed how to gain European recognition for your degree in radiology as well as for your radiology department. Finally, particular attention will be dedicated to the working methodologies of EACCME (European Accreditation Council for Continuing Medical Education) and ACI (Accreditation Council in Imaging) in the framework of CME (Continuing Medical Education) and CPD (Continuing Professional Development) programmes.

***Session Objectives:***
1. To describe the role of the UEMS within the EU.
2. To understand the difference between the ESR and the UEMS.
3. To understand the importance of cooperation between the ESR and the UEMS.

*Author Disclosure:*
**M. Adriaensen:** Other; Board member, UEMS section of Radiology; member, Standards Committee of the European Board of Radiology; member, ETAP 2.0 Scientific Committee.

**A-0213** 08:35
**Putting your interests first: UEMS and ESR advocacy in the EU (part 1)**
P.M. Parizel; *Antwerp/BE*

The ESR was founded in 2005 by merging the European Congress of Radiology (ECR) and the Europan Association of Radiology (EAR) to adequately represent a unified and powerful voice for European radiologists. In recent years, the EU institutions have increasingly shaped policies in the fields of health, research and digitalisation, which has impacted clinical practice for radiologists and biomedical research across the Member States. Under the guidance of the Board of Directors, the ESR closely monitors and assesses EU legislation and policy developments to ensure EU initiatives benefit clinical practice and patient safety. Equipped with a toolbox of instruments, the ESR participates in EU stakeholder consultations, regularly organises policy events and issues statements and position papers to get across the radiologists‘ point of view. Taking into account the complex EU policymaking arena, the ESR has adopted a proactive approach by successfully establishing good working relations with the EU institutions and engaging with other stakeholders active in the fields of health and research. In this respect, the ESR is widely recognised as a major healthcare stakeholder that has successfully promoted the interests of the radiology profession and patient safety at the pan-European level. As collaboration is the key to success at EU level, the ESR is grateful to have found a respected partner in the UEMS to jointly defend the interests of the medical profession and to strive for the highest standards in healthcare, teaching and research.

***Learning Objectives:***
1. To understand the structure of the UEMS and the ESR.
2. To understand the differences between the ESR and the UEMS.
3. To understand the importance of UEMS/ESR political involvement in EU affairs.

**A-0214** 08:40
**Putting your interests first: UEMS and ESR advocacy in the EU (part 2)**
B. Maillet; *Brussels/BE (bernie.mail@skynet.be)*

One of the basic principles of the European Union is Free Movement. Of course, this concerns people, products, funds manpower but also Healthcare and can be found in this particular field in three domains. First the free movement of students organised by the Bologna Process, the free movement of physicians organised by the EU Directive on Professional Qualification Recognition (PQR) and recently the free movement of patients organised by the EU Directive on Patient Safety on Cross Border Healthcare. Concerning the PQR the first initiative was in 1993 with the first version of the Directive that was amended regularly with the last revision in 2013 (EC 2013/55), but the most important version is the EC 2005/35. This Directive recognises automatically the Diplomas of Medicine between the different Member States and also grants recognition for some specialities. The Directives have to be revised every seven years, and the next scheduled revision is planned in 2020, so this would be the ideal moment to try to “fuse” those both specialities and have only “Radiology” mentioned in Annex V and abolish “Diagnostic Radiology”. A joint effort of both the UEMS and the ESR could be considered to achieve this revision.

***Learning Objectives:***
1. To understand the structure of the UEMS and the ESR.
2. To understand the differences between the ESR and the UEMS.
3. To understand the importance of UEMS/ESR political involvement in EU affairs.

**A-0215** 08:45
**To be or not to be at the table: why advocacy matters for radiologists**
K. Riklund; *Umea/SE (katrine.riklund@umu.se)*

ESR and UEMS Radiology section work together for better patient care, harmonised professional and standards. The radiological community os affected by developments in healthcare and financial constraints for healthcare spending together with a growing demand of healthcare combined with an under-representation of the radiologist in decision making and emerging value-based concept. In the talk the need for coordinated actions to an activator for the highest standard of patient care in radiology will be discussed. We will work all through the three levels of the European legislation process and the representation of ESR in EU networks and stakeholder groups.

***Learning Objectives:***
1. To understand the concept of advocacy and the importance of advocacy at the EU level.
2. To understand the three levels of the European Legislation process.
3. To understand the influence of EU directives on national legislation and daily practice.

*Author Disclosure:*
**K. Riklund:** Advisory Board; Swedish Medical Product Agency. Board Member; Dicom Port AB.

**Part 1: What does the EU mean for me? A radiologist's guide**

**A-0216** 08:53
**Creating a European radiology (or healthcare) workforce: the value of qualifications across borders**
H. Käfer[1], M. Frohn[2]; [1]*Vienna/AT*, [2]*Brussels/BE (hatto.kaefer@ec.europa.eu)*

EU Member-States are free to regulate professions and thus restrict access of certain professions on their territory, as long as they respect the principles of non-discrimination and proportionality under EU law. From this derives that EU law does not primarily seek to harmonise professional qualifications and access to professions. This exists only in a few areas, such as transport and health. For doctors, Directive 2005/36/EC on the recognition of professional qualifications foresees minimum harmonisation of training based on years and hours of study and acquired knowledge and skills, which entail an automatic recognition mechanism. This applies to doctors with basic medical education, general practitioners and currently 54 medical specialist categories including radiology and diagnostic radiology, given that the professional is fully qualified in its home Member-State. Evidence of formal qualifications in medical specialist training, which are covered by the automatic recognition system, are listed in point 5.1.3. of Annex V to the Directive. For the remaining medical specialisations, EU law provides recognition mechanism with Directive 2005/36/EC, based on the principle of mutual recognition of professional qualifications. This kind of recognition allows the host Member-State to compare the substance of training and in case of substantial differences, to impose compensation measures in the form of an aptitude test or an adaptation period. Doctors are the most mobile profession in Europe under the Directive when it comes to recognition for the purpose of a permanent establishment with more than 3500 recognition decisions per year throughout Europe, including the EEA and CH.

***Learning Objectives:***
1. To understand the concept of advocacy and the importance of advocacy at the EU level.
2. To understand the three levels of the European Legislation process.
3. To understand the influence of EU directives on national legislation and daily practice.

**A-0217** 09:01
**Brexit means Brexit: radiologists with borders?**
V. Papalois; *London/UK*

"no abstract submitted"

***Learning Objectives:***
1. To understand the concept of advocacy and the importance of advocacy at the EU level.
2. To understand the three levels of the European Legislation process.
3. To understand the influence of EU directives on national legislation and daily practice.

### Part 2: Educating the next generation of radiologists

**A-0218** 09:09
**The benefits of harmonised training for the radiologists of tomorrow**
C. Catalano; *Rome/IT (Carlo.Catalano@uniroma1.it)*

One of the aims of the ESR is to harmonise and standardise training all over Europe. A major effort has been done in the past few years in the preparation of the European Training Curricula and the Undergraduate Training Curriculum, which have recently been updated and received the endorsement of the UEMS. The ETC defined the contents of training and expected learning outcomes of trainees in radiology. The ETC is a living document continuously revised in order to keep up with the developments and knowledge in radiology. The CESMA is an advisory body of the UEMS created to provide recommendation and advice on the organisation of European examinations for European medical specialists. The ESR has therefore developed the EDiR which is available to radiologists and last year radiology residents. It certifies that their level of knowledge and competency is in line with the ETC. The EDiR is an additional qualification of excellence for general radiology, fully endorsed by the UEMS and the ESR. The European Training Assessment Programme (ETAP) has been recently updated to a version 2.0, based on a new platform, which enables a quick and easy online certification process for both applicants and assessors. ETAP 2.0 ensures that the training department meets the quality of standards set by the ESR European Training Curriculum and the UEMS. All these activities of the ESR aim at improving throughout Europe the level of knowledge of Radiologists and Radiologists in training by standardising training.
***Learning Objectives:***
1. To get an overview of the different levels and the changes made in the revised European Training Curriculum in radiology.
2. To learn about the Council for European Specialist Medical Assessment (CESMA).
3. To know about the value of the European Diploma in Radiology (EDiR).
4. To learn about the facts and figures of the European Training Assessment Programme (ETAP) 2.0.

**A-0219** 09:17
**Making your qualifications count at home and abroad: the European Diploma in Radiology and the CESMA**
H.J. Lamb; *Leiden/NL (h.j.lamb@lumc.nl)*

Short informative presentation on the structure, organisation, outcome and future projections for the European Diploma in Radiology (EDiR). Furthermore, short overview of the different levels and the changes made in the revised European Training Curriculum in radiology. Also an introduction to the Council for European Specialist Medical Assessment (CESMA). Finally, facts and figures will be discussed of the European Training Assessment Programme (ETAP) 2.0.
***Learning Objectives:***
1. To get an overview of the different levels and the changes made in the revised European Training Curriculum in radiology.
2. To learn about the Council for European Specialist Medical Assessment (CESMA).
3. To know about the value of the European Diploma in Radiology (EDiR).
4. To learn about the facts and figures of the European Training Assessment Programme (ETAP) 2.0.

**A-0220** 09:25
**ETAP 2.0**
L. Oleaga Zufiría; *Barcelona/ES (lauraoleaga@gmail.com)*

The European Training Assessment Programme (ETAP) is a joint initiative of the European Society of Radiology (ESR) and the European Union of Medical Specialists (UEMS) Radiology section, with the aim of assessing and harmonising the radiology training department programmes in Europe. A qualitative adaptation was made in 2016 modifying the structure of a face-to-face audit to an online audit to facilitate access to a larger number of centres and lighten the work of the assessors, establishing as ETAP 2.0 programme. ETAP 2.0 represents an instrument for the centres to compare the quality of the training and assess the suitability of the program with the European Training Curriculum (ETC). It is strongly linked with the European Diploma of Radiology (EDIR); ETAP 2.0 is a certificate of excellence for training institutions that ensure that their training department meets the quality standards set by the European Society of Radiology and the UEMS. Both initiatives favour the standardisation of the European training, and they both contribute to increase the quality of training across Europe. The differential element of the ETAP 2.0 is its platform, which enables a quick and easy certification process for both applicants and assessors. Representatives of the applicant institutions and the assessors can easily and efficiently store, access and manage all documents and information necessary for the certification process.ETAP 2.0 offers training centres an opportunity to audit their programme as an instrument to check the level of competence, attitude and development of new skills the trainees acquire during the training period.
***Learning Objectives:***
1. To get an overview of the different levels and the changes made in the revised European Training Curriculum in radiology.
2. To learn about the Council for European Specialist Medical Assessment (CESMA).
3. To know about the value of the European Diploma in Radiology (EDiR).
4. To learn about the facts and figures of the European Training Assessment Programme (ETAP) 2.0.
*Author Disclosure:*
**L. Oleaga Zufiría:** Consultant; Telemedicine Clinic.

### Part 3: Staying ahead of the curve with continuing professional development (CPD)

**A-0221** 09:33
**Patient safety and job security for life: CME/CPD in Europe**
P. Ricci; *Rome/IT (paolo.ricci@uniroma1.it)*

CME - Continuing Medical Education - consists of educational activities which serve to maintain, develop, or increase the knowledge, skills, and professional performance and relationships that a physician uses to provide services for patients, the public, or the profession. CPD - Continuing Professional Development - can be defined as the systematic maintenance, improvement and continuous acquisition or reinforcement of the lifelong knowledge, skills and competencies of health professionals. CME-CPD is a clinical and professional duty as well as an ethical obligation for healthcare professionals. It can also be a powerful instrument free from commercial influence in the hands of the medical class which is extremely necessary in the European Union, where healthcare systems became inter-dependent, yet still inhomogeneous, with mandatory and voluntary CME-CPD systems coexisting side by side. At EU-level, the role of CPD to help safeguard patient safety within the context of cross-border mobility has been addressed in several legal instruments: e.g. Council Recommendation on Patient Safety, Directive 2011/24/EU on patients' rights in cross-border healthcare, and Directive 2013/55/EU on the recognition of professional qualifications. The UEMS - European Union of Medical Specialists actively promotes high standards in CME-CPD through the European Accreditation Council for Continuing Medical Education (EACCME), created in 1999 with the aim of encouraging high standards in development, delivery and harmonisation of CME through the international accreditation of events, and the establishment of a “currency” system for the international acceptance of CME credits (ECMEC - 1 ECMEC = 1 hour of CME).
***Learning Objectives:***
1. To learn about the European Accreditation Council for Continuing Medical Education (EACCME).
2. To know about the existence of European CME credits (ECMEC).
3. To learn about the importance of credits in different European countries.

**A-0222** 09:41
**It's easier than you think: the many ways to gain European CME/CPD credits**
M.A. Lucic; *Sremska Kamenica/RS (milos.a.lucic@gmail.com)*

Understanding the tendency of Continuing Medical Education (CME) shift from voluntary to mandatory within Europe, European Union of Medical Specialists (UEMS) and its European Accreditation Council for CME (EACCME) introduced European Continuing Medical Education Credits (ECMEC’s), as CME "tokens" reflecting objectively the CME activity, that facilitate the exchange of CME credits between European countries and comparable systems outside Europe. Considering CME as educational activities which serve to maintain, develop or increase the knowledge, skills, professional performance and relationships that physician/radiologist uses to provide services for patients or profession, ECMEC's are awarded for both live educational events, and e-learning materials, but from recently also for Continuing Professional Development (CPD), defined as the educative means of updating, developing and enhancing how physicians apply the knowledge, skills and attitudes required in their working lives. As CPD incorporates and goes beyond CME, EACCME 2.0, adopted in 2016, enabled EACCME recognition of the several CPD/CME activities, that includes reviewing and/or publishing scientific and educational material, learning by teaching, and examining in UEMS exams, including EDiR exam. Based on the surveys conducted by Accreditation Council in Imaging (ACI) during the last two years, that provided not only valuable information on accreditation systems differences in European countries, but more important, indicated important standpoints of the European radiology community, that include the recognition of ECMEC’s as universal European CME "tokens", and unification of CME/CPD systems within Europe, and shall be acknowledged as the "voice of European radiologists" in future considerations.

***Learning Objectives:***
1. To learn about the European Accreditation Council for Continuing Medical Education (EACCME).
2. To know about the existence of European CME credits (ECMEC).
3. To learn about the importance of credits in different European countries.
*Author Disclosure:*
**M.A. Lucic:** Research/Grant Support; Provincial Secretariat for Science and Technology Development of the Autonomous Province of Vojvodina; Grant No. 142-451-2433/2018.

09:49
**Panel discussion: The state of radiology in the EU: a diagnosis of "the bigger picture"**

**08:30 - 10:00 Room O**

### E³ - ECR Master Class (Cardiac)

### E³ 526

### Cardiac imaging in arrhythmia and sudden cardiac death

**A-0223** 08:30
**Chairperson's introduction**
P. Donato; *Coimbra/PT (donato.pj@gmail.com)*

Computed Tomography (CT) and Magnetic Resonance Imaging (MRI) are used to detect underlying cardiac conditions that can lead to ventricular arrhythmia (VA) and sudden cardiac death (SCD). VA includes a wide spectrum of ECG forms from premature ventricular complex to ventricular fibrillation, and a wide spectrum of clinical presentations, from a total lack of symptoms to cardiac arrest. SCD is the most common consequence of sudden cardiac arrest and is responsible for 50% of all cardiovascular deaths. The risk for VA and SCD vary with different underlying cardiac conditions, and with specific family history and with genetic variants. To prevent SCD is important to identify patients at risk and apply preventive strategies. The risk increases with age, related to the increase of ischemic heart disease, which remains the most common cause of sudden cardiac arrest. There are also various forms of cardiomyopathy leading to cardiac fibrosis or hypertrophy responsible for SCD that can be early depicted by cardiac imaging. AHA 2017 guidelines recommend that in patients presenting with VA who are suspected of having structural heart disease, cardiac MRI or CT can be useful to detect and characterise underlying structural heart disease. The strength of each technique is under study. Imaging arrhythmic patients are challenging due to difficulties on ECG synchronisation. These patients have frequently implanted devices at the time of the MR study, is also important to know the risk that each patient occurs and to know strategies to decrease artefacts caused by the devices.
***Session Objectives:***
1. To learn about existing imaging biomarkers to prevent sudden cardiac death.
2. To understand how to face the challenge of arrhythmia in cardiac imaging.
3. To become familiar with the prerequisites for MR in patients with active implants.

**A-0224** 08:35
**A. The role of CMR in sudden cardiac death**
D. Piotrowska-Kownacka; *Warsaw/PL*

Cardiovascular diseases are responsible for over 4 250 000 sudden cardiac deaths (SCD) each year worldwide, with the risk almost five times higher in men. The major cause of SCD is known or unknown coronary artery disease, but the list of cardiac diseases leading to SCD is much longer especially in the younger population. It includes cardiomyopathies, inherited primary arrhythmia syndromes, inflammatory, rheumatic and valvular heart diseases. The risk stratification for the prevention of sudden cardiac deaths includes invasive and non-invasive diagnostic procedures. Complementarily to echocardiography, cardiovascular magnetic resonance (CMR) is an important imaging technique that enables excellent morphology, function and tissue characterisation of the heart. CMR could be used for diagnostic purposes but also for risk stratification in cases of known cardiac pathology. The lecture will focus on CMR prognostic factors in acute and chronic ischemic heart disease, myocarditis, selected cardiomyopathies and systemic diseases with cardiac involvement.
***Learning Objectives:***
1. To become familiar with the pathophysiology of sudden cardiac death.
2. To introduce cardiac MR biomarkers to prevent sudden cardiac death.
3. To learn how to perform and how to interpret cardiac MR to prevent sudden cardiac death.

**A-0225** 09:00
**B. Preventing sudden cardiac death with CT: pure theory or new diagnostic paradigm?**
K. Nikolaou; *Tübingen/DE (Konstantin.Nikolaou@med.uni-tuebingen.de)*

Sudden cardiac death (SCD) is a major health problem, and there is an urgent need to establish diagnostic tools for primary prevention, e.g. by selecting appropriate patients for implantable defibrillators. Today, diagnostic methods of selecting candidates for preventive measures and therapies still have significant shortcomings. Cardiac CT and Coronary CT Angiography have seen major advancements, e.g., for detecting (malignant) coronary anomalies. But also in the field of myocardial abnormalities such as left ventricular hypertrophy, or in the assessment of vulnerable coronary artery plaques, CT shows great potential, with growing evidence on the use of cardiac and coronary CT for the detection of patients at risk for SCD. Probably in the future, a mixture of established and novel imaging biomarkers for predicting SCD, potentially including several imaging modalities, will be incorporated into clinical guidelines. The goal of this talk is to discuss the current status and future potential of cardiac CT imaging to enhance risk stratification for SCD.
***Learning Objectives:***
1. To learn what to look for in CT to prevent sudden cardiac death.
2. To get an overview about existing evidence of cardiac CT in this indication.
3. To discuss the possible future role of cardiac CT in preventing sudden cardiac death.
*Author Disclosure:*
**K. Nikolaou:** Research/Grant Support; Siemens Healthineers, GE Healthcare, Bayer Healthcare, Bracco. Speaker; Siemens Healthineers, Bayer Healthcare, Bracco.

**A-0226** 09:25
**C. Imaging of patients with arrhythmia and implantable devices**
G. Pontone; *Milan/IT (gianluca.pontone@ccfm.it)*

Evaluation of cardiac arrhythmias using both cardiac magnetic resonance (CMR) and cardiac computed tomography angiography (CCTA) represents an extremely interesting topic in cardiac imaging. CMR allows obtaining data regarding myocardial tissue characterisation as well as correct evaluation of ejection fraction. Information that CMR provides to arrhythmologist can mainly be divided as follow: sudden cardiac death risk stratification, decision-making for cardiac resynchronisation therapy with evaluation of cardiac anatomy, late gadolinium enhancement (LGE) and characterisation of an arrhythmogenic substrate in order to guide ablation of complex arrhythmias. CCTA is mainly used in patients with atrial fibrillation (AF). Indeed, in patients with AF, CCTA allows a detailed evaluation of pulmonary veins anatomy and rule out of coronary artery disease. Furthermore, in patients with AF, CCTA can play a key role for the correct planning of left atrial appendage closure, in particular, if a 3D model is printed. Furthermore, some articles highlight the role of CCTA for accurate definition and location of ventricular arrhythmias. The adverse effect of CMR on pacemaker (PM) and implantable cardioverter defibrillator can be induced by electromagnetic interference or can provide the mechanical effect. Moreover, it is important to consider the heating of cardiac leads. CMR in patients with a cardiac device should take in account safety of patients.
***Learning Objectives:***
1. To learn about tips and tricks for successful cardiac CT in patients with arrhythmia.
2. To learn about tips and tricks for successful cardiac MR in patients with arrhythmia.
3. To become familiar with workflow issues for MR imaging in a patient with an implanted device.
*Author Disclosure:*
**G. Pontone:** Grant Recipient; General Electric, Bracco, Medtronic, Bayer, Heartflow.

09:50
**Panel discussion: Should we screen, who should we screen and how should we screen in order to prevent sudden cardiac death?**

**08:30 - 10:00** **Studio 2019**

### Professional Challenges Session

### PC 5

### Radiology and migrations

**A-0227** 08:30
**Chairperson's introduction**
O. Akhan; *Ankara/TR (akhano@tr.net)*

The total number of asylum seekers who migrate to another country to have a better job or life has increased from 15.000 to almost 1.4 million annually in last two decades in the countries of EU are fleeing from violence, torture, persecution, political or ethnic oppression and poverty. Migration is associated with different cultural, social, political outcome besides the health status of immigrants. The health of immigrants is affected by mainly infectious diseases such as Tuberculosis, HIV, viral hepatitis, Malaria, Schistosomiasis, Echinococcosis, Neurocysticercosis or similar diseases which reflect the epidemiology in the country of origin. The other include psychological problems, cancer, neurological diseases and acute or chronic malnutrition resulting in anaemia, growth disorders, mental and physical development disorders, immunosuppression and bone disorders which are easy to overlook. Therefore, impact of immigration has to be recognised as an important variable on the status of the current health system of the country which hosted the immigrants to improve the status of immigrants' health. Radiological examinations of patients with these diseases play an important role in the diagnosis of most of these diseases. Although these diseases are often incidental radiological findings in the daily practice, most of the radiologists in the western European countries are unfortunately not familiar to the features of these diseases. We should discuss how to raise awareness on migration-related diseases among radiologists. The health issues of immigrants are very challenging to physicians. Awareness of these conditions is mandatory to ensure good clinical practice for these patients' population.
***Session Objectives:***
1. To describe the reasons of migration.
2. To evaluate humanitarian and political consequences of migration.
3. To get an overview on the importance of migration related disease.
4. To understand how we can recognise these diseases on clinical and radiological levels.
5. To raise awareness on migration related diseases among health professionals.

**A-0228** 08:35
**Impact of migration on health: WHO perspective**
S. Severoni; *Copenhagen/DK*

"no abstract submitted"
***Learning Objectives:***
1. To describe the impact and consequences of migration on health as a global problem.
2. To teach how public health has been importantly influenced by human mobility patterns.
3. To discuss how to raise awareness among health professionals to struggle against the diseases related to migration.

**A-0229** 08:53
**Importance of migration for neglected tropical diseases: WHO perspective**
B. Abela-Ridder; *Geneva/CH (abelab@who.int)*

With increasing levels of conflict, civil unrest, poverty and persecution, there is an estimated 68.5 million people currently forcibly displaced worldwide. Malnourished, stressed people with poor housing and sanitation and restricted access to healthcare results in populations at a high risk of disease, especially neglected tropical diseases (NTDs). Global migration is changing the epidemiology of many of these diseases with emergence and re-emergence of NTDs in non- or low endemic countries. The lack of awareness, diagnostics and treatment available in host countries poses a challenge in appropriately diagnosing and treating affected individuals and poses a risk for endemic foci of disease to be established. Outbreaks of dengue, Leishmania and schistosomiasis have been recorded in low prevalence areas where appropriate vectors or intermediate hosts could allow for local transmission. The prevalence of Chagas disease, strongyloidiasis and schistosomiasis in migrant populations ranges from 4.2- 48.5%, 11-56.1% and 5.8 to 44% respectively. Cases of cystic echinococcosis and neurocysticercosis, have also been detected in Europe, with 53% of neurocysticercosis cases attributed to immigrants. Consideration should be given to systematically screen migrant populations on arrival to a host country using standardised screening procedures to reduce the progression and severity of health conditions. It will, however, be important to appropriately manage that individuals are not discriminated against because of a health condition. Better data on disease prevalence and burden in migrant populations will allow increased awareness in host countries, particularly in healthcare practitioners, assure access to timely and appropriate clinical care and management of risk.
***Learning Objectives:***
1. To describe the importance of neglected tropical diseases on the global level emphasising its relation with poverty.
2. To evaluate how migration changes the dimensions of the problem for neglected tropical diseases in Europe.
3. To discuss how to raise awareness for neglected tropical diseases on global and European levels.

**A-0230** 09:11
**Clinical importance of the diseases related to migration**
T. Junghanss; *Heidelberg/DE*

"no abstract submitted"
***Learning Objectives:***
1. To become familiar with the list of diseases related to migration and their clinical findings.
2. To discuss how to think about unusual clinical conditions when facing these patients.
3. To teach how to make differential diagnosis on the clinical ground.

**A-0231** 09:29
**Radiology of the diseases related to migration**
T. Weber; *Heidelberg/DE (tim.weber@med.uni-heidelberg.de)*

Globalisation and migration impact on the spectrum of infectious diseases that health care providers are confronted with. Although a major health care problem in a global context, the majority of these diseases may be uncommon in the autochthonal European population. In several frequently imported infectious diseases, imaging studies play an important role in timely diagnosis and treatment stratification. The migration associated infectious diseases most pertinent for the radiologist include, e.g., cystic echinococcosis, schistosomiasis, and neurocysticercosis as well as tuberculosis and visceral leishmaniasis.
***Learning Objectives:***
1. To learn radiological patterns of diseases related to migration.
2. To discuss how to evaluate unusual radiological conditions of these patients.
3. To teach how to combine radiological and epidemiological features of the patients on the basis of imaging modalities for appropriate differential diagnosis.

09:47
**Panel discussion: How do we raise awareness among health professionals to recognise diseases related to migration? How can we adapt migration and migration related diseases into our education?**

**08:30 - 10:00** **Room E1**

### Breast

### RC 502

### MRI for early detection, staging and management of breast cancer

**A-0232** 08:30
**Chairperson's introduction**
P.A.T. Baltzer; *Vienna/AT (patbaltzer@gmail.com)*

Imaging plays an important role in early detection, staging and management of breast cancer. MRI is the most sensitive method for detection of breast cancer and thus can contribute to all fields of breast cancer diagnosis and treatment. In addition, multiparametric breast MRI provides a number of imaging biomarkers that reveal diagnostic and prognostic information beyond simple cancer detection. Nevertheless, breast MRI has faced several challenges during the past decades due to controversial opinions and contradicting scientific results. Many of these can be attributed to the fact that MRI is more complicated than standard breast imaging methods including newer developments such as digital breast tomosynthesis, ultrasound elastography and contrast-enhanced dual energy mammography. In addition, breast MRI is less readily available, and its application thus needs to be applied to tailored clinical scenarios. One way of overcoming these limitations are simplified and abbreviated protocols that would allow a broader application of MRI, e.g. in intermediate or high-risk screening.

***Session Objectives:***
1. To understand the contribution of MRI to preoperative staging and the context within which it should be recommended.
2. To learn about the different MRI biomarkers and in which clinical setting they are of value.
3. To recognise the steps required to move to abbreviated MRI for high-risk screening.

**A-0233** 08:35
**A. Preoperative staging with MRI: results of preoperative MRI (MIPA) trial**
F. Sardanelli; *San Donato Milanese/IT*

The MIPA study aimed at verifying the impact of preoperative breast MRI. Up to November 2018, over 7,000 patients were recruited in 30 centres. Data from 2,425 patients were: 1,201 (49.5%) received MRI, 1,224 (50.5%) did not. Of these 1,224 MRIs, 210 (17%) were performed for screening (4%) or diagnostic purposes (13%). Of 1,014 MRIs performed as preoperative studies, 59% were ordered by radiologists alone, 32% by surgeons alone; radiologist and surgeons were involved in 68% and 40% of cases, respectively. Mastectomy rate planned at mammography/ultrasound was 185/1,201 (15.4%) in the no-MRI-group, 245/1,224 (20.0%) in the MRI-group (p<0.001). In the MRI group, 21 additional mastectomies (1.7%) were planned after MRI, while 25 patients planned with mastectomy shifted to conservative surgery (CS). Of the 1,004 patients planned for CS before MRI, MRI did not change surgery in 733 (73%), while prompting a wider CS in 143 (12.5%), a less extensive CS in 128 (12.7%). Mastectomy rate was 192/1,201 (16%) in no-MRI-group and 257/1,224 (21%) in MRI-group (p<0.001). Per-patient reoperation rate for close/positive margins were 135/1009 (13.4%) and 80/967 (8%), respectively (p<0.001). Most mastectomies were already planned at mammography/ultrasound, using MRI as a confirmation tool, contributing in determining a lower reoperation rate in women undergoing MRI. Additional mastectomies were compensated by mastectomies shifted to CS and CS surgery was modified by MRI according to disease extent, balancing increased and decreased tissue removal. No increase in tissue removal has been determined by MRI.
***Learning Objectives:***
1. To learn about the evidence for and against the use of MRI in preoperative staging.
2. To understand the background, design and early results of the MIPA trial.
3. To be able to explain the role of preoperative MRI during multidisciplinary tumour board meetings.
*Author Disclosure:*
**F. Sardanelli:** Advisory Board; Bracco, General Electric. Equipment Support Recipient; General Electric. Grant Recipient; Bayer. Research/Grant Support; Real Imaging.

**A-0234** 09:00
**B. MR imaging biomarkers for the clinical setting**
E.A. Morris; *New York, NY/US (morrise@mskcc.org)*

1. To describe MRI biomarkers for breast cancer. 2. To understand the value of biomarkers in clinical practice. 3. To know the new possibilities for the future. Biomarkers - Diagnostic Biomarker - Detection or confirmation of a disease - Risk Biomarker - Identifies women at increased risk of breast cancer - Predictive Biomarker - How is the therapy working? - Prognostic Biomarker (needed more) - Identify cancer aggressiveness relates to patient outcome - MRI BPE. Native fibroglandular tissue will demonstrate variable enhancement patterns and levels of enhancement on breast MRI. The enhancement of the existing underlying fibroglandular tissue has been termed background parenchymal enhancement. As background parenchymal enhancement is related to vascular flow, it has been proposed that this may represent an imaging biomarker of the underlying proliferation of fibroglandular tissue. Investigations have shown that there is an extremely strong association between BPE and risk of breast cancer, at least a strong as the association between mammographic density and breast cancer. As with breast density and distribution of breast parenchyma on mammography, it appears that the background parenchymal enhancement of breast MRI is also extremely variable and women have different patterns and intensity of background parenchymal enhancement. In fact, it has been observed that not all mammographically dense breasts demonstrate increased background parenchymal enhancement. Therefore, it is possible that MRI can further stratify women at high risk for developing breast cancer on the basis of background parenchymal enhancement.
***Learning Objectives:***
1. To learn about the different biomarkers that are available and the evidence for using them in patients with breast cancer.
2. To understand in which clinical settings biomarkers might be of value.
3. To appreciate the newer techniques that are being developed and tested clinically.

**A-0235** 09:25
**C. Screening with abbreviated protocols**
C.K. Kuhl; *Aachen/DE (ckuhl@ukaachen.de)*

Early diagnosis improves the survival of women with breast cancer. Mammographic screening improves early diagnosis of breast cancer. And yet, there appears to be room for improvement. Major shortcomings of mammographic screening are overdiagnosis of prognostically unimportant cancer, as well as underdiagnosis of cancers that are indeed relevant. Failure to detect biologically relevant breast cancer with mammographic screening is driven by host-related factors, i.e. breast tissue density, but also tumour-related factors: Biologically relevant cancers may exhibit imaging features that renders them indistinguishable from normal or benign breast tissue on mammography. These cancers will then progress to become the advanced-stage interval cancers observed in women undergoing mammographic screening. Since breast cancer continues to represent a major cause of cancer death in women, the search for improved breast cancer screening method continues. Abbreviated breast MRI has been proposed for this purpose because it will greatly reduce the cost associated with this method, due to a greatly reduced magnet time (down to 3 minutes), but especially also due to a greatly abridged image interpretation time, i.e. radiologist reading time. This lecture will review the current evidence and presents the EA1141 trial designed to investigate the utility of abbreviated breast MRI for screening average-risk women with dense breast tissue.
***Learning Objectives:***
1. To learn about the evidence for abbreviated MRI and the comparison with standard protocols.
2. To understand the different protocols for abbreviated MRI and the merits of each sequence used.
3. To appreciate the advantages and limitations for abbreviated MRI.

09:50
**Panel discussion: What can we do to improve the adoption of MRI in clinical practice?**

**08:30 - 10:00** **Room E2**

### State of the Art Symposium

### SA 5

### How to face the tsunami of pancreatic intraductal papillary mucinous neoplasia (IPMN)

**A-0236** 08:30
**Chairperson's introduction**
M. Zins; *Paris/FR (mzins@hpsj.fr)*

Intraductal Papillary Mucinous Neoplasm of the pancreas are cystic pancreatic injury, often fortuitous discovered, increasingly recognised in recent years due to improved diagnostic techniques. IPMNs are mucin-producing neoplasms, causing pancreatic duct (main duct and/or branch duct) dilatation. From adenoma to adenocarcinoma, IPNMs present a broad spectrum of development. Accurate initial diagnosis with CT, MRI and EUS and Follow up using mainly MRI are needed.
***Session Objectives:***
1. To briefly introduce the diagnostic challenge in patients presenting IPMN lesions.
2. To learn the main differentials in patients with cystic neoplasms.
3. To understand the imaging strategy when organising follow-up of patients with IPMN lesions.

**A-0237** 08:35
**IPMN: when to watch and when to resect**
W. Schima; *Vienna/AT (wolfgang.schima@khgh.at)*

With the development of thin-slice MDCT and MRI of the pancreas the prevalence of incidentally found pancreatic cysts has skyrocketed. It ranges from 2.6% (MDCT) to 10-45% (MRI) in large series of individuals without the known pancreatic disease. Prevalence of cystic lesions increases with age. The majority of cystic lesions may be IPMN of branch-duct type, although non-neoplastic cysts (in subtle chronic pancreatitis or even simple cysts) may also be found. The presence of IPMN constitutes an increased risk to develop pancreatic cancer. Several guidelines worldwide have addressed this issue by establishing imaging criteria to assess lesions. Most commonly used is the Fukuoka guideline, which defines “worrisome features” and “high-risk stigmata”. Worrisome features include the presence of a cyst ≥3 cm, enhancing mural nodule, thickened cyst wall, and main duct size of 5-9 mm, and rapid growth rate of >5mm/2years. EUS should evaluate these patients. High-risk stigmata (including enhanced mural nodule >5mm, main duct size ≥10mm, or obstructive jaundice in cystic lesion of the head) should prompt resection in surgically fit patients. However, the vast majority of cystic lesions is <3cm and do not show worrisome features. Follow-up schedules have been suggested, stratified according to cyst size. Fukuoka guidelines suggest indefinite F/U, whereas ACR recommendations suggest stop of F/U, if a cyst is unchanged for ten years. In younger patients, an abbreviated F/U MR protocol without IV contrast could save resources. An important consideration should be directed toward clinical consequences of imaging F/U in elderly patients.

***Learning Objectives:***
1. To become familiar with the imaging features of different types of IPMN.
2. To discuss the different international guidelines that define the management of the patients.
3. To understand the multidisciplinary team approach necessary when deciding between conservative or surgical management.

*Author Disclosure:*
**W. Schima:** Speaker; GE Healthcare, Siemens.

**A-0238** 08:58
**Diagnosis of IPMN: the mimickers and how to sort them out**
M.-P. Vullierme; *Clichy/FR (marie-pierre.vullierme@bjn.aphp.fr)*

IPMN develops on the abnormal ductal mucosa. Abundant mucus secretion is related with enlargement of pancreas ducts, main duct and/or branch ducts. Enlargement of the main duct upstream to stenosis could be misinterpreted as IPMN. Stenosis must be ruled. The enlarged ductal lumen should be continuous with the duodenal lumen. When all MPD is enlarged, it remains difficult to locate the exact place of the abnormal mucosa. Because the mucus flows downstream to be excreted in the duodenal lumen, downstream enlargement could appear. Then, for example, IPMN of the tail could be associated with enlargement of the body's MPD, but without IPMN of this body. Enlarged branch duct appears as a round cyst communicating with the main pancreatic duct through an enlarged sinuous branch duct, appearing as a tubular pattern. This communication must be obvious to affirm IPMN. Particularly serious cystadenoma is made of cysts, but without enlarged ducts and without communication. The thickening of the mucosa is often too thin to be visible with CT or MR. Intraluminal nodule could be seen when this thickening is larger. This nodule when present could locate with a good specificity the exact place of IPMN. Desiccated mucus could appear as a lacuna in the lumen, usually with a marked hyposignal with T2, and without enhancement after contrast injection. Chronic pancreatitis could appear upstream to obstructive mucus into the MPD. Ductal and parenchymal abnormalities have the same aspect than with other cause of chronic pancreatitis.

***Learning Objectives:***
1. To highlight the imaging features, which distinguish IPMN from other cystic pancreatic neoplasms.
2. To specifically discuss the differential diagnosis with chronic pancreatitis.
3. To understand the value of the integration of the imaging modalities that define the correct diagnosis.

**A-0239** 09:21
**Follow-up of IPMN: how many MRs do we need?**
R. Pozzi-Mucelli; *Stockholm/SE (raffaella.pozzi-mucelli@sll.se)*

IPMN is the most frequent pancreatic cystic lesion, which is often incidentally detected by CT or MR. Since IPMN has variable malignant potential, it should be followed-up until the patient is considered fit for surgery. MR should be preferred to CT for the surveillance of IPMN, as it does not expose the patient to ionising radiation and it has a higher sensitivity for the identification of mural nodules. Clinicians/surgeons need to tailor the follow-up and its intervals according to several aspects, such as the patient's age and comorbidity, "first-time" detection without previous imaging, presence or absence of risk factors (i.e., diameter of the main pancreatic duct, cyst size, enhancing mural nodules, growth rate), previous pancreatic surgery for IPMN and family history of pancreatic cancer. The surveillance of IPMN might be life-long as this lesion poses the patient at risk of developing an associated invasive carcinoma or a concomitant adenocarcinoma, arising somewhere else in the pancreatic gland. Thus, multiple follow-up MRs are needed, leading to high health-care costs. Therefore, it is essential to customise MR's protocols. We will discuss the indications and pitfalls of extensive protocols (i.e., including MRCP and contrast agent) compared with shorter protocols (i.e., without MRCP and contrast agent).

***Learning Objectives:***
1. To discuss the methods, intervals and length of surveillance in branch ducts IPMN.
2. To understand why MR is an adapted tool for surveillance and which protocol should be proposed.
3. To specifically discuss the development of distinct ductal adenocarcinoma of the pancreas during follow-up of branch duct IPMNs.

**A-0240** 09:44
**Panel discussion: Management of IPMN: what is the Achilles heel of our current concept?**
M. Zins; *Paris/FR (mzins@hpsj.fr)*

Intraductal Papillary Mucinous Neoplasm of the pancreas are cystic pancreatic injury, often fortuitous discovered, increasingly recognised in recent years due to improved diagnostic techniques. IPMNs are mucin-producing neoplasms, causing pancreatic duct (main duct and/or branch duct) dilatation. From adenoma to adenocarcinoma, IPNMs present a broad spectrum of development. Accurate initial diagnosis with CT, MRI and EUS and Follow up using mainly MRI are needed.

**08:30 - 10:00** **Room F1**

### E³ - Rising Stars Programme: Basic Session

### BS 5

### Abdominal emergencies

*Moderator:*
B. Marincek; Kilchberg/CH

**A-0241** 08:30
**Perforation of the GI tract**
V. Maniatis; *Aabenraa/DK (vmaniatis67@gmail.com)*

Perforation of the GI tract represents an emergency and life-threatening condition. Causes of perforation may be traumatic (endoscopy, blunt trauma, ingested foreign body), inflammatory (peptic ulcer disease, diverticulitis, appendicitis, Crohn's disease or other enteritides), bowel ischemia or neoplasms. Clinical diagnosis may be challenging, and patients may be first radiologically evaluated with plain radiographs or ultrasound, although with limited information in cases of perforation. CT scanning is the imaging method of choice in these patients. CT findings of GI tract perforation are divided to direct and indirect. Direct findings include free air or/and oral contrast either intra-eller retroperitoneal. Indirect findings comprise mainly an inflammatory mass surrounding an appendicolith or a radiopaque foreign body. Non-specific findings include bowel wall thickening, mesenteric infiltration, interloop free fluid and abnormal bowel wall enhancement. The sensitivity of CT in diagnosing GI tract perforation is up to 92%. False-positive results usually occur in post-operative patients, where free air as a result of the previous operation can be misdiagnosed as a sign of an anastomotic leak or iatrogenic bowel trauma. CT is also able to depict both the site (with an accuracy of 82-90%) and the cause (in up to 67% of cases) of perforation. It is well documented that CT plays a crucial role in the assessment of acute abdomen, including patients with GI tract perforation, by offering fast and accurate essential information to the clinicians and enabling the correct therapeutic choice.

***Learning Objectives:***
1. To review the aetiologies of the perforation.
2. To present current imaging techniques for evaluation of the perforation.
3. To describe the typical features of the perforation.

**A-0242** 09:00
**Bowel obstruction**
A. Filippone; *Chieti/IT (filipponea378@gmail.com)*

Nowadays imaging has become the primary focus in the treatment of bowel obstruction (BO). Therefore, radiology assumes considerable relevance in assisting the therapeutic decision of the surgeon in cases of BO by addressing the following questions: Is the bowel obstructed? How severe is the obstruction, where is it located, and what is its cause? Is strangulation present? Plain abdominal radiography continues to be the initial examination in these patients due to its wide availability and relatively low cost. However, radiographs are diagnostic in only 50%-60% of cases. Nevertheless, the results of this modality should serve as a basis for triage for further imaging workup and assist in the therapeutic decision. Ultrasound, although not commonly used for the evaluation of BO, may be useful when the obstructed bowel segments are filled with fluid, because allows detecting the level as well as the cause of the obstruction by using the fluid-filled bowel as a sonic window. Multidetector CT scanners with multiplanar reformation capability are significantly more effective in the evaluation of BO. Owing to the early demonstration of strangulation, CT is now considered the best modality for determining which patients would benefit from conservative management and close follow-up and which patients would benefit from immediate surgical intervention. An algorithmic and schematic approach will be proposed for imaging workup and evaluation of patients with BO, based on a review of the literature and the current approach to this entity.

***Learning Objectives:***
1. To review the most common causes of bowel obstruction.
2. To present current imaging techniques for evaluation of bowel obstruction.
3. To become familiar with the typical findings of bowel obstruction.

*Author Disclosure:*
**A. Filippone:** Consultant; BRACCO.

**A-0243** 09:30
**Vascular emergencies**
V.E. Sinitsyn; *Moscow/RU (vsini@mail.ru)*

Abdominal vascular emergencies are life-threating conditions which are very difficult to recognise with the physical examination. Clinical signs may be unspecific and delay with correct diagnosis could lead to catastrophic consequences. Knowledge of the underlying disease and high clinical suspicion help to use modern imaging modalities for diagnosis. Acute vascular emergencies could be arterial or venous. Their major types are active haemorrhage due to rupture or laceration of vessel or occlusion (thrombosis, compression) of the artery or vein. Contrast-enhanced MDCT is a modality of choice for timely diagnosis of acute mesenteric ischemia (arterial or venous). Its diagnostic performance is equal to invasive catheter angiography. The abdominal US also could be used for this purpose. CT with contrast enhancement can see not only blood clots and hematomas but also sites of active bleeding seen as extravasation of contrast media. In case of acute vessel occlusion sign of organ ischemia (malperfusion) could be detected with CT. Diagnostic pitfalls may be related to the small rate of blood extravasation and mucosal bleedings. Late complications of arterial injuries are the formation of pseudoaneurysm and dissection. In case of life-threatening vascular emergencies, different types of radiological interventional procedures such as thrombolysis, embolisation, stenting or grafting could be used for the rapid treatment of the patient without the need for open abdominal surgery.

***Learning Objectives:***
1. To review the most common causes.
2. To present current imaging techniques.
3. To be familiar with the role of interventional radiology.

**08:30 - 10:00 Room F2**

### Special Focus Session

### SF 5b

### Basic interventional radiology for non-interventionalists: let's start with biopsy!

**A-0244** 08:30
**Chairperson's introduction**
J.I. Bilbao; *Pamplona/ES (jibilbao@unav.es)*

Percutaneous biopsy (PB) procedures, which includes both fine needle aspiration biopsy (FNA) and core needle biopsy (CNB), remain essential in the management of oncological patients. In spite of some concerns related with its safety and yield and also with the advenement of liquid biopsy (circulating DNA), PB allows the detailed analysis (i.e., genomics) of heterogeneous tumours allowing a personalised therapeutic approach. This is particularly relevant, for example, in cases in which post-therapeutic imaging (i.e., PET-CT) shows a non-uniform pattern of response and more information is needed from the different parts of a tumour. Therefore, the radiologist that will perform the PB needs to be part of a team in which the individualised management of each patient is being decided. This team (the multidisciplinary tumour board-MDTB) is essential, among other roles, to facilitate decision-making and improve/accelerate the process, to extend treatment options offering cross-speciality knowledge and to sequence therapies by collaboration between equals after the identification of all available options. Within this MDTB radiologists should progress from skilfull-technicians performing procedures to clinical consultants treating patients, participating in the continuity of care, offering evidence, plannifying research and swifting from not just technological aspects to clinical outcomes. Radiologists should be aware of the different levels of sedation/analgesia that are needed depending on the characteristics of the procedure. For PB, in some cases, it would be needed some anxiolysis (minimum sedation) maintaining spontaneous ventilation and normal responsiveness to verbal stimulation.

***Session Objectives:***
1. To learn about the role of percutaneous biopsy in patient management.
2. To explain the crucial role of being part of a multidisciplinary board.
3. To learn about intra-procedure patient analgesia.

**A-0245** 08:35
**How to decide indications and pre-treatment evaluation**
P. Popovic; *Ljubljana/SI (peter.popovic@kclj.si)*

Percutaneous imaging-guided biopsy (PB) is used extensively in daily clinical practice for the pathologic confirmation of certain diseases in the area of thorax, abdomen and musculoskeletal system. Indications for PB include, but are not limited to the following: to establish the nature of diffuse parenchymal disease; to obtain material for microbiological analyses in suspected or known infections; to establish the benign or malignant nature of a suspected tumour; to classify a malignancy (including immunohistochemistry evaluation); to stage a patient with known (or suspected) malignant tumours elsewhere; to evaluate response to therapy or to confirm recurrence and to obtain material for molecular analysis. Patient clinical assessment and preparation are critical for the success of PB. Before the procedure, special attention should be paid to the review of relevant medical history, medications and laboratory data. The evaluation of coagulation status is essential. When possible, antiplatelet/anticoagulation medications should be discontinued before the procedure in particular for biopsies with a moderate or significant risk of bleeding. Procedure planning starts with choosing the most proper imaging modality and technique of access and choice of biopsy needle and device. This is followed by the careful planning of the needle access taking into account the lesion type and location, patients' compliance and technique availability. PB performed by experienced operators are generally safe procedures with a low rate of complications, these still do occur. Therefore a proper and detailed informed consent should be obtained from the patient with presenting the benefits and potential complications of the procedure.

***Learning Objectives:***
1. To become familiar with indications for percutaneous biopsy.
2. To learn what to check before your procedures, taking in mind patients' safety first.
3. To explain how it is possible to obtain a really informed consent.

**A-0246** 08:53
**How to guide your procedure**
L. Crocetti; *Pisa/IT (laura.crocetti@med.unipi.it)*

Once indication to perform a biopsy has been established, the careful review of pre-procedural images allow procedural planning, including the selection of the most proper imaging guidance, patient's position and access routes. Percutaneous needle biopsy (PNB) can be performed under a variety of imaging modalities, including US, CT, MR and fluoro-CT. The selected imaging modality should allow complete visualisation of the target and surrounding relevant anatomy, visualisation of the needle during the procedure, comfortable patient positioning and operator' manoeuvres; finally, after the execution of the procedure, an adequate evaluation of possible complication should be guaranteed. Each imaging modality has its own features that allow the radiologist to choose the most advantageous according to different anatomical compartments. US is widely used as a guidance modality for PNB in solid organs such as liver and kidney. The puncture of lesions located in the lung, mediastinum and bone, as well as of masses in the retroperitoneal and peritoneal spaces is usually performed by CT-guidance. MR guidance can be useful for lesions not detectable with other imaging modalities, provided that special non-magnetic needles are used. Once imaging guidance modality has been chosen, selection of an adequate access route is critical to ensure the success of PNB. Generally, the route should be as short as possible and should avoid all risky structures (lung fissures and bullae, large vessels, bowel, etc.). In specific situations, a longer route is recommended, i.e. in subcapsular liver lesions when a longer tract with intervening normal liver parenchyma reduces the risk of hemoperitoneum.

***Learning Objectives:***
1. To appreciate the importance of pre-procedure planning and selection of image-guidance technique.
2. To learn about advantages and disadvantages of different imaging modalities for guidance.
3. To learn about new techniques and future prospective for improving your results.

**A-0247** 09:11
**How to do your procedure**
P.M. Vilares Morgado; *Porto/PT*

"no abstract submitted"

***Learning Objectives:***
1. To become familiar with needles, and how to handle the biopsy specimen.
2. To learn which device is needed for which patient and lesion based on indication.
3. To highlight how to minimise potential complications.

**A-0248** 09:29
**How to follow-up on your patients**
M. Seidensticker; *Munich/DE (max.seidensticker@med.uni-muenchen.de)*

Biopsy procedures bare - although commonly at a low rate - potentially life threating risks. To know about potential complications and proper patient monitoring is the key to identify the complications and to react accordingly. The lecture will give informations about common and uncommon complications after biopsy procedures, and help will be given to identify the patient at risk and how to avoid/reduce specific complications. Further on, the management of complications will be addressed. Of note, not all complications are avoidable, but proper patient follow-up helps to reduce associated morbidity and mortality.
***Learning Objectives:***
1. To learn how to check your patients in the immediate post-procedure.
2. To become familiar on how to follow-up on patients.
3. To learn about management of post-procedure complications.
*Author Disclosure:*
**M. Seidensticker:** Advisory Board; Siemens, Bayer, Boston Scientific. Research/Grant Support; Bayer, SIRTEX Medical. Speaker; SIRTEX Medical, BTG, Bayer.

09:47
**Panel discussion: Deciding to biopsy within a multidisciplinary board**

**08:30 - 10:00 Room Y**

### Special Focus Session

### SF 5a

### 7 Tesla MR goes clinical

**A-0249** 08:30
**Chairperson's introduction**
S. Trattnig; *Vienna/AT (siegfried.trattnig@meduniwien.ac.at)*

There is growing interest in the ultra-high field (UHF) MRI because of improved clinical results with regard to morphological as well as functional and metabolic capabilities. Moreover, recently one 7T MR system was CE certified, and FDA approved, which raises the question if the huge investments for the installation of a 7T system provide sufficient added clinical value or can even be a game changer in MR. As the signal-to-noise ratio scales supralinearly with the field strength (Bo 1.65) of the scanner, the most obvious application at 7T is to obtain higher spatial resolution in the brain, musculoskeletal system and breast. The dynamic and static blood oxygenation level-dependent contrast increases supralinearly with the field strength, which significantly improves fMRI and SWI in the brain. The better spectral resolution at 7T with higher SNR allows high-resolution MR spectroscopic imaging in tumours and MS. Multi-nuclear clinical applications, such as sodium imaging for the evaluation of repair tissue quality after cartilage transplantation and 31P spectroscopy for the differentiation between non-alcoholic benign liver disease and potentially progressive steatohepatitis, are only possible at ultrahigh fields. In this session from basic information on the benefits of an ultra-high field, advantages of UHF in clinical neuro and MSK applications as well as in the challenging cardiovascular field will be covered.
***Session Objectives:***
1. To understand the tendency to higher static magnetic fields in clinical MRI.
2. To learn about the additional benefits of 7T in Neuroimaging and MSK.
3. To become familiar with challenges and technical solutions in body MR at 7T.

**A-0250** 08:35
**Why is there a benefit to higher static magnetic field in MR?**
P. Jezzard; *Oxford/UK (peterj@fmrib.ox.ac.uk)*

Increasing the static magnetic field strength in an MRI scanner leads to a corresponding increase in signal-to-noise ratio, which can be used in a variety of ways, including improving the spatial resolution of the image, or the spectral separation of an MRS experiment, or the acquisition of time-limited data in less time. Examples of novel scientific insights that have been obtained at ultra-high field include exquisite cortical fMRI, enabling the directionality of input and output flows of information via prior anatomical layer information. There are challenges to realizing high-quality ultra-high field MRI, however, including difficulties in achieving a homogeneous transmit field and hence a uniform image contrast, associated challenges in the amount of tissue RF heating that may result, and difficulties in coping with increased effects from artifacts caused by interfaces between regions of differing magnetic susceptibility, leading to poor static field shim. The talk will overview both the opportunities afforded by ultra-high field MRI and also some of the challenges of realising them.
***Learning Objectives:***
1. To become familiar with scanner and coil technology at ultra-high field.
2. To learn about the benefits of increased SNR and spectral resolution at 7 T.
2. To understand why 7 T is the best for BOLD imaging (fMRI and SWI).

**A-0251** 08:53
**Neuroimaging at 7 T: where does it make the difference?**
J. Hendrikse; *Utrecht/NL (J.Hendrikse@umcutrecht.nl)*

7Tesla MRI in clinical practice has been exploited for high-resolution imaging combining the increased signal to noise ratio (SNR) for an increased contrast to noise ratio (CNR). With this increased contrast to noise ratio, small pathology could be delineated. Examples of this small-sized pathology at the brain tissue level are imaging of the total burden of microbleeds and demonstration for the first time the presence in-vivo of cortical microinfarcts. The knowledge of the cortical microinfarcts at 7Tesla was used for the translation and detection of this cortical microinfarcts at 3Tesla MRI field strength. The detection and characterisation of brain tissue lesions at 7Tesla MRI has been used in dementia patients to unravel for instance the vascular contribution to (vascular) cognitive impairment and ageing in general. In acute (stroke) and chronic cerebrovascular disease or risk factors (hypertension) 7Tesla wall imaging can identify the causes of stroke in an individual patient and quantify the burden of intracranial atherosclerosis. The clear delineation of brain tissue lesions at 7Tesla was also used for the detection and characterisation of multiple sclerosis (MS) lesions including the detection of cortical lesion burden in MS patients. Furthermore, 7Tesla MRI was used for the detection of brain tissue lesions in epilepsy patients especially in epilepsy patients with a negative MRI result at lower MRI field strengths. Finally, 7Tesla has been used in patients with suspected pituitary adenomas with negative MRI results at lower field strengths. 7Tesla MRI may show pituitary adenomas in patients and guide neurosurgeons.
***Learning Objectives:***
1. To learn about the benefits of 7 T for intracranial vessel wall imaging, microbleed detection and microinfarct detection.
2. To become familiar with clinical applications of 7 T MR in brain tumour imaging including pituitary gland imaging.
3. To understand the added value of 7 T MR in MS, epilepsy and dementia.

**A-0252** 09:11
**MSK imaging at 7 T: what are the additional clinical benefits**
S. Trattnig; *Vienna/AT (siegfried.trattnig@meduniwien.ac.at)*

There is growing interest in the use of ultra-high-field (UHF) (7T) for vivo applications in MRI of musculoskeletal tissues. Higher SNR with increasing main magnetic field B0 can be exploited for improving spatial resolution, which allows for examination of small structures which are difficult to see at lower field strength such as the thin cartilage in ankle and wrist joints. Macromolecular content of the connective tissues of joints determines their correct function. Collagen fibre mesh defines the static biomechanical properties whilst glycosaminoglycans define the dynamic biomechanical properties. Early detection of connective tissue microstructure defects is a crucial deciding factor for appropriate treatment or intervention. MRI techniques capable of non-invasive detection of connective tissues macromolecules strongly benefit from ultra-high field MRI. First signs of tendinopathy and meniscus degeneration are associated with the collagen fibre decomposition and can be detected by T2* analysis and high resolution at 7T. The low sensitivity of sodium MRI and the low abundance of sodium require the high SNR of UHF. Several studies have demonstrated sodium MRI as a quantitative marker for cartilage degeneration and monitoring the cartilage transplant maturation by direct quantification of the glycosaminoglycan content. In addition, sodium MRI has been used as a marker for Achilles tendinopathy and treatment monitoring, but also for effects of systemic diseases on joint structures and the possible side effects of drug on tendons. Musculoskeletal MRI at ultra-high field benefits from higher SNR and lower acquisition time, however, optimised protocols and dedicated coils are required.
***Learning Objectives:***
1. To appreciate the important role of coil technology and optimised sequence protocols in MSK at 7 T.
2. To learn about the improvements of morphological MSK at 7 T MRI.
3. To become familiar with the benefits of biochemical MSK using x-nuclei and CEST.

**A-0253** 09:29
**Cardiovascular and abdomen MR at 7 T - still a challenge?**
J. Schulz-Menger; *Berlin/DE (jeanette.schulz-menger@charite.de)*

7Tesla MR has entered the clinical arena in neurology and musculoskeletal imaging, but its capabilities in body imaging including Cardiovascular Magnetic Resonance(CMR) and Abdominal MR (AMR) are still reflected as a challenge. That is caused by the perception of technical challenges as well based on uncertainty regarding clinical need and/or indication. No doubt, the new methodology has always challenges - it is changing our habits. Increasing the field strength comes along with increases in signal- and contrast-to-noise ratio. This benefit is expected to be translated into higher spatial and temporal resolution and faster imaging techniques. Translation into histology like images as well as in the evaluation of pathophysiology are the offers we are getting using 7Tesla. The main technical challenges like field inhomogeneity, lack of coils, gating problems or inconvenient handing are meanwhile nearly solved. Technical improvements by experienced physicists and engineers are still ongoing. That offers completely new insights like sodium-, potassium-imaging as well as cardiac functional de-oxygenation-imaging. A significant step is given by the excellent quality of non-contrast angiography allowing the delineation of small peripheral as well as abdominal vasculature. It was already shown, that CMR is reliable at 7T and has added value by identifying tiny structures like gaps in the myocardium. AMR seems to give the chance to detect small lymph nodes, but MRA is the current focus. There are still challenges at 7T, but solving them will be a significant step towards personalised medicine. Driving this development means establishing the additional value.

***Learning Objectives:***
1. To understand the current challenges in this field at 7 Tesla.
2. To become familiar with dedicated technical solutions at 7 T for patient handling.
3. To learn about the added diagnostic value and unique opportunities in cardiovascular and abdomen MR.

*Author Disclosure:*
**J. Schulz-Menger:** Advisory Board; Bayer, Siemens, circle cvi, SCMR. Board Member; EACVI. Consultant; Bayer. Research/Grant Support; BMBF, DZHK, DHS, HRC.

09:47
**Panel discussion: Will 7 Tesla become clinical routine?**

**08:30 - 10:00 Room D**

### Musculoskeletal

### RC 510

### Imaging of chronic forefoot pain

*Moderator:*
K. Wörtler; Munich/DE

**A-0261** 08:30
**A. Articular disorders**
E.G. McNally; *Oxford/UK*

Forefoot pain has a wide differential diagnosis. Biomechanical causes are common, often related to subtle foot deformity. Other causes include: Biomechanics (Hallux Valgus, MPV, Bunionette, H Rigidus, Hammer/Claw toe, Fallen transverse arch), Bone (Sesamoids, Osteochondritis, Stress, Insufficiency), Joint (RA, Seroneg, Crystal, Infection, Implants), Joint capsule (Capsulitis, Plantar plate, Collateral ligaments).

***Learning Objectives:***
1. To explain the pathophysiology and disease spectrum of articular sources of chronic forefoot pain.
2. To describe the imaging findings of articular disorders that present with chronic forefoot pain.

**A-0262** 09:00
**B. Extra-articular source of pain**
G. Bierry; *Strasbourg/FR (guillaume.bierry@chru-strasbourg.fr)*

After a brief review of forefoot soft tissue anatomy and of the mechanical consequences of the loss of normal foot architecture, we will review the main extra-articular (not related to joint or bone disorders) causes of chronic forefoot pain. The two first, plantar plate injury and Morton neuroma, are thought to be secondary to foot modified strains and are, with fatigue fracture, part of the second ray syndrome. Even if asymptomatic in the vast majority of cases, bursitis will be discussed in the section as well. Last, soft tissue tumours and infection, far less frequently encountered than neuromas and plate injury will be briefly reviewed.

***Learning Objectives:***
1. To explain the pathophysiology and disease spectrum of extra-articular sources of chronic forefoot pain.
2. To describe the imaging findings of extra-articular conditions that are present in chronic forefoot pain.

**A-0263** 09:30
**C. Imaging-guided percutaneous treatment of forefoot pain**
R.L. Cazzato; *Strasbourg/FR*

The forefoot is the most common location for foot pain in adults. Women are more likely to be affected than man, and it has been estimated that forefoot pain causes disabling symptoms in more than 1/3 individuals older than 70. The most common causes of forefoot pain are related to acquired deformities of the toes (e.g. hallux valgus, hammertoes) or of the plantar surface (e.g. corns, calluses), for which a conservative or surgical treatment is routinely proposed and effectively applied. Nevertheless, there are some less common causes of forefoot pain for which percutaneous image-guided interventional treatments have been progressively proposed in the last few years, with results that are encouraging although preliminary in many cases. In such perspective, infiltrative or ablative treatments are becoming increasingly popular to treat degenerative, tumoral or tumour-like painful conditions of the forefoot. Such tendency is mainly related to the main advantages of percutaneous image-guided treatments including their minimally invasive profile accounting for a low post-interventional morbidity rate and a fast convalescence period, and the possibility to be repeated over time.

***Learning Objectives:***
1. To explain the rationale behind the imaging-guided percutaneous treatment of forefoot pain.
2. To describe the imaging-guided percutaneous procedures for the treatment of forefoot pain.

**08:30 - 10:00 Room G**

### EFOMP Workshop

### EF 1

### Big data and the big picture: deep learning in optimisation of medical imaging (part A)

*Moderators:*
J. Damilakis; Iraklion/GR
P. Gilligan; Dublin/IE

**A-0264** 08:30
**Chairperson's introduction**
J. Damilakis; *Iraklion/GR (damilaki@med.uoc.gr)*

Deep learning methods can be used for a large number of tasks in medical imaging. These tasks may cover image production steps such as image reconstruction, dose optimisation, image processing etc. Deep learning can also support research in the field of medical imaging. Machine-learning algorithms and deep-learning methods can be used to develop non-invasive imaging-based biomarkers. Radiomics refers to a method designed to extract a large amount of quantitative and reproducible characteristics from medical images, thereby enabling data mining. Coupled with machine learning methods, radiomics allows for several types of pathologies discovered on radiological images to be automatically classified. Big data and deep learning will be used in everyday medical imaging in the future to improve quality and safety. However, there are several technical, medico-legal and ethical challenges. Medical physicists must be prepared for facing this new technology by updating their training and education programs.

***Session Objectives:***
1. To become familiar with the concept of big data and their management.
2. To discuss the opportunities provided by big data repositories in clinical practice.
3. To understand how big data can support research.

**A-0265** 08:35
**Imaging and dose biobanks**
E. Neri; *Pisa/IT (emanuele.neri@med.unipi.it)*

"no abstract submitted"

***Learning Objectives:***
1. To provide an overview of imaging and dose biobanks.
2. To understand how to gain access to the data.
3. To learn about obstacles in obtaining validated and annotated images and dose data in a systematic fashion.

**A-0266** 09:05
**Statistical methods for analysis of multidimensional imaging data**
K. Van Leemput; *Copenhagen/DK*

This presentation will provide an introduction to two complementary approaches for computationally analysing medical images, namely discriminative vs generative models. The pros and cons of each of these approaches will be highlighted, with a specific emphasis on the role of training data and the (lack of) acquisition standardisation across clinical imaging sites. The presentation will conclude with an example of a generative model for modality-adaptive segmentation of brain tumours and organs-at-risk for radiation therapy planning.
***Learning Objectives:***
1. To learn about the main features of a system for collection and analysis of multidimensional imaging data.
2. To become familiar with different analytical uses of collected imaging data.
3. To understand the difference between the current approaches used in statistical analysis/deep learning in medical imaging.

**A-0267** 09:35
**The use of radiomics in medical imaging**
P. Lambin; *Maastricht/NL*

"no abstract submitted"
***Learning Objectives:***
1. To understand how convolutional neural networks can be used for features extraction in medical imaging.
2. To become familiar with the different capabilities of features extraction tools and their connection with image analysis and interpretation.
3. To identify the possible limitations of radiomics in providing meaningful relationships between features and their clinical interpretation.

**08:30 - 10:00 Room K**

### Chest

### RC 504

### Pulmonary neuroendocrine proliferations and neoplasms

*Moderator:*
M. Silva; Parma/IT

**A-0268** 08:30
**A. Diffuse idiopathic pulmonary neuroendocrine cell hyperplasia (DIPNECH)**
G. Chassagnon; *Paris/FR (gchassagnon@gmail.com)*

Diffuse Idiopathic Pulmonary Neuroendocrine Cell Hyperplasia (DIPNECH) is an under-recognised disease characterised by proliferation of neuroendocrine cells in the bronchial wall. DIPNECH preferentially affects middle-aged women. It is considered a pre-invasive lesion for lung carcinoid tumours and is found in 5.4% of patients undergoing surgical resection for lung carcinoid tumours. Other manifestations of DIPNECH include bronchial obstruction and formation of tumorlets. On computed tomography (CT), an association of mosaic attenuation with multiple small nodules is very suggestive of DIPNECH diagnosis.
***Learning Objectives:***
1. To learn about the diagnostic criteria of this syndrome.
2. To understand the correspondence between imaging and pathology.
3. To know when to suggest this diagnosis on CT.

**A-0269** 09:00
**B. Carcinoid tumours**
H. Prosch; *Vienna/AT (helmut.prosch@meduniwien.ac.at)*

Neuroendocrine tumours of the lung arise from neuroendocrine cells of the bronchial tree and are subdivided into the low-grade typical and atypical carcinoids, as well as the malignant small-cell lung carcinomas (SCLC) and large-cell neuroendocrine lung carcinomas (LCNET). Carcinoids of the lungs are rare tumours comprising only up to 5% of all lung cancers. Most carcinoids (80%) are typical carcinoids, which are primarily located in the lung periphery. Only about 20% of pulmonary carcinoids are atypical, which are more frequently found in the lung core. Since carcinoids originate from the bronchial system, the most common presenting symptoms are attributable to the consequences of obstruction (wheezing, post-obstructive pneumonia, atelectasis) or hemoptysis. While the prognosis for carcinoids is good in most patients, the prognosis for SCLCs and LCNETs is usually very poor. As imaging plays a key role in the diagnosis, staging, and follow-up of neuroendocrine tumours, radiologists need to be familiar with the peculiarities of these tumours.
***Learning Objectives:***
1. To learn about the radiological features of central and peripheral carcinoids.
2. To know when to suspect a carcinoid tumour in case of bronchial obstruction.
3. To learn about the role of FDG and DOTATOC PET/CT in the evaluation of carcinoid tumours.
*Author Disclosure:*
**H. Prosch:** Advisory Board; Boehringer Ingelheim, Roche, MSD, AstraZeneca, BMS.

**A-0270** 09:30
**C. Small cell lung cancer**
M. Das; *Duisburg/DE (Marco.Das@helios-gesundheit.de)*

Small cell lung cancer (SCLC) is one of the most aggressive tumours, with a 5-year survival rate of only 5-10%. Most of the patients present with advanced disease at diagnosis. SCLC is classified by recent TNM classification and UICC criteria, as well as traditional as limited and extensive disease. As SCLC is usually treated by chemotherapy/radiation therapy patients, undergo frequent imaging for response control. The role of radiology is to assess initial diagnosis and extent of disease as well as follow up assessment and tumour response.
***Learning Objectives:***
1. To learn about the common radiological manifestations of small cell lung cancer.
2. To learn how to distinguish between limited or extensive forms.
3. To learn how the TNM staging system should be integrated into the classification of SCLC.

**08:30 - 10:00 Room M 1**

### Special Focus Session

### SF 5c

### MSK intervention: the road from low to no invasion

**A-0271** 08:30
**Chairperson's introduction**
J. Hodler; *Zurich/CH (juerg.hodler@usz.ch)*

Musculoskeletal interventions commonly are performed as an elective procedure. Radiologists, therefore, have a special responsibility to reduce side effects. Correct indication for any procedure, reduction of radiation dose, avoiding medication-related side effects and technically correct procedures are relevant aspects. Technical innovation may assist radiologists in making image-guided interventions safer. These aspects and more will be presented and discussed by experts in their field.
***Session Objectives:***
1. To appreciate the prevalence and importance of complications in MSK interventions.
2. To learn about strategies reducing side effects and complications of MSK procedures.
3. To become familiar with innovations leading to reduced invasiveness in MSK interventions.
4. To appreciate the role of radiologists in developing less invasive interventions.
*Author Disclosure:*
**J. Hodler:** Research/Grant Support; Siemens Healthineers, Guerbet, Bayer.

**A-0272** 08:35
**Avoiding side effects and complications by using MR guided high-intensity focused ultrasound**
A. Napoli; *Rome/IT (alessandro.napoli@uniroma1.it)*

Current treatments for patients with bone metastases include localised therapies (radiation and surgery), systemic therapies (chemotherapy, hormonal and radiopharmaceutical therapies, and bisphosphonates), and analgesics. External beam radiotherapy (EBRT) is the current noninvasive standard for local pain palliation; however, 20%-30% of patients do not achieve symptom relief, and pain may recur in up to 25% of patients following treatment. Magnetic resonance-guided focused ultrasound (MRgFUS) is already clinically approved in the European Union for the palliative care of bone metastases. MRgFUS combines focused ultrasound energy to thermally ablate tissue in combination with continuous MR imaging and thermal feedback. The treatment is noninvasive, does not require ionising radiation, and usually conducted in an outpatient setting. Because focused ultrasound energy is nonionizing, there is no dose limit; treatment can be repeated if needed. The major advantages of MRgFUS include MR-guided three-dimensional visualisation for high-accuracy treatment planning, real-time monitoring of thermal damage in the target zone using MR thermometry and immediate post-treatment assessment of therapy. An additional advantage of MRgFUS is that it can be administered during chemotherapy. MRgFUS ablation is indicated in patients who are considered radiation failures. In our department, we have evaluated the safety and efficacy of MRgFUS treatment for the pain palliation of lesions from different known primary tumours even as a first line modality. In conclusion, MRgFUS ablation is an extremely promising alternative therapy for pain palliation in patients suffering from bone metastases. Focused ultrasound might have a future role in local tumour control.

***Learning Objectives:***
1. To understand the physics of high-intensity focused ultrasound.
2. To understand the role of MR guidance in high-intensity focused ultrasound.
3. To appreciate effects and side effects of high-intensity focused ultrasound.
4. To become familiar with indications for high-intensity focused ultrasound.

### A-0273 08:50

**The 10 most important side effects and complications in MSK interventions**

M. Obradov; *Nijmegen/NL (m.obradov@maartenskliniek.nl)*

The musculoskeletal radiologist has to tailor interventional procedures to a patient's specific anatomy, pathology and comorbidities. Before the start of each procedure, the radiologist has to follow the guidelines to help avoid simple errors. Those guidelines consist of: reading the request, looking at recent radiographs/imaging, patient interview, an explanation of the procedure and obtaining informed consent. The most common general complications during musculoskeletal intervention procedures are haemorrhage, infection and drug-related reactions including complications of intra- and peri-articular steroid and anaesthetic injection. Anticoagulation alone is not a contraindication to most interventional musculoskeletal radiology procedures. The risk of bleeding if therapy is continued against the possibility of a thromboembolic occurring when anticoagulation therapy is withheld or reversed must be weighted. Since musculoskeletal procedures are generally considered clean, the guidelines recommend absolute sterile technique which would include scrub attire, hair coverings and masks in the presence of open instruments/trays, sterile gowns and gloves for participants, use of sterile drapes allowing for generous coverage of the sterile field,minimization of traffic in the procedure area. Adverse events associated with corticosteroid injection can be minimised by ensuring appropriate injecting procedures by a well-trained radiologist and limiting steroids into an annual dose of 3 mg/kg of triamcinolone or its equivalent. An allergic reaction has a higher incidence of local anaesthetics of ester type. Image guidance with smaller gauge needle helps minimise the risk of misadventure and complications. With proper planning and precautions, the consequences of possible complications can be minimised.

***Learning Objectives:***
1. To become aware of potential side effects and complications in MSK interventions.
2. To appreciate the relative importance of side effects and complications in MSK interventions.
3. To recognise the requirements regarding patient information before MSK interventions.
4. To become familiar with the imaging appearance of complications in MSK interventions.

### A-0274 09:10

**Radiation exposure: from reduction to avoidance**

D.J. Wilson; *Oxford/UK (davidwilson.stlukes@btconnect.com)*

The advent of ultrasound for needle guidance has to move the need for radiation exposure in the injection of joints. It also increases the precision as the technique allows real-time confirmation that the injectate is entering the joint. It is also allowed precise placement of needles into tendon sheaths and adjacent to ligaments. There are strong arguments that some injection procedures should only be undertaken using ultrasound guidance. Unfortunately, until recently spine injections including facet joint injections, disc injections, nerve root blocks and procedures at the vertebral bodies have not been practical using ultrasound guidance and required radiation exposure either by fluoroscopy or CT. Now we have ultrasound MRI or ultrasound CT fusion imaging which permits placement of needles and a real-time ultrasound examination with considerable precision and no radiation to the operator, and none to the patient and MRI images are used. Needle tracking techniques allow prediction of the route that the needle will take as well as direct tracking of the needle tip. The only disadvantage of this method it cannot administer contrast agents to identify where injection materials will track. This is a particular disadvantage when will that the needle might be inadvertently placed in a vessel that supplies the spinal cord or the brain. In this presentation, I will present examples of how fusion imaging has changed the preferred technique for complex musculoskeletal injections.

***Learning Objectives:***
1. To learn about the current knowledge in radiation exposure associated with MSK interventions.
2. To understand the need to reduce radiation exposure in MSK interventions.
3. To become familiar with radiation minimisation strategies in MSK interventions.
4. To appreciate alternative image guidance methods in MSK interventions.

*Author Disclosure:*
**D.J. Wilson:** Consultant; St Luke's Radiology Oxford Ltd.

### A-0275 09:25

**Preventing side effects and complications by new procedures: management by radiologists**

A. Bazzocchi; *Bologna/IT (abazzo@inwind.it)*

The endpoint of the “road from low to no invasion” is focused on obtaining the desired effect with no complications or side effects; thus the ultimate purpose is the highest possible therapeutic index. This can be reached by developing new and minimally invasive techniques and tools. The achievement is based on an in-depth knowledge of the procedure itself and on the further investigation about how it works on a biological system. To be able to predict the reaction of the human body is essential. The awareness of different pathophysiological mechanisms involved in processes is also important to stimulate effective and safe approaches and treatment strategies. The management of the radiologist is crucial. The patient needs the radiologist to fully undertake and play this role.

***Learning Objectives:***
1. To become familiar with new methods in MSK interventions.
2. To appreciate solutions to reduce side effects and complications in MSK procedures.
3. To promote the role of radiologist in advancing new technology.
4. To appreciate responsibilities of radiologists in reducing side effects and complications in MSK procedures.

### 09:45

**Panel discussion: How minimal can invasiveness in MSK intervention become?**

**08:30 - 10:00 Room M 2**

### Paediatric

### RC 512

### Intensive care paediatric radiology: the very sick neonate

### A-0276 08:30

**Chairperson's introduction**

S. Stafrace; *Doha/QA (samstafrace@yahoo.com)*

Imaging of neonates in the intensive care setting is somewhat restricted by the fact that these infants are very unstable and best imaged at their bedside, if at all possible. Portable plain films play a vital role - often these neonates have multiple supporting tubes and lines that are prone to displacement and that require correct localisation. Knowing the appropriate landmarks for tube and line positioning and being able to identify pitfalls is very important for the radiologist reporting radiographs in such a setting. Ultrasound is the next big player - mobile, radiation-free, and in the trained hands, often gives the answer serving as a problem solver. Other modalities will come to play once plain films and ultrasound reach their limitations. This session, presented by experts in their field, offers a deep dive into the imaging of the neonate in intensive care, specifically focusing on the brain, the chest and the abdomen.

***Session Objectives:***
1. To learn about the most common neurological emergencies in the intensive care neonates.
2. To understand the wide variety of thoracic devices that may be used in these patients.
3. To discuss the role of US in abdominal emergencies in the critically ill child.

### A-0277 08:35

**A. Neuroimaging in the neonatal intensive care unit**

M.I. Argyropoulou; *Ioannina/GR (margyrop@cc.uoi.gr)*

The imaging modality of choice to evaluate the neonatal brain in the intensive care unit is the Ultrasound (US) coupled with Colour Doppler. State of the art technique should be applied by using the anterior fontanel and accessory acoustic windows. Sectorial and high frequency linear transducers should be used for detailed evaluation of the neonatal brain. Knowledge of the age-related changes of the brain are necessary to assess what is abnormal. The most common abnormalities in premature babies are brain hemorrhagic disease (BHD) and periventricular leukomalacia (PVL). BHD is easily depicted and the different grades can be evaluated as well as complications such as venous infarct and post-hemorrhagic hydrocephalus. The focal form of PVL in early stages appears as heterogeneous hyperechogenicity and later as multiple periventricular cysts of different size. In the early stages of the diffuse form of PVL, US does not show any abnormality, but later on, ventricular enlargement with regular ventricular outlines appears. In full-term babies, hypoxia-ischemia is one of the main morbidities. Dependent on the duration and the severity, US may depict hyperechogenicity of the cortex, hyperechogenicity of the basal ganglia and in the most severe cases heterogeneity along with extensive encephalomalacia lesions. MRI is the gold standard for brain imaging and in neonates can be used to give additional information. MR compatible incubators are useful to provide a safe environment for those fragile babies.
***Learning Objectives:***
1. To learn the most common neurological emergencies in the intensive care patient.
2. To understand the preferential use of US and MRI as imaging techniques.
3. To discuss the key findings that may be useful for differential diagnosis.

**A-0278** 09:00
**B. Chest imaging in the ICU: tubes and catheters**
M.L. Lobo; *Lisbon/PT (mluisalobo@gmail.com)*

A large number of tubes and catheters are required to ensure life support and proper management of the very sick neonate. These include, among others, endotracheal tube, gastric tube, umbilical lines, central venous catheters and peripherally inserted venous central lines and chest drain tubes. A correct position of these devices is crucial to ensure their adequate function and to avoid complications, that can be serious or even fatal. Chest x-ray (or babygram) is the imaging modality of choice to check for the position of tubes, catheters and other devices. A good quality radiography is essential for adequate imaging interpretation; basic simple rules, such as a proper position of the baby and deviation of overlap external lines, should not be overlooked. Additional imaging, such as a lateral film and ultrasound, are helpful in doubtful cases. Knowledge of the optimal position for each device, early recognition of wrong or dangerous positions and their resultant complications, and understanding pitfalls in imaging interpretation (and how to overcome them) are basic requisites that every radiologist should know.
***Learning Objectives:***
1. To learn the crucial role of simple views in critically ill children.
2. To understand the variety of devices that is used in the intensive care unit.
3. To discuss how to avoid the most common pitfalls.

**A-0279** 09:25
**C. The role of imaging in emergencies in the critically ill neonate: abdominal US applications and beyond**
M. Riccabona; *Graz/AT (michael.riccabona@medunigraz.at)*

The objective is to list the various imaging modalities, particularly ultrasound, in imaging emergencies of critically ill neonates at the NICU. Besides refreshing the typical ultrasound approach to urgent neonatal abdominal queries typical findings of common findings in this patient group will be presented; furthermore, restrictions of an ultrasound exam will be discussed. The role of other imaging in these emergency situations will be revisited in the light of patient care, gentle handling, and therapeutic implications in critically ill pre-terms and neonates. Finally, some typical image examples of FAST and other essential emergency US applications at the NICU will be presented. Ultrasound is the mainstay of emergency imaging in the very sick neonate at the NICU, complemented by plain film. Rarely other modalities such as fluoroscopy, CT or MR may become necessary or are feasible.
***Learning Objectives:***
1. To learn about typical body (abdomen, chest and other) emergencies that can be addressed by US in the NICU.
2. To understand some typical findings that may help in the disease characterisation.
3. To appreciate the considerable impact on the prognosis and survival of the critically ill neonate.

09:50
**Panel discussion: Is there a role for FAST scan in the paediatric IC unit?**

**08:30 - 10:00** **Room M 3**

### Oncologic Imaging

### RC 516

### Role of imaging in cancer of unknown primary (CUP)

**A-0280** 08:30
**Chairperson's introduction**
S. Gourtsoyianni; *Athens/GR (sgty76@gmail.com)*

Radiologists involved in CUP MDTs are essentially asked to guide the clinical team to solve the mystery of patients presenting with metastatic disease and an unclear primary, via imaging. The workhorse of oncologic imaging is CT of the torso and based on the spread of disease more dedicated imaging examinations might be requested. PET-CT certainly plays a role in reducing the number of confirmed CUP cases. In addition involved radiologists are able to select the most suitable lesions amenable to biopsy so that tissue diagnosis can be performed. CUP serves as a very good example of rad-path collaboration, as the two disciplines truly join forces for the best possible treatment to be provided. During this session, important issues such as avoiding unnecessary investigations and how to best follow up these patients will be also discussed.
***Session Objectives:***
1. To discuss the role of imaging for the diagnosis of CUP.
2. To review the role of the interventional radiologist in the management of suspected CUP.
3. To learn how to best assess treatment response.

**A-0281** 08:35
**A. MDCT: the imaging workhorse for suspected CUP**
C. Kelly-Morland; *Framlingham/UK (christian.kellymorland@gmail.com)*

Cancer of unknown origin is a heterogeneous subset of metastatic malignancy in which the primary tumour of origin remains elusive despite exhaustive imaging and often invasive investigation. It accounts for approximately 2% of all new cancer diagnoses, and the initial presentation varies widely. This lecture aims to outline common patterns of presentation and present to the audience a systematic approach to imaging evaluation in these patients with examples from a tertiary centre. Issues relating to CT protocoling and multimodality imaging will be addressed with a focus on review areas and important benign mimics of disease. A brief outline of key diagnostic adjuncts including immunohistochemical indices will be provided along with suggestions for disease response assessment methodology.
***Learning Objectives:***
1. To discuss the different patterns of disease spread in CUP.
2. To review optimal protocols and how not to miss important findings.
3. To learn how to best assess treatment response.

**A-0282** 08:58
**B. Role of PET/CT in suspected CUP**
L. Umutlu; *Essen/DE*

"no abstract submitted"
***Learning Objectives:***
1. To review the role of PET/CT in localised versus widespread metastatic disease.
2. To discuss potential false positive and false negative findings.
3. To learn when PET/CT is most appropriate in treatment response assessment.

**A-0283** 09:21
**C. The role of the interventional radiologist**
F. Cornelis; *Paris/FR (francois.cornelis@aphp.fr)*

Interventional Radiology is occupying an increasingly prominent role in the care of patients with cancer, with involvement from initial diagnosis, right through to minimally invasive treatment of the malignancy and its complications. However, approximately 3% to 5% of new diagnoses of cancer are classified as cancer of unknown primary (CUP). Imaging and management of these patients are often frustrating for both clinicians and patients. Adequate diagnostic samples can be obtained under image guidance by percutaneous biopsy and needle aspiration in an accurate and minimally invasive manner. In biopsy planning, modern cross-sectional imaging techniques help define lesion location, accessibility, and suitability for biopsy and aid in ensuring the correct lesion is sampled in the context of multiple lesions. It is important in analysing the patient with CUP to determine where the metastases are and if they have any suggestive imaging features that may allow a tailored search for the likeliest primary site. The advent of PET scanning may have a significant impact in the future, since it allows a survey of the entire body in a single study, and may greatly simplify the search for a primary site in some cases.
***Learning Objectives:***
1. To learn the importance of tissue diagnosis in CUP.
2. To discuss how to choose the optimal target lesion for biopsy.
3. To review the relative risks and benefits in acquiring tissue samples.

09:44
**Panel discussion: How to guide the clinical team to solve the mystery**

**08:30 - 10:00 Room M 4**

### E³ - ECR Academies: Hot Topics in GU Cancer

### E³ 519

### Tumour relapse in gynaecological cancer

**A-0284** 08:30
**Chairperson's introduction**
R.A. Kubik-Huch; *Baden/CH (rahel.kubik@ksb.ch)*

Despite recent advances in diagnosis and therapy of gynaecological cancer, tumour relapse will be seen in about one-third of patients usually within 24 months from the time of diagnosis and often with the dismal prognosis. In the first part of this session, the role of MRI (including functional imaging with DWI and DCE) and PET/CT for the differentiation of postoperative or inflammatory changes and tumour relapse and the best imaging strategy to assess local pelvic recurrence will be reviewed. Defining the treatment of choice for tumour relapse of gynaecological cancer remains a challenging issue, depending on several factors including the type of primary tumour and therapy or the site and extent of recurrence. In radiation-naïve loco-regional recurrence, radiotherapy is usually the salvage therapy of choice. Pelvic exenteration, a radical surgical treatment removing all organs from the pelvic cavity, has emerged as a beneficial option in selected patients with isolated pelvic recurrences. A multimodality imaging approach allows the precise preoperative assessment and therefore individualised tailoring of the surgical treatment, as discussed in the second lecture. Stereotactic body radiotherapy has emerged as an alternative treatment in recurrent disease in the previously irradiated pelvis. CT and MRI fusion techniques exploiting the advantages of both modalities for the planning of the procedure will be demonstrated. The imaging considerations for stereotactic radiotherapy and the potential future role of MR-guided HIFU in a palliative setting will be discussed during the third part of the session.

**A-0285** 08:36
**A. Differentiating relapse from post-treatment appearances**
R. Forstner; *Salzburg/AT (r.forstner@salk.at)*

In Western countries, the incidence of cervical cancer is continuously decreasing. Nevertheless, it continues to be challenging as the peak rate of cervical cancers is at age < 30 years, and an advanced stage is associated with a recurrence rate of more than 30%. The risk of relapse is highest within the first three years, and 90% occur by year five after treatment. The type of treatment, surgery or chemoradiation, mainly depends on the stage and size of a tumour. This results in completely different posttreatment findings. Imaging plays an important role in clinically suspected recurrence, in high-risk conditions and if clinical assessment is inadequate, as in trachelectomy. MRI including DWI and contrast-enhanced techniques is the single best imaging test to assess local recurrence, either central in the pelvis or along the pelvic sidewall. Owing to its higher sensitivity and specificity PET/CT is complemented in suspected recurrence for treatment stratification, particularly to assess the retroperitoneal lymph nodes or presence of distant metastases. However, despite multi-modality approach differentiation of relapse from short-term and long-term complications of radiotherapy, e.g. inflammatory changes, radiation necrosis, extensive fibrosis and fistulation still remains challenging.
***Learning Objectives:***
1. To learn post radiotherapy appearances in cervix cancer.
2. To recognise the appearances of disease relapse.
3. To be familiar with the complications of treatment seen on imaging.

**A-0286** 09:04
**B. Planning exenterative surgery**
H.A. Vargas; *New York, NY/US*

In the past, patients with relapsed gynaecological cancers had limited salvage treatment options and a dismal prognosis. Under those circumstances, imaging detection of recurrence after treatment served the mere purpose of labelling patients with untreatable disease and initiating palliative efforts. More recently, pelvic exenteration has emerged as a valuable option for selected patients, with refinements in surgical technique leading to the reported 5-year overall survival of over 50% for patients with the recurrent gynaecological disease. As such, more precise delineation of location and extent of disease on imaging have become increasingly important. The procedure involves resection of all pelvic viscera involved by disease, including the female genital organs, bladder and/or rectum. It is a radical surgical procedure with a potentially high rate of morbidity and mortality, and therefore careful preoperative planning is essential to optimise the balance between the clinical benefit and potential morbidity. This session will review a multimodality imaging approach to optimise the clinical indications, patient selection and individualised tailoring of this procedure (anterior, posterior, total perineal, total pelvic).
***Learning Objectives:***
1. To learn the important criteria for patient selection.
2. To learn the critical anatomy for surgical planning.
3. To be familiar with the post-surgical appearances and follow-up plan.

**A-0287** 09:32
**C. Salvage treatment with image directed therapy: high-intensity focused US (HIFU) and stereotactic RT**
N.M. deSouza; *Sutton/UK (nandita.desouza@icr.ac.uk)*

Despite surgery and radiotherapy delivered with curative intent in early stage gynaecological cancers, up to 30% of patients recur in the pelvis (10-15%). Treatment of isolated pelvic recurrences depends on previous treatment, site of recurrence, disease-free interval, and patient-related factors (morbidities, performance status). Total pelvic exenteration carries high morbidity. In radiation-naïve, locally recurrent gynaecological cancer, radiotherapy is the salvage treatment of choice. Stereotactic body radiotherapy (SBRT) enables dose escalation while reducing dose to organs-at-risk (OARs). However, to deliver SBRT effectively, accurate delineation of the gross tumour volume (GTV) is vital. Tissue contrast on CT is inadequate where pelvic anatomy is distorted by previous surgery and/or radiotherapy. MRI provides superior soft-tissue contrast for GTV delineation but requires that motion and distortions are recognised and corrected for. Differences in observer interpretation of the tumour outline are greater with CT, while MR does not provide the required electron density information for radiation dosimetry. Therefore, fusion techniques between CT and MRI are frequently employed. Where salvage treatment is not possible, palliative treatment is employed. High intensity focused ultrasound (HIFU) is a non-invasive ablative therapy with the potential to achieve this under MR guidance. Its current worldwide experience is limited. Challenges in delivering this extremely precise ablative technology are related to the depth and site of recurrence, adjacent bowel, bladder and vascular structures, and the thickness of overlying fat and muscle layers, which affect the placement of the ablative focus. Nevertheless, MR guided HIFU is a feasible and viable means of managing these difficult-to-treat patients.
***Learning Objectives:***
1. To be familiar with the imaging considerations for stereotactic radiotherapy in relapsed disease.
2. To be familiar with the concepts of imaging in directing RT treatment plan.
3. To be familiar with the use of HIFU for treatment of pelvic relapse.
*Author Disclosure:*
**N.M. deSouza:** Grant Recipient; Cancer Research UK, EU Framework 7.

**08:30 - 10:00 Room M 5**

### E³ - ECR Academies: Functional Imaging for Disease Management: Research to Medical Practice

### E³ 520

### Functional imaging of the lungs

**A-0288** 08:30
**Chairperson's introduction**
C.M. Schaefer-Prokop; *Amersfoort/NL*

**A-0289** 08:36
**A. Perfusion methods for vascular and airway diseases (CT/MRI)**
M. Rémy-Jardin; *Lille/FR (martine.remy@chru-lille.fr)*

Over the last decade, lung perfusion has progressively left pure clinical research to enter clinical routine, based on the availability of new CT technological advances. This should not lead to forgetting basic approaches proposed with MDCT like the evaluation of dynamic changes within vessels of interest, analysis of bolus speed and propagation time of bolus peak. An important step was the introduction of dynamic CT which allowed evaluation of perfusion changes over time, mostly applied to oncologic indications. Iodine maps resulting from dual-energy CT acquisitions have revolutionised this concept as they can be derived from the same data set as that used for morphological imaging. This combination has mostly been investigated, then applied, in the context of pulmonary vascular diseases, with the objective of improving the diagnostic value of chest CT examinations. It can also be proposed in the evaluation of airway diseases where the combination of perfusion and ventilation is of major interest for the proper understanding of impaired gas exchange. The purpose of this presentation is to highlight some practical issues related to perfusion analysis in clinical situations and to make radiologists familiar with new semiologic features that have to be integrated into daily interpretations.

***Learning Objectives:***

1. To review most useful CT and MRI perfusion methods for lung imaging.
3. To learn about the impact of functional imaging in pulmonary hypertension.
2. To understand the role of perfusion information in airway diseases.

**A-0290** 09:04

**B. Quantitative CT-imaging biomarkers in airways diseases**

H.-U. Kauczor; *Heidelberg/DE (Hans-Ulrich.Kauczor@med.uni-heidelberg.de)*

CT-based quantitative imaging biomarkers in airway disease are related to lung attenuation and airway dimensions. Quantification of low attenuation areas (LAA) reflecting emphysema is based on a threshold value, e.g., 950 HU. Commonly emphysema volume, total lung volume and their ratio called emphysema index (EI) are measured. Another possibility is the percentile defined by the density threshold, below which a defined percentage of lung voxels are distributed, such as the 15th percentile. For the assessment of air trapping expiratory scans with a threshold of -856 HU as well as the ratio of expiratory to inspiratory mean lung attenuation are advised. A more complex approach is the parametric response map (PRM) spatially aligning the expiratory to the inspiratory scan by a deformable volumetric registration process. Then the lung voxels are categorised by using their expiratory and inspiratory attenuation values as normal parenchyma, functional small-airways disease and emphysema. Volumetric data-sets allow for a skeletonised visualisation of the bronchial tree. Quantitative parameters of the airways mainly focus on total diameter, lumen area (LA), wall thickness and wall area (WA) of 1st-8th generation airways as well as the so-called Pi10 which is the square root of the bronchial wall area of a hypothetical bronchus with an internal diameter of 10 mm. An important task is the segmentation of the individual lobes and the detection of the fissures and their integrity. This allows for lobar analysis of LAA as well as patient selection for successful endoscopic lung volume reduction and therapy response assessment.

***Learning Objectives:***

1. To review main functional CT methods of the lungs in airway diseases.
2. To learn about their impact in differential diagnosis.
3. To learn how these methods may help in treatment follow-up.

*Author Disclosure:*

**H.-U. Kauczor:** Research/Grant Support; Siemens, Philips, Bayer. Speaker; Siemens, Philips, Boehringer, Bracco.

**A-0291** 09:32

**C. Lung cancer, tumour heterogeneity and tumour response**

J.M. Goo; *Seoul/US (jmgoo@plaza.snu.ac.kr)*

Malignant tumours are biologically complex and show genomic and phenotypic heterogeneity. Morphologic tumour heterogeneity has been recognised regarding differences in regional tumour cell proliferation, immune infiltration, differentiation status, and necrosis on pathology and differences in attenuation, enhancement, and necrosis on imaging. Recently, understanding of intratumor genetic heterogeneity has been deepened with the progress in deep-sequencing technologies and is closely related with prognostic significance, response to therapy and drug discovery. Imaging can quantify the spatial variation of a tumour by quantifying CT, MR, or PET parameters, which can be used in explaining the tumour blood flow, hypoxia, metabolic and other phenotypic features. These methods can establish whether one tumour is more or less heterogeneous than another and can identify subregions with differing biology. Intratumor heterogeneity is a dynamic process and tends to increase as tumours grow. Heterogeneous tumour frequently shows poor clinical prognosis and is related with emerging treatment-resistant subpopulations of tumour cells after therapy. Although imaging plays a central role in diagnosis, staging, response assessment, and recurrence surveillance in oncologic patients, the assessment has been focused on the measurement of tumour size and average parameter values. In this process, spatially rich information is discarded. There has been considerable effort to extract more information by quantifying overall tumour spatial complexity or identify the tumour subregions. In this lecture, the strengths and weaknesses of methods that measure intratumor spatial heterogeneity and introduce current understanding in intratumor genetic heterogeneity.

***Learning Objectives:***

1. To learn about modern imaging of lung tumours.
2. To understand the role of functional criteria of tumour heterogeneity.
3. To learn about main functional biomarkers of tumour response.

**08:30 - 10:00 Tech Gate Auditorium**

### Special Focus Session

### SF 5d

### Imaging and management of the incidental scrotal lesions

**A-0292** 08:30

**Chairperson's introduction**

M. Bertolotto; *Trieste/IT (bertolot@units.it)*

The widespread use of scrotal US in urology and andrology has caused an increase of incidentally detected testicular lesions. Many such lesions are simple cysts which require neither surgery nor follow-up. When the incidental nodule is solid in nature, it raises the concern for a malignant tumour. Most of these lesions are nonpalpable, presenting as small hypoechoic, asymptomatic nodules with negative markers. In such cases, there is the clinical dilemma of whether to perform orchiectomy or to adopt a more conservative approach. This is justified by the high prevalence of benign nodules presenting in this way, accounting for up to 80% of cases. There is increasing evidence that the multiparametric US, combining the use of grey-scale, Doppler, elastography, and CEUS improves lesion characterisation. Moreover, most of the incidentally detected testicular lesions do not show significant growth during the long-term evaluation and can be safely surveilled with close follow-up. The para-testicular area includes a variety of anatomic structures. US is nearly 100% sensitive for lesion detection, but specificity is lower. The multiparametric US may improve lesion characterisation. However, the overlap of the imaging findings of benign and malignant tumours may always be possible and should be kept in mind. MRI represents a useful supplemental imaging technique in the characterisation of scrotal masses, particularly recommended in cases of nondiagnostic ultrasonographic findings.

***Session Objectives:***

1. To understand the clinical issues about diagnosis and management of incidental scrotal lesions.
2. To understand the role of conventional US modes, CEUS and elastography in scrotal lesion characterisation.
3. To learn how to fit in a multidisciplinary team to manage patients with incidental scrotal lesions.

**A-0293** 08:35

**Incidental testicular lesions: can we characterise them at multiparametric US?**

P.S. Sidhu; *London/UK (paulsidhu@btinternet.com)*

The use of high-resolution ultrasound and the wide availability of this technique has altered the perception of testicular lesions. The traditional clinical view that nearly all focal intra-testicular lesions are malignant is no longer valid, as a large number of incidental intra-testicular lesions are now routinely discovered on ultrasound. The practice of ultrasound is also altering rapidly, without the clinical management keeping pace; the ultrasound techniques of B-mode, colour and spectral Doppler have been strengthened by the addition of contrast-enhanced ultrasound (CEUS) and elastography, further improving diagnostic capabilities. The combination of all these ultrasound techniques is termed multi-parametric ultrasound (MPUS). The ability to detect non-palpable intra-testicular lesions presents a problem for the urologist; should all intra-testicular lesions be managed by orchiectomy as is the tradition. The ability to combine all the various imaging techniques allows a comprehensive assessment of the indeterminate small non-palpable lesion, allowing for the possibility of 'watchful waiting' or 'testis-sparing surgery'. The ability for CEUS to assess the vascularity of the focal lesion is paramount, with an absence of vascularity paramount. The ability to ascertain the presence of vascularity points to a neoplasm and the use of time-intensity curves allows for the assessment of the wash-out curves and possible malignancy. The addition of strain elastography allows for the assessment of the stiffness of the lesion, and the probability of malignancy. With all the aspects of MPUS the ability to be confident in the assessment and the ability to dictate clinical management becomes possible.

***Learning Objectives:***

1. To become familiar with the features at multiparametric US that improve testicular lesion characterisation.
2. To learn about the potential of multiparametric US in imaging testicular lesions.
3. To understand the specific role of conventional US modes, CEUS and elastography in testicular lesion characterisation.

*Author Disclosure:*
**P.S. Sidhu:** Advisory Board; Samsung Medison. Board Member; EFSUMB. Consultant; Itreas Inc.. Equipment Support Recipient; Philips Healthcare, Samsung Medison, GE Healthcare, Siemens Healthineers. Speaker; Philips Healthcare, GE Healthcare, Siemens Healthineers, Hitachi Inc., Samsung Medison, Bracco SpA.

**A-0294** 08:55
**Incidental extra-testicular lesions: can we characterise them?**
M. Secil; *Izmir/TR (mustafa.secil@deu.edu.tr)*

The principal method of examination for the extratesticular structures is an ultrasound (US). The capability of US for detection of lesions is almost 100%. However, the specificity of the method is around 70-90%, depending on the location and character of the lesions. Magnetic resonance imaging (MRI) may be an additional tool for characterisation of these lesions. Incidental non-neoplastic cystic lesions may easily be characterised by the US which includes tunica albuginea, epididymis, and spermatic cord cysts. Spermatic cord lipomatosis and polyorchidism are rare solid non-neoplastic conditions that can be diagnosed by US most of the time, MRI rarely is needed. Fibrous pseudotumor may be characterised by MR with the demonstration of very low signal intensity on T2W images. Incidental neoplastic lesions of benign tumours such as lipoma, adenomatoid tumour and leiomyoma when present with typical features may be diagnosed by the US and/or MR. Papillary cystadenoma having a bunch of grape-like cystic and papillary solid appearance may be diagnostic for von Hippel Lindau disease. The imaging features of most of the solid benign and malignant mesenchymal tumours overlap and render difficulties in characterisation. Malignant mesenchymal tumours of this region rarely present incidentally and are commonly big at the time of diagnosis. They are heterogeneous solid tumours that may contain cystic/necrotic areas and are commonly highly enhancing. Fat content may lead to the diagnosis of liposarcomas, umbilicus type of necrosis of a compact solid lesion may be a clue for leiomyosarcoma but these, of course, are not invariable findings.
***Learning Objectives:***
1. To became familiar with the imaging features of the commonest extra-testicular lesions.
2. To learn about the role of multiparametric imaging (US/MR/CT) in characterisation of extra-testicular lesions.
3. To understand the potential and limitations of imaging in extra-testicular lesion characterisation.

**A-0295** 09:10
**Incidental testicular lesions: the role of MRI and ESUR recommendations**
L. Rocher; *Le Kremlin-Bicêtre/FR (laurence.rocher@bct.aphp.fr)*

The majority of non-palpable testicular small lesions seen on ultrasound are benign. Testis tumour characterisation is still based on Color Doppler US. Leydig cell tumours are the most frequent benign solid lesions with spheric or ovoid slightly hypoechoic pattern, circumferential vascularisation. The presence of microliths, macrocalcifications and hypoechoic areas surrounding the nodule are findings suggestive of the malignant disease. Nevertheless, as the percutaneous biopsy is still forbidden, other modalities as multiparametric MRI including dynamic injection and diffusion are useful to approach the diagnosis and therefore the management which may consist in follow up, tumorectomy, or orchiectomy regarding the supposed diagnosis. Almost non-cystic tumours are in hyposignal on T2, isosignal on T1. Leydig cell tumours demonstrate a high vascularisation (with a higher area under the curve) compared to seminomas. Burned out tumours, which may be underestimated at an ultrasound, show a nodular pattern in hyposignal on T2, defect or weak enhancement, and increased ADC value. Striated pattern are benign. MRI can depict with more sensitivity very small bilateral nodules in case of Leydig cell hyperplasia.
***Learning Objectives:***
1. To become familiar with MR appearance of testicular lesions.
2. To understand when MRI can have a role in the clinical practice for lesion characterisation.
3. To illustrate the ESUR recommendations to help characterisation and management of incidentally detected testicular lesions.

**A-0296** 09:25
**Testicular-sparing surgery: a teamwork of radiologist and urologist**
N. Pavan; *Trieste/IT (nicpavan@gmail.com)*

Nowadays, the clinical and instrumental evaluation of testicular masses consents an accurate selection of patients eligible for the organ-preserving approach. Testis-sparing surgery leads good functional and aesthetic results in patients with benign lesions; it is a safe option for small testicular masses with a reliable pathologist performing frozen section examination and is an important goal in young patients with fatherhood desire. An optimal collaboration between the surgeon and the radiologist is essential to achieve good results.
***Learning Objectives:***
1. To learn about the current EAU recommendations for the diagnosis and management of the incidental testicular lesion.
2. To understand the current trends for the workup of incidental testicular lesion.
3. To become familiar with the value of a multidisciplinary team in planning and performing testicular sparing surgery.

09:40
**Panel discussion: Multiparametric imaging of the testis - how and when can we avoid orchidectomy?**

**10:30 - 12:00 Room A**

### E³ - ECR Academies: Interactive Teaching Session for Young (and not so Young) Radiologists

### E³ 621
### Oncologic imaging

**A-0306** 10:30
**A. Lung cancer: key signs in the new TNM**
A.R. Larici; *Rome/IT (annarita.larici@unicatt.it)*

Lung cancer is a leading cause of cancer-related mortality worldwide. A correct staging is the prerequisite for an adequate management of patients with lung cancer. Recently the 8TH edition of the TNM classification introduced relevant changes of the descriptors T and M. Regarding T, it has been demonstrated by survival analyses that from 1 to 5 cm of diameter every centimetre counts, and those larger tumours are best aligned with either T3 (tumour size of more than 5 to 7 cm) or T4 (tumour size of more than 7 cm). This finding further confirms the common intuition that the larger the tumour, the worse the prognosis. Regarding M, the category of extrathoracic lesions has been distinguished in M1b and M1c to indicate respectively one (oligometastatic disease) and more than one lesion. In this context, it is advisable that radiologists report the number of lesions if only one organ is involved and the number of organs if many. This approach has a relevant clinical impact because oligometastatic disease nowadays is managed and treated differently respect to an extensive M stage disease. Several limitations remain, as the classification of a tumour adjacent to the chest wall, mediastinum and diaphragm, as well as the categorisation of lymphangitic carcinomatosis as an independent descriptor and the evaluation of multiple lung lesions. Certainties and controversies on the topic will be argued in this session, taking into account that cancer staging should be always considered a multidisciplinary process.
***Learning Objectives:***
1. To learn about the new staging system for lung cancer.
2. To highlight the differences in the meaning of CT findings between the new system and the previous one.

**A-0307** 11:15
**B. Incidental findings in oncologic patients**
M.-P. Revel; *Paris/FR (marie-pierre.revel@aphp.fr)*

Incidental findings are unanticipated findings discovered in the course of medical care, which can affect the way the oncologic disease is managed. Among incidental findings in oncologic patients, incidental pulmonary embolism (PE), lung nodules and adrenal masses are frequent findings. Incidental PE is more frequently peripheral, and thus more difficult to diagnose. The recurrence risk in untreated patients is 12%, versus 6% with anticoagulation. Thus, anticoagulant treatment is recommended for cancer-associated incidental PE. The annual incidence of VTE in patients receiving chemotherapy is estimated at 11%. This risk can climb to 20% or higher depending on the type of drug(s) being administered. Incidental PE is not associated with an increased risk of death when adjusted for tumour stage and performance status. Incidental lung nodules are frequently found in oncologic patients. As indicated in the 2005 Fleischner guidelines for management of small pulmonary nodules, patients should be cared for according to the specific clinical situation. Pertinent factors include the site, cell type, and stage of a primary tumour and whether early detection of lung metastases will affect care. Among differential diagnosis of metastasis, Langerhans cell histiocytosis, DIPNECH (Diffuse Idiopathic Pulmonary Neuro Endocrine Celle Hyperplasia) and infectious nodules should be discussed. Regarding incidental adrenal masses, benign adenoma can be diagnosed if the attenuation on unenhanced CT acquisition is below 10 Hounsfield units. If it is not the case, PET CT should be performed and in case of increased FDG uptake, histological assessment is required.

***Learning Objectives:***
1. To recognise the importance of different incidental findings in patients with cancer.
2. To learn how to manage patients with incidental findings.

**10:30 - 12:00** **Room B**

### ESR meets Africa

### EM 1

### Radiology in Africa: facing challenges and opportunities

*Presiding:*
L.E. Derchi; Genoa/IT
H.A. Gharbi; Tunis/TN

### A-0308 10:30

**Chairpersons' introduction (part 1)**
L.E. Derchi; *Genoa/IT (derchi@unige.it)*

Africa is a large continent, with people of different cultures and habits, which may be difficult to understand as a whole. Therefore, a number of speakers from different African regions have been selected to present an overview of Radiology in Africa from a variety of perspectives. They will present topics about professional issues such referral guidelines, radiation protection, use of radiological services and promotion of Radiology in their regions. Furthermore, there will be a discussion on how to develop cooperation between African Radiological Societies and the ESR. This point, specifically, will underline the bilateral nature of the relationship, discussing what ESR can do to help African radiologists as well as on what African radiologists, can offer to European societies.
***Session Objectives:***
1. To give an overview of radiology in Africa.
2. To list the existing cooperation programmes of the European Society of Radiology in Africa.
3. To point out what African radiologists can do for the ESR and what the ESR can do for radiology in Africa.

### A-0309 10:35

**Chairpersons' introduction (part 2)**
H.A. Gharbi; *Tunis/TN (hassen.agharbi@planet.tn)*

Africa is a real mosaïc of 54 different countries with a population of more than 1.200 Million inhabitants, making up 16% of the world's population. The diversity of mosaïc is multifaceted. There are variable socioeconomic levels with GNPs ranging from less than 500 USD, in Somalia to more than 38000 USD in Equatorial Guinea. There are many different cultures and languages, but fortunately English, French, Arab and Portuguese, and internet facilities are widespread, adapted and efficient means of communications. The impact study of medical imaging on a diverse continent is challenging for several reasons. No complete official statistics are available in all countries. The medical imaging market is often very disorganised. There are enormous differences between the different countries and even between different regions in the same country. The number of radiologists varies from less than one radiologist for one million inhabitants, to more than 80 radiologists per one million inhabitants. We can divide the radiological situation in Africa into two regions; the North including Egypt, Tunisia, Algeria and Morocco and South Africa where the situation is more or less acceptable and the centre including 49 countries and 75% of the population where the situation require an urgent solution. In fact, the needs are everywhere and require more or less the same solution from the European Society of Radiology, training, collaborating programs, but Radiation Protection rules must be established and respected so that we can guaranty an efficient and good development of the Radiology.
***Session Objectives:***
1. To give an overview of radiology in Africa.
2. To list the existing cooperation programmes of the European Society of Radiology in Africa.
3. To point out what African radiologists can do for the ESR and what the ESR can do for radiology in Africa.

### A-0310 10:40

**Structured strategies to combat radiation protection challenges in Africa. What can the ESR do?**
D. Husseiny Salama; *Cairo/EG (drdinahusseiny@yahoo.com)*

The advances in health technology continue opening new horizons for the applications of ionising radiation in health care in Africa. This has resulted in an increase in the number of medical procedures, professionals and patients involved, and in the need for structured strategies and a holistic approach towards the full integration of radiation safety and the clinical imaging guidelines in the African health sector. There have been proactive actions in Africa to improve the situation and enhance the implementation of radiation protection in several African countries, however further actions and joint activities are needed to enhance the process. The European Society of Radiology and its Eurosafe campaign have always been at the forefront trying to promote the safe use of ionising radiation in medicine, and this presentation will address strategic planning through which the ESR- African cooperation can act through to impact the future of radiology and radiation-protection in Africa and similar countries in the globe.
***Learning Objectives:***
1. To give an overview on the situation of imaging referral guidelines and clinical decision support (CDS) in Africa.
2. To update on the European societies' activities in the region in regard to guidelines.
3. To give tips from African radiologists on solutions and the way forward.
4. To present the bilateral strategic planning with the ESR to improve the performance of radiation protection.

### A-0311 10:50

**Is imaging underused in Africa? East Africa as an example. Solutions: what can the ESR do?**
S. Vinayak; *Nairobi/KE*

The challenges faced by underdeveloped countries are immense; manpower, education, lack of equipment, maintenance of equipment etc. are just a few examples. Radiology equipment is often unaffordable, so innovative solutions have to be found, tried and tested so that communities can benefit. There are only 500 radiologists serving a population of 200 million inhabitants in Eastern Africa. For ultrasound, WFUMB already has three established COEs (centres of education) providing regular CMEs, training courses and other activities related to education on a regular basis. In addition, Bergen University trains physicians in Addis Ababa. In Khartoum, doctors are being trained to perform abdominal ultrasounds. All these initiatives are in conjunction with MASU & WFUMB. The current use of imaging can be robustly stimulated by using innovative ideas and technology. The WFUMB centre of education (COE) in Nairobi trained midwives to perform basic obstetric ultrasounds on a tablet ultrasound machine with a view to identifying high-risk pregnancies. The scans were transmitted using mobile phone internet technology for validation by radiologists. Imaging is still underutilised in East Africa, and governmental bodies are looking for local solutions to these problems. Help from European societies of radiology can take the form of education and training. How this can be implemented will be articulated in detail. Also, universities of this region can work with radiologists from Europe to carry out research. ESR can help coordinate these activities in a well-structured and robust format that is sustainable.
***Learning Objectives:***
1. To present the current situation in Eastern Africa.
2. To describe what we are doing to promote radiology in Africa.
3. To present what we would like to request from the ESR.
*Author Disclosure:*
**S. Vinayak:** Equipment Support Recipient; Phillips Medical Systems. Grant Recipient; S Vinayak. Research/Grant Support; Phillips Medical Systems.

### 11:00

**Interlude 1: Enjoy the difference between African and European music**
H.A. Gharbi; *Tunis/TN*

### A-0312 11:05

**How to promote radiation protection in West Africa. Needs and expected role of the ESR**
E.H. Niang; *Dakar Fann/SN*

"no abstract submitted"
***Learning Objectives:***
1. To summarise the radiation protection situation in West Africa, discuss workforce aspects and present an overview of educational facilities and maintenance services.
2. To update on the role of European societies in our region, what is done in general and in the field of radiation protection in particular.
3. To explain what we, as African radiologists, can offer to European societies.

### A-0313 11:15

**Most important challenges for imaging in North Africa**
L. Rezgui Marhoul; *Tunis/TN (lamia.rezgui@rns.tn)*

The Maghreb region refers to five countries, three from North West Africa, Morocco, Algeria and Tunisia, also known as the Small Maghreb and two Central Maghreb countries, Mauritania and Libya. It has about 89 million inhabitants. The distribution of medical equipment using ionising radiation in this region is very disparate and depends on multiple factors. The number of radiologists is also very variable from one region to another. Radiation protection has made great strides in some countries, and much work remains to be done in others. Rigorous regulations applied at different levels to guarantee quality and safety in terms of radiation protection exist in some countries, but challenges remain to be faced by others. The Maghreb enjoys a considerable level of development in terms of health development and radiation protection. He remains dependent on deficiencies. These are not all of the same nature and do not require the same solutions.

***Learning Objectives:***

1. To give an overview of the radiology situation in North Africa.
2. To enlist the cooperation programmes between North African societies and the European societies with bilateral exchanges in radiodiagnosis and interventional radiology.
3. To understand what North African radiologists can bring back to the table for European radiology.
4. To propose a plan for the promotion of collaboration between North Africa and ESR.

11:25

**Interlude 2: Enjoy the difference between Arabic and European music**

H.A. Gharbi; *Tunis/TN*

**A-0314** 11:30

**The WHO's programme in Africa: the past, the present and the future**

M.d.R. Perez; *Geneva/CH (perezm@who.int)*

The World Health Organization (WHO) coordinates the international health work with the objective of attaining by all peoples the highest possible level of health. The WHO’s programme of work establishes a “triple billion” target: 1 billion more people enjoying better health and well-being, 1 billion more people benefitting from universal health coverage and 1 billion more people better protected from health emergencies. Medical imaging plays a key role in the achievement of this “triple billion” target because diagnostic radiology and image-guided interventions are linked to the entire healthcare pathway (e.g. health promotion, preventive services, diagnosis, treatment, follow-up, rehabilitation, palliative care). WHO is conducting a Global Initiative on Radiation Safety in Health Care Settings to enhance global access to safe and appropriate use of radiation in health care services. This initiative is currently focused on supporting the implementation of the 10 priorities to improve radiation protection in medicine identified in the Bonn Call for Action. WHO has contributed to the development and launching of the AFROSAFE Campaign to enhance radiation safety and quality in health care in Africa and continues cooperating with local and international partners to support its implementation. A culture of radiation safety and quality can be embedded into policies, processes and institutions as the health care systems grow and develop as it is the case in many African countries. This, which represents at the same time a challenge and an opportunity, can be achieved with good leadership, robust planning and strategic investment.

***Learning Objectives:***

1. To present the WHO's role, objective, core functions, structure and where/how radiology fits into them.
2. To explain the WHO's programmes in Africa and the place of radiology and radiologists in the context of the current priorities in the global public health agenda.
3. To understand the WHO's views about radiation protection in Africa.
4. To present the WHO's radiology programmes/activities in Africa. What has been done? What is currently being done? What is planned in the future?

**A-0315** 11:40

**Panel discussion: Radiology in Africa, reality and dreams**

G. Frija[1], L.E. Derchi[2], H.A. Gharbi[3], D. Husseiny Salama[4], S. Vinayak[5], E. Niang[6], L. Rezgui Marhoul[3], M.D.R. Perez[7], B. Mansouri[8], K.M. Naidu[9]; [1]*Paris/FR*, [2]*Genoa/IT*, [3]*Tunis/TN*, [4]*Cairo/EG*, [5]*Nairobi/KE*, [6]*Dakar Fann/SN*, [7]*Geneva/CH*, [8]*Algiers/DZ*, [9]*Cape Town/ZA (guy.frija@aphp.fr)*

**A-0316/A-0317** 11:55

**Conclusion**

L.E. Derchi; *Genoa/IT (derchi@unige.it)*
H.A. Gharbi; *Tunis/TN (hassen.agharbi@planet.tn)*

Challenges to African radiology are great, but even more significant are the opportunities for growth facing radiologists in this continent. As a matter of fact, the end of this session “ESR meets Africa” is just a beginning. From here reciprocal knowledge and reciprocal cooperation must start, aiming at a strong commitment to excellence and safety and increased availability and use of Radiology worldwide, and especially in Africa.

**10:30 - 12:00** **Room F1**

### E³ - Rising Stars Programme: Joint Session with ESOR

### BE 6

### Radiologic anatomy: head and neck

*Moderator:*
M. Becker; Geneva/CH

**A-0318** 10:30

**Neck spaces**

A. Trojanowska; *Lublin/PL (agnieszka30@yahoo.com)*

A knowledge of neck spaces not only allows for better communication between radiologists and specialists familiar with imaging of this region but also aid in diagnosis as each space has a distinct group of pathologies. It considerably narrows down the differential diagnosis. The anatomy of the neck can be divided into suprahyoid and infrahyoid portions: suprahyoid neck which encompasses the deep spaces between skull base and hyoid bone and infrahyoid neck which lies inferiorly between the hyoid bone and both clavicles. These divisions are arbitrary in there is some continuation of suprahyoid neck spaces into the infrahyoid neck spaces and continuation of some infrahyoid neck spaces into the superior mediastinum.

***Learning Objectives:***

1. To understand the concept of neck spaces and their boundaries.
2. To review the content of each space with emphasis on differential diagnosis.
3. To become familiar with pathological findings typical for each space.

**A-0319** 11:00

**Temporal bone**

J.W. Casselman; *Bruges/BE (jan.casselman@azsintjan.be)*

The anatomy of the external auditory canal, middle ear and bony inner ear is best studied with CT. The highest resolution can be achieved when ConeBeam CT is used, and this technique also has the advantage that there is no quality loss in the axial, coronal and double oblique plane, the planes needed to study the temporal bone anatomy in detail. Double oblique images are indispensable to visualise the stapes, stapes footplate and oval window. Anatomical knowledge is especially needed at those sites where pathology most frequently occurs and changes the anatomy. The footplate, stapes, lenticular and lengthy process of the incus, facial nerve canal, Jacobson’s canal, the scutum, round window, ligaments etc. are some of these structures and they should be studied in detail in every single patient. MR is better suited to demonstrated the anatomy of the membranous labyrinth, inner ear fluid spaces and nerves in the internal auditory canal. Heavily T2-weighted images are best suited to see these structures, but high resolution is needed. Not only the fluid inside the labyrinth must be evaluated but also the normal size of the different nerve branches in the internal auditory canal must be known in order to be able to detect nerve atrophy or hypoplasia. The most important anatomical structures of the temporal bone, as they can be visualised in different planes on (CB)CT and MR will be illustrated in this presentation.

***Learning Objectives:***

1. To know which technique is best suited to visualise specific anatomical structures of the temporal bone.
2. To recognise the clinically most important anatomical structures of the middle and inner ear.
3. To learn how the anatomical structures of the temporal bone look like in the transverse, coronal and double oblique plane.

*Author Disclosure:*

**J.W. Casselman:** Equipment Support Recipient; Cefla s.a. NewTom & Philips Healthcare. Speaker; Cefla s.a. NewTom & Philips Healthcare.

**A-0320** 11:30

**Larynx**

R. Maroldi; *Brescia/IT (roberto.maroldi@unibs.it)*

The larynx is a specialised organ placed at the separation between the respiratory and the digestive tracts. Beyond protecting the airways against food aspiration, it is involved in breathing, swallowing and voiced sounds production. These functions are mostly accomplished by the action of three diaphragms: the epiglottis, the false and vocal cords. All of them, when closed, contribute to seal the larynx during swallowing. When opened, they permit the passage of the air into the trachea. The vibration of the vocal cords (the most caudal of the three sphincters) generates voiced sounds. The vibration is produced by the coordinated action of a group of laryngeal muscles. These muscles are inserted on rigid structures, provided in the larynx by a framework of ossified cartilages: cricoid, thyroid and arytenoid. Cricoid and thyroid cartilage form also a rigid box, a sort of shield. Muscle contraction results in a significant shortening and change of shape. To accommodate these changes, a malleable material (fat) fills the "gap" between muscles and the rigid shield. The lateral "fat-filled-gap" between muscles involved in vocal cord vibration and the cartilage framework is named paralaryngeal space. A midline "fat-filled-gap" separates the epiglottis from the rigid thyroid laminae. CT and MR may precisely delineate the submucosal structures: muscles, fat-filled-gaps (paralaryngeal and pre-epiglottic spaces) and depict un-ossified and ossified cartilages.
***Learning Objectives:***
1. To understand the anatomy and signals of the cartilage framework of the larynx.
2. To learn the surgical subdivision of the paraglottic space of the larynx into key compartments.
3. To learn the anatomy and signals of the muscles within the larynx.

**10:30 - 12:00** **Room G**

### EFOMP Workshop

### EF 2

### Big data and the big picture: deep learning in optimisation of medical imaging (part B)

*Moderators:*
M. Brambilla; Novara/IT
M. Kortesniemi; Helsinki/FI

**A-0324** 10:30
**Chairperson's introduction**
M. Brambilla; *Novara/IT (marco.brambilla@maggioreosp.novara.it)*

The purposes of this session are to provide an overview of the application of deep learning (DL) methods in image optimisation and to understand why DL methods are capable of supporting research. Improvement in radiological optimisation requires patient-specific and indication-specific adjustment of imaging parameters and image analysis methods. DL has been used increasingly in radiology because typical imaging objects such as lesions and organs presented in medical images are in most occasions far too complex to be represented reliably by a certain simple equation and, therefore, robust analysis methods are needed. This session will first introduce an application of DL in the computerised analysis of chest imaging emphasising the complementary role of visual reading and computer-aided diagnosis. The application of DL methods in medical physics such as scatter removal, spatial resolution improvement, noise reduction and dose estimation will be outlined as an example of how such methods can impact on quantitative aspects of medical imaging. The illustration of a paradigm shift: from image quality to care outcome, driven by the introduction of DL methods, will conclude the session together with an analysis of the challenges which must be faced for the introduction of DL in the clinical practice, namely: 1. gaining access to medical archives according to ethical and legislative requirements and 2. obtaining validated and standardized image data in a systematic fashion.
***Session Objectives:***
1. To provide an overview of application of deep learning methods in image optimisation.
2. To understand the need for a deep learning approach in medical imaging.
3. To understand why deep learning algorithms are capable of supporting research.

**A-0325** 10:35
**Computer analysis in chest imaging: from rule-based to machine learning to deep learning**
B. Van Ginneken; *Nijmegen/NL (bram.vanginneken@radboudumc.nl)*

Half a century ago, the term "computer-aided diagnosis" (CAD) was introduced in the scientific literature. Pulmonary imaging, with chest radiography and computed tomography, has always been one of the focus areas in this field. In this study, I describe how machine learning became the dominant technology for tackling CAD in the lungs, generally producing better results than do classical rule-based approaches, and how the field is now rapidly changing: in the last few years, we have seen how even better results can be obtained with deep learning. The key differences among rule-based processing, machine learning, and deep learning are summarised and illustrated for various applications of CAD in the chest.
***Learning Objectives:***
1. To give an overview of application of deep learning methods in the diagnosis of thoracic lesions.
2. To understand issues related to the technical implementation of these methods.
3. To learn about the complementary role of visual reading and computer-aided diagnosis.
*Author Disclosure:*
**B. Van Ginneken:** Founder; Thirona. Grant Recipient; Thirona, Delft Imaging System, MeVis Medical Solutions. Shareholder; Thirona.

**A-0326** 11:05
**Deep learning in CT optimisation**
M. Kachelrieß; *Heidelberg/DE (marc.kachelriess@dkfz.de)*

With the introduction of deep learning in general, and with deep Convolutional Neural Networks (CNN) in particular, machine learning has spread into many medical areas with great success. In particular medical imaging may benefit from the new technology. Important applications such as image analysis, image segmentation and object recognition are well-known and start to become widely available. The applications of machine learning to the field of CT image formation, which describes the process of data acquisition, preprocessing, image reconstruction and post-processing, however, are less known, not as mature and hardly available, yet. Here, the use of machine learning can be mainly categorised into the categories 1) replacement of missing data (sparse view acquisition, limited angle tomography, ···), 2) replacement of time-consuming computations (image reconstruction, scatter prediction, material decomposition, ···), and 3) the incorporation of a priori knowledge (non-contrast CT from contrast-enhanced CT, pseudo CT from MR, ···). This lecture discusses the underlying technology and selected application examples. Not all proposed methods may keep their promises. This may be partially attributed to the fact that the field of deep learning in CT optimisation is rather young and by far not mature. Other methods, in contrast, are highly promising and it appears as if the future will bring CT images at a lower patient dose and with fewer artefacts in clinical routine. Thus, deep learning has the potential to significantly improve and optimise CT image formation.
***Learning Objectives:***
1. To provide the rationale of deep learning methods in image optimisation.
2. To describe application of deep learning methods in medical physics tasks such as scatter removal, spatial resolution improvement, noise reduction, dose estimation.
3. To understand how these methods will impact on quantitative aspects of medical imaging.

**A-0327** 11:35
**From image quality to care outcome**
M. Kortesniemi; *Helsinki/FI (mika.kortesniemi@hus.fi)*

Medical physicists have a long tradition of measuring image quality with objective metrics including contrast, noise and resolution, and their frequency-based derivatives. These methods have supported our main tasks related to quality assurance and optimisation. Along with the technical imaging modality development, the optimisation process has transformed into a more demanding and multi-professional challenge where the image quality metrics should evolve accordingly from technical towards clinical presentation. In the parameter level, this development may include clinical task function and observer-related parameters supplementing the traditional MTF and NPS parameters. New methods enable model observer based detectability and diagnostic accuracy estimates. Ultimately, we should aim beyond the concept of technical quality, to extend our methods and knowledge towards measuring and optimising the diagnostic value in terms of care outcome. Improved optimisation process and more consistent imaging quality (evaluated by target value, its uncertainty and precision) require objective and quantitative connections from diagnostic and technical parameters to clinical outcome parameters. Comprehensive methodology to enable this approach involves combining several types of data together. Deep learning methods - including data quality control and validation - are prerequisites for this kind of data analysis, due to inherent non-linearity of the problem and a large amount of heterogeneous data which is not equitable by traditional methods. Our medical physicist professional role should follow this development and incorporate AI & in-depth learning topics accordingly into our educational programs.
***Learning Objectives:***
1. To introduce the need of measuring the diagnostic value in terms of outcome.
2. To discuss its relevance and limitations regarding the traditional concept of image quality.
3. To introduce the challenges in deep learning based artificial intelligence methods.

**10:30 - 12:00** **Room M 4**

### E³ - ECR Academies: Hot Topics in GU Cancer

### E³ 619

### Tumour relapse in urological cancer

**A-0328** 10:30
**Chairperson's introduction**
V. Logager; *Copenhagen/DK (vibeke.loegager@regionh.dk)*

Relapse of cancer is one of the most difficult tasks. First, you have to know what kind of treatment the patient has undergone? Was it surgical, chemotherapy, radiation or a combination of these? Maybe the patient has undergone some newer minimal invasive therapy that you are not familiar with? The treatments could be in the form of proton therapy, cryotherapy, focused ultrasound or theranostics? There are different organs and different treatments. Nonetheless treated tissue and organs all have ways of reaction that we can get to know and recognise. Within these tissues, we shall find the vital cancer tissue, and this could be relapsing cancer, residual cancer or even a new clone of disease. It is therefore mandatory to learn the imaging strategies in post-treatment follow-up and relapse including the strength and weakness of the different modalities. When relapse has been found, a decision of treatment can be made.

**A-0329** 10:36
**A. Prostate cancer relapse**
V. Panebianco; *Rome/IT (valeria.panebianco@uniroma1.it)*

An elevated serum PSA is a first sign of relapse and imaging is needed to determine the localisation of the recurrence, which may be locally in the prostatic area and/or metastatic. The precise localisation of the local recurrence by imaging is needed if this localisation could change treatment planning. Treatment for prostate cancer can significantly change the appearance of the residual prostate gland or the prostate bed, complicating imaging evaluation. It's necessary to discern post-therapy changes from local recurrence. An overview of current imaging procedures and their performance in local recurrent PCa will be done, focusing on mpMRI. Therefore, mpMRI protocol (consists of T2W images, DWI/ADC and DCE-MRI) and evaluation of the images require specific considerations based on treatment received. For this reason, a standardised method of image assessment is needed to help in image analysis and reporting and to diminish variation in the acquisition, interpretation and reporting of prostate mpMRI in case of local recurrence. The relationships with PSA values will be also discussed. MpMRI can be currently considered as the most reliable imaging biomarker to detect local PCa recurrence in patients with biochemical failure after RP, especially for those PSA values where PET/CT is not recommended (0.2 - 1 ng/mL).
***Learning Objectives:***
1. To learn the follow-up strategy post radical prostatectomy.
2. To recognise relapse and know the patterns of spread.
3. To learn the use of imaging in planning salvage therapy.

**A-0330** 11:04
**B. Non prostate urological cancer relapse**
H.A. Vargas; *New York, NY/US*

Tumour relapse is an undesirable reality in all cancer patients, and short of minimising its occurrence by applying to optimal treatment modality at the time of diagnosis, the best outcomes are achieved by early detection of recurrence and prompt initiation of salvage therapies. Early detection on imaging is facilitated by understanding the expected post-treatment findings and learning common patterns of recurrence for different cancers. In this session, we will review the patterns of recurrence and multimodality imaging appearance of cancers involving the urinary system (kidneys, ureters, bladder).
***Learning Objectives:***
1. To recognise the expected post treatment appearances.
2. To know follow-up strategies for detection of relapse.
3. To learn to recognise pitfalls by case review.

**A-0331** 11:32
**C. Theranostics in urological cancer**
M. Hartenbach; *Vienna/AT (markus.hartenbach@meduniwien.ac.at)*

Emerging techniques and new targets in nuclear medicine have enabled new therapeutic approaches following the very own concept of biomarker imaging and therapy. Performing imaging and therapy using identical biochemical structures and metabolic properties as targets are summarised with the portmanteau-word “theranostics”. But theranostics can be seen in a broader spectrum of applications, especially when focusing on diagnostics that lead to an impact on therapeutic decisions. This lecture will give an overview of biomarker imaging in urological cancer, that has a direct impact on therapeutic decisions as well as treatment with the same target structure focusing on prostate cancer. A prime example is given by the use of bone scintigraphy for Imaging osteoblastic activity which is targeted by 223RaCl2 therapy, proven to provide a survival benefit in castration-resistant prostate cancer patients. Beyond this, PSMA ligand positron emission tomography, as a specific method for imaging the folate hydrolase, has emerged as an almost routinely applied procedure in high-risk prostate cancer primary staging, biochemical recurrence and advanced tumour stages. Recent studies have proven its impact on therapeutic decisions in almost a third of the patients with primary disease and even 75% of the patients with biochemical recurrence who were negative on conventional imaging. Applying the direct theranostic approach in patients who underwent 177Lu-PSMA therapy (RLT), PSMA ligand PET response correlated with overall survival, overcoming the known issues of RECIST criteria. In summary, the theranostic approach enables a more personalised concept with highly specific therapeutic effect and low side effect profile.
***Learning Objectives:***
1. To learn about theranostic options in urological cancers.
2. To understand the advantages and limitations of theranostic approaches.
3. To become familiar with new the indications and outcomes of theranostic approaches.

**10:30 - 12:00** **Room M 5**

### E³ - ECR Academies: Functional Imaging for Disease Management: Research to Medical Practice

### E³ 620

### Functional imaging of the liver

**A-0332** 10:30
**Chairperson's introduction**
L. Martí-Bonmatí; *Valencia/ES (marti_lui@gva.es)*

Functional imaging is the study of human organs and related lesions by the analysis of imaging data. As opposed to structural imaging, functional imaging focusses on revealing biological and physiological activities based on either image features or signal modelling. Results are obtained throughout computer-based image processes for the detection and depiction of specific tissue changes. If successfully implemented, the main advantages of functional imaging will relate to the evaluation of quantitatively measured characteristics, as objective expressions of normal biological and processes, pathological changes, or pharmaceutical responses. The subrogated imaging biomarkers can be shown as features or parameters extracted from medical images, giving information on the regional distribution and magnitude change of the target process. The obtained organs and lesions properties are resolved in space (parametric images and analysis) and time (longitudinal and delta studies). Considered as a non-invasive virtual biopsy, functional imaging and related data will provide fundamental information on the presence, distribution and amount of different pathological changes in diffuse liver diseases and liver tumours. Despite the major progress on their implementation over the last decades, the lack of consistency in the heterogeneous-source medical images has deeply limited replication studies, leading to the failure of most multicentre projects and clinical trials validation. Most efforts are devoted to decrease uncertainty and biases in this precision radiology/medicine approach before they can be used to phenotype, predict treatment and evaluate prognosis. External validation studies. External validation studies are essential before implementing functional imaging in clinical practice and trials.

**A-0333** 10:36
**A. Diagnostic and staging of diffuse liver diseases using functional MRI**
B.E. van Beers; *Clichy/FR (bernard.van-beers@aphp.fr)*

Quantitative MR imaging offers a multiparametric approach to assess the severity of hemochromatosis, nonalcoholic fatty liver disease, chronic viral hepatitis, and cirrhosis. The most validated methods are multi-echo gradient-echo MR imaging to quantify hepatic iron and fat, as well as MR elastography to quantify liver fibrosis, cirrhosis, and potentially liver inflammation. Alternative methods for liver fibrosis staging include apparent diffusion measurements and T1 relaxometry, without or with gadoxetic acid enhancement. The diagnostic performance of these methods should be further compared to that of MR elastography. MR imaging has the potential to become a method of virtual biopsy that may decrease the need for invasive reference examinations in diffuse liver diseases. The integration of ultrasound and MR imaging biomarkers with blood sample biomarkers to further improve the assessment of diffuse liver diseases is a point of current research.

***Learning Objectives:***
1. To review functional MRI approaches of liver diseases.
2. To learn about their impact in characterising diffuse liver diseases.
3. To be aware of validated biomarkers of prognosis.

**A-0334** 11:04
**B. Diagnostic and staging of diffuse liver diseases using multiparametric US**
P.S. Sidhu; *London/UK (paulsidhu@btinternet.com)*

The use of contrast-enhanced ultrasound (CEUS) to assess focal liver lesions is now well established, with superb ability to characterise lesions visually. The ability to measure parameters of the CEUS examination objectively is realised by the fact that the microbubble contrast agent is truly intravascular and has a linear dose response. This differs from the dynamics of other contrast agents used in CT and MR imaging. The drawback of CEUS is the ability to identify the lesion clearly. The use of time intensity curves, particularly calculating washout is undergoing continuing research, and the usefulness is extended to other organs such as the kidney and testis to evaluate different enhancement patterns. Segmentation, colour mapping and use of artificial intelligence will move the usefulness of objective measurements of dynamic CEUS imaging, particularly of liver lesions. The development of ultrasound elastography has allowed the definition of the levels of liver fibrosis, with different techniques available, impacting on clinical management of chronic liver disease. All these new techniques, combined as multiparametric ultrasound (MPUS), as advanced the practice of ultrasound imaging, and is a robust, patient-friendly imaging tool.
***Learning Objectives:***
1. To review functional US approaches of liver diseases.
2. To learn about their impact in characterising diffuse liver diseases.
3. To be aware of validated biomarkers of prognosis.
*Author Disclosure:*
**P.S. Sidhu:** Advisory Board; Samsung Medison. Board Member; EFSUMB. Consultant; Itreas Inc.. Equipment Support Recipient; Philips Healthcare, Siemens Healthineers, GE Healthcare, GE Healthcare, Samsung Healthcare. Speaker; Samsung Medison, Philips Healthcare, Hitachi Inc, Siemens Healthineers, GE Healthcare, Bracco SpA.

**A-0335** 11:32
**C. Characterisation of liver tumours and prediction of tumour response**
B. Taouli; *New York, NY/US (bachir.taouli@mountsinai.org)*

We will discuss the role of multiparametric MRI including T2-weighted imaging (T2WI), diffusion-weighted imaging (DWI) and dynamic post-contrast T1WI imaging for the characterisation of focal liver lesions: to differentiate benign and malignant lesions and characterise subtypes of malignant lesions based on a combination of imaging features. We will also discuss the role of DWI and dynamic contrast-enhanced MRI for assessing tumour response. Finally, we will discuss future directions such as radiomics methods used for assessing liver tumours and tumour response.
***Learning Objectives:***
1. To learn about multiparametric imaging of liver tumours.
2. To understand the role of functional criteria of tumour characterisation.
3. To learn about main functional biomarkers of tumour response.
*Author Disclosure:*
**B. Taouli:** Grant Recipient; Bayer, Guerbet.

**12:15 - 12:45** **Room A**

### Headline Session

### HL 1

### Wilhelm Conrad Röntgen Honorary Lecture

*Presiding:*
L.E. Derchi; Genoa/IT

**A-0336** 12:15
**Oncologic imaging: a new beginning has just begun**
R.G.H. Beets-Tan; *Amsterdam/NL*

"no abstract submitted"
***Learning Objectives:***
1. To know the challenges in cancer management.
2. To understand the role of modern imaging in this management.
3. To learn about future directions in oncologic imaging.

**12:30 - 13:30** **Room C**

### E³ - The Beauty of Basic Knowledge: Pancreas

### E³ 24B

### Chronic pancreatitis

*Moderator:*
C. Matos; Lisbon/PT

**A-0340** 12:30
**How to diagnose and classify**
G. Zamboni; *Verona/IT (gzamboni@hotmail.com)*

Chronic pancreatitis is a chronic inflammation of the pancreas resulting in the destruction of the pancreatic parenchyma, leading to endocrine and exocrine insufficiency. Chronic pancreatitis has many different recognised etiologies: toxic, idiopathic, genetic, autoimmune, recurrent, obstructive. Different pathogenetic theories have been proposed, but the common final pathway is the development of parenchymal fibrosis. Recurrent episodes of clinical or subclinical acute pancreatitis lead to chronic pancreatitis. In the early stages of chronic pancreatitis, imaging has a role in diagnosing the disease, especially with MRI and MRCP, and in identifying a possible aetiology, for example, recognising the presence of variant ductal anatomy, e.g. pancreas divisum. In the more advanced stages, the diagnosis is easy. Imaging has then a role in differentiating between the different morphological types of pancreatitis (macro- or micro-obstructive), in the differential diagnosis, especially with tumours, and in the follow-up.
***Learning Objectives:***
1. To learn about diagnosis of chronic pancreatitis.
2. To understand classification of chronic pancreatitis.
3. To appreciate the role of imaging in the follow up of chronic pancreatitis.

**A-0341** 13:00
**Functional evaluation of chronic pancreatitis**
M.A. Bali; *Brussels/BE (mbali@ulb.ac.be)*

Chronic pancreatitis (CP) is a continuing inflammatory process, responsible for irreversible morphologic changes, pain and exocrine/endocrine impaired function. Parenchymal changes consisting of loss of functional parenchyma and vascularity with fibrotic replacement precede ductal abnormalities. The detection of early stages (the initial parenchymal changes prior to parenchymal/ductal abnormalities not detectable on conventional imaging) and the differential diagnosis between solid focal pancreatic lesions still represent challenges for conventional imaging. Secretin-enhanced MRCP (S-MRCP) allows depiction of ectatic secondary ducts (not visible on conventional unenhanced MRCP) present in early CP. Pancreatic exocrine reserve estimation: differences were reported between severe CP and normal, but not between mild CP and normal. S-MRCP may be useful in differentiating focal CP and pancreatic adenocarcinoma (PAC): the "duct penetrating sign" has been associated more frequently to focal inflammatory mass rather than PAC. New MR innovative technologies such as T1-mapping and MR elastography (MRE) may appreciate parenchymal changes in early CP. T1 relaxation time is a tissue-specific property, independent of imaging parameters: T1 relaxation time seem to be greater in mild CP compared with normal. Fibrotic parenchymal replacement is responsible for increased stiffness which can be detected by MRE prior to ductal and parenchymal abnormalities detectable on conventional imaging. DW-MR imaging allows the evaluation of microscopical properties of the tissue at a molecular level. Several clinical investigations have evaluated quantitative DWI for differentiating pancreatic solid lesions: overlapping ADC and D values have been reported for CP and PAC. More promising seem to be perfusion-related parameters.
***Learning Objectives:***
1. To appreciate functional evaluation of chronic pancreatitis.
2. To learn about the role of imaging in recurrent pancreatitis.

**12:30 - 13:30 Room D**

### E³ - The Beauty of Basic Knowledge: A Survival Guide to Musculoskeletal Imaging

### E³ 25B

### Bone tumours

*Moderator:*
V.N. Cassar-Pullicino; Oswestry/UK

**A-0351** 12:30
**Bone tumours**
K. Wörtler; *Munich/DE (klaus.woertler@tum.de)*

The diagnosis of a bone tumour is based on clinical findings, the age of the patient, the anatomic location of the lesion, its radiologic appearance, and if imaging does not allow for a specific diagnosis, its histopathologic features. Radiography remains the initial imaging modality for evaluation of the location of the lesion with respect to the longitudinal and axial planes of the involved bone, for estimation of its biologic activity by analysing the patterns of bone destruction and periosteal response, and for the depiction of matrix mineralisation. CT is typically used to obtain "radiographic" information in regions of complex skeletal anatomy such as the skull, spine, pelvis and shoulder girdle. MR imaging is best suited to determine the local extent of a bone tumour (local staging), but can also be helpful to narrow the differential diagnosis in specific lesions such as cysts and cartilage-forming tumours. With a clear emphasis on conventional radiography, this course will review the basic imaging features of the most common benign and malignant bone tumours. Important radiographic findings, such as bone destruction patterns, types of periosteal reactions and matrix mineralisation, will be explained step by step in correlation with histopathology as well as advanced imaging techniques.
***Learning Objectives:***
1. To become familiar with the imaging features of benign and malignant bone tumours.
2. To appreciate their imaging characteristic hallmarks on plain film radiography.
3. To learn how best to use imaging modalities in differential diagnosis.

**12:15 - 13:45 Room M1**

### Joint Session of the ESR Ultrasound Subcommittee and the Undergraduate Education Subcommittee

### ESR US SC/UES

### US teaching to undergraduate students: why, how and from whom?

*Moderators:*
C. Nyhsen; Strasbourg/FR
V. Válek; Brno/CZ

**A-0337** 12:15
**Why: US anatomy and pathology teaching, US in clinical practice and as a career choice**
T. Fischer; *Berlin/DE (thom.fischer@charite.de)*

Ultrasound training is time-consuming limiting its presence, especially during medical school curricula. Today, ultrasound is the most used interdisciplinary non-ionizing imaging technique in daily clinical routine. While students have to learn pathology early in their medical curricula, ultrasound should be taught parallel to pathology to create a better understanding of imaging. One major limitation of ultrasound is the accurate image acquisition and interpretation by the use of real-time non-standardized planes (contrarily to standardised planes in CT or MRI). While some organic parts are not visible by ultrasound, the focus of US anatomy and pathology should be on orientation and localisation of the organs or cavities. Therefore, the combination of anatomic structures (in cadavers) and US image fusion with CT or MRI may help to understand the anatomy of organs and landmarks in ultrasound. Focused assessment for sonography in trauma (FAST) represents the most used ultrasound examination. Therefore, US courses of FAST should be offered to each student to learn both anatomical (relationship of organs, landmarks) and technical (knobology, settings) basics of abdominal ultrasound. The introduction of artificial intelligence (AI) in ultrasound during the common years will improve the diagnostic performance in image interpretation. Therefore, radiologists in the future will focus on US-guided interventions and coordinating an interdisciplinary team. While US has to be performed manually, a replacement by robots/AI or sonographers is not predicted. To generate good quality standards in the performance of US, education and practical training by medical professionals are irreplaceable.
***Learning Objectives:***
1. To learn how ultrasound may be used effectively when teaching anatomy and pathology to undergraduate students, including access to hands on practice.
2. To understand how the importance of ultrasound as an imaging modality in daily clinical practice may be highlighted to students.
3. To learn how US teaching can be used to raise students' interest in radiology as a career choice.
*Author Disclosure:*
**T. Fischer:** Advisory Board; Canon, Siemens, Bracco. Consultant; Canon. Speaker; Canon, Siemens, Bracco.

**A-0338** 12:35
**How: US teaching in the radiology department and hands-on classroom teaching using living models and simulators**
M. Bachmann Nielsen; *Copenhagen/DK (mbn@dadlnet.dk)*

The number of medical students in a large university may bring an overwhelming demand for ultrasound equipment and for hands-on training. Students can train by scanning each other but getting enough patients may be difficult. Also, the level of competencies the students should aim for is different in preclinical and clinical part of the study and likely at a lower level than in medical doctors. Normal anatomy and point-of-care ultrasound cases are to be preferred for students. One way to achieve a uniform training for medical students would be to use ultrasound simulators. Some simulators are useful for the initial probe/eye coordination training and for getting a limited overview of simplified abdominal organs while other are fully digitized 3D volumes. Radiologists in training will typically want a more advanced simulator with a higher image quality than students. Available ultrasound simulators are mainly for abdominal examinations. Including US simulators should be based on educational research and a validated test to ensure the quality of learning. Tips for setting up a simulator study room will be given. In Copenhagen we estimate that 30 hours of ultrasound teaching is given during six years in medical school divided among different medical specialties and without a clear curriculum or practical test to ensure competencies. Our experience with offering an eight weeks practical stay in an US department will be given.
***Learning Objectives:***
1. To learn how students may be integrated in clinical US sessions in the radiology department.
2. To understand which different classroom hands on teaching environments can be created, using living models and simulators.
3. To become familiar with the use of simulators in ultrasound, from low cost to the high end spectrum and what the future may offer.

**A-0339** 12:55
**From whom: the importance of radiologists delivering US teaching and staying experts in this field**
K. Rosendahl; *Bergen/NO (karen.rosendahl@helse-bergen.no)*

Traditionally, ultrasound (US) has been an important part of the radiologist's integrated imaging approach. The US findings, together with additional information provided by the patient during the bed-side examination often help tailor further imaging, be it radiography, CT, MRI or nuclear medicine. During the past decades, specialities other than radiology, ranging from trauma surgeons to obstetricians and cardiologists, have taken an interest in ultrasound in order to answer very specific questions during the clinical examination; so-called point-of-care US. While ultrasound technicians and midwives are formally trained to perform the US, standardised training curriculum for clinicians are lacking. As radiologists we are medical expert consultants on imaging, and as such should have a key role in the initial training and setting of standards for diagnostic ultrasound procedures by non-radiologists and medical students. During this lecture, I will discuss how radiologists can continue to be regarded as experts in US through teaching and suggest ways to show students how radiologists provide an effective clinical service in this technique.
***Learning Objectives:***
1. To understand the importance of radiologists maintaining an interest in US and in US teaching in an era where US technology is more affordable and will be used by many specialties other than radiology.
2. To learn how radiologists can continue to be regarded as experts in US through teaching.
3. To become familiar with ways to show students how radiologists provide an effective clinical service in this technique.

13:15
**Panel discussion: How can radiologists use US to teach undergraduate students effectively, within time and cost constraints, whilst promoting their specialty as a career choice?**

**14:00 - 15:30** **Room A**

### E³ - ECR Academies: Interactive Teaching Session for Young (and not so Young) Radiologists

### E³ 721

### Errare humanum est

**A-0356** 14:00
**A. Errors in chest radiograph**
D. Tack; *Baudour/BE (denis.tack@skynet.be; denis.tack@epicura.be)*

Missing lesions on chest radiographs are frequent and the largest source of medico-legal issues. In this course, we report reasons for missing lesions, we distinguish perception and cognitive errors, and we comment on missing nodules, consolidations and infiltrative lung diseases. We provide tips to reduce our error rate, and in particular, we comment on the importance of learning and applying key signs for optimising the detection of abnormalities on both the frontal and the lateral views of the chest.
***Learning Objectives:***
1. To learn to recognise ambiguous signs in plain films.
2. To learn to avoid the most common pitfalls in reading plain chest films.

**A-0357** 14:45
**B. Errors in CT of the chest**
J. Vilar; *Valencia/ES (vilarsamper@gmail.com)*

Observer errors in Chest CT: The aetiology of error in radiology is multi-factorial. In this presentation, I will concentrate on errors attributed to the radiologist. Not seeing a lesion (perceptual error) and not recognising a pathology (interpretative error) are by far the most common causes. In a study of 500 cases, perceptual errors accounted for 80% and 20% were interpretative errors. Perceptual errors occur in CT, as in chest radiographs, despite the fact that CT is a tomographic technique. These errors may be related to technical factors such as noise, resolution, and use of adequate technical protocols including the use and timing of contrast media. Vigilance errors are those due to an insufficient time (reading too fast), satisfaction of search (interrupting vigilance), or inadequate clinical information (ignoring suspicion of pathology). Erroneous interpretation of the findings may be related to ignorance and inadequate information. The radiologist could avoid these errors by applying simple rules such as using checklists, taking enough time to read the studies, comparing with previous examinations, and using the clinical data. In this interactive presentation of cases, I will analyse some common causes of errors in chest CT and offer simple clues to avoid them.
***Learning Objectives:***
1. To learn to interpret ambiguous chest CT signs.
2. To learn to avoid the most common pitfalls in reading chest CT images.

**14:00 - 15:30** **Room M 3**

### ESOR Session

### ESOR

### Artificial intelligence: presented with new lecture formats

*Moderator:*
V. Vilgrain; Clichy/FR

**A-0368** 14:00
**Introduction**
B. Brkljačić; *Zagreb/HR (boris@brkljacic.com)*

In this session, the European School of Radiology (ESOR) will present new lecture formats with an emphasis on artificial intelligence (AI). Three speakers will discuss the concept of AI and machine learning applied in radiology, how neural networks can extract patterns from images and how deep learning can extract radiomics data.

**A-0369** 14:05
**ESOR in action 2019**
V. Vilgrain; *Clichy/FR*

**A-0370** 14:10
**Artificial intelligence (AI) and machine learning (ML) in medical imaging**
W.J. Niessen; *Rotterdam/NL*

"no abstract submitted"
***Learning Objectives:***
1. To define the concept of AI and the underlying techniques that can be applied to radiology.
2. To learn the needs and resources involved in the generation of new machine learning solutions for radiology.
3. To understand how AI can be integrated as a clinical tool to assist radiologists in their daily practice.

**A-0371** 14:30
**Deep learning (DL) in medical imaging**
A. Alberich-Bayarri; *Valencia/ES*

Deep Learning in Radiology can either be applied to reports by deep neural networks (DNN) or to images through convolutional neural networks (CNN). The main applications of deep learning in radiology include image reconstruction, image segmentation, classification, regression, content-based image retrieval and natural language processing of radiology reports, among many others. Specifically, CNN are generally outperforming deterministic computer vision algorithms used for filtering and segmentation. Nowadays, CNN-based algorithms allow for the automated segmentation of organs and tissues within the body with applications like 3D liver segmentation from MR, 3D prostate segmentation from MR, vertebrae detection and labelling from CT, 3D lung and emphysema segmentation from CT, 3D brain lesion detection and segmentation in MR. Deep learning has dramatically changed image analysis pipelines in radiology and has become a key piece in the automation of imaging biomarkers quantification within radiology departments.
***Learning Objectives:***
1. To learn the concept of DL and how neural networks (NN) can extract patterns from images.
2. To understand how DL can extract information from big radiomics data through training/test process.
3. To learn how convolutional NN can automatically extract imaging features.
*Author Disclosure:*
**A. Alberich-Bayarri:** Board Member; QUIBIM SL. CEO; QUIBIM SL. Founder; QUIBIM SL. Shareholder; QUIBIM SL.

**A-0372** 14:50
**My experience: one-year fellowship**
Y.M. Purcell; *Dublin/IE*

15:00
**Awards**

**14:00 - 15:30** **Room M 4**

### E³ - ECR Academies: Chest Imaging

### E³ 722

### Cavitary and cystic diseases of the lung

**A-0373** 14:00
**Chairperson's introduction**
J. Biederer; *Heidelberg/DE (biederer@radiologie-darmstadt.de)*

Air-filled lung lesions are an important diagnostic feature on chest X-ray and CT with a wide range of differential diagnoses. They may result from different conditions such as central necrosis of solid lung lesions and consolidations (malignant or inflammatory), congenital disorders, degeneration (emphysema, bronchiectasis) or trauma. Typically they are an incidental finding and radiology often plays an important role in routing further management of the case. The keys for the most probable diagnosis are on the one hand a precise description for the differentiation of cysts (round, circumscribed, thin-walled space) and cavities (gas-filled space inside a consolidation, nodule or mass) as well as size, profusion and ancillary findings of the lesions on imaging. On the other hand, age, gender, history (tobacco fume exposure) of the patient and other clinical parameters contribute to the interpretation of the case. This session aims to review the diagnostic criteria for air-filled lung lesions by discussing cavitary lesions, cystic lung lesions (focusing on Langerhans cell histiocytosis and lymphangioleiomyomatosis) and their differentials. The attendees will be provided with a practical approach for image interpretation in everyday work based on CT features and clinical background information.

**A-0374** 14:06
**A. Cavitary lung lesions**
A.P. Parkar; *Bergen/NO (apparkar@gmail.com)*

A cavity is defined as “a gas-filled space, seen as a lucency or low-attenuation area, within pulmonary consolidation, a mass, or a nodule”. The wall thickness may vary considerably. There is a continuous transition from cavities to cysts. Cysts are usually thin-walled (i.e., < 2 mm). A wall thickness < 7 mm is highly specific for the benign disease, a thickness >24 mm is highly specific for malignant disease. However, this is not absolute, as thin-walled carcinomas do exist. An additional indicator for malignancy is the lack of perilesional centrilobular nodules around a cavity. The cavity content is unhelpful in differentiating benign and malignant lesions. Wall rim enhancement on contrast-enhanced CT is common in abscesses. A connecting pulmonary artery may be seen in smaller metastases, but not in larger ones, as the larger nodules tend to compress the vessels, so no feeding artery does not always imply benign nodules. The acute onset of symptoms is sometimes helpful to distinguish malignant and non-malignant disease. Hemoptysis is not a useful symptom to differentiate between benign and malignant cavities. Benign diseases may also cause fatigue and weight loss similar to malignancies. Acute onset of fever is usually helpful to distinguish benign disorders from malignant, but pulmonary cancer may cause a super-infection secondary to the tumour. However, the combination of symptoms, laboratory results, past clinical history, and imaging findings leads to recognition of the correct diagnosis. This presentation will guide you through the most commonly encountered cavitary lung in adults.
***Learning Objectives:***
1. To review the diagnostic criteria and differentials of cavitary lung lesions.
2. To learn about the most common causes.
3. To learn about an algorithmic approach to narrow the differential diagnosis.

**A-0375** 14:34
**B. Langerhans cell histiocytosis (LCH)**
A. Devaraj; *London/UK*

The presentation will review the typical and atypical features of Langerhans cell histiocytosis (LCH) on HRCT, and the differential diagnosis when faced with cystic lung disease on CT. The evolution of CT appearances of LCH over time, the complications of LCH, as well as the pathological correlates will be discussed.
***Learning Objectives:***
1. To understand the pathophysiology of LCH.
2. To review key features on CT.
3. To learn about the various stages of the disease.

**A-0376** 15:02
**C. Lymphangioleiomyomatosis**
A. Oikonomou; *Toronto, ON/CA (anastasia.oikonomou@sunnybrook.ca)*

Lymphangiomyomatosis (LAM) is a rare cystic lung disease caused by infiltration of the lung with smooth muscle cells. It occurs in patients with tuberous sclerosis complex (TSC-LAM) and in a “sporadic” form in patients without tuberous sclerosis (S-LAM). S-LAM is seen exclusively in women of reproductive age while TSC-LAM may also be seen in men. Parenchymal lesions in LAM include cysts, which are thin-walled, well-defined, rounded, usually small in size up to 2 cm and may reach large numbers. They have no zonal lung predominance. Nodules are extremely rare in S-LAM and are more commonly seen in TSC-LAM. They may either be solid or ground-glass and usually tiny. They represent multifocal micronodular pneumocyte hyperplasia. Pleural manifestations include chylothorax and pneumothorax. Chylous pericardial effusions may be seen. Recent guidelines for LAM from the European Respiratory Society LAM task force classify LAM as definite, probable and possible. LAM is differentiated by other cystic lung diseases such as Langerhans histiocytosis (PLCH), Birt-Hogg-Dubbe (BHD), lymphocytic interstitial pneumonia (LIP) and amyloidosis. In PLCH the cysts have a bizarre shape, are variable in number and are upper and middle lobe predominant. In LIP the cysts are round and variable in size but usually small and random in distribution surrounded by ground glass opacity. In BHD the cysts are elliptical in shape and have a subpleural, lower zone predominance adjacent to vessels. In amyloid, the cysts are variable in size but usually large and diffusely distributed. Nodules are seen in PLCH and amyloid.
***Learning Objectives:***
1. To learn about the current concepts on pathogenesis.
2. To review the typical and atypical CT features.
3. To learn about the tuberous sclerosis complex.

**14:00 - 15:30** **Room M 5**

### E³ - ECR Academies: Functional Imaging for Disease Management: Research to Medical Practice

### E³ 720

### Functional imaging of the kidneys

**A-0377** 14:00
**Chairperson's introduction**
H.C. Thoeny; *Fribourg/CH (Harriet.thoeny@h-fr.ch)*

Thanks to advances in technology functional imaging of the kidneys allow to assess various physiological and pathophysiological aspects of the kidneys including perfusion, diffusion, glomerular filtration and provides noninvasive information on the oxygen content of the kidneys and also allows assessment of the single kidney noninvasively. Functional imaging in contrast to anatomical imaging allows noninvasive detection of diffuse renal diseases preceding changes in morphology and therefore allowing to start the accurate treatment in a timely manner. Especially in diffuse renal pathologies underlying chronic kidney diseases such as in diabetic patients were serum creatinine is a laboratory parameter which is often decreased in advanced stage disease, for instance, early detection and characterisation of chronic kidney disease might improve prognosis thanks to early treatment. The functional MR imaging techniques applied today include dynamic contrast-enhanced (DCE) MRI, diffusion-weighted MRI (DW-MRI) providing information on the underlying microstructure of the renal parenchyma, Blood Oxygen Level Dependent (BOLD) MRI providing indirect information on oxygen content as well as arterial spin labelling (ASL) proving noninvasive information on renal perfusion. Although all these techniques are promising there are still several challenges to overcome including technical improvements, standardisation, validation of quantitative models, reproducibility studies and clinical applications in larger scale studies as most of these functional techniques, for the time being, are mainly applied in a research setting.

**A-0378** 14:06
**A. MR measurement of split renal function**
D. Mandry; *Nancy/FR (damien.mandry@univ-lorraine.fr)*

Amongst the MRI techniques that carry functional data of the kidney, dynamic contrast enhancement (DCE) is well-established for the assessment of the renal perfusion, especially for the calculation of the split renal function. Its principle is to repeat for several minutes (usually, 6 to 10 min) the same T1-weighted acquisition on the kidneys, following the injection of an exogenous contrast agent. It requires a high temporal resolution for assessment of the vascular phase and preferably a volumic sequence to enable assessment of the whole parenchyma. The first step of the analysis usually is the application of a registration algorithm to correct for breathing-related motions. Then, the different renal compartments have to segmented to measure the parenchymal volume and derive time-intensity curves. Calculation of the split renal function is based either on the comparison of the curves from left and right kidneys, the calculation of the area-under-the-curve, or preferably on the Patlak-Rutland plot method. On top of the absence of ionising radiations, functional MRI provides a morphological analysis of the kidneys as compared to renal scintigraphy, but evaluation of severely dilated kidneys is more challenging.
***Learning Objectives:***
1. To review the principles of functional DCE of kidneys.
2. To learn about post-processing methods.
3. To understand differences with renal scintigraphy.

**A-0379** 14:34
**B. Renovascular diseases (RVD)**
M. Notohamiprodjo; *Tübingen/DE (Mike.Notohamiprodjo@uni-tuebingen.de)*

Renovascular disease, especially atherosclerotic renal artery stenosis, causes gradual deterioration of renal function and thus individual patient outcome. Despite increased awareness due to advances in vascular imaging, renal revascularisation is less commonly performed, likely as a result of several prospective, randomised, clinical trials which fail to demonstrate major benefits of renal revascularisation beyond medical therapy alone. The goal of this lecture is to discuss the available imaging methods to study renal flow and function. Furthermore, we review other non-invasive imaging methods to study the ultrastructure of renal tissue. We also explore the potential of these methods for management of renovascular disease, by triaging patients for revascularisation and to monitor the effects of medical and invasive therapy.

***Learning Objectives:***
1. To review main methods of renal perfusion measurement.
2. To learn about the place of functional approaches in strategy management of RVD.
3. To understand its impact for therapeutic decision.

**A-0380** 15:02
**C. Characterisation of chronic kidney diseases (CKD)**
N. Grenier; *Bordeaux/FR (nicolas.grenier@chu-bordeaux.fr)*

Renal physiology is complex with two compartments functioning under highly different conditions. The cortex is highly perfused with a high level of oxygen whereas medulla shows a poor perfusion level and works under hypoxic conditions. Water movements are multiple along the nephrons to maintain normal homeostasis. Glomerular filtration rate is a major marker of renal function, but its measurement is complex to obtain in clinics. All these parameters can be approached using functional MRI with DCE, DWI, BOLD. Some of these techniques may reflect changes within the extracellular matrix of renal tissue during the process of renal scarring. However, validation of these tools and their impact on patient management still requires further prospective studies.
***Learning Objectives:***
1. To understand the link between functional approach and renal physiological and tissue changes.
2. To learn how functional approach can help in differentiating renal diseases.
3. To be aware of their role in evaluating prognosis of CKD.
*Author Disclosure:*
**N. Grenier:** Advisory Board; Supersonic Imagine, Aix-en-Provence, France. Grant Recipient; Guerbet , GE Healthcare and Bracco.

**16:00 - 17:30** **Room A**

### E³ - ECR Academies: Interactive Teaching Session for Young (and not so Young) Radiologists

### E³ 821

### Gastrointestinal radiology

**A-0390** 16:00
**A. Inflammatory bowel disease**
J. Rimola; *Barcelona/ES (jrimola@clinic.ub.es)*

Bowel imaging had experienced relevant clinical advances in the last decade and represents the first diagnostic tool for assessing bowel inflammation in patients with Crohn’s disease (CD). There is growing evidence that cross-sectional imaging is able to accomplish main endpoints in the diagnosis of CD, including the detection of active disease, especially severe lesions, and the achievement of healing of inflammatory lesions. The identification of structuring and penetrating complications is also a key aspect that has implications in therapeutic management as well as in the determination of bowel damage. The lecture will provide the key points to confidently diagnose activity and complications related to CD.
***Learning Objectives:***
1. To review the spectrum of imaging findings in inflammatory bowel disease, mainly in Crohn's disease.
2. To learn about the management options.
*Author Disclosure:*
**J. Rimola:** Advisory Board; Gilead, Takeda, TiGenix. Consultant; Parexel, Robarts Clinical Trials. Research/Grant Support; Abbvie. Speaker; Janssen.

**A-0391** 16:45
**B. Rectal cancer staging: key findings**
G. Brown; *Sutton/UK*

"no abstract submitted"
***Learning Objectives:***
1. To understand the imaging technique.
2. To identify the key imaging findings.

**16:00 - 17:30** **Room B**

### Special Focus Session

### SF 8a

### Artificial intelligence (AI): our future cannot be predicted, but we have to be prepared

**A-0392** 16:00
**Chairperson's introduction**
G. Langs; *Vienna/AT (Georg.langs@meduniwien.ac.at)*

Machine learning and radiology are rapidly gaining joint momentum as an interdisciplinary research field. After initial expectations start to transition into joint research, it is time for a reality check to understand where we are and which are the most promising and difficult efforts for this emerging interdisciplinary field. The session will give an overview of the current state of machine learning in medical imaging and will explore valuable directions of joint research. Four distinguished speakers from areas covering radiology, machine learning and medical image computing will provide a realistic assessment of where we are, and offer their views about most directions that will have advance both fields having an impact on research in novel biomarkers, and clinical applications. The session will highlight challenges in methodology, the integration of machine learning in radiological routine, and the enablers of successful development of novel and reliable machine learning approaches in medicine. As both fields evolve, and the role of radiology and machine learning is expanding, the session will give a forum for critical, informed discussion.
***Session Objectives:***
1. To learn about the basics of machine learning and deep learning, its current capabilities and limitations and its contribution to research and routine.
2. To understand the current state-of-the-art, where ML/DL techniques struggle and in which directions the major methodological and application advances are being made right now.
3. To appreciate the need for interdisciplinary research and conscious creation of means to exploit the capabilities of AI, and to coordinate development across machine learning and radiology communities.
4. To become familiar with examples of how AI can be used in radiology.
*Author Disclosure:*
**G. Langs:** Founder; contextflow GmbH. Grant Recipient; Siemens, Novartis. Shareholder; contextflow GmbH. Speaker; Siemens, Roche.

**A-0393** 16:05
**Basics of machine learning and deep learning**
B. Van Ginneken; *Nijmegen/NL*

"no abstract submitted"
***Learning Objectives:***
1. To get basic understanding of techniques of machine learning with a focus on deep learning techniques.
2. To understand supervised and unsupervised learning techniques.
3. To know current capabilities and limitations of deep learning methodology in the context of radiology.

**A-0394** 16:20
**AI for lesion detection and characterisation**
B. Stieltjes; *Basle/CH*

"no abstract submitted"
***Learning Objectives:***
1. To see specific examples of AI application in clinical radiology and research.
2. To understand challenges, solutions and pitfalls in translating ML methodology to successful clinical implementation.
3. To learn what AI can add to the management of the radiology department.

**A-0395** 16:35
**Machine learning in medical imaging going forward**
P. Golland; *Cambridge, MA/US*

"no abstract submitted"
***Learning Objectives:***
1. To understand machine learning, where current technology development is heading and how it is aligned with clinical needs.
2. To understand which tasks ML/DL perform well, where the challenges are, where we can expect improvement and what the prerequisites for this are.
3. To learn what machine learning can add to the management of the radiology department.

**A-0396** 16:50
**Which impact does AI have on medicine?**
S.O. Schönberg; *Mannheim/DE*

"no abstract submitted"
***Learning Objectives:***
1. To be prepared for the future role of AI and radiologists, and their interaction, when using AI as a tool for development and clinical routine.
2. To understand how AI will impact the role and tasks of radiologists in the future.
3. To know what the beneficial directions are and how we can facilitate effective joint research at the interface of the involved communities.
4. To try to foresee what the emerging role of AI means for educating the next generation of radiologists.

17:05
**Panel discussion: What is the future role of a radiologist in the diagnostic process?**

**16:00 - 17:30 Room C**

### General Radiography (Radiographers)

### RC 814
### Forensic imaging

**A-0397** 16:00
**Chairpersons' introduction (Part 1)**
A. Dominguez; *Lausanne/CH (Alexandre.DOMINGUEZ@hesav.ch)*

The implementation of radiographers in forensic medicine is becoming more and more widespread. The use of the technical skills of a radiographer increases the quality of services, especially in the field of postmortem imaging angiography. The multi-disciplinary collaboration between pathologists, the radiologists and the radiographers allows the implementation of these new practices. Thanks to the contribution of forensic imaging, the sensitivity of the post-mortem examination within medico-legal investigations are increased.
***Session Objectives:***
1. To provide insights into the role of imaging, and radiographers, in forensic imaging.
2. To appreciate the key aspects of a quality forensic imaging service using angiography.
3. To understand the challenges associated with forensic imaging.

**A-0398** 16:03
**Chairpersons' introduction (Part 2)**
H.T. Patel; *Ahmedabad/IN (hemantmri@gmail.com)*

Forensic Radiology is a segment within the field of medical imaging that focuses on using radiology imaging techniques where evidence may be gathered in a court of law. The use of radiography in support of evidence has a long history dating back to the first few months after Wilhelm Roentgen's discovery in 1895. A bullet seen using an x-ray was used for cases of attempted murder. Pathologists commonly use radiographs when performing autopsies. The images can help them identify elements that are out of place or questionable and then allows them to investigate further. As the radiologic sciences have advanced over the years to include MRI's, CT's, and ultrasound, the accuracy has increased to a much greater degree and has made it even more relevant for investigating crimes and gathering evidence.
***Session Objectives:***
1. To provide insights into the role of imaging, and radiographers, in forensic imaging.
2. To appreciate the key aspects of a quality forensic imaging service using angiography.
3. To understand the challenges associated with forensic imaging.

**A-0399** 16:05
**A. The chain of evidence**
M. Davis; *Dublin/IE (michaela.davis@ucd.ie)*

Diagnostic imaging is a useful tool for and clinical case management. Radiographers by the nature of their work create material which can be potentially used as evidence. This material may be in a variety of formats. It is important that radiographers maintain continuity of evidence within their working practices as the material, may be used by other professionals as part of their decision-making process as well as the courts including both the child and family and criminal courts. The presentation will draw upon examples and discuss potential pitfalls in this area.
***Learning Objectives:***
1. To appreciate the importance of continuity of evidence and record keeping.
2. To learn about essential steps for radiographers to follow.
3. To discuss potential pitfalls when conducting forensic examinations.

**A-0400** 16:28
**B. Post-mortem cardiac angiography**
H. Precht; *Odense/DK (hepr@ucl.dk)*

Computed Tomography (CT) is a widely used imaging tool with high diagnostic accuracy and prognostic value. Cardiac CT Angiography (CCTA) scanning is often used to diagnose cardiology patients as coronary atherosclerosis is a worldwide disease and counts for millions of deaths every year. The CT technology is developing rapidly given new possibilities for optimisation. A CCTA scan can identify elements as coronary stenosis, coronary atherosclerotic-, low attenuation- and spotty calcification plaques, positive remodelling. These components have been considered as important features of coronary plaque vulnerability and instability. As the coronary arteries have an average diameter of 1-4 mm, the CCTA images need to image small details with low attenuation. The CT scanner is challenged to image small details with good contrast and temporal resolution; therefore optimisation of the CT protocol is necessary. New developed CT techniques need to be implemented with caution to ensure the images not change coronary plaques size or content inappropriate. To evaluate new CCTA technique influence of coronary plaques, a correlation with the gold standard of histopathology could give us useful information. To be able to reflect these results to in-vivo CCTA it is important to prepare the heart and arteries with correct pressure, contrast filling in the arteries as contrast/water in the heart chambers. Scattered radiation from the surrounding tissue needs to be included as the most challenging parts is an exact procedure for three-dimensional alignment procedure for PMCCTA and the histopathology data.
***Learning Objectives:***
1. To learn about the development of multiphase post-mortem cardiac angiography (MPMCCTA).
2. To appreciate the benefits and limitations of MPMCCTA examinations.
3. To understand the role of the radiographer in the MPMCCTA.

**A-0401** 16:51
**C. Paediatric forensic imaging**
A.L. Brookes; *Fulwood/UK*

Forensic imaging is the application of any form of diagnostic radiography to the law and is used to provide evidence for a potential criminal investigation. This sub-speciality of radiology is ever expanding, and recent developments have seen forensic imaging utilised and researched more widely, particularly the use of CT in post-mortem settings. Paediatric forensic imaging is the mainstay of routine forensic cases undertaken in imaging departments. Most often, this is the investigation of Suspected Physical Abuse (SPA) in both living and deceased children. In these cases, imaging is used as an adjunct to other evidence collected in the detection of SPA. However, recognition and further prevention of child abuse can be achieved through the use of radiological findings. Nevertheless, forensic imaging in paediatrics extends beyond use for detection of SPA, and in almost all types of forensic imaging, paediatric cases will be found.
***Learning Objectives:***
1. To appreciate the role of the radiographer in paediatric forensic imaging.
2. To understand the challenges associated with conducting paediatric forensic examinations.
3. To discuss the imaging options available for paediatric forensic imaging.

17:14
**Panel discussion: Safeguarding the wellbeing of staff involved in forensic imaging**

**16:00 - 17:30 Room X**

### Multidisciplinary Session

### MS 8b
### Brain imaging in psychiatry

**A-0402** 16:00
**Chairperson's introduction**
J.-P. Pruvo; *Lille/FR (jean-pierre.pruvo@chru-lille.fr)*

This session will introduce the potential of MRI use in Psychiatry. First, the prevalence and burden of the main psychiatric disorders will be presented by Dr Amad. He will notably synthesise the available findings in first-episode of psychosis and the rationale for systematic MRI in this indication. Then, Pr Pruvo will discuss the choice of structural/ functional MRI sequences in this context. He will present a collaborative psychiatry-radiology network in France as proof of concept. Dr Lopes will then develop the perspective of high-field MRI in psychiatry research together with clinical applications based on advanced Machine-Learning adapted to the field of Psychiatry. Finally, Pr Jardri will conclude on applications of image-guided therapy to relieve drug-resistant hallucinations.
***Session Objectives:***
1. To introduce the potential of MRI use in psychiatry.
2. To present the context and introduce the faculty.

**A-0403** 16:05
**The epidemiology of psychiatric disorders: why is MRI needed?**
A. Amad; *Lille/FR (ali.amad@chru-lille.fr)*

The prevalence, severity, and overall burden of morbidity and mortality represented by psychiatric disorders reflect an urgent global public health priority. According to a systematic review of data and statistics from community studies in European Union (EU) countries, the WHO estimated that 27% of the adult population (18-65 years old) had experienced at least one of a series of psychiatric disorders in the past year representing about 83 million people being affected. Despite psychiatric disorders are now defined as brain disorders, the use of brain imaging, especially brain MRI, in psychiatric clinical practice remains limited. Currently, the main indication of brain MRI in psychiatric clinical practice corresponds to the identification of differential diagnoses as many diseases (e.g. brain tumours, neuroinflammatory disorders, aneurysms) can cause and/or be associated to psychiatric symptoms. In this talk, we are going to answer to the question "why MRI is needed in psychiatry", with a focus on the first psychosis episode, by highlighting that considerable research is indicating that the potential uses of brain MRI in clinical practice are numerous for both diagnostic and prognostic purposes but also to guide treatments.
***Learning Objectives:***
1. To know the prevalence and burden of the main psychiatric disorders.
2. To synthesise the available findings in the case of the first-episode of psychosis.
3. To discuss the rationale for systematic MRI in first-episode psychosis.

**A-0404** 16:23
**Strengths of structural and functional MRI in psychiatry**
J.-P. Pruvo; *Lille/FR*

Being able to predict as soon as possible the clinical outcome following a first episode of psychosis (FEP) is crucial, and should allow providing the most appropriate care to patients. Several biomarkers for FEP outcome have already been proposed, including anatomical and functional neuroimaging, neuropsychological and biological biomarkers, but they still lack large-scale validation. In the Hauts-de-France region, the Federation for mental health research (F2RSM) constituted radiologists-psychiatrists pairs and harmonised magnetic resonance imaging sequences to be used in FEP, forming the "PSYMAC clinical network". This procedure also includes follow-up visits to maintain harmonisation over time. The choice of the most adapted sequences to be conducted in this context will be discussed. We expect to later develop and generalise such a network nationwide.
***Learning Objectives:***
1. To discuss the choice of structural/functional MRI sequences in this context.
2. To present a collaborative psychiatry-radiology network in North of France.
3. To present the clinical perspective of the PREDIPSY project.

**A-0405** 16:41
**Advanced MRI and clinical applications: the structured report**
R. Lopes; *Lille/FR (renaud.lopes@gmail.com)*

In this presentation, we will talk about the interest of advanced MRI in clinical applications with a focus on a structured report. First, we will discuss the interest of 3T MRI in a better understanding of the physiopathology of neurological and psychiatric diseases. We will synthesise 5-years of 3T MRI research in biomarkers of these diseases. Next, we will present some studies using ultra-high field MRI in psychiatric diseases and will show the complementarity with 3T MRI. Finally, we will conclude by demonstrating the interests of these biomarkers identified at 3T and 7T MRI in the diagnosis of psychiatric and neurological diseases using artifical intelligence and structured report.
***Learning Objectives:***
1. To synthesise 5-years of 3T-MRI research in psychiatry and neurology.
2. To present the perspective of ultra-high field MRI in psychiatry research.
3. To present clinical applications of machine-learning on MRI-data in the field of psychiatry.

**A-0406** 16:59
**From fMRI capture of hallucinations to innovative image-guided treatments**
R. Jardri; *Lille/FR (renaud.jardri@chru-lille.fr)*

Hallucinations have held the fascination of science since the dawn of humanity. However, their study has become critical with the recent development of functional brain imaging that allowed to explore the neural underpinnings of these experiences. This presentation with briefly summarise the available methods to analyse per-hallucinatory fMRI signal, called "hallucination capture" methods. Recent developments using Machine-Learning will also be presented. Crucially, these methods pave the way to innovative fMRI-guided treatment of drug-resistant hallucinations, including neuro-navigated brain stimulation (e.g. rTMS) and fMRI-based neurofeedback.
***Learning Objectives:***
1. To know the neural bases of hallucinations.
2. To know the different fMRI strategies used to capture hallucinations.
3. To present fMRI-guided treatments of refractory hallucinations.

**A-0407** 17:17
**Multidisciplinary case presentation and discussion**
J.-P. Pruvo; *Lille/FR (jean-pierre.pruvo@chru-lille.fr)*

**16:00 - 17:30** **Room N**

### EuroSafe Imaging Session

### EU 2

### Diagnostic reference levels (DRLs) based on clinical indication

*Moderators:*
G. Frija; Paris/FR
G. Simeonov; Luxembourg/LU

**A-0408** 16:00
**Chairperson's introduction**
G. Frija; *Paris/FR (guy.frija@aphp.fr)*

This session will focus on the concept of clinical diagnostic reference levels (CDRLs) and will provide the European Commission's perspective on the concept. Moreover, the European Commission-funded tender project EUCLID (European Study on Clinical Diagnostic Reference Levels for X-ray Medical Imaging), which is implemented by the European Society of Radiology (ESR), will be presented. EUCLID aims to establish clinical DRLs for the most important, from a radiological perspective, CT and interventional imaging tasks in Europe, and to stimulate national and local efforts in this area. The session will be concluded with presentations on the national experiences of Ireland and Switzerland in the establishment of CDRLs.
***Session Objectives:***
1. To learn about the concept of clinical DRLs (CDRLs).
2. To understand the goals of CDRLs.
3. To understand the methodology of the establishment of CDRs.

**A-0409** 16:05
**European Commission perspective**
G. Simeonov; *Luxembourg/LU (georgi.simeonov@ec.europa.eu)*

The European legislation for radiation protection of patients has been evolving for more than 30 years. The most recent revision - Council Directive 2013/59/Euratom (Basic Safety Standards, BSS) - brings important changes with an implementation deadline of February 2018. In the absence of dose limits and dose constraints, the diagnostic reference levels (DRLs) are the main operational and regulatory tool for optimisation of radiation protection of patients in diagnostic imaging. The BSS directive introduces, inter alia, new requirements for the establishment, regular review and use of diagnostic reference levels (DRLs). Furthermore, the directive expands the application of DRLs to interventional radiology procedures. The European Commission (EC) supports the implementation of legal requirements, e.g. through studies and guidance. The EC guidance on DRLs from 1999 is now largely considered outdated. Guidance on paediatric DRLs was issued in 2018, based on the European PiDRL project. Recent reviews show that, in EU countries, DRLs exist only for a limited number of widely defined imaging procedures without specification of the clinical task/indication. The EUCLID project, to be completed in 2020, is expected to advance the implementation of clinical DRLs. The implementation of the BSS directive remains a national responsibility. Nevertheless, the EC will consider further action to support the implementation of DRLs in clinical practice.

***Learning Objectives:***
1. To learn about the Euratom legal basis for radiation protection of patients and the European Commission's support for implementation of the Euratom requirements for DRLs.
2. To understand the place and the importance of DRLs in the Euratom system for radiation protection of patients.
3. To appreciate the progress made in recent years with respect to DRLs and the key role of Euratom as the driver of progress in this area.

**A-0410** 16:20
**One shoe does not fit all: clinical indication based DRLs**
G. Frija; *Paris/FR (guy.frija@aphp.fr)*

Diagnostic reference levels (DRLs) are an important tool for optimisation. DRLs are usually established for specific anatomical locations. However, different image quality is needed for different clinical indications of the same anatomical area. Thus, the use of DRLs based on clinical indications is suggested instead. Some countries have already established DRLs based on clinical indications, or are in the process of establishing them. This talk will introduce the concept of clinical DLRs (CDRLs) in computed tomography and interventional radiology, and point out the importance of the establishment of local DRLs. It will provide an update on DRLs available in Europe and propose the way forward to promote the establishment of CDRLs.
***Learning Objectives:***
1. To learn about the concept of CDRLs in CT and in interventional radiology.
2. To understand the importance of local DRLs.
3. To appreciate the need to develop local repositories covering the field of quality and safety.

**A-0411** 16:35
**Experience from the EUCLID (European study on clinical DRLs) Project**
J. Damilakis; *Iraklion/GR (damilaki@med.uoc.gr)*

The European Commission launched the ‘European study on clinical diagnostic reference levels for x-ray medical imaging’ (EUCLID) project to provide up-to-date clinical dose reference levels (DRLs). The main objectives of the project are to a) conduct a European survey to collect data needed for the establishment of DRLs for the most important, from the radiation protection perspective, x-ray imaging tasks in Europe and b) specify up-to-date DRLs for these clinical tasks. EUCLID started on August 1, 2017, and the duration of the project is 33 months. A comprehensive review has been carried out to identify the status of existing clinical DRLs for CT, interventional radiology and radiography in Europe and beyond by analysing recent studies, standards and publications. Information about existing clinical DRLs has also been collected from competent national authorities and other organisations involved in the project. A survey has been developed for the collection of data needed for DRLs determination from 14 European countries. Data are collected for 10 CT clinical indications and four fluoroscopically guided interventional procedures. While the hospitals are collecting data and uploading them to a secure platform, literature is reviewed, and different methods of data analysis are investigated by EUCLID experts. Preliminary results will be summarised during this presentation.
***Learning Objectives:***
1. To learn about methodological requirements for establishing DRLs.
2. To learn about the current status of DRLs in adult imaging in Europe and beyond.
3. To understand why clinical DRLs are needed in adult imaging.

**A-0412** 16:50
**Experience with a national survey based on clinical indications**
S.J. Foley; *Dublin/IE (shane.foley@ucd.ie)*

In 2017 an online survey was distributed to all Irish CT centres on behalf of the National Population Dose and Optimisation Sub-committee, which aimed to determine the total collective dose of radiation to patients nationally from the use of CT and to enhance patient safety through promoting the principle of optimisation. The objectives were to identify the median dose administered for the most frequently performed CT procedures in both paediatric and adult services and to inform national diagnostic reference levels (DRLs) for the most common CT procedures. For the first time, DRL data collection requested dose data specific to clinical indications based on a list of the thirteen most common presenting complaints that require a CT imaging procedure. The findings demonstrated that DRLs based on clinical indication, although based on smaller exam numbers, were significantly different from DRLs generated by anatomical area. However, the findings were limited by the interpretation applied to each particular optimisation protocol. This can possibly be overcome by increased standardisation of practices, applying uniform terminology and adopting common information systems.

***Learning Objectives:***
1. To learn about the 2017 Irish national CT survey.
2. To understand DRL differences when derived from clinical indications in contrast to anatomical region.
3. To appreciate aspects for improvement of future surveys.

**A-0413** 17:05
**Update of the national DRLs for CT in Switzerland**
S.T. Schindera; *Aarau/CH (sschindera@aol.com)*

The substantial contribution of computed tomography (CT) to the collective population dose requires efforts to optimise CT radiation doses. Diagnostic reference levels (DRLs) are a useful tool to support this optimisation. Recently, the DRLs for CT have been updated in Switzerland compared to the previous national assessment from 2010. Radiation dose data from 50 CT scanners were collected with locally installed dose management software solutions between 2014 and 2017. Compared to the previous DRLs, a clear trend towards lower doses was observed. The average relative change in CTDIvol was -30% (0% to -47% depending on the protocol) and -22% for dose length product (+20% to -40%). Furthermore, comparing to other European countries, the recently updated national DRLs for CT in Switzerland is lower. The substantial reduction of the DRLs reflects well the efforts of the Swiss radiological community towards the use of dose-efficient protocols.
***Learning Objectives:***
1. To learn about the methodology of updating the national DRLs for CT in the era of dose management software.
2. To learn about the main differences of the updated national DRLs for CT.
3. To understand the impact of the updated national DRLs for CT.

17:20
**Discussion**

**16:00 - 17:30** **Room O**

### Special Focus Session

### SF 8c

### The art to transmit and to receive: how to communicate critical information to our patients

**A-0414** 16:00
**Chairpersons' introduction (Part 1)**
M.H. Fuchsjäger; *Graz/AT (michael.fuchsjaeger@medunigraz.at)*

This session is aimed at all radiologists to learn to understand the power of communication as such, as well as the communication of critical information to our patients. During the last two decades, the term of the invisible radiologist has been coined, something that does not at all reflect the true clinical work of any radiologist which is in many subspecialties very close to our patients. Still, many medical universities do not have communication skills training incorporated into their curricula. In this session stress is laid on the fact that active communication is important, acknowledging that active listening is perhaps the most important part of it. We will learn about the fundamentals of physician-patient communication, the patients’ needs as well as the patients’ perspective of physician-patient communication, as well as strategies to improve our communication with our patients.
***Session Objectives:***
1. To understand the importance of direct communication with patients.
2. To learn about the differences between delivering and receiving critical information.
3. To become familiar with the needs of patients for communication with medical specialists.

**A-0415** 16:03
**Chairpersons' introduction (Part 2)**
C. Loewe; *Vienna/AT (christian.loewe@meduniwien.ac.at)*

The continuous transformation of Radiologists from very technical Physicians far away from patients and clinical decision making towards clinically involved doctors playing a central role in tumour boards and treatment decisions has changed the demands on modern clinical Radiologists. Besides the traditional needs for a highly accurate and precise language for written reports, clinical Radiologists nowadays should also be skilled in direct communication with clinical colleagues and - even more important - with patients. There is an increasing number of situations and occasions where clinical Radiologists are the first ones to inform patients about a possibly unfavourable finding. It is during an ultrasound, but also when it comes to breast care and image guided intervention and therapy when patients are asking questions about their situation. And they do have the right to receive appropriate answers then! Facing the shortage of clinicians in many countries leading to further intensified schedule in usual outpatient services the number of - especially oncological patients - asking for direct communication with the Radiologists right after staging CT is continuously increasing as well. Consequently, Radiologist's are leaving the closed box - and they should find the right empathic words in direct communication. Within this Chairman's introduction, we will recall the need for facing the challenge of direct communication with patients in modern Radiology. Furthermore, we will point out again the possible problems while transmitting critical information's to the patients and how big the difference between delivery and receive might be in this situation.

***Session Objectives:***

1. To understand the importance of direct communication with patients.
2. To learn about the differences between delivering and receiving critical information.
3. To become familiar with the needs of patients for communication with medical specialists.

*Author Disclosure:*

**C. Loewe:** Speaker; Siemens, Bracco, GE Healthcare.

**A-0416** 16:05

**How patients feel about communication**

C. Justich; *Vienna/AT (cjustich@me.com)*

A bigger picture has become the basic requirement in today's medical world. High expectations demanded from physicians have to be met on a professional level and with compassion for the patient. The key to success lies in recognising the importance of communication resulting in a doctor-patient-relationship based on trust. The secret is, how you say what by whom. How: Radiologist with limited time need to find quick and effective ways to communicate, in most cases, they are the first ones to approach a patient (technique examples). Who: Each patient should have one medical leader who guides them. Patients tend to hand over the total responsibility to doctors, and they should be empowered to take it back and to play an active role in their care and healing process. The medical leader has to be the single point of communication to all persons involved. The time between examination and issuing its results must be kept short, the information being provided only after a decision has been made (personal example liver). Today we know, that a positive expectation together with the care of an acknowledged expert, can work better than a placebo. As physicians, you have the power to reduce your patients' stress and anxiety but also to increase them. Statistics are fabulous but be careful about giving prognoses and taking away hope. To set up your wording in the right way will improve the outcome (examples). Making an effort is well worth it, the benefits will be yours.

***Learning Objectives:***

1. To understand the immense impact of communication.
2. To demonstrate how different ways of communication influenced me as a patient.
3. To appreciate benefits of good communication for the physician.

**A-0417** 16:30

**Fundamentals of physician/patient communication**

L. Fallowfield; *Brighton/UK*

***Learning Objectives:***

1. To understand what questions and information are important to patients.
2. To learn some basic principles and techniques of doctor-patient communication.
3. To apply these principles to some common clinical situations.

**A-0418** 16:55

**How can we improve communication with our patients?**

F.J. Gilbert; *Cambridge/UK*

The interaction of radiologist with patient varies in type of examination undertaken. With mammograms, there may be no interaction, with ultrasound it's challenging to avoid communication with the patient who is anxious and will ask questions during the procedure. This is similar to image guided procedures where sometimes informed consent is required. A clear explanation of interventional procedures is essential as patients are giving consent implicitly if no direct consent is being requested. The communication of radiological findings with patients depends on the nature of the discussion. Often when there is an uncertainty of diagnosis, radiological findings are explained together with next steps for patient management. Commonly there is uncertainty about mammographic findings, i.e. microcalcification or asymmetry and further imaging or biopsy is required. The importance of conveying the same message as the clinician in charge of the patient cannot be emphasised enough. Essentially the radiologist must not undermine or contradict the clinician who has to take the ultimate responsibility for the patient. An agreed management plan is important and is often is developed in the Multidisciplinary team meeting (MDT). Patients appreciate clear communication together with a written plan of management. Communication courses give practical tips on effective communication strategies together with advice on how to check whether or not the patient has understood what is being said. An empathetic approach is important in breaking bad news.

***Learning Objectives:***

1. To understand the communication of radiological findings with patients.
2. To discuss communication of uncertainty about radiological findings and why further tests are required.
3. To appreciate the importance of conveying the same messages as the clinician in charge of the patient.

17:20

**Panel discussion: How to convey that we care**

**16:00 - 17:30** **Studio 2019**

### Special Focus Session

### SF 8d

### Non-contrast MR angiography: ready to go?

**A-0419** 16:00

**Chairperson's introduction**

R. Iezzi; *Rome/IT (roberto.iezzi.md@gmail.com)*

MR angiography represents an accepted non-invasive alternative to conventional angiography in vascular imaging. Furthermore, using MR to delineate vascular anatomy has changed dramatically due to continuous technological evolution which provided more advanced equipment and complex software, making it faster with more detailed information obtained. Advantages of MRA over CTA include increased signal-to-noise ratio, easer 3D post-processing, and utility in young patients or in patients who require recurrent follow-up imaging given its lack of ionising radiation. One of the biggest concerns in radiology in recent years is the safety of gadolinium.based contrast agents used in MR imaging. It is well-known that patients with moderate to severe renal insufficiency and vascular or metabolic disorders cannot filter the gadolinium, being at risk for developing the debilitating and possibly life-threatening disease of nephrogenic systemic fibrosis. However, there has been little evidence showing patient safety issues in those with normal renal function. These points will be discussed during the session, also highlighting the renewed interest on non-contrast MRA imaging, obtained with improvements in MR hardware and software, as reported in recent literature, that could overcome MRA. Interestingly, phase-contrast NCE-MRA methods could also offer the potential to provide additional hemodynamic information that currently is obtained using invasive methods.

***Session Objectives:***

1. To understand the role of MR angiography in vascular imaging.
2. To became familiar about the risk of gadolinium injection.
3. To learn about the existing literature on the usefulness of non-contrast MRA imaging.

**A-0420** 16:05

**Non-contrast MR angiography techniques**

T. Leiner; *Utrecht/NL (t.leiner@umcutrecht.nl)*

Magnetic resonance angiography is a highly reliable tool for detection of presence, extent and severity of arterial narrowing in virtually any vascular bed in the body except the coronary arteries. In clinical practice, most radiologists use contrast-enhanced MR angiography (CE-MRA) techniques due to wide availability and ease of use. However, recent advances in scanner hardware and pulse sequence design have led to the development of a set of methods that utilise the intrinsic properties of blood to generate images with high vessel-to-background contrast without the need for an injection of an extrinsic contrast agent. These methods are also known as native or non-contrast MRA (NC-MRA). NC-MRA methods rely on a variety of contrast mechanisms. The most commonly used mechanisms are Flow-independent MRA; non-subtractive inflow-dependent MRA; subtractive 3D MRA, and velocity-selective 3D MRA. In my lecture, I will discuss these mechanisms as well as the evidence for clinical efficacy in various body regions.

***Learning Objectives:***

1. To became familiar with MRI technologies and innovations.
2. To learn about the alternative sequences to study vascular structures.
3. To learn what you need to technically optimise your protocols.

**A-0421** 16:23
**Non-contrast MR angiography for large vessels**
T. Hazirolan; *Ankara/TR (tuncayhazirolan@yahoo.com)*

Determining that gadolinium contrast agents caused NSF and brain accumulation led to the preference of non-contrast MRI applications more frequently. In recent years, there has been an increase in the number of non-contrast MR angiography techniques. The most preferred method in the non-contrast angiographic evaluation of the aorta is the ECG and navigator-gated 3D-SSFP (3D-SSFP)sequence. Protocols that includes cine SSFP, ECG gated 2D black blood TSE, ECG gated white and black blood single shoot sequences, and 3D-SSFP provide a comprehensive assessment of aortic pathologies. In this presentation, advantages, disadvantages and artefacts of non-contrast MRI protocols used in the evaluation of aorta will be discussed.
***Learning Objectives:***
1. To understand the role of MR angiography in aortic imaging.
2. To discuss how non-enhanced MRI sequences could replace gadolinium-enhanced sequences.
3. To learn how to optimise your protocols to avoid the use of intravenous gadolinium.

**A-0422** 16:41
**Peripheral non-contrast MR angiography**
G. Roditi; *Glasgow/UK (Giles.Roditi@glasgow.ac.uk)*

For evaluation of peripheral vascular disease non-contrast, TOF MRA was hampered by long acquisition times plus in-plane flow saturation effects and was soon supplanted by gadolinium-based contrast-enhanced techniques. The extensive craniocaudal coverage and necessity for high spatial resolution required to evaluate the lower limb arteries down to the feet accurately has remained challenging for NC-MRA. Nevertheless, there has been significant progress with both ECG-triggered subtraction FSE (exploiting arterial and venous flow differences, e.g. FBI) and flow-independent bright-blood techniques such as SSFP with IR (NATIVE SPACE, TRANCE) and (ECG)-triggered quiescent interval single shot (QISS) MRA being refined. There is increasing evidence of the accuracy (particularly for QISS); however, the uptake of these techniques has been relatively limited and this presentation will explore the reasons behind this which are in part due to technical limitations & competition from alternative techniques but also more prosaic challenges in the implementations that impact on workflow.
***Learning Objectives:***
1. To understand the role of MR angiography in peripheral disease.
2. To discuss how non-enhanced MRI sequences could replace gadolinium-enhanced sequences.
3. To learn how to optimise your protocols to avoid the use of intravenous gadolinium.
*Author Disclosure:*
**G. Roditi:** Board Member; President-Elect Society of Magnetic Resonance Angiography. Consultant; Canon Medical Research Europe Ltd..

**A-0423** 16:59
**Non-contrast MR angiography in paediatric vascular imaging**
M. Argyropoulou; *Ioannina/GR (margyrop@cc.uoi.gr)*

Contrast-enhanced MR angiography (CE-MRA) has been long considered the modality of choice for vascular imaging in children. The main contraindication of CE-MRA has been the renal failure because of the risk for developing nephrogenic fibrosis. State of the art CE-MRA necessitates lack of motion, a good intravenous access and collaboration of the patient in breath holding, conditions that may be difficult to obtain in young children. Over the last years, there is an increasing concern about the deposition of gadolinium in the brain of pediatric and adult patients leading to an increasing interest in developing new non-contrast-enhanced MRA (NC-MRA) techniques. NC-MRA has been successfully applied in imaging the thoracic and abdominal aorta and its branches. “Black blood” and “white blood” sequences have been used in the evaluation of aortic rings and pulmonary slings. NC-MRA and cine-MRI have been used for the evaluation of coarctation of the aorta and complications such as residual stenosis or restenosis, development of a false aneurysm and aortic dissection. NC-MRA has been successfully used evaluation of aberrant vessels crossing the ureteropelvic junction resulting in hydronephrosis and in the evaluation of the splenoportal vessels. NC dynamic MRA techniques based on arterial spin-labelling can be used in the evaluation of brain vessels offering the possibility to separate arteries from veins and to detect temporal flow differences between the anterior and posterior circulation.
***Learning Objectives:***
1. To learn about the risk of gadolinium injection in the paediatric patient.
2. To discuss the implications of the new knowledge for clinical practice in paediatric radiology.
3. To learn how to optimise your protocols to avoid the use of intravenous gadolinium.

17:17
**Panel discussion: Do we really need gadolinium-enhanced sequences?**

**16:00 - 17:30 Room E1**

### Special Focus Session

### SF 8e

### Breast imaging in pre-menopausal women

**A-0424** 16:00
**Chairperson's introduction**
I. Thomassin-Naggara; *Paris/FR (isabelle.thomassin@tnn.aphp.fr)*

This session will present the physiological changes and benign breast diseases and highlight the challenges and opportunities of breast MR imaging in pre-menopausal women. The objective is to provide an overview of how to manage the enhancement of parenchyma. Then, will be discussed the place of breast screening in pre-menopausal women and present the risk factors that define groups of women that would benefit a breast screening the balance between risk and benefit of radiation dose. Finally, an overview of breast diseases associated with pregnancy will be done in order to learn which imaging techniques could be used to investigate pregnancy-associated breast lesions and breast cancer.
***Session Objectives:***
1. To highlight the different pathologies we may encounter in pre-menopausal women.
2. To present controversies about screening in pre-menopausal women.
3. To become familiar with breast diseases associated with pregnancy.
*Author Disclosure:*
**I. Thomassin-Naggara:** Advisory Board; SIEMENS. Speaker; GE, Samsung, Canon, Guerbet.

**A-0425** 16:05
**Physiological changes and benign breast diseases in young women**
F. Kilburn-Toppin; *Cambridge/UK*

Breast imaging in pre-menopausal women presents particular challenges for the radiologist. Benign breast disorders are much more common in this age group and are multifactorial including genetics and hormone-related causes. Diagnosis of benign disorders is important because they may be accompanied by clinical symptoms, but also to exclude malignancy and lesions of increased risk of cancer which may mimic benign breast disease on imaging. High mammographic density in premenopausal women makes sensitivity limited, and furthermore, the risk of radiation-induced malignancy is much higher in the young glandular breast than beyond the age of 40. Similarly, on ultrasound, a higher proportion of hyperechoic glandular tissue versus hypoechoic fatty tissue can provide a challenge to the interpreting clinician. A limitation of breast MRI in pre-menopausal women is that hormonal fluctuations during the menstrual cycle affect gadolinium uptake in normal breast tissue, which can make MRI examinations challenging to interpret due to false-positive findings, or risk masking malignancy. Studies have suggested performing scans during the follicular phase (day 3-14) to minimise tissue enhancement, and for this reason scheduling of the MRI examination in pre-menopausal women is recommended during the second week of the menstrual cycle. Particular patterns and degree of non-mass enhancement can be associated with benign background parenchymal enhancement, but repeat short interval imaging has been advocated in some circumstances if there is diagnostic uncertainty. Further challenges occur in premenopausal women who lack normal cyclical menses.
***Learning Objectives:***
1. To present the physiological changes and benign breast diseases.
2. To highlight the challenges and opportunities of breast MR imaging in pre-menopausal women.
3. To provide an overview on how to manage enhancement of parenchyma.

**A-0426** 16:30
**Breast cancer screening in pre-menopausal women**
P. Clauser; *Vienna/AT (clauser.p@hotmail.it)*

The risk of developing breast cancer increases with age, and approximately 5% of breast cancers appear before the age of 40. Cancers in young women are often characterised by more aggressive behaviour and a higher recurrence rate. Women with breast cancer below 40 often have a positive family history or mutations. Imaging findings in young women are not significantly different from those in older women. Currently, many programs begin screening mammography at the age of 50, despite the evidence that screening can reduce mortality between 40 and 50 years. In women below 40, cancer detection with mammography is lower, and the risk of false positives is increased. This is related to the lower cancer incidence and the higher breast density, which reduces the sensitivity of mammography. Tomosynthesis can improve cancer detection in women above 40, but due to the use of radiation and the suboptimal accuracy in very dense breasts, it might not be the best screening modality below 40. Ultrasound or MRI have a higher sensitivity in dense breast and could be used for early detection in young women. The costs of the examinations, the number of false positives and the need for a contrast agent make the introduction of these techniques in screening challenging. To optimise early detection, women could be tested for breast cancer risk at an early age, and screening could be optimised based on personal risk. Personalised screening is needed in the follow-up of women after pre-menopausal breast cancer.

***Learning Objectives:***

1. To discuss the place of breast screening in pre-menopausal women.
2. To present the risk factors that define groups of women that would benefit a breast screening.
3. To evaluate the balance between risk and benefit of radiation dose.
4. To learn about the value of tomosynthesis with synth2DMG and MRI in this group of women.

*Author Disclosure:*

**P. Clauser:** Speaker; Invited talks for Siemens Healthcare.

**A-0427** 16:55

**Pregnancy-associated breast cancer (PABC)**

C. Van Ongeval; *Leuven/BE (chantal.vanongeval@uz.kuleuven.ac.be)*

The incidence of PABC is estimated at 1:1000 to 2000 pregnancies. Initially, pregnancy-associated breast cancer (PABC) was defined as breast cancer during pregnancy or within 1 to 2 years postpartum. Because of different physiology and treatment, more appropriate definitions are: BC diagnosed during pregnancy and BC diagnosed in the postpartum period. Correct staging is essential in the selection of treatment. BCP women are almost always present with a palpable mass. Physiological changes of the breast during pregnancy may cause the patient's and doctor's delay. The differential diagnosis ranges from benign lesions (lactating adenoma, galactocele, abscess, cystic disease, fibroadenoma) to malignant epithelial BC and sarcoma or lymphoma. Local staging starts with ultrasound (US) and additional core-needle biopsy of all US abnormalities (distortion, heterogeneity of parenchyma, mass lesion). Additional evaluation of the axilla is mandatory. The physiological changes of the breast composition and lymph nodes during pregnancy and breastfeeding will be discussed. The rate of complication of CNB is low. In case of a histologically proven malignancy, to find additional information on DCIS related microcalcifications, mammography (2 views) is mandatory: radiation safety rules will be described. The sensitivity of MRI during pregnancy may be compromised by physiological changes, but also the prone position may be difficult if pregnancy is farther along. The rules of admission of intermediate and lowest risk gadolinium contrast media are described in the ESUR-contrast-medium-safety protocol. An alternative for Gd-admission is the use of diffusion-weighted(DWI) breast imaging. For systemic staging, whole-body-DWI MRI seems very promising.

***Learning Objectives:***

1. To become familiar with breast diseases associated with pregnancy.
2. To recognise specific characteristics of pregnancy-associated breast cancer.
3. To learn which imaging techniques could be used to investigate pregnancy-associated breast lesions and breast cancer.

17:20

**Panel discussion: At what age should we start to screen women for breast cancer?**

**16:00 - 17:30 Room E2**

### Neuro

### RC 811

### State-of-the-art paediatric neuroradiology

**A-0428** 16:00

**Chairperson's introduction**

C. Calli; *Izmir/TR*

***Session Objectives:***

1. To learn the spectrum of applications and indications for imaging in paediatric neuroradiology.
2. To understand the relevance in order to apply the appropriate image acquisition protocols of disease in the field.
3. To appreciate the clinical relevance of imaging for the diagnostic process of neurological disorders in infants and children.

**A-0429** 16:05

**A. Imaging myelin maturation disorders**

N. Wolf; *Amsterdam/NL*

"no abstract submitted"

***Learning Objectives:***

1. To learn about normal and pathological patterns of myelination.
2. To understand the role of imaging with respect to narrowing the differential diagnosis and supporting the clinical diagnosis.
3. To appreciate the importance of pattern recognition for the diagnosis of myelination disorders in children.

**A-0430** 16:28

**B. Imaging of developmental disorders**

B. Ertl-Wagner; *Toronto, ON/CA (BirgitBetina.Ertl-Wagner@sickkids.ca)*

To understand congenital abnormalities of the brain, it is important to be familiar with the embryologic development. Neuronal proliferation, migration and cortical organisation are important steps in the development of the cortex. Group I disorders of cortical development are disorders of neuronal and/or glial apoptosis or proliferation. Among these are congenital microcephalies (I.A), congenital megalencephalies (I.B), and diffuse or focal cortical dysgenesis or dysplasia (I.C). Microlissencephaly is characterised by a reduced gyration and microcephaly. Hemimegalencephaly is a hamartomatous overgrowth of one cerebral hemisphere or parts thereof. Group II disorders are disorders of neuronal migration. Among these are periventricular (subependymal) heterotopia (II.A), lissencephalies (II.B), focal subcortical heterotopia (II.C), or disorders of terminal migration, e.g. cobblestone lissencephalies (II.D). Heterotopia are defined as areas of grey matter in an ectopic location; they are isointense to the cortex. Group III disorders are disorders of postmigrational development. Among these is polymicrogyria with schizencephaly (III.A), polymicrogyria without clefts or calcifications (III.B), focal cortical dysplasia (III.C), or postmigrational microcephaly. In schizencephaly, there is a cleft that extends from the ependymal to the cortical surface, and that is lined by dysplastic grey matter. In polymicrogyria, there are too many too small gyri and sulci and an irregular grey matter-white matter interface. Important infratentorial congenital abnormalities include Chiari malformations, cystic abnormalities of the posterior fossa / the Dandy Walker spectrum, molar tooth malformations/ Joubert syndrome and dysplastic cerebellar gangliocytoma/Lhermitte-Duclos syndrome.

***Learning Objectives:***

1. To learn about the spectrum of developmental disorders of the brain.
2. To understand the key imaging features that lead to the correct diagnosis.
3. To appreciate the importance of making a correct imaging diagnosis.

**A-0431** 16:51

**C. Imaging in paediatric neuro-oncology**

G. Morana; *Genoa/IT (giovannimorana@gaslini.org)*

Central nervous system tumours are the most common solid neoplasms in children and encompass a wide spectrum of heterogeneous neoplasms, each with its own biology, prognosis, and treatment. Paediatric brain tumours may share similarities with their corresponding adult variants, but incidence rates, locations, molecular and genetic features, and even certain histopathological characteristics are often different and thus may be considered biologically distinct entities. Conventional MRI represents the backbone of brain tumour identification and characterisation but has limitations in distinguishing tumours from tumour mimics, defining tumour grade, evaluating treatment response and predicting patient outcome. Advanced MRI modalities, such as diffusion-weighted imaging, perfusion weighted imaging and magnetic resonance spectroscopy have improved our understanding of brain tumours, overcoming some of the main limitations of conventional MRI and providing additional microstructural, hemodynamic and metabolic information of brain tumours. Beyond MRI, metabolic imaging with PET provides further insights into a tumour biological activity; indeed, PET imaging combined with MRI, either by off-line co-registration or via hybrid PET/MR systems, is emerging as a valuable imaging modality not competing with but rather complementing MRI. Depending on the radiotracer used, different molecular processes can be assessed, and a growing body of evidence supports the promising role of radiopharmaceuticals that target amino-acid transport. The contribution of these methods to the evaluation of paediatric brain tumours is the focus of the present work.

***Learning Objectives:***

1. To learn about the spectrum of neuro-oncological diseases in children.
2. To understand the role of imaging beyond the diagnostic process.
3. To appreciate the increasing clinical relevance of advanced imaging techniques in paediatric neuro-oncology.

17:14

**Panel discussion: Ask the expert: what is relevant for my own daily clinical practice?**

**16:00 - 17:30** **Room F1**

### E³ - Rising Stars Programme: Basic Session

### BS 8

### Renal, adrenal and urinary tract pathologies

*Moderator:*
C.S. Balleyguier; Villejuif/FR

**A-0432** 16:00
**Renal pathologies**
H.C. Thoeny; *Fribourg/CH (Harriet.thoeny@h-fr.ch)*

The most common renal pathologies are focal renal masses (cystic or solid) and are incidentally detected on cross-sectional imaging during the workup of other pathologies. The main task of the radiologist is to categorise these lesions: cystic lesions are stratified according to the Bosniak classification, whereas solid renal masses often correspond to renal cell carcinomas; angiomyolipomas and oncocytomas as benign lesions are less frequent and sometimes show typical imaging findings. Unenhanced CT is the method of choice for the detection of renal calculi and allows to provide information on the size, location and composition of the stone as well as evaluation of upper urinary tract dilatation. Imaging to detect upper urinary tract infection is only performed in case of a search for complications such as abscesses, renal vein thrombosis and pyonephrosis needing intervention. In this context, MRI including diffusion-weighted sequences allows differentiating an abscess from a complicated cyst or a cystic renal cell carcinoma. In addition, imaging of the kidneys is also performed to detect obstruction due to various underlying diseases including intrinsic and extrinsic ureteral pathologies. For the evaluation of most renal pathologies, CT is the method of choice. In pregnant woman and children, ultrasound is preferred, whereas MRI can be helpful as a problem-solving tool to better characterise focal renal masses, to detect diffuse renal pathologies or assess pyelonephritis noninvasively, e.g. in young and/or pregnant woman or children.
***Learning Objectives:***
1. To review the most common pathologies.
2. To present current imaging techniques for evaluation of renal pathologies.
3. To demonstrate the most important imaging findings of the common renal pathologies.

**A-0433** 16:30
**Adrenal pathologies**
M. Stajgis; *Poznan/PL (stajgis@gmail.com)*

Adrenal glands can be affected by a variety of lesions. Still growing number of cross-sectional imaging worldwide has led to an increase in the percentage of incidentally discovered nodules in adrenals. According to the recent data, adrenal masses are seen up to 5-6 % of all abdominal CT examinations, more often in the older population. Therefore a strategy for determining the characteristics of these nodules at possible low cost and high effectiveness is mandatory. The role of diagnostic imaging is to differentiate benign from malignant lesions. Ultrasonography has still the role in the incidental detection of adrenal masses and in monitoring the changes in morphology and size of lesions during treatment or simple observation. CT is recognised as ‘gold standard’ method in evaluation of adrenal pathologies. Due to CT capability in the estimation of the quantity of fat in the defined volume, it is easy to recognise lipid-rich and lipid-poor adenomas, which represent the largest group of adrenal pathology. Multiphase CT protocols in adrenal imaging and some specific calculations based on these techniques will be presented. MR has limited indications in adrenal imaging, this method does not offer additional spatial resolution and is mainly used in patients when iodine contrast media administration is contraindicated. The simple diagnostic imaging algorithm will be presented at the end of the lecture. Some open questions will be addressed to the audience.
***Learning Objectives:***
1. To review the most common pathologies.
2. To present current imaging techniques for evaluation of adrenal pathologies.
3. To demonstrate the most important imaging findings of the common adrenal pathologies.

**A-0434** 17:00
**Ureter and bladder**
M.N. Özmen; *Ankara/TR (mozmen@hacettepe.edu.tr)*

Imaging has a very important role in evaluating the urinary tract. Over the past years, computed tomographic urography (CTU) has become the main imaging modality for this purpose in various clinical settings. Dose reduction techniques have also enabled to obtain CTU’s with very low radiation doses. Several anomalies of the ureter, the muscular conduit between the renal collecting system and bladder, may be seen, including duplication, muscular dysfunction (UPJ-stenosis, primary megaureter) or abnormal termination (ectopic ureteral orifice, ureterocele, vesicoureteral reflux). Intraluminal disease process in ureter might cause obstruction. Ureteral stone is by far the most frequent, with high attenuation values on unenhanced CT and independent of the chemical composition. Size of the stone is predictive of the likelihood of spontaneous elimination. A filling defect is seen on CTU usually raises the suspicion of urothelial neoplasm. Other entities such as a clot, metastasis, pyeloureteritis cystica, and tuberculosis might also (partially) obstruct the ureter and must be considered in differential diagnosis in selected patients. Urothelial cancer has to be considered as a ‘systemic’ disease, with often multiple, synchronous or metachronous lesions. CTU not only depicts the neoplastic lesion but also enables the examination of entire urothelium during staging or follow-up. Several treatment options are available and must be patient-tailored. In the case of hematuria, where CTU is the main diagnostic tool for upper urinary tract evaluation, cystoscopy is the primary diagnostic tool for screening for bladder neoplasms.
***Learning Objectives:***
1. To review the most common pathologies.
2. To present current imaging techniques for evaluation of ureter and bladder.
3. To demonstrate the most important imaging findings of the common ureter and bladder pathologies.

**16:00 - 17:30** **Room F2**

### Emergency Imaging

### RC 817

### Blunt polytrauma: CT protocols, CT interpretation and interventional radiology options

**A-0435** 16:00
**Chairperson's introduction**
K.H. Nieboer; *Brussels/BE (koenraad.nieboer@uzbrussel.be)*

Blunt polytrauma patients. As radiologists, we want the highest standards on imaging, interpretation and treatment for this subgroup. But do we agree on the most optimal CT imaging protocol? Is there a “one scan fits it all whole-body CT scan protocol” solution, or can we use clinical parameters to create subgroup WBCT scan protocols? How do we describe and score solid organ injuries? Do we pay enough attention to intraparenchymatous non-vascular and vascular lesions? How can we optimise our screening technique for these injuries? Who do we call? The surgeons or our interventional radiology colleagues? What is the actual place of IR in the polytrauma setting, what is established and what is new? In this refresher course, we will highlight these questions.
***Session Objectives:***
1. To understand the different CT and contrast protocols that can be performed in blunt polytrauma patients and when to apply them.
2. To be able to quickly identify and classify solid organ injuries.
3. To recognise injuries that can be treated with interventional radiology.
*Author Disclosure:*
**K.H. Nieboer:** Speaker; GE Healthcare.

**A-0436** 16:05
**A. CT protocols in blunt polytrauma**
E. Kashef; *London/UK (elikadoc@icloud.com)*

Trauma is a global pandemic with a high incidence of disability and death. Whole-body Multi-detector Computed Tomography (MDCT) is now the default imaging of choice in the severely injured patient with demonstrable results that early use improves survival. The CT imaging protocol usually includes two separate “dual-phase” arterial and porto-venous phases of the Chest, abdomen and pelvis. Some centres perform an unenhanced CAP CT first, which is becoming less commonplace due to the delay to treatment with little clinical yield. At our centre, patients with blunt or major trauma undergo single-pass biphasic or “combi” CT imaging. However, patients with penetrating injuries or major haemorrhage protocol activation continue to undergo the classic dual-phase imaging. The standard Dual-phase and Combi scan can diagnose or exclude solid organ injury including, liver, spleen, pancreas, kidney injuries as well as hollow viscous injuries which will be shown at this lecture. Imaging in urogenital and Neck injuries require extension or modification of the classic protocols, which can aid in the diagnosis of supra-aortic or non-vascular renal injuries. Follow up imaging in solid organ injury is equally important as delayed bleeding and pseudoaneurysm formation are associated with significant mortality. By integrating radiology into the ABCDE approach of trauma care, we can ensure the correct protocol and imaging pathway is personalised per trauma case while following the most optimal protocol.
***Learning Objectives:***
1. To understand the advantages and disadvantages of different CT protocols adopted in blunt polytrauma patients.
2. To learn the goal in the use of single and dual phase contrast injection protocols in trauma.
3. To become able to incorporate the most suitable CT protocol in defined clinical scenarios.

**A-0437** 16:30
**B. Solid organs injuries: a tailored approach**
M. Patlas; *Hamilton, ON/CA (patlas@HHSC.CA)*

Multi-detector computed tomography (MDCT) is the modality of choice for the comprehensive imaging evaluation of solid organ injuries in clinically stable patients with blunt abdominal trauma. In spite of the high sensitivity of MDCT for detection of vascular injuries in solid organs, in trauma centre audits, the disproportionate number of misses resulting in mortality (a quarter of patients) are caused by delayed intervention for haemorrhage. The presentation will focus on the critical imaging features of blunt solid organ injuries, particularly vascular injuries (haemorrhage, pseudoaneurysms, and arteriovenous fistulae) of solid organs. The presentation will also review numerous challenges in accurate prospective MDCT detection of blunt pancreatic injuries. The role of emergency/trauma radiologist in time management and proper communication of these complex injuries will be elucidated. The misses and misinterpretations related to suboptimal technique and inadequate follow up of critical solid organ injuries will be highlighted. The scenarios requiring interventional radiology and /or surgical intervention will be discussed. Recent radiological and surgical literature will be reviewed.
***Learning Objectives:***
1. To learn about the main traumatic injuries to be detected in solid organs.
2. To understand which crucial findings will change patient management.
3. To understand what surgical and intensive care teams need to know about detected injuries.

**A-0438** 16:55
**C. Interventional radiology in trauma: diagnosis and management**
T. Jargiello; *Lublin/PL (tojarg@interia.pl)*

Blunt trauma may affect any part of the human body. It is devastating because of high kinetic energy usually evoked by motor vehicle accidents or fall from elevations. The most serious conditions are blunt injuries of abdominal solid organs and great vessels. When we consider solid organ blunt trauma we think of the liver, spleen and kidneys. Frequent venous injuries of the liver are usually treated conservatively when stable, and they need surgery in unstable patients. Arterial hepatic trauma is subjected to embolisation. Management of splenic injuries is controversial, but surgeons operate most of them. Renal injuries are less common but when active extravasation happens endovascular therapy it the first-line treatment. Among great vessels, the aorta is the most frequently injured. A spectrum of injury includes aortic transection, false aneurysm and less common dissection. Majority of these injuries are located at isthmus. Aortic root or descending aortic injuries are less frequent as well as iliac or mesenteric transsections. Aortic stent-grafts or covered stents are a very good solution in these cases. Conversely, venous interventions (especially IVC rupture) usually need immediate surgery. Traditionally, endovascular methods were offered to hemodynamically stable patients. Now the role of IR increased greatly, and there are lots of encouraging data of unstable patients treatment. Minimally invasive embolisation and stent-grafting have much to offer as the definitive treatment or as an initial adjunct before surgery. The use of hybrid rooms giving both methods of treatment at the same time is possibly the best choice.
***Learning Objectives:***
1. To learn which vascular injuries in solid organs can be treated with interventional radiology.
2. To become familiar with the imaging findings of great vessel injuries.
3. To understand how vascular injuries can be treated.

17:20
**Panel discussion: What is the best CT protocol in the evaluation of blunt trauma patients?**

**16:00 - 17:30 Room Y**

### Joint Session of the EFRS and EORTC

### EFRS/EORTC

### Radiographers: at the heart of cancer treatment and research

*Moderators:*
C. Beardmore; London/UK
M. Smits; Rotterdam/NL

**A-0439** 16:00
**An overview of the roles of radiographers in research**
J. McNulty; *Dublin/IE (jonathan.mcnulty@ucd.ie)*

Research and evidence-based practice (EBP) underpin modern healthcare and can lead to enhanced patient safety, improved patient outcomes, and efficiencies in service delivery. The contribution of radiographers, academic and clinical, to this evidence through undertaking quality research, and subsequent dissemination is essential and will also serve to raise the profile and standing of radiographers beyond our profession. This applies equally to all three recognised branches of the profession: medical imaging, nuclear medicine, and radiotherapy. Radiographers are much more than facilitators, or data providers, in medical imaging research and some of the leading radiography researchers in the world can be found in Europe. These individuals lead large research groups, successfully compete for national and international research funding, collaborate beyond their profession, and publish in high-impact journals. In 2016, the European Federation of Radiographer Societies (EFRS) published a Statement on Radiography Research which clearly sets out the EFRS position on encouraging, supporting and developing high-quality radiographer-led research in order to strengthen our professional knowledge base. This statement, together with the 2015 Statement on Evidence-Based Practice in the Undergraduate Curriculum, and the European Qualifications Framework (EQF) Level 6(Bachelors) and Level 7(Masters) Benchmarking documents for radiographers, clearly set-out the importance of clear research focus in educational programmes. Radiographers can add value at all stages of the research process and for medical imaging or radiation oncology research to have a true impact and to benefit our patients it must be inclusive and multidisciplinary and span the academic-clinical divide.
***Learning Objectives:***
1. To understand how professional development is supporting the development of radiographer research roles.
2. To consider the opportunities for radiographer research.
3. To gain an insight into the impact of radiographer research in improving patient outcomes.

**A-0440** 16:20
**Establishing the scientific and clinical value of advanced imaging**
M. Smits; *Rotterdam/NL (marion.smits@erasmusmc.nl)*

In this presentation, glioma is used to exemplify the current challenges on oncological imaging. Gliomas constitute a group of tumours with heterogeneous clinical behaviour, with many therapeutic agents under investigation for their effectiveness. The assessment of treatment effect is not without difficulty. It is on the one hand hampered by therapy-related changes which can be indistinguishable from tumour progressions, such as pseudoprogression or radiation necrosis; and on the other hand by a phenomenon called pseudoresponse, occurring in the context of anti-angiogenic treatment, in which contrast enhancement diminishes unrelated to an actual anti-tumoural effect. New, preferably quantitative, markers of response are desperately needed to assess treatment response as accurately and early as possible within the context of a clinical trial. Furthermore, recent insights indicate that the clinical heterogeneity of glioma behaviour can - at least in part - be attributed to the tumour genotype. In the context of clinical trials, it is adamant that patients are correctly stratified according to their tumour genotype, which at this point in time can only be determined from tumour tissue acquired through biopsy/surgery. To avoid such invasive procedures, as well as to obtain a full overview of the tumour and its heterogeneity, and to be able to follow changes over time, advanced imaging can play an important role using a radiogenomic approach. In this presentation, I will discuss the current and potential imaging markers of glioma biological behaviour and response to treatment, and the challenges for implementation in clinical, multicentre trials.

*Learning Objectives:*
1. To understand the importance of imaging in supporting oncology research.
2. To learn about the range of trials evaluating imaging techniques.
3. To appreciate the importance of team working in enabling effective research.

*Author Disclosure:*
**M. Smits:** Other; Independent reviewer (EORTC-1410) | Parexel Intl. Corp. (financial compensation paid to institution).

**A-0441** 16:40
**Fostering excellence in radiation oncology research through a clinical research culture**
H. McNair; *London/UK (Helen.McNair@rmh.nhs.uk)*

Research provides the evidence base for good clinical practice and is an essential component of any health care delivery service. However, conducting research in the clinic by radiography and radiotherapy staff can be difficult. Few research mentors may be available because of the lack of clinical academic positions. Clinical service takes priority when there are staff and time pressure. To create a research culture within these constraints is often difficult but not unachievable. Clinical staff are best placed to pose and answer research questions around the patient pathway and patient technology interface. The first step is to define a research question, consider what needs to be measured and the tools required to measure. The research method may be an audit, service evaluation or trial. Audits are extremely useful tools and are easily accessible to clinical staff. BSc(Hons) educated radiographers, having developed their research skills in training should be ready to engage in research on qualification. Postgraduate courses can also be a driver. Patient and public input (PPI) from idea conception to the dissemination of results is essential. PPI gives insight into the research question from the patient perspective and is essential when developing patient information sheets. To apply for funding output is critical, posters, presentations and papers. Finally, examine the academic pathways of doctors, physicists and other allied health professionals to determine if a similar academic career path can be created for radiography. Radiographers are an essential part of research activity.

*Learning Objectives:*
1. To understand the barriers and enablers which impact upon delivering effective research.
2. To learn how clinical departments can utilise the resources already available with the goal of developing a more confident and competent clinician-researcher culture.
3. To appreciate the importance of patient and professional input into trial design.

**A-0442** 17:00
**Radiation therapy quality assurance (RTQA) in clinical trials: the role of the radiographer/radiation therapy technologist**
M. van Os; *Rotterdam/NL (m.j.h.vanos@erasmusmc.nl)*

The European Organisation for Research and Treatment of Cancer (EORTC) is involved in many multicentre trials in Radiotherapy. It is mandatory that included patients are treated similarly in all internationally participating institutes, and that outcome of the treatment is consistent for analysis. Therefore quality assurance per trial is required checking the equipment and procedures per participating institute. Within the Radiation Oncology Group, this task is assigned to the multidisciplinary RTQA-team consisting of radiation oncologists, physicists and radiotherapy technologists (RTTs). While overall survival or progression free survival are common endpoints to many trials, secondary endpoints often include toxicity. Since radiation induced toxicity is related to delineated volumes, it is of importance that the outlining of the organs at risk (OAR) is performed consistently enabling to ascribe differences in outcome to treatment and discrepancies in OAR delineation. It is also important to be able to disentangle radiation-induced from drug-induced toxicities. However, delineation has proven to show large interobserver variability and especially in delineation of OAR there often is no consensus on clear guidelines. While increasingly more RTTs are involved in the process of treatment planning and volume delineation, the RTT subcommittee initiated the RTQA Delineation Project aiming to develop clear guidelines for OAR delineation in EORTC trials. In this presentation, I will describe the role of RTTs within the EORTC RTQA-group initiating a delineation study on OAR in the male pelvic area and consequently analysing and improving volume definition during two EORTC trials by means of questionnaires and delineation workshops.

*Learning Objectives:*
1. To understand the essential role of the radiographer/radiation therapy technologist in RTQA.
2. To appreciate the potential impact of poor RTQA practices on clinical trials.
3. To consider best practice in multi-centre RTQA for clinical trials.

17:20
**Panel discussion: Preparing our departments for high quality clinical trials**

**16:00 - 17:30 Room D**

### Musculoskeletal

### RC 810

### MR imaging of the knee

*Moderator:*
M. Tzalonikou; Athens/GR

**A-0443** 16:00
**A. Cruciate ligaments: what to know and what to do**
A. Alcalá-Galiano; *Madrid/ES (aalcalagaliano@gmail.com)*

Cruciate ligaments play a key role in knee kinematics and stability. Cruciate-deficient knees are predisposed to early osteoarthritis due to chronic instability. ACL is the most commonly injured major ligament of the knee. PCL lesions are far less common, but more frequently associated with multiligamentous injuries. MRI is the study of choice to evaluate acutely and chronically injured cruciate ligaments, associated injuries and for postoperative imaging following ligament reconstruction. Understanding the variations of their anatomy (double bundle configuration and insertion sites) is key to performing a tailored anatomic reconstruction. Accurate description and grading of cruciate tears and associated injuries are essential for preoperative planning of ligament reconstruction. Primary and secondary signs and indirect imaging findings of acute and chronic ligament injury will be reviewed. Atypical findings and imaging pitfalls will also be discussed. ACL reconstruction is one of the most common orthopaedic procedures, while PCL reconstruction is on the rise. Knowledge of ligament reconstruction techniques allows differentiation of normal postoperative findings from complications, which may be related to graft placement, the graft itself or the donor site. Any standard knee MRI protocol must ensure adequate detection of both soft tissue and bony injury, including the cruciate ligaments. However, protocols may be tailored for specific needs, including oblique acquisitions, 3D or metal artefact reduction sequences to better demonstrate the anatomy and facilitate detection of small tears.

*Learning Objectives:*
1. To describe the normal anatomy and MR imaging appearances of ACL and PCL and discuss pitfalls.
2. To explain the imaging appearances of ACL and PCL pathology and discuss imaging sequences and protocols.
3. To discuss the imaging sequences and protocols relevant to ACL and PCL conditions.

**A-0444** 16:30
**B. Meniscal tears: obvious and subtle**
P. Omoumi; *Lausanne/CH (patrick.omoumi@chuv.ch)*

This lecture will review the basic aspects of MRI of meniscal pathology, including some elementary technical and anatomical considerations, as well as the description of the basic semiology of meniscal tears and their classification. Common pitfalls and errors that need to be avoided will be presented.MRI is the modality of choice for the diagnosis of meniscal pathology as well as treatment planning. A certain number of technical considerations need to be understood by the radiologist. The standard sequence to image the meniscus is intermediate-weighted fast spin-echo sequences, in 2D or 3D. Certain acquisitions parameters may influence the diagnosis of meniscal tears, including the choice of the echo time. Meniscal tears can manifest either as morphological changes or intrameniscal signal changes, with specific diagnostic criteria that will be reviewed. An appropriate terminology, based on standard arthroscopic classifications, needs to be used by the radiologist in order to properly communicate the description of the tear to the referring surgeon. Secondary signs of meniscal tears, including parameniscal cyst and meniscal extrusion, should be used to increase the diagnostic performance for the presence of meniscal tears. Common pitfalls include anatomical variants (i.e. inter-meniscal meniscal and menisco-femoral ligaments, discoid meniscus), as well as other causes for false positive findings such as meniscal flounce, CPPD and meniscal ossicles. Knowledge of common mistakes and patterns of injury helps avoid unnecessary mistakes and improve diagnostic accuracy.

*Learning Objectives:*
1. To describe the anatomy of the menisci and the classification of meniscal tears.
2. To explain unusual imaging appearances of meniscal tears and potential pitfalls.

**A-0445** 17:00
**C. Looking around the corners: posteromedial and posterolateral**
U. Aydingoz; *Ankara/TR (uaydingo@hacettepe.edu.tr)*

Posteromedial and posterolateral corners of the knee (PMC, PLC) can be injured along with anterior (and, less commonly, posterior) cruciate ligament tears. If an injury to the PMC and/or PLC is not properly addressed, cruciate ligament surgery may fail. Although rare, isolated injuries to the PMC and PLC may also occur. Radiologists need to be familiar with the MR imaging appearances of the PMC and PLC structures, and their injury patterns. Semimembranosus is the principal stabiliser of the PMC, while posterior oblique ligament is the main PMC structure that needs repair or reconstruction after an injury to this corner. Popliteus tendon is the key structure that helps in identifying on MR imaging the arcuate and popliteofibular ligaments at the PLC.
***Learning Objectives:***
1. To describe the anatomical structures of the posteromedial and posterolateral corners of the knee.
2. To explain the imaging appearances of pathological conditions that affect the posteromedial and posterolateral corners of the knee.

**16:00 - 17:30** **Room G**

### Special Focus Session

### SF 8f
### The 3D printing lab from bench to bedside

**A-0446** 16:00
**Chairperson's introduction**
F. Kainberger; *Vienna/AT (franz.kainberger@meduniwien.ac.at)*

3D printing has been in use for medical applications since more than 20 years but is currently under strong technological development as the use of machine types for 3D printing labs has become more feasible with improvements in spatial resolution. Radiologists have to define the role of biomedical imaging within this interdisciplinary field, in which advances are mainly driven by surgeons and medical physicists. Current clinical applications of 3D printing are mainly in cardiovascular interventions, maxillofacial, orthopaedic and trauma surgery as well as in health economy. Major goals are the customising surgical devices, personalised surgical training with a focus on complex anatomic situations, and patient empowerment by explaining the procedures with a 3D print. Non-clinical applications are in classic anatomy and related disciplines for educational purposes. With 3D printing, the radiological workflow is changing dramatically, as it is moving from the traditional stepwise referral-report approach to a totally different device-oriented process. There are trends to centralise 3D printing which, on the other hand, have a high potential for personalising the radiological service. As such, 3D printing should be regarded as a holistic approach and substantially influences the personalisation of imaging diagnostics with a prognostic, a preventive and a participatory impact.
***Session Objectives:***
1. To understand the requirements for building a 3D printing lab.
2. To learn the assessment of the impact of 3D printed models for cardiovascular interventions.
3. To understand the value of customising surgical tools and prostheses and to inform the patient.
4. To become familiar with the management changes due to centralised 3D printing.

**A-0447** 16:05
**Creating a 3D printing lab in radiology**
F. Moscato; *Vienna/AT (francesco.moscato@meduniwien.ac.at)*

As 3D printing and its role in medicine receive greater attention, dedicated labs with this focus become more and more common. When planning such a 3D lab an understanding of the variety of different available principles, technologies, methods, and printed material properties is necessary. This technological understanding is complemented by the appreciation of the central role clinical imaging plays. In particular how image planning, resolution and overall image quality impact the whole process of 3D printing. Finally, creating a 3D printing lab takes on the challenges of combining and coordinating knowledge from different technical and clinical disciplines while at the same time guaranteeing strict adherence to medical regulations. In this talk, all these key aspects for the establishment of a 3D printing lab will be discussed drawing upon state-of-the-art knowledge, best practices and examples at our Institution.
***Learning Objectives:***
1. To learn the principles of 3D printing in terms of printer technology, methods, properties of printed material and other requirements when planning a 3D lab.
2. To appreciate and specify the role of radiologists in image planning during the 3D printing process.
3. To understand the correlation of spatial resolution and other image quality parameters among 3D printing, 3D and 2D visualisation.
*Author Disclosure:*
**F. Moscato:** Grant Recipient; Austrian Research Promotion Agency. M3dRES Project (nr. 858060).

**A-0448** 16:23
**Cardiovascular applications of 3D printing**
M. Tam; *Southend/UK (matthewtam2005@gmail.com)*

How to print your first vascular model? I will share personal experience into all steps of the printing process, from patient to model, using aortic aneurysms as cases. The various steps from image acquisition, DICOM editing, segmentation, the creation of a printable mesh or STL file, through to the final print are discussed. What is the current state of the art? A literature review and multi-source update into cardiovascular applications are offered. From bench to beside! Personal insights are offered on the potential value and limitations of embedding 3D printing into a hospital practice from different perspectives - that of a clinician, but also from business and entrepreneurial perspectives.
***Learning Objectives:***
1. To learn the potential applications for aortic and cardiac diseases to facilitate decision making.
2. To appreciate the impact of 3D models for interventional treatment planning.
3. To understand the value of 3D models for diagnosis and treatment in situations with complex anatomy.

**A-0449** 16:41
**Supporting the surgeon with 3D printing**
P. Brantner; *Basle/CH (philipp.brantner@usb.ch)*

3D Printing is becoming an increasingly relevant part of the surgical workflow. It has the potential to improve patient care, shorten operating time and reduce costs in surgical interventions. The lecture will introduce three main concepts with which 3D printing can help the surgeon: anatomical representation, virtual surgery and customised training models. While anatomical models will help surgeons to understand complex anatomy before surgery and allow to pre-contour plates, virtual surgery serves as a template to create patient-specific jigs and guides as well as patient-specific implants. 3D prints can also be used for training purposes allowing residents to become more proficient in common procedures while helping experienced surgeons to prepare for difficult cases. Specific examples will be shown for each concept illustrating how 3D printing can be a radiologic extension to improve patient outcome.
***Learning Objectives:***
1. To learn the standardised and the emerging surgical applications of 3D printing and their dependency on the anatomic complexity.
2. To appreciate the high potential in customizing and shortening the workflow of planning, pre-contouring and conducting surgery.
3. To understand the potential of 3D printing as an extension of diagnostic imaging with view on patient outcomes.

**A-0450** 16:59
**Challenges of centralised 3D printing**
K.A. Eley; *Cambridge/UK (Karen.a.eley@gmail.com)*

Three-dimensional (3D) printing continues to attract considerable attention in the medical community, fuelled by improvements in technology and an associated reduction in costs. However, capital investment for high-end 3D printers (which provide the accuracy required for complex anatomical shapes), combined with the annual maintenance and software licensing fees remain prohibitive for many hospitals, particularly those within the National Health Service (NHS). Housing 3D printing services within the hospital afford a number of advantages, including close communication during image preparation to ensure that the resultant 3D model meets the expectations of the requesting clinician. Whilst individual clinical departments have strived to acquire their own 3D printers. This is not the most cost-effective solution due to replication of costs, including the time commitment for segmentation and post-processing of 3D models. In an attempt to address these issues, we established a fully centralised 3D printing facility employing a full-time dedicated 3D technician. The interdisciplinary approach of centralisation results in a cross-pollination of ideas and provides a centre point of expertise benefitting all specialities across the hospital and wider community. However, centralisation is not without its challenges. The ever-tightening financial constraints of the NHS requires ongoing adaptation and innovation to ensure long-term sustainability.

***Learning Objectives:***
1. To learn the strategies behind a centralisation of 3D printing.
2. To appreciate the potential added value of sharing biomedical print files and other data related to 3D printing.
3. To understand the workflow changes and the potential of quality control and education in treatment planning with 3D printing support.

17:17
**Panel discussion: What are real advantages of 3D printing?**

**16:00 - 17:30 Room K**

### Special Focus Session

### SF 8b

### Challenging HRCT patterns: tips and tricks from the experts

**A-0451** 16:00
**Chairperson's introduction**
J. Babar; *Cambridge/UK (jude_babar@yahoo.co.uk)*

In this session, the focus will be on the central role of HRCT in investigating a broad spectrum of pulmonary diseases. The session aims at demonstrating the pathophysiological correlation of the principal imaging patterns encountered on CT, (airspace disease, nodules and reticulation), and the significance of these patterns in diagnostic decision-making, clinical outcomes and prognosis.
***Session Objectives:***
1. To understand HRCT patterns in relation to the anatomy of the secondary pulmonary lobule and small airways.
2. To revise the causes and pathologies of various HRCT patterns with case based examples and image interpretation pearls.
3. To revise how the pattern and distribution of disease helps in formulating a differential diagnosis.

**A-0452** 16:05
**The mosaic attenuation pattern**
M.-P. Revel; *Paris/FR (marie-pierre.revel@aphp.fr)*

The mosaic attenuation pattern is characterised by a patchwork of regions of decreased, normal and increased attenuation. If there is no asymmetry of vessels among the different regions, the mosaic attenuation pattern usually represents patchy interstitial disease, characterised by focal ground-glass opacity. In this situation, areas of increased attenuation represent the diseased lung whereas areas of lower attenuation represent the normal lung. When there is an asymmetry of vessels among areas of increased and decreased attenuation, this should be termed mosaic perfusion. There are two main causes of mosaic perfusion. The first one is occlusive vascular disease, during which there is a redistribution of the arterial pulmonary blood flow towards lung areas which remain patent. The areas of increased attenuation represent the preserved lung, showing more numerous vessels or vessels of larger size than hypodense areas, corresponding to occluded lung areas. The second cause of mosaic perfusion is an obstructive bronchial disease. There is vasoconstriction in lung areas with abnormal ventilation, causing the redistribution of the pulmonary arterial blood flow towards normally ventilated lung areas.
***Learning Objectives:***
1. To understand the pathophysiology of mosaic attenuation pattern.
2. To revise the common causes.
3. To discuss the common pitfalls in diagnosis.

**A-0453** 16:20
**Inter-and intralobular septal thickening**
N. Sverzellati; *Parma/IT*

A number of linear abnormalities may be evident on HRCT scans, including either interlobular septal lines and intralobular opacities. Interlobular septal thickening is the most diagnostically useful. Thickening of interlobular septa may be seen in conditions associated with dilatation of the pulmonary veins, infiltration of the pulmonary lymphatics, or with infiltration of the pulmonary interstitium by cells, fluid, or fibrosis. Thickened septa should be characterised as smooth, nodular, or irregular. Smooth interlobular septal thickening is commonly seen with pulmonary oedema and lymphangitic carcinomatosis, although nodular interlobular septal thickening is more characteristic of the latter disease. Intralobular interstitial thickening reflects infiltration and thickening of the interstitial framework of the secondary pulmonary lobule and may be caused by pulmonary fibrosis or inflammation in the absence of fibrosis. When underlying fibrosis is present, the reticulation often appears coarse, and traction bronchiolectasis and architectural distortion may also be seen.

***Learning Objectives:***
1. To understand the pathophysiology of this pattern.
2. To revise the common causes.

**A-0454** 16:35
**Approach to nodular pattern**
O. Hamer; *Regensburg/DE (okka.hamer@klinik.uni-regensburg.de)*

Multiple lung diseases present with multinodular densities as the dominant feature. The differential diagnosis can be challenging though is of utmost importance for guiding therapy. In order to determine the correct diagnosis, several aspects should be considered: First, the imaging modality of choice is HRCT. Second, nodule morphology in terms of size, margination and density has to be evaluated. Third, the distribution of nodules relative to the pulmonary lobule must be determined. Here the radiologist should be familiar with three different distribution patterns, namely centrilobular (with tree-in-bud as a subtype of centrilobular), perilymphatic and random. Fourth, collateral HRCT findings, clinical, functional and laboratory data have to be integrated into radiological considerations. When considering these aspects in most cases, a short list of differentials including the most likely diagnosis can be given. The talk will give a review of anatomic considerations regarding the pulmonary lobule. Differences in nodule morphology will be highlighted. A step-by-step analysis of HRCT images in order to determine nodule distribution will be presented. Differential diagnoses will be discussed.
***Learning Objectives:***
1. To understand the pathophysiology of this CT pattern.
2. To revise the common causes.
3. To discuss the common pitfalls in diagnosis.
*Author Disclosure:*
**O. Hamer:** Speaker; Roche, Böhringer-Ingelheim, Novartis, Astra-Zeneca.

**A-0455** 16:50
**Perilobular consolidation**
T. Frauenfelder; *Zurich/CH (thomas.frauenfelder@usz.ch)*

If you search with the search term "perilobular consolidation" in the PubMed, you will find exactly 11 publications. Anatomically the perilobular region is defined as the region bordering the periphery of the secondary lobulus. In the glossary of the Fleischner Society, the perilobular consolidation or pattern is characterised by the alteration of the structures that border the pulmonary lobules (i.e., interlobular septa, visceral pleura, and vessels). The appearance on HRCT is bowed or polygonal opacities with poorly defined margins around the interlobular septa, whereas the thickening is related to the activity of the disease. Perilobular opacities are accompanied by consolidation and/or GGO in the same region. The term has the close relation to organising pneumonia.
***Learning Objectives:***
1. To understand the pathophysiology of this CT pattern.
2. To revise the common causes.

**A-0456** 17:05
**Mixed ground-glass opacification and reticulation**
S.J. Copley; *London/UK*

"no abstract submitted"
***Learning Objectives:***
1. To understand the pathophysiology of this combined CT pattern.
2. To revise the common causes.
3. To discuss the common pitfalls in diagnosis.

17:20
**Questions and panel discussion: The reality of daily practice - what do experts struggle with on interpretation of HRCT?**

**16:00 - 17:30 Room M 1**

### Molecular Imaging

### RC 806

### Merging the best: hybrid imaging

*Moderator:*
G. Antoch; Düsseldorf/DE

**A-0457** 16:00
**A. Hybrid imaging with SPECT/CT**
A. Scarsbrook; *Leeds/UK (a.scarsbrook@nhs.net)*

Latest generation SPECT/CT cameras incorporate multi-detector CT and state-of-the-art gamma camera technology in tandem. These scanners improve the efficacy of a wide variety of nuclear medicine tests by providing more accurate localisation of lesions, exclusion of potentially misleading physiological uptake, characterisation of equivocal or indeterminate activity and detection of additional lesions. In addition, they offer the potential for a more efficient "one-stop-shop" imaging approach. Iterative reconstruction algorithms and faster processing power facilitate radiation dose reduction and increased image resolution. The clinical utility of SPECT/CT is diverse, and a cross-spectrum of applications in musculoskeletal, oncological, cardiological, endocrine, hepatobiliary and GI tract imaging will be presented.

***Learning Objectives:***
1. To learn the basic principles of hybrid SPECT/CT imaging.
2. To understand what complementary information can be provided by SPECT/CT.
3. To learn the clinical applications of SPECT/CT.

*Author Disclosure:*
**A. Scarsbrook:** Grant Recipient; Leeds Hospitals Charitable Foundation (Leeds Cares). Research/Grant Support; Blue Earth Diagnostics.

**A-0458** 16:30
**B. Hybrid imaging with PET/MRI**
F.M.A. Kiessling; *Aachen/DE (fkiessling@ukaachen.de)*

In this talk, an overview of the technological state of the art in PET-MRI, as well as an outlook on emerging new technologies, will be provided. In this context, there will be a brief explanation of new detector set-ups providing higher sensitivity and spatial resolution as well as methods to improve absorption correction and quantification. Furthermore, new PET-insert solutions for Preclinical Research and clinical application will be highlighted. An example of the latter, the concept and initial results for a new PET-insert dedicated to human Breast imaging will be presented. In the final part of the talk, the focus will be set on the medical applications of PET-MRI. In this context, it will be discussed, which applications inevitably demand PET-MRI hybrid imaging. Thus, with this talk, I will try to convince the audience of the high potential and clinical value of PET-MRI and its potential future role in patient management.

***Learning Objectives:***
1. To learn the basic principles of hybrid PET/MRI.
2. To understand what new information can be provided by PET/MRI.
3. To learn about emerging clinical applications of PET/MRI.

**A-0459** 17:00
**C. Hyperpolarised MRI**
F.A. Gallagher; *Cambridge/UK (fag1000@cam.ac.uk)*

There is increasing evidence to support a role for metabolism in tumour development; for example, deregulation of cellular energetics is considered to be one of the key hallmarks of cancer. Changes in tumour metabolism over time are now known to be early biomarkers of successful response to chemotherapy and radiotherapy. There are a number of imaging methods that have been used to probe cancer metabolism: the most widely available is 18F-fluorodeoxyglucose (FDG), an analogue of glucose, used in PET. Hyperpolarised carbon-13 MRI (13C-MRI) is an alternative and emerging molecular imaging method for studying cellular metabolism. This technique allows non-invasive measurements of tissue metabolism in real-time. To date, the most promising probe used in conjunction with hyperpolarised MRI has been 13C-labelled pyruvate: pyruvate is metabolised into lactate in normal tissue in the absence of oxygen, but in tumours, this occurs very rapidly even in the presence of oxygen. Results from many animal models have shown that there is a reduction in the metabolism of pyruvate following successful treatment with chemotherapy. Tumour lactate labelling has also been shown to correlate with the grade of some tumour types. There are now a small number of sites performing human hyperpolarised carbon-13 MRI imaging. This talk will discuss the progress that has been made in this field within the area of oncology and potential clinical applications.

***Learning Objectives:***
1. To learn the basic principles of hyperpolarisation.
2. To understand what new information can be provided by hyperpolarised MRI.
3. To learn about oncological and non-oncological applications of hyperpolarised MRI.

*Author Disclosure:*
**F.A. Gallagher:** Grant Recipient; GSK. Research/Grant Support; GE healthcare, Medimmune.

**16:00 - 17:30** **Room M 2**

### Paediatric

### RC 812

### Predicting fracture risk in children

**A-0460** 16:00
**Chairperson's introduction**
K. Halliday; *Nottingham/UK (kath.halliday@nuh.nhs.uk)*

Assessment of fracture risk in children and babies is of vital importance, not only in those children who are known to have issues with their bone health but also in cases of suspected physical abuse. Issues around bone density and strength often arise both clinically and in court and it is essential that any radiologist involved in the care of children is conversant with new developments in this rapidly changing field. This promises to be an excellent session with an overview of all available techniques.

***Session Objectives:***
1. To learn the diverse factors that may influence fracture risk in the paediatric population.
2. To understand the role of the various techniques in predicting fracture risk in children.
3. To discuss the limitations of the different techniques.

**A-0461** 16:05
**A. Radiographs and DXA**
R.A.J. Nievelstein; *Utrecht/NL*

Fractures are common in children. It is estimated that by the age of 16 years about 50% of boys and 30% of girls will have sustained a fracture, whereas 20% of children will have sustained 2 or more fractures. The major challenge of the clinician lies in the early detection of those children with skeletal pathologies related to an increased risk of fractures. Investigations of those children at risk are aimed to determine whether the child is suffering from an underlying bone fragility disorder and its aetiology in order to guide management. Besides laboratory investigations, radiology plays an important role in the diagnosis and follow up of these children, and Conventional Radiography (CR), as well as Dual-Energy X-Ray Absorptiometry (DXA), are often the imaging techniques of the first choice. This lecture will focus on the role of CR (including Digital X-ray Radiogrammetry (DXR) of the hand) and DXA in the assessment of bone health and prediction of fractures in children. Strengths and limitations of the different techniques will be discussed, and guidelines for its use in daily practice will be presented.

***Learning Objectives:***
1. To learn about the principles for the technique.
2. To understand the potential for use.
3. To discuss the limitations.

**A-0462** 16:23
**B. Quantitative ultrasound**
H.-J. Mentzel; *Jena/DE (hans-joachim.mentzel@med.uni-jena.de)*

Quantitative ultrasound (QUS) of bone is intended for identifying the children who could have an increased risk of osteoporosis in adulthood. QUS is a safe, portable, easy to use and cheap technology for the estimation of bone quality. Measurements can be performed on peripheral skeletal sites (calcaneus, tibia, phalanges), but not on the spine. Several parameters are used to estimate bone elasticity and stability, the speed of sound and broadband ultrasound attenuation. Using age and sex-matched reference values QUS Z-scores can be calculated in children. QUS Z-scores cannot be compared to the golden standard DXA scores. The number of false positives or negatives obtained with QUS depends on the cut-off value utilised. QUS may be a screening method at the moment and can be used to monitor patients previously diagnosed on osteoporosis.

***Learning Objectives:***
1. To learn about the principles for the technique.
2. To understand the potential for use.
3. To discuss the limitations.

*Author Disclosure:*
**H.-J.: Mentzel:** Consultant; Bayer Schering, Siemens. Equipment Support Recipient; Sunlight. Research/Grant Support; Bayer, Bracco, Novartis, Sunlight. Speaker; Bayer, Bracco, Novartis, Pfitzer. Other; Scientific societies (ECR, DRG, GPR, DEGUM, TGRN.

**A-0463** 16:41
**C. Bone quantification with MRI**
C. Rajapakse; *Philadelphia, PA/US*

Millions of people worldwide suffer from bone diseases, predisposing them to fractures and related comorbidities that have devastating consequences. Imaging plays an important role in fracture risk assessment, diagnosis, staging, and treatment monitoring of patients with bone diseases. In particular, the flexibility of MRI has paved the way for non-invasive assessment of bone quality at multiple levels, including trabecular and cortical bone. For example, it is now possible to assess many aspects of bone quality in patients using MRI, including trabecular bone microstructure, cortical bone porosity, bone water content, bone mineral density, marrow composition, and bone strength. Specific MRI pulse sequences used for bone imaging include SPGR, CSI, UTE/ZTE. Many of these new MRI techniques have been developed and applied for assessment of bone quality in adults. This lecture will discuss how pediatric bone imaging could potentially benefit from novel MRI methods.
***Learning Objectives:***
1. To learn about the principles for the technique.
2. To understand the potential for use.
3. To discuss the limitations.

**A-0464** 16:59
**D. CT (including HRpQCT)**
A.C. Offiah; *Sheffield/UK (amaka.offiah@nhs.net)*

The advantages of quantitative computed tomography (QCT) over the more commonly utilised dual-energy x-ray absorptiometry (DXA) include its ability to distinguish cortical from trabecular bone and its ability to assess the geometric properties of the bones using finite element (FE) engineering techniques. QCT using standard clinical scanners may be performed at central (spine) or peripheral (distal radius or tibia) sites. Radiation dose deters the use of central QCT as a primary tool for assessing bone health in children. The maximum resolution of peripheral QCT (pQCT) with standard scanners is approximately 600μm. Dedicated portable pQCT scanners exist, with a resolution of 200μm, and recently, high-resolution pQCT scanners (HRpQCT) have been developed with a resolution around 80μm. This presentation will review the techniques of QCT, pQCT and HRpQCT, comparing the advantages and disadvantages of each in the paediatric population. Using images from healthy children and those with osteopenic conditions for illustration, the talk will cover the parameters that can be determined, including finite element analysis and show how (or if) any of these parameters correlate with bone strength/fracture risk. A few minutes will be used at the end to demonstrate how CT can be used to develop FE models to assess the fracture mechanism. By the end of the talk, delegates will be in a position to develop an informed opinion as to whether CT can be used to predict fracture risk in children.
***Learning Objectives:***
1. To learn about the principles for the technique.
2. To understand the potential for use.
3. To discuss the limitations and disadvantages of this technique.
*Author Disclosure:*
**A.C. Offiah:** Advisory Board; Alexion, BioMarin. Author; Alexion, Pharmagenesis, Merio Biopharma. Consultant; BioMarin. Grant Recipient; Alexion. Speaker; Alexion, BioMarin, InfoMed.

17:17
**Panel discussion: Can we really predict fracture risk in children?**

**16:00 - 17:30** **Room M 3**

### Oncologic Imaging

### RC 816

### Multidisciplinary approach to cancer care

**A-0465** 16:00
**Chairperson's introduction**
R.H. Oyen; *Leuven/BE (Raymond.Oyen@uzleuven.be)*

The multidisciplinary team approach has become widely accepted in the care of cancer patients. Radiologists must be part of the team as an expert consultant in imaging, advising and/or performance of imaging-guided diagnostic and therapeutic procedures, and must contribute to outlining future imaging strategies in the individual patient. Preparation of the cases to be discussed, active participation and continuous education are essential prerequisites for radiologists to become/remain a respected partner of the team. Care for the individual patient is at least as important as care or adherence to guidelines or rules.

***Session Objectives:***
1. To learn about the role of the radiologist in the multidisciplinary team for soft tissue, colorectal and breast tumours.
2. To understand the relevant questions from the team and how they can be answered with imaging.
3. To review current multidisciplinary approaches and potential difficulties.

**A-0466** 16:05
**A. The soft tissues team**
E. Khristenko; *Heidelberg/DE (ekkasatkina@gmail.com)*

The role of radiologists is crucial in diagnosis and differentiation of malignant and benign soft tissue tumours. MRI is the most sensitive tool in local staging and assessment of resectability such as neurovascular encasement, contact between tumour, vessels and nerves. Regrettably, it's not always possible to make a correct assessment of malignancy grade by preoperative imaging, but radiological studies are the best tool for biopsy planning, for CT/US-guided biopsies and incisional/excisional biopsies. The most complicated issue in postsurgical follow up examinations is the ability to distinguish between local recurrence and postoperative or post-radiation changes, which can be very prominent and cause confusion. The important questions to radiologist are the differentiation between tumours and tumour mimics, as the next step to distinguish between benign and malignant tumours and the assessment of resectability. As neoadjuvant chemotherapy is increasingly used for patients with locally advanced and high-risk primary malignancies, also the role of MRI is emerging for response evaluation. The difficulty in the interpretation of imaging and histology in soft tissue tumours, wide selection of treatment modalities and the complexity and intensity of the treatment contribute to the need for systematic multidisciplinary team management. In our institution, we use a personalised, multidisciplinary approach with each case being reviewed on tumour board, which includes orthopaedic oncologist, tumour surgeon, oncologist, pathologist and MSK-radiologist. Plastic or vascular surgeons could be involved if needed. As treatment protocols may vary significantly depending on the correct interpretation of imaging, the role of a radiologist is critically important.
***Learning Objectives:***
1. To learn about the role of the radiologist in the evaluation, treatment planning and follow up of soft tissue tumours.
2. To understand the relevant questions from the team and how they can be answered with imaging.
3. To review current multidisciplinary approaches: how we do it in our institution.

**A-0467** 16:28
**B. The colorectal team**
R.G.H. Beets-Tan; *Amsterdam/NL*

"no abstract submitted"
***Learning Objectives:***
1. To learn about the role of the radiologist in the evaluation, treatment planning and follow up of colorectal tumours.
2. To understand the relevant questions from the team and how they can be answered with imaging.
3. To review current multidisciplinary approaches: how we do it in our institution.

**A-0468** 16:51
**C. The breast team**
C.K. Kuhl; *Aachen/DE (ckuhl@ukaachen.de)*

In breast cancer MDTs, the role of the radiologist is that of a powerful clinical partner. In many, if not most instances, the breast radiologist is the referring physician to the breast surgeon and will know the patient and her family, will have biopsied the patient and discussed the finding with her and will have localised the tumour to enable surgical removal. The radiologist helps with surgical treatment planning by demonstrating local disease extent or will help select patients for neoadjuvant chemotherapy, by providing biopsy of the cancer, or by providing evidence of systemic spread. The radiologist will assess response to neoadjuvant systemic treatment. In patients with therapy-refractory local recurrences or oligo-metastatic disease, the radiologist offers the full range of local percutaneous or trans-arterial treatment strategies to improve local control. With improving methods of radio-genomics, the radiologist will also provide predictive and prognostic information for treatment stratification.
***Learning Objectives:***
1. To learn about the role of the radiologist in the evaluation, treatment planning and follow up of breast tumours.
2. To understand the relevant questions from the team and how they can be answered with imaging.
3. To review current multidisciplinary approaches: how we do it in our institution.

17:14
**Panel discussion: Does the current multidisciplinary approach need changes?**

**16:00 - 17:30 Room M 4**

### E³ - ECR Academies: Chest Imaging

### E³ 822

### CT and intervention in vascular pulmonary diseases

**A-0469** 16:00
**Chairperson's introduction**
J.D. Dodd; *Dublin/IE*

Pulmonary vascular disease covers a wide spectrum of disease entities, many of which have overlapping clinical and imaging features. CT of the pulmonary vasculature has become an integral part of the work-up of such entities. In this session, we will evaluate three distinct disease subcategories of pulmonary vascular disease: pulmonary arterial hypertension, pulmonary vasculitis and hereditary hemorrhagic telangiectasia (Osler-Weber-Rhendu syndrome). The first talk will discuss pulmonary hypertension, the current clinical classification of pulmonary vasculitis, CT signs suggestive of the diagnosis, and CT signs that help discriminate between different causes. The comprehensive nature of CTPA in evaluating the mediastinum, vasculature and lung parenchyma will be shown. The second talk covering pulmonary vasculitis will emphasise the importance of combining CT signs with clinical features and laboratory results since vasculitis may mimic other disorders and sometimes the clinical features can be the major clue. CT findings in granulomatosis with polyangiitis and eosinophilic granulomatosis with polyangiitis will be shown, particularly emphasising the imaging differences between the two entities. The imaging findings of pulmonary haemorrhage will also be shown alongside the commonest causes. Finally, the association between interstitial lung disease and the ANCA associated vasculitis will be discussed. The third talk covering hereditary hemorrhagic telangiectasia will show the typical imaging features of this disease with a particular emphasis on those imaging aspects that determine outcome and treatment strategies. The talk will also cover contemporary aspects of endovascular treatment and their complications.

**A-0470** 16:06
**A. CT imaging of pulmonary hypertension**
N.J. Screaton; *Cambridge/UK (Nicholas.Screaton@papworth.nhs.uk)*

Pulmonary hypertension (PH) typically presents insidiously with non-specific symptoms and is usually progressive with poor outcome independent of aetiology. CT plays a vital role both in suggesting the possibility of pulmonary hypertension, whether initially clinically suspected or not, and in identifying a specific cause of pulmonary hypertension. The causes of pulmonary hypertension can be broadly divided into those affecting primarily the small vessels, PH secondary to left heart disease or chronic lung disease/hypoxia (the most common causes), chronic thromboembolic PH, and multifactorial causes. CT is widely available, inexpensive, and permits a comprehensive assessment of the heart, pulmonary vasculature and lung parenchyma. CT signs such as dilatation of the proximal pulmonary arteries and right heart chambers can be considered as generic features associated with PH of any cause. Signs of a specific cause may lie in the mediastinum (left heart disease, shunt, oesophageal varices, oesophageal dilatation), vasculature (signs of CTEPH, tumour, large vessel vasculitis, fibrosing mediastinitis) or lungs (parenchymal lung disease, mosaic perfusion in CTEPH, signs of a small vessel vasculopathy). CTEPH is a not uncommon sequela of previous acute embolism. If the distribution is proximal, it is potentially cured by surgical pulmonary endarterectomy or by balloon pulmonary angioplasty. Imaging in general and CT in particular play fundamental roles in both identification of CTEPH, its differentiation from acute PE, and in characterising its distribution. Imaging signs in CTEPH can be a subtle and systematic evaluation of a CTPA is essential.
***Learning Objectives:***
1. To learn about the CT signs of pulmonary hypertension (PHT).
2. To become familiar with the current classification of PHT.
3. To be aware of the imaging features suggesting a thromboembolic origin.

**A-0471** 16:34
**B. Evaluating the pulmonary vasculitis**
E. Castañer; *Sabadell/ES (ecastaner@tauli.cat)*

We will emphasise the importance of combine CT signs with the clinical features and laboratory results, as vasculitis may mimic other disorders and sometimes the clinical features are the clue. We will describe the findings in granulomatosis with polyangiitis (GPA) and eosinophilic granulomatosis with polyangiitis, highlighting the differences. Diffuse alveolar haemorrhage (DAH) is one of the manifestations of primary pulmonary vasculitis, among other entities (idiopathic alveolar haemorrhage, collagen vascular diseases, drug reactions, anticoagulation disorders). GPA and microscopic polyangiitis are the most common causes of DAH. Radiologic signs of DAH are nonspecific and variable but must be considered in patients with otherwise unexplained alveolar infiltrates, particularly when seen with new-onset renal insufficiency or a connective tissue disease. Finally, we will mention the association between interstitial lung disease and ANCA associated vasculitis.
***Learning Objectives:***
1. To learn the common imaging features of granulomatosis with polyangitis (GPA).
2. To learn how to differentiate between GPA and eosinophilic granulomatosis with polyangiitis (Churg-Strauss).
3. To learn about the imaging features and differential diagnosis of pulmonary haemorrhage.

**A-0472** 17:02
**C. Rendu-Osler and Behcet's diseases**
B. Peynircioglu; *Ankara/TR (borapeynir@gmail.com)*

Behçet disease is a rare multisystem condition associated with HLA-B51 positivity. Arterial involvement in Behçet's disease (BD) is less common than venous lesions. The most commonly affected arteries are: the aorta, lower extremity arteries, mesenteric, femoral, coronary, renal, subclavian and pulmonary arteries. Behçet-related pulmonary vasculitis is an uncommon and heterogeneous group of conditions, often with associated pulmonary artery thrombus formation. These microthrombi can result in a misdiagnosis of acute pulmonary embolism. Hereditary hemorrhagic telangiectasia also known as Osler-Weber-Rendu syndrome is an autosomal dominant disease that occurs due to vascular dysplasia associated with the disorder in the signalling pathway of transforming growth factor β(TGF-β). The clinical consequence is a disorder of blood vessels in multiple organ systems with the existence of telangiectasia which causes dilation of capillaries and veins, are present from birth and are localised on the skin and mouth, respiratory, gastrointestinal and urinary tract. Many patients with gastrointestinal and other organ manifestations are frequently clinically asymptomatic; therefore, organ screening is essential to avoid later complications and should be performed in centres with particular expertise. Hemoptysis is a life-threatening complication that is likely related to pulmonary artery aneurysm (PAA). Diagnostic imaging is the key to success in the management of these patients pulmonary vasculature. CT is the tool with some known advantages and drawbacks. Vascular interventional radiology may offer effective emergency and or elective therapeutic option. Transcatheter embolotherapy should be considered as the first-line emergency treatment for PAA-related hemoptysis, in association with the immunosuppressive regimen.
***Learning Objectives:***
1. To become familiar with the CT manifestations of these diseases.
2. To identify imaging features determining outcome and treatment strategies.
3. To learn about endovascular treatment modalities and complications.

16:00 - 17:30 Room M 5

### E3 - ECR Academies: Functional Imaging for Disease Management: Research to Medical Practice

### E3 820

### Functional imaging of breast and female pelvis

**A-0473** 16:00
**Chairperson's introduction**
K. Kinkel; *Chêne-Bougeries/CH (karen.kinkel@grangettes.ch)*

The introduction to this functional imaging session in the field of breast and female pelvis will present a short historical overview of what has been developed and accomplished in the last 20 years. Examples of clinical situations where functional imaging is crucial in daily practice are illustrated to raise interest in the three following lectures. Common problems in image characterisation/staging, and solutions detailed in the lectures to overcome these problems are briefly presented.

**A-0474** 16:06
**A. Breast tumours**
J. Camps Herrero; *Valencia/ES (juliacamps@gmail.com)*

Imaging biomarkers (IBM) yield anatomic, molecular or functional characteristics that can be measured as indicators of underlying normal or pathologic biologic processes. IBM can be further divided into quantitative and qualitative, and those that will be the topic of this talk will be the functional quantitative or qualitative IBM derived from image bio-signals (electromagnetic or photonic). These include not only MRI but also mammography and ultrasound. IBM can also be characterised as risk-prediction BM, tumour characterisation and prognosis o therapy (response) monitoring. Risk prediction IBM can be analysed quantitative- or qualitatively in the mammographic breast density or the amount of fibro-glandular tissue or background parenchymal enhancement that can be seen through MRI. IBM of tumour characterisation and prognosis, as well as response evaluation to therapy, are mainly extracted from MRI images that analyse neo-angiogenesis (DCE-MRI), tissue microstructure and cellularity (DWI) and metabolism ((MR spectroscopy). The lecture will focus mainly on these IBM of risk prediction, tumor characterization and response evaluation but will also briefly explain IBM in ultrasound that analyze tissue stiffness (elastography) and angiogenesis (Doppler and contrast-enhanced ultrasound) and will also briefly mention the new IBM (Sodium imaging, spectroscopy with 13P MRSI, Saturation transfer CEST MRI and hyperpolarized MRSI).
***Learning Objectives:***
1. To review actual functional methods of breast imaging.
2. To learn about functional biomarkers of tumour grading.
3. To understand functional approach of tumour response.
*Author Disclosure:*
**J. Camps Herrero:** Consultant; BD BARD.

**A-0475** 16:34
**B. Tumours of the uterus**
E. Sala; *Cambridge/UK (es220@cam.ac.uk)*

The standard MRI uterus protocol includes axial SE T1WI with a large FOV to evaluate the entire pelvis and upper abdomen for lymphadenopathy as well as bone marrow changes; and high-resolution FSE T2WI in the sagittal, axial and coronal planes. Other extra planes and imaging sequences are specific and designed to answer a specific clinical question. Functional MRI sequences such as DCE-MRI and DW-MRI are now part of routine protocols. DCE-MRI is very useful for assessment of the depth of myometrial invasion in endometrial cancer. DW-MRI is now part of routine MRI protocol for evaluation of uterine malignancies. It should be performed at two or more b-values, which include one or more low b-values (50-100 s/mm2) since perfusion contribution to diffusion is then eliminated and a very high b-value (750-1000 s/mm2). Both breath hold and non-breath hold DW sequences can be used. However, the type of DW sequence differs among manufacturers, and the radiologist should be familiar with the strengths and limitations of their own scanners. Combination of DWI with conventional MRI sequences improves lesion detection and radiologist confidence in imaging interpretation. DW-MRI can be useful for accurately determining the depth of myometrial invasion in endometrial cancer. In addition, ADC values are inversely related to the cellularity of tumours which may be useful for distinguishing between benign and malignant tissues and for monitoring tumour response to treatment in cervical cancer.
***Learning Objectives:***
1. To review functional methods for female pelvic imaging.
2. To learn about functional impact for characterising uterine tumours.
3. To be aware of validated biomarkers of prognosis.

**A-0476** 17:02
**C. Ovarian tumours**
A.G. Rockall; *London/UK (a.rockall@imperial.ac.uk)*

Ovarian cancer remains the gynecologic malignancy with the highest mortality. This poor prognosis is partly due to late presentation, with disseminating disease in most cases. Researchers are actively trying to identify methods to improve patient outcomes. Accurate characterisation of adnexal masses has been improved by the development of standardised imaging and reporting methods, both in ultrasound and MRI. MRI characterisation has benefitted by the use of dynamic contrast enhancement of adnexal masses, with the categorisation of curves and the use of diffusion and T2 signal intensities, to allow risk stratification for the likelihood of cancer. Accurate risk stratification should, in turn, allow optimal treatment planning, identifying high-risk tumours for the referral to the gynecologic oncology team and conversely ensuring that low risk, likely benign tumours, are managed appropriately. In patients presenting with disseminated disease, advanced imaging techniques, including diffusion and contrast MRI of the peritoneal cavity, allow better delineation of the extent of disease. In addition, the use of radiometric prognostic vector on CT can predict phenotypically distinct tumours types, with biological and prognostic associations. Current reports of prognostic biomarkers will be discussed as well as the need for robust validation to allow translation into clinical practice.
***Learning Objectives:***
1. To learn how functional methods help in characterising ovarian tumours.
2. To review their role for patient management.
3. To be aware of validated biomarkers of prognosis.
*Author Disclosure:*
**A.G. Rockall:** Speaker; Guerbet.

16:00 - 17:30 Tech Gate Auditorium

### Multidisciplinary Session

### MS 8a

### The "prostate unit" at work

**A-0477** 16:00
**Chairperson's introduction: My PSA is elevated...what now?**
G.M. Villeirs; *Ghent/BE*

An increased or persistently elevated serum PSA can be the first sign of developing prostate cancer. In the modern “prostate unit at work”, multiparametric MRI of the prostate has become an essential tool in the early diagnosis of prostate cancer and in the optimal decision making between the various treatment options.
***Session Objectives:***
1. To understand the role of imaging in the diagnosis and management of prostate cancer.
2. To become familiar with the various treatment options for prostate cancer.
3. To appreciate the role of multidisciplinary discussions.

**A-0478** 16:05
**The radiologist: non-invasive explorer of the prostatic realm**
P. De Visschere; *Ghent/BE (pieter.devisschere@uzgent.be)*

Multiparametric magnetic resonance imaging (mpMRI) is the current state of the art imaging technique used in the assessment of patients with suspected or confirmed prostate cancer (PC). A mpMRI consists of morphological T2-weighted images supplemented with diffusion-weighted imaging and dynamic contrast-enhanced imaging. mpMRI has excellent accuracy for detecting high-grade PC, but low-grade PC may be missed. In patients with elevated PSA, mpMRI may be used as triage test before biopsy: when mpMRI is normal, a biopsy may safely be postponed, but when mpMRI shows a suspicious lesion, a targeted biopsy can be performed. There are three methods for targeted biopsy: cognitive transrectal ultrasound (TRUS) guided biopsy, TRUS-MRI fusion biopsy or in-bore MRI-guided biopsy. Recent large randomised controlled trials have shown that a pathway using mpMRI before biopsy in patients with elevated PSA results in avoiding biopsy in about one-third of men, in reduced detection of clinically insignificant PC and in equal to slightly increased detection of clinically significant PC. mpMRI may also be useful in local staging of patients with biopsy-proven PC and to detect local recurrence in patients treated for PC. Interpretation of mpMRI may be difficult, because some benign conditions such as (granulomatous) prostatitis may mimic PC.

In the radiology report, the PI-RADS score is used to communicate the imaging findings to the clinician.
***Learning Objectives:***
1. To become familiar with the technique of mpMRI of the prostate.
2. To understand the role of mpMRI in the detection of clinically significant prostate cancer.
3. To appreciate the importance of image-targeted biopsy.

**A-0479** 16:25
**The pathologist: guardian of the truth**
S. Verbeke; *Ghent/BE*

Prostate cancer (PCa) is one of the most frequent tumours in men. The diagnosis is based on the microscopic evaluation of prostate tissue. The current standard of care is to obtain at least 10-12 systematic 18-gauge prostate core biopsies. The evolutions in imaging (MRI fusion biopsies) and histological assessment (Grade Group system) have improved the diagnostic and clinical accuracy of this technique. The most common histological type of PCa is acinar adenocarcinoma and accounts for more than 95% of the PCas. Different histological variants have been described which can be of significance due to difficulty in diagnosis (e. g. deceptively benign-looking atrophic variants) and due to prognostic differences (e. g. signet ring-like variants has a worse prognosis). Furthermore, other subtypes such as ductal carcinoma, small-cell neuroendocrine tumours, intraductal carcinoma, urothelial carcinoma, squamous neoplasms, basal cell carcinoma, sarcoma and lymphoma are less common. The worldwide used grading system initially created by Dr. Gleason for PCa is uniquely and solely based on the architectural pattern of the tumour. The grade is defined as the sum of the two most common grade patterns (Gleason score). The grading of PCa was revised in 2014: the initial Gleason patterns 1 and 2 are no longer in use since these patterns are more likely a benign condition. Furthermore, different patterns have been re-categorised, and a set of different grade groups associated with a unique prognosis have been introduced. Hereby, a more accurate grade stratification and simplified categorisation have been accepted which has also implications for treatment.
***Learning Objectives:***
1. To appreciate the importance of good tissue sampling.
2. To become familiar with the different histological subtypes of prostate cancer.
3. To understand the reasons for false positive and false negative results.

**A-0480** 16:40
**The urologist: knight of swords in the prostatic badlands**
N. Lumen; *Ghent/BE (Nicolaas.Lumen@uzgent.be)*

Radical prostatectomy is a standard treatment option for intermediate and high-risk localised prostate cancer and can be an option for locally advanced prostate cancer. When offering radical prostatectomy to the patient, surgical morbidity and functional complications (urinary incontinence, erectile dysfunction) must be taken into account. Appropriate patient selection for radical prostatectomy is thus of utmost importance, and MRI can help to this. Radical prostatectomy obtains the removal of the whole prostatic gland with the prostatic urethra and seminal vesicles. However, some modifications are possible to reduce the functional complications of the surgery. These modifications include bladder neck sparing, nerve sparing, posterior dissection and urethra sparing. MRI can help to decide whether one or more of these modifications are possible for the individual patient.
***Learning Objectives:***
1. To understand the role of imaging in the choice for prostatectomy.
2. To become familiar with open and robotic prostatectomy techniques.
3. To appreciate the importance of imaging in the preservation of clear margins.
*Author Disclosure:*
**N. Lumen:** Consultant; Janssen, Astellas, Bayer. Grant Recipient; Janssen, Bayer, Ipsen. Research/Grant Support; Kom op tegen Kanker, Clinical research fund Ghent University Hospital.

**A-0481** 16:55
**The radiation-oncologist: smooth irradiator of the rogue elements**
V. Fonteyne; *Ghent/BE (valerie.fonteyne@uzgent.be)*

External beam radiotherapy (EBRT) is an established treatment option for prostate cancer (PC) patients. A dose-response relationship for biochemical control is extensively described. The implementation of modern radiotherapy techniques and improvements in patient positioning during treatment enable the safe delivery of 74-78 Gy to the prostate. However, the close vicinity of surrounding organs at risk (OARs), limits further dose escalation to the entire prostate. The incidence of local failure after EBRT is dose-dependent supporting the hypothesis that aggressive tumours are more radioresistant. To avoid excess in toxicity, one can increase the dose exclusively to the area that is at highest risk of local relapse, i.e. the location of the initial tumour site called the dominant intraprostatic lesion (DIL). Theoretically, this might improve local control and positively impact on the risk of developing distant metastases and PC death. Of course, none of these developments are possible without the integration of modern imaging and more precisely magnetic resonance imaging (MRI). The implementation of MRI in the treatment planning has resulted in a refined delineation of the target volume with a clear impact on target volume and enables the precise delineation of the DIL for simultaneous integrated boost. Also, a correct daily positioning is of utmost importance when considering high dose EBRT, again illustrating the benefit of incorporating imaging in the radiotherapy treatment. The combination of an MRI machine with a linear accelerator in one device allows greater precision in cancer treatment and is currently being investigated in clinical practice.
***Learning Objectives:***
1. To become familiar with the various prostate irradiation techniques.
2. To appreciate the need for imaging in radiotherapy planning.
3. To illustrate the importance of imaging in radiotherapy monitoring.

**A-0482** 17:10
**Multidisciplinary case presentation and discussion**
G.M. Villeirs; *Ghent/BE*

The prostate unit is the heart of every multidisciplinary team that treats the man with suspected or confirmed prostate cancer. In this session, the role of all players in the team (radiologist, pathologist, urologist and radiation oncologist) will be explained, with emphasis on the importance of radiology for each of them.

### Friday, March 1

**08:30 - 10:00** **Room A**

### E3 - ECR Academies: Interactive Teaching Session for Young (and not so Young) Radiologists

### E3 921

### Cardiac imaging

**A-0483** 08:30
**A. Grown-ups with congenital heart disease**
A.M. Taylor; *London/UK (a.taylor76@ucl.ac.uk)*

This session will describe how MRI and CT can be used to image adults with congenital heart disease. The common conditions that occur in childhood will be described (acyanotic and cyanotic heart disease) and what operations they undergo that will be important for imaging them when they become adults. Finally, those CHDs that may be diagnosed in adulthood will be described.
***Learning Objectives:***
1. To be familiar with the clinical and radiologic presentations of these patients.
2. To learn about imaging findings and management options.

**A-0484** 09:15
**B. Imaging cardiac valves**
R. Salgado; *Antwerp/BE (rodrigo.salgado@uza.be)*

Valvular heart disease forms an important part of the workload in every cardiology practice. While echocardiography remains a cornerstone in the assessment and follow-up of valvular heart disease, an important role is reserved for MR and CT imaging, although in different contexts. MR, with its ability to investigate the valvular morphology as well as the haemodynamic consequences on the cardiac chambers, forms an important complementary tool to the echocardiographic findings. It provides a reproducible quantification of transvalvular flow and has in recent years gained further importance in tissue characterisation with the introduction of T1/T2 mapping techniques for the detection and distribution of fibrosis. CT has recently gained a prominent place in the non-invasive investigation of the aortic root before transcatheter aortic valve replacement and is also becoming a primary imaging modality in emerging mitral valve interventions. Furthermore, its role in the evaluation of prosthetic heart valves is currently the subject of intense investigation, providing valuable additional information not easily acquired through other imaging methods. During this lecture, the different advantages and limitations of valvular imaging with CT and MR will be discussed, and practical recommendations for clinical practice will be given.
***Learning Objectives:***
1. To understand the impact of imaging.
2. To know the limitations and potential pitfalls of CT and MR techniques.
3. To learn about the imaging technique.

**08:30 - 10:00** **Room B**

### Abdominal Viscera

### RC 901

### Imaging of the complicated postoperative abdomen

**A-0485** 08:30
**Chairperson's introduction**
C.J. Zech; *Basle/CH (christoph.zech@usb.ch)*

Imaging of the postoperative abdomen is a crucial part in the evaluation of postoperative complications and will lead the clinician in the decision for treatment of postoperative complications with either re-laparotomy, interventional-radiology treatment or conservative/medical treatment. Knowledge of the performed surgical techniques and procedures, the normal imaging appearance postoperative and typical findings of frequent complications are inevitable to successfully treat patients with complications. Good interaction between the surgeon and the abdominal radiologist is needed. Nowadays, radiology should be able to diagnose most complications and minimise the number of mere diagnostic laparoscopies to evaluate a complicated postoperative clinical course.
***Session Objectives:***
1. To learn about the most common indications for abdominal surgery.
2. To understand the normal imaging findings in a postoperative abdomen.
3. To describe and identify the most common postoperative complications.

**A-0486** 08:35
**A. Liver**
C. Ayuso; *Barcelona/ES (cayuso@clinic.ub.es)*

Resection is proven to achieve long-term survival in selected patients with primary or secondary focal liver lesions. Advances in surgical techniques including robotic liver surgeries, systemic adjuvant chemotherapy and postoperative intensive care improved the outcome of liver resection. An adequate liver remnant is crucial to guarantee the correct liver function after radical potentially curative liver resection. In borderline cases, portal vein embolisation (PVE), or associating liver partition and portal vein ligation for staged hepatectomy (ALPPS) has been proposed before surgery to increase the liver remnant. Preoperative volumetric analysis of the liver based on imaging plays an essential role in the preoperative workup previous to liver resection. Also, the knowledge of the hepatic vascular anatomy and possible variants of the biliary system are also key aspects to avoid postoperative complications. Imaging tools based on multidetector computed tomography (MDCT) and magnetic resonance (MR) influencing preoperative decisions and surgical approaches will be discussed. The role of different imaging techniques as plain ultrasonography (US) contrast-enhanced ultrasound (CEUS), MDCT and MR for the detection and management of hepatic complications after liver resection or transplantation (infectious, vascular, biliary, parenchymatous (liver rejection) will be analysed.
***Learning Objectives:***
1. To learn about indications for liver interventions and different surgical approaches to focal liver lesions.
2. To understand the role for the different imaging techniques (US, CEUS, CT and MRI) in assessing different types of complications.
3. To appreciate the spectrum of parenchymal, biliary and vascular complications occurring after liver resection or transplantation.
*Author Disclosure:*
**C. Ayuso:** Speaker; Speaker fees and travel grants from Bayer.

**A-0487** 08:58
**B. Pancreas**
R.M. Gore; *Evanston, IL/US (rgore@uchicago.edu)*

Both benign and malignant pancreatic disease carry significant morbidity and mortality. Because of the fastidious nature of the pancreas and its local and regional anatomy, treatment of these disorders also carries significant morbidity. Indeed, perioperative morbidity of pancreaticoduodenectomy is approximately 30-40%. Due to the diversity of pathology, detection and characterisation of postoperative complications can be challenging. Familiarity with the normal imaging appearances of the spectrum of surgical procedures and their complications is vital. In this presentation, the common pancreatic surgical procedures performed for tumour resection (i.e. the Whipple's operation, enucleation, central and distal pancreatectomy, spleen-preserving distal pancreatectomy) and chronic pancreatitis (i.e. Puestow procedure, Beger procedure, Frey procedure) and their normal postoperative appearances are discussed. The common post-surgical complications including pancreatic fistulas and leaks, abscess formation, haemorrhage, and delayed gastric emptying are then described in detail.
***Learning Objectives:***
1. To learn about the various surgical approaches for acute and chronic pancreatitis, benign and malignant pancreatic neoplasms.
2. To understand the profound impact that the partial and complete pancreatectomy have on the adjacent abdominal organs.
3. To appreciate the common postoperative complications of pancreatic surgery.

**A-0488** 09:21
**C. Bowel**
D.J.M. Tolan; *Leeds/UK*

"no abstract submitted"
***Learning Objectives:***
1. To learn about the most common types of major oesophago-gastric, small bowel and colorectal surgical procedures and the expected postoperative anatomy and imaging findings.
2. To understand how to optimise imaging protocols to maximise the opportunity to detect postoperative complications after enteric surgery.
3. To appreciate and identify early and late complications after bowel surgery.

09:44
**Panel discussion: Tips and tricks for my routine clinical practice**

**08:30 - 10:00** **Room C**

### Special Focus Session

### SF 9b

### Clinical simulation and its role in radiography education

**A-0489** 08:30
**Chairpersons' introduction (Part 1)**
L. Oleaga Zufiría; *Barcelona/ES (lauraoleaga@gmail.com)*

There is a diversity of requirements and training schemes for radiographers within European countries. A current challenge is the importance to harmonise and standardise radiography training and accreditation across borders. The digital revolution in Radiology and Radiography continues to advance rapidly. Informatics and information technology evolution are going to substantially reshape the practice of Radiology and Radiography over the next decade. There are a number of interesting developments within informatics, which may have a significant impact on Radiology and Radiography education, and training in the near future. These include the extended functionality of handheld computers, web-based skill and knowledge assessment, standardisation of procedural training using simulated or virtual patients and worldwide online learning. Simulation programmes have proved to be effective in other specialities as a method for teaching and learning. There are mannequin-based simulation programmes, computer-based and simulators for an ultrasound and interventional radiology. Simulation training impacts on the learning curve with numerous benefits for the preparation of radiology/radiography students which will, in turn, benefit the service provided to patients. Simulation provides an option for training, but the place of this within any curricula needs to be carefully determined to promote the highest levels of competency. The education of the future must be a learner-centred education. Trainees must have an active role in the learning process. The teachers are not suppliers of knowledge and information, but organisers of activities and directors of learning experiences.
***Session Objectives:***
1. To review the evolution of training and radiology education in Europe.
2. To evaluate the challenges facing radiology education with the expanding use of informatics.
3. To analyse how clinical simulation methods can transform education in radiology.

*Author Disclosure:*
**L. Oleaga Zufiría:** Consultant; Telemedicine Clinic.

**A-0490** 08:33
**Chairpersons' introduction (Part 2)**
F. Zarb; *Msida/MT (francis.zarb@um.edu.mt)*

Radiology and Radiography education are evolving in line with developments in informatics which has provided a range of new tools for teaching and learning, which are going to substantially reshape the practice of Radiology and Radiography over the next decade. These include the extended functionality of handheld computers, web-based skill and knowledge assessment, standardisation of procedural training using simulated or virtual patients and worldwide online learning. Simulation programs have proved to be effective in other specialities as a method for teaching and learning. Simulation training impacts on the learning curve with numerous benefits for the preparation of radiology/radiography students which will, in turn, benefit the service provided to patients. Simulation provides an option for training, but the place of this within any curricula needs to be carefully determined to promote the highest levels of competency.
***Session Objectives:***
1. To review the evolution of training and radiology education in Europe.
2. To evaluate the challenges facing radiology education with the expanding use of informatics.
3. To analyse how clinical simulation methods can transform education in radiology.

**A-0491** 08:35
**Clinical radiography education across Europe: an overview**
J. McNulty; *Dublin/IE (jonathan.mcnulty@ucd.ie)*

The European Federation of Radiographer Societies (EFRS) represents over 8,000 radiography students through a unique part of pour organisation, namely, the Educational Wing which consists of 61 educational institutions. Education and training are at the heart of several strategic priority areas of the EFRS. The educational aim of the EFRS is to promote and develop all levels of radiography education and research across Europe. Objectives include: the development and advancement of educational standards for radiographer education in Europe; the development of European Qualifications Framework benchmarking documents for radiographers at Bachelors and Masters levels; to research, disseminate and publish materials and knowledge generated by the Educational Wing; and to provide assistance to those institutions wishing to develop or enhance their radiography programmes; and to develop evidence-based practice and radiographer-led research. High educational standards for radiographers are of utmost importance for our profession. This is especially true for the clinical training components of radiography education programmes. However, the clinical components of our curricula present many challenges, some of which were identified through recent EFRS surveys. While similarities exist in the provision of clinical radiography education across Europe, some major differences were also identified in terms of the amount of clinical time in programmes, the supervisory structures, and the governance and oversight of these placements. Professional societies should work collaboratively to establish guidelines for effective clinical placements. This will help ensure our graduates are fit for purpose.
***Learning Objectives:***
1. To present the developments of radiography education.
2. To discuss the current status of radiography education in Europe.
3. To evaluate the challenges and future of radiography education.

**A-0492** 08:50
**Innovation in education: virtual education/computer-based simulator vs patient imaging**
A. England; *Salford/UK (A.England@salford.ac.uk)*

Full-body high-fidelity simulations have formed part of healthcare education for a number of years, especially within the nursing and medicine disciplines. These lifelike mannequins are operated from a computer system with the ability to generate complex events and respond to multiple stimuli. The author, as a radiography educator, has a number of years of experience in integrating patient simulation into radiography training. Such teaching sessions are typically divided into the pre-simulation experience, the simulation and the post-simulation experience. Such an approach is essential to promote realism and also identify feedback and development opportunities. Within the undergraduate radiography programme, a case scenario on contrast anaphylaxis is commonly undertaken using high-fidelity simulation. Within this presentation the contrast anaphylaxis scenario is used as an example of clinical simulation, the advantages and disadvantages of this teaching method, as identified by teaching staff and students, are also discussed. Comparisons are also made with alternative teaching and learning approaches. It is important to stress than simulation is an extremely valuable pedagogical approach, but its merits must be considered amongst the other available teaching and learning methodologies. A diverse curriculum is essential for a high-quality radiography degree programme.
***Learning Objectives:***
1. To demonstrate the benefits of using new teaching approaches.
2. To show how new teaching instruments can improve and shorten the learning curve.
3. To evaluate the pros and cons of radiography education through simulation and patient imaging.

**A-0493** 09:05
**High fidelity: clinical simulation for undergraduate radiography**
A. Louw; *Johannesburg/ZA (amandal@uj.ac.za)*

Within the radiography domain, Simulation-Based Education (SBE) is valued as a pedagogical approach, but the design of simulation experiences, incorporating educational and cognitive theories, still needs exploration to advise best practice guidelines. Educational psychologists indicate that no new knowledge is created in passive manners and they emphasise the need for interaction and stimulation from the environment. Creative teaching methods that capture the interest and imagination of students are also said to stimulate the creative problem-solving ability that radiography students need in their daily working environment - an ability which various researchers point out as lacking among radiography students. SBE offers interaction, stimulation and various opportunities for creativity, but to optimise learning and teaching, educators must also consider the impact of cognitive load, scaffolding and reflection, when they develop simulation experiences. Whereas SBE in the health professions initially developed in response to the awareness that it is unethical to use patients as teaching aids and skills practice commodities, the call for equal and optimal training opportunities that will result in optimal patient centred care now provides further impetus. The increasing authenticity of simulation experiences, however, presents with unexpected ethical issues of concern. Students experience high fidelity manikins often as real-life patients, and the deterioration and demise of these patients can have a major emotional impact on them. Educators should acknowledge that the principle of beneficence applies to both patients and students and must be aware of the possible emotional effect on students and how to manage it.

***Learning Objectives:***
1. To discuss ethics in simulation practices.
2. To provide an overview on how to prepare and execute a simulation experience.
3. To highlight the challenges and opportunities of clinical simulation at the undergraduate level.

**A-0494** 09:20
**How simulation can help prepare students and have a positive impact on interprofessional working**
A. Henner; *Oulu/FI (anja.henner@oamk.fi)*

Interprofessional education is a collaborative approach to develop healthcare students as future interprofessional team members. Interprofessional teams can address complex medical issues. Training future healthcare providers to work in such teams improve healthcare outcomes for patients. With the didactic program, interprofessional collaboration skills, knowledge of professions, patient-centred care, service learning and the impact of culture on healthcare delivery are improved. The community-based experience demonstrates how interprofessional collaborations provide service to patients and how the environment and availability of resources impact one's health status. The interprofessional-simulation experience describes clinical team skills training in both formative and summative simulations. In a simulation-based experience, formative assessment or summative evaluation can be used. Formative assessment fosters personal and professional development and helps participants progress toward achieving objectives. Assessment of student learning in a simulation is no longer a passive activity in which the educator determines how well individual or group performs. Summative evaluation focuses on measurement of outcomes or achievement of objectives and supports the assessment or evaluation of behaviours cognitive (knowledge), affective (attitude), and psychomotor (skills) areas. A successful experience in simulation helps students to understand their professional identity while gaining an understanding of other professional's roles on health care team and offers a safe environment exercise even difficult tasks. Commitment from departments and colleges, diverse calendar agreements, curricular mapping, mentor and faculty training, a sense of community, adequate physical space, technology, and community relationships are critical resources for a successful simulation and positive attitude to work together.
***Learning Objectives:***
1. To demonstrate how interprofessional collaborations improve a service to patients.
2. To describe clinical team skills training in formative summative simulations.
3. To discuss the benefits of student preparation through simulation.

**A-0495** 09:35
**Providing opportunities for practical ultrasound training**
B. Kraus; *Vienna/AT (barbara.kraus@fh-campuswien.ac.at)*

The major challenge in the education of Radiographers in Ultrasound or "Sonographers" all over Europe is, to create a training scenario closely related to clinical reality. To perform Ultrasound in a proper way, hands-on training is mandatory and cannot be replaced by anything else. One option is Skill Lab Training Sessions. Students train on each other to learn and strengthen the major skills by correctly using the Ultrasound probe, implementing the theoretical knowledge about physics and optimization in correctly manipulating scan parameters to adequately demonstrate and be able to visualise anatomical structures in a correct manner. These are the key points in diagnostic ultrasound performance. Other options are different web-based skill and knowledge assessments and simulator-based training settings. Utilities from different providers are for example augmented with real patient scans, including haptic feedback by using a probe, real-time assisted guidance and comprehensive metric-based assessment in one system. Trainers and tutors are able, to upload their own patient scans/cases and share them with other users. The major difference between simulated practice and scanning on each other is the benefit of simulating real clinical case scenarios, including pathological findings during training. However, a balanced combination of hands-on training (Students on each other), simulator-based/web-based training and clinical experience under medical supervision in compliance with all ethical aspects is the ultimate goal in practical ultrasound education.
***Learning Objectives:***
1. To know the role of hands-on training in ultrasonography.
2. To appreciate the value of learning ultrasonography through hands on training.
3. To discuss the opportunities for radiographers training in ultrasound.

09:50
**Panel discussion: Is simulation enough to meet the current challenges facing radiography education? Can simulation replace hands-on patient experience?**

**08:30 - 10:00** **Room X**

### Joint Session of the ESR and ESTRO

### ESR/ESTRO

### Current status and future challenges in MR-integrated radiotherapy

**A-0496**/**A-0497** 08:30
**Chairpersons' introduction**
L.E. Derchi; *Genoa/IT*
U. Ricardi; *Turin/IT*

This session is dedicated to the building of stronger relationships between diagnostic radiology and radiation therapy specialists. Diagnostic radiologists must be aware of the technical advances of radiotherapy and oncology and have to learn how to provide expert support to patients undergoing radiation therapy. Knowledge of technological advances is the first thing to get; changes of our working habits is the second, through the development of new examination techniques to meet the clinical needs of these patients; dialog between the different specialists is the third, and most important one. This session will be repeated at the next meeting of the European Society for Radiotherapy and Oncology (ESTRO). This indicates that both Societies believe that working together, and growing together, is quite important to ensure optimal patient care.
***Session Objectives:***
1. To be aware of the technical advances in radiotherapy and oncology.
2. To know how diagnostic radiologists have to provide expert support to patients undergoing radiation therapy.
3. To understand how to develop strong clinical collaboration between the two specialities.

**A-0498** 08:35
**Clinical status of MR-integrated photon therapy**
L. Boldrini; *Rome/IT (lucaboldrini@hotmail.it)*

The introduction of MR integrated delivery units represents one of the most significant and promising innovations of the last years in radiotherapy delivery technology. The advantages assured by the better visualisation of the therapy volumes allowed by onboard MR imaging and the possibility to use active gating and motion management solutions embedded in this novel workflow need to be further explored and quantified both from a technical and a clinical perspective. The aim of this talk is to describe the current clinical status of the MR-integrated photon radiotherapy and to present the clinical evidence to date available. Its most common applications will be reported, ranging from the patient selection methods to the choice of the most appropriate therapy delivery protocol. Furthermore, the possibility to implement innovative hypothesis-generating approaches (movement management, radiomics, new delivery techniques) in clinical MR-integrated radiotherapy will be presented.
***Learning Objectives:***
1. To learn about the current clinical status of the MR-integrated photon therapy.
2. To understand the most common clinical applications of the MR-integrated photon therapy.
3. To appreciate the possibilities of hypothesis-generating approaches with this new technology (movement management, radiomics, new delivery techniques).
4. To become familiar with the clinical MR-integrated photon therapy workflow, from patient selection to therapy delivery.
*Author Disclosure:*
**L. Boldrini:** Consultant; Varian Medical Systems. Speaker; ViewRay Inc..

**A-0499** 08:55
**Integration of MR and particle therapy: how far are we**
A. Hoffmann; *Dresden/DE (aswin.hoffmann@oncoray.de)*

Precise targeting in proton therapy (PT) is even more important than in conventional photon-based radiation therapy (XT), because PT is more sensitive to anatomical variations than XT. This is due to the steep dose fall-off behind the Bragg peak and to the fact that the range of the proton beam depends on the material composition in the beam path. Especially for moving tumours, these uncertainties currently translate into large safety margins, thus compromising the dosimetric benefit of PT. This urges the need for real-time image guidance during PT dose delivery. Magnetic resonance imaging (MRI) offers real-time image guidance with unparalleled soft-tissue contrast and absence of radiation dose. PT is therefore expected to benefit even more from the integration with real-time MRI than XT. However, so far no hybrid system for MR-integrated PT (MRiPT) has been realised due to a number of hitherto open technological problems. To study the technological feasibility of MRiPT, mutual interactions between the PT and MRI system have to be taken into account. Recent research efforts have realised a proof-of-concept system where first MR images of tissue-mimicking phantoms have been successfully acquired with a clinical in-beam MR scanner during proton beam irradiation. This offers the prospect that the development of a clinical prototype MRiPT system within the next five years should not be considered beyond the realms of possibility. This contribution provides an overview of the current status of MRiPT research achievements and discusses technology issues that need further investigation.
***Learning Objectives:***
1. To learn about the current state-of-the-art in image-guided proton therapy.
2. To understand the rationale for the need to integrate MRI and proton therapy.
3. To become familiar with the technical risks and challenges to integrate MRI and proton therapy.
4. To appreciate which risks and challenges have been overcome by recent research efforts.
5. To discuss the technology issues that need further research.
6. To become familiar with the implications for future developments and clinical treatment workflow.
*Author Disclosure:*
**A. Hoffmann:** Research/Grant Support; Ion Beam Applications, SA, Louvain-la-Neuve, Belgium.

**A-0500** 09:15
**MR-based functional imaging**
R.G.H. Beets-Tan; *Amsterdam/NL*

"no abstract submitted"
***Learning Objectives:***
1. To learn about functional MRI.
2. To appreciate its value as the guidance of radiation treatment.
3. To understand the synergy of an interdisciplinary collaboration between radiologists and radiation oncologists.

**A-0501** 09:35
**Adaptive workflow: current status and challenges**
S. Kharuzhyk; *Minsk/BY*

MRI is used at different stages of oncological patient management including tumour staging, treatment (surgery, radiation therapy) planning and tumour response assessment. Traditional radiation therapy planning workflow integrates high contrast resolution and anatomical details provided by MRI with electron density values obtained from CT. More recently MRI-only radiotherapy planning workflow has been implemented with synthetic CT images generated from MRI data. On-treatment MRI-guidance is a new paradigm of precision radiotherapy that enables a scan-plan-treat approach or adaptive radiotherapy. MRI linac machines integrating clinical MRI scanner with linear accelerator are the current reality in some cancer centres around the globe.
***Learning Objectives:***
1. To understand the role of image-guidance in adaptive radiotherapy.
2. To become familiar with the advantages of MRI guidance compared to CBCT guidance.
3. To learn principles of real-time MRI guidance in radiotherapy.
4. To discuss the current state and further perspectives of MR-guided adaptive radiotherapy.

09:55
**Panel discussion: We need more integration than originally thought: how to get there?**

**08:30 - 10:00 Room N**

### PIER @ ECR Session (Jointly organised with the ESR eHealth Subcommittee)

### PI 1

### Reporting and communication today and tomorrow: challenges to implement structured reporting (RS) and deal with artificial intelligence (AI)

**A-0502**/**A-0503** 08:30
**Chairpersons' introduction**
A. Brady; *Cork/IE (adrianbrady@me.com)*
E. Neri; *Pisa/IT (emanuele.neri@med.unipi.it)*

Structured reporting is rapidly replacing free-text forms of reporting in radiology, in response to the desires of referring clinicians, and as part of ongoing radiology efforts to optimise quality by standardising the format, content and structure of our principal work output, radiology reports. This session will explain how report template structures are developed, how the elements contained within structured reports can be utilised for information extraction and the potentialities for utilising artificial intelligence in supporting radiologists' reporting activity. The value of using structured formats in communicating with referrers will be explained and demonstrated.
***Session Objectives:***
1. To understand what is meant by structured reporting in the context of the likely future of radiology.
2. To appreciate the requirements for and potentialities of radiology structured reporting.
3. To learn about the role of structured reporting in communication with referrers and patients.
*Author Disclosure:*
**E. Neri:** Advisory Board; QUIBIM. Speaker; GE/Healthcare.

**A-0504** 08:36
**Update on developments for structured reporting: Radreport 2.0, TLAP, MRRT**
P. Mildenberger; *Mainz/DE (mildenbe@uni-mainz.de)*

Structured Reporting is in the discussion for many years. There is a broad interest, and consensus to improve quality by providing more precise and focused reports. ESR and RSNA have formed a joint committee (Template Library Advisory Panel, TLAP) to review and validate templates. The development of templates is time-consuming, using approved templates could facilitate the process in a relevant way. Such template, based on the internationally accepted IHE MRRT profile, could be incorporated into radiological information system or dedicated reporting solutions. Especially for epidemiological or research activities, the use of consensual templates could be a huge step forward, because data aggregation would be enabled directly. End of 2018, the new template repository has been launched and is now open for radiologists.
***Learning Objectives:***
1. To learn about new concepts for supporting structured reporting.
2. To appreciate the opportunities of consensual templates based on international standards.
3. To become familiar with the template repository.

**A-0505** 08:52
**The concept of common data elements (CDE) for reporting**
C.E. Kahn; *Philadelphia, PA/US (cekahn@gmail.com)*

Today's radiology reports typically convey the results of imaging studies using unstructured text. This format limits the ability to automatically extract data to support patient care, quality improvement, and medical research. A common data element (CDE) defines the attributes and allowable values of a unit of information. CDEs are "data elements that are collected and stored uniformly across institutions and studies and are defined in a data dictionary." The data dictionary specifies the item's name, data type (e.g., number or text), allowable values, and other attributes. In essence, a CDE is a predefined question and a set of allowable answers to that question. CDEs provide the structure that allows automated systems to extract and exchange key data in a uniform way. This presentation describes the efforts to develop CDEs to support clinical reporting, research, and the application of artificial intelligence systems in radiology.

***Learning Objectives:***
1. To learn about the difference of templates and CDE.
2. To appreciate the flexibility of CDE.
3. To understand how to use CDE in a SR-solution.

**A-0506** 09:08
**Decision support and artificial intelligence (AI) to improve reporting in radiology**
T.K. Alkasab; *Boston, MA/US (TALKASAB@mgh.harvard.edu)*

Artificial intelligence (AI) tools form a natural synergistic pairing with structured reporting (SR) to enable a new, data-enabled paradigm for imaging workflow. As SR modules and templates are defined, they specify a data model for AI tool designers to aim their products at. SR modules also form a natural integration point for the output of AI tools into the clinical workflow. Finally, SR modules can capture feedback from radiologists about AI-generated data elements, which can be fed back to improve the AI tools incrementally. However, in order to realise this vision, the radiologist reporting workflow and toolset will have to re-orient away from prose text creation and more toward data flow. In this new environment, radiologists will review incoming data, augment it through their own interpretation of images, and provide structured data to downstream systems.
***Learning Objectives:***
1. To learn about the next steps for IT-support in reporting.
2. To appreciate the opportunities for quality improvement.
3. To understand the concept of how to implement AI-support in a SR-solution.
*Author Disclosure:*
**T.K. Alkasab:** Other; Nuance Communications.

**A-0507** 09:24
**Communication with referring physicians and patients: what is relevant?**
J.M.L. Bosmans; *Ghent/BE (janbosmans@telenet.be)*

A radiology department creates two products from each patient visit: an imaging study and a report interpretation of the findings. Dimensions of service quality can be defined as reliability, assurance, tangibles, empathy and responsiveness. Multiple surveys have shown that, in case of complex studies, referring clinicians prefer structured reporting (SR) examinations over free text reporting. Moreover, the number of studies reporting the benefits of SR in daily practice is increasing. Despite this, questions have been raised concerning the lack of completeness and flexibility of SR and their influence on radiologists' performance. Patient empowerment is a key element of patient-centred healthcare. Patient empowerment requires that patients have enough information to make decisions concerning their health. Nowadays, as technologists perform the imaging procedure and the radiologist reports the results at another location, the radiologist has become invisible to the patient. In popular media and television shows, radiology is prominently present, but radiologists are virtually absent. A survey among 1146 patients has shown that patients are even ill-informed as to the question whether radiologists are medically qualified and enjoy all the privileges thereof. Despite this, most patients find radiologist consultation beneficial. Patients are comfortable hearing results from the radiologist. Many patients would also like to view their images and receive copies of their reports, potential avenues through which radiologists could add value. It is essential that radiologists learn how to communicate with patients, both directly and through patient-oriented reports.
***Learning Objectives:***
1. To learn about communication pitfalls.
2. To appreciate the potential of SR in communication with referrers and patients.
3. To become familiar with best practice examples.

09:40
**Panel discussion: How to use structured reporting and artificial intelligence in reporting**

**08:30 - 10:00** **Room O**

### Vascular

### RC 915

### Carotid disease 2.0

*Moderator:*
P. Haage; Wuppertal/DE

**A-0508** 08:30
**A. Carotid plaque imaging: tool or fool?**
L. Saba; *Cagliari/IT (lucasabamd@gmail.com)*

Nowadays, stroke represents a critical health problem, and about 25% of all ischemic strokes are due to thromboembolism caused by an atherosclerotic carotid disease. Current guidelines for prevention of stroke in patients with carotid plaques are based on the quantification of the degree of stenosis, and this parameter is currently considered the key element for stratifying the severity of carotid artery atherosclerosis and for the choice of strategies to prevent the occurrence of stroke. The evolution of imaging techniques has allowed for the routine characterization of carotid plaque features and the traditional concept of using degree of luminal stenosis as the sole imaging marker for the selection of the optimal therapeutic approach is challenged by evidence that some types of plaques, so-called “vulnerable plaques”, have a high likelihood to cause ischemic stroke, independent of the degree of stenosis. In this lecture, the first main topic will be to present the radiological method to analyse plaque morphology and recent developments in the assessment of imaging of carotid vulnerable plaque and their potential clinical implications. The second main topic will be the influence of plaque morphology on actual treatment guidelines whereas in the last main topic the possible future role of carotid plaque imaging for treatment decision making will be discussed together with the unmet needs that should guide future research.
***Learning Objectives:***
1. To learn about radiological methods to analyse carotid plaque morphology.
2. To critically review the influence of plaque morphology on actual treatment guidelines.
3. To discuss the possible future role of carotid plaque imaging for treatment decision making.
*Author Disclosure:*
**L. Saba:** Other; Royalties for books from Springer, Oxford University Press, CRC Francis and Taylor.

**A-0509** 09:00
**B. Carotid involvement in inflammatory arterial disease**
M. Edjlali; *Paris/FR*

"no abstract submitted"
***Learning Objectives:***
1. To provide an overview about inflammatory arterial disease.
2. To become familiar with involvement of supraaortic arteries in inflammatory arterial disease.
3. To learn about Radiological diagnosis of supraaortic involvement in inflammatory arterial disease.

**A-0510** 09:30
**C. Carotid stent: medical history or part of the future?**
K. Hausegger; *Klagenfurt/AT (klaus.hausegger@kabeg.at)*

Since carotid artery stenting (CAS) has been introduced as a technique for treatment of extracranial carotid artery stenoses this technique has been compared to carotid endarterectomy (CEA) in several prospective randomised trials. None of these trials could show a clear clinical benefit of one method over the other in the long-term. In the periprocedural phase, minor strokes occurred more often during CAS, whereas myocardial infarcts were seen more often during CEA. Although the overall results of CAS are not inferior compared to CEA, the majority of patients with significant carotid stenoses are treated with CEA. Nevertheless, the tendency towards minimal invasive treatment is still there. With the introduction of mechanical thrombectomy (MTE) as the first line treatment of acute ischemic stroke (AIE) the number of CAS procedures in combination with MTE is increasing, because 10-15% of the patients with AIS have tandem lesions (stenosis of the extracranial internal carotid artery combined with an intracranial large vessel occlusion). There is clear evidence that symptomatic carotid stenoses should be treated. However, there is less evidence for asymptomatic stenoses. Although the historical NASCET and ECAS trials could show a benefit for treatment (CEA) over best medical treatment in the late 1990ies, best medical treatment has significantly improved over time with the availability of effective platelet inhibitors and lipid lowers. Therefore several on-going trials aim the access the indication for invasive treatment (CEA or CAS) of asymptomatic carotid artery stenosis. Future results for CAS may be influenced by better new stent designs.

***Learning Objectives:***
1. To learn about the technique of carotid stent implantation.
2. To review current indications for carotid stent implantation.
3. To discuss the future role of carotid stent implantation.

**08:30 - 10:00** **Studio 2019**

### Special Focus Session

### SF 9a

### Pain palliation in cancer patients

**A-0511** 08:30
**Chairperson's introduction**
A. Gangi; *Strasbourg/FR (Afshin.Gangi@chru-strasbourg.fr)*

The prevalence of pain in newly diagnosed cancer patients is approximately 30% but 75% of cancer patients in the advanced stages of the disease deal with a pain every day. Approximately 10% of patients will have pain that is difficult to manage, and in a small group (2% to 5%), the pain is refractory. Interventional procedures may be indicated in some patients with chronic, refractory, and/or severe pain. This percutaneous image-guided interventions in selected patients offer the potential for improved quality of life, function, and independence. These interventional procedures also broaden the ability of palliative care providers to control pain and limit medication side effects.
***Session Objectives:***
1. To learn about the percutaneous pain palliation techniques in cancer patients.
2. To learn about pain evaluation and clinical selection of the patients.
3. To learn about when to propose these methods during an MDT.

**A-0512** 08:35
**Pain from bone metastases**
G. Tsoumakidou; *Strasbourg/FR (gtsoumakidou@yahoo.com)*

Different interventional radiology procedures can be used to alleviate pain from bone metastases resistant to analgesic therapies (non-opioids, mild and strong opioids). As metastatic bone disease usually represents a complex clinical scenario, a case-based approach is necessary. The origin and type of pain (mechanical or not) should be predefined. The degree of bone destruction, the location of the painful lesion (flat vs long bones, cortical vs medullary lesions) and the presence or absence of cortical rupture and will define the treatment strategy. Cement injection is usually sufficient to treat pain from an impending compression fracture of painful bone tumours involving flat weight-bearing bones without invasion of the surrounding soft tissues. In specific regions, percutaneous bone osteosynthesis (femoral neck, displaced pelvic fractures etc.) can be further proposed. For painful bone tumours with extension into the surrounding soft tissues, thermal ablation (radiofrequency, cryoablation, microwave ablation) is required to treat pain caused by the soft tissue invasion. An additional consolidation technique (percutaneous cement injection for flat bones and surgical stabilisation in cases of long bones) may be also required. In cases of spinal metastasis with epidural extension not responding to EBRT and when surgery is contraindicated, thermal ablation (combined with insulation techniques, temperature and electrophysiologic monitoring) can be proposed in order to limit tumour burden and prevent spinal cord compression. In all cases, the precise clinical evaluation of the patient is mandatory and should include previous treatments, patient-tolerated anaesthesia and life expectancy.
***Learning Objectives:***
1. To learn about the indications and techniques of MSK tumours pain palliation.
2. To learn about patient selection and clinical and imaging follow up.
3. To learn about the indication of consolidation techniques.
4. To become familiar with how to promote interventional procedure in MDT.

**A-0513** 08:58
**Abdominal pain management with minimally invasive techniques**
D.C. Madoff; *New York, NY/US (dcm9006@med.cornell.edu)*

Chronic abdominal pain occurs as a complication of various malignant diseases including pancreatic cancer, and when present may contribute to a lower quality of life and higher mortality. Though various pain management strategies are available as part of a multimodal approach, they are often incompletely effective and accompanied by side effects. Pain originating in abdominal viscera is transmitted via the celiac plexus, an autonomic plexus located in the retroperitoneum at the root of the celiac trunk. Direct intervention at the level of the plexus, referred to as celiac plexus block or neurolysis depending on the injectate is a minimally invasive therapeutic strategy which has been demonstrated to decrease pain, improve function, and reduce opiate dependence. Various percutaneous techniques have been reported, but, with appropriate preprocedural planning, use of image guidance, and postprocedural care, the frequency and severity of complications is low and the success rate high regardless of approach. The main benefit of the intervention may be in reduced opiate dependence and opiate-associated side effects, which in turn improves quality of life. Celiac plexus block and neurolysis are safe and effective treatments for chronic abdominal pain and should be considered early in patients experiencing such symptoms. Other minimally invasive strategies, including catheter placement for malignant bowel obstruction and embolisation for tumour-related pain, will also be discussed.
***Learning Objectives:***
1. To learn about the indication and patient selection.
2. To learn the techniques of pain palliation in abdominal cancer.
3. To learn about patient and clinical follow up, complication management and pain evaluation.
4. To become familiar with how to promote interventional procedure in MDT.
*Author Disclosure:*
**D.C. Madoff:** Advisory Board; Renovorx. Consultant; Guerbet, GE Healthcare, Argon Medical Devices, Embolx, Penumbra.

**A-0514** 09:21
**Pelvic pain management with minimally invasive techniques**
B. Kastler; *Paris/FR (b.kastler@noos.fr)*

Pain is the most fearsome symptom patient cancer and relatives will experience. It is estimated to be present in pelvic pain in a wide-ranging prevalence of 40 % up to 100% in patients with advanced stages particularly. In the end stages of the disease, pain usually becomes refractory to 3rdstep WHO analgesic drugs as strong opioids. To address these pelvic pain issues, various interventional techniques CT-guided techniques can be performed. - Patients with osseous structure involvement (mets or local invasion) usually respond well to analgesic cocktail infiltrations, bone ablation and/or cementoplasty. We will demonstrate how we proceed under local anaesthesia and tumoral block.- Patients with pelvic pain due to non-neural tumours pelvic pain are more difficult to manage. Initially, they suffer from nociceptive pain due to inflammation and irritation of nerves and later on, via traction and destruction of neural structures, neuropathic pain (causalgia and deafferentation). These patients may be treated by blocks and neurolysis: hypogastric plexus aimed procedures (presacral and/or hypogastric nerves) are used to relieve various pelvic cancer pain syndromes. Ganglion impar block to treat lower pelvic or perineal pain, coccydynia, troublesome tenesmus and anorectal pain from local malignancy. Pudendal infiltration in pelvic posterior or sacral involvement by tumours can also be considered. Radiation-induced or surgical pelvic pain patients may also benefit from these techniques.
***Learning Objectives:***
1. To learn about the indication and patient selection.
2. To learn the techniques of pain palliation in pelvic pain in cancer patients.
3. To learn about patient and clinical follow up, complication management and pain evaluation.
4. To become familiar how to promote interventional procedure in MDT.

09:44
**Panel discussion: How to integrate the interventional radiologist in the "palliative care" team?**

**08:30 - 10:00** **Room E1**

### E³ - ECR Master Class (Emergency Imaging)

### E³ 926

### Post-treatment emergencies in oncologic patients

**A-0515** 08:30
**Chairperson's introduction: The role of imaging in the early detection of complications in oncologically treated patients**
D.R. Kool; *Amsterdam/NL*

Oncology patients can present with life-threatening conditions. Those conditions may be caused by local expansion of a primary tumour or metastases, for example, haemorrhage, vena cava superior syndrome or spinal cord compression. Furthermore, complications can be caused indirectly by malignancies, for instance, thromboembolic disease or paraneoplastic metabolic or neurological symptoms. Life-threatening complications can also be an unwanted consequence of tumour therapy. Surgery, radiotherapy, interventional radiology (IR) procedures, chemotherapy and immunotherapy, each have their own set of potential complications that can present in the emergency setting. Hematologic and metabolic complications are mainly diagnosed with clinical and laboratory findings. For other complications, diagnostic imaging is essential for making the correct diagnosis and imaging will influence treatment. Complications of surgery, radiotherapy and IR procedures will mostly be near the treated area. Adverse effects of systemic therapies, chemotherapy and immunotherapy, can occur at remote sites. Complications of oncological treatment can be life-threatening. However, when treated in time they often are reversible. Recognising the imaging findings of complications of tumour therapy timely and accurately is important. Immunotherapy is a more recent treatment, and the adverse effects differ from the more familiar complications of the other tumour therapies. Immunotherapy is increasingly used, and a radiologist will encounter these complications more frequently. In this session complications in the chest and abdomen and of IR procedures will be discussed and their imaging findings will be demonstrated. Interventional techniques used in the treatment of complications of different tumour therapies will be discussed as well.

***Session Objectives:***
1. To learn about different approaches in modern tumour therapy.
2. To understand the radiological appearance of complications following treatment in oncologic patients.
3. To appreciate the role of different imaging modalities in further management of patients.

**A-0516** 08:35
**A. Chest**
H. Prosch; *Vienna/AT (helmut.prosch@meduniwien.ac.at)*

Lung cancer is the most common cause of cancer-related death in Western countries. Treatment in lung cancer is based on three pillars: surgery; radiation therapy; and systemic therapy. Imaging plays a major role in treatment decisions, response evaluation, and last, but not least, also in assessing adverse events following therapy. After surgery, acute (early) complications include pulmonary oedema (hydrostatic, permeability), atelectasis, hemothorax, acute infections, and broncho-pleural fistulas. Acute complications after radiation therapy include mainly early radiation pneumonitis and infections. Following systemic therapy, in addition to infections, non-infectious pneumonitis and sarcoid-like reactions are important complications that must be considered when investigating a patient who presents with acute symptoms.

***Learning Objectives:***
1. To become familiar with the modern approach to malignant chest tumour therapy.
2. To learn how to differentiate clinically important complications.
3. To understand how to look for the early signs of severe and urgent conditions.

*Author Disclosure:*
**H. Prosch:** Advisory Board; Boehringer Ingelheim, Roche, MSD, BMS, AstraZeneca.

**A-0517** 09:00
**B. Abdomen**
R. Basilico; *Chieti/IT (rbasilic@unich.it)*

Despite many improvements in perioperative, post ionising radiation therapy or pharmacological morbidity in abdominal tumour treatments, acute complications after tumour therapies in the abdomen are still common. A number of different therapies, including surgery, by means of a laparoscopic approach or minimally invasive robotic surgery, radiotherapy, chemotherapy and immunotherapy, may be effectively used to treat abdominal tumours. However, these kind of treatments are not devoid of complications and, last, abdominal emergencies may also occur after therapies used for extra-abdominal tumours. Cross-sectional imaging and particularly CT plays a crucial role in the diagnosis and management of most common abdominal emergencies after tumour therapies. The main complications following abdominal, urologic or gynecologic surgery include peritonitis, abscesses, haemorrhage, small-bowel obstruction and anastomotic leaks. Specific complications following hepatobiliary surgery include bile leakage and bile duct injuries: US and MRI with Magnetic Resonance Cholangiopancreatography (MRCP) sequencers are the preferred modalities for assessment of the postoperative biliary tract. Less common postoperative emergencies are vascular complications such as pseudoaneurysms. Small-bowel obstruction caused by radiation enteritis represents one of the most frequent abdominal complications after radiotherapy of prostate and rectal cancer. Whereas, a less known disease such as pneumatosis cystoids intestinalis, may be associated with systemic chemotherapy. Due to the increasing use of immunotherapy for different type of tumours, new inflammatory and immune-related adverse events also occur in the abdomen, and they need to be managed by a multidisciplinary approach.

***Learning Objectives:***
1. To learn about different therapies used in abdominal tumours.
2. To become familiar with possible complications.
3. To understand the effectiveness of imaging modalities in the evaluation of emergent complications.

**A-0518** 09:25
**C. How can interventional radiologists help in the management of oncological treatment complications?**
K.K. Pyra; *Lublin/PL (k.pyra@poczta.fm)*

Patients being treated for cancer frequently experience adverse effects. Interventional radiology plays an important role in the management of oncological treatment complications. Managing these effects improves quality of life and reduces mortality. The most commonly used interventional radiology techniques include embolisation, thermal ablation, vertebral augmentation, cementoplasty, percutaneous internal fixation, drainage of an intraabdominal abscess, radiologically guided peripheral insertion of central catheters, parenteral nutrition, percutaneous transhepatic cholangiography, inferior vena cava filter implantation and many others. IR can manage the majority of procedure-related complications so that surgical reintervention is required in only a small percentage of patients. Even than presurgical endovascular treatment can be invaluable. Blocking pain signal with ablation of the nerve is one of the examples. It is important for clinicians to understand the goals of endovascular treatment and recognise the importance and role of the IR in the management of these patients undergoing oncological treatment.

***Learning Objectives:***
1. To understand the role of interventional radiology in modern tumour therapy and the complications.
2. To be familiar with interventional techniques used in the treatment of complications of different tumour therapies.
3. To learn which tumour therapy complications can be treated with interventional radiology.

09:50
**Panel discussion: What is the impact of complications findings on the continued management of oncologic patients?**

**08:30 - 10:00** **Room E2**

### Neuro

### RC 911

### Update on cerebrospinal fluid (CSF) diseases

*Moderator:*
Z. Merhemic; Sarajevo/BA

**A-0519** 08:30
**A. Imaging strategies for hydrocephalus**
C.A.J. Romanowski; *Sheffield/UK (charles.romanowski@sth.nhs.uk)*

The classical bulk flow hypothesis of CSF production and flow is still widely taught in medical schools and textbooks. In recent years however new scientific evidence has led to some groups challenging this hypothesis. There is now much evidence to support the view that CSF is filtered and reabsorbed across the whole capillary bed of the CNS. In addition, MRI studies have confirmed that CSF flows not simply in a unidirectional manner from the choroid plexus to the subarachnoid space but in a bidirectional manner driven by a systolic expansion of intracranial arteries during the cardiac cycle. This refresher lecture will begin by looking at these new views regarding CSF physiology and flow. The different aetiologies leading to hydrocephalus will be examined. The role MRI in assessing normal CSF flow and disturbances of CSF flow will be examined. This refresher lecture will also include the clinical imaging assessment of the planning and follow-up of modern neurosurgical treatments of hydrocephalus.

***Learning Objectives:***
1. To learn about different types of hydrocephalus and how to distinguish them in imaging.
2. To understand the pathophysiology of CSF circulation.
3. To appreciate MR imaging techniques for diagnosing abnormalities of the CSF flow.

**A-0520** 08:53
**B. Diagnosis and treatment of intracranial hypotension**
E. Papadaki; *Iraklion/GR (fpapada@otenet.gr)*

Spontaneous intracranial hypotension (SIH) usually involves middle age women presented with orthostatic headaches. It is caused by spinal CSF leakage due to dural weakness involving nerve root sleeves and rupture of the protruded arachnoid layer, ventral tears of the dura caused by calcified disc herniations and spiculated osteophytes or CSF-venous fistulas within a spinal epidural vein. Although the International Classification of Headache Disorders (ICHD) provides diagnostic criteria for SIH, based on CSF pressure measurements and imaging evidence of CSF leakage, misdiagnosis is common, and there is not a single diagnostic test that can rule out SIH with certainty. A CSF opening pressure of <6 cm H20 during lumbar puncture facilitates the diagnosis of SIH, however, most patients with SIH have normal CSF pressure. Brain MRI findings include diffuse dural enhancement, brain sagging with descent of the brainstem, the third ventricular floor and the mammillary bodies, narrowing of the interpeduncular and prepontine cisterns and cerebellar tonsillar ectopia, pituitary gland enlargement, dilation of the intracranial venous sinuses ("venous distention sign") and bilateral subdural collections (simple or haemorrhagic), while spinal MRI may reveal epidural collections. For the localisation of the spinal CSF leakage CT myelography (including ultrafast CT myelography and digital subtraction myelography) is considered to be the modality of choice, although MR myelography, even with intrathecal gadolinium, could also be used in certain cases. Nuclear medicine myelography can also be employed. Treatment includes conservative therapy with bed rest, caffeine, and oral hydration, targeted or not epidural patching, or even surgery.

***Learning Objectives:***

1. To learn about the underlying pathophysiology of spontaneous intracranial hypotension (SIH).
2. To understand imaging strategies for this condition.
3. To clarify myths and misperception of intracranial hypotension.

**A-0521** 09:15

**C. RCVS, PRES and others**

F. Bonneville; *Toulouse/FR (bonneville.f@chu-toulouse.fr)*

Reversible Cerebral Vasoconstriction Syndrome (RCVS) and Posterior Reversible Encephalopathy Syndrome (PRES) are disorders of the cerebrovascular autoregulation and are likely to share a common pathophysiology. PRES is mostly due to acute hypertension, that causes blood-brain barrier disruption. It occurs in case of preeclampsia/eclampsia, uremic encephalopathies or drug toxicity such as cyclosporine, tacrolimus or cisplatin. Patients with PRES present with headaches, seizure, altered consciousness or even neurological deficit. Most cases are regressive after blood pressure normalisation or withdrawal of causal agent. CT and MRI typically demonstrate bilateral posterior vasogenic oedema, that involves cortex and subcortical white matter in the temporal, occipital and parietal lobes, as well as in the watershed zones and the cerebellum. Basal ganglia and brainstem may be involved too. Patchy enhancement may be observed in the cortex. Haemorrhage is not rare and may be depicted in the subarachnoid spaces or in the brain parenchyma. CTA and MRA may demonstrate multifocal areas of distal arterial narrowing, a finding similar to RCVS. The latter is a rare disorder characterised by recurrent severe thunderclap headaches, with or without neurological symptoms, and transient narrowing of the cerebral arteries which is reversible within three months. RCVS onset is often associated with vasoactive medications or illicit drugs and the post-partum state. An ischemic or hemorrhagic stroke develops in about one-third of cases.

***Learning Objectives:***

1. To learn about the current controversial mechanism of posterior reversible encephalopathy syndrome (PRES).
2. To understand therapeutic and prognostic implications of PRES and reversible cerebral vasoconstriction syndrome (RCVS) diagnosis.
3. To appreciate imaging features of PRES, RCVS and their differentials.

**A-0522** 09:38

**D. Hypertension-associated brain changes**

A. Krainik; *Grenoble/FR (akrainik@chu-grenoble.fr)*

Acute intracranial hypertension (ICH) is among the most critical emergencies in medicine, which requires active management in neurointensive care and neurosurgery. Increasing pressure within the rigid spaces of the skull may lead to brain herniation through the dural openings, at risk of compression of the central nervous system and cerebral arteries. Besides arterial compression at risk of stroke, the compression of the mesencephalon is at risk of coma, and the compression of the medulla oblongata is at risk of respiratory, cardiac, and blood pressure dysregulations. Thus, careful analysis of the subarachnoid spaces is mandatory in order to identify acute intracranial hypertension that might worsen the short-term prognosis of any expanding lesion. Additionally, daily CSF production is three times its total volume. Thus any acute obstacle on its circulation may also lead to acute ICH. Chronic intracranial hypertension is mostly due to resorption dysfunction of arachnoid granulations and venous drainage into dural sinuses. Because of brain volume changes with ageing, morphological consequences and semiology depend on age, from benign ICH in younger patients with subtle subarachnoid spaces enlargement into the sella and the optic nerve sheaths, to chronic hydrocephalus without intracranial hypertension in elderly.

***Learning Objectives:***

1. To understand the causes of intracranial hypertension.
2. To understand the threats of intracranial hypertension on central nervous system and arterial integrity.
3. To recognise intracranial hypertension and its complications on CT and MR.

**08:30 - 10:00** **Room F1**

### E³ - Rising Stars Programme: Basic Session

### BS 9

### Neurologic emergencies

*Moderator:*
J. Walecki; Warsaw/PL

**A-0523** 08:30

**Haemorrhagic stroke (SAH)**

C. Calli; *Izmir/TR*

"no abstract submitted"

***Learning Objectives:***

1. To review the most common pathologies leading to SAH.
2. To present current imaging techniques for evaluation of SAH.
3. The role of interventional neuroradiology in SAH.

**A-0524** 09:00

**Ischaemic stroke: diagnosis**

M. Taina; *Kuopio/FI*

The five etiological stroke categories are large artery atherosclerosis, cardioembolism, small-artery occlusion, stroke of other determined aetiology, and stroke of undetermined aetiology (cryptogenic). This classification of stroke is based on the patient's clinical status and imaging and laboratory data. With the improved spatial resolution of computed tomography angiography (CTA), CT not only excludes acute intracerebral haemorrhage but also helps to define stroke aetiology, clot location and distribution of compromised circulation. It is pivotal to evaluate the cervicocranial vasculature soon after the onset of acute cerebral ischemia to aid in the determination of the mechanism of the stroke, to help in treatment decisions, and prevent a recurrence. CTA also provides a possibility to evaluate collateral circulation. Potential utility for CT perfusion imaging in acute stroke includes the identification of the irreversibly infarcted core and at-risk brain regions (penumbra) that may be salvageable with successful intra-arterial thrombectomy beyond the standard 4.5-hour window for intravenous thrombolysis. The acute stroke CT protocol including CT, CTA, and CT perfusion helps triage patients with at-risk brain regions to available therapies, such as mechanical thrombectomy, induced hypertension or intensive monitoring of patients who are candidates for craniectomy. Accurate imaging also yields us valuable information for the management of wake-up stroke patients for whom the precise time of onset is unknown.

***Learning Objectives:***

1. To become familiar with the most common aetiologies of stroke.
2. To present current imaging techniques for evaluation of stroke.
3. To recognise the imaging signs of stroke.

**A-0525** 09:30

**Ischaemic stroke: treatment**

T. van der Zijden; *Antwerp/BE (thijsvanderzijden@hotmail.com)*

Acute ischemic stroke is a major cause of death and significant disability worldwide. The main goal in primary acute ischaemic stroke treatment is to achieve as fast as possible reperfusion of the brain area at risk for infarction. Endovascular mechanical thrombectomy has proven to be effective in doing this. Although very fast procedure times with impressive recanalisation rates can be achieved, it is vital to select the correct patients for endovascular treatment in order to avoid futile recanalisation and to prevent additional harm to ischemic stroke patients. Besides proper clinical work-up, adequate neuroradiological work-up should be performed to select the correct patients for recanalisation therapy. Imaging should not only be focused on the exclusion of haemorrhage and/or stroke mimickers and structural imaging of craniocervical arteries but also should give an idea about the extent of potentially salvageable brain tissue versus already infarcted brain. Implementing additional imaging tools, such as perfusion techniques and multiphase CT angiography, might give a better insight of prediction of clinical outcome after a potential recanalisation procedure in a given patient. During the past years, mechanical thrombectomy was mostly done by the use of stent retrievers. Due to ongoing technological developments and experiences on the job, other approaches, such as thrombus aspiration and mixed techniques, has emerged as effective treatment tools as well. Besides knowledge about the criteria and used endovascular techniques, It is also important to recognise the potential complications after mechanical thrombectomy, in order to get the best clinical outcome after treatment.

***Learning Objectives:***
1. To become familiar with the criteria for the endovascular treatment.
2. To present current methods of the endovascular treatment.
3. To recognise complications of the treatment.

**08:30 - 10:00 Room F2**

### Breast

### RC 902

### Screening for breast cancer

**A-0526** 08:30
**Chairperson's introduction**
F.J. Gilbert; *Cambridge/UK (fjg28@cam.ac.uk)*

In 2003 the European Council recommended that mammography is offered two years between the ages of 50-70 years. All countries offer two yearly mammography or digital breast tomosynthesis (DBT) apart from the UK at three yearly. Nine countries start at age 40 or 45 years with 18 commencing at age 50 years. Three countries stop at 64 years, with the majority at stopping at age 69 years although three countries continue until age 74 years. Uptake varies between 37-85%, Latvia and Poland the lowest uptakes and Finland, Ireland and Holland the highest rates. Some countries have adopted DBT while others are conducting large randomised controlled trials to establish the impact, costs and benefits. It is important to ascertain whether or not this will result in a survival benefit and not merely increase overdiagnosis. Some countries offer supplemental screening with ultrasound to women with increased breast density as this increases the likelihood of developing breast cancer by two-fold in the over 50 year age group and increases the chance of cancer being missed or not being detected until the tumour is larger in size. In women at higher family history risk, a multimodal approach is justified. The increased sensitivity of MRI, especially in younger women with denser breasts, means that this more expensive modality is justified where there are more cancers to be found. However, it is important to balance the risks and benefits of MR based screening.
***Session Objectives:***
1. To learn about the strengths and weaknesses of different forms of breast cancer screening.
2. To understand the value of the various screening modalities and how they should be used in practice.
3. To recognise that no single screening strategy serves every patient equally well.
*Author Disclosure:*
**F.J. Gilbert:** Advisory Board; Google DeepMind. Grant Recipient; GE Healthcare, Hologic. Research/Grant Support; Hologic, GE Healthcare, volpara. Speaker; GE Healthcare.

**A-0527** 08:35
**A. Screening with mammography and digital breast tomosynthesis alone**
R.M. Pijnappel; *Utrecht/NL (r.m.pijnappel@umcutrecht.nl)*

Mammographic screening is beneficial to the population. Apart from a reduction in mortality less intense treatment is often possible due to the fact that the tumour burden is lower compared to a non-screening situation. Despite these advantages screening also creates inevitable harm to women. It is the challenge to balance the benefits and harms in order to optimise a screening program. Compared to FFDM tomosynthesis has advantages and disadvantages regarding recall, detection and clinical outcome. The differences between FFDM and tomosynthesis in a screening environment will be highlighted. Errors in screening are inevitable. The main errors are false-positives and false-negative results. The reasons for these errors, consequences for women and possible solutions will be discussed.
***Learning Objectives:***
1. To learn about the benefits and limitations of population-based mammography screening.
2. To become familiar with the advantages and disadvantages of digital breast tomosynthesis.
3. To appreciate sources of error in mammography screening.
*Author Disclosure:*
**R.M. Pijnappel:** Equipment Support Recipient; GE, Hologic, Volpara. Research/Grant Support; Hologic, Bayer.

**A-0528** 09:00
**B. Combined screening with mammography and ultrasound**
T.H. Helbich; *Vienna/AT (Thomas.Helbich@meduniwien.ac.at)*

The sensitivity and specificity of mammography are limited in highly fibroglandular /dense breasts. Digital mammography provides increased sensitivity in young women and those with moderately dense breasts, and digital three-dimensional mammography (Tomosynthesis) promises further improvement. For women with the densest breasts, however, radiography is unlikely to be the optimum solution. MRI, although not affected by breast density, is expensive and access is often limited. Ultrasonography is attractive for breast cancer screening because, likewise, it is not impaired by breast density, and it avoids the use of ionising radiation and the need for breast compression. Nevertheless, enthusiasm for the use of ultrasonography has been limited because its specificity has been much lower than that of mammography, but technical developments have given rise to sharper, more informative images. These improvements foster the use of ultrasound particular in those women with higher breast density. Different trials have been preformed, and promising results have been reported.
***Learning Objectives:***
1. To learn about the added value of ultrasound in screening and its indications.
2. To become familiar with the current level of evidence for screening with ultrasound.
3. To appreciate the role of ultrasound in clinical practice.
*Author Disclosure:*
**T.H. Helbich:** Grant Recipient; EU Grants. Research/Grant Support; Siemens, Guerbet, Bard,.

**A-0529** 09:25
**C. Personalised multimodality screening with MRI**
U. Bick; *Berlin/DE (Ulrich.Bick@charite.de)*

High-risk women with a genetic predisposition will develop breast cancer not only more frequently but also at a much younger age than average-risk women. Traditional population-based mammography screening programs aiming primarily at postmenopausal women are therefore not sufficient as surveillance for these women. Contrast-enhanced breast MRI with its high sensitivity independent of breast density has been shown to be the ideal screening tool for young premenopausal high-risk women. Depending on the age and risk constellation of the individual patient, this can be supplemented by mammography and/or tailored second-look ultrasound. To be effective, high-risk multimodality screening should be offered annually starting at age 25 to 30. However, even annual multimodality screening with MRI may not be sufficient in BRCA1-mutation carriers to detect a sufficiently large proportion of cancers in a curable stage, and risk-reducing mastectomy may be considered as a viable alternative in these patients. Whereas age-specific breast cancer incidence is fairly well established for BRCA1/2 mutation carriers, breast cancer risk prediction in high-risk patients without such a mutation is much more difficult, and substantial variations in calculated risk exist between the currently available models. This is of great importance, as accurate breast cancer risk prediction is vital for correctly tailoring the high-risk screening program. If the observed breast cancer incidence in the screened population is too low, the positive predictive value will decline to unacceptable levels and screening will become ineffective.
***Learning Objectives:***
1. To learn about multimodality screening strategies for high-risk patients.
2. To become familiar with the difficulties in predicting individual breast cancer risk.
3. To appreciate the careful balance of risks and benefits in MRI-based screening.
*Author Disclosure:*
**U. Bick:** Patent Holder; License agreement and Royalties, Hologic, Inc.

09:50
**Panel discussion: What is the best approach to screen for breast cancer?**

**08:30 - 10:00 Room Y**

### E³ - Rising Stars Programme: EFRS Radiographers' Basic Session

### BR 9

### Career planning

**A-0530** 08:30
**Chairperson's introduction: What do new graduates need to look like?**
G. Ioannidis; *Larissa/GR (i.giorgio@yahoo.gr)*

The world of radiology is an everyday evolving science. Its development entangles all those involved in its everyday life. Radiographers are the main characters in the development of radiology. Young graduates who have the basing training from their school start offering their services to patients, although they have the duty to develop their knowledge as well as the science of radiology. This is achieved by participating in specialised training courses, participating in radiology courses, in research protocols as well as enriching their knowledge from other specialities. The most important thing though is to gain experience. Experience is gained from daily contact with radiology. Taking part in an everyday service of a radiology lab confers self-confidence to the radiographer, especially if there is a possibility of attending a large hospital curacy. Feeling confident for his knowledge the radiographer is ready to look for the specialised knowledge in his subject and consequently to develop his speciality. Finds out what he can offer to the other specialities and increases with his knowledge the demands by doctors.

***Session Objectives:***
1. To understand how best to prepare your professional profile.
2. To appreciate the potential for radiographers to work in various jurisdictions.
3. To consider the utility of professional development planning in achieving your professional goals.

**A-0531** 08:35

**The role of preceptorship in first post-radiography positions**

A. Martin; *Manchester/UK (amanda.martin@boltonft.nhs.uk)*

Many radiographer vacancies are filled by newly qualified radiographers who are stepping away from the supportive and safe environment afforded to them as a student, where they are supervised closely and move into clinical practice as an accountable and autonomous practitioner. Confidence levels may be low as they suddenly find themselves being wholly responsible for their decisions for the first time in their radiography journey. Add to this the lack of familiarity in a new department, along with the expectations of staff, the pressure put on the newly qualified radiographer, by themselves or by others, may soon become too much. This may lead to mistakes being made, which could cause patient harm. It could also lead to increased stress levels, periods of sickness or resignation from their post. The provision of a structured support framework for newly qualified radiographers in the first few months of employment is pivotal in their transition into confident and effective practitioners who are likely to stay in post and develop further into valued, experienced and highly skilled radiographers. There are different approaches to preceptorship, but all should share the same key components, leading into the appraisal cycle. The models of preceptorship will be explained before one department approach to preceptorship is discussed.

***Learning Objectives:***
1. To understand the value of preceptorship.
2. To become familiar with common preceptorship programmes.
3. To be aware of how preceptorship programmes can benefits patients, radiographers and health care institutions.

**A-0532** 08:58

**Practising radiography around the world**

C. Buissink; *Groningen/NL (c.buissink@pl.hanze.nl)*

Globalisation and collaboration in an international setting can no longer be ignored. This also applies to the profession of the radiographer. That is why it is important for us as Radiographers to deepen our knowledge and to look for opportunities in the international field. International collaboration has always been strong in research. However, this is not the only place where internationalisation is visible. An international mindset starts at the beginning of a Radiographer career. Therefore, many universities have included internationalisation in their curricula. The first steps could be internationalization@home (Crowther et al. 2001). Students often come into contact with internationalisation through internships, exchange programs or Summer schools. But in recent years, working for shorter or longer periods abroad has also become more common. A difficult challenge is recognition of the diploma, within Europe there are different forms of education and level of education. These are also important discussion points for national and European associations. Working as a radiographer around the world is still quite a challenge. Exchanging experiences is the first step.

***Learning Objectives:***
1. To be aware of the potential to use your radiography qualification in various jurisdictions.
2. To consider barriers to practicing as a radiographer abroad.
3. To appreciate the potential for charitable/voluntary work in developing countries.

**A-0533** 09:21

**The importance of professional registration and CPD**

L.A. Rainford; *Dublin/IE (louise.rainford@ucd.ie)*

Medical imaging is becoming increasingly diverse with the continued development of multiple sub-specialities. The manner in which radiographers work within these sub-specialities and how professional work structures have developed across different countries all place a need on radiographers to continually develop and increase their knowledge and understanding of the areas of imaging they work within. Medical imaging technology and changes in practice alter rapidly. The importance of professional development planning for radiographers will be discussed in maintaining, developing and improving radiographer competencies to ensure optimal practice and service delivery, in our ever-changing and challenging clinical environment. Mandatory or voluntary CPD is becoming a common requirement across European Radiography professional societies. The importance of CPD for radiographers will be discussed, and the EFRS CPD guidelines will be outlined. Profession registration and its importance to Radiography will be discussed in the context of professional society registration and state registration of our profession by government regulators. The benefits and challenges of registration will be outlined and “real world” details will be provided to evidence points addressed in the presentation.

***Learning Objectives:***
1. To understand the importance of professional development planning for radiographers in maintaining, developing and improving radiographer competencies.
2. To be aware of the essential role of CPD and further education for radiographers.
3. To appreciate the importance of professional registration.

09:44

**Panel discussion: My best career advice**

**08:30 - 10:00** **Room D**

### Musculoskeletal

### RC 910

### Inflammatory and infectious diseases of the spine: how to differentiate from degeneration

*Moderator:*
J.L. Bloem; Leiden/NL

**A-0541** 08:30

**A. Spondyloarthritis: a diagnostic chameleon**

V. Zubler; *Zurich/CH*

The group of spondyloarthritides compromises a number of closely related rheumatic diseases with common clinical features: Ankylosing Spondylitis, Psoriatic Arthritis, Arthritis and Spondylitis related to inflammatory bowel disease, and Reactive Arthritis. Patients can also be grouped into two categories based on their predominant clinical presentation: axial or peripheral. Axial Spondyloarthritis is inflammatory arthritis primarily involving the sacroiliac joints and the spine. Presumably, the process of inflammation starts as enthesitis, mainly visible as bone marrow oedema at the insertion of ligaments or tendons into bone. However, bone marrow oedema is an unspecific feature and occurs amongst others frequently in the context of mechanical changes and degenerative processes of the axial skeleton. Imaging is an important component of the classification criteria for axial Spondyloarthritis. Conventional radiography is an essential part of the internationally accepted modified NY criteria for Ankylosing Spondylitis but visualises only the late structural changes of the inflammatory process after years. Magnetic Resonance Imaging can detect the early changes in up to four months after clinical onset. MRI of the sacroiliac joints has been integrated as a key diagnostic criterion of axial Spondyloarthritis according to the Assessment of SpondyloArthritis International Society (ASAS) in 2009.

***Learning Objectives:***
1. To explain the pathophysiology and disease spectrum of spondyloarthritides.
2. To describe the imaging findings of spondyloarthritides.

**A-0542** 09:00

**B. Crystals: may also affect the spine**

P.J. O'Connor; *Leeds/UK (philip.oconnor@nhs.net)*

Crystal deposition in the spine is unusual. The presentation varies from acute pain syndromes to subclinical disease usually detected as an incidental finding on cross-sectional imaging. The pathophysiology, clinical presentation and imaging appearances of crystal disease will be presented. Spinal gout, acute longus colli hydroxyapatite deposition and Crown dens syndrome resulting from either hydroxyapatite or pyrophosphate deposition will be described. Differentiating crystal disease from ligamentous ossification, degeneration and other deposition diseases such as amyloid will be discussed. Gout is a common inflammatory arthritis most commonly seen on men and post-menopausal women. The prevalence of gout is currently increasing over time. Spinal gout typically presents with localised pain, incidental lytic vertebral lesions usually detected on cross-sectional imaging or with symptoms resulting from neurological compromise. Spinal gout most commonly effects the posterior elements of the spine. Crowned dens syndrome results from crystal deposition around the odontoid peg of the cervical spine. It presents with acute neck pain and unless recognised often leads to misdiagnosis and inappropriate treatment. The deposition can be either pyrophosphate or hydroxyapatite crystals with both having identical imaging appearances. A similar clinical presentation is also seen in Acute calcific longus colli hydroxyapatite deposition. This is a rare cause of severe neck pain, dysphagia and odynophagia is often mistaken for other common causes of neck pain. Similar to crowned dens syndrome prompt recognition is important to prevent unnecessary imaging or treatment.

***Learning Objectives:***

1. To explain the pathophysiology and disease spectrum of crystal deposition diseases that affect the spine.
2. To describe the imaging findings of crystal deposition diseases that affects the spine.

*Author Disclosure:*

**P.J. O'Connor:** Advisory Board; uropeean Golf tour medical advisory board. Author; action Editor Grainger and Allison. Research/Grant Support; IHR research grant support.

**A-0543** 09:30

**C. Infection: imaging, indication and techniques for biopsy**

J.-L. Drapé; *Paris/FR (jean-luc.drape@cch.aphp.fr)*

The diagnosis of spinal infections is often made by a combination of clinical symptoms and radiologic abnormalities. Magnetic resonance imaging is the main imaging modality for this diagnosis. Confirmation is based on histopathologic findings and/or identification of pathogens from biopsy specimens or blood cultures. Percutaneous biopsies are widely practiced in this indication, with the more accurate and safer guidance of computed tomography (CT). Two main types of percutaneous biopsies are available: fine needle aspiration biopsy and core needle biopsy. Specific techniques and approaches with varying needle systems are described for each spinal region. It is the procedure of choice in the definitive diagnosis of pathologic lesions of the spine.

***Learning Objectives:***

1. To describe the imaging findings of spinal infection.
2. To explain the biopsy indication and techniques in spinal infection.

**08:30 - 10:00** **Room G**

### Joint Session of the ESR and EFOMP

### ESR/EFOMP

### Medical imaging and emerging issues in occupational radiation exposure

*Moderators:*

M. Brambilla; Novara/IT

W.R. Jaschke; Innsbruck/AT

**A-0544** 08:30

**Is fear of radiation-induced occupational cancer irrational?**

P. Vock; *Spiegel/CH*

Starting with a short overview of the development of occupational dose limits, this presentation will concentrate on the quantitative estimation of the risk of radiation-induced occupational cancer. While facts are often statistically solid in the higher dose range, below around 50 mSv of effective dose the linear-no-threshold (LNT) model of dose-risk relation has been questioned by a number of radiobiological studies; existing epidemiological data, due to limited power, do not differentiate between LNT, non-linear and threshold-based models. In other words, the LNT model might either overestimate or underestimate the risk. Furthermore, even for the same physical dose the gender, age at exposure, the temporal distribution of exposure, whole-body vs regional exposure, and individual radiosensitivity will modify the biological impact and, thus, the cancer risk. Known and unknown influences of these modifications will be discussed. Despite open questions, enough facts are established to give the exposed workers clear rules to protect themselves. The communication, therefore, has to show known and disputed risks and to clarify that a medical worker (above all an interventional radiologist) can avoid a lot of unnecessary exposure and reduce the risk to a low, tolerable level by his/her behaviour and skills that are based on a solid training, and the proper use of technical options of the equipment as well as of protective devices. In conclusion, while professional exposure to ionising radiation asks for our respect and a continuous ALARA culture, nonspecific fear of cancer is not justified.

***Learning Objectives:***

1. To learn how occupational dose limits have been established by ICRP to reduce the probability of stochastic effects.
2. To appreciate the order of magnitude of additional risk of cancer induced by occupational exposures.
3. To understand how these risks should be properly communicated to workers.

**A-0545** 08:50

**Eye lens radiation dose and cataractogenesis**

J. Damilakis; *Iraklion/GR (damilaki@med.uoc.gr)*

Several recent studies have shown a significant association between long-term exposure to low-dose radiation and increased risk of cataract formation. It is evident that the eye lens is more radiosensitive than previously estimated and thus protection of the eyes of occupationally exposed personnel is very important. Radiation-induced opacities and cataracts may take years or decades to develop. It is not entirely clear whether radiation-induced cataracts are stochastic or deterministic effects. However, it is known today that cataracts can occur after receiving radiation doses as low as 0.5 Gy. For this reason, the International Commission on Radiological Protection (ICRP) has accepted the dose threshold of 0.5 Gy for radiation-induced cataracts. The equivalent dose limit for the lens of the eye for occupational exposure has been reduced from 150 mSv per year to 20 mSv per year averaged over defined periods of 5 years, with no annual dose in a single year exceeding 50 mSv. Interventional radiologists may reach cumulative lens doses that put them at risk for developing cataracts. Eye lens dose monitoring methods will be presented and discussed. To keep lens doses as low as possible, exposed personnel must use radio-protective glasses.

***Learning Objectives:***

1. To become familiar with published data on lens opacities among interventionalists.
2. To discuss current methods for monitoring of eye lens doses.
3. To learn how effectively lead glasses protect the eyes of interventional radiologists.

**A-0546** 09:10

**Occupational exposure from interventional radiology procedures: how to measure it, how to reduce it**

W.R. Jaschke; *Innsbruck/AT (werner.jaschke@i-med.ac.at)*

The occupational dose of the Operator in fluoroscopically guided interventions can be assessed by personal dosimetry using thermoluminescence Dosimeters and real-time dosimetry. Personal dosimetry is a very reliable tool to measure personal dose but suffers from the fact that the operator gets the dose information with a time delay of weeks or sometimes even months. Real-time dosimetry is less accurate but provides real-time dose data. Thus, the operator gets immediate feedback on dose rate and how the dose rate changes if certain protective measures are put in place. Real-time dosimetry is, therefore, very important for individual radiation protection and optimisation. Occupational exposure arises mainly from scattered radiation from the patient and the angiographic Equipment. Scattered Radiation is undirected. Thus, radiation protection equipment has to be used to protect the operator. The most important tools are aprons, neck collars, lead glass shields and curtains as well as lead glass goggles. Operators have to be trained how to apply these protective measures most effectively and that they have to be used in every procedure. In addition, decreasing dose to the patient using dose optimised angiographic protocols is another important step to decrease dose to the operator.

***Learning Objectives:***

1. To learn which technical factors contribute to occupational exposures from interventional radiology procedures.
2. To appreciate that optimisation of a patient's exposure is a way to also reduce occupational exposure.
3. To understand additional means to reduce the occupational exposures.

**A-0547** 09:30

**Selection and usage of personal protective equipment in the fluoroscopy and interventional radiology operating room**

M. Brambilla; *Novara/IT (marco.brambilla@maggioreosp.novara.it)*

Workers can be exposed to significant scatter radiation during fluoroscopically guided interventions. Radiation-attenuating personal protective equipment (PPE) includes aprons to protect radiosensitive body organs, glasses to prevent cataract development, thyroid collars to protect a radiosensitive organ, lead equivalent surgical gloves and caps to reduce hands and head exposure. PPE can afford very different degrees of protection of different parts of the body during fluoroscopy and interventional radiology procedures. The concept of dose reduction factors (DRFs), equal to the ratio of the dose to the part of the body with no PPE, divided by that when PPE are worn will be introduced. The typical DRFs provided by the different PPE (Body, Thyroid, Eye lenses, Head, Extremities) will be reviewed. The need of wearing specific PPE will be discussed for the interventionalists, nurses and radiographers. Each worker involved in fluoroscopically guided interventions should wear garments that are appropriately protective for that individual's practice. Review of past personal dosimetry results and consultation with a medical physicist should guide this choice.

***Learning Objectives:***

1. To learn which personal protective equipment is needed for specific radiological practices.

2. To become familiar with occupational dose reduction provided by personal protective equipment.
3. To understand that a correct selection, use and periodic check of personal protective equipment are needed to ensure radiation protection of healthcare personnel.

09:50
**Panel discussion: Has the optimisation of occupational radiation exposure in radiology procedures reached a plateau?**

**08:30 - 10:00 Room K**

### Chest

### RC 904
### Lung nodule management in 2019

**A-0548** 08:30
**Chairperson's introduction**
A. Devaraj; *London/UK*

An overview of recent developments in lung nodule characterisation and management will be provided. Tips on optimal nodule characterisation and measurement will be provided; including how to avoid false positives and false negatives; as well as a brief overview of the appropriate use of lung nodule management guidelines in 2019.
***Session Objectives:***
1. To avoid common errors in nodule categorisation or measurement.
2. To summarise the recent knowledge about nodule management.
3. To understand the place of risk prediction models.

**A-0549** 08:35
**A. Radiological assessment**
T. Frauenfelder; *Zurich/CH (thomas.frauenfelder@usz.ch)*

Lung nodules are common radiological findings in the clinical practice, and the majority of them remain indeterminate at imaging. Determination of lung nodule malignancy is pivotal because the early diagnosis of lung cancer could provide a curative intervention. Therefore the correct interpretation and management of a lung nodule represent relevant issues for radiologists. The probability of nodule malignancy depends on patient risk factors and nodule characteristics (size, morphology, attenuation, growth). In addition, management is substantially influenced by the nodule size, attenuation (solid, ground-glass, part-solid) and growth rate. But in daily life we often struggle by the question: is this a real nodule? And more often: what type of nodule is it, especially if there is a slightly blurry rim? Furthermore, although knowing that perifissural nodules are known to be benign, there are clear definitions what this means. Nevertheless, also here there is no clear black or white. Following the guidelines and definitions, we will establish the characteristics of the different types of nodules.
***Learning Objectives:***
1. To learn how to recognise false nodules.
2. To learn how to classify lung nodules as solid, part solid or non-solid.
3. To be aware of the typical and atypical characteristics of perifissural opacities.

**A-0550** 08:58
**B. Computer-aided diagnosis and AI perspective**
A.R. Larici; *Rome/IT (annarita.larici@unicatt.it)*

Machine learning (ML) describes a subfield of artificial intelligence (AI) in which algorithms are trained to perform tasks by learning patterns from digital data. In classic ML approach, as the ones used in CAD algorithms, programmers define the features that appear distinctive of a disease and mathematic models are trained to identify such features. Newer ML techniques, as deep learning (DL), are focused on a particular outcome (e.g. nodule characterisation), and the distinctive features are identified through a training learning process. ML uses computational models and algorithms that imitate the architecture of the brain (artificial neural networks-ANNs). AI has the potential to replace the tasks of lung nodule detection and characterisation on images, currently performed by radiologists, with the advantage to overcome the subjectivity of human work in a quicker and standardised fashion. AI system also has the potential to automatically integrate multidisciplinary clinical data in the above-described processes and has been applied in the Radiomics analysis. Newer AI approach will increase the accuracy of CAD in lung nodule detection, especially decreasing false-positive results. Using recent CNNs 90% accuracy-one false positive detection per scan has been reported. Until recently only classical CAD-ML approaches were used for nodule characterisation. A recent experience integrating both classic ML and DL systems demonstrated a 0,99 AUC value in the lung nodule malignancy prediction. In this presentation, the most relevant applications of CAD and AI on lung nodule diagnosis and management will be reviewed.
***Learning Objectives:***
1. To review the capabilities and limitation of CAD for the detection of lung nodules.
2. To learn about the performance of AI in the detection and characterisation of lung nodules.
3. To understand the basic concepts of machine learning as applied to imaging.
4. To summarise currently available machine learning techniques in radiology.

**A-0551** 09:21
**C. Management guidelines**
A.A. Bankier; *Boston, MA/US (abankier@bidmc.harvard.edu)*

Pulmonary nodules detected on chest CT examinations often pose a substantial management problem. This presentation will review current guidelines designed to approach this problem. The presentation will also address models for evaluating the risk of a pulmonary nodule to be malignant. Finally, the presentation will discuss the fundamental differences between screening-detected and incidental pulmonary nodules.
***Learning Objectives:***
1. To review the incidentally-found nodule management guidelines.
2. To become familiar with the LungRADS classification of screen-detected nodules.
3. To learn about Brock and Herder and other prediction models for evaluating the malignancy risk.
*Author Disclosure:*
**A.A. Bankier:** Consultant; Spiration, Hummingbird Diagnostics, Daiichi Medical.

09:44
**Panel discussion: What do radiologists need to better manage pulmonary nodules?**

**08:30 - 10:00 Room M 1**

### ESHI$^{MT}$ Session

### ESHI(MT) 2
### Artificial intelligence in hybrid imaging
*Moderators:*
C.C. Cyran; Munich/DE
H.K. Hahn; Bremen/DE

**A-0552** 08:30
**Radiomics+: prediction model using convergent data**
A. Leimgruber; *Lausanne/CH (antoine.leimgruber@hopitalrivierachablais.ch)*

Medical Imaging generates large amounts of data commonly assessed visually using scientific evidence and expert knowledge combined with clinical patient-specific information. Quantitative methods have also been developed in today's multi-modality environment, providing morphologic, functional and biological information. Recent advances in data mining of quantitative image features together with powerful image analytic tools have lead to what is known today as "radiomics". Naturally, radiomics analytic techniques have been welcomed with both enthusiasm and scepticism. The process used in radiomics involves the identification of vast arrays of quantitative parameters within digital images.. The major challenge is to integrate radiomics data with clinical, pathological, and genomic information to decode the different types of tissue biology and specificities of the disease within a patient in a new paradigm that we introduce in this talk as "radiomics+". Imaging is indeed not the only field roaming the uncharted territory of large medical datasets. Pathology, liquid biopsy techniques or genetic analyses all provide an increasing number of molecular biomarkers. Oncology is the large port of entry of this trend given the need to tailor an increasingly diverse array of targeted therapies to the specificities of each patient and the history of each cancer within each individual. Many institutions are in the process of developing analytic personalised oncology programs (APOs). Through this prism and early local and wider APO experience, this talk will endeavour to give credit to both the traditional expert visual assessment and the radiomics approach and analyse the challenges ahead.
***Learning Objectives:***
1. The discuss the omics revolution and the different techniques used in radiomics and radiomics+.
2. To present trends in radiomics.
3. To understand personalised medicine initiatives by the example: where does radiomics+ fit in?

**A-0553** 08:50
**AI and Holomics: predicting the truth from hybrid imaging and non-imaging data**
I. Buvat; *Orsay/FR (irene.buvat@u-psud.fr)*

Hybrid imaging lends itself to the measurement of a large number of radiomic features. These include so-called handcrafted features and deep features derived either from the native images or from parametric images. This wealth of image-based information has a great potential for decision making using modern AI approaches. Yet, it is likely that if images are necessary for in vivo probing of biological mechanisms, they are not sufficient for accurate prediction of the patient outcome that depends on a number of other parameters. This is why radiomic data have to be enriched with other relevant omic or clinical data, yielding the concept of holomics. The integration of that huge diversity of data into accurate and robust models for guiding precision medicine raises a number of new challenges that will be presented and illustrated. Addressing these challenges may actually need rethinking the way we conduct research in imaging as will be discussed. In addition, the overwhelming trend towards AI-based data analysis should not hide the crucial importance of both the quality and the relevance of the input data, whatever they are. Only a clever and thorough use of sensitive and specific images reflecting biological mechanisms in an understandable way and combined with other data that also express phenomena to be accounted for will allow us to approach the truth. In that respect, AI has to be combined with human intelligence to make the most of imaging and non-imaging data in the context of precision medicine.
***Learning Objectives:***
1. To get an overview about shallow and deep learning.
2. To appreciate supervised, unsupervised and reinforcement learning with examples.
3. To learn about Holomics: a holistic approach for precision medicine, concept and examples.

**A-0554** 09:10
**Sharing is caring: on the need for open research data**
O. Ratib; *Geneva/CH*

The developments of imaging biobanks and cloud-based data storage services have radically changed the way we deal with communication and data management in our daily life. On-line storage of medical images is not new; several vendors have offered such services for decades already as part of their commercial solutions. What has really changed these recent years is the emergence of such services for the wide public offering very attractive solutions at a very low cost. In medical applications, however, such systems must comply with strict regulations and guidelines geared toward protecting patient confidentiality and data security. Medical imaging is becoming a major component of the data required in every medical decision in diagnostic, assessment of treatment response, follow-up of disease recurrence and in support for therapeutic and surgical interventions. The wealth of data acquired in clinical routine these days is overwhelming and has not been apprehended yet. The main limiting factor of the development of these new analysis techniques is the lack of sufficiently large sets of structured and well-documented imaging data. There are also major difficulties in the ability to collect these large sets of imaging data due to restrictive regulatory constraints and data protection rules that prevent the usage and exploitation of medical data without formal patient approval. Our presentation will focus on the specific issue of gathering and collecting medical images for the development of large Big-Data repositories for scientific research and review the current challenges that prevent their wide development today.
***Learning Objectives:***
1. To give an introduction to new concepts of Open Research Databases and Big-Data repository and their impact on development of new paradigm of data-driven patient management and personalised healthcare.
2. To understand the underlying challenges and requirements as well as the ethical and legal framework that regulates the setup and usage of large collection of patient data for open research.
3. To review of potential applications in molecular imaging and in support of multi-centric clinical trials.

09:35
**Panel discussion: AI in HI: incremental value or feeding the data explosion?**

**08:30 - 10:00** **Room M 2**

### E³ - ECR Academies: Radiology Leaders' Bootcamp

### E³ 918
### Captain's dashboard

**A-0555** 08:30
**Chairperson's introduction**
S. Morozov; *Moscow/RU (spmoroz@gmail.com)*

A plethora of dashboard’s concepts are thoroughly developed for the general management applications. A basic principle beyond dashboards states that only measurable things can be managed. Hospitals and healthcare systems around the world have developed or purchased and customised dashboards for tracking their patients’ and hospitals’ parameters. Being a captain in a radiology department requires a set of management skills which can be learnt and developed through a career. This session calls together experienced leaders, entrepreneurs and managers who are going to share their insights and practical examples of utilising managerial skills. Basic and advanced techniques to run radiology and healthcare projects through will be presented, including MBO, delegation, PDCA, BSC, KPIs, waterfall, agile, etc.

**A-0556** 08:36
**A. Fundamentals of imaging management and administration**
B. Bijan; *Sacramento, CA/US (drbijanbijan@gmail.com)*

Functioning efficiently, at the level of director/chairperson/executive/chief, requires certain knowledge, skills and experience. Traditionally, the need for such managerial training was less crucial however in today’s rapidly growing imaging universe, having formal training in such areas has become mainstream. Many chairpersons are actively trying to get formal training in management, attending hi-yield management and leadership courses and actively trying to broaden their knowledge, learn new skills and obtain experience. For the sake of time, here at ECR-2019, I will focus my presentation on the crucial areas of the art of building an efficient team, usage of the “Imaging informatics” technology to the max, to understand imaging economy and finance, to understand the concept of “system efficiency”, communication and interaction protocols as well as risk management and the feedback system in place including dashboard monitoring.
***Learning Objectives:***
1. Strategy for a radiologist in a changing healthcare landscape.
2. How to leverage a physician's role by learning managerial skills.
3. Setting a goal, making it doable, prioritising, focusing and choosing what not to do.

**A-0557** 09:04
**B. Being in a driver's seat and managing by objectives**
Y. Menu; *Paris/FR (yves.menu@sat.aphp.fr)*

For medical, technical, regulatory, networking and behavioural reasons, the challenge for managers is to adapt to a rapidly evolving environment. The initial analysis is achieved by different formal or informal tools including interviews, risk mapping and Lean 6 Sigma methods. It identifies gaps between performance and strategic goals, chosen as useful, shared and reachable objectives. Key Performance Indicators (KPIs) are powerful tools that will allow the follow-up of action. They are measuring tools. They are usually related to structure, process or outcome. They follow the SMART method (Specific, Measurable, Assignable, Realistic, Time-related) and measure specific services, like the best use of a CT platform, or Patient wait time. They include and quantify the objective, define the handling person, method of sampling and criteria for success. Balanced scorecards are syntheses aimed at sharing information easily and visually. They summarise KPIs. It is the manager’s preferred dashboard. Balanced scorecards can also be used as a hierarchical presentation of information, with the possibility to access to a “clickable” detailed and analytic level. PDCA (Plan, Do, Check, Act) is a more global tool for management as it highlights strategy (Plan), production (Do), monitoring (Check) and improvement (Act). It helps to classify all management initiatives in a global plan. It is commonly used for certification of a department. Whatever the tool, the manager is still in charge of kick-off initiatives and final decisions. Tools will help him driving the projects and sharing it in and outside the team, but will never be a substitute.
***Learning Objectives:***
1. Definition of a workflow, roles and results.
2. Balanced scorecard, KPIs, dashboard and PDCA instruments for a radiology department.
3. IT solutions for automating and increasing efficiency.

**A-0558** 09:32
**C. Challenging the status quo and making new things happen**
P.M.A. van Ooijen; *Groningen/NL (p.m.a.van.ooijen@umcg.nl)*

Radiology is at the brink of change because of all new developments are shown in Medical Imaging Informatics. For a radiology department to keep up with all developments, quick changes and adaptability of the staff and workflow models is an absolute requirement. Not only for radiologists, but also for radiographers, clinical physicists and other employees. Furthermore, new professional experts will enter the radiology domain such as medical computer scientists, data scientists and technical physicians. All these developments mean that the status quo is heavily challenged and radiology should be prepared to make new things happen. For leaders in radiology, this means that they have to be able to lead those changes against the wind by implementing change management. Furthermore, instruments already common in IT development such as Scrum and Agile will start entering the radiology domain and should be understood by radiology leaders. The overall observed change is that radiology should move (or already moves) into a value-based service using new developments to get to a more patient-centric workflow with extensive integration of the imaging in the patient workflow throughout the health process.
***Learning Objectives:***
1. Leading the changes against the wind: principles of change management.
2. Project management and new instruments, including Scrum, Agile.
3. Value-based service: new frontiers in medical imaging.

**08:30 - 10:00** **Room M 3**

### Paediatric

### RC 912

### Foetal and neonatal imaging pearls

**A-0559** 08:30
**Chairperson's introduction**
E. Vázquez; *Barcelona/ES (evazquez@vhebron.net)*

Fetal MRI has acquired an important role in the diagnostic workup of fetal abnormalities suspected on ultrasound study. Radiologists and clinicians involved in this task should be aware of the uses and limitations of fetal MRI. Suspected CNS disease continues to be the most common clinical indication for this imaging technique. Congenital malformations such as corpus callosum anomalies are among the most common requests, in which MRI can rule out other associated abnormalities, such as sulcation abnormalities or grey matter heterotopia, with high diagnostic accuracy when compared to postnatal findings. In acquired brain damage, the previously normal tissue is destroyed by diverse mechanisms, such as hypoxia, infection, metabolic disease, and even space-occupying lesions. The effect of these insults can be precisely detected with MRI and correlated with postnatal features. This technique can also be of help for evaluating fetal abdominal conditions. For example, ultrasound visualisation of some genitourinary abnormalities may be difficult because of associated oligohydramnios. MRI can accurately delineate the anatomy and identify normal renal parenchyma and cysts. In addition, newer sequences, such as diffusion-weighted imaging, are useful for assessing the normal functional kidney. This course will provide an educational overview of the main techniques and recent technological advances in fetal MRI, applicable in both CNS and body examinations. Finally, a panel discussion will be conducted, focused on overcoming certain challenges in fetal MRI performed for routine and research purposes, including technical and safety concerns.
***Session Objectives:***
1. To learn about the advanced techniques in foetal MRI.
2. To understand some useful applications in brain malformations or acquired injuries.
3. To discuss the main limitations and challenges of foetal MRI.

**A-0560** 08:35
**A. Foetal MRI advanced technics**
G. Kasprian; *Vienna/AT (gregor.kasprian@meduniwien.ac.at)*

A human foetus can be considered as the most “uncooperative” patient. Thus, any “advanced” foetal MR imaging technique has to deal with the problem of unpredictable fetal motion. Since the beginnings of foetal MRI in the 1980ies, the most promising strategy in addressing the problem of foetal motion was shortening the acquisition time of MR sequences. This strategy has been successful for those sequences, which are nowadays regarded as “standard”: T2-TSE, T1SE, SSFP, EPI/T2* and DWI sequences. After shortening Diffusion Tensor Imaging (DTI) sequences to a maximum acquisition time of 2 minutes, it has been possible to reconstruct main sensorimotor and commissural pathways in normal and pathological cases. Postmortem histo-tensor and postnatal DTI studies have recently validated these results. Thus, DTI - commonly regarded as “experimental” in fetal MRI - can even provide clinically important data for our understanding of fetal brain pathologies. If fetal “resting state” BOLD MRI data are processed with meticulous motion, slice timing, bias field and noise correction, connectivity profiles of the fetal brain or the placenta can be generated even in single subjects. Adjusted single voxel spectroscopy sequences can be applied prenatally and may complement standard fetal MRI providing valuable insights into fetal or placental metabolism. Although interpreting the results of advanced fetal MRI techniques may have its uncertainties and risks, careful incorporation of these methods into clinical fetal MRI protocols further strengthens the position of fetal MRI as an important complement to ultrasound in the assessment of fetal pathologies.
***Learning Objectives:***
1. To learn about the principles for advanced techniques in foetal MRI.
2. To understand the potential for their use.
3. To discuss the limitations and disadvantages of these techniques.
*Author Disclosure:*
**G. Kasprian:** Consultant; Bellaria Diagnose. Grant Recipient; Austrian Science Research fund, Nr. I 3925. Speaker; Shire, Biogen.

**A-0561** 08:53
**B. Corpus callosum anomalies: pre- and postnatal correlation**
C. Garel; *Paris/FR (catherine.garel@aphp.fr)*

Corpus callosum (CC) malformations include complete and partial CC agenesis, short CC and CC dysgenesis. They can be isolated or observed in the setting of numerous syndromes. Moreover, pericallosal lipomas or interhemispheric cysts may also be observed in association with CC malformations. The diagnostic contribution of prenatal MRI ranges between 0 and 20% according to the sonographer’s skill and the conditions of the US examination. In about 22.5% of cases, MRI can detect additional abnormalities. The presence of such abnormalities may have a prognostic impact. Disagreement between pre- and postnatal imaging with the postnatal identification of associated cerebral anomalies considered as having a prognostic impact, is observed in about 9%. The prognosis of CC malformations associated with a curvilinear lipoma or with an interhemispheric cyst without cortical anomalies is very good. Except for these two entities, associated CC agenesis usually carries a poorer prognosis than an isolated one. Identification of genes mutations observed in children presenting with CC agenesis with or without intellectual deficiency is currently under development.
***Learning Objectives:***
1. To evaluate the diagnostic accuracy of foetal MRI for diagnosis.
2. To compare pre- and postnatal MRI data.
3. To ascertain the outcome in cases of isolated complete or partial agenesis.

**A-0562** 09:11
**C. Foetal MR imaging of acquired brain pathology**
P.D. Griffiths; *Sheffield/UK (p.griffiths@sheffield.ac.uk)*

MR imaging of the unborn baby’s brain has become an established method of detecting brain pathology ante-natally. Most structural brain abnormalities shown on fetal MR are developmental in nature (e.g. agenesis of the corpus callosum, Dandy-Walker malformation) but there is increasing recognition that some brain abnormalities are ‘acquired’ that is occurring in a brain that was otherwise developing normally (e.g. stroke, trans-placental infections). In this presentation, I will review the types of pathology that can lead to brain injury acquired in utero and present data from the MERIDIAN study that shows the prevalence and range of such abnormalities.
***Learning Objectives:***
1. To learn about the various types of acquired brain pathology in the foetus.
2. To understand the limitations in the comprehension of the underlying cause.
3. To discuss about the best estimates of the prevalence of acquired brain pathology.

**A-0563** 09:29
**D. Pre- and postnatal congenital cystic renal diseases**
M. Cassart; *Brussels/BE (mcassart@his-izz.be)*

The finding of hyperechoïc kidneys in foetuses and children is a challenge for radiologists. It can be simply a normal variant with further resolution or an early sign of congenital renal cystic disease. These renal diseases can be uni or bilateral, isolated or part of polymalformative syndromes. Various entities can be encountered in this context: obstructive uropathies if associated with dilatation of the collecting system, multicystic kidney disease if macrocysts are present. Inherited renal cystic diseases should also be considered: recessive polycystic kidney disease in bilateral undifferentiated enlarged kidneys, dominant polycystic kidney diseases in slightly enlarged well-differentiated kidneys with cortical cysts or renal diseases associated with TCF2 mutations that can have many different aspects. The contribution of imaging can be optimised by the knowledge of the normal sonographic appearance of the kidneys at various ages and the typical patterns of different inherited renal diseases. Syndromes should be suspected in typical renal patterns associated with extrarenal anomalies (central nervous system, limb···) like for example in

Meckel Gruber or Bardet Biedl syndromes. The imaging data should also always be integrated into the familial context (consanguinity) and obstetrical history in case of recurrence. It most frequently allows approaching the specific diagnosis or at least orient the genetic testing to precise the diagnosis and establishes a prognosis.
***Learning Objectives:***
1. To learn about differential diagnosis of hyperechoic kidneys in foetuses and children.
2. To become familiar with the latest classification of inherited renal cystic diseases.
3. To discuss the limitations of the imaging methods for diagnosis.

09:47
**Panel discussion: How to overcome the challenges in foetal MRI in routine and research**

**08:30 - 10:00 Room M 4**

### E³ - ECR Academies: Chest Imaging

### E³ 922

### Chronic obstructive pulmonary disease (COPD)

**A-0564** 08:30
**Chairperson's introduction**
D. Litmanovich; *Boston, MA/US*

**A-0565** 08:36
**A. CT phenotyping and visual assessment**
P.A. Grenier; *Paris/FR (philippe.grenier@aphp.fr)*

COPD is a slowly obstructive airway disorder resulting from an exaggerated inflammatory response to cigarette smoking and air pollution that ultimately destroys lung parenchyma and induces an irreversible reduction in calibre and number of small airways. Both phenomena (emphysema and small airway disease [SAD]) are responsible for airflow limitation. Because often SAD precedes lung destruction, patients having equal impairment of lung function may present different morphologic appearances on CT scans. Although quantitative CT is useful for identifying and sequentially evaluating the extent of emphysematous lung destruction, changes in airway walls and expiratory air trapping, visual assessment of CT scans is important to describe patterns of altered lung structures in COPD. Emphysema is classified as centrilobular (subclassified as trace, mild, moderate, confluent and advanced destructive emphysema), panlobular and paraseptal (subclassified as mild or substantial). Airway disease is commonly found with all forms of emphysema but also commonly occurs in the absence of emphysema as a predominant expression of COPD. The CT features of the bronchial disease include thickening of the walls of segmental and subsegmental airways. CT features of SAD include peripheral centrilobular micronodular opacities (inflammatory SAD) and gas trapping on expiratory CT (obstructive SAD). Associated features may be seen on large airway disease (tracheobronchomalacia, sabre sheath trachea, tracheobronchial outpouching/diverticula). Interstitial lung abnormalities include patchy ground-glass abnormality and/or mid subpleural reticular abnormality. Other associated features include pulmonary arterial enlargement suggesting pulmonary hypertension, and bronchiectasis.
***Learning Objectives:***
1. To learn about imaging-based phenotypes.
2. To understand the limitations of visual assessment.
*Author Disclosure:*
**P.A. Grenier:** Speaker; oxyvie.

**A-0566** 09:04
**B. Quantitative imaging biomarkers**
J.B. Seo; *Seoul/US (seojb@amc.seoul.kr)*

A biomarker is defined as an indicator of a biological state that is objectively measured and evaluated. Biological state includes including, normal biological processes, pathogenic processes, or pharmacologic responses to a therapeutic intervention. Imaging modality can also be used to extract biomarkers. In recent studies on COPD, CT has been accepted as one of the important research tools in evaluating disease severity and characteristics. The extent of low attenuation area and bronchial wall thickening at a segmental and distal level on volumetric CT scan acquired at suspended inspiration state are commonly used as useful imaging markers for evaluating the severity of emphysema and airway wall inflammation, respectively. The clinical values of these two imaging biomarkers are as follows: 1) Many recent studies have proved that the extent of emphysema and bronchial wall thickening are independently related with the degree of airflow limitation, 2) The extent of emphysema is correlated with other clinical parameters such as osteoporosis, exercise capacity, respiratory symptoms and most importantly with BODE index, which is known to be one of the best predictors of mortality, 3) Both parameters may be useful in subgrouping/phenotyping of patients, prediction of treatment response, and prediction of disease progression 4) Both parameters are related to frequency of exacerbation. Furthermore, many additional potential imaging biomarkers have been proposed. They include; (1) assessment of air trapping by direct anatomical matching of inspiration and expiration CT, (2) assessment of peripheral vascular changes in COPD, and so on.
***Learning Objectives:***
1. To understand the role of quantitative imaging in identifying COPD phenotypes.
2. To appreciate the recent development of quantitative imaging methods.
*Author Disclosure:*
**J.B. Seo:** Shareholder; Coreline Soft Co.

**A-0567** 09:32
**C. Is there a role for MRI?**
M.O. Wielpütz; *Heidelberg/DE (wielpuetz@uni-heidelberg.de)*

Magnetic resonance imaging (MRI) has emerged as a new modality for lung imaging only recently, with airway diseases being the most accepted indications for clinical routine imaging. Beyond being a substitute for X-ray and computed tomography (CT), MRI combines morphologic and functional information more consequently than any other technologies. Morphological sequences for proton MRI suitable for airways disease will be introduced, but the focus will be on functional techniques that have been introduced into clinical routine imaging or are most advanced in scientific studies. These are dynamic contrast-enhanced perfusion MRI, T1-mapping with inhalative oxygen, and non-contrast Fourier-decomposition MRI, which allow for a regional analysis of lung function. Further, noble and fluorinated gas MRI will be discussed as advanced scientific methods to study regional lung ventilation. It has been shown that MRI may sensitively detect changes in lung morphology related to large airways diseases such as airway wall thickening, bronchiectasis, mucus plugging or tracheobronchomalacia with lower resolution than CT, but with similar clinical impact. Ventilation abnormalities attributable to small airways disease are closely linked to subsequent perfusion changes, which can be sensitively detected by dynamic contrast-enhanced perfusion MRI. By a combination of morphological with functional techniques, MRI has the potential to specifically differentiate reversible from irreversible lung changes especially in airway diseases such as COPD and cystic fibrosis. This makes MRI an important modality for non-irradiating regional disease monitoring and therapy follow-up. Subsequently, it has now been used as an endpoint in pioneer clinical trials, which will be presented briefly.
***Learning Objectives:***
1. To learn about the morphological and functional evaluation achievable with MRI.
2. To learn about the recent developments in lung MRI.
*Author Disclosure:*
**M.O. Wielpütz:** Advisory Board; Boehringer Ingelheim. Grant Recipient; Boehringer Inhelheim, Vertex.

**08:30 - 10:00 Room M 5**

### Head and Neck

### RC 908

### Head and neck imaging: when does it become abnormal?

*Moderator:*
N. Thieme; Berlin/DE

**A-0568** 08:30
**A. Normative measures in the temporal bone**
F. Veillon; *Strasbourg/FR (Francis.Veillon@chru-strasbourg.fr)*

The normal size of certain parts of temporal bone structures is important to be known for the CT diagnosis of the very frequent minor malformations of the inner ear, the increased thickness of the footplate (chronic inflammation otosclerosis) or the abnormal enlarged facial nerve canal (malformations, tumours). The knowledge of the normal size of the saccule in MRI permits to diagnose its dilation. In CT, the round window (normal width: 1.5 mm in the axial and coronal planes, small from 1.3 mm), the oval window (normal height: 1.5 mm-1.9 mm in the coronal plane, small from 1.3 mm), the normal thickness of the footplate in recent machines: 0.3 mm, increased: 0.5 mm, the normal central islet (surface circumscribed by the lateral semicircular canal in the axial plane, 7-10 mm2, small from 6 mm2), the medial part of the lateral semicircular canal in the axial plane (normal minimum: 1.7 mm, enlarged from 1.8 mm), the modiolus (normal width: 2 mm in the axial plane, small from 1.3 mm, enlarged from 3 mm), the normal aqueduct of vestibule (in the axial plane: 1.5 mm, enlarged: 1,7 mm). The inferior part of the basal turn of the cochlea (normal length: 9 mm, small from 7.3 mm), the geniculate ganglion fossa (3 mm on average, normal limits: 1.8-4.5 mm). The normal saccule in MR coronal view (normal maximal height: 1.6 mm, 1.3 mm on average, normal width: 1.4 mm). You can easily use these measurements in your daily practice.

***Learning Objectives:***
1. To learn about the appearance of the bone in the normal population.
2. To understand the changes in size and shape due to aging.
3. To show some examples of structures which are too large or too small.

**A-0569** 09:00
**B. Normative measures in the orbit**
R. Kohler; *Sion/CH*

In the daily routine, it is not infrequent that the radiologist does not know if his/her observations of the orbit and eye at CT and MRI are in the normal range or a true pathology. Normative measures are objective data that help the radiologist for the diagnosis of many issues but also are useful for the follow-up of some pathologies. A selection of normative measures but also some changes of shape and anatomic variations will be discussed in this presentation according to five main chapters based on the anatomy: Bony orbit: how to define and grade exophthalmos and how to determine hyper-/hypotelorism? Eye: a review of main shape abnormalities. For example, coloboma, microphthalmia and axial myopia will be discussed. Optic nerve and optic nerve sheath: what is their normal size? Hypertrophy, atrophy and tortuosity of the nerve as well as dilatation of the nerve sheath will be exposed. Attention will also be given to two special applications of measurement of the optic nerve: variations in size in case of intracranial hypertension and diffusion-weighted imaging. Extraocular muscles: how to measure them, especially in case of thyroid orbitopathy? Miscellaneous: a short review of abnormalities of the size of the superior ophthalmic vein and lacrymal gland. The normative data covered during this lecture should make the interpretation of cross-sectional imaging of the orbit and eye easier and more confident by giving radiologists a useful “rigid frame” of interpretation.

***Learning Objectives:***
1. To understand differences in the size and shape of the eye.
2. To learn about the normal appearance and anatomic variations of the optic nerve.
3. To discuss normative measures of the soft tissue structures in the orbit.

**A-0570** 09:30
**C. Anatomical variations in the face and neck**
E. Vassallo; *Msida/MT (edithvassallo@gmail.com)*

The head and neck is an anatomical compartment of the body that is fascinating as it is complex. This complexity may, at times, render radiological interpretation daunting. In addition to this, many anatomical variations may be encountered in the head and neck which, unless identified and clearly documented by the radiologist, may lead to undesired (and sometimes catastrophic) outcomes. In this discussion, we present a number of such non-pathological entities, outlining how they may present as pseudolesions or pose added risks of iatrogenic injury at surgery.

***Learning Objectives:***
1. To gain insight into the great variability of head and neck anatomy.
2. To be able to recognise pseudo lesions.

**08:30 - 10:00 Tech Gate Auditorium**

### Genitourinary

### RC 907

### Imaging strategies in renal tumours

**A-0571** 08:30
**Chairperson's introduction**
B. Brkljačić; *Zagreb/HR (boris@brkljacic.com)*

Renal tumours are an important cause of mortality, and timely and accurate diagnosis is crucial for optimal treatment. The session will deal with optimal protocol and medication for CT, CEUS and MRI of renal tumours, with differences at the diagnosis, with the optimal staging protocols and with organ-preserving strategies.

***Session Objectives:***
1. To become familiar with current and emerging renal imaging modalities.
2. To learn about the capabilities of renal imaging in diagnosis and staging of renal tumours.

**A-0572** 08:35
**A. CT, contrast-enhanced ultrasound (CEUS) and MRI: the best out of them**
M. Bertolotto; *Trieste/IT (bertolot@units.it)*

CT is the imaging modality of choice for evaluation of renal masses. The advantages of CT are its widespread availability, high speed of acquisition, high spatial resolution and isotropic imaging. MRI can be a powerful problem-solving tool for lesion characterisation, especially if a small amount of intralesional fat is suspected. The highest contrast resolution provided by MRI and CEUS allows characterisation of lesions with indeterminate enhancement at CT. Like CT, MRI provides excellent anatomic information. Moreover, advanced MRI techniques can provide information about tissue structure and function. The disadvantage of MRI compared to CT is the longer examination time and lesser panoramicity. CEUS lacks panoramicity and suffers from the same technical limitations of conventional US modes. Optimal MDCT protocol for renal masses includes a non-contrast phase followed by postcontrast acquisitions with corticomedullary, nephrographic and delayed phases of enhancement. The nephrographic phase is acquired for assessing the presence of a renal lesion and its enhancement, and is therefore sufficient for detection and characterisation of renal lesions. Corticomedullary and urographic phases are often performed to provide additional information for presurgical planning. Optimal MRI protocol for renal mass evaluation includes TSE T2-weighted imaging, TSE T1-weighted imaging with and without fat suppression, T1-weighted opposed-phase imaging (with in-phase and out-of-phase sequences) for the detection of microscopic fat, DWI, fat-suppressed 3D T1-weighted gradient-echo acquisition before and after administration of intravenous gadolinium-based contrast in corticomedullary, nephrographic and urographic phases, and subtraction imaging.

***Learning Objectives:***
1. To become familiar with the optimal CT and MRI protocols for renal imaging.
2. To understand the best choice of CT, CEUS or MRI according to the clinical need.
3. To illustrate the advantages and disadvantages of CT, CEUS and MRI.

**A-0573** 08:58
**B. Differential diagnosis of renal masses**
N. Grenier; *Bordeaux/FR (nicolas.grenier@chu-bordeaux.fr)*

Imaging is the main source of detection of renal masses. Differentiation between complex cystic and solid masses is not always straightforward and may require several contrast-enhanced methods and DCE-MRI and CEUS are more sensitive for that purpose. Considering cystic masses, Bosniak classification is required. Considering solid masses, characterisation of fat-rich angiomyolipomas is based on plain CT, but fat-poor AMLs can be distinguished from carcinomas by multiparametric MRI only. Multiparametric MRI includes chemical shift gradient echo (GRE) sequences, signal intensity on T2-weighted images, DCEsequences, diffusion-weighted sequences and late contrast-enhanced images. Using different combinations of two or several parameters, now makes it possible to clearly distinguish some renal tumours such as fat-poor AMLs, papillary carcinomas and clear cell carcinomas the later being difficult to separate from oncocytoma when a central scar is absent. A larger validation of all these combinations is still necessary to define those having a clinical significance for routine practice. Percutaneous biopsy remains mandatory before such a validation, as soon as the pathological result is supposed to have an impact on tumour management.

***Learning Objectives:***
1. To learn about the histologic spectrum of renal tumours.
2. To understand the capabilities of imaging for renal tumour characterisation.
3. To become familiar with functional techniques applied to characterise renal tumours.

*Author Disclosure:*
**N. Grenier:** Advisory Board; Supersonic Imagine, Aix-en-Provence, France. Grant Recipient; Guerbet , GE Healthcare and Bracco.

**A-0574** 09:21
**C. Staging and organ-preserving strategies**
P. Asbach; *Berlin/DE (patrick.asbach@charite.de)*

Accurate CT- or MRI-based staging of the two most common malignant neoplasms involving the kidneys, renal cell carcinoma (RCC) and transitional cell carcinoma (TCC), is an important prerequisite for surgical decision making, especially in early stages of the disease. The TNM staging system is particularly different between RCC and TCC regarding the T-stage. Tumour size and invasion of the perirenal fat are important criteria for local staging of RCC, wheres invasion of the muscular layer of the renal collecting system and invasion into the renal parenchyma are important criteria for staging TCC. Different organ-preserving strategies are evolving especially for treatment of RCC (e.g. local excision versus local ablation). Also, important additional information such as detailed vascular anatomy is needed by the surgeon, which has an impact on the respective preoperative imaging protocol.

***Learning Objectives:***
1. To understand the TNM-staging system for renal tumours.
2. To become familiar with optimal staging protocols and imaging findings in the staging of renal tumours.
3. To learn about actual organ-preserving strategies.

09:44
**Panel discussion: How to implement an optimal renal imaging protocol?**

**10:30 - 12:00 Room A**

### ESR meets Italy

### EM 2

### From morphology to function

*Presiding:*
L.E. Derchi; Genoa/IT
R. Grassi; Naples/IT

**A-0579** 10:30
**Introduction: Radiology in Italy**
C. Bibbolino; *Rome/IT (corrado.bibbolino@gmail.com)*

The Italian Society of Medical Radiology (SIRM) counts about 11,000 members. It is Italy's largest scientific society and one of the largest in Europe. Its members include nearly all the radiologists working in Italian hospitals and public or privately owned health facilities, self-employed, residents and retirees. It is organised in twenty regional groups and twenty subspecialty sections. According to the OECD data, Italian Radiology runs more than 2,000 CT scanners and 1,715 MRI scanners, of which only 30% are less than five years old. A recent SIRM census showed that approx. 120,000,000 X-ray, US, CT and MRI examinations are performed every year. Many of the examinations mentioned above are not included in the figures published by the OECD and the Italian Ministry of Health. However, they cause problems of appropriateness and overdiagnosis. Many regional health services have general radiological archiving systems creating important databases. In Italy US investigations are performed by physicians: CT examinations are thus fewer than in countries where US scannings are performed by ultrasound technicians. Mammographic screening covers >70% of the female population between 50 and 70 years whereas screening of the lung and colon is routinely carried out only in some regions. In negotiations with the government and the owners of health facilities, the National Union of Radiologists counting more than 3,500 members represents the Italian radiologists. In 2016, SIRM supported Choosing Wisely by hosting the 3rd World Meeting in its Training Center in via del Cardello 24, Rome, near the Colosseum.
***Session Objectives:***
1. To describe the most important features of Italian radiology.
2. To reflect on new developments of imaging in Italy and Europe.
3. To discuss the role and function of a systemic vision of a radiological society.

**A-0580** 10:35
**Italian emergency network**
V. Miele; *Florence/IT (vmiele@sirm.org)*

Italy's healthcare system is a regionally organised National Health Service that provides universal coverage largely free of charge for Italian citizens. The request for emergency diagnostic and therapeutic services is constantly growing, due to both clinical and epidemiological factors, such as the increasing amount of traumatic events and non-traumatic clinical emergencies. Moreover, there is an overcrowding of the Emergency system, due to the fact that patients with minor health problems are also taken into care, in case of lack of immediate response in the territorial health system. In the care of emergency patients, diagnostic imaging has a very important role, so the majority of patients accessing the Emergency Department performs at least one diagnostic examination, mandatory to decide the care path (immediate treatment, hospitalisation or discharge). The availability of Department of Radiology staffed on a 24-hours a day for emergencies greatly influences the diagnostic performance. Generally, the regional emergency service is organised in specialised centres ("hub"), which are equipped and staffed to provide care for patients suffering from major traumatic and non-traumatic emergencies. In traumatic emergencies, Major trauma Centers (MTCs) are directly connected with peripherals, radially diffused, trauma units ("spokes") that don't provide major trauma care but still play an essential role in less severely injured patients. Despite the longer transport times, this envolves, triage of major trauma patients to an MTC results in a 30% decrease in mortality in the first 48 h compared with transport to a non-MTC, which may be the closest medical facility.
***Learning Objectives:***
1. To review the organisation of the emergency system.
2. To highlight the strengths and weaknesses of the emergency network regarding epidemiological factors, topographic features and territorial distribution of resources.
3. To define the possible areas of clinical improvement and organisational effectiveness of the emergency health system.

**A-0581** 10:55
**Interlude: The sirens: myth or reality?**
R. Grassi; *Naples/IT*

**A-0582** 11:00
**Emergency interventional radiology: brain and body**
G. Carrafiello; *Varese/IT (gcarraf@gmail.com)*

The demand for all types of radiological imaging and intervention on a 24-hour, seven days a week basis has increased significantly in recent years. The term '24-hour radiological imaging services' applies equally to elective and acute services. Interventional techniques are now at the forefront of management of many life-threatening emergencies. Stopping haemorrhage (eg, trauma, gastrointestinal (GI) bleeding, post-partum haemorrhage), thoracic-abdominal aortic aneurysm, traumatic dissection and the complications of Type B dissection, ruptured peripheral aneurysms, acute peripheral and visceral ischaemia, managing sepsis secondary to upper urinary tract and biliary obstruction (often urgent though rarely an emergency), image-guided intervention in subarachnoid haemorrhage and stroke, are the situations where urgent or emergency interventional radiology is indicated. Formal arrangements to secure provision of elective and emergency interventional radiology services are guaranteed in less than 10% of hospitals. These hospitals must be the reference for traumatic patients and stroke. Remnant hospitals should have contacts with closer invertional radiology services.
***Learning Objectives:***
1. To learn about indications.
2. To learn about techniques.
3. To discuss literature and personal experiences.

**A-0583** 11:20
**Interlude: The cyclops: myth or reality?**
R. Grassi; *Naples/IT*

**A-0584** 11:25
**La Radiologia Medica: the role of the journal in an international setting**
A. Giovagnoni; *Ancona/IT (a.giovagnoni@univpm.it)*

La Radiologia Medica is the official Scientific Journal of SIRM founded in 1914 that has inaugurated a new publishing season with the scope of becoming a reference point in the panorama of the major international Journal and a tool for constant and timely updating for members. Starting from January 2014, one hundred years after its foundation, the Journal has renewed its editorial model, equating it with that of major international journals with a monthly frequency in a version online in the English language. The new publishing model, has entered into full acceptance of the SIRM Members and has allowed a series of positive practical repercussions: the reduction of production costs, increase of foreign authorship a greater visibility and penetration within the international scientific / radiological community; greater citability with a consequent positive effect on the value of the IF. The contribution of foreign authors which amounted to 62% of the total submitted manuscripts. The selection of manuscripts is accurate and the percentage of rejection rate, in progressive growth, has reached a value of 67% with a constant increase of the IF which has reached the value of about 1.9. La Radiologia Medica represents one of the most important assets of the SIRM.
***Learning Objectives:***
1. To learn about the organisation of the journal as the official organ of the SIRM.
2. To discuss the strengths and weaknesses of an international, general radiology scientific journal.
3. To consolidate knowledge and to explore future prospects in the international editorial scenario.

11:45
**Panel discussion: How will the radiologists' profession evolve?**

**10:30 - 12:00** **Room N**

### PIER @ ECR Session (Jointly organised with the ESR Audit and Standards Subcommittee)

### PI 2

### Clinical audit: how to deal with the legal and professional requirements

*Moderator:*
G. McGinty; New York, NY/US

**A-0585** 10:30
**Chairperson's introduction**
D.C. Howlett; *Eastbourne/UK (david.howlett@nhs.net)*

This session examines clinical audit and its relationship with the legal and professional requirements of radiological practice, with particular reference to the BSSD, its uptake and what the BSSD and inspection mean for radiology departments. A review and update are provided of currently available audit tools and how they can be used to help implement or enhance audit pathways. A North American perspective is provided discussing peer review, with discussion also of IT and artificial intelligence developments supporting both clinical audit and quality improvement pathways-the potential benefits of peer review and artificial intelligence in the support and enhancement of effective clinical audit programmes are highlighted.
***Session Objectives:***
1. To appreciate the various tools available to facilitate effective clinical audit.
2. To understand the aims and scope of the BSS.
3. To appreciate the potential of peer review and IT/AI to improve quality in radiology now and in the future.

**A-0586** 10:36
**ESR's concept and tools for clinical audit**
K. Drinkwater; *London/UK (karl_drinkwater@rcr.ac.uk)*

The European Society of Radiology's (ESR) concept for clinical audit is a "process of assessing one's practice against defined standards, altering practice if necessary to meet standards, and re-assessing following changes to confirm improvement." The ESR's tool for clinical audit (Esperanto) is designed to facilitate local clinical audit in line with this concept by providing a set of principles, a collection of audit templates and pointers to sources for further information. An effective audit requires repeated cycles, appropriate methodology and an intention from the outset to document the extent of change against standards and resulting health impacts. Such documentation constitutes evidence of safe practice for departments undergoing inspection by regulatory authorities. The audit pathway comprises preparation and planning, measuring performance, implementing change, and sustaining improvement. A wide range of tools have been identified with potential for improving the effectiveness of clinical audit; each of which can be applied at one or more of these stages. Individual tools are used to best effect in combination, guided by a structured framework, and within a team working arrangement. However, knowledge gaps and conflicting evidence make it difficult to predict what works best and when.
***Learning Objectives:***
1. To understand the potential for improving the effectiveness of clinical audit using audit tools.
2. To learn when certain audit tools may be most effective within the audit pathway.
3. To appreciate the differing audit tools currently available.

**A-0587** 10:52
**Overview on adoption of BSS throughout Europe**
A. Brady; *Cork/IE (adrianbrady@me.com)*

The Basic Safety Standard (BSS) which took effect throughout EU member countries in February 2018 imposes certain new practice requirements on radiology departments in the field of radiation safety, and re-emphasises other activities, including the obligation to engage in clinical audit, according to the stipulations of national legislation in each member country. This talk will describe the specific audit-related activities required by the BSS. Information which has been collated by the ESR Audit & Standards Subcommittee and Eurosafe Imaging on BSS adoption, standards already met and audit structures and supports in place will be discussed. The role of the ESR Esperanto Audit support tool in assisting individual departments in meeting their obligations under the BSS will be explained.
***Learning Objectives:***
1. To learn about the legislative requirement in Europe to engage in clinical audit.
2. To appreciate the variations in how this is implemented in different European countries.
3. To understand how this requirement impacts each individual radiology department.

**A-0588** 11:08
**Peer review as key for quality improvement: the US experience**
G. McGinty; *New York, NY/US (geraldinemcginty@gmail.com)*

In response to the IOM report, "To Err is Human", which reported that medical errors accounted for nearly 100,000 preventable deaths each year in the United States alone, the ACR developed the RADPEER program in 2002 on the premise that whenever a radiologist interprets a new imaging study in conjunction with a preceding comparative examination, there is an opportunity to assess the accuracy of the original interpretation. RADPEER is a simple, cost-effective performance tool. The program has evolved both in its operations as well as in the scoring and categories used for reporting. RADPEER is currently the leading method for peer review in the United States. RADPEER is an accepted practice quality improvement method by the American Board of Radiology and can also satisfy hospital and Joint Commission credentialing requirements. Audit systems such as RADPEER have been criticised for not providing opportunities for learning or consideration of contributors to errors. The ACR is currently developing an expanded platform called RADLEARN that will receive images from participants into a registry and will deliver feedback. The anonymised cases submitted can be used for self-study, for teaching and remedial purposes, and to provide content to support internal department reviews especially for smaller practices which might not otherwise encounter such cases.
***Learning Objectives:***
1. To learn about the path to implement peer review.
2. To appreciate the positive effects of peer review.
3. To understand the implications for quality improvements.

**A-0589** 11:24
**Can newer IT-developments including artificial intelligence (AI) help to improve quality in radiology?**
J. Schillebeeckx; *Knokke/BE (jan@schillebeeckx.com)*

Quality in radiology becomes a dominant topic with the impact of the European Directive 2013/59/Euratom and voluntary accreditation platforms like Joint Commission International, Qmentum and others. The compliance with obligatory or voluntary quality standards is often challenged by the search for the up-to-date set of documents and the smooth, flawless integration of processes on the floor. Consequently, the correct documentation often remains hidden, unstructured, incomplete and leads to a non-uniform application on the floor. The interplay between an intelligent "Knowledge" and "Compliance" Management System using artificial intelligence allows hospitals to save time and efforts to link compliance criteria with their specific clinical practice. Smart algorithms are developed to detect key elements in individual accreditation criteria (step I) and in documents ("knowledge", step II) that were initially centralised. Parts of this "knowledge" were automatically retrieved from radiation dose monitoring software (e.g. scheduled/unscheduled maintenance time reports); other parts were detected by the algorithms (e.g. patient identification procedure). A final algorithm enables the matching between found tags per document and individual accreditation questions (such as equipment downtime, available procedures, etc.). An adequate matching process returns a clear overview in the CMS on the accreditation requirements that were met and with which accuracy. In conclusion, artificial intelligence will reduce efforts to detect compliance gaps and, accordingly, will highlight how quality in the radiology department can be improved.
***Learning Objectives:***
1. To learn about basic-concepts for IT requirements for clinical audit.
2. To appreciate the value of IT-support for quality improvement processes.
3. To understand the potential of big-data and artificial intelligence for quality initiatives.
*Author Disclosure:*
**J. Schillebeeckx:** Consultant; Qaelum NV. Shareholder; Qaelum NV.

11:40
**Panel discussion: Why should radiologists care about clinical audit and peer review?**

**10:30 - 12:00** **Room M 2**

### E³ - ECR Academies: Radiology Leaders' Bootcamp

### E³ 1018

### Dream team

**A-0594** 10:30
**Chairperson's introduction**
L. Martí-Bonmatí; *Valencia/ES (marti_lui@gva.es)*

The "Dream Team" term was used for the 1992 USA men's Olympic basketball team, the first American Olympic team to feature active professional players from the National Basketball Association. Even some years before it was used for a group of superheroes in the Marvel Comics universe with different magical energies, surprising powers and marvellous skills. In professional and business developments, great teams are hard to come by. Great team members need to be selected and empowered. To keep abreast of the visionary projects and agreements that keep your radiology department growing and prosperous, there are a number of skills each team member needs to have. From excellent communication skills to expert negotiating tactics, they need to be able to work together to bring in more development and innovative business. Positions to be considered in this Dream Team are Research and Clinical Trials, Education and Certification, Clinical Service and Innovation, IT and Artificial Intelligence, Workflow and Quality, Patient safety and Patient experience, New business and Integrated Diagnosis (radiology, pathology, genetics).

**A-0595** 10:36
**A. How to find the best people and make them a part of a long-time success**
J.H. Thrall; *Boston, MA/US (thrall.james@mgh.harvard.edu)*

Long-term success starts with establishing a favourable culture and work environment and, for radiology, a well-equipped department that will attract the best people. Hiring decisions should balance the desire for the highest intrinsic abilities with good personality traits. After people are hired, they should be mentored and provided regular feedback on professional progress. Further feedback must also include assessment of their behaviours from direct observation (360-degree assessment) and possibly through personality assessment tools such as the Myers-Briggs Type Indicator (MBTI), Enneagram or Hogan Personality Inventory to strengthen their understanding of themselves and their impact on co-workers. Elimination of adverse behaviour-remediation or separation- is critical to achieving a conducive working environment. To maximise and sustain good performance, each person should know his/her roles and responsibilities and those of others. In this regard, the Responsibility Assignment Matrix (aka RACI) approach or variants are useful for unambiguously defining tasks and responsibilities, especially for new initiatives. As people advance in their careers through mentorship and experience, the progressive delegation of responsibilities and leadership opportunities to people with the demonstrated ability is crucial. This creates a stimulus for them, increases departmental bandwidth to get work done and sets the stage for long-term growth. Success in establishing a repeatable virtuous cycle of recruitment, mentorship, feedback, achievement of personal and professional growth and delegation of responsibility with sharing and then the transition of leadership is the key to sustained excellence.
***Learning Objectives:***
1. Defining roles and responsibilities in a team: RACI tool.
2. To know who you are and whom you need (MBTI, enneagram, Hogan).
3. How to delegate without losing quality and time.

**A-0596** 11:04
**B. Leadership in radiology**
M. Forsting; *Essen/DE*

"no abstract submitted"
***Learning Objectives:***
1. Radiologist: a ratio of a manager and a specialist.
2. Psychological aspects of leadership (EQ, enthusiasm, energy, sport, hobby).
3. Tools for organising a team's work, delegating and managing virtual/distributed teams.

**A-0597** 11:32
**C. Leading quality improvement projects**
B. Baeßler; *Mannheim/DE (bettina.baessler@uk-koeln.de)*

Initiating and participating in quality improvement projects often allow to achieve incremental and measurable changes, which make a real difference to patients' experience in a hospital or medical students' learning success. The Model for Improvement helps define what should be accomplished, what change should be made, and what is being measured to know that any change has led to an improvement. Having a clear vision and objectives is of crucial importance, and SMART goals (which are specific, measurable, achievable, relevant, time-bound) will help in achieving continuous improvement. This talk focuses on the basic principles of leading quality improvement projects and shows personal experiences from a quality improvement project in medical education.
***Learning Objectives:***
1. Measuring and managing errors.
2. Peer review and continuous learning.
3. Effective feedback, basics of tutoring and coaching.

**10:30 - 12:00** **Room M 4**

### E³ - ECR Academies: Chest Imaging

### E³ 1022

### Lung cancer in the era of molecular oncology and immune therapy

**A-0598** 10:30
**Chairperson's introduction**
H. Prosch; *Vienna/AT (helmut.prosch@meduniwien.ac.at)*

Lung cancer is the most common cause of cancer-related death in Western countries. In the last several years, a number of new drugs have revolutionised systemic therapy in lung cancer. These new therapies can be divided into two major groups, the targeted therapies and the immunotherapies. Targeted therapies, such as EGFR tyrosine kinase inhibitors, or ALK inhibitors, are a class of drugs that specifically target a well-defined molecular pathway. They have been shown to be more effective than classic chemotherapies in patients who harbour the specific mutation and are associated with fewer toxicities. As these drugs target molecules with a specific mutation, patients who harbour this specific mutation need to be identified. In addition to bronchoscopy, image-guided biopsies are the main modality for obtaining tissue for molecular analysis. Imaging may have a potential role in identifying tumours that harbour a specific mutation, and thus, in guiding further pathologic and genetic work-up. Immunotherapy, however, targets immunological pathways to induce an immunological response against tumours. Immunotherapy has been shown to be a very effective treatment in a subset of patients with non-small cell cancer. Imaging plays a major role in the follow-up evaluation of patients undergoing immunotherapy, as immune reactions must be differentiated from disease progression.

**A-0599** 10:36
**A. Lung adenocarcinomas with EGFR mutations**
M. Lederlin; *Rennes/FR (Mathieu.LEDERLIN@chu-rennes.fr)*

Adenocarcinoma is the most prevalent type of lung cancer, showing a large spectrum of genetics, histologic subtype, CT appearance and prognosis. Activating mutations of EGFR are found in 30%-50% of lung adenocarcinomas in East Asian patients and approximately 15% in Caucasian patients. EGFR mutation status is correlated with nonsmoking status, female sex, lepidic subtype, and a high response rate to EGFR tyrosine kinase inhibitors (TKI). Some CT findings have shown to be associated with EGFR mutation such as nonsolid or mixed ground-glass opacity, air bronchogram, smaller and peripheral tumours. Furthermore, never-smoking patients presenting with diffuse miliary metastatic disease at diagnosis may be diagnosed with adenocarcinoma harbouring EFGR mutation and may show a dramatic response to EGFR-TKI. The most common resistance mechanism to EGFR-TKI is the T790M mutation, against which new irreversible TKIs have been found to be clinically effective, thus increasing demand for rebiopsy in progressive NSCLC to analyse mutational status. Rebiopsies are feasible and informative in most of the patients with acceptable rates of complications. Furthermore, continued EGFR-TKI therapy may be indicated beyond RECIST progression because these tumours grow slowly and some tumour cells remain sensitive to EGFR-TKI. Radiologists should also be aware of the risk of class-effect toxicity of EGFR-TKI, in particular, pneumonitis with an incidence rate of 4-5% in the Japanese population. Finally, European radiologists should keep in mind that a majority of studies dealing with EGFR mutations in adenocarcinomas arise from Asian countries with results that might not be transposable to Caucasian populations.
***Learning Objectives:***
1. To be aware of the importance of detecting epidermal growth factor receptor (EFGR) mutation.
2. To learn about demographic and CT features suggestive of EGFR mutation.
3. To learn about the various initial and follow-up CT features.

**A-0600** 11:04
**B. PD-L1 positive lung tumours**
O.L. Sedlaczek; *Heidelberg/DE (sedlaczek@web.de)*

Immune checkpoint inhibitors are effective in the treatment of many cancers, de-blocking immune pathways; they play an increasing role in the first-line treatment of lung-cancers. This is particularly true when there is evidence for a significant pretreatment tumour lymphocytic infiltration and/or the tumour shows a high expression for PD-L1. As checkpoint inhibitors work through a different mode of action compared to cytotoxic agents, there is a good reason to use therapy response criteria other than RECIST. In contrast to classical chemotherapies anti-tumour response in immunotherapy may take longer, and in the initial phase the response to immune therapies can manifest in a morphologic “progressive disease”, therefore, called “pseudoprogression”. In this situation, early discontinuation of the treatment could be inappropriate, unless PD is confirmed. An initially tolerable PD may even include the detection of new lesions ("unconfirmed progression", iUPD) that may not lead immediately to a discontinuation of the oncologic regiment and has to be reevaluated (iCPD, iSD). The disinhibition of mechanisms normally protecting from autoimmunity and prolonged immunoreactions leads not only to unusual tumour response patterns but also produces atypical toxicities including a broad spectrum of autoimmune diseases. Imaging characteristics of frequent and serious immune-related adverse events will be discussed.
***Learning Objectives:***
1. To know about the impact of programmed death ligand 1 (PD-L1) positivity.
2. To know how to evaluate the tumour response after immunotherapeutics.
3. To be aware of the imaging features of immune therapy complications.

**A-0601** 11:32
**C. ALK-rearranged and other mutations of lung adenocarcinomas**
M. Silva; *Parma/IT (mariosilvamed@gmail.com)*

In the last decade, huge improvements in prognosis and quality of life have been granted by molecular characterisation of non-small cell lung cancer (NSCLC). In particular, Anaplastic Lymphoma Kinase (ALK) and ROS1-positive tumours can now be treated by oral tyrosine kinase inhibitors (TKIs), which allow survival outcomes far beyond it could be expected until a decade ago. Notably, these oncogenes are mostly found in adenocarcinoma histology and in never-smokers (about 60% of all ALK-rearranged NSCLC). Target therapy by TKI improves progression-free survival (PFS) compared with previous reference chemotherapy. In the face of a better disease control by TKI, however, it happens that ALK-positive tumours are prone to driver mutation with resistance to first-line TKI, in the first months of therapy. In clinical practice, diagnostic imaging, notably computed tomography (CT), has a high yield in the management of patients under target therapy. The CT evidence of disease progression, either local or systemic (note: brain metastases are relatively common because first-line TKIs have low trespassing coefficient through the hematoencephalic barrier) is paramount for timely adaptation of therapy. Rapid radiologic progression demands prompt TKI swap towards second-line (e.g. ceritinib, brigatinib, or alectinib) or third-line target therapy (e.g. lorlatinib) or otherwise. Re-biopsy is suggested to pitch the optimal second (or further) line therapy by continuous molecular testing. Adverse events occur in a minority of patients under TKI (1% incidence of lung toxicity). Therapy discontinuation is usually sufficient to reduce toxic effects, with only 3-6% of cases lingering after therapy withdrawal.
***Learning Objectives:***
1. To learn about clinicopathologic features characterising anaplastic lymphoma kinase (ALK)-rearrangement.
2. To learn about other mutations and their implications for management.
3. To see some illustrative cases.

**12:15 - 12:45** **Room A**

### Headline Session

### HL 2

### Josef Lissner Honorary Lecture

*Presiding:*
L.E. Derchi; Genoa/IT

**A-0602** 12:15
**Systemic effects of image guided tumour therapy: have we opened Pandora's box or found the Holy Grail?**
S.N. Goldberg; *Jerusalem/IL (sgoldber@bidmc.harvard.edu)*

Image-guided cancer therapies have matured and gained sufficient clinical acceptance to the point that the relatively new discipline of interventional oncology has joined surgery, radiation, and chemotherapy as the fourth pillar of cancer care. Percutaneous thermal ablation using energy sources, including radiofrequency and microwave to produce heat and cryoablation to produce freezing, is a prominent interventional oncologic technique commonly used to treat a wide range of focal primary and metastatic tumours in the liver, kidney, lung, bone, and elsewhere. Although it has been asserted that tumour ablation is a local therapy, recent research suggests that it can induce systemic effects largely via cytokines and other growth factors. On the one hand, these factors can produce potentially unwanted “off-target” tumorigenesis that, in some cases, may lead to unintended increased cancer progression. Such human-made iatrogenesis could be viewed as an opening of Pandora’s Box. On the other hand, there is ever increasing research documenting that ablation can activate the immune system to induce widespread distant anti-tumoral effects. Leveraging these “abscopal” effects to achieve synergy in combination with recently developed immuno-oncologic therapeutics can be viewed as a Holy Grail. Given this potential double-edged sword, further molecular biologic research is necessary to better understand the mechanisms behind these processes. This will afford better insight as to which pathways are activated in individual patients and ultimately allow us to develop rational strategies to accentuate and more consistently induce positive immune effects, while simultaneously eliminating negative tumorigenic effects to benefit our patients and discipline.
***Learning Objectives:***
1. To appreciate the extent of potentially beneficial and harmful systemic effects of “focal” interventional oncologic therapy.
2. To gain awareness of the molecular mechanisms that cause such systemic effects with an eye toward potentiating them for clinical benefit.
3. To learn how immuno-oncologic techniques can be combined with both percutaneous and transcatheter interventional oncologic therapies to potentially achieve better clinical outcomes.
*Author Disclosure:*
**S.N. Goldberg:** Advisory Board; Xact Robotics, Caesarea, Israel. Consultant; Cosman Company, Cambridge, MA, USA & Angiodynamics, Marlborough, MA, USA.

**12:30 - 13:30** **Room C**

### E³ - The Beauty of Basic Knowledge: Pancreas

### E³ 24C

### Cystic neoplasms

*Moderator:*
T.C. Lauenstein; Düsseldorf/DE

**A-0607** 12:30
**Intraductal papillary neoplasms**
S. Skehan; *Dublin/IE (stephenskehan@me.com)*

Most pancreatic neoplasms with a predominantly intraductal growth pattern are intrapancreatic mucinous neoplasms (IPMNs), which can be classified according to the involvement of the main pancreatic duct or its side branches. There are also much less common solid intraductal tubulovillous neoplasms. IPMNs are characterised by intraductal proliferation of mucin-producing cells arranged in papillary formations. Duct dilatation due to mucin production is the pathological and radiological hallmark. IPMNs demonstrate varying degrees of dysplasia and varying cellular differentiation, with a recognised adenoma-carcinoma sequence. Pancreatic, intestinal and oncocytic cell types are mostly seen in main duct IPMNs, which have a much higher risk of invasive carcinoma than the more common branch duct IPMN, which are usually of gastric differentiation. Branch duct IPMNs are the most common cause of incidental pancreatic cysts. Imaging features such as luminal communication with the main pancreatic duct and pathological/biochemical analysis of cyst fluid can help to distinguish branch duct IPMNs from other causes of pancreatic cysts. Because of the risk of malignant transformation, prolonged imaging surveillance is recommended for individuals with branch duct IPMNs who would be potential candidates for pancreatic surgery. Worrisome features on surveillance include the development of solid or nodular enhancing components, interval enlargement of cysts and dilatation of the main pancreatic duct or common bile duct.

***Learning Objectives:***
1. To learn about classification of cystic pancreatic neoplasms.
2. To appreciate the classification of IPMNs.
3. To understand the diagnostic imaging findings of IPMNs.

**A-0608** 13:00
**Other cystic pancreatic neoplasms**
R. Manfredi; *Rome/IT*

"no abstract submitted"

***Learning Objectives:***
1. To understand diagnostic imaging findings of other cystic neoplasms.
2. To appreciate differential diagnosis of pancreatic cystic lesions.
3. To understand the follow up of cystic pancreatic neoplasms.

**12:30 - 13:30** **Room D**

### E³ - The Beauty of Basic Knowledge: A Survival Guide to Musculoskeletal Imaging

### E³ 25C

### Spinal trauma: how to get it right

*Moderator:*
*V.N. Cassar-Pullicino;* Oswestry/UK

**A-0614** 12:30
**Spinal trauma: how to get it right**
A. Leone; *Rome/IT (a.leonemd@tiscali.it)*

Spinal trauma is an extremely complex event, whose effects and related appropriate treatment choice and timing are classically based on the evidence of the lesions, their anatomic landmarks and mechanisms of injury at diagnostic imaging. The primary aim of this lecture was to describe: 1) range of injuries resulting from high-energy trauma in patients of all ages; 2) indications to the different diagnostic imaging modalities; and 3) imaging findings which have to be looked for in spinal trauma patients, in order to adopt a pattern-based approach for efficient imaging interpretation and communication with all specialists involved in spinal trauma. Radiography and, above all, CT provides most of the information needed for the diagnostic workup; therefore, CT is the preferred imaging modality. MR imaging can provide additional information on the state of paraspinal soft tissues thanks to the high contrast resolution allowing oedema detection; moreover, it is needed to determine the integrity of the discoligamentous complex, contributing to a better evaluation of spine stability, and is thus mandatory in all cases of neurological compromise. In conclusion, the appropriate use of radiography, CT and MR imaging has the potential of correctly identifying the injured spine, allowing early treatment while minimising the risk of a delayed diagnosis.

***Learning Objectives:***
1. To become familiar with imaging features of cervical, thoracolumbar and sacral trauma.
2. To understand the importance of mechanism of injury in the setting of spinal trauma.
3. To appreciate the usefulness of radiography, CT and MR imaging in the detection and evaluation of spinal trauma.

**14:00 - 15:30** **Room C**

### EFRS meets Denmark

### EM 4

### EFRS meets Denmark

*Presiding:*
J. McNulty; Dublin/IE
H. Precht; Odense/DK

**A-0619** 14:00
**Session introduction**
J. McNulty; *Dublin/IE (jonathan.mcnulty@ucd.ie)*

Introduced for the first time for ECR 2013, the ‘EFRS meets’ sessions follow the tradition of the ‘ESR meets’ sessions and give the European Federation of Radiographer Societies (EFRS) the opportunity to highlight the contribution of one of their member societies to the profession of radiography each year. Having met with Spain in the first of these sessions in 2013, the EFRS has gone on to meet with societies and radiographers from Russia, Germany, Sweden, Belgium, Portugal, and Switzerland. The Danish Society of Radiographers, Radiograf Rådet, is a founding member of the EFRS and have been an active member of the EFRS throughout our ten-year history. University College Lillebelt is currently the only Danish educational institution within the Educational Wing of the EFRS and joined in 2009. The Danish Society of Radiographers is extremely active in promoting our profession, advancing education and training opportunities, encouraging radiographers to undertake research at the highest level, and in lobbying for their radiographers. Danish radiographers are currently involved in the EFRS leadership and across a number of our active working groups.

**A-0620** 14:05
**Introduction: Across Denmark**
C. Graungaard Falkvard; *Copenhagen/DK (charlotte@radiograf.dk)*

You will be presented to Denmark as a country with a focus on the good Danish life, Danish culture and the Danish radiography profession. The presentation will also have a focus on the possibilities and struggles in the profession and how to work politically for the profession in Denmark.

***Learning Objectives:***
1. To introduce the EFRS meets session and the involvement of Denmark in the EFRS.
2. To introduce the country of Denmark, Danish life and culture and the Danish radiography profession.

**A-0621** 14:10
**Machine learning: a new aspect of radiography**
L.M. Pehrson; *Copenhagen/DK (Lea.marie.pehrson@gmail.com)*

Machine learning (ML) and deep learning (DL) are becoming established disciplines in the broad field of applying artificial intelligence in analysing and utilising patterns in data sets. As the complexity - as well as the sheer amount of - data increases, applying these patterns to the benefit of, e.g. clinical decision making, becomes increasingly nontrivial. Extraordinary advancements in areas of technology such as high-performance computing have made it possible to attempt solving these problems algorithmically. The purpose of various ML and DL algorithms may be to improve quality, consistency and/or capacity of data interpretation in diagnostics, thus improving diagnostics and treatment decisions to the benefit of clinical outcomes. Considering the implications this may have for the practice of medicine / healthcare. It is important to engage in this area of research from many perspectives. ML is already being applied to the practice of radiology, and the systems being developed today are showing to be robust to real-world conditions. In this discussion, the general concept of AI methods, together with an overview of early results in the field of medical imaging will be introduced. Furthermore, aspects of implementation and its impact will be discussed.

***Learning Objectives:***
1. To recognise Machine learning as a tool for medical image analysis.
2. To learn about the opportunities within machine learning, the challenges and new directions in clinical practice.
3. To acknowledge the potential impact for the patient, radiographer and radiologist.

**A-0622** 14:27
**Improvements in healthcare**
P. Blackburn Andersen; *Kolding/DK (picaandersen@yahoo.dk)*

The number of patients in Danish hospitals increased by 50.000 during the years 2015-2017 while the number of hospital staff increased by 4 in the same period. Furthermore, today´s patients expect a higher level of individualised treatment and care whilst most hospitals struggle with hospital infections, stressed staff and medical mistakes. As we see how time-consuming and expensive medical mistakes are we also recognise improvements that need to happen to increase patient safety, satisfaction and quality of hospital care. Patient involvement and shared decision making have been shown to increase patient satisfaction and safety whilst decreasing medical mistakes. Consequently, patient involvement is fundamental to a continuous and successful improvement process. As radiographers, we encounter a large number of hospitalised patients every day. Naturally, we also see inefficiencies of the hospitalised patients journey to the radiology department. At times we even recognise actual errors made prior to the patient's arrival at Department of Radiology, and this is where radiographers have two choices - do we ignore these errors and inefficiencies because they do not occur in our department, or do we think "patient first" and address the problem to the involved departments? Could we perhaps take it a step further and think "how can we contribute"? By presenting some improvement cases from Kolding hospital, we will see how resourceful and valuable radiographers are to patient safety and satisfaction when improvements are created systematically, vertically and in collaboration with other staff and patients.
***Learning Objectives:***
1. To understand the need for improvement in Danish healthcare (and perhaps European healthcare).
2. To appreciate how radiographers can contribute beyond the radiology department.
3. To acknowledge the need for patient and staff involvement to create improvements.

**A-0623** 14:44
**Technologically mediated patient and radiographer experience**
S. Holm; *Faaborg/DK (suho@ucl.dk)*

The Danish healthcare system has a great focus on patient-centred care in all elements of a patient trajectory. In diagnostic imaging settings, previous qualitative research into patient's experienced care generally investigates verbal and non-verbal communication in the relation between the patients and professionals. The effects that imaging technologies have on these experiences and relations are nonetheless seldom investigated. Yet technological devices and systems play a significant role in shaping human experience, communication and relations in these settings and needs to be addressed. To explore how technologies mediate experience in diagnostic imaging practice, I turn to a particular philosophy of technology, as a theoretical and analytical framework, for studying human and technology relations. Applying concepts developed within this position provides theoretical and methodological means to explore embodied and perceptual lived experience. In particular, postphenomenology reflects on how technological mediation transforms our perceptual experience of our world. This talk suggests that by applying this approach in the unfolding of the specific human dimension of Radiography, we are able to identify important issues that can help professionals to perform improved patient-centred care.
***Learning Objectives:***
1. To understand what is meant by technological mediation.
2. To learn about embodied perception.
3. To explore how technologies mediate experience in radiographic practice.

**A-0624** 15:01
**MR safety**
A.D. Blankholm; *Aarhus/DK (anneblan@rm.dk)*

MR safety is a hot topic amongst MR professionals. Incidents related to MR are rare but can be dangerous. MR safety is related to the three types of fields: main magnetic field, gradient field and the radio frequency (RF) field. The main magnetic field accounts for the projectile effect, torque/rotation and translational forces. The gradient field accounts for the noise and peripheral nerve stimulation. The RF-field accounts for heating. Most MR incidents can be avoided. The key to avoiding MR related incidents is correct planning of the site, awareness about MR safety and MR safety education of internal and external staff. The fact that no databases containing information about the type of implants patients have exists. Often such information in the patient record is inadequate, and the information from the manufacturer is not standardised and can be inadequate. Furthermore, control forms with missing or wrong information is a challenge. A search in a national database reflected few reported incidents but that incidents and near-incidents are happening. A national questionnaire amongst MR operators reflected that more than half of the respondents had been involved in an MR related incident, that was reported to the national database, but one forth had been involved an MR related incident that was not reported. Sixty-one per cent of the respondents indicated that guests in the MR environment are a safety risk. Recommendations for MR safety education of MR safety -officers, -experts and -directors have been published and supported by several organisations and authorities.

***Learning Objectives:***
1. To understand the challenges of MR safety.
2. To appreciate MR safety from the Danish radiographer's perspective.
3. To become familiar with recommendations for MR safety education.

15:18
**Panel discussion**

**14:00 - 15:30** **Room N**

### PIER @ ECR Session (Jointly organised with the ESR Research Committee)

### PI 3

### Professional issues for radiology departments to enable research

*Moderator:*
J.K. Bell; Manchester/UK

**A-0625** 14:00
**Chairperson's introduction**
O. Clément; *Paris/FR (olivier.clement@aphp.fr)*

One of the major issue for academic radiology is to attract the young generation to our discipline in a time of big potential changes in the way we practice with artificial intelligence and the necessity to be visible to the patient. The aim of this session is to explore how research is the main way to follow.
***Session Objectives:***
1. To learn about research in imaging or imaging for clinical research.
2. To appreciate how to attract residents in research.
3. To learn about examples of implementing AI research in radiology.
*Author Disclosure:*
**O. Clément:** Advisory Board; Bayer. Speaker; Bracco, Guerbet.

**A-0626** 14:06
**How to attract residents to research**
H.-U. Kauczor; *Heidelberg/DE (Hans-Ulrich.Kauczor@med.uni-heidelberg.de)*

In the era of fast technological advances in medical imaging, high-quality research plays an essential role in advancing radiology as an independent speciality with its sustained development and radiologists remaining leaders in the field of biomedical imaging. Identifying young radiology residents interested in research, encouraging and supporting them to become a proficient and competitive academic radiologist is an important task for every academic radiology department. It is important that the residents' subspecialty interests are identified early and considered by department chairs for the clinical training and scientific career. In times of ever increasing clinical responsibilities, protected research time for research for residents is crucial. Empowering young residents with an optimal research environment consisting of qualified mentoring, training courses on clinical research methods, study design, biostatistics, human, infrastructure and financial resources and last but not least administrative grant support is of utmost importance. Since the speciality of radiology is tremendously influenced by developments in artificial intelligence, deep learning and other technological advancements, multidisciplinary and integrative research approaches are indispensable. There is no doubt that residents exposed to research will gain experience in critical thinking and leadership in order to pursue a successful career either in academia or many other fields.
***Learning Objectives:***
1. To learn basic concepts for research options for residents.
2. To appreciate the value of research activities in the professional career.
3. To understand the requirements for research environments for residents.

**A-0627** 14:22
**How to organise an imaging department for clinical research**
L.S. Fournier; *Paris/FR (laure.fournier@aphp.fr)*

Radiology and nuclear medicine imaging provide independent and complementary information from clinical examination and biological explorations, such as drug development and validation. In these cases, imaging is not the object of the research but a tool to measure outcomes, therefore its quality must be assured to guarantee the quality of the overall research data. This clinical research occurs on imaging platforms in public or private structures, most of which are part of clinical departments organised to deal with clinical care. There are several challenges to integrate a research among a clinical workflow. First, the volume of imaging dedicated to clinical research is steadily increasing, as imaging is recognised as giving useful biomarkers of disease and drug efficacy. Second, the quality assurance and quality control required are also becoming more stringent. However, there has not been a proportional investment in departments in terms of material and personnel resources. Departments need to organise imaging research units embedded in clinical departments, for quality control, archiving and processing. For this, dedicated personnel needs to be identified, including imaging CRAs and radiographers with training in clinical research, and financial self-sufficiency needs to be reached by including specific overcosts for imaging.
***Learning Objectives:***
1. To learn about concepts for research facilities.
2. To appreciate how to implement quality approaches.
3. To understand the financial background.

**A-0628** 14:38
**How to deal with legal requirements in research activities**
P.M.A. van Ooijen; *Groningen/NL (p.m.a.van.ooijen@umcg.nl)*

One of the major challenges nowadays when performing research is how to comply with the legal requirements that are posed upon the researcher based on the General Data Protection Regulation (GDPR) in Europe. Through the GDPR the rights of the patients or participants in your research are protected, but how to work within this framework and still be able to get your research done might be a challenge. Especially since the GDPR not only restricts the use of prospective data but also retrospective data and the ability to link a number of databases into a new research database. On the other side, open science is gaining interest, and the question is how to make your database open when all these restrictions are in place. In this lecture, we will look into the GDPR and how it affects prospective and retrospective research. Furthermore, we'll discuss how multi-centre studies could be set up with multiple participants from different European countries. Finally, we'll look at some of the concepts and tools that are available to ensure data protection and data security.
***Learning Objectives:***
1. To learn about the relevance of legal framework for research.
2. To appreciate the European perspective for multi-centre projects.
3. To become familiar with best practice concepts for data-protection and data-security.
*Author Disclosure:*
**P.M.A. van Ooijen:** Advisory Board; MedicalPHIT. Board Member; EuSoMII.

**A-0629** 14:54
**How to implement research on artificial intelligence in radiology**
K.J. Dreyer; *Boston, MA/US*

"no abstract submitted"
***Learning Objectives:***
1. To learn about the key-concepts of AI and the challenge for radiology.
2. To appreciate the opportunities for radiologists active in AI-research.
3. To become familiar with new concepts for supporting research on AI in radiology.

15:10
**Panel discussion: Will research in radiology survive?**

**14:00 - 15:30** **Room E1**

### Joint Session of the ESR and BBMRI-ERIC

### ESR/BBMRI-ERIC

### Will the General Data Protection Regulation (GDPR) hamper the secondary use of clinical imaging data for research?

*Moderators:*
E. Steinfelder; Graz/AT
A. van der Lugt; Rotterdam/NL

**A-0630** 14:00
**Why is secondary use of existing imaging data important for research progress?**
E. Steinfelder; *Graz/AT (erik.steinfelder@bbmri-eric.eu)*

There is no medical innovation without biobanking, and there is no biobanking without patients and their consent. In this context, the question of secondary use of samples and data is a very relevant one. BBMRI-ERIC, the world largest biobanking directory, is collaborating with more than 90 public and private health research organisations to provide guidance on how to use the GDPR to both protect patients' rights to privacy and empower medical research. The result of the collaboration will be a Code of Conduct for Health Research, a soft law instrument that research organisations and EU countries can use to better implement the GDPR. One of the main risks is that a piecemeal implementation of the GDPR might affect the European Research Area and obstruct collaboration among European and international research groups, slowing down innovation. Another risk is that the GDPR will negatively impact on the total amount of samples and data shared by biobanks. We know that around 5-10% of samples stored in BBMRI-ERIC biobanks is shared for research purposes. While BBMRI-ERIC is developing a new strategy to increase access, the GDPR can pose new obstacles that will make our final objective, “making new treatment possible”, more time- and resource-consuming.
***Learning Objectives:***
1. To learn about Biobanking and BioMolecular resources Research Infrastructure (BBMRI).
2. To learn what BBMRI can do for you in the GDPR era.
3. To learn about the importance of existing imaging data for research in imaging biomarkers, radiomics and artificial intelligence.

**A-0631** 14:15
**Can I reuse, share or give open access to existing imaging data for research?**
M.T. Mayrhofer; *Graz/AT (michaela.th.mayrhofer@bbmri-eric.eu)*

This presentation will give an overview of the EU General Data Protection Regulation, focusing on imaging data for research. It will discuss privacy principles and concerns of privacy breaches and contextualise it within the policy goal of open access and open science. Thereafter, it will turn to data reuse and data sharing, which are standard practices for research that pose complex challenges upon researchers and research participants alike and include, among others, data security, privacy and good scientific practice: How can data reuse be simplified? How can compliance be demonstrated by controllers and processors towards the regulation? How can open access help to ensure trust by the research participants?
***Learning Objectives:***
1. To learn what you can do with imaging data in the PACS and observational research studies.
2. To learn what you can do with data from investigator initiated or industry sponsored RCTs.
3. To learn how to prevent future problems with re-use of clinical data.

**A-0632** 14:40
**Can I sell or licence imaging data to industry or spin-off companies?**
J.A. Bovenberg; *Aerdenhout/NL (jabovenberg@xs4all.nl)*

My talk will discuss whether and, if so, how medical imaging data can be shared with commercial partners or used by spin off companies. To that end, we will map the various rights vested in these data and examine the various legal bases for sharing. Taking a case based approach, we will specifically explore the various mechanisms to share imaging data with commercial partners outside the EU.
***Learning Objectives:***
1. To understand the issues of sharing data with industry.
2. To learn about proper informed consent for sharing data with industry.
3. To learn whether data can be shared outside the European Union.

**A-0633** 15:05
**How to anonymise imaging data for research?**
B. Gibaud, E. Cordonnier, G. Pasquier; *Rennes/FR (bernard.gibaud@univ-rennes1.fr)*

Secondary use of clinical imaging data is becoming a major topic, especially in the context of the growing needs of training and testing Artificial Intelligence (AI) algorithms. The General Data Protection Regulation (GDPR) which entered in application in Europe in May 2018 enforces strict constraints regarding protection of personal data, especially for sensitive data such as medical images. As a general rule, de-identification is needed to ensure patient confidentiality and can be achieved through anonymisation or pseudonymisation. This presentation will focus on the de-identification of DICOM data. Therefore, the basic principles of DICOM syntax will be recalled, as well as the general organisation of image metadata and pixel data. Then, the principles of DICOM de-identification and re-identification will be introduced (DICOM Part 15 Security and system management profiles). The need for Confidentiality Profiles will be explained, as a means to address the balance between the removal of any information that might directly or indirectly lead to patient identification, and the need to retain information so that the DICOM data remain useful for its intended purpose in the context of secondary use. Examples of such DICOM Confidentiality Profiles will be shown and explained. Several tools implementing DICOM de-identification will be briefly introduced. A concrete use case will be presented to illustrate the use of de-identification / re-identification.
***Learning Objectives:***
1. To learn the difference between anonymisation and pseudonymisation.
2. To become familiar with tools for pseudonymisation.
3. To understand the secrets of the DICOM header.

*Author Disclosure:*
**E. Cordonnier:** Employee; b<>com: develops and sells to industry providers the software development kit, which implements some mechanisms described in the presentation, including some patented features. **G. Pasquier:** Employee; b<>com develops and sells to industry providers the software development kit which implements some mechanisms described in the presentation, including some patented features..

15:25
**Panel discussion: How to overcome the hurdles of secondary use of imaging data**

**14:00 - 15:30 Room M 2**

### E³ - ECR Academies: Radiology Leaders' Bootcamp

### E³ 1118
### Investment strategies

**A-0634** 14:00
**Chairperson's introduction**
E. Kotter; *Freiburg/DE (elmar.kotter@uniklinik-freiburg.de)*

Investment strategies are key to successfully running both a private practice and a radiology department. This session will give insights into the principles of investment strategies for radiology. Participants will be given the keys to understanding how to consider a radiology unit from an economic point of view. The focus will be on the return of investment.

**A-0635** 14:06
**A. Budget: all these numbers**
A. Giovagnoni; *Ancona/IT (a.giovagnoni@univpm.it)*

In simple terms, an operating budget is a projection or a snapshot of the results a practice hopes to achieve or believes it will achieve. The words “hope” and “believe” are used because budgeting is an intuitive process that can be employed for different purposes and executed in various ways. Mapping out a strategic plan involves goal setting, a plan of action to achieve the goals, and monitoring systems to measure the efforts intended to achieve those goals. Operating budgets can tangibly show the results of numerous decisions that have been made and often act as a report card on the success of any given strategy. While hospitals tend to be steeped in the budgeting process, in many radiology practices, budgets are often wrongly considered unimportant or unnecessary. A general feeling that the clinical side of practice lacks control over the business aspects of revenues and expenses can lead to the mindset that there is no reason to project them. While a budget does not define how effective an operation is, without one, operating results are left to chance with no real measure of success or failure. Good budgeting includes both science and intuition in making projections. Practices working with budgets control their destiny better and operate less blindly. This presentation intends to define and illustrate the necessary components that radiology practices must consider as they carve out a budget.
***Learning Objectives:***
1. Why to be aware of financial literacy.
2. Major financial indicators for radiology: to dig into numbers with profit.
3. How to make all these boring numbers work for you and do more with fewer resources.

**A-0636** 14:34
**B. Value-based imaging**
M.G.M. Hunink; *Rotterdam/NL*

Value-based imaging is in vogue. But how do we measure value? For the patient, quality of life and length of life are fundamental. Most of the conveniently-measured patient outcomes are proxies or intermediate outcomes. The value of medical imaging performed for screening, surveillance, diagnosis, or prognosis is a function of pre-test probability of disease, sensitivity and specificity of the imaging test, risk and burden of the imaging test, the gains if the disease is identified, the harms if a non-diseased patient is inadvertently labelled as diseased. The expected value of imaging is largest when the pre-test probability of disease is at the treatment threshold. In treatment planning, the added value of imaging is determined by the net reclassification to a different treatment plan and the net gains from the change in treatment plan. In treatment guidance, the added value of imaging comes from reduced risk and increased effectiveness of the treatment. From the healthcare system perspective, costs are included and should consider not only the imaging tests but also costs of treatment and long-term costs of events during follow-up. From the societal perspective, additionally, productivity gains and losses are important to elucidate in order to fully quantify the value of imaging. Finally, investing in the health, happiness, and skills of our employees is crucial in order to provide value-based care since a competent resilient workforce translates into patient safety and quality of care.
***Learning Objectives:***
1. To understand the value of what we do in radiology.
2. To understand how cost-effectiveness analysis can demonstrate the value in imaging.
3. To recognise how investing in our employees increases the value of what we do.
*Author Disclosure:*
**M.G.M. Hunink:** Advisory Board; EIBIR. Author; Textbook: Decision Making in Health and Medicine. Research/Grant Support; American Diabetes Association.

**A-0637** 15:02
**C. Start-up in radiology**
A. Alberich-Bayarri; *Valencia/ES (alberich_ang@gva.es)*

The main steps when creating a new start-up company in the field of radiology are be presented, including the do's and don'ts from the experience in creating a start-up dedicated to Imaging Biomarkers quantification and artificial intelligence. The topics include bringing to market the developments in research, how to raise funds from zero, deal with investment funds and regulatory aspects. Start-ups must be focused on value, and for that, the hypothesis for the value proposition of the company must be validated from the very beginning. After that validation, the company must dedicate the efforts to be able to demonstrate some traction among stakeholders and potential customers. In parallel, there must be an intense progress in regulatory aspects to reach the clinical market.
***Learning Objectives:***
1. Understanding successful investments: which number you really need to take care of.
2. Designing a standalone imaging centre.
3. Investments: how to find money for new projects in imaging informatics.
*Author Disclosure:*
**A. Alberich-Bayarri:** CEO; QUIBIM. Founder; QUIBIM.

**16:00 - 17:30 Room A**

### E³ - ECR Academies: Interactive Teaching Session for Young (and not so Young) Radiologists

### E³ 1221
### Advances in musculoskeletal techniques: whole-body MR

**A-0646** 16:00
**A. Oncologic application**
F.E. Lecouvet; *Brussels/BE*

This interactive session will illustrate the wide range of indications of whole-body MRI in oncology, the protocols and technical requirements, the contribution of anatomic and functional diffusion sequences and their respective strengths and pitfalls. Latest technical refinements and efforts to decrease examination duration and tailor sequences to the targeted disease will be presented. Simple and more complex cases will highlight how the technique extends the exploration of the body beyond the musculoskeletal system to lymph nodes and visceral metastases screening. Specific cases will show how WB-MRI has become a modality of choice for detecting bone metastases from many cancers and bone marrow involvement by multiple myeloma or lymphoma. The comparison will be provided with bone scintigraphy, CT and PET by the time of lesion detection and for assessment of the response to treatment.
***Learning Objectives:***
1. To become familiar with the technical aspects of whole-body MR.
2. To learn the role of whole-body MR in the management of oncologic patients.

**A-0647** 16:45
**B. Non-oncologic applications**
M. Faruch; *Toulouse/FR (mariefaruch@hotmail.com)*

Thanks to the improvement in technique as the shortening of examination durations, whole-body MR imaging can now be feasible for use in routine clinical practice in numerous skeletal and neuromuscular disorders as the seronegative rheumatologic disease, multifocal ischemic lesions, multifocal eosinophilic granulomas and inflammatory myopathies. Whole-body MR could be helpful for early diagnosis, evaluation of the extent and activity of the disease, and evaluation of therapeutic response. Current MRI protocols usually include anatomic sequences as T1-weighted and water-sensitive sequences as STIR, but could also provide functional DWI sequences.
***Learning Objectives:***
1. To become familiar with the indications for whole-body MR in non-oncologic patients.
2. To learn the role of whole-body MR in the management of non-oncologic musculoskeletal disorders.

**16:00 - 17:30** **Room B**

### GI Tract

### RC 1201

### Imaging the acute abdomen: new insights

*Moderator:*
D.E. Malone; Dublin/IE

**A-0648** 16:00
**A. Acute mesenteric ischaemia**
M. Ronot; *Clichy/FR (maxime.ronot@aphp.fr)*

Acute mesenteric ischemia is defined as inadequate blood supply to the gastrointestinal tract resulting in an ischemic and inflammatory injury that may progress to necrosis of the bowel wall. Prognosis is poor with a mortality rate greater than 95% without treatment, dropping to around 70% when surgical treatment is performed. Contrast-enhanced computed tomography (CT) has become the cornerstone of the diagnosis by showing features of vascular disorders (occlusion and/or insufficient blood supply) and features of intestinal ischemic injury. CT should be performed as rapidly as possible. Imaging-based patient management is required, and multimodal and multidisciplinary management should be introduced. The treatment involves multidisciplinary management by gastroenterologists, vascular and digestive surgeons, cardiologists, intensivists, and diagnostic and interventional radiologists. Based on our experience at a dedicated mesenteric stroke centre, this article gives an overview of the diagnosis of acute mesenteric ischemia. The goal of this presentation is to improve the understanding of the imaging-based diagnosis to further improve the management of this life-threatening condition.
***Learning Objectives:***
1. To learn about the different types and clinical conditions in which acute mesenteric ischaemia occurs.
2. To understand how the imaging features are related to the underlying pathophysiology.
3. To appreciate the role of endovascular interventional procedures in the management of acute mesenteric ischaemia.

**A-0649** 16:30
**B. Low-dose abdominal CT for evaluating suspected appendicitis**
K.H. Lee; *Seoul/US (kholeemail@gmail.com)*

Evaluation of suspected appendicitis in adolescents and young adults is one of few LDCT applications which were validated to be safe and effective regarding clinical outcomes through large randomised clinical trials. As summarised in recent systematic reviews, high-level evidence has been accumulated for the use of LDCT (down to 2 mSv) in diagnosing appendicitis. However, the adoption of LDCT is disappointingly slow. Given the evidence level, there is no reasonable basis to insist on using radiation dose of multi-purpose abdomen CT for the diagnosis of appendicitis, particularly in adolescents and young adults.
***Learning Objectives:***
1. To emphasise the need for reducing radiation exposure in adolescents and young adults with suspected appendicitis.
2. To critically review published evidence indicating that LDCT is comparable with normal-dose CT for diagnostic performance and clinical outcome.
3. To review LDCT imaging techniques and other practical issues for the successful implementation of LDCT in practice.
*Author Disclosure:*
**K.H. Lee:** Grant Recipient; Korea Health Industry Development Institute, National Research Foundation of Korea, Seoul National University Bundang Hospital, Dasol Life Science, Bracco Imaging Korea, GE Healthcare Medical Diagnostics Korea.

**A-0650** 17:00
**C. Acute colonic diverticulitis**
S. Schmidt Kobbe; *Lausanne/CH (sabine.schmidt@chuv.ch)*

Acute colonic diverticulitis (ACD) frequently occurs in the Western worlds, and the severity ranges from mild, simple inflammation to complicated disease with pericolic abscesses or perforation with feculent peritonitis. Only about 25% of patients show complications during the first acute presentation. Imaging plays a crucial role in the initial diagnosis of colonic diverticulitis as well as for excluding the various differential diagnoses. Depending mainly on the location the latter include epiploic appendagitis, acute appendicitis, ischemic, inflammatory, or infectious colitis, gynaecological diseases, such as tuboovarian abscesses, and infarction of the great omentum. Computed tomography (CT) is the most commonly performed imaging modality in an emergency to detect and to confirm ACD although false negative results may occur in 2-21% of cases. Ultrasonography has been known for a high specificity for ACD, but results remain very operator-dependent. Magnetic resonance imaging is still not a first-line emergency modality, but very sensitive for fistulous complications. The most specific imaging finding is the inflamed diverticulum (arrowhead sign), often with pericolic stranding and colonic wall thickening. Complications of ACD may be regional, such as adjacent abscesses and fistulae, or distant, such as thrombophlebitis or organ abscesses, ideally demonstrated by CT with intravenous contrast medium injection. Treatment of colonic diverticulitis has evolved away from immediate surgery towards more conservative and minimally invasive strategies. Today, small abscesses are managed conservatively, while abscesses of >3-4cm require percutaneous drainage, often conducted by radiologists. Surgery has become less and less straightforward but considered as second-line treatment after previous imaging-guided intervention.
***Learning Objectives:***
1. To review the typical and atypical imaging features of acute colonic diverticulitis and their influence on patients' management.
2. To become familiar with the complications and the most important differential diagnoses of acute colonic diverticulitis.
3. To understand the importance of imaging-guided interventions for the management of complicated acute colonic diverticulitis.

**16:00 - 17:30** **Room C**

### ISRRT meets Africa

### EM 5

### Radiographers' challenges offering imaging services in Africa

*Presiding:*
D. Newman; Fargo, ND/US
P. Gerson; Paris/FR

**A-0651/A-0652** 16:00
**Chairpersons' introduction**
D. Newman; *Fargo, ND/US*
P. Gerson; *Paris/FR (philippe.gerson@htd.aphp.fr)*

This paper is about ISRRT involvement in Africa for the past 24 years in more than 10countries. Beginning in 1994 in Tanzania. ISRRT has organised several workshops and congresses in this continent especially in French-speaking African countries. The involvement has contributed to help countries to set up associations and establish a huge network in Africa.
***Session Objectives:***
1. To recognise the demographics and patient accessibility to health services of a particular African country.
2. To understand the infrastructure of the imaging health services and their contribution to the primary and hospital health services to sustain the population and individual health.
3. To appreciate the radiographers' effort to keep up to date with evidence-based practice in imaging services.
4. To become familiar with the radiology education system and lifelong learning opportunities for radiographers practicing in Africa.

**A-0653** 16:08
**Nigeria. Healthcare services in Nigeria: the radiographers' opportunities and challenges**
E. Olasunkanmi Balogun; *Lagos/NG (kanmibalo@yahoo.com)*

Radiography is an art and science of the application of both ionising and non-ionising radiation in diagnostics and treatment of diseases when medically indicated. The study of radiography dates back in Nigeria, first as hospital-based training after which foreign examinations were taken. These examinations were conducted by the college of radiographers, London, United Kingdom. The certificates evolved from MSR, DSR, to DCR In the year 1965 the first indigenous school was established in Lagos (Circa) and then another in Ibadan in the year 1972. The bachelors of Science (BSc) in Radiography started in the year 1983 and has spread over the years with seven universities offering radiography at a first-degree level and three at postgraduate levels. Training of manpower is the bedrock of better practice, therefore, the increase in training schools brought about an increase in manpower and translated to better practice across the country. The status of being a developing Nation as well as a Small non-influential market brought great challenges to the availability and maintenance of infrastructure. Recently the Nigerian government introduced the public, private, partnership (PPP) model of funding which has helped with increasing access to very expensive but needed equipment. There are a few challenges with the Scope of practice, hands-on training and limitations by government policies. Radiography training and practice in Nigeria is progressing and still an arrowhead even in Africa as a continent. However, Research funding opportunities are required.

***Learning Objectives:***

1. To recognise the demographics and patient accessibility to healthcare services in a densely populated country as Nigeria.
2. To understand the infrastructure of the imaging health services and their contribution to the primary and hospital health services to sustain the population and individual health.
3. To appreciate the radiographers' effort to keep up to date with evidence-based practice in imaging services.
4. To become familiar with the radiography education system and lifelong learning opportunities for radiographers practicing in Nigeria and its environs.
5. To look at the challenges of team work/professional rivalry/nomenclature even in the face of infrastructural challenges.

**A-0654** 16:26

**South Africa. The South African radiographer: button pusher or creative thinker?**

H. Friedrich-Nel; *Bloemfontein/ZA (hfried@cut.ac.za)*

In 2014 two of the Universities of Technology in South Africa implemented four-year degrees in radiography. The Health Professions Council of South Africa (HPCSA) and specifically the Professional board for Radiography and Clinical Technology (RCT) guided this process. Previously the education and training model was a two-year hospital-based diploma and a three-year bachelor's degree or diploma. Six years after the first universities implemented the revised education and training model, the question lingers: Do universities deliver radiography graduates with the ability to work independently, think critically and creatively? Recently the HPCSA updated the scope of practice of a radiographer to align this with the four-year bachelor's degree education and training model to offer expanded opportunities to the radiographer who wishes to engage in lifelong learning opportunities. In South Africa, it is a statutory requirement for a health care practitioner to register with the HPCSA. To maintain his or her HPCSA registration each registered healthcare practitioner (radiographer) must pay a yearly registration fee and earn 30 continuing education units (CEUs). Five CEUs must be collected in the category ethics, patient-centred care and patient rights.

***Learning Objectives:***

1. To learn about the radiography education models in South Africa.
2. To become familiar with continuing education requirements and registration.
3. To share the revised scope of practice.

**A-0655** 16:44

**Côte d'Ivoire. Professional practice of radiology and imaging in Africa: radiographers for more commitment and responsibility in patients' safety**

K.B. Yao; *Abidjan/CI (kwame_boniface@yahoo.fr)*

Radiology is the pathway to clinical settings and Diagnostic Imaging. It is a powerful tool in medicine, and its influence is expected to increase for years to come. Besides, the growth of modern imaging depends on advances in technologies, and the professional practice includes the development of radiographers' skills as well as the implementation of radiation safety culture. However, in Africa, hundreds of hospitals and institutions do not have the possibilities to perform the most fundamental imaging procedures, for lack of appropriate technologies, and skills. Moreover, setting a careful balance between the benefits and the risks related to patients' exposure is a challenge. Despite the recurrent shortage, African Technologists keep on struggling to bridge the gap. The methodology of approach consists in covering the state of art of professional practice and safety, revealing the main gaps and actions carried out to bridge them up and, the perspective and operational plan for a better view on the future. As deliverable outcomes, different projects carried out by radiographers and results are presented, and their impact on professional practice and safety discussed. They are essentially made up by the development of homemade equipment for professional practice purpose, training project to strengthen radiographers' skills. The global situation of practice is characterised by a gap between international standards and local settings. Therefore, radiographers are urged to demonstrate more commitment and responsibility in the management of their activities. Besides, ISRRT's support is very much appreciated and exchange between technologists, through international congress is strongly encouraged.

***Learning Objectives:***

1. To reveal the challenges faced by African radiographers in practicing their profession.
2. To learn about the different actions carried out by radiographers for radiographers within Africa, in seeking alternatively affordable means of practicing their profession.
3. To appreciate the ISRRT's initiatives and capacity building projects to provide sustainable support to radiographers in Africa.
4. To understand the clues set up by ISRRT regional officers' to improve patient care.

**A-0656** 17:02

**Kenya. Discovering Kenyan radiographers: past, present and future**

C. Muchuki; *Nairobi/KE (cmuchuki@gmail.com)*

Kenya is a country in East Africa with a coastline on the Indian Ocean with a population of 49 Million. It's a home to wildlife and Maasai Mara Reserve known for its annual wildebeest migrations. The Kenyan radiographers are governed by Society of Radiography in Kenya (SORK) a registered professional body whose overall aim is to promote, support, regulate the professional standards of practice, conduct, and ethics of radiographers. Currently, they are 1200 registered radiographers and 300 radiologists in the country. Kenya medical training college offers Diploma in imaging courses for three years. Additional training is Bachelor of Radiography program an integrated academic and clinical course of 4 years duration. The Kenyan radiological system is continuously innovating. Previously, radiographers would process the films in a darkroom, now we have jumped into a new era. The workflow is greatly facilitated by automatic machine-facilitated workflow processes, and Patients receive digital imaging. Kenya's health care system is structured in a step-wise manner so that complicated cases are referred to a higher level. Gaps in the system are filled by private and church-run units. There are three major national hospitals in Kenya, where Kenyatta National Hospital is the largest Referral Hospital in Kenya and the entire East and Central Africa. It caters for 80,000 in-patients, >500,000 out-patients annually with a bed capacity of 1800. It offers diagnostic imaging service to >300 patients daily. Concerning radiation safety, AFROSAFE campaign addresses issues arising from radiation protection in medicine in Africa.

***Learning Objectives:***

1. To understand the current situation and trend of radiographers in Kenya.
2. To understand the Kenyan health system.
3. To become familiar with Kenyan radiation safety issues; Afro safe Kenyan chapter.

17:20

**Panel discussion**

**16:00 - 17:30** **Room N**

### EuroSafe Imaging Session: jointly organised with the ISR and the IOMP

### EU 3

### Improving radiation protection in medical imaging in low- and middle-income countries: past actions and future directions

**A-0657/A-0658** 16:00

**Chairpersons' introduction**

L. Donoso; *Barcelona/ES (ldonoso@clinic.ub.es)*
M.M. Rehani; *Boston, MA/US (madan.rehani@gmail.com)*

The international professional societies have the mandate and responsibilities to extend outreach to low and middle-income (LMI) countries. In this respect, both the International Organization for Medical Physics (IOMP) and International Society of Radiology (ISR) join hands to assess and improve the situation of radiation protection in medical applications of radiation. This session involves important stakeholders like the International Atomic Energy Agency (IAEA), World Health Organization (WHO) and representative of an important region-Africa. The session will deliberate not only on actions taken, result achieved but also on a vision for the future. There have been important developments through IAEA in LMI countries with the support of WHO, IOMP and ISR such that reasonable information is available on radiation doses to patients in various imaging procedures dominantly in CT and interventional procedures and diagnostic reference levels (DRLs) are available from some of the LMI countries. There are few situations where patient doses are on higher side. However, the information comes largely from few major centres in each country (lacking in breadth), and depth of penetration of radiation protection actions is lacking. The session will identify actions that can be taken to improve the situation to cover the breadth and depth in LMI countries.

***Session Objectives:***
1. To learn about the situation of medical imaging and radiation protection in low- and middle-income countries.
2. To understand the past challenges and possibly future ones in the area of radiation protection in medical imaging in low- and middle-income countries.
3. To learn about the activities of worldwide organisations to support low- and middle-income countries.

**A-0659** 16:05
**The International Society of Radiology's (ISR) vision**
G. Frija; *Paris/FR (guy.frija@aphp.fr)*

Universal health coverage includes health promotion, preventive services, diagnostics, and medicines for communicable and non-communicable diseases. Regarding diagnostics, a global plan for imaging is needed, covering equipment, manpower, and regional governance. Quality and safety need to be addressed in each aspect. The Quality and Safety Alliance of the International Society of Radiology (ISRQSA) is working closely with the WHO and IAEA to improve radiation protection in low-, and middle-income countries (LMICs). It acts as a convener of radiation safety campaigns across the globe and encourages campaign creation in regions where none currently exist. Following the need for a multi-stakeholder approach to radiation protection, the ISRQSA seeks collaboration with related international organisations, including IOMP and ISRRT. The ISR recently defined its forthcoming work plan with the WHO, which will focus, among others, on the following areas: advocating the development of a global plan for equipment and manpower, also in LMICs; awareness raising and advocacy activities towards the implementation of the International Radiation Basic Safety Standards (BSS) and Bonn Call for Action; facilitating access to imaging referral guidelines and providing expertise to support implementation particularly in LMICs; fostering teamwork approaches and risk communication; and safety and quality in the medical use of non-ionizing radiation. In addition, the ISR is involved in the Lancet Oncology Commission on medical imaging and nuclear medicine with the aim of increasing global awareness of the importance of cancer imaging and improving access to imaging.
***Learning Objectives:***
1. To learn about the ISR's radiation protection policy.
2. To appreciate actions which are currently developed.
3. To understand the challenges of low- and middle-income countries to improve radiation protection.

**A-0660** 16:20
**Patient doses in large part of the LMI countries and way forward: The International Organization for Medical Physics' (IOMP) vision**
M.M. Rehani; *Boston, MA/US (madan.rehani@gmail.com)*

The radiation doses to patients in diagnostic imaging examinations and optimisation of dose with image quality is something akin to medical physicists. Medical physicists have published hundreds of papers covering data from more than 80 LMI countries. IOMP collated the data and information on patient doses pertains to computed tomography (CT) dominantly, but also for interventional procedures, mammography and other radiographic imaging. The patient dose information from Eastern European countries is more than other regions like Asia, Africa and Latin America, primarily because of Euratom. The past challenges pertained to crossing the threshold of dose assessment, shift from machine focus to patient focus, whereas future challenges pertain to creating focus on the protection of the individual patient. A large part of the data from LMI countries comes through the work of the IAEA. IOMP plays a role in creating agenda; organising training events; motivating professionals to work, produce results and publish them; disseminating the work; act as an expert for international organisations and provide leadership in creating and propagating outreach programs. Further, IOMP identifies the changing scenario and create a vision for professional colleagues with an emphasis on LMI countries. IOMP works with international organisations like IAEA and WHO and professional bodies.
***Learning Objectives:***
1. To learn about the current situation of patient doses in a large part of the world.
2. To appreciate the challenges and needs based on experience gained.
3. To understand the needs that will impact future actions.

**A-0661** 16:35
**The International Atomic Energy Agency's (IAEA) approach**
D. Gilley; *Vienna/AT (D.Gilley@IAEA.org)*

The International Atomic Energy Agency's mission is to assist its Member States, in the context of social and economic goals, in planning for and using nuclear science and technology for various peaceful purposes and facilitates the transfer of such technology and knowledge in a sustainable manner to developing the Member States. Challenges in low and middle income have been identified, and the IAEA is achieving positive results in radiation protection with the support of local radiology professionals. The IAEA, through the radiation protection of the patient's unit and technical cooperation, have seen improvements in patient safety activities at the local, national and regional levels. The success of these efforts would not be possible without the strong commitment of local radiology professions who support the IAEA goal of bringing the beneficial and safe uses of nuclear and radiology technology to all of society.
***Learning Objectives:***
1. To learn about regional challenges in radiation protection in healthcare.
2. To appreciate the efforts professionals are demonstrating in achieving progress in radiation protection in a resource-limited region with the help of the IAEA.
3. To understand the effects this has on the overall improvement of radiation protection of patients and workers.

**A-0662** 16:50
**The World Health Organization's (WHO) approach**
M.d.R. Perez; *Geneva/CH (perezm@who.int)*

The UN Member States made a commitment towards 17 Sustainable Development Goals (SDGs) by 2030: the SDG #3 aspires to "ensure healthy lives and promote well-being for all at all ages". Half the world lacks access to essential health services. Many countries have adopted national commitments to achieve universal health coverage (UHC), which means ensuring that all people have access to quality essential health services they need for their health and well-being without incurring financial hardship. This presents a unique opportunity for joining global efforts towards achieving UHC and, in this context, ensuring safety and quality in medical imaging services will be central to the success of these efforts. Indeed, both diagnostic radiology and image-guided interventions are linked to health promotion, preventive services, diagnosis, treatment, follow-up, rehabilitation and palliative care. A culture of radiation safety and quality can be embedded into policies, processes and institutions as the health care systems grow and develop as it is the case in low and middle-income countries. This represents at the same time a challenge and an opportunity and requires strong leadership, robust planning and strategic investment. This presentation will summarise WHO's views on the importance of safety and quality in medical imaging for globally advancing patient care, describe the roles and responsibilities of different stakeholders in strengthening radiation protection in health care and identify challenges and opportunities for enhancing radiation protection in medical imaging in low- and middle-income countries.
***Learning Objectives:***
1. To learn about WHO's views on the importance of safety and quality in medical imaging for globally advancing patient care.
2. To identify challenges and opportunities for enhancing radiation protection in medical imaging in low- and middle-income countries.
3. To understand the role and responsibilities of different stakeholders in strengthening radiation protection in health care.

**A-0663** 17:05
**Africa's vision to improve radiation protection**
B. Mansouri; *Algiers/DZ (boudjema.mansouri@gmail.com)*

Radiation protection: Needs and challenges in Africa The burden from communicable and non-communicable diseases, including the socio-economic impact of these, has adversely affected development in Africa due to a poor or absent legislative and regulatory framework for radiation protection in many countries, an inadequate awareness of the radiology safety policies an insufficient awareness about radiation doses and the associated risks in the other health professions , an Inequitable distribution of radiation facilities, equipment and skilled personnel , and the financial and political constraints. The continent's health care system faces big challenges to match health workforce supply and demand. Contemporary discussions in radiation protection entail a systematic articulation of the African health system as well as an explanation of how the professionals apprehend reality and interpret their experiences. To avoid to be left behind the progress and the international actions for safety is recommended, to provide an efficient response for a good medical practice, ensuring that the benefits outweigh risks in all radiological medical procedures using customized systems adapted to the heterogeneous African context The establishment and implementation of regulations to standardize with the development of policies, guidelines the practice of radiation is required and need to be adapted to the specific status of the health system in Africa. It is requested to all the stakeholders and the international organisations to develop national and regional action plan constructed from current discussions about conceptions in African thought and realities ensuring Africa's patients the same quality and safety.
***Learning Objectives:***
1. To understand the African gaps and to identify the challenges with the existing opportunities.
2. To learn about the implementation of a safety culture in medical radiation protection in Africa and the support provided by international organisations, like IAEA and WHO, and professional societies, like ISR and ESR.

3. To learn about the Afrosafe campaign, in particular about the lessons from the past and the envisaged programme for the next three years 2019-2021.

17:20
**Panel discussion: What are the main obstacles for the safe use of imaging in low- and middle-income countries?**

**16:00 - 17:30** **Room O**

### E³ - ECR Master Class (Vascular)

### E³ 1226

### Cone-beam, 4D and more: new diagnostic tools for vascular diseases

*Moderator:*
F. Fanelli; Florence/IT

**A-0664** 16:00
**A. The role of intra-procedural perfusion assessment in peripheral arterial disease**
J.A. Reekers; *Amsterdam/NL (j.a.reekers@amc.uva.nl)*

Non-invasive measurement of blood vessel flow can be performed in many ways. The most used modalities to measure flow are Doppler flow imaging and MRA. The restriction of these flow measurements is that only intravascular flow can be measured while more than 80% of the organ perfusion flows through the microcirculation. To investigate the perfusion of an organ we need to know the total blood flow through that organ and the tissue volume because perfusion is the volume of blood flowing through certain mass (or volume) of tissue per unit time. If we measure true perfusion in a volume of tissue, we measure foremost the flow through the microcirculation of the tissue. With specially designed DSA 2D perfusion acquisition software on the X-ray system, the DSA data are processed instantaneously, allowing for immediate evaluation and comparison. The time taken for contrast to pass through the arterial vasculature and to enter into the venous system is the duration of the maximal analysis possible on the tool. Improvement of tissue perfusion after revascularisation could be a parameter for success and might be a predictor for outcome. Several competing mechanisms play a role in the final interpretation of the perfusion data. The value of each mechanism depends on the clinical situation and the intervention performed. The value of intra-procedural perfusion assessment will be discussed.
***Learning Objectives:***
1. To understand the technique of intra-procedural perfusion assessment.
2. To learn how to target peripheral revascularization therapy based on perfusion assessment.
3. To discuss the value of using intra-procedural perfusion assessment on the outcome of endovascular therapy for peripheral artery disease.

**A-0665** 16:30
**B. CT 4D imaging after (T)EVAR**
R. Schernthaner; *Vienna/AT (Ruediger.schernthaner@meduniwien.ac.at)*

Endovascular Aortic Repair (EVAR) has evolved into a successful treatment option for aortic aneurysms and dissections; however, the possibility of remaining endoleaks after the procedure requires continuous imaging follow-up of these patients. However, the reliable classification of different endoleak types is sometimes challenging, especially in patients with complex, customised stent-graft designs. By adding time as the 4th dimension, 4D CT angiography allows the visualisation of blood flow within the aorta and its branches. Thus, 4D CT angiography facilitates a more detailed analysis of endoleaks and their underlying cause. However, 4D CT angiography requires thorough planning by a vascular radiologist before and during the examination to achieve optimal visualisation at the lowest possible exposure to ionising radiation and contrast material following the ALARA principle. In addition, it is important to understand that 4D CT angiography cannot replace regular imaging follow-up, but should only be used as a supplemental technique in uncertain cases.
***Learning Objectives:***
1. To understand the challenges of imaging follow up after (T)EVAR.
2. To become familiar with the technique of 4D CT.
3. To learn how to establish treatment recommendations based on 4D CT results after (T)EVAR.
*Author Disclosure:*
**R. Schernthaner:** Advisory Board; Siemens Healthineers. Research/Grant Support; Siemens Healthineers.

**A-0666** 17:00
**C. How cone-beam CT can change your practice in interventional radiology**
R. Uberoi; *Oxford (Raman.Uberoi@ouh.nhs.uk)*

C-arm cone-beam computed tomography (CBCT) is a new imaging technology that enables acquisition of cross-sectional imaging with modern angiographic systems equipped with a flat panel detector. Volumetric tomographic images can be combined and co-displayed with conventional 2D angiographic imaging and dedicated software used to plan treatment, navigate/position the catheter or device, monitor the treatment, and assess the final result or verify margins. This technique has the potential to improve outcomes in a wide range of IR treatments. An excellent example of the huge potential benefits of CBCT is in the field on interventional oncology. CBCT with its 3D nature, soft tissue contrast, and with the post-processing software is superior to DSA in lesion detection and tumour-feeding vessel identification. In addition Dual Phase CBCT, where a bi-phasic CBCT is acquired using a single contrast injection allows increased tumour detection versus single phase CBCT alone comparable to gold standard contrast-enhanced MDCT and MRI in lesion detection and in predicting therapy response. Similarly, for Selective internal radiation therapy (SIRT) CBCT also has several potential advantages. Angiographic work-up is required to identify hepatoenteric arteries, originating from the hepatic arteries, defining the vascular territory of all targeted hepatic arteries; and identifying the tumoral lesions within these vascular territories. CBCT can demonstrate extrahepatic contrast enhancement in 52 % of cases, in 33 % of cases these additional CBCT observation scan lead to additional coil embolisation and/or change in catheter position.
***Learning Objectives:***
1. To understand the technique of cone-beam CT.
2. To learn about the role and applications of the cone-beam in the angio suite.
3. To discuss the influence on daily clinical practice in interventional radiology.
*Author Disclosure:*
**R. Uberoi:** Advisory Board; Vascutek, Merit. Investigator; Vascutek, EKOS. Research/Grant Support; Gore, Vascutek, Cook. Speaker; Vascutek.

**16:00 - 17:30** **Studio 2019**

### ESR Ultrasound Subcommittee Session

### US 2

### Tips and tricks for abdominal ultrasound

*Moderator:*
S. Delorme; Heidelberg/DE

**A-0667** 16:00
**Doppler imaging**
F. Calliada; *Pavia/IT (fabrizio.calliada@gmail.com)*

Doppler, it's undoubtedly a not new imaging technique, but an adequate knowledge of the basic physics and the scanner tuning's it's essential to avoid errors and misunderstanding. In the first part, the presentation will cover the principal sources of error that should be avoided during a Doppler examination and the major tuning tricks needed for an optimal and successful examination. In the second part, we will try to become familiar with new Doppler imaging techniques dedicated both to very low-velocity microvascular flow and to high-velocity complex flow representation.
***Learning Objectives:***
1. To learn about actual indications and applications of Doppler imaging.
2. To show better parameter settings for optimal technical results.
3. To illustrate tips and tricks for technical and clinical successful examinations.
4. To become familiar with the new Doppler imaging techniques and applications.
*Author Disclosure:*
**F. Calliada:** Equipment Support Recipient; Shenzhen Mindray Bio-Medical, Hitachi Medical System Europe, Toshiba Medical System Europe. Research/Grant Support; Bracco Imaging Milan. Speaker; Hitachi Medical System Europe, Shenzhen Mindray Bio-Medical.

**A-0668** 16:20
**CEUS**
M. D'Onofrio; *Verona/IT (mirko.donofrio@univr.it)*

Contrast-enhanced ultrasonography (CEUS) is a safe and accurate imaging method to evaluate the vascularity of abdominal organs. CEUS improve the ultrasound characterisation of tumoral masses. CEUS should be performed when possible immediately after the US detection of indeterminate mass in abdominal organs. CEUS is accurate in the characterisation of neoplastic lesions such as liver metastases and pancreatic ductal adenocarcinoma. The use of CEUS in studying focal liver and pancreatic lesions found at the US, especially in the same session of ultrasound examination is, therefore, recommendable to promote faster diagnosis. In particular, liver metastases detection could be improved by the use of contrast-enhanced ultrasound in respect to basal examination. Actual indications and applications of CEUS are presented in official published guidelines. Every CEUS examinations should be performed with better parameter settings for optimal technical results.

***Learning Objectives:***
1. To learn about actual indications and applications of contrast-enhanced US (CEUS).
2. To show better parameter settings for optimal technical results.
3. To illustrate tips and tricks for technical and clinical successful examinations.
4. To become familiar with the new CEUS techniques and applications.

*Author Disclosure:*
**M. D'Onofrio:** Advisory Board; SIEMENS. Consultant; SIEMENS, BRACCO, HITACHI. Speaker; SIEMENS, HITACHI, BRACCO.

**A-0669** 16:40
**Elastography**
D.A. Clevert; *Munich/DE (Dirk.Clevert@med.uni-muenchen.de)*

In the daily clinical routine characterisation of focal lesions using native B-mode ultrasound and colour-Doppler can be difficult or insufficient. Therefore, additional diagnostic information must be taken into consideration, using ultrasound-elastography it is possible to evaluate and characterise tissue properties and focal lesions regarding their stiffness to gather additional information on a non-invasive basis. Regarding the liver, elastography techniques can also be used for the evaluation of fibrosis/cirrhosis. Nowadays, elastography has been implemented into every modern ultrasound system and has been established as a supplementary examination technique to the conventional ultrasound techniques.

***Learning Objectives:***
1. To learn about actual indications and applications of elastography.
2. To show better parameter settings for optimal technical results.
3. To illustrate tips and tricks for technical and clinical successful examinations.
4. To become familiar with the new elastography techniques and applications.

*Author Disclosure:*
**D.A. Clevert:** Speaker; Bracco, Siemens, Philips, Samsung.

**A-0670** 17:00
**Fusion imaging**
C. Ewertsen; *Copenhagen/DK (caroline.ewertsen@dadlnet.dk)*

Image fusion software is available on most high-end ultrasound systems, and several publications for different clinical applications are available. Ultrasound images can be fused with images from CT, MRI or PET/CT, which enables the user to target biopsies or verify the nature of inconspicuous lesions. In order to fuse the images, a co-registration or alignment must be made. This can be done manually, by choosing common points or planes in the different datasets, or automatically by the system by recognition of pixel intensities in the images. This applies for some systems, but not for all modalities. The theory behind these different co-registration methods will be covered as well as their accuracy. Furthermore, examples of clinical applications will be demonstrated. The technique is more time consuming than conventional B-mode ultrasound, but time spent decreases with increasing user experience. Depending on the body size of the patient and the organ of interest patient positioning should be identical to when the previously recorded data set was recorded, but this is not mandatory. However, for liver applications, accuracy of the co-registration may improve if this is taken into account. Also identical in- or expiration may improve accuracy. Real-time image fusion enables real-time assessment of lesions in several anatomical regions.

***Learning Objectives:***
1. To learn about actual indications and applications of fusion imaging.
2. To show better parameter settings for optimal technical results.
3. To illustrate tips and tricks for technical and clinical successful examinations.
4. To become familiar with the new fusion imaging techniques and applications.

17:20
**Panel discussion: When do we need additional imaging techniques and how could we include them in our workflow?**

**16:00 - 17:30 Room E1**

### Breast

### RC 1202

### Minimally-invasive local treatment of breast cancer: the time is now

**A-0671** 16:00
**Chairperson's introduction**
A. Athanasiou; *Athens/GR (aathanasiou@mitera.gr)*

Breast cancer management is evolving towards "less-is-more". Image-guided percutaneous biopsies provide accurate histologic diagnosis thus replacing open surgical biopsies in more than 90%of cases. Breast conservation therapy is the standard treatment for early-stage breast cancer. Sentinel lymph node biopsy has replaced axillary lymph node dissection in many cases. Is treating early breast cancer without surgery the next challenge? For liver metastases, treatment by means of ablative techniques has widely replaced surgery. Is there any place for minimally invasive treatment of early breast cancer? During this course, three main procedures will be presented, and current indications, advantages and disadvantages will be discussed. They include radiofrequency ablation, high intensity focused ultrasound ablation, and cryotherapy ablation. They may offer effective tumour management in selected cases (small<2cm tumours, elderly patients) and provide treatment options that are oncologically safe and cosmetically acceptable. By means of excessive local heating or freezing under imaging guidance, these procedures can cause cell death and tumour destruction. Surgical excision remains the standard local treatment of breast cancer. However, these procedures may represent an interesting alternative of successful treatment in selected cases. Large prospective trials should evaluate the efficacy, cost-effectiveness, cosmetic results and long-term outcome of minimally, imaging-guided local treatment compared with the traditional surgical approach.

***Session Objectives:***
1. To learn about HIFU, radiofrequency ablation and cryotherapy that challenge traditional surgical excision in the management of breast cancer.
2. To become familiar with the role of imaging in using these new technologies.
3. To understand the potential advantages and disadvantages for each of these techniques.

**A-0672** 16:05
**A. High-intensity focused ultrasound (HIFU) therapy**
F. Pediconi; *Rome/IT (federica.pediconi@uniroma1.it)*

Surgical treatment of breast cancer has changed over time, evolving from radical mastectomy to more conservative approaches. This has been possible thanks to technical advantages in the field of diagnostic imaging that allowed the early diagnosis of breast cancers with very small dimensions. Mini-invasive technologies (radiofrequency ablation, cryoablation, etc.) can preserve the original breast volume avoiding glandular resections and surgical scars and ensuring at the same time complete tumour ablation. Ablation with high-intensity focused ultrasound (HIFU) is based on the use of an extra-corporeal ultrasound transducer that selectively destroys target tissue avoiding thermal damages to surrounding structures. The technique can be performed under ultrasound or magnetic resonance (MR) guidance.MR guidance offers several advantages that improve the safety and efficacy of the procedure: a visualization of the planned US beam during each phase of the procedure, a real-time monitoring of the progressive temperature increase within the target tissue and surrounding tissues, an accurate treatment planning, an evaluation of the treatment efficacy thanks to the use of intravenous gadolinium-based contrast agent. HIFU ablation of breast cancer is a new and promising technique that deserves large interest in the field of clinical research in order of its potential application in the clinical practice.

***Learning Objectives:***
1. To learn about the basics of HIFU therapy.
2. To become familiar with the different types of imaging guidance.
3. To appreciate its role in treating benign and malignant lesions.

**A-0673** 16:30
**B. Radiofrequency ablation therapy**
B. Brkljačić; *Zagreb/HR (boris@brkljacic.com)*

Radiofrequency ablation (RFA) is promising, but quite rarely used minimally invasive modality to treat small breast cancer in patients in whom general anaesthesia is contraindicated or who refuse surgery. In most studies, cancers were surgically excised shortly after RFA. In very few studies, RFA was used as the only treatment modality, instead of surgery. The procedure is most conveniently performed under ultrasound guidance, in local analgesia, which allows constant contact with the patient during the procedure. Precise preprocedural imaging is crucial and should include contrast-enhanced MRI in addition to mammography and ultrasound. Preprocedural core biopsy is mandatory, with precise assessment of the tumour type, grade, and immunohistochemical features. Typical US findings of hyperechogenicity of ablated mass are noted during the procedure. Postprocedural mammographic and MRI findings are characteristic and will be presented. The complete ablation can be achieved in small T1-2 N0 M0 breast cancers that present as masses of maximum 2-3 cm in diameter, with the sufficient distance from the skin and pectoral muscle to avoid the thermal lesion of these tissues. In larger lesions, only partial ablation may be achieved. Only solitary, unicentric, invasive ductal cancers, preferably ER/PR positive should be treated. Invasive lobular cancers should not be treated with RFA. Our results will be presented in a small group of patients who were treated with RFA, and who refused surgery, had a contraindication to general anaesthesia and opted for RFA. The technique, preprocedural, intraprocedural and postprocedural imaging findings, as well as the long-term result will be presented.

***Learning Objectives:***
1. To learn about how radiofrequency works.
2. To become familiar with its use in clinical practice.
3. To appreciate the advantages and disadvantages.

**A-0674** 16:55
**C. Cryotherapy**
M.H. Fuchsjäger; *Graz/AT (michael.fuchsjaeger@medunigraz.at)*

Cryotherapy is a new, minimal-invasive image-guided treatment option for breast tumours. A specific 12-17G probe is applied into a tumour. Argon gas making use of the Joule-Thomson effect cools down the needle tip to minus 187 degrees centigrade. An ice ball covering lesion and an adequate safety margin is formed. Coagulative necrosis of the tumour cells after two freezing cycles is the result. Cryoablation for breast tumours can be performed under US, CT or MRI guidance. Several studies showed an overall success rate of more than 90%. Indications for cryotherapy are small tumours, contraindications to general anaesthesia or increased risk of complications, support of standard therapies and palliative approach. Cryotherapy can be performed under local anaesthesia on an out-patient basis with a potential better cosmetic outcome than standard surgical therapies. Minimal-invasive therapies ask for a paradigm-shift as the eradicated tumour is left in situ and resection with clear margins will not be proven histopathologically, but functionally by MR imaging. It is important to emphasise that radiology is not aiming to take over therapy of breast cancer patients but to help with innovative, less invasive treatment options as a member of a multidisciplinary team. Close cooperation with our clinical partners (surgery, gynaecology, oncology, radiation therapy, etc.) is the key to success and avoidance of turf battles. The goal for the future should be minimal-invasive ablation therapy for breast cancer as a valid therapeutic option.

***Learning Objectives:***
1. To learn about the cryotherapy technique.
2. To become familiar with its use in clinical practice.
3. To appreciate its role in treating benign and malignant lesions.

17:20
**Panel discussion: Will we still need surgery for local therapy of breast cancer in the future?**

**16:00 - 17:30 Room E2**

### Neuro

### RC 1211

### Inflammatory and infectious CNS pathology

**A-0675** 16:00
**Chairperson's introduction**
E.T. Tali; *Ankara/TR (turgut.tali@gmail.com)*

Modern life, particularly the influence of globalisation, immigration and expanding tourism, facilitating transportation between the continents, thereby increasing the social-personal relationship that may be the cause of increased infections. The brain has some unique peculiarities like the absence of lymphatics, lack of capillaries in the subarachnoid space, and presence of cerebral spinal fluid (CSF), which is an excellent culture medium for dissemination of infectious processes, in the subarachnoid space and into the ventricular system. Infections of the central nervous system(CNS) frequently present diagnostic and therapeutic challenges.CNS infections are not frequent when encountered, require prompt diagnosis and initiation of specific treatment often are necessary to allow the best chance of recovery without sequelae. The prognosis depends on rapid identification of the site of the inflammation and pathogen. Most CNS infections can be treated successfully as long as they are detected early. Delayed diagnosis remains a major cause of disability. It is particularly unfortunate if a rapidly progressive but treatable disease remains undetected until irreversible damage is caused. Improvements in diagnostic imaging, in particular, computed tomography (CT) and magnetic resonance imaging (MRI) have greatly facilitated the diagnosis and treatment of intracranial and spinal infections.

***Session Objectives:***
1. To understand the role of imaging in the diagnosis and monitoring of inflammatory and infectious diseases of the central nervous system.
2. To learn basic principles of the use of imaging in neuroinfection and neuroinflammation.
3. To appreciate the added value of imaging in addition to the clinical findings and laboratory tests including CSF analysis.

**A-0676** 16:05
**A. Autoimmune encephalitis**
P. Demaerel; *Leuven/BE (philippe.demaerel@uzleuven.be)*

Autoimmune Encephalitis (AE) is emerging as a more common cause of encephalopathy than previously thought. AE remains a difficult diagnosis. The presence of antibodies (Ab) in the CSF confirms the diagnosis, but it can take several weeks before the results become available and a negative result does not exclude AE. Therefore, MR plays an important role in these patients, who often present with nonspecific neurological symptoms. Subacute encephalopathy, new onset seizures or psychiatric symptoms should raise the suspicion of a possible AE. AE consists of a large number of Ab-related diseases. Neuromyelitis Optica and ADEM are typical examples of AE with anti-glial Ab. The diseases with anti-neuronal Ab are usually divided into three groups: (1) Ab to cell-surface antigens, (2) Ab to intracellular synaptic proteins and (3) Ab to intracellular antigens. The imaging-related findings will be reviewed in this course. The most common imaging finding consists of uni- or bilateral FLAIR hypersignal in the hippocampus and/or amygdala. It is important to emphasise that these signal changes can be very subtle. Extratemporal lesions in the cerebral cortex, the grey nuclei, the cerebellum and the brainstem have been reported too and can support the clinical suspicion of AE. Brain MR can also remain normal, and FDG-PET is certainly recommended in these patients. Finally, central nervous system involvement can be observed in several autoimmune diseases. The MR findings can sometimes be very specific. This will be illustrated in, e.g. Susac syndrome, Rasmussen encephalitis, granulomatous angiitis and neurosarcoidosis.

***Learning Objectives:***
1. To learn about the imaging pattern of autoimmune encephalitis.
2. To understand the limited role of conventional MRI and the need for advanced imaging techniques in the diagnostic process and for follow-up purposes.
3. To appreciate the role of imaging in a multidisciplinary and multimodal approach.

**A-0677** 16:28
**B. Infectious encephalitis**
A. Zimny; *Wroclaw/PL (abernac@wp.pl)*

Infectious encephalitis is an inflammation of the brain which can be caused by different pathogens. Majority of them are viruses, less frequently bacteria, fungi and parasites. Immunocompromised patients or people travelling to endemic regions may develop encephalitis due to less frequent pathogens. Adult patients with encephalitis present with acute onset of fever, headache, confusion, and sometimes seizures. Encephalitis may be accompanied with the involvement of meninges (meningoencephalitis) or spinal cord (encephalomyelitis). It is a life-threatening condition, and prompt diagnosis is crucial. Diagnosis may be very challenging and is typically based on symptoms and supported by blood tests, analysis of cerebrospinal fluid and imaging. Brain CT is often a first line imaging method in acute settings, but MRI is a method of choice in the initial diagnosis and follow-up of patients with encephalitis. MR imaging is also used to rule out other important differential diagnoses (vascular, tumoral, inflammatory). The role of different MR sequences including DWI, SWI, perfusion, spectroscopy and post-contrast images will be discussed as well as a significance of a good knowledge of the MRI patterns of brain involvement which can be helpful in suspecting a particular pathogen. Infectious encephalitis is a heterogeneous entity which requires a comprehensive and multidisciplinary approach that takes into account individual clinical, microbiological, and radiological features of each patient.

***Learning Objectives:***
1. To learn about the correct choice of imaging modalities and image acquisition parameters for the detection and monitoring of infectious diseases of the central nervous system.
2. To understand the benefits and challenges of image pattern recognition for diagnostic purposes.

3. To appreciate the heterogeneity of the disease spectrum and challenges to interpret imaging findings in the context of the clinical presentation and possible comorbidities.

**A-0678** 16:51
**C. Inflammatory and infectious myelitis**
M.M. Thurnher; *Vienna/AT (majda.thurnher@meduniwien.ac.at)*

Transverse myelitis (TM) is an “umbrella term” used to describe an inflammatory disorder of the spinal cord that can be idiopathic or associated with the central nervous system (CNS) autoimmune inflammatory diseases, connective tissue autoimmune diseases, or post-infectious neurological syndromes. Myelitis can present either as monophasic or recurrent disease. The most common monophasic diseases causing TM are idiopathic transverse myelitis and acute disseminated encephalomyelitis (ADEM), whereas recurrent disorders include multiple sclerosis (MS), neuromyelitis optica spectrum disorder (NMOSD), vasculitis (Behcet disease, SLE, Sjögren syndrome) and neurosarcoidosis. The recent discovery of multiple novel neural-specific autoantibodies accompanying autoimmune and demyelinating disease has improved the current understanding and classifications. This lecture will provide an overview of radiological, clinical, serological, and prognostic differences of inflammatory and infectious spinal cord diseases.
***Learning Objectives:***
1. To learn about the spectrum of infectious diseases of the spinal cord level and their most characteristic imaging features.
2. To understand the difficulties in image acquisition and image interpretation.
3. To appreciate the clinical relevance of early diagnosis and therapeutic intervention.

17:14
**Panel discussion: Ask the expert: is imaging the key diagnostic modality for an early and specific diagnosis of infectious diseases, leading to a better functional outcome?**

**16:00 - 17:30** **Room F1**

### E³ - Rising Stars Programme: Basic Session

### BS 12

### Paediatric oncology

*Moderator:*
L.-S. Ording Müller; Oslo/NO

**A-0679** 16:00
**The most common brain tumours**
E. Vázquez; *Barcelona/ES (evazquez@vhebron.net)*

Brain tumours are the most common group of solid tumours in the paediatric age. Clinical onset commonly includes signs and symptoms related to increased intracranial pressure, gait disorders, or cranial nerve deficits. Although usually insidious, acute presentations due to stroke or obstruction of cerebrospinal fluid flow can occur. Certain previous histological classifications, such as that of medulloblastoma, are being replaced by new classifications based on genetic characteristics, which enable better prediction of tumour aggressiveness and help to better guide therapy according to the specific tumour type. Magnetic resonance (MR) imaging has an important role in tumour characterisation, surgical planning and follow-up, but structural or anatomic brain MRI is often limited. Advanced MR techniques, including diffusion-weighted imaging, diffusion tensor imaging, functional MRI, perfusion imaging, and spectroscopy, help to improve the accuracy on tumour malignancy and risk in initial diagnosis and to facilitate therapy effectiveness monitoring and diagnosis of possible recurrence during tumour follow-up. The main imaging clues of the most common brain tumours (astrocytoma, ependymoma, and medulloblastoma) in children will be presented in a didactic manner in this talk, together with the updated MR imaging protocols for diagnostic approach and therapeutic monitoring.
***Learning Objectives:***
1. To present current imaging techniques and describe the typical features of astrocytomas.
2. To present current imaging techniques and describe the typical features of medulloblastomas.
3. To present current imaging techniques and describe the typical features of ependymomas.

**A-0680** 16:30
**The most common chest tumours**
A.S. Littooij; *Utrecht/NL (alittooij@hotmail.com)*

Malignant thoracic tumours may arise from the chest wall, pleura, lung parenchyma or mediastinum. When a chest mass is discovered on a conventional chest X-ray, cross-sectional imaging is often required to accurately localise and characterise the mass in order to narrow the differential diagnosis. Magnetic resonance imaging is preferred for mediastinal and chest wall lesions, whereas CT is commonly used for imaging of pulmonary lesions. The location of the lesion is important in determining the differential diagnosis: Mediastinum: tumours in the mediastinum are best characterised by the compartment in which they arise. Tumours arising from the anterior mediastinum are most commonly due to lymphoma or leukaemia followed by germ cell tumours. Lesions in the middle mediastinum are usually seen in association with anterior mediastinal lesions. The tumours of the posterior mediastinum are usually of neurogenic origin with neuroblastoma being the most common. Lung: in children, most pulmonary malignancies are metastatic in nature. Primary pulmonary malignant neoplasms are rare in children. The majority of pulmonary lesions are congenital or inflammatory lesions. Chest wall: the most common malignant chest wall tumours include Ewing’s sarcoma and rhabdomyosarcoma and occur in children older than seven years. Ewing’s sarcoma classically causes a permeative lytic expansive lesion of the rib with bone destruction, periosteal reaction and soft tissue mass. When the soft tissue component is massive, bone involvement may be very subtle. This lecture will highlight the most common malignant thoracic tumours with emphasis on those with characteristic imaging features.
***Learning Objectives:***
1. To present current imaging techniques and describe the typical features of malignant mediastinal tumours.
2. To present current imaging techniques and describe the typical features of pulmonary tumours.
3. To present current imaging techniques and describe the typical features of chest wall malignancies.

**A-0681** 17:00
**The most common abdominal tumours**
S. Franchi-Abella; *Le Kremlin-Bicêtre/FR (stephanie.franchi@bct.aphp.fr)*

Wilms Tumor or Nephroblastoma is the second most common abdominal and renal paediatric tumour. The aims of imaging are to establish the local tumour extension, the lymph nodes involvement, the presence of bilateral disease, the vascular invasion and the presence of metastases (mostly in the lungs). Both MR and CT can be performed for the abdominal extension. CT is standard for pulmonary metastases. US-Doppler can be helpful for the assessment of vascular extension in addition to CT or MR. Lymphoma is the third most common paediatric neoplasm. Non-Hodgkin lymphoma (NHL) commonly involves extra-nodal sites. Patients typically present with widespread disease. CT and MRI are used for staging. FDG-PET/CT plays a role to evaluate the extension of the disease and the response to the treatment. Neuroblastoma is the most common abdominal paediatric solid tumour accounting. The adrenal gland is the most common site of origin (50 %). The aim of imaging is the tumour staging prior to surgery and chemotherapy. The International Neuroblastoma Risk Group staging system is now based on the presence of “image-defined risk factors” (IDRFs) which allow children to be assigned to specific risk groups at the time of diagnosis. IDRFs include vascular invasion, invasion of adjacent solid organs, soft-tissue structures and extension into the spinal canal. CT and MRI are used. MRI is more sensitive for characterising bone marrow involvement, extension to the central nervous system. MIBG scintigraphy or SPECT/CT allows identification of both primary tumours and metastatic disease including bone marrow.
***Learning Objectives:***
1. To present current imaging techniques and describe the typical features of Wilms' tumour.
2. To present current imaging techniques and describe the typical features of non-Hodgkin's lymphomas.
3. To present current imaging techniques and describe the typical features of neuroblastomas.

**16:00 - 17:30** **Room F2**

### Emergency Imaging

### RC 1217

### Why do I miss fractures in emergency?

**A-0682** 16:00
**Chairperson's introduction**
S. Wirth; *Munich/DE (stefan.wirth@med.uni-muenchen.de)*

Fractures are the most common type of missed injuries, especially at the lower extremity and in particular, the foot. Although there are limitations, Radiography is the imaging standard for fracture detection. Typical reasons for overlooking fractures are missed soft tissue signs, insufficient imaging parameters or views, satisfaction of search, small fracture extent or missing displacement of fragments. Appropriate strategies to reduce the number of missed fractures are the correlation of findings with clinical features, knowledge of difficult regions and technical limitations, re-assessment in cases of ongoing pain or disability- if necessary by using cross-sectional imaging, checklists, appropriate communication/ information flow.
***Session Objectives:***
1. To learn the typical constellations and findings of missed fractures.
2. To understand the potential complications resulting from missing fractures.
3. To appreciate direct and indirect fracture signs with different imaging modalities.

**A-0683** 16:05
**A. Missed fractures in children**
K.J. Johnson; *Birmingham/UK (karl.johnson@bch.nhs.uk)*

Traumatic injury and subsequent fracturing are one of the commonest reasons that children attend hospital and undergo some form of imaging. While the majority of injuries are easily detected, in some instances, a fracture may be missed. Broadly speaking, missed fractures occur in children for three reasons, namely; Perception - the fracture was not seen Interpretation - an abnormality was seen but reported as something else Occult injuries - the fracture is present but not visible on the imaging. The fact that the paediatric skeleton is developing and not fully ossified can lead to confusion between normal development and pathological changes. In addition, the plastic nature of a child’s bones and the presence of growth plates means that the fracture patterns are seen different from those in adults. This presentation will discuss the commonly missed fractures that occur in childhood. There will be a discussion on how to avoid perception errors. There will be a review of those anatomical areas in which interpretation errors commonly occur, such as the elbow and wrist, typically due to confusion with normal variations in the growing skeleton. It will highlight those occult injuries in which additional or follow up imaging is useful.
***Learning Objectives:***
1. To become familiar with the commonly missed fractures in childhood.
2. To understand the choice of the best-suited imaging modality.
3. To learn about atypical imaging findings in different clinical scenarios.

**A-0684** 16:30
**B. Missed fractures in adults**
S. Döring; *Brussels/BE (seema.doring@uzbrussel.be)*

Missed fractures are a common problem in the emergency department. Poor X-ray exposure factors, improper positioning, an inadequate number of views are common technical reasons for missed fractures. Subtle non-displaced fractures, inadequate experience, misinterpretation as normal variants, difficulty in the visualisation of fractures due to severe arthrosis or osteopenia, satisfaction of search (SOS) and working under duress in the emergency are important contributory factors. Commonly missed fractures in upper extremity include posterior shoulder dislocation,non-displaced greater humeral tuberosity, radial head, non-displaced radial styloid, scaphoid and hook of hamate fractures. In the lower extremity, femoral neck, Segond, osteochondral fracture of talar dome, lateral and posterior talar process, anterior calcaneal process, Lisfranc dislocation, os peroneus fractures can be easily missed. Apart from long-term pain and morbidity, missed fractures can lead to serious consequences such as osteoarthritis, non-union, delayed union, malunion, avascular necrosis and heterotopic ossifications. Reduction in misdiagnosis of fractures can be achieved by continued education of technical staff to improve X-ray quality. Similarly, continued education and supervision of younger less experienced radiologists can be very helpful. Systematic evaluation of the X-rays, avoiding SOS, knowledge of injury mechanisms and corresponding expected fracture patterns, knowledge of indirect signs of fractures, requesting complementary CT/MRI in negative X-rays with a high degree of suspicion and follow-up repeat X-rays when required are important measures that experienced radiologists employ to decrease misdiagnosis of fractures.
***Learning Objectives:***
1. To become familiar with the most commonly missed fractures in adult patients.
2. To understand which additional information will influence the choice of imaging modality.
3. To learn about atypical imaging findings in adult patients after trauma.

**A-0685** 16:55
**C. Missed musculoskeletal injuries in whole-body trauma CT**
A. Platon; *Geneva/CH (alexandra.platon@hcuge.ch)*

Whole-body usually consists in an initial unenhanced cerebral CT, followed by a helical, single-step continuous acquisition from the vertex to the symphysis pubis, after iv contrast injection. The rate of missed injuries at initial CT reading, reported in the literature, ranges between 2.4% and 47%; more than 50% of them involve the musculoskeletal system. The majority of missed musculoskeletal lesions involve the spine, followed by the skull, shoulder girdle, ribs and pelvic ring. Missed injuries are usually subtle, are frequently located close to other well identified major lesions, and their rate is correlated to the severity of trauma. Based on the literature data, the wide majority of missed musculoskeletal lesions have no impact on the patients’ mortality. However, they may have functional consequences on the outcome of the patients. Multiplanar reconstructions must be systematically obtained to detect subtle fractures and, in the spine, to demonstrate a fracture extension into the posterior vertebral elements, which will turn a stable compression fracture into an unstable lesion. The rate of subtle missed musculoskeletal injuries can be reduced by searching associated indirect CT signs. For instance, pneumocephalus or fluid content in paranasal sinuses are indirect signs of skull (or face) fracture; vocal cord hematoma may suggest the presence of a laryngeal injury and a thickening of the prevertebral stripe a vertebral fracture; the widening of the discal space, an interspinous widening or a misalignment of articular facets at CT will mandate an MRI to look for ligamentous injuries.
***Learning Objectives:***
1. To become familiar with the most commonly missed musculoskeletal injuries in patients after polytrauma.
2. To understand the clinical impact of missed subtle injuries on clinical outcome of trauma victims.
3. To be familiar with less typical imaging findings in musculoskeletal injuries.

17:20
**Panel discussion: How to reduce the rate of missed fractures most effectively and efficiently**

**16:00 - 17:30** **Room Y**

### E³ - Rising Stars Programme: EFRS Radiographers' Basic Session

### BR 12

### Clinical dilemmas in radiography: what graduates need to know

**A-0686** 16:00
**Chairperson's introduction: What are the current issues in radiography?**
J. Santos; *Coimbra/PT (joanasantos@estescoimbra.pt)*

Technology evolution plays a central role in medical imaging diagnosis and treatment. Different imaging modalities, hybrid technologies, new image detectors, high computing capabilities, new image processing tools are being developed daily. Radiographers are integral members of medical imaging and radiotherapy teams delivering high-quality clinical care. Radiographer’s knowledge skills and competencies (KSC) are defined in guidelines. However, radiography courses have different models across Europe, and the definition of professional expertise levels varies per country. Essential tools must be obtained during the graduation. The development of the critical thinking skills of students is mandatory to meet the challenges of daily clinical practice. To be updated radiographers must undertake Continuing Professional Development (CPD) appropriate to their practice. CPD is crucial to preserve and demonstrate continuing competence with autonomy and responsibility. Radiographers developed their clinical practice in a variety of complex procedures improving outcomes for patients without neglecting patient care. The challenge is to obtain proper education and training to effectively link graduation and clinical daily practice.
***Session Objectives:***
1. To understand the current issues facing new graduates.
2. To consider the development needed to become an effective practitioner.
3. To appreciate the changing face of radiographic practice.

**A-0687** 16:05
**Issues surrounding consent**
E. Dodd; *Bolton/UK (emma.dodd@boltonft.nhs.uk)*

The potential for a patient centred approach to all diagnostic imaging begins from the moment a patient arrives within the Radiology department. The prime opportunity for attaining the required trust and rapport with the patient is during the consent process. The complexities of gaining such consent can be influenced by a wide range of varying factors including age, gender, culture and capacity all of which require a very different approach to ensure that informed consent is definitively attained. The presentation of challenges associated with consent may be overt or have the potential to be relatively subtle, and so the radiographer must be aware of both the values of the individual patient and also the requirements of the radiographic examination in order to meet the delicate balance that these potentially opposing concepts present. The increasing pace, expectations and evolution of the role of the radiographer lead to this perpetual dilemma which must be sensitively and adequately broached to avoid the potential for litigation and overall patient dissatisfaction. Student and qualified radiographers alike must ensure that informed consent is achieved by employing a systematic approach alongside appropriate problem solving and open discussion to allow for transparency and candour during every patient encounter.
***Learning Objectives:***
1. To understand the current issues surrounding consent within radiographic practice.
2. To become familiar with methods needed to ensure correct consent for radiographic procedures.
3. To discuss the changing role of the radiographer with regards to patient consent.

**A-0688** 16:28
**Communication and compliance difficulties in imaging**
S. Kada; *Bergen/NO (sundaran.kada@hvl.no)*

Compliance with advice and instructions in the medical imaging department is important because we frequently use ionising radiation in our daily work and have long waiting lists for specialist examinations. Not adhering to instructions may result in repeating of the examination, thereby, increased waiting times for other patients and an additional radiation dose for the patient. Noncompliance can occur at any stage of the medical process - for example, failure to attend for outpatient appointments is regarded as a specific form of noncompliance. Knowledge and understanding of the various types of non-compliance would allow the formation of strategies to tackle them effectively. It is important to understand why patients do not comply with the information they are asked to follow. Compliance is generally seen to improve when patients are given more information about their state of health, investigations and treatment. Studies have shown that increasing the amount of information for the patient not only improves satisfaction but also empowers the patient and increases compliance. Affective and cognitive empathetic communication styles have a significant positive relationship with both patient satisfaction and compliance. However, providing information to certain groups of patients (people with dementia) is a challenge for healthcare professionals. Due to the projected increase in the percentage of the population suffering from dementia, who are frequently referred to imaging departments, it is suggested a CPD program (with theoretical knowledge and practical training of dementia) be provided to all radiographers.
***Learning Objectives:***
1. To be aware of communication and compliance issues within radiology.
2. To discuss methods for improving communication and compliance.
3. To appreciate the need for CPD in areas such as dementia, obesity.

**A-0689** 16:51
**Professionalism in an era of social media (part 1)**
R. García Gorga; *Sabadell/ES (rodrigogg@hotmail.es)*

Social media has become a useful tool for health professionals. The most frequent advantage of using social media is receiving news and updated information about research, and new technologies. However, professionals are getting more involved in other ways of using social media; for instance, for getting in touch with other colleagues and for sharing opinions about different topics related to our profession. These discussions, often motivated by some publication, are an excellent intellectual exercise that helps us stay updated and in touch. Many radiographers have taken a step forward. Social media has served to narrow the gap between professionals and patients, which is an excellent way to provide first-hand information. The close contact with individual patients and patient's associations has another utility; make our profession visible and showing the valuable role of radiographers. In order to use social media with these objectives, it is necessary to identify ourselves adequately using a clear profile. In our opinion, it is mandatory to review the quality of the content we share and maintain a proactive and generous attitude. In our experience, the benefits far outweigh the efforts. It is also a work that can be done as a team. As a result, we identify ourselves more intensively with our profession and contribute to generating a public profile of it. Social networks should be seen as a meeting point between people. It is at this point that we understand the relevance of these tools for those of us who must be health agents.
***Learning Objectives:***
1. To learn about professionalism in social media by showing activity models of radiography professionals in social media.
2. To appreciate the impact of digital information on the profession of radiography.
3. To understand professionalism in social media through the proposal of quantitative and qualitative research models.

**A-0690** 17:02
**Professionalism in an era of social media (part 2)**
M.A. de la Camara; *Talarrubias/ES*

Professionalism in Social Media implies using digital tools to innovate processes and products and keep up with the digital development. One of the keys to professionalism in Social Media is to incorporate the evidence-based radiology resource in order to evaluate the behavioural impact (qualitative and quantitative). This is done by mastering a specific metric and its research with tools provided by Digital Marketing on each of the Social Networks and Digital Platforms used. Examples of shared and connected knowledge of professional practices in various Social Networks and platforms will be presented, and the case of the creation of audio-visual content in Spanish (videos on YouTube) that, through digital narrative, the informative and formative context, and the measurement of quantitative and qualitative data, we can identify better non-clinical communicative practices in the performance of CT and MRI tests, addressing the problem of claustrophobia, and improving the professionalism of the radiographer before the patient in radiology.
***Learning Objectives:***
1. To learn about professionalism in social media by showing activity models of radiography professionals in social media.
2. To appreciate the impact of digital information on the profession of radiography.
3. To understand professionalism in social media through the proposal of quantitative and qualitative research models.

17:14
**Panel discussion: Promoting high-quality patient-centred practice**

**16:00 - 17:30** **Room D**

### Musculoskeletal

### RC 1210

### The radiological investigation of musculoskeletal tumours

**A-0691** 16:00
**Chairperson's introduction**
F.M.H.M. Vanhoenacker; *Antwerp/BE (filip.vanhoenacker@telenet.be)*

Plain films are generally the initial imaging modality in the detection and characterisation of bone tumours, as they accurately depict matrix, cortical permeation or disruption, and periosteal reaction. However, the sensitivity of this technique for the detection of small lesions is limited. CT can be used for characterisation in anatomically complex areas, such as the spine, pelvis and skull. The CT appearance is similar to plain radiographs. Due to radiation restraints, CT is not recommended in children nor for evaluation of the appendicular skeleton in adults. Conventional MRI may be of additional help in narrowing the differential diagnosis of bone tumours. Soft Tissue Tumours (STT) based on imaging remains even more limited, and histology is usually required for a definitive diagnosis. Ultrasound is mostly nonspecific but may be used for superficially located cystic STT. On MRI, analysis of multiple parameters (shape, the presence of signal voids,fluid-fluid levels, SI, intratumoral necrosis, multiplicity, pattern/degree of enhancement) yields the best results. The highest confidence is reached in benign lesions, such as lipomas, vascular lesions, benign neural tumours, periarticular cysts, hematomas, PVNS, GCTTS, and abscesses. The major role of MRI consists - however- of local tumour staging and monitoring of treatment. Recently, the European Society of Musculoskeletal Radiology (ESSR) has published guidelines for detection, characterisation, and referral pathway of musculoskeletal tumours based on conventional imaging. The specific aim of this session is to discuss the role of recent advances in ultrasound technology, advanced CT and MR techniques and hybrid imaging in the imaging evaluation of musculoskeletal tumours.

***Session Objectives:***
1. To describe how to differentiate tumours from other non-tumoural pathological conditions.
2. To discuss the value of imaging modalities in this field.
3. To explain how to determine resectability and extension of the tumour.

**A-0692** 16:05
**A. Radiographs and ultrasound**
L.M. Sconfienza; *Milan/IT (io@lucasconfienza.it)*

Besides advanced techniques, such as computed tomography and magnetic resonance imaging which are important in the workup of musculoskeletal tumours, plain films and ultrasound still play a crucial role. Plain films represent the cornerstone in the evaluation of bone tumours, as they allow for detecting the presence of a bone lesion and giving important information about characterization of the lesion such as the presence and type of periostal reaction, the pattern of bone destruction, the lytic or sclerotic appearance of lesion, the presence of soft tissue involvement or associated findings such as pathological fractures. However, plain films have limitations in particularly complex anatomical locations (e.g., spine and pelvis) and in the evaluation of soft tissue tumours not involving the bone. Ultrasound has limitations in the evaluation of bone tumours, as the ultrasound beam can minimally cross the bony cortex. However, ultrasound may detect the presence of a periostal reaction in early stages and may be used to assess the involvement of surrounding soft tissues and neurovascular bundles by bone tumours. On the other hand, ultrasound is accurate in the detection and evaluation of soft tissue tumours, being able to correctly evaluate the size and the relationship of the mass with the surrounding structures. The use of supplementary tools, such as power Doppler, contrast-enhanced ultrasound, and elastography has been proved to be useful in increasing the diagnostic performance of ultrasound in certain conditions. However, ultrasound may be limited by low contrast resolution, small field of view, and deep lesions.
***Learning Objectives:***
1. To discuss the value of radiography and US in the diagnostic work-up of MSK tumours.
2. To explain the most recent advances and trends in the development of US technology, including contrast-enhanced US and elastography.
*Author Disclosure:*
**L.M. Sconfienza:** Speaker; Fidia Pharma Group, Abiogen. Other; Travel grants from Abiogen, Bracco Imaging Italia.

**A-0693** 16:28
**B. MRI and whole-body MRI**
S.L.J. James; *Birmingham/UK (stevenjames@nhs.net)*

This presentation aims to review a number of aspects of MR imaging related to primary bone tumours. Initially, a review of a basic MR protocol will be performed to establish a minimum requirement to adequately image this type of pathology. The role of some more advanced techniques including chemical shift imaging, diffusion-weighted imaging, MR spectroscopy and perfusion MR imaging will also be discussed. Finally, the role of whole-body MRI will be described. It should be noted that the focus of this talk will be on primary bone tumours and not on the use of these techniques in metastatic disease, myeloma or lymphoma.
***Learning Objectives:***
1. To describe how to perform an advanced clinical MR protocol for MSK tumours.
2. To explain the potential of new MR techniques.
3. To discuss the impact of MR imaging and whole-body techniques in MSK tumour imaging.

**A-0694** 16:51
**C. CT and hybrid imaging**
T. Bäuerle; *Erlangen/DE (tobias.baeuerle@uk-erlangen.de)*

For diagnosis, staging and follow-up of musculoskeletal tumours, CT and the hybrid techniques PET/CT and PET/MRI offer a broad spectrum of methods. On the morphologic level, bone destruction ranging from osteolytic to -blastic lesions and adjacent soft tissue tumours, e.g. in Ewing sarcoma or lytic bone metastases, are captured by CT. Beyond morphology, PET radiopharmaceuticals including [18]F-fluorodeoxyglucose ([18]F-FDG) or [18]F-sodium fluoride ([18]F-NaF) display tumor and bone metabolism, respectively. Whereas the soft tissue tumours of rhabdomyosarcoma or bone marrow involvement in multiple myeloma is assessed by [18]F-FDG, osteoblastic activity is captured using [18]F-NaF for example in sclerotic metastases. Due to the excellent sensitivity of PET, information on the molecular level is available when administering tumour-specific radiotracers such as the [68]Ga- or [18]F-labeled prostate specific membrane antigen (PSMA) ligands for prostate cancer bone metastases. This lecture will summarise the use of CT and the hybrid imaging techniques PET/CT and PET/MRI in primary and secondary musculoskeletal tumours as well as in hematologic disease affecting bone. Current state-of-the-art techniques on the morphologic, metabolic and molecular level will be reviewed, including an outlook on future developments, particularly for hybrid imaging.
***Learning Objectives:***
1. To discuss the role of CT and hybrid imaging in the evaluation of MSK tumours.
2. To describe how hybrid imaging allows the intrinsic combination of functional and anatomical image information.
3. To explain how future developments of novel PET tracers and integrated PET/CT and PET/MRI may impact the management of MSK tumours.

17:14
**Panel discussion: Guidelines for, and the role of, imaging techniques in the management of musculoskeletal tumours**

**16:00 - 17:30** **Room G**

### Special Focus Session

### SF 12

### IT-security and GDPR

*Moderator:*
O. Ratib; Geneva/CH

**A-0695** 16:00
**Chairperson's introduction**
E. Kotter; *Freiburg/DE (elmar.kotter@uniklinik-freiburg.de)*

The introduction of the GDPR has raised many questions among radiologists and hospital administrators. Standard procedures of data management had to be modified, and IT systems had to be modified to adopt. This session will give an overview of GDPR, will summarise the practical aspects of GDPR and patient consent and highlight the special aspects of using mobile devices.
***Session Objectives:***
1. To give an overview on the GDPR and patient consent.
2. To understand practical aspects of GDPR and patient consent.
3. To know security aspects specific to mobile devices and social media.
*Author Disclosure:*
**E. Kotter:** Advisory Board; Agfa.

**A-0696** 16:05
**Understanding the key points of GDPR**
C.D. Becker; *Geneva/CH*

The General Data Protection Regulation implies several new rules for the protection of the confidentiality of patient data, access of data for patients, processing, as well as technical and organisational safeguards.Explicit informed consent is necessary prior to processing and communication of imaging data. Patients have the right to access their image data and to obtain copies of their data ("data portability"). Data must be rectified in case of errors, and the patient and/ or supervising authority must be informed in the case of inadvertent or voluntary inappropriate disclosure of patient data ("data breach"). Derogations may be defined for special purposes, including research or public health, and special guidelines may be defined by national law.
***Learning Objectives:***
1. To give an overview of the GDPR.
2. To understand the key points of GDPR.
3. To review what is important for radiological practice.

**A-0697** 16:28
**Issues related to patient consent to allow access to the data**
O. Ratib; *Geneva/CH*

The developments of imaging biobanks for research and development of analysis tools and machine learning techniques must comply with strict regulations and guidelines geared toward protecting patient confidentiality and data security. The recent legal frameworks and regulatory data protection rules prevent the usage and exploitation of medical data without formal patient approval. Traditional informed consent principles used in medical research require that the patient is informed of the purpose and goals of the research performed with the data collected. This defeats the basic principle of deep learning and Big-Data analytics which looks for random patterns and correlations without a specific pre-established hypothesis. To overcome this dilemma, regulatory bodies, government agencies and academic experts in ethics have promoted new concepts of innovative legal frameworks that will allow and regulate the ability of each individual to contribute to the development of Big-Data collections of images while complying with fundamental ethical principles and regulatory directives. Our presentation will focus on the different technical and practical issues of implementing adequate patient consent and data protection strategies for the development of imaging biobanks repositories for scientific research and data mining.

***Learning Objectives:***
1. To understand the difference between general consent and informed consent.
2. To learn how to manage patient consent.
3. To understand special aspects of data access in the context of scientific data.

**A-0698** 16:51
**Security aspects when using mobile devices and/or social media**
E.R. Ranschaert; *Tilburg/NL (ranschaert@telenet.be)*

Medical specialists and radiologists are using mobile devices to share and exchange medical information and images with other healthcare professionals. Usually, they need advice regarding a diagnosis or treatment, sometimes in an acute setting. Popular messaging services such as WhatsApp are frequently used for such purpose. Transmission of patient data with mobile devices and messaging services, however, does have several risks and limitations, mostly related to the security and privacy of patients, but also from an ethical and legal point of view. Some questions need to be answered: is this type of communication unsafe and/or illegal, and if yes, why? Is this type of communication compliant with the GDPR legislation? What safe and secure options are available for sharing patient information on mobile devices and with messaging services? In this session, these issues will be discussed in more detail.
***Learning Objectives:***
1. To understand the vulnerability of data on mobile devices and in social media.
2. To learn how to protect data on mobile devices.
3. To learn how to protect data and identity in social media.

17:14
**Panel discussion: Data protection: benefit or burden?**

**16:00 - 17:30 Room K**

### Chest

### RC 1204

### Fibrotic lung diseases

**A-0699** 16:00
**Chairperson's introduction**
S.R. Desai; *London/UK*

***Session Objectives:***
1. To understand the implications of diagnosing UIP.
2. To be aware of the various fibrotic entities prognosis.
3. To re-evaluate the links between smoking and fibrosis.

**A-0700** 16:05
**A. Idiopathic pulmonary fibrosis**
N. Sverzellati; *Parma/IT*

Idiopathic pulmonary fibrosis (IPF) is the most common and lethal form of idiopathic interstitial pneumonia. Two guidance documents for the diagnosis of IPF have been recently published by international experts representing major respiratory and radiological societies. The documents have reached similar conclusions and recommendations, with only a few differences. The critical role of high-resolution computed tomography (HRCT) has been emphasised, proposing new HRCT diagnostic criteria. In particular, the role of HRCT has been expanded to permit diagnosis of IPF without surgical lung biopsy in select cases when HRCT shows a classic or probable UIP pattern. Additional investigations, including surgical lung biopsy, should be considered in patients with either clinical or HRCT findings that are indeterminate for IPF. A multidisciplinary approach is particularly important when deciding to perform additional diagnostic assessments, integrating biopsy results with clinical and CT features, and establishing a working diagnosis of IPF if lung tissue is not available.
***Learning Objectives:***
1. To learn about the updated criteria for IPF diagnosis.
2. To understand the implication for patient management.
3. To review illustrative cases with pathological assessment.

**A-0701** 16:28
**B. Lung fibrosis in connective tissue and granulomatous diseases**
P.-Y. Brillet; *Bobigny/FR (pybrillet@yahoo.fr)*

The objective of the course is to review the main CT features of connective tissue diseases and sarcoidosis. The role of the radiologist in case of pulmonary fibrosis will be discussed (evaluation of severity, progression of disease, diagnosis of complications). Connective tissue diseases are a diverse group of immunologically mediated systemic disorders that includes systemic sclerosis and antisynthetase syndrome. Involvement of the respiratory system is common in connective tissue diseases and can affect every part of the lung: pleura, alveoli, interstitium, vessels, lymphatic tissue, and large and/or small airways. Interstitial lung disease (mainly nonspecific interstitial pneumonia, but also usual interstitial pneumonia, organising pneumonia and lymphoid interstitial pneumonia) and pulmonary arterial hypertension are the main causes of mortality and morbidity. Sarcoidosis is a systemic disease of unknown cause that is characterised by the formation of immune granulomas in various organs, mainly the lungs and the lymphatic system. In up to 20%, patients develop fibrotic lung disease, which can be associated with potentially life-threatening manifestations. Three main CT patterns can be observed: bronchial distortion with or without fibrotic masses, Hilo-peripheral lines and honeycombing. Complications of fibrotic pulmonary sarcoidosis include pulmonary hypertension and chronic Aspergillus disease, with hemoptysis.
***Learning Objectives:***
1. To review the CT features of systemic sclerosis.
2. To learn about CT manifestations of antisynthetase syndrome.
3. To learn about lung fibrosis characteristics in sarcoidosis.

**A-0702** 16:51
**C. Other causes of lung fibrosis**
C.P. Heussel; *Heidelberg/DE*

Drug-induced lung disease is an underestimated cause of morbidity in 5-10% of drugs like Amiodarone, Bleomycin, Busulfan, Methotrexate. Other drugs with potential lung toxicity include Cytarabine, Etanercept, NSAR, Azathioprine, Nitrofurantoin, Amphotericine B, Sulfonamide, Sulfasalazin, Statins, TNF-a-Blockers, anti-CD20, INF-a, Cetuximab, Bevacizumab, Alemtuzumab, Trastuzumab, Tacrolimus, Everolimus, Sirolimus. A suspected drug can be checked at www.pneumotox.com and sum up to >450 drugs. Risk factors include rheumatoid arthritis and chronic inflammatory bowel disease, and previous irradiation. Lung toxicity becomes more frequent nowadays in tumour therapy using recent biologicals that might cause autoimmune diseases. Pneumonitis has resulted therein in 5%. Time-point of pulmonary affection is extremely variable between days and several years after exposure. Patterns include alveolitis, COP, and NSIP. Radiation toxicity is related to the irradiation field, however, COP patterns typically occur adjacently to this field. Time-point varies between 6 weeks after the end of irradiation and might be later than 1 year thereafter. Patterns include alveolitis, COP, and NSIP. Smoking-related lung disease include, besides of emphysema, the mainly or partially reversible respiratory-bronchiolitis interstitial lung disease (rb-ILD), desquamative interstitial lung disease (DIP), Langerhanscell histiocytosis (LHC), and in combination of lung fibrosis and emphysema the respective irreversible syndrome (CPFE) which suffers from a worse prognosis than emphysema alone.
***Learning Objectives:***
1. To learn about drug-induced lung fibrosis.
2. To review CT features of post-radiation fibrosis.
3. To learn about smoking-related lung fibrosis.
*Author Disclosure:*
**C.P. Heussel:** Board Member; ECIL-3, ECCMID, EORTC/MSG. Consultant; Schering-Plough, Pfizer, Basilea, Boehringer Ingelheim, Novartis, Roche, Astellas, Gilead, MSD, Lilly, Intermune, Fresenius. Employee; • Head of Diagnostic and Interv Radiology with Nuclear Medicine, Thoraxklinik Heidelberg • Member of the German Center for Lung Research. Grant Recipient; Siemens, Pfizer, MeVis, Boehringer Ingelheim, German Center for Lung Research. Patent Holder; Method and Device For Representing the Microstructure of the Lungs. IPC8 Class: AA61B5055FI, PAN: 20080208038, Inventors: W Schreiber, U Wolf, AW Scholz, CP Heussel. Shareholder; GSK. Speaker; Gilead, Essex, Schering-Plough, AstraZeneca, Lilly, Roche, MSD, Pfizer, Bracco, MEDA Pharma, Intermune, Chiesi, Siemens, Covidien, Pierre Fabre, Boehringer Ingelheim, Grifols, Novartis Basilea, Bayer.

17:14
**Panel discussion: What is the radiologist's role in the evolving spectrum of pulmonary fibrosis?**

**16:00 - 17:30** **Room M 1**

### Oncologic Imaging

### RC 1206

### Translating functional and molecular imaging in oncology

**A-0703** 16:00
**Chairperson's introduction**
R.J. Méndez; *Madrid/ES (ramiro.mendez@outlook.com)*

Different functional imaging techniques are currently able to in vivo assay many biological features of cancer, and they can be used in the detection, characterisation and follow-up of neoplasms. The non-invasive monitoring of the molecular and physiologic changes offers clear advantages for preclinical studies of the disease and the development and analysis of new therapies. Many of these functional imaging techniques and imaging biomarkers even being proved useful in cellular or animal cancer models, will never be brought into a clinical scenario for diagnosing cancer patients, for therapy tailoring or follow-up. To cross this translational gap the imaging technique must be reproducible; it has to be available using clinical equipment, the imaging biomarker should prove its validity for cancer detection, characterisation, prognosis or monitoring. Then, cost-effectiveness would also be considered. All these steps are part of a long road for translating functional and molecular imaging techniques from laboratory research to the comprehensive clinical management of cancer patients. In this session three different functional/molecular imaging techniques used in oncology will be presented, describing biological processes that can be assessed with each technique and analysing how to translate this basic knowledge to benefit cancer patients in a standard clinical practice.
***Session Objectives:***
1. To understand the basic concepts of functional and molecular imaging methods in oncology.
2. To appreciate the challenges in translating novel functional and molecular imaging methods.
3. To learn approaches to enhance clinical translation of functional and molecular imaging methods.

**A-0704** 16:05
**A. Functional cancer imaging with MRI**
S. Gatidis; *Tübingen/DE*

Medical imaging plays an increasingly important role in diagnosis, risk stratification and response assessment in oncology. While morphological assessment of tumour size and localisation is a prerequisite for oncologic imaging, the concept of personalised and precise medicine demands further information on tumour biology for the guidance of the therapeutic decision. MRI is the most versatile clinical imaging modality providing both, morphologic and functional information about tissues and organs in vivo and is thus an effective method for functional cancer imaging. This presentation will give an overview of MR-based methods of functional cancer imaging and their technical principles. Clinical applications of MR-based functional cancer imaging will be summarised, and methods for advanced analysis of complex imaging data will be discussed.
***Learning Objectives:***
1. To learn the basic principles of functional cancer imaging with MRI.
2. To understand the biological process that can be assessed using functional MRI.
3. To learn about clinical applications of functional MRI in oncology.

**A-0705** 16:28
**B. New PET tracers in oncology**
M. Herranz; *Santiago de Compostela/ES (michel.herranz.carnero@sergas.es)*

Advances in imaging modalities both anatomical (MRI, CT, US) and functional (PET, SPECT, fMRI) has provided with a new tool for earlier and more accurate diagnoses, and for personalised treatments follow-up. Positron Emission Tomography (PET), currently always combined with an underlying anatomical technique such as CT or MR, is a noninvasive molecular imaging technique that enables visualisation, measurement and quantification of metabolic events by detecting the distribution of molecules (targeting agent) bound to a positron-emitting compound, chemical complex known as a radiotracer. Development of new specific and sensitive radiotracers, nowadays, targeting practically any biological process (transporter, receptor, nucleic acid, enzymes, and many other molecular targets), is generating huge scientific and clinical interest. Currently, not only cancer but multiple pathologies including cardiovascular and neurodegenerative, are being visualised with specific radiotracers. The huge variability of the chemistry behind the synthesis, the different radionuclides (positrons-emitting-compounds beyond 18F: 11C, 13N, 15O, 68Ga, 62Rb, 89Zr) allow almost infinite combinations for the majority of the processes that occur into the cell, getting closer and closer to a more specific, and more personalised medicine. Here we review the biological bases of different pathological processes and the way they can be visualised with different radiotracers, their clinical utility and future developments.
***Learning Objectives:***
1. To learn the basic concepts in the development of new PET tracers.
2. To understand the biological process that can be assessed using novel PET tracers.
3. To learn about clinical applications of new PET tracers in oncology.

**A-0706** 16:51
**C. Optical imaging in cancer**
D. Razansky; *Zurich/CH (daniel.razansky@uzh.ch)*

Microscopy has been a major optical imaging tool since the seventeenth century. Optical imaging is still a rapidly emerging field with remarkable new approaches continuously emerging to improve on the capabilities and application potential; ultimately impacting biological discovery and healthcare. A significant role in these developments has played the discovery, development and propagation of fluorescent proteins and probes, as well as bioluminescence, to in-vivo imaging applications. Linked to this progress is the ability to visualise disease features and biomarkers with high versatility. The recently introduced multi-spectral optoacoustic tomography methods are poised to revolutionise optical imaging by delivering high-resolution visualisation of structural, functional, metabolic and molecular information deep from optically opaque living tissues. These modalities are uniquely endowed with rich and label-free hemodynamic contrast, excellent spatial and temporal resolution, centimetre-scale penetration into living tissues, and versatile exogenous contrast approaches. State-of-the-art handheld and intraoperative imaging solutions are further transforming medical diagnostics by offering a new level of precision in non-invasive clinical observations of patients, demonstrating high diagnostic efficacy in a number of indications, including skin and breast lesions, cardiovascular and inflammatory diseases and metastatic lymph node detection.
***Learning Objectives:***
1. To learn the basic concepts of optical imaging in oncology.
2. To understand the biological process that can be assessed by optical imaging.
3. To learn about clinical applications of optical imaging in oncology.
*Author Disclosure:*
**D. Razansky:** Shareholder; iThera Medical GmbH.

17:14
**Panel discussion: Key competences to close the translational gap more efficiently**

**16:00 - 17:30** **Room M 2**

### E³ - ECR Academies: Radiology Leaders' Bootcamp

### E³ 1218

### Secrets of radiology communications with patients and referring physicians

**A-0707** 16:00
**Chairperson's introduction**
M. Fatehi; *Tehran/IR (fatehi@irsr.org)*

While the referring physicians are looking for a detailed but concise description of patient’s problem enriched by recommendations for the next step or action, the patients and their loved ones, look for an ordinary explanation of the situation in daily life language. The effectiveness of a professional report is based on not only the imaging aspect of the procedure including anatomy, pathology and physical principles of image acquisition but also mechanisms of decision making in the clinical discipline of the referring physician. In other words, the knowledge requirements of the successful radiology reports are not limited to medical imaging but to the subspecialty profession of the physician. It is not possible to ask patients to avoid reading their professional radiology interpretation but obviously, the report may increase their anxiety. So we need new tools and channels of communicating with patients. The major challenge is to find the best way to inform patient without additional workload and without breaking principles of the medical discipline.

Friday

**A-0708** 16:06
**A. Communication is all that really matters**
J.A. Verschakelen; *Leuven/BE (johny.verschakelen@uz.kuleuven.ac.be)*

A fluid transfer of information among radiologists, referring physicians and patients is essential for timely care, patient satisfaction and optimal health outcomes. As radiology practices become larger and more specialised, communication should be efficient and streamlined. While the introduction of sophisticated systems like computerised order entry, voice dictation systems and picture archiving and communication systems have certainly improved the information transfer, they may come at the expense of personal interaction with patients, referring physicians and medical colleagues. As the practice of radiology is evolving to become more patient-oriented and more multidisciplinary with the radiologist having an important clinical value in the care team, it is obvious that adequate personal communication is essential. Each person has a style of communication that is unique and refers to the choices made when communicating with others. Differences in communication style can be a barrier to effective communication. So it is important to identify its own personal communication style, to identify the barriers one might face when communicating with other styles and learn to enhance communication with others no matter what communication style they have.
***Learning Objectives:***
1. Patients, patients' organisations and referring physicians: "know your client" in radiology.
2. Communications styles for building trust and the role of informed consent
3. Psychology tips and tricks for effective communications and negotiations.

**A-0709** 16:34
**B. Marketing channels for radiology promotion**
D. Pinto dos Santos; *Cologne/DE (daniel.pinto-dos-santos@uk-koeln.de)*

As radiologists (or any other physician) we are constantly marketing ourselves and our practices, whether we are aware of it or not. In this talk, an overview is given about the various channels by which we are already marketing radiology and how to improve those. Also, some channels that traditionally receive less attention are discussed. The 5 P's mnemonic of marketing (product, price, placement, promotion and people) will be introduced to further explain some fundamentals of marketing. These fundamentals naturally have some overlap with the five dimensions of service quality (reliability, assurance, tangibles, empathy and responsiveness) as proposed by Parasuraman et al. which will also be reviewed. To conclude, potential implications on current clinical practice will be discussed, with a special focus on how feedback from patients and referring physicians can be gathered as well as how the radiological report (especially structured reports and lay-language reports) can contribute to improving the perception of radiology.
***Learning Objectives:***
1. Traditional and new marketing channels in healthcare.
2. Multi-media, multifaceted and lay-language reporting: is there a way to change the format of radiology reports?
3. Referring physicians' and patients' feedback as a driver of quality improvement.

**A-0710** 17:02
**C. Personal-branding and promotion for a radiologist**
S. Morozov; *Moscow/RU (spmoroz@gmail.com)*

Current social media channels provide unparalleled possibilities for a wide promotion of healthcare services and professional expertise. Potential favourable outcomes include continuous communications within and outside a professional community, wider promotion of new medical technologies, and quicker distribution of professional news. Relatively low signal-to-noise ratio is a significant limitation of social media's effectiveness. Moreover, care should be taken to avoid misinterpretation of the superficial and brief information. A promotion and communication activity allows to achieve the expected result if it follows a pre-defined plan with an agile modification as it is realised. The major definitions to be preliminary made are the following: mission, executive strategy, target audience, key messages, tools and tactics, metrics, budget, schedule and amendments. The expected SMART result is a major differentiator of leisure social activities from a coordinated art of actions. Careful planning and execution do not exclude a personal touch in the communications. Moreover, a combination of personal and professional messages allows to effectively reach out to a wider audience. Recommendations on the content: at least 20% of personal messages, at least 20% of videos, description, hashtags, geo-tags, and face-tags. Publications about events, meetings with interesting people, resumes of books, teamwork reports, and personal motivations sources are the most attractive. Although social activities are attractive and joyful, their amount should be limited to less than 5% of daily working time. Core professional activities including clinical, research, administrative, teaching, and writing are the most valuable.
***Learning Objectives:***
1. A personal touch in healthcare marketing and challenges of "Group Practice" in developing a brand.
2. The best recipe for building a radiologist's personal brand to be known internationally.
3. Radiologist's role in an early diagnostics promotion and education of patients.
*Author Disclosure:*
**S. Morozov:** Advisory Board; Agfa, Philips.

**16:00 - 17:30** **Room M 3**

### Oncologic Imaging

### RC 1216

### Oncological imaging: where are we now?

**A-0711** 16:00
**Chairperson's introduction: What are the problems of morphologic evaluation**
E. De Kerviler; *Paris/FR (eric.de-kerviler@sls.aphp.fr)*

Since the 1980s, there have been many criteria to assess response of cytotoxic agents in solid tumours: WHO, SWOG, RECIST··· These various response criteria have evolved over a period of time to establish their consistency in the assessment of efficacy in various solid tumours. Using these criteria, the primary endpoint is a significant decrease in the overall tumour burden, with thresholds for a response depending on the method used. For years, the main debate was therefore around the use of unidimensional versus bidimensional measurement of target lesions, the number of target lesions, and the assessment of non-target lesions. In the era of precision oncology, there has been a marked growth of targeted therapies and immunotherapies. Now there is evidence that single morphological response criteria may not be good enough. Evaluating early responses to precision therapy is essential for "go" vs. "no-go" decisions for these expensive molecularly targeted drugs. In the near future, it is clear that CT perfusion, functional MRI and molecular imaging will offer new perspectives in the assessment of the therapeutic response of non-cytotoxic drugs. More than ever before, imaging will play a prominent role in the management of cancer patients.

**A-0712** 16:05
**A. CT perfusion techniques**
H. Schöllnast; *Graz/AT (helmut.schoellnast@medunigraz.at)*

CT-Perfusion imaging is a functional imaging tool which enables qualitative and quantitative evaluation of tumour perfusion and, therefore, assessment of tumour-related angiogenesis. Clinical applications for CT-perfusion imaging have mainly evolved to include lesion characterisation, prediction of prognosis, prediction of response to therapy and assessment of response to local or systemic therapies. Perfusion values have shown significant differences when comparing normal tissue with tumours and between benign and malignant lesions, although there is overlap between benign and malignant lesions. The degree of tumour perfusion is potentially associated with tumour aggressiveness as tumour-related angiogenesis determines the ability of tumours to metastasise and, therefore, affects prognosis. On the other hand, hypoxia within a tumour reflected by restricted perfusion Limits the effect of chemotherapy and radiotherapy, potentially allowing for prediction of response. Both conventional chemotherapy and targeted therapies such as angiogenesis inhibitors may affect tumour vascularisation. In contrast to conventional chemotherapy, which mainly shows a cytotoxic effect on the tumour, angiogenesis inhibitors show rather a cytostatic effect. Traditional tumour size-based response criteria may underestimate the response to angiogenesis inhibitors in the early course of treatment due to stable tumour size despite effective therapy. In contrary, CT-perfusion imaging allows detection of a decrease in tumour perfusion which precedes decrease in size. Use of different models for Perfusion calculation such as compartmental model and deconvolution model, use of different CT-perfusion protocols including contrast media injection protocols and inter- and intraobserver variability when using Perfusion software limit use of CT-perfusion imaging in clinical Routine.
***Learning Objectives:***
1. To learn about current approaches for CT perfusion.
2. To understand the basic principles behind each technique.
3. To discuss radiation exposure from CT perfusion techniques.
4. To appreciate the clinical usefulness of these techniques in routine clinical practice.
*Author Disclosure:*
**H. Schöllnast:** Speaker; Invited Speaker for Canon Medical Systems (formaly Toshiba Medical Systems) in the past.

**A-0713** 16:28
**B. Functional MRI techniques**
V.J. Goh; *London/UK (vicky.goh@kcl.ac.uk)*

It is recognised that cancer is a biologically heterogeneous disease. Thus a 'one size fits all' management approach may not bring about the best outcome for the individual patient. Oncologic therapies have evolved substantially over the last two decades providing cancer patients with a greater opportunity for a cure. This personalised approach has also required a paradigm shift in oncologic imaging to capture this biological heterogeneity and improve patient triage. MRI provides a unique opportunity to combine high contrast and spatial resolution imaging of tumour morphology with physiological imaging of water diffusion, vascularisation and oxygenation status. At initial diagnosis and staging this may allow tumours to be phenotyped better and during therapy, for tumours to be assessed more comprehensively, particularly where therapy is cytostatic and will not result in significant size change. In this lecture, the different functional imaging techniques that can be applied in clinical practice will be discussed, and evidence for practice highlighted. The potential of integrated PET/MRI will also be considered.
***Learning Objectives:***
1. To learn about different functional MR techniques such as diffusion and perfusion.
2. To understand the basic principles behind each technique.
3. To appreciate the clinical usefulness of these techniques in routine clinical practice.
*Author Disclosure:*
**V.J. Goh:** Research/Grant Support; Siemens Healthcare.

**A-0714** 16:51
**C. Assessment by molecular imaging**
J. Grimm; *New York, NY/US (grimmj@mskcc.org)*

Functional imaging in oncology-beyond-morphology basically describes molecular imaging - i.e. imaging and measurements of biological processes at the cellular and molecular level. This means predominantly clinical PET imaging with FDG. However, a multitude of other tracers allows for the detection of additional structural or functional entities, e.g. the imaging of PSMA in prostate cancer to detect disease; or the imaging with amino acids, reflecting higher amino acid metabolism in brain tumours. However, existing tracers can also be used to explore new signatures, such as positron lymphography with 18F-FDG to characterise sentinel lymph nodes. While most of the clinical molecular imaging is done using Pet (or SPECT) imaging, newer modalities are becoming more prominent, such as optoacoustic imaging or optical imaging. Optoacoustic imaging describes "listening to the light", i.e. the detection of absorbers of light with ultrasound. This can be used, e.g. to detect melanoma metastases. Optical imaging uses either fluorescently labelled agents (requiring excitation light) or radiotracers for Cerenkov luminescence imaging, the optical imaging of radiotracers, which does require radiotracers but no incident excitation light.
***Learning Objectives:***
1. To learn about the basic concepts in molecular imaging.
2. To understand how molecular imaging might be integrated into patient care.
3. To discuss clinical examples of where molecular imaging is already established in clinical practice.
*Author Disclosure:*
**J. Grimm:** Advisory Board; LightPoint Medical. Board Member; World Molecular Imaging Society. Grant Recipient; NIH. Research/Grant Support; NIH. Shareholder; Nortis Bio. Other; sponsored research (TargImmune).

17:14
**Panel discussion: Where are we using functional evaluations in clinical practice?**

**16:00 - 17:30 Room M 4**

### E³ - ECR Academies: Chest Imaging

### E³ 1222

### The heart between the lungs

**A-0715** 16:00
**Chairperson's introduction**
J.E. Wildberger; *Maastricht/NL (j.wildberger@mumc.nl)*

Modern high-end CT scanners with faster scan acquisition times allow for routine assessment of cardiac pathologies, even on a standard chest CT. Relevant information regarding the heart can be retrieved, and incidental cardiac findings will play a major role for patient management in the future. Plain CT allows for fat distribution as well as for coronary calcium analysis. On contrast-enhanced scans, coronary arteries and myocardial changes can be assessed. During this session, three esteemed speakers will present an overview of the diagnostics and the clinical impact of various cardiac pathologies on standard thoracic CT scans.

**A-0716** 16:06
**A. Cardiomyopathies**
D.J. Murphy; *Dublin/IE (murphy.84@gmail.com)*

Cardiomyopathies are a heterogeneous, important group of diseases with significant morbidity and mortality, and can be classified into five major subtypes based on morphology (hypertrophic, dilated, restrictive, arrhythmogenic and unclassified). Diagnosis is based on a composite of clinical history and examination, laboratory and genetic testing, electrocardiography and cardiac imaging. Echocardiography is the first-line imaging test for the evaluation of cardiac structure and function, with cardiac magnetic resonance imaging (CMR) the gold standard. The computerised tomography (CT) has a growing role in this area. ECG-gated cardiac CT is a well-established test for coronary artery imaging, but it also allows an excellent opportunity to assess for structural cardiac abnormalities, such as asymmetric septal hypertrophy in hypertrophic cardiomyopathy, or left ventricular dilatation in dilated cardiomyopathy. The CT features of the common cardiomyopathies will be reviewed with a comparison to CMR, along with a revision of normal cardiac chamber anatomy. The improved temporal resolution of modern CT scanners has reduced the deleterious effect of cardiac motion artefact on routine non-ECG gated chest CT, allowing an opportunity for the detection of cardiac structural abnormalities. Standard non-ECG gated chest CT has a reported sensitivity of 68% in the detection of cardiomyopathies, and the most reliable imaging features of cardiomyopathies on standard chest CT, as well as potential pitfalls, will be reviewed.
***Learning Objectives:***
1. To review the normal anatomy on standard chest CT.
2. To learn about the CT signs of various cardiomyopathies.
3. To learn about CT performance for the diagnosis of cardiomyopathy.

**A-0717** 16:34
**B. Coronary artery disease**
M. Williams; *Edinburgh/UK*

The heart and coronary arteries can be identified on imaging performed specifically to assess the heart and also on imaging of the chest performed for other reasons. Normal imaging coronary artery anatomy will be reviewed. Coronary artery calcification (CAC) is a marker of atherosclerotic plaque burden. CAC identified on electrocardiogram-gated computed tomography (CT) is associated with an increased risk of cardiovascular morbidity and mortality. Randomised controlled trials have shown that management guided by CT coronary angiography can improve outcomes for patients with suspected coronary artery disease. CAC can also be identified on non-gated CT performed for other reasons. Calcium scoring or ordinal assessment of CAC on non- gated CT correlates with results from gated imaging. CAC on non-gated CT is associated with increased mortality in a variety of diseases, including patients with respiratory diseases. Evidence of previous myocardial infarction can also be identified on non-gated CT with features including mural thinning, fatty infiltration and complications such as aneurysms.
***Learning Objectives:***
1. To review the normal coronary artery anatomy.
2. To learn about calcium scoring on non-gated CT.
3. To learn about the CT signs of healed myocardial infarction.
*Author Disclosure:*
**M. Williams:** Consultant; GE Healthcare.

**A-0718** 17:02
**C. Fat and calcium in the heart**
F. Pontana; *Lille/FR (Francois.pontana@chru-lille.fr)*

Population ageing and other risk factors are not only associated with calcification of coronary arteries but are also likely to induce changes in the cardiac structure. These changes can be evaluated on a standard chest CT without cardiac synchronisation. Calcifications of the cardiac valves may be a sign of valvulopathy. Aortic valve calcium scoring by CT is useful to estimate aortic stenosis severity when echocardiography is not feasible or not conclusive. Myocardial calcification may be due to abnormalities of calcium homeostasis or calcium accumulation in necrotised cardiac myocytes. Pericardial calcification is a sign of constrictive pericarditis. Physiological age-related fat deposits within the myocardium or the atrial septum are frequent and must not be misreported as pathological. Benign or more rarely malignant cardiac tumours may present fatty component. More recently epicardial adipose tissue seems to be a new cardiac risk factor quantifiable by imaging.
***Learning Objectives:***
1. To learn about physiologic changes.
2. To review the main causes of fat and calcium.
3. To learn how to differentiate pathologic from physiologic images.

**16:00 - 17:30 Room M 5**

### Head and Neck

### RC 1208

### Post-treatment imaging of the head and neck

**A-0719** 16:00
**Chairperson's introduction**
H.B. Eggesbø; *Oslo/NO (h.b.eggesbo@medisin.uio.no)*

Head and neck cancer in the Western countries accounts for 3-4% of all cancers. Worldwide incidence is estimated to 500,000, and in Europe to 250,000. There are three times as many men as women that are affected. Squamous cell carcinoma constitutes for more than 80%. At the time of the diagnosis, one-third is staged I/II, and two-thirds staged III/IV. The recurrence rates for stage I/II is relatively low, and 5-year-survival is 70-90%. Hence, the risk of a secondary primary cancer after radiation therapy (RT) may be higher than the risk of recurrence. For stage III/IV, the local recurrence rates vary from 25 to 80%, and metastases from 10 to 30% and the 5-year-survival is 10-55%. Main treatment options for stage I/II are surgery and/or RT and for stage III/IV chemo- and RT. Regardless of choice of treatment, all tissues undergo major changes that challenge the post-treatment imaging interpretation for all modalities. This session will focus on expected changes after RT and the most frequent surgical procedures. Then, when and how to perform post-treatment imaging, in order to depict early cancer recurrence.
***Session Objectives:***
1. To become familiar with different methods of treatment.
2. To understand post-surgical and post-RT complications.
3. To know how to follow-up with patients in order to depict early recurrence.

**A-0720** 16:05
**A. Normal findings after radiotherapy**
R. Hermans; *Leuven/BE (Robert.Hermans@uzleuven.be)*

After radiotherapy, imaging may be used to monitor tumour response, and to detect recurrent or persistent disease before it becomes clinically evident. Early tumour recurrence may be difficult to distinguish from treatment-induced tissue changes. Therefore, the expected imaging changes after radiotherapy of head and neck cancer should be well known. These tissue changes depend on the radiation dose, the irradiated volume, and the time elapsed since treatment. Reticulation of fat layers, retropharyngeal space oedema, postirradiation sialadenitis, lymphatic tissue atrophy, and thickening of the pharyngeal and laryngeal structures may be seen. These expected tissue changes often appear symmetrical, unless asymmetric radiation portals were used. However, at the site of a bulky tumour, scar tissue may cause an asymmetric appearance also after successful treatment. Therefore, obtaining a baseline CT or MR study after therapy for a neoplasm with high risk for recurrence is recommended, best 3-6 months post-treatment. By comparing subsequent studies with this baseline study, tumour recurrences or treatment complications can be detected with more confidence. Some recommend PET-CT as the initial baseline study, as this technique has a high negative predictive value; the positive predictive value is lower, as therapy-induced inflammatory changes may cause false-positive results. Tissue necrosis is a rare complication of radiotherapy in the head and neck region, usually appearing late after treatment. Both soft tissue, cartilage and bone necrosis may be encountered; differentiation from tumour recurrence at an early stage may be difficult. Other long-term complications may occur, such as the development of a secondary tumour.
***Learning Objectives:***
1. To get acquainted with changes in tissues during and after radiotherapy.
2. To understand expected changes after radiotherapy in CT and MR imaging.
3. To recognise treatment complications from expected tissue changes after radiotherapy.

**A-0721** 16:28
**B. Normal findings after surgery**
A. Trojanowska; *Lublin/PL (agnieszka30@yahoo.com)*

Nowadays, the management of head and neck cancer involves multidisciplinary evaluation and treatment, which usually includes surgery, radiation therapy and chemotherapy. The various approaches to surgical resection and tissue reconstruction, the types of neck dissection, different radiation therapy techniques and the addition of concurrent and neoadjuvant chemotherapy regimens may complicate imaging findings. Differentiating post-treatment changes from tumour recurrence with the use of different imaging modalities is challenging because of the presence of altered anatomy secondary to resection, flap reconstructions, oedema, inflammation and the presence of scar tissue. Therefore, it is essential to be familiar with normal findings after surgery and radiotherapy, to distinguish these characteristics from tumour recurrence and treatment-related complications.
***Learning Objectives:***
1. To get acquainted with the most frequent surgical procedures in the head and neck.
2. To understand how to evaluate post-surgical patients.
3. To learn how to assess microvascular flaps.

**A-0722** 16:51
**C. Treatment monitoring for early detection of recurrence**
A.D. King; *Hong Kong/CN*

Head and neck tumour recurrence occurs in 15-50% of patients with squamous cell carcinoma treated by chemoradiotherapy, the majority are in-field and detected either on the post-treatment assessment or within 2-3 years. MRI and CT aim to identify recurrence and also avoid unnecessary biopsy or surgery. Size criteria for recurrence include a primary focal mass or node (short axis) ≥1cm, or < 75%-90% reduction in nodal size, but size has limitations especially in the assessment of HPV+ nodal disease. The main advantages of MRI over CT are the T2 weighted and diffusion-weighted images (DWI). Tumour recurrence tends to show T2 intermediate signal intensity and restricted diffusion (b-1000 high + ADC map low signal intensity), compared to inflammation (high signal intensity) or mature scar tissue (low signal intensity) on the T2 images and ADC maps. Contrast-enhancement tends to be moderate in recurrence compared to mature scar tissue (mild/absent) and granulation tissue/inflammation (marked), while DCE-MRI parameters show earlier and more intense contrast-enhancement in recurrence compared with benign posttreatment change. DWI is complementary to FDG-PET, helping to reduce the FDG-PET false positive results for recurrence caused by inflammation. False negative findings arise from microscopic disease such as that found at tumour margins or in pre-treatment occult nodal metastases, and also from small/infiltrating tumours mixed with benign post-treatment change. False positive findings arise from post-treatment changes, especially granulation tissue/inflammation, a sterile component of necrotic node (ADC map low signal intensity) and complications such as radionecrosis, radiation-induced tumours and granulomatous polyps.
***Learning Objectives:***
1. To get acquainted with the problem of recurrence in imaging studies.
2. To understand how to estimate signs of recurrence in CT and MRI.
3. To learn about false positive and false negative findings.

17:14
**Panel discussion: What are the challenges in differentiating post-treatment changes from tumour recurrence?**

**16:00 - 17:30 Tech Gate Auditorium**

### Genitourinary

### RC 1207

### Contrast media guidelines

**A-0723** 16:00
**Chairperson's introduction**
H.S. Thomsen; *Herlev/DK (henrik.thomsen@regionh.dk)*

The document ”summary of product characteristics (SPC)” is the legal document that directs the use of drugs, including contrast agents. It is approved by the European Medicines Agency (EMA) and/or the national authorities. It tells us for which examination that you may use the agent/medium based on the studies which the Marketing Authorization Holder (MAH) did prior to the application. Generally, the SPC for gadolinium-based agents are much detailed than that for iodine- or barium based media. However, no SPC cover all aspects. It is here where we need guidelines. Clinical practice guidelines are a key component for medicine, as they provide evidence-based recommendations for physicians and other health care professionals about the management of care for patients with diseases or other clinical conditions. However, all aspects of radiology are not covered by evidence-based knowledge. This applies also to contrast media/agents. Despite the first case of post-contrast acute kidney injury (PCAKI) was reported in 1954, the majority of recommendations dealing with PCAKI is not evidence-based, but eminence based. Generally, despite the fact that contrast agents/media are very safe drugs, adverse reactions occur. They may be acute, late and very late. Through guidelines we want reduce the rate of all adverse reactions. This session is devoted to the most recent developments with regard to 1) PC-AKI, 2) Contrast media and myeloma and 3) non-renal acute adverse reactions.

***Session Objectives:***
1. To learn about emergent issues regarding safe use of contrast media.
2. To understand the mechanisms underlying acute reactions and renal function deterioration after contrast media injection.
3. To provide evidence-based recommendations for safe use of contrast media and for the management of acute reactions.

**A-0724** 16:05
**A. Post-contrast acute kidney injury (PC-AKI)**
A.J. van der Molen; *Leiden/NL (molen@lumc.nl)*

The prevalence of Contrast-Induced Nephropathy, or more appropriately termed Post-Contrast Acute Kidney Injury (PC-AKI), has in recent times been the topic of intensive research. For intravenous CM administration large propensity score based studies showed a much lower frequency than was previously taught. Therefore, the Contrast Media Safety Committee of ESUR has recently systematically reviewed the existing literature and updated their guideline on Prevention of PC-AKI. The recommendations of this update will be explored. Also, results of a follow-up survey study among ESUR members into the barriers and facilitators of the implementation of this guideline in practice will be briefly shown.
***Learning Objectives:***
1. To understand the definition of PC-AKI.
2. To learn about the possible causes for PC-AKI.
3. To review current evidence regarding PC-AKI.
*Author Disclosure:*
**A.J. van der Molen:** Author; Incidental, ESUR CMSC Safety Book. Speaker; Incidental, Guerbet and Canon Medical Systems in past.

**A-0725** 16:30
**B. Iodine-based contrast media in myeloma patients**
F. Stacul; *Trieste/IT (fulvio.stacul@aots.sanita.fvg.it)*

Many radiologists and clinicians still consider multiple myeloma (MM) and monoclonal gammopathies (MG) a contraindication for using iodine-based contrast media. The ESUR Contrast Media Safety Committee performed a systematic review of the incidence of post-contrast acute kidney injury (PC-AKI) in these patients. A systematic search in Medline and Scopus databases was performed for renal function deterioration studies in patients with MM or MG following administration of iodine-based contrast media. Data collection and analysis were performed according to the PRISMA statement 2009. Eligibility criteria and methods of analysis were specified in advance. Cohort and case-control studies reporting changes in renal function were included. Thirteen studies were selected that reported 824 iodine-based contrast medium administrations in 642 patients with MM or MG, in which 12 unconfounded cases of PC-AKI were found (1.6%). The majority of patients had intravenous urography with high osmolality ionic contrast media after preparatory dehydration and purgation. In conclusion, MM and MG alone are not risk factors for PC-AKI. However, the risk of PC-AKI may become significant in dehydrated patients with impaired renal function. Hypercalcaemia may increase the risk of kidney damage and should be corrected before contrast medium administration. Assessment for Bence-Jones proteinuria is not necessary.
***Learning Objectives:***
1. To learn about the relationships between iodine-based contrast media and myeloma.
2. To understand the mechanisms of AKI in myeloma patients.
3. To review current evidence regarding risk of PC-AKI in myeloma patients.
*Author Disclosure:*
**F. Stacul:** Consultant; Bracco International.

**A-0726** 16:45
**C. Ongoing evidence for acute adverse reactions**
O. Clément; *Paris/FR (olivier.clement@aphp.fr)*

Contrast media (CM) use may lead to immediate hypersensitivity (IH) reactions, which should be distinguished from other adverse effects (pain, the sensation of heat, altered taste) and numerous pretreatment protocols have been implemented, but with uncertain efficacy. Different mechanisms were hypothesised to account for this until new diagnostic tests demonstrated an allergic mechanism in some patients through elevated tryptase and histamine concentrations and positive skin tests. Differentiating allergic from non-allergic IH is crucial, as allergy means immune memory and systematic recurrence at re-administration of the responsible or related agents (cross-reactivity), whereas non-allergic IH depends on direct toxicity. Numerous studies have been published over the last few years demonstrating these new pathophysiological pathways, both for iodinated and gadolinium-based agents, originating from European and Asian investigators. As a result, a clear modification of the clinical guidelines on hypersensitivity reactions is being witnessed, with an emphasis on skin testing for patients having presented immediate or delayed reactions. American (ACR Manual on Contrast Media Version 10.2), and European guidelines (ESUR, French, Spanish, British, German) are going to be compared on different aspects of hypersensitivity to contrast media : -pathophysiology - risk factors - skin testing - biological testing (histamine, tryptase and other mediators) - provocation tests. The practical implications in terms of department organisation and medical responsibilities will be discussed.
***Learning Objectives:***
1. To learn about the acute adverse reactions for contrast media.
2. To understand the current evidence regarding risk factors.
3. To illustrate the first-line treatment of acute adverse reactions.
*Author Disclosure:*
**O. Clément:** Advisory Board; Bayer. Speaker; Bracco, Guerbet.

17:10
**Panel discussion: Towards a safer use of contrast media**

### Saturday, March 2

**08:30 - 10:00** **Room A**

### E³ - ECR Academies: Interactive Teaching Session for Young (and not so Young) Radiologists

### E³ 1321

### Writing reports: a survival guide

**A-0732** 08:30
**A. Lung nodule: managing uncertainty**
M. Prokop; *Nijmegen/NL (mathias.prokop@radboudumc.nl)*

"no abstract submitted"
***Learning Objectives:***
1. To identify the key imaging findings.
2. To become familiar with the new Fleischner guidelines.

**A-0733** 09:15
**B. Interstitial lung disease: searching for clarity**
N. Sverzellati; *Parma/IT*

Diffuse interstitial lung diseases (DILDs) are an important but difficult group of disorders. High-resolution computed tomography (HRCT) is at the centre of the assessment of patients who have a suspected DILD. The term DILD comprises more than 200 separate disease entities. However, their pathological and HRCT patterns are relatively restricted compared to their number. This is an essential concept for understanding either the basics of the interpretation of HRCT scans or the need for a multidisciplinary discussion for securing the correct diagnosis. Having an organised approach is important for the efficient and accurate interpretation of HRCT scans. An example of this approach is a systematic sequential analysis of all the anatomical compartments of the thorax (e.g. airways, followed by the lung parenchyma, the pleura itself, and the mediastinum). Each finding should be further described concerning size, shape, location within the lungs, and relationship to any normal surrounding structures. Radiologists are to recognise basic HRCT patterns and key HRCT signs that may link abnormal imaging patterns to lung diseases. Some of these are specific to a disease, whereas others help narrow the differential diagnosis. Recognising these imaging patterns and HRCT scan signs are thus very important for the differential diagnosis.
***Learning Objectives:***
1. To become familiar with the differential diagnosis.
2. To identify the key imaging findings.

**08:30 - 10:00** **Room B**

### Special Focus Session

### SF 13a

### Radiology and pathology: will we ever be (digitally) married?

**A-0734** 08:30
**Chairperson's introduction**
L. Donoso; *Barcelona/ES (ldonoso@clinic.ub.es)*

Pathology and radiology are the core for clinical diagnosis, yet the workflows of both specialities remain in separate "silos," with no direct linkage between their reporting systems, even when both departments belong to the same host institution. Because both radiologists' and pathologists' data are essential to making correct diagnoses and appropriate patient management and treatment decisions, this isolation of radiology and pathology workflows can be detrimental to the quality and outcomes of patient care. With the information technologies advances currently occurring in healthcare, there is a clear opportunity to develop an integrated diagnostic reporting system that supports both specialities and, therefore, improves the overall quality of patient care. In this session, we will review the potential benefits of merging both specialisations and the most relevant technical and organisational factors involved in the integration process.
***Session Objectives:***
1. To learn about digital pathology.
2. To understand the differences with digital radiology.
3. To appreciate the opportunities of a possible integration.

**A-0735** 08:34
**Are digital pathology and AI the definitive factors for the merging?**
P.J. van Diest; *Utrecht/NL*

"no abstract submitted"
***Learning Objectives:***
1. To learn about key aspects in the implementation of digital pathology.
2. To understand the AI impact in pathology.
3. To appreciate the opportunities of a common workflow in the imaging domain.

**A-0736** 08:58
**How would the integration work? (Part 1)**
P.J. van Diest; *Utrecht/NL (P.J.vanDiest@umcutrecht.nl)*

Where radiology has been digital for decades, pathology is at the brink of the digital revolution. Around the world, several labs have turned to full digital diagnostics, and many more labs are working on it. This offers new possibilities for integration of radiological and pathological diagnostics, as the first step towards integrated diagnostics. Both specialities will benefit from seamless access to each other's images. This presentation will provide a visionary overview of such benefits, as well as the first impressions from the integration of digital radiology and digital pathology at the University Medical Center Utrecht.
***Learning Objectives:***
1. To understand the difficulties of the professional integration.
2. To become familiar with the common workflow.
3. To appreciate the disease-specific needs for radiology-pathology integration.
*Author Disclosure:*
**P.J. van Diest:** Advisory Board; Sectra and Philips.

**A-0737** 09:10
**How would the integration work? (Part 2)**
W.B. Veldhuis; *Utrecht/NL*

"no abstract submitted"
***Learning Objectives:***
1. To understand the difficulties of the professional integration.
2. To become familiar with the common workflow.
3. To appreciate the disease-specific needs for radiology-pathology integration.

**A-0738/A-0739** 09:22
**What difference would there be for patients?**
R. Palmqvist; *Umea/SE (richard.palmqvist@umu.se)*
K. Riklund; *Umea/SE (katrine.riklund@umu.se)*

Diagnosis, staging, treatment evaluation and follow up is managed with a combination of patient information sources. Imaging with radiology and nuclear medicine and pathology are the main contributor of information during the entire lifespan of a disease. The already done digital transformation of radiology has meant higher availability to the examinations. The images are accessible when and where they are needed. The consequence of this has been an increased exposure of images to the patients, to the treating clinician and others. The conversion to digital pathology will have the same positive consequences but also with another added value - the combination of digital radiology and pathology in the multidisciplinary meeting room, when meeting the patient, for teaching and evaluation - all information accessible when and where needed. Radiology is often used to determine the site for biopsy or cytologic examination, but the radiologist does not routinely receive the results. The opposite counts for the pathologist that rarely take part in the radiology findings. The novel possibility to have accessibility to both radiology and pathology will be an important base not only for more accurate interpretations but also for continuous education. Examples of how radiologists and pathologists can make use of the digital development in radiology and pathology to make the best for the patients will be discussed at the session.
***Learning Objectives:***
1. To learn about clinical efficiency improvement.
2. To understand the impact on research and innovation.
3. To appreciate the improvement in patient care.
*Author Disclosure:*
**K. Riklund:** Advisory Board; Swedish medical product agency. Board Member; DicomPort AB.

09:46
**Panel discussion: What benefits are there to merge the two specialties?**

**08:30 - 10:00** **Room C**

### Professional Challenges Session

### PC 13

### Working in radiology: burnout and bore-out or engaged and passionate?

**A-0740** 08:30
**Chairperson's introduction (part 1)**
M.G.M. Hunink; *Rotterdam/NL (m.hunink@erasmusmc.nl)*

Symptoms of chronic stress and burnout occur in approximately half of all healthcare professionals leading not only to diminished health and quality of life among the professionals themselves but also to concerns about patient safety, quality of care, patient outcomes, professionalism, and sustainability of our healthcare systems. Radiologists rank in the top 10 of medical specialists with burnout, and they are among the most unhappy physicians. Key symptoms of burnout are emotional exhaustion, a detached, callous, cynical attitude, and a feeling of personal inadequacy. Chronic stress and burnout are due to an imbalance between stressors and resources. Stressors include ambition, perfectionism, self-criticism, lack of self-care, 24/7 electronic connectivity, interpersonal conflicts, employer demands, litigation, digitalisation, the complexity of care, and financial concerns. Resources include resilience training, healthy lifestyle, social support, meaningful activities, effective communication, and efficient work processes. To combat burnout among radiologists and radiographers, and thereby improve the quality of care, effective interventions are needed at both the organisational and individual level. In this session we will discuss the signs and symptoms of chronic stress and burnout, the factors that lead to the problem, and preventive measures that can be taken that will lead to active work engagement, meaningful work, and well-being among professionals working in radiology.
***Session Objectives:***
1. To learn to recognise the symptoms of chronic stress and burnout.
2. To understand what factors contribute to chronic stress and burnout among radiologists and radiographers.
3. To learn what preventive measures can be taken to prevent chronic stress and burnout among radiologists and radiographers.
4. To appreciate what is needed to achieve work engagement, meaningful work and well-being among radiology staff.

*Author Disclosure:*
**M.G.M. Hunink:** Advisory Board; EIBIR. Author; Textbook: Decision Making in Health and Medicine. Research/Grant Support; American Diabetes Association.

**A-0741** 08:33
**Chairperson's introduction (part 2)**
E. Metsala; *Helsinki/FI (eija.metsala@metropolia.fi)*

Work wellbeing is essential to keep on working in fast developing professions such as radiology. Most of the factors contributing work well-being and job-tenure and vice versa to burnout and willingness to change workplace are the same for all the healthcare staff. However, there are differences amongst professional groups. In the light of the current studies work autonomy, job design, leadership and possibilities for professional development as well as possibility to fluently combine family life, hobbies and work e.g. by the means of autonomous work time planning seem to be associated with low levels of stress, work satisfaction and commitment to ones' workplace amongst radiographers. Commitment to ones' workplace predicts job tenure. Having low pay, too little time for patients, working under tensed circumstances. Insufficient resources and amount of employees, as well as administrative pressures, cause stress and dissatisfaction amongst radiographers. Also, one big issue affecting all healthcare staff work circumstances if technological development, e.g. fast coming of Artificial Intelligence coming as a part of our work environment. About all these, we hear more in the upcoming session.
***Session Objectives:***
1. To learn to recognise the symptoms of chronic stress and burnout.
2. To understand what factors contribute to chronic stress and burnout among radiologists and radiographers.
3. To learn what preventive measures can be taken to prevent chronic stress and burnout among radiologists and radiographers.
4. To appreciate what is needed to achieve work engagement, meaningful work and well-being among radiology staff.

**A-0742** 08:35
**A personal story**
M.F. Berger; *Nottwil/CH (markus.berger@paraplegie.ch)*

In this talk, I shall present my episode of burnout that I experienced about seven years ago, from a first-person point of view: early warning signs, failed attempts at self-help, sudden break-out of symptoms, medical workup, interventions and, finally, resolution and consequences. Mindfulness played a major role in my recovery. I shall describe my personal experience with mindfulness and explain how the practice not only can help you cope with stress in your private and professional life but how it may actually make you a better radiologist.
***Learning Objectives:***
1. To learn to recognise the early symptoms of burnout in oneself.
2. To understand how life events and work stress in radiology contribute to burnout.
3. To appreciate the challenges in dealing with burnout.
4. To understand what is needed to prevent chronic stress and burnout.
5. To appreciate the value of mindfulness in being a better radiologist/radiographer.

**A-0743** 08:53
**The role of technology in the job satisfaction and well-being of radiology workers**
S. Aarts; *Eindhoven/NL (s.aarts@fontys.nl)*

The rate of the pace at which technological developments are introduced in healthcare is enormous; especially people working in technology-driven professions are confronted continuously with technological innovations. Unfortunately, technological developments are not always positively perceived by healthcare professionals. For example, the occurrence of 'power supply issues' and lack of knowledge of the technology at hand, can make health professionals sceptical about using new technology. For a technological application to be adequately implemented, health care professionals need to see the benefits of such an application and should feel competent to use it. To date, the role technology plays in the work of health professionals is intensively studied. Research shows that aspects such as 'responsibility' and 'privacy' are important for healthcare professionals when technology is involved: they feel it as their responsibility to safely, efficiently and effectively use the available technological devices and applications. They expressed the need to understand the background and principles of the technological applications they use, rather than 'push a button'. Hence, technology affects multiple aspects of the day-to-day profession of radiology workers, which, in turn, affects job satisfaction and well-being. We will discuss how the role of technology is perceived by health care professionals, especially by radiographers, nuclear medicine technologists and radiation therapist. In addition, several barriers to the implementation of new technological developments will be brought forward during this talk. Moreover, possible solutions for these barriers will be discussed to maintain high job satisfaction and well-being.
***Learning Objectives:***
1. To understand how technology is perceived by radiographers, nuclear medicine technologists and radiation therapists.
2. To learn about the role of technology in the well-being of these healthcare professionals.
3. To learn which factors should be addressed if a new technology is introduced on the work floor in order to acquire/maintain a high job satisfaction and well-being.
4. To understand how the role of technology in the future is perceived by these healthcare professionals and how these ideas/opinions may influence their work, job satisfaction and well-being.

**A-0744** 09:11
**Dealing with daily battles: factors that lead to chronic stress and burnout**
A. Montgomery; *Thessaloniki/GR (antmont@uom.gr)*

Today all evidence in regard to causal factors associated with burnout points to the direction that burnout is an organisational, rather than an individual problem, rooted in issues related to the working environment and organisational culture. Burnout should be viewed as an obvious outcome of systems that are developed within medical education and fostered all through the career of physicians. Burnout, in terms of exhaustion, cynicism, and inefficacy, are experienced by individuals, but this focus on the individual can cause us to forget that it's a shared experience in response to common job stressors meaning that we should frame it as a systems problem, and not simply as an individual one. Thus, we need to move the focus away from a physician-centric approach towards what can be done for the modern healthcare setting to enhance the ability of doctors to thrive. In other words, it is time to move from the burnt out physician to the resilient health care organisation.

***Learning Objectives:***
1. To learn about factors that lead to chronic stress and burnout among healthcare professionals.
2. To appreciate that preventive intervention should be setting-specific and context-dependent.
3. To become familiar with possible interventions at the organisational and individual level.

**A-0745** 09:29
**From chronic stress and burnout to work engagement, meaningful work and employee well-being**
J. Hakanen; *Helsinki/FI*

***Learning Objectives:***
1. To learn what preventive measures can be taken to prevent chronic stress and burnout.
2. To shift the focus from prevention of chronic stress towards positive work experience.
3. To appreciate what is needed to achieve work engagement, meaningful work and well-being.

09:47
**Panel discussion: Achieving well-being among radiology staff**

**08:30 - 10:00** **Room N**

### EuroSafe Imaging Session

### EU 4

### Advanced clinical dosimetry in interventional radiology

**A-0746** 08:30
**Chairpersons' introduction (part 1)**
W.R. Jaschke; *Innsbruck/AT (werner.jaschke@i-med.ac.at)*

The term “clinical dosimetry” in scientific literature was until recently, mainly used for radiotherapy, but is now also applied to diagnostic and interventional procedures although the term “dosimetry for imaging in clinical practice” including patient and staff dosimetry would be more appropriate. Several publications of the International Commission on Radiological Protection (ICRP) recommend that patient protection and occupational protection be managed in an integrated approach. First, interventional radiologists have to learn which parameters of the DICOM dose report are reliable indicators for patient dose. Second, they have to understand that occupational dose is mainly due to scattered Radiation originating mainly from the patient. Thus, patient dose and occupational dose are somewhat related to each other, especially in fluoroscopy guided interventional procedures. New electronic personal dosimeters and automatic registry systems for patient and staff doses allow access to detailed information on doses derived from all the radiation events (fluoroscopy, cine, DSA and CBCT runs). Real-time information on occupational doses (and dose rates) during interventional procedures inside the catheterisation rooms are also available in addition to the patient dose information and skin dose maps. Such information allows active optimisation strategies during and after the interventional procedures. The new interventionists should be trained on the basic dosimetry aspects to properly use the information available inside the catheterisation room during the procedures, and apply it to improve their clinical practice and the radiation safety aspects. Medical physics experts should be an integral part of the optimisation process.
***Session Objectives:***
1. To learn about the clinical value of dosimetry.
2. To understand the technique of dosimetry and the implementation of real time personal dosimetry.
3. To appreciate the impact of dosimetry for dose management in interventional radiology.

**A-0747** 08:33
**Chairpersons' introduction (part 2)**
E. Vaño; *Madrid/ES (eliseov@med.ucm.es)*

The term “clinical dosimetry” in scientific literature was until recently, mainly used for radiotherapy, but is now also applied to diagnostic and interventional procedures although the term “dosimetry for imaging in clinical practice” including patient and staff dosimetry would be more appropriate. Several publications of the International Commission on Radiological Protection (ICRP) recommend that patient protection and occupational protection be managed in an integrated approach. This approach, especially relevant in fluoroscopy guided interventional procedures, has also been included in the recent European Directive on Basic Safety Standards. New electronic personal dosimeters and automatic registry systems for patient and staff doses allow access to detailed information on doses derived from all the radiation events (fluoroscopy, cine, DSA and CBCT runs). Real-time information on occupational doses (and dose rates) during interventional procedures inside the catheterisation rooms are also available in addition to the patient dose information and skin dose maps. Such information allows active optimisation strategies during and after the interventional procedures. The new interventionists should be trained on the basic dosimetry aspects to properly use the information available inside the catheterisation room during the procedures, and apply it to improve their clinical practice and the radiation safety aspects. Continuous comparison of the current patient doses with diagnostic reference levels is now possible. The introduction of automatic patient dose registries (with proper validation of the dosimetric data) will help optimise radiation protection and support to decide which patients could require a clinical follow-up for potential radiation skin injuries.
***Session Objectives:***
1. To learn about the clinical value of dosimetry.
2. To understand the technique of dosimetry and the implementation of real time personal dosimetry.
3. To appreciate the impact of dosimetry for dose management in interventional radiology.

**A-0748** 08:35
**The basis of clinical dosimetry in interventional radiology**
K. Bacher; *Ghent/BE (Klaus.bacher@ugent.be)*

Interventional radiology procedures can be linked to high patient radiation exposures. In order to be able to avoid deterministic (skin) effects and to evaluate the potential risks for radiation-induced cancer and genetic effects, accurate dosimetry in needed. First of all, dosimetric indexes such as dose area product and kerma at reference point are important quantities as they are measures during the fluoroscopy procedures and stored in a DICOM Radiation Dose Structured Report format. These dosimetric indexes are a useful tool for optimisation and the generation of diagnostic reference levels, but they don't describe the actual patient dose. Skin dose can be directly measured by means of small dosemeters, but the latter technique is cumbersome and time-consuming. The generation of skin dose maps, to identify peak skin doses during/after the procedure can overcome these issues. Accurate patient-specific dosimetry requires detailed information concerning patient characteristics and all irradiation events. Nowadays, modern systems allow for the collection of most of the required data. In this lecture, an overview of various dosimetric quantities will be presented, together with their use in clinical dosimetry and optimisation.
***Learning Objectives:***
1. To learn how to assess dose to patients and staff.
2. To appreciate the importance of dosimetry for optimisation.
3. To understand the potential pitfalls in clinical dosimetry.

**A-0749** 08:45
**Real time staff dose monitoring**
F. Vanhavere; *Mol/BE (fvanhave@SCKCEN.BE)*

Staff working in interventional procedures are classified as occupationally exposed workers. Their doses need to be monitored by personal dosimetry. This dosimetrical monitoring is done mostly by passive dosimeters. These have to be sent back after a wearing period of mostly one month so that the results are normally only available to the staff weeks or even months after the exposure. Using active dosimeters would be beneficial to the workers because they can get immediate feedback from their exposure and thus apply better the ALARA principle. Several types of active dosimeters can be used in interventional procedures. But when using active devices, it is important to verify that their characteristics are good enough for use in the specific hospital fields. Especially the low energies, high angles and the pulsed aspect of the X-ray fields can give problems for many active dosemeters. A series of tests in realistic hospital fields have been done within the EURADOS WG12, and the results of these tests and the limitations of the active dosimeters will be reported. But not only active dosemeters are being further developed and used, but also computational methods are more and more used to visualise the radiation fields around the patient. If this can be made available to the workers in real time, this will also be a good ALARA tool. It is possible to go even a step further and determine the doses to the staff with purely computational methods.
***Learning Objectives:***
1. To understand how on-line dosimetry can help in radiation protection of medical staff in interventional procedures.
2. To learn how the ALARA principle (as low as reasonably achievable) can be applied for medical staff in interventional procedures.
3. To learn about the current status of real-time dosimetry.
4. To become familiar with the tools, which are available for radiation protection of medical staff in interventional procedures.

**A-0750** 09:05
**Real time patient dose monitoring**
R.M. Sánchez Casanueva; *Madrid/ES*
*(robertomariano.sanchez@salud.madrid.org)*

Some interventional procedures may require large fluoroscopy times, many DSA images and/or several procedures on the same patient to address complex lesions. In such cases, the radiation dose to patients' skin has to be monitored in real time and in case of high radiation doses, to propose a follow-up for potential skin injuries. In the case of repeated procedures, information about the radiation received in previous interventions may be necessary to manage the radiation dose and avoid potential skin injuries. The management of radiation dose will need several physical quantities as fluoroscopy time, number of images, air kerma at the patient entrance reference point, kerma area product and peak skin dose. An overview of the relevance of those quantities and their units will be provided. Modern C-arms offer some tools to monitor patient doses, from the simplest consisting in an indicator of fluoroscopy time and number acquired images, through the amount of radiation delivered by the X-ray tube, to the most complex skin dose calculators that provide quite accurate dose maps in patients' skin in real time or at the end of the procedures.
***Learning Objectives:***
1. To learn about the motivation for real-time patient dosimetry.
2. To understand quantities and units to monitor patient dose.
3. To learn about prototypes and systems currently available.

**A-0751** 09:25
**Dose management of patients and staff in interventional radiology**
J. Damilakis; *Iraklion/GR (damilaki@med.uoc.gr)*

Fluoroscopically-guided interventional procedures are associated with the relatively high patient and occupational doses mainly due to high utilisation, long fluoroscopy time and a large number of cine acquisitions. Optimisation of protection is essential to reduce patient dose while keeping image quality at acceptable levels. Advanced dosimetry methods will be presented and discussed. Optimising patient dose will result in a decrease in scatter radiation to the staff. To decrease the patient dose, operators should increase the distance of the X-ray tube from the patient as much as practicable and decrease the distance of the imaging detector. To avoid radiation overexposure accidents in interventional suites, interventional radiologists in cooperation with medical physicists should establish standard clinical protocols for each specific type of procedure performed. Cumulative absorbed dose to the skin should be limited to the minimum necessary for the clinical task. To decrease occupational radiation doses, exposed personnel must use protective garments, i.e. a lead apron to protect body trunk, leaded gloves, radio-protective glasses and a thyroid shield. Interventional radiologists should keep hands outside the primary beam. If hands are inside the beam, the automatic exposure control will increase exposure parameters and, therefore, radiation doses to both patient and staff will increase. Radiation dose to staff is reduced drastically when ceiling-suspended protective lead shielding or mobile floor shielding is used.
***Learning Objectives:***
1. To learn about radiation doses and risks associated with fluoroscopically-guided procedures.
2. To describe the actions that should be taken to enhance patient and staff safety during interventional radiology procedures.
3. To learn parameters that can be modified to decrease patient dose without compromising image quality.

09:45
**Discussion**

**08:30 - 10:00** **Room O**

### Vascular

### RC 1315

### US and vascular disease: a perfect match

*Moderator:*
V. Bérczi; Budapest/HU

**A-0752** 08:30
**A. Abdominal aorta**
D.A. Clevert; *Munich/DE (Dirk.Clevert@med.uni-muenchen.de)*

Abnormalities of the abdominal aorta may represent a diagnostic challenge in patients both with acute and chronic clinical symptoms. In addition to the examination using colour-coded duplex ultrasound, contrast-enhanced ultrasound (CEUS) with low-mechanical-index (low MI) may contribute to achieving a precise diagnosis. CEUS is a new and promising method in the diagnosis and follows up of aortic and visceral artery lesions. Colour code duplex ultrasound and CEUS with SonoVue® allow for rapid and noninvasive diagnosis, especially in critically ill patients since these methods can readily be applied bedside. In this RC, the contribution of colour-coded duplex ultrasound and CEUS as compared to Multi-Slice Computed Tomography Angiography (MS-CTA) in various pathologies of the abdominal aorta will be addressed.
***Learning Objectives:***
1. To learn how to perform the examination and its role in diagnostic assessment.
2. To learn about US findings in AAA treatment planning and post-treatment evaluation.
3. To appreciate the role of CEUS and technological innovations in routine practice.
*Author Disclosure:*
**D.A. Clevert:** Speaker; Bracco, Siemens, Philips, Samsung.

**A-0753** 09:00
**B. Upper and lower limb: arterial district**
B. Brkljačić; *Zagreb/HR (boris@brkljacic.com)*

B-mode ultrasound enables evaluation of arterial anatomy and of the vessel wall, detects early atherosclerotic changes, and allows detection of stenosis and occlusions, but tends to overestimate the degree of stenosis and should not be used for stenosis grading. Colour duplex Doppler ultrasound (CDUS) enables fast visualisation of the vessel to be examined. It demonstrates haemodynamic changes, including velocity increase. Underestimation of disease is likely if only colour is used and the precise assessment of haemodynamic disturbances is impossible without a spectral analysis. In general, CDUS has a high diagnostic accuracy, it is cheap and widely available, and there is no exposure to ionising radiation and no contrast media injection. However, the examination is time-consuming and requires high operator dependence. The thorough understanding of haemodynamics and physics is needed for adequate interpretation of Doppler findings. The factors influencing spectral morphology will be discussed in the lecture and the different types of spectra presented. Waveform changes will be presented in stenosis and occlusion. The typical findings of pseudoaneurysms and arteriovenous fistulas will be presented. Findings in acute occlusion and collateral flow will be discussed, both for native arteries and bypass grafts. The role of ultrasound in endovascular recanalisations will be discussed, both in planning and performing of the procedure and in the follow-up. Several clinical examples will be presented.
***Learning Objectives:***
1. To understand how to perform the examination and its role in diagnostic assessment.
2. To understand US findings for diagnosis and follow-up.
3. To underline tips and tricks to start your activity.

**A-0754** 09:30
**C. Ultrasound as guidance for vascular interventions**
W.K. Matzek; *Vienna/AT (Wolfgang.Matzek@meduniwien.ac.at)*

While there is a long tradition of blind puncture of arterial vessels which works quite well in many cases, ultrasound-guided puncture might be advantageous in certain situations. The main reasons for ultrasound-guided puncture are, e.g. unusual puncture site, severely calcified wall, avoidance of double-wall puncture, non-palpable vessel, or small vessel diameter. Eventually, ultrasound-guided arterial puncture can aid the less experienced interventionist at finding appropriate access to the vessel. Vascular malformations are congenital anomalies of veins, arteries, capillaries, lymphatic vessels, or a combination of these. These lesions become clinically apparent at almost any age, starting with newborns. Most of the venous, arteriovenous, and lymphatic lesions, simple or combined, can be treated using interventional techniques and ultrasound guidance contributes significantly to the success of this treatment. A couple of selected cases and treatment options will be presented. Varicose veins can be treated using different strategies. Ultrasound-guided foam sclerotherapy will be discussed, as well as endogenous ablation therapy using radiofrequency and laser ablation.
***Learning Objectives:***
1. To understand the value of ultrasound guidance for arterial puncture.
2. To learn about the possibilities of ultrasonographic guided therapy of AVMs.
3. To become familiar with endovenous treatment of varices.

**08:30 - 10:00** **Studio 2019**

### Special Focus Session

### SF 13b

### Cutting edge imaging and minimally invasive interventions of the spine

**A-0755** 08:30
**Chairperson's introduction**
J. Van Goethem; *Antwerp/BE (johan.vangoethem@uantwerpen.be)*

This session will present the latest developments in spine imaging. The topics that have been chosen cover a wide range of frequently occurring spinal pathology. The best and most renowned speakers will bring you up-to-date on the newest developments in the field. These will be presented in a way to make it easy to implement them in your daily practice.
***Session Objectives:***
1. To become familiar with state-of-the-art spinal imaging and minimally invasive spine interventions.
2. To learn about advanced imaging of the spine.
3. To learn how to improve on spinal imaging.

**A-0756** 08:35
**Imaging strategies in spine and spinal cord trauma**
P.C. Maly Sundgren; *Lund/SE (Pia.Sundgren@med.lu.se)*

Spinal injuries to the spinal column or spinal cord are devastating for the patient, their family and costly for the society. Depending on the degree of trauma, the age and condition of the patient, clinical, and neurological symptoms different imaging strategies are suggested. Different imaging rules and schemes have been suggested where the degree of trauma and the patients symptoms play an important role in the imaging decision making. The presentation aims to focus on when and how to image and present the differences that can be seen in injuries between the pediatric and the adult population.
***Learning Objectives:***
1. To review the imaging strategy in spinal trauma.
2. To know the most optimal imaging techniques in spinal trauma.
3. To learn about new developments in imaging spinal trauma.

**A-0757** 08:53
**Advanced imaging of spinal cord lesions: do we need DWI, perfusion and spectroscopy?**
M.M. Thurnher; *Vienna/AT (majda.thurnher@meduniwien.ac.at)*

Despite its high sensitivity but low specificity, magnetic resonance imaging (MRI) is the modality of choice for diagnosis of spinal cord diseases. Spinal cord examination is one of the most challenging MR examinations from a technical, interpretative, and differential diagnostic standpoint. In practice, there are no satisfactory ways to distinguish among different forms of myelitis and neoplastic conditions when conventional MR sequences are used. In the last decade, several indications for diffusion-weighted MR imaging (DWI) and diffusion tensor imaging (DTI) in the spine have been reported. DTI could provide reliable postoperative evaluation and analysis for cervical spondylotic myelopathy patients. DTI has been proven useful for differentiation of ependymoma and astrocytoma of the spinal cord. This lecture will review advantages and disadvantages of the advanced MR sequences (such as DWI, DTI, Perfusion) when used for the spine. Possible pitfalls, helpful clinical information, and recognition patterns will be discussed.
***Learning Objectives:***
1. To understand the indications for advanced imaging in spinal cord lesions.
2. To differentiate spinal cord lesions.
3. To learn the different imaging patterns of spinal cord lesions.

**A-0758** 09:11
**Spinal CSF leaks: the precise "roadmap" for clinicians**
J.S. Ross; *Phoenix, AZ/US (Ross.Jeffrey1@mayo.edu)*

Spontaneous intracranial hypotension caused by spinal CSF leaks can be a frustrating diagnosis from both diagnostic and therapeutic standpoints. While the classic finding is an orthostatic headache which may be exacerbated with a cough, sneezing or valsalva, a wide variety of other symptoms can be attributed to the disorder including photophobia, imbalance, hearing abnormalities and mental status change. Underlying etiologies may be a dural tear from osseous spur or disc, meningeal diverticulum or CSF-venous fistula. Up to 1/3 of patients may have no aetiology defined. The workup of these patients may vary widely and can include multiple imaging modalities and strategies ranging from nuclear medicine, CT, MR, and myelography. Our workup includes an initial evaluation with contrast-enhanced brain MR and epidural blood patch X2. If the blood patch fails, then a complete spine MR is performed looking for an epidural fluid collection (fast leak). If a fast leak is discovered, then additional dynamic imaging is performed (either dynamic myelography or dynamic CT myelography). If no extradural fluid is identified (presumed slow leak), then routine CT myelography is performed. If this routine study is negative, then MR myelography is performed with attention to defining a CSF-venous fistula.
***Learning Objectives:***
1. To review the most optimal imaging strategy in spinal CSF leaks.
2. To interpret different imaging studies in spinal CSF leaks.
3. To learn the value of different imaging techniques in detecting spinal CSF leaks.

**A-0759** 09:29
**Cutting edge minimally invasive spine interventions**
L. Manfrè; *Catania/IT (lmanfre@me.com)*

During the last decade, we attended an incredible improvement in spine biomechanics knowledge and powerful diagnostic tools, as well as the development of a new generation of “minimally invasive” devices and “covert surgery” procedures, that changed our mind about who, when and how to treat a patient affected by spine disease. The use of X-ray and/or CT-guided procedures give Radiologists a wide range of new possibilities in treating the spine, reducing the risk of side effects and complications in comparison to conventional “open” surgery, lowering the economic costs of the procedures as well as rehab time, avoiding the risk of general anesthesia, as the most of the procedures can be performed in mild sedation. Last, but not least, CT-X ray guided procedures allows significant cost reduction for the Health Care System, as smaller medical staff is needed, with no operating room occupation as well as beds/patients rate reduction, a must in our money-saving critic times. Interventional Radiology, however, cannot progress without an advanced knowledge of Diagnostic Radiology of the Spine, that is fundamental when a decisional route must be proposed to the patient. We will analyse all the most recent CT-guided technique in the treatment of tumours, degenerative and traumatic disease of the Spine, treated with fully CT-guided techniques in simple analogue-sedation.
***Learning Objectives:***
1. To review the current state-of-the-art in minimally invasive spine interventions.
2. To learn how to choose an optimal minimally invasive treatment in low back pain.
3. To learn about new interventional spine techniques available in low back pain.

09:47
**Panel discussion: How can we make spinal imaging better?**

**08:30 - 10:00** **Room E1**

### Breast

### RC 1302

### New mammography: digital breast tomosynthesis and future techniques

**A-0760** 08:30
**Chairperson's introduction**
E.M. Fallenberg; *Munich/DE*

Early detection of breast cancer through X-ray mammography (MG) has been shown to reduce mortality; however, the method is limited by a decreased sensitivity and specificity particularly in young patients and women with radiographically dense breasts due to tissue overlying and masking tumours or architectural distortions. The introduction of full-field digital mammography gave the possibility to develop further technical methods, to overcome these limitations. Some of these are tomosynthesis, contrast-enhanced mammography and contrast-enhanced tomosynthesis as well as most recently also breast CT. In this talk will be presented the background of 2Dmammography, tomosynthesis and contrast-enhanced mammography techniques and breast CT. Potential advantages and disadvantages will be indicated, and a sample of clinical cases will be presented to illustrate how the different techniques contribute to the detection of lesions.
***Session Objectives:***
1. To learn about 2D mammography and digital breast tomosynthesis.
2. To recognise the advantages and disadvantages of digital breast tomosynthesis compared to 2D mammography.
3. To understand the future potential of new breast imaging techniques in lesion assessment.

*Author Disclosure:*
**E.M. Fallenberg:** Advisory Board; Guerbet, GE, Transsonic. Board Member; EUSOBI, DRG. Research/Grant Support; Siemens, GE, Guerbet, Bayer. Speaker; Siemens, GE, Guerbet, Bayer.

**A-0761** 08:35
**A. Should we abandon 2D mammography?**
S. Zackrisson; *Malmö/SE (sophia.zackrisson@med.lu.se)*

Full-field digital mammography (FFDM), also called 2D mammography, with flat panel detectors, has been broadly used in clinical routine for about a decade. Even if there has been advances in the technical development of the digital detectors, the detectors are not the main limiting factor in breast imaging; the challenge is the anatomical noise, i.e. dense breast tissue in the optimisation of breast cancer detection. FFDM is a 2-dimensional modality and inherent in that is that dense breast tissue may cause overprojection and hide or simulate the presence of a tumour on these images even if several views are acquired. Digital breast tomosynthesis, DBT is a 3D mammography technique that, to a large extent, can suppress the confounding effect of the overlapping tissue. DBT is a strong competitor to 2D mammography both for screening and clinical examinations. This presentation will cover 2D mammography in terms of technical background and optimisation of image quality. Furthermore, it will discuss the advantages and disadvantages of 2D mammography compared to DBT and evaluate if there are situations where 2D mammography still could be the preferred method if DBT is transitioned into screening and clinical practice.
***Learning Objectives:***
1. To understand the technique of 2D mammography and what the limits could be.
2. To learn if there are ways to improve the image quality of 2D mammography.
3. To understand when 2D mammography is better than digital breast tomosynthesis.

*Author Disclosure:*
**S. Zackrisson:** Speaker; Siemens Healthcare AG, Astra Zeneca.

**A-0762** 09:00
**B. Clinical validation of digital breast tomosynthesis and results in the last 10 years: where do we stand?**
P. Skaane; *Oslo/NO (per.skaane@outlook.com)*

Digital breast tomosynthesis (DBT), an advanced mammographic technique, has the potential to improve two inherent limitations of conventional digital mammography (DM): The poor sensitivity in women with dense breasts (caused by superimposed parenchyma, “masking effect”) and the low specificity (caused by parenchyma simulating masses, “pseudotumors”). In the diagnostic setting, DBT can replace supplemental views for evaluation of indeterminate noncalcified lesions, “downgrade” indeterminate (BI-RADS 3) lesions, and “upgrade” other equivocal masses indicating the need for biopsy. Evaluation of cancer extent and multifocality is better demonstrated, and preoperative staging is improved. Retrospective US screening studies comparing DM versus DM+DBT have shown significantly lower recall rates for DBT and often increased cancer detection rates. Lack of increased cancer detection in some US studies might have been caused by study design and lack of power. Prospective European screening trials comparing DM versus DM+DBT have all demonstrated a significantly higher cancer detection rates for DBT. Challenges regarding the implementation of DBT in screening include a number of projections, reading time, and women with very dense breasts. The increased radiation dose problem using DM+DBT is solved by synthesised two-dimensional images (SM), created by summing and filtering the reconstructed tomosynthesis slices. Several studies have shown that SM+DBT have comparable results with DM+DBT regarding the recall and cancer detection rates, and SM will replace conventional DM in screening. Studies on cost-effectiveness, potential overdiagnosis, and subsequent interval cancer rates may be required to determine long-term benefits of DBT screening.
***Learning Objectives:***
1. To become familiar with the technique of digital breast tomosynthesis.
2. To understand the results of digital breast tomosynthesis in the screening and diagnostics settings.
3. To know the evolution of digital breast tomosynthesis in screening organisation.

*Author Disclosure:*
**P. Skaane:** Grant Recipient; Per Skaane received funding for additional case interpretations (overtime reading) during the Oslo Tomosynthesis Screening Trial ) OTST) from Hologic, Inc..

**A-0763** 09:25
**C. Contrast-enhanced mammography and other developing techniques**
E. Giannotti; *Nottingham/UK (Elisabetta.Giannotti@nuh.nhs.uk)*

Switching from analogical to digital technology has lead breast imaging to a new era where it has been possible to develop and introduce in the clinical practice new techniques such as tomosynthesis, CT mammography, contrast-enhanced spectral mammography (CESM), CAD and, eventually, advanced breast biopsy techniques, blurring the lines between diagnosis and treatment. CESM combines a iodinate contrast agent with the standard mammographic technique providing not only a morphological but also a functional examination of the breast, being able, in a similar fashion as MRI does, of depicting the enhancement related to the neoangiogenesis of tumour growth. CESM has been shown from literature studies to be a valuable tool in diagnosis and staging of primary breast cancer with a better diagnostic accuracy compared to conventional mammography, especially in women with dense breasts. It is also being explored the potential value of CESM as a screening tool. Further development of digital techniques is ready to be developed and implemented in breast imaging. In the near future, the introduction of Artificial Intelligence (AI) could let mammogram to become a tool of precision medicine. In fact, mammography could be used not only in a diagnostic setting but also as image-driven risk assessment tool and tumour biomarkers in order to move to a personalised screening approach and personalised treatment.
***Learning Objectives:***
1. To become familiar with further developments in digital mammography, such as contrast-enhanced mammography and contrast-enhanced digital breast tomosynthesis.
2. To understand the role of these new techniques in detection and characterisation of breast lesions.
3. To learn about the new potential of combining mammography with molecular imaging, optical imaging and texture analysis.

09:50
**Panel discussion: Mammography, digital breast tomosynthesis and contrast-enhanced mammography: where will we stand in 10 years?**

**08:30 - 10:00** **Room E2**

### Neuro

### RC 1311

### Altered mental state

**A-0764** 08:30
**Chairperson's introduction**
M.A. Van Buchem; *Leiden/NL*

***Session Objectives:***
1. To learn about the complex spectrum disease entities presenting with cognitive decline.
2. To understand the role of standardised imaging and reporting in the context of diagnosis and monitoring of patients with neurocognitive disorders.
3. To appreciate importance of multimodal imaging approaches in the diagnosis of neurocognitive disorders.

**A-0765** 08:35
**A. MRI in the diagnosis of cerebral amyloid angiopathy (CAA)**
J. Linn; *Dresden/DE (Jennifer.Linn@uniklinikum-dresden.de)*

Cerebral amyloid angiopathy (CAA) is defined as the deposition of β-amyloid in the walls of cortical and leptomeningeal cerebral vessels. It is very common in the elderly population with prevalence rates up to 60% and associated with Alzheimer´s disease. Intracerebral lobar microhemorrhages (ICH) are the most devastating presentation of CAA and the main cause of morbidity and mortality. However, in addition, CAA patients can also suffer from transient neurological symptoms and/or progressive cognitive impairment ultimately resulting in dementia. Besides ICH, typical MRI signs of CAA include multiple cerebral microbleeds in a cortical-subcortical localisation, focal subarachnoid haemorrhage, cortical superficial siderosis (cSS), silent cortical infarcts, white matter hyperintensities, and enlarged perivascular spaces. Evidence of previous ICHs and/or (disseminated) cSS represents an important risk factor for recurrence of CAA-related intracranial haemorrhages. To date, there exist no diagnostic test that allows the definite diagnosis of CAA related haemorrhage during life. The “classic” and the “modified” Boston criteria estimate the likelihood of the presence of CAA in vivo based on the pattern of intracranial hemorrhagic lesions on neuroimaging studies.
***Learning Objectives:***
1. To recognise cerebral amyloid angiopathy in imaging studies.
2. To understand the pathophysiology of the diagnosis.
3. To know the clinical consequences of the diagnosis.

*Author Disclosure:*
**J. Linn:** Author; Springer, Thieme Publishers. Speaker; Bayer Healthcare.

**A-0766** 08:58
**B. Imaging in an altered mental state**
J. Boban; *Novi Sad/RS (jasmina.konstantinovic@gmail.com)*

Altered mental state (AMS) represents a group of clinical symptoms associated with different degrees of mental functioning disorder, including cognitive, attention, arousal disorders and decreased level of consciousness. Clinical entities that are endorsed by this term are divided into brain death, coma, vegetative state/unresponsive wakefulness state, minimally conscious state, acute confusional state and Locked-in syndrome. A separate entity included in the definition is dementia from the point of mild cognitive impairment to manifest dementia. Aetiology of these conditions is different and varies greatly, including trauma, infectious diseases, metabolic disorders, psychiatric diseases, psychoactive substances and other, less common causes. The diagnosis of AMS is based on clinical observation and standardised neurobehavioral assessments. A focused clinical examination remains essential in distinguishing between separate entities, including testing of sleep-wake cycles, awareness, motor skills, auditory function, visual function, communication and emotional integrity. Since the rate of misdiagnosis reaches 40%, imaging studies are a complementary tool. Classic diagnostic modalities were computed tomography (CT) and magnetic resonance imaging (MRI), especially useful in the setting of trauma, infection and metabolic disorders. However, emerging new techniques, such as functional MRI and PET scan show promising results in defining etiological factor and neuropathological substrate in AMS. The aim of this lecture would be to present the main differential diagnosis in AMS and to summarise current diagnostic possibilities in different clinical settings.
***Learning Objectives:***
1. To learn about the role of imaging in the diagnosis of neurocognitive diseases: from the exclusionary approach to the inclusionary approach.
2. To understand the challenges when diagnosing neurocognitive disease in a very early stage using imaging.
3. To appreciate the complexity of differential diagnosis of neuroimaging in cognitively affected individuals and the crucial need for other biomarkers to support the clinical diagnosis.

**A-0767** 09:21
**C. MRI in the diagnosis of Alzheimer's disease**
M. Vernooij; *Rotterdam/NL (m.vernooij@erasmusmc.nl)*

This lecture will discuss the use of magnetic resonance imaging in current clinical practice for the diagnosis of Alzheimer's disease. It will tap into characteristic imaging features of AD on MRI, as well as most important differential diagnoses (depending on patient characteristics). Furthermore, future developments in imaging that may alter clinical practice, such as the use of volumetry, are discussed.
***Learning Objectives:***
1. To learn about the imaging features and possible comorbidities of Alzheimer's dementia.
2. To understand the complexity of differential diagnosis.
3. To appreciate the crucial, and so far unmet, medical need to use quantitative MRI techniques in the diagnosis of Alzheimer's disease.

09:44
**Panel discussion: Ask the expert: how can I standardise my image reading and reporting in order to support the clinical process in cognitively impaired patients?**

**08:30 - 10:00** **Room F1**

### E³ - European Diploma Prep Session

### E³ 1323

### Gastrointestinal and abdominal

**A-0768** 08:30
**Chairperson's introduction**
C. Stoupis; *Männedorf/CH (c.stoupis@spitalmaennedorf.ch)*

One of the most important focus of current abdominal imaging is not only to detect abnormalities but being able as well to characterise lesions in the parenchymal organs, facilitating the final diagnosis, noninvasively. Knowledge of anatomy, embryology, physiology and pathology of the liver, pancreas and spleen is essential to understand, diagnose and differentiate congenital, inflammatory, neoplastic and other miscellaneous lesions of those upper abdominal organs. Similar issues apply to the gastrointestinal tract, specifically with regard to pathologies affecting not only the lumen but the wall also and beyond, areas not accessible by endoscopy. In this session, the role of multimodality imaging will be discussed (including hybrid imaging), in order to demonstrate the radiological features of common abdominal diseases, including emergency situations, targeting to the specific diagnosis in acute and chronic abnormalities and displaying the crucial role of radiology in the personalised management of each patient, facilitating on this way not only the diagnosis but enabling as well an efficient and specific therapy.
***Session Objectives:***
1. To understand the typical imaging features of benign and malignant lesions of the hepatobiliary system.
2. To learn the typical imaging features of benign and malignant lesions of the pancreas and spleen.
3. To become familiar with the methodological basis and to differentiate typical features in imaging examinations of the gastrointestinal tract.
4. To understand the role of different imaging modalities including hybrid imaging in diagnosing and staging neoplasms, gastrointestinal and abdominal organ systems.

**A-0769** 08:36
**A. Hepatobiliary system**
I. Santiago; *Lisbon/PT (ines_agp_santiago@hotmail.com)*

An overview of the anatomy, normal variants and congenital disorders of the hepatobiliary system will be provided, followed by the discussion of the imaging features of the most relevant benign and malignant diseases of the biliary tract. Primary and secondary imaging features of acute and chronic diffuse liver diseases will be briefly discussed. The underlying causes and imaging features of the most frequent and relevant benign and malignant focal liver lesions, including cysts, haemangiomas, adenomas, focal nodular hyperplasia, hepatocellular carcinomas and metastases will also be addressed.
***Learning Objectives:***
1. To learn the anatomy, normal variants and congenital disorders of the hepatobiliary system.
2. To become familiar with the primary and secondary imaging features of acute and chronic diffuse liver diseases.
3. To understand the causes and imaging features of benign and malignant focal liver lesions, including cysts, haemangiomas, adenomas, focal nodular hyperplasia, hepatocellular carcinomas and metastases.
4. To learn the various causes and imaging features of benign and malignant diseases of the biliary
tract and gallbladder.

**A-0770** 09:04
**B. Pancreas and spleen**
W. Schima; *Vienna/AT (wolfgang.schima@khgh.at)*

In general, contrast-enhanced MDCT is the primary tool of pancreatic imaging, and MRCP is very valuable for assessment of the ductal system. Important anatomic variants and anomalies of the pancreas include pancreas divisum, and annular pancreas is, which are the result of either failure of fusion or rotation of the pancreas anlagen during the fetal period. Both anomalies may result in significant morbidity. Ductal adenocarcinoma is by far the most common malignant tumour of the pancreas, with approximately 80% of patients having non-resectable (advanced) disease at the time of diagnosis. In patients with equivocal CT findings, contrast-enhanced MRI is the technique of choice for visualisation of small tumours. A wide differential diagnosis exists for cystic masses: from (non-neoplastic) benign pseudocysts to benign, borderline and invasive malignant neoplasms. Contrast-enhanced MRI is the best modality for characterisation of lesions and to guide follow-up in patients. Acute pancreatitis is classified as interstitial edematous or necrotising pancreatitis according to the revised Atlanta classification, which has brought consistent terminology for fluid collections and complications of the disease. Imaging of splenic masses is one of the less esteemed tasks in abdominal imaging. Imaging appearances of many splenic lesions are non-specific and may overlap, which makes non-invasive characterisation quite challenging. However, imaging findings of the most important benign lesions such as hemangiomas, hamartomas, and abscesses should be familiar. If the noninvasive characterisation of focal splenic lesions is not possible, US-guided core needle biopsy may be helpful, which can be performed with low risk of complications.
***Learning Objectives:***
1. To understand the anatomy, normal variants and congenital disorders of the pancreas.
2. To become familiar with the causes and imaging features of benign and malignant pancreatic tumours.
3. To understand the imaging features of acute and chronic pancreatitis and its potential complications.
4. To learn the causes and imaging features of focal and diffuse splenic abnormalities.
*Author Disclosure:*
**W. Schima:** Speaker; GE Healthcare, Siemens.

**A-0771** 09:32
**C. Imaging of the gastrointestinal tract**
D.M. Lambregts; *Amsterdam/NL (doenja.lambregts@gmail.com)*

In this session, a brief overview will be provided of the main imaging anatomy and imaging manifestations of the most important pathologies of the gastrointestinal tract, including acute and chronic inflammatory conditions, bowel obstruction and ischemia and the most important GI tumours. This session serves as a preparation for the European Diploma.
***Learning Objectives:***
1. To become familiar with the anatomy, normal variants and congenital disorders of the oesophagus, stomach, duodenum, small bowel, colon, rectum and anal canal.
2. To understand the imaging features of appendicitis, diverticulitis, tumour stenosis, ileocolic intussusception, colonic fistula, paracolic abscess, epiploic appendagitis, intraperitoneal fluid collection, colonic pneumatosis and pneumoperitoneum.
3. To learn typical radiological manifestations of inflammatory bowel diseases, infection, bowel obstruction and bowel ischaemia.
4. To become familiar with the staging of tumours of the gastrointestinal tract, including features that indicate nonresectability, and to understand the role of different imaging modalities including hybrid imaging in diagnosing and staging.

**08:30 - 10:00** **Room F2**

### State of the Art Symposium

### SA 13

### Cardiac: recalling the forgotten structures

**A-0772** 08:30
**Chairperson's introduction**
C. Peebles; *Southampton/UK (Charles.Peebles@suht.swest.nhs.uk)*

Cardiac imaging techniques tend to focus on the Left ventricle, coronary arteries and cardiac valves, particularly in the adult patient. This reflects the prevalence and significance of disease affecting these structures in the general population. This session will focus on areas of anatomy, and their functional significance, that is often forgotten in the primary cardiac analysis yet may the cause of significant pathology. This introduction will provide an overview of the imaging techniques available.
***Session Objectives:***
1. To become familiar with normal cardiac anatomy and variants outside the cardiac chambers.
2. To understand the important interactions between ventricular chambers and their adjacent structures.
3. To learn what findings are significant and when to report them.
4. To appreciate the strengths and weaknesses of cross sectional imaging techniques when applied to the structures below.

**A-0773** 08:35
**Hide and seek: the pericardium**
A. Nchimi Longang; *Luxemburg/LU (alainnchimi@gmail.com)*

Pericardial diseases encompass a broad spectrum for which delayed diagnosis and inappropriate management are associated with high morbidity and mortality, whereas appropriate treatment may dramatically relieve patient symptoms. Imaging is often necessary to evaluate pericardial fluid collections, calcifications, thickening, hyperemia, masses and constriction. The current presentation will emphasize the definition of the pericardial syndromes and the specific advantages and weaknesses of CT and MRI in evaluating pericardial diseases and differentiating normal from abnormal findings.
***Learning Objectives:***
1. To learn the normal appearances of the pericardium on CT and MRI.
2. To understand the basic spectrum of disease process that affect the pericardium.
3. To be aware of the physiological effects of pericardial disease on cardiac function.

**A-0774** 08:52
**Lost chamber: the right ventricle**
G. Bastarrika; *Pamplona/ES (bastarrika@unav.es)*

Even if traditionally the right ventricle (RV) has been the neglected side of the heart, the role of this cardiac chamber in evaluating different cardiovascular, as well as pulmonary diseases, is being increasingly recognized, including pulmonary arterial hypertension, ischemic heart disease, chronic obstructive pulmonary disease, arrhythmogenic RV cardiomyopathy and congenital heart disease. Currently, echocardiography and magnetic resonance imaging (MRI) are the imaging modalities of choice to assess RV morphology and function. Recently, the added value of multidetector computed tomography (MDCT) with its latest advances, i.e. spectral imaging, is being emphasised. In this lecture, the complementary role of different imaging modalities to appraise the RV and pulmonary circulation will be underlined. Further, imaging features of most common diseases involving the RV as well as prognostic markers related to the RV will be reviewed.
***Learning Objectives:***
1. To learn the normal appearances of the right ventricle on CT and MRI.
2. To differentiate the appearances and physiology of RV volume loading and RV pressure loading.
3. To be aware of adverse prognostic markers related to the RV.
*Author Disclosure:*
**G. Bastarrika:** Grant Recipient; Guerbet. Speaker; General Electric, Siemens.

**A-0775** 09:09
**Friend or enemy: epicardial fat**
K. Gruszczynska; *Katowice/PL (kgruszczynska@poczta.onet.pl)*

Epicardial Fat Tissue (EFT) is the layer of adipose tissue located within the visceral pericardium, directly on the myocardium, around coronary vessels. Paracardial Adipose Tissue (PAT), is located outside the parietal epicardium, in the mediastinum. EFT is metabolically active visceral fat. Under physiological conditions, its function includes myocardial energy supply, lipid-storage, thermoregulation and endocrines secretion. There has been growing interest among researchers in EFT recently, as cardiovascular disease remains the main cause of death in the developed world and due to the epidemy of obesity. The evidence shows the association between EFT and cardiovascular risk factors: obesity, metabolic syndrome, hypercholesterolemia and also coronary atherosclerosis burden. Studies are suggesting that EFT can act as an independent predictor of CAD risk or for adverse coronary events. Traditionally, EFT thickness has been measured by ECHO. However, CT and MRI offer the possibility of the non-invasive quantification of EFT, and the dedicated software already exists. Although EFT is subject to research in diagnostic imaging, it is also useful to recognise its anatomical variants as they could mimic the disease. To sum up, the presentation will give the possibility get to know current opinions on the role of EFT in asymptomatic and symptomatic population and present its anatomy, variants and methods of measurement to practising radiologists.
***Learning Objectives:***
1. To understand the difference between epicardial and pericardial fat.
2. To learn the methods of quantifying epicardial fat.
3. To be aware of the up-to-date evidence relating to cardiovascular risk and the extent of epicaridal fat.

**A-0776** 09:26
**Wrong ways: anomalies of cardiac veins and venae cavae**
M. Hrabak Paar; *Zagreb/HR (maja.hrabak.paar@mef.hr)*

The normal venous anatomy includes drainage of the superior and inferior caval veins and coronary sinus (CS) into the right atrium (RA), and drainage of all pulmonary veins into the left atrium (LA). The most common anomaly of superior vena cava (SVC) is persistent left SVC that usually drains into the CS and can make detection of central venous catheter position more difficult. Rarely the persistent left SVC drains into the LA causing the right-to-left shunt. Most common anomalies of the inferior vena cava (IVC) include left or double IVC, less commonly azygos continuation of the IVC can be observed. Anomalous pulmonary venous return (APVR) is commonly partial with one or two pulmonary veins draining into the SVC, IVC or RA; it can be asymptomatic and is frequently incidentally found during thoracic imaging. Partial right-sided APVR can be associated with a sinus venosus atrial septal defect (ASD). Total APVR is cyanotic congenital heart disease with all pulmonary veins draining into systemic veins, RA or CS, and can be divided into supracardiac, cardiac and infracardiac types. The depiction of pulmonary venous anatomy using CT or MRI is commonly performed for the planning of pulmonary vein isolation in patients with atrial fibrillation. CT mapping of the cardiac venous system is especially important for planning of left ventricular pacing in cardiac resynchronisation therapy. Unroofed CS is a rare form of ASD with a communication between CS and LA. Understanding of venous anomalies is essential for their optimal interpretation and avoidance of diagnostic pitfalls.
***Learning Objectives:***
1. To learn the normal anatomy of pulmonary venous drainage, common anomalies and their clinical significance.
2. To learn about the normal anatomy of systemic venous drainage, common anomalies and their clinical significance.
3. To learn the normal anatomy of coronary venous drainage, common anomalies and their clinical significance.

09:43
**Panel discussion: Which test would you use, MRI or CT?**

**08:30 - 10:00 Room Y**

### E³ - Rising Stars Programme: EFRS Radiographers' Basic Session

### BR 13

### Radiography research: a "how to" guide

**A-0777** 08:30
**Chairperson's introduction: Why is radiography research important?**
D. O'Leary; *Newcastle/UK (desiree.lemiereoleary@gmail.com)*

Radiography as a profession is a young entity, and indeed, in some countries, radiography is not yet recognised as a professional vocation. With the professional registration of radiographers comes the requirement to ensure that we as radiographers are evidence-based practitioners. It is essential in our profession that we strive to ensure that our practice in this extremely technological area of para-medicine, is continually investigated for the benefit of the operators, the public and the most of all, the patient. In the United Kingdom, the number of active research radiographers is low with only around 0.1% of all registered radiographers with doctoral level education. The number of research-active radiographers worldwide is less established but is thought to be lower than 0.1% of practitioners. The number of radiographer published research is increasing; however, the number of authors for these publications are not, again showing that the number of active research radiographers is low. Indeed, the number of radiographer abstracts being presented for this Congress are increasing annually, but these appear to be from a small number of researchers in multiple areas. It is past time that radiographers, radiology managers, and educators take research in radiography more seriously and ensure that this profession thrives on evidence-based practice.
***Session Objectives:***
1. To become familiar with common ethical issues arising in radiography research.
2. To understand the value of undertaking research in radiography.
3. To identify good practices for developing and presenting radiography research studies.

**A-0778** 08:35
**How to avoid ethical issues**
P. Bezzina; *Msida/MT (Paul.bezzina@um.edu.mt)*

As professionals, ethical codes guide on how we should behave in our practice and as individuals in the society that we live in. The first modern code governing ethics in research was developed during the Nuremberg trials in response to the abuse performed during medical experimentation. Various published reports about abuses were the motivating factors that led to the development of legislation on ethical principles and regulations when conducting research. These regulations may be seen as the boundaries that direct us in specific situations to help us make decisions and guide our behaviour while conducting research. Policies and procedures are also generally linked to cultural values and may be explicit to a particular time and are subject to change as attitudes and values evolve in today's society. Conflicts may arise while performing research and these have to be addressed while being respectful to the participants at all times. It is crucial for researchers to be knowledgeable of any pitfalls that may inhibit the undertaking of the study. Research ethics committees have been set up to ensure that researchers follow procedures during the collection of data so that participants willingly consent to take part and are aware, and know of the associated risks and benefits. These committees also need to ensure that legislation is being respected and adhered to. If integrity is not maintained, public confidence and belief in findings will be lost. Therefore, it is important to be transparent, competent, and follow ethical procedures when conducting research.
***Learning Objectives:***
1. To understand the importance of ethics in medical research and the role of research ethics committees.
2. To become familiar with common ethical pitfalls in imaging research.
3. To appreciate strategies for avoiding ethical problems in imaging research.

**A-0779** 08:53
**How to critically appraise a research article**
J. Thompson; *Salford/UK (j.d.thompson@salford.ac.uk)*

When planning a research project, it is vital that a good appreciation of the existing literature is gained. Performing a literature search prior to beginning your research enables you to realise trends, understand the gaps in the existing literature, and limitations in the existing knowledge base. Article critique is a core skill for evidence-based practice and it is important to have the skills and knowledge to successfully critique research articles. The different elements of a research paper will be discussed, helping you understand what information you should expect to find, and which aspects of a research paper require close inspection. Good critique is should always be positive and negative, and this session will help you understand the implications of positive and negative aspects of a research article on the overall outcomes and meaning of the research.
***Learning Objectives:***
1. To appreciate the role of the literature review in the research process.
2. To identify strengths and weaknesses in each section of a research report.
3. To critically appraise the overall value of a research report.

**A-0780** 09:11
**How to write a good scientific abstract**
L. Ribeiro; *Faro/PT (lpribeiro@ualg.pt)*

The abstract is the "door" to your scientific publication, and when written in the right way, will open the desire of the reader to continue reading your full work. Although the main objective for the author is that the number of persons who read the publication will be all interested by the scientific topic, he must write the abstract as the "big picture" in 250 words in order to sell his work. To achieve this goal, it is needed to choose the words that are capable of creating a desire of continue reading, but also with scientific content and respecting the format covenanted by the rigours scientific community, in other words, sell the work within the rules. In this talk, we will try to use the precise and right words to "How to write a good scientific abstract" according to a scientific structure and with the right message of your work. The measure of the success of your abstract is given by the equivalent number of readers of the full work. To conclude we hope to see you all at this talk, and at the end of that fulfil your expectations, because it is the most important!
***Learning Objectives:***
1. To understand general qualities of good scientific abstracts.
2. To be aware of typical formats of scientific abstracts.
3. To consider tips for success.

**A-0781** 09:29
**How to produce a high-quality scientific or educational poster**
C. Malamateniou; *London/UK (christina.malamateniou@kcl.ac.uk)*

Communication and dissemination of research in an ethical and systematised manner is as important as the research itself. One of the most common, most direct and commonly encountered ways to share data is the scientific poster. There are specific rules about the structure, the format, the presentation devised each time by the respective scientific event at which the poster is submitted and presented. However, there are also some generic rules universally applicable about structure, content and presentation of scientific posters that this talk will delve into. Once these rules adhere to the poster can fulfil its aim, which is to communicate the findings of research and stimulate discussion. Many research collaborations have started from a well-organised poster, and it is considered the predecessor of scientific presentations and research papers. It remains one of the most versatile means of disseminating evidence-based practice.
***Learning Objectives:***
1. To become familiar with poster formats and layouts.
2. To consider good practices in poster design.
3. To appreciate important considerations when designing a poster.

09:47
**Panel discussion: Ask the experts**

**08:30 - 10:00 Room D**

### Musculoskeletal

### RC 1310

### Imaging of forgotten small joints

*Moderator:*
R. Sutter; Zurich/CH

**A-0782** 08:30
**A. Sterno-clavicular joints: from trauma to inflammation**
A.-G. Jurik; *Aarhus/DK*

The sternoclavicular joints (SCJs) are the only synovial joints between the upper extremities and the body. They accompany arm movements and move slightly during respiration, resulting in an almost continuous strain on the joints. The SCJ surfaces are incongruent, consisting of a relatively small area of the medial clavicle articulating with the manubrium sterni and the adjacent cartilage of the first rib, but the joint space contains a stabilising intraarticular disc. Furthermore, the SCJ is stabilised by strong surrounding ligaments. Due to the SCJ anatomy, traumatic joint dislocations can occur in connection with shoulder or chest trauma. It is especially important to detect posterior clavicular displacement, which can potentially cause vascular damage. Lesion of the intraarticular disc may be common, but difficult to detect without high-resolution MRI. The stabilising ligaments surrounding the SCJ imply that the sternoclavicular region contains many areas of fibrous tissue inserting into bones (entheses), areas which may be involved by inflammatory disorders, predominantly seronegative spondyloarthritides. Most other joint disorders can also occur in the SCJ, predominantly osteoarthritis due to the joint strain. Detection of SCJ infection is particularly important due to a potential spread to the mediastinum. The SCJs are difficult to visualise by radiography; cross-sectional imaging by ultrasonography, CT, MR or radionuclide imaging is needed for appropriate visualisation. The lecture will encompass illustrations of characteristic imaging features of the most frequent SCJ disorders applying appropriate imaging strategies depending on the suspected disorder.
***Learning Objectives:***
1. To explain the pathologic conditions that involve the sterno-clavicular joints.
2. To describe the imaging findings of abnormalities that involve the sterno-clavicular joints.

**A-0783** 09:00
**B. Symphysis pubis and its surroundings**
A. Kassarjian; *Pozuelo De Alarcón/ES*

The symphysis pubis and its surroundings are a common source of groin pain in athletes. This presentation will discuss the complex anatomy of the symphysis and the para-symphyseal region with an emphasis on MR anatomy. Furthermore, common causes of symphyseal and para-symphyseal groin pain in athletes will be illustrated.
***Learning Objectives:***
1. To explain the pathologic conditions that involve the symphysis pubis and its surroundings.
2. To describe the imaging findings of abnormalities that involve the symphysis pubis and its surroundings.

**A-0784** 09:30
**C. Proximal tibio-fibular joint: a cause for lateral knee pain**
A. Cotten; *Lille/FR (anne.cotten@chru-lille.fr)*

Although rare, various disorders of the proximal tibiofibular joint have been reported. They should be kept in mind in the evaluation of lateral knee pain. They include traumatic subluxation or dislocation, atraumatic instability, congenital or acquired synostosis, osteoarthritis, rheumatic disease, ganglion or synovial cysts, synovial chondromatosis, pigmented villonodular synovitis and hypomobility of the joint. The peroneal nerve can be at risk with pathologies of this joint, either by compressive effect or formation of an intra-neural ganglion. After a reminder of the normal anatomy of this joint, emphasis will be put on the imaging features allowing depiction and assessment of the various disorders affecting this joint, which should not be forgotten any more.
***Learning Objectives:***
1. To explain the pathologic conditions that involve the proximal tibio-fibular joint.
2. To describe the imaging findings of abnormalities that involve the proximal tibio-fibular joint.

**08:30 - 10:00** **Room G**

### Physics in Medical Imaging

### RC 1313

### Dose management in paediatric radiology

**A-0785** 08:30
**Chairperson's introduction**
C. Saidléar; *Dublin/IE (colm.saidlear@cuh.ie)*

The important concept of dose management, specifically in paediatric radiology, is now mainstream following two decades of campaigns by various stakeholder organisations and advocacy groups championed by radiologists, radiographers and medical physicists. The initial drive was to tackle high dose procedures such as CT and interventional radiology. This has now expanded into all diagnostic modalities that use ionising radiation. The IAEA Safety Report No. 71 (2012) highlights and requires the ICRP (2007) concepts of justification, optimisation and risk to be addressed in paediatric radiography, interventional radiology, CT and neonatal imaging, the focus of this refresher session. This report was a tipping point for dose management in paediatric radiology and has led to a significant body of work to implement these concepts in radiology departments and practices across the world. Audit and dose tracking mechanisms are key to this process. Recent initiatives and publications such as the 'The Gentle Way' by the IDoR (2015), RP 185 (European Guidelines on Diagnostic Reference Levels for Paediatric Imaging) by the European Commission (2018), along with enactment into law in member states of the strict requirements in the Council Directive 2013/59/Euratom (Basic Safety Standards) for DRL's and the tracking of same, is timely for the next steps. They provide guidelines, benchmarking tools, meaningful processes and data, which are required to assist all stakeholders, including manufacturers, to obtain the best possible outcome for paediatric patients, that is, diagnostic quality images from a paediatric optimised modality at the appropriate dose.
***Session Objectives:***
1. To become familiar with modern dose management methods in paediatric radiology.
2. To understand the important aspects of paediatric dose management.
3. To appreciate the current trends and limitations.

**A-0786** 08:35
**A. The special case of the paediatric patient: risks and justification**
C. Owens; *London/UK (owensc@gosh.nhs.uk)*

Advances in imaging technology have revolutionised our ability to produce exquisite images in children. We have a huge potential armamentarium and hence a duty of care to the child and parents to choose the correct examination which answers the posed clinical question, with the least exposure to radiation. Choice of the correct modality and optimisation of the machinery for use in children to obtain the best image quality is paramount, especially when using CT scanning, which has the relatively highest radiation burden. We will illustrate with examples comparing older and newer models of CT to compare doses with image quality.
***Learning Objectives:***
1. To learn about risks and justification techniques in paediatric radiology.
2. To understand how these methods are applied in a clinical setting.
3. To appreciate the benefits of new information and the current limitations.

**A-0787** 08:58
**B. Optimisation and technology in paediatric projection radiography, interventional and CT scanning**
V. Tsapaki; *Athens/GR (virginia@otenet.gr)*

Radiological imaging has added incredible value in paediatric patient clinical diagnosis and treatment especially in the last decades with the help of digital technology, multidetector-row computed tomography (CT) and the use of angiography systems. The latest United Nations Scientific Committee on the Effects of Atomic Radiation (UNSCEAR) 2008 report states that approximately 180 million radiological examinations are performed on children and of these 18 million are CT exams. At the same time, all data show that the steadily increasing use of ionising radiation examinations and specifically the high dose procedures such as CT and Interventional techniques) over the last years, result in progressively increasing of population radiation dose. Various studies in the recent literature have reflected a lack of attention to the potential hazards for children or to the need for reducing dose according to the body size of a small patient. It is well known that children are more sensitive to radiation than adults due to longer life expectancy and due to developing and growing tissues. They consequently have more years in which cancerous changes might occur. For all the above reasons continuous need for dose optimisation is needed. The lecture will present a summary of practical ways for radiation dose optimisation in projection radiography, interventional and CT scanning together with the latest trends in technology.
***Learning Objectives:***
1. To learn about the optimisation of techniques in paediatric radiology.
2. To understand how these methods are applied in a clinical setting.
3. To understand the new information that is available and the current limitations.

**A-0788** 09:21
**C. Optimisation in the neonate**
H. Delis; *Vienna/AT (hdelis@gmail.com)*

"Neonates are not little children" one could say paraphrasing the quote "Children are not little adults", that is frequently used to highlight the special attention required in paediatric medicine. With birth weight often even below 1 kg for premature babies, and with a number of examination that can sometimes reach to tens, neonates are a very sensitive group of patients and their particularities and needs in terms of medical imaging are such that deserve a more scrutinised approach to optimisation. Although computed tomography is also used for neonates, it is the traditional radiographic projection imaging that is leading the list of examinations. Radiographs are not typically obtained for the healthy and full-term infant, but they are used for newborns with a spectrum of conditions and especially in the neonatal intensive care unit. Modern tools, such as the utilisation of dose management systems, to extensively review local practices, and iterative reconstruction algorithms that can limit the dose in CT, can provide a very good platform for optimisation. However, traditional optimisation methods, such as tube voltage, filtration and collimation, are of paramount importance to make sure that little patients are handled with the respective care needed.

***Learning Objectives:***
1. To learn about dose management techniques in neonatal radiology.
2. To understand how these methods are applied in a clinical setting.
3. To understand the new information that is available and the current limitations.

09:44
**Panel discussion: Paediatric dose management: are we doing enough for the next generation?**

**08:30 - 10:00** **Room M 1**

### Joint Session of the ESR and EORTC

### ESR/EORTC

### Imaging in oncological trials

*Moderator:*
M. Smits; Rotterdam/NL

**A-0789** 08:30
**Chairperson's introduction**
N.M. deSouza; *Sutton/UK*

This educational session of imaging in oncological trials will explore the role of imaging biomarkers as end-points in oncology trials. It will underline the importance of joint organisational working and collaboration for a successful outcome. Designing trials that assess the validity and utility of imaging biomarkers will be discussed using treatment of oligometastatic disease as an exemplar. The use of imaging biomarkers specific to immunotherapy and derivation of quantitative biomarkers from radiomics platforms will be addressed.
***Session Objectives:***
1. To highlight the collaboration between EIBALL and the EORTC imaging group.
2. To introduce the need for radiologists to learn about oncological trials beyond RCTs and how such knowledge can be used to initiate and advance oncological imaging research.
3. To appreciate the impact imaging has on patient stratification and response assessment.
4. To learn about the requirements for and potential of radiomics in oncological imaging research.
*Author Disclosure:*
**N.M. deSouza:** Grant Recipient; Cancer Research UK, EU Framework 7.

**A-0790** 08:35
**Trial design and imaging end-points**
L. Collette; *Brussels/BE (Laurence.collette@eortc.org)*

Nowadays the trial designs that are used for the evaluation of anti-cancer treatments range from the simplest, the traditional single-arm or 2-arm randomized designs addressing a specific histology, to much more complex designs that either address multiple (targeted) treatments for a given cancer type (“umbrella trials”) or assess treatments across histopathological tumour types ("basket trials"). We will discuss the use and characteristics of these designs. Images play an important role in the assessment of the treatment effect through either qualitative or quantitative measures of change over time. We will discuss some quality requirements that need to be met to ensure that the information they convey results in an accurate evaluation of the trial endpoints. Finally, we will give some thoughts on how the images collected for the study may be exploited to meet additional research objectives.
***Learning Objectives:***
1. To learn about the several types of trial design, including RCT, umbrella and basket trials.
2. To appreciate the different requirements of imaging end-points for different trials and phases.
3. To understand how imaging data acquired in the context of different trials can be used for additional research questions.

**A-0791** 08:55
**Modern imaging-based trials: focus on oligometastatic disease and evaluation of metastatic directed therapies**
F.E. Lecouvet; *Brussels/BE*

Patients with oligometastatic disease (OMD) have controllable symptoms, and cures are theoretically possible. Technical improvements in surgery and radiotherapy have introduced the option of metastasis-directed therapies (MDT) as an adjunct or alternative to the standard of care systemic therapies. Standard imaging methods recommended by current guidelines often have insufficient diagnostic accuracy for a reliable diagnosis of OMD. Modern imaging methods using positron emission tomography /computed tomography (PET/CT) with a tumour specific radiotracers, and increasingly whole-body magnetic resonance imaging (WB-MRI) with diffusion-weighted imaging (DWI), allow an earlier and more precise identification of metastases. This lecture discusses the evidence and offers recommendations for the implementation of standard-of-care (Response Evaluation Criteria in Solid Tumours measurements on CT, MRI and bone scintigraphy) and advanced imaging modalities for identifying and following patients with OMD in the most frequent cancers. The presentation suggests clinical algorithms for integrating modern imaging methods in the care pathway at the various stages of these cancers in order to identify OMD. Clinical trials utilising modern imaging methods are proposed for evaluating the needs for a reliable identification of OMD and the benefits of metastasis-directed therapies.
***Learning Objectives:***
1. To understand how imaging modalities may affect patient selection.
2. To appreciate how imaging modalities may affect response assessment.
3. To learn how to design a trial evaluating the best imaging strategy according to the objective.

**A-0792** 09:15
**Assessing treatment response in the era of immunotherapy**
C. Caramella; *Villejuif/FR (Caroline.CARAMELLA@gustaveroussy.fr)*

Immunotherapy has rapidly and profoundly changed the management of many cancer patients. The high level of disease control rate has led to multiple approvals around different types of cancers, mainly in the metastatic situation, but is going to also win new indications in neoadjuvant and adjuvant therapy. The radiological evaluation of patients under immunotherapy is challenging because of the random occurrence of a new pattern of response named “pseudoprogression”, that is (mis)classified by RECIST 1.1 as a progression. Consequently, iRECIST new guidelines were proposed by the RECIST working group in order to clarify and standardise how progressions in trials investigating immunotherapies should be assessed. In particular, a disease described as progressive by RECIST rules, should be named as an immune unconfirmed progressive disease (iUPD) and therefore require a subsequent evaluation in order to confirm -or not- the progression.
***Learning Objectives:***
1. To learn about new immunotherapies.
2. To appreciate new patterns of response and progression observed under immunotherapy.
3. To learn about the specific criteria derived from RECIST (iRECIST), harmonising data collection in clinical trials.
4. To learn about future functional imaging techniques which could help to better understand effects of immunotherapies.
*Author Disclosure:*
**C. Caramella:** Consultant; BMS, Roche, MSK, Amgen.

**A-0793** 09:35
**Radiomics in oncology trials**
P. Kickingereder; *Heidelberg/DE (philipp.kickingereder@med.uni-heidelberg.de)*

Magnetic resonance imaging plays a key role for diagnosis and treatment monitoring of brain tumours and novel imaging techniques that specifically interrogate aspects of underlying tumour biology and biochemical pathways, have great potential in neuro-oncology. This presentation focuses on the emerging role of radiomics and radiogenomics in establishing the diagnosis, for monitoring treatment response and for predicting prognosis in brain tumour patients.
***Learning Objectives:***
1. To become familiar with the radiomics process.
2. To learn what is important in data collection and curation for radiomics.
3. To understand how radiomics can lead to a new biomarker discovery.

09:55
**Panel discussion: What is the future of image-aware clinical trials? Challenges and opportunities**

08:30 - 10:00 Room M 2

### E³ - ECR Master Class (Paediatric)

### E³ 1326

### Juvenile idiopathic arthritis (JIA)

*Moderator:*
K. Rosendahl; Bergen/NO

**A-0794** 08:30
**A. Conventional radiography: still a helpful method?**
S.C. Shelmerdine; *London/UK (susie_c_s@yahoo.co.uk)*

Juvenile Idiopathic Arthritis (JIA) is a chronic, rheumatic illness that is associated with a high risk of long-term physical disabilities. Radiography can help identify changes related to JIA, which are primarily those of chronic, destructive changes seen in late disease, but it can also provide indirect information on soft tissue inflammation (such as peri-articular increased soft tissue density or the presence of a joint effusion). In the initial assessment of a child with joint pain, radiography is also useful to rule out other differential diagnoses such as neoplasms, posttraumatic changes and developmental defects. In this lecture, we will focus on the typical findings of joint inflammation and destruction seen in a variety of commonly affected joints. We will also touch upon common pitfalls and how such features may overlap with normal findings of skeletal maturation. We conclude by reviewing benefits and drawbacks of using radiography as a follow-up imaging tool and how this modality compares to other radiology techniques for JIA imaging.
***Learning Objectives:***
1. To learn about the features of joint inflammation on plain radiographs.
2. To understand the role of this technique in the diagnosis and follow-up of children with JIA.
3. To appreciate the strengths and limitations of conventional radiography compared to other modalities in JIA.

**A-0795** 09:00
**B. Ultrasound for detecting and grading of inflammation in JIA**
L. Tanturri de Horatio; *Rome/IT*

"no abstract submitted"
***Learning Objectives:***
1. To learn about normal features vs pathology on ultrasound of joints in children.
2. To understand the role of ultrasound in diagnosing and grading of joint inflammation.
3. To appreciate the research-based evidence of ultrasound.

**A-0796** 09:30
**C. MRI and role of contrast in the assessment of synovitis**
L.-S. Ording Müller; *Oslo/NO (lilsofie.ording@googlemail.com)*

MRI scanning is the current gold standard modality for imaging synovitis and tenosynovitis in patients with Juvenile idiopathic arthritis. Enhancement of the synovium after gadolinium injection is traditionally regarded as mandatory in the assessment of synovitis, both for diagnosis and grading, and to distinguish between active and inactive disease. There have been huge advances in medical treatment of JIA over the last two decades, and disease remission is now the ultimate goal for all patients. This increases the role of imaging to detect early signs of disease or relapse and subtle response to treatment. Several research groups work on establishing scoring systems to create objective imaging measures of disease activity in JIA. However, repeatability in the differentiation between normal synovial enhancement and inflammation and of the grading of contrast enhancement in early disease is rather poor. The timing of imaging post contrast injection has shown to play an important role in the assessment and grading of synovitis, and dynamic contrast-enhanced might be a better method to improve quantification of synovitis. New focus on potentially harmful effects of Gd-injection forces us to search for alternative methods in synovitis imaging. The diffusion-weighted imaging (DWI)-derived apparent diffusion coefficient (ADC) seems to be higher in active JIA and may serve as a non-invasive imaging biomarker for JIA in the future. In this lecture, current knowledge on the role of contrast in synovitis assessment, challenges in the standardisation of imaging, potential alternative methods for detection and grading of inflammation in JIA will be presented.

08:30 - 10:00 Room M 3

### Head and Neck

### RC 1308

### Differential diagnoses you don't want to miss

*Moderator:*
E. Arkink; Reykjavik/IS

**A-0797** 08:30
**A. Differential diagnoses of nose and paranasal sinus lesions**
S. Connor; *London/UK (sejconnor@gmail.com)*

Sinonasal pathologies will be categorised and discussed as inflammatory/infectious processes, benign tumours, malignant tumours and miscellaneous sinonasal lesions. CT is the initial imaging study performed for an inflammatory disease to establish the extent and to provide an anatomical road-map for surgery. Important anatomical variants should be reported as they are predictive of disease patterns and impact on surgical approaches. “Red flag” imaging features such as isolated polyps, bony erosion, extra sinus extension and skull base defects should be sought on paranasal sinus CT, as these will indicate further evaluation with MRI or endoscopic correlation. Additional inflammatory processes such as polyps, retention cysts, mucoceles, silent sinus syndrome, rhinolith and odontogenic sinusitis may be recognised on CT. Potential extra-sinus extension of sinonasal infection, including that resulting from invasive fungal disease, requires additional evaluation with MRI to demonstrate orbital, intracranial and deep face involvement. Benign tumours such as inverted papilloma, odontogenic lesions and juvenile angiofibroma may present with typical and sometimes pathognomic appearances on CT and MRI, but imaging also provides useful information on the extent of the abnormality. MRI and CT are complementary in the assessment of sinonasal malignant lesions. MRI, in particular, is vital, to assess for direct skull base, perineural, orbital and deep facial extension. Optimised MRI protocols enable delineation of a tumour, and the most useful MRI sequences will be discussed. Miscellaneous entities such as developmental sinonasal abnormalities and destructive midline pathologies also require pattern recognition on CT and MRI for their differential diagnosis.
***Learning Objectives:***
1. To describe disease patterns in nose and paranasal sinus CT.
2. To learn when additional imaging is mandatory.
3. To provide tools to narrow down the list of differential diagnoses in sinonasal MR studies.

**A-0798** 09:00
**B. Differential diagnoses of paediatric neck lesions**
P.A. Caruso; *Boston, MA/US (pcaruso@partners.org)*

We will review those pediatric head and neck lesions, neoplastic, malformative, and inflammatory where consideration of the differential diagnosis is key to avoid missing critical pathology. As we review these lesions, we will see how taking the time to consider the differential diagnoses particular to the pediatric population can inform our search strategies, protocols, and interpretation. We will review the pertinent embryologic and anatomical considerations as well for each case.
***Learning Objectives:***
1. To become familiar with the normal development of facial and neck structures.
2. To discuss which imaging modality to use: US, CT or MRI?
3. To understand the typical imaging appearance of congenital neck lesions.

**A-0799** 09:30
**C. Differential diagnoses of soft tissue masses in adults**
D. Farina; *Brescia/IT (davide.farina@unibs.it)*

Soft tissue masses of the supra and infrahyoid neck are a rather heterogeneous group of tumours, classified by WHO in nine categories, based on their histologic differentiation: adipocytic, fibroblastic or myofibroblastic, fibrohistiocytic, smooth muscle, skeletal muscle, vascular, pericytic, and chondro-osseous tumours, and tumours of uncertain differentiation. Based on their clinical behaviour and history such tumours may be described as benign, malignant or intermediate, the latter further subclassified as locally invasive or metastatizing at distant sites. The US is generally the first imaging step in infrahyoid neck lesions; MDCT or MRI are mandatory in suprahyoid masses but are also needed to better define the deep extent and anatomic relationships of infrahyoid tumours. In many cases, imaging findings are overlapping and insufficient for tumour characterisation; nonetheless, some specific clues may orient the differential diagnosis. The site of origin of the lesion is probably the first brick in the wall. Therefore knowledge of the spacebased neck anatomy is an essential prerequisite. Patient's age, size and number of lesions, presence and pattern of calcifications are useful additional details. Some specific density or signal intensity patterns may cut the list of differentials, whereas the potential role of the additional information provided by DWI-MRI or dual-energy CT is far from being fully elucidated. However, it is clearly assumed that imaging diagnosis does not replace pathologic assessment, which in a significant number of cases can be accurately obtained with FNA.

***Learning Objectives:***
1. To become familiar with the anatomy.
2. To learn which imaging technique to use.
3. To understand the typical imaging appearance of soft tissue masses.

**08:30 - 10:00 Room M 4**

### E³ - ECR Academies: Chest Imaging

### E³ 1322

### Pleural neoplasms

**A-0800** 08:30
**Chairperson's introduction**
F. Gleeson; *Oxford/UK (fgleeson@mac.com)*

Pleural disease is a common manifestation of both intra- and extrathoracic disease and may be either benign or malignant. Although pleural effusions are common, pleural malignancy is less common and often diagnosed late. This session will discuss both primary pleural malignancy - mesothelioma, and metastatic disease, most commonly adenocarcinoma, and benign pleural tumours, including pleural fibromas. We will discuss their presentation, the use of different imaging modalities including PET/CT and the methods available to obtain a tissue diagnosis. The session will start with a case presentation which will illustrate the importance of considering a pleural aetiology in the differential diagnosis of patients presenting with an abnormal CXR.

**A-0801** 08:36
**A. Malignant mesothelioma**
I. Hartmann; *Rotterdam/NL (i.j.c.hartmann@gmail.com)*

Malignant pleural mesothelioma (MPM) is the most common primary malignant tumour of the pleura and the second most common pleural malignancy after metastatic disease. MPM has a poor prognosis and limited treatment options. Imaging plays a central role in the detection, diagnosis, staging, and response assessment of MPM. Although multimodality imaging, especially PET/CT, is increasingly used, contrast-enhanced CT alone is often sufficient. MPM has a unique morphology and growth pattern, making the image interpretation frequently a challenge. The aim of the presentation is to provide an overview of the most common and less frequent imaging findings of MPM, staging of MPM according to the 8th TNM classification, the role of PET/CT in the management of MPM, and the revised, modified RECIST 1.1 guidelines for MPM response assessment.

***Learning Objectives:***
1. To learn about the various presentation of malignant mesotheliomas.
2. To learn about the role of PET/CT.
3. To learn about the modified RECIST criteria.

**A-0802** 09:04
**B. Pleural carcinomatosis of thoracic and extra thoracic origin**
C. Beigelman; *Lausanne/CH*

Pleural metastases are the most common malignant pleural tumours, usually originating from an adenocarcinoma of the lung, breast, ovary, and stomach. Pleural malignancy may also be related to lymphoma, a thymoma or may be of unknown origin. Typical features of malignancy include circumferential thickening, nodular thickening, thickness greater than 1 cm, and involvement of the mediastinal pleura. A pleural effusion is commonly seen, sometimes without any associated pleural thickening. Multiple pleural-based nodules or focal pleural thickenings, as well as a solitary mass, may also be observed. Focal pleural thickenings (PT) should not be confused with normal structures, typical pleural plaques, PT related to previous tuberculosis, silicosis, or other rarer conditions. Importantly, in case of known or suspected malignancy, the presence of postero-basal PT in supine examination requires an additional low dose acquisition on prone position. Indeed, such thickenings may be reversible, therefore excluding pleural metastasis. Any atypical shape, location or change of a PT should suggest a pleural metastasis in a context of malignancy. In all cases, a careful analysis of other CT findings, previous imaging studies and clinical history are determinant for the final diagnosis of pleural metastasis. Beside contrast-enhanced MDCT, the major imaging modality in this setting, there is an undeniable role of PET imaging. In particular, by using the latest equipment allowing a spatial resolution of 3 mm, a high metabolic activity in the absence of prior thoracocentesis may help to strongly suggest malignancy faced with minor or subtle anomalies. Illustrative cases will be reviewed.

***Learning Objectives:***
1. To review the CT signs of pleural carcinomatosis.
2. To become familiar with the signs of dry pleural dissemination.
3. To review illustrative cases.

*Author Disclosure:*
**C. Beigelman:** Speaker; Gilead; Astra Zeneca; Boehringer.

**A-0803** 09:32
**C. Other pleural and juxta pleural malignancies**
A. Snoeckx; *Antwerp/BE (annemie.snoeckx@uza.be)*

Pleural malignancies, other than mesothelioma and metastases, are very rare. According to the latest World Health Organization Classification, these tumours can be further divided into lymphoproliferative disorders and mesenchymal tumours. The latter includes malignant solitary fibrous tumour, desmoid-type fibromatosis, sarcomas, desmoplastic round cell tumour,... .Pleural lymphoma can be primary or secondary. Primary lymphoma is a very rare entity that can be seen in the setting of HIV with primary effusion lymphoma or associated with pyothorax. Imaging findings are nonspecific, but an isolated pleural lesion is unlikely to represent lymphoma. Secondary pleural lymphoma is most commonly non-Hodgkin lymphoma and mainly seen in association with other types of thoracic involvement. Pleural solitary fibrous tumours are malignant in up to one-third of cases. On CT these well circumscribed pedunculated large masses are heterogeneous. Calcifications and associated pleural effusion are less common. Desmoid tumours appear as well-defined isodense to hypodense masses on CT. Contrast enhancement is variable and central necrosis is usually absent. Whereas chest radiographs can be the initial study for detection, Computed Tomography remains the imaging modality of choice for primary assessment of pleural masses. Magnetic Resonance Imaging and Positron emission tomography may have a role in further characterisation, defining local tumour extension and staging.

***Learning Objectives:***
1. To learn about the CT features of solitary fibrous tumours.
2. To know when to suspect a desmoid tumour.
3. To learn about pleural lymphoma.

**08:30 - 10:00 Room M 5**

### Transatlantic Course of ESR and RSNA (Radiological Society of North America): Sports Imaging

### TC 1328

### Shoulder sports injuries and postoperative MRI

*Moderators:*
L.W. Bancroft; Orlando, FL/US
A.J. Grainger; Leeds/UK

**A-0804** 08:30
**A. Shoulder injuries in the throwing athlete**
L. Steinbach; *San Francisco, CA/US*

Overhead throwing athletes acquire adaptations to the extremes of motion in the dominant shoulder. These changes may aid in pitching, throwing or serving velocity, however for some athletes they might eventually result in an inability to throw with the same velocity. These injuries and adaptations involve capsule, labrum, rotator cuff and biceps tendons, muscles, nerves, and bones. This presentation will review the biomechanics of throwing forces as they relate to the shoulder using baseball as an example. The MR imaging characteristics of the resultant changes in the rotator cuff, superior labrum and osseous structures will also be highlighted.

***Learning Objectives:***
1. To understand the biomechanics of throwing forces as they relate to the shoulder.
2. To become familiar with rotator cuff and labroligamentous injury patterns caused by overhead sports.

**A-0805** 09:00
**B. Postoperative shoulder MRI after instability surgery**
L.W. Bancroft; *Orlando, FL/US (laura.bancroft.md@flhosp.org)*

Purpose: To become familiar with the expected and abnormal MR imaging findings after labral repair, capsular shift/capsulorrhaphy, remplissage and Latarjet/Bristow procedures. Methods and Materials: MR imaging will be used to demonstrate the various normal and abnormal imaging appearances after shoulder instability surgery. Results/Conclusion: Labral re-tear will be evident as contrast or joint fluid extension into linear or complex tear cleft, absent/truncated/fragmented labrum, or labral displacement from an anatomic location. Capsular shift results in smaller capacity joint and sometimes irregular capsular nodularity. Complications of capsulorrhaphy include capsular tears and subluxation of the humeral head. Postoperative MR imaging can evaluate healing after combined remplissage and Bankart repair for moderate size, engaging Hill-Sachs lesions. Laserjet and Bristow procedures may be performed in patients with recurrent dislocations and glenoid deficiency. Incorporated bone will yield non-anatomic glenoid configuration, and complications include non-union, fatty degeneration of subscapularis muscle, and osteoarthrosis.

***Learning Objectives:***
1. To become familiar with the expected and abnormal MR imaging findings after labral repair.
2. To learn about the postoperative imaging features after capsular shift/capsulorrhaphy.
3. To appreciate normal imaging and complications after remplissage and Laterjet/Bristow procedures.

*Author Disclosure:*
**L.W. Bancroft:** Author; Lipipincott. Speaker; World Class CME.

**A-0806** 09:30
**C. Interactive case discussion (Part 1)**
L. Steinbach; *San Francisco, CA/US (lynnesteinbach1@gmail.com)*

This interactive session will showcases that relate to the shoulder in the throwing adolescent. This age group has additional problems related to the stress of throwing. These abnormalities were not discussed in the lecture on the throwing shoulder of the adult. Many of the cases relate to stress on the physical plates around the shoulder.

***Learning Objectives:***
1. To appreciate pathologic and normal developmental changes in skeletally immature throwing athletes.
2. To learn how to differentiate normal and failed labral repairs with MRI.
3. To consolidate the knowledge gained from the session with interactive cases of pre- and postoperative shoulder MRI.

**A-0807** 09:45
**C. Interactive case discussion (Part 2)**
L.W. Bancroft; *Orlando, FL/US (laura.bancroft.md@flhosp.org)*

To engage in interactive case discussions with the expected and abnormal MR imaging findings after labral repair, capsular shift/capsulorrhaphy, remplissage and Laterjet/Bristow procedures. An interactive case discussion will be used to demonstrate the various normal and abnormal imaging appearances after shoulder instability surgery. Labral re-tear will be evident as contrast or joint fluid extension into linear or complex tear cleft, absent/truncated/fragmented labrum, or labral displacement from the anatomic location. Capsular shift results in smaller capacity joint and sometimes irregular capsular nodularity. Complications of capsulorrhaphy include capsular tears and subluxation of the humeral head. Postoperative MR imaging can evaluate healing after combined remplissage and Bankart repair for moderate size, engaging Hill-Sachs lesions. Laterjet and Bristow procedures may be performed in patients with recurrent dislocations and lenoid deficiency. Incorporated bone will yield non-anatomic glenoid configuration, and complications include non-union, fatty degeneration of subscapularis muscle, and osteoarthrosis.

***Learning Objectives:***
1. To appreciate common patterns of athletic injury in the shoulder.
2. To learn how to differentiate normal and failed labral repairs with MRI.
3. To consolidate the knowledge gained from the session with interactive cases of pre- and postoperative shoulder MRI.

*Author Disclosure:*
**L.W. Bancroft:** Author; Lippincott. Speaker; World Class CME.

**08:30 - 10:00** **Tech Gate Auditorium**

### Genitourinary

### RC 1307

### Imaging in pregnancy

**A-0808** 08:30
**Chairperson's introduction**
G. Masselli; *Rome/IT (gabriele.masselli@uniroma1.it)*

Modalities that do not use ionising radiation, such as US and MR imaging, should be the preferred examinations for evaluating a pregnant patient. Ultrasound is currently the standard approach for the initial evaluation of fetal anatomy and maternal conditions during pregnancy since it allows a real-time examination and is widely available and cost-effective. Magnetic resonance imaging, due to its morphological and functional capabilities, can greatly improve the diagnostic performance of US during pregnancy. Determining the cause of acute abdominal and pelvic pain in pregnant women can be difficult because of the multiple confounding factors found in normal pregnancy. Pelvic ultrasound may be of limited value due to the altered body habitus, a small field of view and the presence of interfering overlying structures. MR imaging is extremely accurate in identifying both obstetric and non-obstetric causes and should be used when ultrasound findings are nondiagnostic or equivocal. Imaging of the placenta can have a profound impact on patient management, owing to the morbidity and mortality associated with various placental conditions. Placental conditions affecting the mother and fetus include molar pregnancies, placental hematoma, abruption, previa, accreta, vasa previa, chorioangioma, and retained products of conception. Although uncommon, abnormalities of the placenta are important to recognise owing to the potential for maternal and fetal morbidity and mortality.

***Session Objectives:***
1. To understand how to safely perform imaging procedures in pregnant women.
3. To become familiar with the most relevant pathological conditions in pregnancy.
2. To learn about the indications of imaging procedures in pregnancy.

**A-0809** 08:35
**A. Safety issues in pregnancy: what radiologists need to know**
C. Bourgioti; *Athens/GR (charisbourgioti@yahoo.com)*

Imaging of the pregnant patient is challenging as it involves both the mother and the fetus. A number of medical, ethical or legal issues is likely to be raised. Potentially, all available imaging modalities may be applied to the evaluation of the pregnant patient; however, confusion regarding the safety of the mother and the fetus often results in unnecessary avoidance of useful diagnostic tests, especially those using ionising radiation. Sonography is the frontline imaging method for gravid patients, and its use for diagnostic reasons practically lacks any adverse effects. In the case of equivocal sonography, MRI (3T or less) can be used judiciously, regardless of gestational age, providing that the information gained is likely to offer medical benefit. When sonography or MRI are not feasible or less suitable, including cases with severe trauma or suspected pulmonary emboli, ionising radiation studies can be performed after a careful risk-benefit assessment, always keeping the radiation dose to the fetus as low as reasonably achievable (ALARA principle). The absorbed doses for diagnostic purposes are usually less than 50mGy and considered safe for the fetus; however, when CT is to be performed, implementation of dose reduction techniques or abdominal shielding is advised. Administration of iodinated contrast media intravenously is recommended only under special circumstances, and, neonatal thyroid function should be checked soon after birth. Currently, paramagnetic contrast injection is strongly discouraged, unless the potential benefit for the mother overcomes the potential risks of long-term exposure of the developing fetus to gadolinium.

***Learning Objectives:***
1. To learn about safe radiology practices and procedures in pregnant women.
2. To understand how to minimize the radiation burden in pregnant women.
3. To learn how and when to use contrast media in pregnancy.

**A-0810** 08:58
**B. Imaging acute abdomen in pregnancy**
M. Weston; *Leeds/UK (michael.weston2@nhs.net)*

There are causes of pain that are specific to pregnancy and causes of pain in other organs that may present during pregnancy. Pregnancy-related pain may relate to ectopic pregnancy, fibroid degeneration, placental abruption or uterine dehiscence. Acute conditions may occur in any other organ during pregnancy just as they may in the non-pregnant patient, but commonly seen conditions are appendicitis, pyelonephritis, renal colic and adnexal torsion. Complications post delivery primarily relate to either bleeding or sepsis. Ultrasound and MR form the mainstay of imaging in the pregnant patient, and the talk will concentrate on the appearances in these modalities. However, sometimes it can be better to use a test such as CT, with which the radiologist is familiar despite the perceived risks of ionising radiation, as the risk of misdiagnosis is reduced.

***Learning Objectives:***
1. To become familiar with the different causes of acute abdominal pain in pregnancy.
2. To learn about imaging features of the different causes of acute abdominal pain in pregnancy.
3. To learn how to use different imaging modalities in the setting of acute abdominal pain in pregnancy.

**A-0811** 09:21
**C. Imaging the placenta**
P. Soyer; *Paris/FR*

The placenta can be imaged with ultrasound and MRI for routine assessment, computed tomography for acute complication such as placental abruption, and angiography, should further embolisation be considered for hemorrhagic complication. The placenta can be involved by physiological and morphological abnormalities and may have an abnormal location. Rarely, the placenta can be involved by gestational trophoblastic diseases (hydatidiform mole, invasive mole and choriocarcinoma). Retained products of conception are rarely diagnosed with imaging because obstetricians systematically perform a uterine revision. In vivo assessment of placental vascularisation is routinely performed with Doppler ultrasound but 3D imaging and functional MRI can be used to scrutinise placental functions. Decreased maternal blood to the placenta is associated with preeclampsia and intrauterine growth restriction. Each imaging technique has intrinsic advantages that are counterbalanced by limitations. Placental location is best depicted with MRI. Regarding abnormally invasive placenta (AIP), MRI is used as a second line examination and several MRI findings are associated with AIP with various degrees of sensitivity and specificities, but the added value of MRI by comparison with ultrasound alone is not well established. Functional MRI (arterial spin labelling, blood oxygen level-dependent and oxygen-enhanced, diffusion-weighted MRI) helps estimate the placental function that are related to vascularisation, oxygenation, and metabolism. Current evidence does not support the use of gadolinium-based contrast agent.
***Learning Objectives:***
1. To understand the appearance of the placenta with different imaging modalities.
2. To become familiar with the imaging appearance of the commonest pathological conditions of the placenta.
3. To discuss the added value of cross-sectional imaging in the evaluation of placental abnormalities.

09:44
**Panel discussion: How should we image the pregnant woman, and when?**

**10:30 - 12:00 Room A**

### E³ - ECR Academies: Interactive Teaching Session for Young (and not so Young) Radiologists

### E³ 1421

### Genitourinary radiology for the general radiologist

**A-0820** 10:30
**A. Cystic pelvic masses: differential diagnosis and management**
O. Nikolic; *Novi Sad/RS (nikolic.olivera@gmail.com)*

Cystic pelvic masses are frequent in women and are related to the female reproductive system (gynecologic lesions-usually of ovarian origin). Genital fluid masses in men are less frequent. Moreover, a variety of fluid-filled lesions unrelated to the genital organs may be seen in both sexes, normally associated with the urinary system, gastrointestinal system or miscellaneous group of lesions. The aim of this talk is to present the imaging features of different cystic pelvic entities and their differential diagnoses and to learn the practical workflow in everyday clinical practice. Different types of cystic pelvic masses may have similar imaging characteristics, and therefore radiologic evaluation may be of limited diagnostic use. To avoid misdiagnosis, it is very important to understand the relationship of a mass with its anatomic location, to identify the ovaries at imaging in female patients and correlate imaging findings to the clinical history and laboratory findings.
***Learning Objectives:***
1. To learn the imaging characteristics of the different entities.
2. To become familiar with the practical workflow in everyday clinical practice.

**A-0821** 11:15
**B. Gynaecological emergencies**
M. Weston; *Leeds/UK (michael.weston2@nhs.net)*

Gynaecological emergencies may present with abdominal distension, pain, bleeding or sepsis. Normal physiological changes that might mimic disease are discussed. This presentation will show the common imaging features of ectopic pregnancy, ovarian cyst accidents, hyperstimulation, adnexal torsion, fibroid degeneration, and pelvic inflammatory disease. The appropriate use of US, CT and MR scans will be discussed.
***Learning Objectives:***
1. To become familiar with the differential diagnosis.
2. To identify the key imaging findings.

**10:30 - 12:00 Room X**

### ESR Patient Advisory Group (ESR-PAG)

### PA 1

### Communicating the role of the radiologist: best practices to manage patient expectations

**A-0822/A-0823** 10:30
**Chairpersons' introduction**
N. Bedlington; *Vienna/AT*
N.I. Traykova; *Plovdiv/BG (nikoletatraikova@gmail.com)*

The relationship between the radiologist and patient representative groups is of paramount importance to ESR. A radiologist’s visibility is far too often insufficient to patients due to lack of communication, as a result of understaffed departments, overworked radiologists and not enough time for them to properly communicate with patients. ESR is committed to cooperating with ESR PAG to improve radiologists’ communication skills, to effectively promote their role to the patients and to make the former better understand and appreciate patients’ needs. ESR is also committed to maintaining and improving all aspects of patients-radiologist relationships in the modern high tech radiology department.
***Session Objectives:***
1. To explain to radiologists how to effectively communicate their roles to their patients.
2. To identify room for improvement in patient communication based on experience reports by patient representatives.
3. To discuss approaches on how to maintain a good patient-health professional relationship in the high-tech radiology department.

**A-0824** 10:40
**Explaining the radiologist's role to patients**
A. Brady; *Cork/IE (adrianbrady@me.com)*

Historically, radiologists have often worked,, undercover“. The perception has been created that radiologists are often , the doctors’ doctor“, and that we have little or no engagement directly with patients. This is neither desirable nor true. Nonetheless, patients often do not know if they’ve engaged with a radiologist, and may not understand our role and responsibilities. Conversely, radiologists may not understand the main concerns of patients with whom we interact, given that most of the information provided to us to assist us in caring for patients is delivered from a third party, the referrer. One of the responsibilities of a radiologist which is often underfulfilled is that of communicating with patients. This takes many forms, including explaining the reason for an investigation, what a patient will experience, what will happen after the investigation, and what the findings mean. This talk will explore how these pieces of information may be communicated, and how mutual understanding between patients and radiologists may be improved, for the benefit of all.
***Learning Objectives:***
1. To outline the role of the radiologist in patient care.
2. To consider how best to explain the radiologist's input in diagnosis and care to patients.
3. To understand how radiologists and patients may communicate for the benefit of patients.

**A-0825** 11:00
**The patient perspective: sharing expectations and experiences (part 1)**
B. Bauer; *Abensberg/DE (birgit.bauer@manufaktur-fuer-antworten.de)*

People diagnosed with a disease like MS or other chronic conditions experience a lot of different exams. One of those is an MRI. I will talk about the information given to patients and what they need and what they have to understand when they sign the information leaflet. With my own story and a lot of research, I will bring up the patient perspective about the needs in information and understandability.
***Learning Objectives:***
1. To understand language used within the radiology department and patients' needs to effectively communicate with the radiologist.
2. To learn about programmes that help patients to better understand the illness and medical imaging procedures used in the hospital.
3. To understand both the patient and radiologist's perspective in order to further empower the patient.

*Author Disclosure:*
**B. Bauer:** Board Member; ESR PAG. Speaker; diverse Patient Organisation / Pharma Company.

**A-0826** 11:10
**The patient perspective: sharing expectations and experiences (part 2)**
E. Briers; *Hasselt/BE (erikbriers@telenet.be)*

The patient entering the "Medical imaging - radiology department" normally has some knowledge on the way he is there. He has been referred by another clinician for some imaging procedure. This referring clinician may or may not have taken the time to inform the patient on the upcoming procedure. Because of the "may or may not" it would be prudent for the department to ask the patient: "Do you know why you are here?" this could be the starting point of supplementary information. It is possible that the clinician explained that the procedure would help come to a final diagnosis or on the success or progress of treatment. It is not certain that the patient is also informed on the procedure sensu stricto, entering the confined space of a CT or MRI equipment, the use of contrast and much more. There is almost always extra information needed. At the moment the patient enters the department, the moment to choose is over, but if choices can be made it is important that the patient is involved in this decision. Due to time constraints in the department, the waiting time before the actual procedure seems to be the moment when patients could be given extra information, in print or on dedicated screens. If the intervention is special (biopsies) the information should be given to the patient while he is receptive and before any anaesthesia is applied. Any-way the provided information should be adapted to the personal learning capabilities of the patient(s).
***Learning Objectives:***
1. To outline patient expectations in terms of communication and radiology procedures within the hospital department.
2. To learn about practical solutions in hospital departments to meet patients' demands for involvement in decision-making regarding the care pathway.
3. To understand how to effectively communicate with patients through face-to-face interaction and online reports.
*Author Disclosure:*
**E. Briers:** Advisory Board; Requite project. Board Member; Us Too Belgium. Speaker; None for Pharmaceutical Companies. Other; Member of EAU Guidelines Committee on Prostate Cancer, Alternate Patient Member Committee Of Advanced Therapies - EMA.

**A-0827** 11:20
**Best practices: tools to optimise communication with patients**
L. Robinson; *Gatley/UK (leslierob10@gmail.com)*

This presentation will consider an effective communication from the standpoint of partnership working; that is partnerships between patients, the public and practitioners. Historically, communication in radiography has been predicated on a paternalistic model; i.e. the practitioner determines what it is that the patient needs to know. This is at odds with current health policy on partnership working and shared decision-making where there should be "no decision about me without me". In 2018, the UK Society and College of Radiographers convened a group of patients and practitioners to address this mismatch. The outcome was a set of Guiding Principles, written in the patient voice, which establishes what patients perceive to be effective communication and partnership working within diagnostic imaging and radiotherapy services. The presentation will discuss these Guiding Principles focusing in on what patients have told us they want in terms of effective communication.
***Learning Objectives:***
1. To learn about effective communication and useful information for patients.
2. To learn that an overload of information is equally detrimental to a safe feeling as no information.
3. To understand how patient summaries and online reports can empower patients and contribute to shared decision-making.

11:40
**Panel discussion: How to maintain the human touch in the radiology department in an era of rapid technology adoption**

**10:30 - 12:00** **Room F1**

### E³ - European Diploma Prep Session

### E³ 1423
### Neuro

**A-0828** 10:30
**Chairperson's introduction**
C. Calli; *Izmir/TR*

***Session Objectives:***
1. To learn relevant imaging and interventional algorithms and important imaging features of neurovascular disorders of the brain and spine.
2. To understand imaging features and prognostic implications of tumours of the brain and spine.
3. To become familiar with the role of different imaging modalities including hybrid imaging in diagnosing disorders of the central nervous system.

**A-0829** 10:36
**A. Congenital and white matter disorders of the brain**
A. Rossi; *Genoa/IT (andrearossi@gaslini.org)*

In this lecture I will review the basic concepts about brain maturation and myelination, and how to set up a baseline MR study of the brain in the pediatric age group. I will also examine some frequent pitfalls in pediatric neuroimaging, focussing on a frequent finding: pineal gland cysts. Then, I will address the main congenital malformations of the brain, highlighting the role of MR with diffusion tensor imaging (DTI) in their understanding and classification. I will also examine the principal neurocutaneous syndromes, such as Neurofibromatosis type 1 and 2, tuberous sclerosis, Sturge-Weber syndrome, and von Hippel-Lindau disease. Finally, I will elucidate a few basic concepts about leukodystrophies, focusing on pattern recognition based on MR, and understanding how to differentiate them from acquired white matter disorders, notably acute disseminated encephalomyelitis.
***Learning Objectives:***
1. To understand the development, normal anatomy and normal variants of the brain.
2. To become familiar with common congenital disorders of the brain and neurocutaneous syndromes.
3. To learn imaging features and differential diagnoses of white matter disease, inflammation and neurodegeneration.

**A-0830** 11:04
**B. Neurovascular disorders and trauma of the brain**
M. Vernooij; *Rotterdam/NL (m.vernooij@erasmusmc.nl)*

This lecture is part of a series of lectures aimed at preparation for the neuroradiology part of the EDiR examination.
***Learning Objectives:***
1. To become familiar with the normal anatomy and normal variants of the craniocervical arterial and venous system.
2. To learn the causes and imaging features of stroke, haemorrhage and other common vascular lesions of the brain and their relevance to interventional neuroradiology.
3. To understand the imaging features of traumatic injury to the brain.

**A-0831** 11:32
**C. Tumours of the brain and spine**
M.M. Thurnher; *Vienna/AT (majda.thurnher@meduniwien.ac.at)*

The fifth edition (2016) of the WHO Classification of Tumors of the CNS is the worldwide standard for classifying and grading brain neoplasms. Standardised MR brain tumour protocol is crucial for the preoperative evaluation and interpretation of postoperative changes. Brain tumour imaging objectives include the diagnosis of a brain tumour and the ability to distinguish it from non-tumoral lesions, assessment of histological grade of the tumour, delineation of the tumour borders and extension, differentiation between a tumour and peritumoral oedema, and finally the evaluation of possible recurrence and therapy-induced phenomena. In this lecture, two major issues will be discussed: the value of different conventional and advanced MRI techniques in evaluation of CNS tumours; most common spine tumours not to miss.
***Learning Objectives:***
1. To understand the normal anatomy and normal variants of the spine, spinal cord and nerve roots.
2. To learn imaging features of benign and malignant tumours of the neurocranium.
3. To become familiar with the imaging features of benign and malignant tumours of the spine.

**10:30 - 12:00** **Room Y**

### RTF - Radiology Trainees Forum

### TF 1

### Highlighted Lectures

*Moderators:*
A. Svare; Riga/LV
T. Teneva; Varna/BG

### A-0832 10:30

**Spinal emergencies: a look beyond trauma**
D. Ivanova; *Varna/BG (dari.ivanova@gmail.com)*

Nontraumatic spinal emergencies present a wide range of conditions resulting in acute and/or rapid development of neurological symptoms from the spinal cord. The clinical presentation can be very unspecific, and imaging plays a pivotal role in getting the right diagnosis. The causes of nontraumatic spinal emergencies can roughly be classified into two major groups. The group of intrinsic and non-compressive causes, such as spinal cord ischemia and myelitis, and the group of extrinsic and compressive causes, such as spontaneous extramedullary haemorrhages, spondylodiscitis and epidural abscess, metastatic and malignant entities etc. Most of the cases of compressive spinal cord damage present neurosurgical emergencies and their outcome depend heavily on the timely and correct diagnosis and treatment. MRI is the preferred modality for evaluation of patients with nontraumatic spinal emergencies, and an appropriately tailored and timesaving protocol is always crucial. As an adjunct to MRI, CT and conventional X-ray are very helpful when bone and calcium-containing structures have to be assessed, and ultrasound has a special role in establishing the diagnosis in small children.
***Learning Objectives:***
1. To learn about the various causes, both compressive and non-compressive, of non-traumatic spinal emergencies.
2. To become familiar with the imaging features of different non-traumatic spinal emergencies.
3. To appreciate the role of different imaging modalities in the diagnosis and differential diagnosis of non-traumatic spinal emergencies and the importance of choosing the right imaging protocol.

### A-0833 11:00

**Acute aortic syndrome: intimate knowledge through intimate case**
G.I. Kirova-Nedialkova; *Sofia/BG (gal.kirova@gmail.com)*

Acute Aortic Syndrome (AAS) is a highly lethal disease responsible for a wide range of clinical manifestations. In any given patient, the particular outcomes effects are related to the pattern and extent of the aortic wall injury, and, in the long term, the ability of the aorta to compensate circulatory pressure forces. It is therefore essential for radiologists to understand the characteristic appearance of the different aspects of AAS while recognising and reporting relevant imaging features, which would affect the patient's management. Using a case-based approach, the lecture will provide essential knowledge in evaluating, diagnosing, reporting and therapeutic planning of patients with signs and symptoms of the acute aortic syndrome.
***Learning Objectives:***
1. To focus on the appropriate imaging technique in case of acute chest pain.
2. To guide radiologists through an assessment of the characteristics of acute aortic dissection, penetrating atherosclerotic ulcer and intramural haematoma.
3. To focus on some details that could influence the selection of a therapeutic approach.
4. To foster a critical thinking approach based on specific features of the disease process.

### A-0834 11:30

**Getting it right: how to report an imaging study before the interventional radiology procedure**
R. Dezman; *Ljubljana/SI (rok.dezman@gmail.com)*

Interventional radiology is a medical speciality which provides minimally invasive image-guided treatment of disease. The rapid development and of the speciality has led to the growth of the number and complexity of the procedures, and a consequent subspecialization of interventional radiologists was a logical result. Diagnostic radiologists, however, need to be aware of interventional radiology procedures, know their basic principles, indications and importance of diagnostic follow-up. This lecture will present the importance of a quality report of imaging studies prior to the interventional procedure. The most common interventional procedures that diagnostic radiologists encounter will be presented with focus emergency interventional procedures. Pearls and pitfalls of reporting prior to the intervention and the follow-up studies will be presented. The importance of follow up imaging of the common elective procedures will also be presented.
***Learning Objectives:***
1. To present core procedures of interventional radiology.
2. To provide tips for reporting imaging studies before potential interventional radiologic procedures.
3. To appreciate the importance of collaboration between diagnostic radiology and interventional radiology.

**10:30 - 12:00** **Room M 4**

### Pros & Cons Session

### PS 1427

### US before CT in the acute abdomen?

### A-0835 10:30

**Chairperson's introduction**
J. Stoker; *Amsterdam/NL (j.stoker@amc.uva.nl)*

Acute abdominal pain can be caused by a myriad of diseases. Clinical history and physical examination have substantial limitations in differentiating between urgent and non-urgent conditions and even more in making an accurate diagnosis. Ultrasound and computed tomography are therefore mainstays in the diagnostic workup of these patients. Magnetic resonance imaging is used to a more limited extent. There are different approaches to using ultrasound and computed tomography, taking advantage of each technique's strength. A conditional diagnostic strategy with ultrasound in all patients and computed tomography only in those with negative or inconclusive ultrasound has been advocated as being the most accurate and effective approach. Others have encouraged widespread use of computed tomography as a one-stop-shop technique or have advocated a diagnostic approach tailored to the individual patient or suspected disease. The pros and cons of different diagnostic strategies will be discussed.
***Session Objectives:***
1. To be familiar with the role of clinical findings in the management of acute abdomen.
2. To understand the role of imaging in the management of acute abdomen.

### A-0836 10:35

**A. Acute abdomen: US first!**
J.B.C.M. Puylaert; *The Hague/NL (dr.jbcmpuylaert@wxs.nl)*

CT advantages over US in the diagnosis of the acute abdomen: CT is extremely rapid, the actual costs of CT are probably lower than a time-consuming US examination, CT images are not disturbed by gas and bone, while obesity is even an advantage; producing the CT images is not operator-dependent and CT can be reviewed at a later point in time and also from a distance by means of teleradiology. Finally, CT images are easier understood and accepted by clinicians than US images are. US advantages over CT: US has an image definition in the loose range which is much higher. US is more interactive: patient's history, as well as painful area or palpable mass, can be correlated with the US findings. The US shows peristalsis, pulsations and blood flow. The US shows the effects of respiration, Valsalva manoeuvre, gravity and compression with the probe, allowing to assess whether organs as bowel and gallbladder are soft or rigid.US allows easy puncture of intraperitoneal fluid. US in acute abdomen is performed with graded compression. US examination should be symptom-directed and requires communication with the patient. A US finding may lead to a specific question to the patient and v.v. information provided by the patient may lead to a specific US search for pathology. In patients with an acute abdomen, the entire abdomen should be examined, i.e. from the axilla to the groin. The final US report should be integrated with the clinical findings, laboratory data, CT-scan and possible other radiological examinations.
***Learning Objectives:***
1. To be familiar with the optimal technique and optimal application of US in acute abdomen.
2. To learn the strengths of US in common diseases causing acute abdomen.
3. To understand the arguments why US should be performed as initial imaging technique.

### A-0837 11:00

**B. Acute abdomen: CT of course!**
M. Laniado; *Dresden/DE (micahel@laniado.de)*

The acute abdomen is a potentially life-threatening situation that requires an immediate diagnosis of the underlying cause. The optimal CT technique is to use contrast-enhanced CT at least in the portal-venous (PV) phase. If vascular pathologies or haemorrhages are suspected, plain scans and arterial phase imaging are mandatory in addition to the PV phase. Oral contrast is not recommended. Considering the top ten diseases in patients presenting with an acute abdomen (i.e. acute appendicitis, acute cholecystitis, ileus, gynaecological diseases, acute pancreatitis, urological diseases, perforated viscus, carcinoma, diverticulitis), CT is an accurate technique to either confirm or exclude the working diagnosis of the emergency physician. The strengths of CT in common diseases causing acute abdomen is the ease with which indicative findings are shown such as calcified and even uncalcified stones, gas in the abdominal cavity, the bowel wall or mesenteric/hepatic blood vessels, peri-inflammatory tissue stranding and abscesses, the transition point from bowel distension to collapsed bowel, blood vessel occlusion and compromised organ perfusion, etc. CT should be performed as an initial imaging technique in cases of the acute abdomen because of the widespread 7/24 availability in emergency departments, the speed of image acquisition, the robustness even in uncooperative patients, the standardised imaging protocols and low operator dependence, and the reliability in the discussion of findings with referring physicians. In addition, the diagnosis of acute abdomen is increasingly made in elderly patients making radiation exposure a less significant issue. In conclusion, CT should be used first in cases of acute abdomen.

***Learning Objectives:***
1. To be familiar with the optimal technique and optimal application of CT in acute abdomen.
2. To learn the strengths of CT in common diseases causing acute abdomen.
3. To understand the arguments why CT should be performed as initial imaging technique.

**A-0838** 11:25
**Discussion**
M.M. França; *Porto/PT*

**10:30 - 12:00** **Room M 5**

### Transatlantic Course of ESR and RSNA (Radiological Society of North America): Sports Imaging

### TC 1428

### Wrist and foot/ankle sports injuries

*Moderators:*
L.W. Bancroft; Orlando, FL/US
A.J. Grainger; Leeds/UK

**A-0839** 10:30
**A. Soft tissue wrist injury in the athlete**
C.W.A. Pfirrmann; *Zurich/CH*

Wrist injuries account for 5 % of sports injuries. In the young athlete, fractures are the most common injuries. The hand and wrist are the most common sites for fracture in the young athlete. Physeal injuries are typical overuse injuries in gymnasts. Chronic stress reactions with a widening of the growth plate are seen in the distal radial and less common in the ulnar growth plate. Injuries to the TFCC in the athlete occur in acute trauma and with overuse. TFCC injuries are an important cause for ulnar-sided wrist pain. The differential diagnosis includes ulnar styloid impaction syndrome, ulnar impingement syndrome and tenosynovitis of the extensor carpi ulnaris tendon. Injury to the interosseous ligaments may lead to carpal instability. Chronic injury of the intrinsic or extrinsic ligaments of the wrist may cause ganglion cyst formation.

***Learning Objectives:***
1. To learn about the patterns of injury seen at the wrist in athletes.
2. To understand the advantages and disadvantages of different modalities for imaging the athlete's wrist.
3. To recognise the imaging appearances of cartilage and ligamentous injury at the wrist.

**A-0840** 11:00
**B. Multimodality imaging of foot and ankle injuries in the athlete**
A.J. Grainger; *Leeds/UK*

Ankle injuries are common in many sports, and the complicated anatomy of the ankle joint can be challenging the reporting radiologist. The ankle joint itself is a synovial hinge joint, but the important movement for ankle function also occurs at the joints of the hind and midfoot which are also susceptible to injury. In addition to conventional radiographs, CT, MRI and ultrasound all have important roles to play in the diagnosis of foot and ankle injuries in the athlete. The ligamentous and tendon structures about the ankle are generally superficial in nature and readily amenable to assessment with ultrasound where assessment can be enhanced due to the dynamic capabilities of the technique. While MRI also demonstrates these structures, it has advantages for assessing deeper joint structures such as the chondral surfaces and bones. The complex 3d anatomy of the foot and ankle means that conventional radiographs can struggle to demonstrate bone injury which means CT also has an important role to play. This lecture will focus on the use of these imaging modalities for the assessment of acute and chronic ligamentous and tendon injury. Emphasis will be put on the mechanisms of injury and how they determine the resultant patterns of injury and imaging appearances.

***Learning Objectives:***
1. To appreciate the different and often contributory roles that imaging modalities have in the foot and ankle.
2. To recognise the most common ligamentous and tendon injuries in the ankle.
3. To understand how common patterns of injury relate to the mechanisms involved.

**A-0841** 11:30
**C. Interactive case discussion (Part 1)**
C.W.A. Pfirrmann; *Zurich/CH*

This interactive teaching session will show various cases related to upper extremity sports injuries. Case discussion about soft tissues injuries of the wrist such as TFCC lesions and other wrist and hand injuries in athletes will be done interactively. The differential diagnosis and diagnostic challenges, as well as pitfalls, will be discussed.

***Learning Objectives:***
1. To become familiar with the techniques available and imaging appearances of wrist athletic injury.
2. To become familiar with the techniques available and imaging appearances of foot and ankle athletic injury.
3. To consolidate the knowledge gained from the session with interactive cases of wrist, foot and ankle athletic injury.

**A-0842** 11:45
**C. Interactive case discussion (Part 2)**
A.J. Grainger; *Leeds/UK*

Cases will be presented with the opportunity for audience response highlighting and consolidating ideas presented in the preceding lecture. Abstract for that Lecture: Ankle injuries are common in many sports, and the complicated anatomy of the ankle joint can be challenging the reporting radiologist. The ankle joint itself is a synovial hinge joint, but the important movement for ankle function also occurs at the joints of the hind and midfoot which are also susceptible to injury. In addition to conventional radiographs, CT, MRI and ultrasound all have important roles to play in the diagnosis of foot and ankle injuries in the athlete. The ligamentous and tendon structures about the ankle are generally superficial in nature and readily amenable to assessment with ultrasound where assessment can be enhanced due to the dynamic capabilities of the technique. While MRI also demonstrates these structures, it has advantages for assessing deeper joint structures such as the chondral surfaces and bones. The complex 3d anatomy of the foot and ankle means that conventional radiographs can struggle to demonstrate bone injury which means CT also has an important role to play. This lecture will focus on the use of these imaging modalities for the assessment of acute and chronic ligamentous and tendon injury. Emphasis will be put on the mechanisms of injury and how they determine the resultant patterns of injury and imaging appearances.

***Learning Objectives:***
1. To become familiar with the techniques available and imaging appearances of wrist athletic injury.
2. To become familiar with the techniques available and imaging appearances of foot and ankle athletic injury.
3. To consolidate the knowledge gained from the session with interactive cases of wrist, foot and ankle athletic injury.

**12:15 - 12:45** **Room A**

### Headline Session

### HL 3

### Luigi Oliva Honorary Lecture

*Presiding:*
L.E. Derchi; Genoa/IT

**A-0847** 12:15
**Contrast enhanced ultrasound in paediatrics: ready for clinical practice?**
P.S. Sidhu; *London/UK (paulsidhu@btinternet.com)*

Ultrasound is gentle and patient-friendly imaging technique, with the ability to image in real time, with superb resolution, and the advantage of being less expensive than other imaging techniques. An ultrasound machine is the ultimate ‘plug-and-play’ modality. The uniqueness of real-time, safe, portability makes this an ideal solution for imaging in children. The child is reassured by the presence of the examiner explaining, the parent comforting, and the ability to move and question all the time aids the child's tolerance of the imaging procedure. Many areas of the body are inaccessible to an ultrasound examination, where other imaging techniques are superior. Nevertheless, when ultrasound visualises an organ, it has the best resolution of all the techniques. This is particularly true for abdominal work in the child, where often the body habitus allows for a detailed examination. Ultrasound has long been the poor relation with respect to contrast examinations, often incorrectly a non-contrast ultrasound in the adult is compared unfavourably with the contrast-enhanced CT. Microbubble ultrasound contrast agents have been in clinical practice in adults for 20 years, most used off-licence in areas of need, revolutionising many aspects of the diagnostic capabilities of an ultrasound examination. The introduction of contrast-enhanced ultrasound (CEUS) has been led by a number of pioneers, recognising the usefulness in aiding diagnosis and management with minimal morbidity to the child. This lecture will outline the pathway of CEUS in children to the sanctioning by the FDA.
***Learning Objectives:***
1. To learn about the application of CEUS in children.
2. To understand the legal implications of using CEUS off-label in children.
3. To appreciate the potential of combining ultrasound with CEUS in assessing the child.
4. To become familiar with the techniques and applications of CEUS.

*Author Disclosure:*
**P.S. Sidhu:** Advisory Board; Samsung Medison. Consultant; Itreas Inc. Speaker; Samsung Medison, Philips Healthcare, Bracco SpA, Hitachi Inc, Siemens Healthineers, GE Healthcare.

**12:30 - 13:30 Room C**

### E³ - The Beauty of Basic Knowledge: Pancreas

### E³ 24D

### Solid pancreatic neoplasms

*Moderator:*
I. Lupescu; Bucharest/RO

**A-0852** 12:30
**Diagnosis**
M. Zins; *Paris/FR (mzins@hpsj.fr)*

CT remains the standard of reference technique for diagnosis and staging of pancreatic ductal adenocarcinoma (PDA). Most PDA appear as a hypodense mass at pancreatic or portal phase CT with associated dilatation of the bile ducts and/or the pancreatic ducts. Pancreatic MRI is a solving tool that helps in the diagnosis of the isodense lesion at CT and in the assessment of liver metastases. Differential diagnosis of Solid pancreatic tumours includes a Neuroendocrine tumour (NET) of the pancreas. Typical Pancreatic NETs appear as hypervascularized tumours in the arterial phase using CT or MR.
***Learning Objectives:***
1. To learn about diagnostic imaging findings of solid pancreatic neoplasms.
2. To understand treatment planning.
3. To appreciate differential diagnosis of solid pancreatic neoplasms.

**A-0853** 13:00
**Staging**
N. Kartalis; *Stockholm/SE (nikolaos.kartalis@me.com)*

Pancreatic cancer is a disease with a very dismal prognosis. The 5-year survival is around 5% and has been stable over the last decades. The only potentially curative treatment is surgical resection combined with adjuvant and/or neoadjuvant therapy. However, at diagnosis, less than 20% of patients have a resectable tumour, while the rest have tumours that are locally advanced and/or have distant metastases. In order to improve survival, there is a need to increase the number of patients that undergo surgery. However, for the surgical removal to be meaningful, the goal is to achieve complete macroscopic and microscopic removal of a tumour (R0-resection). For the identification of patients who will benefit from surgery, the so-called "resectability status" was developed. It is based on the presence and extent of tumour contact with the major peripancreatic vessels such as portomesenteric axis (PV/SMV), hepatic artery, celiac artery and superior mesenteric artery. Depending on the presence and degree of the circumferential tumour-vessel contact the tumours are classified in one of the three categories: resectable, borderline resectable and unresectable. The criteria of the various existing classification systems will be discussed and their differences outlined. Furthermore, potential areas of improvement will be analysed and, finally, the role of CT, MRI and PET-CT in the evaluation of these patients will be presented.
***Learning Objectives:***
1. To learn how to stage pancreatic adenocarcinoma.
2. To understand resectability criteria.
3. To appreciate the role of imaging in treatment planning.

**12:15 - 13:45 Room X**

### EDiR Session

### EDiR

### EDiR: an instrument to develop excellence in your career

**A-0848** 12:15
**Chairperson's introduction**
L. Oleaga Zufiría; *Barcelona/ES (lauraoleaga@gmail.com)*

The European Diploma in Radiology (EDIR) examination is designed to test knowledge, skills and competence in anatomy, pathophysiology, imaging procedures, physics and management in general radiology. The exam is divided into three parts: a knowledge test with Multiple Response Questions (MRQs) and Short Cases (SCs) and the Clinically Oriented Reasoning Evaluation (CORE) where problem-solving skills are tested. The EDIR teaser that will be presented during the session includes examples of the three different parts of the exam simulating the real exam. The aim is to get the possible candidates to become familiar with the structure and degree of difficulty of the exam, including some tips and tricks to succeed.
***Session Objectives:***
1. To present the structure of the EDiR examination.
2. To describe how to prepare for the EDiR examination and the resources available.
3. To detail the CORE examination.
4. To learn the importance of structured reporting for the CORE examination.

*Author Disclosure:*
**L. Oleaga Zufiría:** Consultant; Telemedicine Clinic.

**A-0849** 12:20
**EDiR teaser**
L. Oleaga Zufiría; *Barcelona/ES (lauraoleaga@gmail.com)*

Objective structured Clinically Oriented Reasoning Evaluation (CORE) examination is a standard method of testing the knowledge in clinical core competencies. Web-based CORE examination represents a simulation of radiological practice in which candidates face a complete case that must be resolved in each of the areas of sub-specialisation as if they were in a real situation. It is crucial for EDIR candidates to be aware of the structure of the exam, establishing a method of reading the images in a structured way. A broad spectrum of educational materials and structured reporting products are readily available online for trainees to prepare for CORE exam. The first step, in the CORE examination, is to detect the disease. It is important to systematically review the whole picture to avoid perception errors, including " satisfaction of search errors". Next step is to create a list of the most relevant findings; it is essential to be consistent, starting from those that respond to the clinical question. The third step is to prepare a preliminary differential diagnosis, and suggest, if necessary, the most appropriate imaging modality to further study the patient, and finally define the most likely diagnosis. The practice of making structured reports with a specific script can be perfectly combined with the methodology needed to make a correct CORE exam and succeed. The systematic use of structured reports helps in the standardisation; it is a useful tool that serves as a guide for candidates facing the CORE exam.
***Learning Objectives:***
1. To review some practical cases: multiple response questions, short cases and CORE case.
2. To learn new online resources for the examination.
3. To answer any questions from the audience.

*Author Disclosure:*
**L. Oleaga Zufiría:** Consultant; Telemedicine Clinic.

**A-0850** 12:50
**CORE examination (Part 1)**
F. Saez; *Barakaldo/ES (fersaez@yahoo.com)*

Objective structured Clinically Oriented Reasoning Evaluation (CORE) examination is a standard method of testing the knowledge in clinical core competencies. Web-based CORE examination represents a simulation of radiological practice in which candidates face a complete case that must be resolved in each of the areas of subspecialization as if they were in a real situation. A broad spectrum of educational materials is readily available online for trainees to prepare for CORE exam. In order to properly pass the exam, it is necessary to know the structure and establish a method of reading the images that allow the candidate, in a structured way, to answer the questions that are asked.
***Learning Objectives:***
1. To have a look at an action plan for the CORE examination.
2. To better understand the CORE section.
3. To become aware of essential hints for success in the CORE section.
4. To become familiar with structured reporting as a guide for the CORE examination.
5. To understand how to make structured reports.

**A-0851** 13:15
**CORE examination (Part 2)**
L. Oleaga Zufiría; *Barcelona/ES (lauraoleaga@gmail.com)*

Objective structured Clinically Oriented Reasoning Evaluation (CORE) examination is a standard method of testing the knowledge in clinical core competencies. Web-based CORE examination represents a simulation of radiological practice in which candidates face a complete case that must be resolved in each of the areas of sub-specialisation as if they were in a real situation. It is crucial for EDIR candidates to be aware of the structure of the exam, establishing a method of reading the images in a structured way. A broad spectrum of educational materials and structured reporting products are readily available online for trainees to prepare for CORE exam. The first step, in the CORE examination, is to detect the disease. It is important to systematically review the whole picture to avoid perception errors, including "satisfaction of search errors". Next step is to create a list of the most relevant findings; it is essential to be consistent, starting from those that respond to the clinical question. The third step is to prepare a preliminary differential diagnosis, and suggest, if necessary, the most appropriate imaging modality to further study the patient, and finally define the most likely diagnosis. The practice of making structured reports with a specific script can be perfectly combined with the methodology needed to make a correct CORE exam and succeed. The systematic use of structured reports helps in the standardisation; it is a useful tool that serves as a guide for candidates facing the CORE exam.
***Learning Objectives:***
1. To have a look at an action plan for the CORE examination.
2. To better understand the CORE section.
3. To become aware of essential hints for success in the CORE section.
4. To become familiar with structured reporting as a guide for the CORE examination.
5. To understand how to make structured reports.
*Author Disclosure:*
**L. Oleaga Zufiría:** Consultant; Telemedicine Clinic.

13:40
**Panel discussion**

**12:30 - 13:30** **Room D**

### E³ - The Beauty of Basic Knowledge: A Survival Guide to Musculoskeletal Imaging

### E³ 25D

### Arthritis: an imaging approach

*Moderator:*
V.N. Cassar-Pullicino; Oswestry/UK

**A-0854** 12:30
**Arthritis: an imaging approach**
F. Kainberger; *Vienna/AT (franz.kainberger@meduniwien.ac.at)*

The current diagnostic concept is to embed imaging in the assessment systems in rheumatology. This means that imaging should contribute to the detection of early arthritis and to the quantification of abnormalities with prognostic impact. With the combinations of joint pain, laboratory indicators of inflammation and imaging features of tendovaginitis, joint effusion and/or bone marrow inflammation subforms of early arthritis may be defined. Synovitis is today regarded as part of a systemic autoimmunologic disease, and certain phenotypes can be classified with relation to the fibroblastic response and different interleukin expression leading to a destructive-erosive, a sclerotic-proliferative or a mixed imaging appearance. With US and radiography, followed by MRI, the patterns of arthritis and their differentiation from normal anatomic structures can be assessed and displayed in dedicated structured reporting templates. The differential diagnosis of autoimmune-mediated arthritis includes crystal-induced arthropathies, erosive osteoarthritis, posttraumatic and other forms of secondary joint inflammation.
***Learning Objectives:***
1. To become familiar with the current diagnostic concepts in the imaging of arthritis.
2. To understand the pathology of arthritis and its imaging phenotypes.
3. To appreciate the value of radiography, ultrasound and MRI.

**14:00 - 15:30** **Room A**

### E³ - ECR Academies: Interactive Teaching Session for Young (and not so Young) Radiologists

### E³ 1521

### Breast imaging

**A-0855** 14:00
**A. Diagnosis and management of common ductal and nipple-areolar-complex (NAC) abnormalities**
S. Perez Rodrigo; *Madrid/ES (drasilviap@gmail.com)*

The nipple-areolar complex lesions deserve particular consideration. They are a group of entities that arise in this specific area. It may be affected by normal variations in embryologic development and breast maturation. Besides, abnormal processes can be found, and benign and malignant pathology can be seen in this region. Eczema, mastitis, abscesses, adenomas, papillomas and duct ectasia should be considered as benign processes. On the opposite, Paget disease, in situ carcinoma, invasive carcinoma and lymphoma should be included as malignant processes. The radiologist should be aware of the clinical manifestations of these entities (inversion, retraction, palpable mass, nipple discharge, skin changes etc.). We should also keep in mind the different radiological findings and their peculiarities. We should be aware of the different imaging techniques and which of them is better for reaching a specific diagnosis. Mammography, ultrasound, galactography, ductoscopy and MRI can be useful in the diagnosis of these lesions. A multimodal diagnosis is required, and a multidisciplinary approach is recommended to correct treatment.
***Learning Objectives:***
1. To learn about NAC and ductal anatomy, and pathology.
2. To become familiar with the practical workflow in everyday clinical practice.
*Author Disclosure:*
**S. Perez Rodrigo:** Advisory Board; BD Bard. Speaker; BD Bard.

**A-0856** 14:45
**B. Techniques, artefacts and pitfalls in breast MRI**
R.M. Trimboli; *Milan/IT (trimboli.rm@gmail.com)*

Over the last three decades, magnetic resonance imaging (MRI) has dramatically entered the clinical field of detection and management of breast cancer (BC). Thanks to the introduction of contrast-enhanced (CE) sequences it gained very high sensitivity and intrinsic multiparametric nature. A modern, robust protocol is essentially composed of an unenhanced T2-weighted sequence; T1-weighted sequences acquired before and after intravenous administration of gadolinium-based contrast agent (GBCA) at a dose of 0.1 mmoL/kg/body weight; and diffusion-weighted imaging (DWI). A fat suppression with fat saturation, inversion recovery or alternatively, Dixon method, should be added to T1w and/or T2w scans. Non-uniform magnetic field and patient motion may reduce image quality potentially rendering an image or study non-diagnostic. Artefacts in breast MRI may be grouped under two broad categories: patient-related such as positioning, motion, and susceptibility and technical artefacts such as wraparound, chemical shift, and misregistration. Radiologists should be aware of and identify common artefacts to minimise potential negative effects on image interpretation and patient experience. Close attention must be played to injection of contrast material, the timing of the examination, lymph node evaluation, extra-mammary findings and kinetic assessment for avoiding pitfalls that can make interpretation of breast MR images challenging and lead to misdiagnosis. Recognising pitfalls associated with breast MR imaging is necessary for appropriate and accurate interpretation. Finally, empathic patient management has been reported as the most important factor in the overall quality of the examination.
***Learning Objectives:***
1. To describe the basic techniques for breast MRI (including DWI).
2. To illustrate patient-related and technical artefacts, and pitfalls in breast MR.

**14:00 - 15:30** **Room B**

### E³ - ECR Master Class (Abdominal Viscera)

### E³ 1526

### Dual-energy CT of the abdomen: the time is now

*Moderator:*
J. Sosna; Jerusalem/IL

**A-0857** 14:00
**A. Basic principles and different approaches**
L.S. Guimarães; *Toronto, ON/CA (luis.s.guimaraes@gmail.com)*

Dual-energy CT (DECT) refers to the use of CT data from two different energy spectra for differentiating and classifying tissue composition, in addition to displaying anatomy and pathology. DECT data can be obtained using various hardware solutions (dual source, fast kVp switching, 2-rotation fast-kV-mA switch, dual layer detector and split filter). An explanation of how each of these solutions works, as well as the advantages and disadvantages of each, will be presented. Abdominal DECT has as main advantages the ability to identify/quantify certain materials (iodine, calcium, urate, etc.), as well as to increase the possibility of performing low kV imaging in a wider range of patients, which in turn is associated with an increased conspicuity of enhancing structures and lesions. The background and reasons for each of these advantages will be reviewed. Several challenges remain for full implementation of Dual Energy CT in routine clinical abdominal practices. These challenges, including the absence of standardized post-processing (which may be time-consuming), lack of well-defined advantageous routine applications and limitations in terms of patient size/radiation dose will be discussed.
***Learning Objectives:***
1. To learn about the types of dual-energy scanners and principles of dual-energy CT.
2. To understand issues of radiation dose and image quality in comparison with single-energy CT.
3. To appreciate the possible advantages of this technology with its many post-processing applications.

**A-0858** 14:30
**B. Applications for genitourinary system**
H. Ringl; *Vienna/AT (helmut.ringl@meduniwien.ac.at)*

Dual-energy CT is increasingly becoming an important diagnostic tool for the non-invasive assessment of the genitourinary system, with a wide range of applications: The use of dual-energy CT with optimised spit-bolus protocols enables considerable dose savings by eliminating two scan phases. However, using virtual non-contrast images, this protocol provides the same information as an unenhanced, an arterial, a venous, and a urographic phase. Virtual non-contrast images and iodine maps enable better characterisation of renal cysts with dense content, which often pose a diagnostic dilemma in single-energy CT, and which might require MRI or CT follow-up examinations. The reconstruction of low kV mono-energetic images results in a higher iodine contrast, and, therefore, in better lesion conspicuity in the kidneys and the bladder wall. Dual-energy CT enables the quantification of iodine-uptake, and may, therefore, be used for therapy monitoring of cancer of the urinary tract, and the material decomposition algorithms of dual-energy CT are able to discriminate urate from non-urate stones in the case of nephrolithiasis in the clinical routine, making this method a game-changer in therapy.
***Learning Objectives:***
1. To learn about the role of dual-energy CT in the genitourinary system.
2. To understand the value of determination of renal stone composition with dual-energy CT.
3. To appreciate the ability of dual-energy CT to exactly quantify the iodine uptake in renal lesions.
*Author Disclosure:*
**H. Ringl:** Research/Grant Support; Siemens Healthcare.

**A-0859** 15:00
**C. Applications for abdominal organs**
M. Karcaaltincaba; *Ankara/TR (musturayk@yahoo.com)*

Dual-energy CT (DECT) is increasingly used for abdominal applications. DECT can be performed by dual-source CT, fast kVp switching and dual-layer detector CT. The major advantage of DECT is the availability of virtual non-contrast (water) images in every patient. Spectral CT images can allow diagnosis of a low amount of fat (normally invisible on CT) within lesions similar to in-out of phase T1-weighted MR images. DECT can allow diagnosis of calcification or haemorrhage within lesions encountered during routine abdominal CT. Low kV images obtained by DECT is more sensitive to hepatic and pancreas hypervascular lesions compared to standard kV images. DECT can also be helpful in the differentiation of colonic polyp/mass from stool detected during routine abdominal CT. In the future, all CT scanners will be most likely produced by DECT capability.
***Learning Objectives:***
1. To learn about the current applications of dual-energy CT in evaluating the abdominal viscera.
2. To understand the role of dual-energy CT for characterisation of incidental lesions discovered during routine abdominal CT.
3. To appreciate how dual-energy CT increases sensitivity for detecting both hypervascular and hypovascular liver and pancreatic lesions.
*Author Disclosure:*
**M. Karcaaltincaba:** Speaker; Bayer, Pfizer, Philips Healthcare, GE Healthcare.

**14:00 - 15:30** **Room C**

### EFRS Workshop: in association with EIBIR and the Radiography Journal

### EFRS WS

### Growing radiography research

*Moderators:*
J. McNulty; Dublin/IE
J.M. Nightingale; Sheffield/UK

**A-0860** 14:00
**Radiography research: where should we be in 2030? (part 1)**
L.A. Rainford; *Dublin/IE (louise.rainford@ucd.ie)*

The scope of medical imaging and its many sub-specialities has grown substantially over the past two decades. How medical imaging is employed by medical specialities, outside radiology has also altered the clinical use of imaging across medical centres and primary care settings internationally. Healthcare systems are developing to meet the changing needs of patients. There is a need for an evidence base of practice across all medical imaging activity to ensure optimal use. As multiple medical specialities, e.g. cardiology, rheumatology, orthopaedics, emergency medicine clinicians, to name a few, are involved in imaging on a daily basis, the scope of potential research activity is immense. Additionally, there is an increased use by other healthcare professionals such as physiotherapy and midwifery. An overview of this changing landscape will be discussed.
***Learning Objectives:***
1. To explore the changing landscape in medical imaging and how this might influence our research.
2. To consider the future radiography profession, what it might look like and where it might lead our research.
3. To propose some research targets for our profession to work towards over the next decade.

**A-0861** 14:08
**Radiography research: where should we be in 2030? (part 2)**
G. Paulo; *Coimbra/PT (graciano@estescoimbra.pt)*

There is no doubt that the only way to develop a professional field of knowledge is through research and Radiography is even more dependent on it, due to the exponential technological development of its field. In fact, we need to stop, watch and listen, to understand where these new technological roads will take us. One thing is certain: our profession will not be the same in 2030 and universities demonstrate some difficulties in anticipating what the future will bring, to be able to adapt the education models to incorporate the changes arising. Technology is taking over most of the decisions we have to make in our daily clinical practice, and therefore we have an obligation to, through research, develop new competencies in the fields where technology will never take over our role: It's “The human touch”.Radiographers will have to understand that patient safety (understood in a broad concept) and communication skills are the main pillars of our profession and developing them is the only way to keep our profession alive. Therefore, there is an urgent need to bring together professional societies and universities on a global summit, to establish an international strategic research agenda for radiography, as an instrument to develop our profession, anticipating the impact of the new technological advancements in our field of knowledge.
***Learning Objectives:***
1. To explore the changing landscape in medical imaging and how this might influence our research.
2. To consider the future radiography profession, what it might look like and where it might lead our research.
3. To propose some research targets for our profession to work towards over the next decade.

**A-0862** 14:16
**Radiography research: where should we be in 2030? (part 3)**
H. Precht; *Odense/DK (hepr@ucl.dk)*

To support radiography research growth in the future different several elements require consideration. First radiography research should align with modern hospital structures and practices, with a focus on multidisciplinary cooperation to produce new knowledge. Radiography research needs to broad in activity to include, for example, cardiology, forensic medicine, midwifery, machine learning, robot technology, in addition to traditional radiography research subjects. Universities and colleges with Radiography training programmes need to support and advance research activity. Realistic targets could be that Radiography programmes are involved in research work within a specific time-frame, over the next five years to develop research networks. The OPTIMAX model demonstrates a good model for collaborative research; this can be developed nationally between colleges or on a larger scale across colleges in different countries. Staff research portfolios are important and should be public, to highlight the activity and cooperation within our profession. It is important to note that promoting radiography research is to the benefit of patients and is essential to maintain high standards of patient management.
***Learning Objectives:***
1. To explore the changing landscape in medical imaging and how this might influence our research.
2. To consider the future radiography profession, what it might look like and where it might lead our research.
3. To propose some research targets for our profession to work towards over the next decade.

**A-0863** 14:24
**The clinical research radiographer: essential for our profession or facilitating the research of others?**
B. Snaith; *Wakefield/UK (bev.snaith@midyorks.nhs.uk)*

Radiography is developing a research community, supporting the profession to gain capability in research skills. The majority of this activity is taking place in academia, yet research is required to develop evidence-based practice. Radiographers are supporting the delivery of research projects through clinical activity, recruitment and support, but are yet to develop clinical independence in research. This presentation will consider the challenges and opportunities for the development of clinical researcher roles, skills and activities. Using case studies examples from practice will explore the challenges and identify future strategies to support personal and professional development.
***Learning Objectives:***
1. To understand the scope of a research radiographer in clinical practice.
2. To learn how the role can improve clinical research capacity and capability and clinical-academic collaboration.
3. To appreciate the difference between research facilitation and the radiographer as a clinical researcher.

**A-0864** 14:42
**Turning your research idea into reality: opportunities for European funding**
P. Zolda; *Vienna/AT (pamela.zolda@myesr.org)*

Many national funding schemes do not support a cross-border approach, which limits the scientific collaboration of European research groups. Thus researchers rely on European Union funding sources as provided by Horizon 2020, the largest EU research and innovation programme. However, the programme has become highly competitive, and often even high-quality project proposals cannot be funded. Additionally, successful projects are facing the challenge of navigating through the rules of large EU projects while simultaneously carrying out innovative research with partners from across Europe. Consequently, multidisciplinary and multinational consortia require professional support for proposal preparation and project management. The European Institute for Biomedical Imaging Research, EIBIR, is a non-profit organisation founded by the European Society of Radiology and supports researchers and industry partners in the coordination of biomedical imaging research. EIBIR offers expert advice, professional project management and coordination, as well as dissemination services for international collaborative research projects. The EIBIR services also include advice on funding opportunities and proposal writing support by an experienced team with knowledge of the European Commission's requirements. Through EIBIR`s large landscape of network members, shareholder organisations, industry partners and media contacts the conducted research is widely and rapidly communicated. EIBIR is currently a partner and/or coordinator of seven Horizon 2020 projects and relieves researchers of the administrative burden, allowing them to focus on the scientific aspects and thereby ensuring the best project outcome. All services are free of charge for active EIBIR network members and can be used for a moderate annual fee.
***Learning Objectives:***
1. To learn about EIBIR and its services for researchers.
2. To understand project management and dissemination activities of European projects.
3. To appreciate how EIBIR can support your research project.

**A-0865** 15:00
**Research is nothing without effective dissemination**
F. Zarb; *Msida/MT (francis.zarb@um.edu.mt)*

Research is the contribution to the body of knowledge in a particular area/expertise. Research findings should be disseminated, shared and made available to fellow professionals and others for these findings to have an overall beneficial effect. There are a number of ways for disseminating research findings such as through peer-reviewed publication, conference presentations, posters etc. This presentation aims to highlight the importance of research finding dissemination for both the individual researcher/practitioner and to the clinical/academic departments and countries they represent. The dissemination process may not always seem easy and straightforward, requiring a commitment and perseverance in the work it entails. However, the benefits and satisfaction associated with research dissemination outweigh the hard work involved.
***Learning Objectives:***
1. To explore the importance of dissemination at the local, national and international level.
2. To explore the importance of publishing for clinical and academic departments.
3. To consider the benefits and limitations of publishing in different journal formats.

15:18
**Panel discussion: What are the biggest barriers to growing radiographer-led research?**

**14:00 - 15:30** **Room N**

### EuroSafe Imaging Session

### EU 5

### Getting the balance right: radiation risk and imaging benefit in paediatric procedures

**A-0871** 14:00
**Chairperson's introduction**
C. Owens; *London/UK (owensc@gosh.nhs.uk)*

This important session takes a multidisciplinary approach on team efforts to facilitate the best use of expensive medical imaging equipment. We will attempt to ask and answer questions about our role in helping to optimise CT technology for best use in children, including our role as radiologists in dose reduction and adaption to the use of lower dose techniques and post-processing methods which assist with noise reduction.
***Session Objectives:***
1. To learn about risk and benefits in imaging children.
2. To understand the importance of an integrated approach from radiology and medical physics.
3. To appreciate a multidisciplinary approach to imaging optimisation and radiation protection.

**A-0872** 14:05
**Guidelines for the use of conventional radiographs in children and adolescents**
R. Seuri; *Helsinki/FI (raija.seuri@hus.fi)*

Conventional radiography, usually nowadays either CR or DR is the most often used medical imaging modality. The rapid development of other modalities - some more sensitive, some without the burden of ionizing radiation - has changed the use of conventional radiography. Also, the wide postprocessing possibilities and the even more sensitive detectors have challenged our knowledge and practise in optimisation. Evidence-based information on the use of radiological modalities and different examinations, also conventional radiography, is widely available. But even when following the most recent literature we must keep in mind that adult rules do not always apply to paediatrics. The digital imaging techniques of computed radiography (CR) and direct radiography (DR) have changed optimization considerably. They give the possibility of diagnostic imaging with very low patient dose, but at the same time the dose is not any more visually seen in the image. This has created the problem of possible ”dose creep”. Even if the dose is transferred to the image display, systematic dose evaluations are needed to verify and maintain the level of dose optimization. The use of pre-set protocols is usually not possible in paediatric imaging. Deep understanding of the postprocessing systems is not easily available during the optimisation process. The key point is close co-operation between professionals. Wide variation in imaging practices and the parameters used have been noticed in Europe. Lack of written guidelines means variation in both image quality and patient exposure.
***Learning Objectives:***
1. To learn about the current developments in conventional radiography.
2. To understand the strengths and weaknesses of each technique.

**A-0873** 14:25
**Computed tomography: are we doing enough?**
E. Castellano; *London/UK (elly.castellano@rmh.nhs.uk)*

Optimal CT imaging of children requires the concerted effort of manufacturers, radiologists, radiographers and physicists. Only by working together as a multidisciplinary team can images of adequate quality be produced at appropriate radiation doses. Recent developments in CT scanner technology that particularly benefit children, such as lower tube voltages, faster scanning, improved detector design and iterative reconstruction, are reviewed in this talk. The impact of technology on patient dose can be determined using dose management systems. This lecture considers how such a system, in combination with the PiDRL guidelines, can become a valuable tool for benchmarking and monitoring evolving clinical practice. Notwithstanding, dose indicators such as dose-length-product can be misleading when scanning children, so the need for better radiation risk estimates remains; recent advances in CT dosimetry will also be considered. The role of the radiologist is vital in maintaining the balance between radiation dose and image quality. Radiation dose reduction without regard to image quality poses a greater clinical risk than a radiation risk and may precipitate a backlash from clinicians. This lecture suggests ways in which the radiologist can work with radiographers and physicists to define image quality requirements and thus complete the optimisation loop.
***Learning Objectives:***
1. To learn about the current status of CT systems.
2. To understand the strengths and weaknesses of each CT type.
3. To appreciate the importance of dose optimisation and an active physics programme.

**A-0874** 14:45
**Dose reduction strategies in paediatric PET/CT and PET/MR examinations**
J. Schäfer; *Tübingen/DE (juergen.schaefer@med.uni-tuebingen.de)*

High cumulative effective doses (ED) by repetitive PET/CT have been described ranging from 6-400 mSv, whereas the CT proportion is up to 80 % of the total dose. Usually, the administered tracer dose is adapted to body weight according to published recommendation (e.g., dosage card of the EANM), and therefore relatively standardised. However, the kind of CT acquisition is quite variable reaching from CT used only for attenuation correction (AC) over a reduced dose CT for anatomical allocation to whole-body contrast-enhanced diagnostic CT plus an additional CT for AC which reveals the highest radiation of all variants. According to these purposes (also as a combination), the scan-parameters must be optimised using automatic exposure control (AEC). Depending on the patient size and the CT mode, AEC achieves appropriate dose reduction up to 40% by maintaining image quality. Overall in comparison to PET/CT, PET/MRI has shown to be equivalent concerning the PET component. PET/MRI allows for a reduction in radiation dose by replacing CT with MRI making it particularly suitable for pediatric applications. A dose reduction of up to 80% has been demonstrated in individual cases. Moreover, also the administered dose of the PET tracer can be reduced due to both, the higher sensitivity of PET detectors in PET/MR scanners and the longer PET data acquisition times compared to PET/CT. Thus, ED of PET/MR below two mSv are realistic if a local PET/MR is performed (e.g., in inflammatory bowel disease) further reduction is possible.
***Learning Objectives:***
1. To understand the real doses involved in PET/CT and PET/MRI.
2. To appreciate relative risks of multiple examinations and highlight benefits of the different imaging protocols.
*Author Disclosure:*
**J. Schäfer:** Research/Grant Support; German Children's Cancer Charity, Siemens Healthineers - Siemens Healthcare, Bayer AG, Philips Healthcare. Speaker; Philips Healthcare.

**A-0875** 15:05
**Radiation safety in interventional procedures in children**
C. Granata; *Genoa/IT*

Interventional Radiology (IR) encompasses a wide range of techniques, including both cardiac and non-cardiac procedures. During the last decade, there has been a significant increase in IR procedures in children, many of them delivering high radiation doses, without any other treatment option. The few published studies in children mostly concern single centre experience and confirm the very high variation of doses among different centres due to child size, complexity of conditions, technique adopted, experience of medical staff, and available equipment. Furthermore, the underlying disease may require repeated procedures causing a higher cumulative dose. Diagnostic Reference Levels (DRLs) are excellent tools for technique optimisation. Nevertheless, due to the reasons detailed above, national DRLs have not been established in any European country for any IR procedure in children so far. Therefore, a cohesive effort is needed with the initial aim to establish local DRLs at least in the less complex procedures, characterised by a smaller number of variables. Subsequently, local DRLs from different centres should be compared and analysed to better understand the reasons of the large differences in administered doses and to be able to decide if national or even European DRLs for IR procedures in children are feasible and appropriate.
***Learning Objectives:***
1. To learn about current practices in interventional radiology in children.
2. To understand the range of doses across centres.
3. To appreciate the importance of a cohesive approach.

15:25
**Discussion**

**14:00 - 15:30 Room O**

### Cardiac

### RC 1503

### Cardiac imaging in structural heart disease

**A-0876** 14:00
**Chairperson's introduction**
L. Natale; *Rome/IT (luigi.natale@unicatt.it)*

Structural heart diseases refer to non-coronary cardiovascular processes and related interventions. The term was first used in 1999 Transcatheter Cardiovascular Therapeutics meeting. A huge amount of efforts have been done in last two decades, embracing recognition of disease, the underlying pathophysiologic mechanisms, the developments of imaging techniques and catheter-based techniques and devices, resulting in improvement of diagnosis and treatment of structural heart diseases. Main examples of structural heart diseases are congenital (ASD, PFO, VSD, PDA), thrombi source assessment and treatment (left atrial appendage and ventricular aneurysm closure), valvular diseases and hypertrophic obstructive cardiomyopathy. Three final considerations are mandatory: first, the need for a multidisciplinary time, due to the different expertise needed; second, the volume of procedures, that is increasing but is still much lower than coronary interventions; third, the huge technical differences in procedures with consequent further subspecialties needs.
***Session Objectives:***
1. To become familiar with the concept of "structural" heart disease in modern cardiovascular medicine.
2. To get an overview about the minimally invasive treatment possibilities in structural heart diseases.
3. To learn about the requirements and possibilities of modern cardiac imaging in structural heart diseases.

**A-0877** 14:05
**A. CT-guided planning of minimally invasive procedures**
R. Salgado; *Antwerp/BE (rodrigo.salgado@uza.be)*

Transcatheter valve replacement and repair techniques have recently enjoyed significant widespread clinical attention, offering new therapeutic options for certain patient populations with functional aortic and mitral valve disease which were previously unable to undergo surgical valve replacement. While most of the attention has focused on the treatment of severe aortic valve stenosis, intensive research is currently underway investigating the possibilities of transcatheter mitral valve repair and even replacement. While initial trials relied on echocardiography for pre-procedural assessment of the aortic root components, CT has rapidly become the imaging modality of choice for a correct anatomical evaluation. The intrinsic 3D nature of CT offers unparalleled non-invasive access to investigated anatomy, delivering the needed accurate measurements to further optimise the eligibility for and the choice of transcatheter valve best suited for a specific patient. During this lecture, we will further highlight the role of CT in this pre-procedural setting, with attention to the relevant anatomy, the scan protocol and the necessary information that has to be delivered in order to achieve maximal procedural success.

***Learning Objectives:***
1. To learn about the requirements prior to minimally invasive valvular repair and other diseases.
2. To outline the most appropriate imaging protocols.
3. To gain insights into future developments in devices and minimally invasive treatments.

**A-0878** 14:28
**B. Defining the optimal time to treat valvular heart disease: role of MR**
J. Mascherbauer; *Vienna/AT*

"no abstract submitted"
***Learning Objectives:***
1. To outline the clinical problem and to approach treatment decision-making in valvular heart disease.
2. To become familiar with the MR derived imaging biomarkers in valvular heart disease.
3. To discuss the possible future role of MR in outcome prediction in valvular heart disease.

**A-0879** 14:51
**C. Follow-up after minimally invasive valvular repair**
H. Alkadhi; *Zurich/CH (hatem.alkadhi@usz.ch)*

Minimally invasive valvular repair has become a well-established alternative for patients with severe valve disease being considered at high or prohibitive surgical risk. Several devices are currently under preclinical and clinical evaluation. In this lecture, the various devices are being described, along with postinterventional imaging of the devices and their complications. Main challenges and future perspectives on this emerging field are discussed.
***Learning Objectives:***
1. To become familiar with the appropriate imaging technique after minimally invasive valvular repair.
2. To learn about the different devices currently used in treatment of structural heart disease.
3. To become familiar with the normal outcomes and most common procedural complications.

15:14
**Panel discussion: How to face the challenges of the increasing demand for imaging evaluation in structural heart disease**

**14:00 - 15:30 Studio 2019**

### Special Focus Session

### SF 15a

### Everything you always wanted to know about metabolic bone disease (but were afraid to ask)

**A-0880** 14:00
**Chairperson's introduction**
A. Bazzocchi; *Bologna/IT (abazzo@inwind.it)*

Despite appearances, bone is a very active organ. The metabolic life of bone is frantic and fully involved in human physiology and pathophysiological processes. Cross-talks (interactions) between bone and other organs (and between bone and other MSK system tissues) allow for energy exchanges and modulation of functions. Bony structure and composition are dynamically changed, and pathological processes can result in bone healing or lead to permanent alteration and eventually dysfunction and failure. Radiologists are not always completely aware of the importance of bone metabolic conditions.
***Session Objectives:***
1. To become familiar and get answers to the most frequently asked questions regarding findings that could be interpreted or misinterpreted as metabolic bone disease, with a special emphasis on bone marrow patterns.
2. To become familiar with DXA-based body composition assessment and understand the potential applications of recently developed DXA-based software.
3. To become familiar with the typical and atypical features of insufficiency fractures.
4. To learn about texture analysis and its application to bone architecture assessment.

**A-0881** 14:05
**Could "that" be metabolic?**
M.P. Aparisi Gomez; *Valencia/ES (pilucaparisi@yahoo.es)*

The bone marrow is one of the largest organs in the body. Its composition varies physiologically through life, in the context of normal maturation (red to yellow conversion) and also in response to situations of need (yellow to red conversion). The bone marrow is present in the background of every MR examination performed, and sometimes its appearances can pose a challenge to interpretation. In addition to this, opportunistic diagnosis of pathology showing as changes in the bone marrow should be taken advantage of by every radiologist. In this lecture, we will start by reviewing the histology of the normal bone marrow and its expected age-related appearances. We will review the causes that trigger changes in the appearances of the bone marrow, in a first instance addressing the most common physiological and benign situations, with a rationalisation for these. We will also review the pathological conditions involving the bone marrow, and resulting imaging signs. A special emphasis will be made on changes triggered by metabolic diseases. The second half of the lecture will focus on establishing the ideal structured way to interpret appearances of bone marrow on MRI, with a review of the different additional imaging tools, mainly MRI-based, to orientate diagnosis. The radiologist should develop a rational, structured way to assess bone marrow appearances on MRI, and become familiar with bone marrow patterns and their significance, in the context of known pathology but also to raise awareness on the possibility of underlying processes and orientate diagnosis.
***Learning Objectives:***
1. To learn about the variety of different physiological and pathological bone marrow patterns.
2. To understand the pathophysiological mechanisms behind changes in the bone marrow and how they translate into imaging findings.
3. To become familiar with the most frequently asked questions regarding findings that could be interpreted or misinterpreted as metabolic bone disease.

**A-0882** 14:23
**Unrevealed fractures: from missed diagnosis to opportunistic screening**
C.R. Krestan; *Vienna/AT (christian.krestan@meduniwien.ac.at)*

This lecture focuses on occurrence, imaging and differential diagnosis of insufficiency fractures. These fractures occur when normal stress is exerted on weakened bone. The morphologic features and the most common sites of insufficiency fractures and their clinical implications are discussed. A large proportion (up to 1/3 or more) of fractures are asymptomatic and do not come to clinical attention. There is a chance for opportunistic identification of insufficiency fractures including vertebral fractures from various imaging modalities including radiographs, computed tomography, magnetic resonance imaging, and PET- scans performed for other clinical indications. Fractures will often be missed on axial scans; thus midline sagittal reformatted images from computed tomography which include the spine in the field of view have the greatest potential for opportunistic detection. It may be a challenge for the radiologist to detect and diagnose insufficiency fractures as well as to differentiate them from malignant fractures. The different modalities have a broad range of sensitivity in detecting insufficiency fractures. Dual-energy Multidetector CT can depict bone marrow oedema and thus has the ability to differentiate acute from old fractures at different body sites. PET-CT and PET-MRI with hybrid-scanners combine different radiological modalities with the properties of tracers. Hybrid imaging has been the upcoming modality for the differentiation of benign from malignant fractures. The lecture also focuses on the importance of the accurate reporting of fractures to avoid underreporting, as vertebral fractures and fractures at other sites are powerful predictors of future fracture risk.
***Learning Objectives:***
1. To become familiar with the typical features of insufficiency fractures.
2. To understand why some types of insufficiency fractures can be missed.
3. To appreciate the value of opportunistic diagnosis and the potential implications on patient management.

**A-0883** 14:41
**DXA: a much more powerful imaging tool than you thought**
C. Messina; *Milan/IT (carmelomex@gmail.com)*

Dual-energy X-ray absorptiometry (DXA) is the mainstay in clinical practice to evaluate bone mineral density (BMD) in patients with suspected osteoporosis. Nevertheless, DXA can be now considered a multi-parametric tool, being able to offer several other diagnostic possibilities. Among these, one of the most promising is the use of DXA body composition (BC), a technique that is able to quickly assess BC parameters such as lean and fat mass, with very low radiation dose. There is, therefore, a growing interest in DXA BC for the emerging aspects of sarcopenia and osteosarcopenia. DXA can be used for the diagnosis of prevalent and incident vertebral fractures using vertebral morphometry, which showed to be comparable to classic radiographic morphometry analysis. In addition, DXA morphometry is computer-assisted, which helps the radiologist to better fracture detection. The lecture will also cover the clinical utility of Trabecular Bone Score (TBS), a textural index based on grey-level variations that can be obtained from the lumbar spine DXA. Studies in postmenopausal women showed that TBS is associated with vertebral, hip and major osteoporotic fracture risk, but the software also has limitations that can limit its applicability.
***Learning Objectives:***
1. To learn about vertebral morphometry with DXA.
2. To become familiar with DXA-based body composition.
3. To understand the applicability of a recently developed DXA-based software, the "trabecular bone score" (TBS).
4. To understand the technical principles and the clinical utility of hip structural analysis (HSA), which uses DXA data to derive measurements of bone geometric properties at the proximal femur.
*Author Disclosure:*
**C. Messina:** Grant Recipient; Abiogen, Bracco, Sandoz Travel Grant.

**A-0884** 14:59
**The bone naked: texture analysis**
C.M. Phan; *Paris/FR (catherine.phan@sat.aphp.fr)*

Texture analysis is a technique that allows quantification of the spatial arrangement of pixel intensities by using statistical evaluation of image intensities in a region of interest and can be performed with different imaging modalities. Texture analysis is able to detect distinct quantitative differences in tissues, which may not be depicted using a pure visual assessment alone. Texture analysis is an image processing that extracts texture descriptors from an image thereby allowing the mathematic detection of subtle CT attenuation or MRI signal intensity changes amongst image pixels. Most studies have used CT texture analysis in patients with neoplasms to differentiate benign from malignant lesions, to assess tumour grade, and to predict survival. In vivo bone texture analysis is a new application for the assessment of skeletal integrity in different diseases (primary or secondary osteoporosis, haematological diseases). The current features of texture analysis will be reviewed as well as the limitations of this imaging processing.
***Learning Objectives:***
1. To learn about texture analysis and its application to bone architecture analysis.
2. To understand how texture analysis could be used in differentiating control subjects with normal bone density from patients with osteoporosis and patients with hematological diseases such as multiple myeloma.
3. To appreciate the limitations of this imaging processing.

15:17
**Panel discussion: "What lies ahead...?" The future of diagnosis of metabolic bone disease in clinical practice**

**14:00 - 15:30** **Room E1**

### Special Focus Session

### SF 15b

### Imaging in Meniere's disease: what to believe and how to interpret

**A-0885** 14:00
**Chairperson's introduction**
J.W. Casselman; *Bruges/BE (jan.casselman@azsintjan.be)*

For many years Meniere's disease remained a clinical diagnosis which could not be confirmed by imaging. The role of imaging was to exclude other causes like VIIIth nerve schwannoma, labyrinthitis etc. The underlying mechanism of endolymphatic hydrops was already confirmed by Harold Schuknecht on histologic sections (1965, guinea pigs). The imaging diagnosis of endolymphatic hydrops only became possible with the advent of better MR systems/techniques and after it was discovered that there was a selective enhancement of the perilymph spaces after intratympanic or intravenous gadolinium administration. Since then more and more is discovered, and we already realise that endolymphatic hydrops is not only found in patients with Meniere's disease but can be found in many other patients (e.g. BPPV, intralabyrinthine schwannomas, liquor hypotension etc.). During this Special Focus Session, the pathophysiology and clinical presentation and findings in Meniere patients will be discussed explaining why and in which patients imaging is needed. The therapeutic options will also be discussed and especially the possibilities once the imaging diagnosis is available. The state of the art imaging techniques will be shown, and the correlation of the imaging findings and clinical presentation will be presented. The today available MR classifications for endolymphatic hydrops will also be discussed. Many other lesions can mimic Meniere's disease, and many other causes of endolymphatic hydrops exist, these will also be illustrated. Finally, it is important to understand what the clinicians can do with this information and how it will or already has changed the treatment of Meniere patients.
***Session Objectives:***
1. To learn about clinical presentation and treatment strategies in Meniere's disease.
2. To discuss state-of-the-art imaging for the evaluation of endolymphatic hydrops.
3. To understand how to interpret MR hydrops imaging.
*Author Disclosure:*
**J.W. Casselman:** Equipment Support Recipient; Philips Healthcare. Speaker; Philips Healthcare.

**A-0886** 14:05
**Meniere's disease: the clinician's perspective**
A. Zarowski; *Antwerp/BE (andrzej.zarowski@me.com)*

Menière's Disease (MD) is characterised by a specific set of symptoms comprising vertigo spells with a duration of at least 20 minutes usually accompanied by low-frequency perceptive hearing loss, tinnitus and pressure sensation in the ear. The severity and natural history of MD is highly unpredictable, with often prolonged periods without any symptoms. The fear of getting an acute vertigo spell at virtually any moment of daily activities makes MD very annoying to patients and creates significant behavioural changes. Histologically all patients with MD show an increased volume of the membranous labyrinth (endolymphatic space) that is morphologically described as an endolymphatic hydrops (EH). It is however not known if the occurrence of EH is due to endolymph overproduction, malabsorption or other disorder of endolymphatic homeostasis. Or is it merely an anatomical malformation that needs an additional trigger to become symptomatic. One of the most important problems in MD is that until now there have been no objective criteria available for precise definition of MD. Diagnosis is typically based on mere anamnestic criteria and is not accurate. In clinical practice, many different balance problems show similar symptomatology and are classified incorrectly as MD. There have been numerous endeavours to find objective methods allowing for the precise definition of the definitive MD, but they were not successful. Medical imaging was until now only used for exclusion of other pathologies. The development and availability of the specific MRI hydrops protocols initiate a whole new era in the diagnosis and treatment of MD.
***Learning Objectives:***
1. To discuss the pathophysiology of Meniere's disease.
2. To learn about the clinical presentation and diagnostic pitfalls.
3. To become familiar with the therapeutic options for patients with Meniere's disease.

**A-0887** 14:15
**MR hydrops imaging: how to do it?**
A. Attye; *Grenoble/FR (aattye@chu-grenoble.fr)*

Within the inner ear, there are two distinct compartments filled with endolymph and perilymph. The accumulation of endolymph fluid is called "endolymphatic hydrops" (EH). Two alternative routes for contrast media administration have been assessed to identify EH: intratympanic (IT) and intravenous (IV). The main advantage of an IT injection is a higher perilymphatic contrast, particularly in the basal turn if the cochlea, although previous studies suggest this method can cause local toxicity in animal models. In addition, cases of bilateral disease require a double IT injection and an MR scan waiting time of 24 h before imaging acquisition. In contrast, the intravenous method requires a shorter waiting time, which has been evaluated as optimal in the 4-6 hours interval. More recently, the effect of varying the contrast media molecule on normal inner ear structures enhancement has been evaluated, and the use of 3D-FLAIR sequences with constant flip angle proposed are more sensitive than those with a variable flip angle for the hydrops protocol. Finally, radiologists must be vigilant to other inner ear diseases, such as perilymphatic fistulae, Minor syndrome, vestibular neuritis or intralabyrinthine fistulae, which may mimic Meniere's disease symptoms.
***Learning Objectives:***
1. To review different MRI techniques for investigation of endolymphatic hydrops.
2. To present tips and tricks for state of the art imaging today.
3. To discuss how to exclude other pathology mimicking Meniere's disease.
*Author Disclosure:*
**A. Attye:** Research/Grant Support; Guerbet and Bayer Manufactory.

**A-0888** 14:40
**MR hydrops imaging: how to interpret it?**
B. De Foer, A. Bernaerts; *Antwerp/BE (bert.defoer@gza.be)*

Delayed intravenous gadolinium-enhanced 3D FLAIR MRI is able to demonstrate endolymphatic hydrops in patients with Menière's disease. Various semiquantitative grading criteria have been described. A three-stage grading system for vestibular and cochlear hydrops is most frequently used. In normal circumstances, a barely visible non-enhancing cochlear duct is seen in the enhancing scala vestibuli and scala tympanic. Grade 1 cochlear hydrops is defined as a mild dilatation of the non-enhancing cochlear duct into the scala vestibuli with partial obliteration of the scala vestibuli. In grade 2 cochlear hydrops, the scala vestibuli is uniformly obstructed by the distended cochlear duct. In the vestibule -in normal cases- one can clearly discriminate the non-enhancing saccule and utricule in the enhancing vestibule. A grade 1 vestibular hydrops presents as distention of the endolymphatic space of the saccule and utricule, with the enhancing perilymphatic space still visible around it. In grade 2 vestibular hydrops, the saccule and utricule are extremely distended without any visible surrounding enhancing perilymphatic space. Other signs have been described in patients with Menière's disease such as the more pronounced cochlear perilymphatic enhancement and the obliteration of the endolymphatic sac. In patients with definite Ménière's disease, the degree of MR morphological hydrops severity correlates significantly with impairment of hearing function and sacculus function. However endolymphatic hydrops is not only seen in patients with Menière's disease. It has also been reported in a variety of other disease entities such as sensorineural hearing loss, otospongiosis and superior semicircular canal dehiscence.

***Learning Objectives:***

1. To get acquainted with the imaging appearance of endolymphatic hydrops.
2. To correlate imaging findings with clinical presentation.
3. To learn about other causes of endolymphatic hydrops.

**A-0889** 15:05

**MR hydrops imaging: what are the benefits for the surgeon and the patient?**

A. Zarowski; *Antwerp/BE (andrzej.zarowski@me.com)*

Diagnosis of Menière's Disease (MD) significantly influences the emotional and social wellbeing of affected patients. There are different conservative and invasive treatments available for MD, but their value is still questionable. Therapeutic results show large variability and are very difficult to differentiate from placebo effects, which play an important role in the treatment of MD. Lacking objective diagnostic criteria for a precise definition of MD is one of the most important problems for validation of any particular treatment in a placebo-controlled trial. When the study group and the control group comprised patients with an incorrect diagnosis, it becomes very difficult to demonstrate any significant differences between these groups. Since the presence of the endolymphatic hydrops (EH) is the necessary underlying condition for the diagnosis of definite MD, the new MRI diagnostics have dramatically changed the clinical approach to patients with MD by an improved definition of the affected patients. This game-changer gave rise to the re-evaluation of the efficacy of the historically available therapeutic options. In spite of this revolution in MD imaging, some unanswered questions still have to be addressed in future research. Is EH acquired or it is a congenital anatomical malformation? How many patients in the general population show asymptomatic EH? Is EH the necessary and sufficient condition for the development of MD symptoms, so that all patients with a radiologically diagnosed EH will eventually develop symptoms of MD? Does the localisation or the radiological grade of EH correlate with the clinical appearance of MD?

***Learning Objectives:***

1. To recognise the value of endolymphatic hydrops imaging.
2. To appreciate the impact of imaging findings on therapeutic strategy.
3. To discuss patient outcome after different treatment strategies.

**A-0890** 15:15

**Panel discussion: Did you pick up the essentials?**

J.W. Casselman; *Bruges/BE (jan.casselman@azsintjan.be)*

The audience will be able to test their knowledge about Meniere's disease by answering multiple choice questions. They will be able to answer by using an electronic voting system (using their own smartphone/tablet/laptop). The purpose of these questions is to evaluate what the participant picked-up about the "clinical presentation of Meniere's disease, treatment options, MR technique, mimicking pathology, MR diagnosis and classification, other causes of endolymphatic hydrops and the impact of the MR diagnosis on therapeutic strategy and treatment". The answers will be analysed and discussed by the presenters.

**14:00 - 15:30** **Room E2**

### Special Focus Session

### SF 15c

### Abdominal emergencies: advanced imaging in daily routine?

**A-0891** 14:00

**Chairperson's introduction**

M. Scaglione; *Castel Volturno/IT (scaglionefun@gmail.com)*

This special focus session focuses on new imaging developments and advanced approaches to the acute abdomen. As we all know, in the contest of emergency "time" to reach the exact diagnosis and effective management is crucial for every acutely ill patient. In this context, MDCT has deeply changed the classic clinical paradigm and currently represents an "ideal tool" regarding sensibility, specificity, and accuracy. However, the use, overuse and, in certain circumstances, abuse of MDCT mainly related to the continuously increasing number of patients coming to every emergency department, has actually led to a tremendous radiation dose exposure growth worldwide which has reasonably raised worrying levels. For this reason, it is important to know if there are valuable imaging alternatives in the radiology portfolio and to understand how and in which context these new technologies can be appropriately used.

***Session Objectives:***

1. To understand how new technological developments can be used in the emergency setting.
2. To learn specific applications of advanced imaging to investigate abdominal emergencies.
3. To understand how advanced imaging can help to reduce radiation exposure.

**A-0892** 14:05

**Routine CT vs dual energy CT in acute abdominal pain: should we change our scan protocol?**

E. Danse, E. Coche; *Brussels/BE (etienne.danse@uclouvain.be)*

The objective of this presentation is to discuss the optimal scenarios when a routine CT examination requested for acute abdominal conditions is replaced by a dual energy CT acquisition. CT protocols have to be cautiously adapted regarding the clinical context. Concerning the delivered radiation, an adaptation of mAs and the use of iterative reconstructions have to be applied systematically. When renal function is impaired, the volume of contrast medium can be reduced with preservation of adequate contrast enhancement. Virtual non-contrast series, iodine maps, Zeff maps and virtual monoenergetic reconstruction images can be generated automatically and send to the PACS, with a direct impact on the storage capacity of the system. Additionally, the radiologists have to adapt the image analysis process, because new data sets are available (low and high mono-energy images, iodine uptake measurement, Z effective values). In our daily routine, these parameters contribute to optimal imaging reporting and better care to the patients.

***Learning Objectives:***

1. To learn about potential dual-energy CT applications in the acute abdomen (biliary stone, bowel wall enhancement, etc.).
2. To understand how virtual non-contrast images might replace the pre-contrast phase.
3. To become familiar with the available material decomposition reconstructions, image optimisation possibilities and radiation exposure concerns.

**A-0893** 14:30

**CT scan vs MRI in acute abdominal pain: who do we have to send to the magnet?**

I. Millet; *Montpellier/FR (milletingrid@wanadoo.fr)*

Acute abdominal pain results from a wide range of different pathologies. CT is the main imaging modality used in the emergency context due to its availability and its high diagnostic performances. However, it remains an ionising radiation procedure with a potential long-term risk of cancer, and iodinated contrast material is often required, potentially affecting renal function. MRI image quality has been greatly improved in the last few years, with sequences allowing to cover a large volume with good spatial resolution, even in freely breathing patients. MRI has a better sensitivity than CT to detect little biliary stones, a better contrast resolution to characterise the content of the peri-pancreatic collections, a better assessment of pelvic inflammatory disease complications. Moreover, MRI is obviously useful to investigate abdominal pain when CT is not recommended because of ionising radiation risk (pregnant or young patients) or iodinated contrast material allergy. Indeed, MRI can be diagnostic without exogenous contrast material. The protocol can now be tailored to cover both the upper abdomen and the pelvis in less than 10 min. Optimised T2-weighted images with and without fat suppression are mandatory, in association with optimal T1-weighted sequences performed with Dixon techniques.
***Learning Objectives:***
1. To learn about the superior MRI performance in diagnosing specific pathologies such as acute biliary disease, pancreatitis and gynaecological emergencies.
2. To understand that MRI should be used as an alternative to CT for investigating abdominal pain in pregnant, paediatric or young adult patients.
3. To become familiar with new MRI sequences that allow shorter examination times.

**A-0894** 14:55
**CEUS in abdominal inflammatory and infectious conditions (-itis and abscesses)**
T. Fontanilla; *Madrid/ES (fonteche@gmail.com)*

Many abdominal emergencies, including abdominal inflammatory conditions, are evaluated with ultrasound. Contrast-enhanced ultrasound further improves ultrasound effectiveness in this setting. CEUS of inflammatory conditions in any location depends on the presence of liquefied component and its proportion to the phlegmonous component; in the number and thickness of the septa and in the thickness of the wall also. Phlegmonous component enhances in the arterial phase and washes out quickly. The liquefied component does not enhance at all. Abscess walls and septa enhance in arterial phase and wash out quickly or slowly. Pyelonephritis lesions typically enhance initially less than the adjacent parenchyma, quickly enhance similarly the parenchyma and enhance less to it in late parenchymal phase, in which lesions are best depicted. Considering gallbladder, appendix, bowel in diverticulitis or intestinal inflammatory disease, thickening of the wall with arterial phase enhancement happens, except in gangrenous areas. If there is perforation, gas and/or peritoneal fluid and/ or complication with an adjacent phlegmon or abscess occur. CEUS aids in distinguishing phlegmon from an abscess, in measuring the size of the abscess and in defining its internal structure, which influence directly in the therapeutic choice: drainage and antibiotics versus medical treatment alone. CEUS also provides a valuable tool in the follow-up of the lesions: size diminishment, reduction in wall enhancement, and even use of endocavitary ultrasound contrast to check the patency or location of drainage tubes. CEUS findings and usefulness, as well as technical tips to optimise imaging, will be explained with different cases.
***Learning Objectives:***
1. To learn the CEUS image findings of acute inflammatory conditions and their complications (gallbladder, kidney, inflammatory bowel disease and complicated diverticulitis).
2. To become familiar with the indications, advantages and limitations of CEUS.
3. To review technical issues from a practical standpoint.

15:20
**Panel discussion: New techniques in emergency radiology: where are we now and where are we going?**

**14:00 - 15:30 Room F1**

### E³ - European Diploma Prep Session

### E³ 1523

### Principles of imaging and radiation protection

**A-0895** 14:00
**Chairperson's introduction**
C.J. Caruana; *Msida/MT (carmel.j.caruana@um.edu.mt)*

Medical imaging devices are instruments which measure a physical property of tissue voxels (e.g., linear attenuation coefficient in CT). This is crucial to keep in mind as the quantitative view of medical images gains traction among the radiology community. The 3D matrix of voxel values are then visualised as a grey scale (or colour) image by means of a Look-UP-Table (LUT). By good image quality, one means that the device can demarcate the voxels properly (for high spatial resolution) and that the physical property values of the voxels have been measured accurately (inaccuracy renders the image unusable) and with a low uncertainty (for high contrast resolution). On the other hand the higher the precision with which we want to measure the values the higher is the patient dose in CT or the SAR level in the case of MRI. The optimization which is the creation of the right level of image quality whilst avoiding unnecessary patient risk is therefore crucial. Staff safety also needs careful attention. In these presentations we will present the principles of image formation, image quality, basic optimisation and protection of patient and staff from ionising radiation and other physical agents (e.g., magnetic field and gradient fields in MRI) for ETC Levels I and II.
***Session Objectives:***
1. To understand the technical and methodological principles of computed tomography.
2. To understand the technical and methodological principles of magnetic resonance tomography.
3. To learn the principles of radiation biology and radiation protection.

**A-0896** 14:06
**A. Principles of computed tomography**
M. Kortesniemi; *Helsinki/FI (mika.kortesniemi@hus.fi)*

Computed tomography (CT) is an imaging method producing 3D image data of the patient. Like any other x-ray modality, the contrast in the CT image is based on the x-ray attenuation. The physical interactions behind the attenuation also produce radiation exposure to the patient. The majority of the cumulative radiation exposure in radiology is produced by CT. Therefore, proper justification and optimisation of CT studies are essential factors in diagnostic radiology. The CT image data is acquired from the patient with a rotating x-ray tube and detector; both mounted on the opposite sides of the rotating gantry structure. In a multi-slice CT scanner (MSCT), a large number (up to thousands) of raw-data projections are measured from different angles, representing the net attenuation of x-rays across the patient. These raw-data projections are then used to calculate the image slices - or rather the volume - representing the 3D attenuation map of the patient. Thus, the CT image reconstruction is an inverse problem, deducing the 3D image from the raw-data “shadow” signal. Mathematical image reconstruction methods have traditionally been based on filtered back-projection, enhanced in the past decade with iterative reconstruction techniques, and recently starting to utilise even deep-learning (a type of artificial intelligence). Modern CT imaging enables very fast, high resolution and wide coverage scanning, acquiring up to 640 axial slices per rotation in axial scan mode, and even 73 cm length per second with high pitch helical scan with a dual-source CT scanner. Development of CT is continuing strong.
***Learning Objectives:***
1. To understand the physical basis of image formation of computed tomography and of the physics of helical, multidetector and dual-source CT.
2. To learn the scale of Hounsfield units and the principle of window centre and width.
3. To become familiar with modern CT technology.
4. To understand the principles of optimising CT protocols with a focus on patient dose reduction.

**A-0897** 14:34
**B. Principles of magnetic resonance imaging**
D. Sappey-Marinier; *Lyon/FR (Dominique.Sappey-Marinier@univ-lyon1.fr)*

After a rapid survey of Nuclear Magnetic Resonance (NMR) discoveries through the numerous Nobel prices, the fundamental principles will be presented through the quantum aspects of the spin states of hydrogen nucleus, the most targeted candidate in MRI, and the use of three magnetic fields, the static B0 field, the resonant B1 field, and the gradient G, for spin polarization and excitation, and space encoding for image reconstruction, respectively. Relaxation processes, called T1 and T2, will be described to understand the signal measurement and to explain their role in image contrast using a standard spin-echo sequence. Conventional MRI sequences, including T1, T2, T2* tissue contrasts, will be presented to provide an initial comprehension of MRI interpretation, to learn about typical artefacts, and to understand safety issues in MRI.
***Learning Objectives:***
1. To become familiar with the physical basis of image formation in MRI including the principles of pulse sequences and relaxation times.
2. To become familiar with the typical appearance of tissues, organs and main pathological processes of the most commonly used sequences in MRI, including T2-weighted sequences, T1-weighted sequences, STIR sequences, FLAIR sequences, other inversion recovery sequences, T2*- / susceptibility-weighted sequences and MR angiography sequences.
3. To learn typical artefacts on MR imaging and to discuss their respective causes.
4. To understand absolute or relative contraindications against MR imaging and safety issues in the MR environment with regard to patients and staff.

**A-0898** 15:02
**C. Radiation protection**
J. Damilakis; *Iraklion/GR (damilaki@med.uoc.gr)*

The use of ionising radiation for medical imaging requires careful thought so that expected benefits outweigh potential radiation-induced risks. This presentation will provide an overview of medical radiation protection. Specifically, it will cover the main interactions of x-rays and gamma rays with matter and will explain why the photoelectric effect and Compton scatter are the most common effects in diagnostic imaging. The basic principles of medical radiation protection as outlined by ICRP will be described, and the importance of patient and staff dosimetry in diagnostic and interventional radiology will be explained. Methods and tools will be presented that can be used to limit patient dose. Paediatric patients are more sensitive to radiation than adults. The exposure of pregnant patients to medical X-rays is often a complex case and involves emotionally sensitive issues for both prospective parents, radiologists and referring physicians. A program for dose management of pregnant and paediatric patients requiring diagnostic and interventional procedures will be described. Fluoroscopically-guided interventional procedures are associated with relatively high occupational doses. This presentation will give key points to remember for staff dose management in fluoroscopy and interventional radiology procedures.

***Learning Objectives:***
1. To understand the phenomena of x-ray interaction with matter and their effect on image quality and dose.
2. To provide an overview of patient and staff dosimetry in diagnostic and interventional radiology.
3. To understand the basic principles of radiation protection, as outlined by the ICRP (International Commission on Radiological Protection).
4. To become familiar with the concepts and tools for dose management in radiology with regard to adult and paediatric patients.

**14:00 - 15:30** **Room F2**

### Special Focus Session

### SF 15d

### Navigating tumour phenotype with advanced imaging analysis

**A-0899** 14:00
**Chairperson's introduction**
E. Sala; *Cambridge/UK (es220@cam.ac.uk)*

***Session Objectives:***
1. To become familiar with the concept of tumour microenvironment and habitat imaging.
2. To learn what information habitat imaging and radiomics can provide on tumour heterogeneity.
3. To understand how machine learning and artificial intelligence can be used in radiomics.

**A-0900** 14:05
**Understanding tumour micro environment: the role of habitat imaging**
J. O'Connor; *Manchester/UK (james.o'connor@manchester.ac.uk)*

Tumours are complex 3D structures at the anatomical, physiological and metabolic levels. Imaging methods, such as CT, MRI, PET, SPECT and ultrasound can map the spatial variation in these scales. In particular, imaging can detect sub-regions with differing anatomical or microenvironment signatures, which have been referred to as ‘tumour habitats’. It is hypothesised that these habitats identify a tumour with good/poor prognosis or good/poor response to targeted therapy. In this talk, we will consider strategies and tools for identifying these habitats. We will evaluate the evidence that such approaches provide useful information to guide decision-making that merit inclusion in healthcare systems.

***Learning Objectives:***
1. To understand the concept of tumour microenvironment.
2. To become familiar with habitat imaging analysis tools.
3. To learn what information habitat imaging can provide on tumour heterogeneity.

**A-0901** 14:30
**Unravelling tumour heterogeneity using radiomics**
L.S. Fournier; *Paris/FR (laure.fournier@aphp.fr)*

Radiomics is a new 'data-driven' approach for extracting large sets of complex descriptors from routine (or not) clinical images. Advanced methods of image processing are applied to images to extract a large number of descriptors, such as texture analysis from histograms, co-occurrence matrices, fractal analysis, etc. This large set of data can be analysed using bio-informatics and bio-statistics methods then correlated to outcomes, such as treatment response or survival. The purpose of radiomics is to mine images for information on underlying biology, such as gene expression profiles (called radiogenomics), molecular profiles, expression of receptors, microenvironments such as immune cell infiltration or angiogenesis. Texture parameters more specifically are considered to reflect tumour heterogeneity. Radiomics allows discovery of new potential biomarkers, but it is then important to demonstrate their link to tumour biology.

***Learning Objectives:***
1. To learn the definition of radiomics and radiomics tools available.
2. To understand what information radiomics can provide on tumour heterogeneity.
3. To understand how the radiomics features may relate to tumour biology and microenvironment.

*Author Disclosure:*
**L.S. Fournier:** Grant Recipient; Fondation pour la Recherche Médicale, RIHDO consortium (Philips Healthcare, Ariana Pharma, Evolucare). Research/Grant Support; Invectys, Novartis. Speaker; Janssen, Sanofi.

**A-0902** 14:55
**Role of machine learning (ML) and artificial intelligence (AI) for quantitative radiomics**
W.J. Niessen; *Rotterdam/NL (w.niessen@erasmusmc.nl)*

Radiomics techniques aim to establish a relation between a combination of imaging features and clinically relevant outcomes. Radiomics has primarily been applied in the oncology domain and owing to its popularity for different types of tumours radiomic signatures have been developed. Still, there are large challenges in order to bring radiomics to daily clinical practice. Challenges include the robustness and generalisability of radiomic signatures, especially in view of the large heterogeneity in imaging hard- and software, and image acquisition parameters. In this presentation, I will present the basics of a radiomics pipeline. Also, methods for the automatic optimisation of all the parameters in a radiomics pipeline are addressed. Finally, examples of this approach are shown on a variety of applications, including grading of prostate cancer, and characterisation of low-grade gliomas and liver tumours.

***Learning Objectives:***
1. To understand how machine learning can be used to extract radiomic features.
2. To learn how artificial intelligence can be used in radiomics.
3. To become familiar with available artificial intelligence algorithms that can be used to integrate radiomics with other patient data.

*Author Disclosure:*
**W.J. Niessen:** Consultant; I am Scientific Lead at Quantib BV. Shareholder; I am founder and shareholder of Quantib BV.

15:20
**Panel discussion: Can advanced imaging analysis and imaging integration with omics add significant value to standard clinical and genomic data?**

**14:00 - 15:30** **Room D**

### Vascular

### RC 1515

### No time to lose: aortic disease, revisited

*Moderator:*
M. Krokidis; Cambridge/UK

**A-0907** 14:00
**A. Diagnosis and treatment of abdominal aortic aneurysms**
F. Wolf; *Vienna/AT (florian.wolf@meduniwien.ac.at)*

Abdominal aortic aneurysms (AAA) are relatively rare. The annual incidence of new AAA diagnoses is approximately 0.4 to 0.67 % in Western populations. Nevertheless, when radiologists report CT examinations, they will not so uncommonly find pathological changes in the abdominal aorta with a range from simple wall irregularities to large abdominal aortic aneurysms. For radiologists, it is crucial to detect, classify and report aortic aneurysms. It seems to be very complicated to measure an aortic aneurysm in the right way and decide if a stentgraft treatment is possible or not and what kind of stentgraft would be the best. But with a few tips and tricks provided in this lecture, it is relatively easy to decide if an aneurysm has to be treated and if a standard stentgraft would be possible or not. The lecture will end up with different other AAA treatment possibilities beyond the standard AAA stentgrafts - anchors, snorkels, branches and chimneys. For diagnostic radiologists, it makes no sense to plan such complicated procedures - but it is really important to know what is technically possible in order to describe these stentgrafts with its possible complications in the right way in follow-up CT examinations.

***Learning Objectives:***
1. To learn about the definition and classification of abdominal aortic aneurysms.

2. To understand the relevant information and measurement to plan an endovascular aortic repair.
3. To get familiar with the different possibilities of endovascular treatment including anchors, snorkels, branches and chimneys.

**A-0908** 14:30
**B. Acute diagnosis and imaging in aortic dissection**
R. Iezzi; *Rome/IT (roberto.iezzi.md@gmail.com)*

Aortic dissection is the most common acute emergency condition of the aorta, often resulting in the death of the patient. The overall outcome is determined by the type and extent of dissection and the presence of associated complications; therefore, evaluation of the entire aorta, branch vessels, and iliac and proximal femoral arteries is recommended to aid in treatment planning. Early diagnosis and treatment are essential for improving the prognosis. Patients may present with the classic history of acute onset of tearing central chest pain that radiates to the back. Stanford type A dissection involves the ascending thoracic aorta, and the dissection flap may extend into the descending aorta. Type A dissections account for 60%-70% of cases, requiring urgent surgical intervention to prevent extension into the aortic root, pericardium, or coronary arteries. If untreated, type A dissections are associated with a mortality rate of over 50% within 48 hours. Stanford type B dissection involves the descending thoracic aorta distal to the left subclavian artery and accounts for 30%-40% of cases. Management takes the form of medical treatment of hypertension unless there are complications due to an extension of the dissection. CT imaging of the aorta is fast and widely available, which are the important features in making an accurate diagnosis quickly in unstable patients. Multidetector CT allows imaging of the entire aorta with rapid acquisition and data reconstruction to provide prompt and accurate diagnosis and to help identify relevant complications that may have an impact on treatment and management.
***Learning Objectives:***
1. To learn about definition and classification of aortic dissections and subtypes.
2. To understand the importance of accurate diagnosis for appropriate treatment planning.
3. To appreciate the need for acute diagnosis and treatment indication.

**A-0909** 15:00
**C. Endovascular treatment in aortic dissection**
J.P. Schäfer; *Kiel/DE (jp.schaefer@rad.uni-kiel.de)*

Acute aortic dissection represents a life-threatening condition, which must be diagnosed immediately. CTA is considered the imaging modality of choice, offering all relevant information on the pathoanatomy with highest spatial resolution. Stanford classification is used to distinguish between type A- and B-dissections, whereas the left subclavian artery represents the border between the two types. Actually, surgical repair is the method of choice and indicated in type A-dissection, and endovascular repair is the method of choice in type B-dissection if indicated. Type B-dissection may be uncomplicated or complicated. For uncomplicated type B, best medical treatment is the method of choice, and it is defined by no further symptoms, relief of symptoms and absence of additional dissection associated findings. For complicated type B, endovascular repair including a variety of interventions is the method of choice, and it is defined by mesenterial, renal, peripheral and spinal malperfusion, progressive dissection, aneurysm forming, uncontrollable hypertension, rupture, progressive periaortic and pleural haemorrhage, severe hypotension and shock. Regarding symptom onset and imaging-based diagnosis, type B-dissection is classified as acute (<2 weeks), subacute (2-8 weeks), and chronic (>8 weeks). Endovascular repair usually includes prosthesis placement in the descending aorta, to seal the proximal entry tear. This excludes the perfusion of the false lumen along the covered aortic segment and restores the blood flow into the true lumen, maintaining and improving the visceral and peripheral perfusion. Additionally, target visceral artery stenting, membrane fenestrating or embolizing may be indicated. Protocolled CTA follow-up is mandatory.
***Learning Objectives:***
1. To learn about endovascular treatment possibilities for aortic dissections.
2. To understand the role of radiology in modern treatment of aortic dissections.
3. To appreciate the need to combine the radiological information with the clinical situation.

**14:00 - 15:30** **Room G**

### Physics in Medical Imaging

### RC 1513

### Demystifying MRI: things you always wanted to know

*Moderator:*
I. Seimenis; Alexandropolis/GR

***Session Objectives:***
1. To learn about the basic concepts of NMR and relaxation.
2. To understand how MR images are produced.
3. To learn about standard MRI pulse sequences.

**A-0910** 14:00
**A. Basic MR: the building blocks of pulse sequences**
A. Webb; *Leiden/NL (a.webb@lumc.nl)*

This lecture will start by covering the basic principles of magnetic resonance: the net magnetisation produced within a patient, why a radiofrequency pulse has to be applied, and how the MR signal is detected. Pulse transmission and MR signal detection are then linked to the body coil and receive array coils used in a conventional MRI setup. Next, the dependence of the signal on the T1, T2 and T2* relaxation times will be explained. The sequences necessary to introduce T1 (inversion-recovery), T2 (spin-echo) and T2* contrast into the signal will be covered. Finally, the reasons why different tissues have different relaxation times will be discussed, as well as how pathology can alter these relaxation times.
***Learning Objectives:***
1. To learn about how NMR signals are produced.
2. To understand the basic concepts of relaxation.
3. To learn about the operation of inversion-recovery and spin-echo pulse sequences.

**A-0911** 14:30
**B. MR imaging basic concepts: how to turn signals into images**
D.J. Lurie; *Aberdeen/UK (d.lurie@abdn.ac.uk)*

To generate images in MRI, NMR signals must be "labelled" with their location. Three techniques of spatial encoding are employed, all of which use the magnetic field gradient. By sending electrical current (hundreds of amperes) through a gradient coil, a magnetic field is produced whose strength which varies linearly with the position inside the scanner bore. In frequency-encoding, the NMR signal is recorded while a field gradient is applied. Since the magnetic field varies with position along the gradient direction (e.g. X), the NMR resonant frequency (Larmor frequency) is a function of position, so the detected signal contains a range of frequencies; analysing the frequency content generates a one-dimensional projection of the water-distribution within the patient. A method called phase-encoding is employed in the second in-plane dimension (e.g. Y); here, the gradient is pulsed on and off prior to measurement of the signal, affecting the phase of the NMR signal as a function of position. Finally, the slice itself is defined using selective-excitation, in which the radiofrequency pulse used for NMR excitation is specially-shaped and is applied in the presence of a field gradient perpendicular to the slice plane (e.g. along Z). To generate data for an image of NxN pixels, the pulse sequence is usually applied N times, varying the phase-encode gradient amplitude with each repetition. A two-dimensional Fourier transform of the raw data matrix yields the MR image, which can be encoded with NMR parameters (T1, T2, diffusion etc.) information to assist diagnosis.
***Learning Objectives:***
1. To learn how magnetic field gradients encode spatial information.
2. To understand the main ways in which field gradients are used.
3. To appreciate the basic concepts of data collection and image reconstruction in MRI.
*Author Disclosure:*
**D.J. Lurie:** Grant Recipient; Recipient of research funding from GE Healthcare (support of PhD studentship).

**A-0912** 15:00
**C. Practical MRI: a toolkit of standard MR pulse sequences**
G. Hagberg; *Tübingen/DE (gisela.hagberg@tuebingen.mpg.de)*

The MR-sequence is an essential tool to measure MR-properties in tissues, increased contrast between these and quantify relevant data. Besides furnishing the desired tissue-derived MR signal, the sequence must allow spatial encoding to take place as efficient as possible to enable patient compliance, and high-quality MRI at the same time. Starting with a brief introduction regarding MR-tissue properties and spatial encoding in k-space, I will describe the fundamental MR toolkit, based on single echo spin-echo (SE)

and gradient-echo (GE) and show how these are extended to different multiple-echo regimes. Basic image contrast and how imaging parameters: repetition time, TR; echo time, TE; flip angle, FA; inversion delay, diffusion gradients, etc.; influence contrast will be described. The possibility to further enhance imaging by adequate spin-preparation: inversion or magnetisation transfer pulses etc.; will also be discussed. Imaging speed can be achieved while remaining in the single echo regime by faster RF pulsing (shorter TR) giving valuable information linked with magnetic susceptibility like in the GE-based FLASH method, based on spoiling of unwanted echoes. Another possibility is to use multiple (spin) echoes to read out different k-space lines, and hereby speed up spatial sampling, like in the SE-based method RARE. An alternative possibility that further extends available image contrast is to retain the full magnetisation in GE-based sequences and acquire images in the different steady-state-free-precession regimes. The latter techniques have gained momentum through the advent of magnetic fingerprinting and are evolving into another fundamental part of the standard MRI-toolkit.
***Learning Objectives:***
1. To learn about common types of MR pulse sequence.
2. To understand the difference between gradient-echo and spin-echo.
3. To appreciate the factors influencing choice of pulse sequence.

**14:00 - 15:30** **Room K**

### Chest

### RC 1504

### Back to basics: how to interpret a chest radiograph?

**A-0913** 14:00
**Chairperson's introduction**
S.P.G. Padley; *London/UK*

***Session Objectives:***
1. To increase the radiologists' confidence in chest radiography reading.
2. To recognise and interpret the typical abnormalities.
3. To understand the limitations of chest radiography.

**A-0914** 14:05
**A. A chest radiography reading guide**
N. Howarth; *Chêne-Bougeries/CH (nigel.howarth@grangettes.ch)*

The presentation will provide a reading guide for the frontal and lateral chest radiograph. The most useful signs in chest radiology will be introduced, using side-by-side plain film and CT imaging to help understand the imaging features. Although the clinical value of the chest X-ray remains undiminished, errors of interpretation of the chest X-ray remain one of the most frequent causes of malpractice issues. The skills required for accurate interpretation of the chest radiograph will be explored. The objective is to help you improve your performance in plain film interpretation of the chest.
***Learning Objectives:***
1. To learn about chest radiograph quality criteria.
2. To learn about the normal features.
2. To learn which difficult areas to concentrate on.

**A-0915** 14:18
**B. Alveolar, interstitial and nodular syndromes**
F. Molinari; *Lille/FR (francescomolinari.dr@gmail.com)*

Many acute or chronic diseases of the lung may manifest at imaging with the appearance of an alveolar, interstitial or nodular syndrome. All of these imaging syndromes are well known to thoracic radiologists and can be detected at chest radiography. Especially when interpreted in the correct clinical setting, alveolar, interstitial or nodular opacities can provide hints to the diagnosis. In this presentation, the typical imaging signs that allow to make a diagnosis of an alveolar or interstitial disease or suspect the presence of pulmonary nodules will be reviewed by using anatomic and imaging correlation between chest radiograph and CT.
***Learning Objectives:***
1. To learn about alveolar opacities characteristics.
2. To learn how to recognise the presence of interstitial changes.
3. To understand the chest radiograph limitations for lung nodule detection.

**A-0916** 14:31
**C. Lobar atelectasis**
D. Tack; *Baudour/BE (denis.tack@skynet.be)*

Lobar collapse or closure of a pulmonary lobe is associated with a loss of volume and results in reduced or absent gas exchange, a condition where the alveoli are deflated down to little or no volume. This presentation aims to review the radiographic and CT signs of lobar collapse, or loss of volume, and associated abnormalities when for example the cause of the collapse is a bronchial obstruction of a mass. Differentiation of lobar collapse and consolidation will be discussed. Distinct entities will be imaged such as aerated lobar collapse and chronic lobar collapse without mass-like consolidation.
***Learning Objectives:***
1. To review the signs of lobar atelectasis on frontal chest radiograph.
2. To learn about the complementary role of lateral view.
3. To explain the differences with other causes of lung opacity.

**A-0917** 14:44
**D. Pleural syndrome**
A.P. Parkar; *Bergen/NO (apparkar@gmail.com)*

Chest radiographs are particularly useful to diagnose pneumothorax, albeit smaller ones may be missed on radiographs. Pneumothorax is commonly seen as an area of hyperlucency with lack of vessels in the apical and the lateral regions on the photograph performed standing or sitting. Pneumothorax in the supine position is best seen as the deep sulcus sign as the air moves anteromedially and subpulmonic. Pneumothorax can be spontaneous (either primary or secondary) or iatrogenic. It is important to recognise the “do-not-miss” state of tension pneumothorax, which can be fatal if left untreated. A partial pneumothorax is seen when part of the lung is still adherent to the chest wall. It is, however, important to differentiate it from bullous lung disease, which sometimes may be difficult on radiographs, and may require a CT for a definite diagnosis.
***Learning Objectives:***
1. To learn how to identify partial pneumothorax.
2. To be able to identify signs of compressive pleural effusion.
3. To be aware of the limitations of bedside chest x-ray for pneumothorax detection.

**A-0918** 14:57
**E. Mediastinal syndrome**
M. Occhipinti; *Florence/IT (mariaelena.occhipinti@gmail.com)*

Chest radiography has been traditionally used to evaluate the chest, including lung parenchyma, airways, and mediastinum. Despite the wide use of computed tomography imaging, chest radiography still plays a fundamental role in the detection and characterisation of mediastinal lesions through the use of mediastinal lines, stripes, and interfaces. Lines and stripes are formed by air outlining thin or thick intervening tissue on both sides, respectively. Interfaces are formed when structures of different densities contact with one another, such as in the azygoesophageal recess. The recognition and understanding of the anatomic basis of these mediastinal lines, stripes, and interfaces along with their normal and abnormal appearances allow radiologists to develop an appropriate differential diagnosis of mediastinal lesions before obtaining additional information by using chest computed tomography or magnetic resonance imaging.
***Learning Objectives:***
1. To review the normal mediastinal lines.
2. To learn about the normal and abnormal mediastinal contour.
3. To know how to use the silhouette sign to localise mediastinal opacities.
*Author Disclosure:*
**M. Occhipinti:** Consultant; Imbio LLC. Grant Recipient; Menarini Foundation.

15:10
**Panel discussion: Why does the chest radiograph remain essential in the context of expanding cross-sectional imaging?**

14:00 - 15:30 Room M 5

### Transatlantic Course of ESR and RSNA (Radiological Society of North America): Sports Imaging

### TC 1528

### Knee sports injuries and postoperative MRI

*Moderators:*
L.W. Bancroft; Orlando, FL/US
A.J. Grainger; Leeds/UK

**A-0919** 14:00
**A. Sports-related injuries of the knee: what does the orthopaedic surgeon need to know?**
T.T. Miller; *New York, NY/US (millertt@hss.edu)*

The anatomy of the normal ACL and menisci will be reviewed, followed by a discussion of the appearances of various abnormalities of the ACL and menisci, as well as mechanisms of injury and injury patterns. Associated injuries will be discussed. Recommendations will be made for imaging the postoperative meniscus.
***Learning Objectives:***
1. To learn about what to include in knee MRI dictation templates.
2. To understand the most common sports-related injury patterns in the knee.
3. To appreciate which specific orthopaedic indications should prompt additional information in the dictation.

**A-0920** 14:30
**B. ACL reconstruction and cartilage repair**
C. Weidekamm; *Vienna/AT (weidekamm@icloud.com)*

ACL reconstruction aims to stabilise the knee and prevent chondral and meniscal injuries, which are sequelae of anteroposterior translation and are associated with early osteoarthritis. The idea of the double-bundle ACL graft was to restore normal joint kinematics by anatomic reconstruction of the anteromedial and the posterolateral bundle of the original ACL. This was expected to improve clinical outcomes and restore anterior and rotational knee stability. The single-bundle technique, however, causes less osseous defects and is still a popular technique. Complications, such as ACL graft failure, impingement, cyclops lesion, arthrofibrosis, and patellar inferior syndrome, are discussed. The second part of this presentation will illustrate cartilage repair techniques and imaging findings. The radiologist must be familiar with the different cartilage repair procedures and characteristics in cartilage imaging to evaluate long-term progression or failure. Abnormal postoperative findings include hypertrophic filling, incomplete integration of the transplant into the surrounding cartilage, or subchondral defects, osteophytes, cysts, and persistent bone marrow oedema and joint effusion.
***Learning Objectives:***
1. To review the common and uncommon ACL reconstruction techniques.
2. To appreciate the expected and abnormal MR imaging findings after ACL reconstruction.
3. To understand common cartilage repair techniques, and corresponding normal and abnormal postoperative MRIs.

**A-0921/A-0922** 15:00
**C. Interactive case discussion**
T.T. Miller; *New York, NY/US (millertt@hss.edu)*
C. Weidekamm; *Vienna/AT (weidekamm@icloud.com)*

Postoperative imaging after ACL or cartilage repair is indicated in patients with ongoing pain/instability or repetitive injury. Radiography remains the initial imaging modality; however, a further assessment with CT or MRI is recommended. With a clear emphasis on MRI, we will review normal postoperative findings and complications after ACL reconstructions and cartilage repair. The case discussion will cover the most significant pathologies and pitfalls, and normal postoperative findings will be illustrated.
***Learning Objectives:***
1. To appreciate common patterns of athletic injury in the knee.
2. To become familiar with the diagnostic features of failed ACL reconstructions, intact and failed cartilage repair.
3. To consolidate the knowledge gained from the session with interactive cases of pre- and postoperative MRI.

16:00 - 17:30 Room A

### E³ - ECR Academies: Interactive Teaching Session for Young (and not so Young) Radiologists

### E³ 1621

### Paediatric radiology for the general radiologist

**A-0923** 16:00
**A. Pitfalls in paediatric chest and abdomen**
M. Raissaki; *Iraklion/GR (mraissaki@yahoo.gr)*

A pitfall is defined as a likely mistake or problem in a given situation, an unexpected danger or difficulty. In pediatric radiology, two of the most demanding modalities include chest and abdominal radiography as well as ultrasonography. Chest and abdominal radiography carry numerous pitfalls due to superimposition of different structures into a two-dimensional image. Pitfalls can be related to the technique itself, especially in uncooperative patients. Knowledge of anatomy, physiology of respiration and basic radiographic principles aid in the identification of abnormalities and increase the sensitivity of the tests. Pediatric ultrasonography can be fun, nevertheless thoroughness, appropriate technique, attention to details and avoidance of satisfaction of search syndrome is always important. Knowledge of developmental changes and the entities unique to children is essential for the pediatric and general radiologist to avoid significant interpretive errors. Consideration of history and appreciation of age and the clinical condition of the patient are vital components of clinical pediatric radiology.
***Learning Objectives:***
1. To review the basic principles of the paediatric chest radiographs through common mistakes.
2. To emphasise pitfalls when imaging the paediatric abdomen.
3. To learn to avoid the most common pitfalls.

**A-0924** 16:45
**B. Paediatric MSK imaging: normal variants or real injuries?**
F. Saez; *Barakaldo/ES (fersaez@yahoo.com)*

The skeleton of a child is a developing system with a variety of changing normal appearances. Imaging studies, especially plain films, are requested for many clinical reasons, and the radiologist is in the position to determine if an image is a normal finding or we are dealing with a lesion. The way physis and epiphysis grow, ossify, and fuse constitutes a great source of physiologically bizarre appearances, which the radiologist must be familiar with. This talk will concentrate on the plain film diagnosis of some of the most common musculoskeletal variants. Other imaging modalities will also be shown when appropriate for the case. Irregularities, asymmetries, partial fusions, hypo- or hyper-dense bone areas, accessory bones, prominent normal structures, external artefacts, and potential fracture lines are the most often encountered pseudolesions. A defective radiological technique may also be potentially misleading. Patient age, the location of the supposed “abnormality” and lack of significant local symptoms are key factors. Usually, plain films, correlated with regional clinical findings, are the only imaging method that is required. However, in certain doubtful situations, ultrasound, CT, MRI, Bone Scan, or even biopsy, may be needed to reach the right diagnosis. Unnecessary overuse of these imaging modalities and the subsequent family anxiety that ensues from this overuse should be avoided with careful analysis of the X-ray and clinical findings.
***Learning Objectives:***
1. To become familiar with the normal variants.
2. To identify the key imaging findings to differentiate between normal variants and disease.
3. To learn how to integrate age, location and clinical history with the radiological features before establishing a diagnosis.

16:00 - 17:30 Room B

### GI Tract

### RC 1601

### Diagnosis and management of acute pancreatitis

*Moderator:*
C. Triantopoulou; Athens/GR

**A-0925** 16:00
**A. The 2012 revised Atlanta Classification: what has really changed?**
T.L. Bollen; *Nieuwegein/NL (tlbollen@hotmail.com)*

The lexicon of acute pancreatitis (AP) has changed since the publication of the 2012 Revised Atlanta Classification (RAC) which replaces the 1992 Atlanta Classification. The RAC makes a distinction between necrotising pancreatitis and interstitial pancreatitis, depending on the presence or absence of tissue necrosis, respectively. Necrotising pancreatitis is subdivided into three forms: pancreatic parenchyma alone (very rare), peripancreatic tissues alone (extrapancreatic necrosis or EXPN), or combined necrosis (most common). Associated collections are either termed acute necrotic collections (ANC) (incomplete wall) or walled-off necrosis (WON) (completely encapsulated). Fluid collections in interstitial pancreatitis are termed acute peripancreatic fluid collection (APFC) (incomplete wall) or pseudocyst (completely encapsulated). The distinction between the various subtypes of AP has implications for prognosis. Interstitial pancreatitis has a mortality of less than 3% and can virtually always be treated conservatively. Pancreatic necrosis, particularly in the presence of infection, is associated with mortality rates as high as 25%, and surgical management may be required. Patients with EXPN have a significantly better prognosis than those with combined necrosis if necrosis remains sterile, but have a similar outcome in case of infected EXPN. For complete reporting in AP, it is essential to describe the presence of pancreatic necrosis (site and extent) and presence of pancreatic collections with all its features (location, shape, size, homogeneity, degree of encapsulation, and presence of haemorrhage or gas bubbles). Associated findings, such as the presence of pleural effusion, ascites, vascular abnormalities, extrapancreatic parenchymal, biliary, and gastrointestinal complications should be evaluated as well.
***Learning Objectives:***
1. To be aware of the new terminology for the evaluation of acute pancreatitis.
2. To learn about how to report imaging findings.
3. To understand the relationship between imaging findings and prognosis.

**A-0926** 16:30
**B. Role of imaging in diagnosis of acute pancreatitis: new trends**
G. Zamboni; *Verona/IT (gzamboni@hotmail.com)*

Imaging has an important role in the management of acute pancreatitis, first of all in the differentiation between edematous and necrotising pancreatitis. In order to be accurate in this differential diagnosis, imaging - usually contrast-enhanced CT- must be performed with the appropriate timing: the optimal interval between symptoms onset and imaging is 72 hours. Before this, there is a risk of not recognising necrosis, thus misclassifying the disease. CT can recognise the presence of necrosis and define if it is parenchymal only, extra-parenchymal only or both parenchymal and extra-parenchymal. This has important implications, because parenchymal necrosis may lead to ductal disruption, resulting in the disconnected duct syndrome or in collections that are replenished with pancreatic juice with amylase. Imaging has an important role in defining the presence of collections and in classifying them according to the Revised Atlanta classification: a differentiation between fluid and necrotic collections should be made, in order to plan the best treatment for the patient, including collection drainage or surgery when indicated.
***Learning Objectives:***
1. To learn about the proper timing of imaging studies in acute pancreatitis.
2. To learn about the imaging manifestations of necrotising pancreatitis.
3. To understand the different nature of fluid collections with regard to further therapy.

**A-0927** 17:00
**C. Intervention radiology in acute pancreatitis: why, when and how**
M.M. Maher; *Cork/IE (m.maher@ucc.ie)*

Acute pancreatitis may have a benign course with minimal abdominal pain and hyperamylasemia or may be life-threatening, usually due to the development of infected pancreatic necrosis, haemorrhage and/or multisystem organ failure. Fortunately, 70-80% of patients with acute pancreatitis have a benign self-limiting course. In the initial management of acute pancreatitis, assessment of metabolic disturbances and systemic organ dysfunction is critical. In more severe cases, cross-sectional imaging (Ultrasound, Computed Tomography and MRI) plays a prominent role in the assessment of the degree of severity and/or development of complications. It is important that imaging is performed strategically in patients with severe acute pancreatitis as findings on imaging studies frequently impact management and play a central role in determining the appropriate timing of surgery and image-guided interventions. In this presentation, we will review imaging findings in severe acute pancreatitis and specific imaging findings associated with common complications. The question of when, where and why interventional radiology plays a role in the management of severe acute pancreatitis will be discussed. In addition, we will briefly discuss how common and novel interventional techniques may be used optimally to improve patient outcome.
***Learning Objectives:***
1. To learn indications for interventions and type of complications that requires treatment.
2. To understand how likelihood of success of these interventions vary with severity.
3. To review management of local complications of pancreatitis by interventional radiology.

16:00 - 17:30 Room C

### Joint Session of the ESR and EFRS

### ESR/EFRS

### Patient safety in medical imaging

*Moderators:*
A. Brady; Cork/IE
J. McNulty; Dublin/IE

**A-0928** 16:00
**EuroSafe Imaging and the European Basic Safety Standards Directive: progressing patient safety**
G. Frija; *Paris/FR (guy.frija@aphp.fr)*

EuroSafe Imaging is the European Society of Radiology's flagship initiative for promoting quality and safety in medical imaging. Launched in 2014, EuroSafe Imaging leads numerous activities to support and strengthen medical radiation protection across Europe through the implementation of the 13 actions of its Call for Action 2018. Among other activities, EuroSafe Imaging has provided guidance and practical tools to support compliance with the European Commission Directive 2013/59/Euratom ("Basic Safety Standards Directive"), which lays down basic safety standards for protection against the dangers arising from exposure to ionising radiation. It entails substantial new requirements in various areas, e.g. improving the protection of patients in medical imaging procedures, improving the protection of workers, and improving the protection of the public in particular from natural radiation sources. The Directive entered into force in February 2018. This talk will present the EuroSafe Imaging initiative, its achievements to date and future activities. Moreover, it will provide an overview of the BSS Directive and its contribution to promoting patient safety in medical imaging.
***Learning Objectives:***
1. To explore the aims and objectives of EuroSafe Imaging and its achievements to date.
2. To appreciate aspects of the Basic Safety Standards Directive, which will further progress patient safety.
3. To consider what the future holds for EuroSafe Imaging.

**A-0929** 16:16
**Patient safety: beyond radiation protection**
K. Azevedo; *Faro/PT (kbazevedo@ualg.pt)*

Patient safety is a broad field of knowledge and intervention and can be divided into three domains namely: structural factors; processes; and outcomes. Medical imaging services receive a considerable number of patients every day that undergo several number of different procedures. It is important to highlight that each procedure has a potential to cause harm to the patient and this potential is present even before the first contact with the patient and ends after the last contact with the patient. It is clear that the leading objective of an imaging service is to diagnose and treat the patient, but at the same time do no needless damage. To increase patient safety the first step is to make it an institutional cultural theme. Beyond radiation-related issues, patient safety can be affected by the importance given by the management to this subject, communication, feedback and non-punitive response to error, adequate staffing, infection control, adequate continuous professional development, handoffs and transitions, and teamwork in the imaging service and with other units, such as inpatient wards, and the imaging services. The radiographer and the radiologist are key elements in the medical imaging services. To receive and prepare the patient, to perform the exams, from their programming, their execution, their quality evaluation and to diagnose and treat the patient, there are several factors that may influence patient safety, not only at the imaging service level but also at the whole healthcare institution level.
***Learning Objectives:***
1. To appreciate the scope of patient safety related to medical imaging services.
2. To consider a selection of examples of patient safety beyond radiation protection, optimisation and justification.
3. To explore the important roles of radiographers and radiologists in working collaboratively to best ensure patient safety.

**A-0930** 16:32
**Value-based imaging and patient safety**
A. Brady; *Cork/IE (adrianbrady@me.com)*

The value-based healthcare concept is growing in importance, as part of a trend away from concentration on the delivery of volume-based services, and towards concentration on the specifics of delivering value-based care to individuals and the population as a whole. The components of what constitutes value differ among physician, provider and patient groups, and thus the parameters of value in healthcare are not fixed. The original model of value-based healthcare began consideration of value at the point when a patient received a diagnosis; the impact of radiology was effectively excluded, given that much of the work of radiology departments are geared towards arriving at a diagnosis in the first instance. This talk will focus on this "classical" model, and explore how and why it should be varied to include the impact of radiology on delivering value-based healthcare to patients. The impact of considering the value of radiology in patient care regarding ensuring patient safety will be explored.
***Learning Objectives:***
1. To learn about the developing model of value-based healthcare.
2. To appreciate how and why radiology should be included in this model.
3. To understand how this may impact upon patient safety.

**A-0931** 16:48
**Patient safety related education and training**
J. McNulty; *Dublin/IE (jonathan.mcnulty@ucd.ie)*

Patient safety is a major priority for all healthcare professions and one of the biggest areas of public focus. There is no question that embedding the theme of patient safety in undergraduate medical and radiography curricula, together with making it a core part of all postgraduate or specialist education and training, has the potential to improve patient safety. While there has been a lot of focus on this in terms of other professional groups, there is a lack of information regarding the inclusion of patient safety topics within undergraduate radiography curricula or indeed within radiology training programmes. Radiographers and radiologists encounter numerous patient safety issues which stretch far beyond the area of radiation protection. The responsibilities of radiographers and radiologists overlap substantially across most areas; thus team-working within the medical imaging environment is key to ensuring a safe environment for our patients. Recent evidence from the European Federation of Radiographer Societies (EFRS) demonstrated that patient safety is a deeply embedded concept within undergraduate radiography curricula across Europe, however, variations, some of which are significant, do exist in terms of the topics covered, the teaching and assessment methods, and the depth or level in which subjects are taught. This presentation will explore aspects of patient safety-related education and training for radiographers and will highlight the current opinions of the ESR and EFRS in this regard.
***Learning Objectives:***
1. To emphasise the importance of education and training in progressing patient safety in medical imaging.
2. To consider the current status of patient safety in radiography and radiology curricula.
3. To appreciate the activities of the EFRS and ESR in the area of patient safety education and training.

**A-0932** 17:04
**Patient perspectives on patient safety in medical imaging**
E. Briers; *Hasselt/BE (erikbriers@telenet.be)*

Safety for patients in medical imaging is linked to the department of "Medical Imaging - Radiology". It depends on several elements, there is the "why" they are there, the "what" will happen, the "how" does this work and the other personal but important aspects to be in a new environment. Safety has by itself different "imaging" aspects, and there is the radiation, the eventual contrast, the small space, the length of time of being unable to move and others, all of these are important but shall be offset versus the "why" the patient is there. The patient will balance some of the known facts (to him) and associated safety risks versus the risks of having a disease that may or may not cause him a big quality of life loss. The risk taken should be in relation to the expected benefit. A repeated CT-scan for lung cancer looks quite different from a CT-scan for a common cold which no-one would do. The information that the patient needs to be able to weigh the risks versus the benefit should come from multiple sources, the medical team is multi-professional, and all can contribute. They should explain each for his part how the (not yet confirmed) disease could evolve and what the benefit of the final diagnosis would be. On the risk side, careful listening to the patient and taking note of previous experiences in imaging and beyond, allergies, psychological issues with confined spaces, being very young or old, all matter and can influence the procedure taken.
***Learning Objectives:***
1. To understand what patients feel about patient safety in medical imaging.
2. To appreciate the value of active engagement with patients to enhance and promote patient safety.
3. To consider initiatives to implement patient-engagement at the institutional or national level.
*Author Disclosure:*
**E. Briers:** Advisory Board; Requite project. Board Member; Us Too Belgium. Speaker; None for Pharmaceutical Companies. Other; Member of EAU Guidelines Committee on Prostate Cancer, Alternate Patient Member Committee Of Advanced Therapies - EMA.

17:20
**Panel discussion: The role of the ESR and the EFRS in promoting patient safety**

**16:00 - 17:30** **Room N**

### EuroSafe Imaging Session

### EU 6

### CT radiation risk in children: an overview

**A-0933** 16:00
**Chairperson's introduction**
E. Cardis; *Barcelona/ES*

***Session Objectives:***
1. To learn about the radiological protection issues related to CT scanning in paediatric populations.
2. To understand the state-of-the-art in radiation induced detriment from scanning.
3. To appreciate the need for optimisation in paediatric CT scanning.

**A-0934** 16:05
**A literature update**
H. Ducou Le Pointe; *Paris/FR (hubert.ducou-le-pointe@aphp.fr)*

Ultrasound and MRI are the preferred techniques in paediatrics; nevertheless, the use of computed tomography remains essential. In normal use, deterministic effects of ionising radiation are not to be feared (organ doses reach up no more than to tens milligrays). Cancer is the main stochastic effect of ionising radiation. Pediatric population is a subject of concern due to high tissue radiosensitivity and their long life expectancy. Model-based risk estimations predicted cancer risk associated with pediatric CT scan. Epidemiological studies seem to show a positive association between radiation dose from CT scans and the incidence of leukaemia and cancers. These results are questioned due to small sample size and methodological biases (lack of information about the indication of CT scans, reverse causation, risk factor confounding). Results of a large (950 000 children) European epidemiologic study called EPI-CT (EPIdemiological study to quantify risks for paediatric Computerized Tomography and to optimise doses) are expected soon.
***Learning Objectives:***
1. To learn about the nature of the risk of CT for children.
2. To understand how the risk is evaluated.
3. To appreciate the difficulty to draw conclusions based on the literature analysis.

**A-0935** 16:25
**Ongoing studies and first results of the European EPI-CT study**
E. Cardis; *Barcelona/ES*

"no abstract submitted"
***Learning Objectives:***
1. To learn about the largest epidemiological study (1 million patients) of the health impact of CT radiation in young people and ongoing complementary studies.
2. To understand strengths and limitations of the study and to discuss the results.
3. To appreciate the study's implications for optimisation in paediatric imaging.

**A-0936** 16:45
**Communicating benefits and risks to parents and carers**
C. Granata; *Genoa/IT*

In the last two decades, the role of CT in paediatric imaging has strengthened, thanks to better image quality and shorter duration of the exam. However, concerns about the exposure of children to the relatively high CT radiation dose have been increasingly raised. Consequently, health care providers and imaging professionals sometimes must face parents' negative perception of radiation risk-benefit ratio. Experts and the public perceive the risk differently. A one-in-a-million risk is perceived as a low risk by an expert, whereas parents may perceive that the "one" could be their loved ones. Therefore, it should be ensured that patients, parents, and carers receive the information they need about CT radiation risks in a way they can understand. When establishing a dialogue in a clinical setting, it is important to talk slowly, use plain language, analogies, and metaphors, repeat key messages, and encourage questions. The expected outcome of the dialogue is that parents/patients trust health care professionals, as they have understood that the benefits of a necessary CT scan always outweigh any associated risk.
***Learning Objectives:***
1. To learn how to establish a dialogue in a clinical setting with parents and carers.
2. To understand goals and challenges of communicating risks and benefits of CT studies in children.
3. To appreciate the importance of parent and caregiver-centred communication.

**A-0937** 17:05
**Daily practical challenges in CT for children**
C. Owens; *London/UK (owensc@gosh.nhs.uk)*

Children pose a particular challenge as patients in CT, as they are small, mobile and particularly radiation sensitive. Therefore it is important to tailor the examination, customising for each child. Optimising image quality is vital with attention to detail paramount. There are various tips and tricks in terms of immobilisation and distraction devices and important procedure such as ensuring that the child is in the epicentre of the scanner which has a very important role in good image acquisition.
***Learning Objectives:***
1. To learn about the potential challenges and obstacles faced when performing CT in children.
2. To understand how to tackle these challenges.
3. To appreciate the tips and tricks which can assist in acquisition of diagnostic CT images in children.

17:25
**Questions and answers**

**16:00 - 17:30** Room O

### Cardiac

### RC 1603

### Acute chest pain and cardiac imaging

*Moderator:*
P. Croisille; Saint-Etienne/FR

**A-0938** 16:00
**A. Acute chest pain: who should see the patient first, the doctor or the CT department?**
M. Gutberlet; *Leipzig/DE*

"no abstract submitted"
***Learning Objectives:***
1. To become familiar with existing clinical triage scores in acute chest pain and their strengths and weaknesses.
2. To learn about the potential of cardiac CT in the management of acute chest pain patients.
3. To outline recent updates and future developments for triaging patients with acute chest pain.

**A-0939** 16:30
**B. Myocardial perfusion in acute coronary syndrome (ACS): necessity or luxury?**
R. Vliegenthart; *Groningen/NL (r.vliegenthart@umcg.nl)*

Myocardial perfusion imaging (MPI) has a role in the triage setting of acute chest pain patients without ST-elevation myocardial infarction. MPI is used to confirm or exclude myocardial ischemia/infarction and estimate prognosis, in case of low or intermediate risk of an acute coronary syndrome (ACS). In patients with prolonged chest pain, normal/non-diagnostic ECG, and initial negative blood biomarkers, rest MPI has been demonstrated to improve patient outcomes. A normal rest MPI result has a high negative predictive value for ACS and events. After symptoms have resolved, use of stress MPI is to be preferred. There is increasing evidence that MPI can also have value in the diagnostic management of patients suspected of unstable angina or non-ST-elevation myocardial infarction. When used appropriately, non-invasive imaging can reduce the number of missed diagnoses, and guide the management of patients with suspicion of ACS. This lecture covers the imaging techniques used to assess myocardial perfusion, including nuclear imaging, magnetic resonance imaging, and computed tomography perfusion. Contemporary guidelines and current use of MPI techniques in acute chest pain will be discussed.
***Learning Objectives:***
1. To introduce the acquisition techniques of myocardial imaging: nuclear imaging, MRI, CT.
2. To become familiar with the challenges in using myocardial perfusion in the acute setting.
3. To discuss the possible role of perfusion imaging in acute coronary syndromes.

**A-0940** 17:00
**C. Late gadolinium enhancement (LGE) and more: the role of MRI in and after ACS**
J. Bremerich; *Basle/CH (jens.bremerich@usb.ch)*

Imaging plays a critical role in acute coronary syndrome (ACS). After initial treatment, noninvasive imaging is used to guide therapy further. This paper shall review current diagnostic algorithms with respect to strengths and limitations as well as future directions. In ACS, coronary catheter angiography is considered the first line imaging modality to confirm acute coronary artery disease with the advantage to perform angioplasty within a single procedure. Magnetic resonance imaging (MRI) plays a limited role in acute diagnostic workup but is of utmost importance for further therapy guidance. Cardiac MRI provides an assessment of viability in coronary artery disease for planning revascularisation. Moreover, alternative diagnoses such as myocarditis may be confirmed when coronary angiography did not confirm the initially suspected acute coronary syndrome. Myocarditis and infarction may present in a similar fashion including chest pain, ECG abnormalities and elevated markers of myocardial damage such as troponin. Cardiac MRI is a powerful tool to evaluate prognostic indicators such as the presence and size of infarction, myocardial haemorrhage or microvascular obstruction, myocardium at risk and it is the gold standard for myocardial function. Moreover, MRI shows macrostructural damage such as aneurysm formation, perforation, or papillary muscle rupture. Cardiac MRI is useful to exclude other causes of acute chest pain such as aortic dissection or intramural hematoma. Although catheter angiography remains the first-line modality in ACS, MRI provides added value to establish an alternative diagnosis and to guide further therapy.
***Learning Objectives:***
1. To become familiar with the technique of late gadolinium enhancement.
2. To learn about quantitative assessment of LGE.
3. To learn about the predictive power of LGE in ACS.

**16:00 - 17:30** Studio 2019

### State of the Art Symposium

### SA 16

### Breast cancer screening and treatment: how much is too much?

**A-0941** 16:00
**Chairperson's introduction**
G. Forrai; *Budapest/HU (forrai.gabor@t-online.hu)*

Breast screening is a well designed and scientifically proven, evidence-based procedure. However, some scientists question the current method, raising some arguments regarding overdiagnosis and overtreatment. In this State-of-the-Art Symposium, we will analyse these problems, based on epidemiological and statistical data. New approaches, e.g. active monitoring as a treatment option would be discussed - in order to attempt to decrease unnecessary surgeries.
***Session Objectives:***
1. To understand the epidemiological issues of screening.
2. To understand that there are lesions not to be recalled from screening.
3. Learn about 'active monitoring' as a new treatment option.

**A-0942** 16:05
**Overdiagnosis in breast cancer screening: the epidemiologist's view**
S.W. Duffy; *London/UK (s.w.duffy@qmul.ac.uk)*

Overdiagnosis in cancer screening is essentially an epidemiological concept. It is defined as cancer which would not have been diagnosed in the patient's lifetime if screening had not taken place. This definition has no reference to pathology or treatment. Estimation of overdiagnosis is complicated by underlying trends in incidence taking place independently of screening and by lead time. In this presentation, we discuss the information required for reliable estimation of benefits of screening in terms of a breast cancer mortality reduction and the opportunity for less aggressive therapy. We propose a method of estimation of overdiagnosis, based on relative risk of cancer by time since the last screen, which addresses the lead time issue and provides the opportunity for sensitivity analyses to explore a range of potential underlying incidence estimates. We demonstrate the method's use on NHS Breast Screening Programme data to estimate the total excess of breast cancers due to overdiagnosis, and the excess or deficit, due to screening, of cancers receiving radical treatment (mastectomy and/or chemotherapy). We consider these estimates in the context of the mortality benefit associated with mammographic screening.
***Learning Objectives:***
1. To learn about estimation of overdiagnosis with minimal assumptions.
2. To understand the potential biases in estimating favourable and unfavourable effects of screening.
3. To appreciate the connection between the epidemiological definition of overdiagnosis and its clinical implications.
4. To understand the arguments for and against organised screening programmes.

**A-0943** 16:28
**What can the radiologist do to limit overdiagnosis?**
R.M. Pijnappel; *Utrecht/NL (r.m.pijnappel@umcutrecht.nl)*

Screening has been proven to be beneficial to women who do attend all rounds in a screening program. Nevertheless, screening has some limitations. The balance between harm and benefits is delicate. To detect all cancers, the test used for screening should be as sensitive as possible. Despite this, there will always be a gap between test sensitivity and program sensitivity. The difference between test and program sensitivity will be explained during the session. The optimal test for breast cancer screening is under debate. FFDM and Tomosynthesis (DBT) compete to be the best test. The advantages and disadvantages of both FFDM and DBT will be discussed. Recall rate plays an important role in relation to program sensitivity. This relationship will be explained. This explanation will give insight to the policy not to recall every lesion and therefore accept false-negative results. Apart from limiting overdiagnosis in a screening environment, radiologist play an important role in overdiagnosis in the assessment of recalled cases. Ultrasound of the breast and especially ultrasound of the axilla constitute an important part of this overdiagnosis. The optimal timing for performing an ultrasound of the axilla in the workflow is defined by recall rate, a positive predictive value of the recalled lesion, tumour board availability and other logistics items. It is more complex than the BI-RADS score alone. By using the optimal screening test, controlling the recall rate and optimising the assessment of recalled cases, radiologists can play an important role in minimising overdiagnosis.
***Learning Objectives:***
1. To estimate test sensitivity in relation to programme sensitivity in screening.
2. To learn what the optimal test for population based screening is: FFDM or tomosynthesis in relation to overdiagnosis.
3. To understand that there are lesions not to be recalled from screening.
4. To learn the place of the ultrasound of the axilla: in BI-RADS 4, 5 or never?
*Author Disclosure:*
**R.M. Pijnappel:** Equipment Support Recipient; GE, Hologic, Volpara. Research/Grant Support; Bayer, Hologic.

**A-0944** 16:51
**Are there breast cancers we do not have to treat?**
M.G. Wallis; *Cambridge/UK (matthew.wallis@addenbrookes.nhs.uk)*

Not all breast cancers are the same. IHC subtypes replaced grade and now 12 genetic subtypes of breast cancer all of which have different prognosis and respond differently to treatment. Treatment de-escalation is not new, simple mastectomy has replaced Halstead mastectomy, and now conservation with radiotherapy is routine. Side effects of current treatment are physical, cosmetic and psychosocial, ranging from death from catastrophic sepsis to lymphoedema, chronic pain and musculoskeletal symptoms. Number Needed to Treat quantifies the potential for, but trials are needed to identify which individual woman can safely avoid some or all treatment. It is difficult to recruit to these trials because public risk perception does not match reality as shown by the increasing numbers of bilateral mastectomies in the United States. The concept of low-risk cancer or the need for less treatment is a difficult explain in the face of advertising and fundraising campaigns frequently centred around fighting cancer or the new wonder drug. This is led for calls to relabel lower risk cancers with terms like IDLE. Radiology is changing surgical paradigms. Atypia is managed by vacuum excision, there are trials of no surgery for low-risk DCIS and investigating the role of needle biopsy to identify women with a complete pathological response to NAC who might not need surgery. For those women with low-risk disease, we need to offer and make clear that careful radiological monitoring within a trial is an active treatment option not just 'being abandoned to follow up'.
***Learning Objectives:***
1. To review side effects of current treatments.
2. To be able to discuss what lesions we could consider reducing treatment burden.
3. To learn about radiological options to the current surgical treatment paradigm.
4. To learn about 'active monitoring' as a new treatment option.
*Author Disclosure:*
**M.G. Wallis:** Investigator; Chief investigator LORIS trial Grant for UK HTA.

17:14
**Panel discussion: Which one is the real villain, overdiagnosis or overtreatment? For what reason breast screening and diagnostics is blamed?**

**16:00 - 17:30** **Room E1**

### E³ - ECR Master Class (Musculoskeletal)

### E³ 1626a
### State-of-the-art imaging of postoperative joints

*Moderator:*
M.F. Reiser; Munich/DE

**A-0945** 16:00
**A. Postoperative shoulder**
C.W.A. Pfirrmann; *Zurich/CH*

Imaging of the postoperative shoulder is challenging because of various surgical techniques, distorted anatomy and the presence of artefacts because of metallic implants. The most commonly performed surgical procedures include rotator cuff reconstruction, instability surgery, and joint replacements. The radiologists should have knowledge of these techniques. In this presentation, these surgical techniques, the normal imaging findings after these procedures and the abnormal findings as well as imaging strategies will be discussed.
***Learning Objectives:***
1. To explain the most frequently used surgical techniques for glenohumeral instability, subacromial decompression, rotator cuff repair and arthroplasty, and their imaging appearance.
2. To describe potential postoperative complications.

**A-0946** 16:30
**B. Postoperative knee**
E.H.G. Oei; *Rotterdam/NL (e.oei@erasmusmc.nl)*

In this masterclass, we will review the indications for the most commonly performed surgical techniques in the field of meniscal repair, knee ligament reconstruction, and knee cartilage repair, followed by a brief explanation of the surgical procedure. We will also highlight recent advances in joint preserving procedures around the knee. Of each discussed technique, the normal postoperative appearance on imaging will be presented, with an emphasis on MR imaging. This is followed by a discussion on the assessment of potential complications. Among the discussed topics will be complications after ACL reconstruction (tunnel positions, graft re-rupture, impingement, arthrofibrosis, cyclops lesions), assessment of meniscus re-tear after surgical repair, and assessment of the success after cartilage repair procedures.
***Learning Objectives:***
1. To explain the most frequently used surgical techniques for meniscal repair, ligament reconstruction and cartilage repair.
2. To describe potential postoperative complications.

**A-0947** 17:00
**C. Postoperative ankle and foot**
M.J. Ereño Ealo; *Galdacano/ES (mjereno@gmail.com)*

Foot and ankle traumatic pathologies are frequent conditions. Diagnosis, treatment and subsequent management of foot and ankle traumatic pathologies are important to avoid big deformities. The most current and innovative techniques of internal fixation and their applications will be shown.

Very different injuries and cases will be detailed: surgical management and complications of high-energy fractures, tibia pilon fractures; calcaneus, talus and Lisfranc lesions, diabetic (Charcot) foot, Morton´s Neuroma, hammer toe, tarsal coalition, adult acquired flatfoot. Also, patients with arthroplasty, arthrodesis and triple fusion and ankle replacement should be recognised.
***Learning Objectives:***
1. To explain the most frequently used surgical techniques for osteosynthesis, instability, tendon repair and arthroplasty, and their imaging appearance.
2. To describe potential postoperative complications.

**16:00 - 17:30** **Room E2**

### Neuro

### RC 1611

### The degenerative cervical spine

*Moderator:*
N. Chidambaranathan; Chennai/IN

**A-0948** 16:00
**A. Degenerative uncovertebral and facet disease**
J. Van Goethem; *Antwerp/BE (johan.vangoethem@uantwerpen.be)*

Neck pain is a common problem with many possible causes. The facet joint and the uncovertebral joint are frequently involved in degenerative cervical spine disease. It is important to learn how to differentiate normal and asymptomatic changes that occur with age from abnormal findings that are causing neck and/or arm pain. I will demonstrate the use of plain film, CT, SPECT and MRI in diagnosing an offending uncovertebral or facet joint. Many of these offending joints can be targeted specifically, leading to easy and fast pain reduction in many patients with specific neck pain.
***Learning Objectives:***
1. To learn about the physiological and pathophysiological degeneration of the cervical spine.
2. To understand the role of imaging in the diagnosis and clinical decision making in the degenerative cervical spine.
3. To appreciate the clinical relevance of imaging findings in the degenerative cervical spine.

**A-0949** 16:30
**B. Cervical spinal stenosis and cervical spondylotic myelopathy**
A.S. Gersing; *Munich/DE*

Degenerative changes of the cervical spine may be caused by mechanical overload or may occur during the ageing process. Patients with spinal stenosis may present with clinical symptoms ranging from neck pain to spastic paraparesis. Imaging is crucial in these cases in order to identify the cause of these clinical symptoms and to select the appropriate treatment option. Spinal stenosis is caused by certain pathological features that occur during the degenerative process, e.g. marginal osteophytes of the vertebrae, hypertrophy of the ligaments and intervertebral disc degeneration with herniation. Radiographs, CT as well as CT-myelography and MR imaging are the common imaging modalities used for the assessment of the severity of the spinal canal stenosis. Next to the assessment of soft tissue structures and osseous structures of the vertebrae, especially T2-weighted MR imaging is crucial for the assessment of signal changes within the myelon. One of the most important aspects assessed with MR imaging is the differentiation between signs of acute myelopathy and myelomalacia, caused by irreversible damage of the spinal cord. These aspects are of importance for the selection of the adequate treatment option, especially in order to decide between conservative or surgical treatment.
***Learning Objectives:***
1. To learn about the pathophysiology and imaging findings in spinal stenosis and cervical spondylotic myelopathy.
2. To understand the relation between imaging findings and clinical presentation.
3. To appreciate the importance of imaging findings and the clinical presentation with respect to possible treatment options.

**A-0950** 17:00
**C. The postoperative cervical spine**
S. Looby; *Dublin/IE*

Imaging is a routine part of follow up for the postoperative cervical spine. A knowledge of the surgical techniques and/or the hardware used for cervical surgical and instrumentation procedures is very helpful in understanding the postoperative imaging appearances. A knowledge of the imaging modalities and when to use them is required. Ways to reduce metal artefact in imaging should be implemented. Radiography provides information on cervical spine fixation or metallic hardware placement. CT provides information on cervical spine alignment, hardware placement and integrity, and bone graft incorporation or complications. MRI is helpful for complications of surgery not directly related to hardware. These include signs of CSF leakage, pseudomeningocoele, epidural or other haemorrhage and infection. In the context of the degenerative cervical spine, MRI shows residual or recurrent disk osteophyte complex. Long-term sequelae of cervical spine fusion such as hardware failure, advanced degeneration, post laminectomy spondylolisthesis and epidural and other scar tissue are detected by follow up imaging. The entity of the failed back surgery syndrome needs to be considered with the postoperative cervical spine and what to look for in imaging it. The combination of understanding the surgical techniques used, using the correct imaging modality for the patient symptoms and having a systematic approach to the imaging evaluation, renders imaging of the postoperative cervical spine safe, practical and useful.
***Learning Objectives:***
1. To learn about the imaging findings and pitfalls of postoperative cervical spine imaging.
2. To understand the heterogeneity of imaging findings and their clinical relevance.
3. To appreciate the importance of standardised imaging, interpretation and reporting of postoperative imaging findings in the cervical spine.

**16:00 - 17:30** **Room F1**

### E³ - European Diploma Prep Session

### E³ 1623

### Chest

**A-0951** 16:00
**Chairperson's introduction**
J. Vilar; *Valencia/ES (vilarsamper@gmail.com)*

In this session, the speaker, all of them experts in thoracic imaging will approach the main aspects of chest radiology related to the European Training Curriculum, levels I and II. These topics are the objective of preparation for the European Diploma examination. In the examination, there will be questions related to knowledge, skills and decision making that the authors of the three lectures will offer to the audience. Please pay attention to basic facts, differential diagnosis, the value of imaging techniques, diagnostic clues, and management issues related to the topics of the session.
***Session Objectives:***
1. To understand the most important signs in chest imaging.
2. To learn the imaging features of benign and malignant lesions of the lung.
3. To become familiar with the imaging appearance of common lesions of the mediastinum, pleura and chest wall.
4. To understand the role of different imaging modalities including hybrid imaging in diagnosing and staging neoplasms of the chest.

**A-0952** 16:06
**A. Fundamentals of chest imaging**
M. Occhipinti; *Florence/IT (mariaelena.occhipinti@gmail.com)*

Chest imaging remains one of the most complicated subspecialties of diagnostic radiology. The fundamentals in the interpretation of chest imaging are the knowledge of the normal anatomy as well as the radiographic signs. Anatomy of the respiratory system and its variants in the different imaging techniques will be reviewed, including chest radiography, CT and MRI. Monitoring and support devices such as different types of tubes and lines will be shown along with their correct position in the chest. Chest radiography signs are helpful in establishing a particular diagnosis of chest diseases. Therefore, their recognition and understanding is of particular interest for general and subspecialised thoracic radiologists. During the presentation, many radiographic signs will be discussed, including silhouette sign, air bronchogram, air crescent sign, cervicothoracic sign, gloved finger sign, golden S sign, deep sulcus sign, *coeur en sabot* sign, doughnut sign, double density sign, hilum convergence sign, hilum overlay sign.
***Learning Objectives:***
1. To learn the anatomy and normal variants of the respiratory system, heart and vessels, mediastinum and chest wall and to confidently identify these on radiographs, CT and MRI.
2. To gather an in-depth understanding of the most common chest radiography signs (including silhouette sign, air bronchogram, air crescent sign, cervicothoracic sign, tapered margins, gloved finger sign, golden sign, deep sulcus sign).
3. To learn the appearance and correct position of monitoring and support devices (tubes and lines).

**A-0953** 16:34
**B. Inflammation and tumours of the lung**
A. Nair; *London/UK*

"no abstract submitted"
***Learning Objectives:***
1. To understand the imaging features and differential diagnoses of diffuse infiltrative and alveolar lung disease and atelectasis.
2. To become familiar with thoracic diseases in immunocompetent, immunocompromised and post-transplant patients.
3. To become familiar with the differentiation of solitary and multiple pulmonary nodules, benign and malignant neoplasms, hyperlucencies and their potential aetiology and evaluation.
4. To understand the role of different imaging modalities including hybrid imaging in diagnosing and staging neoplasms of the chest.

**A-0954** 17:02
**C. Mediastinum, pleura and chest wall**
A.P. Parkar; *Bergen/NO (apparkar@gmail.com)*

The normal appearance of the diaphragm is usually similar on both sides. The diaphragm may be elevated on either side due to abdominal tumours, paresis of the phrenic nerve, traumatic rupture, or subpulmonic pleura effusion. The pleura may be thickened due to effusion, fibrosis with or without calcifications or due to malignancy. The chest wall has a varying normal appearance according to the shape of the rib cage and sternum. The mediastinum is a complex anatomic area which is affected by changes in vascular system and lymphadenopathy. In addition, various diseases like infections and tumours can be seen in the mediastinum. The postoperative chest radiograph is sometimes challenging to read because it usually is done in a supine position and only a front image is performed. It is important to recognise “do-not-miss” pathology such as pericardial fluid, tensions pneumothorax, or malpositioned tube and central lines.
***Learning Objectives:***
1. To become familiar with the imaging features of common pathologies of the diaphragm, pleura and chest wall on radiography, CT and MRI of the chest.
2. To learn the imaging features and causes of mediastinal and hilar diseases.
3. To understand the imaging features of disorders of the pulmonary vascular system and great vessels.
4. To learn the typical imaging features of the postoperative chest.

**16:00 - 17:30** **Room F2**

### New Horizons Session

### NH 16

### The tumour board of the future

**A-0955** 16:00
**Chairperson's introduction**
M. Prokop; *Nijmegen/NL (mathias.prokop@radboudumc.nl)*

***Session Objectives:***
1. To learn how to structure a tumour board for maximum efficiency and effectiveness.
2. To learn how to incorporate modern Information management tools for a multidisciplinary meeting.
3. To understand current risks and problems and how they affect the role of the radiologist.
*Author Disclosure:*
**M. Prokop:** Research/Grant Support; Canon Medical systems, Research grant, Siemens Healthineers, Research grant. Speaker; Bracco, Personal fee, Bayer, Personal fee, Canon Medical Systems, Personal fee, Siemens Healthineers, Personal fee. Other; Thirona, Departmental spinoff, Mevis Medical Solutions, Departmental Licence agreement.

**A-0956** 16:06
**How to set up and run a multidisciplinary meeting**
C. Dromain; *Lausanne/CH (Clarisse.Dromain@chuv.ch)*

Since few years, evidence has shown that multidisciplinary tumour boards (MTB) improve diagnostic accuracy, adherence of clinical practice guideline and clinical outcomes. However, new factors impact the efficiency of MTB including oncologic disease becoming more and more complex, the increased number of oncologic patients, multiplicity of new therapeutic options and new diagnostic approaches, the development of network operation and the need for multidisciplinarity apart from oncology. The efficiency of MTB will depend upon the limited available resources especially for transverse specialities (diagnostic radiology, interventional radiology, nuclear medicine, pathology) and the need for high quality required in highly specialised medicine. In addition to its primary goal, optimal treatment management in complex cases, MTB also have an education and a social value role. Key elements for running an effective MTB are the selection of patients, preparation of cases, which questions to ask the MTB, the presence of the referring clinician, availability of all findings (clinical, images, pathology etc.), appropriate equipment for images display and direct reporting of the MTB decision. Other parameters of importance are the date and location of the MTB, the role of the chairman, the delay between the announcement of the cases and the MTB, the order of cases presentation and the link with other TBMs. Good running of MTB also need the availability of images with PACS for review, availabilities of histopathological information and availability of clinical and other information. Projection equipment is also needed to display imaging +/- pathology slide.
***Learning Objectives:***
1. To learn how to best structure a multidisciplinary meeting to avoid wasting time.
2. To appreciate the various roles in a multidisciplinary meeting and how they influence meeting organisation.
3. To learn how to integrate modern tools for improved data collection, communication and evaluation in tumour boards.

**A-0957** 16:24
**Preparation for tumour boards: how to increase efficiency of the radiologist?**
D. Regge; *Turin/IT (daniele.regge@ircc.it)*

Radiologists are pivotal for the success of MTBs. The probability of reaching a treatment decision for a patient is largely increased when radiologists participate in the meeting, and patient survival improves among several types of cancer. However, MTBs add on to radiologist’s workload significantly due in particular to the time required to review cases prior to meeting. Increasing efficiency in the preparation of tumour boards will impact favourably on radiologist’s workload, increase efficiency and reduce costs for healthcare providers. The lecture aims to provide practical tips and knowledge on technical advancements that may support the radiologists in preparing for MTBs.
***Learning Objectives:***
1. To learn how effectively prepare for a multidisciplinary tumour board meeting.
2. To appreciate the clinician's perspective and understand the expectations towards the radiologist.
3 To learn how to concisely and efficiently communicate relevant findings.
*Author Disclosure:*
**D. Regge:** Author; Springer. Consultant; im3D. Research/Grant Support; Sanitas. Speaker; GE Healthcare, GE Medical Systems.

**A-0958** 16:42
**Physical or virtual multidisciplinary meetings?**
E. Neri; *Pisa/IT (emanuele.neri@med.unipi.it)*

"no abstract submitted"
***Learning Objectives:***
1. To understand the trade-offs of physical and virtual meetings.
2. To learn how to best set up remote access to a multidisciplinary meeting.
3. To understand how to organise and structure regional multidisciplinary tumour board meetings.

**A-0959** 17:00
**Maximising the added value of tumour boards**
C.D. Becker; *Geneva/CH*

Organ- or disease- specific multidisciplinary tumour boards play a key role for the selection of appropriate modern treatment for patients with neoplastic disease. Radiologic subspecialty expertise is essential and required for both diagnosis and treatment. In the age of personalised medicine, a precise diagnosis must include many different aspects, including morphologic and functional, metabolic, molecular and genetic elements, quantification and individual longitudinal follow- up. Closer integration of all these findings will be required in the future in order to offer the best possible treatment to all patients. Minimally invasive vascular or percutaneous image- guided interventional techniques are gaining increasing importance in tumor treatment. These techniques may be used for curative, adjuvant or palliative indications, and either in an alternative or adjunctive - sequential fashion for a variety of neoplastic diseases. Therefore it is also important that interventional radiology has its place in the multidisciplinary organisation, including decision making at tumor boards.
***Learning Objectives:***
1. To understand the role and challenges of institutional multidisciplinary guidelines including imaging.
2. To learn about integrating diagnostic information before tumour board meetings.
3. To learn how to include the role of interventional radiology in tumour board meetings.

17:18
**Panel discussion: How to strengthen the role of the radiologist in multidisciplinary tumour boards while keeping workload under control**

**16:00 - 17:30** **Room Y**

### E³ - Rising Stars Programme: EFRS Radiographers' Basic Session

### BR 16

### Radiation protection: all you need to know

**A-0960** 16:00
**Chairperson's introduction: Evolving radiographers' role in radiation protection**
B.R. Mussmann; *Odense/DK (bo.mussmann@rsyd.dk)*

The system of radiological protection is built on science, experience and ethics reflected in the basic principles in ICRP103, i.e. optimisation of protection, dose limitation and justification. Radiographers are important stakeholders in the maintenance of the system, both legally, ethically and practically. The session reviews different aspects of radiation protection that may form the basis of further development of radiographers' roles and responsibility.
***Session Objectives:***
1. To understand radiographers' current legal responsibilities regarding radiation protection.
2. To consider the role radiographers play in application of the justification principle.
3. To review recent optimisation techniques for projection radiography.
4. To appreciate how radiographers can best communicate risk-benefit information to patients.

**A-0961** 16:05
**The Basic Safety Standards Directive: early experiences of implementing BSS**
S.J. Foley; *Dublin/IE (shane.foley@ucd.ie)*

The European Commission issued its most recent radiation protection Directive (2013/59/Euratom) in December 2013 which repealed five other Directives (including 97/43/Euratom) and had a deadline for transposition by all Member States of the EU by February 2018. Therefore this presentation will review the European-wide adoption of the new Directive, which takes into account new recommendations from the ICRP in light of new scientific evidence and is hoped will strengthen the culture of radiation safety throughout Europe. While the Directive remains faithful to and maintains the already well-accepted pillars of radiation protection - justification, optimisation and dose limitation, these are further strengthened in this Directive. Further new requirements under the Directive will also be discussed, including obligations related to recording dose information and communicating benefit/risk to patients. Additional responsibilities have also been designated to a number of professionals which will impact the work of both radiographers and radiologists alike, so all imaging staff need to be aware of its implications. This will ensure the true ethos of the Directive can be effectively implemented into routine clinical practice. Finally, some potential challenges for practical adoption of the Directive will be addressed.
***Learning Objectives:***
1. To familiarise attendees with the European BSS Directive (2013/59).
2. To discuss the additional responsibilities of radiographers and radiologists under the Directive.
3. To understand how the implementation of the BSS Directive may affect practice.

**A-0962** 16:23
**Enhancing the culture of justification**
K.B. Lysdahl; *Kongsberg/NO (Kristin.Bakke.Lysdahl@usn.no)*

Justification of referrals is a primary principle in radiology, with impact within and beyond radiation protection. The practice of justification influences the quality of patient care, the professionals' working conditions and the allocation of healthcare resources. Still, reported frequencies of unjustified examinations to indicate implementation challenges, explainable by the complexity of the principle. Justification of an examination demands an overview and balancing of a number of relevant benefits and risks, based on outcome predictions. Moreover, the people involved may appraise the benefits and risks differently, and the distribution of responsibility and power in the decision-making process may not be clear. The presentation will address the potential for radiographers to contribute to justified examinations. They can contribute by vetting the amount and quality of information in the referrals, by providing supplementary information, and by authorising referrals according to guidelines. The possibilities, preconditions and scope of the radiographers' contribution will vary according to cultural and organisation aspects, training and regulations, locally and nationally. Some lessons can be learned by the radiographers' perceived role in justification, reported in the literature. Initiatives from the radiation protection environment to improve adherence to the principle of justification are summarised in the key concepts of Awareness, Appropriateness and Audit. The presentation will consider elements of the AAA approach, and offer reasons why increased involvement by radiologist and radiographers are called for. Finally, initiatives particularly relevant for the imaging departments will be highlighted, including guideline implementation, education, clarification of roles and collaboration in the referral process.
***Learning Objectives:***
1. To appreciate the key role of justification within radiation protection.
2. To be familiar with the role radiographers can play in justification.
3. To consider initiatives for improving adherence to the principle of justification.

**A-0963** 16:41
**Opportunities for optimising dose in digital radiography**
P.H. Hogg; *Salford/UK (P.Hogg@salford.ac.uk)*

From a dose optimisation perspective, general descriptions of image quality that are divorced from the abnormality to be considered are likely to give less accurate and possibly unreliable results. With a range of perspectives in mind, this presentation offers a reflective critical commentary on contemporary image quality definitions in relation to the abnormality to be considered. Building on this, a definition of clinical image quality is then proposed which considers the purpose of the imaging examination; image quality definitions such as this would likely result in 'personalised' optimisation which should result in more valid and reliable dose optimisation outcomes. With a personalised definition in mind contemporary visual and physical approaches to image quality assessment, in pure and modified forms, will be considered in relation to optimisation and the abnormality to be considered. The presentation will then examine potential pre-emptive personalised image quality assessment optimisation approaches that might be implemented before exposure; post-acquisition personalised image quality assessment will also be examined similarly. The presentation will conclude by offering practical steps which might be taken to optimise images, with dose and personalised image quality in mind. Some consideration will be given to the role of Artificial Intelligence (AI) could play in the assessment of personalised image quality in optimisation, with or without human observers as part of the interpretation process.
***Learning Objectives:***
1. To discuss the limitations of image quality as a parameter for use in optimising dose in medical imaging examinations.
2. To discuss a range of visual and physical techniques that can be used to assess medical image quality.
3. To discuss a range of practical measures which might be implemented to minimise radiation dose whilst maintaining a level of image quality that is fit for purpose.

**A-0964** 16:59
**The radiographers' role in benefit-risk communication**
J. Portelli; *Msida/MT (jonathan.portelli@um.edu.mt)*

By now all EU Member States should have implemented the requirements specified in the BSS Council Directive 2013/59/EURATOM into their respective national legislation, thereby ensuring that relevant practices abide by the revised basic safety standards for the protection against dangers arising from exposure to ionising radiation. In effect, apart from the specific responsibilities relating to the justification and optimisation of each medical radiation exposure, referrers and/or practitioners are now legally obliged to provide each patient or their representative with adequate information relating to the benefits and risks associated with the radiation dose to be received from a proposed medical imaging procedure that involves radiation. While referrers and practitioners may have already been satisfying this 'new' responsibility in their daily practice, the fact that this is now specifically defined within regulations should oblige more health professionals to be attentive to fulfil this responsibility as effectively as possible. This lecture will, therefore, seek to outline and emphasise the key role radiographers play in benefit-risk communication. It will highlight the importance of radiographers being knowledgeable of radiation-related concepts, as well as the associated benefits and risks of medical imaging procedures they perform. Additionally, the need for radiographers to develop and have effective communication skills will also be emphasised, since these are necessary to allow them to convey adequate information in accordance to the needs and preferences of different patient groups and their families, as well as to other health professionals that may be involved in the patient's care.
***Learning Objectives:***
1. To understand new legislative requirements under the BSS Directive.
2. To be aware of the key role radiographers play in benefit-risk communication.
3. To understand key principles for appropriate benefit-risk communication.

17:17
**Panel discussion: Teamwork in radiation protection**

**16:00 - 17:30 Room D**

### Musculoskeletal

### RC 1610

### The old spine: challenges of imaging and treatment

*Moderator:*
R. Pedersen; Toensberg/NO

**A-0969** 16:00
**A. Degeneration of the old spine: relevance of findings and differential diagnosis**
V.N. Cassar-Pullicino; *Oswestry/UK (Victor.Pullicino@nhs.net)*

With increasing age degeneration of the spine is inevitable with a tendency to affect all segments from cervical to lumbosacral junction. The underlying pathophysiology is related to the failure of the supporting osteo-ligamentous structures which can manifest itself in an isolated fashion, involving contiguous levels or affecting non-contiguous segments. Failure of the three articulations at each discovertebral motion segment (disc and facet joints) predisposes to micro-movement and a cascade of disc degeneration and facet degeneration promoting disc herniation, facet joint hypertrophy and spondylolisthesis or retrolisthesis. It is, however, important to note that despite visualisation of these structural changes on imaging, one cannot correlate irrespective of severity, associated clinical findings. However, these pathophysiological processes are not uncommonly symptomatic with patients presenting with spinal pain, radiculopathy or myelopathy. The emphasis of this talk is to demonstrate these degenerative processes and their complications ranging from spinal stenosis which can be at multiple sites, to deformity which can be incapacitating and difficult to treat. The combination of advanced degeneration with deformity can alter the balance of the spine and promote unusual biomechanical forces which can overwhelm the bone strength of the vertebral bodies which in the presence of metabolic disease results in bone failure producing destructive discovertebral changes within the spine.
***Learning Objectives:***
1. To explain the pathophysiology of degenerative changes in the spine.
2. To describe the imaging findings of degenerative diseases of the spine.

**A-0970** 16:30
**B. Fractures: bone fragility in the elderly, assessing osteoporosis and bone quality and differential diagnosis**
G. Guglielmi; *Andria/IT (giuseppe.guglielmi@unifg.it)*

Osteoporosis is still considered one major public health problem, dealing with vertebral fractures and hip fractures; also, it is largely known that mortality is also increased after vertebral fractures. Currently, the diagnosis of osteoporosis, measured by dual-energy x-ray absorptiometry (DXA), is based on areal bone mineral density (aBMD), but osteoporotic fractures can also occur when aBMD is still above the pathologic range. Several imaging modalities, such as computed tomography (CT) scans and magnetic resonance imaging (MRI), have been used to improve bone assessments and its relation to fracture risk prediction. Among these the most applied is quantitative CT (QCT) scans, that can measure volumetric BMD (vBMD) and trabecular bone score (TBS), attempting not only to measure the "bone quantity" but also the "bone quality", by means of the study of microarchitecture, structure, bone turnover and bone strength.MRI is also being studied as a tool to assess vertebral bone density based on bone marrow fat content; in fact, vertebral osteoporosis is characterised by thinning trabecular and cortical bone and an increase in fat. A comprehensive assessment of fracture risk requires data on bone density, fracture history, and bone quality, which can be provided by several imaging modalities, each with its advantages and disadvantages. Clinically, DXA remains to most widely available and used technique to assess BMD as it is the only validated method to diagnosis osteoporosis by WHO T-score criteria. The role of densitometers has expanded to include VFA and TBS, though accessibility to these add-on tools is still limited.
***Learning Objectives:***
1. To explain the pathophysiology of osteoporosis in the spine.
2. To describe the imaging findings of spinal osteoporosis and its consequences.

**A-0971** 17:00
**C. Interventional radiology: is there still a place for vertebroplasty and kypho-/stentoplasty**
C. Binkert; *Winterthur/CH (christoph.binkert@ksw.ch)*

The first reports of vertebroplasty were published in the late 1980s. Over the years the technique of polymethylmethacrylate injection under fluoroscopic guidance hasn't changed much. In clinical practice, vertebroplasty became an established therapy for painful mostly osteoporotic fractures. Randomised trials against conservative therapy have consistently shown a benefit for vertebroplasty- the latest study was the VAPOUR trial. On the other hand, vertebroplasty wasn't superior to a sham procedure in three large trails the latest being VERTOS IV. In all sham trials patients benefitted from the intervention (vertebroplasty and sham procedure), so the question is why the sham procedure worked. Despite these trials, vertebroplasty is still offered to many patients suffering from painful vertebral body fractures because of general good pain relief, high patient satisfaction and very few complications. As with many IR procedures, appropriate patient selection is important. Besides the clinical exam (tenderness on percussion) and history (sudden onset of different pain), an MRI should be performed beforehand. Best outcomes were observed with fractures dark on T1 and bright on T2 fatsat/STIR. For pain relief, vertebroplasty seems sufficient. If height restoration is intended kyphoplasty (balloons) or stentoplasty (balloon expandable stents) can be used. The concept of the stent is to avoid recollapsing after deflating of the balloon. For a successful height restoration, the fracture needs to be mobile, which is typically the case within the first 10-14 days.
***Learning Objectives:***
1. To explain the procedures of vertebroplasty and kyphoplasty.
2. To discuss the current role of vertebroplasty and kyphoplasty in spinal fractures.
*Author Disclosure:*
**C. Binkert:** Advisory Board; Merit Medical. Grant Recipient; Abbott; Philips.

**16:00 - 17:30 Room M 1**

### Joint Session of the ESR and ESHI$^{MT}$

### ESR/ESHI(MT)

### Hybrid and translational imaging: the bigger picture

**A-0972** 16:00
**Chairpersons' introduction (part 1)**
O. Ratib; *Geneva 14/CH*

Hybrid imaging combining molecular imaging (PET or SPECT) with anatomical and functional imaging (CT and MRI) offers a scope of new clinical applications. The development of new radiotracers for PET imaging opens a new perspective for more objective evaluation of the efficacy of new treatments. The emergence of hybrid PETMR imaging offers new perspectives for functional hybrid imaging and tissue characterisation. The combination of both modalities improves the accuracy and reproducibility of diagnostic procedures.
***Session Objectives:***
1. To illustrate examples of clinical relevance for hybrid imaging.
2. To highlight the translational aspects of hybrid imaging.
3. To introduce the session faculty and objectives of the presentations.

**A-0973** 16:03
**Chairpersons' introduction (part 2)**
C.C. Cyran; *Munich/DE (clemens.cyran@med.uni-muenchen.de)*

The concomitant acquisition of morphological, functional and molecular information using hybrid imaging techniques allows for a high diagnostic accuracy in the detection of tumour manifestations and the timely differentiation of responders from non-responders using different tracers and in various tumour entities. Clinically available tracers such as [18F]-FDG, [68Ga]-DOTA-TATE/-TOC, [18F]-PSMA und [18F]-DOPA allow for the visualisation of glucose metabolism, tumour receptor and protein expression, or enzymatic activity, depending on the indication and tumour entity. Modified criteria for monitoring response to molecular anti-cancer agents are increasingly integrating functional imaging information (Cheson criteria, PERCIST) to improve the standardised and reproducible assessment of therapy response by imaging studies and their application as qualified imaging biomarkers. An array of novel radiotracers for PET and SPECT imaging is under translational development for a broad range of applications in imaging of neurodegenerative, cardiovascular and oncological diseases. The theranostics concept of tracers such as DOTA-TATE and PSMA for radionuclide therapy in metastasised cancer patients has shown success in selected patient groups and may serve as a blueprint for future application of novel compounds in oncological therapy.
***Session Objectives:***
1. To illustrate examples of clinical relevance for hybrid imaging.
2. To highlight the translational aspects of hybrid imaging.
3. To introduce the session faculty and objectives of the presentations.

**A-0974** 16:05
**Prostate-specific membrane antigen (PSMA) PET/MR: guiding clinical management of prostate cancer**
P.A.T. Baltzer; *Vienna/AT*

Prostate-specific membrane antigen-based PET has rapidly changed the way prostate cancer is diagnosed. It provides a highly accurate tool to detect, stage and grade prostate cancer and can further be used to guide and monitor treatment. It further allows a theranostic approach to treat prostate cancer. On the other hand, MRI provides the to date most accurate tool to image-based prostate cancer detection in the prostate. In addition, its superior soft tissue contrast is considered to be superior to CT in many respects. Whether PET-MRI is indeed superior to PET-CT regarding the staging of prostate cancer is a matter of debate, and empirical data regarding this topic is sparse. The talk will address the learning objectives given below.
***Learning Objectives:***
1. To learn about the requirements in clinical management of prostate cancer.
2. To understand the potential role of PSMA PET/MR in the management of prostate cancer.
3. To learn about the advantages of the hybrid PET/MR technique in this setting.
4. To become familiar with typical findings and pitfalls of hybrid PSMA PET/MR in the evaluation of prostate disease.

**A-0975** 16:30
**On the horizon: novel PET tracers with translational potential**
G. Cook; *London/UK (Gary.Cook@kcl.ac.uk)*

There are few hallmarks of cancer that we cannot exploit with imaging. In particular, there are several PET and SPECT probes that can measure abnormal metabolic processes (e.g. 18F-FDG, 99mTc-sestamibi), proliferation (e.g. 18F-FLT, 18F/11C-choline), receptor overexpression (e.g. 68Ga-dotatate, 68Ga-PSMA, apopotosis (e.g. 18F-ML10), hypoxia (e.g. 18F-MISO, 64/62Cu-ATSM) and angiogenesis (e.g. 89Zr-bevacizumab, 18F-RGD), amongst others. While some of these tracers are useful for diagnosis and phenotyping, many are of interest for predicting early treatment response, especially in the setting of targeted biologic therapies where an early reduction in tumour volume is not expected and where morphologic CT and MRI are therefore limited. Of current interest is the evaluation of response to immunotherapies that may cause pseudoprogression caused by immunocellular infiltration before subsequent tumour shrinkage. In this situation, early pseudoprogression may also be seen with an 18F-FDG PET and hence the interest in more specific tracers such as 89Zr or 99mTc-PDL1 antibody. While PET and SPECT probes are invaluable in reporting on underlying biological tumour characteristics, both CT and MRI provide valuable supportive information in hybrid imaging scanners allowing anatomical reference as well as some functional measurements, e.g. cellularity with DWI and perfusion with DCE MRI / pCT. The ultimate goal of predicting which patients will respond to which treatment, or at least to determine non-response as soon as possible, is not fully achieved in the clinic with imaging as yet but some areas are now contributing to clinical practice, e.g. 18F-FDG PET/CT to guide treatment escalation/de-escalation in lymphoma.
***Learning Objectives:***
1. To become familiar with future targets in oncologic hybrid imaging.
2. To learn how hybrid imaging can support the oncologist in diagnosis and therapy response assessment.
*Author Disclosure:*
**G. Cook:** Consultant; Nanomab. Research/Grant Support; Serac HC, Nanomab.

**A-0976** 16:50
**Advances in theragnostics: monitoring radionuclide therapy**
H. Ilhan; *Munich/DE (Harun.Ilhan@med.uni-muenchen.de)*

The use of radiolabeled compounds for molecular imaging and therapy represents two pillars of nuclear medicine and serves as a perfect example of the theragnostic concept for decades. In recent years hybrid PET/CT imaging using Somatostatin-Receptor- (SSR-) and Prostate Specific Membrane Antigen- (PSMA-) targeting Peptides established as an indispensable tool in the management of Neuroendocrine Tumor (NET) and Prostate Cancer (PC) patients. The selective uptake and accumulation pattern paved the way for the therapeutic application of beta- and alpha-emitting compounds. FDG-PET and the implementation of the PERCIST criteria have demonstrated value in the evaluation of therapy response in tumours with high metabolic activity. However, up to date little is known about the value of specific PET-compounds that are used in the theragnostic concept of radionuclide imaging and therapy. In clinical practice, response assessment and therapy monitoring are still based on morphological criteria, whereas the value of PET-derived information remains unclear. Currently, SSR- and PSMA PET imaging has not yet been validated for therapy monitoring in NET and PC patients. Nonetheless, response evaluation to radionuclide therapy based on PET-information has been proposed, however, mainly in retrospective single-center studies with low patient numbers. Current imaging guidelines state that the value of PET imaging in the assessment of response to radionuclide therapy remains unclear as decreased PET-tracer uptake after radionuclide therapy does not necessarily indicate response and might be attributed to other factors such as dedifferentiation. However, recent data indicate that there might be a high value of PET-imaging for response assessment.
***Learning Objectives:***
1. To learn the principles of radionuclide imaging and therapy in the concept of theragnostics.
2. To understand the differences and common ground between morphological and molecular imaging.
3. To discuss new approaches for response assessment in radionuclide therapy.
*Author Disclosure:*
**H. Ilhan:** Advisory Board; Bayer. Speaker; Bayer, Ipsen.

17:10
**Panel discussion: Hybrid imaging beyond FDG and theranostics: is it a clinical reality?**

**16:00 - 17:30** **Room M 2**

### Professional Challenges Session

### PC 16

### How to organise nationwide ischaemic stroke care (and make multidisciplinary teams work)

**A-0977** 16:00
**Chairperson's introduction: Stroke care in Austria: small country, small challenge?**
C. Loewe; *Vienna/AT (christian.loewe@meduniwien.ac.at)*

The recent developments in the mechanical treatment of ischemic stroke provide fantastic chances to patients but also cause great challenges to the healthcare providers. These interventions are highly specialised but not very frequent, whereas the training and the continuous “maintenance” of skills for Interventionalist’s represent a critical issue in the establishment of this service. Different approaches to this problem have been developed and propagated, and some of them should be discussed during this session. Furthermore, some relevant questions will be raised including how many Neurointerventionalist’s are needed? How many centres are needed? Is a multidisciplinary group combining all available forces preferable compared to highly experienced specialists from only one medical subspecialty? Should the patient be moved to the Center where the specialist is on duty, or should the specialist travel around? Beside these potentially obvious questions, it should also be discussed what kind of organisation model could be attractive in the future for the young generation for doctors, and how we can ensure that our Strokes will be treated accordingly in the future. Even if this session possibly cannot answer all these questions, the awareness about these issues should be increased, and possible ways for possible solutions should be presented. As an introduction to the topic and the idea of this session, the Austrian situation will be presented exemplifying that different approaches to the same challenge are tried out even in a rather small Country like Austria.
***Session Objectives:***
1. To discuss the challenges in establishing and running a nationwide ischaemic stroke care.
2. To realise the importance of multidisciplinary team work for successful stroke care service.
3. To learn about possible training, education and certification in ischaemic stroke care.
*Author Disclosure:*
**C. Loewe:** Speaker; Siemens, Bracco, GE Healthcare.

**A-0978** 16:05
**What you should know being involved in a nationwide ischaemic stroke service**
P. Papanagiotou; *Bremen/DE (papanagiotou@me.com)*

The recent EVT RCTs made convincingly clear that a paradigm shift in the organisation of acute stroke care pathways is urgently needed. In the prehospital phase, the definition of patient triage and transfer protocols are important. There are two options for the patient transfer protocol in the acute stroke setting: in the first, a patient who is triaged as potentially EVT eligible is transferred directly to a Comprehensive Stroke Center where EVT could be offered. In the second option, a patient is transferred initially to a Primary Stroke Center where IVT is offered if the patient is eligible and then transferred further to a Comprehensive Stroke Center where EVT may be offered if indicated. In the in-hospital phase, an optimised stroke management protocol is required. Improvements in in-hospital stroke management can translate into faster treatment. Every stroke centre should have an optimised stroke management protocol to reduce the “door-to-treatment” time. An on-demand, 24-7-365, stroke reperfusion service, is also required. Emergency services are instructed to bring patients with a suspected stroke directly to the stroke treatment room, which should be located in proximity to the CT scanner. After the neurological examination, an unenhanced CT scan, CTA and CT perfusion are performed. Within the 4.5-h time window, patients are treated with IV t-PA immediately. If an occlusion of large intracranial vessel is found, the decision to perform endovascular therapy is based on the clinical condition of the patient, the time window, and the imaging criteria of the CTA and CT perfusion.

***Learning Objectives:***

1. To provide an overview about recent data/guidelines about endovascular stroke treatment.
2. To get familiar with the currently accepted/recommended treatment options.
3. To give an outlook about ongoing trials.

**A-0979** 16:23

**Do you like to travel? Move the patient or the doctor?**

J.-P. Pruvo; *Lille/FR (jean-pierre.pruvo@chru-lille.fr)*

Neuro-interventional mechanical thrombectomy is now standard of care for acute ischemic stroke from large vessel occlusion (LVO) and may be extended beyond the traditional 6-hour time window in patients that are properly selected by neuroimaging. Although ideally patients should be transferred to a higher standard, comprehensive stroke centres for intervention, whether on IV t-PA (“Drip and Ship”) or not, such transfers delay treatment by an average 100 mins, significantly reducing the chance of a good outcome. In an effort to reduce delays to intervention, a recently reported alternative whereby neuro-interventionalists are transported by taxi to perform treatment in primary stroke centres (“Drip and Drive”) has shown feasibility and effectiveness. Worldwide, health care systems are currently evaluating various ways to improve access to emergent stroke care for eligible patients, including training more specialists, telemedicine models, extending the workforce, etc... Guiding principles for patient safety include the accuracy and reproducibility of neuroimaging process leading to indication to treat, reduction of unexpected sources of delay or failure to treat, i.e. adequate training of staff, successful parallel processing, availability of materials, adequate quality and performance of interventional equipment, safety of femoral puncture and closure technique, availability of neurosurgical backup in case of major complication, and adequacy of post-thrombectomy neurological care. Ultimately, whether the patient or the doctor is “shipped” to the site of intervention, attention to quality remains the best guarantee of good outcomes.

***Learning Objectives:***

1. To provide an overview about institutional requirements for a successful endovascular stroke care.
2. To discuss possible solutions including moving the patient to an interventional centre or move the doctor to the patient.
3. To outline the "ideal world" scenario.

**A-0980** 16:41

**Are you experienced, trained or certified? Certification, subspecialisation, specialisation: friend or foe of the patient?**

A. Berlis; *Augsburg/DE*

"no abstract submitted"

***Learning Objectives:***

1. To get an overview about different certification programmes in interventional stroke treatment.
2. To discuss the risks and benefits for the patients of dedicated certifications, ensuring continuous service for the future.
3. To get familiar with possible future concepts of training and education.

**A-0981** 16:59

**Do you need help? Train the cardiologist, neurosurgeon, interventional radiologist**

T.J. Popiela; *Krakow/PL (msjpopie@cyf-kr.edu.pl)*

In Poland, despite the lack of refunds from the National Insurance Company (NIC) between January 2012 and December 2016, a total of 586 mechanical thrombectomies (MT) in acute ischemic stroke with the use of new generation devices were performed in 24 centres. In 78% of cases, the MT teams were composed of a neurologist and an interventional neuroradiologist. In the remaining, the neurointerventionist was a neurosurgeon (3%), a vascular surgeon (1%), or intervention cardiologist (1%). Only 15 of these centres worked 24h/7 days a week, and only 2 performed over 40 procedures each. According to the regulation of the Ministry of Health (MH) of October 16, 2018, apart from (neuro) interventional radiologists, also neurosurgeons, neurologists, cardiologists, angiologists and vascular surgeons were admitted to performing the MT procedure. The minimum requirement was to perform "at least five intracranial vessel treatment procedures performed alone or in the presence of a Proctor who performed at least 50 intracranial procedures". A Pilot Program was launched in 7 centres currently performing most MT procedures. In each of these centres, there are min. three teams that can work on a continuous basis on call, 24h / 7 days a week. In most of these centres, procedures are performed by experienced (neuro) interventional radiologists. Each of the participating centres is obligated to conduct training for doctors of other specialities. After finishing the Pilot Program, a network of 17-20 centres capable of performing MT procedures will be created covering the entire territory of Poland.

***Learning Objectives:***

1. To provide an overview about the situation in Poland: who is doing the endovascular stroke service and who will do it in future?
2. To outline possible problems to ensure continuous service 24/7.
3. To discuss the role of interventional (neuro) radiologists as trainer for other disciplines.

17:17

**Panel discussion: The output (outcome) counts, not the input (specialisation)**

**16:00 - 17:30 Room M 3**

### E³ - ECR Master Class (Chest)

### E³ 1626b

### Patterns of pulmonary toxicity

*Moderator:*

I.E. Tyurin; Moscow/RU

**A-0982** 16:00

**A. Drug-induced lung disease**

C.M. Schaefer-Prokop; *Amersfoort/NL*

"no abstract submitted"

***Learning Objectives:***

1. To learn about the various CT patterns of lung injury.
2. To understand the role of imaging in their recognition.
3. To discuss differential diagnoses.

**A-0983** 16:30

**B. Smoking-related lung disease**

S.R. Desai; *London/UK*

"no abstract submitted"

***Learning Objectives:***

1. To learn about the classification of smoking-related lung diseases.
2. To understand the most common CT patterns.
3. To highlight newly-recognised entities.

**A-0984** 17:00

**C. Inhalation: lung injury beyond smoking**

J.A. Verschakelen; *Leuven/BE (johny.verschakelen@uz.kuleuven.ac.be)*

Non-smoking related inhalation lung injury is usually secondary to chronic or repetitive occupational or environmental exposures but may also result from inhalation of smoke or of chemical products of combustion, a condition that is mostly associated with significant morbidity and mortality. Every year new agents causing pulmonary injury are described, and although many are isolated cases, others prove to be clustered or repetitive events with more or less specific imaging features. The clinical and radiological presentation of inhalational lung injury depends on one hand on the chronicity of inhalation, the amount and phase of the inhaled agent and its toxicity to the lung, and on the other hand on the reaction of the lung to this injury. This presentation will focus on the CT features seen after non-smoking related lung injury caused by direct chemical toxicity to the bronchial tree and lungs and by chronic or repetitive inhalation of particulates and organic dusts. Attention will also be given to the inflammatory syndrome seen in hypersensitivity pneumonitis.

***Learning Objectives:***

1. To learn about the various causes of environmental lung diseases.
2. To understand the changes related to acute lung injury.
3. To describe the long-term sequelae of inhalation lung injury.

**16:00 - 17:30** **Room M 4**

### Multidisciplinary Session

### MS 16

### How to deal with the epidemics of thyroid nodules?

**A-0985** 16:00
**Chairperson's introduction**
A.S. Germano; *Amadora/PT (asgermano@gmail.com)*

Recently, concerns regarding possible overdiagnosis and overtreatment of thyroid carcinomas have been raised. They are based on an exponential rise in the incidence of thyroid carcinomas in developed countries (the "epidemics" of thyroid nodules), concerning mainly small well-differentiated carcinomas, along with a low and stable mortality rate. Potential reasons for these epidemics include the unveiling of a pool of subclinical tumours due to increased availability and access to health care units, increase in the number and improved sensitivity of imaging procedures, with consequent detection of incidentalomas in CT, MR, Doppler, and PET examinations, exhaustive histological examination of thyroid specimens, and screening for thyroid nodules. The coexistence of factors responsible for a true increase in thyroid carcinomas is also possible, namely radiation exposure, smoking, obesity, and iodine intake status. High-resolution ultrasound and ultrasound-guided fine needle aspiration are the best tools to identify and characterise thyroid nodules. However, it is unfeasible to biopsy every single detected nodule. To address this problem, different guidelines on how to deal with thyroid nodules have been published worldwide, and are continuously being updated. Likewise, a uniform system for reporting thyroid cytology, the Bethesda system, which includes an attributed risk of malignancy and a management recommendation for each diagnostic category, was created. Different approaches have been attempted to decrease the epidemics: a proposal of active surveillance rather than surgery for microcarcinomas in selected cases; a nomenclature revision of the encapsulated thyroid carcinoma; an update of the Bethesda system, multidisciplinary tumour boards, with clinicopathologic case discussion.
***Session Objectives:***
1. To understand the concept of the world epidemics of thyroid nodules.
2. To learn about the reasons for this epidemic.
3. To be updated on current guidelines as to how to deal with thyroid nodules.
4. To explain the indeterminate diagnosis on cytology and histology.
5. To discuss approaches to reduce/stabilise the incidence of thyroid nodules detection.

**A-0986** 16:05
**The endocrinologist perspective**
I. Sapinho; *Amadora/PT (inessapinho@sapo.pt)*

We will begin this multidisciplinary session by discussing the true impact of the widespread use of imaging techniques, combined with increased medical surveillance and access to health care services in the generation of the epidemic of thyroid nodules. Ultrasound studies of asymptomatic individuals have reported an incidental detection of 35 to 67%. The important issue is the concerns with malignancy and hyperactivity. So, we will discuss the diagnostic pathway of a patient with a thyroid nodule. Nowadays, thyroid cancer is one of the most frequent endocrine malignancies, with a higher prevalence in females. It's, already, every third cancer in females in most countries, like in Portugal. However, overdiagnosis is also an issue because its occurrence rate has extraordinarily risen in the two past decades, whereas mortality rates have not changed. Finally, we will discuss the rare emergencies related to thyroid nodules, paying special attention to the preoperative and postoperative causes.
***Learning Objectives:***
1. To understand the causes of high prevalence of thyroid nodules worldwide.
2. To outline the diagnostic pathway of a patient with a thyroid nodule.
3. To be aware of the problem of overdiagnosis.
4. To recognise the rare emergencies related with thyroid nodules.

**A-0987** 16:20
**The radiologist role**
A.S. Germano; *Amadora/PT (asgermano@gmail.com)*

High-resolution ultrasonography is, without a doubt, the best imaging technique to detect and characterise thyroid nodules. It is also the best way to guide fine-needle aspiration biopsy of thyroid nodules and lymph nodes. However, it is neither possible nor desirable to biopsy every detected nodule, because the procedure is not exempt from complications, the percentage of indeterminate cytology results is not negligible, and most thyroid carcinomas are well-differentiated and have an indolent course. The challenge is to identify the very rare/aggressive malignant nodules among the very common benign ones. Radiologists are partially responsible for the epidemics of thyroid nodules. To achieve Excellency in the evaluation of thyroid nodules, a radiologist needs, beyond a top ultrasound equipment and acquisition of diagnostic and interventional skills, to be updated on the nuances of the current guidelines regarding ultrasound malignancy risk stratification of thyroid nodules. The 2017 EU-TIRADS will be emphasised. It is also fundamental, in order to appreciate uncertainties in cytology and pathology results, to recognise the recent changes in the World Health Organisation classification of tumours of endocrine organs, the modifications of the staging system for differentiated thyroid cancer, presented in the 8th Edition of the American Joint Committee on cancer, and also the recent updates on the Bethesda System for reporting thyroid pathology. Regarding microcarcinomas (<1cm), active surveillance is being considered as an alternative to surgery. To be implemented, patient preferences need to be taken into account, together with an adequate clinical setting, available resources, and medical expertise.
***Learning Objectives:***
1. To become familiar with the current guidelines for ultrasound malignancy risk stratification of thyroid nodules-emphasis on the EU-TIRADS.
2. To learn how to deal with the incidentally detected thyroid nodules in other imaging modalities.
3. To understand the concept of "active surveillance" of thyroid nodules.

**A-0988** 16:35
**The surgeon intervention**
M. Allen; *Amadora/PT (mallen@hospitaldaluz.pt)*

The Surgeon´s intervention (or not) in the patient with nodular thyroid disease is to be taken as a part of a treatment plan. Three topics will be addressed. To know when to send a patient to surgery. Sending patients to an Endocrine Surgeon doesn't necessarily mean "send a patient to surgery". Depending on the clinical presentation, echographic features and cytology result in a decision as to be achieved. From the clinical evaluation, compressive symptoms like neck or swallowing discomfort, hoarseness, history of thyroid cancer and mediastinal goitres are to be considered. The importance is to have a team that works together and decides the best treatment to a given patient. To understand what the surgeon needs to know before performing a thyroidectomy. The surgeon needs precious information from the Endocrinologist, the Radiologist, the Cytopathologist, the ENT preoperative evaluation, but above all, he/ she needs to understand the Patient expectations from treatment and Balance risk/benefit of a given surgical strategy. Total thyroidectomy and Unilateral lobectomy, have a low incidence of complications, but regardless of the surgical skills and annual case-load, they are not ZERO. To be aware of potential surgical risks and complications. Intra and Post-operative complications and ways to anticipate, prevent and deal with them will be addressed. From bleeding, lesion of recurrent and superior laryngeal nerve, hypoparathyroidism, surgical site infection, to Surgical scar issues. When a surgical procedure goes according to plan and has no complication, the only thing that is left is a scar. Hopefully scarcely identifiable.
***Learning Objectives:***
1. To know when to send a patient to surgery.
2. To understand what the surgeon needs to know before performing a thyroidectomy.
3. To be aware of potential surgical risks and complications.

**A-0989** 16:50
**The pathologist assessment**
P. Borralho; *Amadora/PT (pnunes1@campus.ul.pt)*

Fine needle aspiration (FNA) plays a central role in distinguishing benign thyroid nodules that can be managed conservatively from those with suspicious or malignant features requiring further management. However, its performance needs to be selective, since systematic FNA of all nodules, regardless of the size or appearance, is superfluous and may lead to unnecessary diagnostic surgery. A standardised report of thyroid ultrasound (US) findings facilitates correlation with cytology, enhancing the ability to reach a precise diagnosis. The Bethesda System for reporting thyroid cytology (TBSRTC ) was a very important development, creating a uniform system for reporting thyroid cytopathology and recognising its limits. Each six-diagnostic category is associated with a specific risk of malignancy (ROM) and a respective clinical management recommendation. An updated TBSRTC recently published includes advances in the molecular diagnosis of thyroid nodules and the noninvasive encapsulated follicular variant of papillary thyroid carcinoma (FV-PTC), renamed as noninvasive follicular thyroid neoplasm with papillary-like nuclear features (NIFTP) which, together with well-differentiated thyroid tumours of uncertain malignant potential (WD-TUMP) represents a true ''gray zone'' of ''follicular patterned'' thyroid lesions, that needs to be characterized in order to outright the diagnosis of carcinoma and avoid unnecessary aggressive treatment. Papillary microcarcinoma of the thyroid (PMCT) contributes to the epidemics of thyroid nodules. PMCT is incidentally found in otherwise healthy humans by ultrasonography and FNA, but surgical therapy is only required for patients who have high-risk factors, such as clinically apparent lymph node metastasis and massive extra-thyroid extension.
***Learning Objectives:***
1. To appreciate the utility of conjoint ultrasound/cytology evaluation of thyroid nodules.
2. To understand the cytology diagnosis limits: The Bethesda System.
3. To learn about the indeterminate histology diagnosis: WDT-UMP and NIFTP.
4. To recognise the pathologist contribution to the epidemics: the incidentally detected microcarcinomas.

**A-0990** 17:05
**Multidisciplinary case presentation and discussion: "The thyroid-team decision: when to follow and when to go beyond the guidelines"**
A.S. Germano; *Amadora/PT (asgermano@gmail.com)*

In the same manner, as a multidisciplinary board meeting, selected cases of both straightforward and challenging "real-life" cases, regarding patients with thyroid nodules, will be presented and discussed. The "thyroid-team", comprising an endocrinologist, a radiologist, a surgeon, and a pathologist, will present thyroid nodules that are unmistakably benign or malignant, but also pseudo-nodules, incidentally detected thyroid nodules, those with indeterminate cytology and/or histology results, microcarcinomas, metastatic lymph nodes with no evident thyroid nodule detected, and radiology-cytology discordant cases. Distinctive patient features, including age, symptoms, and co-morbidities, will be fitted into the decision-making process.
***Learning Objectives:***
1. To learn the rationale for multidisciplinary decision-making in standard situations.
2. To discuss challenging diagnosis cases.

**16:00 - 17:30 Room M 5**

### Transatlantic Course of ESR and RSNA (Radiological Society of North America): Sports Imaging

### TC 1628

### Musculoskeletal interventional procedures

*Moderators:*
L.W. Bancroft; Orlando, FL/US
A.J. Grainger; Leeds/UK

**A-0991** 16:00
**A. Diagnostic and therapeutic injections in the athlete: pearls and pitfalls**
P. Peetrons; *Brussels/BE (ppeetrons@his-izz.be)*

The major pitfall in musculoskeletal ultrasound-guided procedures is the incorrect diagnosis. Many patients are now referred to the interventional radiologist from other institutions or private practice. Among them, the radiologist in charge of the interventional procedure discovers incomplete or entirely wrong diagnoses. It is then mandatory to get all Imaging and clinical information before doing the procedure. The preparation of the procedure includes in these cases a full study of the images performed elsewhere and a full new ultrasound investigation. A typical example for this is the asking to perform PRP in a rotator cuff for a so-called "partial rupture" which is in fact a "full thickness tear" and not a "intratendinous interstitial tear"The second pitfall is a misunderstanding of the ultrasound possibilities of covering the lesion with the probe AND a safe route for the needle. An example of this is to inject a tear in the distal biceps brachii tendon for PRP or needling treatment. The so-called Cobra position could not demonstrate the tear if too proximal, and from the anterior approach, the tendon is covered by the artery and the nerve, making the procedure irrealistic. The second part of the lecture describes the different techniques of injection: direct or indirect, with different positions and angles of the needle, emphasising the search for the best approach, not only the safest but the one showing best the needle and the target. Some examples will be given involving upper and lower limbs tendon, ligament and joint injections.
***Learning Objectives:***
1. To become familiar with the most common requests and indications for sports-related injections.
2. To learn about technical considerations for performing MSK injections.
3. To understand reasons to delay injections or avoid certain injectables.
*Author Disclosure:*
**P. Peetrons:** Consultant; Canon Medical.

**A-0992** 16:30
**B. Injectables, percutaneous tendon fenestration and tenotomy: clinical outcomes and current evidence**
J.A. Jacobson; *Ann Arbor, MI/US (jjacobsn@umich.edu)*

For joint abnormalities and tendinopathy, there exist many percutaneous treatment options. Anaesthetic agents are often combined with corticosteroids to inject joints and bursae for diagnostic and therapeutic purposes. All anaesthetic agents are cytotoxic to chondrocytes and synovial cells to some degree. Corticosteroids may be used to decrease inflammation within a synovial space. The use of corticosteroids to treat tendinopathy is counterintuitive, as inflammation is not present, injection into tendon causes tenocyte death, the analgesic effect of corticosteroids is short-lived, and the tendon pathology is not treated. Several ultrasound-guided tendon treatments can be used for tendinopathy. One treatment is tendon fenestration or tenotomy, where a needle is passed through the abnormal tendon segment repeatedly to break up the degenerative process, induce bleeding and inflammation, and initiate tendon healing. Anti-inflammatory medication should be avoided so as not to interfere with the healing response. Another procedure is the injection of autologous whole blood while fenestration, which increases growth factors and other substances to promote tissue healing. Since the majority of growth factors are stored within platelets, the injection of platelet-rich plasma during fenestration has also been used. With this technique, the autologous whole blood is centrifuged to concentrate the platelets for injection. All three of these percutaneous tendon treatments have been shown to be effective, although it is controversial which technique is best. There are more controversial percutaneous tendon treatments, such as injection of mesenchymal stem cells, human amniotic membrane, and deer antler velvet.
***Learning Objectives:***
1. To be aware of the indications and benefits of available injectables used to treat sports-related injuries.
2. To learn about technical considerations for performing tendon fenestration and tenotomy.
3. To become familiar with the current evidence on results of MSK procedures in the literature.
*Author Disclosure:*
**J.A. Jacobson:** Advisory Board; Philips. Author; Elsevier. Consultant; BioClinica.

**A-0993** 17:00
**C. Interactive case discussion (Part 1)**
P. Peetrons; *Brussels/BE (ppeetrons@his-izz.be)*

Interactive voting will be the main goal of this section lecture. Some cases will be presented to the audience, summarising the previous lecture on pitfalls and techniques of interventional procedures in musculoskeletal ultrasound. Ten questions will follow, performing a good summary about what is important to remember in this field.
***Learning Objectives:***
1. To learn the targeted approach to injecting joints, ligaments, tendons and tendon sheaths.
2. To appreciate pitfalls to avoid in MSK procedures for treatment of sports-related injuries.
3. To understand evidence-based data on various MSK procedures in order to give patients realistic expectations after treatment.
*Author Disclosure:*
**P. Peetrons:** Consultant; Canon Medical.

**A-0994** 17:15
**C. Interactive case discussion (Part 2)**
J.A. Jacobson; *Ann Arbor, MI/US (jjacobsn@umich.edu)*

The purpose of the interactive case discussions is to emphasise the teaching points made in the prior lecture reviewing injectables, percutaneous fenestration, and tenotomy. One topic to be addressed will be the imaging findings and potential treatment algorithm for greater trochanteric pain syndrome. In this clinical scenario, a true distended and inflamed bursa is not common, and the primary pathology relates to gluteal tendinopathy. Before pursuing an interventional procedure, conservative management such as proper eccentric physical therapy should be exhausted. The next consideration could be an injection of corticosteroid in the subgluteus maximus (or trochanteric bursa) for the primary purpose to provide temporary pain relief so that the patient can tolerate eccentric physical therapy. The corticosteroid injection is not used as an anti-inflammatory agent as true inflammation is not present, and the pain relief is typically short-lived. If the patient again fails eccentric physical therapy, then the patient may be offered percutaneous fenestration, autologous whole blood injection, or platelet-rich plasma injection. While all three have been shown to be effective, it is still unclear which treatment is best. The dramatic differences in cost should be a consideration when selecting the tendon treatment.

***Learning Objectives:***
1. To learn the targeted approach to injecting joints, ligaments, tendons and tendon sheaths.
2. To appreciate pitfalls to avoid in MSK procedures for treatment of sports-related injuries.
3. To understand evidence-based data on various MSK procedures in order to give patients realistic expectations after treatment.
*Author Disclosure:*
**J.A. Jacobson:** Advisory Board; Philips. Author; Elsevier. Consultant; Bioclinica.

**16:00 - 17:30** **Tech Gate Auditorium**

### Genitourinary

### RC 1607

### Renal transplantation

**A-0995** 16:00
**Chairperson's introduction**
T. El-Diasty; *Mansoura/EG (teldiasty@hotmail.com)*

A kidney was the first and is the most frequently transplanted organ. Imaging evaluation of potential renal donors is crucial to detect renal and extrarenal abnormalities that might preclude donation or lead to alteration of the surgical approach. Despite improved surgical techniques and transplantation management, complications do occur and, if left untreated, may lead to catastrophic consequences. Vascular complications include renal artery stenosis and renal artery and renal vein thrombosis. Urologic complications include urinary obstruction and leak, and peri-transplant fluid collections. Nephrogenic complications include acute tubular necrosis, graft rejection, chronic allograft nephropathy, and neoplasm. Early diagnosis and treatment of these complications are essential to prevent graft failure and other significant morbidities to the patients. Ultrasound can accurately depict and characterise many of the potential complications of renal transplantation and increasingly magnetic resonance imaging and computed tomography also facilitate this role. In addition, interventional radiologic techniques allow nonsurgical treatment. This refresher course highlights the role of imaging in the selection of kidney donors, the imaging features of renal transplantation complications and their interventional management.
***Session Objectives:***
1. To become familiar with the role of imaging in the selection of renal donors.
2. To understand the role of imaging in transplant dysfunction and other abnormalities.
3. To illustrate the capabilities of Interventional Radiology in renal transplantation.

**A-0996** 16:05
**A. Preoperative evaluation of donors (cadaveric or living)**
C. Nicolau; *Barcelona/ES (cnicolau@clinic.ub.es)*

Living renal donor transplantation is the best option for recipient and graft survival. Imaging evaluation is crucial for selecting proper donors, adequate kidneys and the best surgical approach. In this lecture, we will review the current role of CT / MR techniques in the selection process of renal donors and the CT / MR acquisition protocols of renal donors including the use of multiplanar reconstructions such as maximum intensity projections and three-dimensional volume-rendering. Moreover, we will describe the main reportable findings of the CT/MR studies. Accurate anatomic information about the donor´s renal parenchyma, renal vasculature and collecting system should be provided to ensure a successful outcome for renal transplantation.
***Learning Objectives:***
1. To learn about CT/MR techniques in evaluation of cadaveric and living renal donors.
2. To learn about the role of imaging in the selection process of renal donors.
3. To understand the role of image post-processing in planning transplantation.

**A-0997** 16:28
**B. Multiparametric US for diagnosis and intervention**
D.Y. Huang; *London/UK*

"no abstract submitted"
***Learning Objectives:***
1. To be familiar with the appearance of renal transplant dysfunction at multiparametric US.
2. To understand the role of US modes in other renal transplant abnormalities, such as stone disease, tumours and inflammatory or ischaemic changes.
3. To understand the limitations of US modes in renal imaging and intervention.

**A-0998** 16:51
**C. CT/MRI/angiography for diagnosis and intervention**
A. Coutinho Santos; *Lisbon/PT (a.icoutinho.santos@gmail.com)*

Renal transplantation is the treatment of choice of patients with end-stage renal disease. The detection, accurate diagnosis, and timely management of complications of renal transplantation are crucial to avoid graft loss and mortality, and this assessment may involve a multiplicity of imaging modalities. Although ultrasound is the first-line technique used, CT and MR provide excellent anatomic detail and are often helpful, especially when ultrasound findings are inconclusive. CT accurately characterises the size and location of peritransplant fluid collections (e.g., seromas, hematomas, lymphoceles, urinomas) and has a high sensitivity in the detection of calculi. Moreover, it allows percutaneous drainage or aspiration of fluid collections and abscesses and biopsy of neoplastic lesions. CT angiography and increasingly MR angiography have replaced conventional angiography in vascular complications. The last remains the diagnostic reference standard and is used to guide endovascular treatments (e.g., percutaneous angioplasty in renal artery stenosis). However, CT angiography implies radiation exposure and iodinated contrast material is potentially nephrotoxic, which may be contraindicated when renal function is decreased. MR angiography overcomes these limitations, especially with non-contrast-enhanced techniques, and also assures a global evaluation of the renal transplant and peritransplant region with excellent tissue differentiation. With regard to neoplastic complications, both CT and MRI are superior to ultrasound for staging of renal transplant tumour and evaluation of lymphadenopathy or metastases. In addition, some image-guided interventional procedures allow nonsurgical treatment, for example, thermal ablation is a safe and effective therapy in de novo renal allograft renal cell carcinomas.
***Learning Objectives:***
1. To learn about the CT/MRI appearance of renal transplant dysfunctions.
2. To understand the role of CT/MRI/angiography for interventional procedures.
3. To understand the role of CT/MRI/angiography modes in other renal transplant abnormalities.

17:14
**Panel discussion: Could imaging reduce the need for renal graft biopsy?**

### Sunday, March 3

08:30 - 10:00 Room A

### E3 - ECR Academies: Interactive Teaching Session for Young (and not so Young) Radiologists

### E3 1721

### Head and neck imaging

**A-0999** 08:30
**A. Cystic neck lesions**
A. Borges; *Lisbon/PT (borgalexandra@gmail.com)*

Cystic and cyst-like lesions are commonly found in the head and neck and have a unique differential diagnosis based on the patient's age and precise anatomic location. Whereas in children, most are of embryonic/developmental origin, in adults infectious/inflammatory and cyst-like lesions predominate. Imaging plays an important role in the differential diagnosis and treatment planning, by showing the exact anatomic location, relationship with adjacent structures and architectural features and by guiding tissue/fluid sampling for cytological and biochemical analysis. Ultrasound is the preferred modality to assess superficially located lesions. It is free of ionising radiation and provides real-time guidance for tissue sampling. CT or MRI may be required for deep-seated lesions, to assess the deep extent of transpacial cysts or to refine the differential by showing other associated findings. Radiologists should be aware of potential cyst mimics: Very homogeneous lesions which can have a pseudocystic appearance (such as nodal involvement by lymphoma) and solid lesions with extensive cystic/necrotic degeneration (such as lymph node metastasis from HPVp16+ squamous cell cancer or papillary thyroid carcinoma and cystic degeneration of a schwannoma). As these cyst-like lesions are more often found in older patients, a fine needle aspiration cytology is mandatory in adult patients presenting with cystic lesions in the neck. Cytological analysis after cytospin, flow-cytometry and biochemistry analysis of aspirated fluid can further, refine the diagnosis. In this interactive session, we will review cystic neck lesions using a site-specific approach and will highlight the added value of biochemical analysis in the differential diagnosis.
***Learning Objectives:***
1. To become familiar with the differential diagnosis.
2. To know the usefulness of biochemistry of FNA in the differential diagnosis.

**A-1000** 09:15
**B. Non-traumatic head and neck emergencies**
A. Rovira-Cañellas; *Barcelona/ES (alex.rovira@idi.gencat.cat)*

A wide variety of non-traumatic head and neck conditions often present at the emergency departments. CT is in most cases the first-line imaging modality, although, MRI plays an important secondary role in some cases. Radiologists must be able to know and recognise the imaging findings of most common head and neck acute conditions, which usually require a rapid intervention to avoid severe permanent damage or death. These conditions mainly include infectious process and various types of neoplasms involving the oral cavity, pharynx, deep spaces of the neck, the orbital cavity and cervical vessels and nodes. Familiarity with these conditions will facilitate early and accurate diagnosis, a proper assessment of the extent of disease, and the identification of potential complications. In this interactive session different non-traumatic head and neck conditions, which present emergently, will be presented, with a focus on radiological evaluation and interpretation.
***Learning Objectives:***
1. To become familiar with the most common cause of emergencies.
2. To learn the imaging criteria for differentiation.

08:30 - 10:00 Room B

### Abdominal Viscera

### RC 1701

### Hepatocellular carcinoma: from diagnosis to treatment

**A-1001** 08:30
**Chairperson's introduction**
G. Brancatelli; *Palermo/IT (gbranca@yahoo.com)*

Hepatocellular carcinoma (HCC) is the second leading cause of cancer death worldwide. In this session, the imaging criteria to diagnose HCC noninvasively will be presented, along with the benefits of using LI-RADS terminology, interpretation, and reporting for both clinical care and research. The newest LI-RADS and concurrent AASLD updates will be discussed. The typical and atypical appearance of HCC will be shown, along with common mimickers and useful tips of differentiation of focal hepatic nodules in the cirrhotic liver. The role of the available interventional techniques in treating patients with HCC at different stages will be reviewed. Finally, through the presentation of practical cases, the panel will discuss how to improve a patient's experience and prognosis.
***Session Objectives:***
1. To become familiar with the international guidelines for HCC diagnosis.
2. To understand the typical and atypical appearance of HCC.
3. To learn why, when and how to treat HCC with interventional techniques.
*Author Disclosure:*
**G. Brancatelli:** Advisory Board; Guerbet. Speaker; Bayer, Guerbet.

**A-1002** 08:35
**A. How to diagnose HCC: new guidelines**
K. Fowler; *San Diego, CA/US (k1fowler@ucsd.edu)*

This session will focus on the imaging diagnosis of hepatocellular carcinoma in patients at risk. The attendees will learn about Liver Imaging Reporting and Data System (LI-RADS) approach to HCC diagnosis. New updates in 2018 and changes to the existing categories and approach will be discussed. The performance of LI-RADS in practice and potential regional differences in applicability will be highlighted.
***Learning Objectives:***
1. To learn the imaging features and criteria for HCC worldwide.
2. To understand the algorithmic approach to imaging diagnosis (LI-RADS).
3. To review emerging evidence, recent changes to diagnostic criteria and future directions.

**A-1003** 08:58
**B. Unusual appearances of HCC and lesions that simulate HCC: can we increase diagnostic confidence?**
J.M. Lee; *Seoul/US (jmsh@snu.ac.kr)*

Hepatocellular carcinoma (HCC) poses a burden on global health. It has become the fifth most common malignant neoplasm worldwide. As HCC typically has a poor prognosis with a 5-year survival rate of only 28.6%, it is of paramount importance to achieve the earliest possible diagnosis of HCC and to recommend the most up-to-date optimal treatment strategy to increase the survival rate of patients who develop this disease. HCC is commonly diagnosed using dynamic CT and/or dynamic MRI without histological confirmation, on the basis of characteristic arterial enhancement and portal venous or delayed phase washout. HCC usually presents with typical imaging characteristics but at times can present with a broad spectrum of atypical appearances. Familiarity with unusual presentations and their imaging findings is critical to ensuring prompt, accurate diagnosis and treatment. Moreover, while imaging techniques have markedly improved in detecting small liver lesions, they often identify incidental benign liver lesions and non-hepatocellular malignancy that can be misdiagnosed as HCC. The common mimickers of HCC in the cirrhotic liver include nontumorous arterioportal shunts, rapidly enhancing hemangiomas, intrahepatic mass-forming type cholangiocarcinoma (CC), combined HCC-CC, metastases, angiomyolipomas, focal inflammatory liver lesions, and focal nodular hyperplasia-like nodules. Among them, it is essential to recognise the suggestive imaging findings for intrahepatic CC or nonhepatocellular malignancies as the management of those tumours is largely different from that of HCC. Recognition of the typical imaging findings of common HCC mimickers can reduce false-positive HCC diagnosis.
***Learning Objectives:***
1. To demonstrate atypical appearance of hepatocellular carcinoma at cross sectional imaging.
2. To illustrate common mimickers of hepatocellular carcinoma in cirrhotic liver.
3. To provide useful tips of differentiation of focal hepatic nodules in cirrhotic liver.
*Author Disclosure:*
**J.M. Lee:** Research/Grant Support; GE Healthcare, Samsung Medison, Guerbet, Bayer Healthcare. Speaker; Bayer Healthcare, Philips Healthcare, Siemens Healthcare.

**A-1004** 09:21
**C. Interventional techniques (ablation, TACE, radioembolisation): when and how to treat**
R. Duran; *Lausanne/CH (rafael.duran@chuv.ch)*

The incidence of hepatocellular carcinoma (HCC) has been rising worldwide over the last decades and is expected to increase in the coming years. The treatment of HCC is complex and requires a multidisciplinary approach. Treatment allocation and prognostic prediction are mainly based on the Barcelona Clinic Liver Cancer (BCLC) staging system; patients are classified into five stages (0, A, B, C and D). Patient selection is crucial and involves, in particular, the assessment of the liver function, tumour extension and performance status although surgical treatments (resection and liver transplantation) are considered the mainstay of HCC treatment, in fact, 80% of patients present with disease that is not amenable to surgery. Interventional radiology is playing a major role in the management of these patients and provides patient-tailored therapies. Image-guided percutaneous ablation (radiofrequency ablation / microwave ablation) is the most frequently used therapeutic strategy in patients with limited disease (BCLC 0 (single tumor <2cm) and (1-3 tumors <3 cm) A). Transarterial chemoembolization is the gold standard for patients with multifocal disease (BCLC B). Yttrium-90 radioembolization is a recognised treatment option for locally advanced disease (BCLC B-C) and is challenging the hegemony of chemotherapy (sorafenib, lenvatinib, regorafenib, cabozantinib) which is the standard treatment in advanced-stage disease (BCLC C).

***Learning Objectives:***
1. To understand the role of each interventional technique depending on the patients' characteristics and HCC stage.
2. To review the different interventional techniques available for HCC management.
3. To review the patients' outcomes associated with interventional treatment of HCC.

09:44
**Panel discussion: How to improve a patient's experience and prognosis**

**08:30 - 10:00 Room C**

### General Radiography (Radiographers)

### RC 1714

### Ultrasound: continuing to make waves

*Moderators:*
T. Herlihy; Dublin/IE
S.B. Grover; New Delhi/IN

***Session Objectives:***
1. To review technological changes in an ultrasound that facilitates advanced scanning.
2. To understand the current best use of ultrasound in early pregnancy.
3. To appreciate the emerging role of ultrasound in biomechanics and elite sport.

**A-1005** 08:30
**A. Technology advances: from 3D to elastography**
M.R.V. Pedersen; *Vejle/DK (malene.roland.vils.pedersen@rsyd.dk)*

New ultrasound technologies are becoming more and more used in clinical practice. 3D ultrasound scan, ultrasound contrast enhancement, elastography, and 2D Shear Wave elastography are some of the new techniques. 3D ultrasound is being used in different organs and can help diagnose. And this technology is becoming more and more used. An overview of the two main elastography technologies will be reviewed. Elastography - both Strain and Shear Wave elastography are being more and more used in the daily clinical practice. But is this elastography technology reproducible? Is elastography hype or real? What affect the elastography measurements? Are the measured values valid? How many measurements are sufficient? Which organs can be examined with elastography? Measurements reported in meter per second versus kPa - what to choose. Elastography is very often used to distinguish benign from malignant lesions. But can elastography differ between malignant and benign lesions? Studies comparing elastography in tumours have shown good reproducibility. Some of the future advances in ultrasound are 2D Shear Wave elastography, but also ultrasound in combination with other modalities such as MRI have been published recently.

***Learning Objectives:***
1. To review the technological advances underpinning 3D ultrasound scanning.
2. To understand the technical basis and clinical applications of elastography.
3. To become familiar with the future advances in ultrasound scanning.

**A-1006** 09:00
**B. Early pregnancy guidelines**
S. Riaz; *Manchester/UK (s.riaz@salford.ac.uk)*

Early pregnancy patients typically present with clinical symptoms including, previous ectopic, pain, bleeding, hyperemesis and trauma. The scan aims to exclude ectopic pregnancy, missed, threatened, or incomplete miscarriage and to determine the presence/absence of fetal heart pulsations and the presence of a fetal pole and yolk sac. The gestational sac is the first sign of early pregnancy on ultrasound and can be seen with a transvaginal ultrasound scan at approximately 3-5 weeks gestation. Transvaginal scanning is the gold standard in early pregnancy, however as it is an internal procedure it can be declined, this along with early gestational age and a high BMI can lead to misdiagnosis. Machine quality and user competence can also limit the quality of the scan, and this should be taken into account when scanning early pregnancy. Taking all these factors into consideration optimal scanning and current guidelines are needed to make an accurate diagnosis.

***Learning Objectives:***
1. To review current guidelines and indications for early pregnancy scanning.
2. To understand essential requirements for optimal imaging.
3. To become familiar with pitfalls in imaging in early pregnancy.

**A-1007** 09:30
**C. Role of ultrasound in biomechanics and sports science**
R. Santos; *Coimbra/PT (rutemartinssantos@gmail.com)*

In the last years, the use of ultrasound (US) for the study of muscle-skeletal function has grown considerably. The reasons for such growth include the ability to study muscle morphology and its mechanical properties at relatively low cost and accessibility. The continuous improvement of US equipment, including the use of multifrequency probes and the development of new US techniques, such as elastography have contributed to make US an important method in muscle function research. Numerous studies have proven the feasibility of US for studying muscle function in non-pathological conditions. The assessment of parameters such as muscle thickness and echo-intensity allows studying muscle function and muscle morphological responses and adaptations to a variety of stimulus, in particular to muscle disuse and resistance training. Recent developments in US have enhanced our ability for studying muscle tissue mechanical properties in vivo, including changes in muscle stiffness associated with stretching and contraction. Measuring muscle stiffness allows a better understanding of muscle function as a monitor of muscle status, and it can be an indicator of the length of the muscle, as well as of its contraction status. The potential of this technique is enormous and has enabled researchers in many fields, including biomechanics and muscle physiology to probe the function of complex multiarticular muscle groups in passive and active conditions. Nevertheless, it's important to have a great knowledge of anatomy, physiology and be familiar with US technique in order to produce images of high quality and with a higher viability.

***Learning Objectives:***
1. To review the current role of ultrasound in elite sport.
2. To understand the challenges when using ultrasound for evaluating human performance.
3. To become familiar with best practice guidelines imaging in sport / biomechanics.

**08:30 - 10:00 Room X**

### Joint Session of the ESR and ESMRMB

### ESR/ESMRMB

### A new era of gadolinium-less MR

*Moderators:*
D. Sappey-Marinier; Lyon/FR
M.J.P. van Osch; Leiden/NL

**A-1008** 08:30
**Radiomics and multiparametric MRI approaches for a better contrast in cardiac and prostate applications**
B. Baeßler; *Mannheim/DE (bettina.baessler@uk-koeln.de)*

Over the past decades, the technical developments in MRI have enabled the clinical application of multiparametric imaging approaches, which allow for a combined assessment of different morphological and functional tissue characteristics. For multiparametric prostate MRI as well as for cardiac MRI, debates are ongoing whether this allows us to fully replace gadolinium-based examinations by native multiparametric approaches. In addition, the emerging field of radiomics, which means the extraction of a large number of mathematically or statistically derived quantitative features from medical images, thus delivering mineable datasets, delivers additional potential novel imaging biomarkers for non-contrast assessment of prostate cancer and myocardial diseases. The talk will focus on basic concepts, current debates as well as potential hurdles hindering the translation of multiparametric imaging approaches and radiomics into routine clinical practice.

***Learning Objectives:***
1. To learn which gadolinium-free quantitative techniques exist for cardiac and prostate MR imaging.
2. To understand the potential and limitations of quantitative, gadolinium-free parametric imaging and radiomics.
3. To appreciate the link between parametric MR imaging and radiomics and to show potential clinical applications of gadolinium-free techniques in cardiac and prostate MRI.

**A-1009** 08:55
**From native T1 to arterial spin labelling MRI in the heart**
F. Kober; *Marseille/FR (frank.kober@univ-amu.fr)*

This talk will give an overview of quantitative T1 mapping techniques used in cardiovascular MR. It discusses challenges with absolute T1 quantification related to the methods and to the heart itself. Because the blood signal is for some methods among the contributors to the measured absolute T1 value, there is a link between observed T1 and myocardial perfusion. Arterial spin labelling (ASL) as a non-contrast perfusion MRI method for the heart will be introduced, and several past and present approaches will be presented showing their advantages and drawbacks. The challenges of cardiac ASL compared with ASL in other organs will be outlined. It should also become clear in this talk why cardiac ASL has been frequently used in small animals, whereas human cardiac applications are still scarce.
***Learning Objectives:***
1. To learn how T1 is quantified and mapped in the heart and how T1 quantification is linked to perfusion.
2. To understand how arterial spin labelling acquisition is performed in the heart and why it is more challenging than in the brain.
3. To appreciate what these techniques may do better than those based on contrast agents and what they probably won't do.

**A-1010** 09:20
**Brain perfusion and angiography using ASL in comparison with gadolinium based techniques**
H.R. Jäger; *London/UK*

"no abstract submitted"
***Learning Objectives:***
1. To learn about the different non-enhanced MR angiography techniques used for imaging the extra-
and intra-cranial vessels and about the principles of brain perfusion imaging with arterial spin labelling (ASL).
2. To appreciate the differences between static and time-resolved MR angiography of the intra-cranial vessels and the importance of post-labelling delay in ASL perfusion imaging.
3. To understand the clinical applications of TOF MR angiography, ASL angiography and ASL perfusion imaging techniques and their advantages and limitations.

09:45
**Panel discussion: Is it feasible to apply gadolinium-free techniques in clinical practice?**

**08:30 - 10:00** **Room N**

### EuroSafe Imaging Session

### EU 7

### Artificial intelligence and radiation protection

**A-1011** 08:30
**Chairpersons' introduction (part 1)**
G. Frija; *Paris/FR (guy.frija@aphp.fr)*

Artificial intelligence (AI) is a vehicle for improving patient outcomes, and thus it will change the practice of radiology in the next few years. AI will be a method of demonstrating the clinical value of radiology and will allow for faster and more accurate image assessment and hence diagnosis. AI will also support the training of radiologists, improve clinical knowledge, and contribute to research in medical imaging. Furthermore, AI could have knock-on impacts on radiation protection by reducing the incidence of unnecessary procedures, reducing the doses administered, increasing the image quality, and improving patient safety in general. The transformative potential of AI for the radiology profession cannot be ignored: rather practitioners must be prepared to embrace it. This session will provide an overview of how AI can enhance radiation protection in medical imaging.
***Session Objectives:***
1. To understand the mechanisms of how artificial intelligence (AI) can help to reduce necessary doses for imaging procedures relying on ionising radiation.
2. To appreciate which AI methods in medical imaging might be helpful for reducing dose for especially sensitive body regions or highly susceptible patients in general.
3. To learn how results from AI evaluation of radiographic images might help to influence decisions on the most appropriate methods or radiation protection measures on an individual patient basis in an early phase of patient diagnosis and therapy.

**A-1012** 08:33
**Chairpersons' introduction (part 2)**
C. Hoeschen; *Magdeburg/DE (christoph.hoeschen@ovgu.de)*

Using artificial intelligence in medical imaging for radiation protection purposes has three main pillars. The first pillar is that artificial intelligence can be used for investigating parameters of medical imaging procedures based on ionising radiation like image quality evaluation, exposure description and prediction of optimal imaging parameters in such sense depending on patient characteristics and diagnostic task. The second pillar is that artificial intelligence can be used to optimise image quality per dose based on advanced methods for image reconstruction, scatter reduction and thus noise and artefact reduction. The third pillar is the characterisation of patient radiation sensitivity and susceptibility for choosing the right radiation therapy or the optimal imaging procedure on individual patient base. Such radiation protection related aspects will be described in the session, and the difference will be explained in the introduction by the chairs.
***Session Objectives:***
1. To understand the mechanisms of how artificial intelligence (AI) can help to reduce necessary doses for imaging procedures relying on ionising radiation.
2. To appreciate which AI methods in medical imaging might be helpful for reducing dose for especially sensitive body regions or highly susceptible patients in general.
3. To learn how results from AI evaluation of radiographic images might help to influence decisions on the most appropriate methods or radiation protection measures on an individual patient basis in an early phase of patient diagnosis and therapy.

**A-1013** 08:35
**Artificial intelligence: a tool for quality and safety improvement in radiation protection**
G. Frija; *Paris/FR (guy.frija@aphp.fr)*

The use of artificial intelligence (AI) in medicine has attracted enormous attention, and there is a lot of research going on to implement AI in imaging. Current applications include, for example, the detection of breast cancer on mammography images, the detection of lung nodules in CT scans, and detecting pneumonia in chest x-rays. In addition, applications beyond image interpretation are on the rise. The development of AI tools in medical imaging will certainly also have an input on medical radiation protection. However, currently, there is hardly any research work on the use of AI as a tool for quality and safety improvement in imaging, although there are a number of potential applications that could help reduce doses for imaging procedures relying on ionising radiation, especially for sensitive body regions or highly susceptible patients in general. Algorithms can be used to improve image quality and dose optimisation in CT. AI-based organ recognition combined with dose estimation algorithms can provide patient-specific organ doses. This talk will present current trends of AI in radiology and provide examples how these AI systems could contribute to strengthening medical radiation protection for the benefit of patients.
***Learning Objectives:***
1. To learn about current trends of AI in imaging.
2. To understand how these trends could improve quality and safety.
3. To appreciate how AI could affect radiation protection.

**A-1014** 08:55
**Artificial intelligence for scatter reduction and optimising imaging procedures**
C. Hoeschen; *Magdeburg/DE (christoph.hoeschen@ovgu.de)*

Medical imaging based on ionising radiation is strongly suffering from two major drawbacks which result in the need for exposure levels mainly to patients but also to staff in interventional procedures higher than it might be necessary from the physical imaging process. This is the case since noise and artefacts are two of the most relevant factors deteriorating sufficient image quality for diagnostic or interventional purposes. Noise and artefacts both are strongly related to scatter that is generated in the patient body during the interaction of the X-rays with the material. Therefore ways will be discussed how the scatter contribution of the X-rays caused by patients can be estimated and thus be reduced by methods based on artificial intelligence as they have been shown by various groups and its advantages and disadvantages will be highlighted. By such scatter reduction better image quality can be gained and thus exposure levels can be reduced. In addition, this talk will briefly introduce another concept to use x-rays as efficient as possible based on optimising the imaging geometry based on artificial intelligence approaches.
***Learning Objectives:***
1. To learn about actual scatter reduction techniques in imaging based on ionising radiation, their advantages and drawbacks as well as the potential of optimising imaging trajectories.
2. To understand how AI-based procedures can provide a fast solution for scatter estimation and reduction as well as for image trajectory determination.
3. To appreciate how AI can help to reduce patient dose in medical imaging with easy to implement applications.

**A-1015** 09:15
**Artificial intelligence for intelligent reconstruction methods for radiation protection measures**
C.T. Whitlow; *Winston-Salem, NC/US*

"no abstract submitted"
***Learning Objectives:***
1. To discuss radiation exposure due to computed tomography associated with common diagnostic tests and in the setting of screening exams.
2. To describe efforts aimed at reducing radiation dose and improving image quality via conventional approaches (e.g. iterative reconstruction).
3. To introduce methods for improving image quality via novel artificial intelligence (AI)-based approaches, with quantitative results characterising noise reduction and example cases from clinical application.

**A-1016** 09:40
**Using artificial intelligence for optimising procedures reflecting radiosusceptibility of patients**
C. Hoeschen; *Magdeburg/DE (christoph.hoeschen@ovgu.de)*

Radiation protection of patients is an important task in general. It has been found out that it might be even more relevant to certain patients than to others since, obviously, not all patients are prone to negative effects of ionizing radiation in the same way. Such differences are described as individual radiation sensitivity if one refers to short-term effects, especially so-called tissue reactions and as individual radiation susceptibility if one refers to long-term effects like especially cancer induction. Obviously, it is even more important to protect especially the healthy tissue of those patients who are more radiation sensitive or susceptible than those who are not. Some of the potential reasons for individual radiation sensitivity or susceptibility will be described as well as indications that and how specific analysis of imaging data could provide insights into such individual patient configuration and why this might be helpful for radiation protection. Methods based on artificial intelligence would be most promising for this task. There are also medical imaging applications with pretty high doses and especially pretty localized high doses like in interventional procedures. Thus, it would be feasible to optimize not only radiation therapy for individuals that are very sensitive but also for example interventional procedures. For planned CT examinations, it might be better to think about low-dose procedures or even of a replacement of the procedure by means of MRI if feasible in patients with extremely high risk. However, there are some ethical aspects of such approaches.
***Learning Objectives:***
1. To learn about radiosusceptibility and why it is important to look for it in images.
2. To understand what role AI could play in the future for optimising procedures, especially interventional procedures, from the point of view of radiation protection.
3. To appreciate the potential for new research topics bridging the gap between AI and radiation protection with respect to the individual patient.

09:50
**Discussion**

**08:30 - 10:00** **Room O**

### Cardiac

### RC 1703

### Dead or alive: imaging of myocardial viability

**A-1017** 08:30
**Chairperson's introduction**
C. Peebles; *Southampton/UK (Charles.Peebles@suht.swest.nhs.uk)*

Myocardial viability is central to the investigation and management of ischaemic heart disease (IHD). The viable myocardium is defined as 'dysfunctional myocardium that maintains the potential to return to normal function' e.g. it is alive and not dead. Dysfunctional, but viable myocardium is the end result of an ischemic myocardial injury that can either be acute (stunning) or chronic (hibernating). In the former, a single acute ischaemic injury causes temporary dysfunction that will recover after the restoration of myocardial blood flow, but there may be a delay of weeks or months for this to occur. Hibernation is the end result of chronic or repetitive ischaemia causing downregulation of myocyte metabolism and function, which will again recover the following revascularisation. Accurate identification of viable myocardium is critical, as revascularisation of non-viable myocardium confers the risks of revascularisation without benefits of functional recovery. Conversely, failing to revascularise viable myocardium that is ischaemic may place the patient at increased risk. Two general approaches to identifying viable myocardium are the functional improvement of a regional wall motion abnormality during low dose stress or identification of myocardial scar that involves less than the full thickness of a myocardial segment. The first approach is adopted by stress echo and dobutamine stress CMR. The second approach requires the use of extracellular contrast agent and is usually performed by CMR with late gadolinium enhancement or CT with iodinated contrast and delayed imaging. This session will compare the standard techniques, and discuss their pros and cons.
***Session Objectives:***
1. To understand the concept of myocardial viability.
2. To understand the current state-of-the-art to image myocardial viability.
3. To outline the role of echocardiography, SPECT, CT and MRI.

**A-1018** 08:35
**A. MRI**
T. Leiner; *Utrecht/NL (t.leiner@umcutrecht.nl)*

Ischemic cardiomyopathy (iCMP) is defined by the presence of flow-limiting stenoses in at least two coronary arteries and a left ventricular ejection fraction of 40% or less. In patients with iCMP, the amount of viable myocardium directly affects the short- and long-term outcome. Myocardial viability refers to those cardiomyocytes that reside in a suboptimal functional state but are alive as defined by cellular and metabolic function. These myocytes can potentially regain full functionality, resulting in improved cardiac systolic function with a combination of revascularisation and pharmacotherapy. Cardiac magnetic resonance imaging (CMR) is well-suited for the identification of both viable myocardium as well as 'dead' scar tissue and can be used to assign patients to the appropriate therapeutic strategy with high confidence. Because of its high spatial and temporal resolution, CMR can identify the presence, extent and transmurality of left ventricular scar tissue as well the resulting functional impairment. In current clinical practice, two strategies are used, late-gadolinium enhancement (LGE) to detect the presence and extent of scar tissue; and low-dose dobutamine stress imaging (DS) to detect the extent of functional impairment. Both methods are complementary, but LGE is often used alone because transmurality of scar expressed as a percentage of LV wall thickness has been shown to strongly correlate with the possibility of functional recovery. Promising alternative methods for detection of presence and extent of myocardial scar are T1-mapping and algorithmic analysis of myocardial signal intensity on standard cine images.
***Learning Objectives:***
1. To learn about how MR should be performed to assess viability.
2. To understand the strengths and weaknesses of MR compared to other techniques.
3. To learn how to report MRI for viability assessment.
*Author Disclosure:*
**T. Leiner:** Author; DoRadiology - Imaging Anatomy. Grant Recipient; Bayer Healthcare, Philips Healthcare. Speaker; Bayer Healthcare, Philips Healthcare.

**A-1019** 08:58
**B. Hybrid imaging**
F. Caobelli; *Basle/CH (federico.caobelli@usb.ch)*

The concept of myocardial viability in coronary artery disease (CAD) is derived from diverse studies, wherein an irreversible left ventricular contractile dysfunction was demonstrated even if revascularisation occurred. Many controversies still exist on the most appropriate method to assess the presence and extent of viable myocardium. Stand-alone modalities provide important data about myocardial viability, but all these techniques suffer from several limitations. As such, new methods combining different information from different modalities for the investigation of myocardial viability are highly warranted. This constitutes the fundamental basis for the development of hybrid imaging, consisting in the association of different imaging techniques, where both modalities equally contribute to image information, allowing for a comprehensive evaluation. An example of such an approach is the combination of computed tomography coronary angiography (CTCA) and myocardial perfusion imaging either with single-photon emission computed tomography (SPECT) or with Positron emission tomography (PET). Other possible applications include a combination of CTCA and cardiac magnetic resonance imaging (cMR). These approaches are expected to have several potential applications in the next future. Concurrently, new molecular Imaging probes may provide invaluable tools for the guidance and monitoring of therapies. This lecture will discuss the actual state of the art of cardiac hybrid Imaging as well as potential future applications.
***Learning Objectives:***
1. To learn about how hybrid imaging should be performed to assess viability.
2. To understand the strengths and weaknesses of hybrid imaging compared to other techniques.
3. To understand how hybrid imaging could be developed in clinical practice.

*Author Disclosure:*
**F. Caobelli:** Grant Recipient; Mallinckrodt (2014). Speaker; Siemens (2015 - 2017).

**A-1020** 09:21
**C. CT**
F. Bamberg; *Tübingen/DE (fabian.bamberg@uni-tuebingen.de)*

In the field of cardiac CT, substantial research efforts have recently been focused on the development of different approaches to obtain information on myocardial viability. Despite a relatively low level of scientific evidence, these approaches are currently entering the clinical arena given its high relevance for therapeutic decision-making. Analogous to the presence of late gadolinium enhancement on MRI, cardiac CT allows for the detection of delayed iodinated contrast enhancement on delayed CT acquisitions. However, from a clinical perspective, CT imaging protocols are generally combined with myocardial perfusion imaging at rest and stress. In general, such a protocol includes as a first step perfusion imaging followed by a second step that includes a low-radiation scan for the detection of delayed imaging. Further, perfusion imaging by CT can be conducted as a “single-shot” acquisition with or without dual energy or as a dynamic acquisition during rest or stress conditions. The talk will provide an overview of currently available approaches and protocols, highlighting limitations, and review the level of evidence pertaining to the role of CT for assessment of myocardial viability.
***Learning Objectives:***
1. To learn about the potential of CT to assess viability.
2. To understand the strengths and weaknesses of CT imaging compared to other techniques.
3. To understand how to report CT for viability assessment.
*Author Disclosure:*
**F. Bamberg:** Research/Grant Support; Siemens Healthineers, Bayer Healthcare. Speaker; Siemens Healthcare, Bayer Healthcare.

09:44
**Panel discussion: What imaging test in which patient?**

**08:30 - 10:00 Studio 2019**

### Special Focus Session

### SF 17

### Late effects in survivors of childhood cancer

**A-1021** 08:30
**Chairperson's introduction**
C. Owens; *London/UK (owensc@gosh.nhs.uk)*

This session will focus on the optimistic advances in cancer treatment which has resulted in much better survival rates. However, there is considerable morbidity with the more aggressive treatments used which result in late effects within many organ systems. This session will focus on the important role we have as radiologists in identifying, diagnosing and following up the complications of previous cancer treatments in childhood. We will focus on an organ-based approach to diagnosing the sequelae of childhood cancer treatments.
***Session Objectives:***
1. To learn about the cost of survival in long term survivors of childhood cancer.
2. To understand therapy related pathologies which are sequelae of cancer treatment in childhood.
3. To appreciate these disorders and to help to make early diagnoses in order to minimise long term sequelae.
4. To become familiar with the human cost of cancer survival and the effects on the daily lives from survivors of childhood cancer.

**A-1022** 08:37
**Cardiothoracic complications**
A. Secinaro; *Rome/IT (aurelio.secinaro@opbg.net)*

New advances in cancer diagnosis and treatment have increased survival rates in patients with cancer. The growing number of cancer survivors’ results in a higher prevalence of cardiothoracic complications from cancer treatment. There is evidence of a significant incidence of cardiovascular complications among adult survivors of a variety of childhood and adolescent cancers years after treatment. Survivors are significantly more likely than siblings to report congestive heart failure, myocardial infarction, pericardial disease or valvular abnormalities. Chemotherapy-induced cardiac dysfunction is a major contributor to adverse morbidity and mortality rates in cancer patients. Both clinical symptoms and the traditional left ventricular ejection fraction may lack sensitivity as measures of cardiotoxicity. Radiation-induced coronary artery disease has to be considered a possible cause of myocardial infarction. Novel cardio-thoracic imaging techniques such as Cardiac Magnetic Resonance and Cardiac CT can identify a high prevalence of cardiac disease among adult survivors previously undiagnosed with cardiac disease, as 2D echocardiography demonstrated limited screening performance; in particular, detailed and accurate assessment of vascular anatomy (including coronaries), myocardial tissue characterisation and biventricular volumes and global systolic function is now non-invasively achievable. In addition, chest CT is more sensitive than chest radiographs in detecting post-irradiation changes in the lungs as it is able to early detect linear or reticular “structured” opacities related to lung fibrosis.
***Learning Objectives:***
1. To outline chemotherapy related pathology which are sequelae of cancer treatment in childhood.
2. To illustrate with state-of-the-art imaging.
3. To summarise what the radiologist needs to know and how to find it.

**A-1023** 08:55
**MSK**
I.-M. Noebauer-Huhmann; *Vienna/AT (iris.noebauer@meduniwien.ac.at)*

Adult patients who had suffered from cancer during childhood may develop various late sequelae during their lifetime. Cancer- or treatment-related developmental abnormalities may occur in the growing skeleton and can cause deformities and preterm degeneration. A thorough assessment of the present clinical condition, the past medical history, and the altered post-surgical anatomy are essential for the evaluation of follow-up imaging. Also, radiation and chemotherapy lead to the typical time-dependent bone marrow and soft-tissue alterations. Those changes must be differentiated from the recurrent or metastatic disease. Hormone-related skeletal alterations and complications indicative of altered bone stability or decreased osseous blood supply must be detected early. To date, amputation can often be avoided when limb salvage is feasible, through wide tumour resection and reconstruction with modular endoprostheses. While adequate local and systemic tumour control rates can be achieved with those megaprostheses, various complications may still occur. Detailed knowledge of the imaging appearance of such complications, which have been categorised according to the ISOLS classification system, is indispensable. Clear imaging strategies and systematic evaluation algorithms can help radiologists to reliably assess the late post-therapeutic sequelae and to detect potential complications in survivors of childhood cancer.
***Learning Objectives:***
1. To discuss clinical manifestation of late effects to the MSK system following chemotherapy.
2. To illustrate and suggest best methods of radiological follow-up with case studies.
3. To suggest an algorithm to help radiologists to recognise these findings.

**A-1024** 09:13
**Neuro**
R. Gunny; *London/UK*

"no abstract submitted"
***Learning Objectives:***
1. To describe how brain development may be affected by CNS malignancies and therapies used to treat them.
2. To categorise the types of abnormalities.
3. To illustrate these complications.

**A-1025** 09:22
**Fertility issues: male and female (Part 1)**
L. Rocher; *Le Kremlin-Bicêtre/FR (laurence.rocher@bct.aphp.fr)*

The survival rate for children with cancer is about 80 %. Infertility remains one of the most common complications. However, infertile patients with previous cancer treatment require the same complete biological and imaging screening compared to other patients: the infertility status may have other and curative explanations. In women, decreased fecundity is a late effect that arises after chemo- or radiotherapy, as it has been shown that pregnancy is less likely to occur in survivors who have received hypothalamic/pituitary radiation, ovarian/uterine radiation, or treatment with high doses of alkylating agents. Furthermore, the number of eggs in their ovaries may be reduced to such an extent that they will reach menopause much earlier than the average age of 51 years. Follicle counts represent ovarian reserves and can be evaluated with echography. Oocytes make an anti-Mullerian hormone (AMH), which can be measured in blood. In males, these treatments may damage spermatogenesis leading to impaired fertility. Semen analysis and hormonal status are non-invasive methods for estimation of male fecundity. Low levels of Inhibin B and high levels of FSH reflect the impaired spermatogenesis. Testicular hypotrophy with coarse/striated echogenicity reflects those damages. Leydig cell hyperplasia with a multinodular pattern may rarely occur. Methods for fertility preservation need to be suggested before treatment. Concerning pre-pubertal children, testicular tissue or ovarian cryopreservation may be performed with efficient results much advanced in girls compared to boys. Concerning post-pubertal children, oocyte cryopreservation is privileged in girls and sperm conservation is the simplest method in boys.

***Learning Objectives:***
1. To describe the potential effects that chemotherapy has on fertility in males and females.
2. To illustrate with case studies.
3. To discuss how we can help to overcome these issues using radiology.

**A-1026** 09:31
**Fertility issues: male and female (Part 2)**
C. Berger; *Saint-Etienne/FR (claire.berger@chu-st-etienne.fr)*

In Europe, 300 000 individuals had cancer during childhood and survived. Infertility remains one of the most common complications. However, infertile patients with previous cancer treatment require the same complete biological and imaging screening compared to other patients: the infertility status may have other and curative explanations. In women, decreased fecundity is a late effect that arises after chemo- or radiotherapy, as it has been shown that pregnancy is less likely to occur in survivors who have received hypothalamic/pituitary radiation, ovarian/uterine radiation, or treatment with high doses of alkylating agents. Furthermore, the number of eggs in their ovaries may be reduced to such an extent that they will reach menopause much earlier than the average age of 51 years. Follicle counts represent ovarian reserves and can be evaluated with echography. Oocytes in follicles make an anti-Mullerian hormone (AMH), which can be measured in blood. In males, these treatments may damage spermatogenesis leading to impaired fertility. Semen analysis and hormonal status are non-invasive methods for estimation of male fecundity. Low levels of Inhibin B and high levels of FSH reflect the impaired spermatogenesis. Testicular hypotrophy with coarse/striated echogenicity reflects those damages. Leydig cell hyperplasia with a multinodular pattern may rarely occur. Methods for fertility preservation need to be suggested before treatment. Concerning pre-pubertal children, testicular tissue or ovarian cryopreservation may be performed with efficient results much advanced in girls compared to boys. Concerning post-pubertal children, oocyte cryopreservation is privileged in girls and sperm conservation is the simplest method in boys.

***Learning Objectives:***
1. To describe the potential effects that chemotherapy has on fertility in males and females.
2. To illustrate with case studies.
3. To discuss how we can help to overcome these issues using radiology.

09:49
**Panel discussion: How may paediatric radiologists recognise, monitor and help to minimise the late effects in survivors of childhood cancer?**

| **08:30 - 10:00** | **Room E1** |
|---|---|

### Breast

### RC 1702

### Diagnosing ductal carcinoma in situ (DCIS)

*Moderator:*
C. Van Ongeval; Leuven/BE

**A-1027** 08:30
**A. New radiologic-pathologic knowledge on DCIS**
A. Frigerio; *Turin/IT (alfonso.frigerio@gmail.com)*

A modern rethinking of ductal carcinoma in situ must take off from the awareness of the utter inadequacy of the terminology currently in use. Already in 1976, Jensen, Rice and Wellings remarked that "ductal carcinoma in situ of the human breast is of lobular (acinar) origin". While the latest (2012) WHO Classification decided to omit the name 'ductal' from the definition of the most common type of invasive breast carcinoma, it still fails to recognise the inconsistency of this descriptor for its intra-epithelial counterpart. It is time to move to a new inter-disciplinary approach where a mutually respectful interaction between radiologists and pathologists should take full advantage of new imaging techniques and new pathologic methods to achieve better characterisation of the different types of breast lesions and their clinical-biological potential. A new classification and terminology based on the site of tumour origin (acinar vs ductal), as best displayed by diagnostic imaging paired with modern pathologic techniques as large-format histologic sections, should open the way to a better understanding of the lesion biology, eventually improving treatment planning decisions. This lecture will include a discussion of different subtypes of in situ lesions; neoductgenesis as an important feature that should be recognised and reported; the potential of mammographic tumour features as synergistic prognostic factors alongside classical pathological and modern molecular patterns. Overcoming long-lasting barriers to effective communication would be the pre-requisite to a new professional setting where all specialists involved mutually enhance the benefits of their clinical knowledge.

***Learning Objectives:***
1. To learn about different types of DCIS.
2. To become familiar with the risk of developing cancer.
3. To appreciate radiologic-pathologic correlations.

**A-1028** 09:00
**B. Imaging DCIS**
J. Camps Herrero; *Valencia/ES (juliacamps@gmail.com)*

In this presentation, common radiologic manifestations of DCIS in mammography and ultrasound will be reviewed, taking into account that microcalcifications are the most common mammographic manifestation of DCIS. Types of microcalcifications with the highest PPV will be reviewed, as well as uncommon radiological findings (distortion, asymmetry or spiculated lesions without associated microcalcifications). The importance of the anatomical lobule and macroscopic anatomy (large-format histology) cannot be overstressed as it is, together with the sick lobe theory, the best way to understand the distribution of DCIS lesions in all radiological modalities, especially in MRI. Breast MRI is the most sensitive technique for DCIS, MRI can detect up to 48% of cases not detected by mammography, especially in non-calcifying lesions (around 25% of DCIS). The most common MRI appearance of DCIS will be discussed (non-mass lesion in 60-80% of the cases, nodular mass in 14-41%) emphasising that morphological signs are more important than kinetics in DCIS, as well as the non-specific value of DWI. Overlap of imaging features with benign disease or high-risk or B3 lesions is an important issue because it is the cause of false positive findings, potential signs to differentiate between all entities will be reviewed.

***Learning Objectives:***
1. To become familiar with the imaging appearances of DCIS on mammography, ultrasound and MRI.
2. To understand the evidence on MRI for evaluating DCIS.
3. To appreciate the overlap of imaging features of DCIS and benign disease.

*Author Disclosure:*
**J. Camps Herrero:** Consultant; BD BARD.

**A-1029** 09:30
**C. Diagnosing DCIS with biopsy**
M.G. Wallis; *Cambridge/UK (matthew.wallis@addenbrookes.nhs.uk)*

Although a proportion of DCIS (particularly low grade) is found in association with other a benign lesions, the majority present with either micro-calcification on mammography or non-mass like enhancement on MRI. Currently, we have no imaging tests that can reliably determine benign from atypia from cancer, so tissue diagnosis is mandated. Surgical biopsy has been replaced by image guided needle biopsy. The challenge for the operator is accurate targeting and obtaining adequate volume to give the pathologist the best chance of making a correct diagnosis. Given accurate targeting then the of the volume of tissue taken becomes a balance of cost vs benefit. Relatively small volumes of tissue are needed to confirm the benign disease, but the accurate exclusion of an invasive component is better with a larger size. It is clear from long-term follow-up of DCIS that conventional treatment fails the 15 to 20 % who develop invasive disease, a few of whom then die from breast cancer. There is a larger group that never progress is within their lifetime which means that we have either cured them or just overtreated them. Traditional pathology and genetics suggest that there is a low-risk group that either never progresses or if they do they develop low-risk invasive disease. To date, the majority of studies trying to determine which high-grade DCIS will progress have been underpowered and lack validation in independent cohorts, so none are in clinical practice. Three international trials are investigating operative management vs active monitoring for low-risk DCIS.

***Learning Objectives:***
1. To learn about the indications for biopsy when DCIS is suspected based on imaging.
2. To become familiar with the advantages and disadvantages of different biopsy techniques.
3. To appreciate the risk of overdiagnosis and overtreatment with DCIS.

*Author Disclosure:*
**M.G. Wallis:** Investigator; Chief investigator LORIS trial Grant for UK HTA.

**08:30 - 10:00** **Room E2**

### Neuro

### RC 1711

### Diffuse low-grade gliomas: new things you should know

*Moderator:*
I.N. Pronin; Moscow/RU

**A-1030** 08:30
**A. Molecular basis for classification, treatment and predicting outcome in low-grade gliomas**
V.C. Keil; *Bonn/DE (Vera.Keil@ukbonn.de)*

Diffuse low-grade gliomas (LGG) are glial tumours with a high degree of diversity on a histological and genetic level. Their histopathology and genetics determine indeed the course of the disease. Will the lesion remain stable for decades or will it progress dynamically both regarding volume and malignancy? The individual answer to this question determines therapeutic decisions, follow-up planning and therapy monitoring. Neuropathologists define the exact diagnosis of a tumour. But neuroradiologists can make use of multiple MRI and PET imaging tools to non-invasively approximate the histology of a lesion and in consequence its potential for dedifferentiation. While “conventional” MR imaging techniques are still the basis of glioma assessment, more recent quantitative methods expand our insights about a lesion and can provide greater certainty about a presumed diagnosis. DSC-MRI, DCE MRI, ASL, MR spectroscopy and many more imaging techniques of the quantitative and molecular imaging spectrum have become essential diagnostic pillars in glioma assessment. Molecular imaging is however not yet at a point that unequivocal scientific results rendered watertight imaging biomarkers to rely on in LGG assessment. There are conflicts about the additive value of multiple imaging techniques, the reproducibility of results, histopathological correlation and also feasibility in clinical practice. This opening presentation provides an overview of the most widely used techniques in molecular imaging and their current benefits and limitations for a noninvasive LGG assessment in contrast to standard, non-quantitative MRI.
***Learning Objectives:***
1. To learn about the role of imaging in the context of histopathology regarding the prediction of outcome.
2. To understand the role of conventional and quantitative beyond diagnosis.
3. To appreciate the translational approach of diagnosis and monitoring of glial tumour based on the histopathological background.

**A-1031** 09:00
**B. Imaging patterns suggestive of different (molecular) subtypes of low-grade gliomas**
M. Smits; *Rotterdam/NL (marion.smits@erasmusmc.nl)*

Primary brain tumours are histopathologically subtyped into World Health Organisation (WHO) grades I to IV, according to - increasing - degrees of malignancy. These grades provide prognostic information and guidance on management, such as radiotherapy and chemotherapy after surgery. Despite the confirmed value of the WHO grading system, a multitude of studies and prospective interventional trials indicate that tumours with identical morphological criteria, i.e. of the same WHO grade, can have highly different outcomes. To personalise brain tumour management, we need additional diagnostic markers that can differentiate tumours beyond the current morphological WHO grading system. Molecular markers can distinguish subtypes of tumours within the same morphological type and WHO grade, and are therefore of great interest for personalised medicine. Recent genomic-wide studies have resulted in a far more comprehensive understanding of the genomic alterations in gliomas, and the suggestions of a new molecularly based classification. MR imaging phenotypes can serve as non-invasive surrogates for tumour genotypes and as such provide important information on diagnosis, prognosis, and, eventually, personalised treatment. The newly emerged field of radiogenomics links specific MR imaging phenotypes with gene expression profiles. In this presentation I will discuss the three best known tumoural genotypes with prognostic and - potential - therapeutic consequences: 1. isocitrate dehydrogenase (IDH) mutation, 2. 1p19q deletion, and 3. methylguanine methyltransferase (MGMT) promoter methylation. I will give an overview of the known and potential MR imaging features of these genotypes, and their value and validity in a clinical context.
***Learning Objectives:***
1. To learn about whether there is a change of pattern recognition based on conventional and quantitative imaging approaches.
2. To understand the heterogeneity of low-grade gliomas based on histopathology and imaging.
3. To appreciate the perspectives and challenges of diagnostic imaging in phenotyping low-grade gliomas.
*Author Disclosure:*
**M. Smits:** Other; Independent reviewer (EORTC-1410) with Parexel Intl. Corp. (financial compensation paid to institution).

**A-1032** 09:30
**C. Advanced imaging in low-grade gliomas**
N. Bulakbasi; *Mersin/TR (nail.bulakbasi@kyrenia.edu.tr)*

Low-grade gliomas (LGGs) are typically IDH mutant, diffusely infiltrating, slow-growing gliomas having moderately increased cellularity generally without mitosis, necrosis and microvascular proliferation. Supra-total” resection of LGG significantly increases overall survival by delaying malignant transformation compared with a simple debulking. The exact diagnosis is crucial for treatment planning. Data from meta-analysis support the addition of diffusion and perfusion-weighted MR imaging and MR spectroscopy (MRS) in the diagnosis of suspected LGGs. Typically, LGGs have lower cellularity (ADC), angiogenesis (rCBV), capillary permeability (Ktrans), and mitotic activity (Cho/Cr ratio) than those of high-grade gliomas (HGGs). The identification of 2-hydroxyglutarate in MRS with spectral editing gives a clue about its IDH status. A gradual decrease in minimum ADC values and increase in Cho/Cr ratio and maximum rCBV values and the emergence of lactate and free lipid at MRS in time, are consistent with the tumour progression and the poor prognosis. Small tumour diameters, artefacts due to bone or air interfaces, vascular structures, hemorrhagic, cystic/necrotic and calcific changes in the tumoral area can cause critical distortions in quantifying parameters. So, the selection of a proper ROI avoiding these kinds of areas is critical to obtain proper quantified results. The quantitative multiparametric MR imaging of LGGs either can improve the diagnostic accuracy of their differential diagnosis or can assess their prognosis.
***Learning Objectives:***
1. To learn about the spectrum and diagnostic value of the available advanced imaging techniques in neuro-oncology.
2. To understand the added value and possible pitfalls using quantitative MRI techniques in addition to conventional MRI.
3. To appreciate the importance of standardisation of advanced MR image acquisition in the clinical practice of neuro-oncology.

**08:30 - 10:00** **Room F1**

### E³ - European Diploma Prep Session

### E³ 1723

### Musculoskeletal

**A-1033** 08:30
**Chairperson's introduction**
F. Saez; *Barakaldo/ES (fersaez@yahoo.com)*

The musculoskeletal system is one of the important areas which form part of the European diploma in radiology. All radiologists should understand and become familiar with the imaging appearances of the different pathologies that may involve the MSK system. This session will be very valuable in this regard, as the speakers will deal with most of the MSK topics which are part of the European training curriculum, levels I and II.
***Session Objectives:***
1. To understand typical and atypical imaging features of traumatic disorders of the musculoskeletal system.
2. To learn typical imaging features of benign and malignant bone tumours.
3. To become familiar with the imaging appearance of degenerative and inflammatory disorders of the musculoskeletal system.

**A-1034** 08:36
**A. Traumatic disorders of the musculoskeletal system**
L. Meacock; *London/UK (lisameacock@nhs.net)*

Musculoskeletal trauma is regularly encountered in the Emergency Department. Certain injuries have challenging imaging appearances or may have associated soft tissue injuries the Radiologist should be alert to. Failure to recognise or misinterpretation of these conveys significant risks. This case-based lecture will focus on imaging characteristics of common and the more challenging appendicular musculoskeletal injuries, with an emphasis on appropriate imaging and understanding of the underlying mechanism of injury.
***Learning Objectives:***
1. To gain appreciation of typical fracture patterns and their accompanying soft tissue injuries.
2. To develop an understanding of the underlying mechanisms of injury in appendicular trauma and correlate with imaging findings.

3. To understand the benefits and disadvantages of different imaging modalities in assessing trauma involving the skeleton and soft tissue in differing age groups.

**A-1035** 09:04
**B. Bone tumours**
M.-A. Weber; *Rostock/DE (marc-andre.weber@med.uni-rostock.de)*

Primary bone tumours are categorised according to their tissue of origin into cartilage, osteogenic, fibrogenic, fibrohistiocytic, haematopoietic, vascular, lipogenic tumours and several other tumours like Ewing sarcoma and giant cell tumour. Also, they are classified into benign, malignant and semi-malignant, as well as tumour-like lesions. They are rare, but found on radiographs during an investigation of a painful skeletal region or incidentally. The radiograph is the first method to distinguish benign from malignant lesions: at first by analysing the aggressiveness of a lesion according to the classification of Lodwick and second by analysing the mineralisation of the tumour matrix. The matrix may be osteolytic, osteoblastic, or mixed: osteolytic with matrix mineralisation. Based on the Lodwick classification an overview of the three main types of bone destruction patterns visible on radiographs will be given with many examples: type 1: geographic (with a: well-defined border with sclerotic rim, b: well-defined border without sclerotic rim, c: ill-defined border); type 2: geographic with moth-eaten or permeated pattern; type 3: small, patchy, ill-defined areas of lytic bone destruction with moth-eaten or permeated pattern. Periosteal reactions are also indicators of lesion aggressiveness and will be demonstrated. MRI is the best additional imaging modality for local staging, as it allows for accurate assessment of the extent of the disease and the effect of the tumour on the surrounding structures including the joint, neurovascular structures, muscle compartments and skin. MRI also gives details of the extent of the compartmental involvement to help complete excision of the tumour.
***Learning Objectives:***
1. To learn the typical imaging features of common bone tumours.
2. To understand the typical imaging features of "don't touch" ("leave-me-alone") lesions.
3. To become familiar with the imaging manifestations of haematological disorders.

**A-1036** 09:32
**C. Degenerative and inflammatory disorders of the musculoskeletal system**
J.-B. Pialat; *Lyon/FR (jean-baptiste.pialat@chu-lyon.fr)*

Imaging findings of degenerative disorders of the joints are often found, but the correlation to clinical symptoms is not straightforward. After a descriptive approach of radiographic, computed tomography (CT) and magnetic resonance imaging (MRI) semiology, the clinical relevance of these findings will be discussed in the light of actual evidence. Considering the spine, degenerative disorders of lumbosacral and cervical levels are the most clinically relevant. All structures are affected with a variable association of ageing disk degeneration and bulging, bony spurs of vertebral bodies, and osteoarthritis of the face joints (zygarthrosis). These lead to an extensive range of local and radicular pain, or less frequently, to a progressive compression of the spinal cord or of the cauda equina roots resulting in specific neurologic deficits. The thoracic spine is usually considered less often in the spectrum of symptomatic degenerative disorders, but imaging findings are common. These degenerative disorders have to be considered as potential factors in the genesis of pain in patients with ageing static impairments focussing on destabilisation due to vertebral compressive fractures. A comprehensive approach to imaging manifestations of infection, inflammation and metabolic diseases of the musculoskeletal system will end the lecture. Particular focus will be made on the role of radiography, CT, MRI and nuclear medicine techniques to explore these pathologies. An emphasis will also be given to specific findings, which will guide the radiologist in his diagnosis, and will help him rule out some erroneous aetiologies.
***Learning Objectives:***
1. To understand the imaging presentation of degenerative disorders of the joints and to appreciate their clinical relevance.
2. To learn the imaging features and clinical features of degenerative disease of the spine, disc and facet joints.
3. To become familiar with the typical imaging manifestations of infection, inflammation and metabolic diseases of the musculoskeletal system.

**08:30 - 10:00** **Room F2**

### Emergency Imaging

### RC 1717

### Small bowel obstruction: improve your reading and reporting competences

**A-1037** 08:30
**Chairperson's introduction**
S. Romano; *Naples/IT (stefromano@libero.it)*

The small bowel obstruction represents one of the most common causes of the acute abdomen in patients arriving in the Emergency Departments. The introduction to the session presentations will be focused on a brief excursion on definitions and signs and on the main role of the radiologist involved in reporting the exams in giving essential and important information to the clinicians and surgeons requested for a correct therapeutic planning on the patient. The role of the MDCT and of the DECT will be also shortly noted and considered.
***Session Objectives:***
1. To learn how to confidently diagnose small bowel obstruction.
2. To understand how to identify the transition point rapidly.
3. To become familiar with the signs of ischaemia.

**A-1038** 08:35
**A. Step 1: confirm the mechanical small bowel obstruction (SBO)**
I. Millet; *Montpellier/FR (milletingrid@wanadoo.fr)*

A small bowel obstruction is defined by an intestine transverse diameter greater than 25mm. It can be due either to a focal obstacle on the bowel that prevents the normal alimentary bolus flow or to a global stop of the bowel peristalsis. It is then very important to differentiate those two types of SBO, respectively a mechanical SBO from a functional ileus because they don't have the same management nor prognosis. We used the term "mechanical" SBO when there is a transition zone between dilated upstream loops and collapsed downstream ones. To localise precisely the transition zone, multiplanar reconstructions and particularly the coronal view can be very helpful. The faeces-sign, defined as gas bubbles mixed with particulate matter in dilated segments of small bowel, when present (about 40% of cases) can help to identify the transition zone. However, the faeces-sign should not be used to guide treatment decisions.
***Learning Objectives:***
1. To differentiate a functional ileus from a mechanical SBO.
2. To know the diagnostic value of the feces-sign for depicting the transition zone.
3. To become familiar with multiplanar reconstructions for finding the transition zone.

**A-1039** 09:02
**B. Step 2: identify the SBO cause**
J.R. Olalla Muñoz; *Murcia/ES (olalla.jos@gmail.com)*

Small bowel obstruction (SBO) is a common clinical syndrome accounting for 12-20% of hospital admissions of acute abdominal pain and supposes a leading cause of surgical emergencies. Adhesions, together with hernias and malignancies, entail more than 80% of SBO aetiologies and remain the major cause of post-operative SBO. Current evidence-based guidelines establish CT the preferred imaging technique for the diagnosis of adhesive SBO (ASBO), especially in doubtful cases to exclude other cause of obstruction. The diagnosis of ASBO is primarily one of exclusion because adhesive bands are not generally seen at conventional CT; only an abrupt change or beak at the site of obstruction combined with kinking and tethering of adjacent bowel suggests the diagnosis. Recently, CT has been proved to be accurate as a patient-selection tool into operative versus nonoperative treatment groups. Several CT findings like the location of the transition zone, the presence of faeces sign, beak sign, whirl sign, a closed-loop configuration, and a high-grade SBO must be assessed because they are related to the success or failure of nonsurgical treatment. Other causes of localised mural thickening in the transition zone are Crohn disease, neoplasia, intussusception, radiation, hematomas, endometriosis. Finally, gallstone ileus, foreign bodies, or bezoar constitute rare causes of SBO but with an easier radiological diagnosis.
***Learning Objectives:***
1. To know the CT findings allowing adhesive bands to be diagnosed.
2. To know the list of SBO causes with bowel wall thickening at the transition zone.
3. To become familiar with some rare causes of SBO.

**A-1040** 09:29
**C. Step 3: look for bowel ischaemia**
R. Smithuis; *Leiderdorp/NL (rsmithuis@cistron.nl)*

The most common cause of small bowel obstruction is an adhesion. An adhesion can obstruct the bowel in two ways. The most common is when the adhesion crosses the bowel leading to a simple obstruction. Ischemia will be an uncommon finding since the bowel proximal to the obstruction has a normal vascularisation. Things are quite different when a loop of small bowel crawls under an adhesion. Due to peristalsis more bowel may pass under the adhesion until it gets stuck resulting in a closed-loop obstruction. This always leads to venous obstruction and strangulation with ischemia. In any small bowel obstruction, it is the job of the radiologist to determine on CT whether it is a simple obstruction for which conservative treatment is the best option or a closed loop obstruction which needs immediate surgery. Closed loop obstruction can be diagnosed by looking for a cluster of bowel loops with mesenteric oedema and two points of obstruction. CT-scanning is done in the late arterial phase after delivery of intravenous contrast at a rate of 5 ml/sec for optimal bowel wall enhancement. Do not give oral contrast.
***Learning Objectives:***
1. To optimise the CT protocol for assessing bowel wall enhancement.
2. To learn how to diagnose a closed-loop mechanism.
3. To know the CT findings for diagnosing ischaemic complication.

09:56
**Panel discussion: How important is the role of CT in deciding on operative or non-operative patient management?**

**08:30 - 10:00** **Room Y**

### E³ - Rising Stars Programme: EFRS Radiographers' Basic Session

### BR 17

### The future of radiology: what it could mean for new radiography graduates

**A-1041** 08:30
**Chairperson's introduction: What will radiology departments look like in the future?**
G. Paulo; *Coimbra/PT (graciano@estescoimbra.pt)*

It’s well known that the technological evolution and new scientific progress have increased the organisational complexity of healthcare. One of the major contributors to this complexity increase is the influence of medical imaging technology development. There is no doubt that modern healthcare is every day more and more dependent of medical imaging diagnostic and therapeutic procedures, which dramatically contributes to the increase of hospital efficiency, providing more effective and less invasive patient care. The development of medical technology, together with the several pressures on health systems are the potential drivers for the exponential increase of the number of imaging procedures (but not always followed with the necessary increase of radiographers and radiologists). Although this might be seen, in general, as a very positive aspect, reality shows that medical imaging is being seen more as a commodity and therefore there is an urgent need to develop a new paradigm for medical imaging departments, to maintain our identity. The development of a European Radiology Model, based on strategic management tools and professional role development are the key points to maintain the medical imaging procedures under the supervision of the radiological family and increase the visibility and the importance of radiology in health systems, based on a value-based healthcare model. Appropriateness criteria combined with audit and accreditation are the key to the development of a modern European radiology model.
***Session Objectives:***
1. To consider the drivers for change within radiology departments.
2. To appreciate the brief history of evolution within radiology departments.
3. To consider how radiology departments may look in future years.

**A-1042** 08:35
**Future role extension for radiographers**
H.H. Hjemly; *Oslo/NO (Hakon@radiograf.no)*

Development of the professional role of radiographers in the Nordic region has been substantial since the profession was established in the early 70s. In parallel with the development and academising of radiography education, there has been a gradual change in the tasks previously performed by other groups, as well as the continuous supply of new tasks as a result of the significant advances in computing and technology in diagnostic imaging and radiation therapy. My presentation will focus on the current status of the scope of practice for radiographers in the Nordic region, educational possibilities and projects. Based on research done by The Nordic Institute for studies in Innovation Research and Education (NIFU) I will present recommendations on role Development, drivers and barriers for radiographers to take greater responsibilities in diagnostic imaging.
***Learning Objectives:***
1. To understand the current status of role extension for radiographers.
2. To be aware of the international drivers for role extension within radiographic practice.
3. To appreciate the possible development options for radiographer role extension.

**A-1043** 08:53
**Evolving imaging technologies**
J.G. Stowe; *Dublin/IE (john.stowe@ucd.ie)*

With the explosion of Artificial Intelligence (AI) a review of what AI is and is not is highly appropriate at this time. We sometimes ascribe too much value and trust to that ‘intelligence' and a reminder that computers are essentially counting machines should be first and foremost. Computer-aided detection (CAD) is not new but with advances in the use of neural networks and machine learning we can produce creative and successful advances in this area. In the newest generation AI offerings, what we are talking about is advanced pattern recognition, and we must be careful that bias in the training of the 'machine learning’ does not result in flaws in what could otherwise be pattern recognition that is equivalent to if not superior to humans. A review of the number and frequency of press releases and publications is a testament to the growth and importance of this technology. Disseminating of diagnostic imaging information is also a challenge and an area of continuing research and development. There is a tendency to use software that is traditionally intended for social media use however this brings into question ethics, regulatory compliance and governance. Software platforms are evolving to provide credible and secure repositories for the storage, control and tracking of the way we disseminate information rather and we must be cognizant of the issues, and acceptable practice of the management of this data in this new age of evolved data protection regulation as accountability has never been higher.
***Learning Objectives:***
1. To be aware of impending developments with regards to imaging equipment.
2. To consider the potential role for artificial intelligence (AI) within imaging.
3. To appreciate the future role of social media and computing within radiology departments.

**A-1044** 09:11
**Changing the delivery of radiology, responding to the needs of our patients**
K.G. Vikestad; *Oslo/NO (karivi@oslomet.no)*

The continuous emerge of new technologies; together with increasing age of patients and number of examinations being performed represents a big challenge within radiology. This has led to delays in the interpreting and reporting of x-rays, meaning that it takes a long time from examinations is being performed until reports with the results of the patients' x-rays are ready for the referring doctor. Congestion of unreported x-rays represents one of the biggest challenges within the delivery of radiology services today. This could result in delays in patients receiving important treatment, thus putting patients at risk. Shortage of radiologists is also contributing to delays in the delivery of radiology services. Further, with the advanced technology within radiology may lead to radiologists prioritise the more advanced examinations, meaning that the skeletal x-rays are given less priority. Educating and training radiographers to perform reporting, particularly skeletal reporting, is one way to reduce the congestion of unreported x-rays. Radiographers are experts in viewing x-rays, and with proper education and training, they could perform reporting at a comparable level to the radiologists. Reporting radiographers could thus solve the bottleneck of radiology service delivery, resulting in effective and correct reporting of x-rays and shorter response time for patients.
***Learning Objectives:***
1. To be aware of the current challenges facing radiology service delivery.
2. To consider the opportunities available for developing and improving the delivery of radiology services.
3. To appreciate the needs of patients when developing a radiology service.

**A-1045** 09:29
**What will radiology look like in 30 years?**
C. Beardmore; *London/UK (CharlotteB@sor.org)*

The provision of high quality efficient personalised services for patients is the priority for imaging and diagnostics services, with services focused around the patient; this will continue to be our goal. A team working utilising all the available proven technologies will be essential, much of the technical aspects of the role will be automated; radiographer practitioners will need to offer flexibility, creativity and continually adapt their role as robotics capability continues to advance rapidly. Research must continue to be a core part of the radiographer's professional role in order to ensure that the rapidly changing innovations/robotics can be researched and translated into practice efficiently, where there is evidence to demonstrate a benefit for patients. The development of the radiography profession must, therefore, adapt to the changing context of this environment but ensure that research skills remain embedded within the professional education and training in order to ensure that the patients receive personalised care including the changing technological capabilities of equipment including Artificial intelligence and precision medicine. Robotics and increasing availability of vast amounts of data will enable ongoing change. The radiographers' role will change, but the radiographer must remain the patient facing carer across the service supporting patients during their diagnosis and onto their next steps along their personalised pathway of care. Their role needs to be considered within the context of the entire imaging team to ensure that the development of their skills and competencies effectively enable delivery of excellent patient care.

***Learning Objectives:***
1. To understand the key drivers for change within radiology.
2. To understand the possible areas for development over the next 30 years.
3. To appreciate how radiology may look in 2049.

09:47
**Panel discussion: Development needs for the future radiographer**

**08:30 - 10:00 Room D**

### Musculoskeletal

### RC 1710

### Elbow imaging: from detailed anatomy to pathology

*Moderator:*
I. Sudoł-Szopińska; Warsaw/PL

**A-1046** 08:30
**A. The medial and lateral epicondyle**
M.O. De Maeseneer; *Brussels/BE (michel.demaeseneer@uzbrussel.be)*

Bony landmarks at the lateral epicondyle include the tubercles, intertubercular sulcus, supracondylar ridge, and epicondylar ridge. Tendons include the brachioradialis, ECRL, ECRB, EDC, EDM, ECU and anconeus. Ligaments include the radial collateral ligament, annular ligament and LUCL. Pathological conditions include ligament tears, tendinosis, tendon tears and posterolateral instability. Bony landmarks at the medial epicondyle include the tubercles, intertubercular sulcus, epicondylar face and supracondylar ridge. Tendons include the pronator teres, FCR, Palmaris longus, FCU, FDS. Ligaments include the different components of the UCL. Pathological conditions include ligament tears, tendinosis, tendon tears, and VEO. Understanding bony landmarks at the epicondyles help understand tendon and ligament changes. Pathological conditions are then more easily assessed including tendinosis, tendon tears, ligament tears, VEO and posterolateral instability.

***Learning Objectives:***
1. To explain the anatomic considerations and pathophysiology of abnormalities that involve the medial and lateral epicondyles of the humerus.
2. To describe the imaging findings of abnormalities that involve the medial and lateral epicondyles of the humerus.

**A-1047** 09:00
**B. Biceps and triceps**
A. Tagliafico; *Genoa/IT (atagliafico@sirm.org)*

The biceps brachii muscle consists of two heads, the short head and the long head. The two muscle bellies can have some degree of interdigitation proximal to the distal tendon, but they have two separate tendons, one for each muscle at the radial tuberosity insertion. The tendon of the short head attaches distally and slightly anteriorly at the radial tuberosity, whereas the tendon of the long head attaches more proximally. Distal biceps insertion is reinforced by a thin fibrous structure called lacertus fibrosus. Common lesions of the biceps tendons at the elbow include complete ruptures with retraction of the muscle belly or partial tears with different imaging appearance. Partial tears can involve the short or the long head of the biceps brachii tendon. Imaging can shift the therapeutic management from surgical to conservative especially in patients with partial tears. Other disorders related to the distal biceps brachii muscle include impingement and bicipito-radial bursitis. Brachialis muscles injuries are extremely rare. Triceps tendon ruptures can be acute traumatic or more commonly chronic overuse causing degenerative changes to the insertion. Rupture of the distal triceps tendon is uncommon and it may be unrecognised on clinical examination. The most common disruption is an avulsion from the osseous tendon insertion. MRI and US are useful in preoperative planning because it shows whether the rupture is complete or partial. Sometimes, triceps muscle may be a factor contributing to ulnar nerve luxation at the elbow.

***Learning Objectives:***
1. To explain the anatomic considerations and pathophysiology of abnormalities that involve the biceps and triceps brachii.
2. To describe the imaging findings of abnormalities that involves the biceps and triceps brachii.

**A-1048** 09:30
**C. Plica and cartilage**
L. Cerezal; *Santander/ES (lcerezal@gmail.com)*

Synovial plicae are folds of synovial tissue, remnants of embryonic septae of the normal articular development. Plicae have not well-known function and are usually asymptomatic. Chronic inflammation secondary to direct trauma, repetitive sports activities or surgical procedures of the elbow affects the elasticity of the synovial folds and can become symptomatic, sometimes termed elbow synovial fold syndrome, or posterolateral impingement. With elbow motion, the thickened fibrotic plicae can irritate the synovium, leading to inflammatory synovitis and radial head or capitulum articular cartilage wear. The painful snapping of the elbow joint is the most common clinical manifestation. This condition is not uncommon and commonly misdiagnosed as lateral epicondylitis. MRI and CT or MR arthrography are useful tools in the diagnosis of elbow synovial fold syndrome and for exclusion other causes of lateral elbow pain and snapping. Cartilage injuries usually affect the weight-bearing joints, such as the hip and knee and the elbow is one of the least affected joints. Chondral injuries of the elbow usually occur secondary to a previous injury such as elbow dislocation, fractures or chronic mechanical overload in manual workers, throwing sports or people using crutches and wheelchairs. Morphological MRI allows a precise assessment of macroscopic lesions of the articular cartilage. Furthermore, advanced MRI techniques also enable evaluation of the biochemical or ultrastructural composition of articular cartilage and detect early chondral damage.

***Learning Objectives:***
1. To explain the anatomic considerations and pathophysiology of abnormalities that involve the elbow plicae and articular cartilage.
2. To describe the imaging findings of abnormalities that involve the elbow plicae and articular cartilage.

**08:30 - 10:00 Room G**

### Physics in Medical Imaging

### RC 1713

### Dose reduction and image quality implications of iterative image reconstruction in CT

**A-1049** 08:30
**Chairperson's introduction**
K.N. Bolstad; *Bergen/NO (kirsten.bolstad@helse-bergen.no)*

Ever since the development of the first CT, the quest has been to get as much information as possible, in the shortest amount of time as possible. In the early days of CT, the reconstruction time per image was limited by computer power and effective reconstruction algorithms. The filtered back projection algorithms (FBP) were a compromise between reconstruction speed and image noise. Iterative reconstruction algorithms (IR) takes into account a mathematical assumption of the system which results in less image noise but with a longer reconstruction time compared to FBP. Today, with more powerful and faster computers IR is a part of the daily routine. The noise reduction can either be used to improve image quality at the same dose or maintain similar image quality at a lower dose, typically 25-40% dose reduction compared to FBP. Recently, more advanced model-based iterative reconstruction algorithms have become commercially available with a reconstruction time suitable for the clinical routine. These algorithms take into account a mathematical model of the entire system, from the focal spot size and x-ray spectrum to the detector system, resulting in a nearly “noise-free” image. The dose saving potential for these newer algorithms is up to 80-90% compared to FBP. However, the application of IR does not come without a cost. Even though the image noise is reduced, the appearance of the image may change. Each vendors IR has a different appearance and different dose saving potential. Radiologists need time to get used to the new appearance.

***Session Objectives:***
1. To learn about the origins of dose reduction using iterative image reconstruction in CT.
2. To understand dose reduction using iterative image reconstruction in CT.
3. To learn about solutions and workarounds.

**A-1050** 08:35
**A. Basics of iterative image reconstruction in CT**
M. Kortesniemi; *Helsinki/FI (mika.kortesniemi@hus.fi)*

Computed tomography image reconstruction is a mathematical process where the raw data projections (sinogram data) acquired during rotational CT scan exposure are transformed into volumetric (3D) image data. As such, reconstruction is an inverse problem, with no direct analytical solution. Traditional algorithms (filtered back-projection) approximate the true acquisition method and are prone to artefacts and noise. Iterative reconstruction methods approach the final solution gradually in steps and may take quantum statistics, physical properties and limitations of image acquisition more faithfully into account. Thus, a more accurate and correct outcome may be achieved. However, more detailed modelling of the actual CT acquisition system, physics, optics and object also increase the calculation time and related CPU requirements. Especially, the forward projection phase (calculation of simulated raw-data) on each iteration step is computationally intensive. More optimised reconstruction techniques with clinically acceptable reconstruction times are under active research. As the main diagnostic benefit, higher image quality in terms of lower noise and artefacts can be achieved by using iterative methods, and/or a lower radiation dose. However, users should be aware that current iterative reconstructions potentially alter the image texture in different clinically relevant contrast and detail levels. Therefore, sufficient clinical image quality should be verified when applying iterative techniques on new exam indications and protocol optimisation.
***Learning Objectives:***
1. To learn about the basic aspects of iterative reconstruction.
2. To learn about potential dose reduction via iterative reconstruction.
3. To compare iterative reconstruction with other techniques.

**A-1051** 08:58
**B. Iterative image reconstruction in clinical practice: dos and don'ts**
H. Alkadhi; *Zurich/CH (hatem.alkadhi@usz.ch)*

The application of Computed Tomography (CT) imaging is paralleled by concerns regarding its potential risks. These concerns have led to the development of a set of strategies for optimising and lowering the radiation dose of CT. Of these techniques, iterative reconstruction algorithms represent a powerful tool, because they allow lowering the dose at a constant noise level and image quality. However, caution must be taken when iterative reconstruction algorithms are being used, and radiation dose can be lowered only to a level where information in the CT images are not lost. This presentation reviews the clinical challenges of using iterative reconstruction techniques in CT imaging, with a focus on low radiation dose examinations. The benefits and potential pitfalls of iterative reconstruction techniques are discussed in this presentation.
***Learning Objectives:***
1. To understand the radiologist's requirements for image reconstruction.
2. To learn about current best practice in image reconstruction for clinical CT.
3. To learn about the potential benefits and pitfalls of using iterative reconstruction in clinical CT.

**A-1052** 09:21
**C. Image quality assessment of iterative reconstruction: pitfalls and future directions**
C. Walsh; *Dublin/IE (clwalsh@stjames.ie)*

Iterative reconstruction (IR) algorithms offer the potential to provide diagnostic quality scans at a lower dose. IR is significantly different to the previously used Filter Back Projection technique, both in the way images are reconstructed, and in how the software is implemented on CT systems. Manufacturers have different versions of IR software, new IR products appear on the market at a relatively fast rate, and some versions have a high degree of user configurability. To exploit the potential dose savings, centres need confidence that diagnostic image quality is maintained as dose decreases. While dose measurements are comparatively straightforward, the standard physical image quality tests have limitations when assessing clinical image quality, particularly for IR images. For example, non-anthropomorphic test objects have limited clinical relevance or may interact sub-optimally with software designed to produce anatomical images. Some image quality metrics have a technical requirement that systems are linear shift invariant, which is not met by nonlinear IR algorithms. This presentation reviews the pitfalls of using existing image quality tests and overviews some of the developments which provide improved utility for assessing and optimising iteratively reconstructed images. These include developments to standard image quality measurements, and task-based approaches using model observers. The practical aspects of implementing image quality controls in an increasingly sophisticated technological environment are considered. The role of clinical assessment of image quality, and whether the analysis of ‘big data’ can contribute to IQ assessment in the future, is also discussed.
***Learning Objectives:***
1. To learn about basic image quality metrics employed in CT.
2. To understand why image quality assessment is difficult with iterative reconstruction.
3. To learn about up-and-coming methods for image quality assessment.

09:44
**Panel discussion: How low can we go?**

**08:30 - 10:00** **Room K**

### Chest

### RC 1704

### Thoracic emergencies

*Moderator:*
B. Ghaye; Brussels/BE

**A-1053** 08:30
**A. Chest trauma**
M. Brink; *Nijmegen/NL (M.Brink@rad.umcn.nl)*

Chest injuries are important contributors to mortality in major trauma patients. Although chest X-ray can be used as a screening tool in patients with life-threatening airway-breathing or circulatory problems, MDCT is the modality of choice to detect and characterise thoracic injury. MDCT with intravenous contrast has a very high diagnostic accuracy for predominantly pulmonary and vascular injuries. In this session, we will discuss three entities with high impact on patient morbidity and mortality. Pulmonary contusion is a commonly reported entity in high-energy trauma patients but is frequently confused with other causes of pulmonary opacification in trauma patients. Diaphragmatic ruptures need immediate operative intervention, but these injuries are rare and can be missed at MDCT. Finally, major aortic injury has very high mortality if left untreated, and should immediately be recognised. However, accurate diagnosis and adequate treatment depend on appropriate patient selection for MDCT, type and aspect of aortic injuries, scanning technique, and knowledge of mimickers of aortic injury.
***Learning Objectives:***
1. To describe CT features of pulmonary contusion.
2. To learn about signs of diaphragmatic rupture.
3. To know when to suspect and how to confirm aortic injury.
*Author Disclosure:*
**M. Brink:** Grant Recipient; Canon Medical Systems. Speaker; Canon Medical Systems Europe.

**A-1054** 08:53
**B. Haemoptysis**
M.-P. Revel; *Paris/FR (marie-pierre.revel@aphp.fr)*

Haemoptysis represents a potentially life-threatening condition which requires urgent management and care. It is defined by the expectoration of blood during coughing, originating from the lower respiratory tract. The immediate risk is airway compromise in case of severe haemoptysis, whereas small haemoptysis can be less urgently managed. There are various definitions of severity, from 100 mL to 1 L of blood expectorated in 24 hours. The severity also depends on the underlying cause and cardiopulmonary status of the patient. For severe hemoptysis, it is essential first to localise the bleeding side, which can be shown by chest x-ray, demonstrating alveolar opacities of the right or left lung. If not, CT scan will help to localise the bleeding side by showing ground-glass opacities in non -dependent lung areas. Most bleedings are of systemic origin, involving the bronchial arteries or other enlarged systemic arteries, which can be treated by embolisation. Contrast-enhanced CT is recommended in stable patients, to help to depict the normal or ectopic origin of the bronchial arteries. More rarely, bleeding is due to damaged pulmonary arteries, showing false aneurysms of mycotic or traumatic origin or tumoral invasion. In exceptional cases, bleeding can be due to the bronchial fistulization of an aortic aneurysm, with a very poor prognosis.
***Learning Objectives:***
1. To learn about the main causes and mechanisms of massive haemoptysis.
2. To review the role of CT in the assessment of site and cause of bleeding.
3. To learn about the bronchial artery anatomy assessment on CT.

**A-1055** 09:16
**C. Diagnosing PE**
G. Aviram; *Tel Aviv/IL (aviramgalit@hotmail.com)*

Pulmonary embolism (PE) is a life-threatening diagnosis, which has an increased incidence with ageing. Since it has a non-specific clinical presentation, the initial approach when acute PE is suspected should be clinical pre-test probability assessment, using validated clinical decision rules.

If PE is "unlikely", negative D-dimer excludes PE. Recently the D-dimer cutoff level was adjusted to age. When PE is “likely” or if D-dimer levels are elevated above the new threshold, the patient should be referred for CT pulmonary angiography (CTPA). Technological advancements to improve CTPA quality with reduced radiation doses and contrast volume include lowering the kVp, noise reduction strategies, and spectral CT. Though CTPA is highly accurate, diagnosing isolated subsegmental embolism often poses diagnostic and therapeutic dilemmas. Acute PE patient's management can vary between admission to intensive care unit to immediate discharge for home treatment. It should be based on clinical, laboratory and imaging evaluation. The diagnostic CTPA data can contribute to immediate risk stratification. There are various CT parameters for PE severity assessment, among them, increased right to left ventricular diameter ratio, reflux of contrast to the IVC, and reduced left atrial volume, which will be discussed.

***Learning Objectives:***
1. To learn about optimising CT angiography indications.
2. To be aware of the causes of imaging inconclusiveness.
3. To learn how to identify signs of acute right ventricular dysfunction.

*Author Disclosure:*
**G. Aviram:** Research/Grant Support; My institution received a research grant from Philips Health Care.

**A-1056** 09:39
**D. Vascular intervention in thoracic emergencies**
I. Bargellini; *Pisa/IT (irenebargellini@hotmail.com)*

Over the past years, interventional radiological procedures have become essential for the prompt management of life-threatening thoracic vascular emergencies, thanks to their low invasiveness and increased availability. According to the European guidelines for the treatment of aortic diseases, Thoracic Endovascular Aortic Repair (TEVAR) represents the first-line treatment modality for the management of several thoracic aortic emergencies, including traumatic and spontaneous ruptures, complicated type B dissections, intramural hematomas and penetrating ulcers. In fact, the high technical success rate and the low morbidity and mortality rates enable prompt management in the emergency setting and do not preclude future surgical procedures, if needed. Likewise, bronchial and non-bronchial arteries embolization has become the treatment of choice for severe hemoptysis, after failure of medical and endoscopic treatment, with an immediate success rate > 70%. Safety of embolisation is guaranteed by superselective catheterisation of the bleeding vessels and by correct choice of the most adequate embolic agent. Finally, over the past years, catheter-directed thrombolysis and/or thrombus debulking have been proposed as safe and effective treatment options in high- and intermediate-high risk patients with pulmonary embolism. Specifically, the 2014 European guidelines consider catheter-direct revascularization as an alternative to surgical embolectomy for high-risk patients in whom full-dose systemic thrombolysis is contraindicated or has failed. For the success of all these procedures, however, it is essential for interventional radiologists to be adequately trained and skilled and to work within dedicated multidisciplinary teams, to share decisions and manage possible complications.

***Learning Objectives:***
1. To learn about bronchial embolisation for life-threatening haemoptysis.
2. To review endovascular procedures for management of severe PE.
3. To understand the role of the interventional radiologist in aortic trauma management.

**08:30 - 10:00** **Room M 1**

### E³ - ECR Master Class (Hybrid, Molecular and Translational Imaging)

### E³ 1726a
### Quantitative imaging in oncology

**A-1057** 08:30
**Chairperson's introduction**
J. O'Connor; *Manchester/UK (james.o'connor@manchester.ac.uk)*

Images are inherently heterogeneous. In oncology, there is much current interest in quantifying this spatial variation and using this information to derive ‘heterogeneity biomarkers’. At the within tumour level, these biomarkers tend to either identify sub-regions of a tumour that are markedly abnormal or characterise how heterogeneous each individual tumour is. Current research studies are establishing whether or not such biomarkers have value in monitoring tumour response to therapy, or in determining prognosis and prediction of outcome. In addition, alternate approaches compare the variation between multiple lesions in one individual and how this may inform personalised medicine. In this session, various quantitative imaging approaches are discussed that give rise to heterogeneity biomarkers, with emphasis on their current uses, pitfalls and future directions.

***Session Objectives:***
1. To understand the impact of tumour heterogeneity on diagnosis and treatment.
2. To learn the basics of quantifying heterogeneity in tumours.
3. To review the future impact of imaging heterogeneity in tumours.

**A-1058** 08:35
**A. Intra- and intertumoural heterogeneity and the impact for cancer diagnostics**
M. Eisenblätter; *Münster/DE (eisenblaetter@uni-muenster.de)*

Malignant tumours are biologically complex systems with spacially variable gene expression patterns, consecutively variable biochemistry, histopathology and macroscopically variable structure. In the tumour microenvironment, genetic variability of tumour cells meets external stressors and the host immune system. The result is a regional heterogeneity of stromal architecture, biological activity and expression of, e.g. chemokines and growth factors. Consecutively, the development of tumour vasculature, nutrient supply, cell growth and death etc. exhibit significant, regional variation. This variation occurs within a single tumour lesion, between a primary tumour and metastasis and of course and pronounced between tumour lesions - even of the same tumour type - in different individuals. Generally, a high degree of heterogeneity is associated with poor prognosis and is at the same time increasingly relevant for therapy selection and monitoring, due to ever more specific therapy approaches. Various imaging approaches allow for visualisation of spatial heterogeneity in tumours. However, recorded and documented information is often limited to lesion size and macroscopic heterogeneity. In this context, relevant information, silently acquired even during clinical standard examinations, is ignored and dismissed unused. A rigorous analysis of all radiologic imaging data can help to discover this hidden information and provide additive information on tumour biology, relevant for tumour therapy. This talk is intended as an overview of the different levels of tumour heterogeneity, experimental and established imaging approaches to assess tumour heterogeneity and the possibility to provide adequate biomarkers for state of the art tumour therapy by integrated image data analysis.

***Learning Objectives:***
1. To learn about tumour heterogeneity.
2. To review current strategies to explore biological heterogeneity.
3. To understand how this heterogeneity impacts diagnosis and treatment.

**A-1059** 08:53
**B. Quantitative image biomarkers for targeted tumour therapies**
R. García Figueiras; *Santiago de Compostela/ES (roberto.garcia.figueiras@sergas.es)*

Clinical imaging systems are a significant source of non-invasive imaging biomarkers (IBMs) that may reflect important biological properties of cancers. IBMs can provide quantitative information on tumour hallmarks and can be used to evaluate tumour heterogeneity. They may also help us to understand the mechanism of action of therapies and their effects on tumour microenvironment, offering objective measures of change in response to therapy. This presentation will explain the biologic basis for a number of commonly available IBMs obtainable clinically. Beside this, we´ll illustrate how quantification of IBMs is undertaken and provide the validation of such measurements. Finally, the role of IBMs for the assessment of therapy response will be reviewed.

***Learning Objectives:***
1. To learn how heterogeneity can be quantified from images.
2. To understand the main classes of heterogeneity on imaging.
3. To review limits and pitfalls of features extraction.

**A-1060** 09:11
**C. From quantitative imaging to radiomics and deep learning**
H.K. Hahn; *Bremen/DE*

"no abstract submitted"

***Learning Objectives:***
1. To learn how to optimise the acquisition of images for quantitative imaging.
2. To understand image processing which may impact quantification.
3. To become familiar with the concepts behind quantification including machine learning approaches.

**A-1061** 09:29
**D. Imaging heterogeneity and genomic variability in ovarian cancer**
E. Sala; *Cambridge/UK (es220@cam.ac.uk)*

Cancer is caused by genetic (DNA) and epigenetic alterations and frequently arises as a clonal growth from a founder cell. The subclonal heterogeneity provides the basis for inter-metastatic heterogeneity which is of utmost clinical importance. New tumour sampling techniques and circulating tumour DNA methods may allow for more comprehensive evaluation of clonal composition.

As both primary tumours and metastatic lesions are spatially and temporally heterogeneous, they would require multiple biopsies to extract and analyse small portions of tumour tissue, which still doesn't allow for a complete characterisation of the tumour genomic landscape. Therefore, imaging has great potential for a comprehensive evaluation of the entire tumour burden in ovarian cancer as it is noninvasive and is already often repeated during treatment in routine practice, on the contrary of genomics or proteomics, which are still challenging to implement into clinical routine. While initial retrospective studies linking phenotype with genotype in ovarian cancer have shown a high prognostic power, they do not provide any spatial information as quantitative imaging features are generated and averaged over the entire tumour assuming that tumours are heterogeneous but well mixed. This approach ignores spatial heterogeneity readily apparent on imaging. Indeed recent genomics work has highlighted the presence of intratumor variation in gene mutation and expression. However little effort is any has been put into integrating imaging, histopathology and genomics and thus there is a clear need for well designed prospective studies focused on the meaningful integration of phenotype and genotype rather than genomics in isolation.

***Learning Objectives:***

1. To learn about the application of imaging tumour heterogeneity in ovarian cancer.
2. To understand how heterogeneity on imaging may relate to biological heterogeneity.
3. To become familiar with concepts such as imaging phenotypes and signatures.

09:47

**Panel discussion: What can we quantify and why is it essential?**

**08:30 - 10:00 Room M 4**

### Multidisciplinary Session

### MS 17

### Multidisciplinary approach to the diabetic foot

**A-1062** 08:30

**Chairperson's introduction**

E.N. Brountzos; *Athens/GR (ebrountz@med.uoa.gr)*

Diabetes mellitus (DM) has become a global epidemic. According to the World Health Organization, the number of people with diabetes increased from 108 million in 1980 to 422 million in 2014, while the worldwide prevalence of diabetes in 2013 was 8.3%. Moreover, 1.6 million deaths were directly caused by diabetes in 2016, as DM is a major cause of cardiovascular events and advanced peripheral arterial disease leading to limb amputations due to diabetic foot ulceration (DFU). The management of DFU requires a comprehensive multidisciplinary approach which should include radiologists, diabetologists, vascular surgeons, interventional radiologists. Prompt differential diagnosis between pure neuropathic or mixed neuro-ischemic DFU is essential for limb salvage, as major amputation rates are particularly high without rapid revascularisation. Following the clinical assessment, imaging is vital in order to verify the presence and assess the magnitude of arterial disease but also to evaluate soft tissue and bone infections. Once the diagnosis of vascular involvement is set, and the appropriate drug therapy has been administrated, revascularisation by open surgical bypass or peripheral endovascular procedures should be offered immediately, to enable fast wound healing and avoid infection that would compromise the limb. Both surgical and endovascular treatment options result in high limb salvage rates at long-term follow-up, and the decision between the two methods should be case-sensitive. Recent advancements in endovascular procedures have significantly broadened the indications and increased their efficacy. Following successful reperfusion, proper wound care, drug therapy and strict clinical and imaging follow up are fundamental for limb salvage.

***Session Objectives:***

1. To appreciate the magnitude of the diabetic foot as a societal problem.
2. To understand the role of radiological imaging and interventional radiology in the diagnosis and treatment of the diabetic foot.
3. To learn the role of surgical treatment of the diabetic foot.

**A-1063** 08:35

**Diabetic foot: a societal problem**

V. Lambadiari; *Athens/GR*

"no abstract submitted"

***Learning Objectives:***

1. To appreciate the scope of the problem.
2. To learn the prevalence of the arterial ulcers and the amputation risk.
3. To learn the prevention and care of foot ulcerations.

**A-1064** 08:50

**Imaging evaluation of the diabetic foot**

O. Papakonstantinou; *Athens/GR (sogofianol@gmail.com)*

Soft tissue and bone infections are common complications in diabetic foot that can be underdiagnosed leading to delay of treatment and, finally, amputation. Imaging findings and techniques, potential and limitations of plain radiographies, CT, US, scientigraphy, and, with a special focus, MRI will be discussed, aiming at the differential diagnosis between soft tissue and bone infection, between neuropathic osteoarthropathy and osteomyelitis as well as identification of complications such as abscesses, fractures and infarcts. Subsequently, we will touch upon the capabilities of high-resolution MR imaging techniques to provide an accurate mapping of the run-off vessels, which will eventually determine which patients further need DSA and therapeutic interventions.

***Learning Objectives:***

1. To learn how to image soft tissue and bone infections.
2. To learn how to image arterial perfusion: CTA and MRA.
3. To learn when to use DSA.

**A-1065** 09:05

**Interventional radiological treatment (IR)**

S.C. Spiliopoulos; *Athens/GR (stavspiliop@med.uoa.gr)*

Endovascular treatment is today an established option for both critical limb ischemia (CLI) and lifestyle limiting intermittent claudication in subjects with diabetes mellitus (DM). Particularly in patients with tissue loss, prompt revascularisation is highly important to improve limb perfusion, facilitate wound healing and attain limb salvage avoiding major amputation-associated morbidity and mortality. The technological advancements in the field of endovascular devices, as well as the continually increasing experience in endovascular techniques, have broadened the indications of endovascular therapies and have made possible the successful treatment of even small-vessel infrapopliteal and inframalleolar arterial disease (PAD), typical in subjects with diabetic foot disease. Angioplasty and stenting are the main tools for the management of the peripheral arterial disease. Limb salvage rates reported in the literature reach up to 90% at ten years follow up highlight the long-term efficacy of endovascular treatment. However, mid-term loss of patency resulting in clinical relapse and frequent re-interventions is the most significant drawback of endovascular treatment. Drug-eluting technologies- mainly drug-eluting stents and drug-coated balloons- significantly improve patency rates and clinical outcomes according to data from multiple multicenter randomised trials, while new technologies such as percutaneous atherectomy and shock-wave lithoplasty are currently under investigation. Tissue perfusion modalities in order to evaluate and monitor the outcomes of endovascular procedures are currently under development, while the impact of angiosome-guided revascularisation and pedal arch reconstruction on wound healing requires further investigation. Well-designed, multicenter, prospective trials are necessary to investigate the impact of novel endovascular technologies in PAD patients with DM.

***Learning Objectives:***

1. To learn when interventional treatment is indicated.
2. To learn the tool kit.
3. To learn the results of IR treatment.

**A-1066** 09:20

**Surgical management of the diabetic foot**

G. Geroulakos; *Athens/GR (ggeroulakos@med.uoa.gr)*

Limb loss is a significant risk in patients with diabetic foot ulcers. It is estimated that 15% to 20% of patients with diabetes will develop foot tissue lesions ranging from simple cellulitis through ulcer and abscess to osteomyelitis and gangrene. About 15% to 20% of patients with foot ulcers will need an amputation. The surgical strategy has several aims. The first step is to control the infection by a combination of appropriate antibiotic therapy with drainage of pus, removal of necrotic tissue and minor amputation if needed. The second step involves assessment of the limb ischemia. Lack of arterial blood flow reduces the supply of oxygen and nutrients, the delivery of antibiotic agents and impedes wound healing. Although arterial disease in diabetic patients typically affects the distal vessels, preoperative imaging often reveals multi-level occlusions and diffuse disease, which necessitates challenging distal revascularisation either endovascularly or by surgery. Endovascular revascularisation is more appropriate in patients with relatively focal disease in arteries above and below the knee. Bypass to the tibial or pedal vessels with autogenous vein is the gold standard for patients with diabetes and complex tibial disease and has greater durability than endovascular procedures. Primary amputation is indicated in case of overwhelming infection that threatens the patient's life, when tissue necrosis secondary to arterial disease has destroyed the foot or when a prolonged course of treatment is associated with a poor prognosis for a successful outcome.
***Learning Objectives:***
1. To learn what the goal of surgery is in treating the diabetic foot infections.
2. To learn how to prevent osteomyelitis.
3. To learn when to amputate and to what extent.

**A-1067** 09:35
**Multidisciplinary case presentation and discussion**
E.N. Brountzos; *Athens/GR (ebrountz@med.uoa.gr)*

Diabetes mellitus (DM) has become a global epidemic. According to the World Health Organization, the number of people with diabetes increased from 108 million in 1980 to 422 million in 2014, while the worldwide prevalence of diabetes in 2013 was 8.3%. Moreover, 1.6 million deaths were directly caused by diabetes in 2016, as DM is a major cause of cardiovascular events and advanced peripheral arterial disease leading to limb amputations due to diabetic foot ulceration (DFU). The management of DFU requires a comprehensive multidisciplinary approach which should include radiologists, diabetologists, vascular surgeons, interventional radiologists. Prompt differential diagnosis between pure neuropathic or mixed neuro-ischemic DFU is essential for limb salvage, as major amputation rates are particularly high without rapid revascularisation. Following the clinical assessment, imaging is vital to verify the presence and assess the magnitude of arterial disease but also to evaluate soft tissue and bone infections. Once the diagnosis of vascular involvement is set, and the appropriate drug therapy has been administrated, revascularisation by open surgical bypass or peripheral endovascular procedures should be offered immediately, in order to enable fast wound healing and avoid infection that would compromise the limb. Both surgical and endovascular treatment options result in high limb salvage rates at long-term follow-up, and the decision between the two methods should be case-sensitive. Recent advancements in endovascular procedures have significantly broadened the indications and increased their efficacy. Following successful reperfusion, proper wound care, drug therapy and strict clinical and imaging follow up are fundamental for limb salvage.
***Learning Objectives:***
1. To learn how to clinically diagnose the diabetic foot.
2. To appreciate the role of imaging.
3. To understand how to select the patients for medical, interventional radiological and surgical treatment.

**08:30 - 10:00 Room M 5**

### Head and Neck

### RC 1708

### Imaging of eye and orbital pathologies

*Moderator:*
M.M. Lemmerling; Ghent/BE

**A-1068** 08:30
**A. Traumatic lesions of the eye and orbit**
A. Ailianou; *Neuchâtel/CH*

In this lecture, the commonest types of traumatic injuries of the eye and the orbit will be reviewed. The role of CT and MRI in the emergency setting will be emphasised when dealing with fractures and soft tissue injuries. The most common types of facial fractures involving the orbit (blow-out fractures, tripod fractures, Le Fort type II and III, latero-facial fractures, as well as pan-facial fractures) and their radiologic features will be mentioned. A systematic discussion will include associated injuries of the ocular muscles, globe, lacrimal sac, vascular structures, the base of the skull and dura, as well as cranial nerves. Emphasis will be given to what the clinicians need to know and how to report the radiologic findings in a structured way.
***Learning Objectives:***
1. To become familiar with imaging features of orbital fractures.
2. To learn about traumatic lesions of the eye and orbital soft tissues.
3. To review acute and chronic complications after orbital trauma.

**A-1069** 09:00
**B. Infection and inflammation in the eye and orbit**
K. Erb-Eigner; *Berlin/DE (Katharina.Erb@charite.de)*

Infectious and inflammatory diseases of the eye and orbit play a major role in ophthalmology. However, diseases manifesting posterior to the globe are not detectable by ophthalmoscopy, and cross-sectional imaging is often necessary for diagnostic work-up. Radiologists should be familiar both with the anatomy of the eye and orbit and with the imaging features of the most common infectious and inflammatory diseases. Magnetic resonance imaging (MRI) has excellent soft tissue contrast and is the first choice method in most cases. This Refresher Course will present typical MR examination protocols and outline the benefits of multiparametric imaging. Basic clinical features and typical imaging findings in infectious and inflammatory disorders of the orbits including optic neuritis, abscesses, sarcoidosis and idiopathic inflammatory disorders will be discussed. Other topics include emergency findings which require immediate communication and what to report to clinicians to achieve the best outcome for patients.
***Learning Objectives:***
1. To become familiar with infections and inflammation in different orbital structures.
2. To discuss imaging features of infection and inflammation in the eye and orbit.
3. To learn how to reach the final diagnosis.
*Author Disclosure:*
**K. Erb-Eigner:** Consultant; Author of Atlas on Orbital MRI at Pantavision.

**A-1070** 09:30
**C. Benign and malignant masses of the eye and orbit**
P. De Graaf; *Amsterdam/NL (p.degraaf@vumc.nl)*

Ocular and orbital masses in adults and children represent a spectrum of (rare) benign and malignant lesions that can be challenging to diagnose and treat. Most patients with intra-ocular masses are diagnosed on clinical grounds by means of extensive clinical investigation by the ophthalmologist. High-resolution MRI has emerged as an important imaging modality for pretreatment assessment, i.e. confirmation of diagnosis and staging disease extent. The role of CT is limited. Orbital mass lesions, usually presenting with proptosis and/or visual decline, need to be analysed by MRI in order to characterise the type of lesion. The most important intra-ocular and orbital mass lesions will be shown, and specific signs on imaging will be reviewed.
***Learning Objectives:***
1. To discuss advanced imaging techniques in tumoural orbit lesions.
2. To review intraocular tumours.
3. To become familiar with intraorbital masses.

**08:30 - 10:00 Tech Gate Auditorium**

### E³ - ECR Master Class (Genitourinary)

### E³ 1726b

### Prostate MRI: the accreditation issue

**A-1071** 08:30
**Chairperson's introduction**
J. Richenberg; *Brighton/UK (jonathan.richenberg@bsuh.nhs.uk)*

It is widely accepted that multi-parametric MRI (mpMRI) is 'good' in prostate cancer. But what does 'good' mean? How do we measure 'good'? And how do we make 'good' universal? This session considers a strategy to rapidly roll out mpMRI across Europe without sacrificing quality. Before acceding to the clamour for mpMRI, radiologists, urologists and oncologists need to acknowledge that mpMRI is complex: to acquire and to interpret pre-biopsy mpMRI means that radiologists become the praetorian guard of prostates pre-biopsy implies a 'yes'/'no' approach and specialists are more prepared to make such decisions (less PIRADS 3) The first talk explores optimisation of images and proposes standards on acquisition, the second talk explores the benefits of a multidisciplinary approach to prostate cancer diagnosis, the place of specialist centres and the role of the radiologist within this setting; the third talk addresses some of the aspects that surround the accreditation of individuals and centres in the reporting of mpMRI including the concept of on-going performance assessment. In this new era of cardiac imaging, virtual colonoscopy and now radiologically driven prostate cancer diagnosis, accreditation and standard setting becomes crucially relevant, and controversial as doubtless the panel discussion at the session's end will demonstrate!

***Session Objectives:***
1. To understand the emerging role of the multidisciplinary approach for prostate cancer.
2. To discuss the role of the radiologist in the multidisciplinary approach to prostate cancer.
3. To discuss the accreditation and certification issues for prostate imaging.

**A-1072** 08:35
**A. Prostate MRI: minimum and optimal requirements**
N.N.

***Learning Objectives:***
1. To understand the minimum requirements for multiparametric MR of prostate cancer.
2. To understand how to optimise MR imaging technique at 3T and 1.5T.
3. To review sequence parameters and scan optimisation for prostate imaging.
*Author Disclosure:*
**P. Puech:** Consultant; GE ultrasound.

**A-1073** 08:58
**B. Towards a European accreditation of prostate imaging centres**
V. Logager; *Copenhagen/DK (vibeke.loegager@regionh.dk)*

Imaging in itself is nothing. It is in the context of diagnosing and treating the patients that imaging becomes valuable and important. To ensure the best use of expensive equipment when finding the correct diagnose with the least delay, it is necessary to ensure high quality. The multidisciplinary team approach has the potential to ensure this. The expert panel discussions aim to give the optimal treatment to every patient at the right time. Also, an information feedback loop is arranged in this forum, when imaging, histology and patient outcome are held up against each other. Despite guidelines, quality in imaging is not guaranteed. It is necessary to ensure reproducibility and homogeneity in imagining of technical parameters, in scanning procedures, reading and reporting. Reference, specialist prostate centres could be a way to go. High-end centres that are involved in international research with a high volume of examinations, in co-operation with the clinicians, pathologists and oncologists, will gain substantial experience. This includes doing multidisciplinary team meetings. By having a reference centre, it could be possible to measure any institutions image quality and report validity against the international standards of care. To make this possible, it is necessary to agree upon suitable and measurable indicator parameters on quality. In order to know that, what you do, is in line with the standard of care level, we must decide how we most reliable can measure our contribution, from referral to examination and further to the finalised report.
***Learning Objectives:***
1. To learn about the role of the radiologist in the multidisciplinary approach for prostate cancer.
2. To discuss the requirements for a specialist prostate centre, with emphasis to the imaging issue.
3. To understand quality indicators for prostate MRI.

**A-1074** 09:21
**C. Towards a certified radiologist**
H.C. Thoeny; *Fribourg/CH (Harriet.thoeny@h-fr.ch)*

MRI of the prostate is increasingly performed in many centres before a biopsy. On one hand, this allows performing targeted biopsies in case of detection of a suspicious lesion and on the other hand to avoid biopsy in case of a normal prostate MRI. The correct diagnosis is therefore of utmost importance. False positive results lead to unnecessary biopsies including side effects, unnecessary fear and increasing costs, whereas false negative results lead to underdiagnosis of significant prostate cancers. A correct diagnosis is crucial, and besides correct image interpretation, the radiologist needs an in-depth understanding of prostate pathologies and knowledge of the potential treatment options. Strong collaboration in the multidisciplinary tumour boards together with urologists, pathologists, oncologists and radiation therapists with regular feedback is the prerequisite for quality control of the radiologist. Furthermore, level III European Training Curriculum for prostate imaging defines the expected knowledge of the radiologist performing prostate MRI. In many European countries certified prostate cancer centres exist, however, the exact role of the radiologist has to be defined and is becoming increasingly important. The European Society of Urogenital Radiology (ESUR) provides yearly specific teaching courses on prostate imaging allowing continuous education. In line with breast units, the certified radiologists performing prostate MRI need a certain number of exams every year, the exact minimum number has to be defined based on future studies as well as an evaluation based on feedback from pathology.
***Learning Objectives:***
1. To understand the need for a certified radiologist in a prostate unit.
2. To illustrate the level III European Training Curriculum for prostate imaging.
3. To discuss how to certify radiologists for prostate imaging.

09:44
**Panel discussion: Prostate units: is the radiologist in or outside the core team?**

**10:30 - 12:00** **Room A**

### E³ - ECR Academies: Interactive Teaching Session for Young (and not so Young) Radiologists

### E³ 1821
### Imaging of the brain

**A-1075** 10:30
**A. Stroke mimics**
M.M. Thurnher; *Vienna/AT (majda.thurnher@meduniwien.ac.at)*

The correct and prompt diagnosis of ischemic stroke and/or stroke mimics is crucial to assist clinicians in proper triaging and therapy decisions. A stroke mimic represents a non-stroke disorder with a presentation suggestive of acute ischemic stroke. The list of possible stroke mimics consists of seizures and postictal phenomena, headaches, metabolic disturbances, toxicities, and space-occupying lesions. A variety of non-neurological conditions such as systemic infection, cardiovascular events, and psychiatric issues may also masquerade as acute ischemic strokes. In this lecture, key-imaging features of stroke mimics will be discussed in detail.
***Learning Objectives:***
1. To become familiar with the different entities that can mimic a stroke.
2. To know the usefulness of perfusion studies in the differential diagnosis.

**A-1076** 11:15
**B. Acquired toxic-metabolic encephalopathies**
K. Karli Oguz; *Ankara/TR (karlioguz@yahoo.com)*

Acquired toxic-metabolic encephalopathies encompass a wide range of causes including metabolic causes like hypo-/hyperglycemia, hepatic and uremic encephalopathy, or osmotic demyelination, hypertension, exposure to toxic substances, or drug abuse. Clinical history is extremely important in the evaluation of the patient. Best recognised on MRI, these disorders tend to show bilateral and symmetrical involvement of the deep grey matter and cerebral white matter. Some peculiar locations, DWI features and clues from proton MR spectroscopy will guide the radiologists on the way to the diagnosis. In this session, participants will review and practice the imaging features of the most common acquired toxic-metabolic encephalopathies with representative cases.
***Learning Objectives:***
1. To review the most common causes.
2. To learn the MRI and CT appearances of these lesions.

**10:30 - 12:00** **Room B**

### ESR meets Pakistan

### EM 3
### The role of radiology in major healthcare challenges faced by Pakistan
*Presiding:*
M.H. Akram; Islamabad/PK
L.E. Derchi; Genoa/IT

**A-1077** 10:30
**Introduction**
M.H. Akram; *Islamabad/PK (drhamidakram@gmail.com)*

Infections and malignancies are two major health challenges being faced by Pakistan. Amongst the infections, though gastroenteritis, pneumonia, typhoid and malaria are not uncommon, the major challenges are hepatitis and tuberculosis mainly due to their chronic nature, a variety of presentations, complications and lengthy management. Regarding malignancies; though in Pakistani women the incidence of breast cancer is one of the highest in Asia, the most common cancer which involves a reasonable proportion of both sexes is cancer of the lip and oral cavity. To deal with these three major diseases, radiology is playing a crucial role, not only in diagnosis and early detection of complications but also in the management.

***Session Objectives:***
1. To learn about oral cancer in Pakistan and the role that radiology plays in its management.
2. To appreciate the role of radiology in living donor liver transplant in advanced cases of chronic hepatitis.
3. To understand the variety of radiological findings in tuberculosis.

**A-1078** 10:35
**Imaging of oral cancer**
N. Ud Din; *Lahore/PK (najam200@gmail.com)*

The actual burden of head and neck cancer in Pakistan is 18.74% of all new cancers. The incidence of oral cancer was highest in males, averaging 11% in 2012. The social and cultural habits across different provinces of the country seem to determine the increasing variable incidence of head and neck cancer in Pakistan. The obvious high risk is associated with the use of betel, areca and chewable tobacco besides family history, smoking habits, occupation and socioeconomic status. In a cross-sectional study, up to 40% of the participants were chewing at least one of these items on a daily basis. The bulk of the oral cancers were along the alveolus. Economic losses related to head and neck cancer in Pakistan totalled US$16.9 billion in 2010, equivalent to 0.26% of the region's economic output. Estimated direct cost of treatment of disease caused by smoking was Rs.68 billion in the year 2000. The economic consequences of head and neck cancer in South Asia are significant. Multidisciplinary management is critical in addressing this burden. The bulk of our patients present with advanced, stages three and four diseases. Staging, treatment planning, follow up and surveillance rely on imaging with CT/MR and PET/CT routinely. Nutrition, speech, hearing, swallowing therapy, rehabilitation support and palliative care are all challenges. Advanced disease at presentation, poor performance status and suboptimum compliance besides limited financial support undermine the treatment outcomes. Approximately 40% of these patients with head and neck tumours die within one year in spite of definite curative treatment.
***Learning Objectives:***
1. To understand the peculiar causes of high incidence of oral cancer in different regions of Pakistan.
2. To become familiar with the spectrum and scale of advanced disease in Pakistan.
3. To appreciate the variation in imaging and therapeutic practices across Pakistan.

**A-1079** 10:55
**Interlude: Beautiful Pakistan: from sea to sky-high mountains**
A. Majeed; *Islamabad/PK*

**A-1080** 11:00
**Role of radiology in developing a living donor liver transplant programme**
A.I. Rana; *Islamabad/PK (atifrana1@gmail.com)*

Pakistan has one of the highest prevalence rates for viral hepatitis in the world. In a country of approximately 200 million people and a 7% combined burden of hepatitis B virus (HBV) and hepatitis C virus (HCV), it was imperative to have indigenous liver transplantation (LT) program in Pakistan. Living donor liver transplant was initiated in 2012 in our centre, and so far, more than 650 transplants have been performed. Radiology has been at the forefront in this endeavour. Our contribution includes preoperative donor work up with a CT to calculate liver attenuation index, liver volumetric analysis as well as delineation of vascular anatomy for surgical planning. All patients undergo an MRCP as well for biliary anatomy. In the post-operative period, both recipient and donor may require imaging to look for complications such as post-operative collection, vascular complications, or biliary strictures. The most common recipient complication in our patients is a biliary stricture, occurring in about 22% of patients. Our interventional radiologists play an important part in treating these as well as other complications such as post-operative collections, portal vein stenosis or arterial complications.
***Learning Objectives:***
1. To learn about steps in setting up a successful living donor liver transplant program in Pakistan.
2. To understand requirements of preoperative imaging of potential donors.
3. To learn about various postoperative complications in transplant recipients and role of diagnostic as well interventional radiology in managing them.

**A-1081** 11:20
**Interlude: Philanthropic cyber-knife facility in Pakistan**
T. Mahmood; *Karachi/PK*

**A-1082** 11:25
**Many faces of tuberculosis**
U. Siddique; *Peshawar/PK (ummara_81@hotmail.com)*

Tuberculosis (TB) is one of the biggest health issues, and in Pakistan, around 430,000 people contract TB with about 70,000 deaths attributed to it, every year. Pakistan ranks 5th globally among the 22 high TB burden countries, contributes an estimated 43% of the disease in the Eastern Mediterranean region and has the fourth highest prevalence of multidrug-resistant TB globally. TB can involve any organ of the body, e.g. respiratory, cardiac, central nervous, musculoskeletal, gastrointestinal and genitourinary systems. Timely diagnosis is paramount since delayed treatment leads to severe morbidity. Therefore radiological workup is necessary for diagnosis. TB can present with a variety of radiological findings. In complicated TB, owing to delayed diagnosis, clinical and radiologic features of TB may mimic malignancy. Although in many cases biopsy or culture specimens are required to make a definitive diagnosis, it is imperative that radiologists understand the typical distribution patterns and imaging manifestations of TB in various organ systems. The goal of this talk would be to delineate the classical imaging findings in many presentations of TB like a stroke with tuberculomas, sciatica in caries spine, chronic diarrhoea in ileocecal TB, constrictive pericarditis causing congestive hepatopathy, cervical adenitis mimicking metastasis and vague pelvic pain in TB related to pelvic inflammatory disease. It is important to define a novel checklist to confirm TB presenting with different faces on imaging. Improvements in TB management can be made by raising the index of suspicion, having better knowledge of specific radiological patterns of disease and extensive screening studies.
***Learning Objectives:***
1. To learn about the prevalence of tuberculosis in Pakistan.
2. To understand the variety of radiological findings in tuberculosis, its diagnosis and complications.

11:45
**Panel discussion: Why are oral cancer, chronic hepatitis and tuberculosis more common in Pakistan, and in which scenarios is the role of radiology crucial in the management of these diseases?**

**10:30 - 12:00 Room F1**

### E³ - European Diploma Prep Session

### E³ 1823
### Breast

**A-1083** 10:30
**Chairperson's introduction**
M.H. Fuchsjäger; *Graz/AT (michael.fuchsjaeger@medunigraz.at)*

The European Diploma Prep Session on Breast aims to provide a preparation for future European Diploma in Radiology (EDiR) candidates. Topics are defined by the European Training Curriculum (ETC), and sessions are held in close cooperation with the European Board of Radiology (EBR). A short briefing on the EDiR examination as well as 2 sample questions (1 MRQ and 1 Short Case) and 1 sample case of the CORE examination (Clinically Oriented Reasoning Evaluation) will be presented in order to get the audience acquainted with the format. These three cases will include lesion detection and characterisation with mammography, ultrasound of the breast and breast MRI. The content of the session which will provide an overview on mammography, breast cancer diagnosis and interventions as well as multimodality imaging of the breast reflects ETC Level I - 'first three years of learning' and ETC Level II - 'Fourth and fifth years of training (general radiologist standard)' learning objectives.
***Session Objectives:***
1. To understand the methodological principles of mammography.
2. To learn the mammographic appearance of benign and malignant lesions of the breast.
3. To become familiar with the imaging appearance of benign and malignant breast lesions.

**A-1084** 10:36
**A. Fundamentals of mammography**
F. Kilburn-Toppin; *Cambridge/UK*

Mammography is the gold standard for breast cancer detection both in the symptomatic and screening population. This talk will first review the anatomy of the breast in relation to mammographic appearances, allowing accurate interpretation of imaging findings. The importance of mammographic technique will be discussed, highlighting the importance of both compression and positioning of the breast for detection of malignancy. The use of additional mammographic views will be reviewed with case examples, and important artefacts will be covered. Finally, the use of mammography in screening will be covered, with European recommendations for high-quality screening and recommendations for radiology practice. The controversy about the use of mammographic screening regarding reduction in mortality and overdiagnosis and overtreatment will also be discussed.

***Learning Objectives:***
1. To understand the anatomy, normal variants and abnormalities of the female breast.
2. To become familiar with the technical aspects of diagnostic mammography, especially in regard to dose and image quality.
3. To become familiar with the principles of current practice and risk/benefit analysis in breast cancer screening.

**A-1085** 11:04
**B. Breast cancer diagnosis and interventions**
C. Van Ongeval; *Leuven/BE (chantal.vanongeval@uz.kuleuven.ac.be)*

The breast composition and appearance of benign diseases and breast cancer at mammography, ultrasound and MRI will be discussed by the presentation of a correctly structured report of the imaging and interventional procedures. Personal and family history impacts the decisions made during an exam and should be reported. Description of the density according to ACR BI-RADS® lexicon (a,b,c,d) indicates the mammographic (Mx) sensitivity. Description of all Mx abnormalities (asymmetry, mass lesion, architectural distortion and microcalcifications) includes position, size, distance to the nipple, relation to other lesions in the breast. Palpable mass and/or Mx abnormality are investigated with ultrasound (US) of the whole breast and axillary (level 1,2,3) region. The US report includes breast composition, location, description of shape, margins, size of the lesion and evaluation of the lymph nodes(LN), and comparison with previous exams. Depending on the suspiciousness and type of lesion, additional core-needle or vacuum-assisted (ultrasound, stereotactic, MRI guided) biopsies need to be done (assessment categories BI-RADS®lexicon). If needed the lesion(s) should be marked with a clip. The three main indications of the additional use of breast magnetic resonance imaging (MRI) are the pre-operative extent of disease evaluation in the ipsilateral and contralateral breast (i.e. lobular carcinoma, dense breast, young women, discordant Mx/US information), neo-adjuvant therapy evaluation, screening of gene mutation carriers. An assessment report integrating the findings on MX, US, MRI and the second look US, is preferred. Communication with the referring physician and surgeon on the radio-pathological results and eventually use pre-operative localisation technique is advised.
***Learning Objectives:***
1. To learn the different presentation of normal breast patterns and the appearance of common benign diseases and of breast cancer at mammography, ultrasound and MRI.
2. To understand principles and basic application of standardised diagnostic categorisation systems such as the ACR breast imaging reporting and data system (BI-RADS®).
3. To become familiar with indications, contraindications and technical aspects of image-guided interventional breast procedures (fine needle aspiration, core needle biopsy, vacuum-assisted biopsy, presurgical localisation).

**A-1086** 11:32
**C. Advanced imaging of the female breast**
E.M. Fallenberg; *Munich/DE*

"no abstract submitted"
***Learning Objectives:***
1. To understand the role of advanced imaging techniques in evaluation of the breast.
2. To use the added value of new techniques for lesion classification in mammography, ultrasound and MRI.
3. To recognise the major imaging issues for common indications of breast imaging.

**12:30 - 13:30 Room C**

### E³ - The Beauty of Basic Knowledge: Pancreas

### E³ 24E

### Pancreatic adenocarcinoma mimickers

*Moderator:*
A. Ba-Ssalamah; Vienna/AT

**A-1087** 12:30
**Autoimmune pancreatitis**
R. Pozzi Mucelli; *Verona/IT*

Autoimmune pancreatitis (AIP) represents a distinct form of chronic pancreatitis. Histologically, AIP is characterised by a dense lymphoplasmacytic infiltrate of mainly CD4+ T lymphocytes and immunoglobulin G4 plasma cells, located around the pancreatic ducts with mass-forming regions of fibrosis. AIP has been classified into focal or diffuse forms. The differential diagnosis between focal AIP and pancreatic adenocarcinoma represents a medical need since AIP responds to steroid therapy and surgery should be avoided. CT and MRI findings of AIP are characterised by an enlargement of the gland, either focal or diffuse. At CT the affected areas are isodense before contrast enhancement, hypodense (due to hypervascularity) in the arterial phase with the progressive increase in density in the venous and late phases following contrast administration. The involved areas appear hypointense on T1-weighted MR images, mild hyperintense on T2-weighted images with reduced diffusion at DWI. The lesion appears hypovascular during the arterial phase, with progressive enhancement and delayed retention of contrast in the venous and late phases. MRCP is able to assess the involvement of the pancreatic duct system and these features are important in the differential diagnosis with the pancreatic adenocarcinoma in which the main pancreatic duct is characterised by single short stenosis, with marked dilation of the upstream ductal system. In cases in which AIP involves the head of the pancreas, dilatation of the common bile duct and the intrahepatic ducts can be seen.
***Learning Objectives:***
1. To learn about autoimmune pancreatitis.
2. To understand imaging findings of pancreatic adenocarcinoma mimickers.
3. To appreciate differential diagnosis criteria with pancreatic adenocarcinoma.

**A-1088** 13:00
**Paraduodenal pancreatitis**
G. Morana; *Treviso/IT (gmorana61@gmail.com)*

Paraduodenal pancreatitis is a form of chronic pancreatitis involving the duodenal wall in the region of the minor papilla and is considered a distinct entity with peculiar pathological findings. The frequent presence of the so-called heterotopic pancreatic tissue in the duodenal wall may reflect the incomplete involution of the dorsal pancreas in this region and contribute to an obstruction of the outflow in this area. Histologically there is thickening and scarring of the duodenal wall that extends to the adjacent pancreatic tissue and/or cystic changes in the duodenal wall. Occasionally some of the cysts may have a diameter of several centimetres. The clinical profile of PP is middle-aged men, heavy drinkers and smokers with painful pancreatitis, associated with vomiting and weight loss due to duodenal stenosis and impaired motility. The most characteristic imaging finding at CT and MRI is a sheet-like mass between the head of the pancreas and the C-loop of the duodenum (the so-called groove). The mass is hypodense/hypointense to the pancreatic parenchyma. Contrast-enhanced dynamic images show a delayed and progressive enhancement in the late phase that reflects the fibrous nature of the tissue. Cystic lesions are well depicted in the groove or the duodenal wall, especially in T2-weighted images. In early stage MPD is normal; in advanced cases, pancreatic head or entire gland are involved, showing findings of chronic obstructive pancreatitis, with ductal dilatation. There is an absence of vascular encasement, with a leftward displacement of the gastroduodenal artery without obstruction.
***Learning Objectives:***
1. To learn about autoimmune paraduodenal pancreatitis.
2. To understand imaging findings of paraduodenal pancreatitis.
3. To appreciate differential diagnosis criteria with pancreatic adenocarcinoma.

**12:30 - 13:30 Room D**

### E³ - The Beauty of Basic Knowledge: A Survival Guide to Musculoskeletal Imaging

### E³ 25E

### Acute trauma: patterns in the peripheral skeleton

*Moderator:*
V.N. Cassar-Pullicino; Oswestry/UK

**A-1089** 13:30
**Acute trauma: patterns in the peripheral skeleton**
J. Teh; *Oxford/UK (jamesteh1@googlemail.com)*

A systematic approach to analysing trauma imaging of the peripheral skeleton is presented. Typically, imaging begins with x-rays. A step-wise approach may reveal subtle abnormalities that should prompt further action. For example, the presence of a Second fracture on x-ray suggests an ACL injury and thus should lead to an MRI scan. It should be recognised that in trauma, certain injuries cannot occur in isolation; therefore a dislocated radial head on elbow x-ray should prompt imaging of the whole forearm, as Monteggia fracture-dislocation may be present. Understanding mechanisms of injury allow the radiologist to predict patterns of injury. So if bone bruising is present on MRI in the lateral femoral condyle and medial patella, a lateral patellar dislocation should be suspected, and a specific search for an injury of the medial retinaculum should be made. Conversely, recognising classic patterns of injury can allow the mechanism of injury to be deduced. This lecture will focus on classical patterns of acute trauma in the peripheral skeleton.
***Learning Objectives:***
1. To become familiar with the imaging manifestations of common important injuries in the upper and lower limb.
2. To understand the underlying mechanism that result in combination of injuries.
3. To learn how to best employ imaging modalities in their diagnosis.

**14:00 - 15:30** **Room A**

### E³ - ECR Academies: Interactive Teaching Session for Young (and not so Young) Radiologists

### E³ 1921

### Paediatric brain imaging

**A-1101** 14:00
**A. Head and neck emergencies in children**
A. Rossi; *Genoa/IT (andrearossi@gaslini.org)*

Children presenting with acute disease involving the head and neck often display an aspecific clinical picture characterised by pain, fever, malaise, and local or generalised swelling. Regional involvement ranges from the orbits to the paranasal sinuses, temporal bone, skull base, craniocervical junction, upper airway, and neck. Emergent presentations in the head and neck compartment can be grossly categorised into traumatic and non-traumatic, the latter comprising infectious, inflammatory, congenital, and neoplastic conditions. Imaging studies play a fundamental role in the diagnosis of these conditions and provide a basis for subsequent management, including the identification of situations that may dictate immediate surgical treatment. The choice of the most appropriate imaging method is significantly influenced by the patient's clinical conditions and stability (or lack thereof). Although both ultrasound and X-rays often are used, this presentation will mostly focus on findings at CT and MRI.
***Learning Objectives:***
1. To become familiar with the different entities.
2. To identify the key imaging findings.

**A-1102** 14:45
**B. Acute neurological child beyond trauma**
A. Sánchez-Montañez; *Barcelona/ES (anchelsanchezmed@gmail.com)*

This presentation reviews the brain imaging findings of the most common acute neurological non-traumatic disorders in pediatric patients. From the clinical point of view, a wide differential diagnosis of diseases that can occur abruptly in the emergency room is discussed. The most frequent acute symptomatology and manifestations are seizures, headache, irritability, lethargy, paresis, fever, nausea and vomiting. Beyond trauma, there are other entities to consider when a pediatric patient has an acute neurological clinical presentation. Among them, vascular aetiology stands out, whether in the form of an arterial stroke, venous thrombosis or vascular malformation. Other important causes are infections, drugs and their side effects, tumours, metabolic and demyelinating diseases. Neuroimaging techniques include computed tomography (CT), magnetic resonance (MR) and, even cerebral ultrasound in neonates and infants. The combination of basic neuroimaging methods and other advanced techniques such as angiographic and perfusion studies, allow proper differential diagnosis, correct management of the acute neurological onset in children and its subsequent follow-up.
***Learning Objectives:***
1. To become familiar with the most common cause of acute neurological emergencies.
2. To learn the imaging criteria for differentiation.

### B

### Scientific Sessions (SS)
### My Thesis in 3 Minutes (MY)
### Clinical Trials in Radiology (CT)

Presentation numbers are prefixed by the letter B.

Sessions and abstracts are listed by days.

**Wednesday, February 27.... 186**
**Thursday, February 28....... 281**
**Friday, March 1.................. 367**
**Saturday, March 2.............. 413**
**Sunday, March 3................ 489**

### Wednesday, February 27

**08:30 - 10:00** **Coffee & Talk 3**

### Interventional Radiology

### SS 109

### Vascular interventions in visceral arteries and veins (part 1)

*Moderators:*
M. Tsitskari; Athens/GR
N.N.

**B-0001** 08:30
**Quantification of perfusion reduction by using 2D parametric parenchymal blood flow following partial spleen embolisation in patients with hypersplenism**
T. Meine[1], S.K. Maschke[1], M. Kirstein[1], J. Renne[1], T. Werncke[1], C. Dewald[1], F. Wacker[1], B.C. Meyer[2], J.B. Hinrichs[1]; [1]*Hannover/DE*, [2]*Berlin/DE* *(Meine.Timo@mh-hannover.de)*

**Purpose:** To analyze the feasibility of 2D Parametric Parenchymal Blood Flow (2D-PPBF) for the quantification of perfusion reduction following partial spleen embolization (PSE).
**Methods and Materials:** 15 PSE procedures in 14 patients with hypersplenism were included (2015-2018). Outcome was measured via platelet count before and after PSE. To quantify changes in spleen perfusion following PSE using 2D-PPBF, the acquired digital subtraction angiography (DSA) series were post-processed. A reference region-of-interest (ROI) in the splenic artery and a target ROI outlining the embolization territory of the splenic tissue were placed in corresponding areas on DSA pre- and post-PSE. The Wash-In-Rate (WIR), the 50%-to-Peak ($Peak_{0.5}$), the Arrival-to-Peak ($Peak_{Arrival}$), the Mean-Transit-Time (MTT) and the Area-Under-the-Curve-per-ROI-Area (AUC) were assessed and the ratios to the reference ROI were calculated. Mean and standard deviation of the ratios were determined. Comparisons between pre- and post-embolisation data were made using a Wilcoxon signed-rank test.
**Results:** Platelet count increased significantly following PSE from 41.200 ± 19.468 to 103.066 ± 70.350 platelets/microliter (p=0.02). In the embolized splenic territory, the pre-/post-embolisation 2D-PPBF parameter changed significantly: WIR 1.05 ± 2.18 / 0.09 ± 0.07 (p=0.03), $Peak_{0.5}$ 3.30 ± 0.88 / 4.28 ± 1.18 (p=0.011), $Peak_{Arrival}$ 1.95 ± 0.77 / 3.20 ± 1.45 (p=0.027), MTT 4.82 ± 1.23 / 6.39 ± 1.79 (p=0.005) and AUC 0.81 ± 0.79 / 0.13 ± 0.08 (p=0.01).
**Conclusion:** 2D-PPBF is an objective approach to quantify the perfusion reduction of embolized splenic tissue following PSE. It may be used to assess angiographic outcome and set angiographic endpoints for PSE.

**B-0002** 08:38
**Role of multidetector CE-CT before transcatheter endovascular embolisation in active arterial bleedings**
A. Borzelli[1], A. Paladini[2], F. Amodio[1], E. Cavaglià[1], R. Niola[1]; [1]*Naples/IT*, [2]*Novara/IT (antonio.borzelli@libero.it)*

**Purpose:** Identify the role of CE-CT in active bleedings' detection according to clinical evidence and bioumoral parameters of patients.
**Methods and Materials:** 1480 CE-CTs were performed between June 2014 and June 2017 for suspected active bleeding (median patient age 45; 902 M, 578 F) in emergency setting, due to clinical and laboratory evidence of serum haemoglobin value lowering.
**Results:** In 793 CE-CT cases revealed active bleeding followed by angiography. In the remaining 687 cases, CT was negative, but due to clinical and laboratory values worsening, in 89 of them angiography was performed, revealing active bleeding in 65 patients; the latter owing to pseudoaneurysms, intracavitary bleedings or through percutaneous drainages. Among the 793 CT-positive patients, in 64, angiography did not reveal active bleedings and 28 of these were patients affected by DIC or wide small and diffuse bleedings, while in 36, CT revealed small active bleedings not detectable by the following angiography. In the last year (June 2017-February 2018) we considered CT-false-positive patients and in 31 of them angiography was not performed for wide small and diffuse bleedings or for small bleedings detected by CT, preferring clinical and laboratoristic follow-up. In 2 cases, owing to worsening of symptoms and laboratory parameters, angiography was performed, showing active bleeding followed by successful endovascular embolisation.
**Conclusion:** Multidetector CE-CT showed elevated accuracy in revealing active bleedings, but a correct assessment of clinical and laboratory conditions of patients is essential in the presence of mismatch between imaging findings and clinical conditions. In particular, in cases of wide small and diffuse bleedings and very small focal bleedings, only strict clinical and laboratory follow-up is appropriate, to evaluate later the necessity of diriment angiography.

**B-0003** 08:46
**Selective intraarterial embolisation as a treatment for haemorrhoid pathology: preliminary study**
M. Ferrer Puchol[1], A. Gregorio[2], E. Esteban[1], F.J. Blanco[1], V. Primo[2], L. Andreo[2], M. Villalta Santamaria[1], R. Ramiro[1], J. Solaz Solaz[1]; [1]*Alzira/ES*, [2]*Denia/ES (lolesferrer@ono.com)*

**Purpose:** To present patients with bleeding haemorrhoids treated by selective intraarterial embolisation of superior rectal artery (SESRA).
**Methods and Materials:** We present twenty-one patients with disabling chronic rectal bleeding, evaluated by a proctologist, who indicated SESRA as the best therapeutic alternative. The mean age was 60.5 years (45-81). The stage of the haemorrhoidal disease was II/III. They were not suitable for other treatments. They were admitted the same day as embolisation. The inferior mesenteric artery was catheterized using a visceral catheter and superior rectal arteries were catheterized with microcatheter (2.7F). The occlusion of rectal arteries was made by PVA particles (300-500 microns in size) and coils 2-3 mm and particles, verifying the absence of distal flow. All patients were discharged at 24 h. After one month, the surgeon evaluated the patients clinically and with anoscopy.
**Results:** In 19/21 cases, the embolisation was successful, one patient presented inferior mesenteric artery spam and the procedure was suspended and other suffered infrarenal aortic aneurysm avoiding the catheterization. Clinical success was 19/21, follow-up: 2-29 months. One patient presented pain (VAS 3) that disappeared after 5 days. The rest did not experience any discomfort after the procedure. One patient presented rectal bleeding and prolapse, and he is pending surgical revision. Anoscopy performed at 1 month showed significant improvement in haemorrhoids. No complications were detected.
**Conclusion:** The initial results suggest that SESRA is safe and well tolerated and with advantage of absence of anorectal trauma. Additional studies were needed to evaluate treatment´s efficacy.

**B-0004** 08:54
**Post-TIPS encephalopathy: are there any predictive factors?**
C. Perazzini[1], P. Fonio[2], A. Doriguzzi Breatta[3], D. Righi[2], D. Rossato[2], G. Gandini[2]; [1]*Clermont Ferrand/FR*, [2]*Turin/IT*, [3]*Castelnuovo Don Bosco/IT* *(chiara.perazzini@alice.it)*

**Purpose:** To evaluate hepatic encephalopathy (HE) after trans-jugular intrahepatic porto-systemic shunt (TIPS) related to: age, international normalized ratio (INR), creatinine, bilirubin, platelets, portal thrombosis and model for End-of-stage Liver Disease score.
**Methods and Materials:** Retrospective study of 118 patients (53 males and 65 females, mean age 54 y/o) that from January 2008 to June 2013 underwent TIPS and a 6 and 12 months follow-up. HE staged according to West Haven criteria and simplified in moderate and severe has considered. Continuous variables tested for normality with the Shapiro-Wilks test. Comparisons employed non-parametric tests for k distributions and Anova test for $n > 2$ correlated distributions. Categorical variables arranged in r × c tables studied with the Chi-square or with Fisher's exact test. Statistical significance set at two-tails $p < 0.05$. Analysis carried out with source software.
**Results:** The overall HE incidence after 6 moths is 36%: 29/118 patients present a moderate HE, 13/118 a severe one, 74/118 are not affected. Any statistical significance exists between biographical data, INR, creatinine, bilirubin, platelets, portal thrombosis and HE. Portal thrombosis has protective effect. At 12 months, 9/118 patients underwent liver transplantation, 2/118 died. 107/118 remaining have been re-evaluated: any statistical significance exists between biographical data, INR, creatinine, bilirubin, platelets and the incidence of HE. INR, lower bilirubin and a higher platelet are associated with a lower probability of HE.
**Conclusion:** INR, lower bilirubin, higher platelet count, without statistical significance and portal thrombosis, are associated with a lower HE incidence 12 months after TIPS.

**B-0005** 09:02
**Retrograde stent implantation of the celiac artery via the pancreaticoduodenal anastomosis**
F. Pedersoli, P. Isfort, M. Zimmermann, M.F. Schulze-Hagen, S. Keil, C.K. Kuhl, P. Bruners; *Aachen/DE (fpedersoli@ukaachen.de)*

**Purpose:** The antegrade recanalization of a high-grade stenosis of the celiac artery directly from the aorta may present sometimes a challenge. In fact, in the absence of vascular stump ("flash-occlusions") or pronounced calcification of the aortic wall, the trial may remain frustrated. Therefore was investigated the possibility of a retrograde alternative in the case of difficult antegrade access from the superior mesenteric artery, via the inferior and superior pancreaticoduodenal artery, the gastroduodenal artery, and the common hepatic artery.

**Methods and Materials:** We performed a retrospective analysis of all patients, who underwent a stent implantation in the celiac trunk from 01/2010 to 12/2017. The data on the indication to the intervention, the access, the material used, the stent model and the follow-up were collected.
**Results:** In 34 patients was performed a stent implantation into the celiac trunk. The antegrade approach was successful in 30 patients (88%). An antegrade passage of the occlusion or stenosis was not possible in 4 (12%) patients. In these cases, a 5F catheter was placed in the proximal superior mesenteric artery. Then the stenosis could be overcome with 2.4 F microcatheter and micro-wire. Subsequently, the tip of the micro-wire was captured in the aorta with a snare and pulled out in the femoral introducer sheat with a rendezvous technique and then used as a guide for the antegrade implantation of a balloon-expandable stent into the celiac trunk.
**Conclusion:** Retrograde access to the celiac trunk by pancreaticoduodenal arcade represents a valid alternative if antegrade access is not possible

**B-0006** 09:10
**High-flow priapism: colour Doppler findings and embolisation**
G. De Magistris, F. Pane, F. Corvino, F. Giurazza, M. Coppola, F. Amodio, M. Silvestre, E. Cavaglià, R. Niola; *Naples/IT (giuseppedemagistris74@gmail.com)*

**Purpose:** To retrospectively evaluate the efficacy of colorDoppler detectability and the safety of transcatheter arterial Embolization for High-flow Priapism
**Methods and Materials:** Over a 8-year period, 10 patients (mean age 32 years; range 13-65 years) admitted to our department with traumatic high-flow priapism, were reviewed. The patients were evalueted with clinical and imaging findings (colorDoppler ultrasonography and Angiography). Diagnostic Angiography demonstred fistulas between the cavernosal artery and the corpora cavernosa, these communications were embolized with coils and gelatin sponge. A colorDoppler follow-up was peformed at 2 weeks and 3-6 months after procedure
**Results:** At the admittance, colorDoppler detected pseudoaneurysms/fistulas in 9 patients. In 8 of 10 patients a single embolization was sufficient for complete resolution, 2 patients had recurrence of Priapism (confirmed by colorDoppler Imaging) in the subsequent 1- 3 weeks and required a second procedure. 9 patients were embolized with coils and one with spongel. At 3-6 months follow-up, colorDoppler US confirmed the absence of fistula and recanalization of the embolized cavernosal artery. All patients had normal erectile funcion at 6 months
**Conclusion:** ColorDoppler is useful to detect fistulas and to monitor the effectiveness of embolization. Superselective transcatheter embolization of the internal pudenda artery is efficacy, compared to other techniques, and has a high rate of preservating erectile function

**B-0007** 09:18
**Role of c-arm computed tomography-assisted adrenal vein sampling in the assessment of adrenal hypersecretion syndromes**
A. Pisano, M. Tipaldi, G. Orgera, F. Laurino, A. Laghi, M. Rossi; *Rome/IT (andrea.pisano21@gmail.com)*

**Purpose:** Adrenal Vein Sampling (AVS) is a gold standard procedure in the diagnostic work up of adrenal hypersecretion syndromes. However, it is burdened by high failure rates due to technical issues and in particular for the right vein catheterization. Therefore, we investigated the impact of C-arm Computed Tomography-Assisted AVS (C-AVS) in comparison to standard AVS.
**Methods and Materials:** From September 2010 to January 2018, a total of 40 procedures were retrospectively reviewed: 23 AVS (group A) and 17 C-AVS (group B). Overall, 36 patients (90%) had primary hyperaldosteronism clinical features, and 4(10%) suffered from ACTH-independent Cushing's Syndrome with bilateral macronodular hyperplasia.
All patients were infused with 50 microL/h cosyntropin 30 minutes prior the procedure.After catheterization of the right and left adrenal vein, C-arm-CT was performed in group B during CE selective injection, to confirm correct catheter position. Technical success was defined by adequate bilateral sampling, intended as a selectivity index (SI) >3.
**Results:** Technical success was obtained in 52,2% of group A (12pt), while in 47,8% of the cases sampling did not prove diagnostic. On the other hand, technical success was achieved in 82,4% of group B (14pt), whereas 11,7% of them did not meet SI criteria and 1 procedure was aborted due to contrast-media reaction.Technical success was significantly higher in group B than in group A (82,4% vs. 52,2%; p-value <0.05). There was no procedure-related complication.
**Conclusion:** Despite limited data, C-AVS improved the overall success rate of AVS, as it proves crucial for an optimal catheterization of adrenal veins.

**B-0008** 09:26
**Long-term follow-up of giant visceral artery aneurysms endovascular treatment: a single centre experience**
M. Tipaldi[1], M. Pignatelli[1], G. Orgera[1], F. Laurino[2], A. Laghi[1], M. Rossi[1]; [1]*Rome/IT*, [2]*Eboli/IT (tipaldi.andrea@gmail.com)*

**Purpose:** Purpose of this study is to assess the immediate and long-term safety and effectiveness of Giant visceral artery aneurysms (visceral aneurysm> 5cm) endovascular treatment in a single center case series.
**Methods and Materials:** Eight cases of GVAAs (7M, 1F) have been treated in our institution, between August 2006 and June 2017, and were retrospectively reviewed. Embolization was performed with coiling and/or fragment of guidewires and/or embolic agents and/or stents.Technical success was defined as the correct exclusion of the sac at the last angiogram, while clinical success was defined as the complete relive of clinical symptoms, if previously present.Follow-up was performed with CT angiography at 1,3, 12 and 24 months or phone survey if patients were referred to other centers.
**Results:** Technical and clinical success were obtained in 100% of the cases. One minor complication, consisting in an asymptomatic ischaemia of the spleen, and one major complication, consisting in a 4cm splenic abscess concurrently with a small infarctual area, occurred. The median time follow-up was 48 months (range 6 to 84 months) and demonstrated the correct exclusion of the sac in 100% of the cases. One patient died five years after the treatment for causes not related to the procedure.
**Conclusion:** The first line treatment of visceral artery aneurysms is, nowadays, the endovascular one rather than open surgery. Our series have shown that this indication, thanks to the low morbidity and mortality rates associated with, should be expanded even for GVAAs.

**B-0009** 09:34
**Transjugular portal vein stenting: an original approach for palliative treatment of portal vein stenosis/occlusion in neoplastic patients**
A. Mazzaro[1], F.L. Gatti[1], R. Bettini[1], N. Mandruzzato[2], A. Contro[1], G. Mansueto[1]; [1]*Verona/IT*, [2]*Trieste/IT (andrea.mazzaro1558@gmail.com)*

**Purpose:** To evaluate risks and benefits of portal vein stenting as a palliative procedure in a pool of oncologic patients.
**Methods and Materials:** Between April 2014 and August 2018 we treated 12 patients with neoplastic portal vein stenosis/occlusion performing portal vein stenting. In 5 of them was used a transhepatic access and in 7 a transjugular access. Portal stenting has less than 200 cases reported in the literature, most of them with transhepatic access. Transjugular access is an original/unique variant. To evaluate the procedure we considered technical and anatomical success, procedural complications and mortality, 30 days mortality, modifications in clinical and laboratory parameters (haemoglobin, haematocrit, albumin, ALT, bilirubin and INR). Technical success was assessed with intra-procedural portography while complications and anatomical success with follow-up MDCT.
**Results:** In all the procedures technical and anatomical success was achieved with no intraprocedural or early mortality. Two patients treated with transhepatic access developed complications (hepatic abscess and hepatic haematoma). No patient with transjugular access developed complications. All patients showed improvement of clinical conditions and of laboratory parameters (four or more of the six parameters considered) in 30 days, even those who developed complications.
**Conclusion:** Portal vein stenting in oncologic patients was performed safely, it resolved portal vein's stenosis/occlusion leading to a relief of portal hypertension-related symptoms and an improvement in the quality of life. In our experience transjugular access is less invasive and reduces the risk of hepatic bleeding, however it has to be performed by TIPS procedure expert operators.

**B-0010** 09:42
**Use of Onyx (ethylene vinyl alcohol copolymer) in haemorrhage embolisation procedures**
R. Marcello, G. Assegnati, A. Di Blasi, F. Cortese, D. Konda, E. Pofi; *Rome/IT (robermarcello@gmail.com)*

**Purpose:** The purpose of our study was the evaluation of the effectiveness and reliability of the "Onyx" device (ethylene vinyl alcohol copolymer) in the treatment of bleeding by trans-catheter embolization technique.
**Methods and Materials:** 14 Patients in our retrospective analysis underwent transcatheter embolization for bleeding control. All patients underwent diagnostic angiography with trans-femoral access to confirm the findings obtained with US and ce-TCMS investigation work-up.The coaxial technique was always applied in performing all procedures with a 5 Fr diagnostic catheter (Sim 1 Imager, Boston Scientific, USA) combined with a microcatheter 2.7 Fr. (Progreat Terumo, JAP).In all cases "Onyx" (ethylene vinyl alcohol copolymer) was used as embolization material. The source of the bleeding was duodenal ulcer in 5 cases, 2 gluteal haematomas, 1 haemorrhoidal pseudoaneurysm, 2 intraparenchymal renal aneurysms in one case, 1 vascular malformation of the gastric fund, 1 haemorrhage after hip prosthetic replacement, 1 renal angiomyolipoma, 1 abdominal wall haematoma and 1 retroperitoneal haematoma.
**Results:** Prompt cessation of bleeding was achieved in all patients. No immediate or late complications were observed with regards to all procedures carried out. No unwanted embolization of other vascular areas was observed.
**Conclusion:** According to our retrospective analysis, the reliability and effectiveness of "Onyx" (ethylene vinyl alcohol copolymer) as embolization material emerged during many bleeding trans-catheter embolization procedures in different anatomical sites.

**B-0011** 09:50
**Tips and tricks of emergency TIPS in patients affected by severe portal hypertension**
A. Borzelli[1], A. Paladini[2], F. Amodio[1], E. Cavaglià[1], R. Niola[1]; [1]*Naples/IT*, [2]*Novara/IT (antonio.borzelli@libero.it)*

**Purpose:** To evaluate the main causes of failure in TIPS performed in emergency setting in critical patients affected by severe portal hypertension.
**Methods and Materials:** Between Jan 2014 and Jan 2018, we retrospectively considered 72 TIPS performed in emergency setting in critical patients (medium patient age 52; 45 M, 27 F) with severe portal hypertension and affected by variceal bleeding (42), refractory ascites with hepatorenal syndrome (15)and hepatic coma (15). In all cases, we used an e-PTFE-covered stent and in all cases we employed a US-assisted technique to reach the portal vein system once catheterised one of the hepatic veins.
**Results:** In 12 cases, TIPS was not possible. In 7 patients, we observed a marked distortion in liver anatomy due to chronic liver disease with loss of normal anatomic relationship between portal vein system and hepatic veins, which were too thin. In 3 cases, due to anatomic distortion, it was possible to reach only a small peripheral portal vessel, with subsequent impossibility to make the shunt; in 1 case, we noticed a iatrogenic lesion of portal vein during the procedure with subsequent thrombosis of the stent; in 1 case, malpositioning of the stent with early thrombosis not solved by proximal and distal extension of the shunt.
**Conclusion:** The main cause of technical success of emergency TIPS performed in critical patients with severe portal vein hypertension is the marked anatomical distortion of the liver, in particular, its vascular anatomy. It is necessary to reduce the failure rate, to employ a US-assisted approach and, if required, a percutaneous sentinel catheter into the main branch of portal vein, to guide the radiologist during the procedure.

**10:30 - 12:00** **Room B**

### Artificial Intelligence and Machine Learning

### SS 205

### Intelligent dose and quality management

*Moderators:*
M. Fatehi; Tehran/IR
V. Suomi; Turku/FI

**B-0012** 10:30
**Application of artificial intelligence-based image optimisation algorithm in aorta CTA with low tube voltage and reduced contrast medium volume**
M. Wang, Y. Wang, Y. Wang, M. Yu, M. Wang, Z. Jin; *Beijing/CN (hwjwm@163.com)*

**Purpose:** To evaluate the impact of artificial intelligence (AI)-based image optimization algorithm on improving aorta computed tomography angiography (CTA) image quality (IQ) at 80 kVp tube voltage and 40 mL contrast medium (CM) compared with a conventional iterative reconstruction (IR) algorithm at 120 kVp.
**Methods and Materials:** Sixty patients referred for aorta CTA examination were assigned to one of two groups at random on NeuViz 128 CT. Group A underwent an 80 kVp protocol with 40 mL CM (320 mg I/mL) and divided into two subgroups according to reconstruction algorithm (IR for group A1 and further AI-based image optimization for group A2). Group B was scanned with the standard 120 kVp, 80 mL CM and IR algorithm. The quantitative assessment of IQ included aorta attenuation, noise, signal-to-noise ratio (SNR) and contrast-to-noise ratio (CNR). A five-point scale (5-excellent,1- low) was used by two radiologists independently for qualitative image analysis.
**Results:** The image noise was significantly reduced while SNR and CNR were significantly increased in the order of group A1, B and A2 (all P<0.001). Compared with group B, the subjective IQ score of group A1 was significantly lower (P = 0.03) while that of group A2 has no significant difference (P = 0.926). The effective dose and CM volume of group A were reduced by 79.18% and 50%, respectively than that of group B.
**Conclusion:** The AI-based image optimization for aorta CTA with low kV and reduced CM produced IQ comparable to conventional aortic CTA protocol.

**B-0013** 10:38
**Real-time patient-specific CT dose estimation for single- and dual-source CT using a deep convolutional neural network**
J. Maier, S. Dorn, E. Eulig, S. Sawall, M. Kachelrieß; *Heidelberg/DE (joscha.maier@dkfz.de)*

**Purpose:** Common dosimetric quantities in CT such as the CTDIvol or the DLP do not appropriately represent the actual patient dose. More sophisticated methods are not real-time capable. Therefore, we propose the deep dose estimation (DDE), a deep learning-based approach to estimate patient dose distributions in real time.
**Methods and Materials:** The gold standard to calculate patient-specific dose distributions is to perform a Monte Carlo (MC) simulation that models the physics of CT dose deposition. Being computationally expensive, MC cannot be applied in real time. To overcome this drawback without losing accuracy we developed DDE: a deep convolutional network for CT dose estimation. DDE uses a U-net architecture that takes a two-channel input consisting of a CT volume and a first-order dose estimate volume, which can be calculated analytically at the cost of a forward projection. Using this input, DDE is trained to reproduce MC dose distributions. Here, we generalized DDE to dual-source CT and demonstrate its potential to derive accurate dose distributions.
**Results:** Applied to test data, DDE yields dose estimates that differ by less than 3% on average from the ground truth MC simulation. Our experiments demonstrate that DDE applies similarly to single- and dual-source scans, different anatomical regions, different shaped filters, different tube voltages and tube currents.
**Conclusion:** This study demonstrates the potential of deep convolutional neural networks to derive accurate CT dose estimates. Once trained, a 256×256×48 voxel volume can be processed with DDE in 250 ms while achieving the same accuracy as MC simulations.

**B-0014** 10:46
**Dose team: an estimate of radiation dose reference levels for CT exams**
M. Montesano, M. Rengo, S. Badia, N. Panvini, S. Picchia, D. Bellini, I. Carbone, A. Laghi; *Latina/IT (marta.mon@hotmail.it)*

**Purpose:** The aim of the study was to identify radiation dose reference levels for CT exams in our institution.
**Methods and Materials:** A Dose team was established (two radiologists, a TSRM, a radiology resident, the risk manager, the medical director and the medical physicist) whose first objective was to evaluate the quality of the images on 1000 oncological follow- up and to assess the amount of dose delivered through dedicated dose monitoring software (DoseWatch, GE Healthcare). The Dose length product (DLP) has been evaluated for the different anatomical regions for head, neck, thorax, abdomen-pelvis and thorax-abdomen-pelvis. The quality of the images in the different anatomical districts was subsequently assessed.
**Results:** The quantitative analysis allowed to identify the average DLP for the different anatomical districts. The mean dose for the different anatomical districts was for the brain (570.35 mGy-cm ± 121.32), for the neck (211.58 mGy-cm ± 93.12), for the thorax (160.91 mGy-cm ± 41.81), for the abdomen-pelvis (425.93 mGy-cm ± 122.90) and for the thorax-abdomen-pelvis (685.89 mGy-cm ± 240.04). In all anatomical districts a good image quality was obtained.
**Conclusion:** The dose monitoring system and the establishment of a Dose team made it possible to identify radiation dose reference levels for CT for individual anatomical districts.

**B-0015** 10:54
**Clinical indications for computer tomography (CT) diagnostic reference levels (DRLs)**
S. Yu, P. Chu, R. Smith-Bindman; *San Francisco, CA/US (bill.chu@ucsf.edu)*

**Purpose:** To contribute to the creation of CT radiation dose benchmarks for specific clinical indications for adults (>=15 years). We report the distribution of radiation dose metrics based on CT scans submitted in 2016-2017 to a large international dose registry comprised of 160 institutions from 7 countries. The registry was supported through the US National Institutes of Health (NIH) and Patient Centered Outcomes Research Institute (PCORI).
**Methods and Materials:** We describe median (target) and 75th percentile (benchmark) doses for CT dose index volume (CTDIvol) and dose length product (DLP) by three clinical indications from the European Study on Clinical DRLs (EUCLID).
**Results:** Sample sizes: sinusitis N=28,946, cervical spine/trauma N=83,919, pulmonary embolism N=83,882. CTDIvol target (50%) and benchmark (75%) values (in mGy), respectively: sinusitis (14 and 20), cervical spine/trauma (18 and 26), pulmonary embolism (10 and 15). Corresponding DLP values (in mGy-cm) were: sinusitis (235 and 339), cervical spine/trauma (878 and 1390), pulmonary embolism (388 and 596). Sinusitis scans were 68% lower in both CTDIvol and DLP compared to routine head scans. Pulmonary embolism scans were 23% higher in CTDIvol and 8% higher in DLP compared to routine chest scans.
**Conclusion:** Dose metrics from large multi-center studies can help create representative DRLs that can be used for dose optimization and institutional evaluation of CT radiation doses specifically for clinical indications to know if their doses routinely exceed these benchmarks. Having clinical indication DRLs at institutions can lead to indication-specific dose-optimized protocols.

**B-0016** 11:02
**Automatic noise and vessel density measurement for CT angiography quality assurance**
S. Zargaran, F. Wozniak, M. Dewey; *Berlin/DE*

**Purpose:** This work aims to automate the vessel density and noise measurements in coronary CT angiography as part of quality assessment. So far this is done manually by measuring the mean (density) intensity and standard deviation (noise) within a uniform and consistent region of about 1 square cm containing contrast agent (i.e. aorta).
**Methods and Materials:** We used 567 coronary CT scans from the randomized DISCHARGE trial across 26 sites. We initially applied the Canny edge detection and then used Hough transform to find circles with pre-specified radii which should correspond to the aorta. Additional heuristics regarding the expected position along or aorta were also applied to eliminated obvious errors. Thereafter density and noise were measured within the circles.
**Results:** For evaluation, automated measurements that differ from the manual measurements up to a certain threshold, were marked as correct. As such, noise difference tolerances of 5, 10 and 15 HUs led to a noise accuracy of 47%, 67% and 78%, respectively. Similarly, density difference tolerances of 25, 50 and 75 HUs led to a density accuracy of 71%, 90% and 96%. Finally, the combined (noise,density) difference tolerances of (5,25) , (10,50) and (15,75) led to accuracies of 37%, 61% and 75%.
**Conclusion:** An automatic method for quality assurance of noise and vessel density in coronary CT angiography was feasible and has the potential to reduce time and manual effort.
*Author Disclosures:*
**M. Dewey:** Author; editor of Cardiac CT, published by Springer, and offers hands-on workshops on CT imaging (www.ct-kurs.de). Research/Grant Support; FP7 Program of the European Commission for the randomized multicenter DISCHARGE trial (603266-2, HEALTH-2012.2.4.-2), grant support from the Heisenberg Program of the DFG (DE 1361/14-1), Digital Health Accelerator of the Berlin Institute of Health, Institutional master research agreements exist with Siemens Medical Solutions, General Electric, Philips Medical Systems, and Toshiba Medical Systems.. Speaker; ecture fees from Canon Medical Systems, Guerbet, Cardiac MR Academy Berlin, and Bayer.

**B-0017** 11:10
**Impact of ESR iGuide in the diagnostic workflow of paediatric patients with abdominal pain**
M. Gabelloni, C.A. D'Amore, R. Morganti, L. Faggioni, C. Spinelli, E. Neri; *Pisa/IT (mgabelloni@sirm.org)*

**Purpose:** To determine the impact of the ESR iGuide clinical decision support system in reducing the rate of inappropriate emergency paediatric diagnostic examinations.
**Methods and Materials:** 352 paediatric patients referred to Emergency Department of BLINDED Hospital between 2011 and 2016 were retrospectively evaluated. All patients had presented with abdominal pain. Clinical data were entered into the ESR iGuide system to compare appropriateness scores of suggested versus actually performed examinations, and the agreement between them was assessed using Cohen's k coefficient.
**Results:** Out of all ultrasound examinations performed, only 70% (255/352) were considered appropriate by ESR iGuide. Computed tomography (CT) was suggested as appropriate in 70% (249/352) of patients, but only 2 of them went ahead with it. Two other patients underwent CT, although it would have been designated as inappropriate according to ESR iGuide. Abdominal X-ray was suggested as appropriate in 253 patients and performed in 18 of them. Four other patients underwent abdominal X-ray when suggested as inappropriate by ESR iGuide. Finally, 9 patients underwent chest X-ray, which was suggested as appropriate by ESR iGuide in 5 patients. Magnetic resonance imaging and scintigraphy were not assessed because unavailable in our emergency department. The agreement between imaging examinations suggested by ESR iGuide and those actually performed was poor (Cohen k less than 0.2).
**Conclusion:** Strict adherence to the ESR iGuide system would lead to a significant increase in the number of emergency paediatric CT examinations performed instead of ultrasound studies, potentially resulting in higher patient radiation exposure and overall costs.

**B-0018** 11:18
**Semi-supervised multi-to-one style transfer to improve the quality of CT images**
B. Ren[1], L. Li[2], S. Wang[2], C. Xia[2]; [1]*Amherst, MA/US*, [2]*Beijing/CN (xchen@infervision.com)*

**Purpose:** The quality of CT images depends on various factors, such as radiation dose, convolution kernel and resolution. CNN-based methods are sensitive to small variations of image quality, i.e., small perturbations in noise level, texture, contrast, brightness, etc.
**Methods and Materials:** To address this problem, we proposed an image standardisation method which is capable of transforming images from different domains to one target domain, without its domain information and without changing its content. The model first decomposes an input image into content and style latent codes, which are independent of one another. The codes are able to reconstruct the original image or standardise the image to the target domain by replacing the style code. For the target domain, we reduced its style code variance and took the mean of style codes for standardisation. A domain discriminator was also applied to determine whether two images belong to the same domain.
**Results:** To verify effectiveness of the proposed method, our model was trained in a semi-supervised manner. For training, each supervised step updates the model with paired images from collaborating hospital followed by one unsupervised step which learns from unpaired data provided by Infervision. With the same experimental setting, the false positive (fp/np) was reduced to 2.17 from 2.62 after standardisation on 310 test cases, while recall only decreases slightly from 82.4% to 80.9%.
**Conclusion:** In our attempt to address the issue by a cycleGAN-inspired unsupervised model, results have shown a significant improvement of image quality and detection performance on the transformed images.
*Author Disclosures:*
**L. Li:** Employee; Employee of Infervision. **S. Wang:** Founder; Co-founder of Infervision. **C. Xia:** Board Member; Board Member of Infervision.

**B-0019** 11:26
**Protocol repetition in whole-body MR imaging within a large population-based cohort study: behavior of radiologic technologists and potential of automated image quality assessment**
C. Schuppert[1], R. von Krüchten[1], J.G. Hirsch[2], D.C. Hoinkiss[2], S. Selder[3], O. von Stackelberg[1], H.-U. Kauczor[1], F. Bamberg[4], C.L. Schlett[1]; [1]*Heidelberg/DE*, [2]*Bremen/DE*, [3]*Munich/DE*, [4]*Freiburg/DE*

**Purpose:** We aim to assess the frequency of protocol repetition in whole-body MR imaging within the multi-center German National Cohort (NAKO) in consideration of local, staff-dependent and technical influences. We further intent to determine its effect on scan time, and whether or not automated image quality assessment is able to predict protocol repetition.
**Methods and Materials:** All subjects enrolled in the MR substudy of the NAKO until December 31, 2016, were included in the analysis (n=11,347). Whole-body imaging was performed at five different sites, employing a uniform set of twelve protocols. All acquisitions were carried out by trained radiologic technologists (RT), whose decisions for protocol repetition were made without supervision or technological advice. Image quality parameters were derived automatically from the acquired images.
**Results:** RT acquired at least one repeat protocol in 12% (n=1,365) of subjects. The frequency of repetition differed across protocols ($p<0.0001$) as well as across sites (range: 5.28%-24.34%, $p<0.0001$), and varied over time ($p<0.0001$). The mean total scan time of 62.6min increased by 4.8min (95%CI: 4.5-5.2min) in subjects with protocol repetition(s). Several automatically derived image quality parameters were retrospectively predictive for protocol repetition, particularly image sharpness and signal-to-noise ratio, although their predictive value was not uniform for all protocols.
**Conclusion:** MR protocol repetitions are remarkably prevalent even in the highly standardized and static setting of a large cohort study. Automated image quality assessment shows predictive value for the RT's decision to perform protocol repetitions and has potential to improve time- and cost-efficiency of MR imaging studies.

**B-0020** 11:34
**Preliminary results of a neural network for the automatic detection of chest X-ray incorrect exposure**
R. Casale; *Portogruaro/IT (robby_82@hotmail.it)*

**Purpose:** To assess the accuracy of a convolutional neural network (CNN) for the automatic detection of incorrect underexposure in chest X-ray.
**Methods and Materials:** A dataset of n=260 chest X-rays was evaluated, n=128 X-rays with correct exposure and n=132 with incorrect x-ray exposure (underexposure). For the neural network training were used 203 X-rays (100 correctly exposed X-rays + 103 underexposed X-rays) using Inception_v3 architecture. Subsequently, testing was performed with the remaining images (28 correctly exposed X-rays + 29 underexposed X-rays). Then sensibility,

Wednesday specificity, receiver operating characteristic curve (ROC) and area under ROC (AUC) were computed.
**Results:** The neural network correctly classified 27 of 29 underexposed X-rays and 23 of 28 of correctly exposed X-rays. Sensibility and specificity were 0.93 and 0.82, respectively; AUC value was 97.17% (95% CI: 93.11%-100%).
**Conclusion:** Preliminary results demonstrated that neural network has the potential to distinguish between underexposed X-rays and those correctly exposed with an accuracy of 88%. The goal is to supply auxiliary and reproducible information to assist radiologists and technologists when chest X-ray quality is less obvious.

**B-0021** 11:42
**Patient-centric initiative to reduce radiation dose to paediatric patients in computer tomography for improved patient safety in a tertiary care facility in Pakistan**
M. Yusuf; *Karachi/PK (mohammad.yusuf@aku.edu)*

**Purpose:** An initiative to reduce radiation dose to pediatric patients during Computed Tomography's chest and abdomen related procedures based on international best practices for improved safety.
**Methods and Materials:** An assessment was carried out to determine the level of radiation dose being employed for pediatric patients for CT chest and abdomen examinations and was also compared to the international recommendations. The Informatics team covered all domains from technical to educational to address the higher radiation dose. Internationally defined best practices and radiation dose reference levels were identified. CT modalities were configured for appropriate reduced dose protocols, technologists were educated, radiologists were consulted on the image quality and reduced radiation dosage, an in-house system was developed to extract, process and archive the radiation dose results of each procedure and finally produce meaningful reports.
**Results:** The initiative managed to reduce the pediatric Chest and Abdomen procedure's average CT radiation doses by 43% and 62% respectively compared to the average radiation dose that was delivered in procedures prior to the initiative. This also resulted in bringing the dose levels within the reference levels defined by international bodies. For comparison, ICRP publication 87 reference levels were utilized.
**Conclusion:** Adopting a patient centric approach in a proactive manner, the team was able to assess and identify an area that had a direct bearing on patient care and was able to plan and implement an environment that provided significant reduction in the radiation dose being administered to pediatric patients during CT chest and abdomen examinations.

**B-0022** 11:50
**Quality assessment of structured multi-parametric MRI reports of the prostate based on RADLEX mapping of urosurgical key information content**
M.E. Maros[1], F. Siegel[1], B. Kämpgen[2], P. Sodmann[2], W. Sommer[3], S.O. Schönberg[1], T. Henzler[1], C. Groden[1], H. Wenz[1]; [1]*Mannheim/DE*, [2]*Rimpar/DE*, [3]*Munich/DE (matt.maros@gmail.com)*

**Purpose:** The Prostate Imaging Reporting and Data System (PI-RADS v2) was developed to provide imaging and reporting standards of multi-parametric prostate MRI (mpMRI). Urologist rely heavily on radiological reports when planning prostate biopsies. We investigated whether 1) mapping urosurgically relevant key content to RADLEX terms might be feasible to assess radiological report quality with regard to clinical usability 2) and compared it to a fully-automated guideline-based quality assessment.
**Methods and Materials:** A single center retrospective cohort study of 1028 consecutive patients (01/2017-09/2018; mean age 67.2,range 22-89 yrs) with suspected prostate pathology and mpMRI were retrieved from local RIS archives. All reports were generated using a structured reporting tool (www.smart-radiology.com). Independent blinded urologist reviewed 299 mpMRI reports and defined biopsy relevant key information content (KIC). An automatic, cross-lingual mapping of German reports, clinical KIC, and PI-RADSv2 guidline to RADLEX terms was performed using a proprietary information extraction software (www.empolis.com). Then these RADLEX converted texts were compared using free web-tool (radreport-query.com) generated cosine similarty index and Wilcoxon rank-sum test statistics.
**Results:** RADLEX mapping of urologist-defined key content identified 267 while PI-RADSv2 guideline revealed 248 terms. The identified terms showed a highly significant correlation ($p<0.001$). RADLEX similarity scores of the reports were significantly higher ($p<0.001$) when compared to urosurgical KIC (mean = 0.24, 0.11-0.33) than with PI-RADS guideline content (mean = 0.18,0.06-0.28).
**Conclusion:** Biopsy-relevant key RADLEX content could serve as an important quality measure of mpMRI reports of the prostate to improve communication with urologists and to support their planning of invasive interventions.

*Author Disclosures:*
**B. Kämpgen:** Employee; is an employee of Empolis Information Management GmbH. **P. Sodmann:** Employee; is an employee of Empolis Information Management GmbH. **W. Sommer:** Founder; is a co-founder of Smart Reporting GmbH.

**10:30 - 12:00** **Room C**

### Radiographers

### SS 214

### Professional issues in radiography

*Moderators:*
V. Logager; Copenhagen/DK
B. Snaith; Bradford/UK

**B-0023** 10:30
**Moving beyond the bookshelf: an investigation into the use of social media journal clubs in radiography practice**
S.N. Mullen[1], M. O'Connor[2]; [1]*Galway/IE*, [2]*Dublin/IE (sineadnemullen@gmail.com)*

**Purpose:** The introduction of mandatory continuous professional development (CPD) by CORU in October 2015, has increased demand on Radiographers to participate in CPD activities. Social media and digital communications have become prominent features of society and popular methods of communication and information sharing. Therefore, it is important to assess whether such technologies can contribute to CPD in Radiography.
**Methods and Materials:** A Facebook journal club was established to investigate the opinions of Radiographers regarding the use of social media facilitated journal clubs in contributing to CPD. Four teaching hospitals were chosen at random to participate. Six Radiographers from each centre were invited to participate. Two peer reviewed articles were discussed throughout the study. An online survey was used to establish the opinions of Radiographers.
**Results:** A response rate of 79% was obtained. Radiographers felt positive towards the use of social media as a tool in contributing to CPD. There was a slight disparity in opinion between newly qualified Radiographers and more senior members of the team, with newly qualified radiographers afraid of appearing "unprofessional" if found using social media in the department, and senior members being more open towards the use of social media for CPD purposes.
**Conclusion:** Respondents felt positive towards the use of social media facilitated journal clubs. Advantages of social media facilitated journal clubs are convenience and increased interaction with others. Limitations of social media facilitated journal club use in Radiography are a perceived poor professional reflection and the encroachment into an already compromised personal life.

**B-0024** 10:38
**Written patient information in radiology: an Irish perspective**
S.S. Azman, E. McDermott, M.-L. Ryan; *Dublin/IE (marielouise.ryan@ucd.ie)*

**Purpose:** Provision of information before patients attend their radiology examination plays an important role in enhancing patient experience and ensuring consent. Patient information leaflets (PIL) are used to support the verbal consultation between the patient and radiology team. This project aimed to ascertain whether public and private radiology services across Ireland use PILs for general, CT and ultrasound examinations, at what point PILs are given to patients and also to analyse the information included.
**Methods and Materials:** All radiology departments in Ireland were contacted (n=52). The manager of each participating department (n=23) was interviewed to understand at what point PILs are given to patients, and PILs were then collected from the participating departments. Analysis on 34 PILs across the three modalities was conducted under the following sub-headings- general content; radiology-related content; PIL design.
**Results:** 65% of the 23 participating facilities distribute PILs, with 35% being in the form of a written letter in advance of the examination. 74% send PILs by post while the remaining 26% use mixed methods depending on the modality and patient type. Each PIL varied in terms of the amount, depth and categorization of information. The most common information included is the nature of the procedure, the experience during the procedure and result availability after the examination.
**Conclusion:** There is a need to standardise PILs used in Irish radiology departments as both the method and timing of distribution and the information is not consistent, thus potentially impacting the knowledge imparted on the patient.

**B-0025** 10:46
**Influences on radiographers' choice of immobilisation methods in paediatric radiological examinations**
S. Christie, C. Ng, C. Sá dos Reis; *Perth/AU (claudia.sadosreis@curtin.edu.au)*

**Purpose:** To identify the main influences on radiographers' decisions about immobilisation methods in paediatric radiological examinations.
**Methods and Materials:** Australian and New Zealand radiographers were recruited to complete an online questionnaire identifying their use of different immobilisation methods and the influences on their choices. Follow-up interviews were conducted to further explore the findings. Institutional approval was obtained. Closed-end questions were analysed using frequencies and non-parametric tests (Fisher's exact and Mann-Whitney U), while content analysis was applied to open-end questions and interview data.
**Results:** 65 completed questionnaires were received; 7 respondents also participated in interviews. Parental holding was the most likely method to be used (96.9%), but psychological methods (87.7%) were considered preferable as using less force and causing less distress to children and parents. Mechanical and chemical methods were not often used. Participants sought to adapt their immobilisation practice to each situation, often using the child's age as a guide. Existing informal workplace training was perceived as adequate but not optimal. Opinions were divided over whether introducing written guidance would limit radiographers' ability to adapt to different situations.
**Conclusion:** Immobilisation is a case-by-case activity which requires radiographers to seek a balance between different factors, consistently constrained by examination type and resource availability. Patient age was the most commonly considered factor, although this was acknowledged to be an imperfect guide. Short placements at paediatric institutions are recommended as a useful way of improving immobilisation education. Increased support from governing bodies is also required.

**B-0026** 10:54
**Informed consent in diagnostic imaging: an evaluation of current practice**
A. Ramlaul; *Hatfield/UK (a.ramlaul@herts.ac.uk)*

**Purpose:** It is normally assumed that all attending patients have given their informed consent for imaging investigations, although variations may exist in the form in which this is provided. The purpose of this study was to conduct an audit of current practice regarding gaining informed consent within NHS diagnostic imaging departments throughout the United Kingdom.
**Methods and Materials:** A cross-sectional survey was conducted involving lead superintendent radiographers within NHS sites in Scotland, Wales, England and Northern Ireland. The survey explored methods in which information on radiological examinations and procedures was disseminated to patients; radiographer practices with regard to gaining informed consent; and staff knowledge of consent policies. Responses were analysed using descriptive statistics and outcomes were matched according to guidelines provided by the Health and Care Professions Council (HCPC) and College of Radiographers (CoR).
**Results:** The survey yielded a response rate of 46% (220/478). The findings demonstrated mixed awareness of policy documents, together with variations in the methods used for disseminating information to patients and gaining informed consent.
**Conclusion:** Some radiographers appear to have a cursory knowledge of consent policies. There is a lack of standardisation regarding practices for disseminating information to patients as well as for gaining informed consent from patients. The study highlights a possible need for more explicit guidance for gaining informed consent, to enhance patients' clinical outcomes and reduce the likelihood of litigation.

**B-0027** 11:02
**Perceptions about the practice of seeking informed consent in paediatric imaging**
J. Portelli[1], J. McNulty[2], P. Bezzina[1], L.A. Rainford[2]; [1]*Msida/MT*, [2]*Dublin/IE (jonathan.portelli@um.edu.mt)*

**Purpose:** To investigate health professionals' and parents' perceptions about the practice of seeking informed consent for paediatric imaging examinations.
**Methods and Materials:** This research is part of a larger multi-phase study performed at a large general hospital in Malta, which also serves as a primary paediatric referral centre. Following institutional board approval, a purposive sample of radiographers, radiologists, paediatricians, emergency physicians and parents were invited to attend for an audio-recorded semi-structured interview with the primary author. Participants were asked questions about different aspects related to the imaging of paediatric patients, including their thoughts and opinion about the practice of seeking informed consent for such examinations.
**Results:** Nineteen health professionals and seven parents willingly took part in the study. Following thematic analysis of the transcribed interview data, one of the dominant themes that emerged highlighted a general misconception that informed consent was primarily sought in paediatric imaging so as to provide a legal safeguard to the respective health professionals against any possible future medical liability issues that could arise. Furthermore, parents generally expressed feelings of heightened anxiety and concern when referring physicians and/or radiographers asked them to provide their written consent, with many interpreting this to mean that the imaging examination would involve higher risks for their child.
**Conclusion:** The findings highlight a need for relevant authorities to address the noted misconceptions and raise awareness about the true purpose of informed consent, which is to ensure that patients and/or their representatives are provided with relevant information that empowers their health decisions.

**B-0028** 11:10
**Radiographers and scientific research: the Italian experience**
M. Zanardo[1], A. Capaldo[1], M. Codari[1], D. Catania[1], P. Cornacchione[2], S. Durante[3]; [1]*Milan/IT*, [2]*Rome/IT*, [3]*Bologna/IT (moreno.zanardo@unimi.it)*

**Purpose:** Research plays a fundamental role in the development and growth of radiographers' skills. The aim of this study was to investigate how radiographers are currently involved in scientific research and how to increase their involvement in this field.
**Methods and Materials:** The Italian Federation of Scientific Radiographers Societies (FASTeR) proposed a national online survey. The questionnaire was composed of multiple-choice questions, using a 5-point Likert scale to identify radiographers' involvement (1 no involvement, 5 active participation) and skill level (1 no formation, 5 adequate formation).
**Results:** We obtained 315 participants (159 male), mean age of 40±12, 51 (16%) with a master's degree and 293 (93%) workers. Only 13 (4%) radiographers feel themselves adequately trained on research methodology during their qualification, with a mean evaluation of 2.6±1.0. Only 26 (8%) were actively involved in research activities 2.4±1.3. Only 10 (3%) radiographers did not consider research important for their work and professional growth 4.1±1.0, and 23 (7%) affirmed they did not have time for research, while 194 (62%) radiographers were fascinated by participating in research projects. Most of radiographers lack basic knowledge about Evidence-Based Medicine and scientific database (Pubmed/Medline or EMBASE). Continuing Medical Education (CME) courses were the most desirable instrument to improve research methodology, with both theoretical and practical sections 4.2±1.0. Limitations in conducting research projects were a poor knowledge of English and limited involvement by other professional figures.
**Conclusion:** To improve radiographers' involvement and skills in research, research methodology CME courses with theoretical and practical section should be activated.

**B-0029** 11:18
**The construction of care in CT**
R.K. Forton[1], M. Hardy[2], A. Sargeant[2]; [1]*Norwich/UK*, [2]*Bradford/UK (rachael.forton@nnuh.nhs.uk)*

**Purpose:** Patient-centred care and the 'patient voice' are core components of UK healthcare policy and practice guidance. This study explored how care is perceived and experienced within the high-technology environment of CT.
**Methods and Materials:** A grounded theory (GT) methodology using semi-structured interviews obtained primary data from CT radiographers and patients. Recruitment was performed at a 1200-bed teaching hospital over a 6-month period.
**Results:** The patient-radiographer relationship and the radiographer's role in providing care within CT are complex and multifaceted. Both patients and radiographers perceive CT imaging as an integral part of the overall patient care and treatment pathway. As such, the act of being imaged is perceived as a care process. While image acquisition is recognised as a task-orientated and technical process, the human element of providing care is cognitive, dynamic and responsive to individual need. Importantly, patient confidence in the care received was influenced by the radiographer's ability to build a trusting relationship and display technical competence. This in turn facilitated active compliance resulting in a technically accurate examination. Despite previous literature suggesting that technical environments create a barrier to care, patients within this study confirmed that radiographers provide care commensurate to the nursing ideals represented by the 6Cs (care; compassion; competence; communication; courage; commitment).
**Conclusion:** A new model of care encompassing both technical components and patient centeredness has been constructed and will be presented. This model promotes a new vision of patient-centred care based on care perceptions within high-technology environments.

**B-0030** 11:26
**Mobile radiography services can improve the utilisation of diagnostic imaging among nursing home residents**
E. Kjelle, K.B. Lysdahl, H.M. Olerud; *Kongsberg/NO (elin.kjelle@usn.no)*

**Purpose:** To determine utilisation of diagnostic imaging among nursing home residents and the impact of mobile radiography services on utilisation rates.

**Methods and Materials:** The number of examinations made on nursing home residents in 11 hospitals in Norway was collected for the year 2015, which involved five hospitals with mobile service facilities and six without. The data included anatomical region/organ/organ system, modality, and place of examination (hospital or nursing home). Using the 24,805 nursing home beds in the included areas as a proxy for residents, the utilization rates were compared in areas with and without mobile radiography services, using the chi-squared test.
**Results:** A total of 11,066 examinations, 0.45 per nursing home bed, was carried out. Of these, 87% were plain radiographs, 8% were CT scans and 4% were ultrasound examinations. In areas with mobile radiography services, the proportion was 50% per bed, compared to 36% in areas without, $\chi^2$ (df=1, n=11,066)=470.39, p+<0.001.
**Conclusion:** Mobile radiography services increase the overall utilization of examinations among nursing home residents significantly. Nonetheless, the rates of diagnostic imaging are still low compared to the rates for the Norwegian population in general, which is 0.9 examinations per person per year. This study clearly indicates an under-utilisation of diagnostic imaging among nursing home residents and suggests extended use of mobile services to be one possible remedy. This study has been submitted to BMC Geriatrics in 2018.

**B-0031** 11:34
**Burnout level of personnel working in radiology departments in Hungary**
D. Sipos[1], A. Pandur[2], A. Kedves[1], V. Varga[2], M. Csima[1], I. Repa[1], Á. Kovács[1]; [1]*Kaposvár/HU*, [2]*Pécs/HU (cpt.david.sipos@gmail.com)*

**Purpose:** Burnout phenomenon among healthcare professionals is the current problem. Burnout may have wide negative impact that can undermine personal and professional life of the individual, also the quality and the effectiveness of the healthcare system.
**Methods and Materials:** Radiology department workers who were members of the Hungarian Society of Radiographers were invited to participate in our online survey during June to September 2018. Maslach Burnout Inventory (MBI) and SF 36 survey was used to measure burnout levels and participants health status. Self-made questionnaire was made to determine demographic attributes. All data were analysed using SPSS V 24.0. Descriptive statistics, independent samples t test, ANOVA, Kruskal Wallis analysis were performed to examine the relationship between given demographic characteristics and the three dimensions of burnout at the significance level of p<0.05.
**Results:** Total of 404 radiology department workers participated in the survey with the average age of 40.08 years (SD12.18; 22-70). The sample had high mean burnout score for emotional exhaustion (34.28; SD 12.98) and depersonalisation (12.81; SD 6.62) compared to MBI norms. Personal achievement mean (41.03; SD 8.70) showed higher mean compared to MBI norms. Educational level, years spent in health care system, current financial situation had significant influence for all three dimensions of burnout (p<0.05). Type of workplace had significant impact on emotional exhaustion (p=0.001).
**Conclusion:** Our study indicates that high number of radiology department workers are experiencing occupational burnout at emotional exhaustion and depersonalisation dimension. These results vary according to demographic and work-related factors.

**B-0032** 11:42
**VERTIM: pilot study evaluating influence of adding nature images in imaging departments' waiting rooms, according to different aesthetic approaches, on patients' perceived anxiety**
M.P. Loiseau Audirac, C. Bader, C. Castermans, I. Quintana; *Bordeaux/FR (loiseau.marie19@yahoo.fr)*

**Purpose:** Study about influence of physical environment of imaging departments' waiting rooms on pre-examination patients' perceived anxiety. The main objective is to estimate the difference in state anxiety, perceived by patients in 3 types of waiting room environment "Nature GREEN", "Nature SEA", "Nature ZEN" compared to a reference environment "Standard waiting room" and to evaluate emotional perception and satisfaction of patients for each environment.
**Methods and Materials:** 400 patients' anxiety score was measured by STAI-Y trait and state scales after 12 minutes' immersion time. Emotional perception for environment was evaluated by "Scale of the perceived attractiveness" of Sophie Rieunier and satisfaction of patient with digital analog scale.
**Results:** The "Nature GREEN" environment is the only one where it is observed a significant 10% decrease in state anxiety score compared to the reference group "Standard waiting room". This is also where patients' satisfaction score is the highest. Affective perception scores for environment are better in "Nature GREEN" and "Nature SEA".
**Conclusion:** "Nature GREEN" environment has proven its effectiveness in reducing patients' pre-examination anxiety in imaging department. A multi-centre study would be needed to establish evidence-based recommendations for care space design.

**B-0033** 11:50
**Value-based practice (VBP) training for radiographers**
R.M. Strudwick[1], A. Newton-Hughes[2], S. Gibson[3], J. Harris[4], M. Gradwell[3], E. Hyde[5], J. Harvey-Lloyd[1], T.J. O'Regan[6], J. Hendry[7]; [1]*Ipswich/UK*, [2]*Salford/UK*, [3]*Canterbury/UK*, [4]*Guildford/UK*, [5]*Derby/UK*, [6]*Lancashire/UK*, [7]*Kingston/UK (r.strudwick@uos.ac.uk)*

**Purpose:** Value-based practice (VBP) is the consideration of a patient's values in decision-making. VBP takes into account and highlights what matters to the patient. By patient values we mean the unique preferences, concerns and expectations each patient brings to a practice encounter which must be integrated into clinical decisions for the patient[1].
**Methods and Materials:** A small team made up of radiography educators have adapted materials from a VBP handbook, originally developed for medicine. The handbook, conceived by Professor Bill Fulford and Dr. Ashok Handa[2], has been adapted for use by diagnostic and therapeutic radiographers. The scenarios included in the text have been piloted with radiographers and undergraduates at study days and in teaching sessions. We are grateful to the participants for their input. Raising the awareness of values is essential to enable contemporary person-centred care. Sustainable implementation, however, depends on a whole-systems approach where patients are put at the centre of service delivery. The handbook introduces the concept and provides examples of individual values.
**Results:** We have facilitated two successful study days focused on VBP in radiography and teaching sessions with student radiographers.
**Conclusion:** Our aim has always been to share this material; we advocate that all radiographers must gain an understanding of VBP and adopt the approach in practice. The handbook acts as a method of raising awareness. The result is the beginning of VBP conversations in radiography; when embedded, VBP will provide assurance that we put the patient as the centre of everything we do.

**10:30 - 12:00** **Room X**

### Vascular

### SS 215

### Carotid plaque and neurovascular events

*Moderators:*
D.L. Tarnoki; Budapest/HU
N.N.

**B-0034** 10:30
**Comparison of ultra-high frequency US with constrast enhanced MR Angiography and conventional US in the evaluation of carotid atheromatous plaques**
R. Picascia[1], D. Berritto[2], C. Liguori[3], M. La Porta[4], A. Reginelli[5], R. Grassi[3]; [1]*Villaricca/IT*, [2]*Acerra/IT*, [3]*Naples/IT*, [4]*San Severo/IT*, [5]*Torre Annunziata/IT (robertopicascia@gmail.com)*

**Purpose:** to compare diagnostic capabilities of an innovative ultra high frequency ultrasound (HFUS) system with conventional-US and Contrast Enhanced MR Angiography (CEMRA) in the evaluation of atherosclerotic carotid plaque in order to categorize stable vs unstable atheromas.
**Methods and Materials:** 16 carotid plaques (11 patients), causing at least a 30% luminal stenosis, were analyzed. Patients underwent to: Color-Doppler US, HF US and CEMRA. Parameters evaluated were: • Qualitative: Presence/absence hyperechoic spots and of hypoechoic core; fibrous cap (FC) delineation• Quantitative: main plaque and lipid core diameters; fibrous cap thickness
**Results:** HFUS was able to discriminate qualitative characteristics in all plaques. Quantitative values showed significant differences: using HFUS bigger dimensions were measured (mm 4.7-7.8, 6.7-8.7, 3.7-5.8 on three axes), as the lipid core (mm 0.8-2.1, 0.8-1.5, 1.2-2.4). FC thickness showed specific values on High Frequency US (0.10-1.5 upward; 0.45-1.7 downward) while it was clearly assessable only in 3 patients at conventional US. MRI showed always the FC but a precise dimension was never assesable.
**Conclusion:** HFUS is a reliable technique in defining qualitative and quantitative carotid plaque features, highlighting dimensions of the plaque, size of the lipid component, and thickness of the fibrous cap, known as most important predictors of atheroma vulnerability. Moreover this new technique has good reproducibility, fast speed of execution and good spatial resolution, in the absence of ionizing radiation.

**B-0035** 10:38
**Contrast-enhanced perfusion patterns in predicting stroke in vulnerable plaque: a cohort study of carotid stenosis in Chinese patients**
H. Yunqian, J. Jiang, M. Chen; *Shanghai/CN (18721619656@163.com)*

**Purpose:** To investigate the correlation between contrast-enhanced ultrasound perfusion patterns of carotid artery vulnerable plaque and the degree of carotid stenosis, which provides the basis for early diagnosis of cerebral ischaemic stroke.
**Methods and Materials:** From March 2018 to June 2018, 202 patients with carotid plaque who underwent CEUS were enrolled in this study. The patients who did not undergo CTA or DSA examination within 1 weeks after CEUS examination were excluded. The stenosis of carotid artery was determined by CTA or DSA as gold standard. Finally, 80 patients were analysed (35 patients in the stenosis group and 45 patients in the non-stenosis group). The appearance of enhancement in plaques were observed and the enhancement level of the plaques was divided into I-IV grades according to enhanced ultrasound findings of carotid plaques. The patients were divided into plaque base entering mode and plaque surface entering mode by the CEUS perfusion pattern when the contrast agent enters the plaque. The correlation between the CEUS perfusion pattern and the degree of carotid stenosis was analysed.
**Results:** The enhanced level of carotid plaque between the stenosis group and the non-stenosis group showed statistically significant difference. The plaque of the stenosis group and the non-stenosis group entered the plaque with different patterns, and the difference was statistically significant.
**Conclusion:** There remains a significant correlation between the enhancement level of carotid plaque and the degree of carotid stenosis, and the different CEUS perfusion patterns are closely correlated with the degree of carotid stenosis.

**B-0036** 10:46
**Is automatic plaque quantification reliable: an analysis of spectral multienergy CT**
G. Corrias[1], G. Micheletti[2], J. Suri[3], G.M. Argiolas[2], P. Lucatelli[4], L. Saba[1]; [1]*Cagliari/IT*, [2]*Monserrato/IT*, [3]*Denver, CO/US*, [4]*Rome/IT (corriasgmd@gmail.com)*

**Purpose:** To compare semi-automatic plaque quantification on different energy levels of DECT to inform standardization efforts for single-energy protocols where difference is to be avoided, as well as to explore opportunities in multi-energy protocols where differences are intended.
**Methods and Materials:** Retrospective study enrolling 36 patients who underwent DECT of the carotids. Seven energy levels were reconstructed (keV: 40, 66, 70, 76, 86, 100, 120). Plaque subcomponent analysis was performed for each energy separately, without modifying lumen and wall segmentations using commercially available software (vascuCAP, Elucid Bioimaging).
**Results:** Normality of each energy was tested using the Kolmogorov-Smirnov Z test. Since most were not distributed normally, a Wilcoxon test was performed to check differences with a p value <0.05. Differences of component volumes were in the order of the reproducibility coefficient (RDC) at the closest energy levels (calcium 66keV vs 70keV : Z value:-2.1; p≤0.05; LRNC 66keV vs 70keV; Z value:-2.5, p≤0.05; Matrix 66keV vs 70keV:-2.3, p≤0.05), and documented that differences increased with difference in energy.
**Conclusion:** Plaque component analysis has been known to provide important prognostic information on plaque progression and adverse acute cardiovascular events. We established that standards efforts should specify differences of no greater than ±2 keV for single-energy protocols, and have established base data on which exploiting intended differences might be of value were they to be processed by algorithms designed to exploit these differences beyond what single energy may reasonably be expected to do, such as speculated vs. dense calcium, or differing stages of IPH, for example.

**B-0037** 10:54
**Carotid plaque instability and neovascularisation assessment using contrast-enhanced ultrasound and superb micro-vascular imaging**
M. Radzina, A. Lioznovs, A. Jukna; *Riga/LV (lioznov1@gmail.com)*

**Purpose:** There are several causes for cerebral ischaemia and if risk factors are diagnosed timely, the risk of stroke can be significantly reduced. One of the main risk factors for cerebral ischaemia is the unstable atherosclerotic plaque in the arteries. The purpose of the study was to analyse multi-parametric US techniques ability to confirm the plaque's instability more precisely than the baseline investigation of Duplex US.
**Methods and Materials:** In prospective research conducted in the period from 2017 to 2018, were included 22 patients with unstable plaque which were detected with duplex, every patient was analyzed with CEUS, SMI and CTA methods. The results were histologically proven in 8 cases.
**Results:** Unstable plaque was diagnosed in 22 patients using duplex and in 21 patients using CT. Comparing both methods statistically significant correlation was found (rs = 0,781; p = 0,0001), The neovascularization was diagnosed in 13 (59.1%) patients by CEUS - in 6 (46.2%) plaques showed neovascularization grade 1 and in 7 (53.8%) plaques were detected grade 2. Using SMI method neovascularization were found in 12 (54.5%) patients, with statistically significant correlation between CEUS and SMI (r s = 0,801; p = 0,0001). Histology showed vasa vasorum presence in all CEUS positive cases from 2-23 microvessels with positive tendency (p>0.05) within endarterectomy material and significant difference with positive correlation to stenosis grade (70-90%) (p=0.03)
**Conclusion:** New ultrasound methods such as CEUS and SMI provide additional information on detecting aterosclerotic plaque instability and vasa vasorum with positive correlation to stenosis grade.

**B-0038** 11:02
**Early embolisation after carotid artery stenting: role of MR-DWI as pre-procedural predictor and discriminant between intra- and post-procedural events**
F. Guarasci, R. Faletti, M.A. Ruffino, M. Fronda, M. Gatti, G. Varetto, D. Righi, P. Rispoli, P. Fonio; *Turin/IT (guarasci.fab@gmail.com)*

**Purpose:** To evaluate the incidence of intra- and early post-procedural micro-embolism during Carotid Artery Stenting and the role of DW-MRI in the characterization of carotid plaque activity.
**Methods and Materials:** This prospective study included 50 patients who underwent CAS with two different mesh-covered stents. All patients had carotid plaque DW-MRI pre-procedure (except for patients with re-stenosis after endarterectomy, 13/50, 26%), brain DW-MRI pre-proc., at 1h, 24h and 30 days post-proc., clinical evaluation and Doppler US pre-proc., at 24h and 30 days post-proc. Imaging analysis (quantitative and qualitative) was performed in a double-blinded fashion by two radiologists.
**Results:** No statistically significant differences between the two type of stents both in the incidence at 1h (p=0.23), 24h (p=0.36) and in the volume of new brain lesions at 24h (p=0.27). A total of 34 new lesions in 19 patients (38%) were found: 4 (11.8%) at 1h, 30 (88.2%) at 24h post-proc. The 30-day DWI-MR showed complete resolution of all lesions and no evidence of new lesion. The incidence of new lesions at 24h resulted higher in patients with positive carotid plaque DW-MRI (12/16, 75%, vs. 0/21, 0%, with a LR+ of 6.25), results paralleled by the ADC value (0.83+/-0.21 vs 1.42+/-0.52).
**Conclusion:** The majority of early asymptomatic brain lesion did not occur during the procedure but in the ensuing 24h. A positive pre-proc. carotid DW-MRI was associated with an increased incidence of micro-embolizations after CAS.

**B-0039** 11:10
**Ultrasonographic evaluation of changes in carotid arteries in patients with hypertension after a stroke**
P. Gac, E. Trzmielewska, M. Poreba, G. Mazur, M. Sobieszczanska, R. Poreba; *Wrocław/PL (pawelgac@interia.pl)*

**Purpose:** The aim of study was to estimate the frequency of ultrasound-assessed changes in carotid arteries in patients with hypertension after a stroke.
**Methods and Materials:** 111 patients were qualified for the study. They represented two study groups: patients with arterial hypertension after a stroke in their medical history (group A, n=59) and patients with arterial hypertension without stroke in the medical history (group B, n=52). In the studied group an ischemic stroke was observed in 88.1%, and a hemorrhagic stroke- in 11.9%. Ultrasound examination of carotid arteries was carried out in all patients.
**Results:** Significantly higher IMT (intima-media thickness) was observed in group A in comparison to group B (1.09±0.23 mm vs.0.87±0.31 mm; p<0.05). The remaining parameters of ultrasound examination of carotid arteries didn't differentiate group A from group B. The type of stroke, the time elapsed since the stroke and the number of ischemic events didn't determine statistically significant differences in ultrasonography of carotid arteries in contrast to the effectiveness of blood pressure control. In group A, patients with well-controlled hypertension were characterized by lower IMT than patients with non-controlled hypertension (0.95±0.24 mm vs. 1.24±0.21 mm; p<0.05). The regression analysis showed that higher BMI and higher blood glucose concentrations are independent risk factors for higher IMT, while the use of β-blockers is an independent protection factor against higher IMT.
**Conclusion:** In the studied group of patients with hypertension after a stroke, the IMT value in ultrasound examination of carotid arteries may constitute an indicator of high prognostic significance.

**B-0040** 11:18

**Comparison between high-frame rate vector flow and computational flow dynamics in the carotid bifurcation: evaluation of a protocol study**

A. Goddi[1], T. Frattini[1], M. Bozzetto[2], C. Bortolotto[3], L. Milan[1], D. Zatelli[2], R. Novario[1], F. Calliada[3], A. Remuzzi[2]; [1]*Varese/IT*, [2]*Bergamo/IT*, [3]*Pavia/IT* *(chandra.bortolotto@gmail.com)*

**Purpose:** Combining 2D ultrasound High-frame rate Vector Flow (HiFR-VF) and 3D Computational Fluid Dynamics (CFD) on the carotid bifurcation (CB) may allow the clinical validation of a new approach to obtain a comprehensive assessment of the blood flow field. This initial study aimed at ensuring the effectiveness of a protocol designed for a future multicentric prospective study.
**Methods and Materials:** A CB with severe stenosis at B-mode and Doppler US was enrolled. HiFR-VF provided the 2D velocity vectors from multidirectional plane waves and allowed the quantification of the complex flows and wall shear stress (WSS) measurements at different locations. The 3D model of the diseased vessel was reconstructed starting from a 3D FFE MRI multi-chunk sequence that makes use of the inflow effects of blood to visualize vessels. Subsequently, CFD simulation of the CB was performed and the flow field was characterized using velocity streamlines, while reciprocating disturbed flow was described using the oscillatory shear index. HiFR-VF and CFD findings were compared to analyze the ability of HiFR-VF in identifying areas of vascular wall exposed to oscillatory WSS.
**Results:** WSS findings with HiFR-VF showed good agreement with the CFD simulation results. In particular, the areas of abnormal WSS obtained with the two modalities, matched.
**Conclusion:** Although WSS can only be estimated in a 2D plane with HiFR-VF, nonetheless oscillating WSS can be detected and correlate with the CFD results. Preliminary findings show that the study protocol was correctly designed and can be used for prospective clinical investigation.
*Author Disclosures:*
**A. Goddi:** Consultant; Dr Goddi is a consultant for Esaote, Shenzhen Mindray Bio-Medical Electronic Co, and SuperSonic Imagine. **C. Bortolotto:** Consultant; Dr. Bortolotto is a consultant for Bracco Imaging and Doc Congress. **F. Calliada:** Consultant; Prof Calliada is consultant for Hitachi Medical Systems Europe, Shenzhen Mindray Bio-Medical Electronics Co, and Toshiba Medical Systems Europe.

**B-0041** 11:26

**Carotid intima-media thickness (CIMT) as a diagnostic marker of transient ischaemic attacks (TIA)**

D.J. Petrovic; *Belgrade/RS (dusanpetrovic736@gmail.com)*

**Purpose:** To establish the correlation between CIMT and TIA and the correlation between the quantitative and qualitative characteristics of the plaque in the wall of the carotid arteries and the occurrence and type of TIA.
**Methods and Materials:** 120 patients with TIA were tested by duplex ultrasonography and compared to the control group (n=25) compatible in age and gender. Among the ultrasonic parameters, the following ones were examined: CIMT, the presence and size of the plaque, and the presence of ulceration on the free surface of the plaque. Group of patients with TIA was divided into subgroups based on aetiopathological mechanism (emboligenic, haemodynamic and coagulopathy). The Student's t test and Pearson $\chi^2$ test were used for statistical analysis.
**Results:** Statistically, CIMT was significantly higher (p<0.05) among the patients with haemodynamic and emboligenic TIA in comparison with the control group. The frequency of the plaque larger than 70% was statistically significantly higher (p<0.01) among the patients with emboligenic and haemodynamic TIA in comparison with the control group. Regarding the frequency of ulceration, the difference between the group of patients with emboligenic TIA and the control group was highly statistically significant (p<0.01).
**Conclusion:** CIMT and the size of the plaque (larger than 70%) correlate with the occurrence of haemodynamic and emboligenic TIA. The presence of ulceration on the free surface of the plaque correlates with the occurrence of emboligenic TIA. Early diagnosis of carotid artery disease is of paramount importance from the aspect of stroke prevention, and ultrasound plays a pivotal role therein.

**B-0042** 11:34

**Ultrasound estimation of carotid intima media thickness: semiautomatic versus manual measurement**

G. Lastella, F. Wiedenmann, C.B. Monti, F. Secchi, M. Alì, F. Sardanelli; *Milan/IT (giulia.lastella@unimi.it)*

**Purpose:** To evaluate and compare manual and semiautomatic carotid intima-media thickness (CIMT) assessment.
**Methods and Materials:** Ultrasound B-mode images of 200 consecutive patients who underwent color-Doppler of supra-aortic trunks with RS80A Prestige, Samsung Healthcare from 5/2017 to 12/2017 were retrospectively evaluated. For each patient, CIMT was measured on the same image of the right common carotid artery, selected by an experienced (≥5 years) radiologist. Two different readers performed all measurements using both the manual and the semiautomatic method, randomly ordered sessions, separated by at least one week, the least experienced reader (2 years) repeating all measurements 4 weeks apart. Images were graded for quality by considering the quality index (QI), ranging from 0.00 to 1.00 automatically provided by the software.
**Results:** When all 200 cases were included, inter-reader reproducibility was significantly higher (*p*=0.010) for manual CIMT (bias=81%,-0.017 mm, coefficient of reproducibility(CoR)=0.143 mm) than semiautomatic CIMT (bias=75%,-0.001 mm,CoR 0.170=mm), while intra-reader reproducibility did not differ significantly(*p*=0.847). When only cases with IQ ≥0.75 were considered (n=158), inter-reader reproducibility was significantly higher (*p*=0.006 for semiautomatic CIMT (bias=86%,0.001 mm,CoR=0.092 mm) than manual CIMT, while intra-reader reproducibility was not significantly different (*p*=0.772). Concordance between semiautomatic and manual CIMT was fair (κ=0.341) with an agreement of 80% on the whole sample, and fair (κ=0.341) with an agreement of 80% in cases with IQ ≥0.75.
**Conclusion:** To achieve the best accuracy of semiautomatic CIMT, high quality index should be sought. Nevertheless, semiautomatic CIMT assessment with AutoIMT cannot yet substitute manual calculations in the attribution of cardiovascular risk.

**B-0043** 11:42

**Comparative study of common carotid intima-media thickness in type 2 diabetics and non-diabetics in Port Harcourt, Nigeria**

O. Moemenam[1], R. Njeze[2], E. Okwudire[1], U. Ezenwugo[1]; [1]*Port Harcourt/NG*, [2]*Enugu/NG (meetle1@gmail.com)*

**Purpose:** Intima media thickness (IMT) is a measure of atherosclerotic burden in diabetes mellitus (DM), and measurement of carotid IMT has been used as a surrogate for atherosclerosis in less accessible coronary and cerebral arterial systems. We aimed to compare carotid intima-media thickness (CIMT) in type 2 diabetics with controls and correlate CIMT with age, sex, BMI and duration of disease.
**Methods and Materials:** This was a cross-sectional study of 350 Type 2 diabetics and 350 controls recruited by systematic sampling between February 2015 to June 2016 in Port-Harcourt, Nigeria. CIMT was measured by ultrasound 1cm proximal to the carotid bulb and data was analyzed using statistical package for social sciences (SPSS) version 20, at significance level of p<0.05.
**Results:** Type 2 diabetics had significantly higher CIMT than non-diabetics (1.09±0.25mm vs 0.94±0.24mm, p =0.001). There was significant correlation between CIMT and age in type 2 diabetics (r=0.53, p=0.001), controls (r=0.76, p=0.001), and with duration of DM (r=0.194, p=0.001). Negative correlation between CIMT and BMI in the type 2 diabetic participants (r= -0.913, p=0.001) was observed. CIMT was significantly higher in male compared to female diabetics (p=0.001). There was no significant difference in the prevalence of carotid plaques between type 2 diabetics and non-diabetics (22.8% vs 19.4%; p=0.32) or between left and right carotid arteries.
**Conclusion:** CIMT was greater in diabetics compared with controls, and correlated with age in both groups. CIMT may be used as a measure of atherosclerotic disease in our environment.

**B-0044** 11:50

**Weight loss in obesity, impact on carotid intima media thickness and cardiovascular risk factors: a longitudinal study**

B. Domènech-Ximenos, V. Cuba Camasca, J. Daunis-i-Estadella, S. Pedraza Gutierrez, J.M. Fernández-Real, J. Puig Alcàntara; *Girona/ES (bl.domenech@gmail.com)*

**Purpose:** Obesity increases the burden of atherosclerosis and, therefore, represents a risk factor for clinical cardiovascular disease. Intima media thickness (IMT) is a noninvasive marker of subclinical atherosclerosis. It remains unclear whether weight loss in obese subjects is accompanied by a reduction in consistent with a lowering of risk of cardiovascular events. We evaluated the effects of bariatric surgery and of a conservative approach (diet and exercise) on carotid IMT and cardiovascular risk factors in obese subjects.
**Methods and Materials:** We studied 163 individuals [a control group of 75 healthy participants (40 women, age 42 years (31-51), body mass index (BMI) 24.33 (22.23-27.2)) and 88 obese subjects (52 women, age 46 (38-52), BMI 43.37 (40.18-47))]; 22 (25%) obese subjects underwent bariatric surgery. We recorded weight, BMI, blood pressure, cholesterol, triglycerides, fasting glucose, insulin, and insulin resistance index and calculated Framingham Risk Scores (FRS). Carotid IMT was evaluated by ultrasound. The impact of weight loss on carotid IMT at 2-year follow-up was evaluated with regression models.
**Results:** At 2-year follow-up after bariatric surgery, BMI had decreased from 45.45 to 27.75 kg/m2 (p<0.001) and FRS decreased from 15 (10-16) to 13 (8-15) (p=0.035). Mean carotid IMT decreased from 0.85 ± 0.13 mm to 0.65 ±

0.05 mm (p<0.001), a reduction of 0.005 mm per kg of weight lost. Conservative approach did not impact cardiovascular risk markers.
**Conclusion:** Weight loss after bariatric surgery substantially reduces carotid IMT values consistent with a lowering in risk of cardiovascular events.

**10:30 - 12:00** **Room N**

### Musculoskeletal

### SS 210a

### Haematological, metabolic and endocrine diseases

*Moderators:*
G.K.O. Aström; Uppsala/SE
E.H.G. Oei; Rotterdam/NL

**B-0045** 10:30
**Incidence of vertebral fractures in patients with β-thalassaemia major in treatment with oral bisphosphonate**
F. Pellegrino, M. Pontrelli, R. Mungari, M. Fortini, M.R. Gamberini, M.C. Zatelli, M.R. Ambrosio, M. Giganti; *Ferrara/IT (fabiopellegrino07@gmail.com)*

**Purpose:** To analyze the efficacy of oral bisphosphonate therapy (alendronate and risedronate) in patients with β-Thalassaemia evaluating incidence of vertebral fractures (VFs) and bone mineral density (BMD) values.
**Methods and Materials:** 60 patients (31 M, 29 F) with β-thalassaemia major of average age 35.02 ± 6.32 afferent at the center of Ferrara were selected retrospectively with age> 25 years, at least 1 between reduced BMD (Z-score<-2 premenopausal women and men aged <50), osteoporosis (T-score <-2.5), ≥ 1 fragility fracture. We analyzed BMD values, thoracolumbar radiographs for VFs evaluation (according Genant classification) and biochemical data at 2, 3 and 5 years.
**Results:** The mean duration of therapy was 6.23 ± 1.93 years. We observed an improvement from baseline of lumbar spine BMD of 4% to 2 yr of treatment, 2% to 3 yr and 3% to 5 yr. BMD is increased by 1% in the femur (F) and 2% at the neck of the femur (FN) at 2 yr of treatment, at 3 yr + 1% (F) and 0% (FN), at 5 aa -3% (F) and -2% (FN). Incidence of new vertebral fractures was of 27%.
**Conclusion:** Therapy was useful in rising BMD at the lumbar level with an improvement of Z and T-score and in stabilizing BMD at the femoral level (F and FN) in the first 3 yr of therapy with a slight reduction at 5 yr, but VFs in these well-treated patients were still common and morphometry measurement remains a useful test in surveillance of these patients.

**B-0046** 10:38
**Lumbar spine bone mineral density Z-score discrepancy by DXA and QCT in thalassaemic major patients**
F. Pellegrino, L. Perrucci, M. Pontrelli, M.R. Gamberini, M. Fortini, I. Gagliardi, M.C. Zatelli, M.R. Ambrosio, M. Giganti; *Ferrara/IT (fabiopellegrino07@gmail.com)*

**Purpose:** Dual X-ray absorptiometry (DXA) is the most common mode of bone mineral density (BMD) evaluation in young adults with b-thalassaemia major (TM). In addition to DXA that determines an areal BMD, Quantitative Computerised Tomography (QCT) also detects volumetric BMD which is independent of bone size. Our aim is to evaluate concordance of BMD values in thalassaemic patients obtained by QCT and DXA.
**Methods and Materials:** We enrolled 15 patients with TM from the Hospital of Ferrara who underwent both a DXA scan of lumbar spine in regular follow-up and an unenhanced CT, performed for other causes, within 4 months. CT images of lumbar spine were evaluated with a quantitative dedicated post-processing software calculating volumetric BMD for L1-L4 vertebrae. BMD values of both methods were expressed as Z-scores and T-scores and the results were correlated.
**Results:** Of 10 patients (6 males < 50 aged, 4 premenopausal woman), the overall prevalence of severely low bone density (Z-score ≤-2) was 9/10 (90%) by DXA and 3/10 (30%) by QCT with concordance in 4/10 patients (40%). In all 10 patients of this group QCT Z-score was higher than DXA Z-score with significant difference(p-value=0.002). 5 patients(>50 years old) showed T-score ≤-2,5 correspondent to osteoporosis both on DXA and QCT with 100% of concordance.
**Conclusion:** Our data show a discrepancy in lumbar BMD Z-scores by DXA and QCT in thalassaemic patients suggesting that DXA BMD could be falsely low in young subjects with short bones and QCT could be more accurate to assess it.

**B-0047** 10:46
**Opportunistic osteoporosis screening using hydroxyapatite-specific vertebral bone mineral density measurements derived from dual-layer CT**
J.H.W. Bodden, F. Roski, J. Hammel, K. Mei, T. Baum, A. Laugerette, E.J. Rummeny, A.S. Gersing, B.J. Schwaiger; *Munich/DE (jannis.bodden@tum.de)*

**Purpose:** To evaluate the applicability of in vivo hydroxyapatite (HA)-specific bone mineral density (BMD) measurements based on non-contrast-enhanced phantomless dual-layer spectral CT (DLCT).
**Methods and Materials:** BMD were obtained using spectral information from DLCT as well as with quantitative CT (QCT) examining a spine phantom with three artificial vertebral bodies with known HA densities, while simulating different patient positions and grades of obesity. Furthermore, HA-specific BMD values were determined using non-contrast routine DLCT in 174 vertebrae (33 patients; 66±18 years; 33% women; 45% with prevalent fractures) and the results were compared with QCT-based BMD. The discriminative power of HA-specific BMD for differentiating subjects with versus without osteoporotic fractures was analysed using ROC analyses, also for an extended cohort of 79 patients (66±18 years; 52% women; 38% with prevalent fractures).
**Results:** HA-specific BMD measurements in the phantom were more accurate than QCT, particularly when simulating obese patients. In vivo, strong correlations were shown between DLCT and QCT ($r$=0.987, $p$<0.001) and a high agreement in a Bland-Altman plot was observed. ROC areas under the curve (AUC) were 0.889 for QCT- and 0.878 for DLCT-based BMD in 33 patients. The AUC for the extended cohort was 0.858 for HA-specific BMD measurements, with an optimal cut-off at 81 mg/ml.
**Conclusion:** In vivo, DLCT-based HA-specific BMD measurements showed comparable results to QCT measurements as well as for distinguishing patients with vertebral fractures and those without. This suggests that HA-specific BMD measurements obtained from phantomless DLCT may be used for opportunistic osteoporosis screening.

**B-0048** 10:54
**Consistency of bone mineral density assessments between dual-energy CT and quantitative CT**
M. Chen, H. Yuan; *Beijing/CN (chenmin@bjmu.edu.cn)*

**Purpose:** To evaluate the consistency of bone mineral density (BMD) measurements between a phantomless dual-energy CT scanner and conventional quantitative CT (QCT) in vitro.
**Methods and Materials:** QCT and dual-energy CT scans of 23 fresh sheep vertebrae were performed on the same CT scanner which generated dual-energy images from rapid kilovoltage switches (80kv-140kv). A fixed tube voltage of 120kV was applied for QCT scans with the bone equivalent phantom placed below the samples. Trabecular BMD analyses of QCT images were performed using QCTpro software. The dual-energy CT images were processed to generate calcium hydroxyapatite (HAP) density images based on HAP-water material pairs. HAP densities of the same regions were measured. Pearson correlation, paired t test, and intraclass correlation coefficient (ICC) were applied for consistency assessment.
**Results:** BMD measurements of QCT ranged from136 mg/cm$^3$ to 486 mg/cm$^3$, and the BMD measurements of DECT ranged from 128mg/cm$^3$ to 419mg/cm$^3$. BMD derived from DECT images were significantly lower than that of QCT (p<0.001), while they displayed a strongly linear correlation (r=0.989, p<0.001). The ICC between the two measurements was 0.974 (p<0.001).
**Conclusion:** In vitro, the HAP densities acquired by dual-energy CT displays a highly linear correlation with BMD measured by QCT of the same specimens. Although the results from dual-energy CT were not completely same as QCT, the phantomless dual-energy CT has the potential to be applied for BMD assessment clinically.

**B-0049** 11:02
**Metacarpal bone diameter increases constantly, but escalated resorption of the inner surface at menopause explains decreased bone mineral density - a study in 1492 adult women**
C. Samuelsson, J. Kälvesten, M. Wilczek, T.B. Brismar; *Stockholm/SE (carlsamuelson@gmail.com)*

**Purpose:** To assess how bone morphology and mineralization changes with age in women.
**Methods and Materials:** The non-dominant hand was analyzed with digital X-ray radiogrammetry (DXR) in 1,429 women aged 40-74 years undergoing mammography screening twice with 18 to 24 months interval, depending on age. Yearly change in bone parameters was calculated by averaging the individual changes.
**Results:** Both the inner and outer diameter of the metacarpals increased with age while DXR bone mineral density (DXR-BMD) decreased. There was a continuous increase in outer diameter in adult age while there was a five-fold increase in yearly inner diameter growth and DXR-BMD loss during 48 to 53

years of age. In the age-interval 40-47 the change in W and ID was on average 4.65um and 8.8 um /year respectively, compared to 1.9 um and 49 um /year after 53 years of age.
**Conclusion:** The faster decrease in DXR-BMD observed during and after menopause is caused by resorption of the inner cortical surface, increasing the inner diameter that no longer is matched by the increase in outer diameter. To our knowledge it has not previously been shown how individual bone diameter changes with age in adults. The automatized analysis and high reproducibility of DXR-BMD made that analysis possible in a great number of subjects despite a short time-interval. We speculate that most bones in the human body grow in the same pattern as observed in the metacarpals, partly explaining the decreasing BMD at older age.
*Author Disclosures:*
**J. Kälvesten:** Employee; Previous employee at Sectra, that produces the DXR software.

**B-0050** 11:10
**Sarcopenia and osteoporosis in patients with cancer diagnosis: evaluation with CT**
S. Ivanoski[1], V. Vasilevska Nikodinovska[2]; [1]*Ohrid/MK*, [2]*Skopje/MK*
*(slavcoivanoski@gmail.com)*

**Purpose:** To evaluate the prevalence of sarcopenia in patients with and without diagnosis of malignant tumour. To assess the relationship between sarcopenia and osteoporosis in patients with malignancy.
**Methods and Materials:** 70 patients age ≥65 (mean age 71.5), 33 male and 37 female, that underwent abdominal CT were included in the study. Half of patients (N=35) had diagnosis of malignant tumour, majority colorectal cancer (37%). Trabecular bone mineral density at level of L1 vertebral body, in upper vertebral body part, using single slice technique, was measured for detecting osteoporosis. Density and cross-section area of psoas muscles at L3 vertebral body mid level were measured for diagnosing sarcopenia. Cut-off values for CT detection of sarcopenia and osteoporosis established by previous researches were used. Chi square test, Pearson correlation, and linear regression analysis were performed.
**Results:** 40% of patients with diagnosis of malignant tumour had muscle density diagnostic for sarcopenia, compared to 11.4% in patients without cancer. Differences for sarcopenia, assessed by psoas muscle density values, between patients with malignancy and non-malignant group were statistically significant ($\chi^2$=6.06, p=0.016). Osteoporosis values weren't significantly different between groups ($\chi^2$=0.06, p=0.811).There was moderate correlation between bone density and psoas muscle density in patients with malignancy (r=0.441, p<0.05). Regression analysis showed sarcopenia was positively correlated with age (odds ratio=1.169, p=0.036) and malignancy (odds ratio=0.117, p=0.08).
**Conclusion:** Sarcopenia is significantly higher in patients with diagnosis of cancer, compared to patients without malignancy diagnosis. Sarcopenia is associated with osteoporosis in malignancy and should be mentioned in abdominal CT report.

**B-0051** 11:18
**Changes in bone marrow fat upon dietary induced weight loss**
M. Spurny, Y. Jiang, R. Schübel, T. Nonnenmacher, C.L. Schlett, O. von Stackelberg, H.-U. Kauczor, T. Kuhn, J. Nattenmüller; *Heidelberg/DE*
*(manuela.spurny@med.uni-heidelberg.de)*

**Purpose:** Bone marrow fat (BMF) is an ectopic fat storage depot connecting metabolism and bone health. Evidence on changes in BMF with weight loss is limited. Therefore we aimed to analyze changes in BMF content following dietary induced weight loss.
**Methods and Materials:** Magnetic resonance imaging data of the HELENA-trial, an RCT among 143 non-smoking, obese participants (BMI at baseline between 25 and 40 kg/m$^2$, 50% female), were used to quantify BMF content before and after a 12 week dietary intervention phase. Regions of interest (2cm$^2$) were evaluated manually on a post-processing software (OsiriX, Pixmeo SARL, Bernex, Switzerland) using the proton density fat fraction map, based on mean counts from L1 and L2. The study cohort was divided into weight loss quartiles based on changes in body weight between baseline and week 12.
**Results:** Average BMI at baseline was highly similar across the weight loss quartiles (Q1:32.1±4.1kg/m$^2$; Q2: 31.1±3.7kg/m$^2$; Q3: 30.9±3.4kg/m$^2$; Q4: 31.5±3.7kg/m$^2$). Diet induced weight loss varied between 0.0±0.2% for Q1 to -11.3±0.6% for Q4. Relative changes in BMF were 0.7±2.4%, -3.5±2.0%, -1.4±2.4%, and -12.7±3.3% for Q1 to Q4. Across all four weight loss quartiles and for the two group comparison Q1 vs. Q4 there was a significant difference (p<0.01) for changes in BMF. For the two group comparisons Q1 vs. Q2 and Q1 vs. Q3 there were no significant differences (p>0.05) observed.
**Conclusion:** Our data suggest that BMF levels decrease with weight loss. Further research will unravel the association between the amount of weight loss and BMF.

**B-0052** 11:26
**Fat mass does not increase the precision error of trabecular bone score measurements**
C. Buonomenna[1], C. Messina[1], S. Gitto[1], A. Biacca[2], D. Albano[3], L.M. Sconfienza[1]; [1]*Milan/IT*, [2]*Novara/IT*, [3]*Palermo/IT*
*(buonomenna.ciriaco@gmail.com)*

**Purpose:** We investigated the in-vivo effect of increasing BMI and waist circumference on TBS precision error.
**Methods and Materials:** A population of postmenopausal Caucasian women was distributed in three different BMI (normal, overweight, class I obesity), plus two further groups based on waist circumference (WC) diameter (≤88 cm and >88 cm respectively). In-vivo precision error was calculated on 30 consecutive subjects that were scanned two times, with patient repositioning. Coefficient of Variation, percent least significant change (LSC%) and reproducibility were calculated according to the International Society for Clinical Densitometry guidelines.
**Results:** Ninety-five women aged 66 ± 10 (mean ± standard deviation) were included. No significant differences were found for BMD and TBS precision errors, respectively, when comparing BMI groups and WC groups. BMD reproducibility ranged from 95.9% (BMI >30 kg/m$^2$) to 97.5% (BMI <25 kg/m$^2$). TBS reproducibility ranged between 95.8% (BMI= 25-29.9 kg/m$^2$, WC >88 cm) and 96.6% (BMI <25 kg/m$^2$). With the exception of obese group, a significant difference was found between BMD and TBS reproducibility, being that of TBS slightly lower than BMD. A significant decrease of TBS was found between normal and obese subjects, as well as between WC groups; BMD variations between groups were not statistically significant.
**Conclusion:** TBS precision error is not affected by BMI and WC differences. TBS reproducibility showed to be slightly lower than that of BMD, but this difference was mitigated in obese patients. A negative association was found between the amount of fat mass and TBS mean values.
*Author Disclosures:*
**C. Messina:** Grant Recipient; Abiogen Travel Grant. **L.M. Sconfienza:** Grant Recipient; Abiogen Travel Grant.

**B-0053** 11:34
**Dual-energy computed tomography in calcium pyrophosphate deposition: initial clinical experience**
J. Legrand[1], T. Pascart[1], L. Norberciak[1], F. Becce[2], J.-F. Budzik[1]; [1]*Lille/FR*, [2]*Lausanne/CH (legrand.julie@ghicl.net)*

**Purpose:** To determine the dual-energy computed tomography (DECT) attenuation properties of meniscal calcifications in calcium pyrophosphate deposition (CPPD) in vivo, and assess whether DECT was able to discriminate meniscal CPP deposits from calcium hydroxyapatite (HA) in subchondral and trabecular bone.
**Methods and Materials:** Patients with clinical suspicion of crystal-related arthropathy and knee DECT scans were retrospectively assigned to CPPD (n=19) or control (n=21) groups depending on the presence/absence of chondrocalcinosis on DECT. Regions of interest were drawn in meniscal calcifications, non-calcified menisci, as well as subchondral and trabecular bone. Five DECT parameters were obtained: CT numbers (HU) at 80 and 140 kV, dual-energy index (DEI), electron density ($\rho_e$), and effective atomic number ($Z_{eff}$). The four different knee structures were compared using mixed linear models.
**Results:** Meniscal calcifications (n=89) in CPPD patients had mean±SD CT numbers at 80 and 140 kV of 257±64 and 201±48 HU, respectively; with a DEI of 0.023±0.007, and $\rho_e$ and $Z_{eff}$ of 140±35 and 8.8±0.3, respectively. Meniscal CPP deposits were readily distinguished from calcium HA in subchondral and trabecular bone (p≤0.01), except at 80 kV separately (p=0.28). $Z_{eff}$ and $\rho_e$ both significantly differed between CPP deposits and calcium HA in subchondral and trabecular bone (p<0.0001).
**Conclusion:** This proof-of-concept study shows that DECT can discriminate meniscal CPP deposits from calcium HA in subchondral and trabecular bone in vivo, paving the way for the biochemical signature assessment of intra- and juxta-articular calcium crystal deposits.

**B-0054** 11:42
**Retrospective study of foot and ankle dual-energy CT in gouty arthritis patients without first metatarsophalangeal joint uric acid crystal deposition**
H. Yang[1], Y.-S. Hong[2], J. Gong Yong[3], Y.K. Lee[1], S.K. Kim[1], J.H. You[1], E. Park[1]; [1]*Jeonju/KR*, [2]*Gwangju/KR*, [3]*Chonju/KR (yphot@naver.com)*

**Purpose:** To evaluate the accuracy and which DECT findings are characteristic of diagnosing foot and ankle gout in patients without uric acid crystal deposition in first metatarsophalangeal joint (MTPJ).
**Methods and Materials:** Among 97 foot and ankle DECT obtained from January 2016 to September 2017, 27 foot and ankles without first MTPJ green pixelation were enrolled. Two blinded musculoskeletal radiologists evaluated the images and classified the examination findings as positive or negative for the presence of uric acid crystals. Readers also assessed following results of green pixelation: volume, site, size, shape, vanishment after increase minimal HU from 130 to 150. Reference standard was the results of joint aspiration and over one year clinical follow up. Logistic regression analyses were conducted to identify which findings were significantly associated with gout.
**Results:** The sensitivity and specificity of DECT without first MTPJ green pixelation was 0.96 and 0.67. The mean volume of green pixelation was $1.7cm^3$. All false negative patients had submillimeter artifact. The DECT findings showed significant differences between artifact and gout with respect to shape, vanishment after increase minimal HU from 130 to 150, site (p<0.05). Multivariate logistic regression showed correlations between artifact and shape (non-oval, sharp, flat, odd ratio 27.4), and vanishment after increase minimal HU (odd ratio 19.2).
**Conclusion:** The specificity of DECT of foot and ankle without green pixelation in first MTPJ is unsatisfactory due submillimeter artifact. Green pixelation with non-oval, sharp, flat shape and which vanishes after increased minimal HU in post-processing may associated with artifact.

**B-0055** 11:50
**7T T2*-relaxometry detects early tendon degeneration in patients with diabetes mellitus type 1 (DM1) and shows correlations with BMI and age**
M. Zalaudek[1], W. Marik[1], V. Juras[1], M. Riedl[1], B. Hager[1], O. Bieri[2], X. Deligianni[2], M. Weber[1], S. Trattnig[1]; [1]*Vienna/AT*, [2]*Basle/CH* *(martin.zalaudek@meduniwien.ac.at)*

**Purpose:** T2*mapping at 7T was used to investigate alterations in tendon composition and their relation to demographics of patients with DM1.
**Methods and Materials:** Eighteen DM1 patients (11f/7m, 28.25±7.14y, BMI 22.3±2.28) were enrolled together with eighteen sex, age and BMI matched healthy controls (11f/7m, 27.35±5.93y, BMI 21.61±2.67). MRI was performed on a 7T whole body scanner with a dedicated multichannel receive proton knee coil. FS-PDw images were acquired for morphological evaluation of patellar tendon. A variable TE sequence (10 TEs from 1.21-24.48ms) for mono-exponential T2*mapping. Differences in T2*values between diabetics and controls were compared. Sensitivity/specificity was evaluated. T2*values were correlated with BMI and age.
**Results:** On PDw images, tendons appeared unremarkable in all participants. Mean T2*values in patients and controls were 1.29±.41ms and 0.73±.14ms, respectively (p<0.001). AUC was 0.92 (95%CI, 0.82-1), a cut-off set to 0.89ms showed a sensitivity of 88.9% and specificity of 99.44%. BMI and T2* values correlated strong/statistically significant in diabetics (r=0.62, p<0.01) and weak in controls (r=0.26, p=0.3). Age and T2*values correlated weak in diabetics (r=0.255, p=0.31) and strong/statistically significant in controls (r=0.6, p<0.01).
**Conclusion:** Although inconspicuous in routine MRI, tendons of DM1 patients exhibited altered T2*values with a high diagnostic sensitivity/specificity. Furthermore, T2*values of DM1 tendons appear to be influenced by BMI. Age did not influence T2*values in DM1 patients - however, it did in controls. This may reflect a stronger impact of higher body weight to the deteriorated tendon tissue and a partial abolition of age related tendon degeneration in DM1 patients.

**10:30 - 12:00** **Room O**

### Musculoskeletal

### SS 210b

### Musculoskeletal tumour imaging

*Moderators:*
A. Isaac; London/UK
N.N.

**B-0056** 10:30
**Predictive value of MRI radiomic features in differentiating lipoma from atypical lipomatous tumour**
F. Acquafredda[1], Y. Abdelhafez[2], M. Zhang[3], M. Guindani[4], S. Lee[4], D. Sarohia[2], R.D. Badawi[2], L. Nardo[2]; [1]*Brescia/IT*, [2]*Davis, CA/US*, [3]*Montreal, QC/CA*, [4]*Irvine, CA/US (francesco.acquafredda@gmail.com)*

**Purpose:** To explore the predictive role of shape and texture features extracted from MR images for differentiating lipoma from atypical lipomatous tumour (ALT).
**Methods and Materials:** With Institutional Review Board approval, the PACS databases of multiple institutions were searched for the terms “lipoma”, “liposarcoma” and ”lipomatous”. 3251 subjects were found and screened for pathologically proven surgically resected lipoma or ALT and pre-operative MRI study with axial T1 sequence for lesion segmentation. LifeX software was used to extract 3 shape features (volume, sphericity and compacity) and 32 texture indices from 4 texture matrices: grey-level co-occurrence matrix (GLCM), grey-level run length matrix (GLRLM), grey-level zone length matrix (GLZLM) and neighborhood grey-level different matrix (NGLDM). Receiver-operating characteristic (ROC) analysis was used to calculate the area under the curve (AUC) and 95% confidence interval (CI) for each feature.
**Results:** The study included 97 patients (median age 58 years [range: 32-92], 55 women), 25 ALTs and 72 lipomas. Several shape and texture features were significantly predictive of pathological diagnosis (p<0.001). AUC values were as follows: compacity 0.83 (0.75-0.91), sphericity 0.63 (0.51 - 0.76), NGLDM busyness 0.77 (0.66-0.88), GLZLM grey-level non-uniformity (GLNU) 0.77 (0.66-0.88), run-length non-uniformity (RLNU) 0.75 (0.63-0.86), GLRLM GLNU 0.74 (0.63-0.85) and RLNU 0.74 (0.63-0.85). In multivariate analysis, compacity, busyness and GLRLM RLNU were independent predictors for pathology with odds ratio (95%CI) of 2.2 (1.5-3.2; p<0.001), 1.7 (1.1-2.7; p=0.018) and 1.0 (1.0-1.0; p=0.025), respectively.
**Conclusion:** Radiomic features extracted from MR images, specifically compacity and busyness, could help differentiating lipoma from ALT.
*Author Disclosures:*
**R.D. Badawi:** Investigator; United Imaging Healthcare. Research/Grant Support; United Imaging Healthcare.

**B-0057** 10:38
**To biopsy or not to biopsy? A quantitative approach differentiating subfascial lipoma from atypical lipomatous tumour/well-differentiated liposarcoma on MRI**
R. Donners, D. Harder; *Basle/CH (ricardo.donners@usb.ch)*

**Purpose:** To provide MRI-based quantitative decision support when to biopsy subfascial lipomatous tumours.
**Methods and Materials:** MRI of 100 histopathologically proven subfascial lipomas (n=75) and atypical lipomatous tumours/well-differentiated liposarcomas (ALT/WDL) (n=25) were reviewed retrospectively. Exclusion criteria were non-lipomatous soft tissue components, suprafascial and intraabdominal location. Ratios derived from region of interest-based signal intensity (SI) measurements of tumour and adjacent fat on T2 fat-saturated (FS) images were calculated (=mean $SI_{tumour}$/mean $SI_{adjacent-fat}$). Univariate regression analyses were applied and a p value <0.01 deemed significant. The discriminatory ability was assessed by ROC curve analyses. Interreader agreement was evaluated by calculation of intraclass correlation coefficients (ICC). Tumour size, location, septation and patient demographics were noted.
**Results:** T2 FS ratios performed well discriminating lipoma from ALT/WDL (p<0.001, area under the ROC curve (AUC) =0.89, cutoff =1.22, specificity =88%, sensitivity =80%). A T2 FS ratio >1.37 indicated ALT/WDL with 95% specificity and 64% sensitivity. Interreader agreement was excellent (ICC =0.924). ALT/WDL was significantly larger than lipoma (p <0.001, AUC=0.84, cutoff =11 cm, 92% specificity, 72% sensitivity). Combination of a T2 FS ratio <1.22 and size <11 cm as decision support would have spared 61% of lipoma patients from biopsy (combined specificity=99%, sensitivity=58%). Qualitative imaging parameters and patient demographics did not differ significantly (each p>0.022).
**Conclusion:** Lipomatous subfascial tumours with a T2 FS ratio <1.22 and size <11 cm are most likely benign and may not need biopsy. Tumours with a T2 FS ratio >1.37 should be biopsied even when <11 cm.

**B-0058** 10:46
**Diffusion-weighted imaging combined with dynamic contrast-enhanced sequences in MRI as a predictor factor of malignancy in non-fatty soft tissue tumour**
C. Martin[1], L. Lassale[1], N.-E. Regnard[1], F.P. Kuhn[2], F. Larousserie[1], V. Audard[1], D. Biau[1], J.L. Drape[1], A. Feydy[1]; [1]*Paris/FR*, [2]*Zurich/CH* *(chachmartin@hotmail.com)*

**Purpose:** To study the diagnostic performance of DWI criteria and DCE-MR qualitative and semi-quantitative criteria for distinction between malignant and benign soft tissue tumors.
**Methods and Materials:** We consecutively included 38 patients with soft tissue tumors, 15 benign and 23 malignant (biopsy proven), all had DWI and DCE-MR sequences after gadolinium injection with the same parameters. ADC values and ratio of ADC (tumor/muscle) were compared between the benign and malignant groups. Type of enhancement curve, slope of the curve, peak intensity and peak time were also compared between the two groups. Diagnostic performance of each criterion was tested.
**Results:** Mean ADC values and ADC tumor/muscle ratios were significantly lower in the malignant tumor group, respectively 972 versus 1472 $mm^2/s$ (p = 0.003) and 0.810 versus 2.38 (p = 0.003). ADC value analysis allowed distinction of malignant tumors with an acceptable diagnostic performance (AUC=0.772). No significant difference was found for slope (p = 0.33), peak intensity (p = 0.4) and peak time (p = 0.525) in the two groups. Type 5 curves were statistically more frequently found in the malignant tumor group (p = 0.024).

**Conclusion:** Analysis of ADC values and ADC tumor/muscle ratios was useful for the distinction between benign and malignant tumors. The qualitative and semi-quantitative analysis of perfusion parameters did not allow distinction between benign and malignant tumors.

**B-0059** 10:54

**The diagnostic value of shear wave elastography for the differentiation of benign and malignant soft tissue tumours**

M. Ozturk, A.V. Polat, Y.S. Baris, M.B. Selcuk; *Samsun/TR (dr.mesutozturk@gmail.com)*

**Purpose:** Our aim was to assess the diagnostic value of shear wave elastography (SWE) for the differentiation of benign and malignant soft tissue tumours.
**Methods and Materials:** Institutional ethics committee approval and informed consent from each participant were obtained for this prospective study. Fifty patients with soft tissue tumours (28 men and 22 women; mean age ± SD: 44.02 ± 19.13 years) were examined with SWE using virtual touch tissue imaging and quantification (VTIQ) method between May 2016 and June 2018. Five region of interest boxes were randomly placed on the lesion and tissue stiffness was measured as shear wave velocity (SWV) in meter per second (m/s). Mean SWV, maximum SWV, minimum SWV, and the difference between maximum and minimum SWV were used to compare the stiffness of the benign and malignant lesions.
**Results:** Thirty-one benign and 19 malignant soft tissue tumours were diagnosed. Mean SWV, maximum SWV, minimum SWV and the difference between maximum and minimum SWV values for malignant lesions were 3.00 m/s, 3.54 m/s, 2.65 m/s and 0.89 m/s, respectively. Mean SWV, maximum SWV, minimum SWV and the difference between maximum and minimum SWV values for benign lesions were 3.29 m/s, 3.91 m/s, 2.85 m/s and 0.89 m/s, respectively. No statistically significant difference was found between benign and malignant lesions for the measured SWV values (p = 0.353, p = 0.418, p = 0.327, and p = 0.385, respectively).
**Conclusion:** SWE did not contribute to the differentiation of benign and malignant soft tissue tumours.

**B-0060** 11:02

**Do aggressive radiologic features of synovial sarcoma correlate with pathologic grading? A retrospective study**

T.S. Al Kuhaimi, A. Aljefri, M. Alshaikh, S. Almalki, S. Bauones; *Riyadh/SA (T.S.ALKUHAIMI@GMAIL.COM)*

**Purpose:** To study the imaging characteristic signs of aggressive behaving synovial sarcoma (SS) in relation to the pathologic types and grading to look for statistical significance using a radiologic-pathologic correlation.
**Methods and Materials:** After institutional review board approval, a monocentric retrospective study was undertaken on 15 (9 Males & 6 Females; mean age of 35) histopathologically-proven SS with MRI images between January 2012 and December 2017 at King Fahad Medical City in Riyadh. Data was collected on patient's demographics and the imaging features on multiple imaging modalities, in particular MRIs which were reviewed by 2 musculoskeletal radiologists. Pathology slides were reviewed by a musculoskeletal pathologist. Data were analysed descriptively and statistically.
**Results:** Among multiple aggressive imaging findings of SS studied (haemorrhagic pockets, peri-lesional oedema, muscle and bone invasion, neurovascular bundle invasion, triple sign, bowl of grapes sign), the only statistically significant imaging finding that correlates with the histopathological high grade (grade 3) of SS is the neurovascular bundle invasion (P <0.05). No statistically significant correlation (P value varies between 0.135 and 0.853) between other imaging characteristics and histopathologic types of SS was found. No statically significant correlation between tumour size and its probability to metastasize (P 0.516). However, large tumour size (>5cm), intralesional haemorrhage, bone invasion are frequently encountered imaging findings in high-grade and metastatic SS. Most common metastatic site is pleuro-pulmonary (81%).
**Conclusion:** Neuro-vascular bundle invasion could help predict patient histopathological grade and thus aid in disease prognostication.

**B-0061** 11:10

**Performance of 3D texture analysis for tissue characterisation and differential diagnosis of osteosarcoma**

C.S. Lisson[1], C.G.S. Lisson[1], R. Mayer-Steinacker[1], A. von Baer[1], T.F.E. Barth[1], M. Baumhauer[2], A.J. Beer[1], M. Beer[1], S.A. Schmidt[1]; [1]*Ulm/DE*, [2]*Heidelberg/DE (catharinavonend@gmail.com)*

**Purpose:** Differentiation between osteosarcomas and other common bone tumours such as chondrosarcoma, Ewing's sarcoma and chordoma can be a significant radiologic-histologic challenge because osteosarcomas represent a highly heterogeneous tumour group. The clinical relevance is that a distinction is important since, depending on the diagnosis, there are different therapeutic approaches with regard to (neo-)adjuvant chemo-/radiotherapy. This study evaluates the performance of MRI-based 3D texture analysis (TA) in comparison to CT-based TA for the differentiation of osteosarcoma.
**Methods and Materials:** 132 patients were retrospectively evaluated: 64 with contrast-enhanced CT, 28 with non-contrast CT and 40 with contrast-enhanced MRI. All patients had a histopathological diagnosis based on the surgical specimen. TA was performed on CT/MR images obtained for routine purposes using the research software MINT, a spinoff from the DKFZ, which allows and post-process 3D measurements. Kruskal-Wallis test and Dunn-Bonferroni post hoc test were performed to identify the most discriminative texture features (kurtosis, entropy, skewness, MPP, UPP, uniformity).
**Results:** In MRI, the TA skewness in the T2-weighted sequences significantly differentiated osteosarcoma from chondrosarcoma (p = 0.002), Ewing's sarcoma (p = 0.037), and chordoma (p = 0.006). In contrast-enhanced CT, the TA entropy and MPP could significantly discriminate between osteosarcoma and chondrosarcoma (p = 0.002 and p = 0.001), Ewing's sarcoma (p <0.0005 and p <0.0005) and chordoma (p = 0.038 and p = 0.002). In non-contrast CT, osteosarcoma could not be differentiated.
**Conclusion:** Tumour heterogeneity quantified by MRI- and contrast-enhanced CT-based 3D texture analysis has the potential to differentiate osteosarcoma from chondrosarcoma, Ewing's sarcoma and chordoma.

**B-0062** 11:18

**Diagnostic value of MR imaging with metal artefact reduction sequences in local recurrence of malignant bone tumour after joint replacement**

H. Wang, Y. Lu; *Shanghai/CN (shinianxin@sjtu.edu.cn)*

**Purpose:** To investigate the diagnostic value of MR imaging with metal artefact reduction sequences in local recurrence of malignant bone tumour after joint replacement.
**Methods and Materials:** 94 cases who were pathologically diagnosed with malignant bone tumour underwent clinical and imaging follow-up after joint replacement. All cases received MR scans with metal artefact reducing syngo WARP sequences and FSE sequences. The sensitivity, specificity, and consistency rate of syngo WARP sequences and FSE sequences were compared for diagnosing local recurrence of malignant bone tumours. The *Kappa* test was used to assess the consistency of syngo WARP and FSE sequences with pathology in diagnosing recurrence respectively. *ICC* evaluated the consistency of MR images with pathology in measuring the volume of recurrent tumour.
**Results:** 35 of the 94 cases were pathologically or clinically diagnosed as local recurrence after joint replacement. Local recurrence mainly presented as soft tissue masses and bone destruction on MR images. The sensitivity, specificity, coincidence rate and *Kappa* value of syngo WARP sequences in the diagnosis of local recurrence of malignant bone tumours were 94.3%, 94.9%, 94.7%, and 0.887, which were higher than FSE sequences. Compared with pathology, the ICC of MR images in measuring recurrent tumour volume was 0.961.
**Conclusion:** The syngo WARP sequences can significantly reduce metal artefacts in scanning area, and diagnose local recurrence. It also has a good consistency with pathology in evaluating the lesion volume, thus it's recommended for the standard evaluation of postoperative bone tumour.

**B-0063** 11:26

**Denosumab response evaluation in primary osseous giant cell tumours with 18F-FDG PET/CT**

A. Kesari[1], S.S. Swamy[1], S. Sampangi[1], I. Desai[1], M. Ashok Kumar[2], P. Asokan[1], K. Kallur[1]; [1]*Bangalore/IN*, [2]*Bikaner/IN*

**Purpose:** To assess the response of primary osseous giant cell tumor to denosumab with FDG PET-CT.
**Methods and Materials:** We present retrospectively evaluated 90 patients of giant cell tumor treated with denosumab in the setting of recurrence, high surgical risk, high grade lesions and monotherapy. Although most of the patients required surgery (extended curettage, en bloc resection and prosthetic reconstruction, arthrodesis, etc.), in two patients with recurrent GCT of the metacarpal and two patients with GCT of pelvis, denosumab was used as monotherapy. Patients were followed with PET-CT with a mean of 2 years (Range: 1-5 years).
**Results:** PET-CT evaluation showed reduced activity in 81% of the patients and 63% of the Campanacci grade III patients on plain radiographs showed down grading to grade II following denosumab therapy. 77% of the patients showed histological evidence of interval reduction in grade of tumor.
**Conclusion:** Response to denosumab is noted in the form decrease in metabolic activity, ossification of the lesional soft tissue component and lytic bone. The reduction in metabolic activity was more prominent than regression in size of the primary lesion. Thus PET-CT adds to imaging response assessment in osseous GCT.

**B-0064** 11:34
**Comparison of image quality and radiation dose between combined ATCM and FTC technique in whole-body low-dose multidetector CT in the diagnosis of multiple myeloma**
M. Calvo Imirizaldu, A. Ezponda Casajús, I. Soriano, A. Paternain Nuin, M. Elorz, J.D. Aquerreta Beola; *Pamplona/ES (mcalvoi@unav.es)*

**Purpose:** This study aimed to compare image quality and radiation dose of a combined automatic tube current modulation (ATCM) technique with those of a fixed tube current technique (FTC) in whole-body low-dose multidetector CT (WBLDCT), in the diagnosis of Multiple Myeloma.
**Methods and Materials:** 28 consecutive patients (15 men, mean age 63,8±11,3 years) underwent unenhanced WBLDCT for evaluation of Multiple Myeloma using a third-generation dual-source CT scanner (192-slice) with dedicated tin filter, from August-2017 to September-2018. All acquisition parameters were identical in both techniques (100 kV, tin filtration) except for tube current: 18 patients were performed with ATCM and 10 patients with FTC (200 mAs). We recorded objective image noise in muscles at different anatomic levels and in the liver parenchyma, radiation dose (DLP, mSv) and body mass index (BMI, kg/m2). The Mann-Whitney *U* test was used for statistical analysis.
**Results:** No statistical differences were observed in the BMI estimated in both groups (p=0.94). Signal-to-noise ratio was higher in the ATCM group at scapular and pelvic girdles compared to FTC, with statistically significant differences (p=0.014; p<0.001 respectively). Objective image noise values at the upper neck and in the abdominal region found no significant differences between both techniques (p=0.191 and p=0.408). The effective radiation dose with the ATCM technique was 2.91 vs 1.49 with the FTC (p<0.001).
**Conclusion:** WBLDCT with ATCM technique achieves diagnostic image quality with better signal-to-noise ratio in some compromised areas as the scapular and pelvic girdles, while maintaining acceptable low radiation dose levels.

**B-0065** 11:42
**Collagen-bound and water-bound water longitudinal relaxation time by ZTE imaging as biomarker for radiation-induced bone changes in mouse model**
V. Phi Van, I. Grgic, A. Boss, M. Pruschy, M. Wurnig; *Zurich/CH (valeriedoan.phivan@usz.ch)*

**Purpose:** Collagen-bound water (CBW) and pore water (PW) components of cortical bone reflect bone porosity and organic matrix density. Our study evaluates the longitudinal relaxation time of those two compartments as biomarkers for radiation-induced bone damage.
**Methods and Materials:** ZTE images of mouse femur were acquired at 4.7T with 14 different inversion times (0-2,600 ms) before radiation and 3 weeks/3months after radiation at 20 Gy by the xrad system 320. Longitudinal relaxation time of the two compartments CBW, PW and CBW fraction (cbwf) for each timepoint was computed by a bi-exponential fitting. µCT imaging were performed at the same timepoints to validate bone porosity.
**Results:** Baseline measurements displayed a mean T1cbw of 245 ± 110ms, a mean T1pw of 2076 ± 623ms and a cbwf of 6.9 ± 3.4%. 3 weeks after radiation, both T1 relaxation time (CBW 269 ± 89ms, p value = 0.12; PW 2204 ± 575ms, p value=0.09) and cbwf (6.4 ± 3.7%) stayed stabile. After 3 months, T1 relaxation time significantly increases (T1cbw 432 ± 89ms, T1pw 3423 ± 543ms) towards the baseline. Contrarily, cbwf decreased significantly (3.7 ± 2.1%). µCT-derived bone porosity parameter showed good correlation with both T1 relaxation time (pw: r=0.68, cbw:r=0.63) and cbwf (r=0.77).
**Conclusion:** Longitudinal relaxation time of CBW and PW components are sensitive towards bone porosity changes after radiation and may be useful as non-invasive tool for radiation-induced bone damage.

**B-0066** 11:50
**Radiologic predictors of survival in soft tissue sarcoma**
J. Kavanagh, J. Wunder, P. Ferguson, R. Mohankumar; *Toronto, ON/CA (jokavana@tcd.ie)*

**Purpose:** To evaluate the MRI features associated with survival after preoperative radiotherapy in soft tissue sarcoma.
**Methods and Materials:** A review of a prospectively collected database was performed for patients treated for extremity STS. Pre- and post-radiotherapy MRI were reviewed by two fellowship-trained musculoskeletal radiologists. Maximal tumour diameter and volumes were measured on T2-weighted sequences and the change in high T2 signal was estimated as a percentage. High T2 signal was defined as signal intensity similar to an adjacent vessel or fluid. Tumours were divided into partial response (PR), stable disease (SD) or progressive disease (PD). Local recurrence-free survival (LRFS), metastasis-free survival (MFS) and overall survival (OS) were examined using the Kaplan-Meier method and compared with log-rank test.
**Results:** 309 studies were available to review. There was an increase in tumour volume after RT with a mean volume increase of 132cm3 (3190cm$^3$-6087cm$^3$). 117 tumours showed a partial response, 86 tumours were classified as stable disease and 106 tumours as progressive disease. Of the 166 patients assessed for T2 change 106 showed no change, 26 showed a reduction in T2 signal and 34 showed an increase in T2 signal. PD by RECIST had a worse MFS (p<0.001) and OS (p<0.001). Tumours that demonstrated an increase in T2 signal post-radiotherapy had an increased MFS (p=0.035) but no difference in LRFS or OS (p=0.079).
**Conclusion:** PD after radiotherapy is associated with worse MFS and OS. Increase in percentage T2 signal is associated with increase MFS but no change in OS.

**10:30 - 12:00** **Coffee & Talk 2**

### Abdominal Viscera

### SS 201a

### Focal liver lesions: characterisation

*Moderators:*
R. Albazaz; Leeds/UK
M.A. Bali; Brussels/BE

**K-01** 10:30
**Keynote lecture**
D. Weishaupt; *Zurich/CH*

**B-0067** 10:39
**Classification of malignant and benign liver tumours using a radiomics approach**
M.P.A. Starmans, R.L. Miclea, S.R. van der Voort, W.J. Niessen, S. Klein, M.G. Thomeer; *Rotterdam/NL (m.starmans@erasmusmc.nl)*

**Purpose:** Primary solid liver lesions are in clinical practice manually scored by radiologists in benign or malignant based on MRIs. As the interpretation of these MRIs is challenging and observer dependent, we present a radiomics approach as an objective alternative.
**Methods and Materials:** T2-weighted MRIs and pathology were gathered from 119 non-cirrhotic patients with solid primary liver tumours. The malignant class consisted of HCCs, the benign class of FNHs and HCAs. The phenotype was obtained through pathology. The resulting dataset originated from 54 different hospitals, showing heterogeneity in the imaging protocols. Lesions were delineated by a radiologist. Within these regions, radiomics features quantifying shape, intensity, texture and orientation were extracted. Several patient characteristics were added as semantic features. Radiomics was performed using harmonised adaptive workflow optimisation including various feature selection, oversampling and machine learning approaches. Evaluation was implemented through a 100x random-split cross-validation, with 80% of the data for training and 20% for testing. Performance was given in 95% confidence intervals (CIs). For comparison, the tumours were manually scored by a radiologist.
**Results:** The AUC, sensitivity and specificity were, respectively (0.86, 0.99), (0.58, 0.89) and (0.85, 0.98) for radiomics and 0.93, 0.93 and 0.70 for the radiologist. The positive class consisted of the malignant tumours.
**Conclusion:** The AUC of the radiomics approach is comparable to the radiologist. However, the sensitivity is lower than the radiologist but the specificity higher. Hence, radiomics is a promising alternative or complementary approach in distinguishing malignant from benign primary liver tumours in non-cirrhotic patients.

**B-0068** 10:47
**The histology of the right parafissural liver parenchyma: collagen is the basis for a hypoattenuating pseudolesion on CT**
W.M. Klein[1], L. Sonnemans[1], S. Franckenberg[2], D. Gascho[2], M. Prokop[1], W.H. Lamers[3], J.P.J.M. Hikspoors[3], M. Thali[2], P. Flach[4]; [1]*Nijmegen/NL*, [2]*Zurich/CH*, [3]*Maastricht/NL*, [4]*St. Gallen/CH (willemijn.klein@radboudumc.nl)*

**Purpose:** CT images of a healthy liver may show a hypoattenuating area on the right side of the falciform ligament, often noticed in the portovenous enhanced phase, and interpreted as a pseudolesion or a malignancy. The purpose of this study was to evaluate the histology of the parafissural liver parenchyma in healthy livers, and find clues for aetiology.
**Methods and Materials:** Biopsies of the right and left parafissural liver parenchyma were performed either during autopsy or postmortem CT. Histology was examined for the amount of collagen and fat. Left and right side parenchymas were compared using t tests and correlated to CT attenuation.
**Results:** 52 cadavers were included. Mean CT attenuation at the right side of the ligament was 44.9 HU (SD16.7) and left side 46.4 HU (SD16.3) (p 0.32). Excluding the fibrotic (>2% collagen) and steatotic (>5% fat) livers (n=36), significantly more collagen content was present at the right side: mean 0.71% (SD 0.49), compared to left side 0.51% (SD 0.30) (p 0.024), with equal fat content. CT attenuation was correlated to age (Pearson -0.412, p 0.004), not to collagen or fat content.
**Conclusion:** The right parafissural liver parenchyma contains more collagen and an equal amount of fat compared to the left side in healthy livers. We suggest that the high collagen on the right side is a consequence of normal embryological development with disadvantageous supply to the side that is prone to becoming a pseudolesion.

**B-0069** 10:55
**Focal peliosis hepatis: imaging features of magnetic resonance imaging with diffusion-weighted imaging**
W. Mingliang, M. Zeng; *Shanghai/CN (wang.mingliang@zs-hospital.sh.cn)*

**Purpose:** To investigate the MRI and DWI (diffusion-weighted imaging) features of focal peliosis hepatis (focal PH).
**Methods and Materials:** The clinical and MRI data of 19 cases diagnosed as focal peliosis hepatis by pathology were retrospectively analysed. MR scan of the upper abdomen was performed preoperatively in all patients. The imaging features of the lesions were analysed, including number, location, size, shape, signal intensity, enhancement pattern, vessels within and around lesions, and perfusion disorders of hepatic parenchyma. The ADC values of the lesions and adjacent hepatic parenchyma were measured, and the differences were explored statistically.
**Results:** In all 24 lesions, 22 lesions were located in the right lobe and 2 lesions in the left lobe. The median size was 24.4±17.2mm (7.5-72 mm ). On T1WI, 21 lesions showed slight hypointensity, 1 lesion showed slight hyperintensity and 2 lesions were isointense; all 24 lesions showed slight hyperintensity on T2WI, and isointensity or slight hyperintensity on DWI. The mean ADC value was $(1.511\pm0.415)\times10^{-3}$ $mm^2/s$ in the lesions and $(1.769\pm0.690)\times10^{-3}$ $mm^2/s$ in the adjacent hepatic parenchyma, which showed no difference between the two groups (P>0.05). On dynamic MR images, 20 lesions showed gradually filling enhancement, 4 lesions showed markedly persistent enhancement. Punctiform or filiform vessels were found in 9 lesions. Adjacent hepatic perfusion disorders showed in 8 lesions.
**Conclusion:** The MRI performance of focal peliosis hepatis had a certain characteristic. MRI combined with diffusion-weighted imaging could help to make diagnoses.

**B-0070** 11:03
**Negative biopsy of focal hepatic lesions: decision tree model for patient management**
F. Vernuccio, M. Rosenberg, M. Meyer, R.C. Nelson, D. Marin;
*Durham, NC/US (federicavernuccio@gmail.com)*

**Purpose:** To investigate patient- and procedure-related variables affecting the false-negative rate of US-guided liver biopsy, and to develop a standardized patient-tailored predictive model for the management of negative biopsy results.
**Methods and Materials:** We retrospectively included 389 patients (mean age 62 years ± 12) undergone US-guided liver biopsy of 405 liver lesions between January 1, 2013 and June 30, 2015. We collected multiple patient- and procedure- related variables. By comparing pathology reports of biopsy and the reference standard (further histology or imaging follow-up), the biopsy results were categorized as true-positive, true-negative and false-negative. Diagnostic accuracy and diagnostic yield were measured. Univariate and multivariate analysis was performed to identify variables predicting false-negative results. A standardized patient-tailored predictive model of false-negative results based on a decision tree was fitted.
**Results:** Diagnostic accuracy and diagnostic yield were 93.8% (380 of 405) and 89.4% (362 of 405), respectively. The false-negative rate was 6.5% (25 of 387). Predictive variables of false-negative results at univariate analysis included body mass index, lesion size, sample acquisition techniques, and immediate specimen adequacy. The only independent predictors at multivariate analysis were patient age and Charlson Comorbidity Index. By combining lesion size and location with patient age and history of malignancy, we developed a decision tree model that predicts false-negative results with high confidence (up to 100%).
**Conclusion:** False-negative results are not negligible at US-guided liver biopsy. The combination of selected lesion- and patient-specific variables may help predicting patients with false-negative results where aggressive management may be warranted.

**B-0071** 11:11
**Enhancement pattern of hepatocellular adenoma (HCA) on MR imaging performed with Gd-EOB-DTPA vs other Gd-based contrast agents (GBCAs): an intraindividual comparison**
R. Cannella[1], G. Brancatelli[1], B. Rangaswamy[2], M.I. Minervini[2], A.A. Borhani[2], A. Furlan[2]; [1]*Palermo/IT*, [2]*Pittsburgh, PA/US (rob.cannella@libero.it)*

**Purpose:** To conduct an intraindividual comparison of the enhancement pattern of HCA on dynamic MRI study obtained following the injection of Gd-EOB-DTPA and other GBCAs.
**Methods and Materials:** This is a retrospective, IRB-approved study conducted in a single institution. A search of medical records between 2008 and 2017 revealed 17 patients (all females) with at least one pathologically proven HCA who underwent liver MRI with Gd-EOB-DTPA and another GBCA (Gd-BOPTA, n=14; Gd-DTPA, n=1; Gd-DTPA-BMA, n=1; Gd-BT-DO3A, n=1) within 1 year. Enhancement of each lesion on hepatic arterial (HAP), portal venous (PVP), 2-minute and 4- to 5-minute phases was qualitatively evaluated by two abdominal radiologists. Lesions were categorised as hyper-, iso- or hypointense compared to the surrounding liver parenchyma. The presence of a peripheral pseudocapsule was also recorded. The differences in lesion enhancement were assessed using the Wilcoxon signed rank test. A p value <0.05 was considered statistically significant.
**Results:** The final population included 35 HCAs (83% inflammatory subtype). There was no significant difference in lesion size (P=0.708) and enhancement on HAP (P=0.317). HCAs showed more frequently hypointensity on PVP (5/35 vs 1/35, P=0.046), 2-minute (13/35 vs 1/35, P=0.001) and 4- to 5-minute (P<0.001) images obtained after injection of Gd-EOB-DTPA compared to those obtained after other GBCAs. A pseudocapsule was more frequently noted after administration of Gd-EOB-DTPA (13/35 vs 1/35, P=0.001).
**Conclusion:** Enhancement pattern of HCA differs significantly after the injection of Gd-EOB-DTPA compared to other GBCAs. Lesion hypointensity on PVP, 2-minute and 4- to 5-minute images are more frequent when using Gd-EOB-DTPA.
*Author Disclosures:*
**G. Brancatelli:** Advisory Board; Guebert. Speaker; Bayer and Guerbet.
**A.A. Borhani:** Consultant; Guebert. Other; Elsevier/Amirsys. **A. Furlan:** Research/Grant Support; General Electric. Other; Elsevier/Amirsys.

**B-0072** 11:19
**Multi-arterial phase EOB-MRI: different contrast enhancement pattern of focal nodular hyperplasia and hepatocellular carcinoma**
S. Fiore, R. Faletti, F. Gentile, L. Pavan, F. Guarasci, M. Gerboni, M. Donalisio, M. Gatti, P. Fonio; *Turin/IT (fyorello@gmail.com)*

**Purpose:** To characterise the enhancement pattern of FNH and HCC with triple-phase arterial MRI acquisition.
**Methods and Materials:** Study population included 52 patients who had arterial enhancing hepatic lesions (41 HCC and 31 FNH). All patients underwent triple-phase arterial MRI acquisition using hepatocyte-specific agents (Gd-EOB-DTPA). Images were reviewed by two radiologists in consensus: contrast enhancement ratios (CER), liver-to-lesion contrast ratios (LLCR) and signal intensity (SI) were measured. Lesions were categorised based on the peak of LLCR into the following groups: 1) angiographic, 2) early arterial and 3) late arterial. Data were analysed using Wilcoxon signed-rank test.
**Results:** There was no difference in CER between FNH and HCC patients (p>0.09). $LLCR_{FNH}$ were significantly higher than $LLCR_{HCC}$ in angiographic phase (p=0.01), whereas there was not difference in the other phases (p=0.20 and p=0.82, respectively). Moreover, in HCC group there was a significant increase of LLCR between early and middle phases (p=0.009) whereas in FNH group there was a decrease between the middle and the late phases (p=0.004). This behaviour was paralleled by the subgroup divisions: 17 (55%) FNH were classified as the 1$^{st}$ group, 11 (35%) as the 2$^{nd}$ and only 3 (10%) as the 3$^{rd}$; on the other hand, the HCC were homogeneously distributed (34%, 32% and 34%).
**Conclusion:** This study highlights the different enhancement patterns of HCC and FNH: FNH resulted more conspicuous in the angiographic phase and the best phases for optimal visualisation of FNH were the early and middle arterial phases, whereas for HCC were the middle and the late arterial phases.

**B-0073** 11:27
**Intra-individual comparison of contrast enhancement rates of focal liver lesions in DCE-MRI with gadoxetic acid vs gadobutrol**
N.A. Bünting, A. Barabasch, C.K. Kuhl; *Aachen/DE (nbuenting@ukaachen.de)*

**Purpose:** Gadoxetic acid is widely used not only for hepatobiliary phase imaging, but also as regular extracellular contrast agent for dynamic, contrast-enhanced (DCE) MR-imaging of focal liver lesions. We aimed to systematically investigate the utility of gadoxetic acid for this purpose, in comparison to gadobutrol-enhanced MRI obtained in the same patients.
**Methods and Materials:** 71 consecutive patients underwent DCE-MRI according to a standardized protocol on two different occasions, once with gadoxetic acid and once with gadobutrol. 26/71 patients had enhancing focal liver lesions not receiving treatment in between MRI studies. A T1-weighted 3D dynamic GRE sequence was obtained before and at defined intervals in the arterial, portal venous and equilibrium phase after i.v. application of gadoxetic acid or gadobutrol. ROIs were carefully placed in the identical enhancing position of each focal liver lesion in order to calculate and compare enhancement rates and lesion-parenchyma-contrast.

**Results:** Mean enhancement rates of focal liver lesions were significantly higher for gadubotrol compared to gadoxetic acid for all dynamic phases: Arterial phase 170% vs 61%, portal venous phase 250% vs 134% and equilibrium phase 216% vs 118% (each p<0.001). The lesion-parenchyma-contrast in the corresponding phase of DCE-MRI was also significantly higher for gadobutrol in each dynamic phase: arterial phase 1,5 versus 1,2, portal venous phase 1,1 versus 1,0, equilibrium phase 1,2 versus 0,9 (each p≤0.03).
**Conclusion:** Compared to gadobutrol, gadoxetic acid is associated with significantly lower enhancement of focal liver lesions. This will impair the detection and characterization of focal liver lesions, especially those with arterial enhancement.

**B-0074** 11:35
**A predictive model for early recurrence of hepatocellular carcinoma after tumour resection based on whole lesion MR imaging radiomics features**
Z. Zhang, H. Jiang, L. Cao, Z. Ye, J. Chen, B. Song; *Chengdu/CN (zhangzhen950708@163.com)*

**Purpose:** To prospectively develop and validate a prediction model for early recurrence (<1 year) of hepatocellular carcinoma (HCC) using whole lesion radiomics features on preoperative gadoxetic acid-enhanced MR images.
**Methods and Materials:** 114 patients (79 in the training cohort and 35 in the validation cohort) with surgically confirmed HCC were enrolled in this IRB-approved study. Three-dimensional whole-lesion regions of interest were manually delineated along the tumour margins. Radiomics features were selected to build a radiomics signature using least absolute shrinkage and selection operator(LASSO)method in the training cohort. Qualitative radiological features were evaluated by two independent reviewers. Independent risk factors were identified and combined to establish a clinical-radiological model. Finally, a combined prediction model comprising the radiomics signature and all risk factors was constructed. All models were developed in the training cohort with multivariate regression analysis, and their diagnostic performances were measured by receiver operation characteristic (ROC) and decision curves and validated in an independent validation cohort.
**Results:** Fifty early recurrences (49.1%) were confirmed by imaging follow-up. 14 radiomics features were selected to construct the radiomic signature. The radiomics signature showed comparable performance with the clinical-radiological model in the training cohort (AUC=0.842 vs 0.885;P>0.05) and the validation cohort (AUC=0.837 vs 0.880;P>0.05). The combined prediction model outperformed both the radiomics signature and the clinical-radiological model in predicting early recurrence in the training and validation cohorts (AUC=0.942 and 0.890, respectively) with incremental clinical usefulness.
**Conclusion:** Prediction model, based on multi-sequence whole lesion MRI radiomics features, demonstrated potential in the preoperative prediction of early recurrence for HCC.

**B-0075** 11:43
**Using MRI or CT for preoperative evaluation of HCC makes the clinical outcome different**
Y.-C. Chou; *Tainan/TW (yichenchou@hotmail.com)*

**Purpose:** The recurrent rate of hepatocellular carcinoma (HCC) after treatment is high, at least partly because detecting small HCCs can be challenging before treatment. We hypothesized that different imaging modalities or using different contrast agents may affect the clinical outcome.
**Methods and Materials:** We retrospectively analyzed 698 patients with HCC (BCLC 0, A, B) underwent curative treatments, i.e. the operation or/and radiofrequency ablation between 2011 and 2017. There were 170 patients receiving MR with the liver-specific contrast agent (Gd-EOB-DTPA, Primovist), 111 patients having MR with nonspecific contrast agents, 188 patients having contrast-enhanced CT. Biological markers were also analyzed. After curative treatment, patients received imaging follow-up annually. Multivariate analysis and propensity score matching were used. The overall survival and tumor-free survival were calculated using the Kaplan-Meier method and compared between groups using the log-rank test.
**Results:** There was no significant difference in AJCC stage, Child-Pugh score, albumin-bilirubin (ALBI) score between CT and MR groups. However, the overall survival and tumor-free survival were significantly higher in MR groups than the CT group, with p-value < 0.0001 and 0.0006, respectively. The overall median survival for CT and MR group were 3.3 and 4.0 years; and the median tumor-free survival for CT and MR groups were 1.8 and 2.4 years, respectively. The difference in clinical outcome between using liver-specific and non-specific MR contrast was not statistically significant.
**Conclusion:** Using contrast-enhanced MRI as the preoperative imaging modality for patients with HCC provides significantly better clinical outcomes than using dynamic CT.

**B-0076** 11:51
**Outcomes of Yttrium-90 radioembolic treatment for large monofocal hepatocarcinoma in BCLC-A stage patients: a single-centre experience**
T. Gorgatti, A. Vit, M. Sponza, V. Gavrilovic, A. Pellegrin, F. Rosella, R. Girometti; *Udine/IT (tommaso.gorgatti@yahoo.it)*

**Purpose:** There is no definite treatment for Barcelona Clinic Liver Cancer (BCLC) stage-A patients with large (>5 cm) monofocal hepatocarcinoma (HCC). We evaluated the outcomes of transarterial radioembolization (TARE) in this setting.
**Methods and Materials:** We retrospectively reviewed the clinical history of thirty subjects with BCLC stage-A patients with large monofocal HCC in whom TARE was offered as initial treatment between 2005-2017. TARE was performed by a pool of four interventional radiologists. Post-procedural follow-up was performed with multidetector computed tomography (MDCT) or magnetic resonance imaging (MRI) according to the Response Evaluation Criteria In Solid Tumors (RECIST) version 1.1 and/or modified-RECIST. We used Kaplan-Meier and Cox regression analyses to determine overall survival (OS) and time-to-progression (TTP), entering baseline clinical and laboratory features.
**Results:** Patients showed age ≥70 years in 16/30 cases (53%), male gender in 25/30 cases (83%), and predominant alcoholic- or HCV-related cirrhosis (22/30;73%). Median OS was 28 months (range 10.7-45.3). Child-Pugh score B7 was the only independent predictor of shorter OS (p<0.05, HR: 67.00). There was a trend toward shorter survival in larger tumors (p=0.098, HR: 3.85) and increased Model for End-Stage Liver Disease (MELD) score (P=0.066, HR: 4.60). Median TTP was 18 months (range 0.0-37.2). Multivariable analysis showed no independent predictors, despite a trend towards shorter TTP for higher MELD score (P=0.075, HR: 5.80) and previous liver-directed therapy (P=0.059, HR: 13.16).
**Conclusion:** TARE is a valid therapeutic option in BCLC stage-A patients with large monofocal HCC, with less satisfying results in patients with higher Child-Pugh and MELD scores.

**10:30 - 12:00** **Room E1**

### Breast

### SS 202

### Mammography and screening

*Moderators:*
A.M.J. Bluekens; Breda/NL
V. Lehotská; Bratislava/SK

**B-0077** 10:30
**Influence of annual reader volume on performance in a mammography screening program with independent double reading and consensus of digital mammograms**
S.R. Hoff[1,2], T. Myklebust[2,3], S. Hofvind[3]; [1]*Trondheim/NO*, [2]*Aalesund/NO*, [3]*Oslo/NO (solveig.roth.hoff@helse-mr.no)*

**Purpose:** To study the influence of annual screen-reader volume on radiologists' performance in a mammography screening program using independent double reading with consensus of digital mammograms.
**Methods and Materials:** We collected retrospective data from 2,373,433 digital mammograms read by 121 radiologists in BreastScreen Norway from 2006-2016. Logistic regressions with robust standard errors were used to explore how sensitivity, false-positive rate (FPR), accuracy (sensitivity/FPR) and screening-cancer detection rate (SDC) were related to annual reading volume.
**Results:** In the range from 100 to 10,000 annual screen readings, sensitivity increased from 87 to 89%. For higher volumes, there was a decline in sensitivity to 75% at 18,000 annual readings. The FPR declined from 6.7% at 100 annual readings to 4.5% at 4000, to 4.0% at 10 000 and to 3.0% at 18 000 annual readings. Accuracy increased with 50% from 100 to 4000 annual readings, with 15% from 4000 to 10,000 and with 12% from 10,000 to 18,000 annual readings. The SDC was 4.8/1000 at 100 annual readings and 5.1/1000 at 10,000 annual readings. For higher volumes, the SDC rate declined to 3.2 per 1000 at 18,000 annual readings.
**Conclusion:** Our study indicated that increasing annual reader volume had only a minor effect on sensitivity or SDC rate, except for a decline in performance for readers with extremely high annual volumes. The FPR improved with increasing annual reader volume, most markedly up to an annual volume of 4000 readings.

**B-0078** 10:38
**Conversion to digital mammography had no impact on invasive grade 3 cancer detection in the English NHS breast cancer screening programme: analysis of 11.3 million screening tests**
R.G. Blanks[1], M. Wallis[2], R. Alison[1], J. Jenkins[3], O. Kearins[4], J. Patnick[1], R.M. Given-Wilson[3]; [1]*Oxford/UK*, [2]*Cambridge/UK*, [3]*London/UK*, [4]*Birmingham/UK (rosalind.given-wilson@stgeorges.nhs.uk)*

**Purpose:** To describe and model the impact of conversion from film screen mammography (FSM) with full-field digital mammography (FFDM) in the English NHS breast cancer screening programme.
**Methods and Materials:** Annual screening data (KC62 returns) for each of the 80 units over the seven years from 2009/10 to 2015/16 were used to examine the impact of changing from FSM to FFDM. Regression models were used to estimate percentage and absolute change in detection rates.
**Results:** The recall rate to assessment (approximately 3.9%) was almost unchanged by the introduction of FFDM. After conversion to FFDM the overall cancer detection rate rose from 6.95 to 7.95 per 1000 screens, 14% (11%-17%) p<0.001. High-grade DCIS detection rate rose from 0.71 to 1.00 per 1000, 39% (28%-50%) p<0.001. Invasive grade 1 and 2 detection rate rose from 4.30 to 4.93, per 1000: 14% (10%-18%) p<0.001. Invasive grade 3 detection did not change from 1.20 to 1.22, per 1000: 2% (-5%-9%) p=0.53. The magnitude of the effect for high-grade DCIS and grade 1 and 2 invasive cancers was greater for first (prevalent) screen (age 45-52 years). Grade 3 detection rates were not affected by screen type.
**Conclusion:** Digital mammography has increased the overall sensitivity of screening by increasing the cancer detection rate at the same recall rate as film screen and, therefore, improved the effectiveness of the screening process. However, there has been no increase in the detection of potentially life-threatening grade 3 cancers.

**B-0079** 10:46
**The mammographic features of 13,213 women with screen-detected non-invasive breast disease**
M.G. Wallis[1], K. Clements[2], B. Hilton[2], J. Litherland[3], A.J. Maxwell[4], N. Sharma[5], A. Thompson[6]; [1]*Cambridge/UK*, [2]*Birmingham/UK*, [3]*Glasgow/UK*, [4]*Manchester/UK*, [5]*Leeds/UK*, [6]*Houston, TX/US (matthewwallis492@btinternet.com)*

**Purpose:** To describe the mammographic features of screen-detected carcinoma in situ and atypical hyperplasia in the United Kingdom (UK) Breast Screening Programme.
**Methods and Materials:** The Sloane project is a prospective audit of women with screen-detected non-invasive breast cancer and atypia. Screening units in the UK were encouraged to register patients and complete forms documenting imaging features, surgical management, pathology and radiotherapy (if applicable). Data were collected from 1 April 2003 to 24 October 2018.
**Results:** 14,057 women are registered from 82 of the 90 UK screening units to date. 13,213 have a complete pathology record: 11,335 DCIS and 1,878 'atypia'. Overall, micro-calcification was the predominant radiological feature in 11,087 (83.9%) and was present in 11,638 (88.1%). The presence of micro-calcification was directly related to 'grade'. FEA 80.6%, ADH 72.1%, low-grade DCIS 79.1%, intermediate 84% and high-grade DCIS 94.5% (trend for DCIS grade Chi$^2$ 522.9 df=2, p<0.0001). Of those women with calcification, casting/linear calcification increases with 'grade': FEA 8.8%, ADH 12.5%, low-grade DCIS 19.4%, intermediate 30.6%, and high-grade DCIS 50.6%. A mass was the predominant feature in 16.8%, 17.6%, 19.3%, 14.4%, and 4.1%, respectively. After calcification, parenchymal distortion was the predominant radiological feature in 14.2% (125 of 878) cases of LISN compared to 3.3% of the other pathologies combined.
**Conclusion:** As the 'aggressiveness' of non-invasive screen detected cancer/atypia increases, the proportion of women presenting with calcification (and linear casting morphology) and the proportion with a mass as the predominant radiological feature decreases.

**B-0080** 10:54
**Can we optimise the recall rate and reduce overdiagnosis? An analysis of 11.3 million screening tests from the English NHS breast cancer screening programme**
R.G. Blanks[1], R.M. Given-Wilson[2], S. Cohen[2], J. Jenkins[2], J. Patnick[1], R. Alison[1], M.G. Wallis[3]; [1]*Oxford/UK*, [2]*London/UK*, [3]*Cambridge/UK (matthewwallis492@btinternet.com)*

**Purpose:** To model the association between cancer detection and recall rates to understand the optimal balance of harm and benefit.
**Methods and Materials:** Non-linear and linear regression models were used to examine and model the association between recall rate and cancer detection rate using annual screening programme information for the 80 English breast screening units (11.3 million screening tests) supplemented by previously published data from the Dutch screening programme.
**Results:** Low recall rates are associated with low cancer detection. As recall rates rise, the model indicates that the cancer detection rate for invasive cancers and high-/intermediate-grade DCIS reaches a near-plateau above which almost all recalls are false positive. The cancer grade predicts the point the recall rate reaches plateau. For incident screens the recall rate above which almost no additional cancers are found is 2.5% for both grade 3 and high-grade DCIS. 3.9% for grade 2 and 5.2% for grade 1. However, for low-/intermediate-grade DCIS (LIG) detection rate has no discernible plateau with detection rate increasing linearly at a rate of 0.12 (prevalent) and 0.18 (incident) per 1000 for every 1% increase in recall rate.
**Conclusion:** Our model predicts that there is an optimum range for recall that maximises detection of life-threatening cancers, whilst minimising harm (in England this is between 4.6% and 7% at prevalent screen and between 2.6% and 4% at incident screens).

**B-0081** 11:02
**Additional breast cancer detection at screening mammography through quality assurance sessions between screening radiographers and coordinating screening radiologists**
A.M.P. Coolen[1], B. Korte[2], L.E.M. Duijm[3]; [1]*Tilburg/NL*, [2]*Eindhoven/NL*, [3]*Nijmegen/NL (a.coolen88@hotmail.com)*

**Purpose:** To determine the number and characteristics of cancers additionally detected through quality assurance sessions between screening radiographers and coordinating screening radiologists.
**Methods and Materials:** We included a consecutive series of 431666 biennial screening mammograms, obtained at a Dutch breast cancer screening region between January 1, 2009 and July 31, 2016. Each screen was double read by 2 certified screening radiologists and the radiographers were encouraged to classify each obtained mammogram according to BI-RADS. At regular 6 week intervals the group of screening radiographers discussed with a coordinating screening radiologist the mammograms of women that were not recalled by the screening radiologists but considered suspicious by the radiographers. The coordinating radiologist then decided for each case whether secondary recall was indicated. During 2-year follow-up, we obtained data on radiological and pathological outcome of all recalled women.
**Results:** Altogether, 13175 women were recalled (recall rate: 3.1%), of which 2940 were diagnosed with breast cancer (6.8 cancers detected per 1000 screens). A total of 82 women (0.6% of recalls) experienced a secondary recall after the quality assurance sessions, of which 26 (31.7%) proved malignant. These 26 cancers comprised 8 ductal carcinoma in-situ (low grade: 1; intermediate grade: 7; high grade: 2) and 18 invasive cancers (≤10 mm: 1; 11-20 mm: 13; >20 mm: 4. B&R I: 9; B&R II: 7; B&R III: 2).
**Conclusion:** About 1% of the cancers are detected through the quality assurance sessions. A majority of these cancers is invasive and >10 mm and therefore probably not reflecting overdiagnosis.

**B-0082** 11:10
**Two decades of biennial screening mammography: trend in the incidence of delayed breast cancer diagnosis after repeated recall for the same mammographic abnormality**
B. Korte[1], J.R.C. Lameijer[1], L.E.M. Duijm[2]; [1]*Eindhoven/NL*, [2]*Nijmegen/NL (lemduijm@hotmail.com)*

**Purpose:** Multidisciplinary meetings and dedicated radiological modalities (e.g., MRI, tomosynthesis) are increasingly used for the evaluation of recalled women. We determined the trend in the incidence of delayed breast cancer diagnosis after repeated recall for the same abnormality at screening mammography.
**Methods and Materials:** We included all women aged 50-75 years who underwent screening mammography in a Dutch breast cancer screening region between July 1, 1996 - June 30, 2006 (cohort I) and between July 1, 2006 and June 30, 2016 (cohort II). Data were collected on radiologic procedures and histopathology for all recalled women, with a follow-up period of at least 2 years. For each woman with a repeated recall and histological confirmation of breast cancer at the latest recall, two radiologists independently determined whether the woman had been recalled for this lesion at a previous screen.
**Results:** A total of 1411 recalled women were diagnosed with breast cancer among the 280184 screens of cohort I, resulting in a cancer detection rate (CDR) of 5.0 per 1000 screens. In 28 of these women (2.0%), a repeated recall for the same mammographic abnormality proved malignant. Among the 507828 screens of cohort II, 3504 recalled women had breast cancer, with a CDR of 6.9. In 71 (2.0%) women, a repeated recall for the same screening abnormality showed malignancy.
**Conclusion:** No decline in the proportion of women with a >2 year delay in breast cancer diagnosis was observed. The workup of recalled women in the Dutch breast cancer screening programme needs improvement.

**B-0083** 11:18

**Frequency and outcome of bilateral recall at screening mammography**

J.R.C. Lameijer[1], J. Nederend[1], A.C. Voogd[2], V.C. Tjan-Heijnen[2], L. Duijm[3]; [1]*Eindhoven/NL*, [2]*Maastricht/NL*, [3]*Nijmegen/NL (joostlameijer@gmail.com)*

**Purpose:** To determine the frequency of bilateral recall at screening mammography and compare outcome with that of unilateral recall.
**Methods and Materials:** We included a consecutive series of 197,566 screening mammograms obtained between January 1, 2014 and January 1, 2017. During 2-year follow-up, clinical data were collected of all recalls. Screening outcome parameters were determined for women with unilateral and bilateral recall.
**Results:** A total of 5,629 women were recalled (recall rate 3.0%), of which 153 (2.6% of recalls) comprised a bilateral recall. Biopsy was more frequently performed in women with bilateral recall compared to unilateral recall (P<0.001). The proportion of DCIS among screen detected index cancers (lesion with highest BI-RADS) was comparable for unilateral and bilateral recall (P=0.3). Invasive index cancers after bilateral recall showed a worse tumour grading than those after unilateral recall (P=0.04). The proportion of lymph node positive invasive cancers was comparable for both groups (P=0.7), as well as hormone receptor characteristics of bilateral breast cancer after unilateral and bilateral recall. There was no difference in the proportion of true positives after unilateral versus bilateral recall (P=0.8). Unilateral recall showed a better PPV for biopsy (P=0.01).
**Conclusion:** Bilateral recalls comprise a small proportion of all recalls. After bilateral recall, biopsy is more frequently performed compared to unilateral recall, with a better PPV of biopsy after unilateral recall. Tumour characteristics of index cancers and bilateral breast cancers are comparable for unilateral and bilateral recall, except for a worse tumour grading of invasive index cancers after bilateral recall.

**B-0084** 11:26

**Solitary dilated duct visualised by mammography: are we correctly classifying according to BI-RADS lexicon 5th edition?**

V.J. Ayres[1], E.F. Fleury[2], L. Ramalho[2], L.d. Pompei[2]; [1]*Santo André/BR*, [2]*São Paulo/BR (ayresve@gmail.com)*

**Purpose:** To determinate the incidence, pathological significance and risk factors associated with the presence of solitary dilated duct visualised at mammography.
**Methods and Materials:** Prospectively evaluation of consecutive mammography was performed in a breast cancer control center, in accordance with local ethical approval. Patients with solitary dilated duct (SDD) at mammography were referred to additional second-look ultrasonography (US). SDD with intraductal components were submitted to percutaneous biopsy, following anatomopathological correlation. Exclusion criteria considered patients previously submitted to breast surgery.
**Results:** In the period from March 17, 2016 to March 10, 2017, 9,035 mammographic exams were included, 8,125 (90%) screening and 910 (10%) diagnostic exams. 135 SDD (1.49%) were identified at mammography and 94 (1.04%) second-look US were performed. Of these, 22 revealed intraductal components and had percutaneous biopsy performed. No cancer was found at biopsy results. The most prevalent histological findings were: 8 papillomas and 8 fibrocystic changes. Risk factors for mammographic SDD with statistical significance (p<0.05), using *T test* were: breast density pattern “A” or “B”, breastfeeding, pregnancy, hormone replacement therapy and papillary discharge. Main mammographic and US findings associated with second-look US papilloma diagnostic with statistical significance (p<0.05), using *Chi Quadrado* test: suspicious calcification, duct diameter > 3.0mm at US, intraductal mass and hard lesion at elastography.
**Conclusion:** SDD at mammography benefits from second-look US for analysis of intraductal content’s, which often presents benign findings. When intraductal mass was found, the main result was papilloma. We propose that SDD at mammography should be classified as BI-RADS category 0.

**B-0085** 11:34

**Outcome of microcalcifications classified as BI-RADS 3, 4a and 4b in patients with and without a history of breast cancer**

V. Bertani[1], M. La Grassa[2], L. Balestreri[3], N. Berger[4], M. Urbani[3], K. Lang[5], A. Boss[4], T. Frauenfelder[4], M. Marcon[4]; [1]*Udine/IT*, [2]*Pordenone/IT*, [3]*Aviano/IT*, [4]*Zurich/CH*, [5]*Malmö/SE (magda.marcon@usz.ch)*

**Purpose:** To compare the outcome of microcalcifications classified as BI-RADS3, 4a and 4b at mammography in patients with a history of breast cancer to that of women undergoing mammography screening.
**Methods and Materials:** 176 patients(mean age 61 years) with microcalcifications classified as BI-RADS3, 4a and 4b, without sonographic correlation, whom had undergone vacuum-assisted biopsy (VAB) between Oct 2016-Oct 2017 were retrospectively included. The agreement imaging/pathologic result from VAB was verified and for BI-RADS4b lesions and B2 pathologic result excisional biopsy was performed. For each BI-RADS category, pathologic results from VAB in case of B2 diagnosis and from surgical specimens in all the other cases were compared between patients with a history of breast cancer(group A, n=45) and women undergoing screening(group B, n=131). Positive predictive values (PPVs) were compared using Fisher’s exact test.
**Results:** A total of 73 lesions were classified as BI-RADS3(41.5%), 57 BI-RADS4a(32.4%) and 46 BI-RADS4b(26.1%). The overall PPV for BI-RADS 3, 4a and 4b were 5.5%(4/73), 10.5%(6/57) and 58.7%(27/46). The PPV for BI-RADS3 lesions in group A and B were 13.0%(3/23)and 2.0%(1/50), respectively(p=0.089). The PPV for BI-RADS4a lesions in group A and B were 15.4%(2/13)and 9.1%(4/44), respectively(p=0.611). The PPV for BI-RADS4b lesions in group A and B were 77.8%(7/9) and 54.0%(20/37), respectively(p=0.270).
**Conclusion:** The likelihood of malignancy in cases of microcalcifications classified as BI-RADS 3, 4a and 4b at mammography tends to be higher in women with a history of breast cancer than in women undergoing screening, the difference was however not statistically significant.

**B-0086** 11:42

**Architectural distorsions with microcalcifications: a mammographic malignancy predictor of B3 lesions?**

I. De Serio, K. Jerman, V. Londero, C. Zuiani; *Udine/IT (lsdeserio@gmail.com)*

**Purpose:** Breast borderline lesions (B3) demonstrates variable (9%-35%) malignancy upgrade rate on surgical excision (SE). Our purpose was to evaluate the existence of a mammographic (Mx) finding able to predict the likelihood of malignancy on SE of Mx only B3 lesions, without US correlate, that underwent tomobiopsy-guided vacuum-assisted biopsy (VAB).
**Methods and Materials:** Between March 2015-March 2018 we have retrospectively analyzed 88 B3 lesions (10 papillary lesions [PL], 44 radial sclerosing lesions [RSL], 13 atypical ductal hyperplasia [ADH], 21 lobular neoplasia [NL]). Mx findings were divided into 4 categories: microcalcifications (MI), architectural distorsions (DA), masses (MA) alone-associated to other Mx findings, and architectural distorsions with microcalcifications (DA+MI). The relation between Mx findings and malignancy upgrade rate on SE was evaluated with exact Fisher test.
**Results:** An overall upgrade rate of 16% (14/88) was observed for presence of malignancy at SE (9 intraductal carcinomas G1-G2, 2 intraductal carcinomas G3, 1 invasive ductal-lobular carcinoma G2 and 2 invasive lobular carcinomas G2). Mx findings were MI on 32 cases (36%), DA on 31 cases (35%), MA on 14 cases (16%) and DA+MI on 11 cases (13%). A statistically significative association was found between malignancy upgrade rate and lesions having DA+MI as Mx finding (p=0.01). None significant association was found in the other Mx findings. Positive predict values were 10% for PL (1/10), 14% for RSL (6/44), 31% for ADH (4/13) and 14% for NL (3/21).
**Conclusion:** A high statistically significant likelihood of upgrade was found for B3 lesions that Mx present as DA+MI.

**B-0087** 11:50

**False-positive recall in screening mammography: pitfalls of challenging mammogram**

F. Leone[1], M.A. Orsi[1], M. Cellina[1], A. Presazzi[2], D. Mariani[1], G. Oliva[1]; [1]*Milan/IT*, [2]*Pavia/IT (federica-leone@live.it)*

**Purpose:** to review the most common feature in false positive recall in order to suggest a strategy for assessment of challenging screening mammogram
**Methods and Materials:** We reviewed a retrospective cohort of 7230 patients involved in Mammogram Screening program between November 2015 and September 2018. We included the false positive recall which at second level exam have been classified as BIRADS 1 or 2. We evaluated two different groups according to agreement recall (AR) or disagreement recall(DR).We compared the differences among tumoral imaging features between the two groups and considering also the overall findings prevalence in screening recall (SR)
**Results:** We obtained a final cohort of 1157 suspicious findings whose 416(35,9%) have been in AR group and 741(64,1%) in DR group. In the AR group we found, as single or one of multiple features in suspicious lesion, 287(69%) mass, 26 (6,25%) calcifications, 78(18,75%) architectural distortion and 25(6%) asymmetric density; in the DR group we detected 578(78%) mass, 89(12%) architectural distortion, 43(6%) asymmetric density, 31(4%) calcifications. Differences in tumor imaging features between the two groups were statistically significant (p<0.05)
**Conclusion:** Our results show how mass is the most common feature in false positive recall with no changes between one or two readers. Calcification, when classified as BIRADS 2, is a rare challenging feature, mostly due to the breast radiologist's expertise. Our study proves how Screening Program is a good tool to avoid false positive recall for architectural distortion and asymmetric density, considering the higher prevalence of these findings in recall group

**10:30 - 12:00** **Room E2**

### Neuro

### SS 211a

### Neurooncology: glioma

*Moderators:*
N.N.
N.N.

**K-02** 10:30
**Keynote lecture**
J.-F. Meder; *Paris/FR*

**B-0088** 10:39
**Wavelet parameter maps of perfusion-weighted MRI correlate to tumour vascularity and cell proliferation in glioblastoma**
T. Huber[1], L. Rotkopf[1], S. Bette[1], C. Preibisch[1], J. Gempt[1], T. Pyka[1], K. Thierfelder[2], B. Wiestler[1]; [1]*Munich/DE*, [2]*Rostock/DE* *(Thomas.Huber@med.uni-muenchen.de)*

**Purpose:** Wavelet-based reconstructions of dynamic susceptibility contrast (DSC) perfusion weighted imaging (wavelet-PWI) are a new and elegant way of vascular visualization. Wavelet-PWI yields maps with a clear depiction of hypervascular tumour, as recently shown. The aim of this study was to show if the wavelet-PWI power spectrum signal in tumour tissue is associated with tumour vascularity and cell proliferation in glioblastoma multiforme (GBM).
**Methods and Materials:** For this IRB-approved study 12 subjects (63.0+/-14.9y; 7m) with histologically confirmed GBM were included. Target regions for biopsies were prospectively marked on contrast-enhancing GBM regions as seen on preoperative 3T MRI T1-weighted images. During subsequent neurosurgical tumour resection 27 targeted biopsies were taken intraoperatively from these target regions. All specimens were analysed for the endothelial cell marker CD31 and the proliferation marker MIB-1 and correlated to the wavelet-PWI power spectrum signal derived from dynamic susceptibility contrast (DSC)-MRI.
**Results:** Wavelet-PWI maps could be successfully calculated in 12/12 patients within less than 3 minutes. There was a strong correlation between wavelet-PWI power spectrum signal (median=4.41) and conventional relative cerebral blood volume (median=5.97 ml/100g) in Spearman's rank-order correlation (κ=.83, p<.05). The wavelet-PWI power spectrum signal showed a significant correlation to CD31 dichotomized to no or present staining in a logistic regression model (p<.05) and a significant correlation to MIB-1 in a nonlinear generalized model (p<.05).
**Conclusion:** The wavelet-PWI power spectrum signal derived from existing DSC-MRI data might be a promising new surrogate for tumour vascularity and cell proliferation in GBM.
*Author Disclosures:*
**T. Huber:** Consultant; Consultant for Smart Reporting GmbH.

**B-0089** 10:47
**Noncontrast ASL-perfusion in pre-surgical differential diagnosis of brain gliomas**
A. Batalov, N. Zakharova, E. Pogosbekyan, L. Fadeeva, A. Baev, S. Goryaynov, A. Kosyr'kova, A. Potapov, I. Pronin; *Moscow/RU* *(batalov89@gmail.com)*

**Purpose:** To assess the tumour blood flow (TBF) in the supratentorial brain gliomas by ASL-perfusion in comparison with the histopathological characteristics and 5-ALA intraoperative fluorescence.
**Methods and Materials:** The study group included 186 patients (97 female, 89 male, avg. 45 ± 15 years) with primary supratentorial gliomas: 47 - low-grade (LGG) and 139 - high-grade (HGG: 50-grade III, 89-grade IV). Patients were examined on a 3T MR-scanner. The pseudo-continuous ASL (pcASL) technique was used to determine TBF. TBF was normalized regarding to intact white matter (nTBF). In 66 of 186 patients tumour resection was performed using fluorescence-guided technology. All diagnoses were confirmed histopathlogically.
**Results:** TBF and nTBF in the groups of LGG and HGG were significantly different (p<0.001). TBF in the group of LGG was 31.55±15.75 ml/100g/min, nTBF was 1.69±0.82. In the group of HGG, the TBF was 154.63±92.86 ml/100g/min, nTBF was 8.36±5.30. The sensitivity and the specificity of ASL in diagnosis of LGG and HGG were 82.7% and 95.7%, accordingly, AUC 0.946, cutoff-64 ml/100g/min. TBF and nTBF in groups of GrIII and GrIV didn't show statistically significant difference (p=0.08). Nevertheless, we found a significant difference in TBF and nTBF between fluorescent and non-fluorescent gliomas (p<0.05). The sensitivity and specificity of pcASL in predicting fluorescence in gliomas was 77% and 71%, accordingly, AUC-0.722, cutoff-3.3.
**Conclusion:** pcASL is a reliable quantitative technique for the differential diagnosis between LGG and HGG. pcASL can be used in predicting intraoperative fluorescence in gliomas. The study was supported by RFBR №18-29-01018
*Author Disclosures:*
**A. Batalov:** Research/Grant Support; Russian Foundation for Basic Research (grant № 18-29-01018). **N. Zakharova:** Research/Grant Support; Russian Foundation for Basic Research (grant № 18-29-01018). **L. Fadeeva:** Research/Grant Support; Russian Foundation for Basic Research (grant № 18-29-01018).

**B-0090** 10:55
**Lesion and lesion habitat analysis on magnetic resonance imaging: an imaging biomarker for tumour grade and 1p19q deletion status**
A. Alvi[1], F. Mubarak[1], A. Alvi[2], S. Enam[1], M. Shahzad[1]; [1]*Karachi/PK*, [2]*Lahore/PK (aamnaalvi@gmail.com)*

**Purpose:** The purpose of the study is to grade gliomas and predict 1p19 q deletion status on the basis of MRI characteristics. Hypothesis: low-grade gliomas are more heterogeneous, less well circumscribed, have low mean ADC values, usually in frontal and parietal lobes and are 1p/19q codeleted.
**Methods and Materials:** Retrospective analysis of 97 patients (53 male, 44 female; age 20-70 years) with oligodendroglioma grade II and anaplastic oligodendroglioma grade III. Associations of 1p/19q deletion status and grading with imaging characteristics were assessed. Multivariable logistic regression models were used to assess the association of grading and 1p/19q deletion with imaging characteristics and results were summarised with odds ratios and 95% confidence intervals. P values of .005 were considered statistically significant. Statistical analysis was done using SPSS® v. 19 (SPSS Inc., Chicago, IL).
**Results:** Thirty-one of 97 patients had 1p/19q codeleted tumours (21= Grade II, 10= Grade III). They were mostly in frontal and parietal lobes showing heterogeneity, ill-defined margins and low ADC values (mean/SD: 1185.3/283.4). 66 patients did not show 1p/19q codeletion. They had circumscribed borders, mostly in temporal lobe and insula showing homogeneity and relative high ADC values (mean/SD: 1784.2/648.7).
**Conclusion:** Heterogeneous signal characteristics and less well circumscribed borders on T1, T2 and SWI with lower mean ADC values are features of low-grade gliomas and these are usually 1p19q codeleted tumours. Dealing with gliomas, MRI characteristics can predict tumour grade and 1p19q deletion status. In addition, this evaluation is recommended for better treatment planning and effective patient management and prognostication.

**B-0091** 11:03
**Changes in high-grade gliomas after different kinds of radiation therapy depending on IDH status during chemotherapy in combination with bevacizumab**
A.V. Smirnova[1], O.V. Lukina[1], A. Kuzmin[1], A. Tkachev[2], N. Plakhotina[1], M. Cherkashin[1], M. Anishkin[1]; [1]*St. Petersburg/RU*, [2]*Volgograd/RU* *(smirnova_alina@bk.ru)*

**Purpose:** Identification of consistent patterns for accurate MRI-based assessment of recurrence in patients with high-grade gliomas (HGG) treated with radiotherapy, chemotherapy and bevacizumab, taking IDH1 status into account.
**Methods and Materials:** 31 patients with HGG (Group A included 18 patients with IDH1-mutant tumour, Group B included 13 patients with IDH1-wild-type) treated with radiotherapy, chemotherapy (temozolomide and subsequently irinotecan) in combination with bevacizumab were prospectively studied between 2016 and 2018. Changes on T1CE, T2, flair, PWI and 11C-methionine PET/CT before radiotherapy, and after 4, 8, 12 and 16 months were evaluated. Tumour volume estimation was performed with GammaPlan workstation 10.1.
**Results:** The earliest features of recurrence were registered at 8 months after radiotherapy for IDH1-wild-type and 12 months for IDH1-mutant tumours during continued systemic therapy, and in 96.8% of cases were in a form of an increase in T2/Flair anomaly with unconvincing methionine uptake (1.4 or less). At the same time, T1CE showed no signs of pathological contrasting and PWI with no increase in CBV also. Only subsequent observations of T2/Flair hyperintense volumes revealed higher methionine uptake values (1.8 to 4.0) and increase in CBV.
**Conclusion:** Wild-type gliomas are characterized by an earlier onset of progression as opposed to IDH-mutant gliomas. Well-timed identification of recurrence during bevacizumab-containing therapy is challenging due to decrease in contrasting, blood flow and moderate methionine uptake, and can be suspected by T2/Flair. To identify continued tumour growth, it is necessary to take into account all available data to start anti-relapse therapy as early as possible.

**B-0092** 11:11

**Novel imaging signs in detecting the 1p 19q non-co-deleted IDH wild and 1p 19q non-co-deleted IDH mutant lower grade gliomas**

A.N. Kamble[1], A. Kamble[2]; [1]*New Delhi/IN*, [2]*Mumbai/IN*
*(akshaykumar.kamble92@gmail.com)*

**Purpose:** Lower grade glioma are classified based on genetic markers into subgroups, one being 1p19q -co-deletion, this subgroup would include both IDH mutants, IDH wild type of gliomas.
**Methods and Materials:** Total 330 lower grade glioma cases from cancer imaging archive (TCIA) were analysed 171 from TCGA-LGG collection and 159 from LGG-1p19qDeletion collection. Two signs (Sign A and Sign B) were then applied to the whole collection. Confusion matrices and ROC curves were plotted for Sign A, Sign B and T2-FLAIR mismatch sign using 1p 19 q non-codeletion, IDH wild and mutants from TCGA genetic database were taken as a gold standard. These novel signs were validated using LGG-1p19qDeletion collection(n=159 cases).
**Results:** With TCGA data Sign A had 88.19% sensitivity, 63.56% specificity, 87.5% precision, 81.87% accuracy (p value<0.0001) Sign B had 41.09% sensitivity, 91.11% specificity, 92.98% precision (p value <0.0001), T2-FLAIR mismatch sign had a poor 26.11% sensitivity and 96.77% specificity in detecting 1p 19q non-co-deleted gliomas (p value<0.0001). There was a good intra-observer variability with Sign A (κ=0.669, ) and Sign B (κ= 0.691) in TCGA-LGG collection. Validation cohort of TCGA-LGG collection showed Sign A had 87.72% sensitivity, 69.61% specificity, 91.03%of NPV (p value <0.0001), while Sign B had sensitivity of 71.93%, specificity of 90.1%, 80.39% precision, 83.54% accuracy (p value<0.0001).
**Conclusion:** The sign A has good sensitivity and specificity; while sign B has good specificity to identify the 1p 19q non-co-deleted gliomas containing both IDH mutants and IDH wild types of tumour in lower grade gliomas.

**B-0093** 11:19

**Diffusion kurtosis imaging parameters predicting survival in integrated molecular subtypes of diffuse glioma: an observational cohort study**

J.-M. Hempel, N.-C. Nüßle, C. Brendle, B. Bender, G. Bier, M. Skardelly, H. Richter, J. Schittenhelm, U. Ernemann, U. Klose; *Tübingen/DE*
*(nils-christoph.nuessle@student.uni-tuebingen.de)*

**Purpose:** To assess the predictive value of preoperatively assessed diffusion kurtosis imaging (DKI) metrics as prognostic factors in the 2016 World Health Organization Classification of Tumors of the Central Nervous System integrated glioma groups.
**Methods and Materials:** Seventy-seven patients with histopathologically confirmed treatment-naïve glioma were retrospectively assessed between 08/2013 and 10/2017 using mean kurtosis (MK) and mean diffusivity (MD) histogram parameters from DKI, overall and progression-free survival, and relevant prognostic molecular data (isocitrate dehydrogenase [IDH]; alpha-thalassemia/mental retardation syndrome X-linked, [ATRX]; chromosome 1p/19q loss of heterozygosity). The optimal cutoff-values of the metric variables were determined using receiver operating characteristic (ROC) analysis. Univariate survival data were assessed using the Kaplan-Meier method. A multivariate Cox proportional hazards model was performed on significant results from univariate analysis.
**Results:** There were significant differences in overall and progression-free survival between patient age (p=0.001), resection statuses (p=0.002), WHO glioma grades (p<0.0001), and integrated molecular profiles (p<0.0001). Survival was significantly better in patients with lower MK and higher MD values (p=0.009) in gliomas without chromosome 1p/19q LOH (p<0.0001) and those with retained ATRX expression (p=0.008).
**Conclusion:** Patient age and MK from DKI from DKI are relevant factors for preoperatively predicting overall and progression-free survival. Regarding the molecular subgroups, they are unfavourable prognostic factors in gliomas without chromosome 1p/19q LOH and those with ATRX retention.
*Author Disclosures:*
**J. Schittenhelm:** Research/Grant Support; Else Übelmesser Foundation (grant no. 30.19845).

**B-0094** 11:27

**Altered whole brain connectivity as a potential marker of disease burden in glioma patients**

S. Stoecklein[1], V. Stöcklein[1], F. Schöppe[1], N. Thon[1], F.-W. Kreth[1], B.B. Ertl-Wagner[2], J.-C. Tonn[1], H. Liu[3]; [1]*Munich/DE*, [2]*Toronto, ON/CA*, [3]*Boston, MA/US (sophia.stoecklein@med.uni-muenchen.de)*

**Purpose:** In glioma patients tumour cells spread far beyond the lesion that is detected by conventional MRI. Assessing non-lesional brain in glioma patients could provide crucial information about disease burden. We developed an individual measure of altered functional connectivity based on resting-state functional MRI (rsfMRI) and related this marker to WHO grade, IDH mutation status, neurocognitive performance and overall survival.
**Methods and Materials:** 40 patients with suspected de novo glioma were prospectively included and rsfMRI data were obtained. We evaluated the abnormality of functional connectivity by comparing each patient's data to normative data obtained from 1000 healthy individuals. Abnormality was quantified at each voxel, resulting in an individual measure for abnormality (abnormality index, ABI). Statistical analysis was conducted adjusting for tumour volume, age and SNR of rsfMRI data.
**Results:** ABI maps reflected the macroscopic tumor, but also displayed alterations in non-lesional brain tissue. On a quantitative level, ABI was associated with WHO grade, both when summarized in the lesional and non-lesional hemisphere (<0.01,respectively). ABI was increased in patients with IDH-wildtype gliomas, with strongest effects in the non-lesional hemisphere (p<0.01). Association with neurocognitive performance was strongest in the lesional hemisphere (p<0.005). 8/38 patients died within the follow-up period, showing a trend towards association of overall survival with ABI (p=0.12).
**Conclusion:** ABI captures widespread connectivity changes in glioma patients on an individual level. ABI reflects tumour biology and correlates with neurocognitive performance and might be associated with overall survival. Individual ABI maps might therefore proof as a useful complement to conventional structural MRI.

**B-0095** 11:35

**GLINT: GlucoCEST in neoplastic tumours at 3T - first-in-man studies of GlucoCEST in glioma patients**

T. Lindig[1], M. Zaiss[1], K. Herz[1], A. Deshmane[1], B. Bender[1], X. Golay[2], K. Scheffler[1]; [1]*Tübingen/DE*, [2]*London/UK*
*(tobias.lindig@med.uni-tuebingen.de)*

**Purpose:** Dynamic glucose enhanced (DGE) CEST imaging has almost only been shown at ultra-high field (UHF) due to low effect size. First results in brain tumour patients of a DGE CEST method with fast 3D imaging developed for clinical field strength are shown herein.
**Methods and Materials:** CEST saturated images at different frequency offsets were acquired at 160 time points before, during and after a glucose injection (0.3 mg/kg) with 6.3s temporal resolution (Total: 16:45 min) to detect accumulation in the brain. Two glioblastoma (IDH wild-type, unmethylated MGMT promoter) patients (1: male, 70y, 2: female, 75y) and 3 healthy controls were scanned at a clinical 3T System. DGE contrast images were analysed by subtracting each image from a pre-injection baseline image: $\Delta DGE(t)=DGE_{baseline} - DGE(t)$.
**Results:** In the high-grade glioma (1), glucose uptake in the Gadolinium enhancing region could be detected approximately 4 minutes after injection with a maximum increase of $\Delta DGE=0.51\pm0.078$, whereas a contralateral white matter ROI was barely affected ($\Delta DGE=0.07\pm0.085$) at the same time point. The second glioma (2), with the same histology and grading, showed very little gadolinium enhancement as well as no significant detectable DGE effect. Healthy controls did not show any significant DGE contrast.
**Conclusion:** We demonstrated that stable dynamic glucose enhanced imaging can be accomplished at clinical field strength using optimized saturation and readout parameters. First results are promising, and indicate that glucoCEST corresponds more to the disruptions of the blood-brain-barrier with Gadolinium uptake than to the molecular tumour profile or tumour grading.

**B-0096** 11:43

**Preoperative resectability estimates of non-enhancing glioma by neurosurgeons and a resection probability map**

E.J. Hendriks[1], S. Idema[1], A.H. Zwinderman[1], F. Barkhof[1], W.P. Vandertop[1], E. Mandonnet[2], H. Duffau[3], M.S. Berger[4], P.C. De Witt Hamer[1]; [1]*Amsterdam/NL*, [2]*Paris/FR*, [3]*Montpellier/FR*, [4]*San Francisco, CA/US*
*(hendriks.eef@gmail.com)*

**Purpose:** Preoperative interpretation of resectability of diffuse non-enhancing glioma is primarily based on individual surgical expertise, to identify patients who benefit most from resective surgery. Here, we compare the agreement between observed resections and preoperative estimates of neurosurgeons and a resection probability map (RPM).
**Methods and Materials:** 234 consecutive patients were included from two neuro-oncological centres, who had resective surgery with functional mapping between 2006 and 2012 for a supra-tentorial non-enhancing glioma. Extent of resection (EOR) and residual tumour volume (RTV) were segmented and an RPM was constructed in standard brain space. Three junior and three senior neurosurgeons estimated EOR and RTV, blinded for postoperative results. We used Bland-Altman analysis to determine the agreement between the estimations and receiver operating characteristic analysis to calculate the diagnostic accuracy of the neurosurgeons and the RPM to predict observed resections.
**Results:** Preoperative estimates of resection results by junior and senior neurosurgeons were significantly biased towards overestimation of EOR (4.2% and 11.2%) and underestimation of RTV (4.3 and 9.0 mL), whereas estimates of the RPM were unbiased (-2.6% and -0.2 mL, respectively). The limits of agreement were wide for neurosurgeons and for the RPM. The RPM was significantly more accurate in identifying patients in whom an EOR>40% was observed than neurosurgeons.
**Conclusion:** Neurosurgeons estimate preoperative resectability before surgery of a non-enhancing glioma rather accurate - with a small bias, and imprecise - with wide limits of agreement. An RPM provides unbiased resectability estimates, which can be useful for surgical decision-making, planning and education.

**B-0097** 11:51
**Radiomics MRI phenotyping with machine learning to predict the grade of lower grade gliomas: a study focused on non-enhancing tumours**
S. Yoon, Y. Park, Y. Choi, S. Ahn, J. Chang, S. Kim, S.-K. Lee; *Seoul/KR*

**Purpose:** To assess whether radiomics features derived from multiparametric MRI can predict the tumour grade of lower grade gliomas (LGGs; WHO grade II and grade III) and the non-enhancing LGG subgroup.
**Methods and Materials:** Two-hundred and four patients with LGGs from our institutional cohort were allocated to training (n = 136) and test (n = 68) sets. Postcontrast T1-weighted images, T2-weighted images, and FLAIR images were analysed to extract 250 radiomics features. Various machine learning classifiers were trained using the radiomics features to predict the glioma grade. The trained classifiers were internally validated on the institutional test set, and externally validated on a separate cohort (n = 99) from The Cancer Genome Atlas (TCGA). Classifier performance was assessed by determining the area under the curve (AUC) from receiver operating characteristic analysis. An identical process was performed in the non-enhancing LGG subgroup (institutional training set, n = 73; institutional test set, n = 37; and TCGA cohort, n = 37) to predict the glioma grade.
**Results:** The performance of the best classifier was good in the internal validation (AUC 0.85) and fair in the external validation (AUC 0.72) to predict the LGG grade. For the non-enhancing LGG subgroup, the performance of the best classifier was good (AUC 0.82) in the internal validation, but poor in the external validation (AUC 0.68).
**Conclusion:** Radiomics feature-based classifiers may be useful to predict LGG grades. However, radiomics classifiers may have limited value when applied to the non-enhancing LGG subgroup in an external cohort.

**10:30 - 12:00** **Room F2**

### Emergency Imaging

### SS 217

### Best of emergency radiology (part 1): from mass causality incidents to incidental findings and errors

*Moderators:*
F.G. Mück; Munich/DE
M. Stajgis; Poznan/PL

**B-0098** 10:30
**Mass casualty incidents: are you ready? An evidenced-based radiology MCI protocol template**
J.W. Ryan, P.J. MacMahon, F. Bolster; *Dublin/IE (jamesryannchd@gmail.com)*

**Purpose:** Mass casualty incidents (MCIs) are increasing in frequency across Europe. Radiology plays a critical role in appropriate patient triage during an MCI. Major incident planning (MIP) can help optimize radiology's response with the goal of improving patient outcomes. Historically, radiology is often underrepresented or excluded from MIP. Our goal was to identify key topics that should be addressed by radiology during MIP, thus promoting MCI awareness and education amongst radiologists.
**Methods and Materials:** A review of the literature was performed. The advanced search builder on PubMed was utilized. Search terms "radiology" and "mass casualty incident" were used in the primary search filter. External MCI protocols from level 1 trauma centres were obtained on request and reviewed.
**Results:** 27 relevant studies were identified on the primary search of PubMed and review of references. Trauma guidelines from the Royal College of Radiologists and NICE were also reviewed. Key topics include patient identification strategy, scan ordering, patient tracking, MCI scan protocols, result communication, PACS failure contingencies, scalability of response and individualized radiology staff action cards.
**Conclusion:** All radiology departments should partake in MIP and in MCI simulation to help maximize patient throughput and improve triage efficiency in the event of an MCI. We have provided an evidenced-based radiology MCI protocol template for use by radiologists during MIP.

**B-0099** 10:38
**Whole-body trauma CT for critical and mass casualty incident patients: how fast can we go**
L.F.M. Beenen, M.J. Scheerder, D. Bekebrede-Kauffman, N.H.J. Lobe; *Amsterdam/NL (l.f.beenen@amc.uva.nl)*

**Purpose:** In trauma, every second counts: time = life. Whole-body computed tomography (WBCT) for trauma patients can achieve both significant time and survival benefits. Faster radiological diagnosis could be lifesaving, particularly for haemodynamically critical or MCI patients. Hence, high efficacy of the scanning workflow is paramount. However, detailed time claims of different scan setups are not known. The aim of our study was to investigate the impact of different WBCT procedures on trauma CT times.
**Methods and Materials:** Adult anthropomorphic phantoms were used to evaluate 6 different scan protocols in a trauma exercise, with 5 simulation tests for each protocol. Tested workflow settings were: single vs double scout acquisitions, acquisition direction, non-enhanced vs contrast-enhanced, and fixed-delay or bolus triggering contrast administration. Several start and end time points were measured for the complete trauma radiology period: scout acquisitions, CT acquisitions, and reconstruction times, respectively.
**Results:** Of the total trauma radiology time, up to 83% consisted of non-radiation time. Scout acquisition times claim between 7 and 24% of the examination time. Acquisition times of scouts were close (range 70-93%) to total CT acquisition times. The fastest protocol was a non-enhanced one-stop WBCT protocol, costing in mean only 79 seconds to completion (p<0.001). For contrast-enhanced scans, a fixed delay split bolus protocol could present all axial images 159 seconds after start of imaging.
**Conclusion:** Scout acquisition and non-acquisition times are significant time-limiting factors in WBCT. Optimising examination times of WBCT protocols can contribute to faster trauma radiology workflow.

**B-0100** 10:46
**Whole-body CT using biphasic injection protocol with adaptive statistical iterative reconstruction-V (ASiR-V) in multi-trauma patients: impact on dose reduction and image quality**
A.H. Elmokadem[1], E.A. Ibrahim[2], W.A. Gouda[3], A. Abdel Razek[1];
[1]*Mansoura/EG*, [2]*Kuwait/KW*, [3]*Menoufia/EG (walaagoda@yahoo.com)*

**Purpose:** To evaluate dose savings and image quality after implementing adaptive statistical iterative reconstruction-V (ASiR-V) on a revised protocol for whole-body CT (WBCT) for trauma patients.
**Methods and Materials:** One hundred multi-trauma patients were scanned using a 256-section multidetector CT system (GE Healthcare Revolution system). They were randomized into two groups using two different scanning protocols. Group (A) (n=50, age 32.48±8.09) underwent conventional protocol including unenhanced scan for brain and cervical spines, then an contrast-enhanced arterial-phase of the thorax and abdomen followed by a venous and delayed scans of the abdomen and pelvis. Group (B) (n=50, age 35.94±13.57) underwent biphasic injection protocol including unenhanced scan for brain and cervical spines, followed by a one-step acquisition of the thorax, abdomen, and pelvis following a biphasic injection, the examination was ended by delayed phase for the abdomen and pelvis. All examination were done under 50 % ASiR-V. Image count, radiation dose, total acquisition time, mediastinal artifacts were compared between the groups. Two radiologists independently graded image quality from 1 to 5. In addition, contrast enhancement was measured in the pulmonary artery, aorta, inferior vena cava, portal vein, liver, spleen, and kidneys.
**Results:** The mean (±SD) dose length product for group (A) was 2202.3 ± 271.8 mGy*cm and markedly higher when compared to group (B) (p < 0.001) which was 1485.8 ± 489.2 mGy*cm. Protocol B gave a dose reduction of 32.5% and 7.7 % time reduction. The HU values of the aorta & liver were significantly higher in group (A) while kidneys values were higher in group (B). There was no significant difference between the image quality scores for both groups, however group (A) scored higher grades.
**Conclusion:** Implementing ASiR-V algorism into biphasic CT protocol markedly reduced radiation with maintenance of accuracy and image quality.

**B-0101** 10:54
**Diagnostic performance of CT and the use of GI contrast material for the detection of hollow viscus injury after penetrating abdominal trauma**
S. Thorisdottir[1], G. Oladottir[1], M. Nummela[2], S. Koskinen[3]; [1]*Stockholm/SE*, [2]*Helsinki/FI*, [3]*Huddinge/SE (sigurveig.thorisdottir@sll.se)*

**Purpose:** To assess the diagnostic performance of CT regarding hollow viscus injury (HVI) from penetrating abdominal trauma.
**Methods and Materials:** Retrospective analysis of patients with penetrating abdominal trauma during 2013-2016. Data from Karolinska Trauma Registry, medical records, and from local PACS were reviewed. CT and surgical findings were compared.
**Results:** Of 636 patients with penetrating trauma, 177 had abdominal trauma (mean age 34 yrs, range 16-88 yrs; M 163, F 14), and 155 (85%, 155/177)

were imaged with CT on arrival. 128 (83%) were stab wounds and 21 (14%) gunshot wounds. 6 patients had miscellaneous trauma mechanisms. Thereof 47 (47/155; 30%) had emergent surgery after CT imaging. Two patients were imaged using oral, rectal and i.v. contrast, 23 with rectal and i.v. contrast, and 22 with i.v. contrast only. Surgery revealed HVI in 26 patients. CT had an overall sensitivity of 69.2%, specificity 90.5%, PPV 90.0% and NPV 70.4%. CT with oral and/or rectal contrast (n = 25) had sensitivity of 66.7%, specificity 71.4%, PPV 85.7% and NPV 45.5%. CT with i.v. contrast only (n = 22) had sensitivity 75.0%, specificity 100%, PPV 100% and NPV 87.5%. Difference in sensitivity between the oral and/or rectal contrast group and i.v. contrast only was not statistically significant (p=1.00).
**Conclusion:** Stab wounds were the most common cause of penetrating abdominal trauma. The overall sensitivity and specificity of CT in detecting HVI were 69.2% and 90.5%, respectively. The use of oral and/or rectal contrast yielded similar sensitivity to the use of i.v. contrast only.

**B-0102** 11:02
**Evaluation of a CT-based scoring system regarding the prognosis of blunt splenic and liver injuries: a single-centre analysis with 720 patients**
D. Morell Hofert, M. Fodor, E. Braunwarth, M. Haselbacher, T. Schmid, M. Blauth, E.-M. Gassner, S. Stättner, F. Primavesi; *Innsbruck/AT (Dagmar.Morell@i-med.ac.at)*

**Purpose:** Non-operative management (NOM) of blunt splenic and hepatic injuries has been getting increasingly common. Next to clinical parameters, CT-based grading systems are habitually used as screening tools in early management. Usually the AAST classification is used. Shortly, another scoring system, which contemplated contrast-media extravasation, was introduced for spleen injuries (CTSI). This study validates this system for spleen injuries and proposes/validates an adapted classification for liver injuries.
**Methods and Materials:** Retrospective analysis of patients with traumatic blunt liver/spleen lesions in the MUI from 2000 to 2016. CT imaging on admission was reevaluated by two radiologists, using the AAST and the CTSIs. Both classifications were examined regarding their capability to predict the necessity of operative treatment, the failing of NOM and the in-hospital-mortality.
**Results:** In total, 720 patients were analysed (median 32; 230 female), 276 with spleen, 364 with liver and 80 with combined injuries. The total mortality was 5.6%. 160 patients had severe (grade 4-5) lesions according to AAST, with a mortality of 6.0%/5.3% (severe/mild liver injuries, p=0.790) and 10.5%/3.2% (spleen injuries, p=0.008). When using CTSI, 33 patients with liver and 87 patients with spleen lesions had severe injuries (≥4a), associated with a higher in-hospital-mortality: 12.1%/4.9% for liver (p=0,095) and 10.3%/3.0% for spleen injuries (p=0.005). Both classifications showed a high-significant tendency (p < 0.001) of severe lesions more often requiring first-hand operative treatment and were equally good for predicting NOM failure.
**Conclusion:** The CTSI is able to predict in-hospital mortality and proved efficient as a management indication tool and as a predictor for NOM failure. Therefore, it should substitute the AAST classification as the gold standard.

**B-0103** 11:10
**Characteristics and predictive factors of delayed events in patients of blunt splenic trauma designated for non-operative management**
Y.-C. Wong, L.-J. Wang, C.-H. Wu; *Taoyuan/TW (ycwong@cgmh.org.tw)*

**Purpose:** We compared patients with delayed events and immediate events who required splenic angioembolisation (SAE) for salvaging failures of non-operative management (NOM) after blunt splenic trauma.
**Methods and Materials:** From 2012 to 2017, 161 patients of blunt splenic trauma who were designated for NOM and treated with SAE at our institution were identified. Delayed event occurred at least 4 days after trauma, whereas immediate event occurred within 24 hours. We excluded 11 patients whose events fell between 1 and 4 days. The final inclusion was 150 patients (38 women, 112 men) with median of 33.0 (IQR 21, 47) years. Records were retrospectively reviewed for clinical and CT characteristics. Comparisons between delayed and immediate events as well as predictive factors for delayed events were computed.
**Results:** Delayed events occurred in 23 (15.3%) patients and were significantly associated with less vascular injuries on CT (3.5% vs. 96.5%), smaller CT score (3.3 vs. 5.2) and smaller hemoperitoneum score (2.9 vs. 4.3). Significant predictive factors for delayed events were CT score, hemoperitoneum score and initial platelet count. Among 23 delayed events, paired T test showed a worse CT score (3.2 vs 4.8; p=0.001) and lower haemoglobin (11.9 to 10.1 g/dL; p=0.004) on follow-up CT examinations and haemograms. Patients with immediate events had a significant longer length of stay after SAE than patients with delayed event, 12.7 days vs. 9.4 days.
**Conclusion:** Delayed events of blunt splenic trauma were associated with CT findings. The significant predictive factors were CT score, hemoperitoneum score and initial platelet count.

**B-0104** 11:18
**Can MR be useful in the follow-up of blunt liver and spleen injuries: preliminary results**
F. Iacobellis, M. Di Serafino, T. Cinque, C. Stavolo, L. Romano; *Naples/IT (iacobellisf@gmail.com)*

**Purpose:** In haemodynamically stable patients, the standard of care for blunt liver and spleen injuries is the non-operative management. In our Institution, MR was introduced for the follow-up of haemodynamically stable patients with blunt liver and spleen injuries. The aim is to describe the imaging protocol, the main findings and the potentialities in the use of MR in these patients.
**Methods and Materials:** From May 2018, patients with blunt liver and spleen injuries without MR contraindications, after admission CT, were followed up by MR. The imaging protocol was tailored to each patient. Post-contrast sequences were acquired especially in the early follow-up and in high grade injuries. In suspected biliary complications, MR cholangiographic sequences were added, and hepatobiliary-specific contrast agent (gadobenate dimeglumine) administration with delayed acquisitions was used.
**Results:** Seventeen patients with spleen (6 pts.) and liver (11 pts.) injuries of low (10 pts.) and high (7 pts.) grade, were followed-up by MR. The signal behaviour of the healing lesions was documented and described. In 4 patients, liver intraparenchymal collections were detected, MR allowed to characterize them and to examine the biliary ducts communication. In 1 case was observed an intraparenchymal pseudoaneurism of the spleen that was subsequently embolized.
**Conclusion:** MR may constitute a useful alternative to CT to follow-up patients with blunt liver and spleen injuries considering the panoramicity, the high resolution and the lack of ionizing radiation. In this preliminary experience, MR allowed to identify and monitoring, in a less invasive way, vascular and parenchymal liver and spleen injuries.

**B-0105** 11:26
**Repeated imaging work-up in revisiting emergency patients: analysis of clinical and radiological risk factors**
Y. Ahn, G.-S. Hong, K. Park, C.W. Lee; *Seoul/KR (foryourmiracle@gmail.com)*

**Purpose:** To analysis risk factors to cause repeated imaging in revisiting emergency patients.
**Methods and Materials:** A retrospective review identified 12,357 patients returning to emergency room (ER) within 7 days after performed CT or MRI at the first visit and discharged from the ER between 2005 and 2013. Among them, 1250 patients underwent repeated image workup for the same or different part of the body. The patients were divided into two groups according to the revisiting time after discharge: early revisiting group (≤ 72 hours) and late revisiting group (> 72 hours). The factors to cause re-performing the imaging are classified as follows through medical record and image review: 1) radiological factor (RF) (misdiagnosis vs. inappropriate reporting turn-around time [R-TAT]); 2) non-radiology clinician's factor (CF) (mistriage); and 3) patient's factors (PF) (disease or symptom progression).
**Results:** The repeated imaging was performed in 10.12% of revisiting patients. The ratios of RF and CF in early revisiting group were significantly higher from those in late revisiting group (13.50% vs. 8.29% and 12.29% vs. 6.76%, respectively) ($P < 0.001$). In the radiological factors, there was significant difference between early revisiting group (inappropriate R-TAT 64.04%; and misdiagnosis 35.96%) and late revisiting groups (inappropriate R-TAT 30.61%; and misdiagnosis 69.38%) ($P < 0.001$).
**Conclusion:** RC and CF are the major causes of repeating imaging in early revisiting group than late revisiting group. In radiological factors, inappropriate R-TAT is the main cause in the early revisiting group and misdiagnosis in the late revisiting group.

**B-0106** 11:34
**Incidental findings in whole-body CT: a retrospective analysis in over 1000 resuscitation room patients**
S. Sudarski, T. Henzler, D. Schäfer, T. Viergutz, T. Terboven, S.O. Schönberg, H. Haubenreisser; *Mannheim/DE (sonja.sudarski@medma.uni-heidelberg.de)*

**Purpose:** Whole-body computed tomography reveals beyond findings related to the suspected diagnosis often additional incidental findings. Aim of this investigation was the assessment of these findings in whole-body CT scans of patients admitted via the resuscitation room after suffering potential major trauma or life-threatening medical conditions.
**Methods and Materials:** Patients admitted via the resuscitation room were retrospectively included if they had received a whole-body computed tomography scan at admission. The final cohort consisted of 1165 patients (1038 trauma and 127 internal-neurological patients). Whole-body computed tomography reports screened for incidental findings. These findings were then classified as either clinically relevant or not.
**Results:** 465 incidental findings were reported in a total of 293 patients (25.1%). Relevant incidental findings could be detected in 5.8% of the study patients (68/1165). The group of internal-neurological patients was older than the trauma patients (61.6 years vs. 45.5 years). The rate of relevant incidental findings in the internal-neurological group was more than twice as high as in the trauma group (11.0% vs. 5.2%). Yet, in the relatively young trauma group one in 20 patients showed an incidental finding classified as clinically relevant as well.
**Conclusion:** Incidental findings are reported in ¼ of whole-body CT scans of patients admitted to the resuscitation room. About 6% of all patients had incidental findings being rated as clinically relevant. In the internal-neurological older group of patients the rate of incidental findings was doubled compared to the younger trauma group.

**B-0107** 11:42
**Diagnostic error in emergency studies: an analysis of explanatory variables**
F. Facal de Castro[1], R. Mirón Mombiela[1], J. Vucetic[1], J. Mota Martinez[2], E. Arana Fernandez De Moya[1], D. Dualde Beltran[1]; [1]*Valencia/ES*, [2]*Barcelona/ES (facalrayos@hotmail.com)*

**Purpose:** To determine the discrepancy rate between the first and second readings. To estimate the clinical significance of errors. To identify latent factors associated with a higher incidence of errors. To discover weak points in the radiological work that can be solved by training or quality control measures.
**Methods and Materials:** Retrospective observational analysis of diagnostic discrepancies in emergency studies of adult patients from 37 health centres detected after a second reading at the company Iberorad SL from 2014 to 2016.
**Results:** We found a diagnostic discrepancy rate of 1.4% in emergency studies. The average rate of emergency studies with clinically significant discrepancies is 27.4% of total discrepancies. A statistically significant association was noticed with the diagnostic discrepancy, in decreasing order of magnitude, of the following: availability of previous imaging studies; average level of concordance between area of expertise of the radiologist and modality of the study; insufficient clinical information; non-use of speech recognition systems; low level of concordance between area of expertise of the radiologist and modality of the study; study of high complexity; body studies; use of a single monitor to visualise images; and MSK/spine studies.
**Conclusion:** The diagnostic discrepancy rate during the period analysed is similar to that reflected in the medical literature. Most discrepancies are not clinically significant. There are multiple factors associated with diagnostic error. The intensity in which each of these is associated with diagnostic error is different and variable determined by the rest of the characteristics present in the diagnostic process.

**B-0108** 11:50
**Would it be safe to have a dog in the MRI scanner before your own examination?**
A. Gutzeit[1], J.M. Fröhlich[2], F. Steffen[1], D.-M. Koh[3], C. Orasch[1]; [1]*Lucerne/CH*, [2]*Zurich/CH*, [3]*Sutton/UK (agutzeit2000@gmail.com)*

**Purpose:** To determine whether it would be hygienic to evaluate dogs and humans on the same MRI scanner.
**Methods and Materials:** We compared the bacterial load in colony-forming units (CFU) of human pathogenic microorganisms in specimens taken from 18 men and 30 dogs. In addition, we compared the extent of bacterial contamination of an MRI scanner shared by dogs and humans, with two other MRI scanners used exclusively by humans.
**Results:** Our study shows a significantly higher bacterial load in specimens taken from men's beards compared with dogs' furs ($p = 0.036$). All of the men (18/18) showed high microbial counts, whereas only 23/30 dogs had high microbial counts and 7 dogs moderate microbial counts. Furthermore, human pathogenic microorganisms were more frequently found in human beards (7/18) than in dog fur (4/30), although this difference did not reach statistical significance ($p = 0.074$). More microbes were found in human oral cavities than in dog oral cavities ($p < 0.001$). After MRI of dogs, routine scanner disinfection was undertaken and the CFU found in specimens isolated from the MRI scanning table and receiver coils showed significantly lower bacteria count compared with "human" MRI scanners ($p < 0.05$).
**Conclusion:** Our study shows that bearded men harbour higher burden of microbes and more human pathogenic strains than dogs. As the MRI scanner used for both dogs and humans was routinely cleaned after animal scanning, there was substantially lower bacterial load compared with scanners used exclusively for humans.

**10:30 - 12:00** **Room Y**

### Interventional Radiology

### SS 209

### Vascular interventions in oncology

*Moderators:*
R. Duran; Lausanne/CH
N.N.

**B-0109** 10:30
**Operator learning curve for transradial liver cancer embolisation: implications for the initiation of a transradial access programme**
F. Carchesio, R. Iezzi, A. Posa, G. Veltri, A. Gasbarrini, R. Manfredi; *Rome/IT (francesca.carchesio@gmail.com)*

**Purpose:** To analyze transradial approach (TRA) learning curve on patients undergoing hepatic chemoembolization, investigating the relationship between procedural volumes and various benchmarks of procedural success.
**Methods and Materials:** We enrolled sixty consecutive patients who received two unilobar hepatic chemoembolization within a 4-week interval performed by a single interventional radiologist, highly-trained in conventional transfemoral procedures (TFA), but without any previous practical experience in TRA procedures and with a preliminary 2-days theoretical training only. We divided the study population, prospectively consecutively random-enrolled, into 3 groups: A (case 1 to 20), B (case 21 to 40), and C (case 41 to 60), using all TFA procedures performed by the same operator in the same series of patients as the control group. Primary endpoint was to analyze the relationship between procedure volumes and benchmarks of procedural success, such as safety, and procedural variables, including fluoroscopy time, and radiation dose, to define the optimal procedural learning curve.
**Results:** Technical success was obtained in all patients, without needing any switch from radial to femoral access. No major complications were registered without significant differences in terms of minor complications between the three subgroups of patients. An association between incremental TRA volume and reduction decreasing of preparation, puncture, fluoroscopy and total examination times was demonstrated, with values significantly higher only for group A (first 20 patients).
**Conclusion:** TRA is safe and feasible for transarterial hepatic chemoembolization. Operator proficiency improves with greated TRA experience, with a threshold needed to overcome the learning curve represented by 20 cases.

**B-0110** 10:38
**Transarterial chemoembolisation with degradable starch microspheres (DSM-TACE) as second-line treatment in HCC patients dismissing or ineligible for sorafenib**
G. Veltri, R. Iezzi, A. Posa, F. Carchesio, A. Gasbarrini, R. Manfredi; *Rome/IT (Giuseppeveltri1701@gmail.com)*

**Purpose:** To evaluate safety, feasibility and effectiveness of transarterial chemoembolisation with degradable-starch-microspheres (DSM-TACE) in the treatment of patients with advanced HCC dismissing or ineligible for multikinase-inhibitor chemotherapy administration (Sorafenib) due to unbearable side effects or clinical contraindications.
**Methods and Materials:** 40 consecutive HCC patients who underwent DSM-TACE via lobar approach were prospectively enrolled. Tumor response was evaluated on MD-CT based on mRECIST criteria. Primary endpoints were safety, tolerance and overall disease control (ODC); secondary endpoints were progression free survival (PFS) and overall survival (OS).
**Results:** Technical success was achieved in all patients. No intra/peri-procedural death/major complications occurred. No signs of liver failure or systemic toxicity were detected. During the mean follow-up of 18.2 months an ODC of 52.5% was registered. Progression free survival was 6.4 months with a median overall survival of 11.3 months.
**Conclusion:** DSM-TACE seems to be a safe and effective second-line treatment for HCC patients ineligible for Sorafenib administration or dismissing it due to progressive disease or unbearable side effects.

**B-0111** 10:46
**Same-day workup angiography and Yttrium 90 microsphere radioembolisation: feasibility and efficacy of hepatic flow redistribution**
A. Ezponda Casajús, M. Calvo Imirizaldu, M. Morales, I. Vivas Perez, M. De La Torre, M. Rodríguez-Fraile, B. Sangro, J.I. Bilbao Jaureguizar; *Pamplona/ES (aezponda@unav.es)*

**Purpose:** To assess the feasibility and efficacy of performing vascular flow redistribution and transarterial radioembolisation with 90Y-microspheres (90YRE) for hepatic malignancies in a single-day session.

**Methods and Materials:** From November 2015 to September 2018, in 16 patients undergoing same-day workup angiography, cone-beam CT (CBCT), $^{99m}$TC-MAA SPECT-CT and 90YRE, accessory tumour arteries were embolised with microcoils. Perfusion of the lesions in the redistributed and non-redistributed segments was qualitatively compared by assessing the distribution of $^{99m}$TC-MAA. Within 18 hours following treatment, a PET/CT was performed.
**Results:** Sixteen patients were treated for primary (n=11) and secondary hepatic malignancies (n=5). Flow redistribution was achieved by embolisation of intrahepatic (n=12) and extrahepatic branches (n=4). In most cases, CBCT provided additional information of arterial tumour perfusion. In all patients, the $^{99m}$TC-MAA-SPECT/CT showed uptake in the redistributed tumoural areas. The mean lung shunt fraction (LSF) ratio was 7.10%±0.056. Of the 16 patients, 8 received segmental injections to both lobes, 5 to the right lobe and 3 to the left lobe. The median doses administered to the tumour and normal hepatic tissue were 141Gy (range 29-576Gy) and 40Gy (range 5-315Gy), respectively. The 90Y-PET/CT images showed accumulation of microspheres over the tumoural territory, both in the redistributed and non-redistributed tumoural segments, with a mean 90Y activity of 1.57±0.7 GBq. The mean total procedure time was 392 minutes (range 204-532).
**Conclusion:** Same-day embolisation of extra and intrahepatic branches (to redistribute intrahepatic flow patterns to the tumour) and 90YRE procedure is feasible and effective.

**B-0112** 10:54
**Locoregional heparinisation for patients with acute deep venous thrombosis of lower extremity: safety and efficacy evaluation**
Y.-D. Xiao; *Changsha/CN (xiaoyudong222@csu.edu.cn)*

**Purpose:** To assess efficacy and safety of locoregional heparinisation in patients with acute deep venous thrombosis (ADVT) of lower extremity.
**Methods and Materials:** A total of 10 patients with unilateral ADVT who were contraindicated for thrombolysis and systemic anticoagulation were enrolled in the study. The popliteal vein was punctured, and an 8-F sheath was inserted. After confirming the diagnosis and extent of the DVT, suction thrombectomy was performed, and then, a multiple infusion side-hole catheter was advanced to the iliac vein. Through the infusion catheter, 500 IU/h heparin mixed with normal saline was infused using a micropump. Blood samples were taken every 4 hours from both cubital vein and popliteal vein to measure the activated partial thromboplastin time (aPTT), which was to adjust the heparin dose. The symptoms and the complications of patients were evaluated.
**Results:** Our results revealed that for the blood sample from the cubital vein, the aPTT maintained at 30s-45s, while for the blood sample from the popliteal vein, the aPTT maintained at 60s-90s. With the dissolution of thrombus, the aPTT from cubital vein and the popliteal vein tended to be converging. One month later, the symptoms related to ADVT were significantly improved in eight patients. Repeat venography showed that vascular completely recanalisation observed in two patients, partial recanalisation in seven patients. No severe complications such as intracranial bleeding occurred.
**Conclusion:** Locoregional heparinisation is a safe and effective way for patients with ADVT who is contraindicated to thrombolysis and systemic anticoagulation

**B-0113** 11:02
**Response to SIRT (selective internal radiation therapy) in patients affected by unresectable multifocal intra-hepatic cholangiocarcinoma: results of a preliminary study**
A. Paladini[1], D. Beomonte Zobel[2], G.E. Vallati[2], G. Pizzi[2], F. Cappelli[2], A. Borzelli[3], E.M. Amodeo[2]; [1]*Novara/IT*, [2]*Rome/IT*, [3]*Naples/IT (andreapaladini1988@gmail.com)*

**Purpose:** The aim of this study is to demonstrate the results in terms of overall survival (OS) and response to treatment in a cohort of 43 patients (pts) affected by unresectable multifocal intra-hepatic cholangiocarcinoma (ICC) and treated with SIRT.
**Methods and Materials:** Between 2011 and 2018, we treated 43 pts affected by multifocal ICC with SIRT. Response to treatment was evaluated after 2 TC performed at 3 and 6 months.
**Results:** The 3- and 6-month radiological evaluation was performed on 40 pts with the analysis of complete (CR) or partial (PR) response to treatment, stability (SD) or progression disease (PD). Response to treatment (CR + PR) was 70% according to mRECIST and 60% according to the EASL criteria. The median OS was 17.9 months (range: 14.3-21.4 months). A shorter OS was found in pts treated with medical or surgical therapies before SIRT: 16 versus 52 months of the pts treated only with SIRT. We reported a greater correlation between OS and CR and PR according to the mRECIST and EASL criteria compared to RECIST 1.1 criteria. Treatment was well tolerated and no mortality was seen in the first 30 days post-treatment. No peri-procedural complications were documented.
**Conclusion:** SIRT documented excellent results in terms of efficacy in pts affected by ICC and portal thrombosis also. It is a safe and repeatable treatment and, therefore, it is a valid loco-regional therapy in pts affected by unresectable ICC.

**B-0114** 11:10
**Locoregional treatments for unresectable early stage HCC in patients with high-risk for intraprocedural bleeding: is single-step combined therapy safe and feasible?**
G. Veltri, R. Iezzi, A. Posa, F. Carchesio, A. Gasbarrini, R. Manfredi; *Rome/IT (Giuseppeveltri1701@gmail.com)*

**Purpose:** To assess feasibility and safety of a single-step combined therapy with radiofrequency ablation and transarterial chemoembolization (RFA+TACE) in patients with hepatocellular carcinoma (HCC) and uncontrolled coagulopathy. To compare the effectiveness of this approach with TACE alone performed in a control group.
**Methods and Materials:** From January 2010 to June 2017, 143 consecutive cirrhotic patients with single HCC < 8 cm were enrolled in an observational prospective single-center study and divided, according to coagulation tests, into 3 groups (A, low risk; B, intermediate and C, high risk of bleeding). Feasibility and safety of a single-step combined treatment (RFA followed by TACE) were evaluated in terms of technical success rate, incidence of peri-procedural complications and laboratory values variations. Efficacy was evaluated comparing tumour response on 1 month CT in group C and in a control group, composed by 16 patients with severe coagulopathy and single HCC < 8 cm, underwent TACE in a previous period performed by the same operator.
**Results:** Technical success was obtained in all patients, without major complications. Incidence of minor complications was significantly higher in group C after RFA, successfully treated with subsequent TACE without differences between pre- and post-procedural laboratory values. The complete response rates on 1-month follow-up were similar in the 3 groups whereas was significantly higher when compared group C with control TACE Group (p<.001).
**Conclusion:** Single-step RFA plus TACE allows to expand the indication for percutaneous thermal ablation to previously contraindicated cases due to procedural high-risk of bleeding complications improving patient outcome.

**B-0115** 11:18
**Effectiveness and safety transarterial embolisation with bleomycin-loaded PLA/PLGA microspheres for the treatment of vascular malformation using a porcine spleen model**
F. Nurili[1], T. Acar[2], S. Derman[2], I. Caymaz[2], S. Özkanli[2], A. Bas[2], I. Yurdaisik[2], H. Cakiroglu[2], O. Aras[1]; [1]*New York, NY/US*, [2]*Istanbul/TR (fuatnurili@gmail.com)*

**Purpose:** To prospectively study the effectiveness and safety of bleomycin-loaded PLA/PLGA microparticles in the porcine spleen model.
**Methods and Materials:** Bleomycin-loaded PLA/PLGA microspheres were manufactured using the double emulsion solvent evaporation method. The porcine spleen was chosen as a vascular malformation model and eleven pigs were assigned to either the embolisation group (n-7) or control group (n-4). Selective angiography was performed in all animals and bleomycin-loaded PLA/PLGA microspheres (800 -1000 micron in diameter) were administered into the splenic artery in the embolisation group. 0.5mg/kg of bleomycin was used per procedure. After 30 days, porcine spleens were acquired to evaluate for the volumes of organ, presence or absence of microspheres, intraluminal reaction to bleomycin, vessel wall changes and extent of sclerosis.
**Results:** Newly manufactured microsphere formulations showed sustained long-term in vitro release performance. Transcatheter arterial embolisation was technically successful in all animals. Toxicity monitoring showed no haematologic, pulmonary, hepatic or renal toxicities. Microspheres induced significant shrinkage in volume of spleens. The mean spleen volume after embolisation was significantly (P<0.001) reduced compared with the mean volume for the group control. Pathology showed apoptosis/vasculitis of endothelial cells and disconfiguration of the spleen without systemic side effect in the experimental group.
**Conclusion:** Embolisation of the spleen with bleomycin-loaded microspheres with slow release properties can induce strong sclerosis of the spleen locally and promote apoptosis and vasculitis of splenic endothelial cells without systemic side effects. This study shows that transcatheter embolisation with bleomycin-loaded microspheres has the potential to be an alternative treatment for vascular malformations in humans.

**B-0116** 11:26
**Low pretreatment neutrophil to lymphocyte ratio predicts better survival in uveal melanoma liver metastases undergoing hepatic chemoperfusion**
J.M. Ludwig, O. Drescher, J. Haubold, H. Richly, M. Forsting, Y. Li, A. Wetter, S. Bauer, J. Theysohn; *Essen/DE (johannes-maximilian.ludwig@uk-essen.de)*

**Purpose:** To evaluate neutrophil to lymphocyte ratio (NLR) as a pretreatment prognostic factor in patients with unresectable uveal melanoma liver metastases and limited extrahepatic disease undergoing a structured transarterial hepatic chemoperfusion (THC) protocol.
**Methods and Materials:** 56 patients (44% male; median age: 61 years) first treated between 01/2014-10/2015 were assessed retrospectively. A median of 3 (range: 1-11) THC sessions were performed starting with melphalan which was replaced by Fotemustine when progressing. Pretreatment factors were assessed within 1 month prior to start. Kaplan-Meier for median overall survival in months (OS; 95%CI) and cox proportional hazard model for uni- (UVA) and multivariate (MVA) analyses (hazard ratio; 95%CI) were performed.
**Results:** Median OS of the study cohort was 7.7 (6.3-10.9) months. Low pretreatment NLR (≤3.5) was associated with prolonged OS (11.1;7.1-20.6) versus high NLR (>3.5) (6.5; 3.5-7.8), p=.0035. In addition to low NLR (0.4; 0.2-0.75, p=.0045) UVA identified serum lactate dehydrogenase (LDH) ≤2 upper level of normal (ULN) (0.26; 0.13-0.5, p<.0001), gamma-glutamyl transferase (GGT) ≤3ULN (0.3; 0.15-0.6, p=.0004), aspartate aminotransferase (0.27; 0.11-0.7, p=.007), c-reactive protein ≤ULN (0.31; 0.15-0.59, p=.0006), and liver metastasis ≤6cm (0.29; 0.15-0.53, p=.0002) as predictors for prolonged OS. MVA confirmed low NLR (0.36; 0.14-0.83, p=.018), LDH (0.25; 0.08-0.83, p=.03), GGT (0.2; 0.05-0.77, p=.016) and liver metastasis ≤6cm (0.15; 0.06-0.37, p<.0001) as independent predictors. Absolute NLR change ≤1.5-fold within 1 month prior to first THC was associated a lower hazard ratio (0.4; 0.17-0.85, p=.018) in all patients but only proved significant in the low NLR subgroup (12- vs. 6.4-month median OS, p=.019).
**Conclusion:** Low pretreatment NLR is an independent predictor for prolonged OS in patients with uveal melanoma liver metastases treated with THC.

**B-0117** 11:34
**Transvenous pulmonary chemoembolisation (TPCE) for palliative and neoadjuvant treatment of primary lung malignancies**
A.I.A. Mekkawy[1], A.A. Hassan[1], M. El-Sharkaway[1], H.M. Kamel[1], D.B. Thabet[1], N.-E.A. Nour-Eldin[2], N.N.N. Naguib[2], T.J. Vogl[2]; [1]*Asyut/EG*, [2]*Frankfurt a. Main/DE (time_dr@yahoo.com)*

**Purpose:** To assess the response, mean time to progression and mean survival time for patients with primary lung cancer treated with TPCE in palliative and neoadjuvant intent.
**Methods and Materials:** This study included 56 patients (mean age 61.6 ±9 years; 27 females, 29 males) with an unresectable primary lung tumour, who failed or refused systemic chemotherapy, and underwent repetitive TPCE, between 2008 and 2017. Bilateral lung involvement was seen in 39.3% of patients and the median number of lung nodules/patient was 2.5. Regional delivery of the chemotherapeutic agents was performed after catheterization of the tumor-supplying pulmonary artery, followed by embolization with Lipiodol and microspheres. Patients who underwent subsequent ablation represented the neoadjuvant group (n=18). The response according to the revised Response Evaluation Criteria in Solid Tumors (RECIST 1.1) was evaluated and survival parameters were statistically analyzed.
**Results:** After evaluation of the tumour response; partial response was achieved in 14.3% (n=8), stable disease in 58.9% (n=33) and progressive disease in 26.8% (n=15). The estimated mean survival time and mean time to progression were 15.8 ± 1.7 and 9 ± 1.3 months, respectively. The mean survival time was significantly (P < 0.05) longer for the neoadjuvant group (22.3 ± 2.8 months), compared to the palliative group (11.6 ± 1.8).
**Conclusion:** TPCE could be a promising palliative treatment with acceptable survival for patients with primary lung cancer who failed or are not eligible for other treatment options. It, also, has a neoadjuvant potential when combined with ablation therapy.

**B-0118** 11:42
**Predictors for refractoriness and survival after transarterial chemoembolisation among hepatocellular cancer patients: outcomes from a novel southeast Asian cohort**
R. Kuhn, G. Ignacio, J. Jamias; *Quezon City/PH (rudolfvkuhn@yahoo.com)*

**Purpose:** Patients with hepatocellular carcinoma become refractory to repeated sessions of transarterial chemoembolisation. Predictors associated with refractoriness and survival are, however, poorly defined and lack consensus. The aim of this study was to identify predictors associated with overall survival and refractoriness of HCC patients undergoing repetitive TACE. The secondary aim was to validate the ART score, neutrophil-lymphocyte ratio and radiologic response using the mRECIST and CHOI criteria for the first time in a southeast Asian setting.
**Methods and Materials:** The clinical and laboratory characteristics and radiologic response of 39 patients treated with repetitive conventional TACE from January 2012 to June 2018 were analysed in a retrospective cohort.
**Results:** The median overall survival of patients was 23.2 months and overall mortality was 36%. Multivariate Cox regression analysis revealed that Child-Pugh score (hazard ratio=3.47, p=0.044), AST (HR=7.6, p=0.021), tumour size (HR=5.47, p=0.033), progressive disease using CHOI criteria (HR = 5.47, CI 1.15 - 25.99, p = 0.033), neutrophil-lymphocyte ratio (HR=1.25, p=0.049) and nodular enhancement on follow-up CT imaging (HR=1.98, p=0.034) were independent risk factors for poor survival. Multivariate analysis also showed that ALT (p=0.005), enhancement (p=0.003), Child-Pugh score (p=0.010), and progressive disease using CHOI criteria (p=0.022) were predictive of TACE refractoriness/failure.
**Conclusion:** Predictors for poorer survival and TACE failure/refractoriness were identified. A rational treatment strategy and the decision to switch therapy should be individualised considering the patient's clinical condition and aforementioned clinical and laboratory parameters and response on radiologic follow-up.

**B-0119** 11:50
**Radiation dose reduction during angiographic adrenal vein sampling using a new imaging technology**
C. Spink, M. Avanesov, T. Schmidt, M. Grass, G. Schoen, G. Adam, H. Ittrich, P. Bannas; *Hamburg/DE (c.spink@uke.de)*

**Purpose:** To compare the patient radiation doses during angiographic adrenal vein sampling (AVS) before and after an imaging processing technology upgrade.
**Methods and Materials:** In this retrospective single-center-study, cumulative air kerma (AK), cumulative dose area product (DAP), fluoroscopy time and total contrast agent volume were gathered from 70 patients during AVS. 35 procedures were performed before and 35 after an imaging processing technology upgrade from Philips Allura Xper to Philips AlluraClarity. Mean values were calculated and compared using two-tailed t tests. DSA image quality was assessed independently by two blinded readers using a four-rank Likert scale (1=acceptable; 4=excellent) and compared using the Wilcoxon signed-rank test.
**Results:** Using the new imaging technology a significant reduction in DAP (235.1 vs. 170.1 Gycm$^2$, p=0.01) and a significant reduction in AK (1.7 vs. 1.1 Gy, p=0.01) could be achieved. The number of exposure frames (143 vs. 132 frames, p=0.65), fluoroscopy time (42.3 vs. 36.5 min, p=0.47) and amount of contrast agent (179.5 vs. 198.1 ml, p=0.31) did not differ significantly between the two groups. There was no significant difference regarding image quality after the technology upgrade (3.25 vs. 3.32, p=0.68).
**Conclusion:** The new angiographic noise reduction technology significantly reduces radiation dose while maintaining adequate image quality in patients undergoing AVS.
*Author Disclosures:*
**T. Schmidt:** Employee; Philips Healthcare. **M. Grass:** Employee; Philips Research.

**10:30 - 12:00** **Coffee & Talk 3**

### Head and Neck

### SS 208

### Thyroid gland

*Moderators:*
E. Scapin; Monserrato/IT
A. Singhal; Gurgaon/IN

**K-03** 10:30
**Keynote lecture**
P.-Y. Marcy; *Ollioules/FR*

**B-0120** 10:39
**Association of the four international guidelines for thyroid nodule management to surgical and treatment outcomes in patients with small papillary thyroid carcinoma**
J. Lee, J. Kwak, E.-K. Kim, H. Moon, V.Y. Park, J. Yoon; *Seoul/KR (easywg@yuhs.ac)*

**Purpose:** To evaluate the role of four international guidelines for thyroid nodule management in predicting pathologic staging and treatment outcomes for patients with small conventional papillary thyroid carcinoma (PTC).
**Methods and Materials:** From 2007 to 2015, 775 patients who had been surgically treated for conventional PTC measuring 1-2cm were included. Each nodule was categorized according to the FNA guidelines of the 2015 American Thyroid Association (ATA) guidelines, the Korean Society of Thyroid Radiology guidelines (K-TIRADS), the European Thyroid Association guidelines (EU-TIRADS), and the American College of Radiology Thyroid Imaging Reporting and Data System (ACR TIRADS). Multivariate regression analysis was used to analyse predictors for central or lateral cervical lymph node (LN) metastasis, and recurrence/persistence or distant metastasis in patients with small thyroid cancer.
**Results:** Microlobulated or irregular margins, or not parallel shape had higher rates of central lateral LN metastasis (all *P*<0.05). Presence of microcalcifications had higher rates of central or lateral LN metastasis (*P*<0.05). FNA indication of the four guidelines was significantly associated with central LN metastasis (all *P*<0.05). FNA indications of the ATA guidelines, EU-TIRADS, and ACR TIRADS were significantly associated with the presence of lateral LN metastasis (all *P*<0.05). Size, the presence of central LN metastasis, and the extrathyroidal extension were significantly associated with recurrence/persistence (all *P*<0.05). FNA indications of the four international guidelines did not show significant association with recurrence/persistence or metastasis (all *P*>0.05).
**Conclusion:** FNA indication of the EU-TIRADS and ACR TIRADS can be used in deciding upon surgical extent for patients with small PTC.

**B-0121** 10:47
**Efficiency of biopsy criteria in thyroid nodules: the 2017 European Thyroid Imaging Reporting and Data System vs the 2014 American Thyroid Association management guidelines**
V. Perez Riverola, F. Bosch Barragan, M. Prenafeta Moreno, J. Cabero Moyano, I. Capel Flores, F. Guirao Garriga, S. Barcons Vilaplana, A. Rovira Gols; *Sabadell/ES (vperez@tauli.cat)*

**Purpose:** To determine the diagnostic efficiency and assess malignancy risk stratification of the 2017 European Thyroid Imaging Reporting and Data System (EU-TIRADS) in comparison to the 2014 American Thyroid Association (ATA) management guidelines.
**Methods and Materials:** Retrospective study reviewing all fine-needle aspiration biopsies (FNAB) of thyroid nodules done following the ATA criteria at our centre from January 2015 to December 2017. We evaluated the ultrasound characteristics of all the nodules and regrouped them according to the EU-TIRADS. The EU-TIRADS criteria for FNAB are more restrictive than those of the ATA criteria in the very low risk (EU-TR2), low-risk (EU-TR3), and intermediate-risk (EU-TR4) groups; the criteria in the high risk (EU-TR-5) is the same in both systems. We analysed FNABs done under the ATA criteria that did not meet the EU-TIRADS criteria and calculated the rate of malignancy for each subgroup in each system.
**Results:** Of 891 thyroid nodules biopsied, EU-TIRADS criteria for biopsy were unmet in 235 (26.4%): 54 (23%) in EU-TR2, 143 (60.8%) in EU-TR3, and 38 (16.2%) in EU-TR4. FNABs were inconclusive in 39; in the remaining 196, only 4 (2%) were malignant. No EU-TR2 nodules were malignant. Only 1 EU-TR3 nodule was malignant (a noninvasive follicular thyroid neoplasm with papillary-like nuclear features). There were 3 EU-TR4 malignancies: 1 invasive follicular carcinoma, 1 classical papillary carcinoma and 1 follicular variant papillary carcinoma.
**Conclusion:** Using EU-TIRADS would have decreased the number of FNABs by 26.4%; only 2% of malignant nodules would have been missed.

**B-0122** 10:55
**Radiological-pathological correlation of thyroid nodule ultrasound and cytology using the TIRADS and Bethesda classifications**
S. Aslan, S. Ozdemir; *Tokat/TR (serdaraslan28@hotmail.com)*

**Purpose:** To compare the Thyroid Imaging Reporting and Data System (TIRADS) of classifying thyroid nodules with the findings on fine-needle aspiration cytology (FNAC) reported using the Bethesda System.
**Methods and Materials:** A prospective analysis of 150 patients was performed comparing thyroid nodule ultrasound findings based on the TIRADS classification to the FNAC report based on the Bethesda Classification. TIRADS 1 and biopsy-proven malignancy were excluded. Benign-appearing nodules were reported as TIRADS 2 and 3. Indeterminate or suspected follicular lesions were reported as TIRADS 4, and malignant-appearing nodules were reported as TIRADS 5. All the nodules were performed FNAC, and TIRADS findings were compared to Bethesda Classification.
**Results:** Of the 150 patients, 77 were TIRADS 2, 28 were TIRADS 3, 18 were TIRADS 4 and 27 were TIRADS 5. The probability of a malignant FNAC (Bethesda V-VI) in TIRADS 2, 3, 4 and 5 classes were 0%, 7.1%, 16.6% and 81.4%, respectively. The benign FNAC (Bethesda II) in TIRADS 2 was 100%, while for TIRADS 3, 4 and 5 were 75%, 27.7% and 4.6%, respectively. Of the 27 patients that were TIRADS 5, 20 patients had biopsy-proven cancer (74% concordance), but 7 were benign (false-positive sonographic impression). Overall concordance rate with FNAC was 92%, and sensitivity, specificity and negative predictive value were 88%, 90.4% and 95%, respectively.
**Conclusion:** Our study shows a fairly good correlation of thyroid ultrasound reporting using the TIRADS classification with the Bethesda Classification of FNAC.

**B-0123** 11:03
**Combination of ultrasound elastography with TI-RADS in the diagnosis of small thyroid nodules (<10 mm): a new method to increase the diagnostic performance**
Y. Du, C. Ji, Y. Wu, X. Gu; *Shanghai/CN (duyanran44@126.com)*

**Purpose:** To evaluate the diagnostic performance of a new method of combined ultrasound elastography (UE) and thyroid imaging-reporting and data system (TI-RADS) in the differential diagnosis of small thyroid nodules.
**Methods and Materials:** Ultrasonography, TI-RADS, elasticity imaging, virtual touch tissue imaging (VTI) and virtual touch tissue quantification (VTQ) features of 142 thyroid nodules (maximum diameter ≤10 mm according to conventional ultrasound measurement) confirmed by surgery or FNA were retrospectively analysed. Different elastographic methods in small benign and malignant thyroid nodules were compared. The diagnostic efficiency of three adjustment methods of TI-RADS classification and UE (VTI and/or VTQ) was compared.
**Results:** There were 70 benign thyroid nodules and 72 malignant thyroid nodules. The differential ability of UE to diagnose the small benign and malignant thyroid nodules alone is not better than that of conventional ultrasound TI-RADS classification. The sensitivity of conventional ultrasound TI-RADS classification was higher than that of VTI and equal to VTQ (91.67% vs 83.33%, 91.67%), while the specificity of VTI was much higher than that of TI-RADS and VTQ (91.43% vs 75.71%, 60.00%). The diagnostic sensitivity and specificity of TI-RADS plus UE (VTI + VTQ) were 94.44% and 87.14%, respectively. TI-RADS classification combined with UE (VTI + VTQ) was the highest diagnostic efficiency.
**Conclusion:** TI-RADS classification is the basis for the diagnosis of small thyroid nodules. The combination of TI-RADS with VTI and VTQ can significantly improve the differential diagnosis of small benign and malignant thyroid nodules. It may provide a new and reliable method for the clinical diagnosis of small thyroid nodules.

**B-0125** 11:19
**Prediction of Bethesda category of thyroid nodule with specific sonographic findings**
F. Kulali, M. Demir, A. Semiz-Oysu, Y. Bukte; *Istanbul/TR (ftkulali@gmail.com)*

**Purpose:** Our purpose was to depict specific sonographic characteristics of thyroid nodule for each cytological Bethesda category for planning patient management.
**Methods and Materials:** The Institutional Review Board approved this retrospective study, and informed consent was waived. A total of 1488 patients who had undergone ultrasound guided fine-needle aspiration biopsy (FNAB) were included. There were 1260 women (85%) and 228 men (15%) with a mean age of 49 years (range: 12 - 88 years). Sonographic features (the largest diameter, contour, echogenicity, existence of halo, multiplicity, solid/cystic nature, presence of calcifications, lymphadenopathy and thyroiditis findings) were recorded. The relationship between sonographic findings and cytological results was investigated for each Bethesda category.
**Results:** A total of 1488 patients were enrolled in our study. Among Bethesda category 2 nodules, ratios of iso-/hyper-echogenicity, well-defined contour, solid plus cystic component, thin halo and multiplicity were higher than those of Bethesda 3, 4, 5 and 6 categories (p<0.05). Nodules with thick halo (7/75, 9.5%) and lymphadenopathy (4/75, 5.5%) were only observed in Bethesda category 6 nodules. Ill-defined/irregular contour was mostly seen in Bethesda category 5 (5/7, 71%) and category 6 (34/75, 45%) nodules (all *p*<0.05). Solid composition and single nodule were more frequent in Bethesda category 6. There were no statistical difference about the existence of micro-calcification or thyroiditis between Bethesda categories (*p*>0.05).
**Conclusion:** Prediction of Bethesda category of nodule with sonographic findings is possible in some Bethesda categories. Specific sonographic findings can decrease the number of unnecessary FNABs.

**B-0126** 11:27
**On-site evaluation microscopic evaluation of unstained slides to assess adequacy of thyroid fine-needle aspiration cytology**
N.M.H. Nik-Hussin, M. Robinson, K. Kenyon, M. Brown, P.-.T. Diep, M. Atwan; *Lancaster/UK (nik3278@hotmail.com)*

**Purpose:** On-site evaluation of thyroid fine-needle aspiration cytology (FNAC) on stained slides is a proven way to reduce inadequate samples. However, staining requires additional time, specialist staff and reduces clinic throughput. Our institution utilises a unique approach to evaluating adequacy of FNAC by assessing unstained slides. This study aimed to evaluate our practice of assessing adequacy of ultrasound-guided fine-needle aspiration biopsy of thyroid lesions using contemporaneous microscopy on unstained, air-dried samples.
**Methods and Materials:** Retrospective review of cytopathology and radiology reports on 139 consecutive patients who had undergone FNAC sampling between September 2015 and August 2016 at a regional clinic for suspected thyroid cancer. Cytology reports use the Royal College of Pathologist 'Thy' classification system. The samples were obtained and slides prepared by 3 histopathologists and 1 radiologist using a standardised technique. Each operator evaluates the unstained samples for adequacy and performs up to 3 needle passes if no adequate sample was seen on microscopy.
**Results:** Using our method of assessing unstained thyroid fine-needle aspiration cytology biopsy, our service was able to achieve a collective adequacy rate of 93.9% (range 90-100%). This adequacy rate is higher than that is cited in the literature in studies where on-site evaluation of adequacy is not utilised. Our adequacy rate is comparable to studies evaluate stained fine-needle aspiration samples, but without the time penalty required to stain the samples.
**Conclusion:** On-site evaluation of thyroid FNAC on unstained slides is a practical and efficient way of achieving a high adequacy rate.

**B-0127** 11:35
**Malignancy risk of thyroid nodules with isolated macrocalcification**
H.Y. Gwon, D.G. Na, W. Paik, S.J. Yoon; *Gangneung/KR*

**Purpose:** This study was performed to investigate the incidence and malignancy risk of thyroid nodules with isolated macrocalcification.
**Methods and Materials:** From January 2011 to June 2018, a total of 3061 consecutive patients with 3852 thyroid nodules (≥1cm) underwent ultrasonography (US)-guided fine-needle aspiration (FNA). We retrospectively reviewed all US images of those nodules to determine nodules with isolated macrocalcifications. We assessed the incidence, malignancy rate, and size distribution of thyroid nodules with isolated macrocalcifications. The nodule size was categorised as 3 groups (group 1: 1-1.4cm, group 2: 1.5-1.9cm, group 3: ≥2cm).
**Results:** Isolated macrocalcification was found in 38 (1.2%) of 3061 patients. Among 38 nodules with isolated macrocalcifications, the final diagnosis was achieved in 30 nodules and seven malignant tumours (6 conventional type and 1 follicular variant type papillary carcinomas) were diagnosed by surgery (n=6) and FNA (n=1). The malignancy rate of isolated macrocalcification was 23.3% in 30 nodules with final diagnoses and 18.4% in all nodules. The size of isolated macrocalcifications was group 1 (n=27, 71.1%), group 2 (n=8, 21%), and group 3 (n=3, 7.9%). The mean size of malignant tumours was 1.5 ± 0.4 mm and the tumour size was group 1 (n=3, 43%), group 2 (n=3, 43%), and group 3 (n=1, 14%). Among 6 malignant tumours with surgical pathology diagnoses, the extrathyroidal extension was found in 4 (66.7%) and lymph node metastasis in 2 (33.3%) tumours.
**Conclusion:** Thyroid nodule with isolated macrocalcification (≥1cm) was found in 1.2% of our cohort patients and showed an intermediate malignancy risk (at least 18.4%).

**B-0128** 11:43
**Role of acoustic radiation force impulse (ARFI) in differentiating benign and malignant thyroid nodules**
S.K. Suganchand Rikabchand; *Jodhpur/IN (Sunilmed@gmail.com)*

**Purpose:** The purpose of the study was to assess the role of shear wave elastography in differentiating benign and malignant thyroid lesions with pathological correlation in Indian population
**Methods and Materials:** 246 thyroid nodules in 198 patients presenting with thyroid lesions were included in the study. All the patients were subjected to B-mode ultrasound and elastography using shear wave with Virtual Touch Imaging (VTITM) (Siemens Medical Solutions USA, Inc., PA, USA) and Virtual Touch Quantification (VTQTM) (Siemens Medical Solutions USA, Inc., PA, USA) before ultrasound guided Fine Needle Aspiration Cytology was performed. The obtained data was compared and analyzed using an appropriate statistical test to check the validity of elastography in determining the nature of the neoplasm.
**Results:** Of the 246 thyroid nodules 158 were benign and 88 were malignant as evidenced by FNAC results. VTI was 93.7% sensitive and 90.7% specific in detecting benign thyroid nodules whereas 90.9% sensitive and 94.9% specific in detecting malignant thyroid nodules. VTQ was 92.5% sensitive and 87.4% specific in in detecting benign thyroid nodules whereas 89.1% sensitive and 96.1% specific in detecting malignant thyroid nodules.
**Conclusion:** VTI and VTQ both are very useful tools in diagnosing the nature of thyroid neoplasm. Also VTQ helps in providing quantitative values to differentiate benign and thyroid nodules thus can be used in accurate diagnosis. 2D Ultrasound features with elastography can be used in accurate diagnosis of benign or malignant thyroid nodules thus improving patient compliance by reducing invasive procedures determining the nature of neoplasm.

**B-0129** 11:51
**Thyroid nodules: diagnostic performance of ATA and TIRADS on risk stratification for malignancy**
B.C.S. Rabelo, F. Tinoco Alvim de Souza, P. Ramos Botelho Antunes, E. Carvalho de Siqueira, M. Alvares de Campos, L. Vianna Cancado, A. Vianna Cançado, M. Barbosa Alvares; *Belo Horizonte/BR*

**Purpose:** We aimed to access the performance of Thyroid Imaging Reporting Data System (TIRADS) and 2015 American Thyroid Association (ATA) on malignancy risk stratification, by comparing both ultrasound classification systems with fine needle aspiration (FNA) histologic data.
**Methods and Materials:** We retrospectively reviewed exams of 190 patients. Thyroid ultrasound images of each patient and nodule were categorized based on TIRADS and ATA. Both classifications systems were initially compared with each other, and then matched with FNA histologic data classified with Bethesda system. Location and size of each nodule were also verified.
**Results:** Of the 190 evaluated patients (87.4% women), the mean age was 53,7 years (51,12-55,02), the mean size of nodules were 17,87mm (17,07-18,67). More nodules were solid and isoechoic (49.5% and 36.8% respectively). There was a strong correlation between TIRADS and ATA classifications (r=0,860 p<0,0001). When compared with histological analysis, TIRADS AND ATA had a weak correlation. TIRADS r=0,179 p=0,023; ATA r=0,170 p=0,031.
**Conclusion:** Our study showed that TIRADS and ATA had similar diagnostic accuracy in the evaluation of thyroid nodules ultrasound, but both classifications were shown to overestimate the presence of malignant neoplasms.

**10:30 - 12:00** **Room G**

### Physics in Medical Imaging

### SS 213

### CT image quality and optimisation

*Moderators:*
S. Edyvean; London/UK
E. Samara; Sion/CH

**B-0131** 10:30
**Image quality evaluation of ultra-low dose chest CT using six iterative reconstructions**
M. Afadzi, K. Fosså, H. Andersen, T.M. Aaløkken, A.C.T. Martinsen; *Oslo/NO (merafa@ous-hf.no)*

**Purpose:** To assess the effect of six different iterative reconstruction (IR) algorithms on image quality of ultra-low-dose Chest CT using two phantom sizes.
**Methods and Materials:** A Kyoto Kagaku Lungman phantom (with and without extensions) was scanned on four high-end CT systems using fixed tube voltages (80 or 140 kVp) and the lowest mAs on each scanner, resulting in different CTDIvol of 0.1-0.2 mGy (80 kVp) and 0.3-1 mGy (140 kVp) . Images were reconstructed with the IR available on the scanners. Image quality parameters were evaluated for all reconstructions.
**Results:** All IR algorithms improved quantitative image quality parameters for both phantom sizes with increase in dose. Model-based iterative reconstructions (MBIR) improved contrast-to-noise ratio of lesions more than the statistical algorithms at the same dose level. Hounsfield units measured in the smaller phantom were within acceptable range (≤ 6 HU) for all IR at 140 kVp. All algorithms showed lower noise power spectrum (NPS) peaks at 80 kVp than 140 kVp for the bigger phantom. In comparison with the statistical techniques, MBIR algorithms showed lower NPS peaks at 80 kVp. The FIRST algorithm showed higher (p ≤ 0.004) noise texture deviations (NTD) at 140 kVp than 80 kVp; however, NTD of IMR was not dose dependent(p = 0.172). Lower peak frequencies of NPS and higher NTD values indicate coarser and blotchier noise texture respectively.
**Conclusion:** MBIR techniques enhanced quantitative image quality parameters at ultra-low doses more than the statistical techniques but affected the image texture to a higher degree.
*Author Disclosures:*
**M. Afadzi:** Other; Oslo University Hospital has research collaboration with GE Healthcare and Siemens Healthcare.

**B-0132** 10:38

**Evaluating the use of a noise index to identify outliers in CT examinations**

A.S.L. Dedulle, S. Rahimzadeh, N. Fitousi, H. Bosmans, J. Jacobs; *Leuven/BE (niki.fitousi@qaelum.com)*

**Purpose:** To evaluate which outliers would be identified if multi-parameter alerting would be implemented, such as the combined use of patient size, $CTDI_{vol}$ and a noise index for clinical chest CT examinations.
**Methods and Materials:** Fifty thorax CT examinations (Siemens Somatom Force, Germany) with the same study protocol were used. The water equivalent diameter (WED) of the patients, the $CTDI_{vol}$ of each series and other important parameters (e.g. vertical offset) were extracted by the dose management system DOSE (Qaelum, Belgium). The global noise level (GNL) was calculated for expiration series with the lung (Br59d) kernel (Christianson et al. Medical Physics 2015).
**Results:** The WED range was 20cm to 32cm and the correlation between WED and $CTDI_{vol}$ was significant (p<0.01). Three outliers were observed. Two of them had a vertical offset of 3cm to 4cm closer to the tube. Literature indicates this leads to a higher $CTDI_{vol}$ by 10% to 30%. The third outlier had a wrong positioning (arms next to body). When the GNL was added as the third dimension, another exam with very high noise was found even though it was not initially identified as outlier. This case had a vertical offset of 4.4cm away from the tube, which caused a decrease of $CTDI_{vol}$, thus high GNL. The correlation between the WED and GNL was significant (p<0.01), as well as between the $CTDI_{vol}$ and GNL (p<0.05).
**Conclusion:** Introducing an automated image quality index can detect extra and relevant outliers when compared to a simpler dose versus patient size analysis.
*Author Disclosures:*
**A.S.L. Dedulle:** Employee; Qaelum NV. Research/Grant Support; VLAIO grant No. HBC.2016.0233. **N. Fitousi:** Employee; Qaelum NV. **H. Bosmans:** Board Member; Qaelum NV. Founder; Qaelum NV. **J. Jacobs:** CEO; Qaelum NV. Founder; Qaelum NV.

**B-0133** 10:46

**Improved grey-white matter separation by amplitude/power coefficients derived from spectral analysis of dual-energy CT**

I. Leichter, B. Fialkoff, Y. Uziel, E. Ben-David, Z. Romman, J. Sosna, J.M. Gomori; *Jerusalem/IL (gomori@cc.huji.ac.il)*

**Purpose:** In conventional CT there is an overlap in the HU levels of grey matter (GM) and white matter (WM). Our aim was to use dual-energy CT (DECT) to improve GM/WM separation.
**Methods and Materials:** DECT reconstructs virtual mono-energetic (VME) images, which allow for spectral analysis. Three pairs of regions-of-interest (ROI) in GM and WM were marked in VME images of 33 normal subjects, at different brain locations. Spectral-attenuation curves describing the mean HU versus energy, for series of VME images in the range 40-100keV, were generated for GM-ROIs and WM-ROIs and fitted to power curves. An algorithm was developed to generate for each pixel in the image a spectral-attenuation curve, and calculate the amplitude/power coefficients of the fitted power-curve. Based on the amplitude/power coefficients, the algorithm characterized each pixel as GM or WM and created a GM-WM map by superimposing them in different colors over the original image.
**Results:** The mean HU of GM-ROIs was higher than for WM-ROIs, but their ranges completely overlapped. In contrast, the resulting amplitude coefficient of the fitted power curve for GM was significantly higher than for WM, with a large separation ($GM_{min}$ - $WM_{max}$) between their ranges. The power coefficient was negative for GM and positive for WM with a substantial separation between them. The significant GM/WM separation was also confirmed visually and allowed creation of more accurate grey/white matter maps.
**Conclusion:** Amplitude/power coefficients derived from DECT allow better GM/WM separation than conventional CT. The possible clinical applications of this technique need further study.
*Author Disclosures:*
**Z. Romman:** Employee; Employee.

**B-0134** 10:54

**Changes on noise power spectrum by advanced CT image reconstruction techniques**

T. Pan, R. Vikram; *Houston, TX/US (tpan@mdanderson.org)*

**Purpose:** We use noise power spectrum (NPS) to evaluate noise reduction by ASir and ASir-V of GE, SaFire and Admire of Siemens, and PixelShine of AlgoMedica based on deep learning.
**Methods and Materials:** The homogeneous module of ACR CT phantom was scanned for NPS. The baseline filtered back-projections (FBP) were from the standard kernel on GE, Hr44f on Force and J40f on AS+. For noise reduction, all ASir, ASir-V, SaFire, Admire, and PixelShine settings were compared. Centroid frequency ratios (CFR) between the NPS centroid frequencies of noise reduction and FBP were compared. A smaller CFR means more image blurring. Noise magnitude ratios (NMR) between the areas under the NPS curves of noise reduction and FBP were compared. A smaller NMR means more noise reduction. An ideal reconstruction shall maintain CFR of close to 1 and low NMR of close to 0.
**Results:** For the same noise reduction NMR=0.6, PixelShine (0.97) has the least frequency shift (CFR closer to 1), followed by Safire (0.95), ASir-V (0.91), Admire (0.88), and ASir (0.86) . For the same centroid frequency shift of CFR=0.95, PixelShine (0.44) has the most noise reduction, followed by Safire (0.60), ASir-V (0.74), Admire (0.78), and ASir (0.82).
**Conclusion:** PixelShine (noise reduction based on deep learning) has the least centroid frequency shift for the same amount of noise reduction or the most noise reduction for the same amount of centroid frequency shift. For the same centroid frequency shift, ASir-V reduces more noise than ASir; and SaFire reduces more noise than Admire.
*Author Disclosures:*
**T. Pan:** Consultant; Bracco Diagnostic Inc.

**B-0135** 11:02

**Patient size-dependent ultralow-dose data completion scan in a whole-body photon-counting CT scanner**

S. Dorn[1], S. Sawall[1], J. Maier[1], C. Polster[2], S. Faby[3], M. Uhrig[1], H.-P. Schlemmer[1], M. Kachelrieß[1]; [1]*Heidelberg/DE*, [2]*Munich/DE*, [3]*Forchheim/DE (sabrina.dorn@dkfz.de)*

**Purpose:** To find the minimum possible dose of a data completion scan (DCS) on a photon-counting (PC) CT prototype.
**Methods and Materials:** The SOMATOM CounT (Siemens Healthineers, Germany) CT system features a PC detector with 27.5 cm field of measurement (FOM). To correct for truncation artefacts, the additionally available 50-cm FOM conventional detector subsystem can acquire a DCS. Two anthropomorphic phantoms (liver, thorax) of three sizes (small, medium, large) were scanned with 120 kV at 210 $mAs_{eff}$ with the PC subsystem. For each phantom eight DCS were acquired (120 kV) ranging from 200 to 7 $mAs_{eff}$. To further lower the dose also 100 and 80 kV were used. CT value consistency in PC images after detruncation across DCS dose levels was quantitatively assessed.
**Results:** Minimum achievable DCS dose was 0.13 mGy (32cm CTDIvol), corresponding to about 0.06 mSv. No significant shift in CT values was observed between PC images with minimum dose DCS and PC images with high-dose DCS for small and medium phantom size. For the large phantom size, we observed a shift for the minimum dose DCS of about -3 HU. Setting tube voltage to 100 kV resulted in a deviation of 0.1 HU. We achieved an 8.7-fold dose reduction compared to previously proposed DCS protocols [JCAT 40(4):663-669, 2016].
**Conclusion:** DCS settings of 80 kV, 7 $mAs_{eff}$, are sufficient to provide satisfactory data completion of PC images. With larger patients, the tube voltage needs to be increased. DCS dose is small compared to conventional CT scans.
*Author Disclosures:*
**C. Polster:** Employee; Siemens Healthineers, Germany. **S. Faby:** Employee; Siemens Healthineers, Germany.

**B-0136** 11:10

**Optimising CT acquisition parameters for the quantification of lung emphysema**

G. Van Gompel, S. Mahmoudi, C.C. Brussaard, J. De Mey, N. Buls; *Brussels/BE (Gert.vangompel@uzbrussel.be)*

**Purpose:** To optimise image reconstruction settings for improved and reproducible lung emphysema quantification.
**Methods and Materials:** A dedicated technical phantom was composed using lung tissue equivalent material (Leeds, LN330) in a water-filled cilindric tank. CT scans of the phantom were acquired on a Philips ICT scanner at 100 kVp and CTDIvol from 0.4-17 mGy. Images were reconstructed with FBP, iDose and IMR with a selection filters, and the noise power spectrum (NPS) and task transfer function (TTF) were calculated. The emphysema quantification efficiency (EQE) is defined as the percentage of correctly identified pixels (emphysema versus no emphysema) in the images after applying a threshold at -950HU. The EQE dependence of hole size in noise-free conditions was investigated by applying the TTF-derived point spread function on a virtual phantom image with increasing hole size.
**Results:** Image resolution and noise in terms of TTF and NPS improve, and the EQE increases as the complexity of the iterative reconstruction and the hardness of the filter kernel increase, e.g. EQE=96% for IMR sharp plus versus 91% for FBP-B, at 2.2 mGy and 2mm diameter. For decreasing emphysema spot diameters, the EQE has a decreasing trend: e.g. FBP-L EQE=99% at 10mm and EQE=91% at 1mm diameter.

**Conclusion:** The quantification of lung emphysema is impacted by the spatial resolution and noise properties of the reconstruction methods; advanced iterative reconstruction methods in combination with harder filters are preferred. Moreover, lung emphysema consisting of numerous small spots tends to be underestimated in CT images.

**B-0137** 11:18
**Automated image quality assessment in CT using a 3D printed lung vessel phantom and structural similarity index analysis**
I. Hernandez-Giron[1], R. Schippers[2], J.M. den Harder[3], G.J. Streekstra[3], W.J.H. Veldkamp[1]; [1]*Leiden/NL*, [2]*The Hague/NL*, [3]*Amsterdam/NL* *(irene.debroglie@gmail.com)*

**Purpose:** Image quality is frequently assessed using geometric phantoms and simple image quality metrics such as contrast-to-noise ratio and noise power spectrum. Extrapolating these technical results to patient images is not straightforward. There is a need for metrics to analyze image quality in closer relation to clinical practice, including patient's anatomy and radiologists' clinical tasks. We used a 3D printed anthropomorphic lung vessel phantom combined with a structural similarity index metric to analyze, as proof of principle, the effect of dose on CT image quality.
**Methods and Materials:** A 3D printed anthropomorphic phantom, mimicking the human lung vessels distribution (8mm≥diameter≥0.25mm, VisijetEX200 material, 10×15×2.5cm$^3$), inserted in a PMMA thorax-shaped holder (300×200×29mm$^3$) was scanned 10 times (high-resolution-thorax protocol, Aquilion-ONE-Genesis (Canon Medical Systems)), varying dose [$CTDI_{vol}$=0.2-0.4-0.9-2.6-5.2-10.9 mGy]. Image quality was assessed with the structural similarity index (SSIM:0-1 range, 1 is best), based on the assumption that the human visual system extracts structural image information. Each acquired set was compared to two references: a high dose image volume (39.2 mGy) and the phantom design file.
**Results:** When the high dose image set was the reference, image quality deteriorated as dose decreased [SSIM:(0.986±0.2%;10.9mGy)-(0.972±0.4%;5.2mGy)-(0.951±0.9%;2.6mGy)-(0.879±1.5%;0.9mGy)-(0.809±3.5%;0.4mGy)-(0.720±2.9%;0.2mGy)]. SSIM values were slightly lower when the phantom design file was used as reference, for all dose levels: [SSIM:(0.915±1.5%;10.9mGy)-(0.907±1.9%;5.2mGy)-(0.891±1.4%;2.6mGy)-(0.835±2.9%;0.9mGy)-(0.777±3.6%;0.4mGy)-(0.706±2.4%;0.2mGy)].
**Conclusion:** CT image quality can be assessed using a 3D printed anthropomorphic phantom and the structural similarity index, showing image quality improvement (SSIM increase) with increasing dose, as a proof of principle. This approach has the potential to be used in protocol optimization.
*Author Disclosures:*
**I. Hernandez-Giron:** Research/Grant Support; CLUES project (Project Number 13592). NWO Open Technologie Programma, Canon Medical Systems: Ultra-low dose chest CT project.

**B-0138** 11:26
**Sparse-sampling computed tomography (SpSCT) for the detection of pulmonary embolism**
A. Sauter[1], F.K. Kopp[1], J. Dangelmaier[1], D. Deniffel[1], F. Meurer[1], R. Bippus[2], E.J. Rummeny[1], P.B. Noel[1]; [1]*Munich/DE*, [2]*Hamburg/DE* *(andreas.sauter@tum.de)*

**Purpose:** Evaluation of sparse sampling computed tomography (SpSCT) regarding subjective and objective image criteria for the detection of pulmonary embolism (PE) at different dose levels.
**Methods and Materials:** Computed tomography pulmonary angiography (CTPA) scans of 20 clinical patients were used to obtain simulated low-dose scans (100%-50%-25%-12.5%-6.3%-3.1%). 6 levels with full sampling (FS) resulted. From these data, every second (2-SpSCT) or fourth (4-SpSCT) projection was used to obtain sparse sampling scans. 300 image sets (20 patients, 6/5/4 levels of FS/2-SpSCT/4-SpSCT) resulted and were evaluated by four blinded radiologists regarding subjective image quality and diagnostic performance. Additionally, CNR was evaluated.
**Results:** Sensitivity was 100% with 2-SpSCT and 4-SpSCT at the 25% and the 12.5% dose level for all localizations of PE (one subgroup 98.5%). SpSCT showed higher values for sensitivity, specificity, accuracy and the area-under-the-curve at all dose levels compared to FS. Subjective image quality (IQ) was significantly higher for 4-SpSCT compared to FS at all dose levels with a mean IQ of 3.8 at the 12.5% dose level (4=good IQ). 4-SpSCT showed significantly higher IQ compared to 2-SpSCT at the 12.5% dose level. No examination was rated as non-diagnostic at this dose level when using 4-SpSCT (2-SpSCT: 0.5; cases FS: 1.5 cases).
**Conclusion:** Via SpSCT, a significant dose reduction down to a 12.5% dose level (mean effective dose 0.38 mSv in the current study) for CTPA is possible while maintaining full diagnostic confidence. Hereby, 4-SpSCT is superior to 2-SpSCT at lower dose levels.

**B-0139** 11:34
**Application of preset adaptive statistical iterative reconstruction-V in dual-enhanced abdominal CT**
Y. Chai; *Zhengzhou/CN (chaiyarucyr@163.com)*

**Purpose:** To analyse the effect of preset ASIR-V on image quality and radiation dose in dual-enhanced abdominal CT and to investigate the optimal ASIR-V in clinic.
**Methods and Materials:** 180 patients were collected prospectively. All patients underwent two-phase enhanced abdominal CT (120 kVp, NI 10) and were randomly divided into 6 groups (A-F, n=30 each). In group A-F, 0%-50% preset ASIR-V (10% interval) was applied, respectively. Qualitative parameters (subjective image quality, diagnosis confidence and radiation dose) and quantitative parameters [image noise, CT number and contrast to noise ratio (CNR)] were measured and compared among the groups using ANOVA or Kruskal-Wallis H test.
**Results:** The CTDIvol decreased with the preset ASIR-V percent increasing. The effective radiation dose had significant difference among groups (P<0.001), and the ED of group B-F dropped by 10.8%, 21.7%, 31.2%, 44.9% and 61.9%, respectively, compared with group A. Group E showed the optimal image quality (all P<0.01) and diagnosis confidence (all P<0.01) in all phases. The image noise and CT number of liver, pancreas and muscle had no difference among groups. The CNRs had no significant difference except liver and portal CNR in PVP.
**Conclusion:** In abdominal CT, 40% preset ASIR-V can provide the best image quality and reduce radiation dose by 44.9%.

**B-0140** 11:42
**Comparison of 90 and 180 micron resolution cone-beam CT scans in patients with artefact causing root canal filling material**
A. Patney, M. Murugavel, V. Mahajan; *New Delhi/IN (vidur@mahajanimaging.com)*

**Purpose:** Many dental patients have root-canal filled teeth which cause artefacts on a Cone-beam CT (CBCT) scan. We studied the effect of voxel size on image quality of CBCT scans in presence and absence of root canal filling material.
**Methods and Materials:** CBCT scans of 30 patients, 15 with (group-1) and 15 without (group-2) root canal filling, having both 90u and 180u voxel size were obtained. In group-1, patient scans had root canal filling material and no other artefact causing material in field of view. In group-2, patient scans had no artefact causing material. In all scans, CNR (contrast-to-noise ratio) was calculated by determining the mean and standard deviation of grey values on DICOM data, adjacent to root canal filling material (group-1)/central region of scan volume (group-2) and also determining the grey values at edge of the scan volume (both groups). CNRs of the scans were analysed by Student's t-test.
**Results:** Significant difference (paired t-test, p=0.018) was seen in CNR between the 90u and 180u scans of the same patient in group-1. Significant difference (p=0.135) was not seen in CNR between the 90 and 180u scans of the same patient in group-2.
**Conclusion:** In presence of root canal filling material, the image quality of 90 microns scan is significantly better than that of 180 microns scan. However, in absence of artefact causing material, the image quality is not affected significantly by voxel size, even though it is better in 90 microns scan.

**10:30 - 12:00** **Room K**

### Chest

### SS 204

### Lung cancer: guidelines, screening and nodule management

*Moderators:*
F.B. Demirkazik; Ankara/TR
P. Franchi; Teramo/IT

**B-0141** 10:30
**Outcomes of long-term interval rescreening with low-dose CT for lung cancer in different risk cohorts**
A. Lam, R. Aggarwal, G. Liu, J. Kavanagh; *Toronto, ON/CA (jokavana@tcd.ie)*

**Purpose:** We hypothesize that the incidence of screen-detected lung cancer (LC), in participants with previously negative scans, will be highest in the cohort with the highest baseline risk score.
**Methods and Materials:** Individuals with negative baseline screening results from the Princess Margaret International Early Lung Cancer Action Program prior to 2009 underwent low-dose CT rescreening from 2015 to 2018.

Wednesday

Individuals were contacted in order of decreasing risk, as determined by the Prostate, Lung, Colorectal, and Ovarian Cancer Screening Trial's PLCOM 2012 6-year LC risk-prediction model, and then categorized into three risk cohorts according to their baseline risks. The incidence of LC in each risk cohort was determined and compared. Chi-square testing was used for categorical variables and one-way ANOVA on ranks was used for continuous variables.
**Results:** Of the 1261 participants we attempted to recontact, 359 patients returned for a rescreening scan (mean of 7.6 years between scans). Participants were divided into low- (<2%), moderate- (≥2%-<3.5%), and high-baseline risk (≥3.5%) cohorts. On average, those in the high-risk cohort were older (66 vs 62 and 59 years) and had a greater smoking history (54 vs 47 and 29 pack-years) when compared to the moderate and low-risk cohorts, respectively. The incidence of cancer in the high-risk cohort was significantly higher than in the moderate-risk cohort (11% vs 1.7%, p=0.002).
**Conclusion:** There was a significantly higher incidence of LC in the high-risk cohort than in the moderate-risk cohort. The cut-point between the high and moderate risk was determined to be ≥3.5% 6-year baseline risk.

**B-0142** 10:38

**Applying British Thoracic Society 2015 compared to Fleischner Society 2017 recommendations for lung nodule management leads to different discharge and PET-CT referral rates**

L. Royle, M. Anjari, S. Sasikumar, R. SinhaRay, S. Mak, M. Subesinghe, D. Murphy, R. Breen, A. Nair; *London/UK (leanne.royle@nhs.net)*

**Purpose:** To compare agreement, and rates of discharge and referral for PET-CT between the British Thoracic Society 2015 (BTS) and Fleischner Society 2017 recommendations (FS) for pulmonary nodule management.
**Methods and Materials:** In 138 patients [89 (64.5%) ever-smokers] with fully investigated CT-detected incidental pulmonary nodules [median average diameter 7mm, 8 (5.8%) malignant], we retrospectively modelled four management strategies: combined volume- and diameter-based BTS (BTSvol), diameter only-based BTS (BTSdiam), diameter-based FS where ever-smokers were considered high risk (FSsmoking), and diameter-based FS where a Brock model score of ≥5% was considered high risk (FSBrock). We compared agreement using the weighted kappa statistic ($\kappa_w$) and rates of discharge and referral for PET-CT using McNemar's test.
**Results:** Agreement between the BTS and FS strategies was good ($\kappa_w$ 0.66-0.72). However, significantly more patients were discharged using BTSvol [35(25.4%)] compared to BTSdiam [14(10.1%), p<0.0001], and FSsmoking [18(13.0%), p=0.0015], but not compared to FSBrock [37(26.8%), p=0.81]. Conversely, significantly more patients were referred for immediate PET-CT using BTSvol [21(15.2%)], compared to FSsmoking [7(5.1%), p=0.0026] and FSBrock [8(5.8%), p=0.0002]. 4/21(19%) nodules referred for immediate PET-CT using BTSvol were malignant, and none were referred for immediate PET-CT using FSsmoking or FSBrock.
**Conclusion:** Volume-based BTS management can (1) reduce nodule follow-up rates compared to diameter-based BTS and diameter-based, smoking-stratified FS management, and (2) result in higher referral for immediate PET-CT, allowing earlier detection of malignant nodules. However, using risk stratification in the FS with the Brock model, rather than smoking status alone, can yield similar discharge rates to BTS volume-based management.
*Author Disclosures:*
**A. Nair:** Board Member; Medical advisory board member for Aidence BV. Research/Grant Support; Proportion of funding for consultant post from the Department of Health NIHR Biomedical Research Centres Funding Scheme. Other; No conflicts relevant to this submitted work.

**B-0143** 10:46

**Low-dose computed tomography (LDCT) screening reduces overall and lung cancer specific mortality beyond five years**

U. Pastorino[1], M. Silva[2], F. Sabia[1], S. Sestini[1], M. Boeri[1], G. Sozzi[1], N. Sverzellati[2], A.V. Marchianò[1]; [1]*Milan/IT*, [2]*Parma/IT (alfonso.marchiano@istitutotumori.mi.it)*

**Purpose:** To assess the long-term benefit of lung cancer screening (LCS) by low-dose computed tomography (LDCT), notably the overall and lung cancer (LC) specific mortality beyond 5 years and until 10 years.
**Methods and Materials:** The Multicenter Italian Lung Detection (MILD) trial prospectively enrolled 4,099 participants,randomised to either LDCT arm (n=2,376) or control arm (n=1,723); 39,293 person-years of follow-up were accumulated between 2005 and June 2018. The primary outcomes were 10-year overall and LC specific mortality. A landmark analysis was used to test the specific long-term effect of LCS beyond 5 years, notably by selective exclusion of events that occurred < 5 years. Cumulative mortality was evaluated using Kaplan-Meier estimator and differences among groups were tested using Log-rank test, adjusted for sex, age and pack-years. The prognostic value of assigned arm in predicting mortality was investigated by Cox's proportional-hazard's regression adjusted for the above variables.
**Results:** In the whole 10-year LCS, LDCT arm showed a protective non-statistically significant trend for reduction of overall mortality (HR: 0.80, 95% CI 0.62 to 1.03) and a significant 39% reduced risk of LC mortality (HR 0.61, 95% CI 0.39 to 0.95),compared to the control arm.The landmark analysis beyond the 5$^{th}$ year of screening showed significant reduction of mortality in the LDCT arm: 32%reduction of overall mortality (HR: 0.68, 95% CI 0.49 to 0.94),58%reduced risk of LC mortality (HR 0.42, 95% CI 0.22 to 0.79).
**Conclusion:** LDCT screening reduces lung cancer mortality and overall mortality, especially in the long-term range between 5 and 10 years.

**B-0144** 10:54

**Lung cancer CT screening performance at a large hospital system in the US: our 5-year experience showcases the need for better systems of care**

E.J.M. Barbosa Jr.[1], A. Kolansky[1], X. Sui[2], J. Jiang[1]; [1]*Philadelphia, PA/US*, [2]*Beijing/CN (eduardo.mortani@gmail.com)*

**Purpose:** Lung cancer low-dose CT screening (LCS) has been shown to decrease mortality in national trials in the United States and Europe. We assessed performance metrics of our LCS program and patient compliance to screening recommendations.
**Methods and Materials:** We retrospectively identified all patients who underwent LCS from 2014 to 2018, via our EMR. We recorded patient demographics, lung cancer related and incidental findings, lung-RADS category, outcomes, and compliance with screening recommendations.
**Results:** 1373 patients were screened during a 5-year interval, of which 260 (18.9%) returned for follow-up scans (87.7% had two, 2.3% had three, and 0.07% had four). Average age was 65.3, (51.5% men, 48.5% women), average smoking history was 51.4 pack-years (SD=19.8). 34/260 (13.1%) had positive scans, of which 22/260 (8.4%) were lung-RADS category 3, 3/260 (1.1%) were 4A, 5/260 (1.9%) were 4B, and 4/260 (1.5%) were 4X. 2/260 were diagnosed with lung cancer on follow-up. Among patients who had multiple scans, only 14.5% obtained follow-up within a month of the recommended time. Of those who also had positive screening scans, 34.4% followed up on time, 43.8% followed up late, and 21.8% were lost to follow-up.
**Conclusion:** Our rate of positive LCS exams is slightly higher than Lung-RADS prediction (13.1% vs 9%), however most were not confirmed as lung cancers, leading to an estimated false positive rate as high as 94%. Moreover, adherence to LCS recommendations is very low, but increases with positive exams (14.5% vs 34.4%). Healthcare systems should devise strategies to address these problems.

**B-0145** 11:02

**Lesion measurement on a combined "all-in-one" computed tomography window on chest scans: effect on intra- and interobserver variability**

A. Snoeckx[1], J. Cant[2], C. Franck[1], K. Carpentier[1], E. Luyckx[1], S. Nicolay[1], M.J. Spinhoven[1], A. Van Hoyweghen[1], P.M. Parizel[1]; [1]*Edegem/BE*, [2]*Mortsel/BE (annemie.snoeckx@uza.be)*

**Purpose:** Historically, chest CTs are viewed in several window settings to evaluate specific anatomical structures and regions. A newly developed imaging processing technique fuses these conventional windows into a single "All-In-One" (AIO) window. Previous research has shown that lesion detection on this AIO-window is as good as on conventional window settings. This study aims to evaluate consistency of CT measurement of lesions on this novel AIO-window, compared to conventional window settings.
**Methods and Materials:** In this retrospective study, 368 measurable lesions of various size, origin and lesion sharpness were measured on CT images by 6 radiologists with 3 different levels of expertise. All lesions were measured twice on both the AIO-window and conventional window settings, with an interval between repeated measurements of at least one week. Intraclass correlation coefficients were used to assess intra- and interobserver variabilities.
**Results:** Overall intra-observer agreement for the AIO-window and conventional window settings was 0.986 (95% confidence interval (CI): 0.983-0.989) and 0.991 (95%CI 0.989-0.993), respectively. For interobserver agreement this was 0.982 (95%CI 0.979-0.985) (AIO) and 0.979 (95%CI 0.957-0.982) (conventional). Intra- and interobserver agreement differed by size, sharpness and reader experience for both AIO and conventional windows. Measurement variability decreased with increasing lesion size. Regarding sharpness, inter- and intra-observer agreement ranged from 0.986-0.989 (AIO) and 0.985-0.992 (conventional) for well-defined lesions and from 0.978-0.983 (AIO) and 0.974-0.991 (conventional) for ill-defined lesions.
**Conclusion:** Overall intra- and interobserver variability rates were similar for the AIO-window and conventional windows; lesion measurement on the AIO window seems to be reliable and reproducible.
*Author Disclosures:*
**J. Cant:** Employee; AGFA Medical Imaging.

**B-0146** 11:10

**Comparison of computer-aided semi-automated volumetry with manual volumetry of part-solid pulmonary nodules**

S. Werner[1], R. Gast[1], R. Grimmer[2], M. Horger[1]; [1]*Tübingen/DE*, [2]*Forchheim/DE (sebastian.werner@med.uni-tuebingen.de)*

**Purpose:** To test whether computer-aided volumetry (CAV) can compete with manual volumetry performed in part-solid pulmonary nodules.
**Methods and Materials:** 66 part-solid pulmonary nodules were retrospectively identified in 34 thin-slice unenhanced Chest-CTs of 19 patients. CAV and manual volumetry was carried out for the solid and subsolid part. Smooth and sharp kernels were used for CAV. Manuel volumetry was carried out by two radiology residents using the smooth kernel. Ground truth was determined by an experienced radiologist in consensus with one of the residents. Accuracy of CAV and manual volumetry was compared. Consistency of repeated CAV measurements was determined and compared between usage of the soft and hard tissue kernel.
**Results:** Accuracy of manual volumetry/CAV was 79%-80%/77% for the solid part and 73%-76%/67% for the subsolid part. Consistency of CAV was high for the solid part and low for the subsolid part with >90% and 19 % of measurements lying in the correct ground truth range, respectively. Comparison of CAV consistency between soft and sharp tissue kernel showed slightly better results for the sharp kernel which for the solid part yielded volumetric deviations from the ground truth of <5%/<1% in 48/45 of 54 lesions compared to 46/43 of 54 lesions for the soft kernel.
**Conclusion:** CAV of part-solid lung nodules delivers comparable and reproducible accuracy for the solid and subsolid component compared to manual volumetry. Measurement consistency of CAV was high for the solid part and poor for the subsolid part showing slight improvement when using a sharp tissue kernel.
*Author Disclosures:*
**R. Grimmer:** Employee; Siemens Healthineers.

**B-0147** 11:18

**Pulmonary nodule size measuring difference: comparison between machine-learning based auto-measurement on CT images and gross specimens through surgery**

X. Luo, M. Zhang, J. Ding, M. Chen; *Beijing/CN (archaeopteryxdin@163.com)*

**Purpose:** This study was aimed to evaluate the accuracy of machine-learning in measuring the size of pulmonary nodules by comparison between the auto-segmented size with those manually measured from gross specimen in surgery.
**Methods and Materials:** 73 patients (age: 59.5±9.9 years, F=52, M=21), who underwent preoperative CT-guided pulmonary nodule location, were enrolled. The slice thickness of CT images were 1.0 mm. All the images were analysed through machine-learning model for pulmonary nodules. With auto-segmentation, nodule sizes were measured automatically. Preoperative medical adhesive was injected adjacent to the targeted nodule. In surgery, gross specimen of nodules were measured manually. Paired t test was performed to compare the difference.
**Results:** (1) 102 nodules were studied, with 61 GGOs, 30 sub-solid and 11 solid nodules; 24 were confirmed benign, including hamartoma, organized pneumonia, fibrosis, granuloma and lymphoid hyperplasia; the rest were 12 AAHs, 21 AISs, 4 MIAs, 12 IACs and 29 nodules with other malignancy. (2) Among all nodules, the average auto-segmented maximum dimension was 10.8±6.2mm; in gross specimen, it was 9.4±5.7mm (p<0.0001). Measurement difference was observed in GGOs (p=0.0001). However, it was not significant in sub-solid and solid nodules (p=0.1962,p=0.0885). (3) Compared according to different pathology, measurement difference was not significant in benign lesions or AAHs (p=0.3099,p=0.2247). While adenocarcinoma-related nodules were showing different (p<0.0001). However, the difference was not significant in MIAs or IACs.
**Conclusion:** The auto-segmented nodules were measured about 15% larger than those in gross specimens. The causes might connect with nodule's components.

**B-0148** 11:26

**The differential diagnosis of benign and malignant solid pulmonary nodules by CDT-VIBE early dynamic contrast-enhancement of MRI**

H. Zhang, L. Xu; *Dazhou/CN (zhangheng_DZCH@yahoo.com)*

**Purpose:** The purpose of this study was to assess the Imaging characteristics of SPNs (solid pulmonary nodules) by CDT-VIBE early rapid multi-phase dynamic enhanced scanning.
**Methods and Materials:** In this retrospective, institutional review board-approved study, 20 patients (with d≧1cm and solitary SPN confirmed by pathology or clinical treatment) scanned on a 3.0-T magnetic resonance system (Skyra; Siemens) were divided into benign nodule group (n=9) and malignant nodule group (n=11). The CDT-VIBE ( TR = 4.1ms,TE = 1.33ms,Flip angle = 9°,Field of view = 340mm X 255mm,Matrix = 288 X 162 ) was started 20 seconds after the injection of 0.1 mmol/kg Gd-DTPA. Within 26 seconds, 14 high spatial resolution (1.2 X 1.2 X 3 $mm^3$) data sets were acquired. The CDT-VIBE images were evaluated independently by 2 blinded, experienced radiologists with regard to image quality and measuring signal strength of different points in time.
**Results:** In all patients, CDT-VIBE measurements were successfully acquired and the image quality was diagnostic (Figure 1). As shown in Figure 2, in the multi-phase rapid scan of 20-46s, the signal intensity values of the malignant nodule group were greater than 250; while the signal intensity values of the benign nodule group were less than 230, the difference between the two groups was statistically significant (p < 0.05).
**Conclusion:** Compared with the traditional DCE-MRI, CDT-VIBE sequence of the rapid multi-phase dynamic enhanced scan of 20-46s is of great significance in the differential diagnosis of benign and malignant solid pulmonary nodules.

**B-0149** 11:34

**Application of dual-layer detector spectral CT in differentiating benign and malignant lung nodules**

Q. Wen, Y. Hou, Y. Ma, Q. Ma, X. Lu; *Shenyang/CN (2609579904@qq.com)*

**Purpose:** To quantitatively observe the value of dual-layer detector spectral CT (DLCT) in differentiating malignancy and benignity of solitary pulmonary nodules.
**Methods and Materials:** Ninety-one patients with solitary pulmonary nodules proved by pathology underwent contrast-enhanced CT scan in artery phase (AP) and venous phase (VP) with DLCT. The patients were classified into two groups (malignant group, n=51; benign group, n=40). The slope of spectral HU curve ($\lambda_{HU}$) was calculated from the spectral curve ($\lambda_{HU}$ = $|CT_{40\ keV}$ - $CT_{80\ keV}|/40$). Iodine concentrations (IC) were derived from iodine-based material decomposition CT images and normalized to the IC in the aorta (NIC). Normalized arterial enhancement fraction (NAEF) was calculated by $NIC_{AP}$ /$NIC_{VP}$. T test was performed to compare quantitative parameters among the two groups. Receiver operating characteristic (ROC) curve analysis was performed to assess the differential diagnosis performance.
**Results:** NIC, $\lambda_{HU}$ and NAEF of malignant group were all higher than those of benign group (P<0.05). In the benign and malignant groups, the NIC were 0.09±0.04 and 0.15±0.05, respectively, in the AP, and 0.22±0.06 and 0.36±0.10, respectively, in the VP. The $\lambda_{HU}$ were 1.17±0.49 and 1.75±0.63, respectively, in the AP and 1.33±0.49 and 2.09±0.63, respectively, in the VP. The NAEF were 0.374±0.126 and 0.374±0.126, respectively. ROC analysis showed that the area under the curve (AUC) of the NIC in VP was largest ($AUC_{NIC}$=0.90) among all parameters with sensitivity and specificity values were 84.7% and 90.8%, respectively.
**Conclusion:** DLCT imaging with quantitative parameter provides more promising value for distinguishing malignant nodules from benign ones.

**B-0150** 11:42

**Automated CT texture analysis of lung cancer FDG PET-CT data using a novel software tool: preliminary results**

G.M. McDermott[1], M. Hayball[2], B. Ganeshan[2], Z. Riaz[2], A. Scarsbrook[3]; [1]*Leeds/UK*, [2]*Cambridge/UK*, [3]*York/UK (garry.mcdermott@nhs.net)*

**Purpose:** To explore the utility of automated software (TexRAD lung) for extracting texture features from routinely acquired fluorine-18 fluorodeoxygluose (FDG) positron emission tomography-computed tomography (PET-CT) datasets in lung cancer patients.
**Methods and Materials:** 107 patients with lung cancer who underwent pre-treatment FDG PET-CT at a single centre were studied. TexRAD lung was applied to all PET-CT datasets with standard settings: 40% fixed threshold for PET region detection, CT region threshold -50 to 150 Hounsfield Units, filter levels (0, 2mm, 3mm) and minimum single slice area of 200$mm^2$. Auto-contoured FDG-avid regions were transposed to corresponding CT images. Texture parameters were then extracted from a region of interest encompassing the maximal cross-sectional area of tumour on a single axial CT slice, using a filtration-histogram technique. Kaplan-Meier analysis (Mantel-Cox log-rank) was used to assess correlation between texture parameters and overall survival.
**Results:** The software was unable to process some cases due to unusual chest anatomy, confluent benign tracer activity adjacent to the tumour or small tumour size which left 74 studies for analysis. The CT texture kurtosis, extracted with a 3-mm filter, was a statistically significant predictor of overall survival (p=0.0255) at an optimised cut-off level (>=0.47512 indicates poor prognosis, median survival of the poor prognostic group was 1.1yrs vs. good prognostic group at 3.03yrs).
**Conclusion:** Texture features (kurtosis) extracted from routinely acquired FDG PET-CT data using automated software may help predict overall survival of patients with lung cancer. Region extraction methodology warrants further assessment to account for certain patient-specific features.

*Author Disclosures:*
**M. Hayball:** Board Member; Director, TexRAD Limited Director, Cambridge Computed Imaging Ltd. **B. Ganeshan:** CEO; CEO, TexRAD Ltd Director, Feedback plc Director, Stone Checker Software Ltd Director, Prostate Checker Ltd. **Z. Riaz:** Employee; Product manager, Cambridge Computed Imaging Ltd.

**B-0151** 11:50
**Can dual-energy CT replace perfusion CT for the diagnosis of the solid pulmonary nodules?**
B. Zhu, T. Jiang, P. Peng, M. Li; *Beijing/CN (zhubl2017@163.com)*

**Purpose:** To determine the correlation and diagnostic value between dual-energy CT (DECT) (70/Sn150) and low-dose volume perfusion CT (VPCT) parameters in patients with solid pulmonary nodule.
**Methods and Materials:** All patients provided written informed consent. Thirty-three patients (mean age 57 years±7) with solid nodules who underwent same-day DECT and VPCT were enrolled. All CT findings were histologically confirmed. Exploration the correlation and comparison the diagnosis value between VPCT parameters and DECT parameters. According to the ROC analysis of solid pulmonary nodules to determine the optimal diagnostic cut-off value, and pairwise compare the area of ROC curves. The effective radiation doses of the VPCT and DECT scan protocols were recorded. Pearson R, t test and Mann-Whitney U test were used.
**Results:** The effective radiation dosage of the VPCT and DECT were 3.8 mSv, 0.32±0.10mSv, respectively. Significant correlations were found between iodine concentrate (IC) from DECT and VPCT parameters (P<0.05): blood volume (BV), blood flow (BF), flow extraction product (FED), pulmonary nodule enhancement peak (PPnod) and the ratio of PPnod to the aortic enhancement peak. The sensitivity and specificity of IC were 87.50%, 66.70%, which slightly lower than that of BV (100%, 75.00%), FED (94.12%, 81.25%) and PPnod (100%, 81.25%). The area of ROC from combination of PPnod and IC was significantly higher than those of IC (p=0.038).
**Conclusion:** Iodine parameters from DECT are significantly correlated with VPCT parameters, IC combine PPnod from VPCT and VPCT parameters are helpful to the differential diagnosis of benign and malignant for SPNS.

**10:30 - 12:00** **Room M 1**

### Genitourinary

### SS 207

### Prostate cancer

*Moderators:*
P. Puech; Lille/FR
N.N.

**K-04** 10:30
**Keynote lecture**
R.H. Oyen; *Leuven/BE*

**B-0152** 10:39
**The influence of background signal intensity changes on cancer detection in prostate MRI**
A.M. Hötker[1], E. Dappa[2], Y. Mazaheri[3], B. Ehdaie[3], J. Zheng[3], M. Capanu[3], H. Hricak[3], O. Akin[3]; [1]*Zurich/CH*, [2]*Mainz/DE*, [3]*New York, NY/US (andreas.hoetker@usz.ch)*

**Purpose:** To develop a scoring system for background signal intensity (BSI) changes on prostate MRI and assess these changes' influence on cancer detection.
**Methods and Materials:** This IRB-approved, HIPAA-compliant, retrospective study included 418 prostate MRI examinations in 385 men, who subsequently underwent MRI-guided biopsy. The Likert score for the suspicion of cancer assigned by the primary radiologist was extracted from the original report and histopathological workup of the biopsy cores served as the reference standard. Two readers assessed the amount of BSI changes on T2-weighted sequences and assigned a pre-defined BSI score (BSIS) of 1-5 (1=poor, extensive changes; 5=excellent, no changes). The sensitivity and specificity of Likert scores for the detection of prostate cancer and clinically significant cancer (GS ≥ 3+4) were estimated in and compared between subgroups of patients with different BSI scores (BSIS 1-2; BSIS 3; BSIS 4-5).
**Results:** Inter-reader agreement on BSIS was substantial (kappa: 0.783). Sensitivity for prostate cancer detection increased when BSI scores were better (i.e., higher) (reader 1: 0.412 to 0.714; reader 2: 0.526 to 0.728; p≤0.007, both readers). In the detection of significant cancer (Gleason score ≥ 3+4), sensitivity also increased with higher BSI scores (reader 1: 0.5 to 0.824; reader 2: 0.627 to 0.857; p≤0.028), though specificity decreased significantly for one reader (0.667 to 0.377; p=0.009).
**Conclusion:** Background signal intensity changes on T2-weighted images significantly limit prostate cancer detection. The proposed scoring system could improve the standardization of prostate MRI reporting and provide guidance for applying prostate MRI results appropriately in clinical decision-making.

**B-0153** 10:47
**Validation of the PI-RADS 2 size criterion for the prediction of significant prostate carcinomas: are adjustments to the 1.5-cm threshold warranted?**
S. Mahjoub[1], A. Baur[1], J. Pfeil[1], C. Lee[2], M. Rudolph[1], M. Haas[1], H. Cash[1], B. Hamm[1], T. Penzkofer[1]; [1]*Berlin/DE*, [2]*Singapore/SG (penzkofert@gmail.com)*

**Purpose:** To critically evaluate the 1.5-cm size threshold employed in PI-RADS v2.0 between scores 4 and 5 for the prediction of clinically significant (≥3+4) prostate cancers in a large cohort.
**Methods and Materials:** 293 patients with systematic and MRI/TRUS fusion biopsy and confirmed prostate cancer were evaluated. Histopathology was matched to the MRI images, lesion sizes were measured on all available sequences and tabulated against the Gleason grade. ROC-AUC and Youden Index analyses were performed to assess the size criterion's current performance and potential adjustments.
**Results:** 311 lesions were matched to histopathologic findings. Separated by initial tumour zone 59 3+3, 52 3+4, 1 3+5, 27 4+3, 64 4+4, 12 4+5, 1 5+3, 5 5+4 and 1 5+5 tumours were analysed in the peripheral zone, and 35 3+3, 20 3+4, 18 4+3, 18 4+4, 3 4+5, 3 5+5 tumours were included from the transition zone, 10 lesions were equivocal with respect to their zone and excluded. AUCs were higher in PZ-lesions (T2: 0.68, DWI: 0.73, ADC: 0.68 and DCE: 0.78) compared to TZ tumours (T2: 0.63, DWI: 0.63, ADC: 0.66 and DCE: 0.48). When optimizing the threshold using the maximum sum of sensitivity/specificity approach (Youden Index), a threshold of 14 mm in the peripheral zone (DWI), and a threshold of 21 mm TZ lesions (T2) yielded the highest separation.
**Conclusion:** The size criterion had a greater discriminatory power in the PZ than the TZ. Slightly lowering the PZ threshold to 14mm and increasing the TZ threshold to 21mm could potentially lead to a better separation of significant from non-significant tumours.
*Author Disclosures:*
**H. Cash:** Speaker; Hitachi. **B. Hamm:** Other; Siemens Healthineers, Bayer Healthcare.

**B-0154** 10:55
**Prostate cancer risk stratification using biparametric MRI and clinical parameters improves selection of biopsy naïve men for prostate biopsies**
L. Boesen[1], F.B. Thomsen[1], N. Nørgaard[1], V. Logager[2], R. Bisbjerg[1], I. Balslev[1], H. Jakobsen[1], H.S. Thomsen[1]; [1]*Herlev/DK*, [2]*Copenhagen/DK (lars.boesen@dadlnet.dk)*

**Purpose:** To develop a novel predictive model based on biparametric (bp) MRI findings and clinical parameters for detection and ruling out significant prostate cancer (sPCa) in biopsy-naïve men using results from modern biopsy techniques as standard reference.
**Methods and Materials:** 876 biopsy-naive men with suspicion of PCa (PSA<50, cTstage<T3) underwent pre-biopsy prostate bpMRI (T2W and DWI) followed by 10-core standard biopsy (all men) and MRI-TRUS fusion targeted biopsies of bpMRI-suspicious lesions (score ≥3). Various prediction models based on bpMRI and clinical parameters (age, rectal examination [DRE], prostate-specific-antigen [PSA], $\text{prostate}_{\text{volume}}$, $\text{PSA}_{\text{density}}$) were conducted and compared using area under the curve (AUC) and decision curve analysis. Reference standard was combined biopsy results and sPCa was any core with Gleason grade group≥2.
**Results:** Overall, sPCa was detected in 350/876 (40%) men; median [IQR] age and PSA were 65 yrs [60-70] and 7.3 ng/ml [5.5-10.6]. A model that combined bpMRI suspicion scores with age, DRE and $\text{PSA}_{\text{density}}$ in an advanced imaging model achieved the highest discriminatory power on ROC curve analysis (AUC 0.89) and showed good calibration on internal bootstrap validation. The clinical performance of this model was superior (highest net benefit) on decision curve analysis and a biopsy risk threshold ≥20% for the model reduced 42% of biopsies and 50% of insignificant PCa diagnosis, while missing only 7% of GGG≥2 PCas.
**Conclusion:** A predictive multivariable model based on bpMRI imaging and clinical parameters significantly improves risk stratification of sPCa in biopsy-naïve men and could be used for clinical decision-making to avoid unnecessary prostate biopsies.

**B-0155** 11:03
**Restriction of diffusion and tumor aggressiveness: a non applicable correlation for transition zone prostate cancer**
F. Cornud, A. Jemal Turki, M. Barral, P. Soyer; *Paris/FR (matthiasbarral@gmail.com)*

**Purpose:** To show that the restriction of diffusion (ADC) and cell differentiation (Gleason score) are not correlated in transition zone prostate cancers

**Methods and Materials:** 79 patients with an index tumor developed in the transition zone (TZCa, n=37) or peripheral zone (PZCa, n=42) had an MRI at 3T or 1.5T+endorectal probe before biopsy and radical prostatectomy within three months after biopsy. ADC measurement was performed by two independent readers to assess the interreader agreement. A correlation coefficient was calculated between the ADC and the Gleason score according to the zonal origin of the tumour.
**Results:** Between the two groups, the mean age (67±6 vs 63±5), PSA level (9.3±4 vs 8.9±4ng/ml), prostate volume (41±17 vs 55±26ml) were comparable (p>0.05). The large tumor MRI axis was higher in TZCa (16±7 vs 11±6mm, p=0.0007). The ADC value of Gleason 3+3 tumor score of TZCa was significantly lower than in the PZ (728±52 vs 861±121 10-6mm²/s, p=0.0014), and was comparable to that of tumors with >3+4 score in both zones (TZ: 647±124 ; PZ : 646±124 10-6mm²/s). The correlation coefficient between Gleason score and ADC was 0.32 (95%CI[0.01-0.46], p=0.19) in TZCa of 0.45 in PZCa (p=0.0027, 95%CI[0.66-0.17].
**Conclusion:** The relationship between restriction of diffusion (ADC) and cell differentiation (Gleason score) cannot be applied for TZCa, given the high epithelial volume of low-grade TZCa,;This finding has implications for the interpretation of targeted biopsy Gleason score of a TZCa for treatment selection.

**B-0156** 11:11
**How to improve the equivocal category PIRADS score 3? Quantitative multiparametric MRI assessment of prostate cancer with histopathology correlation**
G. Barchetti, R. Campa, M. Pecoraro, F. Barchetti, C. Catalano, V. Panebianco; *Rome/IT (giovanni.barchetti2@gmail.com)*

**Purpose:** To evaluate quantitative mpMRI in characterization of “equivocal” lesions according to Prostate Imaging-Reporting and Data System (PI-RADS), using histopathology as a reference standard.
**Methods and Materials:** Three groups of patients were identified. Group A: 103 patients with mpMRI before radical prostatectomy. Group B: 100 patients with negative mpMRI and follow-up. Group C: 92 patients with mpMRI-targeted biopsy for one or more PI-RADS 3 lesions. All PI-RADS 3 lesions were quantitatively assessed to obtain ADC, normalized ADC (nADC) and Ktrans. Differences in ADC, nADC and Ktrans between PI-RADS 3 lesions and normal tissue or index lesion were evaluated. ROC curves analysis was performed.
**Results:** 49 PI-RADS 3 lesions in group A were malignant and had lower ADC (5,95E-010 vs 9,98E-010, p < 0,001) and higher Ktrans (0,48 vs 0,24, p < 0,001) compared to normal tissue and similar ADC, nADC and Ktrans compared to the index lesion. 53 PI-RADS 3 lesions were benign: they had higher ADC (8,75E-010 vs 5,38E-010, p < 0,001) and nADC (0,79 vs 0,46, p < 0,001) and lower Ktrans (0,26 vs 0,48, p < 0,001) compared to the index lesion and similar ADC and Ktrans compared to normal tissue. Comparable results were obtained in groups B and C. ADC, nADC and Ktrans had an area under curve of 73%, 90% and 67% in discriminating PI-RADS 3 lesions harbouring clinically significant prostate cancer (csPCa) from benign tissue or non-csPCa.
**Conclusion:** quantitative mpMRI metrics (ADC, nADC and ktrans) can discriminate csPCa from non-csPCa in PI-RADS 3 lesions.

**B-0157** 11:19
**Predicting clinically significant prostate cancer in PI-RADS 3 lesions: a retrospective high-volume centre study**
I. Hermie[1], J. Van Besien[1], P. De Visschere[2], G. Villeirs[1]; [1]*Ghent/BE*, [2]*De Haan/BE (isabeauhermie@gmail.com)*

**Purpose:** To investigate which clinical and radiological characteristics can predict clinically significant prostate cancer (csPCa) in PI-RADS (Prostate Imaging Reporting and Data System) 3 lesions on mpMRI (multiparametric prostate MRI).
**Methods and Materials:** A retrospective study was performed on PI-RADS 3 lesions from the period March 2015 to August 2017 in a single high-volume centre. Clinical data such as PSA and the presence of csPCa (Gleason score >= 3+4) were collected from hospital records. Apparent diffusion coefficient (ADC) of the PI-RADS 3 lesion, the contralateral side of this lesion and the internal obturator muscle was calculated on diffusion-weighted imaging sequences. Univariate and multivariate Cox regression analyses was performed to identify risk factors for csPCa.
**Results:** One hundred and thirty-one patients with PI-RADS 3 lesions on mpMRI were examined. In 83 patients (63%) biopsies were performed. For peripheral zone lesions, 37.5% of csPCa was detected. In univariate logistic regression analysis, a higher median PSA density (p=0.009) and in multivariate logistic regression a lower median prostate volume (p=0.025) and a lower ratio of ADC of the PI-RADS 3 lesion on ADC of the contralateral prostate (p=0.003) significantly predisposed for csPCa. Depending on the combination of these parameters, a high sensitivity (100%) or a high specificity (96%) for csPCa could be reached but no combination had both high sensitivity and specificity.
**Conclusion:** PSA density, prostate volume and the ratio of ADC of the PI-RADS 3 lesion on ADC of the contralateral prostate have the potential to predict csPCa in PI-RADS 3 lesions.

**B-0158** 11:27
**Improved diffusion weighted imaging of the prostate: RESOLVE and ZOOMit in comparison to standard EPI sequences**
M. Klingebiel, L. Schimmöller, T. Ullrich, M. Quentin, C. Arsov, G. Antoch, H.-J. Wittsack; *Düsseldorf/DE*

**Purpose:** This study retrospectively evaluates objective image quality (IQ), subjective IQ, and diagnostic performance of diffusion-weighted imaging (DWI) in prostate MRI at 3T comparing high resolution RESOLVE (REadout Segmentation Of Long Variable Echo trains), ZOOMit (selective field-of-view) and standard EPI DWI sequences within the same patients.
**Methods and Materials:** Thirty-six consecutive patients (70±8 years; median PSA 9.1 ng/ml; IQR 6.3-13 ng/ml) with multi-parametric MRI at 3T including RESOLVE, ZOOMit, and EPI DWI with b0,500,1,000 plus calculated b1500 and subsequently histopathologically proven prostate cancer (PCA) by targeted and systematic MR/US-fusion biopsy were included from 03/2016 to 12/2017. Signal intensities (SI) of PCA and benign tissue (peripheral and transition zone; PZ, TZ) in ADC, b1000, and b1500 were analyzed for each respective DWI sequence. Endpoints were comparison of signal-to-noise ratios (SNR), contrast-to-noise ratios (CNR), and subjective IQ (5-point scale).
**Results:** SI and SNR differed significantly for RESOLVE compared to ZOOMit and EPI DWI (p<0.01). CNR was significantly better for RESOLVE for high b values (b1500; p<0.01) and was best in differentiation of PCA from normal tissue in the PZ. CNR of ADC is not significantly different for the three compared DWI sequences (PZ: p=0.34 and TZ: p=0.48). Subjective IQ was significantly better for RESOLVE compared to both ZOOMit and EPI (p<0.01).
**Conclusion:** RESOLVE was superior to ZOOMit and EPI DWI regarding subjective and objective imaging parameters in depiction of PCA. ZOOMit and EPI are more or less comparable to each other. Thus, RESOLVE improves DWI in mp-MRI and might facilitate PCA detection.

**B-0159** 11:35
**Prostate cancer treated with irreversible electroporation: MRI-based volumetric analysis and oncological outcome**
F. Giganti[1], A. Stabile[2], S. Giona[3], C. Orczyk[1], C.M. Moore[1], C. Allen[1], A. Kirkham[1], M. Emberton[1], S. Punwani[1]; [1]*London/UK*, [2]*Milan/IT*, [3]*Frimley/UK (giganti.fra@gmail.com)*

**Purpose:** To assess multiparametric magnetic resonance imaging (mpMRI) characteristics in prostate cancer (PCa) before and after irreversible electroporation (IRE) and to investigate their correlation with the presence of post-operative recurrence.
**Methods and Materials:** MpMRI was performed in 30 men with PCa prior to treatment, after 10 days and at 6 months. An additional scan at 1 year was available for 18 men. Two radiologists assessed retrospectively the following parameters by planimetry: tumour volume, necrotic volume (early post-treatment scan) and residual fibrosis. Residual tumour/recurrence were defined as suspicious areas within the treatment field scored ≥ 4 on a 1-to-5 scale. Oncological outcome was also assessed.
**Results:** Median follow-up was 15.5 months. At early mpMRI, 6 men were undertreated and showed mpMRI recurrence after 6 months. At 1-year, three additional men had recurrence. Overall, 4 men were retreated. Five men did not receive any further treatment. Median time to re-treatment was 15 months. Median pre-treatment lesion volume was 0.65cc, 0.66cc and 0.43cc on the different mpMRI sequences (T2, DWI and DCE). Median necrotic volume was 10.77cc. Median overall residual fibrosis volumes were 0.84cc and 0.95cc at 6-month and 1-year mpMRI. Pre-treatment, necrotic and residual fibrosis volumes were significantly different (p < 0.001). Pre-treatment tumour volumes on diffusion-weighted imaging and necrotic volumes were correlated ($r$= 0.18; p = 0.02).
**Conclusion:** MpMRI is a feasible imaging modality to visualise the IRE ablation effects in men with PCa. MpMRI parameters (tumour, necrotic and fibrosis volumes) are promising tools to assess the efficacy of IRE in PCa.

**B-0160** 11:43
**Is DCE useless in early detection of prostate cancer? The analysis of quantitative parameters might help radiologists to decide if findings mildly restricted on ADC map should be sampled**
M. Pecoraro, R. Campa, G. Barchetti, V. Salvo, C. Catalano, V. Panebianco; *Rome/IT (pecoraro.martina1@gmail.com)*

**Purpose:** The aim is to evaluate whether the DCE can improve tumour detection, applying the quantitative analysis of pharmacokinetic parameters of lesions not having a clearly positive ADC map.
**Methods and Materials:** Among 1236 men who underwent mpMRI for PCa suspicion from 2015 to 2018, we retrospectively enrolled 86 patients treated with radical prostatectomy and 86 patients suspected of harbouring PCa with at least one negative systematic prostate biopsy, at least two negative mpMRI exams and a minimum follow-up of 48 months, as “control group” (Group C). Exams were performed at 3T with a PI-RADSv2-compliant protocol. Quantitative analysis was performed, computing both pharmacokinetic parameters (k-trans, k-ep and ve) and ADC values.
**Results:** Mean ADC value of 82,634mm$^2$/s (95%CI= 77,564 to 88,236), mean k-trans of 0,325min$^{-1}$ (95%CI= 0,278 to 0,372), mean k-ep of 0,568min$^{-1}$ (95%CI= 0,466 to 0,640) and mean ve of 0,77 (95%CI= 0,65 to 0,89). Results were stratified into two groups: lesions with a normalised ADC≤0,62 (Group A) 72% and lesions with a nADC>0,6 (Group B) 28%. The difference of Ktrans values of the groups was not significant (p=0,08), however a statistical difference was found comparing the normalised Ktrans (nKtrans) of healthy prostate tissue with the one of PCa lesions, both separately and together (p<0,05).
**Conclusion:** It seems reasonable to adopt the nKtrans in the decision-making process of performing a prostate biopsy, particularly for those radiologic findings having a nADC>0,6. The omission of DCE would decrease PCa detection, especially when the ADC map cannot be unequivocally interpreted.

**B-0161** 11:51
**Impact of dynamic contrast-enhanced (DCE) MRI on prostate cancer detection: is it worth the effort?**
L. Schimmöller, F. Ziayee, N. Laqua, T. Ullrich, M. Klingebiel, M. Quentin, G. Antoch, C. Arsov; *Düsseldorf/DE*

**Purpose:** While current PIRADS implies a multiparametric (mp) MRI for prostate cancer (PCa) detection many authors discuss DCE only for individual cases. Adverse reactions, additional costs and efforts, and limited value represent main reasons. This study investigates the influence of DCE on PIRADS classification.
**Methods and Materials:** 66 consecutive patients with verified PCa after mp-MRI from 04/-12/2017 have been retrospectively included. PIRADS classifications for T2, DWI and DCE sequences on lesion and patient level have been correlated with histopathological findings. Situations in which DCE could influence the classification were considered: As substitution/aid for non-diagnostic DWI or in peripheral zone (PZ) PCa with a PI-RADS of 3 in DWI so that DCE could upgrade to PI-RADS 4.
**Results:** Of all 159 PCa-lesions, 128 (81%) were positive on DCE. 29 patients (44%) where classified PIRADS 5 without relevant influence of DCE, 34 were PI-RADS 4, and 3 were PI-RADS 3. 43 lesions (27%) were located in TZ. 15/159 (9.4%) in 6/66 patients (9.1%) or 5/75 PI-RADS 4 lesions (20%) in 6/34 PI-RADS 4 patients (18%) were upgraded due to DCE. Of those, 3 (4.5%) patients had clinically relevant PCA (Gleason score ≥7).
**Conclusion:** DCE seems to be very useful for confirming PCa, which may help inexperienced readers. However, in a high portion of PIRADS 5 and PI-RADS 4 lesions the benefit of DCE is minor. Only few lesions with clinically relevant PCA would have been missed in consecutive patients without DCE. Therefore, the general application of DCE might be reformed.

**10:30 - 12:00** **Room M 2**

### Paediatric

### SS 212

### Paediatric cardiothoracic imaging

*Moderators:*
L. Riera; Barcelona/ES
T. Yalynska; Kiev/UA

**B-0162** 10:39
**Ultrasound as an alternative for plain radiography in the detection of the endotracheal tube position in neonatal intensive care unit patients**
R.H. Hashem, R.H. Tomerak, A.M. Tosson, A. Abdelmoneim; *Cairo/EG (rania.hachem@yahoo.com)*

**Purpose:** To detect the role of ultrasound and its accuracy in the detection of the endotracheal tube position in the neonatal intensive care unit (NICU) and to compare it to the gold standard plain radiography.
**Methods and Materials:** Totally 100 intubated neonates were enrolled in the study over one-year period in the NICU .They all underwent high-resolution ultrasound using linear probe (5-8 MHz) on the neck and upper chest in LS to determine the tip of the ETT position, 2-point distance from the arch of the aorta was calculated. Plain radiography of chest and lower neck in AP position performed on the same day was collected. The head was kept in the sniffing position during both procedures.
**Results:** The ultrasound was able to identify the tip of the ETT in 100% of patients. There was statistically significant relation between the ultrasound and the chest radiography to determine the position in the NICU (P value less than 0.001).
**Conclusion:** Visualization of the ETT tip and its distance of 0.6 to 1.2 cm from the upper border of the arch of the aorta by ultrasound using linear high-frequency probe suggest correct position . So high-resolution ultrasound is helpful in identification of the level of the ETT tip, in neonates in the NICU.

**B-0163** 10:47
**Lung ultrasound, a safe & useful tool for neonatal with respiratory problems monitoring**
S. Foutzitzi, S.P. Deftereos; *Alexandroupoli/GR (foutzita@gmail.com)*

**Purpose:** The diagnosis and management of respiratory diseases (RD) is of great importance in Neonatal Intensive Care Unit (NICU). Chest X-Ray (CXR) and computed tomography are usually the appropriate tools for RD diagnosis (but with radiation and neonatal transportation). The safety of lung ultrasound (LUS) led to the increasing use of them in diagnosis and in every day follow-up of RD.
**Methods and Materials:** 104 consecutive neonates from Neonatal Intensive Care Unit (NICU) with a gestational age from 25 Weeks(Wk)+5 days(d) to 40Wk were included in this study. 29.80% were premature(<29 Wk). Preterm were 45.20%(30-34Wk) and 8,32%(35-37 Wk +6d). 58.65% were reveal respiratory distress syndrome(RDS), 7.7%(from seventeen full-term) transient tachypnea of the new born(TTN), 6.25% pneumothorax and 4.68% bronchopulmonary dysplasia. Used criteria were the presence of A-Lines, “sliding sign”, “pleural line” sign, presence of subpleural opacities and the B-lines "density".
**Results:** According to these: 2 pleural fluids (3.12%), 3 pneumothoraxes (4.68%), 4 neonates with TTN(6.25%) were diagnosed. RDS-severity was estimated by B-Lines density(normal/medium/white lung). LUS follow-up showed improvement in 47(73.43%). With the use of LUS we had a decreased CXRs up to 32,1% in comparison with NICU-CXRs in the same period for the last three years.
**Conclusion:** CXR remains the method of choice in RD diagnosis with the disadvantage of radiation. LUS is convenient, noninvasive, free of radiation method with the potential to become a tool for bedside dynamic respiratory monitoring. LUS is a useful tool in NICU reliable, easy to use and reproducible method for the follow-up of illness neonate.

**B-0165** 11:03
**Comparison of Iodixanol 320, Iomeprol 350 and Iopromide 370 effectiveness in CT cardiothoracic angiography in infants**
M. Pop; *Targu Mures/RO (pop.marian@gmail.com)*

**Purpose:** To compare the effectiveness of Iodixanol 320, Iomeprol 350 and Iopromide 370 in CT angiography of infants with congenital cardiovascular anomalies.
**Methods and Materials:** From February 2015 to March 2017, 65 consecutive CT cardiothoracic infants angiographies, performed in a single centre with a 64-slice scanner, using power injector and bolus tracking technique were retrospectively evaluated by an expert radiologist that analysed the axial images to assess the enhancement of great vessels using ROIs placed at the level of main pulmonary artery bifurcation. Age, sex, BMI, clinical indication, contrast medium type, iodine concentration, contrast volume, flow rate, and Hounsfield units of aorta and main pulmonary artery were recorded.
**Results:** The median patient age was 43 days (IQR 11.5:147.5 days), with a M:F ratio of 0.8 and a median BMI of 12.81 kg (IQR 11.5:14 kg). The average volume of contrast (and average flow) was 8.37 ml (1.04ml/s) for Iodixanol 320, 8.2 ml (0.94ml/s) for Iomeprol 350 and 7.39 ml (0.83ml/s) for Iopromide 370. The average enhancement of aorta was 337 UH when using Iodixanol (320 mg Iodine/ml), 627 UH when using Iomeprol (350mg iodine/ml) and 470 UH when using Iopromide (370 mg Iodine/ml); the difference being statistically significant (p<0.05) when comparing Iodixanol with either of the others. Similar results were obtained when assessing main pulmonary artery enhancement.
**Conclusion:** In infants undergoing cardiovascular thoracic CT angiography, the contrast volume and flow are inherently limited; however, Iodixanol 320 provides 45 to 28% less great vessel enhancement than other contrast media.

**B-0166** 11:11
**The difference of left ventricular myocardial strain in isolated LVNC and dilated cardiomyopathy with hyper-trabeculation: structure, functional and risk factors analysis**
H. Fu, R. Xu, Y. Guo, Z. Yang, H. Liu; *Chengdu/CN (fuhang66666@163.com)*

**Purpose:** This study was to evaluate the difference of myocardial strain in children and adult patients with iLVNC and dilated cardiomyopathy hyper-trabeculation by CMR tissue tracking, and further to explore the prognosis impact of different degree of hyper-trabeculation.
**Methods and Materials:** Eighty-nine DCM patients with hyper-trabeculation, 23 LVNC subjects (13 children and 11 adult subjects) and 44 healthy subjects (17 children and 27 adults) and were included. The degree of LV trabeculation was assessed by NC/C ratio for the CMR criterion. The myocardial structure, function and strain were measurement. Tissue-tracking analysis included LV

global peak strain (PS) and peak displacement (PD) in three directions of longitudinal, circumferential and radial. The clinical symptoms and outcome were record.
**Results:** For the strain of GPS and GPD in three directions of longitudinal, circumferential and radial, children/adult with iLVNC had significantly lower strain compared with healthy subjects, respectively (all p < 0.0017); and adult with iLVNC were lower than children. Conversely, no significant difference in strain parameters were observed between adult iLVNC patients and DCM patients with hyper-trabeculation. LV strain parameters were associated with increased indexed LV EDV, ESV, diastolic-mass and decreased EF (absolute R: 0.396-0.908; all p < 0.05).
**Conclusion:** Left ventricular myocardial functional impairment and strain changes were detected in iLVNC patients, especially being already noticeable in the children. With the age growing, the strain of iLVNC patients were lower, but the hyper-trabeculation may have no obviously effect on strain in DCM patients contrast to iLVNC patients.

**B-0167** 11:19
**Radiation exposure of thoracic computed tomography in paediatric patients: can we always achieve a sub-mSv scan in a clinical setting?**
K. Bubel[1], K. Altmeyer[2], J. Pfeifer[1], H. Abdul-Khaliq[1], T. Krenn[1], A. Massmann[1], A. Bücker[1], P. Fries[1]; [1]*Homburg/DE*, [2]*Saarlouis/DE (katharina.bubel@gmx.de)*

**Purpose:** To retrospectively analyze radiation exposure of thoracic CTs in pediatric patients in a clinical setting using a third generation dual source scanner (Siemens, Force) and to identify risk factors preventing sub-mSv examinations.
**Methods and Materials:** In 144 patients (age: mean+/-SD: 6.1+/-5.9yrs; range: 0-18yrs) a total number of 197 examinations of the thorax were performed for different clinical purposes. We acquired a high-pitch protocol using automatic exposure control (CareDose4D, CareKV, Siemens) in the majority of cases. Scans were performed unenhanced, contrast-enhanced (with or without ECG-synchronization) or with tin-filter technique. We retrospectively evaluated dose length product (DLP) and calculated effective doses (E) thereof using age and kV specific conversion factors.
**Results:** Examinations were performed for cardiovascular (n=114), and non-cardiovascular (n=83) indications (inflammation, tumor and congenital lung disease). Mean+/-SD radiation exposure for all examinations was E(all)=0.78+/-0.72mSv with a range of 0.03-3.85mSv. In 42/197 cases (21%) radiation exposure was above 1mSv. Here, patients were significantly older (11.23+/-5.5yrs vs. 3.9+/-4.9yrs, p<0.001) potentially resulting in higher kV-settings (n=33/42 with tube voltage >70 kV). 7 patients presented with metallic implants (pacemaker, stents, coils). 7 scans were performed with retrospective ECG-gating and 3 patients were obese. Radiation exposure was significantly lower in scans with tin-filter technique (0.17+/-0.14mSv; p<0.001) as compared to cardiovascular (0.82+/-0.68mSv) or non-cardiovascular CTs (0.95+/-0.74mSv)
**Conclusion:** Thoracic CTs in pediatrics can be acquired routinely with a radiation exposure below 1mSv using automated exposure control for a broad spectrum of clinical indications. Risk factors for higher doses include age, metallic implants, obesity and retrospective ECG-gating.

**B-0168** 11:27
**Fontan-operated adolescents have increased hepatic fibrotic markers assessed by ultrasound shear wave elastography and MR T1 mapping**
C. de Lange, K. Thrane, K. Thommassen, K. Rydén Suther, L.-S. Ording Müller, O.M. Geier, G. Døhlen, R. Almaas, T. Møller; *Oslo/NO (charlotte.delange@medisin.uio.no)*

**Purpose:** To investigate magnetic resonance (MR) T1 mapping with extracellular volume fraction (ECV) and ultrasound shear wave elastography (SWE) in adolescents with Fontan circulation, as a surrogate measure of hepatic fibrosis.
**Methods and Materials:** Hepatic and splenic native T1 times and ECV were prospectively measured with a modified look-locker inversion recovery sequence and stiffness measured with SWE. Results were compared to a control group and correlated to clinical and haemodynamic information from cardiac catheterization.
**Results:** Thirty-nine Fontan patients aged 16.7±0.6 years and 15 controls aged 19.2±1.2 years were included. Hepatic native T1 times were increased in patients (n=31) 776±48 ms compared to controls 632±52 ms (p<0.001) as well as ECV 48.4±5.7% vs 36.8±3.7%, respectively (p<0.001). Splenic native T1/ECV was comparable between groups (p=0.17 and p=1.0, respectively). For SWE, liver stiffness/velocity was measured to 1.9±0.1 m/s in patients (n=38) vs 1.2±0.1 m/s in controls (p=<0.001). SWE correlated only with ECV (R=0.6, p=0.001) in patients, but was without association with T1 and ECV in controls (R=0.3, p=0.3/R=0.2, p=0.4, respectively). There were no associations between hepatic T1/ECV and SWE with central venous or ventricular pressures or to slightly abnormal liver function.
**Conclusion:** Fontan patients have elevated MR and SWE biomarkers suggestive of diffuse hepatic fibrosis and/or congestion. However, no hepatic failure or correlation to central venous pressure was found. MR T1 mapping and SWE revealed only partial correlation thus opposing an interchangeable use of the two techniques in monitoring hepatic fibrosis. Longitudinal studies are required to test the significance of our findings.

**B-0169** 11:35
**Diagnostic utility of 3 Tesla thorax magnetic resonance imaging in HIV-positive children**
P. Rana[1], K.S. Sodhi[2], A. Bhatia[2], A.K. Saxena[2], D. Suri[2], S. Singh[2]; [1]*Dwarka/IN*, [2]*Chandigarh/IN (pratyaksha_25@yahoo.com)*

**Purpose:** To evaluate the role of 3T thorax MRI in HIV-positive children by keeping CT chest as the gold standard.
**Methods and Materials:** Twenty-five children with confirmed HIV-positive status having pulmonary complaints, and referred for CT chest were included in this prospective study. Thorax MRI was performed in all children using T2 TSE, BTFE, MVXD, mDIXON, STIR and DWI sequences. The images were evaluated for various pulmonary and mediastinal findings. The sensitivity, specificity, PPV, NPV of MRI were calculated. The kappa test of agreement was used to assess the agreement between MRI and CT findings.
**Results:** Sensitivity, specificity, PPV and NPV of MRI was 100% for detection of nodules >4mm, pleural effusion and lymphadenopathy. For consolidation/collapse, sensitivity and specificity was 93.8 and 88.9%, respectively. The sensitivity for detection of bronchiectasis and GGOs was 75%, while specificity was 100% and 94.1%, respectively. Nodules <4mm were not well detected on MRI with sensitivity and NPV of 35% and 23.5%, respectively. The kappa test showed perfect agreement (*k*-1) for detection of nodules >4mm, pleural effusion and lymphadenopathy, with almost perfect agreement (*k*-0.82) for the detection of consolidation/collapse.
**Conclusion:** Amongst the sequences used, T2 TSE sequence was the best pulse sequence to detect parenchymal and mediastinal abnormalities out of all MR pulse sequences. Diagnostic yield was poor with DWI and STIR sequence as compared to T2 TSE sequence with no additional benefit. Common findings such as consolidation, pleural effusion, nodule >4mm and lymphadenopathy were well demonstrated on MRI.

**B-0170** 11:43
**Assessment of lung volume and central airways dimension using MRI in children: a comparison with plethysmography, CT and PFT**
Y. Wang[1], C. Yong[1], P. Wielopolski[2], E.C. van der Wiel-Kooij[2], H.A. Tiddens[2], P. Ciet[2]; [1]*Yinchuan/CN*, [2]*Rotterdam/NL (p.ciet@erasmusmc.nl)*

**Purpose:** To test weather MRI measurements of lung volume and central airways dimensions correlate to validated pulmonary function tests parameters in children with cystic fibrosis (CF), asthma and healthy controls, and to assess capacity to detect trapped air (TA).
**Methods and Materials:** Spirometer-controlled MRI was performed with a breath-hold 3D-SPGR sequence at end-inspiration and expiration using a 3.0 T scanner in 37 subjects, including 12 CF patients (age 11.3 ±2.4), 12 asthma patients (12.9 ±2.8) and 13 healthy volunteers (12.3 ±1.7). We measured lung volume (in ml) and central airways dimensions (area and diameters in trachea and main bronchi every cm). We compared total lung capacity (TLC) and residual volume (RV) measured with MRI and with body pletysmography. We also correlated MRI airway dimensions to forced expiratory flow in 1 sec (FEV1) and $TA_{MRI}$with TA pletyshmography and Forced expiratory flow (FEF25-75). Mixed model analysis was used as statistic.
**Results:** Lung volume and TA measured with MRI significantly correlated with plethysmography and CT measurements (in CF group) (p=0.0006). TA was significantly higher in CF group (p=0.01) than healthy control, and almost significantly higher in the asthma group (p=0.057). There were no differences in TLC between patients' groups, while RV tended to be higher in CF (p=0.058). No difference in central airways dimensions was detected between the groups. MRI parameters had high correlation with FEV1.
**Conclusion:** Lung MRI is a feasible method to evaluate lung volume and central airways dimensions in children with respiratory disorders.
*Author Disclosures:*
**P. Ciet:** Speaker; Vertex Pharmaceutical.

**B-0171** 11:51
**Image quality and incidental findings of chest MRI in a large paediatric population-based study**
A. Pittaro[1], L. Duijts[2], P. Wielopolski[2], M.W. Vernooij[2], A. Van der Lugt[2], V.W. Jaddoe[2], M.M.P.C. Kemner-van de Corput[2], H.A. Tiddens[2], P. Ciet[2]; [1]*Padova/IT*, [2]*Rotterdam/NL (p.ciet@erasmusmc.nl)*

**Purpose:** To describe image quality (IQ) and incidental findings (IFs) of chest MRI in children participating in a population-based prospective cohort study.
**Methods and Materials:** Two end-inspiratory (INSP) and -expiratory (EXP) breath-hold chest-MRI scans were performed in 2498 healthy children using a 3D-SPGR sequence (voxel-resolution=2mm isotropic) in a 3Tesla scanner. IQ was assessed visually using a 5-point scale from poor (score 1) to excellent

(score 5). Imaging artefacts included: motion, wrap, ghosting, low SNR. IFs were classified by trained raters as potentially clinically relevant or non-clinically relevant. Descriptive statistical analysis was used to assess IQ, IFs and artefacts. Significant differences between IQ-INSP and IQ-EXP were assessed with Wilcoxon test.
**Results:** 47 children had missing data. Final analysis included 2451 children (median age 9.9 years, range 9.5-11.9). Median IQ was good to excellent 4.5 (Interquartile Range, IQR= 4-5). IQ was mostly affected by motion artefact (31.9%), fat ghosting (7.9%) or both (6.3%). 1.7% of the cohort subjects had clinically relevant IFs, 45% had non-clinically relevant IFs. Clinically relevant IFs included pulmonary nodules (diameter>10 mm, 0.04%), severe tracheomalacia (collapse>70%, 0.3%), severe trapped-air (>25% lung lobe volume, 0.9%), atelectasis/consolidation (>10 lung lobe volume, 0.08%) and congenital abnormalities (i.e. sequester, 0.38%). Non-clinically relevant IFs were: mild trapped-air (23.8%), atelectasis (15.4%) and mild tracheomalacia (4.5%).
**Conclusion:** Chest MRI provides good quality images, and potentially clinical incidental findings are rare. However, trapped-air, atelectasis and mild tracheomalacia are common non-clinically relevant "incidental" findings on chest MRI in healthy children.
*Author Disclosures:*
**P. Ciet:** Speaker; Vertex Pharmaceutical.

**10:30 - 12:00** **Room M 3**

### Oncologic Imaging

### SS 216

### Multiparametric imaging for pelvic cancers

*Moderators:*
M. Otero-García; Vigo/ES
B.M. Schaarschmidt; Düsseldorf/DE

### **B-0172** 10:30

**Classification of prostate cancer on multiparametric MR imaging using 3D convolutional neural network**

N. Aldoj, S. Lukas, M. Dewey, T. Penzkofer; *Berlin/DE*
*(nader.aldoj@charite.de)*

**Purpose:** To present a novel single-pipeline, fully automated approach for prostate cancer classification based on multiparametric magnetic resonance (MR) imaging using a 3D convolutional neural network (CNN).
**Methods and Materials:** Two hundred patients (provided by PROSTATEx challenge) with a total of 318 lesions for which histological correlation was available were analysed. A novel CNN was designed, trained, and validated simultaneously using three MRI sequences as input (T2-weighted, apparent diffusion coefficient (ADC), and diffusion-weighted images). The network consists of 12 convolutional layers organized in seven blocks with intermittent skip connections of concatenated feature maps and two fully connected and one output layer at the end. The model was trained and validated using 8-fold cross-validation.
**Results:** In terms of detection of significant prostate cancer defined by biopsy results as the reference standard, the 3D CNN achieved an average area under the curve (AUC) of the receiver operating characteristics of 0.82 with the best model having an AUC of 0.87, 68.9% sensitivity, and 88.8% specificity. This performance is comparable to that reported for experienced radiologists (AUC of 0.83) using the prostate imaging reporting and data system (PI-RADS). Lesion size had no effect on the network's performance.
**Conclusion:** The diagnostic performance of the 3D CNN in detecting clinically significant prostate cancer is characterized by a good AUC, moderate sensitivity, and high specificity.

### **B-0173** 10:38

**Ovarian cancer staging and resectability assessment: prospective comparison between diffusion-weighted MRI and MSCT**

A.E. Solopova, A.M. Sdvizhkov, A.D. Makatsaria; *Moscow/RU*
*(dr.solopova@mail.ru)*

**Purpose:** to evaluate the diagnostic potential of diffusion-weighted MRI sequences (DW-MRI) compared with multispiral computed tomography (MSCT) for ovarian cancer (OC) staging.
**Methods and Materials:** 78 patients with suspected OC underwent preoperative examination, including abdominal/pelvic MSCT and DW-MRI, using a 1.5T scanner with b-factors of 0, 1000 s/mm$^2$. Using the obtained data, the peritoneal cancer index (PCI) was calculated and ROC curves for DW-MRI vs MSCT were created. The imaging-based diagnoses were compared with surgical staging and histopathological examination.
**Results:** The following stages of OC were determined: 6 (7.7%) IV; 37 (47.4%) IIIC; 25 (32%) IIIB; 10 (12.8%) IIIA. DW-MRI was markedly superior to MSCT in detecting of tumor implants in the large intestine mesentery (83.8% and 37.8%, respectively), into the small intestine serosa (79.7% and 31.8%), and the para-aortic lymph nodes (71.4 and 35.7%). The specificity were similar (80,4% - DW-MRI and 81,6% - MSCT). The peritoneal cancer index (PCI) calculated from the DW-MRI results correlated with the surgical staging data better than based on MSCT (r=0.814 for DW-MRI, r=0.625 for MSCT). Based on the obtained data, the ROC curves were plotted to show the AUC of 0.926 and 0.693 for DW-MRI and MSCT, respectively with overall sensitivity for correct staging of 83% for DW-MRI and 61% for MSCT. A decrease in ADC values was found in secondary tumor foci (1.09±0.28 for primary tumor, 0.810±0.26 - omentum, 0.750±0.23 × $10^{-3}$ mm$^2$/s - peritoneal implants).
**Conclusion:** For the assessment of ovarian cancer spread, DW-MRI is significantly more sensitive than MSCT.

### **B-0174** 10:46

**Multiparametric MRI in prostate cancer: a radiomic study applied to different diffusion and perfusion models**

D. Grimaldi, S. Monti, C. Rinaldo, V. Brancato, M. Aiello, G. Di Costanzo, C. Cavaliere, A. Ragozzino, M. Salvatore; *Naples/IT*
*(carlocavaliere1983@yahoo.it)*

**Purpose:** To evaluate radiomics features extracted from 2D region and 3D volume of interests on diffusion weighted imaging (DWI), diffusion kurtosis imaging (DKI), and Tofts modelling (TM) of perfusion maps between prostate cancer (PCa), benign prostatic hyperplasia (BPH), and benign peripheral zone (PZ).
**Methods and Materials:** 75 foci of PCa, 100 BPH nodules, and 100 benign PZ from 75 patients who underwent multiparametric MRI of prostate were investigated. MRI was performed with a 3T mMR Biograph scanner. DWI was performed using 7 b-values (0-2500), and non-Gaussian diffusion coefficient (D), deviations from normal distribution (K), and classical apparent diffusion coefficient (ADC) maps were generated. DCE-MRI parameters (Ktrans, ve, and kep) were also determined. First-order statistical features were extracted from T2w MRI, DWI, and DCE-MRI maps on both 2D and 3D segmentations. A logistic regression classifier was used to identify features discriminating clinically significant tumours (PIRADS 4-5), and was evaluated by area under the receiver-operating characteristic curve (AUC).
**Results:** Radiomic features that were identified as useful for PCa detection differentiated PCa from BPH or benign PZ, independently from the segmentation method employed (2D vs 3D). When comparing classification achieved with features extracted from different diffusion and perfusion parameters, a statistical difference was detected only among diffusion features in PCa. When classification was performed using a multivariate model, the classifier identified DKI features as the best predictors for prostate tumour (AUC > 0.85).
**Conclusion:** A classifier that includes features extracted by DKI significantly improves the accuracy of cancer detection.

### **B-0175** 10:54

**Associations between whole tumour histogram analysis parameters derived from ADC maps and expression of EGFR, VEGF, Hif 1-alpha Her-2 and Histone 3 in uterine cervical cancer**

H.-J. Meyer, P. Gundermann, A. Höhn, G. Hamerla, A. Surov; *Leipzig/DE*
*(hans-jonas.meyer@medizin.uni-leipzig.de)*

**Purpose:** Diffusion-weighted imaging (DWI) is able to predict microstructure in tissues and can be quantified by apparent diffusion coefficient (ADC). The present study sought to correlate ADC histogram-based parameters with different histopathological parameters in uterine cervix squamous cell carcinoma.
**Methods and Materials:** 18 female patients (age range 32-79 years) with squamous cell cervical carcinoma were retrospectively enrolled. In all cases, pelvic MRI was performed with a DWI (b values 0 and 1000 s/mm²). Histogram analysis was performed as a whole lesion measurement, calculating several percentile, kurtosis, skewness and entropy. Histopathological parameters included expression of EGFR, VEGF, Hif1-alpha, Her2 and Histone 3. Spearman's correlation coefficient was used to analyse associations between investigated parameters.
**Results:** The investigated ADC histogram showed a good interreader variability, ranging from 0.705 for entropy to 0.959 for ADCmedian. EGFR expression correlated statistically significant with several histogram parameters. The highest correlation was observed for p75 (p=-0.562, P=0.015). There were several correlations with histone 3, the highest with p25 (p=-0.610, P=0.007).
**Conclusion:** Histogram analysis showed a good interreader agreement. ADC histogram parameters can reflect expression of EGFR and histone 3 in squamous cell carcinomas of the cervix, but not expression of VEGF, Hif1-alpha and HER 2.

**B-0176** 11:02
**IVIM MRI: a better tool to assess myometrial invasion in endometrial cancer**
R. Balaji; *Chennai/IN (ravikanthbalaji@gmail.com)*

**Purpose:** Myometrial invasion is a major prognostic factor in endometrial malignancy. The aim of the current study was to investigate diagnostic value of IVIM MRI in peritumoural zone for assessing infiltration of endometrial cancer and comparing with T2 weighted images.
**Methods and Materials:** 68 patients with biopsy proven endometrial cancer were included in the study. Patients underwent MR imaging on a 1.5 Tesla scanner (Achieva, Philips Healthcare, Best, The Netherlands). Imaging sequences included T1, T2, regular diffusion and IVIM performed with 11 b values (0 to 1200 $mm^2$/s). Regions of Interest (ROI) were drawn in the peritumoural zone where the tumour was in proximity to the myometrium. IVIM derived parameters f (perfusion fraction), D (true diffusion) and D* (pseudo diffusion) were obtained. The signal changes and values were compared with corresponding T2 weighted images.
**Results:** The primary tumour in the endometrial cavity had high f and low D values. Similar values were obtained in peritumoural zone and points of infiltration. In 24 patients with only breach of junctional zone and no obvious myometrial invasion seen on T2WI, IVIM parameters were altered with increased f values and low D values compared to normal myometrium.
**Conclusion:** f and D values obtained by IVIM MRI in peritumoral zone could accurately predict depth of myometrial invasion.

**B-0177** 11:10
**Multiplatform reproducibility of DCE-MRI enhancement curves for adnexal masses: quality assurance study for multicentre MROC trial**
A.S. Mehdi, S. Islam, N. Bharwani, S. Sudderuddin, A.G. Rockall; *London/UK (aia.s.mehdi@gmail.com)*

**Purpose:** Dynamic Contrast Enhanced MRI (DCE-MRI) is a useful tool in characterising ovarian tumours; Semi-quantitative DCE-MRI metrics separate the relative enhancement curves of lesions into 3 types; benign (1), indeterminate (2) and malignant (3), using myometrium as internal reference. This technique is being used in the NIHR-funded multicentre study in ovarian cancer (MROC). The purpose of the current study was to assess the agreement of categorisation of these enhancement curves between different platforms and readers.
**Methods and Materials:** 58 females (mean age 54 ±16) underwent pelvic DCE-MRI for characterisation of adnexal masses. The inclusion criteria were presence of solid tissue in lesion and a uterus in situ; yielding 26 lesions for final analysis. DCE-MRI relative time intensity curves were generated for each lesion using 3 applications: Olea sphere 3.0, Osirix DCE plugin 2.2 and Mint Lesion. These were generated from regions of interest drawn on the lesion and outer myometrium. Each curve was categorised as type 1, 2 or 3 by three Consultant Radiologists specialising in gynaecological imaging. Inter-observer agreement for each platform and intra-observer agreement was assessed using a Fleiss kappa score.
**Results:** The inter-observer kappa score ranged from 0.65-0.70 demonstrating strong agreement. The intra-observer kappa score across different platforms ranged from 0.23-0.47 demonstrating fair to moderate agreement.
**Conclusion:** Semi-quantitative DCE-MRI enhancement curves for adnexal lesions demonstrate good reproducibility between readers for a given platform but are less reproducible across different platforms. This indicates a need to develop robust semi-quantitative parameters that may be validated across different platforms.

**B-0178** 11:18
**Texture analysis of multiparametric MRI and association with tumour grading in cervical cancer**
J.A.U. Perucho, E.Y.P. Lee, R. Du, V. Vardhanabhuti, K.W.H. Chiu, E.M.F. Wong; *Hong Kong/HK (jperucho@hku.hk)*

**Purpose:** To explore efficacy and generalizability of texture analysis of multiparametric magnetic resonance imaging (mpMRI) in discriminating histopathological characteristics of cervical cancers in a two-centre setting.
**Methods and Materials:** 130 cervical cancer patients were retrospectively reviewed for pre-treatment diffusion-weighted (DWI) and standard T2-weighted (T2W) abdominopelvic MRI in two centres (centre 1 n=100; centre 2 n=30). Biopsies of each patient were acquired to determine histological subtype and tumour grading. Volumetric regions of interest (VOI) were placed to encompass the entirety of primary tumours on T2W and DWI parametric maps. 144 texture features were calculated using pyradiomics. Redundancy analysis was used for feature reduction while goodness-of-fit measures were used for feature selection. Logistic regression was used to build predictive models with centre 1 serving as the training set and centre 2 serving as an external testing set.
**Results:** In the first centre 80 patients had squamous cell carcinoma (SCC) while 20 had adenocarcinoma (ACA), and 42 patients had well or moderately differentiated tumours (G1/G2) while 58 had poorly differentiated tumours (G3). In the second centre, 25 patients had SCC while 5 had ACA, and 14 had G1/G2 tumours while 16 had G3 tumours. Redundancy analysis demonstrated that only 15% of features were independent predictors. In discriminating histological grading, only one texture, $T2W_{kurtosis}$, achieved reasonable goodness-of-fit and moderate discriminative performance (internal accuracy: 0.64, external accuracy: 0.71). In discriminating histological subtype. Texture features had poor discriminative performance for tumour grading.
**Conclusion:** $T2W_{kurtosis}$ was moderately discriminative in differentiating tumour grading in cervical cancer.

**B-0179** 11:26
**The utility of histogram analysis of whole tumour apparent diffusion coefficient map and MRI volumetry in differentiating low and high grade endometrial cancer**
W.M. Fathallah, M. Habeeb, M.E. Ghanim, M.E. Bandar; *Kuwait/KW (waeldr@hotmail.com)*

**Purpose:** To test the diagnostic performance of histogram analysis of apparent diffusion coefficient (ADC) map and tumor volumetry for differentiating low from high grade endometrial cancer.
**Methods and Materials:** Fifty eight patients with histologically proven endometrial cancer who had preoperative MRI and surgery were retrospectively included in this institutional review board approved study with waiver of informed consent. Histogram data derived from regions of interest (ROIs) manually drawn on all slices of the ADC map including areas of necrosis. Histogram parameters (ADC mean, standard deviation, skewness, kurtosis and $5^{th}$-$95^{th}$ percentiles), tumor volume (TV), uterine volume (UV) and tumor/uterine ratio (T/U ratio) were correlated to tumor histological grade.
**Results:** High grade endometrial cancers demonstrated significantly higher ADC maximum and standard deviation (p<0.001), $75^{th}$ (p=0.008), $90^{th}$and $95^{th}$ (p<0.001) percentiles values than low grade tumors. T/U ratio and TV were significantly higher for high-grade neoplasms (mean T/U of 0.31 for G3, 0.12 for G2 and 0.07 for G1; p<0.001 for G1 vs G2 and G3). Rest of parameters showed no significant correlation with tumor grade. Cutoff values of T/U of 0.11, TV of 12.9 mL , $95^{th}$ percentile of $1.334x10_{-3}$mm2/s , $90^{th}$ percentile of $1.259x10_{-3}$mm2/s , $75^{th}$ percentile of $1.022x10_{-3}$mm2/s , ADC standard deviation of $0.206x10^{-3}$mm2/s and ADC maximum of $1.688x10^{-3}$mm2/s enabled to distinguish G1 from G2-G3 tumors with sensitivity 94.7%, 84.2%, 68.4%, 68.4%, 68.4%, 78.9% and 84.2%, specificity 82.1%, 74.4%, 82.1%, 89.7%, 66.7%, 74.4% and 71.8% and accuracy 86.2%, 77.6%, 77.6%, 82.7%, 67.2%, 75.9% and 75.9% respectively.
**Conclusion:** Histogram analysis of ADC map and MRI volumetry are useful in differentiating low from high grade endometrial cancer.

**B-0180** 11:34
**Ovarian cancer neoadjuvant treatment response evaluation: value of diffusion-weighted MRI**
A.E. Solopova, S.N. Gurov; *Moscow/RU (dr.solopova@mail.ru)*

**Purpose:** To evaluate the capability of diffusion MR-sequences (DW-MRI) in the assessment of neoadjuvant chemotherapy treatment (NACT) response of ovarian cancer (OC).
**Methods and Materials:** 72 zones of interest in 25 patients with disseminated OC, who underwent pelvic and abdominal MRI to clarify the tumor spread, the issue of NACT and after 1 and 3 courses in the evaluation of its effectiveness, were available for analysis after the 3-d course of NACT. MR-study was performed on 1.5 T MRI, using protocol: T2-,T1-, Dynamic 3D FS, DWI with b-factors 0, 200, 400, 600, 1000 m/$mm^2$, with apparent diffusion coefficient (ADC) estimation. The basic treatment response evaluation was performed in accordance with RECIST 1.1 criteria.
**Results:** The average ADC values in the primary tumour before treatment were significantly higher than in omental and peritoneal implants (p=0,024 and p=0,029, respectively). A comparative assessment of ADC before and after treatment revealed significant increase after 3 courses of chemotherapy (p=0,012), while improvement after 1 course in the primary tumour and differences in ADC before and after treatment in the omental and the peritoneal implants are less pronounced and insignificant (p=0,84; 0,71; 0,59 respectively). Information on evaluation of residual tumour and the degree of therapeutic MRI pathomorphosis were: sensitivity - 89,6%, specificity - 81,3%, and accuracy - 87,1%.
**Conclusion:** MRI DWI is informative adjunct in NACT treatment response evaluation in patients with advanced OC. Significant differences in the mean ADC were obtained for the solid component of the primary tumours, indicating their greater sensitivity to NACT.

**B-0181** 11:42
**Pre- and post-MRI risk-stratification reduces prostate MRIs, biopsies and overdetection in prior negative biopsy men**
F.-J.H. Drost, J.F.M. Verbeek, A.R. Alberts, M.J. Roobol, I.G. Schoots; *Rotterdam/NL (i.schoots@erasmusmc.nl)*

**Purpose:** To assess the diagnostic performance of prostate cancer (PCa) detection by incorporating Rotterdam Prostate Cancer Risk Calculator (prostatecancer-riskcalculator.com) in pre- and post-MRI diagnostic setting.
**Methods and Materials:** In 146 consecutive men with prior negative biopsy, mpMRI with blinded standard biopsies (SBx) and MRI-targeted biopsies (MRI-TBx) to PIRADS ≥3 lesions was performed "by default". Various diagnostic strategies w/o MRI(-TBx), SBx, and risk-calculation (pre-MRI and post-MRI) were evaluated to PCa outcomes (Grade Groups [GG]), quantified by detection rates between diagnostic strategies (McNemar-test, p<0.05=*).
**Results:** MRI was negative in 63/146 (43%) men, potentially saving 43% biopsies in the MRI-TBx strategy. PI-RADS 3, 4 and 5 coincided with GG≥2 PCa in 4/23 (17%), 18/33 (55%) and 24/26 (92%) men, respectively. MRI-TBx versus default (MRI-TBx+SBx) reduced 23/40 (58%)* GG=1 but missed 4/49 (8%) GG≥2 PCa. Pre-MRI risk-stratification identified 53/146 (36%) men at low-risk, potentially saving 36% MRIs and biopsies. Pre-MRI risk-based MRI-TBx+SBx versus default (MRI-TBx+SBx) reduced 10/40 (25%)* GG=1 PCa but missed 7/49 (14%) GG≥2 PCa. Post-MRI risk-stratification identified 40/146 (27%) men at low-risk, potentially saving 27% biopsies. Post-MRI risk-based MRI-TBx+SBx versus default (MRI-TBx+SBx) reduced 7/40 (18%)* GG=1 and missed none (0%) GG≥2 PCa.
**Conclusion:** In prior negative biopsy setting, pre-MRI risk-stratification indicating MRI(-TBx) and SBx, may potentially result in saving one-in-three MRIs, with reduced detection of GG1 PCa, at the expense of missing limited GG≥2 PCa. Post-MRI risk-stratification, i.e. (all men undergo MRI) indicating TBx and SBx, may potentially result in saving one-in-four biopsies, not compromising any GG≥2 PCa detection.

**B-0182** 11:50
**DW-MRI can be used to predict complete cytoreduction and survival in colorectal peritoneal carcinomatosis patients using leading predictive models**
M.P. Engbersen, I. Van 't Sant, W.J. Van Eden, J.D.R. Velzing, N.F. Kok, D.M. Lambregts, R.G.H. Beets-Tan, A.G. Aalbers, M.J. Lahaye; *Amsterdam/NL (m.engbersen@nki.nl)*

**Purpose:** This study demonstrates the performance of diffusion-weighted MRI to predict complete cytoreductive surgery (CRS), disease-free survival and overall survival by using 5 different predictive models for HIPEC candidates with colorectal cancer.
**Methods and Materials:** Between February 2016 and October 2017, patients with colorectal peritoneal carcinomatosis considered for CRS/HIPEC who underwent DW-MRI for preoperative staging were included. DW-MRI images were evaluated, retrospectively, to determine the PCI. Relevant clinical parameters were obtained from patient files. Five predictive models were used; the Peritoneal Surface Disease Severity Score (PSDSS), Region Count, Simplified Peritoneal Cancer Index (SPCI), Peritoneal Cancer Index (PCI), and the Colorectal Peritoneal Metastases Prognostic Surgical Score (COMPASS). The performance of the predictive models was assessed by receiver operator characteristics (ROC) analysis and Cox-proportional analysis.
**Results:** Ninety-one patients were included. The mean age was 62.30 (±10.93) and 49% (45/92) of the patients were female. Fifty-three (58%) patients underwent successful CRS/HIPEC. For 38 patients, CRS/HIPEC was deemed infeasible, mostly due to extensive peritoneal carcinomatosis (23/38). ROC curve analysis for predicting a complete cytoreduction showed AUCs for PSDSS, region count, SPCI, PCI and COMPASS of 0.79, 0.81, 0.82, 0.85 and 0.86, respectively. PSDSS, region count, SPCI, PCI and COMPASS showed significant hazard ratios for overall survival of 1.19, 1.69, 1.22, 1.12 and 1.02. None were found for disease-free survival.
**Conclusion:** DW-MRI seems to be able to select patients with colorectal peritoneal carcinomatosis eligible for CRS/HIPEC. All five predictive models are predictive of complete cytoreduction and overall survival, not of disease-free survival.

**10:30 - 12:00** **Room M 5**

### Neuro

### SS 211b

### Vascular diseases and the brain

*Moderators:*
N.N.
M.M.A. Zaitoun; Zagazig/EG

**B-0183** 10:30
**MRA vs DSA based models of cerebral aneurysms: how do the corresponding CFD derived flow fields compare?**
M. Mantatzis, K. Thanosoulis, A. Theodoridis, J. Mokali, C. Gianniotis, I. Seimenis; *Alexandroupoli/GR (mmantatz@med.duth.gr)*

**Purpose:** Computational fluid dynamics (CFD) simulations can be used to study the blood flow dynamics in cerebral aneurysms (CAs). This study aims at comparing the CFD derived flow fields in matching CA models determined by magnetic resonance angiography (MRA) and digital subtracted angiography (DSA).
**Methods and Materials:** Three-dimensional (3D) models of the same CAs were reconstructed from corresponding MRA and DSA data. Following similar meshing, the two models were used to calculate CA haemodynamic parameters, such as the wall shear stress (WSS) and the static pressure. The flow fields were analyzed computationally using the 3D Navier-Stokes equation for laminar flow of incompressible Newtonian fluid. Various inlet conditions were considered.
**Results:** The gross morphologies of the CA models reconstructed from DSA and MRA were comparable, although the two respective aspect ratios differed by about 12%. However, the intrinsically high spatial resolution of DSA seems to result in an elaborate and accurate morphological characterization of the CA that requires less user interference. The 3D model determined from DSA resulted in generally higher WSS and static pressure values compared to those obtained with the MRA based model. Minimum WSS values differed by up to 28% in the two models. Nevertheless, local minima were found at the same CA locations.
**Conclusion:** CFD-derived local values of WSS, a parameter which has been proposed as predictive of the risk for CA rupture, may be dependent on the imaging modality and protocol employed for the reconstruction of the 3D aneurysmal model.

**B-0184** 10:38
**The mechanism of cerebral aneurysms and treatment effect on the genesis**
B.-L. Gao; *Zhengzhou/CN (browngao@163.com)*

**Purpose:** To investigate the mechanisms of cerebral aneurysms and treatment effect on the mechanisms.
**Methods and Materials:** 724 patients with cerebral aneurysms were investigated. The angiographic data of 140 patients were processed for computational fluid dynamics (CFD). Different therapies were analysed for their effects on the mechanisms of aneurysms.
**Results:** 1018 cerebral aneurysms were detected. 717 aneurysms involved a branch (70.4%) and 301 aneurysms were located on a vascular curve (29.6%). On the wall where a cerebral aneurysm was generated, two peak values and one minimal value were demonstrated on the curve of wall shear stress while one peak value was revealed on the total pressure curve. Moreover, cerebral aneurysm occurred on one of the shear stress peaks. Stenting can straighten the arterial bends and change the vascular bifurcation angle, revising the profile of shear stress and total pressure and the distance between two peaks of shear stress and thus making the haemodynamic stresses focused more on the divider of vascular bifurcation. Coiling or surgical clipping alone did not alter the arterial shape and the profile of shear stress and total pressure, without affecting the haemodynamic stresses for aneurysm initiation.
**Conclusion:** Cerebral aneurysms are initiated at sites with both high wall shear stress and total pressure, and stenting especially Y-shaped stenting can decrease the haemodynamic stresses for aneurysm initiation while coiling alone or surgical clipping do not. Stenting with Y configuration is a better choice of treatment than coiling or surgical clipping in managing the genesis of cerebral aneurysms.

**B-0185** 10:46

**Evaluation of haemodynamic and morphological biomarkers to assess the rupture risk of intracranial aneurysms using magnetic resonance fluid dynamics and computational fluid dynamics**

M.G.R.S. Perera[1], H. Isoda[1], T. Mizuno[1], M. Terada[2], T. Naito[2], C. Tanoi[2], T. Izumi[1], H. Sakahara[3], S. Naganawa[1]; [1]*Nagoya/JP*, [2]*Iwata/JP*, [3]*Hamamatsu/JP (mg.roshani@d.mbox.nagoya-u.ac.jp)*

**Purpose:** The purpose of this study was to evaluate in-vivo haemodynamic and morphological biomarkers using magnetic resonance fluid dynamics (MRFD) and computational fluid dynamics (CFD) in order to assess the rupture risk of intracranial aneurysms.
**Methods and Materials:** 41 intracranial aneurysms (33 unruptured, 8 ruptured) were analyzed using MRFD and patient specific MR-based CFD. Two morphological and ten haemodynamic biomarkers were calculated and evaluated for significance with respect to rupture. Mean values of biomarkers for the ruptured and unruptured groups were compared and the existing correlations between CFD based and MRFD based biomarkers were assessed.
**Results:** Four haemodynamic biomarkers, average oscillatory shear index (MRFD, p=0.012; CFD, p=0.041), maximum oscillatory shear index (MRFD, p=0.027; CFD, p=0.016), average relative residence time (MRFD, p=0.027; CFD, p=0.024), maximum relative residence time (MRFD, p=0.045; CFD, p=0.024) were statistically significantly different between the 2 groups with respect to both MRFD and CFD analyses. Inflow concentration index (p=0.024) achieved statistical significance in MRFD analysis whereas spatially and time averaged wall shear stress (p=0.024) was significantly different in CFD analysis. Receiver operating characteristic analysis revealed AUC values greater than 0.731 for all the significant biomarkers. None of the morphological biomarkers, namely aspect ratio and size ratio exhibited statistically significant differences. Moderate to very strong, positive monotonic correlations were observed between CFD and MRFD with respect to the significantly different biomarkers.
**Conclusion:** Oscillatory shear index, relative residence time, inflow concentration index and time averaged wall shear stress could be potential haemodynamic biomarkers for intracranial aneurysm rupture risk assessment.

**B-0186** 10:54

**4D CT-angiography as a predictor of growth of intracranial aneurysm**

E. Grigorieva[1], V. Krylov[2], V. Lukyanchikov[2], N. Polunina[2]; [1]*Fryazino/RU*, [2]*Moscow/RU (iara333@yahoo.com)*

**Purpose:** The prevalence of unrupted aneurysms in population consist of 2.8%, about 30% among them are multiple, giant or partly thrombosed aneurysms, which need more accurate assessment because of high risk of neurological deficit and adverse outcome.
**Methods and Materials:** In 2012-2017, 4D CT angiography was performed on 16 patients with unruptured aneurysms from 3mm to 35mm, 2 patients with partially thrombosed aneurysms. 4D CT angiography was performed with ECG synchronization followed the reconstruction of the R-R interval resulting in a pulsation video sequence of the aneurysm wall. We considered any asynchronous pulsation of the aneurysmatic wall to be haemodynamically significant. All patients were observed during 4 years using CT-angiography.
**Results:** 11 of 16 patients showed the uniform pulsation of the aneurysmal sack, with the stable size over time. In 3 patients (18,75%) the nonsynchronous pulsation of one wall was noted, with an increase in the size of aneurysms by 1-1.5 mm per year and the formation of diverticula at the level of maximum wall pulsation. In 2 patients with partially thrombosed aneurysms the wall pulsation was too weak to be analyzed.
**Conclusion:** 4D CT angiography may provide additional functional information not only about aneurysm wall pulsation but about the tendency of hasty growth that is one more factor of risk of aneurysmal rupture.

**B-0187** 11:02

**Risk evaluation of malignant intracranial hypertension development in children with severe traumatic brain injury**

I. Melnikov, M. Ublinskiy, O. Lukovkina, M. Akhlebinina, T. Akhadov; *Moscow/RU (iliamed@inbox.ru)*

**Purpose:** To determine the potential of DWI in assessing the risk of developing malignant intracranial hypertension in children with severe TBI.
**Methods and Materials:** We retrospectively evaluated clinical and MRI data of 36 pediatric patients with severe TBI. The severity of clinical condition of each patient was evaluated with the use of the Glasgow Coma Scale (GCS). Parenchymal ICP gauge placement was performed in all patients for adequate ICP monitoring. Patients were categorized into three groups: (1) high ICP managed conservatively; (2) malignant ICP managed with DC; (3) normal ICP. Four pairs of symmetrical ROIs were manually drawn on ADC maps, that were grouped for analysis of deep white matter of frontal, temporal, parietal and occipital lobes. All ROIs excluded areas that appeared abnormal on T2WI.
**Results:** Average ADC values in the deep white matter of frontal lobes were significantly increased in children with severe TBI with following DC (851.5+/-54.3x$10^{-3}$ $mm^2$/sec) compared to those with severe TBI and conservatively controlled ICP (756.4+/-40.5x$10^{-3}$ $mm^2$/sec; $p<0.05$).
**Conclusion:** Assessment of DWI and ADC values in severe pediatric TBI is a potential tool for evaluating the risk of malignant ICP development. Early identification of children at high risk for this complication may assist in earlier aggressive (surgical) clinical management of pediatric TBI patients.

**B-0188** 11:10

**Association of amygdala volume and markers of cardiovascular disease**

S. Grosu[1], R. Lorbeer[1], F. Bamberg[2], C.L. Schlett[3], M. Heier[1], S. Rospleszcz[1], A. Peters[4], B.B. Ertl-Wagner[5], S. Stöcklein[1]; [1]*Munich/DE*, [2]*Freiburg/DE*, [3]*Heidelberg/DE*, [4]*Neuherberg/DE*, [5]*Toronto, ON/CA (sergio.grosu@med.uni-muenchen.de)*

**Purpose:** Recent publications suggest a link between amygdala activity measured by PET and cardiovascular disease. Data generated from animal models show that stress leads to larger amygdala volumes. Therefore, we investigated the association between amygdala volume determined by magnetic resonance imaging (MRI) and MRI-based surrogate markers for cardiovascular disease.
**Methods and Materials:** 237 subjects of the KORA study cohort without prior cardiocerebrovascular disease underwent comprehensive 3T MRI assessment to characterize amygdala volume and imaging-based markers of cardiovascular disease (carotid plaque, media wall thickening, myocardial mass, myocardial late gadolinium enhancement, left ventricular function). Amygdala volume was automatically segmented (based on FLAIR images) and corrected for total intracranial volume. Logistic regression analyses of amygdala volume and MR-derived cardiovascular parameters were conducted while controlling for age and sex as well as cardiovascular risk factors, including diabetes, blood cholesterol levels, hypertension, smoking and obesity.
**Results:** Among 237 subjects (mean age: 56.2±9.1, 57% males), the average amygdala volume was 3.04±0.24 ml. The prevalence of MR-based markers of cardiovascular disease was moderate (carotid plaque 20.8%, myocardial late gadolinium enhancement 2.9%). Mean media wall thickening was 0.75± 0.11 mm, mean myocardial mass was 72.9±15.5 g/m2, mean ejection fraction was 69.2± 8.1%. Logistic regression analyses showed no significant association of amygdala volume and any of the cardiovascular parameters ($p >> 0.05$, respectively).
**Conclusion:** In this reference population, MR-based amygdala volume was not associated with markers of cardiovascular disease, potentially indicating an independent role in risk prediction.

**B-0189** 11:18

**Imaging requests, stroke incidence and contributing factors after open-heart surgery**

L. Melazzini, M. Codari, G.D.E. Papini, C. Beretta, M. Ranucci, F. Secchi, F. Sardanelli; *Milan/IT (lucamelazzini@yahoo.it)*

**Purpose:** Stroke is a critical complication of cardiac surgery. In spite of the adoption of neuroprotective strategies, incidence for postoperative stroke remains around 5% for combined cardiac procedures. In this light, we conducted an internal investigation on brain imaging requests, stroke incidence and contributing factors after open-heart surgery at our institution.
**Methods and Materials:** 1014 adults who underwent open-heart surgery between January 2017 and December 2017 were retrieved from our institutional database. Among them, we identified those who underwent head CT or MRI within 7 days from surgery. Their radiological reports were examined to identify cases of stroke. Demographics, anaesthesiological statistics and clinical data were extracted from patients' records and statistically processed to find contributing factors for stroke.
**Results:** 55 patients (5.42% of the study sample) had brain imaging performed within one week after cardiac surgery. Among them, 53 patients underwent head CT scans while two had brain MRI scans too. 18 patients (1.78% of the study sample; PPV of brain scans=32.73%) were found to be radiologically positive for stroke. Stroke was deemed as ischaemic in 17 patients, the one remaining being ischaemic with haemorrhagic transformation. Significant correlations were found between stroke occurrence and previous transient ischaemic attack (p=0.036), diabetes (p=0.001), combined cardiac procedure (p=0.039), use of circulatory arrest ($p<0.001$), creatinine (p=0.029), extracorporeal circulation time (p=0.003) and clamp time (p=0.007).
**Conclusion:** Our results slightly overcome those previously reported in the literature. Given this, stroke persists as a fearsome complication of cardiac surgery, where timely neuroimaging requests play a vital role.

**B-0190** 11:26
**Arterial spin labelling MRI in evaluating perioperative cerebral perfusion of carotid endarterectomy with different post labelling delays: comparison with CT perfusion imaging**
H.M. Xu, Y. Liu, H. Yuan; *Beijing/CN (84498843@qq.com)*

**Purpose:** Taking CT perfusion imaging as a reference standard, to find the appropriate perioperative PLDs which can reflect the accurate cerebral hemodynamics changes after CEA using arterial spin labeling.
**Methods and Materials:** Fourteen patients diagnosed as carotid artery stenosis and scheduled for CEA were recruited. All patients were scanned with two PLDs 3D-ASL (PLD=1.5s and PLD=2.0s) and CTP about 1 week before and after CEA respectively. CBF values were measured respectively in the same position of surgical side and the other side before and after surgery on CBF maps of 3D-ASL and CTP. To calculate perioperative changes of relative CBF values on CTP and 3 different PLD combinations of ASL: perioperative PLD=2.0s, perioperative PLD=1.5s or preoperative PLD=2.0s and postoperative PLD=1.5s.
**Results:** The ICC between these three different PLD combinations of ASL (preoperative PLD=2.0s and postoperative PLD=1.5s; both PLD=1.5s; both PLD=2.0s) and CTP were 0.139, 0.875 and 0.856 respectively. It indicates that the consistency of the first set--preoperative PLD=2.0s and postoperative PLD=1.5s--with CTP was poor, and the consistency of the latter two groups with CTP were good and have no statistically difference. While compared to ASL with PLD of 2.0s, ASL with PLD of 1.5s would underestimates the CBF values since the prolonged arterial transient times.
**Conclusion:** The PLD of arterial spin labeling should be consistent before and after CEA. Compared to ASL with PLD of 1.5, ASL with PLD of 2.0 is a more appropriate to evaluate hemodynamic changes around carotid endarterectomy.

**B-0191** 11:34
**Accuracy of intracerebral haemorrhage volume calculation: comparison between manual volume segmentation, ABC/2 and sABC/2**
A.V. Marinescu, R. Radu, E. Terecoasa, A.N. Marinescu, C. Tiu, A. Nicula; *Bucharest/RO (andrei.victor.marinescu@gmail.com)*

**Purpose:** Accurate estimation of intracerebral haemorrhage (ICH) volume is essential for adequate management decisions. The aim of the study was to determine the accuracy of the ABC/2 and sABC/2 methods relative to manual volume segmentation (MVS) method for estimating ICH volumes.
**Methods and Materials:** Clinical and neuroimaging data were prospectively collected for 47 patients with ICH. Haematoma volume was measured separately by a single examiner using ABC/2, sABC/2 and Carestream MVS. Agreement between these methods was evaluated using Bland-Altman plots and MVS was considered the reference method.
**Results:** Mean patient age was 69.7 ± 11.3 years and 55.3% were males. Median ICH volumes assessed with the MVS, ABC/2 and sABC/2 methods were 12.2 mL, 6.9 mL and 10.7 mL respectively. ICH volumes were systematically underestimated by ABC/2 with a mean difference of -7.6 mL (95% CI -10.6 to -4.47 mL) and systematically overestimated by sABC/2 with a mean difference of 3.6 mL (95% CI 0.31 to 6.9 mL). The variability between the methods increased with larger ICH volumes and therefore, we created second Bland-Altman plots using the geometric means, which showed an ABC/2 to MVS ratio of 0.66 and a sABC/2 to MVS ratio of 1.05. In our cohort, ICH volume percentage difference using ABC/2 and sABC/2 is not expected to exceed 33.72% and respectively 16.9% of the MVS volumes.
**Conclusion:** Absolute haematoma volumes might vary depending on the technique. Further work is needed to identify the most suitable methods for ICH volume measurement and to estimate their clinical impact.

**B-0192** 11:42
**Effect of prolonged acquisition intervals on CT brain perfusion analyses**
F. van Ommen, F. Kauw, E. Bennink, J.W. Dankbaar, H.W.A.M. De Jong; *Utrecht/NL (f.vanommen@umcutrecht.nl)*

**Purpose:** Axial coverage in CT perfusion (CTP) imaging can be increased with toggling table techniques. This, however, prolongs the acquisition interval (AqI) up to 4 seconds. This study investigates the influence of prolonged AqI on quantitative perfusion maps and infarct volumes.
**Methods and Materials:** Quantitative perfusion values (CBF, CBV and MTT) of 25 CTP scans, with an AqI varying from 2-5 seconds, were compared to the 1-second AqI. A commercial method (ISP) (brain perfusion, singular value decomposition algorithm, Intellispace Portal9, Philips Healthcare) and an academic method (NLR) were used for generation of perfusion maps. Infarct core volumes were determined using a CBV threshold and penumbra volumes using a CBV and relative MTT threshold. All AqI-imaging results were qualitatively compared by visual inspection.
**Results:** An AqI-dependent bias was observed although not significant for CBV values up to 4 seconds AqI. Regarding MTT and CBF, ISP provided significantly different values for all AqIs, whereas NLR did not. Infarct core volumes were significantly lower (<8%) for ISP and significantly higher (>29%) for NLR for AqIs of ≥3 seconds. Penumbra volumes were significantly larger (>33%) with ISP for AqIs of ≥3 seconds and for NLR significantly larger (>20%) at AqI of 3 and 4 seconds. Visually, summary maps for ISP and NLR showed minor differences in diagnostic quality up to 4-second interval.
**Conclusion:** An AqI-dependent bias was observed in the quantitative perfusion parameters. The summary maps with intervals up to 4 seconds are of diagnostic quality, suggesting that CTP imaging with a toggling table is feasible.
*Author Disclosures:*
**J.W. Dankbaar:** Grant Recipient; STW: 14732. **H.W.A.M. De Jong:** Grant Recipient; STW: 14732.

**B-0193** 11:50
**Completeness of trauma head computed tomography reports: possibilities for improvement using machine learning**
N. Schmidt, I. Nesic, M. Moor, J. Cyriac, D. Zumofen, B. Stieltjes, K. Blackham; *Basle/CH*

**Purpose:** Regular reevaluation of trauma head CT report quality is of great importance but cannot be performed manually. Here we test a Machine learning (ML) approach for automated completeness detection in reports.
**Methods and Materials:** Literature and neurosurgical suggestions were combined to establish vital items required in a report: intracranial bleeding (ICB), fracture, signs of hydrocephalus, midline position, and vessel status were selected. All CT reports from the last 8 years were retrieved and labelled. ML model creation was done with Python 3.6 with the scikit library. We applied Support Vector Machines (SVM) for item detection. Positive Predictive value (PPV), Sensitivity and F1-score were calculated in comparison to hand labelled reports.
**Results:** 1906 reports were retrievable from our RIS. 146 reports had to be excluded due to missing labelling. 100 of the mentioned reports were also analyzed manually for direct comparison. The results of the SVM indicated the presence of a single critical finding ranging from 63.9% (vessel status) to 92.9% (ICB). In the manual analysis, this range was between 67% (midline) and 99% (ICB). The difference between the manual and the automated detection rate was only statistically significant (p< .5) for ICB and vessels.
**Conclusion:** SVM is suitable for the estimation of level of completeness reports in an automated fashion. It can be implemented as a quality control system and can be trained and applied to any report type. Structured reporting may help to improve the substantial level of incompleteness described here.

**10:30 - 12:00** **Sky High Stage**

### Clinical Trials in Radiology

### CT 2

### Clinical Trials in Radiology 1

*Moderators:*
M. Dewey; Berlin/DE
V.P. Jackson; Tucson, AZ/US

10:30
**Primary stenting of superficial femoral artery in patients with intermittent claudication has durable effects on health-related quality of life at 24 months: results of a randomised controlled trial**
H. Lindgren[1], A. Gottsater[2]; [1]*Helsingborg/SE*, [2]*Malmö/SE (2hanslindgren@gmail.com)*

**Ethics committee approval:** Written informed consent was obtained from all patients. The study was approved by the Medical Ethics Committee of Lund University (Dnr 2009/478).
**Methods and materials**: One hundred patients with stable IC due to SFA disease from seven Swedish hospitals treated with best medical treatment (BMT) were randomised to either the stent (n=48) or the control (n=52) group. Change in HRQoL 24 months after treatment was a primary outcome measure.
**Results:** Significantly better SF-36 Physical Component Summary (mean difference =5, 1; P=.024) and physical domain scores Physical Function (P=.012), Bodily Pain (P=.002), General Health (P=.037), and EQ5D (P=.010) were reported in intergroup and intragroup comparisons between the stent and the control group.
**Limitations:** The long inclusion period and large number of screening failures due to the strict inclusion and exclusion criteria might have created a bias in patient selection
**Conclusion:** In patients with IC caused by lesions in the SFA, primary stenting compared to BMT alone was associated with significant durable improvements in HRQoL, ABI, and walking distance.

**Funding for this study:** This study was supported by grants from the Gorthons Foundation, the Ernhold Lundström Foundation, Regional research funds and funds at Skåne University Hospital, the Albert Påhlsson Foundation, the Hulda Ahlmroth Foundation, Mediel AB, and from the Swedish state under the LUA/ALF agreement.

10:39
**Discussant:**
T.J. Kroencke; *Augsburg/DE (thomas.kroencke@klinikum-augsburg.de)*

10:45
**Ultrasound-guided vs computed tomography-guided renal mass biopsy: a prospective randomised study**
H.M. Farg, A. Abdelhamid, M. Zakaria, M. El-Ksas, A. El Mahdy, M. Abd El-Hameed, T. El-Diasty; *Mansoura/EG (hashim2008_mf@yahoo.com)*

**Purpose:** The purpose of this study was to compare ultrasound (US) vs computed tomography (CT) guidance for renal mass biopsy.
**Ethics committee approval:** The study was approved by the local ethics committee. Written informed consent was obtained from all participants.
**Methods and materials:** This was a single-centre prospective randomised trial comparing US vs CT guidance for renal mass biopsy. Between June 2016 and May 2018, eligible patients with renal masses were randomly assigned in a 1:1 ratio to perform either US- or CT-guided biopsy using 18-gauge automated device. The primary outcome was to evaluate the diagnostic success rate in both groups. Secondary outcomes included total procedure time, total punctures, and complications.
**Results:** A total of 82 patients (men, 57%; mean age, 59.2 years) were included; 41 in each group. The diagnostic success rate was higher in the US group (90%) compared to the CT group (83%); (p <0.001). Four of the patients undergoing US-guided biopsy required re-biopsy compared to 7 patients in CT group; (p <0.001). US significantly shortened the total procedure time (150 seconds) compared to CT group (330 seconds). The complication rate was higher in the CT group (22%) than in the US group (12%). There were no procedure-related deaths in either group.
**Limitations:** Failed biopsy is not usually related to the image guidance procedure and it may be related to sampling error resulting from tumour haemorrhage or necrosis.
**Conclusion:** Ultrasound-guided renal mass biopsy has better diagnostic accuracy, less procedural time and lower complication rate than that of CT guidance.
**Funding for this study:** No funding from industry and/or a funding agency.

10:54
**Discussant:**
N.N.

11:00
**Self-compression mammography in clinical practice: a randomised clinical trial compared to standard compression mammography**
V. Iotti, M. Guberti, L. Canovi, P. Giorgi Rossi, M. Bertolini, R. Vacondio, P. Pattacini; *Reggio Emilia/IT (valentina.iotti@ausl.re.it)*

**Purpose:** Self-compression for mammography may help reaching better compression, with a potential improvement of image quality and reduction of dose. To test the efficacy of a self-compression device, compared to radiographer-lead compression, to reduce the average-glandular-dose without affecting image quality. Secondary outcomes are pain, compression, thickness, time to perform mammography. Here we present preliminary results.
**Ethics committee approval:** Approved by the provincial Ethical Committee. All participants signed a written informed consent form.
**Methods and materials:** Women presenting for mammography for breast-cancer follow-up, symptoms, opportunistic screening or familial-risk were asked to participate in this prospective randomised trial and randomised to self-compression or to radiographer-compression. Anxiety was measured before mammography; pain (visual-assessment-scale) and discomfort were measured after. Image quality was assessed blindly by two independent radiologists and two radiographers. Room-in/room-out time has been measured.
**Results:** From January2018 to July2018, 496 women were enrolled, 245 and 251 in self- and radiographer-compression arm, respectively. Image quality (radiologists' judgement p=0.62; radiographers' judgement p=0.34, slightly better in self-compression for both), dose (0.94 vs. 0.96mGy, p=0.38) and pain (3.8 vs. 4.0, p=0.50) were similar in the two arms; compression was higher in self- than radiographer-arm (114 vs. 102daN, p<0.00005), with a small impact on thickness (46.4 vs. 48.1mm, p=0.13). Moderate/severe discomfort was reported by 7.9% vs 9.9% (p=0.46). Time was slightly higher 11.5 vs.10.2m (p=0.06). Results were similar in women with higher anxiety level.
**Limitations:** Screening population was not included.
**Conclusion:** Self-compression has no impact on dose, pain and discomfort, even if increases compression.
**Funding for this study:** Sustained by the institutional funds of the Reggio Emilia Local Health Authority–IRCCS.

*Author Disclosures:*
**V. Iotti:** Speaker; GE-Healthcare. **L. Canovi:** Speaker; Healthcare.
**R. Vacondio:** Speaker; GE-Healthcare. **P. Pattacini:** Speaker; Healthcare.

11:09
**Discussant:**
A. Oktay; *Izmir/TR (aysenur.oktay@ege.edu.tr)*

11:15
**MRI vs mammography screening for high-risk women: an RCT - the influence of density and effectiveness**
A. Geuzinge[1], S. Saadatmand[1], E. J. J. Rutgers[2], R.M. Mann[3], D. de Roy van Zuidewijn[4], H. Zonderland[2], R. Tollenaar[5], M.B.I. Lobbes[6], M. Ausems[7], M. van 't Riet[8], M. Hooning[1], I. Mares-Engelberts[1], E. Luiten[9], E. Heijnsdijk[1], C. Verhoef[1], N. Karssemeijer[3], J. Oosterwijk[10], I.-M. Obdeijn[1], H. de Koning[1], M. Tilanus-Linthorst[1]; *[1]Rotterdam/NL, [2]Amsterdam/NL, [3]Nijmegen/NL, [4]Leeuwarden/NL, [5]Leiden/NL, [6]Maastricht/NL, [7]Utrecht/NL, [8]Delft/NL, [9]Breda/NL, [10]Groningen/NL (madeleinetilanus@hotmail.com)*

**Purpose:** American guidelines advise women with a familial breast cancer risk MRI-screening, but European mammography. FaMRIsc is the first randomised controlled trial comparing both strategies.
Ethics committee approval: The IRB of ErasmusUniversityMC approved FaMRIsc. All participants gave written informed consent.
**Methods and materials:** From January 2011 until December 2017, women with a cumulative lifetime risk (CLTR) of ≥20% without a BRCA1/2 mutation, aged 30–55 years, were in twelve Dutch centres randomised into two arms: A. yearly MRI-screening, clinical breast examination (CBE), and biennially mammography or B. yearly mammography and CBE. Primary outcomes were number, size and nodal stage of the detected breast cancers. Results were also stratified by mammographic density.
**Results:** More breast cancers were detected in the MRI-arm (N=675) compared to the Mx-arm (N=680) (41 vs 14, p<0.001), after on average 4.3 screening rounds per woman. In the MRI-arm invasive cancers were smaller (median size 8 vs 17 mm, p=0.006) and less often node positive (20% vs 71.4%, p=0.019). Sensitivity hardly differed (95.1% vs 92.9%, p=1), but false positive rate was higher in MRI-arm (160 vs 89.9/1000 woman-year at risk p<0.001). All tumours ≥T2 were in the two highest density categories. In ACR density A-C MRI detected more small tumours than mammography. Results of incident rounds and cost will be discussed.
**Limitations**: are discussed
**Conclusion:** More cancers were detected in the MRI-arm than in the mammography-arm and in a relevantly earlier stage also at low density. We will discuss at what cost.
**Funding for this study:** This study was supported by ZonMw, The Dutch Cancer Society, A Sisters Hope, Pink Ribbon, Stichting Coolsingel.

11:24
**Discussant:**
R. Schulz-Wendtland; *Erlangen/DE (ruediger.schulz-wendtland@uk-erlangen.de)*

11:30
**Variability in cancer-positive predictive value using PI-RADS v2: a multi-institutional study conducted by the SAR Prostate Cancer Disease Focused Panel (aim 2)**
A. C. Westphalen[1], A. B. Rosenkrantz[2], C. McCulloch[1], J. Anaokar[3], S. Arora[4], N. Barashi[5], J.O. Barentsz[6], T.K. Bathala[7], L. Bittencourt[8], M. T. Booker[9], V.G. Braxton[4], D.D. Casalino[5], S.D. Chang[10], F.V. Coakley[11], R. Dhatt[10], S.C. Eberhardt[12], B.R. Foster[11], A. Froemming[13], J.J. Futterer[6], M.R. Gertner[14], L.M. Gettle[15], S.Ghai[14], R.T. Gupta[16], M.E. Hahn[9], R. Houshyar[17], C. Kim[18], C.K. Kim[19], C. Lall[17], D. Margolis[2], S.E. McRae[7], A. Oto[5], R. Parsons[3], N.U. Patel[20], T.J. Polascik[16], J.B. Starcevich[12], B. Spilseth[21], V.S. Tammisetti[7], S.S. Taneja[2], S. Verma[22], C.A. Warlick[21], A.R. Weinberger[5], J. Yu[18], R.J. Zagoria[1]; *[1]San Francisco, CA/US, [2]New York City, NY/US, [3]Philadelphia, PA/US, [4]Nashville, TN/US, [5]Chicago, IL/US, [6]Nijmegen/NL, [7]Houston TX/US, [8]Rio de Janeiro/BR, [9]San Diego, CA/US, [10]Vancouver, BC/CA, [11]Portland OR/US, [12]Albuquerque, NM/US, [13]Rochester, MN/US, [14]Toronto, ON/CA, [15]Madison, WI/US, [16]Durham, NC/US, [17]Orange, CA/US, [18]Richmond, VA/US, [19]Seoul/KR, [20]Aurora, CO/US, [21]Minneapolis, MN/US, [22]Cincinnati, OH/US (antonio.westphalen@ucsf.edu)*

**Purpose:** To determine the variability of positive predictive value (PPV) across centres according to PI-RADS v2 scores.
**Ethics committee approval:** IRB-approved, HIPPA-compliant study, with waiver of signed informed consent.
**Methods and materials:** This retrospective included 3,479 men who underwent prostate mpMRI at 26 DFP-member expert centres from several countries and had a lesion classified as PI-RADS ≥ 2. Exams scored as PI-RADS 1 (i.e. no visible lesion) were not included. MR-targeted biopsy results were the standard of reference. PPV were derived from generalised estimating equations regression to estimate variability across centres.

Wednesday

**Results:** Across all centres, the PPV for PI-RADS score ≥ 2 was 33.8% (range: 12.9%, 68.6%), for PI-RADS ≥ 3 was 37.5% (range, 14.7%, 68.6%), and for PI-RADS ≥ 4, 48.4% (26.4%, 75.9%). The PPV stratified by PI-RADS scores were as follow: score 2=6.9% (range, 4.2%, 50.0%); score 3=15.3% (3.2%, 54.0%), score 4=38.4% (17.7%, 70.0%), and score 5=71.9% (40.0%, 97.1%).
**Limitations:** Outcomes based on biopsy modalities that are different and not perfect.
**Conclusion:** Enormous variation in the PPV exists across expert centres, and the overall PPV among these centres was alarmingly low. Based on the results, a PPV ≥ 50% for PI-RADS v2 score ≥ 4 is proposed as a performance standard, with centres below this benchmark warranting quality improvement efforts. Many of the participating centres did not achieve this threshold.
**Funding for this study:** University of California San Francisco, Department of Radiology and Biomedical Imaging Seed Grant # 16-43.
Author Disclosures:
**A.C. Westphalen:** Advisory Board; 3D Biopsy LLC. Research/Grant Support; GE Healthcare. **A.B. Rosenkrantz:** Other; Thieme Medical Publishers.

11:39
**Discussant:**
H.Y. Kressel; *Boston, MA/US (hkressel@rsna.org)*

11:45
**Role of PI-RADS v2 in clinical models: a multi-institutional study conducted by the SAR Prostate Cancer Disease Focused Panel (aim 3)**
A.C. Westphalen[1], A.B. Rosenkrantz[2], C. McCulloch[1], J. Anaokar[3], S. Arora[4], N. Barashi[5], J.O. Barentsz[6], T.K. Bathala[7], L. Bittencourt[8], M.T. Booker[9], V.G. Braxton[4], D.D. Casalino[5], S.D. Chang[10], F.V. Coakley[11], R. Dhatt[10], S.C. Eberhardt[12], B.R. Foster[11], A. Froemming[13], J.J. Futterer[6], M.R. Gertner[14], L.M. Gettle[15], S. Ghai[14], R.T. Gupta[16], M.E. Hahn[9], R. Houshyar[17], C. Kim[18], C.K. Kim[19], C. Lall[17], D. Margolis[2], S.E. McRae[7], A. Oto[5], R. Parsons[3], N.U. Patel[20], T.J. Polascik[16], J.B. Starcevich[12], B. Spilseth[21], V.S. Tammisetti[7], S.S. Taneja[2], S. Verma[22], C.A. Warlick[21], A. R. Weinberger[5], J. Yu[18], R.J. Zagoria[1]; [1]*San Francisco, CA/US,* [2]*New York City, NY/US,* [3]*Philadelphia, PA/US,* [4]*Nashville, TN/US,* [5]*Chicago, IL/US,* [6]*Nijmegen/NL,* [7]*Houston TX/US,* [8]*Rio de Janeiro/BR,* [9]*San Diego, CA/US,* [10]*Vancouver, BC/CA,* [11]*Portland OR/US,* [12]*Albuquerque, NM/US,* [13]*Rochester, MN/US,* [14]*Toronto, ON/CA,* [15]*Madison, WI/US,* [16]*Durham, NC/US,* [17]*Orange, CA/US,* [18]*Richmond, VA/US,* [19]*Seoul/KR,* [20]*Aurora, CO/US,* [21]*Minneapolis, MN/US,* [22]*Cincinnati, OH/US (antonio.westphalen@ucsf.edu)*

**Purpose:** To determine if PI-RADS v2 improves the prediction of high Gleason score prostate cancer beyond PSA, PSA density (PSAD), and clinical stage (T1C versus T2/T3).
**Ethics committee approval:** IRB-approved with waiver for signed consent.
**Methods and materials:** This retrospective study included 3,479 men who underwent prostate MRI at one of 26 institutions in the USA, Canada, Brazil, South Korea, and Netherlands. Up to 4 lesions/case were classified using PI-RADS. Cognitive, fusion, and in-bore MR-targeted biopsies were performed. Data was collected using REDCap. The impact of PI-RADS on clinical variables was determined using logistic regressions derived area under the ROC curves. Confidence intervals were calculated using cluster-corrected (to account for multiple lesions/person) bootstrapping.
**Results:** PI-RADS score, logPSA, logPSAD, and palpable nodule were predictors of GS≥3+4 on uni- (all P<0.001) and multivariate analyses (palpable nodule=0.03, others P<0.001). The PI-RADS AU-ROC was 73.9% (72.6%, 75.3%). Adding PI-RADS to the clinical model raised the AU-ROC from 60.7% (59.1%, 62.2%) to 80.8% (79.5%, 82.2%). The differences between the AU-ROC of the PI-RADS and combined model (6.8%; 5.8%, 7.9%), and of the clinical and combined model (20.1%; 19.0%, 21.4%) were statistically significant (P < 0.001).
**Limitations:** All centres are expert sites, so results may not apply to all locales.
**Conclusion:** PI-RADS predicts GS≥3+4 prostate cancer better than clinical variables, but its performance may be enhanced by knowledge of PSA, PSAD, and/or presence of palpable nodule.
**Funding for this study:** UCSF Department of Radiology Seed Grant # 16-43.
*Author Disclosures:*
**A.C. Westphalen:** Advisory Board; 3D Biopsy LLC. Research/Grant Support; GE Healthcare. **A.B. Rosenkrantz:** Other; Thieme Medical Publishers.

11:54
**Discussant:**
E. Rud; *Oslo/NO (erikrud@yahoo.no)*

**10:30 - 12:00** **Tech Gate Auditorium**

### Cardiac

### SS 203

### Imaging before and after aortic valve repair

*Moderators:*
C. Celeng; Utrecht/NL
N. Gagarina; Moscow/RU

**K-06** 10:30
**Keynote lecture**
J.-N. Dacher; *Rouen/FR*

**B-0194** 10:39
**Gender-specific reference values of thoracic aortic growth**
L.R. Bons[1], Z. Sedghi Gamechi[1], K.F. Kofoed[2], J.H. Pederson[2], R.P.J. Budde[1], M. de Bruijne[1], J.W. Roos-Hesselink[1]; [1]*Rotterdam/NL,* [2]*Copenhagen/DK (l.bons@erasmusmc.nl)*

**Purpose:** Longitudinal information about individual aortic growth in the general population is scarce. The aim of this study is to present reference values for thoracic aortic growth among men and women.
**Methods and Materials:** Participants of the Danish Lung Cancer Screening Trial with two non-contrast computed tomography at least one year apart were included. The study consisted of current and former smokers aged 50-70 years. The diameters of the ascending aorta (AA) and descending aorta (DA) were measured at the pulmonary bifurcation level with an automatic tool. Mean annual aortic growth and 95th percentile (95th) was calculated between the first and last scan during follow-up. Associations between aortic growth and baseline parameters were investigated using multivariable linear regression analysis.
**Results:** A total of 1987 participants (56% men, mean age 57.4±4.8 years) were included. During a median follow-up of 4.0 years, the AA growth was similar in men (mean 0.12±0.31, 95th 0.47 mm/year) and women (mean 0.11±0.29, 95th 0.42 mm/year). Also the DA growth was not significantly different between men (mean 0.10±0.30, 95th 0.43 mm/year) and women (mean 0.13±0.27, 95th 0.42 mm/year). Larger AA growth was independently associated with increased height, lower weight and smaller AA baseline diameter, while larger DA growth was associated with increased height, absence of antihypertensive medication, larger AA Agatston scores and smaller DA baseline diameter.
**Conclusion:** This longitudinal population-based study shows that ascending and descending aorta growth is 0.1 mm/year. We found no difference in thoracic aortic growth between men and women.
*Author Disclosures:*
**L.R. Bons:** Founder; Dutch Heart Foundation (2003B179). **Z. Sedghi Gamechi:** Founder; the Iranian Ministry of Science, Research, and Technology (MSRT), the Netherlands Organisation for Scientific Research (NWO), the Danish Ministry of Interior and Health.

**B-0195** 10:47
**Aliased flow planimetry by phase-contrast CMR imaging for grading of aortic stenosis**
C. Mantini[1], F. Ricci[1], D. Mastrodicasa[2], D. Rotondo[1], A.R. Cotroneo[1], S. Gallina[1]; [1]*Chieti/IT,* [2]*Stanford, CA/US (cesare.mantini@gmail.com)*

**Purpose:** The aim of this prospective, observational, cross-sectional study was to: I) test the accuracy and precision of a new, simple, non-invasive CMR technique to measure the effective orifice area (EOA) based on aliased orifice area(AOA) planimetry by low-VENC phase-contrast CMR imaging; II) investigate the relationship and the diagnostic agreement of AOA with: valve area planimetry by bSSFP-CMR images($GOA_{cmr}$), standard EOA obtained with continuity equation by TTE ($EOA_{echo}$) and gold standard hybrid EOA.
**Methods and Materials:** We prospectively enrolled 22 consecutive patients (14 men, mean age 70 ± 9 years)with mild, moderate and severe aortic stenosis(AS) and 6 age-and sex matched healthy controls undergoing TTE and CMR imaging on the same day.We performed a comprehensive analysis of the agreement and correlation among i)AOA planimetry by low-VENC phase-contrast CMR imaging, ii)GOA valve planimetry by CMR, iii)EOA by TTE(continuity equation method), and iv) multimodality hybrid EOA ($EOA_{hybrid}$), as obtained by substituting the LVOT area by CMR into the Doppler continuity equation.A phase-contrast slice was prescribed through the tips of aortic valve leaflets, then by setting the VENC value down to40-50 cm/s,with NEX of 5 and free-breathing acquisition, an aliased flow signal was detected.

**Results:** We observed excellent pairwise correlation among AOA, $EOA_{hybrid}$,GOA and EOA (P<0.001), AOA yielding the highest grade of correlation with $EOA_{hybrid}$ ($R^2$=0.987, P<0.001).At exploratory reclassification analysis, 2 patients with severe AS by AOA and 3 patients with severe AS by EOA were reclassified downward to moderate AS by $EOA_{hybrid.}$
**Conclusion:** AOA planimetry by low-VENC phase-contrast imaging is a simple, reproducible, accurate and precise technique for the grading of AS.

**B-0196** 10:55
**CT as a one stop shop for TAVI-Planning: do we still need an additional invasive coronary angiography?**
R.F. Gohmann, C. Krieghoff, P. Seitz, C. Lücke, P. Hartung, M. Abdel-Wahab, D. Holzhey, H. Thiele, M. Gutberlet; *Leipzig/DE*

**Purpose:** To evaluate the suitability of CT coronary angiography (cCTA) to exclude significant CAD during pre-TAVI-evaluation.
**Methods and Materials:** Two-hundred consecutive patients undergoing pre-TAVI-evaluation (97 female; mean-age 79.6±7.4 years) were retrospectively included. All patients were examined with a standard protocol consisting of a retrospectively gated CT scan of the heart, immediately followed by a high-pitch scan of the vascular access route utilizing a single bolus of 70 ml iodinated contrast-medium. No betta-blockers or nitrates were applied. Heart-rate and heart-rate-variability during the scan were 73.2±19.3 and 22.6±32.8 beats-per-minute; attenuation at the ascending was 456.6±142.5 HU. Images were evaluated for significant CAD (stenosis ≥50%); examinations where stenoses could not be excluded were red as positive. Routinely all patients received invasive coronary angiography (ICA) 79.5% (159/200), which was omitted if renal function was impaired significantly and no significant stenosis could be identified on cCTA.
**Results:** cCTA was negative for significant CAD in 44.0% (88/200). Sensitivity, specificity, NPV, PPV and accuracy were 96.3%, 62.0%, 94.2% 72.0%, 79.3%, respectively. The significant stenoses additionally identified on ICA were either located in side-branches (2/3) or the distal LAD; 2/3 had no identifiable plaque on cCTA.
**Conclusion:** cCTA and pre-TAVI-evaluation can be performed jointly with no need for additional contrast medium or medication. cCTA is able to exclude significant CAD in a relatively high proportion of this high-risk collective. Thereby cCTA can reduce the number of ICA and total amount of contrast-medium applied, making pre-procedural-evaluation for TAVI-Planning safer for elderly patients with a high incidence of nephropathy.

**B-0197** 11:03
**Reduced dose, reduced contrast single-acquisition TAVI imaging using novel contrast protocol and spectral low-mono-energetic imaging: a proof of concept study**
P.A. Ball; *Belfast/UK (peterandrewball@me.com)*

**Purpose:** To investigate whether a low-dose low-contrast protocol can still produce excellent diagnostic images with spectral CT
**Methods and Materials:** 20 sequential patient referred for pre-TAVI-gated CT planning were imaged using a novel protocol. 50ml Ioversol 300mg/ml was initially injected at 5ml/s using linear bolus reduction (reducing to 4.2ml/s). A delay of 2 seconds was built in to the protocol prior to a second injection of 20ml of contrast at 4ml/s. The initial gated CTCA was performed using a retrospective acquisition protocol with reconstructions at both 40 and 75% (systolic and mid-diastolic phase). Run off was assessed using mono-energy reconstructions of the aorta iliac and femoral vessels. Doses and contrast administration volume were compared with the previous acquisition protocol along with image quality and reproducibility (patients were compared on a like-for-like basis for patient habits, length of scan, etc.).
**Results:** There was a significant dose reduction from an average of 1750 DLP to an average of 460DLP (p<0.01) This is primarily due to the avoidance of overlapping low-pitch acquisition of the abdominal structures. The image quality of the gated study for annular measurement and run off was assessed by a 5-point Likert scale by two experienced cardiothoracic radiologists. There was a excellent level of agreement of image quality (k 0.76).
**Conclusion:** The protocol using low-mono-energetic CT images provides excellent image quality with a significantly reduced radiation and contrast dose in high-risk patients undergoing TAVI.

**B-0198** 11:11
**Low iodine, fast kV switching dual energy thoraco-abdominal CT angiography for planning of transcatheter aortic valve implantation (TAVI): a pilot feasibility study**
L. Faggioni, M. Gabelloni, M. Bianchi, E. Neri, D. Caramella; *Pisa/IT (lfaggioni@sirm.org)*

**Purpose:** To evaluate the image quality of thoraco-abdominal fast kV switching dual energy CT angiography (DE-CTA) examinations performed for TAVI planning using a low iodine protocol and reconstructed at different monochromatic energy levels.
**Methods and Materials:** Forty nonobese patients with severe aortic stenosis candidate to TAVI underwent thoraco-abdominal DE-CTA for preprocedural assessment of vascular access routes on a high definition, 64-row fast kV switching DECT scanner. All patients received an average volume of 35mL (30-40mL) of an iso-osmolar, moderate concentration (320mgI/mL) iodinated contrast medium. Intra-arterial density was measured on 40keV, 50keV, 60keV, 70keV and 80keV monochromatic images at the same levels inside the ascending thoracic and infrarenal abdominal aorta. The monochromatic dataset with the best overall visual image quality was generated, and the occurrence of extravascular incidental findings was recorded.
**Results:** Intra-arterial density and signal-to-noise ratio increased monotonically with decreasing energy levels, ranging from 150±38 HU and 6.10±1.78 at 80keV to 620±164 HU and 9.97±3.70 at 40keV, respectively. Image noise was highest at 40keV (66±16 HU) and lowest at 70keV (24±6 HU). Monochromatic datasets between 50keV and 60keV yielded an intra-arterial density consistently above 200HU (217-576 HU), along with the best balance among vascular enhancement, noise, and blooming artefacts from densely calcified plaques. Incidental findings occurred in 17/40 patients (42.5%), of which 4 (3 advanced cancers, 1 pulmonary embolism) significantly altered patient management.
**Conclusion:** Pre-TAVI thoraco-abdominal DE-CTA is feasible using a low iodine, fast kV switching approach. 50keV to 60keV reconstructed monochromatic images provide the best overall diagnostic quality.

**B-0199** 11:19
**Psoas muscle cross-sectional areas and aortic valve calcification in patients enrolled for transcatheter aortic valve implantation (TAVI)**
G. Spadarella, L. Bonomo, C.B. Monti, F. Sardanelli, F. Secchi; *Milan/IT (gaia.spadarella@gmail.com)*

**Purpose:** To evaluate the correlation between psoas muscle cross-sectional areas (PCSA) as a marker of central sarcopenia and aortic valve calcification.
**Methods and Materials:** 77 patients from 2015 to 2017 were retrospectively collected from our pre TAVI registry. In the abdominopelvic CT scan, the right and left PCSA was calculated as well as the ratio between the mean cross-sectional area of the psoas muscles and the L4 vertebral body immediately below the origin of the posterior elements. In the thoracic ECG-trigger CT scan aortic valve and anulus calcification (AC) were measured as calcium in $mm^2$ (ACmm) or as Agatson score (ACA). Spearman test was used to test the correlation between these two variables.
**Results:** Of the 77 patients the mean age was 81±8.2 years (82±7.5 in female and 79±8.61 in males). PCSA was 0.71±0.20 overall and 0.79±0.22 in males and 0.63±0.13 in females (p<0.01). ACmm was 1869±1419 overall and 1999±1171 in males and 1736±1640 in females (p=0.12). Similarly, as expected, ACA was 2404±1777 overall and 2544±1395 in males and 2261±2109 in females (p=0.07). No significant correlation was found between PCSA and ACmm and ACA for all patients (r=-0.154 p=0.19; r=-0.148 p=0.215 respectively). A significant negative correlation was found in male patients between PCSA and ACmm and ACA (r=-0.341 p=0.04; r=-0.351 p=0.03). No correlation was observed in female patients for PCSA and ACmm and ACA (r=-0.27 p=0.11; r=-0.29 p=0.09).
**Conclusion:** PCSA can be a useful tool to evaluate cardiovascular risk in male patients undergoing TAVI procedures.

**B-0200** 11:27
**Radiation dose and the repeatability of measurements of dimensions of the aortic valve in multidetector computed tomography performed routinely in the qualification procedure for TAVI**
B. Kedzierski, P. Gac, M. Glosna, R. Poreba, K. Pawlas; *Wroclaw/PL (bkedzierski@wp.pl)*

**Purpose:** The purpose of the study was to evaluate the relationships between the radiation dose and the repeatability of aortic valve dimensions measurements in multidetector computed tomography (MDCT) performer in the qualification for transcatherer aortic valve implantation (TAVI).
**Methods and Materials:** The study was conducted on a group of 60 patients who underwent the MDCT examination in the qualification for TAVI. The radiation dose was expressed using the computed tomography dose index volume (CTDIvol) and the dose length product (DLP). The coefficient of variation (CV) of each aortic valve dimensions measurement was defined as standard deviation of measurements/average value of measurements *100%, based on the measurements performed independently by two radiologists.
**Results:** The highest CV of measurement occurred for the distance between the outlet of the left coronary artery and the aortic ring (9.24±5.17%). The lowest CV of measurement was observed for the aortic bulb average dimension (2.39±1.72%). A significant negative correlation was observed between DLP and CV of the aortic ring minimum dimension measurement (r=-0.25, p<0.05). Regression analysis showed that lower doses of DLP are an independent factor associated with a higher CV of measurement of the minimum dimension of the aortic ring.

**Conclusion:** Due to the demonstrated some relationships between the radiation dose in the MDCT examination performed in the qualification for TAVI and the repeatability of aortic valve dimensions measurements, it is postulated that attempts to perform examination using lower radiation doses should be followed by a subsequent control of the repeatability of aortic valve dimensions measurements.

**B-0201** 11:35
**Dysfunction and thrombosis of aortic valve prostheses in patients after TAVR in computed tomography**
L. Lehmkuhl[1], J. Grimm[2], I. Diamantis[3], M. Wutschke[2], K. Lenk[2], M. Franz[2], C. Schulze[2], U.K. Teichgräber[2]; [1]*Bad Neustadt/DE*, [2]*Jena/DE*, [3]*Weimar/DE* *(lukas.lehmkuhl@campus-nes.de)*

**Purpose:** Aim of the study was to determine the incidence of dysfunction and thrombosis of aortic valve prosthesis in patients after TAVR and to systematically evaluate the prosthesis position and deployment in CT.
**Methods and Materials:** A total of 60 consecutive patients after TAVR (33 Edwards Sapien 3, 22 CoreValve EvolutR and 5 ACURATEneo) were included. All patients received echocardiography and cardiac CT during the 30-day follow-up. The post-TAVR CT protocol included multiphasic cardiac CT (256 Slice GE Revolution, ECG triggering). For image analysis, MPRs were prepared parallel to the aortic valve prosthesis and LVOT axis in cine mode. The following parameters were assessed: Degree of prosthesis deployment (min./max. Diameter, ≥0.9 = circular deployment), prosthesis position in relation to aortic annulus and coronary ostia, motility of the cusps and cusp thrombosis.
**Results:** One third of the prostheses (20/60) were not circularly deployed, the mean degree of development was 0.91 ± 0.06 (0.75-0.99). In 18/60 cases (30%) there was a partial overlap of the left and right coronary ostium, of which both ostia were affected in 8/18 cases. Limited motility of the cusps as well as partial thrombosis could be observed in 6/60 cases (10%), motility restrictions without thrombosis did not occur. The cups thromboses had not previously been detected in echocardiography.
**Conclusion:** In addition to echocardiography, post-TAVR-CT detects a high incidence of motility restriction and partial thrombosis of the prosthesis. Moreover, prosthesis deploiment and position deviates in a relevant percentage from the theoretically optimal implantation result.

**B-0202** 11:43
**Biological versus mechanical aortic valve prostheses: an in vitro 4D flow MRI comparison of haemodynamics**
T.H. Oechtering, A. Panagiotopoulos, M. Sieren, M. Scharfschwerdt, B. Fujita, S. Ensminger, H.-H. Sievers, J. Barkhausen, A. Frydrychowicz; *Lübeck/DE* *(thekla.oechtering@uksh.de)*

**Purpose:** In vitro 4D flow MRI evaluation of haemodynamics distal to mechanical and biological aortic valves.
**Methods and Materials:** Five valve prostheses with 21mm outer diameter [BV= biological valves: Perimount Magna Ease (Carpentier-Edwards), Trifecta valve (Abbott), Tribio (protoype); MV= mechanical valves: trileaflet valve (prototype), On-X (CryoLife)] were scanned at 3T in a flexible silicone aortic phantom. A home-built piston pump pumped blood mimicking fluid at physiological pressure settings with 60bpm. Quantitative haemodynamic parameters were analysed using GTFlow (v.3.1.13, GyroTools, CH), average results ± 2SD from twelve healthy volunteers (VOL, 55±6 years) served as normal reference values. Semi-quantitative grading of visualized secondary flow patterns was achieved on a 0-3 scale.
**Results:** Absolute stroke volumes in MV and BV were within normal limits (VOL=105±25ml, MV=99±11ml, BV=111±3ml) confirming near-physiologic settings in the phantom setup. In contrast to VOL and MV, there was a pronounced central ejection jet distal to BV that hit the outer curvature of the ascending aorta where peak velocities were higher in BV=170±22cm/s compared to VOL=91±13 cm/s and MV=90±24cm/s. MV had more pronounced sinus vortices while there were no sinus vortices distal to BV (median[25%/75%]: VOL=1[1,2], MV=3[3,3], BV=0[0,0]). While VOL and MV developed few or no secondary flow patterns, respectively, there were large secondary helices and vortices distal to all BV.
**Conclusion:** Biological valves showed disturbed flow with the ejection jet colliding with the outer curvature. Whether this increases the risk of aneurysm formation through wall shear stress changes remains speculative. In contrast, mechanical valves' haemodynamics resembled those of healthy volunteers.
*Author Disclosures:*
**B. Fujita:** Equipment Support Recipient; Abbott. **H. Sievers:** Patent Holder; Trileaflet and Tribio valve.

**B-0203** 11:51
**Aortic valve bypass surgery in severe aortic valve stenosis: insights from cardiac and brain magnetic resonance imaging**
C. Mantini[1], M. Caulo[1], D. Mastrodicasa[2], D. Marinelli[1], A. Tartaro[1], A.R. Cotroneo[1], G. Di Giammarco[1]; [1]*Chieti/IT*, [2]*Stanford, CA/US* *(cesare.mantini@gmail.com)*

**Purpose:** To investigate and describe the distribution of aortic and cerebral blood flow (CBF) in patients with severe valvular aortic stenosis (AS) before and after aortic valve bypass (AVB) surgery.
**Methods and Materials:** We enrolled 10 consecutive patients who underwent AVB surgery for severe AS. Cardiovascular magnetic resonance imaging (CMR) and brain magnetic resonance imaging were performed as baseline before surgery and twice after surgery. Quantitative flow measurements were obtained using 1.5-T magnetic resonance imaging (MRI) scanner phase-contrast images of the ascending aorta, descending thoracic aorta(3 cm proximally and distally from the conduit-to-aorta anastomosis), and ventricular outflow portion of the conduit.The evaluation of CBF was performed using 3.0-T MRI scanner arterial spin labelling (ASL) through sequences acquired at the gray matter,dorsal default-mode network,and sensorimotor levels.
**Results:** Conduit flow,expressed as the percentage of total antegrade flow through the conduit, was 63.5±8% and 67.8±7% on early and mid-term postoperative CMR, respectively (P<.05). Retrograde perfusion from the level of the conduit insertion in the descending thoracic aorta toward the aortic arch accounted for 6.9% of total cardiac output and 11% of total conduit flow. No differences were observed between preoperative and postoperative CBF at the gray matter,dorsal default-mode network,and sensorimotor levels (P = .394).
**Conclusion:** After AVB surgery in patients with severe AS,cardiac output is split between the native left ventricular outflow tract and the apico-aortic bypass,with two-thirds of the total antegrade flow passing through the latter and one-third passing through the former.In our experience, CBF assessment confirms that the flow redistribution does not jeopardize cerebral blood supply.

**10:30 - 12:00** **The Church**

### GI Tract

### SS 201b

### New developments in anorectal imaging

*Moderators:*
F. Iacobellis; Naples/IT
A. Maier; Vienna/AT

**B-0204** 10:30
**Correlation between T2WI texture features of primary tumour and nodal metastasis in rectal cancer**
I. Yang, B. Wu; *Chengdu/CN (lqyang95@163.com)*

**Purpose:** To explore the potential relationship between nodal metastasis state and the texture parameters of T2WI in primary rectal cancer.
**Methods and Materials:** 69 rectal cancers which directly received radical surgeries between July 2017 and June 2018 were enrolled retrospectively. Axial T2WI were loaded into the texture analysis software, and whole-tumour ROIs were manually drawn by two radiologists independently, then texture parameters were automatically generated. Pathological results of nodal stage were the reference standard. Interobserver agreement was evaluated. The difference of texture parameters between nodal positive (N+) and negative (N-) groups were compared, diagnostic performance and the correlation with nodal state were analysed.
**Results:** The interobserver agreement was moderate to excellent for texture parameters (ICC= 0.555-0.998). The kurtosis (P=0.002), skewness (P<0.001) and energy (P=0.009) of primary tumour were significantly higher in N- group (n=47), and the entropy (P=0.041) was higher in the N+ group (n=22). The respective AUCs of kurtosis, skewness, energy and entropy in diagnosing nodal metastasis was 0.732, 0.78, 0.652 and 0.636. Both kurtosis and skewness showed better diagnostic performance than energy and entropy (all P<0.05). With a cut-off value of 1.97 and 1.148 of kurtosis and skewness, they revealed sensitivities of 68.2% and 95.5%, specificities of 76.6% and 51.1%, respectively. Both kurtosis and skewness had significant negative correlations with nodal state (rs=-0.375 and -0.453, respectively).
**Conclusion:** Kurtosis and skewness of primary tumour on T2WI correlated with nodal state in rectal cancer. Texture analysis of T2WI may give additional information on assessing the nodal metastasis in rectal cancer.

**B-0206** 10:38
**Quantitative elastography of rectal lesions: the value of shear-wave elastography (SWE) in identifying benign and malignant rectal lesions**
T. Li; *Chengdu/CN (635598031@qq.com)*

**Purpose:** We evaluated the diagnostic value of shear-wave elastography (SWE) in identifying benign and malignant rectal lesions.
**Methods and Materials:** Reviewed of 96 lesions, endorectal ultrasound (ERUS) and SWE examination were performed prior to operation. Elasticity parameters including mean elastographic index (Emean), maximum elastographic index (Emax), minimum elastographic index (Emin) were analysed. Correlation between elastographic parameters and histopathological results were studied. Interobserver and intraobserver agreements were analysed.
**Results:** Of the 96 rectal lesions, 72 were malignant and 24 were benign lesions. Compared with ERUS, ERUS with SWE achieved higher sensitivity (93.0% vs. 88.9%), specificity (83.3% vs. 79.2%), PPV (94.4% vs. 92.7%), NPV (80.0% vs. 70.4%), and overall accuracy (90.6% vs. 86.4%). In ROC curve analysis, Emean and Emax achieved larger the area under the curve (AUC), 0.92 for Emean and 0.91 for Emax, respectively. The optimal cut-off value was 61.3KPa for Emean (sensitivity, 88.9%; specificity, 87.5%), 63.4KPa for Emax (sensitivity, 94.4%; specificity, 83.3%). We found a kappa value of 0.83 (95% CI:0.72-0.95) for ERUS and 0.90 (0.81-0.99) for ERUS with SWE of differential diagnosis in two observers. The intraclass correlation coefficient for the intraobserver variability of Emean in malignant lesion, benign lesion, surrounding normal rectal wall in malignant and normal rectal wall in benign were 0.91 (0.86-0.94), 0.94 (0.88-0.97), 0.92 (0.88-0.95) and 0.89 (0.77-0.95), respectively.
**Conclusion:** SWE is a promising tool that yields additional valuable quantitative data to ERUS examination in rectal lesions. The cut-off value 61.3KPa for Emean may serve as a complementary tool for diagnosis of rectal lesions.
*Author Disclosures:*
**T. Li:** Author; Man Lu.

**B-0207** 10:46
**MRI texture analysis in predicting lymph nodal yield after neoadjuvant chemoradiotherapy in locally advanced rectal cancer (LARC)**
M. Panzeri, A. Di Chiara, A. Palmisano, A. Esposito, P. Passoni, A. Del Maschio, F. De Cobelli; *Milan/IT (panzeri.marta@hsr.it)*

**Purpose:** Overall survival in patients treated with neoadjuvant chemotherapy has been proved to be significantly influenced by nodal status, but all imaging techniques have low accuracy in prediction of the lymph node involvement. Aim of our study was to assess the value of MRI-derived lymph node texture features in the prediction of lymph nodal yield after chemotherapy.
**Methods and Materials:** 58 pts with locally advanced rectal cancer underwent 1.5T MRI before the beginning of CRT (preMRI) and at the end of CRT (postMRI). Multiplanar high-resolution axial-T2w sequences and DWI sequences were acquired. A mean number of 8 pathologic lymph nodes (range 3-19) per patients was found at MRI and a total of 174 lymph nodes were segmented on T2w (VT2) and b:1000 (Vb1000) images. A volumetric histogram analysis of T2 and ADC was performed. Pathological lymph nodal status after chemotherapy was evaluated on surgical sample.
**Results:** At histopathology, 43 patients were pN0 after neoadjuvant chemotherapy, 7 pts were pN1 and 8 pts were pN2. At univariate analysis a significant difference was found between the standard deviation (SD) of T2 values at pretreatment MRI of pN0 patients vs N+ ($p<0.01$). At multivariate analysis, SD of T2 at preMRI was associated with pN0 ($p<0.034$).
**Conclusion:** The evaluation of lymph node heterogeneity at pre-treatment assessed through texture analysis-derived T2-SD may increase MR accuracy in the prediction of nodal involvement at final histology.

**B-0208** 10:54
**Multislice spiral CT images combined with CEA, lymphocyte and neutrophil ratio predict recurrence and metastasis postoperative of rectal cancer**
D.H. Yuan; *Kunming/CN (921574199@qq.com)*

**Purpose:** To exploring the earlier predictors of postoperative recurrence and metastasis of rectal cancer and make a model.
**Methods and Materials:** Retrospective collection patients with rectal cancer underwent surgical resection and pathological diagnosis of 400's from September 2013 to September 2014 The patients have had imaging examination(CT), serum tumour markers, blood routine follow-up for 3 years at least. Statistics in patients with preoperative CT examination in the lesion size, postoperative 3, 6, 12 month serum carcinoembryonic antigen (CEA), CA242, CA199, peripheral blood lymphocyte and granulocyte ratio of conventional. Using Cox proportional hazards model , X-tile software combining Kaplan - Meier curves are stratified analysis to set up normogram model and use ROC curve detected this model.
**Results:** Preoperative CT imaging lesion size (P = 0.000, HR = 0.000, 95% CI: 1.015 1.033), postoperative 1 year lymphocyte by neutrophils ratio (P = 0.028, HR = 0.028, 95% CI: 0.917 3.832), postoperative 1 year CEA value (P = 0.000, HR = 0.000, 95% CI: 1.002 1.005) and as a significant biomarker to predicting recurrence and/or metastasis of postoperative rectal cancer, The stratified threshold of the lesion size Cut-Point in CT images of patients with rectal cancer was 18.75 $cm^3$, and the CEA Cut-Point after surgery was 16.97 ng/ml. Normogram model predict recurrence and metastasis postoperative of rectal cancer AUC is 0.939.
**Conclusion:** Preoperative CT images in the lesion size and the preoperative 1 years CEA values can predict postoperative recurrence and metastasis of rectal cancer.
*Author Disclosures:*
**D.H. Yuan:** Author; Cheng de Liao.

**B-0209** 11:02
**The value of MRI in the evaluation of peri-anal fistula**
A.M.J. Al-Mosawe, M.A.K. Al-Jiboori; *Baghdad/IQ (Drammaralmosawe@gmail.com)*

**Purpose:** To study MRI role in evaluating perianal fistula and the value of using intravenous gadolinium in the determination of precise tract pathway, extensions and other associated pathologies.
**Methods and Materials:** Cross-sectional analytic study was conducted on 32 patients in the MRI department of AL-Imamain AL-Kadhemain medical city, Baghdad, from November 2015 to December 2016. Axial and coronal T2 WI with and without fat suppression and T1 FS before and after contrast administration were performed. The type of fistula, location of internal opening, associated abscesses or sinus tracts were all evaluated. Significance and diagnostic accuracy of each sequence were documented in relation to surgical results.
**Results:** The most common type of fistula encountered was the intersphincteric type, seen in 21 patients (66%); of those, 16 fistulas (50%) were grade I and 5 fistulas (16%) were grade II. Transsphincteric fistulas were seen in 9 patients (28%), 2 of them (6%) were grade III and 7 fistulas (22%) were grade IV. Two patients (6%) had extra-sphincteric type. T2 TSE, T2 TSE FS and T1-weighted FS post-contrast sequences all show significant correlation with surgical results (p value less than 0.05) while the highest significance was obtained by the post-contrast sequence (p value of 0.00001). The highest accuracy was with the use of T1-enhanced FS sequence (98.8%) followed by 87% for the T2FS and only 57% for the T2 TSE.
**Conclusion:** Axial and coronal enhanced fat-suppressed T1 provides the highest accuracy and significance with surgical data, therefore, decreasing the incidence of postoperative complications.

**B-0210** 11:10
**Structured vs narrative reporting of pelvic MRI in perianal fistulising disease: impact on clarity, completeness, and surgical planning**
O. Tuncyurek, A. Garces-Descovich, E.E. Duran, T.E. Cataldo, V.Y. Poylin, K.J. Mortele; *Boston, MA/US (ozum.tuncyurek@gmail.com)*

**Purpose:** To evaluate clarity and usefulness of MRI reporting of perianal fistulising disease using a structured disease-specific template versus narrative reporting for planning of disease treatment by colorectal surgeons.
**Methods and Materials:** In this HIPAA-compliant, IRB-approved study with waiver of informed consent, a structured reporting (SR) template for perianal fistulising disease MRIs was developed in collaboration between colorectal surgeons and abdominal radiologists. The study included 45 consecutive patients who underwent pelvic MRI for perianal fistulising disease prior to implementation of SR, and 60 consecutive patients who underwent pelvic MRI for perianal fistulising disease after implementation of SR. Objective evaluation of the reports for the presence of 12 key features was performed, as also subjective evaluation regarding the clarity and completeness of reports, and impact on treatment.
**Results:** More key features were absent in narrative reports [mean: 6.3±1.8 (range 3-11)] than in structured reports [mean: 0.3±0.9 (range 1-5)], (p=<0.001). The use of SR also increased the percentage of completeness (72.5% to 88.3% for surgeon 1, and 61.2% to 81.3% for surgeon 2; p=0.05 and 0.03, respectively), helpfulness in surgical planning (7.1±1.5 to 7.6±1.5 for surgeon 1, and 5.8±1.4 to 7.1±1.1 for surgeon 2; p=0.05 and p<0.001, respectively), and clarity (7.6±1.3 to 8.3±1.1 for surgeon 1, and 5.2±1.4 to 7.1±1.3 for surgeon 2; p=0.006 and p<0.001, respectively) of the reports.
**Conclusion:** Structured MRI reports in patients with perianal fistulising disease miss fewer key features than narrative reports. Moreover, SRs were described as more complete and clear, and more helpful for treatment planning.

**B-0211** 11:18
**MR fistulography with percutaneous instillation of ultrasound gel and its role in the preoperative mapping: our experience**
G. Fontanella, M. Mancinelli, S. De Lucia, A. Festa, R. Villanacci, C. Manganiello; *Benevento/IT (giovanni.fontanella@hotmail.com)*

**Purpose:** The aim of this study is to show the role of MR fistulography (MRF), performed after percutaneous instillation of ultrasound gel, in the preoperative mapping of perianal fistula, underlying its various advantages.
**Methods and Materials:** 68 patients were selected from September 2017 until September 2018 for pre-operative fistular mapping among those with viable percutaneous orifices. After a basal scan (22 min), we instilled 3-10 cc of ultrasound gel per orifice, then re-scanned using a T2w, fsT2w and DWI protocol. Each case was reviewed through standard reporting by experienced radiologists, comparing either standard MR or MRF reports with the surgical findings. Surgeons in our institution are currently operating on the basis of MRF reports.
**Results:** MRF has shown to be a well-tolerated, faster technique (mean scan time 16 mins), superior to standard MR, allowing the detection of 94 tracts (versus 82 of basal MR). MRF is highly congruous with surgical findings, with specificity and sensitivity in the definition of fistular grade close to 100%, thus resulting in a significant drop of fistular relapses in one year: 2.89% vs 22.43% (from Sept 2016 to Sept 2017). Specificity, sensitivity and accuracy of detection of internal openings range between 90 and 97.5%, data comparable to basal MR.
**Conclusion:** MRF with percutaneous instillation of ultrasound gel is a more accurate, well-tolerated, faster technique for pre-operative mapping, allowing better depiction of lesser tracts and, in our institution, significant drop in fistular relapses.

**B-0212** 11:26
**Introducing 3D modelling of MRI in preoperative mapping of perianal fistula: how it could help the surgeons?**
M.S. Roman, M.Y. Awadallah, A.F. Ahmed, F.S. Fawzi, H.A.S. Shawali, R.F. Elsayed; *Cairo/EG (drmarysalah@yahoo.com)*

**Purpose:** To study the merits of 3D modelling MRI in enhancing preoperative interpretation of perianal fistulous tracts by the surgeons.
**Methods and Materials:** Fifty patients underwent 2D MRI fistulography and reported by an experienced radiologist of 20 years to assure accuracy. Based on 2D report, 3D model was achieved by manual segmentation and 3D reconstruction. A second radiologist and a surgeon both were blinded to 2D MRI findings, were asked to report on internal opening, side branches, abscess collections and supralevator extensions. The data of the 2$^{nd}$ radiologist and the surgeon were compared individually to the 2D MRI findings by the expert radiologist to detect the degree of diagnostic agreement.
**Results:** Comparing 3D MRI findings by the surgeon to 2D findings, the highest and lowest sensitivities were the supralevator extension (100%) and abscess collection (50%), respectively. The highest specificity were internal opening, supralevator extension, abscess collection (100%), the lowest was side branches (88.57%). The highest diagnostic agreement was supralevator extension (100%), the least was internal opening and side branches detection (80%). Comparing second radiologist report to the 2D findings, the highest sensitivity was supralevator extension, abscess collections (100%), the lowest was internal opening (76.32%). The highest specificity was internal opening, supralevator extension (100%). The lowest was side branches (91.43%). The highest and least diagnostic agreements were supralevator extension (100%) and internal opening (80%), respectively.
**Conclusion:** 3D model MRI yielded the highest detection rate for the supralevator extension, an important cause of postoperative recurrence, followed by abscess collection.

**B-0213** 11:34
**Perianal fistula mapping at 3T: volumetric vs conventional MR sequences**
M. Cerit, A.Y. Oner, A. Yildiz, E. Cindil, H.N. Sendur, S. Leventoglu; *Ankara/TR (mahinurp@yahoo.com)*

**Purpose:** To evaluate volumetric 3DSPACE T1, 3DVIBE T1 sequences with conventional 2D T1 and T2 FSE sequences in assessing perianal fistulas.
**Methods and Materials:** 23 patients with perianal fistula were included in this prospective study and underwent pelvic MR at 3T including contrast-enhanced fat suppressed 3DSPACE T1W, 3DVIBE T1W, axial and coronal 2DT1W together with 2DT2W sequences in the axial and coronal planes. Acquisition times were recorded. Results were compared with postsurgical findings defined as the gold standard. Performance of each sequence was evaluated in terms of image quality, presence of artefacts, lesion conspicuity, fistula type, presence of abscess, visibility of internal opening and number of internal opening.
**Results:** Both 3D VIBE-3D SPACE turned out to be the best sequence to determine the fistula type and the best performer in terms of image quality, artefacts, determining locations of internal orifices. Number of internal opening were correctly detected in 23 (100%) patients for 3D SPACE and VIBE sequences, in 17(73,9%) patients on CE-T1WI and in 7(30,4%) patients on T2WI. When combined, T2 and T1W sequences correctly detected internal openings in 17(73,9%). The lesion conspicuity was higher for 3D SPACE and 3DVIBE sequences compared to other 2D sequences (P<0,05). Overall acquisition time for each 3D sequences were shorter compared to 2D sequences combined.
**Conclusion:** 3D SPACE and VIBE sequences offer better diagnostic accuracy, in a shorter scanning time in perianal fistula evaluation when compared to T1WI and T2WI sequences.

**B-0214** 11:42
**Anal fistula: the role of MR imaging in preoperative evaluation**
D.T. Vo, C.C. Phan, L.T.T. Nguyen, H.M.D. Le, T.T. Nguyen; *Ho Chi Minh City/VN (duc.vt@ump.edu.vn)*

**Purpose:** To describe the MR imaging features of anal fistula and evaluate the role of T2W TSE and post-contrast FS T1W TSE gadolinium sequences in detecting the characteristics of the fistula tract.
**Methods and Materials:** 367 patients with anal fistulas, diagnosed and treated at HCMC-UMC between 1/1/2016 and 1/31/2018, were included in this study. This study was a retrospective analysis. We compared the imaging features with the surgical findings.
**Results:** The mean age was 39.3 years (range, 12 - 84 years), male/female=9/1. A total of 411 fistulas were found at surgery. Agreement between the surgical findings and MR imaging for the classification of the primary track, detecting secondary track was very good with ķ = 0.89 (0.84, 0.93), 0.94 (0.90, 0.97), respectively. The sensitivity and specificity of MR imaging in correctly detecting internal openings were found to be 99% and 85.2%, respectively; for abscess, it was 100% and 100%, respectively. Post-contrast FS T1W was very good in detecting abscess; differentiating between abscess and inflammation, active inflammatory components with scars.
**Conclusion:** MRI was highly accurate in the assessment of surgically important parameters of anal fistula. With the very good agreement between the surgical findings and MR imaging for characterisation of perianal fistulas, MR imaging was considered the reliable technique for establishing preoperative fistula map.

**14:00 - 15:30** **Room X**

### Vascular

### SS 315

### Advanced vascular imaging: cerebral and peripheral arteries

*Moderators:*
H.A. Deutschmann; Graz/AT
N.N.

**B-0215** 14:00
**Branching pattern of the cerebral arterial tree**
J. Helthuis[1], T. van Doormaal[1], B. Hillen[2], R. Bleys[1], A. Harteveld[1], J. Hendrikse[1], A. van der Toorn[1], J.J. Zwanenburg[1], A. van der Zwan[1]; [1]*Utrecht/NL*, [2]*Nijmegen/NL*

**Purpose:** Quantitative data on branching patterns of the human cerebral arterial tree are lacking in the 1.0-0.1 mm radius range. We aimed to collect quantitative data in this range, and to study if the cerebral artery tree complies with the principle of minimal work (Law of Murray).
**Methods and Materials:** To enable easy quantification of branching patterns a semi-automatic method was employed to measure 1294 bifurcations and 2031 segments on 7T-MRI scans of two corrosion casts embedded in a gel. Additionally, to measure segments with a radius smaller than 0.1 mm, 9.4T-MRI was used on a small cast section to characterize 1147 bifurcations and 1150 segments. Besides MRI, traditional methods were employed. 733 bifurcations were manually measured on a corrosion cast and 1808 bifurcations and 1799 segment lengths were manually measured on a fresh dissected cerebral arterial tree.
**Results:** Data showed a large variation in branching pattern parameters (asymmetry-ratio, area-ratio, length-radius-ratio, tapering). Part of the variation may be explained by the variation in measurement techniques, number of measurements and location of measurement in the vascular tree.
**Conclusion:** This study confirms that the cerebral arterial tree complies with the principle of minimum work. These data are essential in the future development of more accurate mathematical blood flow models.

*Author Disclosures:*
**J. Helthuis:** Grant Recipient; Netherlands Organisation for Health Research and Development (ZonMw), project number 11.631.0005. **J.J. Zwanenburg:** Grant Recipient; European Union's Seventh Framework Programme (FP7/2007-2013) / ERC grant agreement n°337333.

**B-0216** 14:08
**High-resolution MR image of intracranial blood vessels wall using 3D VISTA sequence**
L. Zheng, Q. Wang, Z. Wang; *Shanghai/CN*

**Purpose:** To introduce the technique of using high-resolution three-dimensional volumetric isotropic TSe acquisition (HR-3D-VISTA) for magnetic resonance (MR) imaging of intracranial blood vessel wall.
**Methods and Materials:** Institutional review board approved this study. From November 1, 2015 to December 31, 2016, 143 cases underwent 3D VISTA MR image of intracranial blood vessels wall using PHILIPS INGENIA 3Tesla MR Scanner. The scan parameters are as follows: TE 21ms, TR 425 ms, matrix 332X332, and voxel 0.6x0.6 x 0.8, and number of slices 120. Then image qualities of non-contrast and contrast-enhanced 3D VISTA (CE-3D-VISTA) images were evaluated and lesion type were analysed. Additionally, 3D-VISTA images were compared with conventional time of flight MR angiography (TOF MRA) and digital subtraction angiography (DSA).
**Results:** The techniques of HR-3D-VISTA finished intracranial blood vessels wall imaging in six minutes for one case. The images of HR-3D-VISTA can well show normal and lesion features of vessels wall. The CE-3D-VISTA image has high image quality than those images of non-contrast 3D-VISTA. 3D VISTA image provide more information for either positive or negative cases with TOF MRA and DSA examination.
**Conclusion:** 3D VISTA MR image of vascular wall technique is a suitable method for intracranial blood vessels, and can play an important role in suspicious cases with vascular diseases by combination with TOF MRA and DSA in clinical practice.

**B-0217** 14:16
**Development and validation of prediction model based on hemodynamic derived from computed tomography angiography for rupture risk of small unruptured intracranial aneurysms**
Z. Shi, G. Chen, C. Zhou, G. Lu, L. Zhang; *Nanjing/CN (fmrishiz@126.com)*

**Purpose:** The risk factors of small intracranial aneurysms (sIAs) (≤ 5mm) rupture remain unclear and this study aimed at determining the risk factors and developing a prediction model of rupture risk of unruptured sIAs.
**Methods and Materials:** The patients with sIA diagnosed by both computed tomography angiography and invasive cerebral angiography were included from January 2010 to December 2016 and were randomly separated into training and validation cohort. A model was developed based on patient characteristics, anatomical characteristics and hemodynamics information by multivariable analysis. The area under ROC curve (AUC) was used to present the diagnosis performance and the model was further assessed in the validation cohort which was randomly selected from the entire dataset and the procedure repeated for 4 times. Random forests were implemented for features selection.
**Results:** The ratio of training cohort to validation cohort was 3 and age, sex, location, HBP, cvWSS, cvOSI, inflowconcentration, flowimpingement and flowstability (all p<0.05)were independently associated with rupture status in multivariate logistic regression in the four groups. The mean AUC of the models was 0.918 and application of the model in the validation cohorts still performed excellently with the mean AUC of 0898. Ten variables passed the feature selection and comprised cvOSI, flowimpingement, size and so on.
**Conclusion:** sIAs have distinct risk factors of rupture, and age, location, OSI, and inflowconcentration et al. were independently associated with sIAs rupture status. We demonstrated that our model can provide reliable quantitative individual risk assessments for sIAs and may help choose the optimum management.

**B-0218** 14:24
**One-stop coronary CTA combined with carotid and cerebrovascular CTA using wide-detector CT: comparison with conventional protocol**
P. Han, W. Li, Z.-G. Yang, K. Diao, R. Shi, Z. Li; *Chengdu/CN (peilhan@foxmail.com)*

**Purpose:** To determine the value of “one-stop” coronary CT angiography (CCTA) combined with carotid and cerebrovascular CT angiography (CCCTA) in single contrast injection using 16 cm wide-detector CT.
**Methods and Materials:** Fifty-six patients with suspected coronary, carotid and cerebrovascular disease were prospectively and randomly divided into Group A (n=36, combined CTAs) and Group B (n=20, conventional protocol: CCTA, followed by CCCTA after contrast re-injection). CTA examinations were performed with contrast dose at 0.7 ml/kg. We compared the contrast dose, CT examination time and contrast-to-noise ratio (CNR), as well as the CT values and standard deviations (SD) of atherosclerotic plaques and arteries between groups A and B.
**Results:** The contrast dose was significantly smaller in group A than in group B (44.6±5.9ml vs. 91.9±11.8ml, *p*<0.001), while examination time was significantly reduced in group A than in group B (6 min vs. 13 min). Both groups achieved satisfied image quality at all examined vessels (no significant difference in CT values at all regions between the two groups) and were available for diagnosis, despite the CNR of coronary arteries were higher in group B (28.34±6.21 vs. 35.95±8.53*, p* < 0.001). Nevertheless, group B also demonstrated a prominently higher mean SD than group A.
**Conclusion:** Combined cerebral, carotid and coronary CTA could save scan time and reduce contrast dose, without impairing image quality. It provides a promising simplified work-flow in clinical practice.

**B-0219** 14:32
**Curved surface reformations - diagnostic accuracy and time-efficiency of a new peripheral CTA reformation technique**
D. Toth, J. Kittinger, K. Lampichler, C. Loewe, R. Schernthaner; *Vienna/AT (daniel.toth@meduniwien.ac.at)*

**Purpose:** Peripheral CTA has evolved into an accurate modality for the assessment of peripheral arterial occlusive disease (PAOD). However, due to the limitations of available angiogram-like reformations such as Maximum Intensity Projections, the evaluation of axial and coronal multiplanar reformations (MPRs) is still required to reach sufficient accuracy. Recently, a centerline-based reformation technique called curved surface reformation (CSR) was developed. The purpose of this pilot trial was to assess the diagnostic accuracy and time efficiency of CSRs compared to standard MPR for peripheral CTA, with intra-procedural DSA as the standard of reference.
**Methods and Materials:** 21 consecutive patients (7 females, mean age 67 years), who underwent peripheral CTA prior to endovascular treatment of PAOD, were retrospectively included. 21 arterial segments were defined in each leg; for each segment, the presence of haemodynamically relevant stenosis (≥70%) was assessed on CSRs and MPRs independently, with DSA as gold standard. Time for image analysis was recorded.
**Results:** 395 out of 882 arterial segments were depicted with DSA and available for analysis. Using MPRs, CTA yielded a sensitivity of 88%, a specificity of 94% and an accuracy of 93%. Using CSRs, sensitivity, specificity and accuracy increased to 95%, respectively (p<0.01, p=0.84 and p=0.09, respectively). The mean reading time decreased significantly from 8’44’’ to 3’15” (p<0.01).
**Conclusion:** CSR is a new centerline-based CTA reformation technique that seems to allow accurate and fast assessment of PAOD without the necessity to review MPRs. Further studies with larger cohorts and several readers are required to confirm this finding.

**B-0220** 14:40
**TSH suppressive therapy aggravates arterial inflammation: an $^{18}$F-FDG PET study in thyroid carcinoma patients**
E. Boswijk, K.J.C. Sanders, E. Broeders, M. de Ligt,
W.D. Vanmarkenlichtenbelt, F. Mottaghy, P. Schrauwen, J.E. Wildberger,
J. Bucerius; *Maastricht/NL (eboswijk@gmail.com)*

**Purpose:** We aimed to investigate the influence of hypothyroidism and TSH-suppressive therapy on arterial inflammation in vivo, using $^{18}$F-fluorodeoxyglucose (FDG) positron emission tomography (PET)/CT.
**Methods and Materials:** Ten well-differentiated thyroid carcinoma patients underwent an $^{18}$F-FDG PET/CT during post-thyroidectomy hypothyroidism and during TSH-suppressive therapy after $^{131}$I (radioiodine) ablation therapy. We analysed $^{18}$F-FDG uptake in the carotids, aortic arch, ascending, descending, and abdominal aorta. Target-to-background ratios (TBRs) corrected for blood pool activity were established for all arterial territories. Additionally, we measured $^{18}$F-FDG uptake in the spleen, bone marrow and multiple adipose tissues. The Wilcoxon sign rank test was used to compare TBRs between hypothyroidism and TSH-suppression. TBRs from both periods were further compared to that of historic control subjects without known thyroid disease with a Mann-Whitney U test.
**Results:** There was a trend towards higher arterial TBRs during TSH-suppressive therapy than during hypothyroidism ($TBR_{max}$all vessels = 1.8 and 1.6, respectively, p=0.058). In concurrence with this, we found increased CRP levels after levothyroxine treatment (CRP = 4.8 and 2.9 mg/L during TSH-suppressive therapy and hypothyroidism, respectively; p=0.005) and increased activity in the bone marrow ($TBR_{max}$2.0 and 1.7 during TSH-suppressive therapy and hypothyroidism, respectively; p=0.014). An exploratory comparison with controls without known thyroid disease also showed a trend towards higher arterial TBRs during TSH-suppressive therapy ($TBR_{max}$all vessels = 1.8 and 1.6 for TSH suppressive therapy and euthyroidism, respectively; p=0.055).
**Conclusion:** Arterial inflammation is increased in patients during TSH-suppressive therapy. This could indicate that TSH-suppression renders patients at an increased risk of atherosclerotic disease.

**B-0221** 14:48

**Comprehensive validation of two accelerated 4D-flow MR sequences at 3T: a phantom study**

S. Ebel[1], L. Hübner[1], B. Köhler[2], S. Kropf[2], B. Preim[2], B. Jung[3], M. Grothoff[1], M. Gutberlet[1]; [1]*Leipzig/DE*, [2]*Magdeburg/DE*, [3]*Bern/CH*
*(sebastian.ebel@icloud.com)*

**Purpose:** To assess for the first time 4D-flow measurement accuracy (flow volume and peak velocity) of different accelerated MR sequences using a constant and pulsatile flow phantom at 3T in comparison to a standard 2D-flow MR sequence.
**Methods and Materials:** The two accelerated 4D-flow MR sequences (GRAPPA2- and k-t-GRAPPA5) were evaluated regarding accuracy of calculated flow volumes, flow velocities, reproducibility, dependency on proper planning of the measuring plane and the encoded velocity ($V_{enc}$). The calculated flow volumes and peak velocities of the phantom were used as the standard of reference. The MR-flow analysis was performed using the custom-made software "Bloodline".
**Results:** No significant differences were found between the 2D- and both accelerated 4D-flow MR sequences and the pump reference regarding flow volumes and flow velocities in continuous and pulsatile flow. An excellent correlation (r=0.99-1.0) to the standard of reference and excellent reproducibility of measurements (r=0.99) could be achieved in all used sequences. An up to two-and-a-half-times overestimated $V_{enc}$ had no impact on the accuracy of flow measurements. However, misaligned measuring planes led to dramatically reduced 2D-flow accuracy while both 4D-flow measurements were not affected by any misalignment.
**Conclusion:** Both accelerated 4D-flow MR sequences demonstrated equal accuracy compared to 2D-flow measurements and better accuracy in any case of plane misalignment. The highly accelerated, faster k-t-GRAPPA5-sequence revealed comparable results to the GRAPPA2 and should, therefore, be used preferentially whenever possible.
*Author Disclosures:*
**S. Ebel:** Grant Recipient; DFG GU 777/4-1 – AOBJ 629068.

**B-0222** 14:56

**Validation of CT imaging characteristics to differentiate intimal from medial calcifications in peripheral vessels and its application in a non-vascular cohort**

L.C.D. Konijn[1], P.A. de Jong[2], H. van Overhagen[1], W.P. Mali[2]; [1]*The Hague/NL*, [2]*Utrecht/NL (l.konijn@hagaziekenhuis.nl)*

**Purpose:** Recently, histologic studies showed that calcifications in the wall of peripheral vessels are not only caused by atherosclerosis in the intimal wall, but also by medial wall calcifications. This might play a role in the development of critical limb ischemia. The aim of this study was to validate a CT imaging score to differentiate intimal from medial wall calcifications in the peripheral vascular beds of this non-vascular cohort.
**Methods and Materials:** A recently developed histologically validated CT scoring system was used to assess calcifications in eight different vascular beds.
**Results:** 204 patients were included (age 61 (range 22-90; male 52%). A high overall prevalence was seen (abdominal aorta 69.1%, iliac 65.2%, femoro-popliteal 52.5%, crural 42.2%). However, calcifications in the upper extremity were scarse (brachial arteries 2.9%, radio-ulnar 1.5%).
Overall, we could see that clusters of CT characteristics that have been validated for intimal (dotted, thick and patchy) or medial calcifications (circular, thin and continuous) indeed correlate strongly between each other. The highest correlation coefficients were found in the crural arteries (medial 0.79, 0.85, 0.76; intimal 0.39, 0.88, 0.76) and slightly less strong correlations for the femoro-popliteal arteries (medial 0.51, 0.78, 0.66; intimal 0.49, 0.58, 0.62).
**Conclusion:** This study strongly suggests that there is indeed a medial and intimal pattern that can be differentiated on CT scan, especially in the leg, with both a different pattern of development. Further studies are needed to explore the on-going process of calcification and how this plays a role in (peripheral) arterial disease.

**B-0223** 15:04

**Virtual monochromatic images in low-tube current dual-energy spectral imaging combined with adaptive statistical iterative reconstruction V in head CT angiography**

T. Song, Z. Li; *Chengdu/CN (407145908@qq.com)*

**Purpose:** To analyse the value of combining the VMS image and ASiR-V in low-tube current dual-energy spectral imaging in head CTA.
**Methods and Materials:** An anthropomorphic angiographic head phantom and 40 patients were examined on a revolution CT with spectral imaging mode at two different tube currents. Images of different energy levels in the low-tube current group were reconstructed with the combination of filtered back projection (FBP), 20%, 40%, 60% and 80% ASiR-V; VMS images at 70keV in the routine tube current group were reconstructed with FBP only.
**Results:** VMS images at 40-55keV yielded a higher CNR compared with 70keV and the subjective scores at 55keV and 60keV were higher than others(*P*<0.05). The SNR and CNR in the low-tube current group with ASiR-V60% and ASiR-V80% were higher than that of the FBP, ASiR-V20% and ASiR-V40% images in the same group (*P*<0.05).The SNR and CNR of the simulated cerebral vessels and subjective scores in the patients' groups of 55keV VMS images in the low-tube current group with ASiR-V60% and ASiR-V80% were higher than that of the 70keV FBP images in the routine tube current group (*P*<0.05). The radiation dose in the low-tube current group was 39% lower than in the routine tube current group.
**Conclusion:** VMS images in low-tube current spectral imaging combined with ASiR-V can significantly reduce radiation dose and ensure image quality in head CTA. The 55 keV VMS images with ASiR-V60% and 80% provide higher image quality.

**B-0224** 15:12

**Comparing the diagnostic accuracy of $^{18}$F-FDG-PET/CT, contrast-enhanced CT and combined imaging in patients with suspected vascular graft infections**

L. Husmann, M.W. Huellner, B. Ledergerber, A. Anagnostopoulos, P. Stolzmann, B.-R. Sah, I.A. Burger, Z. Rancic, B. Hasse; *Zurich/CH*
*(lars.husmann@usz.ch)*

**Purpose:** To evaluate the diagnostic accuracy of positron emission tomography/computed tomography with $^{18}$F-fluorodeoxyglucose (PET/CT), contrast-enhanced CT (CE-CT), and a combined imaging approach (CE-PET/CT) in patients with suspected vascular graft infection (VGI).
**Methods and Materials:** PET/CT and CE-CT were performed prospectively in 23 patients with suspected VGI. Diagnostic accuracy for PET/CT was assessed using previously suggested cut-off points for maximum standardized uptake values ($SUV_{max}$). Using a new 4-point scale for visual grading, two readers independently assessed the diagnostic accuracy of CE-CT and combined CE-PET/CT. Microbiological culture, obtained after open biopsy or graft explantation, and clinical follow-up of the patients served as the standard of reference.
**Results:** Sensitivity, specificity, negative predictive value (NPV), positive predictive value (PPV), and accuracy of PET/CT for the diagnosis of VGI were 100%, 50%, 100%, 72.2%, and 78.3%, respectively. Respective values for CE-CT were 100%, 50%, 100%, 72.2%, and 78.3% for reader 1, and 92.3%, 80%, 88.9%, 85.7%, and 86.9% for reader 2; while respective values for combined CE-PET/CT were 100%, 70%, 100%, 81.3%, and 86.9% for reader 1, and 100%, 80%, 100%, 86.7%, and 91.3% for reader 2.
**Conclusion:** The diagnostic accuracy of combined CE-PET/CT in patients with suspected VGI is very high. The combination of the high sensitivity of PET/CT in detecting metabolically active foci in infection and the high specificity of CE-CT in detecting anatomic alterations appears to be the reason why combined imaging outperforms stand-alone imaging in diagnosing VGI and may be supportive in future decision-making of difficult cases of suspected VGI.

**B-0225** 15:20

**Inter-scanner and inter-software comparison of quantitative 4D flow MRI**

T.H. Oechtering[1], A. Nowak[1], M. Sieren[1], J. Graessner[2], H. Kooijman-Kurfuerst[2], J. Barkhausen[1], A. Frydrychowicz[1]; [1]*Lübeck/DE*, [2]*Hamburg/DE (thekla.oechtering@uksh.de)*

**Purpose:** To compare quantitative 4D flow MRI of healthy volunteers acquired on scanners of different vendors and evaluated with two commercially available software solutions to underline the technique's applicability.
**Methods and Materials:** Thoracic aortic 4D flow MRI was conducted in five healthy volunteers (27±3 years, 1 female) on 3T MRI scanners ($MRI_1$=Ingenia, Philips; $MRI_2$=Skyra, Siemens) using identical scan parameters per guideline recommendations. The ten datasets were evaluated with software $SW_1$=GTFlow (v3.1.13, Gyrotools, CH) and $SW_2$=cvi42 (v5.9.2, Circle Cardiovascular Imaging, CAN) for net stroke volume ($SV_{net}$), peak flow ($Flow_{max}$), maximum velocity ($Vel_{max}$), and maximum area ($Area_{max}$) on six predefined contours. For these parameters, mean absolute and relative errors with SD and Bland-Altman plots were calculated. Inter-scanner comparison was performed for $SW_1$ and $SW_2$ separately. Inter-software comparison was independent of scanner type.
**Results:** Inter-scanner comparison: acquisitions were performed in 14±2min ($MRI_1$) and 17±7min ($MRI_2$; p=0.69). Average differences were small for $SV_{net}$ ($SW_1$: 0±10ml; $SW_2$: -1±10ml). MRI2 underestimated $Flow_{max}$ by 4±12% ($SW_1$: 24±52ml/s, $SW_2$: 27±56ml/s), $Vel_{max}$ by 18±20cm/s ($SW_1$) and 8±20cm/s ($SW_2$), and $Area_{max}$ by 53±142mm² ($SW_1$) and 41±320mm² ($SW_2$). Inter-software comparison: $SW_2$ was underestimating $SV_{net}$ by 8±8ml, $Flow_{max}$ by 37±28ml/s (=9±6%), and $Vel_{max}$ by 10±16cm/s (=7±11%) compared to $SW_1$. In contrast, $SW_2$ overestimated $Area_{max}$ by 100±227mm² compared to $SW_1$. Differences in $Area_{max}$ were smaller when excluding the blurry aortic bulb contour.

Wednesday

**Conclusion:** Quantitative 4D flow MRI results, especially SV, seem comparable between different 3T scanners. However, it is important to focus on one software because inter-software variability was higher than inter-scanner variability.
*Author Disclosures:*
**J. Graessner:** Employee; Siemens. **H. Kooijman-Kurfuerst:** Employee; Philips.

**14:00 - 15:30** **Room N**

### Hybrid Imaging

### SS 306

### Clinical applications of hybrid imaging

*Moderators:*
A. Scarsbrook; Leeds/UK
R. Woitek; Cambridge/UK

### B-0226 14:00

**Modulation of PSMA expression by androgen deprivation therapy (ADT): serial PSMA PET in men with hormone sensitive and castrate resistant prostate cancer commencing androgen blockade**

C. Yin[1], A. Joshua[1], M. Crumbaker[1], L. Emmett[2]; [1]*Darlinghurst/AU*, [2]*Sydney/AU (charlotte.yin@gmail.com)*

**Purpose:** Prostate specific membrane antigen (PSMA) can be effectively targeted for both PET and therapy purposes in the management of prostate cancer (PCa). We aim to evaluate the effect on PSMA PET of androgen blockade (AB) in men with measurable metastatic disease commencing ADT (hormone sensitive cohort) or a novel antiandrogen (enzalutamide or abiraterone) in the castrate resistant state.
**Methods and Materials:** 15 men underwent serial PSMA PET scans at baseline, Day 9, 18 and 28. COHORT 1: 8 men commencing ADT with LHRH agonists. COHORT 2: 7 men commencing novel antiandrogen. Visual and quantitative image analyses was performed using MIM® software.
**Results:** Cohort 1 : A mean 25% reduction in SUVmax by Day 9 with an associated marked PSA response in 100% of men. Subsequent rise in PSMA intensity at some sites of metastatic disease at days 18 and 28 in subset of men. Cohort 2: All men demonstrated an increase in intensity of uptake by Day 9 unrelated to change in PSA. Relatively homogeneous responses at Day 9 in all men but subsequent heterogeneity in treatment response on Day 18 and 28 scans. This was most marked in Cohort 1, where there was an increase in SUVmax in individual lesions despite an overall reduction in tumour volume. This suggests PSMA-PET may be able to identify phenotypes of early castrate resistance prior to PSA, predictive biomarker potential.
**Conclusion:** PSMA is a manipulable receptor with rapid dichotomous in vivo response to AB dependent on PCa phenotype.

### B-0227 14:08

**68 GA PSMA vs whole body diffusion weighted MR imaging in staging of high risk prostate cancer**

M. Logudoss, A. Chellathurai; *Chennai/IN (drlmkmdrd@gmail.com)*

**Purpose:** The purpose of the study is to compare the sensitivity and specificity of 68 GA PSMA and whole body diffusion weighted MR imaging in staging of high risk prostate cancer.
**Methods and Materials:** 68 patients with high-risk prostrate cancer were included in the study. Inclusion criteria included biopsy proven prostate cancer with PSA more than 20 ng/ml and Gleason score more than 7. All patients underwent 68 GA PSMA and whole body diffusion weighted imaging within 1 week duration. Two radiologists read the modalities independently and the results were compared for staging of nodal and distant metastases.
**Results:** The mean age of the patients was 63 years and the mean PSA value was 15 ng/ml. The median Gleason score was 8. Regional nodal metastases were detected by 68 GA PSMA in 48 patients and by DWI in 45 patients. Non-regional nodal metastases were detected in 18 patients by 68GA PSMA and in 17 patients in DWI. Skeletal metastases were detected in 24 patients by both modalities. Both detected hepatic metastasis in one patient. Additionally 68GA PSMA detected pulmonary metastasis in one patient, which was missed by DWI. Statistically it was found that the efficacy of whole body diffusion weighted Imaging was almost equal to that of 68GA PSMA (p<0.05).
**Conclusion:** Whole body diffusion weighted imaging has sensitivity and specificity almost equal to 68GA PSMA in the detection of nodal and distant metastases and can be used with Multiparametric imaging of the prostate for complete TNM staging of prostate cancers.

### B-0228 14:16

**Performance of $^{68}$Ga-PSMA-11-PET/MRI guided biopsy to detect significant prostate cancer**

I.A. Burger, D.A. Ferraro, J. Müller, U. Mühlematter, M. Messerli, P. Stolzmann, N.J. Rupp, O.F. Donati, D. Eberli; *Zurich/CH (irene.burger@usz.ch)*

**Purpose:** MRI guided biopsy significantly improved the detection rate of prostate cancer (PCa) in patients with elevated PSA compared to transrectal biopsy. However, still around 22% of patients with significant PCa will not be detected with multiparametric MRI. We investigated if $^{68}$Ga-PSMA-11-PET/MRI could be used for targeted biopsy.
**Methods and Materials:** We performed an interim analysis of the first 25 patients from a prospective trial. Patients with an elevated PSA were referred to $^{68}$Ga-PSMA-11-PET/MRI before template and image targeted biopsy using the BiopSee® system. All cancer lesions with a Gleason Score (GS) of 3+3 or more were assessed. $^{68}$Ga-PSMA-11 accumulation was quantified in the cancer area using $SUV_{max}$. Significant PCa was considered any GS 4+3 or more, or 3+4 with more than 6 mm core length. Correlation between GS and $^{68}$Ga-PSMA-11 uptake was evaluated using Pearson-Correlation.
**Results:** 17 of 25 (68%) patients had significant PCa on template biopsy. $^{68}$Ga-PSMA-11-PET scans were positive in 16 of them with a median $SUV_{max}$ of 7.8(range 5.2-19.3). The single PET negative lesion had a GS 3+4, with a core length of 6 mm. Two areas were considered suspicious for cancer on PET with mild $^{68}$Ga-PSMA-11 uptake ($SUV_{max}$ 5.5 and 5.1). The patient based positive predictive value was 89%, with a sensitivity of 94%. There was a significant correlation between GS and $SUV_{max}$(C=0.64, p=0.001).
**Conclusion:** $^{68}$Ga-PSMA-11-PET/MRI has the potential to detect significant PCa and can be used for image fusion based biopsy guidance, with the potential to decrease the number of biopsies and associated complications.
*Author Disclosures:*
**I.A. Burger:** Grant Recipient; Sick legat and Iten-Kohaut foundation. Research/Grant Support; GE Healthcare.

### B-0229 14:24

**PSMA expression heterogeneity of the primary tumour on IHC correlates with the detection rate of $^{68}$Ga-PSMA-11-PET in patients with biochemical recurrence**

D.A. Ferraro, J.H. Rüschoff, J. Müller, U. Mühlematter, M. Messerli, M. Hüllner, D. Eberli, N.J. Rupp, I.A. Burger; *Zurich/CH (daniela.ferraro@usz.ch)*

**Purpose:** Around 10% of prostate cancers (PCA) have no increased PSMA expression, therefore a small fraction of patients have negative PSMA-PET scans, despite high PSA values. Currently it is not possible to discriminate those patients before the PET scan. We aimed to investigate if the PSMA expression of the primary tumour predicts PSMA-PET detection.
**Methods and Materials:** 36 patients underwent radical prostatectomy between 2007-2016 and were referred to $^{68}$Ga-PSMA-11-PET for biochemical recurrence, with up to two scans if the first was negative. Lesions suspicious for PCA recurrence were quantified using $SUV_{max}$. The dominant tumour on prostatectomy samples was stained for PSMA. PSMA expression on immunohistochemistry was quantified using a three-tiered system (1+weak, 2+moderate, 3+strong) and the percentage of PSMA negative tumour area. Differences between positive and negative exams were assessed with a t-test.
**Results:** 43 $^{68}$Ga-PSMA-11-PET scans were available, median PSA value of 1.69 ng/ml (range 0.05-405.7ng/ml). 30 (69%) PET exams were positive with a median PSMA uptake of $SUV_{max}$ 9.55 (range 2.6-128). Comparing the positive and negative $^{68}$Ga-PSMA-11-PET scans with immunohistochemistry results: the mean membranous PSMA expression was 2.5±0.5 for the positive and 2.2±0.7 for the negative scans (p=0.101), while the negative tumour area showed a significant difference with mean 9%±17% and 34%±38% (p<0.001) for positive and negative scans, respectively. All three cases with a PSA value above 2ng/ml and negative PET examinations had PSMA-negative tumour areas of 80%.
**Conclusion:** Tumour negative areas on PSMA immunohistochemistry of the primary tumour could predict negative $^{68}$Ga-PSMA-11 PET scans.
*Author Disclosures:*
**I.A. Burger:** Grant Recipient; Grant Recipient; Sick legat and Iten-Kohaut foundation. Research/Grant Support; GE Healthcare.

### B-0230 14:32

**Influence of segmentation techniques on volumetric and textural metabolic parameters of primary tumours on 18F-FDG PET-CT imaging**

M. Novikov, T. Babkina; *Kiev/UA (nicknovi@gmail.com)*

**Purpose:** To evaluate the dependence of metabolic volumetric parameters and metabolic textural indices of primary malignant tumour on image segmentation techniques in 18F-FDG PET/CT imaging in order to select more stable or robust parameters.

**Methods and Materials:** Metabolic PET/CT images were retrospectively analyzed in 50 patients with head and neck and cervical carcinomas. Four different segmentation techniques (fixed SUV2.5 threshold, liver pool threshold, 41%SUVmax threshold and ITK-SNAP algorithm) were utilized to segment primary tumour metabolic volumes with consequent computation of volumetric (SUVmean, MTV and TLG) parameters and textural indices (GLCM Homogeneity, GLCM Entropy, GLRLM SRE, GLRLM SRE, GLZLM HGZE, GLZLM LGZE, Skewness, Kurtosis, Entropy, Energy, Sphericity and Compacity) inside generated volumes.
**Results:** Among computed parameters only two textural indices (GLCM Homogeneity and GLRLM SRE) showed no statistically significant difference, depending on segmentation technique (p<0.05), analyzing both head and neck and cervical carcinomas groups independently and whole study population, with all other indices, especially volumetric, showing significant dependence on segmentation technique used. Additionally, one of the stable textural indices (GLCLM Homogeneity) showed an ability to differentiate high grade (Grade 3) poorly-differentiated tumours from Grade1+2 tumours with relatively high specificity (84.4%) but low sensitivity (44.4%) according to ROC analysis.
**Conclusion:** Metabolic image segmentation technique influence significantly volumetric metabolic indices, unlike selected textural indices, which also show an ability to represent tumour biologic characteristics, providing an opportunity to further investigate them as a potential prognostic biomarker, more stable or robust, compared to volumetric metabolic parameters.

**B-0231** 14:40
**Inflammation focus search with 18F-FDG-PET/MRI: comparative or additive value of PET and MRI**
A. Örnek[1], L. Umutlu[1], J. Kirchner[2], B. Reichardt[1], K. Herrmann[1], V. Ruhlmann[1]; [1]*Essen/DE*, [2]*Dusseldorf/DE (ahmet.oernek@uk-essen.de)*

**Purpose:** To evaluate the comparative or additive value of 18F-FDG PET and MRI for identifying the aetiology of inflammation of unknown origin.
**Methods and Materials:** A total of 24 patients (13 m, 11 w, age 42±23 [8-82] y) with suspicion of an inflammation focus due to laboratory inflammation markers (increased CRP, leukocytes) or fever and up to now non-leading conventional imaging underwent a whole-body PET/MRI. Image analyses included the detection and localization of pathologically (focal) increased tracer uptake in PET including determination of SUVmax using VOI technique and evaluation of the contrast enhancement and diffusion restriction (ADC values) of abnormal lesions in MRI. Descriptive analysis included mean values, standard deviation and range. PET/CT, clinical, and radiological follow-up as well as histopathology served as standards of reference.
**Results:** In 21/22 patients in accordance to the reference standard the PET/MRI contributed to the diagnosis of a (focal) pathological aetiology of the inflammatory disease. In PET all pathological foci showed a moderately to significantly increased FDG uptake (SUVmax 5.5 ± 3.3, range 1.4-14.2). The MRI satisfactorily allows the localization of the findings, but only in 16/21 a corresponding contrast-enhancement and in 9/21 a corresponding diffusion restriction could be found. 2/21 patients showed neither a contrast-enhancement nor a diffusion restriction, but only an increased FDG uptake.
**Conclusion:** Integrated 18F-FDG-PET/MRI shows high potential in identifying the aetiology of inflammation of unknown origin. The MRI satisfactorily allows the localization of the findings, but a significant higher detection rate could be found in PET compared to MRI.

**B-0232** 14:48
**Is PET MRI valuable in inflammation or fever of unknown aetiology?**
T. Rohan, T. Andrasina, A. Litavcova, T. Juza, J. Zavadil, S. Richter, V. Valek; *Brno/CZ (rohan.tomas@fnbrno.cz)*

**Purpose:** To evaluate the benefit of PET/MR imaging in diagnostic process of patients with inflammatory disease of unknown aetiology.
**Methods and Materials:** 51 patients were selected for PET/MR imaging due to inflammation of unknown aetiology, these represent 2.5% of all PET/MR examinations (total of 2056 examinations, Signa, GE) acquired in tertiary referral hospital since 7/2016. All patients underwent intensive investigation lasting at least 20 days (screening x-rays, abdominal ultrasound, dedicated CT or MRI focused in clinical suspect). Clinical (fever, artralgia, weight loss, night sweating) and laboratory (CRP, leukocytes) were monitored and correlated with PET/MR findings. Based on suspected aetiology patients were divided into 3 groups - fewer of unknown origin (26), unknown infection (13) and rheumatic disease (12). Patients were followed up until the aetiology was diagnosed or symptoms faded away.
**Results:** In 26 (51%) patients diagnosis was proved, in 22 (43%) patients symptoms disappeared, in 3 patients symptoms persisted without explanation. PET/MR strongly supported or changed the diagnosis in 42% of cases (70% in subgroup of rheumatoid infections). As most important marker of conclusive PET/MR was shown artralgia and elevated level of CRP at the time of examination, which were present in 54% of diagnosed cases. PET/MR proved diagnosis of rheumatoid disease in 18 patients (50% without any clinical suspicion), overall sensitivity was up to 89% and specificity 100%, negative predictive value was 85%.
**Conclusion:** PET-MRI is a useful tool in the evaluation of inflammatory disease of unknown aetiology especially in patients with suspected rheumatoid disease.

**B-0233** 14:56
**Role of 18 F FDG PET-CT in documenting the disease burden in brain, spinal and lymph node tuberculosis**
K.B.K. Rangan, S. Gambhir; *Lucknow/IN (kasturi.rangan123@gmail.com)*

**Purpose:** Demonstrating the potential use of F18 FDG-PET/CT in documenting disease burden in Brain, Spinal, Lymphnodal Tuberculosis. Also, to monitoring follow up and to establish a therapeutic end point.
**Methods and Materials:** Total of 67 patients(~46years), of which 29 Spinal tuberculosis and 23 Brain tuberculosis and rest were lymph nodal TB. Standard [18F]FDG- PET/CT imaging was done at baseline and its results with compared with site specific MRI. On follow up only [18F]FDG-PET/CT was done at 2, 6 months. Based on the metabolic response in the follow up scans, subjects were categorized as progressive disease(PD), stable disease(SD), partial(PR) or complete metabolic response(CMR).
**Results:** Baseline 18F-FDG PET/CT revealed good agreement between the two modalities (Kappa-0.809) and also revealed additional findings like diffuse spinal uptake(4), lymph nodes(18), lung lesions(19), psoas abscess(8), some special cases such as aortic arch aneurysm and osteomyelitis. In Follow-up study, 30 subjects underwent 2nd scan, among which 7 had CMR, 20 had PR and 3 with PD. Third follow up was done in 8, among which 4 had CMR, 2 had PR and 2 with PD.
**Conclusion:** 18-F-FDG-PET/CT can be one shop stop modality in assessing the disease burden at the start of the treatment, can play pivitol role in individualizing management, monitoring disease status and help in guiding the physicians to reach therapeutic end point. Acknowledgement: IAEA, Coordinated Research Project E-15021

**B-0234** 15:04
**Optimising PET/CT paediatric imaging for patient dose reduction**
H. Kertész, T. Traub-Weidinger, I. Rausch, T. Beyer, J. Cal-Gonzalez; *Vienna/AT (hunor.kertesz@meduniwien.ac.at)*

**Purpose:** To evaluate the potential effective dose (ED) reduction by using advanced image reconstruction methods in pediatric imaging with [18F]FDG PET/CT.
**Methods and Materials:** Data from fifteen pediatric patients with epilepsy were collected. Patients underwent [18F]FDG-PET/CT examination with injected activities of 4.2±1.9 MBq/kg. The patient ED was estimated from the CT and PET acquisitions. The $ED_{CT}$ was defined as the product of the Dose-Length Product (DLP) and CT dose conversion factor ($k_{CT}$). The DLP was derived from the scan length and CT Dose Index ($CTDI_{vol}$). The $ED_{PET}$ was calculated as the injected activity and PET dose conversion factor ($k_{PET}$). The $k_{CT}$ and $k_{PET}$ were obtained from the ICRP-103 publication. The reduced PET injected activity was simulated with reducing the number of counts to 75%, 50%, 35%, 20% and 10% of the total counts. Reconstructions were performed applying OSEM, Maximum-A-Posteriori (MAP) reconstruction with median-root prior (MAP-MRP) and MAP with quadratic prior (MAP-QP). As figure of merit the target-to-background ratio, noise in the uniform region were calculated.
**Results:** The $ED_{total}$ was 5.0±1.3 mSv ($ED_{PET}$=4.7 mSv, $ED_{CT}$=0.3 mSv). Relative deviations below 10% were obtained for count reductions down to 50% applying the reconstruction methods OSEM+4mm filter and MAP-MRP. Using 50% of counts ED for PET could be reduced from 4.7 to 2.4 mSv. This results in a reduction of the total ED up to 53%.
**Conclusion:** A potential reduction of the injected PET activity up to 50% can be achieved without compromising the selected image quality parameters by applying a suitable reconstruction method.

**B-0235** 15:12
**First report from breath-holding high-resolution high-sensitivity SiPM PET/CT: are lung nodules getting more active?**
M. Jreige, M. Meyer, G. Allenbach, M. Pappon, M. Nicod Lalonde, N. Schaefer, J.O. Prior; *Lausanne/CH (mario.jreige@chuv.ch)*

**Purpose:** Accurate pulmonary nodules characterization is highly important considering its impact on management and prognosis. The latest PET/CT scanners combing ultra-dynamic range Silicon-photomultiplier detector technology (SiPM), faster time-of-flight and breath-holding (BH) maneuver may allow better visualization and uptake quantification of smaller lesions. Herein, we investigate the feasibility of 20-second-BH-PET/CT acquisition as compared to the standard free-breathing (FB) and its effects on morphologic and metabolic lung nodule characterization.
**Methods and Materials:** We retrospectively analyzed all PET/CT examinations from 06-10/2018 acquired on a Siemens Biograph Vision 600 with a lung dedicated BH-acquisition in addition to standard FB-acquisition. Only nodules considered highly suspicious for malignancy were included and characterized morphologically (size) and metabolically (presence/absence of uptake, SUVmax, SUVmean, MTV and TLG) on both acquisitions.
**Results:** From the 22 reviewed studies, 11 nodules reported as highly suspicious in nine patients were included. The mean nodule size(mm), SUVmax(g/ml), SUVmean(g/ml), MTV(cm3) and TLG(g*cm3/ml) were 11±4.3, 4.5±3.4, 2.5±1.8, 0.7±1.1 and 2.6±4.9 in FB and 11.6±3.9, 7.7±5.7, 3.3±2, 1±1.2 and 4±7.3 in BH, respectively. Only 72% of the nodules were visible on FB-acquisition compared to 100% on BH, with a trend for a statistically significant difference (p=0.062).Concordance analysis showed a statistically significant increase of 68% of the SUVmax value in BH compared to FB.
**Conclusion:** BH with high-resolution, high-sensitivity PET/CT allowed to detect higher metabolic activity in lung nodules categorized as highly suspicious, in comparison to FB. This increased accuracy in activity detection may help to increase the sensitivity and specificity of malignant nodule characterization.

**B-0236** 15:20
**PET/CT imaging of the novel molecular probe $^{11}$C-aminoglycerol for the diagnosis of liver fibrosis**
X. Chen, J. Xin; *Shenyang/CN (986107759@qq.com)*

**Purpose:** In this study, we utilized amino glycerol as the precursor to synthesize a novel molecular probe [$^{11}$C]AR, which the preclinical performance of liver fibrosis diagnosis was evaluated with in vivo PET/CT imaging.
**Methods and Materials:** We developed a fully automatic synthesis procedure for the preparation of [$^{11}$C]AR with improved synthesis efficiency and radiochemical purity. The distribution of [$^{11}$C]AR was investigated on PET/CT scanner using a rat model. The dynamic PET/CT scans were performed on the control group (n=6) and model group (n=29), which the model group was divided into three stages (S1, S2+S3, S4) according to the stages of liver fibrosis. The images were reconstructed and the region of interest (ROI) in the liver area was selected within 20 mm$^2$. One-way analysis of variance and independent sample t test were used to analyse the difference of SUVmax among the groups in a series of scanning time points.
**Results:** The fully automatic synthesis of [$^{11}$C]AR was successfully achieved with a radiochemical purity and a synthesis efficiency at least 98% and 50%, respectively. The uptake of [$^{11}$C]AR in the model group was significantly lower than that in the control group at all the time points (P<0.05). There were significant differences in the SUVmax between the S1 and S4 groups at 150s, 5min, 10min, 15min and 25min (P<0.01).
**Conclusion:** We demonstrated a new C-11 radiolabeled amino glycerol PET/CT imaging probe [$^{11}$C]AR for diagnosing and determining liver fibrosis stages, which shows potential clinical implications for noninvasive diagnosis of liver fibrosis.

**14:00 - 15:30** **Room O**

### Musculoskeletal

### SS 310

### Upper extremity imaging

*Moderators:*
I. Boric; Zabok/HR
L. Lassalle; Paris/FR

**K-07** 14:00
**Keynote lecture**
A. Blum; *Nancy/FR*

**B-0237** 14:09
**Ultrahigh-field MR microscopy of the upper extremity of the chicken in vivo throughout the in ovo period**
M. Jäschke, K. Thierfelder, B. Klaan, T. Stahnke, T. Lindner, A. Wree, M.-A. Weber, S. Langner; *Rostock/DE (malte.jaeschke@med.uni-rostock.de)*

**Purpose:** Ultrahigh-field MRI (UHF-MRI) with an in-plane spatial resolution of less than 100 µm is known as MR microscopy (MRM). This technique provides highly resolved non-invasive anatomical images and allows longitudinal assessment of embryonic avian development. The aim of the present study was to evaluate the feasibility of in vivo anatomical MRI assessment of the developing upper extremity of the chicken in ovo.
**Methods and Materials:** Thirty-eight fertilised chicken eggs were examined at 7 Tesla (ClinScan, Bruker BioSpin, Germany) acquiring high-resolution T2-weighted anatomical images with an in-plane resolution of 78 x 78 µm. To reduce motion artefacts, the eggs were moderately cooled before and during MR imaging. Two eggs were imaged daily for the entire developmental period, and 36 eggs were examined pairwise at only one time point of the embryonic period. Development of the upper extremity was anatomically and quantitatively assessed. Chondrification on MRI was correlated with histological examination.
**Results:** MRM allowed identification from embryonic stage D5 onwards. First chondrification of the upper extremity was visible at stage D7 and differentiation of the forearm was possible from stage D9 with excellent correlation to histology. Repeated cooling and MRM had no influence on the development of the chicken.
**Conclusion:** MRM allows in vivo assessment of embryonic development of the upper extremity of the chicken in ovo without affecting normal development. The method provides non-invasive anatomical information supplemented by quantitative evaluation. With increasing availability of ultrahigh-field MR systems, this technique may provide a noninvasive complementary tool in experimental musculoskeletal radiology.

**B-0238** 14:17
**MR quantification of the fatty fraction from IDEAL IQ sequence in the assessment of muscle atrophy in rotator cuff tears**
O. Seraydarmansour, Z. Taher Rakei; *Tehran/IR (mansour.omid@yahoo.com)*

**Purpose:** To assess the degree of muscle atrophy and fatty infiltration within supraspinatus muscle in patients with full-thickness rotator cuff tears using a T2*-corrected IDEAL IQ sequence.
**Methods and Materials:** Among the patients who were referred to our imaging centre for shoulder MRI, 45 patients with full-thickness rotator cuff tears were included in group A and 21 normal patients were included in group B. The mean age of the patients was 63 years (age range, 82-41 years). Necessary information for our study including age, sex, weight, the time of trauma and the type of work and physical activity was identified through questionnaires. All MRI examinations were performed with a 1.5 Tesla MRI unit (Explorer, GE Healthcare) using a four-channel coil. All subjects underwent the standard shoulder MRI protocol including proton density fat-saturated fast spin echo in the oblique coronal, axial and sagittal planes. T1-weighted fast spin echo in the oblique coronal and sagittal planes. We also performed T2* correction IDEAL IQ sequence in the oblique sagittal plane. Fat-signal fraction from the mapping images was measured by drawing a circular ROI on the cross section of supraspinatus muscle belly on the Y-view.
**Results:** Fat-signal fraction from the T2*-corrected IDEAL IQ sequence was significantly higher in group A than group B.
**Conclusion:** MRI quantification of fat fraction by using a T2* corrected IDEAL IQ sequence is a valuable method for detecting the fat infiltration in supraspinatus muscle after tearing the rotator cuff.

**B-0239** 14:25
**Evaluation of glenoid labral tears: comparison between dual-energy CT arthrography and MR arthrography of the shoulder**
G. Foti[1], M. Catania[2], A. Beltramello[1], G. Carbognin[1]; [1]*Negrar/IT*, [2]*Verona/IT (gfoti81@yahoo.it)*

**Purpose:** To compare the diagnostic accuracy of dual-energy computed tomography (DECT) arthrography and magnetic resonance arthrography (MRA) of the shoulder in depicting glenoid labral tears.
**Methods and Materials:** This prospective study included 26 consecutive patients studied with DECT (80 kV and tin filter 150 kV) and MR arthrography. DECT data were postprocessed on a dedicated offline workstation (SyngoVia®) using a three-material decomposition algorithm. Two experienced radiologists, blinded to surgical data, evaluated the presence of labral tears on DECT maps and on MRA. Surgical findings served as standard of reference. Diagnostic accuracy values of the DECT and of the MRA and inter-observer and intra-observer agreements were assessed. A value of p<0.05 was considered statistically significant.
**Results:** MRI revealed the presence of labral tear in 19/26 patients (73.1%), with 8 tears of antero-inferior labrum, 10 tears of superior labrum and 1 tear of posterior labrum. The sensitivity, specificity, PPV and NPV and accuracy of DECT arthrography and MRA were 89.5, 100, 100, 77.7 and 92.3%, and 94.7, 85.7, 94.7, 85.7 and 92.3%, respectively. Using McNemar's test, the difference of accuracy between DECT arthrography and MRA was not significant (p=0.45). The inter-observer and intra-obsever agreements were near perfect (k=0.82 and k=0.86, respectively).
**Conclusion:** DECT represents a reliable imaging tool for demonstration of glenoid labrum tears.

**B-0240** 14:33
**Value of 3D-multi-echo-data-image-combination (MEDIC) for evaluation of SLAP lesions at 3T-MRI of the shoulder with arthroscopic correlation**
F. Wuennemann, C. Rehnitz; *Heidelberg/DE (felix.wuennemann@med.uni-heidelberg.de)*

**Purpose:** To prospectively evaluate the ability of 3D-Multi-Echo-Data-Image-Combination (MEDIC) compared to high resolution 2D-proton-density weighted fat-saturated (PDfs) sequence at 3T-MRI in detecting superior labral anterior to posterior (SLAP) lesions using arthroscopy as gold standard.

**Methods and Materials:** 17 consecutive patients (mean age 51.6±14.8 years, 11 men) with shoulder pain underwent 3T-MRI including 3D-MEDIC and 2D-PDfs followed by arthroscopy. Presence or absence of SLAP lesions were evaluated using both sequences by two independent raters with 3 and 14 years of experience in musculoskeletal MRI. During arthroscopy SLAP lesions were classified according to Snyder by an orthopaedic shoulder surgeon. Sensitivity and specificity of 3D-MEDIC and 2D-PDfs for detection of SLAP lesions were calculated using arthroscopy as gold standard.
**Results:** Arthroscopy revealed SLAP lesions in 13/17 patients. Using 3D-MEDIC SLAP lesion were diagnosed in 14/17 patients by rater 1 and in 13/17 patients by rater 2. Using 2D-PDfs SLAP lesions were present in 11/17 and 12/17 patients for rater 1 and 2, respectively. Sensitivity/specificity of 3D-MEDIC was 100%/50% for rater 1 and 100%/67% for rater 2, respectively. Using 2D-PDfs sensitivity/specificity was 91%/83% for rater 1 and 100/83% for rater 2, respectively. The combination of 2D-PDfs and 3D-MEDIC increased specificity from 50% to 83% for rater 1 and from 67% to 100% for rater 2.
**Conclusion:** Using arthroscopy as reference, 3D-MEDIC exhibits high sensitivities in detection of SLAP lesions with moderate specificity. The combination of 3D-MEDIC and 2D-PDfs markedly increased specificity and may therefore be recommended for shoulder imaging with respect to SLAP lesions.

**B-0241** 14:41
**Evaluation of bone loss in shoulder instability using on track/off track method: value of 3D MR arthrography in standard and ABER position**
C. Gianneramo, F. Bruno, E. Cannizzaro, P. Palumbo, S. Iafrate, S. Mariani, F. Arrigoni, A. Barile, C. Masciocchi; *L'Aquila/IT*
*(camillagianneramo@gmail.com)*

**Purpose:** To evaluate the value of the 3D MR Arthrography (MRA) in standard and ABER (Abduction-External Rotation) position in identifying engaging lesions in patients with anterior shoulder instability.
**Methods and Materials:** We retrospectively evaluated 28 patients (17 men and 11 women mean ages 28.3 years range 19-43) with anterior shoulder instability and standard MRI evidence of glenoid bone loss and/or Hill Sacks lesion. All patients were submitted to 3D-CT of the shoulder and 3T-MR Arthrography using a three-dimensional (3D) isotropic PD sequence performed in standard and ABER position. Two double-blind radiologists calculated for each patient the glenoid track and Hill Sacks interval using the “on track/off track” method to identify “engaging” and “non engaging” lesions on CT and MRA exams. The intra and inter-observer agreement were calculated. Results were also compared to arthroscopic findings.
**Results:** Using the “on track/off track” method, 19 bone loss were identified as “non-engaging” lesion and 9 were classified as “engaging” lesions with an “almost perfect” inter-observer concordance both for glenoid track and Hill Sacks interval measured on CT and MRA. Intra-observer agreement was 0.906 for 3D-CT and 0.713 for MRA. ABER position showed weak positive predictive value but strong negative predicted value to detected engaging lesions.
**Conclusion:** MRA has an excellent diagnostic accuracy in identifying engaging lesions. The ABER position could represent an added value in evaluation of engaging and non engaging lesions on patients with anterior shoulder instability.

**B-0242** 14:49
**Ability of T2 mapping at 3T MRI for biochemical assessment of normal and damaged glenoid cartilage of the shoulder: an arthroscopy controlled prospective study**
C. Rehnitz, F. Wuennemann; *Heidelberg/DE*
*(Christoph.Rehnitz@med.uni-heidelberg.de)*

**Purpose:** To prospectively evaluate T2 mapping at 3T MRI of the shoulder regarding its ability to assess healthy glenoid cartilage and focal cartilage damage using arthroscopy as the gold standard.
**Methods and Materials:** 18 consecutive patients (mean age 52.4±14.72 years, 12 men) with shoulder pain underwent T2 mapping at 3T MRI with subsequent shoulder arthroscopy. Cartilage-sensitive morphologic sequences were used to detect normal cartilage and focal cartilage lesions. Then T2 values of normal and damaged cartilage were assessed using region-of-interest analyses by two independent raters. Interrater and intrarater correlation coefficients (ICCs) were calculated. Cut-off values and their sensitivities/specificities including 95% confidence intervals (CI) for the detection of cartilage damage were assessed using arthroscopy as reference. $P<0.05$ was considered significant.
**Results:** Mean T2 value of arthroscopically proven healthy cartilage was 23.9±5.99 ms. In 5 patients, a focal cartilage damage was present exhibiting significantly higher ($p<0.01$) T2 values of 44.7±3.7 ms. A complete data separation was present as the maximum T2 value for normal cartilage (27.3) was lower than the minimum value in damaged cartilage (40.7). Therefore, all cut-off values between 27.3 and 40.7 ms resulted in a sensitivity and specificity of 100% (95%CI: 47.8%-100.0%). Interrater agreement was almost perfect (ICC 0.94-0.95) with moderate intrarater agreement (ICC 0.37-0.67).
**Conclusion:** Using arthroscopy as the gold standard, T2 mapping was able to detect and quantify healthy and damaged glenoid cartilage. The presented cut-off values allowed for a perfect discrimination. T2 mapping may be used as an additional tool to evaluate the biochemical cartilage integrity at the shoulder.

**B-0243** 14:57
**Extremity cone-beam CT of forearm and hand: evaluation of image quality, metal artefacts and radiation dose**
C. Polkowski, I. Yel, M.C. Langenbach, B. Kaltenbach, T.J. Vogl, K. Eichler; *Frankfurt a. Main/DE*

**Purpose:** To investigate the impact of different scan protocols in latest generation cone beam CT on image quality, metal artefacts and radiation dose.
**Methods and Materials:** In a prospective study scans of the same cadaver forearm and hand with a distal radius plate were performed to achieve highest intra-individual comparability. 55 consecutive scans were performed on a latest generation cone beam CT scanner for extremity imaging using different combinations of tube voltage and tube current-exposure time product. Effective radiation dose was calculated on the basis of measurements of seven dosimeters in and on the arm. Image quality and metal artefacts were rated by five different readers independently in a blinded evaluation using a 5-point Likert scale (from 1 indicating non-diagnostic to 5 for excellent image quality).
**Results:** The calculated effective dose for various settings ranged from 0.011 mSv (manufacturer's suggested protocol: 90 kV, 5.0 mA) with an average overall subjective rating of 4.3 and a subjective rating with focus on metal artefacts of 3.8 to 0.003 mSv (70 kV, 2.0 mA) with an average subjective rating of 3.5 and a rating of 3.2 focusing on metal artefacts.
**Conclusion:** Subjective image quality, especially focusing on metal artefacts, is maintained at a moderate to good level despite the use of protocols with significant dose reduction compared to manufacturer's suggested standard protocol.

**B-0244** 15:05
**Physeal changes in youth gymnasts: a three-dimensional assessment of stress-related volume increase of the distal radial physis using magnetic resonance imaging**
R.B.J. Kraan, L. Kox, M. Mens, M. Maas; *Amsterdam/NL*
*(r.b.kraan@amc.uva.nl)*

**Purpose:** Gymnasts are prone to injuries of the distal radial physis as a result of excessive stress applied to the wrist during gymnastics. This can cause damage to the metaphyseal vascularisation and absent or delayed endochondral calcification, with subsequent widening of the cartilaginous part of the physis. This study aims to evaluate stress-related volume changes of the distal radial physis in symptomatic gymnasts, asymptomatic gymnasts and non-gymnasts.
**Methods and Materials:** Symptomatic gymnasts with suspected distal radial physeal injury, asymptomatic gymnasts and non-gymnasts (all <18 years) were included. Volume measurements were performed by creating three-dimensional reconstructions of the distal radial physis on coronal three-dimensional water-selective cartilage MRI images.
**Results:** Between June 2015 and August 2018, 27 symptomatic gymnasts, 18 asymptomatic gymnasts and 24 non-gymnasts were included. No significant difference in median skeletal-age was found (13.1, 11.9 and 13.3 years for symptomatic-, asymptomatic- and non-gymnasts). Median physeal volume was significantly increased in symptomatic (971 $mm^3$, IQR 787-1237) and asymptomatic gymnasts (951 $mm^3$, IQR 871-1004) compared to non-gymnasts (646 $mm^3$, IQR 538-795). Inter- and intrarater reliability of volume measurements were excellent. Of the 10 participants with the highest physeal volumes, nine were symptomatic gymnasts.
**Conclusion:** Increased volume of the distal radial physis is a sign of physeal stress and can be present in both symptomatic and asymptomatic gymnasts. Especially gymnasts with suspected physeal injury showed more extreme volume increases. Volume assessment of the distal radial physis may, therefore, be a valuable addition in the (early) diagnostic workup and follow-up of suspected physeal stress injuries.

**B-0245** 15:13
**Dual-energy CT in the diagnosis of occult acute scaphoid injury: a direct comparison with MRI**
C. Xie, S. Ather, R. Mansour, F. Gleeson, R. Chowdhury; *Oxford/UK*
*(chengxie@doctors.org.uk)*

**Purpose:** Suspected occult scaphoid injuries are a common clinical issue and they require further imaging (CT/MRI) for diagnosis. We investigating the use of dual-energy computed tomography (DECT) for the detection of acute bone marrow oedema/fracture of the scaphoid compared to MRI.
**Methods and Materials:** Prospective study of 17 adult patients who presented acutely (without prior injury) to the emergency department with clinically suspected occult scaphoid fracture and had MRI of the wrist were recruited to have DECT (GE Revolution CT). Two experienced musculoskeletal radiologists independently reviewed the DECTs (blinded from MRI results) for acute bone oedema on the GE AW2.0 Analysis Station using the calcium/water filters. The statistical difference of MRI and reviewers' detection of acute bone oedema (1= present, 0= absent) was performed using Friedman test.
**Results:** MRI showed acute scaphoid fracture/oedema in 11/17 patients. Reviewer A identified acute oedema in 10/11 patients, and Reviewer B identified 7/11 patients that were positive on MRI. On DECT, both reviews did not identify the mild oedema seen on MRI. However, there was no statistically significant difference in oedema detection on MRI and the reviewers on DECT (p-value 0.73). In addition, the reviewers identified more cortical fractures (7 fractures associated with the acute scaphoid oedema) than the MRI.
**Conclusion:** DECT has the capability to detect acute scaphoid oedema and more cortical fractures compared to MRI. However DECT was less sensitive in the detection of mild acute scaphoid oedema, and this was the main limitation of the calcium/water filters.

**B-0246** 15:21
**Trapezial ridge fractures: an underappreciated cause of post-traumatic wrist pain with normal initial radiographs.**
B. Gibney, M.C. Murphy, D. Ahern, A. Moughty, E. Kavanagh, D. Hynes, P.J. MacMahon; *Dublin/IE (bgibney1@gmail.com)*

**Purpose:** Fractures of the trapezium are rarely diagnosed on plain radiographs after acute wrist trauma. High-resolution cross-sectional imaging identifies fractures of the trapezium as the most common radiographically occult carpal bone fracture. We review the fracture frequency, mechanisms and patterns of trapezium fractures.
**Methods and Materials:** Cone-beam CT was performed in patients with suspected radiographically occult radiocarpal fracture following acute injury. The frequency of carpal bone fractures was assessed and compared.
**Results:** 67 radiographically occult wrist fractures were identified with CBCT in 117 patients with acute trauma and negative radiographs. The trapezium was the most frequently fractured carpal bone, making up 29% of carpal fractures. 79% of trapezium fractures involved the volar ridge.
**Conclusion:** Fractures of the trapezium in acute wrist trauma are much more common than described in the literature. Awareness of the types and mechanisms of fracture is important. Cross-sectional imaging should be considered in all cases of post-traumatic wrist pain with negative radiographs.

**14:00 - 15:30** **Room E1**

### Breast

### SS 302

### Digital breast tomosynthesis (DBT)

*Moderators:*
J. Etxano; Vitoria/ES
P.L. Moyle; Cambridge/UK

**B-0247** 14:00
**Digital breast tomosynthesis (DBT) in the assessment of false positive recalls in a single-centre breast screening programme**
M. Orsi, F. Leone, M. Cellina, G. Oliva; *Milan/IT (m.orsi83@gmail.com)*

**Purpose:** To evaluate the power of DBT in revealing false positive mammographic findings in a single centre 2D-mammography-based screening programme.
**Methods and Materials:** Between September 2015 and February 2018, 843 consecutive recalled cases diagnosed BI-RADS 1 or 2 after clinical examination, DBT and ultrasound at second level screening, were retrospectively evaluated by a breast radiologist to assess whether the DBT taken alone was able to exclude with certainty the suspicious findings pointed out at first level screening mammography.
**Results:** We analyzed 843 cases: in 64% (537) DBT alone was enough for the diagnosis, without the need to perform ultrasound, in 30% (254) ultrasound was needed for the diagnosis; in 6% (52) DBT was considered not helpful or misleading.
**Conclusion:** Our results demonstrate that DBT is an effective tool for the correct interpretation of false positive mammographic findings at second level screening in 94% cases and DBT alone is enough for the diagnosis in more than a half cases; moreover, our results indirectly confirm that the implementation of DBT in the first level should reduce unnecessary recalls. DBT is a powerful tool to reveal false positive mammographic finding and should be increasingly used in the screening setting to reduce false positive recalls.

**B-0248** 14:08
**Stratifying tomosynthesis screening results by age groups and by first or subsequent screening**
G. Romanucci[1], F. Caumo[2], M. Zorzi[2], P. Bricolo[1], L. Cugola[1], S. Brunelli[3], R. Mariotto[1], S.A. Montemezzi[1], F. Fornasa[1]; [1]*Verona/IT*, [2]*Padua/IT*, [3]*Peschiera/IT (giovitta@libero.it)*

**Purpose:** To examine the outcomes of a breast cancer screening program based on digital breast tomosynthesis (DBT) plus synthesized two-dimensional (2D) mammography stratified by age groups.
**Methods and Materials:** from April 2017 to March 2018, 22906 women (50-74 years) underwent DBT + synth2D as a screening test. The results - stratified by age groups and by first or subsequent screening - were examined by calculating the proportions associated with each outcome.
**Results:** the detection rate (DR) was 9.17‰ (210/22906) for total screening, significantly lower at the subsequent screening (6.85‰ (110/16065)) than at the first screening (14.6‰ (100/6841)) (p<0.001). Regarding the results stratified by age groups, the DR was 6.1‰ (36/5901) in the 50-54 age group, 6.6‰ (25/4378) in the 55-59 age group, 9.3‰ (45/4838) in the 60-64 age group, 12.1‰ (56/4638) in the 65-69 age group, and 14.0‰ (44/3150) in the 70-74 age group. The decrement in cancer DR was highest in the 70-74 age group (from 14.6‰ at the first screening to 9.8‰ at subsequent screening). Furthermore, the positive predictive value at recall was significantly higher for the 70-74 age group (31.0‰, 44/142) vs. all the other age groups (14.9‰, 166/1113) (p>0.001).
**Conclusion:** the DR for cancer of DBT increases with increasing age; our results also highlight the effectiveness of DBT in terms of cancer detection for women older than 70 years.

**B-0249** 14:16
**Stratifying tomosynthesis screening results by breast density**
G. Romanucci[1], S.A. Montemezzi[1], S. Brunelli[2], P. Bricolo[1], L. Cugola[1], F. Caumo[3]; [1]*Verona/IT*, [2]*Peschiera/IT*, [3]*Padua/IT (giovitta@libero.it)*

**Purpose:** To assess the highest detection rate (DR) of tomosynthesis (DBT) compared to digital mammography (FFDM) in a screening population, with better performance in dense breasts.
**Methods and Materials:** from April 2015 to March 2017, 34071 women (50-69 years) underwent DBT + synth2D as a screening test. The results - stratified by breast density, age groups and radiological features - were compared with those of a historical court that in the previous two years (April 2013 - March 2015) performed the screening test with FFDM, through a retrospective analysis.
**Results:** DR was 9.2 / 1000 (315/34071) in the study group versus 5.2 / 1000 (152/29360) in the historical court (95% CI 4.4-6.1) with an incremental cancer DR of 4.0 / 1000 (95% CI 2.7 to 5.4), P <0.001. Regarding the results stratified by breast density: in non-dense breasts, DR was 8.7 / 1000 (247/28390) for the study group versus 5.1 / 1000 (122/23860) in the historical court; in high density breasts, DR was 12/1000 (68/5681) for the study group versus 5.6 / 1000 (31/5500) for the historical court. The incremental cancer DR was higher in high density breasts with a difference of 6.4 / 1000 (95% CI; 2.7 to 5.4) and improved detection of opacities (72.2% for DBT + synt2D versus 55.1% for FFDM).
**Conclusion:** DBT is more sensitive than FFDM in both adipose and dense breasts with more than double detection in the latter group.

**B-0250** 14:24
**Cancer detection rate, ductal carcinomas in situ and advanced breast cancers in the 2$^{nd}$ round of a population-based screening with tomosynthesis and synthetic mammography**
F. Caumo[1], G. Gennaro[1], G. Romanucci[2], M. Zorzi[1], C. Fedato[3], S.A. Montemezzi[2]; [1]*Padua/IT*, [2]*Verona/IT*, [3]*Venice/IT (francesca.caumo@iov.veneto.it)*

**Purpose:** To compare cancer detecton rate and rates of ductal carcinomas in situ (DCIS) and advanced breast cancers (T2+) found in the first and second rounds of a population-based screening with tomosynthesis (DBT) and synthetic mammography (SM).
**Methods and Materials:** 16,666 women were recruited between Apr 2015 and Mar 2016 for screening with (DBT+SM) in the Verona screening program. 16,065 of them were rescreened two years later for a second round with the same screening test. Cancer detection rate (CDR), rate of DCIS, and rate of T2+ cancers were compared in the two groups. A P value below 0.05 was considered statistically significant.

**Results:** 155 cancers were found in the first round and 117 in the second, leading to a CDR of 9.3/1000 versus 7.3/1000. The decrease of CDR was statistically significant (P = 0.045). DCIS were 19 and 17 in the two rounds, while T2+ cancers were 19 and 13, respectively. DCIS rates were comparable in the two rounds (1.14/1000 vs. 1.10/1000, P = 0.82). T2+ rate decreased from 1.14/1000 in the first round to 0.81/1000 in the second round, but the difference was not significant (P = 0.34). Finally, the invasive cancer rate moved from 8.2/1000 to 6.3/1000, producing a statistically significant difference (P = 0.039).
**Conclusion:** There was no significant change in detection rates of both DCIS and advanced breast cancers. The reduction in cancer detection rate at the second screening round confirmed that screening with tomosynthesis early detected small, invasive breast cancers.

**B-0251** 14:32
**Does breast tomosynthesis increase cancer detection in a screening population with clinically elevated breast cancer risk?**
M. Keupers[1], S. Woussen[1], S. Postema[2], H. Westerlinck[3], G. Zhang[1], L. Cockmartin[1], H. Bosmans[1], C. Van Ongeval[1]; [1]*Leuven/BE*, [2]*The Hague/NL*, [3]*Heverlee/BE (sofie.woussen@gmail.com)*

**Purpose:** Adding digital breast tomosynthesis (DBT) to full-field digital mammography (FFDM) increases breast cancer (BC) detection rate (BCDR) in population screening. This trial investigates the impact of adding DBT to FFDM in screening women with clinically elevated BC-risk.
**Methods and Materials:** This prospective single-institution study included 431 asymptomatic women, aged 40-69, with clinically elevated BC risk who underwent screening with 4-view FFDM + DBT in a single compression (Hologic Selenia dimensions) and additional ultrasound and MRI if IBIS risk score >30%. Four radiologists evaluated density, BIRADS, lesion type and localisation, first for FFDM and second for FFDM+ DBT.
**Results:** Breast density C/D was found in 85% of the women. BCDR (invasive + in situ) was 9.3/1000 for both FFDM and FFDM+DBT. No additional cancers were found by adding DBT nor any of the detected cancers in FFDM were missed with FFDM+DBT. Moderate inter-reader agreement for BIRADS was found for both study arms (ICC FFDM: 0.43 and ICC FFDM+DBT: 0.45). Further analysis with recall majority vote of the 4 readers showed no significant differences in recall rate (McNemar p=1) when adding DBT. Additional US and MRI resulted in a higher BCDR (18.6/1000) with detection of occult invasive cancer, papillary DCIS and non-calcified DCIS, suggesting that non-x-ray or contrast techniques are necessary for BC-detection in this subgroup of patients.
**Conclusion:** In contradiction to screening trials, addition of DBT to FFDM did not increase BCDR for this clinically elevated BC risk screening subgroup; other modalities (i.e. ultrasound, MRI) were necessary to find additional cancers.

**B-0252** 14:40
**Efficiency of mammography, tomosynthesis and breast MRI in detecting malignant extension to the nipple area complex in patients with multifocal or extensive breast DCIS**
R. Capocci[1], V. Tacher[2], R. Bosc[1], J.-L. Totobenazara[1], H. Kobeiter[1], A. Luciani[1], E. Meyblum[2], T.H. Dao[1]; [1]*Créteil/FR*, [2]*Paris/FR (romain.capocci@gmail.com)*

**Purpose:** Ductal carcinomas in-situ (DCIS) are frequent breast cancers with many indications for mastectomy. Cosmetic results are important in this oncological population, increasing conservative surgeries (skin-sparing (SSM) and nipple-sparing mastectomies (NSM)). Our objective was to evaluate the efficiency of digital mammography (DM), tomosynthesis (TS) and breast magnetic resonance imaging (MRI) in detecting tumor extension to the nipple-areolar-complex (NAC) in patients with multifocal or extensive breast DCIS.
**Methods and Materials:** Thirty-four DCIS patients, retrospectively included between May 2011 and November 2017, underwent either SSM or NSM. Two radiologists (one junior, one senior) independently measured the minimal tumor-to-NAC distances, on available prior-to-surgery DM (front, oblique or side views), TS and subtracted post-injection T1-MRI sequences for inter-rater kappa correlation calculation. Measurements were compared to distances evaluated on the surgical specimen (NAC invasion was considered if this distance was under 10mm).
**Results:** Nineteen (56%) SSM and 15 (44%) NSM were performed, with pathological invasion of the NAC in five NSM patients (26%) and ten SSM patients (67%). Using the minimal tumor-to-NAC distance (on DM, TS and MRI), sensibility and specificity for NAC-invasion detection were 67% (IC:95-[0.62-0.71]) and 58% (IC:95-[0.53-0.63]), respectively (cut-off of 10 mm). Using MRI (cut-off of 10mm), sensibility and specificity were 58% (IC: 95-[0.53-0.63]) and 46% (IC:95-[0.41-0.61]), respectively. The inter-reader correlation was 0.76 using the minimal tumor-to-NAC distance based on all available images, and 0.60 using only MRI.
**Conclusion:** Our study suggests that the minimal tumor-to-NAC distance in DCIS could be predicted on multimodality image analysis.

**B-0253** 14:48
**Comparison between rapid unenhanced MRI protocol (RU-MRI) and digital breast tomosynthesis (DBT) in detecting breast cancer in a screening-like population**
V. Marconi, M. Lorenzon, R. Girometti, L. Di Mico, F. Bondini, A. Bracciani, C. Zuiani; *Udine/IT (vale.marconi27@gmail.com)*

**Purpose:** To compare the accuracy of RU-MRI and DBT, in a simulated breast-cancer screening scenario.
**Methods and Materials:** A retrospective analysis was performed on patients who underwent DBT and 1.5T-MRI over 2016-2017 in our Institution. Cancer beyond T1-stage, previous breast surgery and examinations after neoadjuvant chemotherapy were exclusion criteria. Four readers, blinded to clinical history and histology-pathology, reviewed first DBT, then RU-MRI (including only DWI and T1-w sequences) in separate reading sections, reporting lesions using BI-RADS score. Cases with BI-RADS ≥3 assignment were considered as “Screening Recall” (SR). Pathology and negative follow-up served as reference standard. Receiver operating characteristics analysis and k-statistic were used to assess the accuracy of both methods and interobserver agreement in assessing cases as SR. P-values <0.05 were considered statistically significant. A "per-breast" evaluation was performed.
**Results:** A total of 118 breasts (patient’s age: range 31-79; mean 57) were included. Prevalence of disease was 32% (38/118). Readers showed 89.5%, 92.1%, 84.2%, 84.2% sensitivity for DBT, and 92.1%, 84.2%, 92.1%, 84.2% for RU-MRI; the specificity was 81.2%, 78.7%, 90%, 72.5% for DBT, and 92.1, 84.2, 92.1, 84.2% for RU-MRI. The differences between AUCs for two methods, for all readers, were not significant (p>0,05), despite a slight improvement in specificity with RU-MRI. Interobserver agreement was good for RU-MRI (range 0.618-0.783), and moderate to good for DBT (range 0.452-0.718).
**Conclusion:** RU-MRI and DBT are comparably accurate in detecting cancer in screening-like scenario, with acceptable interobserver agreement. Both methods might be the subject for further research in a screening population.

**B-0254** 14:56
**Synthesized mammography: is it as good as digital mammography to find microcalcification?**
V.L.N. Omura; *Sao Paulo/BR (vivinakamura@yahoo.com.br)*

**Purpose:** To compare synthesized mammography (SM) plus digital breast tomossynthesis (DBT) versus conventional full-field digital mammography (DM) alone for detection of microcalcification.
**Methods and Materials:** This prospective multireader study included 119 cases: 94 consecutive diagnostic mammography cases that were submitted to stereotaxic biopsies for suspicious microcalcifications, from July 2017 to January 2018, and 25 additional screening mammograms without microcalcifications (controls). All cases had DM, SM and DBT images. The images were randomly analyzed by two independent radiologists that reported the presence of suspicious microcalcification, using the BI-RADS lexicon. Sensitivity, specificity, positive and negative predictive values were calculated for SM plus DBT and for DM alone. Interreader agreement was calculated with Cohen’s kappa values. Receiver operator curve analysis was performed.
**Results:** Agreement between SM plus DBT and DM was susbstantial (Cohen’s k-coefficient = 0,78 ± 0.09) .When comparing DM versus SM plus DBT prevalence, sensitivity, specificity, positive (PPV) and negative predictive values (NPV) and area under roc curve (AUC) was 74,0%, 93,8%, 85,5%, 94,8%, 82,8%, 0,896, respectively. When comparing SM plus DBT versus DM, prevalence, sensitivity, specificity, PPV, NPV and AUC was respectively 73,0%, 94,8%, 82,8%, 93,8%, 85,5%, 0,888. And finally, when comparing radiologist A versus radiologist B, and radiologista B versus radiologista A, prevalence, sensitivity, specificity, PPV, NPV and AUC was, respectively, 75,0%, 94,9%, 95,0%, 98,3%, 86,4%, 0,950 and 72,0%, 98,3%, 86,4%, 94,9%, 95,0%, 0,923.
**Conclusion:** Synthesized mammography plus DBT showed similar performance to digital mammography for the detection of microcalcification, confirming its potential as an alternative to digital mammography.

**B-0255** 15:04
**A new Fourier-based model observer for image quality evaluation in the central slice of simulated breast tomosynthesis images**
C. Balta[1], I. Sechopoulos[1], R. Van Engen[1], M. Broeders[1], W.J.H. Veldkamp[2], I. Reiser[3]; [1]*Nijmegen/NL*, [2]*Leiden/NL*, [3]*Chicago, IL/US (c.balta@lrcb.nl)*

**Purpose:** To investigate the appropriateness of using a novel Fourier-based prewhitening 2D model observer (FPMO) to quantify image quality in a simulated small-scale breast-structure phantom.
**Methods and Materials:** An *in silico* breast tissue model based on mammographic power-law noise was used to generate 1000 virtual 3D volumes of $256^3$ voxels representing breast-like structures. Soft-edged spherical masses with diameters of 4 mm and 16 mm were inserted at the center of the volumes, with different contrast levels. The central slice of each volume was scored by the FPMO by computing a test statistic using a pre-whitened signal template. This template was computed in the Fourier domain and incorporated the mammographic noise-power spectrum of the breast-like background. The decision variable from the signal-present and signal-absent images was used to estimate the detectability index (d').
**Results:** d' varied between 0.43-2.21 for the 4 mm mass and between 0.66-3.41 for the 16 mm mass. In both cases, d' increased linearly with contrast ($R^2$ = 0.9938 and $R^2$ = 0.9985, respectively) for signal amplitude varying between 2 and 10.
**Conclusion:** As expected, the detectability of masses increased with signal size and linearly with the contrast of the spherical masses. However, being Fourier-based, this FPMO can be expanded to any signal shape, making it possible to use just one MO in image quality assessment of multiple tasks. After further validation, this proposed FPMO could be valuable in assessing clinical image quality objectively in 2D mammography images and potentially in tomosynthesis image stacks.
*Author Disclosures:*
**I. Sechopoulos:** Research/Grant Support; Siemens Healthcare, Canon Medical Systems.

**B-0256** 15:12
**Digital breast tomosynthesis with photon-counting technology: preliminary data from a clinical comparison study**
E. Venturini, C. Losio, M. Rodighiero, S. Tacchini, M. Panzeri, S. Ravelli, E. Schiani, P. Panizza; *Milan/IT (venturini.elena@hsr.it)*

**Purpose:** To compare the image quality and the Average Glandular Dose (AGD) of a new Photon-Counting (PC) tomosynthesis prototype with a commercially available tomosynthesis system.
**Methods and Materials:** After informed consent, women older than 40 years with highly suspicious breast finding at clinical/imaging evaluation (BIRADS 4c/5) underwent bilateral 2-view tomosynthesis with a conventional system and the PC prototype, before breast biopsy. Three readers with different experience in breast imaging independently compared the 3D and the synthetized 2D (s2D) images giving clinical (BIRADS) and conspicuity scores. The density was evaluated according to the ACR classification. The AGDs of the 2 systems were compared.
**Results:** Between 12/2016 and 10/2017 17 women (mean age 71 years) were enrolled. The density score was A-B for 9 women, C-D for the other 8 women. In 15 exams, at least one suspicious finding was detectable by both systems: 11 masses, 1 focus of microcalcifications, 3 foci of microcalcifications with associated mass, 2 distortions. Two exams were negative in both systems due to high breast density and small cancer size. All findings were confirmed as malignant. Comparing the 3D images, the conspicuity of findings was scored as equal or better for PC respectively in 95% observations, while comparing the s2D images the conspicuity of findings were scored as equal or better for PC respectively in 85% observation. The mean AGD of PC tomosynthesis was 50% lower than the other system.
**Conclusion:** PC tomosynthesis performs as well as a conventional system with a drastic reduction of the AGD.
*Author Disclosures:*
**E. Venturini:** Other; Hologic, Philips. **P. Panizza:** Speaker; Philips. Other; Hologic.

**B-0257** 15:20
**Affirm Prone Biopsy System 2D/3D™: tomosynthesis in the vacuum-assisted prone stereotactic biopsy**
C. Depretto[1], D. Baldari[2], F. Cartia[1], C. Ferranti[1], G. Scaperrotta[1]; [1]*Milan/IT*, [2]*Naples/IT (gianfranco.scaperrotta@istitutotumori.mi.it)*

**Purpose:** To assess the diagnostic accuracy in terms of detection rate, methodology and dose of Affirm Prone Biopsy System™ (3D stereotactic biopsy) compared to the traditional 2D prone stereotactic biopsy system MultiCare™ (Hologic Inc).
**Methods and Materials:** In our prospective observational study, we enrolled 270 consecutive patients over 2 years who undergo stereotaxic needle biopsies. 135 procedures were performed with a traditional 2D approach (control arm) and 135 procedures were performed in 3D tomosynthesis (study arm). All samples were taken with the Eviva® 9G vacuum-assisted system. Thanks to tomosynthesis technology, it was possible to biopsy 10 parenchymal distortions (7.5%) not clearly visible in 2D, otherwise destined for surgical excisional biopsy.
**Results:** The total number of exposures per procedure, total duration and mean glandular dose (AGD) were compared. The procedure performed with the MultiCare ™ system involves 10 exposures while with the Affirm only 7, since scouting and targeting (+15 °/-15 °) take place in a single acquisition. The reduction in the number of exposures allows a reduction of the average procedure time of about 25% and also a reduction of the AGD by about 50%.
**Conclusion:** Affirm Prone Biopsy System™ has enabled us to accurately manage all lesions highlighted in tomosynthesis, particularly parenchymal distorsions. We observed a reduction in the number of exposures (30%), in the total duration of the procedure (25%) and in the AGD (50%), which make this system more performing and accurate compared to a traditional 2D prone system.

**14:00 - 15:30** **Room E2**

### Neuro

### SS 311

### Stroke: interventional

*Moderators:*
F. Bozzetti; Parma/IT
I.Q. Grunwald; Southend-on-Sea/UK

**K-08** 14:00
**Keynote lecture**
K.-O. Løvblad; *Geneva/CH*

**B-0258** 14:09
**Validation of automated ASPECTS software for detection of early ischaemic brain changes on non-contrast CT scans**
L. Wolff[1], O. Berkhemer[2], A. van Es[1], T. van Walsum[1], W. Van Zwam[3], D.W. Dippel[1], C.B. Majoie[2], A. Van der Lugt[1]; [1]*Rotterdam/NL*, [2]*Amsterdam/NL*, [3]*Maastricht/NL (l.wolff.1@erasmusmc.nl)*

**Purpose:** In the Alberta Stroke Program Early Computed Tomography Score (ASPECTS), 10 brain regions are dichotomously scored on presence of ischaemic stroke damage. However, considerable inter- and intra-reader variability exists, even for expert readers. We evaluated computed ASPECTS (c-ASPECTS 2.0.1, Frontier, Siemens Healthineers, Forchheim, Germany) in comparison to expert readers.
**Methods and Materials:** Each included baseline non-contrast CT-scan (5.0 mm slice thickness) from the MR CLEAN trial (n=446) was evaluated by three expert readers for manual ASPECTS. 2 observer agreement for ASPECTS-regions (normal/abnormal) were used as ground truth for training (20%) and testing (80%). A c-ASPECTS region score specificity of ≥90% was used to determine the software threshold (relative density difference between affected and contralateral region). Sensitivity, specificity and receiver-operating characteristic curves were calculated. Thereafter, we calculated ICC[1,1], agreement per region and trichotomized ASPECTS (0-4, 5-7, 8-10) between c-ASPECTS and expert readers, and between the expert readers in the test set.
**Results:** A subset (n=442/446) was included. In the training set (n=88), a threshold of 5.6-7.3% was found for a specificity of ≥90%, resulting in a sensitivity and area under the curve of 47-51% and 0.794-0.809. In the test set (n=354) the corresponding results were 90-93%, 42-51% and 0.794-0.811, respectively. Interobserver analysis resulted in an ICC of 0.486, regional agreement of 78-86% and trichotomized ASPECTS agreement of 58-77%. Comparison of c-ASPECTS with expert readers resulted in an ICC of 0.485, regional agreement of 70-77% and trichotomized ASPECTS agreement of 55-77%.
**Conclusion:** The performance of c-ASPECTS is similar to expert readers.
*Author Disclosures:*
**A. Van der Lugt:** Research/Grant Support; Siemens Healthineers.

**B-0259** 14:17
**Non-contrast ASPECTS region density predicts final infarction in acute ischaemic stroke**
W.G. Kunz[1], L.T. Rotkopf[1], D. Puhr-Westerheide[1], F. Dorn[1], S. Tiedt[1], F.A. Wollenweber[1], K.M. Thierfelder[2], A. Kemmling[3], P. Reidler[1]; [1]*Munich/DE*, [2]*Rostock/DE*, [3]*Münster/DE (wolfgang.kunz@med.lmu.de)*

**Purpose:** To determine if automated non-contrast CT (NCCT) density measurements in acute ischaemic stroke provide information on the development of final infarction.
**Methods and Materials:** We selected 121 patients with middle cerebral artery stroke due to large vessel occlusion out of a consecutive cohort. Densities in Alberta Stroke Program Early CT Score (ASPECTS) regions were quantified as average Hounsfield Unit (HU) values using automated segmentation. Relative HU (rHU) values were calculated dividing absolute regional densities of ischaemic by non-ischaemic hemispheres. Final infarction was defined visually per ASPECTS region in a dichotomised fashion and as total volume. A composite-rHU-score incorporating values from all ASPECTS regions weighted by regional relevance was calculated. Receiver-operating-characteristic curve (ROC) analysis was performed to calculate area-under-the-curve (AUC) values. Linear regression analysis was used for multivariable adjustment.

**Results:** Median visual ASPECTS on NCCT was 8 (interquartile range: 6-9). Automated density measurements were feasible in all 121 patients. rHU values yielded significant regional classification of final infarction in ROC analyses for all ASPECTS regions except M3 and M6. Best classifications were achieved for lentiform (AUC=0.810, p<0.001), caudate (AUC=0.777, p<0.001), and insula (AUC=0.764, p<0.001). The composite-rHU-score was independently associated with final infarction volume (β=-0.353, p<0.001), outperforming visual ASPECTS assessment (β=-0.190, p=0.062) in regression analysis. The regression coefficients indicating the effect size were most pronounced in patients that received no reperfusion therapy (β=-0.582, p<0.001).
**Conclusion:** Automated NCCT density measurements identify ASPECTS regions that develop final infarction after stroke. The composite-rHU-score outperformed visual ASPECTS interpretation in the prediction of final infarction volumes.

**B-0260** 14:25
**Accuracy of virtual monochromatic images for the detection of early brain ischaemia on dual-energy CT**
D. Dodig[1], N. Bartolovič[1], S. Kovacic[1], D. Miletic[1], Z. Rumboldt[2]; [1]*Rijeka/HR*, [2]*Charleston, SC/US (doris_5na5@yahoo.com)*

**Purpose:** Reconstructing virtual monochromatic images (VMI) on dual-energy CT (DECT) influences tissue contrast-to-noise ratio and artefacts. The sensitivity of standard head CT for acute brain infarct is in the range of 30-70%. We hypothesised that VMI would allow for a more sensitive and accurate detection of early brain ischaemia.
**Methods and Materials:** Consecutive patients presenting with acute stroke 1-4.5 hours after symptom onset were scanned with DECT. VMI ranging from 40 to 190 keV with a 10 keV increment were reconstructed on a dedicated workstation. Two readers jointly evaluated presence of ischaemic lesions on polychromatic images (representing standard CT) and all VMI reconstructions. The level of detectability between polychromatic and best VMI reconstruction was then compared for each lesion. Follow-up imaging served as the standard of reference. McNemar's group of tests was used for statistical analysis.
**Results:** So far sixteen patients have been included in the study. VMI was more sensitive (63-94%) compared to polychromatic reconstruction (44-81%), however the observed difference was not statistically significant in this small sample. One brainstem infarct was seen on VMI only and one lesion was not detected at all. Eighty percent of the ischaemic lesions were better delineated on VMI reconstructions (p=0.0015). The study is ongoing and these preliminary results are currently being tested on a larger cohort.
**Conclusion:** Detection of early ischaemic stroke appears to be more accurate with VMI than with standard head CT.

**B-0261** 14:33
**Added value of susceptibility-weighted imaging for the prediction of hemorrhagic transformation in patients with acute ischaemic stroke treated by mechanical thrombectomy**
N. Ben Daamer, A. Benaissa, J.P. Le Faucheur, F. Faugeras, S. Benadjaoud, E. Kalsoum, T. Tuilier, H. Hosseini, J. Hodel; *Paris/FR*

**Purpose:** Using susceptibility-weighted imaging (SWI), we aimed at assessing the correlation between the prominence of cerebral veins (PCV) and the occurrence of hemorrhagic transformation (HT) in a cohort of consecutive patients with acute ischaemic stroke (AIS) treated by mechanical thrombectomy (MT).
**Methods and Materials:** 75 patients with AIS treated by MT underwent MRI at 3T including SWI. The presence of HT was systematically assessed using a dual-energy CT (DECT) scan 48 hours after MT. Using SWI, two blinded neuroradiologists analyzed independently: (i) the number of microbleeds (MBs), (ii) the presence of PCV. The extent of PCV was evaluated using a three-point scale: "1": 33% or less, "2": 66% or "3": the entire middle cerebral artery (MCA) territory. Modified Rankin scale (mRS) at three months was available for all patients.
**Results:** According to DECT, HT was observed in 22/75 patients. Inter-observer agreement for the presence and extent of PCV was good (respectively κ=0.83 and κ=0.79); excellent for the number of MBs (κ=0.86). No correlation was found between the number of MBs and HT (p= 0.85). The occurrence of HT was significantly associated with a small extent of PCV (rated"1": 33% or less of MCA territory) on SWI (p=0.019). There was no correlation between the SWI findings (i.e. PCV and MBs) and mRS (p>0.30).
**Conclusion:** Evaluation of PCV extent on SWI may potentially help to predict the occurrence of HT in patients with AIS treated by MT.

**B-0262** 14:41
**Incremental value of CT perfusion for outcome prediction in acute cerebellar stroke**
M.P. Fabritius[1], P. Reidler[1], K. Thierfelder[2], M.F. Froelich[1], L. Rotkopf[1], D. Puhr-Westerheide[1], W.G. Kunz[1]; [1]*Munich/DE*, [2]*Rostock/DE (matthias.fabritius@med.uni-muenchen.de)*

**Purpose:** The clinical diagnosis of cerebellar stroke is challenging. Most frequently applied CT imaging lacks diagnostic sensitivity and fails to provide reliable prognostic information. We aimed to identify imaging parameters that predict outcome in cerebellar stroke patients using multiparametric CT including whole-brain CT perfusion (WB-CTP).
**Methods and Materials:** We selected all subjects with cerebellar WB-CTP perfusion deficits and follow-up-confirmed infarction from a cohort of 3,648 consecutive patients with suspected stroke who underwent WB-CTP. Posterior circulation Acute Stroke Prognosis Early-CT Score (pc-ASPECTS) was determined to assess ischaemic changes on non-contrast CT (NCCT), CT angiography source images (CTA-SI), and on parametric WB-CTP maps. Cerebral blood flow (CBF) deficit volume on WB-CTP imaging as well as the final infarction volume (FIV) on follow-up imaging were quantified. Discharge modified Rankin Scale (mRS) scores were assessed. Regression analyses were performed.
**Results:** 60 patients fulfilled the inclusion criteria. NCCT parameters showed no significant association with FIV. CTA-SI imaging and CBF deficit volume was significantly associated with FIV in univariate linear regression analysis. Only the association of CBF deficit volume was confirmed in a multivariate model adjusted for age, sex, pc-ASPECTS on NCCT and CTA-SI and admission National Institutes of Health Stroke Scale (NIHSS) score (ß, 0.706; p<0.001). Univariate logistic regression showed an association between CBF deficit volume and discharge mRS≤2 (odds ratio [OR], 0.941; p=0.017). This association was not significant after multivariate adjustment, outperformed by NIHSS (OR, 0.776; p=0.009).
**Conclusion:** In contrast to traditional CT imaging parameters, WB-CTP contains prognostic information for morphologic outcome prediction in patients with acute ischaemic cerebellar stroke.

**B-0263** 14:49
**Clinical outcome after thrombectomy in stroke patients with premorbid modified Rankin scale scores of 3 and 4: a cohort study with 136 patients**
F. Seker, J. Pfaff, S. Schönenberger, C. Herweh, S. Nagel, P.A. Ringleb, M. Bendszus, M.A. Möhlenbruch; *Heidelberg/DE*

**Purpose:** In this study, we aimed to analyse clinical outcome after mechanical thrombectomy (MT) in patients with premorbid mRS 3 and 4, because there is currently no data on this patient group.
**Methods and Materials:** Between January 2009 and November 2017, all patients with premorbid mRS 3 or 4 undergoing MT due to anterior circulation stroke were selected. Good outcome was defined as a clinical recovery to the status before stroke onset, i.e. equal premorbid mRS and mRS at 90 days. Besides, mortality at discharge and at 90 days was analysed.
**Results:** 136 patients were included, of which 81.6% presented with premorbid mRS 3 and 18.4% with premorbid mRS 4. 24.0% of patients with premorbid mRS 4 achieved clinical recovery compared to 20.7% of patients with premorbid mRS 3 (P = .788). However, proportion of hospital mortality and mortality at 90 days was non-significantly, but markedly higher in patients with premorbid mRS 4. Multivariate analysis identified low NIHSS (OR 0.92, 95% CI 0.85 - 0.99, P = .040), high ASPECTS (OR 1.45, 95% CI 1.02 - 2.16, P = .049) and TICI 2b-3 (OR 7.11, 95% CI 1.73 - 49.90, P = .017) as independent predictors of good outcome.
**Conclusion:** Good outcome in patients with premorbid mRS 3 and 4 is less frequent compared to premorbid mRS 0-2. Nevertheless, about 20% of the patients return to their premorbid mRS, which may justify endovascular treatment. Most important predictor of good outcome is successful recanalization.

**B-0264** 14:57
**Collateral blood supply role in acute stroke outcomes**
M. Radzina; *Riga/LV (mradzina@gmail.com)*

**Purpose:** Collateral blood supply status is an independent determinant of the clinical outcome, good collateral flow is a factor for favourable clinical outcome and its deficit is considered to be an adverse factor. Early multimodal computed tomography (CT) imaging gives us an insight in various patterns of this parameter and can provide correlation between CT brain perfusion and collateral flow.
**Methods and Materials:** All 105 patients underwent prospective multimodal imaging (CT, CTA, CT perfusion) with pretreatment imaging for evaluation of collaterals on CTA and brain perfusion by CT perfusion imaging in acute stroke patients within 6-8h after onset.45 patients showed good collateral pattern while 40 patients showed poor collateral pattern and 10 patients showed absent collateral pattern.All patients were treated by mechanical thrombectomy.

**Results:** There was statistically significant difference among collateral groups in outcomes by mRs 0-2 in 90 days (p=0.0001).As well as the difference maintained unchanged in significance in the actively treated group by mechanical thrombectomy vs bridging treatment group (p=0.0001).Strong correlation was seen in the size of the lesion (CT perfusion ASPECTS scoring)) and collaterals (p=0.001) with 3.2 times better chance for good outcome if 2 factors were combined.
**Conclusion:** Collaterals can be used as imaging biomarker in prediction of acute stroke outcome independently from treatment selection. Size of the lesion and collaterals should be used as combined set of parameters in prediction of outcome.

**B-0265** 15:05
**Drip, ship, retrieve and leave: a national experience in the management of patients within regional hospitals post endovascular thrombectomy for acute large vessel ischaemic stroke**
E. Griffin, M. Sheehan, D. Brennan, S. Looby, S. Power, A. O'Hare, P. Brennan, J. Thornton; *Dublin/IE (emma.griffin.2@ucdconnect.ie)*

**Purpose:** To report the experiences, outcomes and complications post thrombectomy for patients transferred from and repatriated back to non-endovascular centres with acute large vessel ischaemic stroke. This protocol is known as Drip, Ship, Retrieve and Leave.
**Methods and Materials:** A retrospective review of a prospectively maintained database was performed to identify all patients who were transferred from an outside institution and followed this model from January 2016-December 2017. Key parameters recorded included the presenting National Institute of Health Stroke Score (NIHSS), the location of large vessel occlusion, the administration of intravenous thrombolysis, the degree of reperfusion achieved (Thrombolysis In Cerebral Infarction (TICI) score) and the 90 day modified Rankin Scale (mRS). We recorded any complications encountered.
**Results:** A total of 357 patients were deemed eligible for inclusion with a median age of 69 years (20-96). The median NIHSS at presentation was 15(1-43). All participants had an occlusion confirmed on CT angiography with 92.4% (n=330) in the anterior circulation and 7.6% (n=27) in the posterior circulation. IV thrombolysis was administered in 200/357 patients (56%). Reperfusion was deemed successful in 91% (n=325) with a TICI score of 2a-3, with only complete failure of reperfusion (TICI 0) in 5% (n=17). The median 90 day mRS was 3 with 45% (n=159) of patients having a mRS of ≤2. The 90 day mortality (mRS=6) for this cohort was 21% (n=72).
**Conclusion:** This model is an effective means at managing patients within a national stroke service. It doesn't negatively affect patient morbidity or mortality rates.

**B-0266** 15:13
**Circle of Willis anatomy does not influence the results of mechanical thrombectomy with balloon guide catheter**
A. Velasco González, N. Münnich, T. Rusche, B. Buerke, D. Görlich, C. Sauerland, W. Heindel; *Münster/DE (Aglae.VelascoGonzalez@ukmuenster.de)*

**Purpose:** It has been hypothesised that the suction power through the balloon guide catheter (BGC) might be influenced by the presence of communicant arteries. Our aim was to analyse their effect on the recanalisation rates and the duration of mechanical thrombectomy (MT) performed with a BGC.
**Methods and Materials:** A total of 200 consecutive patients treated with MT through a BGC for acute ischaemic stroke in the anterior circulation were included in this analysis. The anatomy of the ipsilateral circle of Willis (CoW) was determined from the ipsilateral angiographic images and correlated with the features in the CTA. The duration of the procedure, degree of recanalisation, number of passes, and type of SR used were recorded. In the same way, the assumption of clot migration during MT necessitated retrospective evaluation of the CTA.
**Results:** The most common anatomical variation of the CoW was the presence of an anterior communicant artery (ACoA, 76.5%, 153/200). An ipsilateral complete CoW was observed in 22.5% of cases (45/200). A mTICI3 was achieved in 64% of cases (128/200) and mTICI3/2b in 87.5% of cases (175/200). Communicant arteries did not have any effect on the angiographic results. Migration into a new territory occurred in six cases. The presence of the ACoA was an independent factor for protection against migration into a new territory (OR, 0.050, 95%CI, 0.004-10.667).
**Conclusion:** The effectivity of the BGC for MT is not altered by the presence of communicant arteries. Inverted flow through the ACoA could be a factor to prevent migrations.

**B-0267** 15:21
**External referrals for endovascular thrombectomy: our experience in a regional stroke centre and insights into service improvements**
E. Griffin, N.C. Adams, R. Motyer, T. Farrell, A. O'Hare, S. Looby, S. Power, P. Brennan, J. Thornton; *Dublin/IE (emma.griffin.2@ucdconnect.ie)*

**Purpose:** To report experiences at a regional/acute stroke centre of external referrals for endovascular thrombectomy (EVT) in patients with suspected large vessel occlusion (LVO).
**Methods and Materials:** We accept referrals from 24 external centres throughout the Republic of Ireland. Data was prospectively collected on consecutive referrals for EVT during two separate 4 month periods in 2017 and 2018. Several data points were collected at the time of referral including NIHSS scale, IV thrombolysis, imaging time, ASPECTS score and occlusion site. Reasons for not transferring patients and reasons for not performing EVT were recorded.
**Results:** 262 phone referrals were made during the study period. 159 patients (61%) were transferred to our institution and 136 patients had EVT (52%). Twenty-three patients were unsuitable for EVT on arrival due to vessel recanalisation (35%), poor ASPECTS score (30%), no intracranial occlusion (13%), clinical improvement (13%) or haemorrhage on repeat imaging (9%). 103 patients (39%) were deemed ineligible for EVT. Reasons for not accepting patients for EVT included lack of intracranial occlusion (59%), ASPECTS ≤5 (23%), mild or improving symptoms (11%), distal occlusion (4%), and poor baseline (3%). Median NIHSS was significantly lower in the not transferred group (14v7). Median door to imaging times and onset to arrival times were longer in the not transferred group.
**Conclusion:** These data provide valuable insights into the service provision of a comprehensive stroke network with low rates of futile transfer. Access to Neuroradiology and specialised stroke assessment is crucial to optimising patient selection and stroke services.

**14:00 - 15:30** **Room F2**

### Emergency Imaging

### SS 317

### Acute abdomen

*Moderators:*
A. Blanco Barrio; Murcia/ES
D.D. Cokkinos; Athens/GR

**K-09** 14:00
**Keynote lecture**
M. Scaglione; *Castel Volturno/IT*

**B-0268** 14:09
**A comparative study 2007-2015 on acute appendicitis: evolution in use by diagnostic imaging techniques**
M.A. Depetris, L. Ibañez Sanz, E. Martinez Chamorro, S. Borruel Nacenta; *Madrid/ES (marinadepetris1976@gmail.com)*

**Purpose:** To analyse the evolution in the use by diagnostic imaging techniques in acute appendicitis and its impact in the quality of diagnosis process, as negative appendectomy rate is a health care quality indicator.
**Methods and Materials:** This is a retrospective study that included all patients in whom appendectomies had been performed in 2007 and 2015. The data were obtained from pathology reports, surgical protocols and radiology reports. They were compared between the two periods, by assessing imaging test that were performed and their diagnostic quality.
**Results:** The previous data of 2007 were assessed and compared with 2015. Both samples included 394 patients (2007) and 280 (2015). In 120 (30%) patients 2007 vs. 37 (13%) patients 2015, any imaging test was done. There was a drop in the negative appendectomy rate from (38) 9.6% to (11) 3.92%. Gangrenous appendicitis rate showed an increased from (46) 11% to 21%(61). There was an increase of abdominal ultrasonography performed (78.5% vs 54.6%), and also ultrasonography (US)+computer tomography (CT) performed (9% vs. 4.8%). There was just one case in which only TC was done at 2015, whereas in 40 patients (10%) in 2007. Ultrasonography predictive positive value (PPV) showed a slight increase from 92 to 97% and ultrasonography and TC together showed a PPV of 100%. Average hospital stay decreases from 4.6 to 2.8 days.
**Conclusion:** The use of imaging techniques in acute appendicitis has increased, and there was an improvement in health care quality indicators. However, perforated appendicitis does not decrease.

**B-0269** 14:17
**Acute appendicitis: appendicocecal angle, morphological variations detected on CT imaging**
S. Evrimler, H. Aydın; *Isparta/TR (drsehnaz@gmail.com)*

**Purpose:** We aimed to investigate whether there was an association between appendicocecal angle, localisation, length of the appendix and acute appendicitis. To the best of our knowledge, there has been no study investigating the relationship between appendicocecal angle and appendicitis development.
**Methods and Materials:** 226 Abdomen CT scans, ( n=116 normal, and n=110 histopathologically proven appendicitis) performed for prediagnosis of acute appendicitis between 2016-2018, were evaluated retrospectively. Diameter-length-localisation of the appendix, appendicocecal angle, contrast enhancement, mesenteric stranding, peritoneal thickening, lymphadenopathy, appendicolith, complication, primary/secondary classification were evaluated. Localisation of appendix was classified in 8 groups, as follows; 1:pre-ileal, 2: post-ileal, 3: promontoric, 4: pelvic, 5: subcecal, 6: prececal/paracolic, 7: retrocecal, 8: subhepatic.
**Results:** Contrast enhancement ( %100), and mesenteric stranding (94.5%) were the most observed parameters. There was no significant relationship between appendicitis and the lenght (p=0.885) or the localisation of appendix. (p=0.231) Pelvic was the most common localisation. Mean of the appendicocecal angle for appendicitis group, and normal group was 98.19±41.89, and 85.45±43.31, respectively. There was significant difference for appendicocecal angle between appendicitis and normal group(p= 0.028). ROC analysis showed cut-off value of 104.5°, AUC=0.585 (0.510-0.659); p=0.017) (sensitivity 45.45%, specificity 69.82%, accuracy 57.96%). We didn't observe a significant difference for appendicocecal angle in complication development. Localisation showed significancy only for pre-ileal localisation (p=0.030). On the other hand, length was significantly shorter in complicated cases (p=0.024).
**Conclusion:** Appendicocecal angle is the only significant factor amongst angle, length and localisation of the appendix, and can be an aetilogical factor in appendicitis development.

**B-0270** 14:25
**Conditional CT strategy effectiveness in diagnosing acute appendicitis**
R. Luksaite, R. Kliokyte, A. Samuilis, T. Poskus; *Vilnius/LT (rluksaite@gmail.com)*

**Purpose:** To present the results of two retrospective studies done in University Hospital Santaros Klinikos analyzing diagnostic accuracy results before and after the application conditional CT strategy and to compare their diagnostic accuracy.
**Methods and Materials:** Two retrospective analyses of adult patients who were admitted in Emergency room at University Hospital Santaros Clinics with suspected acute appendicitis were done: first study analysed group of 554 patients who from 2008 to 2013 underwent operation for suspected acute appendicitis and the second study included group of 459 patients who underwent operation for suspected acute appendicitis from 2016 to 2018 after implementation of conditional CT algorithm. The results of both algorithms were compared and the positive and negative effects of new diagnostic algorithm were evaluated.
**Results:** In the first study negative appendectomy (NA) rate was as high as 22.9 %. In Conditional CT strategy group the amount of NA was 0.8%. Increase in usage of imaging was noticed: ultrasound form 75 % up to 97 % and CT from 3.4% up to 25 %. Ultrasonography detected inflamed appendix in 67,5% of these cases, CT scan detected acute appendicits in 30%. The sensitivity and specificity of ultrasound and CT scan was.
**Conclusion:** Although applying conditional CT strategy in acute appendicitis diagnostic protocol reduces the amount of negative apendectomies, it increases exposure to ionising radiation, and unnecessary CT scans rate. Taking in to account that potential patient population includes mostly young adults, some new alternatives could be a field for a further search.

**B-0271** 14:33
**Accuracy of grey-scale ultrasound in correctly identifying acute appendicitis in comparison with surgical outcome**
K.M.B. Hassan[1], A.U. Slehria[1], T. Baqir Hassan[2]; [1]*Rawalpindi/PK*, [2]*Okara/PK (baqar78@hotmail.com)*

**Purpose:** To validate ultrasonographic diagnosis of acute appendicitis using surgical outcome as the gold standard.
**Methods and Materials:** Cross-sectional validations setting and duration: Radiology Department, AFIRI Rawalpindi from 1st March 2013 to 31st August 2013. All the patients were referred to the sonography section of Emergency Radiology Department for suspected diagnosis of acute appendicitis. All patients of suspected appendicitis had ultrasound of abdomen. The sampling technique used was consecutive non-probability. Sonographically suspected cases of acute appendicitis resulted in appendicectomy of the patient. Patients were operated by conventional method of appendicectomy. Results regarding appendix by ultrasonography and surgical outcome were recorded on the proforma. Data entry and analysis was done using SPSS v21.
**Results:** A total of 160 patients were included in the study. Mean age of patients was 21.39±4.332 years. There were 77 (48.1%) male and 83 (51.9%) female patients. Clinically, there were 126 (79%) patients positive for appendicitis and on ultrasound findings 121(76%) patients had appendicitis. Surgical outcome showed 125 (78%) patients as positive. Sensitivity and specificity of ultrasound for the diagnosis of appendix was 87.20% and 65.71%. While positive predictive value and negative predictive value of ultrasonography was 90.80% and 58.97%, respectively. Overall diagnostic accuracy of ultrasound was 78.12%.
**Conclusion:** In patients who present with clinically suspected acute appendicitis, imaging is vital and ultrasound can be a good, cheap, readily available and preferred imaging technique to confirm or support the clinical diagnosis and avoid unnecessary and erroneous surgeries especially in females.

**B-0272** 14:41
**Diagnostic performance of abdominal ultrasound in right-sided acute colonic diverticulitis**
F. Pellegrino, G. Tralli, G. Di Stefano, C. Tartari, S. Tartari, M. Giganti; *Ferrara/IT (fabiopellegrino07@gmail.com)*

**Purpose:** To assess value of abdominal ultrasound in patients with suspected right-sided acute symptomatic diverticulitis in comparison with supplementary CT.
**Methods and Materials:** We retrospectively analysed 124 patients from Emergency Department (mean age of 66 ± 18 years) with final diagnosis of acute diverticulitis at discharge. For each patient were registered diagnostic-therapeutic pathway, elective pain location and simple or complicated diverticulitis features with sonography and CT, assessing value of initial US through head-to-head comparison with CT results.
**Results:** Of the 124 patients with diverticulitis 30 underwent directly to CT and were excluded. Among 94 patients with initial sonography and subsequent CT examination within 24 hours, US was positive in 45/94 patients (true positive) and negative in 49/94 (false negative), with diagnostic accuracy of 48%. Sonography diagnosed correctly 15/32 cases of uncomplicated diverticulitis and 30/62 complicated diverticulitis with diagnostic accuracy respectively of 47% and 48%. Regarding location of pain, US was positive in 12/35 patients with right lower quadrant pain and in 33/59 with left lower quadrant pain showing diagnostic accuracy of 34% and 56%.
**Conclusion:** Our data show that US in emergency setting is less reliable for diagnosing right-sided colonic diverticulitis in patients with RLQ pain.

**B-0273** 14:49
**Clinical significance of bedside ultrasonography and second-look ultrasonography in pediatric ileocolic intussusception**
M. Park, C. Lee, G.-S. Hong; *Seoul/KR (lpk1102@gmail.com)*

**Purpose:** The purpose of this study is to identify the performance characteristics of bedside ultrasonography which is performed by clinicians (B-USG), and the clinical significance of the second-look ultrasonography performed by radiologists (R-USG) for the diagnosis of pediatric ileocolic intussusception.
**Methods and Materials:** From October 2013 to December 2017, the patients who visited pediatric emergency department (PED) and underwent ultrasonography by radiologists for evaluating intussusception were included. The included patients were divided into two groups: group A, the patients in whom first-line B-USG were performed at PED, followed by second-look R-USG; and group B, the patients in whom R-USG were performed without B-USG. We compared the ratio of confirmed ileocolic intussusception between the two groups. The sensitivity and positive predictive value of B-USG were calculated using the result of R-USG as the gold standard.
**Results:** A total of 262 patients (mean age, 4.3 years old) were included: 108 patients in group A; and, 154 patients in group B. The ratios of the patients in whom ileocolic intussusception were confirmed were significantly different between group A (47.2 %, 51/108) and group B (28.6 %, 44/154) ($p < 0.05$). In group A, the sensitivity and positive predictive value were 98.0 % (50/51) and 58.8 % (50/85), respectively.
**Conclusion:** The B-USG is highly sensitive for the diagnosis of pediatric ileocolic intussusception. However, due to its low positive predictive value, the second-look ultrasonography by radiologists can improve the diagnostic accuracy, thus reduce unnecessary radiation exposure caused by fluoroscopic reduction.

**B-0274** 14:57
**Is it possible to establish a clinical prediction rule for computed tomography assessment in suspected bowel obstruction at the emergency department?**
R.E. Pacios Blanco, A. Vicente Bártulos, B. Manuel Fernández, E. Cobo Reinoso, A. Muriel García, A. López-Frías López-Jurado, N.A. Almeida, J.I. Gallego Rivera, L. Gorospe Sarasua; *Madrid/ES*

**Purpose:** Bowel obstruction accounts for 5%-15% of hospital admissions for the evaluation of acute abdominal pain at the emergency department. Mortality rates can be significant without early surgical management. Computed tomography (CT) is currently the most sensitive imaging method for the diagnosis. The main objective of this study is to establish a clinical prediction rule to improve the adequacy of CT requests in suspected acute bowel obstruction.
**Methods and Materials:** Scientific evidence to better match the CT requests to the suspicion of acute bowel obstruction was evaluated in the previous multicentre trial MAPAC/FIS13/00896-FIS13/01183/ERDF (European Regional Development Fund), in which an algorithm-based radiological approach was developed for clinical use. We reviewed medical records of 422 patients with abdominal CT scan performed between September 2016 and March 2018 requested under suspected bowel obstruction using the previous algorithm. A prediction rule was developed by use of recursive partitioning based on significant factors after univariate analysis.
**Results:** Abdominal pain was the most reported symptom (91.9%). 50.1% of abdominal CT scans were positive for bowel obstruction and 23.1% revealed other acute diseases. Significant factors to predict bowel obstruction were constipation (p=0.018); abolished bowel sounds (p=0.009) and personal history of cancer (p=0.046). The prediction rule could rule out bowel obstruction with a sensitivity of 80.1% (95% CI, 75.3-84.4 %) using the factors previously mentioned.
**Conclusion:** We developed a prediction rule for CT assessment in suspected bowel obstruction based on significant clinical and radiological features. Prospective validation is needed in other settings. To the best of our knowledge, no clinical prediction rule on this issue has been published.

**B-0275** 15:05
**Fast track CT in patients that referred to emergency major abdominal surgery**
C. Huang[1], C.A. Mardal[2], L.T. Tengberg[2], M. Bay-Nielsen[2], B.B. Bertelsen[2]; [1]*Slagelse/DK*, [2]*Hvidovre/DK (chenxihuang@msn.com)*

**Purpose:** Patients with abdominal pain that referred to emergency major abdominal surgery have a high risk of mortality. To improve the postoperative survival, a standardised, multidisciplinary consultant-led perioperative protocol was introduced at Hvidovre Hospital, including fast track CT diagnostics and surgery. The effort of this showed almost 30 % reduction of 30-day mortality. The present study reports the feasibility of fast track CT and its diagnostic accuracy compared to the intra-operative findings.
**Methods and Materials:** Single-centre intervention study in an unselected consecutive cohort. We included adult patients with the suspicion of (non-traumatic) major abdominal pathology requiring immediate surgery. All patients were included from Hvidovre Hospital (Denmark) from 2013 to 2015, by senior surgeons at the emergency department. In this multidisciplinary effort, the radiological contribution consisted of a fast track abdominal CT scan (predominantly with IV contrast) and rapid reporting within 2 hours of inclusion. Clinical interventions include surgery within 4 hours and perioperative optimization by anaesthesiologists.
**Results:** A total of 371 patients were included. Rapid reporting were achieved for all patients. The CT scans detected a high proportion of the pathologies found at the subsequent surgery with an overall accuracy of 90%, including detection of bowel obstruction and perforated hollow organs at accuracy of 93% and 94%, respectively.
**Conclusion:** The fast and accurate diagnosis provided by CT scan enabled the clinicians to make immediate and educated decisions. The significant reduction of 30-day mortality supports the important role of CT scans and rapid reporting in this multidisciplinary cooperation.

**B-0277** 15:21
**Comparing emergency department disposition of abdominal tomogram to abdominal x-ray**
J.Y. Lee, S. Sabbah, P. Rogalla; *Toronto, ON/CA (Jason.Lee2@uhn.ca)*

**Purpose:** The aim of this study was to analyze the impact of replacing abdominal x-rays (AXR) with abdominal tomograms (AT, ultra-low dose abdominal CT) in the emergency department (ED) with respect to ED length of stay (EDLOS), time-to-ED disposition (TEDD) and time-to-inpatient bed (TIPB).
**Methods and Materials:** A new protocol at our institution has replaced AXR in the ED with AT. All patients with non-traumatic acute abdominal pain in ED referred to AXR were automatically converted to AT. A total of 581 AT patients between 2/1/2017-5/31/2017 and 471 AXR patients between 2/1/2016-5/31/2016 were available for analysis. Duplicate entries of 52 for AT and 99 for AXR were excluded. Thus, a total of 529 AT and 372 AXR patients were analyzed. The TEDD was defined as triage time-to-patient disposition where patient may be admitted or discharged from ED, and TIPD was the time from patient disposition to inpatient bed. EDLOS was defined as the sum of TEDD and TIPD. Timestamps were extracted from the hospital's electronic patient record database.
**Results:** Between the AT and AXR groups, there was no statistical difference between gender (47% vs. 46% male, p=0.8) and age (64.7 vs. 63.3, p=0.5). Statistical difference was not seen with respect to EDLOS (16.3h vs.17.3h, p=0.3), TEDD (8.2h vs. 8.2h, p=0.9) and TIPB (14.0h vs. 11.9h, p=0.06)
**Conclusion:** The conversion of abdominal x-rays to abdominal tomogram for non-trauma emergency department indications did not significantly alter the emergency department length of stay, time to emergency department disposition or time to inpatient bed.

**14:00 - 15:30** **Room Y**

### Interventional Radiology

### SS 309

### Vascular neurointerventions

*Moderators:*
J. Caroff; Le Kremlin-Bicêtre/FR
K. Zelenak; Martin/SK

**B-0278** 14:00
**Initial results of management for acute ischaemic stroke due to large vessel occlusion by direct aspiration first-pass technique at Cho-ray hospital**
T.H.N. Nguyen, P.V. Le; *Ho Chi Minh City/VN (drtuaninr@gmail.com)*

**Purpose:** The development of new revascularization devices has improved recanalization rates, time and clinical outcomes. A direct aspiration first-pass technique (ADAPT) has been introduced as a simple and fast method for achieving good recanalization and clinical outcomes using large bore aspiration catheter for the treatment of acute ischaemic stroke (AIS) due to large vessel occlusion.
**Methods and Materials:** Retrospective analysis of a database of patients undergoing stroke with ADAPT technique at Cho-ray Hospital, from January 2017 to June 2018. Variants evaluating the efficacy and safety evolved: recanalization rates (thrombolysis in cerebral infarction (TICI) score), time to recanalization, complications and clinical outcomes (modified Rankin scale (mRS) score) at the 90-day follow-up.
**Results:** Forty-three patients suffering an AIS treated with ADAPT had initial mean NIHSS score of 17.2 and improved to a mean NIHSS score of 8.7 at discharge. TICI 2b/3 recanalization was achieved in 36/43 (83.7%) patients. The average time to recanalization was 31.7 minutes. 55.8% patients (24/43) presented good clinical outcomes (mRS 0-2), and mRS 3-5 was reported in 13/43 (30.2%) patients. The mortality rate was 6/43 (14%) during follow-up.
**Conclusion:** The ADAPT technique is a fast, simple, safe and effective method with high recanalization rates and good clinical outcomes for management of acute ischaemic stroke with the latest generation of large bore aspiration catheters.

**B-0279** 14:08
**Double aspiration thrombectomy in endovascular recanalisation of acute ischaemic stroke patients**
M. Voormolen, T. van der Zijden, O. D'Archambeau, F. Van den Bergh, A. Mondelaers, C. Loos, P. Vanacker, I. Baar, L. Yperzeele; *Antwerp/BE (maurits.voormolen@uza.be)*

**Purpose:** Endovascular recanalisation by thrombectomy in patients with acute ischaemic stroke is effective and safe. To improve procedure and clinical outcomes, combination of stent-retriever thrombectomy and catheter thrombo-aspiration might increase first-pass recanalisation rate with more complete recanalisation. Our goal was to assess efficacy of endovascular recanalisation by catheter thrombo-aspiration (TA) combined with stent-retriever thrombectomy with simultaneous aspiration on TA catheter and balloon guiding catheter (double aspiration thrombectomy (DAT)) in patients with acute intracranial thrombo-embolic occlusions.
**Methods and Materials:** Between January and August 2018, in our hospital 37 DAT endovascular recanalisation procedures as first-pass attempt were performed in patients with acute ischaemic stroke of anterior circulation, involving 16 female (43%) and 21 male patients. Mean patient age was 71 years (range 38-92). Mean NIHSS was 17 (6-25). Occluded arteries were distal internal carotid (27%), M1 (57%), M2/M3 (5%) and tandem lesion (11%).

**Results:** Recanalisation by DAT was achieved in 34 procedures (92%). First-pass recanalisation rate was 65%. Mean number of passages was 1.4 (range 1-3). Complete recanalisation (TICI 3) was seen in 65%, near complete (TICI 2B) in 27%, fair recanalisation (TICI 2A) in 3% and no recanalisation (TICI 0) in 5%. Median time from groin puncture to recanalisation was 33 minutes (16-120). No major procedure-related complication occurred. Median NIHSS at 24 hours was 4 (1-20). Median mRS at 3 months after stroke was 2 (0-6).
**Conclusion:** First results of double aspiration thrombectomy in patients with acute ischaemic stroke suggest high rates of complete first-pass recanalisation.

**B-0280** 14:16
**Haemorrhagic transformation after stroke: interrater and intrarater agreement**
A. Guenego[1], M. Piotin[2], R. Blanc[2], R. Fahed[2]; [1]*Toulouse/FR*, [2]*Paris/FR (adrienguenego@gmail.com)*

**Purpose:** Haemorrhagic transformation (HT) is a complication of stroke that can occur spontaneously or after treatment. We aimed to assess the interrater and intrarater reliability of HT diagnosis.
**Methods and Materials:** Studies assessing the reliability of the European Cooperative Acute Stroke Study (ECASS) classification of HT or of the presence (yes/no) of HT were systematically reviewed. Eighteen raters independently examined 30 post-thrombectomy computed tomography scans selected from the aspiration vs. stentriever (ASTER) trial. They were asked whether there was HT (yes/no), what the ECASS classification of the particular scan (0/HI1/HI2/PH1/PH2) was and whether they would prescribe an antiplatelet agent if it was otherwise indicated. Agreement was measured with Fleiss' and Cohen's kappa statistics.
**Results:** The systematic review yielded 4 studies involving few (≤3) raters with heterogeneous results. In our 18-rater study, agreement for the presence of HT was moderate (κ=0.55, 95%CI [0.41-0.68]). Agreement for ECASS classification was only fair for all 5 categories, but agreement improved to substantial (k=0.72, 95%CI [0.69-0.75]) after dichotomising ECASS into 0/HI1/HI2/PH1 versus PH2. The interrater agreement for the decision to reintroduce antiplatelet therapy was moderate for all raters, but substantial among vascular neurologists (κ=0.70 [0.57-0.84]).
**Conclusion:** The ECASS classification may involve too many categories and the diagnosis of HT may not be easily replicable, except in the presence of a large parenchymal haematoma.

**B-0281** 14:24
**Endovascular treatment of patients with acute ischaemic stroke in posterior circulation: single-centre experience**
A. Klepanec, J. Harsany, M. Mako, M. Hoferica, J. Cisar, M. Cabukova, M. Rusina, G. Krastev; *Trnava/SK (andrej.klepanec@gmail.com)*

**Purpose:** To evaluate the safety and efficacy of endovascular treatment of patients with acute ischaemic stroke in posterior circulation.
**Methods and Materials:** During the study period from October 2015 till January 2018, 248 patients with acute ischaemic stroke and large vessel occlusion were treated with endovascular thrombectomy in comprehensive stroke centre, of whom, 42 patients were endovascularly treated for acute ischaemic stroke in posterior vertebrobasilar circulation.
**Results:** Median age of patients was 72 years (44-86) and NIHSS 15 points (3-36). Median door to image was 16 minutes (0-63), door to needle 49 minutes (25-85), door to puncture 137 minutes (50-310), puncture to start of revascularisation 15 minutes (5-45), puncture to revascularisation 33 minutes (9-135) and onset to puncture 280 minutes (58 - 1130). After the procedure, successful revascularisation TICI 2b/3 was achieved in 80% of patients. Symptomatic intracranial haemorrhage was present in 2 patients. At 3-month follow-up, favorable outcome with mRs 0-2 was in 37.5% patients, mRs 3-5 in 20% and mRs 6 in 42.5%.
**Conclusion:** Endovascular treatment of patients with acute ischaemic stroke in posterior vertebrobasilar circulation is a safe and effective treatment.

**B-0282** 14:32
**Comparison of mechanical thrombectomy in single- vs. biplane angiosuites**
A. Guenego[1], R. Fahed[2], M. Piotin[2], R. Blanc[2], F. Bonneville[1], P. Mosimann[3], C. Cognard[1]; [1]*Toulouse/FR*, [2]*Paris/FR*, [3]*Essen/DE (adrienguenego@gmail.com)*

**Purpose:** To assess differences in radiation, contrast exposures, procedure duration and outcomes between cerebrovascular mechanical thrombectomy (MT) procedures performed in single-plane (SP) or biplane (BP) angiosuites.
**Methods and Materials:** Consecutive patients treated by MT from four centres between January 2014 and May 2017 were included. Patients and MT characteristics (including type of the angiosuite [SP/BP], recanalisation score, modified Rankin Scale at 3 months, complications, scopy duration, procedure duration, dose-area product (DAP), kerma and contrast load) were assessed. Multivariate analysis were performed (with Bonferroni correction) to compare angiosuites regarding MT efficacy and safety, patient radiation and contrast exposure, and fluoroscopy duration.
**Results:** Within four centres, 906 patients underwent a MT (576 on a biplane angiosuite, 330 on a single plane). After multivariate analysis, BP angiosuites significantly decreased contrast load (100 vs 200mL, 50% lower, relative effect 0.75 (CI: 0.67-0.84), p<0.0001) and fluoroscopy duration (22 vs 27min,19% lower, relative effect 0.83 (CI: 0.74-0.94), p=0.0001) compared with SP angiosuites. There was no difference regarding procedure duration, radiation doses, rate of successful recanalisation, outcome or procedural complications. Interestingly, performing a pre-intervention diagnostic cerebral angiogram before MT significantly increased procedure duration (46 vs 40min, 15% increase, p=0.05), DAP (161 vs 122 Gy.cm², 33% increase, p<0.0001), and contrast load (180 vs 80mL, 125% increase, p<0.0001).
**Conclusion:** Our study shows that BP angiosuites significantly decreased iodine contrast exposition. Furthermore, decision to realise a pre-intervention diagnostic cerebral angiogram before a MT should be clinically motivated to avoid any useless increase in radiation, contrast load and procedure duration.

**B-0283** 14:40
**Impact of haemoglobin levels and anaemic state in the prognosis of patients at three months after mechanical thrombectomy for acute ischaemic stroke**
J.N. Pinto Ramos[1], J. Silva[2], P. Calvão-Pires[1], I. Gil[1], J.P. Marto[1], S. Calado[1], C. Branco[1], J.T.C. Baptista[1], G. Branco[1]; [1]*Lisbon/PT*, [2]*Covilhã/PT (joaonpramos@gmail.com)*

**Purpose:** Those anemic and with lower levels of hemoglobin at admission seem to fare worse in the long-term when compared to non-anemic patients regarding acute ischemic stroke (AIS). We hypothesize hemoglobin and anemia play a lesser role in AIS patients treated with endovascular treatment, potentially reflecting a selection bias.
**Methods and Materials:** Electronic health records were used to collect clinical data as a retrospective analysis of consecutive cases of patients (n=72) that underwent mechanical thrombectomy as part of the acute phase treatment for AIS, in Centro Hospitalar Lisboa Ocidental from 01-01-2016 to 30-06-2018. Good prognosis at 3 months was defined as modified Rankin score (mRs) 0-2 (vs mRs 3-6). We performed 2 logistic regressions: one using the numerical value of hemoglobin at admission; another with anemia at admission as a condition, as defined per the World Health Organization criteria. Dependent variable was defined as good prognosis at 3 months, with adjustment for age, sex, NIHSS at admission, thrombolysis, clinic-to-groin-puncture time and variable of interest.
**Results:** 44.4% of patients had good prognosis at 3 months. Mean value of hemoglobin was 13.34 ± 1.69 g/dL, and 22.2% of patients were anemic. Neither hemoglobin (OR 1.045 [0.747;1.472], p=0.796) nor anemic condition (OR 0.831 [0.227;2.921], p=0.774) seemed to have a significant impact in good prognosis at 3 months.
**Conclusion:** Contrary to most literature regarding AIS in general, hemoglobin levels and anemic condition do not seem to be independent predictors of good prognosis at 3 months in patients with AIS that have undergone mechanical thrombectomy.

**B-0284** 14:48
**Direct puncture of the carotid artery for thrombectomy in acute stroke patients: a single centre experience**
M. Baptista, C. Pinheiro, I. Ribeiro Fragata, R. Carvalho, J. Pamplona, A.P. Nunes, J. Reis; *Lisbon/PT (marianabah@gmail.com)*

**Purpose:** In the late twenties, Egas Moniz introduced cerebral angiography by injecting a radio-opaque medium directly in the cervical carotid artery. The femoral Seldinger technique later replaced direct carotid access. We aimed to prove that in particularly difficult anatomical configurations of the aortic arch, percutaneous access through the carotid artery may be justified.
**Methods and Materials:** Retrospective review of clinical and imaging data of stroke patients, submitted to Digital Subtraction Angiography (DSA) at Hospital São José by direct carotid artery puncture, between January 2014 and September 2018.
**Results:** A total of 9 patients, referred to our hospital for large vessel occlusion stroke, were submitted to DSA by direct carotid artery puncture. Significant arterial tortuosity and difficult anatomy did not allow selective catheterization through femoral access. Mechanical thrombectomy with aspiration was performed in 6 of them, with successful recanalization (TICI 3). In one patient, by the time the vessel was reached, spontaneous recanalization had occurred. In one patient, by the time the vessel was reached, spontaneous recanalization had occurred. In two patients it was not possible to reach the occluded vessel. Four of these patients were also submitted to intravenous thrombolysis. No major complications occurred.

**Conclusion:** At a time when endovascular treatment is part of the guidelines for the treatment of large vessel occlusion strokes, direct carotid artery puncture may offer patients with no femoral access or difficult anatomy a safe alternative to achieve timely recanalization.

**B-0285** 14:56
**Computational fluid dynamics in patients treated with flow-diverter devices**
T. Baptista; *Lisbon/PT (jtbaptista@gmail.com)*

**Purpose:** To simulate, using computational fluid dynamics (CDF), the haemodynamic conditions in pretreatment aneurysms and to identify if flow characteristics are of assistance in predicting whether a specific aneurysm is more prone to thrombosis after flow-diverter (FD) placement, and if are there any haemodynamic indices that correlate with the regions which thrombose first.
**Methods and Materials:** Seven internal carotid artery aneurysms were modelled from 3D pretreatment angiographic studies. Patients were treated using FD and were followed up by digital subtraction angiography and contrast-enhanced 3D time of flight magnetic resonance angiography during 2 to 12 months. Images were segmented using Amira 5.3.3 and @neuFuse 7.3TS software and flow was simulated using ANSYS CFX 13.0 software.
**Results:** Follow-up showed that a smaller (paraophthalmic) aneurysm was still patent at 6-month follow-up imaging whilst in the same vessel a giant cavernous lesion had already been excluded. CFD analysis demonstrated that the paraophthalmic aneurysm was under higher values of wall shear stress (WSS) and that a substantial portion of flow coming from the larger aneurysm was producing a flow impingement area with high values of WSS, low values of oscillatory shear index (OSI) and positive values of aneurysm formation indicator (AFI). Thrombus formation within the aneurysm sac initiated in regions of high OSI and negative values of mAFI.
**Conclusion:** In this study, we observe that CFD may be able to help distinguish between those aneurysms likely to occlude more rapidly and those that may undergo delayed occlusion. Initial regions of thrombosis may also be predicted.

**B-0286** 15:04
**Endovascular treatment of intracranial carotid sidewall aneurysms by flow diversion: a single centre's experience and comparison of Fred and Pipeline stents**
C.A. Gundogmus, D. Tureli, R. Asadov, S. Sabet, N. Andac, F. Baltacioglu; *Istanbul/TR (cagundogmus@gmail.com)*

**Purpose:** To retrospectively evaluate feasibility and efficacy of FRED and Pipeline stents in endovascular treatment of challenging intracranial carotid sidewall aneurysms.
**Methods and Materials:** One hundred and seventeen patients with a total of 151 sidewall aneurysms were included. Patients received either dual anti-platelet therapy (Aspirin and Clopidogrel) or Prasugrel as monotherapy. Patient follow-up was done by DSA.
**Results:** Mean patient age was 50 (ranged 29-76). Eighty-three (54.9%) aneurysms were treated with FRED and 68(45.1%) were treated with Pipeline stents. 3 cases were bleeding aneurysms (2 FRED, 1 Pipeline). Stent deployment failure occurred with both Pipeline(n=2) and FRED(n=4) stents; which in turn were corrected by removal and stent replacement (FRED n=2, Pipeline n=1), balloon dilatation (FRED n=2) . In one case no further action was needed due to adequate contralateral supply via anterior communicating artery. Acute in-stent thrombosis was seen in 4 patients(FRED n=2, Pipeline n=2); 2 treated with Tirofiban and additional stenting, 2 treated with Tirofiban only; no long term sequelae were seen. One patient with giant aneurysm treated with FRED and fully packing with coils haemorrhaged post-intervention and died 10 days later. Follow-up periods ranged between 6 and 63 months (average 36 months). Four patients were lost to follow-up. Complete occlusion was observed in 89.4% of aneurysms (FRED n=68, 88.3%; and Pipeline n=58, 90.6%).
**Conclusion:** Treatment of challenging intracranial carotid sidewall aneurysms with flow diversion using FRED and Pipeline stents is feasible and effective with both stents showing comparable and promising results in short, mid and long-term follow up.

**B-0287** 15:12
**Flow diverters in the treatment of intracranial aneurysms: experience at our institution**
I. Andres Cano; *Cadiz/ES (ignacioanca@gmail.com)*

**Purpose:** Endovascular treatment of intracranial aneurysms has become an alternative to surgical clip ligation. Flow diverters induce disruption of flow near the aneurysm neck, inducing thrombosis into the aneurysmal sac while preserving physiological flow in the parent vessel and adjacent branches. Our goal is to study the demographics, indications of treatment and the outcomes of the patients who were under the procedure with the experience of our institution.
**Methods and Materials:** Retrospective study aiming to identify patients treated with flow diverters between 2007 and 2018 in our hospital.
**Results:** We identified 22 patients (17 females) and 24 aneurysms. The average age of the patients was 51 ± 13.4 years (range 11-71). Ten aneurysms were located on the left, four aneurysms were in the midline and the remaining 10 aneurysms were on the right. There were three complications, one of which led to death. Four of the aneurysms presented with mass effect symptoms and the remaining were incidental.
**Conclusion:** Flow diverters were placed in large and complex intracranial aneurysms. Flow diversion was a safe and highly effective technique for treatment of aneurysms of the anterior and posterior circulation. Only 1 of the 22 patients was discharged with poor outcomes and another one died. 90% of our patients (20 out of 22) were discharged asymptomatic. At follow-up, a high proportion of aneurysms treated with flow diverters achieved complete obliteration.

**B-0288** 15:20
**Balloon-assisted coil embolisation and large stent delivery for cerebral aneurysms with a new generation of dual-lumen balloons**
A. Guenego[1], J.-B. Zerlauth[2], R. Meuli[2], R. Chapot[3], P. Mosimann[3];
[1]*Toulouse/FR*, [2]*Lausanne/CH*, [3]*Essen/DE (adrienguenego@gmail.com)*

**Purpose:** Dual-coaxial lumen balloon micro-catheters that can serve as a remodeling device and through which small stents can be delivered. We report a series of a new dual-lumen balloon catheter with parallel lumens enabling enhanced inflation and deflation properties and through which larger stents may be deployed, including flow diverters (FD).
**Methods and Materials:** All aneurysms that were treated with a Copernic2L (COP2L) dual-lumen balloon catheter at a single institution between February 2014 and December 2016 were assessed. Patient, aneurysm, procedural characteristics, clinical and angiographic follow-up.
**Results:** A total of 18 aneurysms in sixteen patients (14 women) were treated with the COP2L. Mean aneurysm height x width and neck size were 1.1 (min 0.5; max 2.1) and 3.3 mm (min 1; max 6.3), respectively. The COP2L was used for balloon-remodeled coiling exclusively in two aneurysms; coiling and FD stenting in eight; coiling and braided stent delivery in three; coiling, braided and FD stenting in one and for FD stenting without coiling in four (stenting alone). There were three technical complications (3/16, 18.7%), including a perforation and two thromboembolic asymptomatic events that were rapidly controlled with the COP2L. There was no immediate or delayed morbidity or mortality.
**Conclusion:** According to our initial experience, the COP2L is a new type of dual-lumen balloon catheter that appears to be safe and effective for balloon or stent-assisted coiling of cerebral aneurysms and may be used to optimise stent-wall apposition.

**14:00 - 15:30** **Coffee & Talk 3**

### Head and Neck

### SS 308

### Temporal bone

*Moderators:*
K. Markiet; Gdansk/PL
B. Ozgen Mocan; Chicago, IL/US

**B-0289** 14:00
**Comparison of lesion detection and image quality between non-EPI diffusion weighted imaging and multi-shot EPI diffusion weighted imaging of cholesteatoma**
M. Wiesmüller, W. Wuest, R. Heiß, M.S. May, M. Uder, F. Laun; *Erlangen/DE (marco.wiesmueller@uk-erlangen.de)*

**Purpose:** Recently introduced multi-shot echo planar (MS-EPI) diffusion weighted imaging (DWI) techniques can provide high resolution images with low geometric distortion. Non-echo planar imaging techniques (Non-EPI) are known to have a very high sensitivity and specificity in the diagnosis of cholesteatoma. Our aim was to evaluate the diagnostic performance of a MS-EPI DWI sequence and to compare it with a Non-EPI DWI sequence.
**Methods and Materials:** We prospectively included 30 patients for examination on a 1.5 T MRI using a dedicated study protocol consisting of a Non-EPI sequence (HASTE, hDWI) and a MS-EPI sequence (readout-segmented sequence, rsDWI). In 25 patients diagnosis of cholesteatoma was histopathologically confirmed. Each dataset was independently evaluated by two radiologists. Sensitivity and specificity were calculated for hDWI an rsDWI. Image quality was rated as overall image quality, presence of artefacts, lesion visibility and subjective diagnostic confidence on five point Likert scales.

**Results:** Sensitivity and specificity, lesion visibility and subjective diagnostic confidence were significantly higher in hDWI. Presence of artefacts, not affecting the accuracy of diagnosis, was comparable between hDWI and rsDWI. Slightly higher overall image quality was found in rsDWI.
**Conclusion:** MS-EPI sequences provide high overall image quality and low artefacts. However, non-EPI DWI sequences showed superior diagnostic accuracy for cholesteatomas.

**B-0290** 14:08
**Value of four stage vestibular hydrops grading and asymmetric perilymphatic enhancement in the diagnosis of Ménière's disease on MRI**
A. Bernaerts[1], R. Vanspauwen[1], C. Blaivie[1], J. van Dinther[1], A. Zarowski[1], F. Wuyts[1], F.E. Offeciers[1], J.W. Casselman[2], B. de Foer[1]; [1]*Antwerp/BE*, [2]*Bruges/BE (Anja.Bernaerts@gza.be)*

**Purpose:** There is still a clinical-radiologic discrepancy in patients with Menière's disease (MD). Therefore, the purpose of this study was to investigate the reliability of current MRI endolymphatic hydrops (EH) criteria according to Baráth in a larger study population and the clinical utility of new imaging signs such as a supplementary fourth low-grade vestibular EH, the degree of perilymphatic enhancement (PE) and endolymphatic sac enhancement (ESE) in patients with Menière's disease (MD).
**Methods and Materials:** This retrospective study included 148 patients with probable or definite MD according to the 2015 Barany criteria who underwent a 4-hour delayed intravenous Gd-enhanced 3D-FLAIR MRI between January 2015 and December 2016. Vestibular EH, vestibular PE, cochlear EH, cochlear PE, and ESE were reviewed twice by 3 experienced readers. Cohen's Kappa and multivariate logistic regression were used for analysis.
**Results:** The intra- and inter-reader reliability for the grading of vestibular-cochlear EH and PE was excellent (0.7 < kappa < 0.9) except for the ESE (kappa 0.5). The 2 most distinctive characteristics to identify MD are cochlear PE and vestibular EH which combined gave a sensitivity and specificity of 79.5% and 93.6%. By addition of a lower grade vestibular EH, the sensitivity improved to 84.6% without losing specificity (92.3%). Neither ESE, cochlear EH or vestibular PE showed added value.
**Conclusion:** MRI using vestibular-cochlear EH and PE grading system is a reliable technique. Cochlear PE assessment in combination with a 4-stage vestibular EH grading system gives the best diagnostic accuracy to detect MD.

**B-0291** 14:16
**Teaching temporal bone anatomy CT scan as a psychomotor skill: a quasi-experimental study**
S. Sefidbakht[1], B. Bijan[2], P. Keshavarz[1], M. Amini[1], R. Jalli[1], F. Zarei[1], H.R. Abbasi[1], P. Iranpour[1]; [1]*Shiraz/IR*, [2]*Sacramento, CA/US (keshavarz.p2390@gmail.com)*

**Purpose:** To present a teaching method for the radiology residents the cross-sectional anatomy of the temporal bone as a psychomotor skill using predetermined points of transition between orthogonal planes as a roadmap.
**Methods and Materials:** 21 radiology residents entered the study. We provided a teaching scheme based on the Gagne's theory which consisted of 6 steps; gaining attention, informing the learner of the objective,stimulating recall, presenting the stimulus material, providing learning guidance, eliciting performance, providing feedback, assessing performance, enhancing retention and transfer. For the main teaching session, the conceptualization, visualization, verbalization steps of teaching psychomotor skills was employed by guiding the residents systematically using the 3D cursor to move and transit between orthogonal planes for the predetermined anatomical points. The residents were evaluated before and after the training sessions using objective structured clinical examination (OSCE), resident satisfaction and educational goal achievement questionnaires.
**Results:** Mean age was 27.9 ± 8.6 (range from 26-38) years, 14 (66.6%) men and 7 (33.4%) women. We observed that the OSCE score improved significantly after the twenty-minute session (2.57 ± 1.62 *vs.* 8.02 ± 2.43; *p<0.001*). Mean resident satisfaction score measured by standardized questionnaires was 7.63 ± 2.86 /10. Subjectively the residents assessed their ability to identify the anatomical learning objectives after the mini-sessions to have significantly increased(4.33 ± 1.22 *vs.* 8.27 ± 2.46; *p=0.021*).
**Conclusion:** Interpretation of cross-sectional images as a psychomotor skill, may be an effective teaching method. Using a standardized approach and defining predetermined transition points moving between orthogonal planes enhances learning.

**B-0292** 14:24
**Ultra-high-resolution CT for temporal bone imaging: a comparison to cone-beam CT**
T. Yamashiro, M. Tsubakimoto, Y. Xu, Y. Morita, H. Tomita, S. Murayama; *Okinawa/JP (clatsune@yahoo.co.jp)*

**Purpose:** Ultra-high-resolution CT (U-HRCT) scanners, equipped with an advanced detector that receives X-ray signals at 0.25 mm resolution in all three directions can provide more detailed imaging findings than conventional CT scanners. The purpose of this study was to compare the image quality of U-HRCT scans to cone-beam CT (CBCT) scans for temporal bone imaging.
**Methods and Materials:** Fifteen subjects (age range, 18-70 years) underwent both U-HRCT and CBCT on different dates to assess various unilateral ear diseases. It was confirmed that no diseases existed in the contralateral (normal) ear. On the axial and coronal views of the U-HRCT scans (pixel size, 0.16 mm; slice thickness, 0.25 mm for axial and 0.16 mm for coronal views) and the CBCT scans (pixel size, 0.13 mm; thickness, 0.5 mm), the image quality of small structures in the normal ear, such as the tendon of the stapedius muscle and the crura of the stapes, were assessed by three radiologists using a 5-point scale. Comparisons of the scores between the U-HRCT and CBCT were made by Wilcoxon matched-pair test.
**Results:** For all 8 small structures, the scores of the U-HRCT were significantly higher than those of the CBCT (*P*<0.001). Particularly, the visibility of the crura of the stapes, the chorda tympani, and the tendon of the tensor tympani muscle was significantly better on the U-HRCT than the CBCT.
**Conclusion:** The U-HRCT can provide more precise anatomical information of small structures in the temporal bone than the CBCT.
*Author Disclosures:*
**T. Yamashiro:** Grant Recipient; Canon Medical Systems. **M. Tsubakimoto:** Grant Recipient; Canon Medical Systems. **S. Murayama:** Grant Recipient; Canon Medical Systems.

**B-0293** 14:32
**Visualisation and image quality of the chorda tympani nerve in temporal bone: comparison with conventional CT and ultra-high-resolution CT**
M. Fujiwara[1], Y. Watanabe[1], T. Fujiwara[2], C. Matsuo[2], H. Takahashi[1], H. Tanaka[2], Y. Ohta[1], T. Imai[1], N. Tomiyama[2]; [1]*Osaka/JP*, [2]*Suita/JP (m-fujiwara@radiol.med.osaka-u.ac.jp)*

**Purpose:** The aim of this study was to compare visualization and image quality of the chorda tympani nerve (CTN) between conventional CT (c-CT) and ultra-high-resolution CT (u-HRCT) .
**Methods and Materials:** Retrospective visual assessment of 23 CTNs from consecutive 22 patients who underwent both u-HRCT and c-CT for follow- up. U-HRCT scan was performed by a u-HRCT scanner (Aquilion Precision, Canon Medical Systems, Japan). C-CT was reconstructed separately on the left and the right using the following parameters: FOV120mm, 512 matrix sizes, in-plane resolution 0.23mm, slice thickness 0.50 or 0.625mm, and bone kernel. U-HRCT was also reconstructed separately on one side with FOV120mm, 1024 matrix sizes, in-plane resolution 0.12mm, slice thickness 0.25mm and we used two kinds of kernels (FC80(bone), FC15(soft tissue)). Two neuroradiologists evaluated three portions of CTN (posterior canaliculus, tympanic segment, and anterior canaliculus) and scored them on a 4-point scale (1 = no visible, 4 = entire clearly visible).
**Results:** The visual score of the posterior portion was 3.4±0.7 with c-CT and 3.9±0.4, 3.9±0.4 with u-HRCT (FC80 and FC15) and statistically significant difference between c-CT and u-HRCT was observed (p = 0.044). The tympanic segment of CTN was 1.7±0.6 with c-CT and 2.6±0.5, 2.6±0.5, respectively, with u-HRCT (FC80 and FC15). This segment is significantly more clearly visualized with u-HRCT than c-CT (p < 0.01). The anterior portion of CTN was 3.1±0.7 with c-CT and 3.4±0.6, 3.4±0.6, respectively, with u-HRCT (FC80 and FC15) and significant difference was also observed (p = 0.018).
**Conclusion:** U-HRCT delineates CTN more clearly than c-CT.
*Author Disclosures:*
**Y. Watanabe:** Grant Recipient; Canon Medical Systmes.

**B-0294** 14:40
**Comparison between high-resolution 3D-REAL-IR and 3D-FLAIR sequences in the assessment of endolymphatic hydrops in 3 Tesla**
V.M. Suarez Vega[1], F.M. Caballeros Lam[1], P. Domínguez Echávarri[2], R. Garcia de Eulate[2], G. Gallardo Madueno[1], A. Alonso-Burgos[1], C. Sobrido[3], B. Alvarez de Sierra[1], N. Perez[2]; [1]*Madrid/ES*, [2]*Pamplona/ES*, [3]*Vigo/ES (proksua1@hotmail.com)*

**Purpose:** Describe the two sequences used in the assessment of endolymphatic hydrops (EH): 3D-REAL-IR and 3D-FLAIR. Compare the sequences in terms of the imaging findings to best determine their application in clinical practice.
**Methods and Materials:** Fifteen patients (5 females, 10 males) diagnosed of probable or definite Ménière's disease (MD) -according to the Amended 2015 Criteria of the American Academy of Otolaryngology- underwent 3 Tesla MR imaging 4 hours after the intravenous injection of Gadolinium. Items such as affected side´s inner ear, hearing loss and/or vertigo were collected. The presence of vestibular and/or cochlear EH was assessed in both 3D-REAL-IR and 3D-FLAIR sequences. A neuroradiologist, blind for the final diagnosis and clinical data, evaluated the images.
**Results:** All six patients diagnosed with definite MD showed signs of EH in 100% of affected ears and in 4 out of 6 (66%) not affected ears. In the remaining 9 patients with probable MD, 3 of them showed no signs of EH in any sequence and 3 of them signs of EH only in 3D-REAL-IR sequence. *Vestibular* EH detection rates were 58,6% in 3D-REAL-IR sequence and 40,7% in 3D-FLAIR sequence, and *cochlear* EH detection rates were 24% and 11% respectively. In 6 patients both sequences matched the same result, suggesting the 3D-REAL-IR sequence greater degree of severity.
**Conclusion:** Both sequences are suitable in the assessment of EH. However, 3D-REAL-IR has higher detection rates in both vestibular and cochlear EH. We suggest that 3D-REAL-IR might be superior in the diagnosis of MD.

**B-0295** 14:48
**Measurement of the depth of facial nerve at the level of stylomastoid foramen using MR imaging in Bell's palsy**
H. Karaca[1], L. Soydan[2], F. Baskak[2], A. Sever[2]; [1]*Elazig/TR*, [2]*Istanbul/TR (fulyabaskak@hotmail.com)*

**Purpose:** To investigate whether the depth of the facial nerve from the skin surface as it exits the stylomastoid foramen differs between paralyzed and unaffected sides in patients with Bell's palsy.
**Methods and Materials:** Forty-three patients (23 females, 20 males; mean age 43.8±15.2 years) diagnosed with Bell's palsy between January 2014 and June 2017 were retrospectively reviewed and those who had a cranial MR imaging performed within 10 days upon admission to hospital were included in the study. The axial postcontrast CUBE sequence was utilized for measurement of the facial nerve depth. Age, gender, and body mass index (BMI) as well as concomitant chronic diseases, were also noted. The severity of facial paralysis was graded using the House-Brackmann (HB) scoring system.
**Results:** The facial nerve depth was significantly lower on the paralytic side compared to the unaffected side (32.9±5.4 mm vs. 36.9±5.1 mm, respectively; p=0.001). The facial nerve depth on the paralytic side was also lower in female patients than the male patients (31.2±4.6 mm vs. 34.7±5.7 mm, respectively; p=0.03). However, the facial nerve depth on the paralytic side was not correlated with patients' age (r=0.288; p=0.610), BMI (r=0.215, p=0.166), and HB scores (r=0.031; p>0.05).
**Conclusion:** In study cohort of patients with Bell's palsy, the facial nerve in the paralytic side is located more superficially as it exits the stylomastoid foramen when compared to the contralateral side. Therefore, the depth of facial nerve may potentially play a key role in the etiology of Bell's palsy, which should be further evaluated.

**B-0296** 14:56
**Mastoid pneumatisation in children before CI: a risk factor for complications?**
I. Burck, M. Goldenko, M.H. Albrecht, A. Lehn, T. Stöver, M. Harth, J.L. Wichmann, B. Kaltenbach, T.J. Vogl; *Frankfurt a. Main/DE (iris.burck@kgu.de)*

**Purpose:** To evaluate preoperative mastoid air cells (MAC) pneumatisation calculated by 3D-CT volumetry in correlation with complication rate after cochlear implantation (CI) in infants and children.
**Methods and Materials:** MAC volume of 105 patients (mean age 10.41mo±11.36; 37 female, 41 male) with a total of 186 CI was evaluated by CT-based volumetry. Thresholds were defined to determine 3 different parts of MAC volume: osseous, aerated and soft tissue opacification. 8 groups were classified by varying patient age at CT evaluation: 0-3, 4-6, 7-9, 10-12, 13-24, 25-36, 37-48, 49-60mo. Postsurgical follow-up time was set for 2 years.
**Results:** 80 patients were evaluated after exclusion of 25 patients because follow-up time was exceeded. Randomized one implanted ear per patient was evaluated. No significant difference was found between right and left MAC volume (p=0.74), as well as between sexes (p=0.30). 12 patients had complications in follow-up. Group 2 (n =40) showed the highest absolute complication rate (n=7). Complications occurred in groups 1, 2 and 7. No significant correlation was seen between volume-impaired MAC and increased complication rate in total (p=0.79). There was no significant correlation between insufficient aerated MAC and complication rate. 17 patients showed opacified MAC, but without significant correlation with increased complication rate (p=0.27). Patient's age at surgery showed significant impact on complication rate (p=0.049), more complications occurred in younger children.
**Conclusion:** Analysis of preoperative data to identify potential risk factors may reduce post-CI complications. But insufficient MAC pneumatisation seems not to be a potential risk factor for complications.
*Author Disclosures:*
**M.H. Albrecht:** Speaker; Received speaker fees from Siemens and Bracco. **J.L. Wichmann:** Employee; Employee of smart reporting. Speaker; Received speaker fees from Siemens and GE.

**B-0297** 15:04
**Research software in cochlear duct length estimation, Greenwood frequency mapping and electrode array length selection**
A. Dhanasingh; *Innsbruck/AT (anandhan.dhanasingh@medel.com)*

**Purpose:** Cochlear size varies among the population bringing the necessity for electrode arrays available in various lengths. This research software helps in the estimation of the patient's cochlear duct length (CDL) which is then used for the selection of the correct length electrode array matching the patient's cochlear size and as well in getting the patient-specific cochlear frequency map.
**Methods and Materials:** Visual Studio Express 2012 for Windows Desktop is used in the architecture of this research software. The basal turn diameter of the cochlea ("A" value) is retrieved from the pre-operative CT image of the temporal bone. "A" will be taken as the input for the CDL equations proposed by Alexiades et al. for estimating CDL for various insertion depths. Greenwood's equation is used in combination with the CDL for the full length of the cochlea in getting patient-specific frequency map.
**Results:** This research software with the help of the "A" value as input, with button clicks, provides patient-specific CDL for various insertion depths and Greenwood's frequency map. Users have the choice to select any electrode array of their choice and place it under the frequency map to see how good it fits to that particular patient's cochlea along with the possibility to drag and move electrode array mimicking post-operative actual electrode insertion depth.
**Conclusion:** This research software simplifies the overall process of CDL estimation and in getting the patient-specific cochlear frequency map.

**B-0298** 15:12
**MRI in unilateral Meniere's disease: the value of post-contrast cochlear perilymph signal intensity**
L.M.H. de Pont[1], J. van Steekelenburg[1], A. van Weijnen[1], O.D. Vijlbrief[2], H.M. Blom[1], S. Hammer[1]; [1]*The Hague/NL*, [2]*Almelo/NL (l.depont@hagaziekenhuis.nl)*

**Purpose:** Meniere's disease (MD) is characterized by endolymphatic hydrops (EH), although the diagnosis relies on clinical criteria. MR imaging recently became available to detect EH in vivo. However, not all patients with definite MD show EH on MR imaging. The purpose of this study was to evaluate additional value of cochlear perilymph signal intensity ratio (SIR) in patients with suspected MD.
**Methods and Materials:** 3T MR imaging was performed in patients with definite MD, probable MD and patients with other vertigo-associated inner ear disorders. Delayed contrast-enhanced 3D high-resolution FLAIR images of the inner ear were obtained. SIR was calculated based on ROI measurements in the basal cochlear turn. Data were compared using paired t tests.
**Results:** 420 ears of definite MD patients, 54 ears of probable MD patients, and 134 ears from patients with other inner ear disorders were included. EH was present in symptomatic ears in 94%, 45% and 6%, respectively. The affected ears in unilateral definite and probable MD showed higher SIR compared with their asymptomatic side (both P<0.001). No significant differences were detected in patients with other inner ear disorders. Six (5%) patients with unilateral definite MD showed no EH on MRI, whereas the SIR was higher in affected ears (P<0.05). In unilateral probable MD, 11 patients (41%) were classified as having no hydrops and showed no significant SIR asymmetry.
**Conclusion:** Cochlear SIR asymmetry can be used as an additional parameter in discriminating MD from non-MD, which is particularly of value in clinical probable MD.

**B-0299** 15:20
**Double-center study on textural differences between cholesteatoma and middle ear inflammation in non-enhanced high-resolution computed tomography**
C.T. Arendt[1], D. Leithner[1], M.E. Mayerhöfer[2], L. Lenga[1], C. Czerny[2], I. Burck[1], T.J. Vogl[1], R. Schernthaner[2]; [1]*Frankfurt a. Main/DE*, [2]*Vienna/AT (crt.arendt@gmail.com)*

**Purpose:** To investigate whether radiomic features extracted from unenhanced CT can be utilized for differentiation between cholesteatoma and middle ear inflammation (MEI), and to compare CT data from two different centers/scanner in that regard.
**Methods and Materials:** Ninety-five patients (45 with cholesteatoma and 50 with MEI) were included retrospectively. At center A, 45 patients (20 with cholesteatoma, and 25 with MEI) were examined using a 512x512 matrix; whereas at center B, 50 patients (25 with cholesteatoma, and 25 with MEI) were scanned using a 768x768 matrix. Radiomic features (histogram, co-occurrence and run-length matrix, absolute gradient, autoregressive model, Haar wavelet transform) were extracted from axial CT sections using manually defined regions of interest. POE+ACC (probability of error and average correlation) coefficients were used to select subsets of the 10 best radiomic features for differentiation between cholesteatoma and MEI. Linear discriminant analysis followed by k-nearest neighbor classification (using leave-one-out cross-validation) was used for classification, with histology obtained from surgery specimens serving as the reference standard.
**Results:** Using pooled data from both centers, only 59/95 cases were correctly classified (accuracy, 62.11%). Separately for data from center A, 31/45 cases (accuracy, 68.9%) were correctly classified, whereas separately for data from center B, 42/50 cases (accuracy, 84.0%) were correctly classified.
**Conclusion:** Radiomic features extracted from unenhanced CT may be useful for differentiation between cholesteatoma and middle ear inflammation when high-resolution CT data are utilized. Pooling of radiomic data extracted from CT datasets with different resolutions does not appear to be meaningful without further post-processing.

**14:00 - 15:30** **Room D**

### Artificial Intelligence and Machine Learning

### SS 305

### Machine learning: chest and cardiac

*Moderators:*
C.L. Schlett; Freiburg/DE
N.N.

**B-0300** 14:00
**Deep learning reconstruction for calcium scoring reduces radiation dose while maintaining accuracy of Agatston scores**
C. Steveson[1], J. Schuzer[2], T. Acharya[3], K. Bronson[3], S. Rollison[3], W. Bandettini[3], S. Shanbhag[3], M.Y. Chen[3]; [1]*Kanagawa/JP*, [2]*Vernon HIlls, IL/US*, [3]*Bethesda, MD/US (chloe.steveson@medical.canon)*

**Purpose:** Assessing coronary artery calcium (CAC) is a valuable tool in individualizing cardiac risk assessment. Deep convolutional neural network image reconstruction can reduce image noise and enable radiation dose reductions. The aim of this study is to investigate whether the use of a deep learning reconstruction algorithm allows for reduced radiation dose CAC scanning.
**Methods and Materials:** With institutional ethics approval, 40 consecutive patients underwent EKG-gated coronary calcium score scans (120kV, 0.275s rotation speed) with 1) standard clinical dose with filtered back projection (FBP) and 2) reduced radiation dose levels with deep learning reconstruction. Agatston score was calculated on a per patient basis using standard techniques and 130 HU threshold and interpreted by 2 independent readers blinded to clinical data and reconstruction techniques. Signal-to-noise was calculated for each reconstruction. Data was analyzed using linear regression.
**Results:** Patients averaged 63 ± 15 years old, 45% were male, and the median Agatston score was 61.7 (interquartile range 0-315, entire range 0-4121). The median radiation exposure was 93% lower for the reduced (0.05 mSv, interquartile range 0.03-0.08) verses standard dose scans (0.88 mSv, interquartile range 0.63-1.22). There was excellent agreement (R=0.99) between deep learning reconstruction applied to low radiation dose images and standard dose clinical FBP Agatston scores. Signal-to-noise for low dose deep learning images was comparable to standard dose FBP images (2.16 vs. 2.45, respectively).
**Conclusion:** Deep learning reconstruction in conjunction with 93% radiation reduction has an excellent correlation with standard conventional coronary artery calcium scoring.
*Author Disclosures:*
**C. Steveson:** Employee; Canon Medical Systems Corporation. **J. Schuzer:** Employee; Canon Medical Systems Corporation. **M.Y. Chen:** Research/Grant Support; Canon Medical.

**B-0301** 14:08
**Coronary CT angiography derived plaque quantification with artificial intelligence CT fractional flow reserve for the identification of lesion-specific ischaemia**
P. von Knebel Doeberitz[1], T. Pannell[1], M. van Assen[2], M.H. Albrecht[3], C.N. De Cecco[1], T. Duguay[1], R.R. Bayer[1], C. Tesche[4], U.J. Schoepf[1]; [1]*Charleston, SC/US*, [2]*Groningen/NL*, [3]*Frankfurt a. Main/DE*, [4]*Munich/DE (p_knebel@hotmail.de)*

**Purpose:** We sought to investigate the diagnostic performance of coronary CT angiography (cCTA)-derived plaque markers combined with deep machine learning-based fractional flow reserve (CT-FFR) to identify lesion-specific ischaemia using invasive FFR as the reference standard.
**Methods and Materials:** 84 patients (61±10 years, 65% male) who had undergone cCTA followed by invasive FFR were included in this single-center retrospective, IRB-approved, HIPAA-compliant study. Various plaque markers were derived from cCTA using a semi-automatic software prototype and deep machine-learning based CT-FFR. The discriminatory value of plaque markers and CT-FFR to identify lesion-specific ischaemia on a per-vessel basis was evaluated using invasive FFR as the reference standard.
**Results:** 103 lesion-containing vessels were investigated. 32 / 103 lesions were haemodynamically significant by invasive FFR. In a multivariate analysis (adjusted for Framingham risk score), the following markers showed predictive value for lesion-specific ischaemia (odds ratio [OR]): lesion length (OR 1.15, $p$=0.037), non-calcified plaque volume (OR 1.02, $p$=0.007), Napkin ring sign (OR 5.97, $p$=0.014), and CT-FFR (OR 0.81, $p$<0.0001). A receiver-operating characteristics analysis showed the benefit of identifying plaque markers over cCTA stenosis grading alone, with AUCs increasing from 0.61 with ≥50% stenosis to 0.83 with addition of plaque markers to detect lesion-specific ischaemia. Further incremental benefit was realized with the addition of CT-FFR (AUC 0.93).
**Conclusion:** Coronary CTA-derived plaque markers portend predictive value to identify lesion-specific ischaemia when compared to cCTA stenosis grading alone. The addition of CT-FFR to plaque markers shows incremental discriminatory power.
*Author Disclosures:*
**C.N. De Cecco:** Consultant; Bayer, Siemens Healthineers. **U.J. Schoepf:** Consultant; Bayer, Guerbet, HeartFlow Inc., Siemens Healthineers. Research/Grant Support; Astellas, Bayer, General Electric, Siemens Healthineers.

**B-0302** 14:16
**DeformationNet: unsupervised deep learning co-registration for ventricular segmentation of MR images**
B. Norman[1], M. Simonovsky[2], F. Lau[1], S. Sall[1], D. Golden[1], A. Hsiao[3]; [1]*San Francisco, CA/US*, [2]*Dobris/CZ*, [3]*San Diego, CA/US (berk.norman@arterys.com)*

**Purpose:** We propose a deep learning framework, DeformationNet, which allows for unsupervised image co-registration of perfusion MR images and semi-supervised ventricular segmentation capable of transferring across multiple time points.
**Methods and Materials:** Using a previously trained ventricular segmentation network that was trained on cine SSFP images, inference is first run on all time points in a perfusion scan. A heuristic based on the inferred segmentation probability maps is then used to select the perfusion image with the “best” target segmentation mask. DeformationNet is trained to predict dense deformation fields (DDF) between two perfusion MR images in order to register the moving image onto a fixed image. The predicted DDFs are then used to warp the best target ventricular segmentation of the moving image onto all other timepoints (the fixed images).
**Results:** Median Dice values on the test set were 0.855 (IQR values: 0.698-0.899) for LV endo and 0.879 (IQR values: 0.715-0.925) for LV epi. Average computation for calculating the DDFs for an entire perfusion volume was approximately 7 seconds on a Nvidia P100 GPU (compared to traditional iterative co-registration methods which can take in excess of one hour).
**Conclusion:** We propose DeformationNet, an approach using unsupervised neural networks that can quickly produce deformation fields between new sets of images. These can be leveraged to perform image co-registration and transferring of segmentation masks or anatomical landmarks across multiple image frames. This approach may be faster and more accurate than iterative registration methods and can improve the workflow of perfusion analysis.
*Author Disclosures:*
**B. Norman:** Employee; Arterys. Patent Holder; Arterys. Shareholder; Arterys. **M. Simonovsky:** Patent Holder; Arterys. **F. Lau:** Employee; Arterys. Patent Holder; Arterys. Shareholder; Arterys. **S. Sall:** Employee; Arterys. Patent Holder; Arterys. Shareholder; Arterys. **D. Golden:** Employee; Arterys. Patent Holder; Arterys. Shareholder; Arterys. **A. Hsiao:** Founder; Arterys. Shareholder; Arterys.

**B-0303** 14:24
**Radiomic analysis of coronary CT angiography images can identify invasive and radionuclide imaging markers of coronary plaque vulnerability**
M. Kolossvary[1], J. Park[2], J.-I. Bang[2], J. Zhang[2], J. Lee[2], J. Paeng[2], T. Kubo[3], B.-K. Koo[2], P. Horvat-Maurovich[1]; [1]*Budapest/HU*, [2]*Seoul/KR*, [3]*Wakayama/JP (marton.kolossvary@gmail.com)*

**Purpose:** Identification of invasive and radionuclide imaging markers of coronary plaque vulnerability by a single, widely available non-invasive technique may provide the opportunity to identify vulnerable plaques and vulnerable patients in broad populations. Radiomics extracts quantitative metrics from radiological images which describe the heterogeneity and spatial complexity of lesions to create big-data datasets. Our aim was to assess whether radiomic analysis of coronary CT angiography images outperforms conventional clinical assessment to identify invasive and radionuclide imaging markers of plaque vulnerability.
**Methods and Materials:** We assessed seven conventional plaque features and 935 radiomic parameters from coronary CT angiography (CTA) images of stable angina patients who in addition underwent sodium fluoride positron emission tomography ($NaF^{18}$-PET), intravascular ultrasound (IVUS) and optical coherence tomography (OCT). For diagnostic accuracy, area under the receiver operating characteristics curve (AUC) was calculated using 5-fold cross-validation with 1000 repeats. We calculated the two-sided Wilcoxon signed-rank test to compare the distribution of AUC values resulting from the repeated cross-validations.
**Results:** In total, we analysed 44 plaques in 25 patients with all modalities. Radiomic analysis of coronary CTA images outperformed conventional qualitative and quantitative metrics to identify attenuated plaque by IVUS, thin-cap fibroatheroma by OCT and metabolically active plaques on $NaF^{18}$-PET (AUC: 0.72 vs. 0.59; 0.80 vs. 0.66; 0.87 vs. 0.65; p<0.001 all; respectively).
**Conclusion:** Radiomic analysis of coronary CTA images identified invasive and radionuclide imaging markers with good to excellent diagnostic accuracy. Radiomic analysis of coronary CTA images may provide an inexpensive, widely available non-invasive solution to identify vulnerable plaques and, therefore, improve cardiovascular risk stratification.
*Author Disclosures:*
**M. Kolossvary:** CEO; Pictologics Ltd.. Founder; Pictologics Ltd. **P. Horvat-Maurovich:** Founder; Pictologics Ltd.

**B-0304** 14:32
**Prediction of the clinical outcome of chest ground glass area using a radiomic approach**
N. D'Amico, G. Valbusa, E. Grossi, D. Fazzini, A. Malasevschi, F. Rigiroli, S. Papa; *Milan/IT (NataschaClaudia.D'Amico@cdi.it)*

**Purpose:** The purpose of the study is to analyse chest CT images with a first occasional finding of ground glass (gg) and predict, through a radiomic approach, the evolution of the gg area after at least one year.
**Methods and Materials:** 79 patients with a first occasional report of gg in chest CT and a CT image acquired after one year were included in the study. Images were acquired on 64-slice dual source CT (Siemens Somatom) with 3 mm slice thickness. A semi-automatic segmentation of the gg areas was carried out by a resident radiologist and controlled by two expert radiologists, using 3DSlicer image analysis software. For every patient, 696 features were extracted and calculated using the IBEX platform. Clinical data, such as age and gender, were added and analysed with a machine learning (ML) algorithm based on an evolutionary optimization to build a prognostic model of the gg areas.
**Results:** After one year, out of the 79 patients, 19 showed a size increase, and 60 patients were completely negative, i.e. gg areas naturally disappeared (45%) or remained stable (55%). Histological information has confirmed the malignity of 15 of the 19 suspicious patients. The ML algorithm selected 82 features out of 699 including intensity and texture-based features. A KNN classifier based on the 82 features was identified as the best performing ML approach and showed sensitivity and specificity of 89.4% and 91%, respectively.
**Conclusion:** This study demonstrated the feasibility to predict clinical evolution of a gg area, with 90% accuracy.
*Author Disclosures:*
**E. Grossi:** Consultant; Bracco Imaging S.p.A.

**B-0306** 14:40
**Image quality comparison between iterative reconstruction methods and AI-based reconstruction for CT chest**
P. Rogalla[1], B.E. Hoppel[2], M. Masakazu[3], J. Zhou[3], C. Farrell[1], S. Kandel[4]; [1]*Toronto, ON/CA*, [2]*Delafield, WI/US*, [3]*Vernon Hills, IL/US*, [4]*Berlin/DE (Patrik.Rogalla@uhn.ca)*

**Purpose:** To evaluate a new artificial intelligence reconstruction method based on convolutional neural network to improve image quality in thoracic CT compared to two standard of care reconstruction methods, which use iterative reconstruction.
**Methods and Materials:** Under ethical approval, 48 projection datasets from a lung screening trial (120kV, 50mA, 0.5s rotation time, 0.5*80 collimation; Canon Aquilion Genesis) were reconstructed in 5 series: hybrid-iterative IR (AIDR) and forward-projection based IR (FIRST), both with body and lung settings, and AI. All images were reconstructed with 1 mm slice thickness and presented as 5 on 1 (random order, no image annotation) on a 4K monitor. By using forced ranking, 2 readers evaluated the series in the categories: conspicuity, thoracic noise texture, mediastinum quality, mediastinal texture, artefacts, and overall appeal. Overall image quality was graded on a Likert scale (1=excellent, 10=low quality).
**Results:** AI reconstruction was overwhelmingly preferred over all other reconstructions by readers in all patients and in all categories (p<0.001) except for artefacts where AI ranked 2$^{nd}$ behind AIDR body with an average rank of AIDR body/AI in was 2.21/1.70 p>0.05. The average overall appeal ranking/Likert score for AIDR body, lung, FIRST body, lung and AI was 3.88/5.71, 3.11/4.84, 4.91/7.04, 2.15/3.56 , 1.00/1.91 respectively, p<0.001. Kappa for forced ranking k=0.90 and Likert score k=0.78.
**Conclusion:** AI reconstruction shows improved image quality in thoracic CT for lungs and mediastinum when compared to both clinically established iterative methods and may eliminate the need for separate reconstructions for lungs and soft tissues.
*Author Disclosures:*
**P. Rogalla:** Research/Grant Support; Canon Medical. **B.E. Hoppel:** Employee; Canon Medical. **M. Masakazu:** Employee; Canon Medical. **J. Zhou:** Employee; Canon Medical. **C. Farrell:** Employee; Canon Medical.

**B-0307** 14:48
**Unsupervised machine learning for the identification of CT imaging markers of progression in idiopathic pulmonary fibrosis**
J. Pan[1], J. Hofmanninger[1], S. Röhrich[1], F. Prayer[1], M. Holzer[1], N. Sverzellati[2], M. Weber[1], H. Prosch[1], G. Langs[1]; [1]*Vienna/AT*, [2]*Parma/IT (jeanny.pan@meduniwien.ac.at)*

**Purpose:** To identify imaging markers of progression in chest CT scans of patients with idiopathic pulmonary fibrosis (IPF) and compare the performance of the algorithm to chest radiology experts.
**Methods and Materials:** 228 chest CT scans of 80 IPF patients were divided into 114 pairs consisting of two examinations of the same patient at different points in time (T1 baseline and T2 follow-up). Non-rigid registration was performed and lungs were segmented automatically. Unsupervised machine learning was used for textural feature extraction and generation of 20 stable lung pattern clusters. Each voxel was assigned to one cluster, volume differences between T1 and T2 were calculated for each cluster. Results were used to train a Random Forest Classifier to predict the sequence of scans. For evaluation leave-one-out cross-validation was performed and global volume fraction change of each cluster per scan was analysed, with a subsequent comparison of two radiologists' performance.
**Results:** Six out of 20 clusters were identified as predictor of disease progression, used by the classifier to accurately determine the sequence of CT scans in 87 out of 114 pairs (76%). The performance of the classifier was comparable to the expert radiologists (E1: 83% and E2: 67%). The overlap of computational model errors with instances of expert errors were 25.92% (E1) and 66,67% (E2).
**Conclusion:** By applying an unsupervised machine learning approach, we were able to identify six stable CT-patterns predicting disease progression in patients with IPF. The resulting algorithm showed similar accuracy to two chest radiology experts.
*Author Disclosures:*
**J. Pan:** Research/Grant Support; Boehringer Ingelheim.

**B-0308** 14:56
**Imaging biomarkers for the differentiation of benign and malignant small solitary pulmonary nodules smaller than 1 cm based on CT texture analysis**
J. Ji[1], M. Chen[1], X. Hu[1], W. Ye[1], C. Chen[1], P. Pang[2], C. Lu[1], Q. Weng[1], H. Wang[1]; [1]*Lishui/CN*, [2]*Hanghzou/CN (lschrjjs@163.com)*

**Purpose:** To investigate the potential imaging biomarkers for predicting the benign and malignant small pulmonary nodules (PNs) (≤1cm) based on CT texture analysis.
**Methods and Materials:** 89 PNs (≤1cm) from 89 cases were included, 51 nodules were diagnosed with adenocarcinoma, and 38 had benign PNs diagnosed with inflammation or infections. The AK (Analysis Kit, GE Healthcare) software was used to manually delineate the volume of interest of the lesions and extract a total of 396 quantitative texture parameters. The statistical analysis was performed with R software. The PNs were randomly divided into training set (n=59) and validation set (n=30). All pre-normalized (Z-score) feature values were dimension reduction by LASSO algorithm and selected the most useful features in training set. The selected imaging features were then combined into Rad-score, which was further tested by the ROC curve in training set and validation set.
**Results:** Four characteristic parameters (ClusterShade_ AllDirection_ offset4_SD, ShortRunEmphasis_ angle45_ offset1, Maximum 3DDiameter, SurfaceVolumeRatio) were further selected by LASSO ($p$<0.05). The above four parameters as a cluster of imaging biomarkers were used to form the Rad-score, the area under the ROC curve in the training set was 0.792 (95% CI: 0.671, 0.913), and the sensitivity and specificity were 86.10% and 65.20%. The area under the ROC curve in the validation set was 72.9% (95% CI: 0.545, 0.913), and the sensitivity and specificity were 86.70% and 60%.
**Conclusion:** The cluster of imaging biomarkers can reliably predict benign and malignant small solitary pulmonary nodules (PNs) (≤1cm).

**B-0309** 15:04
**Multi-parametric radiomics in low-dose CT for discrimination between emphysematous and non-emphysematous lung tissue**
Y. Nagaraj, M.D. Dorrius, M. Rook, Q. Li, J. Guo, R. Vliegenthart, M. Oudkerk, P.M.A. van Ooijen; *Groningen/NL (y.nagaraj@umcg.nl)*

**Purpose:** Emphysema is part of the Chronic Obstructive Pulmonary Disease (COPD) spectrum and known for its high prevalence and mortality rate worldwide. The appearing lung tissue destruction can be detected on Low-Dose Computed Tomography (LDCT) as decreased lung density and architectural changes. However, the latter is difficult to quantify so far. In this study, we evaluated radiomics features using Generalized Matrix Learning Vector Quantization (GMLVQ) to discriminate between emphysema and non-emphysematous lung tissue using a relevance feature ranking classifier.
**Methods and Materials:** 64 LDCT scans were randomly selected from a large lung cancer screening trial. Independently, three radiologists selected a total of 419 free form Regions of Interest (ROI) from these scans. Every ROI consisted of three consecutive non-overlapping 2D slices. 300 ROIs were classified as emphysematous. ROIs were used to extract radiomics features, which were ranked based on relevance factors from GMLVQ. We used feature selection techniques to decrease redundancy between features before classification.
**Results:** We extracted 1008 features including Gray-Level Co-Occurrence Matrix (GLCM), Gray-Level Run-length Matrix (GLRL), first order histogram features, shape descriptors and image filters. Relevance feature ranking from GMLVQ, which can be related to pairwise correlations between features, resulted in a maximal area under the curve of 0.95 for discrimination between emphysematous and non-emphysematous lung tissue.
**Conclusion:** Multi-parametric radiomics with GMLVQ methods can discriminate between lung tissue with and without emphysema. Potentially, it can be a valuable tool for characterization of emphysema.

**B-0310** 15:12
**Evaluation of a novel fully automatic, artificial intelligence-based diameter measurement algorithm for the thoracic aorta**
M. Pradella[1], T.J. Weikert[1], S. Rapaka[2], R. Kärgel[3], G. Sommer[1], A.W. Sauter[1], J. Bremerich[1], B. Stieltjes[1], P. Brantner[1]; [1]*Basel/CH*, [2]*Princeton, NJ/US*, [3]*Forchheim/DE (maurice.pradella@usb.ch)*

**Purpose:** Thoracic aortic dilatation can only be detected by imaging and is an important risk factor for potential dissection/rupture. Manual diameter measurements are time consuming and error prone. We compared the performance of a novel, fully-automatic, artificial intelligence-based radiology assistant (AI-RA) with radiologists' performance.
**Methods and Materials:** 20 patients with suspected aortic dilatation with ECG-gated, contrast-enhanced CT of the thoracic aorta were analysed retrospectively. Three radiologists (experience: 2, 4.5, 8 years) performed centerline-based measurements of maximum diameters twice at multiple levels according to current ESC guidelines (including aortic sinus (AS), ascending aorta (AA)) using syngo.via (Siemens Healthineers, Germany). Measurement time was recorded. The AI-RA (Siemens Healthineers, Germany) applies multiscale deep reinforcement learning for landmark detection and a deep, convolutional image-to-image network for segmentation. Resulting diameter measurements of the aorta were compared to manual assessment.
**Results:** Average time for manual full aortic measurement was 4:49min±1:55min. The mean average difference of absolute diameter measurements (radiologists vs. AI-RA) at AS and AA was +2.2mm and -1.4mm, respectively. Standard deviation (SD) of radiologists was ±1.6mm (AS) and ±1.5mm (AA) and 0.0mm at both locations for AI-RA. Within-subject coefficient of variation (WSV) for radiologists was 5.0-38.0% (AS) and 2.6-17.1% (AA). WSV of the AI-RA measurements was 0%.
**Conclusion:** The current version of the AI-RA finds plausible diameters comparable to the absolute value and variance range of manual measurements, with neither manual interaction nor introduction of user-based variance. The promising results of this initial study will be fortified in a larger scale study to evaluate solution stability.
*Author Disclosures:*
**S. Rapaka:** Employee; Siemens Healthineers. **R. Kärgel:** Consultant; Siemens Healthineers.

**14:00 - 15:30** **Room G**

### Physics in Medical Imaging

### SS 313

### CT: cardiovascular, calcium, and iodine

*Moderators:*
S. Maguire; Dublin/IE
N.N.

**B-0311** 14:00
**Detectability of small coronary calcifications: a comparison between dual-layer and spectral photon-counting CT**
N.R. van der Werf[1], M. Vonder[2], R.W. van Hamersvelt[1], M. Greuter[2], S.A. Si-Mohamed[3], P. Douek[4], L. Boussel[3], T. Leiner[1], M.J. Willemink[5]; [1]*Utrecht/NL*, [2]*Groningen/NL*, [3]*Bron/FR*, [4]*Lyon/FR*, [5]*Menlo Park/NL (nrvdwerf@gmail.com)*

**Purpose:** For spectral photon-counting computed tomography (SPCCT), small detector elements are necessary to reduce pile-up effects, which result in a substantial increase in spatial resolution. We hypothesize that SPCCT will, therefore, improve detectability of small structures, such as small calcifications in the coronary arteries. The objective of this study was to compare detectability of small calcifications between dual-layer computed tomography (DLCT) and SPCCT.
**Methods and Materials:** A cylindrical phantom containing 100 small cylindrical calcifications (0.5 - 2.0 mm; 90 - 540 mg HA/cm$^3$) was scanned on a clinical DLCT and a prototype SPCCT system. Raw data were acquired at 120 kVp with 147 and 100 mAs for DLCT and SPCCT, respectively, with five repetitions. The smallest available slice thickness was used, 0.67 mm and 0.25 mm for DLCT and SPCCT, respectively. Pixel spacing was 0.49 mm for DLCT and 0.25 mm for SPCCT. Detectability was defined as voxels with ≥130 Hounsfield units (HU) in at least three acquisitions using ImageJ.
**Results:** For SPCCT, 85% of the calcifications were detectable, while only 31% of the calcifications were detected with DLCT. Calcifications measuring 0.5 mm and with a density of ≥190 mg HA/cm3 were all visible with SPCCT while the calcifications smaller than 1.0 mm were not detectable with DLCT, independent of their density.
**Conclusion:** SPCCT is superior in the detection of small calcifications with an increase in detectability of 174% in comparison to DLCT, notably for the calcifications of 0.5 mm with a density of 190 mg HA/cm$^3$ or more.

**B-0312** 14:08
**Stack transition artefact removal (STAR) in cardiac CT with automatic parameter selection**
S. Lebedev[1], E. Fournie[2], K. Stierstorfer[2], M. Kachelrieß[1]; [1]*Heidelberg/DE*, [2]*Forchheim/DE (marc.kachelriess@dkfz.de)*

**Purpose:** To remove stack transition artefacts, i.e. misalignments between sub-volumes (stacks), in cardiac CT reconstructions.
**Methods and Materials:** Phase-correlated reconstructions, e.g. from prospectively/retrospectively gated data, may be affected by stack transition artefacts if the heart is imaged in multiple steps while irregular motion (e.g. cardiac arrhythmia, breathing) is present. The stacks overlap longitudinally by a few mm. However, the consistency in the overlap region may suffer from stack-to-stack heart motion. The redundant information in the overlap region can be used to determine deformation vector fields (DVFs) between stacks. We developed a symmetric Demons algorithm that applies deformations symmetrically in both overlapping stacks. The DVF is expanded to non-redundant areas via interpolation. The DVF smoothness is controlled with one parameter. The registration is initially applied with minimal DVF smoothing to estimate the required deformation magnitudes. Based on the latter, a proper smoothing parameter is set, ensuring a realistic DVF that does not introduce distortions. Five retrospectively gated cardiac, patient data sets acquired with a Somatom Definition AS+ (Siemens Healthineers, Forchheim, Germany) and standard partial scan reconstructions were used to evaluate STAR.
**Results:** STAR considerably improved image quality. Discontinuities, e.g. cuts/breaks in coronary arteries and cardiac valves, were removed or considerably reduced. The automatic parameter selection adjusted the DVF smoothing for individual registrations and overall yielded better results compared to selecting one constant parameter.
**Conclusion:** STAR is able to consistently remove stack transition artefacts. The adaptive parameter selection prevents the introduction of unnatural distortions, therefore improving the robustness of the new algorithm.
*Author Disclosures:*
**S. Lebedev:** Employee; Siemens Healthcare GmbH. **E. Fournie:** Employee; Siemens Healthcare GmbH. **K. Stierstorfer:** Employee; Siemens Healthcare GmbH.

**B-0313** 14:16

**Accurate non-invasive assessment of carotid in-stent restenosis by dual-layer spectral CT**

I. Leichter, E. Ben-David, J. Menat, A. Peretz, Z. Romman, J.M. Gomori, J. Sosna; *Jerusalem/IL (gomori@cc.huji.ac.il)*

**Purpose:** Beam-hardening artefacts in CT imaging of metallic carotid stents reduce visibility of plaque. Our aim was to accurately assess, non-invasively, in-stent stenosis, using iodine imaging by spectral-detector CT (SDCT).
**Methods and Materials:** Virtual mono-energetic images containing iodine solutions were reconstructed at 65keV and 200keV, using SDCT. A spectral map was generated reflecting the relationship between gray-level values of iodine solutions in the two mono-energetic images. An iodine imaging algorithm was developed to mark all pixels fulfilling the spectral map equation and to accurately highlight the contrast-filled lumen. The algorithm was tested on a water-equivalent phantom with plastic tubes containing an iodine solution and surrounded by metallic conichrome stents of 0.2 mm thickness. The tubes of 1.5 mm and 0.75 mm wall thickness mimicked moderate (61%) and mild (34%) in-stent stenosis. The algorithm was used, retrospectively, to quantify restenosis in 2 patients, one with facial numbness, and the other with symptoms of a left CVA.
**Results:** In conventional images of the phantom, due to beam hardening, mild restenosis could not be visualised, and the moderate restenosis was overestimated by 8%. Iodine imaging demonstrated and quantified the mild and moderate restenosis more accurately with deviations of 5.9% and 0.6%, respectively. In the patient with facial numbness, the algorithm measured a restenosis of 79%. In the other patient, interpreted by a radiologist with moderate stenosis, the measured restenosis was 48%.
**Conclusion:** Carotid in-stent stenosis in high-attenuation metallic stents can be more accurately evaluated using iodine imaging produced by spectral dual-layer detector CT.
*Author Disclosures:*
**Z. Romman:** Employee; Employee.

**B-0314** 14:24

**Quantification of metal artefacts in CT imaging: methodological comparison of different techniques and correlation to visual perception**

N. Grosse Hokamp[1], B. Eck[2], J. Holz[1], F. Siedek[1], D. Pinto Dos Santos[1], D. Maintz[1], S. Haneder[1]; [1]*Cologne/DE*, [2]*Cleveland, OH/US (nils.grosse-hokamp@uk-koeln.de)*

**Purpose:** Several techniques aiming to reduce metal artefacts in CT imaging (MAR) have been proposed. To quantify MAR, numerous quantification techniques have been suggested (MAR-QT). We compared available MAR-QT to visual perception of artefacts to establish a standard for artefact quantification in CT imaging.
**Methods and Materials:** 5- and 10-mm Titanium rods were examined in a rectangular water phantom with 25 different scanning and image reconstruction parameters to get a reference database of different types and extents of artefacts. MAR-QT have been identified in the literature, including HU measurements, computation of percent integrity uniformity (PIU) and techniques in the frequency domain (FFT). In total, 38 MAR-QT were implemented in the Matlab environment. To establish a reference ranking, 4 radiologists separately evaluated every image against each other twice using an in-house developed software (2400 comparisons = 2 x 4 readers x 300 comparisons). Rankings were combined to obtain a reference ranking reaching from best to worst image. Intraclass-correlation coefficients (ICC) indicated intra-/inter-reader agreement. The reference ranking was compared to MAR-QT results to identify suited and less suited approaches. Kappa-statistics were used to evaluate agreement between quantitative methods and visual perception.
**Results:** Intra-/inter-reader agreement of visual artefact perception were excellent (ICC 0.85-0.92). No quantitative method was able to depict the exact ranking of visually perceived artefacts. The methods that showed best correspondence were PIU and FFT based.
**Conclusion:** Artefact quantification in CT is challenging. We propose two methods that show best correlation with visual artefact perception and suggest using these to allow comparison between studies.
*Author Disclosures:*
**N. Grosse Hokamp:** Speaker; Philips Healthcare. **D. Maintz:** Speaker; Philips Healthcare.

**B-0315** 14:32

**Influence of iterative model reconstruction level for measurement accuracy of the coronary stenosis caused by non-calcified plaques using low-dose CCTA: a phantom study**

L. Gao, Y. Hou, Y. Ma, L. Yang, X. Lu; *Shenyang/CN (1242179174@qq.com)*

**Purpose:** To evaluate the accuracy of low-dose CCTA with different iterative model reconstruction (IMR) levels in quantitative assessment of the coronary stenosis caused by non-calcified plaques using a pulsatile phantom of coronary arteries.
**Methods and Materials:** A pulsating cardiac phantom mounted with 4 different inter-diameter (2.5, 3.0, 3.5, 4.0 mm) artificial vessels were performed low-dose scan (100 kV, automated tube current modulation; Dose Right Index 13) on 256-slice CT with prospective ECG-gated mode. For each diameter artificial vessels, there were three stenosis degrees (75%, 50% and 25%) with non-calcified plaques (CT value is 20 HU) scanned at heart rate of 60 bpm. For each vessel, the material was arranged ladder like in the iodine-enhanced lumen (350±5 HU in 100 kV) to simulate different stenosis severity. Images were reconstructed with IMR (cardiac-routine IMR-1, IMR-2, IMR-3). The stenosis degree was calculated [1-(luminal stenotic diameter/luminal normal diameter)] and compared with the actual values.
**Results:** For the measured stenosis degrees of non-calcified plaques, there was statistical difference among different IMR levels and the actual value ($P<0.05$), and the measured values were smaller than the actual value. The stenosis degree with IMR-1 was closer to the actual value than those with IMR-2 and IMR-3. At the same IMR level, the measured stenosis degrees had no significant difference among different vessel diameters ($P>0.05$). For severe artery stenosis, the degree of underestimation was larger.
**Conclusion:** Different IMR levels with low-dose CCTA tend to underestimate stenosis despite of the diameters, IMR level 1 can better estimate the stenosis rate. The severity of the stenosis had a significant effect on the underestimation rate.

**B-0316** 14:40

**Iodine quantification accuracy in dual-source dual-energy CT using default parameters and patient-specific calibrations**

S. Dorn, S. Sawall, J. Maier, M. Kachelrieß; *Heidelberg/DE*

**Purpose:** To evaluate the influence of the relative contrast media ratio (RelCM) on iodine quantification accuracy in dual-source dual-energy CT (DS-DECT).
**Methods and Materials:** The ratio of high and low iodine contrast is denoted as RelCM and is an input parameter to the standard iodine map algorithm. To evaluate the influence of default vs manual RelCM calibrations on iodine quantification accuracy, two anthropomorphic phantoms (liver, thorax) of three phantom sizes (small, medium, large) are equipped with vials of iodine concentrations between 2.5 and 30 mg/mL and measured with a DS-DECT (SOMATOM Definition Flash, Siemens Healthineers, Germany). Acquisitions are performed using the tube voltage combination 80 kV/140 kV+Sn. A default value for $RelCM_{def}$ of 3.01 is used as preconfigured by the vendor. The patient-specific $RelCM_{pat}$ is obtained by manually placing calibration ROIs inside the vials.
**Results:** The patient-specific $RelCM_{pat}$ is on average 5.9% (small), 5.0% (medium) and 11.5% (large) smaller compared to $RelCM_{def}$ for both phantoms. Using $RelCM_{pat}$, the relative error of measured to true iodine concentrations is minimized from 16.9% to 7.4%, 16.8% to 9.9% and 13.7% to 7.6% for the liver phantom and from 7.0% to 2.4%, 6.7% to 4.5% and 8.3% to 2.4% for the thorax phantom. We achieve an accuracy improvement of 64.6%, 50.4% and 60.6% across all concentrations.
**Conclusion:** Manual calibrations result in a smaller RelCM and also in more accurate iodine concentrations. Therefore, a prior-based automatic calibration might be useful to increase iodine quantification accuracy while keeping manual user interactions at a minimum.

**B-0317** 14:48

**Effect of dose and iterative reconstruction on coronary calcium scores at different heart rates for dual-layer CT**

N.R. van der Werf[1], M. Vonder[2], R.W. van Hamersvelt[1], M.J. Willemink[3], M. Greuter[2], T. Leiner[1]; [1]*Utrecht/NL*, [2]*Groningen/NL*, [3]*Menlo Park/NL (nrvdwerf@gmail.com)*

**Purpose:** For a novel dual-layer CT (DLCT) conventional data are acquired by combining the output of both detector layers. The purpose of the current study is to systematically assess the influence of hybrid and model-based IR in combination with radiation dose reduction on coronary calcium score (CCS) in DLCT.
**Methods and Materials:** In the centre of an anthropomorphic thorax phantom an artificial coronary artery (38.5 mg mgHA - 196±3 mgHA/$cm^3$) was translated (0 to 30 mm/s or 0 to 75 bpm). A routine clinical calcium scoring protocol was used, with three repetitions per HR. Radiation dose was reduced by decreasing mAs by 38% and 75% from the baseline value. Raw data were reconstructed with hybrid iDose (levels 0 to 7) and model-based IMR (levels 1 to 3). CCS was quantified as mean and standard deviation Agatston score (AS).
**Results:** At reference dose with iDose level 0, AS was 113±9, 105±10, 101±23, and 59±9 for 0 to 75 bpm, respectively. AS increased at decreased radiation dose and decreased for increased HR. For all HR at reference dose, AS decreased up to -17% for iDose, while they increased up to 24% for IMR. At 75%-reduced radiation dose, AS decreased for both iDose and IMR levels.

**Conclusion:** Application of different types and levels of IR substantially influenced the CCS. Overall, AS decreased with increased IR levels and HR. A trend of increased CCS was found for IMR at reference dose, and reduced CCS at low dose, regardless of HR.

**B-0318** 14:56

**Characterisation of iodine quantification for a fast-kVp switching dual energy CT system**

F. Emiro, P. De Marco, D. Origgi; *Milan/IT (francesca.emiro@ieo.it)*

**Purpose:** To assess the accuracy of iodine quantification in a GE Discovery 750HD CT.
**Methods and Materials:** A PMMA CTDI oval phantom (inserts: 1center-4periphery), and a homemade phantom with 6 inserts of different diameters (15-to-6 mm) placed in a NEMA IEC PET body phantom filled with water, were used. Phantoms with inserts filled at different iodine Ultravist® concentrations (3.7-7.4-11.1 mg/ml) were scanned at 3 different dose levels (12-21-32 mGy) and images were reconstructed as Iodine density maps with FBP and 5 different Iterative Reconstruction levels (ASiR 20-40-60-80-100%) with 2.5 mm slice thickness. For each reconstruction 10 slices were analyzed with Imagej and statistical analysis was performed with ANOVA.
**Results:** Iodine concentration measured at the center was significantly lower (p<0.05) than at the periphery (differences ranged from 0.4 to 0.8 mg/ml). Passing from 12 to 21 mGy, iodine quantification was significantly higher: 35%-27%-22%, corresponding to an absolute variation in the range 1.3-2.4 mg/ml, for 3.7-7-4-11.1 mg/ml, respectively (p<0.05). Conversely, no significant differences were observed from 21 to 32 mGy. For all dose and concentration levels, iodine quantification was higher for higher diameters: relative variations were in the range 10-30%, corresponding to absolute variations in the range -0.9÷3.3 mg/ml, depending on dose level and iodine concentration. While iodine quantification shows dependence on position, dose and insert's diameter, no impact for the reconstruction choice (Iterative vs FBP) was observed.
**Conclusion:** Iodine quantification should be assessed carefully in clinical practice, especially in studies that aim to evaluate possible threshold between healthy and pathological tissues.

**B-0319** 15:04

**Does iodine CNR improve when switching from today's energy integrating CT to tomorrow's photon-counting CT?**

S. Sawall[1], S. Dorn[1], J. Maier[1], S. Faby[2], M. Uhrig[1], H.-P. Schlemmer[1], M. Kachelrieß[1]; [1]*Heidelberg/DE*, [2]*Forchheim/DE (stefan.sawall@dkfz.de)*

**Purpose:** To measure the iodine contrast-to-noise-ratio (CNR) improvement obtained with whole body photon-counting (PC) CT compared to a conventional energy-integrating (EI) CT detector as a function of patient size and tube voltage.
**Methods and Materials:** Images of two anthropomorphic phantoms (thorax, liver) of different sizes (small, medium, large) equipped with vials containing different iodine concentrations were acquired at the SOMATOM CounT (Siemens Healthineers, Germany) CT system using tube voltages of 80 kV, 100 kV, 120 kV, and 140 kV. CNR is evaluated in reconstructions obtained using the EI detector, the PC detector using a single bin [20, max] keV and in reconstructions obtained by statistically optimally weighting acquisitions with two bins. The bins were equidistantly placed in the spectrum, e.g. [20, 50] keV and [50, 80] keV for a tube voltage of 80 kV. Iodine CNR improvement factors, i.e. CNR of the PC relative to the CNR of the EI measurement, are reported.
**Results:** In general, iodine improvement factors increase with increasing tube voltage for all patient sizes. In particular, if only one bin is used the improvement is up to 23% (small: 6%-12%, medium: 5%-23%, large: 4%-23%) and up to 37% if an optimal weighting of two bins is performed (small: 9%-16%, medium: 11%-29%, large: 8%-37%).
**Conclusion:** We find an improved iodine CNR of PC compared to EI. In particular, medium and large sized patient acquired at higher tube voltages benefit from PC and a possible dose reduction.
*Author Disclosures:*
**S. Faby:** Employee; Siemens Healthineers.

**B-0320** 15:12

**High concentration contrast medium injection protocol in combination with monochromatic imaging for dose reduction in spectral coronary CT angiography**

P. Liu, Y. Wang, Z. Jin, J. Cao, L. Lin, Y. Yi; *Beijing/CN (liupeijun_yu@163.com)*

**Purpose:** To evaluate the feasibility of high concentration contrast media (HCCM) combined with monochromatic images to reduce contrast medium dose in CCTA.
**Methods and Materials:** 56 patients with suspected coronary artery disease were randomly separated into two groups. All patients were scanned with dual-layer detector CT(IQon CT, Philips). Patients were either injected with 18 ml HCCM (400mgl/mL) at 2ml/s (group A) or 45 ml contrast medium (370mgl/mL) at 4ml/s (group B). Monoenergetic images (40 to 100 keV with 10 keV increment) were reconstructed for group A, conventional polyenergetic images was reconstructed for group B. Region of interest was placed on aorta root,LM, distant segment of LAD, LCX and RCA to measure the attenuation and noise, SNR and CNR were calculated. Image quality was assessed by two reviewers independently with a 4-point scale.
**Results:** In group A, the CT Value, SNR and CNR on 40keV were significantly higher than those on other monochromatic images sets and conventional images(P<0.01), which also were superior to group B(738.95±184.70HU vs 408.95 ± 57.92HU; 23.32±7.32vs11.30 ± 2.33 and 29.29±8.00 vs 14.41 ± 2.56 )(each P<0.01). In group A, the image noise on 40keV monochromatic images was significantly higher than the other keV set(P<0.01), which also were superior to group B(32.70 ± 6.64 vs 36.96±4.52,P<0.05). The subjective image quality score of 40 keV in group A was better than that in group B(P<0.05).The CTDI, DLP and ED had no significant difference between two groups(each P>0.05).
**Conclusion:** 40 keV monochromatic images combined with HCCM is feasible in CCTA study.

**B-0321** 15:20

**Optimal iodine CNR in a whole body photon-counting CT scanner**

S. Sawall[1], S. Dorn[1], J. Maier[1], S. Faby[2], M. Uhrig[1], H.-P. Schlemmer[1], M. Kachelrieß[1]; [1]*Heidelberg/DE*, [2]*Forchheim/DE (stefan.sawall@dkfz.de)*

**Purpose:** To evaluate the iodine CNR improvements obtained with photon-counting (PC) CT compared to using a conventional energy-integrating (EI) CT detector.
**Methods and Materials:** Images of two anthropomorphic phantoms (thorax, liver) of different sizes (small, medium, large) equipped with vials containing different iodine concentrations were acquired at the SOMATOM CounT (Siemens Healthineers, Germany) CT system, a prototype DSCT scanner housing an EI and a PC detector. mAs-matched acquisitions were performed at 120 kV with effective mAs-values ranging from 75 to 400 mAs using the EI detector, the PC detector with one bin [20, 120] keV, and the PC detector with two bins [20, 70] keV and [70, 120] keV with statistically optimal bin weighting. Image noise and iodine-water contrast is measured and CNR is evaluated.
**Results:** PC with one bin shows an increased CNR of up to 30% compared to EI (small 19%, medium 30%, large 17%). Comparing the optimally weighted two-bin PC images with EI shows an increased CNR of up to 37% (small 27%, medium 37%, large 25%). In comparison to EI acquisitions, this corresponds to a dose reduction of up to 41% for standard PC acquisitions (small 30%, medium 41%, large 26%) and 47% if an optimal bin weighting is used (small 38%, medium 47%, large 36%).
**Conclusion:** Iodine CNR using a PC detector is significantly higher compared to measurements acquired with an EI detector. If acquisitions with two energy bins are performed, CNR can be further increased.
*Author Disclosures:*
**S. Faby:** Employee; Siemens Healthineers.

**14:00 - 15:30** **Room M 1**

### Genitourinary

### SS 307

### Prostate cancer and renal imaging

*Moderators:*
P. De Visschere; Ghent/BE
T. El-Diasty; Mansoura/EG

**B-0322** 14:00

**Multi-parametric MRI in patients with low-to-moderate risk of prostate cancer for the differentiation of adenocarcinoma and prostatitis**

S. Ken, E. Lacroix, D. Portalez, R. Aziza, J. Gilhodes, T. Brun; *Toulouse/FR (lacroixemmanuel31@hotmail.fr)*

**Purpose:** To distinguish between biopsy-confirmed prostatic adenocarcinoma (PCa) and prostatitis (PT) with imaging biomarkers derived from multi-parametric MRI.
**Methods and Materials:** Nineteen patients with low-to-moderate risk of prostate cancer underwent mpMRI on a 1.5T scan. Anatomical T2-weighted, diffusion (11b values 0-800s/mm$^2$) and T1-weighted perfusion series were acquired. Regions of interest (ROI) were contoured on computed b2000 maps and reported on Ktrans, D and ADC modalities. Distributions of voxel values inside the ROIs were compared to contralateral normal-appearing tissue (NT). Volume repartitions according to quartiles were compared between the biopsy-confirmed groups of PCa (N=12) and PT (N=7).
**Results:** PCa group: median ADC and D parameters (10$^{-3}$mm$^2$/s) inside the ROIs were significantly lower than in the NT: 1.2[0.7-1.6] vs 1.5[1.2-1.7] (p=0.0022) and 1.0[0.6-1.5] vs 1.3[1.0-1.6] (p=0.0022), respectively. Median

Wednesday voxel intensity of computed b2000 (s/mm$^2$) was significantly higher: 38.2[31.9-87.1] vs 27.6[18.9-77.8] (p=0.0037). Ktrans values were not significantly different between ROIs and NT. PT group: median ADC and D were significantly lower: 1.3[1.0-1.5] vs 1.5[1.2-1.6] (p=0.0280) and 1.1[0.8-1.3] vs 1.3[0.9-1.5] (p=0.0180), respectively. Median b2000 and Ktrans values were significantly higher inside the ROIs: 46.5[27.3-83.8] vs 38.7[21.6-70.2] (p=0.0425) and 0.3[0.1-1.2] vs 0.2[0.1-0.5] (p=0.0280), respectively. Volume of ADC values in the 1$^{st}$-3$^{rd}$ quartiles range was significantly lower in the PCa group compared to the PT group: 0.1%[0.0-0.4] vs 0.3%[0.1-0.4] (p=0.0312). Volume of low Ktrans (<1$^{st}$ quartile) was significantly higher in the PCa group: 0.3%[0.1-0.5] vs 0.1%[0.0-0.2] (p=0.0075).
**Conclusion:** Percentage volume distribution of ADC and Ktrans was able to distinguish between adenocarcinoma and prostatitis.

**B-0323** 14:08

**Prostate cancer and benign tissue response to hypofractionated SBRT monitored by multi-parameteric MRI**

M.J. Zelefsky, K. Zakian, A.G. Wibmer, N. Tyagi, M. Kollmeier, M. Hunt, E. Sala, H. Mejias Vargas; *New York, NY/US (zelefskm@mskcc.org)*

**Purpose:** To explore the role of serial mp-MRI for evaluation of tumour response after stereotactic radiosurgery (SBRT) for localized prostate cancer (PC).
**Methods and Materials:** 30 patients with organ-confined PC with dominant lesions ≥ 0.5 cm on MRI were enrolled on a prospective IRB-approved study. All patients received 40 Gy in 5 fractions of SBRT. A baseline MRI was performed and post-treatment MRIs at 3, 6, 12, 18 and 24 months. At 24 months a repeat prostate biopsy was performed to assess local control and correlate with imaging response. A 3.0 Tesla Philips Ingenia was used with a MRI protocol using standard anatomical $T_1$ and $T_2$-weighted imaging as well as DCE MRI multi-b-value diffusion-weighted imaging for additional quantitative imaging. Tumour Region of Interest (ROI) identification was obtained by two GU expert radiologists who contoured tumour, peripheral zone, transition zone (TZ).
**Results:** In all cases perfusion and diffusion-related parameters changed with time and tended to converge with benign tissue values no later than at 12 months. On T2-weighted images the dominant nodule demonstrated significant tumour shrinkage by 3 months and disappearance in >90% of cases by 6 months. Diffusion coefficients significantly decreased post-SBRT providing earlier information than the post-treatment PSA status which continued to decline during the 2-year post-treatment period.
**Conclusion:** mp-MRI assessment after SBRT demonstrated significant tumour responses at 3 months after treatment far earlier than standard assessment tools such as PSA. Longer follow up will be needed to determine whether MRI initial responses could predict long-term local treatment failures.

**B-0324** 14:16

**Evaluation of dynamic contrast enhancement in PIRADSv2: a report from the Gothenburg 2 screening study for prostate cancer**

J. Wallström, K. Geterud, K. Kohestani, R. Godtman, A. Socratous, M. Hellström, J. Hugosson; *Göteborg/SE (jonas@wallstromdiagnostik.se)*

**Purpose:** In the prostate imaging reporting and data system (PIRADSv2) dynamic contrast enhancement (DCE) has the limited role of upgrading peripheral zone lesions from PIRADS 3 to PIRADS 4 in case of positive DCE. The effect of applying or omitting the upgrading is assessed for PIRADS 3-5 in the Gothenburg2 screening study for prostate cancer (PC).
**Methods and Materials:** The Gothenburg 2 is a long term study inviting 40000 men to PSA-testing followed by multiparametric prostate MRI at 3T in case of elevated PSA. MRI:s from 1,010 men were prospectively classified according to PIRADSv2. All men with PIRADS 3-5 had prostate biopsies. The detection rates of all tumors and Gleason score (GS) ≥7 tumors respectively were calculated for PIRADS 3-5 and PIRADS 4-5 with and without DCE-upgrading.
**Results:** A total of 371 MRI:s were scored PIRADS 3-5 with positive biopsies in 188 men (51%) including 85 GS ≥7 tumors (23%). With DCE-upgrading 262 men were scored PIRADS 4-5 and 162 of these had positive biopsies (62%) including 79 GS ≥7-tumors (30%). Omitting DCE-upgrading a total of 190 MRI:s were scored PIRADS 4-5 and out of these 128 men had positive biopsies (67%) including 73 GS≥7-tumors (38%).
**Conclusion:** Applying the PIRADSv2 criteria for DCE resulted in a lesser proportion of GS≥7 PC in PIRADS 4-5 but a slightly higher total number of GS≥7 tumors. With DCE-upgrading and a biopsy indication cut off at PIRADS 4 the number of biopsies would be reduced by 29% however missing 7 % of GS≥7 tumors.

**B-0325** 14:24

**Predicting clinically significant prostate cancer of the transition zone using multiparametric MRI and quantitative radiomic analysis**

M. Pecoraro, S. Cipollari, R. Campa, G. Barchetti, C. Catalano, V. Panebianco; *Rome/IT (pecoraro.martina1@gmail.com)*

**Purpose:** To implement a quantitative radiomic approach to develop a machine learning classifier based on multiparametrc MRI (mpMRI) images of the prostate, capable of distinguishing clinically significant and non-clinically significant prostate cancer (PCa) of the transition zone.
**Methods and Materials:** Ninety-two patients with elevated PSA who underwent mpMRI were included in the study. Inclusion criteria were: PI-RADS score assessment of 3 or higher for lesions within the transition zone, a subsequent TRUS-MRI targeted fusion biopsy and no prostate biopsies or interventions in the previous 6 months. Lesions within the transition zone were manually segmented by a trained urogenital radiologist on the T2-weighted images and on the ADC maps using 3D Slicer, yielding a Volume of Interest (VOI) for each lesion. A radiomic approach was implemented in Python to extract features from the VOIs that correlated with the presence of histologically-proven clinically significant PCa. Extracted features were then selected on univariate analysis and subsequently fed to a machine learning random forest classifier using the R statistical software package. Statistical analyses, including sensitivity, specificity, accuracy and ROC analysis, were performed on the trained classifier.
**Results:** Of the 92 patients 39 (42%) were positive for clinically significant PCa at histopathology. Radiomic analysis calculated 368 quantitative features for each VOIs. A predictive model using the features selected on univariate analysis achieved a sensitivity of 0.74, specificity of 0.68 and overall accuracy of 0.71.
**Conclusion:** The machine learning classifier based on mpMRI radiomic analysis showed higher accuracy than radiologist assessment in detecting clinically significant PCa.

**B-0326** 14:32

**Radiological prediction of the chance of post-RALP urinary continence on preoperative MR prostate imaging can substantially alter patients' choice of treatment**

S. Heijmink, E. van Muilekom, C. Tillier, N. Grivas, H. van der Poel; *Amsterdam/NL (swheijmink@live.com)*

**Purpose:** Prostate cancer patients are often presented with treatment choices that are equal in oncological outcome but different considerably in morbidity, such as the risk of post-treatment urinary incontinence. The purpose of the study was to examine the influence of the preoperative radiological assessment of the chance of urinary continence after robot-assisted laparoscopic radical prostatectomy (RARP) by means of MRI on the patients' choice of treatment.
**Methods and Materials:** Prospectively, a total of 65 patients with prostate cancer that had preoperative MRI and were referred for RARP were included in the study. In all patients the membranous urethral length and interlevator distance were prospectively assessed. From this, the chance of urinary continence after RARP (continence prediction, Cpred) could be estimated by a previously published prediction model (Grivas et al. Neurourol and Urodyn 2018). Patients were subsequently counselled about the surgery and the predicted chance of continence was presented to them. Their final treatment decision was recorded.
**Results:** Of the 65 patients, 14 (21%) decided to change their preferred choice of treatment from RARP and instead opted for radiation therapy. The average Cpred for the patients that kept RARP with their preferred treatment and those that changed their preferred treatment into radiation therapy was 63% and 42%, respectively (p=0.02).
**Conclusion:** The patients' choice of whether or not to undergo RARP for prostate cancer appeared to be influenced by the predicted chance of urinary continence based on preoperative MRI. This tool may aid patients in better treatment decision making and avoid morbidity.

**B-0327** 14:40

**Value of dynamic contrast enhanced (DCE) MR imaging in PI-RADS 4 patients**

L. Schimmöller, F. Ziayee, T. Ullrich, N. Laqua, G. Antoch, P. Albers, C. Arsov; *Düsseldorf/DE*

**Purpose:** To assess the impact of dynamic contrast-enhanced imaging (DCE) in mp-MRI on prostate cancer (PCa) detection in a large patient cohort assigned to PI-RADS category 4.
**Methods and Materials:** This prospective, single centre cohort study includes 193 consecutive patients with PI-RADS assessment category 4 after mp-MRI (T2WI, DWI, DCE) at 3T with targeted and systematic biopsy as reference standard. Prostate cancer detection rates were compared either in a multiparametric setting or without the effect of DCE in a biparametric setting.

**Results:** PCa detection rate was 62% (119/193) including 48% (92/193) clinically significant PCa (csPCa; Gleason score ≥3+4=7). 38 of these 193 patients (20%) had peripheral lesions upgraded from DWI PI-RADS category 3 to an overall PI-RADS category 4 due to positive DCE findings. Of these 38 patients 18 had PCa including 14 with a GS≥7. Thus 15% (18/119) of the patients with any cancer and 15% (14/92) of the patients with a GS≥7 PCa were detected only based on additional DCE Information.
**Conclusion:** DCE allows detection of a significant number of mostly csPCa that would have been missed with a biparametric protocol. The current PI-RADS decision rules regarding upgrading PI-RADS-3-lesions to overall category 4 due to positive DCE imaging are useful for PCa detection.

**B-0328** 14:48
**Evaluation of periprostatic neurovascular fibres before and after radical prostatectomy by means of 1.5 T MRI diffusion tensor imaging**
V. Di Paola[1], L. Russo[1], M. Miccò[1], M. Sbarra[1], R. Pozzi Mucelli[2], R. Manfredi[1]; [1]*Rome/IT*, [2]*Verona/IT (lucarusso.md@gmail.com)*

**Purpose:** To evaluate if diffusion tensor imaging (DTI) is able to detect changes of periprostatic neurovascular fibres (PNFs) before and after radical prostatectomy (RP), and their relationship with post-surgical urinary incontinence and erectile dysfunction.
**Methods and Materials:** 22 patients (mean age 62.6 years) with biopsy-proven prostate cancer underwent 1.5T DTI before and after RP. The number, fractional anisotropy (FA) values and length of PNFs before and after RP were compared using Student's t-test. Each patient filled out two questionnaires before and after RP, one for the evaluation of urinary continence (ICIQ-SF) and one for the evaluation of erectile function (IIEF-5). The number ratios, FA values and length of PNFs before and after RP (DTI B-A RATIOs) and ratios between scores obtained before and after RP for both ICIQ-SF and IIEF-2 (ICIQ-SF B-A RATIOs and IIEF-2 B-A RATIOs) were calculated to perform Kendall's τ-test between them.
**Results:** There was statistically significant decrease of the number of PNFs after RP at base, midgland, and apex ($p < 0.01$) and of FA values at midgland ($p < 0.05$), with positive statistically significant correlation between the DTI B-A RATIOs of the number of PNFs and IIEF-2 B-A RATIOs ($p<0.05$, $\rho=0.47$).
**Conclusion:** DTI was able to detect that the decrease of the number of the PNFs after RP was statistically related to post-surgical erectile dysfunction ($p < 0.05$). This study demonstrates that 1.5T MRI DTI is a reproducible technique in detecting the changes of the PNFs induced by RP, with high interobserver agreement.

**B-0329** 14:56
**Calculation of total kidney volume (TKV) in autosomal dominant polycystic kidney disease (ADPKD): comparison of 3 different methods**
S. Paolicelli[1], R. Virelli[2], L. Coi[1], S. Matino[1], M. Telegrafo[3], A. Stabile Ianora[1], L. Gesualdo[1], V. Bevilacqua[1], M. Moschetta[1]; [1]*Bari/IT*, [2]*Matera/IT*, [3]*Triggiano/IT (simonapaolicelli@gmail.com)*

**Purpose:** TKV in ADPKD represents an important biomarkers of disease progression, consequently its correct measurement is crucial. The aim of our study is to compare 3 methods of measuring TKV using MR images to identify the most efficient one.-mTKV: manual method. Kidney's contour was manually traced by free-hand on all slices and then the sum of the values of the respective each slice area is multiplied by the slice thickness used.- TKVel: ellipsoid method. TKV was estimated using the ellipsoid formula (Vol = Length x Width x Depht x π / 6).- TKV3D: three-dimensional method. TKV is computed using a software that allows automatic calculation of renal volume (VitreaFX software).
**Methods and Materials:** 50 patients with ADPKD underwent MR examination and obtained a series of images according to axial and coronal planes acquisition. The TKV was calculated using mTKV, TKVel and TKV3D by 2 radiologists. Accuracy, time required, inter- and intra- rater agreement were evaluated comparing different methods.
**Results:** TKV3D method is very reliable if compared with TKVel (RMSE%= 4,1%vs14,2%), showed excellent inter (CV= 1,7% and 2,2% vs 7,8% and 6,4% ) and intra (CV= 2,6%vs14,2%) rater agreement with a time required only slightly greater (7 min vs 4 min).
**Conclusion:** The use of the Vitrea FX software for the measurement of TKV represents a good alternative to the ellipsoid method because, even if the time required is only slightly higher, it should be preferred for its reliability and less variability between the two operators.

**B-0330** 15:04
**Development of a CT diagnostic prediction model for diagnosis of pyonephrosis**
L.H.Q. Chin, K.H. Lee, H. Ng, M. Cheung, Y.L. Li, H.T. Lau, D. Tse; *Hong Kong/HK (chin.leanne@gmail.com)*

**Purpose:** Pyonephrosis is a urological emergency necessitating prompt recognition and intervention. Often clinically nonspecific especially in elderly or immunocompromised patients, mimickers such as pyelonephritis with non-obstructive hydronephrosis also have overlapping features on computed tomographic (CT) imaging. We aim to develop a CT scoring system to diagnose pyonephrosis accurately.
**Methods and Materials:** This is a retrospective review of 63 patients (22 diseases, 41 controls) who underwent CT within 1 week prior to percutaneous nephrostomy (PCN) from 2008-2015. Imaging parameters included attenuation of renal pelvis and parenchyma (HU), pelvic wall thickness, perinephric strand extent, perinephric strands and renal fascia thicknesses, obstructing stone, fluid-debris level and degree of hydronephrosis. Scores were validated with 42 patients (18 diseases, 24 control) requiring PCN from 2016-2018. Independent predictors defined using multivariate binary logistic regression for development of scoring system and Youden's index (J) for cut-off.
**Results:** Pelvic wall thickness, renal fascia thickness, attenuation of pelvic content and parenchyma were significant independent predictors and selected for scoring system 0 to 13 (pelvic wall thickness: 4 points; parenchymal attenuation >30HU: 4 points; pelvic content attenuation >15HU: 2 points; renal fascia thickness >3mm: 3 points.) Receiver operating characteristic (ROC) analysis revealed area under curve (AUC) 0.87 (95% CI, 0.77-0.98). Scores 6 or lower had 82.6% (95% CI 66.2-92.0%) negative predictive value (NPV), and score 7 or higher had 73.7% (95% CI 55.27-86.4%) positive predictive value in diagnosing pyonephrosis.
**Conclusion:** Combined CT imaging parameters can be used to distinguish pyonephrosis from mimickers, facilitating patient triage for emergency intervention.

**B-0331** 15:12
**Correlation between single-kidney glomerular filtration rate (GFR) with ultra-low-dose CT perfusion and 99mTc-DTPA renal scan in healthy and tumour-bearing kidney**
F. Wang, Q. Wang; *Jinan/CN (200062001264@sdu.edu.cn)*

**Purpose:** To validate a method for calculating single-kidney GFR based on ultra-low-dose renal volume perfusion CT (VPCT).
**Methods and Materials:** Thirteen patients with unilateral renal tumour were scanned for VPCT, using a MDCT. The dynamic perfusion scan of the whole kidney was initiated with a 5-s delay after contrast agent was given intravenously, consisting of 36 acquisitions with a total scan time of 117s. For 0-30s, 30-90s, and 90-117s, the acquisition interval was 1.5s, 4.5s and 9s, respectively, with tube voltage 80 kVp and current 70 mAs. CT-GFR=Vol(excr)×HU(excr)-Vol(precontrast)×HU(precontrast), then divided by (area under aortic TDC), then multiplied by (1-hematocrit). The single-kidney CT-GFR and 99mTc-DTPA-GFR were compared with paired *t* test and Pearson correlation analysis was performed. Renal function is classified as normal, mild, moderate and severe impairment in two groups and agreement was evaluated by kappa test.
**Results:** There was no statistically significant difference between CT-GFR and 99Tcm-DTPA-GFR ($t$=-1.533, $P$=0.151; $t$=1.791, $P$=0.099; z=-0.33, $P$=0.741) in 13 tumour-bearing kidneys, 13 healthy and all 26 kidneys. Between two groups, a moderate correlation ($r$=0.402, $P$=0.324) in 13 healthy kidneys and strong correlation ($r$=0.790, $P$=0.062; $r$=0.614, $P$=0.019) in 13 tumour-bearing and all 26 kidneys were found. CT-GFR and 99Tcm-DTPA-GFR were normal in 18 kidneys, of moderate impairment in 1 kidney, mild impairment in 5 and 4 kidneys, respectively, and 99Tcm-DTPA-GFR was severe impairment in 1 kidney. A fair agreement was revealed between two groups ($k$=0.49). Effective radiation dose of CT-GFR was only 14.17±0 mSv.
**Conclusion:** CT-GFR bears the potential as a functional assessment tool in kidney, especially in tumour-bearing kidney.

**B-0332** 15:20
**CT urography for examining the upper urinary tract in case of haematuria: time to reconsider?**
E. Rud, T. Flatabø, E. Baco, G. Sandbækk; *Oslo/NO (erik.rud@ous-hf.no)*

**Purpose:** Three-phase CT urography (CTU) is the gold standard for evaluating the upper urinary tract in patients with haematuria. The aim of this study was to evaluate the detection rate of upper urinary tract cancer, and second to determine the visibility of tumour on each phase constituting a CTU.
**Methods and Materials:** This quality control analysis included all patients referred to CTU after a negative cystoscopy during 2016 and 2017. The CTU results were dichotomized as negative or positive and follow up biopsy results were used as reference standard. Two reviewers retrospectively evaluated the tumour visibility of each CT sequence.
**Results:** Three hundred and seventy-six CTUs were performed in 376 patients with haematuria after a negative cystoscopy. The mean age was 62 years (95% CI: 60-64, range 18-96), 67% (253) were males and 33% (123) were females. Macroscopic and microscopic haematuria occurred in 87% (327) and 13% (49), respectively. Eleven cases of upper urinary tract cancer were detected in 10 patients (2.7%), all found in patients with macroscopic haematuria. Transitional cell carcinomas were found in the renal pelvis and ureter in three (0.8%) and four (1.1%) patients, respectively. Renal cell carcinomas were detected in four patients (1.1%). All cases of upper urinary tract cancer were visible on the nephrographic phase for both reviewers.
**Conclusion:** The prevalence of upper urinary tract cancer in patients with haematuria is low, and all cases were seen on the nephrographic phase. This suggests that the CTU protocol may be simplified without reducing the diagnostic accuracy.

**14:00 - 15:30** **Room M 2**

### Paediatric

### SS 312

### Paediatric abdominal imaging

*Moderators:*
H.-J. Mentzel; Jena/DE
F. Prayer; Vienna/AT

**B-0333** 14:00
**Imaging follow-up in patients with biliary atresia after Kasai portoenterostomy**
R. Cuocolo, M. Caruso, F. Di Dato, C. Mollica, V. Romeo, G. Vallone, M. Petretta, R. Iorio, S. Maurea; *Naples/IT (renato.cuocolo@gmail.com)*

**Purpose:** Biliary atresia (BA) is a congenital, progressive fibro-obliteration of the biliary tree, usually treated surgically with Kasai porto-enterostomy (KP). Clinical status and laboratory values influence patient long-term prognosis; furthermore, prognostic value of patient age at time of KP is currently debated. Aim of our study was assessing imaging findings at follow-up ultrasound (US) and magnetic resonance (MRI) in relation to clinically expected patient prognosis and age at the time of KP.
**Methods and Materials:** Retrospectively, 25 patients treated for BA with KP with post-operative US and MRI exams were enrolled, divided into two groups based on clinical and laboratory parameters which correlated with a better (group A, n=15) or worse expected prognosis (group B, n=10). Uni- and multi-variate statistical analyses were used to evaluate imaging findings in both groups and in relation to the age of each patient at the time of KP.
**Results:** Splenomegaly and enlarged portal vein diameter, on US and MRI, as well as irregular hepatic margins on US were significant ($p < 0.05$) predictors of clinical prognostic grouping. At multi-variate analysis, splenomegaly resulted the only significant variable. None of the imaging parameters was significantly different in relation to patient age at the time of KP.
**Conclusion:** Imaging follow-up by US and/or MRI may contribute to assess BA patients treated with KP in agreement with the clinical outcome status; conversely, patient age at the time of KP did not influence clinical outcome status nor imaging findings, suggesting a limited value of this prognostic parameter.

**B-0334** 14:08
**Usefulness of mono-, bi-, and stretched-exponential model diffusion-weighted imaging for the differentiation of biliary atresia and non-biliary atresia in liver MRI**
J. Kim, M.-J. Kim, M.-J. Lee, H. Yoon, H. Shin; *Seoul/KR (jisooo714@yuhs.ac)*

**Purpose:** To know the usefulness of mono-, bi-, and stretched-exponential diffusion-weighted imaging (DWI) for the differentiation of biliary atresia (BA) from non-BA in liver MRI.
**Methods and Materials:** Patients, who underwent liver MRI with suspicion of BA from November 2017 to September 2018, were retrospectively included and divided as BA and non-BA groups. Liver MRI included DWI using 10 b values at 1.5T. Free-hand ROIs were drawn in the two representative axial images. From mono-exponential model, apparent diffusion coefficient1 (ADC1) using 10 b values and ADC2 using two b values (0, 800s/mm2) were obtained. From bi-exponential model, true diffusion coefficient (D), pseudo-diffusion coefficient (D*), and perfusion fraction (f) were obtained. From stretched-exponential model, distributed diffusion coefficient (DDC) and diffusion heterogeneity index (α) were obtained. Parameters were compared between two groups and diagnostic performances were assessed.
**Results:** Among total 17 patients (BA=12, non-BA=5), ADC1, ADC2, D*, f, and DDC values were significantly lower and α value was significantly higher in BA group. The diagnostic performance of all parameters except for D* and α values was good to excellent and area-under-the-curve (AUC) value of ADC2 was 0.925 (95% confidence interval 0.781-0.987).
**Conclusion:** Children with BA had significantly lower ADC1, ADC2, D*, f, DDC values and higher α values in the liver than in non-BA group. ADC value obtained with two b-values could sufficiently aid the differentiation of BA and non-BA with excellent diagnostic performance.

**B-0335** 14:16
**Technical failure of echo-planar imaging MR elastography in paediatric liver**
J.-K. Kim, M.-J. Kim, H. Yoon, M.-J. Lee, K. Han, H.-J. Shin; *Seoul/KR (kyem88@yuhs.ac)*

**Purpose:** The purpose of this study was to assess the factors influencing technical failure of echo-planar imaging (EPI) MR elastography (MRE) in the paediatric liver.
**Methods and Materials:** In this retrospective study, 240 patients (≤20 years old) who underwent EPI MRE at 3-T system from April 2015 to November 2017 were included. Failure group was defined as no pixel value on elastogram map with a confidence index higher than 95%. Fat fraction and T2* values obtained from 3D volumetric multi-echo gradient sequence MRI were compared between success and failure groups. Logistic regression test using unbiased odds ratio from Firth's penalized maximum likelihood estimation method and ROC curves were obtained to analyse the potential predictive factors for EPI MRE failure.
**Results:** Technical failure rate of EPI MRE in paediatric liver was 2.5% (6 of 240). MR fat fraction and T2* value were significantly lower in the failure group ($p < 0.05$). In logistic regression analysis using unbiased odds ratio (OR), decreased T2* value was the only independent factor influencing MRE failure (adjusted OR 0.758, 95% confidence interval 0.650, 0.883). Using ROC curve analysis, T2* value equal to or less than 3.8 msec showed 100% sensitivity and specificity for predicting EPI MRE failure.
**Conclusion:** Technical failure rate of EPI MRE in paediatric liver was low (2.5%). T2* value (≤3.8 msec) was the only independent factor associated with failure of EPI MRE in paediatric liver. Increased body mass index or severe fatty liver was not associated with technical EPI MRE failure.

**B-0336** 14:24
**Diagnostic value of ultrasound in paediatric veno-occlusive disease (VOD) after HSCT using the European Society for Blood and Marrow Transplantation (EBMT) criteria**
S. Im, H. Chung, S. Kim, H. Park; *Seoul/KR (fllsm@hanmail.com)*

**Purpose:** The EBMT has recently announced new diagnostic criteria for paediatric VOD after HSCT. We compared the diagnostic value of US in VOD using Seattle criteria and EBMT criteria.
**Methods and Materials:** Ninty-six US exams of 61 paediatric patients who had grey-scale and Doppler US within 60 days of HSCT were studied retrospectively. Diagnosis of VOD was done clinically in both Seattle criteria and EBMT criteria. Portal vein velocity (PVV), peak systolic velocity (PSV) of hepatic artery (HA), resistance index (RI) of HA, hepatic vein waveform, gallbladder wall thickening, periportal edema, splenomegaly and ascites were recorded and their relationship with VOD was analysed. A scoring system was developed, appointing two points for PVV <10 cm/s and one point for each of PSV of HA >90 cm/s, RI of HA >0.75, GB wall thickening > 4mm, and presence of ascites.
**Results:** The incidence of VOD was 44.3% (27/61) in Seattle criteria and 59.0% (36/61) in EBMT criteria, with all the 9 additionally diagnosed patients in EBMT grade 3 or 4. Five of them had late-onset VOD. In both criteria, non-VOD and VOD group showed significant difference in PVV, PSV of HA, gallbladder wall thickening and ascites. All patients with PVV less than 10 cm/s were in EBMT grade 3/4. The scoring system showed slightly better performance in EBMT criteria, with slightly better AUC (0.78 vs. 0.74), specificity (74.2% vs. 69.8%) and PPV (85.2% vs. 74.1%).
**Conclusion:** Grey-scale and Doppler US is useful in diagnosing paediatric VOD in the new EBMT criteria.

**B-0337** 14:32
**Comparison of suprapubic vs. transurethral bladder access in voiding cystourethrography**
W. Schlötelburg, M. Kriedemann, C. Wirth, T.A. Bley, S. Veldhoen; *Würzburg/DE (schloetelb_W@ukw.de)*

**Purpose:** To compare suprapubic access (SPA) versus transurethral catheterization (TUC) in voiding cysturethrography (VCUG) in paediatric male patients.
**Methods and Materials:** Within a 5-year period 311 VCUG performed in male patients under 12 years of age were retrospectively analysed regarding bladder access method, procedural switch rate, complication rates, radiation parameters and examination quality. TUC was performed in 213 patients (mean age, 1.45±2.35yrs), 98 received SPA (2.46±3.12yrs). Complication rates were graded in minor (prevesical contrast leakage, premature termination of the examination) and major complications (fever, UTI). Examination quality was assessed based on satisfactory acquisition of fluoroscopic images using a four-point Likert scale: perfect (AP bladder projections with low and maximum filling, AP abdominal projections before and after micturition, dynamic lateral voiding phase projection) good (single missing AP projection), moderate (two missing AP projections) and inadequate (missing lateral voiding phase).

**Results:** In 4% of all SPA examinations a method switch to TUC was necessary. Minor complication rate was 1.8% for TUC and 33.7% for SPA (p<0.001). Major complication rate was 0.9% for TUC and 2% for SPA (p=0.79). Mean fluoroscopy time and mean radiation dose were significantly lower in TUC (TUC, 26±19s / 0.06±0.12dGy / cm$^2$; SPA, 38±33s / 0.17±0.29dGy / cm$^2$; p=0.01/0.001). There was no significant difference regarding the contrast media amount needed (TUC, 62±40ml; SPA, 66±41ml; p=0.32). Regarding the examination quality, there was no significant difference in the two groups (p=0.95): Full diagnostic quality was achieved in 88% of TUC and SPA examinations.
**Conclusion:** TUC allows for significantly lower rates of radiation exposure and periprocedural complications.

**B-0338** 14:40
**Evaluation of the split-bolus single-pass contrast CT protocol in paediatric trauma**
T. Melis[1], B. Gottfriedova[1], R. Bruna[1], M.J. Willemink[2], M. Koci[2]; [1]*Prague/CZ*, [2]*Stanford, CA/US (tomas.melis@fnmotol.cz)*

**Purpose:** The Royal College of Radiologists (RCR) recommends a split-bolus single-pass CT-protocol for evaluation of paediatric trauma. Due to the current lack of data, we assess the feasibility of our adaptation of so-called "Camp bastion" protocol.
**Methods and Materials:** We retrospectively included 102 paediatric trauma-patients who underwent a split-bolus single-pass trauma-CT between February 2016 and August 2018. Contrast-enhancement was measured in the aorta, portal vein, pulmonary trunk, and spleen. Attenuation, contrast-to-noise ratios (CNR) and subjective evaluation of spleen-enhancement homogeneity were compared among four weight groups with 20 kg increments (W1-W4, respectively).
**Results:** Median aortic attenuation was significantly higher (p<0.05 ) in low weight-groups of 0-20kg (441[291-535] Hounsfield units (HU)) and 21-40kg (277[222-313] HU) compared to the high weight-groups of 41-60kg (182[151-247] HU) and 61-80kg (191[153-229] HU). Similar results were found for portal vein (W1-W4: 285[249-361]; 236[208-263]; 203[163-217]; 196[175-230] HU), and pulmonary trunk (W1-W4 388[262-581]; 250[229-330]; 180[158-225]; 173[153-210] HU, respectively. Sufficient aortic enhancement of 185HU was not achieved in 25% of the scans. Despite similar noise-levels (p=0.14), CNR was significantly higher (p<0.05) in low weight-groups (W1 15.0; W2 9.8) compared to high weight-groups (W3 6.7; W4 6.0). Spleen inhomogeneity was observed in 54%; 41%; 8%; 25% (W1-W4) of cases (p=0.006), respectively.
**Conclusion:** Evaluation of the RCR-adapted paediatric trauma protocol showed relatively poor vascular-enhancement in the highest weight-group and high vascular-enhancement in the lowest weight-group. Spleen-homogeneity and angiographic-enhancement were insufficient in a substantial number of cases. Our study indicates that further refining of the split-bolus single-pass CT protocol for children is essential.

**B-0339** 14:48
**The effect of renal function on the intracranial gadolinium deposition in young rats**
J. Kim[1], J. Lee[2]; [1]*Cheon-An/KR*, [2]*Seoul/KR (torannip@gmail.com)*

**Purpose:** To assess the effect of renal function on gadolinium deposition in brain after gadolinium-based contrast agents (GBCAs) injections in young rats.
**Methods and Materials:** A total of 39 young rats were divided into 6 groups according to the renal function (normal or decreased) and injected GBCAs (linear or macrocyclic GBCA or saline). Ten GBCA injections were performed over 2 weeks at a dose of 0.9mmolGd/kg. T1-weighted magnetic resonance image and T1 mapping were performed on a 7 Tesla scanner. Qualitative and quantitative analyses of T1 signal intensity ratio of deep cerebellar nucleus(DCN) to cerebellar cortex or pons, and quantitative analysis of T1 value of DCN were performed.
**Results:** Decreased renal function group with linear GBCA injection was the only group showed statistically significant increase of T1 signal intensity ratio of DCN/cerebellum on week 2 ($P$ = 0.043), T1 signal intensity ratio of DCN/pons on both weeks 2 and 4 ($P$ = 0.043), decrease of T1 value of DCN on weeks 2 and 4 ($P$ = 0.043) compared to baseline. In rats with decreased renal function with both linear and macrocyclic injections, T1 value of fourth ventricle was markedly decreased at week 2 and week 4 ($P$ = 0.043).
**Conclusion:** Repeated administration of both linear and macrocyclic GBCAs to young rats with decreased renal function caused delayed excretion of contrast material from ventricles. Rats with decreased renal function with linear GBCA injections, not with macrocyclic GBCA, showed T1 hyperintensity and decreased T1 value in DCN, suggesting deposition of gadolinium.

**B-0340** 14:56
**The difficulties of childhood hepatocellular carcinoma MRI diagnosis**
M. Gonzalo Carballés[1], L. Riaza Martin[1], A. Coma[2], A. Castellote Alonso[1], G. Guillén Burrieza[1], L. Riera[1], J. Molino Gaete[1], J. Piqueras Pardellans[1], E. Vázquez[1]; [1]*Barcelona/ES*, [2]*Lleida/ES (martagonzalo1991@gmail.com)*

**Purpose:** Hepatocellular carcinoma (HCC) is an aggressive hepatic neoplasm that rarely affects pediatric population (<0.5%). The pathophysiology of HCC is not clearly understood; cirrhosis is a predisposing condition. Most pediatric HCC arise de-novo without underlying liver abnormalities, but it is also found, the coexistence of HCC with another hepatic tumors "nodule inside a nodule". This study was performed to illustrate the characteristic MRI findings and patterns of HCC in the pediatric population and discuss the differences from the typical imaging features described in adults.
**Methods and Materials:** Between December 2001 and September 2018 a total of 10 children had been diagnosed as having HCC in our Hospital. We reviewed our imaging database and selected those patients with HCC pathologically proven that had an abdominal MRI done before treatment. The outcome could be analyzed retrospectively in 5 of those patients.
**Results:** The median age at diagnosis was 10.4 years and the male/female ratio was 4:1. Two patients had predisposing conditions (40%) one had tyrosinemia type 1 and hepatitis C virus and the other Abernethy type II Syndrome. The typical MRI imaging pattern of HCC (T1WI hypointensity, T2WI hiperintensity, early arterial enhancement and venous phase washout) was only seen in one patient (20%). One patient had a coexistent hepatoblastoma (20%) and two of the patients had a malignant transformation of an adenoma (40%).
**Conclusion:** This study illustrated that most of pediatric HCC do not show the typical MRI patterns and coexist with other hepatic tumors, conditions that make its diagnose more difficult.

**B-0341** 15:04
**Role of whole-body magnetic resonance imaging in identifying potential diagnostic clues in children with fever of unknown origin**
F. Sertorio, S. Signa, M. De Cesari, M.B. Damasio, G.M. Magnano; *Genoa/IT (fiammetta.sertorio@gmail.com)*

**Purpose:** To evaluate the ability of WBMRI to identify significant potential diagnostic clues (PDCs) in paediatric patients presenting with fever of unknown origin (FUO), recurrent fever and a non-specific inflammatory clinical picture (IUO, Inflammation of unknown origin). To define if an evaluation of WBMRI using a predefined checklist increases the power of this diagnostic tool.
**Methods and Materials:** We retrospectively collected cases of paediatric patients who underwent WBMRI between January 2010 and December 2015 for these indications: i) FUO, ii) recurrent fever, iii) IUO. All studies have been evaluated twice, the second time according to a predefined checklist, considering the involvement of bone, bone marrow, subcutaneous tissue, muscle, tendons, fascia, spine, lung, parenchyma, lymph nodes, paranasal sinuses, nervous system, joint and vessels looking for PDCs.
**Results:** 102 patients were considered: 24 with FUO, 27 with recurrent fever, 51 with IUO. The mean age of WBMRI execution was 9 years. PDCs were identified at the first evaluation in 76/102 cases and in 29/102 cases the identified PDC was useful for the diagnosis. Blind re-evaluation of WBMRI allowed the identification of additional PDCs in 56 cases (12 of them previously negative). In 10 cases the PDC found contributed to the final diagnosis. After the whole diagnostic work-out a final diagnosis was achieved in 42 patients (41%).
**Conclusion:** WBMRI is a non-radiating accurate method to detect diseases through the body. It can be a powerful diagnostic tool in patients with FUO, recurrent fever and IUO. A predefined checklist increases its diagnostic power.

**B-0342** 15:12
**MRU for diagnosis of obstructive pathology of the upper urinary tract**
L. Delfino, M. Damasio, L.E. Derchi; *Genoa/IT (delfino.lorenza@gmail.com)*

**Purpose:** We report ureteral anomalies distal to the uretero-pelvic junction (UPJ) detected via MRU subjected to surgery. MRU sensitivity and specificity in presurgical diagnosis are explored. We also determinate US-MRU agreement.
**Methods and Materials:** MRU performed between 2007 and 2017 have been retrospectively analysed. Children with megaureter, DDPU, retrocaval ureter, mid-ureteral stenosis, ureterocele and ectopic ureter were identified. Patients' demographics, prior US, MRU data and surgical findings have been assessed. Patients with severe systemic malformation syndromes, UPJ obstruction or previous reflux treatments have been excluded.
**Results:** We analysed US, MRU and surgical findings of 46 patients (22 boys, 24 girls) with a median age at the diagnosis, 1.1 years (6 months - 4.0 years). The gap between US-MRU was 38 days (range 1-83) and between MRU surgery 3.2 months (0.8-5.9). The pathology was bilateral in 9 (19.6%), unilateral in 37 (80.4%): 13 (35.1%) on the right and 22 (64.9%) on the left side. On 63 urinary tracts examined, MRU compared to surgical data (gold

Wednesday standard) showed a sensitivity of 98.0%, specificity of 78.6%, VPP of 94.1%, VPN of 91.7%, with a diagnostic odds ratio of 176.0 (CI 95%: 16.7-1856.8). On 82 urinary tracts examined, US between MRU showed a sensibility of 61.2%, specificity of 54.5%, VPP of 66.7%, VPN of 48.6% with an unsatisfactory odds ratio of 1.9 (CI 95%: 0.8-4.6).
**Conclusion:** MR urography is highly accurate in the assessment of the ureteral anomalies distal to the UPJ with a sensibility and specificity higher than US.

**B-0343** 15:20
**Pancreas iron overload and fat infiltration in paediatric beta-thalassemia major patients**
J. Huang[1], Q. Yang[1], Z. Cheng[1], X. Chen[2], T. Yu[1], J. Zhong[1], Y. Su[1], H. Guo[3], B. Liang[1]; [1]*Guangzhou/CN*, [2]*Zhanjiang/CN*, [3]*Beijing/CN (huangjw25@mail2.sysu.edu.cn)*

**Purpose:** To determine the iron and fat content (FC) in the three different pancreas regions (head, body, and tail) in paediatric thalassemia major (TM) patients.
**Methods and Materials:** In 90 paediatric TM patients (10.7±3.1years), pancreatic iron (T2*) and FC were assessed in pancreas head, body and tail, respectively, and were compared with fasting glucose. Iterative decomposition of water and fat with echo asymmetry and least-squares estimation (IDEAL) imaging was used to quantitatively measure FC.
**Results:** Positive linear correlations were found in both T2* and FC among pancreas head, body and tail ($r$=0.911, 0.932, 0.930, respectively, for T2*, $r$=0.876, 0.810, 0.913, respectively, for FC, all with $p$<0.001), no significant regional difference was found (F=0.253, 0.259, $p$=0.776, 0.772, respectively). Pancreas FC was negatively correlated with pancreas T2* ($r$=-0.895, $p$<0.01). The scatter plot showed that 63% patients with pancreas iron overload (PIO) had elevated FC, while all (100%) patients with elevated FC had PIO. Receiver operator characteristic analysis (area: 0.904, 0.897, respectively, both $p$<0.05) identified patients with elevated fasting glucose at a pancreatic R2* cut-off level of 268 1/s (sensitivity at 75%, specificity at 91.2%), at a pancreatic FC cut-off level of 0.18 (sensitivity at 75%, specificity at 88.2%).
**Conclusion:** In paediatric TM patients, fatty replacement is accompanied with pancreas iron overload, but lags behind the latter. Iron overloading and fat infiltration were homogeneously distributed in pancreas head, body and tail in children. Both pancreas iron overload and fat content were correlated with fasting glucose, and could be a predictor of the latter.

**14:00 - 15:30** **Room M 3**

### Oncologic Imaging

### SS 316

### Myeloma imaging: latest trends

*Moderators:*
N.N.
N.N.

**B-0344** 14:09
**Quantifying the change of total tumour volume in whole-body MRI over time improves risk stratification of smouldering multiple myeloma patients**
M. Wennmann[1], L. Kintzelé[1], M. Piraud[2], J. Hofmanninger[3], B. Menze[2], H.-U. Kauczor[1], J. Hillengass[4], G. Langs[3], M.-A. Weber[5]; [1]*Heidelberg/DE*, [2]*Munich/DE*, [3]*Vienna/AT*, [4]*Buffalo, NY/US*, [5]*Rostock/DE (kintz_l@hotmail.com)*

**Purpose:** The purpose of this study was to improve risk stratification of smouldering multiple myeloma patients, introducing new 3D-volumetry-based imaging biomarkers derived from whole-body MRI.
**Methods and Materials:** Two hundred and twenty whole-body MRIs from 63 patients with smouldering multiple myeloma were retrospectively analysed and all focal lesions >5mm were manually segmented for volume quantification. The imaging biomarkers total tumour volume, speed of growth (development of the total tumour volume over time), number of focal lesions, development of the number of focal lesions over time and the recent imaging biomarker '>1 focal lesion' of the International Myeloma Working Group were compared, taking 2-year progression rate, sensitivity and false-positive rate into account.
**Results:** Speed of growth, using a cutoff of 114mm$^3$/month, was able to isolate a high-risk group with a 2-year progression rate of 82.5%. Additionally, it showed by far the highest sensitivity in this study and in comparison to other biomarkers in the literature, detecting 63.2% of patients who progress within 2 years. Furthermore, its false-positive rate (8.7%) was much lower compared to the recent imaging biomarker '>1 focal lesion' of the International Myeloma Working Group.
**Conclusion:** Therefore, speed of growth is the preferable imaging biomarker for risk stratification of smouldering multiple myeloma patients.

*Author Disclosures:*
**J. Hillengass:** Advisory Board; Amgen, BMS, Celgene, Janssen, Novartis, Takeda. Consultant; Amgen, Celgene. Research/Grant Support; Celgene, Sanofi. Other; Amgen, BMS, Celgene, Janssen, Novartis, Takeda.

**B-0345** 14:17
**Intravoxel incoherent motion imaging (IVIM) assessment of bone marrow infiltration in multiple myeloma**
R. Balaji; *Chennai/IN (ravikanthbalaji@gmail.com)*

**Purpose:** To study the use of intravoxel incoherent motion (IVIM) diffusion imaging in assessing extent of marrow involvement in multiple myeloma before therapy and correlation with histology.
**Methods and Materials:** 75 patients with biopsy-proven myeloma were included in the study. All patients underwent whole body diffusion imaging using DWIBS (diffusion-weighted whole-body imaging with background signal suppression ) sequence on 1.5 Tesla Philips Achieva scanner. The marrow lesions (> 1cm) in the iliac bones were identified for IVIM study. Images were acquired in an axial plane with 11 b values: 0, 20, 30, 50, 80, 100, 200, 400, 800, 1000 and 1200 s/mm$^2$. The IVIM parameters (perfusion fraction [f], molecular diffusion coefficient [D], and perfusion-related D [D*]) and apparent diffusion coefficient (ADC) were obtained for marrow and focal lesions . The molecular diffusion coefficient and ADC values were correlated with degree of plasma cell infiltration.
**Results:** D and f values positively correlated with degree of bone marrow involvement. Multiple myeloma is classified into 3 stages, I < 20% plasma cells, II 20 to 50% and III more than 50%. D values showed greater correlation with stage of involvement (p<0.05) D (x 10-3 mm$^2$/s) in stage III 0.5 8 ±0.21, in stage II 0.80±027, in stage I 1.1 ±0.22. f values were higher in stage III rather than stage I (p<0.08).
**Conclusion:** IVIM MR diffusion imaging may be used to predict degree of marrow infiltration by plasma cells in multiple myeloma and thereby provide pre-biopsy staging.

**B-0346** 14:25
**Response assessment in multiple myeloma using intra-voxel incoherent motion (IVIM) diffusion MRI: a newer paradigm**
R. Balaji; *Chennai/IN (ravikanthbalaji@gmail.com)*

**Purpose:** To evaluate the performance of intra-voxel incoherent motion (IVIM) model of diffusion imaging in assessing treatment response in multiple myeloma.
**Methods and Materials:** Hundred patients with biopsy-proven multiple myeloma on appropriate chemotherapy were imaged before start of treatment and at 2 points during treatment - 1 month and 3 months of therapy. Imaging was performed on Achieva 1.5 Tesla scanner (Philips Healthcare, Best, The Netherlands) using a 16-channel pelvic torso coil. Lesions in iliac bones or sacrum were analyzed. Images were acquired in an axial plane with 11 b values (0 to 1200 s/mm2 ). IVIM parameters (perfusion fraction [f], molecular diffusion coefficient [D], and perfusion-related D [D*]) and apparent diffusion coefficient (ADC) were obtained.
**Results:** D values (P 0.004) were the most sensitive indicator for response. D values increased in the first month of therapy and continued to rise at 3 months. f values (P 0.05) decreased after one month showing further decrease at 3 months. ADC values derived from mono-exponential equation also showed elevation after therapy.
**Conclusion:** IVIM-derived parameters may be useful in evaluating response of patients with multiple myeloma to appropriate therapy thereby obviating need for invasive marrow biopsies.

**B-0347** 14:33
**Full-body 150-kV spectral-shaping low-dose CT in multiple myeloma follow-up: evaluation of image quality and radiation dose**
A.M. Bucher[1], A. Brendlin[2], J.E. Scholtz[1], M. Beeres[1]; [1]*Frankfurt a. Main/DE*, [2]*Tübingen/DE*

**Purpose:** Spectral-shaping by tin-filtration allows for radiation dose savings in computed tomography. Our aim was to evaluate a low-dose protocol using spectral-shaping for multiple myeloma (MM) follow-up in comparison to a low-dose 120-kV protocol.
**Methods and Materials:** Forty CTs from twenty patients (64.4±12.5years; female/male: 11/9) with clinically indicated follow-up of MM were included. All patients had a full-body CT using low-dose 120-kV/60-mAs protocol ($CT_{120}$) and on follow-up a tin-filtered low-dose 150-kV/60-mAs protocol ($CT_{150}$) for intra-individual comparison. Dose-length-product (DLP) was recorded. Signal-to-noise ratio was calculated for the gluteus maximus muscle ($SNR_M$). To assess beam hardening, average muscles density from ROIs of artefact-prone regions (sternocleidomastoid, subscapular and lumbar muscles) were subtracted from reference muscles of the same height ($BH_{HU}$). Subjective image quality for bone, and soft-tissue, and presence of artefacts were rated (5-point Likert-scale) by two independent investigators. We used paired

Wednesday student *t*-test, Wilcoxon signed-rank test and Cohens-Kappa-statistic.
**Results:** On average $CT_{150}$ had 49% less radiation exposure compared to $CT_{120}$ (323.5±79.2 mGycm vs. 661.4±78.2mGycm; *p*=0.001). $SNR_M$ ($CT_{150}$, 0.97±0.68; $CT_{120}$, 0.54±0.31; *p*=0.003) and $BH_{HU}$ ($CT_{150}$: 2.4±2.1; $CT_{120}$: 3.5±1.2; *p*=0.001) were significantly higher with spectral shaping. Subjective image quality was rated higher in $CT_{150}$ compared to $CT_{120}$ (5 [IQR: 4-5] vs. 4 [IQR: 3-4]; *p*=0.016). Inter-observer agreement was excellent (κ=0.886). Impairment of diagnostic confidence for bone through artefacts was lower in $CT_{150}$ (p=0.014).
**Conclusion:** Spectral-shaping allows for full-body CT at 49% lower radiation dose compared to a 120kV low-dose protocol, while improving image quality. It may be an alternative for follow-up CT in MM.

**B-0348** 14:41
**Spatial heterogeneity in growth dynamics and local volumetric biomarkers for risk stratification and dissemination behaviour in smouldering multiple myeloma**
M. Wennmann[1], L. Kintzele[1], T. Hielscher[1], G. Langs[2], H.-U. Kauczor[1], M. Merz[1], J. Hillengass[3], B. Menze[4], M.-A. Weber[5]; [1]*Heidelberg/DE*, [2]*Vienna/AT*, [3]*Buffalo, NY/US*, [4]*Munich/DE*, [5]*Rostock/DE* *(mwennmann@hotmail.de)*

**Purpose:** To assess spatial heterogeneity in growth dynamics between different tumour sites, and to determine site-specific tumour characteristics for risk assessment and prediction of appearance of new focal lesions in smouldering multiple myeloma patients.
**Methods and Materials:** From a cohort of 63 patients with overall 220 whole-body MRI examinations during follow up, local tumour volumes of 151 focal lesions in 29 patients and local growth dynamics of 146 focal lesions in 27 patients were determined using manual volumetry. Volume and speed of growth of the largest focal lesion and speed of growth of the fastest growing focal lesion were used as biomarkers for risk stratification and dissemination behaviour, and their performance was compared using Cox regression and Harrell's C-Index.
**Results:** Seventy percent of patients with >=2 focal lesions show a difference of at least factor 5 in local speed of growth between different tumour sites. All local volumetric biomarkers allow significant risk stratifications (p<0.001), with local speed of growth showing the best prognostic discrimination (C-index 0.798). Local speed of growth correlates significantly with future appearance of new focal lesions (HR=1.44; p=0.026).
**Conclusion:** Growth dynamics between tumour sites differ substantially in the majority of patients and indicate differences in local biology. Local volumetric tumour assessment performs excellent regarding prognosis assessment and can be used in upcoming Radiomics approaches or to choose biopsy sites. The correlation between local speed of growth and future appearance of new focal lesions supports the thesis that local growth dynamic correlates with advance of tumour biology.
*Author Disclosures:*
**J. Hillengass:** Other; Regarding the submitted work: none; outside the submitted work: Amgen, BMS, Celgene, Janssen, Takeda, Novartis.

**B-0349** 14:49
**Rapid study assessment in follow-up whole-body computed tomography in patients with multiple myeloma using a dedicated bone subtraction software**
M. Sieren, F. Brenne, A. Hering, M. Schenk, T.H. Oechtering, J. Barkhausen, A. Frydrychowicz; *Lübeck/DE (malte.sieren@uksh.de)*

**Purpose:** Reading follow-up whole-body computed tomography (CT) examinations in patients with multiple myeloma is a lengthy and demanding process. In line with smart reporting approaches, the aim of this study was to evaluate the diagnostic accuracy and benefit of a software for motion-corrected assisted bone lesion change detection.
**Methods and Materials:** 20 patients (12m,65±9y) receiving a total of 40 low-dose whole-body CT examinations for follow-up evaluation of myeloma bone disease were identified from our imaging archive. The local ethics committee waived consent for this analysis. Bone subtraction maps were calculated from 2mm CT images using a novel, non-linear deformation algorithm applying a point-wise spatial alignment between current and prior CT images. Image presentation used colour coding to enhance lesion perceptibility. Reading time, number of correctly assessed lesions, and resulting disease classification were compared to a standard PACS reading software by two readers following a standardized protocol (reader A: 3y of experience, reader B: 1y of experience). De novo clinical reading by a senior body radiologist with 10y of experience served as reference standard.
**Results:** For both readers, reading time was significantly lower using subtraction maps (reader A: 441±135s, reader B: 1100±397s) compared to standard reading (reader A: 645±162s, reader B: 1652±426s), both p<0.001. Lesion detection rate for subtraction mapping/conventional reading was 92%/78% for reader A and 87%/67% for reader B. All patients were correctly classified by both methods (7x progressive disease, 6x stable disease, 7x remission).
**Conclusion:** Motion-corrected bone subtraction maps significantly speed up reading time and are slightly superior compared to conventional CT reading regarding lesion detection.

**B-0350** 14:57
**Whole-body magnetic resonance imaging (WBMRI) for the assessment of response to treatment in multiple myeloma patients after autologous haematopoietic stem cell transplantation**
N. Lutsik, L. Mendeleeva, M. Solovev, G. Yatsyk, S. Kulikov, V. Savchenko; *Moscow/RU (natalia.parubenko@gmail.com)*

**Purpose:** To determine the differences in bone marrow lesions and value of apparent diffusion coefficient (ADC) detected by whole-body MRI in patients with multiple myeloma (MM) before and after autologous haematopoietic stem cell transplantation (auto-HSCT).
**Methods and Materials:** 18 patients with MM (8 male and 10 female) aged 37 to 66 (median 55) were enrolled in a prospective study in the period from February 2017 to May 2018. WBMRI was carried out with a Philips Ingenia 1.5 T before and 100 days after auto-HSCT. DWI with background body signal suppression (DWIBS) sequences (b values: 0, 500, 1000 mm²/sec) were qualitatively analysed, along with T1 spine echo and short tau inversion recovery T2-weighted images, to evaluate bone lesions. The bone marrow infiltration lesions (≥ 5 mm) were counted and ADC values were calculated and compared before and 100 after auto-HSCT. ADC values were detected in 6 patients. Descriptive, regression and variance analyses were conducted with SAS 9.4.
**Results:** WB-MRI after auto-HSCT demonstrated a significant relative reduction of the number of lesions by an average of 16±0.2% (p=0.007), a significant relative reduction of the tumour volumes by an average of 23±0.26% (p=0.03) and non-significant mean reduction of ADC values by an average of 11±0.13% (p=0.08).
**Conclusion:** WB-MRI with DWIBS sequences, by evaluating posttreatment changes of numbers, total lesion volume and ADC values, might represent a complementary diagnostic tool in the assessment of response to treatment in MM patients after auto-HSCT. For more reliable decrease of ADC values, more patients are needed.

**B-0351** 15:05
**Comparison of 18F-FDG PET/CT and whole-body MRI in assessing newly diagnosed myeloma**
O.A. Westerland, A. Amlani, M. Siddique, M. Streetly, I. El-Najjar, G. Cook, V.J. Goh; *London/UK (olwenwesterland@gmail.com)*

**Purpose:** Advanced imaging including whole-body (wb) MRI and 18F-FDG PET/CT are now recommended by the International Myeloma Working Group for assessing myeloma. It remains unclear which modality should be used first-line. We aimed to compare the diagnostic performance of 18F-FDG PET/CT and wbMRI.
**Methods and Materials:** Following IRB approval, 25 patients (12 male; mean±SD = 61±10 years) with newly diagnosed myeloma underwent 18F-FDG PET/CT and wbMRI. Following a 6hr fast, 400 MBq $^{18}$F-FDG was administered intravenously. At 60 +/-5 mins post injection, imaging was acquired from vertex to feet (GE Discovery PET/CT). wbMR included T2-HASTE, T1-DIXON, DWI (B=50 and 900s/mm$^2$) and post-contrast T1-DIXON sequences from vertex to knees(1.5T Magnetom Aera, Siemens HealthCare, Erlangen, Germany).Images were reviewed by two experienced radiologists and the presence/absence of disease and number of focal lesions recorded. Diagnostic performance was compared to clinical parameters including bone marrow aspirate (reference standard).
**Results:** The sensitivity of wbMRI for diagnosing myeloma was 88%, compared with 84% for 18F-FDG PET/CT (84% for the CT component alone; 48% for PET component alone for detection of bone lesions). In 20/25 patients (80%) wbMRI detected a greater number of bone lesions compared with the CT component of 18F-FDG PET/CT whilst in 3/25 patients (12%) a greater number of lesions were detected on CT compared with wbMRI. 8% of cases were concordant.
**Conclusion:** wbMRI and 18F-FDG PET/CT have a comparable sensitivity for detection of myeloma, however wbMRI detects a greater proportion of focal bone lesions compared with 18F-FDG PET/CT.

**B-0352** 15:13
**Distinct bone marrow pattern in MR imaging of advanced systemic mastocytosis is associated with poor prognosis**
P. Riffel, M. Jawhar, K. Gawlik, H.J. Michaely, W.-K. Hofmann, S.O. Schönberg, A. Reiter; *Mannheim/DE* *(Philipp.Riffel@medma.uni-heidelberg.de)*

**Purpose:** Systemic mastocytosis (SM) is a rare myeloproliferative neoplasm characterized by accumulation of mast cells (MCs) in various organ systems (e.g. bone marrow, BM). The aim of this study was to compare the BM pattern of indolent SM (ISM, normal life expectancy) and advanced SM (advSM, median survival 2-4 years) using whole-body magnetic resonance imaging (WB-MRI).
**Methods and Materials:** 115 patients with SM (ISM: 37, smouldering systemic mastocytosis (SSM): 9, advSM: 69) underwent a wb-MR-exam including sagittal T1 and TIRM sequences of the spine. Image evaluation included pattern and extent of pathologic BM in the spine and the extremities (including osteolysis and vertebral fractures).
**Results:** 89% of patients with ISM revealed a normal BM. 89% of patients with SSM revealed a pathologic BM signal of the spine. 96% of patients with advSM revealed a pathologic BM signal of the spine (activated BM 62%, diffuse sclerotic BM 25%, small-spotted BM 9%). Osteolysis was not seen in any patient. Vertebral fractures were found in ISM (19%) and advSM (19%). MC burden (MC infiltration in BM, serum tryptase, KIT D816V allele burden) was significantly higher in advSM patients with activated/sclerotic BM compared to normal/small-spotted BM with a significantly lower median overall survival.
**Conclusion:** Pathologic bone marrow changes of the spine in patients with SM are indicative for diagnosis of SSM or advSM. In patients with advSM, activated or diffuse sclerotic bone marrow pattern is associated with a more aggressive form based on clinical, genetic and outcome parameters.

**B-0353** 15:21
**Lack of clinically meaningful anatomical variations in the bone marrow ADC values in diffuse myeloma allows untargeted sampling to confirm disease burden**
A. Keeling[1], D.-M. Koh[2], S. O'Connor[1], K. Boyd[1], M. Kaiser[1], M. Blackledge[2], C. Messiou[1]; [1]*London/UK*, [2]*Sutton/UK (archie.keeling@nhs.net)*

**Purpose:** To compare the regional ADC values of bone marrow in the spine and pelvis of patients with diffuse multiple myeloma and their correlation with indices of disease burden.
**Methods and Materials:** The whole-body DWI studies of 50 patients with biopsy positive and imaging confirmed diffuse myeloma were retrospectively reviewed. In each patient, three regions of interest (ROIs) were drawn over four anatomical sites (right ilium, left ilium, L3 vertebrae, T6 vertebrae) on the ADC maps and the average value recorded at each site. The ADC values were compared using the Wilcoxon signed-rank test with p-value < 0.0125 (Bonferroni correction) taken as statistically significance. The average ADC values of all sites in each patient was correlated with marrow cellularity and proportion plasma cell at histopathology, as well as the international staging system (ISS) scores.
**Results:** There was no significant difference in the median ADC value of bone marrow in the left ilium (754), right ilium (733) and T6 (701) but was significantly lower in L3 (636). However, the variance of ADC values across sites were within limits of measurement repeatability (15%). No correlation was found between per-patient average ADC values with marrow cellularity (p=0.7), proportion plasma cells (p=0.2) or ISS scores (p=0.5)
**Conclusion:** There is no clinically meaningful difference in the marrow ADC values across anatomical sites in diffuse myeloma. Untargeted bone marrow biopsy is likely to be representative in this patient group.

**14:00 - 15:30** **Sky High Stage**

### Oncologic Imaging

### MY 3

### Oncologic Imaging

*Moderators:*
M. D'Anastasi; Msida/MT
A. Laghi; Latina/IT

**B-0354** 14:00
**MRI-based texture analysis has the potential to predict response to transarterial radioembolization at baseline**
R.P. Reimer[1], P. Reimer[2], A. Mahnken[3]; [1]*Cologne/DE*, [2]*Karlsruhe/DE*, [3]*Marburg/DE (robert.reimer@uk-koeln.de)*

**Purpose:** The purpose of this study was to investigate, whether magnetic resonance imaging (MRI)-based texture analysis (TA) has the potential to predict response to transarterial radioembolization (TARE) in patients with liver metastases.
**Methods and Materials:** Forty patients (23/17, male/female; mean age 64.2 years) with liver metastases underwent dynamic contrast-enhanced (DCE)-MRI and hepatocellular phase MRI with gadoxetic acid (Primovist®, Bayer Healthcare) after TARE (mean 2.1 days). Response to TARE was evaluated on follow-up imaging (median follow-up, 224 days) based on Response Evaluation Criteria in Solid Tumors 1.1 using a region of interest (ROIs)-based method. Response evaluation and TA was performed using the software mint Lesion™. Results of TA (mean, standard deviation (sd), skewness, kurtosis, entropy (e), uniformity (u)) of the ROIs at baseline were compared between responders and non-responders using Mann-Whitney-U test. Receiver operating characteristics including the area under the curve (AUC) were calculated.
**Results:** 12 patients (30%) were responders and 28 (70%) non-responders. Comparing responders and non-responders, median sd (53.4vs.34.0) and u (0.0071vs.0.0106) at arterial phase, as well as median e (7.35vs.6.85) and u (0.0081vs.0.0106) at hepatocellular phase were significantly different (p<0.05). The AUCs for sd and u at arterial phase were 0.72 (p<0.05) and 0.70 (p>0.05). The AUCs for e and u at hepatocellular phase were 0.74 and 0.72 (p<0.05).
**Conclusion:** Texture analysis at arterial phase and hepatocellular phase MRI has the potential to predict response to transarterial radioembolization at baseline. The results indicate higher tumor heterogeneity of the liver metastases at baseline in responders.

**B-0355** 14:04
**Apparent diffusion coefficient percentage as an early predictor for evaluation of trans-catheter arterial chemoembolisation efficacy in hepatocellular carcinoma**
R.M. Mousa[1], F. Fawzy[2], H. Tantawy[1], T. Sobhy[1]; [1]*Zagazig/EG*, [2]*Sharkia/EG (Rolamousa@zu.edu.eg)*

**Purpose:** To emphasize the role of calculating the percent of change in apparent diffusion coefficient value (%ADC) in evaluating the treatment response of hepatocellular carcinoma (HCC) after transcatheter arterial chemoembolization (TACE) as an early predictor of residual tumour.
**Methods and Materials:** Thirty six patients with 44 hepatocellular carcinoma (HCC) lesions which were all treated with TACE. All lesions were evaluated by conventional MRI, diffusion weighted imaging (DWI), and ADC value measurement before treatment for baseline characterization, then four weeks after TACE to evaluate its efficacy. Results were compared with triphasic study (CT or MRI) done at the same time as a standard of reference.
**Results:** Significantly higher ADC values were found in lesions that were completely embolized rather than the incompletely embolized ones with residual tumoural tissue. The mean ± standard deviation (SD) of lesions before treatment was $1.23 \pm 0.24 \times 10^{-3}$mm²/s. One month after TACE, completely embolized HCC lesions had an increase in mean (±SD) ADC to $1.44 \pm 0.26 \times 10^{-3}$ mm²/s (*P*=0.004), while incompletely embolized HCCs increased to $1.33 \pm 0.24 \times 10^{-3}$ mm²/s (*P*=0.41). Completely embolized lesions also had significantly larger increase in %ADC than incompletely embolized ones (22.8% versus -21.2%respectively, p=0.001). On comparing %ADC before and after treatment with the standard of reference (triphasic CT or MRI), %ADC had 96% sensitivity, 100%specificity, and 94.7% accuracy (%ADC threshold was set at 8.5% based on ROC curve analysis).
**Conclusion:** Percentage ADC represents a reliable early predictor of successful management of HCC by TACE and allows early detection of residual tumoural tissue.

**B-0356** 14:08
**Can intravoxel incoherent motion diffusion-weighted imaging be use for preoperative assessment of microvascular invasion in hepatocellular carcinoma**
Y. Wei, B. Song, H. Jiang, J. Chen; *Chengdu/CN (drweiyi057@163.com)*

**Purpose:** To prospectively evaluate the potential role of intravoxel incoherent motion diffusion-weighted imaging and conventional radiologic features for preoperative prediction of MVI of HCC.
**Methods and Materials:** 115 patients with 135 newly diagnosed were evaluated. For all examinations, studies were carried out by using a 3.0 T MR system. IVIM was performed by using an echo-planner imaging sequence with respiratory gating, twelve b values of 0, 10, 20, 40, 80, 100, 150, 200, 400, 600, 800 and 1000 sec/mm2 were obtained. All the IVIM images were analysed by two radiologists independently. Freehand region of interest (ROI) was used to outline the tumour on the original IVIM image (b=400 sec/mm2), and try to avoid the necrosis and haemorrhage The ADC, ADCslow, ADCfast and f values were automatically calculated. Univariate and multivariate logistic regression analyses were used to screen the independent risk factors of MVI, receiver operating characteristics (ROC) curves were drawn and to determine the optimal cut-off value.
**Results:** Features significantly related to MVI of HCC at univariate analysis were reduced ADC (odds ratio, 0.341, 95% CI: 0.211-0.552; P<0.001) and ADCslow (odds ratio, 0.141, 95% CI: 0.067-0.299; P<0.001). At multivariate analysis, only ADCslow was the independent risk factor for MVI of HCC. The mean ADCslow value for MVI of HCC showed an area under ROC curves of 0.815 (95% CI: 0.740-0.877) with the optimal cutoff value of 0.868.
**Conclusion:** The results of the preliminary study have demonstrated that the decreased ADCslow value was independent risk factor for predicting MVI of HCC.

**B-0357** 14:12
**Liver surface nodularity score as a new predictor of overall survival in patients with HCC treated by TACE**
A. Abou Elkassem[1], A.M.K. Abdel Aal[1], K. Mahmoud[1], A. Gunn[1], S. lirette[2], A. Smith[1]; [1]*Birmingham, AL/US*, [2]*Jackson, MS/US (akamel@uabmc.edu)*

**Purpose:** To assess the accuracy of the CT-based liver surface nodularity (LSN) score for predicting overall survival (OS) in patients with hepatocellular carcinoma (HCC) treated with trans-arterial chemo-embolisation (TACE).
**Methods and Materials:** For this retrospective study, a search of the electronic medical records was performed to identify adult patients with HCC treated by TACE within 6 months of a contrast-enhanced abdominal CT. The LSN score was measured using custom software while blinded to clinical data. Age, gender, race, bead size, Barcelona Clinic Liver Cancer (BCLC) stage of HCC (which includes tumour burden/extent, Child-Pugh score, and performance status), time to progression (TTP), and OS were extracted from the electronic medical records. Predictors of TTP and OS were evaluated by Cox-proportional hazards ratio.
**Results:** A total of 165 patients were included with BCLC stage A (N=46), B (N=62), C (N=46), and D (N=11). The mean (standard deviation) of the LSN score was 3.10 (0.71). The median TTP and OS were 10.2 and 21.4 months. None of the variables were predictive of TTP (p>0.2 for each). Predictors of OS included the LSNscore (HR: 1.41; 95%CI: 1.06, 1.88; p=0.019) and age (HR: 1.03; 95%CI: 1.00, 1.05; p=0.029), but not the BCLC stage of HCC or any of the other variables (p>0.10). For each unit increase in the LSN score, the risk of death increases by 41%.
**Conclusion:** The CT-based LSN score was an accurate independent predictor of OS in patients with HCC treated by TACE.
*Author Disclosures:*
**A. Smith:** Owner; Liver Nodularity LLC. Patent Holder; Liver Nodularity LLC.

**B-0358** 14:16
**Diffusion-weighted imaging of colorectal liver metastases: prospective evaluation of apparent diffusion coefficient in correlation with histologic findings**
M.F.A. Almeida, R. Cruz, M.D.F.S. Begnami, P.N.V.P. Barbosa, A.G.V. Bitencourt, F.J.F. Coimbra, R. Chojniak; *Sao Paulo/BR (mf_arruda@yahoo.com.br)*

**Purpose:** To prospectively correlate apparent diffusion coefficient (ADC) values of colorectal liver metastases with the degree of viable tumour, necrosis and fibrosis.
**Methods and Materials:** Thirty-seven patients with colorectal liver metastases were included. All patients had preoperative MRI scans at Signa HDx 1.5 Tesla GE Healthcare, including DWI sequences with b values of 0-200 and 0-600 sec/mm$^2$. All patients underwent surgical resection, with systematic mapping of the surgical specimen for radiopathological correlation. A total of 92 metastases detected in the DWI sequences were found to be adequately correlated with the macroscopic mapping and were submitted to standard histological analysis to evaluate the percentage of viable tumour, necrosis and fibrosis. ADC values were calculated using post-processing workstation. Spearman correlation, chi-square, Kruskal-Wallis and Mann-Whitney tests were performed.
**Results:** Twenty patients were male (54%) and the mean age was 56.4 years (age range, 32-78). Only 5 patients did not perform preoperative chemotherapy (13.5%). MRI lesions ranged from 0.7 to 9.0 cm (mean, 2.3 cm). Mean ADC values obtained with ROI in the entire lesion and ROI in 50% of the lesion area were respectively: 1.69 and 1.35 (x10$^{-3}$/ mm$^2$) in DWI b200; 1.33 and 1.20 (x10$^{-3}$/mm$^2$) in DWI b600. ADC values on DWI b600 showed a significant correlation with percentage of viable neoplasm, use of target therapy and number of QT cycles.
**Conclusion:** The lesions with the highest percentage of viable tumour had lower ADC values, indicating that DWI sequence can be used to evaluate cellularity and tumour viability of colorectal liver metastases.

**B-0359** 14:20
**Comparison of intravoxel incoherent motion DWI, diffusion kurtosis imaging, and conventional DWI in predicting the chemotherapeutic response of colorectal liver metastases**
H. Zhang, W. Li, T. Tong; *Shanghai/CN*

**Purpose:** The study aimed to compare the performance of pre-treatment intravoxel incoherent motion DWI (IVIM-DWI), diffusion kurtosis imaging (DKI), and conventional DWI for predicting the chemotherapeutic response in patients with colorectal liver metastases (CRLMs).
**Methods and Materials:** Forty consecutive patients with unresectable CRLMs who underwent the standard first-line chemotherapy regimen were prospectively collected. All the images were collected on a MAGNETOM Skyra 3T MR scanner. The response was assessed by response evaluation criteria in solid Tumours (RECIST Version 1.1) after receiving two cycles of chemotherapy. The parameters were compared between the responding group (complete and partial response, n=15) and non-responding group (stable and progressive disease, n=25).
**Results:** Lower baseline ADC, $D_{slow}$, D (P = 0.001, <0.001, and =0.003 respectively) and higher baseline K (P = 0.002) were independently associated with a good response to chemotherapy with area under the ROC curves (AUCs) of 0.845, 0.832, 0.819, and 0.787, respectively. The best predictive parameter was ADC (AUC = 0.845, sensitivity = 73.3 %, specificity = 84.0 % and cutoff value ≤ 1106.85 x 10$^{-6}$ mm²/s). The statistically different factors were fitted by the logistic regression and a predictive factor (PRE1) was generated. The AUC of the PRE1 reached 0.867.
**Conclusion:** Our study showed that pre-treatment IVIM-DWI ($D_{slow}$), DKI (D and K), and conventional DWI (ADC) all demonstrate good diagnostic performance in predicting the chemotherapeutic response of patients with CRLMs, which indicates that the functional MRI parameters might provide additional information for predicting the response of CRLMs.

**B-0360** 14:24
**Diffusion kurtosis imaging-derived histogram metrics for prediction of KRAS/NRAS/BRAF mutation in rectal adenocarcinoma**
Y. Cui[1], Z. Zhuo[2], X. Yang[1], R. Zhang[1]; [1]*Taiyuan/CN*, [2]*Beijing/CN (yanfen210@126.com)*

**Purpose:** To evaluate the potential role of DKI-derived parameters by using histogram analysis derived from whole-tumor volumes for prediction of KRAS/NRAS/BRAF mutations status in patients with rectal adenocarcinoma.
**Methods and Materials:** 152 consecutive patients who underwent MRI examination with rectal adenocarcinoma were retrospectively evaluated. The parameters D, K and conventional ADC were measured using whole-tumor volume histogram analysis. Student's t-test or Mann-Whitney U-test, receiver operating characteristic curves, and Spearman's correlation were used for statistical analysis.
**Results:** All the percentiles metrics of ADC and D values were significantly lower in the mutated group than those in the wild-type group, except for the minimum value of D, while K-related percentiles metrics appeared higher in the mutated group compared with those in the wild-type group. Regarding the comparison of diagnostic performance of all the histogram metrics, $K_{75th}$ showed the highest AUC value of 0.866, and the corresponding sensitivity, specificity, PPV and NPV were 67.57% and 92.31%, 89.29% and 75.0%, respectively.
**Conclusion:** DKI metrics with whole-tumor volume histogram analysis, especially the $K_{75th}$ parameters, yield preferable AUC and specificity for predicting KRAS/NRAS/BRAF mutations than ADC and D, and thus may potentially serve as an optimal imaging biomarker for the prediction of KRAS/NRAS/BRAF mutations for guiding targeted therapy.

**B-0361** 14:28
**Prediction of loco-regional treatment failure after chemo-radiotherapy in anal cancer using FDG PET/CT-derived feature analysis**
P. Brown[1], J. Zhong[1], G.M. McDermott[1], M. Fronza[1], A. Gilbert[1], A. Appelt[1], D. Sebag-Montefiore[1], S. Currie[1], A. Scarsbrook[2]; [1]*Leeds/UK*, [2]*York/UK (peter.brown30@nhs.net)*

**Purpose:** Chemoradiotherapy (CRT) is the primary treatment of loco-regional disease in anal squamous cell carcinoma (ASCC). Loco-regional treatment failure (LRF) can occur due to sub-optimal therapy response. Tumour phenotyping using radiomic analysis may help identify tumours less likely to fully respond. The study purpose was to develop and validate a prognostic model in patients with ASCC incorporating pre-treatment FDG PET/CT-derived features.
**Methods and Materials:** Consecutive patients with ASCC (n = 292) treated with curative-intent CRT at a large tertiary referral centre were studied. After exclusions (small tumour size, incomplete follow-up data, tumour excision pre-PET/CT), 199 patients were analysed; separated into training (n = 145) and validation (n = 44) cohorts. Tumour segmentation and radiomic feature (RF) extraction was performed using specialised software (LIFEx, www.lifexsoft.org) on baseline PET/CT imaging. Cox regression modelling was performed. Primary outcome was LRF, (median follow-up 31.0 months, IQR 16.5-55.5 months).
**Results:** 37 (25.5%) and 12 (27.3%) patients in the training and validation cohorts had LRF within the treatment field. Univariate analysis demonstrated metabolic tumour volume (MTV), SUVmax, 8 CT-derived and 7 PET-derived RFs (first- and second-order parameters) were statistically significant (p < 0.05) for LRF prediction. Conventional discriminators (T, N, M stage, age, sex and histological grade) were not significant in predicting LRF.
**Conclusion:** PET/CT-derived RFs helped predict ASCC patients at higher risk of LRF and may offer additional benefits over conventional imaging and demographic factors. This shows promise for more effective risk stratification and personalisation of CRT regimens in the future.

**B-0362** 14:32
**Increasing reader confidence in cancer imaging using an advanced DWI modification: a prospective evaluation**
C. Dreher[1], T. Kuder[1], S. Windhaber[1], F. König[1], D. Paech[1], R. Gnirs[1], T. Benkert[2], H.-P. Schlemmer[1], S. Bickelhaupt[1]; [1]*Heidelberg/DE*, [2]*Erlangen/DE (c.dreher@dkfz-heidelberg.de)*

**Purpose:** Oncological imaging commonly includes Diffusion Weighted Imaging (DWI) sequences to identify and characterize suspicious lesions. However, image quality can be challenged by the limited SNR of DWI, especially in abdominal examinations. This prospective study investigated an optimized DWI in oncological imaging for improved detection of malignant abdominal lesions.
**Methods and Materials:** This IRB-approved, prospective study included 59 patients (mean age: 57 years, male/female: 22/37). Oncological follow-up MRI-examinations of the abdomen were performed with a 1.5T MRI scanner (AERA, SiemensHealthineers, Germany) with both a standard epi-DWI ("routine-DWI") and an oncologically optimized prototype DWI ("oncoDWI") with b=0,900s/mm². Diagnostic confidence for characterization/detection of suspicious lesions was evaluated by two independent readers using a 5-point Likert Scale. Statistics included Wilcoxon signed rank tests, interreader agreement was analyzed by kappa-statistic (p<0.05).
**Results:** Sixty-one lesions were detected (20 hepatic, 16 lymphatic, and 25 lesion of other origins). Reader confidence for characterization/detection of malignant lesions was significantly improved using the oncoDWI (4.6±0.5) as compared to the routine-DWI (3.7±0.7) (p<0.001). The increased confidence of lesion recognition in the optimized DWI remained significant in all subgroups of hepatic, lymphatic and lesions of other origins (p<0.002) with an overall good interreader agreement (kappa = 0.744) (p<0.001).
**Conclusion:** An oncologically optimized DWI sequence increased the reader confidence for lesion characterization/detection in oncological abdominal MRI both with regard to screening and follow-up examinations while preserving a high interreader agreement.
*Author Disclosures:*
**T. Benkert:** Employee; Siemens Healthineers. **S. Bickelhaupt:** Speaker; Siemens Healthineers.

**B-0363** 14:36
**Preoperative prediction of regional lymph node metastasis in thoracic oesophageal carcinoma with 18F-FDG PET/CT and contrast-enhanced CT texture analysis: a nomogram model**
Z. Xiaochun, W. Shuxia; *Guangzhou/CN (zhangxiaochun579@163.com)*

**Purpose:** To develop and validate a nomogram model for preoperative prediction of regional lymph node metastasis in thoracic oesophageal carcinoma.
**Methods and Materials:** The nomogram model was developed in a primary cohort which consisted of 391 regional lymph nodes from 95 patients with pathologically confirmed thoracic oesophageal carcinoma from January 2015 to December 2016. All patients underwent preoperative 18F-FDG PET/CT and contrast-enhanced CT scans. SUVmax of regional lymph nodes were collected. The texture features of lymph nodes were extracted from enhanced CT images. Lasso regression was used for data dimension reduction, feature selection, and texture feature model building. Multiple logistic regression analysis was used to develop the predictive model. We integrated the SUVmax information with the texture information to develop a nomogram model. A internal validation cohort consisted of 252 lymph nodes from 60 patients in 2017. The calibration and discrimination of the model were assessed.
**Results:** SUVmax and 12 texture features were associated with lymph node metastasis (P <0.05 for both primary and validation cohorts). Predictors of the nomogram model was constructed. The model showed good discrimination(area under ROC curve: 0.87) and good calibration (P value of Hosmer-Lemeshow test: 0.48) in primary cohort. Application of the model in validation cohort could also obtain good discrimination (area under ROC curve: 0.91) and good calibration (P value of Hosmer-Lemeshow test: 0.41).
**Conclusion:** This study combined glucose metabolism information and texture features to develop a nomogram model, which could be used to predict regional lymph node metastasis in patients with thoracic oesophageal cancer.

**B-0364** 14:40
**Prospective evaluation of modified DWI MRI for improved image quality and tissue differentiation in oncological follow-up examinations**
C. Dreher[1], T. Kuder[1], S. Windhaber[1], F. König[1], D. Paech[1], R. Gnirs[1], T. Benkert[2], H.-P. Schlemmer[1], S. Bickelhaupt[1]; [1]*Heidelberg/DE*, [2]*Erlangen/DE (c.dreher@dkfz-heidelberg.de)*

**Purpose:** Diffusion weighted imaging (DWI) is an important part of oncological magnetic resonance imaging (MRI) examinations. This study aims at prospectively evaluating an oncologically adapted DWI sequence for improved image quality and tissue differentiation in patients with abdominal malignancies.
**Methods and Materials:** This IRB-approved, prospective in-vivo and ex-vivo study included phantom measurements using a dedicated phantom (PVP-K30) and 52 prospectively acquired oncological abdominal MRI examinations (mean age: 57 years; male/female: 21/31) using a 1.5T MRI scanner (AERA, SiemensHealthineers, Germany) including both a standard epi-DWI ("s-DWI"; b-values 0,900s/mm²) and an oncologically adapted, prototype optimized DWI ("opt-DWI"; epi-DWI with advanced processing, b=0,50,900,1500s/mm²). Apparent-signal-to-noise-ratio (SNR) was quantitatively evaluated, image quality and tissue differentiation parameters were rated by two independent, blinded readers using a 5-point Likert-Scale. Statistics included Wilcoxon-signed-rank test and kappa statistic (p<0.05).
**Results:** The DWI-phantom demonstrated a significantly optimized contour sharpness and inlay differentiation for the opt-DWI. Apparent-SNR of b900-DWI MRI of the right/left hemiabdomen was significantly increased in opt-DWI (mean: 227±250/150±192) vs. s-DWI (mean: 37±17/40±23) (p<0.001). Image quality parameters (contour sharpness right liver, left liver and pancreas) were significantly increased in opt-DWI vs. s-DWI (p<0.001). Tissue differentiation for the upper abdominal organs and retroperitoneal structures was significantly increased in opt-DWI vs. s-DWI (p<0.001). Interreader reliability test showed good agreement (kappa=0.754) (p<0.001).
**Conclusion:** This prospective study demonstrates the superiority of an oncologically optimized DWI over standard DWI for abdominal oncological imaging. Assessment of anatomical structures including the retroperitoneum and reader confidence was significantly improved using the novel approach in oncological follow-ups.
*Author Disclosures:*
**T. Benkert:** Employee; Siemens Healthineers. **S. Bickelhaupt:** Speaker; Siemens Healthineers.

**B-0365** 14:44
**USPIO contrast agents for detecting cancer: which one works best?**
L. Müller, U. Keber, A. Mahnken, R. Mandic, M.-L. Martini, P. Seyfer; *Marburg/DE*

**Purpose:** Differences regarding physical and chemical properties of ultrasmall superparamagnetic iron oxide (USPIO)-MRI contrast agents influence their uptake, biodistribution and clearance. These characteristics could affect their ability to distinguish between malignant and benign lesions. The purpose of our study was the comparison of four USPIOs to find a suitable agent for possible future clinical practice.
**Methods and Materials:** This study was performed on forty athymic nude mice and approved by the local animal care committee. Hela cells and intramuscular inflammation were implanted into the hind thighs. On a 7T-scanner imaging was performed precontrast and serially 2, 4 and 24 hours after injecting USPIOs. Following USPIOs were used: Feraheme (AMAG Pharmaceuticals Inc., Waltham, MA, USA), P 904 (Guerbet Group, Villepinte, France), FeraSpin XS-Type and FeraSpin M-Type (Miltenyi Biotec GmbH, Bergisch Gladbach, Germany). Pre- and postcontrast differences of SI, SNR and CNR in tumour and inflammation were measured and analysed for significance using Wilcoxon-signed-rank tests and receiver-operating characteristic curves with Venkatraman's tests. Correlation between MR images and histologic slices stained with hematoxylin-eosin, Prussian blue and MAC 3-antibodies was performed.
**Results:** All four contrast agents tested were significantly able to distinguish between cancer and inflammation (p< .05). No significant differences were noticed between the different contrast media regarding their ability to do so (p> .05).
**Conclusion:** The four USPIOs tested show no significant difference regarding their ability in distinguishing between malignant lesions and inflammations. Therefore, USPIOs with similar physical and chemical properties could be used for this purpose in future clinical practice.

**B-0366** 14:48
**Beyond lymph nodes: 18F FDG PET CT in detection of usual and unusual sites of extra nodal lymphoma**
M. Abdel-Kawi, M. Nasr, A. Ibrahim; *Cairo/EG (moustafa.kawi@gmail.com)*

**Purpose:** To compare between PET/CT and contrast-enhanced computer tomography (CE CT) in the detection of extra nodal involvement in lymphoma and to correlate between $SUV_{max}$ of the extra nodal lesion and the hottest LN.
**Methods and Materials:** 100 patients with pathologically proven lymphoma underwent whole body 18F-FDG PET/CT and CECT scan. Images were compared regarding the ability of detection of extra nodal lymphomatous sites. *Kappa* agreement was used to find the degree of agreement between both modalities. *Pearson's* correlation was used for correlating SUVmax of the extra nodal lesions and hottest LN. The degree of FDG uptake was correlated with histopathological type.
**Results:** There was a poor agreement between PET/CT and CE CT in the detection of extra nodal sites *(k=0.32)*. There was a significant positive moderate correlation between SUVmax of the extra nodal lesions and hottest LN(r=0.45). PET/CT study resulted in up staging of 10% and down staging of

5% of cases.
**Conclusion:** FDG PET/CT scan enables better detection of extra nodal lymphomatous sites that show normal morphology at CECT. It may differentiate lymphomatous infiltration from benign causes of increased FDG avidity, which provide proper disease staging.

**B-0367** 14:52
**Evaluation of STIR, T1 DIXON "in and out of phase" and DWI sequences, to propose a standardised protocol of the MRWB in patients affected by myeloma**
A. Trisoglio, E. Soligo, S. Berardo, L. Sukhovei, S. Bor, G. Buoni, A. Stecco, A. Carriero; *Novara/IT (berardo.sara@gmail.com)*

**Purpose:** The International Guidelines include MRWB (MR-Whole Body) in the management of patients with plasma cell disorders, but there is no standardized protocol to follow. The aims of the study are: 1) to evaluate the effectiveness of STIR, T1DIXON and DWI, 2) to understand if there is a different ability to diagnose lesions in the patient before and after therapy, 3) to propose a protocol as short as possible.
**Methods and Materials:** Two radiologists retrospectively reviewed MRWB belonging to 112 patients with myeloma. Inclusion criteria: qualitative suitability of the sequences and presence of an exam after to be used as "control". Exclusion criterion: widespread infiltration. The statistical analysis (Friedman Test and the Sign test) was carried out by considering all patients and then before and after therapy. The "error" of the sequences and the correlation of the two readers with the ICC (Intraclass Coefficient Correlation) were calculated.
**Results:** There is no statistically significant difference in finding myelomatous lesions using only DWI (with ADC) compared to the simultaneous use of STIR or DIXON. No statistically significant differences were found even between patients before and after therapy despite the signal "change" of the lesions.The agreement is excellent for all sequences with average ICC of 0.99(0.95-1).
**Conclusion:** We propose the MRWB protocol: sagittal T1 and STIR on the column + coronal DIXON + DWI (ADC). If the DIXON sequences are not available: sagittal T1 and STIR on the column + coronal STIR + DWI (ADC). Both coronal sequences do not add diagnostic value.

**B-0368** 14:56
**Optimisation of prostate cancer radiotherapy management with MR**
F. Currò[1], M.C. Ricciardi[2], R. Danieli[1], M.F. Cavallaro[1], P. Martingano[1], M. Bertolotto[1], F. Stacul[1], M.A. Cova[1]; [1]*Trieste/IT*, [2]*Padua/IT (francescacurro86@gmail.com)*

**Purpose:** To evaluate the diagnostic accuracy of MR in local staging of prostate cancer and in risk category assessment*;* to demonstrate the role of MR in radiation therapy planning regarding target volume evaluation.
**Methods and Materials:** From January 2015 to June 2018, 109 patients with biopsy-proven prostate cancer addressed to radiotherapy were retrospectively identified; all patients underwent MR for local staging. The correlation between clinical stage and MR stage has been evaluated by Cohen's kappa weighted test; the McNemar test was used to analyze the differences in risk category assessment using T parameter as independent variable. The Wilcoxon test was used to determine the differences of radiation treatment volumes obtained on CT and on CT/MR fusion imaging.
**Results:** The agreement between MR and clinical staging was fair (0.38 , CI 95% 0.20-0.56); the different staging resulted in a statistically significant difference in risk category assessment ($p < 0.003$). Finally, the radiation treatment volumes obtained on CT/MR fusion imaging was significantly smaller than the corresponding value obtained on CT ($p < 0.001$).
**Conclusion:** MR has been proven accurate in the management of patients with prostate cancer candidate to radiotherapy. The use of CT/MR fusion imaging allowed a better evaluation of radiation treatment volumes improving therapy efficacy.

**B-0369** 15:00
**US and CEUS in ovary assessment of a murine model: preliminary findings on protective role of gonadotropin releasing hormone analogue from chemotherapy-induced ovarian damage**
A. Colarieti, M. Venturini, L. Perani, A. Esposito, A. Del Maschio, F. De Cobelli; *Milan/IT (colarieti.anna@hsr.it)*

**Purpose:** The gonadotoxic effect of chemotherapy (CT) can lead to infertility in female cancer survivors. There is controversial evidence regarding the protective role of gonadotropin-releasing hormone analogue (GnRH-a) on CT-induced ovarian damage. First aim of this experimental study on a murine model was to characterize ovaries in normal conditions; second aim was to obtain preliminary findings on anatomical/vascular ovarian changes induced by GnRH-a.
**Methods and Materials:** US and CEUS (Vevo-2100-Visualsonics, 40-20-MHz probes) were performed (day 0, 10, 20) comparing two groups of female BALB/c mice (n=10/group) in prone position respectively treated with subcutaneous injection of GnRH-a, Decapeptyl (DECA-treated-mice) and of PBS (Control-untreated-mice). Main US (ovary-diameter, dominant-follicle-diameter) and CEUS (peak-enhancement, wash-in-area-under-the-curve) parameters were investigated.
**Results:** Ovaries were ultrasonically identified and CEUS analysis was successfully performed in all cases with 100% of technical success. At day 20, a statistically significant increase of the dominant-follicle-diameter and reduced vascularization in DECA-treated-mice compared to Control-untreated mice was recorded.
**Conclusion:** Ovaries can be accurately studied in the murine model in prone position with US and CEUS. GnRH-a seems to exert a protective role on ovaries although further studies combining GnRH-a and chemotherapeutic agents will be needed to obtain more translational information useful for clinical practice.

**B-0370** 15:04
**DWI predicts surgical outcome in advanced ovarian cancer**
H. An, E. Lee, J.A. Perucho, K.W. Chiu, M. Chu, K.Y. Tse; *Hong Kong/HK (610779446@qq.com)*

**Purpose:** To evaluate the values of DWI-derived tumour burden of peritoneal carcinomatosis (PC) and its biology in determining the surgical outcome in advanced ovarian cancer (AOC).
**Methods and Materials:** Thirty-four AOC patients were prospectively recruited with pre-surgical DWI ($b$=0, 400, 800 s/mm$^2$). Tumours with intermediate apparent diffusion coefficient (ADC) segmented by k-means clustering were used for solid tumour volume (STV) calculation discarding fatty, fibrous or cystic tissues. A functional peritoneal cancer index (fPCI) was generated by adding up all STVs from 13 abdominopelvic regions with additional points if porto hepatis, mesentery or bowel serosa were involved. Mean ADC was measured from 3 selected lesions with the largest STVs. At surgery, the surgical PCI (sPCI), surgical duration and surgical outcome (complete cytoreductive surgery or with residual disease) were recorded. Mann-Whitney *U* test, Pearson correlation ($r$) and logistic regressions were performed.
**Results:** Patients who achieved complete cytoreductive surgery had lower fPCI (4.30 vs 10.18) and sPCI (5.70 vs 12.00) but higher ADC (0.97 vs 0.73) than patients with residual disease ($p < 0.005$). fPCI was significantly correlated with sPCI ($r$=0.899, $p$<0.001) and surgical duration ($r$=0.528, $p$=0.001). fPCI, sPCI and ADC ($r$=0.507, 0.529, -0.562) were significantly correlated with residual disease ($p$<0.005). Both fPCI and ADC were significant in univariate and multivariate analyses ($p$<0.05; accuracy=82.4%, sensitivity=72.7%, specificity = 87.0%).
**Conclusion:** Pre-surgical fPCI and ADC were significant predictors for complete cytoreductive surgery in AOC.

**B-0371** 15:08
**Accuracy of MDCT and laparoscopy in the prediction of peritoneal carcinomatosis index score in primary ovarian cancer**
S.A. Ahmed, D. Fouad, G.S. Seifeldein, H.A. Taleb, A. Yehia, N.A.A. El Malek, D. Badary; *Assiut/EG (Doaafouad11@gmail.com)*

**Purpose:** To compare accuracy of MDCT and laparoscopy in preoperative prediction of peritoneal carcinomatosis index score compared with surgery.
**Methods and Materials:** fifty ovarian cancer patients underwent CT, laparoscopy, and laparotomy, peritoneal deposit site and size were documented in each region using Sugarbaker's method by dividing the abdomen into 13 regions, scores calculated based on lesion size (LS) and summed up (range 0-39), calculation of total PCI (peritoneal cancer index) was done radiographic, laparoscopic and compared to surgery.
**Results:** Exploratory laparotomy detected peritoneal deposits in 560/650 regions, the remaining 90 regions were free . CT and laparoscopy over all sensitivity 95%,98.4% specificity 88%,81.7%, PPV 98%,97%, NPV 75%,89.3%, and accuracy 94 %,96% respectively. The mean PCI in 0-8 abdominopelvic regions by CT and laparoscopy was (12.11 ± 7.04 (range 0-25) and (11.96 ± 6.46 (range 0-26), respectively. While in 9-12 regions mean PCI was 4.55 ± 1.90 (range 0-6), 3.95 ± 1.09 (range 0-6), respectively. However, CT is more accurate in upper abdomen and laparoscopy is more accurate in pelvic and small intestinal regions, no statistically significant difference regarding total PCI score .
**Conclusion:** CT and laparoscopy are of comparable accuracy regarding peritoneal deposits detection and final PCI scoring, although CT is less accurate than laparoscopy in regional detection and scoring at the pelvic and small intestinal regions, standardized dedicated MDCT protocol with experienced radiologists in addition to routine use of a standardized PCI form may provide better comprehensive multi-regional analysis for better patient outcome.

**B-0372** 15:12
**IVIM-DWI MRI in meningiomas: a tool to detect early microstructural changes during and after proton-therapy**
M.V. Raciti[1], A. Franconeri[1], G. Buizza[2], G. Fontana[1], G. Viselner[1], A. Iannalfi[1], E. D'Ippolito[1], L.M. Farina[1], L. Preda[1]; [1]*Pavia/IT*, [2]*Milan/IT*
*(mariavittoria.raciti@libero.it)*

**Purpose:** To investigate the early microstructural changes of meningiomas during and after proton-therapy through quantitative analysis of Intravoxel Incoherent Motion-Diffusion Weighted MRI (IVIM-DWI MRI) parameters.
**Methods and Materials:** Since January 2017,20 patients with meningioma were enrolled for proton-therapy. Morphological and DWI-MRI(7b-values: 0,50,100,150,200,400,1000[s/mm$^2$]) for a simplified IVIM were acquired before the 1$^{st}$(t0), at the 10$^{th}$ (t10), 20$^{th}$ (t20), 30$^{th}$ (t30) treatment fraction, 3 and 6 months after the end of the therapy(1$^{st}$-2$^{nd}$ follow-up). Tumour contours were drawn on T2Wimages and registered to DW-MRI. Apparent diffusion coefficient maps(ADC), true diffusion (D), pseudodiffusion (D*) and perfusion fraction(*f*) coefficients from IVIM were obtained for all time-points. Statistical analysis was performed using pairwise Wilcoxon test(α=0.05).
**Results:** Statistically significant differences were found for ADC, D and D* median values between every intra-treatment time-point and 1$^{st}$-2$^{nd}$follow-up. Both ADC and D tumours median values resulted progressively higher from t0 to 2$^{st}$ follow-up; especially, comparing D intra-treatment median values, significant differences were found between t0 and t20(p=0.03), and between t0 and t30(p=0.02). *f* didn't show significant changes at any time-points, but increased its mean and median values at 2$^{nd}$ follow-up. Tumour volume decreased from t0 to 1$^{st}$ follow-up in 12 patients;three of them showed tumour volume reduction greater than 28% and the highest increment of median D from t0 to t30.
**Conclusion:** IVIM-DWI in meningiomas detects early microstructural changes during proton-therapy and potentially predicts tumour response in follow-up. A future goal is to represent microstructural tissue heterogeneity through parametric response mapping, conceiving MRI as an"in-line" image-biomarker to guide individualized treatments and early treatment adaptation,accounting for heterogeneity and response variability.

**B-0373** 15:16
**Predictive role of apparent diffusion coefficient values from diffusion weighted sequences in patients with sacral chordoma treated with carbon ions radiotherapy alone**
S. Casale[1], M. Fanizza[1], C. Paganelli[2], G. Viselner[1], E. Turpini[1], M. Fiore[1], I. Fumagalli[2], F. Valvo[1], L. Preda[1]; [1]*Pavia/IT*, [2]*Milan/IT*
*(casale.silvia88@gmail.com)*

**Purpose:** To evaluate if baseline Apparent Diffusion Coefficient values (ADC) from Diffusion Weighted sequences could predict response to treatment in Patients with sacral chordoma not suitable for surgery treated with carbon ions radiotherapy (CIRT) alone compared with volume modifications.
**Methods and Materials:** Patients included had sacral chordoma histological diagnosis, they had one cycle of CIRT alone and a minimum of 12-months follow-up. All patients underwent MRI before treatment (baseline), every three months in the first two years after treatment and every six months afterwards. For each MRI, lesions volume were obtained and median, kurtosis and skewness ADC values were analyzed within the whole lesion volume. Lesions presenting a volume increase more than 20% from the baseline were considered as progression of disease (PD), lesions presenting a volume reduction of at least 30% were considered as a response disease (RD); lesions between those two limits were considered as stable disease (SD).
**Results:** 60 lesions in 59 patients with sacral chordoma were studied (range follow-up 12-60 months). 6 lesions were categorized as PD, 40 RD and 14 SD. PD showed significant higher baseline median ADC values (mm²/s) (p=0.015) compared with both RD and SD (1659vs1253vs1266). PD showed more negative skewness values (-0.1vs0.26vs-0.03) compared with both the other groups, not significantly different (p=0.2).
**Conclusion:** preliminary results suggest that baseline ADC values could predict response to treatment with CIRT, in particular to detect potential non responder patients.

**14:00 - 15:30** **Tech Gate Auditorium**

### Cardiac

### SS 303

### Benefits of cardiac MR mapping for myocardial characterisation

*Moderators:*
N.N.
N.N.

**B-0374** 14:00
**Quantitative cardiac magnetic resonance in the detection of early cardiac transplant rejection - MRI compared with endomyocardial biopsy**
M.W. Raudner, A. Wielandner, K. Uyanik-Ünal, M.-E. Stelzmüller, A. Zuckermann, C. Loewe, D. Beitzke; *Vienna/AT*
*(marcus.raudner@meduniwien.ac.at)*

**Purpose:** Cardiac transplantation is a lifesaving procedure for patients with end-stage heart failure. As acute rejection is a severe complication patients have to undergo invasive endomyocardial biopsy to rule out rejection episodes. This study is focused at comparing extracellular volume (ECV), T1 and T2 mapping via cardiac magnetic resonance imaging (CMR) with results endomyocardial biopsy at early post-transplant follow-ups to provide reference values for rejecting and non-rejecting patients.
**Methods and Materials:** Forty-five patients underwent endomyocardial biopsy three months after cardiac transplantation alongside CMR on a 1.5T system for evaluation of graft rejection and function. Left ventricular function (LVEF) was derived from short axis steady-state free precision (SSFP) sequences, myocardial ECV (%) was calculated using T1 pre- and postcontrast maps at three short axis slices using a 16-segment model. T2 maps were acquired from identical planes for oedema assessment. CMR findings were compared with histology based on ISHLT guidelines.
**Results:** Myocardial biopsy showed no signs of rejection (0A/0R) in thirty-nine patients and mild rejection (1A/1R) in six patients. Mean LVEF was normal in both groups (64.1% vs. 72.7%; p=.082). Mean ECV% (31.8% vs. 31.8%; p=.980), T1 in ms (1063.0 vs. 1079.1; p=.418) and T2 in ms (51.2 vs. 53.0; p=.137) did not differ significantly between patients with no or mild rejection.
**Conclusion:** ECV is known to be increased in post-transplant patients. In the reported patient cohort, there was no significant difference in the observed quantitative CMR markers in patients with early mild vs. no transplant rejection, showing comparable normal values.

**B-0375** 14:08
**Evaluate diffuse myocardial characteristics in rabbit model based on daunorubicin-induced cardiotoxicity by CMR native T1 mapping imaging**
H. Liu, R. Xu, Y. Guo, Z. Yang, L. Chen, C. Fu, X. Li; *Chengdu/CN*
*(liuhuiimage@qq.com)*

**Purpose:** This study aimed to quantitatively evaluate diffuse myocardial characteristics of daunorubicin-induced cardiotoxicity in rabbit model, and further to explore the heart injury of different cumulative dose and time point.
**Methods and Materials:** Twenty-two adult New Zealand rabbits were injected with daunorubicin weekly for 2-4 weeks (8 subjects with 4mg/kg for 2 weeks, 8 subjects with 4mg/kg for 4 weeks and 6 subjects with 3mg/kg for 4 weeks) and 9 normal rabbit were enrolled. According to the cumulative dose, all modelling rabbits were divided into three groups (mild,20-29mg; moderate, 30-39mg and high, 40-49mg). Cardiac structure, function, mass, and native T1 values measurement were based on cine imaging and pre-contrast T1 mapping.
**Results:** There were four rabbits (18.2%) occurred LVEF decreased among all of modelling rabbits. The mean native T1 values on CMR in rabbits with 4mg/kg weekly at 2 and 4 weeks were significantly higher than those measured before modelling (native T1 values: 1243.90±39.90ms, 1271.18±98.09ms vs. 1115.91±54.95ms; all p < 0.017), and T1 values of 4 weeks modelling rabbits higher than that of 2 weeks. All modelling rabbit had higher T1 values then normal subjects, the high dose group have higher T1 values than moderate dose group (native T1 values: 1297.75 ± 83.16ms vs. 1220.17± 77.85ms) but there is no significant difference between mild and moderate group.
**Conclusion:** Native T1 mapping could early detection diffuse myocardial injury cause by daunorubicin, the higher cumulative dose and longer time may have more severe outcome. And further monitor study is ongoing.

**B-0376** 14:16
**Myocardial characterisation using CT extracellular volume in swine acute myocardial infarction model: comparison with CMR T1 mapping and histology**
H. Xu, Y. Guo, L. Xie, R. Xu, M. Yang, H. Li, H. Fu, C. Fu; *Chengdu/CN (xuhuayan89@sina.com)*

**Purpose:** This study sought to evaluate the myocardial damage in acute myocardial infarction (AMI) of swine model using the revolution computed tomography (CT) extracellular volume (ECV), and further comparing with the cardiac magnetic resonance(CMR)-T1mapping and histology.
**Methods and Materials:** 10 AMI swine models were generated by left descending coronary artery occlusion with surgery. Pre-contrast plain and post-contrast-enhanced CT were scanned within 24 hours after surgery. And CMR pre-/post-contrast T1 mapping using MOLLI sequence on 3T MR scanner (Skyra, Healthcare, Erlangen, Germany) were performed after CT examination (GE, Revolutions, 256 rows). CT-ECV (Ziostation, Version 2.9.2.2, Japan), CMR native T1 and ECV (cmr42, version 5.6.4; Canada) were computed and compared.
**Results:** A total of 160 AHA segments were analysed, in which, 36 segments were found infarcted. CT-ECV as well as native T1, CMR-ECV were all increased in infarct segments comparing with remote non-infarct segment (native T1:1476±50ms vs. 1170±46 ms; CMR-ECV:38.35±4.77% vs. 21.21±3.56%, p <0.05 ). However, post-T1 were decreased for the contrast short T1 time (p<0.05). CT and CMR-T1 mapping had strong correlation in measuring infarct size (r=0.78, p<0.001). Linear correlation showed that CT-ECV correlated well with CMR-ECV. In addition, Bland-Altman reveals good agreement for evaluating both infarct size and ECV value (bias: 1.6±11). Histology verified that the size of infarction well corresponded to CT and CMR.
**Conclusion:** CT-ECV was well related with and showed good agreement with CMR-ECV and histology. In AMI swine model, CT-ECV were usefully for measuring ECV fraction of myocardial damage in the infraction area.

**B-0377** 14:24
**Feasibility of T1-rho mapping as endogenous contrast agent to detect myocardial fibrosis**
C. Celeng, M. Froeling, T. Leiner; *Utrecht/NL (celengcsilla@gmail.com)*

**Purpose:** The aim of this study was to assess the feasibility of native T1-rho mapping as a possible endogenous contrast agent to detect interstitial myocardial fibrosis.
**Methods and Materials:** In this prospective study we enrolled 219 patients (87 female, 132 male; mean age 48±2) referred for the evaluation of suspected or known non-ischemic cardiomyopathies. All patients underwent T1-rho mapping on a 1.5T clinical MR system. To evaluate the ability of T1-rho to detect myocardial fibrosis native and post contrast T1 maps were acquired and the extracellular volume (ECV) was calculated. After the acquisition was completed all images were transferred to a dedicated workstation for motion correction and standardized automatic processing. Spearman correlation coefficients were calculated to determine the correlation between ECV and whole heart T1-rho values, native T1-values and post-contrast T1-values.
**Results:** T1-rho and ECV maps were successfully obtained in 211 patients of which 189 datasets were of sufficient quality for automatic processing. Mean T1-rho relaxation time was 63.9±4.9 ms; mean native T1 was 1093.6±55.7 ms; mean post-contrast T1 was 390.3±57.6 ms; ECV values ranged between 5-47% (25.2±8.0). A strong and significant correlation was found between T1-rho relaxation time and ECV fraction (Spearman coefficient: 0.63, p<0.001). Weak correlations were found between native T1 and ECV (0.31, p<0.001) and post-contrast T1 and ECV (-0.26, p<0.01).
**Conclusion:** T1-rho correlates well with ECV, which indicates that it has the ability to provide information on diffuse myocardial fibrosis without the need for injection of contrast agents.

**B-0378** 14:32
**Myocardial inflammation in DCM and mapping: an elusive connection**
F. Hahn[1], D. Fleischmann[1], M.C. Halfmann[1], H.-P. Schultheiss[2], K.-F. Kreitner[1], P. Wenzel[1], T. Emrich[1]; [1]*Mainz/DE*, [2]*Berlin/DE (fhahn@uni-mainz.de)*

**Purpose:** The purpose of this study was to evaluate non-invasive detection of immunohistochemically established myocardial inflammation in non-ischaemic dilated cardiomyopathy (DCM) using T1 and T2 mapping.
**Methods and Materials:** The study population consisted of 59 retrospectively identified patients with DCM phenotypes who underwent endomyocardial biopsy (EMB) and cardiac magnetic resonance (CMR) imaging. EMB served as the gold standard in all patients. Inflammation in EMB was grouped according to the number of LFA1-positive lymphocytes, CD3-positive lymphocytes, and MAC1-positive macrophages. A control consisted of a matched group of healthy volunteers with CMR imaging (n=58). All imaging was acquired with a 3T Siemens Prisma® Scanner. T1 and T2 maps were created and analysed using dedicated cardiovascular software (Circle CVI®). Global and segmental mapping values as well as septal measurements and tissue heterogeneity parameters were recorded.
**Results:** Mean global T1 and T2 mapping values of DCM phenotype patients were significantly higher compared to healthy volunteers (both p<0.001). However, the presence of EMB-proven myocardial inflammation within the DCM group did not correlate with T1 and T2 mapping values when regarding global (p>0.4 and p>0.6 resp.), septal (both p>0.8) or maximum segmental values (p>0.1 and p>0.9 resp.).
**Conclusion:** T1 and T2 mapping parameters were not able to reliably detect myocardial inflammation in DCM phenotypes. The reasons for this behaviour are currently not fully understood. Possible explanations could be different aetiologies of inflammation, sample errors by EMB within the inflammation free-DCM group as well as high inter-individual differences in mapping values.

**B-0379** 14:40
**T1 and T2 mapping assessment of chronic myocarditis vs dilated cardiomyopathy, hypertensive heart disease and healthy controls**
M.T.A. Wetscherek[1], W. Rutschke[2], C. Frank[3], M. Grothoff[2], P. Lurz[2], H. Thiele[2], M. Gutberlet[2], C. Lücke[2]; [1]*Cambridge/UK*, [2]*Leipzig/DE*, [3]*Halle/DE*

**Purpose:** To evaluate T1 and T2 relaxation times (referred to as T1 and T2) and extracellular volume fraction (ECV) in chronic myocarditis vs dilated cardiomyopathy (DCM), hypertensive heart disease (HHD) and healthy controls.
**Methods and Materials:** Retrospective analysis of 71 consecutive cases that underwent cardiac magnetic resonance (CMR) as part of a full diagnostic workup for suspected myocarditis. All patients had a presentation at >14 days after initial symptoms. 13 cases were excluded due to previous myocardial infarction or technical aspects. The CMR protocol included native T1 and T2 mapping, and post-contrast T1 mapping. The analysis of all cases was done by two readers.
**Results:** 27 patients were diagnosed with chronic myocarditis, 21 patients with DCM and/or HHD, and 10 subjects were included in the control group. All mapping parameters (cut-off values: T1 1070ms, T2 54ms, ECV 30%) yield good diagnostic performance in the assessment of chronic myocarditis with the best Sensitivity/Specificity/Accuracy of 93/80/88 for ECV, followed by 70/80/74 for T2, and 52/88/69 for T1. Significant difference was found between patients with DCM/HHD and the control group regarding T2 and ECV, but not for T1. No significant difference was found between the chronic myocarditis group and the DCM/HHD group. All mapping parameters achieved excellent interobserver agreement, with correlation coefficients ICC>0.977, p<0.001, the highest achieved for ECV (ICC=0.994).
**Conclusion:** Mapping parameters have the potential to aid reaching a definite diagnosis of chronic myocarditis, but clinical-radiological correlation remains critical in its differentiation from other generalized chronic myocardial processes, such as DCM and HHD.

**B-0380** 14:48
**The impact of myocardial thickness in hypertrophic cardiomyopathy using combination of CMR native T1 and T2 mapping**
L. Huang, L. Ran, L. Xia, R. Han; *Wuhan/CN*

**Purpose:** The aim of this study was to investigate the abnormal changes of different hypertrophic myocardial segments in HCM using the combination of native T1 and T2 mapping.
**Methods and Materials:** Forty-seven patients with HCM and 19 healthy volunteers underwent cardiac magnetic resonance imaging (CMR). Imaging protocols included short-axis cine, native T1 and T2 mapping sequences. Native T1 and T2 values, and left ventricular end-diastolic wall thickness (EDTH) were measured for each myocardial segment. The differences in native T1 and T2 values, and EDTH were compared between normal myocardial segments of the healthy volunteers and hypertrophic myocardial segments of HCM patients. The disparity of hypertrophic segments with different severities was also evaluated.
**Results:** Native T1 and T2 values were significantly elevated in non-hypertrophic segments and hypertrophic segments of HCM patients (both P<0.001). Compared to mild and moderate hypertrophic segment, native T1 value of severe hypertrophic segments in HCM was significantly elevated (P<0.05, respectively), but the degree of myocardial thickening reduced (P<0.05, respectively). T2 value of mild hypertrophic was significantly lower than moderate and severe hypertrophic segments (P<0.05, respectively).
**Conclusion:** Native T1 and T2 values were elevated in non-hypertrophic segments of HCM. It is more sensitive for T2 value to detect abnormal changes than native T1 in mild hypertrophic segments in HCM. Native T1 and T2 values had linear correlation with EDTH, and may provide potential incremental value to EDTH for risk stratification and prognosis in HCM.

**B-0381** 14:56
**Assessment of diagnostic performance of cardiac magnetic resonance (CMR) mapping parameters in acute myocarditis (AM) patients**
D. Palumbo[1], A. Palmisano[1], R. Faletti[2], G. Benedetti[1], M. Gatti[2], A. Del Maschio[1], F. De Cobelli[1], A. Esposito[1]; [1]*Milan/IT*, [2]*Turin/IT* *(palumbo.diego@hsr.it)*

**Purpose:** Our aim was to evaluate diagnostic accuracy of CMR mapping parameters in patients with acute myocarditis (AM).
**Methods and Materials:** Between October 2016 and June 2018 we enrolled 33 patients (39 ± 12.4yrs; 18M, 15F) with clinical suspicion and bioptical confirmation of AM who underwent CMR within three weeks from symptoms onset and 40 healthy controls (37.6±11.8yrs; 22M, 18F). CMR protocol included evaluation of native T1 mapping, T2 mapping and Extra Cellular Volume (ECV), each one then analysed in order to assess mean global, basal, mid ventricular, apical and segmental (AHA) values.
**Results:** AM patients demonstrated mean global native T1 mapping, T2 mapping and ECV values significantly higher in comparison with healthy individuals (1103.79ms [1068.6 - 1202.88] vs. 1023.48ms [963.39 - 1108], p<.0001; 53.95ms [51.92 - 58.74] vs. 46.79ms [43.10 - 49.62], p<.0001; 30.28% [27.12 - 32.1] vs. 25.88% [22.63 - 28.78], p=0.05). By means of ROC analysis we then identified as best diagnostic performance threshold for native T1 mapping a value of 1045.45ms (AUC: .949; Se: 90.6%; Sp: 87.5%), for T2 mapping a value of 49.51ms (AUC: .982; Se: 92.9%; Sp: 100%) and for ECV a value of 26.7% (AUC: .894; Se: 80%; Sp: 100%). Thus, above these threshold values, CMR mapping parameters were highly suggestive of active disease.
**Conclusion:** CMR mapping parameters demonstrate an optimal diagnostic performance, with high sensitivity and specificity, in predicting patients with AM.

**B-0382** 15:04
**Evolution of myocardial oedema of acute and subacute myocardial infarction verified in swine model: assessed by native T1 mapping and T2 mapping**
H. Liu, Y.-K. Guo, H. Xu, Z.-G. Yang; *Chengdu/CN (liuhuiimage@qq.com)*

**Purpose:** To dynamically investigate the evolution of myocardial oedema in acute and subacute myocardial infarction (MI) using CMR native T1 mapping and T2 mapping technique.
**Methods and Materials:** 10 acute MI swine model were generated by left descending coronary artery occlusion with surgery. All swine model were performed CMR scanning (MEGNETOM Skyra, Healthcare, Erlangen, Germany) including native T1 and T2 mapping in 24 hours and 7 days. Native T1 mapping were used Modified Look-Locker inversion recovery (MOLLI) sequences. The infarct sizes, native T1 value and T2 value were measured and compared in acute and subacute period in MI swine. Mann-Whitney U test and Pearson correlation coefficient were used for statistical analysis.
**Results:** For MI sizes, there is no difference in acute and subacute MI indicated by both native T1 mapping (p=0.421) and T2 mapping images (P=0.69). Although, the native T1 values of infarction area showed no significant (p=0.22) difference, the T2 values in subacute MI increased compared to acute MI (p=0.032). In addition, the native T1 and T2 value of both MI had close correlation (r=0.718, P=0.019).
**Conclusion:** Compared with the acute phase, the degree of myocardial oedema was determined more serious in subacute stage. CMR T2 mapping technique can determine severity of myocardial oedema caused by acute and subacute myocardial infarction.
*Author Disclosures:*
**H. Liu:** Founder; National Natural Science Foundation of China (81471721, 81471722, 81641169, 81771887 and 81771897), Program for New Century Excellent Talents in University (no: NCET-13-0386), Program for Young Scholars and Innovative Research Team in Sichuan Province (2017TD0005) of China.

**B-0383** 15:12
**Long-axis T1 and T2 mapping in Takotsubo cardiomyopathy: trash or treasure?**
T. Emrich, R.M. Brumberg, P. Wenzel, S. Lyschik, F. Jungmann, S. Rueppel, C. Düber, K.-F. Kreitner; *Mainz/DE (tilman.emrich@gmail.com)*

**Purpose:** Takotsubo cardiomyopathy (TTCM) is a regional disease affecting either the apex, the mid and/or the base of the heart. Long-axis views allow the illustration of these regions in one view. For scientific purposes, short-axis slices are used typically to measure absolute relaxation times in cardiac diseases. The purpose of this retrospective study was to establish the role of long-axis imaging with T1 and T2 mapping in TTCM.
**Methods and Materials:** We compared CMR and ventriculography of 41 consecutive patients with TTCM. TTCM-involved segments and regions were defined as segments with wall motion disturbances and absence of ischaemia. With a dedicated cardiovascular software (Circle CVI®, Calgary, Canada), T1 and T2 maps were created, TTCM-involved and not-involved segments analysed and mean absolute relaxation times recorded.
**Results:** Mean relaxation times significantly distinguished involved from not-involved segments, e.g. TTCM involved: 1408 ± 151 ms (T1 map, 4-chamber view) versus TTCM not-involved: 1296 ± 105 ms; p < 0.001 for all. These results were also achieved in 2-chamber views. Furthermore, T1 and T2 mapping were equally useful for demonstrating myocardial oedema in long-axis views of TTCM patients.
**Conclusion:** Myocardial T1 and T2 mapping is a powerful tool for the detection of myocardial oedema. In segmental and/or regional-dominated diseases such as TTCM, long-axis imaging is a valuable tool to demonstrate the extent of involved segments in one view.

**B-0384** 15:20
**Performance of native T1 and T2 mapping cardiovascular magnetic resonance to detect myocardial oedema in patients with end stage renal disease**
W. Peng, H. Xu, Z. Li, C. Xia, Y. Guo; *Chengdu/CN (yimaerlin@163.com)*

**Purpose:** To evaluate the performance of quantitative native T1 and T2 mapping to identify myocardial oedema (ME) in patients with ESRD.
**Methods and Materials:** Seventy ESRD patients and 16 age-gender-matched healthy volunteers were prospectively enrolled and underwent CMR. All the parameters from CMR, including native T1,T2,T2 SI ratio and myocardial strain, were measured and compared. ROC analysis was performed to determine whether T2 values could be used in discriminating ME between ESRD patients and normal.
**Results:** The global T2 values of ERSD patients without diffused LGE and with diffused LGE were higher than normal controls (43.69±3.62, 41.82±3.43, 38.79±3.69ms, respectively, all P<0.05). The global T1 values of ERSD patients with diffused LGE was highest among three groups (1356.79±40.08ms,1321.02±56.65, 1286.12±52.60, respectively, all P<0.05),but no statistical difference were found between normal and patients without LGE. The LVEF, GRS, GCS and GLS of patients with diffused LGE were significantly decreased (all p <0.05). No statistical difference were found T2 SI ratios among three groups (P=0.86). By ROC analysis, T2 values exhibited a higher diagnostic accuracy for detecting ME(AUC= 0.846; 95% CI: 0.715 to 0.932, Criterion>41.9448, SE 73.5%(55.6-87.1%) SP 87.5%( 61.7-98.4%) )than T1 values or T2 SI ratio(all p<0.05).
**Conclusion:** ME and MF were existed in ESRD patients and had a negative effect on cardiac function. T2 mapping seems to be superior comparing with native T1 and T2 values for assessing ME in ESRD patients . The threshold values of T2 values may be used as indicators for differentiating ME between ESRD patients and normal.

**14:00 - 15:30** **The Church**

### Abdominal Viscera

### SS 301

### Biliary imaging: what next?

*Moderators:*
N.A. Courcoutsakis; Alexandroupolis/GR
S.R. Marticorena Garcia; Berlin/DE

**B-0385** 14:00
**Balanced steady-state free precession MRCP is a robust alternative to respiration-navigated 3D turbo-spin-echo MRCP**
F.C. Hasse, B. Selmi-Özer, C. Rupp, H.-U. Kauczor, T. Weber; *Heidelberg/DE (felix.hasse@med.uni-heidelberg.de)*

**Purpose:** Despite synchronization to respiration, respiration-navigated (RN) 3D turbo-spin-echo MRCP is limited by susceptibility to motion artefacts. Aim of this study was to assess quality of pancreaticobiliary duct visualization of a non-RN MRCP alternative based on balanced steady-state free precession imaging (BSSFP) with overlapping slices compared with RN MRCP.
**Methods and Materials:** Prospective study on 50 consecutive patients receiving MRCP at 1.5T without final diagnosis of pancreaticobiliary duct disease. Intraindividual comparison of coronal RN MRCP (thickness, 1.5 mm; interval, 1.5 mm) with combined coronal (thickness, 4 mm; interval, 1.6 mm) and transverse (thickness, 6 mm, interval, 2.4 mm) BSSFP MRCP. Image quality was scored for 6 pancreaticobiliary duct segments (3 pancreatic, 3 biliary) using a 6-point scale (0, not depicted, 5, entirely depicted with excellent details). A score of 2 or lower was defined to indicate insufficient duct visualization. ANOVA tests were used for statistical group comparison.
**Results:** Overall duct visualization was scored with 4.0±1.0 for RN MRCP (pancreatic, 3.6±1.3; biliary, 4.5±1.0) and 4.7±0.4 for combined coronal and transverse BSSFP MRCP (pancreatic, 4.5±0.7; biliary, 4.9±0.2), respectively (p<0.001). The number of segments visualized insufficiently was lower for BSSFP MRCP (n=6/300) than for RN MRCP (n=56/300, p<0.001). Segments visualized insufficiently in RN MRCP had a mean score of 4.0±1.0 in BSSFP MRCP. Mean acquisition time was 49% shorter for combined coronal and transverse BSSFP MRCP (100.2s±0.4s) than for RN MRCP (198.0s±98.7s).
**Conclusion:** Non-RN BSSFP MRCP with overlapping slices is a fast and robust alternative to RN MRCP for pancreaticobiliary duct visualization.

**B-0386** 14:08
**Can two breath-hold 3D-MRCP replace the conventional 3D-MRCP?**
M. He, H. Xue, Z. Jin; *Beijing/CN (377174431@qq.com)*

**Purpose:** To prospectively evaluate and compare the image quality and diagnostic performance of three 3D MRCP protocols including Compressed-Sensing (CS), Gradient Spin Echo (GraSE) and navigated-trigger(NT) MRCP.
**Methods and Materials:** A total of 64 patients suspected with duct-related pathologies were prospectively enrolled and done the three MRCP protocols randomly. The acquisition time was recorded and compared by paired t test. The imaging quality(IQ) was subjectively evaluated by 2 radiologists independently. The Wilcoxon signed rank test was used to compare IQ scores. The diagnosis performance was evaluated by the AUC value and compared by McNemar's test. The inter-observer agreement was evaluated by linearly weighted kappa coefficients.
**Results:** Compared to NT-3D-MRCP, the acquisition time of CS-BH-3D-MRCP and GraSE-3D-BH-MRCP was significantly decreased (both $P < 0.01$), while the IQ of them was significantly improved (4.53 ± 0.72 and 4.37±1.10 vs. 3.75 ± 1.18, both $P < 0.05$). There is no significant difference in both scan time and imaging quality($P > 0.05$) between these two breath-hold (BH) MRCP. The AUC was 0.811 with NT-MRCP, 0.892 with CS-MRCP and 0.845 with GraSE-MRCP (all $P > 0.05$). The diagnosis performance of the combination of CS-MRCP and GraSE-MRCP (AUC=0.935) was significant better than that of the NT-MRCP ($P < 0.05$). The subjective evaluation all reached good to excellent level of agreement ranging from 0.623 to 0.945.
**Conclusion:** Compared to NT-MRCP, CS-BH-3D-MRCP and GraSE-3D-BH-MRCP can provide better image quality, while decreasing the scan time significantly. When combine these two BH-3D-MRCP, the diagnosis performance was significantly better than that with the NT-MRCP.

**B-0387** 14:16
**Role of dynamic contrast-enhanced MRI in early detection of gallbladder carcinoma**
N. Yadu, S. Saxena; *Lucknow/IN (n_yadu@yahoo.com)*

**Purpose:** Gall bladder carcinoma is a rare disease with notoriously high mortality rates. Gall bladder wall morphology and enhancement pattern on dynamic contrast enhanced MRI can be useful in differentiating early gall bladder carcinoma (stage T2 or lower) from benign mimics.
**Methods and Materials:** Dynamic contrast enhanced VIBE sequences of upper abdomen along with MRCP images were obtained from 30 patients. All dynamic contrast enhanced MR images were evaluated for the delineation of gallbladder wall morphology and enhancement pattern. Statistical analysis was done using Statistical Package for Social Sciences version 15.0 or above. Measure of agreement between imaging findings and histopathological/surgical staging was expressed in terms of measures of agreement (κ, kappa statistic).
**Results:** MRI diagnosis was made in 30 cases. The sensitivity, specificity, positive predictive and negative predictive values were 95.7%, 85.7%, 95.7% and 85.7% respectively. The accuracy of test was 93.3%. Early enhancement persisting into delayed phase was the predominant pattern in malignant pathology (78.3%) while early enhancement with subsequent wash out was more common in histologically benign cases (71.4%). Statistically, the enhancement pattern differed significantly for the two diagnoses (p=0.023). On evaluating the measure of agreement between MRI and histopathology it was found to have almost perfect agreement, which was also significant statistically (κ=0.814; p<0.001).
**Conclusion:** Dynamic contrast enhanced MRI was found to be an accurate and reliable method for diagnosis of early Gall Bladder carcinoma. It helped to justify wall thickening with early enhancement persisting into delayed phase as the diagnostic criteria for malignancy.

**B-0389** 14:32
**Utilisation of virtual non-contrast images derived from dual-energy CT in the evaluation of biliary stone disease: should we obtain true non-contrast images?**
J. Bae, D. Lee, I. Joo, S. Jeon, J.K. Han; *Seoul/KR (jaeseok.bae@gmail.com)*

**Purpose:** To compare the virtual non-contrast (VNC) images acquired through dual-energy computed tomography (DECT) with the true non-contrast (TNC) images in the detection of biliary stones and assess whether VNC images can replace TNC images.
**Methods and Materials:** Between March 2017 and April 2018, we retrospectively enrolled 75 patients with suspicious biliary disease who underwent dual-source DECT and surgery and/or endoscopic intervention within 2 months from the CT. The sensitivity and specificity for detecting gallstone and common bile duct (CBD) stone were compared between the VNC and TNC using McNemar test. In addition, the image quality and acceptability of VNC as replacement for TNC were assessed.
**Results:** In our study, 37 patients had gallstones, 2 had CBD stones, and 6 had both gallstone and CBD stones. For detection of gallstones, the sensitivity and specificity were 90.7% (39/43) and 87.5% (28/32), respectively, for the TNC images, and 88.4% (38/43) and 90.6% (29/32), respectively, for the VNC images. With respect to CBD stones, the sensitivity and specificity were 87.5% (7/8) and 98.5% (66/67), respectively, for the TNC images, and 75.0% (6/8) and 100% (67/67), respectively, for the VNC images. There was no significant difference in the sensitivity and specificity between each image set (P>.05). The VNC images were considered as complete replacements for the TNC images in 72 and 71 patients by the two reviewers.
**Conclusion:** The VNC images acquired through DECT were comparable to the TNC images for the detection of biliary stones and may replace the TNC images.

**B-0390** 14:40
**Diagnosis of biliary stone disease: compressed-sensing-accelerated 3D-MRCP versus conventional MRCP**
X. Zhao[1], M. Huang[1], J. Zhu[2], J. Cheng[1]; [1]*Zhengzhou/CN*, [2]*Beijing/CN (weybey@163.com)*

**Purpose:** To evaluate the image quality and diagnostic performance of a prototype 3-dimensional magnetic resonance cholangiopancreatography (3D-MRCP) with compressed sensing (CS) in diagnosing biliary stone disease.
**Methods and Materials:** This retrospective study recruited 62 patients (M:F=27:35; age, 56.9 ± 16.4 years) who underwent 3D-MRCP on a 3T MAGNETOM Prisma (Siemens Healthcare, Erlangen, Germany). Biliary stone disease was confirmed for all patients by subsequent procedures within two weeks. 3D protocols included conventional MRCP (c-MRCP), navigator-triggered MRCP with CS acceleration (NT-CS-MRCP), and 3D breath-hold CS-MRCP (BH-CS-MRCP). 2 radiologists independently rated image quality and duct visibility. Presence of stones in extrahepatic duct (EHD), gallbladder (GB) and intrahepatic duct (IHD) were evaluated based on coronal source images. Receiver operating characteristic (ROC) analysis was used to calculate the area under the curve (Az value),sensitivity, and specificity of biliary stone disease diagnosis in each biliary duct location.
**Results:** Mean acquisition times of BH-CS-MRCP (17s) and NT-CS-MRCP (207s) were remarkably shorter than c-MRCP (455s, both $P<0.001$). Overall image quality and duct visualization were not significantly different among the 3 protocols (all $P>0.05$), except that NT-CS-MRCP and c-MRCP depicted both pancreatic duct and bilateral second IHD better than BH-CS-MRCP (all $P<0.05$). The diagnostic performances of all three examinations were high for detecting EHD stones, GB stones, and IHD stones, but did not have significant differences (mean Az value: 0.84~0.99). Inter-observer agreement was fair to perfect.
**Conclusion:** NT-CS-MRCP and BH-CS-MRCP can provide comparable image quality and diagnostic performance to c-MRCP for biliary stone diseases, with significantly shorter acquisition time.

**B-0391** 14:48
**Differentiating and segmenting isoattenuating gallstones from bile with dual-layer spectral CT: an ex vivo phantom study**
T.C. Soesbe, M.A. Lewis, Y. Xi, T. Browning, L. Ananthakrishnan, R.E. Lenkinski, J.R. Leyendecker; *Dallas, TX/US (todd.soesbe@utsouthwestern.edu)*

**Purpose:** Develop a dual-energy CT (DECT) method for visually segmenting isoattenuating gallstones from bile and compare it to other DECT methods using an ex vivo phantom reader study.
**Methods and Materials:** Gallstones from 105 cholecystectomy patients were placed inside 120mL vials containing 5% ox-bile. The vials were scanned in a water-filled phantom with dual-layer DECT. From conventional CT, 30 isoattenuating gallstone vials (gallstone diameters 4.3 to 24.7mm) and 30 bile-only controls were selected. Six readers evaluated DECT images for the presence of isoattenuating gallstones on conventional, virtual non-contrast, monoenergetic 200keV, monoenergetic 40keV, and segmented images. The segmented images were created using a 2D-histogram of Compton and photoelectric attenuations and custom ROI software.
**Results:** Segmented images gave the highest mean intrareader (88.1±7.6%) and interreader agreements (first reading 88.2%, second reading 93.6%), as well as the highest AUC (0.99, 95%CI (0.97, 1.00), all adjusted P values <0.02) for all 30 gallstones. No significant difference was found between the segmented and monoenergetic images for gallstones with mean diameters >9mm (both adjusted P values >0.05). For gallstones with mean diameters <9mm, the segmented images had the highest AUC (0.99,95%CI (0.97, 1.00) all adjusted P values <0.007). The pathology versus segmented image gallstone size mean difference was -0.6mm (LOA 2.6 to -3.8mm).

**Conclusion:** Isoattenuating gallstones can be segmented from bile ex vivo with improved detection (especially those <9mm diameter) using dual-layer DECT and 2D-histograms of Compton and photoelectric attenuations. Detecting isoattenuating gallstones in vivo with this method could reduce the need for secondary imaging in symptomatic patients with suspected biliary calculi.

**B-0392** 14:56

**Predicting difficulties on preoperative sonographic evaluation in scheduled laparoscopic cholecystectomy in patients with acute calculous cholecystitis**

T.-K. Kim, H. Han, J. Park, H. Kim; *Daejeon/KR (goodseven@naver.com)*

**Purpose:** The aim of this study was to evaluate the role of preoperative sonography in predicting potential technical difficulties that can be encountered in scheduled laparoscopic cholecystectomy in patients with acute calculous cholecystitis.
**Methods and Materials:** After reviewing the medical records from January 2014 to December 2016, 759 patients meeting the inclusion criteria were enrolled in the final study. The number of the gallstones, the maximum diameter of the gallstones, presence of the sludge in the GB, the thickness of the GB wall, presence of the fluid at the GB fossa, the maximum diameter of extrahepatic duct (EHD), presence of CBD stone, and presence of GB polyp were estimated in preoperative sonography. The duration of operation, the grade of GB inflammation, the grade of adhesion to peritoneal cavity, and the presence of conversion to open cholecystectomy were estimated. The difficulty score was measured by 4 parameters in surgery.
**Results:** Presence of the sludge in the GB, the thickness of the GB wall, presence of the fluid at the GB fossa, the maximum diameter of EHD, presence of CBD stone were statistically significant in simple univariate analysis (p<0.05). Only presence of the fluid at the GB fossa and the thickness were statistically significant in multiple regression analysis (p<0.01).
**Conclusion:** In scheduled laparoscopic cholecystectomy, the difficulty of surgery can be predicted by preoperative US findings. Presence of the fluid at the GB fossa and the thickness of the GB wall are most predictable factors of surgical difficulty.

**B-0393** 15:04

**Portal vein and ligamentum teres variations in diverse gallbladder locations: is the left-sided gallbladder always a high-risk feature for pre-interventional assessment?**

H.-Y. Lin, R.-C. Lee, H.-H. Yen, C.-A. Liu, J.-L. Chen; *Taipei/TW (b101096132@tmu.edu.tw)*

**Purpose:** To emphasise the rare condition of the presence of right-sided ligamentum teres (RSLT) with a normal gallbladder location and to determine if the left-sided gallbladder (LGB) is associated with portal vein variations despite the location of ligamentum teres (LT).
**Methods and Materials:** We reviewed CT studies between June 2005 and July 2018 and excluded data with cholecystectomy (n =10,276). For prevent missing data of rare conditions, the studies were divided into 2 groups first: gallbladder in typical right side location (RGB; n =10,248) and LGB (n =28); than divided into 2 subgroups: LT in typical left side location and RSLT. Each patient with RGB and RSLT was matched with 5 patients with RGB and LT in typical location by sex and age. Outcome was the portal vein anomalies (PVA).
**Results:** The prevalence of LGB was 0.27%, consistent with previous studies. There were 20 patients (71.4%) with RSLT and 8 patients (28.6%) with typical LT location. The prevalence of PVA was 75% (15/20) and 25% (2/8), respectively, and was statistically significant (OR 9.00; 95% CI 1.36-59.78, P = 0.023). The prevalence of RSLT was 0.06% (6/10,248) among all RGBs. The prevalence of PVA was 83% (5/6) in patients with RGB and RSLT and 17% (5/30) in the sex- and age-matched patients with RGB and typical LT location, respectively. The result of conditional logistic regression was significant (OR 5.00; 95% CI 1.45-17.27, P = 0.011).
**Conclusion:** The LGB is not always accompanied with the high-risk feature of RSLT and vise versa.

**B-0394** 15:12

**Prediction for early recurrence of intrahepatic mass-forming cholangiocarcinoma: qualitative and quantitative MRI combined with prognostic immunohistochemical marker**

L. Zhao, X. Zhao, X. Ma; *Beijing/CN (zlyjdzhaoli@163.com)*

**Purpose:** To investigate the predictive performance of qualitative, quantitative MRI features and prognostic immunohistochemical marker for early recurrence (ER) of intrahepatic mass-forming cholangiocarcinoma (IMCC).
**Methods and Materials:** Forty-seven patients with pathologically proven IMCC were enrolled in this retrospective study. Preoperative contrast-enhanced MRI and immunohistochemical stainings of EGFR, VEGFR, P53 and Ki67 were performed in each patient, respectively. The clinicoradiologic and pathological risk factors of ER were identified using univariate analysis. Texture analysis (TA) was performed based on four MR sequences including T2-weighted imaging (T2WI), arterial phase (AP), portal venous phase (PVP), and delayed phase (DP) imaging. A clinicoradiologic-pathological (CRP) model, a TA model, and a combined model were developed, and the receiver operating characteristics (ROC) curve was used to explore their predictive performance to stratify ER.
**Results:** Enhancement pattern and VEGFR showed significant differences between the ER group and non-ER group (P=0.001 and 0.034, respectively). And the TA model based on AP, PVP, and DP images presented superior area under curve (AUC) (0.889, 95%CI: 0.783-0.996) among seven TA models, with sensitivity and specificity of 0.938 and 0.839, respectively. And the combined model contained enhancement pattern, VEGFR and texture parameters showed preferable predictive performance of ER compared to TA model or CRP model alone, with an AUC, sensitivity and specificity of 0.949, 0.875 and 0.774, respectively.
**Conclusion:** Combined model was the superior predictive model of ER compared with TA or CRP model alone. Combining qualitative, quantitative MRI features and VEGFR may be useful for predicting ER and thus helpful to guide personalized treatment in patients with IMCC.

**B-0395** 15:20

**Overrepresentation of uncommon biliary confluence types in hilar cholagiocarcinoma patients**

M. Shorikov, O.N. Sergeeva, D. Frantsev, M. Lapteva, E. Virshke, B. Dolgushin; *Moscow/RU (mshorikov@gmail.com)*

**Purpose:** The purpose of the study is to compare distribution of biliary confluence types in hilar cholagiocarcinoma patients and those with unremarkable bile ducts.
**Methods and Materials:** Biliary confluence types were retrospectively evaluated in 203 hilar cholangiocarcinoma patients ('study group', male/female=108/95, age range 21-84) and 204 patients with unremarkable bile ducts ('control group', male/female=95/109, age range17-81) at MRCP (all patients) and direct cholangiography (141 study group patients) by 2 radiologists (Cohen Kappa=0.7, differences were resolved by consensus). Nakamura classification was used for confluence typing: I-right biliary duct is present; II-independent drainage of right sectoral ducts to confluence, III-independent drainage of the right posterior duct into common hepatic duct, IV-drainage of the right posterior duct into left bile duct, V-other, right bile duct is absent. Group differences were evaluated with Chi-square and Pearson exact test criteria.
**Results:** While control group had confluence type distribution close to one described in literature patients in Klatskin tumour group had a significantly different distribution type(p<0.001). Type I was presented in 75% of the patients in control group and in 30% in Klatskin tumour group, other types respectively: II-9% and 24%, IV-14% and 40%, III-0.5% and 1.7%, IV-14% and 40%. Statistical and biophysical models showed: uncommon confluences have a larger area of bile ducts where the tumour may originate (10% of difference) and are under higher pressure that is described in animal models as an oncogenic factor for cholangiocarcinoma.
**Conclusion:** Uncommon hepatic ducts confluence types are significantly more often present in hilar cholagiocarcinoma patients.

**16:00 - 17:30** **Coffee & Talk 2**

### Abdominal Viscera

### SS 401

### Liver and biliary MRI: protocols and technical frontiers

*Moderators:*
C.S. Reiner; Zurich/CH
M. Ronot; Clichy/FR

**K-11** 16:00

**Keynote lecture**

M. Ronot; *Clichy/FR*

**B-0396** 16:09

**T1 mapping for liver function evaluation in gadoxetic acid-enhanced MR imaging: comparison of look-locker inversion recovery and B1 inhomogeneity-corrected variable flip angle method**

J.E. Kim[1], H. Kim[1], D. Choi[1], D. Nickel[2]; [1]*Jinju/KR*, [2]*Erlangen/DE (wldmsrla80@hanmail.net)*

**Purpose:** To compare look-locker inversion recovery (LLIR) and B1 inhomogeneity-corrected variable flip angle T1 mapping methods for estimation of liver function and prediction of hepatic insufficiency and decompensation on gadoxetic acid-enhanced MR imaging.
**Methods and Materials:** In this retrospective study, 248 patients with normal liver function, chronic liver disease, or cirrhosis underwent gadoxetic acid-enhanced liver MR imaging, including T1 mapping at 10 and 20 min hepatobiliary phase (HBP) by using both methods. T1 relaxation times of liver (T1Liver-pre, T1Liver-post) and spleen (T1Spleen) were correlated between two methods. ΔT1Liver ([T1Liver-pre - T1Liver-post]/T1Liver-pre), adjusted T1Liver ([T1 Spleen - T1 Liver-post]/T1Spleen), and functional liver volume-to-weight ratio (liver volume on volumetric T1 map/[T1Liver-post times patient's weight])) were calculated. The diagnostic performance of T1 parameters and predictive performance of models (serum marker, serum marker plus T1 parameter) were compared.
**Results:** T1Liver-post showed a strong correlation (r = 0.93, p < 0.001) between two methods but were significantly different. For depicting cirrhosis, LLIR-adjusted T1Liver at 10 min HBP showed the highest performance (p < 0.025). For predicting hepatic insufficiency and decompensation, LLIR-adjusted T1Liver (Akaike information criterion [AIC], 58.37; C-index, 0.867) and LLIR-T1Liver-post (AIC, 48.82; C-index, 0.885) at 10 min HBP showed the best performance, respectively, when added to serum albumin level.
**Conclusion:** T1Liver-post showed a strong correlation between two methods but with significant differences. T1 mapping using LLIR at 10 min HBP with obtainment of adjusted T1Liver and T1Liver-post may be the best approach for estimation of liver function and prediction of hepatic insufficiency and decompensation.

**B-0397** 16:17
**Dual-arterial phases and split-bolus intravenous contrast injection: reduction of transient respiratory motion artefact on gadoxetate disodium-enhanced MRI of the liver**
E.-S. Cho, J.M. Choi, J.H. Kim, J.-S. Yu, J.-J. Chung; *Seoul/KR (jjondol@yuhs.ac)*

**Purpose:** To determine whether dual arterial phases and split-bolus contrast injection could reduce transient respiratory motion artefact during second arterial phase on gadoxetate disodium-enhanced MRI of the liver.
**Methods and Materials:** One hundred eighty patients underwent gadoxetate disodium-enhanced MRI of the liver with dual hepatic arterial phases. The first arterial phase was performed after injection of 5mL gadoxetate disodium and the additional second arterial phase was performed after injection of 4 mL gadoxetate disodium after 3 minute-delayed transitional phase. Each arterial phase was acquired using a CAIPIRINHA VIBE sequence. The enhancement of focal hepatic lesion on each arterial phase was assessed using subtraction images. The motion artefact on both arterial phases was assessed by two observers using a five-point scale of 1 (no motion) to 5 (non-diagnostic).
**Results:** Nineteen out of 180 patients (10.6%) had severe motion artefact (mean score, 4.4) on the first arterial phase. Fifteen of them had no or mild motion artefact (mean score, 1.3) on the second arterial phase. In these 19 patients, respiratory motion artefact on the second arterial phase was significantly lower than that of the first arterial phase (p<0.05). The enhancement of focal hepatic lesions was not significantly different between both arterial phases (p>0.05).
**Conclusion:** Respiratory motion artefact was significantly reduced on the second arterial phase, compared to the first arterial phase. Dual arterial phases and split-bolus gadoxetate disodium injection technique could help evaluate the arterial enhancement of focal hepatic lesion in patients with transient respiratory motion artefact.

**B-0398** 16:25
**Whole-body R2s mapping to quantify tissue in iron storage organs: associations with HFE-genotype**
J.-P. Kühn[1], T. Kacprowski[2], A. Röhnert[3], V. Hietschold[3], R. Bülow[1], D. Fedders[3], R.-T. Hoffmann[3], M.-L. Kromrey[1]; [1]*Greifswald/DE*, [2]*Munich/DE*, [3]*Dresden/DE (kuehn@uni-greifswald.de)*

**Purpose:** To investigate the relation between HFE genotypes and the phenotype of tissue iron overload using measurement of transverse relaxation rate (R2s) in whole body magnetic resonance imaging (MRI).
**Methods and Materials:** 483 volunteers, 214 women, mean age 59.3±27.7 years underwent MRI including a five-echo gradient-echo sequence for whole body coverage. Whole body R2s maps were calculated and R2s values were determined for liver, spleen, pancreas, heart, and bones. Volunteers were genotyped for HFE mutation. R2s values of iron storage organs were compared between volunteers with at least one and volunteers with none mutation.
**Results:** In 301 subjects without any mutation in HFE genotypes; R2s [mean s-1±standard deviation] were assessed for liver: 33.4±12.7, spleen: 24.1±13.8, pancreas: 27.2±6.6, heart 34.1±14.2 and bone: 69.3±21.0. 182 participants had at least one mutation in HFE gene. Results with cases of heterozygous/homozygous for the types of mutation were for rs74315324: 0/0, rs74315324: 132/9, rs41303501: 0/0, rs1800562: 33/1 and rs1800730: 11/0; respectively. Participants with at least one mutation in the HFE gene did not show any significant difference in R2s values for any of the organs after correction of multiple testing: liver 35.6±11.8, $p_{BH}$=0.09; spleen: 23.2±13.8, $p_{BH}$=0.36; pancreas: 25.6±6.1, $p_{BH}$=0.08; heart: 32.7±11.8, $p_{BH}$=0.36 and bone: 69.2±19.9, $p_{BH}$=0.98.
**Conclusion:** Whole body R2s mapping is an approach to quantify tissue iron in iron storage organs of the whole body. Unspecific mutation of HFE genotype (i.e. at least one mutation in rs74315324, rs1800562, rs1800730) seems not to affect the phenotype of tissue iron overload.

**B-0399** 16:33
**Optimised imaging of the lower abdomen and pelvic region in hepatocyte-specific MRI: evaluation of a whole-abdomen first-pass shuttle protocol**
U. Fehrenbach[1], U.L. Fahlenkamp[1], J. Kahn[1], A. Baur[1], M. Pavel[2], D. Geisel[1], T. Denecke[1]; [1]*Berlin/DE*, [2]*Erlangen/DE (uli.fehrenbach@charite.de)*

**Purpose:** Gadoxetic acid (Gd-EOB) has shown its advantages in hepatic tumour evaluation besides its disadvantages in extrahepatic staging due to its short haemodynamic interval. This results in the dilemma of which contrast agent to choose for whole abdominal MRI. The aim was to evaluate the achievable contrast of a shuttle protocol for Gd-EOB MRI with accelerated 3D-T1w-sequences enabling complete dynamic liver imaging plus first-pass pelvic imaging.
**Methods and Materials:** 74 patients with abdominal neuroendocrine neoplasms were scanned with a protocol including a first-pass pelvic ($pelvic_{FP}$) sequence in Gd-EOB MRI. Acquisition of this $pelvic_{FP}$ sequence was between portal venous and venous liver phase. Pelvic vessel and tissue enhancement were compared to a commonly acquired late/transitional phase sequence. In the same patients, liver enhancement was compared to a standard Gd-EOB protocol (n=55) as well as to vessel contrast in previously acquired MRI with extracellular contrast medium (ECCM) (n=14).
**Results:** Pelvic vessel and lymph node enhancement showed significantly higher signal intensities (SI) in $pelvic_{FP}$ than in late phase sequences with Gd-EOB (p<0.001). Liver enhancement showed no significant differences compared to the standard protocol. Vessel enhancement in Gd-EOB $pelvic_{FP}$ showed significantly higher SI than an equilibrium phase sequence with ECCM (p=0.003 to 0.04).
**Conclusion:** Including a $pelvic_{FP}$ sequence between portal venous and venous liver phases is technically feasible and achieves improved contrast in the pelvis in Gd-EOB MRI. There was no quality loss in liver enhancement compared to a regular Gd-EOB liver examination. Gd-EOB $pelvic_{FP}$ vessel enhancement was not inferior to an equilibrium phase with ECCM.

**B-0400** 16:41
**Should multiparametric MRI with semi-quantitative perfusion replace triphasic CT in follow-up post TACE**
N. Taha, D. Zidan, E. Geneidi; *Cairo/EG (nohataharadio@gmail.com)*

**Purpose:** To evaluate the role of multiparametric MRI and compare it with triphasic CT in follow up post TACE
**Methods and Materials:** Fifty-four patients were included in this study, all the patients were known to have hepatocellular carcinoma for which they underwent transarterial chemoembolization. Follow up was done clinically by measuring alpha-foetoprotein level and radiologically by triphasic CT and multiparametric MRI. Mp-MRI sequences included were T2 and STIR WIs, T1 inphase and outphase WIs, DWI (b value 0, 200, 800), dynamic T1 thrive including early arterial, late arterial, portovenous, venous and delayed phases. Semi-quantitative perfusion images and curves were done. Confirmation of MRI findings was referred to the typical features of residual lesions by MRI criteria , correlation with alpha-foetoprotien level and follow-up.
**Results:** Thirty-two patients showed well-embolized lesion in CT with residual lesion on MRI. Six patients showed well-embolized lesion in CT and MRI, eight patients showed well-embolized lesion in CT and MRI with another HCC, which was not appreciable by CT, six patients showed residual lesion by both CT and MRI studies and two patients showed residual lesion by CT with negative mp-MRI study. Mp-MRI showed 97.96 % sensitivity, 85.71 % specificity, 98% positive predictive value, 84.5 % negative predictive value and 96.4% accuracy compared to 32% sensitivity, 75% specificity, 61% positive predictive value, 11.1 % negative predictive value and 26.4 % accuracy in detecting residual/recurrent and denovo lesions post TACE.
**Conclusion:** Multiparametric MRI should replace triphasic CT in the assessment of post -TACE patients.

**B-0401** 16:49
**Assessment of whole-body fat in magnetic resonance imaging as a new approach in imaging of metabolic disorders**
J.-P. Kühn[1], R. Bülow[1], D. Fedders[2], D. Seppelt[2], R.-T. Hoffmann[2], M.-L. Kromrey[1]; [1]*Greifswald/DE*, [2]*Dresden/DE (kuehn@uni-greifswald.de)*

**Purpose:** To determine whole-body fat volume using confounder corrected chemical shift encoded magnetic resonance imaging (CSI-MRI).

**Methods and Materials:** Four hundred sixty-seven volunteers aged 36 to 87 years (260 women) underwent a whole-body MRI including five-echo chemical shift encoded sequences acquired in 5 steps. After confounder correction, whole-body proton-density fat fraction was acquired. Based on tissue fat content, we developed a post-processing algorithm for segmentation of fat compartments. Using this algorithm, total body fat volume (TAT), subcutaneous fat volume (SAT) and visceral fat volume (VAT) were assessed. Robustness of the algorithm was tested by evaluation of inter- and intra-observer reliability (n=73 participants) by two observers using a Bland-Altman analysis. Further, TAT, SAT, and VAT were correlated with BMI, gender, and age.
**Results:** Intra-observer reliability (mean bias (%) ± standard deviation; TAT/SAT/FAT: -0.1±0.3 / 0.4±1.0 / 0.2±0.4) and inter-observer reliability (TAT/SAT/FAT: 0.0±0.3 / 0.9±1.4 / 0.4±0.8) was small. TAT, VAT and SAT increased significantly with rising body BMI, p≤0.001. Although SAT and VAT demonstrated significant gender-related differences (p=0.001), no significant differences were seen for TAT (p=0.21).
**Conclusion:** CSI-MRI is an approach to assess whole-body fat volume and fat compartments. TAT, SAT, VAT are associated in changes in BMI. The technique may offer the possibility of advanced imaging in metabolic disorders.

**B-0402** 16:57
**The feasibility of diffusion-weighted magnetic resonance imaging of the liver using multiplexed sensitivity encoding**
Y. Kim, M.-J. Kim, N. Seo; *Seoul/KR (cookieks35@gmail.com)*

**Purpose:** To assess the feasibility of multiplexed sensitivity-encoding (MUSE) technique for high-resolution diffusion-weighted magnetic resonance imaging (DWI) of the liver.
**Methods and Materials:** Thirty-eight patients with 31 lesions who underwent liver MRI with conventional DWI and respiratory-triggered MUSE-DWI at a 3.0-T system for the investigation of suspected hepatic pathology or oncological follow-up were included. Qualitative parameters were independently evaluated by two radiologists blinded to the protocol, and quantitative parameters were calculated based on region of interest measurements. Per-patient parameters were compared between the two sequences using Wilcoxon signed-rank test and paired t test, and per-lesion parameters were compared by linear mixed model.
**Results:** Both reviewers found better overall image quality, sharper liver contour, reduced cardiac motion and susceptibility artefacts, and improved lesion conspicuity in MUSE-DWI (reader average $P < 0.05$). However, ghosting by gallbladder, artefact by pericardial fat, and chemical shift artefact reduced the image quality (reader average $P < 0.01$) in MUSE-DWI. Lesion characterization results were comparable in both sequences. Signal-to-noise ratio (SNR) of the liver was significantly higher (mean, 9.62 vs. 7.37; $P < 0.001$), while lesion to non-lesion contrast-to-noise ratio (CNR) was lower (least square means, 21.68 vs. 28.79; $P = 0.023$) in MUSE-DWI.
**Conclusion:** MUSE-DWI can provide better image quality in terms of distortions, comparable image quality for lesion characterization, and improved SNR of the liver. However, some artefacts can interfere with image interpretation in MUSE-DWI, and CNR was lower than conventional DWI.

**B-0403** 17:05
**Evaluation of liver MRI examinations with two dosages of gadobenate dimeglumine: a retrospective blinded intra-individual study**
M. Kamali, S.E. Clarke, A.F. Costa; *Halifax, NS/CA (mh870250@dal.ca)*

**Purpose:** There is discrepancy in the literature regarding the optimal dose of gadobenate dimeglumine for liver MRI. This study evaluated the quality of liver MRIs performed in the same individual using two dosages.
**Methods and Materials:** This retrospective intra-individual study was performed with ethics approval. Patients who underwent liver MRIs between Sept 2015-May 2017 (0.06 mmol/kg) and May 2017-September 2018 (0.10 mmol/kg) were included. Regions of interest were drawn over the aorta, portal veins and liver on unenhanced, arterial, portal venous and delayed phases; relative enhancement values were compared (paired t-tests). Two blinded radiologists graded the arterial and portal venous sequences of each MRI from 1-4 (1=suboptimal, 2=adequate, 3=good, 4=excellent); grades were compared (Wilcoxon's signed rank test). Blinded radiologists also graded each MRI pair from 1-5 (1=substantially inferior, 2=slightly inferior, 3=equivalent, 4=slightly improved, 5=substantially improved). Inter-reader agreement was assessed (kappa statistic).
**Results:** 60 patients were included. Relative enhancement increased significantly with the higher dose for all structures on all phases (p<0.05). Grades 1-4 were assigned to 18,72,65 and 81 low-dose sequences, and 17,57,68 and 94 high-dose sequences, respectively; for both radiologists and both post-contrast phases, grading was better for the high-dose MRIs (p<0.05). Inter-observer agreement was fair (K range, 0.22-0.37). Compared to the low-dose MRIs, the number of high-dose MRIs graded 1-5 were 9,31,97,90 and 11; inter-observer agreement was moderate-fair (K=0.48 and 0.36 for arterial and portal venous phases, respectively).
**Conclusion:** A higher dose of gadobenate dimeglumine results in increased relative enhancement and improved qualitative assessment of imaging quality for liver MRI.
*Author Disclosures:*
**S.E. Clarke:** Research/Grant Support; Dr. Sharon Clarke has a research agreement with GE Healthcare, however this did not impact our study in any way.

**B-0404** 17:13
**Clinical evaluation of 3D magnetic resonance cholangiopancreatography using three different methods: feasibility of compressed sensing and/or parallel imaging**
M.-L. Kromrey[1], S. Funayama[2], D. Tamada[2], S. Ichikawa[2], H. Onishi[2], U. Motosugi[2]; [1]*Greifswald/DE*, [2]*Chuo/JP*
*(marie-luise.kromrey@uni-greifswald.de)*

**Purpose:** To evaluate the diagnostic quality of 3D MRCP acquired with respiratory triggering compared to breath-hold techniques using compressed sensing (CS) and parallel imaging (PI) in a clinical setting.
**Methods and Materials:** In 93 patients (45 men, mean age 69.7±9.3 years) 3 types of 3D MRCP were performed: breath-hold MRCP with CS and PI reconstruction (BH-CS-MRCP) and PI reconstruction only (BH-PI-MRCP) additionally to respiratory triggered MRCP with navigator echoes (Nav-MRCP) on a 3T scanner. Visualization of common bile duct (CBD), common hepatic duct (CHD), central right and left hepatic duct (cRHD, cLHD), peripheral right and left hepatic duct (pRHD, pLHD), cystic and pancreatic duct and overall image quality was rated on a four-point scale. For quantitative analysis, relative duct-to-periductal contrast (RC) of three main biliary segments was calculated.
**Results:** Acquisition time was accelerated in breath-hold MRCP (23s each vs 1min 29s in Nav-MRCP). Mean grading (Nav/CS/PI) for CBD (2.74/2.87/2.94), CHD (2.82/2.92/3.00), cRHD (2.75/2.85/2.98), cLHD (2.75/2.85/2.92) and cystic duct (2.22/2.34/2.42) was higher in BH-CS- as well as BH-PI-MRCP, whereas Nav-MRCP showed higher grading in the peripheral segments (pRHD: 2.24/2.01/2.12; pLHD: 2.23/2.02/2.13). Overall image quality of Nav-MRCP (2.91±0.7) was not different from BH-PI-MRCP (2.92±0.6) (p=0.163), but higher than that of BH-CS-MRCP (2.80±0.7) (p=0.031). RC for CS-MRCP and PI-MRCP were lower than for Nav-MRCP.
**Conclusion:** Breath-hold 3D MRCP was feasible using PI and CS showing comparable overall image quality and visualization of the greater duct system to Nav-MRCP. The methods are suitable for revised MRI protocols notably in patients with irregular respiratory cycle.

**B-0405** 17:21
**Histogram analysis of stretched-exponential model diffusion-weighted imaging for staging of liver fibrosis: a feasibility study**
F. Hu, B. Song, R. Yang, M. Wang; *Chengdu/CN*
*(yingxianghu_cmc@163.com)*

**Purpose:** To determine the feasibility of stretched-exponential model diffusion-weighted imaging based on histogram analysis for staging of liver fibrosis in patients with chronic liver diseases, with histopathologic findings as the reference standard.
**Methods and Materials:** 56 consecutive patients with chronic liver diseases underwent liver DWI using nine b values at 3.0T. Histogram metrics including mean, standard deviation, skewness, kurtosis, minimum, maximum, range, interquartile (IQ), and percentiles (10, 25, 50, 75, 90th) were extracted from apparent diffusion coefficient (ADC), distributed diffusion coefficient (DDC) and intravoxel heterogeneity index (α) maps. Liver fibrosis and inflammation were evaluated using the METAVIR scoring system. The relationship between histogram metrics and histopathological features was evaluated by Spearman correlation and multiple regression analysis. Receiver operating characteristic curve analyses were further performed for staging liver fibrosis.
**Results:** At multiple regression analysis, liver fibrosis independently influenced with histogram metrics of DDC (mean, $50^{th}$ and $75^{th}$ percentiles; all <0.001) and α ($50^{th}$ percentile and skewness ). Inflammation was not associated with histogram metrics. The mean, $50^{th}$ and $75^{th}$ percentiles of DDC and $50^{th}$ percentile, skewness of α provided good to excellent diagnostic performance for identifying significant fibrosis (F2 or higher) and for diagnosing cirrhosis (F4), with AUCs ranging from 0.752 to 0.936 (all P <0.001). For identifying significant fibrosis, the highest AUC was 0.885 for skewness of α; for diagnosing cirrhosis, the highest AUC was 0.936 for $75^{th}$ percentiles of DDC.
**Conclusion:** Stretched-exponential model DWI based on histogram analysis can be used to stage liver fibrosis and provides more quantitative imaging biomarkers.

16:00 - 17:30 Room K

### Radiographers

### SS 414

### MRI advances

*Moderators:*
I.M. Björkman-Burtscher; Gothenburg/SE
C. Malamateniou; London/UK

**B-0406** 16:00
**Quantitative tissue-tracking and T1 mapping cardiac magnetic resonance (CMR) in active acromegaly**
P. Liu, Y. Wang, J. Cao, L. Lin; *Beijing/CN (liupeijun_yu@163.com)*

**Purpose:** To evaluate myocardial strain and extracellular volume of left ventricular (LV) in patients with active acromegaly by 3T Cardiac Magnetic Resonance.
**Methods and Materials:** 23 patients (mean age 44±16 years, 9 males) with active acromegaly and 15 healthy controls (mean age 44±13 years, 7 males) were included.All participants underwent a 3.0T (MAGNETOM Skyra, Erlangen, Germany) CMR examination including native and 15-20min post-contrast T1 mapping imaging in identical short axis 2-chamber slices of basal, middle and apical LV level. Feature-tracking analysis included assessment of peak strain of the short-axis slice in longitudinal, circumferential and radial direction of left ventricle (LV).Native T1 value, ECV and strain in of basal, mid and apex slice, left ventricular mass index (LVMI) and left ventricular ejection fraction (LVEF) were measured via CVI42 software (Circle Cardiovascular Imaging, Canada).
**Results:** In acromegaly group, LVMI (60 ±26.1vs.47.5± 6.9 g/m2, P< 0.05) and ECV in basal slice (30.3 ±7.2vs.24.7± 6.9, P<0.05) increased significantly, while longitudinal strain of apex slice (-19.17±5.01vs. -22.20±2.37, P<0.05) decreased compared to control group. Average per-slice Native T1 and ECV in mid and apex slice, circumferential and radial strain in basal, mid and apex slice showed no significant differences between acromegaly and healthy controls.
**Conclusion:** Compared to healthy controls, acromegaly patients had increased LVMI, increased ECV in basal slice and decreased longitudinal strain of apex slice by CMR, which might indicate heart abnormity in early periods with normal LV systolic function.

**B-0407** 16:08
**The assessment of carotid plaque morphology using magnetic resonance imaging and computed tomography as biomarkers of carotid plaque vulnerability**
N. Giannotti, P. Kelly, S. Foley, G. Horgan, E. Kavanagh, M.J. O'Connell, J. McNulty; *Dublin/IE (nicola.giannotti@ucd.ie)*

**Purpose:** Looking beyond the degree of carotid stenosis and exploring plaque morphology based on the lipid-rich necrotic core (LRNC), fibrous tissue, and calcification, is an emerging approach for the assessment of plaque vulnerability/risk of future ischaemic cerebrovascular events. Validation studies are necessary to establish the utility of different imaging techniques to support clinical decision making. This study explored the correlation between plaque morphology assessment from computed tomography angiography (CTA) and magnetic resonance imaging (MRI) datasets.
**Methods and Materials:** A sub-group of patients (n=22) from a larger cohort study were included. Inclusion criteria were speech/motor transient ischaemic attack or stroke (Rankin≤3) <72hours; ipsilateral carotid stenosis ≥50%; age≥50 years; carotid MRI and CTA performed. Semi-automated measurements of plaque morphological features were performed using TeraRecon iNtuition™ and MRI-Plaque View2.1. The Wilcoxon signed-rank, Spearman's correlation, and Bland-Altman plots were used to test for statistical differences between CT and MRI, the level of agreement, and for proportional bias, respectively.
**Results:** Results showed a significant agreement in LRNC measurements between CT and MRI (p<0.001). Spearman's test confirmed excellent agreement for calcified area between the two imaging modalities (p<0.001). The absence of proportional bias was confirmed with Bland-Altman plot for lipids and calcium, p=0.54 and p=0.33 respectively. No significant differences were found between CTA and HRMRI measurements of other morphological plaque features.
**Conclusion:** Results of this study found that CT and MRI plaque measurements demonstrate significant agreement for LRNC and calcification. Therefore, if confirmed in larger studies, either modality may be recommended for such analysis.
*Author Disclosures:*
**N. Giannotti:** Research/Grant Support; Health Research Board (IE) CSA/201227, IIRRT Student Research Grant 2015.

**B-0408** 16:16
**Clinical impact of combined compressed sensing and sensitivity encoding on three-dimensional magnetic resonance cholangiopancreatography at 1.5 T**
D. Morimoto, T. Hyodo, M. Itou, H. Fukushima, K. Kamata, M. Takenaka, M. Kudo, K. Miyagoshi, K. Ishii; *Ōsakasayama/JP (morimoto-d@med.kindai.ac.jp)*

**Purpose:** To assess the utility of combined compressed sensing and sensitivity encoding (CS-SENSE) applied to three-dimensional (3D) magnetic resonance cholangiopancreatography (MRCP) at 1.5 T.
**Methods and Materials:** This prospective study was approved by our institutional review board, and all patients provided written informed consent. Fifty-one patients (23 men and 28 women; mean [±SD] age, 68±13 years) underwent 3D navigator triggered MRCP with SENSE alone ($MRCP_{sense}$) and CS-SENSE ($MRCP_{csnt}$), and 3D breath-hold MRCP with CS-SENSE ($MRCP_{csbh}$) at 1.5 T. The acquisition time and relative duct-to-periductal contrast ratios (RCs) of the right and left hepatic ducts, and common bile duct, respectively, were quantitatively obtained for each of the three MRCP methods. The visualisation of bile and pancreatic duct; background suppression; artifacts; and overall image quality were evaluated on 5-point scale. The Friedman test was used to compare the results of $MRCP_{sense}$, $MRCP_{csnt}$, and $MRCP_{csbh}$. A *p*-value < .05 was considered statistically significant.
**Results:** Mean acquisition time of $MRCP_{sense}$, $MRCP_{csnt}$, and $MRCP_{csbh}$ was 348, 143, and 18 seconds, respectively (*p*<.001 in all comparisons). The RCs of $MRCP_{csnt}$ and $MRCP_{csbh}$ were lower than $MRCP_{sense}$ (*p*<.001 for both). All qualitative scores were not significantly different between $MRCP_{sense}$ and $MRCP_{csnt}$. The visualisation scores of bile ducts in $MRCP_{csbh}$ were inferior compared with those of $MRCP_{sense}$ and $MRCP_{csrt}$ (*p*<.05), while those of pancreatic duct were not significantly different compared with $MRCP_{csnt}$.
**Conclusion:** $MRCP_{csnt}$ provides comparable image quality to $MRCP_{sense}$ in about half the acquisition time. $MRCP_{csbh}$ could be a supplemental technique for pancreatic duct.

**B-0409** 16:24
**Quantitative diffusion-weighted knee MR imaging and ADC mapping in healthy volunteers**
A. Alqattan, A. McGee, E. Delahunt, S. Eustace, J. McNulty, A. Cradock; *Dublin/IE (abrar.alqattan@ucdconnect.ie)*

**Purpose:** This healthy volunteer study forms part of a larger clinical study exploring knee bone bruising at sites associated with anterior cruciate ligament(ACL) injury using diffusion-weighted imaging(DWI). The aim was to analyse diffusion characteristics of normal cancellous bone in the mid-lateral femoral condyle and posterolateral tibial plateau.
**Methods and Materials:** Fourteen healthy subjects (18-35 years) without knee pain or trauma were scanned using the routine knee MRI protocol and an optimised axial-DWI sequence with multiple b-values. Thirty circular, $50mm^2$, regions-of-interest (ROIs) were positioned for each case and included locations associated with ACL-related bone bruising (anterior, medial, and lateral ROIs in the medial and lateral femoral condyles (MFC, LFC), medial and lateral tibial condyles (MTC; LTC). ADC values were calculated for each ROI using b-values of 50 and $800s/mm^2$.
**Results:** Mean ADC values for the ROIs in the femoral condyles ranged from $0.318x10^{-3}mm^2/sec$ ($SD=0.117x10^{-3}$) to $0.375x10^{-3}mm^2/sec$ ($SD=0.135x10^{-3}$) (posterior and medial ROIs in LFC respectively). For ROIs in the tibial condyles, the range was $0.233x10^{-3}mm^2/sec$ ($SD=0.072x10^{-3}$) to $0.434x10^{-3}mm^2/sec$ ($SD=0.147x10^{-3}$) (anterior and posterior ROIs in LTC respectively). Overall, ADC values for the distal femur were $0.311x10^{-3}mm^2/sec$ ($SD=0.109x10^{-3}$) and $0.288x10^{-3}mm^2/sec$ ($SD=0.129x10^{-3}$) for the proximal tibia.
**Conclusion:** Measurement of ADC values, in association with DWI sequences, can be used to identify ADC ranges associated with normal/ healthy cancellous bone. These ADC values may then be compared to ADC values in subjects with ACL-related bone bruising in the distal femur and proximal tibia, which may provide additional insights into understanding injury severity and patient recovery.

**B-0410** 16:32
**Evaluation of hippocampus volume in the elderly people by magnetic resonance imaging: comparison of two segmentation methods**
P.A.T. Gamboa[1], M.C. Ribeiro[2], A.P.L. Macedo[2]; [1]*Vila Franca de Xira/PT*, [2]*Lisbon/PT (patricia24.gamboa@hotmail.com)*

**Purpose:** The total volume of the hippocampus has been linked to cognitive function and to the risk of dementia. However, being a complex structure, the role of sub-regions of cognition and the risk of dementia remain unexplored. With the age increasing there is a significant loss of neurons in the encephalic mass, in general, and particularly in the hippocampus. The segmentation of images is one of the most important tasks in the evaluation of this change.

**Methods and Materials:** The aim of this study was to measure and compare the volume of hippocampus Left (HL) and Right (HR) in 100 elderly subjects (≥65 years old). The images obtained retrospectively by MRI T1W, were followed by post-processing through FreeSurfer® (non-operator dependent) and through OsiriX® (operator dependent).
**Results:** The values obtained by FreeSurfer® are higher than OsiriX® (LH=3995,850 mm3 and RH=3501.850mm3) respectively. The lowest value obtained in this study is assessed trough OsiriX® in the RH corresponding to a 3481,200mm3. The difference between the LH and RH is higher with any software, corresponding to 470,350mm3 and 308,100mm3 respectively, however, trough Wilcoxon test these differences aren't significant (p>0,05). In both systems, between genders, the males obtained a highest values (average=4000mm3) than the females(3500mm3).
**Conclusion:** The obtained values with manual procedures are in agreement to the other identical studies concerning anatomy, measures and software used.The results show that the software used can produces different results. Even having higher time consuming, the manual methods are yet considered the more reliable and preferred by the physicians.

**B-0411** 16:40
**Prediction of type 2 diabetes mellitus using noninvasive MRI quantitation of visceral abdominal adiposity tissue volume**
M. Wang, S.-T. Feng, Z.P. Li; *Guangzhou/CN (king_6509@sina.com)*

**Purpose:** To investigate the quantitation of visceral adipose tissue volume (VATV) in patients with type 2 diabetes mellitus (T2DM), impaired glucose tolerance (IGT) and normal glucose tolerance (NGT) using MRI to assess the role of non-invasive imaging in predicting the presence of T2DM.
**Methods and Materials:** Forty-eight patients were included and scanned with iterative decomposition of water and fat with echo asymmetry and least square estimation-image quantification (IDEAL-IQ) sequence. VATV was obtained at the level of second and third lumbar vertebra (VATV L2, VATV L3) and the sum (total VATV) was computed. Proton-density fat fraction (PDFF) of pancreas and liver was measured. ROC curve and binary logistic regression model analysis were employed to evaluate their ability in predicting the presence of T2DM.
**Results:** The values of VATV L2, VATV L3 and total VATV of the T2MD were higher than those of the IGT and NGT. There was no difference in the values of VATV L2, VATV L3, and total VATV between IGT and NGT. The ROC curve analysis showed the areas under curve for VATV L2, VATV L3 and total VATV were 0.76, 0.80 and 0.80 in predicting the presence of T2DM, failed to predict the presence of IGT and NGT. Binary logistic regression model analysis revealed that only VATV L3 was independently associated with incident of T2DM and the sensitivity, specificity and accuracy were 80.00%, 88.20%, and 84.40%, respectively.
**Conclusion:** Compared to hepatic PDFF, pancreatic PDFF, VAVT L2 and total VATV, VAVT L3 was a better predictor for T2DM.

**B-0412** 16:48
**Comparison of MRI acoustic noise levels in different types of sequences**
V.M.F. Silva, I. Ramos, J. Moreira, M. Marques; *Porto/PT (vitorsoft@gmail.com)*

**Purpose:** This study aimed to evaluate acoustic noise levels (equivalent continuous sound level - dB(A) and peak sound pressure level - dB(C)) of different sequences used in a 1.5T and a 3T scanner.
**Methods and Materials:** This study was performed in MRI Department of Centro Hospitalar São João, Oporto, Portugal.A Bruel & Kaejer sound level meter, model 2250, was used. The sequences used on this study were: spin-echo (SE), gradient-echo (GE), inversion-recovery (IR) and echo planar (EPI). 2D and 3 D sequences were used also. These sequences were performed in brain, spine, abdominal and knee MRI.
**Results:** In 1.5T scanner, values of all types of sequences reached to higher values when compared to 3T scanner.It was found statistically significant differences (p<0.05) in: a) the peak sound pressure level between SE and EPI sequences; b) the two acoustic noise parameters evaluated between IR and EPI sequences. Statistically significant differences were found also when comparing 2D and 3D sequences for both parameters evaluated.
**Conclusion:** This study showed that patients and sometimes workers are exposed to relatively high acoustic noise levels of acoustic during MRI procedures. However, those levels do not exceed exposure the limit values, but several of them are near the limits imposed in Portuguese legislation. Radiographers have to take into account safety decisions related to MRI acoustic noise, when it concerns to optimization of sequences' acquisition time (acoustic noise exposure), specially those that almost reach the values imposed in legislation, such as EPI and 3D sequences.

**B-0413** 16:56
**Investigating the impact of fragrance on patient anxiety during MRI scans**
A. Formosa, C. Portanier Mifsud, J.L. Portelli, P. Demicoli; *Msida/MT (altheaformosa3@gmail.com)*

**Purpose:** Despite its benefits, magnetic resonance imaging (MRI) may induce anxiety in some patients. This study aimed to investigate whether patient anxiety can be reduced using lavender fragrance during brain/IAMs MRI scans.
**Methods and Materials:** This experimental study adopted a randomised control-group pretest-posttest design. Patients referred for a brain/IAMs MRI were randomly appointed into one of six scanning sessions forming part of this study. These sessions, consisting of 10 patients each, were then randomly assigned as being control or experimental. Convenience sampling was used to recruit the first 20 willing participants for each group of the study. All patients were scanned in accordance with routine MRI protocols; however, a lavender fragrance was additionally released during the 'experimental' sessions. Each participant was asked to complete the State-Trait Anxiety Inventory (STAI) before and after their MRI scan.
**Results:** The paired samples T test showed a statistically significant decrease between mean pre-test and post-test state anxiety scores in patients of both groups. The mean state anxiety score for control-group patients decreased from 49.9 to 45.1 (p=0.044), whereas that for experimental-group patients decreased from 38.4 to 31.4 (p=0.018). While sample size may have influenced findings, the greater reduction in mean anxiety score (7.0) observed for experimental-group patients was not found to be statistically significant when using the independent samples T test (p=0.534).
**Conclusion:** While fragrance-infused MRI sessions may help decrease anxiety levels in patients, the small sample size of this study necessitates further investigation into how fragrance-infused sessions may impact patients' experience.

**B-0414** 17:04
**Accessibility for patients with cardiac device implantable electronic to magnetic resonance exams: a national survey**
D.M.L. Miranda[1], B. Mateus[2], C.A. da Silva[3], R.P. Almeida[4], A.P. Ribeiro[5], C.T. Brites[6], A.F. Abrantes[4], L. Ribeiro[2]; [1]*Jeddah/SA*, [2]*Amadora/PT*, [3]*Evora/PT*, [4]*Faro/PT*, [5]*Miraflores/PT*, [6]*Carcavelos/PT (duchamiranda@gmail.com)*

**Purpose:** Portuguese population with cardiovascular implantable electronic devices (CIEDs) increased in the last years. According to with Portuguese national registry on cardiac electrophysiology, in 2014, were implanted 1256 (one hundred and ten per million) cardioverter-defibrillator in registered centers. The accessibility, for this patient's, to any type of magnetic resonance (MR) study is an issue for MR major centres. This survey allows us to understand the difficulties from MR centres to satisfy this patient's needs.
**Methods and Materials:** An online survey was sent to radiology or/and cardiology departments from national centres with MR, to determine the accessibility for patients with CIEDs (pacemakers and ICD). A twenty questions survey of closed response questions plus some limited free text answers was used.
**Results:** Departments are aware of MR conditional devices, but only a small percentage offers MR scans to patients with CIEDs. The major issue for all centres is the lack of support from cardiology/electrophysiology and safety evidence. The exams average in this centres is 1 to 10 scans with CIEDs per year and (90%) of them are performed in 1.5T equipment.
**Conclusion:** With the increase of CIEDs, the accessibility to a MR decrease drastically for this patient group. This survey shows that in some Portugal regions it is impossible to perform a MR. The patient needs to drive more than 200km to do it. However, the majority of MR centres mentioned that training with applied practice, guidelines and closer collaboration with local cardiologist might increase the confidence of the professionals to perform those exams.

**B-0415** 17:12
**OLEA/NOVA+ SyntenticMRI application on workflow in pelvis MRI studies**
A. Attanasio, G. Corrias, L. Saba; *Cagliari/IT (tonyattan@gmail.com)*

**Purpose:** The MRI acquisition protocol in standard practice for rectal cancer has an approximate duration of 30 minutes. The purpose of this study is to define a radiographer workflow in order to substitute standard protocol with syntetic MRI (sMRI) and to see if there are any differences in terms of time savings.
**Methods and Materials:** We examined 20 patients with double bolus of Hyoscine-butylbromide injection using an MRI scan with a field of 3 Tesla. The sequences used for our purpose are [T2]-multi-TE-FSE2D and [T1]-MP2RAGE-(3D), the images obtained have been reconstructed on the dedicated workstation with post processing commercial software (Olea Sphere 3.0, Olea Medical, Paris) in order to obtain T1, T2 and fat saturated images.

**Results:** Adding synthetic MRI sequences, would increase the duration of the protocol of 6:53 minutes. The post processing phase showed that 36 seconds are needed to view T1 & T2 maps and 60 seconds are needed to generate T1W, T2W, STIR images. All the images generated would be ready on PACS after 1:58 minutes. However, substituting standard T1, T2, STIR sequences with a synthetic MRI protocol would allow for a shortening of the total acquisition time down to 24 minutes of acquisition time.
**Conclusion:** sMRI reduces the acquisition time of pelvic region MRI of 6 minutes. Another study is currently in progress to understand if these sequences are clinically equivalent to their standard counterparts.

**B-0416** 17:20
**MRI principles and technical issues: where do European radiographers study from?**
M. Zanardo[1], F.M. Doniselli[1], C.B. Monti[1], S. Durante[2], L.M. Sconfienza[1], F. Sardanelli[1]; [1]*Milan/IT*, [2]*Bologna/IT (moreno.zanardo@unimi.it)*

**Purpose:** To analyse where and how MRI principles and technical issues are investigated by radiographers and radiographers in training (RGt).
**Methods and Materials:** We proposed an online survey with 8 multiple-choice questions. We investigated how often radiographers have doubts about MRI principles or technical issues, where and what kind of information they search for, in which language and on which websites. $\chi^2$ test was used.
**Results:** We obtained 300 answers (228 radiographers, 72 RGt, from 9 European countries). Radiographers and RGt mostly search for information both in English and in their native language (66% vs 72%, respectively), with no statistically significant difference (*p*=0.590). No differences were also found in frequency of consultation: radiographers and RGt frequently or often (64% vs 48%, *p*=0.485) have doubts about MRI principles. Post-processing details are equally searched for by radiographers and RGt (23% vs 13%, *p*=0.249) as well as advanced/new techniques (54% vs 50%, *p*=0.713). Clinical protocol settings are borderline significantly more searched by RGt than radiographers (64% vs 44%, *p*=0.054) as well as basic sequences details (36% vs 22%, *p*=0.088). Regarding source of information, radiographers and RGt mostly use internet (74% vs 81%, *p*=0.404) and books (46% vs 56%, *p*=0.343). Radiographers and RGt equally refer to mriquestions.com (43% vs 53%, *p*=0.303) for both technical and clinical issues, radiopaedia.org, (36% vs 47%, *p*=0.227), and mrimaster.com (25% and 14% *p*=0.149).
**Conclusion:** Radiographers and RGt frequently search for information about MRI principles and technical issues with slight differences regarding web sources, clinical protocol settings and MRI sequences.

**16:00 - 17:30** **Sky High Stage**

### Chest

### MY 4

### Chest

*Moderators:*
S. Toledano-Massiah; Paris/FR
M.O. Wielpütz; Heidelberg/DE

**B-0417** 16:00
**Comparison of therapeutic outcome prediction among dynamic perfusion area-detector CT, dynamic perfusion MRI and FDG-PET/CT in small cell lung cancer patients with limited disease**
Y. Ohno[1], Y. Fujisawa[2], M. Yui[2], Y. Kishida[1], S. Seki[1], T. Yoshikawa[1], T. Murakami[1]; [1]*Kobe/JP*, [2]*Otawara/JP (yosirad@kobe-u.ac.jp)*

**Purpose:** To directly compare the capability for therapeutic outcome prediction among dynamic contrast-enhanced (CE)-perfusion area-detector CT (ADCT) and CE-perfusion MR imaging (MRI) and FDG-PET/CT in small cell lung cancer (SCLC) patients evaluated as limited disease (LD).
**Methods and Materials:** Forty-three consecutive pathologically diagnosed SCLC patients assessed as LD underwent PET/CT, dynamic CE-perfusion ADCT and MRI, chemoradiotherapy, and follow-up examination. In each patient, therapeutic outcomes were assessed as therapeutic effect, disease-free interval and overall survival. Then all patients were divided into two groups as follows: 1) responder (CR+PR: n=33) and 2) non-responder (SD+PD: n=10) groups. In each patient, total perfusion (TP) and tumour perfusions from pulmonary (TPp) and systemic (TPs) circulations calculated by dual-input maximum slope method from dynamic ADCT and MRI data and $SUV_{max}$ on PET/CT were assessed at targeted lesions. Then final values were determined as average values from all targeted lesion. To compare the capability for distinguishing two groups, all indexes as having significant difference were assessed by ROC analysis. Finally, disease-free and overall survivals between responders and non-responders assessed by each index were compared by Kaplan-Meier method followed by log-rank test.

**Results:** Area under the curves (Azs) of TPs (ADCT: Az=0.92, MRI: Az=0.92) were significantly larger than that of SUVmax (Az=0.73, p<0.05). Disease-free survivals of responder were significantly longer than that of non-responder by TPs on ADCT (p<0.05) and MRI (p<0.05).
**Conclusion:** Dynamic CE-perfusion ADCT and MRI have better potential for predicting therapeutic outcome than PET/CT in SCLC patients with limited disease.
*Author Disclosures:*
**Y. Ohno:** Research/Grant Support; Canon Medical Systems Corporation.
**Y. Fujisawa:** Employee; Canon Medical Systems Corporation. **M. Yui:** Employee; Canon Medical Systems Corporation. **S. Seki:** Research/Grant Support; Canon Medical Systems Corporation. **T. Yoshikawa:** Research/Grant Support; Canon Medical Systems Corporation.

**B-0418** 16:04
**Correlation between pulmonary function tests, histogram-based analysis indexes and visual HRCT scores in a population of IPF patients**
F. Tiralongo[1], M. Palermo[1], D. Falsaperla[1], A. Stefano[2], S.E. Torrisi[1], P. Foti[1], A. Basile[1], C. Vancheri[1], S. Palmucci[1]; [1]*Catania/IT*, [2]*Segrate/IT (tiralongofrancesco91@hotmail.it)*

**Purpose:** To investigate relationship between visual HRCT score and Pulmonary Function Tests (PFTs), and between histogram-based HRCT analysis and PFTs.
**Methods and Materials:** 33 IPF patients having two HRCT (baseline and follow-up) and two PFTs - collected within three months from HRCT - were retrospectively investigated. A HRCT visual score was calculated quantifying the overall extent (percentage) of normal attenuation, reticulations, honeycombing and traction bronchiectasis measured at three HRCT levels. Histogram-based analysis was performed providing HRCT indexes - represented by kurtosis, skewness, High Attenuation Areas (HAA) and Fibrotic Areas (FA). Strength of association between HRCT visual scores and PFTs values, and between HRCT indexes and PFTs values was investigated using Pearson correlation; multiple linear regression model was performed to investigate variance between HRCT indexes and PFTs values, and between the latter and HRCT visual scores.
**Results:** Fair correlation between HRCT fibrosis score and FVC was found (r=-0.2212); moderate negative correlation was observed between average HRCT fibrosis score and DLCO (r =-0.58). Correlation between HRCT indexes and PFTs was stronger than visual assessment: coefficient r were 0.5762 between kurtosis and FVC, 0.5268 between kurtosis and DLCO, -0.5477 between HAA and FVC, -0.4186 between HAA and DLCO, -0.5035 between FVC and FA, and -0.3836 between DLCO and FA. Multiple regression analysis for FVC, DLCO and HRCT fibrosis score revealed a coefficient of determination $R^2$ equal to 0.3456; $R^2$-adjusted reported a value of 0.30.
**Conclusion:** Histogram-based indexes provided mild-to-moderate correlation with PFTs - whereas visual HRCT assessment reported lower and controversial relationships.

**B-0419** 16:08
**Radiomics machine learning classifiers for predicting Ki-67 proliferation index in non-small cell lung cancer**
P. Rong, Q. Gu, Z. Feng; *Changsha/CN (729570216@qq.com)*

**Purpose:** To develop radiomics classifiers for predicting the expression level of Ki-67 in non-small cell lung cancer (NSCLC) noninvasively and to evaluate the increment compared with the subjective imaging feature classifiers.
**Methods and Materials:** Non-enhanced CT images and tumour Ki-67 expression levels were acquired from 245 patients. A lesion volume of interest (VOI) was manually delineated and radiomics features were extracted by MaZda software from original CT images. A random forest feature selection algorithm (RFFS) was used to reduce features. Six kinds of machine learning methods were used to establish predictive classifiers including radiomics classifiers, subjective imaging feature classifiers and the combined classifiers. The performance of these classifiers was evaluated by the average area under the curve (AUC), and the performance between different classifiers was compared with the Delong test.
**Results:** Among the 245 patients, 117 had high Ki-67 expression, and 128 had low Ki-67 expression, respectively. The differences in gender, age and pathological type between the two groups were statistically significant (all P < 0.05). The radiomics random forest classifier achieved the best performance in predicting the Ki-67 expression level (AUC=0.776, SEN=0.726, SPE=0.661); this classifier performed better than the subjective imaging feature classifiers (AUC=0.625, SEN=0.780, SPE=0.417; P<0.05). The combined classifiers did not improve the predictive performance (AUC=0.780, SEN=0.752, SPE=0.633; P>0.05).
**Conclusion:** The radiomics machine learning classifier based on CT radiomics features is helpful to predict the expression level of Ki-67 in patients with NSCLC and can provide a non-invasive technique for assessing the proliferation of NSCLC.

**B-0420** 16:12
**Quantitative perfusion MRI of the lung in COPD: the problem of short-term repeatability**
A. Ter-Karapetyan[1], M.O. Wielpütz[2], H.-U. Kauczor[2], S. Triphan[2];
[1]*Yerevan/AM*, [2]*Heidelberg/DE (alvardterkarapetyan@gmail.com)*

**Purpose:** 4D perfusion magnetic resonance imaging (MRI) with intravenous injection of contrast material allows for a radiation-free assessment of regional lung function. It is, therefore, a valuable method to monitor response to treatment in patients with chronic obstructive pulmonary disease (COPD). This study was designed to evaluate its potential for monitoring short-term response to hyperoxia in COPD patients.
**Methods and Materials:** 19 prospectively enrolled COPD patients (median age 66 years) underwent paired dynamic contrast-enhanced 4D perfusion MRI within 35min, first breathing 100% oxygen ($O_2$) and then room air (RA), which was repeated on two consecutive days. Post-processing software was employed to calculate mean transit time (MTT), pulmonary blood volume (PBV) and pulmonary blood flow (PBF), based on the indicator dilution theory, for the automatically segmented whole lung and 12 regions of equal volume.
**Results:** Comparing $O_2$ with RA conditions, PBF and PBV were found to be significantly lower at $O_2$, consistently on both days ($p<10^{-8}$). Comparing day 2 to day 1, MTT was shorter by (0.59±0.63)s ($p<10^{-8}$), PBF was higher by (22±80)ml/min/100ml ($p<3\cdot10^{-4}$), and PBV tended to be lower by (0.2±7.2)ml/100ml (p=0.159) at both, RA and $O_2$, conditions.
**Conclusion:** The measurement setup did not reproduce the established hypothesis that hyperoxia increases lung perfusion, instead showing an opposite effect. This shows that the quantification of 4D perfusion MRI based on current mathematical approaches is limited by residual circulating contrast material in the short term and, for COPD patients, even the next day due to limited clearance.

**B-0421** 16:16
**The role of 4D flow MRI in evaluating the outcome of balloon pulmonary angioplasty**
W. Sun, H. Ota, S. Chibana, H. Sato, K. Sugimura, K. Takase; *Sendai/JP (sunwenyu910920@gmail.com)*

**Purpose:** To access the value of haemodynamic features of patients with chronic thromboembolic pulmonary hypertension (CTEPH) before and after balloon pulmonary angioplasty (BPA) using 4D flow MRI.
**Methods and Materials:** Thirty consecutive patients (mean age, 68.4 ± 7.9-year-old, female, 25) with inoperable CTEPH underwent serial MR scans before and after BPA. Haemodynamic features extracted from 4D flow MRI include maximum energy loss (max EL), maximum wall shear stress (max WSS) map and maximum backward flow ratio (BFR) in the systolic phase in the pulmonary trunk. The ratio of the pulmonary trunk to the ascending aorta (PA/AA ratio) and right ventricular (RV) volumetry were evaluated using cardiac cine MRI. Mean pulmonary arterial pressure (mPAP) was obtained by right heart catheterization. Pearson's correlation coefficients were evaluated between mPAP and MRI-derived parameters before BPA and between their differences (ΔmPAP and ΔMRI-derived parameters) before and after BPA. Statistical significance was assumed for $P < 0.05$
**Results:** The mean of mPAP was significantly decreased after BPA (39.0 ± 9.3 mmHg vs 25.0 ± 6.8 mmHg, p<0.01). Significant correlations with pre-BPA mPAP were observed in BFR (r=0.42, p=0.02) and max-WSS (r=-0.37, p<0.05). Significant correlations with ΔmPAP were observed in ΔPA/AA ratio (r=0.402, p=0.03), ΔBFR (r=0.57, p<0.01), Δmax EL(r=0.38, p=0.04), Δmax WSS (r=-0.47, p<0.01). ΔRV end-systolic volume index was marginally correlated with ΔmPAP (r=0.36, p=0.052)
**Conclusion:** Haemodynamic parameters evaluated using 4D flow MRI can reflect the improvement of mPAP after effective BPA and can be used for the management of patients with CTEPH treated by BPA.

**B-0422** 16:20
**Inspiratory/expiratory 3D pulmonary MRI with UTE: capability for clinical stage classification in smokers as compared with thin-section CT**
Y. Ohno[1], M. Yui[2], Y. Kishida[1], S. Seki[1], T. Yoshikawa[1], T. Murakami[1];
[1]*Kobe/JP*, [2]*Otawara/JP (yosirad@kobe-u.ac.jp)*

**Purpose:** To prospectively and directly compare the capability for pulmonary functional loss assessment and clinical stage classification between inspiratory and expiratory 3D MR imaging with ultra-short TE (IS/ES UTE-MRI) and thin-section CT in smokers.
**Methods and Materials:** Eighteen consecutive smokers underwent IS/ES UTE-MRI, thin-section CT and $\%FEV_1$. All smokers were classified into three stages ('Without COPD', 'Mild COPD', 'Moderate COPD') according to the GOLD guideline. From IS/ES UTE-MRI data in each smoker, signal intensity (SI) change ratio within the lung was computationally generated by pixel by pixel analyses. Then ROIs were placed over the lung on all slices, and averaged to determine mean SI change ratio in each subject. On quantitative CT in each subject, percentage of low attenuation area within entire lung (LAA%) was also measured. Then SI change ratio was correlated with LAA%. To compare the capability for pulmonary functional loss assessment, both indexes were correlated with each pulmonary function parameter. Furthermore, both indexes were compared four clinical stages by Tukey's HSD test.
**Results:** SI change ratio had significant and good correlation with LAA% (r=-0.86, p=0.003). SI change ratio and LAA% were correlated with $\%FEV_1$ (SI change ratio: r=0.91, p=0.0006; LAA%: r=-0.91, p=0.0006). SI change ratio had significant difference between 'Without COPD' group and others (p<0.05), although LAA% had significant difference between 'Moderate COPD' group and others (p<0.05).
**Conclusion:** Inspiratory/expiratory 3D MR imaging with UTE has a potential for pulmonary functional loss assessment and clinical stage classification in smokers as well as thin-section CT.
*Author Disclosures:*
**Y. Ohno:** Research/Grant Support; Canon Medical Systems Corporation. **M. Yui:** Employee; Canon Medical Systems Corporation. **S. Seki:** Research /Grant Support; Canon Medical Systems Corporation. **T. Yoshikawa:** Research/Grant Support; Canon Medical Systems Corporation.

**B-0423** 16:24
**Diagnostic value of quantitative dual-source CT dual-energy iodine maps combined with morphological CT features in assessing histological subtypes of lung cancer**
X. Xu, X. Sui, W. Song, L. Song, Y. Huang, Z. Jin; *Beijing/CN (xxllpositive@163.com)*

**Purpose:** To investigate the clinical usefulness of quantitative dual-energy CT (DECT) iodine metrics combined with morphological CT features in distinguishing lung cancer subtypes.
**Methods and Materials:** Consecutive patients suspected with lung cancer were prospectively enrolled and underwent DECT prior to biopsy or surgery. Tumour histological subtypes were determined in 110 patients. Two radiologists interpreted CT morphologic features and measured DECT parameters in a consensual manner. Multinomial logistic regression models were applied to evaluate the accuracy of DECT parameters and CT features in discriminating lung cancer subtypes.
**Results:** Histology revealed adenocarcinoma in 48, squamous cell carcinoma (SCC) in 36 and small cell lung cancer (SCLC) in 26 patients. In analysis of CT features, tumour diameter, distribution, spiculation, pleural retraction, vascular involvement, confluent mediastinal lymphadenopathy, encasement of mediastinal structures and enhancement heterogeneity showed statistical difference (all *P*s<0.05). Iodine density and iodine ratio were different among three lung cancer subtypes ($H$=16.817, $P$<0.001; $H$=20.338, $P$<0.001). Iodine density of adenocarcinoma and SCC was (1.50±0.80) mg/ml and (1.40±0.40) mg/ml, respectively, higher than the (1.20±0.40) mg/ml for SCLC (*P*s<0.01). Iodine ratio of adenocarcinoma and SCC was (16.10±7.02)% and (15.05±4.62)%, higher than the (11.55±3.15) mg/ml for SCLC (*P*s<0.01). No significant difference of DECT parameters was observed between adenocarcinoma and SCC. Accuracy of the model based on CT features was 69.1% and accuracy of the model based on CT features combined with DECT parameters was 80.9%.
**Conclusion:** Quantitative DECT metrics were different among adenocarcinoma, SCC and SCLC; when combined with morphological CT features, higher diagnostic performance can be achieved.

**B-0424** 16:28
**Ultra-high-resolution CT vs state-of-the-art area-detector CT: can higher spatial resolution improve nodule detection capability at same radiation dose in chest nodule phantom study?**
D. Takenaka[1], K. Fujii[2], N. Sugihara[2], S. Seki[3], T. Yoshikawa[3], Y. Ohno[3];
[1]*Akashi/JP*, [2]*Otawara/JP*, [3]*Kobe/JP (yosirad@kobe-u.ac.jp)*

**Purpose:** To determine the influence of higher spatial resolution for nodule detection capability on newly installed ultra-high-resolution CT (UHR-CT) as compared with state-of-the-art area-detector CT (ADCT) in chest nodule phantom study.
**Methods and Materials:** A commercially available chest phantom with simulated subsolid and solid nodules, whose diameters were 4, 6, 8, 10, 12 and 14mm at upper, middle and lower lung field levels, were scanned by standard-dose UHR-CT (Aquilion Precision, Canon Medical Systems) and ADCT (Aquilion ONE, Canon) three times. In this study, UHR-CT applied 0.25mm×160 detector collimation, and ADCT applied 0.5mm×80 detector collimation, and CTDIvols of UHR-CT and ADCT were 9.3mGy and 9.0mGy. Then UHR-CT data were reconstructed as 0.25mm, 0.5mm and 1mm thicknesses. On the other hand, ADCT data were reconstructed as 0.5mm and 1 mm thicknesses. To assess image quality and probability of nodule presence, two chest radiologists were visually assessed, and each final score was determined by the consensus of two readers. To evaluate image quality difference and nodule detection capability among all protocols, Wilcoxon's signed rank tests and ROC analyses were performed. Finally, detection performance was compared among all protocols by McNemar's test.

**Results:** There were no significant differences of image quality and area under the curve among all protocols (p>0.05). Sensitivity of UHR-CT reconstructed as 0.25mm was significantly higher than that of ADCT reconstructed as 0.5mm thickness with 512 matrix (p=0.03).
**Conclusion:** Higher spatial resolution on UHR-CT has a potential to improve nodule detection capability as compared with ADCT in chest phantom study.
*Author Disclosures:*
**K. Fujii:** Employee; Canon Medical Systems Corporation. **N. Sugihara:** Employee; Canon Medical Systems Corporation. **S. Seki:** Research/Grant Support; Canon Medical Systems Corporation. **T. Yoshikawa:** Research/Grant Support; Canon Medical Systems Corporation. **Y. Ohno:** Research/Grant Support; Canon Medical Systems Corporation.

**B-0425** 16:32
**The utility of the ultra-high-resolution CT to evaluate the detailed bronchial tree for COPD**
D. Takenaka, A. Higashida, Y. Kono, T. Hashimoto, H. Maeda, S. Sakamoto; *Akashi/JP (daisuket@hp.pref.hyogo.jp)*

**Purpose:** The purpose of this study was to compare the availabilities to image peripheral bronchus as a tool of COPD evaluation on CT between conventional high-resolution CT (HRCT) and the ultra-high-resolution CT (UHRCT).
**Methods and Materials:** Twenty-five consecutive patients who underwent CT examination were enrolled retrospectively. All CT studies were performed by using ultra-high-resolution CT machine (Aquilion Precision; Canon Medical Systems, Otawara, Japan). All CT examinations were performed with 160-detector row CT scanner; 120kVp, auto-mAs, 1792 channels, collimation 0.25 mm x 160 rows, 1024 matrix. HRCT images were reconstructed with 1mm slice, 1mm interval, 512 x 512 matrix. UHRCT images were reconstructed with 0.25 mm slice, 0.25 mm interval, 1024 x 1024 matrix. Each bronchial tree was defined by Synapse Vincent (Fujifilm Medical; Tokyo, Japan) and evaluated the percent of wall area (WA%), and luminal area (LA) through $3^{rd}$ to $9^{th}$ generation. The WA%s and LAs of the proximal bronchus ($3^{rd}$ and $4^{th}$ Generation) and the peripheral bronchus ($5^{th}$ and more) were statistically compared by using Wilcoxon rank sum test. P value less than 0.05 was considered to be significant in all statistical analyses.
**Results:** The p-values of WA% of proximal and peripheral bronchus were 0.08 and 0.03, respectively. The p-values of LA were 0.10 and 0.00, respectively. Thus, the UHRCT images revealed more detailed visualization of peripheral bronchus to evaluate WA% and LA for evaluation of regional COPD change.
**Conclusion:** The UHRCT image has improved the detailed measurement of peripheral bronchial tree compared to conventional HRCT image.

**B-0426** 16:36
**Late phase lung perfusion by dual energy CT: image quality and clinical findings compared to arterial perfusion**
M.S. May, I. Prael, R. Heiß, M. Kopp, M. Uder, W. Wuest; *Erlangen/DE (matthias.may@uk-erlangen.de)*

**Purpose:** Arterial Dual-Energy-CT (aDE) is an established technique for evaluation of pulmonary perfusion. The aim of this study was to assess the image quality and clinical findings in the portal-venous-phase (vDE).
**Methods and Materials:** vDE-CT was performed in 94 patients using a dual source scanner (70/Sn150kV, 560/140ref.mAs). Pulmonary triggered aDE-CT from the chest served as reference (n=95). Iodine maps were calculated and attenuation was measured in the pulmonary trunk and the aorta. Subjective overall diagnostic image quality was assessed on a five point Likert scale. Image artefacts were classified into five groups and evaluated on a dichotomous scale. Pathological findings were correlated with the anatomical image datasets.
**Results:** No significant difference was found for iodine uptake of the lung parenchyma in both groups (vDE: 23±13HU and aDE: 22±14HU), but iodine attenuation in the aorta and the pulmonary trunk was lower in vDE. Overall perfusion image quality was comparable (p=0.394). Rotation and streak artefacts were found in most of the patients in at least one location. Dual source artefacts were found in only few patients in both groups (vDE 5%, aDE 7%). Recess artefacts were higher (vDE 61%, aDE 23%) and subpleural artefacts were only observed in vDE (17%). Pathological findings were found in 20% of all vDE patients and in 35% of all aDE patients including areas of hyper- (35/30%) and hypoattenuation (65/70%).
**Conclusion:** vDE provides robust image quality of lung perfusion. Relative iodine uptake is increased, suggesting a pooling effect. Different appearance of artefacts should be known for interpretation.
*Author Disclosures:*
**M.S. May:** Speaker; Siemens Healthcare GmbH. **R. Heiß:** Speaker; Siemens Healthcare GmbH. **M. Kopp:** Speaker; Siemens Healthcare GmbH. **M. Uder:** Speaker; Siemens Healthcare GmbH. **W. Wuest:** Speaker; Siemens Healthcare GmbH.

**B-0427** 16:40
**Computed tomography pulmonary angiography for acute pulmonary embolism: prediction of adverse outcome and 90-day mortality in a single test**
N. Akhoundi, T. Faghihi Langroudi; *Tehran/IR (neda.akhoundi@sbmu.ac.ir)*

**Purpose:** Pulmonary embolism (PE) is a potentially fatal cardiopulmonary disease; therefore, rapid risk stratification is necessary to make decisions of appropriate management strategies. The aim of this study was to assess various CT findings to find new predictors of adverse outcome and mortality.
**Methods and Materials:** The study retrospectively enrolled 104 patients with acute PE. Patients were categorised into 4 groups: group 1: patients who experienced adverse outcome, group 2: patients who died within 30 days, group 3: patients who died between 30 and 90 days, group 4: patients who survived without experiencing adverse outcome. Comorbidities such as ischaemic heart disease (IHD) were obtained from their medical records. Patients' CT angiography were reviewed for recording variables such as main pulmonary artery diameter (MPA), RV/LV ratio. Patients death till three months of diagnosis of PE had been registered. Logistic regression analysis was done to find predictors.
**Results:** 16 patients experienced adverse outcome, 10 deaths occurred within 30 days and 5 deaths were between 30 and 90 days. Based on multiple logistic regression, RV/LV ratio (OR 11.54; 95% CI 1.16 to 114.35; p=0.037), LV diameter (OR 0.813; 95% CI 0.681 to 0.969; p=0.021), right-sided pulmonary infarction (OR 0.111; 95%CI 0.017 to 0.730; p=0.022) are predictors of mortality in 30 days. RV/LV ratio of 1.19 could successfully discriminate patients who died within 30 days and who did not.
**Conclusion:** RV/LV ratio, LV diameter, right-sided pulmonary infarction, assessed with helical CT, can help predict mortality in 30 days.

**B-0429** 16:44
**Reduced- and no-dose thin-section radiologic examinations: comparison of capability for nodule detection in patients having pulmonary nodules**
Q. Peng, W. Tang, N. Wu, Y. Huang, H. Ouyang, B. Wu; *Beijing/CN (meetsumay@hotmail.com)*

**Purpose:** To evaluate the capability of pulmonary MR imaging with zero echo time (ZTE) in pulmonary nodules assessments,using low-dose computed tomography (LDCT) as the reference standard.
**Methods and Materials:** Institutional review board approval and informed consent were obtained. Eight-nine consecutive patients (51 males: mean age, 69 years and 38 females: mean age, 71 years) with various pulmonary nodules were examined with chest LDCT and pulmonary MR imaging with ZTE. The interval between two examinations was within two weeks for each patient. Lung images were visually scored by two experienced radiologists who assessed the probability of nodule presence in comparison with LDCT. To compare nodule detection capability of the two methods, consensus for performances was evaluated by means of kappa statistics and $\chi^2$ test, and receiver operating characteristic analyses were used to compare diagnostic performance of both methods.
**Results:** There was no significant difference ($F$ = 0.75, $P$ = 0.56) in figure of merit between methods (LDCT, 0.84 Vs. MR imaging with ZTE, 0.86). Intermethod agreements between ZTE MR imaging and LDCT were significant ($0.70 \le \kappa \le 0.98$; $P < 0.001$). Areas under the curve for pulmonary nodules on LDCT were significantly larger than those on MR imaging with ZTE (P= 0.02).
**Conclusion:** Pulmonary thin-section MR imaging with ZTE was useful in nodule detection, and it is considered at least as effective as low-dose thin-section CT.

**B-0430** 16:48
**Assessment of EGFR mutation status in patients with non-small-cell lung cancer using different iodine quantification methods in dual-energy CT: areal vs volumetric analyses**
X. Xu, X. Sui, L. Song, Y. Huang, Z. Jin, W. Song; *Beijing/CN (xxllpositive@163.com)*

**Purpose:** To investigate the feasibility of areal and volumetric iodine quantification metrics with dual-energy CT in predicting EGFR mutation status of non-small cell lung cancer (NSCLC) and the correlation between the two methods.
**Methods and Materials:** 58 untreated NSCLC patients who underwent DECT in an arterial phase before biopsy or surgery were prospectively enrolled. For each lesion, areal iodine content (IC) and normalized iodine content (NIC) with maximum area among slices and volumetric IC and NIC of the whole tumour were evaluated by two radiologists. In the volumetric analysis, tumour was further sectioned, volumetric parameters of every segment and peel were noted. The diagnostic performances of areal and volumetric iodine metrics in characterizing EGFR mutations were compared and the two iodine quantification methods were correlated with each other.

**Results:** EGFR mutations were found in 28 of 58 patients. $NIC_{areal}$ of EGFR mutant group was higher than that of wild-type group ($t$=2.869, $P$=0.006). $NIC_{volumetric}$ of the inner segment and peel in EGFR mutant group was higher than that in wild-type group ($t$=2.415-2.699, $P$=0.009-0.019). However, volumetric parameters of the total lesion and marginal part showed no significant difference between the two groups. ROC curve analysis revealed that diagnostic performance of $NIC_{areal}$ (AUC=0.693) was similar to that of $NIC_{volumetric}$ of inner segments and peels (AUC=0.655-0.677). $NIC_{areal}$ was strongly correlated with inner segmental $NIC_{volumetric}$ (r=0.818-0.821).
**Conclusion:** Areal and volumetric iodine metrics were applicable in predicting EGFR mutation status, the maximum area iodine quantification was suggested in clinical use due to its convenient access.

**B-0431** 16:52
**Systematic review and meta-analysis of randomized trials on the impact of lung cancer screening by low-dose computed tomography in populations highly exposed to tobacco**
A. Sadate, B. Occean, A. Larbi, P. Fabbro-Peray, J. Beregi; *Nîmes/FR (alexandre.sadate@gmail.com)*

**Purpose:** Lung cancer (LC) has the highest cancer mortality worldwide with poor prognosis. Screening with low-dose computed tomography (LDCT) in populations highly exposed to tobacco has been proposed to improve LC prognosis. Our objective is to perform a systematic review and meta-analysis to evaluate the efficacy of screening by low-dose computed tomography (LDCT) compared to any other intervention in populations who reported tobacco consumption for more than 15 years on LC and overall mortality.
**Methods and Materials:** We searched randomized controlled trials (RCTs) studying screening by LDCT compared with any other intervention with data on LC-specific and all-cause mortality from inception until the 19th of February 2018 using Medline and Cochrane Library databases. Publication selection and data extraction were made independently by two double-blind radiology residents. Two groups of meta-analysis were created with either long or short follow-up.
**Results:** Six RCTs were included in the meta-analysis. In the short-follow-up group, a non-significant excess of overall mortality was observed in the screening group (RR= 1.26, 95% CI: 0.78-2.04). In the long-follow-up group, a significant relative reduction of overall mortality of 6% (RR= 0.94, 95% CI: 0.89-0.99) and a significant relative reduction of LC-specific mortality of 14% was observed in the screening group (RR= 0.86, 95% CI: 0.77-0.96) compared with the control group. The limitations were the heterogeneity of interventions and the over-representation of one study in the long-follow-up group.
**Conclusion:** In populations highly exposed to tobacco, screening by LDCT reduces LC and overall mortality over the long term.

**B-0432** 16:56
**Application of LDCT and Lung-RADS classification in lung cancer screening of the urban population in China**
Q. Meng; *Zhengzhou/CN (mengke436@163.com)*

**Purpose:** To evaluate the application of LDCT and Lung-RADS classification in lung cancer screening in healthy people.
**Methods and Materials:** 8850 volunteers were selected and screened by LDCT from November 2013 to December 2017. All volunteers were divided into high-risk group (n=1339), medium-risk group (n=6344) and low-risk group (n=1167); male group (n=4900) and female group (n=3950); less than 40-year-old group (n=110), 40-45-year-old group (n=794), 45-50-year-old group (n=1633), 50-55-year-old group (n=2565) and over 55-year-old group (n=3858). Grade 3 +4 in the Lung-RADS classification was positive. The Lung-RADS classification of nodules and lung cancer incidence between different groups was compared.
**Results:** According to the classification of Lung-RADS, 590 cases of positive nodules were screened at baseline, and the detection rate was 6.67%, in which the difference between male and female was statistically significant (7.5% vs. 5.6%, p=0.00).The positive rate of pulmonary nodules gradually increased with age in each group, and the difference of nodules classification in each group was statistically significant, p <0.05, the incidence of lung cancer in 40-45-year-old group was significantly higher than that in other groups, and the difference was statistically significant. The difference in nodule classification between the three groups was statistically significant, p <0.05. The incidence rate of lung cancer in high-risk group was significantly more than 3 times higher than that of medium-risk group and low-risk group (2=7.337, p=0.026).
**Conclusion:** LDCT combined with Lung-RADS classification is suitable for lung cancer screening in healthy people, especially in the high-risk group of over 40 years old.

**B-0434** 17:04
**Single- and dual-energy CT pulmonary angiography using second- and third-generation dual-source CT systems: comparison of radiation dose and image quality**
L. Lenga[1], C. Booz[1], J.L. Wichmann[1], J.-E. Scholtz[1], S.S. Martin[2], T.J. Vogl[1], M.H. Albrecht[1], I. Yel[1]; [1]*Frankfurt/DE*, [2]*Charleston, SC/US (lukas.lenga@gmail.com)*

**Purpose:** To evaluate radiation exposure and image quality in matched patient cohorts for CT pulmonary angiography (CTPA) acquired in single- and dual-energy mode using second- and third-generation dual-source CT (DSCT) systems.
**Methods and Materials:** Two-hundred patients (100 men and 100 women) with suspected pulmonary embolism who had undergone clinically indicated CTPA in pulmonary arterial contrast phase were retrospectively enrolled. Patients were equally assigned to one of the four study groups (each with n=50), and matched by gender and body-mass-index. CTPA was performed with vendor-preset second-generation DSCT (group A, 100-kV-SECT; group B, 80/Sn140-kV-DECT) or third-generation DSCT (group C, 100-kV-SECT; group D, 90/Sn150-kV-DECT) scanners. Radiation metrics were recorded using a normalized scan range of 27.5 cm. For objective image quality assessment dose-independent figure-of-merit (FOM) contrast-to-noise ratios (CNR) were calculated. Three blinded radiologists evaluated overall image quality, reader confidence and image artefacts subjectively using five-point Likert-scales.
**Results:** Effective radiation dose calculations revealed no significant differences between SECT and DECT acquisition for each scanner generation (P≥0.353). The mean effective radiation dose was lower for third-generation groups C (1.5±0.8 mSv) and D (1.5±0.7 mSv) compared to second-generation groups A (2.5±0.9 mSv) and B (2.3±0.6 mSv) (all with P≤0.012). Highest FOM-CNR measurements were observed for third-generation DECT (P≤0.032). Ratings for subjective image quality, reader confidence and image artefacts showed no significant differences among the four groups (P≥0.158).
**Conclusion:** Dual-energy CTPA can be performed with both second- and third-generation DSCT systems without detrimental effects on image quality or radiation dose in comparison with SECT.
*Author Disclosures:*
**J.L. Wichmann:** Other; Wichmann JL received speaker fees from GE and Siemens. **M.H. Albrecht:** Other; Albrecht MH received speaker fees from Siemens and Bracco.

**B-0435** 17:08
**Percutaneous needle biopsy for lung lesions focused on needle angle and lesion depth: 14-year results**
J. Lee, I. Lee, J. Kim; *Anyang/KR (jeehyeon@hallym.or.kr)*

**Purpose:** To evaluate the effects of needle angle and lesion depth on complications and technical success of percutaneous needle biopsy (PCNB) for intrapulmonary lesions.
**Methods and Materials:** From 2004 to 2017, total of 689 cases of PCNB were retrospectively reviewed. The angle and the depth were intensively calculated for each case. The angle was subdivided into three categories on the basis of 30 degrees (0-30, 31-60, 61-90). The depth was documented as a continuous variable. The pathologic results and complications were evaluated to assess relationship with the angle and the depth of PCNB by using univariate analysis. Complications were divided into minor and major complications according to severity and necessity for additional procedure.
**Results:** The overall success rate was 93.1% (642/689). Technical success showed no significant correlation with both needle angle (P=0.568) and lesion depth (P=0.144). The overall complications rate was 17.9% (123/689) with 15.7% for minor complications and 2.2% for major complications. No significant correlation was found between complications and the angle (P=0.101). However, complications was influenced by the depth (P<0.01; mean lesion depth ± SD for no complications, minor complications and major complications are 1.49 ± 1.65, 3.39 ± 1.71, and 4.72 ± 1.54, respectively).
**Conclusion:** Needle angle showed no significant relation with technical success and complications. Lesion depth also had no effect on technical success. However, the depth was strongly correlated with the severity of complications after PCNB.

**B-0436** 17:12
**Mare Nostrum and tuberculosis: the role of chest x-ray and clinical-laboratory data among Mediterranean migrants on landing**
G. Scavone[1], M.V. Raciti[2], M.C. Calcagno[1], D.C. Caltabiano[1], G. Galvano[1], A. Scavone[1]; [1]*Catania/IT*, [2]*Pavia/IT (mariavittoria.raciti@libero.it)*

**Purpose:** To report the experience of the Italian centre most involved in clandestine landings, end point of arrival for Mediterranean migrants and bridge to Europe. To determine the still relevant importance of chest x-ray in diagnostic setting of migrants suspected for tuberculosis (TB) infection, comparing radiological findings with clinical-laboratory data.

**Methods and Materials:** From 2010 to 2016, 15115 migrants, mainly from sub-Saharan Africa, have landed at the Catania port. They underwent an initial clinical evaluation on the dock looking for two conditions: fever>37°C and cough with or without sputum. 510 migrants had fever, of these 191 with cough were considered suspect for TB and were carried into the high bio-containment unit: 149 (cough without sputum) were studied with chest x-ray, 42 (cough with sputum) were subjected to GeneXpert (GX), a rapid laboratory test for TB.
**Results:** 25TB pulmonary infections were diagnosed (13.1% of TB suspected, 4.9% of feverish patients, 0.16% of migrants), 19 (76%) of these diagnosed by chest x-ray (16 miliary, 2 with consolidations, 1 with cavitations) and 6 (24%) by GX. 14 suspected patients presented non-TB-related pathology diagnosed by x-ray, 13 lobar and atypical pneumonia, and 1 pleurisy.
**Conclusion:** Mediterranean migratory flows have re-introduced TB and timely diagnosis on landing is mandatory. In symptomatic migrants, chest radiography is the first line technique that allows a timely diagnosis of active or latent tuberculosis and a prompt treatment, preventing the infection spread in the country of landing and in Europe. GX can detect the TB infection but chest x-ray is still necessary to detect GX-negative pulmonary infections.

**16:00 - 17:30** **Tech Gate Auditorium**

### Genitourinary

### SS 407

### Prostate imaging: new diagnostic tools

*Moderators:*
N.N.
J.J. Fütterer; Nijmegen/NL

**B-0437** 16:00
**Prostate imaging self-assessment and mentoring (PRISM): a prototype self-assessment scheme for radiologists**
Y. Chen[1], E. Michalopoulou[1], A.G. Gale[1], T. Barrett[2], P. Haslam[3], A. Patel[4], H. Ahmed[5]; [1]*Loughborough/UK*, [2]*Cambridge/UK*, [3]*Newcastle Upon Tyne/UK*, [4]*Stevenage/UK*, [5]*London/UK (Y.Chen@lboro.ac.uk)*

**Purpose:** The PRISM scheme is a web-based self-assessment and continuous learning platform designed for radiologists across the UK to develop their expertise in interpreting prostate mpMRI images before biopsy, so that they build the confidence to recommend for some men to avoid an immediate biopsy.
**Methods and Materials:** The PRISM Expert Reference Group (ERG) facilitated by Prostate Cancer UK and consisting of expert radiologists, computer scientists and several members of a wider mpMRI clinical expert group was established. Qualitative data regarding software structure and application design were collected, while a focus group moderator generated interviews to capture deeper information on image examination and reporting processes. A collection of validated mpMRI cases acquired from the PROMIS Trial team was used to evaluate participants' image interpretation performance. Different potential variations of an initial conceptual model of the envisioned application were developed and circulated around the ERG. Further face-to-face meetings and interviews were conducted with consultant radiologists to gain their feedback and finalise the application. The final design was then generated and coded.
**Results:** To date, the system has been developed and tested. It is now being piloted in specific sites across the UK and the data resulting from these pilots will be analysed and presented at the conference. We understand at this time no meaningful data can be usefully presented in this abstract of work in progress.
**Conclusion:** This work is at the stage of data collection and will complete at the end of 2018. All data will be presented at ECR 2019.

**B-0438** 16:08
**Use of kZ space for sub-mm through-plane resolution in T2-weighted 2D spin-echo prostate MRI**
S. Kargar[1], S. Riederer[1], E. Borisch[1], A. Froemming[1], R. Grimm[1], A. Kawashima[2], B. King[1], E. Stinson[1]; [1]*Rochester, MN/US*, [2]*Scottsdale, AZ/US (riederer@mayo.edu)*

**Purpose:** To describe how sub-mm through-plane resolution can be obtained with multi-slice kZ-processed data for improved sharpness in prostate T2SE MRI.
**Methods and Materials:** Through-plane resolution of multi-slice MR images is typically no finer than several mm, as limited by the slice profile. Here we describe a method for obtaining sub-mm resolution by compensation for slice profile, done in combination with fine slice-to-slice spacing. The multiple slices are transformed to kZ space for reconstruction. The time for acquiring more slices is accounted for in part by reduced signal averaging. The method was evaluated in 16 subjects for whom prostate MRI was indicated. A reference (TR/TE 3000/100, 28 3.0-mm-thick slices, 4:14 scan time, 0.75 x 0.75 x 3.00 $mm^3$ resolution) and the proposed (TR/TE 3000/100, 78 overlapped 3.0-mm-thick slices, 6:38 scan time, 0.75 x 0.75 x 1.00 $mm^3$ final resolution) axial scans were performed. Images from the two were compared blindly by three experienced uroradiologists using five evaluation criteria of sharpness (prostate capsule, zonal differentiation, structures within the peripheral and transition zones, seminal vesicles) and artefact. Individual acquisitions were also assessed for overall image quality.
**Results:** The proposed method was superior for all five sharpness-related criteria (p<0.04, Wilcoxon signed rank test) but inferior for artefact (p<0.02). Both T2SE scans were rated highly for overall image quality.
**Conclusion:** Multi-slice axial T2SE images can be acquired with modest overlap and reconstructed to provide 1-mm slice resolution for prostate MRI. The resultant images provide improved sharpness of clinically relevant structures.

**B-0440** 16:16
**MRI of the prostate at 3.0T with and without endorectal-coil: two-centre comparison in kiwi-fruit and patients**
U. Mueller-Lisse, S. Murer, J. Scheidler, M. Kuhn, A. Meister, M.K. Scherr, U.L. Mueller-Lisse; *Munich/DE (ullrich.mueller-lisse@med.uni-muenchen.de)*

**Purpose:** To test by means of the kiwi-fruit phantom and validation in patients with benign prostate hyperplasia (BPH) if bi-parametric 3.0T-MRI provides equivalent signal-to-noise-ratio (SNR) and contrast-homogeneity without and with ERC, including both full-field-DWI and reduced-field-DWI.
**Methods and Materials:** ERC/noERC axial T2-weighted (T2WI, TR/TE 7,500/98-101 ms) and diffusion-weighted (full-field-DWI 380x297 mm2, reduced-field-DWI 149-179x71-73 mm2, TR/TE 4,500-5,500/61-74 ms) MR-images were compared for SNR and anterior-posterior contrast in green “Hayward” kiwi-fruit (Actinidia deliciosa, 105-115 g). Ethics-committee-approved retrospective validation in benign-prostate-hyperplasia-patients (negative prostate-biopsy, PIRADSv2 ≤2, age+/-standard-deviation, 62+/-8 years, PSA, 6.3+/-2.4 ng/ml, prostate-volume, 91+/-51 cm3, noERC, n=10, centre 1, ERC, n=11, centre 2) involved identical prostate-MRI-protocols. Two-tailed Wilcoxon-matched-pairs-signed-rank-tests were significant for p<0.05.
**Results:** SNR(noERC) in peripheral kiwi-fruit tissue was higher posteriorly (median/minimum,maximum, 41/22,91) than anteriorly (40/21,87) in T2WI (0.010<p<0.025), but not in full-field-DWI, reduced-field-DWI, and mono-exponential ADC-maps (10/4,17-80/13,126 versus 11/6,20-83/8,137, 0.05<p<1.0). SNR(ERC) was higher posteriorly in T2WI, full-field-DWI and reduced-field-DWI (17/7,74-39/8,80 versus 6/2,72-25/14,64, p<0.001-0.05), but not in ADC-maps (26/17,47-61/8,104 versus 22/15,45-56/7,121, 0.05<p<1.0). Anterior-posterior contrast in kiwi-fruit was higher for ERC than noERC in DWI-sequences (p<0.001-0.05). SNR was higher in reduced-field-DWI than in full-field-DWI for both noERC (median, 15-85 versus 10-37) and ERC (9-61 versus 6-39) in kiwi-fruit and patients.
**Conclusion:** Study results in both kiwi-fruit and patients imply that SNR is similar in noERC and ERC in a bi-parametric prostate-MRI-protocol, image-homogeneity is higher for noERC, and SNR is higher in reduced-field-DWI than in full-field-DWI.

**B-0441** 16:24
**T2-mapping with simulated T2-echo - a diagnostic alternative for 3-Tesla prostate MRI?**
J. Pfeil[1], C. Lee[2], M. Haas[1], M. Taupitz[1], B. Hamm[1], P. Asbach[1]; [1]*Berlin/DE*, [2]*Singapore/SG (julian.pfeil@charite.de)*

**Purpose:** T2-mapping sequences provide quantitative information and also allow the reconstruction of a simulated T2-weighted image which could be used instead of the standard T2-weighted fast spin-echo sequence to save scan time. The aim of this study was to evaluate the image quality and diagnostic utility for PI-RADS scoring of a T2-mapping sequence with simulated T2-echo in comparison to a standard T2-weighted fast spin-echo sequence for prostate MRI.
**Methods and Materials:** In this IRB approved prospective study on 40 patients a multi-echo T2-mapping sequence was added to the routine 3-Tesla prostate MRI protocol. Three readers separately reviewed sets of images blinded to the sequence technique. Reader preference for either the standard T2-sequence or the simulated T2-sequence was compared using the Z-test and the intra-observer agreement for assigning PI-RADS scores was calculated for each reader using the Cohen kappa ($\kappa$).
**Results:** All 3 readers preferred the standard T2-sequence over the simulated T2 -sequence (p<0.05). All 3 readers showed moderate intra-observer agreement between the standard T2- and simulated T2-sequence regarding the assigned PI-RADS scores (R1 $\kappa = 0.58$, R2 $\kappa = 0.44$, R3 $\kappa = 0.43$).
**Conclusion:** T2-mapping with reconstruction of a simulated T2-sequence shows potential for including quantitative sequences into routine prostate MR protocols but image quality is not equivalent to standard T2 fast spin-echo Imaging.

*Author Disclosures:*
**J. Pfeil:** Research/Grant Support; Siemens Healthineers. Other; Siemens Healthineers. **C. Lee:** Research/Grant Support; Siemens Healthineers. Other; Siemens Healthineers. **M. Haas:** Research/Grant Support; Siemens Healthineers. Other; Siemens Healthineers. **M. Taupitz:** Research/Grant Support; Siemens Healthineers. Other; Siemens Healthineers. **B. Hamm:** Research/Grant Support; Siemens Healthineers. Other; Siemens Healthineers. **P. Asbach:** Research/Grant Support; Siemens Healthineers. Other; Siemens Healthineers.

**B-0442** 16:32

**Assessment of diagnostic yields of significant prostate cancer by altering the prostate-specific antigen density thresholds for biopsy according to MRI findings**

L. Boesen[1], V. Logager[3], N. Nørgaard[1], R. Bisbjerg[1], I. Balslev[1], K.-C.D. Thestrup[1], H. Jakobsen[1], H.S. Thomsen[1], A.R. Padhani[2]; [1]*Herlev/DK*, [2]*Northwood, Middlesex/UK*, [3]*Copenhagen (lars.boesen@dadlnet.dk)*

**Purpose:** To assess the influence of prostate-specific antigen density (PSAd) on the diagnostic yields of a negative-, equivocal- and positive biparametric (bp) MRI, to detect and rule out significant prostate cancer (sPCa).
**Methods and Materials:** Prostate bpMRI (T2W and DWI) was performed in 808 biopsy-naive men with clinical suspicion of PCa (PSA<20 ng/ml, cTstage<T3). BpMRI findings were stratified into negative/equivocal/positive for sPCa suspicion using modified PI-RADS. All men underwent standard- and targeted biopsies of bpMRI equivocal/positive lesions. PSAd thresholds were assessed according to bpMRI findings using sPCa detection rates (Gleason grade group≥2) from combined biopsies as reference. Net benefits and decision curve analyses were compared to determine the optimal biopsy strategy.
**Results:** Overall, any and sPCa was detected in 460/808 (57%) and 283/808 (35%) men with median age and PSA [inter-quartile range] of 65 years [60-70] and 6.9 ng/ml [5.4-9.5], respectively. PSAd significantly influenced the diagnostic yield of sPCa stratified by bpMRI suspicion. For biopsy risk-thresholds ranging from 10-30%, the best strategy was restricting biopsies to men with positive bpMRIs or PSAd ≥0.15 ng/ml/cc for equivocal and ≥0.20 ng/ml/cc for negative bpMRIs. This reduced the number of men requiring biopsies by 45% (363/808), over-diagnosis rate of insignificant cancers by 51% (90/177), while missing only 7% (20/283) of men with sPCas.
**Conclusion:** Altering the PSAd threshold according to bpMRI suspicion and restricting biopsies to men with positive bpMRIs or PSAd ≥0.15 for equivocal- and ≥0.20 for negative bpMRIs was the best biopsy strategy in biopsy-naïve men, effectively balancing risks and benefits.

**B-0443** 16:40

**Natural history of prostate cancer on active surveillance: stratification by MRI using the PRECISE recommendations in a UK cohort over 11 years**

F. Giganti[1], A. Stabile[2], V. Stavrinides[1], C. Orczyk[1], S. Punwani[1], C. Allen[1], A. Kirkham[1], M. Emberton[1], C.M. Moore[1]; [1]*London/UK*, [2]*Milan/IT (f.giganti@ucl.ac.uk)*

**Purpose:** To compare the PRECISE score (a 1-5 Likert scale for the likelihood of radiological change of prostate cancer over time; 1: regression, 3: stability and 5: definite stage progression) with clinical progression in men on an MRI-led active surveillance (AS) protocol.
**Methods and Materials:** Men on AS for low or intermediate risk prostate cancer who had had two or more MRI scans between April 2006 and September 2017 were included. A total of 150 men (535 scans) were re-reported by a dedicated radiologist to give a PI-RADS v2 score for each scan, measurement of all lesions and a PRECISE score. Clinical progression was defined by histological progression to ≥ Gleason Grade Group 3 and/or initiation of active treatment. Tumour growth rate between serial scans was calculated.
**Results:** Freedom from clinical progression at 12, 24 and 60 months for PRECISE 1-2 was 100% at all time points; for PRECISE 3 it was 98.4%, 96.7%, 96.7% and for PRECISE 4-5 it was 98.5%, 92.4%, 67.1%, respectively. There was a significant difference between PRECISE 1-2 vs 4-5 and PRECISE 3 vs 4-5 (p<0.001). Fifty-six men (37%) had a visible lesion on all scans, and their tumour growth rate (%) by planimetry was higher with higher PRECISE scores (all p<0.05). Limitations include the fact that serial biopsies were not routinely performed and that the biopsy approach varied (standard vs targeted).
**Conclusion:** Radiological stability (PRECISE 1-3) in men on AS is associated with a high rate of freedom from clinical progression.

**B-0444** 16:48

**Orthopaedic metallic artefact reduction is indispensable for CT evaluation of the urinary tract after hip replacement**

P.N. Kaposi, T. Youn, P. Borsos, P. Magyar, V. Bérczi; *Budapest/HU (kaposipal@gmail.com)*

**Purpose:** The metallic hip prosthesis (TEP) causes extensive artefacts on CT scans. Orthopaedic metallic artefact reduction (O-MAR) may augment evaluation of the bladder and ureter after TEP.
**Methods and Materials:** 30 patients with 41 TEPs were retrospectively enrolled in the study. CT examinations were performed on an Ingenuity Core 64 scanner (Philips Healthcare, the Netherlands) with diagnostic protocols. Series were reconstructed both with a body kernel (NOM) and O-MAR from the same acquisitions. CT densities were measured in freehand regions of interest (ROIs) drawn around the ureters and in ROIs inside the bladder at both sides. Four visibility levels were used for semi-quantitative assessment of image quality.
**Results:** The average (μ) density of 41 prosthesis side ureters was significantly lower on NOM (μ=-94.76 HU ± SD 150.48HU) than on O-MAR images (μ=-13.40HU ± SD 36.37HU; p<0.0004). The TEP side of the bladder (μ=-138.62 HU ± SD 182.64 HU vs. μ=-35.55 HU ± SD 40.21 HU; p<0.0003) was also darker on NOM images. 53.7% of the TEP side ureters was obscured on NOM series compared to 4.9% on O-MAR. A linear-by-linear test found significantly improved conspicuity of the ureters with O-MAR across all levels (p<0.001). The diagnosis was altered in four cases (13%). In two cases ureter stones, in one case a bladder stone and a bladder tumour were discovered with O-MAR.
**Conclusion:** O-MAR significantly improves the conspicuity of the bladder and ureters after TEP, and it can be indispensable for the detection of otherwise invisible lesions adjacent to metallic implants.

**B-0445** 16:56

**Wide-detector axial scan on revolution CT for combining application of ASIR-V and ASIR on unenhanced abdominal CT in vivo: the image quality and radiation dose evaluation**

Z. Zhu, X.-M. Zhao, C. Zhou; *Beijing/CN*

**Purpose:** To investigate the performance of combining adaptive statistical iterative reconstruction v technique (ASIR-V) and ASIR on non-contrast abdominal computed tomography (CT).
**Methods and Materials:** 141 patients (21-78 years; 78 male, 63 female) were enrolled. The scan protocol from group A to E were 0%, 20%, 40%, 60%, 80% ASIR-V respectively in 256 row wide-detector Revolution CT and F with no ASIR-V in 64-detector Discovery 750 HD CT. Each group was divided into 5 subgroups with 0%, 20%, 40%, 60%, 80% ASIR, respectively. Image noise was measured in spleen, aorta and muscle. The CT attenuation and image noise were analysed using the student *t*-test.
**Results:** With increased ASIR-V (from group A to E), the image noise decrease except group B in aorta and muscle ($Noise_B > Noise_A$, $P_{muscle\ A\&B}$=0.233, $P_{aortaA\&B}$=0.796). With fixed ASIR-V (same group) in three organs, the image noise decrease when ASIR increasing (subgroups 20% to 80%).With fixed ASIR (same subgroup) in three organs, the image noise increase when ASIR-V increasing except subgroup ASIR 20%-80% in group D and E in aorta, subgroup ASIR 40% in group B and C in muscle. The CTDIvol and DLP from group A to E decreased with significant difference when ASIR-V increased. The CTDIvol and DLP in group F are higher than group A with significant difference.
**Conclusion:** Simply applying either ASIR-V or ASIR is good for the image quality of non-contrasted abdominal CT scan. While applying both, the image quality deserved no further improvement but with some certain compromise.

**B-0446** 17:04

**Evaluation of a compressed-sensing accelerated high-resolution VIBE sequence for an improved morphologic and functional assessment (“one-stop shop”) of the uterus: initial analysis**

D. Hausmann[1], S. Ignjatovic[1], D. Kreul[1], M. Klarhöfer[2], M. Nickel[3], B. Kiefer[3], R.A. Kubik-Huch[1]; [1]*Baden/CH*, [2]*Zurich/CH*, [3]*Erlangen/DE (danielhausmann@hotmail.com)*

**Purpose:** To compare a prototype compressed-sensing-accelerated VIBE (csVIBE) with variable temporal resolution (range: 4-12 seconds) with a conventional VIBE (convVIBE) and to explore the benefits of additional quantitative perfusion analysis.
**Methods and Materials:** So far, 30 datasets (15 csVIBE/15 convVIBE) were independently assessed by two experienced readers in this IRB-approved study. All patients were examined on state-of-the-art 1.5T clinical MRI scanners (MAGNETOM Avanto Fit/Aera, Siemens Healthcare, Erlangen, Germany). Mann-Whitney U tests were performed to compare image quality (IQ), delineation of structures (DoS), artefacts (A) and diagnostic confidence (DC), which were rated on Likert-type scales (IQ/DoS/DC: 1 (non-diagnostic)-5 (perfect); A: 1 (no artefacts)-5 (severe artefacts)). For perfusion analysis a

Tofts model was used. Ktrans was measured in cervix (Cx), junctional zone (Jz) and myometrium (Mm). These values were compared between first (fp)/second phase (sp) of the menstrual cycle and between pre- and postmenopausal (post) women using t tests. Interobserver agreement was assessed.
**Results:** Ratings for IQ/DoS/DC of csVIBE (4.3±0.7/4.7±0.5/4.6±0.8; κ:0.8/0.2/0.7) and convVIBE (4.1±0.6/4.3±0.7/4±0.7; κ: 0.5/0.7/0.9) were generally high, while A was rated low (cs/convVIBE: 2±0.8/2.2±0.6; κ: 0.6/0.3) (p≥0.05). Ktrans in Jz was (not significantly) higher in fp than in sp and post (0.43 versus 0.19/0.24; (p=0.32)). Values were similar independent of cycle phase and menopausal status in Cx/Mm ((Cx (fp/sp/pm): 0.16/0.12/0.11; Mm (fp/sp/pm) 0.26/0.24/0.15); p≥0.05).
**Conclusion:** csVIBE is useful to morphologically and functionally assess the uterus in a single acquisition at a similar IQ compared to convVIBE. Perfusion analysis and multiple-phase imaging may have the potential to differentiate between various uterine pathologies.
*Author Disclosures:*
**D. Hausmann:** Equipment Support Recipient; Siemens Healthcare.
**B. Kiefer:** Employee; Siemens Healthcare.

**B-0447** 17:12

**Dual-energy CT dose reduction for GU CT while preserving diagnostic performance**

I. Duba[1], A. Khandelwal[2], J. Weaver[2], S. Leng[2], A. Ferrero[2], M. Wells[2], J.G. Fletcher[2], C. McCollough[2], A.F. Halaweish[2]; [1]*Malvern, PA/US*, [2]*Rochester, MN/US (Khandelwal.ashish@mayo.edu)*

**Purpose:** To investigate the diagnostic accuracy of low dose radiation protocols for urological dual-energy (DE) CT applications.
**Methods and Materials:** All data were acquired using a 3rd generation dual-source CT. Accuracy of reduced dose DECT renal stone classification was evaluated, as a function of phantom size and dose, by scanning 88 stones of known composition at 100%, 75%, 50% and 33% of routine dose. Findings were confirmed by measuring stone identification accuracy in reduced dose (via noise insertion) patient DECT's. Dose reduced CT urograms (CTU) were evaluated in 5 CTU cases at similar dose levels. Mixed kV, virtual non-contrast, iodine map and 50 keV monoenergetic images were generated for the full and dose reduced CTUs. Mixed image & 50 keV CNR values were compared across doses and diagnostic quality assessed by a uroradiologist.
**Results:** Phantom validation demonstrated up to 70% & 50% dose reduction for lateral widths <41cm and ≤45cm respectively, while maintaining 100% and 98% stone characterization accuracy respectively. Using this validation, clinical protocols were adjusted accordingly to account for new dose reductions. Iodine CNRs in 50keV low dose CTU images were an average of 82%, 101% and 126% of the full dose values for 33%, 50% and 75% of full dose, respectively. Radiologist review of CTU images found that all 75% dose exams and 2/5 of 50% dose exams were diagnostically acceptable.
**Conclusion:** Optimization of routine DECT acquisitions is achievable at lower dose levels. Continued optimization of CTU exams are ongoing using increased IR strengths and more reader validation.
*Author Disclosures:*
**I. Duba:** Employee; Siemens Healthineers. **C. McCollough:** Research/Grant Support; Siemens Healthineers. **A.F. Halaweish:** Employee; Siemens Healthineers.

### Thursday, February 28

08:30 - 10:00 Room N

### Abdominal Viscera

### SS 501

### Diffuse and focal pancreatic disease

*Moderators:*
E. Amosova; Moscow/RU
A. Taibbi; Palermo/IT

**B-0448** 08:30
**Value of static first-pass dual-energy CT perfusion analysis in patients with acute pancreatitis**
I. Yel[1], L. Lenga[1], J.L. Wichmann[1], M.H. Albrecht[1], T.J. Vogl[1], S.S. Martin[2]; [1]*Frankfurt a. Main/DE*, [2]*Charleston, SC/US (Dr.IbrahimYel@gmail.com)*

**Purpose:** The aim of this study was to investigate the correlation and discriminative diagnostic accuracy of dual-energy CT (DECT)-derived iodine uptake and fat fraction analysis in patients with acute pancreatitis of varying severity.
**Methods and Materials:** In this retrospective study, 53 patients with acute pancreatitis were included, who had undergone portal-venous-phase DECT examinations of the abdomen. Three blinded readers independently evaluated all cases using the modified CT severity index (CTSI) taking pancreatic inflammation, necrosis, and extrapancreatic complications into account. In addition, readers performed region-of-interest (ROI) measurements on DECT iodine perfusion images in the inflammatory pancreatic parenchyma. The correlation between disease severity and iodine uptake, as well as fat fraction values was investigated. The optimal discriminative cut-off iodine uptake value to diagnose severe acute pancreatitis was determined using receiver operator curve (ROC) statistics.
**Results:** Mean CTSI was 5.6 ± 3.2. A significant correlation was found between CTSI scores and mean iodine uptake (Pearson's correlation coefficient r = 0.657; p<0.01). An iodine uptake threshold of ≤ 1.7 mg/ml was found to indicate a severe acute pancreatitis with a sensitivity of 86.7% and specificity of 87.5%. Fat fraction analysis revealed no significant correlation in patients with CTSI scores ≤ 2 (p=0.278). In patients showing CTSI scores > 4, however, a moderate correlation was found between mean fat fraction values and disease severity (r = 0.482; p<0.05).
**Conclusion:** First-pass perfusion analysis in contrast-enhanced DECT shows high diagnostic accuracy to diagnose severe acute pancreatitis. Furthermore, iodine uptake directly correlates with CTSI scores.
*Author Disclosures:*
**J.L. Wichmann:** Speaker; Siemens, GE. **M.H. Albrecht:** Speaker; Siemens.

**B-0449** 08:38
**Diagnostic efficacy of acute necrotising pancreatitis on early CT scan and correlation with laboratory exams**
Y. Song, H. Park, M. Yu, Y. Kim, S. Jung, H. Jeon; *Seoul/KR (thdgustjs88@gmail.com)*

**Purpose:** To investigate the diagnostic efficacy of early and short-term follow up (<2 weeks) CT scan in necrotizing pancreatitis based on revised Atlanta classifications and to correlate it with laboratory exams.
**Methods and Materials:** From Sep 2007 to Aug 2018, a total of 210 patients (147 men and 63 women, mean age 54.9 years) with acute pancreatitis who underwent initial contrast enhanced CT at admission and a short term(within 2 weeks) follow up CT were included. Two abdominal radiologists performed a consensus review of two sets of CT in each patient, and determined the type of acute pancreatitis as interstitial oedematous pancreatitis (IEP) or necrotizing pancreatitis(NP) according to the revised Atlanta classifications. Values of laboratory exams including pancreatic enzymes and C-reactive protein were recorded and compared between the IEP and NP groups at each time point using t-test.
**Results:** The time interval between symptom onset and initial CT scan was 0-3 days (mean 1 day). The type of acute pancreatitis at admission was IEP (n=166) or NP(n=45). In IEP group, 53(31.9%) patients were diagnosed as NP at short term follow up CT(mean 6.6 days, range 1-13 days) (IEP-NP), while stationary or improved IEP was in remaining 113(68.1%) patients (IEP-IEP). At the time of initial CT, serum C-reactive protein was significantly higher in IEP-NP group (5.46±9.14mg/dl) than in IEP-IEP group (4.01±7.22 mg/dl) (P=.042).
**Conclusion:** The diagnosis of necrotizing pancreatitis can be overlooked on early CT scan(within 3 days after symptom onset). Serum C-reactive protein may be helpful in predicting the progression of IEP into NP in the early phase.

**B-0450** 08:46
**Relapse of type 1 autoimmune pancreatitis after steroid therapy: imaging findings and risk factors**
L. Zhu[1], T. Denecke[2], P. Asbach[2], B. Hamm[2], H.-D. Xue[1], Z.-Y. Jin[1]; [1]*Beijing/CN*, [2]*Berlin/DE (zhuliang_pumc@163.com)*

**Purpose:** To evaluate the imaging pattern of relapsed autoimmune pancreatitis (AIP) after corticosteroid therapy (CST), and to identify clinico-radiological risk factors for relapse.
**Methods and Materials:** Radiological and clinical database (2012-2018) of AIP patients treated and followed up for more than 18 months at our institution was retrospectively reviewed. Pattern of pancreaticobiliary manifestations and other organ involvement (OOI) were recorded. Univariate and multivariate analyses were conducted to identify risk factors predicting relapse.
**Results:** Among 103 AIP patients, 44 (42.7%) experienced relapse. The median time interval between initial attack and relapse was 17 months (range 4-61 months). Pancreas involvement was less frequent at relapse compared to initial attack (81.8% vs 100%, p=0.003) and pancreas size was smaller (mean size of pancreatic head, body and tail was 27.6, 17.3 and 20.2 mm at relapse, in contrast to 34.5, 25.0 and 27.3mm at initial attack, all (p<0.01). Patients who experienced relapse had higher frequency of extra-pancreatic bile duct (ExPBD) involvement at initial diagnosis compared to non-relapsed patients (52.3% vs. 25.4%, p=0.023). During relapse, extra-pancreatic bile duct involvement became even more frequent (59.1%) and more extensive. Univariate analysis identified male gender, initial serum IgG4 level, OOI, ExPBD involvement and kidney involvement as risk factors for relapse. Multivariate analysis revealed exPBD involvement as a significant independent predictor of relapse (hazard ratio 1.976, 95% confidence interval 1.149-3.570, p=0.023).
**Conclusion:** During AIP relapse pancreas was less involved and smaller, whereas bile duct and kidney were more involved. ExPBD involvement at initial diagnosis was a significant risk factor for relapse.

**B-0451** 08:54
**Early prediction of the severity of acute pancreatitis using radiologic and clinical scoring systems with classification tree analysis**
A. Ko, H. Park, E. Lee, H. Choi, J. Do, S. Park, B. Choi; *Seoul/KR*

**Purpose:** This study was to develop a decision tree model for the early prediction of the severity of acute pancreatitis (AP) using clinical and radiologic scoring systems.
**Methods and Materials:** For this retrospective study, 192 patients with AP who underwent CT less than 72 hours after symptom onset were divided into two cohorts: a training cohort (n = 115) and a validation cohort (n = 77). Univariate analysis was performed to identify significant parameters. For early prediction of disease severity, a classification tree analysis model was constructed using significant scoring systems shown by univariate analysis. To assess the diagnostic performance of the model, we compared the area under the ROC curve (AUC) with each selected single parameter and their diagnostic performance in the validation set.
**Results:** The Acute Physiology and Chronic Health Evaluation (APACHE)-II score, bedside index for severity in acute pancreatitis (BISAP) score, extrapancreatic inflammation on CT (EPIC) score, and Balthazar grade were included in the CTA model. In the training set, our CTA model showed a higher AUC (0.853) than that of each single parameter (all, p > 0.0125) while achieving specificity (100%) higher than and accuracy (94.8%) comparable to each single parameter (both, p < 0.0125). The CTA model also achieved similar diagnostic performance to the training set with an AUC of 0.833.
**Conclusion:** Our CTA model consisted of clinical and radiologic scoring systems and may be useful for the early prediction of the severity of AP and identification of high-risk patients who require close surveillance.

**B-0452** 09:02
**Preoperative prediction of pancreatic neuroendocrine tumours' grade: the added value of contrast-enhanced ultrasound with quantitative analysis**
Y. Dong, X. Han, D. Yang, W. Lou, Q. Zhang, W.-P. Wang; *Shanghai/CN (dr_mimi@163.com)*

**Purpose:** To evaluate whether the contrast-enhanced ultrasound (CEUS) with quantitative analysis could effectively predict tumour grade of pancreatic neuroendocrine tumours (pNETs) before operation.
**Methods and Materials:** From January 2016 to October 2018, twenty cases of pathologically confirmed pNETs were included. All the patients were confirmed by histopathology after surgery (n=18) or endoscopic -guided fine-needle biopsy (n=2). CEUS were performed before operation or biopsy. An ACUSON S2000 OXANA US system (Siemens Medical Solutions, Mountain View, CA, USA), equipped with a C6-1 transducer was used. Time intensity curves were created and quantitative indexes of CEUS were analysed. CEUS quantitative indexes were compared to histopathological features using the Mann-Whitney U test. Diagnostic accuracy was assessed by ROC-AUC analysis, sensitivity and specificity were assessed for each quantitative index.
**Results:** After injection of ultrasound contrast agents, most pNET lesions displayed focal hyperenhancement (85%) in the arterial phase. During the late phase, most pNET lesions were hyper- (80%) or iso-enhancing (20%). Liver metastases were detected in 3 patients during the late phase scan of the whole liver. Among all CEUS quantitative indexes, area under curve (AUC) was significantly higher in G2 and G3 pNETs tumours with ROC-AUC 0.723, sensitivity 80.7% (95% CI: 61.3-90.5) and specificity 64.1% (95% CI: 40.9-81.7).
**Conclusion:** Preoperative CEUS with perfusion analysis might be helpful in predicting tumour grade and liver metastases of pNETs. AUC might be the potential accurate parameter for identification of pNETs with malignant behaviour.

**B-0453** 09:10
**Diagnostic performance of computerised 3D CT texture analysis of pancreas for the assessment of patients with diabetes**
S. Jang[1], J. Kim[1], S.-Y. Choi[2], S. Park[1], J. Han[1]; [1]*Seoul/KR*, [2]*Gyeonggi-do/KR (junemiru@gmail.com)*

**Purpose:** To evaluate the diagnostic performance of computerised 3D CT texture analysis of the pancreas for assessing diabetes.
**Methods and Materials:** Among 2,493 patients with diabetes, 39 patients with type 2 diabetes (T2D) and 12 patients with type 1 diabetes (T1D) who underwent CT using two, selected CT scanners, were enrolled in our study. We compared these patients with age, body mass index- (BMI), and CT scanner-matched normal subjects. Computerised texture analysis for the entire pancreas was performed by extracting 17 variable features. A multivariate logistic regression analysis was performed to identify the predictive factors for diabetes and its subtypes. A receiver operator characteristic (ROC) curve was constructed to determine the optimal cut-off values for significant variables.
**Results:** In diabetes, the mean attenuation, standard deviation, variance, entropy, homogeneity, surface area, sphericity, discrete compactness, grey-level co-occurrence matrix (GLCM) contrast, and GLCM entropy showed significant differences ($P < .05$). Multivariate analysis revealed that a higher variance (adjusted OR, 1.002), sphericity (adjusted OR, $1.649\times10^4$), GLCM entropy (adjusted OR, $1.057\times10^5$), and lower GLCM contrast (adjusted OR, 0.997) were statistically significant differentiators of diabetes ($P < .05$). The mean AUCs for each feature were 0.654, 0.689, 0.620, and 0.613, respectively ($P < .05$). In the subgroup analysis, a larger surface area (adjusted OR, 1.000; $P = .025$) was a significant predictor for T2D.
**Conclusion:** Computerised 3D CT texture analysis of the pancreas could be helpful for predicting diabetes. A higher variance, sphericity, GLCM entropy, and a lower GLCM contrast were the significant predictors of diabetes.

**B-0455** 09:18
**DWI and dynamic MRI in the study of intraductal papillary mucinous neoplasms (IPMNs): pathological comparison with tumour grade correlation**
G. Giannotti, G. Tedesco, A. Beleù, G. Rizzo, N. Cardobi, R. De Robertis Lombardi, M. D'Onofrio; *Verona/IT (gabriele200590@gmail.com)*

**Purpose:** Evaluation of mural nodules (MNs) and septa of IPMNs through diffusion-weighted imaging (DWI) and contrast-enhanced magnetic resonance (CE-MR) for the characterization of tumour grade.
**Methods and Materials:** 96 patients with pancreatic IPMNs were included in the study. All patients had histological diagnosis after surgical resection (between 2012 and 2017) and a pre-operative MR exam. Histological grade of dysplasia for each lesion was available in all cases. There were 37 mild, 10 moderate, 24 high-grade dysplastic lesions and 25 ductal adenocarcinomas. MR exams were retrospectively and separately reviewed by two radiologists, blinded to surgical and pathological results. A third experienced radiologist played the role of judge in case of doubtful findings or conflicting analyses. The enhancement after i.v. injection of Gd and DWI signal intensity of mural nodules and cystic septa of IPMNs were judged.
**Results:** The degree of dysplasia was divided into two groups: mild-moderate and high-ductal adenocarcinoma. The Pearson's chi-squared test was used to verify the statistical correlation between imaging findings and IPMN histology. There was a statistically significant difference ($p<0.05$) between mural nodule enhancement and degree of dysplasia and DWI signal intensity of mural nodules and degree of dysplasia. Instead, there was no statistical significant difference ($p>0.05$) between septal enhancement and degree of dysplasia and DWI signal intensity of septa and degree of dysplasia.
**Conclusion:** Enhancement and DWI signal intensity of MNs can be used as parameters to predict the degree of dysplasia of IPMNs.

**B-0456** 09:26
**Solid pseudopapillary tumour of the pancreas: correlation of MR imaging appearance with biological behaviour**
W. Mingliang, M. Zeng; *Shanghai/CN (wang.mingliang@zs-hospital.sh.cn)*

**Purpose:** To describe MR imaging features of pancreatic solid pseudopapillary tumours (SPTs) and to identify MR imaging features in predicting biological behaviour.
**Methods and Materials:** Clinical, pathological and MR imaging data of 47 patients (8 men and 39 women; age range 13-58 years) with pathologically proven SPTs were retrospectively reviewed. Image analysis included tumour location, diameter, shape, margin, signal intensity on precontrast images, enhancement pattern, pancreatic duct dilatation, secondary pancreas atrophy and extrapancreatic spread. According to pathological biological behaviour of the tumour, all cases were divided into two groups (invasiveness group and non-invasiveness group). For data analysis, the comparison between subgroups was performed with Student's *t* test. Categorical variables were tested using $\chi^2$ test or Fisher's exact test.
**Results:** 47 lesions were found by surgical specimen analysis in all patients. All lesions were correctly identified and located with MRI. The tumours originated from pancreatic head and neck (n=17), body and tail (n=28), and extrapancreatic area (n=2). 22 lesions were found in invasiveness group and 25 lesions in non-invasiveness group. The median maximum diameter of SPTs in invasiveness group was 5.99±3.08 cm, and 4.8±2.28 cm in non-invasiveness group. Significant differences in age, tumour shape and tumour margin were found between two groups ($P<0.05$). There were no significant difference found in gender, tumour location, cystic changes, intratumoural haemorrhage, enhancement pattern, pancreatic duct dilatation, secondary pancreas atrophy and extrapancreatic spread.
**Conclusion:** MR imaging features such as tumour shape and tumour margin may preoperatively suggest the biological behaviour of SPTs, assisting decisions about treatment.

**B-0457** 09:34
**Solid pseudopapillary neoplasms of the pancreas: clinicopathological and radiological features according to size - 20-year experience from a high-volume centre**
R. De Robertis Lombardi[1], A. Grecchi[2], M. Catania[2], A. Beleù[2], G. Rizzo[2], M.C. Ambrosetti[2], M. D'Onofrio[2]; [1]*Peschiera del Garda/IT*, [2]*Verona/IT (annamaria.grecchi88@gmail.com)*

**Purpose:** To analyse and correlate clinicopathological and radiological features of resected solid pseudopapillary neoplasms of the pancreas according to their size.
**Methods and Materials:** Clinicopathological and radiological features of resected solid pseudopapillary neoplasms (SPN) of the pancreas over a 20-year period were retrospectively analysed. For the purpose of statistical analysis, tumours were divided into three groups according to their size (≤30 mm, 31-50 mm, and >50 mm). Clinicopathological and radiological features were compared among groups using Kruskal-Wallis and Fisher's exact tests.
**Results:** Between January 1997 and December 2017, the study population consisted of 106 patients, with a median age at diagnosis of 31 years (range 7-68). Patients with small tumours (≤30 mm) had a significantly higher age at diagnosis compared with the other groups of patients (p=.038). Large tumours (31-50 and >51 mm) were more frequently located in the pancreatic body/tail compared with tumours ≤30 mm (p=.008). No other significant differences were found between the clinicopathological features of the three groups of patients (all p>.05). Most tumours presented a mixed solid and cystic appearance (54.7%), with well-defined margins (87.7%). Tumours ≤30 mm were significantly more frequently entirely solid (53.8%) compared with larger tumours (p=.028). The rate of incorrect preoperative diagnosis was higher in tumours ≤30 mm compared with larger tumours, albeit without significant differences between groups (p=.561).
**Conclusion:** Malignancy in solid pseudopapillary neoplasms is not correlated with tumour size; tumours ≤30 mm may present atypical imaging features, which may overlap with those of other solid tumours of the pancreas.

**B-0458** 09:42
**Risk assessment for pancreatic fistula after pancreaticoduodenectomy with preoperative computed tomography**
R. Menghini, G. Zamboni, A. Cybulski, R. Valletta, G. Marchegiani, G. Mansueto; *Verona/IT (rossella.menghini@gmail.com)*

**Purpose:** to evaluate the predictive value of preoperative CT features for the risk of postoperative pancreatic fistula.
**Methods and Materials:** we included 58 Patients who underwent preoperative MDCT and pancreaticoduodenectomy in our institution. The Patients were divided in 2 groups in according to clinical data: 29 patients with postoperative pancreatic fistula (POPF) (group A), and 29 patients without POPF (group B). One reader reviewed the CTs and measured at the planned resection plane the main pancreatic duct (MPD) diameter, the density of the parenchyma in the different enhancement phases and the parenchymal thickness. The difference in attenuation between the venous and arterial phase was calculated as a surrogate for parenchymal fibrosis. Body composition was analysed by calculating visceral adipose tissue area (VAT), subcutaneous adipose tissue area (SAT), and skeletal muscle area at the L2-level using ImageJ software. Retrorenal fat thickness and psoas density were also measured. Fisher's exact test was used for categorical variables and Student's t-test for continuous variables.
**Results:** MPD diameter was 3,2±3,1 mm in group A and 6,9±3,3 mm in group B (p<0,0001). The mean attenuation difference between venous and arterial phase was -15,86 HU in group B and 0,89 HU in group A (p=0,0034). No significant difference was observed between groups for all the other parameters.
**Conclusion:** fibrosis, expressed by increasing enhancement of the normal pancreatic parenchyma at the planned resection plane, and MPD diameter may predict the occurrence of pancreatic fistula after pancreaticoduodenectomy.

**B-0454** 09:50
**Comparison of reduced field-of-view (rFOV) and full FOV (fFOV) diffusion-weighted imaging (DWI) in the assessment of insulioma: image quality and WHO grading**
M. He, H. Xue, Z. Jin; *Beijing/CN (377174431@qq.com)*

**Purpose:** To prospectively compare the imaging quality (IQ) of reduced field-of-view (rFOV) and full FOV (fFOV) DWI in detecting the insulioma and correlate the ADC value with WHO classification.
**Methods and Materials:** 48 patients suspected insuliomas underwent pancreas MRI with two DWI sequences. Two radiologists independently assessed the IQ with 4-point scale. The SNR and CNR were evaluated. The Wilcoxon signed rank test was used to compare IQ scores, CNR and SNR. The ADC value of the tumour and the parenchyma were compared by paired-t test between the two sequences. The association between ADC value and WHO classification was assessed by the Spearman correlation analysis. The inter-observer agreement was evaluated by linearly weighted kappa coefficients for IQ and ICC for ADC values.
**Results:** Thirty-six patients with 36 tumours and 21 histological results (G1: n=11, G2: n=10) were enrolled. The IQ score(3.64±0.487 vs. 3.310 ±0.577), SNR (22.520±8.690 vs. 10.284 ±3.321) and CNR (3.454±2.642 vs. 1.327±2.801) were significantly higher in rFOV DWI than in fFOV DWI (all P< 0.05). There is no significant difference in the ADC values with rFOV and fFOV DWI of the tumour (1.106±0.181 vs. 1.100±0.171×$10^{-3}$ $mm^2/s$) and the parenchyma (1.320±0.205vs.1.371±0.223×$10^{-3}$ $mm^2/s$). There is no correlation between the WHO grading and the ADC values of rFOV (r= 0.024) or fFOV (r= 0.142). Inter-observer agreement was good to excellent for IQ (rFOV:0.733-0.882, fFOV: 0.611-0.889) and ADC values (rFOV:0.757-0.915, fFOV: 0.709-0.790).
**Conclusion:** Compared with fFOV DWI, rFOV DWI provides significant better IQ in detecting insulioma. No correlation between ADC value and WHO grading was found in this study.

**08:30 - 10:00** **Sky High Stage**

### Breast

### MY 5

### Breast

*Moderators:*
E.M. Fallenberg; Munich/DE
R.M. Trimboli; Milan/IT

**B-0459** 08:30
**How preoperative sentinel lymph node contrast-enhanced ultrasound helps intraoperative sentinel lymph node biopsy in breast cancer: initial experience**
M. Lu; *Chengdu/CN (graceof@163.com)*

**Purpose:** To evaluate the value of sentinel lymph node contrast-enhanced ultrasound (SLN-CEUS) and surface tracing for the biopsy of intraoperative sentinel lymph nodes (SLN).
**Methods and Materials:** Between June 2015 and December 2017, 473 patients with early invasive breast cancer were recruited. Patients received an intradermal injection of microbubble contrast agent around areola the day before surgery. The locations and sizes of lymphatic channels (LCs) and SLNs were marked on the body surface using gentian violet. Then injection of double blue dye was performed half an hour before the operation. We compared the pathway of LCs and the location of SLNs obtained from SLN-CEUS and blue dye during an operation.
**Results:** Among the 473 patients, the mean number of LCs and SLNs detected by SLN-CEUS was 1.42 and 1.72, respectively, and the coincidence rate was 98.1% compared to blue dye during operation. The medium distance of SLN to skin measured by preoperative CEUS and blue dye was 1.95 ± 0.69cm and 2.03 ± 0.87cm, *P* =0.35. There were three enhancement patterns of SLN, including homogeneous enhancement, inhomogeneous enhancement, and no enhancement in our research, with the sensitivity, specificity, negative predictive value, and positive predictive value of SLN-CEUS for the diagnosis of SLN being 96.75%, 91.80%, 88.17% and 97.82%, respectively.
**Conclusion:** SLN-CEUS with skin marking can identify the pathway of LCs and the location of SLN, measure the distance of SLN to skin, and determine if SLN is metastatic. SLN-CEUS can be used as an effective complement of the blue dye method.

**B-0460** 08:34
**Contrast-enhanced spectral mammography: is it useful in predicting invasiveness in suspicious breast micro-calcifications?**
M. Gomaa, A.F.I. Moustafa; *Cairo/EG (mohammedgomaa555@yahoo.com)*

**Purpose:** To assess the ability of contrast-enhanced spectral mammography for the prediction of invasiveness of suspicious malignant micro-calcifications.
**Methods and Materials:** 150 cases were enrolled for analysis including suspicious malignant microcalcifications on mammogram with no related mass and with pathological proof. The microcalcification morphology and associated enhancement were reviewed according to BIRADS lexicon with results of pathology reviewed and classified into benign, atypical and malignant with evaluation of invasive components.
**Results:** Of the 250 with microcalcifications 130/250 cases with associated mass were excluded. 30/120 cases were benign and 90/120 cases malignant. 17/90 cases were diagnosed as invasive carcinomas and 63/90 DCIS. Out of the 63 patients with DCIS 15/63 cases were low grade, 7/63 cases intermediate grade and 51 cases were high grade (29 with micro-invasion). Pathological enhancement was associated with all 17 cases with invasive cancers, 50/51 cases of the high-grade DCIS and 4/7 of the intermediate invasive cases. No pathological enhancement was elicited with benign masses, all low-grade DCIS cases and 3/7 of the intermediate DCIS. Diagnostic performances of CESM in prediction of high grade DCIS or invasive cancer were sensitivity 98.5%, specificity 81.8% and accuracy 87.5%. The diagnostic performances of CESM in prediction of high grade in DCIS patients were sensitivity 98%, specificity 81.8% and accuracy 93.1%.
**Conclusion:** CESM has a pivotal role in assessment of suspicious micro-calcifications as the presence of associated non-mass enhancement is an indicator of invasive malignancy or high-grade DCIS. Lack of enhancement is favourable to diagnose non-malignant lesion or non-invasive DCIS.

**B-0461** 08:38
**Residual fibroglandular tissue (RGT) following unilateral and bilateral prophylactic mastectomies in BRCA 1/2 germline-mutation carriers**
B. Krug, O. Grinstein, W. Malter, B. Hanstein, F. Siedek, R. Schmutzler, D. Maintz, K. Rhiem, M. Hellmich; *Cologne/DE (Barbara.krug@uk-koeln.de)*

**Purpose:** The oncologic risk resulting from residual fibroglandular tissue (RGT) has not been sufficiently investigated in women carrying pathogen *BRCA 1/2*-germline mutations. Therefore, the presence of RGT was retrospectively evaluated in a unicentre approach.
**Methods and Materials:** All women with PM between 2006 and 2016 and at least one postoperative breast MRI were extracted from the centre's register. 2 radiologists evaluated the index breast MRIs independently using a structured questionnaire. Different assessments were assessed in consensus. The volume of RGT was quantified electronically.
**Results:** 169 women (338 breasts) were eligible. 67 patients had bilateral PM (41.2%) and 102 had unilateral PM and contralateral curative mastectomy (58.2%). RGT was found in 128 mastectomies (37.9%). 68 of the 128 breasts with RGT (53.1%) were related to bilateral PM, 37 (28.9%) to unilateral PM and 23 (18.0%) to curative mastectomy (p<0.0001). RGT was predominantly unifocal and retromamillary. The RGT volume was 6.7±11.9 ml in bilateral PM, 2.5±1.6 ml in unilateral PM and 3.2±1.9 ml in curative mastectomies. There was a positive correlation between preoperative breast volume and RGT in 75 cases with preoperative MRI (p<0.001) and a tendency towards a higher number of RGT in surgery units with <20 PM compared to those with >40 PM (p=0.033). The thickness of residual subcutaneous fat tissue correlated with RGT in non-retroareolar areas. 2 patients developed breast cancer 2 and 5 years after PM.
**Conclusion:** The radicalness of mastectomy may be influenced by the surgeon's and the patient's expectations regarding the aesthetic appearance of the breasts after PM.

**B-0462** 08:42
**Factors that influence performance of the contrast-enhanced ultrasound prediction model in 1023 breast lesions: a multi-center study**
L. Tang[1], Y. Chen[1], Z. Du[1], Z. Zhong[1], Q. Chen[2], J. Luo[2], L. Yang[3];
[1]*Fuzhou/CN*, [2]*Sichuan/CN*, [3]*Kunming/CN (cxxxmx@fjzlhospital.com)*

**Purpose:** To investigate the influence of patient and lesion characteristics on the diagnostic performance of contrast-enhanced ultrasound (CEUS) model in breast lesions.
**Methods and Materials:** We conducted a multicenter study with eight participating medical centers in China, which compared 1,023 breast lesions that were categorized as breast imaging reporting and data system (BI-RADS) 4 or 5, to evaluate a newly established CEUS-based diagnostic model, and to compare its results with patients' pathological outcomes. Univariate and multivariate logistic regression analyses were conducted to determine the influence of clinicopathological characteristics on the performance of CEUS model.
**Results:** Overall diagnostic results for CEUS model in diagnosis of breast lesions were sensitivity: 89.4%, specificity: 65.3%, positive predictive power: 66.4%, and negative predictive power: 88.9%. Logistic regression analysis showed that patients' age (odds ratio [OR]: 0.673; $P$=0.033), lesion maximum diameter (LMD; OR: 0.983; $P$=0.038), and distance from the lesion's deep edge to the pectoralis muscle (DtPM; OR: 1.093; $P$<0.001) were independent influencing factors. The model's diagnostic accuracy was greater for patients older than 35 years than younger patients ($P$=0.005), for LMD > 20 mm than LMD < 10 mm (OR: 1.61), and for DtPM < 3.05 mm than DtPM > 3.05 mm (OR: 1.81).
**Conclusion:** The application of CEUS model for breast lesions is promising. However, its diagnostic performance is influenced by patients' age; LMD and DtPM. Consideration of influencing factors is required to optimize clinical use of the CEUS model.

**B-0463** 08:46
**Effect of breast-conserving surgery with radiotherapy on the apparent diffusion coefficient of fibroglandular tissue at MRI**
N. Radovic, G. Ivanac, I. Biondic Spoljar, E. Divjak, I. Dumic Cule, B. Brkljacic; *Zagreb/HR (niko.radovic@gmail.com)*

**Purpose:** To determine whether breast-conserving surgery (BCS) with radiotherapy (RT) is associated with different apparent diffusion coefficient (ADC) values of fibroglandular tissue (FGT) when comparing the treated breast to the contralateral side on MRI.
**Methods and Materials:** This retrospective study included 108 women who underwent clinical MRI exams following unilateral BCS with RT. Diffusion-weighted imaging was performed at 1.5T using b = 0, 400, and 800 s/mm$^2$. ADC of FGT was calculated separately for each breast using manually drawn regions of interest which excluded areas containing any lesions. Fat-suppressed T2-weighted images were evaluated in order to investigate the presence of diffuse treatment-related breast oedema. Association of previous treatment with FGT ADC and association of treated-to-untreated ADC ratio with treatment-related oedema were assessed using two-tailed t-tests. Relationship between treated-to-untreated ADC ratio and time elapsed since completion of RT was assessed by calculating Pearson's correlation coefficient.
**Results:** Mean ADC value of FGT was higher on the treated side (1.995 ± 0.351 mm$^2$/s) compared to the contralateral breast (1.709 ± 0.309 mm$^2$/s)(p < 0.001). Treated-to-untreated ADC ratio was higher in patients showing diffuse post-treatment oedema (1.311±0.213) than in cases where it was absent (1.117±0.184)(p < 0.001). There was a moderately negative correlation between treated-to-untreated ADC ratio and time passed since completion of RT (R = - 0.442, p < 0.001).
**Conclusion:** Follow-up of BCS with RT is associated with increased ADC values of FGT, which could result in a greater contrast between normal breast parenchyma and recurrent cancer on quantitative diffusion-weighted MRI.

**B-0464** 08:50
**Relevance of arbitration in the management of discrepant screening mammography: Trieste's Breast Unit experience in 2013-2014 biennium**
C. Sachs, F. Giudici, M. Bortul, F. Zanconati, C. Gasparini, M. Assante, M. Tonutti, M.A. Cova; *Trieste/IT (cami_sachs@hotmail.it)*

**Purpose:** The introduction of digital mammography caused an increased rate of second level recalls. Even with the use of digital mammography (since November 2012) Trieste Breast Unit Recall Rate (RR) is one of the lowest in Italy. For this reason the role of the third reader in reducing recalls has been analyzed.
**Methods and Materials:** Mammography of all women adhering to screening between 2013 and 2014 biennium was included. Definition of discrepant and concordant reading number and arbitration outcome were obtained by using MR_mammo_report software. The discrepant cases were read by an "expert" third reader. Arbitrate impact, particularly recalls rate reduction and detection rate (DR) modification, was valuated.
**Results:** 95% of 21006 double readings were concordant (310 positive - 19740 negative). 956 readings (5%) were discordant. Arbitrate evaluation of discordant cases classified 536 negative mammograms (55%) and 420 positive (45%). Decreased recall rate was proved using a third reader (6.03% without arbitrate to 3.49% with arbitrate, p<0.001). Diagnosed carcinomas were 148 (DR =7%), of these 106 (72%) from concordant readings, 40 (27%) for positive arbitrate and 2 (1%) after negative arbitrate (interval cancers). Discordant readings showed a lower positive predictive value (PPV) compared to concordant ones (4.4% vs 34.2%, p<0.001).
**Conclusion:** Contribution by an "expert" third reader to keep RR into the European standards has been fundamental in our experience. Future purpose: to evaluate arbitrate cost savings, including women's behaviour for a spared useless recall.

**B-0465** 08:54
**Technique and diagnostic performance of contrast-enhanced spectral mammography: a systematic review**
A. Cozzi[1], M. Zanardo[1], C.B. Monti[1], L.A. Carbonaro[2], R.M. Trimboli[2], F. Sardanelli[1]; [1]*Milan/IT*, [2]*San Donato Milanese/IT (andrea.cozzi@gmail.com)*

**Purpose:** To perform a systematic review on technique and diagnostic performance of contrast-enhanced spectral mammography (CESM).
**Methods and Materials:** A literature search was performed using MEDLINE/EMBASE for studies investigating CESM. For each included article, we extracted: study design; contrast agent (CA) type, concentration, dose and flow rate; use and dose of a saline flush; total examination time (TET), first acquisition delay (FAD); order of views; sensitivity and specificity.
**Results:** Of 184 initially retrieved articles, 60 were included, totaling 8,225 examinations. Design was prospective in 31/60 studies (52%). Fifty-two studies reported CA type and concentration, iohexol 350 mgI/mL being the most used (16/52 studies, 31%). CA dose and flow rate were reported in 53/60 (88%) studies, with 1.5 ml/kg at a 3 ml/s flow rate in 38/53 studies (72%). Saline flush was used in 20/60 (33%); only 11/20 (55%) reported the dose (10-30 mL). A TET <10 minutes was reported in 39/40 studies (98%), 120 seconds FAD in 44/54 (81%). Order of views was reported in 29/60 (48%) studies; mediolateral-oblique view of the affected breast was firstly acquired in 9/29 (43%). Sensitivity was ≥ 90% in 31/38 (82%) studies while a specificity ≥ 70% was provided in 19/31 (61%).
**Conclusion:** As it was for breast MRI, CESM has high sensitivity and acceptable (but more variable) specificity. An agreement was found for some technical parameters but no dose finding study has been performed. Protocol standardization should be pursued to build a more robust evidence for this new contrast-enhanced breast imaging modality.
*Author Disclosures:*
**F. Sardanelli:** Advisory Board; Bracco, General Electric. Grant Recipient; Bayer, Bracco, General Electric, Real Imaging. Speaker; Bracco, General Electric, Siemens.

**B-0466** 08:58
**Development and validation of nomograms predictive of axillary nodal status to guide surgical decision-making in early-stage breast cancer**
J. Li, W. Ma, Y. Wu, L. Li; *Guangzhou/CN (lijiao@sysucc.org.cn)*

**Purpose:** To develop and validate nomogram models using preoperative noninvasive imaging parameters with clinical-related variables to predict the extent of axillary nodal involvement for stratifying treatment options.
**Methods and Materials:** From May 2007 to December 2017, 397 patients who underwent preoperative routine and dynamic contrast-enhanced MRI for clinical T1-2/N0 breast cancer with clinically negative axilla were enrolled. Patients with data on AUS were included in the study. MR imaging descriptors were retrospectively collected according to BI-RADS (5th edition). Based on logistic regression analyses, nomograms summarizing the strength of the associations between the predictors and each nodal status endpoint were developed. Predictive performance was assessed using the area under the receiver operating characteristic curve (AUC). Bootstrap resampling was performed for internal validation.
**Results:** Age, MRI descriptors (mass margin, lymph node margin, hilum and symmetry), AUS descriptors (cortical thickening and hilum), vascular invasion and HER2 were identified as predictors of any nodal disease. Nomograms that included age and imaging parameters showed good calibration and discrimination; AUC of was 0.809 (0.756, 0.863) for negative axilla (N0) versus any lymph node metastasis, 0.749 (0.675, 0.823) for one or two positive nodes versus N0, and 0.874 (0.819, 0.929) for at least three positive nodes versus two or fewer metastatic nodes. Additionally, the predictive ability of the three nomograms which added vascular invasion and HER2 was significantly elevated.
**Conclusion:** The preoperative nomograms facilitate patients to choose no axillary staging, sentinel lymph node biopsy or completion axillary lymph node dissection according to the burden of axillary nodal metastasis.

**B-0467** 09:02

**Ultrasound-guided vacuum-assisted resection: could it be enough for the diagnosis and treatment of intraductal papilloma?**

Z. Wang; *Beijing/CN (wzllg@sina.com)*

**Purpose:** To evaluate whether ultrasound-guided vacuum-assisted excisional biopsy (VAB) could be enough for the diagnosis and treatment of intraductal papilloma, and to evaluate the lesion characteristics and histologic features affecting the complete resection rate.
**Methods and Materials:** Between March 2008 and October 2016, 2816 patients underwent US-guided VAB. 101 (3.6%) were diagnosed with intraductal papilloma pathologically. The accurate rate and the complete resection rate of intraductal papilloma were evaluated by surgical biopsy or follow-up US. The follow-up period ranged between 14 months and 72 months; meanwhile, lesion characteristics were analysed.
**Results:** 83 (82.2%) were benign papilloma and the recurrence was observed in 8 cases. 2 were intraductal papilloma accompanied by invasive carcinoma. 16 (15.8%) were with signs of atypical hyperplasia by VAB. In them, one intraductal papilloma accompanied by severe atypical hyperplasia underwent surgery, and was diagnosed as invasive carcinoma. 4 cases accompanied by atypical hyperplasia demonstrated atypical intraductal hyperplasia by surgery. 3 cases demonstrated no atypical lesions by surgical pathology. The accurate rate of VAB was 99.0% (100/101). The complete excision rate of intraductal papillomas by VAB was 87.1% (88/101). Intraductal papilloma with largest diameter < 1 cm, with clear margin, without branch involvement or calcification had a significantly higher complete excision rate.
**Conclusion:** VAB is an effective method for the diagnosis of intraductal papilloma. If benign character was demonstrated pathologically, surgery may be avoided but regular follow-up is recommended. If histopathological examination confirms a papilloma with moderate to severe atypical hyperplasia, it was strongly recommended for surgical excision.

**B-0468** 09:06

**Diffusion kurtosis imaging (DKI) in breast cancer: diagnostic accuracy of DKI-derived values in characterization of breast lesions and correlation with prognostic factors**

H. Ahmad Ainuddin, S.K. Kirat Singh, K. Rahmat, M. Ramli Hamid, F. Fadzli, H. Ahmad Ainuddin; *Kuala Lumpur/MY*

**Purpose:** To evaluate the diagnostic performance of diffusion kurtosis imaging (DKI) in the characterization of breast lesions and study the correlation with cancer histological grade.
**Methods and Materials:** A total of 28 women with histologically proven 21 malignant and 28 benign lesions had undergone MRI with DKI/ DWI between September 2017 and October 2018. The imaging findings, DKI (at b : 0-2800) and quantitative DWI/ADC (b: 800)-derived parameters were analysed using Pearson and ANOVA tests.
**Results:** The mean kurtosis ( MK) demonstrated significantly higher values in malignancy (0.86 ± 0.18 ) compared to benign lesions (0.63 ± 0.22 ) (p<0.001). The ADC and mean diffusivity (MD) values were significantly lower for malignancy (ADC: 0.73($10^{-3}$ $mm^2$/s) ± 0.26 and MD: 1.21($10^{-3}$ $mm^2$/s) ± 0.40) compared to benign lesions (ADC: 1.43($10^{-3}$ $mm^2$/s) ± 0.31 and MD: 1.70($10^{-3}$$mm^2$/s) ± 0.39) (p<0.001). A cut-off MK/MD value of 0.74/1.39($10^{-3}$ $mm^2$/s) provided sensitivity of 81%/93% and specificity of 75%/81% with area under curve (AUC) of 0.82/0.83, respectively. ADC showed highest sensitivity/specificity (82%/100%) using a cut-off value of 1.12($10^{-3}$ $mm^2$/s) with AUC of 0.96. In lower tumour grades, MK (1.00 ± 0.22, grade 1) was significantly higher and ADC (0.46($10^{-3}$ $mm^2$/s) ± 0.29) was significantly lower compared to higher grade ( MK:0.70 ±0.14, ADC: 0.88($10^{-3}$ $mm^2$/s ± 0.26, grade 3) (p<0.05).
**Conclusion:** DKI provides additional functional information and is useful in the characterisation of breast lesions. The higher MK, lower diffusivity and lower ADC values showed strong correlation with malignancy.

**B-0469** 09:10

**Can 18 F-FDG PET/CT predict invasive components in DICS of breast diagnosed by needle biopsy? Comparison with variable imaging modalities including mammography, USG and MRI**

N. Choi[1], Y. Kim[1], N. Jung[1], B. Kang[2], J. Lee[2]; [1]*Bucheon/KR*, [2]*Seoul/KR (yossi6876@gmail.com)*

**Purpose:** The purpose of this study was to determine whether the 18F-fluorodeoxyglucose (FDG) positron emission tomography/computed tomography (PET/CT) predicts the underestimation of invasive cancer in patients with biopsy-proven DCIS.
**Methods and Materials:** This study retrospectively reviewed 167 cases in 162 patients with biopsy-proven DCIS who underwent preoperative 18F-FDG-PET/CT between April 2008 and September 2015. The clinicopathological and imaging findings were compared between subgroups divided by the presence of invasion and maximum standardized uptake value (SUVmax), respectively. The correlation of tumour characteristics with underestimation of invasive component was performed. The receiver operating characteristics (ROC) analyses were performed for each imaging modality to predict the invasion.
**Results:** Final pathology was confirmed as pure DCIS in 120 and DCIS with invasion in 47 cases. The age, SUVmax, semiquantitative and visual analyses at 18F-FDG-PET/CT and pathologic tumour size were significantly different between DCIS with invasion group and pure DCIS group. High SUVmax was significantly associated with age, the primary tumour size on MRI, visual detection of primary tumour and axillary lymph node on 18F-FDG-PET/CT, the presence of invasion and pathologic tumour size. The underestimation of invasive cancer was significantly correlated with age (p=0.0138), semiquantitative and visual analyses on 18F-FDG-PET/CT (p =0.0001 and p=0.0028) and tumour size (p=0.0026). The area under the curve (AUC) on ROC analysis was 0.541 for mammography, 0.550 for US, 0.500 for MRI, 0.670 for semiquantitative analysis and 0.620 for visual analysis on 18F-FDG-PET/CT.
**Conclusion:** The 18F-FDG-PET/CT can be a useful imaging tool to predict the upgrade of DCIS to invasive cancer.

**B-0470** 09:14

**Reliability of contrast-enhanced spectral mammography (CESM) in the pre-surgical evaluation of breast cancer: CESM vs histological maximum diameter**

P. Tonelli, C. Boeri, D. Cirone, S. Bianchi, V. Miele, J. Nori Cucchiari; *Florence/IT (paolina.tonelli@gmail.com)*

**Purpose:** The first aim is to assess the reliability of CESM in pre-surgical measurement of breast cancer (BC), compared to the histological size on the surgical piece. The secondary aim was to analyse what factors influence the gap, between CESM and histological measurements.
**Methods and Materials:** We retrospectively evaluated 167 women with histologically proven (in situ or invasive) BC who underwent pre-operative CESM, from from 1.1.2016 to 31.1.2017. Pearson's correlation coefficient was calculated between CESM size and histological size. Further analyses to evaluate factors influencing the discrepancy between the CESM measurement and histological measurement were conducted.
**Results:** CESM measurements showed a strong, consistent correlation with the histological findings (correlation coefficient = 0.852). A slight CESM size overestimation was detected. A gap of 10 mm between CESM diameter and histological diameter was assumed as a critical cut-off. A prevalence of invasive ductal carcinoma associated with an in situ ductal carcinoma was found in cases with difference between CESM size and histological size out of the cut-off. This type of cancer has a mass+non-mass enhancement.
**Conclusion:** These results encourage the use of CESM in pre-surgical staging of BC. The ability to measure non-mass enhancement extension affects the greater discrepancy between the two measurements.

**B-0471** 09:18

**Impact of 18F-FDG-PET/CT using time-of-flight in evaluating axillary lymph node metastases of breast cancer**

M. Mori, T. Fujioka, J. Tsuchiya, K. Kubota, U. Tateishi; *Bunkyo-City Tokyo/JP (m_mori_116@yahoo.co.jp)*

**Purpose:** To evaluate the performance of preoperative N-staging assessment in breast cancer by 18F-FDG-PET/CT using three-dimensional time-of-flight (3D-TOF).
**Methods and Materials:** Sixty-three women with breast cancer (mean age 60 years; range 30-84 years) underwent PET/CT scan with 3D-TOF reconstruction system (Celesteion, Canon Medical Systems). For axillary lymph nodes, visual assessment, short axis diameter, and the maximum standardized uptake value (SUVmax) were compared with pathological diagnosis.
**Results:** There were 61 patients with invasive breast carcinoma (mean invasive diameter 18.9 mm; range 2-90 mm) and 2 patients with non-invasive carcinoma. Axillary lymph node metastases were histologically confirmed in 10 of 63 patients (16%). By visual assessment, there were 8 true positives, 10 false positives, 43 true negatives, and 2 false negatives, respectively. Sensitivity, specificity, positive predictive value (PPV), negative predictive value (NPV), and accuracy were 80%, 77%, 40%, 95%, and 78%, respectively. Histological lymph node metastases were significantly associated with short axis diameter of the lymph node (7.2 vs. 6.1 mm, p = 0.03) and SUVmax of the lymph node (1.60 vs. 0.64, p = 0.02). The area under ROC curve of SUVmax was larger than that of the short axis diameter, but there was no significant difference (0.885 vs. 0.742, p=0.0829).
**Conclusion:** Both visual assessment and SUVmax measurement showed good results in axillary lymph node evaluation. 18F-FDG-PET/CT using 3D-TOF achieved high accuracy in N-staging of breast cancer.

**B-0472** 09:22

**The development of a novel manganese dioxide-based nanoparticles and real-time monitoring of its anticancer effect by MRI**

X. Zhou, B. Xiao, H. Hu, J. Tang; *Zhejiang/CN (misaki20@163.com)*

**Purpose:** Facile synthesis a manganese dioxide ($MnO_2$)-based MRI contrast agent and in vivo evaluation of its photothermal breast cancer therapy.
**Methods and Materials:** Bovine serum albumin (BSA)-$MnO_2$-melanin-like-polymer nanoparticle (BMD NP) was obtained via $KMnO_4$-initiated in situ dopamine-polymerization-induced self-assembly in the presence of BSA, and its size and $r_1$ relaxivity were carefully measured. Mice were injected with 4T1 breast cancer cells for MR imaging. Images were acquired before injection and at 30, 60, 90, and 120 min post-injection and then signal-to-noise ratio (SNRpost/SNRpre) at the tumour ring was quantitatively calculated. Mice bearing same tumours were randomized into the following four groups: BMD NP with/without photothermal therapy; PBS with/without photothermal therapy as control groups. The tumour sizes were measured and calculated every other day.
**Results:** BMD NP had h a diameter of 60 nm and a high $r_1$ relaxivity of 38.14 $mM^{-1}$ $s^{-1}$, which was incredibly 9 times higher than those of clinically used contrast agent (Gd-DTPA). In vivo, an obvious SNRpost/SNRpre at the tumour margin showed 1.54 times that of contralateral tissue at 30 min post-injection, which provided detailed imaging features for precise diagnosis differentiation. The group treated with both nanoparticle and irradiation showed complete ablation without recurrence in 20 days, and the tumour-free period up to 60 days, exhibiting excellent therapeutic efficacy in comparison with the other three groups.
**Conclusion:** The BMD NP having excellent MRI contrasting capability and PTT efficacy plus its facile synthesis was a very promising theranostic agent for MRI-mediated photothermal anti-cancer therapy.

**B-0473** 09:26

**Survey: imaging surveillance among Belgian breast clinics for primary locoregional breast cancer in non-high-risk women**

J. Swinnen, M. Keupers, R.M. Prevos, S. Postema, M. Lavens, T. Thywissen, P. De Beul, J. Soens, C. Van Ongeval; *Leuven/BE (jeroen.swinnen@gmail.com)*

**Purpose:** To assess imaging surveillance among Belgian breast cancer (BC) centres for primary locoregional breast cancer in non-high-risk women, compared to the international practice guidelines.
**Methods and Materials:** An anonymous e-mail checkbox survey was sent to the coordinating radiologists and clinicians of all Belgian BC centres, 01/2017-06/2017. All departments received telephone follow-up. For different treatment subgroups, respondents were asked their preferred imaging modalities, onset, frequency, intermediate frequency alteration and termination of imaging surveillance.
**Results:** Survey was completed by 55/98 (56%) radiologists and clinicians from 37/49 centres (76%). After radiation therapy, 17 (31%) respondents commenced imaging follow-up after a 6-month and 18 (33%) after a 12-month imaging delay. If no adjuvant radiation therapy, 48 (87%) respondents started imaging follow-up 12 months after diagnosis. After sentinel breast-conserving surgery, 55 (100%) and 51 (93%) respondents performed annual radiographic and ultrasound screening, respectively, with 21 (38%) routinely implementing tomosynthesis. Imaging of the reconstructed ipsilateral mastectomy site was performed by 12 (22%) respondents. Annual MRI surveillance was not recommended by 52 (95%) respondents. For BIRADS density C/D breasts, 15 (27%) respondents performed annual MRI. Intermediate alteration of annual imaging frequency was recommended by 17 (31%), with 13 (76%) respondents recommending consecutive biannual imaging. Imaging was not adjusted according to tumour subtype by 47 (85%) respondents.
**Conclusion:** Most Belgian BC centres perform routine ultrasound screening, although controversial among international guidelines. Use of MRI is limited to specific indications, according to the guidelines. Breast tomosynthesis is emerging as a routine surveillance tool despite performance remaining unclear.

**B-0474** 09:30

**Combining ultrafast TWIST VIBE Dixon sequences with diffusion-weighted imaging: a clinically applicable evaluation tool with high accuracy for suspicious masses in breast MRI**

S. Ellmann, E. Wenkel, S. Peter, M. Dietzel, E. Weiland, R. Janka, T. Bäuerle, M. Uder; *Erlangen/DE (stephan.ellmann@uk-erlangen.de)*

**Purpose:** To develop an evaluation tool for classification of suspicious masses in breast MRI when using TWIST VIBE Dixon (TVD) dynamic sequences combined with diffusion-weighted imaging (DWI), and compare it to a tool based on conventional dynamic contrast enhancement (DCE) and DWI. As ultrafast TVD sequences offer the potential to shorten breast MRI protocols, diagnostic accuracy might be hampered. A special focus was thus to maintain high diagnostic accuracy in lesion classification.
**Methods and Materials:** 65 patients received breast MRI between 02/2014 and 04/2015, with 83 reported lesions (60 malignant, 23 benign). Inclusion criteria were suspicion of breast cancer or pre-therapeutic staging. Patients with non-mass-enhancements only were excluded. The protocol consisted of our institute's standard protocol complemented by ultrafast prototype TVD sequences. The apparent diffusion coefficient (ADC) and the peak enhancement of the TVD sequences were used to calculate a generalised linear model (GLM) for malignancy prediction. A second model was calculated using ADC and DCE curve types. Generalisability was ensured by applying leave-one-out cross validations. For easy application of the GLMs, nomograms were created.
**Results:** The GLM based on ultrafast TVD sequences and ADC performed comparably accurate to the model based on conventional DCE and ADC (sensitivity 93.3% vs. 93.3%, specificity 91.3% vs. 87.0%, PPV 96.6% vs. 94.9%, NPV 84.0% vs. 83.3%; no significant differences).
**Conclusion:** This study presents a method to reduce breast MRI examination time while maintaining diagnostic accuracy with ultrafast TVD sequences. The presented GLMs can easily be applied in clinical routine using the supplied nomograms.
*Author Disclosures:*
**E. Wenkel:** Advisory Board; Siemens. **E. Weiland:** Employee; Siemens Healthineers. **R. Janka:** Advisory Board; Bracco, Siemens. **T. Bäuerle:** Advisory Board; Bracco. **M. Uder:** Advisory Board; Bracco, Siemens.

**B-0475** 09:34

**Accuracy comparison of breast cancer detection based on microcalcifications between full-field digital mammograms and synthetic 2D mammograms using a deep convolution neural network**

M. Gurumoorthy[1], S. Kaur[1], N. Kwan Hoong[2], S. Soo[1], K. Rahmat[1], C. Hongmin[3]; [1]*Kuala Lumpur/MY*, [2]*Petaling Jaya/MY*, [3]*Guangzhou/CN (mahen123owen@hotmail.com)*

**Purpose:** To retrospectively compare the accuracy of breast cancer detection based on the analysis of microcalcifications between full-field digital mammograms (FFDM) and synthetic 2D mammograms (C-View) using a deep convolution neural network (DCNN) computer model.
**Methods and Materials:** With institutional ethics committee approval, we retrospectively collected FFDM and digital breast tomosynthesis (DBT) cases using Hologic DBT system. The results of 67 consecutive breast biopsies performed for suspicious microcalcifications via stereotactic-guided core biopsy or surgical excision in 2016 were analysed (22 malignant, 45 benign). Image datasets which consisted of craniocaudal and mediolateral (MLO ) views (FFDM and C-view ) were analysed in a blinded manner using DCNN and provided a probability of malignancy. The DCNN uses probabilistic relationships between breast disease and mammography findings to predict malignancy. The accuracy of DCNN in FFDM images and DCNN in C-view images are compared.
**Results:** DCNN use in FFDM achieved an accuracy of 56.9%, which was very similar to the accuracy of DCNN in C-view which was 56.34%, in predicting breast cancer malignancy.
**Conclusion:** The analysis indicated that the detection accuracy based on microcalcifications between FFDM and synthetic 2D mammogram was comparable. The utilisation of C-view in this clinical setting hence can be considered.

**B-0477** 09:42

**Initial experience of an online mammographic screening test**

M. Iodice[1], B. Brancato[2], L.A. Carbonaro[1], A. Bert[3], C. Perassi[3], C. Zuiani[4], F. Sardanelli[1]; [1]*Milan/IT*, [2]*Florence/IT*, [3]*Turin/IT*, [4]*Udine/IT (maiodice@gmail.com)*

**Purpose:** To evaluate the performance of breast radiologists in an online mammographic screening test and their acceptance of it.
**Methods and Materials:** A mammographic test was composed by 132 screening mammograms with about 1/3 positive cases. Thresholds were set at 62% of sensitivity and 86% of specificity, resulting from 3/4 of 17 expert radiologists exceeding. Test was passed when both thresholds were achieved. The test was free and disposable online for all radiologists members of Italian College of Breast Radiologists (ICBR) by SIRM; no dedicated workstations but average personal computers were required.
**Results:** Five months after the test was published, 488 radiologists signed up for it (27% of ICBR members), 194 (41%) completed it and 88 (45%) passed. Overall 44/194 radiologists (23%) passed the test at the first time: 67/194 radiologists (35%) repeated the test at least once, and 44/67 (66%) of them passed it. Median sensibility resulted 62% and median specificity 86%. Radiologists who passed the tests read a higher number of mammograms per year (median mammograms per year 2528 vs 1090, P<0.001), and more likely work in organised screening programs (40.3% vs 21.0% radiologists who do not work in organised screening programs, P<0.001).

**Conclusion:** With the exception of few local courses with an included final test, no mammographic screening test has been available for Italian radiologists to challenge themselves so far. The SIRM mammographic online test was well accepted by radiologists and the initial results showed a balanced rate of tests passed.

**B-0478** 09:46

**Imaging characterisation of breast desmoid tumours**

M. Le Boulc'h[1], A. Lusque[1], C.S. Balleyguier[2], C. Dratwa[3], P. Cherel[4], M. Kind[5], C. Vaysse[1], S. Le Guellec[1], B. Boulet[1]; [1]*Toulouse/FR*, [2]*Villejuif/FR*, [3]*Paris/FR*, [4]*Saint Cloud/FR*, [5]*Bordeaux/FR (morwenn.le.boulch@gmail.com)*

**Purpose:** Breast desmoid tumours (BDT) are very rare myofibroblastic tumours, locally aggressive without metastatic potential, with high rate of recurrence. The diagnosis, based on core needle biopsy (CNB), could be very difficult, especially for sporadic tumours without CTNNB1 mutation. Their radiologic patterns have not been studied much and are closed from breast cancer radiologic patterns even though treatments differed. For BDT, watch and wait is the front line approach.
**Methods and Materials:** 34 patients with 36 pathologically confirmed BDT were included in this national French multicentric retrospective study. Radiological examinations were reviewed by an expert.
**Results:** Median presentation age was 46 years, mostly women (91.2%), and 25.7% had prior history of breast surgery or biopsy. Mammography visualised the tumour in 14/14, tomosynthesis 1/1, ultrasound in 26/27, magnetic resonance imaging (MRI) in 26/26 BDT in whom it has been performed. In mammography, BDT were spiculated masses or architectural distortions. Ultrasonography interest was to permit CNB needed. MRI helped for diagnosis and preoperative staging. In MRI, BDT were masses, spiculated (92.3%), with spicules touching skin and/or major pectoralis muscle (87.5%), with a fascial tail sign (26.9%). They had early enhancement, persistent on later slides. Sporadic BDT without CTNNB1 mutation had demographic, mammographic and MRI-specific patterns.
**Conclusion:** It is the biggest BDT study ever published. Spiculated breast mass could be BDT, moreover, if some spicules touch skin and/or major pectoralis muscle, or if it owns a fascia tail sign. Persistent enhancement on MRI later slides permit to distinguish BDT from breast cancer.

**10:30 - 12:00** **Room C**

### Radiographers

### SS 614

### Fine-tuning CT

*Moderators:*
D. Biddle; Cambridge/UK
M. Raissaki; Iraklion/GR

**B-0479** 10:30

**Application of low-radiation and low-contrast dose protocol combined with high-strength ASIR-V algorithm in 'one-stop' coronary and carotid and cerebrovascular CT angiography**

W. Li, Y. Zhigang, P. Han, Z. Li; *Chengdu/CN (452766554@QQ.com)*

**Purpose:** To investigate the value of low-radiation dose and low-contrast agent dose (double-low) protocol combined with high-strength adaptive statistical iterative reconstruction (ASIR-V) algorithm in the 'one-stop' coronary and carotid and cerebrovascular CT angiography.
**Methods and Materials:** 80 patients with suspected cardiovascular and cerebrovascular disease were randomly divided into two groups. In both groups, coronary CT angiography (CCTA) scan was immediately followed by carotid and cerebrovascular CT angiography (CCCTA). Group A (n=42) used double-low protocol: CCTA: 80 kV, 550 mA, 80% ASIR-V; CCCTA: 80 kV, 500 mA, 70% ASIR-V, with a 0.6 ml/kg contrast dose. Group B (n=38) used routine-dose protocol: CCTA: 100kV, 500 mA, 50% ASIR-V; CCCTA: 100 kV, 450 mA, 50% ASIR-V, with a 0.8 ml/kg contrast dose. The subjective image quality was assessed using a 4-point system (4=best). Contrast-noise-ratio (CNR) for vessels was calculated to evaluate the objective image quality of CTA. The dose length product of both groups was recorded and the effective dose was calculated.
**Results:** There was no significant difference in the objective and subjective (A vs. B: 3.50±0.68 vs. 3.60±0.73, P=0.54) image quality for these images between the two groups. The group A had 25% less contrast dose (42±6.69 ml vs. 56±7.56 ml) and 70% less overall radiation dose (1.03±0.28 mSv vs 3.38±0.80 mSv) than group B (both P <0.001).
**Conclusion:** The 'one-stop' cardiovascular and cerebrovascular CTA using double-low technique combined with higher strength ASIR-V algorithm can effectively reduce the radiation and contrast dose without loss of image quality compared with the routine-dose CTA protocol.

**B-0480** 10:38

**Assessment of coronary in-stent stenosis using high-resolution scan mode and dual-energy CT: a phantom study**

M. Svanteson, M. Afadzi, A.C. Martinsen, N.E. Kløw, E.K. Lysvik, K. Bjørklund, T.R. Enden; *Oslo/NO (monasvanteson@gmail.com)*

**Purpose:** To assess image quality of an in-stent stenosis model comparing single energy with and without novel high-resolution (HiRes) scan mode and dual energy.
**Methods and Materials:** An in-stent stenosis model with a straw containing a 3.5-mm expanded coronary stent was placed within an anthropomorphic chest phantom. The stenosis was created with lip-balm (-120HU) imitating a soft plaque. The lumen was filled with contrast (300HU). Five scans were performed; 100 and 120 kV -/+ HiRes, and dual energy reconstructed to monochromatic images at 50, 68, 74, 90, 100, and 130 keV. Reconstructions with standard and detail filters were performed for all scans plus high-definition (HD) filters for HiRes scans. Two radiologists blinded to scan parameters evaluated the reconstructions in a random order. Stent diameter, stenosis grading, and image quality on a 5-point scale (stent and plaque visualization) were assessed. Noise texture deviation (NTD) and noise power spectrum (NPS) were assessed using 5 images for all reconstructions.
**Results:** The mean overall image quality (stent+plaque score) for 100kV, 100 kV+HiRes, 120 kV, 120 kV+HiRes, and dual-energy scans were 5.50±0.7, 6.75±0.4, 6.25±1.1, 7.0±0.0 and 4.2±1.1, respectively. The mean underestimation of stent diameter with various filters was 27.6±0.1% for standard, 23.9±0.1% for detail, 17.5±0.0% for HD-standard, and 16.5±0.0% for HD detail. There was no difference in NTD between reconstructions (p=0.22). The HD detail filter had the highest NPS peak frequency, indicating finer image texture.
**Conclusion:** This phantom study indicated that high-resolution CT with HD-detail resulted in the best image quality for assessment of in-stent stenosis.

**B-0481** 10:46

**Patient habitus and renal volume have no effect on a reduced patient-specific contrast media administration during renal CTA: impact on contrast media, radiation dose and image quality**

C. Saade; *Beirut/LB (charbel.saade@aub.edu.lb)*

**Purpose:** To investigate the effect of patient kidney volume, patient habitus and contrast media volume (CMV) on renal artery and parenchymal opacification using a patient-specific contrast formula during renal CTA (rCTA).
**Methods and Materials:** IRB approval for this retrospective study was obtained. RCTA was performed on 101 patients with suspected renal disease. Patients were assigned to a patient-specific contrast formula protocol that is based on patient cardiovascular dynamics. Mean renal volume was quantified using computer-aided software. Multivariate analysis compared renal and contrast media volumes, and effective radiation dose. For each patient, the arteriovenous contrast ratio (AVCR), corticomedullary contrast ratio (CMCR) and contrast-to-noise ratio (CNR) were calculated employing a Student's t test. Regression analyses were performed between each of the CMV groups: 1: <37mL, 2: >37-<50mL and 3: >50mL. Receiver operating (ROC) and visual grading characteristics (VGC) measured the confidence intervals and image quality, respectively.
**Results:** There was no significant difference in patient demographics and radiation dose between each CMV group (p>0.05). Increased mean opacification of the renal arterial vasculature (≥232.34±56.19), parenchyma; anterior cortex (≥101.57±33.62) and medulla (≥49.60±19.30), and posterior cortex (≥98.67±33.98) and medulla (≥47.59±21.01) were above these thresholds (p>0.05). Mean AVCR and CMCR had no statistical significance (p>0.05) between each kidney with a strong correlation of opacification difference between the renal cortex and medulla in each kidney (r=0.993) and renal artery and vein (r=0.946). Both VGC and ROC demonstrated increased confidence in image quality (p<0.03) and pathology (p<0.0001) detection, respectively.
**Conclusion:** Patient habitus and kidney volume have no statistical significance in CMV reduction when employing a patient-specific contrast media formula during rCTA.

**B-0482** 10:54

**The study of usefulness of blending injection method in dynamic liver CT scanning with reduced contrast medium**

J. Kawashima, F. Washizuka, H. Nakano, Y. Shimada; *Tokyo/JP (junji.kawashima@med.toho-u.ac.jp)*

**Purpose:** Currently, there are concerns about contrast CT examinations leading to CIN in patients with CKD (chronic kidney disease). Patient with CKD are required to reduce the contrast medium. However, the injection time is fixed in dynamic CT scan. Therefore, reducing the amount of contrast medium causes a reduction in the injection pressure, which disrupts the bolus injection. In this study, we investigated effect of blending injection guaranteeing normal injection pressure.

Thursday

**Methods and Materials:** The subject is a patient who did dynamic liver CT scan. The normal case is 600 [mgI/kg] but patient with CKD case is 480[mgI/kg]. Patients with CKD were randomly assigned to the following 2 groups: 1. single injection (injection of contrast medium only); 2. blending injection (blend of contrast medium and saline). From the obtained image, comparison was made with normal cases, single injection, blending injection. For the study items, the CT numbers of each scan phase were compared.
**Results:** The variability of the CT number decreased in each scan phase. The medium value of the liver in the portal phase was 103.0HU for single injection and 117.5HU for blending injection. Addition of the saline solution significantly increased the CT number. Blending injection resulted in an increase in volume, so injection pressure could be maintained and significantly increased CT number.
**Conclusion:** Blending injection significantly improved the contrast effect in dynamic liver CT scan.

**B-0483** 11:02
**Post-mortem computed tomography angiography (PMCTA): optimising a targeted PMCTA protocol**
C. Robinson, A. Deshpande, G. Rutty, B. Morgan; *Leicester/UK (claire.robinson@uhl-tr.nhs.uk)*

**Purpose:** Post-mortem computed tomography angiography (PMCTA) is essential to diagnose coronary artery disease as a cause of death. A technique was developed using negative contrast (air) and positive contrast (PC) (clinical contrast) to delineate soft plaques and calcified stenosis. The behaviour of the contrasts, together with factors that may influence the efficacy of PMCTA, has been explored and an optimized protocol has been developed.
**Methods and Materials:** 200 cases were selected from our database of PMCT cases that had successful PMCTA. Each scan was evaluated to determine the order in which the contrast was administered, the degree of vessel filling (classified as 0%, 1-25%, 25-50%, 50-75% and 75-100%) and factors that may have affected the PMCTA.
**Results:** 8 cases with pathologically occluded vessels were excluded. 192 cases were included in the analysis (111 male, mean age 71.1 years (range 18-99)), 102 had the negative contrast first protocol. Using PC first achieves filling of the left anterior descending and circumflex arteries in all cases and only 2% of right coronary arteries failed to fill; a greater percentage of cases achieve more than 50% filling and more clots are removed from the aorta. Multiple injections of contrast achieve better filling of all vessels and increases confidence in diagnosing pathologies. Rolling the deceased into the right decubitus position prior to the third run removes all but 0.5% of persistent clots.
**Conclusion:** A targeted PMCTA protocol of PC first with rolling prior to the third injection achieves the optimal imaging and highest diagnostic potential of PMCTA.

**B-0484** 11:10
**Effect of the scan mode and focal spot size on airway measurement for ultra-high-resolution computed tomography**
R. Mikayama, T. Shirasaka, H. Yabuuchi, Y. Sakai, T. Kojima, M. Kondo, H. Yoshikawa, T. Kato; *Fukuoka/JP (mt_mika_jan_5@yahoo.co.jp)*

**Purpose:** The large inaccuracies were confirmed for small airway measurements in conventional computed tomography (CT). Ultra-high-resolution CT (UHRCT) might improve airway measurements accuracy using precise scan modes with minimal focal spot. The purpose of our study was to assess the effects of the scan mode and focal spot size on airway measurements for UHRCT.
**Methods and Materials:** The COPDGene phantom II containing a plastic tube simulating airway inner diameter of 3 mm and wall thickness of 0.6 mm with rotation at 30 degrees was scanned with super-high-resolution (SHR, beam collimation of 0.25 mm × 160 rows) and high-resolution (HR, beam collimation of 0.5 mm × 80 rows) modes for UHRCT. Each examination was performed with small (0.4 × 0.5 mm) and large (0.6 × 1.3 mm) focal spots five times. The wall area percentage (WA%) was calculated as the percentage of total airway area occupied by airway wall. Statistical analysis was performed to compare the measurement errors for WA% among each scan mode and focal spot size using Tukey-Kramer method ($p < .05$).
**Results:** The measurement errors of WA% at SHR mode were 9.8 ± 1.5% with small focal spot and 18.8 ± 0.4% with large focal spot. The measurement errors of WA% at HR mode were 13.3 ± 0.6% with small focal spot and 21.4 ± 0.8% with large focal spot. There were significant differences in all pairings of the scan mode and focal spot size ($p < .05$).
**Conclusion:** SHR mode with small focal spot could improve airway measurement accuracy at UHRCT.

**B-0485** 11:18
**Organ-based tube current modulation in chest CT: effect on radiation dose and noise**
B.R. Mussmann[1], S. Deppe[2], P. Marschall Skov[1], S. Foley[3], H. Precht[4]; [1]*Odense/DK*, [2]*Kolding/DK*, [3]*Dublin/IE*, [4]*Middelfart/DK (peter.skov.madsen@gmail.com)*

**Purpose:** Organ-based tube current modulation (OBTCM) is designed for anterior dose reduction in CT. This study assessed the dose reduction capability of using organ dose modulation from two vendors at a range of kVp settings in chest CT. The secondary purpose was to assess noise between dose-modulation systems.
**Methods and Materials:** A Lungman phantom with thermo-luminescent dosimeters circumferentially attached around the phantom surface was scanned with and without OBTCM at 80-135/140 kVp using a Toshiba Aquilion Prime and a GE Revolution CT scanner. Equivalent dose was measured. Noise was measured in ROIs in the vertebral body, pulmonary trunk and in free air anterior to the phantom at the level of the carina. Comparisons were performed using Wilcoxon's signed-rank test.
**Results:** Using GE, dose reductions between 1.1 mSv (12%) and 1.56 mSv (24%) ($p<0.01$) were found in the anterior segment and no differences were found posteriorly and laterally. Total dose reductions between 0.64 (8%) and 0.98 mSv (13%) were found across kVp levels ($p<0.01$). With Toshiba anterior dose reductions of 6 to 7% and total dose reduction of 0.34 to 0.76 mSv across kVp levels ($p<0.02$) were found. A dose difference of 60% between right and left lateral positions was found with and without OBTCM ($p<0.0001$). Between OBTCM and non-OBTCM mean noise change across scanners and ROI positions was not clinically relevant, i.e. ranging from -0.6 to 1.5 HU.
**Conclusion:** Both OBTCM systems are capable of anterior and total dose reduction with slightly altered noise at all kVp levels.
*Author Disclosures:*
**P. Marschall Skov:** Author; Bo R. Mussmann, Svea Deppe Mørup, Shane Foley, Helle Precht.

**B-0487** 11:34
**Computed tomography (CT) imaging in weight-bearing positions: the positives and challenges in an institutional trial**
G. Bengo; *Singapore/SG (galileojrbengo@yahoo.com)*

**Purpose:** In orthopaedic practice, conventional (CT) studies are routinely performed for evaluation of lower limb injuries,bony deformity & evaluation of bony alignment for pre-operative planning. Cone-beam CT has allowed studies to be performed while patients weight-bear. The benefits and challenges of CBCT compared to conventional CT were reviewed in this study.
**Methods and Materials:** Weight-bearing lower extremity was performed with a CareStream OnSight 3D Extremity System in 6 months. The mean CTDIvol and mean DLP from every study was recorded for comparisons to similar studies performed at conventional CT for age and gender-matched patients. A post-procedure patient satisfaction survey and technologist was administered to evaluate patients'qualitative and quantitative experiences in CBCT unit. Feedback on image quality and diagnostic value was obtained.
**Results:** A total of 20 patients were scanned in a weight-bearing position in the CBCT unit. Compared to conventional CT,there was reduction of mean CTDIvol ($P<0.05$) and mean DLP ($P<0.05$).The patient survey showed positive overall experience of scan performed and levels of comfort. There was no compromise in image quality in all studies despite of significant reduced doses. Ability to perform weight-bearing studies allows accurate detection of injuries, with surgeons citing Lisfranc fracture-dislocation injuries.
**Conclusion:** The ability to perform weight-bearing studies at reduced doses without compromising image quality was the main advantage of CBCT. Other benefits includes patient comfort, a positive patient experience and enhanced workflow among technologist. Overall, there is potential for CBCT to serve as a lower dose and lower cost alternative to conventional CT in imaging.

**B-0488** 11:42
**Evaluation of usefulness of virtual calcium scoring in dual-energy cardiac CT**
H. Han; *Seoul/KR (gum790311@hanmail.net)*

**Purpose:** This paper evaluates the reliability of virtual noncontrast (VNC) images and virtual mono-chromatic(VMC) images derived from dual-energy CT for coronary artery calcium scoring, in order to decrease patient exposure radiation dose and increase the efficiency of the procedure.
**Methods and Materials:** 60 patients,They were divided into two groups depending on whether calcium in the coronary artery visible in images viewed with the naked eye. Calcium scoring of Lesions, Voxel, Volume and Agatston Score was done on TNC , VNC and VMC images. An independent t-test of a parametric test was used for statistical analysis with SPSS.
**Results:** In the group showing no visible calcium in the coronary artery, there were significant differences between TNC and VNC images ($p<0.001$). On the other hand, there were no significant differences between TNC and VMC

images in Lesions (p>0.605), Voxel (p>0.406), Volume (p>0.521), and Agatston Score (p>0.072). In the group showing visible calcium in the coronary artery, there were no significant differences in Volume (p>0.136) and Agatston Score (p>0.716), but there were significant differences in Lesions (p<0.001) and Voxel (0.046). Significant differences between VNC and VMC images of were found in all aspects.
**Conclusion:** The results of this study to evaluate TNC, VNC, and VMC in dual-energy CT for coronary artery calcium scoring were unexpected. However, if software that can distinguish calcium is developed in the future, it is expected that it will contribute to increase the efficiency of the procedure and reducing the exposure dose of the patient.

**B-0489** 11:50
**Patient perceptions of radiographer communication skills in CT examinations**
L. Ribeiro[1], A.F. Abrantes[1], K.B. Azevedo[1], O. Lesyuk[2], S.I. Rodrigues[1], R.P. Almeida[1], A.C. Viriato[3], M.V. Reis[1]; [1]*Faro/PT*, [2]*São Brás de Alportel/PT*, [3]*Lagos/PT (oksanalesyuk@gmail.com)*

**Purpose:** The improvement of results in healthcare through the transmission of information to the patient within a relation of empathy and trust is already a verified hypothesis. Healthcare professionals should base themselves on interpersonal competence throughout their daily work routine, to promote quality in medical imaging, patient safety and technical excellence. The aim of this study was the exploration of patient's perceptions regarding the performance of radiographer in terms of interpersonal communication skills during CT procedures.
**Methods and Materials:** The instrument used was the questionnaire "Communication Assessment Tool" (Makoul et al. 2007) adapted to the professional reality of the radiographers. A total of 98 valid questionnaires (including 15 questions with a five-point Likert scale) from patients aged between 18 to 85 years old. The paper-based instrument was delivered and filled by the patients after the performance of CT procedures in two public hospitals.
**Results:** The internal consistency of the questionnaire was excellent (Cronbach's alpha = 0.949). Highest ratings were for radiographer behavior items, such as "greet the patients" (4.48), "respect for the patients" (4.63) and "time available to patient-centered care" (4.41). Lowest ratings were "encouraged to make questions" (3.77), "involved patients in decision-making process" (4.03) and "talk with patients about the next following steps" (3.70).
**Conclusion:** Radiographers communication skills were evaluated with good levels of patient confidence with the radiological examinations. Despite the overall positive results, this area of health service delivery must be accorded the attention it deserves to continually improve on patient satisfaction through improved communication.

**10:30 - 12:00** **Room X**

### Vascular

### SS 615

### Contrast medium: dose, timing and alternatives

*Moderators:*
N.N.
S. Tresoldi; Milan/IT

**B-0490** 10:30
**Using the lean body weight (LBW) instead of total body weight (TBW) to dose the iodinated contrast medium (ICM) for abdominal CT: a randomised controlled trial (RCT)**
M. Zanardo, F.M. Doniselli, G. Di Leo, A. Esseridou, S. Tritella, C. Mattiuz, L. Menicagli, F. Sardanelli; *Milan/IT (moreno.zanardo@unimi.it)*

**Purpose:** Due to low diffusion in fat and high in muscles, ICM could be more appropriately dosed on LBW instead of TBW, a hypothesis to be verified on liver contrast enhancement (LCE) in the portal venous phase.
**Methods and Materials:** Ethics Committee-approved RCT involved patients scheduled for contrast-enhanced abdominal CT. Patient LBW was estimated using a balance with bioelectrical impedance analysis. Patients were randomly assigned to the LBW-group, administered 0.65 gI/kg of LBW, or to the TBW-group, administered 0.40 gI/kg of TBW, as controls. Enrolled patients with liver disease or deemed to receive a too low or high ICM dose were excluded. Levene's test for variances and $\chi^2$ test were used.
**Results:** We enrolled 315 patients. After 57 exclusions for protocol violation, 258 were assigned to LBW-group (n=119) or TBW-group (n=139). Age was 65±12 years in LBW-group and 67±12 years in TBW-group (*p*=0.068), TBW 70±13 kg for both (*p*=0.635), LBW 51±11 kg and 51±10 kg (*p*=0.562), body mass index 25±4 kg/m$^2$ for both (*p*=0.866); injected ICM (ml) 85±18 and 83±16, respectively (*p*=0.437). LCE (HU) was 41±9 in LBW-group and 40±7 in TBW-group, with means not significantly different (*p*=0.659), but with different variances (*p*=0.004). Suboptimal LCE (<40HU) was found in 76 (55%) patients in the TBW-group and in 64 (54%) in LBW-group (*p*=0.886), but no repeat examination was needed.
**Conclusion:** ICM doses of 0.65 gI/kg of LBW or 0.40 gI/kg of TBW lead to a comparable diagnostic LCE with a slightly larger variation for the latter, thus negating the study hypothesis.

**B-0491** 10:38
**High-pitch, low-kVp computed tomography for ruling out pulmonary embolism with 17-mL contrast media**
H. Al-Ubeidy, M. Alshamari, J. Widell, T. Eriksson, M. Lidén; *Örebro/SE (matsliden@yahoo.com)*

**Purpose:** The purpose of this phase 4 clinical trial (EudraCT 2015-004657-40) was to perform CT pulmonary angiography (CTPA) for ruling out pulmonary embolism (PE) using a minimal amount of iodinated contrast media.
**Methods and Materials:** 47 patients (25 women) referred for contrast-enhanced chest CT were included in the study. All participants underwent the same study-specific CTPA in addition to the chest CT. The participants received 80 mg I/kg body weight iohexol contrast media using a preparatory saline bolus, a dual-flow iohexol/saline bolus and a saline flush, and a scanner protocol with 80kVp dual-source high-pitch mode. Three readers independently assessed the diagnostic quality for ruling out PE on the three-point scale non-diagnostic, adequate or good-excellent image quality. Additionally, the pulmonary arterial opacification was measured.
**Results:** On average, the patients received 16.8 mL (range 12-20 mL) iohexol 350 mg I/mL. Mean patient weight was 71 kg, range 50-85 kg. Identically for all readers, PE was detected in 1/47 participants. The median number of examinations visually scored concerning PE as good-excellent was 47/47 (range 44-47); adequate 0/47 (0-3) and non-diagnostic 0/47 (range 0-0). The proportion of adequate or better examinations for all readers was 47/47, 100% (95% C.I. 92-100%). The mean attenuation±standard deviation in the pulmonary trunk was 327±71 HU, range 174-515 HU.
**Conclusion:** Diagnostic CTPA with 17-mL contrast media is possible using low-kVp, high-pitch and carefully designed contrast media administration, which can be useful to reduce the risk of nephrotoxic complications.

**B-0492** 10:46
**Determining optimal scan delay in indirect CT venography: time to peak attenuation correlates with cardiac output and heart rate**
T.T. Tran[1], C.H. Kristiansen[1], F. Haidl[1], S. Roy[1], J.C. Lindstrom[1], H. Ashraf[2], P. Lauritzen[3]; [1]*Lørenskog/NO*, [2]*Rasta/NO*, [3]*Oslo/NO*

**Purpose:** Determine whether scan delay 120s is optimal for indirect-CT venography (CTV).
**Methods and Materials:** Twenty-four patients with clinical suspicion of deep-vein thrombosis were prospectively included. CTV was performed with fixed scan delay 120s after injection. Single scans of popliteal vein were obtained at 30s intervals from 30s to 210s to determine time-to-peak-attenuation (TPA). Cardiac output (CO) and heart rate (HR) were recorded continuously/non-invasively using a photoplethysmography-device (NexfinHD, BMEYE, Amsterdam). Attenuation measured at specified sites in inferior vena cava, and each external iliac/common femoral/popliteal veins. Associations between CO and TPA and HR and TPA were estimated by Spearman's rank correlation and linear regression.
**Results:** Median CO=7.8L/min (range: 4.1-11.5), median HR=72.4bpm (range: 58.4-102). Mean TPA=159s (95%CI: 141,176). TPA varied inversely with both CO and HR. Correlation-coefficients between TPA and CO, and between TPA and HR were 0.52 (p=0.009) and 0.41 (p=0.047), respectively. Regression-coefficients were 10.4 for CO and 2.1 for HR. This indicates 74s variation in optimal scan delay from highest to lowest CO. In 63% TPA exceeded scan delay of 120s. At 120s mean attenuation was appreciably higher than essential for diagnostic purposes in larger central veins: 140HU (95%CI, 128, 153) in inferior vena cava, 129HU (95%CI, 118, 139) in external iliac veins, 127HU (95%CI, 115, 139) in common femoral veins and 117HU (95%CI, 105, 129) in popliteal veins.
**Conclusion:** At 120s majority of patients had not reached peripheral venous peak attenuation, although attenuation was higher in larger central veins. Lengthening and individually tailoring scan delay to CO may be helpful.

**B-0493** 10:54

**Real-time patient-specific scan initiation for CT of the abdominal aorta: impact on image quality**

F.R. Schwartz[1], J. Ramirez Giraldo[1], R. Gutjahr[2], D. Boll[3], D. Marin[1], L. Hurwitz[1]; [1]*Durham, NC/US*, [2]*Nürnberg/DE*, [3]*Basle/CH* *(fidesreginadorotheaann.schwartz@duke.edu)*

**Purpose:** To assess the impact of real-time modulation of scan initiation based on patient-specific haemodynamics on contrast enhancement of the abdominal aorta on MDCT.
**Methods and Materials:** This is a HIPAA-compliant, IRB-approved QI project. Exams were performed on a DS-MDCT scanner (120 kVp, 65-75 mL of contrast, 4 ml/sec). Contrast administration was monitored in the descending aorta for the abdominal aortic (AA) and the thoracoabdominal (TAA) protocols. The delay prior to initiation of the diagnostic scan was modulated by a real-time patient-specific (RTPSM) software, incorporating time to threshold and the slope of the enhancement curve. 100 patients (cohort 1) were scanned using RTPSM triggering of scan delay for abdominal aortic protocols. A reference cohort of 30 patients (cohort 2) was identified with exams on the same scanner (preceding 12 months), using a fixed diagnostic delay (FD). Patient demographics, average aortic HU values and coefficients of variance (COV) at 1-mm increments from the diaphragm through the right common femoral artery were collected. Descriptive statistics and Student's t tests were applied.
**Results:** Cohort 1 and 2 had 73% TAA and 27% AA exams. The average scan delay was significantly longer for TAA (12.3 vs 11 sec; $p<0.01$) and AAA protocols (11.5 vs 8 sec; $p<0.01$) using RTPSM compared to FD. Average HU values were significantly higher in the RTPSM cohort (406.6 ± 37.9 vs 371.9 ± 38.4 HU; $p<0.01$) and COV was significantly reduced using RTPSM (9.5 vs 11.6%; $p<0.01$).
**Conclusion:** RTPSM results in higher HU values and better contrast homogeneity throughout the abdominal aorta.
*Author Disclosures:*
**F.R. Schwartz:** Research/Grant Support; Siemens Healthineers, Grant to department of Radiology. **J. Ramirez Giraldo:** Employee; Siemens Healthineers, Forchheim, Germany. **R. Gutjahr:** Employee; Siemens Healthineers, Forchheim, Germany. **L. Hurwitz:** Research/Grant Support; Siemens Healthineers, Grant to department of Radiology.

**B-0494** 11:02

**Patient-specific scan delay for optimised venous triggering in CT abdomen**

W. Coudyzer[1], L. Cockmartin[1], D. Konings[1], T. Busselot[2], D. Bielen[1], D. Vanbeckevoort[1], H. Bosmans[1]; [1]*Leuven/BE*, [2]*Brussels/BE* *(Walter.Coudyzer@uzleuven.be)*

**Purpose:** While the start of arterial phase contrast-enhanced CT scans is personalized to the patient's contrast bolus passage using automated triggering, this procedure is not available for venous phase scans. Fixed scan delays are used. To optimize the contrast injection scheme for venous phase abdominal CT scans, the parameters of impact on venous signal enhancement have been investigated using manually triggered acquisitions.
**Methods and Materials:** Venous phase CT abdomen scans of 100 patients (61male/39female) were acquired on a Siemens Definition Flash CT using a Nemoto injector with iCalc option. The venous signal enhancement was monitored from a region of interest in the hepatic parenchyma. Scans were started manually, immediately after peak enhancement. Registered patient parameters included gender, weight, age, blood pressure and heart rate. Linear regression analysis and Pearson correlations were performed to study their impact on peak enhancement time.
**Results:** Observed peak enhancement times in males and females were significantly different (mean difference 9.1±2.7). Neither heart rate nor blood pressure correlated with observed peak enhancement times. Age had a limited impact depending on the gender (male: $r=0.32$, $p=0.012$; female: $p=0.86$) while body weight (male: $r=0.45$, $p<0.0001$; female: $r=0.76$, $p<0.0001$) was significant. From this analysis, a reference scan delay of 95s for a 65kg female and 104s for a 75kg male could be increased with 7s/10kg and 3s/10kg extra body weight respectively.
**Conclusion:** The fixed scan delay for venous phase CT could be improved by including gender and weight parameters. A candidate time scheme can now be tested in practice.
*Author Disclosures:*
**H. Bosmans:** Founder; Co-founder Qaelum NV. Research/Grant Support; The medical physics team has research agreements with Siemens-Healthineers and GE Healthcare.

**B-0495** 11:10

**To determine the image quality of non-linear blending (NLB) technique in aortic angiography acquired by novel injection protocol compared with linear blending (LB) technique**

L. Xing; *Zhengzhou/CN (278459366@qq.com)*

**Purpose:** To determine the image quality of non-linear-blending (NLB) technique in aortic angiography acquired by novel injection protocol compared with linear-blending (LB) technique.
**Methods and Materials:** 30 patients were prospectively enrolled in the study. Third-generation dual-source scanner (SOMATOM Force) were utilised with tube voltage 70/sn150 kVp. The dose of contrast medium was tailored to the patients' body weight as 0.5 ml/kg with the injection duration defined as examining time (scanning time + time delay after achieving the trigger threshold value).LB image series with weighting factor of 0.5 were compared with non-linear blended image series. The difference in the vessel enhancement , signal-to-noise-ratio (SNR) and contrast-to-noise-ratio (CNR) were evaluated using paired t-test and Mann-Whitney U test. Overall image quality was also rated for both groups. And the consistency of the scores was analysed by Kappa test.
**Results:** The average effective radiation dose was 4.83 mSv. Mean contrast volume was 34.12 ml(350mgI/ml). The average injection rate was 3.24 ml/s. The degree of the enhancement, SNR and CNR of the vessel in NLB group were significantly higher than LB group ($p<0.05$), while the enhancement of soft tissue was of no difference in both groups ($p>0.05$). The rated scores for the image quality of two groups achieved good consistency (kappa=0.708, $p>0.05$). And the scores of NLB group increased 0.55.
**Conclusion:** Low ionised-radiation dose dual-energy CT aortic angiography with novel injection protocol of low-contrast medium and low-flow rate might be feasible when using NLB technique, as it improve the image quality compared to LB technique.

**B-0496** 11:18

**Systematic accuracy assessment of a real-time automated patient specific bolus tracking delay calculation algorithm**

R. Gutjahr[1], G. Jost[2], H. Pietsch[2], B. Schmidt[3], T. Flohr[3]; [1]*Nürnberg/DE*, [2]*Berlin/DE*, [3]*Forchheim/DE*

**Purpose:** To evaluate a fully scanner-integrated automated patient specific bolus tracking delay calculation algorithm utilizing real time monitoring measurements on a realistic circulation phantom.
**Methods and Materials:** Dynamics of contrast media (CM) were mimicked using a circulation phantom with a heart rate of 80bpm and blood pressure of 120/80mmHg. Iodinated CM was injected with flow rates (FR) of 2, 4, 6mL/s while the amount of CM (40mL) was maintained. Measurements were acquired using a perfusion scan protocol (100kVp, 50mAs, 0.1s/image). The mean enhancement was subsampled in three cycle times (CyT, 1, 1.5, 2s) until a threshold CT-values were exceeded (100 or 150HU). The time to peak enhancement was predicted and retrospectively compared for different FR and CyT.
**Results:** At a AT CT-value of 100HU the calculated delay in the ascending aorta (AA), descending aorta (DA), suprarenal aorta (SRA) and external iliac artery (EIA) showed a deviation to the time to peak of 0.18±0.7s at a CyT of 1s, 0.92±0.5s at a CyT of 1.5s, and 0.53±0.5s at a CyT of 2s. Comparing the time to peak enhancement at the AA using low/mid/high flow rates the deviations were 0.51±0.44 s at a CyT of 1s, 0.53±0.66s at a CyT of 1.5s, and 0.71±1.13s at a CyT of 2s. The suggested delay times ranged from 3.5 to 14.9s. An overall average deviation of 1.2±1.0s was observed.
**Conclusion:** The calculated bolus delay times achieved very good accuracy in predicting the time of peak enhancement. This facilitates optimal usage of CM injections and eventual CM or radiation dose reduction.
*Author Disclosures:*
**R. Gutjahr:** Employee; Siemens Healthcare GmbH. **G. Jost:** Employee; Bayer HealthCare Pharmaceuticals LLC. **H. Pietsch:** Employee; Bayer HealthCare Pharmaceuticals LLC. **B. Schmidt:** Employee; Siemens Healthcare GmbH. **T. Flohr:** Employee; Siemens Healthcare GmbH.

**B-0497** 11:26

**3D quantitative analysis of the aorta and pulmonary artery on non-contrast CT**

Z. Sedghi Gamechi[1], A.M. Arias-Lorza[1], J. Holst Pedersen[2], K.F. Kofoed[2], D. Bos[1], M. De Bruijne[1]; [1]*Rotterdam/NL*, [2]*Copenhagen/DK* *(z.sedghigamechi@erasmusmc.nl)*

**Purpose:** Accurate measurements of the size and shape of the aorta and pulmonary arteries are important as risk factors for cardiovascular diseases and Chronic Obstructive Pulmonary Disease (COPD). A fully automatic method is proposed to extract volumes and assess diameters.
**Methods and Materials:** Non-ECG gated, non-contrast chest CT scans were used from 365 participants (mean age 57.4, 49.9% female) of the Danish Lung Cancer Screening Trial. The aorta and pulmonary arteries were manually

Thursday segmented on 25 scans and the diameters of the ascending aorta (AA) and pulmonary artery (PA) at the pulmonary bifurcation level were measured in axial slices on 200 scans. Our automatic algorithm extracted the seed points and a landmark at the pulmonary bifurcation level by multi atlas registration and then traced the centerlines with a minimum cost path. Subsequently, an optimal surface was extracted around the centerline. Diameters were measured in axial slices to compare with manual measurements. Repeatability of diameter measurements was evaluated on 140 baseline- one-year follow-up scan pairs.
**Results:** Segmentation overlap between manual and automated segmentation was 0.95 ± 0.01 for aorta and 0.92±0.02 for pulmonary arteries. The intra-class correlation between manual and automatic AA, PA, and PA:AA ratio were 94%, 87%, and 86% and repeatability was 96%, 92%, and 89%, respectively.
**Conclusion:** Our full automatic method can assess the diameters of the aorta and pulmonary arteries reliably in non-ECG gated, non-contrast CT. This method may replace time-consuming manual segmentations in large-scale studies and, eventually, in clinical practice.

**B-0498** 11:34
**Magnetic particle imaging: artefact-free imaging of the in-stent lumen of coronary stents**
F. Wegner, T. Friedrich, A. von Gladiss, T.M. Buzug, J. Barkhausen, J. Haegele; *Lübeck/DE (franz.wegner@uksh.de)*

**Purpose:** Cardiovascular magnetic particle imaging (MPI) is a tracer-based method that visualizes the spatial distribution of superparamagnetic iron oxide nanoparticles with magnetic fields. In MRI and CT artefact-induced artificial lumen narrowing limits the assessment of the in-stent lumen. This study aims to investigate the potential of MPI for imaging of the in-stent lumen.
**Methods and Materials:** We evaluated eight commercially available coronary stents of different dimensions (diameter: 3 - 4 mm) and materials (stainless steel, platinum-chromium) implanted in silicone vessel phantoms. Prior to imaging, signal-to-noise ratio (SNR) of the stents and the tracer (Resovist diluted 1:100) were calculated separately from the MPI signal. Imaging of eight stented phantoms and a non-stented reference phantom for each diameter (all filled with tracer-solution) was performed in a preclinical MPI-Scanner (Bruker-Biospin, Ettlingen, Germany). For comparison, the stents were also visualized by MRI (Ingenia 3 T, Philips, Hamburg, Germany) and CT (Somatom Definition AS, Siemens, Erlangen, Germany).
**Results:** SNR values of the stents were in the range of background noise. SNR of the tracer-solution was 436 and thus suitable for image reconstruction. It was possible to visualize all of the stented vessel phantoms with MPI. The reconstructed MPI images did not show any stent artefacts, whereas MRI and CT images revealed multiform artefacts and significant artificial lumen narrowing in all devices.
**Conclusion:** MPI is able to visualize the in-stent lumen of coronary stents without any artefacts. Thus, MPI may overcome the disadvantages of MRI and CT concerning the visualization of the in-stent lumen.

**B-0499** 11:42
**Magnetic particle imaging for pulmonary circulation time measurements**
M.G. Kaul, J. Salamon, C.S. Jung, H. Ittrich, M. Graeser, G. Adam, K. Peldschus; *Hamburg/DE (mkaul@uke.uni-hamburg.de)*

**Purpose:** Magnetic particle imaging (MPI) is a new imaging modality providing a high temporal resolution scanning magnetic particle distributions in 3D. The purpose of our study was to determine the time of the passage of a bolus of superparamagnetic nanoparticles through the lungs of mice.
**Methods and Materials:** Nine mice were imaged by 7T MRI (ClinScan, Bruker) to localize heart and lungs. Mice were transferred on a MR/MPI-compatible couch to a MPI scanner (Philips/Bruker). An own-developed receiver coil for mice was used. During the measurement sequence, a bolus of 1 µL of a high concentration SPIO (Perimag, Micromod) was injected into the tail vein. The temporal resolution for a 3D dataset was 21 ms. MRI and MPI images were co-registered to identify the left and right ventricles. A model function was fitted to each pixel time series extracting the time of arrival. Next, regions of interest were drawn in the left and right ventricles and the time difference of the arrival times of left and right ventricle was determined.
**Results:** In all ten mice, the injection was successful generating short bolus profiles. The difference of the bolus arrival times were 0.9 +/- 0.1 s.
**Conclusion:** Due to its high temporal resolution, MPI is able to image the passage of a bolus from the right ventricle through the lungs to the left ventricle. The technique might be used to depict pathologic changes. In combination with the estimation of the cardiac output the blood volume of the lungs can be measured.

**B-0500** 11:50
**Pre-TAVR scanning by a comprehensive contrast-free cardiovascular magnetic resonance protocol: comparison with contrast-enhanced computed tomography**
M.J. Pamminger, G. Klug, S.J. Reinstadler, M. Reindl, C. Kremser, C. Kranewitter, B. Metzler, W.R. Jaschke, A. Mayr; *Innsbruck/AT (Mathias.Pamminger@tirol-kliniken.at)*

**Purpose:** To compare an entirely non-enhanced cardiovascular magnetic resonance imaging (MRI) protocol with contrast-enhanced computed tomography angiography (CTA) for prosthesis sizing and access guidance in transcatheter aortic valve replacement (TAVR) evaluation.
**Methods and Materials:** 25 patients (mean age 82.6 ± 4.6 years, 14 female [56%]) with severe aortic stenosis referred for TAVR evaluation underwent non-contrast three-dimensional (3D) 'whole heart' MRI for aortic root measurements as well as non-enhanced quiescent-interval single-shot (QISS) MR angiography for evaluation of aortoiliofemoral access routes. CTA was performed in 20 (80%) patients.
**Results:** Aortic root geometry assessed by 3D ‘whole heart’ MRI showed strong to very strong correlations (r = 0.606 to 0.874, all p<0.005) compared to CTA. QISS and CTA based measurements of aortoiliofemoral vessel diameters correlated moderately to very strong (r = 0.555 to 0.816, all p<0.011) with good to excellent inter-observer reliability (ICC = 0.81 to 0.999, all p<0.0001) regarding MRI measurements. Mean diameters of infrarenal aorta, common iliac arteries and common femoral arteries differed significantly (bias -0.44 to -0.96 mm, p = 0.026 to <0.0001) between the two modalities. However, inter-method agreement for the decision of transfemoral accessibility was strong (κ = 0.828, p = 0.004).
**Conclusion:** MRI 3D ‘whole heart’ and QISS angiography provide contrast-free evaluation of aortic root and access routes in TAVR patients with moderate to strong correlations compared to CTA measurements. Moreover, decision for transfemoral access strategy showed strong agreement between both modalities, highlighting this MRI protocol to might obviate the need for contrast administration in pre-TAVR scanning.

**10:30 - 12:00** **Room N**

### Hybrid Imaging

### SS 606

### Advanced MR methods in experimental radiology

*Moderators:*
N.N.
I. Rausch; Vienna/AT

**B-0501** 10:30
**Is ADC a predictor of hypoxia in ER+ breast cancer? A study using $^{18}$F-FMISO PET-MRI**
J.C. Carmona-Bozo, R. Woitek, A.J. Patterson, G. Baxter, C. Caraco, E. Provenzano, T.D. Fryer, M.J. Graves, F. Gilbert; *Cambridge/UK (jcc83@cam.ac.uk)*

**Purpose:** To investigate the relationship between ADC and hypoxia in ER+ breast cancers using combined $^{18}$F-FMISO-PET and DWI-MRI.
**Methods and Materials:** Imaging was performed on patients with biopsy-confirmed ER+ breast cancer prior to treatment. Following injection of 300 MBq $^{18}$F-FMISO and a 2-hour uptake period, a 60-min PET/MR scan was performed on a GE SIGNA PET/MR scanner. PET data was analysed by $K_i$ from Patlak analysis with a scaled population-based arterial input function. Tumour hypoxic fractions were calculated as the percentage of voxels with $K_i$ values >2SD of the mean of normoxic muscle. Mean ADC values were calculated from DWI using b-values 0 and 900 s/mm$^2$. The group median ADC value was used to partition tumours into high and low diffusion groups.
**Results:** 15 patients/16 lesions were assessed (ductal: n=10; lobular: n=2; other: n=3; HER2+: n=4 HER2-: n=11). Hypoxia was identified in 50% of the lesions, with hypoxic fractions ranging between 1-58%. Higher mean hypoxic fractions were observed in grade 3 (10.5%) vs grade 2 (5.8%) cancers (p=0.620) and lower median ADC values were found in grade 3 vs low-grade (0.83 vs 1.10 respectively; p=0.160). In addition, lesions in the low diffusion group (ADC≤1.05 mm$^2$/s) demonstrated a higher median hypoxic fraction (4.4%) than tumours in the high diffusion group (0.3%), although this difference was not statistically significant (p=0.957). There was an inverse heterogeneous relationship between mean tumour $K_i$ and ADC ($\rho$=--0.262; p=0.327).
**Conclusion:** Low ADC may be associated with a higher degree of hypoxia in ER+ breast cancer.

**B-0502** 10:38
**Proton MR spectroscopy: a non-invasive tool for the detection of ovarian cancer**
P.N.S. Fahmy, A. Elkalawy, M. Gomaa, S.A. Mansour; *Cairo/EG (nashaat.peter.med@gmail.com)*

**Purpose:** To assess the role of MR spectroscopy (MRS) - as it is a non-contrast-based MR sequence - in the screening of malignancy in ovarian masses.
**Methods and Materials:** This prospective work included 230 females who had 245 adnexal/ovarian masses. Tumours were spotted by preliminary pelvic ultrasound. Masses were assessed by MRI, multi- or single-voxel spectroscopy. Patients' spectra were assessed for peaks of lactate (Lac, 1.31 ppm), lipid (Lip, 1.33 ppm), N-acetyl aspartate (NAA, 2.0 ppm), acetone (A, 2.05 ppm), choline (Cho, 3.23 ppm) and creatine (Cr, 3.4 ppm). The mean values of (Cho/Cr) ratios were performed by a semi-quantitative approach. The operative pathology served as the standard of reference.
**Results:** Cho peak twofold higher than the average noise level was detected in 72% of the malignant and only 5.4% of the benign masses with an accuracy of 83%. Adding lactate to the choline enhanced the accuracy to 93%. The mean Cho/Cr ratios of the malignant ovarian masses (2.8) were significantly higher than that of the benign ones (1.2). We used a ROC curve to determine the best cut-off value (1.7) for the mean Cho/Cr ratio to discriminate malignancy with sensitivity: 81.2%, specificity: 93.3%, PPV: 92.9%, NPV: 82.4% and accuracy: 87.1%.
**Conclusion:** The simultaneous presence of choline and lactate peaks in MR spectroscopy examination of the ovarian masses minimizes the overlap between benign and malignant categories. N-acetyl aspartate and acetone are the specific metabolites in diagnosing complex cystic masses.
*Author Disclosures:*
**P.N.S. Fahmy:** Author; Sahar Mansour, Mohammed Gomaa, Abdelhamid Elkalawy.

**B-0503** 10:46
**MR monitoring of cytolysis and inflammatory tumour microenvironment after oncolytic adenovirus injection in a novel transgenic pancreatic adenocarcinoma mouse model**
F.M. Kubicka[1], B. Fleischmann-Mundt[2], P.M. Kazmierczak[1], M.J. Schneider[1], J. Brooks[2], M. Meier[2], J. Ricke[1], F. Kühnel[2], C.C. Cyran[1]; [1]*Munich/DE*, [2]*Hannover/DE (felix.kubicka@gmx.de)*

**Purpose:** To investigate if a novel pancreatic cancer model (EP-PDAC) is suitable for preclinical monitoring of virotherapy using MRI.
**Methods and Materials:** 3 weeks after pancreatic electroporation (EP) with oncogenes (KRasG12V, Akt2, Cre-p53fl/fl) in mice (n=116), tumour growth was assessed by inspection and histology. Tumour-related complications (ileus, peritonitis, bleeding, invasive growth) were investigated in relation to tumour size. Tumour growth and complications were additionally examined (n=30) by T2-weighted MSME sequence (TR/TE=1238/11, FOV=25.6x25.5 mm) on a 7T small animal MRI. Of these, mice (n=5) were treated at day 22 post-EP by 100 µl intratumoural hTert-adenovirus injection (1x10E9 ifu), or NaCl (n=5, control group) and reexamined by MRI (day 25 post-EP). The extent of the oncolytic-necrotic area was measured by manual MRI-3D-rendering. Results were validated with histological investigations of inflammation (CD45-immunohistochemistry), virus-induced lysis (HE), and fibrosis (Sirius-red).
**Results:** 116 mice reliably developed solitary tumours until day 21 (diameter=1.3-10 mm), which were histologically and MRI-morphologically (n=30) authentic to human PDAC. In contrast to controls, lower T2-signals were detected after virotherapy, histopathologically (HE and CD45) corresponding to oncolytic-necrotic tissue reaching a maximum-ratio of 1-3 regarding intact EP-PDAC using MRI-3D-rendering. Rim-fibrosis was only detectable by Sirius-red staining in all virus-treated, but not in saline-treated mice. MRI and histopathology represented PDAC-characteristic complications (ileus, peritonitis and bleeding) with high, significant correlations to tumour size ($p=0.03$, $\rho=+0.85$) with the exception of invasive growth ($p=0.09$, $\rho=+0.52$).
**Conclusion:** The EP-PDAC is well suited for preclinical evaluation of oncolytic virotherapy by MRI and allows monitoring of microenvironmental changes as validated by immunohistochemistry.

**B-0504** 10:54
**Assessment of tumour hypoxia response to sorafenib in rabbit $VX_2$ liver tumour xenografts by tissue-oxygen-level-dependent MR imaging**
S. Qin, X. Li, Z. Qi, Y. Mei, X. Quan; *Guangzhou/CN (qinshuping1226@163.com)*

**Purpose:** To investigate the alteration in tumour oxygen inhalation and hypoxia levels of rabbit liver $VX_2$ tumour before and after the application of sorafenib, using tissue-oxygen-level-dependent (TOLD) MR.
**Methods and Materials:** 26 $VX_2$ tumours were implanted in the livers of 13 New Zealand white rabbits, which were incubated for control and treated group (5 rabbits) were given a week of sorafenib (20mg/kg/d) at 3weeks after implantation. TOLD was performed on a 3.0T MR scanner before and after 30min of oxygen inhalation, using an animal coil. The longitudinal relaxation rate ($R_1=1/T_1$) was calculated. R1 difference ($\Delta R_1=R_1$ (O2)-$R_1$ (air)) was measured for whole lesion (ΔR1whole) and non-necrotic area (ΔR1non-necrotic), and the corresponding relative change ΔR1%whole and ΔR1%non-necrotic. Immunohistochemical analysis of hypoxia-inducible factor-1αexpression of $VX_2$ liver tumour was graded from H0 to H4.
**Results:** No significant differences were found between control and treated group at week 3 in different ΔR1. Significant differences (P< 0.01) were found in ΔR1 between treated group and control group at week 4. Significant differences were also detected in treated group before and after application of sorafenib. There was no significant difference in the pathological grade of HIF-1α between the treated and the control group after a week of sorafenib (P>0.5), but the HIF-1α-positive cell expression rate of treated group was significantly (P<0.05) higher than control group (week 4).
**Conclusion:** Our results show the potential of TOLD as a non-invasive technique to detect early tumour hypoxia response in rabbit VX2 liver tumour receiving sorafenib.

**B-0505** 11:02
**Amide proton transfer (APT) MRI is a predictor of survival and progression in high-grade glioma patients**
D. Paech, C. Dreher, S. Regnery, S. Goerke, A. Unterberg, W. Wick, M. Bendszus, M. Ladd, H.-P. Schlemmer; *Heidelberg/DE (d.paech@dkfz.de)*

**Purpose:** The purpose of this study was to investigate the predictability of patient overall survival (OS) and progression-free survival (PFS) in newly diagnosed high-grade glioma (HGG) patients using relaxation-compensated chemical exchange saturation transfer (CEST) MRI at 7 Tesla (7T).
**Methods and Materials:** Twenty-six patients with newly diagnosed high-grade glioma (WHO grade III-IV) were included in this prospective ethical committee-approved study. CEST MRI was performed at a 7T whole-body scanner. Predictability of patient OS and PFS were assessed employing relaxation-compensated CEST MRI contrasts by means of amide proton transfer (APT, downfield rNOE-suppressed APT = dns-APT) and relayed nuclear Overhauser effect (rNOE/NOE) imaging. Furthermore, OS and PFS predictability were assessed for clinical parameters (age, treatment, and O6-methylguanine-DNA methyltransferase (MGMT) promotor methylation status) and diffusion-weighted imaging (apparent diffusion coefficient). OS and PFS analyses were performed according to the Kaplan-Meier method with two-sided log-rank statistics.
**Results:** Relaxation-compensated APT imaging was a significant predictor for patient OS (p=0.009) and PFS (p=0.012). Median OS/PFS of patients with low tumour signal intensities ≤4.23% (signal median) was 411/293 days compared to 292/112 days for patients with increased APT values. Strongest association with PFS was found for the dns-APT metric (p=0.009). Among the tested clinical parameters, patient age (older/younger 57 years=median, p=0.005) and treatment (surgery vs. no surgery, p=0.018) were significant for OS.
**Conclusion:** Relaxation-compensated APT MRI serves as a predictor of overall survival and progression-free survival in newly diagnosed, previously untreated glioma patients and may, therefore, help to customize glioma patient treatment and response monitoring in the future.

**B-0506** 11:10
**Early response assessment of glioma patients to definitive chemoradiotherapy using chemical exchange saturation transfer imaging at 7 Tesla**
J.-E. Meissner, A. Korzowski, S. Regnery, S. Goerke, J. Debus, M. Ladd, H.-P. Schlemmer, S. Adeberg, D. Paech; *Heidelberg/DE (d.paech@dkfz.de)*

**Purpose:** Patients with newly diagnosed inoperable glioma receive chemoradiotherapy (CRT). Standard response assessment in neuro-oncology (RANO) takes a minimum of 4 weeks after the end of treatment. In this longitudinal study, we assessed whether chemical exchange saturation transfer (CEST) MRI enables earlier assessment of response to CRT in glioma patients.
**Methods and Materials:** Twelve glioma patients that underwent definitive CRT were included in this study. Three longitudinal CEST MRI measurements were performed for each patient at 7T - the first before, the second immediately after completion of CRT and the third as a 6 week follow-up. The relaxation-compensated relayed nuclear-overhauser-effect CEST signal (rNOE) and the downfield-rNOE-suppressed amide proton transfer (dns-APT) CEST signal were investigated. Additionally, choline-to-N-acetyl-aspartate ratios (Cho/NAA) were evaluated using single-voxel $^1$H-MRS in six of these patients. Performance of obtained contrasts was analysed in assessing treatment response as classified according to the updated RANO criteria.
**Results:** The rNOE signal significantly separated stable and progressive disease directly after therapy (post-treatment normalized to pre-treatment mean±SD: $rNOE_{responder}=1.09\pm0.11$, $rNOE_{non\text{-}responder}=0.87\pm0.09$, p=0.015). In contrast, no significant difference could be observed between both groups when assessing the normalized dns-APT ($dns\text{-}APT_{responder}=0.95\pm0.38$, dns-

$APT_{non\text{-}responder}$=0.97±0.48, p=0.95). In the smaller MRS sub-cohort, normalized Cho/NAA decreased in therapy responders ($Cho/NAA_{responder}$=0.632±0.007, $Cho/NAA_{non\text{-}responder}$=0.946±0.124, p=0.070).
**Conclusion:** rNOE mediated CEST imaging at 7T allowed for discrimination of responders and non-responders immediately after end of CRT, additionally supported by $^{1}$H-MRS data. This is at least 4 weeks earlier than the standard clinical evaluation according to RANO. Therefore, CEST MRI may enable early response assessment in glioma patients.

**B-0507** 11:18
**A GPC3-specific aptamer-mediated MR probe for hepatocellular carcinoma**
M. Zhao; *Shanghai/CN*

**Purpose:** To construct and test a HCC-targeted MR probe based on a GPC3-specific aptamer (AP613-1) with ultrasmall superparamagnetic iron oxide (USPIO).
**Methods and Materials:** Oleic acid-coated USPIO nanoparticles were modified with amino polyethylene glycol on the surface. Amino groups of the USPIO nanoparticles were reacted with carboxyl group of 5' carboxyl modified AP613-1, forming an aptamer-mediated USPIO (Apt-USPIO) probe. The material characterizations of this probe including TEM, zeta potential, dynamic laser scattering and magnetic behavior were measured. The targeting efficiency and MR imaging performance of Apt-USPIO were evaluated both in vitro and in vivo with USPIO alone as a control. The cytotoxicity and biocompatibility of Apt-USPIO and USPIO were analysed by cell counting kit-8 tests in vitro and animal experiments in vivo.
**Results:** TEM imaging revealed the Apt-USPIO nanoparticles were spherical and well dispersed. Specific uptake of Apt-USPIO in Huh-7 cells could be observed by Prussian blue staining test, but no uptake of USPIO could be found. In vitro phantom T2-weighted MR imaging showed a significant decrease of the signal intensity in Apt-USPIO-incubated Huh-7 cells compared to USPIO-incubated Huh-7 cells. In vivo T2-weighted MR imaging showed significantly negative enhancement in the Huh-7 tumours enhanced with Apt-USPIO whereas no enhancement was found with USPIO alone. Excellent biocompatibility of Apt-USPIO and USPIO was also demonstrated.
**Conclusion:** In this study, a molecular MR imaging probe which was highly specific to GPC3 on HCC is successfully prepared. Our results validated the targeted imaging effect of this Apt-USPIO probe in vivo for GPC3-expressing HCCs in xenograft mice.

**B-0508** 11:26
**Evidence from in vivo MRI: ferritin heavy chain and interferon-β gene-modified mesenchymal stem cells transplanted peritumorally inhibit malignant gliomas**
J. Mao, J. Shen; *Guangzhou/CN (707982289@qq.com)*

**Purpose:** In our previous study, we demonstrated that ferritin heavy chain (FTH) gene-based MRI could be used to track the tumour tropism of MSCs to orthotopic gliomas in vivo. However, the tumour tropism of MSCs showed no impact on the progression of gliomas. The purpose of this study was to determine the therapeutic effect of up-regulation IFNβ secretion of MSCs on gliomas, to monitor the biological behaviors of transplanted MSCs, and to determine the best transplantation method.
**Methods and Materials:** Rat MSCs were transduced with a lentiviral vector overexpressing FTH-IFNβ gene. The feasibility, efficacy and biological safety of this genetic modification were investigated in vitro. Intra-arterial, intertumoural and peritumoural injections of FTH-IFNβ transgenic MSCs (FTH-IFNβ-MSCs) were performed in rats bearing intracranial orthotopic C6 gliomas. The progression of gliomas, as well as the location, migration and survival of transplanted FTH-IFNβ-MSCs were tracked using in vivo MRI and confirmed by histology.
**Results:** The transduction efficiency achieved 73.59 ± 0.28%. FTH-IFNβ-MSCs can overexpress FTH and IFNβ effectively and safely. The FTH-IFNβ-MSCs were detected as hypointense signals on T2- and T2*-weighted images on a 3.0 T clinical MRI. FTH-IFNβ-MSCs could be monitored by MRI until 7 days after intra-arterial and intratumoural injection, and 11 days after peritumoural injection. The tumour growth rate of peritumoural injection group was significantly lower than other groups ($P$<0.05).
**Conclusion:** FTH-IFNβ-MSCs transplanted peritumourally can effectively inhibit intracranial orthotopic C6 gliomas in rats, providing a new idea for stem cell-based therapy for malignant gliomas.

**B-0510** 11:42
**Detection of gut inflammation with MR and PET imaging in the insect Manduca sexta: A new screening system for effectors and inhibitors of gut inflammation**
A.G. Müller[1], F.H. Müller[2], M. Hentschel[3], C.-R. von Bredow[1], Y. von Bredow[1], T. Trenczek[1]; [1]*Giessen/DE*, [2]*Neustadt/DE*, [3]*Berne/CH*
*(anton.g.mueller@allzool.bio.uni-giessen.de)*

**Purpose:** This study aims to propose and validate MR and PET imaging features of gut-inflammation in the insect Manduca sexta. The Epithelial structure and intestinal innate immune response in M.sexta are functionally and mechanistically comparable to humans, making M.sexta a valuable model to study the innate part of gut inflammation. This, together with the cost-effective raring and the large, cylindrical gut of M.sexta larvae will provide a quick and easy system to screen for new effectors and inhibitors of gut-inflammation for pharmaceutical and agricultural purposes.
**Methods and Materials:** We established Gd-BOPTA-MR and $^{18}$F-FDG-PET, as well as fusion MRI-PET imaging, methods firstly applied to insects, to detect gut-inflammation. Bacillus thuringiensis-infected (n=20) animals were used as a positive control and compared to healthy animals (n=20). We tested contrast-enhanced, T1-weighted(CHESS/Fat-Sat) gut wall thickness and area, gut wall signal-enhancement, as well as the contrast-enhanced gut diameter and SUVMax as MR and PET diagnostic features.
**Results:** Bt-fed animals showed a significantly lower survival (Log-rank/Gehan-Breslow-Wilcoxon test,p=≤0.0001) compared to control animals. Control and Bt-infected animals differed significantly (p≤0.0001) in each diagnostic finding. All diagnostic features were excellent or good with ROC-areas of 0.96-0.8 and strongly correlated to each other (r=0.5-0.75). We propose contrast-enhanced gut wall thickness with a threshold of 2.05mm and a sensitivity and specificity of 90% as a key diagnostic finding of gut-inflammation in M.sexta. Finally, we compared and validated the empirical MRI resolution using gut phantoms (SD=0.078mm).
**Conclusion:** The M.sexta screening system is a quick and easy tool that allows screening for new effectors and inhibitors of gut-inflammation.

**B-0511** 11:50
**The experimental study of prostate cancer imaging using PSMA targeting ultrasound/MRI dual modality nanobubbles**
Y. Zhu, Y. Chen, W. Guan; *Shanghai/CN (tttmax2004@126.com)*

**Purpose:** The prostate specific membrane antigen (PSMA) is broadly overexpressed on prostate cancer (PCa) cell surfaces. The aim of this study is to develop dual modality contrast agents for PSMA targeted ultrasound/magnetic resonance imaging (US/MRI) dual modality imaging.
**Methods and Materials:** The nanobubbles, characterised by strong penetrating power and stable performance, were served as the US imaging components of the dual modality contrast agents. SPIONs were encapsulated in the polymer shell of the nanobubbles to produce T2-negative contrast enhancement. The polypeptide CQKHHNYLC was conjugated to the nanobubbles for targeting PSMA. The SPIONs loaded nanobubbles (SPIONs/Polypeptide-PLGA NBs) were prepared by a modified double emulsion method. Series of in vitro and in vivo experiments were employed to evaluate the characterisation, targeted dual modality imaging ability, biodistribution and toxicity of SPIONs/Polypeptide-PLGA NBs.
**Results:** The average size of SPIONs/Polypeptide-PLGA NBs was 415nm, spherical shaped, uniformly distributed with superparamagnetic characteristic. SPIONs/Polypeptide-PLGA NBs provided positive contrast enhancement on ultrasound images and negative contrast enhancement on T2 MRI images. In in vivo dual modality imaging, the SPIONs/Polypeptide-PLGA NBs provided specific dual modality contrast on both US and MRI images by targeting PSMA. The NBs was breakdown and metabolised mainly in the reticuloendothelial system and showed no obvious toxicity in vivo.
**Conclusion:** SPIONs/Polypeptide-PLGA NBs could specifically enhance both ultrasound and MRI images via binding with PSMA expressed on prostate cancer cells. Our work provides new strategy for PCa detection, and further broadens the application of nanobubble in cancer imaging.

**10:30 - 12:00** **Room O**

### Musculoskeletal

### SS 610

### Chronic inflammatory diseases

*Moderators:*
M. Boesen; Copenhagen/DK
M. Revelli; Reggio Emilia/IT

**B-0512** 10:30
**Muscle involvement in juvenile localised scleroderma: do we need a volumetric approach?**
S.K.J. Flores Quispe[1], A. Cavaliere[1], F. Zulian[1], M. Weber[2], M. Zuliani[1], R. Stramare[1], E. Quaia[1], C. Giraudo[1]; [1]*Padua/IT*, [2]*Vienna/AT* *(flo_silvia@hotmail.it)*

**Purpose:** To perform a qualitative and quantitative MR-based muscle assessment in juvenile localised scleroderma patients (JLSp) including volumetric analyses.
**Methods and Materials:** JLSp referring to our pediatric unit who underwent to at least one MR scan at diagnosis including axial T2w fat-sat and axial T1w of both extremities were examined. The last available follow-up MR, satisfying the above-mentioned criteria, was also included. Fatty atrophy (Mercury Scale) and oedema (5 points scale) were assessed. Muscle area (MA) performing a ROI at the most affected level, and muscle volume (MV) were measured on the injured (i) and healthy contralateral (hc) extremity (Student's T-test,$p<0.05$). To assess the repeatability two radiologists performed all measurements independently (intraclass correlation coefficient, ICC).
**Results:** Fourteen JLSp (9 females; mean age 7.1±3.6yrs) met the inclusion criteria and 23 MR examinations (nine at follow-up) were evaluated. Muscle oedema was detected in 12 JLSp (mean 1.4±1.3) whereas fatty replacement was identified in one case (stage 2). At diagnosis MA and MV were significantly lower on the injured side (MAi 31.5±17.9 vs MAhc 38.6±22.7, $p<0.009$; MVi 553±330 vs MVhc 652±432, $p<0.033$). At follow-up, muscle oedema decreased in 6 JSLp (mean 1.2±1.4), and no significant difference between the two extremities occurred for MA or MV ($p>0.05$, each). All measurements showed high ICC (>.750, each).
**Conclusion:** In JLSp oedema and significant hypoplasia, rather than atrophic changes, occur at diagnosis and decrease after treatment. This evidence demonstrates the importance of muscle assessment in JLSp but without the necessity of a complex volumetric approach.

**B-0513** 10:38
**MRI evaluation of ankle joint in juvenile idiopathic arthritis**
P. Rampal[1], N. Sachdev[1], A. Taneja[2]; [1]*Delhi/IN*, [2]*New Dehli/IN* *(parikha2993@gmail.com)*

**Purpose:** To evaluate changes in ankle joint in patients of Juvenile Idiopathic Arthritis by MRI and to correlate these changes with the clinical scores which indicate disease severity.
**Methods and Materials:** Study design - Prospective observational study. Inclusion Criteria - 30 patients less than 16 years of age with a clinical diagnosis of Juvenile Idiopathic Arthritis as per the International League of Rheumatology definition with clinically involved ankle joint(pain/ swelling) shall be included in the study. Exclusion criteria - Any Patient with contraindication to MRI .They are subjected to contrast enhanced MRI to look for bone marrow edema, erosions, synovial thickening and enhancement and cartilage destruction and these findings are graded according to the Juvenile Arthritis MRI Score(JAMRIS). This score is compared with the Juvenile Arthritis Disease Activity Score (JADAS - 27) which is calculated using various clinical and biochemical parameters.
**Results:** The sensitivity of clinical diagnosis of ankle joint arthritis is 80% and specificity is 92% when compared with the MRI findings. Patients with higher JADAS tend to have more severe MRI findings in terms of synovial thickness, cartilage surface area involvement thereby making a higher JAMRIS.Bone marrow edema did not show a significant correlation with the disease severity of the patient.Patients on treatment may show clinical improvement early but their MRI findings take weeks to show any significant change.
**Conclusion:** Juvenile Arthritis MRI Score (JAMRIS) could be used to indicate the level of disease activity and thereby indicate prognosis in JIA patients.

**B-0514** 10:46
**Ultra-low-dose CT detects synovitis in patients with suspected rheumatoid arthritis**
T. Diekhoff, S.-T. Ulas, D. Poddubnyy, U. Schneider, S. Hermann, R. Biesen, G.R. Burmester, B. Hamm, K.-G.A. Hermann; *Berlin/DE* *(torsten.diekhoff@gmail.com)*

**Purpose:** To prove feasibility and diagnostic accuracy of contrast-enhanced ultra-low-dose CT (ULD-CT) for inflammatory soft tissue changes (synovitis, tenosynovitis and peritendonitis) in patients with arthritis of the hand.
**Methods and Materials:** In this institutional review board-approved study, 36 consecutive patients over the age of 50 with suspected rheumatoid arthritis underwent ULD-CT (estimated radiation exposure <0.01 mSv) and MRI of the hand with weight-adapted intravenous contrast administration. ULD-CT subtraction and MR images were assessed for synovitis, tenosynovitis and peritendonitis by three readers using a modified rheumatoid arthritis MRI score (RAMRIS). Patients were asked which modality they would prefer for future examinations. Sensitivity and specificity of ULD-CT for detection of inflammatory changes were calculated using MRI as standard of reference. The sum scores were correlated using Pearson's r.
**Results:** All 36 patients showed synovitis in MRI. ULD-CT had 69% sensitivity on the patient level and 65% on the joint level with 87% specificity. Sensitivity was higher in patients with more severe inflammation (80% for MRI RAMRIS >1). There was almost perfect correlation between the modified RAMRIS sum scores of ULD-CT and MRI (Pearson's $r=0.94$). Regarding preferences for future examinations, 85% preferred ULD-CT over MRI. ULD-CT detected more differential diagnoses than MRI (8 vs. 2/12).
**Conclusion:** Contrast-enhanced ULD-CT of the hand allows for depiction of soft tissue inflammation at the hand and can be achieved using very low radiation exposure (<0.01 mSv). ULD-CT may evolve to a fast and comfortable alternative to MRI, although it is not as sensitive as MRI for detecting mild disease.

**B-0515** 10:54
**Prevention of the progressive biochemical cartilage destruction under methotrexate therapy in early rheumatoid arthritis**
M. Frenken, D. Abrar, A. Ljimani, P. Sewerin, M. Klingebiel, G. Antoch, C. Schleich; *Düsseldorf/DE (miriam.frenken@med.uni-duesseldorf.de)*

**Purpose:** Objective of the study was to investigate biochemical cartilage composition under methotrexate (MTX) therapy and to intra-individually assess the impact of inflammation severity on cartilage composition using dGEMRIC MRI in patients with early rheumatoid arthritis (eRA).
**Methods and Materials:** dGEMRIC of MCP joints of the index and middle finger of 28 patients from the AthroMark cohort were examined prior to MTX-therapy as well as after 3 and 6 month. OMERACT RA MRI score and clinical parameters (CRP and DAS28) were registered at any time point. Each patient's second and third MCP joints were dichotomized into the joint with more severe synovitis versus the joint with less severe synovitis according to the RAMRIS synovitis subscore.
**Results:** MCP joints with more severe synovitis ('bad joints') demonstrated significantly lower dGEMRIC values compared to MCP joints with less severe synovitis ('good joints') at time-points 0 and 3 months ($p = 0.002$; $p = 0.019$, respectively). After 6 months of MTX therapy no significant difference of dGEMRIC index was found between good and bad joint ($p = 0.086$).
**Conclusion:** Under MTX therapy, biochemical cartilage integrity remains stable; no further cartilage destruction occurred if patients are treated early in the course of the disease. This might be explainable through reduced inflammation on joint level. In addition, six months of MTX therapy triggered an alignment of dGEMRIC index of MCP joints with initially severe synovitis and less severe synovitis in an intra-individual assessment.

**B-0516** 11:02
**Rheumatoid arthritis magnetic resonance imaging score predicts therapy response**
D.B. Abrar, M. Frenken, Y. Klosterkemper, G. Antoch, C. Schleich; *Düsseldorf/DE (danielbenjamin.abrar@med.uni-duesseldorf.de)*

**Purpose:** The aim of the study was to assess the performance of the RA magnetic resonance imaging (MRI) scoring system (RAMRIS) in combination with serum biomarkers to predict response to methotrexate (MTX) treatment in therapy-naive patients with early RA using high-field MRI.
**Methods and Materials:** 28 patients with RA were prospectively assessed with baseline 3-T MRI of the clinical dominant hand, 3 and 6 months after MTX. The patients met the 2010 American College of Rheumatology/European League Against Rheumatism (EULAR) criteria. RAMRIS and serum biomarkers consisting of various proteins including receptor activator of nuclear factor-kB ligand (RANKL) were obtained. Remission or treatment response was defined according to EULAR. To adjust for intrapersonal correlation, generalised linear mixed models were used.

**Results:** Treatment response at 3 months was associated to low RAMRIS erosion subscores and low total RAMRIS scores (p = 0.019 and 0.03, respectively). Remission at 6 months was associated to low RANKL levels (p = 0.033). Response at 3 and 6 months was predicted more accurately with the inclusion of total RAMRIS score, RAMRIS synovitis subscore at the second metacarpophalangeal (MCP) joint, or a combination of the two (p value likelihood ratio test = 0.035, 0.035, and 0.041, respectively). Remission was more accurately predicted with inclusion of RANKL, with no significant predictive effect of MRI.
**Conclusion:** Baseline total RAMRIS can predict EULAR response. RAMRIS synovitis subscore at the second MCP joint and RANKL are associated with response and remission, respectively.

**B-0517** 11:10
**The value of the simplified RAMRIS-5 in early-RA patients under methotrexate therapy using high-field MRI**
M. Frenken, D. Abrar, A. Ljimani, P. Sewerin, G. Antoch, C. Schleich; *Düsseldorf/DE (miriam.frenken@med.uni-duesseldorf.de)*

**Purpose:** The aim of the study was to evaluate a simplified version of the Rheumatoid Arthritis Magnetic Resonance Imaging Score (RAMRIS) to five joints of the hand (RAMRIS-5) in patients with early rheumatoid arthritis (RA) before and after the initiation of a methotrexate (MTX) therapy using high-resolution, 3 Tesla (T), magnetic resonance imaging (MRI).
**Methods and Materials:** 28 RA patients according to 2010 ACR/EULAR criteria (Ø 56.8 years (range 39 - 74)), seropositive, disease duration < 6 months (range 2 - 23 weeks) were prospectively assessed with baseline investigation including clinical assessment (DAS-28 and CRP) and 3T MRI of the clinical dominant hand, as well as 3 and 6 months after starting MTX therapy. MRI scans were analysed according to RAMRIS and the simplified RAMRIS-5.
**Results:** DAS-28, CRP, RAMRIS and RAMRIS-5 decreased significantly after initiation of a MTX therapy. There was a strong correlation between RAMRIS-5 and RAMRIS at baseline (r=0.838; p=<0.001) and follow-up (3 months: r=0.876; p= <0.001; 6 months: r=0.897; p=<0.001).
**Conclusion:** 3T MRI-based RAMRIS-5, the simplified version of the well-established RAMRIS is a resource-saving, appropriate alternative not only for patients with established but also with early RA. Regarding its shorter expenditure of time, there may be a high potential for using RAMRIS-5 in daily clinical practice to detect and monitor RA.

**B-0518** 11:18
**Silent progression in patients with rheumatoid arthritis: is DAS28 remission an insufficient goal in RA?**
D.B. Abrar, M. Frenken, Y. Klosterkemper, G. Antoch, C. Schleich; *Düsseldorf/DE (danielbenjamin.abrar@med.uni-duesseldorf.de)*

**Purpose:** Remission is arguably the ultimate therapeutic goal in rheumatoid arthritis (RA). Applying modern strategies, clinical remission can be achieved in a substantial number of patients with early RA (ERA). We, therefore, investigated the value of MRI for the detection of radiological progression in patients with DAS28 improvement and/or clinical remission.
**Methods and Materials:** Data sets of 80 RA patients (according to 2010 ACR/EULAR criteria), who fulfilled the following criteria, were retrospectively analysed: availability of two consecutive MRI scans (low-field MRI, follow-up interval 1 year) of the clinically dominant hand and wrist, and the presence of DAS28 (CRP) scores at both time points, which was used to assess disease activity.
**Results:** 71 of the 80 investigated patients presented a numerical improvement of DAS28 (CRP) after 12 months (DAS28 (CRP) T0 average (Ø) 4.96, SD 1.2; DAS28 T4 (12 months) Ø 2.6, SD 1.0), 73% of them also improved in the RAMRIS-Score, while 24% demonstrated an increase despite DAS28 improvement and 3% showed equal values. 48% of patients who improved in the DAS28 reached remission. 41% of these patients had an increase in the RAMRIS Erosion-subscore after 12 months. When considering EULAR response criteria (non-response (n = 7), moderate response (n = 19), good response (n = 45)), an increase of erosions was found in 71.4% of non-responders, 52.6% of moderate responders, and 31.1% of good responders after 12 months, all compared to baseline.
**Conclusion:** Up to 40% of patients in this study demonstrated a progressive erosive disease detected by MRI despite DAS28 improvement or EULAR remission.

**B-0519** 11:26
**Is an abbreviated version of psoriatic arthritis magnetic resonance imaging score (PsAMRIS), PsAMRIS-6, applicable for detection and monitoring of disease-related joint changes?**
D.B. Abrar, M. Frenken, P. Sewerin, G. Antoch, C. Schleich; *Düsseldorf/DE (dba0501@gmail.com)*

**Purpose:** Aim of this study was to evaluate a simplified version of the OMERACT psoriasis arthritis magnetic resonance imaging score (PsAMRIS) reduced to six joints of the clinically dominant hand in patients with psoriatic arthritis (PsA) before and after treatment escalation to a TNF-α inhibitor using high-resolution, 3-Tesla (T) MRI.
**Methods and Materials:** 17 patients with PsA according to the CASPAR classification criteria (mean age 53.2, ± 11.6, minimum/maximum 26/72 years) who qualified for TNF-α inhibitor due to inadequate csDMARD treatment response were prospectively assessed by both baseline investigation and 6 months after escalation using 3T MRI of the clinically dominant hand. Scans were analysed using the OMERACT PsAMRIS and the simplified PsAMRIS-6 (DIP 2-5 + PIP 2 + MCP 2).
**Results:** We found a strong correlation between OMERACT PsAMRIS and the simplified PsAMRIS-6 at baseline (r= 0.871; p < 0.0001) and after six months (r= 0.894; p< 0.00001). Evaluation time was significantly (p < 0.001) reduced from 306.2 to 112.4 seconds at baseline and from 321.25 to 120.5 seconds at follow-up.
**Conclusion:** The simplified PsAMRIS-6 is a time- and resource-saving modification of the OMERACT PsAMRIS. The PsAMRIS-6 is a reliable diagnostic tool for detecting morphological changes and therapy monitoring in PsA.

**B-0520** 11:34
**Diffusion-weighted magnetic resonance imaging in axial spondyloarthritis patients and healthy subjects: inter-MRI reliability and relation to gender, age and inflammation**
J.M. Møller[1], M. Østergaard[1], H.S. Thomsen[1], I.J. Sørensen[2], O.R. Madsen[3], S.J. Pedersen[2]; [1]*Herlev/DK*, [2]*Glostrup/DK*, [3]*Gentofte/DK (jakob.moeller@regionh.dk)*

**Purpose:** To examine test-retest reliability, age and gender influence on spinal DWI and correlation with presence/absence of bone marrow oedema (BME) in SpA patients and healthy subjects.
**Methods and Materials:** In a test-retest setup 25 SpA patients (12 males) and 24 healthy subjects (11 males) were MRI examined at 1.5T twice with a mean interval of 6.8 days (SD 0.9). A sagittal STIR and a single-shot echo-planar-imaging DWI sequence were performed on Th8-L5. On the midsagittal STIR-image, the disco-vertebral unit (DVU) was divided into 4 quadrants by a vertical line through the midpoint of the vertebral bodies. BME was scored present/absent per quadrant (total score range 0-4). On ADC maps six 40mm$^2$ circular ROIs were measured in each DVU (anteriorly, centrally and posteriorly in each vertebral endplate) and mean ADC (µmm$^2$/s) was calculated. All measurements were performed on the central sagittal slice. The inter-MRI agreement was measured by intra-class correlation coefficient (ICC). Variation was also tested by unpaired t tests and ANOVA.
**Results:** Inter-MRI ICCs ranged from 0.66 at T9/T10 to 0.94 at L2/L3. Mean ADC (SD) in females and males was 378(156) and 242(100), p<0.01. Mean ADC (SD) in females <50y was 412(149) and >50y 258(114), p<0.01. For BME scores 0, 1, and 4 in the midsagittal slice, mean ADC (SD) was 298(136), 296(138), and 828(406), respectively.
**Conclusion:** The inter-MRI agreement of ADC measurements by DWI sequences in the lower thoracic/lumbar spine was high. ADC depends on age and gender and the degree of inflammation.

**B-0521** 11:42
**Axial spondyloarthritis: dual-energy virtual noncalcium CT for detection of bone marrow oedema in the sacroiliac joints**
H. Wu, G. Zhang; *Guangzhou/CN (15876530875@163.com)*

**Purpose:** To determine the diagnostic performance of dual-energy CT for the detection of bone marrow (BM) oedema in patients with sacroiliitis of axial spondyloarthritis (axSpA) using a virtual noncalcium (VNCa) technique.
**Methods and Materials:** Forty-seven consecutive patients with 55 sacroiliitis were studied between April 2016 and December 2017. All patients underwent dual-energy CT and 3-T MRI. Two independent readers visually evaluated all sacroiliac joints for the presence of abnormal BM attenuation on VNCa images using a four-point classification system (0 = no oedema, 3 = severe oedema). MR images served as the reference standard. CT numbers on VNCa images were performed a quantitative analysis.
**Results:** In the visual analysis of dual-energy VNCa images, the respective sensitivity, specificity, accuracy, positive predictive value, and negative predictive value for detecting BM oedema for reader 1 and reader 2 were 87.3% and 92.7%, 94.1% and 91.2%, 89.9% and 92.1%, 96.0% and 94.4%, 82.1% and 88.6%, respectively. The interobserver agreement was excellent

(κ= 0.81). CT numbers obtained from VNCa images gradually increased from no oedema to severe oedema ($P < 0.001$). ROC analysis of CT numbers revealed an area under the curve of 0.930 and 0.908 for reader 1 and reader 2. A cutoff value of -33.4 HU provided overall sensitivity, specificity and accuracy of 89.9%, 82.9%, and 86.5% for the detection of oedematous sacroiliac joints.
**Conclusion:** Visual and ROI-based analyses of dual-energy VNCa images had excellent diagnostic performance for detecting different extents of BM oedema in sacroiliac joints in patients with axSpA.

**B-0522** 11:50
**Muscle and pulmonary involvement in patients with antisynthetase syndrome: two aspects of the same disease or two different conditions? Preliminary results of an MR and HRCT study**
A. Fraia[1], E. Zanatta[1], M. Weber[2], E. Balestro[1], R. Stramare[1], A. Doria[1], C. Giraudo[1], E. Quaia[1]; [1]*Padua/IT*, [2]*Vienna/AT (annasara.fraia@gmail.com)*

**Purpose:** To assess the relationship between muscle and pulmonary involvement in patients with antisynthetase syndrome (ASS) at diagnosis.
**Methods and Materials:** Treatment-naïve patients with ASS who underwent MR of the upper and lower limb and HRCT (6 months maximal interval) were retrospectively investigated. Fat muscle infiltration (FMI) and muscle oedema (ME) were assessed using the Goutallier classification (grade 0-4) and a four grades score (0=no edema; 1=slight-interfascicular; 2=slight-intrafascicular; 3=moderate-intrafascicular), respectively. In each pulmonary lobe, the extension of ground-glass opacities (GGO) and interstitial thickening (II) was assessed by the Kazerooni score (0-100%), whereas a modified-Bhalla score was applied for consolidations (Co) (0=absent;1=subsegmental;2=segmental-lobar) and bronchiectasis (Br) (0-3, considering the ratio with vessels). The relationship between muscle involvement and the most frequent pulmonary findings, considering the maximum values (max), was assessed (Spearman coefficient).
**Results:** Eight patients (56±16 yrs, 7 female) matched the inclusion criteria. All patients showed FMI (mean±SD 1.26±0.82, max=3), and ME (mean±SD 0.79±0.74, max=3). GGO (mean±SD 9.12%±8.28%, max=40%) and II (mean±SD 6.72%±7.4%, max=30%) were the most severe pulmonary findings affecting all patients. Co (mean±SD 0.02±0.16, max=1) and Br (mean±SD 0.3±0.46, max=1) occurred in one and five patients, respectively. No significant correlations emerged ($r_{FMIvsGGO}$=0.301,p=0.469 and $r_{FMIvsII}$=0.477,p=0.232), even if a reciprocal trend occurred for ME ($r_{MEvsGGO}$=-0.273,p=0.513 and $r_{MEvsII}$=-0.326,p=0.431).
**Conclusion:** It is well-known that lung interstitial disease may precede, concur or follow the onset of myositis and our findings further suggest that pulmonary and muscle involvement are fully independent. Future studies on a larger population are expected to better characterize this evidence and its clinical implications.

**10:30 - 12:00** **Studio 2019**

### Oncologic Imaging

### SS 616a

### Advances in imaging of liver malignancies

*Moderators:*
L. Grazioli; Brescia/IT
A. Luciani; Créteil/FR

**B-0523** 10:30
**Towards a new model for therapy monitoring of PRRT $^{177}$Lu-DOTATATE treatment of liver metastases in PNET patients**
O.J. Pettersson, K. Fröss-Baron, J. Crona, A. Sundin; *Uppsala/SE (olofjp@gmail.com)*

**Purpose:** Monitoring of cancer treatment by contrast-enhanced CT (CECT), applying RECIST 1.1 criteria, is less suitable for neuroendocrine tumours (NETs) which, when responding, tend to show stable disease and not response. Consequently, new methods are needed to classify those that show stable disease on RECIST. Changes in arterial tumour attenuation and contrast enhancement may earlier reflect therapy effect than decreased tumour diameter.
**Methods and Materials:** 47 patients with metastatic pancreatic NETs (PNETs) undergoing peptide receptor radiotherapy (PRRT) with $^{177}$Lu-DOTATATE underwent CECT at baseline, mid-treatment (cycles 3-5) and at follow-up, 3 months after the last PRRT cycle. In each patient, the liver metastasis at baseline-CT with the highest arterial attenuation was identified and the fold changes in arterial tumour attenuation, contrast-enhancement and transversal tumour area between CT at baseline, mid-treatment and follow-up were calculated. Data were correlated to best response, time to best response and progression-free survival.
**Results:** As an internal control, it was first tested and established that the tumour attenuation was not related to the attenuation of the abdominal aorta. The mean arterial attenuation decreased 19 HU from baseline to follow-up (p<0.05) and between mid-treatment and follow-up (p<0.05) as did the transversal tumour area between baseline and follow-up (p<0.05). A 17 HU increase (p>0.05) of the contrast enhancement from baseline to mid-treatment correlated with time to best response ($R^2$=0.36, p=0.0003) and to progression-free survival ($R^2$=0.27, p=0.0008).
**Conclusion:** Changes in arterial tumour attenuation and contrast enhancement, in addition to lesion size (RECIST 1.1), may add information on therapy response to PRRT.

**B-0524** 10:38
**Early prediction of response of CT textural analysis in unresectable liver metastases of colorectal cancer treated with Cetuximab chemotherapy**
E. Raimondi[1], S. Picchia[1], K. Kouvelakis[1], K. Khan[1], D. Cunningham[1], D.-M. Koh[2], M. Bali[1]; [1]*London/UK*, [2]*Sutton/UK (dr.edoardo.raimondi@gmail.com)*

**Purpose:** To explore changes of CT texture analysis (CTTA) metrics in unresectable liver metastases (LM) in CRC patients treated with Cetuximab.
**Methods and Materials:** After IRB approval, baseline and first-assessment (FA) contrast enhanced CT images of 30 CRC-LM patients treated with Cetuximab monotherapy were retrospectively reviewed. Regions of interests were drawn around up to two target hepatic metastases to record volume and area-derived first-order LM CTTA-metrics, and their percentage change from baseline to FA. Results were compared between responders (Complete Response + Partial Response) and non-responders (Stable disease + Progressive disease) defined at best-response according to RECIST 1.1 throughout treatment.
**Results:** Of the 30 patients, 16 were responders (R) and 14 non-responders (NR). At baseline no differences were observed between R and NR. At FA, in R compared to NR, average area-CTTA metrics showed lower entropy (6.06 vs 6.36; p=0.006) and higher uniformity (0.02 vs 0.014; p=0.002). R showed percentage change in area-entropy of -2.08% and volume-entropy of -1.89% significantly different (p < 0.05) from NR who showed +1.09% area-entropy and +0.91% change in volume-entropy. The percentage changes in area-uniformity and volume-uniformity in R was +8.10% and +8.09% compared with -4.51% and -3.92% in NR (p>0.05).
**Conclusion:** Differences were observed for area and volume-entropy and uniformity between R and NR. These metrics could be used as predictive imaging biomarker of response. Further studies should confirm these data and identify a threshold for early prediction of response.

**B-0525** 10:46
**CT-texture analysis of liver metastases in PNETs vs NPNETs: correlation with histopathological findings**
I. Martini[1], M. Polici[1], M. Zerunian[1], F. Landolfi[1], F. Panzuto[1], M. Rinzivillo[1], B. Annibale[1], A. Laghi[2], E. Iannicelli[1]; [1]*Rome/IT*, [2]*Latina/IT (isabella.martini88@gmail.com)*

**Purpose:** To compare CT-texture analysis and CT features of liver metastases in pancreatic neuroendocrine tumours (PNETs) and in non pancreatic neuroendocrine tumours (NPNETs) according to tumour grading.
**Methods and Materials:** Contrast-enhanced CT images of liver metastases in 23 patients with PNETs and in 25 patients with NPNETs were analysed with 3D CT texture analysis (parameters evaluated: mean attenuation, standard deviation, skewness, kurtosis, entropy, mean of positive pixels and Tx_sigma) at baseline, arterial and portal phase; delta enhancement of the lesions was also calculated. The CT exams were performed before the beginning of any medical treatment. All patients presented a well differentiated tumour according to WHO classification (G1 and G2). Data were analysed with Kaplan Meyer's test.
**Results:** Among CT texture analysis, in a comprehensive comparison between PNETs and NPNETs the parameter "Skewness" was significantly higher in NPNETs (p-value<0.05). This data was confirmed in subgroup comparisons evaluated in portal phase (NPNETs G1 vs PNETs G1, NPNETs G2 vs PNETs G2, and NPNETs G1+G2 vs PNETs G1+ G2). The parameter "Mean" was significantly higher in PNETs in comparison to NPNETs (p-value=0,0066). Among CT features the "delta enhancement" was significantly higher (p-value<0.05) in PNETs in two comparisons: PNETs G1 Vs NPNETs G1 and PNET Vs NPNETs.
**Conclusion:** These results demonstrate significant differences between CT texture parameters of liver metastases in PNETs and NPNETs, and highlight the diversity of the two groups of NETs. These findings, in future, could be used as an "imaging biomarker" to predict therapy response.

**B-0526** 10:54
**Preoperative staging of colorectal liver metastases: is MRI of the liver needed for the surgical planning?**
P. Boraschi, F. Donati, F. Pacciardi, R. Cervelli, G. Tarantini, L. Urbani, M. Castagna, D. Caramella, F. Falaschi; *Pisa/IT (p.boraschi@gmail.com)*

**Purpose:** To evaluate the efficacy of multiparametric 3T MR imaging in the preoperative staging of colorectal liver metastases in patients previously undergone to neoadjuvant chemotherapy (nCT).
**Methods and Materials:** Fifty-eight patients affected by colorectal cancer and focal liver lesions who received preoperative nCT and underwent parenchymal-sparing liver surgery were retrospectively included. All the patients performed multiphasic contrast-enhanced CT (CE-CT) and multiparametric 3T MRI within one month before surgery. At MRI unenhanced (T1- and T2-weighted), DWI with multiple *b*-values (150, 500, 1000, 1500 sec/mm²) and Gd-EOB-DTPA-enhanced T1-weighted LAVA-flex sequences ( including both dynamic and hepato-biliary phase) were obtained. All CT and MR examinations were reviewed by two observers in conference in order to identify and characterize focal liver lesions. CT and MRI findings were related with histopathology, which was our gold standard. Only benign lesions at intraoperative ultrasound remained un-resected and underwent imaging follow-up.
**Results:** A total of 370 hepatic lesions were detected: 292 metastases (79%) and 78 benign lesions. At MRI 359/370 (97.0%) lesions were detected, whereas at CT 245/370 (66.2%) were identified. The sensitivity, specificity, PPV, NPV of MRI and CT metastases characterization were 98.9%, 97.3%, 99.3%, 96.0%, and 87.6%, 41.3%, 82.7%, 51.0%, respectively. The higher yield of MRI could improve the surgical planning in 37 out of 58 patients (63.8%) compared to CE-CT.
**Conclusion:** Multiparametric 3T MRI may provide better diagnostic performance than CE-CT for the identification and characterization of colorectal liver metastases before surgery. It can also potentially improve the surgical planning in over 60% of patients.

**B-0527** 11:02
**CT texture analysis to predict response to target therapy of hepatic metastases from colorectal cancer**
C. Maglia[1], L. Pusceddu[1], V. Vani[2], G. Giannetto[1], V. Romano[1], A. Defeudis[1], V. Giannini[1], S. Mazzetti[1], D. Regge[1]; [1]*Candiolo/IT*, [2]*Turin/IT (claudiomaglia@hotmail.it)*

**Purpose:** In colorectal cancer 50% of patients develop liver metastases (mCRC). Chemotherapy and surgery in oligo-metastatic cases are the therapeutic standard, but this strategy is not resolutive in most cases. Differentiating non- and short-term responders from potentially "cured" patients will spare needless toxicity. In this study we aimed to use CT texture analysis (CTTA) to identify specific imaging biomarkers for mCRC, able to predict patient's response to therapy and overall survival (OS).
**Methods and Materials:** 23 patients with amplified HER2 mCRC received anti-HER2 treatment and underwent CT examination every 8 weeks, until disease progression. CT scans were segmented to extract all liver metastases. CTTA was performed on each segmented area, evaluating 34 parameters. Both mono- and multi-parametric analysis were assessed to identify features most correlated to therapy response. We also performed OS analysis, considering good survivor those with OS>9 months.
**Results:** In 23 patients we found 124 metastases, 55 of which were classified as responder and 69 as non-responder. Nine parameters reached statistical significance in the mono-parametric analysis (best AUC=0.67, p=0.001), while "cluster prominence" and "sum entropy" predicted OS with AUC equal to 0.78 and 0.83, respectively. In the multivariate regression to predict therapy response, ten parameters were considered (AUC=0.82, sensitivity=82%, specificity=72%). The regression model for OS analysis included two variables ("cluster prominence" and "dissimilarity"), reaching sensitivity and specificity of 83% and 82%, respectively.
**Conclusion:** CTTA is a potential biomarker to predict treatment response, possibly saving non-responder patients from toxicity. CTTA could give indications on patients OS, without additional tests.

**B-0528** 11:10
**Histopathological growth patterns of colorectal cancer liver metastases: predictive value of MRI morphological features**
P.A. Bonaffini[1], A. Salman[1], A. Lazaris[1], G. Zu-Hua[1], P. Metrakos[1], H. Vargas[2], C. Hoeffel[3], B. Gallix[1], C. Reinhold[1]; [1]*Montreal, QC/CA*, [2]*New York, NY/US*, [3]*Reims/FR (pa.bonaffini@gmail.com)*

**Purpose:** To retrospectively assess the ability of MRI morphological features to predict histopathological growth patterns (HGP) of colon cancer liver metastases (CRCLM).
**Methods and Materials:** Forty-tree patients with 69 CRCLM were evaluated, with the following criteria: MRI <3 months prior to surgery, CRCLM HGP subtype on pathology (desmoplastic/non-desmoplastic); correlation between MRI w.r.t. location/size and final pathology. Desmoplastic HGP has a better prognosis, potentially benefiting from anti-VEGF-A treatment. Four independent readers, blinded to HGP, evaluated five features per lesion: (1) border regularity (BORDER); (2) peripheral rim presence-thickness (RIM); (3) septa presence-thickness (SEPTA); (4) peri-tumoural liver enhancement (PL); (5) kinetic rim enhancement pattern (KIN RIM ENHANC). A binary score (1 desmoplastic, 0 non-desmoplastic) was given for each parameter and final impression. Descriptive statistics on rater agreement was presented. Univariate logistic regression was used to evaluate the desmoplastic predictability classification.
**Results:** A total of 44/69 (64%) lesions had a confirmed desmoplastic pattern, 25 non-desmoplastic. The MR morphological classification predicted the desmoplastic pathology outcome for all reviewers with a 70-90% accuracy. All reviewers consistently flagged two features as more important predictors of desmoplastic HGP: PL and KIN RIM ENHANC, with an odds ratio (OR) range of 3-9 each. Two reviewers achieved an OR of 5 using RIM. However, the prediction inference was highly consistent when all five features classified a desmoplastic HGP (OR=8.93;95%CI=1.80-44.3).
**Conclusion:** Peritumoural liver signal intensity and kinetic rim enhancement pattern, followed by peripheral rim morphology, are the main MRI morphological features that might non-invasively predict CRCLM desmoplastic HGP, improving treatment stratification.

**B-0529** 11:18
**Volumetric vs bidimensional CT assessment of sarcopenia in patients candidate for liver transplantation**
P. Barbieri, A. Cina, E. Amodeo, R. Manfredi, G. Marrone; *Rome/IT (pierluigi.barb@gmail.com)*

**Purpose:** Sarcopenia is a crucial issue in clinical management. Our aim was to evaluate the prognostic value of a CT bidimensional measure (muscular area at inferior border of L3) and a new proposed volumetric evaluation to identify sarcopenia before transplant, their correlation with post LT mortality and to identify optimal threshold for the tridimensional analysis.
**Methods and Materials:** We evaluated 50 pre-transplant CT scans (venous phase) of adult patients with cirrhosis who underwent LT between 2016 and 2018. We performed both standard bidimensional and volumetric analysis. Images were analyzed with Volume Viewer software (GE Medical Systems). We included a volume from iliac crests to the base of the heart, excluding the visceral content by segmentation. Tridimensional analysis was performed indexing total volume by height squared ($cm^3/m^2$).
**Results:** A Cox proportional regression-model was employed for post-LT survivability analysis. A significant difference (*p<0.05*) was found in population survivability by adopting the lower quartile as cut-off for L3-SMI values, showing thresholds of ≤ 37.9 $cm^2/m^2$ for women and ≤ 39.4 $cm^2/m^2$ for men ([HR] 4,3 [95% CI 1.4-13.2], p = 0.01). Furthermore, also tridimensional values reflected a difference in survivability above and below 25th percentile, thus identifying new potential thresholds of ≤583.7 cm3/m2 for women and ≤629.9 $cm^3/m^2$ for men ([HR] 7 [95% CI 2.3-21.6], p = 0.001).
**Conclusion:** Volumetric measurements appeared to be a reliable predictor in identifying subjects with high post-LT mortality, and thus should be further investigated to evaluate a potential superior prognostic value compared to standard bidimensional analysis.

**B-0530** 11:26
**Prevalence and prognosis of intrahepatic cholangiocarcinoma (ICC) with radiological enhancement patterns that mimic hepatocellular carcinoma (HCC)**
R. Muglia, L. Viganò, N. Gennaro, L. Samà, F. Colapietro, G. Torzilli, L. Di Tommaso, M. Colombo, L. Solbiati; *Milan/IT (riccardo.muglia@humanitas.it)*

**Purpose:** To analyse the radiological enhancement patterns of ICC and their association with prognosis, with particular interest to lesions mimicking HCC.
**Methods and Materials:** We retrospectively evaluated all consecutive patients affected by ICC undergoing surgery between 2007 and 2017, whereas those with mixed HCC-ICC were excluded. Two expert radiologists reviewed the arterial and portal/late phases of preoperative CT or MRI. Full-nodule hyper-enhancement in arterial phase and hypo-enhancement in portal/late phase was classified as "HCC-like pattern". Imaging of ICCs with HCC-like pattern was reviewed by an additional radiologist blinded to clinical data.
**Results:** We reviewed imaging of 92 patients (mean age 68 yrs; males 49%, cirrhosis 20%), including multiphase CT in all and MRI in 87 (95%). Sixty-six (71%) tumours showed arterial hyper-enhancement, categorized as full-nodule (12/66, 18%), >25% of nodule (20/66, 30%) and peripheral (34/66, 52%). The remaining ICCs were either iso-enhancing (5%) or hypo-enhancing (24%). Among 12 ICCs with full-nodule arterial enhancement, 4 were hypo-enhancing in portal/late phase (HCC-like pattern), including 1 cirrhotic patient. Overall, 4/94 (4%) ICCs (1/18 cirrhosis, 6%) showed a HCC-like pattern, accounting for misclassification as HCC on imaging review. After a median follow-up of 29 months, 3-year survival was 62%. All patients with HCC-like pattern ICC are alive and disease free (median 64 months). Peripheral rim enhancement was associated with a worse prognosis (48% versus 70%; p=0.150).
**Conclusion:** ICC can be misdiagnosed as typical HCC in 4% of cases, showing a favourable survival. ICCs with peripheral rim enhancement are associated with the worst prognosis.

**B-0531** 11:34
**The role of dynamic contrast-enhanced MRI analysis of perfusion changes in hepatocellular carcinogenesis**
A.M. Elzeneini[1], M.I. Yousef[2], M.M. Refaat[2]; [1]*Cairo/EG*, [2]*Benha/EG* *(dr.elzeneini@gmail.com)*

**Purpose:** To evaluate the functional role of DCE-MRI analysis of perfusion changes in multi-step hepatocarcinogenesis.
**Methods and Materials:** 53 patients with liver cirrhosis having 87 hepatic lesions (14 DNs, 52 de novo HCCs, 16 recurrent HCCs, 2 malignant PV thrombi, 3 periablation hyperemic rims) and 42 patients without cirrhosis (30 positive for viral hepatitis, 12 negative) all underwent 3T multiphase DCE-MRI assessment. Maximum relative enhancement (MRE), area under curve (AUC), relative area under curve (r AUC), wash-in ratio (WIR), wash-out ratio (WOR), time-to-arrival (T0), and time-to-peak (TTP) semi-quantitative measurements were analysed across groups. Multiphase mean time-%relative enhancement curves were also portrayed.
**Results:** Comparing perfusion metrics across different pathologies of liver cirrhosis revealed the correlation of haemodynamics with multi-step hepatocarcinogenesis and grades of cirrhosis. Significant performances were highest between DNs and HCCs (MRE 0.88, WIR 0.84, WOR 0.78, TTP 0.74, AUC 0.73, T0 0.70) compared to those between cirrhotic liver and DNs (MRE 0.74, T0 0.62). Least performances were between de novo and recurrent HCCs (TTP 0.66, r AUC 0.66, MRE 0.63). Performances between non-cirrhotic and cirrhotic livers (WOR 0.81, TTP 0.80, r AUC 0.63) surpassed those among different grades of cirrhosis (highest was between non-tumoural and tumoural cirrhosis: TTP 0.74, MRE 0.68, WIR 0.65, WOR 0.65).
**Conclusion:** DCE-MRI haemodynamic metrics promise potential usefulness as non-invasive biomarkers in the assessment of liver cirrhosis, characterization of cirrhotic nodules, and evaluation of multi-step hepatocarcinogenesis.

**B-0532** 11:42
**Is CT-texture analysis an useful instrument to predict aggressive behaviour of intrahepatic cholangiocarcinoma?**
A. Fighera, G. Cardano, A. Mazzaro, G. Zamboni, A. Ruzzenente, G. Mansueto; *Verona/IT (alessandrofighera@gmail.com)*

**Purpose:** To assess the value of CT-texture analysis in predicting disease aggressiveness of resected intrahepatic cholangiocarcinoma.
**Methods and Materials:** we analyzed the texture of the main lesion in the preoperative multiphasic CT of 28 patients with histologically confirmed intrahepatic cholangiocarcinoma. Texture analysis was performed using LIFEx software on the largest slice of the lesion in each contrast-enhanced phase (baseline, arterial, portal and 8-minute delayed phase). First order texture histogram parameters (skewness, kurtosis, entropy) were related with tumour size, presence of macroscopic vascular invasion, satellite lesions, histologically confirmed metastatic lymphnodes, tumour recurrence and overall survival. Comparison was made with an unpaired t-test. For categorical variables, the median value was used to divide the patients into two groups (long axis: 56,5 mm; overall survival: 10,5 months).
**Results:** entropy values in the delayed phase were significantly different in lesions <56,5 mm or ≥56,5 mm (3,15±0,08 vs 3,44±0,1; p=0,037), and in single or multiple lesions (3,18±0,07 vs 3,50±0,12; p=0,042). Although differences for all the other indexes were not statistically significant, we noticed a trend to less uniformity in the portal and delayed phase for lesions with metastatic lymphnodes or overall survival <10,5 months after surgery.
**Conclusion:** delayed phase entropy is significantly higher in larger lesions and in tumours with satellite lesions. Additionally, lesions with higher entropy show a trend to earlier recurrence/mortality. These could depend on the presence of higher cellularity and necrotic areas in more aggressive tumours, causing inhomogeneous enhancement.

**B-0533** 11:50
**Imaging response assessment and outcomes in hepatocellular carcinoma after stereotactic body radiotherapy: iRECIST as a potential substitute for traditional criteria**
F. Vernuccio[1], D. Godfrey[2], M. Meyer[3], H.V. Williamson[2], D. Niedzwiecki[2], J. Ronald[2], M. Palta[2], D. Marin[2]; [1]*Palermo/IT*, [2]*Durham, NC/US*, [3]*Mannheim, NC/US (federicavernuccio@gmail.com)*

**Purpose:** To investigate whether, compared to traditional criteria, modified RECIST 1.1 for immune-based therapeutics (iRECIST) improves prediction of local tumour control and survival in patients with hepatocellular carcinoma (HCC) treated with stereotactic body radiotherapy (SBRT).
**Methods and Materials:** 56 HCC lesions (mean size 3.1 cm) treated with SBRT in 45 patients (mean age 67 years) were retrospectively included. Each patient underwent CT/MR exam prior to SBRT and at least once after SBRT. Best overall response was categorized using RECIST 1.1, iRECIST, WHO, mRECIST and EASL criteria. Lesions were then classified as local tumour control (i.e., stable disease, partial or complete response) or local treatment failure (i.e., progressive disease) by each tumour response criteria. Proportions of local tumour control were compared using McNemar's exact test. The 1-year overall survival by each tumour response criteria was estimated using the Kaplan-Meier method.
**Results:** The local tumour control rate was 85.7% by iRECIST, 80.4% by RECIST 1.1, 67.9% by WHO, 76.8% by mRECIST and 71.4% by EASL. The local tumour control rate by iRECIST was significantly higher compared to WHO, mRECIST, and EASL (p values: 0.0010, 0.0391, 0.0074, respectively). The 1-year survival rate for patients with local tumour control was higher (though not statistically significant) for the iRECIST (86.4% [90% CI, 73.7-93.3]) compared to the other tumour response criteria.
**Conclusion:** iRECIST may provide more robust interpretation of HCC response after SBRT, yielding improved prediction of local tumour control and 1-year survival rates compared to traditional criteria.

**10:30 - 12:00** **Room E2**

### Neuro

### SS 611
### MS and demyelinating disorders

*Moderators:*
A.M. Koç; Izmir/TR
T.A. Yousry; London/UK

**B-0534** 10:30
**Cranial nerve enhancement in multiple sclerosis (MS) is associated with younger age at onset and more severe disease**
L. Haider[1], E.C. Wei-Shin[1], E. Olbert[2], S. Mangesius[3], A. Dal-Bianco[1], F. Leutmezer[1], D. Prayer[1], M. Thurnher[1]; [1]*Vienna/AT*, [2]*Tulln/AT*, [3]*Innsbruck/AT (l.haider@live.at)*

**Purpose:** The overall frequency of cranial nerve (CN) pathology and its relation to brainstem lesion formation on magnetic resonance imaging (MRI) in multiple sclerosis (MS) is uncertain. Our purpose was to determine the frequency of cranial nerve enhancement (CNE) on MRI scans and its association with structural imaging findings and clinical outcome measurements.
**Methods and Materials:** We retrospectively analysed 651 MRI scans from 183 randomly selected MS patients. Visually screened and signal intensity measurement-confirmed frequencies of CNE (CN III-XII) on post-contrast T1-weighted MRI images were compared to lesion counts and the MS severity score (MSSS).
**Results:** CNE was present in 8.2% of the analysed MS patients (oculomotor nerve: 1.1%, trigeminal nerve: 2.7%, abducens nerve: 2.2%, facial and vestibulocochlear nerve: 1.6%, vagal nerve: 0.5%). From these, 13% suffered from repeated episodes of CNE and 27% exhibited a CNE duration of >12 months. Median age at MS onset was lower in patients with CNE, 23 vs. 28, p=0.049. MSSS, 5.15 vs. 0.88 (p=0.019), median T2 brainstem, 1 vs. 0 (p=0.041) and total intracranial contrast enhancing lesion counts, 2 vs. 0 (p=0.000), were higher in patients with CNE, compared to age-, disease duration and gender-matched MS patients without CNE.
**Conclusion:** CNE, present in 8.2%, associates with younger age at MS onset, brainstem lesions and more severe disease course.

**B-0535** 10:38
**Multiple sclerosis: evolution of magnetic resonance imaging diagnostic criteria from McDonald 2001 to McDonald 2017, including MAGNIMS 2016**
J.J. Jover Sánchez, J. Díaz Díaz, C. Ferreiro Argüelles, P. Núñez Valentín, E. Rodríguez García; *Madrid/ES (jjjover90@gmail.com)*

**Purpose:** To compare the efficiency of the diverse McDonald criteria and MAGNIMS criteria after a clinically isolated syndrome (CIS) in patients diagnosed with multiple sclerosis (MS).
**Methods and Materials:** This is a retrospective, from 2009 to 2018, evaluation of clinical notes and 1.5 T MRI scans of 73 patients diagnosed with relapsing-remitting MS.
**Results:** Percentage of patients that did not fulfill the different McDonald criteria: 2001 and 2005 criteria-20%, 2010 criteria-11% and only 1% did not fulfill 2017 criteria (1 patient); 11%-MAGNIMS 2016. Diagnosis performing only one MRI study in 3% using McDonald 2001 or 2005 criteria, 32% using 2010 criteria, 47% using MAGNIMS 2016 and 90% using 2017 criteria (47% had dissemination in space demonstrated by MRI and cerebrospinal fluid oligoclonal bands but not dissemination in time). Time between CIS and diagnosis of MS was also longer using McDonald 2001-2010 and MAGNIMS criteria than 2017 criteria. Percentage of diagnosis within the first 6 months after CIS: 2001 criteria-18%, 2005 criteria-21%, 2010 criteria-37%, MAGNIMS 2016 criteria-48% and 83% applying 2017 criteria. Spine MRI was performed in 47% of CIS showing abnormalities in 85%.
**Conclusion:** McDonald 2010 criteria improves the diagnosis of MS compare to McDonald 2001-2005 criteria. Inclusion of symptomatic lesions in the DIT criteria, in MAGNIMS 2016, increased the proportion of patients satisfying the MRI diagnostic criteria for MS in the first MRI. 2017 Revised McDonald Criteria seems to allow an earlier diagnosis. Using these new criteria, most patients presenting with a CIS would need only 1 MRI to fulfill the criteria.

**B-0536** 10:46
**Evaluation of signal intensity in dentate nucleus and globus pallidus after repeated administrations of gadolinium in multiple sclerosis patients**
I. Zorzenon, M. Ukmar, A. Gennari, A. Zdjelar, L. Bottaro, A. Sartori, A. Dinoto, M.A. Cova; *Trieste/IT (irene.zorzenon@gmail.com)*

**Purpose:** The recently described association between repeated administration of gadolinium based contrast agents (GBCAs) and increased signal intensity (SI) in the basal ganglia on T1-weighted unenhanced MR images is subject of debate. Aims of the study were to evaluate in multiple sclerosis (MS) patients SI ratio changes in the dentate nucleus (DN) and globus pallidus (GP) on T1-weighted unenhanced MRI after repeated administrations of GBCAs. We compared patients who received gadobutrol-only with patients who received gadobutrol plus other macrocyclic GBCAs.
**Methods and Materials:** We retrospectively identified 52 MS patients (37 F, 15 M; age 46.2±11 years) who received repeated administrations of GBCAs (median 5, range 3-12) during follow-up MR scans. Baseline and follow-up T1-weighted image series obtained on the same 1.5T scanner were analysed by two readers; ROI measurements of mean SI were performed and SI ratios (DN-to-pons and GP-to-thalamus) differences were calculated fot both gropus. Paired samples T-test was used for the statistical analysis ($p<0.05$).
**Results:** 27 MS patients received gadobutrol only, 25 patients gadobutrol + other macrocyclic GBCAs (gadoteridol and/or gadoterate meglumine). No changes in DN-to-pons and GP-to-thalamus SI ratios have been observed in both groups ($p=0.79$, $p=0.26$). Differences between baseline and follow-up SI ratios did not differ between groups ($p=0.08$, $p=0.37$).
**Conclusion:** Repeated administrations of macrocyclic GBCAs were not associated to SI changes of DN and GP in MS patients, both in gadobutrol only and gadobutrol plus other macrocyclic GBCAs group. Furthermore, SI ratio difference between last and first examinations did not differ between the two groups.

**B-0537** 10:54
**Brain imaging in 43 serologically positive cases of Chikungunya virus infection with neurological complications at our institute during epidemic outbreak**
A. Aryan, K. Sethi; *New Delhi/IN (abhaykaryan@gmail.com)*

**Purpose:** Brain imaging in 43 serologically positive cases of Chikungunya virus infection with neurological complications at our institute during epidemic outbreak.
**Methods and Materials:** Retrospective study of 43 seropositive (PCR positive) cases of Chikungunya virus infection who underwent magnetic resonance imaging (MRI) for neurological complications such as altered sensorium, seizure or stroke-like symptoms from July to November, 2016. The two neonates who were included in the study were maternal seropositive cases. MRI brain findings such as T2W-FLAIR signal and diffusion and gradient images were analysed for pattern of involvement.
**Results:** Patients aged between 5 days to 83 years were included in the study. There were 43 patients, 35 males and 8 females. MRI brain was normal in 13 patients (30%). There were predominantly discrete and confluent supratentorial T2W and FLAIR hyperintense white matter foci in 22 patients (51%) and restricted diffusion was seen in 14 patients (32%). Four patients had infratentorial cerebellar white matter or middle cerebellar peduncle lesions with restricted diffusion. In three paediatric patients including two neonates, pattern of involvement was diffuse white matter changes with restricted diffusion. Haemorrhage in supratentorial white matter was seen in one patient only.
**Conclusion:** Confluent white matter T2 and FLAIR hyperintensity with restricted diffusion were observed in all the paediatric patients of Chikungunya virus fever with neurological complications. Focal and confluent supratentorial white matter T2 and FLAIR hyperintense foci were seen in adult age group with some showing restricted diffusion.

**B-0538** 11:02
**Regional grey matter atrophy by MR volumetry in early relapsing-remitting multiple sclerosis (RRMS) as a predictor of clinical and motor outcome**
E. Kurys-Denis, U. Skrobas, Z. Brudkowska, H. Karakuła-Juchnowicz, W. Krupski, R. Maciejewski; *Lublin/PL (ekurys@gmail.com)*

**Purpose:** Recent MR studies have shown that in MS not only global and white matter (WM) atrophy, but also selective regional grey matter (GM) atrophy appear. We aimed to identify any specific areas of GM volume changes and investigate their importance in correlation to disease length, number of relapses and clinical motor scores in little known early stage of the disease.
**Methods and Materials:** We prospectively recruited 60 patients (46 F, 14 M, aged 34,58 +/- 8,03, median age 35) with RRMS at an early clinical stage (median EDSS score of 1,75). All participants were analysed with EDSS score, 9-peg hole test and 25-feet walk test. The length of the disease and the number of relapses were noted. Brain structures volumetry was done using automatic segmentation technique (Volbrain) with 1,5T MR system acquisition.
**Results:** Decreases of total brain, cerebrum, cerebellum normalised volumes and total WM and both total cerebral and cerebellar WM volumes correlated significantly with the disease length and number of relapses. Only specific sub-cortical GM areas (brainstem, putamen, thalamus, accumbens nucleus) showed significant volume reductions associated with the disease length and the number of relapses. EDSS and clinical motor scores showed significant correlations with the total WM and cerebral WM normalised volumes, as well as with thalamus and amygdala volume decreases.
**Conclusion:** Only sub-cortical part of the GM decreases in the early stage of RRMS. Thalamus volume decrease, followed by amygdala volume decrease seem to be strongly associated with the outcome of EDSS and clinical motor scores.

**B-0539** 11:10
**Automated volumetric assessment of multiple sclerosis disease burden and activity with artificial neural networks**
G. Brugnara, F. Isensee, W. Wick, M. Bendszus, K.H. Maier-Hein, P. Kickingereder; *Heidelberg/DE (gianluca.brugnara@med.uni-heidelberg.de)*

**Purpose:** To evaluate the potential of artificial neural networks (ANN) for automated volumetric assessment of multiple sclerosis (MS) disease burden and activity.
**Methods and Materials:** We used a single-institutional dataset from 334 patients with MS at any disease stage to develop an ANN for automated segmentation of FLAIR and contrast-enhancing (CE) lesions on MRI. Independent testing of the ANN was performed in a single-institutional longitudinal dataset with 82 patients (median number of exams: 3). The performance of the ANN was evaluated by calculating f1 score, positive predictive value (PPV) and sensitivity for the detection of both FLAIR and CE lesions. The f1 score was optimised in the training set and yielded an optimal working point of 14mm3 for CE, and 7mm3 for FLAIR.
**Results:** The total f1 score for the segmentation of FLAIR lesions was 87% (training set) and 88% (test set). The f1 score for the segmentation of CE lesions was 78% (training set) and 80% (test set). The ANN yielded a sensitivity of 76% (160 TP / 52 FN) and a PPV of 85% (160 TP / 29 FP) for detection of CE lesions in the longitudinal test set, and 82% (4873 TP / 1053 FN) and 95% (4873 TP / 235 FP) for detection of FLAIR lesions in the same cohort.
**Conclusion:** Our results highlight the capability of ANN for quantitative state-of-the-art assessment of lesion load on MRI and potentially enable a more accurate assessment of disease burden in patients with MS.

**B-0540** 11:18
**Increased ADC values within normal appearing white matter as a predictor of a poor response to interferon beta therapy in patients with multiple sclerosis in the 4-year follow-up**
J. Bladowska, A. Zacharzewska-Gondek, A. Koltowska, A. Pokryszko-Dragan, S. Budrewicz, M. Sasiadek; *Wroclaw/PL (asia.bladowska@gmail.com)*

**Purpose:** The purpose was to assess the usefulness of apparent diffusion coefficient (ADC) measurements within normal appearing white (NAWM) and grey matters (NAGM) in the prediction of interferon beta (IFN-Beta) treatment response in patients with multiple sclerosis (MS) during 4-year follow-up.
**Methods and Materials:** Ninety-five patients: 29 with MS, who presented relapse during 4 years of treatment (MS1), 35 MS patients without relapse (MS0) and 31 age and sex matched control subjects (CG) underwent plain MR followed by diffusion weighted imaging (DWI) on a 1.5T MR unit. MR examinations were performed before the treatment with IFN-Beta or during the first year of treatment. In DWI the ADC values were measured in 13 regions of interest within NAWM and NAGM.
**Results:** MS1 patients showed significantly higher ADC values in frontal and fronto-parietal WM regions ($p<0.01$) compared to MS0 and CG and additionally in right temporal WM compared to CG. ADC values in both caudate nuclei were significantly lower in MS0 subjects compared to CG. No other statistically significant differences in ADC values between groups were found.
**Conclusion:** Measurements of ADC values within NAWM performed in baseline MRI before or during the first year of treatment may be useful in the prediction of treatment response in MS. Increased ADC values in the frontal and fronto-parietal WM regions can predict a poor response to INF-Beta therapy during the 4-year follow-up. Normal ADC values in these regions and decreased ADC values in both caudate nuclei may predict a good response to treatment.

**B-0541** 11:26
**Quantitative brain lesion distributions in MOG-Ab-positive and AQP4-Ab-positive patients: overlaps and differences**
L. Yang, H. Li, C. Quan, L. Zhou, D. Geng, Y. Li; *Shanghai/CN (mozi001_ren@sina.com)*

**Purpose:** The aim of this study is to quantitatively delineate and compare the brain lesions and their distributions in two subsets of patients with NMOSD phenotype and related disorder: myelin oligodendrocyte glycoprotein antibody positive (MOG-Ab-positive) and aquaporin-4 antibody positive (AQP4-Ab-positive).
**Methods and Materials:** Fifty-seven and thirty-three clinical MR examinations were performed on fifty-two AQP4-Ab-positive and twenty-eight MOG-Ab-positive patients, respectively. Hyper-intense T2 lesions were segmented manually on each axial FLAIR image and the lesion sizes were calculated. Probabilistic lesion distribution maps were created for each group by averaging the lesion images after normalization to standard space. Lobe-wise and voxel-wise quantitative comparisons of the two distributions were performed.
**Results:** We found infratentorial and supratentorial brain lesions in both AQP4-Ab-positive and MOG-Ab-positive patients, with high occurrence frequency and large inter-group overlap mainly in deep white matter (WM). In comparison with AQP4 group, the brain lesions of the MOG-Ab-positive patients had larger size, dispersed distribution, and significantly higher probabilities in grey matter (GM) and juxtacortical WM regions prominently in temporal, limbic and parietal lobes.
**Conclusion:** MOG-Ab-positive and AQP4-Ab-positive group showed similar but quantitatively different brain lesion distribution. These results provide valuable information for imaging-based initial diagnosis and understanding of pathogenesis in the two subsets of patients.

**B-0542** 11:34
**Magnetic resonance imaging findings at optic neuritis of recurrent optic-spinal phenotype, multiple sclerosis in a cohort of Brazilian patients from Rio de Janeiro**
A.C. Brandão Nascimento, A.R. Araujo e Araujo, N.S. Meneguette, M. Papais Alvarenga, C.C.F. Vasconcelos, C.A.M. Bento, P.P. Rafful, L.A. Brandao, R.M. Papais Alvarenga; *Rio de Janeiro/BR (annachrisbrandao@outlook.com)*

**Purpose:** Optic neuritis may evolve into multiple sclerosis and MRI demonstrates lesions in optic nerves. Objective: to study MRI of optic nerves.
**Methods and Materials:** Patients with recurrent optic neuritis (ON) with a good recovery and transverse myelitis (TM) were followed from 2000 to 2015, 10 years of disease and EDSS<3, were classified as "benign"with a recurrent optic spinal phenotype multiple sclerosis (OSMS). A prospective population study was performed on 16 patients OSMS. All patients was negative (AQP4)-IgG and negative (MOG)-IgG. Most patients showed brain and spine lesions met MAGNIMS criteria (2016). For lesions on MRI in sequence, STIR with the optic nerves were divided into three segments: intra-orbital (IO) (25 mm: the longest segment and communication between subarachnoid space around the optic nerve that in suprasellar cistern, canalicular (CAN) (9 mm: as through optic canal and prechiasmatic and chiasmal (CHI) (16 mm: intracranial segment in suprasellar cistern).
**Results:** 2 (12.5%) patients showed normal optic nerves. Lesions on MRI the optic nerves in 9 (56.25%) patients with lesion unilateral, 5 (31.2%) patients with lesions bilateral,14 (87.5%) patients with lesion intra-orbital (IO), 7 (43.7%) showed lesion CAN, 14 (87.5%) IO and 1(6.2%) patient showed lesion prechiasmatic. No patient showed contrast enhancement.
**Conclusion:** Patients with recurrent ON and TM, at least 10 years of disease and low EDSS indicate that optic spinal recurrent phenotype of MS and not a NMO spectrum syndrome. Most patients did not have extensive lesions in optic nerves and visual event good recovery could be classified as a subgruop of MS with a benign course.

**B-0543** 11:42
**Voxel-based analysis of relaxation rates in multiple sclerosis: correlates of cognitive impairment and physical disability**
M. Quarantelli, R. Lanzillo, M. Comerci, M. Cassiano, T. Costabile, A. Prinster, R. Megna, V. Brescia Morra, A. Brunetti; *Naples/IT (quarante@unina.it)*

**Purpose:** Brain tissue relaxation rates have shown significant correlations with clinical data in multiple sclerosis (MS). Aim of this retrospective study was to assess voxelwise the correlation between cortical relaxation rates, obtained by relaxometric segmentation of clinical MRI studies, and both physical and cognitive impairment in MS.
**Methods and Materials:** R1 and R2 relaxation rate maps and GM maps were calculated using a multiparametric relaxometric segmentation procedure, analysing clinical MRI studies in 241 patients with relapsing-remitting MS. Correlation with EDSS and with the percentage of failed tests at a cognitive test battery (Rao's Brief Repeatable Battery and Stroop test, available in 186 patients) were assessed voxelwise, including age, sex, disease duration and voxel GM content as covariates, using the SPM-based "Biological Parameter Mapping" software package.
**Results:** Extensive clusters (p<0.05, corrected for familywise error at cluster level) of inverse correlation between both EDSS and cognitive scores and cortical R1, and to a lesser extent R2, values were present, mostly in the perirolandic cortices for EDSS (p<0.001), and in the hippocampi (p=0.001), precuneus (p<0.001), dorsolateral prefrontal cortices (p<0.001) and cingulate (p=0.003) for cognitive scores.
**Conclusion:** Cortical R1 and R2 values significantly correlate with both physical and cognitive impairment in MS, independent of atrophy, with distinct regional patterns.

**B-0544** 11:50
**Deep grey matter and multiple sclerosis: is there a correlation between MR imaging and disability?**
S. Torlone[1], A. Corridore[1], M. Martino[2], M. Micelli[1], L. Panebianco[1], R. Totaro[1], A. Splendiani[1], C. Masciocchi[1]; [1]*L'Aquila/IT*, [2]*Rome/IT (torlone.silvia@gmail.com)*

**Purpose:** To investigate the association between deep grey matter (DGM) lesions and clinical features in relapsing remitting multiple sclerosis (RR-MS) patients in a long-term follow-up.
**Methods and Materials:** We considered 228 patients with RR-MS, chosen among 460 MS patients of the MS Centre of L'Aquila, with clinical evaluation by EDSS at the onset and during clinical/MRI follow-up. In 45 patients DGM lesions appeared during MRI follow-up. 45 patients without DGM lesions were selected among the 228 RR-SM patients as a control group, according to sex, age, disease duration and EDSS at the onset. In both groups the first and the last MR examination were compared to assess the differences in White Matter (WM) lesion load, cortical lesions frequency and EDSS.
**Results:** WM lesion load and cortical lesions frequency were significantly higher in the group with DGM lesions compared to control group (p=0,005). Although EDSS scores at the onset were similar for both groups, it was increased in patients with DGM lesions (p=0,01). 7 Patients (15,5%) with DGM lesions developed Secondary Progressive MS (SP-MS) with statistically significant frequency compared to control group (p=0,01).
**Conclusion:** A strong correlation between the presence of DGM lesions and EDSS, WM lesion load, cortical lesions frequency and development of SP-MS was observed. EDSS increase was noted at the time of DGM lesions appearance and showed a persistent growth in the follow-up. Future research is needed to improve DGM lesions detection in order to confirm their prognostic value and to help the specialist in therapeutic choices.

**10:30 - 12:00** **Room F2**

### Breast

### SS 602

### Magnetic resonance imaging (MRI)

*Moderators:*
P. Clauser; Vienna/AT
G. Ivanac; Zagreb/HR

### B-0545 10:30

**Dependence of MRI-based size estimates of small invasive breast cancers on patient and tumour characteristics**

W. Sanderink, L.J. Strobbe, P. Bult, I. Sechopoulos, N. Karssemeijer, R.M. Mann; *Nijmegen/NL (wendelien.sanderink@radboudumc.nl)*

**Purpose:** To determine the reliability of MRI tumour size measurements with respect to patient and tumour characteristics in small breast cancers that can be selected for minimal invasive therapy.
**Methods and Materials:** All consecutive cases of cT1 invasive breast carcinomas that underwent pre-operative MRI, treated in two hospitals between 2005 and 2016, were identified retrospectively from the Dutch cancer registry and cross-correlated with local imaging and pathology databases. Concordance between MRI-based measurements and final pathological size was analysed. In addition, the influence of patient (age, WHO performance status) and tumour (location, histological type, grade, molecular subtype) characteristics on the accuracy of the measurement was analysed using the Student's t test, chi-square and multilinear regression analyses.
**Results:** Analysis included 354 cT1 invasive carcinomas in 348 patients (age: 55.1±11 years), including 255 ductal carcinomas, 64 lobular carcinomas and 35 other types. MRI and pathology measurements were available for 318 lesions (36 missing). Mean MRI size was 13.1±4.6 mm and pathology size 12.8±7.5 mm (p=0.573). Pathology tumour size was equal or smaller in 208 lesions (65.4%), and larger in 110 lesions (34.6%). Median MRI underestimation was 4 mm, with 35 lesions (10%) underestimated by more than 5 mm. Neither patient characteristics (p≥0.343) nor tumour characteristics (p≥0.208) were predictive of underestimation of tumour size on MRI.
**Conclusion:** Size measurements of small breast cancers on breast MRI are within 5 mm of pathological size in 90% of patients. However, neither patient nor tumour characteristics predict underestimation, which may hinder patient selection for minimal invasive therapy.

### B-0546 10:38

**Diagnostic accuracy of magnetic resonance (MRI) to predict axillary lymph node metastases in patients with breast cancer: does neo-adjuvant chemotherapy impact accuracy?**

G. Bansal, A. Jaipal; *Cardiff/UK (gjbansal@gmail.com)*

**Purpose:** The aim of this study was to evaluate the diagnostic accuracy of preoperative breast MRI for axillary lymph node (LN) metastases in two groups of patients with breast cancer. Those who received neo-adjuvant chemotherapy (NAC) prior to surgery were compared to patients, which proceeded straight to surgery (non-NAC).
**Methods and Materials:** Between 2011 and 2017, 198 patients with primary breast cancer were divided into two groups (NAC and non-NAC). The preoperative MRI results of axilla were compared to postoperative results in both groups. The axillary LN status (number of abnormal LNs, long axis-short axis ratio, loss of fatty hila and cortical thickness) on MRI was recorded. SPSS (ver 21) was used for data analysis and two-sided p less than 0.05 was taken as statistically significant.
**Results:** There were 112 patients in the non-NAC group and 86 patients in NAC group. Diagnostic accuracy and diagnostic odds ratio (OR) in non-NAC group were 86.3 and 25.9 (95% CI 7.8-86.05) versus 73.8 and 7.2 (95%CI 2.4-16.13) in the NAC group. There was greater correlation between the number of abnormal nodes on MRI and postoperative results in the non-NAC group (*p* less than 0.001). Long-to-short axis ratio of axillary nodes was not significantly different between metastatic and non-metastatic nodes in the NAC group.
**Conclusion:** MRI should be used with caution for the assessment of axillary LNs in patients who received NAC prior to surgery. These results underscore the importance of post-NAC biopsy of clipped nodes and limited sentinel node biopsy.

### B-0547 10:46

**Association between background parenchymal enhancement and tumour response in breast cancer patients receiving neoadjuvant chemotherapy**

F. Petta, R. Rella, A.R. Scrofani, E. Bufi, M. Rosignuolo, M. Romani, P. Belli, R. Manfredi; *Rome/IT (federicapetta@gmail.com)*

**Purpose:** To investigate the association between background parenchymal enhancement (BPE) of the contralateral breast on pre-operative breast magnetic resonance imaging (MRI) and response to neoadjuvant chemotherapy (NAC) in breast cancer patients.
**Methods and Materials:** Two hundreds and twenty-six patients with unilateral invasive breast cancer who underwent breast MRI before and after NAC between 2006 and 2012 were retrospectively analyzed. All images were reviewed by two blinded readers who classified BPE into four categories (1=minimal, 2=mild, 3=moderate, 4=marked) before and after NAC. Cohen's kappa coefficient was calculated to measure the agreement between the two readers. We determined the correlation between BPE and other clinicopathological variables (age, adjuvant therapy, menopausal status, histologic grade, T stage, N stage, histological subtype, molecular subtype, surgical margin status, and mammographic density). Histopathological tumour responses (pathologic complete response, pCR versus non-pCR) were analyzed and a correlation analysis between BPE and tumour response was performed (chi-square test).
**Results:** Cohen's kappa showed substantial agreement (k=0.64) between readers. BPE was significantly higher in premenopausal patients (P=0.004 for reader 1; P=0.011 for reader 2) and in patients with high mammographic density (P=0.04 and P=0.03 reader 1 and reader 2, respectively). Triple negative tumours were more likely to show low BPE (78%, P<0.0001). The pre-treatment BPE was significantly higher in the pCR group than in the non-pCR group (P=0.0377) while there was no significant difference in BPE changes after NAC between the two groups (P>0.05).
**Conclusion:** A higher pre-treatment BPE was related to pCR suggesting a possible role in prediction of response.

### B-0548 10:54

**Association between background parenchymal enhancement in breast MR imaging and outcome of breast cancer patients treated with neoadjuvant chemotherapy**

R. Rella, A.R. Scrofani, F. Petta, E. Bufi, M. Conti, M. Giuliani, P. Belli, R. Manfredi; *Rome/IT (annarita.scrofani90@gmail.com)*

**Purpose:** To retrospectively investigate the association between background parenchymal enhancement (BPE) of the contralateral breast in magnetic resonance imaging (MRI) and long-term outcome in breast cancer patients treated with neoadjuvant chemotherapy (NAC).
**Methods and Materials:** Two hundreds and twenty-two women with invasive breast cancer who underwent breast MRI before and after NAC between 2006 and 2012 were retrospectively analysed. All images were reviewed by two blinded readers, who classified BPE into four categories (1=minimal, 2=mild, 3=moderate, 4=marked) before and after NAC. Patients were grouped according to BPE category (high [moderate or marked] or low [minimal or mild]). Cox regression was used to test associations between BPE, patient and tumour characteristics, recurrence-free survival (RFS) and overall survival (OS).
**Results:** Median follow-up was 59 months. An event occurred in 83 of 222 patients (37%) for RFS and in 27 of 222 patients (12%) for OS. On multivariate analysis, high BPE on pre-NAC MRI (hazard ratio [HR]=0.0737, P=0.0034) and adjuvant chemotherapy (HR=0.5313, P=0.0484) were associated with better RFS. A greater reduction of BPE on post-NAC MRI (HR=26.6275, P=0.0003), menopausal status (HR=2.8290, P=0.0006) and higher nuclear grade (HR=1.6470, P=0.0262) were independent factors associated with worse RFS. BPE on pre-NAC MRI (P=0.022) and reduction of BPE on post-NAC MRI (P=0.009) were the only significant covariates for overall survival.
**Conclusion:** Results suggest that BPE in the contralateral breast is significantly associated with long-term outcome in breast cancer patients treated with NAC.

### B-0549 11:02

**Breast MRI: potential use of unenhanced morpho-functional diagnostic protocols**

M. Nadrljanski, Z. Milosevic; *Belgrade/RS (dr.m.nadrljanski@gmail.com)*

**Purpose:** To test different unenhanced (UE) breast MRI protocols in lesion detection with different b-gradients on diffusion-weighted imaging (DWI) added to unenhanced T2W and T1W series.
**Methods and Materials:** Sixty patients (N=60) were included in the retrospective analysis (1.5T) with histologically proven lesions: malignant (n1=40) and benign (n2=20) and the detection was evaluated with 4 different UE protocols (P1 - 4) with the blinded data: P1 (T2W-STIR, T2W-TSE, T1W-TSE), P2 (P1 + b50, 850), P3 (P2 + b1000), P4 (P3 + b1200). Upon the completion of the study, the full diagnostic protocol (FDP) was unblinded and

Thursday the histologic diagnosis revealed. Performance assessment was performed for all protocols.
**Results:** The average patient age and tumour size in n1 and n2 were similar (64.3+/-4.4 vs. 64.2+/-4.6; 1.39+/-0.46 cm vs. 1.49+/-0.56, p=0.57). In malignant lesion detection (n1), the following sensitivity (Se) was obtained [%]: P1 67.5, P2 80, P3 92.5, and P4 95. In benign lesions (n2), the following Se was obtained [%]: P1 70, P2 80, P3 90, and P4 95. Se of the FDP was 100% in n1 and n2.
**Conclusion:** UE protocols with added DWI with high b values (b 1200, b 1000) contributed to high rate of either benign or malignant lesion detection (Se>=90%). All missed lesions on UE were smaller than 1cm. The difference in Se per protocol between benign and malignant lesion detection was not considered significant (p>0.05). Potential use of UE protocols with high b values may contribute to lesion detection.

### B-0550 11:10

**Non-enhanced breast MRI (DWIBS mammography) for screening women with dense breasts: a feasibility study**

T. Uematsu; *Shizuoka/JP (t.uematsu@scchr.jp)*

**Purpose:** To evaluate clinical feasibility of diffusion-weighted whole-body imaging with background body signal suppression (DWIBS) mammography for detecting mammographically occult breast cancer in women with dense breasts and develop assessment criteria.
**Methods and Materials:** 330 patients with 360 lesions including 34 mammographically occult breast cancers were examined with DWIBS and short T1 inversion recovery (STIR) with 3 T MRI at b values of 0 and 1,000 s/mm$^2$. The scanning time was 8 minutes and 20 seconds. Mammographically occult breast cancers were evaluated for visibility on DWIBS mammography. Each reading time including all 360 lesions was measured, then the signal and morphology on STIR were compared with breast cancers and benign lesions to make the diagnostic criteria. DWIBS mammography was displayed as an inverted grey-scale maximum intensity projection. A lesion detected on DWIBS mammography was considered positive if it showed low signal intensity relative to the background breast parenchyma.
**Results:** DWIBS mammography sensitivity for mammographically occult breast cancer was 91% and the average reading time was 11 seconds. Low/iso-intensity on STIR (p<0.0001), non-mass lesion type (p<0.026), and ill-defined margin (p<0.0001) were significantly associated with breast cancer.
**Conclusion:** Unenhanced DWIBS mammography with STIR has high sensitivity for mammographically occult breast cancer with the shorter scanning and faster reading time; this technique might be useful as a supplemental screening modality for women with dense breasts. A unique area of low signal intensity on DWIBS mammography with low/iso-intensity on STIR should be recalled, especially with ill-defined margin.

### B-0551 11:18

**Rethinking abbreviated breast MRI: potential to skip DCE scans by integrated semi-quantitative T2 and ADC analyses**

M. Dietzel, R. Schulz-Wendtland, E. Wenkel, C. Bielowski, T. Bäuerle, M. Uder, S. Ellmann; *Erlangen/DE*

**Purpose:** To evaluate, whether T2 signal-intensity mapping (T2-SIM) and apparent-diffusion-coefficient (ADC) analysis could allow skipping dynamic-contrast-enhanced scans (DCE) from an abbreviated breast MRI.
**Methods and Materials:** 173 consecutive patients with equivocal breast lesions (BI-RADS IV/V) upon mammography and/or ultrasound received histopathological verification and standardized breast MRI. Protocols included DCE, T2-STIR and SPAIR-DWI sequences according to international standards (B values 50, 400, 800s/mm²). Lesions were defined by regions-of-interest (ROI) by two blinded observers independently (R1/R2: intermediate/no experience in breast MRI). ROIs were automatically copied between the different scans (DCE, T2, DWI). The following parameters were used for lesion characterization: DCE: enhancement characteristics normalized to baseline during the early and delayed phases, T2: signal intensity normalized to noise and background (pectoral muscle), and DWI: mean ADC. Univariate analysis (area under the ROC curve: AUC) was followed by multivariate regression analysis. This allowed to compare integrated assessment of ADC+DCE, ADC+T2 and ADC+DCE+T2 (AUC comparison; alpha<5%). Intraclass-correlation-coefficients/ICC were calculated to assess observer-related bias.
**Results:** There were 176 lesions (malignant: 107/60.8%). Inter-observer variability was "excellent" (ICC: 0.81-0.98). DCE and T2 performed similarly (AUC=0.657/0.669; P=0.9). Both were outperformed by ADC (AUC=0.817; P<0.003). Integration of ADC+DCE and ADC+T2 showed equal results (AUC=0.847/0.833, P=0.45). Use of all three parts of the protocol did not further increase diagnostic performance (ADC+T2+DCE: AUC=0.856; P>0.1).
**Conclusion:** T2 signal-intensity mapping and ADC analysis could allow skipping DCE scans, thus enabling a shorter, saver and cheaper breast MRI examination. This new aspect of abbreviated breast MRI did not sacrifice diagnostic accuracy and should, therefore, be validated in prospective clinical trials.

### B-0552 11:26

**Correlate of perfusion parameters on dynamic contrast-enhanced MRI with bimolecular factors and subtypes of breast cancers**

L. Liu, B. Yin, W. Peng; *Shanghai/CN (llbbsy@163.com)*

**Purpose:** This study aimed to investigate the correlation between the parameters of dynamic contrast-enhanced magnetic resonance imaging (DCE-MRI) and the molecular biological expression of breast cancers.
**Methods and Materials:** This prospective study enrolled a total of 67 consecutive patients with breast carcinomas. DCE-MRI were performed in all patients before surgery. Correlations among perfusion parameters and prognostic factors, including tumour size, oestrogen receptor (ER), progesterone receptor (PR), Ki67, human epidermal growth factor receptor 2 (HER-2), epidermal growth factor receptor (EGFR) expression, CK5/6 and subtypes of breast cancer were analysed.
**Results:** Among these 67 lesions, the mean diameter of tumour was 4.48±1.73cm. There was no correlation between perfusion parameters and lesion size; $K^{trans}$ and Kep were positively correlated with Ki67; the Vp values in negative and positive expression of CK5/6 were statistically different; the mean $K^{trans}$ value of HER-2 overexpression was higher than that of Luminal A and Luminal B; the mean Kep value of HER-2-overexpressing lesions was higher than that of Luminal A. The mean Ve value of triple-negative lesions was higher than that of HER-2 overexpression, Luminal A and Luminal B; the TTP value of HER-2-overexpressing lesions was lower than that of Luminal A and Luminal B; the mean MAX Conc value of triple-negative lesions was higher than that of Luminal B, and the mean MAXSlope of HER-2 overexpressing was lower than that of Luminal A and Luminal B.
**Conclusion:** Perfusion parameters of DCE-MRI can non-invasively evaluate the molecular biological expression and molecular subtype of breast cancer.

### B-0553 11:34

**Can perilesional oedema detected in MRI be considered as a biomarker for breast cancer? The role of T2-weighted sequences**

G. Panzironi, G. Moffa, F. Galati, F. Marzocca, E. Collalunga, F. Pediconi; *Rome/IT (g_panzironi@hotmail.com)*

**Purpose:** To evaluate whether the presence of perilesional oedema on T2-weighted sequences in magnetic resonance imaging (MRI) is correlated with worse breast cancer prognosis.
**Methods and Materials:** From August 2017 to March 2018, 68 patients with biopsy-proven breast cancer underwent breast MRI. All breast MR examinations were performed on 3Tesla scanner using a dedicated protocol, including T2-weighted, DWI and T1-weighted sequences, before and after paramagnetic contrast agent administration (0.2 ml/kg ProHance®, Bracco). 30 patients (mean age of 52 years) with perilesional oedema, detectable as T2-weighted hyperintensity, were enrolled in this study. The patients were divided into 3 groups characterized by different prognostic phenotypes: Luminal A, Luminal B and triple-negative/basal-like, according to molecular profiling (Ki-67 index, hormone receptor status and Her-2 overexpression). Lesion size measurements and histological evaluations were also performed.
**Results:** On histological examination, 40 lesions were detected: 33 (82.5%) were invasive ductal carcinoma (IDC) and 7 (17.5%) were invasive lobular carcinoma (ILC). Tumour mean size was 25.8 mm. The prognostic phenotype of patients was: Luminal A in 6 (20%), Luminal B in 16 (53.3%) and triple negative in 8 (26.7%). Perilesional oedema was significantly correlated with breast cancer with worse prognosis (Luminal B and triple negative) rather than with those with better prognosis (Luminal A) (p < 0.0001, Chi-squared test).
**Conclusion:** Perilesional oedema is significantly associated with more aggressive breast cancer. Therefore, surrounding oedema detected on T2-weighted sequences may be useful and could be considered an additional prognostic indicator in the evaluation of breast cancer.

### B-0554 11:42

**Diagnostic utility of breast DCE-MRI in patients with diagnosis of atypical ductal hyperplasia after percutaneous biopsy**

V. Bertani[1], M. La Grassa[2], L. Balestreri[3], N. Berger[4], M. Urbani[3], T. Frauenfelder[4], A. Boss[4], M. Marcon[4]; [1]*Udine/IT*, [2]*Pordenone/IT*, [3]*Aviano/IT*, [4]*Zurich/CH (magda.marcon@usz.ch)*

**Purpose:** To evaluate the diagnostic utility of DCE-MRI in predicting upgrade to malignancy (UM) after a percutaneous biopsy(PB) diagnosis of ADH.
**Methods and Materials:** In this IRB approved study (January 2016-December 2017) 68 women (median age, 51 years; range 31-73 years) with a PB diagnosis of ADH and subsequently undergoing DCE-MRI at 1.5 T were retrospectively included. PB was performed under stereotactic guidance using 8-11 gauge vacuum-assisted-biopsy (VAB) systems in case of mammography-only findings (40 lesions) or under ultrasound-guidance using 14 gauge core-needle-biopsy (CNB) (28 cases). All cases underwent surgical excision and final histologic diagnosis was recorded. Two radiologists in consensus evaluated: a) maximum diameter (MD) of the lesion in images prior to biopsy b)presence of a MRI-correlation to the lesion. MD and biopsy modality were compared in cases with/without MRI-correlation as well as with/without UM. Comparison between cases with/without MRI-correlation and UM was also performed. Mann-Whitney U test was used to compare median values and $X^2$ to compare proportions.
**Results:** Median MD was 18 mm (IQR 11-30 mm, range 6-63 mm) and 12 mm (IQR 8-20 mm, 6-40 mm) for cases undergoing VAB and CNB, respectively (p=.054). A MRI-correlation was present in 17/40 (42.5%) and in 19/28 (67.8%) which underwent VAB and CNB, respectively (p=.081). 17/68 (25%) had UM: 13/17 (76.5%) low-and intermediate grade DCIS and 4/17 (23.5%) invasive carcinoma. 8/40 (20%) cases after VAB and 9/28(32.1%)cases after CNB had UM (p=0.272). All but two cases with UM had a MRI-correlation (88.2%) and both cases without were classified as low-grade DCIS (MD at surgical excision 3 mm).
**Conclusion:** DCE-MRI can be used to predict UM after a PB diagnosis of ADH and should be used in the lesion assessment to avoid unnecessary surgical procedures.

**B-0555** 11:50
**Usefulness of CAD for diagnostic automated breast US in MRI detected lesions**
Y. Kim[1], N. Choi[1], B. Kang[2], J. Lee[2], N. Jung[1]; [1]*Bucheon/KR*, [2]*Seoul/KR*
*(yumikim17@gmail.com)*

**Purpose:** To evaluate the diagnostic performance using CAD for ABUS and to analyse the characteristics of CAD-detected lesions and the causes of false marks.
**Methods and Materials:** Totally 40 breast cancer patients who underwent diagnostic automated breast ultrasound (ABUS) for detecting multiple suspicious lesions found on MRI were included. We applied the CAD (QVCAD™) to all of the ABUS examinations which were performed after breast MRI. We evaluated the diagnostic accuracy of CAD. Then we analysed the characteristics of CAD-detected lesions and the causes of false-positive, false-negative cases, and false marks.
**Results:** Of the total 122 suspicious lesions found on MRI of 40 patients, 52 daughter nodules near main breast cancer were excluded, and then 71 lesions were analysed. The sensitivity, specificity, PPV and NPV of CAD for ABUS were 75.5%, 90.9%, 94.9%, and 62.5%, respectively. 81.4% invasive ductal cancer was detected by CAD. 85.3% invasive ductal cancer showing mass (exclude non-mass) was detected by CAD. 90.3% invasive ductal cancer, more than 1-cm mass, was detected by CAD. The mean size of true-positive versus false-negative mass was 2.08±0.85cm versus 1.6±1.28cm. False-positive lesion included a sclerosing adenosis and a usual ductal hyperplasia. Additionally, in total 24 false marks, the most common cause was marginal or subareolar shadowing, then 3 simple cysts, a hematoma, and a skin wart. However, false marks did not create confusion determining proper diagnosis.
**Conclusion:** Promising detection sensitivity was obtained from the CAD for ABUS on invasive ductal cancer showing mass which is more than 1cm in size.

**10:30 - 12:00** **Room Y**

### Interventional Radiology

### SS 609

### Vascular interventions in oncology (including radioembolisation)

*Moderators:*
S. Puchner; Vienna/AT
V. Vidjak; Zagreb/HR

**K-12** 10:30
**Keynote lecture**
J.I. Bilbao; *Pamplona/ES*

**B-0556** 10:39
**Multifunctional iron oxide nanoparticle-embedded PVP-HEC microparticles as a drug-eluding embolic material**
F. Nurili[1], G. Bekaroglu[2], S. İsci[2], A. Bas[2], I. Caymaz[2], I. Yurdaisik[2], S. Özkanli[2], H. Cakiroglu[2], O. Aras[1]; [1]*New York, NY/US*, [2]*Istanbul/TR*
*(fuatnurili@gmail.com)*

**Purpose:** To develop MRI-imageable novel HEC-PVP-magnetic embolic particles intended for transcatheter arterial chemoembolisation and magnetic ablation.
**Methods and Materials:** MRI-imageable novel HEC-PVP-magnetic embolic microparticles were prepared from iron oxides linked with PVP using a bridging flocculation process and HEC were incorporated (coated) onto the particles to load doxorubicin (DOX).16 rabbits were used in a rabbit renal embolisation model. Sustained release was accomplished with 1 mg of HEC-PVP-magnetic embolic microparticles containing 16 mcg of DOX. In the chemo-TAE group, embolisation was performed after catheterisation of the renal artery with microcatheters. In the control TAI group, 4 mg DOX alone was injected through the renal artery. Imaging and magnetic ablation were performed after embolisation using a 1.5-T high-field MR scanner equipped with high-performance gradients and fast receiver hardware.
**Results:** All synthesised particles were calibrated in the 40-500-micron range and were easily injectable through a microcatheter. The particles exhibited no cytotoxicity against hFOB cells (P> 0.05). In vivo, the particles were capable of occluding the arteries, and imageable with MRI. In the chemo-TAE group, DOX levels in kidney tissue detected after embolisation were significantly higher than that in the control group(P<0.001). Infarction and necrosis in the renal cortex were observed in the chemo-TAE group and TAE group. The maximum temperature achieved within the rabbit kidney was 48±1°C after 20 min with radiofrequency exposures at 25 kW.
**Conclusion:** These novel microparticles can be loaded with an anticancer drug to be useful for embolisation therapies with traceability and magnetic ablation properties.

**B-0557** 10:47
**Transarterial radioembolisation with Yttrium90 glass microspheres without temporary or permanent occlusion of the cystic artery appears to be safe in terms of adverse effects**
O.M. Topcuoglu, N. Selcuk, B. Sarikaya; *Istanbul/TR*
*(omtopcuoglu@gmail.com)*

**Purpose:** To present the results of transarterial radioembolization (TARE) treatment regarding radiation-induced cholecystitis when the cystic artery take off was noted within the vicinity of the treatment zone.
**Methods and Materials:** Patients with primary or secondary malignant liver tumours treated with TARE, in whom cystic artery was located in the vicinity of the treatment zone on 99-Technetium-MAA angiograms were included in this study. Whole liver dose, tumour dose and healthy injected liver dose, lung dose and if applicable the gallbladder dose were all calculated by using the Medical Internal Radiation Dose (MIRD) formula from SPECT-CT images. Qualitative and quantitative assessment of the gallbladder were performed on SPECT-CT. The observed adverse events were classified according to the National Cancer Institute's Common Terminology Criteria for Adverse Events (CTCAE v5.0).
**Results:** A total of 34 TARE procedures from 29 patients (18 men and 11 women) with a mean age of 65±13.3 years meeting the inclusion criteria, were involved in the current study. The mean tumour dose, healthy injected liver dose, healthy whole liver dose and gallbladder dose were 204.9±66.8 Gy, 70.5±15.7 Gy, 31.1±12.7 Gy and 96.4±53.4 Gy, respectively. The mean follow-up period was 14±5.2 months. Qualitative assessment revealed gallbladder radioactivity on SPECT-CT in 11 (32.3%) patients with 6 mild and 5 moderate-severe radioactivity. There was no detected grade 2 or 3 adverse events.
**Conclusion:** TARE treatment might be safely performed without cystic artery intervention when the cystic artery was located within the treatment zone by means of SPECT evaluation.

**B-0558** 10:55
**Post-SIRT $^{90}$Y-PET/CT showing variable deposition of $^{90}$Y in various liver tumour types**
S. Thoduka[1], S. Mercogliano[1], V. Czernohorsky[1], N. Spahr[2], J.-H. Schierz[1], A. Schenk[3], N. Abolmaali[1]; [1]*Dresden/DE*, [2]*Lübeck/DE*, [3]*Bremen/DE*
*(smita.thoduka@gmail.com)*

**Purpose:** Highly selective uptake of radioactive microspheres is central to effective SIRT. The purpose of our study was to compare the variable selectivity of resin particle deposition, with regard to type of tumour and catheter-position at infusion, in tumour vs. healthy liver tissue as recorded in the post-treatment $^{90}$Y-PET/CT.
**Methods and Materials:** All SIRT procedures between 01/2017 and 08/2018 in the treatment of hepatocellular carcinoma (HCC, n=37), cholangiocarcinoma (CCC, n=13) and metastatic colorectal carcinoma (CRC, n=14) with post-treatment $^{90}$Y-PET/CT were reviewed. Pre-therapeutic gadoxetate late-phase MRI including complete segmentations were registered to post-therapeutic $^{90}$Y-PET/CTs. The selectivity of $^{90}$Y-uptake by the hepatic tumours, as classified into low, moderate and high by an expert of nuclear medicine and radiology, was compared with regard to tumour type as well as catheter-position during infusion. Quantitative radioactivity-uptake in liver and malignant tissue was calculated.
**Results:** 33 out of 64 post-treatment PET/CTs showed highly selective tumour $^{90}$Y-uptake. A high percentage of HCC (54%) and CCC (69%) compared to CRC metastases (28%) showed highly selective deposition. Selective catheter-positioning employed in the treatment of HCC showed a significant (p= 0.012) impact on selective uptake in tumour tissue, whereas catheter-position during the treatment of CCC and CRC showed no significant effect on uptake selectivity.

**Conclusion:** Our study showed the significance of selective catheter-positioning on achieving highly selective uptake in HCC lesions. CCC lesions were well-targeted even without a selective catheter-position. $^{90}$Y-microspheres were comparatively less selective for CRC metastases in the liver. This has possible implications in planning catheter-position during SIRT.

**B-0559** 11:03

**Intrahepatic mitomycin C infusion in liver-dominant metastatic breast cancer: factors influencing the outcome**

B.M. Aarts[1], E.G. Klompenhouwer[1], R.C. Dresen[2], A. Laenen[2], R.G.H. Beets-Tan[1], K. Punie[2], P. Neven[2], H. Wildiers[2], G. Maleux[2]; [1]*Amsterdam/NL*, [2]*Leuven/BE (b.aarts@nki.nl)*

**Purpose:** The aim of this study was to determine the safety and efficacy of Mitomycin C (MMC) infusion in chemo resistant liver dominant metastatic breast cancer patients (LMBC) and to determine factors influencing overall survival.
**Methods and Materials:** We retrospectively analysed 176 LMBC patients treated with MMC infusion between 2000 and 2017. Local response was measured with CT follow-up by RECIST 1.1 criteria after 1-3 cycles. Toxicities were registered by the CTCae version 5.0. Overall survival (OS) and hepatic progression free survival (hPFS) were evaluated using Kaplan Meier methodology. After univariate analysis, a stepwise forward multivariate (MV) prediction analysis was performed to select independent pre-treatment factors associated with OS.
**Results:** RECIST evaluation (n=132) showed a partial response rate of 20%, stable disease of 57% and progressive disease in 23%. Toxicity grade 3 and 4 levels were reported in 17.5%. Median PFS was 5.5 months (CI;4.5-6.8) and median OS was 7.8 months (CI;6.1-9.8). Significant independent baseline predictors of worse OS on MV analysis included amount of prior systemic chemotherapy (HR=1.2 CI;1.1-1.3), prior liver ablation (HR=5.9 CI;1.8-19.4), higher liver tumour burden (HR=2.4 CI;1.5-3.7), elevated levels of bilirubin (HR=2.18 CI;1.3-3.8) and ALT (HR=1.5 CI;1.01-2.09).
**Conclusion:** MMC infusion was safe and effective in LMBC patients. MV analysis showed a worse OS in patients with increased amount of prior systemic chemotherapy, prior liver ablation, higher liver tumour burden and elevated levels of bilirubin and ALT.

**B-0560** 11:11

**Bland embolisation as the sole endovascular treatment for unresectable hepatocellular carcinoma: survival analysis of 230 patients observed for seven years in a single centre**

E. Lanza, I. Bolengo, D. Poretti, R. Muglia, R. Ceriani, V. Pedicini; *Milan/IT (isabella.bolengo@humanitas.it)*

**Purpose:** To assess the overall survival (OS) of patients with unresectable HCC treated with bland embolisation (BE) after a seven-year observation, comparing it with recent chemoembolisation (TACE) literature; to evaluate OS predictors and test whether a modified TAE (TAE-g) with n-butyl-cyanoacrylate changed the OS or procedural time.
**Methods and Materials:** This was a retrospective single-centre study of 230 patients (M:F = 181:49; mean age = 73) who underwent 543 treatments: 228 TAE-g (42%), 227 TAE (42%), and 88 TAEL (TAE + Lipiodol) (16%). TAE and TAE-g were performed using 40µ or 100µ microparticles. We evaluated previous/concurrent percutaneous ablation (PA), Child-Pugh and BCLC scores and survival time from the first treatment. OS and predictors were assessed using log-rank tests and Cox regression analyses.
**Results:** Observation time ranged from 0.1 to 86.7 months; 116 (50%) patients died, 8 (3%) were lost to follow-up, 106 (46%) were still alive at the end of the observation. Median OS was 28 months (survival at 1, 2, 3 years = 85%, 59%, 38%). Significant predictors of OS were the total number of embolisations received (p = 0.035) and having a history of PA (p = 0.024). No differences were noted between TAE and TAE-g.
**Conclusion:** BE performed primarily using small microparticles or secondarily with Lipiodol provided high survival rates in patients with unresectable HCC, higher than that reported by recent TACE trials. The most reliable predictor of survival was PA, followed by the number of endovascular treatments.
TAE and TAE-g provided similar results in OS and procedural time.

**B-0561** 11:19

**Safety and efficacy of small versus large drug-eluting bead transarterial chemoembolisation for hepatocellular carcinoma**

S.Y. Lee, H.-Y. Ou, C.-Y. Yu, Y.-F. Cheng; *Kaohsiung/TW (siehyang418@hotmail.com)*

**Purpose:** To compare the safety and efficacy of 100-300 µm versus 300-500µm drug-eluting bead transarterial chemoembolization (DEB TACE) and to identify predictive factors associated with complete response (CR) in the treatment of hepatocellular carcinoma (HCC).
**Methods and Materials:** This retrospective cohort study enrolled 234 consecutive patients who underwent TACE using 100-300µm DEB (Group A, *n*=75) and 300-500µm DEB (Group B, *n*=159) in a tertiary center between August 2012 and March 2017. The treatment response using modified response evaluation criteria in solid tumours (mRECIST) criteria and adverse events were compared between groups.
**Results:** A total of 704 HCCs in 234 patients were evaluated and the average index tumour size was 3.8cm. The overall CR of all patients was 40.6%. Multivariate analysis demonstrated that CR was associated with tumour size (*P*=0.004), location in the liver (*P*=0.014), lobe involvement (*P*=0.022) and particle size (*P*=0.018). The overall CR rates in Groups A and B were 56.0% and 32.7% (*P*=0.001), respectively. Group A has higher CR than Group B in the subgroup of BCLC B with tumour <3cm (57.9% vs 21.1%; *P*= 0.020) and subgroup of feeding artery ≥0.9mm (55.2% vs 30.9%; *P*= 0.014). There were no differences in complication rates between two groups (44.0% vs 47.2%; *P*=0.531).
**Conclusion:** TACE with 100-300-µm-sized DEB is associated with better response and similar safety compared with 300-500µm, particularly in patients with BCLC B and tumour size <3cm and feeding artery size ≥0.9mm. These findings may help to select an optimal treatment strategy for HCC.

**B-0562** 11:27

**Alternative routes of intraarterial chemoinfusion for intraocular retinoblastoma**

L.S.P. Karanam, S.R. Baddam, S. Honavar, V.R. Palkonda; *Hyderabad/IN (drklsp@gmail.com)*

**Purpose:** Intraarterial chemotherapy (IAC) is a proven effective treatment for retinoblastoma. Direct catheterisation of ophthalmic artery (OA) is not always possible in cases of intraarterial chemotherapy for retinoblastoma. We intend to discuss our experience where we used alternative routes of drug delivery other than direct catheterisation of OA with emphasis on its safety and efficacy in globe salvage.
**Methods and Materials:** 96 eyes underwent 284 sessions of IAC in our institution. In 24 eyes we used alternative routes other than direct OA for drug delivery. We used balloon occlusion technique in 23 sessions, Middle meningeal artery in 32 sessions, middle meningeal artery+OA in 8 sessions, balloon occlusion+OA in 7 sessions for chemoinfusion. Triple-drug regimen with Topotecan + Melphalan + Carboplatin was used in all cases. All the procedures were done under general anesthesia using transfemoral access.
**Results:** 11 patients of group D and 13 patients of group E (International classification of retinoblastoma classification) underwent IAC using alternative techniques. The technique of ophthalmic artery catheterisation is performed by our trained interventional radiologist team safely in all the sessions.The response of the tumour is graded as complete regression, partial regression and no response. In a follow-up period of 2 years, enucleation was done in 4 patients. Complications were all transient.
**Conclusion:** From our experience, it shows that when direct OA catheterisation is not possible, alternative techniques as described in our series can be safely and effectively used to salvage the eyes especially in cases of advanced retinoblastoma.

**B-0563** 11:35

**Retrospective analysis of a cohort of patients affected by unresectable HCC treated with SIRT and liver transplant**

A. Paladini[1], G.E. Vallati[2], G. Pizzi[2], F. Cappelli[2], D. Beomonte Zobel[2], E.M. Amodeo[2], A. Borzelli[3]; [1]*Novara/IT*, [2]*Rome/IT*, [3]*Naples/IT (andreapaladini1988@gmail.com)*

**Purpose:** The aim of our study is to evaluate the radiological and clinical results of SIRT and hepatic transplant on 22 patients (pts) affected by unresectable hepatocarcinoma (HCC). We analysed the role of SIRT as “bridge” or “downstaging” endovascular therapy for pts with intermediate/advanced HCC.
**Methods and Materials:** We evaluated 274 pts affected by unresectable HCC treated with Yttrium-90 resin microspheres between 2008 and 2016. Average delivered dose was 1.6 GBq (1.5GBq-1.9GBq). 22 pts of this cohort had such a good response to treatment (complete or partial) that they became eligible for hepatic transplant. We followed-up pts after SIRT with CT at 1 month, 3 months and 6 months after therapy, and analysed toxicity of SIRT, staging pts from radiological and clinical point of view.
**Results:** 8.02% of pts affected by intermediate/advanced HCC had a successful transplant after SIRT and they are still alive. In 4.4% of cases, SIRT had a downstaging effect on pathology. PR, SD and PD based on mRECIST criteria were evaluated in all pts. Moreover, we evaluated free-survival rate of pts transplanted, and we noticed that it was very similar to free survival rate of pts affected from a low-grade HCC which had an hepatic transplant.

**Conclusion:** SIRT is a very safe and effective loco-regional endovascular therapy. SIRT has a very important role as downstaging and/or bridge therapy in case of intermediate/advanced HCC. Furthermore, pts transplanted after SIRT have the same free survival rate of pts affected by low-grade HCC that had hepatic transplant.

**B-0564** 11:43
**Yttrium-90 radioembolisation in advanced-stage HCC: does portal vein thrombosis affect survival?**
F. Fiore, V. Stoia, F. Somma; *Naples/IT (doc.fiore1959@gmail.com)*

**Purpose:** Portal vein thrombosis (PVT) is generally recognized as a prognostic factor in HCC. Our purpose is to assess and compare the survival of patients with and without PVT, after Y-90 trans-arterial radio-embolisation (TARE) of unresectable HCC, unresponsive to other loco-regional treatments.
**Methods and Materials:** Between November 2005 and November 2012, Y-90 resin-based TARE was performed in 89 patients with unresectable HCC in an IRB-approved prospective protocol. 33/89 patients had PVT, the remaining 56 were either elderly patients, resistant-to-cTACE HCC or underwent TARE as a downstaging therapy. All patients were studied with multi-detector computed tomography (MDCT), angiography, 99m Tc-MAA scintigraphy and liver biopsy. Gastro-duodenal artery was embolised in most cases. Proton-pump inhibitors were administered to prevent gastritis and ulcers. c 2 test with Yates correction and log rank test were used to compare the two proportions and Kaplan-Meier survival curves, respectively.
**Results:** The average activity administered was 1.7GBq. After the treatment, fever and abdominal pain were found in 25 and 16 patients, respectively. No major side effect was observed. According to mRECIST criteria, partial response or complete response was found in 70% of patient three months after the treatment, and in 90.5% nine months after the treatment. No significant difference Yttrium-90 radioembolisation in advanced-stage HCC: does portal vein thrombosis affect survival? found in survival of patients with PVT compared to those without PVT. A complete regression of PVT was observed in more than half patients.
**Conclusion:** Portal vein invasion does not affect survival in advanced stage HCC patients undergoing TARE using Y-90 resin-based microspheres. This procedure helps retracting the portal thrombus.

**B-0565** 11:51
**Embolisation of rabbit renal arteries to test feasibility and effects of CT-visible doxorubicin-eluting montmorillonite microparticles**
F. Nurili; *New York, NY/US (fuatnurili@gmail.com)*

**Purpose:** To develop CT-visible doxorubicin (DOX)-eluting montmorillonite microparticles for transarterial embolisation.
**Methods and Materials:** DOX-loaded montmorillonite particles were synthesised by adsorption method and dispersed in diatrizoate to enable visualisation on CT and to achieve a sustained drug-release profile. Rabbits were used to determine CT imaging properties and DOX release kinetics. Sustained release of the particles into the kidney was accomplished with 1 mg of montmorillonite containing 39.2 ±0.14 mcg of DOX. In the transarterial DOX-loaded montmorillonite embolisation group, embolisation was performed following selection of the renal artery. Control groups were use of DOX and montmorillonite particles alone in separate groups. DOX levels in the blood were periodically measured. Kidney tissue was isolated 24 hours after injection and DOX levels in the tissue were measured.
**Results:** Particles were successfully visualised with CT. Manufactured microparticles were compatible with microcatheters. DOX blood levels in the chemo-embolisation group briefly increased 30 min after embolisation but remained low until 24 hours after embolisation. DOX blood levels in the TAI group rapidly reached a peak more than 30 minutes after injection and then was undetectable at further timepoints. DOX levels in the tissue was significantly higher in the chemo-embolization group compared to the TAI group. Pathology revealed swelling of entire embolised kidney, infarction and necrosis in the renal cortex in the embolisation and chemo-embolisation groups. Vascular injury was observed in only the chemo-embolisation group.
**Conclusion:** CT-visible DOX-loaded montmorillonite maintained high local concentrations but low blood levels of the anticancer drug, showing promise for transarterial embolisation.

**10:30 - 12:00** **Room D**

### Artificial Intelligence and Machine Learning

### SS 605

### Machine learning: musculoskeletal and diverse

*Moderators:*
B. Baeßler; Cologne/DE
R. Blanco Sequeiros; Turku/FI

**B-0566** 10:30
**Deep learning for automatic detection of fractures on chest CT scans after blunt trauma**
L. Yan, X. Chuan, C. Xia, S. Wang, K. Chen; *Beijing/CN (xchen@infervision.com)*

**Purpose:** To evaluate the performance of a deep learning algorithm for automatic detection of rib fractures, scapula fractures and clavicle fractures on chest CT scans after blunt trauma.
**Methods and Materials:** In this retrospective study, we first collected initial chest CT evaluations of 1675 patients (1122 males and 553 females) with blunt trauma (1434 rib fractures, 145 clavicle fractures, 177 scapula fractures) from three collaborating hospitals. Three board-certified radiologists helped to label the three types of fractures on each CT scan. We developed a modified multi-channel deep learning algorithm based on Single Shot Multi-Box Detector and trained the model on all 1675 cases. To evaluate model performance, a test set of 290 chest CT scans (244 rib fractures, 22 clavicle fractures, 35 scapula fractures) from another two hospitals was collected and labeled by the same three radiologists as for the training set.
**Results:** We utilized sensitivity [TPs/(TPs+FNs)] and precision [TPs/(TPs+FPs)] to evaluate the algorithm. For the detection of rib fractures, the algorithm's sensitivity was 94.97% with a precision of 55.72%. For scapula fractures, the sensitivity was 88.89% with a precision of 72.73%. For clavicle fractures, the sensitivity was 91.67% with a precision of 84.62%.
**Conclusion:** Results showed that deep learning could help automatically detect different types of fractures on CT scans with high sensitivity and reasonable precision, especially rib fractures for which missed fractures were not uncommon. We expect such algorithms will improve fracture diagnosis in clinical settings by pre-screening trauma examinations and flagging suspicious cases.
*Author Disclosures:*
**L. Yan:** Employee; Employee, Infervision. **X. Chuan:** Employee; Employee, Infervision. **C. Xia:** Board Member; member of board. **S. Wang:** Founder; co-founder. **K. Chen:** Founder; co-founder.

**B-0567** 10:38
**Whole-body MRI based lesion prediction in multiple myeloma**
R. Licandro[1], J. Hofmanninger[1], M.-A. Weber[2], B. Menze[3], G. Langs[1]; [1]*Vienna/AT*, [2]*Rostock/DE*, [3]*Munich/DE (roxane.licandro@meduniwien.ac.at)*

**Purpose:** The earliest possible detection of bone lesions of Multiple Myeloma (MM) patients is critical as a trigger for treatment and a prime marker of the disease's progression. We use predictive machine learning, to identify a localized risk estimator for future lesion emergence based on pre-stage infiltration patterns observed in Magnetic Resonance Imaging data (MRI).
**Methods and Materials:** $T_1$ weighted whole-body MRI were acquired from 63 patients over several examination points. We propose a lesion risk predictor to identify early signatures of emerging lesions before they reach thresholds for reporting. The method proposed is based on patch-wise training after patient specific accurate image alignment. A predictive random forest was trained to identify bone locations with high-risk of progression to osteolytic lesions.
**Results:** The prediction of emerging lesions (not reported in the observed scan, but in the subsequent), achieves a mean Area Under der Curve of 0.7425 (thorax), 0.769 (legs). Additionally, the predictor was able to identify progressing lesions (which are annotated over all examination scans but increase in size) yielding a mean of 0.7607 and 0.7221, respectively, when using a patch size of 10x4x10 voxels.
**Conclusion:** We propose a local lesion risk predictor for identifying regions in the bone marrow with high risk of disease progression. We showed that the predictor is able to predict future osteolytic lesions and at the same time is able to predict progression of already identified lesions. Our findings indicate hidden imaging markers beyond lesion size, currently used for categorization and risk stratification in MM.

*Author Disclosures:*
**J. Hofmanninger:** Research/Grant Support; Siemens Healthineers, Boehringer Ingelheim. **G. Langs:** Founder; contextflow GmbH. Research/Grant Support; Siemens Healthineers, IBM. Shareholder; contextflow GmbH. Speaker; Roche.

**B-0568** 10:46

**Optimising the dataset for deep learning with x-ray images in musculoskeletal radiology**

M.F. Russe, N. Kocher, E. Kellner, M. Reisert, F. Bamberg, E. Kotter; *Freiburg/DE*

**Purpose:** The datasets for classification tasks in x-ray imaging differ from non-medical tasks due to the standardization of image projections, the grey scale and the similarity in anatomy between the different patients. In this study, the effect of the data split between validation and training data was analyzed for deep learning.
**Methods and Materials:** A dataset of 1900 anterior-posterior images of the wrist was created. The images were classified for fracture or absence of fracture. A test sample with each 50 fracture/no fracture was separated. The remaining dataset contained 450 images with fractures and 1350 without fractures. The training to validation split changed in 6 steps from 50:50% to 90:10%. An established convulsive neuronal network (CNN), GoogLeNet was used. Image distortion to 256*256 pixels was made, but no further data augmentation was performed. The training of the CNN for each split was performed. All datasets were tested on the separate test sample to avoid data leakage.
**Results:** The best overall accuracy is 92% with a Cohen's kappa value of 0.84 at the 80/20 ratio. The overall accuracy and Cohen's kappa for each split are 50/50: 81%, κ0.62; 60/40: 89%, κ0.78; 7030: 90%, κ0.8; 75/25: 91%, κ0.82; 8020: 92%, κ0.84; 9010: 87%, κ0.74.
**Conclusion:** Datasets of x-ray images can be used for the creation of deep learning models with a very good agreement and high accuracy. The ratio of the dataset for training and validation deals a great impact to the result of the performance of the deep learning algorithm.

**B-0569** 10:54

**Predicting the incidence of knee osteoarthritis using deep neural networks: data from the osteoarthritis initiative (OAI) and the multicenter osteoarthritis study (MOST)**

Z. Bertalan[1], T. Paixao[1], R. Ljuhar[1], D. Ljuhar[1], H.-P. Dimai[2], S. Nehrer[3]; [1]*Vienna/AT*, [2]*Graz/AT*, [3]*Krems/AT (z.bertalan@imagebiopsy.com)*

**Purpose:** To evaluate the performance of a deep neural network to predict OA incidence.
**Methods and Materials:** We screened conventional posterior-anterior (PA) knee radiographs of men and women from the Osteoarthritis Inititiatives Study (OAI) and Multicenter Osteoarthritis Study (MOST) database for modality and digitalization artifacts, resulting in a total of 2904 images. These were classified as incident or non-incident over the observation period, based on Kellgren & Lawrence scoring (KL>1). This dataset was split into train- (1451), validation- (581) and test- (872) datasets. Class imbalance between incident/non-incident images was approximately 1:4 and was controlled by oversampling the minority class. We trained an 8-layer convolutional neural network to predict OA incidence on these images, with or without clinical data (such as JSW and Kellgren & Lawrence score). As a comparison, we trained a simple dense neural network that utilizes only this clinical data.
**Results:** A classifier based on radiographs alone achieved an accuracy of 78% on the classification task, with a sensitivity of 67% and a specificity of 81%. In contrast, the CNN trained on the clinical data alone achieved an accuracy of 63%, with a sensitivity of 47% and a specificity of 67%.
**Conclusion:** This study provides strong evidence that applying deep convolutional neural networks has the potential to predict incidence knee-OA based solely on the information contained in a conventional 2D radiograph before any radiographic signs of the disease. Adding additional clinical data could potentially further increase the prediction of OA.
*Author Disclosures:*
**Z. Bertalan:** Employee; IB Lab GmbH. **T. Paixao:** Employee; IB Lab GmbH. **R. Ljuhar:** CEO; IB Lab GmbH. **D. Ljuhar:** Shareholder; IB Lab GmbH.

**B-0571** 11:10

**Automatic MRI-based 3D models of hip cartilage using a 3D U-net-like fully convolutional network for improved morphologic and biochemical analyses**

F. Schmaranzer[1], R. Helfenstein[1], G. Zeng[1], T. Lerch[1], K. Siebenrock[1], M. Tannast[1], Y.-J. Kim[2], G. Zheng[1]; [1]*Bern/CH*, [2]*Massachusetts, MA/US (florian.schmaranzer@insel.ch)*

**Purpose:** The time-consuming and user-dependent post-processing of biochemical cartilage MRI has prevented their widespread use. A time-efficient, fully automated analysis of biochemical 3D images could provide more straightforward and comprehensive information on cartilage thickness, surface area and volume.
**Methods and Materials:** IRB-approved study on 25 symptomatic hips undergoing a contrast-enhanced MRI at 3T including a 3D dGEMRIC sequence (0.8 $mm^3$). Development of a fully automated deep learning-based approach for 3D segmentation of hip cartilage models was based on two steps: (1) 3D training data of hip cartilage was provided by one reader (manual 3D analysis); (2) a deep neural network for fully automated cartilage segmentation (automated 3D analysis) and a software for 3D analysis was developed. dGEMRIC index, cartilage thickness, surface area and volume were measured in the four joint quadrants and compared. Mean average surface distance and mean Dice coefficient were calculated.
**Results:** Regional patterns were comparable for manual/automated 3D methods. Highest dGEMRIC indices were found postero-superiorly (602.1±158.4 ms, 601.8±158.4 ms). Thickest cartilage was found antero-inferiorly (5.3 ±0.8 mm, 4.3±0.6 mm). Smallest surface area was found antero-inferiorly (134±60 $mm^2$, 155±60 $mm^2$). Largest volume was found antero-superiorly (2343±492 $mm^3$, 2294±467 $mm^3$). Mean average surface distance was 0.26±0.13 mm and mean Dice coefficient was 85.7±2.7%.
**Conclusion:** This validation paves way to large-scale use of this method for fully automatic 3D cartilage segmentation for an improved morphological and biochemical analyses of hip cartilage.
*Author Disclosures:*
**F. Schmaranzer:** Grant Recipient; Swiss National Science Foundation. **G. Zeng:** Grant Recipient; Swiss National Science Foundation. **G. Zheng:** Grant Recipient; Swiss National Science Foundation.

**B-0572** 11:18

**Combined traditional image processing and deep learning approach for automated detection of tears of the anterior cruciate ligament: is it the game changer for AI in musculoskeletal MRI?**

V. Venugopal, Y. Mallya, V. Jagannatha, V. Mahajan, H. Mahajan; *New Delhi/IN (vasanthdrv@gmail.com)*

**Purpose:** We propose a novel ensemble approach of traditional image processing combined with deep learning to detect ACL tear on knee MRI.
**Methods and Materials:** FSPD knee MRI images of 66 patients (16 normal, 50 with full- or partial-thickness ACL tears) done on a 3.0 Tesla MRI were extracted, the intensity values of MRI images were standardized by histogram matching followed by intensity normalization image pre-processing techniques. Automatic delineation of tibia, femur and bounding box of the ACL bundle was achieved by a three-dimensional CNN implemented in the research version of HealthSuite Insights (Philips HealthTech). A total of 88 features comprising the first-order statistics and texture measures were computed from Gray Level Co-occurrence Matrix (GLCM) and Gray Level Run Length Matrix (GRLM) of the image volume within the ACL bounding box. Feature subset selection was performed by two-sample t test with statistical significance ($p < 0.05$).
**Results:** The performance of the deep learning approach for segmentation of femur and tibia is evaluated on 20 randomly selected MRI datasets. The ground truth was created by manual segmentation of femur and tibia by a MSK radiologist. The dice score was 0.91 +/- 0.084. The performance of the predictive model for discrimination of ACL tear was assessed by k-fold cross-validation and the accuracy, sensitivity, and specificity were 89.5%, 93.3%, and 81%, respectively.
**Conclusion:** The proposed machine learning technique gave good performance in the delineation of knee anatomical structures and detection of ACL tears in the knee MRI.
*Author Disclosures:*
**Y. Mallya:** Employee; Koninklijke Philips NV. **V. Jagannatha:** Employee; Koninklijke Philips NV.

**B-0573** 11:26

**Ante-mortem chest radiograph retrieval for forensic human identification by deep learning**

Y. Li[1], X. Yang[1], J. Wang[1], H. Xue[1], W.B. Liang[1], Z.H. Deng[1], J. McNulty[2], J.C. Lv[1], L. Zhang[1]; [1]*Chengdu/CN*, [2]*Dublin/IE (291846740@qq.com)*

**Purpose:** Chest radiographs hold potentially high value for forensic comparative radiological identification because of the remarkable number of skeletal features offered by this area and the wide availability of chest radiographs across the population. The purpose of this study was to develop a deep learning retrieval model based on chest radiographs and multi-slice computer tomography (MSCT) chest scans for forensic human identification.
**Methods and Materials:** Data were retrospectively collected from 1052 clinical patients (aged 20-89 years) who had both posterior-anterior (PA) chest radiographs and MSCT scans to develop the model. Before training, the MSCT scans were pre-processed by three-dimensional volume-rendering technique (VRT) to produce the corresponding two-dimensional PA image of the skeletal elements of the chest; the scapulae and clavicles were removed for each case to address potential positional variations. We use a ranking based criterion for evaluation in a test dataset of 263 clinical patients. Mean reciprocal ranking

Thursday

(MRR) was also used to assess the model performance.
**Results:** The model has achieved a top-1 ranking accuracy rate of 78.6%, top-2 of 88.2%; top-5 of 98.1%, and top-10 of 98.9%. The MRR value was 0.869, meaning the relevant target chest radiograph was retrieved at average rank 2.
**Conclusion:** When the MSCT VRT images are used as the query image, the deep learning network model achieves promising results performance, demonstrating the retrieval ability capable of automated searching the ante-mortem chest radiograph based on the post-mortem VRT images of the chest.

**B-0574** 11:34
**Automatic segmentation of the mandible from CT for 3D virtual surgical planning using a convolutional neural network**
B. Qiu, J. Guo, J. Kraeima, R. Borra, M.J.H. Witjes, P.M.A. van Ooijen; *Groningen/NL (qiubj1990@163.com)*

**Purpose:** Segmentation of mandibles from CT scans is crucial for 3D surgical virtual planning such as in planned tumour resections. Segmentation of mandibles in CT scans is a challenging task due to metal artefacts and large variation in the shape and size between individuals, i.e. the presence or absence of teeth. To address this challenge, we propose a convolutional neural network approach for mandible segmentation in CT scans by considering the continuum of anatomical structures through different planes.
**Methods and Materials:** The proposed convolutional neural network adopts the architecture of the U-Net and then combines the resulting 2D segmentation from three different planes (axial, coronal and sagittal) into a 3D segmentation. We used 11 patient CT scans, reconstructed with a kernel of Br64 or I70h(s). The data set is randomly split into the training, validation and the test set, each with eight, two and one cases, respectively. Manual mandible segmentation performed by a single trained researcher is taken as the gold standard.
**Results:** We evaluated the performance of our CNN approach by comparing to that from the gold standard and achieved an average dice coefficient of 88.15% and an average root mean square value of 0.4115 mm by randomly repeating the aforementioned data split procedure four times.
**Conclusion:** An end-to-end approach for automatic segmentation of the mandible from CT scans is proposed. Experimental results demonstrate the effectiveness of the proposed approach in mandible segmentation and its potential employment in 3D virtual planning of mandibular treatment planning.

**B-0575** 11:42
**18F-FDG-PET/CT-based radiogenomic tumour decoding and phenotyping of non-small cell lung cancer**
M. Chodyla, A. Demircioglu, J. Haubold, S. Bogner, K. Herrmann, F. Nensa, L. Umutlu; *Essen/DE (Michal-Kamil.Chodyla@uk-essen.de)*

**Purpose:** The aim of this study was to assess the potential of multiparametric 18F-FDG PET/CT imaging accelerated with machine learning and radiomic algorithms to predict the somatic mutational status as well as N- and M-stage in patients with untreated non-small cell lung cancer.
**Methods and Materials:** 85 patients (mean age 65.85) with histopathological confirmation of primary NSCLC were enrolled in this trial and underwent a whole-body 18F-FDG PET/CT examination. After segmentation of the primary cancer, 3569 textural as well as shape features were extracted from each volume. T-test, F-test, chi-square and randomized logistic regression were used for feature selection. Statistical modeling was performed using Random Forests, Logistic Regression as well as Support Vector Machines . A repeated 5-fold cross-validation was employed for internal validation.
**Results:** Prediction of the somatic mutational status and N-/M-stage obtained an AUC of 0.58 for prediction of KRAS, 0.64 for PDL1, 0.59 for tp53, respectively. Prediction of M-stage was superior when compared to N-stage, yielding an AUC of 0.73 versus 0.62. In all cases Random Forests performed best, while no single feature selection method stood out.
**Conclusion:** Radiogenomic analysis of 18f-FDG PET/CT datasets facilitated only insufficient prediction of the somatic mutational status and N- and M-stage in patients with NSCLC, with M-stage showing best predictive values, thus not serving as a template for non-invasive tumor phenotyping using wide feature vectors extracted from PET/CT data. More advanced methods using deep learning, which promise better predictive performance, should be investigated for a larger patient collective in the future.

**B-0576** 11:50
**Comparison of consistency of nodule detection in repeated chest PA X-ray images between radiologists and deep learning model**
C. Wu, S. Lee, J. Seo, S. Lee, H. Lee, H. Hwang, Y. Cho, N. Kim; *Seoul/KR (njmu_wcj@163.com)*

**Purpose:** To compare nodule detection ability of repeated chest PA from same patient acquired within one week between expert radiologists and deep learning model.
**Methods and Materials:** Totally 260 pairs of repeated chest PAs (116 chest PAs with 128 nodules and 144 chest PAs without nodule, which were confirmed by their chest CT) within 7 days were enrolled in this study. A deep learning model (DLM) using the YOLO v2, fine-tuned with a pre-trained model (DenseNet201), and two board-certificated chest radiologists (R1, R2 with 14 and 13 years' experience) reviewed these chest PAs. The sensitivity, specificity and accuracy were calculated and compared with a nodule-based analysis. Reading time was recorded. The intra- and inter-reader consistencies were evaluated by Cohen's kappa.
**Results:** The sensitivity, specificity, and accuracy of R1 are 0.926, 0.798, and 0.854. While for R2, they are 0.875, 0.831, and 0.850. For the DLM, they are 0.779, 0.866, and 0.827. The Cohen's kappa coefficient of R1, R2 and DLM between repeated chest PAs is 0.578, 0.629, and 0.600. The inter-reader consistency of R1 vs R2, R1 vs DLM and R2 vs DLM is 0.640, 0.501, and 0.488. The average reading time of each case is 18.14 seconds for R1 and 11.66 seconds for R2. While the detection time of DLM is about 0.03 seconds per case.
**Conclusion:** Our DLM has the comparable detection ability with expect radiologists with higher specificity, lower sensitivity. Consistency of nodule detection in repeated chest PAs should be improved for application in routine clinical practice.

**10:30 - 12:00** **Room K**

### Chest

### SS 604

### COPD, emphysema and airways diseases: novel approaches

*Moderators:*
N.N.
J.D. Dodd; Dublin/IE

**B-0577** 10:30
**Dynamic magnetic resonance imaging as an outcome predictor for lung-volume reduction surgery in patients with severe emphysema**
K. Martini[1], C. Caviezel[1], D. Schneiter[1], G. Milanese[2], I. Schmitt-Opitz[1], W. Weder[1], T. Frauenfelder[1]; [1]*Zurich/CH*, [2]*Parma/IT (katharina.martini@usz.ch)*

**Purpose:** We hypothesize a significant correlation between preoperative dynamic MRI and postoperative outcomes measured by pulmonary function tests.
**Methods and Materials:** Dynamic MRI was performed 1 day before and 3 months after lung volume reduction surgery (LVRS). Quantitative measurements were performed on sagittal planes: lung height, anteroposterior diameter, hemidiaphragm height and area as well as perimeter. Additionally, dynamic changes in hemidiaphragmatic area and height were measured. Parameters were normalized by patients' height. Pulmonary function test pre-LVRS and post-LVRS was performed. The cut-off value for treatment benefit was defined as a 30% increase in forced expiratory volume in 1s.
**Results:** Thirty-nine consecutive patients were included. On expiration, lung area on both sides (Pright=0.001 and Pleft=0.016) and anteroposterior diameter of the right lung (Pright=0.003) improved post-LVRS. Dynamic measurements showed differences pre- and post-LVRS on the right lung but not on the left lung (Pright<0.001 and Pleft=0.090). There were no significant changes in lung height for both sides, in hemidiaphragmatic height on the right as well as diaphragmatic area pre- and post-LVRS (P>0.05). Except for hemidiaphragmatic height on the left lung (P=0.039), no significant differences were found on inspiration. Considering the 30% cut-off for treatment benefit, the receiver operating curve analysis indicated a height-normalized expiratory lung area ≥35.793mm$^2$ as the most sensitive preoperative outcome predictor (sensitivity=86%; specificity=61%).
**Conclusion:** Preoperative dynamic MRI can be used as an additional outcome predictor for patient selection in LVRS. A height-normalized total lung area in expiration ≥35.793mm$^2$ correlates with a 30% increase in forced expiratory volume in 1s.

**B-0578** 10:38
**Quantitative assessment of the relationship between spatial distribution of emphysema and pulmonary function in COPD patients by an automatic analysis software**
Q. Yu[1], X. Quan[1], X. Lu[2]; [1]*Guangzhou/CN*, [2]*Shenyang/CN (liudehuayuqing@163.com)*

**Purpose:** To investigate the relationship between spatial distribution of emphysema and pulmonary function in patients with COPD by an automatic analysis software.
**Methods and Materials:** Chest MSCT (end-inspiration) was performed in 119 cases COPD patients (> 40 years old). For the quantitative assessment, the percentage of low attenuation areas(≤-950HU)were automatically calculated for

Thursday each lobe by COPD analysis software (Philips Healthcare). According to the pulmonary emphysema heterogeneity index, the patients were divided into no-emphysema group (LAA%≤1%) and emphysema group (LAA%>1%), and the latter was divided into predominantly upper-lobe subgroup and predominantly lower-lobe subgroup by the formula [HI=($\%LAA_{upper}$-$\%LAA_{lower}$) / ($\%LAA_{upper}$+$\%LAA_{lower}$)]. The differences of pulmonary function parameters which include PFT (FVC%,FEV1%,FEV1/FVC), IOS (Z5,X5,R5%,R20%,Fres), DLco (DLco%) between the 2 groups and the 2 subgroups were compared.
**Results:** Compared with no-emphysema group, PFT and DLco% of emphysema group were higher, while IOS values were lower, and the difference between which were statistically significant(z=-4.353~-2.088, all P<0.05) .Comparing the two subgroups of emphysema group, FEV1%, FEV1/FVC of the predominantly lower-lobe subgroup were slightly lower, while FVC%, IOS, DLco% were slightly higher, and the differences of all lung function indicators between the 2 subgroups were not statistically significant (z=-1.948~-0.284, all P>0.05).
**Conclusion:** COPD software can be used to quantitative analysis of emphysema spatial distribution, which has an effect on pulmonary function. There is no significant difference in lung function between predominantly upper-lobe and predominantly lower-lobe in COPD patients.

**B-0579** 10:46
**Inspiratory/expiratory xenon-enhanced CT with 3D motion analysis: utility for pulmonary functional loss assessment and clinical stage classification of COPD**
Y. Ohno[1], Y. Fujisawa[2], N. Sugihara[2], Y. Kishida[1], S. Seki[1], T. Yoshikawa[1], H. Koyama[3], T. Murakami[1]; [1]*Kobe/JP*, [2]*Otawara/JP*, [3]*Osaka/JP* *(yosirad@kobe-u.ac.jp)*

**Purpose:** To evaluate the utility of 3D lung motion assessment on inspiratory/expiratory xenon-enhanced area-detector CT (Xe-ADCT) for pulmonary functional loss assessment and clinical stage evaluation of COPD.
**Methods and Materials:** 28 consecutive patients with and without COPD underwent inspiratory/expiratory Xe-ADCT examinations as well as pulmonary function tests. Then, all patients were classified by GOLD classification as follows: 'Without COPD', 'Mild COPD', 'Moderate COPD' and 'Severe or Very Severe COPD'. In each subject, xenon ventilation maps such as wash-in (WI), wash-out (WO) and ventilation ratio (VR) maps as well as 3D motion magnitude maps at X-, Y- and Z-axes as well as expansion rate (ER) map were generated by pixel-by-pixel analyses. Then, each regional index was assessed by ROI measurements, and each final value was determined as averaged value. To determine the relationship between Xe- and 3D motion-based indexes, Pearson's correlations were performed. Then, step-wise regression analyses were performed between all indexes and $\%FEV_1$. Finally, discrimination accuracies were performed among all indexes, and combined method by McNema's test.
**Results:** WO had significant and correlations with all motion magnitudes (-0.53<r<-0.39, p<0.05), although VR had significant correlations with same factors and ER (0.37<r<0.49, p<0.05). $\%FEV_1$ was significantly affected by ER as 1st step and VR as 2nd step ($r^2$=0.63, p=0.0003). Moreover, discrimination accuracy of combined method (75 [21/28] %) was significantly higher than that of xenon ventilation-based indexes (53.6 [15/28] %, p=0.03).
**Conclusion:** 3D motion assessment is useful for pulmonary functional loss and clinical stage classification of COPD, when applied with inspiratory/expiratory Xe-ADCT.
*Author Disclosures:*
**Y. Ohno:** Research/Grant Support; Canon Medical Systems Corporation. **Y. Fujisawa:** Employee; Canon Medical Systems Corporation. **N. Sugihara:** Employee; Canon Medical Systems Corporation. **S. Seki:** Research/Grant Support; Canon Medical Systems Corporation. **T. Yoshikawa:** Research/Grant Support; Canon Medical Systems Corporation.

**B-0580** 10:54
**CT measurements of expiratory central airway collapse in COPD patients with suspected tracheobronchomalacia: comparison between transverse and MPR images**
D.M. Tridente[1], B.H. Heidinger[2], D.C. DaBreo[3], A.A. Bankier[1], D. Litmanovich[1]; [1]*Boston, MA/US*, [2]*Vienna/AT*, [3]*Ottawa, ON/CA (dani.tridente@gmail.com)*

**Purpose:** To compare two approaches for measuring the trachea and main bronchi in COPD patients with suspected tracheobronchomalacia (TBM) on CT examinations.
**Methods and Materials:** 97 COPD patients underwent CT imaging with a dedicated airway protocol acquired at inspiration and during dynamic expiration. Transverse images and images perpendicular to the airways' long axis (MPR) were reconstructed. Cross-sectional areas (CSA) were measured in inspiration and expiration at three anatomical levels: trachea, right (RMB) and left (LMB) main bronchus. Decrease of CSA from inspiration to expiration was calculated. Paired t tests and a McNemar test were used for statistical comparison.
**Results:** Mean CSAs were significantly smaller on MPR images than on transverse images at both inspiration and expiration for the trachea (insp: 250±61 vs 282±66, p=.001; exp: 92±52 vs 116±66, p=.005), RMB (insp: 164±39 vs 210±47, p=.001; exp: 52±33 vs 71±43, p=.001) and LMB (insp: 133±31 vs 179±43, p=.001; exp: 50±25 vs 66±37, p=.001). Mean tracheal collapsibility was significantly greater on MPR than on transverse images (63±19% vs 59±20%, p =.001). However, for both RMB and LMB, bronchial collapsibility was not significantly different between transverse and MPR (RMB: 61±26% vs. 62±25%, p=.616; LMB: 61±26% vs. 61±21%, p=.821). Using a threshold of ≥ 80% collapse for TBM diagnosis, more patients were classified with tracheomalacia on MPR than on transverse images without reaching statistical significance (11 vs. 18 patients; 11% vs 19%, p=.174).
**Conclusion:** Using tracheal measurements from MPR rather than from transverse images increased tracheal collapsibility and, therefore, the number of patients with tracheomalacia.

**B-0581** 11:02
**Efficacy of model-based iterative reconstruction in cystic fibrosis assessment on computed tomography**
S. Lin, M. Lin, K. Lau; *Melbourne/AU (Sandraxiaolin@gmail.com)*

**Purpose:** Cystic fibrosis (CF) is a common life-shortening genetic disease predominantly affecting young adults. Accurate radiological assessment of pulmonary disease is vital for disease surveillance and predicting exacerbations, one of the leading causes of morbidity and mortality. We evaluated the efficacy of model based iterative reconstruction (MBIR) constructed non-enhanced ultra-low dose CT chest (ULD-CT) to evaluate CF pathology.
**Methods and Materials:** We compared ULD-CT with chest x-ray and standard adaptive statistical iterative reconstructed (ASIR) non-enhanced low-dose CT (LD-CT). Effective radiation dose was calculated from the recorded dose-length product (DLP) values and compared between the two CT methods. We compared identification of pathology between ULD-CT and chest x-ray. We hypothesized that ULD-CT would be superior to chest x-ray in identification of CF pathology at lower doses than LD-CT.
**Results:** The mean effective radiation dose of ULD-CT was 0.073 mSv, comparable to one chest x-ray which was a 94% reduction compared to LD-CT. Compared to chest x-ray, ULD-CT detected on average, 2.3 more regions of bronchiectasis per study and better delineated varicose and cystic forms of bronchiectasis (p≤0.0001). ULD-CT identified four times more mucous plugging than chest x-ray (p<0.000001) and twice the amount of consolidation (p=0.0002).
**Conclusion:** ULD-CT is superior to chest x-ray in quantifying CF disease and achieves remarkable radiation doses significantly lower than LD-CT, comparable to one chest radiograph. Our results suggest that MBIR constructed ULD-CT is an effective imaging modality for CF surveillance, with potential applications in other disease settings.

**B-0582** 11:10
**Association between small pulmonary vascular area and mild airflow limitation in healthy smokers**
H. Jo, M. Park; *Jinju/KR (inthepresent@hanmail.net)*

**Purpose:** The cross-sectional area (CSA) of small pulmonary vessels can be useful markers to assess early changes of pulmonary vascular alteration in smokers before parenchymal destruction. We investigated the relationship between mild airflow limitation and small pulmonary vascular area in healthy male smokers.
**Methods and Materials:** We enrolled 90 healthy male smokers without COPD from January 2016 to September 2017. All smokers underwent chest computed tomography (CT) and pulmonary function test. We excluded the smokers who had pulmonary disease, cardiovascular disease, or cancer. We measured the small pulmonary vessel area by chest CT. Furthermore, we calculated the percentage of total CSA of less than 5 $mm^2$ divided by the total lung area (%CSA < 5). We evaluated the correlations of %CSA < 5, pulmonary function, and the other clinical variables.
**Results:** The %CSA < 5 was significantly correlated with FEV1/FVC (r = 0.413, p < 0.0001), whereas this was not significantly correlated with FVC and BMI. The pack-years was significantly correlated with FEV1 (r = 0.401, p < 0.001), and %CSA < 5 (r = -0.548, p < 0.001).
**Conclusion:** The small pulmonary vasoconstriction was associated with the amount of smoking and mild airflow impairment in the healthy smoker before lung parenchymal destruction.

**B-0583** 11:18

**Cystic-fibrosis-related lung disease: correlation of HR-CT lung morphology and regional lung function measured by electrical impedance tomography**

U.G. Mueller-Lisse[1], S. Krueger-Ziolek[2], B. Gong[2], H.A. Zimmermann[1], K. Moeller[2]; [1]*Munich/DE*, [2]*Villingen-Schwenningen/DE*
*(ullrich.mueller-lisse@med.uni-muenchen.de)*

**Purpose:** Electrical impedance tomography (EIT) investigates regional lung function. We studied the suitability of EIT to assess the severity of cystic-fibrosis- (CF-) related lung disease when correlated with high-resolution-computed-tomography- (HR-CT-) scoring results.
**Methods and Materials:** With ethics-committee approval, 10 CF-patients (3 female, 7 male, age 35.2±8.4 years, weight 63.9±13.0 kg, height 177.0±10.0 cm, mean±SD) were prospectively enrolled in EIT-measurements during both normal tidal breathing and a forced-vital-capacity- (FVC-) manoeuvre, to determine regional impedance-changes within the lung relating to FVC and forced expiratory volume in one second (ΔIFVC and ΔIFEV1). EIT data were collected both at the 3rd and 5th intercostal space (ICS), with a frame rate of 30 Hz. Pulsatile EIT-signal-components were excluded with a low-pass Butterworth-filter. Lung-areas were defined by pixel-values >20% of the maximum regression coefficient within the functional EIT-image resulting from linear-regression-fitting. Within each thorax-section, median regional ΔIFEV1/ΔIFVC was calculated and correlated with the mean "Total Brody Score" (TBS) deriving from independent retrospective evaluations by two radiologists of available HR-CT-scans.
**Results:** Mean HR-CT TBS-values of CF-patients showed high linear correlation ($r^2$=0.8992) with EIT-derived median regional ΔIFEV1/ΔIFVC obtained within the 5th ICS, and lower linear correlation ($r^2$=0.6872) with EIT-derived median regional ΔIFEV1/ΔIFVC obtained within the 3rd ICS, respectively.
**Conclusion:** Results imply that EIT is suitable to assess the severity of CF-related lung disease. Since HR-CT-based TBS comprises the whole lung, differences in correlations are most likely based on respective lung volumes captured by EIT at different thorax-sections.

**B-0584** 11:26

**Predictive model of lung severity in children with cystic fibrosis using Brasfield and Chrispin-Norman scoring systems**

I. Vasyliv, N. Rohovyk, O. Marushchak, M. Rohovyk; *Lviv/UA*
*(flos.veris@gmail.com)*

**Purpose:** Considering the fact that chest radiography for lung disease is one of the methods used worldwide for the assessment of cystic fibrosis (CF), we propose a modified approach in retrospective analysis and comparison in dynamics the most commonly used scale systems - Brasfield and Chrispin-Norman. The aim of this study was to build a regression model to predict the lung damage severity using Brasfield and Chrispin-Norman scoring systems by evaluation of chest radiographs in children with CF.
**Methods and Materials:** 156 chest radiographs from 51 CF children were taken for the retrospective analysis that consisted of a descriptive part and predicting models. We assigned a unique ID to every patient and also included such features as gender, age when the test was taken, number of tests, scores due to Brasfield and Chrispin-Norman scoring systems.
**Results:** Applying constructed chart to patients' data we detected a correlation between two scoring systems, for example, 2.5 in Brasfield scoring system relates to 3 points in Chrispin-Norman system and 11-18 points in Brasfield system mostly relate to 8-10 points in Chrispin-Norman system. Taking scores of the patient with the ID 38 as an example, we can present a model of linear regression to predict the next possible index in different scale systems.
**Conclusion:** Analysing scores in both Chrispin-Norman and Brasfield scoring systems, we present a regression model to predict a lung severity in CF children (next possible score due to general tendency).

**B-0585** 11:34

**MRI-derived regional flow-volume loop parameters detect early stage chronic lung allograft dysfunction**

T. Moher Alsady, A. Voskrebenzev, M. Greer, L. Becker, T. Kaireit, T. Welte, F. Wacker, J. Gottlieb, J. Vogel-Claussen; *Hannover/DE*
*(moheralsady.tawfik@mh-hannover.de)*

**Purpose:** Chronic lung allograft dysfunction (CLAD) is a major cause for poor long-term survival rates after lung transplantation (LTx). The goal of this study is to examine whether early stages of CLAD could be detected using MRI-derived parameters of regional flow-volume dynamics.
**Methods and Materials:** Sixty-two lung transplantation recipients were included in the study, 29 of which had been diagnosed with CLAD at various stages based on pulmonary function testing. MRI datasets were acquired at 1.5T using a spoiled gradient echo sequence. The datasets were preprocessed and analyzed according to the phase resolved functional lung MRI technique (PREFUL-MRI), resulting in fractional ventilation (FV) maps and regional flow-volume loops (rFVL). Correlation coefficients between rFVL of each lung voxel and the mean rFLV of a healthy region of interest were estimated. The following parameters were calculated: 1) Median and interquartile range of rFVL correlation coefficients, rFVL-CC and rFVL-CC-IQR respectively. 2) FV-based and rFVL-based ventilation defect percentage (FV-VDP and rFVL-CC-VDP).
**Results:** The following rFVL-based parameters discriminated between the presence or absence of early CLAD: rFVL-CC, rFVL-CC-IQR and rFVL-CC-VDP (p<0.003). In accordance with a previous study, FV-VDP could only discriminate between no CLAD and advanced CLAD (p< 0.0001).
**Conclusion:** Using the free-breathing, contrast media-free PREFUL-MRI technique, parameters of regional ventilation dynamics and heterogeneity have been shown to be sensitive for the detection of early CLAD.

*Author Disclosures:*

**J. Vogel-Claussen:** Research/Grant Support; This work was supported by a grant from the German Federal Ministry of Education and Research (IFB-Tx, reference number 01EO1302) and the German Center for Lung Research (DZL).

**B-0586** 11:42

**Dose reduction in CT emphysema densitometry enabled by iterative reconstruction and post-scan noise reduction: a phantom study**

H.J. Wisselink, G. Pelgrim, M. Rook, M. Oudkerk, R. Vliegenthart; *Groningen/NL*

**Purpose:** The aim of this study is to determine the minimum radiation dose required for acceptable image quality for CT emphysema densitometry, using different scan parameters and noise suppression techniques in third-generation dual-source CT.
**Methods and Materials:** The COPDGene phantom was scanned with 16 scan setups (CTDIvol 0.035-10.68 mGy), using CT Force (Siemens Healthineers, Germany) with different kV and mAs settings. Images were reconstructed at 1.0/0.7 mm slice thickness/increment, with three kernels (one soft, two hard), filtered back-projection and three grades of third-generation iterative reconstruction (IR, ADMIRE). Additionally, stand-alone noise suppression software was applied (PixelShine, Algomedica, USA). Outcomes were the overlap in area of the normalized CT density histograms of the emphysema and air inserts, and the radiation dose required for maximum 4.3% overlap, which was defined as acceptable image quality according to QIBA criteria.
**Results:** In total, 384 scans were analyzed. The two hard kernel reconstructions showed more histogram overlap than the soft kernel reconstructions. Overlap was 62.8% for the lowest dose setting. IR and noise suppression reduced this overlap. The correlation between radiation dose and histogram overlap is an inverse exponential ($R^2$>0.95). Intermediate grade IR combined with noise suppression software allowed for 85% radiation dose reduction (to a CTDIvol 0.19 mGy).
**Conclusion:** Noise suppression software, IR, and soft reconstruction kernel allow radiation dose reduction by 85% while still allowing differentiation between emphysema and normal lung tissue in a phantom set-up.

**B-0587** 11:50

**Predictors for bronchoalveolar lavage recovery rates derived from preprocedural quantified computed tomography**

J.F.M. Gawlitza, J.D. Michels, H. Haubenreisser, T. Henzler, M. Borggrefe, S.O. Schönberg, I. Akin, F. Trinkmann; *Mannheim/DE*
*(joshua.gawlitza@medma.uni-heidelberg.de)*

**Purpose:** Bronchoalveolar lavage (BAL) is an important diagnostic method. The percentage of fluid extracted after instillation (recovery rate) is crucial for following diagnostic tests. Currently, no preprocedural predictors for a good recovery rate are known. Therefore, we compared quantified airway parameters from preprocedural lung computed tomography (CT) with recovery rates to find possible predictive markers.
**Methods and Materials:** Recovery rates and patient characteristics were retrospectively acquired from 33 routine procedures. Parenchyma and airway parameters were quantified in preprocedural CT. The bronchial tree was measured in five generations, acquiring eight airway parameters. Recovery rates and patient characteristics were then analysed in context of quantified CT findings.
**Results:** Significant correlations between airway parameters and recovery rate were found for CT parameters of the 3rd or 4th generation bronchus with highest values for percentage wall area (WA%, r=0.56; p<0.01), lumen area (r=-0.47; p=0.01) and mean inner diameter (r=- 0.49; p<0.01). No correlations were found for recovery rates and CT parenchyma parameters. ROC analysis of 4th generation bronchus values yielded an AUC of 0.75 (p<0.01) for distal outer bronchoscope diameter to mean inner diameter ratio. For 3rd generation WA%, we found an AUC of 0.86 (p<0.001). A threshold of approximately 60% recovery rate had 89% sensitivity and 80% specificity to distinguish between patients with good (>50%) and poor (<50%) recovery.

**Conclusion:** Quantified CT parameters, most notably WA% and bronchoscope diameter to inner bronchus diameter, have the potential to identify the ideal location and bronchoscope size for optimal BAL recovery rates in advance, warranting prospective evaluation.

**10:30 - 12:00** **Room M 1**

### Cardiac

### SS 603

### Cardiac MR myocardial feature tracking

*Moderators:*
R. Heiß; Erlangen/DE
R. Salgado; Antwerp/BE

### B-0588 10:30

**Phenotyping diabetic cardiomyopathy in South Asians and Europeans**

E.H.M. Paiman[1], H.J. van Eyk[1], M.B. Bizino[1], I.A. Dekkers[1], P. de Heer[2], J.W.A. Smit[3], I.M. Jazet[1], H.J. Lamb[1]; [1]*Leiden/NL*, [2]*Amsterdam/NL*, [3]*Nijmegen/NL (e.h.m.paiman@gmail.com)*

**Purpose:** To characterize the impact of type 2 diabetes (TD2) on left ventricular (LV) function, myocardial tissue characteristics and aortic stiffness in South Asians and Europeans.
**Methods and Materials:** T2D patients and controls with comparable age and sex distribution, of South Asian and European descent, underwent 3 Tesla cardiac magnetic resonance imaging (MRI) and proton-magnetic resonance spectroscopy ($^1$H-MRS). Differences in cardiac parameters between T2D patients and controls were examined using ANCOVA with adjustment for age, sex, blood pressure and smoking and were reported as mean (95%CI).
**Results:** A total of 131 individuals were included (54 South Asians (50.1±8.7 years, 33% men, 33 patients vs. 21 controls) and 77 Europeans (58.8±7.0 years, 56% men, 48 patients vs. 29 controls)). The transmitral early and late filling rate ratio was lower in T2D patients compared to controls, in South Asians (-0.20 (-0.36; -0.03), *P*=0.021) and Europeans (-0.20 (-0.36; -0.04), *P*=0.017), whereas global longitudinal strain and aortic pulse wave velocity were comparable. South Asian T2D patients had a higher LV mass (+22 g (15; 30), *P*<0.001) with a lower extracellular volume (-1.9% (-3.4; -0.4), *P*=0.013), whilst European T2D patients had a higher myocardial triglyceride content (+0.59% (0.35; 0.84), *P*<0.001) than their control group.
**Conclusion:** Diabetic cardiomyopathy was characterized by impaired LV diastolic function in South Asians and Europeans. Increased LV mass was solely observed among South Asian T2D patients, whereas differences in myocardial triglyceride content between T2D patients and controls were only present among Europeans. The diabetic cardiomyopathy phenotype may differ across ethnicities and tailored management may be required.
*Author Disclosures:*
**E.H.M. Paiman:** Research/Grant Support; 'Cardio Vascular Imaging Group (CVIG)', Leiden University Medical Center (Leiden, the Netherlands).
**H.J. van Eyk:** Research/Grant Support; Roba Metals B.V. IJsselstein (Utrecht, the Netherlands). **P. de Heer:** Research/Grant Support; Dutch Kidney Foundation, Innovation grant PI11.56. **J.W.A. Smit:** Grant Recipient; Novo Nordisk A/S (Bagsvaerd, Denmark). **I.M. Jazet:** Grant Recipient; Novo Nordisk A/S (Bagsvaerd, Denmark). **H.J. Lamb:** Grant Recipient; Novo Nordisk A/S (Bagsvaerd, Denmark).

### B-0589 10:38

**Independent association of extracellular volume with myocardial strain assessed by CMR in subjects without history of cardiovascular disease from the general western population**

C. Storz[1], T. Zitzelsberger[1], S. Rospleszcz[2], K. Nikolaou[1], F. von Knobelsdorff[2], A. Peters[2], J. Schulz-Menger[3], F. Bamberg[1], C.L. Schlett[4]; [1]*Tübingen/DE*, [2]*Munich/DE*, [3]*Berlin/DE*, [4]*Heidelberg/DE (corinna.storz@med.uni-tuebingen.de)*

**Purpose:** To determine the relationship of T1 mapping parameters and myocardial strain, assessed by cardiac magnetic resonance imaging (CMR) in a western general population without prior cardiovascular disease.
**Methods and Materials:** A comprehensive contrast-enhanced CMR protocol was performed. Subjects with impaired ejection fraction (EF)<50% were excluded. Extracellular volume (ECV) was calculated based on T1-mapping in MOLLI sequence. Radial ($E_{rr}$), longitudinal ($E_{ll}$) and circumferential ($E_{cc}$) global strain was derived from cine-SSFP imaging using feature-tracking. Univariate and adjusted analyses were carried out.
**Results:** In 322 participants (56.0±9.2 years, 56.2% males), mean ECV was 24.2±2.9%. ECV was positively correlated with $E_{rr}$ (r=0.18) and negatively correlated with $E_{ll}$ and $E_{cc}$ (r=-0.18 and r=-0.21, respectively; all p<0.003). The associations between ECV with $E_{rr}$ and $E_{cc}$ remained after adjustment for clinical and functional cardiovascular confounders and EF (β=-0.28 and β=0.953, respectively; all p<0.01). We found a significant correlation between ECV and cardiac strain in hypertensive participants, whereas there was no significant interaction effect between ECV and cardiac strain in participants with diabetes.
**Conclusion:** Increased ECV is associated with impaired myocardial strain, determined by CMR, independent of cardiovascular risk factors and EF.

### B-0590 10:46

**Impaired left ventricular function in patients with treatment-resistant arterial hypertension evaluated by feature-tracking myocardial strain CMRI**

C. Ozden[1], M.L. Warncke[1], F. Brunner[1], J. Stárekóvá[1], A. Koops[1], G.K. Lund[1], G.I. Kirova-Nedyalkova[2], G. Adam[1], E. Tahir[1]; [1]*Hamburg/DE*, [2]*Sofia/BG (c.oezden@uke.de)*

**Purpose:** Treatment-resistant arterial hypertension (AHT) is defined as blood pressure above target values despite medication with at least three different antihypertensive drugs including a diuretic, posing a great threat to the hypertensive population. The aim of this study was to investigate the effects of AHT on systolic left ventricular function compared to age- and sex-matched controls by myocardial feature-tracking cardiac magnetic resonance imaging (CMRI).
**Methods and Materials:** CMRI was performed on 28 patients with AHT and 18 controls. Functional and morphological LV parameters were obtained, including end-diastolic volume (EDV) and ejection fraction (EF) using CVi42 (circle cardiovascular imaging). Global peak systolic longitudinal, radial and circumferential myocardial strains were analysed using Segment (Medviso, Sweden).
**Results:** Indexed left ventricular mass (LVMM) was significantly higher in AHT patients compared to controls (81 ±18 g/m$^2$ vs. 61 ±9 g/m$^2$, p<0.0001). Left ventricular EF in AHT patients was similar to EF in controls (61 ±10% vs. 64 ±7%, p=0.309). EDV also did not differ (74 ±16 ml/m$^2$vs. 75 ±12 ml/m$^2$, p=0.774). Global peak systolic longitudinal (-15 ±3% vs. -19 ±2%,p<0.001) and radial LV strains (37 ±11% vs. 48 ±8%, p<0.01) were significantly diminished in AHT patients. Circumferential LV strain did not show significant differences between the two groups (-17 ±5% vs. -19 ±4%, p=0.247).
**Conclusion:** An increase in LVMM in patients with AHT and preserved EF is associated with impaired LV systolic function as indicated by decreased LV systolic strain parameters.

### B-0591 10:54

**Prognostic role of feature tracking in suspected myocardial infarction with non-obstructed coronary arteries (MINOCA): sensitive or senseless?**

T. Leckebusch, P. Wenzel, F. Jungmann, M.C. Halfmann, R.M. Brumberg, C. Düber, K.-F. Kreitner, T. Emrich; *Mainz/DE (leckebuscht@gmail.com)*

**Purpose:** The term MINOCA was established for patients with acute chest pain, elevated troponins and non-obstructed coronary arteries. It is an umbrella term that includes coronary as well as non-coronary causes. The purpose of this study was to investigate prognostic implications of abnormal strain values in MINOCA-patients and its prognostic significance in relation to established risk parameters like EDVI.
**Methods and Materials:** The study comprised CMR examinations of 145 patients. These patients were followed over a median time of 4.2 years. Primary endpoints were defined as MACE including death, stroke, congestive heart failure, recurrent hospitalization or an interventional/surgical procedure. Regarding strain parameters, we compared these patients with a collective of 62 healthy volunteers. A dedicated software (CVI42®, Circle) tracked the movement of endo- and epicardial contours and calculated strain parameters.
**Results:** Based on the strain analysis of healthy volunteers, we set a cut-off value for pathologic strain values at mean value (healthy volunteer) ±2*standard deviation. A significantly higher rate of primary endpoints occurred in patients with abnormal strain values (p<0.05 for all). Kaplan-Meier analyses in MINOCA patients were performed for normal and pathologic groups of strain in normal and abnormal EDVI. After adjusting for EDVI, only LV global radial strain and RV global longitudinal strain were of significant prognostic value.
**Conclusion:** Abnormal strain values are correlated with a higher risk of MACE in patients with MINOCA. After adjustment for normal LV volumes, only LV radial and RV longitudinal strains had a significant effect on the prognosis of MINOCA patients thus outperforming traditional risk factors.

### B-0592 11:02

**Evaluation of left ventricular strain assessment with feature-tracking CMR in STEMI at long term follow-up**

F. Cilia, C. Calvieri, F. Catapano, S. Coco, R.M. Ammendola, G. De Rubeis, N. Galea, M. Francone, C. Catalano; *Rome/IT (fcilia91@hotmail.it)*

**Purpose:** Left ventricular strain assessed by CMR feature-tracking has been recently used to evaluate the benefits of metoprolol administration before primary percutaneous intervention (PCI) at short term follow up (FU). Our aim was to evaluate LV strain assessment at long term FU in STEMI patients.

**Methods and Materials:** This is a pilot retrospective single center study. Twelve acute STEMI patients (aged 55±12 yrs, 100% male) underwent CMR at 1,5 T, at 2-6 days after PCI and after a median follow up of 10 years (IQ 9-11). Feature-tracking CMR analysis was performed on steady-state free precession cine images with a dedicated software (CVI42 v5.3, Circle Cardiovascular Imaging, Calgary, Canada) for both CMR exams.
**Results:** No significant changes in LVEF were observed between the first and the last CMR, while LGE extent significantly reduced (11,7 ±8,4 to 7±4,5, p=0.030) and global longitudinal strain (GLS) significantly improved ( -14±3,3 to -17,9 ± 3,6, p=0.016). At first CMR troponin release and heart rate before PCI correlated with acute GLS (r: -699,p=0.011,r:-678, p=0.045, respectively). Ischaemia duration before PCI correlated positively with global radial strain at long term FU CMR (r:0.729,p=0.017). Interestingly, STEMI who received i.v. nitrates before PCI had no difference in LVEF and LGE extent changes but a greater improvement in GLS at long term follow up (-16±0.8, -21± 0.8, p=0.007).
**Conclusion:** LV strain assessment with feature-tracking CMR adds incremental prognostic information on LV remodelling beyond LVEF. Feature-tracking CMR strain could be a sensitive tool to evaluate long term effect of cardioprotective therapies.

**B-0593** 11:10
**Early detection of left ventricular involvement assessed by 3D CMR feature tracking improves risk stratification in patients with arrhythmogenic right ventricular cardiomyopathy**
X. Chen, L. Li, S. Zhao; *Beijing/CN (cxy0202@126.com)*

**Purpose:** To investigate the diagnostic value of three-dimensional feature tracking cardiac magnetic resonance (3D CMR-FT) for early detection of left ventricular (LV) involvement in arrhythmogenic right ventricular cardiomyopathy (ARVC) patients as well as its prognostic value of predicting adverse outcomes.
**Methods and Materials:** Fifty-eight ARVC patients and 20 healthy control subjects were prospectively enrolled. CMR with late gadolinium enhancement (LGE) and myocardial strain analysis using CMR-FT were performed in all subjects. Global and regional (basal, medial and apical) LV peak strains as well as global longitudinal strain of right ventricle were measured. All patients were followed for cardiovascular events.
**Results:** Of the 58 ARVC patients, 31 (53%) had preserved LV ejection fraction (LVEF ≥50%). In these patients, the LV global longitudinal as well as circumferential, radial and longitudinal strain at the basal level were all significantly reduced compared with the controls (all p < 0.05). During a mean follow-up period of 15.0±7.8 months, 13 (22%) patients experienced cardiac events. The extent of LGE and LV global radial strain (GRS) were significantly associated with adverse outcomes, and LV GRS was the only independent predictor according to multivariate analysis. In patients with preserved LVEF, a basal circumferential strain > -12.77% was significantly associated with outcomes.
**Conclusion:** LV involvement can be identified by 3D CMR-FT in the absence of LVEF reduction. LV GRS was the only independent predictor of adverse outcomes in ARVC patients. Reduced basal circumferential strain was associated with adverse events in patients with preserved LVEF.

**B-0594** 11:18
**CMR tissue tacking assessment of myocardial strain in non-ischaemic dilated cardiomyopathy patients with and without left ventricular hyper-trabeculations**
H. Fu, L. Wen, Y.-K. Guo, Z. Yang; *Chengdu/CN (695868312@qq.com)*

**Purpose:** Our study aimed to evaluate the difference of left ventricular strain and function in non-ischaemic dilated cardiomyopathy (NIDCM) with or without hyper-trabeculations.
**Methods and Materials:** 157 patients of NIDCM were retrospectively enrolled. Patients were divided into two groups according to definition of prominent trabeculations. Cardiac function and myocardial strain were measured based on short-axis, two-chamber and four-chamber cine sequences. Left ventricular ejection (LVEF), peak ejection rate (PER), peak filling rate (PFR) and global peak strain in three directions of radial (GPRS), circumferential (GPCS), longitudinal (GPLS) were obtained.
**Results:** LVEF ( Mean ± SD: 25.9%±10.5%) was smaller than 50% of all 157 patients. Hyper-trabeculations were presented in 86 patients(54.8%). Although LVEF, PER, PFR and GPRS have no statistically significant between the NIDCM patients with or with hyper-trabeculations (p>0.05), for the myocardial strain, the GPCS and GPLS in patients with hyper-trabeculations lower than that of patients without hyper-trabeculations (p=0.040 and P=0.006, p<0.05). GPSR, GPSC and GPSL were mild to moderate correlated with LVEF (correlation coefficients were 0.44, -0.564, -0.321 respectively, p<0.05) in Group A. Despite GPSL was not related to LVEF in patients with hyper-trabeculations, GPSR and GPSL were moderate related to LVEF (correlation coefficients were 0.43 and -0.40, p<0.05).
**Conclusion:** CMR tissue tracking can early detect myocardial strain in NIDCM patients with or with hyper-trabeculations. And patients with hyper-trabeculation may have more severe myocardial deformation. But it's need to do further research to explore whether hyper-trabeculations would affect the prognosis of NIDCM.
*Author Disclosures:*
**Y. Guo:** Founder; Research/Grant Support; National Natural Science Foundation of China (81471721,81771897), Program for Young Scholars and Innovative Research Team in Sichuan Province (2017TD0005) of China.
**Z. Yang:** Founder; Research/Grant Support; National Natural Science Foundation of China (81471722,81771887).

**B-0595** 11:26
**Strain analysis in noncompaction morphology: usefulness in assessing an undefined cardiomyopathy**
E. Cannizzaro, P. Palumbo, C. De Cataldo, A. Corridore, S. Torlone, F. Cobianchi Bellisari, A. Di Sibio, E. Di Cesare, C. Masciocchi; *L'Aquila/IT (estercannizzaro@hotmail.it)*

**Purpose:** To measure left ventricular (LV) myocardial strain with cine-magnetic resonance (MR) imaging to relate LV function to strain values in order to assess the prognostic value of strain analysis and to detect early impairment of the LV function in patients with left ventricular non-compaction (LVNC); to demonstrate that morphology (absence or presence of non-compaction) doesn't influence LV contractility.
**Methods and Materials:** In this retrospective study, 65 patients with a validated diagnosis of LVNC were re-analyzed in order to avoid selection biases and consider only patients with an isolated LVNC (LVNC), obtaining a final population of 21 patients who underwent a post-processing strain analysis. Strain analysis effectiveness' to detect impairment of LV function (<50%) was analyzed by ROC curves and morphology's influence on LV contractility has been analyzed by Cohen's kappa coefficient.
**Results:** In our analysis, we found that global circumferential strain was the most reliable value to detect LV function with a sensitivity of 100% and a specificity of 70,6%. (AUC=91,2%, p<0.001; best cut-off >-13,06). Cohen's kappa coefficient didn't show a concordance between the segmental NC/C ratio and segmental circumferential strain values suggesting a non-significant influence of morphology on LV contractility (k=0,02, 95% CI -0,07 to 0,12).
**Conclusion:** Strain analysis seems to be sensible and specific in assessing LV function and in detecting early patients with a poor or mild-impaired LV contractility. The lack of concordance between the segmental NC/C ratio and the segmental strain values conceivably arise the possibility that morphology doesn't affect the contractility.

**B-0596** 11:34
**Assessment of myocardial deformation in a rabbit model of diabetes mellitus by cardiac magnetic resonance tissue tracking**
Y. Zhang, Z.-G. Yang, Y.-K. Guo, K. Diao, M.-T. Shen, X. Li, K. Xu, H. Yu, C. Xia; *Chengdu/CN (yizhang524@qq.com)*

**Purpose:** To determine the characteristics of myocardial deformation in a diabetes mellitus (DM) rabbit model at different stages by using cardiac magnetic resonance (CMR) tissue tracking.
**Methods and Materials:** Twenty-eight New Zealand white rabbits, randomly divided into the control group, 4-week (n=9), 8-week (n=6) DM groups, induced by Alloxan (80 mg/kg), and the control group(n=8), underwent CMR examination. A series of SSFP cine sequence were used to calculate left ventricle ejection fraction (LVEF). 2D global peak radial strain ($GS_r$) and global peak circumferential strain ($GS_c$) were derived from short-axis slices and 2D global peak longitudinal strain ($GS_l$) was derived from the 4-chamber and long-axis slices. Spearman's rank correlation analysis was performed to determine the correlation between myocardial strain parameters and the stages of DM.
**Results:** All groups had preserved and no statistically different LVEF. The $GS_r$ (19.01±3.92% vs. 27.28±9.04%) and $GS_l$ (-9.91±2.60% vs. -12.92±1.91%) of 8-week group were lower than those of controls (all *P*<0.05), whereas the difference of $GS_r$ and $GS_l$ between the 4-week group and the controls did not reach statistical significance (*P*=0.079 and 0.113, respectively). $GS_c$ showed numeric but no significant difference between either two groups. Additionally, $GS_l$ had a moderate correlation with the stages of DM (r=-0.534, *P*<0.01).
**Conclusion:** DM rabbits demonstrated $GS_l$ and $GS_r$ reduction during the process of DM, even without change in LVEF. Myocardial strains could work as sensitive indices to assess LV myocardial deformation in DM.
*Author Disclosures:*
**Y. Zhang:** Research/Grant Support; National Natural Science Foundation of China (81471721, 81471722, 81641169, 81771887 and 81771897).

**B-0597** 11:42
**Cardiac computed tomography left ventricle myocardial strain analysis**
T. Adla, V. Suchanek, M. Polovincak, L. Miksik, Z. Salagovicova, N. Markova, J. Biskup, M. Rocek; *Prague/CZ (theodor.adla@gmail.com)*

**Purpose:** Myocardial deformation strain analysis is possible from echocardiography and cardiac MRI with different methods. Feature tracking is a relatively new method used for MRI. Purpose of this study was to assess feasibility of using this method for left ventricle (LV) deformation in cardiac CT.
**Methods and Materials:** Ten patients (60 +/- 17 years old) were examined by cardiac CT (Somatom Force, Siemens), retrospective gating with dose modulation (full dose 30 - 70% of RR interval) protocol. Additionally to standard analysis, cine images (20 phases, 5 mm thickness) were reconstructed in 3 long and 3 short axes of LV. Software Segment (Medviso) was used for feature-tracking analysis. Automatic segmentation during heart cycle was assessed visually. Values of global strains were compared with LV ejection fraction.
**Results:** Inaccuracies of propagation of myocardial contour were visible in interventricular septum in cases with low or inhomogeneous right ventricle contrast filling. Global strain values were longitudinal -13 +/- 3%, circumferential -16 +/- 5%, radial from long-axis images 22 +/- 8%, and radial from short-axis images 23 +/-9%. Significant correlations were found between LV ejection fraction and longitudinal strain (R =-0.69, p = 0.03), circumferential strain (R = -0.74, p = 0.01), and radial strain from long-axis images (R = 0.69, p = 0.03).
**Conclusion:** Strain analysis of LV from cine CT images is technically possible using Segment software and some of global strains values significantly correlate with LV ejection fraction. However, good enhancement of the right ventricle is needed.

**B-0598** 11:50
**Two-dimensional and three-dimensional cardiac magnetic resonance feature-tracking myocardial strain analysis in acute myocarditis patients with preserved ejection fraction**
M. Gatti[1], R. Faletti[1], F. Bioletto[1], A. Palmisano[2], G. Benedetti[2], A. Esposito[2], P. Fonio[1]; [1]*Turin/IT*, [2]*Milan/IT (marcogatti17@gmail.com)*

**Purpose:** To explore the potential role of two- (2D) and three-dimensional (3D) cardiac magnetic resonance (CMR) feature tracking (FT) myocardial strain analysis in identifying sub-clinical myocardial dysfunction in acute myocarditis patients with preserved ejection fraction (EF).
**Methods and Materials:** Prospective two centre study-control study. Thirty patients (9 female, 37.2 ±11.8 yrs.) with a CMR diagnosis of acute myocarditis according to the Lake Louise Criteria and preserved EF (>55%) were included in the analysis. CMR data from 24 healthy volunteers (11 female, 36.2±12.5 yrs.) served as control. 2D and 3D LV tissue tracking analysis were performed in a random fashion by two double-blinded operators. Variables were checked for normality and analysed with parametric test.
**Results:** The baseline characteristics of myocarditis patients with preserved EF and the healthy volunteers were perfectly comparable, except for the LV mass index (60±13% vs. 45±11%; p<0.001). The results of the interoperator variability in the 2D and 3D LVCMR FT myocardial strain analysis were p>0.42, ICC>0.80 and $\eta^2$>0.98. There was no statistical difference in 2D and 3D global radial, circumferential and longitudinal strain peak (%) and both systolic and diastolic strain rate (1/s) between acute myocarditis with preserved EF and healthy volunteers (all p=n.s.).
**Conclusion:** There were no difference in 2D and 3D global radial, circumferential and longitudinal strain peak and both systolic and diastolic strain rate of the LV between acute myocarditis patient with preserved ejection fraction and healthy volunteers.

**10:30 - 12:00** **Room M 2**

### Paediatric

### SS 612

### Foetal and obstetric imaging

*Moderators:*
R. Llorens; Valencia/ES
D. Prayer; Vienna/AT

**B-0599** 10:30
**Feasibility of foetal MRI in the evaluation of foetal congenital heart disease**
S.-Z. Dong, M. Zhu; *Shanghai/CN (dongsuzhen@126.com)*

**Purpose:** To evaluate the feasibility of foetal cardiac magnetic resonance (CMR) in the assessment of foetal congenital heart disease.
**Methods and Materials:** This retrospective review included 616 pregnant women referred to a children's hospital for a foetal cardiac MRI from January 2011 to June 2018 due to the finding of a congenital heart disease by echocardiogram (echo) and/or technically limited echo. Screening obstetric ultrasound (US), echo by cardiologists and CMR data of all cases were compared with postnatal diagnoses (postnatal imaging, surgery and/or autopsy). Foetal CMR was performed at 1.5 T or 3.0 T (Discovery 750; GE Medical Systems). Sequences included steady-state free-precession (SSFP), non-gated SSFP cine, single-shot turbo spin echo (SSTSE) and non-gated phase contrast (PC) cine sequences. The CMR images were analysed using an anatomic segmental approach by two radiologists and compared with foetal echo and postnatal findings when available.
**Results:** All 616 cases with congenital heart disease were confirmed by postnatal imaging, surgery and/or autopsy. Obstetric US were correct in 45.8% (282/616), foetal echoes were correct in 83.1% (512/616), CMR were correct in 77.9% (480/616) when compared to postnatal findings. Foetal echoes had a higher specificity for evaluating ventricular size, septal defects and valve anomalies. CMR was more useful in evaluating extracardiac aortic arch, superior vena cava and malposition anomalies, heart diverticulum, and pericardium cyst.
**Conclusion:** Foetal CMR is a promising diagnostic tool for the assessment of congenital heart disease (especially extra-cardiac vascular anomalies) and can be a useful adjunct when foetal echocardiography is limited.

**B-0600** 10:38
**Diffusion-weighted magnetic resonance imaging of the foetal brain in complex congenital heart disease**
S.-Z. Dong, H. Ji; *Shanghai/CN (dongsuzhen@126.com)*

**Purpose:** The aim of this study was to assess whether ADC values in the foetal brain were different in a group of foetuses with complex congenital heart disease (CHD), compared to normal controls.
**Methods and Materials:** Foetal brain diffusion MR imaging was performed in 50 foetuses with complex CHD, Foetal brain MRI examinations with single-shot DWI (b = 0 and b = 700 s/mm²) were performed. ADC values in frontal white matter (FWM), centrum semi-ovale (CSO), thalami (T), basal ganglia and pons were compared with 50 normal controls at the same gestational age.
**Results:** We found no significant differences between gestational age between our 2 groups (CHD 26.6 ± 3.2w and control 26 ±2.8w). All foetal brain morphology and signal were normal. Foetuses with complex CHD had higher ADC values in FWM (2.34 ± 0.18 vs 2.19 ±0.17 $x10^{-3}$ $mm^2$/sec; p<0.05), thalami (2.20 ±0.16 vs 2.06±0.19; p<0.001), centrum semi-ovale (2.34 ±0.19 vs 2.22 ±0.19 x $10^{-3}$ $mm^2$/sec, p<0.05), basal ganglia (2.16 ±0.18 vs 2.03±0.16, p<0.001), and pons (2.14 ±0.18 vs 2.04 ±0.15 10-3 $mm^2$/sec, p<0.05).
**Conclusion:** Foetal diffusion MR imaging is a feasible modality of evaluating abnormal brain diffusivity in foetuses with complex CHD.

**B-0601** 10:46
**Foetal dynamic phase-contrast MR angiography using ultrasound gating and comparison with Doppler ultrasound measurements**
B.P. Schoennagel, J. Yamamura, F. Kording, R. Fischer, G. Adam, H. Kooijman, C. Ruprecht, K. Fehrs, M. Tavares de Sousa; *Hamburg/DE (b.schoennagel@uke.de)*

**Purpose:** To investigate the feasibility of foetal phase-contrast (PC)-MR angiography of the descending aorta (AoD) using an MR-compatible Doppler Ultrasound sensor (DUS) for foetal cardiac gating and to compare velocimetry with Doppler ultrasound measurements.
**Methods and Materials:** In this prospective study 2D PC-MR angiography was performed in ten human foetuses (gestational age 32.4±2.3 weeks) using an MR-compatible DUS for gating of the foetal heart at 1.5T. Peak flow velocities in the foetal AoD were compared with Doppler ultrasound measurements performed on the same day. Reproducibility of PC-MR measurements was tested by repeated PC-MR in five foetuses.
**Results:** Dynamic PC-MR angiography in the AoD was successfully performed in all foetuses using the DUS, with foetal heart rates of 131 - 163 bpm. Time-velocity curves revealed typical arterial blood flow patterns. PC-MR mean flow velocity and mean flux were 19.7±7.9 cm/s and 7.8±3.4 ml/s, respectively. A positive association between PC-MR mean flux and flow velocity with gestational age was obtained (r=0.9, p=0.001 and r=0.65, p=0.06). PC-MR and Doppler ultrasound peak velocities revealed highly significant correlation (r=0.88, p<0.001). Peak velocities were lower for PC-MR compared with Doppler ultrasound (68±26 vs. 95±28 cm/s, p<0.001). Reproducibility of PC-MR was high with no statistical differences (p>0.05).
**Conclusion:** The MR-compatible DUS for foetal cardiac gating allows for PC-MR angiography in the foetal AoD. Comparison with Doppler ultrasound revealed highly significant correlation of peak velocities with underestimation of PC-MR velocities. This new technique for direct foetal cardiac gating indicates the potential of PC-MR angiography for assessing foetal haemodynamics.

*Author Disclosures:*
**B.P. Schoennagel:** Founder; co-founder of northh medical GmbH, the company of the applied Doppler ultrasound gating device. Research/Grant Support; Financial support by the German Research Foundation. Shareholder; shareholder of north medical GmbH. **J. Yamamura:** Founder; northh medical GmbH. Shareholder; northh medical GmbH. **F. Kording:** CEO; northh medical GmbH. Founder; northh medical GmbH. Shareholder; northh medical GmbH. **H. Kooijman:** Employee; Philips healthcare. **C. Ruprecht:** Employee; northh medical GmbH. Founder; northh medical GmbH. Shareholder; northh medical GmbH. **K. Fehrs:** Employee; northh medical GmbH. Founder; northh medical GmbH. Shareholder; northh medical GmbH. **M. Tavares de Sousa:** Founder; northh medical GmbH. Shareholder; northh medical GmbH.

**B-0602** 10:54

**Role of fetal MRI in diagnosis of genitourinary anomalies: is there an added value?**

S. Sefidbakht[1], B. Bijan[2], S. Bagheri[1], Z. Etemadi[1], S. Esmaeilian[1]; [1]*Shiraz/IR*, [2]*Sacramento, CA/US (kmssd87@gmail.com)*

**Purpose:** To retrospectively review fetal MRI's(FMRI) performed over 5 years in our center to establish the added value of FMRI in diagnosis of fetal GU anomalies.
**Methods and Materials:** IRB approved study. Out of771 fetal MRI's performed in our center, 65 were done either primarily to evaluate a GU anomaly seen in ultrasound or an anomaly related to the GUT was incidentally detected. Interpretation of MRI's were done by a single radiologist who was not blinded to the ultrasound results. Final diagnosis was established through phone calls and national registery search. Post-delivery images also surgical notes and biopsy results were obtained when available. The added value of MRI was discussed with a pediatric surgeon, pediatric urologist, perinatologist and radiologist.
**Results:** Final diagnoses included bilateral/unilateral renal agenesis (8/4), multicystic dysplastic kidneys (4), urinary tract obstruction(3), duplicate collecting system with obstruction (2), simple renal cyst (3), adrenal cyst (4), posterior urethral valve (5), crossed fused ectopia (1), autosomal recessive/dominant polycystic kidney disease (3/2), glomerulocystic disease (2), bilateral UPJO and isolated mild bilateral pelvocaliectases, normal outcome (25). FMRIwas considered to have an impact on diagnosis/counseling/management in 26 (confirming presence/absence of kidneys in severe oligohydramnios (8 & 9), confirming probable normal renal function in hyperintense/hyperechoic fetal kidneys (3), multicystic dysplastic kidney diagnosed as pelvocaliectasis in ultrasound(3), crossed fused ectopia (1), megacystis microcolon (1), &bilateral UPJO (1).
**Conclusion:** FMRI had significant additional benefit to ultrasound in congenital genitourinary anomalies which affected either management (27%) or counseling (12%) as a result of more accurate diagnosis.

**B-0603** 11:02

**Abnormalities concomitant with intrahepatic persistent right umbilical vein**

A. Ipek, Ö. Ünal, M. Yildirim Erol; *Ankara/TR (mel-tem89@hotmail.com)*

**Purpose:** Persistent right umbilical vein is an uncommon intrauterine variation. In our study, it was aimed to determine the anomalies associated with intrahepatic persistent right umbilical vein (I-PRUV).
**Methods and Materials:** Prenatal ultrasound results of 43 fetuses with I-PRUV were evaluated retrospectively in 15,632 fetuses who admitted to an obstetric ultrasound between 2010 and 2018, and interviewed with their parents. These fetuses were evaluated in terms of other congenital malformations and accompanying negative results after delivery.
**Results:** 4 of the 43 pregnant women were still pregnant at the end of the study. Additional anomalies detected during prenatal ultrasound: single umbilical artery 4,AVSD 1, tetralogy of Fallot 1, intracardiac echogenic focus 4, ascending aortic dilatation and pulmonary artery stenosis 1, renal pelviectasis 2, bilateral renal agenesis 1, left equinovarus deformity 1, oligohydroamnios 2 and polyhydroamnios 2 fetuses. 1 had multiple anomalies (serious cardiac malformations, neural tube defect, musculoskeletal anomalies,cleft palate and lip etc.) It was learned that 2 fetuses had died intrauterine and 1 died at 3 years old followed by seizures and liver failure.
**Conclusion:** The frequency of I-PRUV is reported in the literature in the range of 1/217-1/1250.The frequency of I-PRUV in this study was 1/363 and was consistent with previous studies. Among the fetuses observed with I-PRUV, the frequency of single umbilical artery was 9.3% (healthy population prevalence was 1% in the literature), and the frequency of cardiac anomaly was 6.9 % (healthy population frequency in the literature was 0.8%). As a result, echocardiography can be used in intrauterine-postnatal period for concomitant cardiac anomaly.

**B-0604** 11:10

**Prenatal MRI assessment of swallowing and pharyngeal structures in foetuses with facial clefts**

S.R. Teixeira, E.R. Arruda, S.M. Saade, O. Jackson, A.N. Pollock, T. Feygin; *Philadelphia, PA/US (teixeiras@email.chop.edu)*

**Purpose:** To determine feasibility of dynamic MRI techniques in assessment of swallowing patterns in foetuses with facial clefts; to assess anatomic variations of nasopharyngeal structures in foetuses with facial clefts in comparison to controls.
**Methods and Materials:** Between 2008 and 2017, foetal MRI, which included dynamic cine sequences, were retrospectively analyzed by 2 paediatric radiologists. Inclusion criteria: suspected foetal facial clefts, >= 19 weeks-of-gestation and technically adequate dynamic MR sequences. The phases of swallowing assessed were: Motion of the foetal lips, tongue, soft palate and hypopharynx. Measurements of the anterior nasal-spine-cranial-cervical junction (ANS-CC), anterior nasal-spine-soft-palate (ANS-SP), presence/absence of velopharyngeal incompetence (VPI) and glossoptosis were recorded. The ANS-CC:ANS-SP ratio was regressed *versus* pathology status, foetal and maternal age. Comparison was done with 35 random controls.
**Results:** Sixty-five cases of foetal facial clefts were included. The chi-square test (equivalent to the two-proportion z-test) revealed a marked difference in the proportion of foetuses exhibiting VPI (55/65=0.85 in the pathological group *versus* 0/35=0 in the control group). The incidence of detected glossoptosis was also significantly higher, in more than 80% of cases. Foetuses with facial clefts had a ANS-CC:ANS-SP ratio 12 percentage points higher than the controls, even after controlling for age effects.
**Conclusion:** Dynamic-cine MRI is a technically feasible and valuable tool in the prenatal assessment of foetuses with facial clefts. It demonstrates statistically significant abnormal patterns of foetal swallowing. Abnormal ANS-CC:ANS-SP ratio may indicate presence of palatal cleft, even in the absence of direct visualization of the cleft on prenatal imaging.

**B-0605** 11:18

**Evaluation of foetal modified myocardial performance index as a predictor of perinatal outcome in gestational diabetes**

R. Khurana, S.K. Bajaj; *New Delhi/IN (rajat3008khurana@gmail.com)*

**Purpose:** To assess foetal cardiac function (both systolic and diastolic) using modified myocardial performance index by Doppler in gestational diabetic mothers in early third trimester (28-32 weeks) and to assess role of this index in prediction of perinatal outcome.
**Methods and Materials:** 1. Study design: Prospective observational study 2. Subjects and Inclusion criteria: 30 singleton pregnant females in third trimester (28-32 weeks) with known history of Gestational Diabetes Mellitus and 30 normal singleton pregnant females in third trimester 3. Exclusion criteria: Multiple pregnancy Intrauterine growth retardation Pregestational diabetes mellitus Congenital anomalies History of cardiac disease or hypertension 4. ULTRASOUND: The Mod-MPI will be calculated in the foetal left ventricle by placing the Doppler sample in the internal leaflet of the mitral valve and estimating the three time periods as ICT (isovolumetric contraction time), IRT (isovolumetric relaxation time) and ET(ejection time). The Mod-MPI is calculated as (ICT+IRT)/ET.
**Results:** The cases had significantly higher median MPI compared to controls, p value<0.0001. 7 abnormal outcomes were recorded in the 30 foetuses in the study group, corresponding to an adverse outcome rate of 23.3% and these foetuses had significantly higher MPI measurements compared to GDM foetuses with normal outcome. The MPI served as an excellent predictor of adverse outcome in GDM foetuses with a total area under the ROC curve of 0.96. MPI z-score greater than 4.0 provided a 100% sensitivity and 80% specificity. No abnormal outcomes were recorded in the control group.
**Conclusion:** MPI has the potential to improve foetal surveillance in gestational diabetes.

**B-0606** 11:26

**What does perinatal autopsy add after antenatal ultrasound and post-mortem MRI investigations**

S.C. Shelmerdine, O.J. Arthurs, A. Wade, R. Jones, W. Norman, A. Taylor, N.J. Sebire, L.S. Chitty; *London/UK (susie_c_s@yahoo.co.uk)*

**Purpose:** To determine the value of perinatal autopsy where non-invasive imaging techniques (post-mortem MRI, PMMR) have been performed in foetuses terminated after antenatal detection of sonographic abnormalities.
**Methods and Materials:** Pre-autopsy PMMR was performed on consecutive foetuses over a 6-year period (2007-2013). Cases were categorised into three groups depending upon whether ultrasound and PMMR findings were concordant, partially concordant or discordant. The yield of new information from autopsy in each of these groups was assessed and diagnostic accuracies for each of five body systems (neurological, cardiac, thoracic, abdominal and musculoskeletal) were calculated using autopsy as the reference standard for both ultrasound and PMMR.
**Results:** Eighty-one foetuses were included. PMMR and ultrasound findings were concordant in 44/81 (54.3%) cases, partially concordant in 26/81 (32.1%) cases, and discordant in 11/81 (13.6%) cases. New information was obtained at autopsy in 19/81 (23%) cases overall, 6/44 (13.6%), 10/26 (38.5%), and 3/11 (27.2%) of the different categories, respectively, The diagnostic accuracy rates for all body systems using ultrasound gave a sensitivity of 76.8% (66.6%, 84.6%), specificity of 92.5% (88.9%, 95.0%) and concordance of 89.0% (85.5%, 91.8%). For PMMR the sensitivity was 79.0% (68.9%, 86.5%), specificity 97.9% (95.5%, 99.0%) and concordance of 93.8% (90.8%, 95.8%). Overall PMMR had a significantly higher concordance rate with autopsy than ultrasound (89.0 vs 93.8%; p<0.001).
**Conclusion:** In the majority of cases, antenatal ultrasound and PMMR were concordant, and there was little added value from autopsy. Where there was partial concordance or discordance, the yield from autopsy was higher.

**B-0607** 11:34
**Reliable evaluation of ADC values in the study of normal foetal brain, using denoising correction: a prenatal MR study**
A. Antonelli, L. Manganaro, M.G. Di Trani, S. Capuani, S. Bernardo, R. Petrillo, C. Catalano; *Rome/IT (amanda.antonelli@hotmail.it)*

**Purpose:** Apparent Diffusion Coefficient (ADC) measurements are useful to evaluate the normal development of foetal Central Nervous System (CNS). Unfortunately, foetal movement artefacts consistently perturb Diffusion Weighted Imaging (DWI) resolution, affecting ADC measurements. Aim of this study was to investigate the potential of denoising DWI to ameliorate the reliability of ADC values in multiple regions of normal foetal brain.
**Methods and Materials:** 30 normal pregnancies underwent foetal MRI at 1.5T, using DW-Spin Echo EPI with b-values 50,200,700 s/mm². Eight regions of interest (ROIs) were manually placed in Frontal White Matter (FWM), Occipital WM (OWM), Thalamus (TH), Basal Ganglia (BG) Cerebellum (CH), Pons, lateral ventricle Cerebral Spinal Fluid (CSF) and amniotic fluid. Raw data were denoised and DWI were segmented to eliminate CSF. Kruskal-Wallis test was used to calculate the differences of ADC values during second and third trimester, with and without DWI pre-processing.Signal-to-Noise Ratio (SNR) was obtained with and without denoising correction to evaluate the reliability of ADC values. Spearman's test was used to calculate the correlation between ADC and Gestational Age (GA).
**Results:** SNR considerably increased and significant differences in ADC values were found with and without correction.ADC values of TH,CH and Pons show a progressive decline in mean diffusivity (p<0.001), while a positive correlation was found between ADC and GA in FWM (p<0.05). In FWM, ADC increases during the second trimester and it decreases in the third trimester. ADC mean values of CH, Pons, FWM and TH are significantly different between second and third trimester (p<0.01).
**Conclusion:** Denoising DWI is an important tool to obtain no-biased ADC values,in order to better characterize normal foetal brain development in second and third trimester,helping also the study of foetal CNS abnormalities.

**B-0608** 11:42
**Assessment of the reproducibility of foetal proton magnetic resonance spectroscopy at different sequence settings**
M. Sokurenko, T. Trofimova, A. Khalikov; *St. Petersburg/RU (maria@sokurenko.name)*

**Purpose:** To evaluate the possibility of obtaining comparable quantitative data of proton magnetic resonance spectroscopy ($^1$H-MRS) of the foetal brain at various stages of pregnancy with different point-resolved spectroscopy (PRESS) sequence parameters.
**Methods and Materials:** Imaging of 70 foetuses' brains (19-39 postmenstrual weeks, normal neurodevelopment) were acquired on 3.0T MRI. Methodology included obtaining T2 SSFSE (TE=70-90 ms, TR=1200) in 3 orthogonal planes; slice thickness 3-4 mm. Then PRESS was applied with different TR/TE parameters: 2000/70 ms and 1500/35 ms. The zone of interest was projected posteriorly from the basal ganglia.
**Results:** The concentration of metabolite (N-acetylaspartate, creatine, choline, inositol) and graphic spectra were obtained. The earliest detection of metabolite concentration was presented at 19 gestational weeks. We did not find a close link between foetal age and the appearance of metabolic concentration. The reproducibility of metabolites was assessed. In the analysis of 70 obtained spectra in 48% of cases it was possible to obtain the values of all 4 metabolites, in 36% - 2 or 3 metabolites, in 16% - 1 or 0 metabolites. Using of PRESS sequence on TR=2000 ms and TE=70 ms has shown higher reproducibility.
**Conclusion:** Using modern 3.0T MR scanners allows to obtain qualitative $^1$H-MR spectra of the foetal brain. Obtaining high-quality graphic spectra of $^1$H-MRS is possible not in all cases and depends on a variety of technical difficulties in conducting the research. Use of PRESS sequence on TR=2000 ms and TE=70 ms has shown higher reproducibility.

**B-0609** 11:50
**Assessment of the dynamics of foetal brain metabolites with proton magnetic resonance spectroscopy**
M. Sokurenko, T. Trofimova, A. Khalikov; *St. Petersburg/RU (maria@sokurenko.name)*

**Purpose:** To evaluate changes of foetal brain metabolite concentrations using proton magnetic resonance spectroscopy ($^1$H-MRS) at various stages of pregnancy with point-resolved spectroscopy (PRESS) sequence.
**Methods and Materials:** Imaging of 70 foetuses' brains (19-39 postmenstrual weeks, normal neurodevelopment) were acquired on 3.0T MRI. Pregnant women were examined in the supine position (feet first) and no contrast agents or sedatives were used. Methodology included obtaining T2 SSFSE (TE=70-90 ms, TR=1200) in 3 orthogonal planes to evaluate anatomic structures and planning the region of interest; slice thickness 3-4 mm. Then applied PRESS with TR=2000 ms and TE=70 ms. The zone of interest was projected posteriorly from the basal ganglia, capturing the thalamus and hypothalamus, its size was 20 mm$^3$.
**Results:** The concentration of metabolites (N-acetylaspartate - NAA, Creatine - Cr, Choline - Cho, Inositol - Ino) and graphic spectra were obtained. Over the gestational period the concentrations of NAA, Cr, NAA/Cho, Cr/Cho were increasing; the concentrations of Cho, Ino, NAA/Cr, Cho/Cr were decreasing. Using PRESS on TR=2000 ms and TE=70 ms has shown high reproducibility.
**Conclusion:** The foetal brain metabolites is changing during pregnancy and this corresponds to brain maturation. This reflects the status of myelination associated with increasing synaptic complexity, increased proliferation and differentiation of cells. Diagnosis of disorders of normal metabolic processes of the foetal brain can be useful in identifying future potential therapeutic strategies.

**10:30 - 12:00** **Room M 3**

### Oncologic Imaging

### SS 616b

### Abdominal tumour imaging: raising the bar

*Moderators:*
D. Caruso; Rome/IT
A. Cieszanowski; Warsaw/PL

**B-0610** 10:30
**Volumetric DCE-MRI radiomics nomogram can predict tumour response in patients with oesophageal cancer treated by neoadjuvant chemotherapy**
J. Qu[1], L. Ma[2], Y. Lu[1]; [1]*Zhengzhou/CN*, [2]*Shanghai/CN (qjryq@126.com)*

**Purpose:** To assess the role of DCE-MRI radiomics nomogram in predicting response to neoadjuvant chemotherapy (nCT) in oesophageal cancer (EC) patients.
**Methods and Materials:** This study prospectively enrolled EC patients with stage cT1N+M0 or cT2-4aN0-3M0. All patients received DCE-MRI within 7 days before the beginning of chemotherapy followed by surgery. nCT response assessment was graded from 1 to 5 according to the tumour regression grade (TRG). Patients were stratified into responders (TRG1+2) and non-responders (TRG3+4+5). According to 10-fold cross-validation, the half of this cohort was set as the training cohort, and the other half was set as the validation cohort. The Spearman, Lasso and PCA regression model was exploited for dimension reduction and selection of the feature space. The discriminating performance was assessed with AUC. Univariable association analysis was adopted for testing the correlation of radiomics signature and response, and DCA was used for comparing three models.
**Results:** All included 82 patients underwent surgery and TRG, and 72 tumour radiomics features were acquired from DCE-MRI. Three most significant radiomics features were identified by Spearman and LASSO, which included Ktransmedian, Vemean, Minintensity, 4 components were identified by PCA, and they were selected to create the radiomics signature significantly associated with response. AUC of radiomics signature performance in the training cohort was 0.855 (95%CI: 0.649-1) by LASSO, and in the validation cohort was 0.821 (95%CI: 0.658-0.985). This model showed good discrimination between responders and non-responders.
**Conclusion:** The study showed DCE-MRI radiomics features could potentially predict response to nCT in EC patients.

**B-0611** 10:38
**Diffusion kurtosis imaging evaluation of histological differentiation and Lauren classification in advanced gastric cancer**
J. Fu, L. Tang, Z. Li, Q. Shi, Z. Yan, X. Li, J. Ji; *Beijing/CN (18410259295@163.com)*

**Purpose:** To investigate the value of diffusion kurtosis imaging (DKI) in assessing different histological type and Lauren classification in advanced gastric cancer.
**Methods and Materials:** This study was approved by the local institutional review board. Fifty-two patients with biopsy-proven gastric cancer were enrolled in prospective study before resection. All DKI images (b=0, 200, 500, 800, 1000, 1500, 2000s/mm$^2$) were blindly determined by radiologists, respectively, using software. The region of interest was manually drawn on the specific slice showing the largest area of tumour. Three parameters of DKI were calculated automatically: apparent diffusion coefficient (ADC), kurtosis (K), and diffusivity (D) value. One-way analysis of variance and receiver operating characteristic curves were used for statistical analysis.
**Results:** Kurtosis was significantly higher in diffuse type (0.741±0.035) than in mixed type (0.629±0.025) and intestinal type (0.571±0.015) of Lauren type ($p < 0.05$). D and ADC were significantly lower in diffuse type than in mixed and intestine type (2.129±0.208 vs. 2.758±0.252 vs. 3.033±0.144; 0.974±0.042 vs. 1.167±0.049 vs. 1.235±0.302, respectively, $p<0.05$). The specificity for the assessment of diffuse type was greater using K (92.30%, cutoff value=0.688) compared with other parameters. There were also significant differences between different differentiations for ADC ($p$=0.000), MD ($p$=0.000), MK ($p$=0.007). AUC curve of ADC, K and D values in diagnosing low-grade gastric cancer was 0.77, 0.68 and 0.70, respectively. When the cutoff values of ADC,K, D values were set to 1.148×10$^{-6}$mm$^2$/s, 0.612, 2.769×10$^{-6}$mm$^2$, sensitivity of 77.30%, 68.20%, 77.27% and specificity of 73.30%, 56.70%, 60.00% were achieved, respectively.
**Conclusion:** DKI parameters exhibit potential predicting the gastric cancer aggressiveness before treatment.

**B-0612** 10:46
**The role of intravoxel incoherent motion and diffusion kurtosis imaging in evaluating pathological complete response to neoadjuvant chemoradiotherapy in rectal cancer**
I. Yang, B. Wu; *Chengdu/CN (lqyang95@163.com)*

**Purpose:** To explore the feasibility of intravoxel incoherent motion (IVIM) and diffusion kurtosis imaging (DKI) parameters in assessing pathological complete response (pCR) to neoadjuvant chemoradiotherapy (CRT) in locally advanced rectal cancer (LARC).
**Methods and Materials:** 32 LARC patients who received neoadjuvant CRT and subsequent surgery were enrolled prospectively. All of them had pre- and post-CRT rectal MRI examinations, including IVIM and DKI sequence with 11 b values. Pathological tumour regression grade (TRG) was the reference standard. Slow diffusion coefficient (D), fast diffusion coefficient (D*), perfusion-related diffusion fraction (f) were calculated with IVIM model. Mean diffusion (MD) and mean kurtosis (MK) were calculated with DKI model. ROIs were manually drawn on three slices with largest tumour on b=800 s/mm$^2$ diffusion imaging, and then were copied to other parameter imaging automatically.
**Results:** pCR (n=6) patients had a significantly higher post-CRT f (P=0.003), MD (P=0.017), as well as higher percentage change of f (P=0.012) and D* (P=0.034) value after treatment than non-pCR (n=26). They showed comparable overall diagnostic performance in selecting pCR with AUCs of 0.808, 0.769, 0.827 and 0.795, respectively. The percentage change of f had a sensitivity of 80.8%, specificity of 83.3%, PPV of 95.55 and NPV of 50%, with the cut-off value of 10.1%. Post-CRT f, MD and percentage change of f and D* also had negative correlations with TRG ($r_s$=-0.416, -0.364, -0.442 and -0.399).
**Conclusion:** Post-CRT f and MD value, and percentage change of f and D* could help to assessing complete responders to neoadjuvant CRT in LARC.

**B-0615** 10:54
**Quantitative magnetic resonance imaging can predict tumour subtype, therapy response and patient survival in pancreatic ductal adenocarcinoma**
G. Kaissis[1], S. Ziegelmayer[1], F. Lohöfer[1], W. Weichert[1], J. Siveke[2], E.J. Rummeny[1], R. Braren[1]; [1]*Munich/DE*, [2]*Essen/DE*

**Purpose:** To evaluate the ADC-map histogram-derived quantitative imaging parameter ADC entropy as a biomarker in pancreatic ductal adenocarcinoma (PDAC).
**Methods and Materials:** We retrospectively analysed 39 cases of resected and 21 cases of metastatic PDAC who underwent clinical 1.5T-MRI assessment. Whole tumour volumes were segmented and quantitative imaging parameters were derived using LifeX. Disease-free (DFS) and overall survival (OS) was compared between patients with ADC entropy above vs. below the median of 6.0. Histopathological heterogeneity and immunohistochemical subtype of tumour samples were determined. The effect of gemcitabine treatment on survival was compared between ADC entropy groups.
**Results:** High ADC entropy led to worse median DFS (8.7 vs. 21.6 months) and OS (12.7 vs. 27.5 months) (N=39, p<0.0001) in the surgical patient cohort. Patients in the high ADC entropy group had a 90% increased risk of high morphological tumour heterogeneity (p=0.09) and all patients with a quasi-mesenchymal molecular tumour subtype (n=9) belonged to this group. Patients with high ADC entropy who underwent gemcitabine treatment (N=25) had significantly improved DFS (5.5 to 9.5 months, p=0.04) and OS (5.5 to 14.6, p=0.01) while gemcitabine had no significant effect on DFS or OS in the low ADC entropy group (N=14). All metastatic PDAC cases belonged to the high ADC entropy group. Gemcitabine-based treatment provided a slight DFS benefit to these patients compared to FOLFIRINOX (11.6 vs. 7.6 months, p=0.15).
**Conclusion:** High ADC entropy predicts worse survival, improved gemcitabine-based chemotherapy response and is associated with increased tumour heterogeneity and the quasi-mesenchymal molecular subtype in PDAC.

**B-1663** 11:02
**Clinical impact of lateral lymph nodes at pelvic MRI in patients with rectal cancer**
I. Blazic, S. Patil, T. Konishi, N.M. Campbell, J. Garcia-Aguilar, M.J. Gollub; *New York, NY/US (ivanablazic@yahoo.com)*

**Purpose:** To determine the relationship between baseline lateral lymph nodes (LLN) size measured on pelvic MRI and clinical outcomes in patients with rectal carcinoma.
**Methods and Materials:** In this IRB-approved, HIPPA compliant retrospective study, we identified 245 patients (157 men, 88 women, median age 53 years) who received neoadjuvant treatment for clinical stage II or III rectal cancer and for whom baseline MR scans were available. Patients underwent surgery (n=216) or nonoperative management (n=29). Lateral node dissection was performed selectively in patients with grossly enlarged pelvic lymph nodes. MRI exams were reviewed for LLN size, characteristics and location by two radiologists blinded to patient outcomes. In a patient-level analysis, LLN size, node roundedness (short-/long-axis ratio ≥ 0.8), border irregularity and heterogeneity were correlated with disease-free-survival (DFS) using the Kaplan-Meier method and log-rank test. LLN was analysed at a literature-based cutoff of 5 mm. The importance of posterior external iliac (PEI) nodes was examined using multivariable Cox regression.
**Results:** A total of 845 lateral lymph nodes were evaluated on baseline MRI images (median 6mm; range 3-17mm). DFS was worse among patients with LLN size 5 mm or greater (p=0.027). Rounded and heterogeneous (p=0.003, 0.035), but not irregularly bordered LLN at baseline was associated with worse DFS. No difference in DFS was observed for PEI node size (p=0.514).
**Conclusion:** Lateral lymph nodes of 5mm or greater on baseline MRI in patients with rectal cancer portends worse survival. Round and heterogeneous nodes were also poor prognosticators.

**B-0616** 11:10
**Investigating blood oxygenation level dependent (BOLD) MRI in small renal masses (SRM), and its correlation with dynamic contrast enhanced (DCE) MRI**
N. Bogveradze[1], D. Prezzi[1], R. Neji[1], C. Kelly-Morland[1], H. Verma[1], K. Lashkhi[2], V.J. Goh[1]; [1]*London/UK*, [2]*Tbilisi/GE (bogveradze.nino@gmail.com)*

**Purpose:** To assess for: 1. Differences in BOLD & DCE MRI parameters between histological groups, including benign & malignant SRM. 2. Differences in BOLD & DCE MRI parameters between SRM & normal renal parenchyma. 3. Within-subject correlations between BOLD & DCE MRI parameters.
**Methods and Materials:** Following ethical approval and informed consent, 21 prospective adults with SRM planned for surgery underwent 3T MRI, including BOLD and DCE MRI in addition to anatomical sequences. Parametric maps (T2*, AUGC$_{60}$, K$^{trans}$, k$_{ep}$ & v$_e$) were analysed by two radiologists in consensus. Differences in imaging parameters were assessed by Kruskal-Wallis and Wilcoxon signed-rank tests. Correlations were assessed using Spearman's rank test. Statistical significance was set at 5%.
**Results:** After technical exclusions (n=3), our cohort consisted of 18 patients with 19 SRM: 11 clear-cell carcinomas (ccRCC), 4 oncocytomas, 3 papillary carcinomas (pRCC) and 1 metanephric adenoma. Mean T2* did not differ significantly between histological groups (P=0.20), but showed consistent values in oncocytomas (median, 30.26 ms; range, 29.22-32.73). Conversely, T2* varied substantially among ccRCC (median, 20.44 ms; range, 9.20-53.08 ms) and pRCC (median, 23.85 ms; range, 5.64-26.62 ms). T2* was lower in SRM than in normal renal parenchyma (P = 0.02). Among DCE MRI parameters, v$_e$ differed significantly between groups (P = 0.03). BOLD and DCE MRI parameters did not show significant within-subject correlations.
**Conclusion:** BOLD MRI provides potentially useful information for the diagnosis of renal oncocytoma, in the context of a multiparametric protocol. It captures information that is not directly correlated to DCE MRI.
*Author Disclosures:*
**V.J. Goh:** Research/Grant Support; Siemens Healthcare.

**B-0617** 11:18

**Does perfusion-metabolic tumour phenotype predict for neoadjuvant therapy response in primary oesophageal tumours undergoing DCE-MRI and $^{18}$F-FDG PET/CT?**

S.J. Withey, K. Owczarczyk, C. Yip, M. Siddique, A. Green, J. Bell, G. Cook, V.J. Goh; *London/UK (samuel.withey@nhs.net)*

**Purpose:** Oesophageal cancer has poor survival rates despite curative treatment. We hypothesized that a low perfusion-high metabolism phenotype may be associated with poorer response to neoadjuvant therapy. We aimed to assess the relationship between tumour perfusion and metabolism with therapy response.
**Methods and Materials:** Following ethical approval and informed consent, DCE-MRI and $^{18}$F-FDG PET/CT were performed prior to neoadjuvant therapy in 24 oeosphageal cancers (18 male; mean age 64 ± 10 years; 21 adenocarcinoma, 3 squamous cell carcinoma). DCE-MRI transfer constant ($K^{trans}$) and $^{18}$F-FDG PET/CT maximum standardised uptake value ($SUV_{max}$) were calculated. Pathological response was defined by Mandard tumour regression grade 1-2; non-response by grade 3-5. $K^{trans}$ & $SUV_{max}$ were compared between pathological responders versus non-responders using Wilcoxon rank test. $K^{trans}/SUV_{max}$ was calculated for each patient and the proportion of responders was calculated for each $K^{trans}/SUV_{max}$ quartile using ChiSquare contingency test. Significance was at 5%.
**Results:** There was no difference in $K^{trans}$ & $SUV_{max}$ between pathological responders and non-responders (p=0.8, 1 and 0.6, respectively). There was no significant difference in pathological response rates between different $K^{trans}/SUV_{max}$ quartiles (50%, 0%, 17% and 33%, respectively; p=0.13), however patients below the 25$^{th}$ percentile (low $K^{trans}$/ high $SUV_{max}$) appeared to have a better response than patients >25% (3/5 versus 3/15, p=0.1).
**Conclusion:** No association was noted between a flow-metabolic phenotype and therapy response in patients. We have, however, highlighted significant variability in response in patients with different flow-metabolic phenotypes that will require characterisation in a larger patient cohort.

**B-0618** 11:26

**Imaging texture features to predict neoadjuvant therapy response in locally advanced rectal cancer**

G. Cappello, V. Doronzio, V. Romano, A. Timpani, G. Desi, F. Cortese, V. Giannini, S. Mazzetti, D. Regge; *Candiolo/IT (giovanni.cappello@ircc.it)*

**Purpose:** Patients with locally advanced rectal cancer (LARC) usually undergo neoadjuvant chemoradiotherapy (CRT) or radiotherapy (RT) alone, followed by surgical resection. In 15-30% of them pathological complete response is reached, therefore a wait-and-see approach could be a viable option. Aim of the study was to predict CRT response in LARC patients before treatment, using texture features derived from MRI and PET acquisitions.
**Methods and Materials:** 47 LARC patients with pre-treatment MRI and PET/CT underwent RT (46-55 Gy, 23-30 fractions) or CRT (RT plus 5-FU or Capecitabine) followed by total mesorectal excision. We considered responders (pR+) patients with complete (tumour regression grade, TRG=1) or near complete (TRG=2) regression; non-responders (pR-) patients with moderate (TRG=3) to no regression (TRG=5). Tumours were semi-automatically segmented on T2-w MRI, ADC maps and PET/CT. From each image first-order and 22 second-order parameters derived from Haralick analysis were extracted. Multivariate logistic regression was performed to identify features most correlated with TRG.
**Results:** 26 patients were classified as pR-, 21 as pR+ after total mesorectal excision. Five parameters were included in the multivariate regression (4 from PET and 1 from MRI), with p=0.002. The area under the ROC curve was 0.83, with sensitivity 75% and specificity 76% in detecting pR+.
**Conclusion:** Texture analysis on pre-treatment imaging could be useful in assessing response to neoadjuvant treatment in LARC patients. If results will be confirmed on larger datasets, the oncological pathway for LARC patients could be personalized delaying or advancing surgery, according to the prediction of treatment response.

**B-0619** 11:34

**CT response evaluation of adrenocortical carcinoma (ACC) during and after chemotherapy: which criteria?**

M. Bertuletti, D. Cosentini, S. Cieno, M. Di Terlizzi, S. Grisanti, B. Frittoli, A. Berruti, R. Ambrosini, L. Grazioli; *Brescia/IT (bertuletti.med@gmail.com)*

**Purpose:** The use of cytoreductive surgery in patients with locally advanced/metastatic adrenocortical cancer (ACC), where chemotherapy is the standard approach, is conditioned by CT evaluation of resectability and response assessment. The purpose of our study was to assess the prognostic impact on progression-free survival (PFS) of three different response criteria (RECIST 1.1, CHOI and tumour volume).
**Methods and Materials:** We retrospectively evaluated MDCT scans of 30 patients with pathologically confirmed ACC. Each examination was performed with a MDCT scanner (either Toshiba Aquilion with 160 detectors array or Philips Brilliance with 64 arrays), before and after i.v. automatic injection of iodinated contrast agents (370 mgI/ml@3ml/s - 1.3 ml/Kg body weight), using a bolus tracking technique and image acquisitions during the arterial and venous phases. Two radiologists performed semiautomatic segmentation of every lesion using a dedicated software (Portal intelliSpace, Philips), to measure lesional longer axis (RECIST 1.1 criteria), attenuation changes (CHOI) and volume changes after therapies.
**Results:** 18 patients underwent cytoreductive surgery, with a median PFS of 15.5 months (6.1 months in patients who did not have surgical approach). A statistically significant correlation was observed between CHOI criteria and PFS (p=0.019), whereas disease response assessed by RECIST 1.1 and volume variations failed to be associated with patient outcome.
**Conclusion:** In our early experience, ACC radiologic response evaluation to chemotherapy using CHOI criteria could represent an additional prognostic factor, especially in patients candidated to surgical approach.

**B-0620** 11:42

**CT-response patterns and the role of CT-textural features in inoperable abdominal/retroperitoneal soft tissue sarcomas treated with trabectedin**

M. Esser[1], C. Kloth[2], W. Thaiss[1], C.P. Reinert[1], J. Fritz[3], M. Horger[1];
[1]*Tübingen/DE*, [2]*Ulm/DE*, [3]*Baltimore, MD/US*

**Purpose:** To evaluate CT patterns and textural features of soft tissue sarcomas following trabectedin therapy as well as their suitability for predicting therapeutic response.
**Methods and Materials:** A total of 31 patients (18 female, 13 male; mean age 58.0 years; range 38-79 years) with sarcoma under trabectedin as a third-line therapy between October 2008 and July 2017 underwent baseline and follow-up contrast-enhanced CT. Response evaluation was based on modified CHOI-criteria and RECIST1.1, classified as partial response (PR), stable disease (SD), progressive disease (PD). For CT-texture analysis (CTTA), mean, entropy and uniformity of intensity/skewness/entropy of co-occurrence matrix (COM) and contrast of neighboring-grey-level-dependence-matrix (NGLDM) were calculated.
**Results:** Following CHOI-criteria, 9 patients achieved PR, 10 SD and 12 PD. RECIST1.1. classified patients into 5 PR, 15 SD and 11 PD. A frequent (n=6/31; 19.3%) pattern of response was tumour liquefaction. In responders, differences in entropy of entropy-NGLDM (p=0.028) and uniformity-NGLDM (p=0.021), in non-responders entropy of average (p=0.039), deviation (p=0.04) and uniformity of deviation (p=0.013) occured between baseline and follow-up. Mean intensity and average were higher when liquefaction occurred (p=0.03; p=0.02), whereas mean deviation was lower (p=0.02) at baseline compared to other response patterns. Differences in mean (p=0.023), entropy (p=0.049) and uniformity (p=0.023) of entropy-NGLDM were found between responders and non-responders at follow-up. For the mean of heterogeneity, a cut-off value was calculated for the prediction of response in baseline CTTA (0.12; sensitivity 89%; specificity 77%).
**Conclusion:** A frequent pattern of response to trabectedin was tumour liquefaction being responsible for pseudoprogression; therefore, modified CHOI should be preferred. Single CTTA feature can be used complementarily for the prediction and monitoring response to trabectedin.

**B-0613** 11:50

**Differentiation of atypical non-functional pNET and PDAC using a CT radiomics-based nomogram**

M. He, H. Xue, Z. Jin; *Beijing/CN (377174431@qq.com)*

**Purpose:** To develop and validate an effective model to differentiate NF-pNET from PDAC and present it in a nomogram.
**Methods and Materials:** Between July 2014 and December 2017, 147 patients (80 patients with PDAC and 67 patients with atypical NF-pNET) with pathology results and enhanced CT were consecutively enrolled and chronologically divided into primary and validation cohort. Three models were built to differentiate atypical NF-pNET from PDAC, including radiomics signature alone, clinico-radiological features alone and their integrated model. The diagnosis performance of the three models was estimated and compared with area under the receiver operating characteristic curve (AUC) in the validation cohort. Nomogram was used to present the model with best performance, and its calibration was also assessed.
**Results:** In the validation cohort, the AUC for the differential diagnosis was 0.884 with the integrated model, which was significantly improved compared with the model based on clinico-radiological features (AUC=0.775, p value=0.004), and comparable with the model based on radiomics signature (AUC=0.873, p value=0.512). The nomogram presenting the integrated model achieved good discrimination performances in both primary and validation cohorts with C-index of 0.960 and 0.884, respectively.

**Conclusion:** The integrated model outperformed the model based on the clinico-radiological features alone and comparable to the model based on the radiomics signature alone in the differential diagnosis atypical NF-pNET from PDAC. The nomogram incorporated radiomics signature, clinico-radiological features can serve as an easy-conduct tool for the differential diagnosis between atypical NF-pNET and PDAC.

**10:30 - 12:00** **Sky High Stage**

### Clinical Trials in Radiology

### CT 6

### Clinical Trials in Radiology 2

*Moderators:*
V.J. Goh; London/UK
U. Mahmood; Oak Brook, IL/US

10:30
**Outcome of pneumatosis intestinalis and porto-mesenteric venous gas: a multicentric study**
T.A. Auer[1], R. Kloeckner[2], D. Pinto Dos Santos[3], M. della Seta[1], D. Geisel[1], B. Hamm[1]; [1]*Berlin/DE,* [2]*Mainz/DE,* [3]*Cologne/DE*

**Purpose:** To evaluate outcome and mortality of the radiological finding pneumatosis intestinalis (PI) and porto-mesenteric venous gas (PMVG).
**Ethics committee approval:** Institutional review board approval of this retrospective study was obtained (EA2/238/17)*
**Methods and materials:** In this retrospective multicentre study design, the radiology information systems of three tertiary care university medical centres searched for CT reports associated with PI. A total amount of 322 patients (132 women, 190 men) with PI were identified. Additionally, presence of "benign PI", PMVG, outcome and mortality (90d-Follow-up) were evaluated. "Benign PI" was defined as conservatively treated patients with radiologically confirmed PI who did not die n within the 90d-follow-up.
Results: Overall mortality for patients with PI was 54.1% within 90d after diagnosis while 53.7% of all patients received surgical treatment. For patients with PI in association with PMVG mortality increased to 68.2% (p < 0.001). Ischemia, obstructive bowel dilatation and/or infection were the most frequent causes of PI. Kaplan-Meier-curve showed a shortened survival for patients with PI and PMVG; Log-Rank-test: p < 0.001 (Median survival: PI: 59d (2.8± SD; 95% CI 54.2-65.3) / PI and PMVG: 40d (3.6± SD; 95% CI 33.2-47.2).
**Limitations:** The limitation of this study is the retrospective and unrandomised character.
**Conclusion:** PI is not strictly associated with ischemia and high rates of mortality. In fact, there are several benign causes or favourable harmless conditions PI is frequently associated with; thus, PI is not inherently connected with an infaust prognosis. Nevertheless, the extension seems to matter as mortality increases significantly when PI comes in association with PMVG.
**Funding for this study:** none

10:39
**Discussant:**
D. Weishaupt; *Zurich/CH (dominik.weishaupt@triemli.zuerich.ch)*

10:45
**Diffusion-weighted MRI in the assessment of nephroblastoma: results of a multi-centre trial**
A.M. Hötker[1], Y. Mazaheri[2], A. Lollert[3], J.-P. Schenk[4], C. Düber[3], P. Mildenberger[3], O. Akin[2], N. Graf[5], G. Staatz[3]; [1]*Zurich/CH,* [2]*New York, NY/US,* [3]*Mainz/DE,* [4]*Heidelberg/DE,* [5]*Homburg (Saar)/DE (andreas.hoetker@usz.ch)*

**Purpose:** To assess the value of diffusion-weighted MRI in the pre-therapeutic evaluation of paediatric renal tumours, including the identification of histological subtypes of nephroblastoma with known adverse outcomes.
**Ethics committee approval:** The institutional review board approved this multicentric study and waived the requirement for informed consent.
**Methods and materials:** This retrospective multicentric study included 122 paediatric patients with 130 renal tumours, who underwent MRI including DWI before neoadjuvant chemotherapy and nephrectomy. Two radiologists independently assessed each tumour volumetrically, and apparent diffusion coefficient (ADC) values were calculated on a voxel-wise basis, including parameters derived from histogram and texture analysis.
**Results:** Inter-reader agreement was excellent (ICC: 0.717-0.975). For both readers, patients with locally aggressive tumour growth (SIOP 3 stage) or with metastases (M1) had significantly lower 12.5th-percentile ADC values (p≤0.028) compared to those with lower-stage tumours, and the parameter Energy differed significantly between patients with M1 and those with M0 status (p≤0.028). Contrast and homogeneity differed significantly between benign nephroblastomatosis and malignant nephroblastoma (p≤0.045, both readers). As compared to all other subtypes, the blastemal subtype demonstrated significantly higher skewness (p≤0.022, both readers) and the diffuse anaplastic subtype demonstrated significantly higher 75th-percentile ADC values (p≤0.042, both readers).
**Limitations:** Given the multicentric retrospective approach, technical differences between the MRI examinations could not be accounted for.
**Conclusion:** Diffusion-weighted MRI may be of value in identifying benign nephroblastomatosis and assessing nephroblastoma subtypes. Therefore, it could play an important role in risk stratification for paediatric patients with renal tumours in the future.
**Funding for this study:** Partly funded by Deutsche Krebshilfe 50-2709-Gr2, EU No 600841 (CHIC project) and NIH P30 CA008748.

10:54
**Discussant:**
K. Rosendahl; *Bergen/NO (karen.rosendahl@helse-bergen.no)*

11:00
**Validation of deep learning algorithms for the detection of critical findings in head CT scans**
S. Chilamkurthy[1], R. Ghosh[1], S. Tanamala[1], M. Biviji[2], N. Campeau[3], V.K. Venugopal[4]; [1]*Mumbai/IN,* [2]*Nagpur/IN,* [3]*Rochester, MN/US,* [4]*New Delhi/IN (swetha.tanamala@qure.ai)*

**Purpose:** To validate a set of deep learning algorithms for automated detection of key findings from non¬contrast head-CT scans: intracranial haemorrhage and its subtypes, calvarial fractures, midline shift and mass effect.
**Ethics committee approval:** Exempt because of retrospective analysis.
**Methods and materials:** We retrospectively collected a dataset containing 313,318 head-CT scans of which random subset (Qure25k dataset) was used to validate and rest to develop algorithms. Additional dataset (CQ500 dataset) was collected from different centres to validate algorithms. Patients with post¬operative defect or age<7 were excluded from all datasets. Three independent radiologists read each scan in CQ500 dataset. Original clinical radiology report and consensus of readers were considered as gold standards for Qure25k and CQ500 datasets respectively. Areas under receiver operating characteristics curves(AUCs) were used to evaluate algorithms.
**Results:** After exclusion, Qure25k dataset contained 21,095 scans(mean-age 43;43% female) while CQ500 dataset consisted of 491 (mean-age 48;36% female) scans. On Qure25k dataset, algorithms achieved an AUC of 0.92 for detecting intracranial haemorrhage (0.90-intraparenchymal, 0.96-intraventricular, 0.92-subdural, 0.93-extradural, and 0.90-subarachnoid haemorrhages). On CQ500 dataset, AUC was 0.94 for intracranial haemorrhage (0.95, 0.93, 0.95, 0.97, and 0.96 respectively). AUCs on Qure25k dataset were 0.92 for calvarial fractures, 0.93 for midline shift, and 0.86 for mass effect, while AUCs on CQ500 dataset were 0.96, 0.97 and 0.92 respectively.
**Limitations:** Inclusion of follow-up scans, low concordance between radiologists for fractures in CQ500.
**Conclusion:** This study demonstrates that deep learning algorithms can identify head-CT scan abnormalities requiring urgent attention with high AUCs.
**Funding for this study:** Qure.ai
*Author Disclosures:*
**S. Chilamkurthy:** Employee; Qure.ai. **R. Ghosh:** Employee; Qure.ai.
**S. Tanamala:** Employee; Qure.ai.

11:09
**Discussant:**
M. de Bruijne; *Rotterdam/NL (marleen.debruijne@erasmusmc.nl)*

11:15
**Patient preference for coronary CT angiography and invasive coronary angiography: results from a randomised controlled trial**
G. Abdala Gehling[1], S. Feger[1], M. Rief[1], M. Laule[1], M. Dewey[1], E. Schönenberger[2]; [1]*Berlin/DE,* [2]*Hanover/DE*

**Purpose:** To compare patient preference for coronary CT angiography (CTA) and invasive coronary angiography (ICA).
**Ethics committee approval:** Institutional review board approval was obtained and all patients provided written informed consent.
**Methods and materials:** 329 patients with stable chest pain, suspected coronary artery disease, and a clinical indication for ICA were randomly assigned to either CTA or ICA at a single centre. Patient preference was assessed using a previously validated questionnaire completed after CTA or ICA. Assessment included preparation for the tests, concern, degree of helplessness, pain, overall satisfaction, and preference for future testing.
**Results:** Out of the patients randomised to CT and ICA, 91% (152 of 167 patients) and 81% (140 of 162 patients, P=0.19 NS) completed the assessment. Patients reported that they felt better prepared for CTA (P<0.001), less concerned (P<0.001) and had a lesser degree of helplessness (P<0.001) than during ICA. Subjective pain measurements were significantly lower for

CTA (6.8±2.3) than for ICA (17.1±3.8; P<0.001). Overall satisfaction was in favour of CTA (P<0.001) and the vast majority of patients (79%) would choose CTA over ICA for future decision making and diagnostic management.
**Limitations:** Incomplete response rate and randomised patient preference assessment.
**Conclusion:** This randomised study shows that patient preference is in favour of CTA when compared with ICA indicating the potential of coronary CTA to be more widely used.
**Funding for this study:** The study sponsor, the German Research Foundation, was not involved in any stage of the study design, data acquisition, data analysis, or manuscript preparation.
*Author Disclosures:*
**M. Dewey:** Grant Recipient; German research foundation. Other; Bayer, Bracco, Cardiac MR Academy, European Commission, European Regional Development Fund, German Foundation of Heart Research, German Federal Ministry of Education and Research, GE Healthcare, Guerbet, Springer, and Toshiba.

11:24
**Discussant:**
J.-N. Dacher; *Rouen/FR (jean-nicolas.dacher@chu-rouen.fr)*

11:30
**Perspectives of radiologists and cardiologists about the severity of health events in the multicentre DISCHARGE trial - on behalf of the DISCHARGE investigators**
V. Wieske, M. Dewey; *Berlin/DE*

**Purpose:** To evaluate physicians' perspectives about the severity of different health events in the context of trial endpoints within the European DISCHARGE trial.
**Ethics committee approval:** Local amendment of the ethics approval for this study was added for the survey if needed due to local standards.
**Methods and materials:** We used the DISCHARGE trial network to evaluate perspectives of physicians from different disciplines on the severity of major adverse cardiovascular events. A questionnaire for identification of doctors at all partner sites was followed by designing a questionnaire about health events severity (scale from 1 to 7). Scheduled physicians were chosen based on the number of available cardiologists and radiologists.
**Results:** A total of 422 physicians from 14 sites were included in this analysis (218 cardiologists, 174 radiologists, 30 other specialists); 63.4 % were male, 36.6 % female doctors. Distribution of severity ranking for cardiovascular death and death of any cause was equal between cardiologists and radiologists. Large discrepancies were observed for hospitalisation for unstable angina which radiologists rating them significantly more severe than cardiologists.
**Limitations:** Only a sub-group of sites (15 of 26) participated in this analysis.
**Conclusion:** Our analysis showed the presence of differences between different medical specialists when rating different health events. This topic is important for patient care and future clinical trial design and thus we will compare the results with the patients perspective as a next step to identify potential differences between the groups.
**Funding for this study:** FP7 Program of the European Commission.
*Author Disclosures:*
**V. Wieske:** Research/Grant Support; FP7 Program of the European Commission, Randomised multi-centre DISCHARGE trial (603266-2, HEALTH-2012.2.4.-2). **M. Dewey:** Research/Grant Support; German Federal Ministry of Education and Research (The terms of these arrangements are managed by the legal department of Charité – Universitätsmedizin Berlin. Other authors declare no competing interests.), Berlin Institute of Health, Institutional master research agreements exist with Siemens Medical Solutions, Philips Medical Systems, and Toshiba Medical Systems, FP7 Program of the European Commission, Randomised multi-centre DISCHARGE trial (603266-2, HEALTH-2012.2.4.-2), European Regional Development Fund, (20072013 2/05, 20072013 2/48). Other; German Heart Foundation/German Foundation of Heart Research, (F/23/08, F/27/10), DFG and BMBF, Joint Program from the German Research Foundation (DFG) and the German Federal Ministry of Education and Research (BMBF) for meta-analyses (01KG1013, 01KG1110, 01KG1210), Hands-on worskhops on cardiovascular imaging, www.herz-kurs.de. Speaker, Toshiba Medical Systems, Guerbet, Cardiac MR Academy Berlin, and Bayer (Schering-Berlex). Author; Springer: Editor of Cardiac CT (Book, 2nd edition).

11:39
**Discussant:**
M. Hrabak Paar; *Zagreb/HR (maja.hrabak.paar@mef.hr)*

11:45
**A multi-stage randomised clinical trial to reduce variation in computed tomography radiation dose**
Y. Wang, P. Chu, S. Yu, R. Smith-Bindman; *San Francisco, CA/US*

**Purpose:** Radiation doses for computed tomography examinations are variable and often higher than necessary for diagnosis, and an RCT was conducted to help sites lower and standardise doses.
**Ethics committee approval:** IRB approved and no informed consent since data received was de-identified after scan was performed.
**Methods and materials:** We created a registry of 1.2 million abdomen, chest, head, and combined chest-abdomen (CAP). CT adult exams collected 11/2015-10/2017 from 101 hospitals. We administered two interventions through stepped-wedge design: simple audit, ranking hospital performance and suggesting improvements, and multicomponent intervention, combining another audit with an 8-week course. To assess dose variance, we fit – for effective dose and CTDIvol – a complex variance mixed linear regression model with the background time and intervention stage as fixed effects of interest and constituents of variance structure. Patient characteristics are added as confounding fixed effects and the machine as a random effect.
**Results:** The variance of effective dose decreased over time for all anatomic areas, though attributability to the intervention varies. Respectively, the multicomponent intervention contributed 6%, 10%, 12%, and 35% dose variance decrease to abdomen, chest, head, and CAP scans, in addition to background time trends. The intervention impact on CTDIvol variance is comparatively modest, with most decrease attributable to background trends.
**Limitations:** All institutions had one type of dose-monitoring software.
**Conclusion:** The dichotomy between strong impact on effective dose variance and comparatively weak impact on CTDIvol variance mirrors the study of impact on dose mean, further insinuating that many dose reductions were achieved by reducing multi-phase scanning.
**Funding for this study:** Funded by PCORI & NIH.

11:54
**Discussant:**
A. Trianni; *Udine/IT (annalisa.trianni@asuiud.sanita.fvg.it)*

**10:30 - 12:00** **Tech Gate Auditorium**

### Genitourinary

### SS 607

### Contrast agents for imaging: not only iodine

*Moderators:*
N.N.
Y. Mytsyk; Lviv/UA

**K-13** 10:30
**Keynote lecture**
F. Stacul; *Trieste/IT*

**B-0621** 10:39
**Macrocyclic or linear MR contrast agent: are there any differences in detection of local recurrence of prostate cancer after radical prostatectomy?**
F. Kossov, B. Olimov, P. Bulychkin, S. Tkachev, G. Akhverdieva, V. Panov, I. Tyurin; *Moscow/RU (fil-doc@yandex.ru)*

**Purpose:** Salvage radiation therapy (SRT) is a basic treatment approach in patients with prostate cancer recurrence (PCaR) after radical prostatectomy (RP). Routine diagnostic techniques (THUS, CT, morphological MRI) have a low accuracy in PCaR detection. Multiparametric MRI (mpMRI) significantly improved the detection of clinical PCaR. The aim is to analyse what is the dominant sequence of mpMRI for PCaR detection in patients after RP? Is the efficiency of the dynamic contrast enhancement (DCE) with different MR contrast agents (MRCA) (macrocycle 1.0 mmol/ml and linear 0.5 mmol/ml) equal?
**Methods and Materials:** 62 men (52-71 years old, median - 64) with biochemical recurrence after RP suspected for PCaR were examined by mpMRI: 1) 33 patients received macrocycle MRCA (gadobutrol, 1,0 mmol/ml); 2) 29 patients received linear MRCA (gadoversetamide and gadodiamide, 0.5 mmol/ml). The response for SRT was declared positive in cases where progressive decrease of PSA was evaluated.

**Results:** DCE was the dominant sequence of mpMRI for PCaR detection.Tumour tissue sizes in patients with clinical PCaR had statistically significant correlation with PSA level (R=0.74; p<0.0008). Macrocycle MRCA was significantly more effective in the detection of small (<8mm) clinical PCaR (p<0,018). Efficacy of PCaR detection (tumour tissue size >8mm) after 1.0 and 0.5 mmol/ml MRCA injection was equal.
**Conclusion:** Macrocycle MRCA with concentration 1.0 mmol/ml is more effective in the detection of more smaller clinical PCaR (<8mm) in comparison with linear MRCA. DCE is the dominant sequence of mpMRI in the detection of PCaR.

**B-0622** 10:47
**Contrast-enhanced ultrasonography (CEUS) surveillance of native kidneys in renal transplant patients: a monocentric experience**
E. Bertelli, S. Agostini, S. Verna, S. Lucarini, E. Savi, L. Mercatelli, P. Carta, S. Serni, V. Miele; *Florence/IT (elena.bertelli3@gmail.com)*

**Purpose:** Renal transplant recipients (RTR) are at higher risk of renal cell carcinoma (RCC) in native kidneys, especially if they are affected by acquired cystic kidney disease (ACKD), so a routine screening is recommended. To decrease the use of contrast-enhanced CT (CECT) and thus avoid the risk of contrast-induced nephropathy in RTR with ACKD, we prospectively studied the usefulness of CEUS in characterizing renal lesions.
**Methods and Materials:** 212 consecutive RTRs underwent routine post-transplant US. ACKD was diagnosed in 68. The patients with questionable US result were enrolled in our study. Native kidneys were studied by CEUS and, in instances of positive CEUS, by CECT. Follow-up (FU) was ≥ 3 years.
**Results:** US identified 38 suspicious or questionable lesions. According to the Bosniak scheme, CEUS classified these lesions as 19 BI, 4 BII, 6 BIIF, 4 BIII and 5 solid. The BIII and solid lesions were further studied by CECT, which confirmed CEUS findings in all the solid lesions. In the 4 BIII, CECT confirmed 2 malignancies, and 2 were degraded to BIIF. No changes were observed in FU lesions. The statistical analysis has shown no difference between the two series. No patient suffered adverse reactions. CEUS decreased by 76% the use of CECT. The limits of our study are the sample size and perhaps the short FU.
**Conclusion:** CEUS is safe and it is a useful tool in screening for the selection of patients who need to be further evaluated by CECT.

**B-0624** 11:03
**Iodine in common contrast-enhanced CT protocols does not affect renal functionin a high-risk population with chronic kidney disease**
M. Koci, A. Graber-Naidich, X.S. Cheng, D. Fleischmann, M.J. Willemink; *Stanford, CA/US (mkoci@stanford.edu)*

**Purpose:** Administration of iodinated contrast is considered a risk factor for post-contrast acute kidney injury (PC-AKI) in patients with chronic kidney disease (CKD). We sought to evaluate the effect of clinically used doses of iodinated contrast administration on the kidney function of high-risk patients.
**Methods and Materials:** We retrospectively identified 362 patients who underwent a contrast-enhanced CT examination of the chest, abdomen, or lower extremities between January 2017 and August 2018, with estimated glomerular filtration rate (eGFR) 30-60 ml/min/1.73m2 in the year before the CT. Contrast volumes (CV), serum creatinine (sCr) before and after CT, were available (at least 14 days before and 48-72 hours after CT). The primary endpoint was PC-AKI, defined as >50% or >0.3 mg/dL increase in sCr after CT. Individual laboratory-defined levels for low, normal, and high sCr values were used to evaluate sCr category changes.
**Results:** Median age (interquartiles) was 66.9 (58.0-75.9) years. Patients with eGFR 30-45 and eGFR 45-60 mL/min/1.73 m2 had similar PC-AKI incidence (10.8% versus 7.7%, respectively; p=0.34) and contrast volume (95 [80-121] ml versus 96 [85-123] ml, respectively; p=0.88). Pre-CT sCr was high in 38.4% (139/361). We observed sCr increase from normal to high in 8.6% (31/362), and sCr decrease from high to normal in 10.8% (40/362). In 8.6% (31/362) of patients sCr increased by 0.3 mg/dL.
**Conclusion:** Our study showed that even in a high-risk population of patients with a substantially reduced kidney function (eGFR<45), the incidence of PC-AKI after contrast-enhanced CT was low and comparable to patients with eGFR>45.

**B-0625** 11:11
**Contrast-enhanced ultrasound (CEUS) efficiency in renal graft complication evaluation**
C.G. García Roch[1], F. García García[1], F.X. Aragon Tejada[2], J. Ciampi Dopazo[1], M. Muñoz Cepeda[1], M. Bernabéu Rodríguez[1], R. Giovanetti González[3], A. Roca[1], P.A. Baron Rodiz[2]; [1] *Toledo/ES*, [2] *Madrid/ES*, [3] *Córdoba/ES (carmen.roch@gmail.com)*

**Purpose:** To evaluate contrast-enhanced ultrasound (CEUS) efficiency in the diagnosis of renal graft parenchymal and vascular complications.
**Methods and Materials:** Retrospective, observational, descriptive study that includes all adult patients transplanted in our centre from January 2011 to January 2015. Patients underwent a first baseline US (B mode and colour and pulsed Doppler US) examination within 24-72 hours post-surgery. In cases with abnormal findings, 2nd generation intravenous US contrast (Sonovue®) was administered as protocolled, to obtain qualitative evaluation of the parenchymal graft and vascular contrast uptake. No complications were observed in any examinations, all of which were performed by the same experienced radiologist.
**Results:** CEUS was performed in 27 patients (out of 131). 6 grafts presented pathological findings in baseline US but CEUS showed no significant alterations. These cases corresponded to exclusively parenchymal involvement: acute rejection in one patient, with histological confirmation, and 5 cases of acute tubular necrosis. A total of 25 complications were depicted in the remaining 21 patients, with infarction, artery stenosis and necrosis being the most frequent. Complementary imaging studies included 10 CT scans and 3 arteriographies, none of which provided additional information to CEUS.
**Conclusion:** Qualitative assessment of the parenchymal uptake of US contrast was insufficient for the diagnosis of ATN and rejection since CEUS in these patients was normal despite presenting anomalies in baseline US. On the contrary, CEUS offers rapid and reliable diagnosis of renal infarction, necrosis, and vascular stenosis or thrombosis with precise detail of location and extension.

**B-0626** 11:19
**Comparison of computed tomography (CT) and contrast-enhanced ultrasound (CEUS) for the management of complex renal cysts: a single-centre experience**
G. Piccoli; *Conegliano/IT (piccoli.gianluca@gmail.com)*

**Purpose:** To compare CT and CEUS Bosniak classification for the assessment of cystic renal masses and to gauge the correspondence between CEUS Bosniak classification and clinical/histological outcome.
**Methods and Materials:** Between March 2017 and February 2018, 35 patients underwent CT and CEUS; 37 complex renal cysts were evaluated.
**Results:** Bosniak score after CEUS was downgraded in 7 patients (6 to Bosniak I); in 18 it was maintained; in 10 upgraded. Eight complex renal cysts underwent surgery: 2 were a simple renal cyst and an oncocytoma (Bosniak IIF and IV after CT, III and IV after CEUS); 6 were malignant: 1 multilocular cystic renal neoplasm of low malignant potential, 1 papillary renal cell carcinoma (RCC) type 1 and 4 clear cell RCC (2 Bosniak IIF, 3 Bosniak III, 1 Bosniak IV after CT; after CEUS 1 Bosniak IIF, 1 Bosniak III, 4 Bosniak IV). Three complex renal cysts were upgraded from Bosniak III to Bosniak IV, all were RCC. Two Bosniak IV cysts were confirmed after CEUS: an oncocytoma and an RCC. Two patients with Bosniak 4 cysts are waiting for surgery. No side effects were recorded after CT or CEUS.
**Conclusion:** CEUS is a useful alternative to CT scan for the assessment of complex renal cysts, in particular, when iodinated CT contrast is contraindicated. CEUS can help to better define Bosniak score and to select patients candidates to end the follow-up or to surveillance. Furthermore, CEUS can reduce the ionizing radiation exposure and the CT scanners workload.
*Author Disclosures:*
**G. Piccoli:** Author; Eugenio Gioulis, Massimo Verdecchia, Marta Veronese, Lorenzo Angelini, Renata Napoli, Francesca Di Cristofano, Federico Petrucci, Salvatore Valerio, Michelangelo Salemi.

**B-0627** 11:27
**Using contrast-enhanced ultrasound to assess diagnostically challenging and indeterminate lesions in native and transplant kidneys**
P. Lee, A. Mehdi, A. Alsafi, C. Harvey, A.K.P. Lim; *London/UK (philippa.lee08@gmail.com)*

**Purpose:** Ultrasound is often the initial modality in detection and assessment of renal lesions which are then commonly characterised further with CT/MR. The use of ultrasound contrast agents (UCAs) allows immediate accurate characterisation without the associated delay, anxiety, cost and potential exposure to nephrotoxic agents which CT/MR entails. This study aims to analyse the role of CEUS in the assessment of diagnostically challenging and indeterminate renal lesions and evaluate if CEUS adds diagnostic value to other imaging modalities.
**Methods and Materials:** We performed retrospective analysis of patients at a tertiary transplant and renal centre in London between 2004 and 2018. 262 patients who underwent CEUS for renal lesions were identified on PACS and their prior and subsequent imaging following CEUS was reviewed and analysed. The role of CEUS diagnostic value was then evaluated and categorised as “diagnosis provided”, “confirmed suspected diagnosis”, “added information only” or “no added value”.
**Results:** CEUS added value compared to the initial US in 82% of cases. 37% of the patients had an initial CT and/or MR. In this group, CEUS added value in 75%. Diagnoses in this group included complex Bosniak cysts, differentiation of cystic and solid lesions, pseudotumours, complicated pyelonephritis/

abscesses, infarction/ischaemia and characterisation of benign/malignant lesions.
**Conclusion:** CEUS adds additional diagnostic value in assessment of focal renal lesions and perfusion abnormalities compared to US and CT/MR. CEUS rivals CT/MR in the characterization of renal lesions and in some cases surpasses them because of real-time imaging and greater spatial and temporal resolution of the microcirculation.

**B-0628** 11:35
**Characterisation of CT-indeterminate renal masses by contrast-enhanced ultrasound and acoustic radiation forced impulse imaging**
S. Kaufmann, W. Thaiss, M. Horger, K. Nikolaou, J. Bedke; *Tübingen/DE*

**Purpose:** To prospectively characterise CT-indeterminate renal masses (CTIRM) using acoustic radiation force impulse (ARFI) elastography and contrast-enhanced ultrasound (CEUS) and to correlate quantitative imaging findings with histopathology or interim follow-up (FU).
**Methods and Materials:** 123 patients with CTIRM (longest diameter <4cm) underwent ARFI and CEUS with CT-image fusion (IF). Exclusion criteria included all contraindications for CEUS and IF. Shear wave velocity (SWV), shear wave ratio (SWR), peak intensity (PE), time to peak (TTP) and wash-in rate (Wi) were quantified. In case of a cystic lesion classified as ≤ Bosniak 2F, follow-up imaging was performed.
**Results:** 77 out of 123 patients underwent surgical resection of a lesion due to suspect imaging findings, whereas 46 patients underwent FU, which did not show upgrading in Bosniak category. Histopathology revealed 58 renal cell carcinomas (5 chromophobe (chRCC), 18 papillary (pRCC) and 35 clear cell (ccRCC)), 10 oncocytomas and 9 non-malignant renal lesions (1 minimal fat AML, 3 focal nephritis and 5 infected cysts). SWV and SWR differed significantly between ccRCC, pRCC, chRCC (p=0.0024, F=13.94) and in SWR also for oncocytoma (p<0.0001, F=14.35). In CEUS, oncocytoma and ccRCC showed significant higher PE values (p<0.0001, F=77.31) as well as higher Wi and lower TTP compared to all other solid lesions.
**Conclusion:** Quantitative CEUS and ARFI imaging can provide relevant information to further characterise CT-indeterminate renal masses to guide urological decision-making and offers the possibility of differentiation between ccRCC from less malignant RCC subtypes and from oncocytoma.

**B-0629** 11:43
**Correspondence analysis between different reference lines used in magnetic resonance defecography of pelvic organs prolapse or the measurement of prolapse of pelvic organs with POPQ**
S. Yévenes Aravena, G. Cárdenas Loguercio, J. Casas Muñoz, F. Araya Campos, C. Garrido Inostroza, V. Manríquez Galán, R. Guzmán Rojas, M. Naser Nassar, D. Castro Caperán; *Santiago/CL (syevenes@gmail.com)*

**Purpose:** The aim of this study was to evaluate which of the reference lines: pubococcygeal (PCL), mid-pubic (MPL) or H (HL), commonly used in Magnetic Resonance Defecography (MRD) for evaluation of pelvic floor dysfunction, has better correspondence with the Pelvic Organ Prolapse Quantification system (POPQ).
**Methods and Materials:** Observational, cross-sectional study. POPQ stage and demographic data was obtained of fifty-six patients in which MRDs was performed. The MRDs were reviewed by four expert radiologist and prolapse was measured using PCL, MPL and HL. A bivariate correspondence analysis and quantification of the inertia of the two dimensions under study was performed between each line and the POPQ.
**Results:** The mean age was 45.2±11.1 years. The analysis of the anterior compartment only obtained an adequate correspondence between PCL and HL on POPQ stages I-II. For the middle compartment there is a slight correspondence between PCL and POPQ stages 0-II, there is also correspondence between the MPL and POPQ stages 0-I, with greater correspondence in patients without prolapse. For the posterior compartment no correspondence was obtained for any of the lines.
**Conclusion:** There is no adequate correspondence between MRD reference lines and POPQ when evaluating the pelvic floor as a whole. When analyzed by compartments, there is better correspondence with PCL and HL in the anterior compartment, and for PCL and MPL in the middle. There is no correspondence for the posterior. These results make us propose that the analysis of MRDs should be done separately, using specific lines for each compartment.

**B-0630** 11:51
**Outcome in 73 patients with benign prostatic hyperplasia treated by an ultrasound guided transperineal laser ablation by interventional radiologists**
G. Patelli[1], F. Besana[1], E. Faietti[1], G. Ori Belometti[1], C. Costa[1], A. D'Alessio[2], G. Mauri[3], C.M. Pacella[4]; [1]*Seriate/IT*, [2]*Zingonia/IT*, [3]*Milan/IT*, [4]*Rome/IT (patellig@yahoo.it)*

**Purpose:** To report results of ultrasound-guided (US-g) Transperineal Laser Ablation (TPLA) in patients with Benign Prostatic Hyperplasia (BPH).
**Methods and Materials:** seventy-three patients (mean age 72.6±10.4years, range 58-93) with obstructive syndrome secondary to BPH, were treated by TPLA using continuous wave (CW) diode laser source at 1064nm (Echolaser SoracteLite, Elesta s.r.l., Calenzano (FI), Italy). Under transrectal US-g, one or two 21G introducer needles for each lobe, according to the prostate volume, were inserted with transperineal approach. Each ablation was performed at 3W power for 1800J energy. A pull back was done case by case according to the prostate volume. Major and minor complications were recorded. The efficacy was evaluated on the changes of means of International Prostate Symptoms Score (IPSS), Quality of Life(QoL), post-void residual (PVR), prostate volume and the ejaculatory function. The mean hospital stay was measured.
**Results:** At a mean follow-up time of 16 months (range 3-45), IPSS improved from 22.9 to 9.6 (*P*<0.001), QoL from 4.5 to 1.2 (*P*<0.001), PVR from 162.3 ml to 64 ml (P<0.001) and prostate volume from 73.47 ml to 50.8 ml (P<0.001). Of 34 patient evaluated for ejaculatory function, 32 (94%) maintained a satisfactory ejaculation. No major complications occurred. The mean operation time was 42.3±8.7min, ablation time 15.6±3.4min, energy deployed 11,540.0±3,680.5J, hospital stay 1.7±0.4days.
**Conclusion:** TPLA is a mini invasive treatment for BPH. This therapeutic approach was safe, efficacious and with a satisfactory outcome.

**10:30 - 12:00** **The Church**

### Head and Neck

### SS 608

### Lymph nodes and parathyroids

*Moderators:*
N.N.
L.M. Lenghel; Cluj-Napoca/RO

**B-0631** 10:30
**Evaluation of acoustic radiation force impulse imaging in the diagnosis of malignant cervical lymphadenopathy**
R. Kamat[1], A. Ramesh[2], K. Nagarajan[2]; [1]*Chandigarh/IN*, [2]*Puducherry/IN (rohanskmt@gmail.com)*

**Purpose:** B-mode ultrasound though useful in the assessment of cervical nodes cannot measure the tissue elasticity. Elastography helps in non-invasive assessment of tissue stiffness. As malignant nodes are generally stiffer than benign nodes, this technique might be beneficial in differentiating them. Acoustic radiation force impulse (ARFI) imaging used in this study is a technique based on shear wave elastography. ARFI could be valuable in the evaluation of cervical lymphadenopathy as a result of its high specificity. Our aims were 1) to assess diagnostic power of ARFI in differentiating benign and malignant cervical nodes and to compare it with B-mode imaging; 2) to compare the shear wave velocities (SWV) between benign and malignant nodes.
**Methods and Materials:** A total of 166 cervical nodes from 166 patients were examined using B-mode, Doppler and ARFI, between October 2015 and April 2017. SWV, virtual touch imaging (VTI) and area ratio (AR) were calculated for each node and the results were compared with FNAC/biopsy.
**Results:** Median SWV of benign and malignant nodes were 1.9 (1.56-2.55) m/s and 6.7 (2.87-9.1) m/s, respectively. A SWV >2.68 m/s helped in identifying a malignant node with 81% specificity, 81.6% sensitivity and 81.3% accuracy. AR and predominantly dark elastograms were useful in identifying malignant nodes. ARFI was found to be inaccurate in tuberculous and lymphomatous nodes.
**Conclusion:** Malignant nodes had significantly higher SWV, AR and darker elastograms compared to benign nodes. SWV had the highest accuracy amongst all the parameters. ARFI imaging accurately identifies the malignant nodes, hence guiding the FNAC/biopsy.

**B-0632** 10:38
**SUV-based lymph node selection for fine-needle aspiration guided by real-time fused ultrasound and FDG PET in head and neck cancer patients**
P. De Koekkoek-Doll, W. Vogel, M.W.M. van den Brekel, L. Smit, R.G.H. Beets-Tan, M. Maas; *Amsterdam/NL (petra.dkd@gmail.com)*

**Purpose:** FDG PET-CT can detect lymph node metastases in head and neck cancer with high sensitivity and ultrasound (US)-guided fine-needle aspiration (FNA) can provide histological proof with high specificity. We assume that real-time fused US+FDG PET-CT for FNA (Fused-FNA) will provide accurate nodal staging. Aim was to determine the optimal $SUV_{max}$ to select nodes for Fused-FNA.
**Methods and Materials:** 128 patients (female 30%, male 70%, age mean 63 and range 32-88) were prospectively included. FDG PET-CT was acquired according to clinical diagnostic protocols. Live US was fused with PET-CT and Fused-FNA was performed using the electromagnetic navigation system Percunav on a Philips EPIQ US machine. FNA took place on nodes with visually increased metabolic activity. The reference standard was cytology from FNA performed on all suspect nodes on US. ROC curves were constructed and area under the curve (AUC) was used to assess diagnostic performance.
**Results:** FDG PET detected a total of 269 suspect nodes in 117/128 (91%) of patients. Live fusion failed in 6 (3%) of these nodes, and Fused-FNA cytology results were inconclusive in 16 (6%) of nodes. In the remaining 247 (94%) FDG-positive nodes 135/247 (55%) Fused-FNA yielded malignant cytology. AUC was 0.887 (95% CI 0,845-0,929). The optimal cut-off for $SUV_{max}$ was established at 4.87, yielding a sensitivity of 90% and specificity of 71%.
**Conclusion:** Selecting nodes with $SUV_{max} \geq 5$ on FDG PET-CT for real-time fusion-guided US-FNA yields a high accuracy for the detection of malignant nodes.

**B-0633** 10:46
**Correlation between preoperative 4D CT parathyroid volumes and perioperative PTH levels**
A. Yates, P. Cromwell, N.M. Hughes, S. Skehan, D. Evoy, E. McDermott, R. Prichard; *Dublin/IE (ayates@tcd.ie)*

**Purpose:** The aim of this study is to examine if there is a correlation between estimated 4D CT volume of a parathyroid gland with perioperative parathyroid PTH level and serum ionised calcium (iCa) levels in patients with a solitary parathyroid adenoma. The secondary aim was to compare the estimated CT parathyroid gland volume against the post operative volume.
**Methods and Materials:** Data was collected from a prospectively maintained database of adult patients who were diagnosed with a solitary PT adenoma and had a pre-operative 4D CT who subsequently underwent a parathyroidectomy over a 5 year period (2012-2017).
**Results:** 58 patients were included in the study. The mean age was 59.84 years. There were 16 men (27.6%) and 42 women (72.4%). The median estimated parathyroid gland CT volume was 0.265cm3 (IQR: 0.150-0.522cm3) while the median post op volume was 0.724cm3 (IQR: 0.399-1.47cm3). The median drop in PTH was 97.9 (IQR: 63.7-158.1ng/L)The correlation between CT volume and PTH drop was 0.474 (P<0.001) using the Spearman's Coefficient.
**Conclusion:** Pre-operative parathyroid weight, as estimated by CT, positively correlates with the post-operative reduction in PTH. Estimating volume using 4D CT imaging is a useful tool in the preoperative workup of parathyroid adenomas.

**B-0634** 10:54
**Evaluation of sensitivity and specificity of elastography for differentiating benign from malignant cervical lymphadenopathy and comparing with B-mode and colour Doppler findings**
K. Mittal[1], V. Bansal[2]; [1]*Delhi/IN*, [2]*New Delhi/IN (kapishmittal1989@gmail.com)*

**Purpose:** To evaluate the sensitivity and specificity of elastography for differentiating benign from malignant cervical lymphadenopathy and to compare its findings with B-mode and colour Doppler findings.
**Methods and Materials:** The present study was conducted on 50 patients with cervical lymphadenopathy. This selection was based on size of the lymph node and other B-mode sonography characteristics. B-mode and colour Doppler findings were recorded. In addition to this, strain elastography was done and five elastography patterns were defined: high elasticity (soft): pattern I represented no or very small hard area while spectrum pattern V presented where almost whole of lymph node showed hard pattern on strain elastography. Additionally, a cine loop of elastography was acquired for calculation of strain ratio using adjacent muscle as reference. A cut-off value of less than two (<2) of strain ratio was used to define benign lymph nodes and more than or equal to two (≥2) was used to define malignant lymph nodes. Final diagnosis of benign versus malignant lymph node was based upon other relevant investigations like FNAC/biopsy findings and/or clinical follow-up after three months.
**Results:** Of all the B-mode parameters, only lymph node border and hilum showed more than 70% accuracy for correctly differentiating benign from malignant lymph nodes while colour Doppler showed 82% accuracy for the same. Elastography pattern and strain ratio achieved 90% and 94% accuracy for correctly differentiating benign from malignant lymph nodes.
**Conclusion:** Elastography pattern and strain ratio are useful in addition to B-mode and colour Doppler ultrasound for the evaluation of cervical lymph nodes.

**B-0635** 11:02
**Diagnostic accuracy of image-guided core needle biopsy of lymphoma suspected patients**
M. Kazemi, M. Mikalani, H. Sharifian, B. Moradi; *Tehran/IR (Ma-kazemi@sina.tums.ac.ir)*

**Purpose:** In this study we aimed to evaluate the efficacy of image-guided core needle biopsy (CNB) for the diagnosis of lymphoma.
**Methods and Materials:** This cross-sectional study during 2016-2018, included 46 consecutive patients with suspicious imaging and clinical findings of lymphoma. They went under image-guided CNB by 16 gauge core biopsy needle. At least 3 cores were obtained from each patient. All the patients were followed at least for 1 year.
**Results:** Pathologic results showed 3 main groups: 1-lymphoma with specific type (35%), 2-lymphoproliferative or lymphocytic and histiocytic infiltration (22%), 3-other pathologies (as other malignancies, infection, reactive) (43%). All the group 2 patients went under surgical excisional biopsy (SEB) that 40% of them showed lymphoma. So in patients with lymphoma 80% were correctly diagnosed by CNB and treated directly. Another remaining 20% were categorized under group 2 and diagnosed by SEB. No patient with lymphoma was missed.
**Conclusion:** Image-guided CNB in patients with lymphadenopathy suspicious for lymphoma, is an effective and safe method as an initial diagnostic tool.

**B-0636** 11:10
**Arterial spin labelling (ASL) MRI to assess nodal metastases in head and neck cancer**
R. Balaji; *Chennai/IN (ravikanthbalaji@gmail.com)*

**Purpose:** To evaluate the feasibility of predicting nodal metastases in head and neck cancers using arterial spin labelling (ASL).
**Methods and Materials:** Twenty-eight patients with various types of locally advanced head and neck cancers (buccal mucosa 14, tongue 10, maxillary sinus 4)were evaluated using ASL on 1.5 T MR scanner (Achieva, Philips Healthcare, Best, The Netherlands) in addition to T2, diffusion and contrast sequences. The ASL maps were fused with fat suppressed T2 weighted images and compared with post contrast T1 weighted images. The primary tumour and suspected nodes were identified on T2 and contrast images and Regions of Interest (ROI) were marked. Necrosis within the nodes that had bright signal on T2WI were avoided. All patients underwent concurrent chemo-radiotherapy after histological analysis.
**Results:** Tumour blood flow (TBF) within suspected nodes showed significant elevation and was comparable with values within the primary mass. The mean TBF in patients within involved nodes from buccal mucosa cancers was 110+/-15.8 mL/100g/min, nodes from primary tongue malignancies 124+/- 22.4 mL/100g/min and nodes from primary malignancies of the maxillary sinus 117 +/- 18.8mL/100g/min.
**Conclusion:** Quantitative assessment of TBF in cervical lymph nodes from primary head and neck cancers is possible with ASL. ASL may be useful for noninvasive assessment of tumour viability in head and neck cancer avoiding use of contrast agents and reducing the danger of allergy and nephrogenic systemic fibrosis (NSF). Fusion of T2WI-FS images would improve morphological description of tumour as ASL images are usually of low spatial resolution.

**B-0637** 11:18
**Role of shearwave elastography in assessment of cervical lymph nodes**
S.K. Suganchand Rikabchand, R. Mohammed Kashif; *Jodhpur/IN (Sunilmed@gmail.com)*

**Purpose:** The purpose of our study was to evaluate diagnostic accuracy of Virtual Touch Imaging Quantification (VTQ) shearwave elastography in differentiation of benign and malignant cervical lymph nodes with pathological results as reference.
**Methods and Materials:** 148 patients with 231 enlarged cervical lymph nodes were examined by elastography and B-mode ultrasonography in our study which was followed up by ultrasound guided core needle biopsy for pathological correlation. The results were compared and analyzed by appropriate statistical test to check the validity of elastography separately and in combination with b-mode characteristics in determining the nature of the lesion.

**Results:** Of 231 lymph nodes 161 were benign and 70 were malignant. The mean shear wave velocity (swv) of the benign nodes (2.5 ± 0.72 m/s) was lower than mean swv of malignant nodes (4.3 ± 1.2 m/s). With a swv cut-off value <3.12 m/s for benign lesion the sensitivity and specificity were 85% and 82% respectively, but when used in combination with b-mode characteristics the sensitivity and specificity increases to 92% and 96% respectively. Swv values >3.12m/s showed 89% sensitivity and 84% specificity in determining malignant nodes and when used in combination with b-mode characteristics the sensitivity and specificity increases to 98% and 92% respectively.
**Conclusion:** VTQ is an excellent tool for analysis for cervical lymph nodes. There is clear difference in swv values between benign and malignant nodes. Thus when values are used in combination with B-mode characteristics of the cervical nodes they can provide results as good as histopathological findings.

**B-0638** 11:26
**Diagnostic performance of computer-aided quantification of intranodal vascularity in differentiating common neck lymph node diseases**
M.T.C. Ying, S.C.H. Cheng, A.T. Ahuja; *Hong Kong/CN (htmying@polyu.edu.hk)*

**Purpose:** Ultrasound is a common imaging modality for the assessment of neck lymph nodes. This study aimed to investigate the value of intranodal vascularity, as evaluated by computer-aided quantification method, in distinguishing common neck node diseases.
**Methods and Materials:** Power Doppler sonograms of 347 patients with palpable neck lymph nodes were acquired (155 metastases, 125 reactive, 44 tuberculous lymphadenitis, 23 lymphoma). Doppler ultrasound images were evaluated using our self-developed computer algorithm and the intranodal vascularity was quantified and expressed as vascularity index (VI). The diagnostic performance of using VI of lymph node in differentiating the four disease groups (metastases, reactive, tuberculous lymphadenitis and lymphoma) was evaluated and compared. The optimum cut-off of VI in distinguishing different groups of lymph nodes was determined using the receiver operating characteristic (ROC) curve.
**Results:** Metastatic and lymphomatous lymph nodes are more vascular and have higher VI than tuberculous and reactive nodes. Among the comparisons of the four lymph node groups, intranodal VI demonstrated the highest diagnostic accuracy in distinguishing metastatic and tuberculous nodes with a sensitivity of 80%, specificity of 73% and area under the ROC curve of 0.82 when using the optimum cut-off VI of 22%.
**Conclusion:** Computer-aided quantification provides objective assessment of intranodal vascularity. Intranodal VI is a useful parameter in distinguishing metastatic and tuberculous lymph nodes, but has limited value in differentiating lymphomatous nodes from metastatic and reactive nodes.

**B-0639** 11:34
**The utility of F18-choline PET/CT for the localisation of parathyroid adenomata in patients with primary hyperparathyroidism (PHP) with negative or equivocal conventional imaging**
R. Ramaesh, G. Browning, S. Wakelin, E. Ward, F. Gibbs, D. Patel; *Edinburgh/UK (rishi.ramaesh@gmail.com)*

**Purpose:** This retrospective study was performed to assess the accuracy of F18-choline PET/CT in localising parathyroid adenomata in patients with PHP with negative or equivocal ultrasound (US), Tc-99m sestamibi, and 4D CT imaging.
**Methods and Materials:** Over a 15-month period, F18-choline PET/CT was performed in 83 patients with negative or equivocal conventional imaging. Of the 29 patients who had undergone surgery; surgical notes, pathology reports and post operative outcomes were reviewed and correlated with imaging and pathological findings.
**Results:** Of the 83 patients, 59 had a positive PET/CT result. Of these, 37 were not detected on ultrasound and 9 were equivocal. Tc-99m sestamibi scan did not locate 47 and was equivocal in 8. SPECT did not localise 47 with 4 equivocal studies. 4D-CT failed to detect 39 of these, with 2 equivocal studies. Of the 29 surgical cases, PET/CT correctly localised the site of the adenoma in 26 patients. There were 2 false-positive cases and one false negative, which was correctly identified on sestamibi imaging. The sensitivity of PET/CT was 96.2%, with a precision of 93%. US detected 7 (24%), Tc-99m sestamibi 4 (14%) and 4D CT detected 6 (17%) cases that were apparent at surgery.
**Conclusion:** In our cohort, F18-choline PET/CT has been shown to identify a significant number of parathyroid adenomata in patients with PHP with negative or equivocal US,Tc-99m or 4D CT and should be performed for preoperative localisation in those patients being considered for surgery with negative or conventional imaging.

**B-0640** 11:42
**Role of multiparametric 4D-MRI in detection of parathyroid lesions in primary hyperparathyroidism**
N. Murugan, D. Kandasamy, R. Sharma, A. Goyal, A.K. Gupta, N. Tandon, S. Vara Taranikanti, S. Agarwal, N. Gupta; *New Delhi/IN (narasiman1149@gmail.com)*

**Purpose:** 4D-CT is the modality of choice for detection of parathyroid lesions but suffers from high radiation dose. This study evaluated the role of multiparametric 4D-MRI in the localization of parathyroid lesions in primary hyperparathyroidism (PHPT).
**Methods and Materials:** In this ethically approved study, 59 patients of suspected PHPT were recruited. 56 patients underwent 4D-MRI on a 1.5T scanner and 3 were excluded because of altered renal parameters. 48 patients underwent surgery and surgical findings and histopathology were used as the reference standard. The MR technique included fat-suppressed T2WI, DWI with three B values (0,400,800) and post contrast T1 dynamic sequences (7 phases)
**Results:** Among 62 histopathologically proven lesions in 48 patients, there were 41 (89.4%) parathyroid adenomas, 20 (32.2%) parathyroid hyperplasias, and 1 (1.6%) colloid nodule. 4D-MRI detected all parathyroid lesions except 2 which were found only at surgery and another lesion which was diagnosed as parathyroid adenoma on imaging but was found to be a colloid nodule. On Fat-saturated T2WI, 54 (91.5%) lesions were homogeneously hyperintense, 4 (6.7%) were heterogeneously hyperintense and 1 (1.3%) was isointense. On high-b value DWI, 56 (94.9%) lesions were hyperintense and 3 (5.08%) were isointense. On dynamic imaging 54/59 lesions showed type-3 enhancement curve, 5/59 showed type-2 curve. Combining the above sequences the accuracy was 95.16%
**Conclusion:** Multiparametric 4D-MRI had high accuracy for localization of parathyroid lesions. 4D-MRI may provide a robust and safer alternative to 4D-CT.

**B-0641** 11:50
**Role of ultrasound elastography in differentiating tuberculosis and malignant lymph nodes**
A.Y.M. Yee[1], R. Norlisah[2], L.S. Chng[3]; [1]*Sungai Buloh/MY*, [2]*Kuala Lumpur/MY*, [3]*Selayang/MY (yeeba1@hotmail.com)*

**Purpose:** To assess the diagnostic accuracy of ultrasound elastography and greyscale features in differentiating TB and malignant lymph nodes.
**Methods and Materials:** 54 patients (7 malignant, 39 tuberculosis, 9 reactive) were examined by both elastography and B mode sonography in this cross sectional study. Elastographic patterns were determined according to the total area and appearance of the hard regions within the lymph node, grade 1 being soft and grade 6 being a hard area occupying the entire lymph node with focal cystic area within.
**Results:** Sensitivity, specificity, positive predictive value and negative predictive value of elastography of 2 and below as well as a strain ratio of 3.475 to diagnose tuberculosis (TB) were 92.3%, 71.4%, 94.7% and 57.1%. It also found that greyscale features of thin echogenic layer and strong internal echoes were only present in TB lymph nodes and not the reactive or malignant lymph node groups.
**Conclusion:** This study shows that the accuracy of ultrasound elastography to differentiate TB from malignant lymph nodes is higher than usual B mode parameters. This can expedite treatment and reduce the need for unnecessary biopsies.

**14:00 - 15:30** **Room B**

### Radiographers

### SS 714a

### Paediatrics and radiation therapy

*Moderators:*
N.N.
E. Farrugia Wismayer; Floriana/MT

**B-0642** 14:00
**Actual dose measurements of assistant during paediatric x-ray examination with a precisely calibrated optically stimulated luminescence dosimeter**
T. Asahara[1], H. Hayashi[2], E. Tomita[2], Y. Mihara[1], S. Goto[2], Y. Kanazawa[1], N. Kimoto[2], T. Asakawa[2], T. Okazaki[3]; [1]*Tokushima/JP*, [2]*Kanazawa/JP*, [3]*Ibaraki/JP (takashi.asahara.111@gmail.com)*

**Purpose:** During paediatric x-ray examination, a patient often needs to be held in position by assistants. It is important to reduce exposure dose to assistant. Currently, their exposure doses are not often monitored by radiation dosimeter at an examination. The aim of this study is to measure the actual dose during clinical examinations.
**Methods and Materials:** The doses to assistants were measured with small-type optically stimulated luminescence dosimeters; the dosimeters were precisely calibrated so as to enable low dose measurement. First, to determine the dosimetric point, a phantom experiment was carried out; 170 dosimeters were attached to the surface of human dummies. Then doses to both hands of assistants during actual paediatric x-ray examinations were measured in 70 cases for 1-35-month-old patients. The typical irradiation conditions were 53 kV and 3.2 mAs. We analysed the relationship between individual dose to assistants' hands and age of patient.
**Results:** The surface dose distributions suggested that dose to hands shows the highest value; therefore, proper dosimetric position was determined to be both hands. From the result of actual dosimetry, the mean doses to right and left hands were 2.06 +/- 0.17 µGy and 2.15 +/- 0.20 µGy, respectively. We found interesting trend that higher doses were observed for lower age patients. We considered that distance from irradiation area contributed to the dose.
**Conclusion:** The actual doses to assistants were measured. To reduce assistants' dose, attention should be paid to the distance between irradiation area and the assistants' hands.
*Author Disclosures:*
**T. Asahara:** Employee; Okayama University Hospital, Japan. **H. Hayashi:** Employee; Kanazawa University, Japan. Grant Recipient; Nagase Landauer, Ltd., Japan, Job Cor., Japan. **Y. Mihara:** Employee; Shimane University Hospital, Japan. **Y. Kanazawa:** Employee; Tokushima University, Japan. **T. Okazaki:** Employee; Nagase Landauer, Ltd., Japan. Research/Grant Support; Kanazawa University, Japan.

**B-0643** 14:08
**Frontiers of periosteal changes: comparison between anthropological data, clinical records and readings of X-ray medical imaging technology**
R.M.C.R. Gaspar[1], A.F. Abrantes[2], V. Matos[1]; [1]*Coimbra/PT*, [2]*Faro/PT (rosa.cristina.ramos@gmail.com)*

**Purpose:** To define protocols of study in radiology for palaeopathology; to improve palaeopathological diagnosis criteria; to identify other pathologies defined so far as "periostitis"; to evaluate the best protocol for the collection on periosteal changes; to develop a data system that gathers and articulates the anthropological evaluations and imagiological descriptions of the collections.
**Methods and Materials:** Our analysis focuses on comparing skeletal findings of the collections in the Research Centre for Anthropology and Health database with a diagnosis of periostitis and the data in the hospital archives of Coimbra Hospital and University that include individuals who have performed a diagnostic bone biopsy and with a positive imaging record of periostitis. Anthropology analysis: observation in ideal conditions. Proposal to classify periosteum changes by Osteoware. Imagiology: systematic analysis by conventional radiology and a complementary study by CT scanner of the lesional area.
**Results:** Expected results are as follows: the determination of specific protocols and guidelines for anthropology will contribute to the differential diagnosis in palaeopathology. The integration of analytical assessments will enrich anthropology, specifically palaeopathology, and will form the basis for future studies. Furthermore, complementing the analysis of this collection with radiology will support the differential diagnosis, rewriting the history of periostitis in Portugal.
**Conclusion:** This study, which desires to be innovative in the Department of Life Sciences, aims to contribute to the body of knowledge of palaeopathology, complementing the field of systematic imaging analysis and thus allowing to correlate findings between anthropology and imaging.
*Author Disclosures:*
**A.F. Abrantes:** Author; Vitor Matos.

**B-0644** 14:16
**Radiation dose associated with patients undergoing upper limb radiographic examination in different patient orientation: a phantom study**
M.M. Abuzaid, W. Elshami, Z. Noorajan; *Sharjah/AE (mabdelfatah@sharjah.ac.ae)*

**Purpose:** The patient orientation during radiography is essential to perform accurate images and apply ALARA principle. There is a common a practice observed by directing the radiation beam towards the patient eye lens and gonads. It was approved that these organs can be affected by relatively low radiation level. The purpose of this study was to investigate the association between the radiation dose received by eye lens and gonads and the patient orientation during upper limb examinations.
**Methods and Materials:** An adult phantom was used to simulate an adult patient and the patient positions during a hand, wrist, and elbow radiographic examination. The initial position was set as the patient facing away from the radiation beam, then placed in a position facing the radiation beam. A Philips X-ray machine was used to generate a series of images, and TLD was used to obtain radiation dose to the eye and gonads.
**Results:** Reduction of the radiation dose was seen when the patient was not facing the radiation beam as well as reduction of the ESD to the eye and gonads area. Results showed significant reduction of ESD and effective dose when the patient is not facing the radiation beam.
**Conclusion:** The orientation does not affect the patient comfort as the patient responses will not change whether the patient is facing or sitting beside the table. This study confirmed the relation between dose reduction and patient orientation and it align with the ALARA principle to able all efforts to minimize unnecessary radiation exposure.

**B-0645** 14:24
**Radiation dose comparison study between conventional methods and slot-scanning system (EOS® system) on paediatric patients with scoliosis**
H. Laitinen, E. Saukko, E. Svedström, O. Pajulo, H. Niiniviita; *Turku/FI (helena_laitinen@hotmail.com)*

**Purpose:** EOS® system is a slot-scanning system (SSS) for orthopaedic use, especially for observing scoliosis. The purpose of this study was to compare radiation doses of paediatric patients in the imaging of scoliosis with computed radiography (CR), digital radiography (DR) and Finland's first, recently installed SSS technique in Turku University Hospital.
**Methods and Materials:** The retrospective study was performed with real patient data (n=244) aged 1-18 years with maximum bodyweight of 100 kg including posterior-anterior or anterior-posterior (PA/AP) and lateral (LAT) views. The data was analyzed with key statistics and presented by average DAP (Dose Area Product) of the whole population and classified to weight groups.
**Results:** The average DAP of SSS (209.2±123.8 mGycm²) was 42 % lower compared with CR (360.9±433.6 mGycm²) and 53 % lower compared with DR (449.3±471.7 mGycm²). When classified to weight groups the DAP of SSS was 47-63 % lower compared with CR and 0-65 % lower compared with DR. The DAP of SSS for 45 kg patient was 81-86 % lower compared with literature.
**Conclusion:** SSS technique exposes to lower radiation doses and should therefore be the preferred method over CR and DR in the imaging of paediatric patients with scoliosis. Radiation doses using SSS in this study were distinctly lower compared to literature. This study indicates that there is a need to set an international diagnostic reference level for scoliosis imaging, as reported dose values vary substantially.

**B-0646** 14:32
**Improvement of safety and image quality with a self-made airway management device for sedation and anaesthesia in paediatric heart CT**
H. Kim, J. Chang, D.-K. Seo; *Seoul/KR (dangkukong@naver.com)*

**Purpose:** The purpose of this study was to improve the young patients' safety and CT image quality by making airway management device for paediatric CT under sedation or general anaesthesia.
**Methods and Materials:** We hollowed out the upper part of solidified polystyrene board into a quadrangle to bend young patients' head back and straighten their upper body. The quadrangle groove was filled with a headrest, which adjusted the height according to the patient's head size. Patient safety during sedation was monitored by anaesthesiologists. The reproducibility of follow-up cardiac CT images was assessed qualitatively by radiologists.
**Results:** Oxygen saturation was raised and maintained by adequate airway positioning with the device. Sedation-related risk of drop in $SpO_2$ was decreased. The frequency of additional treatment due to desaturation was also decreased. It was also easier to position the centre of the small patients with the iso-centre of the scanner. Straight supine posture by the device increased the reproducibility of follow-up heart CT imaging. Shorter scan time improved overall satisfaction of patients, guardians, and radiotechnologists.
**Conclusion:** When performing paediatric CT examinations, it often took 4-5 times longer when they require procedural sedation or general anaesthesia. In these cases, airway management to maintain oxygen saturation is important. Using self-made airway management device, we could improve the patient safety and ensure high image quality, while reducing the amount of sedatives and the examination time required.

**B-0647** 14:40
**Dose optimisation of head CT examinations in paediatrics**
M. Dziedataja; *Riga/LV (madara.dziedataja@gmail.com)*

**Purpose:** The head is the most commonly examined body part for children in all age groups. During the last 5 years, the number of performed head examinations has increased. It is important to be careful when scanning settings to reduce the possible radiation dose as much as possible, without decreasing the quality of the examination.
**Methods and Materials:** A 64 slice Philips Ingenuity scanner was used to scan a head sized cylindrical PMMA dose phantom and a head phantom made from a beavers' skull and gelatin for the purpose of this research. Each phantom was scanned with a different protocol parameter setting of all pre-set age groups to see the difference in image quality, absorbed dose and how much they change by altering the used settings.
**Results:** Gathered results show a gap between the CT calculated radiation dose and the measured absorbed dose as well as the absorbed dose after using AAPM parameters specifically for 64 slice Philips Ingenuity CT scanner. To evaluate image quality changes during the series of scans performed, we replicated the scan of a PMMA phantom on a beaver skull (16cm/diameter).
**Conclusion:** Identical scanning settings cannot be used for everyone due to the large variety in head diameter (12-20 cm), volume and density in all age groups. All age-group protocols are calibrated by the same 16cm/diameter PMMA phantom. Inappropriate protocol settings can lead to poor quality images, that aren't interpretable by radiologists. The bone structures give an insight into the difference made by changing scan settings.

**B-0648** 14:48
**Evaluation of dose to risk organ during brachytherapy for cervical cancer using a novel passive rectum dosimeter**
E. Tomita[1], H. Hayashi[1], T. Asahara[2], Y. Shitakubo[3], K. Sakuragawa[3], H. Ikushima[3], Y. Kanazawa[3], T. Okazaki[4], T. Hashizume[4]; [1]*Kanazawa/JP*, [2]*Okayama/JP*, [3]*Tokushima/JP*, [4]*Tsukuba/JP (emitomita.15@gmail.com)*

**Purpose:** This study aims to confirm the availability of planned dose by measuring the actual dose of rectum during brachytherapy for cervical cancer.
**Methods and Materials:** We measured doses absorbed by patients' rectum (n=18) using a novel dosimeter made from a piece of OSL sheet and a catheter. To obtain three-dimensional images, cone beam CT (CBCT) examination was performed and image-based treatment planning based on TG-43 algorithm was applied. Measured dose was compared with the planned dose calculated by the therapeutic planning system. Then we investigated the correlation of differences between measured and planned doses, treatment planning times and body mass index (BMI).
**Results:** Measured doses were in good agreement with planned dose in most cases. We experienced a rare situation in which the patient urinated after CBCT was taken (n=2). In this situation, the patient's organs will move due to shrinking volume of a bladder. Even if the original therapeutic plan was applied, the difference between actual and planned doses was found to be at most +/-1 Gy. Furthermore, we found that differences in doses do not have a correlation with treatment planning times (r=0.28), but it has a strong positive correlation with BMI (r=0.84). This fact means that the higher the BMI is, the further it is from the assumption of the TG-43 algorithm that assumes the structure of the human body as a water-equivalent medium.
**Conclusion:** Precise dosimetry provided good evidence for dosimetry in brachytherapy. We have evaluated robustness of the planned dose by measuring actual dose.
*Author Disclosures:*
**H. Hayashi:** Employee; Kanazawa University, Japan. Grant Recipient; Nagase Landauer, Ltd., Japan, Job, Cor., Japan. **T. Asahara:** Employee; Okayama University Hospital, Japan. **Y. Shitakubo:** Employee; Tokushima University Hospital, Japan. **K. Sakuragawa:** Employee; Tokushima University Hospital, Japan. **H. Ikushima:** Employee; Tokushima University, Japan. **Y. Kanazawa:** Employee; Tokushima University, Japan. **T. Okazaki:** Employee; Nagase Landauer, Ltd., Japan. Research/Grant Support; Kanazawa University, Japan. **T. Hashizume:** Employee; Nagase Landauer, Ltd., Japan.

**B-0649** 14:56
**Assessing dignity of patients undergoing radiotherapy for pelvic cancer**
M. Calleja[1], M.E. Grech[1], N. Courtier[2], J.G. Couto[1]; [1]*Msida/MT*, [2]*Cardiff/UK (marco.calleja.13@um.edu.mt)*

**Purpose:** Patients undergoing radiotherapy experience physical side effects, but also suffer emotionally, psychologically and socially. All four domains can adversely affect their dignity. The aim of this study was to assess dignity of patients receiving radiotherapy for pelvic malignancies, by identifying the patients' major and minor concerns and comparing any differences in the responses between gender and age groups.
**Methods and Materials:** The study adopted a non-experimental, quantitative and prospective research design. The patient dignity inventory tool (Chochinov et al., 2012) was distributed to 35 patients. This contained 25 statements related to dignity to be rated between 1 ("not a problem") and 5 ("An overwhelming problem"). The target population consisted of adult patients who were in their last two weeks of radiotherapy to the pelvis.
**Results:** The rating of certain domains of patient dignity were statistically higher than others (p=0.001): patients identified 'psychological issues' as the most problematic and 'general support during treatment' the least. Females rated three of the statements related to 'psychological issues' significantly higher than males (p = 0.031, 0.033 and 0.046). Age groups had no significant effect for all of the statements.
**Conclusion:** Patient dignity issues arise mostly from the psychological effects of radiotherapy. In view of this, it is recommended that a psychological assessment of patients is undertaken to identify individuals who require additional psychological support, especially female patients in view of the higher rating in the 'psychological issues domain'. However, these gender differences can be due to resistance by male patients to report psychological issues.

**B-0650** 15:04
**Glioblastoma: 3D-CRT or IMRT/VMAT?**
M.J.V. Pereira, B. Gregório, A. Cavaco, J.P. Figueiredo; *Coimbra/PT (mjvpereira@hotmail.com)*

**Purpose:** The aim was to compare the dosimetric plans with 3D-CRT and VMAT or 3D-CRT and IMRT in patients with GBM submitted to Stupp Protocol.
**Methods and Materials:** The patients selected were submitted to Stupp Protocol and treated with 3D-CRT. 11 patients were planned in IMRT and the other 11 in VMAT, conformed to the same target volume. The aim was to achieve the best coverage with the least organs at risk dose. The evaluation was made by homogeneity, conformity and organ at risk doses.
**Results:** Both special techniques showed a better conformity and homogeneity compared to 3D-CRT. The doses to optical structures were higher in VMAT, except to the right optical nerve and chiasm. The doses to optical structures in IMRT were lower, except to the chiasm. Both special techniques showed a higher dose in 80% of cerebral volume.
**Conclusion:** Better homogenity and conformity in VMAT and IMRT, keeping the organs at risk doses lower or slightly higher than in 3D-CRT, complying with the internationally defined tolerance doses.

**B-0651** 15:12
**Thematic analysis of the competencies of the therapy radiographer practising in the linear accelerator**
J.G. Couto[1], P. McClure[2], S. McFadden[2], P. Bezzina[1], C. Hughes[2]; [1]*Msida/MT*, [2]*Newtownabbey/UK (jose.g.couto@um.edu.mt)*

**Purpose:** In view of the movement of radiography professionals in Europe, radiographers should be trained and competent to practise in any country. The literature identifies various competencies for the radiographers practising on the linear accelerator. The aim of this study was to perform a literature review to identify the competencies required by therapy radiographers across Europe. A thematic analysis of these competencies was subsequently performed.
**Methods and Materials:** A systematic approach was used, where six databases were searched using selected keywords related to "competencies", "radiographer" and "linac". Following the application of inclusion criteria and snowballing, publications were identified, competencies of linear accelerator radiographers were extracted, and a thematic analysis was performed using NVivo (v11.0).
**Results:** 28 publications were analysed and 363 competencies were identified. Thematic analysis resulted in 16 themes and 72 sub-themes that reflects the competencies of therapy radiographers practising on the linear accelerator. Themes identified included specific competencies for the therapy radiographer (e.g. image verification and treatment delivery) and generic competencies which can be performed by other professionals (e.g. patient care and research).
**Conclusion:** This study summarises the key competencies that are documented and required of these professionals across European to safeguard the movement of radiography professionals and subsequent patient safety between countries, these professionals should develop competencies that are standardised across Europe. These themes can provide all European stakeholders with a template of the components that should be included in curriculum design of radiographers' education.

**B-0652** 15:20
**Expectations of radiography students regarding the new undergraduate course in medical imaging and radiotherapy**
L. Ribeiro[1], A.F. Abrantes[1], S. Rodrigues[1], J.P. Arrais[2], R.P. Almeida[1], K.B. Azevedo[1], O. Lesyuk[3]; [1]*Faro/PT*, [2]*Vila Nova de São Bento/PT*, [3]*São Brás de Alportel/PT (oksanalesyuk@gmail.com)*

**Purpose:** To evaluate the expectations of undergraduate radiography students about the creation of the new undergraduate course in medical imaging and radiotherapy (MIR).
**Methods and Materials:** This study was carried out using a self-applied questionnaire to evaluate the students perceptions regarding the skills enhancement in the undergraduate course in medical imaging and radiotherapy. The final sample was composed by a total of 83 radiography students from two different universities. The questions were directed to the sociodemographic data, reasons for choosing the MIR course, the enhancement of skills and the importance of the training process. The data were interpreted and analyzed through descriptive statistics, and correlation tests were performed to evaluate the relationship between variables.

**Results:** The main motivations to choose the degree was "Healthcare related area, enabling greater interaction with patients" (38,8%). Most of students are not satisfied (53,0%) with the new MIR degree and 44,6 % reported that expectations after the frequency in the course slightly coincide with the beginning. Regarding the contribution of the university, 82% of the students answered that the greatest influence to the acquisition of competences is the "Teaching Methodology".
**Conclusion:** The students of the first-year degree have high expectations concerning the new degree and the students who are in transition phase have lower expectations due to less expertise and knowledge in the three different areas (Radiology, Nuclear Medicine and Radiotherapy). In the students perspective, the new undergraduate course will reduce the students professional capacity and competences after their graduation.

**14:00 - 15:30** **Room C**

### Radiographers

### SS 714b

### Radiation dose optimisation in CT and projection radiography

*Moderators:*
L. Bonomo; Rome/IT.
L.J.O.C. Lança; Lisbon/PT

**B-0653** 14:00
**Impact of decentring patients for abdominal CT scan**
S. Moerup[1], H. Precht[2], S. Foley[3], A. Seegert[4], B.R. Mussmann[1]; [1]*Odense/DK*, [2]*Middelfart/DK*, [3]*Dublin/IE*, [4]*Kolding/DK (sdmo@ucl.dk)*

**Purpose:** To evaluate the effect on skin dose and image quality when decentring in the y- and x-directions for abdominal CT scans.
**Methods and Materials:** Patient centring data in CT were collected retrospectively from dose management software (DoseWatch, GE Healthcare) from a single university hospital (n=4 scanners) in 2017. Deviations from the isocentre were categorized for both vertical (±1.5cm, ±3.0cm, ±4.5cm) and horizontal (±1.5cm, ±3cm) planes on 498 patients. An anthropomorphic phantom (PBU-60) was subsequently scanned using each deviation on two scanners (Toshiba Prime, GE HD750) to allow dose and image quality (noise and visual grading assessment) comparisons.
**Results:** Significant skin dose increases (4.3mSv, 50%) were detected anteriorly for both scanners when centred above the isocentre and reductions (2.0mSv, 24%) when scanned below the isocentre. For the HD750, dose decreased up to 8.3mSv in four locations at the -4.5 position. Increased dose were (2.0mSv) noted on the right side with horizontal deviation. With upward vertical deviation noise increased from 13HU (isocentre) to 15HU. No change in standard deviation (SD) was detected with the phantom below isocentre. With left horizontal deviation SD increased up to 14HU while SD decreased to 12HU dextro-laterally. VGA score was lowest at the largest vertical isocentre deviation.
**Conclusion:** Positioning above isocentre may increase noise and skin dose. Similarly, horizontal deviation may increase dose. Subjective image quality is negatively affected by extreme vertical isocentre deviation.

**B-0654** 14:08
**Exploring academic and social factors' effect on CT dose optimisation: a longitudinal study of radiographers from student to first post**
V.T. Major[1], S. Ryan[2], D. O'Leary[3]; [1]*Northwood/UK*, [2]*Hatfield/UK*, [3]*Keele/UK (vicki.major@stricklandscanner.org.uk)*

**Purpose:** We aimed to identify training requirements for UK CT radiographers, and whether social/academic factors in the clinical environment have a bearing on the students' approach toward CT dose optimisation, before and after qualification. The purpose of this study was to a) explore student radiographer's knowledge and experience of dose optimisation within CT scanning; b) measure their emotional intelligence; and c) explore their educational experience.
**Methods and Materials:** A mixed methods approach using convergent parallel methods, integrating and connecting quantitative and qualitative data were acquired simultaneously on two separate occasions. Student radiographers were invited to participate in in-depth interviews at two discrete time points: as final year students and post-employment in their first post. Ethics approval was sought and granted. An emotional intelligence (EI) questionnaire (TEIQue-SF tool) and a pre-validated questionnaire on CT parameters and their influence on both patient dose and image quality were completed and scored.
**Results:** Results were analysed via descriptive statistics and thematic analysis. Six main educational themes were identified: on-the-job training, postgraduate courses, applications training, undergraduate courses, study days and competency assessment. EI did not change over time. Knowledge of CT exposure parameters increased, with a difference in mean scores pre- and post-qualification ($P$= 0.025).
**Conclusion:** In the two-year period from student radiographer to working in their first post, EI remained static although their knowledge of CT dose optimisation increased. The radiographers felt that education was best delivered on the job and supplemented with study days, application specialist talks, medical physics, radiographer and radiologist expert advice.

**B-0655** 14:16
**Is the technology for patient placement in CT really that useful in reducing dose and improving SNR?**
L. Lennon[1], S. Tsui[2], G. Dick[3], J. Hislop-Jambrich[4]; [1]*North Ryde/AU*, [2]*Box Hill/AU*, [3]*Maroondah/AU*, [4]*Notting Hill/AU (LLennon@medical.canon)*

**Purpose:** Contemporary CT scanners employ various technologies to support the minimization of patient dose. Many of these technologies are applied in the background with little or no knowledge of being activated by the operator. Centring of the patient within the bore of the CT however represents an obvious input by the operator, but how much does it impact dose and SNR really? This project aimed to assess the practical utility of the lateral couch centering function on radiation dose and signal to noise ratio (SNR).
**Methods and Materials:** We scanned an anthropomorphic phantom using offset values (lateral and antero-posterior) established from the evaluation of chest and abdominal series performed at 6 sites not employing centering technology. We then assessed the CTDIVOL, DLP and SNR in the liver and mediastinum using a standard clinical scanning protocol. The chest and abdomen were investigated separately using automatic exposure settings.
**Results:** CTDIVOL reduces considerably more with a lowered couch height and lateral scout performed first than a higher position within the gantry. Lateral maladjustment serves to exacerbate the dose differential from the standard isocentre findings, though the actual spread of these values across lateral malalignments was larger for a higher couch gantry position for the CT unit tested. SNR results were in line with the dose delivered.
**Conclusion:** Accurate isocentre positioning is a simple yet highly effective means of achieving dose and image quality optimization.
*Author Disclosures:*
**L. Lennon:** Employee; Canon Medical. **J. Hislop-Jambrich:** Employee; Canon Medical.

**B-0656** 14:24
**The unjustified dose behind cropped radiographs**
J. Guðjónsdóttir, G.A. Hannesdóttir; *Reykjavik/IS (joninag@hi.is)*

**Purpose:** In this study an attempt was made to evaluate how common image cropping, or electronic collimation, is in digital radiography, how large an area of the images is cropped and how high the radiation dose is that corresponds to the cropped area.
**Methods and Materials:** A sample of images was taken from three medical imaging departments. The images were reviewed and, if cropped, the extent was recorded.
**Results:** A total of 1270 images was reviewed. 10,6 % of them had been cropped, 19 %, 7 % and 6 % in sites A, B and C, respectively. 26 % of all chest images were cropped as well as 18 %, 13 %, 10 %, 10 %, 3 % and 2 % of lumbar spine, shoulder, hip, knee, hand and foot images, respectively. The proportion of cropped images was significantly different between sites and between examinations ($p < 0,05$). Considering only the cropped images, the average cropped fraction of each image was from 16,0 % to 36,3 % and the corresponding unjustified dose was estimated to be from 19,0 % to 56,9 % of the dose actually needed for the final image. Averaging the cropped area over all images in the same type of examination showed that up to 4,6 % of the dose in the examinations in the study was unjustified.
**Conclusion:** This study confirms that radiographs are cropped and that the corresponding unjustified dose may be significant. This needs to be considered in the optimization of radiographic imaging procedures.

**B-0657** 14:32
**Scoliosis dose comparison using current clinical protocols**
M. Shanahan[1], M. Geso[2], A. Potter[2], G. Lennie[2]; [1]*Bruce/AU*, [2]*Melbourne/AU (madeleine.shanahan@canberra.edu.au)*

**Purpose:** Radiography is a valuable diagnostic and monitoring tool for patients with adolescent scoliosis. Radiation dose is important as this patient group is statistically more likely to develop cancer during their lifetime. The purpose of this research was to compare entrance surface dose (ESD) and dose area product (DAP) measurements for scoliosis imaging using current clinical techniques for four image acquisition systems: EOS™-normal (EOS-N), EOS™-microdose (EOS-M), DR-standard (DR-S) and DR-low (DR-L) dose.

**Methods and Materials:** EOS™ and Shimadzu RADspeed Auto X-ray coupled with Canon CXDI 70C wireless flat panel detector systems were used. PA and left lateral images were acquired of a RSD anthropomorphic phantom using four image acquisition techniques. Dosimeter measurements were obtained using Deschênes et al. (2010) 13 anatomic locations. Three independent image acquisitions were made for each technique and the resultant ESDs for each location, and DAP measurements were averaged to minimise random error.
**Results:** ESD measurements for EOS-M, EOS-N, DR-L, DR-N ranged from 0-0.09, 0.02-0.57, 0.01-2.48, 0.01-4.54 mGy, respectively, with the highest dose measured on right lateral aspect T10 level. Lower doses were recorded on the breasts (right: 0.01, 0.09, 0.15, 0.29; left: 0.00, 0.04, 0.05, 0.11 mGy). DAP showed similar dose trend across imaging systems (109, 767, 1206, 2380 mGycm$^2$).
**Conclusion:** Direct dosimetry (ESD) allows radiation dose to specific anatomic regions to be measured. DAP provides a general indication of dose. Reviewing ESD for clinical protocols allows identification of potential further dose reduction strategies.
*Author Disclosures:*
**M. Shanahan:** Grant Recipient; Victorian Medical Radiation Practitioners Education Trust. **M. Geso:** Grant Recipient; Victorian Medical Radiation Practitioners Education Trust. **A. Potter:** Grant Recipient; Victorian Medical Radiation Practitioners Education Trust. **G. Lennie:** Grant Recipient; Victorian Medical Radiation Practitioners Education Trust.

**B-0658** 14:40
**Establishment of national diagnostic reference levels for dental radiology**
A.Q.L.C. Brito, R.P.P. Almeida, S. Rodrigues, A.F. Abrantes, L. Ribeiro, P. Sousa, K.B. Azevedo, D.M. Simão; *Faro/PT (andreiabrito8@hotmail.com)*

**Purpose:** Dental radiology is a field where effective dose per exposure is low; however, the frequency of these procedures is high, and professionals must be aware of the dose delivered to the patient. The main goal of this study was to establish the diagnostic reference levels (DRLs) for intraoral and extraoral dental radiology examinations nationwide.
**Methods and Materials:** Entrance surface air kerma (ESAK) and dose-area products (DAP) were determined for a total of 1001 intraoral and 268 extraoral dental equipment's distributed throughout the national territory. The determined values were averaged and compared according to the type of image receptor system, installation duration, and the type and model of dental X-ray machine.
**Results:** For intraoral radiology, the National DRLs were established at the 75th percentile of ESAK values (1.55 mGy). For orthopantomography and cephalometry, DRLs were determined at the 75th percentile of DAP values, corresponding to 107 mGy.cm2 and 80 mGy.cm2, respectively. Most intraoral equipment's has direct-digital receptor systems (81.8%), followed by analog film system (10%) and indirect-digital receptor systems (4.1%). In extraoral X-ray, a total of 28 different equipments was observed. Furthermore, the type and model of the equipment has influence in the radiation dose values.
**Conclusion:** The establishment of DRLs, as a tool for the optimisation of radiological procedures, is a requirement of national regulations. The proposed national DRLs in this study are in accordance with those established by other countries; however, a constant dose optimisation can reduce these values further.

**B-0659** 14:48
**The impact of different exposure factors on radiation dose of lumbar spine in five different sizes phantoms: a factorial design study**
S.J.M. Alqahtani, J.R. Meakin, R.M. Palfrey, K. Knapp; *Exeter/UK (salbeshri@hotmail.com)*

**Purpose:** To investigate the impact of tube potential (kVp), source to image distance (SID) and Copper filtration on dose area product (DAP) on lumbar spine radiographs with different size bespoke phantoms (18, 29, 38, 42 and 46 BMI).
**Methods and Materials:** A factorial design study was conducted using a DR x-ray machine (Multi Fusion Max, Siemens, Germany), and three factors; kVp$^6$ x SID$^3$ x Cu filtration$^3$. The collimation was kept identical across all phantoms. The DAP reading was reported directly from the x-ray image. Analysis of variance was used to investigate the impact of each factor on the DAP.
**Results:** kVp has a significant negative impact on DAP for the 18, 29, 38 and 42 BMI phantoms( $r^2$= 0.53,0. 57, 0.51 and 0.29) but is non-significant for the 46 BMI phantom. SID has no significant impact on the DAP but rather showed a proportional relationship with DAP in the 18 and 29 BMI phantom and inverse relationship with DAP in the remaining 38, 42 and 46 BMI phantoms. The filtration showed a significant negative impact on the DAP across all phantoms ($r^2$=0.40, 0.36, 0.38, 0.48, 0.59).
**Conclusion:** The kVp has the biggest effect on DAP reduction across all phantoms followed by the Cu filtration. The SID can be used especially in the morbidly obese phantoms. These results need to be taken into account with consideration of image quality.

**B-0660** 14:56
**Radiation dose reduction using copper filters**
K. Yagami[1], M. Hirota[1], H. Kato[1], F. Okada[1], S. Suzuki[2]; [1]*Gifu/JP*, [2]*Toyoake/JP (yagami@gifu-u.ac.jp)*

**Purpose:** To measure the patient dose and the scattered radiation dose, when copper filters are added.
**Methods and Materials:** Using a Shimadzu X-ray system, copper filters were added from 0.0mm to 0.5mm at 0.1-mm interval. For each additional copper filters, an anthropomorphic phantom (Alderson Rando Phantom) with thermo-luminescent dosimeters (BeO) positioned was placed at the PA position and exposure was performed under the conditions which had an equivalent transmitted radiation dose and the quality. The SID was set to 140cm, and the irradiation field was from the diaphragm to the symphysis pubis. For each additional copper filter, the entrance surface dose (ESD), the organ dose, the effective dose and the scattered radiation dose were measured.
**Results:** At the copper filter 0.0mm, the ESD was 1460μGy, the lung-absorbed dose was about 30μGy, the liver 190μGy, the ovary 180μGy, and the effective dose was about 130μSv. By adding 0.1-mm copper filter, the ESD was reduced by about 30%, but the organ dose and the effective dose were almost equal within 10%. By adding a 0.5-mm copper filter, the ESD was reduced by about 40%, the organ dose and the effective dose were reduced by about 10%. As for the copper filter addition, the scattered radiation dose fluctuated within about 10%.
**Conclusion:** By adding a copper filter, the dose reduction for ESD was observed as being up to 40%. However, a dose reduction in the organ dose, effective dose, and scattered dose was not much recognized.

**B-0662** 15:12
**Stylised phantom used in PCXMC, the validation against hybrid phantom in obesity**
S.J.M. Alqahtani, J.R. Meakin, R.M. Palfrey, K.M. Knapp; *Exeter/UK (salbeshri@hotmail.com)*

**Purpose:** To investigate the behaviour of the PCXMC stylized phantom compared to a hybrid phantom in obesity.
**Methods and Materials:** Stylized and hybrid phantoms were modified to three different sizes (185.6 cm height with 73 kg, 87 kg and 108 kg weight). The three sizes stylized and hybrid phantoms were exposed to identical dose of area product (DAP) 8 Gy/cm$^2$, in supine lumper spine (AP), with 70 kV, 3 mm Al filtration, and source to skin distance (SSD) of 84.2, 83.3 and 79.4 cm. Correlation of the absorbed doses between determined by PCXMC and CALDose were conducted.
**Results:** CALDose software shows higher absorbed dose (51%) on average across the three different size phantoms compared to PCXMC. Both programs show a negative trend in absorbed dose as the phantom size increase, -24% and -19% on average for PCXMC and CALDose, respectively. The spearman correlations between the estimated absorbed dose by the two software are significant with $P<0.01$, with r= 0.80 in the normal phantom, r= 0.81 in the overweight phantom and r= 0.71 in the obese phantom.
**Conclusion:** PCXMC absorbed dose is lower than CALDose across the different size phantoms. Strong correlations of estimated absorbed dose between the two programs are demonstrated. Both PCXMC and CALDose demonstrated an inverse relationship between absorbed dose and size, which is expected due to the excessive fat acting as a protection layer for the internal organs. This indicates the possibility of using PCXMC in cases of obesity.

**B-0663** 15:20
**Should DR and CR projection radiography have separate LDRLs at a local hospital level?**
A.M. Burke, S.J. Foley; *Dublin/IE (andrea.burke@ucdconnect.ie)*

**Purpose:** DRLs are important optimisation tools for identifying atypical doses either locally (LDRL), regionally or nationally and are set at the 75$^{th}$ percentile of the dose distribution. This study aimed to investigate how CR and DR doses should be accounted in LDRL data for three common radiographic examinations: AP pelvis, AP and lateral lumbar spine, and to determine if DR and CR equipment should have separate LDRLs.
**Methods and Materials:** Twenty patient DAP measurements of each projection were recorded for both DR and CR imaging systems in five Irish hospitals, which were sampled purposively. Dose distributions for CR and DR equipment in each of the hospitals were analysed and compared using parametric ANOVA and independent-samples T test analysis to determine if there is a need for separate LDRLs for CR and DR systems. LDRLs were analysed on a room by room basis and compared with current NDRLs.
**Results:** DR systems deliver on average 46-56% lower doses than CR systems (p<0.05) for AP pelvis (average DR LDRL: 1.66 Gycm², CR LDRL: 2.65Gycm²) and lateral lumbar spine (average DR LDRL: 1.82 Gycm², CR LDRL: 3.25 Gycm²) projections across all sites. For the AP lumbar spine projection, there is an overall average 8% difference between DR (1.65 Gycm²) and CR (1.52 Gycm²) LDRLs, which was not deemed statistically significant

(p>0.05) by the one-way ANOVA test.
**Conclusion:** Separate LDRLs are justified for AP pelvis and lateral lumbar spine projections but not for AP lumbar spine projections and thus facilitate local dose optimisation.

**14:00 - 15:30** **Room N**

### Hybrid Imaging

### SS 706

### Hybrid imaging in oncology: head to toe

*Moderators:*
N.N.
P.M. Kazmierczak; Munich/DE

**K-14** 14:00
**Keynote lecture**
J. Ferda; *Plzen/CZ*

**B-0664** 14:09
**Quantitative 3D analysis of hybrid 18F-FET PET/MR-neuroimaging for differentiation between treatment response and glioma recurrence**
J. Lohmeier, B. Hamm, M.R. Makowski; *Berlin/DE (johannes.lohmeier@charite.de)*

**Purpose:** This cohort study investigated quantitative PET/MR-imaging parameters for differentiation between glioma recurrence and post-treatment related effects (PTRE) using volumetric (3D-VOI) lesion analysis in hybrid contrast-enhanced and 18F-fluoroethyl-L-tyrosine (18F-FET) PET/MR-imaging.
**Methods and Materials:** 45 patients (female/male patients=18/27; age mean±SD, 47±15 a) were retrospectively evaluated regarding glioma recurrence. Histopathology (40%) and conclusive clinical follow-up serial imaging (60%) were used as reference standard (PTRE/recurrence=9/36). PET/MR-imaging was evaluated using semi-automated isocontour (85%) lesion segmentation based on 18F-FET tracer uptake. Standardized-uptake-value (SUV), target-to-background ratio (TBR) and (relative) apparent diffusion coefficient (ADC and rADC) were determined and assessed using receiver operating characteristic analysis (ROC).
**Results:** Cancer relapse showed lower rADCTotal/Vol values than treatment-related changes and presented satisfactory discriminative test performance (AUC±SE=0.744±0.101, p-value=0.016). Quantitative 18F-FET parameters (TBRTotal/Vol, AUC±SE=0.799±0.080, p-value=0.0002; TBRmax, AUC±SE=0.877±0.082, p-value≤0.0001; TBRmean, AUC±SE=0.877±0.082, p-value≤0.0001) facilitated reliable differentiation between recurrence and PTRE. Multiparametric analysis with quantitative parameters from hybrid PET/MR-imaging demonstrated highest diagnostic accuracy (AUC±SE=0.929±0.048).
**Conclusion:** The current study introduced TBRTotal/Vol and rADCTotal/Vol as novel parameters for the differentiation between glioma recurrence and post-treatment related effects. We provided evidence that 18F-FET parameters facilitate reliable classification, which reduced the clinical false positive rate. Additionally, we showed that multiparametric analysis with quantitative parameters from hybrid PET/MR-imaging demonstrated highest diagnostic accuracy, showing a benefit of combining both modalities, which provide distinct information about the underlying physiology.

**B-0666** 14:25
**Prediction of patient outcome in head and neck squamous carcinoma following chemo(radiation): comparison of 4 different FDG PET-CT response assessment interpretative criteria**
J. Zhong, M. Arunsingh, P. Brown, K. Dyker, R. Prestwich, S. Currie, S. Vaidyanathan, A. Scarsbrook; *Leeds/UK (j.zhong.edinburgh@gmail.com)*

**Purpose:** To assess comparative accuracy of fluorine-18 fluorodeoxyglucose (FDG) positron emission tomography-computed tomography (PET-CT) response interpretation criteria (NI-RADS, Porceddu, Hopkins, Deauville) for predicting recurrence in head and neck squamous cell carcinoma (HNSCC).
**Methods and Materials:** All patients with histologically confirmed HNSCC treated at a specialist cancer centre with curative-intent non-surgical treatment between August 2008 and May 2017 were included. Patients underwent FDG PET-CT before and 4 months post-treatment. Metabolic response was assessed using 4 different interpretative criteria (IC) harmonised into 4-point scales (complete response, indeterminate, partial response, progressive disease). IC performance metrics (sensitivity, specificity, positive predictive value (PPV), negative predictive value (NPV), accuracy) were compared.
**Results:** 570 patients (397 oropharynx, 58 hypopharynx, 51 larynx, 64 other/unknown primary). Median age 58 (range 24-84), 431 (76%) were male. 426 patients received chemo-radiotherapy and 144 had radiotherapy alone. Median follow-up was 26 months (range 0-148). 156 patients (27%) progressed during follow-up. All IC were highly accurate for prediction of complete response or disease progression (NPV/accuracy (%)) of primary tumour (NI- RADS 96.3/95.7; Porceddu 96.2/93.6; Hopkins 95.9/93.5; Deauville 96.3/93.7) and nodes (NI- RADS 93.5/93.5; Porceddu 94.3/94.4; Hopkins 91.9/92.3; Deauville 92.9/93.8). PPV was significantly reduced for indeterminate uptake with all IC (mean PPV primary tumour 19.5%, nodes 52.4%). No IC had consistently better performance characteristics for outcome prediction.
**Conclusion:** FDG PET-CT at 4 months post-treatment is highly accurate for prediction of complete response or disease progression. All IC have similar performance characteristics. Individual centres should apply one IC consistently across all patients.

**B-0667** 14:33
**Performance of FDG PET-based textural features for prediction of loco-regional recurrence in advanced larynx and hypopharynx squamous cell carcinoma**
J. Zhong, P. Brown, H.L. Nelstrop, R. Prestwich, G.M. McDermott, S. Currie, S. Vaidyanathan, A. Scarsbrook; *Leeds/UK (j.zhong.edinburgh@gmail.com)*

**Purpose:** To determine whether metabolic tumour parameters and radiomic features derived from fluorine-18 fluorodeoxyglucose (FDG) positron emission tomography-computed tomography (PET-CT) are prognostic predictors in patients with locally advanced larynx and hypopharynx squamous cell carcinoma (SCC).
**Methods and Materials:** All patients with advanced stage larynx and hypopharynx SCC treated at a specialist cancer centre that underwent baseline FDG PET-CT between August 2008 and May 2017 were included. Primary tumour and nodal disease were segmented using LIFEx software (University of Paris-Saclay, France) and radiomic analysis (first- and second-order parameters) was performed which included calculation of metabolic tumour volume (MTV). Lesions less than 64 pixels (5 ml) were excluded. Univariate Cox regression was performed using SPSS Statistics (Version 22, IBM Corp, USA) correlating each tumour characteristic and textural feature to progression-free survival (PFS) with two-tailed significance taken as P<0.05.
**Results:** 101 patients (54 hypopharynx and 47 larynx tumours) with 119 lesions were included (79 tumour and 40 nodes). Median age 62 (range 41-82), 76 (75%) were male. 62 patients received chemo-radiotherapy and 39 had radiotherapy alone. Median follow-up was 26 months (range 0-105). 39 patients progressed during follow-up; 22 at the primary site and 17 within loco-regional nodes. Median loco-regional PFS was 23 months (range 0-105). CT tumour volume (ml), PET MTV and PET-derived grey-level zone length matrix (GLZLM) were independent predictors of PFS (P<0.05). No other features correlated with PFS.
**Conclusion:** Baseline MTV and PET-derived GLZLM are prognostic predictors in patients with locally advanced larynx and hypopharynx SCC.

**B-0668** 14:41
**Clinical impact of breast and whole body simultaneous PET/MRI for staging of breast cancer lesions >2 cm**
V. Romeo, C. Cavaliere, M. Imbriaco, A. Stanzione, R. Cuocolo, A. Pignata, V. Picariello, E. Nicolai, M. Salvatore; *Naples/IT (valeria.romeo@unina.it)*

**Purpose:** To assess the added clinical value of breast and whole body simultaneous 2-(18F)FDG PET/MRI over conventional diagnostic imaging techniques in patients with breast cancer lesions (BCLs) > 2 cm.
**Methods and Materials:** 40 patients with BCLs > 2cm were prospectively enrolled. 38 patients underwent contrast-enhanced breast and whole body simultaneous 2-(18F)FDG PET/MRI; 2 patients prematurely interrupted MRI examinations due to claustrophobia and were therefore excluded. MRI and PET images were evaluated in consensus by a radiologist and a nuclear physician to perform TNM staging. PET/MRI stage was then compared to those obtained using conventional diagnostic imaging techniques (mammography, ultrasound, MRI, chest x-ray and bone scintigraphy) and the possible changes in treatment planning were finally assessed.
**Results:** In 18% (7/38) cases, PET/MRI had a significant clinical impact: 1) changing surgical strategies in two patients due to the exclusion of contralateral BCLs; 2) switching one patient candidate to surgical excision towards neoadjuvant chemotherapy (NAC) due to the detection supraclavicular pathologic lymph-nodes; 3) contraindicating NAC in four patients due to the detection of additional bone and/or mediastinal lymph-node metastases. Finally, the finding of a contralateral BCL in one patient candidate to NAC, even without modifying the therapeutic approach, was crucial in view of the assessment of the response to treatment.
**Conclusion:** Breast and whole body simultaneous PET/MRI examination is useful for staging of patients with BCLs > 2cm, allowing to identify or exclude contralateral BCLs as well as to detect distant metastases that may be missed by conventional imaging techniques.

**B-0669** 14:49

**Can we replace sentinel lymph node resection in breast cancer patients by breast MRI, axillary MRI or axillary $^{18}$F-FDG PET/MRI**

J. Kirchner[1], O. Martin[1], L.M. Sawicki[1], L. Umutlu[2], P. Heusch[1], C. Buchbender[1]; [1]*Dusseldorf/DE*, [2]*Essen/DE (Julian.Kirchner@med.uni-duesseldorf.de)*

**Purpose:** To compare the diagnostic performance of Mamma-MRI, axillary MRI and axillary $^{18}$F-FDG PET/MRI in the detection of lymph node metastases in patients suffering from breast cancer.
**Methods and Materials:** 27 female patients with breast cancer (mean age 54 ± 10 years) with newly diagnosed, histopathologically proven breast cancer were prospectively enrolled in this trial. All patients underwent dedicated prone $^{18}$F-FDG breast PET/MRI and supine whole-body $^{18}$F-FDG PET/MRI. Sentinel lymph node biopsy (SLNB) and/or axillary lymph node dissection was performed in all patients and histopathology served as reference standard. Sensitivities, specificities, PPV, NPV and accuracy regarding axillary lymph node assessment were calculated for dedicated breast MRI, axillary MRI and axillary $^{18}$F-FDG PET/MRI.
**Results:** According to the reference standard lymph node metastases were present in 13 patients with a total of 23 metastases. On a patient based analysis dedicated breast MRI identified 6/13 (46%), axillary MRI 5/13 (38%) and axillary PET/MRI 8/13 (62%) of the patients with a positive nodal status. All modalities revealed on false positive finding (7%). On a lesion-based analysis sensitivity, specificity, PPV, NPV and accuracy were 30%, 90%, 88%, 38% and 50% for breast MRI, 22%, 90%, 83%, 36% and 44% for axillary MRI and 48%, 90%, 91%, 45% and 62% for axillary PET/MRI.
**Conclusion:** Breast MRI or ($^{18}$F-FDG PET/) MRI do not reliably differentiate N-positive from N-negative breast cancer patients. Hence, sentinel lymph node biopsy cannot be replaced by imaging procedures.

**B-0670** 14:57

**Evaluation of a fast protocol for staging patients with bronchial cancer using PET/MRI**

O. Martin[1], L.M. Sawicki[1], J. Kirchner[1], G. Antoch[1], K. Herrmann[2], L. Umutlu[2], P. Heusch[1]; [1]*Düsseldorf/DE*, [2]*Essen/DE (Ole.Martin@med.uni-duesseldorf.de)*

**Purpose:** To evaluate the applicability of a fast MR-protocol for whole-body staging of bronchial carcinoma patients using PET/MR.
**Methods and Materials:** In this prospective study, 52 patients (63.9±8.7years) underwent clinically indicated 18F-FDG-PET/CT and subsequent PET/MRI. For PET/MRI imaging, a fast whole-body MR-protocol was implemented. MRI and PET/MRI datasets were analyzed to identify malignant manifestations. The accuracy for the identification of malignant manifestations was calculated and the tumor stage for each examination was determined. In 26 patients, all available histopathological samples as well as results of prior examinations and follow-up imaging were used for the determination of the reference standard. In the other cases, the results of PET/CT imaging used for reference standard.
**Results:** Both MRI and PET/MRI have a 100% accuracy to correctly identify the extent of the primary (T-stage). In nodal staging and identification of metastases, PET/MRI revealed higher values of diagnostic accuracy than MRI alone (both p<0.05). The values of correct stage did not differ significantly between PET/CT and PET/MRI (93.5% for PET/CT vs. 86.9% for PET/MRI). Average scan duration of whole-body PET/CT and PET/MRI examinations were 17.5±2.3 min and 29.4±3.9 min, respectively. Estimated mean effective-dose for whole-body PET/CT scans were 61.3% higher than for PET/MRI.
**Conclusion:** Using 18F-FDG PET data in addition to whole-body MRI leads to a more accurate evaluation of patients with bronchial cancer. With regard to patient comfort related to scan duration and reduced radiation exposure, fast PET/MRI may serve as a powerful alternative to PET/CT for a diagnostic workup of bronchial cancer patients.

**B-0671** 15:05

**Is there a connection between immunohistochemical markers and grading of lung cancer with ADC and SUV of hybrid 18F-FDG PET/MRI?**

O. Martin[1], P. Heusch[1], J. Kirchner[1], T. Ullrich[1], C. Buchbender[1], F. Nensa[2], K. Herrmann[2], L. Umutlu[2], L.M. Sawicki[1]; [1]*Düsseldorf/DE*, [2]*Essen/DE (Ole.Martin@med.uni-duesseldorf.de)*

**Purpose:** To correlate various prognostically relevant immunohistochemical parameters of primary lung cancer with simultaneously acquired standardized uptake values (SUV) and apparent diffusion coefficient (ADC) derived from hybrid PET/MRI.
**Methods and Materials:** 55 consecutive patients with histologically proven lung cancer (62.5±9.2y) underwent 18F-FDG-PET/MRI. Diffusion-weighted imaging (DWI, b values: 0, 500, 1000) was performed simultaneously with PET acquisition. A region of interest (ROI) encompassing the entire primary tumour was drawn into each patient's PET/MR images to determine the glucose metabolism represented by maximum and mean SUV and into ADC maps to assess tumour cellularity represented by mean and minimum ADC values. Histopathological tumour grading was available in 43/55 patients. In 15 patients, additional prognostically relevant immunohistochemical markers, i.e. pAKTS473, pERK, PTEN and erbB2 were available. Pearson's and Spearman's correlation were used.
**Results:** We found a significant inverse correlation between the ADCmin and SUVmax (r=-0.58,p<0.001) as well as between the ADCmin and SUVmean (r=-0.44,p<0.001). Tumour grading showed a significant positive correlation with SUVmax and SUVmean (r=0.34 and r=0.31, both p<0.05) and a significant inverse correlation with ADCmin and ADCmean (r=-0.30 and r=-0.40, both p<0.05). In addition, erbB2 showed a significant inverse correlation with SUVmax and SUVmean (r=-0.50 and r=-0.49, both p<0.05). The other immunohistochemical markers did not show any significant correlations.
**Conclusion:** The present data show significant correlations between SUV, ADC, grading and erbB2-expression of lung cancer. Hence, 18F-FDG-PET and DWI from hybrid PET/MRI may offer complementary information to the histopathology of lung cancer for the evaluation of tumour aggressiveness and treatment response.

**B-0672** 15:13

**Prediction of overall survival using interim 18F-FDG PET-CT and CT for response assessment of patients with Hodgkin lymphoma treated with immune checkpoint inhibitors**

F.-Z. Mokrane[1], A. Chen[2], B. Zhao[3], S. Ammari[4], L. Schwartz[3], R. Houot[5], L. Dercle[6]; [1]*Toulouse/FR*, [2]*Nanjing/CN*, [3]*New York, NY/US*, [4]*Villejuif/FR*, [5]*Rennes/FR*, [6]*Paris/FR (mokrane_fatimazohra@yahoo.fr)*

**Purpose:** Anti-programmed death 1 therapy triggers new patterns of response and progression in patients with Hodgkin lymphoma. We aimed to predict overall survival (OS) based on interim imaging.
**Methods and Materials:** We retrospectively analysed 61 patients treated by anti-PD1 from 2013 to 2017. Concomitant F-FDG PET/CT and CT scans were acquired at baseline and upon treatment. Three radiologists classified blindly and independently patients as immune-responding or immune-refractory based on the first evaluation, using the International Harmonisation Project Cheson 2014 criteria and the Lymphoma Response to Immunomodulatory Therapy Criteria (LYRIC) (2016 revised criteria). Results were compared imaging assessment to OS. Two interim time points were assessed: interim 1 and 2.
**Results:** After treatment initiation, imaging examinations were acquired at a median time of 3.0 months (n=61 pts) for interim 1 and 6.7 months (n=51pts) for interim 2 evaluations. At the time of analysis, 13 (21%) patients died due to lymphoma progression. Median OS was not reached. The best overall response rate (ORR) with CT1 was 46%, and 59%with PET1. After two years, the OS rates were 50% for progressive disease (PD), 84% for stable disease (SD), 84% for partial response (PR) and 100% for complete response (CR) at PET1. Moreover, PET reclassified patients from SD or PR using CT to CR at interim 1 (31%) and interim 2 (18%).
**Conclusion:** A complete response assessment using first interim PET evaluation is highly predictive of good OS. 18F-FDG PET/CT shows a significant incremental value comparing to CT alone in the identification of these complete responders' high survivors patients.

**B-0673** 15:21

**Comparison evaluation of the diagnostic potential of PET/MRI and PET/CT using mIBG-Iod-124 in neuroendocrine and neuroectodermal tumours**

A. Örnek[1], L. Umutlu[1], J. Kirchner[2], D. Heber[1], K. Herrmann[1], V. Ruhlmann[1]; [1]*Essen/DE*, [2]*Düsseldorf/DE (ahmet.oernek@uk-essen.de)*

**Purpose:** To compare the diagnostic potential of PET/MRI and PET/CT using mIBG-Iod-124 in neuroendocrine and neuroectodermal tumors.
**Methods and Materials:** 20 patients (12 m, 8 w, age 53±16 y) with histopathologically confirmed pheochromocytoma (n=15), paraganglioma (n=2) and ganglioneuroblastoma (n=3) underwent both a whole-body contrast-enhanced PET/CT and PET/MRI approximately 1 day after injection of 48±9 MBq mIBG-Iod-124. Image analyses included the detection and delineation capacity of malignant lesions. Morphological components: transversal maximal tumor diameter and conspicuity (4-point scale (not visible=0 - high contrast=3)). PET images: tumor conspicuity (4-point scale), SUVmax and SUVmean using VOI technique.
**Results:** 58 malignant mIBG-positive lesions (peritoneal and mesenteric n=22, bone n= 15, lymph node n= 8, liver n=8, lung n=2 pleural space n=2, adrenal n=1) were detected with both PET/CT and PET/MRI with strongly positive correlated mean SUVmax (18.3±11.4 vs. 17.5±9.8; Pearson`s correlation r=0.84) and SUVmean (12.1±7.7 vs. 11.1±6.7; r=0.91). Comparable high lesion conspicuity in the PET component of PET/CT (2.9±0.4) and PET/MRI (2.9±0.3; p=0.46) could be assessed with significant higher lesion conspicuity in MRI (2.8±0.6) compared to CT (2.6±0.8; p=0.01). Two small lung lesions were not visible on MRI and five osseous metastases could not be assessed on CT, but showed an unequivocal hyperintensity on MRI.

**Conclusion:** Integrated mIBG-Iod-124-PET/MRI shows high potential as diagnostic procedure in neuroendocrine and neuroectodermal tumors with in comparison to PET/CT comparable detection rate and high lesion conspicuity in PET. A significant higher lesion conspicuity could be found in MRI compared to CT.

**14:00 - 15:30** **Room O**

### Musculoskeletal

### SS 710

### Imaging techniques: trauma and cartilage

*Moderators:*
S. Choudhary; Birmingham/UK
U. Fahlenkamp; Berlin/DE

### B-0674 14:00

**Implementation of an ultra-low-dose CT-protocol for extremities: initial experience with 106 subjects**

Z. Alagic[1], R. Bujila[2], S. Srivastava[2], S.K. Koskinen[3]; [1]*Solna/SE*, [2]*Stockholm/SE*, [3]*Huddinge/SE (zlatan.alagic@sll.se)*

**Purpose:** To assess if ultra-low-dose Computed Tomography (ULD-CT) is a useful alternative to Digital Radiography (DR) in the evaluation of wrist and ankle fractures.
**Methods and Materials:** A ULD-CT protocol on a Revolution™ CT was designed for wrist and ankle fractures. Patients admitted to the emergency department with suspected wrist or ankle fractures were evaluated prospectively. After DR obtained on a Discovery™XR650 radiography system, a ULD-CT was performed. Two readers independently analyzed DR and ULD-CT images, and the results were blinded to the readers. Also, the radiation dose (RD), examination time, and time to preliminary report was compared between DR and CT.
**Results:** In 106 subjects DR and ULD-CT detected 55 and 69 fractures, respectively, corresponding to an odds ratio of 1.7 for ULD-CT vs. DR. Furthermore, ULD-CT provided important additional information about soft tissue in 3 cases (2.8%), non-fracture related findings that explained the symptoms in 10 cases (7.4%), additional fracture-related findings in 22 cases (15.7%) and was able to confirm or rule out suspected fractures on DR in 8 cases (5.6%). The average examination time was shorter for ULD-CT than DR (3.5±1.4 vs. 6.7±3.8 min, $p<0.001$) as well as the average time to preliminary report (22.6±13.6 min vs. 35.6±33.2 min, $p=0.057$). The average estimated effective dose was comparable between ULD-CT and DR (0.66±0.45 µSv vs. 0.51±0.47 µSv).
**Conclusion:** ULD-CT is an excellent alternative to DR for imaging the peripheral skeleton as it provides additional clinically important information at a comparable RD as well as faster examination and reporting times.

### B-0684 14:08

**Tissue stiffness after self-myofascial release in athletes with different experience in foam rolling assessed by quantitative acoustic radiation force impulse elastography (ARFI)**

R. Heiß, I. Mayer, M. Huettel, M.S. May, W. Wuest, M. Kopp, C. Lutter, R. Forst, T. Hotfiel; *Erlangen/DE (Rafael.Heiss@uk-erlangen.de)*

**Purpose:** Self-myofascial release using foam rolling (FR) has been developed into a popular preventive and recovery intervention. However, its effects on target tissue with regard to changes in stiffness properties are still poorly understood. The aim was to investigate the role of foam rolling on muscle and ligament stiffness.
**Methods and Materials:** Acoustic radiation force impulse elastography (ARFI) was performed in 40 volunteers (20 with more than 6 months of experience in FR and 20 without any experience) before and several times (0, 30, 60, 120min, 12h and 24h) after a standardized foam rolling exercise of the lateral thigh. Tissue stiffness was assessed at different compartments of the lateral thigh including superficial and deep muscle tissue (vastus lateralis muscle, VL; vastus intermedius, VM) and connective tissue (distal insertion of the iliotibial band, ITB).
**Results:** Tissue stiffness of the ITB revealed a significant decrease of 13% in experienced athletes at 30 min post-intervention ($p=0.0017$). In non-experienced athletes a 6% increase of stiffness was found at 30 min post-intervention at ITB, which was not significantly different to baseline ($p=0.16$); no significant changes occurred at later time points at ITB. For VLM and VM no significant changes were detected at any time point in both groups.
**Conclusion:** A significant short-term decrease of connective tissue stiffness in experienced athletes is detectable with ARFI.

### B-0675 14:16

**Dual-energy CT for assessment of bone marrow oedema in the acute fracture: a systematic review**

F.C. Müller[1], J.S. Bovin[1], K.K. Gosvig[1], F. Kainberger[2], A. Rodell[3], M. Østergaard[4], M. Boesen[5]; [1]*Herlev/DK*, [2]*Vienna/AT*, [3]*Ballerup/DK*, [4]*Glostrup/DK*, [5]*Copenhagen/DK (felix.c.mueller@gmail.com)*

**Purpose:** The aim of this systematic literature review (SLR) was to investigate dual-energy CT (DECT) for the detection of bone marrow oedema (BME) associated with acute fracture.
**Methods and Materials:** A SLR was done, based on a study protocol in agreement with the PRISM statement, with Medline searched on 01.08.2018. Abstracts were screened by two independent reviewers and studies included based on consensus. Key outcome measures were diagnostic test accuracy, inter-reader agreement, virtual non-calcium (VNCa) values and minimal size for oedema detection.
**Results:** 11 prospective and 8 retrospective studies were identified and investigated the knee, ankle, hip, wrist and spine using scanners from three vendors with different CT and post-processing parameters. Sensitivity was 61%-100% and specificity 70%-100%. Inter-reader agreement was substantial with $\kappa>0.7$ in 11 of 13 studies. In the knee, average VNCa values were -53 to -8 in normal regions and -94 to -58 in oedematous regions. In the spine VNCa values were overlapping between the different studies for vertebra with and without acute fracture. No study investigated minimal detectable oedema size.
**Conclusion:** Results indicate that demonstration of fracture-associated BME with DECT is feasible. Prospective, blinded studies are missing for the wrist and hip. Included knee studies use similar CT-parameters and show high diagnostic test accuracy. Spine studies are technically heterogeneous with high accuracy but inconsistent quantitative results. Bone marrow oedema can be detected on various CT units, but the effect of CT parameters, CT unit and post-processing is still unclear and we suggest that for clinical utilisation and meta-analysis CT parameters should be carefully considered.
*Author Disclosures:*
**F.C. Müller:** Employee; Siemens Healthineers. Research/Grant Support; Innovation Fund Denmark. **A. Rodell:** Employee; Siemens Healthineers.

### B-0676 14:24

**A prospective randomised CT analysis of ankle syndesmosis injuries in Weber B and C type fractures**

A. Favia[1], M. D'addato[2], G. Caliolo[1], C. Campagna[1], V. Favia[1], M. Carrozzo[1], A. Spinarelli[1], A. Scardapane[1], A. Stabile Ianora[1]; [1]*Bari/IT*, [2]*Bisceglie/IT (annafavia@libero.it)*

**Purpose:** To evaluate the role of preoperative computed tomography (CT) scan in patients with programmed stabilisation of ankle syndesmosis injuries in Weber B and C type fractures.
**Methods and Materials:** Between June 2016 and May 2017, 51 patients with Weber fracture type B and C were divided into 3 groups: 14 (27.45%) without preoperative fractured ankle CT scan in Group A, 19 (37.25%) with preoperative fractured ankle CT scan in Group B, and 18 (35.30%) with preoperative bilateral ankle CT scan in Group C. All the patients underwent to stabilisation of syndesmosis and have been subjected to postoperative bilateral ankle CT scan. Clinical outcomes with American Orthopedic Foot and Ankle Society (AOFAS) score at 3, 6 and 12 months were recorded. Seven measurements on postoperative axial CT scan images were confronted between the injured and uninjured ankles to check the accuracy of reduction.
**Results:** At 3 months of follow-up, there was no significative difference between groups ($p= 0.105$). At 6 and 12 months of follow-up, the median AOFAS score was higher in Group C (84.33±1.68 and 93.44± 3.01, respectively) compared to Group B and Group A. The seven variables measured at the CT scan after syndesmotic reduction were statistically different between groups with a better restoration of the tibiofibular distances and the correct ankle anatomy in Group C.
**Conclusion:** Use of the preoperative comparative CT scan of the injured and uninjured ankle gives the surgeon more accurate informations for the reduction and the intraoperative manoeuvers, allowing a better clinical outcome.

### B-0677 14:32

**Reliability of ultrasonography measurement of the anterior talofibular ligament (ATFL) length in healthy subjects, based on examinee experience and patient positioning**

J. Seilern und Aspang, K.-H. Kristen, H. Platzgummer, F. Hartenbach, J. Wiedermann; *Vienna/AT (jesse.seilern@gmail.com)*

**Purpose:** The most common cause of ankle injury is the supination trauma, inflicting a partial or complete rupture of the anterior talofibular ligament (ATFL). Among conventional diagnostic tools and procedures of sports injuries, the method of stress-ultrasonography is reportedly a promising diagnostic tool for examining injuries of the lateral ligaments of the ankle. Preceding studies predominantly examined the comparability of stress-ultrasonography and other established diagnostic tools in terms of efficacy, viability and quality. The purpose of this study was to assess the reliability of stress-ultrasonography of the ATFL based on varying examinee experience and patient positioning.
**Methods and Materials:** Sixteen healthy subjects were examined by four examiners with differing levels of skill and experience in ultrasonography, ranging from laymen to specialist. Measurements were recorded and interrater correlation coefficient (ICC) was applied in four positions, including a neutral position (A), medial rotation (B), plantar flexion (C) and inversion of the foot (D).
**Results:** The correlation of the combined results of all four investigators was 0.333 for position A, 0.386 for position B, 0.320 for position C and 0.517 for position D. The highest ICC (0.811) was recorded between the orthopaedic specialist and the radiology specialist. The lowest ICC (0.299) was recorded between the laymen and the radiology specialist.
**Conclusion:** The reliability of the ATFL examination seems to be exceedingly dependent on the examiner's experience in ultrasonographic (US) diagnostics. Moreover, the positioning described by the European Society of Musculoskeletal Radiology (ESSR) yielded the highest measurement reliability.

**B-0678** 14:40
**Model-based accelerated T2 mapping of sacroiliac joint with 3-T MRI: a preliminary study of Grappatini**
X. Peng, S. Yang, P. Hu, J. Xiao, Y. Pei, W. Liao; *Changsha/CN*

**Purpose:** The objective of this study was to assess the feasibility of GRAPPATINI in the sacroiliac joints.
**Methods and Materials:** The sacroiliac joints of 10 healthy volunteers were imaged by 3-T MRI using routine FSE sequence, multi-slice multi-echo spin echo(MESE)T2-weighted sequence, model-based accelerated T2 mapping (GRAPPATINI). ROIs were drawn in sacral-side and iliac-side cartilage to get T2 values. Intra and interobserver reproducibility of T2 values measured using GRAPPATINI by two radiologists independently were calculated. The consistency of T2 values measured by T2 mapping with MESE and GRAPPATINI was calculated using Bland-Altman. The image quality, SNR and CNR was compared between routine FSE T2W image and synthetic T2W image of GRAPPATINI.
**Results:** Compared to MESE sequence, GRAPPATINI gave a almost 40% reduction of scanning time. Mean (± SD) T2 values were 50.2 ± 5.3 ms and 48.2 ± 6.1 ms for observer 1 and 47.9 ± 5.6 ms for observer 2 measured by GRAPPATINI. The intraobserver intraclass correlation coefficient was 0.78, and the interobserver intraclass correlation coefficient was 0.76. Good agreement between T2 values measured with MESE and GRAPPATINI was identified by Bland-Altman plot. No significant difference for overall image quality, image quality, SNR and CNR was demonstrated between the routine FSE T2W image and synthetic T2W image of GRAPPATINI.
**Conclusion:** Our study shows the feasibility of GRAPPATINI at the sacroiliac joints. Model-based accelerated T2 mapping enable quantitative imaging techniques to proceed in clinical applications.

**B-0679** 14:48
**Feasibility of sub-milliSievert CT of the skeletal pelvis: a human cadaver study**
J.M. Weinrich[1], K.J. Maas[1], J. Stáreková[1], L. Intert[1], S. Sehner[1], M. Regier[2], G. Adam[1], A. Quitzke[1]; [1]*Hamburg/DE*, [2]*Munich/DE*

**Purpose:** To investigate the feasibility of sub-milliSievert (sub-mSv) CT of the skeletal pelvis in human cadavers using a standard-dose (SD) and four different reduced-dose (RD) protocols reconstructed with filtered back projection (FBP) and iterative reconstruction (IR).
**Methods and Materials:** The pelvis of 25 human cadavers was examined using different RDCT protocols with decreasing reference tube currents (RDCT-1: 80 mAs; RDCT-2: 60 mAs; RDCT-3: 40 mAs; RDCT-4: 10 mAs) at 120 kV. A clinical SDCT protocol (100 mAs, 120 kV) served as reference. Raw data were reconstructed using FBP and two increasing levels of IR (IRL4&6). The anterior pelvic ring, acetabulum and posterior pelvic ring including the iliosacral joints were evaluated for image quality and diagnostic acceptability according to a 5-point-scale.
**Results:** Image quality of all anatomical structures was rated as diagnostically acceptable for all protocols reconstructed with IR except for eleven cadavers in RDCT-4. Image quality of reconstructions with FBP was generally rated lower and only diagnostic acceptable for SDCT, RDCT-1 and 2. RDCT-3 with IR was the most reduced dose CT protocol allowing diagnostically acceptable image quality for all anatomical structures in all cadavers. Compared to SDCT, application of RDCT protocols resulted in significantly reduced effective radiation doses (SDCT: 2.0±0.7 mSv; RDCT-1:1.6±0.6 mSv; RDCT-2:1.2±0.4 mSv; RDCT-3:0.8±0.3 mSv; RDCT-4:0.3±0.1 mSv; p=0.001).
**Conclusion:** Diagnostically acceptable sub-mSv CT of the skeletal pelvis is feasible with a reference mAs of 40 at 120 kV using iterative reconstruction. We believe that the presented RDCT-protocol should be implemented in clinical routine as a first-line imaging method.

**B-0680** 14:56
**Quantification and evaluation of pre-post exercise femoral cartilage thickness and T2 changes in ultramarathon athletes**
F. Garcia-Castro[1], J. Catala-March[2], D. Brotons-Cuixat[2], M. Llobet-Llambrich[2], E. Sanchez-Osorio[2], A. Alberich-Bayarri[1]; [1]*Valencia/ES*, [2]*Barcelona/ES (fabiogarcia@quibim.com)*

**Purpose:** Articular cartilage MRI allows the assessment of the integrity of the tissue matrix through the quantification of T2 and thickness. The purpose of this work is to evaluate the variation of these image biomarkers in ultramarathon athletes before and after exercising.
**Methods and Materials:** Thirty-six MRI studies were acquired from both knees of 9 healthy subjects before and after running an ultramarathon (80 km). The MR protocol included a T2 multi-echo sequence with 6 echo times and a high-resolution anatomical sequence (DESS). The multi-echo T2 sequence was registered to the anatomical sequence using an elastic algorithm to achieve spatial consistency. Thickness of the femoral cartilage was calculated using an in-house algorithm based on the distance transform. For the T2-mapping calculation, the voxelwise signal decay across echo times was modelled using a non-linear least-squares fit algorithm. Thickness and T2 parametric maps were aligned to the same spatial reference. Thickness and T2 values were statistically compared before and after exercise, as well as with and without the administration of hyaluronic acid.
**Results:** Significant differences were obtained in the median (p=0.037) and 25th percentile (p=0.017) of the T2 values before and after exercising (higher post-ultramarathon values). In cases with hyaluronic acid, these differences disappeared (p=0.24 and 0.16). There were no significant differences in cartilage thickness.
**Conclusion:** T2-mapping provides information on post-ultramarathon alterations. The hyaluronic acid removed differences in T2 values. Femoural cartilage thickness remained invariant during exercise.

**B-0681** 15:04
**Presence of intravascular contrast material after intraarticular injection for CT arthrography**
C. von Falck, J.B. Hinrichs, F. Wacker; *Hanover/DE (c.v.falck@gmx.de)*

**Purpose:** To analyze the appearence of intravascular contrast material after intraarticular injection for CT arthrography
**Methods and Materials:** A total of 50 CT arthrography examinations were retrospectively reviewed (elbow: 6, wrist: 22, knee: 11, ankle: 8, shoulder: 3). All procedure were conducted under sterile conditions and fluoroscopic guidance according to the institutes standard procedures. The examinations were independently evaluated by two readers for the presence and degree of intravascular contrast enhancement using a 4-point Likert scale.
**Results:** Intravascular contrast material was seen in 72% (36/50) of the examinations. Grade 1 was seen in 16% (8/50), grade 2 in 34% (17/50) and grade 3 in 22% (11/50) of the scans. Only 28% (14/50) of the examinations did not show intravascular enhancement.
**Conclusion:** Intravascular contrast material is commonly seen after intraarticular injection of iodinated contrast agents. This should be considered during pre-interventional risk assessment in patients with allergy to contrast agents or hyperthyroidism.
*Author Disclosures:*
**C. von Falck:** Research/Grant Support; Visage Imaging, Siemens Healthcare. **F. Wacker:** Research/Grant Support; Siemens, Visage Imaging.

**B-0682** 15:12
**Optimising dual-energy CT parameters for virtual non-calcium imaging: a phantom study**
F.C. Müller[1], H. Børgesen[1], K.K. Gosvig[1], A. Rodell[2], C. Booz[3], B. Schmidt[4], B. Krauss[4], M. Boesen[5]; [1]*Herlev/DK*, [2]*Ballerup/DK*, [3]*Frankfurt a. Main/DE*, [4]*Forchheim/DE*, [5]*Copenhagen/DK (felix.c.mueller@gmail.com)*

**Purpose:** Virtual non-calcium (VNCa) images calculated from -energy CT (DECT) data can characterise bone marrow changes associated with fractures and rheumatoid arthritis. We investigated the relationship between dose, tube voltage combinations, pitch, rotation time and reconstruction kernel regarding accuracy and image noise of VNCa images using a bone marrow phantom.
**Methods and Materials:** A bone marrow phantom was developed in-house and imaged using a third-generation dual-source CT scanner with various dose and tube voltage combinations, rotation times, pitch and reconstruction kernels. Accuracy was determined by measuring the mean error between VNCa values in identical volumes of interest in free fluid and fluid in bone and spearman rank correlation coefficients calculated between mean error, image noise (standard deviation of VNCa measurements) and scan parameters.
**Results:** The mean error did not significantly correlate (p>0.05) with tube voltage, dose, rotation time or pitch. Increasing sharpness of the reconstruction kernel correlated with mean error (r=0.83, p=0.015, n(number of experiments)=8). Image noise correlated significantly with tube voltage difference (r=0.96, p<0.001, n=29), dose (r=0.98, p<0.001, n=29) and kernel

Thursday sharpness (r=1.0, p<0.001, n=8) but not with rotation time or pitch.
**Conclusion:** In this study, dose and spectral separation did not affect the accuracy of VNCa images, but correlated with image noise. VNCa reconstructions with similar accuracy and image noise could be achieved using different tube voltage combinations by adjusting the dose. The phantom holds the potential to calibrate image quality across different DECT scanners to be used in multicentre studies of bone marrow changes.
*Author Disclosures:*
**F.C. Müller:** Employee; Siemens Healthineers. Research/Grant Support; Innovation Fund Denmark. **A. Rodell:** Employee; Siemens Healthineers. **B. Schmidt:** Employee; Siemens Healthineers. **B. Krauss:** Employee; Siemens Healthineers..

**B-0683** 15:20
**Importance of minimal HU setting in the foot and ankle DECT: frequency and the common site of submillimeter artefact noted in patients in gout free patients**
H. Yang[1], Y.-S. Hong[2], J. Gong Yong[3], Y. Lee[1], S. Kim[1], J. You[1], E. Park[1]; [1]*Jeonju/KR*, [2]*Gwangju/KR*, [3]*Chonju/KR (yphot@naver.com)*

**Purpose:** To evaluate the frequency of cluster of submillimeter artefact in normal foot and ankle DECT and to compare the difference between two different setting of minimal HU.
**Methods and Materials:** From July to September 2018, 12 normal foot and ankles obtained by DECT were enrolled. Two radiologists reviewed the images and checked if there is a cluster of green pixilation (submillimeter artefact). When it existed, they assessed the volume, site, shape and the width to height ratio of the artefact. Student t-test was used to compared the frequency and volume of DECT with two different minimal HU setting.
**Results:** The cluster of submillimeter was noted at 75% of normal foot and ankle DECT in setting of minimal HU as 130HU. The volume of artefact significantly decreased from 2.03cm$^3$ to 0.3 cm$^3$. These frequency significantly decreased to 33.3% in setting of minimal HU to 150HU. These were most frequent in forefoot. In setting of minimal HU as 130HU, the most frequent anatomic site was FHL at hindfoot level (12/79) and forefoot level (11/79) and, followed by 2$^{nd}$ flexor tendon at forefoot level (9/79). Other additional sites was FDL at hindfoot level, 3$^{rd}$ flexor tendon at forefoot level.
**Conclusion:** Even though the submillimeter artefact decreases with the minimal HU setting from 130 to 150HU, it is not rare. This artefact is common in forefoot, and tends to be flat and wider than tall. Radiologist should be aware of the common site of the artefact including FHL, 2-3$^{nd}$ flexor tendon, and FDL.

**14:00 - 15:30** **Studio 2019**

### Oncologic Imaging

### SS 716

### How to improve bone metastases and osteosarcoma assessment

*Moderators:*
A. Karpenko; St. Petersburg/RU
L. Kintzelé; Heidelberg/DE

**K-15** 14:00
**Keynote lecture**
T. Bäuerle; *Erlangen/DE*

**B-0685** 14:09
**Using radiomics to identify invisible bone metastases on CT in melanoma patients receiving immunotherapy**
Z. Elkarghali[1], T. Nguyen-Kim[2], J. Spekreijse[1], S. Trebeschi[1], J.B. Haanen[1], C.U. Blank[1], W. Vogel[1], R.G.H. Beets-Tan[1]; [1]*Amsterdam/NL*, [2]*Zurich/CH (z.elkarghali@nki.nl)*

**Purpose:** Bone metastases are associated with advanced tumour progression and poor prognosis, however they are not considered measurable under Response Evaluation Criteria in Solid Tumours (RECIST 1.1, modified iRECIST) and PET Response Criteria in Solid Tumours (PERCIST). While visible on PET scan, some metastatic bone lesions are indistinguishable on CT from surrounding healthy tissue. We hypothesize that non-invasive radiomic features extracted from baseline CTs could be used to discriminate between occult lesions and healthy tissue.
**Methods and Materials:** 238 patients with metastatic melanoma (*receiving anti-PD1 immunotherapy*) were retrospectively studied, of whom 61 had PET-confirmed bone metastases. A total of 153 PET-positive/CT-occult lesions were identified (*using the PET scan as benchmark*) and delineated on CT. The healthy contralateral bone within the same image examination was delineated symmetrically for each identified lesion and was defined as a control lesion. Radiomic features were extracted and with a multivariate mixed-model analysis, each of the features were tested on their ability to discern PET-positive/CT-occult lesions from the matched healthy contralateral controls.
**Results:** Of the seventeen non-redundant CT radiomic features, four were found to be predictive of PET-positive/CT-occult lesions. On a coarse scale, more morphological heterogeneity in bone metastases was observed (*coeff.=-0.619, p<0.001*). This difference in heterogeneity becomes less and less prominent at the medium (*coeff.=-2.041, p=0.003*) and fine scale (*coeff.=-0.721, p=0.018*) - suggesting more involvement of larger structures in the bone.
**Conclusion:** Our findings suggest that, with the use of radiomics, discernible morphological differences exist between PET-positive/CT-occult lesions and their healthy controls, specifically in heterogeneity.

**B-0686** 14:17
**Prediction of immune-checkpoint-inhibitor therapy effectiveness by evaluation of bone marrow activity modifications: an FDG-PET/CT study**
G. Ferrero[1], F. Fiz[2], J. Schwenck [3], C. La Fougère[3], C. Campi[4], G. Sambuceti[2], E. Fabbro[5], B. Pickler[3], M. Kneilling[3]; [1]*Pietra Ligure/IT*, [2]*Genoa/IT*, [3]*Tubinga/DE*, [4]*Padua/IT*, [5]*Cuneo/IT (giulio.ferrero@gmail.com)*

**Purpose:** Despite its efficiency, immune checkpoint inhibitor therapy (ICIT) fails in 60-80% of metastatic melanoma patients. Increase in lymphocyte counts has been proposed as an early prognostic marker of ICIT response,but data are inconsistent.Aim of our study was to uncover whether determination of bone marrow(BM) metabolic activity by $^{18}$F-FDG-PET/CT can predict efficient response to ICIT.
**Methods and Materials:** 20 patients, with Stage-IV melanoma(10 responders; 10 non-responders) were retrospectively enrolled.Every subject underwent a whole-body staging PET/CT (PET1) and a second PET/CT (PET2) 118±29 days later,in the course of ICIT. Response was evaluated with PERCIST criteria.A computer algorithm recognized trabecular bone(where BM is housed) in the CT images and extracted the mean SUV from the co-registered PET images;SUV was corrected for background(target-to-background ratio,TBR).Total, axial and appendicular BM was analyzed separately.Bone metastases,previous radiotherapy,high-grade infections and use of BM-stimulating agents were exclusion criteria.
**Results:** After ICIT,whole-body TBR values increased by 0.52±0.64 in responders (p<0.05) and remained stable in non-responders. In axial skeleton TBR increase was 0.5±0.3 in responders (p<0.01) and 0.1±0.16 in non-responders (p=ns). As a consequence,mean TBR increase was higher in responders than in non-responders,both in total and in axial skeleton(p<0.05).Moreover, responders showed a higher baseline TBR in the axial skeleton when compared to non-responders (2.3±0.62 vs 1.61±0.27,p<0.05).
**Conclusion:** Analysis of glucose metabolism by $^{18}$F-FDG-PET/CT in secondary lymphoid organs might be a new sensitive tool in predicting ICIT response.In particular, a higher BM metabolic activity before therapy as well as a higher metabolic increase after therapy was identified in patients who had responded to the ICIT.

**B-0687** 14:25
**Imaging detection and characterisation of bone metastases from neuroendocrine neoplasm with simultaneously acquired Ga-68 DOTATATE PET/MRI**
J. Bai, R. Matthews, L. Bangiyev, A.R. Chimpiri, D. Franceschi; *Stony Brook, NY/US (james.bai@stonybrookmedicine.edu)*

**Purpose:** The purpose of the study is to evaluate imaging characteristics of bone metastases from neuroendocrine tumour (NET) with simultaneously acquired Ga-68 DOTATATE PET-MRI.
**Methods and Materials:** We retrospectively identified 35 patients with NET who underwent combined Ga-68 DOTATATE PET-MRI for tumour staging. Each PET-MRI was evaluated in consensus by a nuclear radiologist, a body radiologist, and a neuroradiologist. 61 lesions from 8 patients demonstrated abnormal osseous uptakes of DOTA consistent with bone metastases. 38 of 61 lesions were located in the spine. MR sequences included axial T1 radial vibe fs, axial T2 haste, and sag T1 Dixon sequences.
**Results:** 32 of 61 osseous lesions with abnormal DOTA uptake demonstrated variable T1 and T2 signal abnormalities. Of the 32 lesions, 26 lesions showed mild hyperintensity on T1 vibe fs. 34 of 38 DOTA-positive spine lesions were detected on Dixon. Among the spine lesions, Dixon demonstrated consistent abnormal signal characteristics with hyperintensity on water sequence and hypointensity on fat sequence. 1 cervical spinal lesion from a patient with concurrent prostate cancer demonstrated mild DOTA uptake, but prominent MR abnormalities. 1 lumbar spinal lesion from a patient with rectal carcinoid cancer also demonstrated mild DOTA uptake, but suspicious MRI features.

**Conclusion:** In this study, Ga-68 DOTATATE was shown to be a highly sensitive molecular marker that can detect bone metastases from NET before signal abnormalities could be identified on simultaneously acquired MRI sequences. The multi-sequential MRI provides incremental diagnostic value to distinguish bone metastases from NET vs bone metastases from other neoplasm and to identify potential complication from bone metastases which would help in clinical management.

**B-0688** 14:33
**Whole-body MRI vs bone scan in the follow-up of oncologic patients**
A. Malich, I. Papageorgiou, D. Wiech; *Nordhausen/DE (ismini.e.papageorgiou@gmail.com)*

**Purpose:** The 99mTc-bone scan (BS) is a standard of diagnosis for the skeletal staging in many oncologic guidelines. However, the number of nuclear medicine departments in Germany is steadily decreasing while MRI devices become more available, fast, precise and affordable. Whole-body MRI (WB-MRI) is a promising radiation-free alternative to bone scan with growing popularity in the oncological practice. We compare the diagnostic efficacy of WB-MRI to BS in a large database from our hospital.
**Methods and Materials:** From a large database of 1797 WB-MRI, we included 285 eligible patients with WB-MRI and BS within a time interval of 12 months. WB-MRIs were evaluated by 2 radiologists blinded to the BS report. Change of the therapeutic decision, confirmation in less than 1 year from the diagnosis or bioptic confirmation served as the gold standard.
**Results:** From the 285 eligible cases a decision agreement between WM-MRI and BS was observed in 222 cases (in 94 cases both methods were true positive and in 128 cases true negative). In 90 cases WB-MRI and BS showed deviating results and the inter-observer agreement was 71.15% with a Cohen´s kappa of 0.42, which speaks for a moderate method agreement. The sensitivity, specificity, PPV and NPV of WB-MRI were 98/85/82/99% and were not significantly different compared to the general WB-MRI metrics in our department.
**Conclusion:** WB-MRI is a radiation-free, reliable and precise bone staging method with equal efficacy compared to the established BS.

**B-0689** 14:41
**Activity concentration in prostate cancer bone metastases predicts the radioisotope therapy outcome: a SPECT/CT study**
G. Ferrero[1], F. Fiz[2], H. Dittman[3], C. La Fougère[3], C. Campi[4], G. Sambuceti[2], S. Sahbai[5], M. Weissinger[3], M. Piana[2]; [1]*Pietra Ligure/IT*, [2]*Genova/IT*, [3]*Tubinga/DE*, [4]*Padova/IT*, [5]*Parigi/FR (giulio.ferrero@gmail.com)*

**Purpose:** 223RaCl2 is the only bone-specific radioisotope therapy that showed a benefit in metastatic castration-resistant prostate cancer (mCRPC) patients' survival.However,a variable percentage of patients progresses in course of 223RaCl2-Therapy.In this study,we applied a computational software analysis to SPECT/CT images from mCRPC patients,to identify automatically the skeletal tumour burden and the activity concentration in bone metastases from prostate cancer and to see if these factors have an impact on the therapy outcome.
**Methods and Materials:** 76 mCRPC patients were retrospectively enrolled.Each patient underwent a whole-body bone SPECT/CT (99mTc-DPD) for staging,then was treated with 6 223RaCl2 applications.A second SPECT/CT was carried out after this therapy:patients with new visible bone lesions were classified as having a progressive disease(PD),patients with clearly diminished uptake in known metastases as having a partial response(PR),the remaining a stable disease(SD).The staging SPECT/CT was analyzed with a segmentation analysis software,which identified the volume occupied by metastases (mVol),the one of normal trabecular bone (bVol) and the mean activity concentration within these two volumes.
**Results:** PD,SD,PR were 21,35,20,respectively.Mean counts in mVol of PD patients were significantly lower when compared to the other two groups (715±190,1058±255 and 975±215 mean counts,for PD,SD,PR, respectively p<0.01).The same pattern(lower mean counts in PD patients) was observed in bVol (p<0.001).Moreover,PD patients had a lower total volume of metastases in comparison with SD and PR patients (p<0.05).
**Conclusion:** Patients with weaker uptake have a worse therapy response; it seems to be associated with a less extended metastatization. These patients may in fact have less tracer-avid foci and their bone lesions could receive a smaller doses during 223RaCl2 therapy.

**B-0690** 14:49
**18F-FDG PET/MRI for therapy response assessment of isolated limb perfusion in patients with soft-tissue sarcomas**
J. Grueneisen[1], B.M. Schaarschmidt[2], M. Chodyla[1], O. Martin[2], Y. Li[1], W. Fendler[1], K. Herrmann[1], M. Forsting[1], L. Umutlu[1]; [1]*Essen/DE*, [2]*Dusseldorf/DE (johannes.grueneisen@uk-essen.de)*

**Purpose:** To assess the diagnostic potential of simultaneously acquired 18F-FDG PET- and MR-derived datasets for therapy response assessment of isolated limb perfusion with melphalan and alpha-TNF (TM-ILP) in patients with soft-tissue sarcomas.
**Methods and Materials:** A total of 45 patients with a histopathologically proven soft-tissue sarcoma manifestation were prospectively enrolled for an integrated 18F-FDG PET/MR examination before (1$^{st}$ scan) and after (2$^{nd}$ scan) TM-ILP. Therapy response was assessed based on different MR- and PET-derived morphological (RECIST 1.1., MR-adapted Choi criteria) and metabolic (PERCIST) criteria. Histopathological results after subsequent tumour resection served as reference standard and patients were categorized as responders/non-responders based on the grading scale by Salzer and Kuntschik.
**Results:** Histopathological analysis categorized 27 patients as therapy responders (Grade I-III) and 18 patients as non-responders (Grade IV-VI). Calculated sensitivities, specificities, positive and negative predictive values and diagnostic accuracies were 22%, 89%, 75%, 43% and 49% for RECIST1.1, 70%, 44%, 66%, 50% and 60% for MR-adapted CHOI criteria and 85%, 78%, 85%, 78% and 82% for the PERCIST criteria. Furthermore, receiver operating characteristic analysis revealed an area under the curve of 0.56 (RECIST), 0.57 (MR-adapted CHOI) and 0.81 (PERCIST), respectively.
**Conclusion:** Our results demonstrate the discrepancy of morphological and metabolic response criteria and underline the superiority 18F-FDG PET over MRI for response assessment of TM-ILP. Considering the essential role of MRI in preoperative diagnostics, integrated 18F-FDG PET/MRI may serve as a useful diagnostic tool for treatment planning and monitoring of initial treatment strategies of soft tissue sarcoma patients.

**B-0691** 14:57
**Histopathologic and radiologic assessment of chemotherapeutic response in osteosarcoma: correlation of ADC with huvos grading**
H.S. Mohanty[1], A.K. Chaturvedi[1], A. Jajodia[1], M. Bind[2], A.S. Rao[1], R. Tripathi[1], V. Mahawar[1]; [1]*New Delhi/IN*, [2]*Bangalore/IN (drmahawarvivek@gmail.com)*

**Purpose:** Histological necrosis, the current standard for response evaluation in osteosarcoma, is attainable after chemotherapy. To establish the role of surrogate markers of response prediction and evaluation using MRI in the pre-surgical assessment.
**Methods and Materials:** In a retrospective analysis MRI images of newly diagnosed osteosarcoma patients who received more than three cycles of neo-adjuvant chemotherapy or high dose methotrexate followed by surgery during 2012-2018 were analysed. All patients had undergone post-chemotherapy conventional, diffusion-weighted and contrast-enhanced MRI. Classifying histological response in post-surgical specimens according to Huvos grading of necrosis as the reference standard, ADC was measured in pre-surgical DW MRI study and compared to it. ADC was measured separately in soft tissues and bony component of tumor or only in the bone/soft tissue whichever feasible in the sample and it was compared with Huvos grading of necrosis.
**Results:** Mean ADC for soft tissue component in 28 samples was 2068.3±382.5 and mean ADC for 55 samples of bony component was 1866.6 ± 357.6. The ADC measured in bony samples of osteosarcoma is significantly correlating with Huvos grading with P value <0.05. However the ADC of soft tissue component is not correlating significantly with Huvos grading.
**Conclusion:** In osteosarcoma, chemotherapy related grade of bony necrosis can be predicted with the help of DW MRI.

**B-0692** 15:05
**Value of the cinematic rendering from CT data in evaluating the relationship between deep soft tissue sarcomas of the extremities and adjacent major vessels**
Z. Li; *Kunming/CN (lizhenhui621@qq.com)*

**Purpose:** To assess the value of cinematic rendering (CR) from volumetric computed tomography (CT) data in evaluating the relationship between deep soft tissue sarcomas (STS) of the extremities and the adjacent major vessels.
**Methods and Materials:** Preoperative contrast-enhanced axial imaging (CEAI) and CT angiography (CTA) with 3D volume rendering (VR) and CR were used to assess adjacent vascular invasion in 43 cases of deep STS of the extremities. All images were evaluated by two musculoskeletal radiologists. The imaging assessments were compared with surgical findings and interpreted as negative (no vascular invasion) or positive (vascular invasion was present).
**Results:** Thirty-four and nine cases were classified as negative and positive, respectively, in surgery. Intra-reader agreement values for the CEAI, VR, and

CR assessments were all excellent (0.984, 0.934, and 0.914, respectively), while the inter-reader agreement for CEAI assessments was greater than that for VR and CR (0.969 vs 0.804 and 0.761). CR showed lower accuracy (0.698), sensitivity (0.778), specificity (0.676), positive predictive values (PPV: 0.389) and negative predictive values (NPV: 0.920) for vascular invasion diagnosis than CEAI or VR; the accuracy, sensitivity, specificity, PPV, and NPV increased to 0.767, 0.889, 0.735, 0.471, and 0.962 for both CEAI and VR. The results were not statistically significant (all P > 0.05).
**Conclusion:** CR shows excellent intra-reader agreement and good inter-reader agreement for assessment of the relationship between tumours and the adjacent major vessels. It has the potential to be used to evaluate vascular invasion in cases of deep STS of the extremities.

**B-0693** 15:13
**Diffusion-weighted MR imaging diagnostic merits in post-therapeutic assessment of musculoskeletal soft tissue sarcoma**
S. Shokry, A.F.I. Moustafa, M.A.M. Hawanna, M.M. Hussein, A. Shokry; *Cairo/EG (drmego@yahoo.com)*

**Purpose:** Assessing the diagnostic merits of DWI with ADC mapping in evaluating tumour response to chemoradiotherapy.
**Methods and Materials:** Study included 36 patients with soft tissue sarcoma, who received chemo-/radiotherapy. Tumour longest dimension according to RECIST 1.1, the longest dimension of the contrast-enhanced portion of the tumour according to (mRECIST), the tumour volume (VOL)($cm^3$) and DWI with ADC values were recorded.
**Results:** ADC values in the non-progressive group were higher than those of the progressive group after neoadjuvant treatment (1.63± 0.42 vs 1.24±0.35) with (P<0.005). ADC variations in the non-progressive group were higher than those of the progressive group (27.09±48.09 vs -3.08±23.5)% with (P<0.05). ADC values after neoadjuvant treatment were negatively related to tumour volume variations (VOL%) after neoadjuvant treatment. ADC variations (ADC%) were inversely correlated with morphologic changes, regardless of the effectiveness of anticancer therapy expressed as changes in tumour size based on (RECIST, mRECIST and three-dimensional volumetric assessment). Linear regression analysis revealed a Pearson correlation coefficient of r=(-0.424 ,-0.478 and -0.479), respectively, with (P<0.005). An increase in the ADC value was not always associated with a reduction of tumour volume. The disease control rate (defined as the percentage of CR+PR+SD patients) was 63.8% and 69.4% according to RECIST1.1 and mRECIST.
**Conclusion:** Quantitative DW imaging after neoadjuvant therapy provides added value in determining treatment response in soft tissue sarcomas. Therapeutic response to neoadjuvant therapy can be underestimated using RECIST 1.1; therefore, the mRECIST should also be considered.

**B-0694** 15:21
**Bone staging of breast cancer with whole-body MRI: using low field strength without enhancer does not mitigate the diagnostic efficacy**
A. Malich, A. Kott, C. Kurrat, J. Feger, I. Papageorgiou; *Nordhausen/DE (ismini.e.papageorgiou@gmail.com)*

**Purpose:** Whole-body magnetic resonance imaging (WB-MRI) is a guideline recommendation for the detection of bone metastasis in breast cancer. In this study, we compare the diagnostic efficacy of 1.5T and 3T WB-MRI as well as the diagnostic benefit from gadolinium enhancement.
**Methods and Materials:** The study was retrospective for 874 patients scanned between 05/2007 and 08/2018. 360 patients were imaged at 1.5T and 514 at 3T with a T1-w FFE and a STIR or a Dixon at the coronal level. The slice thickness was 6mm at 1.5T and 3mm at 3T. In 441 patients, we injected gadoteridol (ProHance) 0.1 mmol/kg. Clinical or bioptic confirmation served as the gold standard.
**Results:** The sensitivity (Se) and specificity (Spe) for 1.5T was 98/91% and the positive predictive value (PPV)/negative predictive value (NPV) was 86/99%. A field strength of 3T showed Se/Spe 100/93% and a PPV/NPV of 83/100%. Binary logistic regression with Fisher's exact test revealed no significant difference between 1.5T and 3T WB-MRI ($P$=0.83). The Se/Spe of WM-MRI (merged 1.5T and 3T) without enhancement was 99/92%, with PPV/NPV 87/99%. Upon administration of ProHance the Se/Spe shifted to 100/93% and the PPV/NPV to 83/100%, overall without statistically significant effect compared to non-enhanced scans ($P$=0.83, Fisher´s exact test).
**Conclusion:** Field strengths of 1.5T or 3T show equal diagnostic efficacy for the diagnosis of osseous metastatic breast cancer with WB-MRI. Gadolinium can be omitted without compromising the WB-MRI diagnostic accuracy.

**14:00 - 15:30** **Room E1**

### Breast

### SS 702a

### Decision tools and artificial intelligence in breast imaging

*Moderators:*
N.N.
T. Sella; Jerusalem/IL

**K-16** 14:00
**Keynote lecture**
F.J. Gilbert; *Cambridge/UK*

**B-0695** 14:09
**Can artificial intelligence identify normal mammograms in screening?**
K. Lång, M. Dustler, V. Dahlblom, I. Andersson, S. Zackrisson; *Malmö/SE (kristina.lang@med.lu.se)*

**Purpose:** To evaluate the effect of using a deep-learning algorithm in mammography screening to identify normal exams.
**Methods and Materials:** 9,588 double-read mammography screening exams including 71 screen-detected cancers and 187 false positives, a sub-cohort from the prospective populations-based Malmö Breast Tomosynthesis Screening Trial, were analysed with a deep-learning-based software. The software is used as a preselection tool that categorizes mammograms with a cancer risk score of 1-10, with 10 indicating the highest risk of malignancy. The effect of excluding cases with low risk scores (≤5) from screen-reading was investigated. A panel of three breast radiologists assessed the radiographic appearance, type and visibility of cancers assigned low risk scores.
**Results:** 5,085 (53.9%) exams, including 8 (11.3%) cancers and 52 (27.8%) false positives had low risk scores. If cases scored 1 were excluded, 1,004 (10.5%) normal exams could be removed, including 6 (3.2%) false positives but no cancers. If cases scored 1-2 were excluded, 1,830 (19.1%) exams could be excluded, including 10 (5.4%) false positives and one cancer, an 8 mm invasive ductal carcinoma. The 8 cancers with low risk scores were all invasive, including the ductal, tubular and lobular type. All, except one, were clearly visible, and the majority (5/8) were spiculated masses.
**Conclusion:** An artificial intelligence system can reduce the screen-reading workload. With further improvement of the software an even greater exclusion of normal mammograms seems possible since the majority of the cancers with low risk scores were clearly visible.

**B-0696** 14:17
**Artificial intelligence software to improve mammography workflow**
A.T. Watanabe[1], R.C. Mayo[2], L.Q. Chang Sen[2], M. Kapoor[2], J. Leung[2]; [1]*Los Angeles, CA/US*, [2]*Houston, TX/US (alyssa90266@gmail.com)*

**Purpose:** To determine whether artificial intelligence (AI)-based software can be used to improve radiologists' workflow efficiency and revenue.
**Methods and Materials:** A retrospective study was performed on a set of 250 2-D Mammograms. The number of cases marked by AI vs traditional CAD, and the number of cases that were recalled were compared. The potential time saved and revenue benefits from use of AI were calculated.
**Results:** 52% of cases had no marks by AI while only 17% were free of CAD marks. Both systems had 100% sensitivity for cancer detection. There were 37% fewer cases marked by AI compared with CAD. Fifteen of the 18 false positive cases had CAD marks whereas only 8 of the 18 were marked by AI. Based on an assumption of 100,000 mammograms per year, extrapolated data shows AI would yield a potential time savings of 64% or 375 hours of radiologist reading time per year based on fewer marked cases. Up to 10% more screening mammograms could be read with the saved time with as much as $1,488,362 per year of increased global revenue based on Medicare Fee schedule rates. In addition, AI software can pre-sort the worklist based on suspiciousness which enhances reader's efficiency and potentially their accuracy.
**Conclusion:** There is potential for decreased reader time, fewer recalls, and increased revenue with AI based software products.
*Author Disclosures:*
**A.T. Watanabe:** Board Member; Chief Medical Officer , Curemetrix.

**B-0697** 14:25

**Artificial intelligence detecting breast cancer on mammography: does breast density play a role?**

A. Rodriguez Ruiz, M. Kallenberg, A. Gubern-Merida, N. Karssemeijer, R.M. Mann; *Nijmegen/NL*

**Purpose:** To analyze the relationship between the breast cancer detection performance of an artificial intelligence (AI) system on mammography and breast density.
**Methods and Materials:** An independent multi-vendor cancer-enriched database of 1397 mammograms was collected across several institutions in Europe (179 with biopsy-verified cancer, 1218 normal with at least two years of negative follow-up). For each mammogram, an AI system computed a score representing the likelihood of presence of cancer (scale 1-10); while the breast density volume fraction (averaged across views) was computed with previously published and validated software. Linear regression analysis was performed for AI score and density. Subsequently, the population was divided into two groups based on the median density value of the population: high-density mammograms (73 cancers, 626 normals) and low-density mammograms (106 cancers, 592 normals). The area under the receiver operating characteristic curve (AUC) of the AI system was compared between the two groups.
**Results:** Median density volume fraction in the population was 0.12 (0.09-0.18). The AI score did not correlate with density ($R^2$ = -0.09), with low AI score categories harboring at same proportions high-density and low-density mammograms. The AUC of the AI system was similar for both groups (0.91 vs 0.89, difference = +0.02, 95% CI = -0.03,0.08).
**Conclusion:** The AI score may be considered as an independent tool to estimate the likelihood of the presence of cancer on mammograms, to stratify screening populations, and to potentially fasten the reading process by reassuring readers on mammograms that are likely normal, irrespective of breast density.
*Author Disclosures:*
**A. Rodriguez Ruiz:** Employee; ScreenPoint Medical. **M. Kallenberg:** Employee; ScreenPoint Medical. **A. Gubern-Merida:** Employee; ScreenPoint Medical. **N. Karssemeijer:** CEO; ScreenPoint Medical.

**B-0698** 14:33

**Improved cancer detection using artificial intelligence: a retrospective evaluation of missed cancers on mammography**

A.T. Watanabe[1], V. Lim[2], E. Weise[3], H. Vu[3]; [1]*Manhattan Beach, CA/US*, [2]*La Jolla, CA/US*, [3]*San Diego, CA/US (alyssa90266@gmail.com)*

**Purpose:** To determine whether an artificial intelligence-based computer-aided detection (AI-CAD) algorithm can be used to improve radiologists' sensitivity in breast cancer screening and detection.
**Methods and Materials:** A blinded retrospective study was performed with a panel of seven radiologists using a cancer-enriched data set from 122 patients that included 90 false negative mammograms obtained up to 5.8 years prior to diagnosis. The mammograms were performed between February 7, 2008 (earliest) and January 8, 2016 (latest) and evaluated using Students T Test statistical analysis.
**Results:** All radiologists showed a significant improvement in their cancer detection rate (CDR) with the use of AI-CAD (p-value=0.0069, Confidence interval = 95%). With the assistance of AI-CAD, the sensitivity of less experienced general radiologists improved to a level higher than a fellowship-trained academic breast imager. The readers detected between 25% and 71% (mean 51%) of the early cancers without assistance. With AI-CAD, the overall reader CDR was 41% to 76% (mean 62%). Overall, there was less than 1% increase in the readers' false positive recalls with use of the AI-CAD.
**Conclusion:** There was a statistically significant improvement in radiologists' sensitivity for cancer detection in this set of primarily false negative mammograms with the benefit of the AI-CAD. The percentage increase in CDR for the radiologists in the reader panel, ranged from 6% to 64% (mean 27%) with the use of AI-CAD, with negligible increase in false positive recalls.
*Author Disclosures:*
**A.T. Watanabe:** Board Member; Chief Medical Officer , Curemetrix. **V. Lim:** Consultant; CureMetrix. **E. Weise:** Employee; CureMetrix. **H. Vu:** Board Member; CureMetrix.

**B-0699** 14:41

**False negative on computer-aided detection application in preoperative automated breast ultrasound of breast cancer patients**

Y. Kim[1], J. Rim[1], S.M. Kim[1], B.L. Yun[1], S.Y. Park[1], H.S. Ahn[2], B. Kim[3], M.J. Jang[1]; [1]*Seongnam/KR*, [2]*Seoul/KR*, [3]*Yongin/KR*

**Purpose:** To analyse the characteristics of ultrasonographic, mammographic, and MRI findings of breast cancers that computer-aided detection (CAD) application missed in automated breast ultrasound (ABUS).
**Methods and Materials:** Total 133 consecutive breast cancer patients (age in mean ± standard deviation, 52.2 ± 10.3 years) who underwent preoperative ABUS were included in our study. Imaging features of mammography, ABUS, and breast MRI as well as pathology results were assessed. Univariable analyses were performed to identify factors associated with missed breast cancers. Multivariable regression analysis was performed using variables which showed a statistical significance in the univariable analyses.
**Results:** The CAD application missed breast cancers in 27.1% (36/133) of our included patients. Missed breast cancers had higher frequency of ductal carcinoma in situ in pathology and the following imaging features in ultrasound: size <1 cm, distance to nipple <5 mm, indistinct margin, and heterogeneous background tissue echotexture. None of the variables assessed in mammography or MRI was associated with missed breast cancers. In the multivariable regression analysis, ductal carcinoma in situ in pathology, size <1 cm, indistinct margin, and heterogeneous background tissue composition were associated with missed breast cancers.
**Conclusion:** Breast cancers missed by ABUS CAD application tended to have size <1 cm, indistinct margin, and heterogeneous background tissue echotexture in ABUS. Understanding the imaging features of CAD application-missed breast cancers would help radiologists to diagnose breast cancer in ABUS more accurately.

**B-0700** 14:49

**Automatic detection of breast lesions on ABUS using a novel transfer learning approach: comparison between three different networks**

J. Santinha[1], A. Vourtsis[2], A. Uysal[3], J. Moreira[1], N. Papanikolaou[1]; [1]*Lisbon/PT*, [2]*Athens/GR*, [3]*Ankara/TR (joao.santinha@research.fchampalimaud.org)*

**Purpose:** To compare three machine learning models that can automatically identify the presence of a lesion on Automated Breast Ultrasound System (ABUS) images.
**Methods and Materials:** Coronal ABUS reconstructions from 195 patients that underwent ABUS examination were randomly selected and utilised in the current study. 130 ABUS images containing lesions were used, while another 130 images were randomly selected from 65 patients with negative exams (2 non-consecutive slices per patient). 60% of the exams were used for training, 20% for validation and 20% as a hold out test data set. 2048 deep features were extracted from each ABUS image using a pre-trained Google's Inception V3 network, the VGG-16 and VGG-19 networks that were trained on ImageNet. A logistic regression algorithm was used to classify ABUS images into normal or abnormal.
**Results:** Testing of the diagnostic performance of the three classifiers was done on 52 images that were kept away from the training-validation phase. Inception V3 network provided with the highest performance (sensitivity, specificity and accuracy were 96.15%, 92.31% and 94.23%, respectively), followed by VGG-16 (sensitivity, specificity and accuracy were 88.46%, 96.15% and 92.31%, respectively) and VGG-19 (sensitivity, specificity and accuracy were 92.31%, 88.46% and 90.38%, respectively).
**Conclusion:** Transfer learning based on Inception V3 offers very high performance for automatic detection of breast lesions on ABUS images.

**B-0701** 14:57

**Automated volumetric analysis of breast cancer vascularisation enables risk stratification in patients scheduled for neoadjuvant chemotherapy**

M. Dietzel[1], S. Ellmann[1], P. Clauser[2], E. Wenkel[1], M. Uder[1], R. Schulz-Wendtland[1], P.A.T. Baltzer[2]; [1]*Erlangen/DE*, [2]*Vienna/AT*

**Purpose:** Volumetric analysis of breast cancer vascularization (VAV) can be automatically performed on a standard pre-therapeutic breast-MRI examination. We investigated whether breast cancer vascularization (VAV) could be used for risk-stratification in patients scheduled for neoadjuvant-chemotherapy using complete response (CR), distant metastasis (MTX) and disease-specific death (DSD) as study endpoints.
**Methods and Materials:** Within this retrospective, IRB-approved study, 77 consecutive patients with primary invasive breast cancer received standard breast-MRI before the initiation of neoadjuvant-chemotherapy according to international recommendations. Diagnostic workup, treatment, and follow-up were done at one tertiary care, academic breast center (CR: 13, MTX: 17, DSD: 14; mean follow-up: 56 months). Based on the MRI-scans, VAV was accomplished by commercially available, FDA-cleared software. DSD, CR, and MTX served as endpoints. Potential of VAV to predict all three endpoints was investigated; the potential of clinico-pathological factors (CPF) to predict DSD and MTX was identified and compared to VAV. Finally, we investigated whether integration of VAV into CPF increased prognostic accuracy (logistic/Cox regression, Kaplan-Meier statistics, ROC analysis, area under the curve/AUC; alpha=5%).

**Results:** VAV was able to predict DSD (AUC= 86.3, hazard-ratio/HR=20.2), MTX (AUC= 80.3), and CR (AUC=79, all P<0.001). CPF enabled the prediction of DSD (AUC= 68.3, HR=2.7; P=0.01) and MTX (AUC= 74.4, P=0.03), yet with a substantially lower accuracy (P>0.04). Combination of VAV and CPF further increased the accuracy (P>0.002) regarding the prognostication of DSD (AUC= 92.2, HR=35.7) and MTX (AUC=85.7%).
**Conclusion:** Automated volumetric analysis of breast cancer vascularization enables non-invasive risk-stratification in patients scheduled for neoadjuvant-chemotherapy.

**B-0702** 15:05
**The Kaiser score, a decision rule based on BI-RADS MRI descriptors: is there an impact of histopathology on the diagnostic accuracy?**
M. Dietzel[1], S. Ellmann[1], E. Wenkel[1], P. Clauser[2], R. Schulz-Wendtland[1], M. Uder[1], P.A.T. Baltzer[2]; [1]*Erlangen/DE*, [2]*Vienna/AT*

**Purpose:** We investigated, whether the diagnostic accuracy of the Kaiser score is influenced by the underlying histopathology.
**Methods and Materials:** Consecutive patients with equivocal breast lesions scheduled for breast MRI (standardized protocols @ 1.5T: dynamicT1-GRE before/after Gd-DTPA [0.1 mmol/kg body weight (BW)]; T2-TSE), with subsequent pathological sampling, were investigated. The Kaiser score was assessed by two experienced radiologists in consensus (blinded to pathology). Diagnostic accuracy of the Kaiser score stratified by the underlying histopathology was investigated (ROC analysis, descriptive statistics, alpha=5%).
**Results:** There were 1080 lesions in 1080 breasts (malignant/benign: 643/437). Histopathology revealed all major subtypes of benign and malignant lesions. Overall accuracy for the differential diagnosis of malignant vs. benign lesions achieved reported values (AUC=0.88). Performance of the Kaiser score regarding invasive cancers (AUC=0.91) was superior to DCIS (AUC=0.73; P<0.001). The Kaiser score performed equally in the assessment of invasive cancers subtypes (AUC=0.92-0.86; P=0.13-0.97). Performance of the Kaiser score in the assessment of papillomas (AUC=86.7) and fibrocystic changes (AUC=88.0) was similar (P=0.57). Yet, the Kaiser score performed better in fibroadenomas (AUC= 91.8; P<0.05). The highest accuracy of the Kaiser score was observed within the differential diagnosis of benign phyllodes tumours vs. malignant lesions (n=10; AUC=98.2; P<0.001).
**Conclusion:** In general, the Kaiser score is independent of the underlying histopathology and reaches high accuracy. Even in the presence of challenging tumours (DCIS), the Kaiser score enabled a precise diagnosis, yet with a slightly lower level of accuracy.

**B-0703** 15:13
**Combining artificial intelligence and MRI in the management of equivocal breast lesions: the perfect couple?**
M. Dietzel, S. Ellmann, E. Wenkel, C. Bielowski, R. Schulz-Wendtland, M. Uder, T. Bäuerle; *Erlangen/DE*

**Purpose:** We evaluated whether combining artificial intelligence (AI) and MRI could be beneficial to the management of equivocal breast lesions.
**Methods and Materials:** 100 consecutive patients were enrolled. All showed equivocal breast lesions (BI-RADS IV) upon conventional assessment (clinical examination, mammography, and ultrasound) and received histopathological verification. Prior to biopsy we performed a standardized breast MRI in each patient. Six agnostic semantic MRI parameters were extracted by two observers independently (R1/R2: intermediate/no experience in breast MRI). These semi-/quantitative parameters have been previously described; they assess geometry, structure, microstructure and vascularity of each lesion. Based on these parameters, AI was used to identify typical MRI thumbprints of certainly benign lesions as defined by a false-negative rate <1%. An AI-algorithm consisting of a polynomial kernel function support vector machine was used for this purpose. This approach has been previously published including, training and evaluation on breast MRI data (n>200). Statistical evaluation included intraclass correlation coefficient/ICC, Mann-Whitney U test and descriptive methods (alpha<1%).
**Results:** 33 lesions were malignant and 67 benign. Agnostic parameters demonstrated excellent inter-observer variability (ICC > 0.8); every parameter showed significant potential to differentiate BI-RADS IV lesions (P<0.001). AI identified typical fingerprints of certainly benign lesions in 31.3% (21/67).
**Conclusion:** Combining artificial intelligence (AI) and MRI could be beneficial to the management of equivocal findings: this perfect couple identified thumbprints of “certainly benign” findings in a substantial proportion of equivocal lesions (31.3%). As such thumbprints showed an extremely low false-negative rate (<1%), biopsy could have been safely avoided in these patients.

**B-0704** 15:21
**Automatic cluster analysis of breast lesions assessed with diffusion-weighted imaging**
J. Schaar, H. Dijkstra, M. Wielema, M. Dorrius, P. Sijens; *Groningen/NL (j.e.schaar@umcg.nl)*

**Purpose:** To optimize and validate an automatic method for cluster analysis of breast lesions to prevent unnecessary biopsies and compare this method with three already existing methods.
**Methods and Materials:** This retrospective consecutive study was approved by the hospital’s institutional review board and informed consent was obtained. 163 breast lesions with known pathology and BI-RADS classification (BI-RADS 2, 3, 4, 5, 6 with 7, 7, 19, 73 and 33 lesions, respectively) were examined (1.5T MRI) with diffusion-weighted imaging using b=0 and 1000s/mm$^2$. The lesions were automatically defined into clusters based on the pixel diffusion values using Matlab. For each cluster mean apparent diffusion coefficient (ADC) and area were calculated. Optimal thresholds and minimal cluster area were determined: the cluster algorithm was optimized in a training set of 24 lesions (12 benign) to yield maximum true negatives and minimal false negatives. The cluster algorithm was validated in a subsequent consecutive set of 139 lesions (23 benign) to obtain sensitivity (Se) and specificity (Sp). The cluster algorithm was compared to three existing methods: the minimal and mean ADC of a lesion and histogram analysis using the 10% lowest ADC pixels.
**Results:** In the training set, optimal thresholds were 1.118x10$^{-3}$mm$^2$/s (cluster algorithm, area>3 pixels), 0.958x10$^{-3}$mm$^2$/s (minimal ADC), 1.705x10$^{-3}$mm$^2$/s (mean ADC) and 1.331x10$^{-3}$mm$^2$/s (lowest 10%). This resulted in the validation set in Se=97.4% and Sp=43.5% (cluster algorithm), Se=95.7% and Sp=30.4% (minimal ADC), Se=93.1% and Sp=43.5% (mean ADC), and Se=97.4% and Sp=21.7% (lowest 10%).
**Conclusion:** Automatic cluster analysis of breast lesion diffusion yields a high sensitivity and can potentially reduce unnecessary biopsies.

**14:00 - 15:30** **Room E2**

### Neuro

### SS 711

### Stroke: diagnostic imaging

*Moderators:*
P. Due-Tønnessen; Oslo/NO
P. Thurner; Vienna/AT

**B-0705** 14:00
**Cerebrospinal fluid volume as a predictor of malignant middle cerebral artery infarction**
F. Kauw, E. Bennink, H.W.A.M. De Jong, L. Kappelle, A.D. Horsch, B.K. Velthuis, J.W. Dankbaar; *Utrecht/NL (f.kauw-3@umcutrecht.nl)*

**Purpose:** To investigate the association between intracranial cerebrospinal fluid (CSF) volume and malignant middle cerebral artery (MCA) infarction.
**Methods and Materials:** Patients with an occlusion proximal to the M3 segment of the MCA were selected from the Dutch Acute Stroke Study (DUST). Admission imaging included non-contrast CT, CT-angiography and CT-perfusion. Malignant MCA infarction was defined as a midline shift larger than 5 mm on follow-up non-contrast CT, which was performed three days after the stroke or in case of clinical deterioration. Clinical characteristics and CT findings were collected. The ratio between CSF volume and intracranial volume (CSF/ICV) was quantified on admission thin slice non-contrast CT. To test the association between CSF/ICV and malignant MCA infarction, odds ratios were calculated for three multivariable models using binary logistic regression. Furthermore, ROC curves were plotted from the models and their AUCs were compared using the DeLong method.
**Results:** Of the 271 included patients, 34 (13%) developed malignant MCA infarction. CSF/ICV was independently associated with malignant MCA infarction in three multivariable models: 1) with age and admission NIHSS (OR 3.3, 95% CI 1.1-11.3, p<0.05), 2) with admission NIHSS and poor collateral score (OR 5.0, 95% CI 2.2-12.4, p<0.001), 3) with clot burden score and poor collateral score (OR 7.3, 95% CI 2.6-22.8, p<0.001). The performance of all models was improved by adding CSF/ICV, but only model 1 improved significantly (AUC 0.787 vs. 0.819, p<0.01).
**Conclusion:** CSF volume is associated with malignant MCA infarction and might be of additional value to clinical prediction models.

**B-0706** 14:08

**Improved diagnostic performance of CT perfusion imaging in acute stroke using advanced noise suppression and Bayesian perfusion algorithms**

E.J. Smit[1], K. Murayama[2], T. Galen[1], S. Pegge[1], L. Kluijtmans[3], Y. Ikeda[4], K. Katada[2], F.J. Meijer[1], M. Prokop[1]; [1]*Nijmegen/NL*, [2]*Toyoake/JP*, [3]*Malden/NL*, [4]*Ōtawara/JP (ewoudsmit@gmail.com)*

**Purpose:** Current CTP techniques have limited diagnostic performance because of moderate image quality resulting from low signal-to-noise ratios. We tested if advanced noise-suppression and Bayesian probabilistic perfusion techniques allow for more reliable diagnosis of ischaemia in acute stroke.
**Methods and Materials:** We evaluated 37 consecutive patients with suspected acute stroke who underwent CTP and DWI within 6 hours after each other. Post-processing for CTP was performed with commercial software using a standard processing technique that uses smoothing filters and single value decomposition (SVD) algorithms and an advanced processing technique that uses a 4D-similarity filter for noise suppression followed by Bayesian perfusion algorithms. Three expert observers rated all images for the presence of a perfusion deficit, diagnostic confidence and image quality on a 5-point Likert scale. MR diffusion-weighted imaging (DWI) served as the reference test.
**Results:** Diagnostic performance was significantly better for the Bayesian-based method compared to the SVD-based method, with area under the ROC curves of 0.88 and 0.72, respectively. At similar sensitivity of 72-73% for detecting DWI infarcts, the Bayesian-based method resulted in a higher specificity with 93% compared to 52% for the SVD-based method. Observers were significantly more confident of their diagnosis using the Bayesian-based method and image quality was rated significantly higher for all perfusion maps.
**Conclusion:** Bayesian-based processing improves the reliability and diagnostic performance of CT perfusion imaging. Using these methods, CT perfusion imaging seems to allow for the reliable diagnosis of acute ischaemic stroke. However, negative perfusion exams still cannot exclude cerebral ischaemia.
*Author Disclosures:*
**E.J. Smit:** Research/Grant Support; Canon Medical. Speaker; Canon Medical. **Y. Ikeda:** Employee; Canon Medical. **M. Prokop:** Research/Grant Support; Canon Medical. Speaker; Canon Medical.

**B-0707** 14:16

**Effect of report length on its clarity in the stroke setting: an AI study**

A. Mereu, L. Saba; *Cagliari/IT*

**Purpose:** To assess whether a longer text report can hide its meaning when a clear message is critical, such in the case of head CT for stroke.
**Methods and Materials:** 300 urgent head CT without contrast free-text reports were retrospectively analysed. All clinical requests suspected acute stroke. Findings were categorized (presence or lack thereof) in acute, such as stroke and haemorrhage, and chronic ones, such as lacunae and leukoaraiosis. Reports' word count was measured. An ensemble of 7 algorithms (based on bagging, boosting, classification trees, maximum entropy, neural networks, random forests and support vector machine) was employed by a supervised AI to predict the findings. Statistical analysis was performed with the multinomial logistic regression and significance was set at $p < 0.05$. All the main tasks involved were handled with the free and open source programming language R.
**Results:** Report length did not correlated significantly with the probability of a correct prediction of acute findings only ($p = 0.5103$), chronic ones only ($p = 0.4258$) or both at the same time ($p = 0.5221$). The whole model was not significant too ($p = 0.4876$).
**Conclusion:** Study results showed that wordy reports did not significantly impair AI predictions. Thus, neither an attentive human reader might be impaired by the amount of information provided.

**B-0708** 14:24

**Evaluation of pial collaterals on multiphase CT angiography: impact on the prediction of infarct size and outcome in acute ischaemic stroke**

E. Puglielli, G. Manente, M. Assetta, P. Pulsone, V. Di Mizio; *Teramo/IT (edopug@hotmail.com)*

**Purpose:** Pial collaterals may vary in CTA single phase (sCTA) depending on whether the CTA is arterial (A), arterio-venous (AV) or venous (V) weighted. We investigate the impact of sCTA weighting on the evaluation of collaterals using the classic MAAS, Tan and Menon methods and their ability to predict infarct size and outcome.
**Methods and Materials:** 162 consecutive patients (mean age of 45 years, April 2009-September 2018) with classical onset, underwent to endovascular therapy, in a single center. Basal CT, CTP and sCTA were used for imaging. The sCTA weighting was determined by comparing the AV ratios between torcula with those obtained from concomitant CTP temporal density curves at the attenuation of the arterial peak or of the venous contrast. Two readers studied infarct volume on imaging of the three collateral scores stratified by sCTA weighing with age, gender, aetiology, symptoms, NIHSS, clot burden score (CBS) and mRS. For primary analysis, we used ordinal logistic regression.
**Results:** Median NIHSS score at presentation was 13 (range 3-36) and the onset to treatment time were 128 (66-181) minutes. sCTA scans were AV-weighted in 105/162 (64.8%) and A-weighted in 57 (35.1%). No association was demonstrated between sCTA weighting and sCTA technique. No association was shown between sCTA weighting, collateral grade and clinical outcome (p<005).
**Conclusion:** After recanalization the sCTA weighting did not significantly impact collateral grade analysis with the three common collateral scores and there is lack of evidence of their ability to predict final infarct size.

**B-0709** 14:32

**Extracted computed tomography angiography measurements as a screening tool for obstructive sleep apnoea in acute stroke patients**

T.J. Jeffery, D. Gibson; *Perth/AU (tim.jeffery@iinet.net.au)*

**Purpose:** Obstructive sleep apnoea (OSA) is reportedly a common co-existent disorder in stroke patients. The presence of OSA could significantly hinder neurological recovery. Radiological correlative measurements on cross-sectional neck imaging correlates characterise both the presence and severity of OSA. There are limited published studies exploring the frequency of these adverse parameters in acute stroke patients. This case series explores the incidence of three robust radiological surrogate measures in routine contrast-enhanced CT imaging of acute stroke patients.
**Methods and Materials:** The imaging for 50 sequential acute stroke presentations from a major tertiary hospital was reviewed. Three measurements were routinely performed on computed tomography extra- and intracranial angiogram (CTA) studies: mandibular plane to hyoid distance (MPH), oropharyngeal length (OPL) and minimal transverse dimension of the retropalatal airway (RPA).
**Results:** Of the 50 cases analysed, 74% had at least one measure exceeding defined thresholds. An inferiorly placed hyoid bone (MPH>18mm) was the most frequent adverse finding at 58%. An elevated OPL and a reduced RPA were less frequent at 42% and 26%, respectively. Only two of the fifty cases were documented as having diagnosed OSA upon discharge.
**Conclusion:** Radiological indicators of sleep apnoea are frequently present in our acute stroke population. The results of this case series suggest that these measurements should be performed routinely on admission CTA studies and be included in radiology reports. This can alert treating teams to consider in patient polysomnography studies for confirmation of OSA and early institution of continuous positive airway pressure support.

**B-0710** 14:40

**Comparison of accuracy for the prediction of infarct tissue volumes between arrival-time insensitive and arrival-time sensitive CT perfusion algorithms**

L. Pennig, F. Thiele, M. Perkuhn, C. Kabbasch, M. Schlamann, J. Borggrefe; *Cologne/DE (lenhard.pennig@uk-koeln.de)*

**Purpose:** Stroke-thrombectomy (ST) can be performed up to 24 hours after onset if a significant CT-perfusion (CTP) mismatch is present. CTP maps can be calculated by arrival-time-sensitive (TSP) and arrival-time-insensitive (TIP) algorithms. This study compared performances of TSP and TIP and investigated optimal parameters of TIP for the prediction of final-infarct-volume (FIV) and penumbra.
**Methods and Materials:** Retrospective study including 54 patients receiving Stroke-CT/CTP-imaging between 2010-2018 with follow-up-imaging as reference standard. CT scans were conducted at 4 different CT-scanners. FIV was obtained using CT and MRI. Patients were stratified as a) not-treated (mTICI 0, n: 25) and b) successful ST (mTICI 2b/3, n: 29). IntelliSpace Portal (Philips, NL) was used to calculate CTP-maps for both TSP and TIP. TSP (default settings) and TIP (full-range of threshold settings for CTP parameters) results were coregistered in 3D with FIV using IntelliSpace Discovery (Philips, NL), and voxel-wise overlap was measured.
**Results:** Baseline characteristics; NIHSS=15.34 (SD±6.8), 67.02 years (SD±13.11), 37/17 m/f, average follow-up-imaging after 2.63 days (SD±2.67). Regarding TIP, penumbra (group A) was best predicted using Tmax (AUC 0.9), FIV (group B), was determined best using relative CBF (AUC 0.8). TIP´s prediction of penumbra and FIV was comparable to previous literature. There was a broad range of thresholds of optimal TIP-settings in both groups.
**Conclusion:** Both algorithms showed a comparable performance, with TIP yielding a slightly better, but statistically non-significant prediction of FIV. TIP showed a broad range of optimal thresholds. Thus, exact thresholds regarding TIP should be evaluated in further studies.
*Author Disclosures:*
**F. Thiele:** Employee; Philips, NL. **M. Perkuhn:** Employee; Philips, NL. **J. Borggrefe:** Speaker; Philips, NL.

**B-0711** 14:48
**Prediction of motor recovery after stroke by assessment of corticospinal tract Wallerian degeneration using diffusion tensor imaging**
R. Elshafey[1], H.S. Darwish[2], H.A. Kamel[1]; [1]*Tanta/EG*, [2]*Ismalia/EG* *(darwish.hoda@yahoo.com)*

**Purpose:** To predict motor recovery after stroke by detection of diffusion tensor (DTI) imaging fractional anisotropy changes of corticospinal tract and correlate findings with clinical scores to provide more effective treatment and rehabilitation.
**Methods and Materials:** 30 patients with cerebral stroke were included, 18 males (60%) and 12 females (40 %); mean age was 58 +/- 11.5 years. The patients underwent conventional MRI, diffusion tensor imaging at admission as well as one month after stroke. Mean diffusivity (MD), fractional anisotropy (FA), fractional anisotropy ratio (rFA) and fibre number (FN) values of corticospinal tract (CST) were calculated at the pons at admission and after one month of stroke. Three-dimensional reconstruction of bilateral corticospinal tract and the structural changes of fibrous bands were observed. Severity of limb weakness was assessed using the motor sub-index scores of the National Institutes of Health Stroke Scale (NIHSS) at admission, and after 1, 6 and 9 months for severity of limb weakness.
**Results:** Significant negative correlation was found between rFA and FN in the ipsilateral CST of the cerebral infarction at the rostral part of pons after one month of infarction and NIHSS score at 6 months ( r= -0.377, P=0.04) ( r=-0.237, P=0.02), respectively, while positive insignificant correlation was observed between MD and NIHSS (r=0.345, P=0.635).
**Conclusion:** DTI is a sensitive tool for the early detection of Wallerian degeneration (WD) in the CST after stroke and can predict motor performance and outcome to provide more effective treatment and rehabilitation and improve quality of life.

**B-0712** 14:56
**Arterial transit artefacts on ASL perfusion MRI in patients with carotid artery stenosis are a better predictor of recent symptoms than degree of stenosis or carotid plaque morphology**
A. Di Napoli[1], S. Cheng[2], G. John[2], M. Brown[2], M. Sokolska[2], H.R. Jäger[2]; [1]*Rome/IT*, [2]*London/UK (adnapoli7@hotmail.com)*

**Purpose:** The most commonly assumed mechanism by which carotid artery stenosis (CAS) causes a stroke or TIA is artery to artery embolism. Carotid plaque imaging allows to assess the component of atherosclerotic plaques and to detect intra-plaque haemorrhage (IPH) ; Arterial Spin Labelling (ASL) is a non-invasive method of assessing haemodynamic effect of CAS and Arterial Transit Artefacts (ATAs) indicate brain regions with delayed arrival of blood. The aim of this study is to identify imaging parameters that distinguish best between asymptomatic and symptomatic patients and to obtain new evidence by which mechanism carotid stenosis causes clinical symptoms.
**Methods and Materials:** 44 patients with cervical ICA stenosis (22 symptomatic and 22 asymptomatic) underwent MRI at 3T including pseudocontinous arterial spin labelling (pACSL) had carotid plaque imaging sequences. Degree of stenosis, plaque morphology, intra-plaque haemorrhage and extent of ATAs were evaluated.
**Results:** ATAs were present only in patients with >70% stenosis (p<0.0001). Presence of symptoms correlated with ATAs (p=0.004) and with their severity (p=0.002), but not with degree of stenosis (p=0.537), plaque morphology (p=0.537) or intra-plaque haemorrhage (p=0.310).
**Conclusion:** The presence of ATAs on ASL imaging correlated best with symptoms in patients with ICA stenosis, suggesting that cerebral haemodynamics play a greater role than previously thought and have to be considered alongside embolic mechanisms.

**B-0713** 15:04
**Clinical decision support based on automated non-contrast CT density measurements in patients with acute ischaemic stroke**
P. Reidler[1], D. Puhr-Westerheide[1], M.P. Fabritius[1], L. Rotkopf[1], D. Apel[1], S. Tiedt[1], F.A. Wollenweber[1], K. Thierfelder[2], W.G. Kunz[1]; [1]*Munich/DE*, [2]*Rostock/DE (Paul.Reidler@med.uni-muenchen.de)*

**Purpose:** To examine the clinical value of automated non-contrast CT (NCCT) density measurements in Alberta-Stroke-Program-Early-CT-Score (ASPECTS) regions to support decision-making in acute ischaemic stroke.
**Methods and Materials:** We included consecutive patients with follow-up-confirmed middle cerebral artery infarction and available NCCT and CT perfusion (CTP) data. ASPECTS region density was assessed using automated software. Relative Hounsfield units (rHU) were defined as ratio of ipsilesional by contralesional region density. Ischaemic core was measured on CTP maps. Regression coefficients from a linear regression analysis on the association of regional rHU and infarction core volume were used as weighting factors to calculate a composite rHU score of all regions. Receiver operating characteristics (ROC) analyses were performed to test this score's discriminative value regarding current thrombectomy selection criteria and subacute stroke complications.
**Results:** In total 121 patients were included. The composite rHU score resulted in significant classification of patients who meet CTP selection criteria of ischaemic core size <70 mL and target mismatch >1.8 (AUC=0.759, p<0.001). The score discriminated between patients with and without subsequent space-occupying oedema development (AUC=0.771, p<0.001). ROC curves are shown in Figure 1. The score could not classify patients by occurrence of haemorrhagic infarction or parenchymal haematoma (both p>0.05).
**Conclusion:** Automated NCCT density measurements have the potential to act as observer-independent imaging biomarkers that could support decision-making in centres without CTP. The composite rHU score allowed classification of patients with current CTP imaging criteria for extended time window thrombectomy selection. The score also identified patients with subsequent space-occupying oedema development.

**B-0714** 15:12
**Pattern of leptomeningeal collateral circulation on perfusion computed tomography in patients with acute ischaemic stroke**
A. Singh[1], U.B. George[2], J.D. Pandian[2]; [1]*Delhi/IN*, [2]*Ludhiana/IN* *(appysingh05@gmail.com)*

**Purpose:** To correlate grade of leptomeningeal collateral circulation with clinical outcome in patients with acute ischaemic stroke.
**Methods and Materials:** Leptomeningeal collateral (LMC) scores were defined for 71 patients presenting within 24 hours of stroke onset on perfusion CT baseline images: 0-absence of vessels; 1- <50%; 2->50-100%; 3-more prominent; as compared with contralateral hemisphere. A score of 0/1 and 2/3 were distributed in poor and good collateral groups respectively. LMC scores were correlated with clinical outcome as early neurological recovery (drop of >/= 4 NIHSS score points at 24 hours) and Modified Rankin score at 3 months (favourable </=2). A p value of <0.05 was taken as significant.
**Results:** 64.7% and 35.3% had good and poor collateral status. No significant difference in early neurological recovery by NIHSS scoring was noted in the two collateral groups; 30.4% and 36% in good and poor collateral group; p value-0.63. A favourable MRS of </=2 was higher in good (59.01%) than poor collateral group (44%), with p value - 0.23. More number of patients had favourable MRS with good (59%) than with poor collaterals (44%). NIHSS score was significantly lower for thrombolysed patients (p value <0.005); however, there was no difference in clinical outcome in two collateral groups. (p value -0.92).
**Conclusion:** Early neurological recovery and long term recovery outcome was favourable in patients with good collaterals, however results were not statistically significant. Though Intravenous thrombolysis resulted in significantly lower NIHSS score, results were not significant between the two collateral groups.

**B-0715** 15:20
**Ultra-low-dose cerebral CT perfusion for the diagnosis of acute ischemic stroke using iterative model reconstruction combined with optimised temporal resolution**
X. Fang[1], R. Wang[1], Y. Liu[1], C. Yuan[1], X. Chen[2], Z. Wang[1]; [1]*Nanjing/CN*, [2]*Shanghai/CN (fmrifangxk@163.com)*

**Purpose:** To evaluate the diagnosis performance of ultra-low dose cerebral CT perfusion (CTP) in patient with acute ischemic stroke using iterative model reconstruction (IMR) combined with low tube voltage.
**Methods and Materials:** 80 consecutive patients with suspected ischemia stroke were enrolled in this study and randomly divided into two groups (n=40 each): Regular dose GroupA, 80kVp, 100mAs, 45 passes were performed with 2 varied intervals (1 second interval for the first 30 passes and 2 seconds for the following 15 passes), reconstructed with iDose(level-3); Ultra-low dose GroupB, 80kVp, 50mAs, 18 passes were performed with 3 varied intervals (2 seconds for the first 3 passes, 1.7 seconds for the next 13 passes and 7.5 seconds for the left 2 passes), reconstructed with IMR(level-1). Perfusion maps of CBF, CBV, and MMTT were created for both groups and values were quantitively compared by using independent t-test. Perfusion maps were qualitatively rated on a 5-point scale (5-excellent, 1-not diagnostic) for both groups and compared by using Mann-Whitney test. Mean CTDIvol and DLP were recorded and compared. Mean effective dose (ED) was estimated and compared.
**Results:** For the overall image quality, no significant quantitative or qualitative differences were found between two groups (all P>0.05). Differences in mean CTDIvol, DLP, and ED between two groups were statistically significant (all P<0.01). There was an 81% ED reduction of Group B (0.662mSv) in comparison to Group A (3.312mSv).
**Conclusion:** Ultra-low dose cerebral CTP can be achieved using IMR in combination with optimized temporal resolution while preserving image quality.

**14:00 - 15:30** **Room F1**

### Breast

### SS 702b

### Breast ultrasound

*Moderators:*
E. Giannotti; Nottingham/UK
M. Marolt Music; Ljubljana/SI

### B-0716 14:00

**Decrease in unnecessary biopsy recommendations by the application of quantitative multiparametric breast ultrasound: a prospective study**

P. Kapetas, P. Clauser, R.A. Woitek, G.J. Wengert, M. Lazar, K. Pinker-Domenig, M. Bernathova, T.H. Helbich, P.A.T. Baltzer; *Vienna/AT (panagiotis.kapetas@meduniwien.ac.at)*

**Purpose:** To evaluate quantitative breast multiparametric ultrasound (mpUS) using B-mode, elastography (Virtual Touch IQ-VTIQ), Doppler and contrast-enhanced US (CEUS) in different combinations of 2, 3 or 4 parameters for the differentiation of benign and malignant lesions and investigate a possible variation according to the reader experience level.
**Methods and Materials:** 124 patients, each with one biopsy-proven breast lesion were included in this prospective, IRB-approved study. Each lesion was examined with B-mode, VTIQ, Doppler and CEUS. Different quantitative parameters were recorded for each modality. 4 readers (2 experienced breast radiologists and 2 residents) independently evaluated B-mode images of each lesion and assigned a BI-RADS score to it. Using ROC curve analysis, the quantitative parameter with the best diagnostic performance for each modality was chosen. The BI-RADS scores of all readers were then combined with the results of the quantitative parameters. Diagnostic performance of mpUS was evaluated with descriptive statistics. Histology was the reference standard.
**Results:** 65 lesions were malignant. MpUS with three parameters (B-mode, VTIQ and CEUS or Doppler) showed the highest diagnostic performance irrespective of the reader experience level (averaged AUC 0.812 vs. 0.789 vs. 0.683 for B-mode, p value=0.0001). All other combinations showed a lower AUC. MpUS with three parameters was able to significantly reduce the number of false-positive biopsy recommendations up to 47% (p<0.0001).
**Conclusion:** Quantitative breast mpUS with 3 parameters (B-mode, VTIQ elastography and CEUS or Doppler) significantly improves the diagnostic performance of B-mode, irrespective of the reader experience level and can obviate unnecessary biopsies.

### B-0717 14:08

**Diagnostic performance of fifth BI-RADS edition ultrasound lexicon for prediction of malignancy in breast masses with histopathological correlation**

K. Gupta[1], T. Chandra[2], B. Venkatesan[3]; [1]*Chennai/IN*, [2]*Orlando, FL/US*, [3]*Pondicherry/IN (hikanika@gmail.com)*

**Purpose:** The fifth edition of Breast Imaging Reporting and Data System (BI-RADS) was released in 2013 with several changes in the descriptors for breast ultrasound. The purpose of this study was to compare the diagnostic performances of fifth BI-RADS edition ultrasound lexicon for prediction of malignancy in breast masses, in comparison with fourth BI-RADS edition.
**Methods and Materials:** Using a prospective study design, 256 solid breast masses were assessed by ultrasound, out of which 174 underwent core biopsy and 160 masses had definitive histopathological results. The ability of individual descriptor of ultrasound in fourth and fifth BI-RADS editions to differentiate between a benign and malignant lesion in the breast was assessed using statistical analysis.
**Results:** The descriptor ‘intraductal calcifications’ was very useful for prediction of malignancy and had the highest sensitivity (97%) among all ultrasound descriptors of fifth BI-RADS edition. The presence of spiculated and indistinct margins were predictive of malignancy- 94.4% of masses with spiculated margins and 100% masses with indistinct margins were found malignant. ‘In rim vascularity’ and ‘internal vascularity’ were found useful in differentiating benign from malignant lesions. ‘Internal vascularity’ depicted a high PPV of 86% in predicting malignancy in a breast mass.
**Conclusion:** Some new descriptors used in fifth edition of BI-RADS, such as intraductal calcifications and internal vascularity, in addition to established descriptors of spiculated/indistinct margins and anti-parallel orientation of the mass result in improved diagnostic performance of ultrasound lexicon in predicting malignancy in breast masses

### B-0718 14:16

**Does establishing preoperative nomogram including ultrasonographic findings help predict the likelihood of malignancy in patients with microcalcifications?**

H. Wang, J. Lai, J. Li, R. Gu, F. Liu, Y. Hu, X. Jiang, J. Mei, F. Su; *Guangzhou/CN (whonglii@163.com)*

**Purpose:** To investigate whether establishing preoperative nomogram including ultrasonographic findings helps predict the likelihood of malignancy in patients with microcalcifications.
**Methods and Materials:** Between May 2012 and January 2017, 475 patients with suspicious microcalcifications detected on mammography (MG) underwent ultrasonography (US). The $\chi^2$ test was used to screen risk factors amongst the variables. Then, a multivariate logistic regression analysis was performed to identify independent predictors of malignant microcalcifications. A mammographic nomogram (M nomogram) and mammographic-ultrasonographic nomogram (M-U nomogram) were established based on multivariate logistic regression models. The discriminatory ability and clinical utility of both nomograms were compared by the receiver operating characteristics curve and decision curve analysis. The calibration ability was evaluated using a calibration curve.
**Results:** Among the cases, 68.2% (324/475) were pathologically diagnosed as breast cancer and 31.8% (151/475) were benign lesions. Based on multivariate logistic regression analysis, age, clinical manifestation, morphology and distribution of microcalcifications on MG and lesions associated with microcalcifications on US were confirmed as independent predictors of malignant microcalcifications. In terms of discrimination ability, the C-index of M-U nomogram was significantly higher than that of M nomogram (0.917 *vs* 0.897, $p$=0.006). The bias-corrected curve was close to the ideal line in the calibration curve. Decision curve analysis suggested M-U nomogram were superior to M nomogram.
**Conclusion:** Combining mammographic parameters with ultrasonographic findings into a nomogram performed better than M nomogram alone, which highlighted the value of ultrasonographic findings for individualized risk prediction in patients with microcalcifications.

### B-0719 14:24

**How should we manage breast ultrasound masses that do not enhance on MRI?**

D. Avendano[1], E.J. Sutton[1], E.A. Morris[1], M.A. Marino[2], P. Gibbs[1], D. Martinez[1]; [1]*New York, NY/US*, [2]*Messina/IT (dlilahh1@gmail.com)*

**Purpose:** Assess incidence of malignant breast masses on ultrasound where magnetic resonance imaging (MRI) correlate does not enhance in women with a new diagnosis of breast cancer.
**Methods and Materials:** IRBA this HIPAA-compliant retrospective study and waived informed consent. Consecutive women identified between June 2017 and March 2018 who a) have new diagnosis of breast cancer b) underwent US where an additional mass (distinct from index cancer) was identified and categorized as either BI-RADS 3/4, and c) underwent bilateral breast MRI where the US correlate didn’t enhance. A benign mass was pathologically proven or defined as showing 2-y imaging stability. A malignant mass was pathologically proven. Sensitivity, Specificity and predictive values were calculated on a per-lesion level by using proportions. Descriptive statistics were also assessed.
**Results:** 230 women (mean age 52) with 309 additional lesions (mean size 9 mm) were identified. Of the 309 breast masses, 140 in 105 women (mean age 54) did not enhance on MRI. The mean size of these masses on US was 16 mm (range 3-74 mm). In 89/140 cases (64%) the patient underwent biopsy and in 51/140 (36%) had 2-year follow-up. None of the 140 masses were malignant, with a NPV of 100%.
**Conclusion:** All breast masses on US that did not enhance on MRI were benign. The results suggest that biopsy or follow-up is not required.
*Author Disclosures:*
**D. Avendano:** Grant Recipient; BCRF Breast Cancer Research Foundation.

### B-0720 14:32

**Contrast-enhanced ultrasound in breast cancer and its role in predicting immunohistochemical subtype: a novel prognostic approach**

S.B. Grover[1], H. Grover[2], P. Jain[1], A.K. Nag[1], A. Katyan[1], N. Antil[1], S.K. Jain[1], A.K. Mandal[1]; [1]*New Delhi/IN*, [2]*Bridgeport, CT/US (shabnamgrover@yahoo.com)*

**Purpose:** Breast cancer prognostication is based on biopsy evaluated receptor subtyping. This study in breast cancer patients aimed to correlate contrast enhanced ultrasound (CEUS) parameters with immunohistochemical (IHC) markers, as a non-invasive alternative for prognosis prediction.
**Methods and Materials:** Fifty consenting female patients with newly diagnosed, biopsy proven breast cancer were included in this prospective institutional review board approved study. CEUS was performed using a 4-9 MHz transducer and both qualitative and quantitative CEUS parameters were recorded. Qualitative parameters included enhancement order, type and margins, and presence of perfusion defects. Quantitative parameters documented were time to peak, mean transit time, peak intensity and area under time intensity curve (AUTIC). IHC parameters including oestrogen receptor (ER), progesterone receptor (PR) and HER2 status were assessed on histopathology. Diagnostic test, Independent t test and receiving operating characteristics (ROC) were used for statistical analyses. p value < 0.05 was considered as statistically significant.
**Results:** Presence of perfusion defects was significantly associated with negative ER and PR status, p<0.05. A higher AUTIC was the only quantitative parameter significantly related to ER and PR negativity, p=0.05, with a ROC cut-off of 566 predicting ER/PR negativity with a sensitivity and specificity of 84.21% and 51.51% respectively. None of the qualitative or quantitative parameters showed association with HER2 status.
**Conclusion:** CEUS has reprised the use of conventional sonography, providing additional tumour characterization, even at the molecular level. Significant CEUS parametric and ER/PR status association can provide accurate non-invasive prognostic information and in future could guide treatment protocol decisions.

**B-0721** 14:40
**Preliminary study of real-time three-dimensional contrast-enhanced ultrasound of sentinel lymph nodes in breast cancer**
M. Lu; *Chengdu/CN (graceof@163.com)*

**Purpose:** To investigate the clinical value of real-time three-dimensional contrast-enhanced ultrasound (3D-CEUS) in the detection of sentinel lymph nodes (SLNs) and drainage lymphatics in breast cancer patients.
**Methods and Materials:** The prospective study was performed in 187 women with pathology-confirmed T1/2 breast cancer between June 2016 and December 2017. 146 in 187 patients were randomly divided into two groups: 73 patients received two-dimensional contrast-enhanced ultrasound (2D-CEUS, 2D-CEUS group), the other 73 patients immediately received three-dimensional CEUS (3D-CEUS, 3D-CEUS group). The number, size, location, enhancement pattern of SLNs and the lymphatic drainage patterns were reviewed; the routes, location of SLNs and lymph channels (LCs) on the surface were marked. All patients underwent blue dye-guided sentinel lymph node biopsy (SLNB) finally.
**Results:** According to the postoperative pathology findings and the blue stained lymphatic drainage routes, the coincidence rates of the 2D-CEUS group and the 3D-CEUS group were 97.3% and 98.6%, respectively (p>0.05); the LN detection rates were 90.4% and 95.6% (p>0.05); the correct diagnosis rates were 88% and 90.8% (p>0.05); the times of operation was 15.42±1.10 and 13.49±0.94 min (P<0.05).
**Conclusion:** Compared to the 2D-CEUS, the 3D-CEUS can show the stereo direction of SLNs and lymph drainage routes including the angle, help the clinicians recognize the spatial location and depth of SLNs and reduce the time of finding the SLNs during operation.

**B-0722** 14:48
**Role of ultrasound in the SOUND trial: can we move away from sentinel lymph node biopsy?**
F. Pesapane, M. Grimaldi, A. Rotili, D. Lepanto, E. Cassano; *Milan/IT (filippopesapane@gmail.com)*

**Purpose:** Our purpose is to evaluate the negative predictive value (NPV) of axillary ultrasound (AUS) in early breast cancer (BC) patients to propose this non-invasive examination as a credible substitute of sentinel lymph node biopsy (SLNB) in the axillary lymph node (ALN) staging.
**Methods and Materials:** We conducted preoperative AUS in 685 consecutive patients enrolled in the SOUND trial, in our institution, evaluating the frequently used signs of suspicious ALN on AUS. NPV of AUS was calculated first considering all false negatives, including micrometastases and isolated tumoural cells (ITC), and then considering macrometastases alone. We used 3 mm as a threshold to assess US-detectable metastases.
**Results:** Preoperative AUS resulted in 4.8% of false positives and 15.5% of false negatives, which is reduced to 8.1% excluding ITC and micrometastases and to 4.9% considering only metastases >3 mm, which are the only lesions that AUS can reliably detect. The NPV was estimated 92% (90-94%, 95% CI) including all cases of positivity to histopathological examination. Excluding ITC and micrometastases, the NPV was 95% (94-97%, 95% CI). Finally, including metastases that can be detected by AUS (namely metastases >3 mm) alone, the NPV was 97% (95-98%, 95% CI). Specificity of AUS in our population was 95% (93-97%, 95% CI).
**Conclusion:** Our results show that in early BC patients, the AUS may represent an effective, non-invasive diagnostic tool for axillary staging, and due to its high NPV, it allows to select women who can benefit from observation only as a treatment approach, replacing the SLNB.

**B-0723** 14:56
**Predictive factors on ultrasound for the early tumour recurrence of breast cancer after breast-conserving treatment**
S.G. Yun, Y.Y. An; *Suwon/KR (sgdream88@gmail.com)*

**Purpose:** To evaluate the clinicopathologic and imaging factors associated with early tumour recurrence in breast cancer patients who underwent breast-conserving treatment.
**Methods and Materials:** We retrospectively reviewed the clinicopathologic data and imaging features (MG, US, and DCE-MRI) of 529 breast cancer patients who underwent breast-conserving surgery and adjuvant therapy at our hospital between January 2009 and December 2014. A total of 36 patients recurred within 3 years after surgery (early tumour recurrence). Risk factors associated with the early tumour recurrence were analysed by univariate and multivariate logistic regression analyses.
**Results:** In univariate analysis, large tumour size (p=0.004), advanced stage (p=0.025), high histologic grade (p=0.002), high nuclear grade (p=0.001), presence of lymphovascular invasion (p=0.044), high Ki-67 proliferation index (p=0.023), Her2-enriched and triple-negative subtypes (p=0.005), ER positive (p<0.0001), and PR positive (p=0.021) were associated with early tumour recurrence. Among imaging characteristics, multifocality (p<0.0001) on US and presence of vessels in the rim on colour Doppler exam (p=0.017) were significantly related to early recurrence. Multivariate logistic regression analysis showed that the multifocality (odds ratio=3.2, 95% CI 0.547-3.803, p=0.003) on US and the vessels in the rim on colour Doppler exam (odds ratio=3.1, 95% CI 1.314-7.352, p=0.0098) were independently associated with early tumour recurrence.
**Conclusion:** The multifocality on US and vessels in the rim on colour Doppler exam are independent factors for predicting the early tumour recurrence of breast cancer after treatment.

**B-0724** 15:04
**The value of automated breast ultrasound (ABUS) coronal view**
S. Schiaffino, A. Tagliafico, L. Gristina, E. Massone, S. De Giorgis, A. Garlaschi, M. Calabrese; *Genoa/IT (schiaffino.simone@gmail.com)*

**Purpose:** To evaluate the value of automated breast ultrasound (ABUS) in women with dense breast, focusing on the potential assessment of coronal images alone, compared to the complete multiplanar (MPR) views.
**Methods and Materials:** From August 2017 to October 2017, consecutive patients with dense breast that referred to our institute for post-mammography ultrasound assessment, pre-operative assessment or follow-up of known benign lesions were invited to perform an additional study with ABUS. Three radiologists, with different levels of experience in breast radiology (5, 15 and 25 years), reviewed the exams twice: the first with the assessment of reconstructed coronal plane alone and the second with complete MPR views. Reporting times, diagnostic performance and interobserver agreement were assessed.
**Results:** 188 women were included in the study, for a total of 67 breast lesions, 25 (37%) malignant and 42 (63%) benign lesions. Compared to MPR, coronal view was associated with lower reading times, respectively, for the three readers: 83±37, 84±43 and 76±30 versus 163±109, 131±57, 151±42 seconds (p<0.035); lower sensitivity: 44.8%, 62.1%, 55.2% vs. 69.0% (p=0.059*)*, 65.5% (p=0.063*)*, 72.4% (p=0.076*)*, respectively; better specificity: 94.1%, 93.7%, 94.2% vs 89.5% (p=0.093*)*, 87.4% (p=0.002*)*, 91.6% (p=0.383*)*, respectively. Agreement between the most and the least experienced rater was fair for categorical variables and significant for continuous.
**Conclusion:** The coronal view allows significantly lower reading times, a valuable feature in the screening setting. The assessment of this plane alone could be considered as "fly through" first assessment, completed with targeted analysis in all the available views.
*Author Disclosures:*
**S. Schiaffino:** Speaker; GE Healthcare. **M. Calabrese:** Speaker; GE Healthcare.

**B-0725** 15:12
**Diagnostic performance of automated breast ultrasound (ABUS) after breast-conserving surgery**
C. Depretto, A. Primolevo, F. Cartia, C. Ferranti, G. Scaperrotta; *Milan/IT (cathie.dp@gmail.com)*

**Purpose:** To assess the non-inferiority of automated breast ultrasound (ABUS) compared to handheld ultrasound (HHUS) in terms of detection rate of local recurrences after breast-conserving surgery (BCS).
**Methods and Materials:** In our study, between April and June 2016, we invited 154 consecutive patients with dense breasts (ACR C or D) coming for scheduled imaging after BCS to undergo ABUS. Mean age was 62 years (range 34-90) and BCS had been performed on average 8 years prior (range 5- 31). ABUS images were analysed by two independent radiologists blinded to conventional imaging.

**Results:** There was interreader agreement between ABUS and HHUS in terms of BI-RADS assessment in 134/154 cases (87%). In this subset, 66 exams were negative (BI-RADS 1), 64 showed benign findings (BI-RADS 2) and 4 cases were suspicious (BI-RADS ≥ 4), subsequently confirmed histologically as malignant. ABUS detection rate for recurrences was equal to HHUS. All the 20 cases of readers' disagreement were small benign findings, not detected by ABUS. 2 further cases were parenchymal distortions detected only by HHUS (one of which resulted negative at biopsy and the other remained unchanged at follow-up after 6 months).
**Conclusion:** ABUS detected all cancers found by HHUS, which suggests its non-inferiority to HHUS in terms of detection rate.

**B-0726** 15:20
**Targeted ultrasound as first-line examination in women with focal breast complaints**
L. Appelman[1], P. Appelman[2], P. Bult[1], R. Mann[1]; [1]*Nijmegen/NL*, [2]*Tienhoven/NL (Linda.Appelman@radboudumc.nl)*

**Purpose:** To determine the relative contribution of ultrasound and mammography for tumour detection in women with focal breast complaints.
**Methods and Materials:** We performed a retrospective evaluation of women who visited our radiology department in 2016. All 482 consecutive women undergoing targeted ultrasound for new focal breast complaints were included, patients undergoing ultrasound for other reasons were excluded. The radiological assessment for mammography and ultrasound was obtained from the reports generated at the time of diagnosis. Ground truth was obtained from histopathological reports when available, or one year of negative follow-up when no biopsy was performed. We compared cancer yield and frequency of false-positive findings between mammography and ultrasound using chi-square and McNemar tests.
**Results:** Based on the targeted ultrasound assessment, 72 biopsies (15%) were performed, of which 47 yielded breast cancer. Forty-five of these cancers were also visible on the concurrent mammogram (p=0.47). Mammography led to the detection of only 1 additional breast cancer in women without cancer at the symptomatic site. In addition, mammography prompted 22 biopsies for additional benign findings. Sensitivity of targeted ultrasound was, therefore, 98% with NPV 99.7%, and for mammography 96% and 99.5% (p=1.0). Mammography also prompted 10 additional biopsies in women with cancer at the symptomatic site, of which 9 proved to be malignant.
**Conclusion:** For initial cancer detection in women with focal breast complaints the interpretation of targeted ultrasound alone by breast radiologists is highly sensitive. In women with cancer at the focal complaint mammography appears useful for staging purposes.

**14:00 - 15:30** **Room F2**

### Emergency Imaging

### SS 717

### Chest emergencies

*Moderators:*
M. Brink; Nijmegen/NL
J.B. Dormagen; Oslo/NO

**K-17** 14:00
**Keynote lecture**
M. Rémy-Jardin; *Lille/FR*

**B-0727** 14:09
**Patient lung volume is significantly related to contrast media volume during CT pulmonary angiography when employing a patient-specific contrast protocol**
C. Saade, F. El-Merhi; *Beirut/LB (charbel.saade@aub.edu.lb)*

**Purpose:** To investigate the effect of patient lung volume and contrast volume on pulmonary artery opacification using a patient-specific contrast formula during pulmonary CTA.
**Methods and Materials:** IRB approved this retrospective study. CTA of the pulmonary arteries was performed on 200 patients with suspected PE using a 256-channel CT and a dual-barrel contrast injector. The contrast media volume was calculated by employing a patient-specific contrast formula. Both contrast media and saline were injected at a flow rate of 4.5 mL/s. The mean cross-sectional opacification profile of eight central and eleven peripheral pulmonary arteries and veins were measured for each patient and arteriovenous contrast ratio (AVCR) calculated for each lung segment. Mean body mass index (BMI) and lung volume were quantified. Receiver operating (ROC) and visual grading characteristics (VGC) measured the confidence intervals and image quality, respectively. Inter- and intra-observer variations were investigated employing Cohen's kappa methodology.
**Results:** The mean pulmonary arterial opacification of the main pulmonary circulation was (343.88±73HU), right lung: upper (316.51±23HU), middle (312.5±39HU) and lower (315.23±65HU) lobes and left: upper (318.76±83HU) and lower (321.91±12HU) lobes. The mean venous opacification of all pulmonary veins was below 182±72HU. Subsequently, the AVCR was observed at all anatomic locations (p<0.0002) where this ratio was calculated. Mean contrast volume of 33±9 mL. Larger lung volumes were significantly correlated to larger volumes of contrast (r=0.89, p<0.03). Inter-observer variation was observed as excellent (κ = 0.71).
**Conclusion:** Increased lung volume is significantly correlated with increased contrast media volume and radiation dose when employing a patient-specific contrast formula.

**B-0728** 14:17
**Age-adjusted D-dimer and risk-stratification in acute pulmonary embolism for reduce the unnecessary CT imaging**
A. Pandur, D. Sipos, B. Banfai, J. Betlehem, B. Radnai; *Pécs/HU (attila.pandur@etk.pte.hu)*

**Purpose:** Pulmonary embolism is connected with high morbidity and mortality. Prognostic assessment is important for the management of patients with pulmonary embolism. To assess the percentage of CTPA which could have been avoided by use of risk-stratification scores with age-adjusted D-dimer testing in patients with suspected PE.
**Methods and Materials:** We conducted a retrospective study of ED patients undergoing CTPA for suspected PE. In our study we analized 659 patient-documentation. Every patient were admitted to the ED and sent for pulmonary CTA. We calculated Wells-score and followed the European Guideline for PE.
**Results:** Our study included 659 patients (407 women, 252 men), admitted to three ED. In the 659 cases all over 105 D-dimer assays, 51 CT angiograms and 212 chest X-ray examinations were carried out rudlessly, which could have mean saved money to the hospitals and less radiation to patients. The age adjusted D-dimer threshold was more specific (70 % versus 60 %) but less sensitive (95% versus 98 %). The sensitivity of the combination (risk-stratification and age adjusted D-dimer test) was 100 %.
**Conclusion:** An age adjusted D-dimer limit has the potential to reduce diagnostic imaging. This is more accurate than a standard threshold of 500 ng/dl. The combination of the risk stratification and the age adjusted D-dimer we can safety diagnose the pulmonary embolism. Finally we can conclude that risk-evaluation in acute PE is indispensable and the appropriate use of guidelines results in lower costs.

**B-0729** 14:25
**Real-time patient specific scan delay in CTA of pulmonary embolism: impact on image quality**
F.R. Schwartz[1], J.C. Ramirez Giraldo[2], R. Gutjahr[3], D. Marin[1], D. Boll[4], L. Hurwitz[1]; [1]*Durham, NC/US*, [2]*Rochester, MN/US*, [3]*Nürnberg/DE*, [4]*Basle/CH (fidesreginadorotheaann.schwartz@duke.edu)*

**Purpose:** To assess whether real-time modulation of scan initiation based on patient specific haemodynamics improves contrast enhancement in the pulmonary arteries when using fluoroscopic triggering in pulmonary embolism (PE) computed tomographic angiogram (CTA) compared to fixed delay time.
**Methods and Materials:** HIPAA-compliant, IRB-approved QI project. Exams were performed on a 3rd generation dual-source MDCT scanner. Contrast administration was monitored in the main pulmonary artery (MPA). The delay prior to initiation of the diagnostic scan was adjusted by a real-time patient specific modulation (RTPSM) software, incorporating time to threshold and the slope of the enhancement curve. 150 patients (cohort 1) were scanned using RTPSM triggering of scan delay in pulmonary embolism CTAs. A BMI and age matched reference cohort of 150 patients (cohort 2) was identified with exams on the same scanner in the preceding 18 months, using a fixed diagnostic delay (FD). Patient demographics and HU values in the MPA, the bilateral posterior lobe segmental and sub-segmental arteries (750 segments per cohort) were collected. Descriptive statistics and student's t-tests were applied.
**Results:** Percentage of females (58.7 vs 61.3%) was similar between cohorts. The average scan delay was statistically significantly longer (9.8 ± 2.4 vs 5 sec; p<0.01) using RTPSM. Average HU values were significantly higher in the RTPSM cohort (409.1 ± 22.2 vs 367.7 ± 20.8 HU; p<0.01) and the number of non-diagnostic segments was significantly reduced in segmental and sub-segmental arteries (-3.6%; p<0.05).
**Conclusion:** RTPSM results in higher HU values and fewer non-diagnostic segments in the pulmonary arteries.
*Author Disclosures:*
**F.R. Schwartz:** Research/Grant Support; Siemens Healthineers, Grant to department of Radiology. **J.C. Ramirez Giraldo:** Employee; Siemens Healthineers, Forchheim, Germany. **R. Gutjahr:** Employee; Siemens Healthineers, Forchheim, Germany. **L. Hurwitz:** Research/Grant Support; Siemens Healthineers, Grant to department of Radiology.

**B-0730** 14:33
**Contribution of CT lung perfusion in diagnosis of acute pulmonary embolism**
A. Oganesyan, E. Mershina, V.E. Sinitsyn; *Moscow/RU (talilen@mail.ru)*

**Purpose:** To study the contribution of CTA iodine maps (IMPS) to the diagnosis of acute pulmonary thromboembolism (PE) and compare the pulmonary perfusion defects severity with other signs and biomarkers of PE.
**Methods and Materials:** 42 patients (f/m - 19/23; age 65 ±10) with suspected PE were performed pulmonary CTA. Single-tube dual-energy scan (DECT) and wide-detector scanner were used. Pulmonary IMPS were reconstructed using DECT (n=29) and lung subtraction (n=13) options. For CTA analysis the number and level of vessel occlusions were marked. Presence of lung perfusion defects on IMPS, signs of right heart failure were studied.
**Results:** CTA signs of PE were found in 42 patients. Defects of lung perfusion on IMPS were present in 83% cases. 6 patients had PE without perfusion defect and signs of right heart overload. The area of lung perfusion defects in IMPS correlated with the Quanadly index (r=0,8, p<0,05) and it had a weak correlation with severity of right heart failure (r=0,3, P<0,05). In 3 patients with segmental and subsegmental PE it was found due to the presence of a defect on IMPS and retrospective analysis of CTA.
**Conclusion:** Addition of IMPS to pulmonary CTA contributes to the improvement of acute PE diagnosis and assessment of its severity. The prognostic value of this biomarker needs to be further studied.

**B-0731** 14:41
**Virtual non contrast imaging of aortic intramural hematoma for replacing the non contrast CT imaging**
S.A. Si-Mohamed[1], N. Dupuis[1], D.C. Rotzinger[2], S. Boccalini[3], Y. Yagil[4], P. Coulon[5], N. Shapira[4], P. Douek[1], L. Boussel[1]; [1]*Bron/FR*, [2]*Lausanne/CH*, [3]*Genoa/IT*, [4]*Haifa/IL*, [5]*Paris/FR (sara.boccalini@yahoo.com)*

**Purpose:** The purpose was to assess *in vitro* and *in vivo* whether Virtual Non-Contrast (VNC) images could replace true non-contrast images (TNC) for intramural hematoma (IMH) diagnostic.
**Methods and Materials:** A phantom of IMH was placed in a semi-anthropomorphic phantom (QRM GmbH, Germany) and imaged on the DLCT (iQonCT, Philips, Israel) at different CTDIvol using iDose 0, 3 and an extension ring. Attenuation, iodine density, image noise, contrast-to-noise ratio (CNR) between the hematoma and lumen were measured. 22 patients with IMH were included. CNR values were compared using t-test. Diagnostic confidence on a 4-point Likert scale was done by two radiologists using a Kruskal-Wallis rank sum, then with inter-agreement analysis using Cohen kappa test.
**Results:** CNR were not significantly different for both phantom sizes ($p$ = 0.78 and 0.65). CNR between blood and hematoma were not lower in VNC compared to TNC images (ΔCNR 95 % CI = - 0.81; 0.85). For both images, higher CTDI*vol* , iDose 3 and smaller phantom were associated with better CNR. In vivo, CNR were found to be significantly different with a higher value for VNC images ($CNR_{TNC}$ = 0.89 ± 0.73 and $CNR_{VNC}$ = 1.04 ± 0.83, p = 0.04). The mean diagnostic confidence scores for the TNC and VNC images were diagnostic to exemplary and not significantly different (3.13 ± 0.74 and 3.22 ± 0.73 respectively, $p$ = 0.56).
**Conclusion:** DLCT offers similar performances with virtual non-contrast images for IMH diagnosis without any compromise in radiation dose or diagnostic image quality.
*Author Disclosures:*
**Y. Yagil:** Employee; Philips Healthcare. **P. Coulon:** Employee; Philips Healthcare. **N. Shapira:** Employee; Philips Healthcare.

**B-0732** 14:49
**Contrast-enhanced US (CEUS) of lung abscess: how it can help**
C.L. Piccolo[1], D. Cozzi[2], M. Trinci[3], M. Galluzzo[3], M. De Cicco[3], V. Miele[3], S. Ianniello[3]; [1]*Aversa/IT*, [2]*Florence/IT*, [3]*Rome/IT (clapiccolo@libero.it)*

**Purpose:** The aim of this prospective study is to evaluate the contribution, in improving diagnostic accuracy, provided by the use of second-generation ultrasound contrast media (Sonovue®) in the diagnosis of lung abscesses.
**Methods and Materials:** At the three emergency departments, 32 patients (28-76 y.o) with signs of fever, cough and chest x-ray positive for limited opacity (suggestive for intrapulmonary abscess), were evaluated from January to september 2018. We evaluated with lung ultrasound (L-US), both the anterior and posterior regions (patients seated or supine our gold standard was contrast medium lung CT; we have described every opacity/abscess with diameters, eco-structural aspect of the wall and of the content, presence of associated pleural effusion. Exclusion criteria: recognition of a not-well delimited consolidation. CEUS was performed immediately after ultrasound with intravenous injection of 2.4 ml of Sonovue® (in 2 boluses) followed by each of 5ml of physiological solution.
**Results:** We identified with L-US 30/32 inhomogeneous sub-pleural round formations (diameters from 3 to 7 cm); we had 2 false-negative exams due to abscesses that did not reach the pleural surface. In 13/32 there was ipsilateral pleural effusion. In 30/32 cases studied with L-US, we performed CEUS which better defined the wall (with intense and persistent contrastographic impregnation) and the content of the abscess.
**Conclusion:** Our limited-experience LUS, integrated with CEUS exam, has shown to contribute significantly in the improvement of diagnostic accuracy and in the best assessment of lung abscesses in patients with clinico-radiological suspect, with interesting perspectives about their follow-up.

**B-0733** 14:57
**Lung ultrasound in the diagnosis of pneumothorax in paediatric stable major trauma: chest x-ray is still useful?**
D. Cozzi[1], C.L. Piccolo[2], M.L. De Cicco[3], M. Trinci[3], M. Galluzzo[3], V. Miele[3], S. Ianniello[3]; [1]*Florence/IT*, [2]*Aversa/IT*, [3]*Rome/IT (dilettacozzi@gmail.com)*

**Purpose:** The aim of this retrospective study is to evaluate the diagnostic accuracy of lung ultrasound in the diagnosis pneumothorax in paediatric stable polytrauma in Emergency room, to try to avoid chest X-ray. All patients underwent multislice computed tomography (MS-CT) exam that represented the "gold standard".
**Methods and Materials:** From January 2017 to September 2018, 46 paediatric patients (mean age 14 yo, range 8-16 yo, 31 males, 15 females) with haemodynamically stable severe trauma (AP > 90 mmHg, injury severity score >15) underwent lung ultrasound. All the exams were performed with portable ultrasound machine in all three emergency rooms, with patients lying on the spinal board. Then all patients underwent a MS-CT immediately after lung ultrasound. No chest x-rays were performed.
**Results:** Among the enrolled patients (46 patients; 92 hemithorax compared), 11/12 pneumothoraces were found with lung ultrasound, while 12/12 were reported with MS-CT; the single false-negative exam reported with lung ultrasound consisted of a pneumothorax with a thickness <5 mm in anterior-inferior para-mediastinal left region. Lung ultrasound did not report any false positive (PPV 100%, specificity 100%, sensitivity 91.6%, NPV 98.7%). Diagnostic accuracy was 98.9%.
**Conclusion:** Our data regarding accuracy of pneumothorax ultrasonographic diagnosis in severe paediatric trauma (within five minutes from arrival) highlighted its important role in the early identification of a potentially lethal pathology, which needs to be diagnosed as soon as possible. Instead of chest X-ray, lung ultrasound represents a promising scenario that could reduce radiation exposure in paediatric patients.

**B-0734** 15:05
**Accuracy of a new system score based on lung ultrasound and clinical data in the decision of a drainage tube necessity in polytrauma patients pneumothorax**
M.L. De Cicco[1], C. Andreoli[1], B. Sessa[2], V. Miele[1], S. Ianniello[1]; [1]*Rome/IT*, [2]*Chieti/IT (marialuisa.decicco@tiscali.it)*

**Purpose:** The aim of this study is to evaluate the validity of a new score system and its utility in the ER to establish the necessity to drainage the pneumothorax diagnosed by ultrasound in adults polytrauma.
**Methods and Materials:** This is a retrospective observational study that involved from January 2015 to June 2018, 315 patients with pneumothorax, evaluated by lung ultrasound lung during primary survey. All ultrasound were performed in ER. It was applied a system score which included the evaluation of the lung point site (parasternal: 1; emiclavear: 2; axillary: 3), the presence of pleural effusion (><300ml: 1/0), pericardial effusion (0/1), heart dislocation (1/0), contusion areas (1/0), F/T ratio (><200ml 1/0). Cut-off established was 6. All patients underwent to MDTC and the results were compared.
**Results:** Among the enrolled patients with pneumothorax 235/315 had a score >6 and the necessity of a drainage was indicated. Of these in 208/235 the necessity was confirmed by CT , while in 27 the patients were just observed and in only two patients during the follow-up the tube was placed. Among the 25 false positives, 12 had an high BMI, 5 had a subcutaneous emphysema, in 8 there was an overvaluation by the US.
**Conclusion:** Our data regarding the validity of a new system score showed its utility in deciding the necessity of a draining tube in polytrauma patients. This score would allow an early diagnosis and a promptly therapeutic choice, avoiding wasting time, essential in patients with many traumatic lesions and above all with serious pneumothorax.

**B-0735** 15:13
**Iatrogenic injuries and misplaced foreign material found in whole-body CT: a retrospective analysis in over 1000 resuscitation room patients**
S. Sudarski, D. Schäfer, T. Henzler, T. Terboven, T. Viergutz, S.O. Schönberg, H. Haubenreisser; *Mannheim/DE (sonja.sudarski@medma.uni-heidelberg.de)*

**Purpose:** Whole-body computed tomography reveals beyond findings related to the suspected diagnosis often additional incidental findings. Aim of this investigation was the systematic assessment of these findings in patients admitted via the emergency room after suffering potential major trauma or life-threatening medical conditions.

**Methods and Materials:** 1362 consecutive patients admitted via the resuscitation area were included retrospectively if they had received a whole-body computed tomography scan at admission. 197 patients were excluded because of missing data, the final cohort consisted of 1165 patients (1038 trauma and 127 internal-neurological patients). Reports from the whole-body computed tomography scans were screened for iatrogenic injuries and misplaced foreign material.
**Results:** In 43 (3.7%) patients a total of 46 iatrogenic injuries or misplaced foreign material were reported. The most common findings were too deeply placed endotracheal tubes. Five transurethral catheters placed in the emergency room were found to be blocked within the urethra.
**Conclusion:** Whole-body CT in our retrospective consecutive cohort of around 1000 patients admitted via the resuscitation area diagnosed iatrogenic injuries and misplaced foreign material in around 4% of the study patients.

**B-0736** 15:21
**Incidental findings in thoracic CTS performed in trauma patients: an underestimated problem**
E.J.M. Barbosa Jr., O. Osuntokun; *Philadelphia, PA/US (eduardo.mortani@gmail.com)*

**Purpose:** Whole-body CT scans are commonly performed to assess trauma patients, and often reveal incidental findings (IF) the patient may be unaware of. We assessed the prevalence, associations and adequacy of follow up of IF.
**Methods and Materials:** We retrospectively identified 1,113 patients who had a chest CT to assess for traumatic injuries (6-years interval). We coded the radiology reports for IF, and queried our EMR regarding clinical history and adherence to follow-up recommendations for IF mentioned in the reports.
**Results:** IF are much more likely (62.2%) to be found in a Chest CT scan than acute traumatic findings (ATF, 32.4%), in patients being evaluated for potential traumatic injuries. 86.4% of patients who had IF also had another relevant diagnosis (RD). Lung nodules were the most common IF (45.7%). A multivariate logistic regression model (MLR) demonstrated accuracy of 89% to predict IF; the 3 statistically significant predictors (p<0.05) were any RD (FDR logworth 68.6), followed by smoking history (29.8) and age (4.1). Radiologists recommended follow-up for IF 53.5% of the time, but only 13.9% of patients ever received a follow-up imaging study or procedure.
**Conclusion:** IF are much more common than ATF, and can be accurately predicted based on MLR utilizing only 3 clinical variables. While radiologists often recommend follow-up for IF in trauma patients, most are never effectively followed up or addressed, leading to increased risk of poor outcomes. Clinicians should be aware of the high prevalence of IF and develop systems for appropriate, evidence-based recommendations and effective management.

**14:00 - 15:30** **Room Y**

### Interventional Radiology

### SS 709

### Vascular interventions in visceral arteries and veins (part 2)

*Moderators:*
D. Cvetko; Zagreb/HR
P. Heiss; Regensburg/DE

**B-0737** 14:00
**Catheter-directed gastric artery embolisation with octreotide acetate-loaded PLA/PLGA (poly(lactide-co-glycolide) acid) microspheres with slow sustained release properties**
F. Nurili[1], T. Acar[2], S. Derman[2], A. Bas[2], I. Caymaz[2], S. Özkanli[2], H. Cakiroglu[3], I. Yurdaisik[2], O. Aras[1]; [1]*New York, NY/US*, [2]*Istanbul/TR*, [3]*Adapazari/TR (fuatnurili@gmail.com)*

**Purpose:** The objective of the present work was to formulate slow and sustained release octreotide acetate-loaded PLA/PLGA (polylactide)(poly(lactic-co- glycolic) acid) microspheres and prospectively test the effects of catheter-directed gastric artery administration of these microspheres on plasma ghrelin levels and body weight in swine. Octreotide is an octapeptide with pharmacologic properties mimicking those of the natural hormone somatostatin. In the GI system it inhibits secretin, gastrin, vasoactive intestinal peptide, motilin and ghrelin. Slow-release local high-dose therapy might significantly decrease all these hormones, especially ghrelin.
**Methods and Materials:** Twelve healthy growing swine were tested. Trans-catheter embolisation with octreotide-loaded microspheres was performed selectively in the gastric arteries that supply the fundus and corpus. Six swine underwent embolisation with octreotide-loaded microspheres, while six control animals underwent a sham procedure with saline. 18 mg of total octreotide per procedure was used .Weight and fasting plasma ghrelin levels were obtained in the animals at baseline and at weeks 1-8. Necropsy was performed to assess mucosal integrity, fibrosis and ghrelin-expressing cells.
**Results:** Ghrelin levels over time was significantly decreased in experimental animals (P<0.001). Weekly ghrelin levels were measured in control and experimental animals as a change from baseline ghrelin values. The average post-procedure weight loss in experimental animals was significantly (P<0.001) higher than that in control animals. The ghrelin-immunoreactive mean cell density was significantly lower in the fundus in the embolised animals compared with control animals.
**Conclusion:** Gastric artery embolisation with biodegradable octreotide-loaded PLA/PLGA microspheres with sustained release properties can significantly suppress ghrelin and significantly affect weight loss.

**B-0738** 14:08
**Prevention of post-partum haemorrhages in patients with morbidly adherent placenta: a monocentric experience**
F. Capasso, M. Coppola, M. Caruso, S. Stilo, A. Stanzione, P. Venetucci; *Naples/IT (francesco.capasso1@gmail.com)*

**Purpose:** To evaluate the performance of various interventional radiology techniques in the prevention of post-partum haemorrhages in patients with MAP.
**Methods and Materials:** Between January and December 2017, 16 pregnant women were referred to our institution due to metrorrhagia and MAP. Among them, 7 underwent cesarean delivery assisted by occlusion balloon catheters located in both hypogastric artery (group 1); in 6 patients an embolisation procedure in leading uterine artery before delivery was performed (group 2); finally, in 3 patients an observational approach was adopted, positioning prevention catheters in both hypogastric artery (group 3). All procedures were performed by mobile C-arm angiograph in gynaecology surgery room. Primary endpoints were haemoglobin reduction and transfusion requirement whereas the secondary ones were hysterectomy rate and hospitalization time.
**Results:** Haemoglobin average reduction was of 0,7 g/dl for group 1 and 2 and 0,9 g/dl for group 3. No blood transfusions were need for patients of group 2 and 3; only one patient of group 1 need transfusion of 1000 mL of blood but her haemoglobin value before delivery was 9,0 g/dL. Hysterectomy was essential in 3 patients of group 1, in 3 patient of group 2 and in 1 patient of group 3. Mean hospitalization time was 7 days.
**Conclusion:** No significant differences were found in both primary and secondary endpoints when comparing the three groups, although our experience might suggest not to adopt an observational approach.

**B-0739** 14:16
**Partial splenic artery embolisation using ethylene vinyl alcohol copolymer in patients with portosplenomesenteric thromboses and portal hypertension**
W. Uller[1], W.A. Wohlgemuth[2], H. Goessmann[1], R. Müller-Wille[1], G. Kirchner[1]; [1]*Regensburg/DE*, [2]*Halle/DE (wibke.uller@ukr.de)*

**Purpose:** Partial splenic artery embolization (PSE) may be a therapy option in patients with symptomatic portal hypertension not eligible for TIPS. The purpose of this study was to evaluate PSE using the permanent liquid embolic agent ethylene vinyl alcohol copolymer (EVOH) for treatment of varices and thrombocytopenia in patients with chronic portomesenteric thrombosis and portal hypertension.
**Methods and Materials:** Data of 11 patients with portal hypertension who underwent PSE with EVOH between 2012 and 2017 were retrospectively analysed. 40-70% of splenic infarction was induced by EVOH. Thrombocyte, leukocyte counts and haemoglobin were measured prior to and 6 months after PSE and follow-up upper gastrointestinal endoscopy was performed. P-values were calculated using the Wilcoxon matched-pairs signed rank test.
**Results:** Causes of portal hypertension included chronic thrombosis of the portal/mesenterial/splenic vein system (n=9) and liver cirrhosis with severe thrombocytopenia (n=2). All patients developed extensive, multiple varices (esophageal, gastric, duodenal). Eight patients suffered from additional hypersplenism. Thrombocytes, leucocytes counts and haemoglobin increased significantly 6 months after the procedure (p=0.0078, p=0.001, p=0.014, respectively). Absence of variceal bleeding and decrease of grade of varices was achieved in 10/11 patients during follow-up (24.7±17.7 months, median 18). All patients developed a mild post-embolization syndrome. Two patients experienced abscesses of the spleen.
**Conclusion:** PSE with EVOH is an efficient technique that decrease varices, variceal bleeding, improves sequels of hypersplenism significantly and prevents recanalization via extra- and intrasplenic arterial anastomoses. The risk of splenic abscess seems to increase with embolization rates of more than 70% of the spleen parenchyma.

**B-0740** 14:24

**Shattered spleen in blunt trauma: clinical results of trans-arterial embolisation, four years' experiences in a single regional trauma centre**

R. Lee, C.H. Jeon, C.W. Kim, H. Kwon, H.M. Cho; *Busan/KR (fkdzja@gmail.com)*

**Purpose:** To evaluate the benefits and risks of splenic artery embolisation (SAE) for patient with high-grade blunt spleen injury (BSI), especially "shattered spleen".
**Methods and Materials:** A retrospective electronic medical record review of all patients undergoing SAE from April 2013 to March 2017 at a Korean regional trauma care centre was performed. Reviewed results included patient demographics, initial and follow-up CT scan results, angiographic findings, embolisation techniques, and clinical outcomes including splenic salvage rate and procedure-related complications.
**Results:** A total of 89 patients were reviewed. CT scan was used to give the American Association for the Surgery of Trauma (AAST) grade of BSI. A total of 31 patients with shattered spleen were identified with a median (SD) age of 42.0 (24.7), including 3 patients of age under 15 years. 28 (90.3%) patients were males and the major mechanism of injury was motor vehicle collision. These patients had mean (SD) Injury Severity Scores (ISS) of 27.0 (9.3); systolic blood pressure of 92.0 mmHg (18.1) and 11 patients were haemodynamically unstable. The rate of successful embolotherapy was 90.3% (n=28) and only 3 patients underwent post-embolisation splenectomy. The spleen salvage rate was 96.4% (n=27). Major complications included rebleeding in 3 embolotherapy patients; 1 splenic abscess. There was no patient with sepsis during follow-up duration (237.0±198.2 days).
**Conclusion:** SAE is a safe and feasible procedure in extremely high-grade BSI and effective for not only haemorrhage control but also spleen salvage.

**B-0741** 14:32

**Preoperative transarterial embolisation using a gelatin sponge for benign prostatic hyperplasia: initial feasibility test**

K.Y. Kim, M.G. Shin; *Jeonju/KR (kky2kkw@gmail.com)*

**Purpose:** To assess the safety and effectiveness of preoperative prostatic artery embolisation (PAE) in relation to the decrease in haemoglobin level, requirement of amount of blood transfusion, hospital days, and procedure-related complications after surgery.
**Methods and Materials:** From the database search, eight patients were identified from May 2017 to September 2018 (embolisation group). For statistical analysis, consecutive patients with large prostate volume (≥ 70 ml) who were operated during the same period without preoperative PAE (non-embolisation group; n=8) were reviewed from hospital record. The outcomes of interest were technical success, complication related to embolisation, haemoglobin reduction, need of blood transfusion, operating time, and hospital admission days after surgery.
**Results:** PAE was technically successful in 87.5% (7/8) patients. Partial success was gained in 1 patient due to prolonged vasospasm of right prostatic artery. The mean haemoglobin reduction was lower in embolisation group compared to non-embolisation group (1.24±0.29 g/dl vs. 2.85±0.45 g/dl, respectively; p=0.021). Median hospital admission days after surgery was 6.5±0.7 days (range, 4-10 days) in embolisation group, and 9.5±1.4 days (range, 2-13 days; p=0.110) in non-embolisation group.
**Conclusion:** Preoperative PAE is safe and may have a preventive effect on blood loss during prostate surgery. It leads to decrease in reduction of haemoglobin without procedure-related complications.

**B-0742** 14:40

**Gastric variceal recurrence after balloon-occluded retrograde transvenous obliteration**

K. Kato, K. Kawashima, T. Suzuki, M. Hamano, S. Ehara; *Morioka/JP (kkato@iwate-med.ac.jp)*

**Purpose:** Balloon-occluded retrograde transvenous obliteration (B-RTO ) is an effective treatment for gastric varices (GV); however, reports on the GV recurrence were limited. Therefore, this study aimed to evaluate the recurrence factors of GV after B-RTO.
**Methods and Materials:** B-RTO was performed in 54 patients with GV between October 2007 and December 2017. GV recurrence was evaluated based on follow-up contrast-enhanced CT (CE-CT) and clinical course. GV recurrence was also compared according to the gastro-renal shunt patency, size of the spleen, and afferent gastric vein classification.
**Results:** The appropriate technique for B-RTO was successfully performed in 53 patients. Complete thrombosis of GV was achieved in 43 patients, partial thrombosis in 6, and non-thrombosis in 4. Among the 43 patients, who had complete obliterated GV, follow-up CE-CT was performed in 34 patients during a mean observation period of 33.3 (range, 3-121) months, which revealed GV recurrence in six patients, and two of them had GV haemorrhage. Developed afferent veins on the recurrent GV were short gastric vein in four patients and left gastric veins in two. Gastro-renal shunt patency was statistically correlated with GV recurrence (p<0.05). However, the size of the spleen and afferent gastric vein classification were not correlated with GV recurrence.
**Conclusion:** Residual gastro-renal shunt seems to cause revascularization of the GV afferent veins, which can result in GV recurrence. Therefore, obliteration of the gastro-renal shunt and GV would support the long-term therapeutic effect of B-RTO.

**B-0743** 14:48

**Trans-arterial embolisation of the prostate with biodegradable flutamide-loaded PLA/PLGA microspheres for benign prostate hyperplasia: a preliminary study in normal swine model**

F. Nurili[1], T. Acar[2], S. Derman[2], A. Bas[2], I. Caymaz[2], I. Yurdaisik[2], H. Cakiroglu[2], S. Özkanli[2], O. Aras[1]; [1]*New York, NY/US*, [2]*Istanbul/TR (fuatnurili@gmail.com)*

**Purpose:** To prospectively evaluate whether transarterial embolisation with biodegradable flutamide-loaded PLA/PLGA microspheres with sustained release properties in the prostate of swine can induce shrinkage in the volume of prostatic tissue. Flutamide is a strong nonsteroidal antiandrogen that acts as selective, competitive and silent antagonist.
**Methods and Materials:** Sustained release flutamide-loaded polylactide/poly(lactic-co-glycolic acid) (PLA/PLGA) microspheres were successfully fabricated using the single emulsion (o/w) solvent evaporation method in our preliminary study. Twelve male swine were randomly assigned to either the experimental group (n-6) or control group (n-6). Selective angiography was performed to deliver microspheres. In the experimental group, 200-700-micron-diameter flutamide-loaded microspheres were used for embolisation. 250 mg of total flutamide was used per procedure. 6 weeks later animals were euthanised for necropsy, the prostates were removed for size measurement and histopathologic examination. Paired Student's t test was used for statistical analysis.
**Results:** All procedures were technically successful. The mean prostate volume after embolisation in the experimental group was significantly (P<0.001) diminished compared with the mean prostate volume for the control group. Microscopic examination showed that normal gland structure was partially replaced by fibrosis and atrophy in the residual gland tissue.
**Conclusion:** Embolisation of the prostatic arteries with flutamide-loaded PLA/PLGA microspheres can induce strong shrinkage of the prostate. This study shows that transarterial targeted embolisation of prostatic arteries with flutamide-loaded PLA/PLGA microspheres with sustained release properties may provide an effective alternative approach to the treatment for benign prostatic hyperplasia in humans.

**B-0745** 14:56

**The application of hepatic artery-targeting guidewire technique combined with indirect portography to transjugular intrahepatic portosystemic shunt**

S. Wang, N. Li, C. Yu; *Tianjin/CN (wase1255@163.com)*

**Purpose:** This study evaluated the value of combined hepatic artery-targeting guidewire technique with indirect portography to transjugular intrahepatic portosystemic shunt (TIPS) for the puncture step.
**Methods and Materials:** We retrospectively reviewed 11 consecutive patients (5 men and 6 women, aged 46-76 years (mean 64 years)) with portal hypertension in whom the TIPS procedure was performed. As the first step in the TIPS procedure in all cases, a micro-guidewire was inserted into the hepatic arterial branch accompanying the portal venous branch through a microcatheter coaxially advanced from a 5-French catheter positioned in the coeliac or common hepatic artery. At the puncture step, the tip of the metallic cannula was aimed 1 cm posterior to the distal part of this micro-guidewire, after which the TIPS procedure was performed. Success rate, number of punctures and complications were evaluated.
**Results:** The TIPS procedure was successfully performed in all 11 patients. The mean number of punctures until success in entering the targeted portal venous branch was 5 (range 1- 14). In 3 patients (27%), the right portal venous branch was entered at the first puncture attempt. The hepatic artery was punctured once in one patient and the bile duct was punctured once in another patient. No serious procedure-induced complications occurred.
**Conclusion:** The TIPS procedure can be accomplished safely, precisely and relatively easily using the hepatic artery-targeting guidewire technique.

**B-0746** 15:04

**What makes a difference between life and death in patients with NOMI? Analysis of clinical data and contrast-enhanced dynamic CT findings before angiography**

R. Miyazawa, M. Kamo; *Tokyo/JP*

**Purpose:** The purpose of this study was to investigate prognostic factors of computed tomography (CT) findings and clinical data in patients with non-occlusive mesenteric ischemia (NOMI).

**Methods and Materials:** This was a retrospective, IRB-approved study, reviewing 21 consecutive patients diagnosed as NOMI on angiography. By chart review, patients were divided into either "survivor" group or "non-survivor" group. Clinical data such as laboratory data or time from taking CT to injecting papaverine were also obtained. Assessment on contrast-enhanced CT images was performed by consensus of two radiologists in following items: defect of mural enhancement, pneumatosis intestinalis, hepatic portal venous gas, paralytic bowel dilatation, bowel wall thinning, and diameter of the vessels.
**Results:** 8 patients belonged to "survivor" group, 11 allocated to "non-survivor" group. Neither of CT findings showed significant difference between alive group and dead group (defect of mural enhancement: 75% and 100% (p=.16), pneumatosis intestinalis: 50% and 45.5% (p=1.00), hepatic portal venous gas: 37.5% and 45.5% (p=1.00), paralytic bowel dilatation: 12.5% and 63.6% (p=.06), bowel wall thinning: 50% and 45.5% (p=1.00)). There was also no significant difference in the diameter of the vessels. Time from taking CT to injecting papaverine was significantly shorter in alive group (207.6 ±126.2 minutes and 578.8 ±506.6) (p=.038). None of laboratory data showed significant difference between each group.
**Conclusion:** It may be difficult to estimate the prognoses of NOMI patients by preceding CT findings or laboratory data. Prompt action to angiography could save lives of the patients.

**B-0747** 15:12
**Temporary balloon occlusion of the internal iliac arteries as a prevention of massive haemorrhage during cesarean delivery among patients with abnormal placentation**
M. Milnerowicz, J. Garcarek, M. Miś, M. Miś, A. Kacala; *Wroclaw/PL (gosia.milnerowicz@gmail.com)*

**Purpose:** To investigate the efficacy and safety of temporary balloon occlusion of the internal iliac arteries (IIAs) in the prevention of haemorrhage during cesarean delivery among patients with placenta previa and placental accretism.
**Methods and Materials:** From 2013 to 2017, 16 patients diagnosed with placenta previa and coexisting placental accretism using ultrasound imaging underwent elective cesarean delivery preceded with temporary balloon occlusion of the IIAs. Depending on penetration of the placenta into the uterine wall, placental extraction with curettage or hysterectomy was performed. Intraoperative blood loss, amount of transfusions, haemoglobin level and procedure complications were analyzed.
**Results:** The mean age was 33.7 years with a mean of 2.9 previous gestations, 80.6% of them were delivered by cesarean. Abnormal placentation was confirmed intraoperatively in all cases. All of the patients had placenta previa and all but one had coexisting placental accretism. 12 patients underwent hysterectomy and the remaining 4 patients required only curettage. Blood loss was assessed by the surgeon in a 3-point scale (low, moderate, significant) and in 13/16 (81.2%) cases was estimated as low.The mean amount of transfused packed red blood cells was 1.1U and haemoglobin drop was 1.7g/dl in the first day postoperatively. One patient underwent nephrostomy due to postoperative urinary stasis. There were no other procedure-related complications, neither maternal nor fetal mortality.
**Conclusion:** Temporary balloon occlusion of the IIAs during cesarean delivery is a safe method and appears to reduce blood loss and transfusion requirements in patients diagnosed with placenta previa and placental accretism.

**14:00 - 15:30** **Room D**

### Artificial Intelligence and Machine Learning

### SS 705

### Machine learning in neuroradiology: vascular and mass lesions

*Moderators:*
T.C. Booth; London/UK
K. Egger; Freiburg/DE

**B-0748** 14:00
**Prospective evaluation of a deep learning algorithm deployed in an urban imaging centre to notify clinicians of head CT scans with critical abnormalities**
S. Tanamala[1], S. Chilamkurthy[1], M. Biviji[2], R. Ghosh[1], P. Rao[1]; [1]*Mumbai/IN*, [2]*Nagpur/IN (swetha.tanamala@qure.ai)*

**Purpose:** Non-contrast Head-CT scans are primary imaging modality for evaluating patients with trauma or stroke. While results of deep learning algorithms to identify head-CT scans containing critical abnormalities has been published in retrospective studies, effects of deployment of such an algorithm in a real-world setting, with mobile notifications to clinician remain unstudied. In this prospective study, we evaluated performance of such an automated triage system in an urban 24-hour imaging facility.
**Methods and Materials:** We developed an accurate deep neural network algorithm that identifies and localizes intracranial bleeds, cranial fractures, mass effect and midline shift (defined as critical abnormalities) on non-contrast head-CT scans. The algorithm is deployed in clinical imaging facility in conjunction with an on-premise module that automatically selects eligible scans from PACS and uploads them to cloud-based algorithm for processing. Once processed, cloud algorithm returns an additional series, viewable as an overlay over the original, and a text notification to radiologist with preview images. Mobile notifications facilitated confirmation of the detected abnormalities. We studied performance of the automated system over 60 days.
**Results:** 194 non-contrast head-CTs were filtered amongst 748 CT images acquired over 60 days. The head-CTs were evaluated by senior radiologist to generate ground-truth. Sensitivity, specificity, AUC and average time to notification of head-CT scans with critical abnormalities were 0.90(95% CI 0.74-0.98), 0.86(0.80-0.91), 0.97(0.92-1.00) and 3.2 minutes respectively.
**Conclusion:** An automated triage system in a radiology facility results in rapid notification of critical scans, with high sensitivity and this may be used to expedite treatment initiation.
*Author Disclosures:*
**S. Tanamala:** Employee; Qure.ai. **S. Chilamkurthy:** Employee; Qure.ai. **R. Ghosh:** Employee; Qure.ai. **P. Rao:** Employee; Qure.ai.

**B-0749** 14:08
**Extracranial determinants of white matter lesion volume: a machine learning-based top 10 list**
S. Grosu[1], S. Rospleszcz[1], F. Bamberg[2], C.L. Schlett[3], A. Peters[4], M. Heier[1], F. Schöppe[1], B.B. Ertl-Wagner[5], S. Stöcklein[1]; [1]*Munich/DE*, [2]*Freiburg/DE*, [3]*Heidelberg/DE*, [4]*Neuherberg/DE*, [5]*Toronto, ON/CA (sergio.grosu@med.uni-muenchen.de)*

**Purpose:** Numerous extracranial, potentially interrelated factors have an impact on the development of white matter lesions (WML). The aim of this study was to train a multifactorial composite score of extracranial parameters using machine learning algorithms to identify the variables that best predict WML volume.
**Methods and Materials:** 400 subjects of the KORA study cohort underwent comprehensive 3T-MRI. Cerebral WML were manually segmented on 3D-FLAIR-images. 90 extracranial parameters including measures of sociodemographics, anthropometrics, diabetes-related issues, behaviour, somatic/depressive symptoms, blood pressure, laboratory values, adipose tissue, medication intake and sleep were collected in a standardized method. To identify relevant predictor covariates associated with WML volume penalized zero-inflated negative binomial (ZINB) regression models based on elastic Net (EN) regularization were calculated.
**Results:** The final study population consisted of 370 participants (58% male; age: 55.72 ± 9.11 years). WML were found in 236 (63.78%) participants (57% male). The EN model was evaluated based on 1000 data splits. The ten variables that were most often selected were age (N=224), controlled hypertension (N=169), HbA1c (N=148), widowed marital status (N=145), prediabetes assessed by OGTT (N=135), antiplatelet medication (N=134), unknown hypertension (N=106), NSAID medication (N=65), physical activity of less than 2h/week (N=46) and alcohol consumption 1g-20g/day (N=46).
**Conclusion:** In this reference population without prior cardiocerebrovascular disease, a systematic machine learning-based analysis of a wide range of extracranial parameters showed that the most powerful determinants for WML volume are age, hypertension, pre-/diabetes, widowed marital status, antiplatelet/NSAID medication, low physical activity and moderate alcohol consumption.

**B-0750** 14:16
**Deep learning for infarct detection and localisation from head CT scans**
R. Ghosh[1], S. Chilamkurthy[1], P. Rao[1], S. Tanamala[1], N. Campeau[2]; [1]*Mumbai/IN*, [2]*Rochester, MN/US (swetha.tanamala@qure.ai)*

**Purpose:** The purpose of this study was to use a deep learning algorithm to detect and localize subacute and chronic ischemic infarcts on head CT scans for use in automated volumetric progression tracking.
**Methods and Materials:** We sampled 308 head CT scans (11840 slices) which were reported with chronic or subacute infarct. The infarcted regions in 11840 infarct-positive slices were marked. We trained segmentation algorithm to predict a heatmap of infarct lesion. The heatmap was used to derive scan level features representative of lesion density and volume to train a random forest to predict scan-level probabilities of chronic infarct. Area under receiver operating characteristics curves (AUC) were used to evaluate scan level predictions.

**Results:** The algorithm was validated on an independent dataset of 1610 head CT scans containing 78 chronic & 9 subacute infarct, 45 chronic ICH, 6 glioblastomas. The distribution of infarct affected territories was - 52.9% MCA, 33.3 % PCA, 9.3% ACA and 4.7% vertebrobasilar territories. The algorithm yielded AUC of 0.8474 (95% CI 0.7964 - 0.8984) for scan level predictions. It identified 8 of 9 subacute infarcts (88.89% recall) and 70 out of 78 chronic infarcts (89.74% recall). The eight missed chronic infarcts constituted of 3 lacunar and 2 hemorrhagic. The volumes of predicted infarct lesions ranged from 1 mL - 526 mL with mean prediction volume as 55.60mL.
**Conclusion:** The study demonstrates the capability of deep learning algorithms to accurately differentiate infarcts from infarct mimics.
*Author Disclosures:*
**R. Ghosh:** Employee; Qure.ai. **S. Chilamkurthy:** Employee; Qure.ai. **P. Rao:** Employee; Qure.ai. **S. Tanamala:** Employee; Qure.ai.

### B-0751 14:24
**Estimation of intracranial haemorrhage volume with the deep learning method**
Q. Gong, Y. Deng, C. Xia, R. Zhang, C. Wu, Z. Wang, Y. Zhang, J. Ma; *Beijing/CN (dr.junma@foxmail.com)*

**Purpose:** Brain haemorrhage is one main cause of stroke. The current standard to estimate bleeding volume is ABC/2, which is an approximation applied to the bleeding regions. We developed a deep learning approach to estimate brain haemorrhage volume by training CNN.
**Methods and Materials:** Our model automatically segments haematoma from brain CT images. It uses DenseNet to extract features from CT images and propagate the features through U-Net for the segmentation. The model was trained on a brain CT dataset that consisted of 3000 brain CT sequences, including 1000 intracerebral haemorrhage (ICH), 1000 extradural haemorrhage (EDH) and 1000 subdural haemorrhage (SDH) cases. The test dataset consisted of 217 brain CT sequences (67 ICH, 87 EDH, and 63 SDH). The gold standard was obtained from manual segmentation by 3 neuro-radiologists with more than 5 years of experience.
**Results:** The ABC/2 method resulted in inaccuracies up to 40 ml. The volume difference between DL model and the gold standard was 1.44±1.91, 1.06±2.04 and 1.44±2.34 ml for ICH, EDH and SDH. Another attending neuro-radiologist performed manual segmentation, and achieved similar performance compared to the DL model. The computation time for the DL model was 4 second per case, as compared to 12 seconds for ABC/s method, and 1 minute by manual segmentation.
**Conclusion:** The results demonstrated that our DL model achieved high accuracy in ICH, EDH, and SDH volume estimation, which significantly outperformed the current standard ABC/2 method. The computational time of the DL model was 4 second, which was 15 times faster than human segmentation.
*Author Disclosures:*
**Q. Gong:** Employee; Infervision. **Y. Deng:** Employee; Infervision. **C. Xia:** Employee; Infervision. **R. Zhang:** Employee; Infervision.

### B-0752 14:32
**Brain disease classification based on routine MRI using a deep learning algorithm**
Y. Liu, P. Gao, Y. Duan; *Beijing/CN (asiaeurope80@gmail.com)*

**Purpose:** To develop and evaluate the efficacy of a deep learning algorithm using clinical routine sequences to classify patients into one of four broad neurological disease classes: normal, tumor, ischemic cerebral vessel disease (ICVD), and multiple sclerosis (MS).
**Methods and Materials:** A deep learning algorithm was trained on MR images from 4507 randomly selected cases restricted to one of the four classes. Each subject's diagnosis was validated by an experienced radiologist in Beijing TianTan Hospital. After training, the model performance was tested in 1167 cases that included the following diagnosis distribution: 212 normal, 739 tumor cases, 111 ICVD, and 105 MS.Sensitivity, specificity, accuracy for classifying each case into its respective disease categories, in a separate test set.
**Results:** Taking the predicted class with the highest probability for each case, the deep learning algorithm achieved an overall accuracy of 94.1%, with a sensitivity and specificity of 85.2% and 95.7%, respectively. Sensitivity for specific classes were: 91.5% (normal), 89.7% (tumor), 83.7% (ICVD), and 76.2% (MS). Specificities for specific classes were 91.3% (Normal), 93.9% (Tumor), 98.4% (ICVD) and 99.0% (MS).
**Conclusion:** The algorithm achieved high sensitivity and specificity across normal cases and several broad classes of neurological disease classes, indicating its potential usefulness in automatic neurological disease detection and diagnosis. Optimization of the algorithm and the extension to other neurological diseases would have to be carried out to enable it to be used in clinical settings to aid radiologists and doctors in their diagnosis of patients.

### B-0753 14:40
**Detection of intracranial haemorrhage on CT of the brain using a deep learning algorithm**
D. Desbuquoit, S. Dekeyzer, J. Huyskens, S. Nicolay, E. De Smet, J. Van Goethem, L. Van den Hauwe, P.M. Parizel; *Edegem/BE (damien.desbuquoit@gmail.com)*

**Purpose:** This prospective study aimed to evaluate the use of a commercially available deep learning algorithm for the detection of intracranial haemorrhage on non-contrast enhanced CT of the brain.
**Methods and Materials:** 500 non-contrast enhanced CT's of the brain performed in June, July and August 2018 were independently analysed on the presence of pathological hyperdensities by a deep learning software package (Aidoc, Tel Aviv, Israel) and a 4th-year radiology resident. Their results were compared to a "gold standard analysis", performed by a senior neuroradiologists with access to clinical information and, when available, previous or follow-up imaging studies.
**Results:** Pathological hyperdensities were present in 134/500 patients, the majority of which were haemorrhages (128/134; 95.5%). Pathological hyperdensities were correctly identified by Aidoc-software in 125/134 cases (93,3%), compared to 133/134 (99.3%) for the resident. Aidoc's false-negative ratio was 9/134 (6.7%). When no pathological hyperdensities were present, the exam was rated negative by Aidoc-software in 345/366 cases (94.3%), compared to 362/366 (98.9%) for the resident. Aidoc's false-positive ratio was 21/366 (5.7%).
**Conclusion:** The use of a deep learning algorithm for the detection of pathological intracranial hyperdensities helped to detect urgent cases more quickly. False positive results occur in a limited number of cases (5,7%) and are mainly due to beam hardening artefacts, hyperdense dural sinuses, or falcine or basal ganglia calcifications. False negatives were slightly more frequent (6,7%) and mainly seen in small or subtle haemorrhages.

### B-0754 14:48
**Development and validation of a deep neural network-based computer-assisted detection system for cerebral aneurysms in CT angiography**
Z. Shi[1], C. Pan[2], C. Zhou[1], D. Fan[2], X. Li[2], G. Lu[1], L. Zhang[1]; [1]*Nanjing/CN*, [2]*Beijing/CN (fmrishiz@126.com)*

**Purpose:** To develop a CAD system for intracranial aneurysms on computed tomography angiography (CTA) images based on a deep convolutional neural network (CNN) and a volume rendering (VR) algorithm, and to demonstrate the usefulness of the system by training and evaluating it using a large dataset.
**Methods and Materials:** There are 209 cases (159 for training and 50 for the validation) with intracranial aneurysms that diagnosed by both computed tomography angiography (CTA) and invasive cerebral angiography in the dataset. The CNN was trained in advance using manually inputted labels in bone-removal images of CT angiography and the corresponding VR algorithm. 3D patches were randomly cropped with or without aneurysms, and kept the ratio between positive and negative about 1.0. The architecture contains an encoder to extract features and a decoder to output a volumetric segmentation. And we added a classification path to determine whether the patch contains aneurysm(s). The diagnostic performance of the system was assessed using Dice coefficient and receiver operating characteristic (FROC) analysis.
**Results:** Our system showed a good performance in detection of intracranial aneurysms with a dice score of 0.65, 92.2% (47/51) of aneurysms with 1.36 false positives per case (FPs/case). At a sensitivity of 73.4%, the number of FPs/case was 0.40.The area under the ROC curve (AUC) is 0.974 for the detection of aneurysm in a voxel.
**Conclusion:** We demonstrated that the combination of a CNN and VR algorithm is useful for the detection of intracranial aneurysms based on the CT angiography images of head.

### B-0755 14:56
**Automated detection of midline shift and mass effect from head CT scans using deep learning**
S. Chilamkurthy[1], R. Ghosh[1], S. Tanamala[1], M. Biviji[2], N. Campeau[3], V. Venugopal[4]; [1]*Mumbai/IN*, [2]*Nagpur/IN*, [3]*Rochester, MN/US*, [4]*New Delhi/IN (sasank.chilamkurthy@qure.ai)*

**Purpose:** Mass effect and midline shift are most critical and time sensitive abnormalities that can be readily detected on head-CT scan. We describe development and validation of deep learning algorithms to automatically detect mentioned abnormalities.
**Methods and Materials:** We labelled slices from 699 anonymized noncontrast head-CT scans for the presence or absence of mass effect and midline shift in that slice. Number of scans(slices) with mass effect were 320(3143) and midline shift were 249(2074). We used these labels to train a modified ResNet18, a popular convolutional neural network to predict softmax based confidences for the presence of mass effect and midline shift in a slice. We modified network by using two parallel fully connected(FC) layers in place of a single FC layer. The confidences at slice-level were combined using random forest to predict the scan-level confidence for presence of mass effect and midline shift. A separate dataset(CQ500 dataset) was collected for the validation of the algorithm. Three senior radiologists independently read each scan in this dataset. Consensus of the readers' opinion was used as the gold standard. We used areas under receiver operating characteristics curves(AUC) to evaluate the algorithm.
**Results:** CQ500 dataset contained 491 scans of which number of scans with mass effect and midline shift were 99 and 47 respectively. AUC for detecting mass effect was 0.92(95%CI 0.89-0.95) and for detecting midline shift was 0.97(95%CI 0.94-0.99).
**Conclusion:** We show that a deep learning algorithm can be trained to accurately detect mass effect and midline shift from head CT scans.
*Author Disclosures:*
**S. Chilamkurthy:** Employee; Qure.ai. **R. Ghosh:** Employee; Qure.ai. **S. Tanamala:** Employee; Qure.ai.

**B-0756** 15:04
**A deep learning method for analysing post-operative MRI scans of glioblastoma patients**
M. Perkuhn, P. Stavrinou, R. Shahzad, F. Thiele, G. Shakirin, D. Garmpis, C. Kabbasch, J. Borggrefe; *Cologne/DE (michael.perkuhn@uk-koeln.de)*

**Purpose:** Cytoreductive surgery is the cornerstone of glioblastoma (GB) therapy and residual tumour volume is considered to be an important outcome measure. The MRI analysis of the contrast-enhancing residual tumour (CE-RT) is challenging e.g. due to small tumour volumes. Detection and segmentation of CE-RT suffers from high inter-rater variability. Our objective was to train and evaluate a deep learning model (DLM) for fully-automatic detection, localisation and segmentation of CE-RT, Oedema (ED) and tumour cavity (TC) on routine postoperative MRI data.
**Methods and Materials:** 94 post-operative GB patients were included. T1, T2, FLAIR, CE T1 sequences were obtained 24-48 hours after surgery. ED, TC and CE-RT were manually delineated by experts (radiologist, neurosurgeon). Ground truth (GT) was obtained by consensus. A DLM for GB segmentation was trained using 5-fold-cross-validation based on transfer learning of a pre-operative model.
**Results:** For CE-RT detection, the DLM achieved 82% sensitivity and 72% specificity (AUC=0.83). ED, TC compartments were detected in all patients. Volume correlations (*R*) were 0.63 (CE-RT), 0.95 (ED), 0.76 (TC). Inter-rater agreement kappa for CE-RT before consensus was 0.43, and 0.54 between the DLM and GT. Automatic segmentation achieved dice coefficients of 0.33±0.23 (CE), 0.67±0.18 (ED) and 0.59±0.21 (TC), outperforming published methods.
**Conclusion:** The proposed fully-automatic approach is robust in detecting CE-RT on routine postoperative data and its accuracy is comparable to inter-rater variability. This makes the method suitable for automatic CE-RT segmentation reviewed by the radiologist in postoperative GB analysis. ED and CE-RT segmentations can also serve as baseline for GB follow-up.
*Author Disclosures:*
**M. Perkuhn:** Employee; Philips Research. **R. Shahzad:** Employee; Philips Research. **F. Thiele:** Employee; Philips Research. **G. Shakirin:** Employee; Philips Research.

**B-0757** 15:12
**A radiomics strategy for amide proton transfer imaging in predicting isocitrate dehydrogenase genotype within grade II/III gliomas**
Y. Han, Y.-Z. Sun, L.-F. Yan, W. Wang, G.-B. Cui; *Xi'an/CN (hanyu0920@163.com)*

**Purpose:** To evaluate the diagnostic performance of radiomics analyses in predicting isocitrate dehydrogenase (IDH) genotype of grade II/III gliomas based on amide proton transfer weighted ($APT_W$) imaging.
**Methods and Materials:** Fifty-nine grade II/III glioma patients with known IDH mutation status were prospectively included (IDH-wild type, 16; IDH mutation, 43). Cases were randomly assigned to the training (n = 49) and test cohort (n = 10). A total of 1044 quantitative radiomic features were extracted from $APT_W$ images. Support vector machine with recursive feature elimination (SVM-RFE) and synthetic minority oversampling technique (SMOTE) were adopted to select the optimal feature subsets and to solve the adverse impact of the imbalance dataset in the training cohort, respectively. The trained SVM prediction model for IDH genotype was validated on both train and test cohort.
**Results:** Our SVM model was established using 19 features selected by SVM-RFE. The AUC and accuracy for IDH genotype on training set were 89.2%, 0.952, while on the testing set were 70%, 0.84, respectively.
**Conclusion:** A radiomics strategy integrating SVM model and APT image features are predictive for IDH genotype in grade II/III gliomas.

**B-0758** 15:20
**BRAIMS: an incoming open source MR imaging dataset for brain instance-aware metastases segmentation**
W.-Y. Guo, S.-Q. Zheng, H.-M. Wu, P.C. Liu, I.-J. Sun, S. Hong, W.-Y. Chung, W.H. Yang, Y.-C. Tu; *Taipei/TW (wyguo@vghtpe.gov.tw)*

**Purpose:** Depicting brain metastases (for number counting and size measuring) is challenging and critical for patient management. A novel dataset for automated instance segmentation of brain metastases and a benchmark model for feasibility evaluation of metastases detection are introduced.
**Methods and Materials:** Brain MR images of 1100 patients (at least, T1-weighted (pre- and post-gadolinium contrast, T1-Gd) and T2-weighted) were acquired for radiosurgery of brain metastasis between 1993 and 2017 in our institute. The current dataset enrolled 308 of them, who had naïve brain metastases. Tumour detection done by an interdisciplinary team served as the ground truth. The BET of FMRIB Software Library was utilized for skull-stripping. Contrast limited adaptive histogram equalization was used for image enhancement for model training. Finally, the benchmark model was based on 2D Mask R-CNN, best known for instance segmentation. Only T1-Gd were used for feasibility evaluation by model.
**Results:** The dataset comprised 308 folders, with each containing three series images in NIFTI format and one segmentation mask volume in 3D TIFF format. Each tumour instance was annotated with an exclusive value. There were 100 tumours of 38 patients in testing set. By patient, the recall of model was 100%. By tumour, the recall was 86%, and the precision was 67.7%.
**Conclusion:** This is a groundbreaking work on dataset and model for instance segmentation and biometric measurements of MR Images. T1-Gd images of brain metastases present clear spatial information and typical texture for automatic tasks. A competition with release of the dataset is planned currently.

**14:00 - 15:30** **Room G**

### Physics in Medical Imaging

### SS 713

### MRI: safety, phantoms and quantification

*Moderators:*
N.N.
D.J. Lurie; Aberdeen/UK

**K-18** 14:00
**Keynote lecture**
A. Webb; *Leiden/NL*

**B-0759** 14:09
**MRI principles and techniques: how and where do radiologists and radiology residents look for?**
F.M. Doniselli, M. Zanardo, L.M. Sconfienza, F. Sardanelli; *Milan/IT (fabio.doniselli@gmail.com)*

**Purpose:** To investigate where and how MRI principles and technical issues are searched by radiologists and radiology residents (RR).
**Methods and Materials:** We proposed a European online survey with 8 multiple-choice questions. We investigated how often radiologists have doubts about MR principles or technical issues, where and what kind of information they search, in which language and in which websites. Chi-squared test was used.
**Results:** We obtained 122 answers (40 radiologists, 82 RR, from 6 different European countries). Radiologists and RR search information in English rather than their native language (75% vs. 68%, respectively, $p$=0.761). RR recognises to have frequently (51%) or often (32%) doubts about MRI principles while radiologists sometimes (45%) or rarely (25%), with a statistically significant difference ($p$=0.002). Post-processing details are equally searched by RR and radiologists (30%, $p$=0.953) as well as advanced/new techniques (49% vs 40%, $p$=0.518). Clinical protocol settings are more searched by RR than radiologists (68% vs 35%, $p$=0.014) as well as basic sequences details (66% vs 10%, $p$<0.001). Regarding source of information, RR uses more internet (90% vs 60% of radiologists, $p$=0.005), books (49% vs 20%, $p$=0.031) or directly ask to same-age colleagues (32% vs 10%, $p$=0.065) or to older colleagues (54% vs 40%, $p$=0.316). Website evaluation described a situation dominated by radiopaedia.org, both for technical and clinical issues (75% and 46%, respectively), mriquestions.com (43% and 30%) and mrimaster.com (16% and 15%).
**Conclusion:** Radiologists and RR search information about MRI principles and technical issues with different frequency. Differences were also found regarding the means, the kind of information searched and the web sources.

**B-0760** 14:17

**Design and testing of a novel complex flow phantom for medical imaging**

S. Ambrogio[1], A. Walker[2], P. Verma[1], A. Narracott[1], J. Fenner[1]; [1]*Sheffield/UK*, [2]*Boroughbridge/UK (simoambr@gmail.com)*

**Purpose:** Calibration and quality control of medical flow imaging scanners is essential and, to that end, a novel, portable, cost-effective, multimodal, complex flow phantom design is presented. This is relevant to recent medical imaging technologies, such as Doppler ultrasound vector flow imaging, which enable real-time quantitative and qualitative analyses of complex blood flow patterns. The phantom has the potential to establish a flow benchmark for comparative studies and for validation of advanced velocity estimation algorithms developed for medical imaging at research level.
**Methods and Materials:** The vortex ring is the reference flow upon which the design of the phantom is based, because it offers patterns that resemble physiological flows and has been demonstrated to be stable, reproducible, predictable and controllable. The phantom is motor-driven and vortex rings with different features can be generated on demand. The design is validated, comparing results obtained from three independent measurements methods, namely optical/video, laser-PIV and pulsed-wave Spectral Doppler ultrasound.
**Results:** Vortex rings with travelling velocities from approximately 1cm/s to 80cm/s and different sizes were produced with a reproducibility typically better than +/-10%. At best, differences of +/-2% were found for peak velocity estimations with laser-PIV and Spectral Doppler. Errors refer to average and standard deviation values calculated on 10 vortex ring acquisitions.
**Conclusion:** Construction and characterisation of an innovative flow phantom, able to produce stable complex flow patterns, was described. The design, currently compatible with Doppler ultrasound modalities, could be adapted for comparative studies with different medical imaging technologies, such as X-rays and magnetic resonance.

*Author Disclosures:*
**S. Ambrogio:** Research/Grant Support; This work is funded by the European Commission through the H2020 Marie Sklodowska-Curie European VPH-CaSE Training Network (www.vph-case.eu), GA No. 642612. **A. Walker:** Owner; Leeds Test Objects Ltd. **P. Verma:** Research/Grant Support; This work is funded by the European Commission through the H2020 Marie Sklodowska-Curie European VPH-CaSE Training Network (www.vph-case.eu), GA No. 642612. **A. Narracott:** Research/Grant Support; This work is funded by the European Commission through the H2020 Marie Sklodowska-Curie European VPH-CaSE Training Network (www.vph-case.eu), GA No. 642612. **J. Fenner:** Research/Grant Support; This work is funded by the European Commission through the H2020 Marie Sklodowska-Curie European VPH-CaSE Training Network (www.vph-case.eu), GA No. 642612..

**B-0761** 14:25

**Phantom for independent global head SAR assessment of MRI-induced temperature change**

G. Oluniran, J. Blackwell, B. Tuohy, N. Colgan; *Galway/IE (g.oluniran2@nuigalway.ie)*

**Purpose:** Underestimation of SAR in MRI poses potential risks to patients and individuals with compromised thermoregulation. EU standardization imposes a head SAR limit of 3.2W/kg and SAR effects are negligible at up to 3W/kg. We developed a phantom and protocol where heating due the RF pulse is measured and verified against scanner-displayed SAR.
**Methods and Materials:** The spherical 3-litre glass phantom comprised of agar (60 g/L), NaCl (10 g/L) and CuSO4 (1 g/L) dissolved in distilled hot water. The phantom was manufactured to achieve thermal equilibrium and T1 properties of the phantom were determined at room temperature using a STIR sequence. A baseline image was acquired using a 2D fast gradient echo. SAR loading is generated with a clinical 3D FLAIR sequence followed by a repeat 2D fast gradient echo. Our phantom is non-perfused, and the period of heating is relatively short. Thus, physiological and conduction effects are ignored, i.e. SAR ≈ $C_{agar}$ ΔT/Δt. Temperature maps were generated using Proton Resonance Frequency Shift (PRF) thermometry. Global SAR was estimated by averaging temperature changes over the whole phantom and compared to the scanner's SAR display. Procedure was repeated for five independent scanners.
**Results:** In the format scanner model/scanner readout [W/Kg]/our calculation [W/Kg]/error [%]: GE 1.5T Signa Explorer/0.42/0.41/2.3, Siemens 1.5T Symphony/1.88/1.90/1.0, Philips 3T Achieva/1.52/1.52/0, GE 1.5T Signa Explorer/0.56/0.55/1.7, Siemens 1.5T Magnetom Sola 1.5/1.49/0.7. Our SAR estimates are in good agreement with scanner displayed data.
**Conclusion:** We have developed a phantom that can independently verify MRI head SAR.

*Author Disclosures:*
**N. Colgan:** Research/Grant Support; The research was funded by Enterprise Ireland. Grant Code: Cf2017 0826 p.

**B-0762** 14:33

**Hotspot temperatures leading to burn in 3T MRI in pigs**

C.-H. Cho[1], C. Grosse-Siestrup[1], G. Brinker[1], J. Nadobny[1], S. Niehues[1], M. Taupitz[1], B. Hamm[1], P. Schlattmann[2]; [1]*Berlin/DE*, [2]*Jena/DE (choch@gmx.ch)*

**Purpose:** Magnetic Resonance imaging (MRI) is based on radiofrequency which induces heat in tissue. Areas of overlapping radiofrequency leads to hotspots and elevated body temperature. The International Electrotechnical Commission (IEC), IEC 60601-2-33:2010 has defined limits of heat exposure, quantified as specific absorption rate (SAR). The aim of this project was to investigate the temperature behavior in vivo online.
**Methods and Materials:** Invasive temperature measurements in pre-calculated hotspots and in the rectum of 20 pigs were performed prior, during, and post radiofrequency exposure in 5 exposure levels (sham, low-2.7W/kg, moderate, high4.8W/kg, extreme 11.4W/kg) in a whole body resonator (60 cm diameter) of a 3T MRI system (123 MHz).
**Results:** Rectal temperatures increased continuously by 2.1±0.9°C independently of the hotspot temperatures which ranged from 40,5°C to 55.9°C. When a core body temperature of 39°C is reached, hotspot temperature may be already 40.5°C within 29 minutes in the low-, 41.6°C within in 20 minutes in the moderate-, and 41.4°C within 21 minutes in the high-exposure group. The hotspot temperature curves showed four characteristic patterns, sinusoid, parabola like, plateau reaching, and linear.
**Conclusion:** SAR is may not be sufficient to define safe temperatures and prevent burns. While the pig model is limited (anatomy, different thermoregulation), our results may improve the understanding of potential burns. Special care is needed for thermoregulatory impaired persons (anesthetized and pharmacologically treated patients, infants and children).

*Author Disclosures:*
**G. Brinker:** Employee; Siemens Medical. **J. Nadobny:** Equipment Support Recipient; Siemens AG.

**B-0763** 14:41

**Accuracy and reproducibility of magnitude-based proton density fat fraction quantification with resolved water-fat ambiguity**

A. Bagur, C. Hutton, M.L. Gyngell, M.D. Robson, M. Brady; *Oxford/UK (Alexandre.Bagur@perspectum-diagnostics.com)*

**Purpose:** Proton density fat fraction (MRI-PDFF) is the leading imaging biomarker for the early diagnosis and monitoring of non-alcoholic fatty liver disease (NAFLD). Typically calculated using chemical shift-encoded (CSE) water-fat separation, it can be complicated by clinical MRI phase images being inaccessible or containing errors. We introduce a confounder-corrected, magnitude-based CSE method that is robust to artefacts and unambiguous over the entire 0-100% MRI-PDFF range.
**Methods and Materials:** The method embodies multi-peak fat spectral modelling and model fitting with multipoint search optimisation. Simulations evaluated the feasibility of the method in multi-echo gradient echo protocols. Publicly available phantom data was used to evaluate bias, linearity and reproducibility of the method across manufacturers, field strengths and acquisition settings. Abdominal human acquisitions at 3T that generated artefacts in our implementation of IDEAL were used to evaluate the robustness of the new method to clinical practice.
**Results:** Excellent MRI-PDFF accuracy and reproducibility observed across manufacturers, field strengths and acquisition settings in the phantom experiment (linear regression: mean r-squared = 0.999, mean slope = 0.995, mean intercept = 0.298% MRI-PDFF). The water-fat ambiguity that typically challenges magnitude-based methods was resolved using our method. Increased robustness to local variability and fat-water swap artefacts was observed in the previously challenging 3T human acquisitions.
**Conclusion:** The introduced magnitude-based MRI-PDFF reconstruction method is accurate and robust across manufacturers, field strengths and acquisition settings. The method shows potential for standardisation and enables increased accessibility to MRI-PDFF in the clinic, notably because it does not require access to phase images.

*Author Disclosures:*
**A. Bagur:** Employee; Perspectum Diagnostics. Patent Holder; Perspectum Diagnostics. **C. Hutton:** Employee; Perspectum Diagnostics. Patent Holder; Perspectum Diagnostics. **M.L. Gyngell:** Employee; Perspectum Diagnostics. Patent Holder; Perspectum Diagnostics. Shareholder; Perspectum Diagnostics. **M.D. Robson:** Employee; Perspectum Diagnostics. Founder; Perspectum Diagnostics. Patent Holder; Perspectum Diagnostics. Shareholder; Perspectum Diagnostics. **M. Brady:** Employee; Perspectum Diagnostics. Founder; Perspectum Diagnostics. Patent Holder; Perspectum Diagnostics. Shareholder; Perspectum Diagnostics.

**B-0764** 14:49
**Quantification of abdominal subcutaneous adipose tissue in MRI of obese patients**
N.P. Linder, K. Solty, A. Hartmann, M. Blüher, R. Stange, H. Busse; *Leipzig/DE (linder@uniklinik-leipzig.de)*

**Purpose:** To evaluate a novel approach for segmentation of abdominal subcutaneous tissue (ASAT) in obese patients. Gantry size often limits full imaging of obese patients. We hypothesized that the equal distribution of subcutaneous adipose tissue on both sides of the body enables ASAT prediction from one-sided segmentation.
**Methods and Materials:** Abdominal MRI was performed at 1.5T using a 2-point Dixon sequence and complete images were segmented within the Dicomflex framework (Stange et al. 2018). After segmentation, the total volumes were divided into half resulting in hemiASAT-R and hemiASAT-R. Results were blotted against the total volume of ASAT (vASAT) and corresponding conversion factors yielded estimated total volume of ASAT (eASAT). Statistical measures of agreement were the coefficient of determination $R^2$ of a linear regression through the origin as well as the standard deviations of the differences between measured and predicted volumes.
**Results:** A total of 26 patients (13 females, mean BMI was 34.0 kg/m²) was identified and all images could be included into the analysis. Mean vASAT was 12,466 (range 7,170 - 23,741) cm³. Correlation between total and half-side ASAT yielded good results ($R^2$>0.99), slightly better for the left side of the body. Mean eASAT was 12,721 (7,491-23,951) cm³. Standard deviations of the differences between vASAT and eASAT were 359 cm³.
**Conclusion:** The observed, equal distribution of ASAT holds promise for the prospective analysis of half-side MRI of subcutaneous adipose tissue.
*Author Disclosures:*
**H. Busse:** Speaker; Siemens.

**B-0766** 15:05
**MR imaging relaxation times of pancreatic tissue measured in vivo at 7.0 Tesla**
M. Damen[1], M.S. van Leeuwen[2], A. Webb[1], D.W. Klomp[2], C. Arteaga de Castro[2]; [1]*Leiden/NL*, [2]*Utrecht/NL*

**Purpose:** Distinction of pancreatitis from pancreatic cancer is challenging with conventional MRI. High field MR offers higher SNR, CNR and resolution than lower fields, potentially offering better diagnostic imaging. However, at 7T image artefacts occur, particularly in the abdomen, ultimately hampering image quality. By using a multi-transmit system, these artefacts can be reduced using optimized channel combination. Though, MR protocols for 7T still need to be optimized. Here, we determine the mean T1 and T2 relaxation times of pancreas at 7T, and optimize T2-weighted protocols for high-resolution imaging of the pancreas.
**Methods and Materials:** 24 healthy volunteers were scanned with a 7T MR system using eight parallel transceiver antennas, each with two additional receive loops, positioned around the abdomen. RF phase shimming was performed to maximize the transmit field in the pancreas region. A Look-Locker-sequence (TFE) was used to obtain images for T1 determination, while T2 was determined from the images acquired with different echo times in one slice. T1 and T2 times were calculated using a mono-exponential fit of the average magnitude signal from an ROI contained in the pancreas. Optimized T1-weighted images were acquired using DIXON sequences, and T2-weighted MRI using multi 2D single-shot TSEs.
**Results:** The average T1 and T2 relaxation times found in healthy pancreas were 896±145ms, and 27±5ms, respectively.
**Conclusion:** We determined the T1 and T2 relaxation times for healthy pancreas at 7T and optimized imaging protocols, that can be used to discriminate healthy from pancreatic cancer tissue based on its T2 differences.

**B-0767** 15:13
**Magnetic resonance 4D-flow derived mean pulmonary arterial pressure at 3T: a validation study**
U. Reiter[1], G. Kovacs[1], C. Reiter[1], C. Kräuter[1], A. Greiser[2], H. Olschewski[1], M.H. Fuchsjäger[1], G. Reiter[1]; [1]*Graz/AT*, [2]*Erlangen/DE (ursula.reiter@medunigraz.at)*

**Purpose:** A close linear relationship between mean pulmonary arterial pressure (mPAP) measured with right-heart catheter (RHC) and duration of vortical blood flow along the main pulmonary artery was reported from 1.5T magnetic resonance 4-dimensional (4D) flow imaging. We hypothesized that this relationship is also valid at 3T, and tested accuracy for the diagnosis of pulmonary hypertension (PH) at 3T in a prospective study.
**Methods and Materials:** 133 patients with known or suspected PH (PH/nonPH, 87/46) underwent RHC and time-resolved, 3-directional phase-contrast imaging covering the main pulmonary artery at 3T. Duration of vortical blood flow along the main pulmonary artery ($t_{vortex}$) were assessed from velocity vector-fields using prototype software (4DFlow, Siemens Healthcare), and were used to determine $mPAP_{MR}=16+0.63 \cdot t_{vortex}$. Relationships between mPAP and mPAPMR were analyzed by correlation and Bland-Altman analysis. The diagnostic performance of $mPAP_{MR}$ to diagnose PH (mPAP≥25 mmHg) was investigated by receiver operating characteristic curve analysis.
**Results:** mPAP and $mPAP_{MR}$ revealed no bias (35±15 mmHg vs. 35±13 mmHg, p=ns), standard deviation of errors was small (4 mmHg), and the correlation between mPAP and $mPAP_{MR}$ was very strong (r=0.96). Area under the curve for the diagnosis of PH using $mPAP_{MR}$ was 0.99. Employing 25 mmHg as cut-off value for PH, $mPAP_{MR}$ predicted PH with a sensitivity/specificity of 0.98/0.89 (confidence intervals, 0.92-1.00/0.76-0.96).
**Conclusion:** Results confirm that the 1.5T relationship between RHC-derived mPAP and duration of vortical blood flow along the main pulmonary artery is also valid at 3T, and that PH can be diagnosed at 3T with equivalent accuracy.
*Author Disclosures:*
**A. Greiser:** Employee; Siemens Healthcare. **G. Reiter:** Employee; Siemens Healthare Diagnostics GmbH.

**B-0768** 15:21
**Contrast-enhanced MR images employed in stereotactic radiosurgery: does susceptibility-related distortion pose a significant problem?**
D.P. Dellios[1], E.P. Pappas[1], I. Seimenis[2], P. Karaiskos[1]; [1]*Athens/GR*, [2]*Alexandroupolis/GR (d.dellios@med.uoa.gr)*

**Purpose:** This study aimed at characterizing and evaluating MR image distortion stemming from susceptibility effects associated with the uptake of gadolinium diethylenetriamine pentaacetic acid (Gd-DTPA), a paramagnetic contrast agent routinely used for target localization and delineation in stereotactic radiosurgery (SRS).
**Methods and Materials:** A custom-made, head-sized phantom incorporating 947 control points and two cylindrical inserts was imaged at 1.5T. The inserts were filled with Gd-DTPA solutions (0-20 mM) to simulate small brain lesions. The read gradient polarity reversal and field mapping techniques were employed to assess total sequence-dependent distortion at the insert locations and $B_0$ inhomogeneity-related distortion, respectively. The net centroid offset stemming from Gd-DTPA susceptibility alone was deduced by subtracting the $B_0$ inhomogeneity-related distortion from the estimated total distortion. The same methodology was applied to Gd-enhanced images with brain metastases.
**Results:** Both techniques provided detailed distortion maps, although the reversed gradient method proved extremely straightforward and efficient. In the phantom study, Gd-DTPA presence significantly affected insert positions, whilst severe warping was observed at specific locations for concentrations >5mM. Under the imaging conditions studied, an average insert centroid offset of 0.068mm/mM (0.204ppm/mM) was estimated. In patient images, the observed total distortion magnitude, ranging from 0.35mm to 0.87mm, was mostly affected by the location dependent relative signs of the $B_0$ inhomogeneity and susceptibility-related distortion components.
**Conclusion:** Target mispositioning in contrast-enhanced MR images could adversely affect the efficiency of SRS treatment planning, especially in locations where Gd-DTPA-induced susceptibility and $B_0$ inhomogeneity add to each other, thus resulting in increased overall distortion.

**14:00 - 15:30** **Room K**

### Chest

### SS 704

### Lung cancer: diagnosis, staging and outcome

*Moderators:*
N.N.
F. Molinari; Lille/FR

**B-0769** 14:00
**Radiogenomics of non-small cell lung cancer (NSCLC): association between CT-based imaging features and EGFR and K-RAS mutations in 122 patients: an external validation**
C. Fanciullo[1], F. De Piano[1], E. De Jong[2], S. Raimondi[1], V. Buscarino[1], S. Rizzo[1]; [1]*Milan/IT*, [2]*Maastricht/NL (cristiana.fanciullo@unimi.it)*

**Purpose:** To validate previously identified associations between semantic radiomic features and clinical features with EGFR/KRAS alterations in patients with NSCLC.
**Methods and Materials:** CT images of 122 patients with NSCLC tested for EGFR/KRAS alterations were included. Clinical and semantic radiomic features were recorded. Univariate and multivariate analyses were performed to look at the associations of the studied features with EGFR/KRAS alterations. Previously calculated model parameters for each gene alteration prediction

Thursday were applied to the validation cohort and ROC (receiver operating characteristic) curves were drawn according to each significant characteristic in the training cohort and the full model. The area under the ROC curve (AUC) with 95% confidence intervals (CI) was calculated and compared between different models.
**Results:** At univariate analysis, EGFR+ confirmed an association with an internal air bronchogram, pleural retraction, emphysema and smoking; KRAS+ with round shape, emphysema and smoking. The AUC (95%CI) for the validation cohort was confirmed to be high for EGFR+ prediction, resulting in 0.82 (0.69-0.95) vs. 0.82 in the training cohort, whereas it was smaller for KRAS+ prediction, resulting in 0.60 (0.48-0.72) vs. 0.67 in the training cohort. Looking at single features in the validation cohort, we found that the AUC for the models including only smoking was similar to that of the full model (including semantic and clinical features) for both gene alterations.
**Conclusion:** This study validated the significant association of internal air bronchogram, pleural retraction, lack of emphysema and smoking with the EGFR+ mutation; round shape and smoking with KRAS rearrangement.

**B-0770** 14:08
**Correlation between EGFR gene mutation and CT, clinical features and pathological subtypes in ground glass lung adenocarcinoma**
L. Kang, X. Zhang, X. Chen, H. Li; *Zhengzhou/CN (18558267@qq.com)*

**Purpose:** To investigate the relationship between epidermal growth factor receptor (EGFR) and CT, clinical features and pathological subtypes in ground glass lung adenocarcinoma.
**Methods and Materials:** 108 Ground Glass lung adenocarcinomas confirmed by pathology were included. All cases were divided into two groups: effective mutation group and null mutation group. The patient gender, age, history of smoking, lesion location, size and CT features( including border, shape, margin, bubble-like lucency, air-bronchogram, pleural retraction, GGO proportion) and histological subtype were assessed. The comparison of size between two groups was performed by rank sum test. Two independent sample T test was used for comparison of age, and the chi square test was used for other indicators. *P*-values <0.05 were considered significant.
**Results:** In 108 cases of glass lung adenocarcinoma, 93 cases of effective mutation group, 15 cases of null mutation group, the mutation rate was 86.1%. The average age of effective mutation group (93 cases) and null mutation group (15 cases) was 49 ± 10.62, 60.75 ± 8.18, respectively. There was significantly different between two groups (*t*=-4.945, *P*<0.05). Patient gender , CT features and histological subtype were not significantly different between the two groups (*P*>0.05).
**Conclusion:** The mutation rate of EGFR gene was higher in patients with ground glass lung adenocarcinoma, and the mutation was more likely to occur in patients with (49 ± 10.62) years old. There was no relationship between EGFR mutation and gender, history of smoking, CT features and histological subtype in ground glass lung adenocarcinoma.

**B-0771** 14:16
**Evaluation of pre-treatment FDG PET-CT-based imaging parameters and textural features as prognostic indicators in non-small cell lung carcinoma**
G.M. McDermott[1], P. Brown[1], A. Scarsbrook[2]; [1]*Leeds/UK*, [2]*York/UK (garry.mcdermott@nhs.net)*

**Purpose:** To determine whether metabolic tumour parameters and radiomic features (RFs) derived from fluorine-18 fluorodeoxyglucose (FDG) positron emission tomography-computed tomography (PET-CT) are prognostic predictors in patients with non-small cell lung carcinoma (NSCLC).
**Methods and Materials:** Patients with NSCLC who underwent FDG PET-CT before lung biopsy and pre-treatment at a single cancer centre were studied. Primary tumour segmentation using semi-automated thresholding based on mean liver standardised uptake value ($SUV_{mean}$) and radiomic analysis (first- and second-order feature extraction) were performed using LIFEx software (University of Paris-Saclay, France). Lesions less than 64 pixels (5cm$^2$) were excluded. Uni- and multi-variate analyses were performed to assess RF prediction of progression-free survival (PFS).
**Results:** 81 patients were included in analysis, mean age 69 (range 41-89), 40 (49%) were male. Tumours were staged T1-T4 (7%, 59%, 10% and 24%, respectively), N0-3 (37%, 15%, 31% and 14%, respectively) and M0-1 (81% and 19%, respectively). Patients were censored at time of recurrence, progression, death or loss-to-follow up, median 10.0 months (IQR: 4.0 - 38.5 months). 51 (63%) patients were censored due to progression/death during follow-up. Metastatic status and 4 CT-derived RFs (CT entropy, CT energy, CT grey-level co-occurrence matrix (GLCM)-homogeneity and CT grey-level run length matrix-short-run emphasis (GLRLM-SRE) were statistically significant predictors (p<0.05) of PFS on multi-variate analysis. Tumour stage, nodal status and all PET-derived RFs were not statistically significant predictors.
**Conclusion:** CT-derived RFs may be better prognostic predictors of outcome than PET RFs in patients with NSCLC.

**B-0772** 14:24
**Value of TSCT features in differentiating pre- and minimally invasive from invasive adenocarcinoma presented as ground-glass nodules smaller than 3 cm**
J. Ji, Q. Weng, H. Wang, S. Fang, X. Wu, M. Chen, Z. Wang, M. Xu; *Lishui/CN (lschrjjs@163.com)*

**Purpose:** To distinguish the invasive adenocarcinoma (IA) from pre-invasive (AIS/AAH) and minimally invasive adenocarcinoma (MIA) appearing as solitary ground-glass nodules (GGNs) less than 3cm by thin-section computed tomography (TSCT) features to guide therapeutic approach.
**Methods and Materials:** A total of 154 lesions histopathologically confirmed with pre-/minimally invasive adenocarcinoma (hereafter pre/MIA) or IA presented as part-solid GGNs (mGGNs) or pure GGNs (pGGNs) were retrospectively reviewed. The TSCT features, including diameter, area, density, shape, vacuole sign, vessels passing, air-bronchogram, margin, and location were compared and assessed thoroughly. Receiver operating characteristic (ROC) analyses were conducted to determine the cut-off values for the qualitative variables and their diagnostic performances.
**Results:** Of 154 nodules, 89 AI, 53 MIA, 8 AIS, and 4 AAH were found. Univariate and multivariate logistic regression of the pre/MIAs and IAs were analysed in mGGNs and pGGNs, respectively. In pGGNs, the significant difference was found in the area (P=0.004, OR=0.124, 95%CI: 0.30-0.515) between pre/MIA and IA groups. In mGGNs, significant differences were found in diameter (P=0.001, OR=0.171, 95%CI: 0.063-0.467), density (P=0.001, OR=0.996, 95%CI: 0.993-0.998) and shape (P=0.102, OR=0.188, 95%CI: 0.25-1.397) between pre/MIA and IA groups. According to the corresponding ROC curve, the optimal cut-off tumour area in pGGNs to differentiate pre-/minimally invasive adenocarcinoma from invasive adenocarcinoma was 0.59cm$^2$. Higher CT values of lesion (≥ -298.5HU) and larger diameter (≥1.45cm) in mGGNs were significantly associated with IAs.
**Conclusion:** Imaging features of TSCT is able to distinguish IAs from pre/MIAs in GGNs and may contribute to guide the clinical management of lesions.

**B-0773** 14:32
**CT bronchovascular bundle thickening sign as a predictor of survival in patients with peripheral small cell lung cancer 3 cm or smaller**
P. Sung, S. Yoon; *Seoul/KR*

**Purpose:** To determine whether bronchovascular bundle thickening (BVB) sign on pretreatment CT images help predict the survival in patients with peripheral small cell lung cancer (SCLC) 3cm or smaller.
**Methods and Materials:** We retrospectively reviewed the pretreatment CT scans of 79 consecutive patients who had pathologically proven peripheral SCLC 3cm or less in diameter (TNM stage I, 21; II, 13; III, 22; IV, 23). The CT characteristics of the nodule and associated findings including the BVB sign were evaluated. We compare the overall survival (OS) and progression-free survival (PFS) using Kaplan-Meier and Cox-regression analysis between the patients with BVB sign and those without it.
**Results:** Among 79 the patients, 34 (43%; 95% CI, 33-54%) had BVB sign. Patients with BVB sign had more frequent mediastinal lymph node metastasis and distant metastasis than those without (50.9% vs 22.7%; *p*=0.041: 60.9% vs 35.7%; *p*=0.049). Those without BVB sign showed better PFS and OS, however they were statistically insignificant (mean, 838 vs 344 days, respectively; *p*=0.058: mean, 1898 vs 1258 days, respectively; *p*=0.120). For 21 patients with TNM stage IA, 16 patients (76%) without BVB sign showed better PFS and OS (mean, 1630 vs 483 days, respectively; *p*=0.023: mean, 2243 vs 1328 days, respectively; *p*=0.038). Multi-variate Cox proportional hazard model showed that BVB sign and associated idiopathic pulmonary fibrosis are poor prognostic factors for PFS (*p*=0.045; HR=5.001; 95% CI, 1.036-24.131: *p*=0.028; HR=10.536; 95% CI, 1.288-86.175).
**Conclusion:** The absence of BVB sign was potentially associated with better PFS in stage IA peripherally located SCLC.

**B-0774** 14:40
**Computerised texture analysis predicts histological invasiveness within lung adenocarcinoma manifesting as pure ground-glass nodules**
Y. Yang, H. Liu; *Shanghai/CN (yang_yang61@126.com)*

**Purpose:** To investigate the value of computerised texture analysis for predicting histological invasiveness of pulmonary adenocarcinoma that manifests as pure ground-glass nodules (pGGNs).
**Methods and Materials:** The study consisted of 138 patients with 142 pathologic analysis-confirmed pGGNs who had undergone computed tomographic (CT) imaging. Each nodule was manually segmented and its computerised textural features were extracted. Seven best features were chosen using hierarchical cluster analysis and the ReliefF method. Multivariate logistic regression analysis was performed to distinguish adenocarcinoma in situ (AIS) and minimally invasive adenocarcinoma (MIA) from invasive adenocarcinoma (IAC). Performance of the logistic regression was evaluated by receiver operating characteristic (ROC) curve analysis.

**Results:** Pathologic analysis confirmed 26 AISs, 71 MIAs and 45 IACs. Seven best features (10 percentile, maximum 3D diameter, surface volume ratio, elongation, maximum probability, large area low gray level emphasis and zone entropy) were chosen using hierarchical cluster and the ReliefF method. Multivariate logistic regression analysis revealed larger maximum 3D diameter, lower surface volume ratio and higher zone entropy as independent differentiators of IACs (adjusted odds ratio [OR]=1.59, P=0.011; OR=0.47, P=0.002; OR=6.78, P=0.001, respectively). The accuracy based on the logistic regression model using these features for differentiating IAC from AIS/MIA reached 78.7% with ROC analysis (AUC, 0.861; sensitivity, 78.0%; specificity, 80.0%).
**Conclusion:** In patients with pGGN, computerised texture analysis has the potential to differentiate histological invasiveness, especially maximum 3D diameter, surface volume ratio and zone entropy are independent differentiators of IACs from AISs and MIAs.

**B-0775** 14:48
**The comparison of hook wire vs coil localisation for video-assisted thoracoscopy surgery**
D. Kim, Y. Song; *Changwon/KR (tobealight@naver.com)*

**Purpose:** A hook wire has been widely used for CT-guided localisation before video-assisted thoracoscopic surgery (VATS). However, microcoils have been suggested to replace wires. The purpose of this study was to compare the efficacy, VATS procedure time, and excised volume of specimens of CT-guided localisation using a hook wire and microcoil.
**Methods and Materials:** The medical records of 106 patients with 110 pulmonary nodules who underwent CT-guided localisation using a hook wire (group A) or microcoil (group B) before VATS performed between March 2013 and January 2017 were retrospectively reviewed.
**Results:** The procedure success rate was 100% in both groups. Dislodgement occurred in four patients in group A and not in group B. Patient pain score was significantly lower for group B than group A (4.0 vs. 6.3; P < 0.001). The VATS success rate was higher in group B than in group A (98.1% vs. 91.1%; P = 0.174). The VATS procedure time was significantly shorter for group B than group A (18.8 vs. 23.6 minutes; P = 0.004). The excised volume of surgical specimens was significantly smaller for group B than group A (8.5 vs. 11.7 $cm^3$; P = 0.043). No major complications related to the localisation procedure were noted in either group.
**Conclusion:** This study showed similar effectiveness of VATS localisation between groups. However, microcoil is superior to hook wire for localisation of pulmonary nodules in terms of VATS procedure time and excised volume of surgical specimens, with the advantages of no dislodgement and less patient pain

**B-0776** 14:56
**Hook-wire localisation vs lipiodol localisation for patients with pulmonary lesions having ground-glass opacity: the LOGIS trial**
J. Hur[1], C.H. Park[1], S.M. Lee[1], J.W. Lee[1], S.H. Hwang[1], J.S. Seo[1], K.H. Lee[1], W. Kwon[2], D.J. Im[1]; [1]*Seoul/KR*, [2]*Wonju/KR (khuhz@yuhs.ac)*

**Purpose:** The aim of this study was to compare the usefulness and safety of the lipiodol and hook-wire localisation techniques.
**Methods and Materials:** This prospective, non-randomised comparative study was conducted between April 2014 and December 2016 at 8 qualifying university teaching hospitals. Two hundred and fifty patients with pulmonary lesions having GGO were included based on the following criteria: pulmonary lesion < 3 cm with GGO >50%; persistence or growth of the lesion during the 3-month follow-up. Patients were assigned in a 1:1 ratio to either the lipiodol (n=125) or hook-wire group (n=125) for preoperative localisation procedures. The primary endpoint was the procedure success rate. Secondary endpoints were procedure-related complication rate, localisation procedure duration, and the resected specimen's safety margin from the lesion.
**Results:** The procedure success rates (hook-wire vs lipiodol group) were 94.40% vs 99.16% (*P*=.084); complications occurred in 53.60% vs 48.33% of patients (*P*=.487). Haemorrhage and dislodgement rates were significantly higher in the hook-wire group (21.6% and 5.6%) compared to lipiodol group (5.83% and 0%) (p<.001, and p=.024, respectively). The lipiodol procedure time was significantly longer than that of the hook-wire technique (20.69±9.34 vs 17.15±7.91 min, *P*=.001). The initially positive surgical resection margin was significantly higher in the hook-wire group (10.53% vs 2.86%, *P*=.048).
**Conclusion:** There was no significant difference in success rate between the hook-wire and lipiodol methods. However, the haemorrhage and dislodgement rates were significantly higher in the hook-wire group, while the hook-wire group showed higher initially positive surgical resection margins.

**B-0777** 15:04
**Diagnostic accuracy of F-18 FDG PET/CT in mediastinal lymph nodal staging of recently diagnosed carcinoma lung**
R. Kumar E, R. Verma, A. Dhamija, E. Belho, V. Gupta, H. Mahajan, V. Mahajan; *New Delhi/IN (ramji2k8@gmail.com)*

**Purpose:** The aim of the study is to evaluate the diagnostic accuracy of FDG PET/CT in mediastinal lymph nodal staging in carcinoma lung patients.
**Methods and Materials:** A total of 120 recently diagnosed lung carcinoma patients, who underwent pre-treatment staging FDG PET/CT between March 2016 and May 2018 were included in the study. Lymph nodes more than 1cm in short axis and SUV max more than 3.0 in F-18 FDG PET/CT scan were considered suspicious for metastasis and correlated with their pathological reports. Clinical TNM staging assessed with PET/CT was compared with pathological TNM staging using appropriate statistical methods.
**Results:** A total of 3117 lymph nodes with a mean of 26 lymph nodes per patient were resected. 179 nodes were reported metastatic, 37 as granulomatous and rest non-specific. FDGPET/CT staged 68 (56.7%) patients as N0, 18 (15%) as N1, 28 (23.3%) as N2, 6 (5%) as N3. The overall per nodal station basis sensitivity, specificity, positive predictive value, negative predictive value and accuracy was 51.2%, 98.3%, 84%, 92.2% and 91.4%. On comparing N staging on PET CT with pathological N staging, 85 (70.8%) patients were staged accurately, 22 (18.3%) were over-staged and 13 (10.8%) were under-staged.
**Conclusion:** FDG PET/CT is an accurate imaging modality for mediastinal lymph nodal staging in carcinoma lung patients. However, in view of low sensitivity due to limited spatial resolution of the PET scanner and and low positive predictive value in granulomatous disease epidemic regions, like India, it is not an alternative to pathological staging.

**B-0778** 15:12
**Dual-energy CT (DECT) lung tumour perfusion: can we distinguish features that can reflect prognosis?**
J. Dewaguet[1], M.-C. Copin[1], J.-B. Faivre[1], V. Deken[1], M. Sedlmair[2], J. Remy[1], M. Remy-Jardin[1]; [1]*Lille/FR*, [2]*Forchheim/DE (martine.remy@chru-lille.fr)*

**Purpose:** To investigate potential relationships between DECT perfusion characteristics and prognostic histopathologic features.
**Methods and Materials:** A two-phase DECT scanning protocol was obtained in the presurgical evaluation of 49 tumours (squamous cell carcinomas: n=12; adenocarcinomas: n=37), including (a) an early phase over the entire thorax (ROI within the ascending aorta) (i.e., intravascular phase of tumoural perfusion); (b) completed by a delayed acquisition over the tumour, 50 s later (i.e., interstitial phase of tumoural perfusion). The first-pass and delayed iodine concentration (IC; mg/mL) and the arterial enhancement fraction (AEF=first pass IC/delayed IC x 100) were calculated over the entire tumour and within the most peripheral 2-mm-thick tumour layer, automatically segmented. The expression of the membranous carbonic anhydrase IX (mCAIX), an immunohistochemical marker of hypoxia, was assessed in tumour specimens.
**Results:** 33 tumours were mCAIX positive (Group 1) and 16 mCAIX negative (Group 2), the former showing a statistically significantly larger volume (p=0.04). At the level of the whole tumour, the delayed IC was significantly higher than that at first pass (median: 1.53 vs 1.4; p=0.04), suggestive of extravascular leakage within the interstitial space; there was no difference in DECT perfusion parameters between the two groups. Compared to Group 2, the outer layer of Group 1 tumours had significantly higher median values of IC (0.53 vs 0.21; p=0.02) and AEF (102.6 vs 65.6; p=0.02) with a trend toward higher delayed IC (0.48 vs 0.39; p=0.35).
**Conclusion:** DECT can provide insight into perfusion characteristics at the level of the tumoural invasion front.
*Author Disclosures:*
**M. Sedlmair:** Employee; Siemens Healthineers. **J. Remy:** Consultant; Siemens Healthineers. **M. Remy-Jardin:** Research/Grant Support; Siemens Healthineers.

**B-0779** 15:20
**The effect of different monochromatic image choices on the accuracy of CT perfusion values for lung tumour in one-stopspectral and perfusion CT scan**
H. Wang, S. Yue, P. Lu, W. Li; *Zhengzhou/CN (1508249322@qq.com)*

**Purpose:** To assess the effect of monochromatic images with different energy levels on the accuracy of CT perfusion data in one-stop spectral and perfusion CT scan.
**Methods and Materials:** One-stop spectral and perfusion CT scan(including whole tumour perfusion CT and whole lung contrast-enhanced CT) was performed in 42 patients with lung tumour. Monochromatic images with energy level from 40 to 140 keV (group A1 to A11) generated from spectral CT were used to reconstruct CT perfusion images with primary perfusion data. CT perfusion parameters including blood volume (BV), blood flow (BF),mean transit time (MTT) and permeability surface (PS) were measured to compare between benign (n=20) and malignant (n=22) tumours by using independent-t test. Receiver operating characteristics (ROC) analysis was performed to calculate cut off values for the differentiation between benign and malignant tumour.
**Results:** BV, BF values of group A1 to A11 and PS values of group A1 to A7 was significantly higher in malignant tumour than in benign tumour. ROC analysis for differentiation between benign and malignant tumours showed larger area under the curve(AUC) of BV, BF value in group A5 and PS in group A6. Sensitivity, specificity were 88.2% and 93.3% respectively at a cut-off value of 6.89 ml/100g for BV and 103.4 ml/(100g*min) for BF in group A5. The cut-off value of PS in group A6 was 10.27 ml/(100g*min) ,generating sensitivity of 83.9% and specificity 73.3%.
**Conclusion:** Use of 80 keV for reconstruction of one-stop spectral and perfusion CT showed higher value of differentiation between benign and malignant lung tumour.

**14:00 - 15:30** **Room M 1**

### Cardiac

### SS 703

### CT-FFR (fractional flow reserve) for assessment of coronary artery disease

*Moderators:*
C.A. Minoiu; Bucharest/RO
R.A.P. Takx; Utrecht/NL

**K-19** 14:00
**Keynote lecture**
G. Pontone; *Milan/IT*

**B-0780** 14:09
**Value of fractional flow reserve computed from triple-rule-out CT angiography data in the emergency department**
S.S. Martin[1], M. van Assen[2], A.M. Fischer[3], C.N. De Cecco[1], M. Bauer[1], A. Varga-Szemes[1], D. Mastrodicasa[1], U.J. Schoepf[1]; [1]*Charleston, SC/US,* [2]*Groningen/NL*, [3]*Mannheim/DE (simartin@outlook.com)*

**Purpose:** The aim of this study was to evaluate the additional value of noninvasive fractional flow reserve (CT-FFR) derived from triple-rule-out (TRO) coronary computed tomography angiography (CTA) data in the emergency department (ED).
**Methods and Materials:** On-site CT-FFR was calculated in 159 of 226 (70%) eligible patients with coronary artery diseases (CAD) who had undergone TRO-CTA. The agreement of CT-FFR (≤0.80) with stenosis on coronary CTA (≥50%), as well as additional cardiac diagnostic testing, was investigated. Furthermore, the predictive value of CT-FFR and CTA for coronary revascularization and major adverse cardiac events (MACE) was retrospectively assessed during a one-year follow-up period.
**Results:** CT-FFR results showed agreement with coronary CTA in 70% (111 of 159) of all cases. CT-FFR (≤0.80) served as a better predictor for coronary revascularization or MACE than assessment of stenosis (≥50%) on coronary CTA (hazard ratio [HR], 3.0; 95% CI, 1.0-9.6 vs. HR, 2.5; 95% CI, 0.8-7.9) (*P* = 0.03). Further diagnostic cardiac testing was performed in 58% (92 of 159) and included SPECT (n = 60), stress echocardiography (n = 31), and stress MRI (n = 1). The agreement with additional diagnostic testing was significantly higher for CT-FFR (63%; 58 of 92) than for coronary CTA results (49%; 45 of 92) (*P*=0.01).
**Conclusion:** CT-FFR derived from TRO coronary CTA was a better predictor for coronary revascularization or MACE and showed better agreement with additional diagnostic testing than significant stenosis on CTA. Therefore, CT-FFR may improve the efficiency of the workup in patients presenting to the ED.
*Author Disclosures:*
**C.N. De Cecco:** Consultant; Guerbet. Grant Recipient; Siemens.
**A. Varga-Szemes:** Consultant; Guerbet. Research/Grant Support; Siemens. **U.J. Schoepf:** Consultant; Bayer, Guerbet. Research/Grant Support; Siemens, Bayer, Astellas, GE.

**B-0781** 14:17
**CT-FFR profiles in patients without coronary artery disease: the effect of location and lumen area**
R.R. Bayer[1], M. van Assen[2], S.S. Martin[1], R.H. Savage[1], R. Steinbach[1], A.M. Fischer[3], L.P. Griffith[1], U.J. Schoepf[1]; [1]*Charleston, SC/US,* [2]*Groningen/NL*, [3]*Mannheim/DE (bayer@musc.edu)*

**Purpose:** To evaluate the influence of measurement location and lumen diameter changes on CT-FFR values in normal patients.
**Methods and Materials:** Patients were included who underwent Calcium scoring (CACS) and CCTA with CT-FFR. Patients were excluded if the CACS was not zero, if there were increased troponin levels or any other cardiac abnormality on the CT-images. On-site CT-FFR was computed for each coronary artery at proximal, mid and distal segments. At each measurement location, the lumen area was measured. CT-FFR was considered positive <0.75. The relationship between CT-FFR and lumen areas were evaluated for each coronary artery and each location.
**Results:** 106 patients were included with corresponding CT-FFR measurements. An overall of 99 (31%) coronary arteries had CT-FFR values <0.75, all at distal locations. In 42 (40%) patients the LAD had distal CT-FFR values <0.75 (mean 0.62 (SD 0.10)). The RCA shows similar results with distal CT-FFR values <0.75 in 65 (61%) patients (0.61 (0.12). The Cx shows only a limited number of patients (16, 15%) with distal CT-FFR values < 0.75 (0.65 (0.09). CT-FFR and lumen area showed good correlation: 0.728 (p-value<0.001) for the LAD, 0.660 (p-value<0.001) for the Cx and 0.712 (p-value<0.001) for the RCA. The lumen areas were not significantly different between the >0.75 and <0.75 group (p-value<0.001).
**Conclusion:** CT-FFR values in patients without coronary artery disease can become positive at a distal location without indicating flow-limiting stenosis independent of lumen area. CT-FFR values measured distal should always be interpreted in combination with the CCTA images.
*Author Disclosures:*
**U.J. Schoepf:** Consultant; Bayer, Guerbet, HeartFlow Inc., Siemens Healthineers. Research/Grant Support; Astellas, Bayer, General Electric, Siemens Healthineers.

**B-0782** 14:25
**High-risk and lipid-rich, but not calcified, plaque predicts ischaemia in non-obstructive lesions (ANOCA): a CTA/CTFFR study**
G. Feuchtner, C. Langer, F. Barbieri, C. Beyer, G.J. Friedrich, F. Plank; *Innsbruck/AT (Gudrun.Feuchtner@i-med.ac.at)*

**Purpose:** ANOCA (angina in the absence of obstructive coronary stenosis) is frequent in patients referred to coronary CTA. The morphological correlate is unclear. Aim was to assess which plaque types by coronary CTA predict ischaemia in ANOCA.
**Methods and Materials:** 106 patients with low-to-intermediate risk referred to 128-slice dual-source CT angiography (atypical chest pain, 89.1%) were included. CTA data were transferred to coronary fluid dynamics (CFD) modelling (Heartflow Inc.); lesion-based and distal $FFR_{CT}$ were calculated. CTA analysis included 1) stenosis severity (CADRADS); 2) plaque composition 1-4 (non-calcified, mixed or calcified) total (SIS), mixed (G-score) plaque burden; 3) High-risk plaque criteria (Napkin-Ring sign, plaque density (HU), positive remodelling, spotty calcification; 4) coronary calcium score; 5) qCTA stenosis.
**Results:** 89 non-obstructive lesions with <70% stenosis (40 high-risk-plaque, 40 calcified and 9 non-calcified) matched for qCTA (%area, %diameter stenosis) were included. In high-risk plaque, lesion-based and distal $FFR_{CT}$ were lower as compared to calcified (p<0.001 and p=0.002), respectively. Prevalence of ischaemia ($FFR_{CT}$<0.8) (25% vs. 2.5%, p=0.007 for lesion based), and 40% vs 17.5% for distal $FFR_{CT}$) was higher in high-risk plaque compared to calcified. Lower plaque density (HU) (p=0.024) predicted ischaemia in low-attenuation plaque (LAP)<300HU, with an strengthened correlation for all lesions (LAP+calcified) (p=0.003) on linear regression analysis. Positive remodelling was associated with lower $FFR_{CT}$ distal (p=.042), and an increasing non-calcified plaque burden (G score) (p=0.008), but not total (SIS) and calcium score.
**Conclusion:** High-risk plaque and and an increasing lipid-necrotic core and total non-calcified plaque burden predict ischaemia in non-obstructive lesions and explain ANOCA, and while an increasing calcium-density acts contrary.
*Author Disclosures:*
**G. Feuchtner:** Research/Grant Support; Heartflow Inc. California USA.

**B-0783** 14:33
**Comparison between stress CT perfusion versus FFRCT in the evaluation of suspected CAD: PERFECTION prospective study**
A. Baggiano[1], M. Guglielmo[1], G. Muscogiuri[1], M. Soldi[1], A. Del Torto[1], A.I. Guaricci[2], M. Pepi[1], G. Pontone[1]; [1]*Milan/IT*, [2]*Bari/IT (andrea.baggiano@gmail.com)*

**Purpose:** The PERFECTION study is a longitudinal, prospective and consecutive cohort study to compare the feasibility and accuracy of FFRCT versus stress-CTP for the diagnosis of functionally significant CAD.
**Methods and Materials:** 147 consecutive symptomatic patients for chest pain referred for clinically indicated ICA plus invasive FFR were enrolled. The primary endpoint was to compare the diagnostic accuracy of cCTA versus cCTA+FFRCT versus cCTA+stress-CTP for the detection of significant CAD defined by ICA and invasive FFR.
**Results:** Rest cCTA was successfully performed in all patients, FFRCT was performed in 143/147 patients, stress-CTP was performed in 144/147 patients. cCTA demonstrated a vessel and patient-based sensitivity (SE), specificity

(SP), negative predictive value (NPV), positive predictive value (PPV) and diagnostic accuracy (ACC) of 99%, 76%, 100%, 61%, 82% and 95%, 54%, 94%, 63%, 74%, respectively. The diagnostic performance of integrated protocol of rest cCTA+FFRCT showed a vessel and patient-based SE, SP, NPV, PPV and ACC of 88%, 94%, 95%, 84%, 92% and 90%, 85%, 92%, 83%, 87%, respectively. Finally, the diagnostic performance of integrated protocol of rest cCTA+stress-CTP showed a vessel and patient-based SE, SP, NPV, PPV and ACC of 92%, 95%, 97%, 87%, 94% and 98%, 87%, 99%, 86%, 92%, respectively. Both FFRCT and stress-CTP significantly improved SP, PPV and overall ACC in both per-vessel and per-patient based model when added to cCTA, while no differences were found between cCTA+FFRCT versus cCTA+stress CTP.
**Conclusion:** Both FFRCT and stress-CTP are valid tools in addition to cCTA to evaluate the functional relevance of CAD.

**B-0784** 14:41
**Coronary computed tomography angiography-derived fractional flow reserve in anomalous origin of the right coronary artery from the left coronary sinus**
C. Tang[1], M. Lu[1], U.J. Schoepf[2], C. Tesche[2], M. Bauer[2], J.W. Nance[2], L. Griffith[2], C. Zhou[1], L. Zhang[1]; [1]*Nanjing/CN*, [2]*Charleston, SC/US (522956587@qq.com)*

**Purpose:** To examine fractional flow reserve derived from computed tomographic angiography (CT-FFR) in patients with anomalous origin of the right coronary artery from the left coronary sinus (R-ACAOS) with interarterial course, its relationship with anatomical features of R-ACAOS with interarterial course on coronary CT angiography (CCTA), and its clinical relevance.
**Methods and Materials:** Ninety-four patients with interarterial R-ACAOS undergoing CCTA were retrospectively included. Anatomic features (including proximal vessel morphology [oval or slit-like], take-off angle, take-off level [below or above pulmonary valve], take-off type, intramural course, %proximal narrowing, length of narrowing, minimum lumen area (MLA) at systole and diastole, and vessel compression index) on CCTA were analysed. Receiver operating characteristic analyses were performed to describe the diagnostic performance in detecting R-ACAOS with interarterial course with CT-FFR ≤0.80.
**Results:** Significant differences were found in proximal vessel morphology, take-off level, intramural course, %proximal narrowing area, MLA at diastole (all $p$<0.05) between normal and abnormal CT-FFR groups. Take-off level, intramural course, and slit-like ostium (all $p$<0.05) predicted lesion-specific ischaemia (CT-FFR ≤0.80) with accuracies of 0.69, 0.71, and 0.81, respectively. Patients with CT-FFR ≤0.80 had a higher prevalence of typical angina (29.4% vs 7.8%, $p$=0.025) and atypical angina (29.4% vs 6.5%, $p$=0.016).
**Conclusion:** Take-off level, intramural course, and slit-like ostium were the main predictors of abnormal CT-FFR values. Importantly, patients with abnormal CT-FFR values had a higher prevalence of typical angina and atypical angina, suggesting CT-FFR as a potential tool to gauge the clinical relevance of patients with interarterial R-ACAOS.
*Author Disclosures:*
**U.J. Schoepf:** Consultant; U. J. Schoepf is a consultant for and / or receives institutional research support from Astellas, Bayer, GE, Guerbet, HeartFlow Inc., and Siemens.

**B-0785** 14:49
**Diagnostic performance of fractional flow reserve derived from coronary computed tomography angiography in myocardial bridging: a comparison with invasive fractional flow reserve**
F. Zhou[1], Y. Wang[2], U.J. Schoepf[3], C. Tesche[4], L. Xu[2], Y. Hou[5], J. Yan[6], J.Y. Zhang[6], L.J. Zhang[1]; [1]*Nanjing/CN*, [2]*Beijing/CN*, [3]*Charleston, SC/US*, [4]*Munich/DE*, [5]*Shenyang/CN*, [6]*Shanghai/CN (nuzhouf@126.com)*

**Purpose:** To investigate the diagnostic performance of machine learning-based coronary CT angiography-derived fractional flow reserve (CT-FFR) to detect lesion-specific ischaemia in myocardial bridging (MB) using invasive FFR as reference standard.
**Methods and Materials:** This retrospective multicentre study included 107 MBs in 106 patients. An FFR value ≤0.80 was considered to be ischaemia-specific. MB was classified as either superficial or deep, while all MB vessels were further divided into 30%-69% and ≥70% groups according to diameter stenosis on invasive coronary angiography. Diagnostic performance of CT-FFR to detect lesion-specific ischaemia was assessed on a per-vessel level, using invasive FFR as reference standard. Pearson's correlation analysis and Bland-Altman plots were used for agreement measurement.
**Results:** Of 107 MBs, 92 were classified as superficial. 49 MB vessels (45.8%) showed ischaemia by invasive FFR. Sensitivity, specificity, accuracy of CT-FFR to detect lesion-specific ischaemia were 0.94 (confidence interval [CI] 0.82-0.98), 0.84 (0.72-0.92), and 0.89 (0.81-0.94), respectively, in all MB vessels. There were no differences in diagnostic performance between superficial and deep MB (all $p$>0.05). The accuracy of CT-FFR was 0.96 (0.85-0.99) in ≥70% stenosis and 0.82 (0.66-0.91) in 30%-69% stenosis ($p$=0.023) with corresponding AUCs of 0.99 (0.91-1.00) and 0.86 (0.74-0.94) ($p$=0.013). Bland-Altman analysis showed a mild systematic underestimation of CT-FFR values when compared to invasive FFR (mean difference=0.014, 95% LoA: -0.12-0.15). The correlation coefficient was $r$=0.77 ($p$<0.001).
**Conclusion:** CT-FFR demonstrated high diagnostic performance for identifying lesion-specific ischaemia in vessels with MB when compared to invasive FFR, regardless of MB depth.
*Author Disclosures:*
**U.J. Schoepf:** Consultant; Astellas, Bayer, General Electric, Guerbet, HeartFlow, Siemens Healthineers.

**B-0786** 14:57
**Coronary CT based on-site FFR might be a useful tool to identify haemodynamically relevant non-culprit lesions in patients who suffered myocardial infarction**
J. Simon, B. Szilveszter, A. Ahres, M. Kolossvary, B. Jablonkai, B. Merkely, P. Andrássy, P. Maurovich- Horvat; *Budapest/HU (juditsimon21@gmail.com)*

**Purpose:** Prior studies demonstrated that CT-derived fractional flow reserve (CT-FFR) has excellent diagnostic accuracy for the identification of ischaemia-causing lesions in patients with stable chest pain. Non-invasive assessment of non-culprit lesions in patients who suffered myocardial infarction (MI) could improve risk stratification and guide therapy.
**Methods and Materials:** We enrolled 33 patients (58.24±9.3years, 33.3% female) who suffered MI with 47 non-culprit coronary lesions. CT-FFR was calculated with an on-site algorithm and values were compared to invasive FFR measured at the index event. FFR value <0.80 was considered haemodynamically significant in both cases.
**Results:** The mean FFR-CT value was 0.85±0.10 while the mean invasive FFR value was 0.82±0.08. The sensitivity, specificity, positive predictive value and negative predictive value of FFR-CT versus invasive FFR was 33.3%, 89.7%, 66.7% and 68.4%, respectively (AUC=0.70). Correlation coefficient between CT-FFR and invasive FFR was 0.43 (p=0.002).
**Conclusion:** The majority of non-culprit lesions of MI patients are in the grey-zone for FFR-CT and therefore the diagnostic performance of FFR-CT for the detection of lesion-specific ischaemia in post-MI patients is moderate. However, the high specificity of FFR-CT in this patient population might be useful test characteristics to identify patients who need revascularization.

**B-0787** 15:05
**The prognostic value of CT myocardial perfusion and CT-FFR for MACE in patients with coronary artery disease**
M. van Assen[1], C.N. De Cecco[2], M. Eid[3], M.J. Bauer[4], M. Scarabello[5], P. von Knebel Doeberitz[6], M. Oudkerk[1], R. Vliegenthart[1], U.J. Schoepf[3]; [1]*Groningen/NL*, [2]*Atlanta, GA/US*, [3]*Charleston, SC/US*, [4]*Munich/DE*, [5]*Milan/IT*, [6]*Heidelberg/DE (m.van.assen@umcg.nl)*

**Purpose:** To determine the prognostic value of dynamic CT perfusion and CT-derived fractional flow reserve (CT-FFR) for the prediction of major adverse cardiac events (MACE).
**Methods and Materials:** Patients from 4 institutes who underwent CCTA and stress dynamic myocardial perfusion CT (CTMPI) were included with a follow-up period of 18 months or until MACE occurred. On-site CT-FFR was computed for each coronary artery. A myocardial blood flow (MBF) index was calculated, for which each vessel territory was normalised to global MBF. The lowest CT-FFR and MBF index was selected for each patient. The prognostic value of CCTA, CT-FFR, and CTP was investigated with univariate and multivariate Cox proportional hazards regression models.
**Results:** 243 vessels in 81 patients were interrogated by CCTA with CT-FFR and 243 vessel territories (1,296 segments) were evaluated with dynamic CTP imaging. Of the 81 patients, 25 (31%) experienced MACE during follow-up. In univariate analysis, a positive index-MBF resulted in the largest risk for MACE (HR 11.4, 95%-CI: 3.4-38.2) compared to CCTA (HR 2.6, 95%-CI: 1.2-5.9) and CT-FFR (HR 4.6, 95%-CI: 2.0-10.5). In multivariate analysis, including clinical factors, CCTA, CT-FFR, and index-MBF, only index-MBF significantly contributed to the risk of MACE (HR 10.1, 95%-CI: 2.1-48.8), unlike CCTA (HR 1.2, 95%-CI: 0.4-3.5) and CT-FFR (HR 2.2, 95%-CI: 0.8-6.4).
**Conclusion:** Our study provides initial evidence that dynamic CTP alone has the highest prognostic value for MACE compared to CCTA and CT-FFR individually or a combination of the three, independent of clinical risk factors.
*Author Disclosures:*
**C.N. De Cecco:** Research/Grant Support; Dr. De Cecco receives institutional research support from Siemens.. Speaker; Dr. De Cecco has received speaker honoraria from Bayer. **U.J. Schoepf:** Consultant; Dr. Schoepf has received consulting fees and or speaker honoraria from Bayer, GE, Guerbet, HeartFlow Inc., and Siemens. Research/Grant Support; Dr. Schoepf receives institutional research support from Astellas, Bayer, and Siemens.. Speaker; Dr. Schoepf has received consulting fees and or speaker honoraria from Bayer, GE, Guerbet, HeartFlow Inc., and Siemens.

**B-0788** 15:13
**A patient specific lumped parametric model based approach to fractional flow reserve derived from coronary computed tomography angiography**
R.W. van Hamersvelt[1], M. Voskuil[1], P.A. de Jong[1], M.J. Willemink[2], T. Leiner[1]; [1]*Utrecht/NL*, [2]*Menlo Park, CA/US (R.w.vanhamersvelt-3@umcutrecht.nl)*

**Purpose:** The purpose of this study was to evaluate an on-site patient specific lumped parametric model based approach to fractional flow reserve derived from coronary computed tomography angiography (FFR-CT) for the detection of hemodynamically significant stenosis.
**Methods and Materials:** Patients who received a coronary computed tomography angiography (CCTA) within 60 days before invasive fractional flow reserve (FFR) were retrospectively selected. Hemodynamically significant coronary artery stenosis was defined as invasive FFR≤0.8. The diagnostic performance of the on-site FFR-CT method was evaluated and compared to that of degree of luminal coronary stenosis assessment on CCTA.
**Results:** In total 57 patients (mean age 58.5 ± 9.2 years, 74% men) and 77 vessels were included. Hemodynamically significant stenosis was present in 37 vessels (48%). FFR-CT showed an improved diagnostic performance compared to degree of stenosis evaluation on CCTA with an area under the receiver operating characteristic curve (AUC) of 0.87 compared to 0.70 (difference in AUC of 0.17, p<0.01), respectively. Sensitivity was similar between FFR-CT (89.2%) and degree of stenosis on CCTA (89.2%), while specificity substantially improved with FFR-CT (77.5% versus 42.5%, respectively).
**Conclusion:** Analysis with an on-site FFR-CT method improved diagnostic performance for the evaluation of hemodynamically significant stenosis compared to stenosis degree evaluation on CCTA.
*Author Disclosures:*
**R.W. van Hamersvelt:** Research/Grant Support; The department of Radiology receives research support from Philips Healthcare. **P.A. de Jong:** Research/Grant Support; The department of Radiology receives research support from Philips Healthcare. **M.J. Willemink:** Research/Grant Support; Philips Research Grant, American Heart Association Grant. Speaker; Philips' Speakers Bureau. **T. Leiner:** Research/Grant Support; The department of Radiology receives research support from Philips Healthcare.

**B-0789** 15:21
**Relationship between coronary CT angiography-derived fractional flow reserve and dynamic CT myocardial perfusion imaging in patients with coronary artery disease**
M. van Assen[1], C.N. De Cecco[2], S.S. Martin[3], A. Fischer[3], R. Steinbach[3], M. Oudkerk[1], R. Vliegenthart[1], U.J. Schoepf[3]; [1]*Groningen/NL*, [2]*Atlanta, GA/US*, [3]*Charleston, SC/US (m.van.assen@umcg.nl)*

**Purpose:** To investigate the correlation between functional parameters describing lesion-specific ischaemia obtained from CT perfusion and CT-FFR at various measurement vessel locations.
**Methods and Materials:** Patients who underwent CCTA with CT-FFR analysis as well as dynamic CTMPI were included. On-site CT-FFR was computed for each coronary artery at proximal, mid and distal segments. Perfusion was analysed per vessel territory by calculating a myocardial blood flow MBF index (MBFi), the ratio between territory MBF and global MBF. Correlations between CT-FFR, MBF, and MBFI were analysed with Pearson's correlation coefficients for all three-vessel territories and for the proximal, mid, and distal CT-FFR locations. Differences in MBFi values between patients with positive (<0.75) and negative CT-FFR values at different vessel locations were evaluated.
**Results:** A total of 100 patients (mean age 61±10 years, 75 male) were included for analysis. The CT-FFR and MBFi values in the left anterior descending territory showed low to moderate correlation (0.36-0.62), with decreasing correlation distally. The CT-FFR and MBFi values from the circumflex territory and right coronary artery territory exhibited the same trend although the mean correlation was lower (0.30-0.40 and 0.28-0.42, respectively). Overall, significant proximal and mid stenosis according to CT-FFR, resulted in a significant decrease in MBF and MBFi values, whereas distal stenosis did not.
**Conclusion:** Our study provides evidence that CT-FFR and MBFi correlate only moderately. Depending on the measurement location of CT-FFR, a significant CT-FFR value reflects a decrease in myocardial perfusion. This effect is less pronounced in distal CT-FFR measurements.
*Author Disclosures:*
**C.N. De Cecco:** Research/Grant Support; Dr. De Cecco receives institutional research support from Siemens.. Speaker; Dr. De Cecco has received speaker honoraria from Bayer. **U.J. Schoepf:** Consultant; Dr. Schoepf has received consulting fees and or speaker honoraria from Bayer, GE, Guerbet, HeartFlow Inc., and Siemens.. Research/Grant Support; Dr. Schoepf receives institutional research support from Astellas, Bayer, and Siemens. Speaker; Dr. Schoepf has received consulting fees and or speaker honoraria from Bayer, GE, Guerbet, HeartFlow Inc., and Siemens.

**14:00 - 15:30** **Room M 2**

### Abdominal Viscera

### SS 701

### CT technical frontiers in liver imaging

*Moderators:*
A. Blachar; Tel Aviv/IL
C. Sofia; Messina/IT

**B-0790** 14:00
**Iodine images in dual-energy CT: benchmarking quantitative iodine concentration values of the healthy liver**
S. Beck, D. Deniffel, I. Riederer, E.J. Rummeny, D. Pfeiffer; *Munich/DE (stef.kaiser@gmail.com)*

**Purpose:** To define quantitative reference values of iodine concentrations in portal venous phase Material Density (MD) iodine images in Dual Energy CT (DECT) for normal liver parenchyma.
**Methods and Materials:** The iodine concentration in the liver parenchyma as well as of the abdominal aorta and main portal vein were obtained in n= 182 patients in MD iodine images, obtained with a dual layer CT scanner. The iodine uptake was evaluated as the absolute iodine concentration and as a blood-normalized value. The influence of age and gender on the iodine uptake was examined.
**Results:** A significant difference could be observed for the absolute liver iodine concentration between genders (male: 1.928 mg/ml, female 2.310 mg/ml), yet not for the blood-normalized iodine concentration (male: 2.133 mg/ml, female 2.097 mg/ml). For the analysis of age, there was a significant negative correlation between the blood-normalized iodine concentrations and the patient age (r= -0.269). Standard iodine values for absolute iodine concentration ranged between 1.022 and 2.834 (male) and between 1.344 and 3.274 (female) and for blood-normalized iodine concentration between 1.451 and 2.783 (± 0.0054 / year).
**Conclusion:** The ranges for absolute iodine uptake vary with the patients' gender whereas the ranges for blood-normalized iodine uptake vary with the patients' age. Standard value ranges for normal quantitative iodine concentration in portal venous phase DECT images were defined for both, absolute and blood-normalized values.

**B-0791** 14:08
**DECT in the diagnosis of the efficiency of chelation therapy in patients with iron overload**
A.M. Titova, V.A. Fokin, G.E. Trufanov, M.O. Ivanova; *St. Petersburg/RU (anisa33@mail.ru)*

**Purpose:** The assessment of liver iron concentration (LIC) by MRI is the most accurate method of controlling chelation therapy in patients with iron overload. However, some patients cannot undergo MRI procedures, liver biopsy is invasive and has several limitations, and measurement of serum ferritin is unreliable. Therefore, DECT as a non-invasive, easily tolerated, low-dose and accurate method for quantitative monitoring of chelation therapy efficiency may be more appropriate.
**Methods and Materials:** We conducted T2* MRI study of the liver in 75 patients with suspected iron overload by 1.5T scanner for LIC assessment. We performed DECT procedures with 80kV and 140kV on the limited area of the liver of the same patient group with the slice thickness of 5mm. We ran a regression analysis to assess the prognostic capabilities of dual-energy ratio, dual-energy index and dual-energy difference (DED) and based on the best indicators, constructed a mathematical model for LIC quantification. A year later, we repeated the same procedures with calculation of dual-energy indicators to 26 of the examined patients receiving iron chelators.
**Results:** DED demonstrated the largest correlation with LIC (r=0.93). Based on the data of 75 patients, we obtained a regression equation. Using the mathematical model of 26 patients after chelation, in all cases using DED, we established the conformity of our findings to the MRI data.
**Conclusion:** DECT with using DED is a useful technique for the quantitative assessment of iron overload in patients who need a control of chelation therapy but cannot undergo MRI procedures.

**B-0792** 14:16
**Measuring liver fat fraction with dual-layer spectral CT material attenuation decomposition (MAD) plots: an iodine-independent method for imaging hepatic steatosis with CT**
T.C. Soesbe, M.A. Lewis, L. Ananthakrishnan, J.R. Leyendecker, Y. Xi, T. Yokoo, R.E. Lenkinski; *Dallas, TX/US (todd.soesbe@utsouthwestern.edu)*

**Purpose:** To determine the accuracy and iodine-independence of MAD plot fat fraction maps. Accuracy was evaluated using an MRI verified phantom, while iodine-independence was evaluated using patients who had contrast-enhanced multiphase CT.
**Methods and Materials:** MAD plot fat fraction accuracy was determined using an anthropomorphic phantom and vials containing different ratios of homogenized bovine liver/lard (0, 25, 50, and 100% fat by volume). The phantom was scanned on a Philips IQon dual-layer CT (120 kVp and 140 kVp) and a Philips Ingenia 3.0T MRI (3x/system) with the MRI fat fraction measurements (mDIXON Quant) used as the reference standard. ROI data from the CT and MRI fat fraction maps were compared to the known fat fraction. For proof of concept *in vivo*, MAD plot fat fraction iodine-independence between pre- and post-contrast dual-layer CT was determined in four patients with varying degrees of hepatic steatosis. Pre- and post-contrast MAD plot fat fraction maps were compared using three ROIs per image, placed in similar liver locations.
**Results:** Bland-Altman analysis showed the mean difference between the known and measured phantom fat fraction percent was CT@120kVp = 0.52±1.77%, CT@140kVp = 0.80±1.94%, and MRI = 1.61±1.23%. Similar Bland-Altman analysis for the *in vivo* liver data showed that the mean difference between the pre- and post-contrast measured fat fraction percent was Venous = 0.21±1.57% and Delayed = -0.61±1.78%.
**Conclusion:** MAD plots provide an accurate method for measuring liver fat fraction with dual-layer spectral CT *ex vivo*, with the potential to do so for *in vivo* contrast-enhanced CT.

**B-0793** 14:24
**Preliminary study for intra-abdominal liquids characterisation based on dual-energy CT**
O. Meyrignac, R. Losco, R. Moreno, A. Sewonu, H. Rousseau; *Toulouse/FR*

**Purpose:** This study aimed for differentiation and characterization of intra-abdominal liquids based on parameters issued from dual-energy CT.
**Methods and Materials:** In vitro, monocentric, prospective study. Forty-six intra-abdominal liquids samples were divided into 5 groups according to their provenance: 12 from proven or strongly suspected infected liquids (I); 7 from bile (B); 6 from serous effusion (SE); 11 from lymphoceles (L); 10 blood samples (S). Acquisition were made on Toshiba One Genesis CT scanner using dual-energy technology with sequential acquisition, with samples placed in a water tank to simulate body surroundings. We analyzed: native densities at 80kV and 135kV, density in virtual monochromatic reconstruction at best CNR (= bCNR), dual-energy dissociation coefficient with water (DCw), atomic Z and electronic density. DCw being defined by the ratio between density differences between sample and water at 30 keV and 135 keV.
**Results:** The overall densities of the S group were significantly higher than the other groups (135kV=55UH, 80kV=58UH, bCNR=56UH; p<0.05). Within these groups, group I had a significantly higher density than all other groups (135kV=28UH, 80kV=35UH, bCNR=35UH ; p<0.05).The DCw of group B was 3.9, significantly higher than the other groups (p<0.05) and DCw of group S, lower than the others (1.5, p<0.05).Averageelectronic density of the S group was significantly higher than that of other groups (3.4810 ^ 23/cm3 p < 0.05).
**Conclusion:** Using dual-energy we can differentiate bile and infected fluids from the other intra-abdominal liquids.

**B-0794** 14:32
**Multi-energy CT quantification of first-pass perfusion in hypervascular liver lesions: potential to differentiate hypervascular metastasis from hepatocellular carcinoma**
B. Kaltenbach[1], J.L. Wichmann[1], I. Yel[1], M.H. Albrecht[1], T.J. Vogl[1], S.S. Martin[2]; [1]*Frankfurt a. Main/DE*, [2]*Charleston, SC/US (benjamin.kaltenbach@outlook.de)*

**Purpose:** To investigate the diagnostic accuracy of first-pass static perfusion quantification for the differentiation of hypervascular hepatic metastasis from hepatocellular carcinoma (HCC) in non-cirrhotic liver patients undergoing multi-energy computed tomography (CT).
**Methods and Materials:** 46 patients (mean age, 64.9±10.1 years; 28 male and 18 female) with either hepatic metastasis due to gastropancreatic neuroendocrine tumour or HCC, who had undergone multi-energy CT of the upper abdomen, were included in this retrospective study. For each lesion, arterial-phase attenuation and fat fraction values, as well as quantitative parameters indicating first-pass perfusion including iodine uptake and normalised iodine uptake (NIU) were measured. Lesion-to-liver-parenchyma ratio (LPR) values were calculated. Histopathology, MRI, and/or PET/CT served as the reference standard for all liver lesions. In addition, the diagnostic accuracy of contrast-enhanced and perfusion analysis for the differentiation of hypervascular hepatic metastasis and HCC was assessed using receiver operating characteristics (ROC) curve analysis.
**Results:** Hypervascular hepatic metastasis and HCC showed significant differences in arterial attenuation (p=0.003), iodine uptake (p<0.001), NIU (p<0.001), and LPR (p=0.003). No significant differences were found for unenhanced attenuation and fat fraction values (p=0.686 and p=0.892, respectively). NIU showed superior sensitivity (100%; iodine uptake, 71%), whereas both iodine uptake and NIU revealed superior specificity (100% and 90%, respectively) compared to LPR (sensitivity, 96%; specificity, 80%) and arterial attenuation analysis (sensitivity, 79%; specificity, 80%) (p=0.016).
**Conclusion:** Quantitative analysis of first-pass perfusion in multi-energy CT can differentiate hypervascular hepatic metastasis from HCC in non-cirrhotic liver, with NIU showing the strongest diagnostic accuracy.

**B-0795** 14:40
**Double low-dose spectral liver CT in patients at high risk of HCC**
J. Yoon[1], E. Lee[1], S.M. Lee[2], W. Chang[3], J. Lee[1]; [1]*Seoul/KR*, [2]*Anyang/KR*, [3]*Seongnam/KR*

**Purpose:** To investigate the clinical feasibility of simultaneous reduction of radiation and contrast doses using spectral CT in a high-risk group of HCC.
**Methods and Materials:** Participants at risk of HCC and body mass index (BMI) less than 30 were enrolled and randomly assigned to standard dose (SD, n=32) and double low-dose (DLD, n=35) groups. Liver CT was performed at spectral CT scanner. DLD group targeted 30% of reduction of radiation and contrast media doses of SD group. Image quality and lesion conspicuity of arterial phase (AP) and portal venous phase (PVP) of FBP images of SD group and 50keV images of DLD group were determined on a five-point scale and compared with each other.
**Results:** Compared with SD group, $CTDI_{vol}$ (8.8±1.7 vs.6.1±0.6, $P$<0.001) and the amount of contrast media (116.9±15.7ml vs. 83.1±9.9ml, $P$<0.001) were significantly lower in DLD group. BMI was not significantly different between the two groups (24.5±2.3 vs. 24.2±2.4, $P$=0.58). Image quality was significantly higher on 50keV images of DLD group than FBP images of SD group (4.0±0.5 vs. 3.1±0.4 on AP and 4.2±0.4 vs. 3.2±0.4 on PVP, $P$s<0.001). Lesion conspicuity was significantly higher on 50keV images of DLD group than FBP images of SD group on AP (1.83 [95% CI: 1.56, 2.11] vs. 2.62 [95% CI: 2.31, 2.93], $P$<0.001) and PVP (1.80 [95% CI: 1.57, 2.02] vs. 2.39 [95% CI: 2.11, 2.67], $P$=0.001).
**Conclusion:** Low monoenergetic image (50keV) was able to provide better lesion conspicuity at low radiation and contrast media doses in patients with BMI < 30.
*Author Disclosures:*
**J. Yoon:** Advisory Board; Siemens Healthcare. Consultant; Samsung electronics. Grant Recipient; Bayer Healthcare. Speaker; Philips Healthcare.
**J. Lee:** Advisory Board; Siemens Healthcare. Consultant; Samsung electronics. Grant Recipient; Bayer Healthcare, Philips Healthcare, Bracco, Starmed, Guerbet. Speaker; Bayer Healthcare.

**B-0796** 14:48
**Estimation of the right posterior section volume in live liver donors: semi-automated CT volumetry in conjunction with portal vein segmentation**
S.Y. Jeong, J. Lee, K.W. Kim, J.K. Jang, H.-J. Kwon, G.W. Song, S.G. Lee; *Seoul/KR (jungsy27@hanmail.net)*

**Purpose:** To determine the accuracy of semi-automated CT volumetry in conjunction with portal vein (PV) segmentation ($RPSV_{SCTV}$) to estimate the volume of right posterior section (RPS) graft compared with intraoperative measured weight (W) in live liver donors.
**Methods and Materials:** The study protocol was approved by the Institutional Review Board at our institution. Among 23 donors who donated RPS grafts for liver transplantation in our institution from April 2003 to August 2016, 17 donors (11 men, six women; age, 31.7±7.7 years) were included. CT scans were acquired within 3 months before liver procurement, and PV anatomy was type I-III. RPS volumes were retrospectively evaluated by $RPSV_{SCTV}$ and by measurement of standard liver volume (SLV) and PV area ratio ($RPSV_{SLV•PVAR}$). RPS volumes were compared with W for correlation coefficients, difference (absolute difference), and percentage deviation (percentage absolute deviation). Regression analysis was performed to identify the method that yielded greatest correlation with W.
**Results:** Mean values of $RPSV_{SCTV}$, $RPSV_{SLV•PVAR}$, and W were 452.4±175.8 (209.0-839.3), 516.54±146.20 (274.06-776.32), and 518.8±122.4 (370.0-789.0), respectively. $RPSV_{SCTV}$ was strongly correlated with W ($r$=0.9414; $P$<.0001), whereas $RPSV_{SLV•PVAR}$ was only moderately did ($r$=0.5899; $P$=.0127). $RPSV_{SCTV}$ showed a significantly smaller absolute difference (35.20±30.82 vs. 104.79±60.27, $P$ =.004) and absolute percentage deviation (6.61±4.90 vs. 19.92±10.37, $P$<.0001) from W, by paired $t$ tests. Equation correlating $RPSV_{SCTV}$ and W was W = -74.7191 + 1.1791 · $RPSV_{SCTV}$ ($R^2$ = 0.8862; $P$<.001).
**Conclusion:** $RPSV_{SCTV}$ is more accurate than $RPSV_{SLV•PVAR}$ for estimating RPS weight in live liver donors, with a mean absolute percentage deviation of 6.61±4.90.

**B-0797** 14:56

**Feasibility of volume index to assess liver volume: comparison of ultrasound and computed tomography**

D. Seppelt[1], T. Ittermann[2], A. Haubold[1], D. Fedders[1], R.-T. Hoffmann[1], C. Kolb[1], J.-P. Kühn[1]; [1]*Dresden/DE*, [2]*Greifswald/DE*
*(danilo.seppelt@uniklinikum-dresden.de)*

**Purpose:** Volume index measurement (VI) is based on simple measurement of liver diameters to estimate liver volume. The purpose was to evaluate the feasibility of VI based either on ultrasound (US) or computer tomography (CT) by comparison with CT based volumetry.
**Methods and Materials:** Fifty-nine patients, 21 women, mean age of 66.8±12.6 years underwent an abdominal CT followed immediately by US of the liver. Liver diameters: anterior-posterior, medio-lateral, and cranio-caudal in their maximum extension were assessed by two observers in CT and US imaging. VI was calculated by multiplication of diameters divided by a constant (3.6). As gold standard served the liver volume determined by a manual segmentation of liver by CT (“true liver volume”). True liver volume and calculated VI determined by CT and US were compared using a Bland-Altman analysis.
**Results:** Liver volumes determined by manual segmentation, CT-based VI, and US-based VI were as follows: 1.500±347cm$^3$, 1.509±432cm$^3$, 863±371cm$^3$. Mean differences of VI between observers were 1.1 (-16.1;18.2)% for CT-based technique, -34.7 (-90.1;20.7)% for US-based VI, respectively. There was an excellent agreement between CT-based VI and true liver volume with a mean bias of 4.4±28.3%, and a high discrepancy for US-based VI; mean bias 58.3±66.9%.
**Conclusion:** Volume index based on measurements of liver diameters is a simple and straightforward approach to estimate true liver volume. CT-based volume index turns out to be a very reliable parameter and can be recommended for clinical usage. Use of US-based volume index should be discouraged from due to its unacceptable low accuracy.

**B-0798** 15:04

**Dose optimisation in multiphasic computed tomography imaging of the liver with high-concentration contrast media**

S. Cavalieri, A. Ferrari, R. Villa, M. Brambilla, A. Stecco, A. Carriero; *Novara/IT (sergiocab451@gmail.com)*

**Purpose:** To reduce radiation dose to patients undergoing multiphasic CT of the liver by exploiting the intrinsic properties of iodine enhancement at low kVp.
**Methods and Materials:** 32 oncology patients underwent multiphasic CT of the liver using a Philips Brilliance iCT scanner and either a standard acquisition protocol (100 kVp in all phases; N=8) or an optimised weight-based acquisition protocol in the arterial phase (80 kVp in patients ≤80 kg; N=16 and 100 kVp in patients >80 kg; N=8). High-concentration contrast agent (Iomeprol 400 mgI/ml) and automatic tube current modulation were used in all patients. The standard dose and the optimised protocols were compared with t test for average $CTDI_{vol}$, liver dose, and peak aortic enhancement measured in the aorta at the level of the coeliac trunk (significance threshold p <0.05).
**Results:** When the optimised protocol was used, patients ≤80 kg showed a significant increase in peak aortic enhancement (507±69 HU vs 324±59HU; p=0,01) and a significant reduction in average $CTDI_{vol}$ (from 12.5±2.6 to 8.3±1.3 mGy; p=0,005) and liver dose (from 17.4±4.1 to 10.9±1.6 mSv; p=0,005) for the arterial phase. As expected, patients ≥80 kg did not show any significant difference in aortic enhancement and radiation dose compared to the standard protocol.
**Conclusion:** Our optimised protocol with use of 80 kVp and high iodine concentration resulted in a substantial improvement of aortic attenuation and radiation dose reduction in patients ≤80 kg.
*Author Disclosures:*
**S. Cavalieri:** Speaker; Bracco Symposium at SIRM 2018. **A. Ferrari:** Speaker; Bracco Symposium at SIRM 2018.

**B-0799** 15:12

**Multidetector liver CT: improved image quality, decreased radiation and contrast media dose with peristaltic contrast media injection system**

C. Saade, S. Khalifeh, L. Karout, L. Naffaa; *Beirut/LB (charbel.saade@aub.edu.lb)*

**Purpose:** To compare hepatic vascular and parenchymal opacification between direct and peristaltic drive contrast media (CM) injection systems and its effect on image quality and pathology detection.
**Methods and Materials:** IRB-approved retrospective study consisted of 182 patients who underwent CT of the abdomen and pelvis in both groups; Group A: employed direct drive injector, and Group B: utilized the peristaltic injector. Quantitative opacification measurements of the vasculature, parenchyma, contrast-to-noise ratio (CNR) and signal-to-noise ratio (SNR) and each hepatic segment (n=8) were compared by employing paired t test and Pearson's correlation. Receiver operating (ROC) and visual grading characteristics (VGC) measured the confidence intervals and image quality, respectively. Reader agreement employed Cohen’s kappa methodology.
**Results:** Mean opacification of the vasculature and liver segments demonstrated no statistical difference between each group. CNR in the liver compared to hepatic vein was higher in Group B (2.17 ±0.82) compared to A (1.83±0.64). SNR in Group B (5.89±1.63 HU) was higher than A (4.95±1.59HU) (p<000.1). Radiation dose and CM volume were significantly lower in Group B than A (p<0.0001). VGC showed no significant difference in qualitative image quality assessment. ROC demonstrated pathology detection in Group B than A (p<0.0001). Inter- and intra-observer variation showed that there is a strong relationship between liver vasculature and parenchymal opacification (Protocol A: κ=0.25-0.76 and Protocol B: κ=0.39-0.91).
**Conclusion:** Significant improvements in quantitative and qualitative assessments of liver pathology can be achieved with low CM and radiation dose when employing peristaltic CM injection system.

**B-0800** 15:20

**A reduced contrast media injection protocol significantly improves the visualisation of liver vasculature at reduced radiation dose during hepatic CTA**

C. Saade, A. Haydar; *Beirut/LB (charbel.saade@aub.edu.lb)*

**Purpose:** To investigate the opacification of liver vasculature and radiation dose by employing a patient-specific (PS) contrast administration protocol during hepatic CTA (HCTA).
**Methods and Materials:** This hybrid retrospective (protocol A) and continuous prospective (protocol B) study was IRB approved. HCTA was performed in 216 consecutive patients, with one of two protocols: protocol A, 100 mL of contrast material injected intravenously; and protocol B, employing the patient-specific contrast media protocol. Each protocol employed contrast material and saline flow rate of 4.5 mL/sec. Attenuation profiles of the hepatic arteries and veins were measured. Effective dose was calculated. Data were compared with the independent sample *t* test. Visual grading characteristic (VGC) analyses were performed.
**Results:** Arterial opacification demonstrated no significant increases in arterial opacification in protocol B, except for right main hepatic artery (p<0.001), segment VI of the right hepatic artery (p<0.036), left main hepatic artery (p<0.002), proximal segment of the gastroduodenal artery (p<0.014). The abdominal aorta and celiac trunk were significantly higher in protocol B compared to A (p<0.007), celiac trunk (p<0.004). In the veins, inferior vena cava, portal (all branches) and proximal splenic veins demonstrated a significant reduction in opacification protocol B compared to A (p<0.001). Effective dose was significantly reduced in protocol B (p<0.001). Contrast media volume in protocol B (57.60±12.25 mL) was significantly lower than in protocol A (*p*=0.001). VGC demonstrated no significant differences between protocols, with reader agreement increasing from moderate to excellent (p<0.001).
**Conclusion:** Employing a PS injection protocol demonstrates significant improvements in the visualization of the liver vasculature at reduced radiation and contrast media dose.

**14:00 - 15:30** **Sky High Stage**

### Artificial Intelligence and Machine Learning

### MY 7

### Artificial Intelligence and Machine Learning

*Moderators:*
M. Dewey; Berlin/DE
M. Zins; Paris/FR

**B-0801** 14:00

**Assistant effects of a computer-aided diagnosis system based on 3D convolutional neural network on LDCT reading**

S. Wang, Z. Wang, S. Zhang, Y. Fu, T. Hu, W. Peng; *Shanghai/CN (shengpingwang2007@126.com)*

**Purpose:** To compare the difference of diagnostic performance between radiologists with and without computer-aided detection (CAD) system based on depth learning algorithm in detecting lung nodules in low-dose computed tomography (LDCT).
**Methods and Materials:** Seventy-nine patients (whether or not lung nodules were found) were enrolled. First, three radiologists randomly read these cases and assessed the risk score without a CAD system. Then, 4 weeks later, the three radiologists read the cases randomly and assessed the risk score again with the aid of a CAD system based on a 3D convolution neural network. The results obtained by one expert chest radiologist with the aid of CAD system is

Thursday used as the ground truth.
**Results:** Number of detected nodules increased in CAD group by 53.22% ($p < 0.01$). The average absolute difference between reader and ground truth was decreased from 0.96 to 0.43 ($p < 0.01$). And the average standard error (SE) of cases between readers decreased from 0.45 to 0.34 ($p = 0.11$) when using CAD system for assistance. The average SE of risk score was from 0.25 to 0.10 ($p < 0.01$) when using CAD system.
**Conclusion:** This study compared the reading results of different radiologists with and without in-depth learning of CAD system. The results showed that with the aid of CAD system, radiologists discovered more lung nodules and obtained better consistency.

**B-0802** 14:04
**Automatic prostate segmentation of magnetic resonance images using convolutional neural networks**
N. Aldoj, F. Biavati, M. Rutz, S. Stober, M. Dewey; *Berlin/DE (nader.aldoj@charite.de)*

**Purpose:** To develop a fully automatic and accurate tool for prostate segmentation using 2D convolutional neural networks.
**Methods and Materials:** A dataset of 188 patients with T2-weighted images was used in this study (PROSTATEx challenge). All T2-weighted axial images (256x256 pixels) were manually segmented and examined by an experienced radiologist. The training set consisted of 143 patients (a total of 2982 slices) and the test set of 45 patients (a total of 912 slices). The developed Dense U-Net was based on the U-Net architecture with six stages. We replaced the classical convolutional layers with a Dense net-like architecture which consists of two small Dense blocks separated by transitional layers and each of which comprised four convolutional operations. Results of the developed network were compared for all test images with the classical U-Net using the dice score.
**Results:** The Dense U-Net achieve an average dice score of 0.89 and a median dice score of 0.90 which was higher in comparison to the classical U-Net with average and median dice scores of 0.885 and 0.89, respectively. Both networks achieved accurate segmentation in the middle region of the prostate with an average dice score of 0.94 for the Dense U-Net and 0.93 for classical U-Net. Both networks resulted in relevantly lower dice scores for the apical and basal region of the prostate (Dense U-Net: 0.72 and 0.79; Classical U-Net: 0.70 and 0.79), respectively.
**Conclusion:** The developed Dense U-Net architecture outperformed the classical U-Net by achieving more accurate prostate segmentation on axial T2-weighted sequences.

**B-0803** 14:08
**Automated x-ray bone segmentation using a conditional adversarial network**
J. Haubold, A. Demircioglu, M. Forsting, L. Umutlu, F. Nensa; *Essen/DE (johannes.haubold@uk-essen.de)*

**Purpose:** To train and evaluate a conditional adversarial network (pix2pix) for automated bone segmentation in x-ray images.
**Methods and Materials:** The bone tissue in 40 x-rays of the left elbow was segmented manually using the 3D Slicer software. The segmentations were used as ground truth for the training of a conditional adversarial network comprising a convolutional and a deconvolutional neural network (pix2pix) on automated bone segmentation. To validate the segmentation efficiency a 5-fold cross validation was performed and therefore the dataset was randomly split into five subsets each containing eight x-rays. In each of five tests the network was trained on 32 images and evaluated on the remaining eight images. Segmentation efficiency was evaluated using the Jaccard similarity coefficient.
**Results:** Overall Jaccard mean was 0.94 ± 0.03 (Jaccard mean for split 1=0.96; split 2=0.96; split 3=0.95; split 4= 0.94; split 5=0.89) showing an overall good similarity of automatically segmented images compared to manually segmented images. Default training epoch value of 100 showed the best Jaccard similarity coefficient compared to 25, 50 and 250 epochs in pix2pix.
**Conclusion:** Using only 40 segmented x-ray images of the elbow a conditional adversarial network could be trained for automated bone segmentation with an overall good similarity coefficient compared to manually segmented images.

**B-0804** 14:12
**Small (< 4 cm) renal masses: convolutional neural network-based deep-learning approach in differentiating fat-poor renal angiomyolipoma from renal cell carcinoma at MSCT**
X. Li, L. Wang; *Nanjing/CN (lixiao19901020@yeah.net)*

**Purpose:** To investigate diagnostic performance using a deep learning method with a convolutional neural network for the differentiation of fp-AML and RCC at dynamic contrast agent-enhanced computed tomography.
**Methods and Materials:** Approved clinical retrospective study from January 2013 to July 2017, patients with fp-AML and RCC were identified from the pathology database. 42 patients with fp-AML (no visible fat at unenhanced CT) and 158 patients with RCC. All patients were examined with a 320-slice dynamic volume CT by using same four phase renal protocol. This study is composed of two stages: A training stage, in which 10-fold validation was performed and a testing stage to evaluate the performance of the models with new pictures to each model. A testing stage which is simply done by loading the testing image dataset into the classifier.
**Results:** The mean AUC for differentiating fp-AML from RCC using model unenhanced, model corticomedullar, model nephrographic, model excretory, model enhanced and model quadruple with the test data sets was 0.64 (95% confidence interval [CI]: 0.58, 0.69), 0.83 (95% CI: 0.81, 0.85), 0.83 (95% CI: 0.81, 0.85), 0.81 (95% CI: 0.79, 0.83), 0.85 (95% CI: 0.84, 0.86), 0.84 (95% CI: 0.83, 0.85).
**Conclusion:** The CNN models except model unenhanced exhibited a high diagnostic performance in distinguishing fp-AML from RCC at dynamic CT. Further research might focus on the application of this tool in other benign renal lesions, such as oncocytomas.

**B-0805** 14:16
**Computed tomography-based radiomics analysis to assess treatment response after perioperative neoadjuvant therapy**
Q. Xu[1], C. Zhou[1], C. Ye[1], G. Tao[1], W. Zhao[1], D. Ren[2], X. Li[2], L. Zhao[2], G. Lu[1]; [1]*Nanjing/CN*, [2]*Beijing/CN (DZ1735002@smail.nju.edu.cn)*

**Purpose:** To assess the value of computed tomography (CT)-based radiomics for diagnosis of good response (GR) to perioperative neoadjuvant chemotherapy (NCT) in patients with locally advanced gastric cancer (GC).
**Methods and Materials:** This retrospective study includes 189 patients (170 in the primary cohort and 19 in the validation cohort) with GC who had a CT scan after NCT between July 2014 and December 2017. Surgical histopathologic analysis was the reference standard for GR. In total, 1232 radiomics features were extracted from portal venous-phase CT of GC for each patient. F test was adopted during feature selection and linear support vector classification was used to build the classifier model. The combination model was implemented within a nested cross-validation structure. The performance of the radiomics model was assessed by its calibration, discrimination, and clinical usefulness with independent validation. Statistical analysis was performed using the Mann-Whitney U test, Chi-square test and logistic regression analysis.
**Results:** The radiomics model consisting of 20 selected features showed good discrimination performance and good calibration. The area under the receiver operating characteristic curve was 0.8354 (sensibility, 0.7559; specificity, 0.7457; accuracy, 0.7521) for the assessment model of GR. Decision curve analysis confirmed the clinical utility of the radiomics model.
**Conclusion:** Contrast-enhanced CT-based radiomics showed good classification performance for preoperative assessment of GR in patients with locally advanced gastric cancer after NCT and has a prospect for application.

**B-0807** 14:20
**A preliminary study of predicting molecular subtypes of breast cancer by the radiomics features of contrast-enhanced ultrasound**
L. Tang, W. Chang, M. Chen; *Shanghai/CN (13661657380@163.com)*

**Purpose:** To analyse the quantitative features of contrast-enhanced ultrasound (CEUS) imaging of breast cancer (BC) through computer image extraction and to explore the preoperative prognosis of different molecular subtypes of BC.
**Methods and Materials:** CEUS images of 189 patients with surgical pathology and molecular typing results of BC were analysed. Through mapping border on the images, the entire internal area of tumour was determined. High perfusion within the lesion area was defined as a high brightness area. Temporal and special features of CEUS images in different areas were extracted by computer and the time-intensity curves (TIC) were drawn. From TIC, the features were extracted such as wash-in rate (WiR), wash-out rate (WoR), rise time (RT), base intensity (BI) and peak intensity (PI) and EI (=PI-BI). The parameters of each area were calculated in different molecular typing groups.
**Results:** The patients' number of Luminal A, Luminal B, HER2+ and triple-negative BC (TNBC) in different molecular subtypes were 46, 75, 37 and 31. The extracted effective features included EI and WoR of internal bright area, WoR and RT of internal overall area. The cutoff value 1.566 of WoR in internal bright area might help to find Luminal A, with specificity in 82.61%. When finding TNBC, the cutoff value of EI at internal bright area was 0.3494; the sensitivity and specificity were 64.52% and 68.99%. When distinguishing between Her2+ and TNBC, WoR of internal overall had a cutoff of 5.7496 and sensitivity of 74.19%.
**Conclusion:** The radiomics features of CEUS could contribute to preoperative prediction of BC molecular subtypes.

**B-0808** 14:24

**The diagnostic value of texture analysis in predicting WHO grades of meningiomas based on ADC maps: an attempt using decision tree and decision forest**

Y. Lu, B. Yin; *Shanghai/CN (06307070008@fudan.edu.cn)*

**Purpose:** The preoperative prediction of the WHO grade of a meningioma is important for further treatment plans. This study aimed to assess whether texture analysis (TA) based on apparent diffusion coefficient (ADC) maps could non-invasively classify meningiomas accurately using tree classifiers.
**Methods and Materials:** A pathology database was reviewed to identify meningioma patients who underwent tumour resection in our hospital with preoperative routine MRI scanning and diffusion-weighted imaging (DWI) between January 2011 and August 2017. A total of 152 meningioma patients with 421 preoperative ADC maps were included. Four categories of features, namely clinical features, morphological features, average ADC values and texture features, were extracted. Three machine learning classifiers, namely classic decision tree, conditional inference tree and decision forest, were built on these features from the training dataset. Then the performance of each classifier was evaluated and compared with the diagnosis made by 2 neuroradiologists.
**Results:** The ADC value alone was not able to distinguish 3 WHO grades of meningiomas. The machine learning classifiers based on clinical, morphological features and ADC value could achieve equivalent diagnostic performance (accuracy 62.96%) compared to 2 experienced neuroradiologists (accuracy 61.11% and 62.04%). Upon analysis, the decision forest that was built with 23 selected texture features and the ADC value from the training dataset achieved the best diagnostic performance in the testing dataset (Kappa 0.64, accuracy 79.51%).
**Conclusion:** Decision forest with the ADC value and ADC map-based texture features is a promising multiclass classifier that could potentially provide more precise diagnosis and aid diagnosis in the near future.

**B-0809** 14:28

**Automated stroke detection and profiling on initial non-contrast CT via machine learning and textural analysis**

J.M.A. Alghamdi, S. Raja; *Riyadh/SA (jmalghamdi@kfmc.med.sa)*

**Purpose:** To explore the utility of machine learning and texture analysis for possible automatic detection and temporal profiling of suspected stroke.
**Methods and Materials:** We reviewed stroke-code patients at King Fahad Medical City in Riyadh, who presented between January 2015 and September 2017. We got a total of 826 patients. After applying the exclusion criteria, we chose 567 patients. Baseline cases were reviewed and staged by 3 radiologists. As a result, we identified subsets of hyper-acute (42), acute (39), and sub-acute (23). Baseline and follow-up CT scans were co-registered on a dedicated review workstation. Evolving stroke on follow-up CTs was contoured on in 3D. The 3D-contours were mirrored on to contralateral normal region. Simultaneously, contours were duplicated on co-registered baseline CTs. Pixel level data from the flagged regions on baseline CT and follow-up were exported to Matlab. Utilizing home-built algorithms, 42 textures were computed. Textural features were analysed in SPSS modeller for classification.
**Results:** Utilizing the 42 textural scalars, among various machine learning algorithms, Decision Trees (C5.1) was found to be the most optimal differentiator between stroke (hyper-acute/acute/sub-acute) versus contralateral normal brain tissue, as well as classifying temporal profiling the stages of stroke. The decision trees were good to excellent in classifying hyper-acute (71%), sub-acute (82%), and modest for acute (60%) stroke.
**Conclusion:** It is feasible to differentiate and chronologically profile stroke utilizing textural analysis and machine learning. We seek to increase the accuracies in a larger series and also utilize additional textures and machine learning algorithms.

**B-0810** 14:32

**A novel improved deep learning framework for liver lesion detection**

Y. Chen[1], J. Ma[2], J. Wang[1], F. Wang[1], S. Feng[1]; [1]*Guangzhou/CN*, [2]*Beijing/CN (chen-yingxi@163.com)*

**Purpose:** Hepatocellular carcinoma (HCC) is the second most frequent cause of malignancy-related death in the world. Early detection of HCC is the key issue in clinical imaging. Computer-assisted diagnosis can help for early detection of lesion in many other organs. There is still lack of an effective deep learning framework for liver lesion detection. This study aims to find out an improved framework for liver lesion detection.
**Methods and Materials:** A new framework has been improved by the following ways: 1) using more numbers of layers in the neural network framework; 2) developing a 2.5D-like structure; 3) using a multi-scale-based deep learning network; 4) fusing the information of multi-phase images. Totally 621 patients were used to train the model, and the training set included 910 hepatic cysts, 495 haemangiomas and 173 HCCs. Other 60 patients were used to test the model, including 98 hepatic cysts, 38 haemangiomas and 30 HCCs. All the images contained tri-phasic sequences. All the lesions were labelled jointly by two experienced radiologists as standard.
**Results:** Before the improvement, the predictive accuracy of hepatic cysts, haemangiomas and HCCs were 69.23%, 64.29% and 70.96%, respectively. After using the improved framework, the predictive accuracy of hepatic cysts, haemangiomas and HCCs rose to 82.76%, 78.98% and 77.88%, respectively.
**Conclusion:** The novel improved deep learning framework has a relative better predictive ability of liver lesion detection, which provides an effective measure for computer-assisted imaging diagnosis. This framework is also helpful for the early detection of HCC.

**B-0811** 14:36

**Evaluation of intra-scanner test-retest variability of machine learning features in low-dose computed tomography of interstitial lung disease**

F. Prayer, S. Röhrich, J. Hofmanninger, A. Willenpart, G. Langs, H. Prosch; *Vienna/AT*

**Purpose:** To investigate the robustness of machine learning imaging features of patients with interstitial lung disease (ILD) in repeated low-dose computed tomography (LDCT) examinations on a single scanner.
**Methods and Materials:** Thirty consecutive ILD patients, after written declaration of consent, received repeated non-contrast LDCT of the chest on a single CT scanner within 15 to 60 minutes. Images of test and retest scans were reconstructed using one- and three-millimeter slice thickness, and soft tissue and lung kernels (i30f, i50f, and i70f). Imaging features used for machine learning-based diagnosis and progression prediction (Haralick texture features) were extracted after isotropic voxel resampling to 0.7mm resolution. Local features were quantized to volume descriptors and used to assess visual similarities between volumes. We evaluated the effect of retest and reconstruction parameters on the visual similarity by varying one parameter while keeping all other parameters fixed.
**Results:** Visual similarity between test and retest yield 0.02 mean (SD 0.01), 0.03 (0.01), 0.46 (0.16), 0.51 (0.02), and 0.12 (0.02) between i30f and i50f kernels, i50f and i70f, i30f and i70f and 1mm and 3mm slice thickness, respectively. Two-sample t tests revealed significantly higher influence of varying reconstruction parameters on visual descriptors compared to test-retest (all $p<4.2e-11$).
**Conclusion:** LDCT imaging features of ILD used for machine learning obtained by repeated scans on a single scanner are reproducible. However, variability is introduced by reconstruction parameters, with slice thickness exhibiting higher influence than reconstruction kernel despite isotropic resampling.

**B-0812** 14:40

**Accurate pulmonary nodule detection in CT images using multi-stage 3D deep convolution neural networks**

X.J. Chen; *Zhengzhou/CN (chenxuejun1967@163.com)*

**Purpose:** To evaluate the application of a new method of computer-aided pulmonary in diagnosing the accuracy and efficiency of lung cancer.
**Methods and Materials:** CT images were acquired from 980 lung cancer patients. Manually marked pulmonary nodule location and pathology results are taken as gold standard. The dataset consists of 7336 nodules in which 3169 are malignant. The detection rate of pulmonary nodules and the malignant prediction rate were evaluated through manual detection and the computer-aided detection. Computer algorithm of computer-aided detection adopted multi-stage strategy. In the first stage, CT images were preprocessed to reduce variances such as CT thickness caused by different manufactures. Second, self-designed neural network mimicking zooming in and out the image was used to extract multi-scale image features and generate region of interest (ROI). Finally, another 3D deep convolution neural network is applied to classify whether the ROI is true or false positive.
**Results:** Compared with the manual detection, the detection of nodules of computer-aided detection was high, which was statistically significant (84.71% vs 95.76%), and false-positive rate of between the computer-aided detection and the manual detection was not statistically significant $P>0.05$. The malignant prediction result of computer-aided detection shows 95.28% recall rate and 98.56% precision rate in patient level, and the malignant prediction result of manual detection shows 94.7% recall rate and 98.00% precision rate in patient level which were not statistically significant $P>0.05$.
**Conclusion:** The multi-stage 3D deep learning method verified by large clinical dataset shows greater efficiency helping detect pulmonary nodules and offer better accuracy.

**B-0813** 14:44

**Deep learning for prostate cancer segmentation and detection: diffusion MRI U-net compared to clinical PI-RADS assessment**

P. Schelb, S. Kohl, J.-P. Radtke, M. Hohenfellner, H.-P. Schlemmer, K. Maier-Hein, D. Bonekamp; *Heidelberg/DE (david@davidbonekamp.de)*

**Purpose:** To compare the performance of DWI-only convolutional neural network (CNN) machine learning with full multiparametric clinical PI-RADS assessment.
**Methods and Materials:** A U-net CNN was trained on ADC maps and b value 1500s/mm2 images from 316 consecutive men with segmentations of clinical PI-RADS lesions yielding Gleason grade group >=2 on targeted biopsy. The CNN was trained on 253 patients (4-fold 3-times repeated cross validation, training/validation=190/63 patients). Ensemble predictions were evaluated on the held-out test set of 63 patients. Radiologist and CNN performances were evaluated on combined extended systematic and targeted biopsy mapping using different net ensemble (CNN) and PI-RADS thresholds.
**Results:** On a per patient basis both, radiologists (at PI-RADS>=3) and CNN (at net ensemble threshold (NET)>=2) achieved sensitivity of 0.96 and specificity of 0.22. Radiologists achieved sensitivity of 0.85 at specificity 0.50 for PI-RADS>=4, while CNN achieved sensitivity of 0.81 and specificity of 0.44 at NET>=6. On a per lesion basis, radiologists achieved sensitivity of 0.63 and specificity of 0.61 at PI-RADS>=3, while CNN achieved sensitivity of 0.52 and specificity of 0.65 at NET>=2. For PI-RADS>=4 radiologists achieved sensitivity of 0.54 and specificity of 0.77, CNN sensitivity of 0.41 and specificity of 0.78 at NET>=6.
**Conclusion:** A U-net CNN using diffusion MRI information only for prostate lesion segmentation and detection demonstrated a promising performance compared to clinical radiologist interpretation. These findings highlight the ability of computer vision to closely model the clinical task with fewer data and encourage development of the method in larger cohorts.
*Author Disclosures:*
**J. Radtke:** Consultant; Saegeling Medizintechnik, Siemens Healthineers. Speaker; Saegeling Medizintechnik. **H. Schlemmer:** Board Member; Curagita. Consultant; Siemens, Curagita, Profound medical, Bayer. Grant Recipient; BMBF, Deutsche Krebshilfe, Dietmar-Hopp-Stiftung, Roland-Ernst-Stiftung. Speaker; Siemens, Curagita, Profound medical, Bayer. **D. Bonekamp:** Speaker; Profound Medical Inc.

**B-0814** 14:48

**A whole-tumour histogram analysis of apparent diffusion coefficient maps obtained using 3.0T MRI for distinguishing uterine endometrial carcinoma from endometrial polyps**

W. Wang, J. Cheng; *Zhengzhou/CN (weijianwang520@163.com)*

**Purpose:** We explored the role of a whole-tumour histogram analysis of apparent diffusion coefficient (ADC) maps for discriminating uterine endometrial carcinoma and endometrial polyps.
**Methods and Materials:** We retrospectively evaluated findings in 40 patients with uterine endometrial polyps and 50 patients with endometrial carcinoma who underwent diffusion-weighted imaging (b = 0,800 s/mm(2) ) at 3T with acquisition of corresponding ADC maps. We derived histogram data from regions of interest drawn on all slices of the ADC maps in which tumour was visualised, including areas of necrosis and haemorrhage in the tumour. We used the Mann-Whitney test to evaluate the capacity of histogram parameters (mean, skewness, kurtosis, 50th, 90th percentiles) to discriminate uterine endometrial carcinoma from endometrial polyps and analysed the receiver operating characteristic (ROC) curve to determine the optimum threshold value for each parameter and its corresponding sensitivity and specificity.
**Results:** Uterine endometrial carcinoma demonstrated significantly higher mean vales of ADC, skewness, kurtosis,50th,90th percentiles than endometrial polyps (P < 0.05). ROC curve analysis of the 90th percentile yielded the best area under the ROC curve (AUC; 0.92), sensitivity of 90%, and specificity of 95%, with a cutoff value of 198.
**Conclusion:** A whole-tumour Histogram analysis of ADC maps might be helpful for discriminating uterine endometrial carcinoma from endometrial polyps.

**B-0815** 14:52

**A computer-aided detection (CAD) system on low-dose chest CT imagesin concurrent-reader and second-reader modes: radiologist performance and reading time**

Q. Hu; *Wuhan/CN (qjhu@outlook.com)*

**Purpose:** To assess the value of a CAD system in second-reader (SR) and concurrent-reader (CR) models for the detection of pulmonary nodules and time efficiency of radiologists on low-dose chest CT images.
**Methods and Materials:** A total of 117 patients with extra-thoracic malignancies, were prospectively included. 9 observers independently evaluated images, first without CAD, then using CAD as SR at both dose settings. Furthermore, 8 observers independently interpreted these datasets thrice, once with CR, twice without CAD, and third with SR. True positive rates(TP), false positive rates (FP) and reading times were recorded. Free-response receiver operating characteristic analysis was also used for the statistical analysis.
**Results:** The sensitivities without CAD for detection of nodules(41.06% and 37.02%) were less than those using CAD as SR(60.57% and 60.39%) in both two dose settings( *P* < 0.001). Reading times were significantly shorter in CR mode (165s) than those without CAD (235s)and in SR mode (294s) (*P*< 0.01). Sensitivity of reading with CAD as CR(64.92%) was significantly higher than reading without CAD (44.71%, p<0.001) and not statistically different in SR mode(66.86%:*P>0.05*) in the low-dose setting. The FOM in the CR mode was statistically significant better than that without CAD(0.58 vs 0.47, respectively; *P* <0.001), and not statistically different compared with using CAD in the SR mode (0.61, *P*=0.259).
**Conclusion:** CAD benefited all readers including small nodules, which both significantly improved diagnostic performance and shortened the reading times, especially for interns, in the low-dose setting.
*Author Disclosures:*
**Q. Hu:** Author; Chong Chen, Shi-chao Kang, Zi-yan SUN, Li-ming XIA.

**B-0816** 14:56

**Reproducibility of fully automated liver volumetric analyses with artificial intelligence: towards laboratory radiology**

D.J. Winkel[1], G. Chabin[2], J. Cyriac[1], B. Stieltjes[1], T.J. Weikert[1]; [1]*Basle/CH*, [2]*Princeton, NJ/US (Thomas.Weikert@usb.ch)*

**Purpose:** Laboratory radiology conveys the vision of a norm-based quantitative disease detection in radiology . Thereto, automated imaging quantification pipelines are mandatory both for large-scale norm-collective creation and disease-related outlier detection. Here, we test such a pipeline in terms of reproducibility and stability using an AI-based algorithm trained for liver volumetric analyses.
**Methods and Materials:** Using a RIS/PACS search engine, we obtained 100 CT datasets with six series for each patient, totaling 600 series: non-contrast (nc), portal venous (pv) and arterial phase (art) with both 1.5mm and 5mm slice thickness. The data were analysed using multi-scale deep-reinforcement learning for 3D body marker detection and adversarial deep image-to-image learning for 3D anatomical structure segmentation. Variations of liver volumes were compared using Cohen's coefficient K. Variations of liver volumes were compared using univariate general linear model analyses with the liver volume as the dependent variable and contrast phase and slice thickness as fixed factors.
**Results:** The liver volumes for the different scan phases, averaged for 1.5 and 5 mm, were 1903±599, 1908±585 and 1928±595 ml (nc, pv, art). The two cycles of analysis yielded identical liver volumes for the given datasets (K = 1). The liver volume was neither dependent on the contrast phase (p = 0.938) nor on the slice thickness (p = 0.605).
**Conclusion:** Fully automated AI-based liver volumetry is a reproducible, stable and precise method. The results of our study provide the necessary confidence for future applications of automated abdominal organ volumetric analyses in the sense of laboratory radiology.
*Author Disclosures:*
**G. Chabin:** Employee; Siemens Healthcare.

**B-0817** 15:00

**Automated detection of lung cancer at ultralow dose PET/CT by deep neural networks: initial results**

M. Schwyzer, D. Ferraro, U. Mühlematter, A. Curioni-Fontecedro, M. Hüllner, G.K. Von Schulthess, P.A. Kaufmann, I.A. Burger, M. Messerli; *Zurich/CH (moritz.schwyzer@usz.ch)*

**Purpose:** The purpose of this study was to assess the accuracy of a deep learning algorithm for automated detection of lung cancer on FDG-PET scans. Additionally, we simulated reduced FDG dose injection and evaluated its impact on the performance of the deep learning algorithm.
**Methods and Materials:** We retrospectively included 50 patients with histologically proven lung cancer and a control group of 50 patients without lung cancer. For deep learning analysis, we performed a binary classification for a total of 3936 slices: Slices, in which the lung tumour is visually present versus slices of locations in the lung where no lung tumour is seen. Besides the standard, full-activity reconstructions, simulated tenfold and thirty-fold reduced dose injection reconstructions were additionally analysed by the deep learning algorithm.
**Results:** The AUC of the deep learning algorithm for the full-activity reconstructions was 0.989, showing no significant difference to the AUC (0.983) of the tenfold reduced dose injection reconstructions. The thirty-fold reduced dose injection reconstruction AUC (0.970) was significantly lower.

**Conclusion:** In this study, we could show that it might be possible to detect lung cancer fully automatically with very low effective radiation doses of 0.11 mSv (3.3% of current clinical routine). Further improvement of this technology might enhance the specificity of lung cancer screening efforts and could lead to new applications for FDG-PET.
*Author Disclosures:*
**M. Hüllner:** Speaker; received speaker's fees from GE Healthcare.
**G.K. Von Schulthess:** Consultant; is a Consultant to GE Healthcare and a Co-Director of IDKD, an educational organization which receives funds from multiple companies. **I.A. Burger:** Speaker; received speaker's fees from GE Healthcare. **M. Messerli:** Speaker; received speaker's fees from GE Healthcare.

**B-0818** 15:04
**Automated lung cancer detection in PET/CT using a deep learning approach**
M. Müller, C.P. Reinert, K. Nikolaou, C. La Fougère, C. Pfannenberg, S. Gatidis; *Tübingen/DE*

**Purpose:** To evaluate the feasibility of automated lung cancer detection in patients undergoing clinically indicated Position Emission Tomography/ Computed Tomography (PET/CT) using a deep learning approach.
**Methods and Materials:** Anonymized FDG-PET/CT data sets of 275 patients with histologically proven lung cancer ("pathologic scans") and 225 patients without a thoracic malignancy ("non-pathologic scans") were retrospectively analyzed. Manual segmentations of tumours were performed using MITK (German Cancer Research Center) and used for evaluation. Sagittal, coronal and transversal maximum intensity projections of PET data were generated prior to further analysis. Two different deep learning approaches were implemented: A seven layer convolutional neural network for classification of examinations into pathologic and non-pathologic and a U-Net architecture for classification and additional spatial segmentation of hypermetabolic cancer lesions. The testing was performed on 50 pathologic and 50 non-pathologic data sets. The classification accuracy was computed as the relative number of correctly classified data sets and the segmentation accuracy was quantified by computing dice coefficients between automated and manual tumour segmentation.
**Results:** The first proposed classifications network was able to distinguish between pathologic and non-pathologic data sets with relatively high accuracy (83 %). Using the U-Net architecture classification accuracy could be improved to 90% and subsequent segmentation accuracy reached 98% dice coefficient. The false positive classification rate dropped to 2%.
**Conclusion:** Neural networks are feasible approaches for automated lung cancer detection in PET/CT. In order to yield clinically usable results and limit the false positive rate, the use of larger training data bases will be helpful.

**B-0819** 15:08
**Non-invasive tumour decoding and phenotyping of cerebral gliomas utilizing multiparametric $^{18}$F-FET PET-MRI and MR fingerprinting**
J. Haubold, A. Demircioglu, M. Gratz, K. Wrede, M. Glas, K. Herrmann, M. Forsting, F. Nensa, L. Umutlu; *Essen/DE (johannes.haubold@uk-essen.de)*

**Purpose:** The introduction of the 2016 WHO classification of CNS tumours has rendered the combined molecular and histopathological characterization of tumours as a pivotal part of glioma patient management. Recent publications on radiogenomics-based prediction of the mutational status have demonstrated the predictive potential of imaging-based, non-invasive tissue characterization algorithms. Hence, the aim of this study was to assess the potential of multiparametric $^{18}$FET-PET MR imaging including MR fingerprinting accelerated with machine learning and radiomic algorithms to predict tumour grading and mutational status of patients with cerebral gliomas.
**Methods and Materials:** 42 patients with therapy-naïve cerebral gliomas underwent an 18FET-PET-MRI examination. Surgical biopsy or resection served for histopathological sampling. To differentiate the mutational status and the WHO grade of the cerebral tumour we trained a support vector machine and random forest with the radiomics signature of the multiparametric PET-MRI data including MRI fingerprinting. As a gold standard, surgical acquired histology and mutational status were used.
**Results:** The 5-fold cross-validated area under the curve in predicting the ATRX mutation was 85.1%, MGMT mutation was 75.7%, IDH1 was 88.7% and 1p19q was 97.8%. The area under the curve of differentiating low-grade glioma vs. high-grade glioma was 85.2%.
**Conclusion:** 18F-FET PET-MRI and fingerprinting enable imaging-based tumour decoding and phenotyping with a good performance in differentiating low-grade vs. high-grade gliomas and in predicting the mutational status of ATRX, IDH1 and 1p19q. These initial results underline the potential of 18F-FET PET MRI to serve as an alternative to invasive tissue characterization.

**B-0820** 15:12
**Deep learning-based automated calcium scoring for cardiac computed tomography**
S.S. Martin[1], M. van Assen[2], A.M. Fischer[3], S. Rapaka[4], P. Sahbaee[1], C. Schwemmer[5], M.A. Gulsun[4], P. Sharma[4], U.J. Schoepf[1];
[1]*Charleston, SC/US*, [2]*Groningen/NL*, [3]*Mannheim/DE*, [4]*Princeton, NJ/US*, [5]*Forchheim/DE (AndreasMarco.Fischer@googlemail.com)*

**Purpose:** The aim of this study was to evaluate a deep learning-based automated calcium scoring application for cardiac computed tomography (CT).
**Methods and Materials:** We analysed an automated calcium scoring application that is composed of multiple deep learning models, including voxel segmentation and computing the likelihood of a voxel being coronary calcium. The software automatically identifies the coronaries and calcified lesions, whereas aortic plaques are excluded in the calculations using a model for aorta segmentation. This algorithm was trained on 2000 annotated electrocardiography-gated cardiac CT scans. Thereafter, the application was evaluated on 263 consecutive patients undergoing cardiac CT. Results were compared to coronary calcium scores (CCS) obtained by standard manual assessment of independent cardiovascular radiologists.
**Results:** Mean CCS revealed no significant differences between the automated algorithm (102.2) and the reference standard (114.2) ($P$=0.156). Moreover, CCS using the automated application showed an excellent correlation with the reference standard (Pearson, r=0.97). Overall, 243 of 263 patients (92.4%) were classified into the correct risk category (0, 1-10, 11-100, 101-400, or >400).
**Conclusion:** Deep learning-based automated calcium scoring for cardiac CT shows a high accuracy compared to reference scores.
*Author Disclosures:*
**S. Rapaka:** Employee; Siemens Healthineers. **P. Sahbaee:** Employee; Siemens Healthineers. **C. Schwemmer:** Employee; Siemens Healthineers. **M.A. Gulsun:** Employee; Siemens Healthineers. **P. Sharma:** Employee; Siemens Healthineers. **U.J. Schoepf:** Consultant; Heartflow, Guerbet, Siemens, Astellas. Research/Grant Support; Siemens, Bayer, GE.

**14:00 - 15:30** **Tech Gate Auditorium**

### Genitourinary

### SS 707

### Prostate and bladder tumours

*Moderators:*
P. Pavlica; Bologna/IT
T. Penzkofer; Berlin/DE

**B-0821** 14:00
**Whole-body MRI for detecting bone metastases in treatment naïve high-risk prostate cancer: cracking nuts with a sledgehammer?**
E. Rud, F. Ottoson, T. Flatabo, E. Baco; *Oslo/NO (erik.rud@ous-hf.no)*

**Purpose:** The aim of this study was to evaluate the anatomical sites of bone metastases in patients with high-risk prostate cancer.
**Methods and Materials:** This retrospective quality control study analysed the prospective results of all whole-body MRIs performed in 2016 and 2017. All patients were treatment naïve, and all had high-risk prostate cancer defined as biopsy Gleason score ≥7b and/or PSA≥20-100 and/or cT≥2c. The MRI sequences included 3DT1, diffusion-weighted images and T2 STIR from C1 to the proximal femur. Results were dichotomized as bone metastasis positive (M1) or negative (M0), and the anatomical sites of metastases were registered. PSA in those with M0 and M1 was compared using Mann-Whitney U test, and the area under the curve (AUC) for PSA was calculated when using M1 as a state variable.
**Results:** 153 patients were included. Median age (interquartile range) was 71 years (64-76) and PSA was 13 ng/ml (7.9-25.0). Gleason score 6: 4 (3%), 7a: 24 (16%), 7b: 38 (25%), 8: 50 (33%), 9: 31 (21%) and 10: 3 (2%), missing in three. Bone metastases were found in 7 out of 153 patients (4.6%, 95% CI: 1.8-9.4), all occurring in the pelvis. The median (IQR) PSA in M0 and M1 disease was 51 (37-71) and 12 ng/ml (7.8-22.5), respectively (p=0.006). All metastases were found in patients with PSA >22ng/ml and Gleason score ≥8. AUC for PSA was 0.917 (95% CI: 0.85-0.99).
**Conclusion:** All cases of bone metastases were found in the pelvis suggesting that whole-body MRI is excessive in patients with high-risk prostate cancer.

**B-0822** 14:08
**Detection of extracapsular extension in high risk prostate cancer patients: multiparametric MRI vs. $^{68}$Ga-PSMA-11 PET/MRI**
U.J. Mühlematter, K. Schawkat, A. Becker, J. Müller, A.M. Hötker, N.J. Rupp, C.S. Reiner, I.A. Burger, O.F. Donati; *Zurich/CH*

**Purpose:** To compare the diagnostic accuracy and inter-reader reliability of multiparametric MRI (mpMRI) and $^{68}$Ga-labeled prostate-specific membrane antigen ($^{68}$Ga-PSMA-11) PET/MRI for detection of extracapsular extension (ECE) in patients with prostate cancer.
**Methods and Materials:** Retrospective analysis of 40 consecutive men that underwent mpMRI and $^{68}$Ga-PSMA-11 PET/MRI within 6 months for suspected prostate cancer that underwent radical prostatectomy between April 2016 and July 2018. Two readers (R1/R2) blinded to clinical and histopathologic findings rated the probability of ECE on mpMRI and PET/MRI in 4 anatomical regions (anterior/posterior on right/left side) using a 5-point Likert-scale. An interval of >3 weeks between readings was maintained to avoid recall bias. Prostatectomy specimen served as reference standard. Accuracy was assessed by calculating the Area under the receiver-operating-characteritics curve (AUC). Sensitivity/specificity was calculated on dichotomized data. Inter-rater reliability was assessed by intra-class correlation coefficient (ICC).
**Results:** Mean age was 63 years (range 51-78). 14/40 (35%) patients had ECE. For mpMRI versus PET/MRI, the region-specific results were as follows: AUC 0.67/0.71 (R1), 0.70/0.77 (R2); sensitivity 0.26/0.37 (R1), 0.47/0.53 (R2); specificity 0.93/0.93 (R1), 0.91/0.89 (R2). For mpMRI versus PET/MRI the patient-specific sensitivity was 0.42/0.55 (R1) and 0.55/0.60 (R2) and specificity was 0.68/0.66 (R1) and 0.62/0.63 (R2). There was no significant difference in ICC of mpMRI (ICC=0.72, CI=0.69-0.75) and PET/MRI (ICC=0.74, CI=0.71-0.77) between the two readers.
**Conclusion:** Our results suggest that $^{68}$Ga-PSMA-11 PET/MR could improve the sensitivity of mpMRI for the detection of ECE in prostate cancer patients.

**B-0823** 14:16
**Prostate cancer local staging: a comparison of short dual pulse and biparametric MRI vs multiparametric MRI**
A. Stanzione, A. Ponsiglione, S. Cocozza, R. Cuocolo, S.G. Picchi, V. Romeo, S. Stilo, A. Brunetti, M. Imbriaco; *Naples/IT (arnaldostanzione@yahoo.it)*

**Purpose:** Local staging plays a pivotal role in the stratification of risk for patients affected by prostate cancer (PCa). Multiparametric MRI (mpMRI) is currently considered the most accurate imaging modality for pre-operative assessment of PCa local stage. Abbreviated unenhanced protocols have been proposed as a cheaper and faster alternative to mpMRI for the detection of PCa. Aim of our study was to evaluate the diagnostic accuracy in PCa local staging of two different abbreviated MRI protocols, in comparison to mpMRI.
**Methods and Materials:** Fifty patients were retrospectively enrolled (35 affected by PCa who underwent radical prostatectomy and 15 patients with negative biopsy results). A short dual pulse (dpMRI) protocol (axial T2-weighted and DWI) and a biparametric (bpMRI) protocol (triplanar T2-weighted and axial DWI) were obtained from the original images of the mpMRI standard protocol. Two experienced and two novice radiologists evaluated each protocol in a blinded, randomised fashion. The radiologists' performances were correlated with the reference standard using a Spearman correlation analysis, and each correlation coefficient was compared between MRI protocols using a paired t test.
**Results:** The correlation analysis showed that all MRI protocols were highly correlated to the reference standard (p≤0.001). A significant difference emerged when dpMRI was tested in comparison with mpMRI (p=0.009), while no differences emerged when bpMRI was compared with the standard mpMRI (p=0.27).
**Conclusion:** The bpMRI protocol appears a feasible tool for assessment of PCa local stage, with a comparable diagnostic performance to the standard mpMRI.

**B-0824** 14:24
**Local staging in prostate cancer (PCa): the role of capsular bulging and the length of capsular contact (LCC) to predict the risk of extracapsular extention (ECE) at final pathology**
G. Stranieri[1], G. Gallo[1], A. De Pascale[1], D. Gned[2], M. Bini[1], D. Basile[1], E. Checcucci[1], F. Porpiglia[1], A. Veltri[1]; [1]*Turin/IT*, [2]*Saint Pierre/IT (giuseppestranieri92@gmail.com)*

**Purpose:** Multiparametric Magnetic Resonance (mp-MRI) had widespread diffusion in preoperative management of PCa in order to guide target biopsy and for local staging. Capsular bulging and $LCC_{max}$ at mp-MRI has shown to be related to ECE. We evaluate the role of capsular bulging and $LCC_{max}$ as biomarkers for predicting ECE.
**Methods and Materials:** We extracted retrospectively patients with PCa from a prospective robot-assisted radical prostatectomy (RARP) database. We included 162 patients who underwent preoperative mp-MRI followed by RARP from January 2016 to December 2017. ECE was assessed by an expert uro-pathologist. One expert reader, blinded from histological response, manually drawn LLCmax and estimated capsular bulging on T2 sequence. $LLC_{max}$≥20mm, $LLC_{max}$≥15mm and capsular bulging was chosen as predictors of ECE. We used chi-square-test (CSQ) and ROC curve for statistical analysis followed by calculation of diagnostic-test values.
**Results:** Among 162 patients, ECE was observed in 97. We evaluated ROC curve for $LCC_{max}$ which showed AUC of 0.8026 (p<0.0001) and best accurancy (ACC) for 19.3mm. Both $LCC_{max}$≥20mm and $LCC_{max}$≥15mm had significative correlation with ECE (p<0.001 at CSQ). $LCC_{max}$≥20mm had specificity (SP) of 0.77 (50/65) and positive predictive value (PPV) of 0.82 (71/86); $LCC_{max}$≥15mm had sensitivity (SN) of 0.89 (87/97) and negative predictive value (NPV) of 0.79 (39/49). Capsular bulging is associeted with ECE (p<0.001 at CSQ) and had SN of 0.86 (84/97) and PPV of 0.79 (84/106).
**Conclusion:** According to our preliminary evaluation both $LCC_{max}$ and capsular bulging at preoperative mp-MRI seems to play a role in predicting ECE.

**B-0825** 14:32
**Multiparametric MRI of the bladder: interobserver agreement and accuracy with the vesical imaging-reporting and data system (VIRADS) at a single reference centre**
G. Barchetti, R. Campa, M. Pecoraro, I. Ceravolo, C. Catalano, V. Panebianco; *Rome/IT (giovanni.barchetti2@gmail.com)*

**Purpose:** To evaluate the accuracy and interobserver variability of the Vesical Imaging-Reporting and Data System (VI-RADS) for discriminating between non-muscle-invasive bladder cancer (NMIBC) and muscle-invasive bladder cancer (MIBC), in patients undergoing multiparametric MRI (mpMRI) before transurethral resection of bladder tumour (TURBT).
**Methods and Materials:** 75 patients referred to our institution for bladder cancer underwent mpMRI before TURBT. All mpMRI were reviewed by two radiologists, blinded to clinical history and histopathology. Correlation between mpMRI diagnosis and histopathology was estimated using the Kendall T. Sensitivity, specificity, positive predictive value (PPV) and negative predictive value (NPV) were calculated for both readers, considering scenario 1 (VI-RADS 3 classified as MIBC) and scenario 2 (VI-RADS 3 classified as NMIBC). The K statistics was used to estimate inter-reader agreement.
**Results:** 53 patients were diagnosed with NMIBC and 22 with MIBC. The correlation analysis yielded the following results for the overall VI-RADS score: T= 0,54 for reader 1 and T= 0,51 for reader 2. Both readers obtained a better sensitivity (91% and 82% vs 82% and 64%) and NPV (96% and 92% vs 92% and 86%) in scenario 1. A lower specificity (91% and 87% vs 96% and 94%) and PPV (80% and 72% vs 89% and 82%) were obtained in scenario 1. Area under curve was 0,918 for reader 1 and 0,897 for reader 2. Inter-reader agreement was good for the overall VI-RADS score (K= 0,731).
**Conclusion:** In conclusion, mpMRI with the use of VI-RADS is accurate in differentiating MIBC from NMIBC.

**B-0827** 14:40
**Magnetic resonance imaging findings differentiate among periurethral bulking agent, urethral diverticulum and periurethral cyst, preventing misdiagnosis**
T. Chulroek[1], D. Wangcharoenrung[1], K. Cattapan[2], K. Hamed[3], A.J. Mitchell[3], M.G. Harisinghani[3], E.J.B. De[3]; [1]*Bangkok/TH*, [2]*Songkhla/TH*, [3]*Boston, MA/US (thitinan.c@chula.ac.th)*

**Purpose:** To evaluate magnetic resonance imaging findings to differentiate periurethral bulking agent, urethral diverticulum and periurethral cyst.
**Methods and Materials:** In this retrospective study, we searched radiologic department database. A total of 50 patients with periurethral cystic lesions and magnetic resonance imaging between 2001-2017 were included with 68 lesions (27 bulking agents, 29 urethral diverticula and 12 periurethral cysts) were found. Two abdominal radiologists blinded to clinical history, independently evaluated T1, T2, and post-contrast images. The readers assessed number, morphological features, location, connection to urethra and mass effect, signal intensity, and enhancement for each lesion. Logistic regression analysis was performed for each univariate significant feature. Operative and pathologic reports were the reference standard.
**Results:** Magnetic resonance imaging findings in bulking agents which helped differentiate from urethral diverticulum were more cases with multiple lesions (P=0.021), upper or upper-mid urethral location (P=0.003), lack of both internal fluid/fluid level (P=0.029) and urethral connection (P=0.038), T1 isointensity, T2 mild hyperintensity compared to muscles but lower T2 signal than urine (P<0.0001). Most cases of urethral diverticula and periurethral cysts were detected at mid and lower urethra. Urethral diverticula were larger than bulking agents and periurethral cysts (P=0.005 & P=0.023) (mean diameter=24, 16, and 15mm, respectively). Most bulking agents (93%) and urethral diverticula (90%) showed mass effect upon urethra while periurethral cysts (75%) did not (P<0.0001).

**Conclusion:** Signal intensity and lesion characterisation on magnetic resonance imaging can significantly differentiate bulking agent from urethral diverticulum and periurethral cyst. These findings may help guide clinicians when considering operative intervention.

**B-0828** 14:48
**MP-MRI can exclude prostate cancer progression in patients under active surveillance**
L. Schimmöller, T. Ullrich, F. Mones, M. Quentin, R. Rabenalt, P. Albers, G. Antoch, C. Arsov; *Düsseldorf/DE*
*(lars.schimmoeller@med.uni-duesseldorf.de)*

**Purpose:** To assess the ability of multiparametric MRI (mp-MRI) of the prostate to exclude prostate cancer (PCa) progression in patients under active surveillance.
**Methods and Materials:** One hundred and forty-seven consecutive patients under active surveillance with known PCa with a Gleason score of 3+3=6 or 3+4=7a were initially enrolled and received mp-MRI (T2WI, DWI, DCE-MRI) of the prostate at 3T. Of these patients, fifty-five received follow-up MRI after a minimum interval of 12 months with subsequently targeted MR/US fusion-guided (FUS-GB) plus systematic transrectal ultrasound-guided (TRUS-GB) biopsy. Primary endpoint was negative predictive value (NPV) of the follow-up mp-MRI to exclude tumour progression. Secondary endpoints were positive predictive value (PPV), sensitivity, specificity, and cancer upgrade after initial mp-MRI.
**Results:** Of 55 patients 28 (51%) had a Gleason score upgrade in the re-biopsy. All of the 28 patients showed findings in the follow-up mp-MRI that were suspicious of tumour progress. 16 of 55 patients (29%) showed signs of tumour progress in the follow-up MRI but had a stable re-biopsy. 11 of 55 patients (20%) showed no signs of progress in follow-up MRI and none of these patients had a Gleason score upgrade in the re-biopsy. NPV was 100%. PPV was 64%. Sensitivity was 100% and specificity 59%.
**Conclusion:** mp-MRI can reliably exclude PCa progression in patients under active surveillance. Over 60% of the patients with signs of tumour progress in follow-up mp-MRI had a Gleason score upgrade in repeat biopsy.

**B-0829** 14:56
**PSAD and ADC values improve the diagnostic accuracy of PI-RADS v2**
A.C. Westphalen, F. Fazel, H. Nguyen, M. Cabarrus, K. Hanley-Knutson, K. Shinohara, P.R. Carroll; *San Francisco, CA/US*
*(antonio.westphalen@ucsf.edu)*

**Purpose:** To determine if PSAD and ADC values improve the accuracy of PI-RADS v2 and identify patients likely to have high-grade cancer on areas without mpMRI visible lesions.
**Methods and Materials:** This is a single-reference-centre, cross-sectional, retrospective study of consecutive men with suspected or known low- to intermediate-risk prostate cancer who underwent 3T mpMRI and TRUS-MRI fusion biopsy from 07/15/2014 to 02/17/2018. Cluster-corrected logistic regression analyses were utilized to predict high-grade prostate cancer (Gleason score ≥ 3+4) at targeted mpMRI lesions and on systematic biopsy.
**Results:** 538 men (median age=66 years, median PSA=7.0 ng/ml) with 780 mpMRI lesions were included. High-grade disease was diagnosed in 371 men. PI-RADS v2 scores of 3, 4, and 5 were high-grade cancer in 8.0% (16/201), 22.8% (90/395), and 59.2% (109/184). ADC values, PSAD, and PI-RADS v2 scores were independent predictors of high-grade cancer in targeted lesions (OR 2.25-8.78; P values < 0.05; AUROC 0.84, 95% CI 0.81-0.87). Increases in PSAD were also associated with upgrade on systematic biopsy (OR 2.39-2.48; P values < 0.05; AUROC 0.69, 95% CI 0.64-0.73). Study limitations include its retrospective single-institution design and use of biopsy rather than prostatectomy specimens of standard of reference.
**Conclusion:** ADC values and PSAD improve characterization of PI-RADS v2 score 4 or 5 lesions. Upgrade on systematic biopsy is slightly more likely with PSAD ≥ 0.15 and multiple small PI-RADS v2 score 3 or 4 lesions.
*Author Disclosures:*
**A.C. Westphalen:** Advisory Board; 3D Biopsy LLC. Research/Grant Support; GE Healthcare. **K. Shinohara:** Advisory Board; 3D Biopsy LLC.

**B-0830** 15:04
**Dynamic contrast-enhanced MRI perspectives in the assessment of aggressiveness of urinary bladder cancer**
R.T. Abouelkheir, M.E. Abou El-Ghar, T. El-Diasty; *El-Mansoura/EG*
*(dr_rasha_taha@hotmail.com)*

**Purpose:** To investigate role of dynamic contrast-enhanced MRI in non-invasive assessment of staging and aggressiveness of bladder cancer correlating with tumour microvessel density measurement as indicator of angiogenesis.
**Methods and Materials:** 81 consecutive patients were referred from our outpatient clinic of Urology and Nephrology Center, Mansoura University, with suspected diagnosis of bladder cancer. Morphologic evaluation, dynamic MRI staging and analysis of "Time- Signal Intensity curves" were done, compared with histopathological staging. Tumour micro-vessel density was identified using CD34 immunoreactivity. Kappa statistics, Spearman's correlation test and ROC curves were used for statistical analysis.
**Results:** High positive correlation was found between dynamic MRI staging and histopathological staging (r = 0.965, p = 0.001), also between the type of time-SI curve and pathological grading (r = 0.727, p = 0.001). High positive correlation was found between the tumour micro-vessel density and pathological staging (r = 0.606, p = 0.001), also between the micro-vessel density and tumour grading (r = 0.773, p = 0.001). Among the 81 cases, 71 cases were pathologically proven as malignant and 10 cases were pathologically proven as cystitis. Dynamic MRI with "Time-SI curve" succeeded in differentiating malignant bladder tumours from cystitis. 58% of malignant tumours showed type III (descending) curve, 31% showed type II (plateau) and 11% showed type I (ascending) curve, depending on tumour stage, grade and angiogenesis. However, cases diagnosed as cystitis had type I curve.
**Conclusion:** Dynamic MRI can increase diagnostic accuracy of bladder tumour staging, angiogenic activity and assessment of tumour aggressiveness; it also can differentiate between malignant bladder tumours and cystitis.

**B-0831** 15:12
**Radiomic analysis of multiparametric MRI for preoperative evaluation of pathological grade in bladder cancer**
H. Wang, F. Zhang, W. Fu, J. Guan, Y. Guo; *Guangzhou/CN*
*(fzhang1105@163.com)*

**Purpose:** To develop and validate a MRI-based radiomics strategy for the preoperative estimation of grading for bladder cancer(BC).
**Methods and Materials:** A total of 100 BC patients were divided into a training set (n=70) and validation set (n=30). Radiomics features were extracted from MR images (including T2WI, DWI and ADC maps) of each patient and 5 feature subsets were constructed. The 5 feature subsets were subjected to least absolute shrinkage and selection operator (LASSO) algorithm respectively and logistic regression (LR) analysis was applied to develop 5 corresponding radiomics models (T2WI_Model, DWI_Model, ADC_Model, Joint_Model and MaxFea_Model). The diagnostic performance of the 5 models was evaluated using accuracy, sensitivity, specificity as well as AUC (area under the receiver operating characteristic curve) in the independent validation cohorts.
**Results:** The accuracy, sensitivity, specificity and AUC of the Joint_Model in both training and validation cohorts was 0.8657, 0.8433, 0.8800, 0.9150 and 0.8333, 0.7692, 0.8824, 0.9276 respectively. The AUC of the remaining four models in training and validation cohorts was 0.8300, 0.8083, 0.8983, 0.9183 and 0.8235, 0.7692, 0.8824, 0.9186 respectively, all of which were significantly lower than that of Joint_Model.
**Conclusion:** The multiparametric MRI based radiomics approach has the potential to be used as a non-invasive imaging tool for preoperative grading in BC. Multicenter validation is needed to acquire high-level evidence for its clinical application.

**16:00 - 17:30** **Sky High Stage**

### Interventional Radiology

### MY 8

### Interventional Radiology

*Moderators:*
N.N.
V. Bérczi; Budapest/HU

**B-0832** 16:00
**Perfusion MRI prediction of progrossion-free survival in patients with hepatocellular carcinoma treat with TACE**
Z. Zhu, X. Zhao, C. Zhou; *Beijing/CN*

**Purpose:** To predict the value of perfusion wieighted imaging (PWI) in progression-free survival (PFS) of hepatocellular carcinoma (HCC) after trascatheter arterial chemoembolization (TACE).
**Methods and Materials:** Ninety patients were enrolled in this study from Sep 2014 to Sep 2017. A total of 10 parameters including 4 clinical indexes: gender, age, size of lesion, tumour thrombus; and 6 functional MRI indexes: pretreatment and posttreatment volume transfer constant ($K^{trans}$), exchange rate constant ($K_{ep}$), and extravascular extracellular space volume ratio ($V_e$) were analyzed by multivariate analysis. Using independent sample t test, ANOVA, the *Kaplan-Meier* survival curve, Log-Rank test and Cox regression analysis to determinate the prognostic value of DCE-MR parameters and clinical indicators.
**Results:** Age, pretreatment $K^{trans}$ and posttreatment $K^{trans}$ were 3 indicators in the prognosis of HCC after TACE ($P_{age}$=0.027, $P_{preKtrans}$<0.001,

Thursday

$P_{postKtrans}$<0.001). Using median as cut-off value, found that age>53 yrs, pretreatment $K^{trans}$>1.165/min and posttreatment $K^{trans}$>1.077/min predicts poor prognosis of HCC. Others (gender, thrombus, size, $preK_{ep}$, $postK_{ep}$, $preV_e$and $postV_e$) showed no statistical significance.
**Conclusion:** Age, pretreatment $K^{trans}$ and posttreatment $K^{trans}$ value can predict the prognosis of TACE treatment for HCC.

**B-0833** 16:04

**Evaluation of mismatch diameter between radial versus distal radial artery in patients suitable for vascular peripheral procedures**

D. Meo, D. Falsaperla, A. Modica, M.C. Calcagno, C. Desiderio, F. Libra, P. Foti, S. Palmucci, A. Basile; *Catania/IT (alessandromodica86@gmail.com)*

**Purpose:** To describe the variability of the diameter of the radial artery (RA) measured at both levels used for percutaneous approach: proximal (whitin 2 cm) to the styloid process (pRA) and distal at the snuff box (dRA) correlating them to cardiovascular risk factors (CRF), sex, and age.
**Methods and Materials:** We enrolled 460 patients (221 female, 239 males) from July to September 2018. The diameter of left and right RA were studied with Ultrasound (US) and Color-Doppler Ultrasound (CD-US). Diameters of pRA and dRA were compared between different sex, age, and CRF(tabagism, hypertension, dislipidemia, BMI>30, diabetes); the possibility of a snuff box approach was also evaluated considering a diameter ≥ of 2mm as a cut-off for the feasibility.
**Results:** The average proximal diameter founded for the pRA were 2.73mm (SD=0.53mm) at the right arm and 2.72mm (SD=0.56mm) at the left arm. The calibre of the dRA was about 32% lower than the proximal one, with an avarage diameter of 2.07mm (SD=0.51mm) and 2.05mm (SD=0.53mm) for the left and right side respectively. Female patients showed smaller diameters than male patients, in particular at the dRA We did not found significative differences related to CFS. Among the 460 patients studied, 360 (66%) were eligible for dRA approach.
**Conclusion:** dRA approach is a relatively new technique with many advantages compared to “traditional radial access"; the evaluation of the feasibility with US and CD-US is a fast and accurate way to decide if the “snuff box approach” can be performed.

**B-0834** 16:08

**Image-guided percutaneous techniques in the treatment of aggressive vertebral haemangiomas: clinical outcomes and therapeutic strategy**

A. Ricoeur[1], J. Caudrelier[2], X. Buy[3], J. Garnon[2], G. Koch[2], R. Cazzato[2], A. Gangi[2]; [1]*Lyon/FR*, [2]*Strasbourg/FR*, [3]*Bordeaux/FR*

**Purpose:** To evaluate the safety and the clinical efficacy of a therapeutic strategy consisting in image-guided percutaneous techniques for treating symptomatic aggressive vertebral haemangiomas (SAVHs).
**Methods and Materials:** This retrospective study identified all the patients affected by SAVHs who had undergone image-guided percutaneous vertebroplasty (PVP) alone or in combination with prior percutaneous intralesional sclerosant instillation (sclerotherapy) in one institution between 1991 and 2016. Fifty-eight patients (39 females and 19 males, mean age: 50.1 years) were included. Imaging studies, pain and neurological evaluations, procedure-related data and complications were assessed. Descriptive statistics, expressed as means and proportions associated to 95 percent confidence interval and standard deviation were used to report the results.
**Results:** Thirty-eight out of 58 patients (65.5%) with back pain and no neurological symptoms had PVP as a single treatment for SAVHs limited to the vertebral body. Technical success was 100%. Clinical success was achieved in 35/38 patients (92.1%, 95% CI[78.6;98.3]) with complete pain relief at last follow-up. Twenty out of 58 patients (34.5%) suffered from back pain and neurological symptoms related to SAVHs with extraosseous extension. Fifteen of them (25.9%) underwent sclerotherapy and additional PVP. Technical success was 93.3%. The 5 remaining patients (8.6%) received combined percutaneous and surgical treatments. Clinical success was achieved in 18/20 patients (90%) with full or almost full neurological recovery and total pain relief.
**Conclusion:** Image-guided percutaneous techniques, including PVP alone, or sclerotherapy followed by PVP, are safe and efficient providing excellent results in back pain and neurological symptoms resolution for the treatment of SAVHs.

**B-0836** 16:16

**Does IV-tPA induce thrombus migration? A retrospective study comparing bridging therapy and thrombectomy alone**

C. Cohen[1], K. Gaillot[2], K. Janot[1], J. Cottier[1], M. Dejobert[1], A.-P. Narata[1]; [1]*Tours/FR*, [2]*La Rochelle/FR (clara.cohen.cc@gmail.com)*

**Purpose:** Thrombus migration (ThrMi) before mechanical thrombectomy (MT) is an epiphenomenon in acute ischaemic stroke (AIS). The aim of this study is to evaluate ThrMi prior MT in a bridging protocol (tPA-MT-group) and stand-alone MT (MT-group).
**Methods and Materials:** 205 consecutive AIS patients treated by MT (tPA and no-tPA) were retrospectively analyzed. Distance between vessel origin and beginning of the thrombus on MRI (3D TOF and/or contrast-enhanced-MRA) and digital-substracted-angiography (DSA) were measured in millimeters using a curve tool and the same anatomical parameters. DSA pixels were converted in millimetres by measuring 3 internal carotid diameters in MRI and determining the ratio from pixels to millimetres.
**Results:** In tPA-MT group(n=129), ThrMi occurred in 36.4%, and in 6.6% of MT group(n=76) (p<0.001, Student t-Test). In tPA-MT group, 27(20.9%) patients had a moderate migration(5-to-10 millimetres), 11(8.5%) patients had a distal migration (>10 millimetres or to another segment) and 9(7%) presented recanalization defined by spontaneous TICI score≥2B. In MT-group, 69 (90.8%) patients had no ThrMi, moderate ThrMi in 6.6%, thrombus' extension in 2.6%; no patient presented distal migration or recanalization. The two groups had the same clinical prognosis (bleeding at 48hours, discharge NIHSS, mRS/death at 3 months). Number of device passes to reach thrombectomy was 1.40 (±1.39) in tPA-MT-group, 1.63 (±1.09) in MT-group, p=0.061.
**Conclusion:** IV thrombolysis seems to promote thrombus migration, present in 36.4% of patients in tPA-MT-group compared to 6.6% of patients in MT-group. This study adds more data concerning IV thrombolysis effects on AIS treatment when MT is also involved.

**B-0837** 16:20

**Focal prostate cancer ablation "out of the rectum and out of MRI": new tools for fusion-guidanceand "super-activesurveillance"**

S. Xu, V. Anderson, M. Li, J. Peretti, C. Garcia, P.L. Choyke, P. Pinto, B. Turkbeyi, B.J. Wood; *Bethesda, MD/US (xus2@cc.nih.gov)*

**Purpose:** To describe novel approaches and tools for fusion guidance for focal prostate ablation outside of the MRI gantry.
**Methods and Materials:** Describe the approach, criteria, workflow, software, and hardware enabling MRI TRUS fusion guided prostate focal laser ablation outside of the MRI gantry, towards an office-based setting. A clinical trial is outlined for approaching focal laser ablation of prostate cancer without requiring MRI. Patients undergo diagnostic MRI only enabling fusion biopsy. Hardware and software tools to enable or facilitate focal laser thermal ablation of prostate cancer targets are identified. Hydrodissection, thermocouples, ultrasound monitoring, and composite ablation planning were implemented in patients. Other tools were deployed in phantoms prior to clinical trial deployment.
**Results:** Early clinical results demonstrate MRI-TRUS fusion system can guide ablation of focal prostate cancer outside of the MRI gantry. Custom software allows for composite laser ablation of targeted tumours via a transperineal approach with or without electromagnetic (EM)tracking. Treatment planning software successfully identifies tissue at risk for under-treatment. Future clinical tools were demonstrated in a custom pelvic phantom, such as augmented reality, an EM-tracked Foley bladder catheter for correction of mis-registration, and a transperineal ultrasound fusion enabling no transrectal imaging or interventions.
**Conclusion:** Novel tools and approaches are demonstrated for fusion guided focal ablation of prostate cancer out of the MRI gantry and out of the rectum. This includes fusion ablation in an ultrasound-guided office-like setting, augmented reality for transperineal interventions, EM-tracked Foley catheter, and transperineal prostate interventions fully outside of the rectum.
*Author Disclosures:*
**S. Xu:** Employee; NIH US Government. Patent Holder; NIH and Philips In Vivo Inc. **V. Anderson:** Employee; NIH. **P.L. Choyke:** Employee; NIH. Equipment Support Recipient; NIH and Philips In Vivo. Patent Holder; NIH and Philips In Vivo. Research/Grant Support; NIH and Philips In Vivo. **P. Pinto:** Employee; NIH. Patent Holder; NIH and Philips In Vivo Inc. Research/Grant Support; Philips In Vivo Inc. **B. Turkbeyi:** Employee; NIH. Patent Holder; NIH and Philips In Vivo Inc. Research/Grant Support; NIH and Philips In Vivo Inc. **B.J. Wood:** Employee; NIH US Government. Equipment Support Recipient; Philips In Vivo Inc. Grant Recipient; NIH. Patent Holder; Patent holder and Royalties for Fusion Biopsy. Research/Grant Support; Intramural Research Program of NIH and Philips In Vivo Inc. Other; Cooperative Research and Development Agreement with Philips In Vivo Inc.

**B-0838** 16:24

**Safety and outcomes of transarterial chemoembolization using small- versus medium-sized drug-eluting beads for unresectable hepatocellular carcinoma**

A.M.K. Abdel Aal, K. Mahmoud, H. El Khudari, N. Aboueldahab, S. Saddekni, A. Gunn; *Birmingham, AL/US (akamel@uabmc.edu)*

**Purpose:** The purpose of this study is to compare the safety and efficacy of using very small size (75um) drug-eluting beads (VS-DEB) with medium-size (100-300µm) drug-eluting beads (M-DEB) in transarterial chemoembolisation (TACE) for the treatment of unresectable hepatocellular carcinoma (HCC).

**Methods and Materials:** We retrospectively reviewed the medical records of patients with HCC between September 2010 and June 2018 who underwent TACE using VS-DEB (n=109) versus M-DEB (n=82). Imaging follow-up was done 4-6 weeks after TACE. Tumour response was measured by m-RECIST criteria. Toxicity was measured according to CTCAE v4.0. Overall and progression-free survival were calculated using Kaplan-Meier curves.
**Results:** The study included 191 patients with mean age of 63.4 (SD=8.6) years. The most common aetiology of cirrhosis was hepatitis C virus (58.1%). There was no difference in the demographics or the BCLC stage between both groups. In BCLC A and B patients, complete response, objective response and disease control was higher in the VS-DEB group (26.8%, 71.8% and 91.6%, respectively) compared to the M-DEB group (3.9% [p=0.001], 45.1% [p=0.003] and 78.4% [p=0.039]). Overall survival in BCLC A and B patients was higher in the VS-DEB group compared to M-DEB (p=0.05). There was no difference in the progression-free survival between both groups. There was no significant difference in grade 3 and 4 toxicity between the two groups.
**Conclusion:** In BCLC A and B patients, the use of VS-DEB offers superior radiologic response and survival advantage compared to M-DEB, without significant increase in toxicity.
*Author Disclosures:*
**A.M.K. Abdel Aal:** Consultant; boston scientific. Research/Grant Support; boston scientific.

**B-0839** 16:28
**MRI-CT fusion imaging before high-selective lumbar sympathicolysis**
B. Reichardt[1], A. Örnek[1], V. Nicolas[2], C. Maier[2]; [1]*Essen/DE*, [2]*Bochum/DE (benjamin.reichardt@uk-essen.de)*

**Purpose:** To reduce side effects and the amount of alcohol applied, highly selective sympathicolysis of individual ganglia is desirable. The sympathetic trunk and individual ganglia are not always visible in CT imaging. We expected from an MRI/CT image fusion before intervention a possibility to identify single ganglia and to lyse them highly selectively.
**Methods and Materials:** 14 patients received a native lumbar MRI (DWIBS, Shinkei, T1, STIR and PDWfs) before sympathicolysis. After successful detection of a single ganglion from the side to be treated, one day later a pre-interventional CT spiral of the lumbar spine in prone position was performed. The CT and the STIR or PDW fs sequence from the MRI data set were transferred to fusion software prior to puncture. After successful matching, highly selective sympathicolysis was performed with 1.5ml 95% alcohol.
**Results:** All interventions were successful and pain reduction was achieved. Apart from dysesthesia of the genitofemoral cutaneous nerve, no side effects occurred. The automated MRT/CT image fusion worked in 12/14 patients, 2 data sets had to be readjusted manually.
**Conclusion:** MRI/CT image fusion before sympathicolysis allows a highly selective intervention at the single sympathetic ganglion sympathetic ganglion, thus reducing the amount of alcohol to be administered and avoiding unwanted side effects. The technique is equally useful for other interventional treatments in which a CT alone is not sufficient.

**B-0840** 16:32
**Lumbar sympathetic trunk in MRI and CT: its visibility and distance to two anatomical landmarks for pre-interventional planning**
B. Reichardt[1], A. Örnek[1], V. Nicolas[2], C. Maier[2]; [1]*Essen/DE*, [2]*Bochum/DE (benjamin.reichardt@uk-essen.de)*

**Purpose:** The lumbar sympathetic trunk's (LST) distance to two anatomical landmarks, the costal process and medial margin of the psoas muscle, was assessed due to its use as landmarks for lumbar sympathetic blocks: the costal process for fluoroscopic guided techniques and the psoas major for CT- and MRI-guided techniques. Based on the measurements, we evaluate the trunk's visibility in MR and CT images for accurate needle positioning.
**Methods and Materials:** Retrospective analysis of 42 CT and MRI dataset before sympathicolysis. LST's distances to the psoas major's medial margin and to the base of the lumbar vertebrae's costal process were measured on the levels L2/3, L3/4 and L4/5. CT protocol: Th12 to S1 (100 kV, 60mAs, 1mm slice thickness). MRI protocol: T1, T2, PDW/STIR, 1mm 3D-DWIBS or 3D-Shinkei with MIP for neurography.
**Results:** LST's mean distance to the psoas major was 0.4 mm at L2/3, 3 mm at L3/4 and 4.6 mm at L4/5. The mean distance to the costal process was 32 mm at L2/3, 34 mm at L3/4 and 33 mm at L4/5. In MR and CT imaging, a structure could be determined as the LST correlating to the measurements with decreasing possible identification from cephalad to caudad levels.
**Conclusion:** The costal process is a usable landmark for fluoroscopic guidance and the psoas major for CT- and MRI-guided techniques. The LST is clearly visible in MR and CT images, which gives both techniques a decisive advantage over fluoroscopy concerning interventions at the LST, due to a visible target.

**B-0841** 16:36
**Bilateral carotid stenting in the same sitting in high-risk patients: a single-centre study**
L.S.P. Karanam, S.R. Baddam, A. Alurkar, V. Pamidimukkala, R. Polavarapu; *Guntur/IN (drklsp@gmail.com)*

**Purpose:** To determine the role of simultaneous carotid artery stenting in high-risk patients with triple-vessel coronary artery disease, and the safety and efficacy of the procedure in these cases.
**Methods and Materials:** The present study is a retrospective analysis of 56 patients, who underwent carotid artery stenting in the same setting in our institution from 2009 to 2018. There were 39 males and 17 females with a mean age of 65 years (53-76 years). Inclusion criteria of the present study were patients with bilateral internal carotid artery stenosis >50% (50-99%) in the symptomatic side and >60% in the asymptomatic side as seen on digital subtraction angiography.
**Results:** Technical success was 100% in our series. Intraprocedural and postprocedural events in the form of hypotension and bradycardia were seen in 23 patients. This was treated with intraprocedural atropine and postprocedural dopamine (5-15 µg/kg/min) or noradrenaline (2-4 µg/min) infusion. One patient suffered from hyperperfusion bleed. 48 patients underwent cardiac bypass surgery after three weeks. No deaths or myocardial infractions occurred either in the postprocedural period (up to 1 month) or on clinical follow-up of 3 and 6 months .
**Conclusion:** Bilateral carotid artery stenting in the same sitting is a safe treatment option even in patients with high-risk factors and can be considered as a safe treatment option in patients with significant bilateral carotid artery disease.

**B-0842** 16:40
**Pneumothorax and pulmonary haemorrhage after CT-guided lung biopsy: incidence, clinical significance and correlation**
V. Sabatino, U. Russo, F.V. d'Amuri, A. Bevilacqua, F. Pagnini, G. Milanese, R. Nizzoli, G. Pedrazzi, M. De Filippo; *Parma/IT (vittorio.sabatino@gmail.com)*

**Purpose:** To evaluate incidence and clinical significance of pneumothorax (PTX) and pulmonary haemorrhage (PH) after CT-guided lung biopsy (CT-LB). To test correlations of PTX and chest tube insertion (CTI) with PH and different imaging and procedural parameters.
**Methods and Materials:** Pre-procedural CT and CT-LB scans of 904 patients were retrieved. Incidence of PTX and of PH (location: type 1 along needle track or type 2 perilesional; severity according to its thickness: low grade <6mm or high grade >6mm) were recorded. CTI was assessed by reviewing medical charts. PTX was considered clinically significant if treated with CTI, PH if treated with endoscopic/endovascular procedure. Evaluated parameters included: nodule-to-pleura distance (mm), emphysema (subjectively quantified in ≤25%, 25-50%, ≥50%) and procedure time (minutes). Binary logistic regression analyses tested whether PH and imaging and procedure parameters were associated with development of PTX and CTI.
**Results:** PTX occurred in 306/904 cases (33.8%); CTI was requested in 18/306 (5.9%). PH occurred in 296/904 cases (32.7%), of which 165/296 (55.7%) type-1 high-graded; no case requested treatment. Type-1 high-graded PH showed a protective effect against PTX and CTI ($OR_{PTX}$=0.340; $OR_{CTI}$=0.177). Nodule-to-pleura distance ($OR_{PTX}$=1.046; $OR_{CTI}$=1.057), emphysema ($OR_{PTX}$=1.330; $OR_{CTI}$=2.074) and procedure time ($OR_{PTX}$=1.018; $OR_{CTI}$=1.037) were associated with an increased risk for PTX and CTI.
**Conclusion:** Pneumothorax and pulmonary haemorrhage have similar incidence after CT-guided lung biopsy. Treatment was required only for pneumothorax (<6% of cases) while pulmonary haemorrhage was not clinically significant. Pulmonary haemorrhage along needle track >6mm may represent a protective factor against development of pneumothorax and against pneumothorax requesting chest tube insertion.

**B-0843** 16:44
**MR imaging-based response evaluation of two different transarterial chemoembolisation protocols using degradable starch micorspheres in therapy of hepatocellular carcinoma**
M.C. Langenbach, M.H. Albrecht, R. Hammerstingl, T. Gruber-Rouh, T.J. Vogl; *Frankfurt am Main/DE (marcel.langenbach@me.com)*

**Purpose:** Therapy response evaluation of transarterial chemoembolisation [TACE] in hepatocellular carcinoma [HCC] comparing two different embolisation protocols, either with Lipiodol or with an additional use of degradable starch microspheres [DSM], based on ADC findings.
**Methods and Materials:** Sixty-one patients (44 men; 17 women; mean age 71 years; range 44-85) with HCC were evaluated in this prospective randomised trial. Treatment protocol included three TACE sessions in 4-week intervals with multiparametric MRI prior and 4 weeks after the last TACE (endpoint). In group 1 (n=30), TACE was performed using Lipiodol only. In group 2 (n=31), Lipiodol was combined with DSM. Differences in tumour volume, diameter, RECIST 1.1 criteria, ADC and survival were analysed and compared using Mann-Whitney U test, Kruskal-Wallis H test and Spearman-Rho.
**Results:** Overall, a decrease in tumour volume and diameter was strongly significant correlated to an increase in ADC values (volume: rho=-0.397, p<0.001; diameter: rho=-0.585, p<0.001) with no differences between the embolisation protocols (p=0.489). Average tumour volume and diameter reduction were comparable between both groups (volume p=0.653, diameter p=0.678). Further, response evaluation according RECIST 1.1 showed no differences (p=0.112). Estimated overall survival was in mean 32.5 months (95% CI: 26.6-38.4), in group 1 29.4 months (95% CI: 21.7-37.1) and in group 2 33.4 months (95% CI: 25.5-41.4) (p=0.844), respectively.
**Conclusion:** An increase of ADC values during TACE is strongly correlates with the local tumour response and thus, may be used as a therapy monitoring factor. Furthermore, additional DSM application showed no significant differences in diameter und volume reduction compared to standard TACE.

**B-0844** 16:48
**The anatomic options of vessels and haemodynamic redistribution of the blood flow at selective intra-arterial chemotherapy (SIAC) in children with an intraocular retinoblastoma**
I. Pogrebnyakov, I. Trofimov, A.V. Kukushkin, E. Virshke, B. Dolgushin; *Moscow/RU (i.pogr@mail.ru)*

**Purpose:** Describing the methodology of a SOAI in organ-preserving treatment of children with an intraocular retinoblastoma and demonstrating the various ways of alkylating agent delivery to a tumour.
**Methods and Materials:** 316 SIAC procedures to 110 children (129 eyes) have been performed from 2011 to 2017. 2 methods of SOAI were applied: 1) the microcatheter technique - superselective catheterization of an eye artery or collateral branches of an ECA at blood flow haemodynamic redistribution; 2) the micro-balloon technique.
**Results:** Technical success was 95.8% (303 procedures). 245 procedures with the use of a microcatheter infusion was carried out in: a. ophthalmica - 196 (80%), a. meningea media - 27 (11%), a. infraorbitalis - 20 (8.2%), a. temp. superficialis - 1 (0.4%), and a. facialis - 1 (0.4%). From 61 procedures with using of micro-balloon 58 were successful. We did not manage to put a balloon more distally than the place of an entry of an eye artery in 3 cases. Unsuccessful attempts - 13 cases: failure of catheterization of a femoral artery - in 3, a kinking/coiling of the ICA - in 3, a vascular collapse as a result of reaction to contrast agent and/or mechanical impact on ICA - in 3, lack of contrasting of a retina - in 3, an occlusion of an ICA - in 1.
**Conclusion:** Use of various techniques for drug delivery to an eye tumour allows to achieve the maximum effect and does not depend on anatomy options and blood flow haemodynamic redistribution in the main vessels of an eye.

**B-0845** 16:52
**The role of selective intraaterial and intravitreal chemotherapy in organ-preserving treatment of the children with an intraocular retinoblastoma**
I. Pogrebnyakov, B. Dolgushin, I. Trofimov, A.V. Kukushkin, E. Virshke; *Moscow/RU (i.pogr@mail.ru)*

**Purpose:** Improving of organ-preserving treatment results of the children with an intraocular retinoblastoma (IRB) with local selective intraarterial (IAC) and intravitreal (IViC) chemotherapy methods.
**Methods and Materials:** 110 children (129 eyes) with a one - and bilateral retinoblastoma were included in the study. All patients/eyes were divided into 2 groups. The 1st group included 99 children/116 eyes who received SIAC as a combined treatment if their previous treatment was not effective enough.The 2nd group included treatment-naïve 11 children/13 eyes with unilateral and bilateral Rbs who received SIAC as the first means of treatment.
**Results:** In the first group, 95 of 116 eyes were saved. The globe salvage by Kaplan-Meier was reached in 94.5% in 1 year, 88.5% in 2 years, 86.5% in 3 years, 82.9% in 4 years and 75.0% from 4th to 6th years. Patients were followed up to 30.3±16.81 month, and 23.74 ±12.45 months was the relapse-free period. In the second group, 12 of 13 eyes were retained. The globe salvage by Kaplan-Meier was reached in 92.3% in 1 and 2 years. Patients were followed up to 13.5±5.3 months, and 23.74 ±12.45 months was the relapse-free period.
**Conclusion:** Local chemotherapy is an effective and perspective organ-preserving method. It can be used as a complex therapy part of advanced and refractory IRB forms and as a monotherapy of the primary identified IRB.

**B-0846** 16:56
**Intermediate-stage HCC, SIRT and balloon-occlusive micro-catheter: the right way?**
A. Paladini[1], G.E. Vallati[2], G. Pizzi[2], F. Cappelli[2], D. Beomonte Zobel[2], E.M. Amodeo[2], A. Borzelli[3]; [1]*Novara/IT*, [2]*Rome/IT*, [3]*Naples/IT (andreapaladini1988@gmail.com)*

**Purpose:** The use of selective internal radiation therapy (SIRT) with 90-Yttrium resin microspheres ($Y^{90}$-ms) for hepatocellular carcinoma (HCC) is increasing. The use of Occlusafe microcatheter (Terumo, Japan) as new balloon antireflux microcatheter for $Y^{90}$-ms infusion is a promising innovative technique potentially improving dose distribution within tumour while sparing normal liver.We evaluated the benefits of using Occlusafe microcatheter in terms of dose distribution as predictor of patients' outcome.
**Methods and Materials:** A prospective enrollment of 10 patients with unresectable HCC who underwent planning pre-SIRT phase was performed. Volumes of the normal liver and tumour were contoured using a software based on pre- and post-treatment CT and SPECT/CT images. Two radiologists performed a qualitative volumetric evaluation in a blinded and randomized fashion using mRECIST parameters. Quantitative analysis of activity distribution based on pre- and post-treatment imaging and dose gradient was performed using a home-made tool for tumour/non-tumour (T/NT) evaluation.
**Results:** Higher T/NT ratio was observed on pre- and post-SIRT images thanks to the use of Occlusafe microcatheter compared with our historical cohort. In particular, the agreement of dose-distribution pre- and post-SIRT was observed in 100% patients, higher than 80% previously reported by our group. The evaluation of the two radiologists, using mRECIST parameters, showed a high dose-outcome relationship.
**Conclusion:** Volumetric and activity distribution measurements of superselective angiography during pre- and post-treatment confirm Occlusafe microcatheter as an improved strategy for increase T-NT ratio and dose distribution prediction in SIRT procedure thus increasing the predictive capability of treatment planning.

**B-0847** 17:00
**Robot-assisted percutaneous placement of K-Wires during minimal-invasive interventions of the spine**
M.H. Albrecht[1], Y. Croissant[1], C. Czerny[1], T.J. Vogl[1], S. Zangos[2]; [1]*Frankfurt a. Main/DE*, [2]*Göppingen/DE (MoritzAlbrecht@gmx.net)*

**Purpose:** To assess the accuracy and time requirements of image-guided percutaneous K-wire insertion in the spine using an advanced robot assistance device for needle guidance and to demonstrate a radiation-free workflow for the physician.
**Methods and Materials:** A planning CT-scan was acquired of a cadaver specimen and analyzed using a 3D-interventional software integrated in the robotic device. The optimal needle path was simulated and the needle holder of the robot was used for guidance during K-wire insertion. Twenty-four K-wires were inserted percutaneously in a transpedicular approach in the following vertebrae: thoracic (T) 2, 7-12 and lumbar (L) 1-5. A post-procedural CT scan was performed to analyse the accuracy of the K-wire insertion.
**Results:** All procedures were carried out without any perforation of the pedicle wall. The mean duration of planning the intervention path was 2:54±2:22min, mean positioning time was 2:04±0:42min and the mean time for K-wire insertion was 2:13±0:54min. In total, the average intervention time was 7:10±3:06min per pedicle. Compared to the planning, the K-wire position showed a mean deviation of 0.5mm in the vertical-axis and 1.2mm in the horizontal-axis. The average intervention path length was 8.1cm.
**Conclusion:** Our findings show a high accuracy in robot-assisted K-wire insertion during spinal interventions without any exposure of the operator to radiation.
*Author Disclosures:*
**M.H. Albrecht:** Speaker; Received speaker fees from Siemens and Bracco.

**B-0848** 17:04
**Predictive $^{99m}$Tc MAA-SPECT/CT dosimetry in patients with locally advanced inoperable hepatocellular carcinoma treated by selective internal radiotherapy with $^{90}$Y resin microspheres**
A.-L. Hermann[1], A. Dieudonné[2], M. Ronot[2], M. Sanchez[2], H. Pereira[1], G. Chatellier[1], E. Garin[3], R. Lebtahi[2], V. Vilgrain[2]; [1]*Paris/FR*, [2]*Clichy/FR*, [3]*Rennes/FR (Annelaure.hermann@gmail.com)*

**Purpose:** To predict survival and tumour response in patients with locally advanced and inoperable hepatocellular carcinoma (HCC) treated by SIRT (yttrium-90 [$^{90}$Y] resin microspheres) using tumour-absorbed-dose calculated prior to therapy.
**Methods and Materials:** Among 184 patients from the SARAH trial who received SIRT, 121 and 109 were included in dose/survival and dose/tumour response analyses, respectively; CT, $^{99m}$Tc-MAA-SPECT/CT and $^{90}$Y SPECT/PET were centralized. Tumour-absorbed-dose was computed using

Thursday

$^{99m}$Tc-MAA-SPECT/CT. Visual agreement between CT-MAA-$^{90}$Y (optimal, suboptimal, not optimal), overall survival (OS) and tumour response on 6-months follow-up CT (RECIST 1.1) were assessed.
**Results:** In the dose/survival population, median OS was 9.3 months (95%CI, 6.7 to 10.7) and median tumour-absorbed-dose was 112Gy (IQR, 67.8 to 220). Patients who received ≥100Gy (n=67) had significantly longer survival than patients who received <100Gy (median 14.1 months; 95%CI, 9.6 to 18.6 *v* 6.1 months 95%CI, 4.9 to 6.8, respectively; *P*< .0001). Among them, those with optimal agreement (n=24) had the longest median OS (24.9 months; 95%CI, 9.6 to 33.9). In the dose/tumour response population, tumour-absorbed-dose was significantly higher in treatments leading to DC *v* no-DC (median 121Gy; IQR, 86.0 to 190 *v* 85.1Gy; IQR, 58.4 to 164, respectively, *P*= .0204), while it was not significant with OR *v* no-OR (*P*= .20). The highest DC rate (77.5%) was observed with tumour-absorbed-dose ≥100Gy and optimal agreement (m=40).
**Conclusion:** Tumour-absorbed-dose computed on $^{99m}$Tc-MAA-SPECT/CT is significantly associated with OS and DC; the most benefit is observed with both tumour-absorbed-dose ≥ 100Gy and optimal visual CT-MAA-$^{90}$Y agreement.

**B-0849** 17:08
**Trans arterial embolization of non-variceal upper gastrointestinal bleeding: is the use of ethylene-vinyl alcohol Copolymer as safe as Coils?**
M. Tipaldi[1], M. Krokidis[2], G. Orgera[1], A. Rebonato[3], C. Ambrogi[1], M. Rossi[1]; [1]*Rome/IT*, [2]*Cambridge/UK*, [3]*Perugia/IT (tipaldi.andrea@gmail.com)*

**Purpose:** The safety of liquid embolics over the conventional coils for the treatment of non-variceal upper gastrointestinal bleeding (UGIB) approach is still unclear. Purpose of this study is to assess the safety of ethylene- vinyl alcohol copolymer (EVOH 6%) over coils in the treatment of UGIB.
**Methods and Materials:** All the upper gastrointestinal tract embolization procedures performed in a single center in a 6-year period were reviewed. Patients embolised with coils (Group A) versus those embolised with EVOH 6% (Group B) were compared. Technical/clinical success, bleeding recurrence, complication and mortality rates were analyzed.
**Results:** A total 71 patients were included in the study (41 Group A and 30 Group B). Coagulopathy was present in 21% of Group A and 46% of Group B patients (p<0.05). Technical and clinical success was 97.6 and 92.7% for Group A, and 100 and 93.3% for Group B respectively, (p>0.05). Ten patients (17% Group A; 10% Group B) rebled within the first 72h and all of them were re-treated successfully with a second embolization. In Group A one major complication (bowel ischaemia) occurred. No complication occurred in Group B. The survival rate in the first 30 days was 90.3% for group A and 90% for group B (p>0.05).
**Conclusion:** This study demonstrated EVOH 6% appears to be as safe and effective as coils in the treatment of non variceal UGIB.

**B-0850** 17:12
**Prospective monocentre pilot study of intrahepatic mitomycin C infusion after radioembolisation with Y-90 in chemo refractory liver dominant metastatic breast cancer patients**
B.M. Aarts[1], E.G. Klompenhouwer[1], R.C. Dresen[2], C. Deroose[2], A. Laenen[2], R.G.H. Beets-Tan[1], P. Neven[2], H. Wildiers[2], G. Maleux[2]; [1]*Amsterdam/NL*, [2]*Leuven/BE (b.aarts@nki.nl)*

**Purpose:** In this pilot study we evaluated the safety of intrahepatic Mitomycin C (MMC) infusion after radioembolization with Yttrium (Y90)-labelled microspheres in chemo resistant LMBC patients.
**Methods and Materials:** Sixteen LMBC patients were included in this pilot study from 2012-2018 and first received Y90. The response after Y90 was evaluated with MRI, PET and laboratory tests. After assessment of no progression of disease, Mitomycin C infusion was administered in different dose cohorts; A: 6mg in 1 cycle, B: 12 mg in 2 cycles, C: 24 mg in 2 cycles and D: max 72 mg in 6 cycles. In cohort D the response was again evaluated after every 2 cycles and continued after assessment of no progression of disease. Toxicities were measured according to common toxicity criteria adverse events (CTCae) version 5.0.
**Results:** Sixteen patients received Y90 treatment. Three patients showed disease progression and 1 patient had serious side effects of Y90, and consequently, were excluded for further intra-arterial therapy. The intended dose of MMC was adjusted in 5 out of the 12 patients due to progressive disease (n=3) and biochemical toxicity (n=2). No grade 3 toxicity levels or higher were reported after MMC infusion. Three grade 2 toxicities were reported consisting of thrombocytopenia (n=1), leukopenia (n=1) and increase in bilirubin levels (n=1).
**Conclusion:** The combined treatment of intrahepatic infusion of MMC after Y90 therapy is safe with a low toxicity profile when MMC is administered in different escalating dose cohorts and adjusted based on clinical, radiological and biochemical parameters.

**B-0851** 17:16
**In the ablation of colorectal cancer liver metastases with a new generation MW system nodule size >2 cm independently predicts worse local tumour progression free survival**
P. Marra[1], A. Della Corte[2], F. Ratti[1], F. Cipriani[1], S. Gusmini[1], M. Salvioni[3], M. Venturini[1], L. Aldrighetti[1], F. De Cobelli[1]; [1]*Milan/IT*, [2]*Vimodrone/IT*, [3]*Bergamo/IT (dellacorte.angelo@hsr.it)*

**Purpose:** In the ablation of colorectal cancer liver metastases (CRLM) high-power microwave generators present theoretical advantages in terms of local tumor control. We aimed to evaluate the outcome of CRLM microwave ablation (MWA) with a new generation system, assessing potential predictors of local tumour progression free survival (LTPFS).
**Methods and Materials:** From November 2014 to June 2018, 31 patients underwent 34 percutaneous, laparoscopic or open MWA procedures for the treatment of 51 CRLM with a 2450MHz/100W generator. Contrast-enhanced CT/MRI were used to assess 1-month complete ablation and LTP at follow up.
**Results:** Technical success and complication rates were respectively 96% and 4.9%. At the time of the analysis 11/51(21,6%) CRLM had locally progressed and overall estimated 1- and 2-year LTPFS rates for all 51 ablated CRLM were 84% and 63%, respectively. At univariable analysis CRLM diameter ≥2cm was the strongest predictor of worse LTPFS with 1-year LTPFS 64% vs 85% (of <2cm nodules), p=0. In a multivariable Cox regression model with CRLM number, RAS mutation, timing of CRLM development, history of post-MWA chemotherapy, number of pre-MWA chemotherapy cycles and time of chemotherapy discontinuation before ablation, nodule proximity to large vessels, energy administration, and side of primary colon cancer, CRLM diameter ≥2cm independently predicted worse LTPFS (HR:28.9, p=0.009).
**Conclusion:** Our preliminary data show that the outcomes of MWA for CRLM with a new generation system are comparable with those reported in the literature and nodule size, with a threshold of 2 cm, represents the strongest predictor of worse LTPFS.

### Friday, March 1

**08:30 - 10:00** **Sky High Stage**

### Musculoskeletal

### MY 9

### Musculoskeletal

*Moderators:*
M. Adriaensen; Heerlen/NL
C. Martinoli; Genoa/IT

### B-0852 08:30

**Perivertebral muscle as index of sarcopenia: comparison with previous muscle imaging and functional indexes**

H.S. Seo; *Ansan/KR (seohs@korea.ac.kr)*

**Purpose:** The area of perivertebral muscle can be easily achieved on abdominal and visceral fat CT scans. The purpose of this study is whether perivertebral muscle can be an index of sarcopenia by comparison with widely used muscle imaging and functional indexes.
**Methods and Materials:** Total 1270 seniors (mean age=63.57±6.94 years (range: 53-83), M:F=69:661) were included. The area of perivertebral muscle was the area of psoas and erector spinae muscles measured at the level of L3-4 on visceral fat CT between -30 and 150 HU. The area of thigh muscle in both sides was measured on CT. Appendicular muscle mass(ASM) was achieved in the mass of the extremities without the bone on DEXA. Right and left hand powers, standard and rapid walking speeds were also measured. The Pearson's correlation was used in statistics.
**Results:** The area of perivertebral muscle was well correlated with the area of thigh muscle(r=0.83, p<.001) and ASM(r=0.83, p<.001), and modified ASM, such as ASM/Ht$^2$(r=0.729, p<.001) and ASM/BMI(r=0.729, p<.001). The area of perivertebral muscle was well correlated with right and left hand powers(r=0.738 and 0.749, p<.001, respectively) and standard and rapid walking speeds (r=0.268 and 0.280, p<.001, respectively).
**Conclusion:** The area of perivertebral muscle on visceral fat CT was well correlated with widely used sarcopenia indexes from muscle imagings and functions. The perivertebral muscle can be a new sarcopenia index and visceral fat CT can supply the information about sarcopenia as well as visceral fat as the risk factor of metabolic syndrome.

### B-0853 08:34

**T2* value analysis of achilles tendons disease with ultra-short echotime (UTE) MRI**

J.-G. Zhang, Z. Li, C. Xia; *Chengdu/CN (zjgscuhx@126.com)*

**Purpose:** To compare T2* value of healthy subjects and patients with achilles tendons (AT) disease by using ultra-short echotime (UTE) MRI, and to analyze the correlation between T2* value and clinical scores.
**Methods and Materials:** 23 patients with AT disease (patients group) and 18 healthy subjects (control group) were enrolled and underwent MR scan with UTE sequence. The T2* values of 3 ROIs in AT were measured on each patient. Independent sample t-test was used to compare the difference of T2* values between the two groups. The correlation of T2* values with the American Orthopaedic Foot and Ankle Society ankle hindfoot scale (AO-FAS) and achilles tendon total rupture score (ATRS) were analyzed.
**Results:** The T2* values were significantly higher in patients group than those in control group (all P<0.05). And there was strong negative correlation between T2* values and AO-FAS or ATRS scores (r=-0.88, P<0.01 and r=-0.91, P<0.01).
**Conclusion:** The T2* values can reflect the degree of oedema in AT, and have strong negative correlation with clinical scores. As an emerging imaging technique, UTE-MRI can not only provide anatomical morphology of AT but also assess AT disease quantitatively, which is helpful to improve the diagnosis of AT disease in early stage.

### B-0854 08:38

**Terahertz time-domain spectroscopy of collagen I and tendon**

J. Yuan[1], L. Yang[2], M. Wang[1], Z. Feng[1], S. Zhang[1]; [1]*Shanghai/CN*, [2]*Zhengzhou/CN (happyyuanjie@163.com)*

**Purpose:** This research studied the spectral characteristic of collagen I and tendon in terahertz (THz) range by the terahertz time-domain (THz-TDS) spectroscopy (THz-TDS).
**Methods and Materials:** Collagen I powder from bovine flexor tendon was obtained from Sigma-Aldrich with purity ≥99%. About 250mg of powder was compressed into a tablet 13mm in diameter. And then we mix collagen with polyethylene (PE) which is transparent in THz frequency region with a vortex mixer. The mixing ratios are 1:4, 2:3, 3:2, and 4:1. The absorption spectra of collagen pellets are measured in the frequency range of 10-100 cm1 using the THz-TDS setup. Bovine tendon was fresh and cut approximately to 1 mm. The tendon dried at room temperature.
**Results:** The vibrational absorption spectrum was calculated based on the semi-empirical theory in the range of 0.1-10THz. The calculated spectrum of collagen I exhibits one absorption peak. The absorption band is 1.16 THz. The absorption bands of tendon are 1.16 THz and 1.43 THz.
**Conclusion:** We demonstrated the use of THz-TDS for measuring absorption spectrum in collagen I and tissues rich in collagen I.

### B-0855 08:42

**Investigating the appropriateness of radiological investigation of lumbar spine and determining relationship between appropriateness and imaging findings in a tertiary hospital**

S. Suganchand Rikabchand; *Jodhpur/IN (Sunilmed@gmail.com)*

**Purpose:** The utilization of radiological investigations is increasing with increase in evidence based medicine and recording of pathology. Investigations though important are sometimes not required as they don't change clinical outcome of disease and thus unnecessary but are still ordered inappropriately. This study aims at studying the appropriateness of radiological investigations ordered in a tertiary hospital.
**Methods and Materials:** We studied all the radiological investigations (Plain radiography, CT and MRI) of lumbar spine during the period January to June 2017. A total of 2416 investigations were ordered, of these 854 were inpatient and 1562 were outpatient. To assess the appropriateness of the investigations ACR Appropriateness Criteria was used.
**Results:** Of 2416 radiological investigations ordered, 1509 were plain radiograph, 749 were MRI and 158 were CT scans. Based on the ACR appropriateness criteria 76% of the radiographs, 54% of MRI and 72% of CT were appropriate. The most common indication for radiograph and MRI was chronic low back pain with radiculopathy. Of the appropriate radiographs (n=1147) ordered 88% had a pathological finding, MRI's (n=405) ordered 78% had a pathological finding and CT's (n=114) ordered 85% had a pathological finding. The most common indication for CT was spine trauma. A Chi Square test correlation of ACR appropriateness criteria and positive finding in a radioloigcal investigation was found to be significant (p<0.03).
**Conclusion:** Based on the results most of the plain radiograph and CT scans ordered were appropriate but the MRI study of the spine was the most misused modality of the all.

### B-0856 08:46

**Identification of Scheuermann's disease with different radiological scoring methods for osteoporotic vertebral fractures: the Rotterdam study**

L. Oei, F. Koromani, S. Breda, S. El Saddy, A. Makurthou, F. Rivadeneira, E. Oei; *Rotterdam/NL*

**Purpose:** Osteoporotic vertebral fractures are a major health issue and should be diagnosed accurately. An important differential diagnosis is Scheuermann's disease. Our aim was to evaluate how different radiological assessment methods perform on independently ascertained cases of radiological Scheuermann's disease.
**Methods and Materials:** Two independent groups of trained research assistants applied the algorithm-based qualitative (ABQ) or software-assisted quantitative morphometry (QM using SpineAnalyzer® software to measure vertebral height ratios, shape and severity of vertebral deformities classified according to Genant). These methods for osteoporotic vertebral deformities were compared on cases of radiological Scheuermann's disease, which were diagnosed by a third group applying the radiological criteria by Sørensen and Sachs. In addition, thoracic spine indexes according to Masharawi were calculated from the raw QM data. Complete data were available for 2,656 lateral spine radiographs from the population-based Rotterdam Study (43% men, age 45-89), an ongoing Dutch prospective cohort study.
**Results:** Radiographic Scheuermann's disease was present in 4.1% (95% CI: 3.3%-4.9%; N=109) of the sample. Of these participants identified with radiographic Scheuermann's disease, QM scored 65.1% (95% CI: 56.2%-74.1%; N=71) as having an osteoporotic vertebral deformity, while ABQ classified only 7.3% (95% CI: 2.4%-12.2%; N=8) of the participants with radiographic Scheuermann's disease as having osteoporotic vertebral fractures. The QM-based Masharawi method was unable to discern between osteoporotic vertebral fractures, Scheuermann's disease and controls.
**Conclusion:** The inability of QM to rule out non-fracture deformities explains in part the higher number of osteoporotic vertebral deformities misclassified when applying QM as scoring method compared to ABQ.

**B-0857** 08:50

**Feasibility study of reducing artifacts of spine metal implants for patients at 3.0T magnetic resonance imaging**

G. Feifei[1], Y. Ge[1], Y. Wei[2]; [1]*Zhengzhou/CN*, [2]*Chengdu/CN (gaofei150@163.com)*

**Purpose:** To investigate the feasibility of reducing artefacts of spine with removal metal artefacts sequence (WARP) at 3.0T MRI.
**Methods and Materials:** 29 patients after spine surgery with mental implants which including 15 cervical and 14 lumbar spine cases were prospectively included. The image quality of WARP sequences and conventional sequences were compared (5 score evaluation scale) as well as the signal to noise ratio (SNR) and contrast noise ratio (CNR) of the image artefacts. The scanning time was recorded. Paired-t test and Mann-Whitney test were used respectively to compare the SNR and CNR, and qualitative scoring between the two sequences. P <0.05 was considered to indicate a significant difference.
**Results:** The image distortion and blur of the WARP sequences were obviously reduced compared with the conventional sequences. The SNR and CNR of the WARP sequences' artefacts were lower than that of the conventional sequences (All P <0.05). The image quality scores of WARP sequences in cervical and lumbar spines[4(3 ~ 5) and 4(3 ~ 5)]were higher than that of conventional sequences'[3(2 ~ 4) and 3(2 ~ 4)] (*P* <0.05). The scanning time of cervical spines in WARP sequence(14 min 9 s)was increased by 64 s(8.2%),and the time of lumbar spines(13 min 41 s)decreased by 9 s(1.1%).
**Conclusion:** The WARP sequences at 3.0 T could effectively reduce the artefacts of metallic prosthesis without prolonging the scanning time at 3.0T MRI.

**B-0858** 08:54

**Noise-optimised virtual monoenergetic imaging of dual-energy CT: effect on metal artefact reduction in patients with lumbar internal fixation**

Y. Zeng[1], K. Deng[2], H. Yang[2], Y. Tan[2], J. Liu[2], D. Geng[1], J. Zhang[1]; [1]*Shanghai/CN*, [2]*Changsha/CN (18211220088@fudan.edu.cn)*

**Purpose:** The purpose of this study was to evaluate the effects of noise-optimised virtual monoenergetic imaging (VMI+) reconstructions on reducing metal artefacts compared to traditional virtual monoenergetic imaging (VMI) and linearly blended (M_0.6) reconstructions in patients with lumbar metal internal fixation in dual-energy CT (DECT).
**Methods and Materials:** Forty patients who underwent DECT were evaluated in this retrospective study. Images were reconstructed with M_0.6 as well as VMI+ and VMI at 10keV intervals from 40keV to 190keV. Attenuation and noise were measured in the hyperdense artefacts, the hypodense artefacts, the spinal canal, the abdominal aorta (AA), and the inferior vena cava (IVC). An artefact index (AI) was calculated. A subjective evaluation was conducted of the metal-bone interface, the surrounding soft tissue, the spinal canal, the AA, and the IVC.
**Results:** The AI values for the hypodense artefacts, the spinal canal, and the IVC were lowest in the 130keV VMI+ series, for the hyperdense artefacts in the 120keV VMI+ series, and for the AA in the 190keV VMI+ series. With the exception of the hypodense artefacts, AI values were lower compared to the M_0.6 images and all of the VMI series. The subjective image quality was greatest at 130keV VMI+ for the metal-bone interface, the surrounding soft tissue, the AA, and the IVC, as well as at 120keV VMI+ for the spinal canal. With the exception of the AA, these rating scores were higher compared to the M_0.6 images and the entire VMI series.
**Conclusion:** DECT with high-keV VMI+ efficiently reduce metal artefacts and show superior image quality in patients with lumbar internal fixation.

**B-0859** 08:58

**Increased lateral meniscal slope is associated with greater risk of lateral bone contusion in noncontact ACL injury**

J. Li, K. Li; *Chongqing/CN (tianwei19900911@163.com)*

**Purpose:** To investigate the effect of an increased lateral meniscal slope (LMS) as measured on magnetic resonance imaging (MRI) on the likelihood of bone contusions in noncontact ACL injury.
**Methods and Materials:** From January 2013 to March 2018, a retrospective review of all ACL reconstruction surgeries was performed. 56 patients were included as study group (ACL+bone contusion group), who were matched to 56 control participants (isolated ACL group). Presence of bone contusion was determined from preoperative magnetic resonance images (MRIs). The LMS, LPTS, MMS and MPTS were measured on the MRI in a blinded fashion. The predictors of lateral bone contusions (LFC, LTP or both) including age, sex, BMI, LMS, LPTS, MMS and MPTS were examined by multivariable logistic regression. Associated findings including concomitant meniscal lesions and intra-articular cartilage damages were also calculated by multivariable logistic regression.
**Results:** The mean LMS in the study group was 6.51°±3.52, which was significantly larger than that in the control group (3.82°±2.70; *P*<0.01). In addition, increased LMS was significantly (*P*<0.01) associated with lateral bone contusions (lateral femoral condyle and lateral tibial plateau) in noncontact ACL injury. However, LPTS, MPTS and MMS were not significantly associated with bone contusions. Moreover, the presence of lateral contusions are associated with concomitant lateral meniscal tears and cartilage damages (*P*<0.01).
**Conclusion:** An increased LMS is associated with increased risk of lateral bone contusions in noncontact ACL injury. LMS could be considered by orthopaedic surgeons as part of the preoperative evaluation of ACL injury.
*Author Disclosures:*
**J. Li:** Author; Ke Li.

**B-0860** 09:02

**Knee MR using a body coil is equivalent to CT in measuring the TTTG distance: removing the systematic bias**

L.U. Aivazoglou, M.K. Toma, P.H.C. Arruda, O.A. Ezzeddine, A. Ormond, M. Nico, F. Silva; *Sao Paulo/BR (lais.epm@gmail.com)*

**Purpose:** To compare measurements related to patellar instability between MR and CT in asymptomatic volunteers, and to evaluate interobserver agreement between MR measurements.
**Methods and Materials:** 17 asymptomatic volunteers were enrolled. A low - dose CT and an MR T1w axial sequence of both knees were performed in full extension, the latter using a body coil. After a training session, two musculoskeletal radiologists evaluated the CT and MR images separately and chose the specific slices to assess the following landmarks: cartilaginous trochlear groove (CTG), bony trochlear groove (TG), center of insertional patellar tendon (PT) and centre of tibial tuberosity (TT). The chosen slices were compared and disagreements were solved in consensus. Then, the chosen slices were superimposed and the following measurements were assessed independently: PT-CTG and TT-TG. Statistical analysis (SPSS 20.0) consisted of intraclass correlation coefficient (ICC) to compare MR and CT measurements and interobserver agreement between MR measurements.
**Results:** Good agreement was observed between CT and MR measurements (p< 0.0001). The mean PT-CTG distance on CT and MR were 17.3±4.2 mm and 16.5±4.1 mm, respectively. When considering TT-TG distance (bony parameters), the mean values were respectively 17.1±4.2 mm and 16.2±3.7 mm. Interrater reliability was excellent for all measurements and for all slice choices on both imaging methods.
**Conclusion:** TTTG measures in MR using a body coil showed excellent reproducibility when compared to CT using both soft-tissue and bony parameters, removing the bias of knee flexion when using the dedicated knee coil.

**B-0861** 09:06

**Quantitative magnetic resonance imaging in patellar tendon-lateral femoral condyle friction syndrome: relationship with subtle patellofemoral instability**

J. Li, H.-T. Yang; *Chongqing/CN (tianwei19900911@163.com)*

**Purpose:** To investigate the correlation of patellar tendon-lateral femoral condyle friction syndrome (PTLFCFS) with subtle patellofemoral instability to explore its pathogenesis.
**Methods and Materials:** One hundred knees of 80 patients with PTLFCFS were analysed retrospectively by retrieving magnetic resonance (MR) imaging data over a 3-year period from our database. Seven quantitative parameters for evaluating patellofemoral stability were measured on MR images, including the Insall-Salvati ratio, tibial tuberosity-trochlear groove (TT-TG) distance, trochlear groove depth, medial trochlear/lateral trochlear length (MT/LT) ratio, medial trochlear/lateral trochlear height (MH/LH) ratio, lateral patellofemoral angle (LPA) & lateral trochlear inclination (LTI) angle. These patellofemoral parameters were compared between the PTLFCFS group & the normal control group (n = 88), & receiving operator characteristic (ROC) curve analysis was conducted to determine the specificity & sensitivity of these parameters.
**Results:** The trochlear depth, MT/LT, LPA, & LTI angle were significantly lower (*P* < 0.001) & the Insall-Salvati ratio was significantly higher (*P* < 0.001) in the PTLFCFS group. However, the TT-TG distance & MH/LH ratio had no significant difference (*P* = 0.231 & 0.073, respectively). The area under ROC curve of the Insall-Salvati ratio, trochlear depth, MT/LT, LPA, & LTI angle were 0.925, 0.784, 0.8, 0.731, & 0.675, respectively. The efficiency of the Insall-Salvati ratio was the highest among those 5 parameters.
**Conclusion:** This study verified the presence of subtle patellofemoral instability by measuring various patellofemoral parameters in patients with PTLFCFS. It confirmed that PTLFCFS is associated with subtle patellofemoral instability & could substantially explain the pathogenesis of PTLFCFS.
*Author Disclosures:*
**J. Li:** Author; Haitao yang.

**B-0862** 09:10

**Clinical utility of fat suppressed 3-dimensional CAIPIRINHA SPACE MRI of the knee in adults**

S. Lee, G.Y. Lee, S. Kim; *Seoul/KR (lish1220@gmail.com)*

**Purpose:** This study was purposed to evaluate the clinical utility of 3-dimensional CAIPIRINHA SPACE MRI of knee compared to 2D conventional method in the symptomatic patients.
**Methods and Materials:** Following internal review board approval and informed consent, in 3 months we enrolled 93 symptomatic patients (37 males and 56 females) who underwent knee MRI with both 2D conventional and 3D CAIPIRINHA methods. Among them, 17 patients had arthroscopic surgery after MRI study. Two radiologists evaluated pathologic MRI findings by scoring systems in each anatomic region. We measured signal to noise ratio (SNR) in 3D CAIPIRINHA MRI. Using intraclass correlation coefficient (ICC), inter-reader agreement, intra-reader agreement and inter-method agreement were applied. In 17 patients who underwent arthroscopy, we evaluated diagnostic performance of both two methods.
**Results:** The 3D CAIPIRINHA MRI had good to excellent inter-reader agreement (ICC, 0.73 - 0.96) and intra-reader agreement (ICC, 0.88-1.0). Inter-method agreement between the 2D conventional and 3D CAIPIRINHA images was also good to excellent (ICC, 0.8-1.0). The diagnostic performance of 3D CAIPIRINHA MRI was equal or better to the one of 2D conventional MRI. The SNR of 3D CAIPIRINHA MRI showed mean, 14.51 and standard deviation, 5.17. Mean scan time of 3D CAIPIRINHA MRI was 4 minutes 43 seconds and the one of 2D conventional method was 17 minutes 27 seconds.
**Conclusion:** In the real practice, the 3D CAIPIRINHA MRI could have equal or better performance compared to 2D conventional MRI, to evaluate pathologic findings of symptomatic patients with faster scan time.

**B-0863** 09:14

**The most accurate measurement of TT-TG distance is through the 2/5 point of femoral trochlear notch**

Y. Qi, Z. Wang; *Tianjin/CN (qywjdyt@126.com)*

**Purpose:** To find a point at femoral trochlear notch to accurately measure tibial tuberosity-trochlear groove distance (TT-TG), and correlate TT-TG with the ratio of lengths of patella ligament and patellar (PLL/PL)and lengths of medial and lateral patellar retinacula ( MPR and LPR), using CT scan and image fusion.
**Methods and Materials:** 60 volunteers (average age, 31.87±7.28y; range,18-40y; men:women, 42:18) underwent CT (GE Discovery CT750) of knee joints, 30 left and 30 right knees, respectively, voltage: 100-120kV, current: 180-230mAs. 5 points were divided equally at femoral trochlear notch on reformatted median sagittal plane. TT-TG was measured on corresponding axial fused images, the distances between the vertical lines of prominent tibial tuberosity and each of the five points at femoral trochlear notch. ANOVA and Mann-Whitney U tests were used to analyse data. PLL/PL was measured on median sagittal image which was the ratio of length of patellar ligament and maximum distance of patella. The lengths of MPR and LPR were measured on axial images of the maximum patella transverse diameters. The correlation between each of 5 TT-TG and PLL/PL and the lengths of MPR and LPR were calculated using Spearman rank correlation tests.
**Results:** The most accurate measurement of TT-TG was the vertical line through 2/5 point of femoral trochlear notch (F=0.668, p=0.615). TT-TG between gender and sides showed no difference (P=0.116, 0.456). There was no correlation of five TT-TG with PLL/PL and the lengths of MPR and LPR (p=0.541-0.909, p=0.068~0.770).
**Conclusion:** The most accurate measurement of TT-TG is the vertical line through 2/5 point of femoral trochlear notch on axial CT fused images, and the distance is irrelevant to PLL/PL and the lengths of MPR and LPR.

**B-0864** 09:18

**Coll-Ha versus bone marrow stimulation for chondral and osteochondral lesion: a 2-year randomized controlled trial**

C. Tamburrano[1], M. Busacca[2]; [1]*bologna/IT*, [2]*Bologna/IT (chiara.tamburrano@libero.it)*

**Purpose:** To assess the benefit provided by a collagen-hydroxyapatite (coll-HA) multilayer scaffold for the treatment of chondral and osteochondral knee lesions.
**Methods and Materials:** The coll-HA scaffold was compared to bone marrow stimulation (BMS) in a multicenter randomized controlled trial: 100 patients affected by symptomatic grade III-IV lesions were evaluated for up to 2 years (51 study group, 49 control group). Primary efficacy measurement was IKDCsubj score at 2 years; secondary were: KOOS, IKDC, Tegner and VASpain scores at 6, 12 and 24 months. Tissue regeneration was evaluated with MRI Mocart score at 6, 12 and 24 months. An external independent agency ensured data correctness and objectiveness.
**Results:** A significant improvement of all clinical scores was obtained in both groups, although no overall significant differences were detected between treatments. The subgroup of patients affected by deep osteochondral lesions (*i.e.* Outerbridge IV and OCD) showed a significantly better IKDCsubj outcome (p=0.036) in the coll-HA group. Severe adverse events were documented in 3 patients in the coll-HA group and in 1 in the BMS group. The Mocart score showed no statistical difference between groups.
**Conclusion:** This biomimetic implant showed no benefit compared to BMS for chondral lesions, but this procedure can be considered safe and a suitable option for the treatment of osteochondral lesions.

**B-0865** 09:22

**Application of simultaneous multi-slice TSE in high-resolution hand and foot imaging**

G. Feifei[1], Y. Ge[1], Y. Wei[2]; [1]*Zhengzhou/CN*, [2]*Chengdu/CN (gaofei150@163.com)*

**Purpose:** To investigate the feasibility of simultaneous multi-slice (SMS) technique in hand and foot imaging by comparing it with the routine TSE sequence and iPAT in regards to imaging time and images quality
**Methods and Materials:** 40 patients underwent MR exams on a MAGNETOM Prisma 3T MR scanner. A sixteen-channel foot/ankle coil and a sixteen-channel hand/wrist coil were used. The MR exam included routine TSE, TSE with iPAT (iPAT-TSE), and a prototype SMS-TSE sequence. The apparent signal-to-noise ratio (SNR) and contrast-to-noise ratio (CNR) were calculated. Image quality was rated by radiologist blindly based on a five-point evaluation. The SNR and CNR were statistically analyzed using LSD-t test. Image quality scores were tested for statistical significance by using Games-Howell test.
**Results:** Compared with the Routine-TSE and iPAT-TSE sequences, the SMS-TSE sequence could greatly shorten the scan time to 55% of the standard TSE. For hand and foot MR imaging, the SNR,CNR of the SMS-TSE sequence are close to the Routine-TSE and slightly higher than the iPAT sequence(p<0.05). No significant difference was observed between the SMS-TSE and Routine-TSE sequence for the SNR. For the hand MR imaging, the image quality scores of the SMS-TSE and Routine-TSE sequences were significantly higher than that of the iPAT sequence(Hand: p=0.005, SMS vs iPAT; p=0.001, Routine vs iPAT; p=0.536, Routine vs SMS).
**Conclusion:** The SMS-TSE sequence can be used to obtain high quality images for the hand and foot at 3T with 45% less acquisition time than the standard TSE sequence.

**B-0866** 09:26

**Cinematic MRI of the wrist: initial study for instability of the wrist in correlation to fluoroscopy and x-ray findings**

B. Reichardt[1], A. Örnek[1], V. Nicolas[2]; [1]*Essen/DE*, [2]*Bochum/DE (benjamin.reichardt@uk-essen.de)*

**Purpose:** Initial study on scapholunary ligamentary instability and ulnar impactiation syndromes in MRI compared to radiography and fluoroscopy. The aim of this study was to develop an MRI protocol for the evaluation of the wrist during continuous active movement to show that dynamic imaging of the wrist is feasible and to show that the resulting anatomical images allow the measurement of metrics commonly used for the dynamic instability of the wrist.
**Methods and Materials:** 10 probands and 10 patients with wrist-pain over the SL-slit or ulna-impaction signs received an initial x-ray in 2 planes as well as instability images with ulnardeviation and "clenched-fist" of both wrists. Additionally fluoroscopy at 7.5f/s with same movements were done. MR-Protocol: 32CHh head coil, standard static MRI were done. Additionally adapted cardiac cine Sequences: single and multislice bSSFP for active MRI with radial/ulnar deviation and "clenched-fist" maneuvers. Evaluation of distal radioulnar joint (DRUJ) congruity, the scapholunate (SL) interval.
**Results:** SL-Slit widening was 4mm (healthy), 9 mm (patients) in ulnar-deviation, 4mm in clenching-fist, 8 mm in patients; correlating to findings in fluoroscopy. DRUJ subluxation ratio were 0.04 (supination), 0.10 (neutral), and 0.14 (pronation) and 0.15, 0.25 and 0.16 in patients. SL interval were 1.43mm (healthy) and 2 (patients) for neutral, ulnar deviation, radial deviation positions, and increased to 1.64 mm (healthy) during the clenched-fist maneuver and 3.1mm in patients.
**Conclusion:** This study demonstrates the initial performance of active-MRI of scapho-lunate instability and ulnar-impaction-syndrome which may be useful in the investigation of dynamic wrist instability.

**B-0867** 09:30

**Comparison of patient satisfaction for wrist examinations in cone beam-computed tomography and multidetector-computed tomography**

J. Neubauer[1], C. Reidelbach[1], E. Kotter[1], S.M. Goerke[2]; [1]*Freiburg/DE*, [2]*Offenburg/DE (jakob.neubauer@uniklinik-freiburg.de)*

**Purpose:** To evaluate and compare patient satisfaction for wrist examinations in cone beam-computed tomography (CBCT) and multidetector-computed tomography (MDCT). Our hypothesis is that patient satisfaction is higher in the CBCT.

**Methods and Materials:** This IRB approved prospective study included patients who were scheduled for a computed tomography examination of the wrist in our institution over a period of 1 year. Patients were randomly assigned to either dedicated musculoskeletal CBCT or MDCT. After the examination, patients were asked to complete a questionnaire that included several questions regarding their satisfaction with the examination. Patients were asked to evaluate on a 6-point Likert scale whether the positioning was comfortable, whether they found the duration of the exam adequate, whether they were able to keep their hands steady during the exam, whether they were in more pain during the exam, and if they were getting intimidated through the scanner. Ratings for the CBCT and MDCT were compared with Mann-Whitney U test. P values <0.05 were considered to denote statistical significance.
**Results:** 108 patients returned the questionnaire, composed of 53 CBCT and 55 MDCT examinations. The patients rated positioning in the CBCT as more comfortable than in the MDCT (<0.001). For the ratings on all other questions, there was no difference between the two modalities.
**Conclusion:** Positioning in the CBCT was perceived as more comfortable than in the MDCT. All other measures of patient satisfaction were rated similarly for both devices.

**B-0868** 09:34
**Diagnostic performance of high-resolution ultrasound in the evaluation of intrinsic and extrinsic ligaments of the wrist in patients with previous trauma or carpal instability**
S. Gitto[1], D. Albano[2], C. Messina[1], V. Chianca[1], S. Rapisarda[1], A. Corazza[3], L.M. Sconfienza[1]; [1]*Milan/IT*, [2]*Palermo/IT*, [3]*Genoa/IT (sal.gitto@gmail.com)*

**Purpose:** To investigate the role of ultrasound (US) in the evaluation of intrinsic and extrinsic carpal ligaments having magnetic resonance arthrography (MRA) as reference standard.
**Methods and Materials:** This prospective study included 19 patients (12M, 7F; mean age 44±19 SD years) referred for wrist MRA and having history of trauma or carpal instability. US examination was performed just before MRA using a 14-6 MHz linear transducer. On both the dorsal and volar sides of the wrist, the intrinsic interosseus and midcarpal, collateral and extrinsic ligaments were evaluated. Ligament thickness was measured and tears were detected. Thereafter, ligament thickness and integrity were blindly assessed on the MRA images obtained with a 1.5-T unit. Ligament detection rate and thickness reproducibility between US and MRA were calculated.
**Results:** On the dorsal side, US detected more ligaments (108/114, 94.7%) than MRA (96/114, 84.2%; P=0.016), while on the volar side the difference was not significant (149/171, 87.1% vs. 156/171, 91.2%, respectively; P=0.296). Ligament thickness reproducibility ranged between 44% (COR=0.9, bias=-0.8, P<0.001) of the volar ulnocapitate ligament and 71% (COR=0.05, bias=-0.1, P<0.001) of the volar scaphotriquetral ligament. Diagnostic performance of US for ligaments where a tear was found was 100% sensitivity, 100% specificity, 100% positive predictive value, 100% negative predictive value, 100% accuracy for the volar and dorsal scapholunate ligaments, and the ulnar collateral ligament; it was 100%, 94%, 50%, 100%, 94%, respectively, for the volar ulnolunate ligament.
**Conclusion:** US has similar diagnostic performance to MRA in the assessment of intrinsic and extrinsic carpal ligaments and ligamentous tears.

**B-0869** 09:38
**Submarine sign of epidermoid cysts: a diagnostic model based on ultrasound feature**
D.H. Lee, S. Kim, A.L. Choi, C. Yoon; *Seoul/KR (edahyunle@gmail.com)*

**Purpose:** To develop a diagnostic model for superficial soft tissue mass with differentiation of epidermoid cyst (EC) based on ultrasound (US) features including a ‘submarine sign’.
**Methods and Materials:** In this retrospective study, patients (n=210) who undertook US for superficial soft tissue mass and surgical excision were included from January 2008 to October 2017. Included patients were split into a derivation (n=114) and validation cohort (n=96) by date of imaging (November 1, 2016). Following US features were analyzed : more than half depth involvement of dermal layer; ‘submarine sign’ (focal protrusion toward the epidermis); posterior acoustic enhancement; posterior wall enhancement; shape, margin, echogenicity, morphological types, vascularity and perilesional fat change. Multiple logistic regression was performed to develop a diagnostic model and constructed it as a nomogram. Then, we assessed the performance of this diagnostic model by discriminatory ability (AUC) and calibration through derivation cohort and validation cohort.
**Results:** More than half depth involvement of dermal layer (OR=2.092; 95% CI: p-value=0.047), submarine sign (OR=7.077; 95% CI: p-value<0.001) and posterior acoustic enhancement (OR=4.251; 95% CI: p-value=0.013) were significantly associated with ECs. The diagnostic model statistically constructed with these features showed good discrimination and calibration ability on both derivation (AUC = 0.831, 95% CI: 0.758-0.904 and Hosmer-Leme show goodness-of fit test, p-value=0.8153) and validation cohort (AUC = 0.865, 95% CI: 0.795-0.933 and Hosmer-Leme show goodness-of fit test, p-value=0.1816).
**Conclusion:** Our diagnostic model and nomogram can be utilized to predict the probability of EC. Also, submarine sign may be helpful image marker for EC.

**B-0870** 09:42
**Machine learning classification of low-grade and high-grade chondrosarcomas based on magnetic resonance imaging-based texture analysis**
S. Gitto[1], D. Albano[2], V. Chianca[1], R. Cuocolo[3], L. Ugga[4], C. Messina[1], L.M. Sconfienza[1]; [1]*Milan/IT*, [2]*Palermo/IT*, [3]*Naples/IT*, [4]*Scafati/IT (sal.gitto@gmail.com)*

**Purpose:** Reliable grading of bony chondrosarcomas is crucial for the clinical outcome, because treatment ranges from intralesional curettage for low-grade neoplasms (G1) to resection or amputation for high-grade tumours (G2-G3). We aim to evaluate the diagnostic accuracy of machine learning (ML) algorithms for discrimination between low-grade and high-grade chondrosarcomas based on texture analysis (TA) parameters extracted from unenhanced Magnetic Resonance Imaging (MRI).
**Methods and Materials:** We retrospectively enrolled 58 patients with histologically proven chondrosarcoma (26 low-grade and 32 high-grade tumours). Patients were randomly divided into training (n=42) and test (n=16) groups for classification model development and testing, respectively. All tumours were manually segmented on T1-weighted and T2-weighted images by drawing a bidimensional polygonal region of interest (ROI). ROIs were used for first order and texture feature extraction on dedicated software, Pyradiomics. For each tumour, different data subsets were obtained by six feature selection methods (C4.5 learner-based, gain ratio evaluator, information gain evaluator, Principal Component Analysis and subset evaluator) and analyzed using 9 ML classification algorithms, evaluating their accuracy for differentiation of low-grade from high-grade chondrosarcoma.
**Results:** A Support Vector Machine correctly classified 75% of chondrosarcomas as low or high grade based on the datasets obtained by the subset evaluator feature selection mode. Specifically, true positive rate was 80% and 67% for identification of high-grade and low-grade tumours, respectively.
**Conclusion:** ML algorithms showed good accuracy for low-grade and high-grade tumour classification based on unenhanced MRI examinations and could prove a valuable aid in preoperative characterization of chondrosarcomas.

**B-0871** 09:46
**Use of MRI for age verification of U-17 footballers: the Ghana study**
B.D. Sarkodie; *Accra/GH (ghana_neo@yahoo.com)*

**Purpose:** A fair playground is absolutely necessary in any age limited sports. Age determination in countries where birth registration is not compulsory can often be difficult making it a challenge to determine ages of people born in such countries. The purpose of this study was to determine the correlation between the chronological ages of under 17 Ghanaian footballers and the FIFA MRI grading.
**Methods and Materials:** The degree of radial epiphyseal fusion was evaluated in 486 male Ghanaian footballers aged 13-16 years over a 4 year period (June 2012-July 2018) using 1.5 T Magnetic Resonance Imaging. The ages of the participants used in the study were those provided by the football players and confirmed with their national passports.
**Results:** Over 49% of these Ghanaian players below the age of 17 years had completely fused radial epiphysis. No significant correlation between the given chronological ages and the degree of fusion was found. The Spearmans correlation was given as (r = 0.068; p = 0.542).
**Conclusion:** There was no correlation between chronological age and degree of radial fusion among Ghanaian players. Normative study among Ghanaian/black African players is long overdue to ensure the U-17 players from these countries are not unfairly disadvantaged.

**10:30 - 12:00** **Room B**

### Genitourinary

### SS 1007

### Prostate biopsy and intervention

*Moderators:*
J. Belfield; Liverpool/UK
L. Schimmöller; Düsseldorf/DE

**K-20** 10:30
**Keynote lecture**
B. Gebauer; *Berlin/DE*

**B-0872** 10:39
**Detection of prostate cancer: comparison between robotic-assisted "in-bore" MRI-guided biopsies and cognitive transrectal ultrasound-guided biopsies**
E. Demozzi[1], F. Cavicchioli[1], M. Hoogenboom[2], M. Catania[3], G. Foti[1], E. Munari[1], S. Cavalleri[1], G. Carbognin[1]; [1]*Negrar/IT*, [2]*Arnhem/NL*, [3]*Verona/IT* *(e.demozzi@gmail.com)*

**Purpose:** To compare prostate cancer detection rate (PCaDR) and clinically significant prostate cancer detection rate (csPCaDR) between magnetic resonance imaging-guided biopsies (MRI-PB) and cognitive transrectal ultrasound-guided prostate biopsies (TRUS-PB).
**Methods and Materials:** For this prospective study, approved by our IRB, between November 2017 and September 2018, 167 men with clinical suspicion for PCa underwent mpMRI and, when suspicious lesion were found (PI-RADS 4 or 5, according to the PIRADSv2 scoring system), MRI-PB (arm A, 86 Patients, 67.6 y, PSA 9.1 ng/mL, PSAd 0.19) or TRUS-PB (arm B, 82 patients, 69.4 y, PSA 11.6 ng/mL, PSAd 0.23) was performed. Clinically significant prostate cancer (csPCa) is defined as Gleason score =or> 3+4.
**Results:** Prostate cancer was diagnosed among 70 patients in arm A (PCaDR 81.4%) and 51 patients in arm B (PCaDR 62.9%; p=0.006). csPCa was diagnosed among 46 patients in arm A (csPCaDR 53.4%) and 15 patients in arm B (csPCaDR 18.5%; p<0.001). Among PI-RADS 4, PcaDR and csPCa was 76.2 (46/59) and 38.9% (23/59) in arm A and 60.0 (36/60) and 16.7% (10/60) in arm B (respectively, p=0.034 and p=0.006). Among PI-RADS 5, PcaDR and csPCa was 92.5 (25/27) and 85.1% (23/27) in arm A and 71.4 (15/21) and 23.8% (5/21) in arm B (respectively, p=0.051 and p=0.001).
**Conclusion:** Robotic-assisted MRI-targeted biopsy can easily be implemented in a general clinical practice and could play a crucial role in the detection of PCa with the goals of reducing the detection of clinically insignificant PCa and maximizing the detection of csPCa.
*Author Disclosures:*
**M. Hoogenboom:** Consultant; SOTERIA MEDICAL CLINICAL SALES ENGINEER.

**B-0873** 10:47
**Is it today acceptable to perform only targeted fusion MR/US prostate biopsy in naïve patients?**
S.R.M. De Martino, B. Corcioni, C. Gaudiano, F. Ciccarese, V. Vagnoni, R. Schiavina, R. Golfieri; *Bologna/IT (sara_demartino@live.it)*

**Purpose:** The recent literature proposes the use of only targeted MR/US fusion biopsy of suspicious lesions (PIRADS>3) in patients without a prior random mapping prostate biopsy. We evaluate our experience in naïve patients who underwent to mpMRI and MR/US fusion prostate biopsy.
**Methods and Materials:** We retrospectively evaluate 56 patients who underwent prostate mpMRI in our Institute, followed by a MR/US fusion biopsy of suspicious lesions completed with random biopsy. 70 lesions were identified and classified according to PIRADSv2 [31 PIRADS 3 (44%); 35 PIRADS 4 (50%); 4 PIRADS 5 (6%)].
**Results:** Of 70 lesions, we found 41 lesions (59%) positive for csPCa (GS>/= 7) and 29 negative lesions (41%). Among negative lesions (GS </=6), 5 of the 29 (17.2%) showed foci of csPCa in the random samples, in particular, 4 of the 20 negative PIRADS 3 (20%) and 1 of 9 negative PIRADS 4-PIRADS 5 group (11.1%).
**Conclusion:** In our experience, we cannot recommend performing only MR/US fusion biopsy in naïve patients due to the significant risk of missing csPCa in the rest of the gland (17.2% of our patients), especially for PIRADS 3 GS 6 lesions of the posterior zone, in which the probability of missing csPCa rises to 20%.

**B-0874** 10:55
**Accuracy of mpMRI/US fusion prostate biopsy in diagnosis of clinically significant carcinomas with histopathological verification**
M. Zagrodzka, M. Lichtarski, A. Lewicki, S. Niezgoda, E. Stachura, J. Brzezinski; *Warsaw/PL (brzezinski.j@gmail.com)*

**Purpose:** The purpose of the study was to establish the success rate of mpMRI/US fusion prostate biopsy in patients with histological confirmation of its result, performed in MR diagnostic centre with fusion biopsies performed on site.
**Methods and Materials:** 86 consecutive patients (aged 42-78 mean 64)with PSA level mean 10.01 (0.96-53.1 ng/ml),mean prostate volume 61.7 ml(13-273 ml), median PSAD 0,13 had mpMRI/US fusion prostate biopsy(with up to 4 target lesions per patient,total 193 lesions assessed)performed after a positive (PI-RADS v.2.0 score 3,4,5)mpMRI. Multiparametric MRI examination and the biopsies were done in MR department(on 1,5T GE HDxt system according to PI-RADS v.2.0 guidelines) specializing in urology and were assessed with regard to the results of the histological examination.
**Results:** Overall 60/193 lesions(31%) had confirmation of clinically significant cancer in the biopsy. In groups with PIRADS score of 5,4,3 the results were respectively 10/12(83%), 31/60(52%) and 19/118(16%).With regard to histopathological findings they were all adenocarcinoma. The remaining findings in all groups were: 8 prostatitis, 88 BPH, 34 prostatitis with BPH, 1 HGPIN, 1 HG with prostatitis, 1 ASAP with prostatitis.
**Conclusion:** Multiparametric MRI/US fusion biopsy is a highly useful diagnostic tool, with marked impact on the success rate of the prostate biopsy in diagnosis of clinically significant cancer. High success rate of cancer detection (83%) can be attributed to precise targeting thanks to biopsy being performed at the same site as the mpMRI examination. There is markedly higher prevalence of prostatitis in lower PI-RADS groups.

**B-0875** 11:03
**Technique and first results: targeted MRI and [$^{68}$Ga]-PSMA PET/MRI ultrasound fusion-guided transperineal prostate biopsies**
N. Guberina[1], L. Püllen[1], T. Stephan[1], J. Grueneisen[1], M. Forsting[1], L. Umutlu[1], H. Reis[1], B. Hadaschik[2], A. Wetter[1]; [1]*Essen/DE*, [2]*Heidelberg/DE*

**Purpose:** The aim of this study was to examine whether patients with elevated PSA-levels benefit from targeted MRI and [$^{68}$Ga]-PSMA PET/MRI ultrasound fusion-guided biopsies, compared to systematic saturation biopsy.
**Methods and Materials:** This retrospective study evaluated 219 patients with increased PSA-level (median PSA 8.3ng/ml) examined according to a multiparametric prostate protocol on a 3T-MR-scanner (PI-RADSvs2). Additionally, 23 patients were examined on an integrated 3T-PET/MRI-scanner. [$^{68}$Ga]-PSMA-11-PET/MR was assessed for functional ($SUV_{max}$) and for morphological variables. All patients underwent systematic transperineal prostate biopsy and targeted-MRI-biopsy using rigid image-fusion (MiM-software, USA) in case of PI-RADS3-5 lesions (median 26 systematic cores and 9 targeted cores). Detection rates for prostate cancer of targeted ultrasound fusion-guided biopsies were compared to systematic prostatic biopsy.
**Results:** Altogether 5979 systematic cores, 1407 MRI-targeted cores and 217 PET/MRI-targeted cores were obtained. Per core targeted MRI ultrasound fusion-guided biopsy (35%, positive target lesions 247/716) and [$^{68}$Ga]-PSMA-PET/MRI ultrasound fusion-guided biopsy (48.5%, positive target lesions 49/101) showed a significantly higher detection rate of prostate cancer than non-targeted systematic biopsies (17.5%, positive systematic cores 492/2800). In the saturation prostatic biopsy histological grading was distributed as follows: grade groups (1) 15.7%; (2) 32.7%; (3) 14.3%; (4) 5.8% and (5) 2.5%. MRI- and PET/MRI-targeted biopsies identified clinically significant prostate cancer with following histological distribution: grade groups (1) 19.7%; (2) 30.1%; (3) 14.8%; (4) 6.6% and (5) 9.5%.
**Conclusion:** The [$^{68}$Ga]-PSMA-PET/MRI ultrasound fusion-guided biopsy is a promising technique for histological tissue verification. It contributes to accurate prostatic biopsies at a considerably lower level of biopsy tissue cores.

**B-0876** 11:11
**A quantitative approach to pinpoint prostate targets in order to reduce the number of TRUS-MRI fusion biopsy cores per patient without affecting the detection rate**
M. Pecoraro, R. Campa, G. Barchetti, V. Salvo, C. Catalano, V. Panebianco; *Rome/IT (pecoraro.martina1@gmail.com)*

**Purpose:** The purpose is to further investigate how many and how larger cores are needed to properly localise and correctly identify the grade of PCa.
**Methods and Materials:** We prospectively analysed 54 patients who underwent radical prostatectomy following TRUS-MRI fusion biopsy, from 2016 to 2018. Two Uro-genital Radiologists used the standard procedure to identify biopsy targets (RADt) and delineated an "ADC-guided phantom target" falling into the standard one (ADCt). They also countered the area of the lesion with the lower normalised ADC value (nADC), minimum 6mm in diameter. So a

Friday correlation of the biopsy and the radical prostatectomy results was made.
**Results:** Index lesion was correctly detected in 78% of cases for both RADt and ADCt. The latter showed a better estimation of the final ISUP classification with a 70% concordance vs 67% of RADt. Both ADCt and RADt overestimated the final ISUP category in 4 cases, RADt caused an underestimation in two. PCa index lesions showed a 0,950cc mean volume and 12,2mm mean diameter, these were found to be 0,435cc and 9,4mm for RADt and 0,268cc and 8mm for ADCt. Mean nADC was 0,61 for PCa, 0,59 for RADt and 0,52 for ADCt.
**Conclusion:** ADC value and dimensional characteristics of PCa were more similar to those of RADt, however there was not a significant difference between ADCt and RADt PCa detection rate. Finally, ADCt showed a finer ISUP category correlation with RP results, allowing the operator to reduce the number of core per lesion, lowering the chances of complications.

**B-0877** 11:19
**Cognitive-MR-US fusion prostate biopsy: which one is worthier to perform?**
R. Turkay, E. Inci; *Istanbul/TR (rustuturkay@hotmail.com)*

**Purpose:** The goal of our study is to compare two prostate fusion biopsy modalities in a selected patient population.
**Methods and Materials:** 100 patients whose psa levels were between 3-10 and had no prior biopsy were enrolled for the study. They were divided randomly into 2 groups. Each group included 50 patients. All patients had PIRADS 3 or 4 or 5 category lesions in their multiparametric prostate magnetic resonance imaging (mp-MRI). Patients with lesion smaller than 5 mm were excluded. Group 1 patients were biopsied with cognitive fusion method and patients in group 2 were biopsied with Toshiba Aplio 500 MR-US fusion biopsy device. Results of histopathologic examinations along with demographic and psa levels were analyzed.
**Results:** We calculated no significant difference between 2 groups in terms of age, psa level and lesion diameter (p= 0.025, p= 0.031, p= 0.040 respectively). In group 1 we detected 38 prostate cancer cases in cognitive fusion biopsy (Gleason scores were 3+3 in 23 patients, 3+4 in 10 and 4+3 in 4 and 4+5 in 1 patient). In group 2 we found 42 prostate cancer cases (Gleason scores were 3+3 in 25 patients, 3+4 in 13 patients, 4+3 in 3 and 4+5 in 1 patient). There was no significant difference between 2 prostate biopsy methods in terms of catching targeted lesions (p=0.032).
**Conclusion:** Our study reveals that in a selected group of patient cognitive biopsy can be alternative for MR-US fusion biopsy method.

**B-0878** 11:27
**Focal cryoablation for recurrent prostate cancer following radiation therapy: assessment of iceball margin and signal intensity of tumour by 3D registration of MRI**
I.I. Oz[1], J.G. Bomers[1], C.G. Overduin[1], S.F. Jenniskens[1], J.P.M. Sedelaar[1], T. Paetz[2], C. Rieder[2], C. Schumann[2], J.J. Futterer[1]; [1]*Nijmegen/NL*, [2]*Bremen/DE (ilkeroz@yahoo.com)*

**Purpose:** To evaluate the effects of iceball margin and T2 signal intensity (SI) of tumour on local outcome of prostate cancer after cryoablation by 3-dimensional (3D) registration of intraprocedural magnetic resonance (MR) images.
**Methods and Materials:** Sixty-one percutaneous MR-guided focal cryoablation procedures were retrospectively reviewed. Intraprocedural MR images were analysed and registered using Software Assistant for Interventional Radiology (SAFIR) software to achieve the assessment of the 3D ablation margin. Local tumour recurrence was defined as evident tumour recurrence on follow-up MR images, positive MR-guided biopsy or biochemical failure without radiological evidence of metastatic disease.
**Results:** The median minimum iceball margin was 2.27 mm (-4.73 to 6.32). The cut-off value for the minimum iceball margin to discriminate local tumour recurrence was 1 mm (p<0.001, AUC = 0.839 (0.722-0.921)). Median tumour volume was 1.22 ml (0.21-11.80) and a significant difference was found in tumour volumes between local control group (n=45) and local tumour recurrence group (n=16) (p=0.049). A significant difference was determined in prior history of androgen deprivation therapy (ADT) administration between local control group and local tumour recurrence group (20% and 50%, respectively; p=0.048). On multivariable analysis, minimum iceball margin (p=0.001) and prior ADT use (p=0.015) remained independent predictors of local tumour progression. Additionally, standard T2-SI value of tumours did not correlate with Gleason score (n=54, p=0.365) and local tumour recurrence (p=0.977).
**Conclusion:** Although 5-10mm iceball margin is preferred to guarantee adequate coverage and treatment, prostate cancer may be effectively treated if iceball margin extends at least 1mm along tumour.

**B-0879** 11:35
**Suspected lesions categorised as PIRADS 3 at multiparametric magnetic resonance (mp-MRI) for prostate cancer (PCa): should we biopsy?**
V. Giasotto, L. Basso, A. Casaleggio, V. Prono, C. Terrone, C.E. Neumaier; *Genoa/IT (Veronicagiasotto@gmail.com)*

**Purpose:** The aim of this retrospective study is to investigate the diagnostic performance of Targeted Biopsy+Standard Biopsy (TB+SB) of lesions categorised as PIRADS 3 at multiparametric Magnetic Resonance (mp-MRI).
**Methods and Materials:** Between March 2017 and August 2018, we enrolled a total of 68 patients suspected to have Prostate Cancer (PCa) on the basis of clinical and laboratory data underwent mp-MRI on 1.5T with Endorectal Coil. All images were evaluated according to PIRADSv2, expressing location of suspected foci based on zonal prostate anatomy. Prostate Volume (PV) and Prostate Specific Antigen density (PSAD) were calculated. All patients underwent TB+SB. All histological results were matched with radiological findings.
**Results:** A total of 79 lesions were identified: 28 PIRADS-3, 37 PIRADS-4 and 14 PIRADS-5. The Detection Rate (DR) of TB+SB was 21,9% for all suspected lesions, 11,8% for PIRADS-3 and 22% for PIRADS-4/5. The DR of SB was 18,7% for all suspected lesions, 10,2% for PIRADS-3 and 16,7% for PIRADS-4/5. The DR of TB was 33,8% for all suspected lesions, 24,3% for PIRADS 3 and 37,8% for PIRADS-4/5. The mean PSAD was 0,08 for PIRADS-3 and 0,2 for PIRADS-4/5.
**Conclusion:** The DR of TB was significantly higher than SB for all lesions sampled, especially for PIRADS-3. The use of PSAD, with clinical and laboratory data, could represent an important adjuvant tool to suggest which lesions scored as PIRADS-3 should be biopsied. Due to the low PPV of PSAD <0,15 reported in literature, biopsy of lesions scored as PIRADS-3 is a cautious approach.

**B-0880** 11:43
**Feasibility of target dependent systematic core biopsy distribution models for transperineal MR/US fusion biopsy**
N.L. Hansen[1], T. Lloyd[2], A. Macchi[3], A. Warren[2], T. Barrett[2], C. Kastner[2]; [1]*Cologne/DE*, [2]*Cambridge/UK*, [3]*Milan/IT*

**Purpose:** To retrospectively model prostate biopsy core distribution with reduced biopsy core numbers and focal distribution using transperineal MR/US fusion guided biopsies.
**Methods and Materials:** Analysis was performed using data from a prospectively completed database of transperineal MR/US fusion guided biopsies between 2012 and 2016. Targeted Biopsy (TB) with 2 cores was taken from each mpMRI lesion followed by 18-24 Systematic Biopsies (SB) from sectors using the Ginsburg protocol. In a data based modelling exercise overall and GS 7-10 prostate cancer detection rate were compared between 2-core TB, 4-core background target biopsy (TB+BSB), 10-20 core saturation target biopsy (Sat TB), and combined TB+SB.
**Results:** 487 men had a single target lesion in MRI followed by combined transperineal targeted and systematic biopsy. TB alone, TB+BSB, and Sat TB detected 71% (246), 78% (269), and 91% (314) of 345 PCa, respectively, and 67% (149), 76% (169), and 91% (202) of 221 GS 7-10PCa, respectively. The best detection rates were found for outer location, Likert 5 tumour probability, prostate volume ≤45 cc, and lesion volume >0.5 cc, leading to detection rates of 98% (44/45) PCa and 94% (34/36) csPCa with 4-core TB+BSB.
**Conclusion:** MRI can give valuable information for which patients a target-dependent systematic core biopsy distribution is possible. A 4-core TB+BSB biopsy model with detection rates of 98% PCa and 94% csPCa may be feasible for large, highly suspicious lesions of those in the outer sectors of small prostates (10% of our study population).
*Author Disclosures:*
**N.L. Hansen:** Grant Recipient; Philips Healthcare Germany. Speaker; Guerbet. **A. Warren:** Research/Grant Support; National Institute for Health Research Cambridge Biomedical Research Centre UK. **T. Barrett:** Research/Grant Support; Cancer Research UK, Engineering and Physical Sciences Research Council Imaging Centre in Cambridge and Manchester, Cambridge Experimental Cancer Medicine Centre. **C. Kastner:** Speaker; Siemens Healthcare, MedCom GmbH.

**B-0881** 11:51
**MRI/US fusion transperineal prostate biopsy versus systematic biopsy for clinically significant prostate cancer detection in patients with negative ultrasound findings**
Y. Chen, Y. Zhu, W. Guan, Y. Qu; *Shanghai/CN (joychen1266@hotmail.com)*

**Purpose:** To assess MRI/US fusion transperineal prostate biopsy versus standard systematic biopsy for clinically significant prostate cancer (PCa) detection in patients with negative US findings.

**Methods and Materials:** A total of 97 men with suspicious multiparametric prostate MRI (PI-RADS category 3 or above) and negative US scheduled for MRI/US transperineal fusion biopsy were enrolled in this prospective study. Transperineal fusion biopsy was performed using Real-time Virtual Sonography system with 2-3 cores sampled from each suspicious lesion. After fusion biopsy, standard 12-core systematic biopsy was performed by another group of physicians who were blinded to MRI results. The diagnostic performance for clinical significant PCa of fusion biopsy was evaluated in comparison with systematic biopsy.
**Results:** The overall cancer detection rate was 49.5% (48/97). PCa was detected by fusion biopsy in 45 patients (46.4%, 45/97) and systematic biopsy in 36 patients (37.1%, 36/97, P=0.035). Among 48 patients with PCa, 44 (91.7%, 44/48) were diagnosed with clinically significant PCa. In comparison with systematic biopsy, the significant PCa detection rate for fusion biopsy was statistically higher (44.3%, 43/97 versus 30.9%, 30/97, P=0.001). MRI / US transperineal fusion biopsy resulted in an additional diagnosis of 14 patients with clinically significant PCa, including 10 patients missed by systematic biopsy and 4 patients undergraded by systematic biopsy. If fusion biopsy was omitted, only one patient with clinically significant PCa would be missed.
**Conclusion:** MRI/US transperineal fusion biopsy can improve clinically significant PCa detection in patients with negative baseline TRUS findings compared with standard systematic biopsy.

**10:30 - 12:00** **Room C**

### Radiographers

### SS 1014

### Melange: nuclear medicine and interventional topics

*Moderators:*
J. Thompson; Salford/UK
N.N.

### B-0882 10:30

**Predictive value of 18F-FDG PET/CT information in the modern 3D-based radiotherapy treatment of head and neck cancer patients**
A. Kedves[1], Z. Dr. Tóth[1], M. Emri[2], D. Sipos[1], V. Koczka[1], I. Repa[1], Á. Kovács[1]; [1]*Kaposvár/HU*, [2]*Debrecen/HU (kedvesandras94@gmail.com)*

**Purpose:** The aim of the study was to evaluate the potential predictive value of the maximum standard uptake value ($SUV_{max}$), lean body mass corrected SUV ($SUL_{peak}$), metabolic tumour volume (MTV) and total lesion glycolysis (TLG) values of the primary tumour in the clinical outcome.
**Methods and Materials:** Retrospective evaluation was performed using PET/CT image datasets of 52 histologically proven head and neck cancer patients, receiving definitive 3D PET/CT fused chemo-radiotherapy (CRT). PET/CT was performed before the start of the CRT and repeated after the completion of therapy clinical for response evaluation. $SUV_{max}$, $SUL_{peak}$, MTV and TLG values of the primary tumour have been assessed before and after the therapy. Two patient groups were created in relation to the presence or absence of viable primary tumour tissue. Group related metabolic parameter evaluation was performed, using the Kruskal-Wallis test.
**Results:** In 28/52 (54%) cases viable residual tumour tissue was detected on the restaging PET/CT image datasets, and in 24/52 (46%) cases complete remissions were found. For therapeutic success prediction assessment, we were unable to find any statistically significant correlation with pre-treatment $SUV_{max}$ and $SUL_{peak}$ values (p=0,44, p=0,33). TLG provided nearly statistically significant differences (p=0,052), and MTV have shown significant differences (p=0,001) between patient groups.
**Conclusion:** According to our results simple metabolic data provided by FDG PET/CT were unable to predict therapeutic response, while complex PET/CT volume information containing parameters proved to be more useful. Survey is planned to be followed with more subject and parameter inclusions.

### B-0883 10:38

**Comparative evaluation of in-house-prepared 99mTc-ethylene-dicysteine-deoxyglucose and 18F-fluorodeoxyglucose in breast cancer**
J. Horn-Lodewyk[1], J. Zeevaart[2], J. Wagener[2], G. Engelbrecht[1]; [1]*Bloemfontein/ZA*, [2]*Pretoria/ZA (jeninehorn@gmail.com)*

**Purpose:** To compare the uptake pattern of in-house-prepared (IHP) 99mTc-ethylene-dicysteine-deoxyglucose (99mTc-EC-DG) with 18F-fluorodeoxyglucose (18F-FDG) in histologically proven breast carcinoma patients.
**Methods and Materials:** Serial whole-body scans were acquired at different time intervals in five breast cancer patients (Stages II-IV). SPECT/CT was performed at 2 hours (h) post-99mTc-EC-DG administration. An average blood time-activity curve was calculated from the pooled blood sample data of five patients at 0-, 1-, 2- and 4 h after 99mTc-EC-DG injection. One-hour post-administration 18F-FDG-PET scans were also acquired. All the scans were qualitatively evaluated by one board-certified nuclear medicine physician. Quantitative comparisons were also made by drawing three-dimensional regions of interest and calculating tumour-to-background (T:B) ratios.
**Results:** The primary breast tumour uptake was visualised with 99mTc-EC-DG and 18F-FDG in five patients. 99mTc-EC-DG tumour uptake in $n$=3 patients was similar to the 18F-FDG uptake. Lower 99mTc-EC-DG was found in $n$=2 compared to 18F-FDG. The blood time-activity curve indicated rapid 99mTc-EC-DG clearance. The T:B ratios of 18F-FDG indicated a mean±SD of 7.29±3.26 and for 99mTc-ECDG 2.49±1.1 for the five patients. A $p$=0.124 indicated non-statistical significant difference between two paired samples.
**Conclusion:** IHP 99mTc-EC-DG SPECT/CT scan has similar diagnostic accuracy compared to 18F-FDG PET/CT for the primary breast tumour. Yet, advantages of 99mTc-EC-DG would be lower cost (no special equipment) as well as lower radiation dose patient and staff. IHP 99mTc-EC-DG has promising uptake properties as a diagnostic tumour-imaging agent and could play a future role in the diagnostic workup and monitoring of these patients.

### B-0884 10:46

**SPECT/CT artefacts: nuclear medicine point of view**
A. Geão[1], E. Pereira[2]; [1]*Lisbon/PT*, [2]*Almada/PT (ana.r.geao@gmail.com)*

**Purpose:** The association of SPECT and CT images can have a synergic effect in Nuclear Medicine imaging. Although, the use of SPECT and CT combined images must be carefully done, due to the several artefacts that may occur and that can diminish the accuracy of this technique.
**Methods and Materials:** The acquisition and processing protocols must be carefully followed, and the acquired images must be reviewed for technical and diagnostic quality before the patient leaves the department.
**Results:** The identification of the artefact and its source (if related to the equipment or to patient characteristics) is very useful in order to prevent or correct the artefact or minimise its effect on image quality.
**Conclusion:** Nuclear Medicine Technologists and Radiographers have an important role on the implementation of strategies to avoid artefacts to occur or to minimise its impact if they are not avoidable. Images must be interpreted by skilled readers who are well aware of the patients' clinical history, using workstations that allow integrated viewing of the functional and na atomic data. In this way, a high quality study will provide useful diagnostic information for further clinical management and patient care.Knowledge and training is very important to enhance image quality and as the quality of SPECT/CT device improves, it is expected that new applications will emerge.

### B-0885 10:54

**Impact of cardiac CT on the use of invasive diagnostic catheterisation in paediatric patients**
C. Griffiths, A.R. Rafferty, R. Allen, P. Noonan; *Glasgow/UK (c.griffiths2@nhs.net)*

**Purpose:** Diagnostic cardiac catheterisation is an invasive procedure used in paediatric patients with congenital heart disease. The investigation requires general anaesthesia (GA), which is associated with neurodevelopmental risks (1). However, non-invasive CT angiography with a cardiac package can also be used and provides detailed information on intra- and extra-cardiac anatomy, coronary arteries, and vascular structures. We investigated whether the introduction of a non-invasive CT scanner with a cardiac imaging package reduced the number of patients undergoing invasive diagnostic cardiac catheterisation procedures.
**Methods and Materials:** A retrospective analysis of patients undergoing diagnostic cardiac catheterisation and CT angiography was conducted for 2 years prior to (2013/14) and after (2016/17) introduction of a cardiac CT scanner at the Royal Hospital for Children, Glasgow. Statistical comparison of the data was undertaken using chi-squared tests.
**Results:** There was a significant difference in the number of diagnostic cardiac catheterisation and CT angiography procedures being conducted pre- and post-introduction of the CT scanner ($X^2$ = 118.09, P < 0.01). Diagnostic CT imaging increased from 85 to 345 cases in 2013/14 vs. 2016/17, whereas cardiac catheterisation procedures decreased from 143 to 88 cases, respectively.
**Conclusion:** Introduction of a CT scanner with a cardiac package resulted in a significant decrease in invasive cardiac catheterization procedures and an associated increase in non-invasive CT imaging. The shift from invasive to non-invasive investigations reduces patient and parental distress, as well as the risks associated with GAs in the paediatric population (2).

**B-0886** 11:02
**Comparison of radiation dose and image quality between an image intensifier and flat panel detector system in ERCP**
E. Saukko[1], H. Niiniviita[1], A. Henner[2], M.T. Nieminen[2]; [1]*Turku/FI*, [2]*Oulu/FI* *(ekaterina.saukko@tyks.fi)*

**Purpose:** In recent years, many conventional image-intensifier (II) fluoroscopy systems have been replaced by flat panel detector (FPD) technology. The aim of this study was to assess the patient radiation dose and image quality in endoscopic retrograde cholangiopancreatography (ERCP) with both types of fluoroscopy systems. ERCP is a gastrointestinal procedure used as a gold standard in the treatment of pancreatobiliary disorders.
**Methods and Materials:** Data related to patient demographics and radiation exposure in ERCP from an II system (n=91) and FPD system (n=101) was retrospectively collected at the same institution. Image quality was evaluated by measuring image noise from regions of interest (ROIs) on ERCP images acquired on each type of fluoroscopy system (n=30 and n=30). These measurements were further used in the signal-to-noise ratio (SNR) and figure of merit (FOM) calculations
**Results:** The median dose area product (DAP), fluoroscopy time (FT) and number of acquired images from II system were 1.55 $Gycm^2$, 1.4 min and 3 images. With FPD system, the median DAP, FT and number of acquired images were 1.72 $Gycm^2$, 1.7 min and 3, respectively. There was no difference in SNR (mean±SD) between systems (II 16.5±5.0 vs. FPD 17.1±7.9). However, the FOM was lower with II system compared to FPD system (1.5±1.1 $l/Gycm^2$ vs. 3.6±6.3 $l/Gycm^2$).
**Conclusion:** The results underline the necessary measures towards the optimisation of patient dose and image quality in ERCP procedures. Slight increase in DAP and FT needs to be further investigated in clinical practice.

**B-0887** 11:10
**Patient radiation dose during coronary angiography and intervention: a multi-centre, retrospective study**
S. Kaartinen, A. Henner, T. Mäkelä; *Oulu/FI (anja.henner@oamk.fi)*

**Purpose:** Radiation dose to patient undergoing coronary angiography procedures is high. In Finland there are diagnostic reference levels for coronary angiographic procedures. This study sought to examine if the reference levels were met in the five hospitals in Northern Finland during the study period and are there differences between different hospitals.
**Methods and Materials:** Data related to radiation exposure, patient demographics and procedural characteristics in coronary angiographic procedures (n=2 901) performed in 2016 in five different hospitals was retrospectively reviewed and analyzed. Patient doses were compared between hospitals, between procedures and to diagnostic reference levels given by STUK.
**Results:** Results of this study indicate that patient radiation doses vary not only between different interventional cardiology procedures but also between the hospitals. Doses given as DAP ($Gy*cm^2$) ranged between 0,00 and 989,69 in all the coronary angiography procedures in all five hospitals and averages (+/-SD) were 39,61 (41,08) for hospital 1, 20,13 (11,44) for hospital 2, 24,74 (19,62) for hospital 3, 15,31 (14,51) for hospital 4 and 38,56 (61,72) for hospital 5. Doses were mostly dependent on the fluoroscopy equipment, patient demographics, procedure and performing physician.
**Conclusion:** There is need to make effort to further minimize the patient doses during coronary angiography and measures should be made to meet the guidelines given by diagnostic reference levels.

**B-0888** 11:18
**Infection control: developing aseptic methods and creating a guideline for interventional radiology unit**
S. Simelius-Nieminen, H. Malmivirta, V. Seiko-Vänttinen, E. Saukko; *Turku/FI (suvi.simeliusnieminen@gmail.com)*

**Purpose:** Aseptic conscience is the base for aseptic procedures. Healthcare-associated infection (HCI) can occur because of microbes in patient, personnel, surfaces or contaminated instruments. WHO estimates that there are 4.5 million HCIs yearly in Europe. Infection control depends on very simple things as good hand hygiene and good aseptic methods in invasive interventions. The aim of this development project was to assess aseptic technique and to create a written guideline for aseptic methods for Interventional Radiology Unit of Turku University Hospital.
**Methods and Materials:** The action research study was carried out with electronic questionnaire and observation of aseptic methods. Electronic questionnaire was sent in September of 2017 to the personnel (n=27) of Interventional Radiology Unit and response rate of 81.5% was achieved. Observation was done from October to November of 2017. In total, 40 interventional procedures were observed and personnel's participation was 100%. The electronic questionnaire and observation material were analysed and similarities with differences were searched using content analysis.
**Results:** Five main themes were highlighted: improving hand disinfection, usage of surgical masks, usage of sterile indicator gloves, usage of chlorine dioxide-based disinfectants and improving antibiotic prophylaxis on bile duct procedures and on nephrostomy patients. Chlorine dioxide-based disinfectant was reclaimed and four other themes were discussed on learning café with personnel. Based on the literature and results, a guideline was created in Interventional Radiology Unit for fluoroscopy-guided procedures.
**Conclusion:** Improvements were done on these themes, but it takes time and effort to successfully implement new ways in practice.

**B-0889** 11:26
**Inoperable patients with chronic thromboembolic pulmonary hypertension: initiation a balloon pulmonary angioplasty programme**
T. Loupa, L. Brites, C.M.D.S. Almeida; *Lisbon/PT (cristindr@gmail.com)*

**Purpose:** Balloon pulmonary angioplasty (BPA) is an emerging treatment for patients with inoperable chronic thromboembolic pulmonary hypertension (CTEPH), however there is a lack of studies in safety and efficacy of BPA for chronic thromboembolic pulmonary disease (CTED) patients.
**Methods and Materials:** Data from all CTEPH patients were collected retrospectively in the centre records. The mean pulmonary artery pressure, the cardiac index and home oxygen therapy were analysed in order to compare patient's progress.
**Results:** BPA sessions were performed in 11 patients (n=14). All patients underwent a comprehensive diagnostic work-up including right heart catheterism at baseline and 24 weeks after intervention. BPA resulted in improvements in World Health Organization (WHO) functional class (3.5 to 2.1). Home oxygen therapy was used (57% to 20%) and a reduction of mean pulmonary artery pressure (59±11 to 46±8 mmHg) and cardiac index (2.3±1.1 to 2.5±1.4 L/min/m², P = 0.92) was verified. Procedure-related adverse events occurred in 20% of the interventions.
**Conclusion:** BPA was an effective therapeutic approach for non-operable CTEPH patients. BPA can be a new treatment option for carefully selected CTED patients.

**B-0890** 11:34
**Dose distribution for vascular procedures at a tertiary training hospital in South Africa**
H. Muller[1], W.I.D. Rae[2], H. Friedrich-Nel[1]; [1]*Bloemfontein/ZA*, [2]*Sydney/AU* *(henramuller@cut.ac.za)*

**Purpose:** Radiation exposure during interventional radiology can deliver skin doses, which can approach the lower limit for biological effects (2Gy). It is thus important that referring clinicians, radiologists, radiographers and patients are aware of these potentially high doses. Interventional procedures are often complicated, requiring long fluoroscopy times. It is, therefore, important to identify specific procedures with an increased risk of radiation injury. The aim of this study was to determine which procedures contribute the highest radiation dose to individual patients and the population in a tertiary training hospital in South Africa.
**Methods and Materials:** The study included 3310 patients undergoing diagnostic and interventional vascular procedures, which involved fluoroscopic x-ray exposure, for a three-year period. Procedural data were entered into a database, which included dose-area products (DAP) and screening times.
**Results:** The maximum dose delivered to an individual patient was 4.16Gy during a renal arteriogram. Doses delivered during transfemoral outflow and endovascular aneurysm repair procedures approached or exceeded the threshold for deterministic effects. Transfemoral outflows and four-vessel angiograms delivered the highest population dose.
**Conclusion:** Specific high-dose procedures were identified which may require patient follow-up to monitor skin effects. Measures to decrease radiation doses and limit radiation effects must be taken especially for the identified procedures. From the study, it is seen that diagnostic procedures are performed more often resulting in a high population dose. Interventional procedures are performed less often but can deliver a high dose to the individual patient due to the complex nature of these procedures.

**B-0891** 11:42
**The establishment of a triage tool for patient stratifications undergoing myocardial perfusion scintigraphy stress tests in Malta**
K. Borg Grima[1], P. Bezzina[1], L.A. Rainford[2]; [1]*Malta/MT*, [2]*Dublin/IE* *(karen.borg-grima@um.edu.mt)*

**Purpose:** Normal myocardial perfusion stress (MPS) scans are associated with less than 1% chance of cardiac events, annually. Within clinical practice it is difficult to identify patients who will not reach 85% or more of the target heart rate during the MPS stress test, potentially undermining the diagnostic capability of the test. A triage tool may provide an alternative way to assess the functional status of a patient before the stress test, aiding to standardise practice across Nuclear Medicine (NM) departments.

**Methods and Materials:** This study consisted of a multi phased approach using several data collection methods, including: an international online survey; 252 retrospective patient file analysis; 6 qualitative interviews with physicians and 300 patient questionnaires. Ethical permission was obtained for each phase.
**Results:** Online survey results highlighted the need for a patient triage tool. In 17 NM centres (n = 43) practitioners were unsure of the presence of stress testing protocols. The final phase linked population identified risk criteria: diabetes (p = 0.012), smoking (p = 0.003) and previous myocardial infarction (p < 0.001), with the results of the interviews, in order to establish through the use of a triage tool, each patient's ability to successfully finish the exercise stress test.
**Conclusion:** Initial findings indicate the proposed triage tool may be an effective method for referring physicians in Malta, to direct patients to the type of diagnostic test suited for their clinical scenario and to the stress test (exercise or pharmacological) that may correctly determine the extent of ischaemia.

**B-0892** 11:50
**Dose reduction in hybrid imaging techniques in nuclear medicine**
A. Geão[1], E. Pereira[2]; [1]*Lisbon/PT*, [2]*Almada/PT (ana.r.geao@gmail.com)*

**Purpose:** Vogel and Wainwright (1969) said "structure without function is a corpse and function without structure is a ghost". Nowadays, hybrid imaging is changing this paradigm. "Nuclear Medicine is evolving into nuclear radiology" and the same procedure is combining functional and structure evaluation. However, hybrid equipments are as challenging as interesting areas of radiation safety for both professionals and patients.
**Methods and Materials:** The frequency of hybrid imaging modalities, like PET/CT and SPECT/CT is increasing because the use of CT in nuclear medicine imaging improves diagnostic accuracy. Hybrid imaging is also increasing patient doses. Huang et al. (2009) points that CT may contribute up to 76% on the total effective dose of a PET/CT, raising special concerns on dose reduction.
**Results:** The first and perhaps most important way to minimize dose comes from deep understanding the equipment. Protocol optimization may enable decreasing the administrated activity of emission image. CT dose can vary widely according to equipments and protocols, defining importance of the appropriate protocol for the scans purpose.
**Conclusion:** Technological improvements in nuclear medicine imaging can influence patient radiation burden significantly: available data on SPECT/PET and PET/CT innovations are mostly linked to improved image quality and/or reduced scanning times, however dose reductions are possible. Available data in CT innovations are often linked to patient dose reduction while maintaining image quality. Nevertheless, a trade-off should be made with respect to image quality improvement, scan time reduction and patient dose reduction.

**10:30 - 12:00** **Room O**

### Musculoskeletal

### SS 1010

### Hip imaging

*Moderators:*
F.B. Ergen; Ankara/TR
V. Njagulj; Novi Sad/RS

**B-0893** 10:30
**Measurement of femoural antetorsion with CT and MR: why trigonometry should be added**
F. König[1], E. Rutz[2], J.A. Jacobson[3], A.L. Falkowski[2], O. Magerkurth[1]; [1]*Baden/CH*, [2]*Basle/CH*, [3]*Ann Arbor, MI/US (fabian.koenig@ksb.ch)*

**Purpose:** To describe a mathematical model allowing transfer of measurements of femoral antetorsion (AT) acquired from axial-oblique-images to the ,,axial method" combining advantages of better visibility of the collum and ability to use the established normal values for assessment of femoural AT.
**Methods and Materials:** Measurement of AT on axial imaging omits the true CCD angle by assuming 90°. Hence, AT is a result of a projection. On oblique-axial-imaging the CCD angle is fully appreciated and anteversion of the collum represents a projection other than that of 90°, inevitably resulting in smaller AT angles. To transfer measurements of oblique-axial-images (OAI), the CCD on true coronal images is assessed (cCCD). Then the angle (oblique-alpha) of the long axis of the collum to the horizontal on OAI is measured. Finally length of the collum (c) from the center of the femoral head to the center of the diaphysis is assessed. By using different right triangles and basic geometry the AT of the femur can be calculated stepwise. Final formula of AT = arctan ((sin(oblique-alpha) x collum length) / (cos(cCCD-90°) x √(collum length$^2$-(sin(obliqe-alpha) x collum lenght$^2$)))
**Results:** This formula provides AT angles equal to those measured on axial images. For example: cCCD=122°; alpha oblique=9°; Collum length: 57mm; Calculated AT=28.457°, Measured AT=28.
**Conclusion:** It is possible to calculate the classic AT angle from measurements on one coronal and one oblique-axial-image with better visibility of the collum. This may increase inter- and intrareader reliability and still allows the use of normal values provided by earlier studies with axial Images.

**B-0894** 10:38
**Failure patterns in femoral head osteonecrosis: a micro-CT study with histological correlation**
C. Mourad[1], C. Galant[2], F. Libert[3], B. Vande Berg[2]; [1]*Beyrouth/LB*, [2]*Brussels/BE*, [3]*Tournai/BE (charbel.j.mourad@hotmail.com)*

**Purpose:** To describe failure patterns of bone and cartilage in osteonecrotic femoral heads using micro-CT with histological correlation.
**Methods and Materials:** Seven resected osteonecrotic femoral heads (6 men and 1 woman) were scanned by using micro-CT (Skyscan 1173, Bruker, Kontich-Belgium; 12 µm isotropic resolution) before histological processing. Fifteen micro-CT images were reformatted to match with fifteen histological slices (9 necrotic and 6 non-necrotic areas). Micro-CT images were analyzed by a musculoskeletal radiologist blinded to histology, to detect cartilage or bone interruption within the non-necrotic specimens and the different layers of the necrotic lesions (hyaline cartilage, the subchondral lamellar bone plate, the subchondral, deep and peripheral trabecular bone of the lesion). The corresponding histological images were independently analyzed by a bone pathologist to asses cartilage and bone interruptions in a similar layer approach.
**Results:** No cartilage/bone interruption was detected in the non-necrotic areas of the specimens at micro-CT and at histology. Within the necrotic areas, bone interruptions were noted in the subchondral bone plate ( 9/9; 100%), subchondral trabecular bone (7/9; 78%),deep trabecular bone within the necrotic lesion (4/9; 44%) and peripheral trabecular bone near the reactive interface (1/9; 11%). All lesions were seen at microscopic analysis. Interruption of the cartilage was detected in 1 specimen at micro-CT and in 4 specimens at histology. A quantitative analysis after segmentation is undergoing.
**Conclusion:** At micro-CT imaging, bone interruptions are found exclusively in the necrotic areas. Superficial layers are more involved.

**B-0895** 10:46
**Image quality of hip MR arthrography with intra-articular injection of hyaluronic acid**
E.A. Nocerino[1], L. Nicosia[2], M. Alì[3], C.B. Monti[3], F. Sardanelli[3], A. Aliprandi[3]; [1]*Cassina de' Pecchi/IT*, [2]*Gallarate/IT*, [3]*Milan/IT (elisabetta.nocerino85@gmail.com)*

**Purpose:** To compare image quality of hip magnetic resonance arthrography (MRA) with intra-articular injection of high-viscosity hyaluronic acid (HA-MRA) to that of hip MRA with intra-articular injection of Gd-based contrast agent (Gd-MRA) in patients with femoral-acetabular impingement (FAI).
**Methods and Materials:** Design: single centre, observational, retrospective, inter-individual, cross-sectional. FAI patients with who underwent hip HA-MRA (3 mL of high-viscosity HA plus 17 mL of saline) were compared with 37 age- and possibly sex-matched FAI patients who underwent hip Gd-MRA (20 mL of 2-mmol/L solution of gadopentetate dimeglumine). Two independent blinded radiologists assessed image quality for all sequences (two-dimensional proton density, non-fat-sat axial, fat-sat coronal and sagittal; three-dimensional dual-echo steady state), using a 5-point Likert scale considering separately cartilage, labrum, as well as round and transverse ligaments. Pearson's $\chi^2$ and Cohen's κ were used.
**Results:** The HA-MRA group was composed of 37 patients (23 males, 14 females; median age 38 years), the Gd-MRA group of 37 patients (21 males, 16 females; median age 38 years), without significant difference for age ($p$=0.937) and sex ($p$=0.636). For both readers, image quality for all the investigated hip structures never resulted significantly different on any of the sequences: labrum ($p \geq 0.340$), cartilage ($p \geq 0.198$), round ligament ($p \geq 0.255$), transverse ligament ($p \geq 0.806$), and capsule ($p \geq 0.314$). Inter-reader agreement ranged from κ = 0.785 to κ = 1.000.
**Conclusion:** Hip HA-MRA provided a performance not significantly different from that of hip Gd-MRA. A perspective for a one-shop-stop combination of MRA and viscosupplementation in FAI is open.

**B-0896** 10:54

**Traction MR arthrography of the hip for characterisation of femoral head necrosis and resulting femoral cartilage damage in patients eligible for joint-preserving surgery**

F. Schmaranzer[1], T. Lerch[1], J.L. Cullmann[1], E. Schmaranzer[2], J.T. Heverhagen[1], K. Siebenrock[1], M. Tannast[1]; [1]*Berne/CH*, [2]*St. Johann/AT (florian.schmaranzer@insel.ch)*

**Purpose:** To assess (1) the location of necrosis, (2) location and pattern of femoral cartilage damage, and (3) diagnostic accuracy to detect femoral cartilage lesions using direct, traction MR arthrography (MRA).
**Methods and Materials:** This is an IRB-approved retrospective study. The institutional database (2016-2018, 360 hips) was reviewed for patients with AVN and complete radiographs and direct, traction MRA of the hip at 3T. Thirty patients were included (mean age 31 ± 9 years, 60% female; ARCO stages (I=30%, II=57%, III=13%). Fourteen (47%) hips underwent joint preserving surgery (10 open, 4 arthroscopic). Traction technique included weight-adapted traction (15-23 kg), a supporting plate to avoid pelvic tilt. Imaging protocol included coronal, axial, sagittal and radial 2D PD-w TSE sequences without fat saturation obtained under traction. Location of necrosis and lesions was described circumferentially and allocated to 4 joint quadrants. We assessed (1) location of necrosis, (2) location and pattern of femoral cartilage damage (normal/delamination/thinning/defect) on traction MRA, and (3) diagnostic accuracy of traction MRA to detect femoral cartilage lesions in the 14 patients (14 x 4= 56 quadrants) undergoing surgery.
**Results:** (1) Necrosis was located most frequently in the antero-superior quadrant (93% of hips). (2) Most frequently femoral cartilage delamination was found in the antero-superior quadrant (87% of hips). (3) Sensitivity was 93% (25/27) and specificity was 100% (29/29) of traction MRA to detect femoral cartilage lesions.
**Conclusion:** AVN predominantly affects the antero-superior quadrant and leads to corresponding femoral cartilage delamination which can be detected accurately using traction MRA.

**B-0897** 11:02

**Postoperative, traction MR arthrography in patients with persisting pain after arthroscopic FAI correction reveals high prevalence of osseous deformities and intra-articular lesions**

F. Schmaranzer[1], T. Lerch[1], K. Siebenrock[1], M. Tannast[1], E. Schmaranzer[2]; [1]*Berne/CH*, [2]*St. Johann/AT (florian.schmaranzer@insel.ch)*

**Purpose:** Patients management after failed arthroscopic FAI surgery is challenging. To assess prevalence of new/residual (1) osseous deformities, (2) intra-articular lesions and (3) progression of osteoarthritis in symptomatic patients undergoing pre- and postoperative MR imaging after hip arthroscopy.
**Methods and Materials:** This is an IRB-approved, retrospective study. From 2010 to 2017, 806 patients underwent arthroscopic FAI correction. Database was reviewed for symptomatic patients with traction MR arthrography (MRA) of the hip obtained before, after hip arthroscopy. 49 patients were included 29±10 years, 67% female. Traction was applied using a MR-compatible traction device. One reader assessed pre-/postoperative images. (1) Acetabular coverage (LCE<25°/>39°=dysplasia/pincer) and Tönnis osteoarthritis (OA) grade were assessed on AP pelvic views. Cam deformity: radial images α>60°. Femoral torsion: low/high torsion: <5°/>30°. (2) Residual tears, retears of the labrum, capsular adhesions/defects. (3) OA progression on MRI.
**Results:** Preoperatively 42 (86%) hips showed deformities: 2 (4%) dysplastic-, 11 (22%) pincer- and 39 (80%) cam deformities. Postoperatively 39 (80%) hips showed deformities: 9 (18%) dysplastic-, 8 (16%) pincer-, 20 (41%) cam deformity, 4 (8%) hips with torsion <5°, 10 (20%) hips with torsion >30°. Postoperatively 14 (29%) cases with residual and 12 (24%) cases with labrum retears were observed. 6 (12%) hips had capsular adhesions and 22 (45%) had capsular defects. Radiographic OA progression was observed in 5 (10%) hips, in 14 (30%) hips on traction MRA.
**Conclusion:** Prevalence of osseous deformities due to over- or undercorrection and intra-articular lesions is high after failed hip arthroscopy. Traction MRA was useful for detection of OA progression.
*Author Disclosures:*
**F. Schmaranzer:** Grant Recipient; Swiss National Science Foundation.

**B-0898** 11:10

**The extension thickness damage (ETD) score: a pre-operative hip MR arthrography-based classification to predict type of labrum surgery**

D. Albano[1], A. Aliprandi[2], M. Brioschi[2], S. Magnani[3], C. Messina[2], S. Sdao[2], F. Randelli[2], L.M. Sconfienza[2]; [1]*Palermo/IT*, [2]*Milan/IT*, [3]*Lodi/IT (albanodomenico@me.com)*

**Purpose:** To develop a new hip MR arthrography (MRA)-based scoring system of labral tears to predict before surgery the treatment option more suitable for labral tears.
**Methods and Materials:** Forty-seven patients (29 males) performed hip MRA for suspicious of femoroacetabular impingement and were afterwards subjected to arthroscopic treatment. Two musculoskeletal radiologists reviewed preoperative examinations and provided the extension thickness damage score based on Extension of tear, Thickness of labrum and type of damage. Chondral lesions grading was based on the arthroscopic findings according to Konan classification. For statistical purposes, patients were divided into two groups, depending on the type of treatment: labral repair or debridement. *U* Mann Whitney, Chi-square, receiver operator curves, and Cohen kappa statistics were used.
**Results:** 35/47 underwent repair, while 12/47 were debrided. In both groups, the median chondral damage was grade III, with no significant differences (p=0.439). The median extension thickness damage score in the repair group (6) was significantly lower (p<0.001) than that in the debridement group (8). The highest diagnostic performance (Area Under the Curve) of extension thickness damage was 0.819. The inter-observer agreement was substantial in the evaluation of Extension (k=0.626) and Thickness (k=0.771), and almost perfect for damage (k=0.827). Higher scores of Extension and Thickness were more frequently associated with debridement (p<0.001;p=0.0016, respectively), with no significant differences on the basis of damage parameter (p=0.284).
**Conclusion:** The MRA-based extension thickness damage score could represent a helpful pre-operative tool, expressing the extent of the damage and its reparability before arthroscopy.

**B-0899** 11:18

**Cam vs pincer femoroacetabular impingement: which type is associated with more hip structural damage? An exploratory cross-sectional study using MRI**

Y. Ragab[1], Y. Emad[1], A. Anbar[1], M. Khalil[2], J. Rasker[3]; [1]*Cairo/EG*, [2]*Stokholm/SE*, [3]*Amsterdam/NL (yragab61@hotmail.com)*

**Purpose:** To investigate the pattern and magnitude of hip structural damage associated with each type of femoroacetabular impingement (FAI) before the development of established hip osteoarthritis (OA).
**Methods and Materials:** Forty-four patients (78 hips) diagnosed with either cam or pincer FAI were consecutively recruited in a prospective cohort study. None of our patients had evidence of osteoarthritis (OA) on the initial plain radiography. All patients had contrast-enhanced MRI and CT scans of the hips. All patients filled in a visual analogue scale (VAS) for pain.
**Results:** The frequency of bone marrow oedema (BME) was 37% in cam FAI and 20.8% in pincer FAI. In cam FAI, BME positively correlated with pain severity as measured by VAS (P<0.0001), cartilage degradation (P=0.001), pseudocysts (P<0.0001), hip effusion (P=0.013) and reactive synovitis (P<0.0001). However, in pincer FAI, BME only correlated with pain severity (P=0.004) and duration (P=0.011) and did not correlate with other MRI signs of structural hip damage.
**Conclusion:** In cam FAI, BME of the femoral head and neck on MRI positively correlated with chondral damage and synovitis, but not in pincer FAI. This correlation suggests that cam FAI might be associated with a worse long-term prognosis. This finding might have an impact on clinical practice and decision-making as it would encourage surgeons to intervene early in cases of cam FAI, thus preventing the possible development of irreversible established hip OA.

**B-0900** 11:26

**MRI hip findings in asymptomatic professional Rugby players, ballet dancers and age-matched controls**

T.N. Blankenstein, A. Grainger, B. Dube, R. Evans, P. Robinson; *Leeds/UK (tomblankenstein@nhs.net)*

**Purpose:** The objective of this study was to investigate the findings in MRI of the hips amongst asymptomatic professional male rugby players and male ballet dancers compared to age- and sex-matched controls.
**Methods and Materials:** After ethics committee approval, 11 professional rugby players, 10 professional ballet dancers and 10 controls completed activity and symptom questionnaires and underwent 3.0T MRI of their dominant hip. Each scan was independently scored by two musculoskeletal radiologists for multiple features, including joint morphology, acetabular labrum appearance, cartilage loss and capsular thickness.
**Results:** Hip questionnaire symptom scores (Hip disability and Osteoarthritis Outcome Score) were significantly higher in rugby players (5/100) and ballet dancers (10/100) compared to controls (0/100) (p<0.05). There was no significant difference in the other questionnaire subscales. The prevalence of labral tears was 87% with no significant difference between groups (p>0.05). The majority of labral tears were full thickness or complex, degenerative type tears. Rates of paralabral cysts were significantly higher in ballet dancers (50%), compared to rugby players (0%) and controls (10%) (p=0.01). Acetabular cartilage loss was present in 54% with no significant differences between groups. Superior capsular thickness was significantly greater in ballet dancers (5.3mm) compared to rugby players (3.8mm) and controls (3.8mm) (p=0.03).

**Conclusion:** Despite the difference in activity between groups, there were equally high rates of labral tears and acetabular cartilage loss, questioning the role which sport plays in the development of these findings and their relationship to symptoms. The focally increased superior capsular thickness in ballet dancers may be an adaptive response to extreme ranges of movement.

**B-0901** 11:34

**3D T1 mapping of hip cartilage: a comparison of a new inversion-recovery-based method with conventional dual-flip angle acquisition**

F. Schmaranzer, T. Lerch, J.L. Cullmann Bastian, K. Siebenrock, M. Tannast, J.T. Heverhagen, M. Ith, B. Jung; *Berne/CH (florian.schmaranzer@insel.ch)*

**Purpose:** Although commonly used for quantitative imaging of hip cartilage, 3D dual-flip angle (DFA) techniques are highly sensitive to flip angle variation (B1 inhomogeneities), even more at 3T. The purpose is to compare precontrast T1 values of hip cartilage using a new inversion-recovery (IR)-based method with conventional DFA acquisition in asymptomatic volunteers.
**Methods and Materials:** This is an IRB-approved study of 18 asymptomatic hips (9 volunteers; mean age 27±2 years, 60% female). Subjects underwent non-contrast, quantitative T1 imaging of hip cartilage at 3T: (1) 3D DFA GRE-based technique (0.9 $mm^3$ isotropic T1 VIBE; acquisition time 8:30 min) including a prescan for B1 correction. (2) 3D dual-IR approach that has been recently introduced in brain imaging (0.9 $mm^3$ isotropic T1 MP2RAGE; acquisition time 7:30 min) in which T1 values are calculated based on two different inversion pulses. Radial images were reformatted for both T1 techniques. Regions of interest were placed manually, based on anatomic landmarks within the cartilage at each hour position of the clock face.
**Results:** Mean T1 values and standard deviation of overall (1488±174ms vs 1036±41ms), anterior (1533±219ms vs 1026±45ms) and posterior (1444±157ms vs 1047±43ms) hip cartilage was higher for the DFA compared to the IR-based method (all p<0.001).
**Conclusion:** Despite the B1 prescan inter-individual differences (standard deviation), T1 values of cartilage were greater with the DFA method compared to the IR method due to the greater flip angle variations at 3 T. Thus, 3D MP2RAGE may provide a more robust alternative for T1 mapping of hip cartilage.
*Author Disclosures:*
**F. Schmaranzer:** Grant Recipient; Swiss National Science Foundation.

**B-0902** 11:42

**Hip arthroplasty and computed tomography of abdomen: a retroperitoneal indicator of post-arthroplasty hip flexor impingement**

M. Alfaqih[1], S. Ali[1], J. Sana Ali[1], P. Chea[1], S. Flacke[1], R. French[2], D. Elentuck[1]; [1]*Burlington, MA/US*, [2]*Durham, NC/US (sarah.m.ali@lahey.org)*

**Purpose:** To evaluate if a tangible association exists between psoas muscle (PM) atrophy with acetabular component version.
**Methods and Materials:** 190 CT scans of abdomen/pelvis in patients with unilateral total hip arthroplasty (THA) were reviewed, culminating a total of 50 patients meeting inclusion and exclusion requirements. The length of the uncovered acetabular component (UAC) was measured. The acetabular version angle was measured on axial (AAV) and sagittal (SAV) reformats. The percentage difference between ipsilateral and contralateral PM in terms of Hounsfield unit (DHU) and area measurement (AM) was assessed. UAC, AAV, and SAV were measured blindly to DHU and AM. Additional analysis was performed by dividing the patients into two groups: group A patients had DHU over 50% and group B patients had DHU less than 50%. UAC, AAV, SAV were compared between groups A and B.
**Results:** Findings revealed a substantial positive correlation between DHU and UAC ($r^2$ =0.8, p<0.001). A negative correlation was demonstrated between AAV, SAV and DHU ($r^2$ = -0.4, p<0.005) and a positive correlation between UAC and AM ($r^2$ =0.5, p<0.001). No correlation was observed between AAV, SAV and AM ($r^2$ = -0.1, p>0.005). Groups A and B included 14 and 34 patients, respectively. Group A was found to have an increased UAC (8mm ±4, p<0.001), decreased SAV (22$^o$ ±10, p<0.01) and decreased ASV (24$^o$±11, p<0.0) compared to Group B (0.08mm±0.5, 32$^o$±11,34$^o$±10).
**Conclusion:** A strong association was found between ipsilateral psoas muscle atrophy in patients with THR who have an increased UAC and decreased AAV and SAV.

**B-0903** 11:50

**The value of the single energy metal artefact reduction algorithm in post-surgery follow-up of patients with hip tumour prostheses**

L. Ding, L. Ma, F.-L. Zhang; *Guangzhou/CN (408021193@qq.com)*

**Purpose:** To evaluate the effect of the single energy metal artefact reduction (SEMAR) algorithm with a 320 Multidetector computed tomography (MDCT) volume scanner in post-surgery follow-up of patients with hip tumour prostheses.
**Methods and Materials:** From November 2015 to September 2018, 103 consecutive patients with a tumour prosthesis of hip joint underwent a 320-MDCT scan. The images were reconstructed using two different methods: iterative reconstruction (IR) alone and IR associated with SEMAR. Four radiologists visually graded the whole image quality at articular level on a 6-point scale from 0 (periprosthetic anatomic structures are completely obscured by metal artefacts) to 5 (periprosthetic structures are recognised with high confidence). Additionally, the readers assessed the presence of periprosthetic complication on a similar 6-point scale from 0 (extensive artefacts, prosthetic complications are unable to be recognised) to 5 (minimal artefacts, prosthetic complications are recognised with high confidence). Wilcoxon matched-pairs signed rank test and Intra-class correlation coefficients (ICCs) were used for the scores of image quality and prosthetic complications.
**Results:** Visualization of periprosthetic structures were significantly improved by the SEMAR algorithm (3.3~4.2 vs. 1.5~2.4, P<0.05). In 64 of 103 patients, prosthetic complications were confirmed by other imaging examination, clinical or pathology. and prosthetic complications. The new algorithm also increased diagnostic confidence of prosthetic complications (4.0~4.4 vs 2.4~3.1, P<0.05). The sensitivity of diagnostic confidence of prosthetic complications was increased (93.2% vs.54.3%, P<0.05).
**Conclusion:** The SEMAR visibly reduces the metal artefact and can increase diagnostic confidence of prosthetic complications in patients with hip tumour prostheses.

**10:30 - 12:00** **Studio 2019**

### Imaging Informatics

### SS 1005a

### Hot topics in machine learning: from radiomics to natural language analysis

*Moderators:*
S.S. Martin; Frankfurt/DE
E. Neri; Pisa/IT

**B-0904** 10:30

**Generation of a curated dataset from unstructured reports using natural language processing, illustrated on CT reports regarding pulmonary embolism**

T.J. Weikert, I. Nesic, J. Cyriac, M. Moor, J. Bremerich, A.W. Sauter, G. Sommer, B. Stieltjes; *Basle/CH (thomasjohannes.weikert@usb.ch)*

**Purpose:** Retrospective classification of exams in positive vs. negative findings is a frequent first step in the development of curated datasets for the training of artificial intelligence algorithms. Given that radiology reports offer this information mostly in a non-structured fashion, we wanted to test the performance of a self-developed NLP-based procedure.
**Methods and Materials:** We downloaded all reports of CT pulmonary angiograms (CTPAs) conducted at our institution in 2016/2017 (n = 2,917; language: German). We then extracted the impression sections. The status (pulmonary embolism: yes/no) was manually assessed by a radiologist. CTPAs with other clinical questions than pulmonary embolism or poor diagnostic quality were excluded. The labelled impression sections from 2017 (n = 1,436) served as ground truth to train a deep neural network for NLP (linear support vector machine classifier). The performance of this network was tested using the exams from 2016 (n = 1,367).
**Results:** Our NLP approach reached a sensitivity of 88.9% and specificity of 96.5%. The positive predictive value was 81.6 and the negative predictive value was 98.1%. In total, the status of 1,303 of 1,365 exams was correctly predicted (accuracy: 95.5%). The cases with wrong classifications had a higher mean word count (40.1) than those with correct predictions (33.5).
**Conclusion:** Our NLP-based approach allows for an automated and highly accurate retrospective classification of CTPA data solely using the unstructured impression section from radiology reports. A higher word count, likely related to a higher number of secondary diagnoses, may be a potential cause of misclassification.

**B-0905** 10:38

**Scanner parameter induced variability of radiomics features in routine chest CT data**

S. Röhrich, J. Hofmanninger, J. Pan, F. Prayer, H. Prosch, G. Langs; *Vienna/AT (sebastian.roehrich@meduniwien.ac.at)*

**Purpose:** To assess the influence of heterogeneous acquisition and reconstruction parameters on radiomic features in pulmonary CT-scans in a large-scale clinical routine population processed by a fully automated unsupervised machine learning (ML) pipeline.

**Methods and Materials:** 4800 patients who received a chest CT-scan at our hospital were included without regard to pathology or indication. An unsupervised ML-approach was applied to identify clusters of patients depending exclusively on imaging features. Prior to processing, images were resampled to an isotropic voxel resolution of 0.7mm. Visual clusters were identified by extracting texture and 3D-SIFT-features within the lung followed by unsupervised dimensionality reduction techniques (deep-stacked-autoencoder) and k-means-clustering. The resulting clusters were compared with regard to different technical parameters (kVp, convolution kernel, slice thickness and scanner type). We performed fisher's exact cell-chi-squared-tests to assess association between cluster membership and technical parameters. In addition, we studied variability in follow-up studies after lung transplantation (LuTX), n=182.
**Results:** Cell-Chi-squared-tests yield p<0.0002 for all 20 parameters after Benjamini-Hochberg-correction for multiple testing, indicating strong associations between visual variation identified by unsupervised ML and technical parameters. There is a smaller variation in the more homogeneous cohort of LuTX-follow-ups, however, we still observed significant associations between technical parameters and visual variation (i.e., kVp, slice thickness, reconstruction kernel and scanner type, all ps ranging from 4.4e-5 to 0.04).
**Conclusion:** The known dependence of radiomics features on technical parameters in CT-scans greatly influences the results of unsupervised ML-approaches, up to a degree where pathological differences in images may become obscured by technical variability.

**B-0906** 10:46
**ePAD: A platform to enable machine learning and AI application development in medical imaging**
D.L. Rubin, M.U. Akdogan, C. Altindag, E. Alkim; *Stanford, CA/US (dlrubin@stanford.edu)*

**Purpose:** To develop a user-friendly tool to enable collaborative, multi-site image annotation and to capture the annotation data in a standards-based format to facilitate radiomics analysis and building deep learning models.
**Methods and Materials:** The electronic Physician Annotation Device (ePAD; http://epad.stanford.edu) is an open source, web-based tool enabling distributed, large scale collection of radiology image annotations. ePAD provides a user friendly interface for image viewing and annotation similar to a PACS, but captures all annotations in the new DICOM-SR/AIM standard object, enabling interoperability of annotations across institutions. ePAD also collects clinical and descriptive information about patients (e.g., diagnoses, imaging observations, and anatomic locations) using AIM templates, and it provides collaboration features such as privileging of access/viewing of annotations and summaries of annotation efforts. ePAD includes tools that leverage the image annotations for creating machine learning models. ePAD is modular and extensible by the community.
**Results:** ePAD is being used by many institutions, currently with 423 users who have created over 50,000 image annotations in 504 projects (on servers that are online, local instances are not included). The platform is particularly well suited for supporting multi-institutional collaborative projects for collecting image annotations in large scale, and it has been recently used for collecting image annotations for The Cancer Genome Atlas (TCGA) project for brain and bladder cancer collaborative groups.
**Conclusion:** The ePAD platform is expected to catalyze image annotation data collection efforts, accelerate collaborations in such projects, and ultimately help lead to machine learning and AI applications to improve radiology.

**B-0907** 10:54
**On the generalisability of deep learning across populations and abnormalities: a case study of the Stanford MURA and CheXNet algorithms**
H. Wang[1], T. Zou[1], C. Xia[1], P. Borrego[2], P. Sousa[2], M. Zhang[3]; [1]*Beijing/CN*, [2]*Barcelona/ES*, [3]*Zhejiang/CN (zhangminming@zju.edu.cn)*

**Purpose:** To investigate the generalisability of deep learning across populations and abnormality locations.
**Methods and Materials:** We re-implemented Stanford ML Group's CheXNet (chest x-ray abnormality detection) and MURA (musculoskeletal abnormality detection) on their original datasets (mainly from American hospitals), and then tested the models on independently collected datasets. We tested three labels (effusion, nodule and pneumonia) of CheXNet on 769 China adults and 200 Spanish children, and tested the MURA model on a Chinese dataset consisting of both MURA's original 7 abnormalities locations (3019 studies: elbow, finger, hand, forearm, humerus, shoulder and wrist) and 5 additional locations (3475 studies: foot, thoracic spine, extremity, sacrum and pelvis).
**Results:** The AUCs of CheXNet on its original American adult test dataset, Chinese adults, and Spanish Children were 0.86 vs. 0.88 vs 0.84 (effusion), 0.84 vs. 0.72 vs. 0.77 (nodule), 0.81 vs. 0.70 vs. 0.57 (pneumonia). Regarding the MURA model, when the original 7 locations were tested, algorithm's sensitivities on the original test dataset and the Chinese dataset were 80.3% vs. 43.9% (elbow), 74.7% vs. 50.0% (finger), 71.9% vs. 53.3% (forearm), 43.9% vs. 19.9% (hand), 86.6% vs. 69% (humerus), 86.3% vs. 69% (shoulder), 75.3% vs. 26.9% (wrist); on the other 5 labels, MURA algorithm's performance was: foot 59.4%, Thoracic-spine 33.9%, extremity 84.1%, Sacrum 70.6%, pelvis 74.0%.
**Conclusion:** Deep learning showed some satisfactory transferability (e.g., effusion and extremity abnormality). Yet, algorithms' overall performance compromised when generalised to different populations; disease complexity (i.e., musculoskeletal abnormalities) and demographic distance (i.e., adults vs. children) may exacerbate the problem.
*Author Disclosures:*
**H. Wang:** Employee; Employee, Infervision. **T. Zou:** Employee; Employee, Infervision. **C. Xia:** Board Member; member of board.

**B-0908** 11:02
**Fully automatic construction of optimal radiomics workflows**
M.P.A. Starmans, S.R. van der Voort, M. Vos, F. Incekara, J.J. Visser, M. Smits, M.G. Thomeer, W.J. Niessen, S. Klein; *Rotterdam/NL (m.starmans@erasmusmc.nl)*

**Purpose:** Radiomics uses medical image features to establish relationships between imaging and clinical data. Many radiomics methods have been described in the literature. However, there is no method that works for all applications. We present a workflow for optimal radiomics classification (WORC) to fully automatically construct an optimal workflow per application.
**Methods and Materials:** WORC states the complete radiomics workflow as a combined algorithm selection and hyperparameter optimisation problem. During training, WORC automatically adapts itself by testing thousands of pseudo-randomly defined radiomics workflows. The best workflows are combined into a single optimal signature. To evaluate WORC, three experiments on different clinical applications were performed: (1) classification of 119 patients with primary liver tumours in benign or malignant on T2-weighted MRIs, (2) prediction of 1p/19q co-deletion in 287 patients with presumed low-grade gliomas on T1- and T2-weighted MRIs and (3) distinguishing liposarcomas from lipomas in 88 patients on T1-weighted MRIs. Ground truth was obtained through pathology. Evaluation was implemented through a 100x random-split cross-validation, with 80% of the data used for training and 20% for independent testing. Performance is given in 95% confidence intervals (CIs).
**Results:** The CIs of the area under the curve, sensitivity and specificity were (0.86, 0.99), (0.58, 0.89) and (0.85, 0.98) for liver tumours (0.74, 0.85), (0.58, 0.76) and (0.72, 0.86) for brain tumours and (0.74, 0.93), (0.59, 0.86) and (0.67, 0.92) for lipomas/liposarcomas.
**Conclusion:** The good results in three different applications demonstrate that WORC is a promising approach for fully automatic construction of optimal radiomics workflows.

**B-0909** 11:10
**Deep DSA (DDSA): learning mask-free digital subtraction angiography for static and dynamic acquisition protocols using a deep convolutional neural network**
E. Eulig[1], J. Maier[1], M. Knaup[1], T. Koenig[2], K. Hörndler[2], M. Kachelrieß[1]; [1]*Heidelberg/DE*, [2]*Nuremberg/DE (elias.eulig@dkfz-heidelberg.de)*

**Purpose:** To provide DSA-like images in the absence of a mask (unenhanced image).
**Methods and Materials:** Conventional DSA selectively displays vessels by subtracting an unenhanced mask image from a contrast-enhanced fluoroscopic image. It thereby relies on the assumption that the static mask, which is typically acquired prior to contrast injection, accurately represents the background anatomy of the patient also during contrast injection. In case of patient, table or C-arm motion this assumption is not fulfilled, however. Therefore, we developed DDSA, a deep convolutional neural network similar to the U-net. DDSA predicts DSA-like images directly from their corresponding contrast-enhanced x-ray images. Supervised training was performed on patient data of different anatomical regions, acquired with a commercial mobile C-arm system. The ground truth was available through conventional DSA. Data were augmented to improve generalisation of the network. Due to its frame-wise applicability DDSA can predict angiograms for static and dynamic data in real-time without the need to acquire masks. DDSA was applied to several clinical test cases with and without patient and C-arm motion.
**Results:** The performance of DDSA was 7 ms for a 1024×1024 x-ray image on a GTX 1080ti GPU. In all test cases, the resulting images are visually similar to conventional DSA even for the challenging case of very thin vessels. Quantitative evaluation on static data showed very small (median absolute relative error: 5.4%) deviation from conventional DSA.
**Conclusion:** Deep DSA has the potential to replace conventional DSA by reducing patient dose and making it applicable to dynamic acquisitions.
*Author Disclosures:*
**T. Koenig:** Employee; Ziehm Imaging GmbH. **K. Hörndler:** Board Member; Ziehm Imaging. CEO; Ziehm Imaging GmbH.

**B-0910** 11:18

**Are radiologists bad teachers for AI algorithms: differences in the interobserver variability between consensus-defined labelling and free labelling of NIH chest x-ray14 dataset**

H. Mahajan, P.K. Ganesan, A. Vaidya, G. Nanda, M. Murugavel, V. Venugopal, M. Barnwal, V. Mahajan; *New Delhi/IN (geetutomar@yahoo.co.in)*

**Purpose:** To assess differences in interobserver variability before and after a consensus-based definition of the NIH Chest x-ray14 dataset labels.
**Methods and Materials:** We randomly extracted 800 x-rays from the NIH chest x-ray14 dataset. They were read by three radiologists (25 years, 20 years and 15 years of experience) in two equally divided batches. Of the 14 NIH labels, atelectasis, consolidation and pneumonia were clubbed under ‘opacity’. The other labels were used ‘as is’. For batch-1, the radiologist assigned labels for 400 x-rays based on their prior understanding of nomenclature. For batch-2, the labels were defined by consensus and assigned on the remaining 400 x-rays. Interobserver variability was assessed (Fleiss bounds) via the Krippendorff's alpha coefficient corrected for chance.
**Results:** Interobserver variability between free and consensus labelling did not vary in general. Opacity, pneumothorax, effusion, nodule/mass and 'no finding' were in the ‘fair to good’ (0.41-0.75) range, while infiltration, emphysema, fibrosis and pleural thickening were in the ‘poor’ (<0.40) bound in both tests. Interestingly effusion and cardiomegaly labelling worsened to ‘poor’ post-consensus. Significantly, no label was in the ‘very good’ (>0.75) category.
**Conclusion:** Training of AI algorithms is advised only on labels with alpha close to 0.75 (example ‘Normal’ vs ‘Abnormal’ or ‘pneumothorax’) when employing a purely image-based interobserver metric as ground truth. More research into the reasons for interobserver variability, and its significance in clinical practice, is needed.

**B-0911** 11:26

**Supervised one-to-one style transfer to improve the quality of CT images**

L. Li[1], B. Ren[2], S. Wang[1], C. Xia[1]; [1]*Beijing/CN*, [2]*Amherst, MA/US (llanqing@infervision.com)*

**Purpose:** The quality of CT is largely affected by attributes of the scan, such as radiation dose, convolution kernel and sharpness. CNN-based technique is susceptible to small perturbations for input. A standardisation is essential for improving the effectiveness and robustness.
**Methods and Materials:** We employed an unrolled proximal primal-dual optimisation scheme, where the operators were replaced with convolutional neural network. It iterates between source-target image pairs to learn the style difference such as noise level and convolution kernel of the commercial scanner. The model was trained on a collection of FDA phantom and data from collaborating hospital, and applied to reconstruct CT scans from our private database. The images were subsequently tested using Infervision AI-CT model with relatively low precision, whose latest version has been commercialised for early lung cancer screening.
**Results:** The reconstructed images achieved SSIM>97% (20-30% improvement), PSNR>39 on validation set, and SSIM>94% (7-12% improvement), PSNR>34 on human phantom test set. The detection result on reconstructed images exhibited significant increase in precision at cost of a modest decrease in recall. The total F1 score was improved by 32% on our private dataset. It was also observed that the lung-to-standard reconstruction was effective regardless of the sharpness attribute.
**Conclusion:** Standardisation of CT images is key to the success of an effective and specialised CNN-based methods. In our attempt to address the issue, results have shown a significant improvement of image quality and detection performance on the reconstructed images.
*Author Disclosures:*
**L. Li:** Employee; Employee of Infervision. **S. Wang:** Founder; Co-founder of Infervision. **C. Xia:** Board Member; Board Member of Infervision.

**B-0912** 11:34

**Opening the "Black Box": radiological insights into a deep neural network for lung nodule characterisation**

V. Venugopal[1], K. Vaidhya[1], V. Mahajan[1], H. Mahajan[1], S. Vaidya[2], A.R. Devalla[3]; [1]*New Delhi/IN*, [2]*Mumbai/IN*, [3]*Bengaluru/IN (vasanthdrv@gmail.com)*

**Purpose:** To explain predictions of a deep residual convolutional network for characterization of lung nodule by analysing heat maps.
**Methods and Materials:** A 20-layer deep residual CNN was trained on 1245 chest CTs from NLST trial to predict the malignancy risk of a nodule. We used occlusion to systematically block regions of a nodule and map drops in malignancy risk score to generate clinical attribution heatmaps on 160 nodules from LIDC-IDRI dataset, which were analysed by a thoracic radiologist. The features were described as heat inside nodule (IH) - bright areas inside nodule, peripheral heat (PH) - continuous/interrupted bright areas along nodule contours, heat in adjacent plane(AH) - brightness in scan planes juxtaposed with the nodule, satellite heat (SH) - a smaller bright spot in proximity to nodule in the same scan plane, heat map larger than nodule (LH) - bright areas corresponding to the shape of the nodule seen outside the nodule margins and heat in calcification (CH).
**Results:** These six features were assigned binary values. This feature vector was fed into a standard J48 decision tree with tenfold cross-validation, which gave an 85% weighted classification accuracy with a 77.8%TP rate, 8% FP rate for benign cases and 91.8% TP and 22.2%FP rates for malignant cases. IH was more frequently observed in nodules classified as malignant whereas PH, AH, and SH were more commonly seen in nodules classified as benign.
**Conclusion:** We discuss the potential ability of a radiologist to visually parse the deep learning algorithm-generated 'heat map' to identify features aiding classification.
*Author Disclosures:*
**K. Vaidhya:** Employee; Predible Health.

**B-0913** 11:42

**Generating dual-energy images from monoenergetic 80 kV images using a conditional generative adversarial network**

A. Demircioglu, J. Haubold, M. Forsting, K. Nassenstein, L. Umutlu, F. Nensa; *Essen/DE (aydin.demircioglu@uk-essen.de)*

**Purpose:** To reduce the image noise and beam hardening artifacts in computed tomography images a conditional generative adversarial network was trained and evaluated.
**Methods and Materials:** In dual-source techniques acquired dual-energy (80+140kV) computed tomography images were retrospectively used to train a conditional generative adversarial network (cGAN), which contains a generative and a discriminative neural network. 80 kV monoenergetic images and 0.5M dual energy images were obtained from 14 patients (4748 images in total) and were used as input data for the cGAN. The trained model was evaluated on another patient (571 images). The quality of the artifact reduction was measured by computing the median of the absolute difference in intensity per pixel relative to the 80kV image. Overall error is computed by average over all test images.
**Results:** The overall error of the cGAN was 0.24 (95 CI%: 0.22-0.27), indicating a very clear reduction of image noise and beam hardening artifacts. None of the test images showed a degradation relative to the 80 kV monoenergetic image. Additional inspection by an expert radiologist showed that the cGAN results were also visually of high quality.
**Conclusion:** Using 4748 training images from 14 patients a conditional generative adversarial network could be trained to generate virtual dual-energy images from 80kV monoenergetic images with an overall good similarity to ground truth 0.5M dual energy beam hardening and image noise reduced images.

**B-0914** 11:50

**Machine learning-based prediction of haematocrit values from native MRI myocardial T1-maps to avoid blood sampling for extracellular volume fraction analysis**

A. Varga-Szemes[1], M. Bauer[2], M. van Assen[3], C.N. De Cecco[1], P. Suranyi[1], L. Griffith[1], U.J. Schoepf[1]; [1]*Charleston, SC/US*, [2]*Munich/DE*, [3]*Groningen/NL (vargaasz@musc.edu)*

**Purpose:** To evaluate a machine learning (ML) algorithm to estimate blood haematocrit levels (Hct) from native MRI myocardial T1-maps to avoid blood sampling prior to cardiac MRI studies involving extracellular volume fraction (ECV) analysis.
**Methods and Materials:** A total of 51 patients (56±13y) who underwent 1.5T cardiac MRI were included. MRI protocol consisted of native (MOLLI 5(3)3) and post-contrast (4(1)3(1)2) myocardial T1-mapping. Native blood T1 values were measured in the left ventricle for Hct estimation. A linear regression (LR) analysis was applied to model the relationship between the image-derived and laboratory Hct (Hct[lab]) values. For the ML approach, 31 additional features based on patient demographics, clinical history, and imaging parameters were extracted and used to train a linear Support Vector Machine.
**Results:** Hct derived by the LR and ML algorithms were 38.7±3.3% and 39.1±3.6%, respectively, and were in good agreement with Hct[lab] (38.7±4.8; P=0.446). The LR approach provided the following model for Hct calculation: Hct[%]=89.8×(R1[native,blood]-19.0). The ML-based Hct showed stronger relationship (r=0.78; p<0.001) to Hct[lab] values than the LR-based Hct (r=0.70; p<0.001). Analysis of the residuals demonstrated an increase in accuracy for the ML approach compared to LR (RMSE 3.07 vs. 3.47). ECV values derived from LR, ML, and lab techniques were in good agreement (38.1±16.9, 37.9±16.8, and 37.9±17.0%, respectively; P=0.475).
**Conclusion:** The ML-based algorithm provides accurate Hct estimation and reliable myocardial ECV calculation, highlighting its potential in clinical workflows to generate ECV without the need for same-day laboratory Hct measurement.
*Author Disclosures:*
**A. Varga-Szemes:** Research/Grant Support; Siemens, Guerbet.
**U.J. Schoepf:** Research/Grant Support; Siemens, Bayer, Bracco, HeartFlow, Guerbet.

**10:30 - 12:00** **Room E1**

### Breast

### SS 1002

### Breast density and risk stratification

*Moderators:*
N.N.
G. Esen; Istanbul/TR

**K-21** 10:30
**Keynote lecture**
A. Vourtsis; *Athens/GR*

**B-0915** 10:39
**Impact of the use of a breast cancer risk assessment tool on the intensive screening of women clinically referred to as intermediate to high risk**
S. Woussen[1], H. Westerlinck[2], M. Keupers[3], R.M. Prevos[4], T. Thywissen[5], C. Van Ongeval[3]; [1]*Harelbeke/BE*, [2]*Heverlee/BE*, [3]*Leuven/BE*, [4]*Meerssen/NL*, [5]*Diepenbeek/BE (sofie.woussen@gmail.com)*

**Purpose:** Screening of women at intermediate risk of breast cancer (BC) requires yearly mammography and US; screening of high-risk women requires additional yearly MRI. Our goal is to verify the subjective interpretation of BC risks against a validated risk-estimation model (IBIS). Thus, to obtain more effective breast prevention strategies.
**Methods and Materials:** We retrospectively reviewed 471 clinically medium- to high-risk women who underwent screening mammography from August 2014 to August 2016. Because of presence of a BC gene mutation (33) or a lack of records (7), 40 women were excluded. Of the 431 remaining patients, 267 (62%) and 33 (14%) received additional screening US and MRI, respectively. Data from each included patient necessary to calculate the IBIS-BC-risk were inserted into the latest version (8.0b) of the risk- evaluation tool.
**Results:** The median age is 51 years and the median IBIS risk score is 23%. There were 6 (14%) breast cancer cases discovered through the screening. The personal lifetime risks are distributed as follows: <17% in 111, 17%-20% in 46, 20%-25% in 99, 25-30% in 71 and ≥30% in 104 women.
**Conclusion:** One fourth of the included patients showed a low IBIS-BC risk (<17%), lifetime risk of BC was clinically overestimated: they should not be screened more intensely. Considering an IBIS score of 25% as reference for breast MRI, 40% of our study group should receive breast MRI, instead of the 14% actually. More effective breast prevention strategies could be obtained by an objectification of the BC risk assessment.

**B-0916** 10:47
**Defining a parsimonious breast cancer risk model to risk-stratify average-risk women for follow-up screening**
M. Abdolell[1], J.I. Payne[1], P. Brown[1], P. Barnes[1], J. Caines[1], K. Tsuruda[2], P. Talbot[1], O. Tong[1], S.E. Iles[1]; [1]*Halifax, NS/CA*, [2]*Oslo/NO (Sian.Iles@nshealth.ca)*

**Purpose:** This study evaluated the consistency and discriminatory power of short-term breast cancer risk models with and without biopsy history (BxHx) within a general screening population.
**Methods and Materials:** All screen-detected breast cancer cases among digitally screened women 40-75 years (2009-2015) within a population-based breast screening program and 3 age- and screen year-matched controls were sampled. Clinical risk factors, fully automated percent mammographic density (PMD), and breast volume assessments were obtained for 1,593 cases and 5,003 controls and used to derive patient-specific risk estimates from a series of logistic regression models. Predictive performance was assessed using area under receiver operator characteristic (AUROC), and agreement between models for assigning women to low (<90th percentile)- versus high (≥ 90th percentile)-risk groups was assessed using weighted kappa.
**Results:** Predictive performance of the multivariate models varied substantially (AUROC: 0.547-0.655). A reduced model with PMD, breast volume, age, family history, and BxHx performed equivalently to the full model that additionally included menopausal status and HRT use (AUROC=0.655 and 0.656, respectively); removing BxHx from the reduced model decreased performance (AUROC= 0.591). Agreement between predicted probabilities of the full versus reduced model classified into low versus high risk was almost perfect (kappa=0.982).
**Conclusion:** A short-term risk model incorporating PMD, breast volume, family history, BxHx, and age may provide a practical solution for risk stratification within a screening population without the need to collect other clinical risk factors that are prone to recall bias and are not always available.
*Author Disclosures:*
**M. Abdolell:** CEO; Densities Inc. **P. Talbot:** Employee; Densitas Inc.

**B-0917** 10:55
**Reproducibility and measurement error in automated breast density assessment**
J.-L. Tham[1], E.F. Harkness[1], K. Stocking[1], A.R. Brentnall[2], J. Cuzick[2], D.G. Evans[1], S.M. Astley[1]; [1]*Manchester/UK*, [2]*London/UK (Sue.Astley@manchester.ac.uk)*

**Purpose:** To quantify the reproducibility and measurement error of volumetric breast density in mammograms.
**Methods and Materials:** We analysed data from the Predicting Risk of Cancer at Screening Study (PROCAS), a large cohort study to estimate risk of breast cancer in women attending routine breast screening. We used data from post-menopausal women who had a repeat mammographic view taken within 28 days of their screen on entry to PROCAS; all mammograms were taken on GE Senographe Essential systems. Volumetric percentage breast density for each mammographic view was assessed using Volpara V1.5.4 and log transformed. Measurement error was assessed by calculating the coefficient of variation and reproducibility assessed using the intraclass correlation coefficient (ICC).
**Results:** 519 repeat views from 419 women were eligible for analysis. The mean age of women was 60 years (range 46-73 years) and the median number of days between images was 18 days (interquartile range 14-21 days). The ICC (95% confidence interval [CI]) was 0.88 (0.84, 0.91), 0.89 (0.85, 0.92), 0.89 (0.84, 0.93) and 0.93 (0.90, 0.95) for the left and right craniocaudal (CC) and mediolateral oblique (MLO) views respectively. The coefficient of variation was 16.7% (15.1, 18.8), 16.4% (14.8, 18.5), 15.8% (13.9, 18.4) and 13.8% (95% CI: 12.1, 16.1) for LCC, RCC, LMLO and RMLO views, respectively.
**Conclusion:** This study found good to excellent reproducibility and a measurement error of 14-17% in assessing volumetric percentage breast density. This should be taken into account when interpreting changes in breast density between successive measurements.
*Author Disclosures:*
**J. Tham:** Other; Volpara software provided free under a research agreement by Volpara Health Technologies (Wellington, New Zealand). **E.F. Harkness:** Other; Volpara software provided free under a research agreement by Volpara Health Technologies (Wellington, New Zealand). **A.R. Brentnall:** Other; Volpara provided free under a research agreement by Volpara Health Technologies (Wellington, NZ). **J. Cuzick:** Other; Volpara provided free under a research agreement by Volpara Health Technologies (Wellington, NZ). **D.G. Evans:** Other; Volpara provided free under a research agreement by Volpara Health Technologies (Wellington, NZ). **S.M. Astley:** Other; Volpara provided free under a research agreement by Volpara Health Technologies (Wellington, NZ).

**B-0918** 11:03
**Factors influencing the disagreement between the automated volumetric breast density and radiologist's visual assessment in assessment of breast density**
H. Lee, J. Moon, J. Hwang, J.-Y. Woo; *Seoul/KR (geenie-m@hanmail.net)*

**Purpose:** To identify the various radiological and clinical factors associated with disagreement between automated volumetric breast density measurement (VBDM) and visual assessment by radiologists in assessment of breast density.
**Methods and Materials:** Three thousand and forty-three women who underwent screening and diagnostic mammography from August 2016 and February 2017 were included. The agreement in breast density between visual assessment by the radiologist based on $5^{th}$ BI-RADS and VBDM (Volpara Version 3.1) were compared using a weighted kappa (*k*) value. The factors including patient age, the purpose of mammography, presence of mass, microcalcifications, macrocalcifications, asymmetry or architectural distortion, a difference in bilateral breast density and BI-RADS final assessment were evaluated using univariate and multivariate analyses.
**Results:** Among 3043 women, 873 (28.7%) showed disagreement. The agreements between visual assessment by radiologist and VBDM were substantial (weighted *k* value = 0.674). Univariate analysis showed patient age (p <0.001), purpose of mammography (p = 0.026) and a difference in bilateral density (p < 0.001) as factors contributing to disagreement. In multivariate analysis, patient age (p = 0.003), presence of mass (p = 0.016) and a difference in bilateral density (p < 0.001) were contributing factors for disagreement.
**Conclusion:** There is substantial agreement in breast density evaluation between VBDM and visual assessment by radiologists. Disagreement between VBDM and visual assessment was related to patient age and a difference in bilateral breast density.

**B-0919** 11:11
**The role of breast density in predicting interval detected breast cancer**
M. Abdolell[1], J.I. Payne[1], P. Brown[1], P. Barnes[1], J. Caines[1], K. Tsuruda[2], P. Talbot[1], O. Tong[1], S.E. Iles[1]; [1]*Halifax, NS/CA*, [2]*Oslo/NO* *(Sian.Iles@nshealth.ca)*

**Purpose:** This study evaluated the predictive performance of short-term interval breast cancer risk models within a general screening population.
**Methods and Materials:** This case control study was performed among digitally screened women aged 40-75 (2009-2015) within a provincial breast screening program in Canada. The sample included all 132 interval breast cancer cases and 885 controls. Interval breast cancer was defined as breast cancer diagnosed after a negative screening examination or after an abnormal screening examination with negative work-up but before the next regularly scheduled screening examination. Data on clinical risk factors including age, breast volume (as a surrogate for BMI), first degree family history, history of breast biopsy, menopausal status, and HRT use were obtained for all subjects. Percent mammographic density (PMD) and breast volume assessments were obtained via automated software (Densitas Inc.). Logistic regression models were used to derive patient-specific risk estimates. Predictive performance was assessed using the area under receiver operator characteristic (AUROC).
**Results:** There was no difference in the average age of cases and controls at 55 and 56 years respectively. The model with PMD alone outperformed a model with all other clinical risk factors combined (AUROC=0.679 vs 0.614, respectively). Adding PMD to a model with all the other clinical risk factors increased the AUROC to 0.716.
**Conclusion:** Percent mammographic density was the most significant predictor of interval detected cancers and was a stronger predictor of interval detected cancers than all other clinical risk factors combined. Model performance needs to be validated using a separate dataset.
*Author Disclosures:*
**M. Abdolell:** CEO; Densitas Inc. **P. Talbot:** Employee; Densitas Inc.

**B-0920** 11:19
**Mammographic parenchymal pattern: correlation with age, breast density and prediction of cancer detection and nodal status in a UK screening population**
L.S. Wilkinson[1], L.M. Warren[2], E. Burnside[3], K. Young[2], C. Patel[4], N.J. Massat[5], J. Myles[5], S. Duffy[5]; [1]*Oxford/UK*, [2]*Guilford/UK*, [3]*Madison, WI/US*, [4]*Kingston/UK*, [5]*London/UK (louise.wilkinson11@nhs.net)*

**Purpose:** To investigate differences in parenchymal pattern (PP) of breast tissue on screening mammograms and evaluate association with screen-detected or interval cancer and node status.
**Methods and Materials:** This case-control study of 1204 women age 50-74 included 302 screen-detected and 297 interval cancers (239 node positive, 360 node negative) and 605 controls. Three readers classified PP grade on prior mammograms from smooth (PP1) to nodular (PP5). Volpara software calculated fibroglandular volume (FGV) and volumetric breast density (VBD) on unprocessed images. Intraclass correlation (ICC) compared readers' PP grades. Trend analysis was performed after one-way ANOVA to test for linear trends between increased PP and mean VBD, and mean FGV. Conditional logistic regression determined whether PP could predict mode of detection (screen detected or interval), and node status (positive or negative).
**Results:** There was good correlation between readers for PP grade (ICC 0.736, 95% CI 0.713, 0.757). Mean VBD did not differ significantly with increased PP grade. Mean FGV linearly increased with PP grade (p<0.0001). The relative risk (RR) of cancer in PP5 vs. PP1 was 2.2 (p<0.01) for screen detected cancer and 2.5 (p<0.01) for interval cancers. In PP5 vs. PP1, the odds ratio (OR) of node positive cancer vs. controls was 1.8 (non-significant, p=0.16) and for node negative cancer was 3.0 (p<0.01).
**Conclusion:** Visual assessment of PP is reproducible. PP grade increases with FGV and age. RR of screen-detected, interval, and node negative cancer significantly increases with PP grade, though the RR of node positive cancer does not.
*Author Disclosures:*
**E. Burnside:** Research/Grant Support; American Cancer Society.

**B-0921** 11:27
**Association between BMI and subsequent breast cancer among women with a prior false-positive without biopsy in BreastScreen Norway**
M. Lilleborge, S. Hofvind; *Oslo/NO (marie.lilleborge@kreftregisteret.no)*

**Purpose:** Evaluate the association between Body Mass Index (BMI) and a subsequent breast cancer, among women with a prior negative recall after additional imaging and ultrasound only (false-positive without biopsy) in BreastScreen Norway.
**Methods and Materials:** BreastScreen Norway is a population based screening program that invites all Norwegian women to biennial independently double-read two-view mammography in birth cohorts corresponding to age 50-69 years. 3,201,915 screens (97% negative and 3% resulting in a recall) were performed among 763,470 women in the study period 1995-2016. We included 55,625 women who experienced a recall concluded negative after additional imaging and ultrasound only. We followed women for invasive breast cancer (ipsilateral and/or contralateral), from false-positive until end of follow-up (31.12.2016, date of death, emigration or diagnosis of breast cancer, whichever came first). Cox regression was used to study how BMI was associated with subsequent breast cancer among women with a prior false-positive without biopsy, adjusted for age.
**Results:** Compared to women of normal weight (BMI 18.5-25), underweight women (BMI <18.5) had a similar long-term risk of subsequent breast cancer (HR=1.07, 95% CI: 0.63-1.83) after a prior false-positive without biopsy. We observed a statistically significant increased long-term risk of subsequent breast cancer (HR=1.25, 95% CI: 1.09-1.43 and HR=1.43; 95% CI: 1.19-1.71) among overweight women (BMI 25-30) and obese women (BMI ≥30).
**Conclusion:** Among women with a prior false-positive without biopsy in BreastScreen Norway, overweight and obese women have a 25% and 43%, respectively, increased long-term risk of a subsequent breast cancer compared to women of normal weight.

**B-0922** 11:35
**The value of automated breast ultrasound added to full-field digital mammography in women with dense breasts**
I. Boca, A. Ciurea, C. Ciortea, A.-S. Berghe; *Cluj-Napoca/RO* *(ioanaboca90@yahoo.com)*

**Purpose:** The objective of our study was to assess the performance of the new imaging techniques: full-field digital mammography (FFDM), 3D mammography and automated breast ultrasound (ABUS) in the detection of breast cancer.
**Methods and Materials:** Two radiologists independently evaluated a total of 127 ABUS acquisitions, the FFDM and 3D mammograms of women with dense breast tissue. During the study no clinical information or patient history was provided to the readers. The results were compared to the gold standard: histopathology for biopsied lesions, HHUS-handheld ultrasound for typically benign lesions (cysts) and follow-up for benign appearing lesions unchanged for at least 2 years.
**Results:** Nineteen breast cancers were proved by biopsy. For FFDM alone the sensibility was 72,7%, the specificity and the positive predictive value was 100% and the negative predictive value was 92,3%. By completing the FFDM with ABUS the sensibility increased to 78,3%, the specificity was 91,8%, the positive predictive value was 69,2% and the negative predictive value was 94,7%. For 3D mammography the sensibility was 100%, the specificity 86,4%, the positive predictive value was 75% and the negative predictive value was 100%.
**Conclusion:** In screening, ABUS added to FFDM compared with FFDM alone, improved reader's detection of breast cancers in women with dense breast tissue, but did not exceed 3D mammography. As a diagnostic method, ABUS associated with FFDM outperformed 3D mammography.

**B-0923** 11:43
**Automated breast ultrasound versus breast tomosynthesis in further evaluation of recalled dense breasts after screening mammograms**
N.H. Said, L. Adel, N.A. Chalabi, R. El Sheikh, H. Aboelmagd; *Cairo/EG* *(norranhussein@yahoo.com)*

**Purpose:** To compare the added value of automated breast ultrasound (ABUS) versus breast tomosynthesis (BT) as diagnostic tools in the work-up of screening detected positive findings in dense breasts recalled from the national screening program.
**Methods and Materials:** After ethics committee approval, and patients' consent, 242 women with dense breasts who underwent screening mammography, and were recalled for suspected positive findings were enrolled in the study. Positive findings included focal asymmetry, mass, distortion, or microcalcifications. All patients underwent both BT and ABUS by two independent breast radiologists and a BIRADS score was given for each modality. BT was performed in CC and MLO views. ABUS images were acquired in anteroposterior, lateral and medial views. Images were interpreted in the coronal 3D view using the survey mode, followed by the transverse and sagittal reconstructed images. Results were compared to pathology and follow-up of negative/typically benign findings
**Results:** Sensitivity, specificity, PPV, NPV, LR positive, LR negative and accuracy of ABUS were 92, 98, 92, 98, 44, 0.08 and 97, respectively, and in BT were 92, 92, 76, 98,12, 0.09 and 92. Agreement by Kappa was 0.896. ABUS and BT both agreed on TP in 43 cases out of 51 proved cancers. There were 4 FN cases in each modality. There were 4 FP by ABUS and 15 by BT. Biopsy was avoided by ABUS in 187 cases and by BT in 176 cases.
**Conclusion:** ABUS has shown a higher accuracy than BT. Its main limitations are microcalcifications and the retroareolar region.

**B-0924** 11:51
**Mammographic screening in male patients at high risk for breast cancer: is it worth it?**
M.A. Marino[1], A. Gucalp[2], B. Bernard-Davila[2], D.M. Keating[2], D. Martinez[2], E. Morris[2], M.S. Jochelson[2]; [1]*Messina/IT*, [2]*New York, NY/US*
*(mariaadele.marino@gmail.com)*

**Purpose:** There is currently no standard of care for screening asymptomatic men who are at increased risk for developing breast cancer. The purpose of this study is to investigate the utility of mammography for breast cancer screening in men at increased risk for breast cancer.
**Methods and Materials:** In this HIPAA compliant IRB-approved single-institution study, mammography, pathology and clinical records on 827 men who underwent mammography between 09/2011 and 07/2018 were analysed via the electronic medical record. 664 of these men presented with masses, pain or nipple discharge and were excluded. The remaining 163 asymptomatic men with family and/or personal history of breast cancer and/or known breast cancer mutation underwent screening mammography and are the subject of this analysis.
**Results:** 163 asymptomatic men 24-87 years (median 66 years) underwent 806 screening mammograms. 125/163 (76%) had a personal history and 72/163 (44%) had a family history of breast cancer. 24/163 (15%) had known mutations: 4/24 (17%) BRCA1 and 20/24 (83%) BRCA2. 792/806 (98%) of screening mammograms were negative (BI-RADS 1 or 2); 10/806 (1.2%) were BI-RADS 3, all of which were subsequently downgraded to BI-RADS 2. 4/806 (0.4%) mammograms were suspicious (BI-RADS 4/5) and all were malignant (mean size 6.25mm, range 4-7mm). There were three T1 and one T4 breast cancer. The estimated cancer detection rate was 4.9/1,000 examinations.
**Conclusion:** Screening mammography in high-risk men yielded a cancer detection rate of 4.9/1000 examinations.
*Author Disclosures:*
**M.A. Marino:** Research/Grant Support; Breast Cancer Research Foundation (PI: Hricak).

**10:30 - 12:00** **Room E2**

### Neuro

### SS 1011a

### Paediatric brain

*Moderators:*
N.N.
M.C. Diogo; Lisbon/PT

**K-22** 10:30
**Keynote lecture**
P.D. Griffiths; *Sheffield/UK*

**B-0925** 10:39
**Prediction of neurodevelopmental outcome in preterm neonates with cerebral MR spectroscopy and DWI using neural-network-based classifiers**
T. Djurdjevic, S. Pereverzyev, L. Lamplmayr, V. Neubauer, U. Kiechl-Kohlendorfer, V. Wallner, A.E. Grams, E.R. Gizewski; *Innsbruck/AT*
*(tanja.djurdjevic@i-med.ac.at)*

**Purpose:** We aimed to evaluate if magnetic resonance spectroscopy (MRS) and diffusion weighted images (DWI) performed in preterm neonates (PNs) at term equivalent age can predict their neurodevelopmental outcome at the corrected age of 12 months using neural-network-based classifiers (NNCs).
**Methods and Materials:** From 309 PNs born before 32 gestational weeks, 214 were excluded because of missing MRS or incomplete data sets. The data sets of 95 PNs were considered for motoric and cognitive development, of whom 8 and 5, respectively were categorized as underdeveloped. Seven potentially relevant metabolite ratios and 2 DWI characteristics were evaluated, each in 6 different areas of the brain. We performed a feature selection algorithm for receiving a subset of those characteristics that we could assume as significant. To reduce bias by unbalanced classes, only PNs that share values of those characteristics with ones that had shown underdevelopment were considered for further calculations. On those smaller sets of PNs, we finally constructed classifiers using NNCs. These classifiers were able to predict underdevelopment in PNs after considering the characteristics selected previously.
**Results:** The constructed NNCs give a 100% accuracy in the case of the motoric underdevelopment. In the case of cognition underdevelopment, we obtain a true positive rate of 100% and a positive predictive value of 83,3 %.
**Conclusion:** MRS and DWI obtained at term equivalent age in PNs allow prediction of their motoric and cognitive development at the corrected age of 12 months. The proposed approach using NNCs promise its use in the clinical practice.

**B-0926** 10:47
**Synthetic MRI detects delayed myelination in preterm neonates**
V. Schmidbauer, G. Geisl, K. Goeral, K. Klebermaß-Schrehof, A. Berger, D. Prayer, G. Kasprian; *Vienna/AT (victor.schmidbauer@meduniwien.ac.at)*

**Purpose:** “Synthetic MRI“ generates different MR contrasts and characterises tissue properties based on one multi-dynamic multi-echo FLAIR sequence (MDME). The aim of this study is to assess the feasibility of “Synthetic MRI“ in the assessment of myelination in term-born (TN) and preterm neonates (PN).
**Methods and Materials:** 30 PN and TN were examined at the approximate due date [gestational age+days to MRI=corrected gestational age (CGA)] using a standardised neonatal MRI protocol (1.5Tesla/T1SE/T2SE/DWI/DTI sequences). MDME sequence (FOV: 200x165x109mm/voxel: 0.9x1x4mm/matrix: 224x158x22slices/TE: 13ms/TR: 3309ms/acquisition time: 5min 22sec.)-based post-processing was performed using “Synthetic MRI“ (Synthetic MR AB, Storgatan 11, Linköping, Sweden, version 8.0.4). Myelination was assessed by scoring seven brain regions on T1/T2 maps generated by “Synthetic MRI“ and on standard T1/T2 images, acquired separately. Analysis of covariance (ANCOVA/covariate: CGA at MRI) was used for group comparison.
**Results:** In 25/30 (83.33%) cases [18 PN (mean gestational age: 178.17d/SD=12.56) and 7 TN (mean gestational age: 279.14d/SD=8.86)] myelination assessment could be performed. T1/T2 maps were generated by “Synthetic MRI“: ANCOVA results showed significantly lower myelination scores in PN compared to TN [T1: F(1, 22)=7.420/p=0.012; T2: F(1, 22)=5.658/p=0.026]. The myelination score showed a positive correlation with the CGA at MRI (T1: r=0.662/n=25/p≤0.001; T2: r=0.676/n=25/p≤0.001). The myelination score based on standard T1/T2 images did not correlate with the CGA at MRI. No significant differences between PN and TN were detectable.
**Conclusion:** Substantial differences concerning the myelination in TN and PN are detectable on T1/T2 maps generated by “Synthetic MRI“. The method allows to detect myelination differences more sensitively and rapidly than standard MR sequences.

**B-0927** 10:55
**Comparison of intracranial volumes of preterm-born infants and age-matched foetuses**
V. Pfahler[1], G.M. Gruber[2], G. Kasprian[2], A. Hilgendorff[1], K. Förster[1], A. Flemmer[1], B.B. Ertl-Wagner[3], D. Prayer[2], S. Stöcklein[1]; [1]*Munich/DE*, [2]*Vienna/AT*, [3]*Toronto, ON/CA*

**Purpose:** The aim of our study was to compare intracranial volume (ICV), brain volume (BV), and volumes of the inner and the outer cerebral fluid spaces (iCSF and oCSF) of preterm-born infants and age-matched foetuses.
**Methods and Materials:** Preterm born infants and foetuses were matched for gestational age (±4 days). Gestational age ranged from 32 to 39 gestational weeks. 23 preterm born infants and 23 age-matched fetuses without reported brain pathology on structural MRI were included in the analysis. Manual segmentation and volumetry was conducted based on axial T2-weighted images of the brain (slice thickness 3 mm), using the itk-SNAP software. ICV, BV and volumes of iCSF and oCSF were compared applying a paired t test at a significance level of alpha=0.05. Statistical analyses were conducted using MATLAB.
**Results:** ICV was significantly smaller in preterm born infants as compared to age-matched foetuses (alpha=0.003, $ICV_{foetal}$=382140 $mm^3$+/-49713, $ICV_{preterm}$=320005 $mm^3$+/-93416). Likewise, preterm born infants displayed significantly reduced BV and oCSF volumes (alpha=0.005 and alpha=0.035, respectively, $BV_{foetal}$=312254 $mm^3$+/-42384, $BV_{preterm}$=259032 $mm^3$+/-83413, $oCSF_{foetal}$=62903 $mm^3$+/-13134, $oCSF_{preterm}$=53381 $mm^3$+/-16732). iCSF volumes did not show a significant difference. Notably, the relation of BV and oCSF volumes to ICV was constant in preterm-born infants and foetuses, meaning that the ratio BV/ICV and oCSF/ICV did not differ significantly in the two groups.
**Conclusion:** Preterm-born infants have significantly reduced ICV, BV and oCSF volumes as compared to age-matched foetuses in utero. The mechanisms underlying these differences and their potential impact on ex utero versus in utero brain development remain to be investigated.

**B-0928** 11:03

**Dentate and basal ganglia T1 hyperintensity after multiple gadolinium administrations and brain microstructure: an in vivo diffusion-weighted MR study in paediatric patients**

P. Gulino[1], E. Salvatore[2], A. Bianchi[1], M. Franzese[2], L. Sali[1], M. Aiello[2], C. Cavaliere[2], M. Salvatore[2], M. Mascalchi[1]; [1]*Florence/IT*, [2]*Naples/IT*
*(pietrogulino@yahoo.it)*

**Purpose:** We explored if visually detectable T1-weighted hyperintensity (T1wH) in gray matter (GM) nuclei of paediatric patients is associated with evidence of local microstructural changes as revealed by diffusion-weighted imaging (DWI).
**Methods and Materials:** Retrospective study on data collected in a paediatric hospital between 2010 and 2017. We included 151 patients who had received 10 or more administrations of gadolinium-based contrast agents (GBCAs) for neuro-oncologic conditions (age <18 years at the time of the last administration) and 55 controls who had not received prior GBCAs. We visually assessed the signal intensity (SI) in T1-weighted images and measured the T1 SI ratio between dentate/pons and pallidus/parietal white matter, caudate/parietal white matter and thalamus/parietal white matter, and the apparent diffusion coefficient (ADC) in the dentate, pallidus, caudate and thalamus. We used non-parametric two-sample Wilcoxon test to evaluate differences of the T1 SI ratio and ADC and performed a multivariate analysis to evaluate the effect of age and of the number of prior GBCAs administrations.
**Results:** T1wH in the GM nuclei was visually detected in 23 of the 151 patients and in none of the controls. The 23 patients had a significantly higher T1 SI ratio in GM nuclei as compared to controls ($p<0.05$). ADC values in GM nuclei were not significantly different in patients with T1wH as compared to controls. Multivariate analysis showed that ADC is associated with age but not with number of GBCAs administrations.
**Conclusion:** T1wH is not associated to local microstructural changes in the mid-term as assessed with DWI.

**B-0929** 11:11

**Gadoterate meglumine, a macrocyclic agent causes gadolinium retention in the brain of children: a case-control study**

E.D. Topcuoglu, O.M. Topcuoglu, A. Semiz Oysu, Y. Bukte; *Istanbul/TR*
*(elifdilaratopcuoglu@gmail.com)*

**Purpose:** To demonstrate whether there was intracranial macrocyclic gadolinium deposition after multiple contrast enhanced MRIs with gadoterate meglumine in paediatric population.
**Methods and Materials:** This retrospective case-control study included children who had at least 3 enhanced brain MRIs. Age- and sex-matched control group with unenhanced brain MRIs were selected for comparison. All patients received gadoterate meglumine intravenously (0.1 mmol/kg). Signal intensity (SI) measurements were made by drawing 6 region of interests (ROIs): dentate nuclei (DN), pons, globus pallidi (GP), frontal white matter (FWM), thalamus (T) and clivus for both groups on unenhanced T1 weighted images. The ratio of those to cerebro-spinal fluid (CSF) were also calculated for standardization. Student t-test was used for comparison of SI and SI ratios. Pearson correlation was calculated for the correlation between the SI and the number of gadolinium administrations.
**Results:** A total of 45 children (age range: 5-17 years; mean, 13.7±3.4 years) were included. A significant difference was detected between two groups for DN/CSF, pons/CSF, GP/CSF, talami/CSF and FWM/CSF ($P<0.001$, $P<0.001$, $P=0.002$, $P=0.002$, $P=0.024$, respectively). There was no significant difference between two groups for clivus and clivus/CSF ($P=0.41$ and $P=0.15$, respectively). A good correlation between the number of gadoterate meglumine administrations and the SI for DN/CSF, pons/CSF, GP/CSF and T/CSF ($r=0.80$, $r=0.73$, $r=0.91$ and $r=0.90$, respectively) was found.
**Conclusion:** A significant T1 SI increase reflecting gadolinium retention in the brain, was detected for children with at least three gadoterate meglumine administrations in this series.

**B-0930** 11:19

**Cavernous sinus enlargement: a novel finding in Sturge-Weber syndrome**

L. Pasquini[1], D. Tortora[2], M. Rossi Espagnet[1], L. Figà-Talamanca[1], F. Manunza[2], C. Occella[2], A. Rossi[2], M. Severino[2]; [1]*Rome/IT*, [2]*Genoa/IT*

**Purpose:** The cerebral venous system is typically involved in Sturge-Weber syndrome and characterized by asymmetrical enlargement. Considering the anatomic centrality of the cavernous sinus and that it is intercalated in the venous network which is commonly affected by Sturge-Weber syndrome, we hypothesized its involvement in the disease and investigated possible clinical correlations.
**Methods and Materials:** Sixty patients with Sturge-Weber syndrome and 84 age-/sex-matched controls were included in this retrospective study. We measured on axial T2WI the left (A), right (B) and bilateral (LL) transverse diameters of the cavernous sinus. We calculated the difference |A-B|, and the “cavernous sinus asymmetry index” as the ratio |A-B|/LL. Finally, we performed a visual analysis to identify patients with asymmetric cavernous sinus enlargement. Differences among groups were assessed by Mann-Whitney U and Jonckheere-Terpstra tests. Clinico-radiological associations were evaluated by Fisher's exact test.
**Results:** All the CS measures were significantly different between SWS patients and healthy controls. The SAI and the left-right difference were significantly higher in SWS patients ($p=0.36$, $p=0.12$). CS enlargement was significantly associated with intraosseous vascular malformations ($p=0.013$) and the presence of ectatic veins ($p=0.002$).
**Conclusion:** The cavernous sinus enlargement occurs not only at the same level of the facial and leptomeningeal capillary malformation, but may also present without other intracranial vascular anomalies and may be related to the blood overcharge or to the recently discovered GNAQ mutation. Further studies are needed to draw definite conclusions regarding this new feature, which should be carefully evaluated in MR scans of patients suspected for SWS.

**B-0931** 11:27

**Pediatric brain: T1-weighted signal intensity of deep brain nuclei increases with age but not with a serial administration of gadoterate meglumine**

P. Pozeg, R. Meuli, J. Forget, P. Maeder; *Lausanne/CH*
*(polona.pozeg@chuv.ch)*

**Purpose:** Current findings on gadolinium retention in the paediatric brain after repeated exposure to macrocyclic contrast agents are inconclusive and possibly confounded by brain maturation processes. We evaluated longitudinal effects of repeated Dotarem exposure (gadoterate meglumine) on the T1-weighted signal intensity (SI) changes in paediatric patients, and assessed the magnitude of age-related increase in T1-weighted SI in a control cohort without prior gadolinium exposure.
**Methods and Materials:** In this retrospective, double-cohort study, we measured SI (native T1-weighted GRE) of multiple deep brain nuclei, normalized by the pons (reference tissue). 24 patients (1-20 years, mean=5.74, SD=4.15), exposed to at least 10 consecutive administrations of exclusively Dotarem, were included in the longitudinal cohort. We analysed SI ratio changes over 10 successive administrations, accounting for age, using linear mixed effects modelling. Secondly, 190 patients, naive to any gadolinium-based contrast agents, were included in the control cohort (age range 1-20 years, 10 patients/bin, bin width: 1 year) to assess the age-dependent SI changes in the ROIs.
**Results:** The number of Dotarem administrations did not significantly affect the SI in any of the ROIs (all $p>.05$), whereas age significantly correlated with SI increase in the globus pallidus and caudate ($p<.05$). This was confirmed with the cross-sectional analyses of the control cohort showing a significant age-dependent SI increase in multiple ROIs, including the globus pallidus and caudate ($p<.05$).
**Conclusion:** Repeated exposure to Dotarem was not associated with brain hyperintensity in the paediatric patients, while age importantly contributed to the SI changes in several deep brain nuclei.
*Author Disclosures:*
**P. Pozeg:** Grant Recipient; Guerbet. **P. Maeder:** Grant Recipient; Guerbet.

**B-0933** 11:43

**MRI findings in gene therapy-treated adrenoleukodystrophy**

P.A. Caruso, K. Buch, C. Corre, F. Eichler; *Boston, MA/US*
*(pcaruso@partners.org)*

**Purpose:** X-linked cerebral adrenoleukodystrophy (ALD) is a genetic disorder caused by mutations in the ABCD1 gene with poor untreated prognosis. Starting in 2014, lentiviral-transfected autologous haematopoietic stem cell transfer (HSCT) was introduced for the treatment of ALD. The purpose of this study is to provide the first description of the time course of intracranial MRI findings in post-lentiviral HSCT treated ALD patients.
**Methods and Materials:** 17 ALD patients who underwent lentiviral gene therapy were included in our cohort. Patients received serial MRI scans at 1, 6, 12, 18, and 24 months s/p HSCT. The clinical neurology severity scale was calculated for each time point. The evolution of the T2 signal abnormality, enhancement, and the Loes score (the standard grading system for cerebral ALD) were evaluated.
**Results:** The clinical ALD severity score stabilised or improved for all patients. The high T2 signal component of the treated ALD increased in 100% of patients at one month and in 80% of patients at all subsequent time points. Enhancement resolved in 40% of patients at one month, and in 100% of patients by 6 months. The Loes score increased an average of 4 points over the 24 months.
**Conclusion:** All ALD patients treated with lentiviral HSCT stabilised clinically, while the T2 signal component of the treated disease extended over the 24-month follow-up period and the enhancement resolved by 6 months.

**10:30 - 12:00** **Room F1**

### GI Tract

### SS 1001a

### Colonic imaging revisited

*Moderators:*
N.N.
L. Curvo-Semedo; Coimbra/PT

**B-0935** 10:30

**Polyp detection rate as a quality measure in CT colonography: analysis of the performance of a CT colonography service using a reduced bowel preparation without dietary restriction**

S. Vicini[1], M. Rengo[2], F. Tiberia[1], D. Bellini[1], G. Trionfera[3], I. Carbone[2], A. Laghi[1], L. Steri[1]; [1]*Latina/IT*, [2]*Rome/IT*, [3]*Valmontone/IT (simone.vicini@gmail.com)*

**Purpose:** To evaluate the performances of a CT colonography (CTC) service using polyp detection rate (PDR) as quality measurement. To stratify results according to patient's age, gender and symptoms.
**Methods and Materials:** We retrospectively analysed 1446 consecutive patients who underwent CTC from July 2015 to September 2018. In all patients a reduce bowel preparation (100 g of Macrogol and 60 ml of hyperosmolar iodinated contrast media for fluid tagging) and no dietary restriction was administered the day before the examination. PDR was calculated, considering only polyps ≥ 6 mm, for the entire population and after the stratification in sub-groups according to age (< 65 vs ≥ 65 yy), gender (male vs female) and symptoms (asymptomatic Vs symptomatic). All positive patients at CTC were subjected to endoscopy to confirm the presence of polyps. Polyps were scored according to the location, dimension and quality of the bowel preparation. Differences between sub-groups were evaluated with Chi-square test.
**Results:** In total 1.446 patients (627/819 M/F, mean age 62.45 ± 14.22 years) were analysed. Bowel preparation was optimal in 1392 patients (96,3%). PDR of total population was 9.19% (133/1446). PDR was significantly higher in older (10.93% vs 7,54%, P = 0.025) and male patients (11.32% vs 7.57%, P = 0.014). PDR was not significantly different between symptomatic and asymptomatic patients (11.29% vs 8.16%, P = 0.052).
**Conclusion:** CTC without diet restriction for bowel preparation is effective in detecting colorectal polyps, with PDR comparable to those previously reported in literature.

**B-0936** 10:38

**Quality of same-day CT colonography following failed optical colonoscopy**

A. Foran, A. O'Shea, M. Morrin; *Dublin/IE (foranat@tcd.ie)*

**Purpose:** To investigate the adequacy of bowel preparation and oral faecal tagging in same day CT colonography following incomplete same-day colonoscopy.
**Methods and Materials:** We conducted a retrospective review of all cases of same-day CTC(single centre) in a one-year period, between January 2017 and January 2018(n= 94). The time interval between initial low-dose, non-contrast pre scan and CTC was then calculated. The colon was the subdivided into 12 sections on subsequent CT colonography; each section was evaluated in prone, supine and, where relevant, in decubitus positions for residual fluid and faeces, completeness of oral tagging and adequacy of distension. The data was the collated across all patients and the median score for each subsection calculated. Over 90% of patients were prepped using Moviprep.
**Results:** The median time to same day CT colonography following low dose non-contrast CT abdomen and pelvis was 4.5 hours (mean 4.4 hours). Overall, the average volume of residual fluid in any given section was just over 25% of the total luminal diameter of that section. Where present, residual fluid and feces was adequately labeled in nearly all patients. The median score for distension for all patients was 3.8(range 0-5), meaning the entire segment of bowel was well distended and visualized.
**Conclusion:** Centres with capacity, ideally should aspire to same day CTC to avoid a second bowel prep for patients. We showed that same day CTC following failed colonoscopy is feasible with excellent image quality when combined with oral contrast labeling.

**B-0937** 10:46

**Role of CT colonography in differentiating sigmoid cancer versus chronic diverticular disease**

R. Valletta[1], N. Faccioli[1], M. Tagliamonte[1], M. Bonatti[2], G. Mansueto[1]; [1]*Verona/IT*, [2]*Bolzano/IT (riccardo.valletta@studenti.univr.it)*

**Purpose:** to retrospectively evaluate morphological findings of chronic diverticular disease and sigmoid cancer at computed tomography colonography (CTC) in order to differentiate them.
**Methods and Materials:** we included in our IRB-approved retrospective study 133 consecutive patients with histologically proven chronic diverticular disease (n = 77) or sigmoid carcinoma (n = 56). Two radiologists retrospectively analyzed CTC studies without awareness of the histological diagnosis. One reader scored each exam according to presence or absence of potential discriminators (length, wall thickness, shouldering phenomenon, thickening type, growth pattern, diverticula, fascia thickening, fat tissue edema, loco-regional lymph nodes, mucosal pattern).
**Results:** the findings that suggest carcinoma diagnosis were: absence of diverticula in the affected segment (sensitivity 87.9%; specificity 90.5%); straight growth pattern (sensitivity 71.4%; specificity 90.9%); shouldering phenomenon (sensitivity 90.5%; specificity 81.8%); complete distortion of mucosal folds (sensitivity 95.2%; specificity 75.8%). Considering mass-like lesions, growth pattern and mucosal folds distortion lose their diagnostic value. The only morphological finding with higher diagnostic value is absence of diverticula in the examined segment; its combination with shouldering phenomenon increases carcinoma diagnosis specificity.
**Conclusion:** carcinoma is best differentiated from chronic diverticular disease in CTC by the absence of diverticula in the affected segment and the presence of shoulder phenomenon.

**B-0938** 10:54

**The diagnosis of internal haemorrhoids from CT colonography has a poor agreement with optical colonoscopy**

L. Lambert, P. Hrabak, A. Novotny, J. Jahoda, A. Burgetova; *Prague/CZ (lambert.lukas@gmail.com)*

**Purpose:** The aim of this study was to assess the diagnostic performance of CT colonography (CTC) in the evaluation of internal hemorrhoids.
**Methods and Materials:** One-hundred-and-five patients who underwent CTC (balloonless rectal tube) for incomplete optical colonoscopy were included in the study. Three gastroenterologists were requested to make a note on the presence of internal hemorrhoids, should they consider requesting a subsequent CTC. For 45 patients, where internal hemorrhoids were reported, a 2:3 age- and a gender-matched cohort of 90 patients with normal findings in the rectum was selected, and images with the endoluminal and transaxial views (supine and prone) of the anal verge were created. Two radiologists and two gastroenterologists evaluated the images for the presence of internal hemorrhoids on a five-point Likert scale and the presence of anal verge prominence, asymmetry, and a cushion-like appearance.
**Results:** The sensitivity and specificity for the detection of hemorrhoids on CTC was 61% (95%CI 53%-68%) and 69 % (95%CI 63%-75%). The specificity was better for the radiologists (81% vs. 63%) and the sensitivity was slightly better for the gastroenterologists (56% vs. 49%). The agreement between the raters in the assessment of the presence of hemorrhoids was 0.61 (95%CI 0.51 to 0.69). Logistic regression identified the prominence of anal verge in supine position (OR 2.7, 95%CI 2.0 to 3.5) as the best predictor of the absence of internal hemorrhoids.
**Conclusion:** Internal hemorrhoids on CTC cannot be distinguished with any certainty. Hemorrhoids should only be suggested as a differential diagnosis of rectal floor findings.

**B-0940** 11:10

**Effectiveness of bowel preparation without diet restriction for optimal quality CT colonography: a validation study**

F. Tiberia[1], M. Rengo[2], L. Steri[1], D. Bellini[1], G. Trionfera[3], I. Carbone[1], A. Laghi[1]; [1]*Latina/IT*, [2]*Rome/IT*, [3]*Valmontone/IT (docfil@hotmail.it)*

**Purpose:** The aim of our study was to investigate whether bowel preparation without diet restriction for CT colonography (CTC) is sufficient to reach the proposed minimum standard rate of adequate bowel preparation (> 90%) and to identify any influencing factors in our population.
**Methods and Materials:** In our retrospective study we enrolled 1446 patients (mean age 62,45+- 14,22 years, 627 men) who underwent CTC after bowel preparation with 100 g of macrogol 60 ml of sodium diatrizoate and meglumine diatrizoate solution, for fluid tagging, the day before the examination. No dietary restrictions were applied. Quality of bowel preparation was assessed with a previously validated 4-point Likert scale evaluating, on a per segment basis, the quantity of tagging and degree of distention. We performed a subgroup analysis to examine the impact of the following factors on bowel preparation: age (> 65 vs <65 years) and diverticular disease.

**Results:** Optimal tagging and distention were rated in 1392 (96,26 %) and 1428 (98,75% ) patients, respectively, resulting in optimal quality of preparation for 1409 (97.44%) patients. Diverticular disease was associated with lower incomplete distention rates (0.7% vs 1.98%, p=0.03) and lower quality of fluid tagging (4.6% vs 2.4%, p=0,03). Patient's age did not affect both tagging (3.5% vs 3.9%) and distention (0.8% vs 1.7%) (p=0.63 and p=0.12 respectively).
**Conclusion:** Our proposed preparation without dietary restriction is effective in providing optimal fluid tagging and bowel distention for optimal quality CTCs. Diverticula disease negatively affects the quality of fluid tagging and degree of bowel distension.

**B-0942** 11:26
**Diagnostic performance of MRI- vs MDCT-categorised T3cd/T4 for identifying high-risk stage II or stage III colon cancers: a pilot study**
S. Shin[1], S. Cho[1], W. Kim[2], H. Kim[1], G.-C. Kim[1], H.-K. Ryeom[1], S.M. Lee[2], K.M. Shin[1]; [1]*Daegu/KR*, [2]*Seoul/KR (ssh-018@hanmail.net)*

**Purpose:** To determine the diagnostic performance of magnetic resonance imaging (MRI)-categorised T3cd/T4 tumours for identifying high-risk stage II or stage III cancer in patients with curatively resectable colon cancer in comparison with that of multidetector computed tomography (MDCT).
**Methods and Materials:** Thirty-eight patients with histopathologically indicated adenocarcinomas prospectively underwent MRI of the colon. Two radiologists independently and retrospectively assessed for T-category, including T3 substage (≤T3ab vs. ≥T3cd). The diagnostic accuracy and inter-reader agreement between assessments using each modality were compared using a pairwise comparison of receiver-operating characteristic curves and a weighted κ statistic, respectively.
**Results:** Twenty-nine patients (76.3%) were histopathologically diagnosed with high-risk stage II or stage III colon cancer. The false-diagnostic-positive rate with MRI was lower than with MDCT (0% vs. 7.9% for reader 1, 2.6% vs. 10.6% for reader 2). The diagnostic performance of MRI was better than that of MDCT across both readers (AUC: 0.707 vs. 0.506 [$P$ = 0.032] for reader 1, 0.651 vs. 0.485 [$P$ = 0.055] for reader 2). Moreover, MRI inter-reader agreement for the assessment of T3cd/T4 was significantly better than that of MDCT (κ=0.821 vs. 0.391 [p.=0.017]).
**Conclusion:** The diagnostic performance of MR imaging of the colon may be better than that of MDCT for identifying high-risk stage II or stage III cases. Given that colon MRI reduced the false-positive diagnosis rate, the usage of MRI-categorised T3cd/T4 diagnoses may prevent neoadjuvant chemotherapy overtreatment, though further studies with a larger sample size are required.

**B-0943** 11:34
**Abdominal calcium score in the evaluation with computed tomography of the risk of leakage of the colon anastomoses**
E. Santonocito, C. Trombatore, M. Travali, S.M. Santonocito, A. Di Cataldo, P.V. Foti, A. Basile, S. Palmucci; *Catania/IT (enzo_san@hotmail.it)*

**Purpose:** Surgery of the abdominal area is burdened by a 4% postoperative mortality, with non-fatal complications in 20-40% of cases. The leakage of surgical anastomosis is one of the complications with the highest rate of morbidity and mortality. The aim of this study is to analyze the correlation between development of complications and degree of atherosclerosis of the abdominal aorta by CT.
**Methods and Materials:** The study was conducted retrospectively, recruiting 57 patients: 30 with anastomotic leakage (cases) and 27 without any postoperative complications (controls). The atherosclerosis degree of the abdominal aorta was quantized in the preoperative CT images through the calculation of the Calcium Score (Agatson method), with identification of the value of AAC (Abdominal Aortic Calcification). The risk of leakage of the surgical anastomosis was analyzed by ROC (Receiver Operating Charatteristic Curve) analysis. The AAC values were compared between the 2 groups by Mann-Whitney U-test.
**Results:** ROC analysis, with a threshold value of AAC ≥ 141 reported an AUC (Area Under Curve) of 0.707, with a 95% confidence interval equal to 0.572-0.820; the sensitivity and specificity values measured were 76.7% and 66.7%, respectively. By comparing the AAC values between cases and controls, the study demonstrated a statistically significant difference (P = 0.003)
**Conclusion:** Definition of the Calcium Score of the abdominal aorta, evaluated by CT, can be considered as one of the morphological predictors of surgical risk of anastomotic leakage; further studies are needed in order to validate the role of the abdominal calcium score in larger series.

**B-0944** 11:42
**Does spectral CT help to predict the severity of ischaemic colitis?**
E. Danse, N. Michoux, A. Kartheuser, O. Dewit, P. Baldin, A. Jouret-Mourin, L. Jacquet, E. Coche; *Brussels/BE (etienne.danse@uclouvain.be)*

**Purpose:** To evaluate the ability of spectral CT to predict the severity of ischaemic colitis.
**Methods and Materials:** 19 patients with a final diagnosis of ischaemic colitis were evaluated with our spectral CT (IQON, Philips Healthcare). The maximal colon wall thickness (mm), UH density on virtual non contrast acquisition ($HU^{VNC}$), on portal phase ($HU^{PORTAL}$), $HU^{PORTAL}/HU^{VNC}$ ratio, Iodine load (mg/ml), Z value were evaluated. CRP, blood lactate level, lactate deshydrogenase level (LDH), leukocyte count were analyzed. The search for differences between evolution (group 1 resolution without surgery, n = 10) and histologically proven necrosis (group 2, n=9) was performed using Mann-Whitney U-test. A multivariable logistic regression analysis followed by a ROC curve analysis was done to identify prognostic factors of necrosis.
**Results:** The $HU^{PORTAL}/HU^{VNC}$ ratio was higher in group 1 (2.3 vs 1.3, $p$ = 0.008). Z and Iodine load were higher in group 1 than in group 2 (Z: 7.97 vs 7.41, $p$ = 0.001, Iodine load: 0.96 vs 0.22 mg/ml, $p$ = 0.001). LDH was significantly higher in group 2 (421 vs 192 UI/L, $p$ = 0.004). At multivariable analysis, the association of CRP and LDH count in predicting the severity of ischaemic colitis demonstrated the highest performance (AUC = 0.986 [0.781; 1.000], Se = 100% [63; 100], Sp = 89% [52; 100], NVP = 100% [63; 100], PPV = 89% [52; 100], $p$ < 0.001).
**Conclusion:** The combination of blood tests and colic wall abnormalities observed with spectral CT can contribute to predict the outcome of ischaemic colitis.

**B-0945** 11:50
**Whole-body MRI: prevalence and extent of asymptomatic diverticular disease and the association of dietary habits, cardiometabolic and constitutional risk factors**
C. Storz[1], T. Rothenbacher[1], S. Rospleszcz[2], J. Linseisen[1], H. Messmann[1], C.N. De Cecco[3], A. Peters[4], C.L. Schlett[5], F. Bamberg[1]; [1]*Tübingen/DE*, [2]*Munich/DE*, [3]*Charleston, SC/US*, [4]*Neuherberg/DE*, [5]*Heidelberg/DE (corinna.storz@med.uni-tuebingen.de)*

**Purpose:** To assess the prevalence and extent of diverticular disease (DD) and the association of cardiometabolic risk factors, dietary habits and constitutional factors assessed by magnetic resonance imaging (MRI) in an asymptomatic general population.
**Methods and Materials:** Participants enrolled in a prospective case-control study underwent a 3 Tesla MRI, including an T1w-VIBE-Dixon sequence. Presence and extent of DD were categorized according to the number of diverticula in each colonic segment. The amount of visceral, subcutaneous and total adipose tissue and the degree of hepatic steatosis (PDFF) were quantified using a multi-echo T1w sequence. Traditional cardiometabolic risk factors were obtained and dietary intake habits were derived using a comprehensive food-intake questionnaire. Univariate and multivariate associations were calculated.
**Results:** Among 393 participants included in the analysis, 42% had DD, predominantly in the left-sided colon. Subjects with DD were older, had significantly higher body mass index as well as higher cholesterol levels and systolic blood pressure (all p≤0.003). In contrast, no significant correlation was found for physical activity, not for alcohol consumption (all p>0.31). Furthermore, subjects with DD had higher amounts of adipose tissue and hepatic PDFF (all p<0.02), and had significantly less intake of vegetables and fiber (OR: 0.73, 95%CI: [0.53, 1.00] and 0.65, 95%CI: [0.46, 0.92], all p<0.02), which was independent of age, sex and mean caloric intake.
**Conclusion:** Assessing DD by whole-body MRI is feasible and demonstrates similar pathophysiological associations as previously reported. MRI as a non-invasive tool provides novel opportunities including identification of high-risk DD phenotypes.

**10:30 - 12:00** **Room F2**

### Emergency Imaging

### SS 1017

### Best of emergency radiology (part 2): from neurological emergencies to penetrating injuries

*Moderators:*
B. Erkan; Istanbul/TR
K. Katulska; Poznan/PL

**B-0946** 10:30
**Is there a clinical need for structured reporting or even more? Results from a clinical questionnaire in the context of head trauma CT**
N. Schmidt, D. Zumofen, A. Jadczak, B. Stieltjes, K. Blackham; *Basle/CH*

**Purpose:** A challenge for the clinician is effective decision-making in the setting of information overload. A superimposed challenge is a radiologic report that is variable in format, length and linguistic style. Structured reporting helps enforce completeness and standardization. To improve the quality and usability of our reports we assessed the referring clinician's needs for trauma head CT.
**Methods and Materials:** A literature search determined several evidenced-based parameters that should affect clinical decision-making. A questionnaire went by email to referring clinicians from emergency room, neurosurgery, otolaryngology, spinal and maxillofacial Surgery. The questionnaire contained calibration questions, opinion questions about the status of the current reports, questions regarding a proposed structured report and communication of findings.
**Results:** 41 respondents completed the questionnaire. 55% read the radiologic impression and 27% the findings on a regular basis. 80% prefer shorter reports and 76% prefer to get structured reports in a table form. 32% wished to receive results other ways than the telephone, for example, a signal on an electronic dashboard. When asked about possible improvements, respondents asked for a link to the images with critical findings, less description of normal findings and continued reporting of all secondary findings.
**Conclusion:** Clinicians prefer structured reports to the narrative style for trauma head CT. There is a clear need for more personalized reports depending on the clinical severity, the clinician's subspecialty and personal preferences. Further developments need to enable other ways to transmit information in a more rapid and personalized format.

**B-0947** 10:38
**Computed tomographic pattern of intracranial injuries among patients with *okada* (motor cycle) road traffic accidents in Southwestern Nigeria**
O.M. Atalabi, O. Akinwumi; *Ibadan/NG (mojisola3t@hotmail.com)*

**Purpose:** To determine pattern and prevalence of intracranial injuries on cranial computed tomography scan in patients with head injury following motorcycle road traffic accident.
**Methods and Materials:** This study was a descriptive cross-sectional study conducted on 190 patients, who presented with head injury at the Accident and Emergency Unit of University College Hospital, Ibadan, following motorcycle road traffic accident in a five-month period and who underwent computerised tomography scan.
**Results:** More than half of the patients (n=59.8%) were within the ages of 20-39 years with a mean age of 33±14.37 years. A total of 183 patients did not wear the crash helmet at the time of injury with 155 patients (n=81.6%) sustaining intracranial injury (p=0.21). Cerebral contusion (n=62.6%), cerebral haematoma (n=31.7%) and cerebral oedema (n=86.8%) were noted in these patients. Intracranial bleed such as subdural, subarachnoid and epidural bleeds accounted for 28.5%, 22.2% and 21.5%, respectively. Only seven patients (3.8%) had intraventricular extension of these bleeds. One hundred and sixty-eight patients (n= 88.4%) had varying types of skull fractures. The significant relationship between the presence of intracranial injury and severity of head injury was demonstrated in this study as the increase in intracranial injuries correlated with severity of head injury from mild to severe (p<0.001).
**Conclusion:** The sharp increase in *okada* (motorcycle) accidents now account for life-threatening intracranial injuries and has become a major public health concern needing enforcement of traffic regulation and safety measures.

**B-0948** 10:46
**CTA evaluation of cervical ICA occlusion vs pseudo-occlusion in major stroke**
S. Molinaro, S. Veglia, O. Davini, C. Ruggieri; *Turin/IT (ilmioalterego52@gmail.com)*

**Purpose:** To evaluate the PPV of CT angiography in distinguishing between thrombotic occlusion and pseudo-occlusion of cervical ICA, using density measures (ROI) in patients with major stroke eligible to endovascular treatment.
**Methods and Materials:** From 353 patients who received a diagnosis of major stroke between Jan 16 and Mar 18, we studied 19 of them with no enhancement of major intracranic vessel and ICA (tandem occlusion) in two-phase CTA scan. Both images and reports were reviewed and three ROI were placed, one in the proximal tract of ICA and two (I and II phase) in the distal one, to assess a correlation between ROI values, angiographic report and CTA.
**Results:** 14 out of 19 patients had a CT diagnosis of occlusion, which was confirmed after in 9 cases with angiography (PPV 64.3%, CI95% 35.1%-87.2%) and 5 of pseudo-occlusion (26%) with regular vessel opacification in angiography in 1 case (NPV 20%, CI95% 0.5%-1.6%). Using the ROI in the II phase allowed to increase the accuracy (AUROC 0.718, CI95% 0.430-1); more specifically, with a 65 HU ROI as cut-point (occlusion <65HU) the PPV was 85.7% (CI95% 42.1% 99.6%) and NPV was 41.7% (CI95% 5.2% 72%).
**Conclusion:** CTA in ischaemic stroke has the role of identifying the region of vasal occlusion. In case of suspect tandem obstruction, visual judgment cannot always distinguish between ICA true occlusion or pseudo-occlusion . The density measurement of the vessel by ROI increases both PPV and NPV and could be used as a new judgement criterion for correct reporting.

**B-0949** 10:54
**Novel rapid imaging protocol of the bilateral MCA territories for thrombectomy in acute ischaemic stroke by old-model 320-row area detector CT**
T. Mori, Y. Tanno, N. Nakai, K. Yoshioka; *Kamakura/JP (morit-koc@umin.net)*

**Purpose:** The 320-row area detector CT (320ADCT) produces large volume data (VD) and image reconstruction of the old model is time consuming. Therefore, 4D-CT angiography (4D-CTA) is not always applicable to pre-thrombectomy. The aim was to design the practical protocol for pre-thrombectomy with any model.
**Methods and Materials:** To avoid subtraction of all images and to shorten image processing time, post-contrast volume scanning (VS) only was performed. VS used 1-s single rotation intermittent dataset scans. The mask was acquired at 1s and beginning 10 s after injection of contrast medium, 20 intermittent volume scans were acquired. Each volume consisted of 120 images, because of not 0.5-mm but 1-mm thickness with z-axis coverage of the not 160 mm but 120 mm (minimal width depending on the model), and total 2520 images were produced. After transferring VD to a workstation (WS), the 8-cm width VD along the middle cerebral artery (MCA) axis was cut out on the WS and 4D-CTA was generated. Examination time and image quality were evaluated.
**Results:** Small VD was transferred quickly to the WS and 4D-CTA of the MCA axis was generated soon and total time until viewing images on PACS was about 5 minutes. Image quality of 4D-CTA was useful in identifying candidates of thrombectomy. Compared to initial protocol of a 0.5-mm thickness with z-axis of the whole brain and subtraction of all images, total operation time became about a quarter.
**Conclusion:** Our protocol can provide practical 4D-CTA of the MCA axis in acute stroke setting.

**B-0950** 11:02
**The Ottawa subarachnoid haemorrhage rule in the decision for acute CT head: external validation in a contemporary European cohort**
R.W. Foley[1], S. Ramachandran[2], A. Akintimehin[3], S. Williams[3], P.A. Kelly[3]; [1]*Bristol/UK*, [2]*Leicester/UK*, [3]*London/UK (rwjfoley@gmail.com)*

**Purpose:** The Ottawa subarachnoid haemorrhage (SAH) rule is a clinical decision tool to aid in the decision for CT Head in Emergency Department (ED) patients with acute non-traumatic headache. The objective of this study was to analyse the performance of the Ottawa rule in a UK cohort.
**Methods and Materials:** Patients undergoing CT Head for the investigation of non-traumatic SAH over a 6-month period at two tertiary centres were assessed. Each patient's Ottawa score was calculated and compared to their final diagnosis. The Ottawa score consists of 6 items, namely age ≥ 40, neck pain/stiffness, witnessed loss of consciousness, onset during exertion, thunderclap headache or limited neck flexion on examination. Analysis was undertaken using R version 3.1.1.
**Results:** The cohort consisted of 366 patients and there were 16 cases of SAH (based on CT findings or xanthochromia in cerebrospinal fluid). The Ottawa rule identified 288 patients requiring CT Head. The sensitivity of the Ottawa rule was 100% (95% CI; 71%-100%) and the specificity was 22% (95% CI; 18%-27%). The negative predictive value was 100% (95% CI; 93%-100%).
**Conclusion:** The Ottawa SAH Rule corrected identified all patients with SAH in this cohort. The use of this rule would allow for an objective triage of patients presenting with acute headache to ED and in 22% of patients CT head could have been avoided. The Ottawa Rule is a useful tool to aid in the decision for CT Head in ED patients under investigation for SAH and should be utilised in clinical practice.

**B-0951** 11:10
**Value of spot sign (SS) on CT angiography (CTA) such indicator of early haematoma expansion in the spontaneous intracerebral haemorrhage (ICH)- some therapeutic considerations**
E. Puglielli, M. Di Bartolomeo, M. Assetta, F. Navarra, M. Di Matteo, M. Galliano, G. Manente; *Teramo/IT (edopug@hotmail.com)*

**Purpose:** Spot sign (SS) on CT-angiography (CTA) are a valid indicator of early haematoma expansion in spontaneous intracerebral haemorrhage (ICH). However, its contribution to outcome have not been well explored.
**Methods and Materials:** We evaluate 93 patients with spontaneous ICH, NCCT and CTA performed on admission within 6h after onset of symptoms, retrospectively from 20013 to 2018. We compare the Blend sign (BS) and black hole sign (BHS) on non-contrast computed tomography (NCCT). Discharge outcome was good (modified Rankin Scale [mRS] 0-3) and poor (mRS 4-6) outcomes. The impacts of SS on outcome were assessed in univariate logistic regression models and the relations with baseline haematoma volume and intraventricular haemorrhage or multivariate for the BS, BHS or SS.

**Results:** Of 93 patients with spontaneous ICH, 11 (11.8%) presented SS. There was a substantial correlation between SS and BS (κ=0.631) and a moderate correlation between SS and BHS (κ=0.345). In the univariate logistic regression the presence of BHS/BS/SS (all *P*<0.001) on admission CT scan were associated with poor outcome. The multivariate analysis identified intraventricular haemorrhage (odds ratio [OR] 2.32 per mL, *P*=0.021), baseline haematoma volume (OR 1.05 per mL, *P*<0.001) and SS on CTA (OR 12.16, *P*<0.002) as independent predictors of poor outcome.
**Conclusion:** Of the CT variables indicating early haematoma expansion, SS on CTA was the most reliable outcome predictor to address for a rapid endovascular treatment. However, some authors give a strong correlation with SS on CTA, BS and BHS on NCCT that can be used for predicting outcome.

**B-0952** 11:18
**Non head-injured paediatrics in the emergency department: clinical predictors of abnormal head CT findings**
J. Wu, H. Huang, M. He, Q. Fan, M. Ma, L. Lin; *Fuzhou/CN (249961548@qq.com)*

**Purpose:** To identify the clinical predictors of abnormal head CT findings of non head-injured paediatrics in emergency department.
**Methods and Materials:** Three years consecutive cases of non head-injured paediatrics (≤14 years old) with head CT acquisitions in emergency department of our hospital from 2015 to 2017 were reviewed retrospectively. Clinical predictors included age, gender, seizure, headache, dizziness or vertigo or ataxia, syncope, vomiting, fever, predisposing conditions (including history of hypoxic-ischaemic encephalopathy, bleeding disorders, malignancy, et al), abnormal physical examinations, altered mental status. Mono factor and multi factors logistic regression analysis were used to identify the independent clinical predictors of abnormal head CT findings of non head-injured paediatrics in emergency department.
**Results:** 22 cases with existed intracranial diseases (5) , unclear medical history records (7) and a history of injury (10) were removed from total 396 cases, and final 374 cases were included. 230 males, 144 females, the average age was (6.9± 4.3) years, the medium age was 7.0 years. 29 (7.8%, 95%CI: 5%-10.5%)of 374 cases with abnormal head CT findings were found. headache (184/49.2%) and seizure (114/30.5%) were the main chief complains. None of abnormal head CT findings in patients with vertigo or dizziness, and syncope were detected. Three independent Clinical predictors of abnormal head CT findings were identified: predisposing conditions (*OR*=12.109, 95%CI:1.96-74.80), abnormal physical examinations(*OR*=3.704, 95%CI:1.17-11.72), altered mental status (*OR*=9.593, 95%CI: 2.80-32.83).
**Conclusion:** Predisposing conditions, abnormal physical examinations, altered mental status were the independent clinical predictors of abnormal head CT findings of non head-injured paediatrics in emergency department.
*Author Disclosures:*
**J. Wu:** Research/Grant Support; Youth scientific research program of Fujian provincial health and family planning commission(N0.2016-1-30).

**B-0953** 11:26
**Contributions of the whole-body CT scan before multi-organ donation in brain dead patients**
F. Leleu, C. De Margerie, C. de Bazelaire, F. Gaudez, C. Lefaucheur, E. De Kerviler; *Paris/FR (florian.leleu.pro@gmail.com)*

**Purpose:** To demonstrate the interest of whole-body CT before multi-organ donation in brain dead patients.
**Methods and Materials:** 240 consecutive patients were referred for CT angiography (CTA) diagnosis of brain death between June 2010 and December 2017. We tried to combine multiphase brain CTA and chest abdomen and pelvis CT in the same session to find contraindication to donation, evaluate the organ morphology and to detect anatomical variants. In addition to the brain evaluation, the CT protocol included an abdominal acquisition without injection, a chest, abdomen and pelvis acquisition at the arterial phase and abdomen pelvis acquisition at portal phase. CT scans were analysed according to a standardized report and compared to postoperative results.
**Results:** 43 out of 240 (17.7%) patients were excluded for organ donation. The first cause (20/43) was the results of the CT scan. In 12 patients (5%), CT revealed a malignant condition. Hepatosteatosis was found in 49 patients, 15 of whom were overweight. A hepatic arterial variant was detected in 51 patients (21%) and renal arterial variant in 86 patients (36%).
**Conclusion:** Whole-body CT performed in brain dead patients allows the detection of contraindications to organ donation. It also allows to analyse the morphology of all organs of interest and can constitute an aid to the surgical procedure by detecting anatomical variants. It is feasible at the same time of brain CTA in countries where this examination is validated for the diagnosis of brain death. Multi-organ donation should no longer be performed without prior whole-body scanning.

**B-0954** 11:34
**Evaluation of a novel 3D-surface reconstruction technique in post-mortem CT after stab attacks: diagnostic accuracy and forensic impact compared to conventional CT and autopsy findings**
J. Böven, J. Boos, R.S. Lanzman, Y. Klosterkemper, P. Kröpil, C. Thomas, G. Antoch, J. Aissa; *Düsseldorf/DE (judith.boeven@med.uni-duesseldorf.de)*

**Purpose:** Evaluation of diagnostic accuracy and forensic relevance of a novel 3D-reconstruction technique (cinematic rendering) used for stab wounds in post-mortem CT compared to conventional CT and autopsy findings.
**Methods and Materials:** Inclusion of six human cadavers undergoing whole body PMCT with blood loss as cause of death in all cases. Two radiologists counted the number of stab wounds in PMCT and the number of the wounds that were detectable in 3D-cinematic-rendering (CR) reconstructions. Afterwards assessment of the total number of stab wounds during autopsy by two forensic pathologists. One forensic pathologist evaluated the CR reconstructions regarding their relevance in forensic interpretation and demonstration on a five-point scale (1: very useful for forensic interpretation and demonstration; 5: not useful).
**Results:** 6 human cadavers had a total number of 99 stab wounds found in autopsy by pathologists. Both PMCT readers detected 59 stab wounds. 56/59 (94,9%) of the stab wounds were found on CR reconstructions. Both, CR and PMCT were significantly inferior compared to autopsy. CR reconstructions were significantly more useful than conventional PMCT (p<0.005) concerning forensic interpretation and demonstration aspects.
**Conclusion:** Photorealistic 3D-reconstructions are declared more useful for forensic interpretation and demonstration compared to conventional PMCT in the diagnosis of stab wounds. A significantly lower amount of stab wounds was found in autopsy performed by forensic pathologists than in PMCT and 3D-CR-reconstructions, however autopsy is still the method of choice in detection of stab wounds. 3D-reconstructions of stab wounds are more useful for demonstration aspects compared to conventional PMCT.

**B-0955** 11:42
**Imaging of penetrating extremity injuries in a Scandinavian level-1 trauma centre: a 4-year analysis of the trauma registry data from the Karolinska trauma centre, Stockholm**
P.M. Cewe Jönsson[1], S. Thorisdottir[2], G.L. Óladóttir[1], S. Koskinen[3]; [1]*Solna/SE*, [2]*Stockholm/SE*, [3]*Huddinge/SE (paulina.cewe-jonsson@sll.se)*

**Purpose:** To analyse incidence, mechanism of injury, imaging approach and clinical outcome in penetrating extremity injuries at a level-1 trauma centre.
**Methods and Materials:** A retrospective cohort study of penetrating injuries between 2013 and 2016 (4 years) to the extremities retrieved from the Karolinska Trauma Registry. Imaging data were accessed through hospital RIS and PACS, and electronic medical records were analysed. Two radiologists collected and analysed imaging data while the third evaluated the subgroup of extremity injuries.
**Results:** Of 636 patients with penetrating injury, 443 had imaging data available. Of these, 36.8% (163/443) had penetrating extremity injuries. This cohort comprised 145 males (89.0%; age range 15-83, mean 31.4) and 18 females (11.0%; age range 18-93, mean 35.7). Main mechanisms of injury were stabbing (SW) 50.3% (82/163) and shooting (GSW) 43.0% (70/163). Half of the injuries were limited to the lower extremities (51.5%, 84/163). Imaging was performed in 85.3% (139/163) of patients. CT angiography (CTA) was the most common imaging modality (93/139; 67%). Arterial damage was present on 44 CTAs (47.3% 44/93). Compared to SW, GSW increased the odds for surgery significantly (OR 2.5 p<0.02). In general, GSW increased the odds for surgery by 7.7 times (p<0.008) compared to SW. 30-day morbidity rate was 11.7% (19/163). Overall mortality rate was 0%.
**Conclusion:** Penetrating extremity injuries are relatively common. In this study, imaging was performed in 85% of patients. CTA was the most common imaging modality. GSW significantly increased the overall odds of surgery compared to SW. No deaths occurred in our patient cohort.

10:30 - 12:00 Room Y

### Interventional Radiology

### SS 1009

### Vascular interventions in the aorta and peripheral arteries

*Moderators:*
R.F. Dondelinger; Liège/BE
F.F. Strobl; Munich/DE

**B-0957** 10:30
**Preoperative tibial artery run off score differences before endovascular or bypass surgery in patients with long popliteal artery lesions**
A. Kratovska, S. Ponomarjova, P. Ivanova; *Riga/LV*

**Purpose:** Objective, quantitative preoperative tibial run-off score (TRS) calculation is not highlighted by guidelines of critical limb ischaemia (CLI) although it plays important role in revascularization treatment method selection (RTMS) for patients with long popliteal artery atherosclerotic lesion. The aim of this study was to analyse association of TRS with RTMS.
**Methods and Materials:** Single centre retrospective case- control study performed in 2016-2017 in tertiary care hospital. 47 patients with infrageniculate bypass surgery (IGBS) and 36 with endovascular therapy (EVT) of femoropopliteal segment (n=83) who met the inclusion criteria were selected. An authors adjusted TRS calculation system (total occlusion=0 point, intact=22) was used to analyse tibial artery status. Age, gender, Rutherford category, femoropopliteal lesion TASC II type, TRS and statistical association of variables with RTMS analysed by SPSS 22 program. $P < 0.05$ considered statistically significant.
**Results:** Mean TRS in IGBS group was statistically significantly higher than in EVT group- 9.074 ±3.887 *vs* 5.7 ±3.852 (p<0.001). In univariate analysis from all the variables analysed age (*t* test, $p$=0.008), gender (Exact Fisher test, $p$ = 0.032),TASC II lesion type (Pearson Chi-Square test, $p$ =0.006) and TRS (*t* test, $p$=0.006) were statistically significantly associated with RTMS. Multivariate analysis ruled out that only TASC II lesion type and TRS are significantly associated with RTMS ($p$<0.001).
**Conclusion:** TRS is a strong predictor of revascularization modality choice in patients with CLI and long popliteal artery lesion and is significantly higher in IGBS group. Objective, quantitative preoperative TRS evaluation could be recommended in a routine practice.

**B-0958** 10:38
**Long-term results of the aortoiliac kissing stents and risk factors of in-stent restenosis**
M. Vértes, I.Z. Juhász, D.T. Nguyen, C. Csobay-Novák, B. Nemes, K. Hüttl, E. Dósa; *Budapest/HU (vertesmiki@gmail.com)*

**Purpose:** To determine the long-term patency of the aortoiliac kissing stents and to identify predisposing factors for the development of in-stent restenosis (ISR).
**Methods and Materials:** A retrospective analysis was conducted of 105 patients (median age 60.9 years; 64 women) with symptomatic aortoiliac occlusive disease who had kissing stents implanted between 2001 and 2015. Patient, vessel, lesion, and stent characteristics were examined. Lesions were TASC A in 52 (49.5%), B in 29 (27.6%), C in 4 (3.8%), and D in 20 (19%) patients. Twenty-five (23.8%) patients had heavily calcified lesions. In all, 210 stents [180 (85.7%) self-expanding and 30 (14.3%) balloon-expandable] were deployed. Follow-up included palpation of peripheral pulses, measurement of ankle-brachial index, and duplex ultrasonography. Mann-Whitney *U*, Fisher's exact, and log-rank tests, as well as Kaplan-Meier, Cox regression, and receiver operating characteristic analyses were used as statistical methods.
**Results:** The median follow-up was 45 months. Primary patency rates were 95%, 93%, 86%, and 77% at 6, 12, 24, and 60 months, respectively. Significant ISR developed in 23 (21.9%) patients. Multivariable analysis showed a longer aortic stent segment to be the only significant determinant of ISR (HR 1.44, 95% CI 1.02-2.01, P=0.035). The primary patency was significantly worse (P<0.001) in patients with longer (>20 mm) aortic stent segments.
**Conclusion:** The kissing stent technique can be performed with good long-term patency rates for the treatment of aortoiliac occlusive disease. Patients whose iliac stents protrude too far into the aorta need closer follow-up.

**B-0959** 10:46
**Comparative assessment of occupational radiation exposure between transbasilic and transfemoral approach during percutaneous retrograde scleroembolisation of left varicocele**
A. Conti, M.C. Calcagno, D. Meo, A. Modica, D. Rizzo, L. Mammino, P.V. Foti, S. Palmucci, A. Basile; *Catania/IT (mc.calcagno@gmail.com)*

**Purpose:** Aim of this study is to compare the occupational radiation exposure difference between transfemoral (TF) or transbasilic (TB) venous access, during percutaneous retrograde scleroembolisation of left spermatic vein.
**Methods and Materials:** In this randomized trial we evaluate 18 males patients (9 via TF and 9 via TB venous access - mean age 29.8 years old, range 18-43 years old), treated, between March and September 2018 (the study is still in progress), by retrograde scleroembolisation of left varicoceles. During the procedures, the occupational exposure is measured using a direct reading integral dosimeter calibrated in HP(10) (personal dose equivalent at the depth of 10 mm). To maximize the statistical coherence and avoiding bias, the procedure has always been performed by the same operator, using the same angiography system, protocol and dosimeter. Data have been collected at the end of each procedure referring to fluoroscopy time, DAP (dose area product) and exposure measured in Hp(10). Statistical analysis has been performed using t-test.
**Results:** Operator occupational exposure in Hp(10) is higher using TF access (63.44±47.24 vs 27.89±17 μSv; p=0.0496). Mean fluoroscopy time(8.311±2.391 vs 5.489±2.045 min; p=0.0161), DAP (92.89±62.69 vs 40.22±22.7 Gycm2; p=0.037), effective dose (E) and eye lens equivalent dose (HE) are higher as well using TF access.
**Conclusion:** TB venous access in the scleroembolisation of left varicocele offers an advantage in terms of better haemostasis, reduced complications, better patient compliance and moreover in terms of lower radiation exposure of the operator and the patient.

**B-0960** 10:54
**Transcatheter arterial embolisation for traumatic thoracic bleeding: 4-years' experiences in a single trauma centre**
C.M. Lee, C.H. Jeon, J.B. Park, C.W. Kim, H. Kwon, H.M. Cho; *Busan/KR (changho.jeon@gmail.com)*

**Purpose:** We aimed to assess the safety and efficacy of transcatheter arterial embolisation (TAE) for thoracic arterial haemorrhage following chest trauma.
**Methods and Materials:** From November 2013 to May 2017, 35 patients were referred to our interventional unit for thoracic arterial bleeding following chest trauma, based on clinical decisions and computed tomography (CT) images. We exclude seven patients expired within one week due to other medical problems. A total of 28 patients (male:female ratio, 21:7; median age, 54 years) who underwent selective TAE of thoracic haemorrhage-culprit arteries were included in this study. Technical and clinical success, complications, and 30-day mortality rate were analysed.
**Results:** In 28 patients who underwent TAE, the main bleeding arteries were in the intercostal artery (n=44), internal mammary artery (n=13), bronchial artery (n=3) and superior and/or lateral thoracic artery (n=2). Gelatin sponge particles (n=39), N-butyl-2-cyanoacrylate (NBCA) (n=15), combination of gelatin sponge particles and microcoils (n=4), polyvinyl alcohol (n=3) and microcoils (n=1) were used as embolic agents. Initial technical success was achieved in all 28 patients, with immediate cessation of bleeding. Four patients showed rebleeding 1-2 days later and underwent repeated TAE with successful result. Clinical success rate was 92.8% (26/28), and two patients underwent thoracotomy for controlling residual bleed. There were no TAE-related major complications infarction or quadriplegia. The 30-day mortality rate was 7.1% (2/28).
**Conclusion:** Our clinical experience suggests that TAE used to control thoracic arterial bleeding following chest trauma is safe and effective as a minimally invasive alternative to surgery.

**B-0961** 11:02
**Vacuum-assisted aspiration thrombectomy for treatment of acute massive pulmonary embolism**
A. Massmann[1], P.M. Lepper[1], P. Fries[1], H. Wilkens[1], A. Buecker[1], B. Huasen[2]; [1]*Homburg/DE*, [2]*Manchester/UK*

**Purpose:** Prospective evaluation of feasibility and safety of vacuum-assisted aspiration thrombectomy (VAST) for percutaneous treatment of acute massive pulmonary embolism (PE).
**Methods and Materials:** In a multi-national cohort of 12 consecutive male patients (median age 54, range 35-80 years) acute massive PE was verified by contrast-enhanced computed tomography and transthoracic echocardiography. Endovascular vacuum-assisted aspiration thrombectomy (Penumbra Indigo XTORQ 8F) was performed during general anaesthesia in all patients after on-set of cardiac shock. Two patients needed extracorporeal membrane oxygenation (ECMO) for cardiac assistance. Anticoagulation in ECMO patients included therapeutic heparinization, otherwise 5.000 I.U. of heparine before and during VAST. Right heart strain, mean pulmonal-arterial pressure (mPAP), peripheral transcutaneous oxygenation, and survival were assessed.
**Results:** Technical success of VAST was achieved in all patients. Mean procedure time (puncture-to-haemostasis) was 112+/-47 (range 55-180) minutes, contrast medium volume 130+/-19 (100-150) ml, thrombectomy aspiration volume 213+/-55 (150-300) ml. mPAP improved from 58+/-7 (52-65) mmHg to 30+/-3 (24-34) mmHg (p=0.0080) and peripheral oxygenation increased from admission 83+/-4 (79-90)% to 96+/-2 (96-99)% postinterventional (p=0.0222). Cardiac imaging studies 24 hours later showed improvement of right to left ventricular quotient from 1.5+/-0.2 (1.24-1.7) to 1.0+/-0.1 (0.86-1.2) (p=0.0031). Follow-up up to 2 years showed no procedure-associated bleeding complication or death.
**Conclusion:** In an international prospective registry of a limited number of patients vacuum-assisted aspiration thrombectomy proved to be a safe and effective option for percutaneous catheter-directed treatment of acute massive pulmonary embolism.

**B-0962** 11:10
**Gemstone Spectral Imaging (GSI) in early CT-follow up after endovascular aortic repair (EVAR)**
H. Muenzfeld, T. Schmidt, H. Posch, B. Gebauer, B. Hamm, G. Böning; *Berlin/DE (hanna.muenzfeld@charite.de)*

**Purpose:** Endovascular aortic repair (EVAR) is one of the most challenging procedures in interventional radiology related to several risks and complications. Next to contrast enhanced ultrasound, CT is used for follow up. We aimed to evaluate the diagnostic accuracy and dose reduction potential of Gemstone Spectral Imaging (GSI) for early CT-follow up after EVAR.
**Methods and Materials:** Preliminarily, 13 cases of GSI CT after EVAR were included. The dual phase protocol includes a dual energy (GSI) arterial phase and a monoenergetic venous phase at an equal scan length. Iodine suppressed images (ISI) and virtual monochromatic images (VMI) were reconstructed from GSI data. Radiation dose was compared intraindividually and to a low dose triple phase protocol, including a pre-non contrast phase. Diagnostic confidence with special regard to endoleak detection was assessed.
**Results:** Preliminary results confirm the diagnostic usability of GSI-CT in early follow up after EVAR with diagnostic confidence levels equivalent to our standard. Intraindividually, GSI technique shows equal radiation exposure compared to monoenergetic images (mean CTDI 8,09±4,5 vs. 8,60±4,1). GSI did not significantly increase mean applied dose compared to the low dose triple phase protocol (mean DLP 1051±389 vs. 842±565 mGy*cm,p>0.2) by replacing the non-contrast phase by ISI.
**Conclusion:** Dual energy GSI CT with a dual phase protocol is feasible for early CT-follow up after EVAR without sacrificing diagnostic confidence and endoleak detection by utilization of ISI and VMIs. GSI does not significantly increase applied radiation dose compared to the monoenergetic phase and to a low dose triple phase protocol.

**B-0963** 11:18
**Prospective single-centre registry of endovascular aortic repair (EVAR) using ultra-low profile altura endograft**
A. Massmann[1], F. Frenzel[1], P. Fries[1], R. Kubale[2], R. Shayesteh-Kheslat[1], A. Buecker[1]; [1]*Homburg/DE*, [2]*Pirmasens/DE*

**Purpose:** To report preliminary prospective single-center experience of endovascular aortic repair (EVAR) using ultra-low profile (14F) Altura (Lombard Medical) stentgrafts.
**Methods and Materials:** During 2016-2018, 31 consecutive patients (3 females) (mean age 73±9;range 57-88 years) underwent elective or urgent (n=2) EVAR of abdominal aortic aneurysms (AAA) (mean diameter 55±4.5 (50-68)mm; angulation 31±24 (0-80°); infrarenal neck length 31±12 (14-55)mm; aorto-iliac aneurysms (n=2; common iliac artery aneurysm diameter 35 and 45mm) and one renal chimney (AAA 53mm) using Altura endografts. Prospective follow-up recording morbidity and mortality outcomes, endoleak and re-intervention included contrast-enhanced duplex-ultrasound and computed tomography.
**Results:** Technical success for device deployment was 100%. Mean procedure time (needle to hemostasis) was 59±13 (40-91)minutes; fluoroscopy time 10±4 (4-18)minutes; dose-area-product 4,131±2,030 (1,377-8,188) µGym2; contrast medium volume 75±29 (40-150) ml. Compared to a per aneurysm characteristics matched patient cohort treated with Medtronic Endurant II, fluoroscopy (median 10 vs. 17minutes p=0.0001); dose-area-product (p=0.0063) and procedure-time (60 vs. 69 minutes; p=0.0477) were significantly lower for Altura. Overall hospitalisation was 6.6±5.2(3-26) days (Intensive care unit 1.4±0.8 (1-4) days). There was no relevant blood loss. Early (≤30days) complications included endoleak type-2 in 30%; one patient experienced mild temporary post-implantation syndrome; 4 patients required additional surgery for symptomatic femoral artery dissection/pseudoaneurysm. During mean follow-up of 5±7(1-25) months freedom from device-related complications, aneurysm-related re-intervention or death was 100%. Aneurysm shrinkage (<5 mm) was seen in 20% of patients.
**Conclusion:** Preliminary experience showed promising short-term results with the low-profile easy-to-use Altura device. Long-term studies are required to assess durability and device-related complications.

**B-0964** 11:26
**Fusion imaging to guide thoracic endovascular aortic repair (TEVAR): comparison of two methods: 2D/3D vs 3D/3D image fusion**
P.-A. Barral, A. Bartoli, P. Piquet, M. Demasi-Jacquier, M. Gaudry, A. Jacquier; *Marseille/FR (pierre-antoine.barral@ap-hm.fr)*

**Purpose:** To compare two-dimensional (2D) versus three-dimensional (3D) image fusion for thoracic endovascular aortic repair (TEVAR) image guidance.
**Methods and Materials:** Between June 2014 and March 2018, all patients who underwent TEVAR were included. TEVAR was performed by a multidisciplinary team in a hybrid suite under image fusion guidance. Two different fusion ,procedures namely 2D/3D and 3D/3D were evaluated in terms of accuracy, X-ray exposure (dose-area product, DAP), volume of contrast medium injected, fluoroscopy time, and procedure time. All aortic and general complications were recorded at one-month follow-up.
**Results:** 42 patients were prospectively included, 26 were performed under 2D/3D, and 16 were performed under 3D/3D. The 3D/3D method allows a more accurate positioning of the aortic mask on the top of the fluoroscopic images (proximal landing zone error vector: 1.68mm±3.1) compared to the 2D/3D (6.6mm±5.8; p=0.008). The 3D/3D image fusion method was associated with significant DAP reduction compared to the 2D/3D method (51 Gy.cm² ± 29 for 3D/3D vs 91.9 Gy.cm² ± 76 for 2D/3D; p=0.01). The volume of contrast medium injected was statistically lower using 3D/3D method versus 2D/3D (47.4 ml±24 vs 113 ml±53; p<0.0001). At one month's follow-up, we recorded 4 complications for 2D/3D and one complication for 3D/3D with no significant differences between the two groups.
**Conclusion:** 3D/3D image fusion is feasible and safe and yields higher image fusion accuracy than 2D/3D image fusion during TEVAR. It helps to reduce contrast volume and irradiation, but does not demonstrate better clinical outcomes at one month.

**B-0965** 11:34
**Distal transradial approach at the anatomical snuffbox for interventional procedures: a single-centre pilot study**
S.-E. Park[1], S. Cho[1], C. Hocheol[2], S. Lee[2]; [1]*Changwon/KR*, [2]*Jinju/KR (uneyes@gnuh.co.kr)*

**Purpose:** Distal radial artery (RA) access at the anatomical snuffbox is a new approach for endovascular procedure. We demonstrate in twenty-one cases the possibility of distal RA access to performed endovascular treatment without any additional equipment or cost. To evaluated the feasibility and safety in a single centre using distal transradial access (TRA) for endovascular treatment.
**Methods and Materials:** Distal TRA was attempted in 21 patients (16 men; mean age, 60.2 years) who underwent 21 consecutive procedures (chemoembolization [n=5], bronchial artery embolization [n=3], renal intervention [n=3], uterine artery embolization [n=1], arteriovenous fistula angioplasty [n=3], subclavian artery stenting [n=3], other embolization [n=3]) from January 2018 to September 2018. Exclusion criteria included procedures requiring sheath > 6 Fr, Barbeau D waveform and distal RA diameter < 2 mm on ultrasound. Patients were evaluated for complications during follow-up (range 14-60 days).
**Results:** Technical success was 95.2% (20/21). There was no major complication. Minor complication (4.8%) included distal RA segmental occlusion (n=1). The failed case required crossover to transfemoral access due to proximal subclavian artery occlusion. Mean haemostasis time is 151.8 minutes (range 120-360 minutes).
**Conclusion:** Distal TRA at the anatomical snuff box was feasible and safe in various diseases over a range of vascular interventions.

**B-0966** 11:42
**Tunnelled central venous catheters (TCVC) in infants using adult peripheral inserted central catheters (PICC) devices: ten years experience**
R. Nourzaie, H. Abbas H.M., L. Monzon, N. Karunanithy, S. Moser, P. Gkoutzios, I. Ahmed, S. Boolkah, A. Diamantopoulos; *London/UK (romman.nourzaie@nhs.net)*

**Purpose:** Tunneled Central Venous Catheters (TCVC) is a reliable venous access in infants (up to 12 months). We present our experience with tunneled adult PICC line devices used in this age group.
**Methods and Materials:** We retrospectively searched our electronic hospital database for a 10 years period collecting data from consecutive pediatrics patients less than 1 year old, who underwent tunneled central venous catheter insertion. The patient's demographics, site of access, and procedure details were recorded where outcomes included technical success and post procedure complications.

**Results:** From April 2008-April 2018 a total of 182 TCVC were inserted in 161 patients (49.7%, n=80 male). Mean age was 100 days (range 0-342) with average weight 4.20 Kg (range 1.80-9.40). The most common indication recorded for insertion was for antibiotics in 41% of cases (n=66) followed by TPN 34.7% (n=56) while other indications were recorded for the remaining cases. Technical success was 99% (n=180). Early complications were seen in 8.8% (n=14) including among others inadvertent line removal in 4.9%, line sepsis in 1.1%, line thrombosis in 1.6% (n=3), and others. Average line functional duration prior to removal was 26 days (range 0-180). The majority of the lines 76.9% lasted for the intended duration of treatment.
**Conclusion:** Tunneled Central Venous Catheters using adult PICC line devices in infants is a safe technique with excellent success rate and minimal complications rates.

**B-0967** 11:50
**Efficacy of drug-eluting stents ELUVIA in treatment of high grade of femoropopliteal lesions**
F. Rosella, T. Gorgatti, A. Vit, V. Gavrilovic, A. Pellegrin, M. Sponza; *Udine/IT (francesco.rosella.md@gmail.com)*

**Purpose:** Preliminar study of efficacy about Drug-Eluting Stents (DES) ELUVIA in 39 cases on 27 patients with high grade of symptoms and femoropopliteal lesions.
**Methods and Materials:** Patients were stratified according to clinical characteristics (age, sex, smoke, diabetes, CKD in HD, dyslipidemia). Lesions were stratified according to TASC II: 3A (7,6%), 2B (5,1%), 27C (69,2%), 7D (17,9%) (average length 95,25mm, DVstd 43,16mm). Primary Outcome was patency at 6, 12 and 18 months during follow up: absence of target lesion revascularization, no significant neointimal iperplasia at B-Mode US and duplex ultrasound peak systolic velocity ratio of ≤2.5, at clinical visit and EchoColorDoppler control.
**Results:** Success rate of procedures was 100%: any vascular complications, neither distal embolic events. The rate of primary patency was of 82% at 6 months, 74% at 12 - 18 months. During follow up occurred 9 restenosis/occlusion, 7 in the first 2 months, 6 TASC C and 1 TASC D, retreated with a success rate of 100%, and rate of secondary patency of 100% at 6 months, 91% at 12 months. Only 2 occurred after sixth month, in patients undergone major amputation (1knee, 1leg).
**Conclusion:** Patency rate was high despite the severity of lesions, and similar to results of studies performed on populations with lower grade of lesions. Study indicates that DES are safe and effective in treatment of high grade of femoropopliteal lesions.

**10:30 - 12:00** **Coffee & Talk 3**

### Head and Neck

### SS 1008

### Head and neck cancer

*Moderators:*
A.D. King; Hong Kong/CN
A. van der Hoorn; Groningen/NL

**K-23** 10:30
**Keynote lecture**
N.I. Traykova; *Plovdiv/BG*

**B-0968** 10:39
**Histogram analysis of CE-MRI for differential diagnosis of pleomorphic adenomas and malignant tumour of parotid gland**
K. Xu, Y. Zhang; *Zhengzhou/CN (109496417@qq.com)*

**Purpose:** To assess the value of whole tumours volume based on enhancement MR histogram analysis in differentiating between pleomorphic adenomas and malignant tumour of parotid gland.
**Methods and Materials:** A retrospective analysis was conducted by brain MRI examination and pathology diagnosis of 64 cases of parotid tumour in our hospital. Among them, there were 41 cases of pleomorphic adenomas, 23 cases of malignant tumour of parotid gland. Respectively, to draw the region of interest (ROI) in the enhancement MR transaxial images of two groups on each layer of tumour level using Mazda software and analyse the whole tumour gray histogram, performing a statistical analysis on the two sets of parameters obtained from histograms to find out statistical significance of each parameter.
**Results:** Through histogram analysis of 12 parameters, these 3 parameters were statistically significant (all P<0.05), including 50%, 90% and 99%, the remaining 9 parameters area, maxnorm, minnorm, mean, variance, skewness, kurtosis, 01%, 10% had no significant difference between the two groups (all P>0.05). Among them, the sensitivity of 90% was 65.9%, the specificity was 73.9%, the area under the curve was 0.703, and the best cut-off value was 236.57, 90% had a high identification efficiency.
**Conclusion:** Histogram analysis of enhancement MR imaging can provide reliably objective basis for differentiating pleomorphic adenomas from malignant tumour of parotid gland. 90% had a high identification efficiency.

**B-0969** 10:47
**The value of high-resolution diffusion-weighted imaging in differentiating lingual squamous cell carcinoma and lingual lymphoma**
B. Wen[1], J. Cheng[1], J. Zhu[2]; [1]*Zhengzhou/CN*, [2]*Beijing/CN (wenbaohong2012@163.com)*

**Purpose:** To investigate the diagnostic value of readout segmentation of long variable echo-trains (RESOLVE) diffusion-weighted imaging (DWI) (b=1000 s/mm$^2$) in the evaluation of lingual squamous cell carcinoma and lingual lymphoma.
**Methods and Materials:** 70 patients with lingual squamous cell carcinoma and 21 patients with lingual lymphoma confirmed by pathology were retrospectively analysed from February 2014 to January 2018; all patients underwent preoperative routine MRI and RESOLVE DWI. The ADC average values of the lesions were calculated; *t* test was used to compare the ADC values of lingual squamous cell carcinoma and lingual lymphoma. Diagnostic performance of ADC value was compared using receiver operating characteristic curves (ROC).
**Results:** The mean ADC value of lingual squamous cell carcinoma was (1.09±0.16) ×$10^{-3}$ mm$^2$/s and lingual lymphoma was (0.64±0.11) ×$10^{-3}$ mm$^2$/s, there were statistically significant differences between them ($t$=12.40, $P$<0.001). The area under the ROC of ADC value differentiating lingual squamous cell carcinoma and lingual lymphoma was 0.999±0.001. Using ADC value ≤0.88×$10^{-3}$mm$^2$/s as the critical value for diagnosing lingual lymphoma and comparing with pathological results, the result obtained had a sensitivity of 100%, specificity of 98.57%, ADC value had high correlations compared with pathological results, and *Kappa* value was 0.970.
**Conclusion:** RESOLVE DWI is quite effective in differential diagnosis between lingual squamous cell carcinoma and lingual lymphoma.

**B-0970** 10:55
**Staging tongue cancer with the 8th edition of TNM: can MR provide a surrogate of the pathologic DOI?**
R. Campora, F.M. Piana Jacquot, I. Zorza, G.M. Agazzi, M. Ravanelli, D. Farina; *Brescia/IT (camporic@hotmail.it)*

**Purpose:** To assess the reproducibility of three surrogate methods of DOI measurements applicable to MR and their correlation with N stage at presentation and follow-up.
**Methods and Materials:** Retrospective analysis on 58 MR scans performed preoperatively in patients affected by SCC of the oral tongue. Two independent operators measured on axial and coronal plane: radiologic tumour thickness (RTT) drawing a line perpendicular to the mucosal surface and reaching the deepest part of the tumour; endophytic tumour (ET) drawing a line between the normal mucosal layers at the extremes of the lesion and from that measuring perpendicularly the tumour depth; reconstructed tumour thickness (TMr), measuring on the unaffected side the distance between normal mucosa and septum, subtracting then the distance between the deepest part of the tumour and the septum. Reproducibility was measured with ICC and Bland-Altman and clinical correlations were studied with Mann-Whitney test.
**Results:** Considering all techniques, ICC showed scored 0.9265 as worst value, Cohen’s k scored 0.6463 as worst value. ET in the coronal plane was the only measurement significantly correlated to N stage (median in pN0 patients 6.7mm, in pN+ 10.6mm p=0.0066) and recurrences (median in R0 patients 5.9mm, in R1 13.9mm p=0.0332).
**Conclusion:** MR measurements showed acceptable reproducibility between two operators with very different experience. ET in the coronal plane appears a viable surrogate to the pathologic DOI.

**B-0971** 11:03
**Parotid tumours differential diagnosis: MRI dynamic contrast-enhanced (DCE) and diffusion-weighted imaging (DWI) compared to fine needle aspiration cytology (FNAC)**
G. Guazzarotti, E. Venturini, R. Mellone, A. De Gaspari, D. Di Santo, A. Del Maschio, F. De Cobelli; *Milan/IT (guazzarotti.giorgia@hsr.it)*

**Purpose:** To determine diagnostic accuracy of combined evaluation of DWI and DCE in the differential diagnosis of parotid tumors, compared to FNAC.
**Methods and Materials:** Patients with parotid lesion underwent preoperative multiparametric MRI(T2,T1,DWI[b:0-1000],DCE) and FNAC. Mean ADC was measured by ROI avoiding necrosis and compared to mean muscle ADC, obtaining ADCratio(rADC). 2 rADC thresholds(identified by ROC analysis) were used to obtain 3 groups: pleomorphic adenomas(PA)/cysts(Cy), lymphomas(L), indeterminate lesions. For indeterminate lesions, Time-Intensity Curves(TIC) were evaluated and classified as type A(time to peak[TTP]≥150s),

B(TTP<150s,washout ratio[WR]≥30%), C(TTP<150s,WR<30%), D(flat); type C were considered malignant. Sensitivity(SE), specificity(SP), positive and negative predictive values(VPP,VPN) were evaluated using histology as gold standard. Diagnostic accuracy of FNAC and MRI were compared.
**Results:** We evaluated 44 benign(19 PA,2 basal cell adenomas[BA],18 Warthin[W],2 IgG4 disease[IG],3 Cy,) and 13 malignant lesions(7 carcinomas[K], 2 metastases[M],4 L). 2 rADC thresholds were identified to distinguish PA/Cy(rADC>1.37; AUC=0.96) and L(rADC<0.57; AUC=0.96) to obtain 3 groups: rADC<0.57(3/4L), rADC>1.37(17/19PA,3/3Cy,1/18W,1/7 K), 0.57<rADC<1.37(17/18W, 2/19AP, 2/2BA, 2/2IG, 6/7K, 2/2M,1/4L). We analyzed TIC for 0.57<rADC<1.37 group, considering type C as malignant (6/6K, 1/2PA, 2/2BA). Combining DWI and DCE we correctly diagnosed 52/57lesions (91.2%):11 True Positives (TP), 2 False Negative (FN),41 True Negatives (TN), 3 False Positives(FP) with 84.6%SE, 93.2%SP, 78.6%VPP and 95.3%VPN. T1&T2 signal and morphology did not allow a better characterization of FP&FN results. 54 patients underwent FNAC, with 8 non-diagnostic finding and 46 adequate specimens:7 revealed malignant cells,39 benign lesion, with 7TP, 1FP, 5FN, 33TN. FNAC correctly discriminated between benign and malignant lesions in 74% cases (40/54), fewer than MRI.
**Conclusion:** DWI and DCE allow to confidently discriminate benign and malignant parotid tumors in preoperative work-out, representing a valid alternative to FNAC.

**B-0972** 11:11
**The value of RESOLVE on 3.0 T MRI for predicting the early efficacy of concurrent radiochemotherapy of advanced stage nasopharyngeal carcinoma patients**
C. Xu; *Zhengzhou/CN (xcm-1977@163.com)*

**Purpose:** To discuss the value of RESOLVE for predicting early therapeutic effect of concurrent radiochemotherapy in advanced stage nasopharngeal carcinoma patients.
**Methods and Materials:** The ADC value and the area of the tumour was measured before treatment and 2 weeks after treatment independently performed by two radiologists with 5 years experiences and the agreement evaluation was performed using ANOVA analysis.The difference between pretreatment ADC value was compared in CR group and non-CR group by independent sample *t* test. ROC curve was drawn and the maximum Youden index value was the cutoff calculating the ADC value and predicting the sensitivity, specificity and area under the curve.
**Results:** ⑴The agreement between 2 radiologist was excellent. The ICC values of the ADC and the area of the tumour before treatment and the area of the tumour after treatment were 0.89,0.92 and 0.95,respectively. ⑵ The pretreatment ADC values of the CR group and the non-CR group were(0.877±0.103×10$^{-3}$)and(0.779±0.078×10$^{-3}$)mm$^2$/s, respectively. There was statistical difference ($t$ value=2.874, $P$ value=0.005). ⑶ ROC curve showed that the sensitivity and specificity of the pretreatment ADC value in predicting CR was 85.2% and 71.0%,with the cut-off value of 0.792×0$^{-3}$mm$^2$/s , and the area under curve was 0.778. ⑷ There was apparently correlation between the pretreatment ADC value and the tumour regression rate(r=0.333,$P$ =0.006).
**Conclusion:** There was important value using the pretreatment ADC value measured by RESOLVE for predicting the early effect of concurrent radiochemotherapy in advanced stage nasopharngeal carcinoma patients.

**B-0973** 11:19
**Impact of radiologic imaging revision in the multidisciplinary management of head and neck cancer patient**
D. Rossi[1], D. Alterio[1], S. Volpe[1], F.A.K. Pounou[1], C. Giannitto[1], M. Ansarin[1], M. Bellomi[1], B.A. Jereczek-Fossa[1], L. Preda[2]; [1]*Milan/IT*, [2]*Pavia/IT (duccio.rossi@ieo.it)*

**Purpose:** To quantify impact of radiologic images revision performed by expert radiologists in the contest of multidisciplinary team (MDT) discussion of head and neck cancers.
**Methods and Materials:** We retrospectively reviewed all cases discussed at MDT meetings from April 2014 to April 2017 and selected only those for whom a radiologic revision was required. Any changes of the radiologic description compared to the original chart was collected and classified as follow: 1) changes of radiological staging 2) changes in treatment strategy cathegorized as “major changes” and as “minor changes”.
**Results:** 540 cases were analyzed. Imaging revision was required at the time of tumour diagnosis in 310 (57.4%) cases and most of patients (69%) had advanced stage (III and IV) tumour. In 262 (48%) cases no changes of the initial radiologic report was performed. In a total of 144 (27%) cases the available imaging was not considered sufficient for a final indication to treatment an further analysis were required. In the remaining 134 (25%) cases, the radiologic revision lead to a modification of either tumor staging (55%) or treatment strategy (45%). Specifically, major and minor modifications were applied in 44 (13%) and 17 (11%) of the considered cases, respectively. Follow-up was available for 118 (86%) cases and showed that MDT decision was correct in 117/118 (99%) cases, with 43 patients having an histological confirmation.
**Conclusion:** Our data strongly confirm the paramount importance to include an expert head and neck radiologist in the core of each Institutional head and neck MDT.

**B-0974** 11:27
**Quantification of heterogeneity to classify benign parotid tumours**
F. Patella[1], M. Sansone[2], G. Franceschelli[1], R. Fusco[2], M. Petrillo[1], F. Pesapane[1], S. Zannoni[1], P. Brambillasca[1], G. Carrafiello[1]; [1]*Milan/IT*, [2]*Naples/IT (battellina@gmail.com)*

**Purpose:** To measure heterogeneity to classify Warthin tumours (WT) and pleomorphic adenomas (PA) of parotid glands by correlating functional IVIM and DCE-MRI histogram parameters with pathology.
**Methods and Materials:** A total of 36 pathologically proven parotid tumours (18 WT and 18 PA) in 25 consecutive enrolled patients were included in this study. For both DCE-MRI and DW-MRI, model-free and model-based parameters were computed voxel-by-voxel on manually segmented VOIs. For each lesion, the following functional biomarkers were considered: Ktrans, kep, vp, MRE, TTP, WIS, WOS, WOR, MRE, f, D, D*. A histogram analysis was performed, identifying for each MRI biomarker central tendency features (mean, median) and heterogeneity parameters (standard deviation, kurtosis, skewness, mad, entropy and energy). The Wilcoxon signed-rank test was used to compare the histogram characteristics in WT and PA. Furthermore, ROC curves and multivariate analysis were employed to identify, respectively, the parameters and the pairs of parameters with the best accuracy.
**Results:** Univariate analysis findings showed that almost all biomarkers exhibit significant difference between PA and WT either for central tendency or heterogeneity parameters (p value <0.05 at Wilcoxon signed-rank test). As regards the ROC analysis, after cross-validation, the highest area under curve (AUC) was achieved using TTP median and skewness and by D* median and entropy. With respect to the multivariate analysis, it seems that no meaningful improvement of classification performance can be obtained using two features.
**Conclusion:** Differential diagnosis of WT and PA may benefit from the use of IVIM and DCE parameters and from the information provided by histogram descriptors.

**B-0975** 11:35
**Lesion-parenchyma ratio of DCE-MRI pharmacokinetic parameters for characterisation of salivary gland tumours**
F. Mungai[1], G.B. Verrone[1], M. Pietragalla[2], L. Bonasera[1], M. Bartolucci[1], V. Miele[3]; [1]*Florence/IT*, [2]*Pistoia/IT*, [3]*Rome/IT (f.mungai@gmail.com)*

**Purpose:** Quantitative data derived from pharmacokinetic (PK) modeling of dynamic contrast-enhanced magnetic resonance imaging (DCE-MRI) are affected by high variability and their clinical use in characterizing tumours is controversial due to low reproducibility. This study introduces a clinically feasible method using DCE-MRI data for characterization of principal salivary gland tumours.
**Methods and Materials:** DCE-MR images were acquired from 60 patients with salivary gland tumours. For each patient, PK parameters of Ktrans, Kep, Ve, Vp, and iAUC (using modified Tofts model) were measured on both the lesion and the normal contralateral salivary gland parenchyma. Lesion-Parenchyma ratio (L/P) for each parameter was then calculated. ANOVA and ROC analysis were performed.
**Results:** Five groups of lesion were identified (reference standard: histopathology): pleomorphic adenomas (n=20), Warthin tumours (n=16), other benign entities (n=4), non-Hodgkin lymphomas (n=4) and malignancies (n=16). Among groups there were significantly different mean values of L/P-Ktrans (p=0.026), L/P-Kep (p=0.019), L/P-Ve (p=0.010), L/P-Vp (p=0.010) and L/P-iAUC (p=0.004). Ratio values higher than 1.0 were found for L/P-Ktrans in malignancies (mean value 1.966); L/P-Ve in adenomas (2.051), other benign entities (1.081) and malignancies (1.591); L/P-Vp in Warthin tumours (1.943) and malignancies (3.797); L/P-iAUC in malignancies (2.106). ROC analysis demonstrated the highest area under the curve (AUC=0.94) for L/P-iAUC, with sensibility and specificity for malignancy, respectively, of 88% and 100% (cut-off value=1.035).
**Conclusion:** The use of lesion-parenchyma ratio of DCE-MRI quantitative data can provide a robust and accurate method for characterization of salivary gland tumours and can be easily implemented in clinical routine.

**B-0976** 11:43
**Theatre radiology: making the inoperable operable with real time, intraoral ultrasound guided trans oral robotic surgery**
A. Jaly, D. Ap Dafydd, V. Paleri, J. Hardman; *London/UK*

**Purpose:** Trans-oral robotic surgery is minimally invasive and has generated promising early results. We illustrate our experience using intra-oral ultrasound, intraoperatively to assess oropharyngeal tumours and guide trans-oral robotic surgery.
**Methods and Materials:** 12 month period: February 2018 to February 2019
Inclusion criteria: Cases suitable for TORS (T1/T2 tumours, recurrences, good mouth opening) Exclusion criteria: Limited mouth opening, high risk anatomy.
All patients assessed whilst under general anaesthesia. A fine, linear intra-operative pencil probe (18 MHz) is applied to the lesion, prior to and/or after introduction of the robotic arms
Ultrasound findings cross-referenced with available pre-operative imaging and collaboratively mapped by the radiologist and surgeon.
**Results:** Good visual correlation of tumour morphology with CT/MR Greater access to the posterior tongue and oropharynx under general anaesthesia. Improved confidence of surgeon in orientating dissection planes. Where contact between the ultrasound probe and posterior oropharyngeal tumours is difficult due to curvature of the tongue, it would be feasible to flood the intubated pharynx with water enabling ultrasound conduction without direct probe contact.Ultrasound of the resected tumour tissue is performed to ensure adequate margins resected.
**Conclusion:** Oncological outcomes of TORS can be comparable to open surgery and transoral laser microsurgery. Added advantages are en bloc resection and inset of free flaps without mandibular split. Less blood loss, shorter patient hospital stay and overall lower complications are seen. The head and neck radiologist plays a vital role using intra-oral ultrasound to guide the surgeons orientation of excision planes with a view to optimising surgical margins.

**B-0977** 11:51
**Usefulness of CT perfusion generated time-density curves and perfusion parameters in the diagnosis and differentiation of parotid neoplasms**
M. Swarup, L. Upreti; *Delhi/IN (sarthak163@gmail.com)*

**Purpose:** To evaluate role of Time-density curves (TDCs) and perfusion values generated from CT perfusion (CTP) in diagnosis and differentiation of parotid neoplasms.
**Methods and Materials:** CTP was performed in 32 patients on a 64 slice MDCT scanner. Data were analysed by deconvolution based software to obtain maps for blood flow (BF), blood volume (BV), mean transit time (MTT), and capillary permeability (PS). Regions of interest were placed through tumour to obtain perfusion values and tissue Time ~ Density curves. TDCs were classified into four types based on time to peak enhancement (TTE) and washout ratio (WR). Mann-Whitney U test was used for comparison of perfusion values.Final diagnosis was established by postsurgical histopathology examination.
**Results:** Study revealed 20 benign and 12 malignant lesions. Among pleomorphic adenomas 8 type A, 3 type C and one type B curve was seen. Two Warthin tumours showed type B curve. Two benign cysts showed type D curve. TDC type was variable in malignant tumours with 2 type A, one type B and 9 type C curves. The malignant tumours showed higher BF, BV and PS values and lower MTT value compared to benign tumours. The BF, BV and PS values ($p < 0.001$, $p < 0.001$, $p < 0.001$) were statistically significant in differentiating pleomorphic adenomas from malignant tumours.
**Conclusion:** TDCs obtained from dynamic CT perfusion of the parotid gland could considerably improve diagnostic accuracy and is helpful in differentiation of various parotid neoplasms complimentary to perfusion values and conventional contrast CT.

**10:30 - 12:00** **Room D**

### Artificial Intelligence and Machine Learning

### SS 1005b

### Machine learning in neuroradiology: diagnostic help and tissue analysis

*Moderators:*
N.N.
J. Hirvonen; Turku/FI

**B-0978** 10:30
**Automatic segmentation for acute ischaemic stroke from DWI using deep convolutional neural networks**
Q. Liu[1], G. Lu[1], X. Cheng[1], Q. Zhang[1], C. Zhou[1], S. Wang[2], K. Liang[2], X. Li[2]; [1]*Nanjing/CN*, [2]*Beijing/CN (349566651@qq.com)*

**Purpose:** DWI hyperintensity region is one of the most important indicators of acute cerebral infarction. Our study is aim at presenting a U-NET based Deep Convolutional neural network for acute ischaemic stroke lesion segmentation in DWI automatically and accurately.The model will lay the foundation for the follow-up research in AI,including lesion measurement, location and automatic recognition of responsible vessels.
**Methods and Materials:** Our study is validated on a dataset comprising clinical acquired DWI from 484 patients(2012-2017). All images are manually tagged . For each 3D slice, we combine the upper and lower slices as a 3-channel input to fuse the 3D information. The network has an analysis and a synthesis path. Each path contains three resolution steps. For the analysis path, each layer has two convolutions followed by ReLU activations. For the synthesis path, each layer consists of an upconvolution and two convolutions each followed by a ReLU activation. MRI equipment type distribution ('SIEMENS': 333, 'GE': 151). Training set included 434 samples,validation set includes 50 patients. The result is mainly evaluated by Dice coefficient.
**Results:** The experiment results show that the algorithm is both reasonable and effective compared with the results of manual annotation. The Dice coefficient is 0.823. (Sensitivity 0.916,Precision 0.850,Accuracy 0.850).
**Conclusion:** We present a deep convolutional neural network for acute ischaemic stroke lesion segmentation in DWI,which do preparations for the follow-up correlation studies. Automatic segmentation can save manpower and material resources for large data annotation. In addition,this model can help beginners to distinguish lesions and artefacts.

**B-0979** 10:38
**Can artificial intelligence improve physician sensitivity in detecting early ischaemic damage on computed tomography?**
J. Chriashkova[1], C. Goncalves[2], M. Aslam[2], S. Perera[2], S. Walter[3], J. Fisher[2], A. Podlasek[2], A.J. Schmitt[4], I.Q. Grunwald[1]; [1]*Chelmsford/UK*, [2]*Southend-on-Sea/UK*, [3]*Homburg/DE*, [4]*London/UK (julija.chriashkova@anglia.ac.uk)*

**Purpose:** ASPECTS (Alberta Stroke Program Early CT Score) is a validated scoring system for assessment of early ischaemic change (EIC) on CT scans. This can be challenging particularly for less experienced clinicians. e-ASPECTS software uses machine learning algorithms to support physicians in detecting EIC using ASPECTS score.
**Methods and Materials:** 26 clinicians (11 radiologists, 6 junior and 7 consultant stroke physicians, 2 non-specialist physicians) independently scored 2560 ASPECTS regions from 64 patients for evidence of EIC on non-contrast CT brain acquired within 4.5 hours of stroke onset. A familiarization training set of 5 patients was used prior to scoring. Images were randomized to manual or software assistance. After two weeks images were rescored using the alternative method. Scorers were blinded to clinical symptoms. Reference standard scores were defined by an independent neuroradiologist with information on clinical symptoms, 24h follow-up, and CT perfusion / MRI scans when available.
**Results:** Mean NIHSS was 11. Mean time to score decreased by 34% (45s, 2:12 to 1:27, mm:ss) using e-ASPECTS assistance. Rater agreement with ground truth was greatest in the radiologist cohort, but performance improved across all clinician categories using e-ASPECTS assistance (radiology kappa: 0.26 to 0.38). Sensitivity to EIC improved by a factor of two across all clinician groups using e-ASPECTS assistance, particularly for less experienced physicians.

**Conclusion:** In acute ischaemic stroke e-ASPECTS assistance increased accuracy and reduced time for detection of EIC. Routine assistance in CT interpretation has the potential to reduce time to treatment and improve accuracy across clinicians and sites.
*Author Disclosures:*
**I.Q. Grunwald:** Shareholder; Co-founder and share holder Brainomix Ltd.

**B-0981** 10:46
**Automated detection and localisation of pneumocephalus in head CT scan**
S. Tanamala[1], S. Chilamkurthy[1], M. Biviji[2], P. Rao[1], R. Ghosh[1]; [1]*Mumbai/IN*, [2]*Nagpur/IN (swetha.tanamala@qure.ai)*

**Purpose:** Pneumocephalus, accumulation of air in intracranial space, can lead to midline shift and compression of brain. In this work, we detail the development of deep learning algorithms for automated detection and localization of pneumocephalus in head-CT scans.
**Methods and Materials:** Firstly, to localize the intracranial space from a given head-CT scan, a skull-stripping algorithm was developed using a randomly sampled anonymized dataset of 78 head CT scans (1608 slices). We sampled another anonymized dataset containing 83 head-CT scans (3546 slices) having pneumocephalus and 310 normal head-CT scans which were randomly sampled to represent natural distribution. These 3546 slices (932 slices had pneumocephalus) were annotated for pneumocephalus regions. Then UNet based deep neural network algorithm was trained on these scans to accurately predict the pneumocephalus region . The predicted pneumocephalus region is refined by removing the regions outside the intracranial space identified by the skull stripping algorithm. The refined pneumocephalus region is then used to extract features. Using these features, a random forest was trained to classify the presence of pneumocephalus in a scan. Areas under receiver operating characteristics curves (AUC) were used to evaluate the algorithms.
**Results:** An independent dataset of 1891 head-CT scans (40 scans had pneumocephalus) was used for testing above algorithms. AUC for the scan level predictions was 0.89. Sensitivity and Specificity of 0.80 and 0.83 respectively were observed.
**Conclusion:** In this work, we showed the efficacy of deep learning algorithms in localizing and classifying the pneumocephalus accurately in a head-CT scan.
*Author Disclosures:*
**S. Tanamala:** Employee; Qure.ai. **S. Chilamkurthy:** Employee; Qure.ai. **P. Rao:** Employee; Qure.ai. **R. Ghosh:** Employee; Qure.ai.

**B-0982** 10:54
**Automatic detection of intracranial calcifications in CT using deep learning**
G. Bortsova, F. Dubost, A. Van der Lugt, D. Bos, M. De Bruijne; *Rotterdam/NL (gerdabortsova@gmail.com)*

**Purpose:** Intracranial carotid artery calcification (ICAC) is a major risk factor for stroke, and might contribute to dementia and cognitive decline. Further research into the relationship between ICAC and neurological diseases is hampered by the time-consuming manual annotation of ICAC lesions. Therefore, we introduce a fully automatic ICAC detection method.
**Methods and Materials:** Non-contrast-enhanced CT scans were performed in 2319 participants of the Rotterdam Study, a population-based cohort study (mean age 69.6(6.8), 51.7% female). Two trained observers annotated the scans by indicating regions of interest (ROI) on the intracranial carotid artery (from the horizontal petrous segment to the circle of Willis) where calcifications were visible. ICAC lesion segmentations were obtained by thresholding these ROIs at 130 HU. We developed a deep learning based algorithm to automatically delineate ICAC lesions in CT scans. The performance was estimated by 10-fold cross-validation.
**Results:** Pearson correlation between ICAC volumes computed from manual and automatic ICAC segmentations was 98%. The interrater agreement was 96%. For patients with ICAC, median sensitivity was 87% (74, 95) and false positive volume (FPV) was 5.4mm$^3$ (1, 19.5). Median FPV to ground truth volume ratio was 0.07 (0.02, 0.2). For ICAC-free patients, median FPV was 1.4mm$^3$ (0.1, 5.5).
**Conclusion:** Our algorithm can be used to automate the time-consuming manual annotation of ICAC in large epidemiological studies, whilst maintaining a comparable level of accuracy. This can facilitate research into causes and consequences of ICAC, which might result in development of new treatments.
*Author Disclosures:*
**G. Bortsova:** Consultant; COSMONiO. Research/Grant Support; Netherlands Organisation for Scientific Research (NWO). **F. Dubost:** Consultant; Quantib BV.

**B-0983** 11:02
**Automated age-specific regional brain atrophy estimation: an integrated workflow**
J. Caspers, B. Turowski, C. Rubbert; *Düsseldorf/DE (Julian.Caspers@med.uni-duesseldorf.de)*

**Purpose:** Estimating deviations of regional brain volume from a patient's normative age cohort is challenging and entails immense inter-reader variations. We propose an automated workflow for age-dependent estimation of brain volume changes relative to a standard population.
**Methods and Materials:** 3D T1w MRIs of 693 healthy subjects aged between 16 and 77 years from the publicly available enhanced Nathan Kline Institute Rockland Sample were preprocessed to generate age- and gender-specific grey-matter (GM) templates. Preprocessing is performed with CAT12 for SPM12 and comprises GM segmentation, normalization to MNI152 and 8mm smoothing. For each age between 18 and 75, voxel-wise mean and standard deviation (SD) maps were generated across all subjects of the respective age ±2 years. To estimate volume changes of an out-of-sample subject, a 3D T1w scan is preprocessed in the same way as for template generation. Then a voxel-wise z-value map is generated from the resulting normalized GM map using the age-dependent mean- and SD-templates. The z-map is transformed into subject space, colour coded and fused with the structural MRI.
**Results:** The proposed workflow can be implemented in a parallelized fashion on a state-of-the-art server to automatically generate individual atrophy maps and send them into the PACS. Processing of one subject is feasible in less than 10 minutes. The colur-coded maps fused with the structural MRI allow for direct semi-quantitative detection of regional brain volume deviations in individual patients.
**Conclusion:** Automated brain volume change estimation as implemented in the presented framework is feasible and could significantly facilitate radiologic workflows.

**B-0984** 11:10
**Radiological reading: man vs machine**
W. Van Hecke[1], A. Ribbens[1], T. Billiet[1], T. Wang[2], L. Ly[2], H.N. Beadnall[2], M.H. Barnett[2]; [1]*Leuven/BE*, [2]*Sydney/AU (wim.vanhecke@icometrix.com)*

**Purpose:** MRI is of paramount importance in the diagnosis and monitoring of patients with multiple sclerosis (MS). Typically, MRI scans are obtained yearly and are crucial to assess disease activity, and therefore therapeutic decision-making, in MS patients. As MS is an inflammatory and neurodegenerative disease, both lesions T2/FLAIR and brain atrophy on T1 should be evaluated. The aim of this study was to assess the quality of clinical radiological reports in patients with MS and to compare these with a radiological structured reporting review and clinically cleared automated software.
**Methods and Materials:** Lesion burden and whole brain atrophy was assessed on baseline MRI scans of 50 MS patients (RRMS or SPMS) in 3 different ways: (1) a standard clinical radiological report, (2) a structured radiological report assessed by a neuroradiologist, (3) quantitative assessment using the CE/FDA cleared icobrain software. A statistical comparison between the three different reporting methods was performed evaluating T2/FLAIR lesion load and brain atrophy.
**Results:** A large discrepancy was found between the radiological report and the expert structured review (i.e. 50% vs 100% of the reports mentioned lesion burden at baseline and 24% vs. 48% mentioned brain atrophy). On the contrary, the expert assessment and the quantitative icobrain results showed a high similarity ($p<0.001$, ANOVA comparing both assessments in terms of small vs. severe lesion load/atrophy).
**Conclusion:** These results demonstrate that automated and clinically cleared MRI software outperforms a standard radiological report in the assessment of disease status on MRI in patients with MS.
*Author Disclosures:*
**W. Van Hecke:** Board Member; icometrix. CEO; icometrix. Founder; icometrix. Shareholder; icometrix. **A. Ribbens:** Employee; icometrix. Investigator; icometrix. Shareholder; icometrix. **T. Billiet:** Employee; icometrix. Investigator; icometrix.

**B-0986** 11:18
**Predicting response to somatostatin analogues in acromegaly: machine learning-based high-dimensional quantitative MRI texture analysis**
B. Kocak, E. Memis Durmaz, P. Kadioglu, O. Polat Korkmaz, N. Comunoglu, N. Tanriover, N. Kocer, C. Islak, O. Kizilkilic; *Istanbul/TR (drburakkocak@gmail.com)*

**Purpose:** To investigate the potential value of machine learning (ML)-based high-dimensional quantitative texture analysis (qTA) against previously suggested relative signal intensity (rSI) evaluation on T2-weighted magnetic resonance imaging (MRI) in predicting response to somatostatin analogs (SAs) in acromegaly patients with pituitary macroadenomas.

**Methods and Materials:** Forty-seven patients (24 responsive, 23 resistant to SAs) were eligible for this retrospective study. Following image processing steps, 828 texture features were extracted from coronal T2-weighted images by manual segmentation of the whole tumour for each patient. Dimension reduction was first done with a reproducibility analysis by two radiologists and then with a wrapper-based algorithm using a nested cross-validation. The macroadenomas were also grouped according to their rSI (hypointense versus others) on T2-weighted MRI. The ML classifiers for qTA and rSI evaluation were k-nearest neighbours (k-NN) and J48, respectively. Reference standard for classifications was biochemical response and resistance. Main performance metric for comparison was area under the curve (AUC).
**Results:** Only 535 out of 828 texture features had excellent reproducibility. Among these, the wrapper-based algorithm yielded 4 textural features. Using qTA, the k-NN algorithm correctly classified 85.1% of the macroadenomas in terms of response to SAs with an AUC of 0.847. Using rSI, the J48 classifier correctly classified 53.1% of the macroadenomas with an AUC of 0.468. There was statistically significant difference between qTA-based classification and rSI-based classification performances (*P*<0.05).
**Conclusion:** The machine learning-based qTA of T2-weighted MRI might be a potential non-invasive tool in predicting response to SAs in patients with acromegaly and pituitary macroadenomas.
The paper related to this abstract has recently been published: Eur Radiol (2018). https://doi.org/10.1007/s00330-018-5876-2

**B-0987** 11:26
**Combining multiple magnetic resonance imaging sequences provides independent reproducible radiomics features**
L. Duron, A. Lecler, D. Balvay, J. Savatovsky, O. Bergès, A. Bouchouicha, O. Galatoire, L. Fournier; *Paris/FR (loic.cmprepa@gmail.com)*

**Purpose:** To evaluate the relative contribution of different Magnetic Resonance Imaging (MRI) sequences for the extraction of radiomics features in a cohort of patients with lacrimal gland tumors.
**Methods and Materials:** This prospective study was approved by the Institutional Review Board and signed informed consent was obtained from all participants. From December 2015 to April 2017, 37 patients with lacrimal gland lesions underwent MRI before surgery, including axial T1-WI, axial Diffusion-WI, coronal DIXON-T2-WI and coronal post-contrast DIXON-T1-WI. Two readers manually delineated both lacrimal glands to assess inter-observer reproducibility and one reader performed two successive delineations to assess intra-observer reproducibility. Radiomics features were extracted using an in-house software to calculate 85 features per region-of-interest (510 features/patient). Reproducible features were defined as features presenting an intra-class correlation coefficient ≥ 0.8 and a concordance correlation coefficient ≥ 0.9 across combinations of the three delineations. Among these features, the ones yielding redundant information were identified as clusters using hierarchical clustering based on the Spearman correlation coefficient.
**Results:** All the MR sequences provided reproducible radiomics features (range 14(16%)-37(44%)) and non-redundant clusters (range 5-14). The highest number of features and clusters were provided by the water and in-phase DIXON T2-WI and water and in-phase post-contrast DIXON T1-WI (37, 26, 26 and 26 features and 14,12, 9 and 11 clusters, respectively). A total of 145 reproducible features grouped in 51 independent clusters were provided by pooling all the MR sequences.
**Conclusion:** All MRI sequences provided reproducible radiomics features yielding independent information which could potentially serve as biomarkers.

**B-0988** 11:34
**Glioblastoma survival prediction using residual tumour volumes detected and segmented by a deep learning model in post-operative MRI**
M. Perkuhn, P. Stavrinou, R. Shahzad, F. Thiele, G. Shakirin, D. Garmpis, M. Schlamann, J. Borggrefe; *Cologne/DE (michael.perkuhn@uk-koeln.de)*

**Purpose:** Cytoreductive surgery is the cornerstone of glioblastoma (GB) therapy and residual tumour volume is considered to be an important outcome measure. Contrast-enhancing residual tumour volume (CE-RTV) on MRI is known to predict survival. However, interpretation of MRI is challenging due to e.g. small tumor volumes and is associated with high inter-rater variability.
Our objective was to evaluate survival prediction of three CE-RTVs, obtained from human readers and a deep learning model (DLM).
**Methods and Materials:** 80 GB patients were retrospectively included, with age=60±12y, Karnofsky performance score (KPS)=85±14. On post-operate MRI, acquired 24-48 hours after surgery, CE-RTV were manually delineated by two experts (radiologist, neurosurgeon). In addition, CE-RTV were segmented by a fully automatic DLM. Impact of CE-RTV on prediction of overall survival (OS) and progression free survival (PFS) was assessed using Cox proportional hazard modelling with censored data. Age and KPS were included as covariates.
**Results:** Median survival was 16.8 months (OS) and 8.8 months (PFS). CE-RTV correlations (*R*) were 0.73 (radiologist-neurosurgeon), 0.67 (radiologist-DLM). CE-RTV were found to be significant predictors of PFS: radiologist (Hazard ratio (HR)=1.1, p<0.02), neurosurgeon (HR=1.1, p<0.02), DLM (HR=1.2, p<0.001), age and KPS were not significant. For OS, only age was a significant predictor (HR=1.03 p<0.03), KPS and CE-RTVs were not significant.
**Conclusion:** Fully automatic quantification of CE-RTV by the DLM was found to be a significant predictor of PFS in post-operative GB, showing the potential of automatic CE-RTV segmentation. For the OS, additional covariates like treatment modalities and performance scores after progression need to be investigated.
*Author Disclosures:*
**M. Perkuhn:** Employee; Philips Research. **R. Shahzad:** Employee; Philips Research. **F. Thiele:** Employee; Philips Research. **G. Shakirin:** Employee; Philips Research.

**10:30 - 12:00** **Room G**

### Physics in Medical Imaging

### SS 1013

### Innovations in imaging: machine learning, quality and new techniques

*Moderators:*
C. Cavedon; Verona/IT
J. Sjöberg; Stockholm/SE

**B-0989** 10:30
**4D-flow-based monitoring of pulmonary pressure in patients with pulmonary hypertension: a feasibility study**
G. Reiter[1], G. Kovacs[1], C. Reiter[1], C. Kräuter[1], A. Greiser[2], H. Olschewski[1], M.H. Fuchsjäger[1], U. Reiter[1]; [1]*Graz/AT*, [2]*Erlangen/DE (gert.reiter@siemens-healthineers.com)*

**Purpose:** Longitudinal haemodynamic follow-up of patients with pulmonary hypertension (PH) is important for individual treatment response monitoring. The purpose of this study was to analyze the potential of magnetic resonance 4-dimensional (4D) flow-derived duration of vortical blood flow along the main pulmonary artery to predict alterations of mean pulmonary arterial pressure (mPAP).
**Methods and Materials:** 29 patients with borderline mPAP or PH repeatedly (2.5±2.3 years) underwent right-heart catheterization (RHC) and near-term, time-resolved, 3-directional phase-contrast imaging at 1.5T or 3T covering the main pulmonary artery. Duration of vortical blood flow along the main pulmonary artery ($t_{vortex}$) were evaluated from velocity vector-fields using prototype software (4DFlow, Siemens Healthcare). The relationship between RHC-derived mPAP differences (dmPAP) and differences of $t_{vortex}$ ($dt_{vortex}$) at baseline and follow-up assessment were analyzed by means of correlation and regression analysis. The diagnostic performance of dtvortex to predict mPAP increases (dmPAP>0 mmHg) or decreases (dmPAP<0 mmHg) was investigated by receiver operating characteristic curve analysis.
**Results:** Mean mPAP at baseline was 44±11 mmHg and 44±14 mmHg at follow-up; dmPAP ranged from -20 to +37 mmHg. dtvortex correlated strongly with dmPAP (r=0.92), and root mean square error of the regression of dmPAP on $dt_{vortex}$ was 5 mmHg. Areas under the curve for predicting increases/decreases of mPAP were 0.95/0.94. The cut-off $dt_{vortex}$=8% resulted in sensitivity/specificity=0.90/0.95 for the prediction of mPAP increases, the cut-off $dt_{vortex}$=0% in sensitivity/specificity=0.94/0.83 for the prediction of mPAP decreases.
**Conclusion:** 4D-flow-derived duration of vortical blood flow along the main pulmonary artery allows accurate mPAP monitoring in patients with PH.
*Author Disclosures:*
**G. Reiter:** Employee; Siemens Healthcare Diagnostics GmbH. **A. Greiser:** Employee; Siemens Healthcare GmbH.

**B-0990** 10:38
**Towards virtual MR imaging: predicting diffusion-weighted brain MR images from T2-weighted images using convolutional neural networks**
V. Mahajan, A. Upadhyaya, A. Venkatraman, V. Venugopal; *New Delhi/IN (vidur@mahajanimaging.com)*

**Purpose:** In a quest for standardisation and speeding up of MR imaging, there is a move towards having a ‘universal’ MRI sequence from which all MR contrasts can be obtained. We propose first-of-its-kind virtual MR imaging where we predict “virtual” diffusion-weighted (DW) images of the brain from axial T2W images using convolutional neural networks (CNN).
**Methods and Materials:** 100 whole-brain MRI scans of patients with no abnormality and 30 with acute infarcts, comprising 25 T2W and DWI (b=1000) slices each, acquired retrospectively, were fed into a 15-layered CNN model with a 75-25 training-validation split. T2W images were assigned as the input to predict DW Images. Binary cross entropy was used as the loss function and

Friday training took 48 hours on an Nvidia 1080ti GPU. For testing T2W, images from 5 independent cases, having 7 T2 hyperintensities with corresponding diffusion restriction (group 1) and 16 with no diffusion restriction (group 2), were also analysed.
**Results:** Binary cross entropy of 0.15 for normal and of 0.11 for infarct cases was obtained. The model took 750ms to produce each image. In the test cases, 6 out of 7 T2 hyperintensities in group 1 showed diffusion restriction and all 16 T2 hyperintensities in group 2 did not show restriction. The one 'missed' lesion in group 1 was 2.5mm in diameter. Blurring of virtual DW images was observed, which did not impede clinical judgement.
**Conclusion:** We demonstrate a novel use-case for deep learning in reducing the MRI exam time and potentially creating a single universal MRI sequence.

**B-0991** 10:46
**An inter and intra patient radiomic study to select the most promising features aiming to precision medicine feasibility**
C. De Mattia, R. Ronza, M. Grassi, M. Pecorilla, P.E. Colombo, S. Siena, A. Vanzulli, A. Torresin; *Milan/IT (cristina.demattia@ospedaleniguarda.it)*

**Purpose:** To characterize with radiomics techniques the pulmonary metastases of 176 patients, distributed in two cohorts, according to primitive cancer. To identify the features (RFs) variability due to image acquisition, reconstruction, contouring, and to lesions morphology.
**Methods and Materials:** First, a standard of CT acquisition and image reconstruction was defined. 357 lesions were contoured using Multi-Modality Tumour Tracking (IntelliSpace Portal 9.0, Philips). For each lesion, 865 RFs were extracted using IBEX platform, v1.0β. For each RF, we evaluated Student t test and relative discrepancy between the two cohorts. For each exam, in all lesions we calculated the relative discrepancy changing scan phase, reconstruction filter, layer thickness and segmentation parameters. Ibex can analyze only the layer where the lesion appears larger (2D) or the entire lesion volume (3D). We compared the two methods.
**Results:** We identified 12 promising RFs (p-value<0.01). Intra-cohort ANOVA test showed no scanner and voltage dependence, excluding images reconstructed with iterative filters. All RFs strongly depend on phase and reconstruction filter, with relative discrepancies over 1000%. Autocorrelation, sum average, sum variance, HGLRE, LRHGLE and SRHGLE are the less dependent on these parameters (relative discrepancy <50%). Segmentation seems to be the less relevant factor on the RFs variability. Generally, RFs more sensitive to lesion morphology are also those more affected by modifications of the above-mentioned parameters. From 3D analysis to 2D, discrepancy can be more than 1000%.
**Conclusion:** A lesion by lesion radiomic characterization is possible, provided that it is obtained with a standardization process of the radiomic workflow.

**B-0992** 10:54
**A novel machine learning approach for assessing osteoporotic fracture risk by texture analysis of femoural neck radiographs**
T. Yeshua, S. Rebibo, K. Sfez, O. Safran, M. Liebergall, I. Leichter; *Jerusalem/IL (Izhak@hadassah.org.il)*

**Purpose:** Bone strength is determined not only by its mineral density but also by the spatial structure of bone trabeculae. Our aim was to asses osteoporotic fracture risk by texture analysis of the proximal femur in routine radiographs, using machine-learning tools.
**Methods and Materials:** 17 radiographs of in-vitro femurs and routine proximal femur radiographs of 44 subjects (15 with osteoporotic fractures and 29 without a fracture) were analyzed. The critical force required to fracture the in-vitro femurs was measured. The gray-levels of all images were automatically balanced to yield equal brightness and contrast. Two regions-of-interest (ROI) for texture analysis were defined, one in the femoral neck, and the other, distal to the lesser trochanter. Five parameters describing the distribution of the gray-levels in the ROIs and 6 Gray-Level Co-occurrence Matrix (GLCM) parameters, describing the spatial allocation of the gray-levels in the ROIs were defined. Support Vector Machine (SVM) with cross-validation was used to train the algorithm and test its results, based on a combination of both ROIs.
**Results:** The algorithm characterized the in-vitro weak and strong bones with an accuracy of 88%. The radiographs of the patients were characterized as osteoporotic or non-osteoporotic with an accuracy of 86%. The most prominent features for estimating fracture risk were the mean gray-level, which is related to bone density, as-well-as smoothness, uniformity and entropy, which are related to the spatial distribution of the trabeculae.
**Conclusion:** Machine-learning tools may be used to analyze routine femoral radiographs and asses fracture risk with high accuracy.

**B-0993** 11:02
**Implementation of channelised hotelling observer (CHO) to assess image quality of GE discovery IGS 740 angiography system**
M. Bertolini[1], V. Trojani[2], A. Nitrosi[1], M. Iori[1], R. Sassatelli[1], O. Ortenzia[3], C. Ghetti[3]; [1]*Reggio Emilia/IT*, [2]*Bologna/IT*, [3]*Parma/IT (marco.bertolini@ausl.re.it)*

**Purpose:** To use the well-known CHO to characterize a novel angiography system (GE Discovery IGS 740) using a contrast-detail phantom simulation in a clinical scatter condition.
**Methods and Materials:** A Leeds TO10 phantom was used. The phantom has 108 details: 12 diameters (size range: 0.25 mm - 11 mm), each with 9 contrasts (declared range: 0.012 - 0.930 at 70 kVp 1.00 with mm Cu filtration). TO10 has been imaged between two 10 cm thick homogeneous solid water slabs. Two FOVs (32 and 20 cm side) were used. Fluoroscopy images were taken using an abdominal protocol at 2 different frame rates (15 fps and 7.5 fps) and at 2 dose levels (low and normal); fluorography images were acquired using an abdominal protocol at 15 fps at 2 dose levels (low and normal). A CHO with 40 Gabor channels was used. An eye-internal noise was added. The model response was compared with human observers for validation. Contrast-detail curves were obtained from the CHO output using a visibility threshold of 75%. Wilcoxon signed rank tests were performed.
**Results:** The CHO model distinguishes between the 2 dose levels (p<0.002). FOVs and frame rates were not statistically significant (p=0.10 and p=0.18). It's worth noting that fluoroscopy with FOV=20 normal dose level (17.6 mGy/min) was not statistically different (p=0.50) respect to fluorography with FOV=32 low dose level (42.1 mGy/min).
**Conclusion:** A model observer is an effective tool to evaluate image quality even in clinical protocols optimization thanks to higher results reproducibility compared with human one.

**B-0994** 11:10
**Effective atomic number image produced with energy-resolved photon counting detector toward the development of next-generation plain X-ray diagnosis**
N. Kimoto[1], H. Hayashi[1], T. Asakawa[1], T. Asahara[2], A. Katsumata[3], Y. Kanazawa[2], S. Yamamoto[4], M. Okada[4]; [1]*Kanazawa/JP*, [2]*Tokushima/JP*, [3]*Gifu/JP*, [4]*Yokohama/JP (natsumi.kimoto.0717@gmail.com)*

**Purpose:** Analysis of X-ray attenuation is fundamental for developing a material identification method. We have developed a procedure to derive an effective atomic number ($Z_{eff}$) image using a prototype energy-resolving photon counting detector. In this study, we aim to verify our procedure for the application to diagnosis using plain X-ray.
**Methods and Materials:** Our method can determine $Z_{eff}$ pixel by pixel. First, the intensity of X-rays before and after penetrating the samples in three energy bins was measured, and the products of linear attenuation coefficient (μ) and material thickness were calculated. Next, beam hardening was properly corrected; the response of a multi-pixel-type CZT detector, energy resolution and charge sharing were also taken into consideration. Then we calculated $Z_{eff}$ by means of our method in which the relationship between μ and $Z_{eff}$ was used. To verify our method, we measured samples of which $Z_{eff}$ was known: acrylic ($Z_{eff}$=6.5) and aluminium ($Z_{eff}$=13) having mass thicknesses of 1, 5, and 10 g/cm$^2$, and dental samples. Finally, we compared the difference between calculated and reference $Z_{eff}$.
**Results:** Our present method can obtain $Z_{eff}$ images with accuracies of Z+/-0.5 regardless of material thickness. Furthermore, we can analyse the fine structure of dental enamel and dentine quantitatively. These results show our method works properly for human body structure material ($Z_{eff}$<20).
**Conclusion:** Using a prototype photon counting detector, we demonstrated the procedure of generating $Z_{eff}$ image. We expected that it has carry-over effect to establishment of new quantitative diagnosis and utilization of plain X-ray to various diseases.
*Author Disclosures:*
**H. Hayashi:** Employee; Kanazawa University. Grant Recipient; JOB CORPORATION, Nagase Landauer. **T. Asahara:** Employee; Okayama University. **A. Katsumata:** Employee; Asahi University. **Y. Kanazawa:** Employee; Tokushima University. **S. Yamamoto:** Employee; JOB CORPORATION. **M. Okada:** Employee; JOB CORPORATION.

**B-0995** 11:18
**K-edge subtraction imaging at the Munich compact light source**
S. Kulpe[1], E. Braig[1], M. Dierolf[1], B. Günther[1], K. Achterhold[1], J. Herzen[1], E.J. Rummeny[2], F. Pfeiffer[1], D. Pfeiffer[2]; [1]*Garching/DE*, [2]*Munich/DE (stephanie.kulpe@tum.de)*

**Purpose:** About one-third of all deaths worldwide can be traced back to cardiovascular diseases. An important interventional radiology procedure for their diagnosis is digital subtraction angiography (DSA). An alternative to DSA is K-edge subtraction (KES) imaging, which has been shown to be advantageous for moving organs and to eliminate image artefacts caused by patient movement. As highly brilliant, monochromatic X-rays are required for this method, it has been limited to synchrotron facilities so far, limiting the feasibility in clinical routine.
**Methods and Materials:** In this study, a filter-based KES method was applied at the Munich compact light source (MuCLS), a compact synchrotron X-ray source based on inverse-Compton scattering, which provides a tunable, quasi-monochromatic X-ray beam. A series of experiments of an excised porcine heart was performed to demonstrate the feasibility of the proposed KES technique. To simulate the situation in a realistic setting, namely ribs of the chest overlying the coronary arteries, a porcine costal arch fixated in formalin was set in front of the heart in the experimental setup.
**Results:** It is shown experimentally that the technique of KES increases the visibility of small blood vessels overlaid by bone structures. Thereby, the contrast-to-noise ratio (CNR) of the KES images was significantly higher than the CNR of the conventional X-ray image.
**Conclusion:** The results show that the quasi-monochromatic spectrum of the MuCLS enables filter-based K-edge subtraction imaging. We believe that KES at a compact synchrotron source can become an important tool in pre-clinical research and possible future clinical diagnostics.

**B-0996** 11:26
**Areal bone mineral density estimation using photon-counting energy-discriminating computed tomography topograms**
T. Nowak[1], B. Schmidt[1], A. Euler[2], N. Saltybaeva[2], T. Flohr[1], H. Alkadhi[2]; [1]*Forchheim/DE*, [2]*Zurich/CH (Tristan.Nowak@gmx.net)*

**Purpose:** Dual-energy X-ray absorptiometry (DXA) is a well-established standard for the diagnosis and monitoring of osteoporosis. DXA provides areal bone mineral density (aBMD) maps which are estimated from two spectrally separate planar x-ray images. With the advent of photon-counting energy-discriminating detectors, perfectly aligned multi-energy topograms with good spectral separation can be acquired in a single scan. We investigated the potential of these topograms for a DXA-like aBMD evaluation.
**Methods and Materials:** A series of topograms of a BMD calibration spine phantom (European Spine Phantom, QRM GmbH, Möhrendorf, Germany) was acquired on a research photon-counting CT scanner (Siemens Healthcare GmbH, Forchheim, Germany). A tube voltage of 140 kV and two energy thresholds of 20 keV and 75 keV were selected. The phantom consists of water-equivalent plastic with diameters of 260 mm (lateral) and 180 mm (anterior-posterior) and holds three anthropomorphic vertebrae, L1-L3, of varying hydroxyapatite content. Collimation and mAs-product were set to 3 mm and 6 mAs. A custom tool was developed to subtract soft tissue and create aBMD density maps from the input topograms. Deviations from the known aBMD values of 0.5, 1.0 and 1.5 g/cm² of the anterior-posterior projections of vertebrae L1-L3 were evaluated.
**Results:** From the generated aBMD maps, mean values for vertebras L1-L3 were extracted and averaged across scans. The resulting values of 0.53 ± 0.10 g/cm², 0.99 ± 0.09 g/cm² and 1.45 ± 0.09 g/cm² for L1-L3 were in good agreement with the ground truth.
**Conclusion:** Our evaluation shows a promising new application for single-scan multi-energy topograms, which might provide an alternative to using dedicated DXA.
*Author Disclosures:*
**T. Nowak:** Employee; Siemens Healthcare GmbH. Shareholder; Siemens Healthineers AG. **B. Schmidt:** Employee; Siemens Healthcare GmbH. Shareholder; Siemens Healthineers AG. **T. Flohr:** Employee; Siemens Healthcare GmbH. Shareholder; Siemens Healthineers AG.

**B-0997** 11:34
**Spectral photon-counting for gout diagnosis in plain radiography: a feasibility study**
F.A. Huber[1], S. Gkoumas[2], T. Thuering[2], F. Becce[3], R. Guggenberger[1]; [1]*Zurich/CH*, [2]*Baden-Dättwil/CH*, [3]*Lausanne/CH (florian.huber@usz.ch)*

**Purpose:** To distinguish crystal suspensions of monosodium urate (MSU) from calcium hydroxyapatite (HA) deposits in plain radiography.
**Methods and Materials:** Three suspensions of either MSU (200mg/ml) or HA (150 and 200mg/ml) synthetic crystals in agar were produced at clinical concentrations, each sealed in industry-standard polystyrene and supported on a thick polymethylmethacrylate (PMMA) plate with equal attenuation to cortical bone. Samples were scanned with both conventional radiography using a vendor microfocus X-ray tube (50kV/300uA) and a spectral photon-counting (PC) detector prototype with four energy thresholds per acquisition set at 15, 25, 30 and 35 keV. Images were then reconstructed for the following energy bins: 15-25, 25-30, 30-35 and 35-50 keV. Material decomposition calibration was performed with chemically pure PMMA and polyvinylchloride slabs of multiple different thicknesses, respectively. Using a custom Python-based post-processing software, effective atomic numbers ($Z_{eff}$) of the respective samples were calculated. All samples were additionally scanned with dual-energy CT (80kV and tin-filtered 150kV) and analysed with a vendor workflow for gout (syngo.via CT DE Gout).
**Results:** Despite equal concentrations, MSU was significantly less attenuating than both HA-samples. MSU and HA suspensions had significantly different $Z_{eff}$ (mean+/-SD: 7.76+/-.26 vs. 9.67+/-.39, p<.001). $Z_{eff}$ of HA was significantly higher than of similar attenuating PMMA plate. $Z_{eff}$ numbers from PC were comparable to dual-energy CT reference values.
**Conclusion:** This feasibility study demonstrates discrimination of MSU from HA crystal suspensions in PC radiography based on $Z_{eff}$ differences. Further studies have to investigate whether this novel technique could replace DECT in vivo.

**B-0998** 11:42
**Automatic analysis and result presentation workflow for diagnostic X-ray quality assurance**
V.-M. Sundell, E. Hippeläinen, J.I. Peltonen; *Helsinki/FI (juha.peltonen@hus.fi)*

**Purpose:** The quality assurance (QA) of diagnostic X-ray systems is regulated with international and national guidelines. The measurement of different radiation and image quality parameters is required in constant intervals. The follow-up of the results requires substantial manual labor in multi-unit radiological department. We have developed an automatic QA analysis and result presentation workflow to enable efficient monitoring of the X-ray equipment performance.
**Methods and Materials:** Monthly DIGRAD phantom images from 63 diagnostic X-ray machines were analysed with the developed workflow. The workflow included the follow-up of exposure (mAs), dose area product (DAP), exposure index (EI), modulation transfer function (MTF), noise-power spectrum (NPS) and contrast-to-noise ratio (CNR). Of the measures, mAs, DAP and EI are extracted from the image header and MTF, NPS and CNR calculated from the image. The images are sent to an image-processing server for analysis and results available in a web based-interface within a few minutes. Additionally, if the results are exceeding predefined error borders, e-mail is automatically sent to QA experts.
**Results:** The developed centralized QA workflow enables an effective follow-up of the diagnostic X-ray equipment performance. The produced data can be utilized to detect possible drifts in the imaging hardware before substantial change in image quality or radiation dose. The results are immediately available for radiographers and QA experts.
**Conclusion:** The presented workflow is providing an effective tool for QA experts to follow the performance of diagnostic X-ray equipment in large radiological department.

**B-0999** 11:50
**Quality controls in ultrasound: a trial to evaluate the obsolescence of ultrasound probes with a low-cost agar gel phantom**
A. Collocola[1], M. Ortino[2], S. Durante[1]; [1]*Bologna/IT*, [2]*Taranto/IT (alessio.collocola@studio.unibo.it)*

**Purpose:** Nowadays ultrasounds are the most installed equipment within the medical operative realities, but that does not fall into precise quality control programs like all other imaging equipment. Considering (Considering the elevated number of exams performed every day, it is essential to set up intuitive programmed quality controls using simple and low-cost accessories.
**Methods and Materials:** Only linear probes with different crystal technology are underwent quality check. In order to do this, a 5% agar gel phantom was created with a set of two modules specifically designed to certify in an effective way, the uniformity, sensitivity, mechanical, electrical and global hygiene of the device.
**Results:** The studied probes tend to lose uniformity and sensitivity even after 3 years with an average of 40 daily examinations and about 8 hours of non-stop operation. The phantom used was homogeneous and isoecogenous in 95% of uses according to the professionals. The equipment was found to be dirty and with mishandled power and network cables in the 70% of cases.
**Conclusion:** Unlike the experts and installers declarations the probes begin to lose uniformity and sensitivity after 3 years from the first date of installation. The absence of a programmed quality control discourages the professionals and it could damage patients over time. However, the use of a low-cost phantom and pre-filled forms could improve the obsolescence of the probes.

**10:30 - 12:00** **Room K**

### Chest

### SS 1004

### Advanced methods for nodule evaluation

*Moderators:*
I. Pulzato; Genua/IT
M. Regier; Hamburg/DE

**K-24** 10:30
**Keynote lecture**
C.J. Herold; *Vienna/AT*

**B-1000** 10:39
**Are the referrals being made for the investigation of pulmonary nodules appropriate according to the BTS guidelines?**
W. Weston; *London/UK (williamweston@gmail.com)*

**Purpose:** The British Thoracic Society (BTS) provide guidelines for the investigation and management of pulmonary nodules (PNs). The Imperial healthcare trust runs a PN multi-disciplinary team (MDT) meeting weekly. The service aims to save time in outpatient appointments and lung cancer MDTs, reduce the number of CT examinations requested, and provide a systematic approach to ensure BTS guidelines are complied with. We aimed to assess if referrals to the PN MDT and their recommendations were in accordance with BTS guidelines.
**Methods and Materials:** We carried out a retrospective study of the referrals and follow-up for patients discussed in the PN MDT between 9/2017 and 4/2018.
**Results:** 154 nodules were referred to PN MDT. 16% of PNs referred to the MDT were discharged without any follow-up. 38% were discharged after CT surveillance or further investigations i.e. PET-CT. 44% of nodules had volumetric analysis performed.
**Conclusion:** The Imperial College Healthcare Trust runs a comprehensive service for the investigation of PNs. Although there is scarcity of published data on this topic, our finding that only 16% of referrals were immediately discharged from the PN MDT shows a good awareness of what requires further investigation. We provided a summary of the BTS management algorithms locally to raise awareness amongst general radiologists of features of benign nodules that do not require referral. We also emphasised the importance of seeking out previous imaging studies for comparison and performing volumetric analysis where possible.
*Author Disclosures:*
**W. Weston:** Consultant; Dr Nicola Strickland, Dr Sarah Sheard.

**B-1001** 10:47
**Accuracy of model-based iterative reconstruction for CT volumetry of part-solid nodule and pure solid nodule at various dose settings: an anthropomorphic chest phantom study**
S.-K. Kim, C. Kim, K.Y. Lee, J. Cha; *Ansan/KR*

**Purpose:** To investigate the accuracy of model-based iterative reconstruction for volume measurement of part-solid nodule (PSN) and pure solid nodule (SN) compared with filtered back projection (FBP) and hybrid iterative reconstruction (HIR).
**Methods and Materials:** Four different SNs (diameters: 3 mm, 5 mm, 7 mm, and 9 mm) and 4 PSNs (3 mm, 5 mm, 7 mm, and 9 mm, inner solid portion diameters; 20 mm, outer ground-glass portion diameters) and chest phantom was used for study. The scanning was repeated 4 times at five radiation dose levels (120 kVp at 100 mAs, 50 mAs, 20 mAs, and 10 mAs, and 80 kVp at 10 mAs). Each CT scan was reconstructed using FBP, HIR (iDose$^4$), and MIR (IMR-R1, IMR-ST1, IMR-SP1). The volume of SNs, and solid/GGO portion of PSNs were measured semi-automatically.
**Results:** The lowest absolute percentage measurement error (APE) of GGO part of PSN were demonstrated in IMR-R1 among the algorithms in all dose level setting (all P<0.05), and the APE of solid portion of PSN in all dose level setting without 80 kVp/10 mAs also showed the significantly lowest value in IMR-R1 among the algorithms (all P<0.05). For SNs, the APEs at 120 kVp/50 mAs, 20 mAs, and 10 mAs were the lowest in IMR-ST1 among the algorithms (all P<0.05, except 120 kVp/20 mAs), and the others were the lowest in IMR-SP1 (all P≤0.01).
**Conclusion:** MIR showed higher accuracy of SN and PSN volume measurements compared with FBP and HIR in most dose settings in this phantom study.

**B-1002** 10:55
**Assessment of predictors for pulmonary subsolid nodule detection in non-lung apex in ultralow dose CT reconstructed with ASiR-V**
Y. Kai, Y. Hui Shu; *Beijing/CN (yekaibysy@bjmu.edu.cn)*

**Purpose:** The purpose of this study was to assess independent predictors for sensitivity of pulmonary subsolid nodule detection in non-lung apex by using ultralow dose CT(ULDCT) reconstructed with ASiR-V.
**Methods and Materials:** 37 patients who underwent chest LDCT with Revolution CT(GE Healthcare, USA) and found subsolid nodule in non-lung apex were additionally scanned with a chest ULDCT. Reference tube voltage and current were set to be 120 kV and smart mAs for LDCT and 120 kV/2.8 mAs for ULDCT. After scanning, LDCT were reconstructed with 50% ASiR-V and 90% ASiR-V for ULDCT. A standard of reference (SOR) was established about the presence, tyes and size of lung nodules on LDCT. Sensitivity of the ULDCT was compared against the SOR. Then, logistic regression was used to test independent predictors for performance of pulmonary nodule detection.
**Results:** The mean effective radiation dose of ULDCT was 0.096±0.005 mSv compared with 0.83±0.32 mSv for LDCT, which was 88% lower. A total of 46 nodules were identified by LDCT and overall sensitivity was 63.04% for ULDCT (29/46). The sensitivity for nodules with diameter greater than 5mm and 6mm, it was 81.8%(27/33) and 92%(23/25), respectively. In multivariate analysis, size and patients' BMI were independent predictors for sensitivity(P<0.05). But when it came to BMI≤30, size was the definitive factor for nodule detection and BMI had little influence on it.
**Conclusion:** For people with BMI≤30, ultralow dose CT reconstructed with ASiR-V enables a high sensitivity for the detection of pulmonary subsolid nodules longer than 6 mm in an extremely low dose.

**B-1003** 11:03
**Comparison of the capability of nodule component measurement and management on follow-up CT examinations between 3D CADv systems with and without CNN**
Y. Ohno[1], A. Yaguchi[2], K. Aoyagi[3], Y. Kishida[1], S. Seki[1], Y. Ueno[1], T. Yoshikawa[1], T. Murakami[1]; [1]*Kobe/JP*, [2]*Kawasaki/JP*, [3]*Otawara/JP (yosirad@kobe-u.ac.jp)*

**Purpose:** To evaluate the utility of 3D computer-aided volumetry (CADv) with convolutional neural network (CNN) for pulmonary nodule management on follow-up CT examinations.
**Methods and Materials:** 139 pulmonary nodules (70 malignant and 69 benign nodules) underwent repeated CT, pathological, and follow-up examinations as well as surgical resections. In this study, CADvs with and without CNN automatically assessed solid, GGO and total nodule (TN) volumes. Then doubling time (DT) was determined from TN volume change by each CADv system. To evaluate the accuracy of volume measurement on each CADv method, solid, GGO and TN volumes within each nodule were measured by 3 different radiologists. Then gold standards were determined by simultaneous truth and performance level estimation method. To determine the utility of CNN, each volume assessed by CADvs with and without CNN was statistically correlated with those of gold standard. Then each volume measurement error was also compared between CADvs with and without CNN by t test. ROC analysis was performed and diagnostic performance of DT was compared with each other by McNemar's test.
**Results:** CADv with CNN (0.82<r<0.92, p<0.0001) showed better correlations with gold standard than that without CNN (0.57<r<0.75, p<0.0001). Measurement errors of GGO and TN by CADv with CNN were significantly smaller than those without CNN (p<0.05). When applied each feasible threshold value, sensitivity of CADv with CNN (92.2%) was significantly higher than that without CNN (83.3%, p=0.004).
**Conclusion:** 3D CADv with CNN is more useful than that without CNN for management of pulmonary nodule on routine follow-up CT examinations.
*Author Disclosures:*
**Y. Ohno:** Research/Grant Support; Canon Medical Systems Corporation.
**A. Yaguchi:** Employee; Toshiba Corporation. **K. Aoyagi:** Employee; Canon Medical Systems Corporation. **S. Seki:** Research/Grant Support; Canon Medical Systems Corporation. **T. Yoshikawa:** Research/Grant Support; Canon Medical Systems Corporation.

**B-1004** 11:11
**Comparing volume doubling time and $SUV_{max}$ for benign and malignant lung nodules**
D.D. Yang, B. Riley, A.N. Tavare, S. Hare, A. Parthipun; *London/UK (ddyang21@gmail.com)*

**Purpose:** To assess the ability of volume doubling time (VDT) and PET/CT avidity to distinguish between benign and malignant lung nodules.
**Methods and Materials:** All patients with lung nodules ≤3 cm who underwent lung biopsy at a single centre between May 2016 and May 2017 were identified retrospectively. Patients with CT and $^{18}$F-FDG-PET/CT scans performed ≥28 days but <2 years apart, both demonstrating the biopsied nodule, were included. VDT was calculated from interval change in longest axial diameter and compared with $SUV_{max}$. Statistical comparisons were performed using t test and chi-squared test.
**Results:** 49 lung nodules were included. 12 nodules were benign, 6 squamous cell lung cancer (SCC), 27 lung adenocarcinoma, 1 small cell lung cancer, and 3 metastatic disease from extrapulmonary primaries. Median VDT was 488 days for benign and 317 days for malignant nodules. Median $SUV_{max}$ was 2.7 for benign and 4.0 for malignant nodules. Comparing benign and malignant nodules, difference in VDT was statistically significant ($p$=0.04), while difference in $SUV_{max}$ was not ($p$=0.16). There was no statistically significant association between VDT (categorised as ≤400 days and >400 days) and $SUV_{max}$ (categorised as >mediastinal blood pool (MBP) and ≤MBP) ($p$=0.72). Comparing adenocarcinoma and SCC, median VDT was 332 and 92 days, respectively, and median $SUV_{max}$ was 3.2 and 9.2, respectively.
**Conclusion:** VDT demonstrated significant difference between benign and malignant nodules, while $SUV_{max}$ did not. Future work will determine an optimal VDT cut-off for predicting malignancy and assess whether a combination of VDT and $SUV_{max}$ can improve accuracy of prediction.

**B-1005** 11:19

**Quantitative analysis of diffusion weighted magnetic resonance imaging in lung nodules characterisation**

A. Sudarkina, A. Dergilev, N. Gorbunov, V. Kozlov; *Novosibirsk/RU (a.sudarkina@mail.ru)*

**Purpose:** To evaluate the contribution of diffusion weighted imaging (DWI) with different types of data acquisition for differentiation of malignant and benign lung nodules.
**Methods and Materials:** 63 patients (mean age of 57.5±15.7 years) with 76 lung nodules (33 benign, 43 malignant; mean size of 2.1±1.1cm) were undergone chest MRI on 1,5T scanner. Two series of DWI (acquired during free breathing and synchronized with breathing) were obtained for all patients. 72 solid lung nodules were included in the study with assessment of Lesion-to-Spinal cord Ratio (LSR) on DWI with high b-factor and apparent diffusion coefficient (ADC) for each nodule in both series of DWI.
**Results:** In benign nodules the mean ADC values obtained from synchronized and non-synchronized sequences were $1.52\pm0.46\times10^{-3}$ $mm^2/s$ and $1.42\pm0.49\times10^{-3}$ $mm^2/s$ respectively, LSR - 0.52±0.49 and 0.39±0.45 respectively. In malignant nodules the mean ADC values obtained from synchronized and non-synchronized sequences were $1.18\pm0.23\times10^{-3}$ $mm^2/s$ and $1.13\pm0.26\times10^{-3}$ $mm^2/s$ respectively, LSR - 1.28±0.64 and 0.9±0.5 respectively. The differences of the mean ADC and LSR in both malignant and benign nodules were significant (p<0.05) in both types of data acquisition. The best area under the curve of 0.826 was obtained for LSR values from synchronized diffusion series (the cut-off value of 0.8 can be used for differentiation malignant and benign nodules with a sensitivity of 80% and a specificity of 72%).
**Conclusion:** Quantitative analysis of DWI is a promising tool which can be used in combination with CT and conventional MRI for noninvasive differentiation of malignant and benign lung nodules.

**B-1006** 11:27

**A feasible method to set coherent image quality between different CT scanners with a general phantom for low dose lung cancer CT screening**

W. Qi, R. Shanshan, Z. Miao, S. Ye, C. Lei, H. Nan; *Beijing/CN (qiweiwei@pkuph.edu.cn)*

**Purpose:** To develop a feasible method to generate adequate image quality for lung cancer CT screening at low radiation dose between different CT scanners with general image quality control phantoms.
**Methods and Materials:** First, the Lungman N1 phantom simulating normal size chest with 4 nodules and the Catphan 500 phantom were scanned with low dose on scanner1 (Philips iCT). The image quality of the simulated nodules with FBP reconstruction were evaluated by two radiologists to select the lowest reference mAs (mAs0) for detecting nodules. Second, the physical parameters were also assessed using the Catphan 500 at the lowest mAs. Third, CT scanner2 (GE Revolution) scanned the Catphan 500 phantom to find the right NI (NI0) for getting the above mentioned physical parameters. Finally, 100 volunteers assigned to two scanners randomly underwent lung cancer screening with either the NI0 or mAs0 setting.
**Results:** 1. The Reference mAs0 was 23mAs@100kV on CT scanner1 and NI=27HU@100kV on CT scanner2. The physical parameters for lung cancer screening with the determined scan techniques were 7 line pairs/cm for the spatial resolution and 15.0mm@1.0% for the low contrast resolution. 3. The image quality of the two groups had similar scores (p>0.05) but with similar radiation doses (P>0.05). All positive nodules (Group 1: 9 solid and 3 ground glass opacity (GGO) nodules, and Group 2: 11 solid and 9 GGO nodules) were detected.
**Conclusion:** Using general quality control phantoms to set coherent image quality between different CT scanners for low dose lung cancer CT screening is feasible.

**B-1007** 11:35

**Understanding temporal enhancement of pulmonary nodule with dynamic contrast enhancement CT and utility of each acquisition**

C. Jokerst[1], M. Gotway[1], D. Panse[2], K. Cummings[1], E. Jensen[1], P. Panse[1]; [1]*Phoenix, AZ/US*, [2]*Albany, NY/US (panse.prasad@mayo.edu)*

**Purpose:** Dynamic contrast enhancement for pulmonary nodule entails image acquisition precontrast,1, 2, 3 and 4 minutes post-contrast and predicts aggressiveness if the nodule enhances >15HU over baseline (Swensen et. al, Radiology 2000; 214: 73-80). However, the temporal enhancement of the pulmonary nodule over this time frame is not well validated especially the necessity of the 3- and 4-minute acquisitions. An IRB-approved retrospective study was conducted to evaluate these temporal characteristics.
**Methods and Materials:** All SPN enhancement CT studies (n=610) successfully completed from 2004 to 2017 were included. The average nodule attenuation (HU) from unenhanced and dynamic contrast-enhanced images at 1, 2, 3 and 4 minutes were recorded. An attenuation difference greater than 15 HU between the unenhanced and any of the enhanced 1, 2, 3 or 4 minutes was considered a positive study. The number of nodules showing >15 HU enhancement only at 3 or 4 minutes but did not enhance at 1 and 2 minutes was also calculated.
**Results:** 61 nodules demonstrated maximal positive enhancement at 1, 86 at 2, 66 at 3, and 69 at 4 minutes. 16 and 18 nodules showed more than 15 HU enhancement only at 3 and 4 minutes, respectively.
**Conclusion:** Nodule enhancement exceeding 15 HU using a dynamic enhancement protocol may occur at any of the 4 time points dictated by the study protocol. Although a large number of nodules demonstrate maximal enhancement at 1 and 2 minutes, maximal nodule enhancement occurring at 3 and 4 minutes is seen in a substantial proportion of patients.

**B-1008** 11:43

**Effect of iterative reconstruction and artificial neural network denoising techniques on lung nodule volumetry in ultra-low dose computed tomography: a phantom study**

D. Han, M.A. Heuvelmans, G. Pelgrim, G. Sidorenkov, P.M.A. van Ooijen, M. Oudkerk, R. Vliegenthart; *Groningen/NL*

**Purpose:** To compare lung nodule volumetry for iterative reconstruction (IR) and non-iterative technique artificial neural network (NiTANN) in ultra-low dose CT (ULD-CT), using low-dose CT (LDCT) with filtered back projection (FBP) as reference.
**Methods and Materials:** Five artificial round solid nodules (volume, 40-85 $mm^3$) placed in a thoracic phantom, were scanned with third generation dual-source CT (Force, Siemens, Germany) with three repositions. A LDCT protocol (120 kVp, 20 ref mAs), and ULD-CT protocol with tin filter (Sn100 kVp, 46 ref mAs) were used, with dose-length product of 33.7 mGycm and 4.9 mGycm, respectively. ULD-CT images were reconstructed with FBP and IR (ADMIRE) level 3 and 5, with a sharp reconstruction kernel without edge enhancement. ULD-CT FBP images were further processed using NiTANN (PixelShine, Algomedica, USA). Semi-automated volumetry (MMOncology, Siemens, Germany) was performed on 120 nodule datasets. Accuracy and variability of nodule volumetry in ULD-CT were compared to LDCT using Wilcoxon signed-rank test with Bonferroni correction and Bland-Altman's analysis, respectively.
**Results:** Nodule volumes for ULD-CT were significantly lower than for LDCT (median difference for ULD-CT/IR3: -2.5 $mm^3$ IQR [7.3], ULD-CT/IR5: -3 $mm^3$ IQR [4.5], ULD-CT/NiTANN: -8 $mm^3$ IQR [6.0]). The relative 95%-limits of agreement were ±20%, ±19% and ±15% for IR3, IR5 and NiTANN, respectively. No significant difference was found when measured volumes were compared to physical volume.
**Conclusion:** An artificial neural network denoising technique showed slightly larger underestimation of nodule volumes than IR in ULD-CT, but tended to have less variability.

**B-1009** 11:51

**Detectability of non-solid pulmonary nodules by deep learning: results from a phantom study**

Q. Li[1], L. Li[2], K. Liu[1], C. Xia[2], Y. Xiao[1], S. Liu[1]; [1]*Shanghai/CN*, [2]*Beijing/CN (925454346@qq.com)*

**Purpose:** To investigate how different nodule size, nodule density, effective dose and reconstruction kernels affect the performance of a deep learning algorithm for the detection of non-solid pulmonary nodules in phantom CT scans.
**Methods and Materials:** CT scans were performed on a chest phantom containing non-solid nodules (diameters of 5, 8, 10, and 12 mm; -630 and -800 HU for each diameter) at two different radiation dose settings ( 4.1mGy and 0.3mGy ). A total of 64 scans were obtained. A deep learning model was developed based on real-patient data (without any phantom data). Evaluation of deep learning model focused on *sensitivity* [TPs/(TPs + FNs)] and *precision* [TPs/(TPs+ FPs)].

Friday

**Results:** Higher nodule density [-800HU: 67.4%; -630HU: 91.2%] and larger nodule size [5, 8, 10, 12mm: 57.8%, 89.1%, 98.6%, 82.0%] were significantly associated with higher detection sensitivity of non-solid nodules. Dose significantly affected both detection sensitivity [low dose vs. ultra-low dose: 90.3% vs. 62.0%] and precision [low dose vs. ultra-low dose: 77.4% vs. 55.9%]. Additionally, dose effect strongly interacted with nodule density; it was more challenging for deep learning algorithm to detect lower density nodules in ultra-low dose scans [-800HU vs. -630HU: 38.7%% vs. 84.7%] than in low dose scans [-800HU vs. -630HU: 84.9% vs. 95.7%].
**Conclusion:** Phantom studies can be applied to systematically evaluate the performance of deep learning algorithms in computer-aided detection tasks.
*Author Disclosures:*
**L. Li:** Employee; Employee of Infervision. **C. Xia:** Employee; Employee of Infervision.

**10:30 - 12:00** **Room M 1**

### Cardiac

### SS 1003

### Cardiac CT in search of coronary artery disease

*Moderators:*
M. Kantarci; Erzurum/TR
M. Krupiński; Krakow/PL

**K-25** 10:30
**Keynote lecture**
M. Williams; *Edinburgh/UK*

**B-1010** 10:39
**Acute chest pain evaluated with coronary CT: validation of new atherosclerotic markers**
M. Fusaro[1], M. Rattazzi[2], C. Nardin[1], M. Tiepolo[1], A. Dorigo[1], G. Tessarin[2], G. Stamati[3], C. Agostini[1], G. Morana[1]; [1]*Treviso/IT*, [2]*Padua/IT*, [3]*Corigliano Calabro/IT*

**Purpose:** The aim of this study is analysing the association between circulating new atherosclerotic markers and the presence of coronary artery disease (CAD).
**Methods and Materials:** Patients with acute chest pain (ACP) and low risk of ACS (normal TnT and ECG) arriving to ED from March 2016 to October 2017 were evaluated with a triple rule-out CT. We measured circulating levels of polymorphonuclear elastase (PMN), lactoferrin, proteinase 3 (PR3) and neutrophil gelatinase-associated lipocalin (NGAL). Patients have been then divided according to the presence/absence of CAD and biochemical/clinic parameters and correlated to CT scan. Statistical analysis was performed with SPSS 24.0.
**Results:** 200 consecutive patients, 86 with CAD (A) and 114 without CAD (B). The two groups showed significant difference in terms of age (A: 57.6±8.0, B: 50.5±9.3, p<0.001) and gender (males: A 79.1%, B 57%, p=0.001). No difference was observed for systolic and diastolic blood pressure, lipid profile and smoke. Circulating levels of PR3, NGAL and PMN appear superimposable in both groups while a significative increase of lactoferrin levels has been observed in group A (A: 128.7±78.0 U/ml, B: 105.5±42.6 U/ml, p=0.014), which remained significative even after adjustment for age, gender, waist circumference, presence of hypertension, presence of diabetes, lipid profile and smoke habit (p=0.015). After exclusion of patients with CAD >50%, the difference remained significant (p=0.033).
**Conclusion:** Circulating levels of lactoferrin are associated with the presence of CAD, independent of traditional cardiovascular risk factors, leading to a more precise stratification of patients with acute chest pain.

**B-1011** 10:47
**HTA of patient management with and without triple rule out CT protocol**
G. Tessarin[1], M. Fusaro[2], C. Bortolanza[2], G. Morana[2], E. Quaia[1]; [1]*Padua/IT*, [2]*Treviso/IT (tessarin.giovanni@gmail.com)*

**Purpose:** Health technology assessment (HTA) of Triple Rule Out (TRO) CT protocol vs standard of care for the management of Patients with acute chest pain, in the the clinical setting of a tertiary referral hospital.
**Methods and Materials:** We evaluated 8 parameters derived from the HTA hospital-based model: general relevance, safety, effectiveness in literature and real life, economic impact, equity, social and ethical impact, management impact. The study consists of 3 phases: parameter prioritization, analysis and conclusions.
**Results:** We identified 1420 expected cases/year for NSTEMI, 426 for pulmonary embolism, 9676 for aortic aneurysm. Both safety and social impact fostered TRO protocol (Likert scale 1 vs 0). Effectiveness, quoted in terms of length of stay in the hospital, was 23.22 hours for TRO vs 30.8 hours for standard of care. Estimated costs were 349.23 € for TRO and 336.65 € for standard of care per Patient. Cost-effectiveness analysis showed a Incremental cost-effectiveness Ratio of -1.66 for the TRO. Both protocols had same equity, legal and organisational impact. Final score was of 0.89 for CT vs 0.74 for standard of care, showing superiority of TRO.
**Conclusion:** HTA analysis showed a better score for TRO, thanks to his safety, effectiveness and good social impact.

**B-1012** 10:55
**Second generation of dual source computed tomography for evaluating coronary artery lesions in Vietnamese paediatric patients with Kawasaki disease**
H. Nguyen, T. Nguyen, P.M. Thong, H.S. Ha, N.K. Viet, L.T.T. Lien, P.B. Ngoc, H.V. Hoa; *Hanoi/VN (thanhhuong.nguyenrad82@gmail.com)*

**Purpose:** To evaluate coronary artery lesions (CAL) in paediatric patients with Kawasaki disease by DSCT 256 detectors then comparing with the measurements of 2D echocardiography (2DE).
**Methods and Materials:** 24 children (21 boys) from with Kawasaki disease underwent DSCT 256 detectors at Bach Mai hospital from December 2015 to July 2018. The average effective dose of DSCT and 4-point subjective imaging quality was collected. The location, number and size of coronary aneurysm were independently evaluated by DSCT and 2DE. Bland - Altman analysis was used to evaluate the agreement of aneurysms measurements between DSCT and 2DE.
**Results:** 96.7% (232/240) coronary artery segments had good imaging quality (score ≤ 3). Average effective dose of DSCT was 1.58 ± 0.56 msV (0.77÷ 3.20). Coronary aneurysm in 1 segment, 2 segments and 3 segments was 80.5%, 16.7% and 2.8%, respectively. The mean ± SD aneurysm diameter measured by DSCT and 2DE was 7.18 ± 2.72mm and 7.16 ± 2.55mm, respectively. The mean ± SD aneurysm length measured by DSCT and 2DE was 13.49 ± 8.65mm and 13.28 ± 8.58mm, respectively. Bland - Altman plot showed a good agreement between DSCT and 2DE.
**Conclusion:** DSCT is a feasible modality with excellent imaging quality and low effective dose exposure for rapid and accurate assessment of CAL in infants and children due to Kawasaki disease.

**B-1013** 11:03
**Randomised trial comparing transdermal with lingual and sublingual nitroglycerin administration for coronary vasodilation in coronary CT-angiography**
J.-E. Scholtz[1], V. Baliyan[2], S. Hedgire[2], N.D. Mercaldo[2], R.A. Takx[3], U. Hoffmann MD MPH[2], T.J. Vogl[4], B. Ghoshhajra[2]; [1]*Frankfurt a. Main/DE*, [2]*Boston, MA/US*, [3]*Utrecht/NL*, [4]*Frankfurt/DE (janerikscholtz@gmail.com)*

**Purpose:** Nitroglycerin increases coronary artery diameter and improves coronary artery evaluation in coronary CT-angiography (CCTA). This study aimed to compare the safety and efficacy of lingual, sublingual and transdermal administration of nitroglycerin in coronary vasodilation.
**Methods and Materials:** This prospective, randomized controlled study included 198 subjects who underwent scheduled CCTA between 6/2017-9/2017. Subjects were randomized to one of three routes of nitroglycerin (0.8mg) administration: tablet(sublingual), spray(lingual), or patch(transdermal). After acquiring ECG-gated non-contrast CT, nitroglycerin was given at least 5 minutes (lingual/sublingual) or 45 minutes (transdermal) before ECG-gated CCTA. Linear quantile mixed-effects models were constructed to quantify relationship between coronary diameter and nitroglycerin administration method. Side effects and vital sign changes were documented. When headaches were present, intensity was scored using a 10-point Likert scale (1-minimal to 10-worst imaginable headache).
**Results:** The studied population had a mean age of 58.1±12.6 years of which 91/198 [46.0%] were female. On average, coronary diameters in CCTA were 1.14 (95% confidence interval: 1.13-1.15) times larger compared to coronary diameters prior to nitroglycerin treatment. Differences between spray (1.15[1.14-1.16], difference: 0.00[-0.02, 0.01], p=0.815) or patch (1.16[1.15-1.17], difference: 0.01[-0.01, 0.03], p=0.201) compared to tablet (1.15[1.14-1.16]) as reference were unable to be detected. Thirty (15.2%) patients reported transient headache with a median intensity of 3 (Q25-Q75: 2-5), 2(2-5), and 2(2-5) for tablet, spray, and patch, respectively (tablet vs. patch, p=0.004).
**Conclusion:** Lingual, sublingual and transdermal nitroglycerin administration had no statistically significant differences in the vasodilatory effect on coronary arteries, with transdermal route being a safe alternative for CCTA.
*Author Disclosures:*
**U. Hoffmann MD MPH:** Research/Grant Support; KOWA, HeartFlow Inc.
**B. Ghoshhajra:** Consultant; Medtronic, Inc., and Siemens Healthcare, Inc.

**B-1014** 11:11
**Comparison of coronary lumen diameter in CT angiography: spray nitroglycerin vs sublingual nitroglycerin tablet administration**
B. Choi[1], K.Y. Lee[1], C. Kim[1], S. Lee[1], B.-K. Je[1], J. Cha[1], H. Yong[2], E.-Y. Kang[2], Y.-W. Oh[2]; [1]*Ansan/KR*, [2]*Seoul/KR (chlqurrudk@gmail.com)*

**Purpose:** To compare the coronary vasodilation effects of spray nitroglycerin (NTG) with those induced by sublingual NTG administration in coronary CT angiography (CTA) and to investigate the effects of spray NTG in elderly patients.
**Methods and Materials:** From June 2016 to March 2018, a total of 1169 CTA using spray NTG (spray group) and 828 CTA using sublingual NTG (sublingual group) were analysed. Our institution used only sublingual NTG tablet for coronary CTA prior to March 2017 and subsequently only used spray NTG according to the hospital policy. Among these, 93 patients underwent CTA twice using both spray NTG and sublingual NTG. Diameters of all measurable segments were measured and compared between spray group and sublingual group. Also, comparison of vessel diameters between spray group and sublingual group in the elderly patients were performed. The intra-individual analysis was also performed in 93 patients.
**Results:** No statistically significant differences were evident between these 2 groups in baseline characteristics. Spray group tends to have more measurable vessels than sublingual group. All coronary segments in spray group were significantly larger than those in sublingual group (all P<0.01). This tendency was also found in the elderly. In the intra-individual analysis, all coronary segments in spray group were significantly larger than those in sublingual group (all P<0.001).
**Conclusion:** Spray NTG is more effective in evaluating coronary arteries in CTAs even in the elderly, resulting in vasodilatation more effectively than sublingual NTG. We recommend spray NTG to be preferred mode of administration at CTA.

**B-1015** 11:19
**Systolic high-pitch coronary CT angiography for evaluation of the coronary arteries in heart transplant recipients**
M. Calvo Imirizaldu, A. Ezponda Casajús, A. García Baizán, J.C. Pueyo Villoslada, G. Rabago, G. Bastarrika Alemañ; *Pamplona/ES (mcalvoi@unav.es)*

**Purpose:** This study aimed to evaluate the feasibility, image quality, and radiation dose of high-pitch coronary CT angiography (CCTA) in orthotopic heart transplant recipients (OHT).
**Methods and Materials:** 22 consecutive OHT (16 men, mean age 62.5±11.8 years, mean BMI 26±4.3 kg/m2, mean heart rate 88.2±13.6 bpm) underwent CCTA to rule out coronary allograft vasculopathy in a high-pitch mode using a third-generation dual-source CT scanner. Data acquisition was triggered at 35% of the R-R´interval. Two independent observers blindly assessed image quality on per-segment, per-vessel, and per-patient basis using a 4-point scale (4-excellent, 1-not evaluative). Scores 2-4 were considered of diagnostic quality. Interobserver agreement on the image quality was assessed with kappa statistics. Radiation dose was recorded for each patient.
**Results:** 322 coronary segments were evaluated. Diagnostic image quality was observed in 98.1% of the segments. Interobserver agreement for image quality assessment was very good on a per-patient (κ = 0.82), per-vessel (κ = 0.83), and per-segment basis (κ = 0.89). Mean image quality score was 3.5±0.74 for the entire coronary tree, 2.7±1.03 for the RCA, 3±0.76 for the LCA, and 2.5±0.8 for the Cx. Per-patient mean image quality score was 2.2±0.79. Estimated mean radiation dose was 0.49±0.32 mSv.
**Conclusion:** High-pitch coronary CCTA in a systolic phase of the cardiac cycle allows achieving diagnostic image quality coronary angiograms in OHT at very low radiation dose.

**B-1016** 11:27
**Automated coronary artery segmentation using a U-net deep learning architecture on cardiac CT**
C. Wald, N. Aldoj, S. Feger, N.-A. Hartmann, S. Lukas, S. Zargaran Tavakoli, M. Dewey; *Berlin/DE (christian.wald@charite.de)*

**Purpose:** Exact segmentation of coronary arteries on cardiac computed tomography (CT) is crucial for accurate evaluation of the presence of coronary artery disease. In an automatic approach we propose a convolutional neural network in order to segment the coronary arteries on cardiac CT.
**Methods and Materials:** We used a total of 22 patients who underwent 320 row coronary CT. For each we obtained a 3D CT volume with 512*512*560 voxels where coronary arteries were manually annotated slice-wise. We developed a neural network of 3D U-net architecture for automated segmentation. Nine of the manually segmented volumes were used for training, three for validation and nine for testing. For computational reasons we used patches with 48*48*48 voxels and included 20,000 for training. We evaluated the model patch-wise on test cardiac CT volumes and reconstructed the coronary arteries of the whole volumes by merging patches.
**Results:** We evaluated the performance of the network by computing the dice score between the manual segmentations and the network output on test volumes. We achieved an average dice score of 0.75±0.04. The test volumes were manually annotated twice and the average inter-reader dice score was 0.77±0.05 which was not significantly different from the network performance (p=0.27). Evaluating the network on the right coronary artery only resulted in an average dice score of 0.75± 0.1 which did not differ significantly from the entire network output (p=0.87).
**Conclusion:** The proposed method of automatically extracting coronary arteries using a 3D U-net is comparable to a human reader.
*Author Disclosures:*
**C. Wald:** Research/Grant Support; Berlin Institut of Health, DFG. **M. Dewey:** Research/Grant Support; European Commission, DFG, Berlin Institut of Health. Speaker; Canon Medical Systems, Guerbet, Cardiac MR Academy Berlin, Bayer. Other; Siemens Medical Solutions, General Electric, Philips Medical Systems, Toshiba Medical Systems.

**B-1017** 11:35
**The optimal monochromatic image level of coronary CT angiography on a dual-layer detector spectral CT with half-dose contrast agent**
X. Huang, Y. Hou, Y. Ma, S. Gao, Z. Jia, X. Lu; *Shenyang/CN (632136789@qq.com)*

**Purpose:** To investigate the optimal monochromatic level of reconstruction images of coronary CT angiography (CCTA) on a dual-layer detector spectral CT(DLCT) with half-dose contrast agent.
**Methods and Materials:** A prospective collection of 200 patients (116 males, 84 females, age 55±11 years) were randomly divided into the routine dose contrast group and half-dose contrast group (both n=100). CCTA was performed on a DLCT with prospective ECG-gated mode and 120 kV. For the routine dose group, polychromatic images with iterative model reconstruction (IMR) (group A) were reconstructed. For the half-dose group, three monochromatic levels of images were reconstructed (group B, 45 keV; group C, 50 keV; group D, 55 keV). Iodixanol 270mgI/ml was used with a dose of 0.8ml/kg and 0.4ml/kg, respectively, for the routine group and half-dose group. Objective indicators (CT values, SD, SNR, CNR), subjective indicators (contrast, sharpness, subjective noise, acceptability) in each group were compared.
**Results:** The CT value, SNR, CNR in group C were higher than those in group A (P<0.001). The SD in group B was larger than that in group A (P<0.001). There was no significant difference between objective scores of group D and group A (P>0.05). There were no significant differences in sharpness, subjective noise, and acceptability between group C and group A (P>0.05), and the contrast was superior to that of group A (P<0.001). The contrast in group B was better than that of group A (P<0.001), but the subjective noise was worse than that of group A (P<0.001). The contrast, sharpness and acceptability of group D were all worse than that of group A (P<0.001).
**Conclusion:** 50 keV monochromatic images of DLCT provide compatible coronary image quality with half-dose contrast agent.

**B-1019** 11:43
**Predictive role of lipid panel and DLCN score in the assessment of coronary artery disease with coronary CT in patients with familial hypercholesterolemia**
A. Ascione, G. De Rubeis, N. Galea, M. Francone, C. Catalano; *Rome/IT (andrea121.ascione@gmail.com)*

**Purpose:** To assess the possibility of predicting presence and severity of coronary artery disease as observed with cardiac CT through lipid panel and Dutch Lipid Clinic Network (DLCN) score.
**Methods and Materials:** Between January 2014 and March 2018, 86 patients (50 males [58%]; mean age 48) affected by Familial Hypercholesterolemia were analyzed through coronary CT. Lipid panels and DLCN scores were completed for all patients. The degree of stenosis for all 17 coronary segments was assessed according to CAD-RADs; total calcium burden was also computed. The predictive model was implemented using linear and logistic regression.
**Results:** Mean values (mg/dL) of the lipid panels were: Total cholesterol 340 ± 88, HDL 60 ± 16, TG 120 ± 56, LDL 257 ± 79; mean DLCN score was 7 before genotyping and 13 after genotyping. Prevalence of significant Coronary Artery Disease (CAD-RADs ≥ 4) was 12,8% (11 out of 86). The average number of lesions detected at pre-contrast CT was 2 ± 5,2, Agatston score 73 ± 257,5. LDL cholesterol, TG and total cholesterol levels were able to predict the Agatston score (r = 0,26 [p = 0,03]; r = 0,4 [p = 0,006; r = 0,24 [p = 0,4]). Only the TG value was able to predict the level of CAD-RADs (r = 0,4 [p < 0,05]); neither DLCN score nor any lipid panel value can predict significant coronary artery disease (CAD-RADs > 4).
**Conclusion:** Lipid panel and DLCN score cannot predict the degree of significant Coronary Artery Disease at cardiac CT.

**10:30 - 12:00** **Room M 3**

### Oncologic Imaging

### SS 1016

### Lung cancer: screening and beyond

*Moderators:*
G. Cicchetti; Rome/IT
X. Montet; Geneva/CH

### B-1020 10:30

**Comparison of RECIST, iRECIST, and PERCIST for the evaluation of response to PD1/PD-L1-blockade therapy in patients with non-small cell lung cancer**

L. Beer, M. Hochmair, A. Haug, D. Kifjak, B. Schwabel, M.E. Mayerhöfer, H. Prosch; *Vienna/AT (lucian.beer@meduniwien.ac.at)*

**Purpose:** The aim of this study was to compare the Response Evaluation Criteria in Solid Tumors (RECIST) 1.1, the immune RECIST (iRECIST) criteria, and the Positron Emission Tomography Response Criteria in Solid Tumors (PERCIST) 1.0 for the assessment of response evaluation in patients with advanced non-small cell lung cancer (NSCLC) treated with PD-1 / PD-L1 inhibitors.
**Methods and Materials:** This prospective study of 42 patients treated with a PD-1 / PD-L1 inhibitor was approved by our Institutional Review Board, and all patients gave written, informed consent. Tumor burden dynamics were assessed on [18F]FDG PET/CT before and after treatment initiation. Immunotherapeutic responses were evaluated according to the RECIST 1.1, iRECIST, and PERCIST 1.0 for the dichotomous groups, responder vs. non-responders. Cohen's κ coefficient, and Wilcoxon´s signed-rank test were used to evaluate concordance among these criteria. We assessed progression-free survival (PFS) and overall survival (OS) using the Kaplan-Meier test.
**Results:** RECIST 1.1 and PERCIST 1.0 response classification were discordant in six patients (14.2% %; κ= 0.581). RECIST 1.1 and iRECIST were discordant in two patients, who evidenced pseudoprogression after treatment initiation. Median PFS, as well as OS, was significantly longer for responders compared to non-responders for all criteria (p<0.001) without any significant difference between the three criteria (p>0.05).
**Conclusion:** RECIST 1.1 and PERCIST 1.0 show only a moderate agreement, but both can predict treatment response to PD-1 / PD-L1 inhibitor therapy. In cases of pseudoprogression, metabolic tumor activity may help to correctly classify treatment response.

*Author Disclosures:*
**L. Beer:** Grant Recipient; Österreichische Nationalbank, Theodor Körner Fond, Krebsforschungsfond der Stadt Wien. **H. Prosch:** Advisory Board; Boehringer Ingelheim, Roche, MSD, BMS, AstraZeneca, Novartis. Speaker; Boehringer Ingelheim, Roche, MSD, BMS, AstraZeneca, Novartis.

### B-1021 10:38

**Imaging programmed cell death ligand-1 (PD-L1) expression in non-small cell lung cancer with $^{99m}$Tc-anti PDL1 sdAb SPECT**

G. Chand[1], Y. Xing[1], C. Liu[1], N. Wong[1], L. Meszaros[1], J. O'Doherty[2], H. Ting[1], J. Zhao[1], G. Cook[3]; [1]*Shanghai/CN*, [2]*Doha/QA*, [3]*London/UK*

**Purpose:** This early phase 1 study aimed to evaluate safety, dosimetry, biodistribution and imaging characteristics of $^{99m}$Tc-anti PDL1 sdAb with single-photon emission computed tomography-computed tomography (SPECT-CT).
**Methods and Materials:** 10 patients with advanced untreated non-small cell lung cancer underwent whole body planar and thoracic SPECT/CT imaging at 1 and 2h post injection after injection of 5.3-11.1 MBq/Kg. 3 patients underwent further imaging at different timepoints for dosimetry calculations. Patients were monitored for adverse events. All patients had immunohistochemistry analysis of %PD-L1 on biopsies of the primary tumours.
**Results:** No adverse events related to $^{99m}$Tc-anti PDL1 sdAb were recorded. Effective dose was 0.009+/-0.001 mSv/MBq. All scans showed an expected biodistribution of PD-L1 including kidneys, spleen, liver, bone marrow and low-level lung activity. SPECT primary tumour to blood pool ratios (T:BP) varied from 1.24 to 2.23 (mean 1.71) at 1h and 1.24-3.12 (mean 2.13) at 2h. Nodal T:BP ratio varied from 1.04 to 2.36 (mean 1.78) at 1h and 1.18-3.4 (mean 2.21) at 2h. Pleural and bone metastases also showed tracer uptake. Heterogeneity between primary tumour and metastatic PD-L1 expression (>25% difference) was present in 4 of 7 patients with nodal metastases.
**Conclusion:** $^{99m}$Tc-anti PDL1 sdAb is safe and is associated with acceptable dosimetry similar to other diagnostic SPECT agents. Biodistribution is as expected for tissues that express PD-L1. Tumour PD-L1 expression and heterogeneity is measurable with better contrast at 2h and correlates with IHC measurements in the primary tumour.

*Author Disclosures:*
**G. Chand:** Employee; NanoMab Technology Ltd. **N. Wong:** Employee; NanoMab Technology Ltd. **L. Meszaros:** Employee; NanoMab Technology Ltd. **H. Ting:** CEO; NanoMab Technology Ltd. **J. Zhao:** Investigator; NanoMab Technology Ltd. **G. Cook:** Consultant; NanoMab. Research/Grant Support; Theragnostics.

### B-1022 10:46

**Non-small-cell lung cancer treated with pembrolizumab: correlation between texture analysis and iRECIST**

M. Zerunian[1], D. De Santis[1], D. Caruso[1], T. Biondi[2], G. Funicelli[1], E. Rosati[1], A. Zucchelli[1], C. De Dominicis[1], A. Laghi[1]; [1]*Rome/IT*, [2]*Belvedere Marittimo/IT (marta.zerunian@gmail.com)*

**Purpose:** To correlate CT-derived texture parameters and iRECIST in patients with advanced non-small-cell lung cancer (NSCLC) treated with pembrolizumab as a first line.
**Methods and Materials:** Twenty patients with advanced NSLC were prospectively enrolled. All participants underwent contrast enhanced CT at baseline and after pembrolizumab. Response to therapy was assessed with iRECIST. An expert radiologist, blind to response to therapy results, manually drew a volume of interest (VOI) of the whole tumour at baseline and after immunotherapy on venous phase by a dedicated software (TexRad). Texture parameters (*Mean, Entropy, Kurtosis, Skewness*,and *MPP*) were extrapolated and reported. Results were correlated with iRECIST and *P* values <0.05 were considered significant.
**Results:** Twelve (60%) patients showed partial response (iPR) while 8 (40%) patients had confirmed progressive disease (iCPD). *Entropy* and *Mean* decreased in patients with PR (*Entropy*: 5.2 ±0.3 vs 4.3 ±0.4, *P*= 0.03; *Mean*:14.7 ±24.9 vs 8.0 ±25.8, *P*= 0.01)and increased in patients with DP (*Entropy*: 4.9 ±0.3vs 5.1 ±0.3, *P*= 0.04; *Mean*:6.3 ±15.8 vs 12.0 ±23.1, *P*= 0.01). The others texture parameters analyzed did not show significant differences before and after immunotherapy (all *P*> 0.05).
**Conclusion:** *Entropy* and *Mean* could be used as imaging biomarkers to predict response to immunotherapy in patients with NSLC. Texture parameters may play a role in the radiologic assessment of advanced NSLC treated with pembrolizumab.

### B-1023 10:54

**RECIST 1.1 evaluations in a phase II clinical trial: does reader expertise represent a risk factor for measure reliability?**

H. Beaumont[1], T.L. Evans[2], C. Klifa[1], S.R. Hong[3], M. Chadjaa[4], Z. Monostori[5], A. Iannessi[6]; [1]*Valbonne/FR*, [2]*Philadelphia, PA/US*, [3]*Seoul/KR*, [4]*Vitry-sur-Seine/FR*, [5]*Budapest/HU*, [6]*Nice/FR (hubert.beaumont@mediantechnologies.com)*

**Purpose:** In imaging-based clinical trials, double reads of imaging data are common practice; therefore, discrepant interpretations can result from these two evaluations. This study analysed discrepancies that occurred between local investigators (LI) and blinded independent central review (BICR). We tested if the LI reader expertise was a potential risk factor in RECIST 1.1 evaluations of progressive disease (PD).
**Methods and Materials:** We retrospectively analysed imaging data from a RECIST 1.1, multi-site, phase II clinical trial of 147 patients with adult small cell lung cancer. All LI and BICR readers were similarly trained prior to the trial. Qualifications of Lis' readers were radiologist, oncologist, clinical research associate (CRA) and sub-investigator. We compared the proportions of adjudication rate triggered by radiologists versus non-radiologists in using R CRAN test for equality of proportions. Reasons for discrepancies were analysed by a third-party reader.
**Results:** A total of 36.7% patient data required adjudication. The reasons for adjudication included differences in 1) reporting new lesions (53.7%), 2) measures of tumour burden change (18.5%), and 3) progression status of non-target lesions (11.2%). The proportion of patient data evaluated by LI readers were radiologist: 79.6% (117/147), oncologist: 13.6% (30/147), CRA 2% (3/147), sub-investigators: 2% (3/147), miscellaneous: 2.7% (4/147). The adjudication rate was not different between radiologists and non-radiologists (p=0.28), neither the adjudication rate due to the appearance of new lesions (p=0.52).
**Conclusion:** In this trial, we did not find that different reader qualifications were associated with significant risk factors.

*Author Disclosures:*
**H. Beaumont:** Employee; Median Technologies. **C. Klifa:** Employee; Median Technologies. **M. Chadjaa:** Employee; SANOFI.

**B-1024** 11:02
**Everolimus-related pneumonitis in advanced neuroendocrine tumours: correlation of radiologic findings and clinical symptoms**
U. Fehrenbach[1], V. Rodriguez Laval[2], M. Pavel[3], I. Steffen[1], A. Enriquez Puga[2], H. Jann[1], T. Denecke[1]; [1]*Berlin/DE*, [2]*Madrid/ES*, [3]*Erlangen/DE (uli.fehrenbach@charite.de)*

**Purpose:** Everolimus, an mTOR inhibitor, is approved for the treatment of advanced pancreatic neuroendocrine tumours (NET). Drug-induced pneumonitis is a major adverse event in everolimus treatment and can lead to determination of the treatment. To evaluate the correlation between clinical symptoms and radiological findings in everolimus-induced pneumonitis in patients with advanced neuroendocrine tumours.
**Methods and Materials:** 90 patients with advanced NET treated with everolimus were included in this retrospective study. All patients received chest CT scans prior to the initiation of everolimus and at a regular interval during the treatment. At the day of the scan, every patient was clinically examined. Cough and dyspnea as the most common symptoms of pneumonitis were scored according to the “Common Terminology Criteria for Adverse Events 3.0”(symptomatic score). Chest CT scans were scored based on the severity of interstitial lung disease (ILD score) and the overall pulmonary extent (pneumonitis extent). Pattern analyses of the pulmonary disease was performed independently by 2 radiologists.
**Results:** Pneumonitis was diagnosed in 18(20%) of the patients. There was no significant correlation between symptomatic score and ILD score or pneumonitis extent. Pattern analyses showed significant results when divided in cryptogenic organizing pneumonia(COP)(n=14) and non-COP(n=4) pattern. Patients with non-COP pattern showed significantly higher ILD scores than with COP pattern(p=0.035).
**Conclusion:** We could show that the severity of CT findings in everolimus-induced pneumonitis does not correlate with the patients’ clinical symptoms. That’s why asymptomatic patients should not ultimately quit therapy in advanced neuroendocrine tumours. CT pattern analysis could help in the prediction of clinical impact.

**B-1025** 11:10
**Differentiation of benign lung nodules and metastases: a machine-learning approach using texture features from spectral detector CT-derived iodine maps and conventional images**
S. Lennartz[1], A. Mager[1], N. Grosse Hokamp[1], S. Schäfer[2], D. Maintz[1], T. Persigehl[1]; [1]*Cologne/DE*, [2]*Heidelberg/DE (simon.lennartz@uk-koeln.de)*

**Purpose:** The diagnostic value of iodine quantification for assessing pulmonary malignancies has previously been investigated mostly with ROI-based iodine quantification. The purpose of this study was to analyze if benign lung nodules (BLN) and pulmonary metastases (PM) can be differentiated with a machine-learning-based classification approach using texture analysis of spectral detector CT (SDCT)-derived iodine maps and the referring conventional images.
**Methods and Materials:** For this IRB-approved study, we retrospectively identified 66 patients who underwent SDCT (IQon, Philips) of the chest: 29 patients with 54 BLN confirmed by prior/follow-up CT (constant in size for ≥6 months) or histopathology and 37 patients with 180 PM verified by histopathology, $^{18}$F-FDG-PET-CT or unequivocal change during treatment (maximum lesion number: 6 per patient). Semi-automatic 3D segmentation of all lesions was performed (mint lesion, Mint Medical). Volumetric attenuation (from conventional images) and iodine concentration (from iodine maps) were acquired. Entropy, kurtosis, mean of the positive pixels (MPP), skewness and uniformity of the positive pixels (UPP) within the volumes of interest were calculated for conventional images and iodine maps. All acquired parameters were transferred to an available machine-learning framework (Matlab, MathWorks).
**Results:** K-nearest neighbour classification method (KNN) with 10 neighbours and Euclidean distance metric yielded the best performance in differentiating BLN from PM with a diagnostic accuracy of 84.2% using cross-validation with a prediction speed of approx. 1200 obs/sec.
**Conclusion:** Based on first-order texture features derived from quantitative iodine maps and conventional images obtained with SDCT, KNN facilitates accurate differentiation of benign lung nodules and pulmonary metastases.
*Author Disclosures:*
**S. Lennartz:** Other; Received travel expense reimbursement from Philips Healthcare. **N. Grosse Hokamp:** Speaker; Philips Healthcare. **S. Schäfer:** Employee; Mint Medical GmbH. **D. Maintz:** Speaker; Philips Healthcare.

**B-1026** 11:18
**Lung cancer screening with low dose computed tomography: the republic of Kazakhstan experience**
Z. Zholdybay, G. Akhmetova, G. Akhmetova, A. Panina, A. Ainakulova; *Almaty/KZ (doctorpanina@gmail.com)*

**Purpose:** To evaluate the initial results of a pilot project of a lung screening program with low dose computed tomography (LDCT) in the Republic of Kazakhstan, discuss the challenges of program implementation.
**Methods and Materials:** 569 patients were screened from July to October 2018. 230 men (40.5%) and 339 women (59.5%), ≥30 years old (smokers, non-smokers, former smokers with no previous history of cancer and healthy individuals). A helical LDCT of the chest was performed (120 kVp, 10mA with images reconstruction at 1-1.25mm overlapping slices). Baseline LDCT data were considered positive if at least one non-calcified nodule 6mm or larger in size, or non-solid nodule 8mm or larger in size was identified. Follow-up monitoring for persons with positive baseline LDCT data were carried out according to Fleischner guidelines.
**Results:** The median age at baseline was 58.5 years (range 35-82). A total of 38 pathological findings were revealed in the lungs. Of these, 22 (57.9%) with obvious malignant signs, 10 (26.3%) ground glass opacity areas and 6 (15.8%) were assessed as probably benign. All nodules were pathologically verified. Thus, from the 32 suspicious nodules in 21 (65.6%) cases - non-small cell lung cancer was identified, in 5 (15.7%) cases - adenocarcinoma, in 6 (18.7%) cases no tumour cells were detected.
**Conclusion:** LDCT lung cancer screening program showed rather high specifity 98,0% with lower sensitivity 81,3%. The average frequency of lung cancer in randomized patient population was 4,6%.

**B-1027** 11:26
**One-year results from the South Wales Rapid Diagnostic Clinic for patients with serious but non-specific symptoms of cancer**
I.A. Siddiqui[1], K.G. Foley[1], B. Palaniappan[1], A. Gibson[2], T. Pearce[2], G.J. Davies[2]; [1]*Cardiff/UK*, [2]*Ynysmaerdy/UK (imran.a.siddiqui@gmail.com)*

**Purpose:** Cancer is a leading cause of death in the UK. Initiatives such as the 2-week wait (2-WW) have been implemented to expedite investigations for patients with red-flag symptoms. However, only 50% of patients diagnosed with cancer have such symptoms. Approximately 20% of patients have serious but non-specific symptoms, such as nausea and weight loss. These patients often experience diagnostic delays. Here, we present results from the first 12 months of a rapid diagnostic clinic (RDC) aiming to investigate patients with serious but non-specific symptoms of cancer.
**Methods and Materials:** This is an ongoing prospective population-based interventional study. Forty-two GP practices covering a population of approximately 301,000 patients were invited to participate. Consultant review was performed in the RDC and appropriate radiological investigations were requested. The CT was performed and reported by a consultant radiologist on the same day. The primary outcome was the conversion rate.
**Results:** In 12 months, 259 patients (median age 69.2 years) were recruited. Twenty-five cancers were diagnosed, a conversion rate of 9.7%. Lung cancer was the most frequent malignancy (n=6). Overall, 76% of patients were stage 4. The most common presenting symptom was weight loss (77%). 138 patients (53.3%) were seen within 1 week of referral.
**Conclusion:** These results highlight the potential benefit of a RDC investigating patients with serious but non-specific signs of cancer. This rate was higher than the USC conversion rate in our health board (3-8%). Such diagnostic interventions should be considered to improve conversion rates in this specific patient group.

**B-1028** 11:34
**Polmonary metastases from pancreatic cancer: morphologic and quantitative differences compared to lung metastases from colorectal cancer**
I. Vicentin, R. Ronza, M. Pecorilla, C. De Mattia, C. Sgrazzutti, P.E. Colombo, A. Torresin, S. Siena, A. Vanzulli; *Milan/IT (ilaria.vicentin@unimi.it)*

**Purpose:** To find out the morphological and quantitative differences between lung metastases from pancreatic cancer (PC) and colorectal cancer (CRC).
**Methods and Materials:** 326 lung metastases derived from PC (160) and from CRC (166) were retrospectively reviewed at CT by 2 radiologists, categorizing them in 3 groups according to their morphology: solid nodule, solid excavated nodule, air space pattern (ground glass lesions and nodules with a halo sign). The quantitative analysis consisted in radiomics workflow: 10 categories of radiomics features were considered, clustered in 5 families (Shape, First Order Statistics, Gray Level Co-occurrence Matrix, Gray Level Run Length Matrix, Neighbour Intensity Difference). A statistical univariate analysis was conducted.

**Results:** Among the 160 PC lung metastases, 43 presented the air space pattern (26.9%), 87 were solid nodules (54.3%) and 32 were solid excavated nodules (18.8%). Among the 166 CRC lung metastases, 140 were solid nodules (84.3%), 20 were solid excavated nodules (12.1%) and 6 nodules presented an air space pattern (3.6%). The statistical univariate analysis showed that radiomics families of First Order Statistics, Gray Level Co-occurrence Matrix, Gray Level Run Length Matrix and Neighbour Intensity Difference were valid predictors for the discrimination between the two cohorts.
**Conclusion:** The difference between the metastatic pattern of PC and CRC is significant since among our cohort, 25 patients with PC presented lung metastases with an air space pattern, whereas only 2 patients with CRC presented the same occurrence. This difference can also be assessed from the quantitative point of view through specific radiomics features.

**B-1029** 11:42
**Difference between measurement of T size at computed tomography and at resected specimen: is it relevant for NSCLC stage?**
D. Colombi[1], S. Chiesa[1], F. Bodini[1], N. Morelli[1], M. Silva[2], N. Sverzellati[2], C. Franco[1], E. Michieletti[1]; [1]*Piacenza/IT*, [2]*Parma/IT*
*(colombidavide@gmail.com)*

**Purpose:** To evaluate the impact of the differences between presurgical contrast enhanced computed tomography (CECT) and gross pathology measurements on the non-small-cell-lung-cancer (NSCLC) T-staging.
**Methods and Materials:** Thirty-eight tumours were evaluated in 34 consecutive patients (8 females; median age: 69 years-old; interquartile range: 66-76) with NSCLC surgically treated. The maximum diameter of the tumour was measured with digital caliper at CECT, using multiplanar reconstruction if necessary. The largest diameter from the pathology report was used. Tumour stage was defined by the 8$^{th}$ edition lung cancer TNM stage classification, for radiological stage (rStage) and pathological stage (pStage). The Wilcoxon test examined the difference between tumour size obtained by CECT and pathology. The concordance correlation coefficient (CCC) was used to explore the agreement between the measurements.
**Results:** The median largest diameter of the tumour on CECT (median size: 30 mm; interquartile range: 19-43) was significantly ($p$=0.02) higher than the median pathology measurement (median size: 25 mm; interquartile range: 15-41). The CCC between the two diameters was poor (CCC: 0.781; 95%CI: 0.624-0.878). Upstaging at rStage was observed in 15/38 (39%) tumours, due to larger T size on CECT in 93% of the cases. Most frequent upstaging occurred from IA1 pStage to IA2 rStage and from IA2 pStage to IA3 rStage (respectively 4/15, 27%).
**Conclusion:** In resected NSCLC, tumour T size as assessed by CECT is significantly larger than the corresponding gross pathology specimen. These differences would imply radiological upstaging in 39% of the cases, especially within subclassification of stage I.

**B-1030** 11:50
**Assessing the precision of an AI-powered algorithm for automatic detection and 3D segmentation of primary tumours in NSCLC**
T.J. Weikert[1], M. Pradella[1], F. Durlak[2], J.I. Sperl[2], B. Stieltjes[1], J. Cyriac[1], J. Bremerich[1], G. Sommer[1], A.W. Sauter[1]; [1]*Basel/CH*, [2]*Forchheim/DE*
*(thomasjohannes.weikert@usb.ch)*

**Purpose:** Manual tumour segmentation is time consuming and cost intensive, but mandatory when it comes to radiation treatment planning, therapy response monitoring or radiomics. The purpose of this study was to assess the performance of an AI-based radiology assistant to automate this task.
**Methods and Materials:** We preselected 75 FDG-PET/CTs for non-small-cell lung cancer (NSCLC) staging from our RIS/PACS archive (all confirmed by histology, solid and peripheral T1/T2 stages). First, all main masses were fully segmented and annotated manually by a radiologist on the CT component. Lesion volumes were calculated. In parallel, the same series were fed into interlinked AI-algorithms applying deep convolutional neural networks for automated detection and segmentation. The time required for both procedures was recorded. The intraclass correlation coefficient (ICC) for the detected volumes as measure of interrater reliability between manual and automatic analyses was calculated based on a single-rater, absolute agreement, 2-way random-effects model.
**Results:** The algorithm correctly detected 75 of 75 main lesions and attributed them to the respective lung lobe. Mean tumour volumes were: 6,810.52±8,353.53 mm$^3$ for manual segmentation versus 7,105.45±8,989.48 mm$^3$ for automated analysis. ICC was 0.956 (95% confidence interval 0.931 - 0.972; p<0.001). Mean processing time of the algorithm was 64±23 seconds compared to a mean of 342±382 seconds for manual tumour segmentation.
**Conclusion:** The algorithm facilitates a fast and reliable detection and 3D segmentation of solid peripheral pulmonary T1 and T2 tumours. Further evaluation regarding the performance on advanced and more challenging tumours (T3/T4, central, subsolid) is warranted.

*Author Disclosures:*
**F. Durlak:** Consultant; Siemens Healthineers. **J.I. Sperl:** Employee; Siemens Healthineers.

**10:30 - 12:00** **Room M 5**

### Neuro

### SS 1011b

### Functional MRI

*Moderators:*
E. Seliverstova; Moscow/RU
A. Yakimov; Moscow/RU

**B-1031** 10:30
**Functional MRI study of language organisation in left-handed and right-handed trilingual subjects**
S. Yazbek, C. Atat, J. Abdel Hay, R. Moussa; *Beirut/LB*
*(chirine.atat@gmail.com)*

**Purpose:** fMRI has replaced WADA testing for preoperative assessment of language lateralization and localization. In Lebanon, most people are trilingual. Selecting the appropriate language to test is, therefore, intricate. Our objective is to compare fMRI maps for all 3 languages in left-handed and right-handed trilingual subjects.
**Methods and Materials:** We included 15 right-handed and 15 left-handed healthy adult volunteers, fluent in Arabic, French and English. We performed fMRI for each volunteer with a visual responsive naming task paradigm repeated for every language and we compared the areas of activation.
**Results:** Language lateralization was identical for the 3 languages in 87% of volunteers. The 4 subjects who demonstrated different lateralization were all left-handed. Broca and Wernicke areas demonstrated a bilateral activation in 88% of the cases, and presented the same anatomical localization for all 3 languages whereas the number and localization of the accessory language areas were different. There was a strong correlation between the global activation index and the chronology of language acquisition in right-handed subjects ($r_s$ = .275, $p$ = .0068) with the last language learned inducing a higher degree of activation.
**Conclusion:** In right-handed trilingual patients, fMRI performed for a single language can accurately determine language lateralization, whereas in left-handed all languages need to be tested. To determine the anatomical localization of accessory language areas, it is preferable to study all 3 languages. If the duration of the fMRI study was to be shortened, a study of the last language learned in right-handed patients could be considered.

**B-1032** 10:38
**Functional network analysis of human language: a novel approach using task-based fMRI**
L. Pasquini, Q. Li, G. Del Ferraro, K. Peck, A.I. Holodny, H. Makse; *New York, NY/US*

**Purpose:** Task-based functional MRI (tb-fMRI) is a powerful method to identify brain areas involved in cognitive performances, such as language production. Pre-surgical planning of brain tumour is routinely accompanied by fMRI investigation to guide the neurosurgeon's understanding of the eloquent brain areas. Although tb-fMRI sheds light on brain areas involved in cognitive processes, it does not elucidate how these areas functionally interact with each other and integrate to form a comprehensive network. Resting-state fMRI on the other hand, has provided information on brain connectivity, but it is less implemented in clinical practice.
**Methods and Materials:** In this work, we employ statistical inference techniques to reconstruct brain networks from tb-fMRI unearthing the functional connectivity among brain areas involved in language performances. We tested this new method on 9 healthy right-handed subjects who performed a language production task.
**Results:** Our results show a robust common functional language network across healthy individuals which entangles the Broca's area, Wernicke's area, supplementary motor area, and pre-motor cortex in the left hemisphere of right-handed subjects. Furthermore, we investigated the functional connectivity of the anatomical sub-parts of the Broca's areas, such as pars opercularis and pars triangularis.
**Conclusion:** This new approach allows to gather valuable information about brain connectivity from the tb-fMRI examination routinely performed in clinical practice. This post-processing technique is general and not limited to language tasks, in addition to healthy controls it can be applied to patients and help understanding critical clinical questions, such as brain functional connectivity for pre-surgical planning.

**B-1033** 10:46

**Altered resting-state functional connectivity of the anterior cingulate cortex in anxious rats post-acoustic trauma**

X.-M. Xu, G.-J. Teng; *Nanjing/CN (xmxu15@163.com)*

**Purpose:** Acoustic trauma (AT) often leads to aberrant neural activity in a distributed brain network that not only includes the central auditory pathway, but also extra-auditory brain regions. Exactly where and how AT-induced cochlear hearing loss affects the central nervous system during the acute and sub-acute periods are poorly understood. We aimed to identify the neural substrate for aberrant patterns that mediat AT-related anxiety.
**Methods and Materials:** Forty-five male Sprague-Dawley (SD) rats were exposed to broadband noise of 122 dB for 2 hours and randomly divided into three groups: 0-day post-noise (N0D-acute period), 10-day post-noise (N10D-subacute period) and controls. Each group had 15 rats. All of them underwent emotion-related behavioural measurements (including open-field test and light/dark box) and resting state blood oxygenation level-dependent fMRI.
**Results:** Behavioural tests revealed that rats were much more anxious in the acute period and sustained little till 10 days later. Using an anterior cingulate cortex (ACC) seed, rats undergoing acoustic trauma demonstrated a large-scale network that had a spatial distribution beyond auditory network during both periods (N0D and N10D). Between-group analysis revealed that N0D rats displayed widespread reductions in functional connectivity, spanning primary somatosensory cortex, medial geniculate body, inferior colliculus, cingulate cortex, area 2, cerebellar lobule comparing with N10D rats and a similar pattern had also occurred in comparison with control group, while the FC increased during the sub-acute period.
**Conclusion:** Taken together, an “acoustic-causing” network accounting for distress, pain and gating of anxiety was proposed.

**B-1034** 10:54

**Reward-related system fMRI activation as a monitoring tool for alcohol abuse rehabilitation**

O. Omelchenko, M. Makarchuk, O. Podkovka, L. Myronyak, V. Rogozhyn; *Kiev/UA (Ol.Omelchenko@Gmail.Com)*

**Purpose:** Early response evaluation is necessary in patients with alcohol abuse rehabilitation. We propose reward-related system activation analysis with fMRI as the rehabilitation monitoring tool in alcohol abuse patients.
**Methods and Materials:** Two groups of subjects were studied using 1.5T CANON Titan. Group 1 consisted of 8 patients (4F, 4M, aged 28-43) with alcohol abuse, undertaking non-pharmaceutical rehabilitation and alcohol drinking refusal for 8 weeks. Group 2 consisted of 10 healthy volunteers (5F, 5M, aged 22-50) without alcohol abuse and normally drinking small amounts of alcohol. Event-related fMRI was used. Visual stimuli of alcohol-related items contrasting with non-alcohol related bottles were used for stimulation. For fMRI we used EPI pulse sequence: TR/TE=3000/60ms. Analysis was made with FSL (Oxford, GB).
**Results:** No alcohol craving was reported by Group 1 patients. No strong emotions were reported by Group 1 patients during observation of alcohol-related stimuli. Activation of the uncus of left hemisphere was shown for Group 1 and 2. Activation of right insula, right dorsolateral prefrontal cortex and left striatum was shown for Group 1. Pronounced activation of right insula, dorsolateral prefrontal cortex and left uncus was found in Group 2. Activation of right nucleus accumbens, left anterior cingulate cortex and motor cortex was shown for Group 2. Thus, absence of activation of reward-related system (nucleus accumbens, anterior cingulate) may be the correlate of successful alcohol abuse rehabilitation.
**Conclusion:** Reward-related system fMRI activation may reward-related as a tool for alcohol abuse patients rehabilitation monitoring.

**B-1035** 11:02

**Brain resting state connectivity in the development of secondary hyperalgesia in healthy men**

M.S. Hansen[1], L. Becerra[2], J.B. Dahl[1], D. Borsook[3], J. Mårtensson[4], A.F. Christensen[1], J.D. Nybing[1], I. Havsteen[1], M. Boesen[1], M.S. Asghar[1]; [1]*Copenhagen/DK*, [2]*Boston, MA/US*, [3]*Charlestown, MA/US*, [4]*Lund/SE (morten.sejer.hansen@regionh.dk)*

**Purpose:** Central sensitisation is a condition in which there is an abnormal responsiveness to nociceptive stimuli. As such, the process may contribute to the development and maintenance of pain. Factors influencing the propensity for development of central sensitisation have been a subject of intense debate and remain elusive. Injury-induced secondary hyperalgesia can be elicited by experimental pain models and is believed to be a result of central sensitisation, potentially reflecting individual levels of central sensitisation. The objective of this study was to investigate possible associations between the size of secondary hyperalgesia area and brain connectivity in known resting state networks.
**Methods and Materials:** We recruited 121 healthy, male participants (age 22, (SD 3.35)) who underwent resting state fMRI. Prior to the scan, areas of secondary hyperalgesia following brief thermal sensitisation (3 min. 45°C heat stimulation on the right thigh) were evaluated in all participants. Data were processed using FSL-Melodic software and analysed using dual regression.
**Results:** 115 participants were included in the final analysis. We found a positive correlation (increasing connectivity) with increasing area of secondary hyperalgesia in the sensorimotor- and default mode networks. We also observed a negative correlation (decreasing connectivity) with increasing secondary hyperalgesia area in the sensorimotor-, fronto-parietal-, and default mode networks.
**Conclusion:** Our findings indicate that increasing area of secondary hyperalgesia is associated with increasing and decreasing connectivity in multiple networks, suggesting that differences in propensities for central sensitisation, assessed as secondary hyperalgesia areas, may be expressed as differences in the resting state central neuronal activity.

**B-1036** 11:10

**Altered cortical morphology of visual cortex in children with intermittent exotropia**

L. Lu, S. Tang, X. Wang, Q. Gong, X. Huang; *Chengdu/CN (1575389273@qq.com)*

**Purpose:** To investigate cortical differences and age-related cortical differences between children with intermittent exotropia (IXT) and normal developmental children (NDC).
**Methods and Materials:** 16 IXT and 16 age-, gender-, handedness-, education- matched NDC were recruited. All participants completed ocular examinations, including visual acuity, cycloplegic refraction, angle of deviation measured by prism and alternate cover test, stereoacuity, and exotropia control using the Newcastle Control Score. All participants underwent high-resolution anatomical magnetic resonance imaging (MRI) using a 3-T MR scanner. FreeSurfer software was used to obtain measures of cortical volume, thickness, and surface area. Statistical differences of MRI measures between IXT and NDC were identified using a least general linear model with the intracranial volume, gender and age as covariates.
**Results:** Compared to NDC, children with IXT showed reduced surface area in primary visual cortex, increased surface area in inferior temporal cortex, increased cortical thickness in left orbitofrontal cortex, right middle temporal and inferior frontal cortex ($p<.05$; Monte Carlo corrected). We also observed significant age-related differences of cortical measures between two groups. Specifically, normal age-related decreases of surface area and cortical volume of superior parietal cortex were disrupted in children with IXT.
**Conclusion:** Quantitative characterization of independent aspect of cortical anatomy in IXT patients, we identified neuroanatomic deficits in visual cortex and association cortices highlighting the widespread impact of altered input to the visual system in IXT.

**B-1037** 11:18

**Preoperative brain mapping: task-based and resting-state fMRI matching**

A. Smirnov, M. Sharaev, T. Melnikova-Pitskhelauri, E. Pogosbekyan, L. Fadeeva, D. Pitskhelauri, I. Pronin; *Moscow/RU (alex.s.smirnof@gmail.com)*

**Purpose:** In the current work, we propose a three-step approach to automatic and efficient functional brain areas mapping as well demonstrate in case studies on three patients with gliomas the potential applicability of constrained source separation technique (semiblind independent component analysis, ICA) to brain networks discovery and the similarity of task-based fMRI (t-fMRI) and resting-state fMRI (rs-fMRI) results.
**Methods and Materials:** Three patients with brain glioma were examined with MRI 3T with t-fMRI and rs-fMRI protocols. Here we used tapping and language tests for t-fMRI, blind and semiblind ICA for both methods t-fMRI and rs-fMRI. Pre-processed fMRI data were passed to GIFT software to create both blind and semiblind spatially constrained ICA for both task-based and rs-fMRI. To measure similarity between spatial maps, we use Dice coefficient, which shows the ratio of overlapping voxels and all active voxels in two compared maps for each patient.
**Results:** RSNs of interest (motor and language) discovered by rs-fMRI highly correlate with t-fMRI reference and are located in anticipated anatomical regions. In general, in comparisons of the functional brain areas obtained with t-fMRI and rs-fMRI, there is a greater specificity of semiblind ICA compared to blind ICA.
**Conclusion:** Our preliminary stage in the study of functional changes in neuronal activity caused by brain tumours showed the degree of concordance between t-fMRI and rs-fMRI as well as produced by blind and semiblind ICA. We hope that further our research will allow predicting the activity of neural network architectures and non-invasive mapping of functional areas for preoperative planning.

*Author Disclosures:*
**A. Smirnov:** Research/Grant Support; Supported by grant №18-29-01032 of Russian foundation for basic research. **M. Sharaev:** Research/Grant Support; Supported by grant №18-29-01032 of Russian foundation for basic research. **T. Melnikova-Pitskhelauri:** Research/Grant Support; Supported by grant №18-29-01032 of Russian foundation for basic research. **E. Pogosbekian:** Research/Grant Support; Supported by grant №18-29-01032 of Russian foundation for basic research. **D. Pitskhelauri:** Grant Recipient; Grant №18-29-01032 of Russian foundation for basic research.

**B-1038** 11:26
**The effect of visual stimulation on the aspartate concentration in activated cortex at 3T**
A. Manzhurtsev, P. Menshchikov, M. Ublinskiy, I. Melnikov, T. Akhadov, N. Semenova; *Moscow/RU (andrey.man.93@gmail.com)*

**Purpose:** Aspartate (Asp) is a neurotransmitter whose levels are reported to be decreased in activated brain zones at 7 Tesla. Previously we demonstrated that Asp can be measured more accurately using MEGA-PRESS pulse sequence at 3T. The fMRS of Asp is performed at 3T for the first time.
**Methods and Materials:** Eleven healthy subjects (19-34 y.o.), Philips Achieva dStream 3T and Head-Neck SENSE coil. Video stimulation: monitor, mirror, 8-Hz flashing checkerboard. The spectroscopy voxel (20x40x30 mm) was located in visual cortex. Asp signal was obtained with $Asp_{MEGA-PRESS}$ sequence: TE=90 ms, TR=2000 ms, NSA=288, 27 ms editing pulses applied at $\delta_{On}$=3.89 ppm and $\delta_{Off}$=5.21 ppm (9.5 min). Spectrum in rest was obtained and then spectrum during visual stimulation. Asp peak was processed in jMRUI (AMARES) annd Asp intensity ($I_{Asp}$) was found. PRESS spectra from OFF-series were compiled; creatine ($I_{Cr}$) was found in LCModel. Asp was normalized on Cr: $I_{Asp}/I_{Cr} = Asp_{Cr}$. For each participant, the relative effect of stimulation on $Asp_{Cr}$ was calculated: rel.$Asp_{Cr}$=$Asp_{Cr}$(stimulus)/$Asp_{Cr}$(rest). rel.$Asp_{Cr}$ was compared with 1 with Mann-Whitney (MW) criterion.
**Results:** The Asp peak at 2.71 ppm has high SNR and is easily approximated by a single peak in AMARES (jMRUI). The statistically significant (*p* value<0.05) decrease of rel.$Asp_{Cr}$ by 4% was revealed.
**Conclusion:** The SNR of Asp peak in $Asp_{MEGA-PRESS}$ 24 mL spectrum is much greater than at 7T without spectral editing and is sufficient enough for the confident quantitative analysis. The decrease of Asp in response to neuronal activation is in good accordance with previous findings at 7 Tesla.

**B-1039** 11:34
**Advanced MR imaging with cognitive and vestibular stimulation in vestibular migraine**
H.S. Çubuk, K. Bicakci, S. Bicakci; *Adana/TR (hazal_selvi@hotmail.com)*

**Purpose:** The aim of the study was to evaluate the changes in cortical activation during 5 different tasks after stimulation of vestibular pathways at patients with vestibular and episodic migraine and to identify metabolic changes possibly be seen in thalamus, that is a key role in vestibular and pain pathways.
**Methods and Materials:** Fifteen patients with vestibular migraine and 15 patients with episodic migraine that had diagnosed at Çukurova University Neurology Department were included into this prospective study. Functional MRI was applied before and after the caloric test. BOLD fMRI data were registered during conjunctive eye movements, cognitive task, visual stimuli with 3 Tesla MR 32-channel head coil. SPSS v20 and SPM software was used for the statistical analysis.
**Results:** Functional MR imaging demonstrated increased cerebellar and insular activation in patients with vestibular migraine following caloric testing during finger-nose testing. During object naming task to activate linguistic-associated cortical areas increased thalamic activation was determined in vestibular migraine patients after caloric testing. In migraine patients, loss of activation in the occipital lobe was observed during visual stimulation after caloric testing.
**Conclusion:** Activation changes in brain regions during vestibular pathway activation and during performing cognitive, different tasks in vestibular and episodic migraine patients provide information about the pathophysiology of disease and functional changes that may occur during an attack in patients.

**B-1040** 11:42
**Long-term reactions to pulsatile tinnitus are featured by weakened short-range functional connectivity within a brain network on the right-sided temporal lobe**
H. Lv, Z. Wang, P. Zhao, Z. Wang; *Beijing/CN (chrislvhan@126.com)*

**Purpose:** Recently, efforts that characterize the brain functional activity features in patients with pulsatile tinnitus (PT) are getting increasing concern. Accumulating evidence indicated aberrant functional connectivity (FC) of right middle temporal gyrus (MTG) in PT patient group with prolonged disease duration. Possible predictive effect of those functional connections may exist.
**Methods and Materials:** To test this hypothesis, resting-state functional magnetic resonance imaging (fMRI) were obtained from 34 patients with recent onset PT (RPTIN), 24 patients with long-term PT (LPTIN) and age-, gender-, education-matched 35 healthy controls were enrolled in this study. Right MTG was set as seed region to investigate the intrinsic FC within the whole brain. Results were further correlated with pulsatile tinnitus characteristics, especially disease duration.
**Results:** Functionally connected with hubs belonging to executive control network, default mode network (DMN) and limbic network, the strength of FC was mainly decreased in patient groups compared with normal controls. Relative to RPTIN and normal controls, LPTIN patients were further characterized by significant decreased FC among several short-range brain regions adjacent to the seed. Lastly, the disease duration showed negatively correlated with decreased FC between the seed and right fusiform gyrus/parahippocampal gyrus, right inferior frontal gyrus as well as right MTG (brain area adjacent to seed region) significantly, indicating a possible predictive effect of those functional connections.
**Conclusion:** Long-term reactions to pulsatile tinnitus mainly involved weakened short-range functional connectivity, especially within a functional network in the temporal lobe on the right hemisphere of the brain.

**B-1041** 11:50
**Functional network-based statistics reveal abnormal resting-state functional connectivity in minimal hepatic encephalopathy**
H.-J. Chen, T.-X. Zou; *Fuzhou/CN (chj0075@126.com)*

**Purpose:** Whole-brain functional network analysis is an emerging methodology for exploring the mechanisms underlying hepatic encephalopathy (HE). This study aimed to identify the brain subnetwork that is significantly altered within the functional connectome in minimal HE (MHE), the earliest stage of HE.
**Methods and Materials:** The study enrolled 19 cirrhotic patients with MHE and 19 controls who underwent the resting-state fMRI and cognitive assessment based on the psychometric hepatic encephalopathy score (PHES). A whole-brain functional connectivity (FC) matrix was calculated for each subject. Then network-based statistical analyses of the functional connectome were used to perform group comparisons, and correlation analyses were conducted to identify the relationships between FC alterations and cognitive performance.
**Results:** MHE patients showed significant reduction of positive FC within a subnetwork that predominantly involved the regions of the default-mode network, such as bilateral posterior cingulate gyrus, bilateral medial prefrontal cortex, bilateral hippocampus and parahippocampal gyrus, bilateral angular gyrus, and left lateral temporal cortex. Meanwhile, MHE patients showed significant reduction of negative FC between default-mode network regions (such as bilateral posterior cingulate gyrus, medial prefrontal cortex, and angular gyrus) and the regions involved in the somatosensory network (i.e., bilateral precentral and postcentral gyri) and the language network (i.e., the bilateral Rolandic operculum). The correlations of FC within the default-mode subnetwork and PHES results were noted.
**Conclusion:** Default-mode network dysfunction may be one of the core issues in the pathophysiology of MHE. Our findings support the notion that HE is a neurological disease related to intrinsic brain network disruption.

**10:30 - 12:00** **Sky High Stage**

### Clinical Trials in Radiology

### CT 10

### Clinical Trials in Radiology 3

*Moderators:*
M. Dewey; Berlin/DE
U. Mahmood; Oak Brook, IL/US

10:30
**Lung cancer risk after baseline round of screening: only 20% of NLST eligibles require annual round**
M. Silva[1], F. Sabia[2], G. Milanese[1], S. Sestini[2], C. Jacobs[3], B. van Ginneken[3], M.Prokop[3], C.M. Schaefer-Prokop[4], N. Sverzellati[1], A.V. Marchianò[2], U. Pastorino[2]; *[1]Parma/IT, [2]Milan/IT, [3]Nijmegen/NL, [4]Amersfoort/NL (mariosilvamed@gmail.com)*

**Purpose:** To calculate the risk of lung cancer (LC) in 1 year and 3 years after baseline low-dose computed tomography (LDCT) in high-risk subjects selected by the National Lung Cancer Screening Trial (NLST) criteria.
**Ethics committee approval:** IRB: ProtINT05/53
**Methods and materials:** Subjects from the Multicentric Italian Lung Detection (MILD) trial were selected according to NLST criteria: age≥55 years and pack-years≥30. Baseline characteristics were: smoking status (former/current), gender, percent of predicted forced expiratory volume in first second (FEV1%pred,90%threshold), Tiffeneau ratio (70%threshold), nodules at

Friday baseline LDCT. The risk of LC in 1 and 3 years was calculated by multivariate models.
**Results:** In 1,248 NLST eligible screenees, LC frequency was 1.2% at 1year, 2.6% at 3years. At 1year, nodule volume on LDCT was the only predictor of LC risk (volume>250mm3, odds ratio (OR) 34.25, p=0.0009). At 3years, the risk of LC was predicted by: nodule volume 113-250 mm3(OR9.52 p=0.01), nodule volume >250 mm3(OR29.07, p<0.001), Tiffeneau≤70% (OR2.08 p=0.0195). A simulation of triennial screening rounds, with selective annual round only for nodule volume ≥113 mm3(19,9% in our population) showed 40% reduction of LDTC through 3 years, and 80% LDCT saving at each annual round.
**Limitations:** Administrative difficulties and lack of funding limited the recruitment (4,099 recruited as compared to 10,000 of original design).
**Conclusion:** Annual round is worthwhile for nodule ≥113mm3 (about 20% in our population). Screening every 3 years can safely reduce the LDCT burden for nodule <113mm3(about 80% in our population).
**Funding for this study:** Grants: Italian Ministry of Health (RF2004), AIRC2004 (IG1227) and AIRC5xmille (IG12162), Fondazione Cariplo (2004-1560) and Lombardy Region (2006-1688).
*Author Disclosures:*
**C. Jacobs:** Research/Grant Support, MeVis Medical Solutions AG.
**B. van Ginneken:** Shareholder; Thirona BV. Other; MeVis Medical Solutions.

10:39
**Discussant:**
H.-U. Kauczor; *Heidelberg/DE (Hans-Ulrich.Kauczor@med.uni-heidelberg.de)*

10:45
**Clinical trials in Radiology and data sharing: results from a survey by the European Society of Radiology research committee**
M. Bosserdt, B. Hamm, M. Dewey; *Berlin/DE (maria.bosserdt@charite.de)*

**Purpose:** To determine the current situation and future directions of trials and data sharing in radiology.
**Ethics committee approval:** not necessary
**Methods and materials:** This survey was conducted between July and September 2018 among European heads of imaging departments and speakers at the Clinical Trials in Radiology sessions at ECR 2015-2018. The survey was approved by the ESR research committee and administered online.
**Results:** The response rate was lower among department heads (27%, 114/428) than among Clinical Trials in Radiology speakers (56%, 18/32; p<0.001). These institutions reported having conducted 429 trials, leading to 332 publications, of which 43% were first and 44% last authorships by those institutions. For future trials, 98% of respondents (93/95) said they would be interested in sharing data, although only 34% had shared data already (23/68, p<0.001). The major barriers to data sharing were data protection (78%, 74/95), ethical issues (49%, 47/95), and the lack of a data sharing platform (49%, 47/95). Of the respondents, 89% believed a platform would facilitate data sharing (85/95 vs. 10/95, p<0.001) and should offer easy data uploading (74%, 70/95), data safety (66%, 63/95), easy communication between providers and re-users (62%, 59/95), and data access policies (56%, 53/95).
**Limitations:** Low response rate.
**Conclusion:** A considerable number of imaging trials are being performed and published by radiologists in Europe while data sharing is hardly taking place, despite great interest: most likely because of data protection and ethical issues, as well as the absence of a data sharing platform.
**Funding for this study:** none

10:54
**Discussant:**
R. Maksimović; *Belgrade/RS (uzica.maksimovic@med.bg.ac.rs)*

11:00
**Carotid stenting with antithrombotic agents and intracranial thrombectomy leads to the highest recanalisation rate in acute stroke patients with tandem lesions**
P. Papanagiotou[1], D. Haussen[2], M. Boutchakova-Meyer[1], F. Turjman[3], J. Labreuche[4], M. Piotin[5], A. Kastrup[1], B. Gory[5]; [1]*Bremen/DE,* [2]*Atlanta, GA/US,* [3]*Bron/FR,* [4]*Lille/FR,* [5]*Paris/FR*

**Purpose:** The aim of this study was to identify the optimal endovascular approach in acute stroke patients with tandem lesions.
**Ethics committee approval:** The study was approved by the local institutional review board.
**Methods and materials:** This is an international, multicentre registry with a total of 482 patients with AIS and tandem lesions. Patients were treated by intracranial thrombectomy as well as one of the following four strategies: 1) acute carotid artery stenting of extracranial ICA with antithrombotic agents; 2) acute carotid artery stenting of extracranial ICA without antithrombotic agents; 3) balloon angioplasty of extracranial ICA; 4) intracranial thrombectomy alone. The main outcome endpoints of the study were the degree of recanalisation and the 90-day clinical outcome. The safety endpoints were symptomatic intracerebral haemorrhage (sICH) and all causes of mortality at 90 days.
**Results:** Using univariate analysis, the rates of successful reperfusion (modified thrombolysis in cerebral infarction 2b-3) and favourable clinical outcome after 90 days were significantly higher after acute carotid stenting with antithrombotic therapy and thrombectomy compared to the group with thrombectomy alone. After adjusting for confounding variables, acute stenting with antithrombotic therapy was independently associated with successful recanalisation (OR 2.4, 95%CI 1.25-4.59; P=0.008). The rates of sICH and 90-day mortality were comparable between all four treatment groups.
**Limitations**: This is a retrospective study with noncontrolled design.
**Conclusion**: Acute stenting of the extracranial ICA with antithrombotic therapy in combination with intracranial thrombectomy is associated with higher recanalisation rates in treatment of acute stroke patients with tandem lesions.
**Funding for this study:** This work was supported by Stryker.

11:09
**Discussant:**
K. Compagne; *Rotterdam/NL (c.compagne@erasmusmc.nl)*

11:15
**Additional MRI screening in women with extremely dense breasts: primary outcome of the first round of the randomised DENSE trial**
M.F. Bakker[1], S. Lange[1], R.M. Pijnappel[1], R.M. Mann[2], C. Loo[3], B. Bisschops[4], M.B.I. Lobbes[5], M.D.-J.F. de Jong[6], K.M. Duvivier[3], J. Veltman[7], W.B. Veldhuis[1], C. van Gils[1], On behalf of the Dense Trial Study Group; [1]*Utrecht/NL,* [2]*Nijmegen/NL,* [3]*Amsterdam/NL,* [4]*Dordrecht/NL,* [5]*Maastricht/NL,* [6]*'s Hertogenbosch/NL,* [7]*Almelo/NL*

**Purpose:** To evaluate the effect of supplemental MRI for women with extremely dense breasts within a population-based screening programme.
**Ethics committee approval:** Approved by Dutch Minister of Health, Welfare and Sport (2011/19); participants gave written Informed Consent.
**Methods and materials:** Between 2011-2015, we randomised 40,373 screening participants (aged 50-75) with a negative screening mammography and extremely dense breasts (ACR category 4 by Volpara software) to (an invitation for) supplemental 3.0-T MRI at 8 sites (intervention arm; n=8,061) or mammography screening only (control arm; n=32,312). The difference in interval cancers, during the two-year screening interval, was investigated by intention-to-treat (ITT) analysis, and by complier-average causal effect (CACE) analysis to account for noncompliance.
**Limitations:** The study was not powered for mortality as outcome; interval cancer rate was used as the best proxy.
**Funding for this study:** Funding sources: UMC Utrecht, ZonMw, Dutch Cancer Society, Pink Ribbon/A Sister's Hope, Bayer AG Pharmaceuticals.
*Author Disclosures:*
**M.F. Bakker:** Research/Grant Support; Bayer AG Pharmaceuticals, Volpara Solutions. **S. Lange:** Research/Grant Support; Bayer AG Pharmaceuticals, Volpara Solutions. **R.M. Mann:** Research/Grant Support; Siemens Healthcare, Seno Medical, Bayer AG Pharmaceuticals, Medtronic, Screenpoint, Identification Solutions. **M.B.I. Lobbes:** Research/Grant Support; Bayer AG Pharmaceuticals. **C. van Gils:** Research/Grant Support; Bayer AG Pharmaceuticals, Volpara Solutions.

11:24
**Discussant:**
P.A.T. Baltzer; *Vienna/AT* (patbaltzer@gmail.com)

11:30
**Detection rate for advanced neoplasia of single round of CT colonography vs three rounds of faecal immunochemical test in population screening for colorectal cancer: the SAVE randomised trial**
L. Sali, L. Ventura, M. Mascalchi, P. Mantellini, M. Zappa, G. Grazzini; *Florence/IT (lapo.sali@unifi.it)*

**Purpose:** To compare a single round of CT colonography (CTC) vs three biennial rounds of faecal immunochemical test (FIT) for colorectal cancer (CRC) screening.
**Ethics committee approval:** Trial was approved by the Ethical Committee of the Local Health Unit of Florence (432/2010) and written informed consent was obtained in all participants.

**Methods and materials:** In this pragmatic randomised trial, 14981 subjects aged 54 to 65 years, living in a district of Florence, Italy, were randomised (1:2) and invited by mail to one of two screening interventions: 1) single round CT colonography; 2) three biennial rounds of FIT. Main outcome was detection rate for advanced neoplasia, defined as the proportion of invitees to screening intervention with screen-detected cancer or advanced adenoma. Secondary outcome was positive predictive value (PPV) for advanced neoplasia.
**Results:** Participants to single CTC were 1286/4825(26.7%) subjects, whereas participants to first, second and third FIT rounds were 4677/9288(50.4%), 4709/8676(54.3%) and 4215/7914(53.3%) subjects. The cumulative detection rate per-invitee for a single CTC round (1.39%; 67/4825) was lower than that for three FIT rounds (1.98%; 184/9288) (p=0.012). However, PPV of single

CTC (53.2%) was higher than that of three FIT rounds (32.4%) (p<0.0001).
**Limitations:** Lack of a screening colonoscopy group as comparator for detection rate.
**Conclusion:** For population screening of CRC, a strategy based on single CTC yields a lower detection rate per-invitee than three biennial FIT rounds but is associated with less unnecessary diagnostic colonoscopies.
**Funding for this study:** This trial was funded by Tuscany Region and by Cassa di Risparmio di Firenze Foundation.

11:39
**Discussant:**
T. Mang; *Vienna/AT (thomas.mang@meduniwien.ac.at)*

11:45
**Cost-effectiveness analysis of computed tomography vs invasive coronary angiography in a randomised controlled trial**
G. Abdala Gehling[1], I. Perez[1], A. Hollingsworth[2], T. Reinhold[1], M. Dewey[1]; [1]*Berlin/DE*, [2]*Oklahoma City, OK/US*

**Purpose:** To conduct a cost-effectiveness analysis of computed tomography (CT) coronary angiography and invasive coronary angiography (ICA) from the perspective of insurance providers.
**Ethics committee approval:** Institutional review board approval was obtained and all patients provided written informed consent.
**Methods and materials:** 329 patients with stable chest pain and suspected coronary artery disease were randomly assigned to either CT or ICA at a single centre. We gathered cost information from hospital billing information and from follow-up questionnaires. Quality of life was assessed with the EuroQoL-5D-3L questionnaire.
**Results:** 273 patients could be included in the cost-effectiveness analysis. There was no significant difference in quality of life before CT and ICA (0.86 vs 0.87 QALY/year; p=0.45) and after a median of 4.43 years of follow–up in the CT and ICA group (0.85 vs 0.86 QALY/year; p=0.97). Average insurance cost per patient for the initial management was 758.45±184.01€ in the CT and 1970.42±133.38€ in the ICA group (p<0.001). Outpatient CT was significantly less costly than inpatient CT (394.85 vs 1501.42, p<0.001). The incremental cost per follow-up year was similar in the CT (1805.76±511.99.€) and the ICA group (1997.31±742.92€; p=0.57).
**Limitations:** Incomplete follow-up questionnaires and limited outpatient procedure cost information.
**Conclusion:** Initial management of patients with stable chest pain using CT is more cost-effective than using ICA while follow-up cost are similar.
**Funding for this study:** German Research Foundation was not involved in any stage of the study design, data acquisition, data analysis, or manuscript preparation.
*Author Disclosures:*
**M. Dewey:** Grant Recipient; German Research Fundation. Other; Bayer, Bracco, Cardiac MR Academy, European Commission, European Regional Development Fund, German Foundation of Heart Research, German Federal Ministry of Education and Research, GE Healthcare, Guerbet, Springer, and Toshiba.

11:54
**Discussant:**
N.N.

**10:30 - 12:00 Tech Gate Auditorium**

### Abdominal Viscera

### SS 1001b

### Hepatobiliary contrast agents: focal and diffuse liver disease

*Moderators:*
A. Torregrosa Andrés; Valencia/ES
F. Vernuccio; Palermo/IT

**B-1042** 10:30
**Gadoxetic acid (GA)-enhanced liver MRI and hepatic haemangiomas (HH): diagnostic performance and impact of dynamic phase on lesion characterisation**
F. Castagnoli[1], M. Grazioli[1], M. Puglia[2], B. Frittoli[1], M. Morone[1], L. Romanini[1], F. Donato[1], R. Ambrosini[1], L. Grazioli[1]; [1]*Brescia/IT*, [2]*Naples/IT* *(francy-cast@hotmail.it)*

**Purpose:** To evaluate diagnostic accuracy of MRI using GA for characterisation of HH, comparing the dynamic phase alone with respect to the whole study. Diagnostic confidence of different experienced radiologists was also tested.
**Methods and Materials:** We prospectively enrolled 124 patients with suspected lesions at CECT/US, excluding hepatopathic patients. Histology/biopsy or lesion stability at follow-up imaging were the standard of reference. A 1.5T scanner was used, with acquisitions of baseline, dynamic and hepatobiliary phase images before and during automatic i.v. injection of GA. Three sets of imaging were reviewed by 3 differently experienced radiologists in 3 sessions (1: baseline; 2: dynamic phase and 3: unenhanced+dynamic phase images). Pearson's Chi-square analysis was used to assess sensitivity, specificity and diagnostic accuracy for the whole examination, for the dynamic study and for each reader and session. Inter-reader agreement was evaluated using Cohen's Kappa.
**Results:** 332 lesions were analysed: 165 HH (49.7%), 91 metastases (27.4%), 3 CC (0.9%) and 76 benign lesions (22%). Sensitivity, specificity and diagnostic accuracy were, for the whole-study evaluation, 99.6%, 99.6% and 99.6% and, for the dynamic study alone, 98.2%, 99.6% and 98.8%, without a statistically significant difference between them (p<0.0001). Excellent interobserver agreement (99% concordance, k=0.98) was observed both for the whole study and for the dynamic phase evaluation (98.8%, k=0.97).
**Conclusion:** The dynamic study alone using GA demonstrated high sensitivity and specificity values. Combined evaluation (baseline+dynamic images) slightly improves diagnostic performance in less experienced readers and in small, non-typical HH.

**B-1043** 10:38
**Gadoxetic acid-enhanced magnetic resonance imaging for prediction of the postoperative prognosis of intrahepatic mass-forming cholangiocarcinoma**
M.-J. Kim, S. Kim, C. An, K. Han, S. Lee; *Seoul/KR (sjlee37@yuhs.ac)*

**Purpose:** To identify imaging markers that independently predict the postoperative outcome of intrahepatic mass-forming cholangiocarcinoma (IMCC) using gadoxetate disodium-enhanced magnetic resonance imaging (MRI).
**Methods and Materials:** Data from 54 patients who underwent preoperative gadoxetate disodium-enhanced MRI and curative surgery for IMCC were retrospectively evaluated. The prognostic power of various imaging and pathological features reportedly associated with recurrence-free survival (RFS) and overall survival (OS) were analysed using Cox regression models. A model combining imaging and pathological features was developed and its performance was evaluated using the Harrell C index and Akaike information criterion.
**Results:** Capsule penetration ($P$ = 0.016) and tumour size ($P$ = 0.015) were independent markers for RFS, while capsule penetration ($P$ = 0.012) and hepatic vein obstruction (HVO, $P$ = 0.016) were independent markers for OS, respectively, in the imaging-based model. Capsule penetration was the only imaging marker identified in the combined prediction model of RFS, and the combined model showed a higher C index and lower AIC value compared with the model based on pathological features alone.
**Conclusion:** The evaluation of capsule penetration and HVO using MRI may improve prediction of the postoperative prognosis of intrahepatic IMCC by incorporating imaging markers with a surgical staging system.

**B-1044** 10:46
**Peritumoural radiomics features from gadoxetic acid-enhanced MR imaging predicting early recurrence of hepatocellular carcinoma**
Z. Zhang, J. Chen, H. Jiang, L. Cao, Z. Ye, B. Song, T. Duan; *Chengdu/CN (zhangzhen950708@163.com)*

**Purpose:** To investigate the ability of peritumoural radiomics features from gadoxetic acid-enhanced MR images for preoperative prediction of early recurrence (<1year) in hepatocellular carcinoma (HCC).
**Methods and Materials:** 39 patients with surgically confirmed HCC were enrolled in this IRB-approved prospective study. A total of 1925 radiomics features within peritumoural region (a 5-pixel-wide radius surrounding the tumour boundary) were obtained from multiple phases (T2-weighted, non-enhanced T1-weighted, arterial phase, portal venous phase and hepatobiliary phase). The most predictive features were selected to build a radiomics signature using the least absolute shrinkage and selection operator(LASSO)algorithm with a 10-fold cross validation applied. Then, a prediction model comprising radiomics signature and predictive clinical variables was constructed using multivariable logistic regression analysis. Receiver operation characteristic (ROC) analysis and decision curve analysis were performed to evaluate the accuracies of the predictive models.
**Results:** Nineteen early recurrences (48.7%) were confirmed by imaging follow-up. A radiomics signature with four radiomics features, including Correlation, InverseDifferenceMoment (IDM) from T2-weighted images and Histogram MinIntensity, IDM from arterial phase images, demonstrated good performance with the sensitivity,specificity and area under the ROC curve of 85%,92% and 0.926, respectively. Multivariate logistic regression analysis identified the radiomics signature (odds ratio[OR]=1.761;p<0.001), microvascular invasion (OR=5.859;p=0.046) and Edmonson-Steiner histologic grade (OR=1.158;p=0.042) as independent predictive factors for early recurrence. The integrated prediction model comprising all predictive factors showed an improved predictive performance than the radiomics signature (AUC=0.975 vs.0.926, p<0.05).
**Conclusion:** Peritumoural radiomics features along with clinical variables had the potential of predicting early recurrence in patients with HCC after resection.

**B-1045** 10:54
**Abbreviated gadoxetic acid-enhanced MRI (AGAM) including second shot arterial phase (SSAP) for hepatocellular carcinoma surveillance**
J. Kim, C. Lee; *Seoul/KR (pridebio@naver.com)*

**Purpose:** To evaluate the feasibility of an abbreviated gadoxetic acid-enhanced MRI (AGAM) including second shot arterial phase(SSAP) for hepatocellular carcinoma (HCC) surveillance.
**Methods and Materials:** A total of 132 patients underwent gadoxetic acid-enhanced MRI using a modified injection protocol for HCC surveillance between July and December 2017. 82 HCCs in 68 patients were initially diagnosed based on the histopathologic result or typical imaging features. Modified injection protocol included routine dynamic imaging after a first injection of 6-mL and SSAP after a second injection of 4-mL. Image set 1 was full original protocol. Image set 2 consisted of T2WI, DWI, hepatobiliary phase, and SSAP (simulated abbreviated protocol). Acquisition time was measured in each image set. The per-patient sensitivity, specificity, PPV, and NPV, and per-lesion sensitivity and PPV were compared between two image sets. Image quality evaluation and visual assessment of vascularity were performed on the original AP, SSAP, and their subtraction images.
**Results:** The acquisition time was significantly shorter in image set 2 than in image set 1 (6.2 vs. 24.4 minutes, p<0.0001). The diagnostic performances were not significantly different between image sets 1 and 2 in two readers (p=0.277-0.814). The mean motion artefact score was significantly lower on the SSAP than on the original AP (1.77±0.74 vs. 2.07±0.84, p<0.001). 75/77 (97.4%) hypervascular HCCs showed hyperintensity on the SSAP and/or second subtraction images.
**Conclusion:** An AGAM including SSAP showed comparable diagnostic performance to a full dynamic MRI with faster image acquisition, fewer motion artefact, and preserved visual arterial hypervascularity for detecting HCC.

**B-1046** 11:02
**Assessment of abbreviated MRI of liver for initial follow-up of patients with liver metastases from colorectal cancer**
M.R. Torkzad[1], A. Riddell[2], D.-M. Koh[1]; [1]*Sutton/UK*, [2]*Surrey/UK (michael.torkzad@gmail.com)*

**Purpose:** To compare abbreviated MRI (ABB) of liver to standard MRI of liver (STD) for follow-up of patients treated for colorectal cancer liver metastases.
**Methods and Materials:** A retrospective analysis of all consecutive patients with colorectal metastases treated at our centre from 2011 until 2017 was done by two senior radiologists blinded to the results of each other. Each radiologist evaluated initially only HBP (hepatobiliary phase images with gadoxetate) and DWI (diffusion-weighted images) images up to b value of 800 (henceforth ABB). Later, the same radiologists assessed STD which included all pre- and postcontrast images, two sets of T2-weighted images with low TE (80-110 ms) and high TE (> 200 ms), double-echo images, as well as ABB sequences. One radiologist also compared surgical planning based on each of the two imaging sets.
**Results:** 114 patients could be included. A high degree of correlation was found between STD and ABB for both radiologists (R = 0.95; Spearman Rho) for the determination of type of response based on RECIST. The number of metastases found by both sets showed a high degree of correlation (R = 0.98; Pearson). Bland-Altman analysis revealed no bias (0.02) for number of metastases. No difference in surgical planning was found between the two sets of images
**Conclusion:** Abbreviated MRI protocol for initial follow-up with only DWI and HBP provides almost the same results as a standard complete imaging set, and can, therefore, potentially reduce imaging and interpretation time and cost.

**B-1047** 11:10
**Preoperative detection of colorectal liver metastases: DWI alone or combined with MDCT is no substitute for Gd-EOB-DTPA-enhanced MRI**
A. Schulz, E.S. Joelsen-Hatlehol, K.W. Brudvik, B. Hanekamp, K.K. Aasand, E. Viktil, C.K. Johansen, J. Dormagen; *Oslo/NO (anselm.schulz@gmail.com)*

**Purpose:** To evaluate if preoperative detection of colorectal-liver-metastases (CRLM) with less resource-consuming DWI alone or in combination with MDCT can compete with gadolinium-etoxybenzyl-diethylenetriaminepentaacetic acid (Gd-EOB-DTPA)-enhanced MRI.
**Methods and Materials:** Forty-four patients received MDCT and Gd-EOB-DTPA-enhanced MRI including DWI prior to liver resection for CRLM. Two radiologists evaluated five image sets each. The DWI-set consisted of diffusion-weighted sequences (b-values 0, 50 and 800s/mm²), ADC-map, coronal, axial T2-weighted single-shot sequences. The DWI-T2F-set contained additionally respiratory-triggered T2-weighted TSE-SPIR sequences. The MDCT-set consisted of precontrast, arterial, portal-venous and late phase, the MDCT-DWI-set additionally of DWI. The CE-MRI-set contained all MRI-sequences. The diagnostic performance of the imaging-sets was based on 299 lesions including 123 CRLM. Histopathology and follow-up were reference standards.
**Results:** CE-MRI-set had highest sensitivity (p≤0.013) with 95.1% compared to 72.4%, 73.2%, 73.2% and 87.0% for DWI-set, DWI-T2F-set, MDCT-set and MDCT-DWI-set. CE-MRI-set had highest sensitivity (p≤0.012) for CRLM <10mm with 87.1% compared to 54.8%, 51.6%, 22.6% and 58.1% for DWI-set, DWI-T2F-set, MDCT-set and MDCT-DWI-set. The MDCT-DWI-set improved sensitivity overall and in size-dependent subgroup analyses compared to MDCT-set (p≤0.031). MDCT-DWI-set showed highest specificity (98.3%) followed by 97.7%, 97.7%, 94.9%, 87.6% for the DWI-set, DWI-T2F-set, MDCT-set and CE-MRI-set (p<0,001).
**Conclusion:** Combination of DWI with MDCT improved sensitivity and specificity compared to each modality alone. DWI might be sufficient as follow-up or screening for primary staging of colorectal cancer in case of negative MDCT. MRI with DWI and Gd-EOB-DTPA showed highest sensitivity for CRLM and should be the preferred preoperative modality when meticulous lesion identification is essential.

**B-1049** 11:18
**Findings of sinusoidal obstruction syndrome on gadoxetic-acid-enhanced MRI in patients with chemotherapy for colorectal liver metastases are poorly reproducible between radiologists**
F. Staal[1], J.B. Houwers[2], B. Heeres[1], M. de Boer[1], D.M. Lambregts[1], M. Maas[1], R.G.H. Beets-Tan[1]; [1]*Amsterdam/NL*, [2]*Maastricht/NL (f.staal@nki.nl)*

**Purpose:** To assess the reproducibility of gadoxetic acid-enhanced MRI (EOB-MRI) to determine the presence and severity of sinusoidal obstruction syndrome (SOS) in patients treated with chemotherapy for colorectal liver metastases (CRLM).
**Methods and Materials:** 32 patients treated with chemotherapy for CRLM (either oxaliplatin based, oxaliplatin based in combination with bevacizumab, or non-oxaliplatin-based treatment), with available EOB-MRI scans in the hepatobiliary phase, were retrospectively included. The presence and severity of SOS were independently scored by three radiologists with varying experience (10-20 years of experience in EOB-MRI), who were blinded to the clinical data, using a 5-point scale (SOS score 0=definitely not present to 4=definitely present). The inter-observer agreement between readers was assessed with a quadratic-weighted kappa.
**Results:** The mean time between chemotherapy completion and EOB-MRI scan was 1.1 (+/-1.4) months. Inter-observer agreement was poor with kappas ranging from 0.25 (95%CI 0.01-0.48) to 0.33 (95%CI 0.08-0.58). More equivocal scores (SOS score = 2) were chosen by less experienced radiologists (6.3% and 43.8% equivocal scores) compared to a more experienced radiologist (3.1% equivocal scores).
**Conclusion:** The assessment of SOS on hepatobiliary phase EOB-MRI in patients treated with chemotherapy for CRLM shows a poor inter-observer agreement for the diagnosis of SOS. This questions the value of hepatobiliary phase EOB-MRI for this purpose.

**B-1050** 11:26
**Enhancement features, blood-based bio-markers and immunohistochemical expression of hepatocyte transporters in patients with non-alcoholic fatty liver disease (NAFLD)**
D.S. Feier[1], N. Bastati-Huber[2], A. Beer[2], S. Pötter-Lang[2], R.M. Fragner[2], A. Ba-Ssalamah[2]; [1]*Cluj-Napoca/RO*, [2]*Vienna/AT (diana.feier@gmail.com)*

**Purpose:** To quantify enhancement features of gadoxetic acid enhanced-MRI and blood-based biomarkers parameters in patients with non-alcoholic fatty liver disease (NAFLD), and correlate them with the immunohistochemical expression of the hepatocyte transporters (OATPB2/8, MRP2, MRP3).
**Methods and Materials:** The local institutional review committee approved this study and waived written informed consent. This was a retrospective study of gadoxetic acid-enhanced 3T MR imaging performed in 46 consecutive patients with NAFLD (mean age (SD), 49.46 (15.52) years. The MR images were analyzed by using the relative enhancement (RLE). NAFLD fibrosis score (NFS) and FIB-4 were used as blood-based biomarkers parameters. Regression analyses were applied to identify variables associated with OATPB2/8, MRP2, MRP3 expression.
**Results:** A statistically significant higher number of patients with NASH had increased lobular inflammation (p<0.0001), ballooning (p<0.0001), liver steatosis (p=0.003) and liver fibrosis (p=0.002) compared to those with simple steatosis. RLE, NFS and FIB-4 were independent predictors of NASH patients with a generated model able to differentiate NASH from simple steatosis patients with a probability of 87% [AUROC=0.87 (95%CI 0.74-0.94)], a sensitivity of 85% (95%CI 70.2-94.3), and a specificity of 75% (95%CI 42.8-94.2). Relative enhancement was positively correlated with the expression of

OATP2/8 expression (r=0.41, p=0.002) and NFS and FIB-4 positively correlated with the expression of MRP2 (r=-0.37, p=0.007).
**Conclusion:** Imaging and blood-based biomarkers parameters used together may discriminate between patients with NASH and simple steatosis.

**B-1051** 11:34
**Postoperative complications after living donor liver transplantation: risk assessment on Gd-EOB-DTPA-enhanced 3-T MR imaging using quantitative-qualitative parameters**
A. Kahraman[1], M. Kolu[2], B. Kahraman[1], Z. Ozdemir[1], L. Karaca[1], S. Yilmaz[1]; [1]*Malatya/TR*, [2]*Sanliurfa/TR (mdasagir2003@hotmail.com)*

**Purpose:** To investigate if Gd-EOB-DTPA-enhanced magnetic resonance imaging (MRI) with measurements of quantitative and qualitative parameters on hepatobiliary phase images can predict the risk of postoperative complications after living donor liver transplantation (LDLT).
**Methods and Materials:** This prospective study included 60 subjects (30 living donor candidates and 30 their recipients). We obtained Gd-EOB-DTPA-enhanced 3T MRI before LDLT in donors and after LDLT in their recipients. MRI parameters including relative liver enhancement (RLE) value, biliary signal value and visual evaluation of liver and biliary enhancement on hepatobiliary phase images were recorded. The patients were followed for postoperative biliary and vascular complications and then grouped according to the presence and absence of complications. The relations between MRI parameters and postoperative complications were analysed statistically. In addition to MRI parameters, the latter also included postoperative laboratory variables.
**Results:** The recipients with postoperative complications had significantly lower mean RLE values and biliary signal values than those without and than donors (p<0.001). Qualitative MRI parameters involving visual assessments of liver enhancement and biliary signal were also significantly different in recipients with postoperative complications compared to donors and patients without postoperative complications (p<0.001). RLE values and biliary signal values were inversely related to the postoperative bilirubin values in recipients (p=0.005, r=-0.496; p<0.001, r=-0.624). There were no correlation between INR values and MRI parameters (p=0.837, p=0.813).
**Conclusion:** The quantitative and qualitative MRI parameters obtained by Gd-EOB-DTPA-enhanced MRI on hepatobiliary phase images can help with the assessment of the risk for postoperative complications after LDLT.

**B-1052** 11:42
**What is the effect of steatosis, iron overload and renal function on the uptake and excretion of gadoxetic acid-enhanced MRI?**
L. Beer, N. Bastati, S. Pötter-Lang, D. Stoyanova, E. Michael, Y. Bican, H. Einspieler, A. Ba-Ssalamah; *Vienna/AT (lucian.beer@meduniwien.ac.at)*

**Purpose:** To explore the effect of steatosis, hepatic iron overload, and renal function on gadoxetic-acid (GA) uptake in hepatobiliary phase (HBP) images in patients with diffuse chronic liver disease (CLD).
**Methods and Materials:** This retrospective IRB-approved study included 265 patients (164 men, 101 female, mean age 54±16 years) with CLD who had undergone GA-enhanced MRI of the liver between 2010-2015. Demographic and clinical data were obtained from our patients' medical history and included age, sex, body mass idex, creatinine, and estimated glomerular filtration rate (eGFR). Relative liver enhancement (RLE), was calculated by two radiologists using unenhanced and GA-enhanced HPB images, 20 minutes after administration of the GA. Hepatic fat fraction (%) was measured on dual--echo gradient-recalled echo. Hepatic siderosis was semiquantiatively assessed on T2-weighted, fat-suppressed images (Grade 1-3). Spearman´s correlation coefficients were used to assess the correlation between RLE and laboratory parameters. ANOVA with Bonferroni-post-hoc-test was used to compare different groups.
**Results:** There was a significant correlation between RLE and eGFR (R=0.225, p<0.001) and siderosis grade (R=-0.210, p=0.001), while hepatic fat fraction did not correlate with RLE (R=0.098, P=0.116). RLE in patients with highly impaired renal function (eGFR<30 ml/min/1.73 m²) was significantly lower than in patients with slightly impaired (eGFR 30-90) or normal kidney function (eGFR >90) (RLE:46%vs.82%vs.89%, p<0.001). In addition, RLE was significantly lower in patients with severe siderosis compared to those with no siderosis (RLE:46%vs.86%; p=0.011).
**Conclusion:** Gadoxetic-acid relative enhancement significantly correlates with renal function; however, it is independent of hepatic fat fraction in patients with diffuse CLD.

**10:30 - 12:00** **The Church**

### Paediatric

### SS 1012

### Paediatric musculoskeletal and oncologic imaging

*Moderators:*
N. Mitreska; Skopje/MK
U. Schwarz-Nemec; Vienna/AT

**K-26** 10:30
**Keynote lecture**
J.M. Patsch; *Vienna/AT*

**B-1053** 10:39
**Making measurements on coronal ultrasound images of infant hips during Barlow manoeuvre: a new and rapid screening method for developmental dysplasia of the hip**
Ü.Y. Ayaz, M.E. Döğen; *Mersin/TR (umityasarayaz@yahoo.com)*

**Purpose:** In order to shorten the examination time for sonographic screening of developmental dysplasia of the hip (DDH), we aimed to obtain the femoral head coverage (FHC) as well as *alpha and beta* angles of the normal infant hips during Barlow maneuver using only the coronal ultrasound (US) images.
**Methods and Materials:** In this prospective study, a total of 150 hips of 75 healthy infants were included. The infants were initially evaluated by US and then clinically followed-up till their hips were proved to demonstrate normal orthopaedic development at physical examination. Median clinical follow-up time was 276 days. US examination of the hip in coronal plane was performed during Barlow maneuver in supine position using a linear-array transducer.
**Results:** The positions of the femoral heads in their acetabuli and the development of hip components (bony acetabulum, cartilaginous labrum) were normal in all the infants. All the hips were stable and classified as Graf type 1 with alpha angles ≥ 60º. Mean FHC with range values for the right and left hips were, 65.9±7.3% (range, 47.8%-80.8%) and 64.4±6.9% (range, 46%-77%), respectively. The difference between the mean FHC of right and left hips was not significant (P>0.05).
**Conclusion:** Our up-to-date FHC values were considered to be more clinically relevant, since the measurements were made during Barlow maneuver. Our newer technique can be used for obtaining FHC, as well as *alpha and beta* angles using the same single coronal US image for each hip which significantly shortens the examination time.

**B-1054** 10:47
**Use of magnetic resonance imaging to predict progression of juvenile idiopathic arthritis in paediatric patients in clinical remission**
F. Sertorio, M. De Cesari, M. Mazzoni, A. Pistorio, A. Urru, F. Magnaguagno, C. Malattia, G.M. Magnano; *Genoa/IT (fiammetta.sertorio@gmail.com)*

**Purpose:** To assess the prevalence of subclinical synovitis as detected by MRI in a cohort of patients with juvenile idiopathic arthritis (JIA) in clinical remission and to evaluate its association with disease flare and structural damage progression.
**Methods and Materials:** All contrast-enhanced MR performed from 2007 to 2015 on patients with JIA in clinical remission were analysed by two independent readers. Joint damage progression was assessed by conventional radiography (CR). Statistical analyses were performed.
**Results:** Ninety patients were included: 14/90 (15.6%) in remission off medication, while 76/90 (84.4%) in remission on medication. 45 patients were assessed by MRI in the wrist, 30 in the hips, 13 in the ankle and 2 in the knee. 57 patients (63.3%) had evidence of subclinical synovitis on MRI. The interobserver agreement for presence/absence of synovitis was good (k=0.74). 43/57 patients (75,4%) with subclinical synovitis experienced a disease flare versus 11/33 patients (33.3%) who had not any synovial inflammation. Radiographic damage progression was assessed by CR in 54/90 patients and was detected in 19/54 patients (35.2%). A significant association between systemic JIA subtype and deterioration of joint damage was found; MRI-detected bone marrow oedema (BMO) score and the baseline radiographic damage score were also significantly related to structural progression.
**Conclusion:** Subclinical synovitis detected by MRI was significantly associated with disease flare, while BMO showed remarkable promise in predicting joint destruction. These findings support the MRI utility for the assessment of JIA patients in clinical remission and may have important clinical implications for their management.

**B-1055** 10:55

**Secondary fractures in paediatric radial head and neck fractures: common association, commonly missed**

A.J. Degnan, V.M. Ho-Fung, J.C. Nguyen, J.T.R. Lawrence, S.L. Kaplan; *Philadelphia, PA/US (degnan.andrew@gmail.com)*

**Purpose:** Radial head and neck fractures are typically viewed as adult-type fractures uncommon in children. The nature and frequency of associated additional fractures are poorly understood.
**Methods and Materials:** Elbow radiographs in 500 consecutive children (<18 years) during a five-year period (January 1, 2013-December 31, 2017) with reports containing terms related to radial head or neck fracture were re-reviewed for fracture identification. Pathologic fractures and fractures sustained prior to the study period were excluded. Review of cases yielded 270 unique patients with radial head and/or neck fractures.
**Results:** Approximately two-thirds (65.6%) of radial head and/or neck fractures occurred in isolation whereas 34.4% of patients had an additional elbow fracture with concomitant olecranon fractures being most common (23.0%), followed by medial epicondyle fractures (4.1%), coronoid fractures (3.3%) and other types of additional fractures. Complete radius fractures were more likely to have an additional fracture than incomplete fractures (OR: 3.30, CI: 1.88-5.78, p<0.001). Significant joint effusion was also associated with the presence of an additional fracture (OR: 2.18, CI: 1.06-4.45, p=0.03). Combined radial head/neck and olecranon fractures occurred in a younger age group (7.32 years, SD: 3.19) than isolated radial head/neck fractures (9.89 years, SD: 3.85), p<0.001. Approximately one-third (32.6%) of additional fractures were not identified on initial radiographs, 39% of which were not visible on initial radiographs.
**Conclusion:** Radial head and neck fractures are underappreciated in the paediatric population. These radius fractures have a substantial association with additional fractures, and secondary fractures are often missed or occult on initial radiographs.

**B-1056** 11:03

**The applicability of the Greulich and Pyle atlas to a modern Irish population**

L.L. Mc Carthy[1], C. Brenner[1], J. Sammon[2], L.D. Spence[2], J. O'Connor[1]; [1]*Dublin/IE*, [2]*Cork/IE (lauraghmccarthy1@hotmail.com)*

**Purpose:** The Greulich and Pyle Radiographic Atlas of the Hand and Wrist is the gold standard in clinical practice for assessment of skeletal development of children from birth to maturity. The literature however suggests that the atlas is not universally applicable to all populations. Given the clinical importance of the Greulich and Pyle atlas and the need to ensure that methods used for age assessment are tested it is imperative that a study be undertaken to determine its appropriateness for use in a modern Irish population.
**Methods and Materials:** A retrospective cross-sectional analysis was performed of hand/wrist radiographs from children presenting to the Accident and Emergency Department of Cork University Hospital between 2008-2013. Radiographs of 444 children (Male: Female 257:187) with an age range of 0-19 years were examined. Observers were blinded to chronological age at the time of assessment of skeletal age. Each radiograph was assessed using the Greulich and Pyle Atlas and a skeletal age assigned. Chronological age was subtracted from skeletal age to obtain the residual difference.
**Results:** Skeletal and chronological age were highly correlated in both males (0.968) and females (0.968) (p <0.01). The mean difference between skeletal age and chronological age was positive in value, indicating that in the current sample skeletal age was advanced compared to chronological age. For males mean skeletal age was advanced by 1.14 (±15.12) months and for females mean skeletal age was advanced by 2.58 (±12.78) months.
**Conclusion:** The Atlas appears to still be applicable to a modern Irish population.

**B-1057** 11:11

**The value of MR spectroscopic bone marrow fat fractions in the assessment of treatment response in patients with Gaucher disease**

A.J. Degnan, V.M. Ho-Fung, D.-J. Wang, C. Barrera, R. Ahrens-Nicklas, C. Ficicioglu; *Philadelphia, PA/US (degnan.andrew@gmail.com)*

**Purpose:** Gaucher disease involves deficiency of β-glucocerebrosidase that results in the accumulation of abnormal macrophages in the spleen, liver and bone marrow. Liver and spleen volumes are currently used alongside laboratory markers for disease severity. It is unclear if these markers are sufficient in assessing overall severity and risk for bone-related complications including impaired growth and osteonecrosis. Magnetic resonance spectroscopy (MRS) measures bone marrow quantitatively with lower fat fractions corresponding to more severe disease.
**Methods and Materials:** Patients with Type I Gaucher disease were imaged using MRI and MRS for liver and spleen volumes as well as fat fraction calculations within the lumbar spine and femoral neck prior to and during enzyme replacement therapy. Healthy control MRS data were included for comparison. Statistical analysis included Mann-Whitney U tests and Pearson correlation.
**Results:** This IRB-approved retrospective study included 6 Gaucher patients (2 female, 4 male; 9.8 years, SD: 5.8) and 6 healthy controls (3 female, 3 male; 11.7 years, SD: 3.7). Fat fractions were significantly lower in patients (femoral neck: 31.9% vs. 62.6%, p=0.020; lumbar spine: 18.5% vs. 35.8%, p=0.019). Organ volumes decreased and fat fractions increased significantly with treatment. Treatment duration correlated with femoral neck fat fraction (Pearson: 0.912, p<0.001), age (Pearson: 0.892, p<0.001) and lumbar spine fat fraction (Pearson 0.689; p=0.007); inverse correlations with organ volumes were not significant.
**Conclusion:** Bone marrow fat fractions in Gaucher disease may better correspond with treatment duration than traditionally monitored organ volumes. Larger prospective study is needed to assess if bone marrow imaging provides additional information in guiding therapy in Gaucher disease.

**B-1058** 11:19

**Non-breath hold dynamic contrast enhanced abdominal and thoracic MRI in paediatric oncologic patients using a GRASP-sequence at 3T**

T. Woerner, P. Raczeck, A. Buecker, G. Schneider; *Homburg/DE (dr.guenther.schneider@uks.eu)*

**Purpose:** One of the few advantages of modern CT scanning over MRI in paediatric imaging is the possibility to perform dynamic CE studies without the need of a breath-hold. However, new MRI techniques like Golden-Angel Radial Sparce Parallel MRI (GRASP) allow for free-breathing dynamic contrast enhanced volumetric imaging. The aim of our study was to evaluate GRASP sequences in evaluation of abdominal and thoracic malignancies in paediatric patients.
**Methods and Materials:** In 32 paediatric patients MRI was performed on a 3T scanner (Siemens,VIDA). Patients below 10 years of age were sedated for imaging studies. MR protocol included non breath-hold unenhanced radial T2w images, radial T1w and T1w fs VIBE sequences and DWI. During contrast administration (0.05 mmol/kg MultiHance,Bracco) a GRASP Sequence with a temporal resolution of 4 - 6 sec and reconstruction of 16 - 26 phases was performed. After CM radial VIBE sequences were repeated. In patients with liver lesions another set of radial VIBE sequences was performed in the biliary phase of CM excretion to further characterise lesions.
**Results:** Dynamic non-breath-hold imaging of the abdomen was successful in all cases and even excellent in thoracic imaging depiction of perfusion characteristics of tumours. Characterisation of liver lesions based on the typical enhancement characteristics was feasible in all cases and in a similar manner renal and adrenal tumours could be evaluated.
**Conclusion:** Non-breath-hold dynamic contrast enhanced imaging is feasible in paediatric patients using GRASP sequence and gives detailed information about perfusion characteristics of abdominal and thoracic tumours without radiation exposure.
*Author Disclosures:*
**A. Buecker:** Grant Recipient; Siemens. **G. Schneider:** Grant Recipient; Bracco, Siemens. Speaker; Bracco, Siemens.

**B-1059** 11:27

**Is TI-RADS useful as an adult ultrasonographic malignancy risk stratification method in pediatric thyroid nodules?**

Y. Durum, V. Ozturk, N. Ersöz, A. Anık, C. Karaman; *Aydin/TR (md.suhaozturk@gmail.com)*

**Purpose:** Data on TI-RADS generally belong to studies performed in adults. Therefore we aimed to evaluate the performance and utility of TI-RADS in the paediatric groups.
**Methods and Materials:** From January 2015 to 2018, 108 nodules were evaluated in 1028 thyroid ultrasound examinations. Images were retrospectively evaluated by two radiologists with 3 and 7 years of paediatric radiology experience, according to TI-RADS classification. Morphological findings of the detected nodules and their histopathological results were recorded. Histopathological findings and at least 6 months of follow-up imaging were taken as reference.
**Results:** 73 patients were female (67.6%). The mean age was 11.4 and the mean nodule size was 7.5 ± 8.3 mm. According to TI-RADS, 41 (38%) were hypoechoic and 5 (4.6%) were very hypoechoic. When margin features evaluated, 85 (78.7%) lesions were smooth, 8 (7.4%) were irregular, According to the histopathologic assessment 100 (95.2%) of the nodules were benign and 5 (4.8%) were malign. 2 nodules were evaluated as non-diagnostic cytology and 1 nodule as suspicious for malignancy. All malign nodules were in the TI-RADS 5 category. The majority of benign nodules (79%) were found in TI-RADS 1, TI-RADS 2 and TI-RADS 3 categories. 80% of the malign nodules were very hypoechoic and taller than wide in shape, also all malign nodules had microcalcifications (p=0.000). The sensitivity of TI-RADS was 100%, specificity was 78.8%, PPD was 19.2%, NPD was 100%.
**Conclusion:** According to our study, TI-RADS system can be used to evaluate thyroid nodules in paediatric patients similar to adults.

**B-1060** 11:35
**Paediatric rhabdomyosarcomas: which type of radiological assessment after induction chemotherapy better predicts the survival?**
G. Orsatti[1], A. Varotto[1], M. Bonzini[2], M. Minotti[2], C. Morosi[2], F. Crimì[1], C. Giraudo[1], E. Quaia[1], R. Stramare[1]; [1]*Padua/IT*, [2]*Milan/IT*
*(giovanna.orsatti@gmail.com)*

**Purpose:** To investigate the prognostic value of radiological response assessment after induction therapy in paediatric rhabdomyosarcomas (pRMS), comparing three different methods.
**Methods and Materials:** This retrospective, bicentric study included 62 non-metastatic pRMS. Two radiologists measured tumour size on pre- and post-treatment MR images using three methods: the maximal diameter, in accordance with the Response Evaluation Criteria in Solid Tumours (1D), the multiplication of the two maximal axial diameters, respecting the World Health Organization criteria (2D) and the multiplication of the three maximal diameters and π/6, following the European paediatric soft tissue sarcoma study group guidelines (3D). Regarding tumour response, each patient was classified as a responder (complete or partial response) or a non-responder (stable or progressive disease) according to each method and its respective therapeutic thresholds. The event-free survival (EFS) and the overall survival (OS) were assessed (Kaplan-Meier plots and log-rank test, $p<0.001$). The inter-observer agreement (concordance correlation coefficient (CCC)) and the inter-method agreement for the therapeutic responses (weighted κ-coefficient) were calculated.
**Results:** As regards the five-year EFS, the 3D assessment showed significantly higher values for responders than non-responders (80.4% vs 22.2%, $p<0.0001$); no significant differences emerged for 1D and 2D methods. The 3D measurements were significantly better predictors of five-year OS (84.1% vs 44.4%, $p=0.0003$). The inter-observer agreement was higher for 3D than 1D and 2D measurements (CCC=0.93, 0.86 and 0.75, respectively); the inter-method agreement for the therapeutic responses was poor (highest value, $k_{1Dvs2D}=0.57$; $p<0.0001$).
**Conclusion:** The 3D assessment of therapeutic response in pRMS seems to provide a better correlation with the clinical outcome than other radiological methods.

**B-1061** 11:43
**Diffusion-weighted magnetic resonance imaging for the evaluation of Wilms tumours: radiological-pathological correlation**
E. Nazarova, N. Merkulov, A. Mitrofanova, G. Tereshenko; *Moscow/RU*
*(evelina.nazarova@fccho-moscow.ru)*

**Purpose:** Wilms tumour (WT), or nephroblastoma is one of the most common extracranial tumours in pediatric population. Different histological types of nephroblastoma impact staging, treatment options and outcome. The aim of the study was to analyze possible correlation between diffusion-weighted imaging (DWI) data and different histological types of WT.
**Methods and Materials:** The study was based on data of 31 pediatric patients with histopathologicaly verified WT. Median age at the time of diagnosis was 5 years (from 1 to 8 years old), male to female ratio was 1:1,3. Imaging data was obtained with 3T MRI (magnetic resonance imaging) scanner. The apparent diffusion coefficient (ADC) value for each tumour was calculated using region-of interest measurements. The mean ADC values and histological types of WT were compared using regression analysis method.
**Results:** Out of 31 patient 6/31 (19%) were low risk, 16/31 (52%) intermediate and 9/31 (29%) high risk. The mean ADC values were 0.34±0.05 ×10-3 mm2/s for low grade WT, 1±0.12 ×10-3 mm2 /s for medium grade WT, and 0.6±0.06 ×10-3 mm2 /s for high grade WT. Intraclass correlation coefficient was 0.93.
**Conclusion:** In our study, we have found a significant correlation between ADC values and histological types of WT. MRI DWI can be a useful tool in the initial assessment and differential diagnosis in patients with nephroblastoma.

**B-1062** 11:51
**Role of intraarterial chemotherapy for retinoblastoma: our experience with review of literature**
L.S.P. Karanam, S. Baddam, V. P, S. Honavar; *Hyderabad/IN*
*(drklsp@gmail.com)*

**Purpose:** To describe the role of ophthalmic artery chemotherapy in 78 children of intraocular retinoblastoma. Technical details, procedural events, complications and outcomes were analysed in this large single-centre cohort.
**Methods and Materials:** From April 2014 to February 2018, 78 patients with age group between 6 months to 83 months underwent IAC at our institution: 45 eyes of group D and 33 eyes of group E. Demographic details, no. of sessions, response to therapy, follow-up were documented in all the cases. We used techniques of direct ophthalmic artery catheterisation, middle meningeal artery cannulation and balloon occlusion methods for the targeted drug delivery with triple-drug regimen (topotecan, melphalan and carboplatin).
**Results:** 78 patients underwent a total of 228 sessions. Complications in the form of transient lid oedema, swelling, loss of eyelashes and conjunctival congestion occurred in 8 patients. Fever with neutropenia occurred in 2 patients. No procedural-related complications such as strokes or femoral artery puncture site haematomas occurred in any patient. Recurrent tumour was seen in 3 eyes, persistent sub-retinal seeds in 2 eyes and persistent vitreous seeds in 1 eye. 15 patients underwent enucleation and globe salvage was achieved in 80.7% of overall patients and 87.3% in treatment-naive eyes in a follow-up of 2 years in 85% of the patients.
**Conclusion:** OAC achieves greater biological effect while minimizing systemic effects and plays important role in globe salvage especially in places with advanced retinoblastoma. It can be done safely and should be considered as first-line treatment with acceptable risks.

### Saturday, March 2

**08:30 - 10:00** **Room K**

### Radiographers

### SS 1314

### Radiographic issues

*Moderators:*
N.N.
B.R. Mussmann; Odense/DK

**B-1063** 08:30
**The impact of imaging receptor technology on workflow, patient dose values and image quality of bedside chest radiography**
J. Santos[1], S. Carmo[2], A.R. Cardoso[2], C.M.D.S. Almeida[2]; [1]*Coimbra/PT*, [2]*Lisboa/PT (joanasantos@estescoimbra.pt)*

**Purpose:** The aim of this study was to compare different image receptor technologies in order to optimise bedside chest radiography workflow and examination dose values.
**Methods and Materials:** The quality control of the portable equipment was verified. Exposure parameters and examination dose values were analysed for standard patients that were submitted to chest radiography using the existing computed radiology (CR) image detector (n=18). Experimental tests with an adult anthropomorphic phantom were performed in order to compare the CR image receptors and a digital radiography (DR) detector (n=12). Dose values (DAPmGy.cm$^2$) and objective image quality was performed (image signal and noise) and new pre-set protocols for DR receptor were defined with clinical approval. Standard patient exposure parameters and examination dose values using DR were analysed for comparison (n=29). Images from patients submitted to chest radiography with both systems were selected (n=18). Three radiologists, using the European Criteria for chest radiography, performed subjective image quality analysis. The time between the radiographers start the examination and image availability on the system was quantify.
**Results:** Experimental phantom tests revealed the possibility to optimised the exposure and reduce dose values in 25%. The images were available in the system 30 minutes before with the DR system. With the new image receptor it was possible to reduce patient dose in 32% with similar image quality.
**Conclusion:** The technology allowed workflow time reduction and examination dose reduction maintaining image quality. The experimental tests and the results discussion contributed to radiographer's faster technology adaption.

**B-1064** 08:38
**The effects of weight-bearing and non-weight-bearing anteroposterior DR of feet in hallux valgus deformity**
H. Zhang, Z. Wang, J. Liu; *Tianjin/CN (15602179624@163.com)*

**Purpose:** To measure and compare the angles about hallux valgus, using weight-bearing and non-weight-bearing anteroposterior DR to provide more reliable data for rectifying hallux valgus.
**Methods and Materials:** During 2016.12-2017.05, 15 patients (20 feet) (men:women=5:10; average age: 60.1±4.3y) with hallux valgus received weight-bearing and non-weight-bearing anteroposterior DR (GE Discovery XR656) before operation. Weight-bearing position: the patients stood on the special device for foot and ankle weight-bearing imaging. Both feet were separated and were equal to shoulder's width. Non-weight-bearing position: the patients sat on the photographic table, with feet close to the table. The X-ray tube inclined 15°cephaladly. The centre line was at the base of the third metatarsal of the foot with hallux valgus, the focus-film distance was 120cm. Voltage: 50KV; current: 4mAs. The hallux valgus angle (HVA), intermetatarsal angle (IMA) between first and second metatarsal bone, distal metatarsal articular angle (DMAA) of first metatarsal were measured using Image pro Plus 6.0. The HVA, IMA and DMAA of weight-bearing and non-weight-bearing groups were compared using paired t test.
**Results:** The HVA, IMA, DMAA in weight-bearing position was 38.18±11.69, 15.64±4.15, 18.23±8.36; in non-weight-bearing position was 35.15±10.77, 12.96±4.05, 15.00±7.96. The HVA, IMA, DMAA in weight-bearing position were larger than that of non-weight-bearing position, showing statistically significant differences (t = 2.98, 6.4, 2.21, P=0.010, P=0.000, P=0.044).
**Conclusion:** The HVA, IMA and DMAA in physiological weight-bearing group are larger than non-weight-bearing groups in patients with hallux valgus.

**B-1065** 08:46
**Dose optimisation of lumbar spine radiographs using a phantom: what are the ideal exposure parameters?**
Z. Lai, C. Sá dos Reis, Z. Sun; *Perth/AU (claudia.sadosreis@curtin.edu.au)*

**Purpose:** To investigate the optimal exposure parameters in lateral lumbar spine radiograph for dose reduction while maintaining adequate image quality.
**Methods and Materials:** 36 images were acquired manipulating exposure parameters: source-to-detector distance (SDD) (100cm, 130cm, 150cm), beam energy (85kVp±10), beam intensity (4.5mAs, 9mAs, 18mAs) and additional copper filter (0mm, 0.1mm, 0.2mm, 0.3mm) to investigate their impacts on effective dose (ED) and IQ. IQ was assessed using objective approach (contrast-to-noise ratio/CNR and magnification) and perceptual approach (6 observers provided IQ score and drawn lines in relevant anatomical structures). The perceptual approach was repeated with two luminance levels (170lux and 25lux). PCXMC software with Monte Carlo simulation was used to estimate ED. Data were analysed using descriptive statistics, Student's t test and intraclass correlation coefficient (ICC) according to the nature of questions.
**Results:** The highest ED (0.022mSv) was found at 100cm, 75kVp, 18mAs and 0-mm copper filter, while the highest CNR (7.23) was achieved at 130cm, 75kVp, 18mAs and 0-mm copper filter. The lowest ED and CNR were generated at 150cm, 95kVp, 4.5mAs and 0.3-mm copper filter. No significant difference was observed between the results for different luminance levels (*p*=0.9). All observers were able to identify relevant anatomical structures in all images. The intra- (0.61-0.79) and inter-observer ICC (0.55-0.82) varied between moderate and good.
**Conclusion:** This study showed that is possible to detect all relevant lumbar spine anatomical structures in lateral radiographs using 150cm, 95kVp, 4.5mAs and 0.3-mm copper filter. These parameters allowed the lowest ED (0.002mSv) and a CNR of 2.13. Further studies should be performed including different body habitus and equipment.

**B-1066** 08:54
**An investigation into the effectiveness of breast and gonad lead shielding in abdominal radiography**
Z. Kierans[1], A. Arrigo[2], J. Kotze[3], L. Van Der Velde[4], H. Zwaagstra[4], K. Gronning[5], M. O'Connor[1], I. Verzuijlen[4]; [1]*Dublin/IE*, [2]*Lausanne/CH*, [3]*Bloemfontein/ZA*, [4]*Groningen/NL*, [5]*Oslo/NO (zoe.kierans@ucdconnect.ie)*

**Purpose:** Lead shielding can be applied to radiosensitive organs to minimise radiation dose and, therefore, the risk of stochastic effects. Using a phantom-based approach, this study assessed whether lead shielding for breast and gonads influences dose in abdominal radiography.
**Methods and Materials:** AP abdominal X-ray examinations were performed on six anthropomorphic phantoms: a neonate, 1-, 5-,10- and 15-year-olds and an adult male phantom. Breast attachments were added to the 15-year-old and adult phantoms to mimic female patients. The radiation dose to the breasts and male gonads, shielded (lead equivalent 0.5mm thickness) and unshielded, was measured using a RADCAL dosimeter. Lead shields applied to these regions were positioned outside the primary beam. Five dose measurements were acquired and averaged for each protocol. Descriptive statistics were used to describe mean dose, standard deviation and percentage dose reduction with shielding. Wilcoxon signed rank tests were used to test significance of differences in organ doses with and without leads.
**Results:** Radiation dose to the breast tissue was reduced by 46-93% across all age groups, with the greatest reduction to breast dose found in the 15-year-old and adult phantoms. In adults, breast dose was reduced by 50.4µGy to 3.3µGy. A lesser dose reduction of 13-50% to the male gonads was achieved with shielding. The dose reduction observed with shielding in each age group was statistically significant (p<0.05).
**Conclusion:** During AP abdominal X-ray examinations, shielding of the breasts and male gonads is recommended to reduce radiation to these radiosensitive regions.

**B-1067** 09:02
**The impact of different exposure factors on image quality of lumbar spine in five different sizes phantoms: a factorial design study**
S.J.M. Alqahtani, J.R. Meakin, R.M. Palfrey, K.M. Knapp; *Exeter/UK (salbeshri@hotmail.com)*

**Purpose:** To investigate the impact of tube potential (kVp), source to image distance (SID) and Copper filtration on image quality score( IQS) of lumbar spine radiographs with different size bespoke phantoms (18, 29, 38, 42 and 46 BMI).
**Methods and Materials:** A factorial design study was conducted with a DR x-ray machine (Multi Fusion Max, Siemens, Germany), using three factors; kVp$^6$ x SID$^3$ x Cu filtration$^3$. All images were subjectively assessed by two experienced reporting radiographers using the European Image Quality Criteria (CEC) with 2 alternative forced choice (2AFC) and a 5 point Likert scale. Analysis of variance was used to investigate the impact of each factor on the IQS.
**Results:** kVp has a significant negative impact on IQS, with $r^2$ decreases as the BMI increase( $r^2$= 0.92, 0.91, 0.87, 0.52 and 0.25). SID has no significant impact on IQS across all phantoms. However, the IQS decreased along with the SID in the 18, 42 and 46 BMI phantoms while increased in the 29 and 38 BMI phantom. The filtration showed a negative non-significant impact on IQS in the first three phantoms. However, in the 42 and 46 BMI phantoms, the filtration has a significant negative impact on the IQS with $r^2$= 0.17 and 0.21 respectively.

**Conclusion:** To achieve an optimal image across the different phantoms, lower kVp settings were the most effective; this might oppose what is in clinical practice. SID could be used in middle BMI range and filtration should be used with caution in morbidly obese range

**B-1068** 09:10

**Lumbar spine imaging: can we reduce the effective and organ dose with the use of PA projection?**

E. Alukic, N. Mekis; *Ljubljana/SI (erna.alukic@zf.uni-lj.si)*

**Purpose:** The aim of the research was to determine, whether the use of PA projection in lumbar spine radiography decreases the entrance surface dose (ESD), effective dose and organ dose to the patient in digital radiography.
**Methods and Materials:** The measurement were performed on 100 patients that were referred to lumbar spine radiography. Patients were randomly divided into two equal groups of 50. One group was imaged in the AP and the other in the PA projection. Body Mass Index (BMI) was calculated for each patient from their weight and height. Also, the Dose Area Product (DAP), exposure index (EXI), tube time-current (mAs), image field size and the source-patient distance were obtained. Both effective dose and selected organ dose calculation were done according to the Monte Carlo simulation with the use of PCXMX 2.0 program and the ESD was calculated from the tube output. The selected organs in the study were the organs that lie in the primary field. Prior to the study the National medical ethic committee approval was obtained.
**Results:** In PA projection, the ESD was significantly reduced, by 33%, effective dose by 53% and the average absorbed organ dose by 64% respectively. There was no statistically significant difference in the BMI and EXI between the AP and PA projection.
**Conclusion:** Based on the results the use of PA projection in lumbar spine imaging should be the first method of choice in general radiography.

**B-1069** 09:18

**Direct projection augmented reality-guided x-ray examination system: a preclinical study**

N.-H. Koo; *Seoul/KR (kunohyun@naver.com)*

**Purpose:** The aim of this study was to evaluate the utility of direct projection augmented reality-guided X-ray examination system.
**Methods and Materials:** An X-ray examination on the chest and abdomen were conducted using Definium 8000 DR syetem and Rando phantom. The resulting images were respectively extracted in the DICOM format through PACS system and were directly projected onto the phantom in the form of an augmented reality using a beam projector. To evaluate the utility of direct projection augmented reality-guided X-ray system, a radiologist and a radiological technologist indicated T3, T9 and L3 spine using metal rods before and after the application of direct projection augmented reality technique. The mean error of the initial indicated position from the center of the reference spine was measured and was compared using independent t-test.
**Results:** Mean errors significantly decreased after the application of direct projection augmented reality (T3:5.02±0.83mm, T9:4.81±0.87mm, L3:4.24±0.74mm) compared to before the application (T3:67.48±7.23mm, T9:78.59±9.83mm, L3:52.37±5.91mm), with statistical significance.(p<.001).
**Conclusion:** It was confirmed that errors in the position significantly decreased when applied with direct projection augmented reality in the X-ray examination. Conclusion, It is expected that a clinical application of direct projection augmented reality-guided X-ray examination based on the study results will be a great help in minimizing re-examination due to position errors and radiation exposure.

**B-1070** 09:26

**Pelvis imaging: achieveing dose reduction with different patient position**

A. Resnik[1], J. Žibert[2], N. Mekis[2]; [1]*Novo mesto/SI*, [2]*Ljubljana/SI (anja.resnik13@gmail.com)*

**Purpose:** The aim of the research was to determine the effect on the radiation dose to the patient and image quality in pelvis radiography when cranially or caudally positioned on the table according to AEC chambers.
**Methods and Materials:** The research was carried out in two parts on two x-rays: Siemens Ysio and Siemens Axiom Luminus. In the first part of the study we measured DAP on the phantom, where 10 images were taken in cranial and 10 in the caudal layout. The second part of the study was expanded to 200 patients, one hundred per each digital x-ray. We measured DAP, field size, height and mass from which we calculated ITM. The effective dose and absorbed dose to selective organs were calculated using PCXMC2.0. The image quality assessment was performed by three experienced radiologists with the use of ViewDEX imaging software. Prior to the study the National medical ethic committee approval was obtained.
**Results:** When patients were caudally orientated on the examination table (Siemens Ysio), DAP value was reduced by 37.7% (p=0.002), average effective dose by 35.7% (p=0.002) and mean absorbed dose to selective organs by 33.4% (p=0.001) while image quality was better by 5.2% (p=0.041). We did not find any significant difference between orientations on Siemens Axiom Luminus due to specific AEC chambers.
**Conclusion:** We conclude that positioning patients caudally (HA) orientated according to AEC chambers is a method of selection.

**B-1071** 09:34

**Geometric efficiency: measuring methods' comparison**

C. Dionisi, M. Coppo, R. Villa, M. Brambilla; *Novara/IT (maga.coppo@gmail.com)*

**Purpose:** To assess the reliability and the uncertainty related to two different methods for geometric efficiency (GE) evaluation.
**Methods and Materials:** CT Scanners: two different multi-detector computed tomography (MDCT) Philips Ingenuity 128 (Philips, The Netherlands) and Philips iCT Brilliance 256 (Philips, The Netherlands) were used for the measurement of GE. Gafchromic films: after a proper calibration in air kerma the Gafchromic films were acquired in free air for different nominal collimations. The geometric efficiencies were obtained from the analysis of the profiles converted in air kerma. CT Dose Profiler: this solid-state detector was scanned in helical mode in free air for different nominal collimations; its dedicated software allows to automatically estimate the effective field width and the geometric efficiency.
**Results:** The analysis of geometric efficiencies obtained with the Gafchromic method underlines a good agreement with the expected values, characterised by a difference lower than 8%. Nevertheless, the accordance decreases for thinner collimations (under 5mm), up to 17%. Similar results were found for the evaluation performed throughout the CT Dose Profiler probe. The probe is designed to measure GE automatically only with spiral acquisitions, making it unable to measure GE for collimations only available on axial mode.
**Conclusion:** This study shows how both methods, through the use of radiochromic films or Dose Profiler, can measure geometric efficiency for both thin and wide beam collimations. Nevertheless, the automatic evaluation performed by the CT Dose Profiler probe makes it feasible for periodically constancy check.

**B-1072** 09:42

**Optimisation of shunt radiography**

A. Bremnes, B. Ween; *Oslo/NO (alexabre@odont.uio.no)*

**Purpose:** Hydrocephalus shunt protocols vary. To maintain high accuracy using flat panel detectors, an investigation of grid versus air gap with respect to image quality and doses was done.
**Methods and Materials:** A phantom study included 15 images. A panel of 16 professionals graded image sharpness subjectively, in grades of lines per millimetre (lp/mm). Dose-area product (DAP) and signal noise ratio (SNR) values were measured. Images represented a grid/non-grid situation, whereas non-grid images were measured with air gaps in steps up to 50cm.
**Results:** Images were rated within the range of 1.2-1.6 lines per mm. Highest spatial resolution represents 1.6 lp/mm, which were rated 99 times (41%) of the total of 240 evaluations for all images. The other ratings (59%) were distributed over a wide area. DAP values showed little variations: 12.6 (+/- 0.4) mGycm² for grid, and 12.6 (+/- 0.2) for air gap, respectively. SNR variations were not important. The results indicate that air gap is preferable over the grid. Longest air gap (50cm) got highest scores, followed by 45 and 40cm.
**Conclusion:** Longer air gaps are better than grid when the aim is to achieve the best image quality without increasing doses.

**B-1073** 09:50

**Optimisation of collimation and its impact on patient dose in radiography of lumbal spine in anteroposterior and lateral projection**

A. Pažanin[1], D. Škrk[2], N. Mekis[2]; [1]*Dubrovnik/HR*, [2]*Ljubljana/SI (apazanin@gmail.com)*

**Purpose:** To determine the change of radiation dose to the patient when the collimation referred in the literature is used for the lumbar spine radiography in AP and lateral projection.
**Methods and Materials:** The study was performed on 90 patients that were referred to the lumbar spine radiography in one of the Croatian hospitals. Patients were randomly divided into two equal groups. First group was imaged with the current collimation protocol and the second group with the collimation mentioned in the literature. For each patient DAP, image field size, source-to-patient distance, weight and height were collected. From that data using the PCXMC Monte Carlo simulation method effective dose and absorbed organ dose were calculated.
**Results:** There was no statistically significant difference in BMI between the group of patients with and without collimation. The reduction of primary field in AP projection when the collimation was used was by approximately 36%(p<0.001) and DAP by 49%(p<0.001). The reduction of the primary field in the lateral projection was 40%(p<0.001) but we didn't find any statistically significant difference in DAP. The effective dose in PA projection was decreased by 50% and average organ dose by 43% respectively. After the

Saturday image quality assessment, it was found that the central ray positioning in the lateral position in group without collimation was incorrect that resulted in lower DAP values, effective dose and organ dose.
**Conclusion:** Based on the results we can conclude that the use of proper collimation and central ray positioning is crucial to obtain ALARA principle.

**08:30 - 10:00** **Sky High Stage**

### Genitourinary

### MY 13

### Genitourinary

*Moderators:*
P. Asbach; Berlin/DE
K. Markiet; Gdansk/PL

### B-1074 08:30

**Amide proton transfer MR imaging for cervical cancer: a preliminary study**

Y. He, C. Lin, Y. Qi, X. Wang, H. Zhou, H. Xue, Z. Jin; *Beijing/CN (ylhe_526@163.com)*

**Purpose:** To evaluate the feasibility of amide proton transfer (APT) MR imaging for detection of cervical cancer.
**Methods and Materials:** Between 10/2017 and 9/2018, 52 patients with pathologically confirmed cervical cancer and 49 healthy volunteers underwent APT MR imaging on a 3T MR scanner (Ingenia CX, Philips Healthcare, the Netherlands). APT values, calculated based on 3D TSE images acquisition with dual RF transmits interleaved labelling. The B0 artefact was corrected via 3 shifted echo times in the 3D TSE sequence; and APT contrast was generated in line. Results were blindly measured by two radiologists for cervical cancer lesions and normal cervical stroma with mean ROI area of 568.7 $mm^2$ and 557.5 $mm^2$ respectively. Inter-observer ICC was computed. Student's t-test was performed to compare the differences of APT values between cervical cancer and normal cervix. ROC analysis was performed to computationally determine the feasible threshold value, sensitivity and specificity.
**Results:** APT values of cervical cancer and normal cervical stroma were 2.716±0.060 and 1.853±0.059 (mean±standard error of mean) respectively with statistical significant difference ($p$<0.0001, 95%CI 0.6931-1.033). Motion artefact was not significant in region of cervix. Inter-observer ICC was 0.988 (95%CI 0.978-0.993). The AUC for differentiating cervical cancer from normal cervical stroma was 0.916. The feasible threshold values was determined as 2.221 with sensitivity of 81.25% and specificity of 83.67%.
**Conclusion:** APT MR imaging is feasible in cervical cancer detection. Cervical cancer showed significant higher APT values than those of normal cervical stroma.

### B-1075 08:34

**Correlation of three-dimensional computerized tomographic renal volumetry with DTPA split renal function in prospective donors for renal transplantation**

G. Choudary, P.G. Kale, S. Peddi; *Tirupati/IN (gpvchowdary777@gmail.com)*

**Purpose:** Evaluation of split renal function (SRF) in living donors is important to ensure that the donor retains the better functioning kidney. The SRF is traditionally measured by means of renal scintigraphy. Recent studies have reported the utility of three-dimensional (3D) computerised tomographic (CT) renal volumetry as an alternative to renal scintigraphy in estimating SRF. The purpose of the current study is to compare the split renal volume (SRV) obtained from 3DCT with split renal function obtained from scintigraphy.
**Methods and Materials:** This is a retrospective study over 5 years that included 23 prospective renal donors who underwent CT and renal scintigraphy. Renal volume was calculated using Siemens syngo volumetry software. Pearson’s correlation coefficient was used to determine the correlation between quantitative data sets.
**Results:** 23 donors were evaluated. The mean body weight and surface area were 57.3±9.3 kgs and 1.56±0.13 $m^2$, respectively. The mean total renal volume was 212.7±38 $cm^3$ and mean split renal volume was 49.76±2.86 and 50.23±2.86 on right and left sides, respectively. No significant correlation was found between uncorrected SRV and SRF. Significant correlation was found between renal volume and function when both were corrected to body surface area (p value <0.0001).
**Conclusion:** Renal volume calculation can be done on CT data set already acquired for the assessment of renal vascular anatomy. The simple volumetric method with 3DCT has the potential to replace renal scintigraphy for the assessment of SRF as a preoperative imaging examination for living-donor transplantation.

### B-1076 08:38

**Prostate magnetic resonance imaging with and without contrast medium: is biparametric MRI sufficient for routine examinations?**

D. Junker[1], F. Steinkohl[2], B. Sztankay[1], U. Nagele[1]; [1]*Hall in Tirol/AT*, [2]*Innsbruck/AT (daniel.junker@tirol-kliniken.at)*

**Purpose:** Biparametric (bpMRI) and multiparametric (mpMRI) magnetic resonance imaging of the prostate were compared to investigate if and how diagnostic accuracy and tumour detection rates of prostate MRI are influenced by omitting dynamic contrast-enhanced imaging (DCE).
**Methods and Materials:** In this retrospective study, the results of bpMRI and mpMRI were compared when using the PI-RADS version 2 scoring system for 236 included patients. The distribution of tumours to PI-RADS score levels, tumour detection rates, diagnostic accuracy, and RoC analysis were calculated and compared to the results of histopathological analysis.
**Results:** The PI-RADS scores were changed in 9.75% of patients when using bpMRI instead of mpMRI, increasing the number of PI-RADS 3 scores by 8.89%. No changes of more than one score level were observed. BpMRI did not show significant differences in diagnostic accuracy or tumour detection rates (AuC of 0.914 vs 0.917 in ROC analysis). Of 135 prostate carcinomas (PCa), 5.93% were downgraded from PI-RADS 4 to PI-RADS 3 when omitting DCE, while 94.07% were scored identically. Changes were observed for only low-grade PCa with Gleason score 6 or 7a.
**Conclusion:** BpMRI did not lead to significant differences in diagnostic accuracy when compared to mpMRI. Accordingly, it seems reasonable to use a biparametric approach for initial routine prostate MRI to reduce risks, examination time and costs without significantly lowering the diagnostic accuracy.

### B-1077 08:42

**Benefit of diagnostic support of PI-RADS assessment by quantitative ADC measurements and radiomics in MR diagnosis of prostate cancer**

P. Schelb, S. Kohl, J.-P. Radtke, M. Wiesenfarth, M. Hohenfellner, H.-P. Schlemmer, K. Maier-Hein, D. Bonekamp; *Heidelberg/DE*

**Purpose:** We investigate the added benefit of quantitative mean ADC (mADC) and radiomic machine learning (RML) when used in combination with clinical decision-making according to PI-RADS.
**Methods and Materials:** Previously published mADC and RML models developed and validated on a cohort of 316 consecutive patients (training cohort, 183 patients; test cohort, 133 patients) have shown increased performance compared to PI-RADS. Here, we combine both models with PI-RADS by allowing the models to downgrade (Design A) or upgrade (Design B) PI-RADS reports. Clinically significant prostate cancer (csPC) was defined as Gleason Grade Group >=2 and a positive MRI as PI-RADS >=4. The McNemar test was used to compare differences in sensitivity and specificity.
**Results:** On a patient basis, compared to PI-RADS, specificity increased from 43% to 65% (mADC, p<0.001) and 62% (RML, p<0.001), while sensitivity decreased marginally from 89% to 87% (mADC, p=1.0) and remained unchanged at 89% (RML) in the test cohort for Design A. For Design B, specificity decreased from 43% to 30% (mADC, p=0.002) and 32% (RML, p=0.004), while sensitivity increased from 89% to 96% for both designs (p=0.25) in the test cohort.
**Conclusion:** Significant diagnostic improvement can be achieved by downgrading clinical PI-RADS reports based on mADC or RML, suggesting an approach to effectively incorporate quantitative and radiomic information into current PI-RADS reporting.
*Author Disclosures:*
**J. Radtke:** Consultant; Saegeling Medizintechnik, Siemens Heathineers. Speaker; Saegeling Medizintechnik. **H. Schlemmer:** Board Member; Curagita. Consultant; Siemens, Curagita, Profound, Bayer. Grant Recipient; BMBF, Deutsche Krebshilfe, Dietmar-Hopp-Stiftung, Roland-Ernst-Stiftung. Speaker; Siemens, Curagita, Profound, Bayer. **D. Bonekamp:** Speaker; Profound Medical Inc..

### B-1078 08:46

**Multiparametric MRI outperforms three popular clinical models in predicting pT3 prostate cancer in patients undergoing radical prostatectomy**

E. Zanelli[1], L. Cereser[1], G. GIannarini[1], V. Ficarra[2], C. Zuiani[1], R. Girometti[1]; [1]*Udine/IT*, [2]*Messina/IT (rgirometti@sirm.org)*

**Purpose:** To compare clinical models (CM), mpMRI alone, and mpMRI plus clinical models (mpMRI-CM) in predicting pathologic stage T3(pT3) in patients with clinically-localized prostate cancer (PCa).
**Methods and Materials:** Over two years we prospectively performed staging 3.0T mpMRI in patients referred to radical prostatectomy for biopsy-proven <cT3 PCa, assessing the risk for extraprostatic extension (EPE) and/or seminal vesicle invasion (SVI) with the Partin tables (PT), Memorial Sloan Kettering Cancer Center nomogram (MSKCCn), and Cancer of the prostate risk assessment (CAPRA) score. Two radiologists, blinded to pathology, reviewed

Saturday mpMRI independently using the prostate imaging reporting and data system version-2 (PI-RADSv2), and assessed EPE/SVI with a PI-RADSv1- and v2-derived score. Receiving operating characteristics and logistic regression analyses were performed to determine the per-patient accuracy of mpMRI vs CM vs mpMRI-CM in predicting pT3 (EPE and/or SVI).
**Results:** We included 73 patients, most with cT1c (67.1%) and/or grading group 1-2 (86.2%) cancers. Median risk for EPE/SVI was 27.3/1.9% (PT) and 47.0/2.0% (MSKCCn); median CAPRA score was 3. On pathology, 32.9% of patients showed pT3. Areas under the curve (AUCs) for pT3 were 0.62 for PT, 0.62 for MSKCCn, 0.64 for CAPRA, and 0.73-0.74 for mpMRI readers (p<0.05). AUCs of mpMRI-CM were similar or lower compared to those of mpMRI alone. mpMRI was the only independent predictor of pT3 (OR 7.40/5.53 for readers 1/2, respectively).
**Conclusion:** mpMRI outperformed CM in predicting pT3. Adding mpMRI to CM did not improve predictive accuracy compared to mpMRI alone, suggesting a stand-alone use for staging PCa.

**B-1079** 08:50
**Do DCE-derived quantitative parameters predict clinically significant prostate cancers? A study on patients referred to radical prostatectomy**
F. Greco, R. Girometti, L. Cereser, G. Como, C. Zuiani; *Udine/IT (rgirometti@sirm.org)*

**Purpose:** To investigate whether dynamic contrast-enhanced (DCE)-derived parameters predict clinically significant prostate cancer (csPCa).
**Methods and Materials:** We retrospectively included patients with biopsy-proven csPCa referred to radical prostatectomy who underwent staging multiparametric magnetic resonance imaging (mpMRI) over a two years period. mpMRI was acquired on a 3.0T magnet with a prostate imaging reporting and data system version 2 (PI-RADSv2)-compliant protocol, including a DCE sequence (44 acquisitions of a 8.8 sec-long T1-weighted fat-satured 3D sequence). Image analysis was performed by two readers in consensus, placing a region-of-interest on (1) non-affected prostate and (2) PI-RADS≥3 findings proven to be a cancer on pathology, measuring the transfer constant ($k^{trans}$), rate constant ($k_{ep}$), extravascular extracellular volume fraction ($v_e$), and fractional plasma volume ($v_p$) (Extended-Toft model). Receiver operating characteristic analysis and logistic regression were run to assess the prediction of csPCa compared to non-affected prostate, on a per-lesion basis.
**Results:** Included were 59 patients with 51 csPCas. Of the investigated parameters, those showing a significant difference compared to non-affected prostate (p<0.01;U Mann-Whitney-test) were $k^{trans}$ (0.50 vs. 0.30 $min^{-1}$) and $k_{ep}$ (1.66 vs. 0.93 $min^{-1}$). Despite $k^{trans}$ and $k_{ep}$ showed an area under the curve of 0.84 (cut-off >0.28 $min^{-1}$) and 0.88 (cut-off 1.18 $min^{-1}$) in assessing cancer, respectively, none of them was a predictor of csPCa in a multivariate model including all DCE parameters and PI-RADSv2-related features (p>0.01).
**Conclusion:** DCE quantitative parameters do not predict pathology-proven csPCa, suggesting they have no diagnostic role at the moment.

**B-1080** 08:54
**Transperineal thermoablation in patients with benign prostatic hyperplasia: our experience**
R. Regine[1], F. Palmieri[2], F. Zanfardino[1], G. Fatigati[1], A. Martino[1], M. Cornacchia[1], P. Cozzolino[1]; [1]*San Giorgio a Cremano/IT*, [2]*Caserta/IT (francescopalmieri@hotmail.com)*

**Purpose:** To evaluate the feasibility and safety of trans-perineal laser thermoablation (TPLT) of the prostate in patients with benign prostatic hyperplasia (BPH).
**Methods and Materials:** Twenty-two patients with age range 53-87 y.o. with obstructive BPH underwent a trans-perineal laser thermoablation treatment . Clinical examination, PSA serum analysis, trans-rectal ultrasound and urodynamic examinations were performed for all patients. Under sedoanalgesia, all patients underwent a TPLT treatment for each prostatic lobe by means of two 21-gauge 15-cm needle connected to 980-nm diode laser (3600 J energy; 4 W power). Every patients used a urinary catheter for 15 days after treatment. Prostate volume and uroflowmetry were measured 1, 3, 6 and 12 months after TPLT.
**Results:** No major complications from TPLT treatment were noted. In particular, no patients reported local, pelvic, or abdominal pain during the procedure or subsequent alterations of defaecation rhythm, ano-rectal/intestinal problems, or haematuria. Prostate volume reduced of 15-20% and of 35-59%, respectively, 1 month and 12 months after TPLT; uroflowmetry results improved in all patients.
**Conclusion:** In our experience, transperineal laser thermoablation in patients with benign prostatic hyperplasia was demonstrated to be a safe, well-tolerated, and repeatable method to treat BPH, reducing prostate volume and improving uroflowmetry results. Using a trans-perineal approach we avoided the complications that a trans-uretral surgery can cause.

**B-1081** 08:58
**Kidney stone composition analysis using machine learning and spectral detector CT: an ex-vivo study**
N. Grosse Hokamp, J. Salem, S. Lennartz, R.P. Reimer, A. Heidenreich, D. Maintz, S. Haneder; *Cologne/DE (nils.grosse-hokamp@uk-koeln.de)*

**Purpose:** Several recent studies suggest using dual energy CT for differentiation of kidney stones by means of HU-measurements on low and high energy datasets; however, most studies focused on single component stones. This study aimed to determine, if machine learning enables a more precise differentiation with respect to the main component on a per-voxel basis.
**Methods and Materials:** 202 stones harvested from patients were scanned in a non-anthropomorphic water phantom using a spectral detector CT scanner. Virtual monoenergetic images (VMI) of 40 keV and 200 keV as well as conventional CT images (CI) were reconstructed. All stones were semi-automatically segmented on CI, the resulting volume of interest was transferred to VMI datasets. All pixel values were exported and served as input functions in an available machine learning framework (Matlab, MathWorks, Natick, USA). The main component of the stone was determined using infrared-spectroscopy.
**Results:** Segmentation resulted in a total of 74.769 voxels, average voxels per stone was 370. An unweighted k-nearest neighbor classification (KNN) with 10 neighbors and Euclidean distance metric performed best. This KNN yielded an overall accuracy of 82.2% using 5-fold cross-validation with a prediction speed of approx. 1200 observations per second. Greatest overlaps were found between stones which's main component was calcified (whewellite, weddellite and brushite).
**Conclusion:** Machine learning enables accurate analysis of kidney stone composition. Even in mixed stones the main component may be predicted.
*Author Disclosures:*
**N. Grosse Hokamp:** Speaker; Philips Healthcare. **D. Maintz:** Speaker; Philips Healthcare.

**B-1082** 09:02
**Comparison between biparametric MRI (bp-MRI) and multiparametric MRI (mp-MRI) for the diagnosis of prostate cancer (PCa): preliminary results of a prospective randomised trial**
G. Giannetto[1], L. Pusceddu[1], V. Doronzio[2], V. Vani[1], V. Romano[3], C. Maglia[4], S. Mazzetti[2], D. Regge[5], F. Russo[4]; [1]*Turin/IT*, [2]*Candiolo/IT*, [3]*Candiolo TO/IT*, [4]*Torino/IT*, [5]*Candiolo-Torino/IT (ggiannetto27@gmail.com)*

**Purpose:** To compare bp-MRI and mp-MRI in a prospective randomised clinical trial including biopsy-naïve subjects at risk for PCa.
**Methods and Materials:** Biopsy-naïve men with increased PSA values (≤15 ng/ml) and negative digital rectal examination were randomised into two arms. Subjects in arm A underwent fast bp-MRI protocol (axial T2W and DWI) with no endorectal coil (ERC) and no iv contrast agent. Men in arm B underwent mp-MRI including T1W, T2W, DWI and DCE with ERC and iv contrast agent administration. After MRI, all subjects underwent biopsy, which was considered the reference standard of this study. Patients with positive MRI and biopsy Gleason Score≥3+4 were defined as true positives.
**Results:** Overall, 143 men underwent MRI examination. 99/143 (69%) were randomised in arm A and 44/143 (31%) in arm B. In the bp-MRI arm, 32/99 (32%) men had a suspicious lesion and in 23 cases biopsy was positive for PCa. In the mp-MRI arm, 23/44 (52%) men were positive at imaging and 15 were PCa at biopsy. In arm A, sensitivity and specificity were 74% and 87%, respectively; in arm B, 88% and 70%, respectively.Considering only PIRADS 4-5 subjects, 19/23 (83%,p=0.35) and 13/15 (87%,p=0.14) were positive at biopsy in arm A and B, respectively.In 7/67 (10%) subjects with negative bp-MRI, a biopsy revealed a clinically significant PCa (csPCa). In 1/21 (5%) man negative at mp-MRI, a biopsy found a csPCa.
**Conclusion:** Mp-MRI has higher sensitivity than bp-MRI in identifying csPCa in biopsy-naïve men, while bp-MRI reveals higher specificity.

**B-1083** 09:06
**Application of intravoxel incoherent motion imaging (IVIM) in patients with hyperuricemia**
Z. Cheng, X. Cai, Y. Feng; *Guangzhou/CN (zhongyuancheng08@163.com)*

**Purpose:** The objective of this study was to evaluate the utility of IVIM parameters to identify the early changes in in patients with hyperuricemia.
**Methods and Materials:** A total of 84 male participants, asymptomatic hyperuricemia (27 cases), gout patients (31 cases) and 26 age- and sex-matched healthy volunteers were enrolled in this study. All subjects underwent IVIM sequence (b=0,30,50,80,100,150,200,300,500,800,1000s/$mm^2$) on a 3.0T MRI scanner. Mean cortical and medullary IVIM parameters (ADC, D, $D^*$ and f values) were calculated by setting multiple regions of interest. Differences in every IVIM parameter among the three groups were assessed. Also, they were correlated with estimated glomerular filtration rate (eGFR).

Saturday

**Results:** The D values in the renal cortex and medulla were significantly decreased from the control group, AH to GA group (p<0.05). The cortical and medullary ADC values were significantly decreased across the three groups, except for the comparison between HA and GA group (p<0.05). The f values of the three groups were decreased in turn, the control group was significantly higher than the HA group and GA (p<0.05), whereas the cortical f values has only difference in the HA group and the GA group (p<0.05); There was no statistically significant difference between the D* values among the three groups. No significant correlation were found between IVIM parameters and eGFR (p>0.05).
**Conclusion:** IVIM demonstrated potential useful to assess early hyperuricemia with renal dysfunction in patients with hyperuricemia. Its evaluation index D value was more sensitive to the early renal function impact by hyperuricemia than eGFR.

**B-1085** 09:10
**Explorative investigation of whole-lesion histogram MRI metrics for differentiating uterine degenerative leiomyomas and leiomyosarcomas**
K. Ma; *Zhengzhou/CN (1322725260@qq.com)*

**Purpose:** The purpose of this study is to assess the utility of texture analysis of multiple MRI sequences for the differentiation of uterine degenerative leiomyomas and leiomyosarcomas.
**Methods and Materials:** 12 leiomyosarcomas and 29 degenerative leiomyomas undergoing MRI before resection were included. DICOM images for the following three sequences were exported for subsequent analysis using the software named Mazda. : ADC map, and delayed axial contrast-enhanced T1-weighted image. Using the software, a Whole-lesion volumes of interest incorporating all lesion voxels on all slices was placed on the largest uterine lesion . The diagnostic performance of histogram metrics was assessed.The ROC curves were compared in pairs by the Delong method , and P < 0.05 was considered statistically significant.
**Results:** For the ADC maps, significant differences were observed for mean, skewness, 10th percentile, 50th percentile, 90th percentile; For T1-weighted contrast-enhanced images, significant differences were observed for mean, standard deviation, 10th percentile, 90th percentile, 99th percentile (p<0.05). ROC analysis indicated that 90th percentile of ADC had the highest diagnostic accuracy , the maximum area under the ROC curve was 0.891 with a specificity of 72.7% and a sensitivity of 81.8%.
**Conclusion:** For whole-lesion histogram metrics obtained on various MRI sequences, 90th percentile of ADC provided the highest performance for differentiating degenerative uterine leiomyomas and leiomyosarcomas. Although these findings require validation in larger studies, they have implications for facilitating improved treatment selection for these two entities.

**B-1086** 09:14
**Imaging surveillance in operable invasive transitional cell carcinoma of the bladder**
S.A. Ahmed, M.M. El Barody, N.A.A. El Malek, M.A. El Salam Ebrahem; *Assiut/EG (Elbarody_radiol@yahoo.com)*

**Purpose:** To evaluate diagnostic accuracy of abdominopelvic ultrasound (US), virtual cystoscopy (VC) and MRI diffusion (MRI DWI) with conventional cystoscopy (CC), in diagnosis of bladder lesions after transurethral resection (TUR).
**Methods and Materials:** Forty-five patients with bladder cancer underwent complete TUR and received chemoradiation schedule, they imaged prospectively by US, VC and MRI DWI followed by CC and histopathology of any detected abnormality. Sensitivity, specificity, positive and negative predictive values and accuracy were calculated and compared to CC and histopathology as gold standard.
**Results:** After median 24 months of follow up, US, VC and MRI DWI detected 23, 28 and 29 lesions, respectively, of the 30 lesions detected by CC. Pathology confirmed local recurrence in 18 lesions (40%) and benign post-operative changes in 12 lesions (27%), while remaining 15 patients were negative. Eighteen lesions (60%) were larger than 1 cm, twenty-three lesions showed polypoidal morphology (77%). Accuracy of US, VC, and MRI DWI in diagnosis of bladder lesions was 73%, 93%, and 93%, respectively (P>0.10).
**Conclusion:** MRI DWI and VC were of comparable accuracy in diagnosing bladder lesions after TUR; US had lowest sensitivity while DW-MRI was more accurate in recurrence characterization in masses less than 1 cm and focal wall thickening.

**B-1087** 09:18
**Contrast-enhanced T1 mapping MR imaging for evaluation of myometrial invasion in endometrial cancer: initial experience**
Y. He, C. Lin, Y. Qi, X. Wang, H. Zhou, H. Xue, Z. Jin; *Beijing/CN (ylhe_526@163.com)*

**Purpose:** To assess diagnostic performance of contrast-enhanced T1 mapping images for evaluation of myometrial invasion in endometrial cancer.
**Methods and Materials:** Institutional review board approval and informed consent were obtained. From 12/2017 to 9/2018, 31 consecutive patients with endometrial cancer, aged 26-72 years (mean age, 51 years), were prospectively enrolled for preoperative evaluation by multi-parameter pelvic MR imaging on a 3T MR scanner (Ingenia CX, Philips Healthcare, the Netherlands). MOLLI sequences (enhanced scheme, 13 seconds) for T1 mapping were appended after clinical routine sequences after contrast Gadopentetate Dimeglumine injection. Two radiologists blindly interpreted myometrial invasion on T2-weighted, DWI, DCE and contrast-enhanced T1 mapping images. Inter-observer agreement was evaluated by Kappa analysis. According to surgical pathologic findings, accuracy assessment, receiver operating characteristic analysis for diagnostic performance was compared.
**Results:** Reader agreement was excellent for contrast-enhanced T1 mapping images (κ>0.75). Diagnostic accuracy of contrast-enhanced T1 mapping to assess existence of myometrial involvement was 0.94, and sensitivity, specificity, PPV, NPV were 1.0, 0.67, 0.92, 1.0 respectively. AUC was 0.833 (95%CI 0.656-0.942), which was significant better than those of T2-weighted, DWI and DCE images (p=0.017). Diagnostic accuracy of contrast-enhanced T1 mapping for evaluating deep invasion of myometrium was 0.94, sensitivity and specificity was 0.83 and 0.96. AUC was 0.897 (95%CI 0.743-0.977), revealing similar diagnostic performance compared with clinical routine sequences (all p>0.05).
**Conclusion:** Contrast-enhanced T1 mapping images could provide elevated accuracy for detecting superficial myometrial invasion. It showed similar diagnostic performance compared with the common sequences in differentiating FIGO IA and IB endometrial cancer.

**B-1088** 09:22
**Evaluation of a semi-automated segmentation tool for volumetric analysis of the renal cortex and medulla in non-contrast T1-weighted MR images**
H. Seuss, R. Janka, P. Dankerl, A. Cavallaro, M. Uder, M. Hammon; *Erlangen/DE (hannes.seuss@uk-erlangen.de)*

**Purpose:** A semi-automated segmentation tool was evaluated for its accuracy regarding the volumetric analysis of the total kidney parenchyma and the differentiation of the cortex and medulla in non-contrast T1-weighted (T1w) magnetic resonance imaging (MRI) scans.
**Methods and Materials:** Two readers performed a semi-automated segmentation of the renal parenchyma in axial, non-contrast-enhanced T1w MRI images. The segmentation was itemized into the total kidney volume (TKV) and cortex volume (CV). The volume of the medulla was calculated. 78 patients (2*78 kidneys) with healthy kidneys that received an MRI for the evaluation of the liver were included into the study. The MRI protocol was adapted to fit the 'German National Cohort' (T1w DIXON gradient echo). For the segmentation of the kidney, we developed a semi-automated threshold-based segmentation tool. Manual segmentation served as the reference standard.
**Results:** There was no significant difference between semi-automatically and manually segmented volumes of the TKV (p > 0.456). The CV, however, differed significantly (p < 0.002). The mean volume for the TKV was 156.0±37.8ml for the right kidney and 162.5±39.8ml for the left kidney. For the CV it was 61.3±23.2ml for the right kidney and 62.8±23.3ml for the left. No significant interreader variability was present.
**Conclusion:** Semi-automated segmentation and volumetric analysis of the TKV in native T1w MRI images deliver accurate and reproducible results. However, the differentiation of the renal cortex and medulla has a high error and can only be used for approximations of the volume.

**B-1089** 09:26
**Correlation of erectile dysfunction after radical prostatectomy with findings of preoperative multiparametric prostate MRI**
M. Sauer, P. Tennstedt, C. Berliner, H. Huland, G. Adam, D. Beyersdorff; *Hamburg/DE (m.sauer@uke.de)*

**Purpose:** To evaluate whether preoperative multiparametric prostate MRI (mpMRI) can help to stratify the risk of erectile dysfunction (ED) after radical prostatectomy (RP) based on specified imaging parameters.
**Methods and Materials:** 102 patients (median age: 63 years) underwent standardized mpMRI at 3T prior to prostatectomy. Erectile function was assessed before and after RP using the Expanded Prostate Cancer Index Composite Questionnaire score (EPIC). Tumour stage, extracapsular extension (ECE), prostate volume, membranous urethra length/angle (MUL/MUA), apex form according to Lee et al., apex infiltration (AI) and PI-RADS scores were determined for every patient's MRI. Additionally, various clinical parameters including age, preoperative EPIC score and the degree of nerve sparing surgery were registered. All parameters were correlated to the postoperative EPIC scores determining the degree of erectile dysfunction.
**Results:** The median preoperative and postoperative EPIC scores for all patients were 79% and 43%, respectively (with 100% representing best functionality). Of all patients 21% demonstrated tumours > T2 with 18%

positive ECE and 75% positive AI. Median PI-RADS score was 4 with dominance of Apex Type C (58%), a median MUL/MUA of 10mm/120° and a median volume of 43 ml. However, none of these imaging parameters correlated to the postoperative EPIC scores (p>.05). Of all clinical parameters, there was significant correlation of preoperative EPIC score (p<.001), and the extent of surgical nerve sparing (p<.05).
**Conclusion:** None of the investigated imaging parameters of mpMRI correlates with the degree of postoperative erectile dysfunction.

**B-1090** 09:30
**Can MRI including DCE and DWI improve characterisation of endometrial carcinosarcoma?**
L. Rivière[1], L. Rossi[1], T. Lam Giang[2], E. Laas[1], G. Bataillon[1], P. Cherel[3], H. Brisse[1], F. Reyal[1], C. Malhaire[1]; [1]*Paris/FR*, [2]*Genève/CH*, [3]*Saint-Cloud/FR* *(luctkd@hotmail.fr)*

**Purpose:** To describe the MRI characteristics of endometrial carcinosarcoma (CS), including dynamic contrast-enhanced (DCE) and diffusion-weighted imaging (DWI), in comparison with those of endometrioid carcinoma (EC).
**Methods and Materials:** In a retrospective study of our institute histopathologic database from 2003 to 2018, patients with CS or EC who received a preoperative MRI were included. Morphological, perfusion (maximum relative enhancement (SIRel)) and wash-in rate (WIR)) and DWI were analysed. The T2 signal heterogeneity, necrosis, haemorrhage, intensity and heterogeneity of enhancement were recorded by two radiologists. Inter-reader agreement was calculated.
**Results:** Ninety-one patients (36 CS and 55 EC) were included. The CS size and cavity dilatation were greater than those of EC (82 mm vs 38mm, p<0.001;43 mm vs 19 mm, p<0.001, respectively). In comparison with EC, CS more often showed heterogeneity in the T2 signal (97% vs 38%, p<0.001; k=0.88), necrosis (70% vs 13%, p<0.001; k=1) and haemorrhage (83% vs 25%, p<0.001; k=0.95). CS was enhanced more often greater or equal to the myometrium (94% vs 44%, p<0.001; k=0.85) and heterogeneously (97% vs 55%, p<0.001; k=0.94) in comparison with EC. SIRel and WIR were greater in CS than in EC (92% vs 63% and 46% vs 36%, p=0.01). CS more often showed a high signal intensity on DWI in comparison with EC (100% vs 65%, p=0.02); however, no significant difference was found regarding the ADC values.
**Conclusion:** A bulky endometrial mass containing necrosis and haemorrhage with an enhancement greater or equal to the myometrium suggests a diagnosis of CS.

**B-1092** 09:34
**Prostate cancer lesion characterisation: rapid acquisition using compressed sensing in combination with semi-automatic lesion segmentation for dual-parameter decision support**
D.J. Winkel, T. Heye, M.R. Benz, B. Stieltjes, D. Boll, V. Hofmann; *Basle/CH* *(verena.hofmann@usb.ch)*

**Purpose:** To investigate the diagnostic performance of dynamic contrast-enhanced (DCE) MRI to detect prostate cancer comparing high-spatiotemporal resolution golden-angle radial sparse imaging (GRASP) with standard-of-care sequences (volumetric interpolated breath-hold examination: VIBE) and to assess whether dual-parameter decision support, using perfusion and diffusion characteristics, can achieve higher diagnostic accuracy compared to single-parameter assessments.
**Methods and Materials:** 101 peripheral zone prostate cancer lesions in 94 patients and a clinical suspicion for prostate cancer were evaluated by 3T MRI. Histopathological confirmation on MRI-TRUS fusion biopsy was matched with normal prostate parenchyma contralaterally. MRI was performed with DWI and DCE using two acquisition schemes: GRASP (temporal resolution 2.5s, in-plane resolution 0.56x0.56mm) or VIBE (temporal resolution 10s, in-plane resolution 1.25x1.25mm). Quantitative perfusion (Ktrans, Kep) and diffusion (ADC) parameters were assessed using univariate general linear model analyses defining processing (planimetry/volumetry), histopathology (normal/pathologic), and sequence (VIBE/GRASP) as independent variables. Receiver operating characteristics (ROC) and accuracies were determined.
**Results:** DCE with GRASP yields diagnostic accuracy comparable to standard-of-care sequences [volumetric: .81 vs .83Ktrans, .79 vs .75Kep] (p>0.05). Single-parameter analyses: ADC outperformed both Ktrans/Kep in the detection of prostate cancer [volumetric: 94ADC] (p<0.001). Dual-parameter analyses: combining ADC with Ktrans or Kep obtained from GRASP [volumetric: .97ADC/Ktrans and .97ADC/Kep], improved performance compared to any single-parameter evaluation and any assessment based on VIBE [volumetric: .94ADC/Ktrans and .94ADC/Kep] (p<0.001). Planimetric analyses of DCE-MRI with GRASP showed similar results.
**Conclusion:** Diagnostic performance of DCE-MRI using GRASP is comparable to standard-of-care sequences. When GRASP is employed in combination with volumetric lesion processing in a dual-parameter discrimination system, it showed better diagnostic performance compared to single-parameter methodologies as well as any evaluation approach using the VIBE sequence.

**B-1093** 09:38
**Pitfalls in reporting fast biparametric magnetic resonance imaging of the prostate: what kind of cancers do we miss? A preliminary retrospective analysis**
L. Pusceddu[1], G. Giannetto[1], V. Romano[1], V. Vani[2], F. Cortese[1], G. Desi[2], S. Mazzetti[1], D. Regge[1], F. Russo[2]; [1]*Candiolo/IT*, [2]*Turin/IT* *(laura.pscd@gmail.com)*

**Purpose:** The aim of the study was to assess the causes of diagnostic failure in the identification of prostate cancer (PrCa) in patients who underwent fast biparametric MRI (bpMRI).
**Methods and Materials:** We retrospectively evaluated data from a randomized trial in which men with increased PSA values (4<PSA≤15 ng/ml) and without previous prostate biopsy were considered for inclusion in a fast bpMRI protocol (axial T2W and DWI with no endorectal coil and no i.v. contrast agent administration). After MRI, all patients underwent biopsy, which was considered as the reference standard. An experienced radiologist reviewed all cases with no suspicious lesion at MRI but with PrCa revealed by biopsy, in order to assess if lesions could be visible but incorrectly interpreted and their characteristics.
**Results:** 23/99 patients included in the study had a positive biopsy; bpMRI did not identify 11/23 cases (47%). Four missed lesions had a biopsy Gleason Score (bGS) 3+3, five had a bGS 3+4 (2/5 with pattern 4<10%) and 2 cases had a Gleason primary 4. At the image review, 4 missed lesions, described as microfoci of PrCa at biopsy (<1 mm), were not visible. Among tumors detected during MRI review, the following characteristics were responsible for the missed identification: triangular or striped shape, proximity to the adenoma pseudocapsule and intermediate ADC values.
**Conclusion:** BpMRI shows some limitations in the identification of tumors with specific MRI features. These missed lesions are usually less aggressive from a pathological standpoint.

**10:30 - 12:00** **Room B**

### Abdominal Viscera

### SS 1401a

### Assessing pancreatic malignancy

*Moderators:*
N. Kartalis; Stockholm/SE
M. Klauß; Heidelberg/DE

**B-1094** 10:30
**Diagnostic accuracy of CT features and CT texture analysis in differentiating focal autoimmune pancreatitis from adenocarcinoma: a retrospective study**
G. Cardano, A. Fighera, A. Campagnola, G. Zamboni, L. Frulloni, G. Mansueto; *Verona/IT (drpeppex@gmail.com)*

**Purpose:** To assess the value of morphological criteria and texture analysis in differentiating between focal autoimmune pancreatitis (AIP) and pancreatic adenocarcinoma (PDAC) at CT.
**Methods and Materials:** We reviewed the multiphasic MDCT scans of 60 patients with solid pancreatic lesions: 30 focal AIP, 30 PDAC. Each scan was evaluated for: lesion size and margins, main pancreatic duct (MPD) intralesional stenosis and upstream dilation, upstream chronic pancreatitis, biliary dilation. Attenuation was measured in the lesion and the unaffected parenchyma in all phases. Texture-analysis of the lesion was performed with LIFEx.
**Results:** Significant differences were observed between AIP and PDAC in unenhanced (p=0,010), arterial (p=0,008) and venous (p=0,006) lesion attenuation, unenhanced normal parenchyma attenuation (p=0,048), biliary dilation (p=0,004), and texture-Entropy in the delayed phase (p=0,00). Univariate logistic regression analysis confirmed significance of lesion density in unenhanced (p=0,014), arterial (p=0,014) and venous phases (p=0,011) as “AIP risk factors”. Upstream MPD dilatation (p=0,004), biliary dilatation (p=0,007), upstream chronic pancreatitis (p=0,01) are “PDAC risk factors”. Multivariate regression analysis confirmed the predictive capacity of upstream chronic pancreatitis (p=0,011) for adenocarcinoma. The whole logistic binomial univariate model showed a significant predictive value for diagnosis of PDAC, with a COX-SELL R2=0,422; the ROC curve confirmed its accuracy, with AUC=0,918 (p=0,037).
**Conclusion:** Upstream MPD and chronic pancreatitis, biliary dilatation, lesion density at unenhanced, arterial and venous phases were statistically significant in differentiating between AIP and PDAC. Progressive enhancement significantly increases the probability of AIP; upstream MPD dilatation, biliary dilatation and chronic pancreatitis increase the probability of PDAC.

**B-1095** 10:38

**Contrast-enhanced ultrasound for differentiation of pancreatic carcinoma (PC) from mass-forming chronic pancreatitis (MFCP)**

P. Gupta, S. Sinha, N. Dhaka, A. Rajwanshi, K. Vaiphei, R. Kochhar; *Chandigarh/IN (pankajgupta959@gmail.com)*

**Purpose:** To evaluate the diagnostic performance of contrast-enhanced ultrasound (CEUS) in the differentiation of PC from MFCP.
**Methods and Materials:** Consecutive adults with a mass in the head of the pancreas underwent a CEUS of the pancreas. Parameters assessed were the pattern of enhancement (hypoenhancing/isoenhancing and complete or incomplete) and dot and lace vascular pattern (defined as visualisation of dot or lace-like vessels within the lesion). Cytology or histopathology was used as the reference standard.
**Results:** Of the 48 patients, the diagnosis of PC was confirmed in 27 (72.9%) and MFCP in 10 (27.1%). Eleven patients were excluded as sampling could not be performed (n=6), and diagnosis other than PC (n=5). There were 23 males and 14 females with a mean age of 55.14 years. Hypoenhancement was documented in 25 patients with PC and only 1 patient with MFCP. Iso-enhancing mass was seen in 9 patients with MFCP and only 2 patients with PC. A complete enhancement was seen in 3 patients with MFCP and only 1 patient with PC. Dot and lace pattern was seen in 20 patients with PC and only 2 patients with MFCP. The sensitivity, specificity, PPV, and NPV of the degree of enhancement for differentiation of PC from MFCP was 96.15%, 81.82%, 92.59% and 90%, respectively. Complete vs incomplete enhancement had a high sensitivity (96.15%) but lower specificity (30%).
**Conclusion:** CEUS is a useful imaging modality in the differentiation of PC from MFCP. Hypoenhancement has the highest diagnostic accuracy among all the CEUS parameters.

**B-1096** 10:46

**Quantitative analysis of arterial-phase MR images in differentiating focal-type autoimmune pancreatitis from pancreatic ductal adenocarcinoma**

J. Kwon, J. Kim; *Seoul/KR (wisewisdom.rad@gmail.com)*

**Purpose:** To assess whether quantitative analysis of arterial-phase MR images is helpful to differentiate focal-type autoimmune pancreatitis (AIP) from pancreatic ductal adenocarcinoma (PDA).
**Methods and Materials:** Thirty-six patients with focal-type (single mass forming) AIP and 72 patients with surgically resected PDA, who underwent dynamic contrast-enhanced MR imaging (DCE-MRI) were included. The signal intensity (SI) of the pancreatic mass and normal parenchyma and the lesion contrast ($SI_{normal\ pancreas}/SI_{mass}$) were measured on each phase of DCE-MRI. The lesion contrast on arterial phase (AP) ($contrast_{AP}$) and the contrast ratio between AP and unenhanced phase (UP) ($contrast_{AP/UP}$) were compared between AIP and PDA groups. The specificity of $contrast_{AP/UP}$ using appropriate cutoff values for the diagnosis of AIP and PDA was compared with those of previously known key MRI features of both diseases.
**Results:** The $contrast_{AP}$ and $contrast_{AP/UP}$ were significantly lower in AIP than in PDA (1.23 vs. 1.84, P<0.001; 0.84 vs. 1.15, P<0.001). The specificity of $contrast_{AP/UP}$ for AIP when using cutoff value <0.9 was 97.2%, which was comparable to those of the key MRI features including non-discrete mass, delayed homogeneous enhancement, halo sign, and duct penetrating sign (94.4%-100%; P≥0.500). The specificity of $contrast_{AP/UP}$ for PDA when using cutoff value ≥1.0 was 97.2%, which was comparable to or slightly higher than those of the key MRI features including target appearance, main pancreatic duct (MPD) abrupt narrowing, upstream MPD marked dilatation, and pancreatic atrophy (88.9%-94.4%; P≥0.375).
**Conclusion:** Quantitative analysis of the lesion contrast on arterial-phase MR images is helpful to differentiate focal-type AIP from PDA.

**B-1097** 10:54

**Comparison of reduced field-of-view and large field-of-view diffusion-weighted 3T MR imaging in the evaluation of solid pancreatic lesions**

P. Boraschi, F. Donati, G. Tarantini, R. Cervelli, F. Pacciardi, C. Lombardo, U. Boggi, F. Falaschi, D. Caramella; *Pisa/IT (p.boraschi@gmail.com)*

**Purpose:** To compare image quality and apparent diffusion coefficient (ADC) values of reduced field-of-view (rFOV) and large field-of-view (lFOV) diffusion-weighted MR imaging (DW-MRI) at 3T-device in the evaluation of solid pancreatic lesions.
**Methods and Materials:** Forthy-eight patients with histologically proven solid pancreatic lesions (ductal adenocarcinoma, n=22; invasive adenocarcinoma accompanying IPMN, n=8; neuroendocrine tumour, n=15; autoimmune pancreatitis, n=3) who underwent MRI at 3T-device (GE DISCOVERY MR750;GE Healthcare) were retrospectively included. In all cases, DW-MRI was performed using both rFOV FOCUS and lFOV respiratory-triggered spin-echo echo-planar sequence, each with multiple *b*-values (0, 500, 1000 sec/mm²). Two radiologists in conference assessed a series of subjective image quality parameters (sharpness; distortion; ghosting, motion and susceptibility artefacts; lesion conspicuity, and overall image quality) of both diffusion-weighted sequences, using a 4-point-scale semiquantitative grading system. ADC values were calculated by another radiologist by drawing the ROI on the central slice of the pancreatic lesion for both rFOV and lFOV DW-MRI (b=500,1000 sec/mm²). Radiologists were blinded to the corresponding patient information, especially lesion location and pathological results.
**Results:** More artefacts were present on lFOV compared with rFOV FOCUS diffusion-weighted images (p<0.01). Lesion conspicuity and overall image quality of rFOV DW-MRI were rated superior than those of lFOV DW-MRI (p<0.01). All ADC values were significantly lower when calculated from rFOV images than lFOV ones.
**Conclusion:** The rFOV DW-MRI provided reduced artefacts, better lesion conspicuity and overall image quality than the lFOV sequence. ADC valued derived from rFOV DW-MRI were significantly lower when compared with those derived from lFOV DW-MRI.

**B-1098** 11:02

**Assessment of pancreatic ductal adenocarcinoma lesion using virtual monoenergetic images from dual-layer spectral detector CT**

Y. Yang, Q. Han, X. Chen, F. YAN; *Shanghai/CN (yyz01a15@rjh.com.cn)*

**Purpose:** To quantitatively and qualitatively assess the image quality of virtual monoenergetic images (VMI) derived from dual-layer spectral detector CT for pancreatic ductal adenocarcinoma (PDA) lesion detection in comparison to 120-kVp conventional polyenergetic images (PI).
**Methods and Materials:** Forty-six patients underwent multiphasic pancreatic imaging protocols for PDA were retrospectively identified. PI were reconstructed at 120kVp, and VMI with spectral reconstruction at 40keV($VMI_{40}$), 50keV($VMI_{50}$), 60keV($VMI_{60}$), and 70keV($VMI_{70}$). CT value and noise were measured in the lesion and normal pancreatic parenchyma, SNR and CNR were calculated for all VMIs and PI. Using a 5-point scale (5-excellent; 1-markedly limited), images were qualitatively assessed by 2 radiologists for lesion detection. Paired t test was used to compare the qualitative measures and Wilcoxon signed rank test was used to compare the qualitative assessment.
**Results:** The mean CT value, noise, SNR and CNR were 76.3±50.4HU, 12.5±1.4HU, 6.1±3.9, 7.8±5.9 for $VMI_{40}$; 61.1±33.5HU, 11.4±1.5HU, 5.4±2.9, 5.4±4.3 for $VMI_{50}$; 52.1±23.4HU, 10.9±1.5HU, 4.9±2.2, 3.8±3.2 for $VMI_{60}$; 46.8±17.8HU, 10.6±1.6HU, 4.5±1.8, 2.8±2.6 for $VMI_{70}$; 46.2±18.4HU, 13.2±2.3HU, 3.6±1.5, 2.2±2.1 for PI. $VMI_{40}$ $VMI_{50}$ $VMI_{60}$ revealed significantly higher attenuation values compared to PI (p<0.01). All VMIs showed significantly lower noise levels compared to PI (all p<0.01). SNR and CNR increased from $VMI_{70}$ to $VMI_{40}$, and all were higher than PI (all p < 0.01). In qualitative assessment, $VMI_{40}$ and $VMI_{50}$ demonstrated better in lesion detection than PI (4.8, 4.5 vs 4.0, P<0.05).
**Conclusion:** $VMI_{40}$ and $VMI_{50}$ showed superior image quality in comparison to conventional images and could provide more diagnostic information for PADC lesion detection.

**B-1099** 11:10

**To provide evidence for early diagnosis of pancreatic cancer: magnetic resonance spectroscopy**

X. Fang; *Shanghai/CN (fx0412@foxmail.com)*

**Purpose:** To analyse the metabolites of tumours and peritumoural pancreatic duct intraepithelial neoplasia (Pan IN) tissue in pancreatic cancer by $^1H$ magnetic resonance spectroscopy ($^1H$ MRS) .
**Methods and Materials:** Before operation, respiration-triggered water suppression point-resolved spectroscopy (PRESS) sequence of $^1H$ MRS was used for the detection of metabolites in tumours and peritumoural Pan IN tissue. This study calculated the ratio of choline-containing metabolite (CCM) peak area to lipid (Lip) peak area (CCM/Lip), cholesterol and the unsaturated parts of the olefinic region of fatty acid (Chol+Unsat) peak area to Lip peak area (Chol+Unsat/Lip), Chol+Unsat peak area to CCM peak area (Chol+Unsat/CCM) in every $^1H$ MRS data. The paired sample *t* test was used for comparing metabolites in tumours and peritumoural Pan IN tissue.
**Results:** 24 patients with pancreatic duct adenocarcinoma were confirmed by histopathology. The ratio of CCM/Lip in tumours $(2.66\pm0.84)\times10^{-1}$ was higher than peritumoural Pan IN tissue $(2.00\pm0.81)\times10^{-1}$. The ratio of Chol+Unsat/Lip in tumours $(3.24\pm1.09)\times10^{-1}$ was higher than peritumoural Pan IN tissue $(2.58\pm0.92)\times10^{-1}$. There were statistically significant differences ($P$<0.05). The ratio of Chol+Unsat/CCM had no statistical difference between tumour tissue and peritumoural Pan IN tissue.
**Conclusion:** $^1H$ MRS can distinguish tumours tissue and peritumoural Pan IN tissue in pancreatic cancer. To provide evidence for early diagnosis of pancreatic cancer.

**B-1100** 11:18
**Evaluation of vascular and local lymph node invasion, resectability of pancreatic ductal adenocarcinoma with spectral detector CT**
Y. Yang, Q. Han, X. Chen, H. Dong; *Shanghai/CN (yyz01a15@rjh.com.cn)*

**Purpose:** The aim of this study was to compare the predictive accuracy of 120kVp conventional dataset and spectral dataset derived from spectral detector CT (SDCT) in predicting vascular and local lymph node invasion in patients with pancreatic ductal adenocarcinoma (PDAC).
**Methods and Materials:** Forty-three patients with surgical and pathologic confirmation of PDAC were retrospectively analysed by 2 radiologists independently. All patients had SDCT examination and two setup datasets were created: 120kVp conventional images and spectral images which included virtual mononchromatic image and iodine density image. Peripancreatic arterial invasion, venous invasion and local lymph node invasion were measured using two setup datasets respectively. NCCN criteria guidelines were used in predicting the correlation between the resectability, surgical grade and pathology.
**Results:** The mean sensitivity(Se), specificity(Sp), positive predictive value(PPV), and negative predictive value(NPV) of 120kVp conventional dataset were as follows: 60.0%, 83.3%, 84.2%, and 62.5% for arterial invasion; 63.0%, 87.5%, 89.5.8%, and 58.3% for venous invasion; 56.7%, 84.6%, 89.5%, and 45.8% for lymph node invasion; those results of spectral dataset were 84%, 88.9%, 91.3% and 80.0% for arterial invasion; 85.2%, 93.8%, 95.8%, and 78.9% for venous invasion; 83.3%, 84.6%, 92.6%, and 68.8% for lymph node invasion. To predict tumour resectability using spectral dataset, Se, Sp, PPV and NPV were 92.1%, 80.0%, 97.2%, 57.1% respectively, and compared with surgery and pathology with good agreement (κ = 0.73-0.78).
**Conclusion:** Spectral datasets improve assessment of arterially invasion, venous invasion and lymph node invasion, predictive resectability in PADC with increasing conspicuity and margin sharpness of the lesion.

**B-1101** 11:26
**Perfusion parameters quantified with perfusion CT in pancreatic adenocarcinoma before and after neoadjuvant chemoradiotherapy: comparison between responders and non-responders**
A. Hamdy, Y. Ichikawa, Y. Toyomasu, M. Nagata, N. Nagasawa, H. Sakuma; *Tsu/JP (ahmed.hamdy@kasralainy.edu.eg)*

**Purpose:** To monitor serial changes of blood flow (BF), blood volume (BV) and permeability-surface area product (PS) before and after neoadjuvant chemoradiotherapy (CRT) in patients with pancreatic ductal adenocarcinoma (PDA) using perfusion CT, and to compare tumour perfusion in responders and non-responders to therapy.
**Methods and Materials:** Perfusion CT before and after CRT was acquired in eighteen patients with pathologically proven PDA who underwent pancreatectomy after completing neoadjuvant CRT. Quantitative maps of BF, BV and PS were generated from 4D perfusion images acquired with dual-source CT. After pancreatectomy, patients were categorised into responders and non-responders according to histopathological grade of response to therapy. Correlations among pre- and post-CRT perfusion, histopathological response, CA 19-9 and RECIST were assessed.
**Results:** Baseline BF was significantly higher in responders compared to non-responders to CRT (48±12.4 vs 34.2±18 mL/100g/min, P= 0.03) while no significant difference was found for BV and PS (BV= 5.2±2 vs 3.7±2.7 mL/100g, P= 0.2 and PS= 26.2±6.9 vs 22.3±13.3 mL/100g/min, P=0.4). In responders, all perfusion parameters increased significantly after CRT (BF= 57.6±16.7 mL/100g/min, P= 0.004; BV=7±2.5 mL/100g, P= 0.02 and PS= 33.4±10.2 mL/100g/min, P=0.03). In non-responders, perfusion increase was not significant (BF= 42.7±14.8 mL/100g/min, P= 0.08; BV=4.9±2.4 mL/100g, P= 0.07 and PS= 27.8±10.4 mL/100g/min, P=0.1). RECIST and CA 19-9 showed no correlation with perfusion parameters and correlated poorly with histopathological response (r=0.2)
**Conclusion:** Quantitative perfusion CT is a promising tool for monitoring response to CRT in patients with PDA. Increased perfusion after CRT and higher baseline BF predict response to CRT.
*Author Disclosures:*
**H. Sakuma:** Grant Recipient; DAIICHI SANKYO COMPANY, LIMITED, Fuji Pharma Co., Ltd., FUJIFILM RI Pharma Co., Ltd., Eisai Co., Ltd..

**B-1102** 11:34
**Perfusion vs metabolism: which functional parameter should we choose in patients with pancreatic adenocarcinoma to predict response to chemoradiotherapy?**
A. Hamdy, Y. Ichikawa, Y. Toyomasu, M. Nagata, N. Nagasawa, H. Sakuma; *Tsu/JP (ahmed.hamdy@kasralainy.edu.eg)*

**Purpose:** Perfusion CT and $^{18}$F-FDG PET/CT are used for functional assessment of pancreatic ductal adenocarcinoma (PDA). We aim to compare the ability of both techniques to predict histopathological response of PDA to neoadjuvant chemoradiotherapy (CRT).
**Methods and Materials:** Perfusion CT and PET/CT were performed before CRT in 14 consecutive patients with biopsy-confirmed PDA who were planned for neoadjuvant CRT and surgical resection. Blood flow (BF), blood volume (BV), and permeability-surface area product (PS) were quantified from dynamic perfusion CT covering the entire pancreas acquired with third-generation dual-source CT. $SUV_{max}$ was determined for estimation $^{18}$F-FDG uptake in PDA. Patients were classified as responders or non-responders to therapy according to the histopathological grade of response obtained after pancreatectomy. Baseline perfusion parameters and $SUV_{max}$ were compared between both groups. Correlations among perfusion, $SUV_{max}$ and histopathological response were assessed.
**Results:** Perfusion: responders showed significantly higher BF than non-responders to CRT (48.3±11.4 vs 32.1±12.5 mL/100 g/min, respectively. P= 0.02). Moreover, BF and BV showed significant correlation with histopathological grade of response (BF: r= 0.70, P= 0.005, BV: r= 0.60, P= 0.03). Metabolism: there was no significant difference in baseline $SUV_{max}$ between responders and non-responders (5.5±3.2 and 6.5±5, respectively, P=0.70). $SUV_{max}$ exhibited only weak negative correlations with perfusion and histopathologic response, without statistical significance (BF: r=-0.22, BV: r=-0.31, PS: r=-0.29, response: r= -0.07).
**Conclusion:** Tumour perfusion estimated with perfusion CT is a better functional imaging biomarker than tumour metabolism estimated with PET/CT for the prediction of histopathological response to therapy in patients with pancreatic adenocarcinoma.
*Author Disclosures:*
**H. Sakuma:** Grant Recipient; DAIICHI SANKYO COMPANY, LIMITED, Fuji Pharma Co., Ltd., FUJIFILM RI Pharma Co., Ltd., Eisai Co., Ltd..

**B-1103** 11:42
**CT texture analysis for detection of tumour recurrence in patients resected for pancreatic adenocarcinoma**
A. Mazzaro, G. Zamboni, A. Fighera, A. Campagnola, G. Mansueto; *Verona/IT (andrea.mazzaro1558@gmail.com)*

**Purpose:** To compare the CT texture analysis features of local tumour recurrence and postoperative fibrosis in patients who underwent resection of pancreatic adenocarcinoma.
**Methods and Materials:** We reviewed the CTs of 61 consecutive patients with resected pancreatic adenocarcinoma that had an early detection of solid hypodense tissue in the surgical bed at the after-surgery follow-up CT. For all patients clinical data and further follow-up CTs were available. Two readers in consensus performed texture analysis of the tissue in the first CT by drawing a ROI in the most representative slice in the venous phase. First-order statistics CT texture data (variance, skewness and kurtosis) were extracted using Mazda software. An unpaired T-test was used for statistical analysis.
**Results:** Among the 61 patients, the further follow-up CTs revealed in 29 the development of local tumour recurrence and in 32 post-surgical scar tissue which remained stable in size and imaging features. Mean kurtosis was 0,459 for patients with local recurrence and -0,126 for patients with post-surgical fibrosis, with a significant difference between the two groups (p<0,0001). Mean variance at the first CT was 315,13 for recurrence and 296,70 for fibrosis, with no significant difference between groups (p=0,55). Mean skewness was 0,096 for recurrence and -0,057 for fibrosis, with no significant difference (p=0,67).
**Conclusion:** Kurtosis appeared to be an effective parameter to differentiate between local recurrence and post-surgical fibrosis in patients resected for pancreatic adenocarcinoma. Other first-order statistics from CT texture analysis do not appear useful in the detection of local recurrence.

**B-1104** 11:50
**Locally advanced and metastatic pancreatic cancer: the role of CT quantitative imaging biomarkers for predicting outcomes of patients treated with chemotherapy**
S.-H. Cheng, H.-D. Xue, Z.-Y. Jin; *Beijing/CN*

**Purpose:** The primary aim of this study was to determine if CT texture analysis measurements of the tumor are independently associated with progression-free survival (PFS) and overall survival (OS) in patients with locally advanced or metastatic pancreatic cancer who were treated with chemotherapy.
**Methods and Materials:** 41 patients with locally advanced or metastatic pancreatic cancer who underwent contrast-enhanced CT before chemotherapy between 2014 and 2017 were analyzed with quantification of mean gray-level intensity (Mean), entropy, mean of positive pixels (MPP), kurtosis, standard deviation (SD), and skewness for fine to coarse textures (spatial scaling factor (SSF) 0-6, respectively). The association between pretreatment and posttreatment texture parameters, as well as *Δ* value (difference between posttreatment and pretreatment texture parameters), and survival time was assessed by using Kaplan-Meier analysis and a Cox proportional hazards model.
**Results:** Multivariate Cox model indicated that tumor size, tumor SD (HR, 0.942; 95% CI: 0.898, 0.988) and skewness (HR, 0.407; 95% CI: 0.172, 0.962) measurements with SSF=3, and tumor SD (HR, 0.958; 95% CI: 0.92, 0.997)

Saturday measurements with SSF=4 were significantly and independently associated with PFS, while tumor size and tumor SD (HR, 0.928; 95% CI: 0.882, 0.976) measurements with SSF=3 were significantly and independently associated with OS. None of the post-therapy or*Δ* in texture parameters had a significant association with OS or PFS.
**Conclusion:** Pretreatment CT quantitative imaging biomarkers from texture analysis are associated with PFS and OS in patients with locally advanced and metastatic pancreatic cancer who were treated with chemotherapy.

**10:30 - 12:00** **Room C**

### Radiographers

### SS 1414

### Ultrasound and computer applications in medical imaging

*Moderators:*
N.N.
J. Reponen; Oulu/FI

**B-1105** 10:30
**Effect of adding charcoal capsule to abdominal ultrasound preparation on image quality**
M. Tomehy, M. Wazan, A. Abdul Jabar, E. Abbass, N. Mishah; *Jeddah/SA (mut315@hotmail.com)*

**Purpose:** Patient preparation before abdominal ultrasound plays a major role to ensure better visualization of organs, but abdominal gases in many patients still exist in large amount causing artifacts; it is important to search for safe, efficient, reliable and cost effective method to improve patient preparation to eliminate abdominal gases.
**Methods and Materials:** Prospective Study on randomly selected sample size of 52 adult patients from different ages and mixed gender. Pediatric, pregnant, abdominal surgery, bedridden, and patients with bowel obstruction were excluded from this study. For accepted patients the routine upper abdominal ultrasound with usual preparation fasting instruction for 8 hours was followed, then same group of patients scanned again after 48 hours and instructed to follow the same standard preparation for but, in addition they were asked to take one capsule of charcoal 8 hours. Then we compared ultrasound image quality in abdominal region for the same patient before and after by focusing on pancreas as a reference.
**Results:** Improvement in pancreas head region was 50%, pancreas neck the improvement was 32.5%, pancreas body 57.45% and 32.5% in the pancreas tail. A significant improvement was notices in overweight, obese patients group by 57.2% while in underweight & normal weight the improvement was 39.05%. The improvement in males 66.9% while 49.9% in females. Overall improvement in pancreas visualization was 63%.
**Conclusion:** Active charcoal can be used to reduce gastrointestinal gases in efficient, low cost, safe and ease of use to improve the visualization of adult abdominal ultrasound.

**B-1107** 10:46
**Sonographic estimation of amniotic fluid volume using the amniotic fluid index and the single deepest pocket in a resource-limited setting**
F.A. Burabe[1], G. Luntsi[1], N.I. Chigozie[2], F. Nkubli[1], J.Z. Dlama[1], P.A. Ogenyi[1]; [1]*Maiduguri/NG*, [2]*Awka/NG (Falmata.Burabe@gmail.com)*

**Purpose:** To determine the normal value of amniotic fluid (AF) volume among pregnant women in a Northern Nigerian population and to determine if there is a relationship between AF index (AFI) and single deepest pocket (SDP) with anthropometric variables.
**Methods and Materials:** A prospective cross-sectional study was conducted among singleton pregnant women at late second and third trimester attending the antenatal clinic in Abubakar Tafawa Balewa Teaching Hospital, Bauchi, from December 2016 to April 2017. The mean AFI and SDP were measured by sonography. Participation was voluntary. Descriptive statistics, i.e. mean, standard deviation, and correlation coefficient, were used for the analysis.
**Results:** A total of 206 women, aged between 18 and 40 years, with fetal gestational ages between 22 and 39 weeks were included in the study. The values for AFI in the study ranged from 12 to 28.7 cm, with a mean value of 19.84 ± 3.64 cm, and SDP ranged from 3.7 to 9.1 cm with a mean value of 6.04 ± 1.12 cm. This study found a weak relationship between the anthropometric variables and AFI and SDP and a strong relationship between AFI and SDP with a correlation coefficient of $R = 0.901$ and $P = 0.014$.
**Conclusion:** The mean values for AF volume using AFI and SDP in the studied population were 19.84 ± 3.64 cm and 6.04 ± 1.12 cm, respectively; a strong positive relationship between AFI and SDP; and a negative relationship between body mass index with AFI and SDP.

**B-1108** 10:54
**Relation between fascia and pain by ultrasound**
R.A. Santos, F. Couceiro; *Coimbra/PT (rutemartinssantos@gmail.com)*

**Purpose:** To compare the degree of fascia change through ultrasound/elastography and the pain scale: to evaluate, compare and standardize fascia changes degree and Visual Analog Scale for Pain.
**Methods and Materials:** Thirty individuals without associated pathology were evaluated. The ultrasound B-mode and elastography quasi-static scans were collected from the intersection point of the fascia through analysis of the myofascial vector of the lateromotion carpus (la-ca) The centre of coordination of these vectors is over the muscle belly of the two extensor carpi radialis muscles. Also all participants were asked, using the Visual Analog Scale for pain intensity, in the same intersession point of the fascia, between “no pain” (score of 0) and “worst imaginable pain” (score of 100). For B-mode images, ImageJ software was used to analyse the thickness and echo-intensity and for elastography images, the color fractions were measured with a custom MATLAB routine.
**Results:** In individuals with a higher pain intensity there were found a higher fraction of blue pixels (harder) ($p<0.001$). There are no significant differences in fascia echo-intensity between the individuals with different intensity pain.
**Conclusion:** As the fascia connects with all structures of the body, an imbalance between its components, easily affects any part of the body, causing discomfort or pain. It's fundamental to find a method that helps in the diagnosis of the fascia changes, it was able to evaluate its characteristics. Elastography becomes an attractive method to better understand the fascia, its characteristics and its changes detection, thus aiding in diagnosis.

**B-1109** 11:02
**Real-time sonography as a teaching aid of anatomy in undergraduate radiography institutions in Northern Nigeria**
M.S. Umar[1], D. Joseph[2], A. Auwal[3], S. Charbel[4], P.A. Ogenyi[1], G. Njokwu[1], M.A. Fati[3], A.S. Moi[3], S.L. Shem[5]; [1]*Bauchi/NG*, [2]*Kano/NG*, [3]*Maiduguri/NG*, [4]*Beirut/LB*, [5]*Gombe/NG (umar.sani888@gmail.com)*

**Purpose:** To assess real-time ultrasound scanning as a teaching aid of human anatomy, and to determine what teaching methods radiography students consider effective for understanding human anatomy.
**Methods and Materials:** One hundred and ten self-administered, structured and pretested questionnaires were distributed to clinical radiography students in Northern Nigeria. The questionnaire consisted of two sections: demographics and preferred methods of delivery of anatomical information. Participation was voluntary. Comparisons among teaching methods were made using repeated measures ANOVA.
**Results:** With Duncan’s multiple range test (MRT), it is clearly shown that 3D radiology imaging differ with mean value (5.2522) followed by computer programs (5.1292), anatomic models (4.7593), lab videos (4.5815), textbooks (4.5358), animal dissection (4.2568), lectures (3.2568) and finally ultrasound scan (3.6087), ($P<0.0001$).
**Conclusion:** This study shows that 3D radiology imaging is the most preferred method of delivering anatomical information and ultrasound scanning is the least preferred method.

**B-1110** 11:10
**Muscular changes with ageing: ultrasound evaluation**
R.A.M. Santos[1], H.I. Ferraz[2]; [1]*Coimbra/PT*, [2]*Peso da Régua/PT (rutemartinssantos@gmail.com)*

**Purpose:** To analyse and characterise the muscle in elderly individuals by ultrasound and to verify muscle changes with age.
**Methods and Materials:** 40 elderly participants, with a mean age of 80 years, were submitted to an ultrasound examination for quadriceps muscle characterisation. The quadriceps muscle was evaluated in a longitudinal view, using the same parameters in all acquisitions. All the images were analysed by Image J software and the results were obtained using SPSS v.24 software. The quadriceps muscle in elderly individuals showed an increase in echo-intensity values and a decrease in thickness values when compared with an young population. In participants with more advanced age the values were more evident.
**Results:** The quadriceps muscle in elderly individuals showed an increase in echo-intensity values and a decrease in thickness values when compared with an young population. In participants with more advanced age the values were more evident.
**Conclusion:** The ageing significantly affects human skeletal muscle architecture. These structural alterations are expected to have implications for muscle function in old individuals.

**B-1111** 11:18

**The role of obstetric ultrasound in low resources setting: pilot survey done in Elgeyo-Marakwet and Kericho county from 2016 March to 2018 December**

K. Clive; *Kericho/KE (kosgeykips@gmail.com)*

**Purpose:** There are vast inequalities across maternal and infant mortality with the developing world accounting for the majority of the burden and within countries rural areas expecting worse outcomes than urban. This review focuses on obstetric ultrasound service and its potential to improve maternal and newborn health in low resource settings. Recent advances in affordability, durability, and portability have brought ultrasound to the forefront as a sustainable and high impact technology for use in developing world clinical settings as well.
**Methods and Materials:** Ultrasound services were introduced at Elgeyo and Kericho county Data sheets for each ultrasound scan performed during routine clinical care were collected and analyzed to determine patient demographics, which ultrasound applications were most frequently used, and whether the use of the ultrasound changed patient management plan
**Results:** Adult women appeared to benefit most from the presence of ultrasound services. During these outreaches a total of 1029 women were scanned. Twenty percent of these scans showed abnormalities. These woman were referred to the nearest health center or hospital where they received more check-ups.
**Conclusion:** We suggest ultrasound is a useful modality that particularly benefits women's health and obstetrical care in the developing world and it feasible,factual and accurate.

**B-1113** 11:34

**An ethnographic study to investigate general practitioner's opinion of ultrasound services in primary care**

M. Boland, L.A. Rainford, C. Byrne, M.-L. Ryan; *Dublin/IE (mairead.boland@ucdconnect.ie)*

**Purpose:** In Ireland, patients may wait 42 weeks for an ultrasound, which is detrimental to patient workflow and delays diagnosis. Research investigating increased imaging access in primary care shows that timely imaging corresponds to decreased unnecessary admissions to Emergency Departments (ED). GP opinions were sought of direct access ultrasound on; waiting lists, patient management, referral pathways and determining GP satisfaction with radiology report content and radiology accessibility for further consultation.
**Methods and Materials:** An ethnographic approach was applied to survey two groups of GPs - one who have direct access to ultrasound within a primary care setting and GPs who refer to regional hospitals with long waiting lists. Semi-structured interviews were completed, themes were identified and GP's responses compared.
**Results:** GPs with direct access (n=12) reported waiting times of 2/3 weeks compared to several months for hospital referrals. This group indicated the benefit of patients remaining within primary care, reporting potential reductions in unnecessary ED and consultant referrals and enhanced management of gynaecological conditions and abdominal presentations. GPs considered patient's preference, time to access services and ease of travel when referring. 50% of GP's with direct access indicated satisfaction with radiology reports and radiology accessibility, the remainder highlighted the importance of radiology guidance within reports.
**Conclusion:** Direct access to diagnostics was evidenced as beneficial to both patients and GPs in reducing waiting times and providing efficient diagnosis. Radiology guidance and consultation availability was highlighted as important for primary care imaging service users to support efficient patient management pathways.

**B-1114** 11:42

**The effect of deep learning reconstruction on calcium scoring**

C. Steveson[1], J.L. Schuzer[2], T. Acharya[3], K. Bronson[3], S. Rollison[3], W. Bandettini[3], S. Shanbhag[3], M.Y. Chen[3]; [1]*Kanagawa/JP*, [2]*Vernon HIlls, IL/US*, [3]*Bethesda, MD/US (chloe.steveson@medical.canon)*

**Purpose:** Assessing coronary artery calcium (CAC) is a valuable tool in individualizing cardiac risk assessment. Deep convolutional neural network image reconstruction has the potential to improve image quality. The aim of this study is to investigate the effect of a deep learning image reconstruction algorithm on Agatston calcium score accuracy and signal to noise ratio (SNR) in CAC scanning.
**Methods and Materials:** With institutional ethics approval, 40 consecutive patients underwent 2 EKG-gated coronary calcium score scans (120kV, 0.275s rotation speed, 100-140mm range, mA was calculated using automatic exposure control). Each scan was reconstructed with conventional filtered back projection (FBP) and deep learning reconstruction. Agatston score was calculated on a per patient basis using standard techniques utilizing a 130 HU threshold. SNR was calculated for each reconstruction. Data was analyzed using linear regression and paired t-test.
**Results:** Patients averaged 63 ± 15 years old, 45% were male, and the median radiation exposure was 0.88 mSv (interquartile range 0.62-1.22). The median Agatston score was 61.7 (inter quartile range 0-315) with clinical FBP reconstruction. Deep learning reconstruction had excellent agreement with FBP (R=0.99) over a wide range (0-4121); however, it underestimated calcium score by 6%. SNR for deep learning reconstruction improved 37.1% compared to FBP (3.24 ± 0.43 vs. 2.46 ± 0.39, p<0.00001).
**Conclusion:** Deep learning image reconstruction has an excellent correlation with conventional FBP reconstruction for CAC imaging at standard radiation doses. The improved image quality and higher SNR may enable lower radiation while maintaining accurate Agatston scores.
*Author Disclosures:*
**C. Steveson:** Employee; Canon Medical Systems Corporation. **J.L. Schuzer:** Employee; Canon Medical Systems Corporation. **M.Y. Chen:** Research/Grant Support; Canon Medical.

**B-1115** 11:50

**Radiographer as RIS/PACS administrator: promoting professional networking and continuing professional education by the society of radiographers in Finland**

H. Syysmäki[1], P. Wood[2], R. Laaksonen-Heikkilä[1]; [1]*Turku/FI*, [2]*Helsinki/FI (hanna.syysmaki@edu.turkuamk.fi)*

**Purpose:** The purpose of this study was to investigate the background information about Finnish PACS and RIS -administrators and what are their needs of professional networking and continuing professional education. This study was an empirical part of the master's thesis performed at Turku University of Applied Sciences.
**Methods and Materials:** A quantitative questionnaire survey was conducted in 2017. The questionnaire developed for the study was sent to all members of the Society of Radiographers in Finland (SORF), but targeted only to radiographers who works as PACS and RIS -administrators. Estimated number of Finnish PACS- and RIS -administrators were 60 persons. In total, 37 responses were received.
**Results:** The results of the questionnaire showed, that there is not enough continuing professional education for PACS and RIS administrators in Finland. In addition, the content of continuing professional education did not adequately support or enhance their professional competence. Professional support works best among the workers who work for the same employer. Networking seems to be valued and therefore respondents were interested in a wider professional network. However, there were also reservations about the benefits of networking through the professional division.
**Conclusion:** Finnish PACS and RIS administrators need more continuing professional education that adequately support and enhance their professional competence. Opportunities for professional support and networking should be developed.

**10:30 - 12:00** **Room N**

### Hybrid Imaging

### SS 1406

### Advanced methods in experimental radiology

*Moderators:*
S. Gatidis; Tübingen/DE
F.M.A. Kiessling; Aachen/DE

**B-1116** 10:30

**Spectral photon-counting CT multi-phase liver imaging with dual contrast agent**

S.A. Si-Mohamed[1], V. Tatard-Leitman[1], P. Coulon[2], P. Douek[1], P.B. Noel[3], L. Boussel[1]; [1]*Bron/FR*, [2]*Suresnes/FR*, [3]*Munich/DE (salim.si-mohamed@chu-lyon.fr)*

**Purpose:** To demonstrate the feasibility of dual-contrast multiphase liver imaging via K-edge imaging using Spectral Photon-Counting CT (SPCCT).
**Methods and Materials:** *In-vivo* experiments were conducted on 4 rabbits after approval by the local ethics committee. Data acquisition was performed at 120 kVp and 100 mA. The protocol was to inject the iodine contrast agent (400 mg/mL, 1.5 mL/kg) 21 seconds before the injection of gadolinium contrast agent (0.5 M, 5 mL/kg). The acquisition was performed 10 seconds thereafter. Conventional HU, and quantitative iodine and gadolinium images were analyzed.
**Results:** SPCCT allowed the discrimination between the two contrast agents; gadolinium led to an enhancement of the arteries and Iodine of the liver parenchyma and portal veins. The attenuation values (HU) and concentrations

(mg/mL) of contrast agents were calculated for the aorta (CT = 1103.1 ± 3.2; gadolinium = 20.5 ± 0.5; iodine = 6.9 ± 0.2), hepatic arteries (CT = 605.6 ± 7; gadolinium = 8.5 ± 1.3; iodine = 0.7 ± 0.2), portal vein (CT = 247.3 ± 3.3; gadolinium = 0 ± 1.3; iodine = 4.2 ± 0.3) and liver parenchyma (CT = 145.3 ± 1.5; gadolinium = - 0.3 ± 0.9; iodine = 2.1 ± 0.1). Finally, the pharmacokinetics of the two contrast agents were analyzed and found to be similar to those expected.
**Conclusion:** SPCCT allows *in vivo* dual contrast qualitative and quantitative multi-phase liver imaging in a single acquisition. A single acquisition has the major advantage of reducing patients' exposure to ionizing radiation.
*Author Disclosures:*
**P. Coulon:** Employee; Philips Healthcare.

**B-1117** 10:38
**Spectral CT of cancer cells with nanoparticles: in vitro and in vivo results**
M. Moghiseh, D. Kumar, R. Aamir, C. Lowe, K. Chitcholtan, P. H Butler, P. Sykes, A.P. Butler, N. Anderson; *Christchurch/NZ (mahdieh_moghiseh@yahoo.com)*

**Purpose:** To determine if spectral CT can identify and quantify gold nanoparticles (AuNPs) targeted to specific cancer cells in vitro and in vivo. The long-term goal is to evaluate tumour response to treatment and to facilitate anti-cancer drug development.
**Methods and Materials:** Initially, two ovarian cancer cell lines (OVCAR5 and SKOV3) were studied. To evaluate the effect of AuNP size, plates of 600,000 of each cell line were incubated with four sizes particles (18, 40, 60 and 80nm) at four concentrations (6.4, 12.8, 25.8 and 38.5μg/mL). The cell plates were imaged with a MARS spectral CT working in clinical x-ray energy range. A second experiment was performed to validate the quantification method in a mouse model. The gold quantification results were confirmed using phantoms containing oil, water, gold chloride, and hydroxyapatite.
**Results:** The first experiment demonstrated that uptake of AuNPs into ovarian cancer cells varies according to particle size and cell type. The best size particle to detect SKOV3 were 18 and 80nm. However, uptake of the same size of particles was very low for OVCAR5 cells. Based on these results, 18 and 80nm particles were injected into the mouse model of ovarian cancer cell (in vitro results are under investigation).
**Conclusion:** This study has established a methodology to quantify drug delivery to specific ovarian cell. This is proof of principle that drug delivery to cells can be measured using non-invasive spectral CT. Our methodology is designed to accelerate advances in cancer research and translate it to human imaging using spectral CT.

**B-1118** 10:46
**Quantification of gadolinium with 3rd generation dual source dual energy CT: role of spectral separation**
A. Agostini, A. Borgheresi, A. Mari, A. Lorenzoni, L. Ottaviani, S. Maggi, A. Giovagnoni; *Ancona/IT (andrea.agostini84@yahoo.it)*

**Purpose:** To evaluate feasibility, accuracy and the role of spectral separation in Gadolinium quantification with a 3rd generation Dual Source Dual Energy CT (dsDECT) on a phantom model.
**Methods and Materials:** Preparation of phantom (Electron Density Model 062M, CIRS, USA) with 5 inserts containing scalar dilutions in distilled water solution (0.00375M- 0.05M) of Gadolinium chelate (Gd-BOPTA 0.5M, MultiHance, Bracco). Scanning parameters: 3rd generation dsDECT (Somatom Force, Siemens Healthineers); preliminary acquisitions at 70, 80, 90, 100, 140, Sn150 kV; Dual Energy acquisitions 70-100/Sn150 kV, 80/140 kV; modulated mA; rotation time 0.5s; pitch 0.7; slice thickness/spacing 2/1 mm. Dual energy Indexes (DEI) were calculated with 5 identical cloned regions of interest (ROI) on each insert on preliminary acquisitions (ImageJ 1.52a, NIH, United States) and linear regression model (MedCalc v12.5, MedCalc Software). Gadolinium densities within inserts were calculated on Dual Energy acquisitions with Liver VNC (Syngo.via Frontier, Siemens Healthineers) and compared with actual Gd concentrations with Bland-Altman Analysis. Non-parametric tests were used with significant p<0.05.
**Results:** Linear regression models provided DEI ranging between 1.64 at 80/140 kV and 2.56 at 70/Sn150 kVp ($R2>0.999$; $p<0.001$). Quantification of gadolinium densities on dual energy acquisitions provided percentage errors of 7%-37% for acquisitions at 70/Sn150 kV and of 16%-76% at 80/140 kV with significant differences (Friedman p = 0.024) and a trend to increase at lower concentrations and spectral separation.
**Conclusion:** Gadolinium quantification is feasible with 3rd generation dsDECT with increasing accuracy at wider spectral separation and higher concentrations.

**B-1119** 10:54
**Quantitative evaluation of nonalcoholic fatty liver disease using normalised local variance US in a rat model**
J. Bae, J. Lee, D. Lee, H. Kim, J. Han; *Seoul/KR (jaeseok.bae@gmail.com)*

**Purpose:** To identify factors that influence the speckle pattern of hepatic parenchyma on ultrasonography (US) using a normalized local variance (NLV) technique in a dietary-induced nonalcoholic fatty liver disease rat model using histopathologic examination and magnetic resonance spectroscopy (MRS) as standards of reference.
**Methods and Materials:** Sixty-four male Sprague-Dawley rats were fed a methionine-choline-deficient diet with variable periods (0, 2, 4, 6, 8, 10, 12 days or 2, 3, 4, 5, 6, 8, or 10 weeks; four rats in each group except for eight rats in 8 and 10 weeks groups). At the end of each diet duration, NLV US and MRS were performed. Thereafter, the rats were sacrificed, and livers were histopathologically evaluated. The Spearman correlation was performed to assess the relationship between the NLV values and steatosis or fibrosis, and multivariate linear regression was used to determine factors for the NLV values.
**Results:** There was a negative correlation between the NLV value and the hepatic steatosis (Spearman ρ, -0.570 and -0.534 for histopathologic examination and MRS, respectively; P < .001 for both). The NLV value and the hepatic fibrosis showed negative correlation as well (Spearman ρ, -0.616; P < .001). Multivariate linear regression revealed that the stage of hepatic steatosis and fibrosis (P = .002 for both) were significant factors affecting the NLV values.
**Conclusion:** The NLV values have a negative correlation with the fat fraction measured at histopathologic examination and MRS. The stage of hepatic steatosis and fibrosis were significant factors that affected the NLV values.
*Author Disclosures:*
**J. Bae:** Grant Recipient; Canon Medical Systems. **J. Lee:** Grant Recipient; Canon Medical Systems. **D. Lee:** Grant Recipient; Canon Medical Systems.

**B-1120** 11:02
**Dual-targeting and execrable ultrasmall SPIONs for T1-weighted-positive MR imaging of intracranial glioblastoma by targeting lipoprotein receptor-related protein**
C. Du, X. Liu, H. Li, L. Yu, D. Geng, Y. Chen, J. Zhang; *Shanghai/CN (1944465685@qq.com)*

**Purpose:** To more precisely delineate the boundary of intracranial glioblastoma pre-surgical operation, a multifunctional-targeted nanoprobe composed of PEGylated ultrasmall superparamagnetic iron oxide nanoparticles (USPIONs) with surface-conjugated Angiopep-2 was successfully stepwise constructed, which efficiently crossed the blood-brain barrier (BBB), targeted the glioblastoma and then generated positive contrast enhancement for $T_1$-weighted MR imaging.
**Methods and Materials:** The targeting capability and especially the biocompatibility/excretion of these ANG-modified MRI nanoprobes were systematically evaluated not only at intracellular level in vitro but also on tumour xenografts in vivo.
**Results:** The relaxivity $r_1$ was calculated to be as high as 4.68 $mM^{-1}s^{-1}$ and the relaxivity $r_2$ was determined to be 15.96 $mM^{-1}s^{-1}$, resulting in a relatively low $r_2/r_1$ ratio ($r_2/r_1 = 3.41$). For targeting ANG/PEG-USPIONs group, the enhanced bright contrast of tumour in $T_1$-weighted images was observed distinctly, and the boundary of tumour was delineated much clearer. The optimal $T_1$-weighted MR contrast enhancement of the tumour was at 1.5 h post-injection of ANG/PEG-USPION nanoprobes, and the enhanced signal intensities of ANG/PEG-USPION nanoprobes were maintained for at least 4 h, which was much longer than commercial Gd-DTPA contrast agents (1 h).
**Conclusion:** The as-constructed dual-targeting ANG/PEG-USPION nanoprobes has relatively high $r_1$ relaxivity and could be utilized as positive contrast agent for MR imaging of intracranial glioblastoma.

**B-1121** 11:10
**EGFR-targeted liposomal nanohybrid cerasomes: theranostic function and immune checkpoint inhibition in a mouse model of colorectal cancer**
Y. Li, Y. Du, X. Liang, H. Xue, Z. Jin; *Beijing/CN (shandongliyuan1988@126.com)*

**Purpose:** Epidermal growth factor receptor (EGFR) is a major target for the treatment of colorectal cancers (CRCs), and programmed death ligand-1 (PD-L1) is an attractive target for CRC immunotherapy.
**Methods and Materials:** Herein, we report the synthesis of porphyrin-containing liposomal nanohybrid cerasomes decorated with cetuximab, an anti-EGFR antibody, and conjugated with IRDye800CW and MRI-contrast DOTA-Gd, to enable *in vivo* tumour detection and photodynamic therapy (PDT). Moreover, PD-L1 was added as an adjuvant therapy. The antitumour efficacy of PDT combined with PD-L1 immunotherapy was assessed.

**Results:** EGFR-targeted nanoparticle showed targeted imaging of tumours. EGFR-targeted PDT combined with PD-L1 immunotherapy was more effective against tumour growth than simultaneous albeit non-targeted nanoparticle delivery with laser irradiation plus PD-L1 immunotherapy.
**Conclusion:** Thus, EGFR-targeted nanoparticle exhibited significant potential toward dual-modality imaging-guided precise PDT, combined with immunotherapy.

**B-1124** 11:18
**Cell death PET imaging of rat liver cirrhosis with $^{68}$Ga-NOTA-PEG$_3$-Duramycin**
S. Shu, X. Xianhong, T. Ganghua; *Guangzhou/CN (15626214090@163.com)*

**Purpose:** The aim was to assess the value of $^{68}$Ga-1,4,7-triazacyclononane-1,4,7-triaceticacid (NOTA)-tripolyethylene glycol (PEG$_3$)-Duramycin ($^{68}$Ga-NOTA-PEG$_3$-Duramycin) positron emission tomography (PET) imaging of liver cirrhosis in a rat model.
**Methods and Materials:** Male Sprague-Dawley rats (160~180g) were used to establish liver cirrhosis rat model via intraperitoneal injection of thioacetamide (TAA), control rats were treated with saline (n=3 per group). At week 10 of TAA administration, PET/CT with $^{68}$Ga-NOTA-PEG$_3$-Duramycin or $^{18}$F-fluorodeoxyglucose ($^{18}$F-FDG) was performed on TAA-induced and control rats. The liver accumulation of $^{68}$Ga-NOTA-PEG$_3$-Duramycin or $^{18}$F-FDG, illustrated as percentage injected dose per gram (%ID/g) of tissue. Liver tissues were harvested and analyzed for histologic examination after PET/CT imaging. Binding assays of $^{68}$Ga-NOTA-PEG$_3$-Duramycin with Juakat cells was preformed. Data were analyzed by Student t test.
**Results:** Binding assays showed that $^{68}$Ga-NOTA-PEG$_3$-Duramycin accumulated in both apoptotic and necrotic cells. After treatment with TAA or saline for 10 weeks, model rats showed a significantly higher liver accumulation of $^{68}$Ga-NOTA-PEG$_3$-Duramycin than control rats (P<0.05). In TAA-treated rats, the liver-to-muscle uptake ratio of $^{68}$Ga-NOTA-PEG$_3$-Duramycin was also significantly higher than that of $^{18}$F-FDG (P<0.05). Over-proliferation of fibroblasts and deposition of collagen was seen in TAA-induced rat liver samples sections via histologic examination. Compared with control rats, TAA-treated rats had higher liver hydroxyproline content (P<0.05). Immunofluorescence staining showed more apoptotic cells in the TAA-treated rats than those in the control rats.
**Conclusion:** $^{68}$Ga-NOTA-PEG$_3$-Duramycin PET/CT imaging could be applied for noninvasive diagnosis of liver cirrhosis in a rat model.

**B-1125** 11:26
**In vivo tracking of tumour-derived exosomes correlates with premetastatic cell infiltration to target-sites of metastasis**
M. Gerwing, V. Kocman, A. Helfen, M. Stölting, L. Greune, A. Schmidt, W. Heindel, M. Wildgruber, M. Eisenblaetter; *Münster/DE (Mirjam.Gerwing@ukmuenster.de)*

**Purpose:** Exosomes, small vesicles carrying inter alia proteins, miRNA and RNA, are important mediators in intercellular communication. The purpose of this project was to assess the *in vivo* biodistribution of exosomes from highly malignant breast cancer cells in comparison to exosomes from the serum of healthy mice, and their effect on the immune cell infiltrate in target organs of metastasis.
**Methods and Materials:** Exosomes were isolated from the tissue culture supernatant of highly malignant 4T1 breast cancer cells or the serum of healthy BALB/c mice. The purity of the isolate was checked by electron microscopy and western blotting. After labeling with the fluorescent dye DiR (750/780 nm), exosomes were injected i.v. into healthy BALB/c mice and their distribution was assessed using fluorescence-reflectance imaging (FRI). After *ex vivo* imaging of the organs, lungs and spleen were stained for FACS analysis of granulocytes, T- and B-cells to identify changes in the immune cell content.
**Results:** The assessment of the *in vivo* distribution of DiR-labelled exosomes with FRI showed exosomes from highly malignant 4T1 cells to preferentially accumulate in the target organs of metastasis, in this case lung, liver and spine (tumour-exosomes vs. serum-exosomes: lung 18.6 vs. 10.4, p=0.01; liver 72.2 vs. 56.5, p=0.02; spine 5.1 vs. 3.5, p<0.01). Furthermore, an increased infiltration of immune cells in these target-organs of metastasis after injection of tumor-derived exosomes was identified.
**Conclusion:** Exosome accumulation, which promotes the infiltration of immune cells in target-organs of metastasis, can be visualized by FRI.

**B-1126** 11:34
**Orthotopic hepatocellular carcinoma: molecular imaging-monitored intratumoural hyperthermia-enhanced direct oncolytic virotherapy**
J. Song[1], F. Zhang[1], J. Ji[1], M. Chen[1], Q. Weng[1], X. Yang[2], M. Xu[1], C. Lu[1], Z. Zhao[1]; [1]*Lishui/CN*, [2]*Seattle, WA/US*

**Purpose:** To validate the feasibility of molecular imaging-monitored intratumoural radiofrequency hyperthermia (RFH)-enhanced direct oncolytic virotherapy for hepatocellular carcinoma (HCC).
**Methods and Materials:** This study included *in vitro*confirmation experiments using luciferase-labeled rat HCC cells and *in vivo*validation experiments on rat models with orthotopic HCCs. Both cells and HCCs in four groups (n=6/group) were treated by: (1) combination therapy of oncolytic virotherapy (T-VEC) plus RFH at 42℃for 30 minutes; (2) oncolytic virotherapy alone; (3) RFH alone; and (4) saline. For *in vitro*confirmation, confocal microscopy andbioluminescence optical imagingwere used to evaluate the cell viabilities. For *in vivo*validation, oncolytic viruses were directly infused into rat HCCs through a multi-functional perfusion-thermal RF electrode, followed by RF hyperthermia. Ultrasound and optical imaging were used to follow up size and bioluminescence signal changes of tumours overtime, which were correlated with subsequent laboratory examinations.
**Results:** For in vitro experiments, confocal microscopy showed the lowest number of viable cells, as well as a significant decrease of bioluminescence signal intensity of cells with combination therapy group, compared to other three groups (P < .001). For in vivo experiments, ultrasound and optical imaging showed the smallest tumour volume, and significantly decreased bioluminescence signal intensity in combination therapy group compared to other three groups (*P*< .05), which were well correlated with pathologic analysis.
**Conclusion:** It is feasible to use molecular imaging to guide RFH-enhanced intratumoural oncolytic virotherapy of HCC, which may open new avenues for preventing persistency or recurrence of RFA-treated intermediate-to-large HCCs.

**10:30 - 12:00** **Room O**

### Musculoskeletal

### SS 1410

### Knee imaging

*Moderators:*
R. Guggenberger; Zurich/CH
V. Vasilevska-Nikodinovska; Skopje/MK

**B-1127** 10:30
**Super-resolution reconstruction of knee MR images**
E. De Smet, B. Jeurissen, J. Sijbers, F. Vanhevel, P.M. Parizel, P. Van Dyck; *Antwerp/BE (elinedesmet@hotmail.com)*

**Purpose:** Super-resolution reconstruction (SRR) combines several distinct low-resolution images into a single high-resolution image. This study explores the feasibility of SRR for isotropic 3D knee MRI and compares this method to direct 3D acquisition.
**Methods and Materials:** Seventeen subjects (3 volunteers and 14 patients with knee pain) underwent 3T MRI with sagittal 2D TSE thick-slices (TR/TE=3080/36ms, voxel size=0.5×0.5×2.0mm$^3$, number of slices=53, scan time=1min55s). The experiment was repeated 7 times, while the slice stack was rotated around the phase encoding direction in increments of 26 degrees (0°, 26°, 51°, 77°, 103°, 129°, 154°). SRR was performed to an isotropic high-resolution grid with voxel size 0.5×0.5×0.5mm$^3$. Sagittal 3D TSE acquisition was also obtained (TR/TE=1300/38ms, voxel size=0.5×0.5×0.5mm$^3$, number of slices=208, scan time=12min42s). Two radiologists evaluated subjective image quality using a Likert scale. Volunteers and a phantom (Newmatic Medical, MI, USA) were scanned twice for quantitative image evaluations. Findings were analysed using the Friedman test (p<0.05).
**Results:** Overall image quality and visibility of anatomic structures were significantly better with SRR than with 3D TSE, exhibiting blurring artefacts and poor low-contrast object detectability (p<0.05). Meniscal lesions were better depicted with SRR (p<0.05), whereas observer ratings for cartilage and ligamentous lesions were similar. Relevant SNRs and CNRs were significantly higher for SRR than for 3D TSE (p<0.05). Clear improvement in resolution achievable by SRR was also confirmed by the edge response curve in the phantom study.
**Conclusion:** SRR of knee MRI is feasible and represents a promising alternative for high-resolution isotropic 3D imaging of the knee.

**B-1128** 10:38
**Identification of bone marrow oedema and osteochondral injuries of the knee: diagnostic accuracy of dual-energy CT and virtual non-calcium techniques**
G. Foti[1], M. Catania[2], A. Beltramello[1], G. Carbognin[1]; [1]*Negrar/IT*, [2]*Verona/IT (gfoti81@yahoo.it)*

**Purpose:** To prospectively evaluate the diagnostic accuracy values of dual-energy computed tomography (DECT) to identify bone marrow oedema and osteochondral injuries of the knee.
**Methods and Materials:** This prospective study included 33 consecutive patients studied with DECT (80 kV and tin filter 150 kV) and MRI with short tau inversion recovery images (STIR) within 7 days. DECT data were post-processed on a dedicated offline workstation (SyngoVia®) using a three-material decomposition algorithm. Two experienced radiologists, blinded to MRI, evaluated the presence of abnormal attenuation of each knee on DECT maps. STIR images served as the standard of reference. Diagnostic accuracy values of the DECT maps and of the CT numbers using receiver operator curves (ROC) and inter- and intra-observer agreements were calculated. A value of $p<0.05$ was considered statistically significant.
**Results:** MRI revealed the presence of bone marrow oedema of the knee in 17/33 cases (51.6%), with 10 osteochondral injuries. DECT numbers were significantly different between positive (mean -5.5 ± 32.6HU) and negative cases (mean -72.4 ± 41.6HU) with a p value <0.001. Using -15HU cutoff to identify bone marrow oedema, the sensitivity, specificity, PPV and NPV and accuracy of DECT were 89.5, 92.9, 94.4, 86.6 and 90.9%, respectively. The inter-observer and intra-observer agreements were near perfect (k=0.82 and k=0.86, respectively).
**Conclusion:** DECT represents a reliable imaging tool for demonstration of bone marrow oedema and osteochondral injuries of the knee.

**B-1129** 10:46
**Dixon imaging of bone stress injury of the knee: comparison to conventional proton density-weighted MR imaging**
A. Chhabra[1], P. Pezeshk[1], R. Dessouky[2], O. Morales[1], J. Shah[1];
[1]*Dallas, TX/US,* [2]*Zagazig/EG*

**Purpose:** Dixon sequence is a chemical shift-based MR sequence that produces four sets of images (water only, fat only, in-phase, and out-of-phase) and is currently being used in various musculoskeletal applications. The aim of this study was to compare the extent of lesion detection and inter-reader performance of Dixon versus conventional proton density-weighted (PDW) imaging in bone stress injuries (BSI).
**Methods and Materials:** In this retrospective cross-sectional study, 32 consecutive BSI were compared on both conventional versus Dixon images. Both sequences were compared in terms of area of bone marrow oedema, inter-trabecular fracture detection, and fracture conspicuity. Inter-reader reliability was also evaluated. p value <0.05 was considered statistically significant.
**Results:** Dixon images showed excellent quality except in two cases with some motion degradation. BSI detection on Dixon water image was equivalent to the routine fat-suppressed PDW sequence. BSI area on water image was not significantly different from the inter-trabecular lesion area on opposed-phase imaging (p=0.9531). Opposed-phase images detected more number of fractures than the water, PDW and in-phase images (p value <0.0001, =0.0008 and <0.0001, respectively) with superior fracture conspicuity than water, PDW and in-phase images (p value <0.0001, =0.0085 and =0.0035, respectively). Inter-reader agreement was excellent for bone marrow area measurements and fair to moderate for fracture detection and conspicuity.
**Conclusion:** Dixon imaging is as sensitive as conventional fat-suppressed PDW imaging of the knee for the identification of bone bruise with superior detection and improved characterization of the inter-trabecular fractures on opposed phase images.
*Author Disclosures:*
**A. Chhabra:** Consultant; ICON Medical, Not related to this abstract. Other; Royalties, Jaypee and Wolters, Not related to this abstract.

**B-1130** 10:54
**Evaluation of MRI findings for association between patella baja and quadriceps fat pad oedema**
S. Sajid[1], P. Nepal[2], S.I. Alam[1], M. Warfa[1], S. Sajid[1]; [1]*Doha/QA,* [2]*Connecticut, CT/US (dia_saji@yahoo.com)*

**Purpose:** Quadriceps fat pad oedema is a finding, commonly overlooked. So far, no study could be found in literature describing the association between patella baja and quadriceps fat pad oedema. The purpose of our study is to determine the association between patella baja and quadriceps fat pad oedema.
**Methods and Materials:** This was an institute ethics committee approved study carried out in tertiary center for Bone and Joint disease at Doha, Qatar. Retrospective case control study was performed between September 2017 to August 2018; selecting 50 patients in each study and control group. MRI findings of 50 patients with Patella Baja were compared with 50 patients with normal patella. Presence or absence of quadriceps fat pad oedema was evaluated in both groups. Patellar position was assessed on T1 weighted sagittal sequence using Insall-Salvati ratio; whereas quadriceps fat pad oedema was evaluated on sagittal fat suppressed Proton Density sequence. Patients with trauma and contracture deformities were excluded from the study.
**Results:** Mean age of patients was 39 years (range 25-56). 82 were male and 18 were female. Indication of performing knee MRI was pain in all cases. Out of 50 cases with patella baja; 41 cases (82%) were found to have significant oedema in quadriceps fat pad. In cases with normal position of patella, only 6 cases (12%) were found to have oedema in quadriceps fat pad.
**Conclusion:** Patella baja is strongly associated with oedema in quadriceps fat pad.

**B-1131** 11:02
**Integrated analysis reveals disturbed metabolic pathway and identifies potential biomarker panel for the prediction of cartilage degeneration**
Y. Hu, H. Tao, S. Chen; *Shanghai/CN (huyiwen2016@163.com)*

**Purpose:** The purpose of this study was to identify potential synovial fluid (SF) biomarkers for diagnosis and monitoring the early course of cartilage degeneration using integrated analysis.
**Methods and Materials:** Cartilage degeneration was surgically induced by anterior cruciate ligament (ACL) transaction in right knee of twelve New Zealand White rabbits. MRI scanning and SF sample collection was performed pre-surgery, and post-surgery at time points of 4, 8 weeks. Ultra-performance liquid chromatography - mass spectrometry combined with multivariate statistical analysis was used to distinguish between samples from ACL-transaction knees and healthy controls. UTE- T2* measurements, pathway analysis and receiver operating characteristic analysis were also performed to select the potential metabolite biomarkers.
**Results:** UTE- T2* values of lateral tibia (TL) cartilage showed significant decreases over the study period. Statistical analysis of metabolic data found a total of 103 identified metabolites were markedly different between the three groups, particularly those related to the lipid and amino acid metabolisms. Furthermore, the SF levels of 24 metabolites were found to be inversely correlated with UTE- T2* values of TL, while hippuric acid positively correlated with quantitative MRI. Among the 25 potential markers, N1-acetylspermidine, 2-amino-1,3,4-octadecanetriol, L-phenylalanine, 5-hydroxy-L-tryptophan, L-tryptophan, hexanoylcarnitine were identified as potential biomarkers with high area under the curve values.
**Conclusion:** Taken together, we identified the six small molecules in SF, which exhibited inflammatory and oxidant-related property, as promising biomarkers to facilitate early diagnosis of cartilage degeneration.

**B-1132** 11:10
**Retrospective comparative study to assess the pitfalls of CartiGram and the complementary role of FSPD in the evaluation of cartilage lesions of the knee joint**
R. Thaha, S. Jogi, S. Rajan, A. Singh, V. Mahajan, A. Mehndiratta, V.K. Venugopal, H. Mahajan; *New Delhi/IN (drsriramrajan@gmail.com)*

**Purpose:** To analyse the common pitfalls in T2 cartilage maps of the knee joint. To compare visualization of normal cartilage and chondral pathology on fat-saturated proton density (FSPD) and T2 CartiGram by contrast ratio measurements.
**Methods and Materials:** The contrast ratio of weight-bearing and non-weight bearing cartilage was calculated on the CartiGram and FSPD images of 54 consecutive knee MRI scans performed on a 3.0 T MRI by selecting one slice each from the lateral and medial compartments and placing 3 ROIs on lateral and 2 on medial compartment. Mean of these T2 values was used to estimate contrast ratio of normal weight-bearing and non-weight-bearing segments on both sequences. The contrast ratio of the chondral lesions and the normal cartilage on both sequences was calculated. The pitfalls of CartiGram and additive value of FSPD were studied.
**Results:** The contrast ratio between weight-bearing and non-weight-bearing cartilage was statistically significantly higher (p < .05) on the CartiGram for normal cartilage. For chondral pathologies, contrast ratio difference was higher on FSPD images, but statistically insignificant (p = 0.91). The common pitfalls encountered on cartigram included 47 studies with joint capsule overlapping cartilage, 10 with cartilage fissures seen only on FSPD images, 28 with wider cartilage lesions on FSPD, and 5 with synovial thickening.
**Conclusion:** Overlapping of joint capsule tissue with posterior femoral cartilage leads to a spurious assumption of intact femoral cartilage on CartiGram. Cartilage fissures and wider cartilage lesions are better seen on FSPD whereas smaller lesions are better seen on T2 CartiGram.

**B-1133** 11:18
**Evaluating variability of T2 values of the cartilage, menisci and muscles around knee joint on CartiGram sequence at 1.5 T and 3.0 T MR**
R. Thaha, S. Jogi, S. Rajan, V. Venugopal, R. Bhattacharjee, A. Mehndiratta, A. Singh, M. Barnwal, V. Mahajan, G. Nanda; *New Delhi/IN (geetutomar@yahoo.co.in)*

**Purpose:** To evaluate the temporal and inter-magnetic field strength variability of T2 values of the cartilage, menisci and muscles of the knee joint using healthy volunteer data.

**Methods and Materials:** T2 CartiGram (CG) sequence of knee joint was performed on four healthy asymptomatic volunteers (age 32±3 years) on 3.0T and 1.5T (GE Healthcare) MRI scanners in addition to the standard MRI. In the first part of the study, for evaluating the same day variability at 1.5T and 3.0T, CG was performed twice with a break of 5 minutes and subject lying in the same position inside the scanner. In the second part, for evaluating inter-day variability on the same scanner, CG was performed twice with a gap of 1 month on 3.0T scanner. From the T2 map, mean values and coefficient of variation were calculated.
**Results:** The intra-day coefficient of variations for the lateral and medial side were 0.49%, 0.77% for the cartilage, 1.57%, 1.60% for muscle and 2.2%, 2.7% for meniscus on 1.5 T whereas the similar values were 0.69 %, 0.6 % for cartilage, 0.66 %, 1.2 % for muscle and 3.0 and 1.8 for meniscus on 3 T.The temporal coefficient of variations for the lateral and medial side were 3.2%, 4.7% for the cartilage, 1.17%, 0.46% for muscle and 7.8%, 4.3 % for meniscus.
**Conclusion:** The intra-day variability of the T2 values was lowest for the cartilage on 1.5 T and 3T scanner whereas the temporal variability was lowest for the muscles.
*Author Disclosures:*
**R. Bhattacharjee:** Employee; Philips Healthcare.

**B-1134** 11:26
**Time-saving opportunities in radiology: accurate structural imaging and $T_2$ mapping of cartilage and meniscus in knee osteoarthritis using a 5-minute DESS MRI scan**
S.M. Eijgenraam[1], A.S. Chaudhari[2], M. Reijman[1], B.A. Hargreaves[2], G.E. Gold[2], E.H.G. Oei[1]; [1]*Rotterdam/NL*, [2]*Stanford, CA/US (s.eijgenraam@erasmusmc.nl)*

**Purpose:** Quantitative $T_2$ measurements and structural assessment of knee osteoarthritis (OA) currently require multiple separate MRI sequences, resulting in long acquisition times. We evaluated a 5-minute double-echo steady-state (DESS) sequence for simultaneous $T_2$ relaxometry of cartilage and meniscus, and structural OA assessment, in patients with no, mild and moderate knee OA.
**Methods and Materials:** We scanned 53 patients using a 5-minute 3D DESS-sequence: 20 patients with no OA (Kellgren-Lawrence grade (KLG) 0), 18 patients with mild OA (KLG2) and 15 patients with moderate OA (KLG3). We performed single-slice $T_2$ measurements of four cartilage and four meniscus ROIs and structural OA evaluation with the MRI Osteoarthritis Knee Score (MOAKS) using DESS with multiplanar reformatting. Statistical testing between KLG for $T_2$ and MOAKS was performed using ANOVA, correlations between KLG and $T_2$ or MOAKS per ROI were assessed using linear regression analyses.
**Results:** In cartilage, mean $T_2$ values were 36.1±4.3, 40.6±5.9 and 47.1±4.3 ms for KLG0, KLG2 and KLG3, respectively (p<0.001). In menisci, mean $T_2$ values of 15±3.6, 17.5±3.8 and 20.6±4.7 ms were found for KLG0, KLG2 and KLG3, respectively (p<0.001). The correlation between KLG and $T_2$ ranged from 0.43 to 0.71 and from 0.39 to 0.51 in cartilage and meniscus ROIs, respectively. The correlation between KLG and MOAKS ranged from 0.50 to 0.62 and from 0.29 to 0.52 in cartilage and meniscus ROIs, respectively.
**Conclusion:** $T_2$ measurements and structural imaging biomarkers of cartilage and meniscus, simultaneously acquired with a 5-minute DESS-scan, can distinguish different stages of OA, making this sequence an accurate and time-saving tool for knee OA research.

**B-1135** 11:34
**Do Ahlbäck scores identify subgroups with different magnitudes of cartilage thickness loss in patients with moderate to severe radiographic osteoarthritis? Data from the OAI**
S. Hangaard[1], M. Boesen[2], H. Bliddal[2], W. Wirth[3]; [1]*Frederiksberg/DK*, [2]*Copenhagen/DK*, [3]*Salzburg/AT (stine.hangaard.01@regionh.dk)*

**Purpose:** To investigate if the Ahlbäck scoring system of radiographic knee osteoarthritis (OA) is able to further subdivide KLG-3 and -4 patients into groups with different sensitivity to change in cartilage thickness over one year in patients with moderate to severe knee OA.
**Methods and Materials:** One-year femorotibial cartilage thickness change was obtained from 3D-cartilage sensitive MRI data in 108 osteoarthritis initiative (OAI) participants with moderate to severe radiographic knee OA KLG-3/4. KLG-scores were available from the public available OAI-database. Ahlbäck scores were performed using the same baseline x-rays. Subregional change in cartilage thickness was assessed after manual segmentation of weight-bearing femorotibial cartilage at baseline and 1 year. Cartilage thickness change was analysed in the entire femorotibial joint (FTJ), the medial compartment (MFTC) and the lateral compartment (LFTC). One-year change was calculated for FTJ, MFTC, LFTC and the location-independent ordered values 1 and 16 (OV1/OV16) representing the subregions with largest loss (OV1) and gain (OV16) within each knee.
**Results:** Of the 108 patients, n=30/78 had KLG-3/4. The corresponding Ahlbäck scores (1-5) were n=30/33/36/9/0. Cartilage thickness changes between Ahlbäck groups showed no statistical significant difference for FTJ, MFTC, LFTC and OV1, but change in OV16 was significantly higher in Ahlbäck-4 knees (p=0.03) compared to Ahlbäck 1-3 knees.
**Conclusion:** Radiographic knee OA grading with Ahlbäck scores was not superior to KLG for prediction of cartilage thickness loss over one year, in patients with moderate and severe knee OA supporting the continuous use of the easier and more widely used KLG in clinical trials.

**B-1136** 11:42
**Association between baseline meniscal extrusion on MRI and long-term incident knee osteoarthritis in two different cohorts**
J. Van der Voet, D. Schiphof, D. Vroegindeweij, S. Bierma - Zeinstra, J. Runhaar, E. Oei; *Rotterdam/NL (javandervoet@gmail.com)*

**Purpose:** Previously, we identified a significant association between meniscal extrusion and short-term incident knee osteoarthritis (KOA). To validate these findings, we evaluated long-term incident KOA in knees with meniscal extrusion, using two cohorts.
**Methods and Materials:** We used data from the PROOF study, which evaluated a high-risk population of overweight women, and the Rotterdam Study (RS), a prospective population-based cohort study. Meniscal extrusion was defined as ≥ 3 mm on MRI. Outcomes were incident radiographic (KL ≥ grade 2) or clinical KOA according to the ACR criteria, assessed at 6.6 years (PROOF) and 5.1 years (RS). With generalized estimating equations, we determined the association of knees with and without baseline meniscal extrusion and incident KOA, adjusted for baseline differences. Furthermore, we computed the population attributable risk percentage (PAR%) of meniscal extrusion.
**Results:** PROOF: 421 knees were available for analysis of which 23% had baseline meniscal extrusion. Incident radiographic KOA was borderline significantly higher in knees with meniscal extrusion compared to those without (OR 1.95, 95% CI 0.95, 3.98 PAR 11%). Incident clinical KOA was significantly higher (OR 2.33, 95% CI 1.19, 4.59, PAR 18%).
RS: 891 knees were analyzed of which 46% showed meniscal extrusion. Incident radiographic KOA was significantly higher in knees with extrusion (OR 2.54, 95% CI 1.12, 5.76, PAR 30%). Incident clinical KOA was borderline significantly higher (OR 2.05, 95% CI 0.9, 4.65, PAR 31%).
**Conclusion:** Meniscal extrusion is significantly associated with long-term incident KOA. A high number of incident cases were attributable to meniscal extrusion.

**B-1137** 11:50
**The prevalence of Baker's cyst in relation to the arrangement pattern between medial head of gastrocnemius tendon and semimembranous tendon**
D.Y. Han, K.N. Ryu, J.S. Park, W. Jin, S.Y. Park, S.J. Yun; *Seoul/KR (koreahdy@naver.com)*

**Purpose:** To investigate the cause of Baker's cyst by analyzing the arrangement pattern that can give much friction between medial head of gastrocnemius tendon (MHGCT) and semimembranous tendon (SMT).
**Methods and Materials:** From August 2017 to February 2018, patients who underwent knee MRI with "Baker's cyst" in PACS were searched. Patients(control group) who did not have "Baker's cyst" in the knee MRI performed in the same period were also searched. For screening purpose, age and gender were adjusted by propensity score matching and 116 patients were selected. For the imaging analysis, the arrangement pattern between MHGCT and SMT was classified into 3 types (type 1: concave SMT, type 2: flat SMT, type 3: round SMT) and type 2 and 3 hypothesized that they would induce baker's cyst better. When imaging analysis was performed, presence of osteoarthritis, major ligaments and tendons tear were also described. Demographic findings was investigated through medical chart review. Univariable logistic regression was performed on all variables and the odds ratio was calculated.
**Results:** The frequency of type 1, 2 and 3 was statistically significant depending on the presence or absence of Baker's cyst(p<0.001). The frequency of type 3(n=33, 70.21%) was the highest in Baker's cyst group and type 1(n=70, 64.81%) was the highest in non-Baker's cyst group. Baker's cyst was more common in type 2(OR: 2.59, CI: 1.30-5.17, p=0.0068) and type 3(OR: 4.11, CI: 1.91-8.83, p=0.0003).
**Conclusion:** The arrangement of MHGCT and SMT is considered to be a factor affecting the formation of Baker's cyst.

10:30 - 12:00 Studio 2019

### Genitourinary

### SS 1407

### Renal and adrenal tumours

*Moderators:*
M.-F. Bellin; Le Kremlin-Bicêtre/FR
S. Ramanathan; Doha/QA

**B-1138** 10:30

**Which is the accuracy for malignancy of contrast-enhanced ultrasound of Bosniak ≥2F cystic renal lesions incidentally found on CT and MRI?**

A. Cretese, G. Como, R. Girometti, L. Cereser, G. Giannarini, C. Valotto, C. Zuiani; *Udine/IT (andrea.cretese@gmail.com)*

**Purpose:** To investigate the accuracy of contrast-enhanced ultrasound (CEUS) in detecting malignancy in incidentally found complex renal cysts (CRC) previously categorized as Bosniak ≥2F.
**Methods and Materials:** We analyzed a retrospective cohort of 18 patients who underwent CEUS between July 2015/July 2018 to assess CRC categorized Bosniak ≥2F after incidental detection on computed tomography (CT) or magnetic resonance imaging (MRI). CEUS was performed by one radiologist with 15 years of experience using a Logiq E9 ultrasound system (GE healthcare, Chicago, USA) and i.v. administration of 2.4 mL INN-sulphur hexafluoride, categorizing CRC according to Bosniak classification. After matching CEUS with the standard of reference (postoperative pathology or imaging follow-up up to 12 months), we calculated per-lesion sensitivity, specificity, positive predictive value (PPV) and negative predictive value (NPV) for malignancy, using a CEUS-based Bosniak ≥3 categorization.
**Results:** CEUS found a total of 20 CRC, categorizing them as Bosniak ≤2F, Bosniak 3, and Bosniak 4/solid in 8/20 (40%), 7/20 (35%), and 5/20 (25%) cases, respectively. Based on CEUS findings, patients were referred to surgery (10/18 cases) or imaging follow-up (8/18 cases), with a final diagnosis of malignancy in 10/20 CRC (50%). Sensitivity, specificity, PPV and NPV of CEUS were 100%, 80%, 83%, and 100%, respectively. CEUS changed initial Bosniak categorization in 7/20 lesions (35%) (p<0.05; McNemar test), including 3 upgraded cases (all malignant on pathology) and 4 downgraded cases (all stable at follow-up).
**Conclusion:** CEUS was accurate in assessing malignancy in incidental CRC, changing initial Bosniak categorization in about one third of patients.

**B-1139** 10:38

**Contrast-enhanced computed tomography (CECT) quantitative radiological parameters to predict outcome in patients with renal cell carcinoma (RCC)**

L. Basso[1], G. Buonomenna[2], A. Sorrentino[3], G. Perugin[1], F. Rosa[1], A. Olivero[1], N. Testino[1], C. Terrone[1], C. Neumaier[4]; [1]*Genoa/IT*, [2]*Avellino/IT*, [3]*Siena/IT*, [4]*Genova/IT (lukabasso89@gmail.com)*

**Purpose:** Visceral and subcutaneous adipose tissue (VAT and SAT) are clinically associated with increased risk of chronic kidney disease (CKD), due to chronic inflammatory response after surgery. The aim of this retrospective study was to evaluate the usefulness of computed tomography (CT) in patients with renal cell carcinoma (RCC) for the evaluation of kidney volumes and its relationship with VAT and SAT in the prediction of a decrease of estimated glomerular filtration rate (eGFR) 12 months after surgery.
**Methods and Materials:** We enrolled 112 patients who underwent radical nephrectomy. Pre-operatory and follow-up eGFR was calculated using the CKD epidemiology study equation. CT performed prior to surgery was evaluated by two expert radiologists. VAT, SAT, total adipose tissue (TAT) and abdominal circumference were calculated on an axial plane passing through umbilical plane. VAT%, tumour volume (TV), functioning resected volume (FRV) and contralateral kidney volume (CKV) were calculated using dedicated segmentation protocol and analysed with a simple linear regression model.
**Results:** Mean BMI was 26.0 kg/m. Mean eGFR was 70.1 ml/min decreasing to 53.68 ml/min at 12 months. Mean VAT was 158.48 cm. Mean SAT was 200.25 cm with a mean VAT% of 44%. Mean CKV was 162.31cm. Mean FRV of 139.96 cm. Pre-op eGFR (p<0.001), BMI (p=0.021), CKV (p=0.02) and %VAT (p=0.007) showed significant correlation with decrease of eGFR 12 months after surgery.
**Conclusion:** Other than the well-known factors predicting the decrease of renal function after nephrectomy, the CKV and VAT% could predict patient outcome in pre-operative evaluation.

**B-1140** 10:46

**Kidney volumes in living donors: does size really matter?**

F.R. Schwartz[1], B. Shaw[1], R. Lerebours[1], F. Vernuccio[2], F. Gonzalez[1], L. Hurwitz-Koweek[1], K. Ravindra[1], D. Marin[1]; [1]*Durham, NC/US*, [2]*Palermo/IT (fidesreginadorotheaann.schwartz@duke.edu)*

**Purpose:** Computed tomography angiography (CTA) is performed before transplant surgery to assure the smaller donor kidney is transplanted and assess for blood supply and possible disease. This study assesses the relationship between renal diameters and renal volumes, as well as the frequency of appropriate renal selection for transplant using conventional renal diameters and the long-term outcome in donors.
**Methods and Materials:** This is a HIPAA-compliant, IRB-approved study. 50 renal donors were identified from 2010-2018 (mean age 40 years; 30 F, 20 M). Renal size was assessed by measuring renal diameters in three dimensions and volume; arterial supply and venous drainage were assessed. Glomerular filtration rates (GFR) were documented before surgery, within 24 hours post-transplant, at 6, 12 and 24 months. Descriptive statistics (means, SD, IQR), paired t tests and Spearman's rank correlations were used for comparisons.
**Results:** The left kidney had a larger average volume (165 vs 154.6 $cm^3$; P<0.01) than the right but craniocaudal lengths were similar (10.9 vs 10.8 cm). 10% of kidneys had two or more arteries, 6% had abnormal venous drainage. GFR recovered from 51.8 mL/min/1.73 $m^2$ directly to 63.9 mL/min/1.73 $m^2$ 24 months after surgery. There was a strong correlation between craniocaudal and laterolateral diameters and volume (P<0.01). The larger kidney was transplanted in 28% of cases which had no statistically significant impact on long-term renal function (P<0.01).
**Conclusion:** Renal volume correlates well with conventional diameters and transplanting the larger donor kidney had no long-term detrimental effect on donor kidney function.
*Author Disclosures:*
**F.R. Schwartz:** Research/Grant Support; Siemens Healthineers, Grant to department of Radiology. **L. Hurwitz-Koweek:** Research/Grant Support; Siemens Healthineers, Grant to department of Radiology.

**B-1141** 10:54

**Native T1 mapping as an in vivo biomarker for the identification of higher grade renal cell carcinoma**

L.C. Adams; *Berlin/DE*

**Purpose:** To identify higher grade clear cell renal cell carcinoma (cRCC) with native T1 mapping and to histologically correlate the results with the collagen volume fraction.
**Methods and Materials:** For this institutional review board-approved, singlecenter prospective study, 68 consecutive patients received abdominal MRI scans at 1.5 T between January 2017 and July 2018, using a modified Look-Locker inversion recovery (MOLLI) sequence. 30 patients with cRCC (20 men, mean age 61.9 years±13.1), who underwent partial or radical nephrectomy and histological grading according to the ISUP classification, and a separate healthy cohort of 30 individuals without renal malignancies or complex cysts (16 men, mean age 59.7±14.6 years), met the eligibility criteria. T1 values were quantitatively measured with region of interest (ROI) measurements in T1 maps. Quantification of the collagen volume fraction was performed on histological sections (picrosirius red staining).
**Results:** Native T1 values were significantly lower for lower grade cRCC (ISUP 1-2) compared to higher grade cRCC (ISUP 3-4) (p<0.001). A cut-off value of 1101 ms distinguished higher grade from lower grade tumours with a sensitivity of 100% (95% CI: 0.69-1.00), a specificity of 85% (95% CI: 0.62-0.97) and an accuracy of 90% (95% CI: 0.73-0.98). Native T1 values were significantly associated with the histological collagen volume fraction (p<0.05). Furthermore, T1 times in the renal cortex, medulla and tumour tissue showed an excellent inter-observer agreement.
**Conclusion:** Native T1 mapping could represent an in vivo biomarker for the differentiation of lower and higher grade cRCC, providing incremental diagnostic value beyond qualitative MRI features.

**B-1142** 11:02

**Reducing radiation dose while maintaining observer performance using prior iterative reconstruction (PIR) in multiphase dual energy renal CT**

P. Navin[1], A.F. Halaweish[2], B. Schmidt[2], B. Kim[2], M. Wells[2], A. Khandelwal[2], T. Moen[2], C. McCollough[2], J. Fletcher[2]; [1]*Galway/IE*, [2]*Rochester, MN/US (paddynavin@yahoo.co.uk)*

**Purpose:** To investigate the dose reduction potential using a novel prior iterative reconstruction (PIR) algorithm in multiphase renal dual-energy (DE) CT.
**Methods and Materials:** Projection data from multi-phase renal DECT examinations was collected and noise inserted (using a validated technique) to simulate 60% dose reduction and reconstructed images were used as input to the PIR algorithm. Three GU radiologists blindly examined datasets (two reading sessions) using either full dose or 40% dose PIR image sets.

Saturday

Probability of malignancy was assessed [0 (no chance) to 100 (definite)] with malignancy assumed at probability ≥ 70. Area under the ROC curve (AUC), sensitivity and specificity were compared on a per-patient and per-lesion basis. CT number accuracy and subjective image quality were assessed (5 point Likert scale).
**Results:** Twenty-three patients had 49 renal lesions (11 solid renal-neoplasms). CT number was nearly identical between techniques. AUC was similar between multiphase routine dose and lower dose PIR [per patient: 0.950 v 0.916 (p=0.356); per lesion: 0.931 v 0.884 (p=0.304)]. Per patient sensitivity was similar (78% routine dose [95% CI 37-90%] v 82% lower dose PIR [CI 35-93%] [p = 0.37]), as was specificity (91% routine dose [CI 51-98%] v 93% lower dose PIR [CI 50-99%] [p > 0.99]), with similar findings on a per lesion level. Subjective image quality demonstrated no statistical difference between the two groups (p = 0.34).
**Conclusion:** PIR is a new reconstruction method for multi-phase CT examinations that promises to facilitate radiation dose reduction by over 50% for multiphase DECT renal exams.
*Author Disclosures:*
**A.F. Halaweish:** Employee; Siemens Healthineers. **B. Schmidt:** Employee; Siemens Healthineers. **C. McCollough:** Research/Grant Support; Siemens Healthineers.

**B-1143** 11:10
**Differentiation of adrenal adenomas from adrenal metastases using single-phased dual energy CT: a comparison to MRI**
M.T.O. Winkelmann, M.N. Bongers; *Tübingen/DE (moritz.winkelmann@med.uni-tuebingen.de)*

**Purpose:** Aim of this study was to evaluate accuracy of dual energy CT (DECT) iodine and fat quantification and virtual-unenhanced imaging (VNC) for differentiation between adrenal adenomas and metastases using MRI as a reference.
**Methods and Materials:** 47 patients with 51 lesions (female:male=29:18,mean age:67 years) having received clinically indicated DECT and MRI (mean interval: benign 2.9 months/ malignant 0.28 months). Quantitative parameters such as VNC values, fat fraction, iodine density and mixed image (CT-mixed) values were collected. MRI in- and opposed phase and contrast enhanced imaging were used as a reference. Mean values of VNC, fat fraction, iodine density and CT-mixed in DECT images were compared between adenomas and metastases using non-parametric tests. Diagnostic accuracy was assessed by calculating receiver operating characteristics (ROC). Results are given as median with interquartile ranges.
**Results:** Iodine density and fat fraction values in DECT showed significant differences between adrenal adenomas (2.4mg/mL [1.7, 2.9]; 31.1% [27.1, 34.6]) and metastases (1.6 mg/mL [0.4, 2.5], 16.6% [12.4, 23.4])(all p ≤ 0.03). Analysis of VNC revealed significant differences between adrenal adenomas (5.4HU[-2.6, 12.7]) and metastases (27.6HU[15.8, 32])(p≤0.0001). No significant differences between adenomas (59.3HU [46.7, 75.4]) and metastases (62.4HU [43.3, 89.2]) were detected in CT-mixed. ROC analysis revealed significantly higher AUC values and increased sensitivity for VNC and fat fraction (AUC = 88%/sensitivity = 71.4%; 88%/92.9%) than for CT-mixed (53.6%/50%; p ≤ 0.005).
**Conclusion:** DECT is suitable for differentiation between adenomas and metastases by quantification of fat fraction and computation of VNC images with high diagnostic accuracy.

**B-1144** 11:18
**Incidental adrenal masses (IAM) at basal CT or enhanced CT: differentiating non-functioning adenomas from tumour lesion using histogram and texture analysis (TA)**
G. Stranieri, A. Priola, D. Basile, S. Priola, A. Veltri; *Turin/IT (giuseppestranieri92@gmail.com)*

**Purpose:** Usefulness of CT histogram and texture analysis to differentiate non-functioning adenomas from malignant lesions in the setting of IAM found at basal CT or enhanced-CT.
**Methods and Materials:** We extracted retrospectively patients who underwent basal CT (IAM: medium density>10HU; % negative pixels<10%) or in portal phase CT (medium density>20HU) from January to December 2017. We included 70 IAM: 46 non-functioning adenomas and 24 malignant lesions. The reference standard was hystological response or their typization with multiphasic enhanced-HCT, chemical-shift magnetic resonance or PET-CT. A commercial software (LifeX) was used to obtain textural parameters and histogram analysis. The results were compared with logistic regression model to estimate discrimination capability.
**Results:** At basal CT, the two groups shown significative differences in minimal value HU (p=0.02) and in LRHGE of GLRLM matrix (p=0.02) with AUROC of 0.822 and 0.856 (LRHGE: sensitivity 79%; specificity 90%). The logistic analysis demonstrated the LRHGE as an indipendent predictive parameter at univariate (Odds Ratio[OR]=1.0001; p=0.04) and multivariate analysis after correcting for age and size.At enanched-CT, the two groups shown significative differences in GLRLM and GLZLM matrices: RLNU and GLNU were the best discriminators (p=0.03, AUROC=0.719, sensitivity 70%, specificity 86%; p=0.04, AUROC=0.719, sensitivity 60%, specificity 79%; respectivly). The logistic analysis found RLNU as the only significative parameter at multivariate analysis (OR=1.0012; p=0.04). The Histogram analysis didn't show difference between groups, either at basal or enhanced-CT (p>0.05).
**Conclusion:** Our study shows that TA could be useful to characterize IAM at CT, avoiding additional risks or costs linked to other studies.

**B-1145** 11:26
**Reassessment the ability of renal CE-CT for avoiding pathological upstaging of clinical T1 to pathological T3 renal cancer in the era of robotic partial nephrectomy**
S. Takahashi[1], Y. Ueno[2], K. Sofue[2], N. Negi[2], K. Kagawa[2], T. Murakami[2], U. Tanaka[2]; [1]*Takatsuki/JP*, [2]*Kobe/JP (satorutakahashi2@mac.com)*

**Purpose:** Tumour invasion into the renal sinus fat or segmental branches of the renal vein are now attracting increasing attention for avoiding upstaging to pT3 and early recurrence of small RCC after partial nephrectomy. We evaluated the ability of multiphasic renal CECT with focusing on the tumour extension into the renal sinus.
**Methods and Materials:** We retrospectively evaluated surgically proven 214 RCCs in 212 patients underwent preoperative multi-phasic thin-slice(≦1mm) CECT on 3rd generation dual-source CT scanner. A board-certificated radiologist blinded to the surgical finding scored probability of sinus fat invasion (SFI), perinephric fat invasion (PFI), and renal vein invasion (RV) using a 5-point scale with evaluating the contour of tumours.
**Results:** 46 RCCs were diagnosed as ≧pT3a (SFI, 13; PFI, 28; RV, 33). Sensitivity, specificity, accuracy and Az-value for diagnosing each pT3-factor were 85%, 95%, 95%, 0.81 for SFI; 71%, 98%, 94%, 0.88 for PFI; and 85%, 85%, 85%, 0.94 for RV; respectively, while overall performance of diagnosing pT3 disease were 89%, 76%, 79%, 0.88. Smooth bulging into the sinus fat was demonstrated in 41% of ≦pT2 tumour and 17% of ≧pT3 tumour, while irregular bulging was found in 19% (32/168) of ≦pT2 tumour and in 78% (36/46) of ≧pT3 tumour.
**Conclusion:** Detailed analysis of thin-slice renal CECT could predict the possible tumour invasion into the renal sinus fat or the peripheral branches of the renal vein, and could be useful for preventing pathological upstaging of cT1 to pT3 in cases of small RCC treated with partial nephrectomy.
*Author Disclosures:*
**S. Takahashi:** Research/Grant Support; Siemens Healthineers.

**B-1146** 11:34
**Low energy imaging for depicting renal venous tumour thrombus on multi-phasic contrast-enhanced dual-energy CT with 3rd generation dual-source CT scanner**
S. Takahashi[1], T. Itoh[2], Y. Ueno[3], N. Negi[3], K. Kagawa[3], K. Sofue[3], U. Tanaka[3], A. Kusaka[3], T. Murakami[3]; [1]*Takatsuki/JP*, [2]*Tokyo/JP*, [3]*Kobe/JP (satorutakahashi2@mac.com)*

**Purpose:** Accurate diagnosis of tumour venous thrombus in the segmental branch is attracting increasing attention for avoiding unanticipated upgrading to pT3 in partial nephrectomy for small RCC. We evaluated the ability of low energy CECT with either low-kVp or low-keV Mono+ image for depicting renal tumour thrombus.
**Methods and Materials:** We evaluated preoperative multi-phasic CECT images at 3rd generation DECT for RCC with surgically proven tumour thrombus (n=36). Mono+ (40 to 190keV) and mixed images were generated for nephrographic phase (NP) and excretory phase (EP) DE acquisition, while arterial phase (AP) images were scanned at single-energy 70kVp. HU values of the renal parenchyma, tumour, tumour thrombus, the renal vessels, and fat were measured. SNR, contrast ratio (CR) and dose-weighted CNR (CNRD=CNR/CTDIvol^0.5) of tumour and tumour thrombus were compared.
**Results:** CTDIvol and DLP were below the national DRL for dynamic body CT. HU values of tumour thrombus varied in cases (AP, 249±116; mixed NP, 189±27; mixed EP 136±21). Although there were no statistically significant differences in CR of tumour thrombus among phases (AP, 0.97±0.06; NP, 0.91±0.06; EP, 0.94±0.06; respectively), CNRD in AP (2.2±1.7) were significantly greater than those in NP and EP (0.17±0.15, 0.41±0.22, 0.22±0.14, respectively). CNRD for tumour thrombus was the highest at 40keV both in NP and EP, and average CNRD for tumour thrombus in NP were statistically significantly greater than in EP (p<.001).
**Conclusion:** 40-keV NP Mono+ images were useful for depicting renal tumour thrombus in multi-phasic DECT, while 70kVp AP images could also satisfactory demonstrate venous thrombus.
*Author Disclosures:*
**S. Takahashi:** Research/Grant Support; SIemens healthineer. **T. Itoh:** Employee; SIemens healthineers.

**B-1147** 11:42
**Efficacy of Raysum image in urolithiasis detection**
K.K. Lau, S. Deb, A. Kuganesan; *Melbourne/AU (kenkplau@gmail.com)*

**Purpose:** Baseline abdominal radiographs (AXR) are commonly performed for computed tomography urinary tract (CTU) proven calculi. Subsequent AXR may be used to assess urolithiasis progression. A virtual ray sum image (RSI) from CTU dataset can be created with a similar AXR appearance. The aim of this retrospective study was to determine the efficacy of the RSI in detecting urolithiasis.
**Methods and Materials:** All consecutive adult patients with urolithiasis positive CTU and an AXR within 24 hours of CTU and similar patient number with negative CTU in 5 month-period were included. Patients who had the baseline AXR > 24 hours after CTU were excluded. AXR and RSI were blindly reviewed by two radiologists. Patient thickness, presence of excess gas/faecal material, calculus size, mean calculus Hounsfield unit (HU) and location were analysed to determine effect on the AXR and RSI.
**Results:** 152 calculi in 50 adult patients were included with RSI sensitivity of 44% (95% CI 36-52) and AXR 30% (95% CI 22-38). There was substantial agreement between the two techniques with Kappa=0.70 (95% CI 0.58- 0.81, p<0.001). No invisible calculus on RSI became visible on AXR. No false positive was seen on RSI. Logistic regression analyses demonstrated calculus size and mean calculus HU significantly affected sensitivities of both RSI and AXR.
**Conclusion:** Raysum Image as a post processed image derived from CTU dataset may remove the baseline AXR in patients with CT proven urolithiasis. This would reduce patient radiation dose and streamline workflow in busy radiology departments.

**B-1148** 11:50
**Diagnostic accuracy of dual-energy CT for renal mass evaluation: systematic review and meta-analysis**
N. Panvini[1], D. Bellini[1], A. Laghi[2], D. Marin[3], B.N. Patel[4], C.L. Wang[5], I. Carbone[1], A. Mileto[5]; [1]*Latina/IT*, [2]*Rome/IT*, [3]*Durham, NC/US*, [4]*Stanford, CA/US*, [5]*Seattle, WA/US (npanvini88@gmail.com)*

**Purpose:** To perform a systematic review and meta-analysis to evaluate the diagnostic accuracy of dual-energy CT (DECT) for renal mass evaluation.
**Methods and Materials:** In March 2018, we searched MEDLINE, Cochrane Library, Embase and Web of Science databases. Analytic methods were based on Preferred Reporting Items for Systematic Reviews and Meta-Analyses. Pooled estimates for sensitivity, specificity, and diagnostic odds ratios were calculated for DECT-based virtual monochromatic imaging and iodine quantification techniques as well as for conventional attenuation measurements from renal mass CT protocols. $I^2$ was used to evaluate heterogeneity. Methodological quality of included studies and potential bias were assessed using items from Quality Assessment Tool for Diagnostic Accuracy Studies-2.
**Results:** Of the 1043 articles initially identified, 13 were selected for inclusion (969 patients, 1193 renal masses). Cumulative data of sensitivity, specificity and summary diagnostic odds ratio were 83% (95%CI: 76-89%; $I^2$: 88.1%), 75% (95%CI: 70-80%; $I^2$: 99.1%), 183.4 (95%CI: 30.7-1093.4; $I^2$: 61.6%) for virtual monochromatic imaging; 99% (95%CI: 97-100%; $I^2$: 17.6%), 91% (95%CI: 89-94%; $I^2$: 84.2%), 511.5 (95%CI: 217-1201; $I^2$: 0%) for iodine quantification, respectively. No significant differences in area under the curve were found between iodine quantification and conventional attenuation measurements ($P$ =0.79).
**Conclusion:** DECT yields high accuracy for renal mass evaluation. Determination of iodine content with the iodine quantification technique demonstrates diagnostic accuracy similar to conventional attenuation measurements from renal mass CT protocols. The iodine quantification technique may be used to characterize incidental renal masses when a dedicated renal mass protocol is not available.

**10:30 - 12:00** **Room E1**

### Breast

### SS 1402a

### Interventional breast radiology and high-risk lesions

*Moderators:*
S. Barter; Bedford/UK
A. Jalaguier; Marseille/FR

**B-1149** 10:30
**Trends in frequency and outcome of high-risk breast lesions at percutaneous biopsy in women recalled at biennial screening mammography**
B. Korte[1], J.D. Luiten[2], L.E.M. Duijm[3]; [1]*Eindhoven/NL*, [2]*Tilburg/NL*, [3]*Nijmegen/NL (bramkorte_@hotmail.com)*

**Purpose:** To determine the incidence, management and outcome of high risk breast lesions among women recalled at biennial screening mammography.
**Methods and Materials:** We included a consecutive series of 376519 screens of women who received biennial screening mammography in a Dutch breast cancer screening region between January 1, 2011 and December 31, 2016. During 1-year follow-up, radiological reports and biopsy reports were collected of all recalled women to determine final outcome.
**Results:** During this 6-year screening period, the proportion of recalled women who underwent percutaneous biopsy remained fairly stable (range: 39.2%-48.1%, mean: 44.2%, 5212/11783), whereas the proportion of high risk lesions at percutaneous biopsy (e.g., flat epithelial atypia, atypical ductal hyperplasia, lobular carcinoma in-situ, papillary lesions) gradually increased from 3.6% (28/775) in 2011 to 9.5% (86/901) in 2016. The proportion of high risk lesions at percutaneous biopsy that underwent a subsequent surgical excision fluctuated between 41.0% (2013) and 65.5% (2014) through the years (mean: 52.5%, 177/337), but the excision biopsy rate for high risk lesions per 1000 screens and per 100 recalls increased from 0.31 (2011) to 0.70 (2016) and from 0.99 (2011) to 2.50 (2016), respectively. The malignancy rate of the excision biopsies ranged from 21.1% (8/38, 2014) to 31.3% (5/16, 2011), with 27.7% (49/177) of all excision biopsies being malignant.
**Conclusion:** The proportion of high risk lesions at percutaneous biopsy almost tripled, with a concommitant increased excision biopsy rate for these lesions per 1000 screens and per 100 recalls. The malignancy rate at excision biopsy remained stable.

**B-1150** 10:38
**External validation of the STAR (suspicion type age response of biopsy) score for risk stratification of B3 breast lesions detected at ultrasound-guided biopsy**
C. Grippo[1], P. Jagmohan[2], A.M. Stoeger[3], P. Kapetas[3], T.H. Helbich[3], P.A.T. Baltzer[3], P. Clauser[3]; [1]*Rome/IT*, [2]*Singapore/SG*, [3]*Vienna/AT (cris.grippo@gmail.com)*

**Purpose:** The STAR (suspicion type age response of biopsy) score is a newly developed risk stratification algorithm to be used as a decision-making tool for the management of breast B3 lesions detected on ultrasound (US)-guided biopsy.The purpose of this study was to investigate the robustness of this score by an external validation.
**Methods and Materials:** We retrospectively reviewed all consecutive histologically confirmed B3 lesion diagnosed at US-guided biopsy at our institution and included all lesions with subsequent open surgery or radiological follow-up 24 months.The STAR score was independently assessed by two breast radiologists (R1, R2) by assigning each feature a score from 0 to 2 (maximum score of 5) and results were tested by ROC-curves. A threshold of≥1 was applied to calculate sensitivity, specificity, positive and negative predictive value (PPV and NPV).
**Results:** 129 B3 lesions were evaluated. Surgery was performed on 117/129 (90.6%) lesions and 11/117 (9,4%) lesions were malignant. Follow-up of remaining lesions was negative. The area under the ROC-curve ranged from 0.75 to 0.79, with no significant difference between the two readers (p=0.5). At a threshold of ≥1, a sensitivity, specificity, PPV and NPV of 90%/90% (R1/R2), 39%/38% (R1/R2), 11%/12% (R1/R2) and 97%/98% (R1/R2) were identified. In both readings, 46 lesions had a score ≤1 and could have avoided surgery. Of these, only one malignant lesion was underdiagnosed (DCIS-G1).
**Conclusion:** The STAR score showed a high NPV upon external validation and therefore has the potential to reduce unnecessary surgeries for US-detected B3 lesions in clinical practice.

**B-1151** 10:46

**Should atypical ductal hyperplasia be surgically excised after percutaneous needle biopsy: systematic review and meta-analysis**

S. Schiaffino[1], M. Calabrese[1], E.F. Melani[1], E. Massone[1], A. Villa[2], G. Di Leo[3], M. Zanardo[3], F. Sardanelli[3]; [1]*Genoa/IT*, [2]*Sarzana/IT*, [3]*Milan/IT* *(schiaffino.simone@gmail.com)*

**Purpose:** To assess the reported underestimation rate (UR) of atypical ductal hyperplasia (ADH) diagnosed percutaneously using surgical excision (SE) or follow-up for conservative management (CM).
**Methods and Materials:** A systematic search was performed in October 2018 using MEDLINE and EMBASE for studies reporting the UR for pure ADH cases diagnosed at percutaneous biopsy. The study was registered on PROSPERO and written following the PRISMA statement. The pooled UR (pUR) was calculated using the random-effect model; subgroup and meta-regression analyses for the assessment of potential UR-predictors. The Newcastle-Ottawa scale and Egger test were used for study quality and publication bias, respectively.
**Results:** Of 521 articles, 93 were analysed totalling 177,001 percutaneous biopsies, 7,425 of them (4.2%) being ADH (5,916 SE, 494 CM). Thirty-seven studies used core-needle, 36 vacuum-assisted biopsy, 17 mixed, 3 did not report; 32 stereotactic guide, 13 ultrasound, 9 MRI, 6 mixed, 33 did not report. Heterogeneity was high ($I^2$=80%). pUR was 27.0% (95% CI 24.2-30.0%), 29.2% (95% CI 26.4% - 32.3%) for SE and 5.5% (95% CI 3.7% - 8.0%) for CM (*p*<0.001). When reported, the proportion of upgrade to invasive cancer versus ductal carcinoma in situ was 19.2%-9.2% overall, 29.2%-8.6% for SE, 5.5%-3.7% for CM (*p*<0.001). Subgroup analysis found significant impact of type of needle (*p*<0.001) and type of imaging guiding the biopsy (*p*<0.001) on the surgical pUR. Quality of studies was low-to-medium. No risk of publication bias (*p*=0.081).
**Conclusion:** The pUR for ADH was 27.0%, higher for SE (29.2%), but clinically relevant (5.5%) also for CM.

**B-1152** 10:54

**Radiologic and clinicopathologic associations with upgrade to malignancy in high-risk breast lesions**

C. You, Y.J. Gu, S. Chen, X.X. Shen, W.J. Peng; *Shanghai/CN* *(youchao8888@aliyun.com)*

**Purpose:** To assess the radiologic and clinicopathologic features of high-risk breast lesions (HRLs) diagnosed with biopsy and further to develop a prediction model to distinguish HRLs that upgrade to malignancy after surgery.
**Methods and Materials:** This retrospective study included 267 patients with biopsy-proven HRLs who performed both mammography (MG) and MRI preoperatively and underwent surgical excision between January 2017 and March 2018. Positive predictive value (PPV) for upgrade to malignancy in all HRLs and specific for each sub-category were evaluated. Multivariate analysis was conducted to identify association between clinical (age, family or personal history of breast cancer), radiological findings before biopsy (lesion size, imaging characteristics and final BI-RADS category of both MG and MRI) and final excision outcome.
**Results:** The overall upgrade rate of HRLs to malignancy was 31.4% (84 of 267). The most common HRL was ADH (58.8%, 157 of 267) and the most common upgrade type of malignancy was DCIS (54.8%, 46 of 84). At multivariate analysis, upgrade was more likely to be seen in mass lesion at MG (OR = 1.69; 95% CI: 1.28, 3.72) and in moderate or marked background parenchymal enhancement (BPE) breast at MRI (OR = 1.25; 95% CI: 2.3, 5.28). The category of BI-RADS 4-5 versus BI-RADS 1-3 was also independently associated with upgrade.
**Conclusion:** The rates of upgrade to malignancy in biopsy-proven HRLs were high, at around 31%. The presence of a mass at MG, moderate or marked BPE at MRI, and BI-RADS 4-5 category may represent useful predictors of upgrade.

**B-1153** 11:02

**Positive predictive value for malignancy of uncertain malignant potential (B3) breast lesions diagnosed on vacuum-assisted biopsy: is surgical excision still recommended?**

F. Ballati, P. Lomoro, M. Lucioni, G. Di Giulio; *Pavia/IT* *(france.balla@hotmail.it)*

**Purpose:** Breast lesions classified as of "uncertain malignant potential" are a heterogeneous group of abnormalities with an increased risk of associated malignancy. Clinical management of B3 lesions diagnosed on vacuum-assisted breast biopsy (VABB) is still challenging: surgical excision is no longer the only available treatment and VABB may be sufficient for therapeutic excision. The aim of the study was to evaluate the positive predictive value (PPV) for malignancy in B3 lesions that underwent surgical excision, identifying possible up-grading predictive factors and characterizing the malignant lesions eventually diagnosed. These results were compared with a subset of patients with B3 lesions who underwent follow-up.
**Methods and Materials:** 1250 VABBs were performed between January 2006 and December 2017 at our centre. 150 B3 cases were diagnosed and 68 of them underwent surgical excision. VABB findings were correlated with excision histology. A PPV for malignancy for each B3 subtype was derived.
**Results:** The overall PPV rate was 28%, with the highest upgrade rate for atypical ductal hyperplasia (41%), followed by classical lobular neoplasia (29%) and flat epithelial atypia (11%). Only two cases of carcinoma were detected in the follow-up cohort, both associated with atypical ductal hyperplasia at VABB.
**Conclusion:** Open surgery is still recommended in case of atypical ductal hyperplasia while, for other B3 lesions, excision with VABB-only may be an acceptable alternative if radio-pathological correlation is assessed, if all microcalcifications have been removed by VABB and if the lesion lacks high-risk cytological features.

**B-1154** 11:10

**Second-line vacuum-assisted excision biopsy with 8G needle for the management of B3 microcalcifications: preliminary results**

C.M. Weiss, E. Di Gaetano, S. Plataroti, E. Cattarin, D. Bragagnolo, G. Morana; *Treviso/IT (cmweiss@me.com)*

**Purpose:** To assess the clinical impact of the new management pathway incorporating a second-line vacuum-assisted excision biopsy (VAEB) with 8G needle for microcalcifications of uncertain malignant potential (B3).
**Methods and Materials:** A retrospective analysis was undertaken of all B3 lesions on first-line vacuum-assisted biopsy (VAB) with 10G needle for microcalcifications between January 2017 and January 2018. Following a VAEB, all cases were discussed at the multidisciplinary meeting (MDM) to decide upon management options, referral for 5 years of annual mammographic surveillance (MS) or surgical biopsy (SB). Outcome measures assessed included final diagnosis, frequency of SB, impact on management and comparison with previous results.
**Results:** In 143 VAB for microcalcifications we observed B3 lesions in 33/143 (23%) patients (mean age 54; range 42-76). 9/13 (27%) patients underwent subsequent SB due to imaging findings and pathological results (e.g. ADH expression in B3), 3/9 with a malignant result. The remaining 24/33 (73%) patients were referred to VAEB: 11/24 (46%) B2 lesions and 13/24 (54%) B3 lesions. After MDM, 21/24 (87.5%) patients with completely removed microcalcifications (11B2; 10B3) were referred to MS and 3/44 (12.5%) undergoing SB received a final benignity diagnosis (3/3; 100%).
**Conclusion:** In our study, the overall malignant lesions' incidence in B3 microcalcifications was 3/33 (9%), similar to 10% (3/29) of the previous period January 2015 to December 2016 in which all VAB B3 underwent SB. The new pathway with second-line VAEB has reduced the number of benign SB by 62% while maintaining the same rate of malignancy. MS results are still ongoing.

**B-1155** 11:18

**Percutaneous ultrasound-guided excision with vacuum-assisted breast biopsy (VABB) system of breast lesions with imaging-histology discordance**

C. Depretto, F. Balestra, F. Cartia, C. Ferranti, G. Scaperrotta; *Milan/IT* *(cathie.dp@gmail.com)*

**Purpose:** To assess the performance of percutaneous ultrasound-guided excision of radiologically-histologically discordant breast lesions as an alternative to surgical excision.
**Methods and Materials:** We retrospectively evaluated 159 breast lesions in 159 patients which undergo a 14G ultrasound-guided biopsy and subsequent percutaneous ultrasound-guided excision with VABB system between January 2015 and April 2018. Lesions, with an average diameter of 10.3 mm (range 3-30 mm), showed suspicious ultrasound imaging (BI-RADS 4) but histological outcome non-malignant: B1 (n = 18 patients), B2 (n = 109) and B3 (n = 32). We assessed: the update rate (UR), the technical success (TS) and the technical efficacy (TE) of the procedure and the delayed false-negative results (DR), with radiological follow-up in the following two years; procedural complications were assessed.
**Results:** The post-VABB histological outcomes were B1 (n = 7), B2 (n = 117), B3 (n = 24) and B5 (n = 11), of which 5 CDI (2 associated to DCIS) and 6 DCIS, all surgically radicalized. The UR was 6.9%. The TS was 100%. The TE was 87.9%. The DR was 0%. There were only 7 minor complications (4.4%): haematomas, seromas and liponecrosis.
**Conclusion:** The study demonstrates that VABB excision can be an effective alternative to surgery for all discordant lesions; it allows to adequately diagnose malignant lesions not identified with the CNB, without determining either diagnostic delays or major complications. Compared to surgical treatment, it has lower costs, more simplified and rapid management and almost no cosmetic impact for patients.

**B-1156** 11:26
**Minimally invasive excision of suspicious breast lesions**
N.M. Abdel Razek; *Cairo/EG (naglaabdelrazek@yahoo.com)*

**Purpose:** To assess the diagnostic efficiency of BLES is a management option for small suspicious breast lesions.
**Methods and Materials:** The study is a prospective one conduced during the period from February 2011 to January 2018. approved by the ethical committee.The study included 435 patients with small suspicious non-palpable breast lesions categorized as BIRADS 4& BIRADS 3 with positive family history for breast cancer. All cases were histopathologically diagnosed and the results were compared to the results of open surgery .
**Results:** 435 suspicious lesions were successfully removed using the BLES,19.3 % (84/435) were benign including fibrocystic disease, sclerosing adenosis ,radial scars and papillomas without atypia.38.2% (166/435)were high risk lesions including ,papillomas with atypia, ALH, ADH & 48.4 %(142/435) were malignant including DCIS, LCIS,IDC&ILC.The maximum size removed was 12 mm .The margin was free in 40/142 (28.2%) and the margin was close in 45/142 (31.7%) & the margin was involved in 57/142 (40.1%)of the malignant cases (all were below 8 mm size . 22/142 proven malignant lesions were exposed to Re-surgery and in 20/142, no surgery after BLES, only radiotherapy and sentinel node diagnosis. BLES is 100% as sensitive as the OB regarding the concordance and sensitivity with no underestimation.
**Conclusion:** BLES could be an efficient management for small breast carcinomas if complete excision with free margin was achieved yet still it is the decision of the MDT.Further group work and multicenter studies are required to set guidelines for its use as an optional management for T1 cancer

**B-1157** 11:34
**Performance of breast lesion excision system (BLES) in a complete removal of small clusters of suspicious microcalcifications proved malignant**
A. Christou[1], V. Koutoulidis[2], D. Koulocheri[2], E. Panourgias[2], C. Zografos[2], G. Zografos[2]; [1]*Brighton/UK*, [2]*Athens/GR (alexandrachristou@gmail.com)*

**Purpose:** To retrospectively assess the role and performance of BLES in excision of small clusters of microcalcifications proved histopathologically to be cancers.
**Methods and Materials:** Between January 2014 and 2016, 90 cases of microcalcifications were diagnosed as cancers in our department through the course of BLES biopsy under stereotactic guidance. 20-mm probe was used in all cases. From these, 54 had an initial mammographic size up to 14 mm and selected for statistical analysis to assess the success of the method in excision. The histopathology result of the BLES specimens were compared with the final surgical result. The mean age of our target population was 58.2 years (st dev=8.2, range 43-79 years).
**Results:** 41/54 were pure DCIS, 5/54 were invasive cancers (IDC, ILC, tubular) and 8 had both invasive (6) or microinvasive (2) and DCIS components. Complete removal was achieved in 31/54 cases (57.4%). The initial mammographic size and the histopathologically free disease margins of BLES specimen were the only statistically significant predictive factors for excision. At a cut off initial size of 5 mm ROC analysis showed specificity 95.7% and AUC 72% and free disease margins revealed specificity 80.6% and AUC 78% (success rate 78.2%, p<0.001). The radiology (absence of calcifications on post-BLES mammogram)-BLES pathology concordance in BLES excision showed sensitivity 89.3%. The underestimation rate was 11.1% (6/54 cases).
**Conclusion:** BLES under stereotactic guidance is an accurate biopsy technique with potential use as a therapeutic tool of selected cases of small cancers expressed as suspicious microcalcifications.

**B-1158** 11:42
**Utility of diagnostic breast excision biopsies during two decades of screening mammography**
J.D. Luiten[1], A.C. Voogd[2], V.C.G. Tjan-Heijnen[2], J. Wesseling[3], E.J.T. Luiten[4], L.E.M. Duijm[5]; [1]*Tilburg/NL*, [2]*Maastricht/NL*, [3]*Amsterdam/NL*, [4]*Breda/NL*, [5]*Nijmegen/NL (jackyluiten@hotmail.com)*

**Purpose:** We evaluated the use and value of breast surgical excision biopsies for diagnostic purposes over the last decades in women undergoing mammographic screening, either as a primary procedure or following an inconclusive percutaneous biopsy.
**Methods and Materials:** All women with an excision biopsy among 777,151 screens, obtained from January 1996 until December 2015, were included.
**Results:** Of 17,001 (2.2%) women referred with screen-detected abnormalities, 1007 (5.9%) underwent excision biopsy. Whereof 550 (54.6%) performed as first diagnostic intervention, decreasing from 4.2 per 1000 screens in 1996-1997 to 0.02 per 1000 screens in 2014-2015. The remaining 457 (45.4%) excision biopsies were performed secondary to pathologic findings at percutaneous biopsy. During 1996-1997, 0.8 secondary biopsies per 1000 screens were performed, decreasing to 0.3 per 1000 in 2006-2007 and afterwards increased to 0.6 per 1000 in 2014-2015 (p=0.005). Of all 457 secondary biopsies, 274 (60.0%), had a benign pathology outcome, increasing from 30.8% in 1996-1997 to 76.3% in 2014-2015. Of the 30 patients with a (pre-) malignant pathology at secondary biopsy in the two most recent cohorts (2012-2015), 11 (37%) showed invasive carcinoma and 19 (63%) DCIS (of which 13 low grade DCIS).
**Conclusion:** Although the use of excision biopsy significantly decreased over the past two decades, we observed a significant increased rate in more recent years. Since the vast majority of currently performed excision biopsies reveals a benign diagnosis or show low grade DCIS, a secondary excision biopsy should only be considered if radiologic surveillance and repeated percutaneous biopsy continues to yield indeterminate results.

**B-1159** 11:50
**A retrospective analysis comparing radioactive seed and wire localisation in breast surgery**
P.G. Kumar, J. Young, H. Chen, Y. Li, Q. Qi, X. Peng, T. Khoury; *Buffalo, NY/US (prasanna.kumar@roswellpark.org)*

**Purpose:** The aim of this study is to compare radioactive seed localization (RSL) to wire localization (WL) in breast surgery to compare positive surgical margins, post-operative upgrade rate. Secondary aim was to analyze the specimen size, volume and operating room (OR) time.
**Methods and Materials:** Out of a total 297 patients, 145 WL and 152 RSL breast excision procedures were collected and analyzed. Surgical, radiological and pathological data for each procedure were collected, including pre-operative biopsy diagnosis, final (post-operative) pathological diagnosis, margin status, upgrade rate, lesion size, OR time usage. The final pathological diagnoses were divided into three categories including invasive carcinoma, ductal carcinoma in-situ (DCIS), and benign/high risk lesions.
**Results:** RSL procedures had a lower positive margin rate than WL procedures (2.6% versus 11%, P=0.005). There were no statistical differences in the specimen volume, lesion size to specimen volume ratio and OR time usage between RSL and WL groups. In a multivariable logistic regression model, the WL procedure had a significantly higher odds of positive margin compared to RSL (odds ratio=3.91, P=0.021) adjusted for the final pathology finding and post-operative upgrade of diagnosis. In addition, our data showed that post-operative upgrade of a patient's diagnosis was significantly associated with increased rate of positive surgical margin (25% versus 5.7%, P=0.016).
**Conclusion:** RSL performed better than WL in regard to lower rate of positive surgical margins and lower post-operative upgrade for both invasive cancers and DCIS. This finding favours increased use of RSL as a replacement of WL in the future.

**10:30 - 12:00** **Room E2**

### Neuro

### SS 1411a

### Brain tumours

*Moderators:*
X. Golay; London/UK
E.J. Hendriks; Amsterdam/NL

**B-1168** 10:30
**Can MR and CT imaging features differentiate benign from malignant meningiomas?**
F. Salah, A. Tabbara, N. Al Arab, A. Sibahi, K. Asmar, H. Tamim, M. Makki, R. Hourani; *Beirut/LB (fatima.a.salah@gmail.com)*

**Purpose:** This study aims to highlight MRI and CT characteristics that plays a valuable role in distinguishing benign from high-grade meningiomas pre-operatively.
**Methods and Materials:** Seventy-one patient with intracranial meningiomas who underwent surgical resection at the American University of Beirut Medical Center between 2008 and 2017 were evaluated for various CT and MR imaging features. The correlation between imaging findings, operative reports and histopathological grading was analyzed via univariate and multivariate logistic regression analysis. We analyzed the relation between ADC ratio and tumor recurrence. MRI accuracy in detecting brain invasion was also studied.
**Results:** Univariate analysis results showed a significant correlation between high grade meningiomas (WHO grade II & III) and several MRI features including tumor size and volume (p=0.002,0.02), heterogeneous enhancement (p<0.0001), presence of intra tumoral necrosis (p<0.0001), ill-defined margin (p=0.003), bone necrosis (p=0.004),and brain invasion(p=0.001). High grade meningiomas proved to have a higher rate of recurrence (p=0.007) and higher incidence of brain invasion in pathological reports (p=0.01). However, no significant correlation was established in multivariate analysis while imposing for tumor volume except for older age and presence of intra tumoral necrosis (p=0.04 and 0.001) respectively. The ADC value was not reliable in predicting the tendency to recur. Studying the accuracy of brain invasion detection by MRI, in comparison to the operative report, showed high specificity (83%) and low sensitivity (31%).
**Conclusion:** MR imaging has a promising role in predicting meningioma grade prior to resection which can directly impact future management protocols regarding surgical planning and complications.

**B-1160** 10:38
**Paediatric cerebellar tumours: how useful is the apparent diffusion coefficient?**
A. Arous[1], E. Jemel[2], M. Krichene[3], O. Zayani[3], M. Mahmoud[2], C. Drissi[2], S. Naji[2], N. Hammami[2], M. Ben Hammouda[2]; [1]*Paris/FR*, [2]*Tunis/TN*, [3]*Sfax/TN* *(eya_d@yahoo.fr)*

**Purpose:** Determination of histological types of paediatric cerebellar tumours determines the prognosis. Diffusion-weighted imaging (DWI), through apparent diffusion coefficient (ADC), allows evaluating water diffusion within tissues which can help to appreciate tumour cellularity. The aim of our study is to evaluate the ability of ADC values to differentiate between the most common cerebellar tumours in children.
**Methods and Materials:** Children operated for cerebellar tumours between 2010 and 2014 were retrospectively included in this study. The minimum ADC value of solid component of tumours ($ADC_{min}$) and the ratio of $ADC_{min}$ compared to normal tissue ($ADC_{ratio}$) were measured by two radiologists blinded to the histopathological diagnoses and compared using one-way analysis of variance (ANOVA). A receiver operator curve (ROC) analysis was also performed to generate optimal cutoffs for which the ADCmin would predict tumour histology.
**Results:** Our study included 31 cerebellar tumour. There were 7 pilocytic astrocytomas (PA), 14 medulloblastomas (MB) and 10 ependymomas (EP). $ADC_{min}$ values and $ADC_{ratio}$ were significantly different in three types of tumours ($p$<0.001). $ADC_{min}$ values were lowest for MB (0.49 ± 0.06) without overlap with $ADC_{min}$ values of EP (0.96 ± 0.12) and PA (1.27 ± 0.18). For the ROC analysis, $ADC_{min}$ ≥1.21 distinguished PA from the other tumours with a sensitivity of 71.4% and a specificity of 100%. MB was discriminated from PA and EP by an $ADC_{min}$ value ≤ 0.7 with sensitivity and specificity of 100%.
**Conclusion:** $ADC_{min}$ and $ADC_{ratio}$ values are reliable parameters to distinguish the most common paediatric cerebellar tumours.

**B-1161** 10:46
**The value of whole tumour volume-based ADC histogram analysis of differential diagnosis in paediatric posterior fossa tumours**
S. Yuanyuan; *Zhengzhou/CN (857435584@qq.com)*

**Purpose:** To study the value of whole tumours ADC histogram analysis of differential diagnosis in two paediatric posterior fossa tumours (ependymoma and astrocytoma).
**Methods and Materials:** A retrospective analysis was conducted by brain MRI examination and pathology diagnosis of 77 cases of posterior fossa tumors in children patients in our hospital. Among them, there were 41 cases of ependymoma (there were 21 males and 20 females ) and 36 cases of astrocytoma (there were 19 males and17 females). To draw the region of interest (ROI) in the ADC MR transaxial images of two groups on each layer of tumour level using Mazda software and analyse the whole tumours gray histogram, performing a statistical analysis on the two sets of parameters obtained from histograms to find out statistical significance of each parameter.
**Results:** Through histogram analysis of 9 parameters, these 6 parameters were statistically significant (all P<0.05), including mean, skewness, Perc.10%, Perc.50%, Perc.90% and Perc.99%, the remaining 3 parameters, variance, kurtosis, Perc.01%, had no significant (all P >0.05).
**Conclusion:** The whole tumours ADC grey histogram analysis of two kinds of paediatric posterior fossa tumours in the differential diagnosis has certain value. It can be used as a new auxiliary method of diagnosis in two paediatric posterior fossa tumours.

**B-1162** 10:54
**Histogram analysis of enhancement MRI may predict relapse of primary CNS lymphoma after surgery-combined first-line chemotherapy**
L. Hao; *Zhengzhou/CN (18339298063@163.com)*

**Purpose:** Primary central nervous system lymphoma (PCNSL) is defined as extranodal lymphoma confined to the central nervous system. Despite the high complete remission rate achieved with aggressive first-line therapy, 10-35% of PCNSL are treatment refractory and 35-60% of patients relapse. Our aim was to verify if T1 enhancement MRI histogram analysis is able to predict the relapse of PCNSL after surgery combined first-line chemotherapy.
**Methods and Materials:** Retrospective analysis of pathologically confirmed 60 cases of PCNSL was enrolled in this study. All patients who had undergone surgery and with histologically proved PCNSL, followed up with polychemotherapy, but suffering from a relapse were consecutively recorded 6 months later. Drawing the region of interest (ROI) on the maximum level of enhanced MR axial image and going on histogram analysis, these two steps are all performed on the software named Mazda. A statistical analysis was performed to find out the characteristics of the significant differences between the relapse and no relapse.
**Results:** Sixteen of 60 enrolled patients (26.7%, 8 men, 8 women, median age 50.5 years) relapsed. Histogram analysis of enhanced MRI included the mean, variance, skewness, kurtosis, and the 1, 10, 50, 90 and 99 percentile characteristics. The mean, variance, 90th and 99th percentile were detected for statistical significance. ROC curve analysis of the 90th percentile yielded the best area under the ROC curve (AUC 0.92), sensitivity of 91%, and specificity of 95%, with a cutoff value of 188.
**Conclusion:** Histogram analysis of T1 enhancement MRI can provide more quantitative information of characteristics, which provides a new way to predict the relapse of PCNSL.

**B-1163** 11:02
**Analysis of clinical, imaging and pathological characteristics in patients with pineal parenchymal tumour of intermediate differentiation**
T. Du, M. Zhu, X. Qi, K. Yao, L. Wang, X. Zhang, J. Hu, J. Gao; *Beijing/CN* *(bridge_tq@163.com)*

**Purpose:** To analyse the features of imaging, pathology and prognosis of patients with pineal parenchymal tumour of intermediate differentiation (PPTID).
**Methods and Materials:** 17 patients with PPTID, 10 male and 7 female (age range 4-65 years), underwent MR scanning and surgery. According to the proposal of the 2016 4th edition of WHO Classification of Tumors of Central Nervous System [1], the cases were graded and followed up.
**Results:** All the patients had supratentorial hydrocephalus, 6 and 11 of whom had tumours growing into aqueduct and squeezing aqueduct, respectively. 13 cases (76.5%) with clear boundaries between tumours and thalamus were observed by MR. The cases were divided into grade II (10 cases) and grade III (7 cases), and into 3 groups by Ki-67 index (≤5%, 6-10% and >10%). Ki-67 index of PPTID III was significantly higher than that of PPTID II ($x^2$=11.102, P=.004). There were 6 cases (35.3%) with recurrence revealed by follow-up, including 2 of grade II and 4 of grade III, 3 (17.6%)of whom died, including one of grade II and 2 of grade III. $x^2$ test on tumour recurrence and ki-67 index showed that patients with high ki-67 index had significantly higher recurrence rate than those with low ki-67 ($x^2$=7.242, P=.027).
**Conclusion:** 76.5% of the tumours had clear boundaries with thalamus, suggesting that most of the PPTID had no obvious peripheral infiltration. That 35.3% of the tumours were observed growing along the aqueduct could be the imaging characteristic of PPTID. The patients with high ki-67 index in PPTID group were more likely to relapse.

**B-1164** 11:10
**Correlation between [$^{11}$C]methionine PET uptake and apparent diffusion coefficient in patients with gliomas**
Z. Savintseva, T. Skvortcova, D.V. Zakhs; *St. Petersburg/RU* *(lycaidas@gmail.com)*

**Purpose:** The aim of the study was to assess the value of apparent diffusion coefficient (ADC) from diffusion weighted imaging and PET/CT with [$^{11}$C]methionine (PET-MET) for glioma grading and to correlate these metrics.
**Methods and Materials:** 26 adults with untreated histologically proven brain gliomas were included. Patients underwent MRI with diffusion sequences and PET-MET. ADC maps and [$^{11}$C]methionine uptake maps were generated for each patient and then coregistrated head-to-head using application software. Metrics evaluation included calculating minimum ADC (ADCmin) and maximum [$^{11}$C]methionine uptake index measured as the ratio of the highest tumour SUV to the that in the intact brain (T/Bmax). Mean value of ADC and T/B were obtained for each tumor on the corresponding MR and PET images. Statistical analysis included Spearman's correlation coefficient and discriminant analysis.
**Results:** T/Bmax and ADCmin values were negatively correlated (-0,85, p<0,05). The localization of maximum [$^{11}$C]methionine uptake and minimal ADC did not match in 30% of cases. T/Cmean and ADCmean also showed significant negative correlation (-0,5, p<0,05). The T/Cmax showed best accuracy to distinguish between low and high-grade gliomas using discriminant analysis. The combination of T/Cmax and ADCmin reached of 100% accuracy.
**Conclusion:** We found a strong negative correlation between T/Cmax and ADCmin. But areas of maximum MET uptake and minimal ADC within a tumour did not always matched. It could be a result of the different biological features visualized by PET-MET and ADC. The complementary use of those two methods could be of interest for the accurate preoperative grading of gliomas.

**B-1165** 11:18

**Association between tumour acidity and hypervascularity within human gliomas using pH-weighted amine CEST-EPI and DSC-perfusion MRI at**

Y. Wang[1], J. Yao[2], C. Raymond[2], B.M. Ellingson[2]; [1]*Beijing/CN*, [2]*Los Angeles, CA/US*

**Purpose:** To investigate the association between image contrast obtained from pH-sensitive amine chemical exchange saturation transfer echo planar imaging (CEST-EPI) and relative cerebral blood volume (rCBV) measurements obtained from dynamic susceptibility contrast (DSC)-perfusion MRI in patients with glioma.
**Methods and Materials:** pH weighting was obtained using CEST-EPI estimation of the magnetization transfer ratio asymmetry ($MTR_{asym}$) at 3ppm and rCBV was estimated using DSC-MRI. The correlation between median [BE1] $MTR_{asym}$ at 3ppm and rCBV within T2 hyperintense lesions was evaluated in a total of 81 patients with histologically proven gliomas (40 WHO IV, 23 WHO III, and 18 WHO II gliomas).
**Results:** Both $MTR_{asym}$ at 3ppm and rCBV within T2 hyperintensity increased with increasing tumour grade. A strong correlation was observed between median $MTR_{asym}$ at 3ppm and rCBV within areas of T2 hyperintensity ($R^2$=0.335, $P$=0.0043). No association was observed between $MTR_{asym}$ at 3ppm and rCBV within areas of necrosis or enhancing tumour.
**Conclusion:** The general degree of tumour acidity, measured with amine CEST-EPI, was correlated with hypervascularity, measured with DSC-MRI. These data confirm a potential association between tumour glycolysis and angiogenesis in human gliomas.

**B-1166** 11:26

**Differentiation of histologically proven intracerebral tumour entities with quantitative iodine mapping in dual-layer computed tomography**

J. Borggrefe, M. Gebest, M. Hauger, C. Kabbasch, M. Schlamann, J. Dörner; *Cologne/DE (Jan.Borggrefe@uk-koeln.de)*

**Purpose:** Dual-Layer computed tomography (DLCT) allows for precise quantitation of iodine content and iodine homogeneity in cerebral tumor volumes with blood-brain barrier disruptions. We used iodine density mapping (IDM) to investigate feasibility for the discrimination of biopsy proven malignant cerebral lesions (MCL).
**Methods and Materials:** Retrospective study of 139 consecutive DLCT scans conducted prior to stereotactic needle-biopsy at one CT scanner (IQON, Philips, NL). Iodine density (ID) and contrast-to-noise-ratio (CNR) were determined in IDM using ROIs in MCL and healthy contralateral white matter. Size and localization of ROIs were kept constant between reconstructions. Standard deviation of IDM was considered representative of iodine inhomogeneity (IDI). ID, CNR and IDI were correlated with histopathology.
**Results:** Mean age was 59.4 (±sd 17.1) years, 52 female patients. There were no significant differences of ID in dependence of age but between genders in low-grade astrocytoma (m;0.42±0.09 (mean±sd) vs. f;0.92±0.16, p<0.002) and lymphoma (m:1.15±0.16 vs. f;1.47±0.19, p<0.05). Lymphomas showed strongest CNR (3.28±1,23) in IDM which was significantly higher than in glioblastoma (2.37±1.55, p<0.005) and metastases (1.95±1.14, p<0.02). Glioblastoma and metastases did not show significant differences. The strong enhancing lesions (lymphoma/metastases/GBM) had higher iodine content than oligodendrogliomas, Grad II and Grad III astrocytoma that showed IDM CNR in the range 1.22-1.27±0.45-0.82).
**Conclusion:** Quantitative IDM appears feasible to discriminate tumor lesions such as lymphomas from all other tumors and GBM from low and intermediate grade astrocytoma. It was not capable to discriminate metastases from GBM. Gender may be a significant confounder of IDM in certain brain lesions.
*Author Disclosures:*
**J. Borggrefe:** Speaker; JB received honorarium for scientific lectures from Philips.

**B-1167** 11:34

**Consecutive acquisition of MRDSA and perfusion MRI in cases of brain metastasis through the addition of a supplementary dose of a Gd-based contrast agent at 3T**

K. Tsuchiya, W. Watanabe, A. Abe, K. Uchida, J. Handa, S. Goto; *Kawagoe/JP (tsuchiyak-kyr@umin.ac.jp)*

**Purpose:** To assess the feasibility and value of MR digital subtraction angiography (MRDSA) combined with perfusion imaging (PWI) by employing a dual injection of a Gd-based contrast agent in the diagnosis of brain metastasis.
**Methods and Materials:** Sixteen patients were imaged using a 3-T scanner. Imaging protocol included conventional pre-contrast T1/T2-weighted (T1/T2WIs), FLAIR, and diffusion-weighted images. After the first injection of gadoteridol at 0.1 mmol/kg, MRDSA and the first post-contrast T1WIs were obtained. After a second 0.1 mmol/kg injection of gadoteridol, PWI and a second post-contrast T1WIs were acquired. PWI maps and MRDSA images were evaluated in terms of lesion contrast using a 3-point scale (1=poor/2=fair/3=good). Furthermore, assessment of whether these techniques added diagnostic information to conventional MR imaging was performed. Finally, contribution of the second injection in terms of number of lesions and their visibility was assessed.
**Results:** In all cases, MRDSA and PWI maps allowed assessment of tumour haemodynamics with average score of 2.9 ± 0.5 and 2.9 ± 0.5, respectively. In 7/16 cases (43.8%), MRDSA and/or PWI provided additional diagnostic information to conventional MR imaging. While the number of detected lesions did not increase after the second injection, the lesion visibility improved in 14/16 cases (87.5%).
**Conclusion:** Consecutive acquisition of MRDSA and PWI by employing a dual single-dose injection of the macrocyclic agent gadoteridol, which can be used in such a cumulative administration scheme in our country in cases of suspected brain metastasis, improves the diagnosis as well as the visualization of brain metastasis at 3T.

**B-1169** 11:42

**The forgotten juxta-sellar mass: ectopic pituitary adenomas**

A. Alagely, L. Okromelidze, G.K. Vilanilam, E.H. Middlebrooks, V. Gupta; *Jacksonville, FL/US*

**Purpose:** To increase awareness of ectopic pituitary adenoma (EPA) presenting as a juxta-sellar mass and review the radiologic features and management strategies.
**Methods and Materials:** This case series details four unique radiographic presentations and key imaging findings of EPA based on their anatomic location: suprasellar, cavernous sinus, sphenoid sinus, and clivus.
**Results:** Clinical and radiological manifestations of EPA are variable. The symptomatology depends on mass effect upon adjacent structures, hormonal activity, and rarely, CSF leakage into the sphenoid sinus. Measurement of hormonal titers can facilitate early diagnosis and avoid unnecessary biopsies and/or surgical intervention. On CT, EPAs are often isodense to gray matter and enhance moderately after intravenous contrast. Evaluation of adjacent bone involvement can also be performed using CT. On MRI, EPAs are often low signal on T1WI and variable signal on T2WI with mild to moderate enhancement after intravenous gadolinium. MRI also best reveals the anatomic location of the lesion relative to the pituitary gland, cavernous sinus, and suprasellar structures.
**Conclusion:** A high index of suspicion of EPA is warranted in the evaluation of juxta-sellar lesions located in the suprasellar cistern, cavernous sinus, sphenoid sinus, and clivus. Laboratory investigation of elevated pituitary hormones may help avoid invasive procedures in establishing the diagnosis, facilitate appropriate management, and achieve better outcomes.

**10:30 - 12:00** **Room F2**

### Breast

### SS 1402b

### Breast imaging biomarkers and radiomics

*Moderators:*
D. Djilas; Sremska Kamenica/RS
I.-A. Gheonea; Craiova/RO

**B-1170** 10:30

**Radiomics based on baseline DCE-MRI is predictive of tumour pathological complete response to neoadjuvant systemic therapy in breast cancer patients**

R. Granzier[1], A. Ibrahim[1], H. Woodruff[1], T. van Nijnatten[1], M. de Boer[1], F. Hulsmans[2], P. Lambin[1], M. Smidt[1], M. Lobbes[1]; [1]*Maastricht/NL*, [2]*Sittard-Geleen/NL (r.granzier@maastrichtuniversity.nl)*

**Purpose:** To evaluate the applicability of radiomics models based on baseline dynamic contrast-enhanced MRI (DCE-MRI) for the prediction of pathologic complete response (pCR) of breast tumours to neoadjuvant chemo(targeted)therapy (NCT) in breast cancer patients.
**Methods and Materials:** Two independent cohorts from 2 centres were used to train and validate a radiomics-based classification model. The training cohort included 102 patients with 129 breast tumours. The external cohort (120 patients, 125 breast tumours) was split into testing (n=63) and validation cohorts (n=62). Patients received NCT and underwent baseline DCE-MRI scan. Tumours were delineated manually by 3 individuals on MRI acquired 2 minutes after contrast injection. Features were extracted using OncoRadiomics software. Overall concordance correlation coefficient was used to assess feature stability among observers. Stable features were ranked and selected via support-vector machine recursive feature elimination. Random forest binary classifications were generated using the training cohort and hyper-parameters tuned on training and test cohorts. Performance of the model was assessed using area under the curve (AUC).

**Results:** 30.2% of tumours in training cohort achieved breast pCR, 23.8% and 32.2% of tumours achieved pCR in test and validation cohorts. 1322 features were extracted, of which 1094 features were stable. The 20 top-ranked features were selected to build the pCR prediction model. The AUC of the model was 0.68 in both training cohort and external validation.
**Conclusion:** Our results demonstrate that baseline DCE-MRI radiomics analysis on pretreatment MRI can be used to predict tumour pCR after NCT. Further studies with larger sample sizes are needed to confirm these results.
*Author Disclosures:*
**H. Woodruff:** Employee; (minority) shares in the company Oncoradiomics.
**P. Lambin:** Advisory Board; Oncoradiomics SA, Convert pharmaceuticals SA. Patent Holder; Two times issued to Oncoradiomics (PCT/NL2014/050248, PCT/NL2014/050728), 1 time issued patent to ptTheragnostic/DNAmito (PCT/EP2014/059089). Research/Grant Support; Varian Medical, Oncoradiomics, ptTheragnostic, Health Innovation Ventures and DualTpharma. Shareholder; Oncoradiomics SA, Convert pharmaceutical SA. Other; three non-patentable inventions (softwares) licensed to ptTheragnostic/DNAmito, Oncoradiomics and Health Innovation Ventures.

**B-1171** 10:38
**Feasibility study of whole breast texture analysis for predicting tumour response to neoadjuvant chemotherapy**
J.-Y. Zhou, H. Dan, G. Cheng; *Shenzhen/CN (aftdoctor@163.com)*

**Purpose:** To investigate the feasibility of whole breast texture analysis in predicting tumor response to neoadjuvant chemotherapy(NAC) for patients with non-mass breast cancer.
**Methods and Materials:** In this institutional review board approved study, 70 patients with non-mass breast cancer between March 2008 to March 2018 were included. Breast magnetic resonance(MR) imaging was performed before treatment(E1), after two cycles of NAC(E2), and after four cycles of NAC(E3, usually before surgery). Two-dimensional computerized analysis was performed and the texture features of the lesion side and the healthy side were obtained. Pathological results obtained by surgery and the Miller-Payne pathological grade was used as the gold standard. These quantitative parameters were analyzed by t-test and least absolute shrinkage and selection operator(LASSO) regression.
**Results:** Pathologic complete response(Miller-Payne grade 4 and 5) was achieved in 20 (28.6%) of 70 cases. Skewness(E3 lesion, E3 lesion/ E3 health, E3 lesion/E1 lesion), kurtosis(E3 lesion, E3 lesion/E3 health, E3 lesion/E1 lesion), consistency(E3 health), entropy(E1 lesion/E1 health), relevance(E2 lesion, E1 lesion/E1 health) showed significant differences in t-test(p<0.05). LASSO regression analysis showed that the whole breast texture features have a good performance in judging tumor response of neoadjuvant chemotherapy.(area under the receiver operating characteristic curve = 0.803).
**Conclusion:** It is feasible to use the whole breast texture analysis as a new method of predicting tumor response to NAC in non-mass breast cancer.
*Author Disclosures:*
**J. Zhou:** Research/Grant Support; This study is supported by an internal research fund of Peking university shenzhen hospital. **H. Dan:** Research/Grant Support; This study is supported by an internal research fund of Peking university shenzhen hospital. **G. Cheng:** Research/Grant Support; This study is supported by an internal research fund of Peking university shenzhen hospital.

**B-1172** 10:46
**Tumour texture parameters of IDC in neoadjuvant chemotherapy: early identification of non-responders on breast MRI**
M. Nadrljanski, Z. Milosevic; *Belgrade/RS (dr.m.nadrljanski@gmail.com)*

**Purpose:** Texture parameters (variance of SI, mean of gradient, variance of gradient, kurtosis of SI, and entropy) were evaluated on baseline MRI and following the 2nd cycle of neoadjuvant chemotherapy (NAC) in patients with IDC, to assess the tumour texture features in early identification of non-responders (NR) to NAC.
**Methods and Materials:** Fifty patients (N=50) were included in the retrospective analysis of baseline and MRI following the 2nd cycle of NAC. Texture parameters were computed and correlated to morphologic features and DWI-ADC in 25 NR with IDC. Additional matched 25 responders (R) served as the control group.
**Results:** Tumour size and ADC did not change significantly in NR after the 2nd cycle of NAC (2.88+/-0.38 vs. 2.76+/-0.36 [cm], p=0.14; 1.01+/-0.14 vs. 1.05+/-0.13 [mm2/s 10-3], p=0.36), tumour texture parameters changed significantly: variance of gradient (346.5+/-12.6 vs. 355.6+/-16.9, p=0.01), kurtosis of SI (1.47+/-0.09 vs. 1.54+/-0.11, p=0.02), entropy LH (60.39+/-4.34 vs. 64.42=/-3.05, p=0.001) and entropy HL (61.02+/-5.51 vs. 65.63+/-3.63, p<0.00001). Variance of SI, mean of gradient, variance of gradient and kurtosis of SI were significantly different between NR and R after the 2nd cycle of NAC (p<0.00001; p=0.0008; p=0.047, p<0.0001).
**Conclusion:** Tumour texture parameters: entropy HL, entropy LH, kurtosis and variance of gradient increase in NR following the 2nd cycle of NAC. Texture parameters (variance of SI, mean of gradient, variance of gradient and kurtosis of SI) significantly differ between NR and R after the 2nd cycle of NAC. Texture parameters may contribute to early identification of NR on breast MRI.

**B-1173** 10:54
**Background parenchymal enhancement on breast MRI in patients undergoing neoadjuvant chemotherapy: is it a biomarker of tumour response?**
C. Costanza[1], G. Mariscotti[1], M. Durando[1], A. Tagliafico[2], L. Bergamasco[1], I. Castellano[1], P. Fonio[1]; [1]*Turin/IT*, [2]*Genoa/IT*
*(dott.claudiacostanza@gmail.com)*

**Purpose:** To evaluate if changes in background parenchymal enhancement (BPE) on breast MRI can be associated to tumour response in patients with locally advanced breast cancer undergoing Neoadjuvant Chemotherapy (NCT).
**Methods and Materials:** A retrospective review identified 55 patients with locally advanced breast cancer who underwent NCT between January 2015 and January 2018. All patients had breast MRI before, during and after NCT. Two dedicated breast Radiologist, blinded to clinical and pathological data, rated BPE level according to BI-RADS criteria (minimum=1, mild=2, moderate=3, marked=4) on pre- and post-NCT breast MRIs. A statistical analysis was performed to compare BPE changes and the pathological response after treatment (complete response versus non-complete response, including partial and non-response).
**Results:** In the study population, tumour size at diagnosis ranged from 2-9cm (mean:3.7cm); 30/55(55%) patients were pre/perimenopausal and 25/55(45%) postmenopausal. There was a complete pathological response post-CTN in 23 patients(42%), partial response in 27(49%) and non-response in 5(9%). At MRI pre-CTN, BPE level was minimum in 15/55 patients(27.6%), mild in 1/55(2%), moderate in 22/55(39.2%) and marked in 17/55(31.4%) and did not significantly (p=0.995) differ based on final pathological response. Post-CTN BPE levels were lower in complete pathologic response cases, although not statistically significant(p=0.885). BPE level decreased after CNT in 21/55(38.2%) patients. The decrease in BPE occurred in 10/23(43%) cases of complete pathological response and in 10/32(31%) cases of non-complete response(p=0.60).
**Conclusion:** Our preliminary results suggest that a decrease in BPE could have a role as a potential biomarker for predicting tumour response in patients undergoing NCT.

**B-1174** 11:02
**A new objective mathematical tool for assessment of response and predicting residual disease in breast cancer using CESM**
M. Gomaa, E. Kamal, R.M. Kamal, S. Mostafa, R. Mubarak, M. El-Adawy;
*Cairo/EG (mohammedgomaa555@yahoo.com)*

**Purpose:** Initiating a new objective mathematical tool for evaluation of response post-neoadjuvant chemotherapy in breast cancer and residual disease using the CESM.
**Methods and Materials:** 42 breast cancer patients were included. All underwent 2 CESM; before and after NAC. We used a mathematical image analysis software (MATLAB and Simulink) (Release 2013b). The proposed technique used 42 CESM images as inputs. The technique consists of 3 main steps 1-preprocessing 2. Extracting the region of Interest (ROI) and 3-Assessment the response to chemotherapy. The technique depends on the analysis of the tumour number of pixels included within the ROI and intensity values to find the percentage between the ROI before and after chemotherapy. We compared the evaluation via mathematical objective technique, RECIST 1.1 & combined response evaluation approach using both RECIST 1.1 in addition to subjective visual evaluation.Results were correlated to postoperative pathology using Miller-Payne grades.
**Results:** Correlation with pathology between the proposed method, RECIST & combined method was 0.89,0.59&0.69 with accuracy 85.7%,54.8%&85.7% respectively. With classification of patients into responder and non-responders, the mathematical evaluation showed higher sensitivity, positive and negative predictive values (100, 97.5,100 % respectively) compared to the evaluation of RECIST method (87.2%, 97.1% 28.6%) and the combined response method (97.4%,97.4% and 66.7%). The pathology showed 39/42 patients were responders. Combined response evaluation approach; 38/39 patients were responders while using RECIST 1.1; only 34 patients and using the proposed method; 39/39 were responders.
**Conclusion:** Mathematical analysis of CESM allows objective evaluation of the response of breast cancer to chemotherapy.

**B-1175** 11:10

**Radiomic signatures derived from diffusion-weighted imaging for the assessment of breast cancer receptor status and molecular subtypes**

D. Leithner[1], J.V. Horvat[2], M. Marino[1], B. Bernard-Davila[1], M.S. Jochelson[1], D. Martinez[1], E.A. Morris[1], S. Thakur[1], K. Pinker-Domenig[3]; [1]*New York, NY/US*, [2]*Sao Paulo/BR*, [3]*Vienna/AT (doris.leithner@googlemail.com)*

**Purpose:** To evaluate the diagnostic performance of radiomic signatures extracted from diffusion-weighted imaging (DWI) for the assessment of breast cancer receptor status and molecular subtypes.
**Methods and Materials:** Ninety-one patients with biopsy-proven breast cancer (luminal A, n=49; luminal B, n=8; HER2 enriched, n=11; triple negative, n=23), who underwent multiparametric 3T MRI were included in this IRB-approved retrospective study. To compare annotation segmentation approaches (a) all lesions were manually segmented on high b-value DWI and propagated to ADC maps and (b) a subgroup (n=79) was directly segmented on the ADC map. Radiomics analysis included first-order histogram (HIS), co-occurrence matrix (COM), run-length matrix (RLM), absolute gradient (GRA), autoregressive model (ARM), discrete Haar wavelet transform (WAV), and lesion geometry (GEO) features. Fisher, probability of error and average correlation, and mutual information coefficients were used for feature selection. Linear discriminant analysis and *k*-nearest neighbor classification with leave-one-out cross-validation were used for pairwise separation of molecular subtypes. Histopathology was the gold standard.
**Results:** The following accuracies >90% were achieved for approach (a) segmented on DWI: luminal B vs. HER2-enriched, 94.7% (based on COM features); luminal B vs. others, 92.3% (COM, HIS); HER2 enriched vs. others, 90.1% (RLM, COM). Higher accuracies were achieved with approach (b) segmentation on ADC maps: luminal A vs. luminal B, 91.5% (COM, WAV); luminal B vs. HER2-enriched, 100% (COM, WAV); luminal B vs. others, 91.1% (WAV, ARM, COM).
**Conclusion:** DWI radiomic signatures enable the assessment of breast cancer molecular subtypes with high diagnostic accuracy. Higher accuracies are achieved when tumour segmentations directly are performed on ADC maps.
*Author Disclosures:*
**M.S. Jochelson:** Other; Received speaker honoraria from GE Healthcare.
**E.A. Morris:** Grant Recipient; GRAIL. **K. Pinker-Domenig:** Grant Recipient; GRAIL.

**B-1176** 11:18

**Radiomic signatures with contrast-enhanced magnetic resonance imaging for the assessment of breast cancer receptor status and molecular subtypes: initial results of a multicenter study**

D. Leithner[1], J.V. Horvat[2], M. Marino[1], B. Bernard-Davila[1], M.S. Jochelson[1], D. Martinez[1], E.A. Morris[1], S. Thakur[1], K. Pinker[3], A. Meyer-Baese[4]; [1]*New York, NY/US*, [2]*Sao Paulo/BR*, [3]*Vienna/AT*, [4]*Tallahassee, FL/US (doris.leithner@gmail.com)*

**Purpose:** To evaluate the diagnostic performance of radiomic signatures extracted from contrast-enhanced magnetic resonance imaging (CE-MRI) for the assessment of breast cancer receptor status and molecular subtypes.
**Methods and Materials:** One-hundred and thirty-two patients with biopsy-proven treatment-naïve breast cancer (luminal A, n=49; luminal B, n=32; HER2-enriched, n=24; triple negative, n=27) who underwent CE-MRI at 3T were included in this IRB-approved HIPAA-compliant retrospective multicenter study. Radiomics analysis of manually segmented tumors included calculation of features derived from first-order histogram (HIS); co-occurrence matrix (COM), run-length matrix (RLM), absolute gradient (GRA), autoregressive model (ARM), discrete Haar wavelet transform (WAV) and lesion geometry (GEO). Fisher, probability of error and average correlation (POE+ACC), and mutual information (MI) coefficients were used for feature selection (optimal subsets of ten features each). Linear discriminant analysis followed by *k*-nearest neighbor classification (with leave-one-out cross-validation) was used for pairwise radiomics-based separation of receptor status and molecular breast cancer subtypes. Histopathology served as the standard of reference.
**Results:** Radiomic signatures yielded the following accuracies >80%: luminal A vs. luminal B, 85.2% (POE/mainly based on HIS and COM features); luminal A vs. HER2-enriched, 84.9% (MI/entirely based on HIS and COM features).
**Conclusion:** Radiomic signatures with CE-MRI enable the assessment of breast cancer receptor status and molecular subtypes with high diagnostic accuracy. In particular, luminal A breast cancers appear to have distinct radiomic signature on CE-MRI.
*Author Disclosures:*
**M.S. Jochelson:** Other; Received speaker honoraria from GE Healthcare.
**E.A. Morris:** Grant Recipient; GRAIL. **K. Pinker:** Grant Recipient; GRAIL.

**B-1177** 11:26

**Radiogenomics of invasive breast cancer: association between ultrasound vascular imaging features and tumour genomic profiles for tumour angiogenesis**

E. Park[1], B. Seo[1], M.-R. Han[2], J. Kim[1], H. Lee[1], K. Cho[2], O. Woo[2], S. Song[2]; [1]*Ansan/KR*, [2]*Seoul/KR (epark100@naver.com)*

**Purpose:** To evaluate the relationship between ultrasound vascular imaging features and genetic alteration of tumour tissue for angiogenesis in invasive breast cancer patients.
**Methods and Materials:** This retrospective study was approved by the institutional review board. Preoperative ultrasound vascular imaging features were correlated with genomic characteristics in 31 invasive breast cancers. We evaluated quantitative and qualitative ultrasound vascular parameters on superb microvascular imaging (SMI) and contrast-enhanced ultrasound (CEUS); parameters of SMI (vascular index, vessel morphology, distribution, and penetrating vessel) and CEUS (time intensity curve parameters and enhancement pattern). Targeted next-generation sequencing of 105 genes was performed for the genomic DNA obtained from breast tumour and normal tissues. Single-variant association test was performed to evaluate relationship between ultrasound imaging features and genomic profiles. We used PLINK to detect the single variants associated with ultrasound features.
**Results:** Our targeted sequencing approach identified that ultrasound vascular features such as penetrating vessel, vessel distribution, vessel morphology and vascular index on SMI, and enhancement degree, enhancement order, area, slope, and time to peak on CEUS were associated with one or more somatic mutations in *APC*, *ATM*, *BRCA1*, *BRCA1*, *CSF1R*, *FGFR1*, *HIF1A*, *KDR*, *MET* and *NTRK1*, with *FGFR1* being the most commonly mutated gene (p<0.05). The PolyPhen-2 analysis revealed that 33% of the identified mutations (39 of 119) were predicted to have damaging effects on protein function.
**Conclusion:** Our preliminary radiogenomic results demonstrate the potential of ultrasound vascular imaging to predict tumour angiogenesis and genetic variants associated with invasive breast cancer in a non-invasive manner.
*Author Disclosures:*
**B. Seo:** Research/Grant Support; This work was supported by Research Grant, Toshiba Medical Systems Korea Co.,Ltd, Guerbet, and Philips healthcare.

**B-1178** 11:34

**Radiomics analysis of breast cancer on digital breast tomosynthesis**

F. Valdora, C. De Luca, B. Bignotti, F. Rossi, J. Matos, M. Calabrese, A. Tagliafico; *Genoa/IT (valdorafrancesca@gmail.com)*

**Purpose:** To analyse Breast Cancers (BCs) on Digital Breast Tomosynthesis (DBT) in women with dense breasts and negative mammography using Radiomics.
**Methods and Materials:** This retrospective study is Institutional Review Board approved. We selected BC patients who underwent DBT evaluation from December 2017 to August 2018. N= 56 women (age range 34-83) with proven diagnosis and with Mammography-Negative Dense Breasts. N=28 controls (age range 40-80) were included. Using a Radiomics approach normal and pathological breast parenchyma were evaluated, and correlations among radiomics features and clinical and prognostic parameters were investigated. Regions of interest (ROIs) were done by two radiologists with different expertises. N=106 Radiomics features were extracted and evaluated. p<0.05 was considered as statistically significant.
**Results:** The median age of the patients was 55 years. After Radiomics feature number reduction (from 117 to 106). 13 out of 106 radiomics features significantly correlated to tumor size; the feature Entropy significantly correlated to histological subtype (r=-0.413; p.002) and with ki67, the proliferation index (r=-0.280; p.004). the feature Energy correlated with Progesteron Receptor status (r=-0.281; p.004). ROC analysis identified seven features belonging to the first order (n=1), gray level Co-occurrence matrix (GLCM) (n=4) and gray level Run Lenght Matrix (glrlm) (n=1) and neighborhood gray tone difference matrix (NGTDM) (n=1) with an area under the curve for diffentiation of normal and pahtological tissue of at least 0.76 (0.75 to 0.86) with p<0.05.
**Conclusion:** The analysis showed a correlation with progesteron receptor and proliferation index. These information could improve understanding of breast cancer through imaging.

**B-1179** 11:42
**Radiomics and deep learning can aid classification of contrast-enhanced spectral mammograms**
Y. van Wijk, A. Jochems, S. Primakov, M.B. Lobbes, P. Lambin, H. Woodruff; *Maastricht/NL (y.vanwijk@maastrichtuniversity.nl)*

**Purpose:** Our aim was assessing the benefit of radiomics in tumor classification on contrast-enhanced spectral mammography (CESM). This could potentially reduce the number of unnecessary biopsies. Hypothesis: radiomics features extracted from delineated masses can help differentiate between benign and malignant tumors as determined by pathology.
**Methods and Materials:** Both low-energy and recombined CESMs were available for 968 patients, as well as golden standard pathology results. We delineated 1135 masses on 562 patients. 1864 radiomics features were extracted from the delineated masses, of which the eight most relevant were selected via correlation bias reduction and recursive feature elimination. The data was balanced based on outcomes using an adaptive synthetic sampling approach, and a random forest model was built to classify pathology as invasive ductal carcinoma (IDC) or non-IDC. The dataset was split into training (795) and validation (340), and model performance was evaluated as the AUC of the ROC.
**Results:** We found that 401 delineations were of IDCs. The most predictive features involved mean, median and 90th percentile of intensity. The model performed with an AUC of 0.86 (95% CI:0.82-0.90) on validation data.
**Conclusion:** The radiomics model was highly predictive in distinguishing between IDC and non-IDC. Future studies will include clinical features and we will validate on external data. We will also compare the model to deep learning methods and physician performance.
*Author Disclosures:*
**P. Lambin:** Grant Recipient; Varian Medical, Oncoradiomics, ptTheragnostic, Heath Innovation Ventures, DualTpharma. Patent Holder; DNAmito. Shareholder; Oncoradiomics.

**B-1180** 11:50
**Texture analysis and machine learning for differentiation between phyllodes tumours of the breast from fibroadenomas in non-contrast MRI**
M. Tsuchiya, T. Masui, M. Katayama, K. Terauchi, M. Sasaki, K. Kawamura, T. Yamada, H. Sakahara; *Hamamatsu/JP (D16018@hama-med.ac.jp)*

**Purpose:** To investigate whether magnetic resonance (MR) features can distinguish phyllodes tumours of the breast from fibroadenomas and assess the feasibility of machine learning based texture analysis (TA).
**Methods and Materials:** This retrospective study included 50 women with histopathologically proven phyllodes tumours of the breast (n=25; benign, 14; intermediate, 8; malignant, 3) or fibroadenomas (n=25) who underwent breast MRI between January 2004 and January 2018. Regions of interest for TA were manually drawn on axial fat-saturated T2-weighted images on the slice displaying the largest cross-sectional area of the tumour. First order, second order, and higher order texture features were extracted from each tumour by using a freely available software package. Texture feature selection based on machine learning analysis were performed for selection features and multiple logistic regression model. The accuracy of TA features for differentiating between phyllodes tumours of the breast from fibroadenomas was evaluated using a receiver-operating-characteristic (ROC) analysis.
**Results:** Five independent texture features allowed for differentiation between phyllodes tumours of the breast and fibroadenomas on fat-saturated T2-weighted images: WavEnLH.s-5, S(0,1) Contrast, S(0,2) Contrast, VertlRLNonUni, S(0,2) SumAverg (WavEnLH.s-5, P=0.017; S(0,1) Contrast, P=0.017; S(0,2) Contrast, P=0.011; VertlRLNonUni, P=0.025; S(0,2) SumAverg, P=0.017). Multiple logistic regression models revealed that the feature of S(0,2) Contrast resulted in the highest accuracy for differentiating phyllodes tumours of the breast and fibroadenomas, with an area under the curve (AUC) of 0.74 with a cut-off ≥4.24 (76.0% sensitivity, 76.0% specificity).
**Conclusion:** This machine learning based TA of non-contrast MR images allows for the distinguish phyllodes tumours of the breast from fibroadenoma.

**10:30 - 12:00** **Room D**

### Artificial Intelligence and Machine Learning

### SS 1405

### Imaging informatics: management and performance analysis

*Moderators:*
C.C. Bortolotto; Pavia/IT
S. Morozov; Moscow/RU

**K-28** 10:30
**Keynote lecture**
L. Donoso; *Barcelona/ES*

**B-1181** 10:39
**Machine learning in radiology management: predicting ED workflow overloads**
O. Pianykh, D. Parke; *Boston, MA/US (opiany@gmail.com)*

**Purpose:** The goal of our study was to investigate whether we can predict ED examination volume surges several hours in advance, so that additional resources can be allocated to keep ED reporting on time.
**Methods and Materials:** Complex and unpredictable ED workflow calls for advanced prediction tools, therefore machine learning was chosen. ED department defined 7 unread CT cases as overload threshold, leading to stress and delays. Therefore we developed a logistic predictive model to forecast the probability of overload several hours in advance. A full year of historical ED data was used to account for seasonal trends. More than 100 predictive features were derived from the HIS data, including current time and date, number of active radiologists, patient and examination counts and types, examination complexity scores, and more. Several machine-learning algorithms were evaluated to find the model with the optimal predictive quality.
**Results:** Random forest model provided the most accurate prediction, 79.5% accurate for 2-hour prediction. These results were found to be very satisfactory and sufficient for decision-making.
**Conclusion:** Large and noise ED workflow data presents a perfect target for machine-learning applications. Workflow overload predictions enable optimized resource management, help avoid stressful overloads, keep critical diagnostic time to the minimum.

**B-1182** 10:47
**Performance of the competence centre for MRI and CT imaging in a network of diagnostic centres**
A. Fedorov, N. Berezina, M. Cherkashin, A. Lavrenteva; *St. Petersburg/RU (fedorov@ldc.ru)*

**Purpose:** To evaluate the effectiveness of a centre of competence (CC) in a network of diagnostic centres of wide geography with remote medical personnel training and a quality control of examinations.
**Methods and Materials:** A common network of clinics has 100 units of multimodal diagnostic equipment in four countries. The CC works with VNC and VPN remote access technologies via secure communication channels with reliable storage of patient's personal data. The CC has three MR and CT technologists, who support each centre in conducting and maintaining the quality of examinations upon request. The specialist of the CC comes to the scanner via remote access and corrects the clinical protocols or solves the problem of visualization after received request.
**Results:** When training medical personnel, a webinar platform is used that supports the function of displaying the presenter's desktop, from which a remote access session is broadcast to one of the scanners where the examination is being conducted. During the commissioning of the CC, 74 scanners were connected in 57 cities. In the first year, the number of online consultations exceeded 400. After 5 years, more than 1,000 consultations were carried out in a year, approximately 4.2 per day. Over the 5 years of the centre's work, positive statistics on research cancellations due to various malfunctions has been noted - from 7% to 3.4%.
**Conclusion:** CC can provide online advice including emergency requests; there are standards of clinical protocols, updates , and developing new scanning techniques in the clinical practice.

**B-1183** 10:55
**Fuzzy PACS: linking large unorganised image and report databases for development and validation of deep learning algorithms**
R. Mukherji, V. Mahajan; *New Delhi/IN (vidur@mahajanimaging.com)*

**Purpose:** Development and validation of deep learning (DL) algorithms for medical imaging requires access to large organised datasets of images and their corresponding reports. Currently, most medical imaging data in the world are unorganised and require images and text reports to be manually linked. An approach for linking medical images and reports of patients, where no unique identifier for linking them exists, is presented.
**Methods and Materials:** A DICOM image database of 311,694 studies and a separate mySQL database with 296,938 reports needed to be matched at study level. No unique identifier existed to link the two databases and not all reports had matching images, and there was only partial overlap between the databases. Additionally, patient names were inexactly entered with varied formats in the two databases making direct matching impossible. Fuzzywuzzy Python library, which incorporates fuzzy string matching, a technique based on Levenshtein distance between strings to estimate text similarity was used to match patient name in the two databases following date and modality-level filters. Four fuzzy matching techniques (simple, partial, token-set and token-sort ratios) were evaluated.
**Results:** Simple, partial, token-set and token-sort ratios gave 4.56%, 46.45%, 57.37% and 7.97% matches of reports, respectively, with 95% match confidence. Token set ratio, which had the highest match percentage, matched 170,336 reports to their corresponding studies.
**Conclusion:** Fuzzy matching is a promising technique to merge independent datasets without unique identifiers, saving thousands of man-hours, critical for development and validation of DL algorithms.

**B-1184** 11:03
**Subspecialisation in radiology: effects on the diagnostic spectrum of radiologists and report turnaround time in a Swiss university hospital**
M.H. Maurer[1], M. de Bucourt[2], A. Berghöfer[2], A. Huppertz[3], A.B. Rosenkrantz[4], F. Streitparth[5], J.T. Heverhagen[1], T. Meyl[1]; [1]*Berne/CH*, [2]*Berlin/DE*, [3]*Potsdam/DE*, [4]*New York, NY/US*, [5]*Munich/DE (martin.maurer@insel.ch)*

**Purpose:** To analyse the changes in radiologists' work profiles and the reporting time after the implementation of a professional subspecialisation in the radiology department of a Swiss university hospital.
**Methods and Materials:** In a retrospective analysis, the overall number of different radiologic examinations performed in the department of radiology of a large Swiss university hospital were documented for 2014 and 2016 before and after the implementation of subspecialised reporting (subspecialities: abdominal, musculoskeletal, cardiothoracic, emergency, and paediatric imaging) in May 2015. For six selected radiologists the number and types of reported examinations as well as the related radiology report turnaround times (RTATs) were analysed in detail and compared between the two 1-year periods.
**Results:** Overall, there was a significant increase of 10.3% in the total number of examinations performed in the whole department in 2016 compared with 2014. For 4 of the 6 radiologists, the range of different types of examinations significantly decreased with the introduction of subspecialised reporting ($p<0.05$). Furthermore, there was a significant change in the subset of the ten most commonly reported types of examinations reported by each of the 6 radiologists. Mean overall RTATs significantly increased for 5 of the 6 radiologists ($p<0.05$).
**Conclusion:** Implementation of subspecialised reporting led to a change in the structure and a decrease in the range of different examination types reported by each radiologist. Mean RTAT increased for most radiologists. Subspecialised reporting allows the individual radiologist to focus on a special field of professional competence but can result in longer overall RTAT.

**B-1185** 11:11
**Operational performance optimisation by means of an imaging analytics platform: a case study**
P. Szatmari[1], P. Pytlewski[2], N. Papanikolaou[3], R. Illing[1]; [1]*Budapest/HU*, [2]*Warsaw/PL*, [3]*Nicosia/CY (peter.szatmari@affidea.com)*

**Purpose:** To optimize operational performance using an advanced imaging analytics platform in combination with lean management concepts.
**Methods and Materials:** Affidea is operating the public hospital of Walbrzych through a public-private partnership scheme, where the MRI scanner was connected to the MR Excellence Platform to monitor in real-time several key performance indicators including number of exams, examination time, non-scanning time and image resolution. Several interventions in the operational level were done following the six sigma and Kaizen philosophy to eliminate waste. The main interventions included elimination of free slots in the daily schedule, continuity of exam bookings, implementation of pre-built monthly scheduler and grouping of similar exams in designated scheduling blocks. All relative inputs were extracted from the MR Excellence Platform.
**Results:** There was a significant reduction in the waiting time to perform an exam from 8 days to 1.78 days after the implementation of the Operational Optimization project, while the average number of daily exams performed raised from 24.5 to 28.4 after the optimization of MRI protocols. All new sequences were evaluated in terms of diagnostic efficiency and image quality and approved by the local radiologists.
**Conclusion:** Imaging analytics-powered operational optimization can result in significant improvement in operational efficiency of an MRI department maintaining high diagnostic standards.
*Author Disclosures:*
**P. Szatmari:** Employee; Affidea BV. **P. Pytlewski:** Employee; Affidea BV. **N. Papanikolaou:** Owner; MRIcons LTD. **R. Illing:** Employee; Affidea BV.

**B-1186** 11:19
**Flagging of pathological head CT interpretations using natural language processing (NLP)**
Y. Barash, T. Levy, Y. Elazar, E. Zimlichman, E. Konen, S. Soffer, G. Guralnik, M.M. Amitai, E. Klang; *Ramat Gan/IL (yibarash@gmail.com)*

**Purpose:** Alerting on pathological findings in imaging reports is a quality measure. We compared two NLP algorithms for flagging pathological head CT reports: the bag of words (BOW) algorithm and the long short-term memory (LSTM) deep learning algorithm.
**Methods and Materials:** Institutional review board approval was granted. The BOW model is used for text classification where the frequency of occurrence of each word is used as a feature for training a classifier. LSTM is a neural network that has some internal contextual cells that act as long-term or short-term memory cells. The output of the LSTM network is modulated by the state of these cells. This can be utilized in NLP as the sequence of words in a paragraph has contextual meaning. We collected consecutive head CT non-English (Hebrew) reports performed in our ER during annual January and February (2013 - 2017). Each report was labeled as either normal or pathologic (e.g. haemorrhage, infarct, mass). All the words in the dataset were tokenized. The BOW algorithm used unigrams and bigrams as features. For the LSTM network, we embedded each word after tokenization, MLP over the last hidden layer and cross-entropy loss for training. The algorithms' performances were assessed using the accuracy metrics.
**Results:** We retrieved 5,890 head CT reports. The incidence of pathological findings was 55%. The algorithm BOW algorithm showed an accuracy of 87.5% and the LSTM algorithm showed an accuracy of 89.1%.
**Conclusion:** The LSTM algorithm showed improved accuracy over the classic BOW algorithm on non-English reports.

**B-1187** 11:27
**Fast displaying time of electronic patient record and PACS sharing system with IHE-XDS/XDS-I and cloud technology**
H. Kondoh[1], T. Kawai[1], M. Mochida[1], T. Yamaguchi[2]; [1]*Yonago/JP*, [2]*Hino/JP (kondoh@med.tottori-u.ac.jp)*

**Purpose:** EPR and PACS sharing system was demanded interoperability and quick viewing even on Internet. We developed that sharing system with IHE-XDS and XDS-I and cloud technology.
**Methods and Materials:** Global standard IHE-XDS and XDS-I was introduced on centre cloud server and thin-client infrastructure with one registry server. It was connected EPR server and DICOM server of regional eighteen hospitals and gathered HL7 based data and DICOM images. It showed the data and images on thin client infrastructure and IP-sec VPN through Internet. The image display time of the sharing system though internet seemed to be faster than intra-hospital PACS with RAID disc and gigabit ethernet. The display times were measured by video data and compared the sharing system access from PC with iPhone tethering and from PC in Heidelberg conference through Internet with intra-hospital PACS.
**Results:** The display times were 0.627 seconds (0.37-0.86) in intra-hospital PACS, 0.228 seconds (0.23-0.27) in the sharing system access with iPhone tethering, 0.250 seconds (0.198-0.297) access from PC in Heidelberg.
**Conclusion:** The thin client system was said to reduce the network load to 1/1000. And Large flash memory dramatically reduced data access in the server. So even if Internet used, the display time was seemed to be faster than intra-hospital PACS. Because the thin client system is sending the subtraction image on same matrix size, there are no data reduction in black and white image.

**B-1188** 11:35
**Blended learning using radiology short video based lecture series improves medical school student performance and satisfaction**
A. Vavasseur, F. Muscari, P. Otal, H. Rousseau, L. Dercle, F. Mokrane; *Toulouse/FR (vavasseur.a@chu-toulouse.fr)*

**Purpose:** A shift in student's behavior has been observed in medical schools. Medical school students (MSs) are *millenniums* and part of a new '*YouTube*' generation. Our purpose is to measure the impact of a combination of educational resources as a blended learning (short video-based lecture (VBL), as part of the flipped classroom) among three promotions of MSs from our university hospital.
**Methods and Materials:** 3 consecutive promotions performed a pre-test, based on abdominal imaging (respectively 102, 93 and 109 students). Then, they had access to 61 VBLs focused on abdominal imaging (total duration of 5h 11m). VBLs were available over a period of 5 months via a dedicated educational online platform. Finally, MSs performed a post-test and a face to face course to correct it. The efficiency of this teaching format was measured quantitatively by a post-test and learner analytics, as well qualitatively (direct learner feedback using student's satisfaction questionnaire).
**Results:** Blended learning combining VBLs and face to face final course significantly improved student results. The average post-test results was 77.2% and significantly higher than the pre-test (53.8%; Student t-test: P<0.001). Satisfaction surveys about this format showed that 99% of students were satisfied or very satisfied by this e-learning training. The amount of videos views was 91 views per student (average) and a total of 27662 views. Moreover, students adherence to this medical education format increased across promotions.
**Conclusion:** The flipped classroom based on short video based lecture attracts medical students, improve their performances in radiology and obtains their adherence.

**B-1189** 11:43
**Automatic segmentation for coronary computed tomography angiography using convolutional neural networks**
J. He[1], Q. Zhou[1], X. Li[2], X. Xu[1], X. Ding[1], X. Zhou[1], M. Zhang[1]; [1]*Hangzhou/CN*, [2]*Beijing/CN (jiafa_he@zju.edu.cn)*

**Purpose:** To present a convolutional neural networks (CNN) for automatic segmentation for Coronary Computed Tomography Angiography.
**Methods and Materials:** 115 patients were examined by 128-slice dual-source CCTA and then the CT images were automatic segmented by CNN technique, of which 91 patients is used for training and 24 for testing. We present a multi-scale 3D convolutional neural networks to segment the coronary artery for automatic segmentation and assisting the diagnosis of coronary artery disease (CAD). Specifically, the multi-scale 3D CNN was firstly utilized to segment coronary artery and aorta from the test dataset. Then a vessel-measurement-based region growing algorithm was proposed to refine the vessel segmentation, especially for the vascular branching, resulting a test Dice coefficient of 0.7833. In addition, several key indicators of coronary artery disease were derived automatically including starting point of artery vessels.
**Results:** Experimental results show that coronary artery and aorta were identified and segmented successfully while the maximum symmetric surface distances are 0.35mm and 0.42mm, respectively. Comparing with most proposed methods, our method is full-automatic and can realize the segmentation without any manual interaction. As for the efficiency, it takes only an average time of 3.4s to segment one volume of the CCTA.
**Conclusion:** In this study, we presented a multi-scale 3D convolutional neural networks and showed the feasibility of the proposed method on segmenting coronary artery from CT.

**B-1190** 11:51
**Deep learning based chest x-ray whole-image search and retrieval**
N. Ramanauskas, J. Dementaviciene, D. Barušauskas, J. Bialopetravicius, J. Armaitis, G. Rimeika; *Vilnius/LT (naglisr@gmail.com)*

**Purpose:** A typical hospital currently possesses more than a decade worth of digital radiological images with their radiological descriptions in the PACS/HIS system. We aimed to create a radiological chest x-ray search system.
**Methods and Materials:** In our solution, a deep learning neural network trained on a large set of radiographs with respective radiological findings is used in order to index the images. This indexing process takes into account not only the pathology, but also the localization of the pathology, and the overall features of the radiograph. In order to quantify the quality of the search system, a radiology resident was presented with a set of 77 challenging chest x-ray images. Each of the reference images was subsequently indexed by the search system, and used to retrieve 10 radiographs from the hospital database of more than 200`000 images. A radiology resident was asked to evaluate and write reports for each of the images initially without assistance. After each report he was asked to review the the returned search matches and modify the report if he found it necessary. We evaluated the initial and the modified reports and extracted statistics.
**Results:** The report was modified after inspecting search matches for 56/77 cases. The impression was modified after inspecting search matches for 50/77 cases. The differential diagnosis was expanded for 28/77 cases.
**Conclusion:** An image search solution for frontal chest x-ray images based on a Deep Learning model has been created.

*Author Disclosures:*
**N. Ramanauskas:** Employee; Oxipit, UAB. Founder; Oxipit, UAB.
**D. Barušauskas:** Employee; Oxipit, UAB. Founder; Oxipit, UAB.
**J. Bialopetravicius:** Employee; Oxipit, UAB. Founder; Oxipit, UAB.
**J. Armaitis:** Employee; Oxipit, UAB. Founder; Oxipit, UAB.

**10:30 - 12:00** **Room G**

### Physics in Medical Imaging

### SS 1413

### Radiation protection technology and management

*Moderators:*
L. Bowden; Dublin/IE
A. Trianni; Udine/IT

**K-29** 10:30
**Keynote lecture**
A. Trianni; *Udine/IT*

**B-1191** 10:39
**A proposed paradigm revision for recurrent radiation safety training**
S. Balter[1], P. Abrams[1], S. Kalra[2], E.M. Leidholdt[3]; [1]*New York, NY/US*, [2]*Philadelphia, PA/US*, [3]*Mare Island, CA/US (sb2455@cumc.columbia.edu)*

**Purpose:** Designing a radiation training curriculum to meet the needs and concerns of clinical audiences can be perplexing. We formulated and tested a new teaching paradigm structured as a slow-motion, one-on-one conversation. We describe the results of a recent seminar that used this paradigm.
**Methods and Materials:** The test was conducted at a recent international interventional cardiology meeting. Topics were staff radiation safety and patient radiation management. Several weeks in advance of the session, two clinical faculty members polled their workplace colleagues and presented key 'audience' questions to two medical physicists. The clinical faculty presented expanded versions of their questions at the session, following which the physicist faculty answered these questions point-by-point. The session started with essential general topics not included in the clinical question list and ended with a discussion between the entire faculty and audience.
**Results:** Essential basic topics (e.g. dose) had to be added. Priority audience concerns about reproductive risks and clinical radiobiology were unanticipated. Enough time was allowed to circulate several versions of the entire training set to the faculty before going live. Faculty interactions resulted in major improvements in the questions, answers, and general topic sections. The audience remained engaged throughout the presentations and discussion and asked many additional questions.
**Conclusion:** This format was highly productive and generated high levels of audience participation. Audience members informally polled after the session felt that many of their a priori concerns were presented and appropriately discussed. The final topic list also provides insights into improvement of formal didactic training.
*Author Disclosures:*
**S. Balter:** Speaker; Mavig. **S. Kalra:** Advisory Board; Boston Scientific. Speaker; Abiomed, Boston Scientific, Phillips/Spectranetics, Abbott Vascular.
**E.M. Leidholdt:** Author; Lipponcott Williams and Wilkins.

**B-1192** 10:47
**The reasons for practitioners' passive approach to informing patients prior to their radiological examinations**
H. Oikarinen, L. Ukkola, A. Henner; *Oulu/FI (helja.oikarinen@ppshp.fi)*

**Purpose:** Despite requirements, there are suspicions that patients do not obtain adequate information from any source in connection with their radiological examinations. The aim was to survey whether practitioners in Northern Finland provide information, and if not, to elucidate the reasons for the passive approach.
**Methods and Materials:** Using a questionnaire, practitioners were asked whether they have informed patients about the necessity or radiation dose and risks of the examination during the past year. In case of a passive approach, the reasons were inquired. In all, 205/361 (57%) practitioners responded to the inquiry: 85% of the respondents were radiographers and 15% radiologists, and 53% were from the university hospital and 47% from other departments.
**Results:** Altogether 43% of the respondents did not inform about the necessity. 87% of them assumed that the referrer had already provided the information. Furthermore, 62% and 56% did not inform about the dose or risks, respectively. Regarding the passive approach to dose information, practitioners supposed that the referrer had already informed, information was not needed or might cause unnecessary fear (41, 47 and 41%, respectively). There were similar reasons for avoiding risk information (63, 37 and 45%, respectively). According to the open question, many practitioners expect patients to ask questions before informing them.
**Conclusion:** Ignorance regarding responsibilities, assumption that patients are not interested and concern about causing unnecessary fear were the main reasons for the passive approach among the practitioners. There is a need to provide them education, practical guidelines and easy-access digital educational material.

**B-1193** 10:55
**A retrospective review of justification of computed tomography examinations in Northern Ireland**
Y. Sullivan, L. Fraser, G. Woodhouse; *Oxford/UK (yvonne.sullivan@phe.gov.uk)*

**Purpose:** Justification is one of the key principles of radiation protection. The most appropriate use of CT relies on many factors which must be considered each and every time a scan is justified. The Regulation and Quality Improvement Authority (RQIA), commissioned this study to assess the justification rate of CT examinations in a single 24-hour period across NHS Trusts in Northern Ireland.
**Methods and Materials:** The study retrospectively reviewed the clinical information within 450 referrals for diagnostic CT scans against published referral guidelines to assess justification. Each referral was independently assessed by two consultant radiologists and where opinions differed, arbitration was carried out. The reviewers were also asked to indicate if an alternative modality would have been more appropriate in any referrals that they deemed unjustified. Results were analysed for variations in justification rate by patient gender, patient age, geographical location and anatomical region scanned.
**Results:** This study found that 94% of the CT referrals reviewed were justified. The number of justified scans varied with regard to the anatomical region being scanned, with the abdomen and pelvis being the only region that demonstrated any statistical significance in the number of unjustified examinations. The highest suggested alternative modality was MRI at 33% of the unjustified referrals.
**Conclusion:** The study compared favourably to a similar study carried out in Sweden from 2009 where the justification rate was 80%. This review shows that conventional systems utilising up-to-date referral criteria and robust justification processes can ensure that inappropriate examinations are rare.

**B-1194** 11:03
**A radiation dose index monitoring (RDIM) systems comparison for CT procedures**
A. Mazzilli[1], A. Loria[1], R. Castriconi[1], T. Lusa Aguero[2], R. Calandrino[1], A. del Vecchio[1]; [1]*Milan/IT*, [2]*Valencia/ES (mazzilli.aldo@hsr.it)*

**Purpose:** Since the issuing of the 2013/59/Euratom directive, several RDIM systems have been developed. The aim of this study was to compare Effective Doses (ED) and Organ Doses (OD) calculated by two different systems for Computed Tomography (CT) procedures.
**Methods and Materials:** Dosimetric data were collected for more than 30 cardiac acquisitions of adult patients performed with a dual-energy CT device. The ED and the OD for each acquisition were calculated by the two most widespread RDIM systems and the differences were evaluated. Both RDIM systems calculations are Monte Carlo (MC) based. The software allow an interactive dose calculation modifying acquisition parameters. In order to perform a more appropriate comparison, ED and OD differences between the two software were analyzed in automatic and interactive mode considering the same acquisition scan-length and target region. For the interactive mode comparison, the organs have been grouped into irradiated, partially-irradiated and not-irradiated.
**Results:** Statistical analysis of automatic and interactive mode yielded an ED median difference of 0.53mGy (25th-percentile: 0.16mGy; 75th-percentile: 2.4mGy) and 0.45mGy (25th-percentile: 0.21mGy; 75th-percentile: 2.11mGy), respectively. The median OD differences for interactive mode showed a similar behavior of the two software for partially-irradiated and not-irradiated organs (1.20mGy and 0.42mGy). For irradiated organs a greater dispersion of the doses (4.56mGy) was found.
**Conclusion:** Although the ED differences were quite small, a great variability in the OD was found, especially for the irradiated organs. Therefore, dose measurements in phantom or independent MC-simulations should be made to validate these kinds of software.

**B-1195** 11:11
**Using an automatic patient dose registry to help in the optimisation of interventional radiology practice**
E. Vano, J.M. Fernandez, R.M. Sánchez-Casanueva, J. Ten; *Madrid/ES (eliseov@med.ucm.es)*

**Purpose:** Obtaining sufficient diagnostic information (image quality) during interventional procedures is a priority, but optimisation also requires considering patient radiation doses. This work presents the experience using a homemade automatic patient dose registry (“Dose On Line for Interventional Radiology” DOLIR) for optimisation.
**Methods and Materials:** The automatic patient dose registry allows collecting data for all the radiation events (when the Radiation Dose Structured Reports are available) and auditing the protocol of the examinations and their different imaging acquisition modes: fluoroscopy, cine, DSA and CBCT events. A continuous comparison (using samples of 30 procedures) with Diagnostic Reference Levels (DRLs) allows suggesting corrective actions when appropriate. The Medical Physics Service carries out periodic validation of patient dose quantities managed by the system.
**Results:** The system used in a large university hospital during the last two years is currently connected to the interventional systems of other 6 hospitals, so as to jointly manage the patient dose values and arrange intercomparison of protocols. The current number of interventional procedures available in the system is about 40.000. Kerma area product, kerma at the patient entrance reference point and some calculated skin dose maps (and peak skin doses) are included.
**Conclusion:** The use of the automatic patient dose registry allowed an easy auditing process of the individual patient dose values (in case of potential skin radiation injuries) and obtaining median patient dose values for groups of procedures (with the same or similar clinical indications). The easier comparison with DRLs helped suggest corrective actions when appropriate.

**B-1196** 11:19
**Update of the diagnostic reference levels for CT in Switzerland using dose management software**
C. Aberle[1], N. Ryckx[2], R. Treier[3], S. Schindera[4], R. Menz[1]; [1]*Basle/CH*, [2]*Lausanne/CH*, [3]*Liebefeld/CH*, [4]*Aarau/CH (roman.menz@usb.ch)*

**Purpose:** To update the previous Swiss national diagnostic reference levels (DRLs) by acquiring big data on CT radiation doses with dose management software.
**Methods and Materials:** CT dose data from 14 radiological institutes in Switzerland, for a total of 50 CT scanners, were collected between 2014 and 2017 through local dose management software. Fifteen different standard CT protocols were compared. Data cleaning steps were adjusted individually for each participating institute and protocol. After data cleaning, 220,269 CT exams were available. The DRLs were calculated as the 75$^{th}$ percentile of median $CTDI_{vol}$ and DLP values per scanner. They were compared to the previous DRLs published in 2010.
**Results:** The DRLs for single acquisitions were 51 mGy ($CTDI_{vol}$, -22%) and 890 mGy cm (DLP, -11%) for CT of the head, 7 mGy ($CTDI_{vol}$, -30%) and 250 mGy cm (DLP, -37%) for CT of the chest, 11 mGy ($CTDI_{vol}$, -27%) and 540 mGy cm (DLP, -17%) for CT of the abdomen and pelvis, 25 mGy ($CTDI_{vol}$, 0%) and 420 mGy cm (DLP, +20%) for CT of the facial bones/sinuses, and 8 mGy ($CTDI_{vol}$, -47%) and 300 mGy cm (DLP, -33%) for CT to exclude pulmonary embolism. The average relative change, compared to the previous DRLs, was -30% for $CTDI_{vol}$ (0% to -47% depending on the protocol) and -22% for DLP (+20% to -40%).
**Conclusion:** The updated national DRLs for CT are substantially lower compared to the previous DRLs demonstrating the efforts of the radiological community in Switzerland to lower CT radiation exposure.
*Author Disclosures:*
**C. Aberle:** Research/Grant Support; Federal Office of Public Health (FOPH), Switzerland. **S. Schindera:** Research/Grant Support; Federal Office of Public Health (FOPH), Switzerland.

**B-1197** 11:27
**Exposure levels from radiology procedures used in myeloma diagnostics**
L. Weber[1], I. Kristiansson[1], M. Hansson[1], R. Siemund[1], H. Geijer[2], M. Geijer[1]; [1]*Lund/SE*, [2]*Örebro/SE (lars.weber@skane.se)*

**Purpose:** Myeloma diagnostics have traditionally been performed using a radiographic skeletal survey. CT scanning has been suggested as a replacement and has been introduced in our institution. Effective doses have been reported in the literature, without correlation with body mass index (BMI). We have evaluated the effective doses imparted to the patients during this procedure.

**Methods and Materials:** Length, weight and dose-length product were collected for each patient and CT examination. Conversion factors based on BMI were generated using Monte Carlo-based simulations on voxel-based phantoms (n=5). For each phantom size a sex- and BMI-specific conversion factor was generated. Subsequently, individual conversion factors were generated through exponential curve fitting. Finally, the effective dose was calculated for each patient based on sex and BMI.
**Results:** The sex-averaged conversion factors for underweight, normal weight and obese patients (BMI = 18, 21 and 25, respectively) were 0.011, 0.011 and 0.010 mSv/mGycm, respectively. The median effective dose for a scan length from the top of the head to the knees was 5.1 mSv (n=256, range = [2.7, 12.6]) for all patients. Dose levels in our institution are comparable with published data but higher than that for conventional skeletal imaging.
**Conclusion:** CT scanning for myeloma diagnostics in our institution showed higher doses than have been published for conventional skeletal imaging but is still comparable to other investigations of this methodology. The advantages in terms of shorter examination times are perceived to be positive for the predominantly elderly patients where dose concerns are of less importance.

**B-1198** 11:35
**A method to optimize DSA clinical protocols**
V. Ravaglia, G. Venturi, N. Scrittori, S. Farnedi; *Ravenna/IT (valentina.ravaglia@auslromagna.it)*

**Purpose:** To optimize DSA vascular protocols using an objective method of evaluation, in terms of a figure of merit (FOM) that correlates patient entrance dose (ED) and image quality.
**Methods and Materials:** Measurements were performed on a Philips AlluraClarity FD20 angiographic system for 7 different clinical peripheral protocols. Exposure parameters and added filtrations F1, F2 and F3 (1 mm Al + respectively, 0.1, 0.4 and 0.9 mm Cu) were automatically selected by AEC. A dedicated DSA phantom with Al strips simulating vessels was imaged and ED was simultaneously measured. To evaluate image quality we measured and averaged the SNR on different subtracted images. A FOM was calculated as the ratio between $SNR^2$ and ED. We also evaluated "low-dose" protocols, obtained adding Cu filters at tube exit.
**Results:** AEC selected only 3 different configurations: $C_1$ (68kV and F2), $C_2$ (80kV and F1) and $C_3$ (65kV and F1) with ED per frame, respectively, 1.93, 2.87 and 2.29 mGy/min. ED per frame for $C_{2\text{-low}}$ (80kV and F2) and $C_{3\text{-low}}$ (65kV and F2) was 1.34 and 1.14 mGy/min. As example, we reported SNR and FOM (nGy/frame) for the thicker strip for clinical and "low-dose" protocols: $C_1$ 1.83 and 1727.7, $C_2$ 2.09 and 1514.0, $C_{2\text{-low}}$ 2.20 and 3603.7, $C_3$ 1.25 and 687.7 and $C_{3\text{-low}}$ 1.42 and 1763.5, respectively.
**Conclusion:** Among clinical protocols, the lower ED and higher FOM was for $C_1$, the unique configuration with F2. Even for other protocols, F2 reduces the dose but not the SNR, resulting in a higher FOM.

**B-1199** 11:43
**Tissue equivalency in radiopaque 3D printing of patient phantoms over a wide range of radiation energies - applications in imaging and dosimetry**
F.B. Schwarz, M. Ziegert, B. Hamm, M. Scheel, P. Jahnke; *Berlin/DE (felix-benjamin.schwarz@charite.de)*

**Purpose:** Radiopaque 3D Printing (R3P) is a method to produce patient specific phantoms with detailed anatomy, but was so far only demonstrated with iodine enhanced ink. The purpose of this work was to further develop R3P towards tissue equivalent phantoms.
**Methods and Materials:** Radiopaque inks were designed corresponding to the attenuation coefficients of human tissues over an energy range of 10 keV to 100 MeV. CT images were printed on single-side adhesive-coated paper with inkjet technology. The printed paper sheets were stacked, bonded together, and laser cut to shape, resulting in stable phantoms. Using a phantom manufactured from a CT data set of a porcine specimen, Hounsfield units (HU) and absolute dose measurements were compared against porcine tissue. In addition, tissue equivalent anthropomorphic breast phantoms were manufactured and evaluated in mammography and digital breast tomosynthesis (DBT).
**Results:** Linear attenuation coefficients of the developed materials had a mean deviation from human tissue by <3% over the entire energy range. HU showed similar energy dependency for the tissue equivalent phantom as for porcine tissue, with low energy influence on soft tissue and decreasing HU with increasing radiation energy for bone tissue. Dose measurements did not differ significantly between the phantom and the porcine sample when HU were adjusted. The breast phantoms provided realistic images and dose values in mammography and DBT.
**Conclusion:** Radiopaque 3D printing with tissue equivalent materials yields realistic patient phantoms for application over a wide range of diagnostic and therapeutic energies, including spectral CT and absolute dosimetry.

*Author Disclosures:*
**F.B. Schwarz:** Grant Recipient; 03EFHBE093. **M. Ziegert:** Grant Recipient; 03EFHBE093. **M. Scheel:** Patent Holder; DE202015104282U1, EP000003135199A1, US020170042501A1. **P. Jahnke:** Grant Recipient; 03EFHBE093. Patent Holder; DE202015104282U1, EP000003135199A1, US020170042501A1.

**B-1200** 11:51
**Should testing for lead contamination be included in lead radiation protection apparel quality control?**
M. Barati[1], R. Illing[2], L. Kardos[2], C. Paraskevopoulou[1], K. Katsari[1]; [1]*Amsterdam/NL*, [2]*Budapest/HU (milan.barati@affidea.hu)*

**Purpose:** Lead Radiation Protection Apparel (RPA) is routinely used for radiation protection of patients, accompanying persons and personnel during diagnostic and interventional procedures. In 2017 an article, 'Lead Aprons are a Lead Exposure Hazard' was published,suggesting the addition of lead dust testing to the quality control (QC) process. The aim of this study was to assess whether this test should be included in our own process.
**Methods and Materials:** The QC program was performed on 212 RPAs from 11 Affidea centers in Hungary. RPA condition was evaluated through visual/tactile inspection and imaging.'Damaged' is defined for aprons with defects >15mm$^2$ on parts shielding critical organs or defects >670mm$^2$ on any other area; collars/gonad shields with defects >11mm$^2$. 'Passed with limitations' includes all RPA with defects not greater than these criteria. RPA with no defects were 'passed'. LeadCheck (3M) colorimetry was used to test for lead dust.
**Results:** 84% RPA 'passed' QC, 9% 'passed with limitations' and 7% were 'damaged'.All RPA 'damaged' and 'passed with limitations'and 15% of 'passed' were tested for lead dust. 43% of 'damaged' RPA, 11% of 'passed with limitations' and 4% of 'passed' were found contaminated. Contaminated RPAs were cleaned, retested and found negative for lead dust.
**Conclusion:** Results prove the importance of lead dust assessment as part of QC. Further investigation of the reappearance of lead dust must be undertaken to establish the frequency of cleaning.

**10:30 - 12:00** **Room K**

### Chest

### SS 1404

### Advanced lung imaging: MRI and artificial intelligence

*Moderators:*
N.N.
J. Coolen; Leuven/BE

**B-1201** 10:30
**Comparison of quantitative regional perfusion-weighted phase resolved functional lung (PREFUL) MRI with pulmonary perfusion DCE-MRI in COPD patients**
T.F. Kaireit[1], A. Voskrebenzev[1], M. Gutberlet[1], J. Freise[1], B. Jobst[2], H.-U. Kauczor[2], T. Welte[1], F. Wacker[1], J. Vogel-Claussen[1]; [1]*Hannover/DE*, [2]*Heidelberg/DE (till.kaireit@me.com)*

**Purpose:** Perfusion-weighted non-contrast enhanced proton lung MRI during free breathing is maturing as a novel technique for assessment of regional lung perfusion, but has yet not been validated in COPD patients. The goal of this study was to determine if phase-resolved functional lung (PREFUL-) MRI correlates with dynamic contrast enhanced (DCE)-MRI as established reference and lung function testing.
**Methods and Materials:** Forty-seven patients with stable COPD were included as a single center subgroup analysis nested in the German COPD cohort study COSYCONET - a prospective, observational, multicenter cohort. For PREFUL-MRI a spoiled gradient echo sequence and for DCE-MRI a contrast-enhanced three-dimensional time-resolved spoiled gradient echo sequence was used at 1.5T. Slices of PREFUL and DCE-MRI were matched using a landmark based approach and co-registered. Perfusion defect percentages (QDP) were calculated for both methods and correlated with each other and lung function testing using Spearman's correlation coefficient (r), spatial overlap metrics and Bland-Altman plot analysis.
**Results:** PREFUL-MRI and DCE-MRI matched spatially with a spatial overlap of 62.2(57.2-67.2)%. QDP were comparable on a global 39.3(31.8-45.5)% vs. 44.7(35.4-50.0)%) as well as on a lobar level, although a systematic overestimation of PREFUL-QDP compared to PBF-QDP mainly in the lower lobes resulted in an overall overestimation for the whole lung with a mean difference of 5%(95% confidence interval [CI]: 3.0%;7.0%). Significant correlations with lung function test parameters was found for both methods, such as PREFUL-QDP vs. FEV1, r=-0.75, P<0.0001.
**Conclusion:** PREFUL-MRI is a promising noninvasive, radiation-free tool for quantification of regional perfusion in COPD patients.

**B-1202** 10:38
**Fully automated 3D quantification of the lung emphysema in COPD using 3D-UTE MRI**
I. Benlala, P. Berger, P.O. Girodet, C. Dromer, J. Macey, F. Laurent, G. Dournes; *Pessac/FR*

**Purpose:** To develop and validate a fully automated 3D quantification of emphysema extent in COPD patients using 3D-MRI with ultrashort echo time (UTE).
**Methods and Materials:** Twenty-nine COPD patients (21 males/ 8 females, age 70+-7 years) and 11 healthy volunteers (10 males/1 female, age 64+-4 years) were prospectively enrolled between 2014 and 2017. All patients underwent pulmonary function test, 3D-UTE MRI at millimetre spatial resolution (voxel size=1mm$^3$) and MDCT scan the same day. Two readers experienced in chest imaging performed the evaluation. 3D automatic quantification of relative low signal area (LSA%) and relative low attenuation area ($LAA\%_{<-950HU}$) were measured as a surrogate for emphysema using 3D-UTE MRI and MDCT, respectively. Skewness of lung MR frequency distribution histogram was also determined. Comparison of means was performed using t-test and correlation using Pearson test. Reproducibility was assessed using Lin's concordance correlation coefficient.
**Results:** Using 3D-UTE, LSA% was significantly higher in COPD patients than in healthy volunteers (p<0.001). Skewness of lung MR frequency distribution histogram was significantly right tailed towards the lowest signal values in COPD (p<0.001). In 29 COPD patients, both LSA% and skewness correlated well with $LAA\%_{<-950HU}$ (r=0.77, p<0.001; r=0.87, p<0.001, respectively) and FEV1 (r=-0.43, p=0.01; r=-0.55, p<0.001, respectively). Intra and inter-observer reproducilbilty of both LSA% and skewness were found perfect using Lin's concordance correlation coefficient ($\rho_c$>0.99).
**Conclusion:** 3D automatic quantification of the lung emphysema is feasible and reproducible using non-contrast enhanced 3D-UTE MRI and may prove useful in the follow-up of COPD patients without radiation exposure.

**B-1203** 10:46
**Comparison of quantitative capability among DWI with FASE and EPI sequences at 1.5T or 3T systems and FDG-PET/CT: for differentiating malignant from benign solitary pulmonary nodules**
Y. Ohno[1], M. Yui[2], H. Lee[3], H. Koyama[4], Y. Kishida[1], S. Seki[1], T. Yoshikawa[1], Y. Kassai[2], T. Murakami[1]; [1]*Kobe/JP*, [2]*Otawara/JP*, [3]*Seoul/KR*, [4]*Osaka/JP* *(yosirad@kobe-u.ac.jp)*

**Purpose:** To directly and prospectively compare the quantitative capability for diagnosis of solitary pulmonary nodules (SPNs) among diffusion-weighted imaging (DWI) with fast advanced spin-echo (FASE) and echo planar imaging (EPI) sequences in a 3T system, DWI with EPI sequence at a 1.5T system and FDG-PET/CT.
**Methods and Materials:** 97 consecutive patients with 129 SPNs underwent DWIs with FASE and/or EPI sequences at 3T and 1.5T systems, FDG-PET/CT, and pathological and/or follow-up examinations. According to final diagnoses, all SPNs were divided into malignant (n=87) and benign (n=42) SPNs. In each lesion, apparent diffusion coefficients (ADCs) from all DWIs ($ADC_{3TFASE}$, $ADC_{3TEPI}$ and $ADC_{1.5TEPI}$) and $SUV_{max}$ were assessed by ROI measurements. To compare all indexes between two groups, Student's t test was performed. Then ROC analyses were performed to compare diagnostic performance among all indexes. Finally, sensitivity, specificity and accuracy were compared among all methods by McNemar's test.
**Results:** There was significant difference of all indexes between two SPN groups (p<0.0001). ROC analyses showed area under the curve (Az) of $ADC_{3TFASE}$ was significantly larger than that of others (p<0.05). Accuracy (AC) of $ADC_{3TFASE}$ (90.7%) was significantly higher than that of others ($ADC_{1.5TEPI}$: 84.4%, p=0.008; $ADC_{3TEPI}$: 82.9%, p=0.002; $SUV_{max}$ 75.2%, p<0.0001).
**Conclusion:** DWIs with FASE sequence has a better potential for quantitative diagnosis of SPNs than DWI with EPI sequence at 1.5T and 3T systems and FDG-PET/CT. FASE sequence would be better to be applied for DWI at 3T system to improve diagnostic performance of SPNs.
*Author Disclosures:*
**Y. Ohno:** Research/Grant Support; Canon Medical Systems Corporation. **M. Yui:** Employee; Canon Medical Systems Corporation. **S. Seki:** Research/Grant Support; Canon Medical Systems Corporation. **T. Yoshikawa:** Research/Grant Support; Canon Medical Systems Corporation. **Y. Kassai:** Employee; Canon Medical Systems Corporation.

**B-1204** 10:54
**Paediatric Hodgkin's and non-Hodgkin's lymphoma on chest MRI: do we really need intravenous contrast agent?**
C.T. Arendt, M. Beeres, D. Leithner, P. Tischendorf, M.C. Langenbach, B. Kaltenbach, T.J. Vogl, T. Gruber-Rouh; *Frankfurt a. Main/DE (crt.arendt@gmail.com)*

**Purpose:** Increasing awareness of potential short- and long-term side effects from gadolinium-based contrast agents has underlined the need for contrast-free MR imaging. The aim of this study was to determine whether the intravenous administration of contrast media is necessary for thoracic imaging in children and adolescents with lymphoma.
**Methods and Materials:** Patients under 25 years of age who underwent histopathological examination of thoracic lymph nodes and at least one chest MRI with unenhanced and contrast-enhanced sequences (Jan 2010 - Oct 2016) were enrolled in this monocentric, retrospective study. Seven different thoracic lymph node stations, including mediastinal, hilar, periclavicular and axillar regions, were evaluated by two readers regarding lesion diameter, number, shape, necrosis and infiltration of surrounding structures. Findings were categorized into suspicious (>1 cm; round; necrosis; infiltration) and unsuspicious.
**Results:** 51 consecutive patients (mean age, 16.0 yrs; age range, 4-23 yrs; 60.8% male) with histologically proven diagnosis of thoracic Hodgkin's (70.6%) and Non-Hodgkin's lymphoma (25.5%) and lymphadenopathy (3.9%) were retrospectively included. 236 examinations were analysed, 36 study participants received additional follow-up studies (average number, 4.7; range, 1-15). Most lymph nodes categorized as suspicious were located in the mediastinum (89.5%). High agreement (lowest κ=0.88) between unenhanced and contrast-enhanced sequences was found for both suspicious and unsuspicious lymph nodes. The only significant difference was observed in sizing mediastinal lymph nodes with a deviation of 1mm (p<0.001; all other p>0.05).
**Conclusion:** Staging and disease surveillance in young patients with thoracic lymphoma can be safely done without contrast agent.

**B-1205** 11:02
**Differentiation of inflammatory andfibrotic ground-glass opacity (GGO) with MRI: a pilot study**
C. Romei[1], L. Turturici[1], L. Tavanti[1], M. Marletta[1], P. Wielopolski[2], S. Colagrande[3], D. Caramella[1], F. Falaschi[1], P. Ciet[2]; [1]*Pisa/IT*, [2]*Rotterdam/NL*, [3]*Florence/IT (chiara.romei@gmail.com)*

**Purpose:** To differentiate fibrotic and inflammatory GGO using T2-weighted (T2-w) and contrast-enhanced MRI (CEMRI).
**Methods and Materials:** After consent, 3 patients with IPF/NSIP underwent CT and MRI on the same day, which were repeated after 6-month-therapy. MRI consisted of 3D-SPGR-PD-w, 2D-PROPELLER, 3D-CUBE-T2-w and CEMRI acquired at 5-10-20 minutes. Two main radiological interstitial lung disease (ILD) features were quantitatively scored with PRAGMA-ILD on MRI: Fibrotic(FL) and Normal Lung(NL) tissue. FL included all fibrosis-related changes (honeycombing, reticulation, GGO). Data were expressed as ml and % total lung volume. MRI data were compared with CT data scored with CALIPER-software and clinical data. Based on T2-w signal intensity (SI) and CEMRI, FL was classified as inflammatory. Statistic included Pearson correlation and Mann-Whitney-U test.
**Results:** MRI-score had excellent correlation with CALIPER (r=0.97, p<0.00001). MRI-score overestimated fibrosis (mean-difference 15.3 % SD 7.5 %) and underestimated normal lung (mean-difference 17.8 SD 7.5) compared to CT. 4/9 ROIs showed significant difference in T2-w SI and were labelled as inflammatory. The mean SI differences FL were 106.8 (SD 32.1) and 2.1 (SD 34.4) for the inflammatory and the stable ROI, respectively. Inflammatory ROIs had higher SI difference than stable ROI between baseline and follow-up (p<0.0001). Two inflammatory areas, as expected in the NSIP, evolved in fibrotic tissue. CEMRI showed rapid peak of enhancement in the FL suspicious for active inflammation.
**Conclusion:** In our pilot study ILD morphological changes can be reliably be quantified with MRI. T2-w SI and CEMRI can identify inflammation area and their evolution during ILD therapy.

**B-1206** 11:10
**What is the contribution of MR spectroscopy in the differential diagnosis of solitary pulmonary masses?**
A. Hekimoglu, O. Ergun, A. Turan, T.T. Turkmenoglu, B. Hekimoglu; *Ankara/TR (azadhekimoglu@gmail.com)*

**Purpose:** The aim of our study was to evaluate the availability of Magnetic Resonance Spectroscopy (MRS), which is a non-invasive method for the differentiation of benign or malignant pulmonary masses.

**Methods and Materials:** A total of 59 patients (45 male, 14 female) with pulmonary masses were included in this prospective study. MRS was applied to the pulmonary lesions of the patients. Cholin (Cho), lipid and H2O parameters were determined in TE 50, 135 and 270 bands in MRS. Afterwards CT-guided percutaneous needle biopsy was performed. According to the biopsy results, pulmonary masses in 25 patients were benign and pulmonary masses in 34 patients were malignant.
**Results:** Cho, Cho/H2O and Cho/lipid levels were significantly higher in TE 50 band in malignant group compared to benign group (p <0.001, p = 0.011 and p <0.001). Cho, Cho/H2O and Cho/lipid levels were significantly higher in TE 135 band in malignant group compared to benign group (p <0.001, p = 0.012 and p = 0.012). Cho and lipid levels were significantly higher in TE 270 band in malignant group compared to benign group (p = 0.050 and p = 0.013).
**Conclusion:** When the other conditions were kept constant, the probability of malignancy was increased significantly 17.381 times (95% CI: 3.779-79.932) in those with Cho levels above 1.65 compared to those with Cho levels below 1.65 in the TE 50 band (p<0.001). In conclusion, MRS can be used in the differential diagnosis of pulmonary masses.

**B-1207** 11:18
**Imaging of thymic lesions in patients with myasthenia gravis: is dual energy CT superior to MRI?**
N. Rajamohan, R. Sharma, K. Devasenathipathy, A. Goyal, A.S. Bhalla, R. Parshad, D. Jain; *New Delhi/IN (naveenrajamohan@gmail.com)*

**Purpose:** To compare imaging parameters of Dual energy CT (DECT) with MRI for differentiating Thymoma from Thymic Hyperplasia (TH) and correlate the imaging findings of thymoma with the WHO grade & Masaoka Koga (MK) stage among patients with Myasthenia Gravis (MG).
**Methods and Materials:** Fifty-four patients of MG were recruited and underwent a single venous phase DECT (n=29), a Dynamic Contrast Enhanced MRI (1.5T, n=41) or both (n=23). Based on the histopathology they were divided into two groups (i) Thymoma (n=27) & (ii) Thymic hyperplasia (n=18), while patients with normal thymus glands (n=9) were excluded. Statistical analysis was performed between the two groups for DECT and MRI.
**Results:** Quantitative MRI parameters including Signal intensity index (SII) and Chemical Shift Ratio (CSR) showed a sensitivity & specificity of 47%/75% and 65%/86% respectively for detecting thymoma, at present published cutoff values (SII<7.77, CSR>0.849). Using ROC analysis, we propose a cutoff for SII<38% & CSR>0.75 which improves the sensitivity and specificity to 65%/68% & 89%/86% respectively. Quantitative DECT parameters using our proposed cutoffs for attenuation on Virtual Non-Contrast image (>33.3 HU) and Venous Phase (>51.3 HU), & fat fraction (<17.8%) showed a sensitivity and specificity of 100%/100%, 89.9%/90.9%, & 100%/100% respectively. DECT was also superior to MRI in predicting WHO grade, however no quantitative parameter on either DECT or MRI could differentiate the MK stages.
**Conclusion:** DECT has the potential to provide equal if not greater information than MRI for characterizing thymic lesions in patients with MG, however further validation is required.

**B-1208** 11:26
**Lung volumes and its association with subclinical impairment in whole-body MR imaging**
R. von Krüchten[1], R. Lorbeer[2], C. Schuppert[1], H. Schulz[3], H.-U. Kauczor[1], A. Peters[3], S. Karrasch[2], F. Bamberg[4], C. Schlett[1]; [1]*Heidelberg/DE*, [2]*Munich/DE*, [3]*Neuherberg/DE*, [4]*Freiburg/DE (rvkruechten@gmx.de)*

**Purpose:** A substudy of 400 whole-body MRI scans from the KORA-FF4 cohort showed good correlation between MRI-derived lung volumes and residual volume derived by pulmonary function tests and chronic obstructive pulmonary disease (COPD). As cardiac dysfunction often accompanies COPD, we aimed to evaluate this relationship using whole-body MRI.
**Methods and Materials:** 400 subjects without cardiovascular diseases from the KORA-FF4 cohort study underwent whole-body MRI. Pulmonary volumes were derived from coronal T1w-sequences. Cardiac function was assessed from cine-SSFP sequences using cvi42, and LV filling rates were assessed using pyHeart (in-house developed). Cardiac function parameters were standardized to body surface area, and its association to lung volume was analyzed using Pearson correlation and multivariate linear regression models.
**Results:** MRI parameters were available for 356 subjects (56.4±9.2 years). Cardiac measurements were within the normal physiologic range, and the mean lung volume was 4.0±1.1L. In a univariate model, left ventricle (LV) and right ventricle (RV) stroke volume were negatively correlated to lung volume. After multivariate adjustment, stroke volume as well as end-diastolic volume from LV (ß=-2.75, p=0.001; ß=-1.71, p=0.001) and RV (ß=-2.14, p=0.02; ß=-1.45, p=0.004) were negatively associated with lung volume. Myocardial mass, peak ejection rate and ejection fraction were not associated with lung volumes. For LV, the early diastolic filling rate was negatively associated with lung volume (ß=-17.3, p=0.006; ß=-11, p=0.08).
**Conclusion:** Using whole-body MRI, we observed an association between lung volume and biventricular end-diastolic and stroke volume, as well as LV filling rates, indicating potential for early detection of subclinical cardiac impairment.

**B-1209** 11:34
**Multi-parametric approach among CEST imaging, DWI and FDG-PET/CT for diagnosis of solitary pulmonary nodule**
Y. Ohno[1], M. Yui[2], Y. Kishida[1], S. Seki[1], T. Yoshikawa[1], T. Murakami[1]; [1]*Kobe/JP*, [2]*Otawara/JP (yosirad@kobe-u.ac.jp)*

**Purpose:** To determine the capability of multi-parametric approach among chemical exchange saturation transfer (CEST) imaging, diffusion-weighted imaging (DWI), and FDG-PET/CT for diagnosis of solitary pulmonary nodules (SPNs).
**Methods and Materials:** 113 consecutive patients with 122 SPNs underwent CEST imaging and DWI at a 3T MR system, FDG-PET/CT, and pathological and/or follow-up examinations. According to final diagnoses, all SPNs were divided into malignant (n=76) and benign (n=46) SPNs. In each patient, magnetization transfer ratio asymmetry ($MTR_{asym}$) was calculated from z-spectra at 3.5ppm in each pixel, and $MTR_{asym}$ map was computationally generated from CEST data. In each lesion, $MTR_{asym}$, apparent diffusion coefficient (ADC) and $SUV_{max}$ were assessed by ROI measurements. To compare all indexes between two groups, Student's t test was performed. Then ROC analyses were performed to compare diagnostic performance among all indexes as well as combined methods. Finally, sensitivity, specificity and accuracy were compared among all methods by McNemar's.
**Results:** Each index had significant difference between malignant and benign SPNs (p<0.05). ROC analyses showed area under the curve (Az) of combined method (Az=0.92) was significantly larger than that of $SUV_{max}$ (Az=0.77, p<0.05). Sensitivity (SE) and accuracy (AC) of combined methods (SE: 85.5 [65/76]%, AC: 85.2 [104/122]%) were significantly higher than those of $SUV_{max}$ (SE: 64.5 [49/76]%, p<0.05; AC: 71.3 [87/122]%, p<0.05).
**Conclusion:** Multiparametric approach among CEST, DWI and PET/CT has a significantly better potential for diagnosis of SPNs than PET/CT.
*Author Disclosures:*
**Y. Ohno:** Research/Grant Support; Canon Medical Systems Corporation. **M. Yui:** Employee; Canon Medical Systems Corporation. **S. Seki:** Research/Grant Support; Canon Medical Systems Corporation. **T. Yoshikawa:** Research/Grant Support; Canon Medical Systems Corporation.

**B-1210** 11:42
**Potential of a machine learning model for dose optimisation in chest CT quality assurance**
C. Rubbert[1], A. Meineke[2], L.M. Sawicki[1], Y. Klosterkemper[1], G. Antoch[1], J. Boos[1]; [1]*Düsseldorf/DE*, [2]*Berlin/DE (Johannes.Boos@med.uni-duesseldorf.de)*

**Purpose:** To evaluate the feasibility of machine learning (ML) for detection of dose optimisation potential in quality assurance of chest CT in a retrospective, cross-sectional study.
**Methods and Materials:** 3,199 chest CTs were used for training and testing of the neural network (01/2016-12/2017, 61% male, 62±15 years, 80/20 split). The model was optimised and trained to predict the volumetric computed tomography dose index ($CTDI_{vol}$) on scan and patient metrics (scanner, study description, protocol, patient age, sex and water-equivalent diameter ($D_W$)). The root mean-squared error (RMSE) was calculated as performance measurement. A validation dataset of 100 separate, consecutive examinations (01/2018, 60% male, 63±16 years) was independently reviewed by two blinded radiologists for dose optimisation potential and used to define an optimal cutoff.
**Results:** RMSE was 1.71, 1.45 and 1.52 for the training, test and validation datasets, respectively. CT scanner and $D_W$ were the most important features. The radiologists found dose optimisation potential in 7/100 of the validation cases. A percentage deviation of 18.3% between predicted and actual $CTDI_{vol}$ was found to be the optimal cutoff. 8/100 cases were flagged by the model (range 18.3-53.2%), including all cases flagged by the radiologists. In the false-positive case, the model predicted a 33% lower $CTDI_{vol}$, which was due to a severe soft tissue emphysema leading to a very small $D_W$.
**Conclusion:** ML can comprehensively detect chest CT examinations with dose optimisation potential; however, a finale human review is mandatory. A deviation of >=18.3% between the predicted and actual $CTDI_{vol}$ seems adequate for CT quality assurance.
*Author Disclosures:*
**A. Meineke:** Consultant; Pulmokard GmbH. Employee; Cerner HS Deutschland GmbH.

**B-1211** 11:50
**AI-powered detection of pulmonary embolism in CT pulmonary angiograms: a validation study of the diagnostic performance of prototype algorithms**
T.J. Weikert, D.J. Winkel, J. Bremerich, B. Stieltjes, A.W. Sauter, G. Sommer; *Basel/CH (thomasjohannes.weikert@usb.ch)*

**Purpose:** To validate the performance of deep convolutional neural networks optimised for the detection of pulmonary embolism (PE) on CT pulmonary angiograms (CTPAs).
**Methods and Materials:** We downloaded all CTPAs performed in 2017 along with the corresponding reports (n = 1,499) from our RIS/PACS archive using an in-house-developed search engine. The reports were manually reviewed by a radiologist. CTPAs with other clinical questions than PE or poor diagnostic quality were excluded. The remaining exams were then classified into positive (n = 232) and negative (n = 1,204) for PE. All emboli in positive exams were labeled by a radiologist using bounding boxes. The data served as ground truth for the validation of prototype algorithms (Aidoc, Tel Aviv, Israel) that had previously been trained on 28.000 independent CTPAs from other centres.
**Results:** Four trained prototype algorithms were tested on our CTPA dataset. The best performing algorithm was a fully convolutional neural network with a backbone based on the Resnet architecture. It achieved a sensitivity of 93% and a specificity of 95%. This corresponds to a positive predictive value of 77%.
**Conclusion:** The best-performing AI algorithm we validated is capable of detecting pulmonary embolism in CTPAs with a high sensitivity and specificity. In a clinical setting, this can complement conventional workflows with a worklist prioritisation and has the potential to improve the quality of healthcare by accelerating the diagnostic process and communication. We plan to further test the algorithm and finally implement it in the clinical routine to perform prospective evaluations.

**10:30 - 12:00** **Room M 1**

### Cardiac

### SS 1403

### Quantification of myocardial perfusion and ECV (extracellular volume fraction)

*Moderators:*
K. Nikolaou; Tübingen/DE
A. Rossi; Milan/IT

**B-1212** 10:30
**Diagnostic accuracy of combined CCTA and low-dose dynamic stress CT myocardial perfusion with a whole-heart coverage CT scanner in patients with suspected CAD**
A. Baggiano[1], M. Guglielmo[1], G. Muscogiuri[1], M. Soldi[1], A. Del Torto[1], A.I. Guaricci[2], M. Pepi[1], G. Pontone[1]; [1]*Milan/IT*, [2]*Bari/IT (andrea.baggiano@gmail.com)*

**Purpose:** Our purpose is to test the diagnostic accuracy of integrated evaluation of cCTA plus dynamic myocardial stress-CTP with low-dose protocol using a whole-heart coverage CT scanner as compared to ICA plus invasive FFR.
**Methods and Materials:** Eighty-five consecutive symptomatic patients scheduled for ICA were prospectively enrolled. All patients underwent rest cCTA followed by stress dynamic CTP with a whole-heart coverage CT scanner (Revolution CT, GE Healthcare, Milwaukee, Wisconsin). Diagnostic accuracy and overall effective dose were assessed and compared to ICA and invasive FFR.
**Results:** The prevalence of obstructive CAD and functionally significant CAD were 69% and 44%, respectively. cCTA alone demonstrated a per-vessel and per-patient sensitivity, specificity, negative predictive value, positive predictive value, and accuracy of 83%, 66%, 89%, 54%, 71% and of 96%, 32%, 86%, 65%, and 68%, respectively. Combining cCTA with stress CTP, per-vessel and per-patient sensitivity, specificity, negative predictive value, positive predictive value, and accuracy were 71%, 86%, 86%, 72%, 81% and 88%, 75%, 82%, 82%, and 82%, with a significant improvement in specificity, positive predictive value, and accuracy in both models. The addition of stress CTP to cCTA improved the area under the curve from 0.82 to 0.87 (p=0.011) and from 0.78 to 0.87 (p:0.011) in a vessel and patient-based model, respectively. The mean ED for cCTA and stress CTP was 2.8 ± 1.2 and 5.3 ± 0.7 mSv, respectively.
**Conclusion:** The addition of dynamic stress CTP to coronary artery imaging by cCTA provides additional diagnostic accuracy with acceptable radiation exposure.

**B-1213** 10:38
**The value of low-dose dynamic CT myocardial perfusion imaging for accurate evaluation of microvascular obstruction in patients with acute myocardial infarction**
J. Zhang; *Shanghai/CN (andrewssmu@msn.com)*

**Purpose:** To investigate the diagnostic performance of quantitative parameters generated from dynamic CT myocardial perfusion imaging (MPI) for the assessment of acute myocardial infarction (AMI) and microvascular obstruction (MVO) with reference to cardiac magnetic resonance (CMR) imaging.
**Methods and Materials:** Patients with AMI and successful reperfusion treatment within 1 week were prospectively enrolled. All patients were referred for CMR imaging and dynamic CT-MPI on the same day. Various quantitative parameters and late iodine enhancement (LIE) were analysed for the evaluation of AMI and MVO with reference to CMR findings.
**Results:** Twenty-seven patients with 442 segments were ultimately included for analysis. The mean radiation dose for dynamic CT-MPI and LIE was 3.3 ± 1.1 mSv and 2 ± 0.6 mSv, respectively. MBF was significantly lower in MVO (+) segments compared to MVO (-) segments and reference segments (23.08±7.95 ml/min/100ml vs. 44.60±14.97 ml/min/100ml and 75.07±7.34 ml/min/100ml, both p<0.001). According to ROC curve analysis, MBF was found to have largest AUC among all parameters for identifying AMI with and without MVO as determined by LGE (AUC=0.941 and 0.996, both p<0.001). The diagnostic accuracy of MBF-based assessment for identifying MVO was 99.2%, which significantly outperformed other quantitative parameters and LIE. There was good correlation between the AMI area and MVO area estimated by MBF and LGE (r=0.95 and 0.99, both p< 0.001).
**Conclusion:** MBF derived from dynamic CT-MPI is accurate and outperforms other quantitative parameters and LIE for diagnosing AMI and MVO. The AMI and MVO area can also be accurately estimated by MBF.

**B-1214** 10:46
**Characteristics of first-pass myocardial iodine distribution as assessed by dual layer CT with different injection protocols**
S. Boccalini[1], S.A. Si-Mohamed[1], M. Matzuzzi[2], B. Plaine[2], D.C. Rotzinger[3], L. Hanquier[2], L. Boussel[1], D. Revel[1], P. Douek[2]; [1]*Bron/FR*, [2]*Lyon/FR*, [3]*Lausanne/CH (sara.boccalini@yahoo.com)*

**Purpose:** To compare iodine distribution in healthy myocardium at first-pass perfusion imaging between two different contrast injection rates using dual-layer CT (DLCT).
**Methods and Materials:** Between January and July 2018, patients with clinically indicated coronary CT were randomized to undergo spectral coronary DLCT-angiography with different injection protocols. The two protocols were: 35mL in patients <80kg and 0.5mL/kg in patients >80 kg (maximum 45mL) at 2.5mL/sec (A) or double contrast dose at 5.0mL/sec (B). Patients with coronary stenosis >50% were excluded. Regions-of-interest were manually drawn on the 16 AHA standard myocardial segments. Iodine concentration was measured on 2-material decomposition (iodine-water) maps in mg/mL.
**Results:** 25 and 28 patients for protocol A and B were included, respectively. With median values of 1.37mg/mL (IQR=0.53) at 2.5ml/sec and 1.74mg/mL (IQR=1.04) at 5.0mL/sec the overall iodine concentration was significantly different (p<0.01). For group A no significant difference was found between the 16 segments (p=0.8), between basal, mid and apical segments (p=0.34) and between coronary territories (p=0.31). In group B the homogeneity of iodine concentration between the 16 segments and basal, mid and apical segments was at the limit (p=0.06 and p=0.07) while being significantly different between the territory of the LCX (median=2.03 mg/mL; IQR=1.06) vs LAD (median=1.63 mg/mL; IQR=0.92) and vs RCA (median=1.69 mg/mL; IQR=1.12) (p<0.01).
**Conclusion:** Iodine concentration in healthy myocardium obtained with DLCT was higher at faster injection rate and greater dose. Iodine myocardial distribution was homogeneous at lower injection rate. Conversely, significant differences between coronary territories at higher flow rates were found.

**B-1215** 10:54
**Feasibility of extracellular volume quantification using dual-energy CT**
M. van Assen[1], C.N. De Cecco[2], P. Sahbaee[2], P. Griffith[2], M.J. Bauer[2], A. Varga-Szemes[2], M. Oudkerk[1], R. Vliegenthart[1], U.J. Schoepf[2]; [1]*Groningen/NL*, [2]*Charleston, SC/US (griffile@musc.edu)*

**Purpose:** To assess the feasibility of dual-energy CT (DECT) to derive myocardial extracellular volume (ECV) and detect myocardial ECV differences without a non-contrast acquisition, compared to single-energy CT (SECT).
**Methods and Materials:** Subjects (n=35) with focal fibrosis (n=17), diffuse fibrosis (n=10), and controls (n=9) underwent non-contrast and delayed acquisitions to calculate SECT-ECV. DECT-ECV was calculated using the delayed acquisition and the derived virtual non-contrast images. In the control and diffuse fibrotic groups, the entire myocardium of the left ventricle was used to calculate ECV. Two ROIs were placed in the focal fibrotic group, one in normal and one in fibrotic myocardium.

Saturday

**Results:** Median ECV was 33.4% (IQR, 30.1-37.4) using SECT and 34.9% (IQR, 31.2-39.2) using DECT (p=0.401). For both techniques, focal and diffuse fibrosis had significantly higher ECV values (all p<0.021) than normal myocardium. There was no systematic bias between DECT and SECT (p=0.348). SECT had a higher radiation dose (1.1mSv difference) than DECT (p<0.001).
**Conclusion:** ECV can be measured using a DECT approach with only a delayed acquisition. The DECT approach provides similar results at a lower radiation dose compared to SECT.
*Author Disclosures:*
**C.N. De Cecco:** Consultant; Guerbet. Research/Grant Support; Siemens.
**M. Oudkerk:** Research/Grant Support; Siemens. **R. Vliegenthart:** Research/Grant Support; Siemens. **U.J. Schoepf:** Consultant; Bayer, GE, Guerbet, HeartFlow Inc., Siemens. Research/Grant Support; Astellas, Bayer, Siemens.

**B-1216** 11:02
**Cardiac CT in the quantification of myocardial extracellular volume fraction (ECV) with a semiautomatic method: comparison with cardiac MR**
D. Vignale, A. Palmisano, A. Del Maschio, F. De Cobelli, A. Esposito; *Milan/IT (vignale.davide@hsr.it)*

**Purpose:** To assess the potential of cardiac CT in the quantification of extracellular volume fraction (ECV), a parameter with increasing diagnostic and prognostic role, in comparison to cardiac MR as the reference standard.
**Methods and Materials:** 14 patients affected by various diseases (3 idiopathic dilated cardiomyopathy, 3 CAD, 2 acute myocarditis, 2 chronic myocarditis, 1 hypertrophic cardiomyopathy, 1 amyloidosis, 2 no overt cardiac pathology) underwent cardiac CT including a delayed low-kilovoltage scan (10 minutes after contrast injection). The same patients underwent cardiac MR at 1.5 T with native and post-contrast T1 mapping. ECV-CT is defined as the quotient between the density difference of myocardium and the density difference of blood pool before and after contrast injection, weighted to the haematocrit. With MATLAB®, basal and late-phase CT images were co-registered to create a map of the heart ECV on a short-axis mid-ventricular slice. ECV-MR is defined as the quotient between the relaxivity difference of myocardium and blood pool before and after contrast injection, weighted to the haematocrit. With the same MATLAB® toolkit, we co-registered T1-native and T1-enhanced MR images to create a map of the heart ECV on the same slice used for CT. ECV-CT and ECV-MR maps were segmented to extract ECV values of LV septum and lateral wall.
**Results:** 28 ECV values were compared. ECV-CT range was 23.4-72.3% (mean 33.1±10.0%). ECV-MR range was 25.3-75.2% (mean 34.2±11.0%). Correlation between ECV-CT and ECV-MR was optimal (r: 0.943, p<0.001).
**Conclusion:** ECV can be semi-automatically extracted from cardiac CT datasets, with a good correlation with cardiac MR.

**B-1217** 11:10
**AI-based prediction of haematocrit values from non-contrast CT imaging data - towards fully automated CT-derived myocardial extracellular volume fraction quantification**
M.J. Bauer[1], M. van Assen[2], C.N. De Cecco[1], J.A. Durden[1], M. Scarabello[3], H.T. Hudson[1], L. Griffith[1], A. Varga-Szemes[1], U.J. Schoepf[1];
[1]*Charleston, SC/US*, [2]*Groningen/NL*, [3]*Milan/IT (email@bauermaximilian.de)*

**Purpose:** Imaging approaches for the quantification of the myocardial extracellular volume fraction (ECV) require the patient's haematocrit as an input variable. We assessed the performance of a fully automated, in-house developed artificial intelligence (AI) approach to predict haematocrit values from multiple patient- and acquisition-related variables in comparison to a linear regression (LR) model employing HU values alone.
**Methods and Materials:** A total of 286 patients that had undergone non-contrast cardiac CT and had haematocrit values determined via blood sampling within 2 days of the scan were retrospectively included. For each case, we measured HU values in the ascending aorta both manually and by a fully automated deep learning (DL) segmentation approach. A LR analysis was employed to model the relationship between HU values and laboratory haematocrit values. In addition, a total of 31 features on patient demographics, clinical history, and imaging parameters were extracted for the machine learning (ML) approach and used to train a linear Support Vector Machine (SVM) employing repeated, nested k-fold cross validation.
**Results:** The HU values obtained by the manual (mean 44.0±3.7) and the DL segmentation approach (mean 44.3±3) were in excellent agreement ($R^2$=.95). The LR approach demonstrated a weak relationship between the model and the laboratory haematocrit values ($R^2$=.46, MAE=2.4%) while the ML approach resulted in a moderate relationship ($R^2$=.59, MAE=1.8%).
**Conclusion:** The use of AI is a promising approach for the calculation of CT-derived haematocrit values that benefits from additional information and comes with immediate potential for fully-automated ECV quantification approaches.
*Author Disclosures:*
**C.N. De Cecco:** Consultant; Guerbet. Research/Grant Support; Siemens.
**A. Varga-Szemes:** Consultant; Guerbet. Research/Grant Support; Siemens.
**U.J. Schoepf:** Consultant; Bayer, Guerbet. Research/Grant Support; Siemens, Bayer, Astellas, GE.

**B-1218** 11:18
**Could "synthetic hematocrit value" has a use on ECV calculating via cardiac MRI? A single centre study**
A. Erdemir, O. Öcal, T. Hazirolan; *Ankara/TR (a.gurkan.erdemir@gmail.com)*

**Purpose:** Currently, myocardial extracellulary volume (ECV) can easily get calculated on cardiac magnetic resonance imaging (MRI), by using patient's hematocrit values acquired via blood sampling. However, sometimes blood sampling can be missed out which makes ECV calculating difficult. Synthetic ECV estimation, by synthetic hematocrit value, may make the ECV calculating process much more appliable and comfortable. The aim of this study is to investigate synthetic hematocrit values' validity on ECV estimating in our center's routine practice.
**Methods and Materials:** Between November 2017 - July 2018, 327 patients with cardiac mri were reviewed. 61 patients who have appropriate imaging and blood sampling included to study. Whole basal, middle and apical segments' native ECV and synthetic ECV values were assessed via cardiac MRI. The results of each segments' synthetic values compared with each segments native values using both "ANOVA" and "Interclass Correlation Coefficent" analysis.
**Results:** The vaules of both "synthetic hematocrit and synthetic ECV" were significantly reliable as compared with the values of both "native hematocrit and native ECV regardless of cardiac segments. (p<0,0001). The results of both synthetic hematocrit and synthetic ECV values had above 0,80 interclass correlation and above 0,95 unstandardized coefficient for each segments.
**Conclusion:** Synthetic ECV values, acquired via synthetic ECV calculation, can be accepted as native ECV values in routine practice, regarding increasing both ECV estimation rates and patients comfort.

**B-1219** 11:26
**Safety and diagnostic accuracy of regadenoson as vasodilator in stress perfusion cardiovascular magnetic resonance**
A. Ezponda Casajús, M. Calvo Imirizaldu, A. García Baizán, A. Paternain Nuin, J.C. Pueyo Villoslada, J. Gavira, G. Bastarrika Alemañ; *Pamplona/ES (aezponda@unav.es)*

**Purpose:** To assess the safety and diagnostic accuracy of regadenoson as vasodilator in stress cardiovascular magnetic resonance (CMR) studies performed in individuals with known or suspected coronary artery disease (CAD).
**Methods and Materials:** We prospectively recruited 217 patients (159 men, mean age 65.±11.9 years, 26.7% diabetic) with known or suspected CAD who underwent stress-CRM. Examinations were acquired in a 1.5-Tesla MR system (MAGNETOM Aera, Siemens Healthineers) using an intravenous bolus of 0.4mg (5mL) of regadenoson. Vital signs were monitored at rest and under stress and clinical symptoms and adverse events induced by the vasodilator were collected. Test results were confirmed by conventional coronary angiography (CCA) when available.
**Results:** Most patients remained asymptomatic (49.3%). The most frequent symptoms were chest pain (18.9%) and dyspnoea (17.1%). All symptoms were transient and well-tolerated. During the stress, the average increase in heart rate (HR) was 24.3±13.7 bpm and the mean decrease in systolic and diastolic blood pressure (SBP and DBP) was 7.0±17.4 mmHg and 4.9±10.2 mmHg, respectively. In the obese (BMI≥30; n=51) a statistically blunted HR (mean difference of -5.9± 2.1; CI-10.1,-1.6;p=0.07) and SBP responses (mean difference of 8.3±2.8;CI 2.8,13.7;p=0.03) were noted. The DM group had a significantly higher HR at baseline (67.7±19.9 vs 62.5±15.5 bpm; p=0.03) and lower increase in HR (19.1±9.3 vs 26.1±14.5 bpm;p=0,001), whereas symptomatic patients showed a greater increase in HR (27.4±11.2 vs 20.6±10.7bpm, p=0.001). Compared with CCA, regadenoson stress-CRM showed 94.9% sensitivity, 68.8% specificity, 88.1% positive and 84.6% negative predictive values to detect significant coronary stenosis.
**Conclusion:** Regadenoson is a well-tolerated and effective vasodilator that can be safely used in stress-CMR.

**B-1220** 11:34
**Prognostic value of myocardial perfusion reserve index in patients with coronary microvascular disease: a multi-centre study**
W. Zhou, M. Chai, W. Wong, P. Yap, Y. Lai, T. Lee, S. Leung, J. Lee, M.-Y. Ng; *Hong Kong/HK (zwl1993@hku.hk)*

**Purpose:** Coronary microvascular disease(CMD) is an established cause of stable chest pain in patients without obstructive coronary artery disease(OCAD). CMD identification by myocardial perfusion reserve index (MPRI) on stress cardiac magnetic resonance (StCMR) has been demonstrated with a cut-off of 1.4. However, the prognostic value of MPRI identification of CMD has not been established.
**Methods and Materials:** 106 StCMR studies of symptomatic patients (50% male) without OCAD performed at three different MRI units since 2009 were analysed retrospectively. OCAD (i.e. >50% narrowing or fractional flow reserve <0.8) was excluded by catheter angiogram or CT coronary angiogram. MPRI was derived semi-quantitatively as upslope ratio of stress and rest myocardial perfusion signal intensity curve corrected for arterial input function. A MPRI <1.4 was classified as having CMD. Primary outcome was a combined major adverse cardiovascular event(MACE) of acute coronary syndrome, heart failure hospitalization and all-cause death.
**Results:** 54.7% patients had CMD. Reduced MPRI was associated with older age (*p*=0.03), female (*p*=0.02), diabetes (*p*=0.01), hypertension (*p*=0.03), and hyperlipidemia (*p*=0.004). Median follow-up was 5.75 years (CI:5.51-6.00) and 12.2% MACEs happened. CMD patients had worse prognosis compared to non-CMD patients (hazard ratio 3.29; CI 1.04-10.4; *p*=0.04). MPRI remained an independent prognostic marker (hazard ratio=0.066; CI 0.01-0.77; *p*=0.03) after adjusting age, gender, BSA, cardiovascular risk factors and ejection fraction.
**Conclusion:** Reduced MPRI is an independent predictor of MACE and provides future therapeutic developments in CMD patients.

**B-1221** 11:42
**Modification of left ventricle myocardial perfusion reserve after the implantation of coronary sinus stent: a stress-rest CMR study**
A. Palmisano, A. Esposito, G. Benedetti, F. Giannini, L. Baldetti, A. Del Maschio, F. De Cobelli; *Milan/IT (palmisano.anna@hsr.it)*

**Purpose:** The treatment of refractory angina is a challenge. The implantation of a hourglass shaped stent (Reducer) in the coronary sinus showed to improve symptoms, but objective data demonstrating the effect on myocardial perfusion reserve are lacking. Aim was to evaluate the modification in myocardial perfusion reserve occurring after Reducer implantation through stress-rest CMR.
**Methods and Materials:** 15 patients underwent 1.5 T CMR and clinical evaluation [Canadian Cardiovascular Society grading of angina pectoris (CCSA Class) and six minutes walking test (6minWT)] before and 3 months after Reducer implantation. CMR examinations included stress-rest first-pass perfusion and LGE. Segmental and global Myocardial Perfusion Reserve index (MPRi) were measured, also accounting for myocardial layer.
**Results:** Thirteen out of 15 patients reported an improvement ≥ 2 CCSA class (p<0.0001) and a 60% increase in the average distance during the 6minWT (p=0,004).Stress-CMR demonstrated an improvement of myocardial perfusion reserve [ischaemic burden per patient from 13% to 10.88%, p=0.0092; median MPRI from 1.2 (0.9-1.5) to 1.3 (1.1-1.7), p<0.0001; number of segments with inducible perfusion defects (IPDs) from 92/240(38%) to 69/240(29%); p<0.001]. MPRI improvement was largely greater in more ischaemic segments at baseline: segments with IPDs vs without IPDs (ΔMPRI: 0.432 vs 0.183;*p*=0.0051) and with baseline MPRI <1.3 vs ≥1.3 (ΔMPRI: 0.355 vs -0.036, p<0.001). Moreover, MPRI improved according with a transmural gradient from epicardium to endocardium (ΔMPRI 0.4057 vs 0.3156, p=0.0282).
**Conclusion:** Clinical benefit from CS Reducer implantation was associated with a significant improvement in myocardial perfusion reserve driven by the more ischaemic segments.

**B-1222** 11:50
**Left ventricular subclinical myocardial dysfunction in uncomplicated type 2 diabetes mellitus is associated with impaired myocardial perfusion**
X. Liu, Z.-G. Yang, Y.-K. Guo, Y. Ren, Y. Gao; *Chengdu/CN (liuxihsd@163.com)*

**Purpose:** This study aims to investigate the association between left ventricular (LV) myocardial deformation and microvascular perfusion using cardiovascular magnetic resonance (CMR) imaging in patients with type 2 diabetes mellitus (T2DM).
**Methods and Materials:** We recruited 71 T2DM patients and 30 healthy individuals; the T2DM patients were subdivided into newly diagnosed T2DM group (*n* = 31, with diabetes for ≤5 years) and longer term T2DM group (*n* = 40, with diabetes >5 years). LV deformation parameters, including global peak strain (PS), peak systolic strain rate (PSSR), and peak diastolic strain rate (PSDR), and myocardial perfusion parameters such as upslope, time to maximum signal intensity (TTM), and max signal intensity (Max SI) were measured and compared among the three groups. Pearson's correlation was used to evaluate the correlation between LV deformation and perfusion parameters.
**Results:** The longitudinal PS and PSSR were significantly decreased in longer term group compared to the newly diagnosed group or normal subjects. Pooled data from T2DM patients showed a decrease in global PDSR compared to healthy individuals, apart from lower upslope and Max SI. Further, TTM was significantly higher in the longer term group compared to the other two groups. We also show a significant correlation between decreased longitudinal PDSR and increased TTX in the T2DM patients (r = 0.515, p < 0.001).
**Conclusion:** Our results imply that a contrast-enhanced 3.0T CMR can detect subclinical myocardial dysfunction and impaired myocardial microvascular perfusion in the early stages of T2DM, and that the myocardial dysfunction is associated with impaired coronary microvascular perfusion.

**10:30 - 12:00** **Room M 2**

### Paediatric

### SS 1412

### Paediatric neuroradiology

*Moderators:*
A. Sánchez Montañez; Barcelona/ES
F.M. Triulzi; Milan/IT

**B-1224** 10:30
**Model-based iterative reconstruction: is it relevant for reducing radiation dose in paediatric head CT?**
P.K. Atri, K.S. Sodhi, A. Bhatia, A.K. Saxena, N. Khandelwal; *Chandigarh/IN (sodhiks@gmail.com)*

**Purpose:** To evaluate the potential of model-based iterative reconstruction (MBIR) technique on dose reduction and image quality in children undergoing computed tomography (CT) head examinations.
**Methods and Materials:** This was a prospective study approved by the institutional ethics committee. Eighty-eight children (age range 5-16 years) with a history of seizures underwent contrast-enhanced CT scan. 41 children underwent CT study according to the MBIR technique while 47 children underwent CT head with the non-MBIR protocol. Images were reviewed by two paediatric radiologists, who were blinded, in a random order. Mean dose-length product, CT dose index (CTDI) volume and mean effective dose was recorded for both groups. Image quality, image noise and diagnostic acceptability of two image sets were also recorded. Student t test, Mann-Whitney U test, kappa test and chi-square tests were used for statistical analysis.
**Results:** In the MBIR group, the mean dose-length product was reduced by 79.8%; the mean CTDI volume was reduced by 88.5%, while the mean effective dose was reduced by 81% when compared to the non-MBIR group. No significant difference was seen in diagnostic acceptability, image noise and image quality between the two groups.
**Conclusion:** Model-based iterative reconstruction technique is highly effective in reducing radiation dose in paediatric head CT examinations without any significant difference in image quality, image noise,and diagnostic acceptability.

**B-1225** 10:38
**A systematic quantitative morpho-volumetric analysis in infants with sagittal craniosynostosis and relationship with the severity of scaphocephalic deformity**
M. Panfili, R. Calandrelli, F. Pilato, C. Colosimo; *Rome/IT (panfilimarco@gmail.com)*

**Purpose:** In Isolated Sagittal Synostosis (ISS) the severity of head-shape deformity can vary significantly among affected individuals. This study aimed to assess skull base morphometry and intracranial volume related to the severity of scaphocephaly.
**Methods and Materials:** 68 infants with ISS were divided in three groups according to the severity of cranial deformity (group I: mild deformity; group II: moderate deformity; group III: severe deformity), by combining two scaphocephaly severity indexes as descriptors of the relation of three morphological measurements (length, width and height). High-resolution CT images were quantitatively analyzed. Dimensions of cranial fossae, supratentorial (ICV) and infratentorial (PCFV) cranial volume, supratentorial (WBV) and infratentorial (PCFBV) brain volume, ICV/WBV ratio; PCFV/PCFBV ratio, supratentorial and infratentorial cerebrospinal fluid volumes (CSF) were calculated.

**Results:** In all groups, anterior and middle skull base length was found increased while posterior hemifossae lengths (MO) were unaffected. In mild group ICV/WBV ratio was significantly different and ICV, WBV and CSF supratentorial volume resulted increased (p<0.05) compared with other groups. In moderate and severe groups FCPV/FCPBV ratio and CSF infratentorial volume resulted reduced (p<0.05); FCPBV was found increased only in the severe subgroup (p<0.05) .
**Conclusion:** This study provides new insights in understanding the compensatory changes that occur in scaphocephalic patients with different degrees of severity. In particular our study suggests that patients with severe scaphocephaly had an earlier loss of secondary compensatory changes with a cranial volume insufficient to accommodate the cerebral growth and this may suggest the need of a prompt cranial expansion.

**B-1226** 10:46
**MRI patterns of brain injury in neonates with pathology-proven placental abruption**
P.A. Caruso, K. Buch, S. Bates, D. Roberts; *Boston, MA/US (pcaruso@partners.org)*

**Purpose:** It is conventionally held that the MRI pattern of brain injury in neonatal hypoxic ischaemic encephalopathy (HIE) in term babies is determined by the duration and severity of hypoxic ischaemic injury (HII). Severe HII results in central patterns of injury (CP) that predilect the perirolandic cortex, basal ganglia, and thalami; moderate HII results in peripheral patterns (PP) that involve watershed territories. Placental abruption (PA), the classic mechanism for acute profound HII, would expectedly produce a CP. The purpose of this study was to determine the frequency of CP in a non-hypothermia-treated neonate (NHTN) cohort with HIE and placental abruption.
**Methods and Materials:** Between 2005 and 2018, 14 NHTN with pathology-proven PA and clinically diagnosed HIE underwent brain MRI. Patterns of injury were classified as CP, PP, and mixed.
**Results:** There were 6 males and 8 females (mean gestational age 36.5 weeks; range 26-41 weeks). Pure CP was identified in zero patients. 5 patients had a mixed pattern of injury. 5 had a normal MRI. 2 patients had a PP. 1 patient showed an isolated venous thrombosis. 1 showed isolated infarct.
**Conclusion:** In our cohort of neonates with pathology-proven placental abruption, the expected central pattern was the least frequently observed. Of the abnormal MRIs, mixed patterns of injury were most frequent, and pure peripheral patterns of injury were observed. Additional patterns with venous thrombosis and infarction were also seen. The results of this study challenge the convention that placental abruption is usually associated with a central pattern of HII.

**B-1227** 10:54
**Frequency of optic nerve involvement in paediatric intracranial germ cell tumours**
K. Buch, O. Rapalino, N. Tarbell, T. Yock, S. MacDonald, P.A. Caruso; *Boston, MA/US (kbuch@partners.org)*

**Purpose:** Of the three most common paediatric sellar/suprasellar brain tumours (germ cell tumours, craniopharyngiomas, and optic pathway hypothalamic gliomas), optic nerve (ON) involvement is traditionally thought to be associated more specifically with optic pathway hypothalamic gliomas. ON involvement may, however, rarely be observed in intracranial germ cell tumours (GCT). ON involvement thus may influence need for biopsy and may impact radiation planning as targeted imaging of the ONs may not be routinely included in pretreatment planning of GCTs. The purpose of this study is to determine the frequency of ON involvement in a large cohort of patients with intracranial GCTs.
**Methods and Materials:** All patients with intracranial pure germinoma and nongerminomatous germ cell tumours (NGGCTs) treated at our institution with proton radiotherapy between 1998 and August 2018 were reviewed. Pre-treatment brain MRIs were evaluated for the presence of ON involvement.
**Results:** 101 patients were identified with intracranial GCTs, including 61 pure germinoma and 40 NGGCTs. Of these patients, six (6%) demonstrated ON involvement. In those with CN involvement, the median age at diagnosis was 15.5 years, most were female (n = 4), and the majority had histologically proven pure germinoma (n = 5).
**Conclusion:** ON involvement may be seen in paediatric GCTs and thus should not be considered a specific feature of optic pathway hypothalamic gliomas. Dedicated orbital and ON sequences should be considered for radiation planning as it is important to identify ON involvement prior to treatment and to adjust radiotherapy field appropriately to ensure full radiation dose.

**B-1228** 11:02
**MRI findings in Christianson syndrome**
P.A. Caruso[1], M. Pescosolido[2], K. Buch[1], E. Morrow[2]; [1]*Boston, MA/US*, [2]*Providence, RI/US (pcaruso@partners.org)*

**Purpose:** Christianson syndrome (CS) OMIM 300243 is an x-linked disorder caused by mutation in the SLC9A6 gene OMIM 300231 characterised by intellectual disability, seizures, ataxia, microcephaly, and ophthalmoplegia. MRI findings in CS have been reported only rarely and as case reports. Our purpose is to present the spectrum of imaging findings in the largest cohort of independent CS pedigrees.
**Methods and Materials:** 14 male patients with the CS phenotype and genetically proven deleterious SLC9A6 mutations were recruited for evaluation. MRIs on all 14 patients were retrospectively reviewed by two neuroradiologists. Images were reviewed for cerebellar pattern of atrophy, cortical, white matter, and nuclear signal abnormality, and supratentorial white matter and hippocampal abnormalities.
**Results:** Cerebellar atrophy was seen in 7/14 (50%) patients with a unique pattern of cerebellar atrophy that predilects the inferior cerebellar lobules, gracilis and biventer (lobules VIIBm, VIIIAm, VIIIBm), and that results in high T2/FLAIR signal in the depths of the cerebellar fissures, in 5/14 (36%) patients. A dragonfly cerebellum was seen in 2/14 (14%). Dentate nuclear abnormal high T2 signal was seen in 6/14 (43%). 9/14 (64%) patients had occipital white matter lesions. Hippocampal abnormalities were seen in 5/14 (36%).
**Conclusion:** Cerebellar atrophy and occipital white matter lesions are frequently seen in CS. The pattern of cerebellar atrophy predilecting the inferior hemispheres seen in 36% of patients presents a unique MRI endophenotype that in the setting of intellectual disability, seizures, and microcephaly is strongly suggestive of CS and may be seen as a potential useful imaging biomarker.

**B-1229** 11:10
**Deep medullary venous thrombosis in neonates correlates highly with the development of shunt-dependent hydrocephalus**
K. Buch, S. Rincon, K. Krishnamoorthy, G. Gomboli, P.A. Caruso; *Boston, MA/US (kbuch@partners.org)*

**Purpose:** Deep medullary venous thrombosis (DMVT) in neonates may lead to shunt-dependent hydrocephalus (ShDH). While 10% of newborns with IVH from germinal epithelial bleeds (GEB) eventually require intraventricular shunts, the frequency of ShDH in neonates with IVH from DMVT remains unknown. Our purpose is to study a cohort of neonates with DMVT to determine if DMVT is a risk factor for ShDH.
**Methods and Materials:** Between 2006 and 2016, 27 neonates were found to have DMVT by MRI. DMVT was scored as mild involving 1-3 central veins, moderate 4-6, or severe, greater than 6. The extent of DMVT, presence of hydrocephalus, IVH, seizures, and frequency of progression to ShDH were recorded.
**Results:** 17 males and 10 females with a mean gestational age of 37 weeks (range 26-41 weeks) were included. The majority of neonates were scanned at 1-10 days of life (average = 10.2 days, range 0-66 days). 12 patients had mild DMVT, 10 moderate, and 5 severe. 21 patients had IVH that ranged from trace to severe. Hydrocephalus was observed in 13/27 (48%) patients. 5/27 (19%) of neonates developed ShDH and 5/21 (24%) of the neonates with DMVT and IVH developed ShDH.
**Conclusion:** The frequency of ShDH in neonates with IVH and DMVT is 24%, over twice as high as the frequency of ShDH in neonates with IVH from GEB. Awareness of this increased risk may indicate closer imaging surveillance in this group of neonates.

**B-1230** 11:18
**Characterising resting-state networks in bilateral severe-to-profound sensorineural hearing loss infants within an early sensitive period**
S. Wang, G. Fan; *Shenyang/CN (jelly_66@126.com)*

**Purpose:** The development of central auditory system has a sensitive period during the first few years of life. This study was aimed to characterize the patterns of resting-state functional network connectivity in infants with bilateral severe-to-profound sensorineural hearing loss (SNHL) within an early sensitive period.
**Methods and Materials:** 36 infants with bilateral severe-to-profound SNHL (mean age = 16 months, range 6-35 months) and 33 age and sex matched healthy controls were recruited for the present study based on referral for clinical MRI and other inclusion criteria. All subjects underwent 3.0T anatomical and resting-state functional MRI. Independent component analyses (ICA) was applied to identify 20 spatially distinct brain networks including auditory, visual, somatosensory, executive control, default-mode networks (DMN), cerebellar, and subcortical networks. Participant-level network maps were obtained using dual-regression and functional network connectivity (FNC) was used to test within- and between- network connectivity differences between patients with SNHL and typically developing infants.

**Results:** We find that infants with SNHL exhibit significantly stronger connectivity within auditory, DMN and executive control network. Trends were also observed for stronger connectivity in the cerebellum network. In addition, we observed significant differences in the amplitudes of between-network correlations, which involved auditory, visual and somatosensory networks.
**Conclusion:** These novel findings provide valuable insights into the characteristics of early brain reorganization and compensatory activation changes in congenital bilateral severe-to-profound SNHL infants within early sensitive period.

**B-1231** 11:26
**MRI in the preoperative diagnosis of medulloblastoma: correlation with molecular subgrouping**
N. Plakhotina[1], E. Vázquez[2], I. Delgado Alvarez[2], A. Sanchez-Montanez Garcia-Carpintero[3], E. Martinez[2], A. Llort[2], G. Burcet Rodríguez[2], J. Camacho[2]; [1]*St. Petersburg/RU*, [2]*Barcelona/ES*, [3]*Tarragona/ES* *(plahotinadezhda@gmail.com)*

**Purpose:** To determine the value of MRI for diagnosing the various molecular subgroups of medulloblastoma.
**Methods and Materials:** A retrospective analysis was performed of MRI findings in 28 patients with medulloblastoma, examined between January 2010 and January 2018. There were 10 (35.7%) female and 18 (64.3%) male patients with a mean age of 4.7 years. All patients underwent MRI of brain, including conventional sequences, DWI, MR spectroscopy, ASL perfusion, and dynamic enhanced perfusion, as well as whole-spine MRI, including sagittal T1 sequences with and without gadolinium enhancement and sagittal DWI. Based on the molecular analysis results, tumors were divided into 3 groups: wingless (WNT), sonic hedgehog (SHH), non-WNT/non-SHH (joining groups 3 and 4) for the purposes of the study.
**Results:** All cases showed a taurine peak at a TE of 135 msec, which was approximately 4.14±2.4. A taurine peak was found at 30-msec TE in only 19 cases (67.8%); hence, we suggest that execution of a long TE is more effective for identifying the main marker of medulloblastoma. As a work in progress, the MRI features obtained using the advanced techniques, mainly perfusion and spectroscopy, are undergoing statistical analysis to determine which parameters are useful for predicting the various medulloblastoma molecular subgroups.
**Conclusion:** Since determination of the molecular subgroup is an important factor for oncologists choosing a treatment strategy with regard to relapses, leptomeningeal distribution, and survival, we consider it important to determine the MRI signs that help in the identification of subgroups before starting treatment in these patients.

**B-1232** 11:34
**The additive value of diffusion-weighted imaging in the initial workup for paediatric head and neck lesions**
A.E. Gabr, A. Nada, M. Gomaa, R.H. Hashem, A. Shokry; *Cairo/EG* *(ahmed_elshenawy.nci@yahoo.com)*

**Purpose:** To assess the role of diffusion-weighted MR imaging and ADC value in the initial assessment of paediatric cases presented with head or neck lesions and its capability for differentiating benign from malignant masses.
**Methods and Materials:** Between March 2016 and October 2017, 56 patients (39 male patients, 17 female patients) presented to the Paediatric Oncology Department of the National Cancer Institute with head and neck lesions. These patients were subjected to conventional MRI to get an accurate characterization of the lesions. Then diffusion-weighted imaging echo planar sequence was added to the conventional MR examination to evaluate the role of DWIs in the diagnosis and characterization of the masses. These lesions were correlated to histopathological findings of the available specimens through image-guided FNAC, true-cut biopsies or surgically excised specimens.
**Results:** This study included 56 patients, their ages range from 1 to 16 years with the mean age of 9.2 years. The mean ADC values of malignant, benign solid and benign cystic lesions were found to be 0.64 X $10^{-3}$ $mm^2$/sec, 1.58 X $10^{-3}$ $mm^2$/sec, and 2.10 X $10^{-3}$ $mm^2$/sec, respectively. ADC value of 1.25 X $10^{-3}$ $mm^2$/sec was used as a threshold value for differentiation between malignant and benign lesions with sensitivity 100% and specificity 94.1%.
**Conclusion:** Our study concluded that DWI is a significant and non-invasive technique that can be reliably used for characterization of and differentiation between malignant and benign lesions of the head and neck regions.

**B-1233** 11:42
**Evaluation of white matter maturation in children with developmental delay using diffusion tensor imaging (DTI)**
A. Yap, K. Rahmat, R. Muridan, P. Seow, C. Fong, L. Ong, L. Tan, N. Ramli; *Kuala Lumpur/MY (katt_xr2000@yahoo.com)*

**Purpose:** This study assessed the white matter maturation using diffusion tensor imaging (DTI) in paediatric patients with developmental delay to imply delayed white matter tract maturation.
**Methods and Materials:** Nineteen developmental delayed children and twenty-five neurodevelopmentally healthy children underwent MRI and cross sectional DTI scans. The developmental assessment of all the subjects was then performed. An automated processing pipeline for the white matter tracts assessment was comprised of DTI data pre-processing, image registration, DTI analysis and evaluation of tracts was implemented. Spearman test was used to establish correlations between the DTI metrics and developmental delay components.
**Results:** The results obtained from the developmental delayed group were compared with age-matched healthy group. Findings showed that majority of the 17 white matter tracts investigated demonstrated significant differences between the two groups. The FA values were lower while MD and RD values were higher in the delayed group. The AD values were slightly varied, where some white matter tracts in the delayed group showed higher and lower values. Correlations were seen between the DTI metrics with the developmental delay components in the white matter tracts such as corona radiate, medial lemniscus, cingulum, optic radiation, superior cerebellar peduncle, superior longitudinal fasciculus and posterior limb of internal capsule.
**Conclusion:** Most of the white matter tracts assessed showed diffusion changes that implied delayed white matter maturation in developmental delayed patients compared to control. The DTI metrics had potential as non-invasive imaging markers for delayed white matter maturation in children with developmental delay.

**10:30 - 12:00** **Room M 3**

### Oncologic Imaging

### SS 1416

### Neurooncology: making the most of quantitative imaging

*Moderators:*
N.N.
E. Papadaki; Iraklion/GR

**B-1234** 10:30
**Enhancement of T1WI histogram in the identification with primary central nervous system lymphoma and glioblastoma**
C. Chen, J. Cheng, Y. Zhang; *Zhengzhou/CN (chrnchrn812@163.com)*

**Purpose:** To investigate the diagnostic value of enhanced T1WI histogram analysis in the identification of primary central nervous system lymphomas and glioblastomas.
**Methods and Materials:** Retrospective analysis of pathologically confirmed 41 cases of primary central nervous system lymphoma and 30 cases of glioblastoma. T1, T2-weighted, and enhanced T1 magnetic resonance images of the two types of tumours were collected. The MaZda was used to extract the region of interest of the tumour. The histogram analysis was performed on the maximal area of interest of the tumour to observe the two types of tumours. The histogram features of the tumour, including the mean, variance, skewness, kurtosis, and the 1, 10, 50, 90, and 99 percentiles, were used to find significant differences between the two kinds of tumours. Statistical analysis was performed using SPSS 21.0 statistical software. The ROC curve was used to analyze the diagnostic efficacy of statistically significant parameters between the two groups of tumours.
**Results:** Among the nine parameters extracted using histogram analysis, the mean, variance, skewness, 1st, 10th, and 50th percentiles were statistically significant ($P<0.05$), while there was no significant difference in kurtosis, 90th percentile, and 99th percentile ($P>0.05$). In these parameters, the skewness was the most effective in identifying primary central nervous system lymphomas and glioblastomas, and the area under the ROC curve was 0.960, and the sensitivity and specificity were 80% and 100%, respectively.
**Conclusion:** Histogram analysis is helpful for the identification of primary central nervous system lymphoma from glioblastoma and the skewness has a high diagnostic efficiency.

**B-1235** 10:38

**T2WI histogram analysis of whole tumour volume for differentiating glioblastoma from primary central nervous system lymphoma**

C. Chen, J. Cheng, Y. Zhang; *Zhengzhou/CN (chrnchrn812@163.com)*

**Purpose:** To study the value of T2WI whole tumor volume histogram analysis in the diagnosis of primary central nervous system lymphoma and glioblastoma.
**Methods and Materials:** Retrospective analysis of 41 cases of primary central nervous system lymphoma and 29 cases of glioblastoma confirmed by pathology in our hospital. T2WI axial images were selected, Mazda software was used to map the regions of interest on each layer of the tumour in both groups, and a total tumour volume histogram analysis was performed to observe the histogram characteristics of the two tumours, including mean, variance, and skewness. The kurtosis, the 10th percentile, the 50th percentile, the 90th percentile, and the 99th percentile identify the significant differences between the two tumours. Statistical analysis was performed using SPSS 21.0 statistical software. The ROC curve was used to analyze the diagnostic efficacy of statistically significant parameters between the two groups of tumours.
**Results:** In the nine parameters obtained from T2WI whole tumour volume histogram analysis, the mean, variance, kurtosis, skewness, 50th percentile, 90th percentile, and 99th percentile were statistically significant (P<0.05). Among these parameters, the variance identification of the primary central nervous system lymphoma and glioblastoma was the most effective, and the area under the ROC curve was 0.884, and the sensitivity and specificity were 69% and 90.2% respectively.
**Conclusion:** The whole tumour volume T2WI histogram analysis is helpful for the identification of primary central nervous system lymphoma from glioblastoma and the variance has a high diagnostic efficiency.

**B-1236** 10:46

**Whole tumour histogram analysis of apparent diffusion coefficient for evaluation of histological grade and BRAF mutation status in pleomorphic xanthoastrocytomas**

J. Yan, J. Cheng, F. Liu; *Zhengzhou/CN (ayyj1986124@126.com)*

**Purpose:** To investigate whether histogram-profiling of apparent diffusion coefficient (ADC) could stratify histological grade and predict BRAF mutation status of pleomorphic xanthoastrocytomas (PXAs).
**Methods and Materials:** The ADC maps of 61 patients with pathologically diagnosed PXAs were used for whole tumour histogram-profiling according to the 2016 WHO classification. The patients were firstly allocated to the grade II and grade III anaplastic PXA groups, and further divided into BRAFV600E-mutated and wild-type groups. Comparative statistics were performed to investigate the association of histogram parameters between groups. Receiver operating characteristic (ROC) curve was applied to decide the accuracy and optimum cut-off value of histogram parameters. Binary logistic regression analysis was used to determine the independent predictors of the BRAFV600E mutation.
**Results:** Various histogram parameters (ADCmean, ADCmax, ADCp10, ADCp25, ADCp75, ADCp90, ADCmedian) were significantly greater in the grade II PXA group than those in the grade III anaplastic PXA group (all P<0.05). ROC analysis revealed that ADCp90 was the most accurate parameter for stratifying histological grade of PXAs, with a cut-off value of $1.34\times10^{-3}$ $mm^2/s$ (sensitivity 0.882 and specificity 0.889). Further, ADCmax and ADCp90 were significantly greater in the BRAFV600E-mutated group compared with the wild-type group (P=0.018 and 0.027, respectively). Binary logistic regression analysis revealed that ADCmax was independently associated with the BRAF mutation status (P=0.030).
**Conclusion:** Whole tumor histogram-profiling of ADC was useful for histological grading of PXAs, rendering ADCp90 as the most accurate parameter with high specificity and sensitivity. Further, ADCmax was the independent predictor for BRAF mutation of PXAs.

**B-1237** 10:54

**Prediction of the BRAF mutation status of pleomorphic xanthoastrocytomas using pre-operative multi-parametric MRI**

J. Yan, J. Cheng, F. Liu; *Zhengzhou/CN (ayyj1986124@126.com)*

**Purpose:** We aimed to predict the BRAF mutation status of pleomorphic xanthoastrocytomas (PXAs) based on pre-operative multi-parametric MRI characteristics.
**Methods and Materials:** A set of 18 clinical and MRI features were evaluated in 33 patients with pathologically-confirmed PXAs for prediction of BRAFV600E mutation. The results obtained for the BRAF-mutated and wild-type groups were compared. Characteristics that were significantly more prevalent (P<0.05) in the BRAF-mutated PXAs were defined as diagnostic features. The receiver operating characteristic curve and binary logistic regression analysis were carried out to determine diagnostic accuracy and optimal cut-off value.
**Results:** A comparison of findings between the groups showed that the cases with BRAF-mutated PXAs tended to be younger (P=0.010), and more often had a history of epilepsy (P=0.047). Though the difference in number was not significant in the two groups, all the multiple lesions were found in the wild-type group. Furthermore, BRAF-mutated PXAs tended to be superficial with obvious meningeal contact (P=0.040), appeared as predominantly cystic with a mural nodule (P=0.024). The mean ADC and normalized ADC ratio were significantly greater in the BRAF-mutated group (P=0.012 and 0.003, respectively). The optimal cut-off values of mean ADC and normalized ADC ratio were $0.86\times10^{-3}$ $mm^2/s$ (sensitivity 0.769 and specificity 0.824) and 1.07 (sensitivity 0.769 and specificity 0.882), respectively. The combination of all these significant features resulted in the highest sensitivity and specificity of 0.923 and 0.882, respectively.
**Conclusion:** The BRAF mutation status of PXAs may be predicted using pre-operative multi-parametric MRI with high specificity and sensitivity.

**B-1238** 11:02

**To study the diagnostic value of adc global gray histogram in differentiating primary central nervous system lymphoma from pleomorphic glioblastoma and single brain metastases**

Z. Ma, J. Cheng; *Zhengzhou/CN*

**Purpose:** To study the diagnostic value of adc global gray histogram in differentiating primary central nervous system lymphoma from pleomorphic glioblastoma and single brain metastases.
**Methods and Materials:** A retrospective analysis in our hospital had brain MRI examination and 95 cases of pathologically confirmed patients, including central nervous system lymphoma 38 cases , 29 cases of glioblastoma , 28 cases of solitary metastases.Three groups of MR ADC axial images were used to draw the region of interest on each level of tumour with MAZDA software, and the gray level global histogram was analyzed. The parameters of the three groups of histograms were tested by kruskal-wallis, and the comparison between groups was tested by bonferroni method. The statistical significance of each parameter was compared.
**Results:** Through histogram analysis of 9 parameters, these 9 parameters were statistically significant (all P <0.05),including mean ,variance , kurtosis, skewness, Perc.01%,Perc.10%,Perc.50% ,Perc.90% and Perc.99%.The sensitivity of perc.50% between glioblastoma and central nervous system lymphoma was 84.21, specificity was 86.21. The sensitivity of mean perc.50% between glioblastoma and single metastatic tumour was 0.90%. The sensitivity of perc.90% perc.99% between the central nervous system lymphoma group and the single metastatic tumour group was 92.86% and the specificity was 63.16.
**Conclusion:** Histogram analysis is helpful for the identification of central nervous system lymphoma and glioblastoma and single metastatic tumour. The perc.50% between glioblastoma and central nervous system lymphoma, between glioblastoma and single metastasis, perc.90% between central nervous system lymphoma and single metastatic tumour has high diagnostic efficacy.

**B-1239** 11:10

**A whole-tumour histogram analysis of apparent diffusion coefficient maps for distinguishing lateral ventricle central neurocytoma from ependymoma**

W. Wang, J. Cheng; *Zhengzhou/CN (weijianwang520@163.com)*

**Purpose:** We explored the role of a whole-tumour histogram analysis of apparent diffusion coefficient (ADC) maps for discriminating lateral ventricle central neurocytoma from ependymoma.
**Methods and Materials:** We retrospectively evaluated findings in 35 patients with lateral ventricle central neurocytoma and 20 patients with ependymoma who underwent diffusion-weighted imaging (b = 0,800 s/mm(2) ) at 3T with acquisition of corresponding ADC maps. We derived histogram data from regions of interest drawn on all slices of the ADC maps in which tumour was visualized, including areas of necrosis and haemorrhage in the tumour. We used the t test to evaluate the capacity of histogram parameters (variance, kurtosis,1th,10th percentiles) to discriminate central neurocytoma from ependymoma and analyzed the receiver operating characteristic (ROC) curve to determine the optimum threshold value for each parameter and its corresponding sensitivity and specificity.
**Results:** Lateral ventricle central neurocytoma demonstrated significantly lower variance, kurtosis,1th,10th percentiles than ependymoma(P < 0.05). ROC curve analysis of the variance yielded the best area under the ROC curve (AUC; 0.83), sensitivity of 80%, and specificity of 82%, with a cutoff value of 2012.
**Conclusion:** A Whole-Tumour Histogram analysis of ADC maps might be helpful for discriminating lateral ventricle central neurocytoma from ependymoma.

**B-1240** 11:18

**Characterisation of peripheral nerve sheath tumours in patients with neurofibromatosis type 1 using diffusion-weighted magnetic resonance imaging**

J. Salamon[1], L. Well[1], K.I. Geier[1], L. Späth[1], M. Kaul[1], J. Herrmann[1], V.-F. Mautner[1], G. Adam[1], T. Derlin[2]; [1]*Hamburg/DE*, [2]*Hannover/DE*

**Purpose:** To evaluate the potential of diffusion-weighted MRI (DW-MRI) for differentiation of malignant and benign peripheral nerve sheath tumors (MPNST/BPNST) in patients with neurofibromatosis type 1 (NF1) using apparent diffusion coefficient (ADC) and intravoxel incoherent motion (IVM) models.
**Methods and Materials:** 26 patients with NF1 were included in this prospective study resulting in n=67 tumors. DW-MRI was performed at 3T (T1wTSE, T1w mDixon with in- and opposed phase, T2wTSE and DWI). DW-MRI consisted of eleven b-values (0-800 s/mm²). A monoexponential function was used to calculate ADC maps and a biexponential function was applied for the IVIM model, generating diffusion coefficient (D) and perfusion fraction (f) maps. Regions of interest (ROI) were manually drawn to determine signal intensities. Mann-Whitney-U, Fisher's exact test, receiver-operating-characteristic (ROC) analyses and spearman correlation were performed. Inter-rater reliability was evaluated.
**Results:** MPNST compared with BPNST were characterized by lower mean ADC values ($ADC_{mean}$ 1.23 vs. 2.09 x $10^{-6}$ mm²/s;p<0.0001) and lower mean diffusion coefficients ($D_{mean}$ 1.03 vs 1.88 x $10^{-6}$ mm²/s;p<0.0001). Perfusion fraction f was significantly higher in MPNST than in PBNST (21% vs 11%, p=0.0003). For both entities, f was higher in the periphery compared to the center of the lesions (p<0.0001). ROC showed good levels of sensitivity and specificity for both ADC and D (sensitivity vs specificity: $ADC_{mean}$ 92%/98%, AUC 0.983; $D_{mean}$ 92%/98%, AUC 0.979). Inter-rater reliability was good to very good (kappa $ADC_{mean}$ 0.898; $D_{mean}$ 0.646).
**Conclusion:** Diffusion weighted imaging allows identification of MPNST in NF1 with high levels of sensitivity and specificity.

**B-1241** 11:26

**ASL perfusion MRI reveals reduced cerebral blood flow in the hippocampal region in patients with former childhood medulloblastoma**

Y. Tanyildizi, A. Kronfeld, M.-A. Neu, A. Wingerter, A. Russo, K. Khawaja, A. Tropine, M.A. Brockmann, J. Faber; *Mainz/DE (y.tanyildizi@gmx.net)*

**Purpose:** The number of long-term survivors of former childhood Medulloblastoma is increasing. Therefore late sequelae of radiochemotherapy (RCT), such as reduced cerebral blood flow (CBF), are gaining importance. In this study changes of cerebral blood flow (CBF) were analyzed using arterial spin labeling (ASL) perfusion MRI.
**Methods and Materials:** Perfusion weighted images of 28 patients (mean age:25.2; range:10-55 years; mean years after primary radio-chemo-therapy 14.9, range:1-45) and 24 healthy subjects (mean age:32; range:22-63 years) were acquired using ASL. For each individual CBF-maps were calculated and brought into MNI-space using SPM12. Mean CBF in grey matter was determined for every group. A voxelwise two-sample t-test was performed (SPM12), and mean CBF was calculated in the regions showing a significant difference and compared on an individual basis.
**Results:** No significant differences regarding mean grey matter CBF-values between the two groups were observed. (patients (healthy):67.0 (66.4) ± 3.09 (1.24) ml/100g/minute, respectively). However, voxelwise t-test revealed a highly significant reduction of CBF in the hippocampal region (patients (healthy):68.1 (80.6) ± 3.95 (2.07) ml/100g/minute, respectively) between the healthy and the RCT group.
**Conclusion:** ASL perfusion MRI can be used as a noninvasive tool for imaging CBF reduction after RCT in patients with former childhood medulloblastoma. A significantly reduced CBF in the hippocampal region, which is known to be sensitive to ionizing radiation, was found .We suggest that ASL Perfusion sequences should be included in the regularly follow up MRI protocols. Furthermore the effects of the reduced CBF in the hippocampal region have to be investigated.

**B-1242** 11:34

**Machine learning-based radiomics to predict molecular subgroups of medulloblastoma**

J. Yan, Z. Zhang, J. Cheng; *Zhengzhou/CN (ayyj1986124@126.com)*

**Purpose:** Novel biological insights have led to consensus of classifying medulloblastoma into four distinct molecular subgroups - wingless (WNT), sonic hedgehog (SHH), Groups 3, and Group 4. We aim to predict molecular subgroups of medulloblastoma by using advanced machine learning approach which extracts important information based on pre-operative MRI radiomics features.
**Methods and Materials:** Thirty-seven patients with medulloblastoma (WNT = 11, SHH = 9, Group 3 = 8 , and Group 4 = 9) were enrolled, and all were scaned by using contrast-enhanced T1WI sequence. A total of 1089 radiomics features were extracted from contrast-enhanced T1WI images. Then, statistically significant different features in comparision among the four groups were selected for the subsequent model training and performance evaluation by employing one way-ANOVA. After feature selection, a support vector machine was used to predict molecular subgroups of medulloblastoma. Finally, predictive performance was assessed by accuracy, precision, recall and F1. To access algorithm generalization, we used a 2-fold cross-validation approach.
**Results:** The molecular subgroups of medulloblastoma can be classified by important radiomics features and machine-learning approaches with a 70% accuracy, a 67% precision, a higher recall of 92%, and a 77% F1.
**Conclusion:** Our findings suggest a great potential to use MR radiomics features-based machine learning method to predict the molecular subgroups of medulloblastoma.

**B-1243** 11:42

**Delayed FDG PET/CT in differentiating true progression from pseudo-progression in glioblastoma treated with chemoradiation**

M.K. Gule-Monroe, M. Chen, J. Li, B.J. O'Brien, C. Chung, S.D. Ferguson, S.S. Prabhu, J.M. Johnson; *Houston, TX/US*

**Purpose:** Delayed 18F-FDG PET has been shown to increase the specificity of PET imaging for cerebral neoplasm. We sought to compare 1-hour and 5-hour imaging post-injection in patients with metastatic brain tumours treated with radiation.
**Methods and Materials:** 27 patients with suspicious enhancement following radiation were identified and imaged with this protocol. Diagnostic confirmation of progressive disease (PD) or radiation necrosis (RN) was by pathology (5 cases) and/or radiographic follow-up (16 cases). Maximum standard uptake values (SUV) were calculated for suspicious areas of MR enhancement (lesion) and compared to normal appearing brain (background) at both time points.
**Results:** 17 patients had pathology and/or imaging follow-up. 12 cases were classified as having PD and 5 as having RN. PET findings were concordant in all 5 cases with pathologic follow-up. PET findings were concordant in 14/16 cases based on radiographic follow-up imaging. The mean L/B for PD at the early time point (1.6±0.7) was significantly lower than L/B for the later time point (2.4±1.1), p=0.0013. The mean L/B for PD was 2.5±1.1 at the later time point compared to 0.8±0.2 for RN (p=0.001). For the earlier time point, L/B for PD was 1.6±0.7, compared to the L/B for RN, which was 0.7±0.2 (p=0.004).
**Conclusion:** L/B ratios at early and delayed time points successfully differentiate between PD and RN in the majority of cases, with significantly greater L/B ratios between TP and RN on delayed imaging versus 1-hour imaging. These results are promising and further investigation is warranted.

**B-1244** 11:50

**Application of histogram analysis of diffusion Kurtosis imaging to tumour grading and tissue heterogeneity**

A. Gao[1], J. Cheng[1], J. Bai[1], Y. Zhang[1], Y. Hong[2], D. Kong[2]; [1]*Zhengzhou/CN*, [2]*Hangzhou/CN (annygao456@163.com)*

**Purpose:** The aim of this study was to evaluate the significance of DKI parameters in grading glioma.
**Methods and Materials:** DKI (541s) and conventional sequence at 3.0T MR scanner (Prisima Siemens Healthcare, Erlangen, Germany). DKI acquires data with 5 b values (0-2500sec/$mm^2$) and 30 directions for every b value. DKI was scanned using a SE-EPI sequence and the parameters were: FOV =220×220$mm^2$, slice thickness = 5mm, slices = 20, TR/TE = 3500/78ms. Matlab platform was used to calculated diffusion parameters Kapp and Dapp value based on TRACEW parameter image.ROIs were manually drawn around the solid part of the tumour. Histogram analysis from these parameters were correlated with glioma grades. Independent-samples T test or Mann - Whitney - Wilcoxon test was used. The parameters with the best percentile from cumulative histograms were identified by analysis of the AUC of the ROC analysis.
**Results:** 25 low grad gliomas and 49 high grad gliomas were observed. Kapp values increased with increasing glioma grade, but the Dapp values decreased. There were significant difference between high and low grade glioma groups on the Kapp value of maximum, mean, standard deviation, 75th percentile, 95th percentile, and the Dapp value of minimum, mean, 25th percentile, 50th percentile, 75th percentile, 95th percentile, skewness coefficients. According to the ROC , the highest AUC were found at Dapp minimum value (AUC,0.829), Kapp 75th percentile value (AUC,0.819).
**Conclusion:** Histogram analysis of DKI parameters from solid part of the tumour data can be a useful method for glioma grading and Tissue Heterogeneity.

**10:30 - 12:00** **Sky High Stage**

### Head and Neck

### MY 14

### Head and Neck

*Moderators:*
M. Lell; Nuremberg/DE
R. Saat; Tallinn/EE

### B-1245 10:30

**Perfusion MRI with high spatial and temporal resolution in the head and neck region using golden angle radial sampling**

A. Tomppert, M.S. May, M. Wiesmüller, R. Heiß, A. Nagel, M. Uder, W. Wuest; *Erlangen/DE (andrea.tomppert@uk-erlangen.de)*

**Purpose:** Conventional perfusion sequences often provide either poor spatial or poor temporal resolution. Our aim was to set up a golden-angle radial sparse parallel (grasp) sequence to overcome this problem in head and neck perfusion MRI.
**Methods and Materials:** We prospectively included 60 patients for examination on a 3 T MRI using the study protocol. Grasp was applied to a volume interpolated gradient echo perfusion sequence (vibe) with 135 measurements and a resulting temporal resolution of 2.5 s and a spatial resolution of 0.94 x 0.94 x 3.00 mm (A). Additional sequences with matching spatial (B: 1 measurement, 145 s, 0.94 x 0.94 x 3.00 mm) and temporal (C: 3 measurements, 2.5 s, 1.88 x 1.88 x 3.00 mm) resolution without grasp were obtained subsequently. Image quality was evaluated as overall diagnostic quality, mucosal and vessel delineation, lesion enhancement and edge sharpness, artefacts and fat suppression quality on a five point Likert scale.
**Results:** Overall diagnostic image quality, vessel delineation and lesion contrast was highest in A. Mucosal fold and edge sharpness of lesions was comparable to B and superior to C. Slight radiating artefacts, not affecting the diagnosis, were found using the grasp technique in A. Pulsation, moving and susceptibility artefacts were comparable to B and C. Quality of fat suppression was very high and comparable to B and C.
**Conclusion:** High temporal and spatial resolutions can be obtained synchronously using the grasp-vibe sequence for perfusion evaluation in head and neck MRI.
*Author Disclosures:*
**M.S. May:** Speaker; Siemens Healthcare GmbH. **M. Wiesmüller:** Speaker; Siemens Healthcare GmbH. **R. Heiß:** Speaker; Siemens Healthcare GmbH. **M. Uder:** Speaker; Siemens Healthcare GmbH. **W. Wuest:** Speaker; Siemens Healthcare GmbH.

### B-1246 10:34

**Preliminary diffusion kurtosis imaging study of head and neck squamous cell carcinomas associated with histologic grades**

T. Su, Y. Chen, X. Chen, T. Zhang, X. Shi, H. Xue, Z. Zhang, Z. Jin; *Beijing/CN (angelasu.love@163.com)*

**Purpose:** To preliminarily investigate the clinical value of diffusion kurtosis imaging (DKI) in head and neck squamous cell carcinomas (HNSCCs), and to evaluate its correlation with different histologic grades.
**Methods and Materials:** Total 22 HNSCCs patients who underwent tumour resection and lymphadenectomy were involved. DKI data were obtained by a single-shot echo-planar imaging sequence with three b values of 0, 800 and 1600 s/mm$^2$. Diffusivity (D) and kurtosis (K) and conventional apparent diffusion coefficient (ADC) were calculated. The MR images were compared with the histopathologic findings. One-way ANOVA, spearman correlation and receiver operating characteristic curve (ROC) analysis were analyzed, with P value of ≤0.05 indicating significance.
**Results:** In correlation analysis, ADC value was negatively correlated with kurtosis (r=-0.511, p=0.003), and positively correlated with diffusivity (r=0.882, p<0.001). And kurtosis value was negatively correlated with histologic grades (r=-0.384, p=0.030). In ROC analysis, AUC of the kurtosis and diffusivity values for differentiating well-differentiated from poorly-moderately differentiated carcinomas were 0.753 and 0.729 separately, higher than that of ADC values. And AUC of combined three parameters together was 0.965, with 94.1% sensitivity and 100% specificity, higher than combinations of either two.
**Conclusion:** In this preliminary study, kurtosis derived from DKI was negatively correlated with histologic grades of HNSCCs. And the relatively high diagnostic accuracy of combined ADC and DKI parameters may be helpful for the preoperative differentiation of histologic grades.

### B-1247 10:38

**Radiohistological comparison study of temporal bone specimen after CI electrode array insertion: is CBCT superior to MSCT?**

I. Burck, S. Schneider, I. Yel, A. Lehn, S. Balster, S. Helbig, T. Stöver, T.J. Vogl, B. Kaltenbach; *Frankfurt a. Main/DE (iris.burck@kgu.de)*

**Purpose:** To evaluate subjective image quality and diagnostic accuracy of cone-beam CT (CBCT) images in temporal bone specimen with implanted CI electrode array in comparison to histological assessment.
**Methods and Materials:** In this retrospective study, two radiologists independently reviewed CBCT (96kV vs. 120kV setting) and CT images of 20 temporal bone specimens after electrode implantation. Qualitative evaluation of bone structures of the otic capsule, inner and outer cochlear wall, lamina spiralis ossea, intracochlear electrode position with regard to lamina spiralis ossea, visualization of single electrode contacts on the array, metal artefacts and overall image quality was performed using a 5-point scale. Intracochlear electrode position was subsequently correlated by histological examination.
**Results:** Radiological assessment of bone structures of the otic capsule, inner and outer cochlear wall, lamina spiralis ossea, electrode position with regard to lamina spiralis ossea, visualization of single electrode contacts on the array, metal artefacts and overall image quality were significantly higher in CBCT at both tube voltages compared to CT (p≤0.025). No significant differences were found between CBCT at 96kV and 120kV (p≥0.532). The mean intraclass correlation was good (0.651). The determination of the intracochlear electrode position with histological correlation was highly correct in 120kV (100%) and 96kV CBCT (97.5%) images, in CT only 77.5% were determined correctly.
**Conclusion:** Our data suggest that CBCT may significantly improve diagnostic accuracy of temporal bone imaging after CI compared to CT, in particular, to determine the intracochlear localization of the implant. There is no significant difference using 120kV or 96kV CBCT protocols for the assessment.

### B-1248 10:42

**Utility of histogram analysis of ADC maps in differential diagnosis of Pleomorphic adenoma and Acinar cell carcinoma originated from parotid gland**

X. Zhang, J. Cheng, K. Ma; *Zhengzhou/CN (zxn9725651@126.com)*

**Purpose:** To explore the diagnostic value of the histogram analysis derived from apparent diffusion coefficient (ADC) maps in the differential diagnosis of Pleomorphic adenoma and Acinar cell carcinoma.
**Methods and Materials:** 22 cases of parotid neoplasms confirmed by pathology who underwent MRI scanning were retrospectively analysed, including 10 cases of Pleomorphic adenoma and 12 cases of Acinar cell carcinoma. Drawing the region of interest (ROI) on the maximal section ADC maps and going on histogram analysis. Performed a statistical analysis on the histogram parameters to find out the characteristics of the significant differences between the two groups. The ROC curve was analysed to determine the optimum threshold value for each parameter and its corresponding sensitivity and specificity.
**Results:** In the 9 parameters which are extracted from histogram, ADC mean, ADC variance, ADC 10th percentile (ADC_10th), the 90th percentile (ADC_90th), the 99th percentile (ADC_99th) have the statistical significance (P < 0.05). ROC analysis indicated that (ADC_99th) had the highest diagnostic accuracy for differentiating the 2 groups, with the area under the curve being 0.891, a cutoff value of 186.50, and a sensitivity of 100.0% and a specificity of 72.7%.
**Conclusion:** The ADC histogram is useful for the differential diagnosis of pleomorphic adenoma and acinar cell carcinoma and can accurately distinguish between them. Histogram analysis of ADC maps can provide reliably objective basis for differentiating pleomorphic adenoma and acinar cell carcinoma.

### B-1249 10:46

**The value of ADC histogram in differentiating nasopharyngeal carcinoma from nasopharyngeal lymphoma**

K. Ma; *Zhengzhou/CN (1322725260@qq.com)*

**Purpose:** The purpose of this study is to assess the utility of texture analysis of apparent diffusion coefficients (ADC) maps for the differentiation of Nasopharyngeal Carcinoma (NPC) and Nasopharyngeal Lymphoma (NPNHL).
**Methods and Materials:** 79 patients with diagnosis of nasopharyngeal malignant tumours including 45 NPCs and 34 NPNHLs in our hospital from June 2015 to March 2018 were retrospectively enrolled in this study. All the patients were proved by pathology and examined by sequence of T1WI, T2WI and DWI . Drawing the region of interest(ROI) on the selected MR images and going on histogram analysis, these two steps were all performed on the software named Mazda. Performed a statistical analysis on the histogram parameters to find out the characteristics of the significant differences between the two groups and ROC were used to describe its efficiency in differential diagnosis.

**Results:** Significant differences were observed for mean, standard deviation, and 10th, 50th, 90th, and 99th percentiles of the ADC values. ROC curve analysis showed that the sensitivity and specificity of these parameters were 62.2% and 82.4%, 88.9% and 64.7%, 100% and 35.3%, 80.0% and 61.8%, 80% and 55.9%, 80.0% and 79.4%, successively, and the areas under ROC were 0.801, 0.813, 0.731, 0.797, 0.795, 0.838, respectively.
**Conclusion:** The histogram analysis of the ADC maps can be used to identify nasopharyngeal cancer and nasopharyngeal lymphomas. Although these findings require validation in larger studies, they have implications for facilitating improved treatment selection for these two entities.

**B-1250** 10:50
**Prognostic value of restaging 18-FDG PET/CT volumetric parameters for post-index irradiation recurrence of head and neck squamous cell carcinoma**
K. Musaieva, B. Musaiev, Y. Kmetyuk; *Kiev/UA (kitlyboo@gmail.com)*

**Purpose:** The purpose of this study was to define the prognostic value of metabolic and volumetric parameters for recurrent HNSCC and the role of salvage re-irradiation in overall survival in this cohort.
**Methods and Materials:** Pretreatment PET/CT scans and after treatment PET/CT scans of 69 patients who underwent salvage treatment for recurrent HNSCC, were retrospectively evaluated. Metabolic response was assessed using PET response criteria for solid tumors (PERCIST). Multiple statistical image features related to the standard uptake value (SUV) were computed: metabolic tumor volume, maximumSUV, meanSUV, total lesion glycolysis. The correlation between the image features and local control and overall survival was calculated.
**Results:** In this study we reported on the utility of PET/CT in survival prognostication for recurrent head and neck cancer. Analysis indicates that whole body metabolic tumour volume WB MTV has potency in estimating the overall survival. High WB MTV (over 21,5 cc) corresponds with poor prognosis and low one-year survival rate (56,0% of patients will not survive over 12 months); on the other hand, one-year survival at WB MTV < 21,5 cc is estimated as 93,2%. This observation remained robust even after accounting for other known prognostic factors including salvage re-irradiation and recurrence location.
**Conclusion:** With this study we established a pre-salvage volumetric cut-off point (whole body metabolic tumour volume WB MTV of 21,5 cc) that predicts one-year survival with high accuracy. A more extensive study is warranted to decide if this cut-off point is acceptable for subgroup of patients who will undergo a salvage re-irradiation.

**B-1251** 10:54
**Update of old thyroid US reports to EU-TIRADS with AI**
A. Mereu, L. Saba; *Cagliari/IT*

**Purpose:** To assess the ability of AI to update old thyroid US free-text reports of patients with Hashimoto's disease to the 2017 European Thyroid Imaging and Reporting Data System (EU-TIRADS) categories.
**Methods and Materials:** 500 thyroid US free-text reports of subjects with Hashimoto's disease were retrospectively assessed. All reports were manually categorized accordingly to the 2017 EU-TIRADS. An ensemble of 7 algorithms (based on bagging, boosting, classification trees, maximum entropy, neural networks, random forests and support vector machine) was employed by an AI to assign a single EU-TIRADS category to each report. To simulate a real world situation, only a randomized sample consisting of 10% (50 reports) of the dataset was provided to the AI to train itself. All the main tasks involved were handled with the free and open source programming language R.
**Results:** A Kappa statistic of 0.402 indicated fairly good predictions for all classes. EU-TIRADS categories 2, 3, 4 and 5 were predicted with overall precision of 0.930, recall of 0.564, F-score of 0.702 and Kappa statistic of 0.566.
**Conclusion:** Study results showed that the AI categorized old reports in accordance with the latest EU-TIRADS classification with fair performance, although provided with only one tenth of the total dataset to train itself. Even if a human second-look is mandatory, that might be a viable approach to update old reports to new risk levels for scopes such as patient recall, modify follow-ups and research.

**B-1252** 10:58
**Application of RESOLVE-DWI ADC maps based on whole-tumour histogram analysis in differentiating common parotid gland tumours**
C. Song, J. Cheng, Y. Zhang; *Zhengzhou/CN (songchengru@126.com)*

**Purpose:** The purpose of this study was to evaluate whether whole-tumour histogram-based analysis of RESOLVE-DWI ADC maps can help in the discrimination of parotid gland tumours (pleomorphic adenoma, Warthin tumour, malignant parotid gland tumour).
**Methods and Materials:** The MR images of 36 patients with a biopsy- or surgery-proven pleomorphic adenomas, 14 patients with Warthin tumours and 19 patients with malignant parotid gland tumours were retrospectively analysed. Histogram-based analysis was performed with the software MaZda. ROIs were drawn on every section of the ADC map containing the tumour, then 12 parameters derived from histogram were calculated. Statistical analysis among the three groups were performed to find out the statistical significance of each histogram parameter. And the differential efficiency of each parameter was determined using a receiver operating characteristic curve (ROC) analysis.
**Results:** Totally 9 parameters (MinNorm, MaxNorm, mean, variance, skewness, Perc.10%, Perc.50%, Perc.90%, Perc.99%) among three groups were statistically significant (P<0.05). Between the pleomorphic adenomas and Warthin tumours, these 9 parameters were of statistical significance. Mean, skewness and Perc.50% revealed high diagnostic efficiency with the areas under the ROC curve (AUC) of 0.976, 0.970, 0.970, respectively. Between the pleomorphic adenomas and malignant parotid gland tumours, also these 9 parameters were of statistical significance. Mean, Perc.10% and Perc.50% revealed high diagnostic efficiency with AUC of 0.851, 0.866, 0.841, respectively. But between the Warthin tumours and malignant parotid gland tumours, only 3 parameters (mean, skewness, Perc.50%) was statistically significant.
**Conclusion:** Whole-tumour histogram analysis of ADC maps are effective in differentiating common parotid gland tumours.

**B-1253** 11:02
**Diagnostic utility of mean peak systolic velocity of superior thyroid artery (STA PSV) in the differential diagnosis of thyrotoxicosis**
P.K. Sarangi, S. Parida, B.K. Mohanty, S. Mangaraj, J. Mohanty, B.M. Swain; *Cuttack/IN (lipu90sarangi@gmail.com)*

**Purpose:** To evaluate the utility of mean peak systolic velocity of superior thyroid artery (STA PSV) in differentiation of Graves' disease (GD) and thyroiditis presenting with thyrotoxicosis.
**Methods and Materials:** A total of 111 patients with newly diagnosed thyrotoxicosis (82 with GD and 29 with thyroiditis) without antithyroid medication or β-blockers intake, history of neck irradiation, neck surgery, radioiodine therapy were included. 45 age and sex matched healthy subjects were included as control. Thyroid function tests (FT3, FT4, and TSH) and serum TSH receptor antibody (TRAb) were done. All subjects underwent a detailed colour Doppler ultrasonography of the thyroid gland and spectral flow analysis of both superior thyroid arteries using 5-12 MHz linear transducer of PHILIPS HD 7 USG machine. Mean STA PSV was obtained by averaging right and left STA PSV. Receiver operating characteristic curve (ROC), sensitivity, and specificity for mean STA PSV was calculated using SPSS 24.0 software.
**Results:** On color Doppler study, the mean STA PSV of patients with GD was significantly higher than thyroiditis and control group [(92.79±44.95 cm/sec, 37.89±15.33 cm/sec, 20.07±6.77 cm/sec respectively (p<0.001)]. Mean STA PSV greater than 54.275 cm/s had 82.9 % sensitivity and 86.2 % specificity in diagnosing Graves' disease. Mean STA PSV greater than 84.925 is 100% specific for GD.
**Conclusion:** The mean STA PSV has high sensitivity and specificity in differentiating Graves' disease from thyroiditis and can be used routinely in clinical practice as a cheap diagnostic tool where serum TRAb test is not available or affordable.

**B-1254** 11:06
**Visualisation of the trigeminal nerve branches on enhanced 3D-SPACE-STIR sequence: initial experience**
C. Chen, H. Haiwei, S. Yunkun, H. Xu; *Xiamen/CN (fjmuccf@163.com)*

**Purpose:** To investigate the value of magnetic resonance (MR) three-dimensional sampling perfection with application optimized enhanceds using different flip angle evolution short time inversion recovery (3D-SPACE -STIR) sequence in detecting the trigeminal nerve branches.
**Methods and Materials:** Eleven patients who underwent non-enhanced and enhanced 3D-SPACE-STIR MR scan were enrolled prospectively. Imaging scores were evaluated by three radiologists according 3 levels. Weighted κ analysis was used to assessment the interobserver variability. Displaying rates of the ophthalmic branch (V1), maxillary branch (V2), mandibular branch (V3) trunk and divisions of V3 were analysed. Signal intensity of the V1, V2, V3 trunk and lateral pterygoid muscle (LPM) were measured to calculate the signal intensity ratio (SIR) and contrast ratio (CR).
**Results:** It showed higher imaging scores (2.85±0.36) on enhanced 3D-SPACE-STIR MR imaging than non-enhanced imaging (1.18±0.39), with excellent interobserver variability. The SIR and CR values of V1, V2 and V3 trunk were higher on enhanced MR imaging. Displaying rates for the three trunks on enhanced MR imaging were 97.73%, 100% and 100%, respectively, while 93.18%, 95.45% and 100% on non-enhanced MR imaging. A similar tendency was observed for the divisions of V3. Displaying rates for inferior alveolar, lingual, buccal, masseteric, auriculotemporal and deep temporal nerves on enhanced MR imaging were 100%, 100%, 86.36%, 97.73%, 100% and 90.91%, respectively, while 100%, 97.73%, 84.09%, 90.91%, 93.18% and 79.55%, respectively, on non-enhanced MR imaging.
**Conclusion:** Enhanced 3D-SPACE-STIR MR imaging demostrates better background suppression and excellent visualization of the trigeminal nerve branches.

**B-1255** 11:10
**Three-dimensional shear wave elastography for differentiating benign from malignant thyroid nodules**
C.-K. Zhao; *Shanghai/CN (798434308@qq.com)*

**Purpose:** To prospectively evaluate the diagnostic performance of three-dimensional (3D) shear wave elastography (SWE) for assessing thyroid nodules.
**Methods and Materials:** A total of 176 surgically or cytologically confirmed thyroid nodules (malignant 63, benign 113) in 176 patients who had undergone conventional ultrasound (US), two-dimensional (2D) and 3D SWE examinations were included in this study. Quantitative elasticity values were computed on 2D and 3D SWE (E_mean, E_max and E_SD of a large region of interest (ROI) and E_mean of a 2 mm ROI). Diagnostic performances of conventional US, 2D SWE and 3D SWE were assessed. The role of 2D SWE and 3D SWE in reducing unnecessary fine-needle aspiration (FNA) for low suspicious nodules was also evaluated.
**Results:** The diagnostic performances in terms of area under the receiver operating characteristic curve (AUC) were 0.612 for conventional US, 0.836 for 2D SWE ($P$<0.001 in comparison with conventional US), and 0.839 for 3D SWE ($P$<0.001 in comparison with conventional US). E_max achieved the highest diagnostic performance in 2D SWE while E_SD in 3D SWE, whereas no significant difference was found between them ($P$>0.05). 3D SWE increased the specificity in comparison with 2D SWE (88.5% vs. 82.3%, $P$=0.039). For the 37 nodules with low suspicion on conventional US, 2D SWE was able to avoid unnecessary FNA in 77.1% (27/35) of benign nodules and 3D SWE further increases the number to 88.6% (31/35).
**Conclusion:** 3D SWE is a useful tool in predicting thyroid nodule malignancy and reducing unnecessary FNA procedures in low suspicious thyroid nodules.

**B-1256** 11:14
**DWI-associated entire-tumour histogram analysis for the differentiation of nasopharyngeal carcinoma from nasopharyngeal lymphoma**
K. Ma, J. Cheng, X. Zhang, C. Song; *Zhengzhou/CN (1322725260@qq.com)*

**Purpose:** To investigate the diagnostic efficiency of DWI using entire-tumour histogram analysis in differentiating the nasopharyngeal carcinoma (NPC) from nasopharyngeal lymphoma (NPNHL) in comparison with conventional ROI-based measurement.
**Methods and Materials:** DW images (b of 0-1000 s/mm2) from pathology confirmed 45 NPCs and 34 NPNHLs were retrospectively collected . The measurement of tumour apparent diffusion coefficients (ADCs) was performed with using histogram-based and ROI-based approach, respectively. The diagnostic ability of ADCs from two methods for differentiating NPC from NPNHL was determined by ROC regression, and compared by McNemar’s test.
**Results:** Histogram-based ADCs (mean, standard deviation, 10th, 50th, 90th, 99th) and ROI-based ADCs (mean) reflected significant difference between NPC and NPNHL (all p values <0.05). Histogram 99th ADCs had dominantly high Az (0.838), Youden index (0.594), and positive likelihood ratio (LR+, 3.89) in differentiating NPC from NPNHL against standard deviation, 10th ADCs, 50th ADCs, 90th ADCs, and ROI-based ADCs. Histogram standard deviation, 10th ADCs and 99th ADCs showed higher sensitivity (80.0%-88.9% vs. 70.6%, $p < 0.05$), but lower specificity (78.6%-79.4% vs. 83.3%, $p < 0.05$) than ROI-based ADCs in differentiating NPC from NPNHL.
**Conclusion:** DWI-associated histogram analysis had higher sensitivity, Az, Youden index, and LR+ for differentiation of NPC from NPNHL than ROI-based approach. It can be speculated that the histogram has certain auxiliary value in clinical diagnosis.

**B-1257** 11:18
**Accessing the skull base: CT-guided submaxillary core needle biopsy for the diagnosis of skull base lesions**
I. Burck, M. Rosenzweig, N.-E. Nour Eldin, H. Kvasnicka, T. Stöver, V. Jacobi, T.J. Vogl; *Frankfurt/DE (iris.burck@kgu.de)*

**Purpose:** To assess the time efficiency and evaluate the diagnostic accuracy of CT-guided submaxillary core needle biopsy (CNB) for the diagnosis of deep-seated head and neck masses.
**Methods and Materials:** Twenty-seven patients with skull base lesions underwent CT-guided submaxillary CNB between 2003 and 2018. All CNB were performed under CT guidance and local anaesthesia using an 18-gauge-needle for biopsy, 2-4 histological specimens were obtained for histopathological analysis. Histological findings were classified as "benign", "suspicious for malignancy" or "malignant". Medical records were reviewed with regard to therapy, in case of operative strategies surgical results, histological findings or in case of chemotherapy or radiotherapy treatment response. Additionally, clinical follow-up was evaluated. Prevalence, CNB-sensitivity and specificity, positive predictive value as well as negative predictive value were calculated.
**Results:** Eighty-nine percent of specimens from CNB were considered accurate, 11% of the histological specimen were non-diagnostic, so re-biopsies were performed in 3 cases. There were no postinterventional complications. All patients could be released from the hospital after at least 8 hours (7.87h±3.4). Histological diagnosis could be delivered within on average 2 days (2.14d±0.7). CNB sensitivity was 90%, specificity was 100%, positive predictive value was 100% while negative predictive value was 94.4%.
**Conclusion:** Based on our findings, we conclude that CT-guided submaxillary CNB is a time-saving procedure that has a high diagnostic accuracy and, therefore, allows for major clinical decisions on the basis of histopathological diagnosis from histological specimen, as well as preventing patients from undergoing open surgical procedure to obtain adequate histological specimen.

**B-1259** 11:26
**3D imaging of the paranasal region with a multipurpose cone beam detector system: image quality and radiation exposure**
S. Ohlmeyer, T. Buder, M. Uder, W. Wuest; *Erlangen/DE (sabine.ohlmeyer@uk-erlangen.de)*

**Purpose:** In times of cost efficiency multi-purpose devices are increasingly offered on the market. Besides x-ray and fluoroscopy, a previously introduced twin robotic x-ray scanner offers a 3D cone beam option. Aim of this study was to evaluate various scan parameters and post-processing steps to optimize image quality and exposure values for imaging of the parasinus region.
**Methods and Materials:** Four human cadaver heads were examined with different tube voltages (90 - 121 kV), dose levels (278 - 2180 nGy) and prefiltration methods (Cu 0.2/0.3 mm, Sn 0.4 mm). Datasets were reconstructed in 0.2-cm slices with and without metal artefact reduction algorithm in three different kernels. In total, 80 different scan protocols and 480 datasets were evaluated. Image quality was rated in 10 anatomic regions on a 5-point Likert scale reaching from 5 excellent to 1 insufficient.
**Results:** For the diagnosis of sinusitis a scan protocol with 121 kV/DL 278/Cu 0.3 mm proved to be sufficient (Score 2). The mean CT volume index ($CTDI_{vol}$) was 1.70 mGy and the mean effective dose 77 µSv. Scan protocols with 121 kV/DL 1090/Cu 0.3 mm were rated sufficient for preoperative diagnostics (Score 3), resulting in a mean $CTDI_{vol}$ of 4.66 mGy and an effective dose of 212 µSv. Metal artefact reduction algorithm was beneficial in all scan protocols.
**Conclusion:** Sinusitis and preoperative planning of parasinus surgery can be performed with low radiation dose and diagnostic image quality with a multipurpose cone beam system. Imaging parameters must be adapted to the clinical indication.

**B-1260** 11:30
**Structural changes of the disc of temporomandibular joint in the first stage of dysfunctions**
D.V. Dushkova, Y. Vasilev; *Moscow/RU (ddushkova@yandex.ru)*

**Purpose:** To assess the structure of the intraarticular disk (meniscus) of the temporomandibular joint (TMJ) in patients with complaints of discomfort during opening of the mouth, as well as in patients undergoing orthodontic treatment.
**Methods and Materials:** The study included 107 patients with symptomatic dysfunction of the TMJ, aged 13 to 68 years. All patients underwent magnetic resonance imaging (MRI) in the position of occlusion, in the open mouth position, as well as a pseudodynamic series of images using high-resolution T1-weighted images (WI) and dynamic T2WI. The studies were performed on a MR-tomograph Siemens Essenza 1.5 T using a head coil.
**Results:** 214 joints were examined, in 127 (63 right and 64 left TMJ) there were no signs of disc displacement, which corresponds to the first stage of dysfunction. Seventy-nine TMJ revealed an asymmetric arrangement of the meniscus bulges: much of it is in the back, the front pole of the meniscus has a flattened shape. The difference in height between the anterior and posterior thickening varied from 35% to 77%. When analysing a series of successive pseudo-dynamic T1WI, the lag of the anterior thickening of the intraarticular disc during movement is tracked in five positions, as well as its deformation between the aspects of the articular tubercle and the head of the condyle.
**Conclusion:** MRI using a static and dynamic protocol with symptomatic dysfunction of the TMJ revealed 62% of the structural changes in the disk without signs of its displacement.

**B-1261** 11:34
**Correlation between the size of the inferior orbital wall defect and treatment tactics in orbital trauma according to the MSCT data**
O. Pavlova, N.S. Serova, D. Davydov, S.K. Ternovoy; *Moscow/RU (dr.olgapavlova@gmail.com)*

**Purpose:** To establish the correlation between the size of the defect within the inferior orbital wall and proper treatment tactics in patients with orbital trauma according to the multi-slice computed tomography (MSCT) data.
**Methods and Materials:** A total of 91 patients with orbital trauma (100%) were admitted to the hospital during the 1-2 day after the injury. MSCT was performed using Toshiba Aquilion One 640. The size of the inferior orbital wall defect was measured using volumetric analysis and calculation of the defect square. According to the acquired size and square, the classification of defects was established: small (below 54 $mm^2$), moderate (from 54,1 $mm^2$ to 117,2 $mm^2$), severe (from 117,3 $mm^2$) and total.
**Results:** According to the classification 9 small inferior orbital wall defects (10%) were revealed, in 19 patients - moderate (21%), in 27 cases - severe (29%) and in 36 patients - total defects (40%). According to acquired data conservative treatment was performed in patients with small defects with additional diagnostic control. Patients with moderate defects underwent implantation of endoprosthesis of inferior orbital wall. Patients with severe and total defects underwent implantation of inferior orbital wall endoprosthesis combined with osteosynthesis fixation within midface structures.
**Conclusion:** The classification of the inferior orbital wall defects according to the MSCT data provide additional diagnostic information about the patients with orbital trauma. Developed classification allows to define the correct treatment tactics in order to select conservative or surgical treatment as well as to establish the correct size of orbital wall endoprosthesis.

**B-1262** 11:38
**Anatomage table: a possible new tool for diagnosis and classification of Le Fort fractures**
Z. Falaschi, S. Cavalieri, I. Valente, G. Mazzucca, A. Carisio, S. Bor, A. Carriero, F. Boccafoschi, A. Stecco; *Novara/IT (zenofalaschi@gmail.com)*

**Purpose:** Anatomage is a virtual anatomic dissection table. Despite it being widely used for students' education, there are still few clinical studies about its usefulness as a diagnostic tool. The purpose of this study is to evaluate Anatomage's capability to improve maxillo-facial CT scan's diagnostic accuracy.
**Methods and Materials:** Ten trauma patients who performed a maxillo-facial CT scan were enrolled. Five patients were diagnosed with a type I, II or III Le Fort fracture, while five had no evident maxillary bones fracture (control group). Data obtained from the exams were uploaded on the Anatomage table for reading. Four different groups of readers were identified: attending doctors, senior residents, junior residents and students. They evaluated CT scans on the radiological workstation (RW) first, and 37 days later reviewed the Anatomage-generated 3D models. Each group included two reciprocally blinded readers. Four parameters were assessed: grade of Le Fort fracture, confidence, anatomic resolution and 3D-model handling. Qualitative parameters were assessed using Likert scale.
**Results:** Intra-reader agreement of Le Fort grade, measured with Cohen's kappa, was above 90% in all readers. Kappa values showed almost perfect agreement in 7/8 readers and substantial agreement in 1/8. Qualitative parameters scores were higher on Anatomage than on RW: Confidence 3.53/4 vs. 3.13/4; Anatomic Resolution 3.51/4 vs. 2.96/4; 3D-Model Handling 3.70/4 vs. 2.79/4.
**Conclusion:** Anatomage Table can be a valid aid in diagnosis and classification of complex maxillo-facial injuries, both for residents, students and attending doctors.

**B-1263** 11:42
**Functional multi-detector computed tomography (FMDCT) for various orbital pathologies**
N.D. Kudryavtsev, M.V. Malakhova, Y.O. Grusha, S.S. Danilov, V.V. Khovrin, K.B. Puzakov, T.N. Galyan; *Moscow/RU (nikita_kudryavtsev@hotmail.com)*

**Purpose:** Modification of methodology of orbital functional computed tomography with the aim to reduce radiation exposure and to increase quality of generated images.
**Methods and Materials:** For modification of the method, 17 patients with thyroid eye disease (TED), orbital tumours and paretic strabismus were chosen. During the study patients moved globes in the following order: center, top, down, left, right and orbits were scanned only after globes were fixed in end points. 5 scans were carried out within one study. Studies were performed with volume mode in 640-MDCT.
**Results:** For patients with TED, alongside with increase of muscle size, we detected evolution of their activity vector, decrease of contractive function, optic nerve compression at the top of the orbit by enlarged muscles. For patients with posttraumatic paretic strabismus, we detected total absence of functional activity of the lateral rectus muscle. In two cases we detected extraconal tumours, slow-moving when the eyeball is in motion and pressing the lateral rectus muscle inwards. In 3 cases, we diagnosed moving intraconal tumours of the orbit that were not intimately connected to the optic nerve and not bringing pressure to it when patient changed point of gaze.
**Conclusion:** FMDCT is a valuable method that allows to reduce radiation exposure (average dose 400-500 DLP), to detail topography of different orbital structures and reveals features of dynamic relationships of soft and bone tissues (including pathologic).

**B-1264** 11:46
**Value of ADC and DCE-MRI in differentially diagnosing of angiomatous meningioma and solitary fibrous tumours/haemangiopericytoma**
C. Chen, C.-.P. Ren, R.-.C. Zhao, J.-.L. Cheng; *Zhengzhou/CN (934717369@qq.com)*

**Purpose:** This study aimed to assess the value of ADC and DCE-MRI in differentially diagnosis of angiomatous meningioma and solitary fibrous tumours/haemangiopericytoma.
**Methods and Materials:** A total of 17 cases of AM and 18 cases of the SFT/HPC confirmed by pathology were analysed retrospectively. All of patients underwent ADC and DCE-MRI. Age, gender, ADC, TIC, SImax, Tmax, MCER and EER were measured and compared AM to SFT/HPC. The diagnostic efficacy of ADC and DCE-MRI in distinguishing AM and SFT/HPC were calculated. Whether or not ADC and DCE-MRI were correlated with age. Two independent samples t tests, t' test, chi-square test, Wilcoxon rank sum test and Pearson correlation were used.
**Results:** The ADC value [( $0.91\pm0.03)\times10^{-3}mm^2/s$], MCER [(304.59±34.43)%] of 17 cases of AM were statistically different from 18 cases of SFT/HPC [$(1.20\pm0.05)\times 10^{-3}mm^2/s$, (179.88±27.54)%] and gender, too ($P<0.05$). The cut-off value of ADC and MCER was, respectively, $1.03\times10^{-3}mm^2/s$ and 226.7% in differential diagnosis of AM and SFT/HPC. There were 6 I type TICs, 10 II type TICs , and 1 III type TICs in 17 cases of AM. There were 5 I type TICs, 9 II type TICs, and 4 III type TICs in 18 cases of SFT/HPC. The age, SImax, Tmax and EER were not statistically different between the two tumours ($P>0.05$). The age of AM and ADC value, age of SFT/HPC and ADC value were all linearly dependent.
**Conclusion:** ADC value of AM is less than SFT/HPCs; MCER of AM is greater than SFT/HPCs, and ADC is more valuable than MCER, and these parameters can help diagnosis.

**10:30 - 12:00** **Tech Gate Auditorium**

### Neuro

### SS 1411b

### Diffusion tensor imaging (DTI)

*Moderators:*
K. Kenigsberg; Minsk/BY
N.N.

**K-30** 10:30
**Keynote lecture**
G. Kasprian; *Vienna/AT*

**B-1265** 10:39
**The assessment of diffusion tensor imaging parameters of visual pathways in patients with primary open-angle glaucoma: preliminary study**
A. Piekarek, P. Podgórski, M. Czarnecki, W. Rydel, A. Turno-Krecicka, M. Misiuk-Hojło, A. Zimny; *Wroclaw/PL (alina1979@op.pl)*

**Purpose:** The aim of our study was to estimate the value of quantitative diffusion tensor imaging of the optic pathways in primary open-angle glaucoma.
**Methods and Materials:** Sixteen glaucoma patients (34-84y; mean 62.8) and 16 healthy age-matched individuals (37-75y; mean 58.7) underwent diffusion tensor imaging of the brain using 1.5T MR scanner (FOV 24, matrix 96x96, b=0, b=1000,15 directions, TE =100, TR=11000, NSA=4, slice thickness=2.5). Based on the Johns Hopkins University single-subject JHU-MNI SS BPM Type II V2.1 atlas for every participant were extracted 12 regions of interest (ROIs) along optic pathways and additional tracts in which measurements of averaged fractional anisotropy (FA) and diffusivity (total diffusivity-trace) automatically were performed. For statistical analysis, Student's t test was indicated ($p=0.05$).
**Results:** For the optic tracts, posterior limbs of internal capsules, right posterior thalamic radiation (including optic radiation), right posterior superior temporal gyrus FA values were significantly lower ($p<0.05$) in glaucoma patients vs. controls. Trace values increased significantly in POAG group for the retrolenticular parts of internal capsule, posterior limb of right internal capsule and right posterior thalamic radiation (including optic radiation) ($p<0.05$). Considering the cingulum, superior longitudinal fasciculus, sagittal stratum and posterior parts of middle and inferior temporal gyrus no significant difference in DTI parameters in both hemispheres was found ($p>0.05$).
**Conclusion:** Diffusion tensor imaging (DTI) can detect glaucomatous optic pathway degeneration and differentiate involved from not involved areas.

**B-1266** 10:47
**Diffusion tensor imaging in trigeminal neuralgia caused with neurovascular conflict and in estimation of long-term results of microvascular decompression**
M. Amelin, J. Rzaev, G. Moysak, A. Tulupov; *Novosibirsk/RU* *(amelin_me@mail.ru)*

**Purpose:** To estimate fractional anisotropy changes in trigeminal nerve root in patients with trigeminal neuralgia, caused by neurovascular conflict before and in long term after microvascular decompression.
**Methods and Materials:** Study included 33 patients (23 female, 10 male, aged 26 to 81 years (mean 60 years,)) with TN, who underwent microvascular decompression. Before microvascular decompression, in a day after it and in a long term after (up to 19 months) diffusion-tensor imaging of trigeminal nerves was performed using 1.5- Tl MR unit Simens Mgnetom Avanto.
**Results:** In all patients in affected side we found statistically significant ($p<10^{-10}$) drop of fractional anisotropy comparing to healthy side. After successful MVD FA on affected side also had difference from healthy side ($p<0.001$). In 6 patients with recurrent pain after MVD FA of trigeminal nerve root in affected side decreased comparing to level before MVD, all of them had a long story of pain and poor FA dynamic right after MVD.
**Conclusion:** Difference of FA between affected and healthy side in patients with TN is related to microstructural changes of affected side. Microstructural recovery changes in trigeminal nerve root in patients with disappearing of pain related with increase of FA and recurrence or persisting of pain related to decrease or absence of FA dynamic comparing to pre-operational data. Application of DTI could be helpful in selection of treatment tactics. Recovering of FA after MVD is an objective criterion of success of MVD.

**B-1267** 10:55
**Diffusion tensor imaging for vascular dementia prediction: threshold level of fractional anisotropy in liable white matter tracts**
I. Levashkina[1], S.V. Serebryakova[1], O.V. Kitaigorodska[2]; [1]*St. Petersburg/RU*, [2]*Kyiv/UA (levashkina.ldc@yandex.ru)*

**Purpose:** To develop criteria of cerebral tracts integrity using DT-MRI to predict vascular dementia. To find the threshold level of fractional anisotropy (FA) in liable tracts to measure dementia risk.
**Methods and Materials:** Characteristics of 235 subjects with diagnosed encephalopathy participated in the study: age 65.29±7.49, 79 subjects with cognitive impairment and 156 with no disorders. DT-MRI was done on 3T Siemens Magnetom (Neuro 3D package). To receive FA in 11 regions of interests, manual free-hand method was applied.
**Results:** Statistically significant ($p\leq0.05$) FA decrease was identified in three regions for cognitive impairment group: front sections of corona radiata (ROI1), inferior longitudinal fasciculi (ROI2), anterior horn of internal capsule (ROI3). ROC analysis showed FA values lower than 0.280 for ROI1, 0.400 for ROI2 and 0.520 for ROI3 mean highest risk of dementia. FA values higher than 0.400 for ROI1, 0.505 for ROI2 and 0.625 for ROI3 mean low risk. FA between threshold levels - higher risk as closer to the low bound. To make the results independent from MRI type and measurement method, authors suggest to use not absolute values of FA, but index ratio of FA in liable ROI to splenium of corpus callosum, which shows no difference for groups with and without cognitive impairment. The index also was calculated for three main ROIs and can be disclosed.
**Conclusion:** Three ROIs, correlated with cognitive disorders risk, were identified. For dementia prediction recommended to use index - FA value in each ROI to splenium of corpus callosum.

**B-1269** 11:11
**Determination of fractional anisotropy cut off values on diffusion tensor imaging in differentiating recurrence versus radiation necrosis in gliomas**
P. Asokan[1], S.S. Shivalingappa[1], A.A. Rao Kesari[1], M. Ashok Kumar[2], I. Desai[1], S. Sampangi[1]; [1]*Bangalore/IN*, [2]*Bikaner/IN* *(asokaprakash@gmail.com)*

**Purpose:** To compare FA values extracted from DTI MR images in differentiating recurrence and necrosis in gliomas. To develop cut off values for tumour recurrence.• To develop cut off values for differentiation of tumour recurrence from radiation necrosis.
**Methods and Materials:** This is a single institutional retrospective study from March 2015 to May 2018, evaluating the role of DTI FA values in differentiating recurrence from radiation necrosis. Patients of all ages with glioma treated with surgery/chemotherapy and radiotherapy who are referred to the radiology department for magnetic resonance imaging evaluation. Total 82 cases are evaluated with DTI FA values.
**Results:** Out of 82 cases, 57 cases are with residual disease, 25 cases are with radiation necrosis. Results between residual and radiation necrosis is highly significant between two groups with $p<0.001$. The mean score of minimum FA ratio in residual disease is 0.26 +/- 0.18 and radiation necrosis is 0.13 +/- 0.055. The mean score of maximum FA ratio in residual disease is 0.15 +/- 0.06 and radiation necrosis is 0.08 +/- 0.030.
**Conclusion:** Hence with MRI Diffusion tensor imaging FA values we can differentiate between residual disease and radiation necrosis and the cut off value for residual disease is between 99 to 182, the cut off values for radiation necrosis is 47 to 80. The FA ratio in residual disease is high ranges between 0.26 to 0.15 and low in case of radiation necrosis is 0.13 to 0.8.

**B-1270** 11:19
**White matter involvement in young non-demented Down's syndrome subjects: a tract-based spatial statistic analysis**
A. Guarnera, A. Romano, M. Moraschi, A. Pierallini, A. Bozzao; *Rome/IT* *(guarneraalessia@gmail.com)*

**Purpose:** Cognitive decline in Down syndrome shows neurodegenerative aspects similar to what is observed in Alzheimer's disease. Few studies reported information on white matter integrity. The aim of this study was to evaluate white matter alterations in young, non-demented Down's syndrome subjects, by means of DTI technique, compared to a normal control group, to define whether these changes are present in asymptomatic subjects.
**Methods and Materials:** The study group consisted of seventeen right-handed subjects with DS and seventeen control subjects. All individuals were examined by MR exam including DTI acquisition (32 non-coplanar directions); image processing and analysis were performed using FMRIB Software Library software package. Finally, the diffusion tensor was estimated voxel by voxel and the fractional anisotropy, mean diffusivity, axial and radial diffusivity map derived from the tensor. A two-sample T test was performed.
**Results:** A FA decrease and a MD, RD and AD increase were found in DS subjects, compared to control subjects, in the region of the anterior thalamic radiation, the inferior fronto-occipital fasciculum, the inferior longitudinal fasciculum and the cortico-spinal tract, bilaterally. In addition, MD, AD and RD showed changes in more white matter tracts, suggesting greater sensitivity for WM damage than FA.
**Conclusion:** We demonstrated white matter changes in specific regions of young, non-demented DS subjects and that MD changes seemed to precede FA ones. These parameters could be used as predictive/early biomarkers of cognitive decline and could have great impact in better adapting the timing of therapies.

**B-1271** 11:27
**Diffusion tensor imaging in intractable unilateral mesial temporal lobe epilepsy**
A.N. Parimalai, A. Chelladurai, M. Logudoss; *Chennai/IN* *(anand7687@gmail.com)*

**Purpose:** To confirm the efficacy of Diffusion Tensor Imaging (DTI) parameters in the diagnosis of unilateral mesial temporal lobe sclerosis (MTS) and to know the extratemporal and contralateral hemisphere involvement in intractable MTS.
**Methods and Materials:** We evaluated 25 patients with intractable unilateral MTLS by using DTI, MRI, clinical and EEG parameters. Out of these 12 were right sided and 13 were left sided MTLS. We compared the mean diffusivity (MD) and fractional anisotropy (FA) values in hippocampus, parahippocampus, fimbriae, fornix middle cerebellar peduncle, corpus callosum, inferior fronto occipital fasciculus, superior longitudinal fasciculus, anterior and posterior cingululum, thalamus, internal capsule, caudate and lentiform nucleus.
**Results:** There was significant decrease in FA values with increased mean diffusivity in ipsilateral hippocampus, parahippocampus, fimbrae and fornix, inferior fronto- occipital fasciculus and posterior cingulated, contralateral hippocampus, parahippocampal white matter, fimbriae ,fornix , middle cerebellar peduncle, corpus callosum ,uncinate fasciculus, inferior fronto occipital fasciculus, superior longitudinal fasciculus, and cingulum.
**Conclusion:** DTI shows microstructural abnormalities beyond the involved hippocampus extending to ipsilateral and contralateral major white matter tracts that is not apparent on conventional MRI. These observations shows epileptogenic focus and spread in the neural network, which may be responsible for secondary generalization.

**B-1272** 11:35
**Excessive alcohol consumption in adolescent men compromises frontal white matter integrity via disruption of myelin (and axons): a combined DTI-metabolic study**
Q. Shen[1], N. Heikkinen[2], O. Kärkkäinen[2], M. Kononen[2], H. Gröhn[2], Y. Liu[2], Z. Zhang[1], T. Tolmunen[2], R. Vanninen[2]; [1]*Changsha/CN*, [2]*Kuopio/FI* *(uuqo@163.com)*

**Purpose:** To investigate influences of subclinical alcohol use during adolescence on WM microstructure and to characterize those with serum metabolic alterations.
**Methods and Materials:** Thirty-five moderate-to-heavy drinkers (15 males, 20 females) and 27 light-drinking controls (12 males, 15 females) were included based on their consumption items of Alcohol Use Disorders Identification Test (AUDIT-C) scores measured at three time points over ten years. Magnetic resonance imaging (MRI) was conducted at study endpoint. Whole brain analysis of fractional anisotropy (FA) was performed using tract-based spatial statistics (TBSS). FA, radial diffusivity (RD), axial diffusivity (AD) and mean diffusivity (MD) values within the significant regions on FA map were computed for subsequent between-group comparisons and correlation analyses with serum metabolite concentrations.
**Results:** Decreased FA was found in moderate-to-heavy drinking men in bilateral genu and body of the corpus callosum, superior and anterior corona radiata, and right inferior fronto-occipital fasciculus (Pfamily-wise-error-corrected<0.05), accompanied by increased RD and a smaller area of reduced AD (P<0.05). No significant difference in FA was detected in moderate-to-heavy drinking women. Among those affected regions in moderate-to-heavy drinking men, DTI metrics correlated with serum level of metabolites (P<0.05) playing roles in energy metabolism, myelination and axonal degeneration.
**Conclusion:** Long-term subclinical alcohol use during adolescence is associated with altered white matter integrity in frontal inter- and intra-hemispheric fibers in men. No such observations were found in women, suggesting different effects of excessive alcohol use on male and female adolescents. The compromised WM integrity in moderate-to-heavy drinking men is correlated to serum metabolic alterations.

**B-1273** 11:43
**Revealing the microstructure abnormalities of spinocerebellar ataxia by fMRI and DKI: from ROI based analysis to a VBA and TBSS study**
H.S. Qiu, J.P. Chu, J. Zhao, M.S. Hu, C. Wu; *Guangzhou/CN* *(handsome_yau@163.com)*

**Purpose:** To investigate potential abnormalities in spinocerebellar ataxia (SCA) patients by fMRI and DKI, and correlate them with the score on the scale for the assessment and rating of ataxia (SARA) of SCA.
**Methods and Materials:** Prospectively involved 25 patients (male:10; mean age: 36.3y) with genetic diagnosis as SCAs (SCA1: 5; SCA2: 2; SCA3:13; SCA6:1; presymptomatic SCA3:4). All patients underwent whole brain fMRI and DKI examinations. DKI (FA, MD, MK, Ka and Kr) parameters in cerebellum and dental nucleus were obtained. Further analyzing the association between whole brain DKI parameters and SARA by TBSS and VBA. In addition, correlations between fMRI parameters (ALFF, fALFF and ReHo) and SARA were analysed by VBA as well.
**Results:** Irrespective of SCAs types and duration, FA of cerebellum was negatively correlated with SARA ($r$ = -0.419, $P$ = 0.03) . Whereas, at VBA, the reduction of FA value was detected in left inferior frontal gyrus and inferior temporal gyrus ($P_{FWE} < 0.05$) while the MK and Kr had significant decreases in the left anterior central gyrus ($P_{FWE} < 0.05$). Furthermore, at TBSS, significantly decreasing FA, MK and Kr values were found in widespread white matter regions, especially in MK and more pronounced were demonstrated in callosum and cerebellar peduncle ($P_{TFCE} < 0.05$). However, there weren't significant associations found among all fMRI parameter and SARA.
**Conclusion:** DKI, especially MK, was superior than fMRI for detecting the severity of SCA, and TBSS was the most sensitive way to reveal the white matter abnormality of SCA.

**B-1274** 11:51
**Cerebral white matter structural integrity in patients with mild cognitive impairment: evaluation with diffusion tensor imaging and morphometric assessment on 3T MRI**
A. Haris Phuah, K. Rahmat, N. Ramli, F.I. Rozalli, F.B. Fadzli, L. Tan, N.I. Saedon, M. Tan; *Kuala Lumpur/MY (katt_xr2000@yahoo.com)*

**Purpose:** To investigate the differences in diffusion tensor imaging (DTI) metrics, morphometry assessment and white matter (WM) integrity between patients with mild cognitive impairment (MCI) and normal healthy control.
**Methods and Materials:** 33 subjects prospectively underwent brain MRI ( 17 MCI and 16 controls) . Using template- based ROI technique to process the DTI, the fractional anisotropy (FA), mean diffusivity (MD), axial diffusivity (AD) and radial diffusivity (RD) values in 50 WM tracts were obtained. Visual rating for WM scoring , WM lesion volume and cerebral morphometric analysis were performed .
**Results:** The MCI group had significantly lower FA and higher MD, AD as well as RD in WM tracts predominantly involving corpus callosum, corona radiata and cingulate gyrus when compared to the normal controls (p<0.05). DTI parameter changes in these areas were also significantly associated with lower MoCA scores (p<0.05). The MCI group had significant higher WM lesion volume. With morphometry analysis, the MCI group had significantly smaller hippocampal volume (p<0.05). Smaller hippocampal volumes and increased WM lesion volumes were significantly associated with poorer MoCA scores (p<0.05). Although there were no significant differences in the Fazekas scores between the 2 groups, a higher Fazekas score was significantly correlated with a lower MoCA score (p<0.05).
**Conclusion:** DTI , morphometric analysis and WM lesion assessments are useful for detecting MCI . DTI is useful in detecting WM tracts compromise seen early on in MCI patients . Early therapeutic interventions could potentially halt the deterioration and prevent the onset of dementia.

**10:30 - 12:00** **The Church**

### GI Tract

### SS 1401b

### Gastric cancer and beyond

*Moderators:*
I. Blazic; Belgrade/RS
C. Dromain; Lausanne/CH

**B-1275** 10:30
**Advanced gastric cancer response to neoadjuvant chemotherapy: is CT texture analysis a reliable imaging biomarker? A multi-centre study from Italian research group for gastric cancer**
L. Di Giacomo[1], G. Bagnacci[1], V. Nardone[2], P. Tini[1], F. Gentili[1], D. Marrelli[1], F. Roviello[1], L. Volterrani[1], M.A. Mazzei[1]; [1]*Siena/IT*, [2]*Naples/IT* *(digileti@libero.it)*

**Purpose:** To evaluate the reliability of CT texture analysis (CT-TA) in predicting histological response to neoadjuvant chemotherapy (NAC) in patients with resectable advanced gastric cancer (AGC) before surgery.
**Methods and Materials:** 70 patients from five GIRCG centres, with a pre-surgical biopsy-proven diagnosis of GC, treated with NAC followed by surgery. All patients were classified as T≥3 or N+(M0) at CT staging and underwent a pre-surgical restaging CT after NAC. The population was divided in two groups as follows: 29 internal patients (Siena University Hospital) and 41 from the other four GIRCG centres. Gross tumour volume (GTV) was segmented on pre- and post-NAC CT by the dedicated contouring software RayStation and TA parameters were extrapolated through LifeX software. A correlation between preselected TA parameters and development of complete pathological response (TRG 1) was searched.
**Results:** TA parameters significantly related to the endpoint were the same for both groups, except for delta Grey-level co-occurrence matrix (GLCM) entropy (p: 0.006 and 0.152) and included post-GLCM contrast (p: 0.017 and 0.001), post-GLCM dissimilarity (p: 0.027 and 0.001), delta entropy (p: 0.002 and p: 0.006), delta GLCM contrast (p<0.001), and delta GLCM dissimilarity (p<0.001). At multivariate analysis only delta GLCM contrast was significant (p: 0.001 and 0.014, Nagelkerke $R^2$: 0.546 and 0.435). ROC curves were generated from the logistic regression of all the cohort of patients (AUC 0.763; standard error: 0.098; p: 0.006).
**Conclusion:** TA could be a reliable tool to select responder patients in a pre-surgical time. Further prospective studies are required to confirm our results.

**B-1276** 10:38
**Diagnostic accuracy of CT for lymph node metastasis in gastric cancer: comparison of spectral parameters developed dual-energy CT and conventional CT**
Y. Chai; *Zhengzhou/CN (chaiyarucyr@163.com)*

**Purpose:** To investigate the optimal diagnostic threshold and accuracy of spectral parameters for metastatic lymph nodes of gastric cancer with dual-energy CT and to compare with conventional CT parameters.
**Methods and Materials:** This study received institutional review board approval and all participants provided written informed consent. 86 patients with gastric cancer underwent preoperative enhanced CT in Discover GSI CT. The spectral parameters (iodine value) and the conventional parameters were measured. The diagnostic efficiency of each factor was assessed using t test and ROC.
**Results:** Among 552 lymph nodes found in CT images, 338 nodes were positive and 214 were negative with pathological results. The results of t test showed that the short diameter, the ratio of short to long diameter, the CT

number and iodine value in AP and VP of positive lymph nodes were higher than those of negative lymph nodes (all $P<0.05$), the areas under curve were 0.600, 0.880, 0.832, 0.755, 0.864, 0.835, respectively. The diagnosis accuracies of iodine value in AP and VP were 86.9%, 82.2%, higher than the CT number in AP and VP (69.9%, 66.9%). Taking the ratio of short to long diameter over 7.25 as optimal diagnosis threshold, the diagnosis accuracy was 75.6%. Combined the ratio of short to long diameter with the iodine value in AP, the diagnosis accuracy was 89.2%.
**Conclusion:** The diagnosis accuracy of dual-energy CT parameters was higher than conventional CT for lymph node metastasis in gastric cancer and could be improved by combining size and spectral CT parameters.

**B-1277** 10:46
**Applying extended texture features to identify quantifiable changes in gastrointestinal stromal tumours undergoing tyrosine kinase inhibitor (TKI) therapy**
K. Ekert; *Tübingen/DE (kaspar.ekert@med.uni-tuebingen.de)*

**Purpose:** Applying extended texture features to identify quantifiable changes in gastrointestinal stromal tumors undergoing tyrosine kinase inhibitor (TKI) therapy. Our aim was to identify quantitative texture features which could be used complementary to modified Choi criteria or even as a substitute helping radiologists to determine response categories in GIST.
**Methods and Materials:** We identified 25 GIST patients with 124 scans, each examined with a standardized CT protocol at one scanner between 1/2014-7/2018. 92 texture features, based on pyradiomics library, were extracted and correlated to response categories as well as vitality of tumor lesions based on mCHOI criteria. Inter-rater testing was performed by a second experienced radiologist. Regression and AUC analysis was performed. First and second order features were included, higher order features were excluded due to the small patient number.
**Results:** Ten variables could be confirmed to be significantly associated with disease progression. Of them, four textural parameters were significantly positively associated with disease progression and negatively with progression free survival (Glcm Id, Glcm Idn, Glrlm and Ngtdm). Single variables were shown to be significantly inferior to the combination of all variables. Gclm Id, Gclm Idn and Glrlm non-uniformity were associated with the number of pre-treatments, Glrlm non-uniformity also with tumor vitality (enhancement) whereas Gclm Idn and Ngtdm coarseness were associated with the number of mutations.
**Conclusion:** CT-texture analysis seems to work well for both assessment of tumor vitality and response to treatment which could therefore be used either complementary to mCHOI criteria or even for automated (e.g. computer-aided) diagnosis.

**B-1278** 10:54
**Small (<5cm) gastric sub-epithelial tumours: identification of gastrointestinal stromal tumours using CT with a practical scoring method**
L. Liu, S. Rao, M. Zeng; *Shanghai/CN (llh9821@163.com)*

**Purpose:** To determine significant CT features that can identify gastrointestinal stromal tumours (GISTs) among small (< 5cm) SETs and to explore a practical scoring method based on these features.
**Methods and Materials:** Forty-two patients with small (< 5cm) gastric SETs (31 GISTs and 11 non-GISTs) from hospital I were included for primary analyses, and 66 (48 GISTs and 18 non-GISTs) from hospital II constituted a validation cohort. Pre-operative CT images were reviewed for imaging features: lesion location, growth pattern, lesion margin, enhancement pattern, dynamic pattern, attenuation at each phasic image and presence of necrosis, superficial ulcer, calcification and peri-lesion enlarged lymph node (LN). Clinical and CT features were compared between the two groups (GISTs vs non-GISTs) and a GIST-risk scoring method was developed at the step of primary analyses, and then its performance for identifying GISTs was tested in the validation cohort.
**Results:** Seven clinical and CT features were significantly suggestive of GISTs rather than non-GISTs: older age (> 46.5 y), non-cardial location, irregular margin, lower attenuation on unenhanced images (<45 HU), heterogeneous enhancement, necrosis and absent of enlarged LN (p<0.05). At validation step, the established scoring method with cut-off score dichotomised into ≥ 4 vs < 4 for identifying GISTs revealed an AUC of 0.86 with an accuracy of 92%, a sensitivity of 100% and a negative predictive value (NPV) of 100%.
**Conclusion:** With a simple and practical scoring method based on the significant CT features, GISTs can be accurately differentiated from non-GISTs.
*Author Disclosures:*
**L. Liu:** Research/Grant Support; National Natural Science Foundation of China (No. 81701656), Key research and development project of Shandong province, China (No. 2018GSF118153).

**B-1279** 11:02
**Prediction of surgery requirement in mesenteric fibrosis on CT using a radiomics approach**
M.P.A. Starmans, A. Blazevic, S.R. van der Voort, T. Brabander, J. Hofland, W.J. Niessen, W.W. de Herder, S. Klein; *Rotterdam/NL (m.starmans@erasmusmc.nl)*

**Purpose:** Mesenteric fibrosis (MF) surrounding a mesenteric mass is a hallmark feature of small intestinal neuroendocrine tumours. Since this can induce severe abdominal complications, prophylactic resection of the primary tumour and mesenteric mass is often recommended. However, there is a need for better prediction of abdominal complications to prevent unnecessary surgery. We present a radiomics approach to identify patients prone to develop complications due to MF.
**Methods and Materials:** CT scans were gathered for 43 patients who had MF, of which 20 required palliative surgery within one year. The resulting dataset originated from 17 different scanners and thereby showed heterogeneity in the imaging protocols. The mass and mesenterium were delineated by a clinician. Within both regions, radiomics features quantifying shape, intensity, texture and orientation were extracted. Patient age and gender were added as semantic features. Radiomics was performed using harmonised adaptive workflow optimisation including various feature selection, oversampling and machine learning approaches. Evaluation was implemented through a 100x random-split cross-validation, with 80% of the data used for training and 20% for independent testing. Performance was given in 95% confidence intervals (CIs).
**Results:** The AUC, sensitivity and specificity were, respectively (0.56, 0.92), (0.43, 0.96) and (0.43, 0.85). The positive class consisted of patients who required surgery.
**Conclusion:** Although the CIs of the performance measures are wide due the small sample size, the results suggest there is a relation between CT imaging features and the need for surgery. Hence, radiomics shows the potential to identify patients prone to develop complications due to MF.

**B-1280** 11:10
**Assessment of metastatic GIST heterogeneity using texture analysis: ADC texture as a potential biomarker of 5-year survival**
J. Fu, F. Mengjie, J. Li, X. Li, L. Tang, D. Dong; *Beijing/CN (18410259295@163.com)*

**Purpose:** To determine if ADC texture features of metastatic GIST are related to 5-year overall survival.
**Methods and Materials:** The study was approved by the institutional review board. This retrospective study included 51 patients selected from 205 consecutive patients from 2006 to 2012. Texture features of the entire maximum metastatic GIST tumour were assessed with DWI (0, 1000 s/mm$^2$) using in-house software. The features were divided into four groups: histogram features, grey-level co-occurrence matrix (GLCM) features and grey-level run-length matrix (GLRLM) features and were extracted from the ROI images in T2, DWI and ADC images. Patients were followed up until death and were censored at 5 years if they were still alive. Kaplan-Meier analysis was performed to determine the relationship. The curves of the high- and low-risk groups were compared using log-rank testing. The prognostic abilities of the predictors were assessed by calculating the concordance probability.
**Results:** All patients had more than five liver metastases. There were 35/56 (68.62%) deaths for five years. Four DWI texture features and three ADC texture features were statistically significant in univariate analysis ($p<0.05$). DWI_L_GLCM_ maximum_ probability (HR2.06,1.36-3.13) and ADC_H_GLRLM_mean (HR2.17,1.46-3.24) and ADC_O_GLCM_ cluster_ shade (HR1.88,1.32-2.67) were identified as representative prognostic indicators. The optimum threshold points of those were 1.19, 1.71 and 2.19, respectively.
**Conclusion:** Metastasis GIST imaging features were independently associated with survival. Worse heterogeneity is associated with poor prognosis.
*Author Disclosures:*
**J. Fu:** Speaker; Fujia.

**B-1281** 11:18
**Lymphadenectomy of small intestine neuroendocrine neoplasias (si-NEN): testing the most recent preoperative classification with contrast-enhanced computed tomography (CT)**
F. Zugni, L. Funicelli, E. Bertani, M. Bellomi; *Milan/IT (fabio.zugni@unimi.it)*

**Purpose:** To test the most recent classification for resectability of si-NENs in a retrospective series of patients operated in our centre (European Institute of Oncology, Milan).
**Methods and Materials:** We blindly analysed the pre-surgical CT of 40 consecutive patients that underwent abdominal surgery for si-NENs between 2008 and March 2018. We assessed the resectability of the lymph node metastases using the classification proposed by Lardière-Deguelte et al. based

Saturday on the degree of involvement of the superior mesenteric artery (SMA). In addition, we evaluated the involvement of the superior mesenteric vein (SMV) and the presence of fibrosis and retraction in the mesentery. We finally analysed the surgical reports to assess whether the primary tumour and all lymph node metastases had been completely resected.
**Results:** Of the three tumours classified as unresectable, none received a complete resection (conversion to explorative intervention). Out of the 37 classified as resectable, 32 received a complete resection. Among the five tumours classified as resectable that did not receive a complete resection, three had CT signs of mesenteric retraction, and two showed an involvement of the SMV that was more severe and proximal than the involvement of the SMA.
**Conclusion:** Using the classification based on the involvement of the SMA we correctly evaluated the resectability of 35 out of 40 tumours (87.5%). In five cases (12.5%), the tumour was incorrectly classified as resectable. Evaluating the severity of involvement of the SMV and the presence of mesentery retraction might be helpful for improving the preoperative assessment of these patients.

**B-1282** 11:26
**Newly appearing hepatic lesions on follow-up CT after curative-intent surgery for colorectal cancer: when and why should we order liver MRI?**
D.K. Kim, C. An; *Seoul/KR (kdk7118@yuhs.ac)*

**Purpose:** To develop a prediction model to assess the likelihood of metastasis for newly appearing hepatic lesions found on computed tomography (CT) after colorectal cancer surgery, and to examine the added value of magnetic resonance imaging (MRI).
**Methods and Materials:** Of 1,676 patients who underwent curative surgery for colorectal cancer between Jan 2008 and Dec 2009, 89 patients with newly appearing hepatic lesions on follow-up CT after surgery (metastasis, n=64; benign, n=25) were included in this retrospective study. Two radiologists analysed the CT features of the hepatic lesions. Clinical information and prospectively written reports of CT and MRI were reviewed. Decision tree analysis and chi-square test were performed.
**Results:** Independent CT features associated with metastasis were spherical shape with circumscribed margin ($P$<0.001), heterogeneous attenuation ($P$=0.024), and bulging appearance ($P$=0.008). In the decision tree, 52 patients with at least one of the CT features or elevated CEA level belonged to the high-risk group. Of non-high-risk patients, 15 with stage 1 cancer or time interval (from surgery to finding new hepatic lesion on CT) > 3 years belonged to the low-risk group. The remaining 22 belonged to the intermediate-risk group. For high-, intermediate-, and low-risk groups, 92.3%, 63.6%, and 13.3% proved to have liver metastasis, respectively. Subsequent MRI was useful in making more correct and confident diagnosis, and detecting additional metastatic lesions.
**Conclusion:** Decision tree model using CT and clinical information, and subsequent MRI could be useful in assessing the likelihood of liver metastasis after colorectal surgery.

**B-1283** 11:34
**Effect of dual-energy CT single-energy imaging on image quality of mesenteric vessels and segmental thickening intestinal wall**
J. Yang, B. He; *Nantong/CN (75002213@qq.com)*

**Purpose:** To evaluate the image quality of mesenteric vessels and thickened intestinal wall in a dual-energy CT single-energy image.
**Methods and Materials:** Retrospective analysis of 30 patients with dual-energy bowel imaging data, using dual-energy mono to reconstruct eight single-energy images of arterial phase, small intestine phase (45keV, 50keV, 55Kev, 60keV, 65Kev, 70keV, 75Kev, 80Kev), compared with 120 kv images, to analyse the SNR, CNR, and image quality subjective scores of mesenteric artery and thickened intestinal wall.
**Results:** The mesenteric artery: the SNR of the 120 kV group was the highest, the SNR of the 60Kev and 65Kev groups was higher than other groups (P<0.05). The CNR of the single-energy group was higher than 120Kvp group, and the CNR of the 60Kev and 45Kev groups was the highest (P<0.05). Subjective scores of all groups were above 4 points (P>0.05). Segmental thickening intestinal wall: the SNR of the 120 kv group was the highest, the SNR of the 50Kev, 55Kev, 60Kev, 65Kev groups was higher than other groups (P<0.05). The single-energy group CNR was higher than 120Kv group, and the 55Kev and 50Kev groups were the highest (P<0.05). The subjective scores of the 45Kev and 50kev images were the highest (P<0.05).
**Conclusion:** The 120kv group image has obvious advantages in SNR, but the single-energy image has obvious advantages in CNR; the 60Kev group image has the best CNR in mesenteric vascular; The 50kev group image can improve the CNR of the intestinal wall of the lesion without affecting the diagnosis.

**B-1284** 11:42
**Peritoneal carcinomatosis extent evaluation: radiologic, laparoscopic and pathologic**
S.A. Ahmed, D. Fouad, H.A. Taleb, D. Badary; *Assiut/EG (Doaafouad11@gmail.com)*

**Purpose:** To evaluate preoperative categorization of peritoneal carcinomatosis by MDCT and laparoscopy compared with pathology and to evaluate its impact on surgical decision-making.
**Methods and Materials:** Forty ovarian cancer patients who underwent preoperative CT and diagnostic laparoscopy were included in the study. Calculation of peritoneal cancer index was done using Sugarbaker's method and then peritoneal carcinomatosis extent was categorized into low, moderate and large. Agreement in general and in each category between CT, laparoscopy, surgery and pathology was assessed using kappa agreement.
**Results:** Complete CRS was performed in 35 and incomplete in 5 patients; preoperative peritoneal carcinomatosis extent categorization in general showed good agreement between CT and laparoscopy (kappa =0.79), between CT and surgery (kappa =0.80), and excellent agreement between laparoscopy and surgery (kappa =0.89) (p<0.001). Agreement between CT and pathology was good (ICC = 0.63) in low-category group and excellent (ICC = 0.93) in moderate and large group (ICC = 0.94) (p<0.02). Agreement between laparoscopy and pathology was excellent in all categories, in low-category group (ICC = 0.91), in moderate group (ICC = 0.90) and in large group (ICC = 0.92) (p<0.04). Agreement between CT and laparoscopy was moderate in low-category group (ICC = 0.60), and excellent in moderate group (ICC = 0.89) and large group (ICC = 0.91) (p<0.01).
**Conclusion:** Although both CT and laparoscopy generally correlate with surgery in preoperative categorization of peritoneal carcinomatosis extent, laparoscopy is more accurate in low category, both accurately reflect disease burden and can be helpful for surgical mapping.

**B-1285** 11:50
**Using 2-mSv appendiceal CT in usual practice for adolescents and young adults: willingness survey of 579 radiologists, emergency physicians, and surgeons from 20 hospitals**
H. Kim[1], K. Lee[1], Y. Ko[2], J. Choi[1], J. Sim[3]; [1]*Seongnam/KR*, [2]*Anyang/KR*, [3]*Seoul/KR (iamjust@naver.com)*

**Purpose:** To survey care providers' willingness to use 2-mSv CT in their usual practice for adolescents and young adults with suspected appendicitis.
**Methods and Materials:** An ethical committee approved this prospective study. We introduced 2-mSv CT in 20 hospitals through a pragmatic clinical trial. At the final phase of the trial, we invited 698 potentially involved care providers in the survey regarding their willingness to use 2-mSv CT. Multivariable logistic regression analyses were performed to identify factors associated with the willingness. Nine months after the completion of the trial patient recruitment, we surveyed whether the hospitals were using 2-mSv CT in usual practice.
**Results:** The analyses included responses from 579 participants (203 attendings and 376 trainees; 221 radiologists, 196 emergency physicians, and 162 surgeons). Regarding the willingness to immediately change their standard practice to 2-mSv CT, 158 (27.3%), 375 (64.8%), and 46 (7.9%) participants responded as "yes" (consistently), "partly" (selectively), and "no", respectively. The willingness varied considerably across the hospitals, but only slightly across the participants' departments or job positions. The willingness was significantly associated with attendings ($p$ = 0.004), intention to maintain the dedicated appendiceal CT protocol ($p$ < 0.001), belief in compelling evidence on the carcinogenic risk of conventional-dose CT radiation ($p$ = 0.028), and hospitals having more than 1,000 beds ($p$ = 0.031). Fourteen of the 20 hospitals kept using 2-mSv appendiceal CT in usual practice after the trial.
**Conclusion:** Most of the care providers were willing to use 2-mSv CT selectively or consistently.

**14:00 - 15:30** **Coffee & Talk 2**

### Musculoskeletal

### SS 1510

### Musculoskeletal interventions

*Moderators:*
A. Alcalá-Galiano; Madrid/ES
T. Kaya; Eskisehir/TR

**K-31** 14:00
**Keynote lecture**
M. Reijnierse; *Leiden/NL*

**B-1286** 14:09
**European multi-centre study on technical success and long-term clinical outcome of radiofrequency ablation for the treatment of spinal osteoid osteoma and osteoblastoma**
T. Beyer[1], C. Rehnitz[2], K.M. Thierfelder[1], C.S.P. van Rijswijk[3], J. Martel Villagrán[4], M. Muto[5], C. von Falck[6], J. Gielen[7], M.-A. Weber[1]; [1]*Rostock/DE*, [2]*Heidelberg/DE*, [3]*Leiden/NL*, [4]*Madrid/ES*, [5]*Naples/IT*, [6]*Hannover/DE*, [7]*Edegem/BE (thomas.beyer@med.uni-rostock.de)*

**Purpose:** To evaluate technical success and clinical long-term outcome of CT-guided radiofrequency ablation (RFA) of spinal osteoid osteomas (OO) and osteoblastomas (OB) in six different European centres.
**Methods and Materials:** A total of 87 patients with the diagnosis of spinal OO (77) or OB (10) were treated with CT-guided RFA, after three-dimensional CT-guided access planning. Patient's long-term outcome was assessed by clinical examination and questionnaire-based evaluation including 10-point visual analogue scales (VAS) regarding the effect of RFA on severity of pain and limitations of daily activities. Clinical success was defined as a reduction of >30% in the VAS score and patient's satisfaction.
**Results:** Overall, RFA was technically successful in 82/87 cases (94.3%) with no major complications; clinical success was achieved in 78/87 cases (89.7%). The OO/OB were localized in the cervical (n=9/3), the thoracic (n=27/1), the lumbar (n=29/4) and the sacral spine (n=12/2). The RFA procedure was performed with a mean of 1.08±0.31 RF-electrode positions. A decrease in severity of pain after RFA was observed in 86/87 patients (98.9%) with a persistent mean reduction of overall pain score from 8.04±0.96 to 1.46±1.95 (p<0.001) after a median follow-up time of 29.35±35.59 months. VAS scores significantly decreased for limitations of both daily (5.70±2.73 to 0.67±1.61, p<0.001) and sport activities (6.40±2.58 to 0.67±1.61, p<0.001).
**Conclusion:** Even in a multi-centric setting, this trial proves that RFA is a safe and efficient method to treat spinal OO/OB and should be regarded as first line therapy after interdisciplinary case discussion.

**B-1287** 14:17
**Who gets kyphoplasties and how much height is restored: a single-centre demographic profile**
P.R. Jayaram, T. Marshall; *Norwich/UK (premruben@yahoo.com)*

**Purpose:** To describe the demographic profile of patients who received kyphoplasties and the degree of height restoration.
**Methods and Materials:** All patients between January 2010 and June 2018 were included. Patients must have reported pain and bone oedema signal changes on MRI to qualify for treatment. The age, gender, indication, DEXA results, and original, pre- and post-procedural height of the target level were recorded. The original height was taken as the mean height of the nearest adjacent normal vertebral bodies. Heights were measured on CT or MRI scans immediately preceding and following the procedure.
**Results:** 114 kyphoplasties were performed (M=52, F=62). The mean age was 66.9 (range 28-89). 48 procedures (42.1%) were performed for osteoporosis, for whom DEXA results were available for 16 (33.3%), showing a mean T score of -1.82 (range -4.3 to 1). A further 48 procedures were performed for malignancy, 15 for trauma (13.2%), and 3 for pain/collapse secondary to a haemangioma (2.6%). There were 61 wedge, 38 biconcave, and 1 posterior fractures. 81 were performed as single-level procedures and 33 were performed as part of a multi-level operation. The mean original height was 29.6mm (range 22-43mm), the mean height lost was 12.4mm (41.4%, range 0-32mm), including 14 without height loss, and the mean height restored was 3.7mm (27.9%, range 0-16mm). No immediate major complications were recorded.
**Conclusion:** Osteoporotic and malignant pathological fractures formed the majority of caseload. Kyphoplasties should focus on other treatment goals, such as pain relief, as only a quarter of height loss was restored.

**B-1288** 14:25
**Long term efficacy of a dorsal indirect approach for CT-guided cervical nerve root injections**
S. Billingsley[1], C. O'Neill[1], D. McKean[2]; [1]*Aylesbury/UK*, [2]*Oxford/UK (sarah.billingsley123@gmail.com)*

**Purpose:** Catastrophic complications have been rarely reported for selective cervical nerve root via an anterolateral transforaminal approach. We assess the short and long term efficacy of a dorsal indirect approach for CT guided cervical nerve root injection.
**Methods and Materials:** We performed a retrospective study of patients who had undergone CT guided nerve root injections for cervical radicular symptoms between March 2012 and July 2018 at our hospital. Spinal needles were advanced to at the dorsal border of the facet joint under CT fluoroscopy guidance. Contrast was injected to demonstrate foraminal flow and a non-particulate corticosteroid dexamethasone preparation was gently instilled. The primary outcomes measures were pain before the procedure then 2, 4 and 8 weeks after the procedure, length of analgesic benefit and complications.
**Results:** Between March 2012 to July 2018, 90 patients underwent CT-fluoroscopic guided nerve root injections at the levels of C2-C8. Complete data was obtained from 23 patients. 54% were female and 46% were male. 76% of these patients reported analgesic benefit with the average duration of analgesic effect lasting 18 months. 3 patients underwent further procedures including discectomy and acupuncture therapy. Patient satisfaction was high and 80% of patients would recommend this procedure to family or friends in a similar clinical situation. The only complication reported was pain on needle insertion.
**Conclusion:** A dorsal indirect approach to nerve root injections is a safe and well tolerated procedure. We report high rates of long term analgesic benefit for the treatment of cervical radicular symptoms.

**B-1289** 14:33
**Morphological changes of disc herniation following CT-guided periarticular infiltration**
S. Talarczyk[1], D.F. Uhlenbrock[1], P. Haage[2], C.A. Stückle[3]; [1]*Dortmund/DE*, [2]*Wuppertal/DE*, [3]*Witten/DE (sarah.talarczyk@rub.de)*

**Purpose:** Aim of this study was to analyse volume reduction of disc herniation in patients with specific low back pain following CT-guided interventional therapy.
**Methods and Materials:** Between 5/2017 and 6/2018 volumetric MRI examinations of seven patients suffering from low back pain were compared before and after CT-guided periradicular infiltration therapy. Under CT control therapy a glucocorticoid, a local anaesthetic and a contrast agent were injected close to the affected nerve root. For the volumetric measurement, a volumetric 3D dataset was employed. The measurement itself was performed with Siemens SyngoVia either with the manual 3D tool by two experienced physicians or with the automated 3D-segmentation tool. The findings were paralleled to an age- and gender-matched control group with comparable disc herniations without therapy. Both got a control MRI with volumetric assessment 6 to 12 months after their first MRI. T testing was used to assess the significance of volume reduction, and a ROC analysis to compare the two measurement tools.
**Results:** Under CT-guided therapy, disc volume was significantly reduced compared to the control group (t=0.02). In the intervention group, disc volume was reduced by an average of 18% whereas the disc volume even increased in the control group by an average of 8%. The measured volume differed significantly between the automated segmentation tool and the manual 3D-segmentation (AUC= 0.5). Consequently, the manual 3D-tool was used for our volume analysis.
**Conclusion:** Periradicular CT-guided infiltration leads to significant volume reduction of herniated discs over time compared to non-treated patients.

**B-1290** 14:41
**Lumbar nerve root blocks using MR: the effectiveness and safety of ultrasound/MRI fusion image guidance**
D.J. Wilson, G.M. Allen, S.A. Bullock, J. Denton; *Oxford/UK (wilsonrad@gmail.com)*

**Purpose:** We compared the outcome of nerve root blocks using ultrasound/MRI fusion image guidance combined with needle tracking to fluoroscopic-guided blocks.
**Methods and Materials:** To assess the efficacy and safety for nerve root blocks in 112 patients using fluoroscopic guidance and 57 patients using fusion guidance. We compared the outcome scores at 24 hours and two weeks. We recorded use of analgesia and the patient's perception of the value of the procedure. We recorded complications, adverse events and duration.
**Results:** Pain diaries were returned by 68% in the fluoroscopy group and 70% in the fusion group. The visual analogue pain score (0 to 10) was reduced at 24 hours by 3.29 (SD 2.35) for the fluoroscopy group and by 3.69 (SD 2.58) in the fusion group, p 0.399. At two weeks the pain reduction was 3.27 (SD 2.57)

for the fluoroscopic group and 4.21 (SD 2.95) for the fusion group p 0.083. There was no significance different in pain response or in the use of analgesics between the two groups. The patient's overall conclusions for fluoroscopy-guided procedures were "very helpful" 20.3%, "a little helpful" 50.6% and "no change" 29.1%. For the fusion-guided injections, they were "very helpful" 47.5%, "a little helpful" 40% and "no change" 12.5%.The fusion-guided procedures took slightly less time to perform. There were no serious complications recorded in either group.
**Conclusion:** Ultrasound/MRI fusion imaging with needle tracking is a safe and effective alternative technique compared to fluoroscopic image-guided injection.
*Author Disclosures:*
**D.J. Wilson:** Advisory Board; Medica Reporting UK Ltd. Board Member; St Luke's Radiology Oxford Ltd. Owner; St Luke's Radiology Oxford Ltd. Shareholder; European Imaging Harley St, London. **J. Denton:** Employee; Manor Hospital Oxford.

**B-1291** 14:49
**Recovering from non-specific low back pain despair: ultrasound-guided intervention in iliolumbar syndrome**
D.K. Singh, S.B. Grover, N. Kumar, B.K. Nayak, S. Tomar, S. Suman, A. Katyan, H. Rajani, R.N. Misra; *New Delhi/IN (dksinghrad@gmail.com)*

**Purpose:** Patients with non-specific low back pain (NSLBP) are normally in despair due to discordance between distressing clinical symptoms and paradoxical normal imaging studies (radiograph and MRI). However, patients of iliolumbar syndrome (ILS) are a potentially recoverable group. This study was, therefore, aimed at assessing the therapeutic role of ultrasound-guided intervention in the latter.
**Methods and Materials:** Forty consenting patients with NSLBP revealing iliolumbar ligament (ILL) thickening >2mm on ultrasound were included in this Institutional Review Board-approved prospective interventional study. Two-stage ultrasound-guided interventions were performed under standard aseptic precaution. In primary diagnostic intervention, 4ml of 0.25% bupivacaine was infiltrated in the ILL. Clinical pain scoring was assessed both pre- and 72 hours post-procedure using visual analog score (VAS). The respondents to the primary intervention were considered to have a confirmed diagnosis of ILS. The latter group was further managed with therapeutic platelet-rich plasma (PRP) injection, undertaken by the standard protocol. The VAS was assessed 6 weeks after the second procedure.
**Results:** Out of 40 patients, 31 (77.5%) were diagnosed with ILS on primary intervention. Of these 31 patients who underwent PRP therapy, 29 (93.5%) showed significant improvement on VAS scale.
**Conclusion:** Ultrasound-guided diagnostic and therapeutic intervention using bupivacaine and PRP, respectively, were found to result in remarkable recovery in ILS group of patients with NSLBP. The potential of further exploiting this management strategy in patients of NSLBP living a life in despair, needs no further emphasis.

**B-1292** 14:57
**Prospective randomised controlled trial comparing a battery-powered and a manual drill in bone biopsy**
C. Tsoi, E.K.C. Law, R.K.L. Lee, A.W.H. Ng, J.F. Griffith; *Hong Kong/HK (caritatsoi@gmail.com)*

**Purpose:** To compare the diagnostic accuracy, pain score, radiation dose, procedure time and complications of a battery-powered drill versus the traditional manual drilling system in CT-guided bone biopsy.
**Methods and Materials:** This was a single-centre prospective randomised controlled trial approved by the institutional review board with signed informed consent obtained. Consecutive patients referred to our centre for CT-guided bone biopsy were recruited from November 2015 to July 2018. Patients were randomised into two groups, namely biopsy performed either with battery-powered drill or traditional manual drill performed or supervised directly by one of three experienced musculoskeletal radiologists. Primary outcomes included procedure time, pain score (visual analogue scale; 0-10), radiation dose metrics (CTDIvol and DLP), diagnostic yield, specimen adequacy and complications .
**Results:** 173 patients were recruited and randomised into a battery-powered drill group (n=93) or manual drill group (n=80). Baseline demographics including age, sex, lesion characteristics and locations were similar between both groups. When compared with traditional manual drill, biopsy with battery powered drill was quicker to perform (12 vs. 19 minutes; p<0.05) with reduced radiation dose (DLP 102 mGy-cm vs. 147mGy-cm; P<0.05), and was less painful (visual analogue scale 2.6 vs. 5.8; p<0.05). Diagnostic yield and specimen adequacy were similar for both methods (battery: 88% vs. manual 83%). No major or minor complication was identified for either group.
**Conclusion:** Battery-powered drill was superior to manual drill for CT-guided bone biopsy with a shorter procedure time, reduced radiation dose and pain score, while achieving a similar diagnostic yield.

**B-1293** 15:05
**Ultrasound-guided distension hydrotherapy with graded physiotherapy in primary adhesive capsulitis: a pilot study**
D.K. Singh, N. Kumar, S. Tomar, B.K. Nayak, S. Suman, A. Katyan, M.K. Mittal, S.B. Grover, R.N. Misra; *New Delhi/IN (drnishithkumar@rediffmail.com)*

**Purpose:** Current treatment options for adhesive capsulitis of shoulder joint are associated with variable results. Our study was aimed to evaluate clinical efficacy of ultrasound-guided distension hydrotherapy followed by physiotherapy in patients with primary adhesive capsulitis.
**Methods and Materials:** 20 patients of primary adhesive capsulitis of shoulder joint consented for distension hydrotherapy were included in this prospective study. Under ultrasound-guided posterior approach, an intra-articular needle was placed in the glenohumeral joint and 4 ml of steroid mixed with local anaesthetic agent was instilled, followed by phasic capsular distension with 30 ml of normal saline. All patients received 4 weeks of physiotherapy 48 hours after distension hydrotherapy. Parameters evaluated before and at 4 weeks post-procedure included visual analogue scale (VAS) pain score and goniometer angle measurement of range of external rotation. Data analysis was done using SPSS version 21.0 and paired t test was applied to determine statistical significance.
**Results:** Mean VAS pain score was significantly reduced following 4 weeks of combined therapy. Pre- versus post-therapy mean (SD) VAS: 8 (0.79) versus 4 (1.16), respectively, p value less than 0.0001. Mean range of external rotation was significantly increased following 4 weeks of combined therapy. Pre- versus post-therapy mean (SD) external rotation angle: 16.05 (7.56) versus 49.20 (11.25) degrees, respectively; p value less than 0.0001.
**Conclusion:** Combined therapy using ultrasound-guided distension hydrotherapy followed by physiotherapy achieved encouraging short-term clinical improvement in our pilot study. Further trials are needed to substantiate long-term prognosis.

**B-1294** 15:13
**Ultrasound-guided percutaneous release of the carpal tunnel: comparison between senior vs junior operator and learning curves: a cadaveric study**
C. Dekimpe; *Nice/FR (dekimpe.chloe@gmail.com)*

**Purpose:** The purpose was to evaluate, on a cadaveric cohort, the feasibility and the learning curve of ultrasound-guided percutaneous carpal tunnel release.
**Methods and Materials:** Fourteen carpal tunnel releases were carried out on un-embalmed cadavers by a senior and a junior radiologist. Procedures were realized with an 18MHz linear probe. An ultrasonographic anatomic evaluation was first performed to detect any anatomical variant. After hydrodissection with lidocaine of the carpal tunnel, a 3-mm hook knife was introduced into the security zone to perform a retrograde section of the transverse carpal ligament (TCL) under ultrasonographic guidance. Anatomic dissection was performed for each wrist. The main evaluation criterion was the complete TCL section. The procedure duration (minutes), skin incision size (millimetres), the integrity of the median nerve, thenar motor branch, and palmar vascular arch were also evaluated.
**Results:** The senior operator was able to perform a complete release after training on 3 specimens and the junior operator after 4 specimens (p > 0.05). In most of the cases when complete release was not achieved, it was due to an incomplete section of distal TCL (10 mm missing section on average). Mean duration time of procedure was 14 minutes (11min senior versus 17min junior, p > 0.05). No damage of the median nerve nor the vascular structure was observed. Mean size of the skin incision was 3mm.
**Conclusion:** The ultrasound-guided percutaneous release of the carpal tunnel demonstrates to be a safe and efficient procedure with a rapid learning curve.

**B-1295** 15:21
**Clinical response in hip osteoarthritis: comparison between intra-articular treatment with hybrid hyaluronic acid (HA) and combined therapy with Platelet-Rich Plasma (PRP) and HA**
I. Capretti, P. Palumbo, S. Iafrate, F. Bruno, F. Arrigoni, S. Mariani, L. Zugaro, A. Barile, C. Masciocchi; *L'Aquila/IT (ilaria_capretti@hotmail.it)*

**Purpose:** To evaluate the clinical outcome in patients treated with intra-articular injection of hybrid HA or combining HA and PRP.
**Methods and Materials:** We included 28 patients (aged between 40 and 70) with moderate-severe grade of hip osteoarthritis, diagnosed by magnetic resonance (MR) exam on 3T MR scanner. Patients with recent trauma or rheumatic or systemic diseases were excluded. Patients received different intra-articular injection in a randomized way: group A (14 patients) were treated with US-guided intra-articular injection of a hybrid formulation of HA; group B (14 patients) treated with a combination of HA and PRP. Clinical outcome and functional evaluation were investigated by means of Visual Analog Scale (VAS) and Harris Hip Score (HHS), before treatment and at six and twelve months.

**Results:** Group A showed a mean VAS value of 8.5 with a reduction of 78.6% at six months and 58.2% after twelve months; mean HHS value was 69 before treatment and 84 and 80 respectively at the follow-up. Group B showed a mean VAS value of 8.7 before treatment and an improvement of 79.3% and 81% at the follow up; mean HHS value of 67 before treatment and 89.1 and 88 at the follow-up. In group B, patients, aged between 40 and 55, had a better result.
**Conclusion:** All patients had good clinical recovery, but younger patients achieved better clinical benefit with HA-PRP therapy; while the patients, aged over 55 years, had similar results in both groups.

**14:00 - 15:30** **Room Y**

### Interventional Radiology

### SS 1509

### Nonvascular interventions in oncology (including kidney)

*Moderators:*
J. Curic; Zagreb/HR
R. Dezman; Ljubljana/SI

**K-32** 14:00
**Keynote lecture**
N.N.

**B-1296** 14:09
**Safety and efficacy of microwave ablation of stage T1 renal cell carcinoma**
K. Mahmoud, H. El Khudari, A. Gunn, E. Keasler, E. Bready, A. Salei, J. Winston, P. Patten, A.M.K. Abdel Aal; *Birmingham, AL/US (akamel@uabmc.edu)*

**Purpose:** To evaluate clinical and technical factors associated with treatment outcomes of microwave ablation (MWA) for renal cell carcinoma.
**Methods and Materials:** We retrospectively reviewed medical records of 29 patients with 31 tumours who underwent MWA for stage 1 RCC between 2008 to 2018 in our institution. Patient demographics, tumour characteristics, technical success defined as absence of residual tumour within 3 months of procedure and complications were reported. The recurrence-free, cancer-specific and overall survival rates were analysed. A univariate analysis was performed to identify any potential predictors of complications, local recurrence, or survival.
**Results:** Mean age of the patients was 64 ±10.6 years and 34.5% of the patients had chronic kidney disease stage 3 at baseline. The median Charlson comorbidity index was 5 (range: 5-12). The median tumour size was 2.7 cm (range: 1.0 - 6.1) with 18 (58.1%) posterior tumours. Stage T1a tumours were seen in 93.5% of patients. Median number of probes was 1 (range:1-3) and biopsy was performed in 22 (72.4%) tumours. Technical success rate was 93.1%. Minor and major complications were seen in 5 (17.2%) and 1 (3.4%) patients, respectively. No local recurrence was reported. The overall survival was 100%, 84.6% and 84.6% at 1, 3 and 5 years. Cancer-specific survival was 100% at 5 years. There were predictors for complications or survival outcomes.
**Conclusion:** Percutaneous MWA is safe and efficacious thermal ablation modality for treatment of stage 1 RCC with acceptable outcomes.

**B-1297** 14:17
**Percutaneous management of Indiana pouch stones: a single-centre retrospective study**
M. Ozturk, J.C. McDermott, P.F. Laeseke, S.Y. Nakada, S.P. Hedican, S. Best, M.G. Kleedehn; *Madison, WI/US (dr.mesutozturk@gmail.com)*

**Purpose:** To present our experience treating reservoir stones in patients with Indiana pouch urinary diversions using a percutaneous approach.
**Methods and Materials:** Seven patients were treated percutaneously for Indiana pouch reservoir stones between 2008 and 2018. The Indiana pouch was filled with dilute water-soluble contrast and was punctured with an 18-gauge needle by an interventional radiologist under direct ultrasound guidance. A wire was inserted through the needle and a balloon set was used over the wire to dilate the tract. A 30 F sheath was placed over the fully inflated balloon into the pouch. A urologist inserted a nephroscope through the sheath and removed the stones. At the end of the procedure, a Foley catheter was left through the access sheath into the lumen of the pouch and was left open to drainage.
**Results:** Mean age of the study population was 47.3 ± 14.7 years (range: 29-69). Five patients had multiple stones and the median stone number was 3 (range 1-8). Mean maximum stone diameter was 24.43 ± 4.89 mm (range: 19-33). All patients underwent technically successful puncture and stone removal of the pouch. Median post-operative hospital admission was 1 day (range: 1-5). The Foley catheters were removed after a median value of 18 days (range: 10-19). No major complications were reported. One patient suffered from flank pain and fever 14 days after the procedure, but responded well to antibiotic treatment.
**Conclusion:** Percutaneous approach for Indiana pouch reservoir stones provides a direct and safe treatment method without significant peri-procedural complications.

**B-1298** 14:25
**Predictors of outcomes of percutaneous cryoablation for renal cell carcinoma**
K. Mahmoud, A. Gunn, H. El Khudari, A. Salei, E. Bready, J. Winston, E. Keasler, P. Patten, A.M.K. Abdel Aal; *Birmingham, AL/US (akamel@uabmc.edu)*

**Purpose:** To evaluate the clinical and technical factors affecting the outcomes of percutaneous cryoablation (PCA) of renal cell carcinoma (RCC).
**Methods and Materials:** The medical records of the patients who underwent PCA for RCC between 2004 and 2018 were retrospectively reviewed and 128 patients were included. Patient demographics, tumour characteristics, technical success defined as absence of residual tumour within 3 months of procedure, and complications were reported. Recurrence-free, cancer-specific and overall survival rates were analysed. A univariate analysis was performed to identify predictors of the outcomes.
**Results:** Mean age of the patients was 65 (SD=11.5) years. Chronic kidney disease at the baseline was seen in 38.3% of patients. The median tumour size was 3.0 (range: 1.2 - 8.7) cm with 73 (57%) posterior tumour location. The study included T1a (69.5%) and T1b (28.9%) tumours. The median number of probes used was 2 (range:1 - 7) and 81 (63.3%) tumours were biopsied. Technical success rate was 90.6%. Minor complications were seen in 26.6% and major in 6.3% of patients. On univariate analysis, tumour biopsy (p=0.019), endophytic/mixed location (p=0.026), nearness to collecting system (p=0.012) and renal sinus involvement (p=0.003) were associated with complications, and the latter two were associated with decreased overall survival (p=0.036 and p=0.051, respectively). None of the variables predict local recurrence. The recurrence-free, cancer-specific and overall survival at 3 years was 66.6%, 100% and 93.4%, respectively.
**Conclusion:** Tumour biopsy, tumour location, nearness to collecting system and renal sinus involvement were predictors of outcomes of PCA of RCC.

**B-1299** 14:33
**Role of R.E.N.A.L. nephrometry and PADUA scoring systems in percutaneous ablation for renal cell carcinoma**
K. Mahmoud, A. Gunn, H. El Khudari, A. Salei, E. Bready, J. Winston, E. Keasler, P. Patten, A.M.K. Abdel Aal; *Birmingham, AL/US (akamel@uabmc.edu)*

**Purpose:** The aim of the study is to investigate the ability of R.E.N.A.L. nephrometry and PADUA scoring systems to predict the complications and survival outcomes in patients with renal cell carcinoma (RCC) who underwent image-guided renal tumour ablation.
**Methods and Materials:** We retrospectively reviewed the medical records of 175 patients who had RCC that was treated with thermal or non-thermal ablation. The data on demographics, modality of ablation and tumour characteristics were reported. The RENAL and PADUA scores were calculated. Technical success and complications were analysed. Kaplan-Meier curves for recurrence-free, cancer-specific and overall survival were obtained.
**Results:** The study included 175 patients with 177 tumours, 104 (59.4%) males, with a mean age of 64.7 (SD=11.5) years. There were 53 (30.3%) patients with CKD stage 3. Mean tumour size was 3.04 (SD=1.3) cm, with 75 tumours (42.4%) located posteriorly. Technical success was seen in 159 (90.9%) patients. Thirty-day complications was 27.9%. Recurrence-free and overall survival at 1 year was 94.8% and 98.5%, respectively. There was no correlation between the RENAL and PADUA scores with the occurrence of complications, recurrence-free survival or overall survival. The nearness to the collecting system and biopsy before the procedure correlated with the occurrence of complications (p=0.03 and p=0.02, respectively).
**Conclusion:** RENAL and PADUA scores do not correlate with the occurrence of complications, recurrence-free or overall survival in patients with RCC treated with percutaneous ablation. The nearness to the collecting system and biopsy correlated with the occurrence of complications.

**B-1300** 14:41
**Modified ABLATE-score (mABLATE): a specific nephrometric score to predict complications and relapses in percutaneous cryoablation of renal lesions**
M. Papa, A. Losa, N. Suardi, G. Agostini, T. Maga, F. Gaboardi, G. Cardone; *Milan/IT (papa.maurizio@hsr.it)*

**Purpose:** RENAL and mRENAL scores have been validated on predicting complications and relapses in percutaneous treatments of renal lesions. However, these scores remain of surgical origin. We aim to build a specific interventional score (mABLATE).
**Methods and Materials:** Taking inspiration from the paper "AJR2014;202:894-903" with some changes based on our practical experience, a score was built trying to take into account the real difficulties faced in our experience. The mABLATE was retrospectively assessed on 60 cryoablations to evaluate its predictive value for complications and relapses. Logistic regression was used to predict complications; Cox regression for relapses; ROC analysis for accuracy.
**Results:** Among 60 renal lesions treated with cryoablation we experienced 3 bleedings with anemia (5%). Among 51 malignant lesions (ML) we experienced 7 relapses (13.7%), with a mean FU of 19,3 months. Mean ML RENAL score: 7.2pts. Mean relapsed RENAL score: 7.71pts. Mean ML mRENAL score: 7.26. Mean relapsed mRENAL score: 8pts. Mean ML mABLATE score: 4.98. Mean relapsed mABLATE score: 8.07pts. The Cox regression analysis for Renal (p=0.35) and mRENAL (p=0.29) showed a lack of predictive value for relapses. mABLATE score was found to be an independent predictor of relapses(HR 1,56; p=0,001). The predictive accuracy based on the ROC analysis of the mABLATE showed an AUC of 89.1%(vs 61,5% for mRENAL and 58,8% for RENAL). In the logistic regression analysis none of the scores showed statistic significance in predicting complications(p>0.05) due to small numbers.
**Conclusion:** mABLATE score showed to be a better predictor of relapses than RENAL and mRENAL.

**B-1301** 14:49
**Safety and outcomes of percutaneous cryoablation for large T1b renal cell carcinoma**
A. Gunn, K. Mahmoud, H. El Khudari, A. Salei, E. Keasler, E. Bready, J. Winston, P. Patten, A.M.K. Abdel Aal; *Birmingham, AL/US (akamel@uabmc.edu)*

**Purpose:** To describe the safety, technical results, and clinical outcomes of percutaneous cryoablation of stage T1b renal cell carcinoma (RCC).
**Methods and Materials:** A retrospective review of the medical records of 36 patients with RCC lesions measuring 4.1-7.0 cm who underwent percutaneous cryoablation from 2004-2018. Patient demographics, co-morbidities, tumour characteristics, technical parameters of the procedure, technical outcomes, complications, and local recurrence rates were recorded. Cancer-specific survival rates were estimated using the Kaplan-Meier method. A multivariate analysis was performed to identify any potential predictors of complications, local recurrence or survival.
**Results:** Mean tumour diameter was 4.9 cm (±1.2), with a median of 3 probes (range: 1-7) used. The median number of procedures required to achieve local control was one (mean: 1.5, range: 1-4). Minor complications not requiring therapy occurred in 15 patients (41.7%). Two major complications (5.6%) included one abscess and an incidence of acute renal failure, both which were appropriately managed without long-term sequelae. No significant change in glomerular filtration rate was seen after ablation (p=0.95). Seven patients (19.4%) had local recurrence, which occurred at a mean of 26.7 months (range: 4.3-52.2). Cancer-specific survivals were 100%, 100%, 100%, and 84.6% at 1, 2, 3, and 5 years, respectively. On univariate analysis, endophytic/mixed tumours (p=0.0014) and nearness to the collecting system (p=0.006) were independent predictors of complications. None of the evaluated variables were able to predict local recurrence.
**Conclusion:** Percutaneous cryoablation is a viable therapeutic option for stage T1b RCC with an overall low rate of major complications.

**B-1302** 14:57
**Comparison of cryoablation and microwave ablation in the treatment of small renal tumours (SRT): technical success, complications and midterm clinical outcome**
M. Panzeri, M. Colombo, M. Papa, G. Cardone, M. Venturini, U. Capitanio, F. Montorsi, A. Del Maschio, F. De Cobelli; *Milan/IT (panzeri.marta@hsr.it)*

**Purpose:** To evaluate feasibility, safety, complication rate and mid-term results of MicroWave Ablation (MWA) with a new high-power system based on Thermosphere™ Technology in the treatment of SRT not eligible for surgery, and to compare these data with those of cryoablation (Cryo).
**Methods and Materials:** From 2014 to 2018, 78 patients with one or more SRT (total 94) underwent percutaneous ablation (Cryo with Galil Medical or MWA with Thermosphere Emprint) under CT/US guidance. CT/MRI follow-up was performed at 1, 6, 12 and 18-24 months. MRENAL, procedural time, technical success, complications (according to the CIRSE classification) and follow-up data were recorded.
**Results:** 49 patients (60 nodules) underwent cryoablation, while 29 patients (34 nodules) were treated with MWA. The median mRENAL score was 7.5 in the Cryo group, 9 in MWA group. No significant difference was found between the two groups in tumour or patient's characteristics. There were 22 complications in the Cryo Group (36%): 18/22 (81%) were grade 1, 2/22 (9%) grade 2, 1/22 (5%) was 3 and 1/22 was 4. In the MWA group 6 complications were recorded (18%), all grade 1 (p=0.85). A generalized linear mixed model adjusted for mRENAL score showed a protective effect against complications of MWA (p=0.74). Median procedural time was 106 minutes for Cryo, 41 minutes for MWA (p<0.0001). During follow-up, residual disease was found in 6/60 patients after cryoablation (10%) and in 3/34 nodules after MWA (9%); no significant difference was found between the two groups.
**Conclusion:** In our series, MWA has proven to be a safe and effective ablative technique and it could be considered not inferior to cryoablation.

**B-1303** 15:05
**Focused ultrasound subthalamotomy in patients with asymmetric Parkinson's disease: a pilot study**
E. De Luis Pastor, M. Del Alamo, R. Martinez Fernandez, J. Mañez Miro, C. Gasca, R. Rodriguez Rojas, J. Pineda Pardo, L. Vela, J. Obeso; *Mostoles/ES*

**Purpose:** Ablative neurosurgery has been used to treat Parkinson's disease (PD) for decades. MRI-guided focused ultrasound allows focal lesions to be made in deep brain structures without skull incision. We investigated the safety and preliminary efficacy of unilateral subthalamotomy by focused ultrasound in PD.
**Methods and Materials:** We performed a prospective, open-label pilot study with ten patients with PD with markedly asymmetric parkinsonism. Enrolled patients underwent focused ultrasound unilateral subthalamotomy. The subthalamic nucleus was targeted with brain images acquired with a 3T MR system. The primary outcomes were safety and a change in motor status of the treated hemibody as assessed with part III of the Movement Disorders Society-Unified Parkinson's Disease Rating Scale (MDS-UPDRS III) in both off-medication and on-medication states at 6 months. Adverse events were monitored up to 48 h after treatment and at scheduled clinic visits at 1, 3, and 6 months after treatment.
**Results:** The most frequent adverse events were transient gait ataxia (related to subthalamotomy) and transient high blood pressure during the procedure. Transient facial asymmetry and moderate impulsivity were also recorded. The mean MDS-UPDRS III score in the treated hemibody improved by 53% from baseline to 6 months in the off-medication state and by 47% in the on-medication state.
**Conclusion:** MRI-guided focused ultrasound unilateral subthalamotomy was well tolerated and seemed to improve motor features of Parkinson's disease in patients with asymmetric parkinsonism. Large randomised controlled trials are necessary to corroborate these preliminary findings and to assess the potential of such an approach to treat PD.

**B-1304** 15:13
**The effect of histogram parameters derived from quantitative T1-perfusion on predicting the high-intensity focused ultrasound outcome of uterine fibroids**
N.M. Duc[1], C. Li[2], H. Quang Huy[1], J. Yang[2], B. Keserci[3]; [1]*Ho Chi Minh City/VN*, [2]*Xi'an/CN*, [3]*Kelantan/MY (bsnguyenminhduc@pnt.edu.vn)*

**Purpose:** To investigate the effect of whole-tumor histogram parameters derived from quantitative dynamic contrast-enhanced MRI (DCE-MRI) on predicting magnetic resonance-guided high-intensity focused ultrasound (MRgHIFU) ablation outcome defined as non-perfused volume (NPV) ratio ≥ 80%.
**Methods and Materials:** Institutional review board approved this prospective study. 28 symptomatic uterine fibroid women (43.3 ± 4.5 years) were evaluated quantitative DCE-MRI prior to MRgHIFU ablation at the Department of Radiology, the First Affiliated Hospital of Xi'an Jiaotong University. The population was divided into 2 groups: group 1 with NPV ratio ≥ 80% (n = 8) and group 2 with NPV ratio < 80% (n = 20). Mann Whitney U test was carried out to compare four histogram parameters: median, mean, skewness and kurtosis between group 1 and group 2. Multiple logistic regression was accessed independent factors in predicting NPV ratio ≥ 80%.
**Results:** Mean NPV ratio was 94.8% ± 4.8 for group 1 and 54.4 % ± 12.8 for group 2, respectively (p< 0.05). Median histogram parameter derived from quantitative DCE-MRI which represented the asymmetry and shape of histogram regarding to whole tumor vascular perfusion of group 1 was significantly lower than group 2 (p < 0.05). Multiple logistic regression manifested that there was only median parameter had significant effect on NPV ratio ≥ 80% (p < 0.05).

**Conclusion:** The achivement of NPV ratio ≥ 80% was feasible in MRgHIFU ablation of uterine fibroids. The median histogram parameter could be served as an effective factor in patient selection criteria for NPV ratio ≥ 80%.

**B-1305** 15:21

**The role of magnetic resonance imaging parameters in predicting the treatment outcome of high-intensity focused ultrasound ablation of adenomyosis**

N.M. Duc[1], B. Keserci[2], H. Quang Huy[1], L. Van Phuoc[1], L. Trong Khoan[3], D. Duc Cuong[4], B. Van Giang[4], V. Dang Luu[4], N. Duy Hue[4]; [1]*Ho Chi Minh/VN*, [2]*Kelantan/MY*, [3]*Hue/VN*, [4]*Ha Noi/VN (bsnguyenminhduc@pnt.edu.vn)*

**Purpose:** To investigate the role of magnetic resonance imaging (MRI) parameters in predicting the treatment outcome of high-intensity focused ultrasound (HIFU) ablation of adenomyosis defined as the immediate non-perfused volume ratio (NPVr).
**Methods and Materials:** 50 women (40.3 ± 6.0 years) with symptomatic adenomyosis underwent MRI-guided HIFU ablation. Multivariate linear regression analyses were carried out in (i) baseline parameter group, (ii) T2 signal intensity (SI) group and (iii) semiquantitative perfusion group to investigate the potential predictors of each group affected based on the NPVr. Based on all the significant screening MRI parameters acquired from the multivariate analyses, generalized estimating equation (GEE) was used to predict the immediate NPVr.
**Results:** The results of multivariate analyses revealed that there were four statistically significant predictors ($p < 0.05$): abdominal subcutaneous fat thickness, T2 SI ratio of adenomyosis to myometrium, relative enhancement ratio of adenomyosis to myometrium, time to peak enhancement ratio of adenomyosis to myometrium. GEE analysis generated linear equation for predicting the immediate NPVr (y) with four statistically significant predictors from multivariate analyses: $y = 165.952 + 0.119x1 - 10.514x2 - 56.177x3 - 39.812x4$, where x1 = abdominal subcutaneous fat thickness, x2 = T2 SI ratio of adenomyosis to myometrium, x3 = relative enhancement ratio of adenomyosis to myometrium, x4 = time to peak ratio of adenomyosis to myometrium.
**Conclusion:** The findings in this study suggest that the outcome of MRI-guided HIFU treatment of adenomyosis based on multivariate analyses and prediction model appears to be clinically feasible.

**14:00 - 15:30** **Room M 1**

### Cardiac

### SS 1503

### Coronary calcium and plaques: diagnosis and prognosis

*Moderators:*
P. Donato; Coimbra/PT
C. Lücke; Leipzig/DE

**B-1306** 14:00

**Presence of coronary artery calcifications on CT is associated with PE-related mortality in patients with acute PE**

B.H. Heidinger[1], D.C. DaBreo[2], B.J. Carroll[3], S.A. Feldman[3], D. Mohebali[3], I. McCormick[3], J.D. Matos[3], W.J. Manning[3], D. Litmanovich[3]; [1]*Vienna/AT*, [2]*Kingston, ON/CA*, [3]*Boston, MA/US (benedikt.heidinger@meduniwien.ac.at)*

**Purpose:** To evaluate the association of coronary artery calcification (CAC) visualised on computed tomography pulmonary angiography (CTPA) at time of acute pulmonary embolism (PE) diagnosis with PE-related mortality.
**Methods and Materials:** We searched for all patients with an ICD-10 code of PE and included those with PE verified on CTPA in this IRB-approved retrospective study. CAC was visually graded by a cardio-thoracic radiologist into four categories: absent, mild, moderate, or severe. PE-related mortality was defined as death related to PE within 30 days of diagnosis. Logistic regression analysis was used to compare the PE-related mortality according to the presence of CAC.
**Results:** In the 479 patients who met inclusion criteria, mean age was 63±16 years, 53% were female, and 47% had a prior smoking history. 253/479 (53%) patients had CAC (mild: 143/479, 30%; moderate 89/479, 19%; severe 21/479, 4%). Overall, 19/479 (4%) patients died, with a PE-related mortality of 2% in those with absent CAC, 5% in mild CAC, 7% in moderate CAC, and 10% in severe CAC. PE-related mortality was higher in patients with any CAC than in those without CAC (OR 3.5 95%CI 1.1-10.7; P=0.028).
**Conclusion:** Among patients admitted for PE, CAC was associated with higher PE-related mortality. Evaluation of CAC on CTPA at the time of diagnosis may be utilized as an additional prognostic parameter and could be used to assist in early risk stratification in patients with PE.

**B-1307** 14:08

**Coronary artery calcification and prediction of mortality in patients with pulmonary embolism**

L. Norton, D. Maguire, K. Mac a' Bháird, G. Cooper, O. Sheerins, M. Adamson, C. Kelly, G. Roditi; *Glasgow/UK (lindsey.norton@nhs.net)*

**Purpose:** To assess thoracic CT features that predict mortality in patients with acute pulmonary embolism (PE) on CT pulmonary angiography (CTPA).
**Methods and Materials:** We evaluated standard CTPA scans of 161 consecutive patients diagnosed with acute PE for signs of right heart strain including right ventricular:left ventricular ratio, SVC cross sectional area, PA trunk diameter (mm), PA trunk area (mm2), Azygous vein diameter (mm), IVC reflux grade and bowing of interventricular septum. Clot burden was assessed using Obstruction index Qanadli score. Coronary artery calcification was assessed using Weston score.
**Results:** Of 161 patients, 34 were deceased within 6 months of presentation. Univariate analysis revealed Age >65 years (p=0.004), PESI score >100 (<0.001) and coronary artery calcification (p<0.001) were strongly associated with increased patient mortality. Troponin >5ng/dl approached significance on univariate analysis (p=0.053). High obstruction index (Qanadli score ≥20) also correlated with increased mortality (p=0.012). Traditional CT indicators of right heart strain were not significantly predictive (Transverse and 4-chamber reformat RV:LV ratio, (p=0.231 and p=0.252 respectively)).
**Conclusion:** Coronary artery calcification is a novel and powerful independent predictor of mortality in patients with acute PE performing better than obstruction index (clot burden) and CT features of right heart strain in our study. These results may reflect reduced capacity of underperfused myocardium to respond to increased right ventricular afterload. The relationship between coronary artery disease and subsequent mortality in patients with PE risk deserves further investigation.

**B-1308** 14:16

**Accuracy and variability of coronary CT angiography with iterative model reconstruction for plaque burden assessment compared with IVUS: a pulsating cardiac phantom study**

Q. Wen, Y. Hou, L. Yang, Y. Ma, X. Lu; *Shenyang/CN (2609579904@qq.com)*

**Purpose:** To systematically evaluate the accuracy and intra-/inter-reader variability of coronary CTA with iterative model reconstruction (IMR) in coronary plaque burden assessment compared with intravascular ultrasound (IVUS).
**Methods and Materials:** A dynamic cardiac phantom mounted with total 12 artificial vessels with 4 different inner diameter(2.5, 3.0, 3.5, 4.0 mm) was scanned on 256-slice CT. Three kinds of coronary plaque were arranged evenly in the iodine-enhanced lumen (350±5 HU at 100 kV) to simulate different stenosis severity (75%, 50% and 25%). CT images were reconstructed with IMR(cadiac routine, level 1). Coronary plaques were categorised into 2 groups: calcified group and non-calcified group. The cross-sectional areas of the lumen ($CSA_{lumen}$) and vessel ($CSA_{vessel}$) were assessed by two radiologists; the value of plaque area (PA) and plaque burden (PB) were calculated.
**Results:** A total of 96 slice (32 calcified and 64 non-calcified) of stenosis were analysed, respectively, in CTA and IVUS image. Average PB assessed by CTA was higher than that of IVUS on the whole (42.49±16.18% vs 40.77±16.15%, P<0.014). There existed a significant correlation between two methods (r=0.839; P<0.001). In the sub-group, CTA overestimated the calcified plaque burden by 3.6±2.0% (P<0.001) while there was no statistical difference in non-calcified group between two methods. There exist great inter-/intra-reader correlation of CCTA (r=0.81, P<0.001; r=0.89, P<0.001), and there was no significant difference between intra-/inter-reader measurement (P>0.05).
**Conclusion:** With IMR, evaluation of plaque burden by CCTA has a low inter-/intra-reader variability and a great correlation with IVUS measurements, while calcified plaque is overestimated by CCTA.

**B-1309** 14:24

**Retrospective evaluation of the accuracy of coronary computed tomography angiography compared to invasive coronary angiography: radiological and therapeutic approach in CAD-RADS 3/V**

C. De Cataldo, A. Corridore, S. Torlone, E. Cannizzaro, P. Palumbo, F. De Matteis, M. Di Luzio, E. Di Cesare, C. Masciocchi; *L'Aquila/IT (camilladeca@hotmail.it)*

**Purpose:** CAD-RADS is an excellent tool finalized to the optimisation of the management of patients affected by coronary artery disease (CAD) undergoing Coronary Computed Tomography Angiography (CCTA). However, it only allows a marginal consideration of plaque characteristics (modifier V). Our study aims to assess the radiological and therapeutic management of CAD patients, classified by CT examination as CAD-RADS 3/V (stenosis of 50-69% with vulnerable plaque).

**Methods and Materials:** We retrospectively evaluated 71 patients who underwent CCTA and were defined as CAD-RADS 3/V because of the display of two or more morphological features (low attenuation plaque, spotty calcifications, napkin-ring sign, positive remodelling). Clinical and therapeutic follow up was performed.
**Results:** A first group of 15 patients was sent to ICA (Invasive Coronary Angiography) with IVUS within three months after CCTA; a second group of 56 patients underwent functional evaluation (Cardiac Magnetic Resonance Stress Imaging with Adenosine) and ICA within a year. The first group displayed an excellent conformity between CCTA and ICA stenosis values (μ=1.03, Coefficient of Variation=12%); in the second, we highlighted a significant discrepancy because of stenosis progression or overlaid thrombosis. A strong correlation was noticed between stenosis entity and morphological features, except for positive remodelling. In the first group, 8 patients underwent PTCA, 50 in the second, 17 of which experienced cardiovascular accident in the period of time between CCTA and ICA.
**Conclusion:** Our experience confirms the importance of a more effective approach to CAD-RADS 3/V patients, for whom a functional evaluation risks to represent a therapeutic delay.

**B-1310** 14:32

**Improving CAD-RADS score with new iterative model reconstruction algorithm in low dose CCTA: inter-observer agreement and comparison with hybrid iterative reconstruction algorithm**

C. Talei Franzesi[1], L. Riva[1], D. Ippolito[1], C. Cangiotti[1], A. de Vito[1], C. Maino[1], M. Porta[1], S. Sironi[2]; [1]*Monza/IT*, [2]*Bergamo/IT (ctfdoc@hotmail.com)*

**Purpose:** To evaluate the inter-observer agreement of the CAD-RADS system and to compare image quality, CNR and SNR between new MBIR (IMR) algorithm and hybrid-iterative reconstruction algorithm (iDose[4]) of low dose cardiac-CT angiography(CCTA).
**Methods and Materials:** Eighty patients were prospectively enrolled in this study. All patients were examined with a 256-slice scanner (iCT-elite,Philips) using low-dose (80-kV, 70mL of CM) CCTA protocol combined with prospective or retrospective ECG-gated technique, reconstructed with iDose[4] and IMR algorithm. Each study was evaluated by 2 readers using the CAD-RADS lexicon based on the degree of maximum coronary stenosis (vessel >1.5 mm in diameter). Coronary arteries and aorta contrast-enhancement(HU), standard deviation(SD), SNR and CNR were measured in the axial native images. Inter-observer agreement of CAD-RADS system and image quality scores(5-point Likert scale) was determined using weighted k-statistic. Inter-observer agreement of mean attenuation values(HU) of coronary arteries and aorta was assessed with linear regression analysis and Bland-Altman plots. Radiation dose exposure(DLP,mGy*cm) and effective dose(ED) were reported for all the examinations.
**Results:** For CAD-RADS agreement, the k-value was very good(0.90). Moreover we found a significant higher value of qualitative analysis, SNR and CNR with the IMR algorithm compared to the iDose[4], due to a lower noise level(p<0.5). The mean DLP measured was 191.44mGy*cm and the mean ED was 2.68mSv.
**Conclusion:** Inter-observer agreement of CAD-RADS was excellent confirming the importance, the feasibility and the reproducibility of CAD-RADS system for CCTA. In addition, we showed a lower noise and higher image quality with IMR algorithm compared to iDose[4].

**B-1311** 14:40

**Reliability of coronary calcium scoring on low-dose chest CT: comparison with ECG-triggered cardiac CT based on third-generation dual-source CT**

C. Xia, A. Alsurayhi, M. Vonder, G. Pelgrim, M. Rook, M. Oudkerk, R. Vliegenthart; *Groningen/NL (c.xia@umcg.nl)*

**Purpose:** To assess the reliability of low-dose chest CT-based coronary calcium score (CS), compared with dedicated electrocardiographically (ECG)-triggered cardiac scans using third generation dual-source computed tomography (CT).
**Methods and Materials:** In the Imaging in Lifelines (ImaLife) study, 646 consecutive participants underwent an ECG-triggered cardiac acquisition immediately followed by a low-dose chest acquisition using CT Force (Siemens Healthineers, Germany). Both cardiac and chest scans were acquired with high-pitch spiral mode and reconstructed with dedicated coronary calcium scoring settings for the purpose of this analysis. Thirty cases were excluded due to cardiovascular interventions or technical issues. CS was measured in Agatston method on both scans using dedicated software (CaScoring, syngo.via, Siemens, Germany).
**Results:** The median age of the participants was 54 years (range 45-63) and 40% were male. A positive CS was observed in 37% of the cardiac acquisitions. Compared with dedicated cardiac scans, the accuracy, sensitivity, and specificity of chest acquisitions in identifying the presence of coronary calcium was 0.97 (95% CI 0.95-0.98), 0.92 (95% CI 0.87-0.95), and 0.99 (95% CI 0.98-1.00), respectively. The reliability of chest scans in CS risk categorization was very high (weighted kappa 0.95). Although the mean difference of CS between two acquisitions was 6.94, the reliability of chest acquisitions in quantifying CS was high (intraclass correlation coefficient 0.994-0.996). The CS correlation between chest scans and cardiac scans was strong ($\tau_b = 0.933$).
**Conclusion:** Coronary calcium can be reliably detected on low-dose chest CT scanning based on third-generation dual-source CT with a sensitivity of 92%.

**B-1312** 14:48

**Accuracy of calcium scoring calculated from contrast-enhanced coronary computed tomography angiography using a dual-layer spectral CT**

J. Nadjiri, G. Kaissis, F. Meurer, K.-L. Laugwitz, A.S. Sträter, D. Pfeiffer, P.B. Noel, E.J. Rummeny, M. Rasper; *Munich/DE*

**Purpose:** The objective of the present investigation was to evaluate the accuracy of virtual non-contrast CACS computed from spectral data in comparison to standard non-contrast imaging.
**Methods and Materials:** We consecutively investigated 20 patients referred to coronary computed Tomography angiography (CCTA) with suspicion of CAD using a dual-layer spectral CT system (IQon; Philips Healthcare, The Netherlands). CACS was calculated from both, real- and virtual non-contrast images by certified software for medical use. Correlation analyses for real- and virtual non-contrast images and agreement evaluation with Bland-Altman-Plots were performed.
**Results:** Mean patient age was 57.7 ± 14 years (n=20). 13 patients (65%) were male. Inter-quartile range of clinical CACS was 0-448, the mean was 334. Correlation of CACS from real- and virtual non-contrast images was very high (0.94); p < 0.0001. The slope was 2.3 indicating that values from virtual non-contrast images are approximately half of the results obtained from real non-contrast data. Visual analysis of Bland-Altman plot shows good accordance of both methods when results from virtual non-contrast data are multiplied by the slope of the logistic regression model (2.3). The acquired power of this results is 0.99.
**Conclusion:** Determination of calcium score from contrast-enhanced CCTA using spectral imaging with a dual-layer detector is feasible and shows good agreement with the conventional technique when a proportionality factor is applied. Our data suggest that radiation exposure can be reduced through omitting additional native scans for patients referred to CCTA when using a dual-layer spectral system without the usual limitations of dual-energy analysis.

**B-1313** 14:56

**Influence of image reconstruction parameters on cardiovascular risk reclassification by computed tomography coronary artery calcium score**

C. Mantini[1], E. Maffei[2], P. Toia[3], F. Ricci[1], A.R. Cotroneo[1], F. Cademartiri[4]; [1]*Chieti/IT*, [2]*Treviso/IT*, [3]*Palermo/IT*, [4]*Rotterdam/NL (c.mantini@rad.unich.it)*

**Purpose:** To investigate the influence of different CT reconstruction parameters on coronary artery calcium scoring (CACS) values and reclassification of predicted cardiovascular (CV) risk.
**Methods and Materials:** CACS was evaluated in 113 patients undergoing ECG-gated 64-slice CT. Reference CACS protocol included standard kernel filter (B35f) with slice thickness/increment of 3/1.5 mm, and field-of-view (FOV) of 150-180 mm. Influence of different image reconstruction algorithms (reconstructed slice thickness/increment 2.0/1.0-1.5/0.8-3.0/2.0-3.0/3.0 mm; slice kernel B30f-B45f; FOV 200-250 mm) on Agatston score was assessed by Bland-Altman plots and concordance correlation coefficient (CCC) analysis. Classification of CV risk was based on the Mayo Clinic classification.
**Results:** Different CACS reconstruction parameters showed overall good accuracy and precision when compared with reference protocol. Protocols with larger FOV, thinner slices and sharper kernels were associated with significant CV risk reclassification. Use of kernel B45f showed a moderate positive correlation with reference CACS protocol (Agatston CCC = 0.67), and yielded significantly higher CACS values (p < .05). Reconstruction parameters using B30f or B45f kernels, 250 mm FOV, or slice thickness/increment of 2.0/1.0 mm or 1.5/0.8 mm, were associated with significant reclassification of CV risk (p < .05).
**Conclusion:** Kernel, FOV, slice thickness and increment are major determinants of accuracy and precision of CACS measurement. Despite high agreement and overall good correlation of different reconstruction protocols, thinner slices thickness and increment, and sharper kernels were associated with significant upward reclassification of CV risk. Larger FOV determined both upward and downward reclassification of CV risk.

**B-1314** 15:04
**Coronary calcium screening with non-gated standard and low-dose chest CT in comparison with ECG-gated calcium scoring CT**
K.N. Zhuravlev, V.E. Sinitsyn, A.V. Shpektor; *Moscow/RU (Kir232@mail.ru)*

**Purpose:** Coronary artery calcification (CAC) is a recognized marker of coronary atherosclerosis. It is frequently detected on chest CT. The purpose of this study was to evaluate the correlation between the coronary calcium scoring values obtained with gated CT and those obtained with non-gated standard and low-dose chest CT.
**Methods and Materials:** 425 consecutive patients were prospectively studied with non-gated chest CT. The standard CT protocol was used in 214 cases, and low-dose one in 211 cases. ECG-gated cardiac CT for CAC scoring was performed in the same patients. Agatston calcium score values and stratification of patients into five risk categories according to calcium score values were compared with the reference data from the gated cardiac CT group.
**Results:** Included in the final analysis were 398 patients. The mean calcium scores were 288.3 for the standard CT protocol and 287.7 for the low-dose CT. The correlation between CAC scores obtained with gated and non-gated CT was high (r=0.977 for standard CT and 0.988 for low-dose CT). It remained high after exclusion from analysis of CAC score values of 0 (r=0.975 and 0.986, resp.). Categorization of patients into the five risk groups according to the calcium score values was also high for the both chest CT protocols (kappa 0.877 and 0.892, resp.).
**Conclusion:** The study shows that analysis of CAC and the risk stratification of the screened patients is feasible with help of non-gated chest CT. These results could be used for combined screening of the lung cancer and coronary atherosclerosis.

**B-1315** 15:12
**Association between coronary artery calcium score and cardiovascular magnetic resonance imaging parameters in patients with end-stage renal disease**
Q. Xie, X. Zhou, L. Lin; *Shenzhen/CN (15800011332@163.com)*

**Purpose:** Whether coronary artery calcium score (CACS) is reliable for risk stratification of cardiovascular disease in patients with end-stage renal disease (ESRD) remains controversial. Our study aimed to investigate the associations between CACS, CMR and clinical parameters in ESRD patients.
**Methods and Materials:** 33 ESRD patients (18 males; mean age 45±11 years) and 43 healthy volunteers (24 males; mean age 43±10 years) underwent CMR examinations. All the ESRD patients underwent CACS scans. CACS, LV function parameters, global LV strains, LV myocardial native T1 and T2 of the mid-cavity slice of all the subjects were measured and compared. The ESRD group were divided into 3 subgroups according to tertiles of the CACS, and their clinical and CMR parameters were compared. Correlations between CACS and the CMR and clinical parameters in the ESRD group were analysed, respectively.
**Results:** LV global longitudinal strain (GLS) was statistically impaired in ESRD group (-14.2±3.2%,) compared with healthy group (-16.5±2.2%, p<0.001). LV GLS were statistically impaired in the upper tertile (-12.3±3.6%) compared with the lower tertile (-15.4±2.5%, p=0.023). CACS was correlated with LV GLS (r=0.41, p=0.018). CACS was strongly correlated with years of chronic kidney disease (r=0.58, p<0.001) and years of dialysis (r=0.53, p=0.002). There was no correlation between CACS and native T1, T2, LV GLS, LVMASS, LVEF (p>0.05).
**Conclusion:** ESRD patients demonstrated impaired LV GLS compared with healthy people. Increased CACS was associated with impaired LV GLS as well as long duration of the chronic kidney disease and dialysis in ESRD patients.

**B-1316** 15:20
**Implications of incidental coronary artery calcification on thoracic computed tomography**
V. Tilliridou, J. Murchison, E.J.R. van Beek, D.E. Newby, M.C. Williams; *Edinburgh/UK*

**Purpose:** Coronary artery calcification (CAC) can be identified on thoracic CT performed for non-cardiac indications. Although CAC is associated with increased mortality, we do not know the implications for management of reporting incidental CAC.
**Methods and Materials:** This audit assessed the images of all patients undergoing contrast or non-contrast CT of the thorax over a 1-month period. Information on cardiovascular risk factors and current medication were obtained from electronic patient records. Ten-year cardiovascular risk was assessed using the ASSIGN score, a cardiovascular risk tool validated for the Scottish population. The presence of coronary artery calcification was recorded and graded on a 4-point scale in each coronary artery, and summed to give a total CAC score (CACS). The presence of aortic and mitral valve calcification was also assessed.
**Results:** The images of 326 patients were assessed (66±14 years, 51% male). CAC was identified in 216 patients (66%), and 47 patients (14%) had severe CACS. Patients with CAC had a higher 10-year cardiovascular risk score (27±15 versus 12±10, p <0.0001); 39 (12%) patients had a 10-year risk of <20% and 10 patients (3%) had a risk of <10%. Age, male gender and smoking were independent predictors of the presence of CACS (p<0.001). Although patients with CAC were more likely to be taking preventative medications (p<0.001), nearly a half (97/216, 45%) were not taking any preventative medication.
**Conclusion:** CAC is prevalent on thoracic CT performed for non-cardiac indications and can identify patients who may benefit from preventative medication despite low-cardiovascular risk scores.
*Author Disclosures:*
**M.C. Williams:** Consultant; GE Healthcare.

**14:00 - 15:30** **Room M 2**

### Paediatric

### SS 1512

### Paediatric radioprotection, techniques and forensic imaging

*Moderators:*
N.N.
J. Piqueras; Barcelona/ES

**B-1317** 14:00
**Evaluation of virtual grid post-processing for paediatric no-grid radiography in an unselected paediatric patient cohort**
M. Beeres[1], J.-E. Scholtz[1], D. Leithner[1], A. Barakat[2], T.J. Vogl[1], T. Gruber-Rouh[1], B. Kaltenbach[1], C. Polkowski[1], A.M. Bucher[1]; [1]*Frankfurt a. Main/DE*, [2]*Liverpool/UK (beeres@gmx.net)*

**Purpose:** Virtual grid post-processing (VGP) allows for the calculation of anti-scatter grid images from digital radiographs taken without a physical anti-scatter grid. Because a physical scatter grid is frequently avoided in the paediatric setting for radiation protection purpose, application of VGP is especially interesting in this population.
**Methods and Materials:** 250 pediatric digital projection radiographs were evaluated with VGP and an intra-individual reference without virtual grid post-processing. Radiographs of the hand (n=100), extremities (n=50) and chest (n=100). Indication for hand radiographs was bone age determination, chest and extremities were taken from with a variety of clinical indications. All images were compared by two readers. Delineation of bone structure, soft tissue differentiation and delineation of foreign material were rated by each reader.
**Results:** Mean age was 7 years. All images were of diagnostic quality. For chest radiographs, a clear benefit was shown for VGP in all three categories in delineation of bone structure (p<0.01), soft tissue differentiation (p<0.01) and delineation of foreign material (p<0.01). Bone delineation for hand and extremities was inferior in RVGP due to loss of trabecular structure (p=0.01). There was no benefit in delineation of osteosynthetic material for VGP in skeletal images (p>0.8). Inter-reader agreement was good (k=0.82).
**Conclusion:** VGP is beneficial in chest radiographs in the pediatric setting, since delineation of ribs, pulmonary vasculature and semitransparent foreign material can benefit from VGP reconstructions. VGP did not show improvements when compared with no-gird hand or extremity radiographs, or when evaluated on osteosynthetic material.
*Author Disclosures:*
**M. Beeres:** Other; Travel Grant: Bayer Vital.

**B-1318** 14:08
**Impact of detector pixel size on clinical image quality of digital neonatal chest X-ray images**
M. Smet, L. Breysem, M. Wang, H. Bosmans, N. Marshall, S. Rodríguez Pérez; *Leuven/BE (Marleen.Smet@uzleuven.be)*

**Purpose:** To compare neonatal chest X-ray clinical image quality (IQ) of digital detectors with different pixel sizes (ps) using visual grading analysis (VGA).
**Methods and Materials:** Over a period of 14 weeks, routine bedside neonatal chest image acquisitions for a given patient were alternated between two CsI-detectors: the Agfa® DX-D 35C (ps=0,125mm) and the novel Agfa® DR10s (ps= 0,148mm). A relative VGA study was performed on 50 chest X-ray image pairs (100 images) of 30 neonates. Two experienced paediatric radiologists and one radiology trainee scored the images following the European quality criteria, with additional questions on tube/catheter visibility. The relative VGA scores were analysed using visual grading characteristics (VGC). Noise and diagnostic acceptability were scored on all images individually using absolute VGA.

**Results:** Area under the VGC curve with associated 95% confidence interval (CI) was 0.54 (0.45-0.62], 0.63 (0.55-0.71) and 0.56 (0.48-0.64) for readers 1, 2 and 3. Only one (experienced) reader scored image quality significantly higher for the small ps detector (i.e. CI excluded 0.5). Using the median score from all readers the AUC= 0.56 (0.48-0.64), i.e. no significant statistical difference existed between the detectors. Intraclass correlation coefficient was 0.64 (95% CI: 0.50-0.76), indicating reasonably good reader agreement. Absolute scores showed similar noise levels for both detectors, with 7 as median value (1-10 scale (10=best)). The three readers classified 98% of images as acceptable for diagnostic use.
**Conclusion:** Clinical IQ for the larger ps detector was not significantly different from the small ps detector in neonatal chest X-ray imaging applications.

**B-1319** 14:16
**Post-mortem ultrasound-guided biopsy for perinatal death investigation: a pilot study**
S.C. Shelmerdine, J.C. Hutchinson, N.J. Sebire, O.J. Arthurs; *London/UK (susie_c_s@yahoo.co.uk)*

**Purpose:** Minimally invasive autopsy methods are becoming more acceptable as they reduce the need for large disfiguring incisions. Laparoscopic approaches and percutaneous 'blinded' biopsies of organs have been trialled, without imaging guidance, with variable success. This study aims to assess tissue yield of post-mortem ultrasound-guided biopsies in a perinatal cohort.
**Methods and Materials:** All perinatal deaths consented for minimally invasive autopsy were included in this prospective study over a 1-year period. Ultrasound-guided biopsies of the liver, both kidneys, both lungs, spleen and myocardium were performed using a 14G cutting biopsy needle via either a periumbilical or subcostal route. A pathologist, blinded to the intended target organ, assessed samples for organ type, sufficient tissue volume for analysis and underlying abnormalities.
**Results:** 12 perinatal cases underwent image-guided organ biopsies, 6 via the periumbilical route and 6 via the subcostal route. Excluding the spleen, the intended target organ was obtained in 80-100% of cases via periumbilical route and 66.7-91.7% via subcostal route. Splenic tissue was obtained in 20% of samples via periumbilical route versus 16.7% subcostal route biopsies. All samples obtained via periumbilical route and 80-100% via subcostal route yielded sufficient tissue for analysis.
**Conclusion:** Post-mortem ultrasound-guided organ biopsies are a feasible technique for obtaining the majority of tissue samples in perinatal death investigation. A periumbilical route resulted in higher target organ yield than a subcostal route, and will be assessed in a larger study for diagnostic accuracy. Splenic tissue remains difficult to sample using this method.

**B-1320** 14:24
**Pediatric Computer Tomography (CT) Diagnostic Reference Levels (DRLs) by age and size**
S. Yu, P. Chu, R. Smith-Bindman; *San Francisco, CA/US (philip.w.chu@gmail.com)*

**Purpose:** Contribute to the creation of pediatric (<15 years) CT radiation dose benchmarks by age and size. We report the distribution of radiation dose metrics based on CT scans submitted in 2017 from a large international dose registry comprised of 139 institutions from 7 countries. The registry was supported through the US National Institutes of Health (NIH) and Patient Centered Outcomes Research Institute (PCORI).
**Methods and Materials:** We describe mean pediatric doses for dose length product (DLP) across institutions in Europe and US. Pediatric age is split into four age categories and dose metrics were categorized by quintiles.
**Results:** We analyzed 3,007 European pediatric CT scans and found the following mean DLP values (in mGy-cm): youngest age group and smallest patient diameter: abdomen (50), chest (36), and head (270); oldest age group and largest patient size: abdomen (278), chest (149), and head (715). We analyzed 57,252 US pediatric CT scans and found the following mean DLP values (in mGy-cm): youngest age group and smallest patient diameter: abdomen (99), chest (42), and head (269); oldest age group and largest patient size: abdomen (514), chest (273), and head (704).
**Conclusion:** Dose among pediatric patients varies greatly by age and size in the United States and Europe. Within the same patient size category, mean DLP in the head, chest, and abdomen can double in dose comparing the highest age group to the lowest age group. A larger and more representative sample is needed to reliably confirm the result dose in European pediatric scans.

**B-1321** 14:32
**Evaluation of ultra low dose CT examinations of the paediatric chest using spectral shaping and an iterative reconstruction algorithm**
M. Wetzl, D. Weinmann, M. Kopp, J. Roth, R. Heiß, W. Wuest, O. Rompel, M. Uder, M.S. May; *Erlangen/DE (matthias.wetzl@uk-erlangen.de)*

**Purpose:** Aim of this study was to evaluate the minimum diagnostic radiation dose in paediatric CT using a step wise approach with spectral shaping and advanced iterative reconstructions.
**Methods and Materials:** 62 paediatric patients with the indication for CT of the chest were examined using a third generation dual source scanner, equipped with a 0.6 mm tin filter. The patients were randomized to different imaging protocols (100Sn kVp, 96/64/32 reference mAs) representing 15%, 10% and 5% of the institutional reference protocol. Images were reconstructed using FBP and different levels of an advanced iterative reconstruction algorithm (ADMIRE). Image noise and subjective image quality were assessed. Radiation doses of CTs were compared to 64 chest radiographs.
**Results:** Mean age was 13.7 years and mean BMI was 18.9 without significant differences in the subgroups. Mean doses were 0.22, 0.15, and 0.07 mSv (p< 0.01) for the CT subgroups and 0.035 mSv for chest radiographs. In all subgroups image noise was reduced up to 53 % (p< 0.01) using ADMIRE level 4, compared to FBP. A sufficiently high subjective image quality was achieved using ADMIRE level 3 in the 96 mAs subgroup and using ADMIRE level 4 in the 64 mAs subgroup. With the 36 mAs protocol, which provided radiation doses comparable to radiographs in two planes, adequate image quality was limited to central lung structures, pleura, and lung nodules using ADMIRE level 4.
**Conclusion:** The combination of latest technological developments allows for high diagnostic image quality in paediatric CT examinations of the chest while approaching radiation doses from radiographs.
*Author Disclosures:*
**M. Kopp:** Speaker; Siemens Healthineers AG. **R. Heiß:** Speaker; Siemens Healthineers AG. **W. Wuest:** Speaker; Siemens Healthineers AG. **O. Rompel:** Speaker; Siemens Healthineers AG. **M. Uder:** Speaker; Siemens Healthineers AG. **M.S. May:** Speaker; Siemens Healthineers AG.

**B-1322** 14:40
**Relative regional air volume change as a biomarker in humidifier disinfectant-associated lung injury in children with normal-looking chest CT**
H. Ko[1], J. Choi[2], K. Chae[3], S. Park[1], J. Jang[3], C. Lee[2]; [1]*Seoul/KR*, [2]*Iowa City, IA/US*, [3]*Jeonju/KR*

**Purpose:** Many routine chest CT scans of the children exposed to humidifier disinfectants (HDs) were normal and could not provide any evidence of humidifier disinfectant-associated lung injury (HDLI). The purpose of the study was to investigate relative regional air volume change (RRAVC) in HD-exposed children with normal-looking chest CT.
**Methods and Materials:** 32 children (11.1±3.6 yrs) with normal-looking chest CT were enrolled in this study. Inspiration-expiration CT image matching via image registration was used to compute RRAVC of acinar scale lung regions. Subjects were divided as diffuse homogeneous (V1, n=14), diffuse heterogeneous (V2, n=10) and increased ventilation in the lower lung (V3, n=8). PFTs including DLco and questionnaire data relevant to HD exposure were obtained.
**Results:** Coefficient of variation (CV) of RRAVC was significantly higher (p<0.05) in V2 (0.830±0.446) than V1 (0.228±0.057) and V3 (0.356±0.093). There was a significantly longer HD exposure in V3 (V1,15.4±8.6; V2,14.7±5.7; V3=29.8±11.4 months; p=0.004), who showed relatively increased ventilation in the lower lobes. No significant difference was found between normal subjects and subjects with decreased DLco and/or FEV1 in HD exposure duration and RRAVC distribution category.
**Conclusion:** Heterogeneity of local ventilation and relatively increased ventilation in the lower lobes can be new characteristics of HDLI-induced pulmonary functional abnormality. RRAVC characteristics may be used as a biomarker to illustrate earlier regional ventilation change for the children who have been exposed to chemical agents by inhalation.
*Author Disclosures:*
**H. Ko:** Grant Recipient; RE201806027, Korea Ministry of Environment(MOE). **J. Choi:** Grant Recipient; RE201806027, Korea Ministry of Environment(MOE), 2016R1D1A1B03936078, National Research Foundation of Korea (NRF) grant, funded by Ministry of Education, Republic of Korea, 2017R1D1A1A09082160, National Research Foundation of Korea (NRF) grant, funded by Ministry of Education, Republic of Korea. **K. Chae:** Grant Recipient; RE201806027, Korea Ministry of Environment(MOE).

**S. Park:** Grant Recipient; RE201806027, Korea Ministry of Environment(MOE). **J. Jang:** Grant Recipient; RE201806027, Korea Ministry of Environment(MOE). **C. Lee:** Grant Recipient; RE201806027, Korea Ministry of Environment(MOE), 2016R1D1A1B03936078, National Research Foundation of Korea (NRF) grant, funded by Ministry of Education, Republic of Korea, 2017R1D1A1A09082160, National Research Foundation of Korea (NRF) grant, funded by Ministry of Education, Republic of Korea.

**B-1323** 14:48

**CT chest under general anaesthetic: is the degree of atelectasis improved by prone positioning?**

A. Kirby, A.J. Quigley, J. Jones, S. McGurk; *Edinburgh/UK (akirby3@nhs.net)*

**Purpose:** Anaesthesia-related pulmonary atelectasis is a common problem when performing paediatric CT imaging under general anaesthetic. This can make interpretation of pulmonary parenchyma more challenging and can lead to both false-positive and false-negative results. Anaesthetic techniques have been proposed to limit the degree of atelectasis but the use of a prone position rather than a standard supine position has not been explored. This study investigates prone positioning versus the standard of supine positioning. The primary outcome measure is the severity of pulmonary atelectasis encountered.
**Methods and Materials:** 109 cases were prospectively and randomly assigned to the comparison (prone n=43) and control (supine n=63) groups. Prone cases were rotated to the standard axial plane and were reviewed independently by two consultant radiologists. Concordance between the two assessors was measured by means of a weighted Cohen's Kappa test. Superiority of comparison versus control group was investigated by means of a Mann-Whitney U test.
**Results:** Inter-observer concordance was found to be 'very good' with a Kappa of 0.868 (95% CI 0.84-0.90). There was found to be no statistical significance between prone and supine imaging in the severity of atelectasis with a p value of 0.57.
**Conclusion:** As no significant improvement was demonstrated between prone and supine positioning, the status-quo of standard supine positioning is suggested. This has both practical and anaesthetic benefits over prone imaging. Further investigation into other avenues of anaesthetic technique, positioning and scan technique may yet yield benefits over current best practice.

**B-1324** 14:56

**PET-MRI is non-inferior to PET-CT in paediatric and adolescent Hodgkin's lymphoma for staging and early response assessment**

M.V. Verhagen, T.A. Watson, D. Neriman, S. Punwani, S. Taylor, S. Daw, A. Shankar, L. Menezes, P.D. Humphries; *London/UK (mvverhagen@gmail.com)*

**Purpose:** Treatment regimens for paediatric Hodgkin's lymphoma (HL) depends on accurate staging and treatment response assessment, for which the reference standard is PET/CT. We hypothesised that PET/MRI is non-inferior to PET/CT for both staging and response assessment.
**Methods and Materials:** 24 patients (mean age 15.4 years, range 8-19.5 years) with histologically proven HL were prospectively recruited in 2015 and 2016. 3 patients did not undergo response assessment PET/MRI, resulting in 24 scans (720 nodal and extranodal sites) at staging, and 21 (630 sites) at response assessment. PET/CT was immediately followed by PET/MRI for both staging and response assessment. Anatomical MRI and PET/MRI were prospectively evaluated by blinded radiologists/nuclear medicine physicians and compared to PET/CT as reference standard. A multidisciplinary panel corrected for perceptual errors to create an enhanced PET/CT reference standard and corrected MRI and PET/MRI data.
**Results:** Corrected staging anatomical MRI sensitivity was 85.8% (95%CI 77.4%-89.9%), specificity 97.9% (95%CI 96.3%-98.9%). Corrected staging PET/MRI sensitivity was 100% (95%CI 96.7%-100%), specificity 99.5% (95%CI 98.36-99.87). Corrected response assessment anatomical MRI sensitivity was 0% (95%CI 0%-48.32%), specificity 99.7% (95%CI 98.7%-99.9%). Corrected response assessment PET/MRI sensitivity was 83.3% (95%CI 36.5%-99.1%), specificity 100% (95%CI 99.2%-100%) Modified Ann-Arbor staging agreement was moderate (k 0.403) for MRI and excellent (k 1.0) for PET/MRI. MRI response assessment disagreed with PET/CT in 6 out 21 patients. Deauville grading agreement between PET/MRI and PET- CT was excellent (k 0.835).
**Conclusion:** PET/MRI is non-inferior to PET/CT for staging and early treatment assessment of HL in adolescents.

**B-1326** 15:12

**Magnetic resonance imaging assessment of iron status in paediatric patients with hematologic disorders**

E. Nazarova, D. Kupriyanov, G. Tereshenko, N. Smetanina, G. Novichkova; *Moscow/RU (evelina.nazarova@fccho-moscow.ru)*

**Purpose:** To introduce MRI T2*-method for iron assessment in parenchymal organs of transfusion-related paediatric patients with hematologic disorders.
**Methods and Materials:** MRI investigation was performed using a 3T MR scanner in 85 paediatric patients aged from 2 to 18. For iron estimation we have used Radial T2*-mapping with free breath technique. T2* values were evaluated by special relaxation maps tools and for liver were correlated with iron concentration measured by liver biopsy immediately after MRI scan.
**Results:** For non-invasive assessment of LIC (liver iron concentration) we have received a calibration curve between the biopsy data and the R2* (R2*=1/T2*) values with correlation coefficient 0.95 ($R^2$=0.89; confidence interval of 87% - 97% with CC= 95%). According to the grades of liver siderosis patients were divided into groups and we have obtained reference T2* values in ms for myocardium (M), pancreas (P) and pituitary gland (PG) : non- iron overload - M > 15 ms, P > 11 ms, PG > 11 ms; mild - M = 11-20 ms, P = 4-25 ms, PG = 11-40 ms; moderate - M = 15-30 ms, P = 2-15 ms, PG = 9-20 ms; severe - M = 10-18 ms, P = 1-10 ms, PG = 7-30 ms.
**Conclusion:** Iron assessment by MRI is a good indicator of iron content in the parenchymal organs.

**B-1327** 15:20

**Diffusion kurtosis imaging (DKI) utility in differentiating low- and high-grade gliomas in paediatric patients: further experience**

I. Voicu, A. Napolitano, M. Vinci, F. Diomedi-Camassei, C. Carducci, A. Carai, A. Mastronuzzi, P. Toma, G. Colafati; *Rome/IT (paul.voicu@hotmail.it)*

**Purpose:** To investigate whether diffusion kurtosis imaging (DKI) can differentiate low- and high-grade gliomas (LGG and HGG) in paediatric patients.
**Methods and Materials:** DKI sequences (30 directions, 3 b values: 0, 1000, 2000) acquired with simultaneous multislice technique (SMS) are part of the diagnostic neuro-oncologic protocol in use in our Institution, performed on a 3T magnet. Fifty consecutive children (median age 8.6 years, 27 males) affected by histologically confirmed gliomas were retrospectively studied. Mean kurtosis (MK), axial kurtosis (AK), radial kurtosis (RK), fractional anisotropy (FA) and apparent diffusion coefficient (ADC) values were calculated and the corresponding maps were obtained. The solid volume of the tumour (VOI) was segmented semiautomatically. Mean and highest 20 percentile (H20%) DKI values in tumour VOIs were calculated for each metric. Based on tumour location, patients were further categorized into brainstem location (bl) or non-brainstem location (nbl). Differences among DKI-derived metrics in VOIs of low and high-grade gliomas (LGG and HGG respectively) in different locations (bl or nbl) were assessed with a MANOVA. Post-hoc analysis was performed with independent sample t tests.
**Results:** after excluding three patients for motion artefacts, the scans of forty-seven children (32 LGG and 15 HGG) were analysed. MANOVA revealed significant differences between LGG and HGG (Pillai's trace <0.0001). Post-hoc analysis revealed all metrics were significantly different between nblLGG and nblHGG, while AK and H20%AK were significantly lower in blHGG compared to blLGG (p<0.008 and p<0.006, respectively).
**Conclusion:** DKI metrics can differentiate low and high-grade gliomas in paediatric patients with location-specific patterns.

**14:00 - 15:30** **Room M 3**

### Neuro

### SS 1511

### Neurocognitive impairment and psychiatric disorders

*Moderators:*
N.N.
J. Boban; Novi Sad/RS

**B-1328** 14:00

**Determining the best leader nodes in Alzheimer networks**

A. Moradi Amani[1], L. Meyer-Baese[2], A. Tahmassebi[3], K. Pinker-Domenig[4]; [1]*Melbourne/AU*, [2]*Atlanta, GA/US*, [3]*Tallahassee, FL/US*, [4]*Vienna/AT (lisameyerbaese@gmail.com)*

**Purpose:** Fusing modern network theory and control strategies yields a novel transformational paradigm in dementia research. One research direction is determining the best driver nodes in Alzheimer networks that can be directly manipulated via external inputs to influence the overall network trajectory and simulate the disease progression. Identifying the best driver set in these networks is an unsolved problem in brain connectomics.
**Methods and Materials:** We examine 249 subjects with FDG-PET and T1-weighted MRI images consisting of 68 control, 111 mild cognitive impairment (MCI) and 70 Alzheimer's disease (AD). We consider only 42 out of the 116 from the AAL in the frontal, parietal, occipital and temporal lobes. Different from previous work on controllability of disease networks, we determine the best disease driver nodes based on a centrality measure in the connection graph.
**Results:** We demonstrate the precision of the proposed centrality for correctly determining driver nodes and compare it with heuristic methods including considering hub nodes with maximum degree, maximum betweenness, and maximum closeness. We show the differences in structural and functional brain networks reflecting the changes from controls over MCI to AD, and their locations in the frontal and temporal lobe.
**Conclusion:** We have established a new method to determine the best driver nodes that are influencing the dynamics of the dementia networks. Implicitly, we can gain an understanding of dementia evolution and the subsequent development of therapeutic solutions.

**B-1329** 14:08
**Quantitative MR-phase information enables classification of Alzheimer's disease stages**
H. Indo[1], T. Yoneda[1], N. Kurehana[1], Y. Tatewaki[2], T. Mutoh[2], A. Ishiki[2], N. Tomita[2], H. Arai[2], Y. Taki[2]; [1]*Kumamoto/JP*, [2]*Sendai/JP* *(170w0201@st.kumamoto-u.ac.jp)*

**Purpose:** The purpose of this study was to show quantitative MR-phase corresponding to iron in the amyloid plaque (AP) could classify a group of Alzheimer's disease (AD) patients with high Mini-Mental State Examination (MMSE) score from one of lower score, which would connect to noninvasive image diagnosis of AD.
**Methods and Materials:** AD patients (8 males, 9 females, 79 ± 5 y) with high MMSE score (≥ 25, 10 patients) and low score (≤ 20, 7 patients) were scanned by 3T-MRI with multi-echo GRE sequence ($1^{st}$ TE = 7.3 ms. ΔTE = 8 ms). Phase data was collected from typical cortices (Superior Frontal Gyrus; SFG, Superior Temporal Gyrus, Precuneus, Cuneus) and evaluated by Double Gaussian Analysis already reported (M. Tateishi et al., MRMS, in print) to define a phase corresponding iron in the amyloid plaque. We derived a proportionality coefficient (PC) of the phase with TE as a quantitative metric representing AP accumulation in the cortex we measured. The PC of two groups were statistically evaluated by Welch's t test ($p < 0.01$, significantly different) in all regions.
**Results:** Although subtraction of PC (SPC) between SFG and others were only statistically significantly different, that of other regions did not show it.
**Conclusion:** The SPC may represent difference of iron loading in the AP and relate to clinical evaluation MMSE score. Our study showed the SPC noninvasively and easily derived from MRI data might be a quantitative metric correlated to MMSE score and classify the AD stages.

**B-1330** 14:16
**Altered cortical thickness related to single nucleotide polymorphisms (SNPs) in the major histocompatibility complex (MHC) in antipsychotic-naive schizophrenia**
B. Tao, Y. Xiao, W. Zhang, L. Yao, S. Lui; *Chengdu/CN* *(1945828646@qq.com)*

**Purpose:** To explore the relationship between changes in cortical thickness and single nucleotide polymorphisms (SNPs) in the major histocompatibility complex (MHC) in a group of antipsychotic-naive schizophrenia (AN-SCZ) patients.
**Methods and Materials:** Twenty-five AN-SCZ patients and 51 healthy controls (HCs) participated in the present study. General linear models were used to explore the associations between the average cortical thicknesses of each brain region (N=68) and each of the 11 SNPs in the MHC in the AN-SCZ patients and HCs, respectively. Next, we performed an independent-sample t-tests to investigate whether there were significant decreased cortexes in the AN-SCZ patients compared with HCs in the brain regions that were significantly associated with the SNPs. Finally, we examined the correlation of clinical symptoms with cortical thickness in the above brain areas in the patients group using Pearson correlation tests.
**Results:** Seven of the 11 SNPs within the MHC regions exhibited significant associations with the cortical thickness in the AN-SCZ patients, including rs1635, rs1736913, rs2021722, rs204999, rs2523722, rs3131296 and rs9272105. And the AN-SCZ patients exhibited significant reductions in cortical thickness in the above brain regions, especially the frontal cortex. Furthermore, the left entorhinal region showed a negative correlation with PANSS activation scores in the AN-SCZs ($r=-0.601$, $P=0.03$).
**Conclusion:** The present study provided evidence for the potential effect of MHC risk variants on cortical thickness deficits in AN-SCZ.

**B-1331** 14:24
**Drug effects on brain structure in acute and chronic schizophrenia**
J. Zeng, W. Zhang, Y. Xiao, J. Liu, B. Tang, G. Fu, S. Lui; *Chengdu/CN* *(jiaxinzengscu@163.com)*

**Purpose:** The study aims to reveal the effects of antipsychotics on brain structure in schizophrenia patients. We intend to find out the brain regions that are vulnerable to antipsychotics throughout the acute and chronical treatment stage in a large cohort of first episode drug-naïve schizophrenia patients.
**Methods and Materials:** Diagnosis of schizophrenia were determined using the Structured Interview for the DSM-IV (SCID-P).T1 images were acquired from 179 first episode drug-naïve schizophrenia patients(M/F=77/102), 41 six-week follow-ups(M/F=16/25), 71 one-year follow-ups(M/F=26/45) and 36 two-year follow-ups(F/M=14/22). Grey matter volume was obtained using voxel based morphometry. The FreeSurfer software package was performed to acquire the cortical thickness and surface area. General liner modal was carried out through each comparison between first episode drug-naïve schizophrenia patients and six-week, one-year and two-year follow-ups after treatment using age and gender as covariates. After false discovery rate correction, common brain regions which differ in drug-naïve schizophrenia patients and treated patients through the three comparison were extracted.
**Results:** Grey matter volume showed common altered brain regions in right and left thalamus in first episode drug-naïve schizophrenia patients compared with six-week, one-year and two-year follow-ups, respectively. Besides, grey matter volume decreased in all treated groups compared with drug-naïve schizophrenia patients at baseline in right and left thalamus. There are no overlap brain regions in cortical thickness and surface area between those groups after correction.
**Conclusion:** Thalamus is vulnerable to antipsychotics in not only acute phase but also chronical phase, which reveals the pharmacology and physiology mechanism of drugs on schizophrenia.

**B-1332** 14:32
**Heterogeneity of brain structure alterations in patients with never-treated first episode schizophrenia**
Y. Xiao, J. Zeng, G. Fu, B. Tang, C. Yang, S. Lui; *Chengdu/CN*

**Purpose:** Although schizophrenia is a heterogeneous clinical syndrome, one important question that remains largely unanswered is whether the complex and subtle deficits revealed by MRI could be used as objective biomarkers to resolve neurobiological heterogeneity within this disorder.
**Methods and Materials:** To address this question, 163 drug-naïve first-episode schizophrenia (FES) patients and a confirmation data set of chronic-treated patients (n=133) were recruited. High-resolution anatomic data were acquired and processed via Freesurfer software to obtain cortical thickness, surface area and cortical volume measurements. Subsequently, a density peak-based clustering algorithm was employed to classify schizophrenia into subtypes with distinct neuroanatomical patterns.
**Results:** We found three subtypes of neuroanatomic alterations that were also observed in the confirmation sample. Subtype 1 showed mainly increased surface area and cortical volume in left inferior parietal, superior frontal, superior temporal and right fusiform cortex than healthy controls. Subtype 2 showed subtle cortical alteration. Subtype 3 showed mainly decreased surface area and cortical volume in left precentral, inferior temporal, right superior parietal and rostral middle frontal cortex.
**Conclusion:** Current findings, in a sample of never-treated FES patients, demonstrated three subtypes of patients with distinct patterns of grey matter alteration. Further, the three subtypes' pattern were confirmed by another data set of chronic-treated patients, suggesting the subtyping defined by FES structural MRI are relatively stable after illness onset, which could help to compressively explain the complex and heterogeneous findings of schizophrenia.

**B-1333** 14:40
**An amplitude of low-frequency fluctuation study in first-episode drug-naive patients with major depressive disorder**
X. Hu, X. Hu, H. Li, L. Zhang, L. Lu, X. Bu, S. Tang, Q. Gong, X. Huang; *Sichuan/CN (820546310@qq.com)*

**Purpose:** The purpose of the current study is first to investigate the alterations of amplitude of low-frequency fluctuation (ALFF) in first-episode drug-naive MDD patients with relatively short illness duration and then to explore whether these changes could be reversed by antidepressant treatment.
**Methods and Materials:** Thirty first-episode, drug-naïve MDD patients (mean illness duration = 14 weeks) and 52 healthy controls (HC) were scanned with resting-state functional magnetic resonance imaging to obtain the ALFF within the whole brain. After MRI examination, antidepressant treatment was applied for all patients. After 6 weeks, 9 follow-up MDD patients were scanned again. Voxel-based analysis of ALFF maps were compared between pre-treatment MDD and HC using two-sample t test. Correlations between ALFF and HAMD score or illness duration were conducted using multiple regression. Paired t

Saturday test was used to compare ALFF maps between post-treatment MDD and pre-treatment MDD.
**Results:** Compared with HC, pre-treatment MDD patients had increased ALFF in the left dorsal anterior cingulate cortex (dACC) and inferior posterior lobe of cerebellum ($p < 0.05$, FWE corrected). The ALFF of right dorsolateral prefrontal cortex (rDLPFC) was negatively correlated with HAMD score in MDD patients ($r = -0.591$). Post-treatment MDD patients had significantly decreased ALFF in the left dACC compared to pre-treatment MDD patients ($p < 0.05$, FWE corrected).
**Conclusion:** Our study demonstrates that left dACC, cerebellum and rDLPFC paly an important role in the early course of MDD without interference of antidepressants and the left dACC may be a potential target for antidepressants.

**B-1334** 14:48
**Role of MR functional connectivity in discriminating cognitive impairment in BD**
M.M. Eid, M.H. Othman, S. Abdelal, H.A. Hasan, H.E. Khakifa, S. Sayed; *Asyut/EG (Mido_eid1985@yahoo.com)*

**Purpose:** Disorders in functional communication have been suggested for the cognitive contribution and emotion deficit observed in bipolar disorder (BD). Technical contact between cortical prefrontal cortex (mPFC) and other brain regions may be particularly abnormal. The aim of this study was to describe the dynamics of time to Default Mode network connection (DMN) status in BD and to study its association with perception.
**Methods and Materials:** Our study included , euthymic BD (n = 16) and sound healthy comparison (HC, n = 25) participants performed resting-state functional MRI, we used of high resolution sequences extracted from the human neural network project, and completed neural measures to address operational function. A dynamic functional connectivity approach was used to measure DMN correlations in each participant, with other brain areas of interest as mPFC , posterior cingulate cortex (PCC), and para hippocampal region. To do description of temporal dynamics between these regions and other DMN nodes using subsets of the time series.
**Results:** We found marked decreased (marked variability ) dynamic functional connectivity between mPFC and PCC , in the bipolar group compared to healthy controls . On the other hand we found apparent connectivity between DLPFC ,brainstem and right amygdala in the Bipolar group with more dynamic range in compare to the control group.
**Conclusion:** The fluctuation in the resting-state functional connectivity may be an index of the interlinkage flexibility that decreases in BD and link to ongoing cognitive disability during periods of euthymia.

**B-1335** 14:56
**Spectrum of autoimmune encephalitis: establishing imaging patterns on FDG PET/CT for specific antibody sub-types**
V. Gupta, R. Verma, R. Ranjan, E. Belho, H. Mahajan; *Delhi/IN (ethelbelho@gmail.com)*

**Purpose:** To establish specific imaging patterns on $^{18}$FDG PET/CT in different antibody sub-types of autoimmune encephalitis.
**Methods and Materials:** A total of 35 serologically proven cases of autoimmune encephalitis that underwent F-18 fluoro-deoxy glucose (FDG) PET/CT scan were included. The patterns of FDG uptake in different antibody subtypes were recorded and comparison with normalised data was attempted. The areas of hypo-/hyper-metabolism that were two standard deviations from the mean were considered as abnormal. The patients were also analysed based on the Z score surface maps of the 3D stereotactic surface projections (SSP) image and regional Z scores were evaluated. Post-treatment follow-up scans were also acquired.
**Results:** All the patients had an abnormal pattern of $^{18}$FDG uptake, both on visual inspection and semiquantitative analysis. Voltage-gated potassium channel (VGKC) complex receptor antibody encephalitis patients were found to have typical areas of hypermetabolism in bilateral medial temporal regions and bilateral basal ganglia with relative global hypometabolism in rest of the cortical and subcortical structures. The subjects with N-methyl-D-aspartate-receptor antibody (NMDAR-Ab) encephalitis showed variable degrees of focal hypermetabolism in bilateral fronto-temporal regions, basal ganglia, thalami and cerebellar hemispheres with inhomogeneous parieto-occipital hypometabolism. The post-treatment FDG PET/CT scans showed reversal to normal metabolism in the corresponding areas.
**Conclusion:** $^{18}$FDG PET/CT scan may serve useful as a non-invasive diagnostic modality in the early diagnosis and management of patients with clinical suspicion of autoimmune encephalitis.

**B-1337** 15:12
**Central nervous system (CNS) imaging findings in granulomatous polyangiitis (GPA), IgG4-related (IgG4-RD) and Erdheim-Chester diseases (ECD): comparison and pictorial essay**
S. Gerevini, E. Bozzolo, R. Cao, E. Della Torre, V. Canti, G. Cavalli, M. Lanzillotta, L. Dagna, A. Falini; *Milan/IT (robertacao1@gmail.com)*

**Purpose:** Background: GPA, IgG4-RD and Erdheim-Chester diseases are immune disorders characterized by systemic inflammatory infiltration. Aim: to investigate and compare radiological differences of CNS involvement in these different forms of disorders and revision of the literature.
**Methods and Materials:** We retrospectively reviewed neuroradiological studies of patients with a diagnosis of GPA, IgG4-RD or ECD, presenting an involvement of the CNS. Specific involvement of different structures of brain, paranasal sinuses and of dural layers was classified according to location and imaging findings and compared between the three groups.
**Results:** 29 patients with GPA, 15 with IgG4-RD and 17 with ECD were enrolled. In GPA, paranasal sinus involvement was always observed (29/29) with a maxillar predominance (28/29. A lower rate of sinus infiltration was observed among IgG4-RD (11/15) and ECD (2/17) patients. Orbital involvement was present in all the groups but in ECD patients with an intraconical component (9/9, whereas in GPA and IgG4 with an extraconical predominance (6/6 and 7/8, respectively). In IgG4 patients, dural layers showed a basal involvement (6/6, whereas in ECD distribution involved falx and convexities. In ECD patients, 9 presented with white matter lesions, and 4 with a hypophysary involvement whereas fewer cases of cerebral lesions were observed in the other 2 groups (3 in GPA and 1 in IgG4).
**Conclusion:** Despite a common definition of CNS location of involvement of these groups of diseases according to the literature a direct evaluation showed some differences that should be considered for a differential diagnosis.

**B-1338** 15:20
**Tissue sodium concentration (TSC) in normal brain: preliminary study**
P. Wawrzyniak, B. Bobek-Billewicz, A. Hebda, A. Awramienko, P. Mazgaj; *Gliwice/PL (pawel.wawrzyniak@io.gliwice.pl)*

**Purpose:** Purpose of this work was to establish database of tissue sodium concentration (TSC) in normal brain of healthy volunteers. TSC can be used as a marker of tissue viability in stroke or radiation therapy monitoring.
**Methods and Materials:** This study was approved by local bioethics committee. 35 volunteers were scanned with 23Na protocol in the span of year (10.2017-10.2018), from this pool 29 studies were of acceptable quality. Volunteer mean age was 37 years (min 22 and max 64). Volunteer acquisitions were acquired in one scanner session which lasted approximately 1 hour. Two bottles with 70 and 20 mmol/l Na concentration were placed inside coil for calibration. One scanner session consisted of 23Na GRE acquisition (radial 3D GRE, TR/TE 100/2.87 ms, FA 90 deg, NeX 23, FoV 300x300 mm, matrix 64x64 which gives 4.7x4.7x4.7 mm voxel) performed twice, MPRAGE (1x1x1 mm), SPACE-FLAIR and Resolve-DTI. Only 23Na and MPRAGE acquisitions were used. Denoising filtration was done to 23Na and MPRAGE images. MPRAGE were segmented, and for MPRAGE and 23Na normalization and coregistration was also done, all in SPM12. Next 23Na signal intensity from WM, GM and CSF was acquired using masks from MPRAGE segmentation. Than calibration curve was acquired from phantom signal and TSC was calculated from GM, WM and CSF.
**Results:** In healthy volunteers, mean TSCs were GM 33.21 +/- 4.76 mmol/l, WM 28.41 +/- 4.03 mmol/l and for CSF 41.3 +/- 6.69 mmol/l.
**Conclusion:** This work is a base for further papers with sodium imaging in brain lesions.

**14:00 - 15:30** **Room M 4**

### GI Tract

### SS 1501a

### Rectal cancer: what's new?

*Moderators:*
N.N.
D. Miletić; Rijeka/HR

**B-1339** 14:00
**Clinical feasibility assessment of T3 sub-stage in rectal cancer using MRI**
P. Tripathi[1], W. Guo[2], C. Yang[2], B. Rai[1], M. Zeng[2], H. Yu[1]; [1]*Wuhan/CN*, [2]*Shanghai/CN (pratik9_band@hotmail.com)*

**Purpose:** To investigate the reproducibility of the distance of mesorectal extension by tumour invasion in T3 stage rectal cancer by evaluating sub-stages T3a, T3b T3c and T3d individually versus T3ab and T3cd combined together using MRI.

Saturday

**Methods and Materials:** 188 patients with surgically and histologically confirmed T3 rectal cancer who underwent preoperative MRI were enrolled in this study. Two blinded radiologists evaluated the maximum distance of mesorectal extension (mrDME) in T2-weighted image in MRI. The study population was sub-classified into T3a (< 1 mm), T3b (1-5mm), T3c (5-15 mm) and T3d (>15 mm) according to the distance of mesorectal extension by tumour invasion. The inter-/intra-observer data were assessed statistically.
**Results:** Inter-/intra-observer kappa (k) and intraclass coefficients (ICC) between the two groups were very distinct. In the individual group, the inter-observer and intra-observer k for the mrDME were 0.700 and 0.718, respectively; the inter-observer and intra-observer ICC were 0.772 and 0.786, respectively. In the combined group, the inter-observer and intra-observer k for the mrDME were 0.819 and 0.883, respectively; the inter-observer and intra-observer ICC were 0.829 and 0.796, respectively.
**Conclusion:** There was a distinct increase in the kappa (K) and ICC value in the combined group compared with the individual group. This high reproducibility result suggested that it is more reliable to measure T3ab and T3cd combined together than individually. This finding can play a crucial role in the management of rectal cancer and clinical decision-making for non-expert radiologists in a non-academic setting.

**B-1340** 14:08
**Preoperative MR restaging of rectal cancer in UKMMC: correlation with surgical and histopathologic findings**
C. Lee, S. Osman, L. Mazlan, F. Mohammed; *Kuala Lumpur/MY (dr.leechungyuen@gmail.com)*

**Purpose:** To determine the accuracy of restaging MRI in rectal cancer post-neoadjuvant chemoradiotherapy.
**Methods and Materials:** Between November 2016 and May 2018, we did a comparative cross-sectional analysis of 26 patients who underwent restaging MRI following neoadjuvant chemoradiotherapy. 3.0T MRI is used and images were evaluated by an experienced radiologist and on-table intraoperative tumour T staging is determined by an experienced colorectal surgeon. Both were blinded to results from the other as well as histological results. Interobserver variability was determined. Accuracy of restaging MRI was assessed through the comparison of tumour characteristics on MRI with histopathologic outcomes.
**Results:** Restaging MRI correctly predicted tumour T staging in 18 cases, representing an accuracy of 69.2%. Overstaging was more common than understaging. Positive predictive values (PPV) for T0 is 100%. For T1/2, PPVs and NPVs were 66.7% and 94.1%, and for T3/4 they were 93.3% and 90.9%. MRI suggested tumour regression grade did not correspond with histopathologic tumour regression grade. Interobserver agreement is fair to moderate ($\kappa$ =0.371-0.579).
**Conclusion:** Restaging rectal cancer MRI is inaccurate for prediction of tumour T staging, and the interobserver variability is significant.

**B-1341** 14:16
**Magnetic resonance of rectal cancer response to therapy: comparison between 3.0 and 1.5 Tesla**
E. Lucertini[1], M. Zerunian[1], D. Caruso[1], D. De Santis[1], G. Moltoni[1], F. Landolfi[1], T. Biondi[2], E. Iannicelli[1], A. Laghi[1]; [1]*Rome/IT*, [2]*Belvedere Marittimo/IT (elena.lucertini@gmail.com)*

**Purpose:** To evaluate Signal Intensity(SI) differences between 3.0T and 1.5T on T2 weighted(T2w), Diffusion Weighted Imaging(DWI) and Apparent Diffusion Coefficent(ADC) in rectal cancer pre, during and post-neoadjuvant chemoradiotherapy(CRT).
**Methods and Materials:** 22 patients with locally advanced rectal cancer were prospectively enrolled. All patients underwent T2w, DWI and ADC pre, during and post-CRT on both 3.0T MRI and 1.5T MRI. A radiologist drawn regions of interest(ROIs) of the tumor and obturator internus on the selected slice to evaluate SI and relative SI(rSI). Additionally, a sub analysis evaluating the SI before and after-CRT(ΔS.I.pre-post) in complete responder patients(CR) was performed on T2w, DWI and ADC.
**Results:** Significant differences were observed for T2w and DWI on 3.0T MRI compared to 1.5T MRI pre, during and post-CRT(all *P* <0.001), whereas no significant differences were reported for ADC among all controls(all *P*>0.05). rSI showed no significant differences in all the examinations for all sequences(all *P*values >0.05). $\Delta SI_{CR}$ showed significant differences between 3.0T MRI and 1.5T MRI for DWI$\Delta SI_{CR}$(188.39±166.90 vs 30.45±21.73 *P*value 0.0262) and ADC$\Delta SI_{CR}$(-0.58±0.27 vs -0.21±0.24 *P*value 0.0265), while no significant differences were observed for T2w$\Delta SI_{CR}$.
**Conclusion:** T2wSI and DWISI showed significant differences for 3.0T compared to 1.5T in all 3 controls, while ADCSI showed no significant differences on both field strengths. rSI was comparable for 3.0T and 1.5T MRI, therefore rectal cancer patients can be assessed both at 3.0T MRI and 1.5T MRI. However, a significant DWI$\Delta SI_{CR}$ andADC$\Delta SI_{CR}$ on 3.0T might be interpreted as a better visual assessmentin discriminating response to therapy compared to 1.5T.

**B-1342** 14:24
**Prognostic value of tumour regression grade on MR in rectal cancer: a large-scale, single-centre experience**
H. Yoen, S.H. Kim, J.H. Yoon, B.Y. Hur, J.H. Kim, H.E. Park, H. Oh, J.K. Han; *Seoul/KR (zelkova0712@gmail.com)*

**Purpose:** To determine the prognostic value of tumour regression grade on MR (mrTRG) in rectal cancer compared to pathology (pTRG) and to assess the effect of diffusion-weighted image (DWI) on inter-observer agreement.
**Methods and Materials:** We retrospectively enrolled 489 patients (M:F=323:166; mean age, 59.67years) with histopathologically proven rectal cancers who underwent both pre-chemoradiotherapy (CRT) and post-CRT MRI between January 2005 and December 2016. Two radiologists individually determined mrTRG using a 5-point grading system without and with DWI. Two pathologists graded pTRG using a 5-point grading system. For assessment of interobserver variation, intraclass correlation coefficients (ICC) were used. Kaplan-Meier estimation and Cox proportional hazard models were used for survival analysis.
**Results:** According to mrTRG, 48 patients (7 for mrTRG1 and 41 for mrTRG2) were classified as good responders while 355 (130 for mrTRG3, 214 for mrTRG4 and 11 for mrTRG5) as poor responders. By pTRG, 60 patients (6 for pTRG1 and 54 for pTRG2) were classified as good responders while 298 (191 for pTRG3, 96 for pTRG4 and 11 for pTRG5) as poor responders. For mrTRG, ICC between the two radiologists was 0.273 (fair agreement) on MR without DWI and improved to 0.333 when DWI was added. Overall survival (OS) was significantly different according to the age, pre-CCRT variables (EMD and EMVI), post-CCRT variables (tumour size, T and N stages, EMD, extramesorectal node, EMVI) and mrTRG, but not by pTRG.
**Conclusion:** mrTRG better predicts OS than pTRG. The addition of DWI on T2W MR improves the interobserver agreement.

**B-1343** 14:32
**Importance of tumour volume reduction rate on T2w and DWI during and after CRT for prediction of treatment response to CRT in locally advanced rectal cancer (LARC)**
A. Di Chiara, A. Palmisano, G. Della Vecchia, A. Esposito, P. Passoni, C. Fiorino, A. Del Maschio, F. De Cobelli; *Milan/IT*

**Purpose:** A complete response to CRT improve patients' prognosis and may address patients to a conservative management. To MR is asked high accuracy in the prediction of response and in the identification of residual cancer. Aim of this study was to investigate volumetric imaging biomarker prior, during and after CRT derived from DWI and T2w images.
**Methods and Materials:** 43 patients with LARC underwent 1.5T MRI before, during and after CRT. Tumour volume was manually segmented on high resolution T2-weighted sequence (VT2w) and on DWIb,1000 image (Vb,1000) at each time point, then tumour volume reduction rate (TVRRT2w and TVRRb,1000) calculated. After surgery, patients were classified according with Rödel's tumour regression grade (TRG) in: Non Responders (NR:TRG 0,1,2), Partial Responders (PR:TRG3) and Complete Responders (CR:TRG4). Kruskall-Wallis test was used to compare measurements in different classes and multinomial regression analysis for the prediction of classes of response performed.
**Results:** According to TRG: 10 patients were NR,22 PR and 10 CR. TVRRb,1000 resulted significantly different among classes only after CRT (CR: -97%, PR:- 78%, NR:-69%, p=0.0020), with higher reduction in CR respect to PR (p=0.0021) and NR (p=0.0001). At multivariate analysis predictor of CR were TVRRT2w (<-58%, p=0.0028) at mid-MRI and TVRRT2w and TVRRb,1000 at postMRI (<-83% p=0.0090 and <-87%, p=0.0265), with a sensitivity of 91%, 82% and 91% respectively.
**Conclusion:** Tumour volume modification on T2w and b1000 images is effective in the prediction of response to CRT; in particular early modification on T2 images seems useful to predict CR during treatment.

**B-1344** 14:40
**Whole-tumour texture analysis of ADC maps in the prediction of pathological response of locally advanced rectal cancer (LARC)**
A. Di Chiara, A. Palmisano, A. Esposito, P. Rancoita, P. Passoni, A. Del Maschio, F. De Cobelli; *Milan/IT*

**Purpose:** Tumour heterogeneity is an imaging biomarker of aggressiveness. Texture analysis allows to extract features of tumour heterogeneity from conventional images. Aim of our study is to evaluate the role of whole-tumour histogram-based texture analysis of ADC maps in the prediction of pathological response of LARC to chemoradiotherapy (CRT).
**Methods and Materials:** 43 pts with LARC underwent 1.5T MRI before CRT(pre-MRI), during (mid-MRI), and after CRT (post-MRI). Cancer volumes were segmented on ADC maps at each time-point using T2W-images as reference. The following histogram-based parameters were extracted: 25th, 50th, 75th percentile, mean, skewness and kurtosis and their role in the prediction of histopathological response was evaluated with multinomial regression analysis.
**Results:** 11 patients resulted to be complete responders (CR), 22 partial responders (PR) and 10 non-responders (NR). Only at pre-MRI, 3D-ADCmean values were significantly different among the groups (median: NR 1.07x10-3 $mm^2$/s, PR 1.14x 10-3 $mm^2$/s and CR 1.27x 10-3 $mm^2$/s; p=0.0079), resulting higher in CR than in PR and in NR (p=0.0042 and p=0.0029, respectively). At multinomial regression analysis 75$^{th}$ percentile and 3D-ADCmedian at pre-MRI were positive predictor of response (p=0.0247 and p=0.0415, respectively). A 3D-ADC median cutoff-value of >1.198 x10-3 $mm^2$ at pre-MRI resulted best predictor of CR with a sensitivity, specificity and accuracy of 82%, 88% and 86%, respectively.
**Conclusion:** Pretreatment features of ADC distribution values might be an helpful tool in the prediction of tumour response to CRT.

**B-1345** 14:48
**T2-weighted signal intensity (T2SI) textural analysis in an evaluation of neoadjuvant chemoradiotherapy (CRT) response in patients with locally advanced rectal adenocarcinoma**
G. Della Vecchia, A. Di Chiara, M. Panzeri, A. Palmisano, A. Esposito, P. Passoni, A. Del Maschio, F. De Cobelli; *Milan/IT*

**Purpose:** Tumour response to CRT is often characterized by fibrotic evolution associated, however the sole visual assessment is characterized by low sensistivity. Aim of the study was to assess the value of histogram-based analysis of tumor T2SI in the assessment and prediction of complete pathological response in locally advanced rectal cancer (LARC).
**Methods and Materials:** 67 patients with LARC underwent 1,5 T MRI before, during and after CRT. High-resolution multiplanar T2w sequences were acquired. Cancer volume was manually segmented and the histogram analysis of T2SI in the entire lesion was performed with the extraction of the following parameters: 25th, 50th, 75th percentile, range,mean, standard deviation, skewness and kurtosis calculated.After surgery, patients were classified according to Rodel tumor regression grade (TRG) in Non-Responder (NR=TRG0-2), Partial Responder(PR:TRG3), Complete Responder (CR:TRG4).
**Results:** At histopatology 14 patients were NR, 37 PR and 16 CR. Any difference in T2SI texture parameters was found before and during treatment. After CRT, only T2SI mean value was different among classes of response, in particular it was significantly lower in CR compared to other groups (median,IQR: NR 530,7 +/-154,8, PR 526,9 +/- 233,7, CR 359,8+/195,4; p=0,008). Based on ROC analysis, at restaging MRI, a T2SI cut-off value of 363,93 was able to predict CR with an AUC of 72, a sensitivity of 78% and a specificity of 63 %.
**Conclusion:** Histogram-based analysis of whole tumour T2SI might provide a new insight in tumour heterogeneity and be a promising tool in the assessment of tumoural complete response after CRT.

**B-1346** 14:56
**Diffusion and perfusion MR parameters to assess preoperative short-course radiotherapy response in locally advanced rectal cancer**
R. Fusco, V. Granata, B. Pecori, A. Petrillo; *Naples/IT*
*(r.fusco@istitutotumori.na.it)*

**Purpose:** To assess preoperative short-course radiotherapy (SCR) tumour response in LARC by means of Standardized Index of Shape (SIS) by dynamic contrast-enhanced magnetic resonance imaging (DCE-MRI), apparent diffusion coefficient (ADC), intravoxel incoherent motion (IVIM) and diffusion kurtosis imaging (DKI) parameters derived from diffusion-weighted MRI (DW-MRI).
**Methods and Materials:** 34 patients who underwent MRI scans before and after SCR followed by delayed surgery were enrolled. SIS, ADC, IVIM parameters (tissue diffusion (Dt), pseudo-diffusion (Dp), perfusion fraction (fp)) and DKI parameters (mean diffusivity (MD), mean of diffusional kurtosis (MK)) were calculated for each patient. IVIM parameters were estimated using two methods, namely conventional bi-exponential fitting (CBFM) and variable projection (VARPRO). After surgery, the pathological TNM and tumour regression grade (TRG) were estimated.
**Results:** The best results to predict pCR were obtained by VARPRO Fp mean value pre-treatment with AUC of 0.84, a sensitivity of 96.4%, a specificity of 71.4% and an accuracy of 91.2%. The best results to assess after treatment pCR were obtained by SIS with an AUC of 0.89, a sensitivity of 85.7%, a specificity of 92.6% and an accuracy of 91.2%. Moreover, the best results to differentiate after treatment responders vs. non-responders was obtained by SIS with an area under ROC of 0.94, a sensitivity of 93.3%, a specificity of 84.2% and an accuracy of 88.2%.
**Conclusion:** SIS is a hopeful DCE-MRI angiogenic biomarker to assess preoperative treatment response after SCR with delayed surgery. Furthermore, an important prognostic role was obtained by VARPRO Fp mean value pre-treatment.

**14:00 - 15:30** **Sky High Stage**

### Radiographers

### MY 15

### Everyday challenges for radiographers and radiologists

*Moderators:*
J. Santos; Coimbra/PT
N.N.

**B-1350** 14:00
**Impact of body mass index on set-up variations and treatment margins for patients receiving radiotherapy to the prostate**
S. Farrugia, G. van Dijk, S. Mercieca; *Msida/MT*
*(sarah.farrugia.14@um.edu.mt)*

**Purpose:** The aim of the study was to evaluate the impact of body mass index (BMI) on set-up variations and planning target volume (PTV) margins for patients receiving radiotherapy for prostate cancer to ensure accurate treatment delivery.
**Methods and Materials:** The mean random (RV) and systemic set-up variation (SV) in the lateral, supero-inferior and antero-posterior directions were calculated from daily variations of 49 patients, selected through exhaustive sampling. Spearman rank correlation ($r_s$) was used to analyse the correlation between BMI and the SV and RV. The van Herk formula was used to calculate the PTV margin required.
**Results:** The mean SV was 1.4mm, 0.9mm and 1.4mm and the mean RV was of 1.7mm, 1.5mm and 1.8mm in the supero-inferior, lateral and antero-posterior directions, respectively. A significant correlation was calculated between patient BMI and overall set-up variations, with a negative correlation in the supero-inferior ($r_s$: -0.137, p value: <0.001) and the lateral directions ($r_s$:-0.158, p value <0.001), and a positive correlation in the antero-posterior direction ($r_s$: 0.065, p value: 0.020). Correlations between SV or RV and patient BMI were similar in direction, however, weaker and not statistically significant. The PTV margin for the new local protocol was 4.7mm in the supero-inferior direction, 3.4mm in the lateral direction and 4.9mm in the antero-posterior direction.
**Conclusion:** A uniform PTV margin of 5mm is suitable to treat prostate cancer. A positive correlation was noted in the antero-posterior direction for the BMI indicating that for patients with high BMI, reduction of margins needs to be more carefully evaluated.

**B-1351** 14:04
**An augmented patient-specific approach to administration of contrast agent for CT renal angiography**
C. Saade; *Beirut/LB (charbel.saade@aub.edu.lb)*

**Purpose:** Retrospective and prospective study performed on 200 consecutive patients undergoing renal CTA, investigates the opacification of renal vasculature, radiation dose, and reader confidence.
**Methods and Materials:** 100 patients were assigned retrospectively to protocol A and the other 100 were allocated prospectively to protocol B. Both protocols implemented a contrast material and saline flow rate of 4.5 mL/sec. Protocol A utilized a 100mL of low-osmolar nonionic IV contrast material(Ioversol 350 mg I/mL) while protocol B employed a patient-tailored contrast media formula using iso-osmolar non-ionic (Iodixanol 320 mg I/mL).
**Results:** Arterial opacification in the abdominal aorta and in the bilateral main proximal renal arteries demonstrated no statistical significance (p>0.05). Only the main distal renal artery of the left kidney in protocol B was statistically significant (p<0.046). In the venous circulation, the IVC demonstrated a significant reduction in opacification in protocol B (59.39 HU ± 19.39) compared to A (87.74 HU ± 34.06) (p<0.001). Mean CNR for protocol A (22.68 HU ± 13.72) was significantly higher than that of protocol B (14.75 HU ± 5.76 p<.0001). Effective dose was significantly reduced in protocol B (2.46 ± 0.74 mSv) compared to A (3.07 ± 0.68 mSv) (p<0.001). ROC analysis demonstrated significantly higher area under the ROC curve for protocol B (p< 0.0001), with inter-reader agreement increasing from moderate to excellent in renal arterial visualization.
**Conclusion:** Employing a patient-tailored contrast media injection protocol shows a significant refinement in the visualization of renal vasculature and reader confidence at reduced radiation dose during renal CTA.

**B-1352** 14:08
**Improving technical quality in mammography**
V. Risi[1], M. Zanardo[2], R.M. Trimboli[3], C.B. Monti[2], G. Di Leo[3], L.A. Carbonaro[3], F. Sardanelli[2]; [1]*Lumezzane/IT*, [2]*Milan/IT*, [3]*San Donato Milanese/IT* *(verarisi1@gmail.com)*

**Purpose:** To improve the technical quality of mammograms through personal information of radiographers.
**Methods and Materials:** Craniocaudal (CC) and mediolateral oblique (MLO) views of both breasts were evaluated using criteria defined by European guidelines. Presence of the pectoral muscle, nipple in axis and in profile, presence of inframammary angle, and skin folds were evaluated. Each mammography examination was evaluated using a 5-point Likert scale, ranging from 1 (lowest quality) to 5 (highest quality). While the entire group of radiographers was informed about the general performance, each of them received her personal data through a non-public report. Thereafter, the performance of the group was re-evaluated with the same criteria. Data were reported using median and interquartile range (IQR). Mann-Whitney *U*test and $\chi^2$test were used.
**Results:** A total of 156 mammographic studies were evaluated (78 pre-audit and 78 post-audit). Pre-audit CC views had a median score of 4 [IQR 3-4], with 107 (69%) good (score4) to excellent (score5) quality examinations, while post-audit CC views had a median score of 4 [IQR 4-4], with 134 (86%) good to excellent quality examinations ($p$<0.008). Pre-audit MLO views had a median of 4 [IQR 3-4], with 38 (24%) good to excellent quality examinations, while post-audit MLO views had a median score of 4 [IQR 3-4], with 93 (60%) good to excellent quality examinations ($p$<0.001).
**Conclusion:** Technical performance in mammography was significantly improved for both views by a means of personal information of each radiographer about her performance. High-quality MLO views remain a more challenging goal.

**B-1353** 14:12
**Reflective clinical portfolios as a tool to develop competence in radiography students**
M. Morris; *Limerick/IE (mikealamorris07@gmail.com)*

**Purpose:** Reflective clinical portfolios are used in undergraduate health science degree programmes to prepare students for their professional careers by promoting reflective practice and critical thinking. The current study investigated the impact of reflective clinical portfolios on the development of competence in Radiography students and which elements of reflective clinical portfolios students valued most.
**Methods and Materials:** An online survey was disseminated to undergraduate radiography students and recent graduates to gather their opinions on the impact of reflective portfolios on competence development. Recent graduates were defined as those who had been working for less than one year. A stratified sampling approach of second, third and fourth year undergraduates and recent graduates from an Irish University was adopted to represent the population.
**Results:** Tutor feedback and competency assessments were reported to be beneficial in developing competence. Respondents who chose self-reflections as the most beneficial were all in Stage 4 or qualified. Those who ranked this as the least beneficial were in Stage 2 or Stage 3. Modality objective lists were considered unbeneficial by 45% of respondents. The online e-portfolio was chosen as the preferred portfolio format by 62% of respondents. Challenges outlined with portfolio use included lack of guidance and technical problems.
**Conclusion:** There was a general consensus that the reflective clinical portfolio is a valuable learning tool in developing competence during the Radiography degree programme, despite some respondents' dissatisfaction with specific aspects of these tools. Areas for improvement of clinical portfolios have been identified for consideration by educators.

**B-1354** 14:16
**A comparative analysis of local diagnostic reference levels for adult projection radiography of the chest, abdomen and pelvis in Irish centres**
M. Malone, S.J. Foley, L.A. Rainford, R. Toomey; *Dublin/IE* *(mariamalone16@gmail.com)*

**Purpose:** Diagnostic reference levels (DRLs) are important tools for optimisation in identifying atypical doses for standard patients undergoing radiological examinations. This research aimed to determine why local diagnostic reference levels (LDRLs) for adult projection radiography of the chest, abdomen and pelvis vary significantly across Irish centres.
**Methods and Materials:** Stratified sampling was performed to select one site from each of four hospital categories (tertiary referral, regional, general and private). Dose-area product measurements, exposure factors, source-to-image distances and the use of filtration were retrospectively recorded in each site for chest (postero-anterior), abdomen (antero-posterior) and pelvis (antero-posterior) examinations. Systematic random sampling was employed to sample 30 patients from the total number of examinations performed three months prior to the date of data retrieval. The data were utilised to formulate LDRLs based on the 75$^{th}$ percentile of the distribution of dose data. Kruskal-Wallis tests were performed to comparatively analyse the data.
**Results:** Significant variations were observed in LDRLs established for radiographic examinations of the chest ($p$<0.001), abdomen ($p$=0.02) and pelvis ($p$<0.001), with differences of up to 86%, 85% and 130%, respectively, between the departments with the highest and lowest LDRL. The national diagnostic reference level (NDRL) for chest imaging was exceeded in the general department while the NDRLs for the abdomen and pelvis were exceeded in the regional site.
**Conclusion:** Imaging protocols, namely high mAs settings, low kVp settings and lack of additional filtration, can be attributed to the significant variations observed in LDRLs between the sites studied.

**B-1355** 14:20
**Dose optimisation in CT for cocaine body packing: where is the limit of extensive dose reduction?**
J. Aissa, L.M. Sawicki, E. Appel, P. Heusch, C. Thomas, G. Antoch, J. Boos; *Düsseldorf/DE (joel.aissa@med.uni-duesseldorf.de)*

**Purpose:** To evaluate the detection rate and image-quality in CT-body-packer-screening at different radiation dose levels and to determine a dose threshold that enables a reliable detection of incorporated body packs and incidental findings with a maximum of dose saving.
**Methods and Materials:** We retrospectively included 27 individuals who underwent an abdominal CT due to suspected body. CT images were reconstructed at different radiation dose levels of 50%, 10%, 5% and 1%. All 135 CT reconstructions were evaluated by three independent readers. Reviewers determined the presence of foreign bodies and evaluated the image quality using a 5-point ranking scale. Additionally, visualization of incidental findings was assessed.
**Results:** A threshold of 5% (effective dose 0.11±0.07 mSv) was necessary to correctly identify all 27 patients with suspected body packing (p<0.008). Extensive noise insertion to a dose level of 1% (0.02±0.01 mSV) led to false positive solid cocaine findings in three patients. Image quality was comparable between 100% and 50% (p<0.024). The threshold for correct identification of incidental findings was 10% of the initial dose (effective dose 0.21±0.13 mSv).
**Conclusion:** A dose reduction to 0.11 mSv seems sufficient for detection of incorporated cocaine body packets. However, a minimum effective dose of 0.21 mSv seems to be required to properly identify incidental findings.

**B-1356** 14:24
**Evaluation of radiology request forms**
S. Kitalia, J. Sakwa, E. Aswani, L. Kanamu; *Nairobi/KE* *(sheilakitalia@gmail.com)*

**Purpose:** Radiology request forms are essential communication tools used by doctors and clinicians referring patients for radiological investigation. Their importance, however, is highly underestimated.
**Methods and Materials:** Radiology request forms (RRF) formed the study sample. 295 request forms formed the sample size. 37 (12.54%) request forms were excluded because they entailed the use of unconventional papers. During data collection, systemic random sampling method was used and data were collected using observational checklist. The collected data were then analysed using statistical package of social sciences software and presented on pie charts, bar graphs and frequency tables for better understanding.
**Results:** The study revealed that radiology requesting practice is poor. In the 258 radiology request forms, none was completely filled. The least filled field were patients' address (9.63%), referring department (5.12%) and the hospital number (27.04%). The best-filled field in the study was patients' name at 97.33%. Age of the patient was filled in 67.00% of the forms. Date was blank in 88.93% of the request forms. 14.34% of the RRF had not indicated the examination requested. This study revealed that 59.84% of the RRF did not provide patients' clinical history. Only 1.62% of the forms provided patient's LMP. 62.09% of the RRF provided the referring clinician name. 48.98% of the forms had referring clinician's signature.
**Conclusion:** Radiological request forms are often omitting relevant patient information. It is important that clinicians be educated on the value of filling radiology request forms to provide relevant information to aid in proper patient diagnosis.

**B-1357** 14:28
**Obstetric ultrasound - knowledge and perception of pregnant women in Portugal**
R.A. Santos, R. Fernandes; *Coimbra/PT (rutemartinssantos@gmail.com)*

**Purpose:** To evaluate the knowledge and perception of pregnant women on obstetric ultrasound of diagnosis performed during pregnancy.
**Methods and Materials:** The study will be conducted by means of a questionnaire to pregnant women whose pregnancy surveillance is being performed in Portugal. It will be carried out at national level in public and private health units.
**Results:** Pregnant women are expected to have some knowledge about the objective of diagnostic obstetrical ultrasound but a lower level of knowledge regarding the parameters evaluated and safety of the examination and that the influence of the completion of an enlightenment/training session is results of the questionnaire responses.
**Conclusion:** The perception and knowledge of Portuguese pregnant women about diagnostic obstetric ultrasound is not yet studied and fundamental for the effectiveness of diagnosis and follow-up of pregnancy, as well as it is essential that pregnant women have an active participation in the change of the National Health Plan in Portugal, revision and extension to 2020.

**B-1358** 14:32
**Expectant mothers' perception of prenatal sonography in southeastern population in Nigeria**
H.C. Elugwu; *Nnewi/NG (elugwuemeka@yahoo.com)*

**Purpose:** The study was conducted to assess the perception of pregnant women to prenatal sonography and to investigate the factors affecting maternal satisfaction to prenatal sonography in some tertiary institutions in south-eastern Nigeria.
**Methods and Materials:** The study was a cross-sectional study that involved 150 patients. It was conducted by administering semi-structured questionnaires. The questionnaire contained a total of 17 questions consisting of 16 close-ended questions and 1 open-ended question. The respondents were pregnant women who presented for obstetric sonography in Federal Teaching Hospitals and Federal Medical Centers in south-eastern Nigeria. The data were analyzed descriptively using frequency tables and percentages.
**Results:** There was a high indication of clinical use of obstetric ultrasound such as the health and well-being of the foetus (35.3%), foetal age determination, and the expected date of delivery (24.7%). Most respondents (84.6%) perceived prenatal sonography as being necessary, and 66.7% of the patients considered the result of obstetric sonography to be reliable. Majority of the respondents (88.7%) considered the services rendered during their sonographic scan to be satisfactory.
**Conclusion:** Most of the women perceived prenatal sonography as necessary and reliable. The services rendered during sonography were also considered satisfactory by most women; however, most pregnant women did not know about diagnostic ultrasound safety during the prenatal period.

**B-1359** 14:36
**Mobile phones as a potential vehicle of nosocomial pathogens in radiology departments**
T.C.P.L. Guerreiro[1], L. Ribeiro[2], A.F. Abrantes[2], S. Rodrigues[2], R. Raposo[2], F. Soares[2], R.P.P. Almeida[2]; [1]*Santiago Do Cacém/PT*, [2]*Faro/PT (tatianaguerreiro35@gmail.com)*

**Purpose:** Mobile smart phones have become increasingly integrated into the practice of radiographers, and can be colonized by potential bacteria pathogens. For this reason, adherence to hand hygiene by radiographers is crucial to prevent nosocomial infections. The main goal of this study was to assess the Radiographers knowledge about hand hygiene and to investigate the potential level of contamination on their mobile phones.
**Methods and Materials:** A self-applied questionnaire about knowledge and attitudes regarding hand hygiene and the use of mobile phone during clinical practice was applied to 21 radiographers from a public hospital. Their phones were swabbed using a standardized technique to check the types of microorganisms and the amount of contamination of the mobile phones.
**Results:** 85% of radiographers received training about nosocomial infection control, 81% considers hand hygiene important to prevent the nosocomial infections, 100% admits to use mobile phone during the shift and 76% don't have special attention to his mobile phone hygiene. Microorganisms like *Staphylococcus* (positive coagulase and oxacillin resistant) and alpha-haemolytic *Streptococcus* were identified. A fungus suggestive of *Candida Albicans* was also found.
**Conclusion:** This study demonstrates that mobile phones are potentially vehicles for pathogenic bacteria in the radiology departments. Despite the radiographers are aware of the importance of hand hygiene, adequate knowledge and a positive attitude are needed for optimal compliance. Disinfection guidelines utilizing alcohol wipes for regular cleaning of mobile phones should be developed and implemented to reduce the transmission of pathogens.

**B-1360** 14:40
**Diffusion tensor imaging of the growth plate: a preliminary study**
O. Kvist[1], S. Diaz Ruiz[1], T. Dorniok[1], J. Sanmartin Berglund[2], C.-E. Flodmark[1]; [1]*Stockholm/SE*, [2]*Karlskrona/SE (ola.kvist@sll.se)*

**Purpose:** To determine the feasibility of diffusion-tensor imaging (DTI) and tractography of the distal femur for the evaluation of the physis maturation related to age and sex.
**Methods and Materials:** Forty adolescents, 14-17 years old, 5M/5F in each group were examined. Image acquisition using echo-planar DTI was performed on a 3T MRI scanner, 15 directions; b values of 0 and 600 s/mm². Fibre tracks were reconstructed using Philips Fibertrak with ROI in the physis of the distal femur in three separate slices. A minimum fractional anisotropy (FA) of 0.15 and a maximum angle threshold of 30° were used. FA and apparent diffusion coefficient (ADC) of fibre tracts were measured and evaluated against age and sex. The physis was visually graded on the b0 images as open (14, 4F/10M), partly open (13, 4F/9M) and closed (13, 12F/1M) and compared to FA and ADC.
**Results:** FA increased linearly with age (increase 0.1/year from 0.4 to 0.7). For females, plateau was reached at 16. FA values for males were lower (>0.2 before reaching maximum). ADC decreased non-linearly from 0.8·10-3 $mm^2$/s at age 14 to 0.5·10-3 $mm^2$/s at 17. ADC for males was larger than average at all ages with decreasing gender difference with age. Plotting FA and ADC against the grading type showed a linear increase in FA and a linear decrease in ADC, with a smaller gender-related difference (<0.1).
**Conclusion:** FA increases and ADC decreases with progressing physis maturation, earlier for females than for males. FA and ADC correlate well with visual grading.

**B-1361** 14:44
**Comparison of extremity cone beam-computed tomography and multidetector-computed tomography in injured children**
S. Tschauner, R. Marterer, E. Nagy, G. Apfaltrer, G. Singer, M. Riccabona, E. Sorantin; *Graz/AT (Sebastian.Tschauner@klinikum-graz.at)*

**Purpose:** To compare extremity cone beam-computed tomography (CBCT) and multidetector-computed tomography (MDCT) in injured children.
**Methods and Materials:** 61 CBCT examinations were prospectively acquired. 10 MDCT studies were completed in parallel, and 51 were retrospectively matched with MDCTs of the same region, age and sex. 7 study pairs were excluded due to differences in cast or metal implant presence. Optimized exposure protocols were used in both modalities to achieve a realistic comparison. Image quality assessment was performed quantitatively and qualitatively. Moreover, dose records were read out.
**Results:** Objective image quality measurements were superior in CBCT (noise and contrast-to-noise ratio, p<0.001). Subjective image quality ratings revealed more streak artefacts in CBCT (p<0.001), which degraded the CBCT image impressions with varying degrees. Overall, motion artefacts were infrequent, but only seen in CBCT. CT dose index (CTDIvol) was significantly lower in CBCT (p<0.001).
**Conclusion:** CBCT was able to achieve better objective image qualities at lower doses than MDCT in paediatric extremity imaging. However, CBCT image impression was commonly degraded by streak artefacts, which made the modality's advantages partly ineffective.
*Author Disclosures:*
**S. Tschauner:** Equipment Support Recipient; Braincon GmbH & Co KG.
**R. Marterer:** Equipment Support Recipient; Braincon GmbH & Co KG.
**E. Nagy:** Equipment Support Recipient; Braincon GmbH & Co KG.
**G. Apfaltrer:** Equipment Support Recipient; Braincon GmbH & Co KG.
**G. Singer:** Equipment Support Recipient; Braincon GmbH & Co KG.
**M. Riccabona:** Equipment Support Recipient; Braincon GmbH & Co KG.
**E. Sorantin:** Equipment Support Recipient; Braincon GmbH & Co KG.

**B-1362** 14:48
**Artificial intelligence in bone age assessment: accuracy and clinical efficiency of a novel fully automated algorithm for bone age assessment in comparison to the Greulich Pyle method**
C. Booz, I. Yel, L. Lenga, A. Al Kamali, S. Boettger, J.L. Wichmann, T.J. Vogl, M.H. Albrecht, B. Bodelle; *Frankfurt a. Main/DE (boozchristian@gmail.com)*

**Purpose:** To evaluate the accuracy and clinical efficiency of a novel artificial intelligence (AI) system for bone age (BA) assessment compared with the Greulich Pyle (GP) method.
**Methods and Materials:** Radiographs of left hand and wrist of 414 patients (mean chronological age 10.3 years, range 3-17 years) were retrospectively analyzed. Total BA was assessed independently by three blinded radiologists using the GP method. In comparison, an AI system automatically calculated total BA. Reference BA was determined by the consensus of two blinded experienced radiologists (28 and 32 years of experience in paediatric imaging) using the GP method. Root mean square deviation (RSMD), mean absolute difference (MAD), Pearson product-moment correlation and Bland-Altman plot were calculated for statistical analysis.
**Results:** Reference mean total BA was 9.9 years. AI-derived mean total BA was 10.0 years, mean total BA calculated by the three reviewers was 10.2 years. RSMD and MAD were significantly lower between AI-derived BA and reference BA (RSMD 0.2 years, MAD 0.1 years) than between BA calculated by the three radiologists and the reference standard (RSMD 0.7 years, MAD 0.5 years) (all comparisons, *p*<.001). There was significantly higher correlation between AI-derived BA and reference BA (r=0.99) than between BA assessed by the three radiologists and reference BA (r=0.90) (*p*<.001). Mean evaluation times were reduced by 87% using the AI system compared to reviewer-based BA assessments.
**Conclusion:** A novel AI system allows for accurate BA assessment and improves clinical efficiency by reducing evaluation times without compromising the accuracy compared with the GP method.

**B-1363** 14:52
**High-resolution MR imaging of the orbit in patients with retinoblastoma: prospective study correlated with histopathological results**
Y.S. Habib, A. Youssef, H.A. ElKiki, H.A. ElZomor, M.M. Rezk; *Cairo/EG (yomnahabib85@gmail.com)*

**Purpose:** To assess diagnostic accuracy of preoperative magnetic resonance (MR) imaging for the detection of tumour extent in patients with retinoblastoma.
**Methods and Materials:** Fifty-eight eyes in 30 girls and 27 boys with retinoblastoma (mean age at diagnosis was 23 months ±18.9) were reviewed on unenhanced T1-weighted, spin-echo T2-weighted, and gadolinium-enhanced T1-weighted MR images with and without fat suppression. MR imaging parameters such as anterior chamber hyperintensity, involvement of choroid, ciliary body, optic nerve, sclera, orbital fat, and pineal gland were determined.
**Results:** The accuracy of MRI in detection of choroidal invasion is 86.2% with sensitivity, 95.3% and specificity, 60%. Regarding detection of prelaminar optic nerve invasion, MRI has 60% sensitivity, 82.9% specificity, and 75.8% accuracy. Postlaminar optic nerve invasion was detected in 23 eyes with MRI accuracy, 86.2%; sensitivity, 85.2%; specificity, 87%. Of nine eyes with histologically proven scleral invasion, MRI has accuracy, 93%; sensitivity, 55.6%; specificity, 100%. Extraocular fat invasion was suspected on MRI in 5/58 eyes with accuracy, 98.3%; sensitivity, 100%; specificity, 98%. Anterior chamber enhancement correlated well with main MRI and histolopathology findings. Tumour size (maximum diameter in mm) was statistically associated with postlaminar optic nerve invasion (ρ = .002) and choroidal invasion (ρ = .007).
**Conclusion:** MR imaging shows promising role for tumour staging and detection of metastatic risk factors.

**B-1364** 14:56
**Should we still look for a correlation between ADC and SUV in lymphoma: a volumetric [18F] FDG-PET/MR study of paediatric Hodgkin's lymphoma**
M.G. Riga[1], G. Orsatti[1], A. Varotto[1], F. Crimì[1], M. Weber[2], R. Stramare[1], P. Zucchetta[1], E. Quaia[1], C. Giraudo[1]; [1]*Padua/IT*, [2]*Vienna/AT (mariagiovanna.riga@gmail.com)*

**Purpose:** Previous studies showed controversial results about the correlation between metabolic activity and cellular density in lymphomas. Thus, our aim was to investigate the relationship between ADCs and SUVs simultaneously acquired in paediatric patients with Hodgkin's lymphoma (HL) at staging, using a volume-based histogram analysis.
**Methods and Materials:** Paediatric patients with histologically proven HL were enrolled in this prospective study and underwent a whole body integrated 3T [18F]-FDG-PET/MR, including DWI, for staging. PET images were re-sliced according to the ADC maps and, for each patient, the lesion with the largest volume was selected. A volumetric region of interest (vROI) has been then drawn on the DWI images along the margins of the tumour including the entire lesion. The same ROIs were copied on the corresponding PET dataset. Pixel-based SUVs and ADCs were collected from each entire vROI. The Pearson correlation coefficient was applied to assess the relationship between SUVs' and ADCs' mean, median, skewness, and kurtosis.
**Results:** Fifteen paediatric patients (eight female; mean age 13.33±3.53 yrs) have been enrolled. According to Ann Arbor, four patients were stage I, seven stage II and four stage IIIS. The mean (±SD) lesion volume was 46.39±62cm$^3$. No significant correlation between ADCs' and SUVs' mean (r=0.003, p=0.990), median (r=-0.039, p=0.890), skewness (r=-0.116, p=0.682) or kurtosis (r =-0.085, p=0.763) emerged.
**Conclusion:** Our preliminary results showed that volumetric ADCs and SUVs simultaneously acquired are independent biomarkers in paediatric HL at staging. Future studies after chemotherapy are necessary to further assess this evidence and its clinical implications.

**B-1365** 15:00
**Diagnostic value of C-reactive protein and the influence of visceral fat in patients with obesity and acute appendicitis**
A.D. Do Amaral Castro[1], F.A. Sulla Lupinacci[1], S. P.Pribeiro[1], E. Kaiser Ururahy Nunes Fonseca[1], M.R. Peixoto[1], T. Larocca Skare[2], A.T. Sakuma[1], F. Yamauchi[1], A. Tachibana[1]; [1]*São Paulo/BR*, [2]*Curitiba/BR (felipesulla@gmail.com)*

**Purpose:** The purpose of this study is to assess sensitivity, specificity, positive predictive value (PPV), and negative predictive value (NPV) of CRP in patients with AA and their correlation to body mass index (BMI) and body fat composition.
**Methods and Materials:** This is a retrospective study based on clinical records and imaging studies of 191 subjects with histopathologically confirmed AA compared to 249 controls who underwent abdominal computed tomography (CT). Clinical and epidemiological data, BMI, and CRP values were extracted from medical records. CT scans were assessed for AA findings and body composition measurements.
**Results:** CRP values increased according to patients' BMI, with varying sensitivity from 79.78% in subjects with normal or lean BMI, 87.87% in overweight, and 93.5% in individuals with obesity. A similar pattern was observed for NPV: an increase with increasing BMI, 69.3% in individuals with normal or lean BMI, 84.3% in overweight, and 91.3% in individuals with obesity. There was a positive correlation between CRP and visceral fat area in patients with AA.
**Conclusion:** Variations exist for sensitivity, specificity, PPV, and NPV values of CRP in patients with AA, stratified by BMI. An increase in visceral fat area is associated with elevated CRP across the BMI spectrum.

**B-1366** 15:04
**Impact of added CT venography performed in combination with CT pulmonary angiography on the detection of deep venous thrombosis and relevant occult CT findings**
P.I. Douek, D.C. Rotzinger, R. Meuli, V. Dunet, S. Schmidt Kobbe; *Lausanne/CH (pauline.douek@gmail.com)*

**Purpose:** To assess the additional diagnostic value of CT venography (CTV) simultaneously performed with CT pulmonary angiography (CTPA) in the context of thromboembolic disease for the detection of deep venous thrombosis (DVT) and other relevant CT findings.
**Methods and Materials:** Patients older than 49 years referred for suspected pulmonary embolism (PE) from January 2015 to December 2016 and undergoing CTPA combined with CTV were included in this monocentric retrospective study. While one radiologist reviewed clinical records of all patients, two other radiologists blinded to all results independently analysed the CTV images in view of DVT of lower extremities and/or other relevant pathological findings visible, which were registered and classified into non-relevant or major with therapeutic consequences (yet unknown pelvic tumours or inflammation). Interobserver agreement for the detection of DVT was evaluated using the Cohen's kappa coefficient.
**Results:** Among 696 patients, 119 had PE (17.1%) and 54 had DVT (7.8%), of which 16 (2.3%) occurred without concomitant PE. Interobserver agreement kappa between the two readers was substantial (κ=0.78). CTV examinations led to the diagnosis of major incidental findings in 40 patients, of which 8 had PE associated or not with DVT, and 4 had only DVT. Additional CTV lead to a change of therapeutic management in 30 patients (4.3%), consisting of 16 DVTs without PE and 14 relevant pelvic findings with therapeutic consequences.
**Conclusion:** In patients with suspected PE, additional CTV performed with CTPA rarely enables the detection of DVT without concomitant PE or relevant underlying pelvic lesions with therapeutic consequences.

**B-1367** 15:08
**Reliability of on-call radiology residents' interpretations in detecting intracranial aneurysms on 640 slice multidetector computed tomography angiography**
M.B.H. Chaudhry, M. Azeemuddin, M.N. Ahmad, F. Mubarak, T.U. Haq; *Karachi/PK (mustafa.b.chaudhry@gmail.com)*

**Purpose:** To assess interobserver agreement (IOA) between on call resident(OR) and neuroradiologist (NR) in detecting intracranial aneurysms(IA), on 640-MDCTA, in patients referred from emergency department (ED).
**Methods and Materials:** Prospective study was performed between June 2016 to July 2017. Patients with history of vascular pathology, prior intervention or acute head trauma were excluded. Sample size of 71 was calculated, taking previous Kappa(κ) value of 0.84 between NR, for 320-slice MDCTA. All MDCTA were interpreted by senior OR, year 3 to 5 (PGY3 to PGY5). MDCTA were reinterpreted and finalised by NR, following morning.
**Results:** 77 patients, 39(52 %) males and 38(48 %) females were prospectively included. Mean age was 47.7 ±15.2 years. 24(31%) cases were read by PGY3, 32(41%) by PGY4 and 21(28%) by PGY5. 31(40%) cases were finalised by assistant professor, 19(25%) by associate professor and 27(35%) by professor NR. 42 IA in 29(38%) patients were reported by RR, while NR finalised 35 IA in 25(32.5%) patients on following morning. Discrepancy was noted in 4(14%) cases when OR over called 7(16.7%) IA. No IA was missed by OR on MDCTA. Mean height of IA is 8.3±7.3 mm and mean width is 6.9±6.5 mm. Strong IOA between OR and NR(κ = 0.88, CI 95%, p-value <0.001). Stratified κ-value for grade of OR[0.81(PGY3) vs. 1(PGY4) vs. 0.84(PGY5)]. Sensitivity of 640-MDCTA in detecting IA by OR is 1, specificity is 0.92, PPV is 86.2% and NPV is 100%
**Conclusion:** 640-MDCTA is reliable in diagnosing IA, if read by OR, ruling out the need for DSA in ED.

**B-1368** 15:12
**Forensic relevance and diagnostic impact of a novel 3D-reconstruction technique (cinematic rendering) of severe injuries in postmortem computed tomography**
J. Böven, J. Boos, R.S. Lanzman, P. Kröpil, Y. Klosterkemper, C. Thomas, G. Antoch, J. Aissa; *Düsseldorf/DE (judith.boeven@med.uni-duesseldorf.de)*

**Purpose:** Performance and forensic relevance of a novel, photorealistic 3D-reconstruction method used for visualisation of traumatic injuries in comparison to conventional postmortem computed tomography (PMCT).
**Methods and Materials:** In total 38 pathologies from 22 human cadavers undergoing whole-body PMCT after traumatic death were retrospectively analysed. PMCT was performed and all pathologies were reconstructed with 3D-cinematic rendering and CT slices in all 3 dimensions. Images were evaluated according to their expressiveness and judicial relevance by two forensic pathologists using a five-level scale (1: high expressiveness, 5: low expressiveness). In addition, readers decided whether cinematic rendering reconstructions are suitable for judicial reviews. Two radiologists analysed all images due to the detection rate of pathologies in cinematic rendering reconstructions and PMCT.
**Results:** Forensic estimation: mean expressiveness of cinematic rendering reconstructions: reader 1: 2.4±1.1 (range: 1-5); reader 2: 1.9±1.2 (range: 1-5); total: 2.1±1.2 (range: 1-5). Mean expressiveness of conventional PMCT: reader 1: 4.1±1.1 (range: 2-5); reader 2: 3.7±1.1 (range: 1-5); total: 3.9±1.1 (range: 1-5). Concerning forensic interpretation and demonstration aspects results showed that CR reconstructions were significantly more expressive than conventional PMCT (p<0.05). Regarding the radiologic detection rate of all pathologies the evaluation of both radiologists lead to no significant difference between 3D reconstructions and original CT slices for all pathologies.
**Conclusion:** There is no significant difference concerning the detection of pathologies by radiologists between PMCT and cinematic rendering reconstructions. For forensic pathologists, CR reconstructions are more helpful than standard PMCT for correlation with autopsy results and judicial reviews.

**B-1369** 15:16
**Neonatal chest X-ray**
S. Johansen; *Oslo/NO (safora.johansen@oslomet.no)*

**Purpose:** To our knowledge, there is little known about radiation optimization in neonatal chest X-ray imaging. The aim of this study was to consider radiographic techniques, protocols, and radiation dose in four countries to assess possible learning outcomes.
**Methods and Materials:** The protocols and exposure parameters from one hospital in Norway, Portugal, South Africa and Canada were collected and analysed. Dose area product (DAP) and effective dose (ED) from 200 neonatal chest X-ray examination were calculated.
**Results:** There were differences in protocols, field of view (FOV) and radiation dose. The highest and lowest median DAP (dose) belonged to Portugal and Norway, 13.7 and 9.0, respectively. The kV and mAs differed between 50 and 73 and 0.5 and 2.2 between the involved hospitals. The used inherent filter was highest in Norway (2.9mm Al) and lowest in Canada (2.5mm Al). Field of view (FOV) varied remarkably between the involved hospitals. The largest FOV was 891 $cm^2$ in Portugal and the smallest was 90 $cm^2$ in Norway.
**Conclusion:** The results show that the high kVp-low mAs protocol, with careful collimation, gives lower radiation dose and is, therefore, preferable in neonatal chest X-ray imaging. There is a strong need of standardization of neonatal chest X-ray protocol to avoid high dose to this patient group.

**14:00 - 15:30** **Tech Gate Auditorium**

### Abdominal Viscera

### SS 1501b

### Multimodality imaging for assessment of liver function and portal hypertension

*Moderators:*
M. Garbajs; Ljubljana/SI
N.N.

**B-1370** 14:00
**Quantitative assessment of liver function before hepatectomy using two-dimensional shear wave elastography correlated with indocyanine green clearance test**
R. Fu, T. Qiu, Q. Lu, Y. Luo; *Chengdu/CN (448939373@qq.com)*

**Purpose:** To find the possibility of quantitative liver function assessment before hepatectomy using non-invasive two dimensional shear wave elastography (2D-SWE) by analysing the correlation between liver stiffness measured by 2D-SWE examination and the results of indocyanine green (ICG) clearance test, and to determine the cutoff point of liver stiffness value to differentiate normal and abnormal liver function.
**Methods and Materials:** We retrospectively studied 236 patients who were examined by 2D-SWE and ICG clearance test before liver resection. Liver stiffness of background parenchyma and ICG retention rate at 15 minutes (ICG-R15) were noted.
**Results:** Liver stiffness values of ICG-R15 < 10% group(median: 10.0 Kpa, IQR: 7.1-13.5Kpa, range: 4.5-31.0Kpa) were significantly smaller than that of ICG-R15 ≥ 10% group(median: 14.2 Kpa, IQR: 11.3-19.0Kpa, range: 5.3-38.2Kpa), with analysis of Mann-Whitney U test (p < 0.0001, Z=-4.292). Spearman correlation test showed the significant correlation between liver stiffness and ICG-R15(r= 0.427, *p*< 0.0001). Receiver operating characteristic (ROC) curve showed a liver stiffness cutoff point of 11.0 Kpa to determine normal or abnormal liver function, with sensitivity of 84.4%, specificity of 68.0%, and area under the ROC curve (AUC) of 0.742 (*p*< 0.05).
**Conclusion:** 2D-SWE examination exhibits promising performance for quantitative assessment of liver function reserve before hepatectomy, and has good efficiency to differentiate normal and abnormal liver function.

**B-1371** 14:08
**Liver function evaluation with Gd-EOB-DTPA-enhanced MRI: can visual scoring replace signal measurement?**
S. Xie, J. Li, H. Wang, W. Shen; *Tianjin/CN (xiess1989_happy@163.com)*

**Purpose:** To assess liver function with Gd-EOB-DTPA-enhanced MRI using visual score of liver parenchyma and portal vein signal difference at hepatobiliary phase in comparison with relative liver enhancement (RLE).
**Methods and Materials:** This retrospective study included 52 patients (Child-Pugh class A, n=26; B, n=15; and C, n=11). Visual difference in Signal intensity (SI) between liver parenchyma and portal vein at hepatobiliary phase images was evaluated using a 5-point scale (5, liver SI was obviously higher than that of portal vein; 4, liver SI slightly higher than portal vein; 3, liver SI equal to portal vein; 2, liver SI slightly lower than portal vein; 1, liver SI obviously lower than portal vein). Pre- ($SI_{pre}$) and postcontrast ($SI_{20min}$) liver SI were measured using ROI placement. RLE calculated as follow: RLE=$SI_{20min}/SI_{pre}$.
**Results:** Both Visual score and RLE were decreased as the progression of liver function damage. Visual score (Child-Pugh class A, 4.80±0.50; B, 3.47±0.99; C, 2.45±0.93; P<0.001) and RLE (Child-Pugh class A, 2.01±0.31; B, 1.50±0.24; C, 1.30±0.14; P<0.001) had significant differences between different liver function groups. Pairwise comparison showed visual score and RLE had significant difference between Child-Pugh class A and B (P=<0.001, <0.001), A and C (P=<0.001, <0.001), no significant difference between B and C (P=0.233, =0.447).
**Conclusion:** In Gd-EOB-DTPA-enhanced MRI, visual score of liver parenchyma and portal vein signal difference at hepatobiliary phase can be easily used for liver function evaluation, and had similar value to RLE.

**B-1372** 14:16

**Assessing liver function: diagnostic efficacy of parenchymal enhancement and a liver volume ratio of Gd-EOB-DTPA enhanced MRI studies**

D. Ippolito[1], A. Pecorelli[1], L. Riva[2], C. Maino[1], A. de Vito[1], I. Salemi[1], M. Porta[1], C. Talei Franzesi[1], S. Sironi[3]; [1]*Monza/IT*, [2]*Missaglia/IT*, [3]*Bergamo/IT* *(davide.atena@tiscali.it)*

**Purpose:** To assess whether Gd-EOB-DTPA enhanced-MRI study is useful to determine liver function in comparison to Child Pugh (CP), Model for End-stage Liver Disease (MELD) score and biochemical test.
**Methods and Materials:** We retrospectively reviewed all Gd-EOB-DTPA enhanced-MRI studies performed between May 2010 and September 2016. Patients were divided in study and control group according to the presence or absence of liver cirrhosis. Liver volume was calculated using the software Intellispace Portal(Philps) on hepatobiliary phase. The rate of liver-to-muscle ratio was calculated on T1 sequence in portal(irPOR) and hepatobiliary phase(irHEP) and than normalized for liver volume(irPOR/LV and irHEP/LV).
**Results:** 303 Gd-EOB-DTPA enhanced-MRI studies were included in the study. Cirrhosis was present in 175 (57.8%) cases. IrHEP was significantly lower in cirrhotic than in non-cirrhotic patients(0.54±0.28 vs 0.66±0.39,p=0.001). When considering only cirrhotic patients, irHEP significantly correlates with both CP and MELD score (R= -0.316,p<0.0001 and R= -0.170,p=0.010 respectively). The irHEP progressively decreased from CP-A to CP-C (0.59±0.29 to 0.25±0.19,p<0.0001) and from MELD≤10 to MELD 19-24 (0.58±0.29 to 0.45±0.41, p=0.030). Same results were observed when irHEP was normalized for liver volume. Among biochemical parameters total bilirubin, GOT and albumin had the strongest correlation both with irHEP and irHEP/LV (R=-0.278 and-0.285,R=-0.277 and -0.291;R= 0.282 and 0.262 respectively). None correlations were found with irPOR.
**Conclusion:** Gd-EOB-DTPA enhanced-MRI studies significantly correlates with clinical score used to evaluate liver function. In clinical practice this imaging technique could be used to correctly characterize focal liver lesion.

**B-1373** 14:24

**MR relaxometry in chronic hepatitis b and c: the contribution of Molli T1 mapping and T2 mapping in hepatic function evaluation with Gd-EOB-DTPA-enhanced magnetic resonance**

D. Kuru Oz, E. Peker, M. Kul, I. Erden, A. Erden; *Ankara/TR* *(digdem_k@hotmail.com)*

**Purpose:** To investigate the contribution of pre-and postcontrast T1 mapping with Gd-EOB-DTPA, obtained with different MOLLI sequences and T2 mapping to the evaluation of hepatic function in patients with chronic hepatitis B/C (CHB/C).
**Methods and Materials:** A total of 41 patients with CHB/C were included in this study. T1 mapping before (MOLLI5(3)3, 3(3)3(3)5, MOLLI3(2)3(2)5 and MOLLI5(3)3 adjusted for heart rate(ahr)) and 20 min after administration of Gd-EOB-DTPA (MOLLI4(1)3(1)2, MOLLI3(3)3(3)5, MOLLI3(2)3(2), MOLLI4(1) 3(1)2(ahr)) and T2mapping were performed. Pre- and postcontrast T1 relaxation times of the liver (T1 liver), changes between pre-and postcontrast T1 liver (ΔT1liver), adjusted postcontrast T1 liver (postcontrast T1 liver-T1 spleen/T1 spleen) and T2 values were evaluated among Child-Pugh stages. To differentiate Child-Pugh A-B stages ROC analysis was used.
**Results:** Of the 41 patients 29 were in the Child-Pugh A, 12 in the Child Pugh B group. No significant relationship was found between the Child-Pugh stage and precontrast T1-T2 relaxation times in any sequence (p>0.005). Postcontrast T1 mapping, ΔT1 liver and adjusted postcontrast T1 liver values were significantly different between Child-Pugh A-B groups (p≤0.001). For ΔT1 liver the cutoff values, sensitivity, specificity and accuracy were for MOLLI5(3)3-MOLLI4(1)3(1)2; 0.625, 100%,65%,76%, for MOLLI3(3)3(3)5-MOLLI3(3)3(3)5 and MOLLI3(2)3(2)5- MOLLI3(2)3(2)5; 0.643, 100%,65%,76%, for MOLLI5(3)3-MOLLI4(1)3(1)2(ahr); 0.589, 90%,69%,76%, respectively. The cutoff value of adjusted postcontrast T1 liver, sensitivity, specificity and accuracy for MOLLI5(3)3-MOLLI4(1)3(1)2 was -0.558, 90%,88%,89%, respectively. The cutoff values of adjusted postcontrast T1liver were for MOLLI3(3)3(3)5-MOLLI3(3)3(3)5; -0.587, for MOLLI3(2)3(2)5-MOLLI3(2)3(2)5; -0.581, for MOLLI5(3)3-MOLLI4(1)3(1)2(ahr); -0.561, with sensitivity, specificity and accuracy of 88%,88%,88%, respectively.
**Conclusion:** Postcontrast T1 liver, ΔT1 liver and adjusted postcontrast T1 liver may provide additional information regarding the clinical severity in CHB/C patients.

**B-1374** 14:32

**Predicting liver failure after extended liver resection following portal vein embolisation with gadoxetic acid-enhanced MRI**

D. Theilig, I. Steffen, T. Denecke, B. Hamm, D. Geisel; *Berlin/DE*

**Purpose:** Predicting post-hepatectomy liver failure (PHLF) after major liver resection following portal vein embolisation (PVE) by means of sequential gadoxetic acid-enhanced MRI scans.
**Methods and Materials:** 36 patients who underwent extended liver resection following PVE were evaluated prospectively with gadoxetic acid-enhanced MRI scans at set intervals during the course of their treatment, that is, before, 14 days and 28 days after PVE as well as 10 days after extended liver resection. Relative enhancement (RE) and liver volume of the left and right liver were determined. The study population was divided into two groups with respect to signs of PHLF according to the PHLF severity grading system of the International study group of liver surgery. Differences between the two groups were assessed using Mann-Whitney U Test and ROC curve analysis.
**Results:** RE of the left lobe prior to PVE versus 14 days after PVE was significantly lower in patients with PHLF than in those without PHLF (MWU test p< 0.001) and proved to be the best predictor of PHLF in ROC analysis with a AUC of 0.854 (p<0.001) and a cut-off value of -0.044 with a sensitivity of 75.0% and a specificity of 92.6%.
**Conclusion:** Gadoxetic acid-enhanced MRI as an imaging-based liver function test performed before and after PVE can help to predict PHLF. The risk of PHLF can be predicted as early as 14 days after PVE underscoring the hypothesis that it may be feasible to perform extended liver resection earlier after PVE than the current standard.
*Author Disclosures:*
**T. Denecke:** Author; Drs. Geisel, Hamm and Denecke have received travel support and honoraria from Bayer AG. **B. Hamm:** Author; Drs. Geisel, Hamm and Denecke have received travel support and honoraria from Bayer AG.
**D. Geisel:** Author; Drs. Geisel, Hamm and Denecke have received travel support and honoraria from Bayer AG.

**B-1375** 14:40

**Shear wave dispersion slope obtained by shear wave elastography could help detect allograft abnormality after liver transplantation**

D. Lee, J. Lee, J. Bae; *Seoul/KR (dhlee.rad@gmail.com)*

**Purpose:** To evaluate whether shear wave dispersion slope could help detect allograft abnormality after liver transplantation.
**Methods and Materials:** From Jan 2018 to Sep 2018, we prospectively enrolled 93 patients who underwent liver transplantation. Before liver biopsy, shear wave elastography evaluation was done, and both stiffness (kilopascal, kPa) and shear wave dispersion slope ([m/s]/kHz) were obtained. Allograft abnormality was diagnosed by histopathologic examination. Mann-Whitney U test and receiver operating characteristic analysis were used to evaluate relationship between allograft abnormality and parameters derived from shear wave elastography evaluation.
**Results:** Allograft abnormality was detected in 43 patients (43/93, 46.2%): acute cellular rejection, n=27; cholangitis, n=9; reperfusion injury, n=1; reactivation of viral hepatitis, n=2; non-alcoholic steatohepatitis, n=1; alcoholic hepatitis n=1; and chronic rejection, n=2. Mean liver stiffness value with allograft abnormality was 10.4±8.7 kPa, and significantly higher than liver stiffness value without allograft abnormality of 6.4±1.7 kPa (P<0.001). Mean shear wave dispersion slope was 15.7±5.6 [m/s]/kHz in 43 patients with allograft abnormality, and significantly higher than 10.9±2.3 [m/s]/kHz in 50 patients without allograft abnormality. The area under the curve for the prediction of allograft abnormality was 0.853 for shear wave dispersion slope, and significantly higher than 0.744 for stiffness value (P=0.0013). The optimal cut-off value of shear wave dispersion slope to detect allograft abnormality was set at 10.8 [m/s]/kHz with sensitivity of 97.7% (42/43) and specificity of 60% (30/50).
**Conclusion:** Shear wave dispersion slope obtained from shear wave elastography could be useful to detect allograft abnormality after liver transplantation.

**B-1376** 14:48

**Spleen ARFI: a noninvasive tool for detection of oesophagal varices in cirrhotics**

M. Rajasekaran[1], R. Rathinasamy[2], M. Viyannan[2], D. Bala Lakshmoji[2]; [1]*Bangalore/IN*, [2]*Coimbatore/IN (muthusubramanian1989@gmail.com)*

**Purpose:** Current guidelines recommend all cirrhotic patients should undergo upper gastrointestinal endoscopy (UGI scopy) screening for detection of oesophageal varices. This imposes heavy burden on endoscopy units and decreases patient compliance. Thereby, this study was performed to determine the utility of spleen ARFI as non-invasive predictor for the detection of oesophageal varices and to assess the efficacy of spleen ARFI in differentiating low-grade from high-grade oesophageal varices.
**Methods and Materials:** Consecutive 50 cirrhotic patients with EVs and 50 cirrhotic patients without EVs, who had undergone screening UGI scopy were included. B mode ultrasonography and acoustic force impulse imaging (ARFI) elastography was done with Siemens Acuson S2000™/Acuson S3000 ™ ultrasound systems. Demographic, clinical, bio-chemical, ultrasonography and elastography parameters were recorded. Independent sample 't' test and Chi-square test were performed to identify predictors of EVs and high-grade EVs. Cut-offs of significant indicators were determined by ROC analysis.

**Results:** Spleen ARFI cut-off of ≥3.16m/s (AUROC: 0.906) precisely detects EVs with 94% sensitivity and 92% specificity. Diagnostic accuracy of spleen ARFI in detection of EVs is the highest of all the noninvasive parameters analysed in this study. Furthermore, exclusively spleen ARFI cut off of ≥3.29m/s (AUROC: 0.874) can confidentially differentiate low-grade vs high-grade varices with 85% sensitivity and 100% specificity.
**Conclusion:** Spleen ARFI can be used as a noninvasive tool for the detection of oesophageal varices and differentiating high-grade vs low-grade oesophageal varices, with high degree of diagnostic accuracy in cirrhotic patients.

**B-1377** 14:56
**The extent of portosystemic collaterals depends on portal pressure and hepatic dysfunction rather than on PlGF-driven neoangiogenesis**
K. Lampichler, L. Reider, B. Simbrunner, A. Stadlmann, M. Mandorfer, T. Bucsics, R. Paternostro, U. Asenbaum, T. Reiberger; *Vienna/AT (katharina.lampichler@meduniwien.ac.at)*

**Purpose:** Despite a growing body of evidence from experimental studies, the impact of hepatic dysfunction and neoangiogenesis on portosystemic collaterals/shunts (PSS) has yet to be systematically investigated. Placental growth factor (PlGF) is a pro-angiogenic factor that has been linked to liver fibrosis and portal hypertension in animal studies. We assessed the association of PSS with portal pressure, hepatic dysfunction and PlGF levels in patients with chronic liver disease (CLD).
**Methods and Materials:** 107 patients with CLD were prospectively enrolled. The severity of portal hypertension (PHT) was assessed by hepatic venous pressure gradient (HVPG). Hepatic dysfunction was evaluated by ALBI and Child-Pugh score (CPS). PSS were semiquantitatively classified as mild, moderate or severe on contrast-enhanced CT and MRI scans.
**Results:** The extent of PSS was significantly associated with HVPG (mild:12 vs. moderate:19 vs. severe:15 (median; mmHg);p=0,0095), portal vein thrombosis (3,9% vs. 26,3 % vs. 44%;p=0,0002), ascites (25,5% vs. 47,4% vs. 33,3%;p<0,0001), hepatic encephalopathy (13,7% vs. 34,2% vs. 11,1 %; p=0,0337), FIB-4 score (3,9 vs. 5,2 vs. 8,8;p=0,0001) and ALBI score (-2,41 vs. -1,96 vs. -1,9; p=0,0021). There was no significant difference between PlGF levels and extent of PSS. PlGF levels showed positive correlations with HVPG (r=0,2348, p=0,0149) and ALBI (r=0,3144, p=0,001) and increased in parallel to CPS (A:22,09 vs. B:25,12 vs. C:39ng/dl; p=0,0001). PlGF levels were higher in patients with PVT (22,38 ng/dl vs. 27,3 ng/dl; p=0,0163).
**Conclusion:** The extent of PSS is mainly determined by portal pressure, hepatic dysfunction and fibrosis/cirrhosis. PlGF-driven neoangiogenesis was not associated with PSS formation.

**B-1378** 15:04
**A comparative study of sound touch elastography measurement on liver's right lobe and left lobe for diagnosis of chronic liver disease using liver biopsy as gold standard**
I. Gatos[1], P. Drazinos[2], S. Yarmenitis[3], I. Theotokas[1], A. Kanavaki[1], E. Manesis[1], P. Zoumpoulis[2]; [1]*Athens/GR*, [2]*Kifissia/GR*, [3]*Marousi/GR (p.zoumploulis@echomed.gr)*

**Purpose:** Chronic Liver Disease (CLD) is currently one of the major causes of death. Sound Touch Elastography (STE) that is available in Resona 7 Ultrasound (US) device and is similar to Shear Wave Elastography (SWE), seems promising for CLD diagnosis but needs to be validated. Standard STE examination protocol involves measurements on the Right Lobe (RL) when feasible. An alternative measurement on Left Lobe (LL) can be performed but has not been yet evaluated. The aim of this study is to compare the performance of STE on liver's RL and LL in CLD diagnosis using Liver Biopsy (LB) as 'gold standard'.
**Methods and Materials:** 163 subjects, 56 normal (F0) and 107 with CLD (F1-F4), were included in the study. A B-Mode and Elastographic examination was performed on each patient with Resona 7 US device. STE measurements were performed on the RL and LL of each patient. STE measurements on each lobe were compared to LB results (Metavir Stage: F0-F4). Receiver Operating Characteristic analysis was then performed on each set of STE measurements (RL and LL) to obtain best cut-off stiffness values.
**Results:** ROC analysis for each lobe (RL/LL) showed: $Accuracy_{RL}$ / $Accuracy_{LL}$=0.9476/0.9524 for F=F4, $Accuracy_{RL}$ / $Accuracy_{LL}$=0.9476/0.9429 for F≥F3, $Accuracy_{RL}$ / $Accuracy_{LL}$=0.9619/0.9619 for F≥F2, and $Accuracy_{RL}$ / $Accuracy_{LL}$=0.8524/0.8190 for F≥F1. Best cut-off stiffness values for each lobe (RL/LL) were: F=F4: 13.7/14.25 kPa, F≥F3: 9.5/9.45 kPa, F≥F2: 9.15/9.05 kPa, F≥F1: 6.15/6.85 kPa respectively.
**Conclusion:** This study shows that STE LL measurements, when feasible, are reliable for CLD diagnosis and can be used as an RL measurement alternative.
*Author Disclosures:*
**I. Gatos:** Equipment Support Recipient; Mindray is cooperating with Diagnostic Echotomography SA in relation with a Clinical Study. A Resona 7 Ultrasound system has been provided by Mindray in order to carry out this study. **P. Drazinos:** Equipment Support Recipient; Mindray is cooperating with Diagnostic Echotomography SA in relation with a Clinical Study. A Resona 7 Ultrasound system has been provided by Mindray in order to carry out this study. **S. Yarmenitis:** Equipment Support Recipient; Mindray is cooperating with Diagnostic Echotomography SA in relation with a Clinical Study. A Resona 7 Ultrasound system has been provided by Mindray in order to carry out this study. **I. Theotokas:** Equipment Support Recipient; Mindray is cooperating with Diagnostic Echotomography SA in relation with a Clinical Study. A Resona 7 Ultrasound system has been provided by Mindray in order to carry out this study. **A. Kanavaki:** Equipment Support Recipient; Mindray is cooperating with Diagnostic Echotomography SA in relation with a Clinical Study. A Resona 7 Ultrasound system has been provided by Mindray in order to carry out this study. **E. Manesis:** Equipment Support Recipient; Mindray is cooperating with Diagnostic Echotomography SA in relation with a Clinical Study. A Resona 7 Ultrasound system has been provided by Mindray in order to carry out this study. **P. Zoumpoulis:** Equipment Support Recipient; Mindray is cooperating with Diagnostic Echotomography SA in relation with a Clinical Study. A Resona 7 Ultrasound system has been provided by Mindray in order to carry out this study.

**B-1379** 15:12
**The primary study of low-dose 640-slice volume liver perfusion CT: a whole-organ perfusion in patients with cirrhosis of the liver before and after portosystemic shunting**
N. Djuraeva, A. Ikramov, N. Vakhidova, A. Amirkhamzaev, K. Shamirzaev, A. Sultanov; *Tashkent/UZ (sidikovan@yandex.ru)*

**Purpose:** To study and compare the perfusion parameters of the liver in patients with cirrhosis using volumetric low-dose computed tomographic perfusion of the liver before and after portosystemic shunting.
**Methods and Materials:** 50 patients with cirrhosis (28M and 22F) aged 43±1.5 years, weighing 65±3.5 kg were included. The control group without liver pathology comprised 10 people. All patients underwent volume low-dose (16-cm detector width) liver perfusion, a contrast medium content of 40 ml, Kv 100/80, MA 200/150, effective radiation dose (E) of 17 mSv (20±1, 2). E was calculated using DLP (mGy*cm) multiplied by e; e is the dose coefficient for the corresponding anatomical region (0.015mSv/mGy*cm).
**Results:** The parameters of hepatic arterial blood flow (HABF), hepatic portal blood flow (HPBF), hepatic perfusion index (HPI) in the control group were HABF 36 ml/min/100 ml (±5), HPBF 120 ml/min/100ml (±15), HPI 39%±4. Differences of HABF, HPBF, HPI before and after portosystemic shunting were statistically significant (P≤0.05). The parameters of HPI after portosystemic shunting increased from 20% (±7.5) to 27% (±6), HABF from 34.9 ml/min/100 ml (±12.5) to 49.5ml/min/100ml (±15), HPBF decreased from 144.3 ml/min/100ml (±17.5) to 128.3 ml/min/100ml (±15). The width of portal vein after portosystemic shunting was reduced by 16-20% (±3mm), splenic vein increased by 16-20% (±3mm).
**Conclusion:** Volume low-dose (17mSv) CT perfusion of the liver is a non-invasive method that allows quantitative assessment of statistically significant changes (P ≤0,05) in liver perfusion after portosystemic shunting in patients with cirrhosis of the liver.

**B-1380** 15:20
**Comparison of liver stiffness evaluated by shear wave elastography in morbidly obese patients prior to bariatric surgery with liver biopsy**
F. Rajabzadeh, A. Jangjoo, T. Jamialahmadi, L. Goshayeshi, M. Nemati, K. Ghafarzadegan; *Mashhad/IR (farnood@yahoo.com)*

**Purpose:** Non-alcoholic fatty liver disease is a prevalent condition among severely obese patients. It can gradually progress to liver fibrosis. Shear wave elastography has been validated as a noninvasive diagnostic tool for liver stiffness measurement in a wide range of liver disorders associated with fibrosis. However, technical feasibility and accuracy of this method are still under debate in severely obese patients. We aimed to assess the diagnostic accuracy of shear wave elastography (SWE) as a non-invasive tool by comparing it with that of liver biopsy as the standard test in bariatric surgery candidates.
**Methods and Materials:** A total of 111 severely obese candidates for bariatric surgery were recruited. Liver stiffness was measured using SWE fourteen days before liver biopsy. Liver biopsy was taken on the day of surgery. The area under the receiver operating curve (AUROC) was calculated for the staging of liver fibrosis. Liver biopsy was considered as the gold standard.
**Results:** As the fibrosis stage progressed, the median liver stiffness measurement (LSM) increased on 2D-SWE. Patients with a higher fibrosis stage exhibited LSMs with markedly higher median values than those of subjects with less severe fibrosis (all p values < 0.05).
**Conclusion:** Non-invasive LSM with SWE is a viable option in severely obese patients. Using SWE, patients with NASH could be distinguished from those with NAFLD. Finally, SWE may be considered as a diagnostic tool in clinical evaluation before bariatric surgery.

**14:00 - 15:30** **The Church**

### Genitourinary

### SS 1507

### Imaging in pregnancy and female tumours

*Moderators:*
M. Basta Nikolic; Novi Sad/RS
G. Masselli; Rome/IT

**B-1381** 14:00
**MRI diagnostic accuracy of placental adhesion disorders and diagnostic impact on foetal-maternal outcomes in interventional radiology-assisted delivery**
F. Monelli, F. Fiocchi, A. Pecchi, F. Casari, E. Bertucci, E. Petrella, P. Torricelli; *Modena/IT (mofilippo@hotmail.it)*

**Purpose:** Assess accuracy and reproducibility of MRI and specific findings in the diagnosis of invasive placentation (IP) in high-risk patients and to evaluate the impact of interventional radiology (IR) assistance on delivery outcomes.
**Methods and Materials:** 21 patients (mean age 36.05) with clinical/echographic risk factors for invasive placentation underwent 1.5T MRI examination. Images were reviewed by two readers. Gold standard was histology or obstetric evaluation at the delivery. Sensitivity and specificity of MRI findings were calculated, and reproducibility was estimated with Cohen's K-test. Impact of eventual IR assistance was evaluated regarding multiple maternal-child factors during and after delivery.
**Results:** Incidence of IP was 61.90% (of placenta percreta was 14.29%). MRI had sensitivity of 100% (0.7530-1.0000) and specificity of 87.5% (0.4735-0.9968). Gold standard was histology in 10 cases and obstetric evaluation in 11. MRI findings with higher sensitivity were placental heterogeneity, uterine bulging and thinning of the uterine-placental interface. Findings with higher specificity were uterine scarring, placental heterogeneity and myometrial interruption. MRI inter-rater agreement with Cohen's K was 1 (1.0000-1.0000). IR assistance was performed on 11 of the 14 patients diagnosed with IP and it had a positive impact on almost every outcome evaluated with a statistical significance ($p<0.05$) in terms of blood loss, red cell transfusion, APGAR at 1st and 10th minute and risk of being transfused.
**Conclusion:** MRI is an accurate, reproducible and useful tool in prenatal diagnosis of IP in particular to plan a safe and appropriate delivery with eventual IR assistance, which positively affects delivery outcomes.

**B-1382** 14:08
**Role of shear wave elastography of placenta in the prediction of preeclampsia in high-risk pregnancies**
M. Singh[1], T. Singh[2], V. Singla[2], V. Jain[2]; [1]*Kurukshetra/IN*, [2]*Chandigarh/IN (mandeep.virk12@gmail.com)*

**Purpose:** We aimed to compare placental elasticity values between normal pregnancies and preeclamptic pregnancies and evaluate the utility of shear wave elastography of placenta as a predictor for preeclampsia.
**Methods and Materials:** A prospective analytical study was performed with 90 singleton pregnancies. Patients having any of the 7 risk factors for developing preeclampsia [primigravida, past h/o preeclampsia, family history of preeclampsia, h/o pregestational diabetes, chronic hypertension, advanced maternal age (≥40) and BMI ≥26] were enrolled in the study. Shear wave elastography was performed in all patients at 20-24wks gestation and at 34-36wks gestation, at two sites - center and edge of placenta. The patients were divided into two groups - normal pregnancies (group A) or developed preeclampsia (group B). Women with posterior placentation, obstetric disorders other than preeclampsia or multiple gestation were excluded from the study.
**Results:** Shear wave elasticity values at 20-24wks gestation for group B at centre of placenta (21.73 vs 9.72kPa) as well as at edge of placenta (21.6kPa vs 10.15kPa) were significantly higher than those for group A ($P < .05$). Similar results were seen at 34-36wks gestation. The best results were obtained with edge of placenta reading at 34-36wks gestation; using cut-off of 13.1kPa, we attained sensitivity of 95.2%, specificity of 92.8% and diagnostic accuracy of 93.3% for predicting the development of preeclampsia.
**Conclusion:** Patients with preeclampsia have significantly higher stiffness of the placenta. Shear wave elastography is useful to evaluate placental function. Elastography can be used as a supplementary tool to existing methods for prediction of preeclampsia.

**B-1383** 14:16
**Imaging detection of placenta adhesion disorder in patients with placenta previa: comparison between US and MR findings**
A. Volpe, V. Romeo, M.I. Ginocchio, S. Maurea, P.P. Mainenti, M. Petretta, L. Sarno, P. Martinelli, A. Brunetti; *Naples/IT (valeria.romeo@unina.it)*

**Purpose:** The diagnostic performance of ultrasound (US) and magnetic resonance (MR) in the detection of placental adhesion disorders (PAD) in patients with placenta previa (PP) is not currently established; the aim of this study was to compare US and MR imaging findings to detect PAD in patients with PP.
**Methods and Materials:** Fifty-one patients with PP underwent US and MR examinations. The presence of US and MR imaging findings suggestive of PAD was assessed. Penalized maximum likelihood logistic regression was performed in order to identify US and/or MR imaging findings independently associated to PAD, considering histology as standard of reference. Only variables statistically significant ($p<0.05$) at univariate analysis were considered for multivariate analysis. Receiver operating characteristic curve (ROC) analysis was performed and the area under the curve (AUC) was calculated to determine the predictive value of the significant identified variables on imaging studies.
**Results:** At univariate analysis, loss of retroplacental clear space, smallest myometrial thickness and placenta lacunar spaces on US as well as intraplacental dark bands, focal interruption of myometrial border and abnormal vascularity on MR were statistically significant ($p<0.01$); of these, only intraplacental dark bands at MR ($p=0.002$) was independently associated to PAD at multivariate analysis. ROC curve analysis showed an AUC value 0.942 for MR significantly higher as compared to that (0.798) of US ($p=0.02$).
**Conclusion:** MRI demonstrated a significantly higher diagnostic accuracy to detect PAD in patients with PP as compared to US with intraplacental dark bands being the most reliable imaging finding.

**B-1384** 14:24
**MRI criteria and sequence evaluation for the diagnosis of placental adhesive disorder among radiologists with different reporting experience**
J.P. Zawaideh[1], J. Smith[2], A. Bruining[3], P.L. Moyle[2], E. Sala[2], H.C. Addley[2], S. Freeman[2]; [1]*Genoa/IT*, [2]*Cambridge/UK*, [3]*Amsterdam/NL (jeriespz89@gmail.com)*

**Purpose:** To determine the most useful MR sequence to accurately diagnose placental adhesion disorders (PAD). To assess inter-reader differences in accuracy, sensitivity, and specificity for specific MRI characteristics, based on experience.
**Methods and Materials:** Retrospective review of placental MRI of 27 women (17 with PADs) was undertaken by 5 radiologists: 2 junior radiologists: 2- and 5-year MRI-reporting experience; 3 senior radiologists: >8-year experience (although 1 without obstetric-radiological background). All readers evaluated the presence of previously identified MRI features (dark T2W bands, placental heterogeneity, uterine bulging, abnormal vascularity, myometrial thinning, retroplacental dark zone loss, tenting of the bladder and direct invasion of adjacent pelvic structures), scored the overall degree of placental invasion (normal placenta, placenta accreta/increta or percreta) and chose the most useful/diagnostic MRI sequence. For each reader overall accuracy, sensitivity and specificity were calculated.
**Results:** The senior radiologists' accuracy was significantly higher than that of juniors, 83.3% and 67.9%, respectively. Presence of dark T2W bands was the most sensitive criteria for diagnosing PAD (92.9%), followed by uterine bulging (82.7%). Abnormal vascularity, myometrial thinning and loss of retroplacental dark zone manifested high sensitivity for the senior radiologists but demonstrated low sensitivity for the least experienced reader. All readers considered sagittal T2w SSFSE the most useful sequence. Steady-state sequence and DWI were of limited use in diagnosis.
**Conclusion:** The preferred MR sequence to assess PAD was sagittal SSFSE. Overall the most reliable imaging feature is the presence of T2W dark bands. However, the diagnostic value significantly depends upon the reader's experience.

**B-1385** 14:32
**Comparison of diagnostic accuracy between clinical risk factors and MRI to predict placental adhesion disorders in patients with placenta previa**
S. Maurea, M.I. Ginocchio, F. Verde, V. Romeo, P.P. Mainenti, M. Petretta, L. Sarno, P. Martinelli, A. Brunetti; *Naples/IT (valeria.romeo@unina.it)*

**Purpose:** In patients with placenta previa (PP) clinical risk factors have been suggested to predict the occurrence of placental adhesion disorders (PAD). The aim of this study was to assess the diagnostic accuracy of clinical risk factors and magnetic resonance imaging (MRI) to predict PAD in PP.
**Methods and Materials:** Seventy patients with PP who underwent MRI on the basis of ultrasound suspicion of PAD were evaluated. The presence of clinical risk factors and MRI signs suggestive of PAD was assessed. Univariate analysis was performed to identify clinical risk factors and/or MRI signs independently associated to PAD considering histology as standard of reference. Only variables statistically significant (p<0.05) were considered for multivariate analysis. Receiver operating characteristic curve (ROC) analysis was performed and the area under the curve (AUC) was then calculated.
**Results:** At univariate analysis, smoking, previous cesarean section (PCS), intraplacental dark bands (IDB), focal interruption of myometrial border (FIMB) and abnormal vascularity were statistically significant; of these, only IDB (p<0.001) and FIMB (p <0.05) were independently associated to PAD at multivariate analysis. ROC curve analysis showed an AUC value of 0.62 for smoking and PCS and of 0.90 when considering the presence of DB and FIMB at MRI (p < 0.05); an AUC value of 0.92 was observed when the combination of MRI signs along with smoking and PCS was considered (p = ns).
**Conclusion:** Clinical risk factors do not improve the diagnostic accuracy of MRI to predict PAD in patients with PP.

**B-1386** 14:40
**Epithelial ovarian cancer: tumour staging and detection of lymph node metastasis using ADC histogram parameters**
F. Wang, J. Liu, Y. Wang, Y. Zhou, C. Liu, D. Liang, L. Xie; *Beijing/CN (wfeng1223@sina.com)*

**Purpose:** The purposes of this study were to investigate the potential of ADC histogram parameters in epithelial ovarian cancer (EOC) for distinguishing different tumour stages and lymph node status, and to correlate ADC values with p53 and Ki-67 expression.
**Methods and Materials:** 43 patients with pathologically proven EOC underwent preoperative MRI (including DWI). Staging and lymph node status were determined postoperatively. ADC values were calculated using histogram analysis. The following histogram metrics were obtained: mean, median, 10th and 90th percentile. Ki-67 labeling index (LI) was defined as the percentage of positive cells, while p53 expression was categorised as wild-type and mutated p53. ADC parameters were compared among different tumour groups (staging and lymph node status) using one-way ANOVA test and independent sample t test. The latter was also used to compare ADCs between two p53 types, while Pearson correlation analysis was used to correlate Ki-67 LI with ADCs.
**Results:** ADC parameters differed significantly between stage I and II, I and III, I and IV (P<0.05). ADC parameters were significantly lower in lymph node-positive than in lymph node-negative group (P<0.05). There were significant negative correlations between ADC parameters and Ki-67 LI (r was -0.420, -0.356, -0.446 and -0.436 for mean, 10th, 90th and median, respectively, P<0.001). ADCs were all significantly lower in mutated p53 than in wild-type p53 (P<0.001).
**Conclusion:** ADC histogram analysis can help discriminate stage I EOC from advanced stage EOC and detect lymph node metastasis. ADCs were well correlated with Ki-67 LI and may be indicate p53 expression.

**B-1387** 14:48
**A mathematical-descriptor of tumour mesoscopic structure from CT images annotates prognostic and molecular phenotypes of epithelial ovarian cancer**
A.G. Rockall[1], P. Lee[1], H. Lu[1], M.A. Arshad[1], G. Avesani[2], E. Curry[1], A. Thornton[1], F. Kanavati[1], C. Fotopoulou[1], E.O. Aboagye[1]; [1]*London/UK*, [2]*Mantova/IT (philippa.lee08@gmail.com)*

**Purpose:** CT images can be quantitatively analysed to describe intuitive features such as shape and texture. The features generated from such analyses (i.e. texture analysis or radiomic data) have been associated with prognosis and cellular pathways in many cancer types. In this study, we aimed to investigate the clinical value of radiomic data as a potential biomarker in serous ovarian cancer (OC).
**Methods and Materials:** Following ethical approval, we annotated 364 CT scans of women with OC including expert segmentation of disease, clinical and comprehensive molecular information (fresh frozen tissue and blood) with copy number profile, proteomic and molecular subtype of cases. Using TextLab 2.0, 657 quantitative mathematical descriptors were extracted. Using machine learning, we derived a non-invasive summary-statistic of the primary ovarian tumour based on 4 descriptors, which we name 'Radiomic Prognostic Vector' (RPV).
**Results:** RPV reliably identifies the 5% of patients with median overall survival less than 2 years, significantly improves established prognostic methods, and the finding was validated in two independent, multi-centre cohorts. Genetic, transcriptomic and proteomic analysis from two independent datasets elucidate that stromal phenotype and DNA damage response pathways are activated in RPV-stratified tumours, consistent with a biological correlate for RPV. RPV demonstrates a significant difference in outcome in patients undergoing complete cytoreductive surgery.
**Conclusion:** RPV and its associated analysis platform could be exploited to guide personalized therapy of OC and may help inform triage of patients for cytoreductive surgery and tailoring of surgical radicality. It is potentially transferable to other cancer types.

**B-1388** 14:56
**Adnexal cystic lymphangioma in patients with massive leiomyomatous uterus: a not so uncommon finding**
C. Bourgioti, K. Chatoupis, A. Antoniou, C. Tzavara, E. Panourgias, M. Vergetidou, L. Moulopoulos; *Athens/GR (kostas.chatoupis@bayer.com)*

**Purpose:** To investigate any association between the presence of an adnexal cystic lymphangioma (ACL) and an enlarged leiomyomatous uterus.
**Methods and Materials:** A retrospective observational study was conducted by two expert radiologists with consensus using a 10-year MRI database (July 2008-June 2018); eighty-five patients (mean age: 45.5 years) were considered eligible due to the presence of a single (n=31) or multiple (n=54) leiomyomas causing marked uterine enlargement. The association of specific leiomyoma features (maximum diameter,location,number) or uterine volume with the presence of ACL was statistically tested. Chi-square tests and Fisher's exact tests were used for the comparisons of proportions; student's t-tests and Mann-Whitney tests were used for the comparison of continuous variables between subjects with and without ACL.
**Results:** ACL (unilateral=9, bilateral=8) was recorded in 17/85 (20%) of the cases; it was more frequently observed when the largest leiomyoma was located in the uterine fundus (33.3%). Patients with ACL had significantly more leiomyomas (median:6vs2,p=0.001), greater maximum diameter of largest leiomyoma (median:13.3vs7.2cm,p<0.001) and larger uterine volumes (median:676.7vs223.1cm$^3$,p<0.001) compared to patients without ACL. ROC curve analysis for number of leiomyomas showed that the optimal-cut-off for the prediction of ACL was the presence of 5 leiomyomas with 53.8% sensitivity and 84% specificity [area under the curve (AUC)=0.65, 95%CI:0.51-0.83, p=0.049 ); for the uterine volume, the optimal-cut-off was 310cm$^3$ with 71% sensitivity and 75% specificity (AUC=0.79, 95%CI:0.66-0.92,p<0.001).
**Conclusion:** The presence of ACL is significantly associated with increased number of leiomyomas and larger uterine volume; follow-up studies are needed to investigate its clinical significance.

**B-1389** 15:04
**MRI correlates of molecular classification of endometrial cancer: a preliminary study**
M. Bonatti[1], R. Valletta[2], F. Lombardo[2], G. Avesani[3], F. Erdini[1], G. Negri[1], G. Zamboni[2], G. Schifferle[1], G. Bonatti[1]; [1]*Bolzano/IT*, [2]*Verona/IT*, [3]*Mantova/IT (matteobonatti@hotmail.com)*

**Purpose:** To evaluate the MRI features of the different molecular classes of endometrial carcinoma (EC).
**Methods and Materials:** IRB-approved retrospective study. We included 22 consecutive patients who underwent preoperative MRI and hysterectomy for EC in our institution between Jan 2017 and Sep 2018. Two readers independently evaluated 15 qualitative MRI parameters of EC including growth pattern (polypoid/infiltrative), tumour margins (sharp/irregular) and peritumoural T2-hyperintensity (yes/no); discrepancies were solved by consensus. A third reader manually segmented EC on para-axial T2-weighted images and ADC maps using commercially available software (LIFEx); neoplasm volume and radiomic features including first and second order textural parameters were calculated. MRI data were compared with the molecular subgroup (1-4) assigned by the pathologist according to p53 and POLE mutations and microsatellites instability.
**Results:** The pathologist classified 4 EC in group1, 7 in group 2, 8 in group 3 and 3 in group 4. Interobserver agreement in qualitative parameters evaluation was 0.79 for peritumoural T2-hyperintensity, 0.53 for growth pattern and 0.39 for tumour margins. None of the qualitative parameters showed a statistically significant correlation with the molecular subclass of EC. Group 2 ECs were significantly larger than others (median 30.5cc vs 2.1cc). Grey-level co-occurrence matrix homogeneity and grey-level run length matrix of T2-weighted images were significantly higher in group 2 ECs than in others; none of the other first- and second-order textural parameters reached statistically significant differences.
**Conclusion:** MRI showed promising results in differentiating molecular subgroups of EC; larger cohort studies must be performed to reach statistical significance.

**B-1390** 15:12
**The potential of IVIM model as a marker of normal placenta microstructural changes during pregnancy: a foetal MR study**
A. Antonelli[1], L. Manganaro[1], S. Capuani[1], R. Grimm[2], B. Kuehn[2], A. de Rinaldis[2], C. Catalano[1]; [1]*Rome/IT*, [2]*Erlangen/DE (amanda.antonelli@hotmail.it)*

**Purpose:** To investigate the potential of intravoxel incoherent motion (IVIM) model in the quantification of placenta micro-perfusion obtaining apparent diffusion coefficient D, perfusion fraction fp and pseudo-diffusion coefficient D*.
**Methods and Materials:** 35 normal placental MR examination using a clinical 1.5 T scanner (Siemens Healthcare, Erlangen, Germany) were enrolled. The study protocol included a diffusion-weighted spin echo-EPI prototype sequence using 10 different b values (0, 10, 30, 50, 75, 100, 150, 400, 700, 1000 s/mm$^2$). 'Siemens MR Body Diffusion Toolbox' prototype software was used to obtain D, f and D* maps. For each placenta, six ROIs were manually placed on both foetal and maternal side: central ROI (C-ROI), peripheral ROI (P-ROI) and umbilical ROI (U-ROI). Pearson test with Bonferroni correction was performed to study the correlation between D, D*, f and GA, body mass index (BMI) and basal glycaemia (bG).
**Results:** The fraction of perfusion fp in foetal umbilical and in foetal peripheral ROIs are significantly higher (U: p <0.0000049; P: p<0.048) than in maternal umbilical and maternal peripheral ROIs, reflecting that foetal placenta is richer in villi than mother's side. In the foetal side, there are statistically significant differences between umbilical and central ROIs and between umbilical and peripheral ROIs. D and D* showed significantly different values in foetal umbilical and peripheral ROIs. In the maternal side, umbilical fp is significantly higher than peripheral fp.
**Conclusion:** Results are in substantial agreement with physiological placenta micro-perfusion phenomena during gestation. IVIM parameters of non-pathological placenta are powerful and helpful tools able to quantify the physiological microperfusion of placental parenchyma, reflecting its physiological maturation during gestation. In clinical practice, those data will be helpful to better assess placental abnormalities.

**B-1391** 15:20
**The role cerebroplacental ratio in prediction of foetal outcome among patients with pre-eclamsia**
S.F. Kenis, J.B. Igashi, D. Ibe, A. Saliu, N.B. Adams, I. Aliyu; *Zaria/NG (shedrackkenis@yahoo.com)*

**Purpose:** To evaluate the value of cerebroplacental ratio as predictors of foetal outcome among patients with pre-eclampsia and to determine the cut-off birth weight below which brain sparing response occurs.
**Methods and Materials:** Cross-sectional study involving ninety patients with pre-eclampsia. Patients underwent Doppler examination of the umbilical artery and middle cerebral artery. The foetal outcomes were also noted. The sensitivity, specificity, positive predictive value and negative value of the cerebroplacental ratio to predict adverse foetal outcome were computed. The relationship between the cerebroplacental ratio and the birth weight ratio (BWR: birth weight divide by mean birth weight for the same gestational age) was determined.
**Results:** The sensitivity, specificity, PPV and NPV of MCARI/UARI in predicting perinatal outcome were determined. The point of intersection of the curve of the relationship between MCARI/UARI and the BWR with the line for all normal foetuses >=30 weeks (MCAR1/UARI=1.08) corresponds to BWR = 0.78. Above this cut-off, increasing the BWR does not affect the MCARI/UARI, i.e. no variation in cerebroplacental ratio. Below this cut-off, however, the lower the BWR the lower the cerebroplacental ratio (i.e the severer the brain sparing response). The mean MCARI/UARI ratio of foetuses with birth weight ratio <0.78 (0.77±0.32) is significantly lower than the mean MCARI/UARI of foetuses with birth weight ratio of >=0.78 (1.29±0.33).
**Conclusion:** Cerebroplacental ratio shows good test performance in the prediction of perinatal outcome. It appears that birth weight ratio of less than 0.78 is the critical weight below which brain sparing response appears.

**16:00 - 17:30** **Room G**

### Artificial Intelligence and Machine Learning

### SS 1605

### Machine learning: genitourinary and diverse

*Moderators:*
N.N.
M. Benndorf; Freiburg/DE

**B-1392** 16:00
**Can artificial intelligence predict prostate cancer local stage on unenhanced MRI? A preliminary report**
R. Cuocolo, A. Stanzione, A. Ponsiglione, V. Romeo, S. Cocozza, A. Brunetti, M. Imbriaco; *Naples/IT (renato.cuocolo@gmail.com)*

**Purpose:** To identify the best treatment option for prostate cancer patients, an accurate local staging is crucial and currently MRI is the recommended imaging modality. Aim of our study was to assess the performance of different machine learning algorithms applied to texture analysis features extracted from unenhanced MRI in the prediction of prostate cancer local stage using radical prostatectomy specimens as reference standard.
**Methods and Materials:** In all, 39 patients were enrolled in this retrospective study. Manual segmentation of MRI index lesions was performed on both T2-weighted images and ADC maps for texture analysis data collection. Features of first, second and higher order were extracted from both the original images and after filter application (wavelet transform). Combinations of different feature selection methods and machine learning classifiers were tested, and their performance was compared to a baseline accuracy reference (zero rules algorithm).
**Results:** The classifier showing the best performance was the Bayesian Network, using the dataset obtained by the Subset Evaluator feature selection method. It showed a percentage of correctly classified instances of 82%, an AUC of 0.88, a true-positive rate of 0.82 and a true-negative rate of 0.80. These values were found to be significantly higher compared to the ones obtained by the baseline reference algorithm.
**Conclusion:** A combined machine learning and texture analysis approach appears as a feasible tool to predict histopathological local stage of prostate cancer on biparametric MR images.

**B-1393** 16:08
**CT based machine learning radiomics for differentiating tumour grade in clear cell renal cell carcinoma**
X. Yi, Y. Zhang, L. Liu; *Changsha/CN (yixiaoping@csu.edu.cn)*

**Purpose:** To develop and validate CT-based machine learning Radiomics method for preoperative prediction of low and high WHO/ISUP grades in clear cell renal cell carcinoma (ccRCC).
**Methods and Materials:** A single-center retrospective review was conducted on 264 ccRCC patients [training cohort: 199; validation cohort: 65], which were sub-classified into two groups: low-grade (Grade I and II) and high-grade (Grade III and IV). Machine learning Radiomics based on the least absolute shrinkage and selection operator (LASSO) method, Random forest (RF) method, and Support vector machine (SVM) method, was used. The area under the curve (AUC) of operating characteristics (ROC) was used to evaluate their performance.
**Results:** Based on texture features, the SVM classifier (Score 3) yielded an AUC value of 0.8170 (95% CI: 0.7353-0.8987) in the training cohort and 0.8017 (95% CI: 0.6878-0.9157) in the validation cohort. Based on traditional radiological features, a new SVM classifier (Score 6) achieved an AUC of 91.75 (95% CI: 0.8765-0.9585) in the training cohort and 0.8088 (95% CI: 0.7064- 0.9113) in the validation cohort. The SVM 3 method (Score 7), constructed on the basis of Scores 3 and 6, provided an AUC of 0.9235 (95% CI: 0.8646-0.9824) in the training cohort and a AUC of 0.9099 (95% CI: 0.8324-0.9873) in the validation cohort.
**Conclusion:** A machine learning Radiomics method of CT demonstrated improved performance in predicting low or high WHO/ISUP grade for ccRCC preoperatively, which may potentially affect treatment strategies.

**B-1394** 16:16
**Differentiation of clear cell and non-clear cell renal cell carcinomas by all-relevant radiomics features from multiphase CT**
G. Liu, G. Wu; *Shanghai/CN (sdzclgq@126.com)*

**Purpose:** To develop a radiomics model with all-relevant imaging features from multiphasic computed tomography (CT) for differentiating clear cell renal cell carcinoma (ccRCC) from non-ccRCC, and to investigate the underlying molecular basis of the all-relevant imaging features by assessing their possible radiogenomics link with a key ccRCC driver gene, the VHL gene mutation.
**Methods and Materials:** In this retrospective two-center study, 255 patients (188 ccRCC and 67 non-ccRCC) were included. 156 radiomics features were extracted from standardized triphasic CT. Two radiomics models were built using random forest from a training cohort (170 patients), where one model was built with all-relevant features selected by Boruta algorithm and the other with features selected by minimum-redundancy maximum-relevance ensemble (mRMRe) algorithm. The potential radiogenomics association that linked the selected imaging features with the VHL mutation were investigated. All models were tested on an independent validation cohort (85 patients).
**Results:** Correlation with gold standard volume measure was greatest for the automated algorithm (95 % confidence interval for R2 is 0.962 to 0.988), followed by measures of seeding (0.773 to 0.923), ellipse (0.755 to 0.916), area (0.645 to 0.872), diameter (0.491 to 0.803), and perimeter (0.491 to 0.803). Phantom studies on partial volume regions showed the accuracy of the partial volume measurement was 5% (c.f. 13% for repeated area measurements).
**Conclusion:** All-relevant radiomics features in corticomedullary phase CT can be used to differentiate ccRCC from non-ccRCC with high accuracy. These subtype-discriminative imaging features were found to be significantly associated with the VHL gene mutation.

**B-1395** 16:24
**Influence of segmentation margin on machine learning-based high-dimensional quantitative CT texture analysis: an experimental radiomics study on renal clear cell carcinomas**
B. Kocak, E.S. Memis Durmaz, E. Ates, M. Baykara Ulusan, O. Kilickesmez; *Istanbul/TR (drburakkocak@gmail.com)*

**Purpose:** To determine the influence of slight differences in segmentation margin on reproducibility of each step of the machine learning (ML)-based high-dimensional quantitative computed tomography (CT) texture analysis (qCT-TA).
**Methods and Materials:** For this retrospective study, 47 patients with renal clear cell carcinomas (RcCCs) were included from a public database. Two segmentations were obtained by two radiologists for each tumour: (i) contour focused and (ii) margin shrinkage of 2mm. Using PyRadiomics, 828 texture features were extracted from filtered and non-filtered CT images. Interobserver reproducibility was assessed by intraclass correlation coefficient (ICC). Attribute selection was done using correlation-based feature selection. ML classifier was *k*-nearest neighbors. Classifications were performed with and without using synthetic minority oversampling technique (SMOTE). Main performance metric was the area under the curve. Reference standard was nuclear grade.
**Results:** The segmentation with margin shrinkage of 2mm (772 of 828) yielded more texture features with excellent reproducibility (ICC≥0.9) than contour-focused segmentation (714 of 828). The attribute selection algorithms resulted in different subset of texture features for two segmentation data with only one common feature. Either with or without SMOTE, all ML-based models based on contour-focused segmentation (AUC range 0.865-0.984) performed better than those with shrinkage of 2mm (AUC range 0.745-0.887), $p<0.05$.
**Conclusion:** Each step of a ML-based high-dimensional qCT-TA was susceptible to the slight change of 2mm in segmentation margin. Despite yielding less features with excellent reproducibility, use of the contour-focused segmentation provides better classification performance in qCT-TA of RcCCs for distinguishing nuclear grade.

**B-1396** 16:32
**To categorise ovarian epithelial cancers and predict clinical outcome using multiparametric radiomics**
H. Zhang, X. Liu, P. Zhang, G. Zhang; *Shanghai/CN (dr.zhanghe@yahoo.com)*

**Purpose:** To evaluate the ability of multiparametric MRI radiomics in categorising ovarian epithelial cancer and determine whether MRI radiomics are associated with survival in ovarian cancer patients.
**Methods and Materials:** The total 280 patients with pathologically proven OEC were retrospectively included in this study. Axial T1WI, sagittal fat-suppressed T2WI, coronal T2 WI and apparent diffusion coefficient map were chosen as the premium picture for the lesion segmentation. We calculate the diagnostic performance of the signatures derived from MRI radiomics in differentiating both between ovarian benign and malignancies and Type 1 and Type II ovarian epithelial cancers. Least absolute shrinkage and selection operator (Lasso) method is used for feature selection for determining the associations between MRI radiomics and survival data.
**Results:** For the classification between benign and malignancy, MRI radiomics model achieves an area under the receiver operating characteristic curve of 0.97 and accuracy of 0.90 in leave-one-out cross-validation. The result of independent validation cohort is 0.95 of AUC and 0.87 of accuracy. For the classification between Type 1 and Type II subtypes, our method achieves an AUC of 0.86, an accuracy of 0.93 in leave-one-out (LOO) cross-validation and an AUC of 0.85 and an accuracy of 0.84 in independent validation cohort. Patients with higher risk scores are more likely to have poor prognosis (HR = 3.891, $p < 0.000$).
**Conclusion:** Radiomic features extracted from MRI are highly correlated with ovarian cancer classification and prognosis of patients. Multiparametric MRI radiomics can provide ovarian cancer diagnosis and prognosis estimation with high accuracy.

**B-1397** 16:40
**Automated multiregional prostate segmentation in magnetic resonance using deeply supervised convolutional neural networks**
R.L. González[1], V. Venugopal[2], A. Jimenez-Pastor[1], L. Marti-Bonmati[1], A. Alberich-Bayarri[1], M. Barnwal[2], V. Mahajan[2]; [1]*Valencia/ES*, [2]*New Delhi/IN (rafaellopez@quibim.com)*

**Purpose:** A CNN-based automatic prostate segmentation method is proposed, aiming to identify and differentiate central-transitional and peripheral prostate glands as well as seminal vesicles.
**Methods and Materials:** A total of 131 axial T2-weighted MR prostate examinations were acquired in different 3T machines and with different acquisition protocols. The central and peripheral glands and the seminal vesicles were manually labelled in all the acquired T2-weighted series by an expert to train the models. A deeply supervised U-Net-based architecture was used to train this network with the Dice score coefficient as cost function and Adam as optimization algorithm. To maximize the performance of the CNN, a cyclic learning rate was used during the training stage. Also, image processing algorithms were used to further refine the predicted segmentation masks during inference.
**Results:** The clinical validation was performed on a different set of 25 T2-weighted cases from an external centre which was not part of the training set. The segmentation results from the network were compared and corrected by an expert radiologist to match best truth. Finally, the Dice score coefficient between the model's predictions and the expert corrected masks was calculated. The scores for the central-transitional gland, peripheral gland, seminal vesicles and background were 0.92±0.03, 0.90±0.05, 0.91±0.05, and 0.99±0.00, respectively.
**Conclusion:** Fully automated multiregional segmentation of the prostate gland and seminal vesicles can be addressed by deeply supervised CNN. This step will help localizing prostatic lesions and characterizing the pattern of prostatic enlargement.
*Author Disclosures:*
**R.L. González:** Employee; Quibim. **A. Jimenez-Pastor:** Employee; Quibim. **A. Alberich-Bayarri:** Employee; Quibim.

**B-1399** 16:56
**Deep learning with a convolutional neural network for predicting Fuhrman grade of clear cell renal cell carcinoma at MSCT**
X. Li[1], W. Li[2]; [1]*Nanjing/CN*, [2]*Shanghai/CN (lixiao19901020@yeah.net)*

**Purpose:** To investigate diagnostic performance by using a deep learning method with a convolutional neural network for the prediction of histologic grade in clear cell RCC at dynamic contrast agent-enhanced computed tomography.
**Methods and Materials:** Approved clinical retrospective study from January 2013 to July 2017, patients with clear cell RCC were identified from the pathology database. 69 low grade ccRCC patients and 28 high grade ccRCC patients. All patients were examined with a 320-slice dynamic volume CT by using same four phase renal protocol (unenhanced phase, corticomedullary phase, nephrographic phase and excretory phase). The CNN was composed of four convolutional layers, four maximum pooling layers, a flatten layer and three fully connected layers. The process used in building models was consistent with section one. The diagnostic performance of CNN models in differentiating grade of ccRCC were evaluate.
**Results:** The mean AUC fordistinguishing low-grade ccRCC and high-grade ccRCC inmodel unenhanced, model corticomedullar, model nephrographic, model excretory, model enhanced and model quadruple with the test data cohorts was 0.55 (95% CI: 0.49,0.61), 0.77 (95% CI: 0.74,0.79), 0.76 (95% CI: 0.74,0.79), 0.69 (95% CI: 0.66,0.72), 0.78 (95% CI: 0.76, 0.79), 0.75 (95% CI: 0.74, 0.77).
**Conclusion:** Our results show that maybe the CNN models except model unenhanced could be used to differentiate the Fuhrman grade of ccRCC at dynamic CT. This technique requires further validation on a larger scale prior to implementation into clinical practice.

**B-1400** 17:04
**Feature rejection for radiomic analyses based on variations of tumour segmentation masks**
B. Schachtner, M. Ingrisch, E. Gresser, M.J. Schneider, A. Schreier, O. Solyanik, G. Magistro, D. Nörenberg; *Munich/DE (Balthasar.Schachtner@med.lmu.de)*

**Purpose:** Radiomics tools as e.g. pyradiomics can easily produce more than 1000 features, some of which depend strongly on small variations to the boundary line of the segmentation of the region of interest. The approach presented rejects those without the need to specify the target class of the classification problem or using a test-re-test procedure.
**Methods and Materials:** A dataset of 86 patients with prostate carcinoma was used in a radiomic analysis. Segmentations of tumours were performed by expert radiologists and 1482 features were extracted with the pyradiomics package. Variations of these features were calculated using masks dilated and eroded by one pixel in the plane of high resolution. The impact of these variations on the features with respect to the distribution of the features was evaluated using the intraclass correlation coefficient (ICC). The impact on classification performance was tested using a simple machine learning setup with minimum-redundancy maximum-relevancy (mRMR) feature selection and a random forest in cross-validation.
**Results:** In a simple machine learning setup the auROC could be raised by 10 percentage points from not using the feature rejection to using a cut on ICC of 0.83.
**Conclusion:** Feature rejection based on variations of the segmented masks provides a target-class agnostic way to reject unstable features and may help to improve the performance of classification algorithms.

**B-1401** 17:12
**Deep convolutional neural network for histopathological classification of pancreatic neuroendocrine neoplasms**
Y. Luo; *Guangzhou/CN (luoyanji163@163.com)*

**Purpose:** To evaluate the efficacy of deep convolutional neural network (DCNN) for the pathological classification of pancreatic neuroendocrine neoplasms (P-NENs) on contrast agent-enhanced computed tomography.
**Methods and Materials:** 103 patients (poor differentiated [G3], n=19; well differentiated [G1+G2], n=84) preoperatively investigated with multispiral computed tomography (MSCT) and subsequently with histopathologically proven P-NENs were enrolled. The 103 datasets were normalised and augmented by multiple preprocessing techniques (rotated, contrast-enhanced and noise-added images), and were split into training (81.6%), validation (5.8%), and test set (12.6%) with 8-fold cross validation. The DCNN with the residual learning framework (ResNet) was used to classify the images as having manifestations of poor- or well-differentiated P-NENs. The DCNN was composed of 50 convolutional, one maxpooling, one global average pooling and two fully connected layers. Training and testing were performed eight times. Accuracy, sensitivity, and specificity for categorising poor- and well-differentiated P-NENs with DCNN model and the area under the receiver operating characteristic curve for poor- versus well-differentiated P-NENs were calculated.
**Results:** The accuracy, sensitivity, and specificity of classifying poor- and well-differentiated P-NENs were 80.6%, 79%, and 81%, respectively. The area under the receiver operating characteristic curve for differentiating histopathological grading of P-NENs was 0.79.
**Conclusion:** Deep learning with DCNN showed high diagnostic performance in differentiating histopathological classification of P-NENs at dynamic CT images.

**B-1402** 17:20
**Comparison of automated quantitative ultrasound image analysis vs expert scoring systems for prediction of malignancy in thyroid nodules**
A. Galimzianova, T.S. Desser, S. Siebert, S. Syed, A. Kamaya, D. Rubin; *Stanford, CA/US (tsdesser@stanford.edu)*

**Purpose:** To develop a computational framework to predict malignancy in thyroid nodules seen on ultrasound (US) images, and compare it to existing expert scoring systems.
**Methods and Materials:** We collected retrospectively a dataset of US images of 92 biopsy-confirmed nodules. The nodules were manually delineated and two expert radiologists annotated them using the standardized TIRADS lexicon of American College of Radiology. As reference estimators, we implemented five expert scoring systems: ATA, ACR TIRADS, BTA, K-TIRADS and EU-TIRADS. Our automated quantitative method combined computer-extracted features for echogenicity, texture, edge sharpness and margin curvature of thyroid nodules in a regularized logistic regression model. A leave-one-out cross-validation was performed using area under the receiver operating characteristic curve (AUC), sensitivity and specificity metrics.
**Results:** The AUC of our automated method was 0.83 (0.70, 0.95), a performance result similar to the expert scoring systems, with AUC values from 0.68 to 0.83 (p=0.99, DeLong's test). However, our method was superior in that it could spare the need for biopsy in 20 out of 46 benign nodules when following a curative strategy to triage (at sensitivity of 1.00, statistically significantly higher than three other expert systems), or identified 10 out of 46 malignancies when following a conservative strategy (at specificity of 1.00, statistically significantly higher than five other systems).
**Conclusion:** Our automated method predicted thyroid nodule malignancy at the level of five expert systems. It could thus be an objective tool for thyroid nodule triage and may reduce unnecessary thyroid nodule biopsies.
*Author Disclosures:*
**A. Kamaya:** Author; Elsevier.

**16:00 - 17:30** **Room K**

### Chest

### SS 1604

### Inflammatory, fibrotic and infectious lung diseases

*Moderators:*
C. Beigelman; Lausanne/CH
M. Benegas; Barcelona/ES

**B-1403** 16:00
**CT quantification of spatial heterogeneity of boundary between fibrotic and non-fibrotic areas in idiopathic pulmonary fibrosis**
J. Park, J. Jung, S.H. Yoon, H. Hong, J.-H. Yoon, H. Kim, J.M. Goo; *Seoul/KR (et_2004@naver.com)*

**Purpose:** This study aimed to quantify the spatial heterogeneity of boundary between fibrotic and non-fibrotic areas on chest CT in patients with idiopathic pulmonary fibrosis (IPF) for evaluating IPF severity and survival prediction.
**Methods and Materials:** We retrospectively included 93 consecutive IPF patients who underwent baseline thin-section standard-dose chest CT from 2006 through 2016. The non-fibrotic areas were segmented below a threshold value of -500 HU, followed by the extraction of boundary between fibrotic and non-fibrotic areas using an in-house software. Gaussian curvature analysis was performed for calculating histogram features including entropy and uniformity. We analysed the correlation between those CT features and gender, age, and physiology (GAP) score, or CT fibrosis score and evaluated the prognostic value of the features in patients' survival.
**Results:** The boundary curvature between fibrotic and non-fibrotic areas was more heterogeneous in patients with higher GAP score (Spearman $\rho$ for entropy=.320, p=.002; $\rho$ for uniformity=-.337, p=.001). The heterogeneity of boundary curvature was also positively correlated with CT fibrosis score (entropy, $\rho$=.250, p=.016; uniformity, $\rho$=-.269, p=.009). In the univariate Cox-regression analysis, the higher entropy (p=.017), the lower uniformity (p=.009), right-sided skewness (p=.038), and kurtosis (p=.047) were significantly associated with worse survival.
**Conclusion:** The spatial heterogeneity of boundary curvature between fibrotic and non-fibrotic areas was correlated with the GAP score as well as the degree of CT fibrosis, along with predicting survival in patients with IPF.

**B-1404** 16:08
**Fibrotic lung disease in MPO-ANCA positive patients**
B. Del Rio Carrero, P. Luburich Hernaiz, S. Bolivar Cuevas, E. Carreño García, V. Vicens Zygmunt, M. Molina Molina; *Barcelona/ES (belendlrio@gmail.com)*

**Purpose:** Association between fibrotic interstitial lung disease (ILD) and anti-neutrophil cytoplasmic antibody (ANCA)-associated vasculitides has been well stablished. However, while findings shown in eosinophilic granulomatosis with polyangiitis (EGPA) and granulomatosis with polyangiitis (GPA) have been extensively described, those presenting in patients with microscopic polyangiitis (MPA) and in MPO-ANCA positive patients without vasculitis diagnosis are still poorly understood. Our purpose is to describe lung CT findings in patients with MPA and in those with MPO-ANCA positive titres not fulfilling criteria for vasculitis.
**Methods and Materials:** In this retrospective descriptive study we reviewed every patient with a positive MPO-ANCA quantification in our hospital in the last 3 years and selected those with at least one chest CT with acceptable technical quality. From this 69 patients, we excluded 11 diagnosed with GPA and EGPA. Chest CTs of the remaining 58 patients were reviewed buy 2 radiologists establishing findings by consensus. Patients with fibrotic ILD were subdivided according to radiologic patterns of UIP, possible UIP, NSIP and cystic disease not fulfilling all the criteria for UIP.
**Results:** Fibrotic ILD was found in 22 (37.9%) out of 58 patients; 8 (13.8%) presented UIP pattern, 1 (1.7%) presented possible UIP pattern, 3 (5.2%) presented NSIP pattern and 10 (17.2%) presented cystic disease not fulfilling criteria for UIP pattern.
**Conclusion:** Patients with MPO-ANCA positive titres with or without MPA diagnosis frequently present with fibrotic ILD. The most frequent pattern presented was the cystic disease not fulfilling all the criteria for UIP, followed by UIP and NSIP.

**B-1405** 16:16
**To assess clinical and radiological disease severity in a population of IPF patients which differ for the presence of diffuse pulmonary ossification (DPO) on HRCT**
M. Palermo, G. Sambataro, F. Tiralongo, C. Desiderio, D. Falsaperla, S. Torrisi, A. Basile, C. Vancheri, S. Palmucci; *Catania/IT (monica.palermo91@gmail.com)*

**Purpose:** To assess clinical and radiological disease severity in a population of IPF patients which differ for the presence of Diffuse Pulmonary Ossification (DPO) on HRCT.
**Methods and Materials:** This retrospective study was carried out on a 47 IPF patients - 25 with DPO on baseline HRCT (group A, average age 68.5) and 22 (group B, average age 66.3) with no calcifications detected on baseline HRCT. The quantification of lung fibrosis was obtained using a semiquantitative method, based on HRCT fibrosis score. Therefore, the overall extent (percentages) of normal attenuation, reticulations, honeycombing and traction bronchiectasis was quantified on three HRCT slices - selected at different levels (apex, mid-lung and base). Pulmonary Function Tests (PFTs) - acquired nearest to the baseline HRCT - were compared between the two groups, using a t-test; HRCT fibrosis scores were compared using a U-test of Mann-Whitney.
**Results:** PTFs values were not different among the two groups p= 0.22 for FVC and 0.59 for DLCO. Considering HRCT fibrosing scores, the Mann-Whitney U-test revealed no statistical difference (p value of 0.48*)* between group A and B with a median value of 216.5 for DPO patients and a median value of 241.5 for non-DPO patients.
**Conclusion:** Although literature shows that DPO is an important marker of fibrosis, in our preliminary study disease severity seems to be similar in IPF patients with and without DPO. A longitudinal analyse should be invoked to better investigate prognostic value of DPO.

**B-1406** 16:24
**Combined pulmonary fibrosis and emphysema (CPFE): epidemiological and clinical features, visual scoring and quantitative CT assessment in 72 patients**
A. Carnevale, A. Cossu, E. Cotti, M. Graziano, S. Chiarello, M. Giganti; *Ferrara/IT (moni.graziano@gmail.com)*

**Purpose:** To retrospectively evaluate epidemiological, clinical and radiological data of 72 patients exhibiting chest CT features consistent with combined pulmonary fibrosis and emphysema (CPFE); to compare two different quantification methods of lung fibrosis (LF) in these patients: a reader-based visual scoring system and a computer-aided one.
**Methods and Materials:** We retrospectively identified 72 consecutive adult patients (64m/8f) who exhibited CPFE features at chest CT. All patients' data, including smoking habits, professional exposure, autoimmune conditions, were collected. For each study, LF percentage extent was determined, using Horos open-source DICOM viewer, by comparing quantitative assessment (QCT) of LF volume and total lung volume. An experienced chest radiologist, blinded to QCT results, evaluated CT images for the extent of fibrosis, and emphysema separately, by visually estimating the percentage of parenchymal involvement as the mean of five anatomical sections' results. LF and emphysema patterns were also assessed. Statistical analysis was performed through dedicated software (MedCalc).
**Results:** CPFE patients were more frequently males, smokers or former smokers (n=64/66). Female patients were more frequently affected by a rheumatologic condition (p< 0.0001). A strong correlation between QCT-based and reader-based assessment of LF extent was found (r=0.80; p<0.0001).
**Conclusion:** This work confirmed the relevant role of cigarette smoking in the pathophysiology of CPFE, and highlighted the importance of autoimmunity in female subjects. We demonstrated a strong correlation between a LF reader-based scoring system and a computer-based analysis, thus providing the hypothesis that the latter method may be a feasible tool for LF quantification even in CPFE patients.

**B-1407** 16:32
**Can we reduce the radiation dose with wide-volume scan mode in a 320 detector-rows CT for interstitial lung disease?**
A. Abdo, E. Karam, S. Leygnac, T. Henry, K. Haioun, A. Khalil, M.-P. Debray; *Paris/FR (alainabdo7@gmail.com)*

**Purpose:** Wide-Volume (W-V) mode is an alternative to helical mode to explore large anatomic zones with wide-area detector rows CT. Despite its ability to reduce the radiation dose, it is infrequently used for lung diseases. The purpose of this study is to compare image quality and radiation dose of W-V chest CT to those of standard helical CT in the setting of interstitial lung diseases (ILD).
**Methods and Materials:** We evaluated 53 patients referred for follow-up or screening of ILD, requiring prone scan in addition to the routine unenhanced supine scan. Each examination included both helical and W-V mode acquisitions (320x0.5mm or 240x0.5mm collimation, according to the height of the thorax). Comparative analysis was performed by two radiologists blindly to the acquisition mode, including analysis of normal anatomical structures and elementary signs, overall image quality and artifacts.
**Results:** Median CTDI and DLP with W-V (3.1 mGy and 93.2 mGy.cm) were significantly reduced (p<0.0001, Wilcoxon rank test) as compared to helical mode (3.7 mGy and 124.4 mGy.cm), leading to a mean 16% [95% CI 11-20%] and 27% [95% CI 22-30%] reduction of CTDI and DLP, respectively. Image noise and quality were not significantly different between the two modes. Shifting artifact at the junction of two volumes was inconstantly seen in the W-V scans and, when present, did not impair diagnostic quality in 87% of cases.
**Conclusion:** W-V mode allows significant radiation dose reduction as compared to the standard helical mode and could be routinely used for diagnosis and follow-up of ILD.
*Author Disclosures:*
**K. Haioun:** Employee; Employee of Canon Medical Systems.

**B-1408** 16:40
**Applying the low-dose protocol in high resolution computed tomography examination in patients with rheumatoid arthritis**
B. Zwierko, M. Dura, P. Zuchowski, S. Jeka; *Bydgoszcz/PL (p.zuchowski81@gmail.com)*

**Purpose:** In the course of rheumatoid arthritis (RA), some of the most common complications are lung diseases. Presently, the gold standard in lung diagnostics is the high resolution computed tomography (HRCT). The aim of this study is to evaluate the utility of the low dose protocol in HRCT examination of the lungs in patients with RA.
**Methods and Materials:** 35 (28F/7M) patients with RA were enrolled. Average disease duration was 8,8 years. The study lasted 12 months. Only patients with previously diagnosed lung changes via HRCT were enlisted. All patients had already undergone standard HRCT lung examination at baseline and "low dose" protocol examination at 12 months. All exams were evaluated by two radiologists. A single tomograph was used in the study - GE Lightspeed Pro 32.
**Results:** Both radiologists noted a loss in image quality in every exam using the "low dose" protocol. It is the radiologists' opinion that it had no negative impact on the diagnostic quality of the examination. The average value of the Dose-Length Product (DLP) index with the standard HRCT examination was 146 mGy*cm, while with the "low dose" protocol - 53 mGy*cm (significant difference, p < 0,05).
**Conclusion:** The use of the “low dose” protocol in HRCT examination allowed for a moderate reduction of DLP by 64% in comparison with the standard examination protocol. The "low dose" protocol adheres perfectly with the basic principle of patient radiation protection - ALARA.

**B-1409** 16:48
**Feasibility of low-dose CT with spectral shaping and third-generation iterative reconstruction in evaluating interstitial lung diseases associated with connective tissue disease**
X. Xu, X. Sui, L. Song, Y. Huang, Z. Jin, W. Song; *Beijing/CN (xxllpositive@163.com)*

**Purpose:** To investigate the feasibility of low-dose CT (LDCT) with tin-filtration and third-generation iterative reconstruction (IR) in evaluating interstitial lung diseases associated with connective tissue disease (CTD-ILD).
**Methods and Materials:** 53 consecutive adult patients with CTD-ILD underwent regular-dose chest CT (RDCT) at 110 kVp followed by LDCT with tin-filtered 100 kVp. RDCT was reconstructed with filtered back projection (FBP) and advanced modelled iterative reconstruction (ADMIRE), and LDCT with ADMIRE. Image noise, streak artefact, image quality and visualisation of normal and abnormal CT features were evaluated and compared among RDCT ADMIRE, RDCT FBP and LDCT ADMIRE groups.
**Results:** The mean radiation dose of LDCT was reduced to 20% of RDCT. Objective image noise of RDCT ADMIRE (38.08 ± 6.37 HU), LDCT ADMIRE (51.68 ± 9.06 HU) and RDCT FBP (62.09 ± 10.95 HU) increased progressively ($p$ < 0.001 in any two pairs). RDCT ADMIRE significantly improved subjective image noise, streak artefact and overall image quality compared with RDCT FBP and LDCT ADMIRE (*p*s < 0.001), while no significant difference was noted between the latter two groups. All abnormal lung structures were better scored in RDCT ADMIRE compared with RDCT FBP (*ps* < 0.001). LDCT ADMIRE was inferior to RDCT FBP in visualising peripheral bronchi and vessels as well as reticulation (*ps* < 0.001); other normal and abnormal structures were similar between the two groups.
**Conclusion:** LDCT with tin-filtration and third-generation IR was applicable in evaluating CTD-ILD. Image quality was significantly improved after applying ADMIRE algorithm to CT protocols.

**B-1410** 16:56
**HRCT does not offer added discriminatory value for eosinophilic lung diseases in patients presenting to a severe asthma clinic**
E. Robinson, M. Mak, D. Jackson, B. Kent, G. Santis, A. Nair; *London/UK (szemun.mak@gstt.nhs.uk)*

**Purpose:** To assess the frequency and discriminatory ability of HRCT features and diagnostic sensitivity of radiologists for distinguishing allergic bronchopulmonary aspergillosis (ABPA), eosinophilic granulomatosis with polyangiitis (EGPA) and severe asthma only (SevA).
**Methods and Materials:** 64 patients' (21 ABPA, 21 EGPA, and 22 SevA) HRCTs were analysed. Two blinded radiologists scored 30 features per lobe and specified a diagnosis (ABPA/EGPA/SevA). The frequency of variables was calculated. One-way analysis of variance and Chi-squared test were used to assess continuous and categorical variables, respectively. Logistic regression models for diagnosing ABPA and EGPA using potentially statistically significant HRCT variables (LR-CT), including clinically useful variables (LR-All), were assessed with area under the curve (AUC) analysis. Sensitivity and inter-observer agreement for diagnosis were calculated.
**Results:** Bronchial wall thickening was the most frequent finding. Bronchiectasis (Bxt) (upper or proximal) and consolidation distribution did not discriminate between the diagnoses. Serum IgE, Bxt extent and severity, HDP, volume loss, centrilobular nodules, distal mucus plugging, and FEV1/FVC were greater (p<0.05) in ABPA compared to EGPA and SevA. With LR-CT, only greater Bxt severity and HDP predicted ABPA (p<0.001, AUC=0.89, CI 0.78-0.96), only a lower Bxt extent (p=0.03, AUC=0.67, CI 0.53-0.78) predicted EGPA. With LR-All, no variable predicted ABPA, and only a lower serum IgE (p=0.005, AUC=0.76, CI 0.62-0.86) predicted EGPA. Overall diagnostic sensitivity was low (53.1%) with only fair inter-observer agreement (k=0.29).
**Conclusion:** Traditional HRCT distributions of abnormality are not helpful in predicting ABPA or EGPA. No single HRCT variable is discriminatory when clinical variables are accounted for.

**B-1411** 17:04
**Relevance of high-resolution computed tomography in diagnosis of infectious disease versus rejection reaction in patients after lung transplantation with acute respiratory symptoms**
S. Maier[1], A. Ernst[2], U. Sommerwerck[3], G. Weinreich[1], F. Nensa[1], L. Umutlu[1], H. Kuehl[4]; [1]*Essen/DE*, [2]*Neustadt Holstein/DE*, [3]*Wuppertal/DE*, [4]*Kamp-Lintfort/DE*

**Purpose:** Complications after lung transplantation (LTX) are still common. The diagnostic value of chest CT in LTX patients with respiratory symptoms and the possible differentiation between rejection reaction and infection were examined.
**Methods and Materials:** The included patients showed dyspnea, signs of bronchopulmonary infection, worsening of the general condition or decrease of the forced expiratory pressure in 1 second (FEV1). Selected clinical parameters were compared with morphological changes in the HRCT.
**Results:** 60% (198) of patients showed a decrease of FEV1, 30% (94) showed no losses in FEV1. In 10% of patients no spirometry data were available. Comparing the FEV1 with pathologic changes in lung morphology the HRCT showed a sensitivity of 80% at a specificity of 95%, a LR+ of 14,96 and a LR- of 0,22 which indicates a high diagnostic evidence. In 50% (158) of patients a histological examination was performed. In 20% (63) pathological changes were found. In comparison to the histological results the morphologic changes in the HRCT showed a sensitivity of 86% at a specificity of 83%, a LR+ of 5,09 and a LR- von 0,17.
**Conclusion:** Morphologic changes in CT-scans showed in 16 of 20 examined clinical features at least high diagnostic evidence (LR+ 2-5, LR- 0,2-0,5), giving HRCT-exams a high diagnostic value in diagnosis of acute dyspnea in patients after lung transplantation. A specific differentiation between an infectious cause and an immune reaction against the graft associated with acute dyspnea based only on the HRCT findings is not possible.

**B-1412** 17:12
**Clusters of micronodules in active pulmonary tuberculosis: prevalence, patient characteristics, and natural history**
J. Hong, S. Yoon, J. Goo, J.-J. Yim, J.-G. Im; *Seoul/KR (keke4@naver.com)*

**Purpose:** To evaluate the prevalence, patient characteristics, and natural history of clusters of micronodules (CM) in active pulmonary tuberculosis (TB).
**Methods and Materials:** From January 2013 through July 2018, we enrolled a total of 881 consecutive patients with bacteriologically or PCR-proven active pulmonary TB who had a diagnostic chest CT. Patients were categorized into three groups based on whether they have a typical radiologic bronchogenic spread of TB, CM, or both. Among the patients solely having CM, we analysed radiologic findings of incidental pre-diagnostic CT if available, along with those of diagnostic CT.
**Results:** CM was the sole radiologic finding in 23 patients (2.6%) and was in accompany with radiologic bronchogenic spread in 48 patients (5.4%). Compared with patients without CM (830 patients, 91.9%), patients with CM only were less smear or PCR-positive (29.9% vs 5.0%, p<0.01) and higher proportion of male (60.1% vs 82.6%, p<0.01). Mean diameter of CM at pre-diagnostic CT was 4.8±2.7 cm. CM only had paucity-segmental (median 2.0, IQR 1.0-3.0) involvement at diagnostic CT. During the progression, median time to change was 17.0 months (IQR, 8.1-39.4) and bronchial wall thickening (58.7 vs 83.3%), bronchiectasis (58.3% vs 100%), and lobular consolidation (33% vs 83.3%) were additionally found along with the increased extent of CM (median involved segment, 1.0 vs 2.0).
**Conclusion:** TB solely manifesting as CM was found in 2.6% of patients with active pulmonary TB and prevalent in male with less smear or PCR-positivity. During the progression, CM had a unique indolent nature and accompanied by bronchocentric changes.

**B-1413** 17:20
**Correlation of USG diaphragm thickness measurements with pulmonary function and inspiratory strength tests in myotonic dystrophy type 1**
S. Kose, F. Koç, I. Hanta; *Adana/TR (sevgulkarakose@gmail.com)*

**Purpose:** Myotonic dystrophy type 1 (DM-1) is the most common neuromuscular disease in adults and its prevalence is 1/8000.The aim of this study is to evaluate the correlation between diaphragm thickness measured by B mode ultrasound with pulmonary functions tests in DM-1 patients.
**Methods and Materials:** Total number of 31 patients diagnosed with DM-1 followed by the Çukurova University Hospital, Department of the Neurology were included in this study. All patients had full laboratory and genetically confirmed diagnosis. The study designed as a prospective matched case control study. There were 18 healthy patients in control group. Diaphragm thickness was measured in B mode ultrasound with a high frequency linear transducer. Pulmonary function tests and inspiratory strength tests were measured onsupine and sitting position.
**Results:** Mean diaphragm thickness in milimeters at end of the inspiration and expiration for DM-1 disease and control group were 2.16, 1.85, 3.47, and 2.72 consecutively. Diaphragm thickness at end of inspiration and expiration of DM-1 patient group was significantly thinner than control group with p values of <0.01 and <0.01, respectively. There was a positive correlation between diaphragm thickness and pulmonary function tests. Statistical analysis showed also a positive correlation between diaphragm thickness and inspiratory strength tests.
**Conclusion:** Diaphragm ultrasound is an easy, cheap method which strongly and significantly correlates with pulmonary function and inspiratory strength tests in DM-1 patients. Therefore, diaphragm ultrasound may be effectively used in the surveillance of respiratory function.

**16:00 - 17:30** **Sky High Stage**

### Neuro

### MY 16

### Neuro

*Moderators:*
P. Demaerel; Leuven/BE
M. Severino; Genoa/IT

**B-1414** 16:00
**Correlation of cerebrovascular reserve assessed by acetazolamide-stress SPECT with collaterals on arterial spin-labeling MRI in patients with carotid occlusive disease**
H. Kang, Y. Kim; *Seoul/KR (gknroo@gmail.com)*

**Purpose:** We evaluated the correlation between cerebrovascular reserve (CVR) on acetazolamide-stress single photon emission computed tomography (SPECT) brain scans and intracranial collaterals on arterial spin-labeling (ASL) magnetic resonance imaging (MRI) in internal carotid artery (ICA) stenosis.
**Methods and Materials:** 86 patients with ICA stenosis (>70%) underwent cerebral blood flow (CBF) examinations by pulsed ASL brain MRI and SPECT imagings with Tc-99m HMPAO in the resting and after acetazolamide challenge. We observed the presence of intracranial collaterals, which are manifested by arterial transit artefact, on ASL brain perfusion scan. CVR based on rest-SPECT and acetazolamide-stress SPECT was calculated. With acetazolamide stress SPECT, the 82 patients were grouped as either showing or not showing evidence of decreased CVR. We assessed the relationship between reduced CVR and intracranial collaterals shown as ATA on ASL brain perfusion.
**Results:** In 61/86 (70%) of the ICA stenosis patients, ASL showed ATA in ipsilateral to the stenosis. With acetazolamide stress SPECT, the 27/82 (32%) patients showed evidence of decreased CVR. In 45/55 (81%) of the normal

CVR group and 16/27 (59%) of the reduced CVR from the SPECT results, pulsed ASL showed ATA in ipsilateral to the stenosis. Significant relationship was observed between normal CVR group and ATA showing group in ICA stenosis patients on ASL brain perfusion (p=0.035, chi-square test).
**Conclusion:** Our results suggest that ATA with pulsed ASL imaging represents intracranial collaterals and good CVR in the setting of chronic stenosis or occlusion of the ICA.

**B-1415** 16:04

**Long-term neurodegeneration of substantia nigra secondary to ipsilateral infarct is quantified with iron-sensitive imaging and impacts motor outcome**

P.-A. Linck[1], G. Kuchcinski[2], F. Munsch[3], R. Griffier[1], G. Okubo[4], P. Renou[1], V. Dousset[1], I. Sibon[1], T. Tourdias[1]; [1]*Bordeaux/FR*, [2]*Lille/FR*, [3]*Boston, MA/US*, [4]*Kyoto/JP (pierre.linck@hotmail.fr)*

**Purpose:** To demonstrate that long-term neurodegeneration of substantia nigra (SN) secondary to disconnection by a supra-tentorial infarct can be quantified with iron-sensitive imaging and contributes independently to clinical outcome.
**Methods and Materials:** 181 stroke patients (75 striatum infarcts, 106 other locations) were prospectively evaluated at 24-to-72h (baseline) and at one year (follow-up) clinically and with MRI to quantify iron through R2*. SN was segmented to measure an asymmetry index (SN-AI) of R2*. We focused on the $95^{th}$ percentile as a metric of high iron content. $SN\text{-}AI_{95}$ was compared according to infarct location with unpaired *t* test and also regressed with variables expected to influence iron accumulation. Voxel-based analysis was conducted on average R2* maps. We also identified individual voxels whose infarction was significantly associated with high $SN\text{-}AI_{95}$ through voxel-based lesion-symptom mapping (VLSM). Multiple regression models were used to test the independent association between $SN\text{-}AI_{95}$ and clinical scores.
**Results:** Visual inspection, $SN\text{-}AI_{95}$ and average maps consistently showed a delayed increase of R2* within SN that was significantly higher if ipsilateral striatum was involved than if not (p<0.0001). VLSM confirmed that striatum but also insula, internal and external capsules were associated with increased $SN\text{-}AI_{95}$. This association was independent of infarct volume, baseline $SN\text{-}AI_{95}$ and other confounders (β=4.99 [2.94; 7.04], p< 0.0001). Such increase of $SN\text{-}AI_{95}$ was an independent contributor of poor motor outcome (Box-and-Block, p=0.01) but not of cognitive or emotional outcome.
**Conclusion:** Long-term neurodegeneration of SN remotely from an ipsilateral infarct can be quantified with R2* and independently impacts motor outcome at follow-up.

**B-1416** 16:08

**Detection and prevalence of cerebral microbleeds in middle and old age groups: a clinical-based approach**

S.E.E.E. Elbadawi; *Mansoura/EG (Free_s10@hotmail.com)*

**Purpose:** Cerebral microbleeds are increasingly recognized neuroimaging finding. Aim was to examine clinical determinants in middle and old age population (50 years and older).
**Methods and Materials:** 471 individuals underwent brain MRI at 1.5T, including susceptibility weighted imaging (SWI). 36 persons were excluded due to the presence of neoplasms. Associations between hypertension, diabetes mellitus, cognitive impairment and history of ischaemic heart disease or prior cerebrovascular accidents with the presence of CMBs were assessed by logistic regression model.
**Results:** Two groups were enrolled according to the presence or absence of microbleeds; microbleeds group (n=177, 40.7%) (age, 67.5 ± 8.8) and control group (n=258, 59.3%) (age, 66.8 ± 8.4). No significant difference in age categories between the two groups was detected. However, for those aged 80 years or more, a higher proportion fell into microbleeds group (13.6% versus 3.5%). There was a statistically significant higher proportions of male gender (P = 0.005), hypertensives (P = 0.003) and history of CVA (P = 0.016) in the microbleeds group while there was no significant difference regarding history of DM (P = 0.629), IHD (P = 0.445) or cognitive impairment (P = 0.147). Binomial logistic regression was performed. HTN had 2.9 times and patients with history of CVA had 2.5 times higher odds to exhibit microbleeds. The most frequent site of CMBs was lobar.
**Conclusion:** CMBs' prevalence rises with ageing. Confounders including male gender, hypertension and history of CVA strongly correlated with CMBs.

**B-1417** 16:12

**Carotid artery plaque detected on ultrasound is associated with impaired cognitive state in the elderly: a population-based study in Wakiso district, Uganda**

M. Kenneth[1], F. Ameda[2], R.K. Byanyima[2], N. Nakasujja[2]; [1]*Mbarara/UG*, [2]*Kampala/UG (mworozikenneth@gmail.com)*

**Purpose:** Carotid artery disease which includes carotid artery stenosis, plaques, clots and increased intima media thickness has been reported by several studies to be associated with dementia. Dementia is an end stage of usually asymptomatic cognitive impairment. This study set out to determine the prevalence of abnormal carotid ultrasound findings and their association with cognitive function among the adults ≥60 years in Wakiso district, Uganda, in 2018.
**Methods and Materials:** This was a cross-sectional study which included 210 participants. Carotid artery stenosis, presence of plaque, stenosis and intima media thickness were assessed by Doppler ultrasound. Cognitive status was assessed using a Mini Mental State Exam (MMSE) test.
**Results:** The prevalence of plaque was 21.4%. Plaque was associated with an abnormal cognitive function at both univariate and multivariate analyses, respectively, with OR=3.8 (95% CI=1.90-7.54, p value=0.0001) and OR=3.4 (95% CI=1.38-8.15, p value=0.007). The cognitive function was 43.8%, 19%, 34.3% and 2.9% for normal, mild, moderate, and severe status, respectively.
**Conclusion:** The prevalence of carotid artery plaque was high in this elderly population. Carotid artery plaque was associated with abnormal cognitive function.

**B-1418** 16:16

**Are aneurysmal subarachnoid haemorrhage scales applicable to non-aneurysmal subarachnoid haemorrhage?**

D. Páez Granda, M.J. Martínez Cutillas, J. Díaz Pérez, G. Parrilla Reverter, J. Berná-Serna, M. Espinosa de Rueda Ruiz, B. Garcia-Villalba, J. Zamarro Parra, J. Felices Farias; *Murcia/ES (drdiegopaez@hotmail.com)*

**Purpose:** Aneurysmal subarachnoid haemorrhage (aSAH) and non-aneurysmal subarachnoid haemorrhage (naSAH) have a different clinical evolution. The objective of this work is to analyse if a clinical scale used in the evaluation of patients with aSAH (Hunt and Hess (HH)), and a radiologic scale that estimates the risk of vasospasm in cases of aSAH (modified Fisher Scale (mFS)), could be applied in naSAH.
**Methods and Materials:** We reviewed the radiology database and medical records of all patients who presented to our hospital during an 8-year period. The inclusion criteria were patients with spontaneous SAH, negative angio-CT (aCT), and at least one digital subtraction angiography (DSA) of cerebral vessels without disclosed neurovascular pathology. We divided cases in perimesencephalic (pmSAH) and aneurysmal type (atSAH) haemorrhages.
**Results:** One patient of the pmSAH group and 14 with atSAH reached a score >2 in HH scale on admission. The commonest complication was hydrocephalus, observed in 1 patient of the pmSAH group (mFs of III), and 14 of the atSAH group, all of them mFs IV. Delayed cerebral ischaemia (DCI) was the second most frequent complication, detected in 1 case of pmSAH (mFs of III) and 4 from the atSAH group, all of them mFs IV.
**Conclusion:** The rate and type of neurological complications in our cohort of naSAH is different than the reported incidence in aSAH. The categorization of these bleedings in pmSAH and atSAH is a more helpful tool than mFs to predict neurological complications. Worst HH scores are related to the development of these complications.

**B-1419** 16:20

**Lactate-weighted imaging of brain tumours using chemical exchange saturation transfer (CEST) MRI at 7 Tesla**

D. Paech, J.-E. Meissner, M. Wennmann, A. Radbruch, M. Bendszus, W. Wick, A. Unterberg, M. Ladd, H.-P. Schlemmer; *Heidelberg/DE (d.paech@dkfz.de)*

**Purpose:** Cancer cells exhibit up-regulated lactate dehydrogenase (LDH) resulting in increased levels of lactate production in tumours (Warburg effect). The purpose of this study was to investigate lactate levels in tumours of patients with newly diagnosed glioma using Lactate-weighted Chemical Exchange Saturation Transfer (Lactate-CEST) at 7 Tesla (7T).
**Methods and Materials:** Ten patients (5 female, 5 male) with newly diagnosed and histologically proven glioma (8 patients with glioblastoma WHO grade IV and 2 patients with glioma WHO grade II) were enrolled in this prospective IRB-approved study. Lactate-CEST MRI was performed at a 7T whole-body scanner (Magnetom 7T; Siemens Healthcare, Germany) employing a centric-reordered 2D-GRE-CEST sequence (resolution of $1.71x1.72x5\ mm^3$) and a 24-channel head coil. Pre-saturation consisted of 10 Gaussian-shaped pulses (pulse length: tp = 90 ms, duty cycle: DC= 80%, and saturation power B1 = 1.1µT). Magnetization transfer asymmetry, defined as $MTR_{asym}= Z(\Delta\omega = -0.6\ ppm) - Z(\Delta\omega = +0.6\ ppm)$, yields the lactate-weighted MRI contrast. Lactate-w. signals were investigated in three regions of interest (solid tumour, necrosis, contralateral white matter = CLWM) and compared using the Wilcoxon rank-sum test.
**Results:** Lactate values were significantly increased both in solid (3.94 ± 1.22) and necrotic (4.06 ± 1.29) tumour regions compared to CLWM (3.45 ± 1.21) overall patients (p<0.05). There were no statistically significant differences between the solid tumour and necrosis (p>0.05).
**Conclusion:** Lactate-CEST MRI could serve as an additional imaging biomarker providing complementary information about the heterogeneity of tumours, with implications for biopsy targeting, patient therapy and response monitoring.

**B-1420** 16:24

**Analysing the impact of sarcopenia on brain: using correlation between paraspinal muscle and brain volume**

H. Lee, H.S. Seo, Y.H. Lee; *Ansan/KR (kkkmucu@naver.com)*

**Purpose:** Muscle density is one of the important values in assessing sarcopenia, and closely related to skeletal muscle lipid content. We compared paraspinal muscle size and density to volume of variable compartments of brain in elderly, to find a correlation between sarcopenia and brain functions.
**Methods and Materials:** Total 1293 participants (M:F = 620:673, mean age = 63.52±7.16, range 53-82 yrs) were enrolled in the study, and went through visceral fat CT and brain MRI. Size and density of paraspinal muscle were measured at umbilical level cut of visceral fat CT. Each volume of brain compartments was processed by BRAINS Auto-Workup tool. Pearson's correlation and partial correlation were used to reveal the relationship between size and density of paraspinal muscle, and volume of the brain, while control, the covariates including age, sex, BMI, smoking, alcohol consumption, hypertension and diabetes.
**Results:** Pearson's correlation showed positive correlation between paraspinal muscle size, density and virtually all of the volume of brain compartments. On partial correlation, positive correlations were found between paraspinal muscle size and supplementary motor cortex, inferior lat. Ventricle. Besides, positive correlations were found between paraspinal muscle density and grey matter, white matter, cerebrum, cerebellum, frontal lobe and parietal lobe.
**Conclusion:** There was positive correlation on both paraspinal muscle size and muscle density to brain volume, and muscle density is significantly more correlated. Several compartments of brain such as frontal and parietal lobes, which have the main role in executive and cognitive functions, and motor and sensory functions showed relationship with paraspinal muscle density.

**B-1421** 16:28

**Glymphatic pathway of gadolinium-based contrast agents through the brain: overlooked and misinterpreted**

K. Deike-Hofmann[1], J. Reuter[1], D. Paech[1], S. Bickelhaupt[1], R. Gnirs[1], M. Forsting[2], C. Heussel[1], H.-P. Schlemmer[1], A. Radbruch[1]; [1]*Heidelberg/DE,* [2]*Essen/DE (k.deike@dkfz-heidelberg.de)*

**Purpose:** The "glymphatic system" (GS), a brain-wide network of cerebrospinal fluid microcirculation, supplies a pathway through and out of the central nervous system (CNS); malfunction of the system is implicated in a variety of neurological disorders. In this exploratory study, we analysed the potential of a new imaging approach that we designed, delayed T2-weighted gadolinium-enhanced imaging, to visualize the GS in vivo.
**Methods and Materials:** Heavily T2-weighted fluid-attenuated inversion recovery MRI (hT2w-FLAIR) was obtained before, 3h and 24h after intravenous gadolinium-based contrast agent (GBCA) application in 33 neurologically healthy patients. Signal intensity (SI) was determined in various cerebral fluid spaces and white matter hyperintensities (WMHs) were quantified by applying the Fazekas scoring system (FS).
**Results:** Delayed hT2w-FLAIR showed GBCA entry into the CNS via the choroid plexus and the ciliary body, with GBCA drainage along perineural sheaths of cranial nerves, and along perivascular spaces of penetrating cortical arteries. In all sites, a significant SI increase was found 3h and 24h p.i. compared to baseline (p<.01). Furthermore, SI increase and FS were positively correlated in the perivascular spaces 3h p.i. (p<.05).
**Conclusion:** Delayed T2-weighted gadolinium-enhanced imaging can visualize the GBCA pathway into and through the GS. Presence of GBCAs within the GS might be regarded as part of the natural excretion process and should not be mixed up with gadolinium deposition. Rather, the found correlation between WMH, an imaging sign of vascular dementia, and GS functioning demonstrated feasibility to exploit the pathway of GBCAs through the GS for diagnostic purposes.

**B-1422** 16:32

**Arterial spin labelling for the evaluation of immediate postoperative residual lesion in adult brain tumours**

C. Cohen[1], B. Law-Ye[2], D. Dormont[2], M. Sanson[2], D. Galanaud[2], N. Pyatigorskaya[2]; [1]*Tours/FR,* [2]*Paris/FR (clara.cohen.cc@gmail.com)*

**Purpose:** In brain tumours, evaluation of surgical resection on postoperative MRI can be challenging. Our goal was to evaluate the contribution of pseudo-continuous arterial-spin-labelling (pCASL) in the postoperative residual lesion research.
**Methods and Materials:** Sixty-four patients were prospectively included. On the basis of the results of preoperative DSC-PWI assessment, some lesions, such as high-grade gliomas (31), certain metastases (11), and meningiomas (4), were classified as hyper-perfused (HP+ group, n=49); other lesions, such as low-grade gliomas (10) and certain metastases (2), were classified as non-hyper-perfused (HP- group, n=15). To confirm the absence/presence of a residual lesion or disease progression, postoperative MRI including pCASL sequence, and follow-up MRI, were performed within 48 hours and 1-6 months after the resection, respectively. All the images were qualitatively and quantitatively evaluated by two raters. The ASL cerebral blood flow (CBF) was calculated and normalised to the contralateral tissue. The pCASL-CBF maps and contrast enhancement were visually assessed.
**Results:** In HP+group, the mean normalised CBF was 2.21±1.03 or 0.99±0.31 (*p*<0.001, AUC=0.965, cut-off=1.16) for patients with or without residual tumours, respectively. The mean normalised CBF was not discriminative for assessing residual tumours in HP- group (*p*=0.639). Visual CBF evaluation allowed classifying correctly 94.10% patients belonging to HP+ group (sensitivity 94.12%, specificity 86.67%, *p*<0.001). Visual evaluation correlated with contrast-enhancement evaluation and with the mean normalised CBF values (r= 0.5458 and 0.5469, respectively).
**Conclusion:** Qualitative and quantitative ASL evaluation has high diagnostic performance in postoperative assessment of hyper-perfused tumours. In this case, postoperative pCASL may be useful, especially if contrast injection cannot be performed or when contrast enhancement is doubtful.

**B-1423** 16:36

**Arterial spin-labeling and magnetic resonance spectroscopy as imaging biomarkers for the detection of epileptogenic zone in non-lesional focal impaired awareness epilepsy**

R.E. Mohamed, R.M. Dawoud, A.A. Aboelsafa; *Tanta/EG (rany1997@yahoo.com)*

**Purpose:** To recognise the epileptogenic zone in patients with non-lesional focal impaired awareness epilepsy (FIAE) by evaluating the interictal changes in rCBF and cerebral metabolic alterations using PASL-MRI and MV 1H-MRS, respectively.
**Methods and Materials:** 26 patients with non-lesional FIAE (14 females and 12 males) were subjected to PASL-MRI for estimating rCBF and MV 1H-MRS for measuring NAA/Cho, NAA/Cr, NAA/(Cho+Cr), MI/NAA, Glx/NAA and Glx/Cr ratios, in addition to ASL asymmetry index percent (ASLAI%) and percent asymmetry factor (%AF).
**Results:** For the identification of the epileptogenic zone; increased ASLAI% assessed by PASL-MRI (at cut-off value ≥5.96% and AUC of 0.968) showed 96.01 sensitivity, 91.70% specificity and 95.78% accuracy, and increased %AF (at a cut-off value ≥ 9.98% with AUC of 0.983) showed 100% sensitivity, 97.65% specificity and 98.14% accuracy, while decreased NAA/(Cho+Cr) ratio estimated by MV 1H-MRS (at a cut-off value ≥0.59 with AUC of 0.977) showed 100% sensitivity, 96.90% specificity and 97.74% accuracy. Moreover, the combined use of PASL-MRI and MV 1H-MRS yielded 100% sensitivity, 98.45% specificity and 98.86% accuracy.
**Conclusion:** The combined use of PASL-MRI and MV 1H-MRS can be considered as in vivo biomarkers for the proper identification of epileptogenic zone in patients with non-lesional FIAE.

**B-1424** 16:40

**Fully automated segmentation of meningiomas using deep learning on multiparametric MRI: automated segmentation yields accuracies as good as manual interreader variabilities**

K.R. Laukamp, F. Thiele, L. Pennig, R.P. Reimer, G. Shakirin, D. Zopfs, M. Timmer, M. Perkuhn, J. Borggrefe; *Cologne/DE (kai.laukamp@uk-koeln.de)*

**Purpose:** Volumetric assessment of meningiomas is highly valuable for primary assessment and therapy/surgery planning. Further, it is more sensitive for detection of tumor growth than conventional diameter methods and thereby serves as an effective tool in meningioma monitoring. We used a state-of-the-art deep-learning-model (DLM) on routine multiparametric MR-data to investigate performance in automated detection and segmentation.
**Methods and Materials:** 56 MR-datasets (T1-/T2-weighted, T1-weighted contrast-enhanced [T1CE], FLAIR; n=19 from local scanners and n=37 from referring institutions) of n=38 grade I and n=18 grade II meningiomas were included. The DLM was trained on two independent datasets of 249 gliomas and 60 meningiomas. It segments four different tumor classes as defined in BRATS-benchmark. Preprocessing included registration, skull stripping, resampling, and normalization. DeepMedic-architecture (Kamnitsas, 2016) was used as basis. 3D-convolutional-neural-network for segmentation and a 3D-post-processing-step to remove false positives were applied. Detection and segmentation results were compared to manual segmentations from two experienced radiologists in consensus in FLAIR and T1CE tumor volume.
**Results:** 55 of 56 meningiomas were detected correctly by the DLM. Fully automated segmentation correlated very well with manual segmentations: average dice-coefficients were 0.87±0.15 for contrast enhancing tumor volume in T1CE and 0.82±0.12 for total tumor volume (union of tumor volume in FLAIR and T1CE).
**Conclusion:** Deep learning based automated segmentation yielded excellent segmentation accuracies, comparable to manual interreader variabilities, and thereby might improve and facilitate clinical management of this highly frequent tumor entity.

*Author Disclosures:*
**F. Thiele:** Employee; Philips Healthcare. **G. Shakirin:** Employee; Philips Healthcare. **M. Perkuhn:** Employee; Philips Healthcare. **J. Borggrefe:** Speaker; Philips Healthcare.

**B-1425** 16:44
**Imaging the seizure onset zone and fast electrical circuit activity in epilepsy with electrical impedance tomography**
A. Witkowska-Wrobel, K. Aristovich, M. Faulkner, J. Avery, D. Holder; *London/UK (anna.wrobel@ucl.ac.uk)*

**Purpose:** Electrical impedance tomography (EIT) is a new method for imaging internal impedance changes within a subject using EEG electrodes. It could advance the management of epilepsy by imaging changes occurring over milliseconds due to ion channel opening or seconds due to cell swelling and so image seizure onset and propagation in epileptic patients. It requires the use of intracranial electrodes, already used in presurgical evaluation for epilepsy. The feasibility of its use in epilepsy has been evaluated in a modelling study based on intracranial patients.
**Methods and Materials:** The accuracy of seizure onset zone localisation was evaluated using 10M element patient-specific Finite Element Models (FEMs) created from frontal/mesial temporal lobe epilepsy patients with 8-12 depth electrodes inserted. 30 seizures were simulated as resistance changes 1cm in diameter, for either ion channel opening or cell swelling (1/10% change respectively). EIT images were compared to EEG inverse source modelling (EISM) and visual spike recognition (SEEG).
**Results:** Seizure onset localisation was significantly improved with EIT compared to EISM/SEEG. EIT could detect seizures more accurately, independently of orientation, and with broader coverage whereas EISM/SEEG were blind to tangential and distant sources (15/15 vs. 8/15 detection EIT vs. SEEG respectively). The reconstructed localisation error was 46.2 ± 25.8mm (EISM) and 5.2 ± 1.8mm (EIT, n=30, p < 0.01).
**Conclusion:** Results suggest EIT could provide a radically improved method for localising seizure onset by providing functional real-time information. Work is in progress to evaluate its accuracy in anesthetized pigs and in human epilepsy subjects.

**B-1426** 16:48
**Meta-analytic investigations of grey matter alterations in patients with Tourette syndrome**
S. Zhang, W. Wang, X. Su, H. Sun, Q. Yue, Q. Gong; *Chengdu/CN (835408876@qq.com)*

**Purpose:** Tourette syndrome (TS) is a neurodevelopmental disorder characterized by chronic motor and phonic tics. The neuroimaging studies revealed grey matter volume alterations in TS, but the previous findings have not found a common theme with respect to functionally important regions that could drive TS behaviour. The aim of this paper was to find consistent grey matter alterations in TS patients.
**Methods and Materials:** We searched Pubmed, Embase and Web of Science for articles published from inception to October 2018, with the search terms “Tourette syndrom” or “TS” plus “VBM” or “voxel-based morphometry”. After excluding ineligible publications, we identified 10 relevant studies that investigated grey matter alterations in TS, then we performed a meta-analysis using the effect size signed differential mapping method to quantitatively estimate regional grey matter abnormalities in patients with TS.
**Results:** We included 10 studies comprising 342 TS patients and 356 healthy controls in our meta-analysis. The meta-analyses showed robust GMV reductions in the bilateral inferior frontal gyrus (IFG) and increased GMV in the midbrain area in patients with TS.
**Conclusion:** Our findings suggested that the consistent GMV alterations in TS patients were decreased in IFG and increased in midbrain area. The IFG is involved in decision-making through its function in the representation of value and reward. Volume reduction in IFG could explain deficits in the flexible control of behaviour in TS patients. Besides, the midbrain GMV alternations may play a role in triggering specific vocal responses on the basis of the environmental and motivational information.

**B-1427** 16:52
**Grey matter network changes with ageing in a large group of never-treated patients with schizophrenia**
B. Yang, W. Zhang, B. Tao, J. Liu, S. Lui; *Chengdu/CN (1045561481@qq.com)*

**Purpose:** Abnormal structural brain networks are believed to be an important neuropathology of serious mental illness. The knowing of brain network changes with ageing could advance our understanding of these network disturbances and potential pathophysiology of schizophrenia. In the present study, we enrolled a large group of never-treated schizophrenia patients to investigate the pattern of network changes with ageing.
**Methods and Materials:** We stratified 152 never-treated schizophrenia patients into 4 subgroups A1-A4 by age (16-24, 25-34, 35-44,>45), and matched controls for each subgroup were selected from 210 healthy subjects. High resolution T1 images of brain were obtained with a 3.0 T MR scanner (GE), and preprocessed by FreeSurfer. The gray matter network matrices were constructed by correlating the cortical thickness of every pair of regions within AAL brain segmentations across individuals. The network properties including nodal centrality and efficiency were then calculated.
**Results:** Compared to healthy controls, all patient subgroups showed some common network property changes while some distinct changes. The common changes along ageing were nodal centrality loss at regions mainly within default mode network (DMN), control network (CN). More importantly, the network deteriorates with ageing and more nodal centrality decreases at isthmus cingulate, right caudal anterior cingulate were showed at subgroups A2-4, and more abnormality of left precuneus showed at A3-4.
**Conclusion:** The alternations of topological properties within DMN and CN may represent trait-related structural network changes in schizophrenia, while the changes at later ageing may represent illness progression with more-wide spread brain abnormalities.

**B-1428** 16:56
**Role of whole brain diffusion kurtosis neuroimaging in disentangling bipolar depression from unipolar depression**
V. Gupta, M. Maralakunte, S. Grover, C.K. Ahuja; *Chandigarh/IN (muniraju987@gmail.com)*

**Purpose:** Depression is the most common morbidity affecting people worldwide. Fact to be noted is that *not all depressions* are same. Bipolar depression (BD) is frequently misdiagnosed as unipolar depression (UD), leading to inefficient treatment and worst clinical outcome. Purpose of the study is to identify novel neuroimaging markers in differentiating bipolar depression from unipolar depression in the context of depressive episode.
**Methods and Materials:** Depressed subjects with BD (n=21), UD (n=18) and healthy control (HC) (n=20) were subjected for diffusion kurtosis imaging (DKI) evaluation. The diffusivity and kurtosis metrics, such as mean diffusivity (MD), axial diffusivity (AD), radial diffusivity (RD) and fractional anisotropy (FA) measured at b1000, and mean kurtosis (MK), axial kurtosis (AK), radial kurtosis (RK) and kurtosis fractional anisotropy (KF) measured at b2000 value, employing whole brain atlas-based analysis.
**Results:** Bipolar depression subjects exhibited significant diffusivity and kurtosis metric abnormalities involving right external capsule, left amygdala and right hemicerebellum, as compared to UD and HC.
**Conclusion:** Present study is a unique study which explored whole brain DK MR technique to evaluate microstructural changes in BD, UD and HC. It has provided additional insights into the brain areas which might play central role in etio-pathogenesis of depression. Present study is able to find specific imaging markers which potentially differentiate BD from UD.

**B-1429** 17:00
**Brain MRI characteristics in neuromyelitis optica spectrum disorders: a multi-center study in China**
G. Cao, Y. Duan, W. Zhu, Y. Liu; *Beijing/CN (caoguanm@163.com)*

**Purpose:** To investigate the brain MRI features in neuromyelitis optica spectrum disorders (NMOSD) and its clinical relevance in China in a multi-center setting.
**Methods and Materials:** 238 NMOSD patients (202 females, age mean ± SD: 41.5 ± 13.7 years) were recruited from six centers in China, who satisfied the 2015 diagnostic criteria. The brain MR manifestations were classified into 4 types: (1) normal; (2) NMOSD-specific lesions (high AQP-4 regions); (3) multiple sclerosis lesions (satisfying 2015 McDonald criteria); (4) nonspecific white matter change. The lesion probability map was calculated with SPM12 after drawing the ROI. Demographic and cognitive features were analyzed.
**Results:** Eighty-three (86/238, 36%) patients had normal MR imaging findings, and the others (152/238, 64%) had abnormal MRI findings: forty-two patients (18%) with NMOSD-specific lesions distribution in dorsal brainstem, corticospinal tract corpus, callosum and periependymal lesions surrounding the ventricular system; fourteen patients with MS-like abnormalities (6%); and ninety-six patients with nonspecific lesions (40%). Lesion probability map showed the distributions. The average age of patients with specific lesions was smaller than those with nonspecific lesions (36.8 years and 44 years respectively, P=0.005). Patients with abnormal MRI findings (type 2-4) performed worse cognitive performance [Montreal Cognitive Assessment (MoCA) test] than those without brain lesions (P=0.049).
**Conclusion:** In this relatively large NMOSD cohort, nonspecific lesions were the most common findings in NMOSD, and NMOSD-specific lesions were observed in 18% patients. Patients with lesions presented worse cognitive performance than patients with normal MRI.

**B-1430** 17:04

**Less regulated, more segregated ventral visual network in patients with major depressive disorder**

H. Chen, J. Yan, K. Liu, Y. Deng, X. Huang, R. Su, Z. Xiaojun; *Guangzhou/CN*

**Purpose:** Previous studies indicate that major depressive disorder (MDD) can profoundly modify the visual cortices as well as the visuo-attentional systems of brain. However, little is known on the specific pattern of the network-level abnormalities of brain visual system in MDD. In this study, based on a series of previous evidences of visual cortical hyperactivity, we hypothesize that the ventral/dorsal visual network in MDD is possibly characterized as a status of increased autonomy (i.e., a hyperinteractive intra-network configuration, while receiving less outside modulation from attention network).
**Methods and Materials:** RS-fMRI scans were collected from 148 participants, including 78 medication-free MDD patients and 70 matched healthy controls. The dorsal/ventral visual networks were defined based on our previously published brain coordinates from activation likelihood estimation analyses. The static and dynamic network properties were, respectively, calculated and compared between MDD and control groups. Moreover, the inter-network connectivity quantified using the multivariate distance correlation between the dorsal attention network (DAN) and visual/dorsal network was also assessed between two groups.
**Results:** Results indicated that both dorsal ($p$=0.019) and ventral visual ($p$=0.002) networks in MDD were found with significantly increased clustering coefficient. A higher mean variability of dynamic functional connectivity was found in both the networks ($p$=0.002, 0.003, respectively). Moreover, ventral visual network in MDD group showed a decreased inter-network connectivity to DAN compared with control group ($p$=0.018).
**Conclusion:** These evidences may reveal a hyperinteractive, while less regulated, network characteristic of the ventral visual system, thus reflecting an autonomous status under the pathological effect of depression.

**B-1431** 17:08

**Extended memory network degeneration in Parkinson's disease patients with lean muscle loss**

Y.-N. Wu, W.-C. Lin, C.-H. Lu; *Kaohsiung/TW (echo7221@gmail.com)*

**Purpose:** Parkinson's disease (PD) is a common neurodegenerative disease associated with gray matter atrophy. Development of frailty and decrease muscle mass were observed and might relate to increase morbidity in life span. This study aimed to evaluate the relationship of decrease lean muscle mass to gray matter volume (GMV) and other risk factors PD patients.
**Methods and Materials:** Thirty patients with PD (all Hoehn & Yahr =<2) and fifteen healthy volunteers underwent brain and bilateral thigh MRI. For thigh MRI, IDEAL sequence was employed, and the ROI of fat percentage was measured at level of 50% femur length. Voxel-base morphometry (VBM) was used to access regional GMV difference between groups. Further correlation analysis was performed to evaluate the changes between GMV and fatty percentage of bilateral thigh after adjusting for age and gender. Multiple linear regression analysis was used to identify risk factors of decrease lean muscle mass of the thigh in PD.
**Results:** Compared with controls, patients with PD had significantly higher thigh fat percentage and smaller GMV in several brain locations of the extended memory network, including left superior temporal gyrus, right uncus and left inferior temporal gyrus. Further multiple linear regression study suggested that higher fat percentage of thigh can be predicted by female gender, increase disease duration and smaller GMV in left superior temporal gyrus in patient with PD.
**Conclusion:** Patients with PD experience muscle mass loss in thighs, and these alternations are associated with extended memory network degeneration, longer disease duration and female gender.

**B-1432** 17:12

**Dendritic growth and branching of the cortical neurons can be altered by ultrasound exposition**

Z. Ferenczi, T. Papp, B. Szilagyi, P. Szucs, E. Berenyi, Z. Meszar;
*Debrecen/HU (fzsuzsanna.90@gmail.com)*

**Purpose:** Ontogeny of the cerebral cortex requires complex spatial and temporal orchestration of postmitotic neurons for populating this highly organised laminated structure of the brain. After migration and lamination, appropriate dendritic growth and branching are essential for proper circuit formation and correct function of neurons. Coordinated dendritic growth is largely determined not only by intrinsic factors but also by environmental effects. Ultrasound (US) examination is one of the most important and commercial examinations during pregnancy worldwide. US stimuli can modify the number of proliferation of neurons in vivo, but we do not know exactly how this stimuli effects on dendritic differentiation. Thus, we investigated the effect of US stimuli on dendritic development in the frontal cortex and the hippocampus of prenatal mice.
**Methods and Materials:** We labelled cortical plate neurons at E14.5 with GFP by in utero-electroporation which was followed by ultrasound exposition for 10 minutes (frequency: 3.0 MHz, mechanical index 0.9, thermal index: 0. 9) at E18 and then at P3 the newborn mice were sacrificed and their brains were processed for histology. Analysis of hippocampal and cortical pyramidal cells was performed with Imaris and Neurolucida software.
**Results:** During the examination of pyramid cells, there seemed to be a higher branching frequency and there was an increase in spine density as well compared to the neurons of the nontreated animals.
**Conclusion:** Our data suggest that ultrasound has prolonged effects on the morphology of differentiating cortical neurons.

**B-1433** 17:16

**The relationship between cognitive function and magnetic resonance spectroscopy findings in Parkinson disease patients**

M. Sanei Taheri, M. ArabAhmadi, H. Haghighatkhah, M. Ranjbar, M. Amiri;
*Tehran/IR (mehran_arabahmadi@yahoo.com)*

**Purpose:** Cognitive impairment (CI) is one of the most notable disabilities of Parkinson's disease that is associated with lower quality of life. Early detection of CI is, therefore, very important for these patients. The purpose of this study was to examine the relationship between cognitive function and the metabolic data from magnetic resonance spectroscopy (MRS) of the patient suffering from Parkinson's disease.
**Methods and Materials:** We recruited 45 patients with Parkinson's disease and subjects were divided into three groups based on scales for outcome from Parkinson cognition (SCOPA-COG) test. Patients were classified as non-cognitive impairment (NCI; n=15), mild cognitive impairment (MCI; n=15) and dementia (PDD; n=15). All subjects underwent MRI and 1H-MRS techniques and metabolic changes such as NAA/Cr and NAA/Cho ratios, which were measured in the left hippocampal area of the brain.
**Results:** The mean and standard deviation of the NAA/Cr ratio in the three cognitive groups (NCI, MCI, PDD) were (2.51±0.037), (2.50 ± 0.033) and (2.47± 0.025), respectively. ANOVA test showed a significant difference in the three groups. Furthermore, the Scheffé test showed a significant difference between patients in the MCI and PDD groups (p=0.01). There was no significant difference between the non-cognitive impairment and mild cognitive impairment groups (p=0.54). No significant difference was found in NAA/Cho ratio (p=091).
**Conclusion:** A decreasing NAA/Cr ratio has an influence on cognitive function and the development of severe cognitive dysfunction in Parkinson-suffering patients. Furthermore, H-MRS determinant can be useful to evaluate cognition in Parkinson patients.

### Sunday, March 3

**08:30 - 10:00** **Coffee & Talk 3**

### Interventional Radiology

### SS 1709

### Nonvascular liver interventions

*Moderators:*
T. Albrecht; Berlin/DE
T. Andrasina; Brno/CZ

**B-1434** 08:30
**Percutaneous stereotactic CT-based microwave ablation for neuroendocrine liver metastasis**
M.H. Maurer, A. Lachenmayer, G. Beldi, D. Candinas, J.T. Heverhagen, C. Kim-Fuchs; *Berne/CH (martin.maurer@insel.ch)*

**Purpose:** To analyse the success of CT-based percutaneous stereotactic microwave ablation (MWA) for liver metastases from neuroendocrine tumours (NET) in terms of local recurrences or reduction of symptoms in a palliative setting.
**Methods and Materials:** Between 01/2015 and 06/2018, 380 different hepatic tumours were ablated percutaneously in 203 patients. Of these, 18 (4.7%) ablations were done in seven patients with NET liver metastases. Median age was 69 years (54-75 years). Three primary tumours were in the pancreas, four in the small bowel and one of unknown primary. Ablations were either done in a curative setting or as a means of reducing tumour load to treat symptomatic patients.
**Results:** There were no peri-, and postinterventional complications. The first follow up with an MRI was done three months after the intervention, with an overall median follow up of 18.9 months (4.2-33.1).There was no local recurrence in patients treated with curative intent. One out of six patients developed new metastases in the liver and received two further sessions (five lesions) and is now tumour free. The patient with palliative ablation had a significant reduction of symptoms in the follow up.
**Conclusion:** Percutaneous CT-guided MWA for patients with NET liver metastasis is a safe treatment option. Ablations can be used to treat metastasis in a curative setting or to reduce tumour load and symptoms in patients not amenable to definitive surgery. Especially patients with difficult to reach intrahepatic lesions profit from stereotactic guidance.

**B-1435** 08:38
**Minocycline hydrochloride as a soft sclerosing agent for symptomatic simple renal and hepatic cysts: our experience**
A. Paladini[1], D. Beomonte Zobel[2], M. Falcione[2], A. Borzelli[3], C. Di Stasi[2], A. Cina[2], G.E. Vallati[2], G. Pizzi[2], E.M. Amodeo[2]; [1]*Novara/IT*, [2]*Rome/IT*, [3]*Naples/IT (andreapaladini1988@gmail.com)*

**Purpose:** The aim of this paper is to retrospectively evaluate our ten years' experience on the sclerosing procedure of simple hepatic and renal cysts. We used minocycline hydrochloride, an antibiotic drug from the tetracycline family, as sclerosing agent. Through ultrasound-guided percutaneous access, it was injected in the cysts. In conclusion we evaluated efficacy and safety of the procedure.
**Methods and Materials:** We analysed 75 patients (54 patients with 60 renal cysts and 21 patients with 24 liver cysts) with symptomatic cysts. Patients were treated with ultrasound percutaneous drainage of cystic formation and subsequent injection of minocycline hydrochloride into the cyst itself. In larger cystic formulations, the treatment was repeated twice. We performed follow-up with ultrasound 6 and 12 months after procedure.
**Results:** The success rate of this treatment is 100% - in case of liver cysts - and 86% for renal cysts. Among minor complications, 5 patients reported fever (<38 °) in the 24 h following treatment, treated with paracetamol.
**Conclusion:** In conclusion, sclerosing treatment with minocycline hydrochloride is effective and safe, with a low level of complications. Symptoms disappeared in all patients with liver cysts (100% success rate) and 93% of patients with renal cysts.

**B-1436** 08:46
**Reduced periablational inflammation for microwave ablation (MWA) compared to radiofrequency (RF) and irreversible electroporation (IRE)**
M. Kak, N. Goldberg; *Jerusalem/IL (drkakradiologist@gmail.com)*

**Purpose:** To determine the extent of variable post-ablative inflammatory response in the periablational tissue when using microwave (MWA), radiofrequency thermal ablation (RF), and irreversible electroporation (IRE).
**Methods and Materials:** Wild-type C57b 9-10-month old mice (n= 27 for MWA) (total n=192) were anaesthetized and subject to laparotomy to enable direct liver ablation. For MWA (n= 27), 10 W were applied for 12 seconds [AMICA MWA system]; data for RF and IRE collected from other researchers in our unit (Bulvik et al.). Mice were sacrificed at days 1, 3, 7, 14, and 21 to permit analysis of the dynamic inflammatory changes in the border zone. Activated inflammatory cell populations in the border zone were assessed to detect the inflammatory changes in MWA, RF and IRE, respectively. Additionally, cells entering G1 phase (CDC47) were measured, counted and compared among the three methods of ablation in both the periablational and distant regions of the liver.
**Results:** Overall, the border zone was less extensive in MWA (37 μm) (stdev=10.4) than RF (82 μm) (stdev=8.6), and IRE (108 μm) (stdev=10.3) (p<0.01). There were fewer macrophages in the border zone and fewer percentage of hepatocytes cells entering G1 phase (CDC47) in MWA (35%) in the distant liver compared to either RF (44%) or IRE (60%).
**Conclusion:** MWA produces less of an inflammatory response than RF or IRE. Reduced recruitment and activation of these potentially tumorigenic cell populations potentially may lead to reduced systemic effects including development of distant liver tumours after MWA.

**B-1437** 08:54
**Safety and efficacy of IRE after major liver surgery**
A. Barabasch[1], P. Heil[2], M. Distelmaier[1], C.K. Kuhl[1], P. Bruners[1]; [1]*Aachen/DE*, [2]*Essen/DE (abarabasch@ukaachen.de)*

**Purpose:** In patients with tumor recurrence in the liver remnant after liver-surgery, local ablation often is the procedure of choice. IRE is a non-thermal ablation-technique that has shown to avoid damage of vessels and bile ducts, crucial for patients left with a single draining vein, single portal-vein-branch or bile-duct that must remain patent to preserve liver-function. Additionally, these patients exhibit innumerable surgical-clips which might interfere with IRE. We report on our experience with IRE in this situation with regards to efficacy, safety, and liver-function.
**Methods and Materials:** 16 patients were treated with CT-guided-IRE for recurrent primary or secondary liver-malignancies. 13/16 had undergone trisectorectomy, 3/16 had non-anatomic major liver-resection. Distances from the active-tip of the IRE-probe to the nearest surgical-clip were measured on CT-images acquired during IRE. Liver function was assessed by the MELD-score immediately before, 1 and 6 weeks after IRE. Wilcoxon-signed-rank-test was used to investigate changes in MELD-Score.
**Results:** All patients had titanium-surgical-clips in an average distance of 24±21mm (range 0-65mm) from the active tip of the probe. In 8/16 patients, surgical clips were located closer than 20mm. Based on post-interventional-CT and follow-up-MRI, complete ablation was achieved in all cases. Presence of clips did not modulate the ablation zone. No patient exhibited vessel thrombosis. Average MELD-score before IRE was 4.4±4.9, 1 week after IRE 3.9±3.7 and 6 weeks after IRE 4.9±3.8 (p>0.05).
**Conclusion:** IRE is a safe local ablative method for patients after major liver surgery. Surgical clips close to the IRE probes do not interfere with the ablation.

**B-1438** 09:02
**Stereotactic image-guided microwave ablation of hepatocellular carcinoma**
M.H. Maurer, V. Banz-Wüthrich, G. Beldi, C. Kim-Fuchs, D. Candinas, P. Tinguely, J.T. Heverhagen, A. Lachenmayer; *Berne/CH (martin.maurer@insel.ch)*

**Purpose:** To assess the therapeutic success of CT-based percutaneous stereotactic microwave ablation in patients with hepatocellular carcinoma (HCC).
**Methods and Materials:** We retrospectively analyzed a cohort of patients treated with percutaneous stereotactic image-guided microwave ablation for HCC at our institution between 01/2015 and 12/2017. All interventions were performed using CT-guidance with needle trajectory planning by landmark-based registration and an aiming device for precise needle placement.
**Results:** In total 163 interventions were performed in 92 patients (mean age 66 (50-84) years, 81 (83.5%) men). Patients presented on average with one tumor (1-6), a maximum of four tumors were ablated per session. Forty-three patients (44.3%) had one or more previous HCC treatments, 14 (14.4%) were transplanted afterwards. Mean tumor size was 16 mm (5-43 mm), mean ablation time per lesion was 5 (1-24) minutes. Within 30 days, no major or liver-specific complications occurred, six (4.6%) patients had minor (grade 1-2) complications after a total of 131 interventions. Of the 77 patients with a minimum 6-month follow-up after intervention, mean overall survival was 13.9 months after ablation and 23.7 months after initial diagnosis. Local recurrence occurred in 20/131 (15.2%) lesions, but could successfully be re-ablated in 14 cases (12.2%). Tumor size ≥ 3 cm was significantly correlated to local tumor recurrence (p=0.005).
**Conclusion:** Percutaneous stereotactic image-guided microwave ablation is safe and efficient for the treatment of HCC. It might offer a curative treatment approach especially for inoperable and conventionally unablatable lesions by an accurate and precise needle positioning in a minimally-invasive setting.

**B-1439** 09:10
**Comparison between percutaneous and laparoscopic microwave ablation of hepatocellular carcinoma**
A. Della Corte, P. Marra, F. Ratti, F. Giombi, S. Gusmini, M. Salvioni, M. Venturini, L. Aldrighetti, F. De Cobelli; *Milan/IT (dellacorte.angelo@hsr.it)*

**Purpose:** Based on patient and nodule characteristics, some authors favor laparoscopic over percutaneous HCC microwave ablation (MWA), however data are controversial. We compared the two approaches in terms of technical success, complications and local tumor control rates.
**Methods and Materials:** From October 2014 to September 2018, 60 consecutive patients underwent percutaneous or laparoscopic MWA of 68 HCC nodules with a 2450MHz/100W Microwave generator (Emprint, Medtronic). Complete ablation (technical success) and LTP at follow-up were assessed by contrast-enhanced CT/MRI. Forty-five patients (51 HCC nodules) satisfied inclusion criteria (adequate follow-up, complete ablation), of which 27 (29 nodules) underwent percutaneous MWA and 18 (22 nodules) underwent laparoscopic MWA.
**Results:** Technical success and complication rates did not significantly differ between the two groups (p= 0.27 and p=0.15, respectively). 6/45 patients (13.3%) suffered procedure-related complications (CIRSE classification grade-3): 2 cases (abscess, haematoma) in the percutaneous group (7.4%) and 4 (pneumothorax, respiratory failure, fever, portal thrombosis) in the laparoscopic group (22.2%). 5/51 (9.8%) HCC nodules showed local progression with 1- and 2-year LTPFS rates of 93% and 83.6%, respectively. Four LTPs occurred in the percutaneous group (13.7%), while 1 LTP in the laparoscopic one (4.5%) (p=0.27). At logrank analysis, operative approach was not a statistically significant predictor of LTPFS (p=0.47). Subgroup analysis showed that subcapsular nodules had worse LTPFS after percutaneous procedures (1-year LTPFS 100% laparoscopic vs 50% percutaneous, p=0.04).
**Conclusion:** No significant difference in complication rate was found between the two approaches. Laparoscopic MWA should be preferred over percutaneous MWA for treatment of subcapsular HCC.

**B-1440** 09:18
**Value of ultrasound fusion imaging for the thermal ablation of medium-sized hepatocellular carcinoma**
E. Xu, L. Luo, K. Li, Q. Zeng, L. Tan, Y. Long, Q. Huang, R. Zheng; *Guangzhou/CN (xuerjiao@126.com)*

**Purpose:** To investigate the effectiveness and safety of thermal ablation of patients with medium-size hepatocellular carcinoma (HCC) under the assistance of ultrasound fusion imaging.
**Methods and Materials:** From Dec. 2010 to Dec. 2017, 76 patients with 78 medium-size (3-5 cm) lesions of HCC who underwent radiofrequency ablation (RFA) or microwave ablation (MWA) in our institute were included. Ultrasound fusion imaging was used to assist the ablation procedures and assess the technical success immediately, then guide supplementary ablation. Contrast-enhanced CT/MR was performed one month later to assess the technique efficacy. The complication, local tumour progression (LTP) and intrahepatic distant recurrence (IDR) were followed up. The overall survival (OS) was analysed.
**Results:** With the assistance of ultrasound fusion imaging, single electrode or antenna was used in 68.4% (52/76) of patients. According to the ultrasound fusion imaging evaluation, 30.8% (24/78) of lesions received supplementary ablation immediately during the ablation procedure. 71 of 78 liver tumours achieved 5 mm ablative margin. There were no ablation-related deaths, and the major complication rate was 6.6%(5/76). The technique efficacy rate was 100%(78/78) according to the contrast-enhanced CT/MR one month later. Patients were followed up from 6 to 79 months with a median time of 22 months. LTP rate was 5.1% (4/78) and IDR rate was 28.9% (22/76). The 1-, 3-, 5-year OS rates were 95.7%, 89.4%, 80.6%, respectively.
**Conclusion:** With the assistance of ultrasound fusion imaging, thermal ablation of medium-sized HCC is effective and safe which could achieve a higher technique efficacy rate and a lower LTP rate.

**B-1441** 09:26
**Radiofrequency ablation of liver cancers adjacent to the gallbladder without additional invasive measures under contrast-enhanced ultrasound monitoring: a preliminary study**
E. Xu, Y. Long, R. Yan, K. Li, L. Luo, Q. Zeng, L. Tan, M. Zhang, R. Zheng; *Guangzhou/CN (xuerjiao@126.com)*

**Purpose:** The purpose of this study was to investigate the feasibility, safety and efficacy of intra-procedural contrast-enhanced ultrasound (CEUS) monitoring of the radiofrequency ablation (RFA) of liver cancers adjacent to gallbladder (GB) without invasive measures.
**Methods and Materials:** From July 2016 to April 2017, patients with liver cancers adjacent to GB (≤10 mm) who intended to undergo ultrasound-guided RFA without invasive auxiliary means in our hospital were prospectively enrolled. During the RFA procedures, CEUS was employed to evaluate the therapeutic response and the perfusion integrity of the GB wall. The outcomes of GB and liver cancers were followed up and recorded.
**Results:** 23 patients (18 male, 5 female) with 23 liver cancers (mean 18 mm, range 8-34mm) adjacent to GB were enrolled. There were 12 tumours that abutted the GB while 11 tumours located within 10 mm of the GB. After the RFA procedures, intra-procedural CEUS evaluation demonstrated the perfusion of the GB wall was integrated in all 23 patients and technical success rate of RFA was 100% (23/23). According to the contrast-enhanced CT/MR one month after RFA, the technical efficacy rate was 100% (23/23). During the follow-up period (range: 12-23 months, median: 17 months), no local tumour progression occurred in any lesion and no major complications arose in any patient. Overall survival at 1-year was 100%. Thickening of GB wall was detected in 12 patients. Five of them were restored.
**Conclusion:** CEUS-monitored RFA of liver cancers adjacent to GB without invasive auxiliary means was feasible, safe and effective.

**B-1442** 09:34
**Stereotactic image-guided microwave ablation for malignant liver tumours: can computer-assistance broaden treatment eligibility?**
M.H. Maurer, A. Lachenmayer, V. Banz-Wüthrich, S. Weber, D. Candinas, J.T. Heverhagen, P. Tinguely; *Berne/CH (martin.maurer@insel.ch)*

**Purpose:** Treatment success of microwave ablation for liver lesions depends on tumor reachability and accurate ablation probe positioning. We investigated factors influencing targeting accuracy, procedural efficiency and technical success of percutaneous Stereotactic Image-guided MicroWave Ablation (SIMWA) for malignant liver lesions.
**Methods and Materials:** Data from all patients treated with SIMWA from 2015 to 2017 were analyzed retrospectively. A computed tomography (CT)-based navigation system was used for needle trajectory planning, stereotactic needle positioning, validation of needle positions and validation of ablation zones and technical success. Factors potentially influencing target positioning errors (TPE) of positioned ablation needles were analyzed using univariable and multivariable linear generalized estimating equations (GEE).
**Results:** Overall 301 lesions (174 HCC, 87 CRLM, 17 NET, 23 other) were treated in 153 patients. In 25 (8%) lesions multiple parallel needles were placed to create larger ablation zones. Correction of needle position was necessary in 4 (1%) lesions. Median TPE per ablation needle was 2.9mm (0.2-14.1mm) (n=384). Factors significantly influencing TPE were underlying cirrhosis (Mean diff. 0.686, CI 0.212-1.161), trajectory length (0.202, 0.127-0.277) and trajectory angle (-0.085, 0.170-0.000). Subcapsular or superior dorsal lesion location (segments VII/VIII) did not influence TPE. Median time per intervention was 67min (20-253min), and technical success rate was 96% (290/301 lesions).
**Conclusion:** Due to precise trajectory planning and stereotactic needle positioning, SIMWA allows highly accurate and successful targeting of intrahepatic lesions, even for otherwise difficult-to-target tumors. This might allow broadening of treatment eligibility for patients with malignant liver tumors not reachable with conventional image-guidance.

**B-1443** 09:42
**Percutaneous cryoablation in early-stage hepatocellular carcinoma: analysis of local tumour progression factors**
D.K. Kim, K. Han; *Seoul/KR (kdk7118@yuhs.ac)*

**Purpose:** To assess the efficacy and safety of percutaneous cryoablation for early- or very early stage hepatocellular carcinoma and evaluate risk factors for local tumour progression after percutaneous cryoablation.
**Methods and Materials:** In this retrospective study, 34 consecutive treatment-naïve patients were treated with percutaneous cryoablation for three or fewer nodules smaller than 3 cm in size. The safety of percutaneous cryoablation was evaluated by assessing percutaneous cryoablation-related complications and comparing hepatic function before and after percutaneous cryoablation. The efficacy was evaluated by calculating technical success, local tumour progression rates, and disease progression rates. Furthermore, prognostic factors of percutaneous cryoablation for local tumour progression were analysed.
**Results:** The technical success rate was 100%, and complete response was achieved in all patients (100%) by 1 month after percutaneous cryoablation. During a mean 24.5 ± 12.0-month follow-up interval, the incidences of local tumour and disease progression were 11.8% and 38.2%, respectively. The respective cumulative local tumour progression-free and disease-free survival rates were 93.1% and 83.6% at 1 year and 84.0% and 60.7% at 2 years. Hepatic function was normalized within 3 months after percutaneous cryoablation. No major complications occurred other than one case of small haematoma. On multivariate analysis, a minimal ablative margin < 5 mm was a significant risk factor associated with local tumour progression.
**Conclusion:** Percutaneous cryoablation is an effective and safe therapy for patients with early- or very early stage hepatocellular carcinoma. A minimal ablative margin < 5 mm was a significant prognostic factor for local tumour progression.

**B-1444** 09:50
**TRANS-FUSIMO: preliminary in-vivo animal results of MR-guided focused ultrasound of liver under respiratory motion**
C. Gagliardo[1], P. Toia[1], M. Schwenke[2], J. Strehlow[2], L. Cicero[1], G. Cassata[1], A. Melzer[3], T. Preusser[2], M. Midiri[1]; [1]*Palermo/IT*, [2]*Bremen/DE*, [3]*Dundee/UK (cesare.gagliardo@unipa.it)*

**Purpose:** Treating liver tumors using Focused Ultrasound (FUS) is a great challenge. Prior to human applications, an in-vivo animal trial using the TRANS-FUSIMO treatment system (TTS) is ongoing in order to evaluate the safety and the technical efficacy and efficiency of generating predefined necrotic lesions.
**Methods and Materials:** The trial includes a crossbred porcine model of thirty large white swine (all females; 55-85Kg) that will be treated using the TTS under general anaesthesia with intubation. All treatments haven been performed under ventilator-controlled breathing and using an improved non-clinical prototype FUS transducer integrated with a 1,5T MRI unit; a set of interventional flexible coils were used. Before the treatment, a 3D LAVA sequence was scanned; 3D FIESTA sequences were then used for planning. During each sonication, real-time multi reference thermal monitoring was achieved using a 3mm isotropic EPI-GRE slice (8Hz). At the end of the treatment session we injected 2ml/Kg of gadobenate dimeglumine and a 3D LAVA sequence was than scanned to identified any necrotic lesion.
**Results:** The results from the first successfully treated animals will be presented. Liver lesioning was possible during both breath-hold and ventilator-controlled breathing due to the TTS motion compensation algorithm which allows the HIFU electronic steering to be controlled according to the MRI images. During all pre-clinical sessions, the TTS was used, including real-time multi reference thermal monitoring.
**Conclusion:** Although the TRANS-FUSIMO animal trial is still ongoing and subject to further optimizations the TRANS-FUSIMO treatment system is capable of perform liver lesions compensating respiratory motion.

**08:30 - 10:00** **Room M 2**

### Head and Neck

### SS 1708

### Miscellaneous and orbits

*Moderators:*
N.N.
N.N.

**B-1445** 08:30
**Comparison of single shot echo planar imaging and turbo spin echo diffusion weighted imaging of the orbit in patients with uveal melanoma**
E. Gumeler, S. Parlak, G. Yazici, E. Karabulut, H. Kiratli, H.K. Karli Oguz; *Ankara/TR (ekimgumeler@gmail.com)*

**Purpose:** Although orbital diffusion weighted imaging (DWI) has become an essential part of orbital magnetic resonance imaging, susceptibility to magnetic inhomogenities of echoplanar imaging (EPI) DWI has inherent obstacles. This study was conducted to compare single shot (SSh) turbo spin echo (TSE) DWI with SShEPI-DWI in patients with uveal melanoma.
**Methods and Materials:** Both T1, T2 WI and DWI of 32 patients with uveal melanoma were assessed qualitatively including geometric distortion, lesion discrimination and overall quality by two radiologists based on a 4-point scale. Distortion ratio (DR), signal-to-noise (SNR) and contrast-to-noise ratios (CNR) and apparent diffusion coefficient (ADC) measurements for each DWI were noted. General linear model for repeated measurements and Friedman test was applied for comparison. Pearson correlation test was performed to reveal the relationship between T1, T2 and DWI signal intensities.
**Results:** Geometric distortion was higher in EPI-DWI, overall quality was better in TSE-DWI for both reviewers. One reviewer scored EPI-DWI slightly better for lesion discrimination, the other found no significant difference. DR was higher in EPI-DWI; SNR and CNR were better in TSE-DWI ($p<0,05$). Pearson correlation demonstrated negative correlation between T1 and both T2, EPI-DWI and TSE-DWI signal intensities ($p<0,05$ for all). ADC values were also higher in TSE-DWI.
**Conclusion:** TSE-DWI, less sensitive to magnetic field inhomogenities and susceptibility artefacts, may be a good alternative to EPI-DWI for orbital imaging. Negative correlation between T1 versus both T2 and DWI signal intensities is a confounding factor, suggesting that the amount of intrinsic melanin may affect the DWI signal.

**B-1446** 08:38
**MRI in the assessment of orbital invasion: diagnostic accuracy and impact on surgical planning**
G. Nocivelli, M. Ravanelli, M. Ferrari, A. Deganello, R. Maroldi, D. Farina; *Brescia/IT (gnoci89@gmail.com)*

**Purpose:** To assess MRI performance in detecting infiltration of periorbit, extrinsic muscles, apex, nasolacrimal duct (NLD) in patients with nasoethmoidal or maxillary sinus malignancies.
**Methods and Materials:** Retrospective analysis of 103 MRI (SET2, SET1, VIBE, high resolution matrix, slice thickness ≤3mm) in patients with maxillary (71/103) or nasoethmoidal (32/103) cancer. Periorbital invasion was considered when the tumour was abutting the medial/inferior orbital wall using periorbital focal interruption or irregular tumour-to-extraconal fat interface as criteria. Extrinsic muscle invasion was assessed only in tumours infiltrating intra/extraconal fat, using focal replacement/enhancement of muscular fibers as criteria. Criteria for apex invasion were invasion of optic canal or superior orbital fissure. Criteria for NLD were interruption of bony wall or mucosal layer. Imaging findings were compared to retrospectively revised surgical specimens (exenteratio orbitae 50/103, sparing surgey 53/103).
**Results:** In the assessment of periorbital invasion MRI obtained sensitivity 57%, specificity 71%, PPV 74%; pathological evidence of neoplastic necrosis is related to significant increase of MRI accuracy. For extrinsic muscles infiltration MRI obtained specificity 86% and NPV 78%, with sensitivity 58% and PPV 70%. Overall accuracy was 76%, significantly higher in the absence of perineural spread. On apex MRI obtained 100% specificity and PPV, sensitivity 56% and NPV 78%. For NLD sensitivity was 89% and NPV 88%, specificity (41%) and PPV (44%) were lower.
**Conclusion:** Diagnostic performance on periorbital infiltration is limited by microscopic breaches undetectable with MRI, that however generally are treated conservatively. Muscular and apex invasion (decision boundaries for enucleation) is more accurately identified.

**B-1447** 08:46
**The intrinsic iodine content of the thyroid is correlated to the HU value of the thyroid on true non-contrast scans**
A.M.J.L. van Kroonenburgh, S.M.J. van Kuijk, A.A. Jacobi-Postma; *Maastricht/NL (anna.van.kroonenburgh@mumc.nl)*

**Purpose:** Virtual non-contrast (VNC) scans from Dual energy CT are thought to reflect True non-contrast (TNC) images and could therefore be used as a substitute to lower radiation dose. The difference in thyroid imaging however exceeds the normally accepted 15 HU, this is thought to be due to the intrinsic iodine content of the thyroid. We investigated the correlation between Thyroid Stimulating Hormone (TSH) and the density measurements on TNC and VNC.
**Methods and Materials:** In the period from November 2016 until June 2018 we included forty-seven patients who underwent a DECT scan of the head neck region. The correlation of TSH values and density measurements on TNC and VNC, as well as the ΔHU between VNC and TNC were calculated.
**Results:** TSH of patients: mean 2.3, range (0.14-7.1). 35 Patients were euthyroid, 1 hypo- and 4 hyperthyroid. 7 Patients did not have a TSH test prior to the CT scan. Density thyroid VNC: mean 52.2, range (27.8-77.5); Density thyroid TNC: mean 104.0, range (48.0-151.4); deltaHU mean 51.8, range (6.7-110.5). The correlation between TSH and the HU value on TNC scan is 0.32 ($p=0.050$) and for VNC reconstructions -0.08 ($p=0.64$).
**Conclusion:** No correlation between TSH and VNC is found as expected, because the intrinsic iodine is subtracted from the images. The increased organification of iodine due to higher TSH levels is reflected in the density measurements of the TNC. Therefore Δ HU measurements reflect the intrinsic iodine content of the thyroid.
*Author Disclosures:*
**A.M.J.L. van Kroonenburgh:** Author; Institutional grant from Siemens. Speaker; Institutional grant from Siemens. **A.A. Jacobi-Postma:** Author; Institutional grant from Siemens.

**B-1448** 08:54
**Additional diagnostic value of dynamic MRI sequences in patients with temporomandibular joint dysfunction compared to static imaging**
J.-E. Scholtz, D. Günther, T.J. Vogl; *Frankfurt a. Main/DE*

**Purpose:** To evaluate the added value of a dynamic magnetic resonance imaging (MRI) sequence for the assessment of the temporomandibular joint (TMJ) compared to standard static MRI sequences in patients with TMJ dysfunction (TMD).
**Methods and Materials:** In this retrospective study 71 patients with TMD underwent MRI exam. We acquired 5 static T1- and T2-weighted sequences in parasagittal and paracoronal views and one dynamic sequence (trueFISP) in parasagittal view for each TMJ. We evaluated morphology and function of intra-articular structures and rated dynamic images as (1) “more informative”, “equal informative”, and (3) “less informative” compared to static images.

**Results:** Mean age was35±14.7 years and 50/71 (70.4%) were female. 127/142 (89.4%) TMJs were diagnostic. 42 (33.1%) TMJs had no disc displacement (DD), 56 (44.1%) had DD with reduction (DDwR), and 29 (22.8%) had DD without reduction (DDwoR). In 38/127 (29.9%) TMJs, dynamic images were rated "more informative", in 84/127 (66.2%) "equally informative", and in 5/127 (3.9%) "less informative" compared to solely static images for intra-articular structures. Therefore, 27/71 (38.0%) patients benefited from dynamic sequences while dynamic and static sequences were "equally informative" in 44/71 (62.0%) patients. Dynamic images were "more informative" in 23/56 (41.1%) TMJs with DDwR (p<0.001) and in 13/29 (44.8%) TMJs with DDwoR (p=0.007)
**Conclusion:** Dynamic MRI sequences are beneficial for evaluation of morphology and function of the TMJ in patients with disc displacement compared to static sequences.

**B-1449** 09:02

**Pilot study of three-dimensional MRI in the evaluation of eye shape of high myopia and classification of posterior staphyloma**

L. Zheng, L. Zhang, G. Zhang; *Shanghai/CN (zhenglinfeng04@aliyun.com)*

**Purpose:** To study the feasibility of quantitative measurement of shape of high-myopia eyes and classification of posterior staphyloma using 3D MRI.
**Methods and Materials:** 246 eyes with high myopia (Diopter≥-600 and axial length ≥26.0 mm) in 129 patients and 50 emmetropia (Diopters -100 to 100, emmetropic eye group) were included in our hospital. Then using 3D-FIESTA sequence and MR scanner, we acquired 3D image data covering entire orbit and reconstructed with volume rendering by workstation. Next, we evaluated eye morphology, shape and extent of posterior staphyloma and relationship of posterior staphyloma with the optic nerve. In addition, length of anterior to posterior (AP), left to right (RL), and superior to inferior (SI) for every eye were measured and the ratios of RL/AP and SI/AP were calculated. Finally, we classified the posterior staphyloma as broad and narrow type according to shape and extent of staphyloma.
**Results:** Of the 246 high-myopia eyes, 100 eyes showed only axial elongation (axial elongation group), and 146 showed posterior staphyloma (posterior staphyloma group). There was a statistically significant difference in AP line length, ratio of RL/AP and SI/AP (reflected eye ball deformation) among the emmetropic eye group, the posterior staphyloma group and the axial elongation group (P<0.001). The age differences between these three different groups were statistically significant (P<0.001). Of the 146 posterior staphyloma, 111 were wide-type staphylomas and 35 were narrow-type staphylomas.
**Conclusion:** 3D MRI is a useful tool to measure quantitatively the morphology of high-myopia eyes, diagnose and classify the posterior staphyloma.

**B-1450** 09:10

**Mineralisation patterns of laryngeal cartilages: normal variations and their clinical impact**

R.H. Kshirsagar, N. Sable, A. Mahajan, M. Thakur; *Mumbai/IN (rupalikshirsagar90@gmail.com)*

**Purpose:** The aim of our study was to determine the variations in location, distribution, and sex predilection of laryngeal cartilage mineralization, particularly of sclerosis in a population of persons without laryngeal cancer as an aid to understanding the significance of this entity when seen in patients with laryngeal cancer.
**Methods and Materials:** This is a retrospective study of record-based cross-sectional type. A total of 185 subjects were included in the study after application of the inclusion and exclusion criteria. Two independent observers evaluated the CT images. In case of discrepancy, third radiologist evaluated these scans. The cartilages assessed were thyroid, cricoid and arytenoids. Mineralization was categorized as calcification, sclerosis, and ossification. When the cartilage demonstrated soft-tissue attenuation, it was considered as non-mineralized. The frequencies of total, symmetric and asymmetric mineralization of these cartilages were calculated.
**Results:** Asymmetric mineralization of the thyroid, cricoid and arytenoid cartilages was seen in 45.4%, 15.7% and 21.1% of our study population, respectively. Asymmetric thyroid, cricoid and arytenoid sclerosis were seen in 4.3%, 1.6% and 7.6% of study population, respectively. Asymmetric thyroid and cricoid sclerosis was more commonly seen in women than in men while asymmetric arytenoid sclerosis was more commonly seen in men than in women, but the differences were not statistically significant. Unilateral arytenoid sclerosis was seen more frequently on the left side compared with the right.
**Conclusion:** Normal variations in mineralization patterns should be taken into consideration when evaluating CT scans of patients with laryngeal cancer for cartilage invasion to avoid false-positive reads.

**B-1451** 09:18

**Orbital adnexal lymphoma: diagnosis and follow-up with combined [18F]FDG-PET-CT**

U.G. Mueller-Lisse, A. Klingenstein, A. Garip-Kuebler, C. Hintschich; *Munich/DE (ullrich.mueller-lisse@med.uni-muenchen.de)*

**Purpose:** Combined whole-body 18F-fluoro-2-deoxyglucose positron-emission tomography with computed tomography ([18F]FDG-PET-CT) demonstrates location, morphology and metabolism of FDG-avid tumours, such as, e.g., lymphomas. We tested for associations between the maximum of the standardized uptake value (SUVmax for [18F]FDG) and therapy status, lymphoma stage, and histological grading in orbital adnexal lymphoma (OAL).
**Methods and Materials:** With ethics-committee-approval, pre- and post-therapeutic whole-body-[18F]FDG-PET-CT-scans of 21 patients with OAL diagnosed by means of incisional biopsy between July, 2002, and November, 2016, were identified and re-evaluated for SUVmax in respective OAL tumours in a single-armed retrospective single-centre study. Two-tailed Fisher-exact-tests were statistically significant for p<0.05 in contingency tables with SUVmax as the dependent variable and status prior to/after systemic therapy, pre-therapy Ann-Arbor-classification-based lymphoma stage, and histological grading as the respective independent variables.
**Results:** [18F]FDG-PET-CT showed all OAL lesions. Histology identified 18 malignant B-cell-non-Hodgkin lymphomas, two malignant T-cell-lymphomas, and one systemic lymphoma with associated chronic polypoid sinusitis. SUVmax levels of OALs decreased significantly after systemic therapy (p<0.001). Higher-stage OALs showed significantly higher SUVmax levels (p=0.014). There were no statistically significant associations between histological tumour grade and SUVmax level.
**Conclusion:** Whole-body-[18F]FDG-PET-CT in patients with OAL showed significant associations of SUVmax levels in orbital tumours with both tumour stage prior to therapy and therapy status, but not with histological tumour grade. Findings imply that [18F]FDG-PET-CT can be reasonably applied for whole-body staging and follow-up in patients with OAL.

**B-1452** 09:26

**Validation of exophthalmos MRI measurements in patients with Graves orbitopathy, compared to opthalmometry results**

E. Wnuk, E. Maj, A. Jabłońska-Pawlak, O. Rowinski; *Warsaw/PL*

**Purpose:** Although evaluation of the orbital structures using magnetic resonance imaging (MRI) is well described in literature, there is no consensus on which measurement method is the most useful in exophthalmos assessment. The aim of the study is to correlate two MRI methods of exophthalmos measurement with exophthalmometry results and to determine a proper technique of exophthalmos measurement.
**Methods and Materials:** Fifty-four patients (108 orbits) with exophthalmos in the course of Graves orbitopathy were enrolled in the study. Two measurements on axial T2W orbital MRI images were performed by two independent radiologists: the distance from the interzygomatic line to the corneal apex (anterior distance) and to the sclera (posterior distance). Within four weeks an exophthalmometry was performed by ophthalmologist using Hertel exophthalmometer. The interobserver variation was assessed by the Pearson correlation coefficient. Values were analysed using mean and standard deviation and t-student test.
**Results:** On MRI images the mean anterior distance measured by the first observer was 2.06±0.3 cm and by the second observer was 2.06±0.29 cm. The mean posterior distance values were 0.29±0.28 and 0.34±0.28 cm respectively. The mean exophthalmometry result was 2,1±0,33 cm. There was a very high agreement between observers for the anterior (r=0.98, p=0.01) and high for the posterior (r=0.95, p=0.01) distance measurement. Exophthalmometry results strongly correlated with MRI anterior distance measurements (r=0.9, p=0.01).
**Conclusion:** The distance measurement between the corneal apex and interzygomatic line possesses better reproducibility and is directly correlated with Hertel exophthalmometry. This method is sufficient enough in routine practice.

**B-1453** 09:34

**Evaluation of quantitative shear wave sonoelastography of major salivary glands in Sjögren's syndrome**

E. Temel, E. Cindil, S. Ozhan Oktar, N.B. Demir, H.N. Sendur, S. Haznedaroğlu, H. Satis, R. Bilici Salman; *Ankara/TR*

**Purpose:** To investigate the diagnostic performance of the quantitative elasticity values derived from shear wave elastography (SWE) of the involvement of major salivary glands in patients with Sjögren's syndrome (SS).
**Methods and Materials:** A total of 134 patients with SS and 30 healthy volunteers were involved in this study. The same ultrasonography device (Logiq E9, GE Healthcare, Milwaukee, WI, USA) with a linear transducer (9 MHz) was used for the SWE imaging of bilateral submandibular and parotid

Sunday glands in all patients. Mean shear wave velocity (SWV) in m/s was compared between the patients and healthy volunteers using Student's *t*-test.
**Results:** The SWV values of parotid glands did not show a statistically significant difference between the patients with SS and the healthy volunteers (p> 0.05). The SWV values of both submandibular glands showed statistically significant differences between two groups (p< 0.001). The AUROC curves were 0.65 (95% CI: 0.55-0.75) for right submandibular gland and 0.64 (95% CI: 0.55-0.74) for left submandibular gland. The best threshold values were 1.7 and 1.8; sensitivity 63% and 57%, specificity 57% and 67% for the right and left submandibular glands, respectively.The mean SWV values of submandibular glands in both sides showed a moderate correlation with each other (r=0.46, p<0.001) .
**Conclusion:** The steady inclination in technological advances of ultrasound elastography is promising to provide better diagnostic values in process. In current condition, our findings suggest that SWE have a moderate ability to the diagnosis of patients with SS.

**B-1454** 09:42
**Visualisation of the maxillary nerve using a micro-surface coil and three-dimensional double-echo steady state with water excitation sequence**
G. Hong, M. Zou, Z. Yang; *Guangzhou/CN (zoumsh@mail2.sysu.edu.cn)*

**Purpose:** To display the courses, divisions and lengths of main segments of the maxillary nerve using a small surface coil combined with three-dimensional double-echo steady state with water excitation (3D-DESS-WE) sequence.
**Methods and Materials:** The maxillofacial region of 49 volunteers were scanned by a small surface coil combined with 3D-DESS-WE sequence. All 77 nerves were evaluated by two neuroradiologists using a 5-point scale (4, excellent; 3, good; 2, fair; 1, poor; and 0, none). The intraclass correlation coefficient (ICC) was used to access interobserver variability. The lengths of maxillary nerve in pterygopalatine fossa (between foramen rotundum and posterior wall of maxillary sinus) was measured. The signal characteristics of the nerves and adjacent structures were analysed. $SIR_{N/M}$ (signal intensity ratio of the nerve to superior rectus muscle) were calculated.
**Results:** In 49 volunteers (77 sides), all 77 maxillary nerve trunks were displayed (100%). Compared with muscles, nerves showed iso-signal intensity ($SIR_{N/M}$=1.10) on 3D-DESS-WE images. Average subjective scores of all small coil 3D-DESS-WE images were close to 3 points (2.56±1.03), and intraclass correlation coefficient (ICC) between two radiologists was 0.91 (p<0.01). The length of maxillary nerve in pterygopalatine fossa (between foramen rotundum and the posterior wall of the maxillary sinus) was 10.81±1.52 mm.
**Conclusion:** Small surface coil combine with 3D-DESS-WE sequence performed high quality in maxillary nerves MR imaging using MPR, CPR post-processing technology, which are suitable for clinical application.

**B-1455** 09:50
**An audit of the use of ultrasound scanning in investigation of paediatric inflammatory neck masses**
P. Gammack, A. Harper, S. Alexander, J. Jones; *Edinburgh/UK (pauline.gammack1@nhs.net)*

**Purpose:** This audit was carried out to determine whether first-line ultrasound is necessary for diagnosis and management of children presenting with acute inflammatory neck masses, and aid development of a clinical protocol for such patients.
**Methods and Materials:** Data was collected regarding patient age, physical findings, symptom duration, ultrasound findings and management using the online 'TrakCare' patient record system.
**Results:** 73 patients were referred for ultrasound for inflammatory neck masses over 18 months, with an age range 1 month to 12 years 10 months and average age of 3 years 9 months. Of those, 5 (7%) showed an abscess on initial examination and a further 3 showed abscess on subsequent ultrasound. The remaining scans showed either uncomplicated lymphadenitis, lymphadenitis with necrosis/liquefication, soft tissue inflammation or other non-inflammatory masses such as haemangiomas. Abscess had an average symptom history of 5 days, compared with complicated/uncomplicated lymphadenitis which was diagnosed after average 3 days of symptoms. Most had either fluctuance, erythema or heat on examination. 5 patients underwent subsequent incision and drainage, the remainder were treated either with antibiotics or no treatment. Patients aged 12 months or less were more likely to require incision and drainage.
**Conclusion:** Ultrasound may not be necessary for a large proportion of such cases, and particularly where symptoms have been present for less than 3 days, where there is absence of fluctuance, erythema and calor and for older children. The new guideline could have a significant impact on the use of ultrasound in such cases.

**08:30 - 10:00** **Room M 3**

### Oncologic Imaging

### SS 1716

### Which whole-body exam should I choose?

*Moderators:*
N. Bogveradze; Tbilisi/GE
G. Cook; London/UK

**K-33** 08:30
**Keynote lecture**
M.E. Mayerhöfer; *Vienna/AT*

**B-1456** 08:39
**Comparison of $^{68}$Ga-DOTATOC PET-MRI and MRI-DWI for whole body staging of patients with neuroendocrine tumours**
A. Salem[1], J. Grueneisen[1], B.M. Schaarschmidt[2], M. Chodyla[1], O. Martin[2], C. Rischpler[3], M. Forsting[1], K. Herrmann[1], L. Umutlu[1]; [1]*Essen/DE*, [2]*Düsseldorf/DE*, [3]*Munich/DE (ahmed.salem@uk-essen.de)*

**Purpose:** To compare the diagnostic potential of integrated $^{68}$Ga-DOTATOC Positron emission tomography-magnetic resonance imaging (PET-MRI) and diffusion weighted imaging-MRI (DWI-MRI) for whole-body staging of patients with neuroendocrine tumours (NET).
**Methods and Materials:** A total of 43 patients with a histopathologically proven NET (50% G1, 30% G2 and 5% G3) underwent a whole-body $^{68}$Ga-DOTATOC PET-MRI examination 60 minutes after the injection of a mean activity of 60,2 MBq. The MR study protocol comprised the acquisition of a T1w 3D VIBE sequence (pre- and post-contrast), a T2w HASTE and a diffusion-weighted EPI sequence. Two readers evaluated the following datasets 1. whole-body MRI including DWI, 2. $^{68}$Ga-DOTATOC PET-MRI without DWI regarding (1) lesion detection, (2) lesion conspicuity (4-point ordinal scale) and (3) diagnostic confidence (5-point ordinal scale). Wilcoxon's signed-rank test was used to determine the significance of differences between the ratings.
**Results:** $^{68}$Ga-DOTATOC PET-MRI correctly detected 70 malignant lesions. Among them, MRI-DWI enabled correct identification of 59 malignat lesions. Additionally, $^{68}$Ga-DOTATOC PET-MRI exhibited a significant higher conspicuity (PET-MRI: 2.93 ± 0.75; MRI+DWI: 2.27 ± 0.99) and diagnostic confidence (PET-MRI: 4.59 ± 0.60; MRI+DWI: 4.09 ± 0.88) in the detection of malignant lesions (p<0.01). Furthermore, $^{68}$Ga-DOTATOC PET-MRI and MRI-DWI showed an equivalent performance in the detection of benign lesions.
**Conclusion:** Our results demonstrate the superiority of $^{68}$Ga-DOTATOC PET-MRI in detecting malignant lesions when compared to MR-DWI alone, underlining its superior potential for whole-body staging of patients with NETs when compared to MR-DWI.

**B-1457** 08:47
**Whole body low-dose CT-study combined with model based iterative reconstruction algorithm for follow-up of oncologic patients: image quality and dose deduction**
D. Ippolito[1], I. Salemi[1], C. Talei Franzesi[1], C. Maino[1], L. Riva[2], A. de Vito[1], C. Massimo[1], T. Giandola[1], S. Sironi[3]; [1]*Monza/IT*, [2]*Missaglia/IT*, [3]*Bergamo/IT*

**Purpose:** To compare radiation dose and image quality of low-dose CT protocol combined with iterative model-based reconstruction algorithm (IMR) with standard-dose CT approach combined with hybrid-iterative reconstruction algorithm (iDose) for follow-up of oncologic patients.
**Methods and Materials:** Ninety-nine patients with known oncological diseases who underwent, during their clinical follow-up, both low dose CT performed on 256-row scanner,with 100 kV and automated mAs modulation (depending on patient weight),and standard-dose CT performed on 256-row scanner, with 120 kV and automated mAs modulation, were enrolled. Images were reconstructed with IMR for the first CT examination and iDose algorithm for the second CT examination. We evaluated density values in liver and spleen and signal-to-noise ratio (SNR), along with image noise, dose parameters and diagnostic quality with 4-point scale.
**Results:** Noise of images expressed as SD values, measured in liver and spleen, was significantly lower in IMR images (liver 12,09 vs 17,10, p<0,001) whereas SNR was statistically higher (liver 8,55 vs 4,99, p<0,001) compared to iDose reconstruction. Volumetric-Computed-Tomographic-Dose-Index (CTDIvol) and Dose-Length-Product (DLP) were significantly lower in IMR compared to iDose reconstruction (DLP 499,9 vs 872,55 mGy*cm, p<0,001), with overall dose reduction of 42,71%. 4-point scale qualitative analysis did not reveal any significant differences in terms of diagnostic quality (p=0,04).

**Conclusion:** Automatic tube-current modulation combined with IMR algorithm and low kV setting allows dose reduction of 45,72% in whole body CT imaging without loss of diagnostic quality, thus representing a useful diagnostic approach in reducing dose exposure in oncologic patients who undergo several follow-up studies.

**B-1459** 09:03

**F-18 FDG PET/CT compared to Tc-99m MDP Bone scintigraphy in assessment of metastatic osseous disease in patients with breast cancer and the relation to serum CA15-3 and alkaline phosphatase**

H. Nasr[1], N. Alnajashi[2], A. Alqarni[2], H. Farghaly[3]; [1]*Cairo/EG*, [2]*Riyadh/SA*, [3]*Assiut/EG (hatemnasr@gmail.com)*

**Purpose:** To assess F-18 FDG-PET/CT compared to Tc-99m MDP bone-scan in assessment of metastatic osseous-disease in breast-cancer patients and the relation to CA15-3 or alkaline-phosphatase serum levels.
**Methods and Materials:** We reviewed PET/CT and concomitant bone-scans for 37 patients (age 55.38±13.08 years; 36 females) with known metastatic breast-cancer to bone. A visual semi-quantitative metastatic osseous-score was developed after dividing skeleton into 7-zones. Metastatic osseous-score (MS) was calculated for PET/CT (PMS) and bone-scans (BMS). McNemar-test was used to test which study is showing more lesions. ROC analysis used to define the cutoff-values for CA15-3 that best predict additional value for PET/CT over bone scan.
**Results:** Metastatic skeletal lesions were matched in 20 patients (54.1%). In 13 patients (35.1%) more metastatic burden were detected on PET/CT. In 4 patients (10.8%) more intense lesions were detected on bone-scan (p=0.049). Most of additional lesions on PET/CT were osteolytic/predominantly medullary (new lesions). Lesions with higher uptake on bone-scan were sclerotic with low FDG (non-active healed/healing). CA15-3 was positively correlated to PMS (r=0.386;p=0.018) but not to BMS (r=-0.027;p=0.874). Differently alkaline-phosphatase was positively correlated to both PMS(r=0.389;p=0.017) and BMS(r=0.363;p=0.027). Cutoff-value CA15-3 >47U/ml best predicted additional findings on PET/CT compared to bone-scan (sensitivity, specificity, PPV, NPV and accuracy of 61.5%, 79.2%, 61.5%, 79.2% and 73% respectively (p=0.013)).
**Conclusion:** FDG-PET/CT is more sensitive in detecting metastatic osseous lesions and tends to identify new osteomedullary lesions. Higher CA15-3 correlates to metastatic osseous burden on PET/CT but not on bone-scan and predicts more metastatic osseous involvement on FDG-PET/CT compared to bone-scan.

**B-1460** 09:11

**Comparison of capability for TNM stage assessment among whole-body MRI and PET/MRI at different field strength and FDG-PET/CT in non-small cell lung cancer**

Y. Ohno[1], K. Aoyagi[2], M. Yui[2], Y. Kishida[1], S. Seki[1], T. Yoshikawa[1], T. Murakami[1]; [1]*Kobe/JP*, [2]*Otawara/JP (yosirad@kobe-u.ac.jp)*

**Purpose:** To prospectively and directly compare TNM stage assessment capability among whole-body MRI and PET/MRI at 1.5 and 3T MR systems and FDG-PET/CT in non-small cell lung cancer (NSCLC) patients.
**Methods and Materials:** 104 consecutive pathologically diagnosed NSCLC patients (62 men, 42 women; mean age 71 years) prospectively underwent whole-body MRI at 1.5T and 3T systems, integrated PET/CT, and surgical, pathological and/or follow-up examinations. Final diagnoses of T, N and M factors and clinical stage in each patient were determined according to all examination results. Then each factor and clinical stage were visually assessed on both whole-body MRIs, PET/MRIs and PET/CT with contrast-enhanced brain MRI. Kappa statistics were used to determine agreements for assessment of all factors and clinical stage with final diagnoses, and McNemar's test was used to compare each diagnostic accuracy among all methods.
**Results:** On each factor and clinical stage assessments, agreements between all methods and final diagnosis were substantial or almost perfect (0.60<k<0.98). Diagnostic accuracies of N factor and clinical stage on whole-body MRI as well as PET/MRI at both field strengths were significantly higher than those of PET/CT (p<0.05).
**Conclusion:** Whole-body MRIs and PET/MRIs at 1.5T and 3T systems have significantly better potential for N factor and clinical stage assessments than PET/CT in NSCLC patients.
*Author Disclosures:*
**Y. Ohno:** Research/Grant Support; Canon Medical Systems Corporation. **K. Aoyagi:** Employee; Canon Medical Systems Corporation. **M. Yui:** Employee; Canon Medical Systems Corporation. **S. Seki:** Research/Grant Support; Canon Medical Systems Corporation. **T. Yoshikawa:** Research/Grant Support; Canon Medical Systems Corporation.

**B-1461** 09:19

**Does PET/MR improve lesion detection in oncology: a prospective unicentre study in 1003 examinations**

B.M. Schaarschmidt[1], O. Martin[2], J. Kirchner[2], J. Grueneisen[1], P. Heusch[2], M. Forsting[1], G. Antoch[2], K. Herrmann[1], L. Umutlu[1]; [1]*Essen/DE*, [2]*Düsseldorf/DE*

**Purpose:** To investigate if positron emission tomography/magnetic resonance (PET/MR) imaging improves lesion detection and characterization and reduces radiation exposure in oncological tumour staging in comparison to PET/computed tomography (PET/CT).
**Methods and Materials:** A total of 1003 oncological whole-body examinations in 918 patients (mean age 57.8y, 400 female, 518 male) were included in this prospective single-centre study. In all patients, PET/CT was performed prior to PET/MRI. A consensus reading was performed for both examinations by board-certified nuclear medicine physicians and radiologists. Analyses of additional lesions and missed lesions in PET/MR compared to PET/CT as well as indeterminate findings in PET/CT that could be classified by PET/MR were performed. Furthermore, the effective dose of PET/MR as well as full- and low-dose PET/CT was analysed. Lesion detection between PET/MR and PET/CT was compared using McNemar's test. Mean effective doses were analysed using paired t tests. p<0.001 indicated statistical significance.
**Results:** Additional lesions were detected in 26.3% (264/1003) of all examinations by PET/MR. This resulted in a TNM change in 2.9% (29/1003). Only in 2.9% (29/1003) cases, lesions were missed by PET/MR that were visible in PET/CT, resulting in a TNM change in 0.5% (5/1003). Indeterminate findings in PET/CT could be classified by PET/MR in 11.1% (111/1003). Radiation exposure could be significantly reduced by PET/MR by 83.2% compared to full-dose PET/CT and by 36.1% compared to low-dose PET/CT (p<0.001, respectively).
**Conclusion:** In comparison to PET/CT, PET/MR leads to improved lesion detection and reduced radiation exposure, which might be especially beneficial in paediatric patients.
*Author Disclosures:*
**B.M. Schaarschmidt:** Shareholder; Benedikt M. Schaarschmidt is a stockholder for General Electric and was a stockholder for Bayer AG, Siemens AG, Siemens Healthineers AG, TEVA Pharmaceuticals till September 2018.

**B-1462** 09:27

**Prospective comparison of whole-body MRI and $^{68}$Ga-PSMA PET/CT for the detection of biochemical recurrence of prostate cancer**

L.M. Sawicki, C. Buddensieck, J. Boos, J. Kirchner, T. Ullrich, R. Rabenalt, P. Albers, G. Antoch, H. Hautzel; *Düsseldorf/DE (Linomorris.sawicki@med.uni-duesseldorf.de)*

**Purpose:** To assess whole-body MRI (wb-MRI) for detection of biochemical recurrence of prostate cancer (PCa) after curative treatment in comparison to $^{68}$Ga-PSMA PET/CT.
**Methods and Materials:** 30 patients (mean age: 64.9±8.8 years) with newly documented biochemical recurrence of PCa (mean PSA 2.11 ± 1.97 ng/ml) following curative therapy prospectively underwent both wb-MRI including a dedicated pelvic imaging protocol and PET/CT with 167±35 MBq $^{68}$Ga-PSMA within 10.4±9.6 days. PET/CT and MRI were separately evaluated regarding PCa lesion count, type, localization, and diagnostic confidence (3-point scale; 1-3) by two physicians. The reference standard was based on histopathology results, PSA following targeted salvage irradiation, and follow-up imaging. Lesion-based and patient-based detection rates were compared using chi-square test. Differences in diagnostic confidence were assessed by Welch test.
**Results:** A total of 58 PCa lesions were detected in 22/30 patients in the study cohort. $^{68}$Ga-PSMA PET/CT detected 57/58 (98.3%) lesions in 21/30 (70%) patients, while wb-MRI detected 15/58 (25.9%) lesions in 13/30 (43.3%) patients. The higher detection rate of $^{68}$Ga-PSMA PET/CT was statistically significant both on a per-lesion (p=0.001) and per-patient (p=0.039) basis. In 8/30 (26.7%) patients no relapse was detectable with both modalities. Except for one local recurrence in the former prostate fossa that was exclusively detected by wb-MRI, all lesions detected by wb-MRI were also detectable on $^{68}$Ga-PSMA PET/CT. Additionally, $^{68}$Ga-PSMA PET/CT offered superior diagnostic confidence in identifying PCa lesions (2.7±0.6 vs. 2.3±0.6, p=0.011).
**Conclusion:** $^{68}$Ga-PSMA PET/CT significantly outperformed wb-MRI for the detection of biochemical recurrence in PCa patients after curative treatment.

**B-1463** 09:35
**Whole body MR imaging and MR-guided biopsy in initial diagnosis and follow-up of paediatric malignancies: comparison with other imaging modalities**
T. Woerner, A. Buecker, G. Schneider; *Homburg/DE (Tobias.Woerner@uks.eu)*

**Purpose:** 79 paediatric oncologic patients underwent whole body MRI for staging and follow-up. When necessary biopsy of tumours was performed under MR guidance for histologic proof of primary tumours or for proof of recurrence or metastases. Results were compared with established staging procedures incl. PET.
**Methods and Materials:** 389 whole body examinations in 79 patients were performed on either a 1.5 or 3 T scanner (Siemens, Aera/VIDA) 24 lymphoma, 37 solid tumours including neuro-, nephro- and hepatoblastoma as well as 18 pts. with different types of sarcoma were evaluated. Protocol included T1w sequences pre- and post-CM (0.05 mmol/kg BW MultiHance) and in the hepatobiliary phase, DWI, transversal T2w TSE sequences and a coronal whole-body STIR-sequence. If indicated MR-guided biopsy of tumours/metastases or recurrent disease was performed to acquire tissue samples.
**Results:** Differences between MRI and CT were seen regarding the higher number of detected small lung metastases (<3 mm) on CT. Advantages for WB-MRI were seen in follow-up of T-cell Lymphoma, in which MRI could show PET-negative residual disease under chemotherapy and in local staging of sarcoma. MRI facilitated detection of recurrent tumours and restaging in solid tumours, especially in Ewing-sarcoma.
**Conclusion:** WB-MRI can correctly stage and diagnose a variety of malignant tumours in paediatric patients and allows for accurate patient management during therapy including MR-guided biopsy. Advantages of established imaging methods were only seen for detection of small lung metastases on pulmonary CT, however differences did not result in a change of patient management.
*Author Disclosures:*
**A. Buecker:** Grant Recipient; Bracco, Siemens. Speaker; Bracco, Siemens.
**G. Schneider:** Grant Recipient; Bracco, Siemens. Speaker; Bracco, Siemens.

**B-1464** 09:43
**Utility of 3T MRI whole-body DWI in onco-imaging for the detection of metastasis: comparison with PET/CT**
D.H. Vijayakumar[1], M.M. Kulkarni[2]; [1]*Bangalore/IN*, [2]*Mumbai/IN (deepti202@gmail.com)*

**Purpose:** To evaluate the role of whole-body diffusion-weighted MRI in onco-imaging for the detection of metastasis using 3 Tesla MRI and compare its accuracy with PET/CT.
**Methods and Materials:** A prospective study of total of 35 patients with histopathologically proven primary malignancy or metastasis who underwent both whole-body diffusion-weighted MRI and PET/CT within 15 days of each other with no surgical or medical intervention in between. Whole-body diffusion-weighted MRI along with fast m-DIXON sequence was performed on Philips Ingenia 3 T machine. Reformatted and source images were analysed for lesions showing restricted diffusion, suggesting metastasis. Data were tabulated on the basis of presence or absence of metastasis in various regions, number of lesions and correlated with 18 FDG PET/CT.
**Results:** High correlation on Pearson's correlation test and very good agreement on Kappa's test was found between DWIBS and PET/CT in detecting skeletal, lung and liver metastasis. On a case-based analysis, our study showed 94.9% sensitivity and 95.4% accuracy as compared to PET/CT. On a lesion- based analysis, 87.5% sensitivity and 85.8% accuracy was found in whole-body DWIBS as compared to PET/CT.
**Conclusion:** Whole-body DWIBS with a high b value of 1000s/mm $^{2}$ is comparable to the current gold standard in detection metastases, i.e. PET/CT and can be used as a screening modality as it is quick and safe. DWIBS is excellent for the detection of brain metastases, which are generally missed on PET/CT.

**B-1465** 09:51
**Impact of spectral imaging in patients suspected for occult cancer: a study of 93 patients**
M.B. Andersen[1], D. Ebbesen[2], J. Thygesen[3], M. Kruis[4], F.R. Rasmussen[3]; [1]*Roskilde/DK*, [2]*Viborg/DK*, [3]*Aarhus/DK*, [4]*Copenhagen/DK (mibrandersen@gmail.com)*

**Purpose:** To investigate the diagnostic impact and performance of spectral dual-layer detector CT in cancer compared to CE-CT.
**Methods and Materials:** In a national workup program for occult cancer ninety-three patients prospectively enrolled had a contrast-enhanced Philips IQon Spectral CT scan. The readings were performed with and without spectral data available. A minimum of 3 months between interpretations were implemented to eliminate recall bias. The sequence of reads for the individual patient was randomised. Readers were blinded for patient identifiers and clinical outcome. Two radiologists with 9 and 33 years of experience performed the readings in consensus. If disagreement, a third specialist radiologist with 11 years of experience determined the outcome of the reading.
**Results:** Significantly more (probable) cancer findings were identified on the spectral reading (six vs. one, p=0.03). Eight probable cancer findings on CE-CT were excluded as cancer on spectral CT. In thirty-four possible cancer findings, spectral added to the diagnosis in nineteen cases. For the possible cancer findings, spectral information was strongly correlated with a difference between the spectral and conventional reading (P<0.001). Hundred and four benign lesions were found on CE-CT and spectral datasets (multiple lesions per patient could be present). In the conventional readings, the radiologists were entirely certain about the benignity in 60% of the cases and in the spectral readings in 89% of the cases (p<0.0001).
**Conclusion:** In conclusion, we find that access to spectral data adds to find (probable) cancer or exclude the diagnosis. Furthermore, it increases the radiologists' certainty about benign lesions.
*Author Disclosures:*
**M.B. Andersen:** Speaker; Philips, Roche, BMS. **M. Kruis:** Employee; Philips.

**08:30 - 10:00** **Sky High Stage**

### Abdominal Viscera

### MY 17

### Abdominal Viscera and Gastrointestinal Tract

*Moderators:*
L.K. Blomqvist; Stockholm/SE
T.C. Lauenstein; Düsseldorf/DE

**B-1466** 08:30
**Gadoxetic acid-enhanced liver MR can predict tumour recurrence after curative treatment for small single hepatocellular carcinoma**
D. Lee, J. Lee; *Seoul/KR (dhlee.rad@gmail.com)*

**Purpose:** To determine significant predictive factors of tumour recurrence after curative treatment for single nodular HCCs ≤ 3cm.
**Methods and Materials:** This retrospective study was approved by the institutional review board. A total of 373 patients with single nodular HCCs ≤ 3cm who underwent pre-treatment gadoxetic acid-enhanced MR followed by hepatic resection (n=143) or radiofrequency ablation (RFA) (n=230) were included. We analysed their clinicopathological and MR features to determine which features may help predict the presence of microvascular invasion (MVI) or recurrence-free survival (RFS). RFS was estimated using Kaplan-Meier analysis and significant predictive MR findings for RFS were evaluated using Cox-proportional hazard regression model.
**Results:** Non-smooth tumour margins on hepatobiliary phase (HBP) were significantly associated with MVI in 143 patients treated by hepatic resection (P=0.048, odds ratio=2.60 [1.01-6.69]). In addition, non-smooth tumour margins (hepatic resection: P=0.012, hazard ratio [HR]=2.17 [1.19-3.97]; RFA: P=0.019, HR=1.79 [1.10-2.91]) and presence of non-hypervascular HBP hypointense nodules (hepatic resection: P=0.001, HR=3.55 [1.74-7.23]; RFA: P<0.001, HR=2.12 [1.40-3.20]) were significant negative predictive factors of RFS after both hepatic resection and RFA. When high-risk MR findings for recurrence were defined as the presence of non-smooth tumour margins and/or non-hypervascular HBP hypointense nodules, 5-year RFS in patients with high-risk MR findings was significantly lower than in those without after both hepatic resection (31.1% vs. 74.2%, P<0.001, HR=3.56 [1.94-6.55]) and RFA (24.3% vs. 54.6%, P<0.001, HR=2.38 [1.62-3.51]).
**Conclusion:** Non-smooth tumour margins and the presence of non-hypervascular HBP hypointense nodules were significant predictive factors of tumour recurrence after either hepatic resection or RFA.

**B-1467** 08:34
**Radiomics of gadoxetic acid-enhanced MRI: a predictive biomarker for CK19-positive hepatocellular carcinoma**
W. Wang[1], Y. Ding[1], L. Yang[1], M. Zeng[1], S.-X. Rao[1], J. Tian[2]; [1]*Shanghai/CN*, [2]*Xi'an/CN (787356268@qq.com)*

**Purpose:** To develop and validate a radiomics signature-based model derived from gadoxetic acid-enhanced MR images to preoperatively identify cytokeratin (CK) 19-positive hepatocellular carcinoma (HCC).
**Methods and Materials:** A cohort of 227 patients with single HCC was classified into a training set (n=159) and a time-independent validated set (n=68). A total of 647 radiomic features were extracted from multi-sequence MR images separately. The least absolute shrinkage and selection operator regression and decision tree methods were utilized for feature selection and radiomics signature construction. A further multivariable regression model was built incorporating clinical factors and the fusion radiomics signature with significant relation to CK19 expression.
**Results:** In the whole cohort, 57 patients were positive for CK19 and 170 patients were negative for CK19. Based on 11 and 6 radiomics features extracted in arterial phase and hepatobiliary phase images, a fusion radiomics signature achieved a satisfying performance with an AUC of 0.951 and 0.822 in training and validation datasets. The final combined model integrated serum a-fetoprotein levels, arterial rim enhancement pattern, irregular tumour margin and the fusion radiomics signature, with a sensitivity of 0.818 and specificity of 0.974 in the training cohort and that of 0.769 and 0.818 in the validated cohort. The nomogram based on the combined model showed satisfactory prediction performance in training (C-index: 0.959) and validation (C-index: 0.846) dataset.
**Conclusion:** The radiomics signature-based model derived from arterial phase and hepatobiliary phase images of gadoxetic acid-enhanced MR imaging can be a reliable biomarker for identifying CK19-positive HCC.

**B-1468** 08:38
**Intra-individual comparison of the MRI contrast agents gadoxetic acid vs gadoteric acid in liver MRI of patients with HCC and underlying cirrhosis**
M. Della Seta, F. Collettini, M. Schmelzle, N. Raschzok, B. Hamm, T. Denecke; *Berlin/DE (marta.della-seta@charite.de)*

**Purpose:** To the best of our knowledge, the ability of liver-specific contrast agents to produce enhancement characteristics analogous to those obtained with extracellular contrast agents during the early perfusion phases was never confirmed. The aim of our prospective study is to intra-individually compare the two contrast media in patients with HCC and underlying cirrhosis.
**Methods and Materials:** Twenty-four patients with HCC diagnosis underwent two liver MR examinations at 3T, one with gadoteric acid and one with gadoxetic acid. Quantitative evaluation using regions of interest was performed. Our endpoints were the evaluation of peak maximum enhancement between both contrast agents in the lesion with reference to normal tissue, the evaluation of wash-out, with reference to the healthy liver, and the analysis of signal-to-noise and contrast-to-noise values.
**Results:** Twenty-three patients had adequate image quality. Relative peak enhancement was significantly higher (*p*= < 0.001) for gadoteric acid (176.4±95.9; CI: 215.6-137.2) than for gadoxetic acid (101.7±100.8; CI: 142.9-60.5). The wash-out of gadoteric acid was significantly stronger compared to gadoxetic acid (20.5±17.1; CI:27.6-13.5 vs. 7.9±19.4; CI:15.9-0) (*p*=0.006). The CNR after administration of the liver-specific contrast agent and the extracellular contrast agent also showed a significant difference (*p*=0.005). The SNR between the two contrast media did not differ significantly (*p*=0.39).
**Conclusion:** Gadoteric acid shows a significantly higher relative peak enhancement and a significantly stronger wash-out compared to gadoxetic acid. After the administration of the two contrast media the CNR differs significantly as well. No significant difference in SNR was found between the two contrast agents.
*Author Disclosures:*
**F. Collettini:** Advisory Board; Guerbet. **B. Hamm:** Grant Recipient; Guerbet.

**B-1469** 08:42
**MRI of hepatocellular adenomas: is there an additional value of using Gd-EOB for subtype differentiation?**
T.A. Auer[1], C. Grieser[2], D. Geisel[1], B. Hamm[1], T. Denecke[1]; [1]*Berlin/DE*, [2]*Bremen/DE (timo-alexander.auer@charite.de)*

**Purpose:** Morphologic and enhancement characteristics to differentiate among hepatocellular adenoma (HCA) subtypes evaluated with gadoxetic acid-enhanced MRI.
**Methods and Materials:** Sixty-four patients with 100 histopathologically proven HCA examined with gadoxetic acid-enhanced MRI were retrospectively enrolled (standard of reference: surgical resection, n=92; biopsy, n=7). Three radiologists evaluated all MR images regarding morphological features as well as the vascular and hepatocyte-specific enhancement in consensus. Histopathological subgroup analysis was based on the Bordeaux classification (including steatotic (H-HCA), inflammatory (I-HCA), b-catenin (b-HCA) and unclassified (U-HCA) adenomas).
**Results:** Overall, 29 H-HCA (29%), 41 I-HCA (41%), 6 b-HCA (6%) and 24 U-HCA (24%) were present. For differentiation of HCA subtypes, presence of intralesional fat (H-HCA, n=27; I-HCA, n=5, b-HCA, n=1; U-HCA, n=3), atoll sign (I-HCA, n=25) and a central scar (b-HCA) were significant (p<0.001). For hepatobiliary phase, most HCA were visually found being hypointense (overall: 71%; H-HCA = 96.5%; I-HCA = 53.5%; b-HCA = 80%, U-HCA = 66.6%). Relative enhancement was not significant between the different HCA subtypes (p=0.674).
**Conclusion:** Following the Bordeaux classification, typical morphologic MR appearances of the different HCA subtypes are present and reliable. On hepatobiliary phase, most HCA show hypointensity; however, I-HCA is the most heterogeneous subtype and some present with a characteristic patchy uptake pattern. Combining Gd-EOB behaviour, the qualitative MR characteristics and predisposing risk factors increase sensitivity for a more accurate diagnosis. In our collective, sensitivity increased by combining atoll sign and heterogeneous Gd-EOB-uptake in the diagnosis of I-HCA.

**B-1470** 08:46
**Quantification of hepatic steatosis: evaluation of 4 MRI techniques vs reference method by biopsy**
C. Boudinaud, A. Abergel, M. Fontarensky, J. Joubert Zakeyh, L. Boyer, B. Magnin; *Clermont-Ferrand/FR (claireboudinaud@msn.com)*

**Purpose:** Given the growing prevalence of obesity and metabolic syndrome, the management of hepatic steatosis is a major issue. We assessed the quantification of hepatic steatosis using 4 different MRI methods, to determine which was the most correlated to the reference method, that is, histological measurement by liver biopsy.
**Methods and Materials:** Sixty patients requiring liver biopsy in non-tumour tissue, whatever the indication, were included prospectively at Clermont-Ferrand University Hospital between March 2017 and March 2018. In parallel, a liver MRI (1.5T) was performed using 4 different steatosis quantification techniques (3-echo MRImaging, 6-echo MRImaging, 11-echo MRImaging and MRSpectroscopy). Quantitative histological and imaging data were compared. In secondary analyses, we studied the possible influence of alcohol drinking, hepatic iron overload, or the presence of liver fibrosis.
**Results:** All four MRI techniques were found to have an excellent correlation with histological measurement: 3-echo MRImaging (r = 0.852, p < 0.001), 6-echo MRImaging (r = 0.819, p < 0.001), 11-echo MRImaging (r = 0.818, p < 0.001), and MRSpectroscopy (r = 0.812, p < 0.001). The presence of alcohol, iron overload or fibrosis did not interfere with reliability, whichever technique was used.
**Conclusion:** In the evaluation of hepatic steatosis, our study showed a high correlation of the 4 MRI techniques with the histological standard; MRSpectroscopy appearing as the less reliable, 3-echo MRImaging as the highest correlated (this, without compounding factor in a large population).

**B-1471** 08:50
**The value of MRI-PDFF in detecting and grading hepatic steatosis in patients with nonalcoholic fatty liver disease: a meta-analysis**
Z. Ye, X. Xiong, J. Chen, L. Cao, T. Duan, Z. Zhang, B. Song; *Chengdu/CN (948080771@qq.com)*

**Purpose:** To evaluate the diagnostic performance of magnetic resonance imaging-proton density fat fraction (MRI-PDFF) in detecting and grading hepatic steatosis (HS) in nonalcoholic fatty liver disease (NAFLD) patients.
**Methods and Materials:** A comprehensive literature search was conducted to identify studies on the diagnostic accuracy of MRI-PDFF for the assessment of biopsy-proven HS. The degrees of HS were histopathologically defined as mild (>5% steatosis), moderate (>33% steatosis) and severe (>66% steatosis), according to nonalcoholic steatohepatitis clinical research network scoring system. Data were extracted to calculate the pooled sensitivity, specificity, positive and negative likelihood ratios, as well as the area under the summary receiver operating characteristic curve (AUC) in each group.
**Results:** 16 studies with 1277 subjects were included in this meta-analysis. The pooled sensitivity, specificity, positive likelihood ratio and negative likelihood ratio were estimated to be 0.87 (95% confidence interval [CI]: 0.84-0.90), 0.94 (95% CI:0.91-0.97), 12.83(95% CI:5.93-27.78) and 0.13 (95% CI:0.08-0.22) for the detection of mild HS with MRI-PDFF, 0.80 (95% CI:0.76-0.84), 0.91 (95% CI:0.88-0.93), 7.83 (95% CI:5.68-10.80) and 0.21 (95% CI:0.15-0.29) for the detection of moderate HS, and 0.74 (95% CI:0.67-0.80), 0.92 (95% CI:0.89-0.94), 7.05 (95% CI:4.96-10.04) and 0.29 (95% CI:0.18-0.47) for the detection of severe HS, respectively. The AUCs for mild, moderate and severe HS detection were 0.96(95% CI:0.95-0.97), 0.94(95% CI:0.92-0.95) and 0.92(95% CI:0.90-0.94), respectively. Substantial heterogeneity was observed. No publication bias was detected.
**Conclusion:** MRI-PDFF shows high diagnostic accuracy in detecting and grading HS and may serve as a noninvasive tool for evaluation of HS in NAFLD patients.

**B-1472** 08:54

**Dual-energy CT in patients with fatty liver disease: improved assessment of hypoattenuating liver lesions using virtual monoenergetic imaging**

L. Lenga[1], I. Yel[1], C. Booz[1], S.S. Martin[2], J.-E. Scholtz[1], T.J. Vogl[1], J.L. Wichmann[1], M.H. Albrecht[1]; [1]*Frankfurt/DE*, [2]*Charleston, SC/US* *(lukas.lenga@gmail.com)*

**Purpose:** To assess the impact of noise-optimized virtual monoenergetic imaging (VMI+) on delineation and measuring accuracy of hypoattenuating liver lesions in patients with fatty liver disease compared to standard reconstruction.
**Methods and Materials:** Twenty-eight patients with fatty liver disease and hypoattenuating liver lesions who had undergone unenhanced and contrast-enhanced portal-venous dual-energy CT were included in this retrospective study. In addition to standard linearly-blended M_0.6 reformats, noise-optimized virtual monoenergetic images (VMI+) were reconstructed in 10-keV intervals. Contrast-to-noise ratio (CNR) of the liver parenchyma was analyzed and the best VMI+ series was further subjectively investigated. Image quality and lesion margin demarcation were evaluated. Size measurements of all hypodense lesions (n=61) were performed in a predefined sequence independently two times. Inter- and intra-rater agreement was assessed using Intraclass correlation coefficient (ICC).
**Results:** The calculated CNR was greatest at 40-keV VMI+ (5.8±3.8 HU), significantly higher compared to standard reconstructions (3.5±1.5 HU) (P<0.001). Furthermore, 40-keV VMI+ provided the best subjective overall image quality (P≤0.194). Margin demarcation of liver lesions was found to be superior for 40-keV VMI+ compared to standard linearly-blended image series (P≤0.001). Inter-observer agreement for lesion measurements was higher for 40-keV VMI+ reconstructions (ICC, 0.87) vs standard images (ICC, 0.69). Intra-observer agreement was greater for 40-keV VMI+ (mean ICC, 0.94), in comparison with linear-blended reconstructions (mean ICC, 0.82).
**Conclusion:** Noise-optimized VMI+ reconstructions significantly improve contrast and demarcation of hypoattenuating liver lesions in patients with fatty liver disease compared to standard reconstruction, which translates into improved inter- and intra-rater agreement for quantitative size measurements.
*Author Disclosures:*
**J.L. Wichmann:** Other; Wichmann JL received speaker fees from GE and Siemens. **M.H. Albrecht:** Other; Albrecht MH received speaker fees from Siemens and Bracco.

**B-1473** 08:58

**CT-based liver surface nodularity score for noninvasive assessment of clinically significant portal hypertension**

A. Abou Elkassem[1], A.M.K. Abdel Aal[1], A. Gunn[1], K. Mahmoud[1], S. Lirette[2], A. Smith[1]; [1]*Birmingham, AL/US*, [2]*Jackson, MS/US (akamel@uabmc.edu)*

**Purpose:** To evaluate the accuracy of the CT-based liver surface nodularity (LSN) score to detect clinically significant portal hypertension (CSPH) in patients with chronic liver disease (CLD).
**Methods and Materials:** For this retrospective study, we identified 46 adult patients with HCV, alcoholic or NASH compensated CLD and contrast-enhanced abdominal CT imaging within 6 months of hepatic vein pressure gradient (HVPG) measurements obtained between 2004 and 2018. The LSN score was measured using custom software. Total liver volume, liver segmental volume ratio (segments I-III vs. IV-VIII), splenic length/volume, and portal vein width were measured from the CT images. A HVPG ≥10 mmHg was used to define CSPH. The accuracy for predicting CSPH was assessed by receiver operating characteristics area under the curve (AUC).
**Results:** CSPH was present in 63% of patients. In patients with HVPG<10 mmHg vs. CSPH, the average LSN scores were 2.23 vs. 2.55 (p<0.001), average splenic volumes were 426 vs. 736 cc (p=0.031), average splenic lengths were 12.8 vs. 14.9 cm (p=0.037), average FIB-4 indices were 3.17 vs. 5.83 (p=0.038), and average MELD scores were 12.0 vs.16.0 (p=0.041). Other CT metrics did not have a significant difference (p>0.05). The accuracy for predicting CSPH was highest with the LSN score (AUC=0.80) as compared to splenic volume, splenic length, FIB-4 index, and MELD score (AUC=0.69 for each). The probability of having CSPH increased about fivefolds for each standard deviation increase in LSN score (OR: 5.36; 95%CI: 1.69, 17.0; p=0.004).
**Conclusion:** The CT-based LSN score accurately detects CSPH in patients with CLD.
*Author Disclosures:*
**A. Smith:** Owner; Liver Nodularity LLC. Patent Holder; Liver Nodularity LLC.

**B-1474** 09:02

**Clinical significance of pancreatic steatosis: association between pancreatic exocrine insufficiency and organ fat content**

M.-L. Kromrey[1], N. Friedrich[1], R.-T. Hoffmann[2], R. Bülow[1], U. Motosugi[3], J.-P. Kühn[1]; [1]*Greifswald/DE*, [2]*Dresden/DE*, [3]*Yamanashi/JP* *(marie-luise.kromrey@uni-greifswald.de)*

**Purpose:** To investigate the relationship between pancreatic fat content and depression of pancreatic exocrine function.
**Methods and Materials:** In 1,458 volunteers (681 men, mean age 52.8 years) receiving whole-body MRI, pancreatic fat content was quantified by proton-density fat fraction (PDFF). Additionally, hepatic, visceral and subcutaneous fat volumes were determined. Association of organ fat content with exocrine pancreatic function measured by faecal elastase (normal function: >200 µg/g, n=1,319; moderate restriction:100-200µg/g, n=110, manifest restriction: <100 µg/g, n=29) was performed using age, gender and BMI-adjusted linear regression models.
**Results:** Mean pancreatic fat content was significantly higher in subjects with decreased compared to normal faecal elastase levels (9.36±4.95% vs 7.30±3.59%, p<0.01; overall mean: 7.50±3.78%). An inverse association between pancreatic fat and enzyme levels was detected for the whole study population (beta: -7.19 [standard error: 1.39]; p<0.01) as well as in the subgroup with normal function (-4.26 [1.32]; p<0.01), whereas subjects with restriction in exocrine pancreatic function displayed just a trend towards an inverse relation (-1.28 [0.84]; p<0.13). Concerning other fat compartments, linear regression analyses showed no significant associations between hepatic, visceral or subcutaneous fat and faecal elastase.
**Conclusion:** An inverse relationship between pancreatic fat content and faecal elastase levels suggests an association of pancreatic steatosis with a restriction of pancreatic exocrine function.

**B-1475** 09:06

**T1 mapping of autoimmune pancreatitis as a quantitative outcome biomarker**

L. Zhu[1], Z.-y. Sun[1], Z.-y. Jin[1], T.-y. Qian[1], D. Nickel[2], M. Makowski[3], P. Asbach[3], B. Hamm[3], H.-d. Xue[1]; [1]*Beijing/CN*, [2]*Erlangen/DE*, [3]*Berlin/DE* *(zhuliang_pumc@163.com)*

**Purpose:** To investigate the ability of T1 mapping to visualize and quantify the short-term and mid-term improvement of autoimmune pancreatitis with corticosteroid treatment (CST), and to correlate T1 relaxation time of the pancreas with clinical status and serum IgG4 levels.
**Methods and Materials:** The institutional review board approved this prospective study and all patients provided written informed consent. Pancreatic MR, including native T1 mapping, was performed in 33 AIP patients before and during CST, and in 20 patients without pancreatic diseases (as control). T1 relaxation time of the pancreatic head, body and tail was measured separately in each patient. Clinical symptoms and serum IgG4 level of the patients in the mean time of the MR scan were recorded.
**Results:** The T1 relaxation time of AIP was significantly elongated compared to normal pancreatic tissue (1124.5±95.7 ms vs 784.3±41.8 ms, p<0.001). After short-term CST (4 weeks) treatment, the T1 relaxation time of AIP already shortened significantly (957.2±97.3 ms, p<0.001), and after mid-term CST (12 weeks), the T1 relaxation time further shortened towards normalization (844.2±71.6 ms, p<0.001). In 27 AIP patients with elevated serum IgG4 at baseline, T1 relaxation time shows positive correlation with serum IgG4 level (r=0.289, p=0.011). In 6 AIP patients with normal serum IgG4 level at baseline, T1 relaxation time shortening was in accordance with or preceded symptom relief.
**Conclusion:** T1 mapping can be used to evaluate parenchyma inflammation of AIP and quantify response to treatment. It provides a quantitative outcome surrogate for AIP.

**B-1476** 09:10

**Combination of diffusion kurtosis imaging and conventional MRI helps to differentiate fibrotic from inflammatory bowel strictures in patients with Crohn's disease**

J. Du, X. Li, L. Huang, S. Huang, Z. Li; *Guangzhou/CN* *(dujinfangchn@163.com)*

**Purpose:** Distinguishing fibrotic from inflammatory strictures is an important clinical requirement. We evaluated the value of diffusion kurtosis imaging (DKI) in combination with conventional MRI for characterising Crohn's disease (CD) strictures using surgical histopathology as a reference standard.
**Methods and Materials:** 31 consecutive CD patients undergoing conventional MRI and DKI (b values of 0-2000 s/mm$^2$) before surgery were recruited. We observed T2WI hyperintensity and enhancement pattern on conventional MRI, and measured apparent diffusion coefficient (ADC), apparent diffusional kurtosis (Kapp) and apparent diffusion for non-Gaussian distribution (Dapp) on

DKI. A model with combined MRI variables was proposed for characterisation of intestinal strictures, and its performance was validated on 9 new CD patients.
**Results:** Histologic inflammation scores significantly correlated with T2WI hyperintensity ($r$=0.477, $P$<0.001), ADC ($r$=-0.226, $P$=0.044) but not with enhancement pattern ($r$=0.037, $P$=0.746). A strong correlation of histologic fibrosis scores with Kapp ($r$=0.604, $P$<0.001) was found, followed by Dapp ($r$=-0.491, $P$<0.001) and ADC ($r$=-0.270, $P$=0.015). Using T2WI hyperintensity as a predictor, conventional MRI could differentiate mild from moderate-to-severe inflammation with a sensitivity of 0.970 and a specificity of 0.479. Kapp enabled to identify non-mild from moderate-severe fibrosis with a sensitivity of 0.959 and a specificity of 0.781. The MRI strictured classification that combing of T2WI hyperintensity and Kapp moderately agreed with the pathological classification ($\kappa$=0.507, $P$<0.001). In the validation set, there was a moderate agreement between the MRI and the pathological classification ($\kappa$=0.530, $P$<0.001).
**Conclusion:** Combination of DKI and conventional MRI helps to characterise bowel strictures in CD patients.

**B-1477** 09:14
**Perfusion fraction derived from IVIM-DWI may facilitate differentiation of moderate to severe inflammation in ulcerative colitis**
J. Podgorska, K. Pasicz, E. Zagórowicz, A. Mróz, B. Gołębiewski, P. Kus, J. Jasieniak, W. Skrzyński, A. Cieszanowski; *Warsaw/PL (jpodgo@gmail.com)*

**Purpose:** To determine the efficacy of intravoxel-incoherent motion (IVIM) parameters in assessment of inflammatory activity of ulcerative colitis in reference to histopathological examination.
**Methods and Materials:** In this prospective study 20 adult patients with ulcerative colitis underwent 3T magnetic resonance diffusion weighted imaging (DWI) with b-values of 0, 10, 30, 50, 75, 100, 150, 200, 500, 900 s/mm$^2$ within 1-6 days of endoscopic evaluation with biopsies. The inflammatory activity of bowel segments (rectum, sigmoid, descending, transverse, ascending colon) was graded using a six-grade classification system for inflammation. IVIM-derived parameters ($f$, D and D*) calculated from ROIs placed within the bowel wall were correlated with the histopathologic score. T-test was used for comparison and ROC curve analysis was performed.
**Results:** In total 33 bowel segments were analysed. For comparison two data groups were created: inactive or mild activity (grade 0-2, n=23) and moderate to severe activity (grade 3-5, n=11). Statistically significant differences between both groups were found in $f$ (mean = 0.19, SD = 0.09 and mean = 0.28, SD = 0.13, p=0.024; AUC = 0.723, sensitivity 0.82, specificity 0.59, accuracy 0.67 for cut-off value 0,185) and D (mean = 1.34 x10$^{-3}$mm$^2$/s, SD = 0.36x10$^{-3}$mm$^2$/s and mean = 1.07x10$^{-3}$mm$^2$/s, SD = 0.20x10$^{-3}$mm$^2$/s, p=0.0083; AUC = 0.735, sensitivity 0.91, specificity 0.54, accuracy 0.66 for cut-off value 1.242x10$^{-3}$mm$^2$/s). No significant difference was found in D*.
**Conclusion:** IVIM-derived perfusion fraction may facilitate distinguishing moderate to severe inflammation from less active or inactive disease in ulcerative colitis.

**B-1478** 09:18
**Machine learning-based analysis of rectal cancer MRI radiomics for prediction of metachronous liver metastasis**
M. Liang, X. Zhao; *Beijing/CN (lm_1202@163.com)*

**Purpose:** To use machine learning-based MRI radiomics to predict metachronous liver metastases (MLM) of rectal cancer patients at baseline treatment.
**Methods and Materials:** This study retrospectively analysed 108 patients with rectal cancer(54 in the MLM and 54 in the non-metastasis group). Feature selection were performed in the radiomic feature sets extracted from images of T2WI and venous phase(VP) sequence respectively, and the combining feature set with 2058 radiomic features incorporating T2WI and VP sequences with the least absolute shrinkage and selection operator method. Fivefold cross-validation and two machine learning algorithms(support vector machine, SVM; logistic regression, LR) were utilized for MLM predictive model building. The diagnostic performance of the models were evaluated by ROC curves with indicators of accuracy, sensitivity, specificity and area under the curve (AUC), and compared by DeLong test.
**Results:** Five, 8, and 22 optimal features were selected from 1029 T2WI, 1029 VP, and 2058 combining features, respectively. Four-group models were constructed using the 5 optimal T2WI features ($Model_{T2}$), the 8 optimal VP features ($Model_{VP}$), the combined 13 optimal features ($Model_{combined}$), and the 22 optimal features selected from 2058 features ($Model_{optimal}$). In $Model_{VP}$, the LR was superior to the SVM algorithm ($P$=0.0303). The $Model_{optimal}$ using the LR algorithm showed the best prediction performance ($P$=0.0019-0.0081), with accuracy, sensitivity, specificity, and AUC of 0.80, 0.83, 0.76, and 0.87, respectively.
**Conclusion:** Radiomics models based on baseline rectal cancer MRI has high potential for MLM prediction, especially the $Model_{optimal}$ using the LR algorithm. Moreover, except for $Model_{VP}$, the LR was not superior to the SVM algorithm for model construction.

**B-1479** 09:22
**MRI-detected extramural vascular invasion is a strong risk factor in predicting distant metastasis in rectal cancer**
P. Tripathi[1], D. Hu[1], M. Zeng[2], W. Guo[2], S. Rao[2], B. Rai[1]; [1]*Wuhan/CN*, [2]*Shanghai/CN (pratik9_band@hotmail.com)*

**Purpose:** Extramural vascular invasion (EMVI) has been recommended as an independent prognostic factor for poor overall survival rate in rectal cancer. We aimed to evaluate the MRI-detected EMVI (mrEMVI) in predicting (synchronous) distant metastasis in T3 rectal cancer.
**Methods and Materials:** Histopathologically confirmed T3 rectal cancer patients who underwent preoperative MRI without previous treatment were enrolled for this study. Two blinded radiologists evaluated the location of the tumour, the degree of mesorectal extension and mrEMVI. mrEMVI was further categorized into EMVI positive and EMVI negative in T2-weighted image using mrEMVI scoring system. The results along with other clinical characteristics (age, sex, tumour location, MRI detected the distance of mesorectal extension, lymphatic invasion, perineural invasion, mrEMVI score and CEA) were then correlated with synchronous metastases to determine the risk factors.
**Results:** Among 180 patients, 38 patients were confirmed to be mrEMVI positive, 142 patients mrEMVI negative. There were a total of 34 patients with synchronous metastasis in which 25 were mrEMVI positive and 9 were mrEMVI negative. Three factors were significantly associated with synchronous metastasis: mrEMVI (p=0.001; OR=8.665), histopathological lymphatic invasion (p=0.001; OR=12.940) and preoperative CEA (p =0.026; OR=4.124). mrEMVI score 4 was more liable for synchronous metastasis (p=0.044; OR=9.429) than mrEMVI score 3 in rectal cancer.
**Conclusion:** There is an intimate relationship between mrEMVI and synchronous metastasis in rectal cancer. mrEMVI positive is an independent risk factor for synchronous distant metastasis in rectal cancer. mrEMVI score 4 is a strong risk factor for synchronous metastasis than mrEMVI score 3 in rectal cancer.

**B-1480** 09:26
**DWI and high-resolution T2WI-volumetry association with lymphovascular invasion and N-stages in resectable rectal cancer**
X. Chen, H. Li; *Chengdu/CN (20shenbaise@163.com)*

**Purpose:** To determine whether diffusion-weighted imaging (DWI)-volumetry and high-resolution T2-weighted imaging (T2WI) MR-volumetry could predict lymphovascular invasion (LVI) and N-stages in resectable rectal cancer.
**Methods and Materials:** 50 consecutive patients with rectal cancer who underwent radical surgery in 1-week after DWI and high-resolution T2WI were retrospectively identified. Gross tumor volume (GTV) was evaluated on DWI and high-resolution T2WI. Univariate and multivariate analyses were performed to determine whether GTV could predict LVI and lymph node metastasis (LNM). Mann-Whitney U test was performed to compare GTV among N-stages. Cutoffs of GTV were investigated using area under the receiver operating characteristic curve (AUC) analysis for predicting LVI and N-stages.
**Results:** DWI-GTV and T2WI-GTV increased with LVI ($r$=0.750 and 0.710,$P$<0.0001,respectively) and increasing of N stage ($r$=0.780 and 0.755,$P$<0.0001,respectively). Univariate analysis showed DWI-GTV and T2WI-GTV could predict LVI ($P$<0.0001). Multivariate analyses indicated only DWI-GTV as an independent risk factor of LVI ($P$=0.005, odds ratio=1.207) and LNM ($P$=0.005, odds ratio=1.420). The Mann-Whitney U test showed DWI-GTV and T2WI-GTV could distinguish N0 from N1, N0 from N1-2, N0-1 from N2 ($P$<0.0001). DWI-GTV could predict LVI (cutoff,11.05cm$^3$;AUC,0.899), and distinguish N0 from N1 (cutoff,10.86cm$^3$;AUC,0.865), N0 from N1-2 (cutoff,10.46cm$^3$;AUC,0.934), N0-1 from N2 (cutoff,17.7cm$^3$;AUC,0.932). T2WI-GTV could predict LVI (cutoff,13.74cm$^3$;AUC,0.877), and distinguish N0 from N1 (cutoff,12.25cm$^3$;AUC,0.827), N0 from N1-2 (cutoff,13.36cm$^3$; AUC,0.911), N0-1 from N2 (cutoff,20.43cm$^3$; AUC,0.927).
**Conclusion:** High-resolution T2WI-GTV and DWI-GTV of resectable rectal cancer were correlated well with the LVI and LNM, but the latter is a potentially more promising non-invasive technique that can help predict the preoperative LVI and distinguishing N-stages.

**B-1482** 09:30
**Prediction of gastric and esophagogastric junction cancer response to neoadjuvant treatment by functional imaging with PCT**
J. Kruk-Bachonko, W. Krupski, T. Skoczylas; *Lublin/PL (asiakruk1@wp.pl)*

**Purpose:** The Perfusion Computed Tomography (P-CT) exam has shown encouraging results for prediction of tumor response to chemotherapy (CHT) and radiochemotherapy (RCT) in gastric and esophagogastric junction cancer.

We studied time course of perfusion parameters values changes in patients treated with neoadjuvant treatment (CHT and RCT). The aim of this study was to prospectively evaluate these findings by using functional perfusion parameters.
**Methods and Materials:** 59 patients with gastric cancer qualified for systemic treatment (36 patients received chemotherapy and 23 patients received radiochemotherapy) were involved in to P-CT exam. Study protocol involved baseline P-CT exam before neoadjuvant therapy and P-CT after neoadjuvant therapy before surgical treatment. Perfusion-CT exam examined the following parameters: Blood Flow (BF), Blood Volume (BV), Mean Transit Time (MTT) and Permeability Surface (PS). Positive clinical response to neoadjuvant treatment was defined as tumor size reduction 25% or more.
**Results:** Responders showed a higher baseline BF and BV value than non-responders and showed statistically significant (p<0,05) BF and BV decrease after therapy. Linear regression of tumor size reduction and perfusion parameters showed significant positive correlation between responders' PS (p<0,05) value and tumor size reduction after neoadjuvant therapy. Linear regression showed significant negative correlation between the baseline MTT value (p<0,05) and tumor size reduction after neoadjuvant therapy.
**Conclusion:** P-CT reveled a positive prediction value of P-CT in early responders and non-responders patients identification. A full evaluation of gastric cancer treatment should include P-CT exam as a standard procedure.

**B-1483** 09:34
**Added value of MRI to endoscopic and endosonographic response assessment after neoadjuvant chemoradiotherapy in oesophageal cancer: a pilot study**
S.E. Vollenbrock, J.M. van Dieren, F.E.M. Voncken, S.T. van Turenhout, L.L. Peppelenbosch-Kodach, K.J. Hartemink, B.M.P. Aleman, R.G.H. Beets-Tan, A. Bartels-Rutten; *Amsterdam/NL (s.vollenbrock@nki.nl)*

**Purpose:** In order to select oesophageal cancer patients after neoadjuvant chemoradiation (nCRT) for organ-preserving treatment instead of surgery, complete response (CR) assessment must be accurate. As endoscopic and endosonographic assessment with biopsies of the primary tumour area and fine needle aspiration (FNA) of lymph nodes is known to result in a high number of false negatives, our aim is to determine the added value of MRI.
**Methods and Materials:** Twenty-two patients with locally advanced oesophageal cancer underwent MRI (1.5 T, T2W+DWI; b-values 0,200,800 s/mm$^2$), endoscopy with biopsies and endosonography with FNA after nCRT. One radiologist scored MRIs using a 5-point score (1=definitely CR, 2=probably CR, 3=inconclusive, 4=probably residual tumour, 5=definitely residual tumour). Histopathology of the resection specimen was the reference standard (Mandard tumour regression grade 1=pathological CR, 2-5=residual tumour). Sensitivity and specificity of residual tumour detection were calculated for endoscopy+endosonography, and for endoscopy+endosonography including MRI.
**Results:** Three (14%) of 22 patients achieved a pCR. Endoscopy with biopsies and endosonography with FNA found residual tumour in 9 of 19 patients with residual disease (sensitivity 47%). After adding MRI, 17 of 19 residual tumours were assessed correctly (sensitivity 89%). All complete responders had negative endoscopic biopsies (specificity 100%); one was incorrectly assessed as residual tumour on MRI (specificity 67%).
**Conclusion:** The addition of MRI to endoscopic and endosonographic response assessment improves detection of residual tumour after nCRT in oesophageal cancer patients.

**B-1484** 09:38
**Multiparametric combined FDG-PET/CT and MR imaging to predict response to chemoradiotherapy in rectal cancer: whole tumour versus sub-volume analysis**
N.W. Schurink[1], M. Berbee[2], J.J.M. van Griethuysen[1], W. van Elmpt[2], M. Maas[1], M.J. Lahaye[1], F.C.H. Bakers[2], R.G.H. Beets-Tan[1], D.M. Lambregts[1]; [1]*Amsterdam/NL*, [2]*Maastricht/NL (n.schurink@nki.nl)*

**Purpose:** To assess the individual and complementary performance of quantitative parameters from pre-treatment multiparametric MRI and FDG-PET/CT to predict treatment response in rectal cancer.
**Methods and Materials:** A pilot group (n=20) underwent multiparametric MRI (T2W + DWI; b0, 100, 500, 1000) and FDG-PET/CT before neoadjuvant treatment + surgery. Images were anatomically co-registered using rigid + non-rigid registration. Whole-tumour volumes were segmented on T2W-MRI and transferred to the other modalities/sequences; each tumour was additionally divided into 7x7x7mm$^3$ sub-volumes. The following parameters were calculated per-tumour and sub-volume: T2-texture (uniformity/entropy), T2-signal intensity (SI), ADC (mean/max/min/median/10$^{th}$ and 90$^{th}$ percentile), SUV (mean/max/median), and CT-HU. Performance to predict poor response (Mandard TRG3-5) vs. good response (TRG1-2) was calculated using ROC analysis for [1] each individual parameter (whole-tumour), [2] multiparametric combination of the 5 best-performing parameters (whole-tumour), and [3] the proportion (%) of 'poor-response' sub-volumes within the tumour. Sub-volumes were defined as 'poor response' when ≥4 (out of 5) parameters within that sub-volume were indicative of TRG3-5, using a cut-off derived from dichotomisation by median split.
**Results:** Best single predictive parameters were T2-uniformity (AUC 0.78), T2-SI (AUC 0.70), ADCmax (AUC 0.76), SUVmax (AUC 0.78) and SUVmean (AUC 0.73). Combined multiparametric performance (whole-tumour) was AUC 0.91. The proportion of 'poor-response' sub-volumes resulted in AUC 0.74.
**Conclusion:** Multiparametric analysis of quantitative MRI and FDG-PET/CT data has potential added value to predict response to neoadjuvant treatment. If there is a potential benefit for performing sub-volume (or voxel-wise) analysis, this needs to be established by further and larger studies; with our current method, we have so far not demonstrated a clear added value.

**B-1485** 09:42
**Rectal wall T2 relative signal intensity: a non -invasive biomarker of gastro-intestinal (GI) toxicity in patients treated with pelvic radiotherapy (RT)**
G. Della Vecchia, A. Di Chiara, M. Panzeri, A. Palmisano, A. Esposito, P. Passoni, C. Fiorino, A. Del Maschio, F. De Cobelli; *Milan/IT*

**Purpose:** GI toxicity is commonly experienced by patients with pelvic neoplasia treated with RT. A non-invasive biomarker of GI toxicity may help patient's management.In Patients with locally advanced rectal cancer (LARC) treated with chemioradiotherapy, GI toxicity is expected for RT direct on rectal wall. Aim was to evaluate the relative modification of T2w rectal wall signal intensity (T2ratio) during and after treatment in relation to the degree of GI toxicity experienced.
**Methods and Materials:** Sixty-six patients with LARC underwent 1,5 T MRI before, after 6 weeks and at the end of CRT. An ROI was manually drawn on rectal wall and obturator muscle on T2w images at each time point and the relative T2 ratio (SIrectum/SImuscle) calculated.GI toxicity was scored following National Cancer Institute Common Terminology Criteria for Adverse Event in: G0=absent, G1=low, G2=medium, G3=high.
**Results:** 5 patients resulted G0, 28 G1, 25 G2 e 8 G3. T2 ratio was significantly different only at post CRT (median G0=1.17, G1=2.42, G2=2,36, G3=3,31; p: 0.041). A cut-off value of 1.785 was able to predict GI toxicity (G1-3) with 0.82 AUC, 84% and 72% of sensitivity and specificity. A cut-off value >2,675 predicted G3 with 0.75 AUC, 75% sensitivity 60% specificity. No significant difference among T2ratio and GI toxicity was found at before and during CRT.
**Conclusion:** T2 ratio could be a helpful non-invasive biomarker of GI toxicity, that could be apply in other setting of pelvic RT.

**10:30 - 12:00** **Room C**

### Radiographers

### SS 1814

### CT and professional issues in radiography

*Moderators:*
N.N.
J.M. Nightingale; Sheffield/UK

**B-1486** 10:30
**Brain CT with 64- and 16-slice CT scans: comparison of image quality and radiation dose with standard protocols**
M.M. Abuzaid[1], W. Elshami[1], A.A. Sulieman[2], C. Kappas[3]; [1]*Sharjah/AE*, [2]*Alkharj/SA*, [3]*Larissa/GR (mabdelfatah@sharjah.ac.ae)*

**Purpose:** The purpose of this study was to compare radiation dose and image quality of 16- and 64-MDCT scanners during brain procedures.
**Methods and Materials:** CT scan parameters and patient data were collected for 40 patients who underwent routine brain scan protocol. Average values of CTDIvol, DLP and effective dose (ED) were calculated and compared for the two scanners. Three radiologists evaluate independently image quality using a 5-point scale, as well as select a preferable image.
**Results:** The DLP and effective dose values are higher for 64-slice CT (1160 mGy/cm and 2.55 mSv) versus 16-slice CT (670 mGy/cm and 1.47 mSv), The p value was less than 0.05, which is statistically significant. In terms of image quality, 64CT scanners images scored higher than 16CT scanner with average scores of 4.5 and 4.0, respectively. In preferred image selection, radiologists preferred the 64CT scanner images in 15 out of 18 image sets (83%), significance of p < 0.01.
**Conclusion:** The high number of detectors produces high image quality and increases the amount of radiation dose received by the patients. Optimizing of scanning protocol leads to ensure that patient dose is within the level of acceptance limits.

**B-1487** 10:38

**Physicians knowledge and perceptions of radiation dose and inherent risks in computed tomography examinations**

M. Gharbi, F. Aouini, H. Kamoun; *Tunis/TN (manel.gharbi88@yahoo.fr)*

**Purpose:** Integrating CT into routine care has improved patient health care dramatically. Despite these great medical benefits, there is concern about the potential radiation-related cancer risk. Consequently, dose reduction and radiation protection is a topic of scientific concern. The purpose of this study was to evaluate the knowledge of physicians prescribing CT examinations on the radiation protection of patients.
**Methods and Materials:** This descriptive study was performed from the 22nd January to the 12th February 2018 at the military hospital of Tunis. A questionnaire was distributed to all clinician on medical staff. Several questions related to their prescription pattern and their knowledge of radiation protection.
**Results:** We identified 94 physicians. 73 of them (77.7%) responded to our questionnaire. The mean of professional experience was 8 years. 42.5% of clinicians prescribing CT examinations were interns and resident and 57.7% were senior doctors. While 80.8% of physicians claimed that they considered the risks from exposure to ionizing radiation when prescribing a CT examination, only 16.4% informed their patients about those risks. Knowledge of the radiation dose delivered during CT evaluation of the abdomen and pelvis was poorly understood and the risks related to small doses of radiation were grossly underestimated. Finally, only 19.2% of clinicians had received training with regards to radiation protection.
**Conclusion:** In the present study, knowledge gaps concerning computed tomography radiation doses and associated health risks are evident. It seems that only the attendance of a radiation protection course has a positive influence on the physician's level of knowledge.

**B-1489** 10:54

**Adaptive statistical iterative reconstruction for computed tomography of the spine**

M. Tsuda; *Yonago/JP (joey.no.1@hotmail.co.jp)*

**Purpose:** To evaluate the effect of adaptive statistical iterative reconstruction technique for sagittal CT of the spine.
**Methods and Materials:** An improved adaptive statistical iterative reconstruction algorithm (ASiR-V) was used in this study. Sagittal planes of all images were reformatted to 2.0 mm thickness, and were reconstructed using filtered back projection (FBP), blending 50%, and 100% levels of ASiR-V (ASiR-$V_{50}$, ASiR-$V_{100}$, respectively). Subjective image quality was assessed on a five-point scale by three radiographers. We assessed the images of 32 patients. For objective assessment, modulation transfer function (MTF) was measured using a custom phantom to estimate z-axis resolution. Noise power spectrum (NPS) was measured using a cylindrical water phantom and 2.0-mm thick axial images to evaluate image noise.
**Results:** There were significant subjective differences in each reconstruction; ASiR-$V_{100}$ was scored the highest. There were no enhancements in degeneration of z-axis resolution using ASiR-V in all contrast images. NPS curves demonstrated that ASiR-V was less efficient for reducing image noise.
**Conclusion:** ASiR-V effectively improved image noise and image quality for sagittal CT of the spine.

**B-1490** 11:02

**CT scan chest: a new approach to lung cancer**

A. Bernardo, F. Gonçalves, M.O. Fernandes; *Lisbon/PT (abernardo@hospitaldaluz.pt)*

**Purpose:** Demonstrate the evolution of the radiation's dose reduction with the use of new technologies;Compare radiation dose´s applied in TC Thorax screenings with the European Guidelines on Quality Criteria for Diagnostic Radiographic Images (EGQCDRI).
**Methods and Materials:** A sample of 157 cases was used - 122 performed with the *SOMATOM Force CT* (Siemens Healthcare AG) and 35 performed with the *SOMATOM Emotion 16 TC* (Siemens Healthcare AG);*Syngo Plaza* (Siemens Healthcare AG) was used to consult images and the radiation's dose. Results were recorded on Excel sheets, as well as the respective means and the standard deviations;We compared the results obtained with the t-Student statistical test between the TC Low dose's of *SOMATOM Emotion 16* and *SOMATOM Force* using the SPSS program;Radiation dose values were compared between the *TC SOMATOM Force* and the reference values present in the EGQCDRI.
**Results:** It was verified that there is a statistically significant difference between the parameters of radiation doses between the different equipment used (p <0.0001), within a reliable range of 95%, nonetheless maintaining diagnostic quality;When comparing the radiation doses of the *CT SOMATOM* Force with the ones of the EGQCDRI, we can see that they are similar in value.
**Conclusion:** We concluded that new technological approaches about CT have brought a significant radiation's dose reduction, being nowadays equated to the RX Thorax. Several parameters may have an influence on the final image quality and should be adjust to guarantee diagnostic quality on this type of study.

**B-1491** 11:10

**Prophylaxis of contrast-induced nephropathy in computed tomography procedures by use of low-osmolality contrast media and good hydration**

H.H. Mansour; *Gaza/PS (husam-RT2007@hotmail.com)*

**Purpose:** To settle the prophylaxis strategy for reducing the effect of IV contrast on the development of post -CT examination acute injury nephropathy.
**Methods and Materials:** Cross-sectional study for a total of 60 patients (32 males and 28 females) who received iodinated contrast agents during CT procedures over a period of one month. CIN was defined as a relative increase of >25% or an absolute increase of >0.5 mg/dL in serum creatinine levels 3 days post-CT procedure. Patients who undergo intravenous contrast CT procedures were included in this research to ensure good preparation and follow-up.
**Results:** Paired t test was used to study the differences between urea and creatinine pre- and post-contrast media. The test revealed no statistically significant differences between urea pre-CT exam (mean= 35.3, SD = 15.2) and post-CT with contrast media (mean= 33.9, SD = 25.4) with t= 0.52 and p value= 0.608. Moreover, the study shows that no statistically significant differences between creatinine pre-CT test and post-contrast media CT (mean= 0.96, p value = 0.27) and post-CT with contrast media (mean= 0.95, p value =0.48) with t= 0.52 and p value= 0.608.
**Conclusion:** Low-osmolality contrast media and good hydration pre- and post-IV contrast administration are the preventive approaches to a declination of CIN.

**B-1492** 11:18

**Eye lens protection: new perspective**

A.J.C.D. Cunha; *Braganza/PT (altinoc@gmail.com)*

**Purpose:** Cataract is, according to World Health Organization (WHO), an eye lens opacity which prevents clear vision, being responsible for 51% of world blindness, affecting 20 million people (2010). Most of the cases are related to ageing process or it may develop after eye injuries, inflammation, and some other eye diseases.Food concerns is a quite trendy issue nowadays, so it plays an important role on people daily routine, as they seek for a healthy way of life.
**Methods and Materials:** Ionizing radiation (IR) is an important source in the generation of free radicals such as superoxide radical and hydroxyl radical, among the various physical/chemical agents; interacts with cells and produces cytotoxic effects. So IR is a proven human carcinogen and cataractogen. The crystalline lens of the eye is among the most radiosensitive tissues in the body.A literature review demonstrates that evidence and the antioxidant and radioprotective effects of several food components, such as Propolis and its component Caffeic Acid Phenethyl Ester (CAPE).
**Results:** What if we could merge eye lens radiation protection and food? Could it be possible to prevent or reduce radiation-induced cataract by changing food habits or introducing new kinds of foods on our daily routines? Studies shows that after irradiation, cataract rate drop 30 % in Propolis and 40 % in CAPE.
**Conclusion:** In a near future we will prevent the occupational exposure to ionizing radiation with not only individual protection equipment and but also with individual protection food, changing the radiation protection as we know it.

**B-1493** 11:26

**Development of 3D printed quality control tool for X-Ray beam alignment and collimation evaluation**

M.V.L. Oliveira, J. Barros; *Salvador/BR (marcusradiology@gmail.com)*

**Purpose:** To develop a 3D printed low-cost tool for evaluation of X-ray beam alignment and collimation
**Methods and Materials:** The study was divided in two phases: 1) 3D printed prototype development and 2) Comparison with commercial test object. A 3D printer was used for developing two objects, with 40% of infill and printed with two filaments: PLA (Polylactic acid) and ABS (acrylonitrile butadiene styrene). Two X-ray equipment (SIEMENS, model MULTIX B) were used for Beam Collimation and Beam Alignment tests. For validation, it was compared with a commercial Collimator / Beam Alignment Test Tool.
**Results:** The tests performed with the prototype and the standard tool showed deviation of ± 1 cm between the light and radiation field. The Central Ray perpendicularity was evaluated through the coincidence between the rod and the metallic circle. The test of CR alignment held with a standard tool showed the axis perpendicularity of 1.5º while both prototypes presented less than 3º.
**Conclusion:** The prototypes proved to be an effective tool and easy to handle. The variety of printing materials and the ease on filament acquisition reflect the low cost of production.

**B-1494** 11:34
**Portuguese diagnostic reference levels national survey**
J. Santos[1], P.M. Teles[2], M.C. Sousa[1], G. Paulo[1], J. Venancio[2], F. Caseiro Alves[1], A. Figueira[3], P. Rosario[2], P. Vaz[2]; [1]*Coimbra/PT*, [2]*Lisbon/PT*, [3]*Porto/PT (joanasantos@estescoimbra.pt)*

**Purpose:** National survey was carried out in Portugal in order to establish national Diagnostic Reference Levels (DRL's) per for specific radiography and CT examinations involving all the societies and the government agency responsible for radiation issues.
**Methods and Materials:** Exposure parameters and dose values for the most frequent radiography and CT consider the most commune procedure type or clinical information. Data from 10 standard patients per procedure was collected on the participating centres and submitted on the website "NRD Portugal". Equipment brand, technology and quality control details were also obtained.
**Results:** The majority of the centres revealed that equipment quality control was performed by the manufacture (51%). Data from 1590 standard patients submitted to radiography and CT was analysed. DRL's for specific indications were defined for radiography: chest routine, pelvic arthrosis, standing abdominal, lumbar spine without osteosynthesis material; and for CT: head for trauma, head for stroke, chest routine, high resolution chest, abdomen for liver metastases and trunk. Significant differences were founded for head CT trauma and stroke dose values. However no differences were founded in chest CT dose values for routine and high resolution CT.
**Conclusion:** This was the first Portuguese national survey promoted that considers clinical information on the most frequent radiography and CT examinations. As expected CT technology can contribute to high dose reduction. The majority of Portuguese DRL's are according to the literature.

**B-1495** 11:42
**FoRRM: a pilot research mentoring scheme for radiographers in the UK: what have we achieved? An evaluation**
C. Malamateniou[1], F. Mellor[2], R. Appleyard[3], H. McNair[1], K. Knapp[4]; [1]*London/UK*, [2]*Bournemouth/UK*, [3]*Sheffield/UK*, [4]*Exeter/UK (christina.malamateniou@gmail.com)*

**Purpose:** The programme's main aim was to increase research capacity for Radiography in the UK by formal one-to-one mentoring of radiography practitioners/novice radiographer researchers by senior members of the radiography research community.
**Methods and Materials:** Twelve mentoring pairs were recruited, matched and supported by mentoring training and three networking and evaluation events. A steering committee facilitated this scheme. An action research qualitative methodology was used and purposive sampling. Written reflective accounts of all mentees and mentors at the end of the 9-month project were collected and focus group discussion notes were kept. Informed consent and research ethics approval was sought at the beginning of the project. Data was analysed using thematic analysis. Descriptive statistics was also used to highlight overall results on key outcomes. This was a pilot study funded by the Society of Radiographers.
**Results:** The majority of mentoring pairs met their agreed targets; This scheme was able to improve research capacity within radiography in all of the following categories: i) increase research outputs (abstracts, papers, presentations etc), ii) improve career prospects of Radiographer mentees and iii) enhance personal development. Mentees and mentors discussed benefits, opportunities and challenges of the scheme. Important recommendations were discussed for a future launch of the scheme, subject to securing renewed funding.
**Conclusion:** This pilot scheme was the first formal radiography mentoring scheme to help build research capacity in the UK. There have been many benefits for mentees, mentors and the profession and lessons learned, which will hopefully be applied to a future round.

**B-1496** 11:50
**Dementia patient care in the medical imaging department**
R. Challen, L.-F. Low, M.F. McEntee; *Sydney/AU (rachelchallen94@gmail.com)*

**Purpose:** People with dementia have difficulties with memory, executive functions and behaviour which pose a challenge during diagnostic imaging. There is abundant literature on the radiographic diagnosis of dementia; however, there is little research on how to best to care for people with dementia during imaging procedures. The aim of this study is to explore the experiences of dementia care in imaging departments through the perspectives of people with dementia, carers, radiographers and student radiographers.
**Methods and Materials:** This was a cross-sectional qualitative study. Four people with dementia and six carers participated in individual semi-structured interviews; eight academic radiographers and 19 student radiographers participated in focus groups. Interviews and focus groups were transcribed and thematically analysed.
**Results:** Participants described positive and negative experiences during imaging procedures. Common themes existed among people with dementia, carers and radiographers. Findings were (1) people with dementia and carers had negative experiences such as distress and pain; radiographers experienced stigma and violence. (2) Negative experiences during imaging were associated with disrespected personhood, poor communication, insufficient knowledge of dementia, inappropriate time management, overly stimulating physical environments and exclusion of carers. (3) Departmental protocols that contributed to negative experiences included lack of preparation, lack of dementia protocols, and the use of restraints.
**Conclusion:** People with dementia and their carers can experience poor care in imaging departments and radiographers can find it difficult working with people with dementia. Radiographers need training about dementia, imaging services can improve their procedures and environment, and work in greater partnership with carers.

**10:30 - 12:00** **Room X**

### Vascular

### SS 1815

### Imaging of the thoracic aorta

*Moderators:*
P.I. Kalmar; Graz/AT
N.N.

**B-1497** 10:39
**A clinically driven new classification for aortic dissections**
S. Malekzadeh[1], D. Bodenmann[2], A. Topolski[1], A.-M. Jouannic[1], S.D. Qanadli[1]; [1]*Lausanne/CH*, [2]*Frauenfeld/CH (soonazmalakzadeh@gmail.com)*

**Purpose:** To report a new classification for aortic dissections (AD), based on a modified Stanford classification, to consider the aortic arch as a separate entity and integrate patterns influencing treatment strategy notably malperfusion syndrome (MPS). The proposed classification was evaluated in a large population of ADs.
**Methods and Materials:** All patients with proven de novo acute AD who were admitted to our hospital from 2005 to 2017 were included in this study. All pre-therapy CT angiographies were reviewed and reclassified using the new classification based on three types and four subtypes: Type: A, dissection involving at least the ascending aorta; Type B, dissection involving exclusively the descending aorta; and type C, dissection involving the aortic arch with/without descending aorta. Subtype: 0; absence of MPS; 1, dynamic MPS; 2, static MPS; 3, static and dynamic MPS.
**Results:** A total number of 228 consecutive patients were included in the study. According to the new classification, AD were distributed as 153 type A, 50 type B (38B0, 5B1, 6B2, 1B3) and 25 type C (18C0, 6C2, 1C3). The new type represented 11% of all ADs. MPS was present in 28% of type C. Treatment strategies in type C included endovascular interventions and surgery in 32% and 12%, respectively.
**Conclusion:** The new classification is easy to use and feasible in a large group population. It should be helpful driving the decision making process and especially in integrating the latest development in trans-catheter therapies.

**B-1498** 10:47
**Application value of dual-source CT combined with intelligent modulation and iterative reconstruction in aortic dissection imaging**
P. Jin; *Chengdu/CN (pujin_online@163.com)*

**Purpose:** To explore the clinical application value of second-generation dual-source CT combined with intelligent modulation and iterative reconstruction in emergency aortic dissection imaging
**Methods and Materials:** 40 emergency patients with clinical suspected aortic dissection were included in this study.Conventional scanning was performed in the conventional group, and large-pitch intelligent modulation and iterative reconstruction were performed in the control group.The mean CT value, mean noise, signal noise ratio, contrast noise ratio, effective radiation dose, image quality and aortic root image quality were evaluated and analyzed
**Results:** 40 patients successfully completed CT aortic dissection imaging.There was no difference in image quality between the conventional group and the control group(t=1.283,$P$>0.05).The quality of aortic root images in the control group was better than that in the conventional group, and the difference was statistically significant($x^2$=22.556,$P$<0.05).The mean CT value and mean noise of aorta in the conventional group were slightly higher than those in the control group.However, SNR and CNR in the control group were higher than those in the conventional group, and the difference was statistically significant(t=-21.042,-15.924,8.530,11.495,$P$<0.05).The radiation dose of the conventional group was significantly higher than that of the control group,the difference was statistically significant(t=-12.327,P<0.05)

**Conclusion:** The combined intelligent modulation technique and iterative reconstruction technique of dual-source CT large pitch scan can guarantee the image quality and reduce the radiation dose, and can be used as a conventional imaging method of emergency CT aortic dissection.

**B-1499** 10:55
**Role of the cardiosynchronised computed tomography angiography in the dissection of descending thoracic aorta**
C. Ruggieri, S. Veglia, A. Ruffino, G. Ciccone, A. Evangelista, O. Davini; *Turin/IT (chiara_ruggieri@virgilio.it)*

**Purpose:** To identify a possible role of the cardio-synchronized computed tomography angiography (CTA) in the dissection of descending thoracic aorta (DTA).
**Methods and Materials:** We retrospectively analysed 23 exams performed using cardio-synchronized CTA (retrospective gating) in suspected acute aortic syndromes or in chronic thoracic dissections. We measured the total vessel areas (TOTA), and the areas of the true (TLA) and false lumen (FLA) at 2 levels (2 cm below the isthmus and 3 cm above the diaphragm). We made measurements in 2 arterial phases (40% and 75% of the cardiac cycle) and in the venous one (no cardio-synchronized acquisition).
**Results:** In the venous acquisition the average TLA in $mm^2$ (SD) was, respectively, 445 (278) and 410 (267) in the proximal and distal points (intermediate values compared to 40% and to 75% phases). The TLA average (SD) in the 40% and 75% phases was, respectively, 462 (283) and 419 (276) in the proximal site; 436 (269) and 388 (267) in the distal site. So it was significantly larger in the 40% vs 75% phase both in the proximal site (mean difference 42.9, 95% CI: 23.8-62.0, p = 0.0001) and in the distal point (mean difference 47.4, 95% CI: 30.7-64.0), p <0.0001). No significant differences emerged for the TOTA.
**Conclusion:** We demonstrated that intimal flap dynamic and lumen variations (essential aspects in the aortic dissection) are significantly affected by the various phases of the cardiac cycle. These data lead to think a possible role of cardio-synchronized CTA in follow-up of DTA dissection.

**B-1500** 11:03
**Thoracic aortic fluid dynamics in bicuspid valve patients: correlations with valvular morphology using 4D-flow magnetic resonance imaging**
G. Pambianchi, N. Galea, I. Carbone, F. Cilia, F. Catapano, M. Francone, C. Catalano; *Rome/IT (giacomo.pambianchi@gmail.com)*

**Purpose:** Evaluate the impact of bicuspid aortic valve (BAV) morphology on the transvalvular flow pattern, aortic flow turbulence and wall shear stress (WSS), using cardiac magnetic resonance (CMR) and 4D-flow imaging.
**Methods and Materials:** Eighteen healthy BAV patients (aortic diameter≤45mm, lack of severe valvular disease) were studied by CMR on a 3.0T unit (Discovery, GE Healthcare), including cine-MR sequences acquired orthogonal and parallel to the aortic root, contrast-enhanced MR angiography and 4D-flow imaging. In each patient we assessed, valvular morphology (valve phenotype, leaflet length, leaflet asymmetry index), valvular flow characteristics (peak flow velocity, flow jet angle, flow displacement), turbulent flow within the ascending aorta (systolic flow reversal ratio, SFRR) and WSS (total, axial and circumferential) at four different levels of the ascending aorta. All data were correlated by bivariate correlation and linear regression analyses.
**Results:** RL-BAV patients (n: 12), RN-BAV (n: 4) and RN/RL-BAV (n: 2) demonstrated peculiar phenotype-related flow patterns. Direct correlations between leaflets asymmetry index, flow jet angle (r: 0.603, p: 0.008) and flow displacement (r: 0.712, p: 0.001) were observed. Axial WSS was highly related to flow displacement (r: 0.74, p: 0.004) and SFRR (r: 0758, p: 0.001). RN-BAV patients showed the greatest ascending aorta diameters (p: 0.008), higher degree of flow displacement (p: 0.022) and SFRR (p: 0.009).
**Conclusion:** BAV phenotype and leaflets asymmetry are strongly involved in aortic root flow eccentricity generation, in particular, flow displacement and flow angle. These parameters are also correlated to the downstream flow pattern, WSS, SFRR and aortic diameter. A deeper knowledge of the relationship between valve morphology and aortic flow dynamics could ameliorate risk stratification strategy in BAV patients.

**B-1501** 11:11
**Follow-up of thoracic aorta diseases with concurrent evaluation of coronary arteries using wide-detector-coverage CT with low-dose protocol**
A.D. Annoni, M.E. Mancini, A. Formenti, G. Muscogiuri, M. Guglielmo, S. Mushtaq, G. Pontone, M. Pepi, D. Andreini; *Milan/IT (andrea.annoni@ccfm.it)*

**Purpose:** To assess evaluability, image quality, radiation exposure and coronary evaluability of a submillisievert CT angiography (CTA) scan protocol of thoracic aorta in follow-up of patients with thoracic aorta diseases.
**Methods and Materials:** We enrolled 80 patients (32F; BMI 25.4±2.97 $kg/m^2$) referred to our hospital for CTA of the thoracic aorta that underwent CT with 80 kV, new adaptive statistical iterative reconstruction algorithm-V and 40ml of contrast medium. Image quality was assessed using a semi-quantitative four-point Likert scale, by two independent readers. For each patient, attenuation, image noise, contrast-to-noise ratio (CNR) were measured at different aortic levels and at the level of the proximal coronary arteries. Mean effective dose (ED) was calculated.
**Results:** Mean aortic attenuation for ascending aorta, aortic arch and descending aorta were above 600HU. Mean image noise and CNR for ascending aorta, aortic arch and descending aorta were 38.5±6.9, 35.05±7,1 and 36.9±9.1, respectively, and 17.1±3,3, 17.6±2.0 and 16.8±2.5, respectively. Mean luminal attenuation of proximal coronary arteries was above 360 HU. 459 out of 960 coronary segments were classified as good image quality. Coronary evaluability (number of coronary segments evaluable/total number of coronary segments) in a segment-based model was 83% (790/960 segments). Mean ED was 0.81±0.29mSv.
**Conclusion:** 80kV CTA of the thoracic aorta using 160mm detector coverage and ASIR-V allows low-dose good quality exams with complementary coronary tree evaluation. Our results gain in importance considering the use of CT for serial follow-up of aortic diseases.

**B-1502** 11:19
**ECG-gated MR angiography at 3T for follow-up after aortic surgery involving the ascending aorta**
A. Busse[1], C. Neßelmann[1], A. Hoffman[2], P.M. Dohmen[1], M.-A. Weber[1], F.G. Meinel[1]; [1]*Rostock/DE*, [2]*Wuppertal/DE (anke.busse@med.uni-rostock.de)*

**Purpose:** To evaluate the feasibility, image quality and diagnostic yield of ECG-gated MR angiography in the post-surgical follow-up of patients after aortic surgery involving the ascending aorta.
**Methods and Materials:** In this study, we retrospectively analyzed a cohort of 19 patients (median age 60 years, range 38-79 years), who underwent MR angiography for follow-up imaging after replacement of the ascending aorta. Our MRI protocol consisted of an ECG-gated contrast-enhanced MR angiography performed at 3T. Mean examination duration was 24 minutes. All examinations were analyzed by two readers in consensus for image quality at various levels of the aorta on a five-point scale ranging from 1 (non-diagnostic) to 5 (excellent). Diagnostic findings were recorded. We also analyzed whether additional down-stream imaging was performed within one month of the MRI examination.
**Results:** Subjective image quality was rated as “sufficient” (score 3.1 ± 1.0) for the aortic root and as good to excellent for the ascending aorta (score 4.6 ± 0.7), the aortic arch (4.6 ± 0.8), supra-aortic branches (4.7 ± 0.69) and descending aorta (4.6 ± 0.7). In eight cases (42%), a clinical relevant or new pathology was detected. One patient of this study population underwent re-evaluation with CT within one month of the MRI examination after a repeat surgical procedure.
**Conclusion:** ECG-gated MR angiography at 3T is feasible and yields good image quality for the detection of post-operative complications after replacement of the ascending aorta. This technique may serve as alternative to CT particularly in younger patients with repeated follow-up.

**B-1503** 11:27
**Real-time patient-specific scan initiation for CT of the thoracic aorta: impact on image quality**
F.R. Schwartz[1], J.C. Ramirez Giraldo[1], R. Gutjahr[2], D. Boll[3], D. Marin[1], L. Hurwitz[1]; [1]*Durham, NC/US*, [2]*Nürnberg/DE*, [3]*Basle/CH (fidesreginadorotheaann.schwartz@duke.edu)*

**Purpose:** To assess impact of real-time modulation of scan initiation based on patient-specific haemodynamics on contrast in the thoracic aorta on MDCT.
**Methods and Materials:** This is a HIPAA-compliant, IRB-approved QI project. Exams were performed on a DS-MDCT scanner (120 kVp, 65-75 mL at 4 ml/sec). Contrast administration was monitored in the ascending aorta (thoracic aorta, TA) and descending aorta (thoraco-abdominal aorta, TAA). The delay prior to initiation of diagnostic scan was modulated by a real-time patient-specific (RTPSM) software, incorporating time to threshold and the slope of the enhancement curve. 120 patients (cohort 1) were scanned using RTPSM triggering of scan delay for TA and TAA exams. A reference cohort of 30 patients (cohort 2) was identified from the same scanner (preceding 12 months), using a fixed diagnostic delay (FD). Patient demographics, average aortic HU values and coefficients of variance (COV) at 1-mm increments from the aortic valve through the diaphragm were collected. Descriptive statistics and Student’s t tests were applied.
**Results:** Protocol distribution was 61 and 39% (TAA/TA) in cohort 1 and 60/40% (TAA/TA) in cohort 2. Average scan delay was significantly longer for TAA (12.4 vs 11 sec; p<0.01) and TA (12 vs 9 sec; p<0.01) exams using RTPSM. Average HU values were significantly higher in the RTPSM cohort (403.1 ± 31.8 vs 372.4 ± 38.1 HU; p<0.01) and COV was significantly reduced using RTPSM (8.1 vs 11.6%; p<0.01).

**Conclusion:** RTPSM results in higher HU values and better contrast homogeneity throughout the thoracic aorta on MDCT exams.
*Author Disclosures:*
**F.R. Schwartz:** Research/Grant Support; Siemens Healthineers, Grant to department of Radiology. **J.C. Ramirez Giraldo:** Employee; Siemens Healthineers, Forchheim, Germany. **R. Gutjahr:** Employee; Siemens Healthineers, Forchheim, Germany. **L. Hurwitz:** Research/Grant Support; Siemens Healthineers, Grant to department of Radiology.

**B-1504** 11:35
**A preliminary study of CT spectral imaging on patients with aortic dissection using revolution CT**
B. Wen; *Beijing/CN (wwwyyner@163.com)*

**Purpose:** To study the application of CT spectral imaging on patients with aortic dissection.
**Methods and Materials:** 30 aortic dissection patients were studied: 15 patients were scanned by spectral imaging protocol and 15 patients by routine protocol. The spectral imaging series ranging from 40 to 140 keV (10 keV intervals) were reconstructed. The quantitative image quality indices including signal intensity, noise, SNR and CNR of multiple aortic segments were measured and calculated. The virtual non-contrast images were reconstructed using CTA spectral imaging data and were compared with real non-contrast images. Then the subjective evaluation of image quality between the best monochromatic images and routine datasets was performed by two radiologists independently, and the radiation dose was compared in the two groups.
**Results:** 480 aortic segments were evaluated with good consistency of two radiologists. In the spectral imaging series, the 40-70 keV reconstruction showed superior SNR levels (+44.3, +33.7, +22.3,+9.1 %) and CNR levels (+36.5, +24.8, +11.6,+1.2%) compared to routine scans. Combined with the subjective evaluation of true and false lumen displays, the 70 keV reconstruction image was considered as the best monochromatic image. The image quality between spectral imaging and routine scans at nearly all aortic segments had no significant difference. The virtual non-contrast images were comparable to the real non-contrast images. The DLP for spectral imaging was lower than the comparison group.
**Conclusion:** The CT spectral imaging on aortic dissection patient has good clinical application value with reduced radiation dose.

**B-1505** 11:43
**ECG-gated CTA of thorax aorta with extended data postprocessing in ascending aorta aneurism diagnosis**
A. Skripnik, V. Fokin, R. Mironchuk, G.E. Trufanov, O. Moiseeva, O. Irtyuga, V. Uspenskiy, E. Malev, P. Murtazalieva; *St. Petersburg/RU (skripnikalexey@mail.ru)*

**Purpose:** To develop a computed tomography angiography (CTA) scanning protocol with extended data postprocessing for the evaluation of the aortic wall elongation in patients with an expansion of the ascending aorta (AA) of varying degrees.
**Methods and Materials:** 43 patients (26 men) aged 42 to 76 years with a pre-established diagnosis of AA aneurysm were examined. ECG-gated CTA was performed after bolus intravenous injection of 100 ml of contrast agent (iodine concentration 300 mg/ml). The final systole and diastole phases were determined for each study and the maximum aortic diameter (d) and cross-sectional area (S) of the aorta were evaluated.
**Results:** The patients were divided into three groups, according to the AA diameter, measured during the diastole phase. The 1st group included 20 patients with d<45 mm (mean 41.06±1.9 mm), the 2nd group - 9 patients with d 45-50 mm (mean 47.03±1.16 mm); 3rd group - 14 patients with d 50-55 mm (mean 52.78±2.67 mm). Mean values of S in the 1st, 2nd, and 3rd groups were 1289±116, 1703±75, and 2096±249 $mm^2$, respectively. Distensibility (D) in d and S were calculated as: 1. $D_d=(d_{sistolic} - d_{diastolic})/d_{diastolic}$; 2. $D_S=(S_{sistolic} - S_{diastolic})/S_{diastolic}$. $D_d$ of AA in the 1st, 2nd and 3rd groups were 4.88±0.54, 4.73±0.69 and 2.91±0.34 mm, respectively. $D_S$ of AA in patient of the 1st, 2nd and 3rd groups were, respectively, 8.85±0.95, 7.88±1.36 and 4.98±0.78 $mm^2$.
**Conclusion:** Our CTA scanning protocol with extended data postprocessing allows to evaluate the tensile properties of AA wall in different cardiac cycle phases.

**B-1506** 11:51
**Age at aortic coarctation reconstruction is correlated with aortic vessel wall stiffening: an evaluation of pulse wave velocity and wall shear stress from 4D flow MRI**
J.F. Juffermans, I. Nederend, P. van den Boogaard, A.A.W. Roest, J.J. Westenberg, H.J. Lamb; *Leiden/NL (J.F.Juffermans@lumc.nl)*

**Purpose:** Uncertainties exists in whether the abnormal haemodynamic situation in coarctation patients before curative reconstruction causes aortic wall deterioration. Therefore, this study evaluated the effect of age at curative reconstruction on the arterial wall stiffness expressed in pulse wave velocity (PWV) and the 3D wall shear stress (WSS) in CoA patients with curative reconstruction. Additionally, effect of valve morphology was evaluated.
**Methods and Materials:** 21 patients (13.7±2.6 years) were included, including 14 subjects with a bicuspid aortic valve. Mean time after CoA correction is 12.6±3.0 years. For normal PWV comparison, 19 volunteers (13.3±2.8 years) were included. PWV was determined from two high-temporal 1-directional velocity-encoded acquisitions, for the ascending aorta (AO) plus the aortic arch and the descending AO. The peak WSS was determined from 4D flow MRI for the ascending AO, aortic arch and descending AO.
**Results:** There were no significant differences between volunteer and patient for neither the characteristics nor the PWV. Correlations were found between age and PWV in both aortic segments (proximal AO: r=.322, p=.042; descending AO: r=.361, p=.022). For tricuspid aortic valve (TAV) patients, a correlation was found between age at reconstruction and descending AO PWV (r=.796, p=.010).
**Conclusion:** The absence of significant PWV difference between volunteers and patients indicates that curative reconstruction does not result in AO wall stiffening. However, the correlation within TAV patients between the age at curative reconstruction and descending AO PWV suggests that a prolonged period of abnormal haemodynamic exposure may result in increased AO wall stiffening.

**10:30 - 12:00** **Room O**

### Musculoskeletal

### SS 1810

### Knee and muscle imaging

*Moderators:*
M. Nevalainen; Oulu/FI
E. Springer; Vienna/AT

**B-1507** 10:39
**Medial meniscal ossicle is associated with medial posterior root tear and ACL tear: a case-controlled multicentric SIMS study of 42 cases**
A. Caudal[1], G. Lefebvre[2], D. Guenoun[3], G. Corcos[4], J.-F. Nisolle[5], V. Vuillemin-Bodaghi[4], B. Vande Berg[5]; [1]*Nice/FR*, [2]*Lille/FR*, [3]*Marseille/FR*, [4]*Paris/FR*, [5]*Brussels/BE (caudalamandine@yahoo.fr)*

**Purpose:** To test the hypothesis that medial meniscal ossicle is associated with posterior root tear.
**Methods and Materials:** The MRI and available radiographs of knees with a medial meniscal ossicle and of a sex- and age-matched control group were collected from 17 imaging centres. Two readers assessed only all SE T1-weighted images and available radiographs to detect the presence of a medial meniscal ossicle. Consensus reading was used as reference standard for the presence of a meniscal ossicle. Two other readers blinded to the presence of a meniscal ossicle by reading only the fat-saturated MR images assessed the presence of meniscal, ligament and cartilage lesions.
**Results:** After consensus reading, there were 42 knees with a medial meniscal ossicle in 39 men (43.77 yrs +/- 13.59) and 7 women ( 40.8 yrs +/- 6.1). The frequency of posterior root tear in the study group (62% and 76% for R1 and R2) was higher than that in the control group (11% and 4%, respectively) (p<10-4). There was no difference in frequency of medial posterior horn and body lesions. The frequency of ACL but not of MCL/PCL lesions was higher in the study than in the control group (p=0.007 and 0.003). The degree of cartilage degradation of the medial compared to the lateral femoro-tibial joint was higher in the study than in the control group (p=0.04 and 0.02).
**Conclusion:** There is a statistically significant association between medial meniscal ossicle and posterior root tear of the medial meniscus, ACL lesions and medial femoro-tibial cartilage degradation.

**B-1508** 10:47
**Rupture of the anterolateral ligament in complete acute traumatic anterior cruciate ligament tear: new insights into acute pivot shift trauma to the knee**
M.T. Mastio[1], A. Duarte Vallejo[2], J. Raya[1], L. Jazrawi[1], J.T. Bencardino[3]; [1]*New York, NY/US*, [2]*Bogota/CO*, [3]*Great Neck, NY/US (mastio@me.com)*

**Purpose:** To evaluate the prevalence and association of anterolateral ligament (ALL) rupture with other meniscal and ligamentous injuries in patients with complete acute traumatic ACL tears.
**Methods and Materials:** 57 patients M45:F12, mean age 21 (range:13-34) with acute post traumatic ACL rupture who underwent ACLR between 2011 and 2015 were enrolled in this cohort. Preoperative MRI was reviewed by an experienced (20 years) MSK radiologist for: ALL rupture, MM tear, LM tear, posteromedial corner (PMC) injury, posterolateral corner (PLC) injury, MCL tear, and LCL tear. Odds ratios and their 95% confidence interval were used to assess the associations of ALL with other injuries in the knee. To rule out confounding factors we used the Cochran Mantel Haenszel method in an analysis stratified by gender and BMI (normal, overweight).
**Results:** Most commonly reported lesions were of MM 63% and PLC 64%. ALL partial or complete tears and Segond avulsion fracture were reported in 28 patients (49%). Anterolateral lesions were associated with a 10 fold decreased risk of MM tear (odds ratio [OR] = 0.10, 95% [CI]=[0.028, 0.38]) and a 4.6 fold increased risk of LCL tear (OR=4.68, 95% CI=[1.28 17.1]). Neither gender nor BMI were confounding for the associations of ALL with MM and LCL tears. No other statistically significant associations were found.
**Conclusion:** Failure of the ALL during acute traumatic ACL rupture is often associated with tear of the LCL. This may have a protective effect over the MM due to potential "capsular release" during pivot shift trauma.

**B-1509** 10:55
**Healing of the anterior cruciate ligament following primary repair: a longitudinal DTI study**
E. De Smet[1], T. Billiet[2], C. Heusdens[1], P.M. Parizel[1], P. Van Dyck[1];
[1]*Edegem/BE*, [2]*Leuven/BE (elinedesmet@hotmail.com)*

**Purpose:** There is a lack of objective biomarkers to guide the appropriate timing for return to sports after anterior cruciate ligament (ACL) surgery. The purpose of this study was to investigate the ability of diffusion tensor imaging (DTI) to monitor ACL healing following primary repair.
**Methods and Materials:** Twelve patients who underwent primary ACL repair were enrolled prospectively. All patients had clinical and DTI follow-up at 3, 6 and 12 months postoperatively. DTI was acquired at 3T using single-shot SE-EPI (b-values: 0/400/800s/mm$^2$, 10 directions; TR/TE: 1300/45ms; voxel size: 1.5×1.5×6.0mm$^3$; FOV 768×768×60mm). Fiber tractography (FT) was performed to delineate the ACL. The posterior cruciate ligament (PCL) served as a control. Fractional anisotropy (FA), mean diffusivity (MD), axial diffusivity (AD), and radial diffusivity (RD) were calculated within the FT volumes. Data were compared using a linear mixed effects model for all repeated measures, and paired t tests between different time points (p<0.05).
**Results:** All knees were stable at 12 months postoperatively. ACL and PCL FT were feasible at all time points. The ACL showed a significant decrease of FA over time (F=8.29, p=0.0040). Diffusivities of the ACL were most often larger than those in the PCL and showed a decreasing trend over time, reaching significance for AD (MD: F=1.45, p=0.23; AD: F=10.15, p=0.0014; RD: F=0.36, p=0.55). There were no significant changes over time in the PCL.
**Conclusion:** Our study has shown the ability of DTI to quantitatively monitor healing of the ACL repair. The results indicate incomplete healing at 12 months postoperatively.

**B-1510** 11:03
**Can pre-operative MR imaging findings predict early failure following anterior cruciate ligament reconstruction?**
M. Mastio[1], A. Duarte[1], J. Raya[1], L. Jazrawi[1], J.T. Bencardino[2];
[1]*New York, NY/US*, [2]*Great Neck, NY/US (mastio@me.com)*

**Purpose:** To identify markers of early failure of ACL graft reconstruction (ACLR) based on preoperative MRI.
**Methods and Materials:** We identified 26 patients with early ACLR failure (<2Y) who underwent revision between 2011-2015. Exclusion criteria included graft choice other than autografts (n=4) and non available preoperative MRI (n=3). Nineteen patients with early ACLR failure were included. The control group consisted of 38 subjects who underwent ACLR with a minimum of 2 years of clinical FU and no evidence of graft failure matched by age, sex, BMI and graft type. Preoperative
MRI obtained within 8w (range 1-8w) following initial trauma were blindly reviewed by an experienced musculoskeletal radiologist for intra and periarticular lesions including: anterolateral ligament (ALL) injuries (stretch, partial, complete), medial meniscus tear (MM), lateral meniscus tear (LM) tear, posteromedial corner injury (PMC), posterolateral corner injury (PLC) injury, medial collateral ligament tear (MCL), and lateral collateral ligament tear (LCL). Logistic regression analysis was performed.
**Results:** Mean time to ACLR failure was 14mo (range, 1-24mo). Mean FU of those in the control group was 38mo (range, 25-61). MM tear was the best predictor of early ACLR failure (Accuracy=66.7%, p=0.08) followed by LM (p=0.13). MM was a significant predictor of ACLR failure (p=0.02) with OR 4.2 and 95% CI). All other variables were not associated with ACLR failure (p>0.20).
**Conclusion:** MM tears were the best predictor of early failure of ACLR. Thus, preserving the integrity of the MM during ACLR procedure may be crucial in minimizing the risk for early ACLR failure.

**B-1511** 11:11
**Feasibility of lower limb muscle volume estimation during contraction with real-time dynamic MRI: implications to clinical diagnostics**
M. Garetier, R. Grimandi, F. Tissier, C. Pons-Becmeur, D. Ben Salem, B. Borotikar; *Brest/FR (marc.garetier@wanadoo.fr)*

**Purpose:** Ability to determine volume of a contracted muscle has a diagnostic value. We assessed the volume of thigh muscles during contraction on dynamic MRI using a real-time balanced steady-state gradient echo sequence (True-FISP).
**Methods and Materials:** Twelve healthy adult volunteers participated in this study after signing an informed consent. An MRI of the mid-thigh region (Siemens Magnetom Avanto$^{fit}$ 1.5T) was acquired on each volunteer supine, using real-time True-FISP sequence (TR=3.6, TE=1.8, number of slices=5, slice thickness=8mm, size pixel=2.08X1.4, time=13sec, time frames=10) during rest and contraction. During dynamic state, volunteers pressed an inflated balloon (150mmHg) placed under the knee joint to achieve a pressure of 200mmHg monitored on manometer. Rectus femoris (RF), vastus lateralis (VL), vastus intermedius (VI), vastus medialis (VM) and biceps femoris long head (BFLH) muscles were manually segmented in image sets of static state and dynamic state at which pressure reached 200mHg. The segmented cross-sectional areas were used to determine muscle volume within the acquisition field. Static and dynamic state volumes were compared with paired two-sample t test.
**Results:** The mean volume was estimated during rest and contraction, respectively, at 29.16cm$^3$ and 28.15cm$^3$ for RF, 72.22cm$^3$ and 70.9cm$^3$ for VI, 80.63cm$^3$ and 80.27cm$^3$ for VL, 36.25cm$^3$ and 36.69cm$^3$ for VM, 30.77cm$^3$ and 30.66cm$^3$ for BFLH. No significant difference was found for muscle volume between static and dynamic states (p>0.05), except for RF (p=0.005).
**Conclusion:** Volume of thigh muscles can be evaluated during contraction on dynamic MRI with real-time sequence, allowing quantification of the muscle deformation during contraction.

**B-1512** 11:19
**Musculoskeletal elastography: exploration of confounders in a challenging field**
L. Ruby[1], S.J. Sanabria[1], T. Mutschler[1], K. Martini[1], T. Frauenfelder[1], V. Klingmüller[2], M. Rominger[1]; [1]*Zurich/CH*, [2]*Marburg/DE (lisa.ruby@usz.ch)*

**Purpose:** To identify and investigate the quantitative impact of confounders in muscle shear wave elastography (SWE), a challenging field due to anisotropy and nonlinear viscoelasticity of muscle.
**Methods and Materials:** The influence of region of interest (ROI) size and location, excitation pulse, phantom stiffness, muscle fibre orientation and activation state on shear wave velocity (SWV) was assessed with a GE Logiq E9 in three different test objects: an isotropic elasticity phantom with four certified stiffness values (elasticity phantom), explanted porcine muscle (ex vivo porcine muscle) and stretched and relaxed calf muscles of healthy volunteers (volunteers) in parallel and transverse probe direction to the fibres. Regression analysis was performed to estimate the impact of examined confounders.
**Results:** For the elasticity phantom, SWV increased with greater stiffness (p<0.001), yet underestimated the true stiffness of all four nominal stiffness values. In healthy volunteers, the main variance-contributing factors were in order of importance muscle activation state, fibre orientation, horizontal ROI size and insertion depth. For the elasticity phantom, ex vivo porcine muscle and volunteers, a higher insertion depth significantly reduced SWV (p<0.05). For both the volunteers and the ex vivo porcine muscle, parallel compared to transverse probe-fibre orientation (p<0.001), muscle stretch (p<0.01) and an increasing horizontal ROI size (p<0.01) led to significantly higher SWV values. The comparison of different Logiq E9 software versions showed significant differences in SWV only for the ex vivo porcine muscle (p<0.001).
**Conclusion:** ROI location and size, software choice, muscle fibre orientation and activation state are important confounders that significantly influence SWV in muscle.

**B-1513** 11:27
**Quantification of abdominal fat and skeletal muscle tissue at CT: associations between single-slice measurements and total compartment volumes**
A. Faron, J. Luetkens, F.C. Schmeel, A. Sprinkart, D. Thomas; *Bonn/DE (anton.faron@ukbonn.de)*

**Purpose:** Body composition is of great prognostic value in several oncologic diseases as well as cardiometabolic disorders. We aimed to investigate the correlations of skeletal muscle mass and abdominal adipose tissue compartments between volumetric measures and area-based measurements to identify a useful landmark for estimation of total compartment volumes.
**Methods and Materials:** In this retrospective study, volumetric quantifications of paraspinal skeletal muscles (SM) and adipose tissue compartments (visceral adipose tissue, VAT; subcutaneous adipose tissue, SAT) were performed in 50

consecutive patients (26 male; mean age, 63 ± 15 years) who underwent abdominal multislice-CT for diagnostic purposes between 06/2017 and 07/2018. Associations between total volumes of SM, VAT, and SAT with area-based measurements at eight predefined anatomical landmarks (levels B12/L1 to L5/S1; level of the umbilicus, U; level of the radix of the superior mesenteric artery, MSA) were studied using correlation coefficients.
**Results:** Statistical analysis revealed a strong association of area-based measures of adipose tissue compartments obtained at all studied landmarks with total VAT and SAT volume (VAT: all r>0.89, P<0.0001; SAT: all r>0.95, P<0.0001). The strongest associations with total SM volume were found for area-based measurements obtained at L2/3, L3/4, and MSA (r>0.87, P<0.0001) and were further improved by normalization to volume of interest height (r>0.92, P<0.0001).
**Conclusion:** Area-based measurements of SM, VAT, and SAT at several anatomical landmarks are strongly associated with total compartment volumes and, therefore, allow for easy and simultaneous assessment of skeletal mass as well as amount of adipose tissue compartments from a single-slice image.

**B-1514** 11:35
**Distribution patterns of intra- and extra-myocellular fat by magnetic resonance imaging in subjects with diabetes, prediabetes and healthy controls from a population-based cohort**
L.S. Kiefer[1], J. Fabian[1], S. Rospleszcz[2], C. Storz[1], C.L. Schlett[3], F.W. Roemer[4], K. Nikolaou[1], F. Bamberg[5]; [1]*Tübingen/DE*, [2]*Munich/DE*, [3]*Heidelberg/DE*, [4]*Erlangen/DE*, [5]*Freiburg/DE (lena.kiefer@med.uni-tuebingen.de)*

**Purpose:** To determine distribution patterns of total, intra- and extramyocellular lipids (IMCL and EMCL) by magnetic resonance imaging (MRI) in subjects with type 2 diabetes mellitus (T2DM), prediabetes and healthy controls.
**Methods and Materials:** Asymptomatic subjects from the general population were classified with T2DM, prediabetes or healthy controls and underwent multi-echo Dixon MRI (TR 8.90ms, six echo times, flip angle 4°). Total myosteatosis, IMCL and EMCL were quantified as proton-density fat fraction (PDFF) in abdominal skeletal muscle compartments using a standardized segmentation algorithm. Cardiometabolic risk factors were prospectively obtained in a comprehensive health assessment.
**Results:** Among 337 included subjects (mean age: 56.0years, 56.4% males, 14.5% T2DM, 23.4% prediabetes) intramuscular fat content was highest in obese subjects with T2DM ($PDFF_{muscle}$: 15.8%, IQR: 11.9-17.0%; IMCL: 7.7%, ICQ: 6.4-8.9%; EMCL: 7.1%, ICQ: 5.4-9.2%). There were significant differences in both IMCL and EMCL between the diabetes status groups, being lowest in non-obese, healthy controls and highest in obese, diabetic subjects (IMCL: 5.5%, IQR: 4.6-6.5%; 7.7%, IQR: 6.4-8.9%; EMCL: 3.6%, ICQ: 2.4-5.3%; 7.1% ICQ: 5.4-9.2%; respectively, p<0.001). After adjustment for age and gender, the association of IMCL and prediabetes, but not T2DM was attenuated (IMCL: β:0.49, 95%CI: -0.01-0.99, p=0.055; β:1.31, 95%CI: 0.25-2.38, p=0.016; respectively), whereas EMCL remained significantly associated with both prediabetes and T2DM (p<0.001).
**Conclusion:** There are significant differences in $PDFF_{muscle}$, IMCL and EMCL between subjects with T2DM, prediabetes and healthy controls. Patterns of intramuscular fat distribution by MRI may, therefore, serve as promising imaging-biomarkers, specifically in early stages of impaired glucose tolerance.

**B-1515** 11:43
**Assessment of skeletal muscle mass by magnetic resonance imaging in a population-based cohort**
L.S. Kiefer[1], J. Fabian[1], S. Rospleszcz[2], C. Storz[1], C.L. Schlett[3], F.W. Roemer[4], K. Nikolaou[1], F. Bamberg[5]; [1]*Tübingen/DE*, [2]*Munich/DE*, [3]*Heidelberg/DE*, [4]*Erlangen/DE*, [5]*Freiburg/DE (lena.kiefer@med.uni-tuebingen.de)*

**Purpose:** To determine skeletal muscle mass parameters and their associations by magnetic resonance imaging (MRI) and bioelectrical-impedance analysis (BIA) in non-obese and obese subjects.
**Methods and Materials:** Subjects from a population-based cohort underwent anthropometry, BIA (50kHz, 0.8mA) and multi-echo Dixon MRI (TR 8.90ms, six echo times, flip-angle 4°). Abdominal skeletal muscle mass by MRI was quantified as total and fat-free cross-sectional area by a standardised segmentation algorithm and normalised to subjects body height$^2$ (AMMI: abdominal muscle mass index).
**Results:** Among 335 included subjects (median age: 56.0±5.0years, 56.1% males), 95 were classified as obese (BMI≥30kg/m$^2$: 28.4%). AMMI by MRI and BIA-based measures of muscle mass showed a moderate to strong, linear correlation, being stronger in non-obese subjects (non-obese: r<0.743, obese: r<0.562, respectively). $AMMI_{Total}$ was lowest in non-obese females and highest in obese males (2507.5mm$^2$/m$^2$ (IQR 2178.0-2790.6mm$^2$/m$^2$), 3359.8mm$^2$/m$^2$ (IQR: 3170.9-3773.3mm$^2$/m$^2$), respectively, p<0.001). No significant difference was found regarding $AMMI_{Fat\text{-}free}$ between non-obese and obese subjects (1632.3mm$^2$/m$^2$ (IQR 1379.0-2029.3mm$^2$/m$^2$), 1605.1mm$^2$/m$^2$ (IQR 1407.2-2028.3mm$^2$/m$^2$), respectively, p=0.705). Concerning the ratio of normalised fat-free to total abdominal skeletal muscle mass, normal weight males and females had a significantly higher $AMMI_{Fat\text{-}free}/AMMI_{Total}$ compared to obese, male and female subjects (male: 59.9% (IQR 55.0-68.2%) and 57.0% (IQR 47.6-65.5%), p=0.027; female: 55.9% (50.9-62.6%) and 49.1% (44.4-53.5%), p<0.001).
**Conclusion:** $AMMI_{Fat\text{-}free}$, $AMMI_{Total}$ and their ratio by MRI based on a standardised segmentation-algorithm may be valid and robust alternatives to BIA in both non-obese and specifically obese subjects, and may, therefore, serve as promising imaging biomarkers for the simple, however, comprehensive assessment of skeletal muscle mass parameters.

**B-1516** 11:51
**Magnetic resonance spectroscopy in muscle tissue and bone marrow to identify that a body was previously frozen and is meanwhile thawed**
D. Gascho, J. Heimer, M. Thali, N. Zoelch; *Zurich/CH (dominic.gascho@irm.uzh.ch)*

**Purpose:** We investigated the use of non-invasive, in-situ magnetic resonance spectroscopy (MRS) for the identification that a body was previously frozen and is meanwhile thawed.
**Methods and Materials:** Severed hint legs (study group: n=7) of 14 sheep were measured before and several times after storing in a deep freezer (-20°C). The opposite hint legs (control group: n=7) were kept at room temperature and repeatedly measured. A clinical 3 Tesla MR scanner was used to perform 1H-MRS. Single voxel measurements and spectroscopy-based relaxation time measurements in the muscle tissue and bone marrow were conducted. The Shapiro-Wilk test was used to determine if the data sets were normal distributed. The t-test was used to test whether the study group differs significantly from the control group. The same protocol was applied on humans (n=4), who was stored in the deep freezer during the identification process.
**Results:** Significant alterations in the relaxation times of lipids in the bone marrow were observed in the study group after freezing and thawing. The peak are ratio between lipids in the bone marrow allowed for a clear identification of a previous frozen state. These post-thawing effects were also indicated in human bone marrow. Alterations in relaxation times of water and peak area ratios were also observed in the muscle tissue, which described the thawing process in accordance to previous investigations in meat science.
**Conclusion:** Non-invasive in-situ 1H-MRS in yellow bone marrow indicates an approach for the objective detection of a previous frozen state in a meanwhile thawed body.

**10:30 - 12:00** **Studio 2019**

### Imaging Informatics

### SS 1805a

### Machine learning in breast imaging

*Moderators:*
U. Bick; Berlin/DE
O. Diaz; Girona/ES

**B-1517** 10:30
**Autodetection of mass and lymph node in breast mammography images with deep learning method**
S. Liang[1], J. Ma[1], C. Xia[1], Y. Deng[1], R. Zhang[1], Z. Yu[2]; [1]*Beijing/CN*, [2]*Zhengzhou/CN (13673666622@126.com)*

**Purpose:** High-density breast is associated with increased risk of breast cancer and makes lesion detection difficult in mammography images. We developed a deep learning algorithm to automatically detect masses and lymph node abnormalities in mammography and investigated the influence of breast density on algorithm performance.
**Methods and Materials:** A total of 12,510 mammography images were collected from 3,669 patients with both CC and MLO views, of which 2,762 patients came from collaborating hospitals (630 studies for testing, 2,132 for training), 857 patients from the DDSM open dataset (for training), and 50 patients from the INBreast open dataset (for testing). Our algorithm mainly built upon Faster R-CNN. To gain better detection performance, four tricks were applied: image normalisation, data augmentation by flipping, alternating training, and transfer learning.
**Results:** In the hospital testing dataset, mass detection sensitivity and false-positive rate (FPR) were 83.7% and 13.4%, sensitivities across different breast densities (ACR A to D) were 95.9%, 78.7%, 83.9%, and 77.1%, respectively; lymph node detection sensitivity and FPR were 86.4% and 30%, and sensitivities across different breast densities were 83.3%, 90.5%, 87%, and 86.7%. On the INBreast dataset, mass detection sensitivity and FDR were 94.5% and 48% and sensitivities across different breast densities were 97.6%, 92.7%, 100%, 75%, respectively.

**Conclusion:** Our deep learning algorithm achieved sensitive mass and lymph node abnormality detection in mammography, especially for mass detection on breasts of lower densities. The algorithm can assist radiologists with breast cancer screening more precisely in future.
*Author Disclosures:*
**S. Liang:** Employee; Employee of Infervision. **J. Ma:** Employee; Employee of Infervision. **C. Xia:** Employee; Employee of Infervision. **Y. Deng:** Employee; Employee of Infervision. **R. Zhang:** Employee; Employee of Infervision.

**B-1518** 10:38
**Integration of AI into the breast MRI assessment enables objective and accurate diagnosis even by an unexperienced reader**
S. Ellmann, M. Dietzel, E. Wenkel, C. Bielowski, R. Schulz-Wendtland, M. Uder, T. Bäuerle; *Erlangen/DE*

**Purpose:** Interpretation of breast MRI requires high level of training. It is further limited by observer-related bias, particularly among less experienced readers. We investigated, whether integration of artificial intelligence (AI) into the breast MRI assessment enables objective and accurate diagnosis even by an unexperienced reader.
**Methods and Materials:** 173 consecutive patients showing 176 suspicious breast lesions (BI-RADS IV/V: n=100/76) upon complementary assessment (clinical examination, mammography, ultrasound) received standardized breast MRI prior to histological verification. Six agnostic, semi-/quantitative MRI parameters were independently assessed by two observers (R1/R2: intermediate/no experience in breast MRI). Interobserver variability was studied by ICC (intraclass correlation coefficient). AI consisting of a polynomial kernel function support vector machine was applied to differentiate benign vs. malignant lesions based on the agnostic MRI parameters. Results were verified by tenfold cross validation. Diagnostic accuracy was evaluated (benign vs. malignant: area under the ROC curve (AUC), alpha=5%).
**Results:** There were 107 malignant lesions (60.8%). The agnostic parameters showed excellent interobserver variability (ICC: 0.81-0.98) with variable diagnostic accuracy (AUC: 0.65-0.82). Overall performance of AI to differentiate benign from malignant lesions reached AUC=90.1%. Results were integrated into an open-access web application and published online (input: agnostic criteria, output: likelihood of malignancy).
**Conclusion:** Integration of AI into the breast MRI assessment enabled objective and accurate diagnosis even by an unexperienced reader. Results can be easily validated based on our open-access web application.

**B-1519** 10:46
**Texture features from mammographymay predict the characterisation of breast tumours**
Y. Li, Y. Cui, J. Zhu, W. Li; *Jinan/CN (13631697@qq.com)*

**Purpose:** To explore the feasibility and accuracy of extracting texture features from digital mammograms for predicting benign and malignant breast masses using Radiomics.
**Methods and Materials:** 192 patients who diagnosed as breast masses (Benign: 92 Malignant: 100) by mammography were enrolled. All breast masses were classified as BI-RADS 3, 4, and 5, and at last confirmed by histopathology. Lesion area was marked with a rectangular frame on the images at the 5M workstation. The rectangular regions of interest (ROI) were segmented and 456 radiomics features were extracted from every ROI. Extracted features were dimensioned by Lasso algorithm. Post-dimension features were classified using Support Vector Machine (SVM). 70% of the data as a training set and the other 30% as a testing set. The reliability of the Classifier was evaluated by the 10-fold cross-validation. The classification accuracy was evaluated by the accuracy , sensitivity and the AUC.
**Results:** 20 radiomics features that are valuable for diagnosing benign and malignant tumours were screened by Lasso algorithm.10-fold cross-validation show the accuracy was 92.54%,so the model is stable. In the testing sets, Through SVM model the lasso dimension reduction algorithm, the classifier can achieve an accuracy of 91.44% ,a sensitivity of 96.70%,and the AUC of 0.90.
**Conclusion:** Radiomics techniques can be used to extract texture features from digital mammograms for predicting the characterization of breast tumours. This method offer high accuracy and sensitivity,and it is expected to provide additional diagnostic information to the imaging doctors.

**B-1520** 10:54
**Prediction of benign and malignant breast masses using digital mammograms texture features**
Y. Cui, Y. Li, Y. Yin, B. Li, J. Dong, J. Zhu; *Jinan/CN*

**Purpose:** To investigate the feasibility and accuracy of texture features extracted from digital mammograms at predicting benign and malignant breast mass using Radiomics.
**Methods and Materials:** 192 digital mammograms data who diagnosed as breast masses (Benign: 92 Malignant: 100) by mammography were enrolled. Enrol criteria: breast masses classified as BI-RADS 3, 4, and 5 and at last confirmed by histopathology. Lesion area was marked with a rectangular frame on the Cranio-Caudal (CC) and MedioLateral Oblique (MLO) images at the 5M workstation. The rectangular regions of interest (ROI) was segmented and 456 radiomics features were extracted from every ROI. Extracted features were dimensioned by Lasso and Maximum Relevance Minimum Redundancy (MRMR) algorithm. Post-dimension features were classified using Support Vector Machine (SVM). 70% of the data as a training set and the other 30% as a testing set. The reliability of the Classifier was evaluated by the 10-fold cross-validation. The classification accuracy was evaluated by the accuracy and sensitivity.
**Results:** Both the Lasso and MRMR screened 20 radiomics features respectively. 10-fold cross-validation show their accuracy were 92.54% and 87.31%, respectively. In testing sets, Through the lasso dimension reduction algorithm, the classifier achieves an accuracy of 91.44% and a sensitivity of 96.70%. Through the MRMR algorithm, the classifier achieves an accuracy of 91.37% and a sensitivity of 90.00%.
**Conclusion:** Radiomics texture features from digital mammograms may be used for benign and malignant prediction. This method offer better accuracy and sensitivity. It is expected to provide an auxiliary diagnosis for the imaging doctors.

**B-1521** 11:02
**Can image registration support radiologists in breast tomosynthesis image reading?**
M. Costa[1], D. Ermacora[1], S. Pesente[1], G. Simonetti[1], E. Belladonna[2], M. Tonutti[2]; [1]*Udine/IT*, [2]*Trieste/IT (matilde.costa@tecnologieavanzate.com)*

**Purpose:** This work proposes an image registration approach able to correlate corresponding positions between digital breast tomosynthesis (DBT) images and evaluates its effectiveness for simultaneous and synchronized navigation of multiple images.
**Methods and Materials:** The proposed registration method supports the correlation between the following images: 2D-synthetized image and 3D image (2Ds-DBT), where the corresponding depth of a 2D point is identified by an out-of-plane pattern (OPP) detector; 3D image and priors (DBT-priors), where corresponding locations are found matching couple of OPPs with an anisotropic feature descriptor; 3D images of different projections (2-view DBT), where corresponding regions in ipsilateral views are matched combining the OPP detector/descriptor with an elastic breast compression model. The target registration error (TRE, expressed as the average distance with reference positions annotated by experts) is evaluated as registration accuracy estimate.
**Results:** The estimated mean TRE and the percentage of cases below a tolerance (success rate) defined by experts are respectively: 3.4 mm and 97% below 10 mm tested on 215 datasets with a total of 2887 markers for 2Ds-DBT; 6.7 mm and 96% below 10 mm tested on 120 datasets with 980 markers for DBT-priors; 18.3 mm and 96% below 40 mm tested on 101 datasets with 295 markers for 2-view DBT.
**Conclusion:** We showed that the proposed registration algorithms for DBT images lead to a stable synchronization (success rate of 96%) among multiple images with an acceptable average TRE between corresponding regions. This suggests the feasibility of an interactive synchronization tool between images for a straightforward simultaneous multi-view and multi-image DBT reading.

**B-1522** 11:10
**Machine learning can combine B mode ultrasound and strain elastography features to better characterise BIRADS 3 and 4 lesions**
A. Prakash; *Jaipur/IN (dr.aparnaprakash@gmail.com)*

**Purpose:** Machine learning uses knowledge from previous dataset and tries to predict future outcome without assuming the usual statistical distribution patterns in data. We evaluate the feasibility of combining the strain ratio and strain elastography score with b mode ultrasound descriptors in predicting a benign or malignant outcome of BIRADS 3 & 4 lesions.
**Methods and Materials:** IRB approval was obtained and 200 consecutive patients undergoing breast biopsy prospectively included to undergo strain elastography. Of these 37 cases were characterised as BIRADS 3 and 32 as BIRADS 4. A random forest algorithm was trained using the b mode ultrasound descriptors of shape, margin, echogenicity, calcification, acoustic shadowing, surrounding tissue distortion, the final BIRADS description and the Elastography score and Elastography ratio as the predictors and final benign or malignant outcome as the target. A 10 fold cross validation was used because of the small sample size.
**Results:** The machine learning model was able to predict a malignant outcome in BIRADS 3 & 4 lesions with a sensitivity of 91.30% and specificity of 93.33%. The positive likelihood ratio of the lesion turning out malignant was 15.50 when the model predicted a malignant outcome for BIRADS 3 lesion.
**Conclusion:** Machine learning algorithms can combine b mode and sonoelastography imaging finding to predict outcome with a high degree of accuracy. However opacity in the functioning of these algorithms need to be removed before their implementation in clinical utilization.

**B-1523** 11:18
**Computer-aided detection of breast masses in dedicated breast CT images using adaptive parenchyma local search and deep learning**
M. Caballo, J. Teuwen, R.M. Mann, I. Sechopoulos; *Nijmegen/NL*
*(Marco.Caballo@radboudumc.nl)*

**Purpose:** To develop an automated detection system for mass-like lesions in unenhanced breast CT images.
**Methods and Materials:** The proposed system is composed of four main steps. First, the breast parenchyma (including any suspicious mass) is identified using automatic segmentation. Second, a multi-scale candidate selection algorithm based on second-order features is used to discretize the search space. The algorithm identifies possible centers of masses, generating around each center a candidate region whose dimensions adapt to the local size of the parenchyma. Then, a multi-view convolutional neural network extracts texture and shape-related features and classifies these candidate regions (scaled to the same dimensions) as either masses or normal tissue. To handle the skewness in class proportion, the network was trained iteratively by dividing the dataset into multiple batches, each always containing the whole set of masses, and as many different normal examples. Finally, fusion of multi-view information from the detected regions is used for false positive reduction. The system was trained on 75 patient-breast CT images on a patch-basis (43,890 normal, 2,601 masses), and tested on a global-basis on another 20 images.
**Results:** The developed computer-aided detection system has an average detection sensitivity of 90% at 3 false positives per scan.
**Conclusion:** Results indicate that the combination of second-order features and deep learning is a promising approach for breast mass detection in unenhanced breast CT images, and will be further evaluated in future with a larger image dataset.

**B-1524** 11:26
**Artificial intelligence CAD system capability to classify breast density by mammography**
E.F. Fleury, P.E.Z. de Assis, F.A. Azevedo, R. Lotufo; *Sao Paulo/BR*
*(edufleury@hotmail.com)*

**Purpose:** Determine an artificial intelligence CAD system capability to classify breast density by mammography.
**Methods and Materials:** The breast density (BD) pattern of 1.000 mammograms from a retrospective screening setting were determined. The BD were classified using an artificial intelligence CAD system with machine-learning technologies, which classified the breast density according to the BI-RADS 5th edition lexicon, which adopts criteria for evaluating the histogram of fibroglandular density rather than its distribution. The breast density patterns are: a. Almost entirely fat; b. Scattered areas of fibroglandular density; c. Heterogeneously dense; d. Extremely dense. Craniocaudal incidence was opted for analysis because it presented a better contrast due the best compression of the breast compared to the mediolateral-oblique view, and to have fewer structures that contaminate the images, like pectoral muscle. An observer with 16 years of experience in breast imaging has previously classified the mammograms visually, adopting the same criteria used by the software. Agreement between the CAD and the observer was determined.
**Results:** The results show good agreement between the CAD system and the observer to classify the BD according to the BI-RADS lexicon, with agreement of 78.3%.
**Conclusion:** The use of an artificial intelligence CAD system has the potential to be used in clinical practice to classify the BD pattern by mammograms according to the lexicon BI-RADS 5th edition.

**B-1525** 11:34
**Automatic deep learning-based denoising filter for 4D dedicated breast CT perfusion imaging of breast cancer**
M. Caballo, R.M. Mann, I. Sechopoulos; *Nijmegen/NL*
*(Marco.Caballo@radboudumc.nl)*

**Purpose:** To develop a 4D denoising filter for perfusion contrast-enhanced dedicated breast CT imaging.
**Methods and Materials:** Twenty 4D software phantoms were generated from different real 3D patient dedicated breast CT scans using a previously developed algorithm that simulates dynamic contrast enhancement patterns within the different breast tissues and tumours. All phantoms had a temporal resolution of 10s over 5-min scanning, and were corrupted with noise levels corresponding to acquisitions resulting in average glandular doses per 3D scan of 0.95mGy, 0.7mGy and 0.5mGy. A 4D image denoising deep learning algorithm was developed that first removes the quantum noise from the baseline scan (time zero) using a convolutional neural network, and then recovers the noisy perfusion curves using deep pattern recognition networks which recognize and average similar enhancement patterns throughout the image. The algorithm was trained using 8,000 image patches and 50,000 perfusion signals extracted from 13 pairs of 4D phantoms (noisy and noise free), and tested on the remaining 7 on a global basis. Signal-to-noise ratio was calculated both in the baseline scan and on perfusion curves, within homogeneous regions.
**Results:** On average, the signal-to-noise ratio of the baseline scan and of the perfusion curves increased after the filtering (+11.46±1.14dB, +24.34±1.96dB, respectively).
**Conclusion:** The filter was able to increase the signal-to-noise ratio both spatially and temporally. Future work includes evaluating additional perfusion patterns, incorporating more realistic noise and spatial resolution loss models deriving from 4D breast CT image-acquisition simulations, scatter correction, and testing with real patient 4D images.

**B-1526** 11:42
**Ability of an artificial intelligence CAD system to determine quality control of the mammographic study**
E.F. Fleury, P.E. de Assis, F.A. Azevedo, R. Lotufo; *Sao Paulo/BR*
*(edufleury@hotmail.com)*

**Purpose:** Evaluate the ability of an artificial intelligence CAD system to determine quality control of the mammographic study.
**Methods and Materials:** Retrospectively evaluation of mammographic quality pattern of 1.000 consecutive screening mammograms in the cranio-caudal and oblique medio-lateral incidences. The mammograms were classified using an artificial intelligence CAD system with the technology of deep-learning that automated classified the quality of the acquired images according to the following criteria: I. Bad Quality; II. Acceptable Quality; III. Good Quality. An observer with 16 years of experience in breast previously classified the mammograms visually, adopting the criteria proposed by the MQSA (Mammographic Quality Standards Act), which assesses: 1. Positioning; 2. Contrast; 3. Artifacts; 4. Blurring. The agreement was determined between the CAD system and the observer.
**Results:** The agreement between the CAD system and the observer to classify the mammograms quality was good, with 70.0% agreement.
**Conclusion:** The artificial intelligence CAD system has the potential to be used in clinical practice to determine the quality control of the breast mammograms at screening settings.

**B-1527** 11:50
**Scatter radiation correction in digital breast tomosynthesis with a deep learning convolutional neural network**
A. Rodriguez Ruiz[1], C. Fedon[2], K. Michielsen[3], I. Sechopoulos[1]; [1]*Nijmegen/NL*, [2]*Trieste/IT*, [3]*Leuven/BE*

**Purpose:** To develop a deep learning convolutional neural network (CNN) that can model the scatter signal in digital breast tomosynthesis (DBT) projections.
**Methods and Materials:** Homogeneous software phantoms representing different compressed breasts undergoing a cranio-caudal DBT exam were implemented in a Monte Carlo (MC) simulation of a 15° wide / 15 projection DBT scan with $10^8$ photons, yielding the scatter fraction (SF) image estimate of each projection. A CNN was trained to predict these SF images using the MC-simulated SF images of 180 phantoms as ground truth. The mean absolute error (MAE) between the MC-simulated SF and the CNN-predicted SF was computed in 20 other phantoms. The method was evaluated on 140 patient DBT scans (including 20 masses, 22 calcification clusters) by comparing original and scatter-corrected volumes in terms of the signal-difference-to-noise-ratio (SDNR) of the lesions and the area under the receiver operating characteristic curve (AUC) of a commercially-available artificial intelligence CAD system for DBT.
**Results:** The MAE between the CNN-predicted and the MC-simulated scatter images was less than 1%. The CNN generated scatter images up to $10^5$ times faster than MC simulations (0.01 s versus 130 s). After scatter correction, the SDNR of calcifications increased (+25%, P=0.016), while the SDNR of masses (+6%, P=0.455) and the detection accuracy (+0.03 AUC, 95% CI = [-0.12, 0.17]) remained similar.
**Conclusion:** A deep learning CNN can predict the scatter image in DBT projections as accurately as a Monte Carlo simulation but in a significantly lower amount of time.
*Author Disclosures:*
**A. Rodriguez Ruiz:** Employee; ScreenPoint Medical.

**10:30 - 12:00** **Coffee & Talk 2**

### Abdominal Viscera

### SS 1801a

### Diffuse liver disease: fibrosis, steatosis and inflammation

*Moderators:*
T. Denecke; Berlin/DE
M.M. França; Porto/PT

**B-1528** 10:30
**Prospective comparison of transient elastography (1D-TE), shear wave elastography (2D-SWE) and magnetic resonance elastography (MRE) for assessment of liver fibrosis in HCV patients**
J. Matos, I. Mussetto, F. Paparo, S. Perugin Bernardi, M. De Cesari, L. Cevasco, G. Cenderello, L. Bacigalupo, G.A. Rollandi; *Genoa/IT*
*(jfgavinadematos@gmail.com)*

**Purpose:** To assess the agreement among three different stiffness imaging techniques for fibrosis staging in HCV patients. To understand which factors may influence the concordance between the different techniques.
**Methods and Materials:** This was a prospective study. 93 HCV patients were enrolled from March 2017 to September 2018. Each patient was subjected to three techniques performed by blinded operators on the same day. 77 patients had reliable liver stiffness measurements on 1D-TE, 2D-SWE and MRE. Patients were assigned to three different fibrosis groups using kPa cutoffs recommended in a 2015 consensus statement (group 1: no fibrosis, group 2: moderate fibrosis, group 3: advanced fibrosis or cirrhosis). Techniques were confronted (1D-TE vs MRE , 1D-TE vs 2D-SWE , MRE vs 2D-SWE) to assess agreement (kappa Cohen) and kPa value correlation (Spearman's test). Logistic regression was performed to assess factors that determine disagreement.
**Results:** Agreement among all three techniques was observed in 64.9% of patients. Complete disagreement was seen in just 2.6% Inter-rater agreement in assigning correct fibrosis group was good for all three pairs of techniques (1D-TE vs MRE 0.81 , 1D-TE vs 2D-SWE 0.66 , MRE vs 2D-SWE 0.70). Strong correlation of kPa values was observed for all three pairs of techniques. BMI was the only feature associated with disagreement (OR: 1,17; p: 0,02).
**Conclusion:** Tested techniques correctly assign patients to the same fibrosis group in the majority of cases. Good agreement and strong correlation was demonstrated between the different techniques in non-invasive assessment of liver fibrosis in HCV patients.
*Author Disclosures:*
**G.A. Rollandi:** Grant Recipient; GE Healthcare.

**B-1529** 10:38
**Liver stiffness quantification in patients with non-alcoholic steatohepatitis: comparison of shear wave elastography and transient elastography with liver biopsy correlation**
G. Caruana, A. Taibbi, S. Petta, R. Cannella, G. Busè, G. Cutaia, D. Giambelluca, V. Di Marco, T.V. Bartolotta; *Palermo/IT*
*(rob.cannella@libero.it)*

**Purpose:** To assess the accuracy of liver stiffness quantification in patients with non-alcoholic steatohepatitis (NASH) using shear wave elastography (SWE) in comparison with transient elastography (TE).
**Methods and Materials:** This is a prospective study performed in a single institution, including 49 patients with histological diagnosis of NASH. The stiffness of the right liver lobe was measured on the same day with two techniques: TE (FibroScan, Echosens), and SWE (RS80A ultrasound system, Samsung Medison). In the SWE evaluation, 11 patients were excluded due to more than 75% measurements fails. Receiver operating curves (ROC), areas under the ROC (AUROC) and 95% confidence intervals (CI) were calculated to assess the accuracy of TE and SWE for the diagnosis of significant fibrosis (F2-F4) and advanced fibrosis (F3-F4). Spearman's rank coefficient (rho) was used for correlation of TE with SWE. A $p<0.05$ was considered for statistical significance.
**Results:** 38 patients were included in the final population. Overall 24 (63%) patients had significant fibrosis and 17 (45%) had advanced fibrosis. TE and SWE showed an AUROC of 0.711 (95% CI: 0.545-0.877, p=0.032) and 0.729 (95% CI: 0.562-0.896, p=0.020) for the diagnosis of significant fibrosis. The AUROC for the diagnosis of advanced fibrosis were 0.803 (95% CI: 0.648-0.9580, p=0.002) and 0.811 (95% CI: 0.667-0.955, p=0.001) for TE and SWE, respectively. There was a significant correlation between TE and SWE measurements (rho=0.455, p = 0.004).
**Conclusion:** SWE and TE have both a good accuracy, with a significant correlation, for the diagnosis of advanced fibrosis in NASH-patients.
*Author Disclosures:*
**T.V. Bartolotta:** Advisory Board; Samsung Medison. Speaker; Samsung Medison.

**B-1530** 10:46
**Comparison of sound touch elastography (STE) and shear wave elastography (SWE) using liver biopsy as 'gold standard' for chronic liver disease assessment**
I. Gatos[1], P. Drazinos[2], S. Yarmenitis[3], I. Theotokas[1], A. Soultatos[1], E. Panteleakou[1], P. Zoumpoulis[2]; [1]*Athens/GR*, [2]*Kifissia/GR*, [3]*Marousi/GR*
*(p.zoumpoulis@echomed.gr)*

**Purpose:** Chronic liver disease (CLD) is currently one of the major causes of death and the major cause of hepatocellular carcinoma development. sound touch elastography (STE) that is available in Resona 7 Ultrasound (US) device and is similar to shear wave elastography (SWE), seems promising for CLD diagnosis but needs to be validated. The aim of this study is to compare the diagnostic performance between the STE and SWE for CLD assessment, using Liver Biopsy (LB) as "gold standard".
**Methods and Materials:** 290 subjects, 68 normal (F0) and 222 with CLD (F1-F4), were included in the study. A B-Mode and Elastographic examination was performed on each patient with Resona 7 and Aixplorer US devices. The STE (Resona 7) and SWE (Aixplorer) measurements were performed on the Right Lobe (RL) of each patient and were compared to LB results according to the Metavir Classification System (F0-F4). Receiver Operating Characteristic (ROC) analysis was then performed for each of the two methods to obtain best cut-off stiffness values.
**Results:** ROC analysis showed $AUC_{STE}$=0.9741 and $AUC_{SWE}$=0.9854 for F=F4 (Cirrhosis), $AUC_{STE}$=0.9723 and $AUC_{SWE}$=0.9755 for F≥F3, $AUC_{STE}$=0.9675 and $AUC_{SWE}$=0.9662 for F≥F2, $AUC_{STE}$=0.8889 and $AUC_{SWE}$=0.9288 for F≥F1 Fibrosis Stages. Best cut-off stiffness values for each method (STE/SWE) were: F=F4: 12.2/13.5 kPa, F≥F3: 9.5/8.7 kPa, F≥F2: 9.15/8.55 kPa, F≥F1: 6.5/6.05 kPa respectively.
**Conclusion:** Both STE and SWE can differentiate between all Metavir fibrosis stages. SWE seems more reliable in differentiating normals from patients with F≥F1 and Cirrhotic patients (F=F4) but less accurate in diagnosing intermediate stages (F≥F2, F≥F3).
*Author Disclosures:*
**I. Gatos:** Equipment Support Recipient; Mindray is cooperating with Diagnostic Echotomography SA in relation with a Clinical Study. A Resona 7 Ultrasound system has been provided by Mindray in order to carry out this study. **P. Drazinos:** Equipment Support Recipient; Mindray is cooperating with Diagnostic Echotomography SA in relation with a Clinical Study. A Resona 7 Ultrasound system has been provided by Mindray in order to carry out this study. **S. Yarmenitis:** Equipment Support Recipient; Mindray is cooperating with Diagnostic Echotomography SA in relation with a Clinical Study. A Resona 7 Ultrasound system has been provided by Mindray in order to carry out this study. **I. Theotokas:** Equipment Support Recipient; Mindray is cooperating with Diagnostic Echotomography SA in relation with a Clinical Study. A Resona 7 Ultrasound system has been provided by Mindray in order to carry out this study. **A. Soultatos:** Equipment Support Recipient; Mindray is cooperating with Diagnostic Echotomography SA in relation with a Clinical Study. A Resona 7 Ultrasound system has been provided by Mindray in order to carry out this study. **E. Panteleakou:** Equipment Support Recipient; Mindray is cooperating with Diagnostic Echotomography SA in relation with a Clinical Study. A Resona 7 Ultrasound system has been provided by Mindray in order to carry out this study. **P. Zoumpoulis:** Equipment Support Recipient; Mindray is cooperating with Diagnostic Echotomography SA in relation with a Clinical Study. A Resona 7 Ultrasound system has been provided by Mindray in order to carry out this study..

**B-1531** 10:54
**Volumetric iodine density using dual-layer spectral CT for liver fibrosis staging and histopathological correlation**
L. Wang, R. Li, F. Yan, J. Qiang, Q. Han, X. Chen; *Shanghai/CN*
*(15216572203@163.com)*

**Purpose:** To investigate the value of volumetric iodine density (VID) using dual-layer spectral CT for liver fibrosis staging and correlated with histopathological results.
**Methods and Materials:** Twenty rabbit models of $CCl_4$-induced liver fibrosis were established and four untreated rabbits served as controls. All of rabbits underwent four-phasic (noncontrats, arterial phase [AP], venous phase [VP], and equilibrium phase [EP]) enhanced CT using dual-layer spectral CT (IQON). Volumetric iodine density of the liver ($VID_{liver}$) and aorta ($VID_{aorta}$) were derived based on whole-liver and whole-aorta volume measurement. Normalized volumetric iodine density (NVID) were determine as $\Delta VID_{liver}/ \Delta VID_{aorta}$. Fibrosis stage, percentage of sinusoidal area (SA%) and α-SMA staining microvessel area (MVA) were quantified at histopathology. Correlation analysis was performed between NVID and fibrosis stage, SA%, and MVA. Receiver operating characteristic (ROC) analysis was performed for assessing

Sunday diagnostic performance of NVID in detection of clinical significant fibrosis (≥F2) and advanced fibrosis (≥F3) .
**Results:** No significant correlation was identified between either $NVID_{AP}$, $NVID_{VP}$, and fibrosis stage (P>0.05). $NVID_{EP}$ showed significant positive correlation with fibrosis stage (r=0.667, P=0.0004) and SA% (r=-0.603, p<0.001. There was no significant correlation between $NVID_{EP}$ and MVA(r=0.2666, P=0.2079). $NVID_{EP}$ had a sensitivity, specificity, and AUROC of 92.9%, 70.0%, 0.796, respectively, for the diagnosis of ≥F2 fibrosis; and 100%, 73%, and 0.870 , respectively, for the diagnosis of ≥F3 fibrosis.
**Conclusion:** Whole-liver volumetric iodine density using dual-layer spectral CT may be a potential biomarker for liver fibrosis staging.

**B-1532** 11:02
**Reproducibility of shear wave dispersion imaging for evaluation of non-alcoholic fatty liver disease**
J. Yoo, J. Lee, H.-J. Kang, S. Ahn; *Seoul/KR (jeongin527@gmail.com)*

**Purpose:** To evaluate the intraobserver and interobserver reproducibility of shear wave dispersion imaging (SWDI) for noninvasive evaluation of nonalcoholic fatty liver disease (NAFLD).
**Methods and Materials:** This prospective study was approved by our institutional review board and informed consents were obtained from all subjects. In group I, 71 patients with suspected or alleged NAFLD underwent grey-scale ultrasound (US) imaging and two sessions of dispersion imaging by one abdominal radiologist. In group II, 19 asymptomatic volunteers underwent grey-scale US and three independent sessions of dispersion imaging by three abdominal radiologists. The visual grade of hepatic steatosis on grey-scale US was scored as 0 (absent) to 3 (severe) by two independent reviewers. Intra-observer (group I) and inter-observer (group II) reproducibilities of dispersion imaging were assessed using intraclass correlation coefficient (ICC). Independent association of variables with dispersion slope was analysed using a multivariate linear regression model.
**Results:** For intra-observer reproducibility, ICC was 0.955 (95% CI, 0.926-0.973). In the subgroup analysis of group I, higher BMI, higher grade of steatosis on US, presence of LFT abnormality were not associated with reduced ICC values. ICC for inter-observer agreement of three radiologists was 0.800 (95% CI, 0.572-0.916). There were no significant differences in dispersion slope among different visual grades of hepatic steatosis. In the multivariate analysis, elasticity, ALT, and AST were significantly related to the dispersion slope as independent variables (p<0.001, p=0.02, and p=0.03, respectively).
**Conclusion:** Shear wave dispersion imaging showed excellent intra-observer and inter-observer agreement.

**B-1533** 11:10
**Are there different cut-off values for staging liver fibrosis using 2D-SWE implemented on different systems from the same manufacturer?**
I. Sporea, F.B. Bende, A.S. Popescu, R. Sirli, M. Danila; *Timisoara/RO (isporea@umft.ro)*

**Purpose:** To evaluate the range of liver stiffness(LS) cut-off values for predicting different stages of liver fibrosis(LF) for 2D-SWE.GE implemented on 3 different systems from GE Healthcare(LOGIQ E9,LOGIQ S8,LOGIQ P9).
**Methods and Materials:** We re-evaluated three studies performed in our Department evaluating the performance of 2D-SWE.GE using different systems(LOGIQ E9,LOGIQ S8,LOGIQ P9) for predicting different stages of LF, using Transient Elastography(TE) as the reference method. 1.LOGIQ E9 study: included 331 subjects with or without chronic hepatopathies; reliable LSM were obtained with both methods in 303/331(91.5%) subjects; LF distribution using was: F<2: 91/303(30.1%);F=2: 31/303(10.2%);F=3: 37/303(12.2%);F=4: 144/303(47.5%). 2.LOGIQ S8 study: included 179 consecutive subjects, with or without chronic hepatopathies; reliable LSM were obtained in 171/179(95.5%) subjects; LF distribution was: F<2: 51(29.8%);F=2: 40(23.4%);F=3: 31(18.1%);F=4: 49(26.7%). 3.LOGIQ P9 study: included 234 consecutive subjects, with or without chronic hepatopathies; reliable LSM were obtained in 205/234(87.6%) subjects; LF distribution was: F<2: 40/131(30.5%);F2: 24/131(18.3%);F3=22/131(16.8%);F4=45/131(34.4%).
**Results:** The best cut-off values for 2D-SWE.GE for predicting F≥2, F≥3 and F=4 were: 1.For LOGIQ E9: 6.7 kPa(AUC-0.95, Se-92.7%, Sp-85.5%), 8.2 kPa(AUC-0.97, Se-95%, Sp-89.3%),9.3 kPa(AUC-0.96, Se-91.7%, Sp-92.5). 2.For LOGIQ S8: 6.9 kPa(AUC-0.92, Se-85.8%, Sp-90.2%), 8.2 kPa(AUC-0.93, Se-87.5%, Sp-86.8%), 9.3 kPa(AUC-0.91, Se-85.7%, Sp-81.2%). 3.For LOGIQ P9: 6.8 kPa(AUC-0.93, Se-83.5%. Sp-91.2%), 7.6 kPa(AUC-0.94, Se-86.5%, Sp-92.7%), 9.3 kPa(AUC-0.91, Se-75.5%, Sp-92.5%). The LS cut-off range for 2D-SWE.GE implemented on different systems were: for F≥2: 6.7-6.9 kPa, for F≥3 : 7.6-8.2 kPa and for F=4: 9.3 kPa.
**Conclusion:** The LS cut-off values for 2D-SWE.GE implemented on different systems for predicting F≥2, F≥3 and F=4 are quite similar.

**B-1534** 11:18
**Differentiating mild and substantial fibrosis from normal subjects: comparison of diffusion-kurtosis imaging and conventional diffusion-weighted imaging**
S. Xie, Q. Li, H. Qi, Y. Cheng, W. Shen; *Tianjin/CN (xiess1989_happy@163.com)*

**Purpose:** To compare the diagnostic accuracy of liver diffusion-kurtosis imaging (DKI) and conventional diffusion-weighted imaging (cDWI) for differentiating mild and substantial fibrosis from normal subjects.
**Methods and Materials:** Twenty-seven healthy volunteers (S0) and forty-five patients with pathological proved mild (S1) or substantial (S2) liver fibrosis underwent DWI with multiple b values (b= 0, 1,000, 1,500, 2,000 s/mm$^2$). Mean apparent diffusion (MD), mean kurtosis (MK) and apparent diffusion coefficient (ADC) of liver parenchyma were measured and compared between patients with mild or substantial fibrosis and normal subjects. The discriminative abilities of DKI and cDWI parameters were analysed and compared by receiver operating characteristic (ROC) curve analysis.
**Results:** There were significant differences in MD and ADC values between patients with mild or substantial fibrosis and normal subjects (all *P*<0.05). Moreover, MD value was statistically different between S0 and S1 (*P*=0.028), S0 and S2 (*P*=0.005). ADC value was statistically different between S0 and S2 (*P*=0.012). There was no significant difference in MK for all groups (*P*=0.646). In addition, MD and ADC values significant correlated with fibrosis stages (*r*=-0.668, -0.341; all *P*<0.01), and MK have no correlation with fibrosis stages (*r*=0.180; *P*=0.130). AUC for MD and ADC were 0.937 and 0.707 for characterisation of S1-2, 0.817 and 0.658 for characterisation of S2. MD performed better than ADC for the characterisation of S1-2 and S2 (all *P*<0.05).
**Conclusion:** MD value derived from DKI is feasible to differentiate mild or substantial fibrosis from normal subjects, and offer better performance than ADC derived from cDWI.

**B-1535** 11:26
**Liver fibrosis and inflammation: influence on the 3D multi-echo Dixon parameters in patients with chronic liver diseases**
F. Hu, B. Song, R. Yang, M. Wang; *Chengdu/CN (yingxianghu_cmc@163.com)*

**Purpose:** To evaluate the influence of liver fibrosis and inflammation on the 3D Multi-Echo Dixon (3D ME Dixon) parameters in patients with chronic liver diseases and to determine the diagnostic performance of 3D ME Dixon for the simultaneous assessment of liver steatosis and iron overload.
**Methods and Materials:** 99 consecutive patients with chronic liver disease underwent confounder-corrected 3D ME Dixon scan at 3.0T MR scanner. Liver biopsy was reviewed in all cases, grading liver steatosis, siderosis, fibrosis, and inflammation. Spearman correlation and multiple regression analysis were performed to determine the relationship between 3D ME Dixon parameters and histopathological features. Receiver operating characteristic analysis was performed to determine the diagnostic performance.
**Results:** At multivariate analysis, only liver steatosis independently influenced MRI-PDFF values ($R^2$*=0.800, P <*0.001). liver iron overload and fibrosis influenced R2* values ($R^2$=0.631, *P*<0.001). liver R2* values were moderately correlated with fibrosis stages (*r*=0.610, *P*=0.000) in the subgroup with the absence of iron overload. The AUC of MRI-PDFF was 0.989 for the diagnosis of steatosis grade 1 or greater, and 0.986 for grade 2 or greater. The AUC of R2* was 0.815 for identifying iron overload grade 1 or greater, and 0.876 for steatosis grade 2 or greater.
**Conclusion:** 3D ME Dixon can be used to simultaneously evaluate liver steatosis and iron overload in patients with chronic liver diseases, especially for quantification of liver steatosis. However, liver R2* value may be affected by the liver fibrosis in the setting of chronic liver diseases with the absence of iron overload.

**B-1536** 11:34
**2D MRE liver stiffness values are nearly equivalent for three sequence types across four multi-centre drug-development clinical trials**
M.S. Middleton[1], W.C. Henderson[1], A.L. Louie[1], R. Myers[2], S. Djedjos[2], C. Sirlin[3]; [1]*La Jolla, CA/US*, [2]*Foster City, CA/US*, [3]*San Diego, CA/US (msm@ucsd.edu)*

**Purpose:** To demonstrate equivalence of 2D MRE sequences in four multi-center drug-development clinical trials
**Methods and Materials:** MRE liver stiffness is an endpoint in many multi-center drug-development clinical trials. Equivalence across sequence types (GRE, SE, SE-EPI) at 3T has been reported in single-center, single-scanner studies. However, to justify interchangeable use in clinical trials, validation of equivalence is needed across different sites and MR scanner types. To accomplish this, 2D MRE sequence results were reviewed for four multi-center drug-development nonalcoholic fatty liver disease (NAFLD) clinical trials (Gilead NCT02854605, NCT02781584, NCT02856555, and NCT02466516).

Sunday

Liver stiffness differences across these sequences were compared to region-of-interest (ROI) analysis areas, and correlation analysis of liver stiffness differences and analysis areas was performed.
**Results:** GRE, and either SE or SE-EPI sequences were compared for 58 MRs, for two MR manufacturers and four scanner types. Mean GRE liver stiffness was 0.06 kPa less than the mean for SE and SE-EPI sequences (p=0.74). Absolute differences in liver stiffness were weakly correlated with total ROI areas. Absolute differences were greater between GRE and the other sequences for smaller total ROI areas (0.52, 0.41, and 0.29 kPa for <700 Px, 700-2000 Px, and >2000 Px, respectively), but statistical significance was not reached for those comparisons (p>0.05).
**Conclusion:** Liver stiffness values for three 2D MRE sequences show near equivalence for different types of scanners in four large multi-center NAFLD drug-development clinical trials. These data support using these sequences interchangeably in future multi-center clinical trials.
*Author Disclosures:*
**M.S. Middleton:** Consultant; Median, Kowa, Novo Nordisk. Employee; Gilead. Equipment Support Recipient; General Electric, Siemens. Grant Recipient; Gilead, Bayer, General Electric, Siemens. Research/Grant Support; Gilead, Bayer, Intercept, Enanta, Shire, Roche. Shareholder; Pfizer. **R. Myers:** Employee; Gilead. **S. Djedjos:** Employee; Gilead.

**B-1537** 11:42
**Comparison of sound touch elastography (STE) and vibration-controlled transient elastography (VCTE) using liver biopsy as a reference for diagnosis of chronic liver disease**
I. Gatos[1], P. Drazinos[2], S. Yarmenitis[3], I. Theotokas[1], A. Kanavaki[1], E. Manesis[1], P. Zoumpoulis[2]; [1]*Athens/GR*, [2]*Kifissia/GR*, [3]*Marousi/GR (p.drazinos@echomed.gr)*

**Purpose:** Chronic Liver Disease (CLD) is one of the major causes of death nowadays and the major cause of Hepatocellular Carcinoma development. Vibration Controlled Transient Elastography (VCTE), commercially available via Fibroscan ultrasound (US) device, is a well-established imaging method in CLD diagnosis, while Sound Touch Elastography (STE) that is similar to Shear Wave Elastography (SWE), seems promising but needs to be validated. The aim of this study is to compare the diagnostic performance between STE and VCTE using Liver Biopsy (LB) as “Gold Standard”.
**Methods and Materials:** 210 subjects, 60 normal (F0) and 150 with CLD (F1-F4), were included in the study. A B-Mode and Elastographic examination was performed on each patient with Resona 7 and Fibroscan US devices. STE and VCTE measurements were performed on the RL of each patient and measurements were compared to LB (Metavir Stage: F0-F4). Receiver Operating Characteristic (ROC) analysis was then performed for both methods to obtain best cut-off stiffness values.
**Results:** ROC analysis showed $AUC_{STE}$=0.9747 and $AUC_{VCTE}$=0.9808 for F=F4 (Cirrhosis), $AUC_{STE}$=0.9790 and $AUC_{VCTE}$=0.9792 for F≥F3, $AUC_{STE}$=0.9731 and $AUC_{VCTE}$=0.9644 for F≥F2, $AUC_{STE}$=0.8941 and $AUC_{VCTE}$=0.9185 for F≥F1 Fibrosis Stages. Best cut-off stiffness values calculated were: F=F4: 13.7/11.3 kPa, F≥F3: 9.5/8.7 kPa, F≥F2: 9.15/8.2 kPa, F≥F1: 6.15/6.1 kPa respectively.
**Conclusion:** Both VCTE and STE can differentiate between all Metavir fibrosis stages. STE is more reliable in differentiating CLD patients with intermediate fibrosis stages (F≥F2, F≥F2) but is less accurate in detecting fibrosis (F≥F1) and diagnosing Cirrhosis (F=F4) than VCTE.
*Author Disclosures:*
**I. Gatos:** Equipment Support Recipient; Mindray is cooperating with Diagnostic Echotomography SA in relation with a Clinical Study. A Resona 7 Ultrasound system has been provided by Mindray in order to carry out this study. **P. Drazinos:** Equipment Support Recipient; Mindray is cooperating with Diagnostic Echotomography SA in relation with a Clinical Study. A Resona 7 Ultrasound system has been provided by Mindray in order to carry out this study. **S. Yarmenitis:** Equipment Support Recipient; Mindray is cooperating with Diagnostic Echotomography SA in relation with a Clinical Study. A Resona 7 Ultrasound system has been provided by Mindray in order to carry out this study. **I. Theotokas:** Equipment Support Recipient; Mindray is cooperating with Diagnostic Echotomography SA in relation with a Clinical Study. A Resona 7 Ultrasound system has been provided by Mindray in order to carry out this study. **A. Kanavaki:** Equipment Support Recipient; Mindray is cooperating with Diagnostic Echotomography SA in relation with a Clinical Study. A Resona 7 Ultrasound system has been provided by Mindray in order to carry out this study. **E. Manesis:** Equipment Support Recipient; Mindray is cooperating with Diagnostic Echotomography SA in relation with a Clinical Study. A Resona 7 Ultrasound system has been provided by Mindray in order to carry out this study. **P. Zoumpoulis:** Equipment Support Recipient; Mindray is cooperating with Diagnostic Echotomography SA in relation with a Clinical Study. A Resona 7 Ultrasound system has been provided by Mindray in order to carry out this study.

**B-1538** 11:50
**Liver fibrosis staging with Cannon shear wave elastography: comparison with three other non-invasive tools using histologic criteria as a reference standard**
S. Byun, J. Lee, J. Min, K. Shin, S. You, K. Cheon, I. Song, K. Kim; *Daejeon/KR (pureeyes111@cnuh.co.kr)*

**Purpose:** To investigate the value of Cannon shear wave elastography (C-SWE) in the assessment of liver fibrosis and to compare the diagnostic performance of C-SWE with that of other noninvasive tools.
**Methods and Materials:** Sixty-eight patients with chronic liver disease underwent liver stiffness (LS) measurements with C-SWE and elastography point quantification (ElastPQ) in the same session by two observers, respectively. All patients were examined using transient elastography (TE) and serologic test. The inter-observer agreements of both techniques were assessed with intraclass correlation coefficients (ICCs). The diagnostic performance in significant fibrosis (F≥2) and cirrhosis was evaluated using the receiver operating characteristics curve (AUC) analysis with histologic criteria as reference standard.
**Results:** The values with C-SWE were significantly greater than those with ElastPQ ($p$ < 0.001). The LS measurements with C-SWE (r = 0.663 and 0.651) and ElastPQ (r = 0.790 and 0.668) significantly increased as liver fibrosis progressed. The inter-observer agreement was excellent for C-SWE (ICC= 0.842) and ElastPQ (ICC = 0.879). The estimated cut-off values with C-SWE for both observers were 8.88 kPa and 9 kPa to detect significant fibrosis and 13.3 kPa and 10.4 kPa to detect cirrhosis; these were higher than those with ElastPQ. For the prediction of liver fibrosis, C-SWE, ElastPQ, and TE showed comparable high diagnostic performance (range of AUC: 0.802-0.958) and significantly better than that of the serologic fibrosis marker test.
**Conclusion:** C-SWE is a promising noninvasive US-based technique for assessing liver fibrosis and shows high diagnostic performance comparable to that of ElastPQ and TE.

**10:30 - 12:00** **Room E1**

### Breast

### SS 1802a

### Radiation dose, quality assurance, and safety in breast imaging

*Moderators:*
M. Koutalonis; Colchester/UK
I. Kralik; Zagreb/HR

**B-1539** 10:30
**Multiparameter dose monitoring in mammography: the use of combined breast thickness and density determined DRLs**
L. Cockmartin, K. Lemmens, A. Jacobs, J. Binst, H. Bosmans; *Leuven/BE (lesley.cockmartin@uzleuven.be)*

**Purpose:** To propose a method for improved high dose alerts using breast thickness and density driven diagnostic reference levels (DRLs) for quality improvement in digital mammography (DM).
**Methods and Materials:** Screening mammograms were collected on 4 DM systems: Siemens Inspiration, Hologic Selenia Dimensions, Fuji Innovality and GE Essential with in each dataset 8045, 507, 1422 and 1372 images respectively. Breast thickness and mean glandular dose (MGD) were extracted from the dicomheaders (DOSE, QaelumNV) while volumetric breast density (VBD) was assessed using Volpara™. A fixed value DRL (2mGy for 60mm) as well as a DRL curve based on the 95$^{th}$ percentile of the MGD obtained from large patient samples on 71 systems tested in the Belgian breast screening, are plotted on these data. Additionally, data were colorcoded for their Volpara Density Grade (VDG) and a curve fit was applied to the four VDG groups separately. Outliers were defined as exceeding the 95$^{th}$ percentile DRL curve, and then also as > 1mGy distance to the density-determined curve fit.
**Results:** Compared to the use of traditional DRL curves that created 778 (9%) outliers on Siemens, present approach showed only 43 (0.5%) outliers. For Hologic this number dropped to 2 (0.7%), for Fuji Innovalty to 4 (0.3%) and for GE Essential to 2 (0.1%). This dose monitoring approach pointed to occasional problems in the high density groups.
**Conclusion:** Multiparameter dose alerting, using both thickness and density, results in improved selection of cases that need further verification and makes dose monitoring more efficient and relevant.
*Author Disclosures:*
**H. Bosmans:** Founder; Co-founder Qaelum NV. Research/Grant Support; The medical physics team has research agreements with Siemens-Healthineers and GE Healthcare.

**B-1540** 10:38
**Accuracy of mammography dosimetry in the era of the European Directive 2013/59/Euratom transposition**
G. Gennaro[1], S. Bigolaro[1], M. Hill[2], R. Stramare[1], F. Caumo[1]; [1]*Padua/IT*, [2]*Wellington/NZ (gisella.gennaro@iov.veneto.it)*

**Purpose:** To evaluate the parameters affecting mammography mean glandular dose (MGD) in the era of the European Directive 2013/59/Euratom transposition, where accurate and personalised dose estimates are of heightened importance.
**Methods and Materials:** 4028 women who had a mammography examination by one of five mammography units using different detector technologies were included. 16,558 images were processed by a software algorithm that determines breast density (BD) and uses BD to estimate patient-specific MGD. Entrance dose values and half value layers measured for each mammography system within 6 months of imaging were collected to calibrate MGD for equipment performance. Mean MGD values considering BD, without and with calibration data, were compared with manufacturer-reported MGD.
**Results:** MGD estimated using BD is mostly higher than MGD provided by manufacturers for the studied systems, leading to relative mean MGD differences of 6.6%, 15.3%, 4.4%, 13.5%, -1.7%, for the five mammography units. The average differences between measured and manufacturer-provided entrance doses are small for the five units (-1.3%, -1.4%, -2.61%, 1.2%, 2.6%, respectively), so the additional effect of system calibration is minimal. The inclusion of system calibration in patient-specific MGD estimation results in relative mean differences of 1.0%, 9.7%, 5.9%, 10.6%, and -6.7%, compared to manufacturer-reported MGD respectively, although individual MGD differences as large as 44% were observed, which illustrates the importance of ensuring dose accuracy at the patient level.
**Conclusion:** Increasing levels of accuracy in mammography dosimetry are possible in the era of the European Directive 2013/59/Euratom transposition, taking into account patient-specific BD and equipment calibration.
*Author Disclosures:*
**M. Hill:** Consultant; Volpara Solutions Europe.

**B-1541** 10:46
**First clinical application of spiral breast CT with photon-counting detector: patient-specific radiation dose assessment**
N. Saltybaeava[1], M. Marcon[1], N. Berger[1], D. Kolditz[2], C. Steiding[2], A. Boss[1], H. Alkadhi[1]; [1]*Zurich/CH*, [2]*Erlangen/DE (Natalia.Saltybaeva@usz.ch)*

**Purpose:** Dedicated breast CT (BCT) has demonstrated superior image quality compared to digital Mammography (DM) and breast tomosynthesis (BT), however the radiation dose from BCT was higher than the one from DM and BT, especially when referring to the screening settings. The novel BCT scanner equipped with photon-counting detector (PCD) allows for more efficient dose utilization. The aim of the study is to assess the radiation dose from the first clinical application of the BCT with PCD.
**Methods and Materials:** This study included 17 patients underwent breast CT examination on the first clinical BCT equipped with CdTe detector (Nu-view, Erlangen, Germany). Fixed tube voltage of 60kV and tube current of 25mA were used based on preliminary studies. The individual patient DICOM data together with patient-specific scan parameters were used as an input for Monte Carlo (MC) simulations. The accuracy of the MC simulations was validated against the measurements performed on the same BCT system using ionization chamber in 16-cm CTDI phantom. For each patient the average glandular dose (AGD) was calculated based on the individual 3D dose distribution obtained from MC simulations.
**Results:** The MC simulation has shown high accuracy comparing to the direct dose measurements, with the mean difference of 4.4%. The average glandular dose ranged from 4.6 to 6.1 mGy, depending in the breast size. The mean ADG was found to be 5.2 mGy.
**Conclusion:** BCT system with photon-counting detector allows performing breast examinations with full 3D imaging capabilities using the dose comparable to that from two-view DM.
*Author Disclosures:*
**D. Kolditz:** Employee; AB-CT. **C. Steiding:** Employee; AB-CT.

**B-1542** 10:54
**Gadolinium deposits in healthy women undergoing repetitive GDBCA-enhanced breast MRI in the context of high-risk breast cancer screening**
B.I. Bennani-Baiti[1], K.B. Krug[2], D. Giese[2], K. Weiss[2], T.H. Helbich[1], P.A.T. Baltzer[1]; [1]*Vienna/AT*, [2]*Cologne/DE (barbara.bennani-baiti@meduniwien.ac.at)*

**Purpose:** To assess, whether women undergoing contrast-enhanced breast MRI exams in the context of high-risk screening exhibit signal alterations in the dentate nucleus.
**Methods and Materials:** In this IRB-approved prospective study, 41 healthy women (16 with no previous gadolinium-based contrast agent (GDBCA) exposure and 25 that had >5 GDBCA administrations (range 6-25) in the context of high-risk breast cancer screening) underwent 3T MRI of the brain, with a dedicated head coil, including T1 mapping and mpRAGE sequences. T1 times and T1 signal intensities were measured for dentate nucleus (ND) and pons, employing Horos software. Ratios of ND to pons were calculated and further statistical analyses were carried out with SPSS and Medcalc.
**Results:** Spearman's rank correlation revealed no correlation between number of cumulative GDBCA doses and either quantitative T1 times of ND (P=0,4457) or T1 signal intensity ratios of ND to pons (P=0.2885). Similarly, no correlation was found for age and either quantitative T1 times of ND (P=0.1855) or T1 signal intensity ratios of ND to pons (P=0.1653). Multiple regression analysis confirmed these results (P>0.05 for all potential associations of age and cumulative number of GBCA doses with ND T1 times and ND to pons T1 signal ratios).
**Conclusion:** The dentate nucleus does not exhibit altered T1 signals after high-cumulative dosages of macrocyclic GDBCAs in healthy women. This indicates that currently employed macrocyclic GDBCAs do not result in Gd deposits in the brain of healthy women participating in a high-risk screening programme for early breast cancer detection.

**B-1543** 11:02
**Mammography positioning errors: a multi-centre study**
N. Sharma[1], S. Schofield[2], M. Fletcher[1], R.L. Hammond[3], A. N. Larson[3], A.S. Bakken[3], E. Handberg[3], N.-M. Hopland[3], S.E. Iles[2]; [1]*Leeds/UK*, [2]*Halifax, NS/CA*, [3]*Oslo/NO (mo@dal.ca)*

**Purpose:** The objectives of this study are to evaluate agreement between radiologists and radiographers in assessing mammography positioning errors.
**Methods and Materials:** 672 FFDM studies rejected due to positioning errors were independently reviewed by nine radiographers and one radiologist from three breast imaging centres in Canada, Norway, UK. Readers were provided with a PGMI scale and trained on the use of the image quality review tool (Densitas Inc.). Reviewers evaluated studies for positioning errors including PNL, CC exaggeration, nipple position, skin folds, portion cut-off, patient related artefacts, posterior tissue, pectoralis muscle (position, shape, and thickness), IMF, sagging, positioning on image receptor. Interrater agreement overall and by centre was evaluated using weighted Fleiss' Kappa.
**Results:** Agreement between readers on individual positioning errors across all centres was slight to moderate (kappas ranging from 0.09 to 0.49), and was only slight for PGMI score (kappa=0.176). Positioning errors with highest agreement between readers within centres included portion cut-off, patient related artefacts, PNL length difference <10mm, portion cut-off, skin folds (kappa=0.88, 0.61, 0.53, 0.52); other positioning errors generally showed slight to fair agreement (kappas < 0.5).
**Conclusion:** Agreement amongst readers on mammography positioning criteria within and between centres ranges widely; automated image quality assessment may help to improve standardization.

**B-1544** 11:10
**Post-contrast cone-beam breast-CT without the prior pre-contrast scan: can we reduce radiation exposure while maintaining diagnostic accuracy?**
S. Wienbeck, U. Fischer, J. Lotz, J. Uhlig; *Göttingen/DE (susanne.wienbeck@med.uni-goettingen.de)*

**Purpose:** To evaluate whether post-contrast Cone-Beam Breast-CT (CBBCT) alone is comparable to the current standard of combined pre- and post-contrast CBBCT regarding diagnostic accuracy and superior regarding radiation exposure.
**Methods and Materials:** This IRB-approved study included 49 women (61 breasts) with median age 57.9 years and BI-RADS 4/5 lesions diagnosed on mammography/ultrasound in density types c/d breasts. 2 radiologists rated post-contrast CBBCT and pre- and post-contrast CBBCT with subtraction images on the BI-RADS scale separately for calculation of inter- and intraobserver agreement and in consensus for diagnostic accuracy assessment. Sensitivity, specificity and area-under the curve (AUC) were compared via McNemar test and DeLong method, respectively. Subtraction imaging misregistration were measured from 1 (no artefacts) to 4 (artefacts with width >4mm).
**Results:** A total of 100 lesion (51 malignant; 6 high risk; 43 benign) were included. AUC, sensitivity and specificity showed no significant differences comparing post-contrast CBBCT alone versus pre- and post-contrast CBBCT (AUC: 0.84 vs. 0.83, p=0.643; sensitivity: 0.89 vs. 0.85, p=0.158; specificity: 0.73 vs. 0.76, p=0.655). Inter- and intra-observer agreement were excellent (intraclass correlation coefficient ICC=0.76, ICC=0.83, respectively). Radiation dose was significantly lower for post-contrast CBBCT alone versus pre- and post-contrast CBBCT (median AGD 5.9 mGy vs. 11.7 mGy, p<0.001). High-degree misregistrations were evident in the majority of subtraction images (level 1/2/3/4: 16.9%/27.1%/16.9%/39%), in particular for bilateral exams (3.2%/29.2%/8.3%/58.3%).

**Conclusion:** Diagnostic accuracy of post-contrast CBBCT alone is comparable to pre- and post-contrast CBBCT in type c/d breasts, while yielding a significant twofold radiation dose reduction.

**B-1545** 11:18

**Local diagnostic reference levels in mammography: setting a maximum/alarm dose in a diagnostic mammography unit using dose monitoring software**

R.M.A. Hegazy[1], E.A. Aghahowa[2], R. Yasin[3], A. Eduarte[2]; [1]*Cairo/EG*, [2]*Kuwait/KW*, [3]*Menofia/EG (raniahegazy@hotmail.com)*

**Purpose:** To show feasibility of a dose monitoring software to set an alarm level guided by the local DRLs used in mammography above which it will give a red flag so radiation monitoring team will be alerted to a need for revision of the mammography technique.
**Methods and Materials:** This is a retrospective study comprising mammographic imaging data for 300 consecutive breasts of variable compositions randomly selected. Data were collected using "DoseWatch™" by GE. Mean glandular dose (MGD) was recorded in relation to patient age, compressed breast thickness (CBT), percentage of glandular tissue (FGL), projection views and skin entrance dose among other imaging parameters. The formula mean±1.96*SD was used to calculate the MGD range for all patients.The maximum calculated dose was rounded to the nearest whole number.The resultant value was used as an upper threshold above which the dose monitoring software gives a red flag.
**Results:** MGD was calculated for breast thickness above 50 mm in CC, MLO and3D tomography in CC view and was found to be 1.32,1.4 and 1.62 mGy, respectively. Local DRLs for breast imaging was calculated as follows: average MGD CC view= 1.32 minimum=0.74; maximum=2.42; alert level=2 mgy. Average MGD MLO view=1.4 minimum=0.92; maximum=2.42; alert level=2 mgy. Average MGDTomoCC view= 1.62 minimum=0.88; maximum=3.75; alert level=3mGy. Data found were well comparable to internationally established results.
**Conclusion:** A dose monitoring software is an easy and reliable tool to set local DRLs in a busy mammography unit. This can be used to establish local and then national DRLs and to compare with published literature worldwide.

**B-1546** 11:26

**A new technique in mammography: self-compression-survey of patient contentedness**

S. Ulus, O. Kovan, A. Arslan, P. Elpen, M.E. Aribal; *Istanbul/TR (silaulus@hotmail.com)*

**Purpose:** Senographe Pristina (GE Healthcare,Chicago, IL) is a) is a next-generation mammography technology that features a self-compression tool allowing patients to manually adjust the degree of breast compression. Purpose of this study is to evaluate the experience of this technology compared to previous examinations with a survey study.
**Methods and Materials:** The survey studies (12-item questionnaire) subsequent to mammography examinations of 365 patients who underwent screening/diagnostic mammography between April-July 2017 at our centre were reviewed retrospectively. Women who never had a mammography before, who had a previous mammography examination ≥2 years ago or who didn't want to use the self-compression device were discluded. 106 women were included.
**Results:** Patient contentedness was high. 70.8% said it was a better experience compared to previous ones. 84.9% of the participants found the examination comfortable; 75.5% of the participants found the examination more comfortable compared to previous ones. 52.8% of the participants declared they were less anxious compared to previous examinations. 66.9% of the participants declared they found the new design attractive; when compared to previous examinations, 39.7% found it more attractive. 27.3% said the new design decreased anxiety. In the evaluation of impact of patient-assisted compression (PAC) on comfort 80.2% said they found it more comfortable. In the evaluation of impact of PAC on anxiety 64.2% said it decreased anxiety. 72.6% of the participants said the exam was shorter.
**Conclusion:** Self-compression is a useful technique for decreasing pain during mammography examinations resulting in compliance of women with screening guidelines.

**B-1547** 11:34

**Impact of a radiological consultation on the surgical management of breast cancer patients: a retrospective study of 1114 cases**

C. Dratwa; *Paris/FR (chloedratwa@gmail.com)*

**Purpose:** Evaluate the impact of a radiology image consultation on the surgical strategy in patients with a newly-diagnosed breast cancer.
**Methods and Materials:** During 2014, 1114 patients with breast cancers (invasive: 87.5%, in situ: 12.5%) underwent an imaging-surgery consultation. Followings parameters were evaluated: missing data in the initial imaging report, number and pathological results of additional breast lesions detected during this consultation, effects on surgical management, impact on treatment delay and second breast surgeries. Uni- and multivariate analyses were done to identify parameters (clinical, radiological and pathological data) with a significant impact on surgical changes (p< 0.05).
**Results:** BI-RADS category was missing in 4.6% of patients. Cancer size was not evaluated in 126 (11.3%, 63.2% of calcifications vs 3.7% for masses). At consultation, at least one additional lesion was detected in 299 patients (26.8%). Among the 236 patients with percutaneous samplings, 102 lesions were malignant (60 invasives, 42 in situ), 16 at high risk, 114 benign and 4 non-contributive. On 1081 patients with primary surgery, surgical management was modified in 147 (13.6%). Delay for surgery was respected in 95%. Reoperation rate was 7% versus 12.9% in 2013. At univariate analysis, "calcification", BI-RADS categorization omission, unknown size, in situ and lobular subtypes were associated with modified surgical strategies. At multivariate analysis, masses with calcifications and lobular subtype were significantly associated with surgical changes (OR=1.92 95% CI [1.1-3.24]).
**Conclusion:** In breast-cancer patients, imaging consultation modified the surgical management in 13.6% without increasing delay for surgery and with a less re-excision rate.

**B-1548** 11:42

**Improving quality assurance in DBT breast screening by visual search monitoring**

Y. Chen[1], E. Michalopoulou[1], A.G. Gale[1], D. Bernardi[2]; [1]*Loughborough/UK*, [2]*Trento/IT (Y.Chen@lboro.ac.uk)*

**Purpose:** The current research investigates how experienced DBT radiologists visually inspected FFDM cases.
**Methods and Materials:** Seven radiologists interpreted 40 normal, benign or malignant cases. Each case comprised FFDM or C-view, and DBT views. The cases were presented on a workstation with dual 5 MP monitors and a remote eye tracker positioned underneath the workstation recorded the participants' visual search behaviour. Participants used their normal breast screening reporting procedure of examining the cases, together with prior mammographic images, and then examined breasts as FFDM and DBT views. Diagnostic reporting data, visual gaze position data and DBT slice scrolling behaviour data were recorded.
**Results:** Examination of the visual search data indicated that radiologists tended to visually examine large areas of the FFDM views to locate potential suspicious areas and then use the DBT views to 'drill down' across image slices to examine their details. Out of 119 total examinations, 25 cancers were missed on the malignant cases. Scrolling behaviour data demonstrated that individuals who correctly recalled a malignancy spent 56s whilst a missed cancer was evidenced by quickly scrolling through the range of slices where the abnormality existed for only 22 seconds. Participants spent on average 1:10s on successfully recalled malignant cases and 1:08s on malignant cases which were not successfully recalled (two-tail paired t test, p=0.294, n.s.).
**Conclusion:** FFDM images were searched to establish initial potential areas of interest which were then examined in detail using DBT slices. This strategy could improve quality assurance in breast cancer screening trainees.

**B-1549** 11:50

**Diagnostic performance of low dose contrast-enhanced dual energy mammography in comparison to contrast-enhanced MRI**

P. Clauser[1], P.A.T. Baltzer[1], P. Kapetas[1], R.A. Woitek[1], F. Leone[2], K. Pinker-Domenig[1], M. Weber[1], M. Bernathova[1], T.H. Helbich[1]; [1]*Vienna/AT*, [2]*Milan/IT (paola.clauser@meduniwien.ac.at)*

**Purpose:** To compare the diagnostic performance of a low-dose contrast-enhanced dual-energy mammography (CEDEM) system with breast contrast-enhanced magnetic resonance imaging (CE-MRI) in women with unclear or suspicious findings on conventional imaging.
**Methods and Materials:** The ethics committee approved this prospective, monocentric study and all patients gave written informed consent. Women with unclear or suspicious findings on conventional imaging and no contraindications for CEDEM or CE-MRI were invited to participate in the study. Included in the final analysis were women with a histological diagnosis (standard of reference). Three off-site, blinded readers evaluated the images according to BI-RADS lexicon in a randomized order, each in two separate reading sessions. Detection rate, sensitivity, specificity, negative and positive predictive values (NPV, PPV) were calculated and compared with multivariate statistics.
**Results:** Included were 80 patients (mean age 54.3 years, standard deviation 11.2) with 93 lesions (32 benign, 61 malignant). Detection was higher with CE-MRI (92.5%-94.6%) than with CEDEM (79.6%-91.4%, P=0.038). This was related to a higher detection for benign lesions with CE-MRI. No differences in sensitivity (CEDEM 65.6%-90.2%; CE-MRI 83.6%-93.4%, P=0.086) and NPV (CEDEM 59.6%-71.4%; CE-MRI 63.0%-76.5%, P=0.780) were found. Specificity was higher with CEDEM (46.9%-96.9%) than with CE-MRI (37.5%-53.1, P=0.001). PPV was higher with CEDEM (76.4%-97.6%; CE-MRI 73.3%-77.3% P=0.007). Accuracy of CEDEM was as good as CE-MRI (75.3%-76.3%

versus 72.0%-75.3% respectively, P=0.514).
**Conclusion:** CEDEM showed high detection rate for malignant lesions. Sensitivity and accuracy of CEDEM were as good as CE-MRI, with a higher specificity. CEDEM shows the potential to reduce unnecessary biopsy.
*Author Disclosures:*
**P. Clauser:** Other; Invited talks for Siemens Healthcare. **M. Bernathova:** Other; Invited talks for Siemens Healthcare. **T.H. Helbich:** Research/Grant Support; The current study was supported by Siemens Healthcare and Guerbet.

**10:30 - 12:00** **Room E2**

### Neuro

### SS 1811a

### Parkinson and related disorders

*Moderators:*
S. Cocozza; Naples/IT
M.F. Vasco Aragão; Recife, PE/BR

**B-1550** 10:30
**Advanced imaging technique in diagnosis of Parkinson's disease: quantitative MRI mapping of substantia nigra**
M.V.M. Micelli[1], A. Corridore[1], S. Torlone[1], L. Panebianco[1], P. Cerrone[1], M. Martino[2], P. Sucapane[1], A. Splendiani[1], C. Masciocchi[1]; [1]*L'Aquila/IT*, [2]*Rome/IT (micelli.mvm@gmail.com)*

**Purpose:** Parkinson's disease (PD) is characterised by a degeneration of the dopaminergic neurons in the substantia nigra (SN). Susceptibility-weighted imaging (SWI) shows an abnormal signal intensity inside SN-pars compacta(SNpc) in PD patients. The aim of the study is to assess T1- and T2-values inside SNpc in PD patients.
**Methods and Materials:** 35 PD patients and 20 sex- and age-matched subjects, without movement disorders, underwent HR-SWI and a synthetic sequence using a 3.0T MRI scan and a 32 phased-array head coil. MRI software automatically generated quantitative maps and T1-, T2- and proton density values were obtained using a manual region of interest (ROI) on SNpc by two-blinded neuroradiology. Analysis of covariance was conducted to analyse the T1-, T2- and proton density values and interobserver agreement was evaluated by calculating the kappa coefficient from the mean kappa pairwise scores.
**Results:** HR-SWI showed a loss of the swallow-tail sign in all PD patients (15 cases were monolateral); no degenerations were identified in control group. Quantitative MRI mapping showed T2 (ms)- and R2 (1/s)-values to be statistically different between PD and controls (p<0.002), in detail (mean ±SD): R2-values: 15.5 ±1 vs 12.9 ±1 (PD vs controls), T2-values: 67.3 ±7.5 vs 76.8 ±5.1 (PD vs controls). Proton density- and T1-values were not statistically different. Interobserver agreement was higher in quantitative MRI mapping (100%) than HR-SWI (85%).
**Conclusion:** T2-mapping is an objective method in quantifying the hypointensity inside SN. It could discriminate patients affected by Parkinson's disease due to the capability to assess pathological changes inside nigral neurons.

**B-1551** 10:38
**Executive network integrity predicts sarcopenia in patients with Parkinson's disease**
C.Y. Lee, Y.-S. Chen, P.-C. Chen, C.-H. Lu, W.-C. Lin; *Kaohsiung/TW (rose80113@hotmail.com)*

**Purpose:** Sarcopenia in Parkinson's disease (PD) patients is significantly associated with morbidity and mortality as disease progression. However, an analysis of clinical severity and brain change, such as white matter (WM) alteration in PD patients with sarcopenia (PDSa), is still limited. A better understanding of associated factors with sarcopenia could provide a focused screen and early intervention in PD patients.
**Methods and Materials:** 52 PD patients and 19 healthy participants accepted dual-energy X-ray absorptiometry to measure the body composition. Using diffusion tensor imaging, the difference of WM integrity was measured between PDSa and without sarcopenia (PDNSa). Multivariate analysis was performed to explore the relationship between clinical factors, WM integrity and sarcopenia in PD patients.
**Results:** 21 PD patients (40.4%) exhibited sarcopenia. Compared to PDNSa, PDSa had lower fractional anisotropy accompanied by higher radial diffusivity and/or higher mean diffusivity in fronto-striato-thalamic circuits including bilateral cingulum, left superior longitudinal fasciculus, left genu of corpus callosum, and right anterior thalamic radiation, which prominently participate in the executive function. In addition, decreased muscle mass was associated with worse WM integrity in these regions. Further multiple linear regression analysis revealed that worse WM integrity in left cingulum, right anterior thalamic radiation, together with gender (male) were independent risk factors for predicting sarcopenia in PD patients.
**Conclusion:** WM alterations in the executive functional network, such as fronto-striato-thalamic circuits, might be an important risk factor for ongoing sarcopenia in PD. These patterns of WM damage imply the underlying aetiology and provide a marker to predict the development of sarcopenia for comorbidity early prevention.

**B-1552** 10:46
**Treatment of essential tremor (ET) and Parkinson disease (PD) tremor with MRgFUS: preliminary results from 21 patients in a single centre**
F. Bruno, M. Micelli, L. Panebianco, F. Arrigoni, M. Varrassi, A. Catalucci, P. Sucapane, A. Ricci, C. Masciocchi; *L'Aquila/IT (federico.bruno.1988@gmail.com)*

**Purpose:** To report our experience in the treatment of ET and PD tremor using MRgFUS thalamotomy.
**Methods and Materials:** In the period February-September 2018, we enrolled 21 patients (12 males, 9 females, mean age 64.6 years) with disabling and refractory tremor (11 ET, 10 PD tremor, mean duration of symptomatology 10.4 years) who were subjected to unilateral Vim ablation using MRgFUS. Clinical evaluation was performed using the Fahn-Tolosa-Marin scale (FTM) for tremor and the QUEST score for quality of life, assessed before treatment, immediately after treatment and with follow-up at 1 month and 3 months. Instrumental MRI follow-up was performed at the same follow-up periods. Sonication parameters were recorded in all procedures.
**Results:** Treatment was effective (substantial and immediate reduction of tremor) in 20 out of 21 patients (95.2%). FTM scores decreased from mean values of 33.4 before treatment to 14.7 immediately after treatment. In patients followed-up at 1 month (10 patients), the clinical evaluation showed a minimal increase in the mean FTM values due to the reappearance of tremor in 4 patients (mean FTM 16.4 vs. 14.7). At 3 months (6 patients), we found a substantial stability of treatment effects. Quality of life evaluation showed substantial improvement (73.2% reduction of the QUEST scores). Temporary side effects and complications (dysarthria, perioral paresthesias, limb weakness) occurred in 3 patients.
**Conclusion:** In our experience with a relatively small number of patients and a short follow-up, MRgFUS thalamotomy resulted in a safe and effective treatment option for tremor in ET and PD patients.

**B-1553** 10:54
**Functional connectivity alterations of dorsal premotor cortex subregions in Parkinson's disease**
B. Sigl[1], C. Jockwitz[2], C. Mathys[3], S. Eickhoff[2], C.J. Hartmann[1], C. Rubbert[1], B. Turowski[1], S. Caspers[2], J. Caspers[1]; [1]*Düsseldorf/DE*, [2]*Jülich/DE*, [3]*Oldenburg/DE (benjamin.sigl1988@gmail.com)*

**Purpose:** Parkinson's disease (PD) is associated with impairments in motor and cognitive function. Areas of the dorsal premotor cortex (PMd) "6d1-3" are mediators between motor and cognitive networks. We used resting state functional connectivity (RSFC) to investigate PD-related changes of these areas.
**Methods and Materials:** RSFC was assessed in 40 PD patients under dopaminergic medication ("ON") and after >12h withdrawal ("OFF") and in 40 matched healthy controls (HC). Microstructurally defined areas 6d1-3 within PMd were used as seed regions. We tested for group differences in whole-brain RSFC (PD-OFF/ON vs HC) and for correlations with UPDRS-III score.
**Results:** There was RSFC-decline in PD-OFF compared to HC for left area 6d1 and 6d2 with right 6d1 and right dorsomedial prefrontal cortex respectively. The latter normalized under medication. Connectivity increase was found for right area 6d3 with bilateral pre- and postcentral regions in PD-ON. We found negative correlation of UPDRS-III with RSFC of right areas 6d2 and 6d3 with left 6d1 in PD-OFF, and of left area 6d2 with bilateral dorsolateral prefrontal cortex in PD-ON, and a positive correlation for RSFC between right 6d3 and bilateral central region.
**Conclusion:** Distinct RSFC-changes of PMd areas in PD reveal their differential involvement in PD pathology. Connectivity loss within the premotor cortex and with dorsolateral prefrontal cortex are probably directly related to PD symptoms such as motor planning, sensorimotor transformation and cognitive motor control. RSFC-increase of right 6d3 with sensorimotor areas in PD-ON might indicate a dopamine-induced compensation for motor impairment through increased cognitive motor control.

**B-1554** 11:02
**DMN-neuronal activity pattern in patients with PD in early stages**
E. Seliverstova, Y. Seliverstov, R. Konovalov, M. Krotenkova, S. Illarioshkin; *Moscow/RU (eselivers@yahoo.com)*

**Purpose:** To assess pattern of neuronal activity within default mode network (DMN) in patients with Parkinson's disease (PD) in early stages comparing to healthy controls.
**Methods and Materials:** We have examined and compared 3 independent right-handed groups (25 patients with PD in early stage, 15 females, mean age 60.2, in pharmacological treatment; 16 newly diagnosed PD patients, 9 females, mean age 55.3; 21 healthy volunteers, 14 females, mean age 48.2) underwent 1.5 T resting-state fMRI scanning; data were calculated using software GIFT and SPM.
**Results:** We have observed multidirectional changes in neuronal activity within DMN in examination groups of patients, reduction and extension of activation in different DMN areas. But we observed unique equal pattern - significantly greater areas of spontaneous neuronal activity in right/left precuneus [pFWEcorr<0.05; T>5] and significant reduction of activation in the right inferior parietal lobule in both groups of PD patients (in pharmacological treatment and newly diagnosed PD patients) versus healthy volunteers [pFWEcorr=0.037; T=4.7]. This area is involved in visual-spatial perception (disturbed in PD patients); area changes correlated with decrease in MoCa score and increase in UPDRS (p<0.05).
**Conclusion:** We have found certain changes of the activation pattern within DMN in patients with early stage PD, including multidirectional changes in neuronal activity in DMN areas in groups of patients, and same significantly changes in both precuneus and right inferior parietal lobule. Our findings may indicate the ongoing neurodegenerative process, reflect neuroplasticity phenomena and be demonstration of functional intercommunication, and functional connectivity as compensatory mechanisms in neurodegeneration.

**B-1555** 11:10
**Resting state functional MRI connectivity comparison between Alzheimer's disease and Parkinson's disease dementia: a sub-analysis**
P.A. Harman[1], C. Law[2], M. Johnson[3], S. Pardhan[1], I.Q. Grunwald[1], K. Ramavathu[4]; [1]*Chelmsford/UK*, [2]*Stanford, CA/US*, [3]*Southend-on-Sea/UK*, [4]*Westcliff on Sea/UK (Kumar.Ramavathu@southend.nhs.uk)*

**Purpose:** Resting state functional MRI (RS fMRI) has been suggested as a potential biomarker for Alzheimer's Disease (AD). However, there is little research into the functional connectivity (FC) patterns of Parkinson's disease dementia (PDD) and no study has sought to directly compare the two. We present a sub-analysis on a set of patients from a larger study which aims to evaluate the use of RS-fMRI in clinical routine, on a 1.5T MRI.
**Methods and Materials:** As part of an on-going, prospective, observational study, sub-analysis was performed on a small subset of patients. Confirmed PDD patients were age and gender matched to confirmed AD patients. Participants underwent a RS-fMRI scan on a standard 1.5T clinical care MRI and also undertook a neuropsychological battery assessing various areas of cognition. Functional connectivity in regards to clinical characteristics was evaluated along with cognitive profiles.
**Results:** Analysis showed characteristic differences between the PDD and AD group, specifically regarding the retrieval region in the left executive control network. The AD group had increased levels of connectivity between the retrieval region to left insula and prefrontal cortex regions.
**Conclusion:** To date we believe this is the first comparison of FC in AD and PDD. A reduction of connectivity from a seed in the left executive control network could represent a distinguishing feature for PDD groups in RS-fMRI data. It seems feasible to incorporate RS fMRI scanning into clinical routine scanning, even on a 1.5T scanner.

**B-1556** 11:18
**3T susceptibility-weighted imaging and Parkinson's disease: new fields in neuroimaging**
A. Corridore[1], S. Torlone[1], M. Micelli[1], M. Martino[2], L. Panebianco[1], P. Cerrone[1], P. Sucapane[1], A. Splendiani[1], C. Masciocchi[1]; [1]*L'Aquila/IT*, [2]*Rome/IT (antonella.corridore@gmail.com)*

**Purpose:** Aim of the study was to evaluate the morphological features of the Substantia Nigra (SN) in Susceptibility-Weighted Imaging (SWI) sequences in order to assess its reliability in Parkinson Disease (PD) diagnosis and its correlation with neurodegeneration severity.
**Methods and Materials:** We prospectively evaluated 60 consecutive patients with PD diagnosis, who had been admitted at the Neurological Movements Disorders Ambulatory of our Institution in L'Aquila.
Patients underwent standard brain 3T-MR examination supplemented by SWI sequence for the Nigrosome-1 evaluation; a control group of 20 healthy patients was also provided. Two expert neuroradiologists analysed the SWI images in blind and defined a SN degeneration severity score (mild, moderate and severe). A complete evaluation of the motor function by UPDRS, a cognitive evaluation by MoCA and an analysis of non-motor disorders by NMSS were performed.
**Results:** SWI sequences showed accuracy in the disease detection in patients with PD compared to control group ($\chi 2=10.31$; p=0.001). A statistically significant correlation ($\chi 2=8.22$; p=0.016) between laterality of symptoms and contralateral degeneration of SN pars compacta was depicted. Rigidity and bradykinesia showed a non-statistically relevant association with the contralateral SN degeneration degree. The presence of allucinations and their severity seem negatively related to the SN hypointensity measured through SWI sequence (p=0.015).
**Conclusion:** SWI sequence performed in patients with PD represents an efficient support in the differential diagnosis and, potentially, in the monitoring of the disease progression.

**B-1557** 11:26
**Structural integrity impact of focused ultrasound thalamotomy and correlation to behavioural outcomes for tremor disorders**
G. Zur, O. Lesman-Segev, I. Schlesinger, A. Eran, I. Kahn; *Haifa/IL (gil_zur@yahoo.com)*

**Purpose:** To evaluate white matter changes following MRI-guided focused ultrasound (MRgFUS) ablation of the thalamus and correlate it with treatment successes in essential tremor (ET) and Parkinson's disease (PD) patients.
**Methods and Materials:** 22 ET and 17 PD patients, candidates for MRgFUS treatment for medication refractory tremor, were recruited and scanned with diffusion tensor imaging (DTI) ($2\times2\times2$ $mm^3$ resolution, 25 gradients) before the ablation and a day, a week, one-three months, and four months-to-one year following ablation. Diffusivity parameters and fiber tractography measures were extracted. Tremor was evaluated (CRST and UPDRS) at baseline, one month and six months post-ablation.
**Results:** We found a pattern of decline and recovery in diffusivity measures and fiber tractography in the motor thalamus and throughout the dentato-rubro-thalamo-cortical pathway. The ablation core and the pathway between the red nucleus and motor thalamus did not show full recovery in late follow-up scans (P<0.005). Inverse correlation was found between fractional anisotropy (FA) measured in the motor thalamus a day before or a day following ablation and tremor relief ($R^2=0.26$, P<0.02, and $R^2=0.37$, P<0.005, respectively). Long-term tremor relief was seen following the treatment.
**Conclusion:** Long-term white matter damage was found in the ablation core, and in the tract between the thalamus and red nucleus that is in line with the long-term tremor relief, suggesting it may play a role in tremor pathogenesis. Pre-ablation FA values in the motor thalamus correlated with treatment success thus implying its value as a possible biomarker for patient selection.

**B-1558** 11:34
**Lewy body dementia and posterior cortical variant of Alzheimer's disease: distinguishing imaging patterns on FDG PET/CT and $^{99m}$Tc-TRODAT SPECT scan**
V. Gupta, R. Verma, R. Ranjan, E. Belho, H. Mahajan; *Delhi/IN (ethelbelho@gmail.com)*

**Purpose:** To establish differences in imaging patterns of posterior cortical atrophy (PCA), and dementia with Lewy bodies (DLB) on $^{18}$F-FDG PET/CT using $^{99m}$Tc-TRODAT scan as the scintigraphic gold standard. This study aims to identify areas of similarities and differences on $^{18}$F-FDG PET imaging between the two.
**Methods and Materials:** A retrospective analysis of 70 patients clinically suspected of having posterior dementia was done. All patients underwent $^{18}$F-FDG PET/CT scan of the brain and dopamine transporter imaging with $^{99m}$Tc-TRODAT-1 SPECT scan on two consecutive days. The studies were analysed both visually and semi-quantitatively. The patients were divided into possible PCA with TRODAT scan normal (n=38) and possible DLB with TRODAT scan abnormal (n=32). The FDG PET/CT uptake patterns were recorded and areas of hypometabolism that were two standard deviations from the mean were considered as abnormal.
**Results:** All patients had abnormal pattern of FDG uptake on PET. Significantly reduced metabolism was found in parieto-temporal and occipital association cortices and cingulate cortices in PCA patients. DLB patients showed significantly reduced uptake in the visual cortex with variable involvement of parieto-temporal cortices, with significant difference in the regional z scores between PCA and DLB patients. No significant difference was found between z score of occipital association cortex, which showed hypometabolism in both groups.
**Conclusion:** FDG PET may be useful as a non-invasive diagnostic modality in differentiating the two posterior cortical dementias, despite significant clinical and imaging overlap, with or without additional TRODAT scan.

**B-1559** 11:42

**Analysis the changes of white matter structure with tract-based spatial statistics according to diffusion tensor imaging in Parkinson's disease**

C. Chen, J. Cheng; *Zhengzhou/CN (chrnchrn812@163.com)*

**Purpose:** To assess the white matter structural changes in patients with Parkinson's disease (PD), using tract-based spatial statistics (TBSS) approach.
**Methods and Materials:** A total of 88 PD patients and 45 healthy volunteers matched by age and sex were enrolled. DTI were performed on a 3T MAGNETOM Prisma. Data were processed using FSL software. TBSS technique was used to extract the dispersion index values of cellulose skeleton in abnormal white matter,including fractional anisotropy (FA), mean diffusivity (MD), axial diffusivity (L1), and radial diffusivity (L23). Independent sample t-test was performed to compare the differences between the two groups. Pearson correlation analysis was applied to reveal the correlation between diffusion index values and clinical indicators.
**Results:** Compared with healthy volunteers, PD patients presented decreased FA and increased MD, L1 and L23, in the following regions: genu and body of corpus callosum, anterior limb of internal capsule, posterior limb of internal capsule anterior corona radiata, superior corona radiata, left posterior corona radiata and thalamic radiata, right sagittal stratum, external capsule, and superior longitudinal fasciculus. The FA decreased at fornix, while the MD and L23 increases at the anterior limb of internal capsule increased. These findings were positively correlated with disease duration. The L23 of the right anterior limb of internal capsule increased, and it was positively correlated with the unified PD rating scale score.
**Conclusion:** Extensive structural changes in the white matter of PD patients can be characterised by disruption of projection fibres in the descending pathway, mainly descending from the corpus callosum and posterior thalamic radiata.

**B-1560** 11:50

**The value of MR neurography in the differential diagnosis between amyotrophic lateral sclerosis and multifocal motor neuropathy**

M. Kronlage[1], K. Knop[2], D. Schwarz[1], T. Godel[1], S. Heiland[1], M. Bendszus[1], P. Bäumer[1]; [1]*Heidelberg/DE*, [2]*Hamburg/DE*

**Purpose:** Differential diagnosis between amyotrophic lateral sclerosis (ALS) and multifocal motor neuropathy (MMN) may be difficult, which has a strong impact on adequate therapy. The purpose of this study was to assess diagnostic accuracy of MR neurography in differential diagnosis of ALS and MMN.
**Methods and Materials:** Twenty-two patients with ALS and eight patients with MMN underwent MR neurography of upper and lower extremity nerves using high-resolution T2-weighted sequences. MR neurographies of 15 matched healthy subjects served as a control group. Images were independently read by two readers, who rated fascicular lesions and muscle denervation signs and made a final, image based diagnosis while being blinded to the clinical diagnosis. Results were compared with the reference standard of diagnosis according to standard clinical and electrophysiological criteria.
**Results:** Nerves of patients with ALS either appeared normal or showed mild T2w-hyperintensitites without fascicular enlargement, whereas nerve lesions in MMN were characterized by fascicular swellings. Muscle denervation signs were more prominent in ALS compared to MMN. By imaging, 19 of 22 patients with ALS, 7 of 8 patients with MMN and all of the 15 healthy controls were correctly diagnosed, implying a sensitivity of 0.86 (95% CI: 0.67 - 0.95) and a specificity of 1.00 (95% CI: 0.86 - 1.00) for the diagnosis of ALS, as well as a sensitivity of 0.88 (95% CI: 0.53 - 0.99) and a specificity of 1.00 (95% CI: 0.91 - 1.00) for the diagnosis of MMN.
**Conclusion:** MR neurography may assist differential diagnosis of ALS and MMN.

**10:30 - 12:00** **Room F2**

### Breast

### SS 1802b

### DWI, spectroscopy and other advanced breast MR techniques

*Moderators:*
N.N.
N. Radovic; Zagreb/HR

**K-36** 10:30

**Keynote lecture**

P.A.T. Baltzer; *Vienna/AT*

**B-1561** 10:39

**Screening-like single- and double-reading of DWI for breast cancer detection**

F. Pesapane, E. Cassano, A. Rotili, S. Penco, R.M. Trimboli, F. Sardanelli; *Milan/IT (filippopesapane@gmail.com)*

**Purpose:** To test diffusion-weighted imaging (DWI) for breast cancer (BC) detection.
**Methods and Materials:** Single-centre 1.5-T images of 790 breasts in 434 consecutive women (age 49±10, mean ± standard deviation) entered the analysis. Two radiologists with 8- (R1) and 5-year (R2) experience blindly assessed DWI images (b=0 and b=800 s/mm$^2$), with a per-breast recall/no-recall reading. Apparent diffusion coefficient maps were qualitatively evaluated. Double reading (DR) was considered positive even with a single recall. Pathology and follow-up served as reference standard. McNemar and Cohen κ were used.
**Results:** Per-breast prevalence was 13% (101/790): 65 invasive ductal, 9 invasive lobular, 9 invasive ductal-lobular, 8 in situ ductal, 5 invasive not otherwise specified, 5 other type (median size 18 mm, interquartile range 11-25). Sensitivity was 88/101 (87%, 95% CI 79-93%) for R1, 84/101 (83%, 95% CI 74-90%) for R2, and 94/101 (93%, 95% CI 86-97%) for DR (DR versus R1, p=0.031); specificity 639/689 (93%, 95% CI 91-95%), 613/689 (89%, 95% CI 87-91%), and 603/689 (88%, 95% CI 85-90%), respectively (DR versus R1, p<0.001). For cancers ≤20 mm, ≤15 mm, or ≤10 mm, DR sensitivity resulted 60/65 (92%, 95% CI 83-98%), 39/44 (89%, 95% CI 75-96%), and 26/30 (87%, 95% CI 69-96%), respectively. Interobserver agreement was substantial (κ=0.739).
**Conclusion:** In this enriched population, DWI showed a high sensitivity, also for small tumours, favourably comparable with that of screening mammography, with an even acceptable specificity. Prospective studies in non-enriched populations are needed for testing DWI in BC screening.

**B-1562** 10:47

**Diffusion-weighted imaging with background suppression in detection of breast malignancies: a non-invasive, non-contrast and non-radiation technique**

P. Yadav[1], V. Kulkarni[1], Y. Patil[1], S. Harit[2]; [1]*Pune/IN*, [2]*Manipal/IN (yadavpratiksha@hotmail.com)*

**Purpose:** To evaluate the utility of non-contrast MRI sequences with use of 3-D diffusion-weighted imaging and background suppression in differentiating malignant and benign breast lesions. To evaluate the sensitivity and specificity of the non-contrast MRI and DWI in detection of breast malignancies.
**Methods and Materials:** This study was IRB and IEC approved and included 68 breast lesions of 57 cases, suspicious for malignancies on mammography and breast ultrasound. All the cases underwent for breast MRI on 1.5 Tesla machine using dedicated breast coil. Multiplanar localizer was applied with 3mm slice thickness. T1WI, T2WI and STIR in axial plane, STIR coronal and sagittal plane. DWI was done with b value 1500 sec/mm$^2$. Post-processing was done and ADC calculations were also obtained. All the cases were correlated histopathologically.
**Results:** The lesions which showed diffusion restriction considered positive whereas lesions did not show restriction considered as benign. DWI with increased b value demonstrates lesions better with background suppression. In 68 breast lesions, 35 (51.4%) were malignant and 33 (48.5%) benign on DWI. Histopathology revealed 37 lesions (54.4%) were malignant and 31 (45.5%) benign. Sensitivity of DWI was 91.6% (95% CI= 77.5, 98.25), specificity 90.62% (95% CI =74.9, 98.02), PPV 91.6% and NPP 90.6%. Mean ADC of malignant lesions was 0.609 ± 0.35× 10-3 mm$^2$/s and benign lesions was 1.732 ± 0.53 × 10-3 mm$^2$/s.

**Conclusion:** When b value increases, signal intensity of the normal breast decreases which demonstrates improved contrast resolution between malignant lesion and normal breast tissue. It is a very useful non-contrast, non-radiation, non-invasive MRI technique.

**B-1563** 10:55
**Diffusion with very high b-value in breast MRI: preliminary results**
H. Hamri[1], E. Weiland[2], M. Scholer[1], Z. Jolibois[1], C. de Bazelaire[1]; [1]*Paris/FR*, [2]*Erlangen/DE (hamri.hajar@yahoo.fr)*

**Purpose:** To evaluate the diagnostic yield of very high b-value DWI combined with a T2-weighted sequence in breast MRI.
**Methods and Materials:** In this retrospective study approved by our IRB, breast MRI was performed at 1.5T with an 18 channel breast coil (MAGNETOM Aera, Siemens). Protocol included 2D-T1-FSE, 2D-single-shot-EPI-diffusion-SPAIR with a single b-value of 2500s/mm2 (prototype), 3D-T2-SPAIR, and 3D-T1-VIBE-SPAIR dynamic contrast enhanced (DCE) sequences. T1-w, T2-w and T1-w DCE sequences were first analyzed and findings were classified according to the BIRADS lexicon. All findings with high Diffusion signal associated with low T2 signal were classified as suspicious regardless of morphology. All suspicious lesions were biopsied. BIRADS 1-3 lesions had 2 years follow-up. Diagnostic yields were compared using ROC curves.
**Results:** One hundred and twenty-seven patients (mean 53 yo, range 20-93) with 36 breast cancers and 101 benign lesions or normal breast were included. Higher sensitivity but lower specificity were found with the T1-w, T2-w and T1-w DCE sequences compared with DWI and T2-w sequences (0.972, 0.842 versus 0.889, 0.921 respectively). However, the comparison of ROC curves showed no significant difference ($AUC_{T1+T2+T1\ DCE}$=0.907 and $AUC_{DWI+T2}$=0.905, p=0.952). DWI and T2-w sequences missed 4 large cancers (>20mm) because of their intense T2-w signal.
**Conclusion:** Combined analysis of DWI using very high b-value with T2-w sequences reached similar diagnostic yield than standard breast MRI protocol with contrast injection. Further studies combining morphological features and tumor sizes from T1-w and T2-w unenhanced sequences are mandatory.
*Author Disclosures:*
**E. Weiland:** Employee; Employee Siemens Healthcare GmbH.

**B-1564** 11:03
**Evaluation of breast lesions with simultaneous multi-slice DWI in comparison to routine read-out segmented DWI**
W. Sanderink[1], J. Teuwen[1], I. Sechopoulos[1], N. Karssemeijer[1], L. Appelman[1], E. Weiland[2], R.M. Mann[1]; [1]*Nijmegen/NL*, [2]*Erlangen/DE (wendelien.sanderink@radboudumc.nl)*

**Purpose:** To compare conventional readout-segmented diffusion weighted imaging echo-planar imaging (rs-DWI-EPI) to a prototype Simultaneous Multi-Slice single shot-DWI-EPI (SMS-ss-DWI-EPI) at 3T using b-values of 0 and 800 s/mm$^2$, in terms of image quality (IQ), lesion conspicuity, and lesion description.
**Methods and Materials:** From September 2017 to August 2018, 15 women with known breast cancer or suspicious breast lesions were scanned with the conventional rs-DWI-EPI (resolution: 1.2x1.2x5 mm, TA: 4:23 min) and the SMS-ss-DWI-EPI (resolution: 0.9x0.9x4 mm, TA: 2:45 min) during the same clinical examination on a 3T system. In total, 33 lesions (26 malignant, 5 benign and 2 unknown) were detected on the contrast enhanced series. Two dedicated breast radiologists (4 and 10 years of experience with breast MRI), independently scored both sequences for overall IQ (1: extremely poor to 9: excellent). All lesions were also independently evaluated for conspicuity (1: not visible 2: visible if location is given, 3: visible) and a BIRADS score per lesion (1 to 5) was given.
**Results:** IQ was significantly higher for conventional rs-DWI-EPI (mean 5.3 ± 1.9), than for SMS-ss-DWI-EPI (mean 3.6 ± 1.7) (p=0.001). Benign lesions had a similar visibility in both sequences. Malignant lesions were significantly better visible in the SMS-ss-DWI-EPI (11/52 reads) (p=0.015). There was no significant difference in BIRADS scores (p=0.228) between the two sequences.
**Conclusion:** Despite the perceived poorer image quality of the SMS-ss-DWI-EPI sequence, malignant lesions are better visualized using this sequence. The technique might therefore enable lesion detection on unenhanced breast MRI.
*Author Disclosures:*
**E. Weiland:** Employee; Siemens Healthcare.

**B-1565** 11:11
**Diagnostic performance of region of interest methods in diffusion-weighted imaging of breast lesions: a systematic review and meta-analysis**
M. Wielema[1], M.D. Dorrius[1], G.H. De Bock[1], R.M. Pijnappel[2], M. Oudkerk[1], P.E. Sijens[1]; [1]*Groningen/NL*, [2]*Utrecht/NL (m.wielema@umcg.nl)*

**Purpose:** The purpose of this systematic review and meta-analysis was to assess the accuracy of different region of interest (ROI) methods in the discrimination of benign from malignant breast lesions in diffusion weighted imaging (DWI) using the apparent diffusion coefficient (ADC), in terms of sensitivity, specificity and area under the SROC curve (AUC).
**Methods and Materials:** PubMed and EMBASE were searched for studies applying ADC in breast lesions, up to 24-10-2017(n = 2154). Data were pooled based on 4 ROI method categories: ROI1: whole lesion, ROI2: subtracted whole lesion, ROI3: selected circular regions and ROI4: lowest diffusion selection. The more subjective ROI methods (ROI2, ROI3) excluded necrotic, cystic and hemorrhagic areas. Pooled sensitivity, specificity and AUC of ROIs were calculated. To explore heterogeneity, the following covariables were considered: field strength, lowest b-value, image of ROI drawing, pre-or post-contrast DWI, slice thickness and ADC threshold.
**Results:** Forty-nine studies (4958 lesions) were included. Pooled sensitivity, specificity and AUC were: 0.85(0.75-0.91), 0.77(0.60-0.89), 0.89(0.86-0.91) for ROI1; 0.92(0.89-0.94), 0.86(0.82-0.88), 0.92(0.89-0.94) for ROI2; 0.90(0.85-0.93), 0.90(0.83-0.94), 0.95(0.93-0.97) for ROI3 and 0.89(0.83-0.93), 0.83(0.79-0.86), 0.87(0.83-0.89) for ROI4, respectively. Significant heterogeneity was found between studies (p<0.001). None of the co-variables could explain the heterogeneity.
**Conclusion:** Using AUC as an indication of global diagnostic performance, ROI3 exceeded ROI1 and ROI4 (no overlap between ranges). Due to the non-uniformity of the data, however, no single ROI method significantly outperformed in the use of ADC to differentiate benign from malignant. Further standardization of ROI methods is needed to optimize the breast DWI protocol.

**B-1567** 11:19
**Feasibility study of synthetic diffusion-weighted MR imaging in patients with breast cancer by qualitative and quantitative assessment**
B. Choi; *Changwon/KR (iawy82@gmail.com)*

**Purpose:** To investigate the clinical feasibility of breast synthetic diffusion-weighted imaging (DWI) in patients with breast cancer by assessing diagnostic image quality in comparison with conventional DWI.
**Methods and Materials:** Thirty-two patients with breast cancer were assessed using both synthetic and conventional DWI with b values of 100, 800 s/mm$^2$ ($cDWI_{800}$) and 1500 s/mm$^2$ ($cDWI_{1500}$) before surgery. Synthetic DWI with b value of 1,500 s/mm$^2$ ($sDWI_{1500}$) were generated from conventional DWIs at b values of 100 and 800 s/mm$^2$. Image quality assessment was performed using 4-point Likert scale for comparison of $sDWI_{1500}$, $cDWI_{1500}$ and $cDWI_{800}$. For quantitative analysis, contrast ratios (CRs) of cancer and glandular tissue were calculated on each DWI. Interobserver agreement was assessed using weighted kappa statistics and intraclass correlation coefficient.
**Results:** Both $sDWI_{1500}$ and $cDWI_{1500}$ showed better normal glandular tissue suppression and lesion conspicuity than $DWI_{800}$ (P<0.05). Although $cDWI_{1500}$ showed better image quality than $sDWI_{1500}$ (P≤0.005), $sDWI_{1500}$ and $cDWI_{1500}$ showed no significant difference of sensitivity for detecting cancer. CRs of cancer and glandular tissue was greater on $sDWI_{1500}$ than on either conventional DWI (P<0.001).
**Conclusion:** sDWI of the breast using b value 1500 s/mm$^2$ is a feasible option for diagnostic use which can improve conspicuity of cancer with better CRs and background breast tissue suppression compared with cDWI.

**B-1568** 11:27
**MR mammography of suspicious lesions: influence of residual fat signal on diffusion kurtosis imaging**
A. Mlynarska-Bujny[1], S. Bickelhaupt[1], F. König[1], F. Laun[2], W. Lederer[1], H. Daniel[3], S. Delorme[1], H.-P. Schlemmer[1], T. Kuder[1]; [1]*Heidelberg/DE*, [2]*Erlangen/DE*, [3]*Mannheim/DE*

**Purpose:** In diffusion-weighted imaging of breast, not fully suppressed fat can lead to distortion of the quantitative parameters due to the low diffusion coefficient of fat. This work aims to evaluate various fitting models accounting for contamination from residual fat signal.
**Methods and Materials:** 198 patients with suspicious mammography findings and indication for breast biopsy were involved. DWI scans, before the biopsy, were acquired in one of two study centres (Group A - 1.5T Philips Ingenia, 105 patients; Group B - 1.5T Siemens Aera, 93 patients) using 4 b-values (0, 100, 750 and 1500 s/mm$^2$). ROIs were segmented on the highest b-value slice where the lesion was visible. Fat ROIs were segmented on the contralateral breast. The following curve-fitting models have been compared: 1) diffusion kurtosis equation, describing non-Gaussian distribution; 2) empirically adjusted diffusion kurtosis model with inclusion of unsuppressed fat signal; 3) modified

Sunday kurtosis model, with additional factor accounting for signal from fatty area; 4) mono-exponential Gaussian diffusion.
**Results:** We demonstrated by the analysis of ROC curves, based on logistic regression, that the AUC values obtained for *Model2* [0.86 (95% CI 0.80 to 0.91)] and *Model3* [0.86 (95% CI 0.80 to 0.91)] are significantly higher (*p* < 0.03) than AUC values of *Model1* [0.79 (95% CI 0.72 to 0.86)] and *Model4* [0.77 (95% CI 0.70 to 0.84)].
**Conclusion:** Kurtosis-based fitting models, adapted to account for residual fat signal, show the potential to better discriminate between benign and malignant lesions than classical kurtosis-based and pure Gaussian diffusion models.

**B-1569** 11:35
**Characterisation of breast parenchyma and cancer during lactation using diffusion tensor imaging**
N. Nissan[1], E. Furman-Haran[2], T. Allweis[2], T. Menes[3], O. Halshtok Neiman[4], R. Faermann[1], A. Shalmon[5], M. Gotlieb[5], M. Sklair[5]; [1]*Tel Hashomer/IL*, [2]*Rehovot/IL*, [3]*Tel Aviv/IL*, [4]*Kiryat Ono/IL*, [5]*Ramat Gan/IL* *(noam_nissan@hotmail.com)*

**Purpose:** To investigate the utility of DTI parametric maps in characterization of the normal and malignant transformations of the lactating breast.
**Methods and Materials:** This prospective study was IRB approved and all participants signed an informed consent. Since January 2017, 28 lactating patients (median age and lactation duration: 36y and 6m, respectively) were scanned by diffusion tensor imaging (DTI) together with conventional dynamic contrast-enhanced (DCE) breast-MRI protocol at 1.5T. Indications included newly diagnosed breast cancer (n=11) and screening of high-risk patients (n=17). DTI was acquired using 32 directional diffusion gradients and 0.700s/mm$^2$ b values. DTI parametric maps of the principal diffusion coefficients (λ1, λ2, λ3), mean-diffusivity (MD), fractional-anisotropy (FA) and maximal-anisotropy index (λ1-λ3) were generated and analysed at pixel resolution using a dedicated software granted by permission. Based on the DCE subtraction images, DTI's normal and malignant regions-of-interest (ROIs) were delineated and were statistically evaluated.
**Results:** The normal fibro-glandular tissue exhibited bi-modal distribution, characterized by a sub-areolar central area with increased diffusivity and reduced anisotropy as opposed to the peripheral areas with relatively lower diffusivity and increased anisotropy, possibly reflecting the respective changes in lactiferous ducts' diameter. All 11 cancers were detected by DTI maps of λ1, λ2, λ3 and MD in agreement with DCE, exhibiting substantial contrast compared with the ROIs of the apparently normal tissue of the contralateral breast and fibroglandular tissue of healthy high-risk patients (p<0.001, for all).
**Conclusion:** DTI maps enabled the characterization of physiological lactation-induced parenchymal changes as well as malignant transformations, with substantial parametric contrast, improving the tumour's conspicuity as revealed by DCE alone.

**B-1570** 11:43
**Breast 1H-MR-spectroscopy to distinguish benign from malignant breast lesions: an updated systematic review and meta-analysis with clinical implications**
C. Sodano, P. Clauser, P. Kapetas, T.H. Helbich, P.A.T. Baltzer; *Vienna/AT (claudiasodano@hotmail.it)*

**Purpose:** To investigate the diagnostic performance of breast 1H-MR-spectroscopy to distinguish benign from malignant breast lesions and factors influencing sensitivity and specificity by meta-analytic methods.
**Methods and Materials:** A comprehensive database search using predefined search terms was performed for all articles listed till June 2018. Eligible for this study were publications investigating >10 patients at either 1.5T or 3T for the purpose of distinguishing benign from malignant breast lesions. Both single-voxel and multivoxel techniques were eligible. A reference standard by either histopathological examination or follow-up of >12 months was required. Statistical analysis included pooling of sensitivity and specificity and subgroup analyses based on several covariates including acquisition parameters, field strength and publication date.
**Results:** Thirty-three studies, including 1404 malignant and 770 benign lesions were included. Most publications reported only diagnostic indices of the choline resonance and not on other metabolites. Pooled sensitivity and specificity were 81.7% (95%-CI: 76-86.3%) and 82.5% (95%-CI 78.3-86.1%), respectively. While neither publication date nor field strength had an effect on diagnostic performance estimates (P>0.05), echo time (TE) had: studies applying TE≤144 ms reported higher sensitivity (85.6%, 95%-CI: 78.4-90.6%) compared to studies using TE>144ms (74.9%, 95%-CI 63.4-83.8%) while specificity did not differ.
**Conclusion:** The diagnostic performance of 1H-MR-spectroscopy to distinguish benign from malignant breast lesions using the choline resonance seems to be higher using shorter echo times. Whether consideration of multiple metabolites increases the diagnostic performance of 1H-MR-spectroscopy has not sufficiently been investigated yet.

**10:30 - 12:00** **Room Y**

### Interventional Radiology

### SS 1809

### Nonvascular musculoskeletal interventions

*Moderators:*
N.N.
G. Velonakis; Athens/GR

**B-1571** 10:30
**Intraarticular osteoblastomas: from challenging diagnostic entity to minimally invasive treatment with magnetic resonance-guided focused ultrasound (MRgFUS)**
P. Palumbo, F. Arrigoni, C. De Cataldo, M. Ruschioni, L. Zugaro, A. Barile, C. Masciocchi; *L'Aquila/IT (palumbopierpaolo89@gmail.com)*

**Purpose:** Intraarticular osteoblastomas location is very uncommon and presents challenging diagnostic difficulties because of the non-specific feature. The purpose of our study was to evaluate the efficacy of MRgFUS in the treatment of intraarticular osteoblastoma.
**Methods and Materials:** We retrospectively analyzed 6 cases of patients (mean 23,75 years) with an imaging and histological diagnosis of osteoblastomas. All patients were considered eligible for a non-invasive treatment of MRgFUS. Clinical evaluation was performed using VAS score, and a visual score ranging from 0 to 10 was used also to evaluate the functional impairment. Follow-up imaging was performed at 6 and 12 months after the procedure, with both MR and CT exams.
**Results:** Efficacy of procedure was guaranteed by the temperature obtained in all the regions of the lesion, with a mean temperature of 63°C. Non-significant variation in temperature in the remaining intracapsular region was found. Follow-up imaging after 1 year revealed a significant decrease of bone oedema and synovitis in all cases, with concomitant bone densification. No one shows any precocious onset of osteoarthritis. Clinic follow-up showed a significant decrease in VAS, while functional impairment shows a significant trend depending on the onset of symptoms.
**Conclusion:** MRgFUS confirmed its high efficacy in the treatment of intraarticular lesion, demonstrated by the complete resolution of pain symptomatology as well as in bone restitutio-ad-integrum, other then by the lack of signs indicating cartilage damage. Unless otherwise stated, completely non-invasive approach with MRgFUS could be considered as first line treatment of intraarticular bone lesion.

**B-1572** 10:38
**Retrospective multivariate analysis of the outcomes of percutaneous thermal ablation of painful bone metastasis in oncological patients**
G. Bianchi, F. Arrigoni, P. Palumbo, A. Cortellini, L. Zugaro, K. Cannita, C. Ficorella, A. Barile, C. Masciocchi; *L'Aquila/IT (giampaolo.bianchi90@libero.it)*

**Purpose:** The aim of this study is to assess the effectiveness of interventional radiology procedures on quality of life (QoL) in patients with painful bone metastases.
**Methods and Materials:** A retrospective analysis of 27 consecutive advanced cancer patients, who have been treated with ablation( RFA, CA or MWA) for bone metastases (BM) was performed. Pain intensity variation was evaluated with a Visual Analogue Scale(VAS). Median time to opioid dose escalation, median time to radiological progression of the treated BM and median overall survival (OS) were computed.
**Results:** 27 patients were evaluated. Axial bones were involved in 22 patients (81.5%) and 17 patients (63%) had a single bone metastasis. Before treatment, 41.1% of patients had a Performance Status (ECOG-PS) of 0-1, while the 51.9% of ≥ 2.; 40.7% received concomitant bone-oriented treatment (denosumab or zoledronate). 74% of them took concomitant opioid analgesic therapy. Median follow up was 16.5 months. After treatment, 85.2% of patients experienced an improvement in pain intensity (p-value <0,05). Median time to opioid dose escalation was 16.2 months (95%CI: 5.9-26.9; 15 events). Median time to radiological progression of the treated BM was 10.3 months (95%CI:7.6-16.9,16 events) and median OS was 27.2 months (95%CI:8.9-41.2,15 censored patients).
**Conclusion:** Percutaneous CT-guided thermal ablation led to a significant improvement of the QoL in particular in terms of time to opioid dose escalation. Also median time to radiological progression of the treated BM and median OS seems to be very promising compared to the outcome data known in literature about medical therapy alone.

**B-1573** 10:46
**Interventional urethral realignment to posterior urethral rupture accompanied by complex pelvic fracture**
C.M. Lee, C.H. Jeon, C.W. Kim, H. Kwon, H.M. Cho; *Busan/KR*
*(mooya3696@gmail.com)*

**Purpose:** Primary realignment of complete posterior urethral injury is a difficult procedure because of the pelvic bleeding and haematoma formation that occur during complex pelvic fracture. In this article, we report the result of the treatment of posterior urethral rupture by interventional primary urethral realignment performed with fluoroscopy guidance.
**Methods and Materials:** This retrospective study included 10 patients with traumatic posterior urethral injuries who were treated with urethral realignment from November 2016 to May 2018. All 10 patients were men with the median age of 46.0 years. Technical success rate of urethral realignment, required procedure time, duration of urethral catheterisation, and complications after procedure were investigated.
**Results:** Urethral realignment was technically successful in 8 of 10 patients (80%). In 2 of 8 patients, Snare catheter was used for passing through the urethral injury site. The median procedure time was 46 minutes (range, 32-69 min). The mean duration of urethral catheterisation after urethral realignment was 63 days (range, 48-94 d). There were no immediate procedure-related complications, although all 10 patients developed varying degrees of symptomatic urethral stenosis after procedure.
**Conclusion:** Interventional primary urethral realignment is a safe and minimally invasive procedure that can be performed in a patient with posterior urethral rupture accompanied by complex pelvic fracture. Thus, it can be viewed as an effective treatment method that can reduce the frequency of invasive surgical procedures by maintaining urethral continuity early on.

**B-1574** 10:54
**Percutaneous stabilisation of humeral metastatic lesion**
A. Al Raaisi, E. Cormier, E. Shotar, M. Drir, F. Clarencon, J. Chiras; *Paris/FR*
*(alraisi_am@hotmail.com)*

**Purpose:** Bony metastases are very common in patients with advanced cancer and pathologic fractures may occur within either lytic or blastic foci. A pathologic fracture of the humeral neck or shaft is commonly treated surgically employing nails, rods, or plate and screws combined with immobilization using an external brace support. Some patients are treated conservatively because of their poor clinical condition and comorbidities. Percutaneous cementoplasty alone is insufficient for stabilization of the fracture. Percutaneous stabilization using dedicated spindles in the management of hip joint lytic lesions demonstrated its effectiveness. In this study, we thought to evaluate the effectiveness of reinforced cementoplasty with spindles for painful and unstable lesions involving the humerus in terms of pain relief and functional recovery.
**Methods and Materials:** We describe four cases (3 males and one female) of bone malignancies involving the humerus (3 patients with fractures and one patient with no fracture) which were treated by percutaneous insertion of metallic nails through an 8-gauge cannula followed by PMMA bone cement injection in our department from 2016-2018. Procedures were performed under general anaesthesia in two patients and the other two were sedation.
**Results:** All patients gained full restoration of functionality and pain relief without need for further analgesic therapy. No complications were observed during or after the procedures.
**Conclusion:** Percutaneous stabilization of humeral fractures is an original minimally invasive technique providing pain relief and effective bone stability was shown to be safe and effective in the treatment of painful bone metastases refractory to conventional therapy.

**B-1575** 11:02
**Percutaneous intervertebral disc coagulation therapy (PDCT) by plasma light: a new method for the treatment of lumbar and cervical disc herniation**
C. Zini[1], D. Notaro[2], G. Sadotti[2], M. Bellini[2]; [1]*Florence/IT*, [2]*Siena/IT*
*(zini.chiara@gmail.com)*

**Purpose:** To evaluate the effectiveness, to describe the technique and the advantages of percutaneous intervertebral disc coagulation therapy based on plasma thermal reaction (PDCT).
**Methods and Materials:** 44 patients (age range: 18-87 years, mean: 52,7) with contained and extruded symptomatic lumbar (n.48) and cervical (n.6) disc hernias in the absence of free fragments causing radiculopathy and without improvement after 6-week of conservative therapy were enrolled. Pre-treatment discography was performed in all the patients. The PDCT system (PLASMA D30,Jeisys Corporation, Seoul, South Korea) was equipped with a diode laser module and 0.4 cm plasma optical fiber; all the treatments were performed under local anesthesia and premedication with antibiotics therapy using fluoroscopic guidance (Innova 3131iq, General Electric Healthcare, CT, USA). Before the procedure and 3 months later, the MRI of spine and Visual Analog Scale (VAS) and Oswestry disability index (ODI) were accomplished
**Results:** Most patients (n. 36) showed great improvement in symptoms with relevant post-operative VAS scale reduction (p<0,001) and ODI reduction. No major complications occurred. 16 patients referred mild pain or heartburn when the plasma fiber close was to the annulus, symptoms disappeared immediately after treatment.
**Conclusion:** PDCT can be effective, safe and convenient minimally-invasive decompression methods in selected patients.

**B-1576** 11:10
**Percutaneous CT guided RFA treatment of osteoid osteoma in children and adults: it has high success rate in children too**
C. Sahin, Y. Oc, A. Hasanefendioglu Bayrak; *Istanbul/TR*
*(cennetsahin2@hotmail.com)*

**Purpose:** Efficacy and safety of Radiofrequency Ablation (RFA) of osteoid osteoma (OO) are demonstrated in many studies. However, there is a hesitation about the use in the pediatrics. Our purpose is to investigate the efficacy and safety of RFA of OO in adults and pediatrics.
**Methods and Materials:** A total of 114 patients (81 male and 33 female; mean age of 17.9 years; age range 13 month-42 years) had 115 CT-guided RFA between May 2015 and August 2018 with the diagnosis of OO. Of the 114 patients, 63% were children and 37% were adults. The clinical and technical success of the treatment were evaluated by assessing the pain symptom, complication rates and follow-up radiological findings.
**Results:** All the patients had a favorable immediate pain relief in 24 hours after the procedure. Only one patient (15 y-o; male) had pain relapse in 3 months after RFA and a second RFA was performed. During 19 months follow-up he had no pain. The technical success of the procedure was recorded as 100%. The clinical success was 100% in adults and was 99% in pediatric population. No major complication occurred during neither treatment nor recovery period. Seven minor complications (2 were in pediatrics) were noted which were treated successfully.
**Conclusion:** The rapid relief of the pain symptoms, low relapse rate and low complication rates demonstrate the efficacy and safety of RFA of OO in both pediatrics and adults. We think that RFA can safely be used as first choice treatment method in pediatrics too.

**B-1577** 11:18
**Balloon vesselplasty vs percutaneous vertebroplasty in chronic compression fractures of the dorsolumbar spine**
D.N. Srivastava, A. Monga, K. Madhusudhan, S. Malla; *New Delhi/IN*
*(drdeepsrivastava@gmail.com)*

**Purpose:** To evaluate the efficacy of percutaneous vesselplasty in non-traumatic vertebral fractures of the thoracolumbar spine and compare it with percutaneous vertebroplasty.
**Methods and Materials:** 50 patients with chronic vertebral compression fracture (>12 weeks), severe pain (VAS: more than equal to 7) and disability attributable to the vertebral fracture were included and underwent vertebral augmentation procedure out of which 27 underwent vertebroplasty and 23 underwent vesselplasty. Clinical and imaging follow-up was done for the two groups and evaluated for pain, disability scores, increase in anterior vertebral body height and volume of cement injected. Complication rate was also compared in the two groups.
**Results:** Mean decrease in pain score was 4.27 in vertebroplasty group and 4.45 in the vesselplasty group. The mean increase in the physical functionality scores was 27.4 in the vertebroplasty group and 35.9 in the other group which was statistically significant (p=0.005). The mean amount of cement injected was 3.84 ml in vertebroplasty group and 4.68 ml in the other group which was significantly higher (p=0.008). The mean change in anterior vertebral height was 0.63 mm in the vertebroplasty group and 2.47 mm in the vesselplasty group which was significantly higher (p<0.001). There was cement leak seen in 34% patients in the vertebroplasty group which was minor and mainly involved the paravertebral and intradiscal regions. In the other group, no intradiscal leak was seen.
**Conclusion:** Vesselplasty is superior to vertebroplasty in terms of disability scores, increase in anterior vertebral body height, volume of cement injected with low complication rate.

**B-1578** 11:26
**"SpineJack®" percutaneous placement in magerl A2 and A3 traumatic vertebral compression fractures of the thoracolumbar spine**
G. Sadotti[1], D. Notaro[1], C. Zini[2], M. Bellini[1]; [1]*Siena/IT,* [2]*Florence/IT*

**Purpose:** To prospectively evaluate safety and effectiveness of SpineJack® device (SJ) (VEXIM SA Balma - France) to achieve anatomical restoration of traumatic vertebral compression (VCFs) in Magerl A2 and A3 thoracolumbar fractures .

**Methods and Materials:** 18 patients (16 male; mean age 57 years, age range 27-83 years) with traumatic thoracic (N=12) and the lumbar VCFs (N=7) within 3 weeks from the time of injury classified as Magerl type A2.2 (N=2), A2.3 (N=11) and A3.1 (N=6) were enrolled; exclusion criteria were spontaneous/osteoporotic and neoplastic vertebral fractures, posterior wall involvement of more than 1/3 than of the spinal canal. Visual analogue scale (VAS) score and CT and/or MRI has been performed before and 48 hours, 1 and 6 months after procedure. Technical success was defined as correct placement of SJ implant.
**Results:** A total of 11 VCFs has been treated with 100% technical success. 1 patient performed at 2 levels in the same session. No major complications related to procedure were registered; asymptomatic cement leakages occurred in 4 patients along fractures lines. All cases showed relevant improvement of symptoms with preoperatively mean VAS score of 7.4 dropped to 1,05 , 0,22 and 0,11 within 48 hours,1 and 6 months respectively. Mean height lift of 8.5 mm has been registered after procedure; 5 VCFs presented height lifting greater than 11 mm.
**Conclusion:** SJ placement can be effective and safe in traumatic VCFs, leading to immediate and lasting relief of pain and vertebral height recovery.

**B-1579** 11:34
**Intraarticular lumbar facet joint steroid injection vs. medial branch block: which technique ensures better clinical outcome for managing lumbar back pain? A single-centre experience**
A. Izzo, M. Varrassi, F. Bruno, G. Bianchi, F. Cobianchi Bellisari, S. Carducci, A. Giordano, E. Di Cesare, C. Masciocchi; *L'Aquila/IT (izzo@live.it)*

**Purpose:** To compare the clinical outcome of intraarticular lumbar facet joint (LFJ) steroid injection and lumbar medial branch block (MBB) in the management of LFJ pain.
**Methods and Materials:** Fifty-seven patients with LFJ pain were recruited on the basis of clinical evaluation and MR-findings and randomly assigned to one of two groups. Twenty-seven patients (Group A) underwent intraarticular LFJ steroid injection. Thirty patients (Group B) were assigned to lumbar MBB. In both procedures, we injected 1.5mL of Ropivacaine with 2mL of Triamcinolone. We assessed the severity of LFJ pain by VAS Score and the disability grade by RMQ Score, before and at 1, 3, 6 months after treatment.
**Results:** Average values of VAS and RMQ before treatment in Group A were 8.1 and 16.6. The treatment was effective (VAS reduction >50%, RMQ score <9) in 85.2% of patients after 1 month, 77.7% after 3 months, 66.6% after 6 months. Average values of VAS and RMQ before treatment in Group B were 7.8 and 16.1. The treatment was effective in 86.6% of the patients after 1 month, 70% after 3 months, 50% after 6 months.
**Conclusion:** In this study, both intraarticular LFJ steroid injection and MBB significantly relieved LFJ pain and the positive effects of these techniques persisted for at least 6 months after the procedure. Both procedures showed similar effectiveness in the treatment of LFJ pain at 1 and 3 months, however, intraarticular LFJ steroid injection guaranteed a better clinical outcome after 6 months. We believe that both the techniques are strongly effective in LFJ pain treatment.

**B-1580** 11:42
**Fusion imaging of US and MRI coupled with electromagnetic virtual navigation for periradicular infiltrations of steroids in low back pain: pilot experience of a radiation-free procedure**
F. Arrigoni, G. Bianchi, P. Palumbo, S. Mariani, F. Bruno, L. Zugaro, A. Barile, C. Masciocchi; *L'Aquila/IT (arrigoni.francesco@gmail.com)*

**Purpose:** Low back pain is a disabling condition affecting an increasing number of population. If the source of pain is radicular, it can be decided to proceed to an effective symptomatic treatment, the periradicular steroid injection, which requires the accurate positioning of the needle usually obtained by means of CT or fluoroscopy.
**Methods and Materials:** Fifteen monolateral periradicular (L4-L5 or L5-S1) injections were performed using a virtual navigation system able to fuse imaging of the lumbar spine obtained by an isotropic volumetric MRI with a real time US. The system couples the imaging obtained with an electromagnetic guide that allows visualising the needle on MR imaging. Prior to drug injection, final position (FP) of the needle's tip (obtained using virtual navigation) was verified with a single CT slide centred on the needle. FP was correct when the needle's tip was in or strictly close to the neural foramen. An operator trained for using the system performed all procedures.
**Results:** Twelve (80%) FPs were judged correct. Two (13%) FPs were judged inaccurate (incorrect co-registration of MR-US imaging). In one case, it was not possible to reach a correct FP using virtual navigation. No complications occurred.
**Conclusion:** This pilot study demonstrates the feasibility of this innovative technique for spinal infiltrations. Although additional studies are needed, this technique has multiple advantages that include absence of radiation exposure, excellent imaging guidance (MRI virtually navigated system), multiple infiltrations without need for repeat imaging, and no need for CT suite.

**B-1581** 11:50
**Is the anaesthesia a key factor for the good outcome of bone ablation procedures: a review of the last two-year experience in an MSK interventional centre**
A. Izzo, F. Arrigoni, P. Palumbo, F. Bruno, C. De Cataldo, S. Mariani, L. Zugaro, A. Barile, C. Masciocchi; *L'Aquila/IT (izzo@live.it)*

**Purpose:** The aim of our study is to retrospectively review the outcomes of different anesthetic approaches in bone ablation procedures in order to choose the best option.
**Methods and Materials:** We retrospectively evaluated 98 ablations performed in the period between January 2016 and July 2018. Three different anesthetic approaches were used: general anesthesia, sedation and loco-regional anesthesia (Brachial Plexus Block, Spinal Anesthesia). To evaluate the outcomes, three parameters were used: procedural technical success, patient comfort (Scale 1-5) and operator comfort (Scale 1-5).
**Results:** Of 98 ablations, 50 were benign bone lesions, 48 metastatic bone lesions. Overall procedural technical success was 100%. Three cases were of general anesthesia: patient comfort was 5/5 in all cases; operator comfort was in 1 case 5/5, in 2 cases 4/5. Twenty patients underwent sedation: in 3 patients (15%) with benign bone lesion, patient comfort was 1/5 and operator comfort 3/5; in 2 patients (10%) with metastatic bone lesion, patient comfort was 3/5 and operator comfort 4/5. Seventy-five patients underwent loco-regional anesthesia: patient and operator comfort was 5/5 in all cases.
**Conclusion:** General and loco-regional anesthesia proved itself to be effective, but general anesthesia showed an operator comfort (average value 4.3) lower than loco-regional anesthesia. Sedation showed globally a less effectiveness (75%), especially in benign bone lesions, probably because of their important inflammatory component. In our experience, loco-regional anesthesia is the best anesthetic practice for the treatment of bone lesions. Therefore, we feel that the collaboration with experienced anesthetists who know this procedure is essential.

**10:30 - 12:00** **Room D**

### Artificial Intelligence and Machine Learning

### SS 1805b

### Machine learning: radiomics analysis

*Moderators:*
W.J. Niessen; Rotterdam/NL
M.F. Russe; Freiburg/DE

**B-1582** 10:39
**Machine learning-based analysis of CT radiomics model for the prediction of colorectal metachronous liver metastases**
M. Taghavi[1], S. Trebeschi[1], D. Meek[2], D.M. Lambregts[1], C. Verhoef[2], J.B. Houwers[3], U.A. van der Heide[1], R.G.H. Beets-Tan[1], M. Maas[1]; [1]*Amsterdam/NL*, [2]*Rotterdam/NL*, [3]*Maastricht/NL (m.taghavi@nki.nl)*

**Purpose:** To develop and validate a CT-based radiomics model for the prediction of metachronous colorectal liver metastases at primary staging with a machine learning algorithm.
**Methods and Materials:** 91 colorectal cancer (CRC) patients from three centers were included. Two groups were assessed: patients who had no evidence of liver metastasis at primary staging, or during follow-up of at least 24 months (n=67) and patients who had no evidence of liver metastases at primary staging but developed metachronous liver metastases within 24 months after primary staging (n=24). After liver parenchyma segmentation (excluding vessels and benign liver lesions), a large number of radiomics features (n= 7,344) were extracted for each patient by use of an open-source python package (PyRadiomics) on primary staging CT. Patients were divided into training set (n=54) and independent validation set (n=37). To mitigate the risk of overfitting due to the medium sample size, 5-fold cross-validation approach was used for training. Bayesian-optimized random forest with wrapper feature selection was trained on the training set and optimized with sequential model-based optimization.
**Results:** The area under the curve (AUC) of the machine learning-based radiomics model performance in the training cohort was 0.88 (95%CI: 0.85-0.92), and in the validation cohort it was 0.87 (95%CI 0.82-0.91) with sensitivity 0.88 (95%CI 0.80-0.95) and specificity 0.72 (95%CI 0.63-0.82).
**Conclusion:** A machine learning-based radiomics analysis of routine clinical CT imaging at diagnosis could provide valuable biomarkers to predict patients at risk for developing colorectal liver metastases.

**B-1583** 10:47
**In-house automatic liver segmentation from CT based on a deep convolutional neural network with data augmentation**
J. Witowski, A. Złahoda-Huzior, M. Stanuch, A. Grochowska, M. Pędziwiatr; *Krakow/PL (jwitos@gmail.com)*

**Purpose:** To develop and evaluate deep convolutional neural network (CNN) for automatic liver parenchyma segmentation and test differences in training accuracy based on different normalization and augmentation parameters.
**Methods and Materials:** 21 computed tomography (CT) studies including 11,966 slices of patients qualified for laparoscopic liver resection for oncological purposes were used to develop the network. Ground truth segmentation was performed by experienced radiologist. Data was divided in a ratio of 70% to 30% for training and test data sets, respectively. A standard 2D U-Net architecture was employed to the network. We conducted a series of experiments with the same data, modifying z-score normalization range and data augmentation methods. Batch size of 1; 15% validation data split and 70 epochs were consistently used throughout the study.
**Results:** Highest Dice similarity coefficient (DSC) of 0.931 on a test set was achieved for a CNN with normalization in range from -200 to 500 HU and 21 augmented CT volumes created with elastic deformations. CNN with no augmentations or normalization scored 0.799 DSC, while CNN with normalization only improved DSC to 0.895. Augmentations based on rotation and translation only did not improve CNN accuracy.
**Conclusion:** State-of-the-art CNNs are feasible to develop in-house solutions for accurate liver segmentation. Both normalization and elastic transformations were required to achieve best results, with normalization being the most important factor.
*Author Disclosures:*
**J. Witowski:** Consultant; MedApp S.A. **A. Złahoda-Huzior:** Employee; MedApp S.A. **M. Stanuch:** Employee; MedApp S.A.

**B-1584** 10:55
**Development and validation of a dual-energy CT-based radiomic nomogram for preoperative prediction of lymph node metastasis in patients with gastric cancer**
J. Li; *Zhengzhou/CN (lijingqingqing@163.com)*

**Purpose:** To develop and validate a dual-energy CT-based radiomic model for the preoperative prediction of lymph node metastasis (LNM) in patients with gastric cancer (GC).
**Methods and Materials:** A total of 193 pathologically confirmed gastric adenocarcinoma patients who underwent biphasic enhanced scans on a rapid kV-switching DECT were retrospectively enrolled and divided into a training set of 97 patients and a test set of 96 patients. Radiomic features, containing deep learning features and hand-crafted features, were extracted from biphasic and three energy levels (40, 65, 100 keV) images. Quantitative iodine concentration, overall survival (OS) and progression free survival (PFS) were collected. A radiomic predictive nomogram was built by machine learning method in the training set; its performance was evaluated using ROC curve, accuracy, and Harrell's concordance index (C-index) in test set.
**Results:** The dual phasic, triple energy image-based radiomic signatures were significantly associated with LNM ($P<0.001$) and demonstrated good performance for discriminating LNM in the test set with AUCs of 0.773 and 0.752 in the training set. The nomogram that incorporated two radiomic signatures, tumour thickness, and CT-reported lymph node status was significantly associated with LNM status ($P<0.001$), with AUCs of 0.864 in training set and 0.834 in test set. The nomogram also exerted a promising prognostic ability with C-indices of 0.660 ($P$=0.0082) for PFS and 0.710 ($P$=0.0025) for OS.
**Conclusion:** This study provided a new insight into the role of DECT-based radiomic nomogram for the preoperative prediction of LNM and prognosis in GC patients.

**B-1585** 11:03
**Development of a MRI radiomics model for pre-therapeutic evaluation of lymph node metastasis in rectal cancer**
S. Hu, C. Xia, W. Peng, Z. Li; *Chengdu/CN*

**Purpose:** To establish and validate a radiomics model using MRI data for preoperative assessment of lymph node (LN) metastasis in patients with rectal cancer.
**Methods and Materials:** A database consisted of 139 patients suffered rectal cancer with/without LN metastasis was divided into training set (41 patients with LN metastasis) and validation set (18 patients with LN metastasis), using 10-fold cross-validation with proportion 7:3. Surgical histopathologic analysis was the reference standard for LN metastasis. Radiomic features were extracted from MRI T2 weighted imaging (T2WI) by Artificial Intelligent Kit (A.K.). One-way ANOVA, rank-sum test, GLM, Spearman, Lasso model were used for feature selection, data redundancy and dimension reduction. The remaining features were included in the model developed by logistic regression analysis and presented with a radiomics nomogram. The receiver operator characteristics (ROC) analysis was performed to validate the radiomics model curves.
**Results:** In total, 396 features were extracted and 17 selected features was significantly associated with LN status (p<0.05). After data redundancy and dimension reduction, 3 features including ClusterShade_angle135_offset7, LongRunHighGreyLevelIEmphasis_AllDirection_offset1_SD and SurfaceVolumeRatio were used to derive the radscore and develop the prediction model. A nomogram for predicting pathological lymph nodes metastases , with an AUC of 0.769 on the training set and 0.684 on the validation data was successfully plotted.
**Conclusion:** In this study, we established and validated a radiomics model to diagnose LN metastasis in patients with rectal cancer, which can be a useful and convenient tool to facilitate the preoperative individualized assessment.

**B-1586** 11:11
**Deep learning based radiomics and its usage in prediction for metastatic colorectal cancer**
D. Nörenberg[1], T. Huber[1], S. Maurus[1], N. Jäger[1], A. Katzmann[2], A. Mühlberg[2], J. Moltz[3], V. Heinemann[1], J. Holch[1]; [1]*Munich/DE*, [2]*Erlangen/DE*, [3]*Bremen/DE (Dominik.Noerenberg@med.uni-muenchen.de)*

**Purpose:** To compare machine learning techniques for predicting tumor growth and one-year overall survival in patients with metastatic colorectal cancer.
**Methods and Materials:** We employed deep convolutional sparse autoencoders (DCAE) to extract deep features from 473 (tumor growth) / 684 (survival) lesion segmentations of 250 / 192 liver metastases at multiple timepoints in 113 / 104 computed tomography images with contrast of 59 / 45 colorectal cancer patients under first-line therapy after semiautomatic tumor segmentations of colorectal cancer liver metastases and trained a deep convolutional neural network (DCNN) to predict tumor growth and short-term survivors using grouped cross-validation. Metrics were determined using bootstrapping with single lesion segmentations for each patient.
**Results:** The classifiers using the deep learning algorithm performed with the highest AUC for tumor growth and one-year-survival over prediction based on RECIST diameters of liver metastases or Radiomics (0.662/0.691 with deep learning vs. 0.621/0.587 for RECIST and 0.567/0.562 with Radiomics, respectively) while preserving the highest -coefficient (tumor growth: 0.225 vs. 0.157/0.089; survival: 0.236 vs. 0.221/0.167).
**Conclusion:** This study demonstrates a deep learning model for predicting tumor growth and one-year overall survival in metastatic colorectal cancer patients using semiautomatic tumor segmentations of liver metastases derived from contrast-enhanced CT images.

**B-1587** 11:19
**A new approach to predict grade of non-functional pancreatic neuroendocrine tumour ≤2 cm: a radiomics nomogram**
Y. Bian; *Shanghai/CN (bianyun2012@foxmail.com)*

**Purpose:** This study aimed to establish a tumour grade (G) prediction model using radiomics features of multi-slice spiral CT (MSCT) images to accurately predict preoperative grading of non-functional pancreatic neuroendocrine tumours (NF-pNETs) of size ≤ 2 cm.
**Methods and Materials:** The prediction model was developed in a primary cohort that consisted of 69 patients with clinicopathologically confirmed NF-pNETs, and data was gathered from January 2014 to January 2017. Radiomics features were selected using least absolute shrinkage and selection operator (LASSO) logistic regression algorithm. In multivariable logistic regression analysis, a predictive model was developed; radiomics nomogram was built and its performance assessed. Decision curve analysis (DCA) was performed with training set for clinical usefulness of nomogram estimation. An independent validation cohort contained 46 consecutive patients from February 2017 to December 2017. Of 115 NF-pNETs, 33 were low-grade (G1, n=18) and intermediate-grade (G2, n=15) tumours of size ≤ 2 cm.
**Results:** The multivariable logistic regression model included arterial and portal venous phase rad-scores. The individualized prediction nomogram showed good discrimination and calibration in the training and validation sets. An AUC of 0.85 can also revealed good discrimination by the nomogram in tumour size≤ 2 cm set. With threshold probability of >5% for a doctor or patient, DCA indicated more net benefit from radiomics nomogram than from “treat all” or “treat none” strategies.
**Conclusion:** Radiomics nomogram, which incorporates arterial and portal venous phase radiomics scores, is a noninvasive preoperative prediction tool, with favourable predictive accuracy for ≤ 2 cm non-functional pNETs grade.

**B-1588** 11:27
**Development of a multiparametric radiomics combined T2WI and DWI to staging rectal cancer**
D. Wen, C. Xia; *Chengdu/CN*

**Purpose:** To explore whether radiomics analysis in T2-weighted imaging and Readout-segmented echo-planar diffusion-weighted imaging can help to predict the pathologic staging in rectal cancer.
**Methods and Materials:** This study prospectively enrolled 524 patients with rectal cancer, which underwent 3.0T MR examinations with both T2WI and DWI sequences. Patients were graded into staging I-II or staging III-IV in accordance with histopathologic findings after surgery. According to 10-fold cross-validation, 70% of this cohort (367 patients) was set as the training cohort, and the others (157 patients) was set as the validation cohort. 396 radiomics features were extracted from T2WI and DWI. The Spearman, Lasso and PCA regression model was exploited for dimension reduction and selection of the feature space. The multivariable logistic regression analysis was adopted to identify the radiomics signature of pathologic staging. The discriminating performance was assessed with the area under receiver operating characteristic curve . The Mann-Whitney U test was adopted for testing the potential correlation of the radiomics signature and the response in training cohorts, and decision curve analysis (DCA) was used for comparing three models.
**Results:** 14 radiomics features were selected to create the radiomics signature significantly associated with response (P<0.001) after feature dimension reduction. AUC of radiomics signature performance in the training cohort was 0.856, and in the validation cohort was 0.779.
**Conclusion:** Multiparametric radiomics of rectal cancer, which combined T2WI with DWI, showed a fairly high accuracy in staging of I-II or III-IV rectal cancer.

**B-1589** 11:35
**CT texture analysis in liver fibrosis shows a strong correlation with portal phase enhancement and can predict cirrhosis in decision tree model**
P.N. Kaposi, B. Budai, V. Frank, S. Shariati, A. Folhoffer, M. Abonyi, V. Bérczi; *Budapest/HU (kaposipal@gmail.com)*

**Purpose:** CT texture analysis (CTTA) has been successfully used to assess tissue heterogeneity and it may facilitate the diagnosis of liver cirrhosis when utilized in machine learning models.
**Methods and Materials:** CT scans were retrospectively collected from twenty-seven patients with chronic hepatitis. Examinations were performed with either a 16 (11 cases) or a 64 slice (16 cases) scanner (Philips Healthcare, the Netherlands). The stage of liver fibrosis was determined with shear-wave ultrasound elastography. Contrast-enhanced series in the portal venous phase (PVP) were reconstructed to 5 mm slice thickness with no gap. Anatomical liver segments were labeled manually, and 1112 texture parameters (TP) were calculated for each. TPs were used in a hierarchical cluster analysis (HCA), group comparisons, and in a binary tree prediction model (BTM).
**Results:** CTTA was performed on 319 anatomical liver segments. A comparison between the two scanners found a significant difference (p<0.05) in 305 TPs. HCA revealed a uniform pattern, which divided segments into two groups. PVP densities showed a significant difference between the two groups in both the 16 (mean±SEM= 108.9±1.69 HU vs. 96.6±1.16 HU, p<0.001) and the 64 slice (mean±SEM= 101.1±1.24 HU vs. 85.2±1.83 HU, p<0.001) sets. Meanwhile, liver stiffness was not significantly different. A BTM was trained on the 16 slice set, and it could predict cirrhosis in segments of the 64 slice set with 92% accuracy.
**Conclusion:** Contrast enhancement and scanning parameters have a strong influence on TPs. CTTA-based artificial intelligence may achieve success in in the detection of liver cirrhosis.

**B-1590** 11:43
**MR imaging of rectal cancer: radiomics analysis to predict hepatic metastases**
S. Hu, W. Peng, C. Xia, Z. Li; *Chengdu/CN*

**Purpose:** To investigate the value of T2-weighted-based radiomics for prediction of hepatic metastases in patients with rectal cancer.
**Methods and Materials:** 180 patients with rectal cancer underwent magnetic resonance imaging between March 2016 and February 2018 were prospectively included. 98 patients were confirmed to hepatic metastases by biopsy, surgical histopathologic, CT enhanced imaging or follow up. Using 10-fold cross-validation, all patients were divided into training cohort (125 patients) and validation cohort (54 patients) with proportion 7:3. The Spearman, Lasso and PCA regression models were exploited for dimension reduction and selection of the feature space after texture features extracted by Artificial Intelligent Kit. The radiomics signature of liver metastases was developed by multivariable logistic regression analysis . The discriminating performance was assessed with the area under receiver operating characteristic curve (AUC). The Mann-Whitney U test was adopted for testing the potential correlation of the radiomics signature and the response in training cohorts, and decision curve analysis (DCA) was used for comparing three models.
**Results:** A total of 396 features were extracted and two of them were eventually selected to create the radiomics signature significantly associated with response (P<0.001). The radiomics signature showed a good accuracy with an AUC of 0.701 on the training set and 0.652 on the validation data.
**Conclusion:** T2-weighted-based radiomics for prediction of hepatic metastases from rectal cancer was successfully developed and validated. With a good discrimination performance, this radiomics model could play an important role in the preoperative prediction of liver metastases from rectal cancer.

**B-1591** 11:51
**MR imaging radiomics textures for predicting the gene expression in related to recurrence risks of HCC given by GOLM1, RND1 and SETD7**
X. Hu; *Chongqing/CN (xianling_hu@foxmail.com)*

**Purpose:** To investigate whether the combination of radiomics and regression model-based classification can predict gene expression signatures of GOLM1, RND1 and SETD7 of HCC preoperatively.
**Methods and Materials:** 114 pathology confirmed early-staged HCC patients treated with surgical resection were included. All of these patients underwent Gd-EOB-DTPA enhanced MRI examinations. ROIs of the largest slice of each protocol (one unenhanced and 5 enhanced T1-WI MR images) were segmented by graph-based semi-automatic segmentation algorithm using MATLAB. A total of 1650 textures were extracted for each ROI. Three gene expression signatures, GOLM1, RND1 and SETD7 were evaluated using immunohistochemistry. PCA combined with Holm t test was applied to determine the optimal subsets. Logistic regression and ROC were conducted to assess the predictive ability.
**Results:** The multiple linear regression analyses demonstrated significant associations ($R^2$ = 0.14-0.22, P < 0.0006) between features subsets and gene signatures. The yielded ROC curves of each gene signatures with all AUC values (0.76, 0.74, 0.90, 0.90, 0.79, and 0.71 ) show statistical difference from chance. The prediction models for gene signatures SETD7 tumour and ANTL had the best statistical difference with AUC 0.90, positive predictive values (PPV) 100% and true negative rate (TNR) 100%. Models to predict SETD7 tumour achieved the best classification accuracy (ACC) and negative predictive value (NPV) of both 84%, and RND1 ANTL achieved the best true positive rate (TPR) of 93%.
**Conclusion:** The study revealed that quantitative HCC MR imaging radiomics could predict gene signatures for preoperatively assessing the risk of HCC recurrence.
*Author Disclosures:*
**X. Hu:** Author; Chuanming Li, Jian Wang.

**10:30 - 12:00** **Room G**

### Physics in Medical Imaging

### SS 1813

### Radiation dose optimisation in CT

*Moderators:*
K.N. Bolstad; Bergen/NO
G. O'Reilly; Dublin/IE

**B-1592** 10:30
**Influence of arm positioning on radiation dose during combined thorax and liver CT imaging: a phantom study**
O.V. Ivashchenko, J.J. Roelofs, A. van der Most, W.J.H. Veldkamp, I. Hernandez Giron; *Leiden/NL (o.ivashchenko@lumc.nl)*

**Purpose:** Impaired shoulder movement is a common morbidity related to various causes. If required, thorax or liver CT of these patients will be performed with 'arms_down', instead of the standard 'arms_up' setup. Only a few studies investigated the influence of arms position during thorax-abdominal CT, partially due to the high cost of fully articulated anthropomorphic phantoms. Here, the effect of arms positioning on patient dose is investigated using affordable in-house-developed arm extensions for the RANDO-phantom.
**Methods and Materials:** Two anatomically correct phantom arms were constructed as an extension of the RANDO-phantom. Bony parts were created with acrylate-based clay, manually shaped to a typical bony anatomy of the upper limb. Soft tissue was cast on industrial silicon. The attenuation of the phantom materials (Hounsfield units, HU) was compared to patient tissue in CT images. Subsequently, acquisitions were performed selecting the available clinical protocol 'thorax_tumour+liver' in two CT systems: Aquilion-One and Aquilion-Genesis (Canon Medical Systems), with two arm configurations ('arms_up','arms_down') and doses were compared in terms of $CTDI_{vol}$ and dose-length-product(DLP).

**Results:** Attenuation of the arm phantoms [bone: (224±213)HU, soft tissue: (16±29)HU] closely mimicked values of the human arm [bone: (402±359)HU, soft tissue:(12±48)HU]. For the thorax part of the clinical protocol and 'arms_down' configuration, dose increase of 36.5% (DLP↑=23mGy·cm,$CTDI_{vol}$↑=0.7mGy; AquilionONE) and 19.6% (DLP↑=18.1m mGy·cm,$CTDI_{vol}$↑=0.6mGy;AquilionONE GENESIS) was observed, compared to the 'arms_up' setup. For the liver part, corresponding dose increases were 26.6% (DLP↑=25.0 mGy·cm ,$CTDI_{vol}$↑=1.0mGy) and 22% (DLP↑=21.3 mGy·cm,$CTDI_{vol}$↑=0.9mGy).
**Conclusion:** In this phantom study, arms positioning during combined thorax-liver CT significantly influenced patient dose (up to 36%).
*Author Disclosures:*
**I. Hernandez Giron:** Research/Grant Support; CLUES research project (Project Number 13592) funded by the NWO Dutch Organization inside the Open Technology Programme, Research project related to Ultra-low dose thorax CT, funded by Canon Medical Systems.

**B-1593** 10:38
**The effect of scout view length on patient dose from thoracic CT examinations performed with tube current modulation**
K. Perisinakis, A. Tzedakis, N. Ntoufas, M. Velivasaki, J. Stratakis, J. Damilakis; *Iraklion/GR (kostas.perisinakis@med.uoc.gr)*

**Purpose:** To investigate the effect of altering the scanogram boundaries, with respect to a standard region to be imaged, on the modulated mA values in thoracic CT examinations.
**Methods and Materials:** The body region typically imaged in thoracic CT was defined on one adult and four pediatric physical anthropomorphic phantoms. The mean mA per each of the rotations required to image the prescribed image volume was recorded for a modern 128-slice CT system keeping the image volume unchanged but altering the scanogram boundaries symmetrically up to ±10 cm in steps of 2 cm. Monte Carlo methods and five mathematical anthropomorphic phantoms were employed to quantify dose differences between helical CT acquisitions resulting to the same image volume but involving scanograms of different length.
**Results:** mA modulation was found to be significantly affected when scanogram length was altered with respect to the preset imaged volume. The difference in modulated mA was up to 15% with the highest values recorded for regions close to the imaged volume boundaries. The difference in dose to the radiosensitive organs of thorax was found up to 12% with higher and lower value recorded for thyroid and lung, respectively.
**Conclusion:** Scanogram length in thoracic helical CT scans performed with mA modulation should cover the entire volume to be imaged plus the overscanning regions at either side to avoid increase of dose to organs close to image volume boundaries such as thyroid.
This project has received funding from the Euratom research and training programme 2014-2018 under grant agreement No. 755523.

**B-1594** 10:46
**Investigation the causes of overscanning in z-axis in thorax, abdominopelvic and thoraco-abdominopelvic CT examinations**
O. Yar, M.R. Onur, I. Idilman, E. Akpinar, D. Akata; *Ankara/TR (drozanyar@gmail.com)*

**Purpose:** The aim of this study was to determine the frequency of excessive z-axis coverage of single-phase thorax CT, abdominopelvic CT, thoraco-abdominopelvic CT, stone protocol abdomen CT and pulmonary CT angiography examinations and evaluate the factors that may be associated with overscanning in CT.
**Methods and Materials:** Between March 1 and April 1, 2018, 2032 CT examinations performed in 1531 cases were included in our study. According to accepted reference points determined for CT examinations, the lengths of over-scanned z-axis were measured. Excessive scan frequency, the ratio of over-scanned length to the acceptable scan length were evaluated according to the gender, age, type of examination, indication of examination, the time of acquisition of the examination, the presence of some factors that may affect the determination of the scan range in topogram, the throughput of CT equipments and the experience of the CT technicians.
**Results:** While the frequency of over-scanning in z-axis was not significantly different between genders ($p = 0.433$), statistically significant differences in terms of over-scanning in z-axis were obtained between the age groups ($p < 0.001$), types of examinations ($p < 0.001$), indications for examination ($p=0.009$), conditions that may affect determination of the scan range in topogram ($p<0.05$), acquisition time ($p=0.042$) and technician's experience ($p>0.001$).
**Conclusion:** Overscanning in z-axis is a preventable cause of increased radiation dose in CT. Taking measures for preventable causes of high over-scanning will reduce the effective radiation dose of exposed cases.

**B-1595** 10:54
**Comparison of radiation doses imparted during multidetector CT scanners and cone beam computed tomography for the postoperative, spinal fusion assessment**
N. Guberina, M. Forsting, A. Ringelstein; *Essen/DE*

**Purpose:** To assess and compare radiation doses imparted during various, multidetector, single- and dual-source CT scanners (MSCT) as well as cone beam computed tomography (CBCT) for the postoperative examination of spinal fusion of the lumbar spine.
**Methods and Materials:** Radiation doses were assessed during standardized lumbar spine CT protocols at different MSCT ((I) single-source CT scanner Somatom Definition AS+, (II) 2nd generation of dual-source CT scanner Somatom Definition Flash in single-source technique, (III) 2nd generation of dual-source CT scanner Somatom Definition Flash in dual-source technique (all Siemens Healthcare, Erlangen, Germany)) and at the CBCT Ziehm Vision RFD 3D (Ziehm Imaging GmbH, Nürnberg, Germany), (IV) (a) RFD-3D (Standard) and (b) RFD-3D (L-D). Image quality was examined independently by evaluating various important parameters (pedicle placement, quality control of spinal fusion surgery, and the evaluation of the lumbar spine in case of complications) determined on real patients' examinations. Wilcoxon signed rank test was used to determine statistically significant differences ($p < 0.05$).
**Results:** In MSCT setting following radiation doses were assessed ($CTDI_w$; DLP): (I) 8.9mGy; 96.8mGycm; (II) 14.2mGy; 153.4mGycm; (III) 22.0mGy; 236.2 mGycm; in the CBCT setting radiation doses were distributed as follows: (IV) (a) 17.2 mGy; 187.0 mGycm; (b) 13.0 mGy; 143.1 mGycm. Overall, image quality, was evaluated as good for both, MSCT and CBCT examinations.
**Conclusion:** Dual-source MSCT scanning involves higher radiation dose for the postoperative examination of spinal fusion of the lumbar spine. Radiation doses are ower during CBCT and single-source MSCT compared to dual-source MSCT lumbar spine examinations while maintaining sufficient image quality.

**B-1596** 11:02
**Effective dose comparison between cone beam CT (CBCT) and MSCT for three types of head examinations**
D. Buytaert[1], B. Keelson[2], G. Van Gompel[2], J. Casselman[3], N. Buls[2], K. Bacher[1]; [1]*Gent/BE*, [2]*Brussels/BE*, [3]*Bruges/BE (dimitri.buytaert@ugent.be)*

**Purpose:** To (1) measure and compare organ and effective doses (E) for three types of head examinations with CBCT and conventional MSCT, and (2) to establish CTDIvol to E conversion factors for CBCT.
**Methods and Materials:** This experimental study included sinus, temporal bone and mandibula CBCT and CT examinations. Images from an Alderson RANDO anthropomorphic phantom were acquired on a Newtom 5G (Newtom, Verona, Italy) CBCT and GE Discovery 750HD (GE Healthcare, Milwaukee, WI, USA) CT device. The phantom was positioned and scanned with clinical standard of care protocols. Organ doses were measured using 160 calibrated TLD dosimeters, of which 148 were positioned inside the first 10 slices of the RANDO phantom. From these organ doses the E was calculated and CTDIvol to E conversion factors were determined.
**Results:** E values for sinus, temporal bone and mandibula were respectively equal to 105, 238 and 65 µSv on the CBCT device. The respective values calculated for the CT device were 56, 1816 and 743 µSv. The respective CBCT conversion factors from CTDIvol to E were 28.09, 23.90 and 35.46 µSv/mGy, while for CT these conversion factors were respectively equal to 35.47, 34.78 and 56.29 µSv/mGy. For sinus, temporal bone and mandibula the E on CT was respectively a factor 0.53 lower, 7.6 higher and 11.4 higher than on CBCT.
**Conclusion:** The CT sinus protocol resulted in lower E than the protocol on the CBCT, however the CT temporal bone and mandibula protocols resulted in much higher doses than the CBCT.
*Author Disclosures:*
**D. Buytaert:** Research/Grant Support; Newtom. **B. Keelson:** Research/Grant Support; Newtom. **G. Van Gompel:** Research/Grant Support; Newtom. **J. Casselman:** Equipment Support Recipient; Newtom. **N. Buls:** Research/Grant Support; Newtom. **K. Bacher:** Research/Grant Support; Newtom.

**B-1597** 11:10
**Characterisation of patient skin dose in CT imaging**
M. Cabuy[1], G. Van Gompel[2], P.D. Deak[3], J. de Mey[1], N. Buls[2]; [1]*Jette/BE*, [2]*Brussels/BE*, [3]*Berlin/DE (margo.cabuy@outlook.com)*

**Purpose:** Investigate the influence of CT acquisition and geometrical parameters on patient skin dose in CT imaging.
**Methods and Materials:** A Monte Carlo software (ImpactMC) simulating dose distributions was experimentally validated for the scanner geometry (GE Revolution CT). MC experiments were conducted on virtual cylindrical and elliptical phantoms with varying size (diameter 16 and 32 cm; major axis 9.35, 18.7 and 37.8 cm) and position in the CT gantry. In addition, the impact of following acquisition parameters on skin dose were assessed: beam shaping

Sunday filter (Medium and Body); tube voltage (70, 80, 100, 120 and 140 kVp); beam collimation (40 and 80 mm). For each condition, the surface skin dose, normalized by $CTDI_{vol}$, was estimated.
**Results:** The ImpactMC software validation yielded a dose accuracy with variance<5.5%. For each beam shaping filter, a generalized model of skin dose in function of the patient, geometric and acquisition variables was fitted to the data. The local surface skin dose could be accurately (stdev <11%) estimated considering only the distance between skin and isocentre. Knowledge of the actual patient position in the gantry, phantom shape, size, tube voltage and beam collimation has only a minor additional impact on the model accuracy (stdev<3%). For all simulations, an increasing trend in skin dose was observed when the skin approaches the isocentre. The impact of the beam shaping filter is largest at the isocentre (15% difference in skin dose).
**Conclusion:** Skin dose in CT can be estimated based on CT images and $CTDI_{vol}$ of the CT acquisition.

**B-1598** 11:18
**Coronary calcium scoring in CT with a calcium-aware image reconstruction technique: dose reduction by lowering x-ray tube voltage**
R. Booij, R.P. Budde, D. Bos, M. Kremer, M. Dijkshoorn, M. van Straten; *Rotterdam/NL (r.booij@erasmusmc.nl)*

**Purpose:** To assess the accuracy and dose reduction potential of a reconstruction technique that aims at a constant CT number for calcium when varying tube voltage.
**Methods and Materials:** A cardiothoracic phantom was scanned in two sessions. First, with a phantom insert containing calcifications varying in size and density at eight tube voltages (70-140 kV). Tube load was adapted by the scanner to maintain the reference image quality defined at 120 kV. Second, 21 ex vivo hearts were scanned at 120 kV and at an automatically selected tube voltage. Images were reconstructed with the standard calcium score kernel (Qr36) and a calcium-aware kernel (Sa36). Agatston score was determined after manual exclusion of lesions caused by noise.
**Results:** For the phantom insert, CT numbers differed up to 64% for Qr36 and 10% for Sa36 when compared to the reference scan at 120 kV. Similarly, Agatston scores differed up to 40% and 15% for Qr36 and Sa36, respectively. For the ex vivo hearts, the automatically selected tube voltage was always 90 kV instead of 120 kV. Corresponding CTDIvol values were 0.64 mGy and 0.79 mGy, respectively. Agatston scores were up to 300 higher with the new low-dose protocol using Sa36 compared to the reference protocol with Qr36 and 120 kV. However, the clinical impact of this increase seems limited.
**Conclusion:** A calcium-aware image reconstruction technique allows for consistent CT numbers and comparable calcium scores when lowering kV, resulting in a possible dose reduction of up to 19%.
*Author Disclosures:*
**R. Booij:** Other; Departement has a master research agreement with Siemens Healthineers. No funding was received for this topic. **M. Dijkshoorn:** Author; Training Consultant, Siemens Healthineers. No funding received for this topic. **M. van Straten:** Other; Departement has a master research agreement with Siemens Healthineers. No funding was received for this topic.

**B-1599** 11:26
**Dose management in computed tomography using the as-low-as-diagnostically acceptable image quality approach**
G. Widmann[1], G. Degenhart[1], A. Al-Ekrish[2], W. Puelacher[1], G. Feuchtner[1], W.R. Jaschke[1], R. Jacobs[3], R. Pauwels[3]; [1]*Innsbruck/AT*, [2]*Riyadh/SA*, [3]*Leuven/BE (gerlig.widmann@i-med.ac.at)*

**Purpose:** For a specific CT examination, the as-low-as-diagnostically acceptable (ALADA) dose for each manufacturer, scanner, protocol, and dose saving technology is usually unknown. Purpose was to develop an ALADA image quality approach which may guide dose optimisation by comparative image quality phantom testing.
**Methods and Materials:** Spatial resolution, contrast resolution, contrast-to-noise ratio and subjective noise and sharpness scores from a published ALADA clinical craniomaxillofacial trauma reference protocol at $CTDI_{vol}$ 2.66 mGy (80 kV, 40 mA) were compared with the results from two other scanners of a different vendor. Test protocols included fixed settings of 100 kV and 35 mA, 80 kV and 40 mA, and protocol adoptions to achieve doses of around 2.66, and 1.00 mGy. The influence of various sharp and soft kernels, and iterative reconstructions was evaluated.
**Results:** Doses of around 2.6 mGy could provide similar image quality. 3.08 and 3.44 mGy (100 kV, 35 mA) did not provide significant image quality advantages. Doses of 1.73-1.92 mGy (80 kV, 40 mA) had more noise, while 1.00-1.03 mGy also decreased contrast resolution. Only bone kernel H70h showed similar spatial resolution, but had higher noise than the reference bone kernel. Iterative reconstructions reduced noise and improved contrast-to-noise ratios substantially.
**Conclusion:** Similar scanner settings may not provide similar doses. Image quality results are largely influenced by convolution kernel and image reconstruction technology. A phantom-based ALADA image quality approach may facilitate protocol optimisation and ensure that patients receive CT examinations with the lowest diagnostically acceptable radiation dose.

**B-1600** 11:34
**Clinical diagnostic reference levels for CT examinations: a prospective multicentre study**
H. Brat[1], F. Zanca[2], B. Rizk[3], S. Montandon[4], D. Racine[5], E. Meicher[1], D. Fournier[1]; [1]*Sion/CH*, [2]*Heverlee/BE*, [3]*Villars-sur-Glane/CH*, [4]*Gland/CH*, [5]*Lausanne/CH (hugobene@gmail.com)*

**Purpose:** To establish clinical DRLs (CDRLs) according to BMI in a Swiss multicentre setting.
**Methods and Materials:** After protocol harmonization and optimization according to clinical indications and BMI (<25;>25), 5310 abdomen and 1058 chest CT examinations (5 Philips scanners) were prospectively collected from February 2017 to June 2018. Local CDRLs per indication (third quartile of the median dose values of each CT modality) were calculated and compared to the new national DRLs (NDRLs). Mann-Whitney tests and Wilcoxon test were used as appropriate (significant difference: p<0.05).
**Results:** For chest examinations, CDRLs were below or equal to P50 and P75 NDRLs for CTDIvol (range 4.9-6.0 vs 6/7mGy) and DLP (164-209 vs 210/250mGy.cm) in the “all BMI” and BMI<25 groups. For BMI>25 patients, they were higher than P50 and P75 NDRLs for CTDI (range 7.1-7.9 vs 6/7mGy) and DLP (267-321 vs 210/250mGy.cm), suggesting new NDRLs (7mGy, 250nGy.cm) are challenging for overweight population. For BMI< 25 abdomen examinations, local CDRLs were significantly lower than P50 and P75 NDRLs (4.6-7.0 vs 10/11mGy and 226-273 vs 470/540mGy.com). For BMI>25, CDRLs were lower than NDRLs P75 only (7.1-10.6 vs 10/11mGy and 384-465 vs 470/540mGy.cm), indicating that P75 NDRLs are achievable for BMI >25, but too high for BMI<25 patients. For chest, CTDIvol and DLP variability was larger when considering BMI rather than clinical indication; for abdomen a large variability was observed among indications.
**Conclusion:** Addressing patient size is relevant when defining DRLs for both chest and abdomen examinations, as clinical indication seems relevant only for abdomen.
*Author Disclosures:*
**F. Zanca:** Employee; Former GE employee. **S. Montandon:** Employee; Philips Medical Systems.

**B-1601** 11:42
**Monte Carlo simulation of dual-energy CT systems: initial validation of radiation dose measurements**
Y. Toufique, O. Bouhali, J.O. Doherty, J. Goracy, I. Delakis; *Doha/QA (toufique.yassine@gmail.com)*

**Purpose:** Patient dose measurement and diagnostic reference levels are based on computed tomography dose indices (CTDI), a quantity that can be measured using an ion chamber. The aim of this work is the validation of a numerical Monte Carlo (MC) model of the Definition Flash (Siemens) CT scanner using the GATE (GEANT4 application for tomographic emission) toolkit by the simulation of the measurement process. In addition, a MC model of the x-ray tube, within the CT system was validated by comparing the simulated GATE energy spectrum with a calculated spectrum via SpekCalc software.
**Methods and Materials:** Experimental CTDI measurements were made in a PMMA body phantom (32 cm) and head phantom (16 cm) using a 100-mm-long pencil chamber, which represents the absorbed dose along the longitudinal z-axis of the scanner measured during a single rotation of the x-ray source. Measurements were performed at five different positions in the phantom (center, 90°, 180°, 270°, 360°).
**Results:** The comparison between simulated x-ray spectrum and the calculated spectrum shows good agreement. A difference in the K-characteristic x-ray intensity was observed. The relative difference between simulated and measured CTDI values at 70, 80, 100, 120 and 140 kVp was less than 3%.
**Conclusion:** In this study, we demonstrated that GATE can model a complete CT scanner from the x-ray tube to the absorbed dose (CTDI). Future simulation work will investigate CT dose reduction techniques as well as advanced acquisition methodologies.

**B-1602** 11:50
**Radiation burden of patients with hepatocellular carcinoma from multiple TACE and CT procedures**
L. Sukupova; *Prague/CZ (lucie.sukupova@gmail.com)*

**Purpose:** The transarterial chemoembolization (TACE) is a high-dose therapeutic procedure, sometimes performed repeatedly, which is accompanied by multiple CT exams during the treatment and follow-up. The aim of this study was to estimate organ and effective doses from TACE and CT procedures for patients who underwent TACE repeatedly.
**Methods and Materials:** 166 patients underwent 210 TACE during 06/2012-06/2018. 109 patients underwent TACE only once, 40 patients underwent TACE repeatedly (2-5 times). These 40 patients underwent 241 abdomen CT exams (2-11 CT exams) during the treatment and follow-up. The follow-up period lasted up to 4.2 years, median 1.3 years.
The dose estimate of organ and effective doses was performed in PCXMC for TACE and in ImpactDose for CT exams. The actual size, anatomic and dose information was taken from archived images and RDSR or from DICOM headers.
**Results:** The median/maximum/minimum values of the cumulative organ doses from TACE and CT procedures to the most exposed organs were 1.1/0.3/3.2 Gy to adrenals, 1.7/0.5/5 Gy to kidneys and 1.1/0.3/2.7 to the liver. The highest organ doses in a single procedure were 2.8 Gy to kidneys and 1.2 Gy to the liver. The cumulative effective doses were between 80 and 513 mSv, median 210 mSv.
**Conclusion:** Organ doses from a single TACE can reach 2.8 Gy for some organs, but this is still remote from the tolerance doses known from radiotherapy. Higher organ and effective doses increase the risk of stochastic effects, but they may be of lower importance in patients with life-threatening HCC.

**10:30 - 12:00** **Room K**

### Chest

### SS 1804

### Thoracic imaging: new approaches and techniques

*Moderators:*
N.N.
G. Chassagnon; Paris/FR

**K-38** 10:30
**Keynote lecture**
E.J. Stern; *Seattle, WA/US*

**B-1603** 10:39
**Visibility of bronchial artery on virtual monoenergetic image and advanced virtual monoenergetic images**
T. Nomura, T. Niwa, J. Hashimoto, Y. Imai; *Isehara/JP (yh_4amm1208@yahoo.co.jp)*

**Purpose:** To assess the visibility of bronchial artery on virtual monoenergetic image (VMI) and noise-optimized advanced VMI (VMI+).
**Methods and Materials:** Eighty-seven patients with oesophageal cancer were scanned on a third-generation 128-slice dual-source CT system before operation. Tube voltages were set to 90 kVp (tube A) and 150 kVp (tube B). Gantry rotation time, pitch, and collimation were 0.25 s, 0.55, and 192 × 0.6 mm, respectively. Images were reconstructed with VMI and VMI+, energy levels at 40-120 keV in 10 keV increments as well as composite image equivalent to CT at 105 kVp. CT attenuation value and contrast to noise ratios (CNR) of bronchial artery on VMI and VMI+ were compared to those on composite image. Subjective analysis was performed by two radiologists in terms of the visibility of bronchial artery.
**Results:** CT value of bronchial artery on VMI at 40-60 keV, and VMI+ at 40 and 50 keV were significantly higher than that on composite image ($p< 0.05$). CNR on VMI at 40-60 keV was significantly higher than that on composite image ($p < 0.05$); whereas CNR on VMI+ at 40 and 50 keV showed no significant difference to that on composite image. In subjective analysis, both VMI and VMI+ at 40 and 50 keV showed better visualization of bronchial artery than composite image.
**Conclusion:** VMI and VMI+ at low keV may be useful to visualize bronchial arteries.

**B-1604** 10:47
**The value of multiple quantitative spectrum parameters for assessing the lymph node metastasis of lung cancer with dual-layer detector spectral CT**
L. Gao, Y. Hou, Y. Ma, Q. Ma, X. Lu; *Shenyang/CN (1242179174@qq.com)*

**Purpose:** To investigate the value of multiple quantitative spectrum parameters for the metastatic lymph nodes in lung cancer using dual-layer detector spectral CT.
**Methods and Materials:** Thirty-nine patients with lung cancer undergoing dual-phase enhanced scan with dual-layer detector spectral CT were selected. According to pathological findings, sixty-eight lymph nodes were divided into metastatic group (n=37) and non-metastatic group (n=39). Short diameter of lymph nodes, normalized iodine concentration (NIC) and the slope of energy spectrum curve ($\lambda_{HU}$) in arterial (AP) and venous (VP) phase were measured and calculated. Receiver operating characteristic (ROC) curves were drawn to confirm the optimum threshold and diagnostic efficiency.
**Results:** Short diameter of lymph nodes, NIC and λHU in AP and VP of the two groups all showed significant differences ($P$<0.05). Moreover, the optimum diagnostic efficiency of AP NIC in metastatic lymph nodes was the largest ($AUC_{AP\ NIC}$ =0.843 vs. $AUC_{VP\ NIC}$=0.698; $AUC_{AP\ \lambda\ HU}$=0.840 vs. $AUC_{VP\ \lambda HU}$=0.716; $AUC_{short\ diameter}$ =0.736), and its threshold was 0.15mg/dL. The sensitivity and specificity were 89.2% and 69.2%, respectively. When choosing the short diameter of the lymph node ≧1cm as the standard, the sensitivity and specificity were 71.9% and 68.2%, respectively. When the two diagnostic criteria are combined, its sensitivity can be improved to 97.0% and the specificity can be increased to 90.2%.
**Conclusion:** Multiple quantitative spectrum parameters of dual-layer detector spectral CT imaging provide an effective noninvasive method for accurate evaluation of metastatic lymph nodes in lung cancer. Combining the short diameter of the lymph nodes and AP NIC, the accuracy of the diagnosis can be improved.

**B-1605** 10:55
**Using relationship between pulmonary veins and pulmonary nodules to distinguish benign intrapulmonary lymph nodes from lung cancer on CT**
J.L. Barnett[1], I. Pulzato[1], R. Wilson[2], S. Padley[1], A.G. Nicholson[1], A. Devaraj[1]; [1]*London/UK*, [2]*Belfast/UK (ilaria.pulzato@gmail.com)*

**Purpose:** Accurately distinguishing benign intrapulmonary lymph nodes (IPLNs) from small lung cancers is highly desirable in lung cancer screening and for incidentally detected nodules. We aimed to test our hypothesis that IPLNs were more frequently connected to pulmonary veins, compared to lung cancer, and that this could be used to distinguish the two types of nodules.
**Methods and Materials:** Two radiologists reviewed 62 pathologically confirmed benign intrapulmonary lymph nodes and 61 pathologically confirmed small sized (<15mm) lung cancers and assessed the number and type of pulmonary blood vessels arising from, or terminating within, these nodules. Nodule location and outline and shape were also reviewed.
**Results:** Connection to a pulmonary vein was significantly more frequent in IPLNs (93.5%) compared to lung adenocarcinomas (21.3%) [p<0.001]. IPLNs were never (0%) associated with pulmonary arteries, but identified in 55.7% of lung cancer nodules. [p<0.001]. The connection to a pleural surface was present in both IPLNs (38.7%) and lung adenocarcinomas (37.7%) [p=1.0].
**Conclusion:** Connection to a pulmonary vein as opposed to a pulmonary artery can be used in conjunction with other CT signs to identify IPLNs in the setting of small indeterminate pulmonary nodules.

**B-1606** 11:03
**Application of computer-aided diagnosis system based on 3D deep convolutional neural network in lung cancer screening**
Q. Meng, J. Ding; *Zhengzhou/CN (mengke436@163.com)*

**Purpose:** To evaluate the application value of CAD system based on the novel deconvolution rapid residual CNN lung cancer screening of urban China.
**Methods and Materials:** The LDCT imaging data of 8850 lung cancer screening volunteers with 1111 nodules of lung from November of 2013 to December of 2017 were evaluated through visual detection (VD) method, CAD method or VD combination CAD method. The detection rate, the missed diagnosis rate and the number of false-positive rate of nodules were observed.
**Results:** Compared with the VD method, the detection rate of nodules in CAD and VD combined with CAD was significantly increased (80.1% vs 94.2%; 19.9% vs 5.8%, $\chi^2$=101.65, $P$=0.00); the detection rate of nodules in VD combined with CAD method was higher than that of CAD. The number of false-positive nodules detected by CAD method was highest among the three methods. Compared with the VD, the detection rate of lung-RADS classification by CAD and VD combined with CAD was significantly increased ($\chi^2$=25.083, 23.449, p=0.000, 0.000). Compared with the VD, the detection rate of types of nodules by CAD or the VD combined with CAD was statistically significant ($\chi^2$=6.955, 6.821, p=0.031, 0.033). Compared with VD and VD combined with CAD, the positive prediction rate of lung cancer by CAD was significantly reduced, the rate of missed diagnosis and the false-positive rate were significantly increased, and the difference was statistically significant (88.2% vs 94.1%, 94.1%, 9.2% vs 2.1%, 2.1%; $\chi^2$=14.605, 14.693, p=0.002, 0.002).
**Conclusion:** The CAD combined with VD method is the preferred method to use in the large-scale LDCT lung cancer screening of urban China.

**B-1607** 11:11
**Deep learning-based CAD may improve detection of pulmonary nodules while preserving a low false-positive rate**
J. Lieman-Sifry[1], S. Brouha[2], A. Yen[2], E. Weihe[2], K. Jacobs[2], M. Horowitz[2], D. Motiuk[2], A. Hsiao[3]; [1]*San Francisco, CA/US*, [2]*San Diego, CA/US*, [3]*La Jolla, CA/US (jesse@arterys.com)*

**Purpose:** We investigate the rate at which false positives (FP), defined as a nodule detected by deep learning-based computer aided detection (DL-CAD) not noted by any of four general radiologist readers, are overturned upon subspecialty secondary read to be true nodules.
**Methods and Materials:** We train and validate a DL-CAD system on standard-dose and low-dose CT scans. The system consists of a high-sensitivity 2D-U-Net-based network and a 3D-VGG-based classifier to reduce the FP rate. To test the DL-CAD system, nodules were identified on 100 scans selected from the NLST dataset independently by four general radiologists. 3/4 consensus defines a true nodule. Intermediate confidence nodules predicted by the DL-CAD algorithm that were not detected by any of the four general radiologists, which would otherwise be considered “false positives,” were presented to five dedicated thoracic radiologists and two fellows as secondary readers. Each FP was reviewed and radiologists assigned their confidence level (0-10) that the prediction was a nodule worth further investigation in a web-based survey. We define FP with average scores of 5 and above as “overturned false positive” nodules.
**Results:** At operating points of 1.7FP/scan and 0.5FP/scan, 38% of FPs (65/171), and 54% of FPs (26/48) were overturned, respectively. With 260 nodules of 3/4 consensus in the ground truth data, 65 overturned FP suggests that an increase of sensitivity of up to 25% (65/260) could be achieved with use of the DL-CAD system.
**Conclusion:** A substantial fraction of intermediate confidence lesions from DL-CAD may constitute nodules worth further follow-up.
*Author Disclosures:*
**J. Lieman-Sifry:** Employee; Arterys. **A. Hsiao:** Founder; Arterys.

**B-1608** 11:19
**Analysis of pleural effusion dynamics in patients with pulmonary infarction based on CTPA measurements**
M. Kastelic[1], J. Vidmar[2], K.K. Šifer[1], I. Kocijančič[2]; [1]*Celje/SI*, [2]*Ljubljana/SI (marko.kastelic@gmail.com)*

**Purpose:** There is a lack of studies about pleural effusion dynamics in patients with pulmonary infarction. The aim of this study was to investigate the relationship between possible factors contributing to the development of pleural effusion, its size and time course in patients with pulmonary infarctions.
**Methods and Materials:** CTPA studies from 103 patients with pulmonary infarction were retrospectively analyzed along with the following data: patient comorbidities, size of pulmonary infarction, location and size of pleural effusion and the time between the onset of clinical symptoms of pulmonary infarction and CTPA study.
**Results:** A highly statistically significant difference (p = 0.005) was found in the mean size of pulmonary infarction in patients with effusion (34.5 $cm^3$) compared to those without it (14.3 $cm^3$). The size of the effusion peaked between $4^{th}$ - $5^{th}$ day after the onset of clinical symptoms of pulmonary infarction. In the first 5 days after the onset of clinical symptoms, a highly significant correlation (p = 0.001) was found between the size of the effusion and time with an approximate rate of increase in effusion layer of 1.3 mm/12 h.
**Conclusion:** Patients with a pleural effusion are more likely to have a larger pulmonary infarction than those without it. If present, the effusion can be expected to increase in a relatively slow linear fashion in the first 5 days after the onset of clinical symptoms of pulmonary infarction.

**B-1609** 11:27
**Pulmonary arterial enlargement predicts long-term survival in COPD patients**
A. Ezponda Casajús, J. De Torres, A. Alcaide, A. Campo, J. Bertó, J. González, J. Zulueta, G. Bastarrika Alemañ; *Pamplona/ES (aezponda@unav.es)*

**Purpose:** Pulmonary artery enlargement (PAE) is associated with exacerbations in chronic obstructive pulmonary disease (COPD) and with survival in moderate to severe patients. The potential role of PAE in survival prediction has not been compared with other clinical and physiological prognostic markers.
**Methods and Materials:** In 188 patients with COPD, PA diameter was measured on a chest CT. The following clinical and physiological parameters were registered: age, gender, smoking status, pack-years history, dyspnoea, lung function, exercise capacity, body mass index, BODE index and history of exacerbations in year prior to enrolment. Proportional Cox regression analysis determined the best predictor of all-cause survival.
**Results:** During a mean follow-up of 83±42 months, 43 patients died. Age, pack-years history, smoking status, BMI, FEV1%, six-minute walking distance, Modified Medical Research Council dyspnea scale (MMRC), BODE index, exacerbation rate prior to enrolment, PA diameter and PAE (diameter ≥30mm) were associated with survival. In the multivariable analysis, age (HR 1.08; 95% CI 1.03±1.12, p<0.001) and PAE (HR 2.78; 95%CI 1.35±5.75, p = 0.006) were the most powerful parameters associated with all-cause mortality.
**Conclusion:** In this prospective observational study of COPD patients with mild to moderate airflow limitation, PAE was the best predictor of long-term survival along with age.

**B-1610** 11:35
**Synchrotron phase-contrast imaging of the lung: a proof-of-concept study on porcine lungs in a human-scale chest phantom**
W. Wagner[1], F. Wuennemann[1], J. Biederer[2], P. Konietzke[1], W. Stiller[1], M.O. Wielpütz[1], H.-U. Kauczor[1], G. Tromba[3], C. Dullin[4]; [1]*Heidelberg/DE*, [2]*Sehheim-Jugenheim/DE*, [3]*Trieste/IT*, [4]*Göttingen/DE (willi.wagner@med.uni-heidelberg.de)*

**Purpose:** To prove the applicability of in-line free propagation phase-contrast synchrotron tomography on a human scale using a chest phantom with ventilated fresh porcine lungs.
**Methods and Materials:** Freshly excised heart and lung explants were positioned in an ARTIChest chest phantom. Artificial lung nodules were induced by injection of 3% agarose gel, containing either 0.5% or 1.0% of a clinical contrast agent with an iodine content of 300 mg/ml. Local areas of interest were imaged with a pixel size of 100 micrometer at the SYRMEP beamline of the Italian synchrotron light source Elettra and compared to standard imaging using a clinical Philips iCT 256 CT scanner.
**Results:** Synchrotron phase-contrast lung imaging yields a high-resolution depiction of anatomical hallmarks of healthy lungs and artificial lung nodules. Interlobular septa, margins of subsegmental bronchi and pulmonary blood vessels can be visualized in superior detail compared to clinical CT scanners. 33 mGy air kerma were measured using the clinical scanner compared with 13 mGy in synchrotron acquisitions (or 4 mGy with a third of the projections). We achieved a dose reduction by 60 to 88%, respectively.
**Conclusion:** We demonstrate that synchrotron-based local-area free propagation phase-contrast CT can be performed on a human-scale chest phantom prepped with fresh porcine lungs. Moreover, we demonstrated that improved image quality by PBI allows for a more detailed characterization of anatomical landmarks of healthy lungs and the assessment of artificial lung nodules.

**B-1611** 11:43
**Preoperative multi-detector CT scoring criteria for assessing pericardial, lung, and phrenic nerve invasion of thymoma**
S. Lee, S. Yoon; *Seoul/KR (eesanghyup@gmail.com)*

**Purpose:** To devise new preoperative multi-detector CT (MDCT) criteria for pericardial, lung, and phrenic nerve invasion of thymoma.
**Methods and Materials:** We retrospectively reviewed 224 consecutive patients with resected thymoma who underwent a preoperative MDCT scan. Morphologic and ancillary CT findings were evaluated for the invasion of pericardium, lung, and phrenic nerve. Multivariate logistic regression analyses were performed for identifying significant CT findings, and we devised CT scoring criteria for each structural invasion.
**Results:** On multivariate logistic regression, pericardial invasion was associated with pericardial crossing sign (OR, 60.3 [95%CI, 8.3-438.7]; *p*<.001), pericardial tail sign (OR, 42.3 [95%CI, 9.6-187.4]; *p*<.001), and pericardial thickening (OR, 5.2 [95%CI, 1.3-20.4]; *p*=0.019). 2 or higher pericardial invasion score provided the sensitivity/specificity of 76.2%/96.6%. Lung invasion was associated with microlobulation (OR, 33.6 [95%CI, 9.8-114.7]; *p*<.001), ground-glass opacity at surrounding lung (OR, 11.0 [95%CI, 1.4-88.8]; *p*=0.024), and calcification (OR, 3.4 [95%CI, 1.0-11.6]; *p*=0.048). 3 or higher lung invasion score provided the sensitivity/specificity of 69.6%/96.5%. Phrenic nerve invasion was associated with microlobulation (OR, 16.2 [95%CI, 4.5-58.3]; *p*<.001), tumour coverage along the course of pericardiophrenic vein (OR, 8.0 [95%CI, 2.2-29.1]; *p*=0.002), mediastinal fat infiltration (OR, 5.3 [95%CI, 1.7-16.9]; *p*=0.005), and calcification (OR, 3.9 [95%CI, 1.1-13.5]; *p*=0.030). 2 or higher phrenic nerve invasion score provided the sensitivity/specificity of 65.2%/94.0%.
**Conclusion:** Our preoperative MDCT scoring criteria offered reasonable diagnostic accuracies for pericardial, lung, and phrenic nerve invasion of thymoma, which can help accurate assessment of T2 and T3 descriptors.

**B-1612** 11:51
**A reader study in postmortem x-ray dark-field chest radiographs and correlation with conventional x-ray and CT**
A.A. Fingerle[1], F. De Marco[2], K. Willer[2], P.B. Noel[1], A. Sauter[1], D. Pfeiffer[1], E.J. Rummeny[1], J. Herzen[2], F. Pfeiffer[2]; [1]*Munich/DE*, [2]*Garching/DE* *(alexander.fingerle@tum.de)*

**Purpose:** To describe imaging features in x-ray dark-field radiographs, we performed a reader study on postmortem chest x-ray dark-field images to quantify dark-field signal strength, address intra- and interobserver agreement, grade image quality and correlate dark-field signal changes with findings in conventional x-ray and CT images.
**Methods and Materials:** IRB approval was obtained. 9 human bodies (3 female, 6 male, age range 52-88 years) were imaged with an experimental three-grating asymmetric imaging setup at 70 kVp. Chest x-ray dark-field radiographs and conventional x-rays were simultaneously acquired. CT imaging was performed on a 256-slice MDCT (Brilliance iCT) at 120 kVp. 3 readers evaluated dark-field signal strength in 6 lung regions by visual assessment on a 6-point ordinate scale. Intra- and interobserver agreement were assessed. Image quality was graded. Dark-field signal changes were correlated with findings in conventional x-ray and CT images.
**Results:** Dark-field signal strength increases from the apex to the base of the lungs, correlating with the amount of lung tissue. Intra- and interobserver agreement were substantial to very good for grading of both dark-field (r=0.793-0.910) and conventional (r=0.772-0.947) x-ray images. Image quality was graded good (4/6) for dark-field and very good (5/6) for conventional images. Pulmonary infiltrates correlated with areas of reduced dark-field signal.
**Conclusion:** Our results demonstrate that the dark-field signal shows significant differences depending on lung region and existing pathologies. Furthermore, the dark-field signal can be quantitatively correlated to visual assessment, providing further impetus for clinical studies addressing specific diseases of the lungs.

**10:30 - 12:00** **Room M 1**

### Cardiac

### SS 1803

### Advanced cardiac MR for evaluation of left ventricular structure and function

*Moderators:*
N.N.
R.G. Chelu; Rotterdam/NL

**B-1613** 10:30
**Comparison of signal intesity ratio, extent and transmurality between a novel LGE black-blood and standard LGE bright-blood sequences in patients with ischaemic heart disease**
G. Muscogiuri[1], M. Gatti[2], S. Dell'Aversana[3], M. Guglielmo[1], A. Baggiano[1], A. Guaricci[4], M. Pepi[1], G. Pontone[1]; [1]*Milan/IT*, [2]*Turin/IT*, [3]*Naples/IT*, [4]*Bari/IT* *(g.muscogiuri@hotmail.it)*

**Purpose:** To assess the reliability of a novel dark-blood LGE (DBLGE) technique compared to standard bright-blood LGE (SBBLGE) sequence in patients with ischaemic cardiomyopathy.
**Methods and Materials:** This prospective study included 78 patients (63.1 ± 12.6 years, 62 males) with clinical history of ischaemic cardiomyopathy who underwent CMR at 1.5T (Discovery MR450w, GE Healthcare, Waukesha, WI) with postcontrast SBBLGE and DBLGE acquisition. Two observers performed the imaging analysis in a double-blinded fashion. The endpoints were a) qualitative and quantitative analyses of signal intensity ratio (SIR); b) n° segments involved; c) transmurality index (i.e. 0-25%; 25-50%, 50-75% and 75-100%); d) papillary muscle enhancement; e) microvascular occlusion (MVO). A cut-off p value<0.05 was considered statistically significant.
**Results:** There were no interobserver variability (all p>0.05). Subjective image quality in DBLGE compared to SBBLGE was higher for the discrimination between LGE and blood signal (p<0.001), inferior (p<0.001) between LGE and myocardium and similar between blood and myocardium (p=0.56). DBLGE provided higher SIR between LGE and blood signal (1.18±1.15 vs 0.18±0.42; p<0.001), lower SIR between LGE and myocardium (0.91±4.95 vs 1.96±1.64; p<0.001) and between blood and myocardium (-0.26±0.71 vs 1.57±1.26; p<0.001). The n° segments involved was similar (p = 0.08). The transmurality index was inferior for DBLGE (3.09±1.02 vs 3.30±1.11; p=0.007). DBLGE was superior in identifying papillary muscle hyperenhancement (25 vs 17 cases; p<0.001) and inferior in MVO detection (7 vs 12 cases; p<0.001).
**Conclusion:** The DBLGE sequences when compared to SBBLGE provided better contrast between LGE and blood pool, seemed to be superior in identifying papillary muscle hyperenhancement, whereas underestimated the trasmurality extension of LGE and the presence of MVO.

**B-1614** 10:38
**Diagnostic accuracy of single-shot two-dimensional multisegment late gadolinium enhancement in ischaemic and non-ischaemic cardiomyopathy**
G. Muscogiuri[1], M. Gatti[2], S. Dell'Aversana[3], S. Pica[1], M. Guglielmo[1], A. Baggiano[1], A. Guaricci[4], G. Pontone[1]; [1]*Milan/IT*, [2]*Turin/IT*, [3]*Naples/IT*, [4]*Bari/IT (g.muscogiuri@hotmail.it)*

**Purpose:** The aim of this manuscript is to assess the reliability of single-shot two-dimensional multi-slice late gadolinium enhancement ($2D\text{-}MS_{LGE}$) compared to standard single slice two-dimensional inversion recovery segmented gradient echo ($2D\text{-}SS_{LGE}$).
**Methods and Materials:** Sixty-seven patients who underwent clinically indicated cardiac magnetic resonance imaging (CMR) were enrolled. Image quality was assessed using a 4-point scale. Segments positive for LGE were classified as ischaemic or non-ischaemic for $2D\text{-}MS_{LGE}$ and $2D\text{-}SS_{LGE}$. Interobserver and intraobserver variability was assessed with both sequences. The endpoints were a) detection of myocardial segments involved by LGE; b) classification of LGE as ischaemic and non-ischaemic pattern. Sensitivity, specificity, positive predictive value and negative predictive and diagnostic accuracy values were calculated for the two endpoints.
**Results:** $2D\text{-}MS_{LGE}$ and $2D\text{-}SS_{LGE}$ were successfully performed in all patients with comparable image quality (1.56 ± 0.59 vs. 1.54 ± 0.58, p=0.84). For the overall population, $2D\text{-}MS_{LGE}$ correctly identified 1093 out of 1139 myocardial segments involved by LGE [96%; CI95: 95%-97%] as compared to $2D\text{-}SS_{LGE}$. Similarly, $2D\text{-}MS_{LGE}$ correctly identified 1128 out of 1139 [99%; CI95: 98%-99%] and 1108 out of 1139 [97%; CI95: 96%-98%]of non-ischaemic and ischaemic LGE pattern. Interobserver and intraobserver variabilities for quantification of LGE using $2D\text{-}MS_{LGE}$ were, respectively, 0.98 and 0.99. The acquisition time was shorter for $2D\text{-}MS_{LGE}$ as compared to $2D\text{-}SS_{LGE}$ (2.0±0.5 min vs. 6.0±2.0 min, p=0.01).
**Conclusion:** $2D\text{-}MS_{LGE}$ seems to be a reliable tool in both ischaemic and non-ischaemic cardiac disease as compared to $2D\text{-}SS_{LGE}$ with the advantage to require lower scan time and breath-hold.

**B-1615** 10:46
**Left ventricle volumes and function assessment with cardiac magnetic resonance four-dimensional flow imaging**
M. Guglielmo[1], A. Baggiano[1], G. Muscogiuri[1], A.I. Guaricci[2], D. Andreini[1], F. Alamanni[1], M. Pepi[1], G. Pontone[1]; [1]*Milan/IT*, [2]*Bari/IT* *(g.muscogiuri@hotmail.it)*

**Purpose:** Aim of this study is to compare 4D flow, echo and CMR quantification of LV volumes and LVEF using CMR SSFP cine sequences as the reference standard.
**Methods and Materials:** 10 consecutive patients (age: 59 ± 10) with indication to mitral valve plasty (MVP) for severe mitral regurgitation were enrolled. All patients underwent a presurgical assessment of LV end-diastolic (LVEDV), end-systolic (LVESV) volume and LVEF with CMR SSFP cine, 4D flow imaging and TTE. 3 to 5 months after the surgery all the measurements were repeated. Data were analysed using Student's t test, Bland-Altman plots, and intraclass correlation coefficient (ICC).
**Results:** Subjective image quality was lower for CMR 4D imaging compared to SSFP cine imaging. There was a good correlation between CMR SSFP imaging and both TTE and 4D flow imaging measurements. However, higher LVEDV and higher LVESV were found with CMR SSFP imaging as compared to CMR 4D imaging and TTE both at presurgery and postsurgery evaluations. LVEF was comparable between CMR SSFP imaging, CMR 4D imaging (mean difference with CMR SSFP imaging 2.4%) and TTE (mean difference with CMR SSFP imaging 3.5%). Reproducibility for CMR SSFP imaging was excellent. Reproducibility for echocardiography and 4D imaging was moderate.
**Conclusion:** CMR 4D flow imaging without contrast media is a new technique that provides an accurate evaluation of LV ejection fraction with a good correlation with CMR SSFP measurements. However, our data show an underestimation of LV volumes with CMR 4D imaging compared to CMR SSFP assessment.

**B-1616** 10:54
**Comparison of standard 3D LGE imaging with novel short inversion time 3D LGE Imaging in patients after myocardial infarction**
M. Polacin, M. Gastl, I. Kapos, A. Gotschy, J. von Spiczak, H. Alkadhi, R. Manka; *Zurich/CH (Malgorzata.Polacin@usz.ch)*

**Purpose:** Late gadolinium enhancement (LGE) visualizes myocardial scar and fibrosis. After myocardial infarction (MI), subendocardial infarcts can be missed due to poor contrast between the blood pool and subendocardium. The aim of this study was to evaluate the benefit of 3D LGE imaging using an inversion recovery sequence with a fixed, short inversion time (TI =100 ms) (short 3D LGE) over the standard 3D LGE imaging with an adjusted TI (3D LGE).
**Methods and Materials:** 3D LGE and short3D LGE (same spatial resolution 1.2 x 1.2 mm², slice- thickness 8 mm; field of view, 350 x 350 mm²) were acquired in 27 patients with MI (8 female, mean age 64.8 ± 12 years) at 1.5T (Achieva, Philips, Best, Netherlands). Two independent, blinded readers evaluated 459 segments (AHA 17-segment model) using a 5-point Likert scale in terms of scar visibility. Contrast- to- noise ratio (CNR) between scar and blood pool was calculated.
**Results:** 3D LGE showed 98 infarcted segments out of 459 (21.4%), short3D LGE revealed 107 segments (23.3%). Short3D LGE demonstrated a better scar visibility (4.3 vs 2.8, P < 0.01) and better CNR between scar and blood pool (806.7 ± 256 vs. 209.5 ± 149, P < 0.01). Agreement between the readers was moderate for 3D LGE and excellent for short3D LGE (weighted κ = 0.52 vs. 0.78).
**Conclusion:** Short3D LGE provided a very good scar visualization and could be used additionally to standard 3D LGE imaging, especially in patients with subendocardial scarring and suboptimal nulling of the myocardium.

**B-1617** 11:02
**Cardiac magnetic resonance for response prediction to Levosimendan treatment in patients with acute decompensated heart failure prior to cardiac surgery**
A. Wielandner, F. Gremmel, R. Laggner, D. Senn, A. Zuckermann, C. Loewe, D. Beitzke; *Vienna/AT (alice.wielandner@meduniwien.ac.at)*

**Purpose:** Levosimendan may be used in patients with chronic heart failure and acute left ventricular dysfunction to improve left ventricular (LV) function and reduce LV size. So far, the impact of the myocardial composition on therapy response has not been investigated. Cardiac magnetic resonance (CMR) is a well established method to assess both LV function and composition and therefore may be used to predict treatment response to Levosimendan. This retrospective analyses is aimed to evaluate the impact of myocardial scar burden on the therapy response to Levosimendan in patients with acute decompensated heart failure prior to cardiac surgery.
**Methods and Materials:** Forty-one patients underwent CMR scan before and immediately after administration of Levosimendan. LV ejection fraction (LVEF) LV end-diastolic and end-systolic volumes (LVEDV / LVESV normalized to body surface), stroke volume (SV), late gadolinium enhancement (LGE) in % of the LV mass as well as proBNP levels were obtained. Patients were additionally grouped presence and absence of LV scar.
**Results:** There was a decline of LVEF, LVEDV, LVESV, SV and proBNP after Levosimendan administration (mean value before/after: LVEF 28.5 /27.4%, n.s.; LVEDV 131.7/123.6ml/m²,p<0.00; LVESV 95.9/86.64ml/m², p<0.00; SV 35.8 vs 33.2ml/m², n.s.; proBNP 6069/4498pg/mL,p<0.00). No significant correlation between scar burden (%) and LVEDV, LVESV and EF changes could be found. Further, sub-group analysis (presence or absence of scar) revealed no significant differences between the groups (p>0.05).
**Conclusion:** Levosimendan effectively reduces LV size in patients with acute decompensated heart failure. LV scar burden (%) does not influence the therapeutic response.

**B-1618** 11:10
**Noninvasive assessment of myocardial oxygenation after acute myocardial infarction with breath-hold-induced blood oxygen level-dependent imaging**
K. Shi, Z.-G. Yang, Y.-K. Guo, S. Huang, X. Li; *Chengdu/CN (kshi0110@qq.com)*

**Purpose:** Blood oxygen level-dependent (BOLD) imaging is a noninvasive MR technique for determining myocardial oxygenation. In recent years, the safety profile when performing stress status has raised serious concerns in clinical practice. The purpose of this study was to investigate feasibility of breath-hold (BH)-induced BOLD imaging without adenosine administration in acute myocardial infarction (AMI).
**Methods and Materials:** Seven healthy rabbits were treated with different hypoxia-inhaled gases and adenosine administration, whereas the other seven healthy rabbits with different intervals of BH and adenosine administration. Subsequently, nine AMI rabbits received an optimal interval of BH and compared the diagnostic performance with adenosine administration. Myocardial signal intensity (SI) alterations were monitored when inhaled hypoxia gases, BH manoeuvres, as well as adenosine administration, respectively.
**Results:** Myocardial BOLD SI increased as inhaled oxygen concentration decreased. BOLD imaging performed when BH lasted from 30 to 60 seconds yielded similar BOLD SI increase (r ranged from 0.40 to 0.62; all P < 0.05) as compared with adenosine administration. Moreover, BH lasted for 30 seconds seems to be optimal to achieve the diagnostic performance with minimal interval (all P > 0.05). When applied 30 seconds' BH in AMI rabbits, it showed significant effectiveness for assessing myocardial response among infarcted, salvaged and remote myocardium, as compared with adenosine administration (r = 0.89; P < 0.05).
**Conclusion:** BH-induced BOLD imaging achieves similar diagnostic performance for assessing myocardial oxygenation compared with adenosine administration. It is likely a safer and more tolerable alternative method for AMI patients.

**B-1619** 11:18
**Alterations of the mitral valve vortex ring in ischaemic heart disease: an explorative 4D-flow study**
C. Kräuter[1], U. Reiter[1], C. Reiter[1], A. Schmidt[1], A. Greiser[2], M. Masana[3], M.H. Fuchsjäger[1], R. Stollberger[1], G. Reiter[1]; [1]*Graz/AT*, [2]*Erlangen/DE*, [3]*Barcelona/ES (corina.kraeuter@medunigraz.at)*

**Purpose:** Blood flow into the left ventricle is accompanied by formation of a three-dimensional (3D) vortex ring at the mitral valve leaflets, which is considered to support ventricular filling and diastolic function. The aim of this study was to compare vortex ring properties of patients with chronic ischaemic heart disease (IHD) and healthy controls derived from cardiac magnetic resonance four-dimensional phase-contrast (4D-flow) measurements.
**Methods and Materials:** 10 subjects (3 IHD patients and 7 age-matched healthy controls) underwent left heart 4D-flow imaging at 3T. Pre-processing of velocity data as well as automated Q-criterion-based extraction and evaluation of 3D mitral valve vortex rings were performed by prototype (4DFlow, Siemens Healthcare) and in-house software. Vortex ring properties in patients and controls were compared by t-test.
**Results:** In all subjects, vortex rings were present during early and late diastolic filling and dissolved during diastasis. Duration of existence of early diastolic vortex rings was longer in patients compared to controls (226±50ms vs. 150±37ms, p=0.03), whereas their duration of existence did not differ in late diastole (132±27ms vs. 139±20ms, p=ns). Early diastolic peak mean vorticity was lower in patients compared to controls (56±5 $s^{-1}$ vs. 89±14 $s^{-1}$, p<0.01), while its delay to mitral valve opening was longer (170±13ms vs. 96±15ms, p<0.01). Late diastolic peak mean vorticity did not reveal differences (61±7 $s^{-1}$ vs. 74±12 $s^{-1}$, p=ns).
**Conclusion:** Early diastolic mitral valve vortex ring formation differs between IHD patients and healthy controls. Vortex ring properties derived from 4D-flow imaging might represent new metrics of diastolic function.
*Author Disclosures:*
**A. Greiser:** Employee; Siemens Healthcare GmbH. **G. Reiter:** Employee; Siemens Healthcare Diagnostics GmbH.

**B-1620** 11:26
**4D- vs 2D-flow imaging in the evaluation of transmitral velocity profiles: comparison with echocardiography**
C. Reiter[1], C. Kräuter[1], E. Kolesnik[1], G. Reiter[1], A. Greiser[2], A. Schmidt[1], D. Scherr[1], M.H. Fuchsjäger[1], U. Reiter[1]; [1]*Graz/AT*, [2]*Erlangen/DE (Clemens.Reiter@medunigraz.at)*

**Purpose:** Transmitral peak velocity profiles assessed by Doppler-echocardiography represent important markers of diastolic left ventricular function. The purpose of the present study was to evaluate if cardiac magnetic resonance (CMR) four-dimensional phase-contrast imaging (4D-flow) provides better estimates for transmitral velocities than standard two-dimensional phase-contrast measurements (2D-flow) compared with echocardiography, respectively.
**Methods and Materials:** 53 subjects without signs of heart failure (male/female, 22/31; age, 62±8 years) underwent echocardiography as well as CMR whole-heart 4D-flow imaging and prototype navigator-gated through-plane velocity-encoded 2D-flow imaging in a plane positioned at the tips of opened mitral leaflets. Peak early (E) and late (A) diastolic transmitral velocities were evaluated in compliance with the guidelines for echocardiography, with routine software in case of 2D-flow measurements, and with prototype software from maximum velocities within the transmitral inflow tract in case of 4D-flow measurements. Comparisons of CMR derived E, A and E/A ratios with corresponding echocardiographic parameters were performed by means of correlation and Bland-Altman analysis.
**Results:** Echocardiographic peak velocities (E=76±15cm/s; A=73±16cm/s) were underestimated more severely by 2D-flow (E=65±12cm/s, p<0.001; A=55±12cm/s; p<0.001) than by 4D-flow measurements (E=73±14cm/s, p=ns; A=67±13cm/s, p<0.001). Echocardiographic E/A ratio (1.08±0.28) differed from 2D-flow E/A ratio (1.26±0.41, p<0.05), the correlation was strong (r=0.61), and the standard deviation of errors was large (SD=0.33). 4D-flow E/A ratio did not exhibit a bias to echocardiography (1.12±0.27, p=ns), the correlation was very strong (r=0.81), and the standard deviation of errors was smaller (SD=0.17).
**Conclusion:** Compared with echocardiography, CMR 4D-flow is superior to 2D-flow imaging in assessment of transmitral velocity profiles.
*Author Disclosures:*
**G. Reiter:** Employee; Research and Development, Siemens Healthcare Diagnostics GmbH, Graz, Austria. **A. Greiser:** Employee; Siemens Healthcare GmbH, Erlangen, Germany.

**B-1622** 11:42
**Evaluation of the prognostic value of visually detected cardiac MRI findings in asymptomatic diabetics and healthy volunteers**
N. Panagiotopoulos[1], N. Fechtrup[1], T.H. Oechtering[1], U. Roggenbuck[2], K. Nassenstein[2], J. Barkhausen[1], P. Hunold[1]; [1]*Lübeck/DE*, [2]*Essen/DE (nikolaos.panagiotopoulos@uksh.de)*

**Purpose:** To evaluate the prevalence and prognostic value of wall motion abnormalities (WMA), late gadolinium enhancement (LGE), and perfusion defects (PD) in cardiac MRI (CMR) in asymptomatic diabetics (D) and non-diabetics (ND).
**Methods and Materials:** CMRs of 44 diabetics and 228 non-diabetics (64±7y, 32% female, comparable cardiovascular risk) of the Heinz-Nixdorf Recall Study were screened for pathological findings. Primary endpoints (PEP: myocardial infarction, sudden cardiac death) and secondary endpoints (SEP: overall mortality, other vascular diseases) were defined. Distribution of pathologies between diabetics and non-diabetics, and their association with endpoints were analysed using Mann-Whitney-Wilcoxon, Fisher's exact test, and Cox regression.
**Results:** Diabetics showed more often LGE (D=18%, ND=6%; p=0.01), PD (D=23%, ND=9%; p<0.01), and WMA (D=14%, ND=9%; p=0.12) than non-diabetics. During the observation period of 10 years, 8/228 non-diabetics, and 3/44 diabetics reached PEPs, while 40/228 non-diabetics, and 12/44 diabetics reached SEPs. A higher percentage of individuals reaching PEP had LGE than those not reaching PEP (D: 33% vs 17%, p=0.38, ND: 12% vs 6%, p=0.32). Individuals reaching SEP had more often LGE than those not reaching SEP (ND: 13% vs 4%, p=0.02; D: 33% vs 10%, p=0.06). In diabetics, PD were accompanied by a higher incidence of SEP (p=0.03). In non-diabetics, hazard ratio (HR, [confidence interval]) for SEP was increased if LGE or WMA were present ($HR_{LGE}$=2.2 [0.84;5.70], p=0.11; $HR_{WMA}$=1.4 [0.51;3.70]).
**Conclusion:** Diabetics presented significantly more pathological CMR findings than non-diabetics. Despite a follow-up period of 10 years and a sizeable cohort of 272 individuals, the small number of reached endpoints did not permit a precise evaluation of the prognostic value of CMR findings.
*Author Disclosures:*
**N. Panagiotopoulos:** Research/Grant Support; DFG: GZ: PA 2878/1-1 AOBJ: 628773.

**B-1623** 11:50
**Prognostic validity of stress cardiac magnetic resonance (CMR) in the intermediate-long term outcome assessment of known-CAD patients**
P. Palumbo, E. Cannizzaro, A. Corridore, C. De Cataldo, S. Torlone, M. Di Luzio, A. Di Sibio, E. Di Cesare, C. Masciocchi; *L'Aquila/IT (palumbopierpaolo89@gmail.com)*

**Purpose:** The need for a prognostic assessment of CAD patients is continuously increasing. The purpose of our study was to assess the prognostic role of Adenosine Stress CMR compared to CCTA in the outcome of heart patients.
**Methods and Materials:** 116 known-CAD patients who underwent first Coronary CTA and then stress CMR were selected retrospectively, with an average interval of 2.26 ± (1.35) months between the two examination. Two group analysis was performed according to the imaging-findings (stenosis severity, perfusion defect and LGE) and to the patient history (moderate-to-severe atherosclerotic plaques, stenting or CABG). Primary and secondary end-point was defined (MACE and clinical worsening respectively). The mean follow-up was 38.96 ± (19.35) months.
**Results:** Among the imaging-findings, a positive logistic regression association between LGE and MACE (Odds ratio 2.16, p = 0.048) was detected. A significant Log-Rank test was found between positive CCTA and stress MRI findings patients with MACE. Only perfusion defect showed a significant correlation with clinical worsening (Odds Ratio 5.43, p = 0.032), as confirmed by the Log-Rank test which evidence a correlation between clinical worsening with the positive stress MRI alone. No correlation was found between MACE or clinical worsening and patient history.
**Conclusion:** Our study demonstrates the prevalent role of Stress CMR as a predictive prognostic factor in the outcome of heart patient. Stress CMR may be useful in identifying patients at risk of clinical worsening, even in the absence of major events due to an OMT, and in patients with a non-severe CCTA stenosis.

**10:30 - 12:00** **Room M 2**

### Head and Neck

### SS 1808

### Imaging techniques in head and neck radiology

*Moderators:*
N.N.
J. Govindaraj; Chennai/IN

**B-1624** 10:30
**Value of CT image texture analysis in the differential diagnosis of benign and malignant parotid tumours**
L. Yang, T. Jiang; *Beijing/CN (36584269@163.com)*

**Purpose:** To investigate the value of multi-slice spiral CT image texture analysis in differentiating adult benign and malignant parotid gland tumour.
**Methods and Materials:** Retrospective analysis was carried out in 33 patients with pathologically confirmed parotid gland tumour from August 2013 to April 2018. All patients were proven by pathological findings and underwent conventional and contrast-enhanced CT scan before therapy. According to the pathological findings the patients were divided into benign and malignant groups (22 and 11 cases, respectively). Texture features of the lesions were extracted by manually drawing ROI on the maximum level of tumour in conventional and enhanced CT imaging.The CT texture parameters between BPT and MPT groups were compared. ROC curve analysis was performed regarding the statistically significant parameters and the areas under curve (AUC) were calculated.
**Results:** CT texture parameter Cluster Shade, Sum entropy; Gray Level Non Uniformity, Large Area High Gray Level Emphasis, Contrast, Zone Entropy, Gray Level Variance, Sum Entropy; Entropy, IDMN, Sum Entropy, Difference Entropy, Gray Level Non Uniformity Normalized, Small Area Low Gray Level Emphasis were significant difference between BPT and MPT group (*P*<0.05). The best CT texture parameter that differentiated the benign and malignant parotid gland tumour was Sum Entropy of CT venous phase (AUC=0.88, P<0.05). With Sum Entropy≤1.53 as diagnosis threshold in differentiating benign parotid gland tumour, the sensitivity and specificity were 100% and 72.70%, respectively.
**Conclusion:** There was significant difference in CT texture analysis parameters between benign and malignant parotid gland tumours. CT texture analysis has certain clinical application values in differentiating parotid tumours.

**B-1625** 10:38
**Virtual non-contrast reconstructions derived from DECT cannot replace true native scans in head and neck imaging**
A.M.J.L. van Kroonenburgh, S.M.J. van Kuijk, A.A. Jacobi-Postma; *Maastricht/NL (anna.van.kroonenburgh@mumc.nl)*

**Purpose:** Virtual non-contrast (VNC) scans from Dual energy CT are thought to reflect True non-contrast (TNC) images and could therefore be used as a substitute to lower radiation dose. The general accepted difference in HU measurement between VNC and TNC is 15 HU points in order to accept VNC as a substitute for TNC. We investigated whether VNC reflects TNC in the head and neck region.
**Methods and Materials:** From November 2016 until June 2018 forty-seven patients underwent a DECT scan as part of their regular diagnostic work up for primary hyperparathyroidism with TNC, 30 s and 50 s postcontrast DECT scan of the neck. VNC images were calculated from postcontrast scans. Best fit ROIs were placed in thyroid tissue, parathyroid adenoma, lymph node, carotid artery, jugular vein, fat and sternocleidomastoid muscle. VNC densities were compared to TNC. Also difference in VNC 30sec densities and VNC 50sec densities were compared.
**Results:** Differences in mean density between TNC and VNC of the organs were as follows: Thyroid 54,1 HU (p<0,001); parathyroid adenoma 20,2 HU (p<0,001); Lymph node 22,3 HU (p<0,001); Carotid artery 15,5 HU (p<0,001); Jugular vein 14,4 HU (p<0,001); Fat 61,2 HU p<0,001); Muscle 11,8 HU (p<0,001). The mean HU value between 30 sec and 50 sec did not differ more than 5 HU.
**Conclusion:** The mean difference in density measurements exceeds far the limit of 15 HU in thyroid tissue, this possibly reflects the intrinsic iodine of the thyroid. Thus VNC cannot replace TNC in head and neck imaging.
*Author Disclosures:*
**A.M.J.L. van Kroonenburgh:** Author; Institutional grant from Siemens. Investigator; Institutional grant from Siemens. Speaker; Institutional grant from Siemens. **A.A. Jacobi-Postma:** Author; Institutional grant from Siemens.

**B-1626** 10:46
**Machine learning analysis in the prediction of tumour grade and nodal involvement in oropharyngeal and oral cavity squamocellular carcinoma using CT-derived texture features**
V. Romeo, R. Cuocolo, L. Ugga, S. Cocozza, F. Verde, S. Dell'Aversana, A. Elefante, S. Staibano, A. Brunetti; *Naples/IT (valeria.romeo@unina.it)*

**Purpose:** To assess the diagnostic accuracy of CT texture analysis (TA) features in predicting tumour grade and nodal involvement in oropharyngeal (OP) and oral cavity (OC) squamocellular carcinoma (SCC) using a machine learning approach.
**Methods and Materials:** Forty patients with histologically proven OP (n=7) and OC (n=33) SCC were retrospectively evaluated. Lesions were segmented on contrast-enhanced CT images to extract first, second and higher order TA features. Different feature selection methods and machine learning algorithms were tested to obtain the highest percentage of correctly classified instances for prediction of tumour grade (G2 or G3), nodal involvement (presence/absence of metastatic lymph nodes) and N stage (N1 or N2).
**Results:** The feature selection method that performed better was k-nearest neighbor algorithm (k-NN) for all groups. Selected features were 1) root mean square and long run emphasis; 2) cluster prominence, dissimilarity, long-run low-gray-level emphasis (LRLGLE) and long-run high-gray-level emphasis (LRHGLE); and 3) range, mean deviation, surface area, high-gray-level run emphasis, short-run high-gray-level emphasis, LRLGLE and LRHGLE for prediction of tumour grade, nodal involvement and N stage, respectively. The k-NN algorithm performed best in all evaluated tasks, with an accuracy of 91.6 ± 14.04% for grade prediction, 85.5± 19.19% for nodal involvement and 90 ± 19.82% for N stage.
**Conclusion:** Machine learning analysis using CT TA may be reliable in predicting tumour grade and nodal involvement in OP and OC SCC, possibly playing a significant role in defining clinical and surgical strategies.

**B-1627** 10:54
**Added prognostic value of pre-treatment MRI texture analysis in patients with primary nasopharyngeal carcinoma**
J. Fang, J. Mao; *Guangzhou/CN (197284131@qq.com)*

**Purpose:** To determine the prognostic value of pretreatment MRI texture analysis for progression-free survival (PFS) in patients with primary nasopharyngeal carcinoma (NPC).
**Methods and Materials:** Ethical approval by the institutional review board was obtained for this retrospective analysis, and the need to obtain informed consent was waived. This study consisted of 79 patients with primary NPC. Texture analysis of the primary tumour was performed on pretreatment T2-weighted images (T2WIs) and contrast-enhanced T1-weighted images (CE-T1WIs). The Cox proportional hazards model was used to determine the association of texture features, tumour volume and the tumour-node-metastasis (TNM) stage with PFS. Survival curves were plotted using the Kaplan-Meier method. The prognostic performances were evaluated with C-index.
**Results:** Tumour volume (hazard ratio, 1.054; 95% confidence interval [CI]: 1.016-1.093) and CE-T1WI-based uniformity (hazard ratio, 0; 95% CI: 0-0.001) were identified as independent predictors for PFS (P=0.005; P=0.001). Kaplan-Meier analysis showed that smaller tumour volume (less than the cutoff value, 11.699 cm3) and higher CE-T1WI-based uniformity (greater than the cutoff value, 0.856) were associated with improved PFS (P=0.008; P<0.001). The combination of CE-T1WI-based uniformity with tumour volume and the overall stage estimated PFS better (C-index, 0.794; 95% CI: 0.641-0.947) than the tumour volume (C-index, 0.616; 95% CI: 0.463-0.769) or the overall stage (C-index, 0.627; 95% CI: 0.500-0.754) did (P=0.006; P=0.009).
**Conclusion:** A texture parameter of pretreatment MRI, CE-T1WI-based uniformity, can be used to improve the estimation of PFS in NPC patients. Texture analysis of pretreatment MRI has added prognostic value for NPC patients.

**B-1628** 11:02
**Application of the IEC3.1 standard of $CTDI_{100}$ to a supine CBCT device**
B. Keelson[1], D. Buytaert[2], G. Van Gompel[1], J. Casselman[3], K. Bacher[2], Y. De Brucker[1], N. Buls[1]; [1]*Brussels/BE*, [2]*Gent/BE*, [3]*Bruges/BE (bkeelson@etrovub.be)*

**Purpose:** To investigate the feasibility of extending the IEC 3.1 $CTDI_{100}$ as defined by the IAEA to CBCT and compare with device indicated CTDI and an approximation of the "true" CTDI.
**Methods and Materials:** Weighted CTDI (CTDIw) measurements of a 16cm diameter dosimetry phantom were obtained on a Newtom 5G (Newtom, Verona, Italy) CBCT using a 15x5 cm and 15x12 cm field-of-view (indicated as diameter by height).CTDIw following the IEC 3.1 definition was computed by multiplying the CTDIw of a reference collimation (15x5) with the quotient of free-in-air CTDI measurements at the collimation of interest (15x12) and the reference collimation. In addition, to approximate CTDI∞ ("true" CTDI), CTDI measurements were performed in a 32 cm long phantom, constructed as a longitudinal combination of two 16 cm diameter phantoms. For the latter measurements, referred to as CTDI300, an integration length of 30 cm was used i.e. integrated dose over three adjacent positions with the pencil chamber.
**Results:** True $CTDI_w$ values were 22mGy/100mAs and 25,8mGy/100mAs for the 15X5 and 15X12 protocols respectively. These values corresponded to an underestimation by the device Indicated $CTDI_w$ values of 57% and 31% for the 15x12 and 15 x 5 protocols respectively. The IEC 3.1 definition however resulted in underestimations of 18 and 14 % for the 15x12 and 15x5 protocols respectively relative to the true $CTDI_w$.
**Conclusion:** CTDI using the IEC 3.1 method resulted in reasonable deviations from the "true" CTDI and is recommended to allow for comparison of doses between conventional CT and CBCT.

**B-1629** 11:10
**Texture analysis for differentiating between nasopharyngeal cancer and nasopharyngeal malignant lymphoma on unenhanced CT**
H. Tomita, T. Yamashiro, M. Tsubakimoto, G. Iida, S. Murayama; *Nishihara/JP*

**Purpose:** To investigate a usefulness of texture features in differentiating between nasopharyngeal cancer and nasopharyngeal malignant lymphoma (ML) on unenhanced CT.
**Methods and Materials:** Thirty nasopharyngeal tumours including 17 nasopharyngeal cancer and 13 nasopharyngeal ML were identified on 18F-FDG PET/CT. All nasopharyngeal cancer and 7 of 13 nasopharyngeal ML were confirmed by endoscopic biopsy. The remaining 6 patients with ML were diagnosed by lymph node biopsy. On unenhanced CT, 36 texture features were calculated following lesion segmentation on the maximum area. Student's t test and the area under the curve (AUC) were measured to compare values for SUVmax, SUVmean, and 36 texture features. Support vector machine (SVM) was constructed to evaluate diagnostic accuracy and AUC of combinations of texture features with 50 repetition of 5-fold cross validation.
**Results:** Significant differences of SUVmax and SUVmean were not found between nasopharyngeal cancer and nasopharyngeal ML. There were significant differences of texture features as follows: 1 histogram feature ($p$ = 0.0236), 4 grey-level co-occurrence matrix features ($p$ = 0.008-0.03), 2 grey-level run-length matrix features ($p$ = 0.008 and 0.01), 1 neighborhood grey-level different matrix feature ($p$ = 0.006), 2 grey-level zone length matrix features (GLZLM) ($p$ = 0.009-0.012). Maximum AUC of 0.824 were obtained from zone-length non-uniformity in GLZLM. The highest accuracy of the combined texture features using SVM was 79.1% with the AUC of 0.80.
**Conclusion:** Texture features would provide useful information to discriminate between nasopharyngeal cancer and nasopharyngeal ML on unenhanced CT while SUVmax and SUVmean by PET/CT cannot differentiate them.

**B-1630** 11:18
**Dose optimisation in dual-energy CT imaging of the neck: evaluation of acquisition parameters using a 3D-printed patient phantom**
P. Jahnke, B. Hamm, T. Diekhoff; *Berlin/DE (paul.jahnke@charite.de)*

**Purpose:** To evaluate acquisition techniques for dual-energy computed tomography (DECT) of the neck and to deduce optimal scanning parameters for acceptable image quality at minimal dose.
**Methods and Materials:** DECT of the neck was performed with 80 and 135 kVp tube voltage on a 320-row CT, using a radiopaque 3D printed phantom for realistic simulation of a patient contrast CT. Tube currents were varied between 40 and 400 mAs for 80 kVp and 5 and 160 mAs for 135 kVp. Virtual blended images (VBI) and virtual monochromatic images (VMI) were reconstructed with all possible pairings (186 in total). Single energy CT (SECT) was performed with 80, 100, 120 and 135 kVp. Hounsfield units (HU), signal-to-noise ratios (SNR) and contrast-to-noise ratios (CNR) were compared between VBIs, VMIs and SECT. Non-linear regression analysis was performed to deduce optimal DECT settings.
**Results:** 100 and 120 kVe VBI reconstructions overestimated HU slightly (31.68 ± 1.18 HU and 22.03 ± 0.75 HU, respectively). HU of 75 keV VMIs did not differ significantly from 120 kVp SECT. SNR and CNR of VBI were inferior to the corresponding SECT images at similar dose levels. VMI SNR and CNR were highest at 65 keV, but inferior to SECT values at similar dose. Non-linear regression indicates that DECT can achieve approximately 80% SNR and CNR with 120% exposure of the corresponding SECT.
**Conclusion:** Sequential DECT results in significantly inferior image quality per radiation dose compared to clinical standard SECT. Tube currents should be adapted to the desired DECT information.
*Author Disclosures:*
**P. Jahnke:** Grant Recipient; 03EFHBE093. Patent Holder; DE202015104282U1, EP000003135199A1, US020170042501A1.

**B-1631** 11:26
**Dose reduction in CT imaging of the neck - individual performance and synergetic effects of dose reduction techniques in a clinical context**
P. Jahnke, F.B. Schwarz, M. Ziegert, B. Hamm, M. Scheel, T. Diekhoff;
*Berlin/DE (paul.jahnke@charite.de)*

**Purpose:** To systematically evaluate the clinical performance of automated tube potential selection (ATPS), automated tube current modulation (ATCM), iterative reconstruction (IR) and pitch variation for dose reduction in CT imaging of the neck.
**Methods and Materials:** A radiopaque 3D printed patient head and neck phantom was used for realistic simulation of clinical neck imaging. 54 CT acquisitions were performed (80-row CT). Three ATCM settings and three fixed tube currents, ATPS and fixed 120 kVp and three pitch settings were systematically combined. Images were reconstructed with filtered back projection (FBP) and IR. In a first reading session, images were rated by three radiologists with an absolute grading. In a second session, a relative grading was performed by ten radiologists with a five-step rating scale. Dose efficiency of the selected acquisition techniques and synergetic effects were analyzed.
**Results:** Inter-rater reliability was excellent. The highest dose efficiency at acceptable image quality was achieved with ATCM Quality, ATPS, pitch 0.813 and IR. Compared with a clinical standard protocol, the combinations of (ATCM Quality, ATPS, pitch 0.637, IR) and (ATCM Standard, 120 kVp, pitch 0.637, IR) yielded 62% and 58% dose reduction respectively at non-different image quality. ATPS decreased dose more at higher tube currents and was more efficient with IR. FBP required higher tube currents than IR for optimal dose efficiency. Pitch increase added value for IR reconstructed images at fixed tube currents.
**Conclusion:** Optimal acquisition settings depend on the available dose reduction techniques, which contribute unequally to dose reduction in neck CT imaging.
*Author Disclosures:*
**P. Jahnke:** Grant Recipient; 03EFHBE093. Patent Holder; DE202015104282U1, EP000003135199A1, US020170042501A1.
**F.B. Schwarz:** Grant Recipient; 03EFHBE093. **M. Ziegert:** Grant Recipient; 03EFHBE093. **M. Scheel:** Patent Holder; DE202015104282U1, EP000003135199A1, US020170042501A1.

**B-1633** 11:34
**Dual-layer detector CT angiography in preoperative assessment of carotid body tumours: comparison of virtual monoenergetic and polyenergetic images**
Y. Chen, Y. Zhu, H. Liu, K. Xu, S. Yu, T. Su, Z. Zhang, Y. Wang, Z. Jin;
*Beijing/CN (bjchenyu@126.com)*

**Purpose:** The aim was to investigate the ablility of dual-layer CT angiography (DLCTA) in assessment of carotid body tumor (CBT), and comparison of virtual monoenergetic and polyenergetic images.
**Methods and Materials:** Patients with suspected CBT referred to cervicocerebral CTA were retrospectively reviewed. All examinations were performed using a dual-layer detector CT. Polyenergetic images (120keV) and virtual monoenergetic images (40keV to 120keV with a 10 keV increment) were reconstructed. The feeding arteries , Shamblin type were evaluated based on polyenergetic images. Signals (±SD) of the CBT were measured to calculated the SNR. Subjective image quality including visualization of feeding arteries as well as carotid arterial wall were evaluated, using a 4-point Likert scale. Student's t-test and Wilcoxon test were used to determine statistical significance.
**Results:** 17 patients were included into the study. Feeding arteries included external carotid arteries (ECA, n=1), internal carotid arteries (ICA, n=8) and both (n=8). Shamblin type included I (n=3), II (n=5) and III (n=9). SNR in the virtual monoenergetic images were superior to polyenergetic images at kiloeletron volt levels ranging from 40 keV to 50 keV (P<0.05). Visualization of feeding arteries received significantly higher scores at kiloelectron volt levels of 40 keV to 70 keV (p<0.01). Visualization of carotid arterial wall was perceived significantly higher at kiloelectron volt levels of 40 keV to 50 keV (p<0.05).
**Conclusion:** Compared to polyenergetic images, virtual monoenergetic images reconstructed from DLCTA at low keV ranging from 40 to 50 keV improve the objective and subjective image quality of CBT.

**B-1634** 11:42
**Are pre-MRI orbital radiographs always necessary in patients with suspected foreign bodies?**
A. Foran, M.J. Lee; *Dublin/IE (foranat@tcd.ie)*

**Purpose:** To review indications for performing pre-MRI orbital radiograph, to ensure compliance with international guidelines, and to review our protocol for suspected intra-orbital foreign bodies.
**Methods and Materials:** An audit was performed of all pre-MRI orbital radiographs performed between July 2014 and July 2018, positive findings were recorded as well as patients who proceeded to MRI.
**Results:** Between July 2014 and July 2018, 286 patients had pre-MRI orbital radiographs. Reasons included occupational exposure (36), a documented history of foreign body (161) and otherwise unspecified history (89). Only 3 patients overall did not proceed to MRI due to the presence of a radiopaque foreign body within the orbit, all of whom had a documented history of foreign body within the eye.
ACR guidelines recommend pre-MRI orbital radiograph only for patients who have had a history of orbital foreign body for which they sought medical attention. We edited our MR questionnaire to reflect these guidelines. Based on our review of the radiographs of the patients who did not proceed to MRI, we deemed that our radiograph protocol was suboptimal, prompting us to change our radiograph protocol. Our plan following implementation of these changes is to re-audit in 3 months time to assess the number of radiographs performed in the interim.
**Conclusion:** Orbital radiographs were performed in 286 patients, only 3 patients who had an intraorbital foreign body did not proceed to MRI. A new MRI questionnaire reflecting ACR guidelines has been implemented to decrease unnecessary radiographs.

**10:30 - 12:00** **Room M 3**

### Oncologic Imaging

### SS 1816

### Improving the prediction of head and neck malignancies

*Moderators:*
B. Verbist; Leiden/NL
G. Zanirato Rambaldi; Bologna/IT

**B-1635** 10:30
**Microflow and resistance index in suspicious lymph nodes**
P. De Koekkoek-Doll, M.W.M. van den Brekel, W. Vogel, L. Smit, R.G.H. Beets-Tan, M. Maas; *Amsterdam/NL (petra.dkd@gmail.com)*

**Purpose:** Ultrasound-guided fine needle aspiration cytology (FNAC) is one of the most important modalities for nodal staging in head and neck cancer. The US vascularity in metastatic lymph nodes (LN) was reported as a sensitive feature. We aim to improve the sensitivity using non-contrast-enhanced slow tissue flow measurement clinical techniques.
**Methods and Materials:** Prospective study including 123 patients with 217 suspicious nodes. Mean age 62 years (23-87 yrs.). The eL18-4 transducer (Philips) was used for conventional ultrasound (B mode) nodal assessment. Presence of increased peripheral vascularity with microflow imaging (MFI) and measurement of resistance index (RI) values was assessed. FNAC was performed in all nodes and served as the reference standard. Sensitivity and specificity were calculated for capsular vascularisation and the absence of a fatty hilum. Cut-off values for RI were defined.
**Results:** 100/217 nodes were malignant at cytology. 86/106 (86%) of LN with significant MFI were malignant (sensitivity 86%, specificity 83%, PPV 81%, NPV 84%). Absence of a hilum indicated nodal involvement in 83% (80/96) (sensitivity 80%, specificity 86%, PPV 83%, NPV 83%). Combining an absent hilum and presence of MFI correctly predicted 73/81 (90%) malignant nodes (sensitivity 92%, specificity 75%). The RI yielded a sensitivity of 91% and specificity of 50%, with a cut-off of 0.71. In case of RI<0.6, none of the nodes were malignant.
**Conclusion:** Extra-capsular vascularisation (microflow) combined with the absence of a fatty hilum has a high diagnostic performance to detect metastatic nodes.

**B-1636** 10:38
**Ability of computed tomography (CT) radiomic features to differentiate lymphomas from thymic masses in the anterior mediastinum**
M. Kirienko, G. Ninatti, L. Cozzi, N. Gennaro, F. Ricci, C. Carlo Stella, P. Zucali, A. Chiti, M. Sollini; *Milan/IT (gaia.ninatti@st.hunimed.eu)*

**Purpose:** The present study aimed at evaluating the ability of computed tomography (CT) radiomic features to classify anterior mediastinal masses as lymphomas or thymic neoplasms.
**Methods and Materials:** A cohort of 110 patients diagnosed with either thymic neoplasia or lymphoma with at least one mediastinal localization >4 cm in size, and with baseline non-contrast-enhanced CT imaging available at our institution, was retrospectively studied. The cohort was divided into a training and a validation group. Radiomic textural features (n=41) were extracted from manually segmented CT images using LIFEx software. Statistical analysis was performed using the R platform. Combinations of imaging features were used as predictors in linear discriminant analysis (LDA) with backward stepwise variable insertion to classify the lesions as lymphomas or thymic neoplasms. Pathology was used as reference standard. Scoring metrics included analysis of the receiver operating characteristic (ROC) curves in terms of area under the curve (AUC), sensitivity, specificity and accuracy.
**Results:** Fifty-seven patients (27 males, 30 females) were affected by thymic neoplasia (3 thymic carcinoma, 54 thymoma) and fifty-three (20 males, 33 females) by lymphoma (39 Hodgkin's, 12 non-Hodgkin's, 2 ns), median age of 61 and 36 years, respectively. LDA selected 12 CT radiomic features. In the training group, sensitivity, specificity, accuracy and AUC of the model resulted 0.85+-0.06, 0.94+-0.04, 0.90+-0.06 and 0.94+-0.06, respectively; in the validation group, sensitivity, specificity, accuracy and AUC resulted 0.73+-0.07, 0.83+-0.05, 0.77+-0.07 and 0.85+-0.06, respectively.
**Conclusion:** CT radiomic features have the potential to differentiate at diagnosis between lymphomas and thymic masses, sparing the use of invasive diagnostic procedures.
*Author Disclosures:*
**L. Cozzi:** Advisory Board; Varian Medical Systems. **C. Carlo Stella:** Advisory Board; Genenta Science, ADC Therapeutics, Sanofi, Boehringer Ingelheim. Grant Recipient; Rhizen Pharmaceuticals. Speaker; MSD, BMS, Amgen, Janssen, AstraZeneca. **P. Zucali:** Advisory Board; Astellas, Pfizer, BMS, Janssen, MSD, Novartis. Speaker; Astellas, Pfizer, Sanofi, Janssen, BMS, Ipsen, Novartis. **A. Chiti:** Advisory Board; Blue Earth Diagnostics, Advanced Accelerator Applications. Grant Recipient; Sanofi. Speaker; General Electric, Blue Earth Diagnostics, Sirtex Medical System.

**B-1637** 10:46
**Role of diffusion MRI in differentiation of residual/recurrent neck malignancies and post-treatment changes in comparison with histopathology findings**
A. Jajodia, D. Aggarwal, A.S. Rao, R. Aggarwal; *New Delhi/IN*
*(ankushjaj@gmail.com)*

**Purpose:** Role of diffusion-weighted (DW) MR imaging and ADC mapping in differentiating residual or recurrent neck malignancies from post-operative/post-radiation changes with histopathological/cytological correlation and comparison with PET-CT.
**Methods and Materials:** Prospective observational study for a year in 62 post-radiation/post-operative patients suspected to have residual/recurrent tumour of neck with lesion diameter more than 5mm measured on MRI.
**Results:** Mean ADC for recurrent/residual tumours: 1.008 ± 0.220 x 10-3mm$^2$/s - significantly lower than mean ADC value for post-treatment changes of 1.69 ± 0.40 x 10-3mm$^2$/s (p < 0.0001). The overall diagnostic accuracy, positive predictive value (PPV) and negative predictive value (NPV) of the qualitative assessment for the use of DWI in differentiating tumour recurrence from post-treatment changes were 96.6%, 96% and 83.3%, respectively. Upon quantitative analysis of the DW imaging data, a threshold ADC value of 1.3x 10-3 mm$^2$/s used for differentiating between post-treatment changes and recurrent cancers showed the highest combined sensitivity of 94%, specificity of 83.3%, accuracy of 93.6%, positive predictive value of 95.9%, and negative predictive value of 83.3%.
**Conclusion:** DW MRI is a promising non-invasive MRI technique used to differentiate recurrent/residual head and neck malignancies from post-treatment changes based on ADC values. Advantage short scanning time; safely added to standard MRI protocol with minimum patient discomfort. Complementary use of DW and PET/CT imaging may increase diagnostic confidence for differentiating recurrent disease from radiation therapy-induced changes after 6-12 months in post-treatment cases.

**B-1638** 10:54
**MRI-based radiomic to assess lipomatous soft tissue tumours malignancy: a pilot study**
A. Bouhamama[1], B. Leporq[1], F. Lame[2], C. Bihane[1], M. Sdika[1], J.-Y. Blay[1], O. Beuf[1], F. Pilleul[1]; [1]*Lyon/FR*, [2]*Le Gosier/FR*
*(amine.bouhamama@lyon.unicancer.fr)*

**Purpose:** To develop and validate a MRI-based radiomic method to evaluate lipomatous soft tissue tumours malignancy.
**Methods and Materials:** 81 subjects with lipomatous soft tissue tumours including 40 lipomas and 41 atypical lipomatous tumours or well-differentiated liposarcomas with fat-suppressed $T_1$w contrast enhanced MR images available were retrospectively enrolled to constitute the database. For each tumor, 87 radiomic features were extracted. A reduction of learning base dimension was performed from relevancy and reproducibility criteria. Next, to predict malignant lesions, a model was prototyped using a linear support vector machine.
**Results:** After combination with relevancy criterion, 35 features were integrated in the model. To predict malignant tumours, model diagnosis performances were as follow: AUROC = 0.96; sensitivity = 100%; specificity = 90%; positive predictive value = 90.9%; negative predictive value = 100% and overall accuracy = 95.0%.
**Conclusion:** This work demonstrates that it is possible to assess soft tissue lipomatous tumours malignancy with a routinely used MR acquisition in clinical oncology. These encouraging results need to be further confirmed in an external validation population.

**B-1639** 11:02
**MRI histogram and texture analysis as predictors of response to therapy in head and neck malignancies**
R. Balaji, V.K. Anand, G. Krishnamurthi; *Chennai/IN*
*(ravikanthbalaji@gmail.com)*

**Purpose:** To evaluate the use of histogram and texture analysis of metastatic neck nodes from head and neck squamous cell carcinoma (SCC) using fat-suppressed T2-weighted (Fs-T2WI) and diffusion (DW) imaging for the prediction of response to therapy.
**Methods and Materials:** Fifty patients with primary squamous cell carcinomas of head and neck region with metastatic nodes (24 well-, 17 moderately and 9 poorly differentiated SCC) were retrospectively analysed. Quantitative parameters with histogram features (relative mean signal, coefficient of variation, kurtosis and skewness) and grey-level co-occurrence matrix (GLCM) features (contrast, correlation, energy and homogeneity) were calculated using Fs-T2WI and DW data with a manual tumour region of interest (ROI).
**Results:** Relative mean signal and contrast were significantly lower in poorly differentiated SCC (2.3±0.63, 50.1±12.9) compared to both the moderately (4.6±0.81, 79.5±9.8) and well-differentiated SCC (3.1±0.69, 8,7±14.5). The homogeneity in poorly differentiated SCC (2.8±0.1510-1) was higher than that in moderately (2.4±0.1810-1) and well-differentiated SCC (1.9±0.1710-1).
**Conclusion:** Parameters obtained by histogram and texture analysis of Fs-T2WI and DW may be useful for non-invasive prediction of prognosis in metastatic SCC head and neck malignancy.

**B-1640** 11:10
**Lymph node measurement on serial imaging in clinical trials**
M. Bagheri, A. Shafiei, F. Farhadi, A.B. Apolo, L.R. Folio, E.C. Jones, R.M. Summers; *Bethesda, MD/US (mohammad.bagheri@nih.gov)*

**Purpose:** To optimize target node measurement using RECIST-1.1, when they merge or split.
**Methods and Materials:** We identified target nodes either separated from conglomerate nodes (split node) or merged with other nodes (merged node) over time on serial CT in 261 cancer patients enrolled in clinical trials. Average of short axis diameter changes of three other well-circumscribed nodes was measured as ground truth (surrogate nodes). Merged nodes were grouped: One target node merged with adjacent nodes (Group-1) either linearly(1-A), or non-linearly(1-B). Two target nodes merged (Group-2). Correction factors were calculated based on surrogate changes for merged nodes. Short axis of the largest nodal fragment was compared with RECIST and surrogate for split nodes.
**Results:** We identified 46 merged (29 group-1, 17 group-2) and 28 split nodes. Nodal size change per RECIST was similar to surrogates in group 1-A, while there was a significant difference in groups 1-B, 2, and split nodes with surrogates (p<0.001). While all surrogate nodes in group 2 enlarged, RECIST indicated a decreased size in 76% of cases. Furthermore, RECIST indicated an increase in size for 60% of split nodes while their surrogates showed a decrease. Calculated correlation factors for merged lesions in groups 1-A, 1-B, and 2 were 1, 0.75, 1.5 respectively. No significant difference was noted between surrogate measurements and our proposed method of applying a correction factor for merged nodes and measuring the largest fragment when node splits.
**Conclusion:** Our proposed method can improve the accuracy of nodal assessment when they merge or split.
*Author Disclosures:*
**L.R. Folio:** Other; Research agreement with Carestream Health and two unrelated issued patents on image processing/ quantification. **R.M. Summers:** Research/Grant Support; PingAn, NVIDIA. Other; Royalties: iCAD, PingAn, ScanMed, Philips.

**B-1641** 11:18
**Image briefly: the new aphorism for surveillance in testicular cancer**
R. Balaji; *Chennai/IN (ravikanthbalaji@gmail.com)*

**Purpose:** To study the use of limited MR sequences for assessing relapse in surveillance of patients with testicular tumours.
**Methods and Materials:** 130 patients with primary testicular germ cell tumours were included in the study from 2013 to 2018. All patients were followed up every year with MR imaging. Imaging of the abdomen and pelvis covering the inguinal regions was performed using a 1.5T scanner (Achieva, Philips Healthcare, The Best, Netherlands). Axial STIR (respiration triggered) and diffusion sequences (DWIBS free breathing) with b values of 0 and 800 s/mm$^2$ were performed. Imaging was completed in most patients (115) within 12 minutes. 15 patients had breathing difficulties or were claustrophobic and required 15 to 20 minutes.
**Results:** Relapse usually occurred in the first 2 years after therapy predominantly in the retroperitoneal or pelvic nodes. Out of 130 patients, 21 (16%) developed nodal relapse. MR imaging performed with STIR and diffusion sequences identified affected nodes in all patients who then underwent image-guided biopsy and histopathological confirmation.
**Conclusion:** Relapse in testicular cancers almost always involves the retroperitoneal or pelvic nodes. Shortened MR imaging protocols for surveillance in such patients can help replace CT imaging with its attendant radiation hazards.

**B-1642** 11:26
**Conventional ultrasound and ultrasound elastography in non-Hodgkin lymphoma: ultrasound prognostic index compared with the international prognostic index**
J. Jiang, M. Chen; *Shanghai/CN (kkxkk12340@163.com)*

**Purpose:** To explore the predictive value of prognosis for conventional ultrasound (US) and ultrasound elastography (UE) in patients with non-Hodgkin lymphoma (NHL) as ultrasound prognostic index compared with the international prognostic index (IPI).
**Methods and Materials:** A total of 168 patients with NHL were divided into two groups by IPI. The US and UE features of enlarged lymph node in these patients were analysed retrospectively. Ultrasound prognostic index was developed to compare with IPI in complete response (CR) rate.
**Results:** 86 patients with IPI 0, 1, 2 were divided into good prognosis group, while others with IPI 3, 4, 5 into poor prognosis group. Correlation analysis indicated significant correlation between IPI and several US and UE features: long axis diameter (L), short axis diameter (S), hilum status, vascular pattern, elastography score ($P<0.05$). These features were statistically different between two groups ($P<0.05$). Ultrasound prognostic index score was assessed by these features: L>3.2cm, S>1.2 cm, absent hilum, peripheral or mixed vascular pattern, elastography score 3 or 4. The sensitivity, accuracy and AUC value were 80.6%, 72.6% and 0.781 when ultrasound prognostic index≥3 was determined to be the cutoff for poor prognosis. CR rate assessed by ultrasound prognostic index in the low-risk range (ultrasound prognostic index 0 or 1) was better than that assessed by IPI in the low-risk range (IPI 0 or 1).
**Conclusion:** This study suggests the predictive value of US and UE in the prognosis of NHL. Compared with the IPI, ultrasound prognostic index may have a better predictive value in the patients with good prognosis.

**B-1643** 11:34
**Strain elastography ultrasound for lymph node characterisation**
A. Malich, C. Kurrat, D. Predel, N. Bang, I. Papageorgiou; *Nordhausen/DE (ansgar.malich@shk-ndh.de)*

**Purpose:** To evaluate the diagnostic accuracy of strain elastography (SE) in differentiating benign from malignant lymph nodes.
**Methods and Materials:** The study was retrospective for n=53 patients investigated with ultrasound in B-mode and SE in a single session. All patients received either an image-guided stance biopsy or surgical resection, n=30 were found with benign inflammatory changes and n=23 with a neoplastic condition. Applied elastographic metrics: (1) average tumour strain, (2) average reference fat (REF) strain, (3) ratio tumour/REF (strain ratio) and (4) Tsukuba-Ueno scale.
**Results:** The Tsukuba-Ueno class of the malignant lymph nodes was on average score above 3/5 compared to lower than 2 of the benign lesions, p=0.003, Mann-Whitney rank-sum test. The tumour strain and the strain ratio did not differ significantly between benign and malignant lymph nodes, p>0.05, Mann-Whitney rank-sum test. The sensitivity/specificity (Se/Spe) of the Tsukuba-Ueno scale was 61/73% for a cut-off value of 3, and 87/50% for a cut-off of 2. The strain ratio showed a low prognostic value with an area under curve = 0.58. For a cut-off strain ratio 1.63, the Se/Spe was 65/57% (ROC analysis with Youden statistics).
**Conclusion:** Strain elastography is a weak surrogate marker to differentiate between inflammation and lymph node malignancy. The Tsukuba-Ueno scale is the only metric that showed a moderate sensitivity.

**B-1644** 11:42
**Absence of vascularisation in thyroid nodules: new ultrasound feature for stratifying their malignancy risk**
M. Minotti[1], F. De Piano[1], L. Tofanelli[1], G. Giugliano[1], F. Maffini[1], G. Mauri[1], M. Bellomi[1], L. Preda[2], E. De Fiori[1]; [1]*Milan/IT,* [2]*Pavia/IT (laura.tofanelli@unimi.it)*

**Purpose:** To determine if the absence of vascularization in thyroid nodules is an ultrasound feature significantly associated with malignancy.
**Methods and Materials:** This retrospective study evaluated 155 thyroid nodules (46 benign, 109 malignant) in 110 patients according to the following inclusion parameters: solid thyroid nodules, pre-operative US with colour-Doppler evaluation available, surgery performed at our Institution (November 2012- July 2018). One radiologist with 20 years‘ experience scored the nodules according to TI-RADS 2017. Vascularity was also evaluated with colour-Doppler as an additional criteria and classified as: absent, peripheral, mixed, peripheral incomplete, diffusely rough. The association of the absence of vascularization with the risk of malignancy was assessed by sensitivity (SE), specificity (SP), positive predictive value (PPV), negative predictive value (PPN) and diagnostic accuracy (DA).
**Results:** Thyroid nodules without vascularization were 40/155, 2/46 were benign and 38/109 were malignant. The SE, SP, PPV, NPV and DA values related with the absence of vascularization were respectively 35%, 96%, 95%, 38% and 52.9%.
**Conclusion:** This study demonstrated that the absence of vascularization, in addition to TI-RADS classification, could be an useful US feature for better stratify the risk of malignancy in thyroid nodules.

**10:30 - 12:00** **Room M 4**

### GI Tract

### SS 1801b

### Inflammatory bowel disease: what now?

*Moderators:*
N.N.
N.N.

**B-1646** 10:39
**Clinical application of 640 slice spiral CT double low enterography in inflammatory bowel disease**
X. Pan, M. Guo; *Guangzhou/CN (wfbbq@163.com)*

**Purpose:** To investigate the clinical application of 640-slice spiral CT enterography combined with low tube voltage, low contrast injection rate and dose technique in inflammatory bowel disease.
**Methods and Materials:** Forty patients diagnosed as Crohn's disease were randomly divided into two groups. Both groups were scanned by 640-slice CT spiral mode. Double low dose group (group A) was scanned by low dose (100KV), contrast injection rate 2.3 ml/s and low contrast volume 0.8 mL/kg. Routine dose group (group B) was scanned by routine dose (120KV), contrast injection rate 3.5 ml/s and contrast volume 1.2 mL/kg. Computed signal-to-noise ratio (SNR) and contrast-to-noise ratio (CNR) were calculated. Image quality (bowel wall CT value, noise, SNR and CNR), radiation dose and contrast dose were compared and analyzed between the two groups.
**Results:** All patients in double low dose group received volume CT index (CTDIw) about 3.9-8.6 mGy, with an average of 6.3 mGy, significantly less than 25 mGy, and radiation dose significantly reduced ($p < 0.05$). The radiation dose and dose of contrast medium in group A were significantly lower than those in group B ($P < 0.05$). There was no significant difference in image quality scores between the two groups ($P > 0.05$).
**Conclusion:** In CT enterography, spiral scanning combined with low tube voltage, low flow rate and low contrast agent dosage can clearly display the small bowel and its surrounding structures, and reduce the radiation dose and contrast agent dosage (33%) and adverse reactions without significantly reducing the image quality.

**B-1647** 10:47
**Magnetic resonance enterography: is routine administration of gadolinium-based IV contrast really needed for the assessment of Crohn's disease?**
S.J. Sammut, N. Cook, N. Mahmood, R. Balasubramaniam, B. Thomas, A. James; *Stoke-on-Trent/UK (stephen.sammut@uhnm.nhs.uk)*

**Purpose:** MR enterography (MRE) is well established for diagnosing and monitoring Crohn's disease (CD). Standard protocol includes dynamic contrast-enhanced sequences using gadolinium-based contrast agents (GBCAs). GBCAs have recently come under scrutiny for retention in the brain tissue and hence avoidance is desirable. The purpose of this study was to explore if there was a diagnostic benefit of contrast-enhanced MRE (CEMRE) sequences.
**Methods and Materials:** We identified 50 cases for retrospective review performed over 3 months (Dec 2017 - Feb 2018) at our University Teaching Hospital. 10 cases were excluded leaving 40 cases. Initial review only included non-CEMRE sequences. Cases were read independently by radiology fellows and then double-read by consultant GI radiologists. The primary outcome was whether the conclusion differed based on CEMRE. A third radiologist arbitrated differences in opinion.
**Results:** CEMRE added some diagnostic benefit in 5 cases (12.5%). In one case, a third skip lesion was identified. In 3 cases, findings were more conspicuous on CEMRE but the overall conclusion did not change. The final case was felt to be due to poor quality of T2 and DWI sequences. Thus, only in 1 case (2.5%), did the final conclusion change significantly.

**Conclusion:** In most cases CEMRE added no diagnostic value, though more junior radiologists more often found them useful. Avoiding GBCAs increases patient safety, in addition to a cost-benefit and reduced scan time. It is suggested that routine use of GBCA is not required for MRE, but can be used in selective circumstances.

### B-1648 10:55

**Can diffusion-weighted imaging (DWI) in MR enterography discriminate inflammatory strictures from fibrotic strictures in Crohn's disease?**

M. Travali, P. Foti, N.M.G. Ognibene, S. Palmucci, L. Puzzo, G. Inserra, L. Zanoli, S. Parisi, A. Basile; *Catania/IT (mario.travali.a34l@gmail.com)*

**Purpose:** Differentiation between inflammatory and fibrotic bowel strictures has relevant implications in therapeutic management of patients with Crohn's disease. The aim of our study is to retrospectively evaluate the capability of magnetic resonance enterography (MRE) conventional and DWI sequences to differentiate fibrotic strictures from inflammatory ones in Crohn's disease.
**Methods and Materials:** 21 patients with Crohn's disease that underwent surgical resection with pathological confirmation within 3 months from preoperative MRE were retrospectively selected. Two radiologists blinded to histopathology in consensus evaluated the following bowel wall MRE qualitative and quantitative biomarkers at level corresponding to pathological specimens: mural thickness, maximum calibre of pre-stenotic upstream bowel, T1 ratio, T2 ratio, pattern of wall enhancement, DWI apparent diffusion coefficient (ADC). A blinded pathologist scored stricture histological specimens for fibrosis (0-2) and acute inflammation (0-12). MRE findings were correlated with histopathological data representing the reference standard.
**Results:** Quantitative ADC measurements correlated significantly with fibrosis score (P<0.001), whereas no significant correlation was observed between ADC values and inflammation score (P=0.64). Maximum calibre of pre-stenotic upstream bowel correlated significantly with fibrosis score (P=0.0069), but not with inflammation score. T2 ratio correlated significantly with both fibrosis (P=0.023) and inflammation score (P=0.0048). T1 ratio, mural thickness and pattern of wall enhancement showed no significant correlation neither with inflammation nor with fibrosis score.
**Conclusion:** Quantitative ADC measurements from DWI sequences could improve the ability of MRE to noninvasively differentiate fibrotic strictures unlikely to respond to medical therapy, therefore requiring surgical treatment, from inflammatory strictures liable to undergo pharmacological treatment.

### B-1649 11:03

**MRI quantitative parameters of small-bowel perfusion for early diagnosing and assessing activity of Crohn's disease: a preliminary study**

Z. Xianying, W. Yinchen; *Fuzhou/CN (1571476087@qq.com)*

**Purpose:** To explore the relationship between MRI quantitative parameters such as the volume transfer constant ($K^{trans}$), the reflux constant ($K_{ep}$) and the extravascular space volume fraction ($V_e$) and the change of blood perfusion and permeability of Crohn's Disease (CD), and evaluate the value of small-bowel perfusion for early diagnosing and assessing activity of CD.
**Methods and Materials:** 48 patients who underwent bowel examination with magnetic resonance enterography (MRE) were recruited. $K^{trans}$, $K_{ep}$ and $V_e$ of normal and terminal small-bowel were collected and compared. According to Crohn's disease activity index (CDAI), all patients were divided into three groups such as remissive group, mild group and moderate-severe group. The differences of $K^{trans}$, $K_{ep}$ and $V_e$ among the three groups were compared. The correlations between quantitative parameters and CDAI and small-bowel wall thickness were, respectively, evaluated.
**Results:** In 48 patients, $K^{trans}$, $K_{ep}$ and $V_e$ of terminal small-bowel were higher significantly than that of normal bowel (p<0.05) with the most remarkable increase in $V_e$. There were 13 cases of remissive group, 20 cases of mild group and 15 cases of moderate-severe group. $K^{trans}$, $K_{ep}$ and $V_e$ of remissive group were lower than active group (p<0.05). With the progress of CD, $K^{trans}$, $K_{ep}$ and $V_e$ also increased (p=0.000). All quantitative parameters had high positive correlation with CDAI (p<0.05). However, it was not statistically significant in correlation between $K^{trans}$ and small-bowel wall thickness.
**Conclusion:** MRI quantitative parameters of small-bowel perfusion can reflect the change of blood perfusion and permeability of CD, and have a certain value for early diagnosing and assessing activity of CD.

### B-1650 11:11

**High spatiotemporal resolution free-breathing quantitative bowel perfusion imaging**

V.C. Obmann[1], K. Yang[2], N. Seyfried[2], M. Stopchinski[2], K. Wright[2], S. Ghodasara[2], A. Panda[2], Y. Chen[2], V. Gulani[2]; [1]*Berne/CH*, [2]*Cleveland, OH/US (verena.obmann@insel.ch)*

**Purpose:** To use a free breathing 3D ultra-fast DCE-MRI exam (spiral GRAPPA) for the acquisition of high spatial and temporal resolution imaging to provide quantitative perfusion modelling of the entire bowel in patients with Crohn's Disease enabling objective assessment of disease status.
**Methods and Materials:** In this IRB approved prospective study, 11 patients with Crohn's Disease with at least one inflamed bowel segment were included. 50 coronal free-breathing DCE volumes covering the abdomen (temporal resolution 1.6 s, acquisition time 1.5 min) were acquired over a time course of 1.5min with 1.5 mm in-plane resolution and 4 mm uninterpolated slice thickness. Mural multislice ROI were drawn in different segments of the bowel. The single input, single compartment Tofts model was utilized to calculate $K^{trans}$ and $v_E$. A Mann-Whitney-U test was performed to compare $K^{trans}$ and $v_e$ of different segments.
**Results:** Median patient age was 31 years (range 19-77 years, M:F=7:5). $K^{trans}$ and $v_e$ values for segments with inflammation were significantly higher compared to unaffected segments ($\Delta K^{trans}$ = +133% and $\Delta v_e$ = +106%, p=0.007 and 0.004, respectively). Between active and chronic inflammatory changes there were no significant differences (both, p=0.368).
**Conclusion:** A free-breathing 3D quantitative perfusion assessment of the bowel which enables high temporal resolved evaluation of the crucial early arterial portions of the bowel contrast enhancement is presented. This technology should be explored for the assessment of early response to therapy.
*Author Disclosures:*
**K. Wright:** Research/Grant Support; Siemens Healthineers. **V. Gulani:** Research/Grant Support; Siemens Healthineers.

### B-1651 11:19

**Ability of diffusion-weighted magnetic resonance imaging to characterise bowel fibrosis depends on the degree of bowel inflammation**

X. Li, S. Huang, B. Lu, L. Huang, Z. Li, J. Du, Y.-K. Zhong; *Guangzhou/CN (zyingkui@163.com)*

**Purpose:** The ability of diffusion-weighted imaging (DWI) to assess bowel fibrosis in patients with Crohn's disease (CD) remains unclear. Another knowledge gap is whether bowel inflammation could have a confounding impact on the apparent diffusion coefficient (ADC) when detecting fibrosis, given that bowel inflammation and fibrosis are always coexistent. This study assessed the role of DWI in characterisation of bowel fibrosis using surgical histopathology as reference standard.
**Methods and Materials:** Abdominal DWI was performed before elective surgery in 30 consecutive patients with CD. The ADCs in pathologic bowel walls were calculated. Region-by-region correlations between DWI and the surgical specimens were performed to determine the histologic degrees of bowel fibrosis and inflammation.
**Results:** The ADCs correlated negatively with bowel inflammation ($r$=-0.499, $P$<0.001) and fibrosis ($r$=-0.464, $P$<0.001) in 90 specimens; the ADCs in regions of non-fibrosis and mild fibrosis were significantly higher than those in regions of moderate-severe fibrosis ($P$=0.008). However, there was a significant correlation between the ADCs and bowel fibrosis ($r$=-0.641, $P$=0.001) in mildly inflamed segments but not in moderately ($r$=-0.274, $P$=0.255) or severely ($r$=-0.225, $P$=0.120) inflamed segments. In the mildly inflamed segments, the ADCs had good accuracy with an area under the receiver-operating characteristic curve of 0.867 ($P$=0.004) for distinguishing non-fibrosis and mild fibrosis from moderate-severe fibrosis.
**Conclusion:** The ADC only enables accurate detection of the degree of bowel fibrosis in mildly inflamed bowel walls. Therefore, caution is advised when using ADC to predict the degree of intestinal fibrosis.

### B-1652 11:27

**ADC values of DWI and DWIBS in bowel imaging: when they are consistent and when not?**

I. Apine, M. Baduna, R. Pitura, G. Krumina; *Riga/LV (dr.ilze.apine@gmail.com)*

**Purpose:** To assess consistency between ADC-DWI and ADC-DWIBS in different bowel filling degree and bowel content in MRE examinations.
**Methods and Materials:** Patients with no evidence of IBD were scanned with 1.5T MRI system to assess ADC-DWI and ADC-DWIBS in high SI bowel walls at b=800: 1) in small bowels (54 patients) before and after filling with 2.5% mannitol solution, 2) in large bowels (65 patients) at presence of intraluminal faeces, filled with mannitol afterwards. Bowel distention was maintained with 1.5 l of 2.5% mannitol solution orally. Differences between ADC-DWI and ADC-DWIBS were assessed with two-sample t-test.
**Results:** No statistically significant difference was found between ADC-DWI and ADC-DWIBS in small bowel walls filled with mannitol being $1.95\times10^{-3}$ $mm^2/s$ (SD 0.41) and $1.91\times10^{-3}$ $mm^2/s$ (SD 0.52), respectively (p=0.7255) but statistically significant difference of 25.4% was found in empty bowel loops, in DWI being $1.28\times10^{-3}$ $mm^2/s$, SD 0.48, and in DWIBS being $1.02\times10^{-3}$ $mm^2/s$, SD 0.51; p=0.0011. No statistically significant difference between ADC-DWI and ADC-DWIBS was found in large bowel in presence of mannitol being $2.15\times10^{-3}$ $mm^2/s$ (SD=0.42) and $2.06\times10^{-3}$ $mm^2/s$ (SD=0.64) (p<0.3642) whereas statistically significant difference of 23.4% was found in bowel walls in presence of faeces being $1.05\times10^{-3}$ $mm^2/s$ (SD=0.44) for DWIBS and $1.37\times10^{-3}$ $mm^2/s$ (SD=0.4232) for DWI (p<0.0001).

**Conclusion:** ADC-DWI and ADC-DWIBS differ in presence of media of short T1 value (fat around collapsed bowel and high-viscosity faeces) whereas in abundance of long T1 value background (large amount of mannitol) ADC-DWI and ADC-DWIBS are consistent.

**B-1653** 11:35

**Validation of the simplified magnetic resonance index of activity (sMaRIA) for Crohn's disease without using gadolinium**

N. Capozzi[1], A. Fernandez-Clotet[2], I. Alfaro[2], I. Ordás[2], J. Panes[2], J. Rimola[2]; [1]*Bologna/IT*, [2]*Barcelona/ES (nunziacp@gmail.com)*

**Purpose:** To validate a simplified Magnetic Resonance Index of activity (sMaRIA) based on Magnetic Resonance Enterography (MRE), which does not include gadolinium enhanced sequences, for assessing disease activity and therapeutic response in patients with Crohn's disease (CD).
**Methods and Materials:** We prospectively included patients with active CD and at least one intestinal segment with severe inflammation at endoscopy (CDEIS>8.5 or presence of ulcers), requiring treatment with a biological TNF-inhibitor. Patients were evaluated by both MRE and ileo-colonoscopy at baseline and at week 46 (wk-46) of therapy with TNF-inhibitor. Ileo-colonoscopy (CDEIS) was considered the gold standard. The sMaRIA was read using T2-w sequences. Comparison between sMARIA and CDEIS for detecting active/severe lesions and therapeutic response was performed.
**Results:** Data from 43 patients (236 segments; 51 with ulcers) at baseline and from 37 patients (191 segments; 44 with ulcers) at wk-46 were available. The sensitivities and specificities of sMaRIA for detecting active disease at segment level (sMaRIA≥1) were 80% and 95% (AUC 0.88), and for detecting severe inflammation (sMaRIA≥2) were 80% and 93% (AUC 0.88) respectively. The sensitivity and specificity of sMaRIA for detecting mucosal healing at segment level (sMaRIA<2) were 73% and 93%. There were statistical significant changes (p=0.000) in sMaRIA score between baseline and post-treatment in patients with mucosal healing. The correlation of magnitude of changes between CDEIS and sMaRIA was moderate and significant (r=0.72; p<0.001).
**Conclusion:** The sMaRIA is a reliable tool that would avoid the use of gadolinium to detect inflammation and assess therapeutic response in luminal CD.

**B-1654** 11:43

**Correlation analysis between magnetisation transfer ratio with collagen area fraction in the surgical specimen of Crohn's disease**

S. Huang[1], X. Li[1], Z. Fang[1], J. Lin[1], Z. Zhang[2], C.C. Sun[1], S. Feng[1], Z. Li[1]; [1]*Guangzhou/CN*, [2]*Florida, FL/US (codesy@foxmail.com)*

**Purpose:** We aimed to establish a new index in histology, collagen area fraction, to quantify the amount of collagen and to reflect the severity of bowel fibrosis in patients with Crohn's disease (CD), meanwhile for assessing the correlation between magnetization transfer ratio (MTR) and collagen area fraction in the surgical specimen of CD.
**Methods and Materials:** We analysed the histopathological data and MT imaging of 31 patients with CD undergoing surgical resection. The MTR were measured and the most representative sections of resected segments were stained with Masson trichrome. The collagen area fraction derived from image segmentation using Image J software and semi-quantitative histological fibrosis score (0-4) were simultaneously calculated for the same section to evaluate the severity of bowel fibrosis.
**Results:** The collagen area fraction strongly correlated with the histological fibrosis score ($r$=0.733, $P$<0.001). It proved to be more accurate for diagnosing Crohn's disease strictures (AUC =0.815, $P$<0.001) compared with the histological fibrosis score (AUC=0.771, $P$<0.001). High repeatability was observed for the collagen area fraction, with an intraclass correlation coefficient of 0.915. A good correlation between MTR and collagen area fraction was found ($r$=0.643, $P$<0.001). Moderate accuracy of MTR was shown (AUC= 0.766, $P$<0.001) for differentiating a collagen area fraction>0.5 from a collagen area fraction≤0.5 in bowel walls.
**Conclusion:** The collagen area fraction is a reliable index to quantify the severity of bowel fibrosis in the surgical specimen. MTR can be a noninvasive biomarker to detect and grade the bowel fibrosis in patients with CD.

**B-1655** 11:51

**Magnetic Resonance Fingerprinting (MRF) enables simultaneous T1 and T2 mapping of the bowel wall in patients with Crohn's disease**

V.C. Obmann[1], W.-C. Lo[2], N. Seyfried[2], Y. Jiang[2], A. Panda[2], K. Wright[2], K. Ropella-Panagis[2], V. Gulani[2]; [1]*Berne/CH*, [2]*Cleveland, OH/US (verena.obmann@insel.ch)*

**Purpose:** Magnetic Resonance Fingerprinting (MRF) provides simultaneous mapping of T1 and T2 relaxation times in a single acquisition. To evaluate the feasibility of MRF-derived T1 and T2 measurements in the bowel for the evaluation of inflammatory bowel disease.
**Methods and Materials:** 33 patients (19:14 M:F) undergoing MR enterography exams at 1.5T were included in this IRB approved study. 3 axial and 3 coronal 2D-MRF slices were acquired through the bowel using a single breath-hold (24s) MRF-FISP sequence (in-plane spatial resolution 1.6x1.6 $mm^2$). Information from weighted clinical images and endoscopy were used to identify affected segments. On T1 and T2 maps, regions of interest (ROIs) were drawn in the wall of small bowel and colon to assess T1 and T2 relaxation times in the bowel wall for each patient. Mann-Whitney-U and Kruskal-Wallis-Test with Dunn-Bonferoni post-hoc-tests were used to assess differences in T1 and T2 values between unaffected, acutely inflamed, and chronically inflamed bowel.
**Results:** Average age was 32 years (range 12-77 years). There were 11 segments with chronic disease and 16 with acute inflammation. T1 relaxation times allowed differentiation between unaffected segments (1468 ± 314ms, n=47) and inflamed segments (active 1386.0 ± 349.2ms and chronic 1296.8 ± 281ms), p = 0.028. T2 relaxation times further allowed distinction between segments with active (67.5 ± 48.4ms) and chronic fibrotic changes (48.4 ± 21.5ms), p = 0.009.
**Conclusion:** Initial application of MR Fingerprinting to evaluate the bowel wall is presented, with promising results for quantitative differentiation of normal, chronically disease, and acutely inflamed bowel.
*Author Disclosures:*
**W. Lo:** Research/Grant Support; Siemens Healthineers. **Y. Jiang:** Research/Grant Support; Siemens Healthineers. **K. Wright:** Research/Grant Support; Siemens Healthineers. **V. Gulani:** Research/Grant Support; Siemens Healthineers.

**10:30 - 12:00** **Room M 5**

### Genitourinary

### SS 1807

### Testicular, renal and bladder tumours

*Moderators:*
F.M. Drudi; Rome/IT
O.W. Kozak; Gdansk/PL

**B-1656** 10:30

**Testicular cancer follow-up: less is more**

E. Rud, C.W. Langberg, E. Baco, G. Sandbække; *Oslo/NO (erik.rud@ous-hf.no)*

**Purpose:** Patients are routinely monitored for relapse after primary treatment for testicular germ cell cancer. The Swedish and Norwegian Testicular Cancer Group recommends abdominal MRI instead of CT to reduce the radiation dose. The current MRI protocol is designed to screen retroperitoneal lymph nodes, abdominal organs, and the bony structures from the diaphragm to proximal femur. The aim of this study was to evaluate the rate and location of relapse in patients with testicular cancer.
**Methods and Materials:** We included all patients that underwent abdominal MRI after treatment of testicular germ cell cancer during the calendar year 2012 and 2017. MRI reports were classified as negative or positive, and positive reports were cross-checked with follow-up imaging and biopsy results. The location of true positive findings was registered according to anatomical site.
**Results:** During 2012 and 2017, we performed 2315 MRI examinations. Sixteen patients (0.7%, 95% CI: 0.4-1.1) with relapse were detected of which 12 were seminomas and four were non-seminomas. Fourteen (87%) were located in the retroperitoneal lymph nodes, and two occurred in pelvic and inguinal lymph nodes. No metastases occurred in parenchymatous organs or bony structures.
**Conclusion:** All cases of abdominal relapse occurred in retroperitoneal or pelvic lymph nodes. For this reason, a simpler MRI protocol containing fewer sequences can be used instead of screening all the abdominal organs as well as the bony structures.

**B-1657** 10:38

**Diagnostic performance of contrast-enhanced ultrasound (CEUS) in unclear testicular pathology**

M.H. Lerchbaumer, S.R. Marticorena Garcia, M. Kallenbach, B. Hamm, T. Fischer; *Berlin/DE (markus.lerchbaumer@charite.de)*

**Purpose:** Contrast-enhanced ultrasound (CEUS) has been used as an additional imaging technique to conventional ultrasound in order to clarify acute and chronic testicular pathologies. CEUS is easy and fast to perform, overcomes the limitations of gray-scale ultrasonography. The aim of this retrospective single-center analysis is to evaluate the diagnostic performance of CEUS in the assessment of unclear testicular pathology.

**Methods and Materials:** CEUS examinations of patients with unclear testicular pathologies between 2012 and 2018 were retrospectively analysed. Examinations were performed using B-mode, colour Doppler and CEUS after injection of a second-generation blood pool agent (SonoVue®, Bracco, Milan, Italy). Results were interpreted by an experienced radiologist (EFSUMB level 3).
**Results:** 43 patients were examined without any adverse reaction, where out 16 patients presented with a tumour lesion (benign and malign). Median tumour size measured by ultrasound was 17.0 mm (IQR, 9.0-44.8) with a correlation coefficient of 0.917 compared to the tumour size in histopathological report (median size: 22.5 mm; IQR, 14.0-41.8). When matched to the histopathological report and clinical follow up, CEUS represented a sensitivity of 92% (95%-CI, 67-97), a specificity of 90 % (95%-CI, 74-97), a positive predictive value (PPV) of 80% (95%-CI, 55-93) and a negative predictive value (NPV) of 96% (95%-CI, 82-99).
**Conclusion:** CEUS is a feasible tool to differentiate between traumatic and inflammatory testicular pathologies. While differentiation between malign and benign lesions using CEUS due to enhancement is currently difficult, patients with contraindications to other imaging modalities benefit from this method in the daily clinical routine.

**B-1658** 10:46
**The added value of scrotal palpation during testicular ultrasound (US): a technical note**
J.P. Zawaideh[1], C. Trambaiolo Antonelli[1], A. Olivero[1], M. Bertolotto[2], L.E. Derchi[1]; [1]*Genoa/IT*, [2]*Trieste/IT (jeriespz89@gmail.com)*

**Purpose:** Guidelines on performance of scrotal US indicate that “If a palpable abnormality is the indication for the study, this area should be directly imaged”. However, a small lesion may be difficult to identify, even when the patient is asked to indicate where it is located. We herewith underline the beneficial role of scrotal palpation carried out by the radiologist during US examination for scrotal masses.
**Methods and Materials:** We reviewed the US findings in a series of 7 patients in whom US and simultaneous palpation allowed to identify an otherways non-visible lesion and/or helped to recognize its nature.
**Results:** In 2 patients an extratesticular lesion was not detected during the “normal” US and could be seen only through the combination of US and simultaneous palpation. In 5 patients palpation gave more information on the lesion, thus leading to its characterization: in 3 it could be displaced during palpation and recognized as a normal appendix testis (2) or as a pedunculated nodule arising from testicular surface (1); in 2 lesions were seen as freely mobile inside the tunica vaginalis, and recognized as “scrotoliths”.
**Conclusion:** Scrotal palpation carried out by the radiologist during US examination for scrotal masses can increase the diagnostic accuracy of the examination, especially in difficult small lesions.

**B-1659** 10:54
**Focal testicular lesions: multiparametric US features and association with histopathology**
I. Campo, C. Sachs, R. Bussani, M. Bertolotto, M.A. Cova; *Trieste/IT (irenecampo11@gmail.com)*

**Purpose:** Ultrasound features of solid scrotal tumours are often non-specific. US, however, identifies the lesion in the largest number of cases. To illustrate the histopatologic features which determine echogenicity, echotexture, stiffness, and vascularity of different testicular masses.
**Methods and Materials:** 116 testicular lesions investigated with colour Doppler US for which histological specimens were available for review were considered. 68 lesions had also CEUS, 39 elastography. Imaging features were correlated with histological characteristics.
**Results:** Most testicular cancers were hypervascular at colour Doppler interrogation (65/116)and with increased consistency at elastography (24/39). 14/23 hypovascular lesions with small vessels at colour Doppler interrogation were hypervascular at CEUS. Seminomas presented with lower echogenicity compared to other cancers (29/35), mixed tumors were heterogeneous. Irrespective of histotype, abundance of stroma was the main factor determining increased echogenicity. Other factors were hyalization, interleaved necrotic areas and tumor nests, athrophic changes of the surrounding parenchyma. Heterogeous appearance was observed in presence of different histotypes, calcifications, necrotic or fibrotic areas. Lesions were avascular at colour Doppler interrogation if vessels were lacking or were very small. In the latter case, they were vascularized at CEUS. Lesions with abundant stroma were hard at elastography, while necrotic changes caused soft or mixed appearance.
**Conclusion:** The different histological features of testicular lesions determine their sonographic appearance. US is highly sensitive for detection of testicular lesions, but specificity is low. The different US modes, if taken individually, are non-specific but if used together improve lesion characterization.

**B-1660** 11:02
**Apparent diffusion coefficient and magnetization transfer ratio in predicting successful sperm retrieval after mTESE**
A.A. Ntorkou, A. Tsili, A. Goussia, L.G. Astrakas, A. Kaltsas, N. Sofikitis, M.I. Argyropoulou; *Ioannina/GR (alexdorkou@hotmail.com)*

**Purpose:** To assess any probable correlation between apparent diffusion coefficient (ADC) and magnetization transfer ratio (MTR) with the presence of spermatozoa retrieved after microdissection testicular sperm extraction (mTESE) in non-obstructive azoospermia (NOA).
**Methods and Materials:** This retrospective study included 49 consecutive NOA men (mean age: 36.3 years) who underwent scrotal 1.5 T MRI, a day prior to mTESE. Forty five age-matched men (mean age: 35.4 years) were used as controls. The MRI protocol included diffusion-weighted imaging and magnetization transfer imaging. Based on the more advanced histopathologic pattern, NOA testes were classified as follows: group 1, testes with higher Johnsen score (hJS) ≥8; and group 2, testes with hJS <8. The testicular volume (TV), the mean testicular ADC and MTR were measured. Non-parametric statistical tests were used to assess differences in TV, ADC and MTR between: a) NOA men and controls, b) NOA groups and c) NOA testes with positive and negative sperm retrieval.
**Results:** TV ($P < 0.001$) was reduced and both ADC ($P < 0.001$) and MTR ($P = 0.013$) were increased in NOA testes compared to controls. TV ($P < 0.001$) was reduced and ADC ($P = 0.015$) and MTR ($P = 0.003$) were increased in group 2 compared to group 1. TV ($P < 0.001$) was reduced and both ADC ($P = 0.011$) and MTR ($P = 0.045$) were increased in NOA testes with failed sperm retrieval.
**Conclusion:** This preliminary study showed that TV, ADC and MTR may be useful for predicting the outcome of mTESE.

**B-1661** 11:10
**MRI diffusion-weighted and shear wave elastography in testis**
M.R.V. Pedersen[1], P.J. Osther[1], H. Nissen[1], P. Vedsted[2], H. Møller[2], S.R. Rafaelsen[1]; [1]*Vejle/DK*, [2]*Aarhus/DK (Malene.Roland.Vils.Pedersen@rsyd.dk)*

**Purpose:** The purpose was to evaluate ultrasound shear wave elastography in combination with the diffusion MRI in testicles with different tissue characteristics.
**Methods and Materials:** A prospective study, with a total of 132 patients. All patients underwent b-mode ultrasonography including shear wave elastography measurements and an MRI diffusion examination of the scrotum.
**Results:** 53 patients with normal testicular tissue, 53 patients with testicular microlithiasis and 23 patients with malignant testicular tumours and 3 patients with benign testicular tumors were included.
There was a significant differences in stiffness between patients wit testicular microlithiasis (0.78 m/s), normal testicular tissue (0.77 m/s) and patients with a testicular cancer (1.92 m/s) (p<0.001).Similar, there was a statistical significant difference in MRI diffusion values between patients with testicular microlithiasis (0.978) and normal testicular tissue (0.929) and testicular cancers (0.743) (p<0.01).
**Conclusion:** Patients with testicular microlithiasis had no malignant characteristics measured with shear wave elastography or MRI diffusion. Testicular tumors had higher elastography values, and higher MRI diffusion restriction compared to normal testicular tissue. MRI in combination with Shear wave elastography may be a useful tool to differentiate benign from malignant testicular lesions.

**B-1662** 11:18
**Percutaneous ablation of renal cancer: radiofrequency vs. microwave**
I.D. Domínguez Paillacho, J. Mesa Quesada, L.J.J. Zurera Tendero, C. Pulido Carmona, M. Lombardo Galera, M.E. Perez Montilla, J.J. Espejo Herrero; *Córdoba/ES (davko61988@gmail.com)*

**Purpose:** To compare the efficacy and safety of renal percutaneous thermoablation of renal cancer by radio frequency (RF) and microwave (MWs).
**Methods and Materials:** We retrospectively reviewed the malignant renal tumours treated with RF (RITA system) and MWs (AMICA system) in our centre in the last 12 years. Control was performed one month after treatment to check if there was a tumour remnant and follow-up every 6 months to detect recurrence.
**Results:** We identified 54 lesions in a total of 44 patients. 26 patients had histological confirmation by biopsy and in 29 the diagnosis was clinical/radiological. 37 lesions were treated with RF and 17 with MWs. The mean tumour size in the group of MWs was greater than the RF group (36 mm vs 26 mm, respectively, p = 0.001). Tumour rest was identified in 8 lesions treated with RF (for which a second intervention was performed), while all ablations were complete with MWs (p = 0.03). The mean time of follow-up was 24.7 ± 22 months and no tumour recurrence was detected. 4 patients (9%) developed one or more complications: 3 in the RF group (urinary tract lesion, hepatic subcapsular haematoma, wall abscess and intracystic haemorrhage)

and 1 in the group of MWs (retroperitoneal haematoma) (p> 0.05).
**Conclusion:** Percutaneous renal thermoablation is a safe and effective technique. The group of patients treated with MWs, despite presenting a more unfavourable profile, have obtained better results of complete ablation in a single session compared with the RF group.

**B-1664** 11:26
**In vitro and in vivo quantification of urinary stone volume using attenuation threshold-based optimized ultra-low-dose CT method**
N. Rasouly[1], B. Kastler[2], O. Hélénon[2], B. Habib Geryes[2], A. Comte[3];
[1]*Kabul/AF*, [2]*Paris/FR*, [3]*Besançon/FR (nrasouly@gmail.com)*

**Purpose:** To assess in practical clinical settings if an optimised ultra-low-dose protocol is able to follow-up kidney stones of different chemical composition in daily practices using variable threshold-based computed tomography methods.
**Methods and Materials:** 17 stones of different chemical compositions, sizes and shapes were scanned in an anthropomorphic phantom using ultra-low-dose protocol with variable threshold CT method (30%, 40%, 50%, 60% and 70% of stone attenuation), segmented with 0.625-mm-thick sections, tube voltage of 100 KV and tube current of 75 mAs. A statistical analysis was performed to assess bias and precision of this method in comparison with fluid displacement method as a reference standard.
**Results:** The ultra-low-dose CT protocol with variable attenuation threshold and 3 different reconstruction algorithms (standard, soft and bone) is an accurate and precise method in stone volume measurement to compare with fluid displacement method as a reference standard. However, notable differences were only observed between the 'Bone' reconstruction algorithm with a threshold of 30% and the 'Standard' reconstruction algorithm with a threshold of 30% as well as the ‘Soft’ reconstruction algorithm with a threshold of 30%. However, all methods are equivalent in bias and precision.
**Conclusion:** Variable threshold-based CT method which is derived from attenuation of each stone can be used to quantify urinary stone volume even at ultra-low-dose protocol (equal to one abdominal radiograph) with high level of accuracy and precision which is comparable with the fluid displacement method as a reference standard in estimation of kidney stone volume.

**B-1665** 11:34
**Bladder perivascular epithelioid cell tumour: dynamic CT and MRI analyses of 8 cases with 2-year follow-up**
D. Xuesong; *Chongqing/CN (duxs1966@126.com)*

**Purpose:** To evaluate the dynamic computed tomography (CT) and magnetic resonance imaging (MRI) characteristics of bladder perivascular epithelioid cell tumour (PEComa) and to improve the diagnosis of bladder PEComa.
**Methods and Materials:** A retrospective analysis was undertaken of the dynamic CT, MRI characteristics of 8 PEComas diagnosed at histopathology.
**Results:** The age of the patients ranged from 27 to 50 years (mean 36.3 years). There were more women in this group (5/8). Dynamic CT (6 cases) and MRI (4 cases) demonstrated tumours that were of low density or hypointense on T1-weighted imaging (WI) and hyperintense on T2WI; some were isodense with fat (CT: 3/8; MRI: 2/8). The tumours usually had well-defined borders and were of a regular shape (CT: 8/8; MRI: 8/8). Tumour diameters ranged from 1.5 to 12 cm (mean 4.7 cm). Most tumours (CT: 6/8, MRI: 5/8) enhanced heterogeneously and significantly on arterial and venous phases. Tumours appeared slightly hypodense on delayed CT imaging, although some (4/8) had delayed enhancement. The expression rate of HMB-45 (human melanoma black monoclonal antibody) was 100% (8/8).
**Conclusion:** Knowledge of dynamic CT, MRI, and clinicopathological characteristics could help improve the diagnosis of PEComa.

**B-1666** 11:42
**Comparative study of MR urethrography, sonourethrography and RGU with VCUG for diagnosis of male urethral strictures**
P. Joon[1], R. Rastogi[2], A. Agarwal[3], Y. Gupta[4], A. Pushkarna[3]; [1]*Gurugram/IN*, [2]*Greater Noida/IN*, [3]*Moradabad/IN*, [4]*Faridabad/IN (dr.pawanjoon@gmail.com)*

**Purpose:** The aim of study is to evaluate the role of MRU as the potential imaging tool for evaluation of patients with urethral stricture disease and to compare its efficacy to Sonourethrography and RGU with VCUG.
**Methods and Materials:** The study included 50 men who were referred to Radiology department with history suggestive of urethral stricture disease. All patients underwent RGU/VCUG followed by Sonourethrography and MRU prior to the surgical intervention. The length of the strictures, their site, presence or absence of spongiofibrosis and any other associated periurethral pathologies were looked for in all the modalities and results were compared.
**Results:** The mean lengths of short and long segment strictures was 0.96cm +/- 0.42 and 3.57cm +/-1.99 respectively on MRU, 0.90 +/- 0.38 and 3.60 +/- 2.00 respectively on Sonourethrography and 0.84cm +/- 0.42 and 3.69cm +/- 2.03 respectively on RGU/ VCUG (p<0.05). Four patients of anterior urethral strictures were wrongly diagnosed as short segment strictures on RGU but were correctly diagnosed on MRU and Sonourethrography. In cases of anterior urethral strictures, no discordance was found between MRU and Sonourethrography.
**Conclusion:** The study showed that MRU and Sonourethrography were more sensitive to RGU/VCUG in both for the stricture length and associated periurethral pathologies like urethral diverticula and sinus tracts. Also, we were able to have information about presence or absence of spongiofibrosis on MRU and Sonourethroraphy. However, MRU scores over Sonourethrography in that the latter cannot be used to evaluate posterior urethral pathologies, though both being radiation free imaging modalities.

**10:30 - 12:00** **Sky High Stage**

### Cardiac

### MY 18

### Cardiovascular

*Moderators:*
M. Francone; Rome/IT
F. Wolf; Vienna/AT

**B-1668** 10:30
**The forecaster of coronary atherosclerosis: black or white, fat or calcium?**
R. Khurana, A. Yadav, T.B.S. Buxi, K.S. Rawat, S.S. Ghuman; *New Delhi/IN (rishabhkhurana88@gmail.com)*

**Purpose:** Occurrence of first-time coronary artery disease (CAD) events is higher than recurrent CAD events. Over the years, coronary calcium score (CCS) has stood as an important risk factor for screening and prognosticating CAD. However, sometimes patients with no coronary calcium escape CAD detection, and present for the first time with a major adverse coronary event. Hence, there exists a need for improved CAD risk assessment and earlier intervention. Regional intrathoracic fat depots: epicardial fat volume (EFV) and pericardial fat volume (PFV) have been implicated as independent risk factors for CAD. Extracoronary calcium (ECC) at aortic and mitral valves, aortic root and descending thoracic aorta can also be assessed.
**Methods and Materials:** EFV and PFV were quantified in 950 patients using semi-automated technique on 128-slice MDCT coronary angiography and correlated with degree of coronary artery stenosis, CCS estimation, ECC sites and plaque characterization. These correlations were performed in isolation and combination with degree of CAD and the Framingham risk score categories.
**Results:** Fat volumes and CCS were significantly higher across different CAD severity groups. Subset of patients with CCS=0, detected with CAD, had significantly higher volumes of regional fat than those without CAD. Improved CAD detection was seen when EFV was taken into consideration with CCS.
**Conclusion:** Regional intrathoracic fat depots are an independent risk factor for CAD. Although CCS is a primary tool for CAD screening, EFV can possibly predate CCS as a CAD risk predictor. Improved cardiovascular risk profiling is achieved by considering EFV together with CCS.

**B-1669** 10:34
**Treatment of chronic myocardial infarction with unmodified stem cells at point of care: a feasibility study on pigs**
A.S. Haenel[1], M. Ghosn[2], T. Karimi[3], J. Vykoukal[2], D.J. Shah[2], D. Schulz[2], A. Raizner[2], C. Schmitz[4], E. Alt[5]; [1]*Luebeck/DE*, [2]*Houston, TX/US*, [3]*New Orleans, LA/US*, [4]*Munich/DE*, [5]*New Orleans, TX/US (Alexander.Haenel@uksh.de)*

**Purpose:** Numerous studies investigated cell-based therapies for myocardial infarction (MI). The mixed results of these studies have established the need for developing innovative approaches for improving cell-based therapy for MI. We tested the hypothesis that after blocking the left anterior descending (LAD) artery of pigs for 180min at time point T0, the delivery of $18 \times 10^6$ fresh, unmodified autologous adipose-derived regenerative cells (UA-ADRCs) four weeks after the initial infarction (time point T1) into the temporarily blocked LAD vein results in improved cardiac function and structure at ten weeks after T0 (time point T2).
**Methods and Materials:** UA-ADRCs isolated from adipose tissue were characterised by flow cytometry. Either cells or saline as control was retrogradely delivered via an over-the-wire balloon catheter placed in the temporarily blocked LAD vein at T1. Effects of UA-ADRCs were assessed immediately before T1 and at T2 by cardiac magnetic resonance (CMR) using steady-state free precession and late gadolinium enhancement imaging, and post-mortem histologic analysis
**Results:** Blocking the LAD artery for 180 min reduced the mean left ventricular ejection fraction (LVEF) to clinically relevant 35% four weeks after induction of the MI. Unlike the delivery of saline, the delivery of UA-ADRCs showed statistically significant improvements in cardiac structure and function at T2:

Sunday increased mean left ventricular mass (+29%), decreased mean relative amount of scar volume of the left ventricular wall (-21%), and increased mean cardiac output (+37%).
**Conclusion:** The delivery of UA-ADRCs four weeks after MI as performed in this study significantly improves myocardial function and structure.
*Author Disclosures:*
**C. Schmitz:** Consultant; SciCoTec. **E. Alt:** Board Member; Isar Klinikum, InGeneron, Inc.

**B-1670** 10:38
**Prevalence of asymptomatic pacemaker and ICD lead perforation on computed tomography**
F.F. Aloufi; *Riyadh/SA (faisal-aloufi@hotmail.com)*

**Purpose:** The aim of this retrospective study is to measure the frequency of the pacemaker and ICD lead perforation demonstrated on multidetector computed tomography (CT) that was performed for wide varieties of clinical indications.
**Methods and Materials:** This study included 500 patients with (285) pacemaker and (215) ICD implants who were referred for different clinical indications over the last 5 years. CT was reviewed by 2 cardiothoracic radiologists for device and tip position. Perforation is considered when the star artefact which presents the tip is outside the cardiac cavity.
**Results:** 500 patients had right ventricular leads. 17 (3.4%) patients out of 500 patients had a lead perforation. The ICD lead perforation was more common than pacemaker lead perforation. 11 (5%) patients out of 215 versus 6 (2%) patients of 285, respectively. 10 (59%) subjects out of 17 cases of lead perforation were noted in noncardiac CT indications, 2 (12%) subjects out of 17 cases of total lead perforation were noted in CT that were performed for suspicious lead perforation, and 5 (30%) subjects in whom CT was performed for coronary assessment.
**Conclusion:** ventricular ICD leads perforated more frequently than ventricular pacemaker leads. The interpreting physicians must be familiar and report it. The treating physicians should know this incidental finding of lead perforation is common and no further workup is to be requested, especially if the patient is asymptomatic.

**B-1672** 10:46
**Hybrid $^{123}$I-mIBG SPECT/CT cardiac imaging for identification of left atrial ganglionated plexi in patients with atrial fibrillation**
N.A. Nikitin, S.M. Minin, I. Mikheenko, D.V. Losik, S.V. Shaiakhmetova, A.B. Romanov; *Novosibirsk/RU (n_nikitin@outlook.com)*

**Purpose:** Addition of ganglionated plexi (GP) radiofrequency ablation (RFA) to pulmonary vein isolation (PVI) increases the effectiveness of atrial fibrillation (AF) interventional treatment. Solid-state gamma camera, using radiolabelled noradrenaline analogue $^{123}$I-metaiodobenzylguanidine ($^{123}$I-mIBG), has the potential to image small cardiac sympathetic innervation structures located in left atrium (LA) epicardial surface and corresponding to anatomical locations of GP. The purpose of this study is to image and assess the influence of RFA on cardiac sympathetic innervation patterns in AF patients.
**Methods and Materials:** 15 paroxysmal AF patients scheduled for PVI underwent $^{123}$I-mIBG SPECT imaging using a cardiac dedicated solid-state gamma camera. SPECT tomograms were co-registered with cardiac CT tomograms. Discrete $^{123}$I-mIBG uptake areas (DUAs) were identified and marked according to location towards LA walls and PVs ostia. 3D SPECT/CT images with marked DUA sites were imported into CARTO and tested during interventional treatment by high frequency stimulation (HFS). Follow-up $^{123}$I-mIBG SPECT imaging was acquired 5-7 days after interventional treatment.
**Results:** A total of 40 DUAs in LA were identified in 12 patients (3.3±1.49 per patient). Positive HFS response at DUA sites was achieved in 8 (67%) patients. In follow-up images 3 DUAs in the LA were identified (0.25±0.62 per patient; p<0.001 vs baseline). In 10 patients (83%) DUAs were not visualized compared to baseline images.
**Conclusion:** The cardiac sympathetic innervation patterns in LA can be visualized by discrete $^{123}$I-mIBG uptake areas using hybrid SPECT/CT cardiac imaging. RF catheter ablation can precisely and effectively target the identified sympathetic innervation structures in AF patients.
*Author Disclosures:*
**N.A. Nikitin:** Research/Grant Support; Russian Science Foundation (project # 17-75-20118). **S.M. Minin:** Research/Grant Support; Russian Science Foundation (project # 17-75-20118). **I. Mikheenko:** Research/Grant Support; Russian Science Foundation (project # 17-75-20118). **D.V. Losik:** Research/Grant Support; Russian Science Foundation (project # 17-75-20118). **A.B. Romanov:** Research/Grant Support; Russian Science Foundation (project # 17-75-20118).

**B-1673** 10:50
**Assessment of right ventricular deformation in end stage renal disease patients by cardiac magnetic resonance tissue tracking**
Y. Zhang, Z.-G. Yang, Y.-K. Guo, H. Xu, W. Peng, C. Xia, Z. Li; *Chengdu/CN (yizhang524@qq.com)*

**Purpose:** To investigate myocardial deformation of right ventricle and its ability to predict heart failure (HF) in End stage renal disease (ESRD) patients evaluated by cardiac magnetic resonance (CMR) tissue tracking.
**Methods and Materials:** Fifty-seven consecutive patients with ESRD and 21 age- and sex-matched health controls were prospectively recruited and underwent 3.0T CMR examination. A series of SSFP cine sequence, including 8-12 slices of short axis and horizontal 4-chamber were scanned. The global right ventricle (RV) myocardial strain variables were measured. Then, all patients were followed to assess the HF as a cardiac outcome during 11-30 months.
**Results:** ESRD patients with reduced right ventricle ejection fraction (RVEF≤45%) (n=6), global peak longitudinal strain (GLS) was significantly decreased comparing with patients with preserved RVEF (RVEF>45%) (n=51) and normal controls (-14.39±5.99% vs. -20.88±4.83%, -14.39±5.99% vs. -23.56±3.65%; respectively, all P < 0.05). After the follow-up, there were 15 patients suffered from HF and 25 free from. Patients suffered from HF, had decreased GLS compared with normal controls (-20.24±5.65% vs. -23.56±3.65%, P<0.05). In patients free from HF, GLS were lower than that in normal controls but without significant difference (-21.71±4.50% vs. -23.56±3.65%, P=0.301).
**Conclusion:** ESRD patients demonstrated myocardial strain reduction early before they suffer HF, even without change in RVEF. Myocardial strains could work as auxiliary indices to predict RV myocardial deformation in ESRD.
*Author Disclosures:*
**Y. Zhang:** Grant Recipient; National Natural Science Foundation of China (81471721, 81471722, 81641169, 81771887 and 81771897), Program for New Century Excellent Talents in University (No: NCET-13-0386), Program for Young Scholars and Innovative Research Team in Sichuan Province (2017TD0005) of China.

**B-1674** 10:54
**Left ventricular global strain analysis in arrhythmogenic right ventricular cardiomyopathy with or without syncope: a cardiac magnetic resonance imaging study**
M.-T. Shen, Z. Yang, K. Diao, L. Jiang, S. Huang, B. Hu, Y. Guo; *Chengdu/CN (smt1020@qq.com)*

**Purpose:** To clarify the diagnosis value of left ventricular (LV) strain on feature tracking cardiovascular magnetic resonance (FT-CMR) for arrhythmogenic right ventricular cardiomyopathy (ARVC) patients and to explore the correlation between LV systolic function change with syncope in ARVC patients.
**Methods and Materials:** CMR was performed in 24 with definite ARVC (11 with syncope, 13 without syncope), 18 with borderline ARVC and 28 healthy controls. Feature-tracking analysis was applied to cine CMR images on a commercial post-processing image station. LV global longitudinal strain (GLS), circumferential strain (GCS) and radial strain (GRS) were defined as the peak strain value within a cardiac cycle.
**Results:** LV GLS of patients with definite ARVC (-13.05±3.02%), with borderline ARVC (-15.46 ±2.22%) and the controls (-17.35±2.39%) were different among these three groups (all p<0.05). Additionally, in patients with definite ARVC, LV GRS (25.51±5.81% vs 35.89±11.63%), LV GCS (-13.25±2.30% vs -16.35±3.65%), and LV GLS (-11.29±2.40% vs -14.81±2.56% was statistically significant lower in patients with syncope than those without (*p*<0.05, respectively), whereas left ventricular ejection fraction (LVEF) (50.14±11.53% vs 53.49±10.35%, *p*=0.46) and other heart function indexes showed no difference. There was a moderate correlation between the global strains and syncope (GRS: r=-0.41, GCS: r=0.382, GLS: r=0.41; all p<0.001). LV GLS < -13.4% exhibited 79% specificity and 83% sensitivity for predicting the presence of syncope (AUC=0.877).
**Conclusion:** For definite ARVC patients, syncope was correlated with the reduction of LV systolic function. Global strain could differentiate between controls, definite and borderline ARVC, and predict the presence of syncope.

**B-1675** 10:58
**Quantitative T2 mapping for detecting myocardial oedema and its association with myocardial injury biomarkers in acute myocardial infarction swine models**
L. Zhang, Y.-K. Guo, Z.-G. Yang, X.-S. Li, C. Fu, H. Xu, M.-X. Yang, R. Xu, L. Xie; *Chengdu/CN (zhanglu_scu2018@163.com)*

**Purpose:** To investigate the ability of T2 mapping for detecting myocardial oedema in swine models with acute myocardial infarction (AMI), and determine the relationship between T2 mapping and myocardial injury biomarkers.

**Methods and Materials:** The AMI model was induced in 10 swine (surgery of left anterior descending artery occlusion). The cardiac magnetic resonance (CMR) scan was completed within 24 hours post-MI including T2-mapping and late-enhancement with gadolinium (LGE). The T2 value of oedematous myocardium and remote myocardium were obtained by ROI-measurement on T2-mapping images. The infarct size and oedematous size were measured on LGE and T2-mapping images, respectively. We measured myocardial injury biomarkers cardiac troponin I (cTnI), creatine kinase (CK) and creatine kinase isoenzyme MB (CK-MB) at the same time points as the magnetic resonance scanning.
**Results:** The T2 value of oedematous myocardium was higher than that of remote myocardium (54.90 ± 9.64 ms vs. 39.94 ± 6.89 ms; P<0.05). Measured oedematous size was larger than that of infarct size (0.77 ± 0.24 cm² vs. 0.54 ± 0.24 cm²; P<0.05); A strong correlation was demonstrated between the size of oedematous myocardium and cTnI (r=0.897, P=0.039), but the size of oedematous myocardium has no obvious correlation with CK and CK-MB (all p > 0.05).
**Conclusion:** Quantitative T2 mapping is a valuable for the assessment of myocardial oedema that could be a reliable marker to reflect myocardial injury in AMI, and the size of oedematous myocardium would be higher than LGE size.
*Author Disclosures:*
**Y. Guo:** Research/Grant Support; National Natural Science Foundation of China (81471721,81771897), Program for Young Scholars and Innovative Research Team in Sichuan Province (2017TD0005) of China. **Z. Yang:** Research/Grant Support; National Natural Science Foundation of China (81471722,81771887).

**B-1676** 11:02
**Comparison of the incidence of in-stent restenosis and the newly developed plaques after stenting between diabetic and non-diabetic patients: evaluation with DSCT angiography**
R. Shi, Z.-G. Yang, Y.-K. Guo, Q. Zhao, Y. Gao, P.-l. Han; *Chengdu/CN (shirui_1121@qq.com)*

**Purpose:** To evaluate the stents in diabetic mellitus (DM) and non-DM patients and compare the incidence of newly developed plaques in originally plaque-free segments between the two groups after coronary stenting by dual-source CT.
**Methods and Materials:** Thirty-eight coronary artery disease patients, 21 DM patients and 17 non-DM patients, who scheduled for stenting were consecutively included. The patency of the stent, and the distribution, number and types of newly developed plaques in originally plaque-free segments showed on the preoperative and follow-up coronary CT angiography (CCTA) data were determined.
**Results:** A total of 375 originally plaque-free coronary segments and 81 stents (DM vs. non-DM:51 vs. 30) were analyzed. At the follow-up images, 72 stents were patent,9 stents (DM vs. non-Dm: 7 vs. 2, P>0.05) had in stent restenosis (ISR) of less than 50%, and no stent had ISR more than 50% . Thirty-one newly-developed plaques (DM vs. Non-DM: 23 vs. 8, P<0.05) were identified, including 10 calcific plaques, 11 non-calcified plaques, and 10 mixed plaques. There is no statistical difference in the type of new plaques in the two cohorts(P>0.05). But it showed a mild correlation between the presence of plaques and DM (r=0.106, p<0.05).
**Conclusion:** The application of lipid lowering and antithrombotic therapy after stenting may induce the incidence of ISR both in diabetic and non-diabetic patients, but diabetes may be associated with the incidence of newly developed plaques in originally plaque-free segments, suggesting that these segments in diabetic patients need to be paid more attention to in follow-up period.

**B-1677** 11:06
**The correlation between coronary artery stenosis and myocardial perfusion indexes: a prospective “one-stop” 256-row detector computed tomography study**
K. Liu, Z. Li, K. Diao, W. Peng; *Chengdu, Sichuan/CN (634270986@qq.com)*

**Purpose:** To investigate the effect of different degrees of coronary artery stenosis on the transmural perfusion ratio (TPR) in different segments of the left ventricle using combined CT myocardial perfusion imaging (CTP) and CT angiography (CTA) on 256-row detector CT.
**Methods and Materials:** Ninety-four participants (M/F: 58/36, 63.7±6.7 years) were prospectively enrolled and underwent “one-stop” CT examination, from which CTA and CTP imaging were automatically derived. Patients with CAD were further stratified into mild, moderate and severe stenosis group. TPR was calculated using CTP on Ziostation2 (Minato-ku™, Tokyo, Japan). One-way analysis of variance was used to compare TPR between the healthy control and CAD patients of different stenosis severity. Spearman correlation was used to assess the relationship between the degrees of coronary artery stenosis and the TPR of relevant segments.
**Results:** Totally, 1504 myocardial segments were identified using standardized 17 AHA segments model (except the apex). TPR values of segments subtended by the relevant artery were significantly decreased in CAD groups compared with the healthy control group (P<0.05). There was a negative correlation between left anterior descending artery (LAD) stenosis degree and TPR (basal anterior segment: r=-0.466, p=0.009; basal anteroseptal segment: r=-0.519, p=0.003; apical anterior segment: r=-0.439, p=0.015; apical septal segment: r=-0.375, p=0.041).
**Conclusion:** The TPR acquired through the “one-stop” CT protocol was correlated with the corresponding coronary artery stenosis, making this combined CT scan a promising assessment tool for CAD patients to simultaneously quantify the diseased artery and myocardium.

**B-1678** 11:10
**Cardiovascular magnetic resonance early predictors of unfavourable left ventricle remodelling in patients with acutemyocarditis**
A. Palmisano[1], G. Benedetti[1], D. Palumbo[1], G. Peretto[1], R. Faletti[2], S. Sala[1], M. Gatti[2], F. De Cobelli[1], A. Esposito[1]; [1]*Milan/IT*, [2]*Turin/IT (palmisano.anna@hsr.it)*

**Purpose:** Acute myocarditis has an highly variable course ranging from complete recovery to end-stage dilated cardiomyopathy, and predictors of outcome are still largely unknown. Aim of the study was to determine the potential value of short-term CMR for the prediction of adverse left ventricle remodelling.
**Methods and Materials:** From September 2016 to July 2018, 33 consecutive patients with biopsy-proven acute myocarditis underwent baseline CMR (11 ± 8 days after acute clinical presentation) and short-term CMR (65 ± 10 days from baseline MR). Lake-Louise criteria (LL), T1 and T2 mapping were evaluated at each time point, as well as their modification over time. MR parameters were correlated with left ventricle (LV) end-diastolic volume (EDV) and ejection fraction (EF) at short-term CMR.
**Results:** The vast majority of patients (N = 18,54.5%) presented with infart-like clinical presentation (18,54.5%), followed by 9 (27.3%) with heart failure, 6 (18.2%) with sudden cardiac death/arrhythmia. At baseline CMR, LV volume was 135 ml with EF 53% with a significant elevation of both LL criteria (T2-ratio:2.8, Hyperemia. 13%, LGE 6%) and mapping parameters (T1:1103.79, ECV: 31, T2: 53.95) without differences among clinical presentation (p>0.05). At short-term follow-up, a slight recovery of EF was experimented (56%), and all LL and mapping parameters significantly reduced (T2-ratio:2.3, Hyperemia. 7%, LGE 4%, T1:1080, ECV: 28, T2: 51), without differences among clinical presentations. Among all parameters analysed only short-term recovery of T1 mapping was associated to improved LV volume and function (EDV R=0.884, p<0,001, FE%; R= 0,757, p<0,001).
**Conclusion:** Short-term T1 recovery is significantly associated to favourable left ventricle remodelling.

**B-1679** 11:14
**$FFR_{CT}$ vs CAD-RADS™ and its impact on patient management: a retrospective pilot study**
S. Braspenningx[1], C. Van Mieghem[2], P. Vanhoenacker[3]; [1]*Edegem/BE*, [2]*Kortrijk/BE*, [3]*Aalst/BE*

**Purpose:** Only few data exist regarding the impact of $FFR_{CT}$ (coronary computed tomography angiography-derived fractional flow reserve), more specifically the added value of $FFR_{CT}$ compared to the CAD-RADS™ score alone. This study sought to examine in retrospect the impact of $FFR_{CT}$ on further patient management, and to generate hypotheses concerning further prospective studies.
**Methods and Materials:** A total number of 51 patients were included. The sensitivity, specificity, PPV, NPV, LR+ and LR- of two strategies, i.e. CAD-RADS™ score alone vs the combination of CAD-RADS™ with $FFR_{CT}$, to predict the final clinical decision was calculated. Bland-Altman statistics and concordance correlation coefficient were used for concordance of $FFR_{CT}$ and invasive FFR (iFFR) values. ROC curves for sensitivity and specificity of $FFR_{CT}$ vs iFFR were constructed.
**Results:** The combination of CAD-RADS™ with $FFR_{CT}$ had a slightly lower sensitivity than CAD-RADS™ alone (72.22 vs 77.78) but a higher specificity (51.72 vs 17.24), PPV (48.15 vs 36.84) and NPV (75.00 vs 55.56) for predicting the final clinical decision, conservative management or revascularization. The correlation between $FFR_{CT}$ and iFFR results was relatively poor (concordance correlation coefficient 0.29). The Bland-Altman plot indicated a systematic bias with underestimation of $FFR_{CT}$ results by 0.05. The ROC curves showed only moderate sensitivity and specificity.
**Conclusion:** As compared to the CAD-RADS™ anatomical score alone, the combination of CAD-RADS™ with $FFR_{CT}$ proved to be superior for predicting subsequent patient management. When comparing individual $FFR_{CT}$ values with corresponding iFFR data, correlation between these two techniques was rather weak.

**B-1680** 11:18
**Left ventricular function impairment and deformation assessment of post-transcatheter aortic valve implantation (TAVI): a systematic review and meta-analysis**
M.-T. Shen, Z. Yang, K. Diao, Q. Zhao, L. Jiang, S. Huang, Y. Guo; *Chengdu/CN (smt1020@qq.com)*

**Purpose:** The aim of this study was to elucidate changes in LVEF and left ventricular global longitudinal strain (LV GLS) at different follow-up durations after TAVI by a systematic review and meta-analysis.
**Methods and Materials:** Studies that provided both pre- and post-TAVI LVEF and LV GLS were included. The rate of change was defined as the variable difference of post- and pre-TAVI divided by pre-TAVI. The pooled GLS, LVEF and rate of change were computed using a fixed- or random-effect model. Subgroup analysis was performed to find possible sources of heterogeneity.
**Results:** Data were included from 17 studies with a total of 1109 patients, and the follow-up durations ranged from at-discharge to one year. The LVEF and LV GLS improved significantly post-TAVI (LVEF: WMD=3.868%, 95% CI=2.937 to 4.799, GLS: WMD=-1.922%, 95% CI= -2.372 to -1.473, $p$<0.001, respectively). The pooled rates of changes of LVEF and GLS after TAVI at different follow-up assessments were: short-term (<10 day) (LVEF:ES=0.048, 95% CI=0.023 to 0.073, GLS:ES=0.079, 95% CI=0.048 to 0.110); mid-term (1-6 month) (LVEF: ES=0.022, 95% CI=0.010 to 0.034, GLS: ES=0.063, 95% CI=0.044 to 0.082); and long-term (1 year)(LVEF: ES=0.075, 95% CI=0.053 to 0.097, GLS: ES=0.147, 95% CI= 0.114 to 0.179) ($p$<0.001, respectively). No significant evidence of publication bias or sensitivity analyses was observed.
**Conclusion:** LV systolic function evaluated by GLS and LVEF on echocardiography improved significantly after TAVI intervention. GLS was more sensitive and could serve as a potential assessment tool for the efficacy of therapeutic interventions and for predicting patient prognosis and clinical outcomes.

**B-1681** 11:22
**Assessment of left ventricular deformation in restrictive cardiomyopathy with preserved ejection fraction by cardiac magnetic resonance tissue tracking**
S. Huang, Z.-G. Yang, K. Diao, K. Shi, Y. Gao, M.-T. Shen, Y.-K. Guo; *Chengdu/CN (huangshan82833@163.com)*

**Purpose:** To determine left ventricular (LV) deformation in restrictive cardiomyopathy (RCM) using tissue-tracking strain analysis on cardiac magnetic resonance (CMR) imaging and further investigate the relationship between strain parameters and myocardial injury.
**Methods and Materials:** Forty RCM patients (61.6 ± 1.6 years) with preserved LV ejection fraction (LVEF≥45%) and 35 normal controls (51.0 ± 1.6 years) were included. Systolic global radial strain (sGRS), circumferential strain (sGCS), longitudinal strain (sGLS) and diastolic global radial strain rate (dGRSR), circumferential strain rate (dGCSR), longitudinal strain rate (dGLSR) were compared between RCM patients and the normal controls. New York Heart Association functional classes (NYHA) and plasma troponin T levels were recorded for clinical reference.
**Results:** Diastolic strain rates decreased significantly in RCM patients compared with the controls (dGRSR: -0.95±0.10 vs. -2.14±0.10; dGCSR: 0.64±0.06 vs. 1.10±0.04; dGLSR: 0.54±0.04 vs. 0.92±0.04; all $p$<0.001). And despite with preserved EF, RCM patients demonstrated distinctly lower systolic strains (sGRS: 16.78 ± 1.13 vs. 34.61 ± 1.37; sGCS: -11.42 ± 0.59 vs. -19.5 ± 0.45; sGLS: 7.81 ± 0.53 vs. 17.27 ± 0.45; all $p$<0.001). Elevation in troponin level was correlated with more severe NYHA classes (r=0.626, $p$<0.001) and strain parameters including sGRS (r=-0.600, $p$<0.001), sGCS (r=-0.560, $p$=0.001), sGLS (r=-0.410, $p$ < 0.05) in RCM patients. In addition, the presence of atrial fibrillation was associated with enlarged atrial volume (r=0.563, $p$<0.001) in RCM.
**Conclusion:** CMR-based strain imaging can detect both systolic and diastolic function in RCM patients before LVEF decreased. Impaired systolic strain indices are significantly correlated with myocardial injury.

**B-1682** 11:26
**Diastolic dysfunction in competitive triathletes following a strenuous endurance sports event**
J. Stáreková, B. Scherz, M. Avanesov, J.M. Weinrich, K. Müllerleile, U. Radunski, G. Adam, G.K. Lund, E. Tahir; *Hamburg/DE (j.starekova@uke.de)*

**Purpose:** The purpose of this study was to analyse left ventricular (LV) diastolic function by cardiac magnetic resonance (CMR) following an endurance competition in triathletes with (LGE+) and without fibrosis (LGE-).
**Methods and Materials:** 30 asymptomatic male triathletes (45 ±10 years) underwent CMR before and 2.1 ±1.1 hours after competition. To detect fibrosis, late gadolinium enhancement (LGE) imaging was included in the baseline CMR. Diastolic LV function was determined by early peak-filling rates (EPFR) and atrial peak-filling rates (APFR) using cine CMR in baseline and post-race studies.
**Results:** Focal myocardial fibrosis was detected in 10 triathletes. At baseline, APFR was higher in LGE+ compared to the LGE- triathletes (161 ±34 vs. 121 ±30 ml/s/m², P<0.01). While APFR in LGE- triathletes showed a significant increase (121 ±30 vs. 163 ±57 ml/s/m², P<0.001), APFR in LGE+ triathletes remained unchanged before and after the competition (161 ±34 vs. 169 ±50 ml/s/m², P=0.75). EPFR remained unchanged in both groups. There were no significant post-race differences regarding LVEF, LV and RV volumes. However, LGE+ triathletes had higher post-race left atrial volumes (43±9 vs. 34±7 ml/m², P<0.01).
**Conclusion:** Post-race diastolic function in LGE- triathletes was characterised by a significant increase of APFR. In contrast, the LGE+ group did not show any relevant changes coming from already increased baseline values. This suggests exhaustion of left atrial compensatory mechanisms most likely related to impairment of diastolic function in LGE+ triathletes.

**B-1683** 11:30
**Evaluation of coronary arteries with prospectively-ECG-triggered-High-Pitch-CT in patients undergoing TAVI-planning**
P. Seitz, S. Gottschling, M. Abdel-Wahab, H. Thiele, M. Borger, D. Holzhey, R.F. Gohmann, C. Krieghoff, M. Gutberlet; *Leipzig/DE (patrick.seitz@helios-gesundheit.de)*

**Purpose:** To assess image-quality and grade of stenosis of coronary arteries in preprocedural prospectively-ECG-triggered-High-Pitch-CT in patients referred to transcatheter-aortic-valve-implantation (TAVI) in comparison to invasive-coronary-angiography (ICA).
**Methods and Materials:** In 100 patients (54 male, mean age 79±5.9 years) a Dual-Source-CT-Scan (Siemens, Definition FLASH) and an ICA was performed. The cardiac scan was performed in prospectively-ECG-triggered-High-Pitch-Mode after application of 70 ml Imeron400 (Flow 3.5 ml/s). Grade of stenosis was evaluated per patient and per segment with Syngo.via (Siemens, VB10B) and was performed by one and in 1/3 of the patients by two blinded observers. Image quality was assessed using a 4-point- (0-3) and grade of stenosis using a 5-point-scale (0-4).
**Results:** 22% of patients had known coronary heart disease (CHD), in 2 cases a coronary anomaly was detected. Diagnostic image quality (Grade 1-3) could be achieved in 30.3% of segments. Sensitivity (75%), specificity (80.5%), and especially the negative predictive value (NPV) with 98.5% were good in comparison to ICA, but the positive predictive value (PPV) was only 16%. A coronary stenosis >50% could be ruled out completely in all segments for 3 patients, with an excellent NPV and sensitivity (100%), but low PPV (43.6%) and specificity (8.82%). The inter-rater-agreement per patient was excellent (Kappa=1.0), and moderate (Kappa=0,405) per segment.
**Conclusion:** Even within the limitations in patients referred to TAVI (high heart rate, coronary calcifications, etc.), a valid evaluation of coronary arteries was possible in 30% of segments using prospectively-ECG-triggered-High-Pitch-CT with a high sensitivity and NPV, and should always be performed, if the image quality is feasible.

**B-1684** 11:34
**Relationship between epicardial adipose tissue and coronary vascular function in patients with normal myocardial perfusion by 82Rb PET/TC**
S. Dell'Aversana, A. Ponsiglione, C. Nappi, A. Stanzione, M. Puglia, L. D'Acierno, G. Lassandro, A. Cuocolo, M. Imbriaco; *Naples/IT (dellaversanaserena@gmail.com)*

**Purpose:** We assessed the relationship between epicardial adipose tissue (EAT) and coronary flow reserve (CFR) in patients with suspected or known coronary artery disease (CAD) and normal myocardial perfusion imaging (MPI).
**Methods and Materials:** We retrospectively evaluated 272 subjects referred for suspected or known CAD to stress-rest 82RbPET/CT, showing normal MPI. CAC score was measured according to Agatston method; EAT volume (EATv) was measured in $cm^3$ on unenhanced CT. The ln(CAC+1) score and lnEATv transformation were used to reduce heteroscedasticity. Semi-quantitative perfusion scores (SSS, SRS, and SDS) were automatically calculated. Myocardial perfusion was considered normal when the SSS was<3. Absolute myocardial blood flow (MBF) was computed from rest and stress imaging. CFR was defined as the ratio of hyperemic to baseline MBF and considered reduced if <2.
**Results:** 95 (35%) patients showed reduced and 177 (65%) normal CFR. Compared to patients with normal CFR, those with reduced CFR were older (60±11 vs. 67±9, P<0.05) and showed higher ln(CAC+1) (3.9±3 vs. 4.7±3, P<0.05) and lnEATv (4.5±1 vs. 4.7±.1, P<0.05). At univariable logistic regression analysis, age, ln(CAC+1) and lnEATv resulted significant predictors of reduced CFR. At multivariable analysis, only age and lnEATv were independently associated with reduced CFR (HR: 1.05 and 1.89 and 95% C.I: 1.02-1.08 and 1.01-3.54, respectively, P<0.005).

**Conclusion:** In CAD and normal MPI, age and EAT are associated with reduced CFR confirming that cardiac visceral fat may influence coronary vascular function. EAT evaluation may play an important role in identification of coronary vascular dysfunction in patients with normal myocardial perfusion.

**B-1685** 11:38

**Imaging carotid artery-vulnerable plaque with ultrasound and contrast-enhanced ultrasound: correlation of cerebrovascular symptoms with quantitative and multi-parametric indexes**

V. Rafailidis[1], I. Chryssogonidis[1], E. Grisan[2], C. Xerras[1], G.-A. Cheimariotis[1], T. Tegos[1], D. Rafailidis[1], P. Sidhu[2], A. Charitanti-Kouridou[1]; [1]*Thessaloniki/GR*, [2]*London/UK (billraf@hotmail.com)*

**Purpose:** To investigate the diagnostic accuracy of quantitative carotid plaque features and a multi-parametric vulnerability index (VI) for the detection of symptomatic plaques, using ultrasound (US) and contrast-enhanced ultrasound (CEUS).
**Methods and Materials:** This is a cross-sectional observational study of 54 patients (72.2% male, median age 61 years) and 62 carotid plaques (50% symptomatic) examined with US and CEUS. The study lasted for two years (July 2016-July 2018). A VI combined degree of stenosis (DOS), greyscale median (GSM) and a quantitative surface irregularity index (SII) were calculated using both colour Doppler imaging (CDI) and CEUS, thus leading to two VI (VI-CDI and VI-CEUS). The SII was based on manual delineation of plaques with specific software. Mann-Whitney U and T tests were used to compare variables and receiver operating characteristic curves (ROC) analysis for diagnostic accuracy comparison. Level of significance was set at 0.05.
**Results:** Mean DOS of plaques was 68.9% (SD 12.8%). Symptomatic plaques showed significantly higher DOS (p<0.001), lower GSM (p=0.001), higher SII (both with CDI and CEUS - p<0.05) and higher VI calculated both with CDI (p<0.001) and CEUS (p<0.001). The area under the curve (AUC) for the detection of symptomatic plaques was 0.656 for SII-CDI, 0.690 for SII-CEUS, 0.772 for DOS alone, 0.783 for VI-CDI and 0.802 for VI-CEUS. No allergic reaction to contrast agent was noted.
**Conclusion:** The VI has superior diagnostic accuracy to DOS alone for the detection of symptomatic plaques, with best accuracy achieved with the SII calculated using CEUS, thanks to improved plaque surface delineation.
*Author Disclosures:*
**V. Rafailidis:** Grant Recipient; V.R. has received a scholarship for his PhD studies on «Imaging of the carotid vulnerable plaque with contrast-enhanced ultrasound and multi-detector computed tomography angiography» from the, Alexander S. Onassis Public Benefit Foundation. **P. Sidhu:** Grant Recipient; Author P.S. has received lecture fees from Bracco, Siemens, Samsung, Philips, and Hitachi.

**B-1686** 11:42

**Cardiac magnetic resonance with 4D flow imaging for mitral regurgitation severity assessment**

M. Guglielmo[1], G. Muscogiuri[1], A. Baggiano[1], A.I. Guaricci[2], D. Andreini[1], F. Alamanni[1], M. Pepi[1], G. Pontone[1]; [1]*Milan/IT*, [2]*Bari/IT (g.muscogiuri@hotmail.it)*

**Purpose:** The purpose of this study was to compare CMR 4D flow and SSFP imaging for the assessment of MR severity using the degree of left ventricular (LV) remodeling after surgery as the reference standard.
**Methods and Materials:** 10 consecutive patients (age: 59 ± 10) with indication to mitral valve plasty for severe mitral regurgitation were enrolled. MR severity was assessed using both CMR SSFP-PC imaging and CMR 4D flow imaging without the use of contrast agents. The pre-surgical estimate of regurgitant severity was correlated with the postoperative decrease in LV end-diastolic volume.
**Results:** Agreement between CMR SSFP-PC imaging and CMR 4D flow imaging for MR regurgitant volume (RV) was excellent for both pre- (r=0.8, p<0.05, mean difference 5.1mL) and post-surgery (r=0.9, p<0.05) evaluations. There was a strong correlation between post-surgical LV remodeling and MR severity as assessed by CMR 4D flow imaging (r=0.81, p<0.005) that was comparable to CMR SSFP-PC (r=0.78, p<0.005). The average time for MR assessment with CMR SSFP and PC imaging evaluation was 10 minutes, and 2 minutes with CMR 4D flow imaging.
**Conclusion:** CMR 4D flow imaging without contrast agents allows an accurate and quick evaluation of MR regurgitant volume. There is a strong correlation between MR severity assessed with CMR 4D flow imaging and post-surgical LV remodeling. Indeed, CMR 4D flow imaging may represent an alternative method for MR severity assessment.

**10:30 - 12:00** **Tech Gate Auditorium**

### Neuro

### SS 1811b

### Neuroradiology: miscellaneous

*Moderators:*
N.N.
L. Haider; Vienna/AT

**B-1687** 10:30

**White matter hyperintensities volumetric burden in healthy adults: a systematic review and meta-analysis**

L. Melazzini[1], M. Codari[2], M. Zanardo[1], M. Bolchini[1], G.D. Papini[2], F. Sardanelli[1]; [1]*Milan/IT*, [2]*San Donato Milanese/IT (marina.codari@grupposandonato.it)*

**Purpose:** To perform the first systematic review and meta-analysis of available studies reporting volumetric WMHs burden in healthy adults.
**Methods and Materials:** A systematic search on the available literature on this topic published between 2008 and 2018 was performed in September 2018. From each study, data about sample size, patients' age and WMHs burden were retrieved. Using the random-effect model, we calculated the pooled WMH burden, with 95% confidence interval (CI) and modelled standard deviation (SD). Subgroup/meta-regression analyses for assessing age factor and Egger test for publication bias risk were performed.
**Results:** Twenty-eight studies comprising 30 study parts were included in this systematic review and meta-analysis. Final samples comprised 6998 healthy subjects: mean age 44.7- 84.1 years. Mean WMHs volume ranged from 0.27 ± 0.51 cm3 to 15.85 ± 14.57 cm3, resulting in high heterogeneity (I2 = 99.9%, *p* < 0.001). Meta-analysis produced a pooled WMHs volume of 6.484 cm3 (95% CI 5.246-7.722 cm3). Meta-regression analysis between WMHs volume and age showed a positive correlation between WMHs volume and subjects' age, (y = 0.26x - 11.2; R2 = 0.41). Visual inspection of funnel plot showed low risk of publication bias, confirmed by the Egger- test (*p* = 0.929) and by Kendall's tau (*p* = 0.089).
**Conclusion:** Results of our meta-analysis showed high heterogeneity in WMHs burden across the analysed studies. Supporting the impact of age as the main unmodifiable risk factor for WMHs development, a positive correlation between WMHs and age was proved by meta-regression analysis.

**B-1688** 10:38

**Prevalence of incidental intracranial vascular findings in a tri-ethnic population-based study**

R. Rehwald[1], S. Moriconi[2], L. Smith[2], C.H. Sudre[2], M.J. Cardoso[2], J.H. Gillard[1], H.R. Jäger[2]; [1]*Cambridge/UK*, [2]*London/UK (rr538@cam.ac.uk)*

**Purpose:** This study aims to present the prevalence, clinical relevance and updated epidemiology of incidental vascular findings on high-resolution brain magnetic resonance angiography (MRA) in a tri-ethnic UK population-based cardiovascular cohort.
**Methods and Materials:** The trial was approved by the institutional research ethics committee and all participants gave written informed consent. In the prospective inter-ethnic community-based setting of the Southall and Brent Revisited (SABRE) trial, European, South Asian and African-Caribbean study participants were recruited for 3T high-resolution cerebral time-of-flight MRA (visit 3, 2014-2018). All scans were centrally reviewed for incidental vascular abnormalities by two neuroradiologists independently. The population prevalence with 95% CI were then calculated for the incidental findings overall and for each ethnicity, followed by multigroup comparisons.
**Results:** A total of 766 subjects (348 female, 45.4%), aged 37-90 years (mean: 72.2±6.6), had an adequate MR scan performed (European: 326, 42.5%; South Asian: 257, 33.6%; African-Caribbean: 179, 23.4%; Other: 4, 0.5%). In 97 of 766 study participants (12.6% [95% CI: 10.5%-15.2%]) incidental vascular findings have been found, of which cerebral aneurysms (37/766; 4.8% [95% CI: 3.5%-6.6%]) and intracranial atherosclerotic stenoses (ICAS) (35/766; 4.6% [95% CI: 3.3%-6.3%]) were most frequently observed, followed by arteriovenous malformations (15; 2.0% [95% CI: 1.2%-3.2%]). ICAS were significantly more frequently observed in South Asians than in Europeans and African-Caribbean (p = 0.035).
**Conclusion:** The prevalence of incidental cerebral aneurysms in a community-based cohort is higher than previously expected. ICAS prevalence significantly differs by ethnicity and might be targeted by population-specific primary risk factor prevention.

**B-1689** 10:46

**Age and gender effects on brainstem MR planimetry**

S. Mangesius, A. Hussl, C. Müller, F. Krismer, P. Mahlknecht, M. Schocke, E. Gizewski, W. Poewe, K. Seppi; *Innsbruck/AT (stephanie.mangesius@tirol-kliniken.at)*

**Purpose:** MR planimetry of brainstem structures can be helpful for the discrimination of parkinsonian syndromes. The aim of this study was to evaluate age and gender effects on brainstem MR-planimetric measures.
**Methods and Materials:** MR-planimetric measures including brainstem diameters (midbrain, pons, middle and superior cerebellar peduncle) and areas (pontine and midbrain), the ratios derived from both midbrain-to-pons diameters and areas as well as the Magnetic Resonance Parkinson Index (MRPI) were assessed on 1.5 Tesla-weighted MR images in a large cohort of 97 healthy controls (mean age 62.8 years; SD 10.9 years; female-to-male-ratio = 49:48). Planimetric measures were performed on an imaging database program (IMPAX EE). Gender- and age-related differences of all as well as of combined planimetric measurements were calculated using univariate and multivariate GLMs, and repeated for the population relevant for the differential diagnosis of parkinsonism, aged 50 to 80 years.
**Results:** Univariate and multivariate analyses showed significant effects of gender on both pontine measurements ($p<0.05$ for all) with male subjects showing larger measurements, and significant effects of age on midbrain measurements ($p\leq0.001$ for all) as well as on combined measurements and ratios derived from midbrain-to-pontine measurements ($p<0.01$ for all). Intriguingly, there were neither gender nor age effects on combined measurements or midbrain diameter in the population aged 50 to 80 years.
**Conclusion:** Although age and gender might influence single brainstem MR-planimetric measures, our results indicate that there is no need for age- or gender-specific cut-offs for the age group relevant for the differential diagnosis of neurodegenerative parkinsonism.

**B-1690** 10:54

**Automated detection of enlarged perivascular spaces on brain MRI**

F. Dubost, H.H. Adams, P. Yilmaz, G. Bortsova, M. Ikram, W.J. Niessen, M.W. Vernooij, M. De Bruijne; *Rotterdam/NL (gerdabortsova@gmail.com)*

**Purpose:** Enlarged perivascular spaces (PVS) are an emerging biomarker for cerebral small vessel disease. Annotation of PVS is a tedious and error-prone task. An automated method to detect PVS location is introduced.
**Methods and Materials:** T2 weighted MR images (voxel size 0.49 x 0.49 x 0.8 mm3) were acquired from 2200 subjects from a population-based study. An expert rater quantified PVS in the centrum semiovale and basal ganglia (both on a single slice) and, hippocampi and midbrain (whole volume). A weakly supervised deep learning algorithm based on convolutional network regression was developed. The algorithm was trained to predict the PVS count, but also produces high-resolution heatmaps revealing PVS location and size. The algorithm was trained on 1200 scans with PVS count, and evaluated on 1000 MRI scans with PVS dot annotations. Sensitivity and average number of false positives per scan (FPavg) were computed, and compared to intra-rater (n=40) reliability.
**Results:** The agreement between PVS detections and manual annotations reached sensitivity/FPavg of 0.71/1 for midbrain, 0.70/1.1 for hippocampi, 0.57/1.9 for basal ganglia, and 0.51/5.2 for centrum semiovale. The intra-rater reliability for a smaller subset reached sensitivity/FPavg of 0.77/0.4, 0.79/1.1, 0.72/2.1, and 0.55/4.3, respectively.
**Conclusion:** For each region, the automated detection of PVS performs only slightly worse than the level of intra-rater agreement. By integrating PVS locations in the analyses, this method may strengthen large clinical studies of enlarged perivascular spaces.
*Author Disclosures:*
**W.J. Niessen:** Other; Quantib's Chief Scientific Officer.

**B-1691** 11:02

**Preliminary study on changes of oxygen uptake fraction in pilots before and after hypoxia exposure**

J. Liu[1], W.-S. Zhang[2], J.L. Cheng[1]; [1]*Zhengzhou/CN*, [2]*Beijing/CN (tongnuo@yeah.net)*

**Purpose:** The changes of oxygen extraction fraction (OEF) in brain tissue of pilots before and after hypoxic exposure were measured by spin echo EPI sequence on MRI.
**Methods and Materials:** 35 healthy male pilots were scanned with MR spin-echo EPI sequence (parameters: TR/TE=4000ms/103.3ms, layer thickness 5.0mm, layer number 22, FOV=24cm*24cm, matrix Matrix=96*96, excitation frequency NEX=2, average scanning time 3 minutes and 24 seconds), and then inhaled 14.5% oxygen concentration of hypoxic mixture for 10 minutes. The spin echo EPI sequence was scanned again. Two groups of EPI data were processed by GE workstation Functool software, and OEF maps were obtained. One ROI was set in bilateral temporal cortex and one ROI in bilateral occipital cortex, and the OEF values were measured.
**Results:** Compared with before hypoxic exposure, the pulse and oxygen saturation of pilots decreased after hypoxic exposure, and OEF increased in bilateral temporal lobe and right occipital lobe cortex. OEF increased significantly in the right temporal lobe and right occipital lobe cortex, but not in the left occipital lobe.
**Conclusion:** The OEF values of pilots before and after hypoxic exposure could be accurately measured by using spin echo EPI sequence, and the OEF values of right temporal lobe and right occipital lobe cortex were significantly increased after hypoxic exposure.

**B-1692** 11:10

**A resting-state functional magnetic resonance imaging study of hypoxic exposure in pilots**

J. Liu[1], W.-S. Zhang[2], J.L. Cheng[1]; [1]*Zhengzhou/CN*, [2]*Beijing/CN (tongnuo@yeah.net)*

**Purpose:** The objective of this study was to evaluate the basic changes in brain activity in pilots after hypoxic exposure using resting-state functional magnetic resonance imaging (rs-fMRI) method.
**Methods and Materials:** Thirty-five healthy male pilots were subjected to rs-fMRI scans, and fractional amplitude of low frequency fluctuation (fALFF) and regional homogeneity (ReHo) approaches were used to analyze their brain functions before and after hypoxic exposure.
**Results:** Compared to pre-hypoxia conditions, the fALFF was decreased in the bilateral superior temporal gyrus and the right frontal gyrus, and increased in the left precuneus, while ReHo was decreased in the right frontal gyrus after hypoxic exposure.
**Conclusion:** Hypoxic exposure significantly affected the brain functions of pilots, which may have an impact on their cognitive ability. Furthermore, the sensitivity of ReHo was poorer than fALFF for this set of data.

**B-1693** 11:18

**Preliminary study of hypoxic exposure effect on cerebral blood perfusion of pilots using 3D ASL**

J. Liu[1], W.-S. Zhang[2], J.L. Cheng[1]; [1]*Zhengzhou/CN*, [2]*Beijing/CN (tongnuo@yeah.net)*

**Purpose:** The objective of this study was to investigate how the cerebral blood perfusion changed in pilots with hypoxic exposure through the measurement of resting cerebral blood flow (CBF) using a 3D pcASL technique.
**Methods and Materials:** In our study, 35 healthy male pilots were subjected to MRI examinations separately. The low oxygen mixed gas inhaled by participants through a breathing mask was approximate to the air composition at the altitude of 3000m with the oxygen concentration of 14.5%.3D pcASL images were acquired at both pre-oxygen gas inhalation (pre-OL) and post-low oxygen mixed gas inhalation(post-OL).
**Results:** Compared with pre-OL , 3D pcASL scan for pilots post-OL showed lower CBF values in various regions , including bilateral superior temporal gyrus, middle temporal gyrus, lingual gyrus, left inferior temporal gyrus, right middle occipital gyrus, inferior occipital gyrus, fusiform gyrus, cuneus and cerebellum.
**Conclusion:** The cerebral blood perfusion after hypoxic exposure is decreased mainly in the temporal and occipital lobe for the right side, and arterial apin-labelling technique can monitor CBF changes of the pilots in hypoxic exposure.

**B-1694** 11:26

**Evaluating the diagnostic value of different imaging modalities in cerebral venous thrombosis**

O. Shafaat[1], F. Faeghi[2], A. Bagheri[1], M. Rabbani[1], K. Jabbari[1]; [1]*Isfahan/IR*, [2]*Tehran/IR (omid.shafaat@yahoo.com)*

**Purpose:** Cerebral venous thrombosis (CVT) is a rare condition which is hard to diagnose because it has nonspecific symptoms. It can be potentially fatal if it remains undiagnosed and if the treatment is not started promptly. This study was designed to compare different MRI sequences with susceptibility weighted imaging (SWI) for visualization and evaluation of CVT.
**Methods and Materials:** Eighteen patients with CVT and eleven control patients without CVT were analyzed using different imaging modalities including MRI, SWI, and Phase-contrast- magnetic resonance venography (PC-MRV). The MR sequences included: T1-weighted spin echo (SE) imaging, T2-weighted turbo spin echo (TSE), T2-weighted SWI and three-dimensional phase contrast MRV.
**Results:** SWI showed the highest sensitivity, positive predictive value, and negative predictive value for the detection of cortical vein thrombosis (100%) and specificity of all sequences in the diagnosis of cortical veins thrombosis was 100%. The sensitivity of PC-MRV, SWI, T2W, and T1W sequences in the diagnosis of venous sinus thrombosis were 100%, 75%, 50%, and 8.3%, respectively. Specificity and positive predictive value of all sequences in the diagnosis of the venous sinus thrombosis were 100%. The negative predictive value of PC-MRV, SWI, T2W, and T1W sequences in the diagnosis of venous sinus thrombosis were 100%, 78.6%, 64.7%, and 50%, respectively.
**Conclusion:** SWI had a high diagnostic value for diagnosing cortical vein thrombosis. PC-MRV was the superior MRI sequence for detecting venous sinus thrombosis. Besides PC-MRV, only SWI reached a sensitivity of over 50% for venous sinus thrombosis, followed by T2W, and T1W.

**B-1695** 11:34
**Radial diffusivity role in classification of patients with antiphospholipid syndrome and "normal" radiological exams**
S. Sandiramourty, H. Nguyen Troung Thanh, J. Greffier, A. Larbi, J. Frandon, J.P. Beregi, J.-C. Gris, F. Pereira; *Nîmes/FR (sandiramourty.shridevi@gmail.com)*

**Purpose:** Automatically classify patients with Antiphospholipid Syndrome (APS) without neuroradiological manifestations.
**Methods and Materials:** Antiphospholipid Syndrome is defined as an autoimmune disease which is associated to thrombosis with common impact on MRI. However non-thrombolitic APS patients usually present "normal" routine MRI examinations. So there is an interest to find a way to diagnostic them. Diffusion-Tensor MRI (DT-MRI) was performed on 30 women with recurrent pregnancy loss (15 controls [C]; 15 antiphospholipid syndrome patients [APS] with high blood titre of Lupus Anticoagulant or Anti-β2-Gligoprotein-I antibodies). Acceded with Radial Diffusivity (RD), preceding study has demonstrated microstructural brain disruption in APS patient. Here, RD values were extracted within the significant clusters, in which voxels were considered attributes to perform Hoeffding tree classification. This is an incremental decision-tree learning model that assumes the distribution of data don't change over time. Thus, small samples may be enough for optimal splitting attribute.
**Results:** A total of 5225 attributes were found significant to produce 96.67% of accuracy (29 instances). Kappa statistics was 0.93, mean absolute error was 0.03 and the relative absolute error was 6.59%. All but one control subject were correctly classified resulting sensitivity of 1 and specificity of 0.93.
**Conclusion:** Radial Diffusivity index is an efficient attribute to classify patients with Antiphospholipid Syndrome by means of Hoeffding tree algorithms.

**B-1696** 11:42
**MRI alterations in the brachial plexus of ALS patients**
S. Gerevini, F. Agosta, E. Spinelli, P. Scamarcia, N. Riva, E. Pagani, A. Quattrini, A. Falini, M. Filippi; *Milan/IT (gerevini.simonetta@hsr.it)*

**Purpose:** To investigate brachial plexus MRI abnormalities in a large cohort of patients with amyotrophic lateral sclerosis (ALS).
**Methods and Materials:** Brachial plexus MRI scans were obtained from 43 ALS patients and 12 healthy controls. Nerve roots and limb girdle muscles were evaluated for the presence of signal alterations (T2, T1 and STIR) and volume changes. C5, C6 and C7 roots were delineated on axial, T2-weighted volumetric images. Nerve root volumes and T2-signal intensities were measured. Adipose tissue thickness between trapezius and supraspinatus muscles was measured on T1-weighted images. Quantitative measures were compared using Mann-Whitney U tests. Correlations between clinical and MRI features were assessed using the Pearson correlation test.
**Results:** At visual inspection, T2 hyperintensity and volume alterations of C5, C6, C7 nerve roots were observed in ALS patients. Muscle signal alterations and bilateral fat infiltration were also observed. The quantitative analysis showed increased T2-signal intensity in C5, C6 ($p<0.001$) and C7 roots ($p=0.001$, bilaterally) of ALS patients compared with healthy controls. Patients also showed increased C6 and C7 root volumes (p ranging 0.001-0.02). Adipose tissue thickness between trapezius and supraspinatus muscles was increased in patients ($p<0.001$, bilaterally). Disease progression rate measured using ALS Functional Rating Scale correlated with T2-signal intensity of left C6 and right C7 ($r=0.45$, $p=0.03$; and $r=0.50$, $p=0.02$, respectively).
**Conclusion:** T2 hyperintensity and increased volume of brachial plexus roots likely reflect lower motor neuron and axonal degeneration in ALS, and might represent a tool to monitor disease evolution. Muscle structural abnormalities are consistent with denervation atrophy.

**B-1697** 11:50
**Magnetic resonance imaging evaluation of arteritis and other complications in tuberculous meningitis**
S.U.K. Khan; *Gulbarga/IN (mksadaquat@gmail.com)*

**Purpose:** To evaluate the pattern of infarction and arteritis in tubercular meningitis.
**Methods and Materials:** Fifty patients with intracranial tuberculosis were subjected to MRI and MRA evaluation. Clinical signs and symptoms, focal deficitis and CSF findings were recorded. Presence of exudates, infarction, tuberculomas and hydrocephalus were noted. Sites of exudates and infarction were noted. On intracranial MRA narrowing of middle cerebral artery (MCA), anterior cerebral artery (ACA) and posterior cerebral artery (PCA) were recorded. MRA findings were correlated with clinical and MRI findings.
**Results:** Fifty patients of aged 2-65 yrs were included. Meningitis was seen in 24 (48%) cases, tuberculomas in 28 (56)%, hydrocephalus in 10 ( 20%) , arteritis and infarcts were seen in 7 (14%) cases. Majority of cases (70%) of infarcts in our study were located in MCA territory. Basal ganglia was the most common location 5 (71%) followed by cerebral cortex, parietal 4 (57%), frontal region 3 (43.%) and temporal region 3 (43%). On MR angiography MCA was involved in 5 cases, ACA in 4 cases and PCA in 3 cases.
**Conclusion:** MR imaging plays an important role in detection, localisation and charterisation of arteritis and infarction in tuberculous meningitis. Majority of infarcts (71%) were seen in basal ganglia region. MCA territory was involved in majority (70%) of cases. MR angiography demonstrated narrowing due to vasculitis.

**14:00 - 15:30** **Room B**

### Radiographers

### SS 1914a

### Educational issues in radiography

*Moderators:*
J.M. García Santos; Murcia/ES
R. Santos; Coimbra/PT

**B-1698** 14:09
**Understanding diagnostic student radiographer attrition in the UK**
S.J. McAnulla, K.M. Knapp, S. Ball; *Exeter/UK (S.J.McAnulla@exeter.ac.uk)*

**Purpose:** Diagnostic student radiographer attrition is approximately 36%; however, little research has been undertaken on this subject. This study explored risk factors for attrition and strategies that enabled these to be overcome.
**Methods and Materials:** This was a two-phase study. Phase one: data for 579 student diagnostic radiographers (468 completers and 111 non-completers) from three UK universities were analysed. Logistic regression was used to estimate completion based on individual characteristics. Phase two: content analysis of data from an online survey of 168 current UK student diagnostic radiographers.
**Results:** Phase one: attrition was 19% in these data. Increased age, non-A-level entry qualifications and poor academic performance were discriminators for attrition ($p<0.005$). Phase two: the challenges reported by the groups identified as being 'at risk' showed that for mature students, external responsibilities/pressures and financial pressures were likely to be the greatest cause of attrition and for younger students academic difficulty and workload were most significant.
**Conclusion:** Increased age appeared to the greatest risk factor for attrition. The majority of non A-level entry students were mature and different challenges were reported by mature and younger students. For mature students, study may be compromised to accommodate external pressures. For the younger students, the qualifications may be a factor as many reported academic struggles. Poor academic performance may result from both of these situations, increasing the risk of attrition. For the majority, appropriate support was a factor in students' decisions to continue.

**B-1699** 14:17
**Impact of a CT scan simulator on student learning**
G. Photopoulos[1], J.G. Stowe[2], C. Buissink[3], C. O'Halloran[2], M. Quinn[2], F. Tschan[4], R. Verwoolde[3], A. Dyhre Lia[5]; [1]*Halifax, NS/CA*, [2]*Dublin/IE*, [3]*Groningen/NL*, [4]*Lausanne/CH*, [5]*Oslo/NO (gr627731@dal.ca)*

**Purpose:** Radiographers who have a satisfactory understanding of computed tomography (CT) scan parameters positively impact patient dose and image quality. This study aims to assess the effectiveness of an interactive CT simulation tool on student radiographer learning regarding the relationship between CT scan parameters, patient dose, and image quality.
**Methods and Materials:** The sample population (n=30) chosen from a group of European radiography students was divided evenly into a quality control and intervention group. Every participant from each group was administered a questionnaire, designed to measure understanding of different scan parameters' effects on image quality and radiation dose. The intervention group underwent interactive CT training using a CT simulation tool; the quality control group was a baseline and did not receive any teaching. The next day, the questionnaire was re-administered to each participant.
**Results:** T test results demonstrate a statistically significant improvement in mean questionnaire scores for the intervention group increasing from 58.00% to 67.78% ($P=0.0000617$), while the quality control group's scores did not change from 61.56% ($P=1.00$). Further analysis demonstrated that the use of the intervention task significantly improved CT questionnaire scores ($P<0.05$) in both understanding of image quality and patient dose concepts. There was some correlation between time taken to complete the intervention task and improvement in scores, there was minimal correlation with CT experience, country of university, year of study, and time taken to complete questionnaires.
**Conclusion:** The CT simulation tool demonstrates improved student understanding on how CT scan parameters affect patient dose and image quality.

**B-1700** 14:25
**Mapping university skills labs in radiography: students' perspectives**
K. Cronin[1], S.S. Ghotra[2], V. Harsaker[3], M. Gillard[2], J. Fitzgerald[1], I. Kieft[4], H. Sekkelsten[5], O. Reynolds[1]; [1]*Dublin/IE*, [2]*Lausanne/CH*, [3]*Oppegård/NO*, [4]*Groningen/NL*, [5]*Oslo/NO (kevin.cronin@ucd.ie)*

**Purpose:** Establishing an effective theory and practice relationship is necessary for every radiography student. The effectiveness of a skills lab (SL) is paramount to ensure student radiographers are prepared for clinical placement (CP). This study will map the perspectives of radiography students regarding the SL.
**Methods and Materials:** This study was a quantitative one with one qualitative element. A paper-based questionnaire was administered to 26 radiography students from Optimax summer school, who were from seven different countries, and the data were compiled in Excel. The questionnaire contained 3 closed questions concerning demographics, 6 closed questions regarding students' own SL, 3 of which were Likert scale questions, and 1 open question inquiring about how SL could be enhanced, according to students.
**Results:** Students indicated that a competent lab tutor, smaller group size and simulated patient interaction to be important factors in the SL. Additionally, environmental factors (light, temperature) were less important. Students mentioned that their equipment is of a lower standard than CP, but they also said that they feel well prepared for CP. Students found modern equipment not hugely important.
**Conclusion:** Students indicate that theoretical and practical skills labs prepare them well for CP. However, they suggest that a competent lab tutor and additional time are important factors in the SL.

**B-1701** 14:33
**Active learning of exposure technique in digital radiography**
S. Lewis, L.J. Hazell; *Johannesburg/ZA (lynneh@uj.ac.za)*

**Purpose:** With increasing evidence of exposure creep in digital radiography, radiography students' understanding of exposure technique in digital radiography becomes paramount. Developing strategies to enhance this understanding is necessary. The purpose of this study was to explore active learning to assist in the understanding of related exposure creep in digital radiography.
**Methods and Materials:** A demonstration was designed to obtain four x-rays of a phantom hand using a constant kilovoltage, focal film distance, focal spot size, four-sided collimation and using the same computed radiography (CR) cassette. Only the milliampere per second (mAs) was changed for each exposure. Students had to predict the outcome of each exposure prior to the CR cassette being read. After the CR cassette was read, students compared their predicted and the actual outcome. Dose-area products (DAP), exposure indicators and image quality for each exposure were also tabulated. Combining the documented results with theory, students critically evaluated and reported on the effect of mAs in digital radiography.
**Results:** Students observed that at 120 times the acceptable mAs with 144 times the DAP, a post-processed image would still be diagnostically acceptable. Students found that active learning challenged their understanding of exposure creep in digital radiography. Students predicted outcomes of exposure questioned their comprehension of the theory.
**Conclusion:** The demonstration allowed students to critically evaluate exposure technique in digital radiography through active learning. It was found to be effective in the understanding of exposure technique, exposure indicators, dose creep and the wide dynamic range in digital radiography.

**B-1702** 14:41
**Integrating CT simulation into radiography undergraduate training**
J.G. Stowe, C. O'Halloran; *Dublin/IE (caoimhe.ohalloran@ucdconnect.ie)*

**Purpose:** The research will investigate the efficacy of integrating a new open-source CT scan parameter simulator into an undergraduate training programme as an active learning tool. Learning will be assessed across the themes of CT image quality and dose in the context of scan and reconstruction parameters. The use of the CT simulator will allow students to investigate the effects of scan parameter manipulation in a safe environment with no risk to patients.
**Methods and Materials:** A CT scan parameter simulator has been developed through a collaboration effort involving University College Dublin and the Western Norway University of Applied Sciences. This simulator has been successfully piloted as part of the OPTIMAX Research School 2018 in University College Dublin. A statistically significant improvement in performance was found in the pilot study and ethical approval was given to trial it as part of an overall CT training module in an undergraduate Radiography programme. This semester will see the pilot tested on a cohort of over 50 students where its efficacy as part of an overall training module will now be assessed.
**Results:** Data from the results of the simulation active learning intervention will be assessed at the end of November using a paired sample T-test to assess if using the CT scan parameter simulation can generate a significant difference in the learning of CT image quality and dose topics.
**Conclusion:** Results will be available by December 2018 and it is envisaged that this will lead to pan-European trials in 2019.

**B-1703** 14:49
**Peer-assisted learning to improve educational performance in radiography training**
J.G.E. Du Plessis; *Bloemfontein/ZA (duplesj@cut.ac.za)*

**Purpose:** During a visit to Sweden in 2017, peer-assisted learning (PAL) was identified as an ideal strategy to address the shortcomings of supervision during clinical placement.
**Methods and Materials:** To explore the impact of PAL, the first cycle of an action research project was implemented in two undergraduate radiography modules at the Central University of Technology, Bloemfontein, South Africa. PAL was excluded in the first quarter of 2018 in the selected modules. After the first assessment in both modules, 'strong' students were identified to act as the mentors of 'struggling' students. A structured PAL strategy was then implemented during pre-scheduled sessions after each assessment and prior to the following assessment. The performance of mentors and mentees for each consecutive assessment was compared with the performance of the previous assessments to measure the impact of PAL.
**Results:** The results from the implementation of PAL revealed a significant increase in the students' combined average year-end results when compared to the mid-year results. These results also delivered a p value $<0.05$, showing a significant difference between mid- and year-end results. Surprisingly, all mentors also showed an increase in their own year-end results.
**Conclusion:** It is evident from the results that PAL, as an academic advising tool, holds the potential to become an integral part of curricula of programmes including clinical training. Due to the advantages evident from the results, the implementation of PAL in radiography curricula and other health science programmes could assist in enhancing success to previously struggling students.

**B-1704** 14:57
**Factors influencing student radiographers' assessment of chest radiograph image quality**
S. Heitmann[1], R. Toomey[2], M. Chen[3], P. Pettka[1], M. Molehe[4], M. Selau Junior[5], K. Fernandes[6], K. Davies[7], J. Pires Jorge[6]; [1]*Oslo/NO*, [2]*Dublin/IE*, [3]*Groeningen/NL*, [4]*Free State/ZA*, [5]*Florianópolis/BR*, [6]*Lausanne/CH*, [7]*Prosperous/IE (soheitmann@gmail.com)*

**Purpose:** Image quality assessment is a critical part of radiography, with the potential to influence patient radiation dose and outcome. Previous studies have shown cultural influences on image quality assessment. This study aimed to determine whether country of education, percentage of degree completed, or weeks of clinical experience influence student radiographers' decisions to accept or reject chest radiographs, with the goal of understanding what influences might shape attitudes to image quality.
**Methods and Materials:** 23 radiography students from Ireland, Netherlands, Norway and Switzerland were timed while accepting or rejecting 30 chest radiographs on the basis of image quality. Each participant then gave reasons for any rejections. The total time taken, reject rate and reasons for rejection were compared between students in earlier/later stages of their degrees, with more/less clinical experience, and from different countries using Mann-Whitney U and Kruskal-Wallis tests.
**Results:** None of clinical experience completed, percentage of degree completed or country of education influenced time taken to view the images ($p>0.05$). Participants with more clinical experience rejected more images than those with less ($p=0.03$). Swiss students rejected significantly fewer images on the basis of "exposure" than Irish ($p=0.04$) or Norwegian ($p=0.03$) students, although overall rejection rates did not differ significantly between countries ($p>0.05$).
**Conclusion:** Clinical experience influences student radiographers' assessment of chest x-ray image quality in terms of both rejection rates and reasons for rejection of images. Country of education also influenced reasons for rejection. This implies that cultural differences in clinical practice may shape students' behaviours.

**B-1706** 15:13
**The opinions of American radiography educators on lead shielding use in conventional radiography**
A. Wall[1], S.R. Kucharczyk[2], W. Hennessy[3], M. O'Connor[1], J. McNulty[1]; [1]*Dublin/IE*, [2]*Limerick/IE*, [3]*Hamden, CT/US (alyson.wall@ucdconnect.ie)*

**Purpose:** The requirement for lead shielding in conventional radiography has been questioned in recent years with varying protocols employed across clinical sites. A recent European-based study found lack of consensus in radiography educators' opinions concerning lead shielding use. The current work investigates the standpoint held by American educators on the matter.
**Methods and Materials:** An online survey was distributed to 608 radiography programme directors across the United States to investigate their opinions on lead shielding use in conventional radiography. Topics covered included: the literature influencing their teachings; whether shielding use is incorporated into student assessments; and observed practice regarding patient shielding in affiliated clinical sites.
**Results:** From a total of 130 respondents, 122 believed shielding provides the patient with an overall dose reduction with these opinions based on textbooks(60%), journal articles(31.5%) and professional experience(26.9%). Interestingly, 26%(n=34) felt shielding of patients over the age of 50 was unnecessary. Most respondents(73.9%;n=96) place equal importance on breast and gonadal shielding. 70.8%(n=92) indicated that protocols in affiliated clinical sites met the American Registry of Radiologic Technologists and American Society of Radiologic Technologists radiation protection practices, however, 84.6%(n=110) are of the opinion that more emphasis should be placed on breast shielding. 53.8%(n=70) reported that failure to use shielding resulted in automatic failure in clinical assessments while for the remainder, omission is not as severely penalised.
**Conclusion:** There is general consensus amongst American radiography educators that lead shielding of patients is important, which is reflected in their student assessments to varying degrees.

**B-1707** 15:21
**Simulation-based learning to facilitate clinical readiness in diagnostic radiography: a metasynthesis**
L.J. Hazell[1], H. Lawrence[1], H. Friedrich-Nel[2]; [1]*Johannesburg/ZA*, [2]*Bloemfontein/ZA (lynneh@uj.ac.za)*

**Purpose:** The aim of this presentation is to present a metasynthesis to identify the attributes for facilitation of simulation-based learning for clinical readiness in a diagnostic radiography programme in South Africa.
**Methods and Materials:** A qualitative, metasynthesis was undertaken from the existing literature in English, which was retrieved from databases (Medline, CINHAL, Google Scholar and ScienceDirect). The keywords used were simulation-based learning in radiography, simulation-based education in radiography and simulation-based learning in nursing. The large volume of data was reduced to units of meaning. The metasynthesis used the following steps: develop a review question, develop a search strategy, extraction of themes from the literature, and metasynthesis of themes.
**Results:** Themes and categories were developed from the literature. Theme 1: an authentic and realistic situation which is relevant to the development of a professional in the context of the profession. Theme 2: building confidence in a safe, reliable and nurturing environment. Theme 3: active participation in a collaborative process.
**Conclusion:** The metasynthesis revealed three major themes that can be used as a framework to motivate for the use of simulation-based learning in a diagnostic radiography programme to facilitate clinical readiness. The strategies would need to stay true to the framework developed so that simulation-based learning can be designed to fit the contextual needs of this dynamic and unique profession.

**14:00 - 15:30** **Room C**

### Radiographers

### SS 1914b

### Mammography quality assessment

*Moderators:*
N.N.
D. Mizzi; Msida/MT

**B-1708** 14:00
**Radiation risk from digital breast tomosynthesis screening: a comparison with full-field digital mammography**
R.M.K. M. Ali[1], A. England[2], C.E. Mercer[2], A.K. Tootell[2], P.H. Hogg[2]; [1]*Najaf/IQ*, [2]*Salford/UK (P.HOGG@SALFORD.AC.UK)*

**Purpose:** To compare the radiation risk from digital breast tomosynthesis (DBT) screening with that from full-field digital mammography (FFDM) screening.
**Methods and Materials:** To simulate compressed breasts, two perspex-polyethylene breast phantoms were used, one phantom for compressed breast in craniocaudal position and the other for compressed breast in mediolateral oblique. An adult ATOM dosimetry phantom was loaded with high-sensitivity thermoluminescence dosimeters; the phantom was then positioned on Hologic Selenia Dimensions mammographic machine to imitate DBT and FFDM screening. Organ radiation doses were measured from 4-view DBT screening and 4-view FFDM screening. Organ radiation doses were used to calculate effective dose from one screening session and the effective risk that could arise from a range of national screening programmes.
**Results:** Mean glandular dose (MGD) for DBT was 3.6mGy; MGD for FFDM was 2.8mGy. For DBT, other organ (e.g. thymus, lungs, salivary glands, thyroid, contralateral breast and bone marrow) radiation dose was also higher than for FFDM. The use of DBT for breast cancer screening increases the effective dose (E) of one screening session by 22%. E for DBT was 0.44mSv; E for FFDM was 0.34mSv. Similarly, the total effective risk during female's lifetime of different screening programmes was increased by 23%.
**Conclusion:** The use of DBT for breast cancer screening may increase the screening programme achievement and it also increases the radiation dose to screening clients.

**B-1709** 14:08
**What criteria should be considered by radiographers while assessing the image quality of breast implant mammograms?**
C. Sá dos Reis[1], I. Gremion[2], K. Schmidt[2], N. Richli Meystre[2]; [1]*Perth/AU*, [2]*Lausanne/CH (claudia.sadosreis@curtin.edu.au)*

**Purpose:** To identify the most relevant image criteria that can be applied in the analysis of mammography exams performed in patients with breast implants (BI).
**Methods and Materials:** The study was conducted in 2 phases: i) literature review to find critical imaging criteria used to assess mammography exams; ii) assessment of 1207 BI mammograms to identify the appropriate criteria. A threshold of 75% of achievement was the cut-off to consider each criterion as applicable to CC, MLO and ML images. Descriptive statistics, chi-square test, independent t test, Pearson correlation were performed.
**Results:** 24 criteria considering positioning, exposure, sharpness and artefacts were identified during the literature review for normal mammograms. Applying those to BI mammograms, for 37.3% of CC images retroglandular fat was not included. Considering MLO and ML images, the “Pectoral-Nipple-Line” criterion was achieved in 35% of the images. The placement of the implant (retropectoral or retroglandular) or performing Ecklund manoeuver had significant influence on the visible anatomy (p=<0.005). The alignment of the breast in the middle of detector and the detector height (p=<0.001) were considered highly relevant, having also impact on the amount of tissue displayed.
**Conclusion:** Part of the criteria used to assess normal mammograms is not applicable to BI imaging due to implant overlap. The alignment of the image in relation to detector’s centre seems to have an impact on the amount of tissue visible to assess. Further studies are necessary to define how many projections, best technique to acquire the images and suitable quality criteria to adequately assess BI mammograms.

**B-1710** 14:16

**The use of different angulation in mediolateral oblique view based on patient anatomy in mammography**

A. Bedene, E. Alukic, J. Žibert, N. Mekis; *Ljubljana/SI (erna.alukic@zf.uni-lj.si)*

**Purpose:** The purpose of this research was to investigate if the quality of imaging in mammography can be maximized for patients with specific anatomy with the use of alternative (35° or 55°) angulation in mediolateral oblique (MLO) projection instead of standard projection with 45° angle.
**Methods and Materials:** 491 women were included in the study of different constitutional type, who undergone screening mammography at angle of 55° and 35°, and additional imaging (tomography) at an angle of 45°. Slovenian criterion (classification) were used to assess the quality of mammographic images. Three measurements were performed on the mammograms: the width of the pectoral muscle, the retromammary space (fatty tissue) and inframammary part of the breast.
**Results:** When comparing 45° and 55° angle all three measurements (the width of the pectoral muscle, the retromammary space and inframammary part of the breast) were statistically significant in favour of 55° angulation. 35° angle showed more retromammary and inframammary part of breast compared to the standard angle of 45°, both results were statistically significant. There was no statistically significant difference regarding the display of pectoral muscle between mentioned angulations.
**Conclusion:** The results showed that the use different angulation in MLO projection showed more diagnostically important breast tissue. We recommend the use of a 35°angle as more appropriate for patients with longer chest and small breasts and the use of a 55°inclination in short-term chest and large breast examiners.

**B-1711** 14:24

**Indications and the outcome of the mammography at Douala General Hospital**

N. Narcisse Fidele; *Douala/CM (nana_nar6@yahoo.fr)*

**Purpose:** This study aimed to list indications and results of mammography and/or breast ultrasounds at Douala General Hospital to determine the proportion of routine mammographic screening.
**Methods and Materials:** This descriptive cross-sectional study was carried out at Douala General Hospital using pre-established data sheets. The study recruited all patients who met the selection criteria and reported to the radiology and medical imaging department for breast screening using physical examinations, mammography and/or ultrasounds.
**Results:** The study recruited 372 patients, 96.8% of whom were between 40 and 50 years old. The reasons given for the medical consultation were systematic screening (33.01%), pain (27.18%) and lumps (25.24%). Breast examination by inspection was normal in 87.1% of women, and by palpation in 66.7%. Mammography revealed nodular opacities (18.3%), spiculated images (4.3%) and micro-calcifications (3.2%), while ultrasound identified fibroadenomas (16.48%) and cysts (6.18%). Suspicious lesions (ACR 4 and 5) were discovered in 7.6% of cases by mammography and 8.51% of cases by ultrasound. The results indicated that there was no significant association between the use of clinical examination and mammography ($p = 0.754$). The use of clinical examination alone for breast screening may not be sufficient.
**Conclusion:** Our findings indicate that in Cemaroon, the routine screening mammography accounts for less than one-third (33.1%) of all indications. Benign lesions were most common; however, 7.6% and 8.51% of suspicious malignant lesions were observed using mammography and ultrasound, respectively.

**B-1712** 14:32

**Early detection of breast cancer education via interprofessional E-learning: the EBreast project**

E. Metsälä[1], T. Kukkes[2], K.M. Rannisto[3], J. Pires Jorge[4], A. Henner[5], B. Strøm[6]; [1]*Helsinki/FI*, [2]*Tartu/EE*, [3]*Oys/FI*, [4]*Lausanne/CH*, [5]*Oulu/FI*, [6]*Bergen/NO (eija.metsala@metropolia.fi)*

**Purpose:** The project “Education and training in early detection of breast cancer for health care professionals” (EBreast) funded by EU was started in 2015 with the goal of studying and developing health care staff competencies related to early detection of breast cancer.
**Methods and Materials:** An evidence-based developmental method was applied for education and training in early detection of breast cancer for health care professionals (the EBreast project). The learning modules were evaluated according to the relevance of the content, technical quality and achievement of learning outcomes by health care students and professionals in the five European countries.
**Results:** Health care staff (biomedical laboratory scientists, midwives, nurses, public health nurses, radiographers) competency needs related to the breast cancer diagnostic chain focused on an interprofessional way of working and patient communication, on technical performance of mammography, and on related further examinations as well as on quality assessment. To achieve these objectives, the learning material package was developed based on the evidence provided by expert opinion and research results.
**Conclusion:** Teaching multifaceted health care processes such as the diagnostic process of breast cancer interprofessional education is as means of ensuring client-centred high-quality services to the patient. The core benefit of open access E-learning is easily accessible material, regardless of time zone and geography, creating the opportunity for equity in education and harmonized health care practices in Europe.

**B-1713** 14:40

**Acceptable quality dose for mammography: a guide for practitioners in developing countries**

D.Z. Joseph; *Kano/NG (josephdlama@gmail.com)*

**Purpose:** Acceptable quality dose (AQD) is a reference dose value or suggested standard dose that is used as an optimisation tool capable of producing good quality images in diagnostic radiological examination. The purpose of the study is to evaluate the AQD for mammography in a Nigerian Hospital.
**Methods and Materials:** The study is a prospective cross-sectional study conducted in a Nigerian teaching hospital, 30 patients who came for mammography examination were enrolled in the study. Thermoluminescent (TLD-LiF) dosimeter chips were used for dose assessment for cranio-caudal and mediolateral oblique views while image quality was assessed using European guidelines. Analysis was carried out using Statistical Package for Social Sciences version 23.0 Chicago, USA.
**Results:** The result shows an average mean glandular dose (MGD) of 0.63mGy and a mean CBT of 1.95mm. The range of MGD for cranio-caudal view and mediolateral oblique (MLO) were 0.88mGy and 1.67mGy, respectively, and the range ESD for CC and MLO were 0.01-6.03mGy and 0.01-3.47mGy, respectively. Most patients’ examinations were within acceptable quality doses with percentage score from 60% to 80% for doses and image quality assessment using European guidelines. Patient dose increased with increase in compressed breast thickness (CBT).
**Conclusion:** AQD has a number of inbuilt advantages because it considers crucial parameters in radiation dose estimation and useful for the formulation of optimisation guidelines and etiquette for countries and regions globally.

**B-1714** 14:48

**Optimization of radiation doses and image quality for mammography examination**

D.Z. Joseph[1], F. Nkubli[2], N. Christian[3]; [1]*Kano/NG*, [2]*Borno/NG*, [3]*Anambra/NG (josephdlama@gmail.com)*

**Purpose:** The purpose of the study was to evaluate mean glandular dose and compare with reference dose levels and determine the relationship between compressed breast thickness (CBT) and mean glandular dose (MGD). To assess the image quality in comparison with European Guidelines.
**Methods and Materials:** A total number of 30 dose values and mammogram of the patients was evaluated. IAEA dose survey was used for collecting data from records of mammography examination. Information such as patient age, weight, body mass index, compressed breast thickness and technical parameter (kVp, mAs, tube Filtration) and European Commission Image quality template was used for assessing image quality. Mean glandular dose (MGD) was calculated and statistical package for social sciences was used for analysis. Pearson’s correlation was used to determine the relationship between CBT and MGD.
**Results:** The result shows an average MGD of 0.534 mGy and a mean CBT of 1.95 cm. The range of MGD for cranio-caudal (CC) and medio-lateral oblique (MLO) were 0.00-0.91 mGy and 0.00 - 1.67 mGy, respectively, and the range entrance skin dose for CC and MLO were 0.01-6.01 and 0.01-3.47, respectively. A correlation of 0.484 and 0.052 for CC and MLO respectively was gotten between the CBT and MGD. Image quality criteria fulfilled the requirement of European Guidelines with 80% total score.
**Conclusion:** The average of MGD was 0.53mGy. The MGD does not depend on the CBT. The images obtained are within acceptable limit
*Author Disclosures:*
**F. Nkubli:** Author; Flavious Nkubli, Christian Nzotta.

**B-1715** 14:56

**A core curriculum for the breast radiographers working in Italy**

S. Pacifici[1], P. Bindinelli[2], V. Galli[3], A. Giacobbe[1], D. Mariotti[4], S. Pedilarco[5], S. Salimbeni[6], G. Santucci[7], D. Severi[4]; [1]*Rome/IT*, [2]*Verona/IT*, [3]*Modena/IT*, [4]*Forlì/IT*, [5]*Milan/IT*, [6]*Bologna/IT*, [7]*Cesena/IT*

**Purpose:** According with the EU Parliament Resolutions on Breast Cancer, all member States were invited to establish a network of certified and interdisciplinary breast units which meet Eusoma's requirements and quality criteria, in order to ensure that women with breast cancer have the right to be cured in centres that ensure quality and efficacy standards. A characterizing element of the Breast Units is the specialisation of the multi-disciplinary team members. Eusoma guidelines indicate the Radiographer as a core-team member. Actually, due to the lack of a common European training standard, dedicated breast radiographers don't exist in Italy as in many others European countries. These premises have led to the establishment of the Senonetwork's Radiographers Working Group, in order to contribute to the development of breast radiographers and to support their role in the Italian Breast Units.
**Methods and Materials:** Review of the literature.
**Results:** Screening radiographers (mammographer) are distinguished from diagnostic breast radiographers. However, in the perspective of Breast Centres where converge first+second level screening, diagnostic, staging and follow-up, this distinction may be overcome, recognizing a single professional to fit this set-up: the breast radiographer.
**Conclusion:** Core curriculum of breast radiographers must be ensured after the basic training through courses to be carried out at Breast Centres or Regional Reference Centres, which programme is defined according with the EUSOMA recommendations. Senonetwork's Radiographers WorkingGroup, with the Training WorkingGroup and the Senonetwork's representative of radiology, proposes to harmonise the different proposals in order to promote a homogeneous training for the breast radiographers.

**B-1716** 15:04
**Radiographers' competencies for pain management in mammography**
M. Pettersson, E. Metsälä; *Helsinki/FI (eija.metsala@metropolia.fi)*

**Purpose:** Breast cancer is the most common cancer among women. Mammography is the most widespread method in diagnosing breast cancer and it is used as a screening technique as well as for breast cancer controls. It is widely known that mammography can cause pain, fear and anxiety and that can lead to patients avoiding the examination. The aim of this study was to identify radiographers´ pain management competencies and educational needs in mammography and, also to find information to make a local pain management model of care.
**Methods and Materials:** The data for the thesis was collected with an electronic theme writing form from the radiographers (n=12) of the department of interest. Radiographers participating in the study were selected with discretionary sample. The data was analyzed with inductive content analysis. During the analysis was found such information that there was a need for an additional research question.
**Results:** Based on the analysis radiographers´ are aware that mammography can cause pain and they know how to decrease that pain, fear and anxiety. Nevertheless, they don´t always associate their competencies with pain management because of non-medical aspect of care. Radiographers´ feel that they have limited possibilities of pain-relieving during mammography due to the limited time for each patient. The results suggest that radiographers´ knowledge about pain management methods should be improved.
**Conclusion:** Based on the analysis there can be found many educational needs and challenges too. Both educational institution and organization must increase the volume of mammography courses and education.

**B-1717** 15:12
**Women's experience of pain in screening mammography**
T. Wathén, K. Paalimäki-Paakki, A. Henner; *Oulu/FI (anja.henner@oamk.fi)*

**Purpose:** Breast cancer is the most common cancer of women. That is why it is important to detect in early phase with screening. Women are often afraid of mammography due to pain and discomfort. The purpose of this study was to describe women's experience of pain and unpleasant feelings during screening mammography.
**Methods and Materials:** A questionnaire was given after examination to all women participating in screening mammography in one hospital mammography unit during two weeks: one week in November 2017 (older group was invited to screening) and one week in February 2018 (those coming first time). Data were analysed in Excel.
**Results:** Totally 167 (61%) women returned the questionnaire, 45% aged 49 to 54 years and 43% 65 to 69 years. 21% participated first time to screening program and more than half of them were afraid of pain in advance. Half of the participants found the examination to be slightly painful and 10% rather painful. Women want to discuss with the radiographer during the examination: what is happening in examination, importance of the compression, impact to the compression force used and about possible feelings of pain. Discussion and friendliness/politeness decreases women's experience of pain. Women prefer counselling for breathing during the compression and kind words from the radiographer instead of pain medication.
**Conclusion:** Professional radiographer in screening mammography is empathic, peaceful, and kind, listens to the woman and explains the examination process and possibility of pain. It is also important to allow the possibility to ask and tell woman's own opinions and feelings.

**B-1718** 15:20
**Scanxiety in follow-up breast cancer women**
S. Pacifici, D. Giudice; *Rome/IT*

**Purpose:** br />**Methods and Materials:** Review of the literature on both follow-up scans related posttraumatic stress disorders and on the effects of non-empathetic, non-compassionate behaviors.
**Results:** Beside the uncertainty of the test results, the root of scanxiety is thought to be related to the amount of time that elapses between when the test is performed and the results known.However, scanxiety might not be entirely related to the examinations, but also to patient's approach and lack of compassion from healthcare professionals
**Conclusion:** As being diagnosed with cancer is hard enough, we need to do all we can do to make the patient journey less stressful .
Cancer care providers and healthcare professionals would seriously consider scanxiety, as it may even cause patients to delay medical examinations and other critical parts of their cancer treatment or post-treatment plan.Breast radiographer's skills should not just related to physics or image acquisition techniques.
Additional skills related to communication, team roles, efficiency, critical thinking and patient experience are strongly recommended, as they allow us to be able to help patients reduce scanxiety.

**14:00 - 15:30** **Room X**

### Vascular

### SS 1915

### Vascular imaging after stent-graft and stent insertion

*Moderators:*
A.D. Annoni; Milan/IT
M. Müller-Eschner; Mainz/DE

**B-1719** 14:00
**Additive value of dual energy (DE) acquisition in CT follow-up of endovascular aortic repair (EVAR or TEVAR)**
G. Agliata[1], N. Schicci[2], P. Esposto Pirani[1], C. Tagliati[1], A. Giovagnoni[1]; [1]*Ancona/IT*, [2]*Fabriano/IT (agliata.g@gmail.com)*

**Purpose:** Evaluating the added value of DE technique acquisition in early endoleak detection in TEVAR/EVAR CT follow-up.
**Methods and Materials:** 82 patients (55M and 27F; mean age 70±5 years) treated with TEVAR or EVAR, underwent DECT scans using a III generation DECT (Somatom Force, Siemens Healthcare).All the scans were obtained using a standard protocol constituted of an angiographic phase, with a delay assessed through bolus-test technique, and a late DE acquisition with 60 seconds of delay from the angiographic phase. Unenhanced images were obtained from the DE late phase. A radiologist with five year of experience in CT angiography, reviewed the images to detect potential endoleak (and its features, such as localization, extent and supply vessel). Firstly native CT images were evaluated. Then, independently, was performed a post-processing analysis of DE phase dataset and qualitative and quantitative assessment of iodine distribution were performed.
**Results:** Native CT images evaluation allowed detection of 25 endoleaks (30.5%; 44% type I and 56% type II); post-processing evaluation of DE iodine distribution maps allowed detection of 29 endoleaks (35%; 38% type I; 62% type II).
**Conclusion:** DE technique acquisition and post-processing analysis increases CT diagnostic value in EVAR/TEVAR follow-up, allowing demonstration of small endoleaks (especially type II ones) unrecognized in native images evaluation.

**B-1720** 14:08
**Prospective single-centre study to evaluate artifacts using CEUS in follow-up after EVAR: possible adverse effects on endoleak detection in comparison with CTA**
F. Frenzel[1], R. Kubale[2], J. Stroeder[1], A. Massmann[1], P. Jagoda[1], A. Bücker[1], P. Minko[1]; [1]*Homburg/DE*, [2]*Pirmasens/DE (felix.frenzel@uks.eu)*

**Purpose:** The aim of this study is to prospectively detect, categorize and interpret relevance of artifacts of contrast-enhanced ultrasound (CEUS) used in follow-up after endovascular aortic repair (EVAR) for detection of endoleaks and postinterventional control.
**Methods and Materials:** 76 Patients underwent EVAR between 11/2012-06/2017 including complex cases (n=22) with reconstruction of iliac-bifurcation, chimneys as well as mycotic aneurysms using six different stent graft vendors. Patients were followed up (mean 417 days) with CT-Angiography (CTA)

(n=257) and CEUS (n=135). CEUS was performed blinded to the results of CTA using an Acuson S2000 or S3000 (Siemens Healthcare, Germany) and i.v. application of sulphur hexafluoride (SonoVue, Bracco, Italy). Corresponding pairs of CTA /CEUS examinations were reviewed retrospectively a second time by two experienced radiologists.
**Results:** 55 patients (9 females; mean age 72.5±8.4) had 97 simultaneous CTA/CEUS examinations. Artifacts were seen in 59% of cases: 22% contrast-agent related, 22% post-interventional, 9% attenuation artifacts, 3% ultrasound scanner- and 3% stent graft-specific artifacts. Endoleaks were detected by CEUS in 56% and by CTA in 48% of cases. Mismatch in detection of endoleaks was observed in 9%, in 4% related to artifacts in CEUS (1 false-negative and 3 false-positive). In 5%, CEUS proved advantageous over CTA through dynamic assessment of late endoleaks or beam hardening artifacts in CTA caused by embolization materials.
**Conclusion:** CEUS has a high ability to detect endoleaks after EVAR, comparable or even superior to CTA. However knowledge of possible artifacts is essential to interpret imaging during CEUS correctly.

**B-1721** 14:16
**Quantitative analysis of dynamic CTA for the detection of endoleaks after abdominal aorta aneurysm endovascular repair: a feasibility study**
M.J. Bauer[1], F. Lavra[2], M. Scarabello[3], M. van Assen[4], L. Saba[2], C.N. De Cecco[1], U.J. Schoepf[1]; [1]*Charleston, SC/US*, [2]*Cagliari/IT*, [3]*Milan/IT*, [4]*Groningen/NL (email@bauermaximilian.de)*

**Purpose:** To assess the feasibility of quantitative analysis of dynamic computed tomography angiography (dCTA) for the detection of endoleaks in patients who underwent endovascular repair of abdominal aortic aneurysms (EVAR).
**Methods and Materials:** Twenty patients scheduled for contrast-enhanced CT angiography (CTA) of the abdominal aorta post-EVAR were prospectively enrolled. All patients received a standard triphasic CTA protocol followed by a dCTA acquisition. In addition to visual assessment of time-resolved images, the dCTA acquisition enabled reconstructions of color-coded maps depicting blood perfusion (dynamic CT perfusion, dCTP). Observers assessed the dCTA and dCTP images for the detection of endoleaks and indicated their diagnostic confidence based on a modified 5-point Likert scale (1 - certain absence, ···, 5 - certain presence of endoleaks).
**Results:** In total, 9 endoleaks were detected in 7 patients using triphasic CTA as the reference standard. There was complete agreement for endoleak detection between the two techniques on a per-patient basis. Both dCTA and dCTP identified an additional potential endoleak in one patient. Moreover, dCTP demonstrated superior diagnostic confidence for endoleak exclusion compared to dCTA (1.0 [1 - 1] vs 1.5 [1.5 - 1.5], respectively; p <0.01).
**Conclusion:** Quantitative analysis of dCTA data in form of dCTP is feasible and can aid in the detection of endoleaks demonstrating a strong correlation with visual assessment of dCTA images. Moreover, our findings suggest that analyzing dCTP imaging data has the potential to increase the endoleak detection rate compared to the assessment of triphasic CTA images as the current clinical standard method.
*Author Disclosures:*
**C.N. De Cecco:** Consultant; Guerbet. Research/Grant Support; Siemens.
**U.J. Schoepf:** Consultant; Bayer, Guerbet. Research/Grant Support; Siemens, Bayer, Astellas, GE.

**B-1722** 14:24
**Flow volume of the abdominal visceral arteries increase after EVAR treatment**
M. Sugiyama[1], Y. Takehara[2], M. Alley[3], N. Unno[1], K. Katahashi[1], T. Wakayama[4], A. Nozaki[4], S. Naganawa[2], H. Sakahara[1]; [1]*Hamamatsu/JP*, [2]*Nagoya/JP*, [3]*Stanford, CA/US*, [4]*Hino/JP (m-sugi@hama-med.ac.jp)*

**Purpose:** Endovascular aneurysm repair (EVAR) is widely performed to prevent abdominal aortic aneurysm (AAA) from rupture. EVAR may also repair deformed blood pathway and relieve blood flow energy loss caused by non-laminar flow. This may provide more efficient blood delivery to the visceral arteries. In the present study, we aimed to evaluate the hemodynamic changes of the abdominal visceral arteries after EVAR treatment within AAA patients using 3D cine PC MR imaging (4D-Flow).
**Methods and Materials:** 10 patients (67 to 80 y.o.) with AAA underwent 4D-Flow covering whole abdominal aorta within the period of 1 year before and after EVAR treatment. All the MR studies were conducted on 3.0T MR imagers. Time resolved contrast enhanced 3D MR angiography was performed for the segmentation of the aorta. The acquired 4D-Flow data was postprocessed using an flow analysis software (Flova II, R'tech, Japan). The flow volume were measured at the cross sections of the following arteries; celiac artery (CA), superior mesenteric artery (SMA), and renal arteries (RA). Each datasets consisted of flow volume data of 20 phases per cardiac cycle. The flow volume of the visceral arteries at each cardiac phase between pre- and post-EVAR condition were statistically analyzed using Wilcoxon's signed-rank test.
**Results:** Significant increase of the flow volume in CA and SMA were observed at mid-diastole.
**Conclusion:** Flow volume of the SMA and CA increased after EVAR treatment for the AAA patients. This may be reflecting the repair of blood flow passage by EVAR and more efficient blood delivery.
*Author Disclosures:*
**Y. Takehara:** Other; Endowed chair of Nagoya University Graduate School of Medicine. **T. Wakayama:** Employee; GE Healthcare Japan. **A. Nozaki:** Employee; GE Healthcare Japan.

**B-1723** 14:32
**Cumulative radiation dose from contrast-enhanced CT in follow-up after EVAR**
S. Magnani, G. Mauri, G. Di Leo, R.M. Trimboli, D. Poretti, L.M. Sconfienza, F. Sardanelli; *Milan/IT (dr.smagnani@gmail.com)*

**Purpose:** To estimate the average cumulative radiation dose deriving from contrast-enhanced computed tomography (CT) examinations in patients treated with endovascular aneurysm repair (EVAR).
**Methods and Materials:** After Ethics Committee approval, records of 110 patients (105 males, 5 females; age 75±7 years [mean±standard deviation]) who underwent EVAR between March 2000 and May 2008 were retrospectively analyzed. The number of CT scans performed in each patient from March 2000 to May 2015 was retrieved. The mean effective dose deriving from abdominal, thoracic or thoraco-abdominal CT scan was derived from another sample of 30 patients. The patient cumulative effective dose was calculated by summing up all radiation doses from each single examination.
**Results:** During a median follow-up of 25 months (interquartile range [IQR] 10-69 months), these patients underwent a median of 7 CT scans overall; 4 (IQR 2-8) abdominal scans, 2 (IQR 1-4) thoracic scans, and 4 (IQR 2-5) thoraco-abdominal scans. The median cumulative effective dose was 205 mSv (IQR 126-367 mSv) overall; 18 mSv (IQR 9-36 mSv) for thoracic scans, 132 mSv (IQR 66-264 mSv) for abdominal scans, and 160 mSv (IQR 80-200 mSv) for thoraco-abdominal scans. The maximal cumulative effective dose was 1,166 mSv in a patient who underwent 38 CT examinations during a 54-month follow-up.
**Conclusion:** Patients treated with EVAR underwent multiple serial CT follow-up examinations, thus receiving a very high cumulative radiation dose.

**B-1724** 14:40
**Low tube voltage and low contrast media volume multidetector CT angiography in evaluation of abdominal aorta in EVAR follow-up: a comparison study**
E.G.M. Antonuccio, S.J. Vasquez Cuadra, S.P. Pirillo, L. Tipaldi; *Rome/IT (dr.gabrieleantonuccio@gmail.com)*

**Purpose:** To compare ionizing radiation dose delivered, image quality and diagnostic performance of Multidetector CT Angiography (MDCTA) of the abdominal aorta after EVAR and arterial splanchnic vessels in two different acquisition protocols: low tube voltage plus low dose contrast medium (CM) protocol (A) versus conventional protocol (B) in the same patients.
**Methods and Materials:** 40 Patients (26 M; 14 F) referred to MDCTA of the abdominal aorta in follow-up after EVAR, who had undergone conventional protocol (64-slices scanner; 120-kVp, automatic mA, 100mL iomeprol [400mg iodine/mL] @4mL/sec i.v. - B), were enrolled in our single-center prospective randomized study in order to compare conventional protocol to low dose protocol (64-slices scanner; 80-kVp, automatic mA, 40mL iomeprol [400mg iodine/mL] @4mL/sec i.v.- A).
**Results:** No significant differences were noted in terms of image quality with either axial source images, multiplanar reconstructions or volume rendering images; no significant differences were found among the two protocols in terms of noise throughout aortic stent or vessels ramifications features, moreover DLP in protocol B (531,3±67,9 mGy-cm) was about 1/3 compared to protocol A (1536,1±233,1 mGy-cm) (p<0,0005).
**Conclusion:** Reducing simultaneously tube voltage and CM volume determines significative dose reduction to the patient without compromising images quality and diagnostic performance of MDCTA in EVAR follow-UP.

**B-1725** 14:48
**Spectral imaging on a 80 mm wide-detector CT with fast kVp switching for follow-up CT angiography after EVAR planning: image quality, radiation dose and ultra-low volume contrast medium**
P. Hou; *Zhengzhou/CN (houping1221@163.com)*

**Purpose:** To investigate the feasibility of reducing radiation exposure and CM dose in follow-up CTA after EVAR through the joint application of spectral imaging and ASIR-V.
**Methods and Materials:** The conventional protocol with kV Assist scan and CM volume of 60 ml was applied in control group A. The ultra-low dose protocol which performed GSI scan with CM volume of 0.6 ml/kg was utilized in study group B. Group A included TUE images, arterial phase .While in group B,

Sunday

VUE images and 60 kev data with 50%ASIR-V set were automatically reconstructed from AP. CT values and CNR of aorta and its branches for TUE and VUE images and AP images were obtained and compared.
**Results:** No significant difference in image quality was found between TUE 4.32 ± 0.53 and VUE 4.21 ± 0.51 images (P > 0.05).VUE images had higher image noise than TUE images (P < 0.001). For AP images, group B had equivalent subjective scores and CT values as Group A, whereas they had higher or equivalent CNRs. Group B had 30.3% and 21% reductions on CM volume and injection rate, respectively, than Group A. The effective dose for group A was 20.5 mSv ± 4.2 and that for group B was 12.6 mSv ± 2.7.
**Conclusion:** Monochromatic images of 60keV in spectral aortic CTA can minimize the amount of CM and injection rate but without sacrifice image quality, and VUE images as an alternative to TUE images, can provide comparable image quality and reduced the radiation dosage.

**B-1726** 14:56
**Split-filter dual-energy CT: value of advanced monoenergetic reconstructions for the assessment of vessel stents**
E. Appel, C. Thomas, Y. Klosterkemper, J. Aissa, G. Antoch, J. Boos; *Düsseldorf/DE*

**Purpose:** To assess the effect of split-filter dual-energy CT advanced virtual monoenergetic reconstructions (VMI) on in-stent lumen visibility and image quality.
**Methods and Materials:** Nine different peripheral vessel stents were placed in a phantom, filled with iodine and examined using a split filter dual-energy CT scanner. Image reconstructions of linearly mixed images (Au/Sn) for 120kV were performed using three different kernels (I46f, Q33f, D33f). VMI and advanced VMI for ten different virtual photon energy levels (40-130keV) were reconstructed. In-stent lumen diameter, artefact width, image noise and lumen density for all reconstructions were assessed using a semiautomatic software tool. Image quality was assessed through signal-to-noise ratio (SNR) and a 5-point Likert scale (1=poor, 5=excellent). Digital angiography served as reference standard for diameter measurements. Standardization with Z-scores was performed to as assess the optimal VMI virtual photon energy level.
**Results:** Advanced VMI led to the highest overall in-stent lumen diameter at 130keV which was significantly higher than AuSn/I46f (3.7±0.5 vs. 3.4±0.4; p<0.002). SNR was comparable between AuSn/I46 at 120keV and advanced VMI at 70 keV (11.4±1.0 vs. 11.6±1.3). Averaged over all reconstructions, both readers reported significantly higher image quality for advanced VMI compared with conventional VMI reconstructions (3.8±1.1 vs. 2.3±1.1, p=0.005) with optimal subjective image quality for advanced VMI at 80 keV (4.6±0.6). Overall, advanced VMI reconstructions at 70 keV were found to be optimal as determined by the Z-score analysis.
**Conclusion:** Advanced VMI reconstructions at 70 keV are optimal for the imaging of vessel stents in split-filter dual-energy CT.

**B-1727** 15:04
**Comparison of automated software diameter/square detection with radiologist visual assessment for 70% stenosis on coronary CT angiography**
S. Hu, W. Peng, Z. Li; *Chengdu/CN*

**Purpose:** To explore the feasibility of software automated detection of coronary 70% stenosis using diameter/square-method in comparison of manual evaluation.
**Methods and Materials:** Fifty-eight patients both underwent CCTA and invasive coronary angiography were retrospectively included. A binary approach (70% stenosis as a cutoff) was used to grade the stenosis severity. For software, all CCTA data sets were analyzed using GE AW Volume Share 5 (AW4.6) product. Double references were selected and corresponding reference diameter/square values were obtained from an automatic trend analysis of coronary artery. As for human manual analyses, axial images, multiplanar reformations and maximum intensity projections in free double-oblique planes were interpreted. Wilcoxon rank-sum test and McNemar test were performed to compare the differences between two groups.
**Results:** Of the 58 patients (44 men, mean age 65.16 +/- 9.64 years), 179 vessels were analyzed. For both per-patient and per-vessel evaluations, the software square detection revealed best sensitivity (100% and 95.31%, respectively) and negative predictive value (NPV) (100% and 96.91%, respectively). Software diameter detection showed highest specificity (SPE) and positive predictive value (PPV), with SPE of 85.71% and PPV of 90.01% for patient-based analysis, SPE of 93.86% and PPV of 88.71% for vessel-based analysis. The significant differences between human reading and software diameter detection (P=0.013) / square detection (P<0.001) were found.
**Conclusion:** Compared with human diagnosis, both the diameter and square automated detection algorithm have relatively higher accuracy for diagnosing 70% coronary artery stenosis. AW4.6 tool showed an improved evaluation distinguishing the “severe” stenosis that must be implanted stents.

**B-1728** 15:12
**Non-contrast magnetic resonance imaging in the follow-up after endovascular aortic repair**
G. Lastella[1], P.M. Cannaò[2], M. Ali[1], F. Secchi[2], F. Sardanelli[1]; [1]*Milan/IT,* [2]*San Donato Milanese/IT (giulia.lastella@unimi.it)*

**Purpose:** The aim of this study is to define the diagnostic accuracy of noncontrast MRI with CT as reference standard in the follow up of patients treated with endovascular aortic repair (EVAR).
**Methods and Materials:** Forty-two patients after EVAR for aortic and/or iliac arterial aneurysms were prospectively enrolled and underwent MRI and contrast-enhanced CT in the same day between 0,01 and 1,55 years after the implantation. MRI was focused on true fast imaging with steady-state free precession (TRUFI) and half-Fourier-acquisition single-shot turbo spin-echo (HASTE) sequences at the precise level of the vessel were EVAR was implanted. CT was performed before and after iodate contract injection. Two independent observers reviewed MRI to evaluate the presence of hyperintensity near the EVAR. Then, they evaluated in CT images the presence of endoleak and classified them according to Stanford classification in type I (a,b) and II.
**Results:** MRI revealed that 11 patients had no hyperintensity, and no one of these had endoleak in CT images. 31 patients presented hyperintensity in MRI, and 19 of these reveled a presence of endoleak (6 type I, 13 type II). Sensitivity, specificity, positive predictive value and negative predictive value were 100% (19/19), 48% (11/23), 61% (19/31) and 100% (11/11) respectively. Positive likehood ratio was -1.9 and negative likehood ratio was 0.52.
**Conclusion:** The absence of hyperintensity near the EVAR in MRI images can exclude the presence of endoleak; nonetheless the hyperintensity can correlate with the presence of endoleak and require CT with iodate contrast to confirm it.

**B-1729** 15:20
**Evaluation of blood pressure variations on CTA geometries of abdominal aortic aneurysms**
R.C. Aydin[1], F. Bräu[2], M. Notohamiprodjo[3], J.E. Roos[4], S. Leng[5], E.E. Williamson[5], K. Nikolaou[3], C.J. Cyron[6], R.P. Marcus[7]; [1]*Geesthacht/DE,* [2]*Munich/DE,* [3]*Tübingen/DE,* [4]*Lucerne/CH,* [5]*Rochester, MN/US,* [6]*Hamburg/DE,* [7]*Luzern/CH (roland.aydin@gmail.com)*

**Purpose:** To assess the relationship between blood pressure variations and the abdominal aorta aneurysm geometry using CTA imaging and computational simulation of the mechanical properties of the vessel wall.
**Methods and Materials:** Fifty adult patients with known abdominal aorta aneurysms underwent contrast enhanced CT-examinations on a 3rd -generation dual-source- CT (SOMATOM Force, Siemens Healthcare). Blood pressure was measured upon admission as per the standard protocol, during imaging procedure and at same hours at home following discharge. The mean arterial pressure (MAP) values and its maximal variations were recorded. The impact of these changes in vessel diameter and volume was estimated analytically using an idealized cylinder geometry and stiffness parameters from a range of established aortic wall properties. The aneurysm geometry data and the widest MAP differences were submitted to a finite element analysis to model the changes predicted by the analytical estimate.
**Results:** The average difference between MAP readings during scanning and at home was 9.1 mmHg (10.8%) with a maximum of 31.2 mmHg (23.7%). This resulted in an aneurysmal difference in diameter of 0.08-0.22% (0.19-0.63% for the maximum MAP difference).Comparing MAP during the imaging process and at hospital admission, the average relative difference was 10.6% (9.4mmHg), with a maximum of 29.5% (27.7mmHg). The difference in the aneurysmal diameter was 0.07-0.21% (0.18-0.60% for the maximum MAP difference).
**Conclusion:** CTA images of abdominal aorta aneurysms for measurement of its diameter and data from mechanical simulations such as finite elements analysis are insensitive to the timepoint of blood pressure measurement.

**14:00 - 15:30** **Room N**

### Musculoskeletal

### SS 1910a

### Spine imaging

*Moderators:*
T. Akinci D'Antonoli; Basel/CH
A. Feydy; Paris/FR

**B-1730** 14:00

### Machine learning classification of spinal lesions: compared accuracy of texture parameters extracted by different software

V. Chianca[1], D. Albano[1], R. Cuocolo[2], C. Messina[1], S. Gitto[1], A. Corazza[3], I. Merli[1], A. Brunetti[2], L.M. Sconfienza[1]; [1]*Milan/IT*, [2]*Naples/IT*, [3]*Genoa/IT (vitochia@hotmail.it)*

**Purpose:** To compare the accuracy of machine learning (ML) algorithms for classification of spinal lesions based on texture analysis (TA) parameters extracted by different software from unenhanced Magnetic Resonance images (MRI).
**Methods and Materials:** We retrospectively enrolled 146 patients with 146 spinal lesions (49 benign, 57 metastatic and 40 primary malignant lesions) imaged using MRI. Of them, 117 were histopathologically confirmed after surgery while 29 benign lesions were confirmed by follow-up. Patients were randomly divided in training (n=100) and test groups (n=46), respectively for classification model development and testing. Lesions were manually segmented on T1-weighted and T2-weighted images by drawing a bi-dimensional polygonal region of interest. These were used for first order and texture feature extraction on two software, 3D-Slicer heterogeneity CAD module (hCAD) and Pyradiomics. For each of them, different data subsets, obtained by four feature selection methods were analyzed by 9 ML classification algorithms to evaluate their accuracy in identifying benign vs. malignant lesions and benign vs. primary malignant vs. metastatic lesions.
**Results:** In the test group, a random forest algorithm correctly classified 89% of lesions as benign or malignant, based on hCAD TA, while a Support Vector Machine could achieve an accuracy of 87% from Pyradiomics TA. For the classification of benign, primary malignant and metastatic lesions, RF models accurately classified 70% of lesions for both TA software.
**Conclusion:** ML algorithms show good accuracy in spinal lesion classification based on non-contrast MRI exams. Furthermore, feature extraction performed using different software has shown consistent results at subsequent ML analysis.

**B-1731** 14:08

### A triple-classification radiomics model for the differentiation of primary chordoma, giant cell tumour, and metastatic tumour of sacrum based on magnetic resonance imaging

P. Yin, N. Hong; *Beijing/CN (yinping915@pku.edu.cn)*

**Purpose:** To develop and validate a triple-classification radiomics model for the preoperative differentiation of sacral chordoma (SC), sacral giant cell tumour (SGCT), and sacral metastatic tumour (SMT) based on T2-weighted fat saturation (T2w FS) and contrast-enhanced T1-weighted (CE T1w) magnetic resonance imaging (MRI).
**Methods and Materials:** 120 pathologically confirmed sacral patients were retrospectively analysed and divided into a training set (n=83) and a validation set (n=37). Morphology, intensity and texture features were assessed based on formfactor, Haralick, grey-level co-occurrence matrix (GLCM), grey-level run-length matrix (GLRLM) and histogram. Analysis of variance (ANOVA), least absolute shrinkage and selection operator (LASSO), Pearson correlation, random forest (RF), area under the receiver operating characteristic curve (AUC) and accuracy analysis were used to build and validate our model.
**Results:** The median age of SGCT (33.5, 25.3-45.5) was significantly lower than those of SC (58.0, 48.8-64.3) and SMT (59.0, 46.3-65.5) groups ($\chi 2$=37.6, P<0.05). For the differential value, features extracted from joint T2w FS and CE T1w images outperformed those from T2w FS or CE T1w images alone. Compared to CE T1w images, features derived from T2w FS images yielded higher AUC in both training and validating set. The best performance of radiomics model based on joint T2w FS and CE T1w images reached an AUC of 0.773 and an accuracy of 0.711.
**Conclusion:** Our 3.0T MRI-based triple-classification radiomics model is feasible to differentiate SC, SGCT, and SMT, which may be applied to improve the precision of preoperative diagnosis in clinical practice.

**B-1732** 14:16

### Comparison of radiomics nomograms for the differentiation of sacral chordoma and sacral giant cell tumour based on 3D computed tomography and multiparametric magnetic resonance imaging

P. Yin, N. Hong; *Beijing/CN (yinping915@pku.edu.cn)*

**Purpose:** To compare radiomics nomograms based on 3D computed tomography (CT) and multiparametric magnetic resonance imaging (mpMRI) for the preoperative differentiation of sacral chordoma (SC) and sacral giant cell tumour (SGCT).
**Methods and Materials:** 83 SCs and 54 SGCTs patients with histologically proven were retrospectively analysed. All patients underwent CT [CT and CT enhancement (CTE)] or mpMRI [T1-weighted, T2-weighted, diffusion-weighted imaging (DWI) and contrast-enhanced T1-weighted sequences] scans before surgery. We built six models based on each scan and four nomograms based on fused scans combined with or without clinical data. The area under the receiver operating characteristic curve (AUC) and accuracy (ACC) analysis were used to assess the performance of radiomics models. Decision-curve analysis was used to assess the clinical usefulness of nomograms.
**Results:** Age, sex and tumour location had significant difference between SC and SGCT ($t_{age}$=9.00, $\chi^2_{sex}$=10.86, $\chi^2_{location}$=26.20, P<0.01). For individual scan, radiomics model based on DWI features had the highest AUC of 0.889 and ACC of 0.885 in validating set, followed by CT (AUC=0.857, ACC=0.846) and CTE (AUC=0.833, ACC=0.769). For fused scan, CT fused scan had a better AUC of 0.942 and ACC of 0.880, while MR fused scan achieved a lower performance than individual scan. Clinical-radiomics nomogram based on CT fused scans achieved the highest AUC of 0.948 and ACC of 0.920.
**Conclusion:** Radiomics model based on CT and mpMRI has a certain predictive value in distinguishing SC and SGCT, but CT fused features are more recommended. Radiomics nomograms perform better when combined with clinical data.

**B-1733** 14:24

### Texture analysis for quantitative evaluation of central lumbar spinal stenosis

F.A. Huber, S. Stutz, I. Vittoria De Martini, M. Mannil, A.S. Becker, S. Winklhofer, J.M. Burgstaller, R. Guggenberger; *Zurich/CH (florian.huber@usz.ch)*

**Purpose:** To investigate the use of texture analysis (TA) in detection and grading of lumbar spinal stenosis (LSS) in MRI.
**Methods and Materials:** 343 images of 82 patients from a nationwide LSS cohort study, undergoing MRI of the lumbar spine due to neurogenic claudication, with a single-level severe central LSS were included (1-5 images of levels L1-S1 per patient). One expert radiologist as reference standard rated LSS according to a standard four-point (normal-severe) and to eight-point Schizas grading scale additionally evaluating epidural fat. Two independent readers analogously performed qualitative ratings and additionally performed TA in all images by placing two regions of interest (ROI): (1) dural sac only, (2) additionally including epidural fat and bilateral recesses. Interreader agreements for qualitative and quantitative parameters were calculated. TA parameters with intraclass correlation <.75 were excluded for feature reduction. Remaining features were analysed with machine learning (ML) algorithms (Weka 3) for correlation with LSS grades using test and training data sets and 10-fold cross-validation.
**Results:** Qualitative ratings showed moderate reproducibility for both LSS classification systems, but high correlation with cut-off cross-sectional area (CSA)<130mm² for severe LSS (AUC=.722-.916). For both ROIs, ML-evaluation of TA with a decision tree classifier revealed higher performances for LSS grading compared to qualitative assessments using the reference CSA cutoff, respectively.
**Conclusion:** Qualitative grading of LSS is moderately reproducible. ML-supported TA reveals highly reproducible quantitative features that increase diagnostic performance for severe LSS detection, with minor impact of grading score and CSA border definition.

**B-1734** 14:32

### Evaluation of osseous cervical foraminal stenosis in spinal radiculopathy using susceptibility-weighted magnetic resonance imaging

G. Engel, Y.Y. Bender, L.C. Adams, S.M. Böker, U.L. Fahlenkamp, G. Diederichs, M. Wagner, B. Hamm, M.R. Makowski; *Berlin/DE*

**Purpose:** The aim was to evaluate the diagnostic performance of susceptibility-weighted magnetic resonance imaging (SW-MRI) for the evaluation of osseous foraminal stenosis (FS) of the cervical spine compared to conventional MRI-sequences, using computed tomography (CT) as a reference standard.
**Methods and Materials:** Twenty-one patients with radiculopathy of the cervical spine were prospectively included. CT and MRI data sets were available for all patients. As standard of reference, 280 neuroforamina of the cervical spine, including 58 stenosis, were identified on CT-images. T1-, T2-

and SW-MRI were performed. The presence of FS was assessed in all sequences. Sensitivity and specificity were calculated and differences in detection rate and severity scoring of FS were tested. CT was used as reference standard for all analysis.
**Results:** 56 of 58 FS could be correctly identified on SW-MR magnitude images. SW-MRI achieved a sensitivity of 96.6% and specificity of 99.5% for the identification of osseous FS. Conventional T1-weighted MRI sequences achieved a sensitivity and specificity of 43.1% and 100% and T2-weighted MRI sequences a sensitivity and specificity of 65.5% and 99.1%, respectively. The overall detection rate was significantly (p<0.05) higher on SW-MRI. There was no significant difference (p>0.05) in severity scoring compared to CT. T1- and T2-weighted MRI underestimated the degree of osseous FS. Intermodality and interobserver agreement were highest for SW-MRI.
**Conclusion:** SW-MRI enables the detection of osseous FS of the cervical spine in patients with spinal radiculopathy with a higher sensitivity compared to conventional T1- and T2-MRI sequences, with CT as a reference standard.
*Author Disclosures:*
**G. Engel:** Research/Grant Support; Siemens Healthineers AG provided financial and technical support for conducting the study. Siemens Heathineers AG did not have control over the study conduct or data analysis.

**B-1735** 14:40
**Advantages of T2* relaxation time with ultra-short TE on intervertebral disc degeneration**
H. Takashima[1], M. Yoshimoto[1], R. Imamura[1], Y. Akatsuka[1], M. Nakanishi[1], M. Yoneyama[2], M. Hatakenaka[1], T. Yamashita[1]; [1]*Sapporo/JP*, [2]*Tokyo/JP (takashima@sapmed.ac.jp)*

**Purpose:** To investigate the association of intervertebral disc (IVD) degeneration with T2* relaxation time using ultra-short TE (UTE).
**Methods and Materials:** This study included 50 patients (mean age, 52.5 ± 21.8 years) who underwent lumbar routine MR examination using a 3-T MR system (Ingenia, Philips Healthcare). A 3D UTE sequence with fat suppression was used, which was acquired using a phase-encoded stack of spiral trajectory as the first TE and four gradient echoes. TEs were set at 0.16, 4.6, 9.2, and 13.8 ms. Scanning was performed around the lumbar spine using a sagittal slice orientation. We classified the grade of disc degeneration as I-V in the midsagittal section according to the Pfirrmann classification, and T2* was calculated from all TE images at the level of L2/3-L5/S1. We also evaluated the correlation between the T2* of IVDs and Pfirrmann classification.
**Results:** The T2-weighted image-based Pfirrmann classification for our patients was as follows: grade II, 62 discs; grade III, 43 discs; grade IV, 67 discs; and grade V, 28 discs. The T2* of grades II, III, IV, and V was 44.9 ± 17.4 ms, 30.6 ± 13.0 ms, 23.3 ± 10.8 ms, and 13.1 ± 13.6 ms, respectively. T2* RT decreased with increasing grade ($r = -0.724$, $p < 0.01$) and was significantly different between grades II-V ($p < 0.05$).
**Conclusion:** The T2* of IVDs using UTE enabled to identify high IVD degeneration and may, therefore, be useful as a new technique for the MR evaluation of IVD degeneration.
*Author Disclosures:*
**M. Yoneyama:** Employee; Philips.

**B-1736** 14:48
**Molecular lumbar intervertebral disc alterations in patients with leg length discrepancy**
M. Frenken, D. Abrar, M. Knautz, D. Latz, A. Ljimani, G. Antoch, C. Schleich; *Düsseldorf/DE (miriam.frenken@med.uni-duesseldorf.de)*

**Purpose:** Leg length discrepancy (LLD) is a frequent incidental finding during orthopaedic physical examination and could be a predisposing factor for early degenerations of lumbar intervertebral discs or vertebral facet joints. The purpose of this study was to elucidate the effect of LLD on glycosaminoglycan content (GAG) in lumbar discs.
**Methods and Materials:** 11 patients (25.6 ± 4.3 years) with LLD greater than 10 mm and 14 control subjects (23.9 ± 3.5 years) without LLD were examined using a 3T MR scanner. Morphological T2-weighted sequences in sagittal and transversal orientation and Glycosaminoglycan chemical exchange saturation transfer (gagCEST) sequence were performed. Subjects with bulged or herniated discs were excluded.
**Results:** Nucleus pulposus-gagCEST values of L5/S1 disc were significantly lower in patients with LLD compared to control group ($p = 0.0008$). For all other disc levels, no significant difference was found.
**Conclusion:** This study supports the hypothesis that LLD greater than 10 mm could be a predisposing factor for early molecular alterations of lumbar discs of L5/S1. Remarkably, we observed lower gagCEST values of the lumbar disc of L5/S1 caused by LLD even before any morphological pathology could be found.

**B-1737** 14:56
**MR imaging of transitional lumbosacral junction: co-incidental or causative factor for the disc degeneration and herniation**
K.A. Bhagwat, V. Mahadevappa; *Davangere/IN (bhagwatkishan@gmail.com)*

**Purpose:** To identify MR imaging findings and to know the causal relationship between transitional lumbo-sacral junction and degeneration of the lumbar disc. To identify the significance of the transitional lumbosacral junction
**Methods and Materials:** The retrospective study was carried out at teaching hospital. 600 cases, over a period of 18 months, who were referred to the department of diagnostic radiology for MRI of Lumbosacral spine, from the department of Orthopaedics, Neurosurgery and other departments, were included in the study. Inclusion Criteria:- 1. Patients of all age groups. 2. Patients with history of low back pain.
Exclusion Criteria:- Patients with metallic implants, cardiac pacemakers, cochlear implants; who are claustrophobic.
**Results:** In our review of 600 cases, 180 cases were having transitional lumbosacral junction. Out of 180 cases, 91 were male population and 89 were females (M:F Ratio - 1.1: 1). 30 out of 180 cases were of age less than 30 years and the remainder were more than 30 years of age. 128 cases showed sacralization and 52 cases showed lumbarisation.50% of the disc herniations were found to be at L5-S1 intervertebral disc level. In non-lumbosacral transitional vertebral cases, about 53%, 27% and 20% of the disc herniations were found to be at L4-L5, L5-S1 and L3-L4 cases respectively.
**Conclusion:** We conclude that there is a causal relationship between the transitional vertebra and degeneration of the disc immediately cephalad to it, whereasin non-lumbosacral transitional vertebral cases, disc herniations were common atL4-L5 disc levels.

**B-1738** 15:04
**Dual-energy computed tomography detects disk injuries in patients with vertebral fractures: a prospective diagnostic accuracy study**
M. Pumberger, M. Fuchs, N. Engelhard, K.-G.A. Hermann, M. Putzier, M.R. Makowski, B. Hamm, T. Diekhoff; *Berlin/DE (torsten.diekhoff@gmail.com)*

**Purpose:** To evaluate the diagnostic accuracy of dual-energy computed tomography (DECT) in the assessment of disk injury in patients over the age of 50 with vertebral fractures using magnetic resonance imaging (MRI) as the standard of reference.
**Methods and Materials:** The prospective study was approved by the local ethics committee (EA1/372/14), and all patients gave written informed consent. Patients with suspected fracture underwent DECT and MRI of the spine. Three readers scored the DECT collagen maps for the presence vs. absence of disk injury and MR images according to the Sander classification (0-3). Only disks at risk (target disks) were included in the analysis. Sensitivity and specificity were calculated. Fleiss's κ was used for interrater agreement. Attenuation in Hounsfield units (HU) in DECT was compared between affected and unaffected disks. MRI was used as the standard of reference.
**Results:** A total of 295 disks in 67 patients were analysed. DECT had .85 sensitivity and .75 specificity. Sensitivity varied with the severity of disk damage (1: .80, 2: .85 and 3: .98). Fleiss's κ was .41 for MRI and .51 for DECT. HU values measured in the DECT collagen maps were lower for disks with injury compared to normal disks (80.3 ± 35.2 vs 97.9 ± 41.0, p= .0002).
**Conclusion:** DECT collagen maps allow identification of disk injury in elderly patients with vertebral fractures and can yield additional diagnostic information compared with conventional CT.

**B-1739** 15:12
**Dose optimisation in spinal computed tomography for planning of scoliosis surgery**
Y. Klosterkemper, M. Konieczny, T. Hesper, J. Aissa, G. Antoch, J. Boos; *Düsseldorf/DE (Yan.Klosterkemper@med.uni-duesseldorf.de)*

**Purpose:** To assess the potential for dose optimisation in patients with adolescent idiopathic scoliosis (AIS) undergoing preoperative spinal CT.
**Methods and Materials:** Ten patients with AIS (3 male, 7 female, 18±11 years) were included in this prospective study. CT examinations were performed with automated exposure control (mean CTDIvol 4.1±0.9mGy; DLP: 192±50mGyxcm). Dose reduction to 50%, 20 %, 10% and 5% was simulated using dedicated reconstruction software. Two spinal surgeons blinded to the dose level independently and randomly measured the size of each pedicle for screw size selection. Additionally, the confidence in the measurements was assessed (5=very confident in the measurement, 1=measurement cannot be performed with any confidence). Two radiologists rated the image quality for the assessment of bone and soft tissue structures (5=excellent, 1=non-diagnostic). Bonferroni was used to correct for multiple testing (p<0.0125).
**Results:** Pedicle length and width measurements were comparable between 100% and 50% reconstructions (36.4mm/4.1mm vs 36.6mm/4.1mm) whereas both measurements decreased with further dose reduction (20%: 36.1mm/4.1mm; 10%: 35.5mm/4.0mm; 5%: 34.6mm/3.9mm). Confidence in the measurements was excellent at 100% and 50% (all ratings of 5) and decreased with further dose reduction (20%: 4.7; 10%: 3.7; 5%: 2.5). Image quality decreased with decreasing dose (4.9±0.4 for 100 % to 1±0 for the 5% reconstructions; p<0.001, respectively). For bone assessment, image quality was comparable between 100% and 50% reconstructions (4.9±0.4 vs 4.7±0.5).
**Conclusion:** Dose of preoperative spinal CT in AIS patients can be reduced to 50% without impairment of pedicle size measurements or surgeons' confidence in planning the operation.

**B-1740** 15:20
**Radiation exposure during radiographic and computed tomography lumbar spine imaging for adults**
C. Gernhardt, N. Guberina, M. Burggraf, U. Dietrich, J. Theysohn, M. Forsting; *Essen/DE (Christian.gernhardt@uk-essen.de)*

**Purpose:** The purpose of this study was to examine radiation dose levels and image quality of important lumbar spine imaging techniques of major clinical concern.
**Methods and Materials:** In a retrospective study design 41 patients received both (A) radiographic and (B) computed tomography of the lumbar spine. Patients were examined at the single-source CT-scanner SOMATOM Definition AS+, dual-source SOMATOM Definition Flash and SOMATOM Force between March 2015 and March 2018. Important CT parameters and dose descriptors like $CTDI_{vol,}$ DLP and DAP were examined. Additionally, effective and organ doses were calculated using established radiographic conversion factors and Monte Carlo simulation, following IRCP 103 recommendations. Intermodality comparison of radiation exposure and image quality was performed in this ideally matching patient collective.
**Results:** The mean effective dose [median, CI] for a standardised lumbar spine imaging is summarized as follows: (A) 0.27 mSv [0.21 mSv, CI 0.15 - 0.36] and (B) 5.56 mSv [4.90 mSv, CI 3.34 - 6.30]. Image quality on a five-pointed Likert-scale is rated as follows: for (I) delineation of spinal disc herniation (A) 0.8 and (B) 3.6; (II) delineation of spinal canal stenosis (A) 2.8 and (B) 4.1; (III) postoperative control, spinal fusion assessment and exclusion of vertebral body sintering (A) 3.1 and (B) 4.2.
**Conclusion:** This is the first data acquisition of radiation exposure during important lumbar spine imaging techniques in an ideally matching patient collective. The assessment of radiation exposure is crucial for the determination of diagnostic reference levels and choosing the best imaging protocol for clinical questions.

**14:00 - 15:30** **Room O**

### Musculoskeletal

### SS 1910b

### Spine and peripheral nerve imaging

*Moderators:*
N.N.
G. Bodner; Vienna/AT

**K-41** 14:00
**Keynote lecture**
C. Martinoli; *Genoa/IT*

**B-1741** 14:09
**Spinal osseous lesions in tuberosclerosis**
F. Düzgün, G. Yilmaz Ovali, I.S. Örgüc; *Manisa/TR (fatihdzgn@yahoo.com)*

**Purpose:** To characterize bone lesions incidentally detected in abdominal and thorax computed tomography (CT) and magnetic resonance imaging (MRI) scans in tuberosclerosis patients.
**Methods and Materials:** Twenty-three patients were examined retrospectively for the presence of spinal, thorax and abdominal CT or MRI scans performed for other components of tuberosclerosis. Only 7 patients had at least one of the above mentioned studies.
**Results:** All of six adult patients had sclerotic bone lesions on thoracal, lumbar vertebra, sacrum and iliac bones, which distributed around the subcortical bone surrounding the spinal canal in a specific pattern. No sclerotic bone lesion was determined in the pediatric patient. Lesions were mostly located in posterior elements and sacrum was involved in all patients.
**Conclusion:** Recognition of sclerotic bone lesions is useful for supporting the clinical diagnosis of tuberosclerosis and differing from osteoblastic metastasis as well.

**B-1742** 14:17
**Application of 3.0T magnetic resonance diffusion tensor imaging in diagnosis of discogenic low back pain**
S. Tian, H. Yuan; *Beijing/CN (ts@bjmu.edu.cn)*

**Purpose:** The aim of our study is to observe the morphology and integrity of annulus fibrosus by diffusion tensor imaging (DTI), and quantitatively analyse the annulus fibrosus by measuring the apparent diffusion coefficient (ADC) and fractional anisotropy (FA).
**Methods and Materials:** Patients with recurrent low back pain and healthy volunteers were enrolled in the study over a continuous period of time. The ADC and FA values at the posterior margin of the annulus fibrosus were measured by conventional lumbar MR sequence and axial DTI of L3-S1 levels (3.0T). The responsible intervertebral discogenic low back pain segment was determined by discography. The ADC and FA values were measured at the posterior margin of the annulus fibrosus of the segment.
**Results:** 30 subjects in the experimental group and 24 subjects in the control group were included in the study. There was no significant difference in demographic parameters and the area of region of interest (ROI) measured manually between the two groups. The ADC value of ROI in the experimental group was significantly higher than that in the control group (9.84±4.01 vs. 6.10±3.17, p=0.0002), and the FA value was significantly lower than that in the control group (0.231±0.150 versus 0.341±0.201, p=0.007).
**Conclusion:** 3.0T magnetic resonance DTI is instructive for the diagnosis of discogenic low back pain and preoperative localisation of the responsible segment. ADC and FA values can provide objective indicators for intervening intervertebral discogenic low back pain.

**B-1743** 14:25
**Diffusion tensor imaging with quantitative evaluation of sciatic nerve within the pelvis in patients with non-contributory lumbar spine magnetic resonance imaging in radiculopathy**
M. Catania[1], G. Foti[2], A. Beltramello[2], G. Carbognin[2]; [1]*Verona/IT*, [2]*Negrar/IT (id634iff@studenti.univr.it)*

**Purpose:** Diffusion tensor imaging with quantitative evaluation of sciatic nerve within the pelvis in patients with non-contributory lumbar spine MRI in radiculopathy.
**Methods and Materials:** 32 consecutive sciatica patients with negative lumbar MRI were studied between October 2016 and February 2018 with DTI sequence of the pelvis (TR/TE 5800/97 ms; b=1000; slice thickness 3.5 mm; directions=20) using a 1.5T scanner (Siemens Aera). DTI data were post-processed by two radiologists blinded to clinical data. Each radiologist placed two ROI on the nerve roots at three different levels within the pelvis on both sides and the mean value was used for further analysis. Clinical findings served as the standard of reference. Diagnostic accuracy values of the FA numbers using ROC curves and relative AUC were calculated. Inter-observer and intra-observer agreements and continuous and categorical variables were calculated. A value of p<0.05 was considered statistically significant.
**Results:** The lumbar nerve roots were visualized and FA values were calculated in all subjects. The FA values were significantly different between suffering nerve roots (178± 48; range 146-285) and spared side (296±52; range 221-412) with a p value <0.001. The ROC curve analysis revealed an AUC of 0.816 (95% confidence interval: 0.682-0.874). Using a FA of 220 as cutoff to identify suffering nerve roots, the sensitivity, specificity, PPV, NPV and accuracy were 81.8, 95.4, 90.0, 91.3 and 90.9%. The inter-observer and intra-observer agreements were near perfect.
**Conclusion:** DTI can quantitatively demonstrate the presence of suffering sciatic nerve roots within the pelvis.

**B-1744** 14:33
**Preoperative and postoperative strain elastography, and shear wave elastography findings of the sciatic nerve in patients with unilateral lumbar disc herniation**
V. Buruldaу[1], U.O. Çelebi[1], M. Ogden[1], M.H. Akgul[1], A. Dogan[2], M.F. Ozveren[1]; [1]*Kirikkale/TR*, [2]*Kahramanmaras/TR (vedoctor@hotmail.com)*

**Purpose:** The aim of this study was to compare preoperative and postoperative findings of the sciatic nerve using B-mode ultrasound (US), strain elastography (SE), and shear wave elastography (SWE) in patients with unilateral lumbar disc hernia.
**Methods and Materials:** This prospective study was conducted with a pre-test and post-test design. Preoperative and postoperative (one month after surgery) B-mode ultrasound, SE, and SWE findings of the patients who underwent unilateral spinal decompression surgery were compared. The cross-sectional area (CSA) was measured with the direct tracing method using an electronic caliper.
**Results:** A total of 20 patients (9 males, 11 females) with a mean age of 46.2±13.1 years were included. The CSA, diameter, SWE values of the sciatic nerve were significantly higher in the involved side compared to those of the non-involved side (all p<0.05). Blue and blue-green were the most common colour codes in the involved side while green and green-yellow-red were the most common colour codes in the non-involved side. The CSA, diameter, and SWE values of the sciatic nerve decreased after surgery in the involved side (all p <0.05), and no difference was determined in the non-involved side (all p>0.05).
**Conclusion:** Lumbar decompression surgery decreases the sciatic nerve diameter, CSA, and stiffness of the sciatic nerve.

**B-1745** 14:41
**Pilot study on radiomics of peripheral nerves: feasibility of quantitative imaging phenotyping on magnetic resonance imaging**
A. Tagliafico, F. Rossi, F. Valdora, M. Grandis, L. Benedetti, B. Bignotti, A. Schenone, C. Martinoli; *Genoa/IT (federossi0590@gmail.com)*

**Purpose:** To study radiomics features of the peripheral nerve MRI in normal and pathological peripheral nerves.
**Methods and Materials:** We retrospectively reviewed clinical 1.5T MRI data from January 2016 to January 2018 of 20 adult patients (11 men and 9 women; mean age 54.2±3.7) with peripheral neuropathy and 20 age- and sex-matched controls (12 men and 8 women; mean age 56.4±4.9). Radiomic analysis was done on fast spin echo axial T1-weighted images. Regions of interest (ROIs) were done by two radiologists in consensus. N=104 radiomics features were extracted and evaluated. Mann-Whitney U test for unpaired data with 1000 bootstraps samples was used to compare radiomics features of normal and pathological peripheral nerves. Reading time was also estimated.
**Results:** The pathological group included systemic neuropathies (n=8/20), Schwannomas (n=2/20), diabetic neuropathies (n=2/20), entrapments (n= 5/20), post-traumatic/post-surgical neuropathies (3/20). Statistically significant differences (p< 0.05) were found in n=22/104 (21%) features. N=5/22 features were shape based, n=7/22 were first-order features, n= 10/22 features were grey-level run length matrix (Glrlm). Mean time to perform radiomics analysis was 3 (±45 minutes) hours per patient.
**Conclusion:** Radiomics analysis in MRI of peripheral nerves is feasible and found differences between normal and pathological nerves in n=22/104 features.

**B-1746** 14:49
**Diffusion tensor imaging and tractography acquired with magnetic resonance imaging for preoperative assessment of benign peripheral nerve sheath tumours**
B.J. Schwaiger, A.S. Gersing, B. Cervantes, C. Knebel, J. Dangelmaier, D. Pfeiffer, E.J. Rummeny, D. Karampinos, K. Woertler; *Munich/DE*

**Purpose:** To assess the diagnostic potential of fibre tractography and diffusivity analysis based on diffusion-weighted (DW) sequences for preoperative differentiation of benign peripheral nerve sheath tumours.
**Methods and Materials:** In 17 patients with histologically confirmed schwannomas and 5 patients with neurofibromas (mean age 41.9±17.1y, 13 women) 3T MR imaging was performed including a fat-suppressed 3D DW-TSE sequence (TR/TE=1600/33ms; b-values=0;400) comprising 6 diffusion directions for diffusion tensor (DT) imaging analysis. DT were computed from iso-DW images using linear fitting, and fibre tracks were determined. Mean diffusivity was computed from derived Eigenvalues. Fascicle visualization was graded using a four-point scale ranging from poor to very good. Tumour location in relation to nerve fascicles and the number of fascicles were noted. MR imaging findings were compared to analogous intraoperative findings. Agreement of MR and intraoperative findings was calculated using Cohen's kappa.
**Results:** Fascicle visualization was graded as very good/ good in 82%. Schwannomas were significantly more often located eccentrically (94.1%) than neurofibromas (0%; P<0.01). Tractography revealed fascicles to be significantly more often continuous in schwannomas (88.9%) than in neurofibromas (0%; P=0.014). Mean diffusivity in schwannomas ($1.94 \pm 0.40 \times 10^{-3} mm^2/s$) was significantly higher than in neurofibromas ($0.89 \pm 0.21 \times 10^{-3} mm^2/s$; P<0.001). Fascicle courses around the tumour as graded on MR and intraoperatively showed a substantial agreement (κ=0.78).
**Conclusion:** Preoperative diffusion-weighted imaging-based fiber tractography and diffusivity analysis accurately differentiated between schwannomas and neurofibromas and showed a high agreement with intraoperative findings and thus could support individual treatment and surgical planning.

**B-1747** 14:57
**Sonographic mapping of brachial plexus and comparison with magnetic resonance imaging and electrophysiological studies**
A. Agarwal; *New Delhi/IN (ayushagarwal9.8@gmail.com)*

**Purpose:** To compare ultrasonography (USG) with MRI and electromyography/nerve conduction studies (EMG/NCV) for evaluating the brachial plexus pathologies.
**Methods and Materials:** Thirty patients with clinical suspicion of brachial plexus involvement underwent sonography of brachial plexus on both sides. Magnetic resonance imaging (MRI), the gold standard, was done subsequently and compared with sonography findings. EMG/NCV studies were also compared.
**Results:** The findings on USG (e.g. thickened hypoechoic nerves, increased surrounding hyperechogenicity) had excellent correlation with MRI (e.g. thickened nerves with increased surrounding hyperintensity on T2/ STIR images) and EMG/NCV studies. Out of 30 cases, 15 cases had abnormal USG findings and MRI was abnormal in 19 cases. In 4 cases where USG was normal, MRI showed the intradural root avulsions and associated pseudomeningoceles which could not be picked up on ultrasound. These findings correlated with NCV/EMG studies in all the cases (100%).
**Conclusion:** USG is more readily available than MRI and can be done in acute emergency settings. It can also be used for follow-up of patients in brachial plexus injury after initial MRI has been done. Furthermore, USG is the only imaging modality available in patients who have contraindications for MRI . The drawbacks of USG include that it could not demonstrate the intradural root avulsions and associated pseudomeningoceles (in 100% cases). Also, the extent of visualisation of brachial plexus on ultrasound is very good for C5, C6 and C7 roots (100% cases) and limited in C8 root (15 cases: 50%) while T1 root could not be visualised in any case.

**B-1748** 15:05
**High-resolution ultrasound of the superficial sensitive nerves of the arm and the forearm: anatomy, scanning technique and clinical relevance**
R. Picasso[1], F. Zaottini[1], F. Pistoia[1], M. Miguel Perez[2], C. Martinoli[1]; [1]*Genoa/IT*, [2]*Barcelona/ES (riccardo.picasso@gmail.com)*

**Purpose:** The aim of this study was to describe the potential role of high-resolution ultrasound (US) for evaluation of superficial sensitive nerves of the arm and forearm.
**Methods and Materials:** After US-guided percutaneous latex injection, n=3 cadaveric limbs were dissected to identify the course of the medial cutaneous nerve of the arm (MCNA) and the medial (MCNF), lateral (LCNF) and posterior (PCNF) cutaneous nerves of the forearm and determine whether US is able to localize these small nerves. A consecutive series of n=11 patients who had sensory disturbances in the territory of the MCNA, MCNF, LCNF and PCNF were examined with US using a 17-5MHz transducer. One patient had prolonged arm compression by a plaster cast, n=3 underwent a penetrating injury along the nerve course and n=1 had a history of repeated cannulations of elbow veins. Other n=6 patients presented with symptoms of superficial neuropathy in absence of any history of trauma.
**Results:** Cadaveric dissection confirmed that US-guided injections placed latex into and around the MCNA, MCNF, LCNF and PCNF in all specimens. In 10/11 patients, US allowed detection of definite nerve abnormalities. Pathological findings included n=4 fusiform neuromas and/or fibrous encasement, n=3 schwannomas, n=1 neurofibroma, n=1 intraneural ganglion cyst arising from the radiocarpal joint and n=1 mechanical instability at the elbow level.
**Conclusion:** High-resolution US can identify the MCNA, MCNF, LCNF and PCNF and characterize their abnormalities providing unique information about these small nerve branches.

**B-1749** 15:13
**Therapeutic implications of ultrasound of peripheral nerves in leprosy**
Y. Aswani[1], S. Saifi[2]; [1]*Udaipur/IN*, [2]*Mumbai/IN (aswaniyashant@gmail.com)*

**Purpose:** Leprosy and lepra reactions have the same treatment regimen except steroids in the latter. Lepra reactions may be identified on clinical and histopathological grounds. However, they may be clinically silent as well. Neuritis as a result of lepra reactions may cause permanent disability within 24 hours of its onset. The aim of this study is to determine ultrasound criteria to distinguish involvement of peripheral nerves due to leprosy from lepra reactions.
**Methods and Materials:** It was a retrospective analysis of all new leprosy cases between September 2014 and August 2015 at a tertiary care institute. Leprosy was diagnosed on clinical findings and histopathology of skin lesions. However, lepra reactions were diagnosed on clinical basis only. Nerve ultrasound findings of these patients were analysed (transducer: 6.6- 8.4 MHz, Xario, Toshiba). The studied peripheral nerves were bilateral ulnar, median, lateral popliteal and posterior tibial. Nerve involvement was tabulated as thickening (increase in cross-sectional area), internal echotexture (preserved to lost fascicular architecture) and Doppler signals.
**Results:** Of 20 patients, 16 were males. The age range was 16 to 53 years. Ulnar nerve was the most commonly involved nerve. Maximum thickening of the nerves was above the osseofibrous tunnels along their course. Both leprotic nerves and nerves involved by lepra reactions were thickened (p > 0.05) and had change in internal architecture (p > 0.05). Doppler signals were significantly present in lepra reactions (sensitivity: 91.6%; p < 0.05).
**Conclusion:** Nerve ultrasound is an excellent screening modality to differentiate between leprosy and lepra reactions.

**B-1750** 15:21
**Time to replace T2-STIR with diffusion-weighted imaging for visualisation of nerve disorders?**
S. Rajan, H. Mahajan, M. Barnwal, V. Mahajan, V. Venugopal; *New Delhi/IN (drsriramrajan@gmail.com)*

**Purpose:** To determine if diffusion-weighted imaging of nerves can provide additional information or change the diagnosis compared to T2/STIR imaging.
**Methods and Materials:** 88 MRI scans (48 lumbar plexus, 24 brachial plexus, 16 peripheral nerves) performed on a 3.0T MR750w (GE Healthcare, USA) to assess nerve damage/disease were extracted from PACS, anonymised and segregated into scans with T2w STIR (2.5-5mm/0-1mm gap; TR 5150ms, TE 48ms, TI 187-188ms, bandwidth 62.6 kHz, FOV 24x24, matrix 288x128), structural nerve imaging (SNI) and diffusion-weighted (4mm/1mm overlap, TR 8743, TE 63.5, TI 249.5, bandwidth 250kHz, FOV 40x40cm, matrix 64x128), functional nerve imaging (FNI). Scans were read by a senior specialist radiologist (16 years' experience). Visibility of focal signal abnormality, diffuse signal abnormality, nerve fibre continuity and muscular changes were recorded in each scan (present vs absent). Results from both sets of images were compared and the value of functional nerve imaging was evaluated.
**Results:** Overall, FNI changed the diagnosis in 58 (66%) cases compared to SNI. The number of cases where the diagnosis changed in lumbar plexus, brachial plexus and peripheral nerves were 36 (75%), 16 (67%) and 5 (31%), respectively. Findings visualised on FNI but not on SNI were focal signal abnormalities (24/27%) and diffuse signal abnormalities (39/45%). Nerve fibre continuity and muscular changes appeared similar on both with no change reported in 82 (93%) and 85 (97%) scans.
**Conclusion:** Diffusion-weighted imaging of nerves, especially for visualisation of the lumbar and brachial plexus, on 3.0T MR, adds to the diagnosis and could replace T2-STIRw structural imaging.

**14:00 - 15:30** **Studio 2019**

### Imaging Informatics

### SS 1905a

### Machine learning in chest radiology

*Moderators:*
N.N.
A. Farchione; Rome/IT

**B-1751** 14:00
**Evaluation of deep learning software tool for CT-based lung nodule classification**
J.T. Murchison, G. Ritchie, D. Senyszak, E.J.R. van Beek; *Edinburgh/UK (edwin-vanbeek@ed.ac.uk)*

**Purpose:** A new fully automatic deep learning software tool (CAD) was developed to facilitate pulmonary nodule management on chest CT. This study was aimed to assess the clinical performance of the tool for classifying pulmonary nodules.
**Methods and Materials:** A total of 314 Chest CT scans from unique subjects were selected from the NHS Lothian database. A panel of experienced thoracic radiologists reviewed the CT scans for the presence of pulmonary nodules and subsequently determined the composition (solid or sub-solid). A reference standard for the classification of pulmonary nodules was created based on the majority consensus of the panel.
**Results:** A total of 325 solid nodules and 57 sub-solid nodules were included in the reference standard. The sensitivity, specificity, positive predictive value and negative predictive value of CAD for determining the composition of solid nodules were 98.8%, 68.4%, 90.7% and 94.7%, and 68.4%, 98.8%, 94.7% and 90.7% for sub-solid nodules, respectively. The accuracy and kappa of CAD for determining the composition (solid vs sub-solid) of a pulmonary nodule was 94.2% and .77.
**Conclusion:** The CAD software yielded a high accuracy of 94.2% and a kappa score of .77 for determining the composition (solid, sub-solid) of a pulmonary nodule.
*Author Disclosures:*
**E.J.R. van Beek:** Advisory Board; Aidence, IMBIO. CEO; QCTIS. Consultant; Mentholatum. Founder; QCTIS. Research/Grant Support; Siemens.

**B-1752** 14:08
**Improved pulmonary nodule malignancy prediction with surrounding tissues**
K. Liu[1], W. Tang[2], C. Xia[2], Y. Deng[2], R. Zhang[1], L. Fan[1], Y. Xiao[1], S. Liu[1]; [1]*Shanghai/CN*, [2]*Beijing/CN (469884218@qq.com)*

**Purpose:** In this paper, we examined the influence of surrounding tissues on nodule malignancy classification.
**Methods and Materials:** The method combined ResNet and UNet to predict nodule malignancy. Chest CT images containing 942 nodules (325 benign, 617 malignant) were used for training, and another 200 nodules (78 benign, 122 malignant) were collected separately as testing set. The malignancy of all nodules was pathology proven. We resampled the images into 0.6 mm pixel space in all dimensions and processed the images in three ways: (1) small tissue condition, cubes (64*64*64 pixels) with target nodules in the centre; (2) large tissue condition, cubes (96*96*96 pixels) which contained a larger scope of surrounding tissues; (3) without tissue condition, nodules only with no surrounding tissues. We trained and tested three different models with the above data, respectively, and used chi-squared test to compare the results.
**Results:** The presence of surrounding tissues significantly affected prediction accuracy, $\chi^2 = 8.61$, $p = .013$. Specifically, the large tissue condition produced the highest accuracy of 86%, which was significantly higher than the without tissue condition, $\chi^2 = 8.34$, $p = .004$. Moreover, including surrounding tissues significantly improved malignant nodule sensitivity ($\chi^2 = 19.37$, $p < .001$). Yet, whether surrounding tissues were included or not did not affect specificity ($\chi^2 = 1.29$, $p = .52$).
**Conclusion:** Our study showed that including surrounding tissues could improve pulmonary nodule classification, especially the sensitivity of malignant nodules. This could provide new directions to radiomics research, where conventional methods segment nodules only.
*Author Disclosures:*
**W. Tang:** Employee; Infervision. **C. Xia:** Employee; Infervision. **Y. Deng:** Employee; Infervision. **R. Zhang:** Employee; Infervision.

**B-1753** 14:16
**Automated classification of chest x-rays as normal/abnormal using a high sensitivity deep learning algorithm**
V. Venugopal[1], M. Tadepalli[2], B. Reddy[2], A. Modi[2], S. Gupta[1], P. Warier[2], P. Rao[2], H. Mahajan[1], V. Mahajan[1]; [1]*New Delhi/IN*, [2]*Mumbai/IN (drharshmahajan@yahoo.com)*

**Purpose:** Majority of Chest X-Rays (CXRs) performed globally are normal and radiologists spend significant time ruling out these scans. We present a Deep Learning (DL) model trained for the specific use of classifying CXRs into normal and abnormal, potentially reducing time and cost associated with reporting normal studies.
**Methods and Materials:** A DL algorithm trained on 1,150,084 CXRs and their corresponding reports was developed. A retrospectively acquired independent test set of 430 CXRs (285 abnormal, 145 normal) was analysed by the algorithm, classifying each X-Ray as normal or abnormal. Ground truth for the independent test set was established by a sub-specialist chest radiologist with 8 years' experience by reviewing every Chest X-ray image with reference to the existing report. Algorithm output was compared against ground truth and summary statistics were calculated.
**Results:** The algorithm correctly classified 376 (87.44%) CXRs with a sensitivity of 97.19% (95% CI - 94.54% to 98.78%) and specificity of 68.28% (95% CI - 60.04% to 75.75%). There were 46 (10.70%) false positives and 8 (1.86%) false negatives (FNs). Out of the 8 FNs, 3 were designated as clinically insignificant (mild, inactive fibrosis) and 5 as significant (rib fractures, pneumothorax).
**Conclusion:** High-sensitivity DL algorithms can potentially be deployed for primary read of CXRs enabling radiologists to spend appropriate time on abnormal cases, saving time and thereby cost of reporting CXRs, especially in non-emergency situations. More in-depth prospective trials are required to ascertain the overall impact of such algorithms.
*Author Disclosures:*
**M. Tadepalli:** Employee; Qure.ai. **B. Reddy:** Employee; Qure.ai. **A. Modi:** Employee; Qure.ai. **P. Warier:** Employee; Qure.ai. **P. Rao:** Employee; Qure.ai.

**B-1754** 14:24
**Efficacy and clinical and radiological influencing factors on bone suppression by deep learning algorithm in digital chest radiograph**
H. Moon, G.-S. Hong, Y. Byeon, C. Lee; *Seoul/KR (aslhey92@gmail.com)*

**Purpose:** To determine the efficacy and influencing factors on bone suppression by deep learning algorithm (DLA) in the digital chest radiograph (CXR).
**Methods and Materials:** Among 4551 patients who underwent CXR and had bone suppression image (BSI) generated by DLA from 2017 to 2018, 400 patients were randomly selected. Two radiologists rated the bone suppression

Sunday at each of 2400 lung regions in consensus using 5-point scales from no bony change (score 1) to definite bone removal (score 5). Technical success of DLA on bone suppression was defined as mean score ≥ 4. The per-patient efficacy of DLA was correlated with patient's clinical and radiological factors (i.e., patient's age, sex, position, rotation degree, inspiration degree, cardiothoracic ratio [CT ratio], presence or absence of lung lesion, and body mass index [BMI]) using multiple logistic regression model and the per-regional efficacy in 6 regions was compared using ANOVA.
**Results:** The per-patient efficacy of DLA on bone suppression was 67% (n=268/400). Bone suppression of DLA was significantly influenced by patient's position (OR: 1.55, 95% CI 1.14-2.15) and presence of lung lesion (OR: 0.51, 95% CI 0.33-0.80), not by patient's age, sex, rotation degree, inspiration degree, CT ratio, and BMI. Per-regional efficacy was significantly differed according the lung regions ($P < 0.0001$).
**Conclusion:** Patient's position and presence of lung lesion are significant independent influencing factors for BSI generation using deep learning. The efficacy of DLA on bone suppression varies significantly with location.

**B-1755** 14:32
**3D computer-aided CT texture analysis with machine learning: capability to play as second reader for radiological finding assessment in interstitial lung disease**
Y. Ohno[1], A. Yaguchi[2], K. Aoyagi[3], D. Takenaka[4], T. Yoshikawa[1], Y. Kishida[1], S. Seki[1], T. Murakami[1]; [1]*Kobe/JP*, [2]*Kawasaki/JP*, [3]*Otawara/JP*, [4]*Akashi/JP* *(yosirad@kobe-u.ac.jp)*

**Purpose:** To evaluate the capability of a 3D computer-aided CT texture analysis with machine learning to play as second reader for radiological finding assessment in patients with interstitial lung disease (ILD).
**Methods and Materials:** 3D computer-aided CT texture analysis software developed by means of machine learning method was tested by thin-section CTs in 81 ILD patients. In each patient, thin-section CT findings were divided into 7 different radiological findings as follows: 1) normal lung, 2) ground-glass opacity (GGO), 3) reticulation, 4) emphysema, 5) nodular lesion, 6) consolidation and 7) honeycomb. Then total 350 lesions were randomly selected, and classified by this software and two board-certified chest radiologists. In addition, final radiological finding at each lesion was determined by consensus of two readers. To determine the inter-observer agreements among both readers and software, kappa statistics were performed. In addition, agreement and accuracy for radiological finding evaluation were also evaluated between software and consensus results.
**Results:** Inter-observer agreement among both readers and the software were determined as substantial (reader 1 vs. reader 2: κ=0.70, p<0.0001; reader 1 vs. software: κ=0.73, p<0.0001; reader 2 vs. software: κ=0.64, p<0.0001). Agreement between consensus result and the software was also evaluated as almost perfect (κ=0.84, p<0.0001). Accuracy of this software was determined as 79.4%.
**Conclusion:** 3D computer-aided CT texture analysis with machine learning has a potential to play as second reader for radiological finding assessment in interstitial lung disease patients.
*Author Disclosures:*
**Y. Ohno:** Research/Grant Support; Canon Medical Systems Corporation. **A. Yaguchi:** Employee; Toshiba Corporation. **K. Aoyagi:** Employee; Canon Medical Systems Corporation. **T. Yoshikawa:** Research/Grant Support; Canon Medical Systems Corporation. **S. Seki:** Research/Grant Support; Canon Medical Systems Corporation.

**B-1756** 14:40
**Automatic pulmonary nodule detection in low-dose CT scans using convolutional neural networks based on maximum intensity projection**
S. Zheng[1], J. Guo[1], M. Rook[1], X. Cui[1], R.N.J. Veldhuis[2], M. Oudkerk[1], P.M.A. van Ooijen[1]; [1]*Groningen/NL*, [2]*Twente/NL (s.zheng@umcg.nl)*

**Purpose:** Despite their potential benefit, computer-aided detection (CAD) systems are not routinely used by radiologists for pulmonary nodules detection in clinical practice. In radiological evaluation, maximum intensity projection (MIP) improves detection of pulmonary nodules. Our objective was to explore if using MIP images as input data could increase automatic detection of lung nodules by using convolutional neural networks (CNNs).
**Methods and Materials:** The proposed two-dimensional (2-D) CNNs approach utilizes MIP images with different slab thicknesses (5 mm, 10 mm, 15 mm) and 1mm plain multiplanar reconstruction (MPR) images as input, which extracts more discriminative information of nodules from vessels through their morphologies. We used the public LUNA16 dataset collected from seven academic centres to train and test our approach. The dataset consisted of 888 low-dose CT scans with 1186 nodules that were annotated by four radiologists. Nodules larger than 3 mm that were accepted by at least three out of the four radiologists served as the reference standard.
**Results:** Our proposed approach achieved a sensitivity of 95% for lung nodule detection, which was better than the result (86%) obtained from the MPR images alone. Using the thick MIP images helped detection of small pulmonary nodules (3-5mm), while the network trained with MPR images performed relatively better in localizing large nodules (>10mm).
**Conclusion:** The preliminary results show that MIP images can improve the sensitivity of automatic pulmonary nodule detection, which indicates the importance and effectiveness of integrating multi-slab thickness contextual information into 2-D CNNs framework in CT data.

**B-1757** 14:48
**Towards a deep learning model for exhaustive chest x-ray pathology classification**
N. Ramanauskas, J. Dementaviciene, J. Armaitis, D. Barušauskas, J. Bialopetravicius, G. Rimeika, J. Stankeviciene; *Vilnius/LT* *(naglisr@gmail.com)*

**Purpose:** Despite increasing volume of other imaging techniques, chest x-rays (CXRs) still make up a large fraction of all radiological studies. Thus, increasing accuracy and productivity of radiologists reading CXRs remains of paramount importance. However, CXRs constitute a stark challenge for computer-aided diagnosis. This is because (i) CXRs are two-dimensional projections of three-dimensional data; (ii) at least three major different types of CXRs exist (i.e., AP/PA/LAT); (iii) many different radiological findings of the lungs, the heart, and the spine are observed from a single CXR; (iv) in clinical practice, additional information besides the image is used to make radiological conclusions. In this work, we attempt to tackle challenges (i)-(iii) by creating a deep learning (DL) model which takes AP/PA images as input, and outputs scores pertaining to 76 different radiological features and their localizations. We leave (iv) for future work.
**Methods and Materials:** A database of CXR images (n = 301 255) was used. The data was divided into a training set (70%), a testing set (20%), and a validation set (10%). A single convolutional neural network (CNN) based on Inception was used to construct a model for CXR pathology classification.
**Results:** Our model achieves average area under receiver operating curve (AUC) of 93%.
**Conclusion:** We have created a Deep Learning model that covers 76 chest x-ray radiological findings with an AUC value of 93%. This model shows promise for various applications, such as patient triage and pre-generating structured reports.
*Author Disclosures:*
**N. Ramanauskas:** Employee; Oxipit, UAB. Founder; Oxipit, UAB. **J. Armaitis:** Employee; Oxipit, UAB. Founder; Oxipit, UAB. **D. Barušauskas:** Employee; Oxipit, UAB. Founder; Oxipit, UAB. **J. Bialopetravicius:** Employee; Oxipit, UAB. Founder; Oxipit, UAB.

**B-1758** 14:56
**CT-based radiomics predict immune-iherapy response in patients with non-small-cell lung cancer**
S. Wang, J. Gong, H. Yu, W. Peng; *Shanghai/CN* *(shengpingwang2007@126.com)*

**Purpose:** To predict the disease control rate (DCR) to immune-checkpoint inhibitors (nivolummab) therapy for non-small-cell lung cancer (NSCLC) patients, we developed a radiomics analysis approach based on computed tomography (CT) images.
**Methods and Materials:** Twenty-seven staged IV NSCLC patients after failed platinum-based chemotherapy were included. First, the enhanced CT images of these patients were divided into baseline group and one cycle therapy group. Second, we applied a semiautomatic segmentation method to segment the targeted lung nodules and computed 842 quantitative imaging (QI) features from each segmented nodule from CT images. Third, two random forest (RF) classifiers were built using QI features extracted from baseline and therapy CT images, respectively. Then, a Relief feature selection method was used to remove redundant QI features from the initial feature pools. Finally, a leave-one-case-out (LOCO) cross-validation method was applied to train and test the classifiers by using selected features.
**Results:** Areas under receiver operating characteristic curves (AUC) generated by using baseline and one cycle therapy CT scans were 0.81±0.08 (95% CI: [0.60, 0.94]) and 0.68±0.11 (95% CI: [0.44, 0.86]), respectively. The AUC value yielded by baseline group data was significantly higher than one cycle therapy group data.
**Conclusion:** This study demonstrates (1) feasibility of predicting the DCR to immune-therapy by using CT-based radiomics analysis and (2) higher performance by applying baseline group CT scans than using one cycle therapy data.

**B-1759** 15:04
**Evaluation of deep learning software tool for CT-based lung nodule detection**
J.T. Murchison, G. Ritchie, D. Senyszak, E.J.R. van Beek; *Edinburgh/UK (edwin-vanbeek@ed.ac.uk)*

**Purpose:** A fully automatic deep learning software tool (CAD) was assessed for the clinical performance of detecting pulmonary nodules.
**Methods and Materials:** CT studies of 273 patients were selected from the NHS Lothian database based on the inclusion criteria for lung screening (age 50-74 years, current or past smokers). A panel of experienced thoracic radiologists was evaluated for the presence of pulmonary nodules, alternately reading without (UNAIDED) and with (AIDED) CAD results. Majority consensus served as the reference standard. One-tailed Welch's t test was used to compare UNAIDED vs AIDED mean sensitivity at p<0.05.
**Results:** 269 nodules with largest axial diameter between ≥5mm and ≤30mm were included. The sensitivity of UNAIDED and AIDED radiologists was 71.9% (95% CI: 66.0%, 77.0%) and 80.3% (95% CI: 75.2%, 85.0%) (p < 0.01) at an average number of false positives per scan (FPR) of 0.11 and 0.16, respectively. In a stand-alone test, the sensitivity of CAD was equivalent to that of UNAIDED radiologists at a FPR of 0.62 and was equivalent to AIDED radiologists at a FPR of 0.88. At a FPR of 1.0 per scan the sensitivity was 82.3%, outperforming both UNAIDED and AIDED radiologists.
**Conclusion:** The use of CAD yielded a nearly 10% higher sensitivity, compared to readings without CAD, with minimal increase of the false-positive rate. In a stand-alone performance, test the CAD system outperformed radiologists reading with and without the CAD system at an average false-positive rate of 1.0 per scan.
*Author Disclosures:*
**E.J.R. van Beek:** Advisory Board; Aidence, IMBIO. CEO; QCTIS. Consultant; Mentholatum. Founder; QCTIS. Research/Grant Support; Siemens.

**B-1760** 15:12
**A new architecture of the computer-aided system for lung cancer diagnostics**
A.A. Meldo, L.V. Utkin, V.M. Moiseenko, M.A. Ryabinin; *St. Petersburg/RU (Anna.meldo@yandex.ru)*

**Purpose:** To develop a new architecture for computer-aided system in lung cancer diagnostics.
**Methods and Materials:** Open datasets LIDC, LUNA16 for training the system, own dataset LIRA (lung images resource annotated) for testing the system. A new classification approach based on dimension reduction by means of histograms of chord lengths and radiodensities for every segmented lung nodule. A new approach based on applying the Siamese neural network for non-typical lung nodules. Data processing and training the computer-aided system were carried out using a Supercomputer of Peter the Great, St.Petersburg Polytechnic University.
**Results:** The following accuracy measures are obtained by applying the first approach (histograms): sensitivity is 93%, specificity is 97% and accuracy is 95%. Accuracy measures by applying the Siamese neural network are recently investigated. A new dataset LIRA is collected, which contains a set of "typical", "non-typical" lung cancer nodules and "non-cancer" lung nodules.
**Conclusion:** The developed system is effective for detecting the lung nodules. An advantage is the ability of functioning independently on CT equipment. The use of the dimension reduction improves the training and the usage of the system. The proposed Siamese neural network is a perspective tool for differential lung disease detection and allows us to apply small datasets for training.

**B-1761** 15:20
**Fully automatic detection and segmentation of thoracic lymph nodes in MSCT scans of the chest using deep learning**
H. Carolus[1], A.-I. Iuga[2], T. Brosch[1], R. Wiemker[1], T. Klinder[1], D. Maintz[2], A. Höink[2], M. Püsken[2]; [1]*Hamburg/DE*, [2]*Cologne/DE (heike.carolus@philips.com)*

**Purpose:** Detection of malignant lymph nodes (LN) in CT examinations of cancer patients is indispensable for the correct TNM staging, directly impacting therapeutic decisions. Nevertheless, especially mediastinal and hilar LNs can be easily overlooked due to their small size and comparatively low contrast. To overcome this diagnostic challenge, we propose a deep learning algorithm for LN segmentation in chest CT images.
**Methods and Materials:** 89 CT scans from a public CT lymph node database (http://doi.org/10.7937/K9/TCIA.2015.AQIIDCNM) were analysed. Available landmark annotations of 388 LNs were extended to obtain a significantly more comprehensive and high-quality database suitable for algorithmic training. All visible LNs with a short-axis diameter (SAD) of ≥ 5 mm were segmented semi-automatically yielding a set of 4276 mediastinal, hilar and axillary LNs, i.e. 48 LNs per patient on average. Using these data, a fully convolutional net based on 3D fovea patches was trained with 4-fold cross-validation for automatic detection and segmentation of thoracic LNs.
**Results:** For enlarged, clinically relevant LNs (SAD ≥ 10 mm), 78% sensitivity was achieved and 60% for small LNs with 11.8 false-positive detections per volume. No significant performance differences between mediastinal and axillary area were found. In 94.1% of the cases with enlarged LNs, at least one LN is detected as enlarged.
**Conclusion:** Based on the extensive and rigorous annotations, the proposed 3D deep learning approach achieves state-of-the-art performance for enlarged, potentially malignant LNs. This allows this CAD system to support the radiologists, increasing their detection rate of thoracic LNs and accelerating the diagnostic process.
*Author Disclosures:*
**H. Carolus:** Employee; Philips Research. **T. Brosch:** Employee; Philips Research. **R. Wiemker:** Employee; Philips Research. **T. Klinder:** Employee; Philips Research.

**14:00 - 15:30** **Coffee & Talk 2**

### Abdominal Viscera

### SS 1901a

### Diagnosis of hepatocellular carcinoma: the LI-RADS perspective

*Moderators:*
A. Ba-Ssalamah; Vienna/AT
D. Christodoulou; London/UK

**B-1762** 14:00
**Diagnostic accuracy of CEUS LI-RADS in characterisation of small liver nodules in patients at risk for hepatocellular carcinoma**
J. Huang[1], A. Lyshchik[2], J.-W. Li[1], Y. Luo[1], J.-B. Liu[2], Q. Lu[1]; [1]*Chengdu/CN*, [2]*Philadelphia, PA/US (51967689@qq.com)*

**Purpose:** To evaluate diagnostic accuracy of the Contrast Enhanced Ultrasound Liver Imaging Reporting and Data System (CEUS LI-RADS) on hepatocellular carcinoma (HCC) in small focal liver lesions(FLL) in patients at risk for HCC.
**Methods and Materials:** Patients at risk for HCC presenting with FLL≤20mm were enrolled in this study. Each FLL was characterised according to the CEUS LI-RADS and American Association for the Study of Liver Disease (AASLD) 2005 criteria with histology or CE-CT/CE-MRI or more than 12 months' follow-up as reference standard.
**Results:** Totally 175 nodules in 172 patients were included. The mean nodule size was 16.1±3.4mm. Totally, 105 nodules were proved HCC by histology. The sensitivity, specificity, positive predictive value (PPV) and positive likelihood ratio (+LR) of CEUS LR-5 and AASLD criteria for the diagnosis of HCC were 73.3% vs. 88.6% (P<0.05), 95.7% vs. 85.7% (P<0.05), 96.3% vs. 90.3% (P<0.05), 17.1 vs. 6.2 (P<0.05), respectively. The rate of HCC in CEUS LR-4 and CEUS LR-M was 50.0 % and 75.0 %, respectively. All cases of intrahepatic cholangiocarcinoma were characterised as LR-M by CEUS LI-RADS while classified as HCC by AASLD criteria.
**Conclusion:** CEUS LI-RADS performed well in characterisation of small FLLs in patients at risk for HCC. CEUS LR-5 demonstrated exceptionally high specificity, PPV and +LR compared to AASLD criteria for diagnosis of HCC with slightly lower sensitivity. CEUS LR-4 and CEUS LR-M categorisation is highly suspicious for malignancy, but not specific enough for HCC.

**B-1763** 14:08
**Non-invasive imaging diagnosis of hepatocellular carcinoma in patients with both chronic liver disease and a history of extrahepatic primary cancer**
M. Cho, C. An, K. Aljoqiman, M.-J. Kim; *Seoul/KR (chom974@yuhs.ac)*

**Purpose:** To evaluate the accuracies of the diagnostic criteria and various features for differentiating between hepatocellular carcinoma (HCC) and liver metastasis in patients at risk for both of them.
**Methods and Materials:** The study subjects were 59 HCCs and 45 metastases pathologically confirmed between 2008 and 2017 at a single institution in 104 patients with both chronic liver disease (i.e. cirrhosis and chronic hepatitis B) and history of extrahepatic primary cancer. Dynamic contrast-enhanced magnetic resonance imaging or computed tomography images were retrospectively reviewed in consensus by two radiologists, based on Liver Imaging Reporting And Data System (LI-RADS) v2018. Serum tumour markers, tumour multiplicity, and suspicious lymph node metastasis were also evaluated.
**Results:** Major features had sensitivities of 33.9-88.1% and specificities of 76.3-97.8% for HCC. LR-M features had sensitivities of 76.6-80% and specificities of 86.4-93.2% for metastasis. Elevation of tumour markers for metastasis, tumour multiplicity, and suspicious lymph node were more frequently observed in metastasis than HCC (*P* <0.034). The sensitivity and specificity were 69.5% and 95.6% for HCC diagnosis by LR-5, and 85.1% and 89.1% for metastasis by LR-M. One of the two metastases categorized as LR-5 was smaller than 2 cm, and the other was accompanied by tumour multiplicity.
**Conclusion:** LI-RADS features and diagnostic criteria showed high accuracies in patients at risk for both HCC and metastasis. Radiologic and clinical ancillary findings suggestive of metastasis as warning signs may help minimize the risk of misdiagnosis of metastasis as definitely HCC in this patient group.

**B-1764** 14:16
**A radiomics-clinical model to diagnose hepatocellular carcinoma: prospective comparison with the EASL and LI-RADS (v2018) criteria**
H. Jiang, X. Liu, J. Chen, L. Cao, Y. Wei, B. Song; *Chengdu/CN (hanyu_jiang@foxmail.com)*

**Purpose:** We aimed to develop a multidisciplinary radiomics-clinical diagnostic model for hepatocellular carcinoma (HCC) and to compare its accuracy with the 2018 version of European Association for the Study of the Liver (EASL) and the Liver Imaging Reporting and Data System (LI-RADS) criteria.
**Methods and Materials:** Ethical approval by the institutional review board and informed consent were obtained for this prospective study. From July 2015 to April 2018, consecutive patients with chronic liver diseases and suspected liver lesions were enrolled and underwent gadoxetic acid-enhanced magnetic resonance imaging. We constructed a three-dimensional whole-lesion-based radiomics-clinical nomogram with the least absolute shrinkage and selection operator model and multivariate logistic regression analysis. The diagnostic accuracy of the radiomics-clinical model was validated in an independent cohort and compared with the EASL and LI-RADS criteria, as reviewed by two independent radiologists, with receiver operating characteristic analysis.
**Results:** A total of 164 nodules in 161 patients were included (mean size 5.41±2.67 cm, 129 HCCs). The sensitivity and specificity of the radiomics-clinical nomogram were 88% and 92% in the training cohort and 69% and 96% in the validation cohort, respectively. These measures were 96% and 60% for the EASL criteria and 92% and 89% for the LI-RADS criteria, respectively. The areas under the ROC curves of the radiomics-clinical model (0.890) and LI-RADS criteria (0.904) were significantly higher than that of the EASL criteria (0.781) (p<0.05 for both).
**Conclusion:** The radiomics-clinical model demonstrated similar overall accuracy to the LI-RADS criteria with markedly high specificity for HCC in high-risk patients.

**B-1765** 14:24
**Value of targetoid appearance on T2-weighted imaging and signs of tumour vascular involvement for differentiating hepatocellular carcinoma (HCC) from other non-HCC liver malignancies**
R. Cannella[1], T.J. Fraum[2], D.R. Ludwig[2], A.A. Borhani[3], K.J. Fowler[4], A. Furlan[3]; [1]*Palermo/IT*, [2]*Saint Louis, MO/US*, [3]*Pittsburgh, PA/US*, [4]*San Diego, CA/US (rob.cannella@libero.it)*

**Purpose:** To evaluate sensitivity and specificity of targetoid appearance on T2-weighted imaging and features of tumour vascular involvement for differentiating HCC from other non-HCC primary hepatic malignancies.
**Methods and Materials:** This is an IRB-approved retrospective study performed at two large liver transplant centres. The final population included 401 pathologically-proven lesions imaged between 2007 and 2017 with liver protocol contrast-enhanced CT or MRI. The cohort consisted of 172 intra-hepatic cholangiocarcinoma and 91 combined hepatocellular-cholangiocarcinoma, with addition of 138 HCCs for control. Two abdominal radiologists (R1; R2) independently reviewed the imaging studies (119 CT; 282 MRI), recording the presence of targetoid appearance on T2-weighted images and features of tumour vascular involvement including: encasement, attenuation, tethering, occlusion, obliteration, and tumour in vein (TIV). Sensitivity and specificity of each feature were calculated for the diagnosis of non-HCC malignancy. The Cohen k test was used to assess the inter-reader agreement.
**Results:** The sensitivity and specificity of targetoid appearance on T2-weighted images for the diagnosis of non-HCC malignancy were 25.3% and 32.5% (R1; R2) and 98.3% and 97.4% (R1; R2), respectively. Among the features of tumour vascular involvement, those providing the highest sensitivity for non-HCC malignancy were vascular encasement (R1: 33.8%; R2: 38.0%) and obliteration (R1: 26.2%; R2: 32.3%). The highest specificity for non-HCC malignancy was provided by tethering (R1: 100%; R2: 97.1%) and occlusion (R1: 99.3%; R2: 99.3%). The inter-reader agreement was moderate to substantial (k=0.430-0.765).
**Conclusion:** Targetoid appearance on T2-weighted, vascular tethering and occlusion demonstrated strong inter-reader reliability and high specificity for non-HCC malignancy.
*Author Disclosures:*
**A.A. Borhani:** Consultant; Guebert. Other; Elsevier/Amirsys. **A. Furlan:** Research/Grant Support; General Electric. Other; Elsevier/Amirsys.

**B-1766** 14:32
**Imaging features of hepatocellular carcinoma: qualitative and quantitative comparisons between hepatobiliary and extracellular contrast-enhanced MRI**
J. Son[1], S. Hwang[1], M.-.S. Park[1], S. Park[2], M.-.J. Kim[1], J.-Y. Choi[1], K. Han[1]; [1]*Seoul/KR*, [2]*Goyang/KR (hijwson@yuhs.ac)*

**Purpose:** To compare the major imaging features of HCC on MRI with gadoxetate disodium (hepatobiliary agent, HBA) and gadoterate meglumine (extracellular agent, ECA).
**Methods and Materials:** Among surgically proven 185 HCCs in 169 patients who underwent liver dynamic MRI with either HBA (n=120) or ECA (n=49), 55 nodules for each of the two contrast media were matched according to the tumour size, Edmonson grade (major and worst), and gross type on pathologic specimen. For qualitative analysis, two board-certified radiologists independently reviewed dynamic enhancement patterns and capsule appearance. For quantitative analysis, the third radiologist measured signal intensity at each phase by placing the region of interest (ROI) for tumour and normal liver parenchyma. The lesion-to-liver contrast (LLC) and lesion-to-liver contrast enhancement ratio (LLCER) were calculated.
**Results:** On qualitative analysis, delayed phase (DP, 3 min) washout was more frequently seen with HBA (mean=90%, 49.5/55) than ECA (mean=73.6%, 40.5/55) (p=0.026). Capsule appearance was less frequently seen with HBA (mean=37.3%, 20.5/55) than ECA (mean=75.5%, 41.5/55) (p<0.001). On quantitative analysis, the LLC on AP was better with ECA (p=0.003) whereas LLC on DP was better with HBA (p<0.001). The LLCER from unenhanced to AP was higher with ECA (p=0.022) whereas LLCER from PVP to DP was higher with HBA (p<0.001).
**Conclusion:** The detection of DP washout, LLC on DP, and LLCER from PVP to DP was better with HBA, whereas detection of capsule, LLC on AP, and LLCER from unenhanced to AP was better with ECA.

**B-1767** 14:40
**Radiologic-pathologic correlation of non-hypervascular hypointense nodules on gadoxetic acid-enhanced MRI: a multi-centre retrospective observational study**
I. Joo, S. Kim, T. Kang, K. Lee, J. Lee; *Seoul/KR*

**Purpose:** To assess the distribution of pathologic diagnosis of non-hypervascular hypointense nodules (NHHNs) on gadoxetic acid-enhanced MRI (Gd-EOB-MRI) and to determine useful clinical and imaging features for differentiating histologic grades of NHHNs.
**Methods and Materials:** This multi-centre retrospective study included 334 NHHNs of ≤30 mm that were pathologically diagnosed as hepatocellular carcinomas (HCCs) or benign cirrhosis-associated nodules in 298 high-risk patients. Histologic slides of NHHNs were reviewed by central pathologic review to determine the final pathologic diagnosis. Two independent radiologists analysed imaging features, and any discrepancies were resolved by a third reviewer. Clinical and radiologic features were compared according to histologic diagnoses. Logistic regression analysis with generalized estimated equation was performed to find prediction factors to differentiate HCCs from benign nodules.
**Results:** By central pathologic review, NHHNs were confirmed as progressed HCCs in 147, early HCCs or dysplastic nodules (DNs) with HCC foci in 68, high-grade DNs in 92, and low-grade DNs or regenerative nodules in 27. According to the histologic categories, nodules were different in clinical and radiologic features: elevation of serum alpha-fetoprotein (≥100 ng/mL) was more frequently found in patients with HCCs than those with benign nodules, and MR imaging features including hypointensity on precontrast T1-weighted imaging, hypointensity on portal venous phase, hyper-intensity on T2-weighted imaging, restricted diffusion, and well-defined margin were suggestive of HCCs than benign nodules (Ps<0.05).
**Conclusion:** NHHNs on Gd-EOB-MRI correspond to the wide spectrum of pathologic diagnoses from RNs to progressed HCCs. Ancillary imaging features are helpful to differentiate HCC from benign cirrhosis-associated nodules.
*Author Disclosures:*
**J. Lee:** Research/Grant Support; Grants from Bayer Korea Ltd.

**B-1768** 14:48
**Diagnostic performance of hepatobiliary phase hypointensity and major imaging features for the diagnosis of HCC in lesions between 10 and 19 mm, and arterial phase hyperenhancement**
F. Vernuccio[1], R. Cannella[1], M. Meyer[2], K.R. Choudhoury[2], M.R. Bashir[2], A. Furlan[3], D. Marin[2]; [1]*Palermo/IT*, [2]*Durham, NC/US*, [3]*Pittsburgh, PA/US (federicavernuccio@gmail.com)*

**Purpose:** To determine the diagnostic performance of hepatobiliary phase (HBP) hypointensity and major imaging features for the diagnosis of hepatocellular carcinoma (HCC) in lesions with arterial phase hyperenhancement (APHE) between 10 and 19mm categorised as LR-3 or LR-

Sunday

4 in patients at-risk for HCC.
**Methods and Materials:** In this retrospective dual-Institution study, we included 189 APHE lesions between 10 and 19mm - including 135 LR-3 and 54 LR-4 - in 144 consecutive patients (mean age 58 years). A composite reference standard of pathology and imaging follow-up was used. The diagnostic performance - sensitivity and specificity- of HBP hypointensity and LI-RADS major imaging features for the diagnosis of HCC was assessed and compared using a logistic regression model.
**Results:** In the overall included lesions, LI-RADS major imaging features showed a specificity of 95%-100% for the diagnosis of HCC, and HBP hypointensity yielded significantly higher sensitivity (84%) but lower specificity (84%) compared to the major imaging features. In LR-3 APHE observations between 10 and 19mm, HBP hypointensity yielded moderately elevated sensitivity (73%) and specificity (85%).
**Conclusion:** All LI-RADS major imaging features have nearly perfect specificity in APHE lesions between 10 and 19mm, which supports the changes in the LI-RADS v2018 and suggests adding enhancing “capsule” for the upgrade to LR-5 of these lesions. HBP hypointensity significantly improves sensitivity while maintaining moderately high specificity for the diagnosis of HCC in LR-3 APHE lesions between 10 and 19mm, thus justifying the mandatory upgrade of these LR-3 lesions to LR-4.
*Author Disclosures:*
**A. Furlan:** Grant Recipient; research grant from General Electric. Other; book contact from Elsevier/Amirsys.

**B-1769** 14:56
**Predicting progression to hypervascular HCC in hypovascular hypointense nodules on gadoxetic acid-enhanced MR images in patients with chronic liver disease**
W.S.R.V. Teerasamit; *Bangkok/TH (Mojiamp@gmail.com)*

**Purpose:** To identify the patient characteristics and MR imaging features of hypovascular hypointense nodule on hepatobiliary phase gadoxetic acid-enhanced MR imaging in patients with chronic liver disease associated to progression to hypervascular hepatocellular carcinoma (HCC).
**Methods and Materials:** The institutional review board approved this retrospective review of total 40 patients with 60 hypovascular hypointense nodules on hepatobiliary phase gadoxetic-enhanced MR images. Univariate and multivariate Cox regression analysis for the hypervascular HCC development were used to define the variables including initial nodule size, cause of cirrhosis, history of locoregional therapy of HCC, fat-containing, signal intensity on T1W, T2W, portal and equilibrium phases of dynamic phase, and DW images. Furthermore, cumulative percentage incidence of hypervascularity and growth rate was calculated by using receiver operating characteristic (ROC) curve.
**Results:** The prevalence of progression to hypervascular HCC was 45% (27 out of 60). Multivariable Cox analysis of developing hypervascularization was initial nodule diameter more than 1 cm. (P=0.027; HR 2.52; 95% CI: 1.11,5.74) The mean growth rate was significantly higher in subsequent hypervascular nodule than in those without hypervascularization ( P < 0.001). Cumulative risk incidence of hypervascularization at 3, 6, 12, 24 months were 5%, 20%, 35%, 44 %, respectively.
**Conclusion:** Initial nodule diameter more than 1 cm is an independent risk factor for progression to hypervascular HCC in hypovascular hypointensity on hepatobiliary phases in patients with chronic liver disease.

**B-1771** 15:12
**Liver imaging reporting and data system v2018: visualisation rate and diagnostic value of ancillary features favouring malignancy in observations at intermediate and high risk for HCC**
R. Cannella[1], F. Vernuccio[1], D. Marin[2], A. Furlan[3]; [1]*Palermo/IT*, [2]*Durham, NC/US*, [3]*Pittsburgh, PA/US (rob.cannella@libero.it)*

**Purpose:** To assess visualisation rate and diagnostic accuracy of LI-RADSv2018 ancillary features (AFs) favouring malignancy in LR-3 and LR-4 observations on gadoxetate-enhanced MRI.
**Methods and Materials:** In this IRB-approved retrospective dual-institution study, we included consecutive patients at risk for HCC imaged with gadoxetate-enhanced MRI between 2009-2014 fulfilling the following criteria: i) at least one LR-3 or LR-4 ≥1 cm; ii) non-rim arterial phase hyperenhancement; iii) confirmation of benignity or malignancy by pathologic proof or imaging follow-up. Four readers recorded the LI-RADSv2018 AFs favouring malignancy. We compared difference in visualisation rates of AFs between benign lesions and HCCs and calculated sensitivity and specificity of each AF for the diagnosis of HCC. Significance was set at P-value <0.05.
**Results:** 205 observations were selected in 155 patients including 167 (81.5%) LR-3 and 38 (18.5%) LR-4. There were 126 (61.5%) HCCs and 79 (28.5%) benign lesions. A significantly larger number of AFs favouring malignancy was found in LR-3 and LR-4 that progressed to HCC compared to benign lesions (P<.001 and P=.003). The most common AFs favouring malignancy in HCCs included hepatobiliary phase hypointensity (P<.001), transitional phase hypointensity (P<.001) and mild-moderate T2 hyperintensity (P<.001). Sensitivity and specificity of AFs for the diagnosis of HCC were 2.4%-76.2% and 86.1%-100%, respectively. Hepatobiliary phase hypointensity yielded the highest sensitivity but the lowest specificity.
**Conclusion:** LR-3 and LR-4 progressing to HCC have a significantly higher number of AFs favoring malignancy compared to benign lesions. Among all AFs, hepatobiliary phase hypointensity yields the highest sensitivity for the diagnosis of HCC.
*Author Disclosures:*
**A. Furlan:** Research/Grant Support; General Electric. Other; Elsevier/Amirsys.

**B-1772** 15:20
**Response assessment of HCC after locoregional therapy on gadoxetic acid-enhanced MRI: performance of the LI-RADS TR categorisation and added value of ancillary features**
S. Kim, I. Joo, H.-C. Kim, S. Ahn, H.-J. Kang, S. Jeon, J. Han; *Seoul/KR (shrimpkim90@gmail.com)*

**Purpose:** To determine the performance of the liver imaging reporting and data system (LI-RADS) treatment response (LR-TR) categorization on gadoxetic acid-enhanced MR imaging (Gd-EOB-MRI) and to investigate the added value of ancillary features (AFs) to the conventional enhancement-based criteria for detecting viable tumours.
**Methods and Materials:** This retrospective study included 207 patients with Gd-EOB-MRI after locoregional treatment for hepatocellular carcinoma (HCC) and a reference standard for marginal recurrence (viable, n=107; non-viable, n=100). For each treated observation, two independent radiologists assigned treatment response categories (TR-nonviable, TR-equivocal, or TR-viable) according to different criteria: i) LR-TR, ii) TP-included: including transitional phase (TP) for determining washout, iii) AF-applied: applying AFs on hepatobiliary phase, diffusion-weighted imaging, and T2-weighted imaging for the category adjustment. Diagnostic performances of imaging criteria were compared using the McNemar test.
**Results:** For detecting viable tumours, LR-TR-viable resulted in sensitivities of 64.5% and 39.3%, specificities of 98.0% and 98.0%, and accuracies of 80.7% and 67.6% in reviewers 1 and 2, respectively. TP-included viable and AF-included viable showed significantly higher sensitivities (77.6% and 55.1%; 88.8% and 86.0%) than LR-TR in both reviewers (Ps<0.001), while their specificities (98.0% and 96.0%; 97.0% and 96.0%) were comparable to LR-TR (Ps>0.05). In addition, TR-equivocal was less frequently assigned on AF-applied (1.4% and 7.2%) than LR-TR (18.4% and 35.3%) or TP-included (7.7% and 25.7%) in both reviewers.
**Conclusion:** LR-TR on Gd-EOB-MRI provides specific diagnosis of viable tumours but shows limited sensitivity. By applying ancillary features in the category adjustment, more sensitive and confident diagnosis of recurred HCC can be achieved.

**14:00 - 15:30** **Room E2**

### Neuro

### SS 1911a

### Contrast media and advances in neuro MRI

*Moderators:*
N.N.
D. Stojanov; Nis/RS

**B-1773** 14:00
**Comparison of contrast-enhanced modified T1-weighted 3D TSE black blood and 3D MP-RAGE sequences for detection of meningeal enhancement**
M.F. Froelich, R. Pons Lucas, E. Coppenrath, W. Sommer, K.M. Treitl, T. Saam, N.N. Sommer; *Munich/DE*

**Purpose:** To compare a contrast-enhanced modified T1-weighted 3D TSE black blood sequence (T1-mVISTA) with a 3D gradient echo sequence (MP-RAGE) for the detection of meningeal enhancement in patients with suspected meningitis or meningeosis carcinomatosa.
**Methods and Materials:** We consecutively selected subjects with known meningeal affection from 10/2013 to 10/2017 for this retrospective study. All patients underwent T1-mVISTA (0.75-mm isotropic resolution) and MP-RAGE (0.8-mm isotropic resolution) at 3-Tesla after application of contrast agent in random order. Presence of dural and leptomeningeal enhancement, supra- and infratentorial location, side, visual contrast enhancement (VCE), image quality and diagnostic confidence were assessed by an experienced radiologist for both sequences in separate reading sessions three weeks apart. Results were compared utilizing chi-square and Wilcoxon tests for paired two-tailed samples. A p<0.05 was considered statistically significant.

**Results:** A total of thirty patients (12 female, mean age 22 years) were included in the analysis. 11 patients (36.67%) had a suspected infectious aetiology. T1-mVISTA detected either dural or leptomeningeal enhancement in 30 patients (100% sensitivity), while MP-RAGE showed any meningeal enhancement in 24 patients (80% sensitivity). Diagnostic confidence did not differ significantly in patients showing dural/leptomeningeal enhancement in both sequences (all p>0.05). However, a total of 19 patients showed dural enhancement on MP-RAGE while 21 patients were dural-positive on T1-mVISTA (p<0.01). Furthermore, MP-RAGE showed leptomeningeal enhancement in 15 patients, while T1-mVISTA detected leptomeningeal enhancement in 26 patients, including all MP-RAGE-positive patients (p=0.038).
**Conclusion:** T1-mVISTA may offer an increased sensitivity compared to MP-RAGE sequences, especially for leptomeningeal enhancement.

**B-1774** 14:08
**Application of half-dose contrast-enhanced T2-fluid-attenuated inversion recovery sequence in brain metastasis**
T. Jin; *Shanghai/CN*

**Purpose:** To assess the detection rate and image quality of ½-dose CE T2-fluid-attenuated inversion recovery (FLAIR) in intracranial metastases, compared with CE-T1-weighted imaging (T1WI).
**Methods and Materials:** Thirty-three patients with known cancers and brain metastasis underwent pre- and post-contrast sequences including: i) ½-dose CE-T2-FLAIR, ii) ½-dose CE-T1WI, and iii) full-dose CE-3D-BRAVO sequence scan. The optimum scanning time for ½-dose CE-T2-FLAIR was analyzed, and we compared contrast ratio (CR) and the number of parenchymal metastases on three sequences above.
**Results:** One hundred and forty-one ½-dose CE-T2-FLAIR, 88 ½-dose CE-T1WI, and 123 full-dose CE-BRAVO sequence-enhanced lesions were classified as metastases. When metastases were ring-enhanced or solid-enhanced with a diameter of <5 mm, the number of metastases detected in ½-dose CE-T2-FLAIR was higher than that in ½-dose CE-T1WI and full-dose CE-BRAVO. However, if solid-enhanced metastases were ≥5 mm, the number of metastases detected in ½-dose CE-T2-FLAIR was less than that in ½-dose CE-T1WI or full-dose CE-BRAVO. In 77 metastatic lesions which both displayed in three sequences, the median CR was significantly higher using optimized ½-dose CE-T2-FLAIR (in phase 2, CR=71.6) than ½-dose CE-T1WI (CR=11.7, P<0.001) or full-dose CE-BRAVO sequence (CR=18.9, P<0.001).
**Conclusion:** Half dose CE-T2-FLAIR has distinct advantage in detecting brain metastases, especially ring-enhanced or small solid-enhanced lesions.

**B-1775** 14:16
**Is synthetic MRI of the brain feasible in a clinical setting? A comparison with standard protocol**
G. Corrias, A. Balestrieri, L. Saba; *Cagliari/IT (corriasgmd@gmail.com)*

**Purpose:** To investigate Radiologist's satisfaction and image quality using an alternative syntetic MRI (sMRI)protocol in Brain imaging.
**Methods and Materials:** 30 patients were examined using an MRI scan with a field of 3 Tesla and a 32-channels coil. On the top of the standard brain MRI protocol, two additional sMRI sequences were added: [T2]-multi-TE-FSE2D and [T1]-MP2RAGE-(3D). From these two sequences FLAIR, DIR and fat sat images have been reconstructed on the dedicated workstation with a commercially available software (OLEAnova+, Olea Medical, Paris). One radiologists with 6 years of experience graded T1, T2, DIR and FLAIR from the standard and the experimental protocol on a 5 points scale voting image quality, lesion detection and overall satisfaction. Intra-class correlation coefficient (ICC) and repeatability coefficient (RC) were estimated to evaluate agreement between every pair of sequences.
**Results:** The results were grouped in two categories (score 1-3, sufficient; score 4-5, insufficient). Concordance with these two groups of scores was above 80% for almost all sequences analyzed except from FLAIR images.
**Conclusion:** Syntetic MRI would the reduce the acquisition time of a brain MRI of 6min:50sec. Image quality, lesion detection and overall satisfaction are not different between the standard protocol and the reconstructed sequences obtained from sMRI.

**B-1776** 14:24
**Diagnostic efficacy and safety of gadoteridol in neuroradiology: qualitative assessment in a large sample of MRI studies at 1,5T**
A. Del Poggio, C. Pereira, A. Iadanza, A. Falini, N. Anzalone; *Milan/IT (adelpoggio@gmail.com)*

**Purpose:** The suspension of linear gadolinium chelates has restricted the choice in neuroradiological field to the macrocyclic compounds. We prospectively evaluated in a clinical setting the safety and diagnostic accuracy of a macrocyclic compound (gadoteridol).
**Methods and Materials:** From November 2017 to February 2018 a consecutive cohort of patients undergoing to 1.5T neuroradiological magnetic resonance (MRI) with gadoteridol was prospectively enrolled. Image quality and adverse events associated with contrast administration were assessed. Diagnostic performance compared to histological data or to a control MRI was evaluated in a subgroup of patients with neoplastic disease. Inter-reader agreement (kappa) between two expert neuroradiologists was calculated for the diagnosis of malignancy.
**Results:** 244 patients (128M/116F; mean age 53.5±17.2 years) were enrolled. Overall, 122 (50%) were diagnosed with oncological pathology. Other diagnoses were inflammatory (43 [17.6%]), vascular (24 [9.8%]), infectious or granulomatous (9 [3.7%]) or 'other' (26 [6.6%]). 30 patients (12.2%) were negative. In all cases the MRI exam was diagnostic. There were two allergic reactions (0.8%), one moderate/severe, in line with the literature. Diagnostic accuracy for the diagnosis of malignancy among the subgroup with neoplastic pathology was 97.3% (95%CI 92.3-99.1) with sensitivity and specificity of 94.3% (95%CI 80.8-99.3) and 98.7% (95%IC 92.8-99.9), respectively. The inter-reader agreement for the diagnosis of malignancy was excellent (K=0.85 [95%CI 0.76 - 0.95]).
**Conclusion:** Gadoteridol was safe and guaranteed good image quality in a large population. Within the subgroup with neoplastic pathology the diagnostic accuracy for malignancy was 97.3% with sensitivity and specificity of 94.3% and 98.7% respectively.

**B-1777** 14:32
**MRI T1w brain signal intensity and relaxation times in individuals with prior exposure to gadobutrol**
M. Saake, A. Schmidle, M. Kopp, J. Hanspach, T. Hepp, A. Nagel, A. Doerfler, M. Uder, T. Baeuerle; *Erlangen/DE*

**Purpose:** The purpose was to screen a large MRI population for the number of injected doses of gadobutrol in patients with normal and impaired kidney function and to recall selected individuals for assessment of gadolinium brain retention.
**Methods and Materials:** The database of the University Hospital Erlangen was searched for patients who received gadobutrol between 2007 and 2017 and not any other intravenous MRI contrast agent. Patients with at least 5 (range 5-20) contrast-enhanced MRI scans and normal kidney function (group 1) and at least 1 (range 1-9) contrast-enhanced MRI scans and impaired kidney function (group 2) were matched with control patients who received no MRI contrast agent before (groups 3 and 4). All individuals from groups 1-4 were invited. In participants, brain MRI was performed: T1w morphology and T1 mapping.
**Results:** Between 2007 and 2017, over 35.000 patients received up to 26 single doses of gadobutrol only at our institution. Of these, 701, 1239, 94 and 409 patients matched the criteria of groups 1-4, respectively. After recalling 2.444 individuals, we were able to perform brain MRI in 220 individuals (groups 1-4: 76, 84, 25, 35, respectively). No significant differences in T1w signal intensity ratios and T1 ratios were found in the dentate nucleus and globus pallidus between patients of groups 1 and 2 and the matched controls.
**Conclusion:** Based on the measured ratios, we found no statistical evidence for gadolinium brain retention after administration of multiple doses of gadobutrol in patients with normal and impaired kidney function.
*Author Disclosures:*
**T. Baeuerle:** Grant Recipient; Bayer.

**B-1778** 14:40
**Glutamate concentration changes in response to continuous stimulation**
A. Manzhurtsev, P. Menshchikov, M. Ublinskiy, N. Semenova, O. Bozhko, T. Akhadov; *Moscow/RU (andrey.man.93@gmail.com)*

**Purpose:** Functional MRS of glutamate (Glu) is of high interest since it is the main excitatory neurotransmitter. There is a problem of separating Glu and glutamine signals in MRS, especially at 3 Tesla. We used TE average to measure local Glu change in response to continuous visual stimulation at 3T.
**Methods and Materials:** Twelve healthy subjects (18-27 y.o.) were analysed with Philips Achieva dStream 3T and Head-Neck SENSE coil. Video stimulation: monitor, mirror, 8-Hz flashing checkerboard. The spectroscopy voxel (20x40x30 mm) was located in visual cortex. Glu signal was obtained with TE-averaged sequence: TE=35, 45, ..., 185 ms, TR=2000 ms, NSA=16, total averages: 256, t=8.5 min. Spectrum in rest was obtained and then spectrum during visual stimulation. Spectral processing: LCModel, program based on FID-A was written to simulate TEavg brain in vivo spectra; basis set for LCModel was created, Glu/Cr and NAA/Cr values were obtained. The stimulation/rest values on Glu/Cr and NAA/Cr were found and compared with a value of 1 (Mann-Whitney criterion).
**Results:** Glu peak at 2.35 ppm was perfectly fitted by the simulated basis set. The increase (by 5%, p<0.05) of Glu/Cr was revealed, NAA/Cr remained unchanged.

**Conclusion:** For the first time, TEavg was used for functional MRS of glutamate. It allowed to confidently observe the growth of Glu at 3 Tesla during continuous visual stimulation, which is in agreement with published data obtained at 7 Tesla. The stability of NAA in this study (that is often reported to be decreased in neuroactivation) might be the manifestation of neuronal adaptation.

**B-1779** 14:48

**Two postprocessing method of diffusion kurtosis imaging in glioma grading**

A. Gao[1], J. Bai[1], Y. Zhang[1], J. Cheng[1], Y. Hong[2]; [1]*Zhengzhou/CN*, [2]*Hangzhou/CN (annygao456@163.com)*

**Purpose:** To compare histogram analysis and DKE in glioma grading.
**Methods and Materials:** DKI and conventional sequence at 3.0T MR scanner. DKI acquires data with six b values (0-2500 sec/mm$^2$) and uses diffusion encoding in 30 directions, scanned using a SE-EPI sequence. DKE software was used to calculated diffusion parameters FA, MK, and RK and AK. Histogram analysis was based on Matlab platform,which was used to calculated diffusion parameters Kapp and Dapp value based on TRACEW parameter image. Independent-samples T test or Mann-Whitney-Wilcoxon test was used to distinguish glioma grads. The parameters with the best percentile were identified by analysis of the AUC of the receiver operating characteristic ROC analysis.
**Results:** Grade II(n=21), III(n=13) and IV(n=39) glioma were observed. There were significant difference between high and low grade glioma groups by histogram analysis of the Kapp value of maximum, mean, standard deviation, 75th percentile, 95th percentile, and the Dapp value of minimum, mean, 25th percentile, 50th percentile, 75th percentile, 95th percentile, skewness coefficients. According to the ROC , the highest AUC were found at Dapp minimum value (AUC,0.829), Kapp 75th percentile value (AUC,0.819). However, DKE method proved that MK value is significant higher in III grade glioma than it in IIgrade glioma (AUC,0.996). When it comes to III and IV grade glioma, MK was significant higher in IVgrade glioma than III grade glioma(AUC,0.953).
**Conclusion:** MK value, calculated by DKE software, is a helpful parameter for identifying glioma II, III and IV grade.

**B-1780** 14:56

**Open-source software multimodal neuroimage processing for presurgical assessment: benefits and comparison to proprietary software**

P. Donnelly-Kehoe, A.A. Ojeda, G. Pascariello, P.A. Acevedo, M.B. Nallino, A.L. Rodríguez Musso, J. Gomez; *Rosario/AR (ojedaheleg@hotmail.com)*

**Purpose:** Multimodal neuroimage processing allows an optimum presurgical assessment. Structural sequences are used as anatomical guides, fMRI protocols serve to recognize functional regions and DWI reveal the underlying structural connectivity. Proprietary software exhibit several limitations in the processing of each modality and also in the multimodal integration. This work introduces an open-source based toolbox for the multimodal presurgical assessment with state-of-the-art methods.
**Methods and Materials:** We developed pipelines to process structural, functional and diffusion MRI. The structural pipeline included an automatic segmentation of neuroanatomic regions; fMRI included the remotion of movement and non-neural signals based on independent component analysis. The tractography pipeline removed movement and eddy currents and the neuroanatomical segmentation was used for a standardized tract segmentation. Four clinical cases were processed to compare results with the available proprietary software.
**Results:** Results obtained by the proposed method showed to be superior to the ones obtained by the proprietary software. Main advantages were: (i) structural segmentation automatically delimits anatomical structures; (ii) noise and artefacts from fMRI and DWI were successfully removed, improving signal to noise ratio; (iii) anatomical and functional information was included in the segmentation of tracts; (iv) multiples fMRI protocols were combined within the same space.
**Conclusion:** The application in clinical cases showed that our methods allow a more effective presurgical assessment than proprietary software that are commonly used in the clinical practice. In this context, the usage of open-source packages is a better alternative for the neuroimage processing, allowing to know the processes and to improve them.

**B-1781** 15:04

**Clinically-implemented, CAD-assisted decision support system to limit gadolinium injection in patients with multiple sclerosis**

M. Bilello, R. Mattay, A. Mamourian; *Philadelphia, PA/US (Michel.Bilello@uphs.upenn.edu)*

**Purpose:** The growing concern about deposition of free gadolinium in the brain among patients that undergo serial contrast-enhanced MRI studies needs to be addressed. We have implemented a clinical decision support system to limit contrast injections in patients with multiple sclerosis (MS).
**Methods and Materials:** The main idea is to inject only when scans demonstrate new lesions. The rationale stems from a study we recently conducted, which revealed that 1) 50% of scans with new lesions had enhancing lesions, 2) 100% of scans with enhancing lesions had new lesions, and 3) 24% of all scans had new lesions. A major component of the system is a CAD program that detects new brain lesions on T2/FLAIR images. Here is the workflow: the 3D FLAIR sequence used by the CAD program is acquired first. Immediately after completion, our clinical 3D lab runs the CAD program, and calls the MR technologist with the results: if there is at least one new lesion, the technologist is to perform a complete contrast-enhanced scan. If not, only the non-contrast part of the scan gets done.
**Results:** We expect to limit the use of gadolinium injection to about 25% of all our follow-up MS brain scans. With more than 100 such scans per month, we expect to avoid about 75 unnecessary gadolinium injections each month.
**Conclusion:** Our system limits the use of unnecessary gadolinium injections in MS brain scans. This will address the growing concern of patients, who now receive such injections for all their surveillance scans.

**B-1782** 15:12

**Machine learning-based exploration of extracranial determinants of grey matter volume in the KORA-MRI study**

F. Schöppe[1], S. Rospleszcz[1], F. Bamberg[2], C.L. Schlett[3], W. Rathmann[4], K.-H. Ladwig[1], A. Peters[1], B.B. Ertl-Wagner[5], S. Stöcklein[1]; [1]*Munich/DE*, [2]*Freiburg/DE*, [3]*Heidelberg/DE*, [4]*Düsseldorf/DE*, [5]*Toronto, ON/CA (Franziska.Schoeppe@med.uni-muenchen.de)*

**Purpose:** The aim of this data-driven exploratory study was to identify potential extracranial determinants of grey matter volume (GMV) using a machine learning algorithm.
**Methods and Materials:** From the prospective cohort from the Cooperative Health Research in the Region of Augsburg, Germany (KORA FF4 study), extracranial variables (n = 58) were obtained in a standardized way and whole-brain GMV (ratio-corrected for intracranial volume) was derived from magnetic resonance imaging (3T, FLAIR). Using a machine-learning approach, we evaluated an elastic net on 1000 splits of training and testing data to identify the most important extracranial factors of GMV.
**Results:** 293 participants (59% men, 55.4±9.1 years, mean whole-brain GMV 20.5%±1.3) were included in the analysis. 11.9% of the subjects had type 2 diabetes mellitus, 23.2% prediabetes and 64.8% were normoglycemic. From 58 different extracranial variables, four variables were identified to be the most important determinants of whole-brain GMV: age (selected in 1000/1000 splits), glomerular filtration rate (794 splits), diabetes (323 splits) and diabetes duration (122 splits). GMV was lower in participants with diabetes compared to normoglycemic subjects (19.7%±1.6 vs 20.6%±1.2, p<0.001) and decreased with increasing diabetes duration (r = -0.15, p = 0.009). The elastic net model showed better performance compared to a constant linear regression (mean squared error = 1.10 vs. 1.59, p<0.001).
**Conclusion:** Applying a machine learning approach, elastic net identifies extracranial determinants of GMV and provides information on their relative importance. Our results underline the importance of assessing extracranial variables such as diabetes status upon inclusion of subjects in imaging studies.

**B-1783** 15:20

**Virtual monoenergetic images from spectral detector CT (SDCT) enable radiation dose reduction in unenhanced cranial CT**

R.P. Reimer, T.E. Lichtenstein, D. Flatten, D. Maintz, J. Borggrefe, N. Grosse Hokamp; *Cologne/DE (robert.reimer@uk-koeln.de)*

**Purpose:** Recent studies demonstrated that grey-white-matter-differentiation is improved by means of 65 keV-virtual monoenergetic images ($VMI_{65keV}$) as compared to conventional images (CI). Our study evaluated, if this enables a reduction of radiation-dose.
**Methods and Materials:** 115 consecutive patients receiving SDCT of the head between 2/2017 and 6/2017 (age: 55±19yrs, M/W: 56%/44%) were retrospectively included. For routine protocol adjustment, tube current time product was reduced by 10% during the study period, resulting in 60 and 55 patients examined with 320 mAs and 290 mAs, respectively. All other scanning parameters were kept identical (including tube voltage 120 kVp). Regions-of-interest were placed in grey and white matter on CI and copied to identical positions in $VMI_{65keV}$. Contrast-to-noise ratio (CNR) was calculated. Two radiologists, blinded to the reconstruction technique evaluated grey-white-matter-differentiation on a 5-point Likert-scale. Statistical assessment was performed using ANOVA adjusted for multiple comparisons and Wilcoxon test.
**Results:** Irrespective of mAs, noise was significantly lower in $VMI_{65keV}$ as compared to CI ($VMI_{65keV}$/CI, e.g. grey-matter with 290 mAs: 3.1±0.6HU/4.6±0.9HU, p<0.0001). In CI, noise was slightly higher in 290 mAs protocols as compared to 320 mAs (e.g white-matter: 4.9±0.9HU/4.6±0.8HU, p>0.05). Overall, CNR in $VMI_{65keV}$ was superior to CI irrespective of mAs (p<0.0001). Particularly, $VMI_{65keV}$ with 290mAs showed a higher CNR than CI with 320 mAs. Subjective analysis confirmed better grey-white-matterdifferentiation on $VMI_{65keV}$ images, even using 290 mAs.
**Conclusion:** $VMI_{65keV}$ from SDCT allow for radiation-dose reduction in cranial CT. While this proof-of-concept included a radiation-dose reduction of 10%, our data suggest that even greater reduction seems possible.
*Author Disclosures:*
**D. Maintz:** Speaker; Philips Healthcare. **J. Borggrefe:** Speaker; Philips Healthcare. **N. Grosse Hokamp:** Speaker; Philips Healthcare.

**14:00 - 15:30 Room F1**

### Breast

### SS 1902a

### Contrast-enhanced mammography, breast CT, and other new imaging techniques

*Moderators:*
M. Bernathova; Vienna/AT
F. Pesapane; Milan/IT

### B-1784 14:00

**The accuracy of contrast-enhanced mammography: a retrospective multireader study**

M. Calvo Imirizaldu[1], I. González de la Huebra Rodríguez[1], A. Ezponda Casajús[1], J. Etxano[2], A.M. Elizalde Pérez[1], L.J. Pina Insausti[1]; [1]*Pamplona/ES*, [2]*Vitoria/ES (igonzalez.6@unav.es)*

**Purpose:** To compare the accuracy of Digital Mammography (DM) and Titanium Contrast Enhanced Mammography (TiCEM).
**Methods and Materials:** IRB approved study. From October-2017 to June-2018, 80 patients with 120 histologically confirmed lesions were recruited. All of them underwent DM and TiCEM. This is a new technique to perform contrast enhanced mammography using a titanium filter. Three readers, blinded to the final diagnosis, retrospectively evaluated the lesions and classified them into the BI-RADs categories. Reader 1 was an expert with 20-years of experience, Reader 2 was a resident with 3-months of experience and Reader 3 was an intermediate level (3-years). For every case readers assessed the DM images and immediately the TiCEM images. Statistical analysis: ROC curves (STATA 12.0 software) for the complete database and for subgroups (dense and non-dense breasts).
**Results:** Out of the 120 lesions, 41 were benign and 79 malignant (12 DCIS, 5 ILC, 62 IDC). The AUC´s of DM vs TiCEM for Reader 1 were: 0.72 vs 0.86; p< 0.001. For Reader 2 were: 0.63 vs 0.80; p<0.001. For Reader 3 were: 0.70 vs 0.79; p<0.001. These data were similar for dense and non-dense breasts. Comparison between the AUC of DM for Reader 1 (expert) vs AUC of TiCEM for Reader 2 (Resident) found significant differences (0.72 vs 0.80; p=0.04).
**Conclusion:** The accuracy of TiCEM was significantly better for all the readers, in dense and non-dense breasts. The accuracy of a Resident reading a TiCEM study was even better than that of an expert radiologist reading DM.

### B-1785 14:08

**Contrast-enhanced digital mammography, magnetic resonance imaging and mammography alone: a comparison of diagnostic performance in symptomatic women**

W. Tang; *Shanghai/CN (tangwei12@fudan.edu.cn)*

**Purpose:** To compare the diagnostic efficiency of contrast-enhanced digital mammography (CEDM), digital mammography (DM) and magnetic resonance imaging (MRI) in symptomatic women.
**Methods and Materials:** Between March and December 2017, 246 patients with 282 histologically proven lesions all underwent DM, CEDM and MRI. Two radiologists were interpreting all images. The diagnostic performance of each method was assessed by receiver-operating characteristic (ROC) curve. The sensitivity, specificity and accuracy were compared using McNemar's and Fisher's exact test. A Kappa test was used to assess the interobserver agreement.
**Results:** The area under the ROC curve was lower in the group that underwent DM (Radiologist 1 [R1], 0.829; Radiologist 2 [R2], 0.862) than CEDM (R1, 0.927, P=0.0214; R2, 0.910, P=0.0229) and MRI (R1, 0.939, P=0.0006; R2, 0.925, P=0.0009). However, the difference between CEDM and MRI was not significant (R1, P=0.2262; R2, P=0.2843). The accuracy (R1, 73.8%; R2, 72.4%) and sensitivity (R1, 74.9%; R2, 74.2%) of DM were lower than CEDM (accuracy: R1, 89.7%, P=0.001; R2, 90.8%, P<0.001; sensitivity: R1,92.1%, P<0.001; R2, 90.8%, P<0.001) and MRI (accuracy: R1, 91.3%, P=0.001; R2, 92.7%, P<0.001; sensitivity: R1, 94.7%, P<0.001; R2, 95.4%, P<0.001). In contrast, no significant difference was observed between CEDM and MRI (accuracy: R1, P=0.744; R2, P=0.660; sensitivity: R1, P=0.602; R2, P=0.759). The interobserver agreement of each method was excellent (k=0.896, 0.929 and 0.882 for DM, CEDM and MRI, respectively.
**Conclusion:** The diagnostic performance of CEDM and MRI is superior to that of DM in symptomatic women; MRI is slightly better than that of CEDM, but without statistical significance.

### B-1787 14:16

**Evaluation of single-view contrast-enhanced mammography as potential strategy for screening of patient with high breast cancer risk: a non-inferiority retrospective study**

M.B.I. Lobbes, J. Hecker, I. Houben, R. Pluymakers, C. Jeukens, U. Lalji, S. Gommers, J.E. Wildberger, P.J. Nelemans; *Maastricht/NL (marc.lobbes@mumc.nl)*

**Purpose:** Many guidelines recommend screening of high-risk women using breast MRI. Contrast-enhanced mammography (CEM) might be an alternative strategy. However, its radiation dose is higher than mammography. As an alternative reading strategy, evaluation of single-view (1V) CEM exams might be considered instead of double-view (2V) readings to lower total radiation dose used per exam.
**Methods and Materials:** This retrospective observational non-inferiority study evaluates whether use of 1V does not result in an unacceptable worse sensitivity for detecting breast cancer (non-inferiority margin: -10%). All CEM images from May 2013 and December 2017 were included. 1V readings were performed by consensus opinion of three radiologists, with 2V readings being performed after six weeks. Cases were considered ‘malignant’ if the final score was BI-RADS $\geq$4, enabling us to calculate sensitivity, specificity and AUC under the ROC curve. Histopathological results or a minimal follow-up of six months served as gold standard.
**Results:** 368 cases were evaluated. Sensitivity decreased with 9.6% from 92.9% to 83.3% when only 1V was used for evaluation (p=0.0009). The lower limit of the 90% CI around the difference in sensitivity between 1V and 2V readings was -15% and lies below the predefined non-inferiority margin of -10%, and non-inferiority of 1V to 2V reading cannot be concluded. AUC for 1V was 0.861 versus 0.899 for 2V (p=0.0174).
**Conclusion:** 1V evaluations as an alternative reading strategy to the standard 2V evaluations have a worse overall diagnostic performance and it cannot be recommended as an alternative reading strategy to lower CEM-associated radiation exposure.

### B-1788 14:24

**Comparison of the intensity and pattern of enhancement on contrast-enhanced spectral mammography between malignant and benign breast lesions**

Y. Liu, J. Yu, L. Su; *Chengdu/CN (ly0283@yeah.net)*

**Purpose:** To investigate the relationship between histological results and the intensity and pattern of enhancement on contrast-enhanced spectral mammography (CESM).
**Methods and Materials:** Breast CESM was performed on patients with ACR-BIRADS 4 or above by ultrasound. Two experienced radiologists analysed the images and diagnosed together. Regarding pathology results as the gold standard, and an enhancement of breast lesion on CESM as positive result, the diagnostic accuracy of CESM was analysed. The enhancement characteristics of CESM images were qualitatively assessed, including the intensity and pattern of enhancement.
**Results:** 50 cases were enrolled, in which 29 were malignant and the remaining 21 were benign. The performance of CESM was: sensitivity 100%, specificity 38.1% and area under the ROC curve 0.714. All malignant lesions showed enhancement, and most frequently presented as medium or strong enhancement (24). For these with medium or strong enhancement (30), 24 lesions were malignant. There were 13 cases of benign lesions presented as enhancement; and most frequently as weak enhancement (7). There were 17 malignant lesions and 3 benign lesions with homogeneous enhancement, 12 malignant lesions and 9 benign lesions with heterogeneous enhancement. There was significant difference of the degree of enhancement between benign and malignant groups ($P$<0.001), but there was no significant difference of the pattern of enhancement between the two ($P$ = 0.917).
**Conclusion:** The specificity of CESM is limited. Medium or strong enhancement on CESM is related to breast cancer, which may help predict the risk of malignancy for suspicious breast lesions.

### B-1789 14:32

**Propagation-based computed tomography and cone-beam computed tomography of the breast: a comparison of radiological image quality**

S. Tavakoli Taba[1], P. Baran[2], Y. Nesterets[2], S. Pacile[3], S. Wienbeck[4], C. Dullin[4], F. Arfelli[3], S. Lewis[1], G. Tromba[3], T. Gureyev[2], P. Brennan[1]; [1]*Sydney/AU*, [2]*Melbourne/AU*, [3]*Trieste/IT*, [4]*Göttingen/DE (amir.tavakoli@sydney.edu.au)*

**Purpose:** Propagation-based phase-contrast CT (PBCT) and cone-beam breast CT (CBCT) are two cutting-edge techniques for breast cancer imaging. A comparison of the radiological quality of images produced by the two techniques are presented.
**Methods and Materials:** PBCT and CBCT were performed on eight formalin-fixed breast tissue samples which were of different sizes and included various types of tumour. The PBCT experiment was conducted at the SYRMEP beamline of the Elettra synchrotron in Trieste, Italy at standard radiation dose of 6 mGy and low dose of 1.5 mGy. The CBCT scans were collected using a Koning Breast-CT machine (CBCT1000) at the University Medical Center Göttingen, Germany at standard radiation dose of 6 mGy. Six medical imaging experts independently compared the images from the two modalities and rated radiological quality for each set of images using a five-point rating scale. Image quality was analysed using visual grading characteristics (VGC) and the area under the curve ($0 \leq AUC_{VGC} \leq 1$) was calculated to measure the difference in the image quality of the two systems.
**Results:** VGC analysis revealed that the image quality was significantly higher for standard dose PBCT than standard dose CBCT; $AUC_{VGC}$ =0.990 (p≤0.01). Moreover, the image quality was significantly higher for low dose PBCT than standard dose CBCT; AUCVGC =0.812 (p≤0.05).
**Conclusion:** PBCT can lead to improvements in radiological image quality of breast images even at lower radiation doses compared to CBCT. PBCT is expected to become a viable method for early diagnosis of breast cancer in the future.

**B-1790** 14:40
**Dedicated breast CT may prevent unnecessary biopsies in women recalled from mammographic screening**
R.M. Mann, G. van Spijker, M. Caballo, I. Sechopoulos; *Nijmegen/NL (Ritse.mann@radboudumc.nl)*

**Purpose:** To assess the potential of dedicated unenhanced breast-CT (BCT) in the clinical work-up of women recalled from mammographic screening.
**Methods and Materials:** 51 women recalled from screening participated in this ethics board-approved study. All women underwent unilateral unenhanced BCT using a dedicated breastCTsystem (Koning Corp., NY, USA), in addition to the regular clinical work-up (tomosynthesis, and ultrasound for soft tissue findings). BCT images were evaluated by an independent radiologist (BI-RADS ≥3 positive). The potential impact of BCT on sensitivity and positive predictive value for biopsy (PPV3) was assessed using the McNemar test. Subgroup analyses were performed for different types of mammographic findings.
**Results:** Clinical work-up led to biopsy for 26 lesions, (6 malignant, 20 benign, PPV3 23%). BCT was positive in 39 lesions (mean size 11.8 mm, range 3-51 mm), (5 malignant, 34 benign, sensitivity 83%, ns). The missed cancer was located in the axillary tail and therefore outside the BCT field of view. BCT had no impact on the indication to biopsy or not for mass lesions (n=23, 7 biopsied, 4 malignant), with findings being consistent with those from mammography. In patients presenting with calcifications (n=21, 18 biopsied, 2 malignant) BCT was negative in 7 patients with benign lesions, potentially improving the PPV3 for calcifications from 11% to 18% (p=0.023). In women with architectural distortion or focal asymmetry (n=8, 0 biopsied) BCT also dismissed the screening findings.
**Conclusion:** This pilot study suggests that unenhanced BCT may be used to prevent unnecessary biopsies in women recalled for benign calcifications.
*Author Disclosures:*
**R.M. Mann:** Grant Recipient; Siemens Healthineers, Bayer healthcare, Medtronic, Screenpoint medical, Identification solutions **I. Sechopoulos:** Grant Recipient; Koning.

**B-1791** 14:48
**Influence of different examination settings on the imaging of microcalcifications in CBCT**
C. Neubauer, J. Wolf, J. Neubauer; *Freiburg/DE*

**Purpose:** The aim of the study was to evaluate the influence of different examination settings on the imaging of microcalcifications in CBCT.
**Methods and Materials:** 9 imaging phantoms, each with a different number of microcalcifications, were examined in CBCT with 243 different examination protocols. The kV, the mA and the slice thickness were varied. 27 radiologists evaluated 9 examinations of different examination protocols with regard to the number of delimitable microcalcifications and the subjective assessment of image quality. Reference standard for the number of microcalcifications was a consensus reading by two radiologists of mammography and tomosynthesis of the phantoms. A p-value of 0.05 was assumed to be statistically significant.
**Results:** Examination protocols with a slice thickness of 0.1 and 0.2 were associated with more accurate imaging of the microcalcifications and were rated higher in image quality compared to examination protocols with a slice thickness of 0.4 mm. All other parameters showed no influence.
**Conclusion:** For a most accurate representation of microcalcification in CBCT, a slice thickness of at least 0.2 mm should be chosen. The adjustment of kV and mA is not essential.

**B-1792** 14:56
**The feasibility of using clinically compatible grating interferometry mammography in breast cancer diagnosis**
C. Arboleda[1], K. Lang[2], Z. Wang[1], T. Köhler[3], S. Prevrhal[3], C. Leo[4], S. Forte[4], R. Kubik-Huch[4], M. Stampanoni[1]; [1]*Villigen/CH*, [2]*Malmö/SE*, [3]*Hamburg/DE*, [4]*Baden/CH (carolina.arboleda@psi.ch)*

**Purpose:** Grating-interferometry mammography (GIM) is an emerging breast imaging technique able to provide phase-contrast (PC) and dark-field (DF) images that may contain information complementary to conventional attenuation-only (AT) images. The purpose of this work was to study the potential diagnostic value of PC and DF images in mastectomies.
**Methods and Materials:** Fifteen mastectomy samples were measured on a clinic-ready GIM investigational device directly after surgery at a mean glandular dose of 1.8 mGy and 14 sec acquisition time. Three breast radiologists performed a qualitative assessment of the individual appearance of the PC and DF signals for anatomical structures, tumor masses and microcalcifications. The DF signal was further quantified in relation to tumor size and type. Readers also compared tumor visibility in a weighted fusion of all three images (WF) to that in AT images.
**Results:** DF contrast was pronounced for all microcalcifications and tumor masses, with wider contrast range for masses.While anatomical structures did not provide much DF contrast, their edges were enhanced in PC. Some masses also showed PC edge enhancement. WF images showed a slightly improved tumor visualization compared to AT images.
**Conclusion:** In this first feasibility study of clinically compatible GIM, it was found that the DF signal was more pronounced for pathological lesions than for anatomical structures, which could have a diagnostically relevant impact, especially for tumors embedded in dense breast tissue. Fused images, incorporating the edge-enhancing effect of the PC signal, contributed to improved image quality when compared to conventional AT images.

**B-1793** 15:04
**Optoacoustic imaging is helpful in predicting breast cancer molecular subtypes**
B. Dogan[1], G. Menezes[2], R. Butler[3], E. Neuschler[3], P. Lavin[4], R. Aitchison[4], L.F. Tucker[5], P. Otto[2], S. Grobmyer[2]; [1]*Dallas, TX/US*, [2]*San Antonio, TX/US*, [3]*New Haven, CT/US*, [4]*Framingham, MA/US*, [5]*Wirtz, VA/US (giselalgm@gmail.com)*

**Purpose:** To investigate the potential role of optoacoustic imaging combined with conventional grey-scale US (OA/US) in non-invasively diagnosing breast cancer molecular subtypes.
**Methods and Materials:** In this multi-institutional study, 2105 women with a suspicious breast mass underwent pre-biopsy OA/US scans between 12/2012 and 09/2015. Those diagnosed with invasive breast cancer at biopsy were included in this analysis. Seven blinded readers scored the internal (OAINT) and external (OAEXT) OA/US features of identified cancers. The ratio of total internal to total external OA/US features (RInt/Ext) was derived. Tumour hormone receptor (ER and PR), HER-2neu status, and available ki-67 (%) were derived from pathology specimens. Analysis of individual OA/US features and tumour molecular subtypes of Luminal A (LumA), Luminal B (LumB), triple negative (TNBC) and HER2 amplified (HER2-E) was performed using ANOVA.
**Results:** Of 532 patients with available molecular subtypes, 186 (35.0%) were LumA, 244 (45.9%) LumB, 79 (14.8%) TNBC and 23 (4.3%) were HER2+. OAEXT was lower in TNBC compared to LumA (p<0.0001), whereas OAINT was lower in LumA compared to TNBC (p=0.031). The mean RInt/Ext was significantly higher in TNBC (1.7, SD ± 0.7) compared to LumB (1.3, SD ±0.5) and LumA (1.2, SD ± 0.5) subtypes (p<0.0001), but not significantly different from HER2 (1.5, SD ±0.6). RInt/Ext helped distinguish LumA vs LumB (p=0.044), LumA vs HER2+ (p=0.0286), LumA vs TNBC (p<0.0001), and LumB vs TNBC (p<0.0001).
**Conclusion:** Functional OA/US features might help non-invasively distinguish breast cancer molecular subtypes, showing promise as a clinical prognostic tool that can facilitate management decisions.
*Author Disclosures:*
**G. Menezes:** Employee; Part-time employment contract at Seno Medical Instruments. **P. Lavin:** Consultant; Research contract with Seno Medical Instruments to provide study design and analysis services. **R. Aitchison:** Consultant; Research contract with Seno Medical Instruments to provide study design and analysis services. **S. Grobmyer:** Advisory Board; Medical Advisory Board at Seno Medical Instruments.

**B-1794** 15:12
**Optoacoustic imaging features correlate with breast cancer clinicopathological prognostic factors**
B. Dogan[1], G. Menezes[2], R. Butler[3], E. Neuschler[3], P. Lavin[4], R. Aitchison[4], L.F. Tucker[5], P. Otto[2], S. Grobmyer[6]; [1]*Dallas, TX/US*, [2]*San Antonio, TX/US*, [3]*New Haven, CT/US*, [4]*Framingham, MA/US*, [5]*Wirtz, VA/US*, [6]*Cleveland, OH/US (giselalgm@gmail.com)*

**Purpose:** To identify the potential role of OA/US as an indicator for breast cancer clinicopathological prognostic markers of grade, Ki-67 and axillary lymph node (LN) metastasis.
**Methods and Materials:** Patients with primary invasive breast cancer who underwent pre-biopsy OA/US between 12/2012 and 09/2015 in this prospective multi-centre clinical trial were retrospectively reviewed. Blinded 7 readers scored the internal (OAINT) and external (OAEXT) OA/US features of cancers. The ratio of total internal to total external OA/US feature scores (RInt/Ext) was derived. Histologic grade, Ki-67 labeling index (%), and axillary surgical pathology (negative LN, 1-2 positive, or ≥3+ LN) were correlated with OA/US feature scores. ANOVA tests were used to identify the relationship between OA/US features, prognostic factors and metastatic LN groups. Pearson correlation was used to test correlation of continuous variables.
**Results:** Overall, 629 patients with 653 invasive cancers were identified. Increasing Ki-67 correlated with all OAINT (p=0.002) features, the highest correlation being with internal haemoglobin score (p=0.0004). Increasing RInt/Ext correlated with increasing Ki-67 index (p<0.0001), while there was moderate correlation with OAEXT (p=0.03). Grade I cancers had lower OAINT (p=0.0112) than Grade III and lower RInt/Ext (p=0.0033) than Grade II and III cancers. Regarding LN status, both OAINT (p<0.0031) and OAEXT (p<0.0001) were higher in patients with metastatic LN compared to those with negative LN, while significant OAINT (p=0.0082) and OAEXT (p=0.0003) differences were identified between LN negative, 1-2 LN and ≥3+ LN patient groups.
**Conclusion:** OA features correlate with breast cancer clinicopathological prognostic factors of histologic grade, Ki-67, and axillary LN status.
*Author Disclosures:*
**G. Menezes:** Employee; Part-time employment contract at Seno Medical Instruments. **P. Lavin:** Consultant; Research contract with Seno Medical Instruments to provide study design and analysis services. **R. Aitchison:** Consultant; Research contract with Seno Medical Instruments to provide study design and analysis services. **S. Grobmyer:** Advisory Board; Member of the Medical Advisory Board at Seno Medical Instruments.

**14:00 - 15:30** **Room F2**

### Breast

### SS 1902b

### Breast cancer staging, therapy monitoring and recurrence

*Moderators:*
N.N.
M.A. Orsi; Milan/IT

**B-1795** 14:00
**CT staging in newly diagnosed breast cancer: are we adhering to current guidelines?**
N.A. Healy, J.A. Tanner, F. Kilburn-Toppin; *Cambridge/UK (nualahealy@gmail.com)*

**Purpose:** Published guidelines advocate staging CT for Stage III and IV breast cancer only. The aim of this study was to determine if staging CT scans performed in newly diagnosed asymptomatic breast cancer patients adhered to published guidelines. We also aimed to evaluate the workload generated from additional tests ordered based on staging CTs.
**Methods and Materials:** A PACS system search generated all body CT staging scans performed at Addenbrooke's Hospital, Cambridge, from January 2014 to March 2018, which included 'breast' in indication or result. Only asymptomatic patients with newly diagnosed breast cancer who had CT imaging performed within 3 months of surgery were included in the study. Patient demographics, imaging findings and pathology results were retrieved from the hospital EPIC system and PACS. Number of additional tests generated by staging CTs and outcomes of these investigations were recorded.
**Results:** A total of 3001 'breast' CT scans were performed during the time period. Of these, 241 were performed in asymptomatic patients recently diagnosed with breast cancer, with 197 performed pre-operatively and 44 post-operatively. 85 patients (35%) were appropriately staged according to guidelines. Metastases were detected in 27 patients (11%) at diagnosis, 16 (59%) of whom had been correctly referred for staging. 64 patients (30%) who did not have metastasis required additional imaging on the basis of CT findings.
**Conclusion:** Many patients are inappropriately referred for staging CT following diagnosis of breast cancer, generating additional imaging in a third of cases, thus increasing the workload of the radiology department.

**B-1801** 14:08
**Can breast MRI aid in predicting high burden axillary disease in patients diagnosed with breast cancer?**
N. Healy, F. Kilburn-Toppin, M. Wallis; *Cambridge/UK (nualahealy@gmail.com)*

**Purpose:** In cases of low volume axillary disease in breast cancer sentinel node biopsy can be offered over axillary lymph node dissection. The aim of this study was determine if preoperative breast MRI can predict high burden axillary disease on histology (3 or more abnormal nodes) and compare with US findings.
**Methods and Materials:** A search of hospital records retrieved all breast MRIs performed from Jan 2015 - Dec 2017. All MRIs performed for local staging of new diagnosed breast cancer with positive axillae were included. A retrospective review of imaging for all patients with a positive axillary status was performed to determine nodal status on MRI and correlate it with sonographic and pathology results.
**Results:** Of the 1217 breast MRIs, 434 with complete data were performed for local staging of breast cancer. 179 had disease in the axilla, 110 detected pre-operatively and 69 on axillary sampling. 105 of 179 had neo-adjuvant treatment. 23 in the surgery group had 3 or more abnormal lymph nodes on histology; MRI axilla was abnormal in 16/23 (70%) and US abnormal in 13/23 (56%). In the neoadjuvant group 46 had 3 or more abnormal nodes; MRI was abnormal in 39/46 (85%) and US abnormal in 44/46 (96%) cases. These included 19 that had greater than 3 nodes based on fibrosis and MRI predicted axillary disease in 18/19 (95%) cases.
**Conclusion:** MRI can aid in detection of high axillary nodal burden and may be useful in combination with US at identifying a positive axilla pre-operatively.

**B-1796** 14:16
**Clinical value of 18F-FDG PET/CT for staging elderly and young women with preoperative breast cancer**
K. Kubota, T. Fujioka, M. Mori, J. Tsuchiya, U. Tateishi; *Tokyo/JP (kbtmrad@tmd.ac.jp)*

**Purpose:** The purpose of this study was to investigate the clinical value of routine 18F-FDG-PET/CT staging for primary breast cancer in the elderly and young patients.
**Methods and Materials:** Preoperative breast cancer patients under 35 y.o. (young group) and over 75 y.o. (elderly group) who underwent FDG-PET/CT from 2005 to 2017 were recruited. Image findings, clinical stage, the status of lymph node metastasis, the status of distant metastasis and detection of other malignancies were examined.
**Results:** There were 22 patients (stage 0: 3, I: 7, II: 8, III: 3, IV: 1) in the young group and 107 patients (stage 0: 12, I: 58, II: 33, III: 2, IV: 2) in the elderly group. SUVmax of the breast cancer was significantly higher in the young group than the elderly group (6.4 ± 5.9 vs 3.3 ± 3.1, p =0.0005). The sensitivity, specificity, PPV, and NPV for detecting lymph node metastasis in the young group were 85.7%, 100%, 100%, and 90.9%, respectively. The sensitivity, specificity, PPV, and NPV for detecting lymph node metastasis in the elderly group were 31.8%, 98.8%, 87.5%, and 84.8%, respectively. No other malignancy was found in the young group, whereas other malignancies (2 colon cancer, 1 oesophageal cancer, 2 lung cancers) were identified in 5% (5/107) in the elderly group.
**Conclusion:** FDG uptake of the young breast cancer was high, and PET/CT showed a high ability to diagnose lymph node metastasis in young patients. Incidental cancer was found in elder patients. Management of PET/CT according to age may be necessary.

**B-1797** 14:24
**Comparative evaluation of strain ratio on sonographic elastography and T2* values on 3Tesla MRI in differentiating malignant from benign axillary lymph nodes in breast cancer**
S. Sharma, R. Bansal, S. Jain, P. Sethi; *New Delhi/IN (dr.princysethi06@gmail.com)*

**Purpose:** To assess whether strain ratio and T2* values can improve the sensitivity and specificity of differentiating metastatic from benign axillary lymph nodes in breast cancer patients taking histopathology as reference standard.
**Methods and Materials:** The study was done on 43 patients. A multi-echo transverse T2*W MR sequence was obtained with TE= 0.9- 1.5 ms, TR=37.2 ms and flip angle=25°. Sonographic elastography was done using high frequency linear probe (L3-16 MHz). Manual selection of the region of interest was done on suspicious lymph nodes for calculation of T2* values and strain ratio. ROC curves were obtained for various T2* and strain ratio values in comparison to histopathological findings as gold standard.
**Results:** Correlation with histopathology was better with T2* values than strain ratio. The sensitivity and specificity were calculated using cut off values obtained from ROC curve (31.225ms for T2* value and 1.85 for SR) and were 70.37%, 68.75% for strain ratio and 96.29%, 93.75% for T2* value respectively. The positive predictive value and negative predictive value were also assessed, values being higher for T2* than strain ratio. Comparison of areas under ROC curve was statistically significant with p value 0.018.
**Conclusion:** T2* can be used as a potential biomarker for differentiating metastatic from benign axillary lymph nodes owing to its high sensitivity, specificity and relative ease of performance. Quantitative assessment of changes in T2* values may allow more objective analysis of signal changes with significant differences between benign and malignant lymph nodes, even in case of partial infiltration.

**B-1798** 14:32

**Biopsy-proven ductal carcinoma in situ radiographic features and upgrade rates**

L.C.A. Chan, K.H. Wong, P.Y. Tang, K.Y. Tam, K.O. Cheung, C.L. Chiu; *Hong Kong/HK (lokchi327@gmail.com)*

**Purpose:** To review the radiographic presentation of biopsy-proven ductal carcinoma in situ (DCIS) by ultrasound-guided core biopsy or stereotactic-guided vacuum-assisted biopsy and their upgrade rates to invasive carcinoma at surgical excision.
**Methods and Materials:** 142 image-guided core biopsies with the diagnosis of DCIS of 139 patients referred from surgical breast clinic or oncology clinic to a single centre from March 2004 to March 2014, were reviewed. 83 (58.5%) were sonographically visible lesions with or without associated mammographic abnormality and ultrasound-guided biopsy performed. 59 (41.5%) were mammographic detected microcalcification but sonographically occult groups and stereotactic-guided vacuum-assisted biopsy done. Their radiographic features, the biopsy and surgical histopathological results were reviewed.
**Results:** Majority of the sonographically visible DCIS were seen as a mass lesion (94.0%). They were shown on ultrasound mainly as irregular (78.2%), non-circumscribed (71.8%), hypoechoic (91.0%) masses. A small proportion presented as dilated duct (2.4%) or microcalcification groups (3.6%). The mammographic detected and sonographically occult microcalcification groups were predominantly fine pleomorphic (40.7%) or amorphous (55.9%) microcalcification, ranging from 3 to 25 mm (mean: 8.9 mm) in size. There was no significant difference in the DCIS histologic grades between sonographically visible lesions and mammographic detected only microcalcification groups (*p*= 0.93). The subsequent upgrade rate upon surgical excision compared with the sonographic guided core biopsy was significantly higher than of stereotactic-guided vacuum-assisted biopsy (26.7% vs 10.5%, *p*= 0.027).
**Conclusion:** DCIS exhibits various radiological appearance. Upgrade from DCIS to invasive cancer especially based on ultrasound-guided biopsy should be considered in subsequent surgical management.

**B-1799** 14:40

**MRI assessment of axillary lymph nodes after neoadjuvant chemotherapy based on breast cancer subtype**

V. Ruggeri, G. Mariscotti, M. Durando, C. Costanza, L. Bergamasco, I. Castellano, P. Fonio; *Turin/IT (vruggerim@gmail.com)*

**Purpose:** Breast MRI is routinely used to determine residual breast disease after neoadjuvant chemotherapy (NCT) in locally advanced cancer; however, its role in predicting axillary nodal response is controversial. We investigated the role of breast MRI in predicting residual nodal disease after NCT based on cancer subtype.
**Methods and Materials:** A retrospective review was completed using our institutional database, including all patients who underwent NCT from January 2011 to May 2018. Patients underwent breast MRI before, during and after NCT. MRI images were reviewed by two dedicated breast Radiologists, blinded to clinical and pathological results. At MRI post-NCT, lymph nodes reported as larger than 1 cm, exhibiting loss of fatty hilum, thickened cortex, or irregular contour were considered pathologic. Lymph node MRI characteristics were compared with surgical pathologic findings.
**Results:** Axillary MRI images after NCT and pathologic findings were available for 83 patients. Residual nodal disease was present in 43 patients (51.8%), 40 (48.2%) had a complete nodal pathologic response. Sensitivity, specificity and positive and negative predictive values of MRI were 62.8%, 80.0%, 77.1% and 66.7%, respectively. A subset analysis by cancer phenotype demonstrated that, for triple negative subtype, MRI had the highest specificity (100%) and positive predictive value (100%). MRI showed a significantly higher sensitivity in HER2 positive than in HER2 negative cancers (90.0%vs.54.4%,p=0.04), as well as a higher negative predictive value (91.7%vs.58.3%,p=0.03).
**Conclusion:** Our preliminary results could support the use of MRI post-NCT for assessing axillary nodal response, considering that its reliably might vary based on cancer subtype.

**B-1800** 14:48

**Which factors affect the accuracy of MRI in predicting response and estimating residual disease after neoadjuvant chemotherapy?**

H. Ahn, Y. An, J. Baek, Y. Suh, H. Choi; *Suwon/KR*

**Purpose:** To evaluate the diagnostic accuracy of breast magnetic resonance imaging (MRI) in predicting pathologic complete response (pCR) after neoadjuvant chemotherapy (NAC), and to determine the factors affecting the accuracy of MRI in evaluation of residual tumour.
**Methods and Materials:** A total of 88 breast cancer patients who underwent breast surgery after NAC were included in this study. A pCR was defined as the absence of invasive cancer in pathologic specimen, and a clinical CR as the absence of enhancing lesion on post-NAC MRI. The maximal diameter of the residual tumour on post-NAC MRI was compared with the pathologic tumour size. We analysed the accuracy of MRI in predicting pCR, and the factors affecting the size discrepancy of residual tumour between MRI and pathology.
**Results:** The residual tumour size on MRI strongly correlated to the pathologic tumour size (r=0.9, p<0.001), and showed greater accuracy in the patients with concentric reduction pattern (r=0.918, p<0.0001) than the patients with scattered reduction pattern (r=0.816, p=0.0004). The sensitivity, specificity, diagnostic accuracy and area under the curve (AUC) for predicting pCR with MRI were 93.67%, 66.67%, 90.91% and 0.8017, respectively. The size discrepancy was significantly greater in patients with luminal type (P=0.0226), multifocal tumours and non-mass enhancement on pre-NAC MRI (P=0.0467).
**Conclusion:** MRI is an accurate tool for determining pCR and evaluating residual tumour in breast cancer, especially in patients with concentric reduction pattern during NAC. The molecular tumour subtype and lesion morphology on pre-NAC MRI influence the accuracy of MRI in evaluating residual tumour.

**B-1802** 14:56

**Response of breast cancer to neoadjuvant treatment assessed using hyperpolarised $^{13}$C MRI**

R.A. Woitek, M.A. McLean, J. Grist, A. Gill, J. Abraham, F. Gilbert, C. Caldas, K. Brindle, F.A. Gallagher; *Cambridge/UK (rw585@cam.ac.uk)*

**Purpose:** Carbon-13 magnetic resonance imaging ($^{13}$C-MRI) following injection of hyperpolarized [1-$^{13}$C]pyruvate was used to determine early response of breast cancer to neoadjuvant treatment.
**Methods and Materials:** Six patients with treatment naïve breast cancers (4xTNBC, 1xIDC, 1xILC) underwent dynamic $^{13}$C-MRI at 3T (MR750, GE Healthcare) using an 8-channel $^{13}$C breast coil following injection of hyperpolarized [1-$^{13}$C]pyruvate using IDEAL encoded spiral chemical shift imaging. One patient with TNBC was rescanned after 21 days of neoadjuvant chemotherapy (NACT) and another one after eight days of NACT plus a PARP inhibitor (NACT+P).
**Results:** After 21 days of NACT, the summed lactate to pyruvate ratio (LAC/PYR) decreased by 53% alongside a 68% decrease in tumour volume whereas in the patient under NACT+P for 8 days, LAC/PYR increased on spectral (0.068 vs. 0.103) and imaging data (0.038 vs. 0.045) despite stable tumour volume. After seven cycles of chemotherapy, both patients showed complete pathological response. Among all six patients, the summed lactate to pyruvate ratio (LAC/PYR) was significantly correlated with tumour volume (rho=0.810, p=0.022; Spearman's correlation).
**Conclusion:** This study showed that hyperpolarized $^{13}$C-MRI can be used to assess response to NACT alone but might be limited in detecting very early response to NACT+P. With DNA damaging drugs, such as platinum, DNA repair mechanisms are activated and can deplete the tumour cells' NAD(H) pool, which can limit the hyperpolarized label exchange between $^{13}$C-pyruvate and $^{13}$C-lactate. With PARP inhibitors, this depletion may be impaired, preserving the NAD(H) pool maintain a high LAC/PYR ratio at early time points during treatment.

**B-1803** 15:04

**Longterm review of recurrence after breast cancer treatment: data from breast cancer/tumour type analysis**

M. Lavens, H. Wildiers, P. Neven, I. Nevelsteen, A. Smeets, M. Keupers, R.M.A. Prevos, A. Laenen, C. Van Ongeval; *Leuven/BE (matthias.lavens@telenet.be)*

**Purpose:** Imaging follow-up after breast cancer treatment includes a yearly mammography (MG) and according to the European guidelines an ultrasonography (US). Recurrence rates are useful as a predictive value, but could also serve as data for new guideline recommendations.
**Methods and Materials:** 8038 women were reviewed (Jan 2000- Jan 2016) on local (ipsilateral breast and local lymph node metastasis) and contralateral (breast only) recurrence. Women were excluded after detection of distant metastasis. Our pathologists used the St-Gallen-tumour type classification to determine BC subtypes.

**Results:** Sixteen year cumulative incidence of local recurrence was 3.83% for luminal A primary breast cancer subtype, 3.38% for luminal B, 4.78% for Her 2 and 9.73% for Triple Negative (TN) subtype. Extended analysis of TN versus Luminal A showed a four-year hazard ratio (HR) of 4.77 for TN versus 0.51 for luminal A (p<0.001) and an eight-year hazard ratio of 5.14 versus 1.14 (p=0.003). Twelve-year-HR and sixteen-year-HR showed no Triple Negative versus Luminal A difference. Sixteen-year-cumulative-incidence of contralateral recurrence was 5.91% for luminal A subtype and 7.36% for TN (p=0.09). In a sample analysis 88 % of all relapses were detected with the combination of clinical examination, MG and US.
**Conclusion:** A remarkable higher local recurrence rate of primary Triple Negative and Her2 breast tumours should questioning a dedicated imaging surveillance protocol (MG, US, MRI), particularly the first eight year after diagnosis.

**B-1804** 15:12
**Contrast-enhanced mammography screening women after breast-conserving therapy**
D. Avendano[1], M.S. Jochelson[1], K. Pinker-Domenig[2], M.A. Marino[3], J. Gluskin[1], J.S. Sung[1]; [1]*New York, NY/US*, [2]*Vienna/AT*, [3]*Messina/IT (dlilahh1@gmail.com)*

**Purpose:** To investigate the added value of contrast-enhanced mammography (CEM) compared to low-energy mammography in routine imaging of breast cancer patients after breast-conserving therapy (BCT).
**Methods and Materials:** In this HIPAA compliant IRB-approved retrospective single-institution study 427 consecutive asymptomatic breast cancer patients treated with BCT who underwent screening CEM between January 2013 and March 2018 as part of their routine follow up were included. Histopathology was used as the standard of reference.
**Results:** Ten (2.3%) of 427 patients (854 breasts) were diagnosed with breast cancer during follow-up with 10 lesions: eight with an ipsilateral recurrence and two with contralateral cancer. All 10 (100%) cancers were detected with CEM whereas only 5/10 cancers (50%) were seen with mammography. Sensitivity, specificity, and accuracy of CEM was 100%, 98% and 98% compared to low-energy mammography alone which was 50%, 98% and 97%. The combined interpretation of CEM and low-energy mammography achieved sensitivity and specificity of 100% and 98.7% with an improved accuracy of 98.7%.
**Conclusion:** Data indicate that CEM is more sensitive than low-energy mammography for breast cancer detection after BCT in the screening setting. Best results are achieved with the combined interpretation of low-energy mammography and CEM.
*Author Disclosures:*
**D. Avendano:** Grant Recipient; BCRF Breast Cancer Research Foundation.
**M.A. Marino:** Grant Recipient; BCRF Breast Cancer Research Foundation.

**B-1805** 15:20
**Does internal mammary artery irradiation for breast cancer make significant difference in diameters of internal mammary artery? Correlation with computed tomography**
G. Durhan, A. Erdemir, S. Sarı, M. Gültekin, J. Karakaya, M. Akpınar, F. Yildiz, F. Demirkazik; *Ankara/TR (a.gurkan.erdemir@gmail.com)*

**Purpose:** Internal mammary nodal irradiation improves loco-regional control and decreases rates of distant metastasis. However, irradiation accelerates atherosclerosis and may cause stenosis of not only coronary arteries but also internal mammary artery (IMA). IMA has two major importance in patients diagnosed as breast cancer. First, IMA is an excellent conductor for coronary bypass grafting (CABG). Second, IMA is the most common recipient vessel used in free flap breast reconstruction. The aim of study is to investigate radiation effects on internal mammary arteries via computed tomography (CT).
**Methods and Materials:** Between January 2010 and December 2016, 3612 patients with breast cancer treated with radiotherapy (RT) were reviewed. 239 patients who have appropriate imaging and nodal irradiation were included to the study. Bilateral IMA diameter were assessed via thin-section contrast-enhanced CT. The results of ipsilateral IMA with nodal irradiation compared with the results of contralateral non-irradiated IMA via "independent-samples t test" analysis.
**Results:** Diameters of "irradiated IMA" were significantly smaller than "non-irradiated IMA" regardless of right or left (p<0.0001) . All vascular calcifications were found in irradiated side. Radiation dose was not correlated with vessel calibres and calcifications. Although presence of calcification was found in patients having longer time interval between RT and CT, it did not reach statistical significance. Also, time interval between RT and CT was not correlated with calibre.
**Conclusion:** Calibres of IMA are decreased due to RT regardless of radiation dose and time interval. Evaluation of vessels via CT before CABG and flap reconstruction can help surgeon to select proper vessel.

**14:00 - 15:30** **Room Y**

### Interventional Radiology

### SS 1909

### Nonvascular interventions in the lung

*Moderators:*
C. Pusceddu; Cagliari/IT
Z. Tüdös; Olomouc/CZ

**K-42** 14:00
**Keynote lecture**
T.K. Helmberger; *Munich/DE*

**B-1807** 14:17
**Pre-surgical marking under CT guidance of no-palpable, non solid/solid/partially solid pulmonary nodules: when and why to use it**
I. Campo, S. Cernic, F. Arban, R. Pozzi Mucelli, F. Sacconi, M.A. Cova; *Trieste/IT (irenecampo11@gmail.com)*

**Purpose:** To assess the value of pre-surgical CT marking of no palpable, non solid/solid/partially, solid pulmonary nodules to improve the surgical outcome.
**Methods and Materials:** 29 nodules on 23 patients, identified by diagnostic CT/HRCT between January 2013 and February 2018, assessed during multidisciplinary meetings, were considered suspect (after adequate follow-up and/ or in high-risk neoplastic patients) the same were marked with metal landmarks under CT guidance, prior to thoracoscopic surgery. The safety of the procedure, the type of surgery and the histological pattern of the marked lesions were finally evaluated.
**Results:** 29 nodules were evaluated, respectively 12 non-solid, 9 partially solid and 8 solid. The average duration of the marking procedure was about 33,5 minutes and the complications found were all of low clinical impact (pneumothorax and alveolar haemorrhage). 100% of the nodules were localized during the surgery. In 18 cases the type of surgery was thoracoscopic, the average surgical time of about 1 hour and 30 minutes and the average duration of hospitalization of 6,6 days. Out of 29 nodules, 21 were found to be neoplastic, 8 of preinvasive type.
**Conclusion:** Small nodules non-solid, partially solid or solid, considered suspect after multidisciplinary discussion, can be marked safely during the preoperative phase by positioning of metallic markers. This kind of procedure determines an improvement of the surgical outcome (in particular regarding timing and surgical accuracy) and a more precise anatomo-pathological evaluation of the marked lesions.

**B-1808** 14:25
**CBCT-guided hydrogel plug percutaneous deployment before video-assisted thoracoscopic surgical resection**
A. Beneventi[1], N. Casamassima[1], A. Muollo[2], A. Coppola[1], E. Macchi[1], F. Piacentino[1], A. Ierardi[3], F. Fontana[1], G. Carrafiello[4]; [1]*Varese/IT*, [2]*Busto Arsizio/IT*, [3]*Marsala/IT*, [4]*Milan/IT (beneventi.a@gmail.com)*

**Purpose:** Cone Beam Computed Tomography (CBCT)-guided hydrogel plug deployment was recently proposed for prevention of pneumothorax after percutaneous lung biopsies. This is our initial experience of CBCT-guided hydrogel plug positioning for localization of difficult intraoperative lung nodules undergoing video-assisted thoracoscopic (VATS) resection.
**Methods and Materials:** We retrospectively evaluated the medical notes of 27 consecutive patients undergoing VATS lung wedge resection for surgical biopsy or definitive treatment of 28 small pulmonary nodules (malignant, 82%) between October 2017 and July 2018.Difficult intraoperative nodule recognition was anticipated based on lesion size <10 mm, or depth from pleura:size ratio >1, or ground-glass opacity (GGO), or operating surgeon judgement. All lesions were preoperatively marked by CBCT-guided hydrogel plug. Study endpoints were: post-localization pneumothorax frequency; intraoperative nodule localization rate; successful VATS resection rate.
**Results:** Mean size of solid nodules (n=24) and GGOs (n=4) was respectively 10.4+3.4 mm and 16.0+6.2 mm. Only one hydrogel plug marking procedure caused clinically relevant pneumothorax (1/28, 4%). All nodules were intraoperatively localized: 25 (89%) by hydrogel plug; 3 (11%) by palpation and pleural puncture hole visible in case of plug displacement. All nodules were completely excised by VATS, without complications.
**Conclusion:** CBCT-guided hydrogel plug deployment is useful not only to reduce pneumothorax rate, but also to mark challenging lung nodules. Moreover, since this device is used after biopsy procedure, it's not necessary to submit the patients to double puncture.

**B-1809** 14:33
**CT-guided lung biopsy: a simplified classification and scoring system to assess appropriateness of indication according to nodules' 18-FDG uptake, dimension and localisation**
M. Tipaldi[1], G. Orgera[1], F. Laurino[2], E. Ronconi[1], M. Krokidis[3], A. Zolovkins[1], A. Laghi[4], M. Rossi[1]; [1]*Rome/IT*, [2]*Eboli/IT*, [3]*Cambridge/UK*, [4]*Latina/IT (tipaldi.andrea@gmail.com)*

**Purpose:** To develop a simplified classification and scoring system to predict risk of technical failure in patients undergoing CT-guided transthoracic core needle biopsy (TTNB) according to nodules' characteristics and 18-FDG uptake.
**Methods and Materials:** CT-guided lung biopsies, performed in our institution from January 2014 to January 2017, were reviewed retrospectively. Lesions were classified into 3 clusters of low (n=72), intermediate (n=118) and high (n=58) technical difficulty on the basis of nodule diameter, distance from the pleural needle access and basal localisation. Technical success, length of procedure, delivered radiation dose and complications were investigated in each cluster and compared with 18-FDG uptake.
**Results:** Overall technical success was 83% (n =206), 71% (n=196) malignant and 12% (n=30) benign. PET-negative nodules had significant longer procedures (p<0.0001) and delivered dose (p=0.0436) despite fewer successful results (50% vs 92%, p<0.0001) then positive one. Technical failure showed a growing trend from 11% (n=8) in low-difficulty group, 14% (n=16) in the intermediate and 31% (n=18) in the high one, resulting statistically significant (p=0.0043) as well as the increase of procedure length (p=0.0007) estimated delivered dose (p=0.0115) and rate of complications (p=0.0055). High-difficulty PET-negative nodules had the highest technical failure rate amongst all groups (80%), significantly worse than middle- and low-difficulty PET-negative group (p=0.0008).
**Conclusion:** TTNB of high-difficulty PET-negative nodules is associated with very poor technical success rate and, in this group of patients, other strategies should be considered.

**B-1810** 14:41
**Fusion imaging technique in lung biopsy to demonstrate the correlation between standardised uptake value (SUV) and specimen's diagnostic value**
A. Muollo[1], A. Beneventi[2], E. Macchi[2], F. Piacentino[2], C. Floridi[3], F. Fontana[2], G. Carrafiello[4], C. Fugazzola[2]; [1]*Busto Arsizio/IT*, [2]*Varese/IT*, [3]*Perugia/IT*, [4]*Milan/IT (alessandra.muollo29@gmail.com)*

**Purpose:** In the context of a large study about PET/CT-CBCT-guided percutaneous lung biopsies, ten selected patients underwent double bioptic sampling, respectively in the SUV maximum and in the SUV minimum zone of the lesion. Aim of this study was to demonstrate the correlation between SUV value and specimen's diagnostic value.
**Methods and Materials:** Ten patients with voluminous pulmonary masses (diameter>30mm), characterized by homogeneous uptake of 18F-FDG at PET/CT scan, underwent double percutaneous bioptic sampling (SUV maximum and SUV minimum zone) using PET/CT-CBCT guidance. Diagnostic efficacy rate was calculated. The pathologist reported the percentage of neoplastic cells/flogosis/fibrosis for each sample analysed; furthermore, he evaluated the possibility to draw up a biomolecular profile researching antigens and genetic mutations of lung cancer in the two group.
**Results:** 9 lesions were malignant, one case was benign (flogistic). Diagnostic efficacy was equal to 100% (10/10) for the SUV maximum group and 70% (7/10) for the SUV minimum group (p=0,21). In the first group the pathologist found major percentage of neoplastic cells, while in the second group flogosis and fibrosis were predominant. The biomolecular profile was obtained in 100% (9/9) of cases for the first group and in 33,3% (2/6) of the second group (p=0,011).
**Conclusion:** Correlation between SUV value and specimen's diagnostic value was demonstrated. In addition, using fusion imaging PET/CT-CBCT technique for lung lesions biopsy admit to obtain more helpful information from tissue sampling.

**B-1811** 14:49
**The value of core-biopsy of nodules less than 10 mm**
P.S. Sánchez; *Atibaia/BR (penelope.san@hotmail.com)*

**Purpose:** The objective of our study is to determine the value of core-biopsy of nodules less than 10 mm. The diagnostic accuracy, complication rates with haemoptysis, haemoptysis, thoracic drainage and the impact on patients will be reported.
**Methods and Materials:** Retrospective, unicentric study, through the review of charts, images and chest tomography reports in an oncology reference centre. Will be analyzed 40 biopsies per fragment, of pulmonary nodules measuring up to 9 mm(ranging from 5 to 9 mm) guided by computed tomography, using needle 18 or 20 G conducted between January 2014 and December 2018. Consent was completed by all patients or their caregivers. At tomographic images at the time of biopsy were retrospectively reviewed in the standard pulmonary window (width 1500 HU, level 600 HU).
**Results:** The anatomopathological results will be collected and classified as positive or negative for malignancy, of primary or secondary origin and if they were compatible with the histopathology of the surgical piece when feasible. Negative results will be considered as true negatives when there are histological or microbiological confirmations, or if the lesion disappears or reduces in size or remains stable for at least 2 years of follow-up. Growth of the malignant nodule or surgical confirmation of the malignancy will be considered false negative results.The sensitivity, specificity, and accuracy of the core biopsies will be calculated. The positive and negative predictive values will be calculated for nodules with positive and negative results, respectively. Indices of pneumothorax, haemothorax and pleural drainage will be calculated.
**Conclusion:** The study showed that the complication rate was similar to that of nodules larger than 10 mm and the biopsy effectiveness rate was equal to that of the larger nodules.

**B-1812** 14:57
**Percutaneous radiofrequency thermal ablation (RFA) of lung neoplasms using a new 15 G internally cooled jet-tip electrode: preliminary results**
F. Carchesio, R. Iezzi, A. Posa, T. Congedo, G. Veltri, S. Margaritora, R. Manfredi; *Rome/IT (francesca.carchesio@gmail.com)*

**Purpose:** Aim of our study was to assess the safety and feasibility of CT-guided RFA in unresectable lung neoplasms, using a new 15 G internally cooled jet-tip needle.
**Methods and Materials:** 14 consecutive patients with lung neoplasms (< 4cm), both primary and secondary, unsuitable or refusing surgery, underwent percutaneous CT-guided RFA using a 15G electrode with 3-cm exposed tip. Primary endpoints were the incidence and grade of adverse events and technical success; secondary endpoint was complete response determined using CT and/or FDG-PET performed 6 months after procedure.
**Results:** A total of 14 nodules were treated (mean diameter: 28mm; range: 12-38mm). All patients underwent technically successful procedure without neither major complications nor intraprocedural death. Mild pneumothorax not requiring a tube drainage placement was registered in 40% of patients while a mild perilesional haemorrhage was registered in all cases. During the follow-up period, a complete response was obtained in 13 of 14 nodules with one partial response requiring a repeated procedure in one patient.
**Conclusion:** Percutaneous RFA using a 15G internally cooled J-Tip electrode is a safe and feasible treatment for unresectable lung lesions, with a high rate of complete response obtained.

**B-1813** 15:05
**Leaving-tail microcoil localisation for small pulmonary nodules prior to thoracoscopy**
T. Su, L. Jin; *Beijing/CN*

**Purpose:** To describe standardised interventional protocols of CT-guided localising lung nodules with leaving-tail microcoil prior to video-assisted thoracoscopic surgical (VATS) resection.
**Methods and Materials:** This study retrospectively included ninety-six patients who underwent preoperative microcoils localisation towards small pulmonary nodules. The location of pulmonary nodule and proper patient's position for microcoil implantation have been documented. The head of microcoil was pinpointed adjacent to the target nodule while its end tail remained above the visceral pleura. After the implantation, CT scan was conducted to assess the depth of the microcoil and its length outside the pleura. The results of CT were compared with observation by VATS within 24 hours. The complications and success rate of implantation were recorded.
**Results:** CT-guided microcoil implantations were performed targeting 105 pulmonary nodules. The planning access route and implantation site were determined based on the location of the pulmonary nodule, and were followed by an appropriate body position on the CT table. After implantation, CT scan showed the intra-pulmonary depth and extra-pleural length of microcoil were 18.0±6.2 mm (2 to 36 mm), 8.1±6.0 mm (-13 to 26 mm), respectively. During VATS, localisation microcoils from 95 cases (90.5%, 95/105) were visualised that their front-ends were partially inserted and their end tails remained above the visceral pleura. Finally, 104 (99.0%, 104/105) localising pulmonary lesions were successfully resected by VATS and no serious complications occurred in the localisation.
**Conclusion:** According to the location of lesion in the lungs, the standardised microcoil implantation method is the key to successful preoperative localisation.

**B-1814** 15:13

**Retrospective analysis of the role of intraparenchymal blood patching and the rate of pneumothorax after percutaneous CT-guided core lung biopsy**

R.M. Perl[1], J. Hetzel[1], H. Bösmüller[1], C. Kloth[2], M. Horger[1]; [1]*Tübingen/DE*, [2]*Ulm/DE*

**Purpose:** To assess the rate of biopsy-related pneumothoraces after percutaneous CT-guided lung puncture and to evaluate the impact of intraparenchymal blood patching (IBP) and of other tumour and operator-related risk factors.
**Methods and Materials:** Retrospective analysis of CT-guided lung biopsies performed at our institution between 01/2003 and 08/2018. The image data of 868 patients were evaluated, of whom 419 received an IPB. The data were analysed for pneumothorax rates, need for thoracic drainage, other risk factors such as lesion size, lesion depth, location within the lungs, needle calibre, number of samples taken and operators' experience.
**Results:** The pneumothorax rate was significantly lower in the IPB group (10.74%) than in the non-IPB group (15.37%). In both groups, lesions < 3 cm showed a significantly higher rate of pneumothorax (p <0.05); furthermore, pneumothorax rate increased with increasing lesion depth (p <0.001). The rate of pneumothorax was significantly lower (p <0.05) for 17G needles in the IPB group, but not for other calibers. When sampling from the lower lobe, the rate of pneumothorax was significantly reduced by IPB (p <0.001). Only in less experienced operators pneumothorax rate differed from 11.1% with IPB to 24.8% without IPB. When taking >3 samples pneumothorax rate was significantly lower in the IPB group (p <0.01).
**Conclusion:** The use of IPB significantly reduces the incidence of pneumothorax in CT-guided lung biopsies, in particular, for lesions located deeper in the lungs, when more than 3 samples are taken, when samples are taken from the lower lobe or by less experienced operators.

**B-1815** 15:21

**Gelfoam slurry tract embolisation after computed tomography-guided percutaneous lung biopsy: does it prevent major pneumothorax?**

R. Sum, K.K. Lau; *Clayton/AU (r.rm.sum@gmail.com)*

**Purpose:** To assess the efficacy of biopsy tract embolisation using an absorbable gelatin sponge slurry (Gelfoam) in the prevention of major pneumothorax after percutaneous lung biopsy.
**Methods and Materials:** A retrospective analysis was conducted on 273 patients who underwent computed tomography-guided percutaneous lung biopsy over a 9-year period. Smoking status, underlying medical condition, radiological evidence of emphysema, location and size of the lesion, length of biopsy tract, and presence of a post-biopsy pneumothorax on CT and plain radiograph were recorded.
**Results:** Gelfoam was used in 166 patients (mean age 71, range 20-96) and not used in 107 patients (mean age 69, range 27-96). Proportions of patients with radiological evidence of emphysema were similar (63.6% and 67.5% in the non-Gelfoam and Gelfoam groups, respectively). The most commonly used needle size was a 20-gauge coaxial needle. There was no significant difference in pneumothorax complications in either group on immediate post-biopsy CT (p=0.06) and 4 hours post-biopsy plain radiograph (p=0.24). The need for an intercostal catheter to treat a major pneumothorax was significantly higher when Gelfoam was not used (OR=0.1328, p=0.01) - 9 patients (8.4%) in the non-Gelfoam group required an intercostal catheter while only 2 patients (1.2%) required this in the Gelfoam group.
**Conclusion:** There is no significant difference in the rates of developing a pneumothorax when a gelatin sponge slurry was used for biopsy tract embolisation. However, gelfoam reduced the likelihood for necessitating an intercostal catheter for the treatment of a major pneumothorax.

**14:00 - 15:30** **Coffee & Talk 3**

### Cardiac

### SS 1903a

### Cardiac MR in cardiomyopathies: tips and tricks

*Moderators:*
N.N.
E. Pershina; Moscow/RU

**B-1816** 14:00

**Intravoxel incoherent motion diffusion-weighted imaging for the assessment of myocardial fibrosis in hypertrophic cardiomyopathy**

R. Wu, R.-Y. Shi, D.-A. An, C.-W. Wu, B. Chen, L. Wu, J. Xu; *Shanghai/CN (wuyidaoruirui@163.com)*

**Purpose:** To investigate the feasibility and potential of intravoxel incoherent motion (IVIM) to detect the myocardial fibrosis in hypertrophic cardiomyopathy (HCM) patients.
**Methods and Materials:** Fifty-five HCM patients were enrolled and underwent IVIM diffusion-weighted cardiovascular resonance imaging (DW-CMR). Cine, T1 mapping, IVIM DW-CMR and late gadolinium enhancement (LGE) were performed. Strain, native T1, extracellular volume (ECV), parameters derived of IVIM-CMR (D, D* and f) and LGE analyses were segmented into 16 American Heart Association (AHA) segments and the correlations among these parameters were analysed. According to LGE and ECV, positive regions for myocardial fibrosis were defined as the presence of LGE (LGE+) or ECV ≥ 29.6%.
**Results:** D values were significantly increased in both LGE+ (1.89μm$^2$/ms; 1.75μm$^2$/ms, 2.04μm$^2$/ms) and ECV ≥ 29.6% (1.84μm$^2$/ms; 1.69μm$^2$/ms, 1.97μm$^2$/ms) group than LGE- (1.63μm$^2$/ms; 1.56μm$^2$/ms, 1.73μm$^2$/ms) and ECV < 29.6% (1.61μm$^2$/ms; 1.54μm$^2$/ms, 1.68μm$^2$/ms) group. Compared to LGE- and ECV <29.6% group, D* (34.9 ± 5.5 μm2/ms vs 55.2 ± 11.4 μm2/ms, p< 0.001; 37.5 ± 6.9 μm2/ms vs 59.6 ± 9.2 μm2/ms, p < 0.001, respectively) and f values (10.8 ± 1.29% vs 12.5 ± 1.26%, p < 0.001; 10.9 ± 1.1% vs 13.0 ± 1.00%, p = 0.021, respectively) were significantly decreased in both LGE+ and ECV ≥ 29.6% group. Moreover, significant correlations were demonstrated between IVIM-derived parameters and ECV, as well as strain and strain rate.
**Conclusion:** IVIM DW-CMR is sensitive to detect myocardial fibrosis and has potential of characterizing the extent fibrosis in HCM patients.

**B-1817** 14:08

**Analysis of strain values in asymmetrical hypertrophic cardiomyopathy: correlations with wall thickness and extent of focal fibrosis**

C. De Cataldo, S. Torlone, A. Corridore, P. Palumbo, E. Cannizzaro, F. De Matteis, A. Di Sibio, E. Di Cesare, C. Masciocchi; *L'Aquila/IT (camilladeca@hotmail.it)*

**Purpose:** To assess Radial (RS), Longitudinal (LS) and Circumferential (CS) Strain values in patients affected by Asymmetrical Hypertrophic Cardiomyopathy (HCM) and their correlation with wall thickening (WT) and extent of focal fibrosis (LGE%).
**Methods and Materials:** We retrospectively analysed 35 HCM patients with preserved ejection fraction (EF); a control group of 20 healthy patients was provided. We collected Global, Per Planes and Segmental RS, CS and LS values with dedicated software (Cvi42) for each patient. We divided cardiac segments on the basis of WT and LGE%. Statistical analysis with Kruskall-Wallis test, Spearman test and ROC curve was performed.
**Results:** On a per-patient analysis, we noticed a significant impairment of Global Strain values compared to control group (GLS median -13,6% vs -17,14%; GCS median -17,31% vs -18,44%; GRS median 27,42% vs 41,84%) (p=0,04). GRS, GCS and GLS values were positively related to EF but negatively to age and myocardial mass. On a per-segment analysis, we noticed that both hypertrophic and/or enhanced segments and morphologically not affected segments in HCM showed impaired Strain values, with a significant discrepancy between these two groups and compared to control group (p<0,000001), especially for RS values (cut-off=21,77, Sensitivity=75,7%, Specificity=88,75%, AUC=0,858). RS values varied proportionally to WT and LGE%, while LS and CS values were more affected by LGE%.
**Conclusion:** Although our small specimen size, our experience provides impaired Strain values both in involved segments and apparently not involved segments in HCM, demonstrating the entirety of the disease.

**B-1818** 14:16
**MRI characteristics and clinical outcomes of hypertrophic cardiomyopathy with infarct-like late enhancement**
L. Li, S. Zhao; *Beijing/CN (tyyxlilu@126.com)*

**Purpose:** The purpose of our study is to evaluate the prevalence, cardiac magnetic resonance (CMR) features and prognostic value of infarct-like LGE in HCM.
**Methods and Materials:** From 1411 HCM patients confirmed by CMR, 57 patients in group A (age 47.70±13.29 years; 71.9% males) and 413 patients in group B (age 45.65±13.43 years; 68.1% males) were compared in terms of clinical and CMR findings. Event-free survival and Cox regression analyses were also performed in this study.
**Results:** The prevalence of infarct-like LGE was approximately 11.61% (57/491). LVEF was lower in group A than in group B [55.77±16.07 vs 66.90±8.50, p<0.001). Compared to group B, group A had larger LVEDVi (73.28±27.61 vs 64.77±16.79 ml/m2, p=0.027), LVESVi [26.67 (18.35-41.50) vs 20.52 (15.80-25.73), p<0.001] and more extensive LGE extent [18.20% (8.25-28.93) vs 8.51% (5.02-14.90), P<0.001]. Group A showed higher annual cardiovascular (8.71% vs 1.17%, p<0.001), arrhythmic (3.05% vs 0.35%, p<0.001), heart failure (5.66% vs 0.82%, p<0.001) event rate than group B, especially infarct-like LGE located in free wall. In multivariate analysis, LVEF (HR, 0.93; 95% CI: 0.915-0.96, P<0.001), the presence of NTHA III/IV (HR, 2.29; 95% CI: 1.10-4.78, P=0.028) and the presence of infarct-like LGE (HR, 3.69; 95% CI: 1.70-8.00, P=0.001) were identified as independent prognostic risk factors of cardiovascular events.
**Conclusion:** Infarct-like LGE was not rare in HCM. If confirmed in further studies, presence of infarct-like LGE can be used for risk stratification in cardiovascular events of HCM.

**B-1819** 14:24
**Sudden cardiac death (SCD) risk stratification in hypertrophic cardiomyopathy (HCM) patients by cardiac magnetic resonance imaging (CMR) with late gadolinium enhancement (LGE)**
E. Mershina, N. Krylova, V.E. Sinitsyn, N. Poteshkina, M. Balaeva, A. Pylev; *Moscow/RU (elena_mershina@mail.ru)*

**Purpose:** To study the relationship between the functional heart parameters of HCM patients and the extent of LGE by CMR.
**Methods and Materials:** EchoCG and LGE CMR at 1,5T scanner were performed in 44 patients with HCM (55,2±15,8 years old; f/m=22/22). The myocardial mass, the mass of LGE foci, the values of LVEDV, LVESV were obtained. The myocardial performance index (Tei) was calculated by EchoCG.
**Results:** LGE CMR has identified intramyocardial fibrosis in 34 (77,3%) patients. Total number of ventricular extrasystoles (VE) during 24-hour ECG-monitoring was correlated with the extent of myocardial fibrosis (r=0,45, p=0,04). Patients with the presence of LGE had larger LV EDV and larger RV EDV (p=0,02). There was no correlation between LGE presence and LVOT obstruction (p>0,05). A direct relationship was established between the myocardial performance index (Tei) and the presence of myocardial fibrosis: for basal interventricular septum segments r=0,47 (p=0,03), for basal lateral segments r=0,52 (p=0,02); for global LV r=0,54 (p=0,03), for RV r=0,67 (p=0,046). HCM patients with myocardial fibrosis had more pronounced dyspnea (r=0,43, p=0,046) due to myocardial dysfunction. Estimated risk of SCD by ESC calculator was associated with the presence (r=0,35, p=0,02) and severity (r=0,48, p=0,017) of LV myocardial fibrosis.
**Conclusion:** The presence of LV myocardial fibrosis in HCM patients according to LGE CMR was associated with the increase of the LV and RV volumes, myocardial dysfunction, the increase of VE amount and estimated risk of SCD. The global LV index T > 0.5 can predict LV myocardial fibrosis existence in HCM patients.

**B-1820** 14:32
**The diagnostic performance of novel non-contrast markers in discrimination of cardiac amyloidosis and hypertrophic cardiomyopathy on cardiovascular magnetic resonance imaging**
S. Huang, Z.-G. Yang, K. Shi, K. Diao, Y. Gao, Y.-K. Guo, Y. He, S. He, M.-T. Shen; *Chengdu/CN (huangshan82833@163.com)*

**Purpose:** To evaluate and compare the diagnostic accuracy of myocardial contraction fraction (MCF), ejection fraction strain ratio (EFSR) and other deformation-based parameters in differentiating cardiac amyloidosis (CA) from hypertrophic cardiomyopathy (HCM) without using contrast agents on cardiovascular magnetic resonance imaging (CMR).
**Methods and Materials:** We retrospectively analysed CMR scans of patients with biopsy-proven CA (n = 45, 59.2±1.6 years) and HCM (n=45, 50.5±2.1 years), both with preserved left ventricular ejection fraction (LVEF) and moderate increased LV mass. Morphological and functional parameters combined with strain and strain-derived ratios indices were analysed. MCF was calculated by dividing LV stroke volume by LV myocardial volume which was calculated as the ratio of LV mass over 1.05 (myocardial density). EFSR was calculated by dividing LVEF by global longitudinal strain (GLS).
**Results:** The maximum LV mass showed no significant difference between the two groups (CA 112±5.4g vs HCM 122.5±5.0g; *p*=0.16). CA patients had preserved LVEF (56.68 ± 1.01%) but slightly lower than HCM patients (62.51 ± 0.72%) (*p*<0.05). MCF showed the best performance to discriminate CA from HCM (AUC=0.87, 95%CI [0.80-0.94]), followed by EFSR (AUC=0.86, 95%CI [0.78-0.95]) (MCF vs EFSR, *p*=0.87). Individual systolic strains including global radial strain (GRS), circumferential strain (GCS) and longitudinal strain (GLS) had smaller AUCs (GRS: 0.73, 95%CI [0.61-0.85]; GCS: 0.73, 95%CI [0.62-0.85]; GLS: 0.74, 95%CI [0.64-0.86]) than MCF (all *p*<0.05) and EFSR (all *p*<0.05).
**Conclusion:** MCF and EFSR, two useful indices derived from non-contrast cine images, manifest excellent diagnostic performance in differentiating CA from HCM.

**B-1821** 14:40
**Cardiac magnetic resonance in myotonic dystrophy type 1: a contribution to risk assessment**
M. Alì, C.B. Monti, L. Melazzini, B. Fossati, M. Cavalli, R. Cardani, G. Meola, F. Sardanelli, F. Secchi; *Milan/IT (marco.ali90@gmail.com)*

**Purpose:** To evaluate left ventricular extracellular volume (ECV) and strain on cardiac magnetic resonance (CMR) as potential imaging-biomarkers of subclinical cardiac pathology in myotonic dystrophy type-1 (DM1) patients.
**Methods and Materials:** We retrospectively analyzed CMR examinations of DM1 patients with preserved ejection fraction performed between 2014 and 2016. ECV was calculated from native and post-contrast T1-mapping, referring to normal values reported in literature. Global circumferential strain (CS) was calculated and negative non-contrast CMR scans in age- and sex-matched subjects without history of cardiac disease were used as controls.
**Results:** Nine patients and nine controls without significant difference for age (p=0.931) and sex (p=1.000) were analyzed. Global CS was -19.1% (interquartile range [IQR] -20.9%,-15.3% in patients and -21.7% (IQR -22.7%,-21.3%) in controls (p=0.011). Patients had a median global ECV of 32.3% (IQR 28.4%,37.6%). A positive significant correlation between global ECV and CS (ρ=0.733,p=0.025) and a borderline significant correlation between septal ECV and septal CS (ρ=0.667,p=0.050) were found.
**Conclusion:** In DM1 patients with preserved ejection fraction, CMR allowed to detect impaired contractility, correlated with ECV expansion, likely due to subclinical fibrosis. CMR-derived CS and ECV may have a role as imaging biomarkers of myocardial involvement in DM1 patients, with potential prognostic role.

**B-1822** 14:48
**Cardiovascular magnetic resonance (CMR) in patients with Fabry disease**
E.A. Mershina, S.V. Moiseev, V.E. Sinitsyn, P.S. Pilius, A.S. Moiseev, V.V. Fomin; *Moscow/RU (polishka1903@gmail.com)*

**Purpose:** To perform an analysis of the imaging biomarkers found with CMR with late gadolinium enhancement (LGE) in patients with Fabry disease.
**Methods and Materials:** 61 patients (19-69 years, median 37,3: m/f - 45/16) with a confirmed diagnosis of Fabry disease who underwent CMR were included in the analysis. CMR images were obtained with a 1.5T scanner before and after gadolinium injection. The images were acquired in cardiac short-axis and in 4, 3 and 2-chamber orientations. The total mass of the myocardium was calculated including papillary muscles and trabecular layer. The indexed values of EDV, ESV, SV, and EF were obtained. Patterns of LGE were analysed.
**Results:** The patients were divided into 3 subgroups based on their imaging phenotypes: 1) without LV wall thickening; 2) with concentric LV wall thickening; 3) with asymmetric septal hypertrophy (ASH). Hypertrophy of the LV myocardium was detected in 35 patients (59%), 23 of 35 patients had ASH. In 15 of 35 patients (4%) with myocardial hypertrophy, intramyocardial fibrosis was identified with LGE. Most frequently the fibrosis was found in the inferolateral (n=6) and anterolateral segments (n=6). The frequency of myocardial LGE was 24% in men (11/45) and 25% in women (4/16).
**Conclusion:** The results of the study demonstrate that Fabry disease has a number of different phenotypic expressions which are manifested on CMR images both in extent and location of hypertrophy and pattern of myocardial LGE. Combination of radiological and genetic assessments of patients with suspected Fabry disease brings the optimal results.

**B-1823** 14:56
**Role of cardiac magnetic resonance (CMR) imaging for early detection of myocardial involvement in patients affected by Anderson Fabry disease (AFD)**
S. Coco, R. Ammendola, A. Bracci, F. Catapano, F. Cilia, G. De Rubeis, N. Galea, M. Francone, C. Catalano; *Rome/IT (simona.coco@uniroma1.it)*

**Purpose:** Cardiomyopathy is a complication of Anderson-Fabry Disease (AFD) with dramatic impact on morbidity and mortality; medical therapy is recommended in patients with evidence of cardiac involvement. Our aim was to investigate the role of Cardiac Magnetic Resonance (CMR) in early detection of cardiac involvement in AFD at pre-hypertrophic stage.
**Methods and Materials:** 10 biopsyproven AFD patients with normal wall thickness at echocardiography (<11mm) underwent to CMR (1.5 T, Avanto, Siemens, Erlangen, Germany) with following sequence protocol: STIR T2w, cineMR, late enhancement and T1 mapping with MOLLI technique before and 15 minutes after injection of 0.15 mmol/Kg gadolinium (GdDOTA, Guerbet, Paris, France). Indexed LV volumes and mass, native T1 (nT1), extracellular volume fraction (ECV) and tissue tracking parameters were analysed. Results were compared with 10 healthy age and gender-matched volunteers.
**Results:** No significative differences were found in myocardial mass (Mass/BSA: 44,81 vs 47,72 g/m2, p:0,59), ventricular volumes (EF: 58,95 vs 60,62%, p:0,62) and left ventricular myocardial strain (Global radial strain: 48,9 vs 45,7 p:0,45; global circumferential strain: 21,4 vs 19,5 p:0,41; global longitudinal strain: 20,5 vs 19,5, p:0,59) between AFD and healthy subjects. No subjects had shown edema or LGE; nT1 was significantly lower (p<0,01) in AFD patients (933 +/- 22 ms) than healthy volunteer cohort (1010 +/- 16 ms); no significative discrepancy was noted between the two groups in ECV values (23% vs 24,2%, p: 0,23).
**Conclusion:** nT1 value appears the only marker of early myocardial involvement in nohypertrophic AFD patients.

**B-1824** 15:04
**Feature tracking allows detection of diminishing wall motion disturbances in Takotsubo cardiomyopathy**
R.M. Brumberg, T. Emrich, P. Wenzel, S. Benz, R. Kloeckner, M.C. Halfmann, C. Düber, K.-F. Kreitner; *Mainz/DE (tilman.emrich@gmail.com)*

**Purpose:** Wall motion disturbances in Takotsubo cardiomyopathy (TTCM) are often rapidly decreasing in days between diagnosis via ventriculography and CMR imaging. The purpose of this retrospective study was to illuminate the diagnostic performance of cardiac magnetic resonance imaging feature tracking in fading wall motion abnormalities in TTCM.
**Methods and Materials:** We compared cardiac magnetic resonance imaging (CMR) and ventriculography of 41 consecutive patients with TTCM. TTCM-involved regions were defined as regions with wall motion disturbances in ventriculography. With a dedicated cardiovascular software (Circle CVI®, Calgary, Canada), left and right ventricular strain values from TTCM-involved and non- involved region were analysed and recorded.
**Results:** In all patients, there was a mild or marked normalisation of regional wall motion between ventriculography and CMR. Nevertheless, even in the CMR presence of only mild wall motion disturbances, feature tracking parameters of the left ventricle could easily discriminate between TTCM-involved and non-involved segments (e.g. circumferential strain in TTCM-involved region: -14.0 ± 7.9 vs. TTCM-non-involved: -19.2 ± 4.4; p < 0.0001). Interestingly, right ventricular strain values did not differ between involved and non-involved segments (e.g. RV radial strain: p = 0.069).
**Conclusion:** CMR feature tracking is a powerful tool for detection of fading left ventricular wall motion abnormalities in TTCM. Further research in larger cohorts is needed to establish the time relation between diminution of wall motion disturbance and feature tracking parameters.

**B-1825** 15:12
**Functional assessment and prognostic value of cardiac magnetic resonance in arrhythmogenic right ventricular cardiomyopathy**
M.-T. Shen, Z. Yang, K. Diao, L. Jiang, B. Hu, S. Huang, Y. Guo; *Chengdu/CN (smt1020@qq.com)*

**Purpose:** To assess left ventricular (LV) global systolic function using feature tracking cardiovascular magnetic resonance (FT-CMR) in arrhythmogenic right ventricular cardiomyopathy (ARVC) patients and investigate if the LV systolic function abnormalities and clinical characteristics predict adverse outcome in ARVC patients.
**Methods and Materials:** CMR was performed in 40 ARVC patients and 32 healthy controls. Feature-tracking analysis was applied to cine CMR images on a commercial post-processing image station. LV global longitudinal strain (GLS), circumferential strain (GCS) and radial strain (GRS) were defined as the peak strain value within a cardiac cycle. The end-point was a composite of cardiac death, sustained ventricular tachycardia, ventricular fibrillation, and appropriate ICD discharge.
**Results:** LV GRS(35.11 ±11.30% vs 50.84 ± 12.08%), LV GCS(-15.78 ± 3.25% vs -20.18 ±1.88%), LV GLS(-12.79 ±2.70% vs -18.15 ±1.93%, p<0.001 for respectively) were lower in ARVC patients compared with healthy controls. Twenty-seven with Late Gadolinium enhancement(LGE)(67.5%), eight with myocardial fatty infiltration(20%), eleven patients occurred syncope(27.5%), nine with heart failure(22.5%) and nine had Implantable Cardioverter Defibrillator(ICD)(22.5%). Thirteen patients met the end-point over a mean follow-up of 3.6 ±1.7 years. Univariate analysis showed LV GS, clinical and imaging characteristics (concluding the presence of LGE, syncope, heart failure and ICD) correlated with adverse outcome(p<0.005). Logistic regression analysis showed syncope and LV GCS were independent predictors of adverse outcome(p<0.05).
**Conclusion:** Strain analysis by FT-CMR can objectively quantify global left ventricular dysfunction in ARVC patients and can also provides important prognostic information. LV GCS and syncope were independent predictors of cardiac events in ARVC patients.

**B-1826** 15:20
**Magnetic resonance first-pass perfusion imaging for assessment of myocardial microvascular dysfunction in pediatric patients with acute leukemia**
Q. Zhao, R. Xu, Z.-G. Yang, Y.-K. Guo; *Chengdu/CN (zhaoqin626@foxmail.com)*

**Purpose:** Daunorubicin-induced cardiotoxicity remains an important concern in patients with acute leukaemia. This study aimed to clarify the segmental coronary microvascular dysfunction in paediatric leukaemia patients with daunorubicin therapy using cardiac magnetic resonance (CMR) first-pass perfusion imaging.
**Methods and Materials:** Thirty-six patients with leukaemia (25 in treatment group and 11 in non-treatment group) and 9 healthy volunteers (control group) underwent CMR at rest. Perfusion parameters including maximal slope ($Slope_{max}$), time to peak ($T_{peak}$) and peak signal intensity, late gadolinium enhancement (LGE), left ventricular ejection fraction (LVEF) were evaluated and compared by independent-sample t-test or ANOVA test. The spearman correlation test was used to calculate the relationships between perfusion parameters, LVEF , LGE, and cumulative dose of daunorubicin.
**Results:** A total of 720 myocardial segments were analyzed. Compared with non-treatment group, $T_{peak}$ increased in basal segments (24.86±7.29 s vs. 31.27±8.18 s, p < 0.05) and mid-ventricular segments (27.70±6.62 s vs. 33.39±6.61 s, p < 0.05) in treatment group. $Slope_{max}$, peak signal intensity and LVEF were not shown significant difference in the three groups. LGE was identified in 12 patients with daunorubicin therapy. Cumulative dose of daunorubicin (mean 129.25±60.31 mg) was moderately associated with the degree of LGE (r = 0.36, p < 0.05).
**Conclusion:** The segmental myocardial microvascular dysfunction could be early identified by CMR resting first-pass myocardial perfusion, which may contribute for the prevention or timely treatment of daunorubicin-induced cardiotoxicity in paediatric patients with acute leukaemia.
*Author Disclosures:*
**Z. Yang:** Research/Grant Support; the National Natural Science Foundation of China (81771887, 81471722). **Y. Guo:** Research/Grant Support; the National Natural Science Foundation of China (81471721, 81771897).

**14:00 - 15:30** **Room D**

### Artificial Intelligence and Machine Learning

### SS 1905b

### Machine learning: visceral organs and GI tract

*Moderators:*
D. Pinto dos Santos; Cologne/DE
M. Prokop; Nijmegen/NL

**B-1827** 14:00
**Semi-automatic technique for Crohn's disease diagnosis, using a supervised machine learning algorithm**
G. Lo Re, M.C. Terranova, D. Picone, S. Franchini, S. Vitabile, M. Midiri; *Palermo/IT (giuseppe.lore12@gmail.com)*

**Purpose:** Radiological diagnosis of Crohn's disease (CD) may be challenging, mimicking different non-CD ileitis, and requires gastro-intestinal expertise. Our purpose is to develop a new semi-automatic technique for CD diagnosis, using Kernel Support Vector Machine (KSVM).
**Methods and Materials:** Three radiologists without gastrointestinal expertise extracted a dataset, composed of 900 MR enterography studies of 900 different patients (400 affected by CD, 500 not affected), selected for KSVM training and validation. Each patient was "labelled" by 22 determined qualitative features, which are associated with CD or may help in CD diagnosis, according to worldwide literature. At the end, each study was labelled according to histological specimen results (HR). 900 MR enterography studies have been divided as follows: 4/5 (720 patients) with HR labelling were used for KSVM training, and 1/5 (180 patients) without HR labelling were used for KSVM validation. Stratified K(5)-fold cross-validation strategy was used to enhance KSVM classifier reliability to compute and reduce the average error and/or any patient selection bias across all K experiments.

**Results:** The KSVM results were compared with the histological specimen results: sensitivity: 92.80%; specificity: 97.00%.
**Conclusion:** The great advantage is that this method, compared to the traditional reporting, does not require gastro-intestinal expertise and allows an easier differential diagnosis from non-CD ileitis.

**B-1828** 14:08
**CT texture analysis to predict response to target therapy of hepatic metastases from colorectal cancer**
L. Vassallo, V. Giannini, A. Defeudis, S. Mazzetti, G. Cappello, D. Regge; *Candiolo/IT (lorenzovassallo1987@gmail.com)*

**Purpose:** To identify specific imaging biomarkers of hepatic metastases using CT texture analysis (CTTA), able to predict patient's response to therapy and overall survival.
**Methods and Materials:** We exploited the imaging dataset of HERACLES trial (NCT03225937): 23 patients with amplified HER2 mCRC were included in the study. All had received anti-HER2 treatment, and underwent CT examination every 8 weeks, until disease progression. CT scans were semi-automatically segmented to extract all liver metastases. CTTA was performed on each segmented area, computing for each lesion 34 quantitative parameters. Mono-parametric and multi-parametric analysis were assessed to identify features correlated to therapy response. We also performed a correlative survival (OS) analysis, considering subjects with good survival those with OS > 9 months.
**Results:** In 23 patients we found 124 metastases, 55 classified as responding and 69 as non-responding. Nine parameters reached statistical significance in mono-parametric analysis (best AUC=0.67, p=0.001), while in multivariate regression ten parameters were used in the model, achieving AUC equal to 0.82, sensitivity of 82% and specificity of 72%. For OS analysis, 12 patients were "good" and 11 "poor" survivors. In mono-parametric analysis, "cluster prominence" and "sum entropy" predicted OS with AUC equal to 0.78 and 0.83, respectively. The regression model with two variables ("cluster prominence" and "dissimilarity") reached sensitivity of 83% and specificity of 82%.
**Conclusion:** Our study demonstrated CTTA as a potential biomarker to predict response of hepatic metastases to target therapy, possibly saving patients predicted as non-responder from toxicity. Moreover, CTTA could give indications on patients' OS.

**B-1830** 14:16
**Automatic visceral fat characterisation on CT scans through deep learning and CNN for the assessment of metabolic syndrome**
A. Jimenez-Pastor, A. Alberich-Bayarri, F. Garcia-Castro, L. Marti-Bonmati; *Valencia/ES (anajimenez@quibim.com)*

**Purpose:** Metabolic syndrome is a risk factor of developing cardiovascular disease and diabetes. Visceral fat has been recognized as a predictor of metabolic risk factors. The main goal is the fully-automatic segmentation of visceral and subcutaneous fat by the concatenation of different convolutional neural networks (CNN) on Computed Tomography (CT) images.
**Methods and Materials:** A set of 20 CT series from obese patients with metabolic syndrome were collected. In CNN development, 80/20% images was used for training/test, respectively. The axial reconstructed images were manually classified into abdominal or non-abdominal. All these images were used to train a first 15-layer-CNN to classify abdominal CT sections of the full scan. For the fat segmentation, all the abdominal CT images were manually segmented into visceral and subcutaneous fat. A total of 1270 2D-images were used to train two new different encoder-decoder CNN for the automated fat segmentation and volumes quantification.
**Results:** The accuracy obtained on the first CNN for abdominal/non-abdominal images classification was 99.35%. To evaluate the performance of both encoder-decoder-CNN, the DICE coefficient over the validation dataset was calculated, obtaining 94.55% and 98.05% for visceral and subcutaneous fat respectively. Mean relative error between the handcrafted and CNN visceral/subcutaneous fat volumes was 3.01/0.94% and the mean relative error on mesenteric-to-total-fat-ratio was 2.17%.
**Conclusion:** Visceral and subcutaneous fat can be automatically characterized through the segmentation of both fat components by CNNs. This allows the precise measurements of the relative fat fraction and the prediction of metabolic risk factors development on patients with metabolic syndrome.

**B-1831** 14:24
**Application of a clinical decision support system to the imaging pathway of patients with hepatocarcinoma and cholangiocarcinoma**
M. Gabelloni, M. Di Nasso, C.A. D'Amore, R. Morganti, L. Faggioni, G. Masi, E. Neri; *Pisa/IT (mgabelloni@sirm.org)*

**Purpose:** To determine the effectiveness of the ESR iGuide clinical decision support system in reducing the rate of inappropriate diagnostic examinations and the resulting costs for the healthcare system.
**Methods and Materials:** Forty patients (17 affected by hepatocarcinoma and 23 by cholangiocarcinoma, respectively) were retrospectively evaluated. Using ESR iGuide, all patient data (including signs and symptoms and laboratory values) that could have justified imaging were reviewed to calculate the appropriateness score of every imaging examination performed. The agreement between the examinations suggested by ESR iGuide and those actually performed was assessed using Cohen's k coefficient.
**Results:** A total of 98 imaging examinations of the abdomen were performed in 40 patients from year 2013 to 2017. Out of all imaging examinations, 79.6% (78/98) were considered appropriate by ESR iGuide and 20.4% (20/98) were designated as having marginal utility or even not suggested. Out of these latter, 85% were biopsies (17/20), 5% (1/20) abdominal CT, 5% (1/20) barium contrast enemas, and 5% (1/20) colonoscopies, respectively. The agreement between the examinations suggested by ESR iGuide and those actually performed was poor in all cases (Cohen's k less than 0.2). According to the established prices in the BLINDED regional healthcare system, the total cost of the 98 imaging examinations was €14,016, of which 21.6% (€3,033) were related to imaging tests classified as inappropriate by ESR iGuide.
**Conclusion:** The use of ESR iGuide could cut the costs of the healthcare system by reducing the number of inappropriate abdominal diagnostic examinations and potentially improving patients' outcome.

**B-1832** 14:32
**Automated CT abdominal imaging protocol selection using natural language processing and machine learning**
P. Rogalla[1], B.E. Hoppel[2], K. Noro[3], Y. Yamazaki[3], K. Utsunomiya[3], Y. Shimomura[3], S. Sugiyama[3]; [1]*Toronto, ON/CA*, [2]*Delafield, WI/US*, [3]*Nasushiobara/JP (patrik.rogalla@uhn.ca)*

**Purpose:** To develop and evaluate an automatic protocolling engine based on machine learning for predicting the correct imaging protocol in abdominal CT.
**Methods and Materials:** Determining the optimal imaging protocol for patients undergoing abdominal CT has become a challenging and time consuming task in most Radiology Departments Under ethical approval, 110,000 historical imaging requests were extracted from a RIS database containing the executed imaging protocols and associated patient information valuables (eg, history, indication, primary diagnosis, age, free text comment field). 141 features were identified from the valuables, including 100 features extracted from the free text comments using Latent Dirichlet Allocation; (natural language processing). This approach summarizes words as "topics" by finding the repeating pattern of co-occurring terms in a text. Using 141 features as input, the classification model was trained by L1 Logistic Regression predicts the 12 most frequently applied protocols. A 5-fold cross validation was executed using all the imaging requests with an 80/20 split of training to testing.
**Results:** The natural language processing method had an average specificity and sensitivity of 96.1 % and 62.5% compared to L1 Logistic Regression, not using natural language processing with specificity and sensitivity of 96.0% and 39.1%. Evaluation when using highly recommended results increase sensitivity to 81.4% and accuracy of 88.4% when choosing protocol.
**Conclusion:** The use of a natural language processing models with machine learning for protocolling in abdominal CT improves the average sensitivity for picking the correct protocol making it feasible to predict the radiologist's choice correctly in most cases.
*Author Disclosures:*
**P. Rogalla:** Research/Grant Support; Canon Medical. **B.E. Hoppel:** Employee; Canon Medical. **K. Noro:** Employee; Canon Medical. **Y. Yamazaki:** Employee; Canon Medical. **K. Utsunomiya:** Employee; Canon Medical. **Y. Shimomura:** Employee; Canon Medical. **S. Sugiyama:** Employee; Canon Medical.

**B-1833** 14:40
**AI automatic identification of complete- and non- responders using texture analysis of rectal cancer 3T MR images performed before, during and after neoadjuvant CRT**
R. Ferrari, C. Mancini Terracciano, C. Voena, M. Rengo, M. Zerunian, R. Paramatti, R. Faccini, A. Laghi; *Rome/IT (marta.zerunian@gmail.com)*

**Purpose:** To evaluate an Artificial Intelligence (AI) model for classification of Complete (CR) and Non Responders (NR) patients with rectal cancer treated with neoadjuvant Chemo-RadioTherapy (CRT). AI input is based on textural analysis of high resolution 3T MR images performed before, during and after CRT.
**Methods and Materials:** 55 consecutive patients with rectal cancer were prospectively enrolled in this study. Patients underwent 3T T2-weighted (T2w) Magnetic Resonance Imaging (MRI) before, during and after CRT; ROIs around the tumor were manually drawn. All patients underwent complete surgical resection and the specimen was used to evaluate patients' response. Two AI models were built with the most statistically significant features training a Random Forest (RF) classifier on a 28 patients (training cohort). The model performances were estimated on 27 patients (validation cohort) using a ROC curve analysis and a decision curve analysis.

**Results:** Textural analysis points to a lower intra-tumor heterogeneity at the pre- and during-treatment stage for CR patients with a characteristic time evolution of some of the textural features. The obtained AI model shows good discriminatory ability with a ROC AUC of 0.94 (95% CI: 0.89,0.99) in the validation cohort. The discriminatory power of the AI model built for NR discrimination has a ROC AUC of 0.85 (95% CI: 0.80,0.90). Decision curve analysis confirms clinical usefulness of the models.
**Conclusion:** AI models based of textural parameters of MR images of patients with rectal cancer taken before, during and after CRT, show good performances for stratification of response to therapy.
*Author Disclosures:*
**A. Laghi:** Research/Grant Support; Italian Association of cancer research.

**B-1834** 14:48
**Radiomics nomogram for the preoperative prediction of lymph node metastasis in pancreatic cancer**
Y. Bian; *Shanghai/CN (bianyun2012@foxmail.com)*

**Purpose:** To develop and validate a radiomics nomogram for the preoperative prediction of lymph node (LN) metastasis in pancreatic ductal adenocarcinoma (PDAC).
**Methods and Materials:** In this retrospective study, 213 patients with surgically resected, pathologically confirmed PDAC underwent multislice computed tomography (MSCT) within one month before surgery. All patients were divided into a training set (n=185) and a validation set (n=46); The least absolute shrinkage and selection operator (LASSO) logistic regression algorithm was used to select the radiomics features. Multivariable logistic regression analysis was used to develop the predictive model that incorporated the radiomics signature, computed tomography (CT)-reported organs or vascular status, and CT-reported LN status, and a radiomics nomogram was built. The performance of the nomogram was assessed in the training set and validated in the internal independent validation set. Finally, decision curve analysis (DCA) was performed to estimate the clinical usefulness of the nomogram.
**Results:** The multivariable logistic regression model included the radiomics signature, CT-reported organ or vascular invasion status, and CT-reported LN status. The individualized prediction nomogram showed good discrimination in the training set [area under the curve (AUC), 0.9206; 95% confidence interval (CI), 0.88816-0.9597] and in the validation set (AUC, 0.9125; 95% CI, 0.8311-0.9938) and good calibration. DCA demonstrated that the radiomics nomogram was clinically useful.
**Conclusion:** The presented radiomics nomogram that incorporates the radiomics signature, CT-reported organ or vascular invasion status, and CT-reported LN status was a noninvasive preoperative prediction tool with favourable predictive accuracy for LN metastasis in patients with PDAC.

**B-1835** 14:56
**A sparse representation radiomics method based on multi-modality ultrasound images for hepatocellular carcinoma diagnosis and PD1 prediction**
W. Wang, Y. Dong, Z. Yao, Q. Zhang, D. Yang, J. Yu; *Shanghai/CN (puguang61@126.com)*

**Purpose:** Programmed cell death protein 1 (PD1) is of great importance for surveillance and treatment of hepatocellular carcinoma (HCC). An updated radiomics method based on multi-modality ultrasound image is proposed to discriminate HCC and predict PD1 index.
**Methods and Materials:** A total of 65 patients with operation and histopathologically proved HCC were included in the prospective study. Every patient underwent multi-modality ultrasound examination before operation, including B mode ultrasound (BMUS), shear wave elastography (SWE), and shear wave viscosity (SWV) imaging. The radiomics analysis system was built on sparse representation theory (SRT) and support vector machine (SVM) for asymmetric data. With the sparse regulation of the SRT, we try to make accurate diagnosis of HCC and prediction of PD1 before operation. The accuracy, sensitivity, specificity and area under the receiver operating characteristic curve (AUC) were calculated to evaluate performance of radiomics models.
**Results:** Based on radiomics methods, a total of 2560 features were extracted from multi-modality ultrasound images for each HCC lesion. Two radiomics models were built and leave-one-out cross validation (LOOCV) was used to evaluate models. The performance of each model improved as the viscosity modality was included. In LOOCV, the AUC was 0.97 for HCC discrimination (95% CI 0.93 to 0.99) and 0.97 for PD1 prediction (95% CI 0.89 to 0.98).
**Conclusion:** Radiomics analysis based on multi-modality ultrasound images could help to make accurate diagnosis of HCC and prediction of PD1 level before operation.

**B-1836** 15:04
**Dynamic contrast-enhanced CT diagnosis of primary liver cancers using transfer learning of pre-trained convolutional neural network: comparison with radiologists**
K. Oyama, A. Yamada, E. Yoshizawa, F. Ichinohe, D. Komatsu, Y. Fujinaga; *Matsumoto/JP (oyama1988@shinshu-u.ac.jp)*

**Purpose:** To evaluate diagnostic performance of transfer learning (TL) using pre-trained convolutional neural network and 3 phasic dynamic contrast-enhanced CT (DCE-CT) in primary liver cancers.
**Methods and Materials:** We evaluated 215 consecutive patients with histologically proven primary liver cancers including 6 early, 58 well-differentiated, 77 moderately-differentiated, 29 poorly-differentiated hepatocellular carcinomas (HCCs), and 13 non-HCC malignant lesions containing cholangiocellular components. TL using pre-trained convolutional neural network (GoogLeNet) and preoperative 3-phasic DCE-CT images at maximum cross sectional area of lesion was performed. Diagnostic performance (DP = [number of correctly classified cases]/[total number of cases]) was compared with general radiologists (GR) and experienced abdominal radiologists (AR). Correlation between sub-categorical number of training samples and DP was statistically evaluated.
**Results:** Overall DP for classification of histological subtype and differentiation in primary malignant liver tumors on DCE-CT by TL, GR, and AR was 45.9%, 39.1%, and 47.9%, respectively. Sub-categorical DP for well-/moderately-differentiated HCC by TL (50.0%/59.1%) was better than that by AR (48.3%/51.4%). Sub-categorical DP for early HCC/non-HCC malignant lesions by TL (20.0%/0%) was worse than that by GR (33.3%/30.8%). Sub-categorical DP for poorly-differentiated HCC by TL was 16.7%. There is a significant correlation between Sub-categorical DP by TL and sub-categorical number of training samples (r=0.89, p<0.05).
**Conclusion:** TL by pre-trained convolutional neural network may aid diagnosis of primary liver cancers on DCE-CT for GR in future because its diagnostic performance was close to that by AR when sub-categorical number of training samples was large.

**B-1837** 15:12
**Chronic liver disease assessment with a machine learning method using parameters derived from ultrasound B-mode and shear wave elastography examination**
P. Drazinos[1], I. Gatos[2], S. Yarmenitis[3], I. Theotokas[2], A. Soultatos[2], E. Panteleakou[2], P. Zoumpoulis[1]; [1]*Kifissia/GR*, [2]*Athens/GR*, [3]*Marousi/GR (p.zoumpoulis@echomed.gr)*

**Purpose:** Chronic Liver Disease (CLD) is one of the major causes of death and the major cause of Hepatocellular Carcinoma development. Therefore, accurate diagnosis regarding CLD progress is very important. Our aim is to build and train a machine learning model that will estimate the impact of parameters derived from an Ultrasound (US) B-Mode and Shear Wave Elastography (SWE) examination in significant fibrosis (F≥F2) diagnosis using Liver Biopsy (LB) as ‘Gold Standard’.
**Methods and Materials:** Our dataset consisted of 217 individuals (132 F0-F1 and 85 F2-F4). An US examination was performed on all individuals and an LB examination for individuals with fibrosis stage F1-F4. We randomly divided our dataset into training and test samples (70%-30%). For the training sample a 10-fold cross validation (CV) was performed. The SWE measurements of the Liver’s Right Lobe and Left Lobe as well as the existence of Nodularity and the CLD subtype/etiology of the patient were used as inputs for the training and testing of a Neural Network (NN) classifier.
**Results:** The NN classifier had a 10-fold CV accuracy of 96.26% and a 95% Confidence Interval (CI) of 96.18% - 96.35%. The mean accuracy of the classifier on the test samples was 95.56% with a 95% CI of 95.46% - 95.66%. The mean Area Under Curve (AUC) for the test samples was 0.983 with a 95% CI of 0.976 - 0.991.
**Conclusion:** The proposed method achieved high accuracy results and could be used to assist radiologists for CLD assessment in everyday clinical practice.
*Author Disclosures:*
**P. Drazinos, I. Gatos, S. Yarmenitis, I. Theotokas, A. Soultatos, E. Panteleakou, P. Zoumpoulis:** Equipment Support Recipient; Mindray is cooperating with Diagnostic Echotomography SA in relation with a Clinical Study. A Resona 7 Ultrasound system has been provided by Mindray in order to carry out this study.

**14:00 - 15:30** **Room G**

### Physics in Medical Imaging

### SS 1913

### Breast imaging physics

*Moderators:*
G. Gennaro; Padua/IT
E. Keavey; Galway/IE

**K-43** 14:00
**Keynote lecture**
G. Gennaro; *Padua/IT*

**B-1838** 14:09
**Proposal of a breast phantom for dosimetry quality control procedures in digital mammography and digital breast tomosynthesis**
C. Fedon[1], R. Van Engen[1], J. Binst[2], L. Cockmartin[2], D. Dance[3], A. Jacobs[2], R. Longo[4], K. Tri Wigati[2], I. Sechopoulos[1]; [1]*Nijmegen/NL*, [2]*Leuven/BE*, [3]*Guildford/UK*, [4]*Trieste/IT (christian.fedon@radboudumc.nl)*

**Purpose:** To design new quality control (QC) phantoms that properly stimulate the automatic exposure control (AEC) of current digital mammography (DM) and breast tomosynthesis (DBT) systems to prospectively determine the exposure parameters selected for patient breasts of varying characteristics.
**Methods and Materials:** Breast fibroglandular information (e.g. peak and mean density, and mean dense tissue area) was obtained retrospectively from a cohort of about 4000 screening DM images using commercial software (Volpara™). A 2 cm-thick phantom slab was designed and manufactured based on the acquired Volpara™ data and the state-of-art of AEC for all current systems. This *base* slab is composed of polyethylene (PE) (simulating adipose tissue) with a half-ellipsoid inset of polyoxymethylene (simulating fibroglandular tissue). Phantom thicknesses up to 8 cm were obtained by adding 2 cm-thick PE slabs to the *base* slab. The AEC response was tested on systems from several vendors, which utilize different AEC philosophies and techniques. The average glandular dose (AGD) from AEC based phantom acquisitions was compared to AGDs from patients and from the standard homogeneous PMMA phantoms (according to the EU guideline).
**Results:** The mean AGD of the phantom images agrees with the mean patient AGD to within one standard deviation. Results from statistical student t-test show that the new phantom has the potential to better reproduce the exposure parameters used for patients than current homogeneous PMMA phantoms (without the need for spacers).
**Conclusion:** The phantom seems to provide a simple and reproducible QC method to predict the patient AGD using routine clinical settings.

**B-1839** 14:17
**Device-specific reference values for modulation transfer function (MTF) quality control measurements of digital mammography detectors**
K. Wigati[1], H. Bosmans[1], D.S. Soejoko[2], L. Cockmartin[1], A. Jacobs[1], J. Binst[1], K. Lemmens[1], N. Marshall[1]; [1]*Leuven/BE*, [2]*Jakarta/ID (kristinatri.wigati@kuleuven.be)*

**Purpose:** Presampling modulation transfer function (MTF) measurements quantify detector sharpness and are recommended in the EUREF digital mammography quality control (QC) protocol. The full potential from this measurement will only be realized when reproducibility and limiting values are known for the systems in service. This retrospective study produces average MTF data for vendors/devices and assesses MTF measurement reproducibility.
**Methods and Materials:** Our physics QC service routinely measures MTF in two perpendicular directions using an edge test object imaged with 2 mmAl at the tube, grid removed. MTF is calculated from linearized DICOM ‘For Processing’ images and averaged for the two directions, where appropriate. The 50% point of the MTF ($MTF_{0.5}$) and MTF at 5mm$^{-1}$ ($MTF_{5mm-1}$) were collated from seven years of QC reports of 37 digital mammography systems (6 vendors/9 models). For these parameters, difference from test to test, and difference from baseline value were calculated.
**Results:** MTF measurements were found to be reproducible. For example, 5 fault-free Siemens Inspiration systems (14 tests) had a mean $MTF_{0.5}$ of 5.32±0.28mm$^{-1}$ (coefficient of variation (cov) of 5.3%) while $MTF_{5mm-1}$ was 0.53±0.02 (cov of 4.7%). Average deviation of $MTF_{0.5}$ between successive tests (with 95% confidence intervals) was 3.9% (2.6%-5.2%), while for the difference from baseline value, this result was 4.1% (2.6%-5.7%). For comparison, the permitted baseline change is ±10% in the protocol. For all other vendors, similar reproducibility and confidence intervals were found.
**Conclusion:** Average deviation of MTF from baseline is typically <5%, making the MTF a robust means of assessing detector sharpness.
*Author Disclosures:*
**H. Bosmans:** Founder; Qaelum.

**B-1840** 14:25
**Comparative phantom tests of digital mammograms, breast tomosynthesis and synthetic mammograms**
L. Vancoillie, L. Cockmartin, N. Marshall, H. Bosmans; *Leuven/BE (liesbeth.vancoillie@uzleuven.be)*

**Purpose:** To compare digital mammography (DM), digital breast tomosynthesis (DBT) and synthetic mammograms (SM) using the 3D structured L1 phantom with calcification and mass lesion-like inserts via a human observer detectability study.
**Methods and Materials:** Twelve combined DM-DBT phantom images were acquired at automatic exposure controlled dose levels on the GE HC Senographe Pristina, Hologic Selenia Dimensions and Siemens Inspiration systems at respective MGD/image values for DM/DBT: 1.20mGy/1.20mGy; 1.77mGy/2.24mGy; 1.33mGy/2.61mGy. Detectability was assessed via human reading using a 4-alternative forced choice (4-AFC) method. Threshold diameter ($d_T$) of the different inserts was defined as the diameter for 62.5% detected fraction.
**Results:** Microcalcification detectability was better for DM and DBT compared to SM, with p< 0.001 for all systems. Threshold diameter increased from DM to SM by 23 µm, 17 µm and 33 µm for the GE, Hologic and Siemens, respectively. Detection of both spiculated and non-spiculated masses was significantly better in DBT mode compared to SM, while SM in turn outperformed DM. For the 3 vendors, $d_T$ for mass-like lesions increased going from DBT to SM by 2.06 mm, 1.42 mm and 1.11 mm for the non-spiculated masses and by 1.50 mm, 0.05 mm and 1.46 mm for the spiculated masses for GE, Hologic and Siemens respectively.
**Conclusion:** For microcalcification detection, better scores were obtained for DM than for SM, while DBT was significantly better than SM for mass-like lesions, suggesting that SM, in its current stage of development, cannot be recommended as a stand-alone modality for any of the devices.
*Author Disclosures:*
**H. Bosmans:** Founder; Co-founder of Qaelum NV. Research/Grant Support; The medical physics team has a research agreement with Siemens-Healthineers and GE HealthCare..

**B-1841** 14:33
**Image improvement factor (Q) measured for three different antiscatter grids used in mammography imaging**
N. Marshall, H. Bosmans; *Leuven/BE (nicholas.marshall@uzleuven.be)*

**Purpose:** The grid image improvement factor (Q) describes the relative improvement in signal to noise ratio (SNR) for grid use compared to a non-grid technique: a well-designed grid should improve SNR, allowing dose reduction at constant SNR. The Q factor was assessed for grids in three different digital mammography systems.
**Methods and Materials:** Grids from GEHC Pristina (2D and 3D mode), Siemens Inspiration (2D) and Hologic Dimensions (2D) were assessed. The grid primary and total transmissions, $T_p$ and $T_t$, were measured for poly(methyl methacrylate) (PMMA) thicknesses from 20mm to 70mm, using automatic exposure control factors. For $T_t$, the PMMA was positioned on top of the breast support and imaged with a large field of view. $T_p$ was measured with PMMA at the x-ray tube, with a 10mm collimated beam. Q was computed as $Q = T_p^2/T_t$.
**Results:** For GEHC Pristina, Q increased from 0.774 at 20mm to 1.367 at 70mm PMMA. For the same thickness range, Q ranged from 0.835 to 1.259 for Siemens Inspiration, while for Hologic Dimensions, Q varied from 0.866 to 1.333. Reproducibility error was 0.6%. At 45mm PMMA, equivalent 53mm breast thickness, Q was 1.140, 1.076 and 1.136 for GEHC Pristina, Siemens Inspiration and Hologic Dimensions, respectively.
**Conclusion:** The Q factors show a dose-equivalent SNR gain by 4-9% for PMMA thicknesses above 40mm, suggesting grid use is beneficial above this thickness. Benefit can reach 26-37% for the thickest breasts (70mm PMMA, 90 mm breast equivalent). Assessment is straightforward and should be part of commissioning tests.
*Author Disclosures:*
**H. Bosmans:** Founder; Co-founder Qaelum NV, Belgium. Research/Grant Support; The medical physics team has a research agreement with Siemens-Healthineers and GE HealthCare.

**B-1842** 14:41
**Analysis of noise decomposition results in mammography quality control: is quantum noise the dominant noise source?**
K. Wigati[1], H. Bosmans[1], D.S. Soejoko[2], K. Lemmens[1], J. Binst[1], A. Jacobs[1], L. Cockmartin[1], N. Marshall[1]; [1]*Leuven/BE*, [2]*Jakarta/ID (kristinatri.wigati@kuleuven.be)*

**Purpose:** The EUREF QC protocol for digital mammography describes decomposition of noise, measured in homogeneous images into electronic, quantum and structured noise terms: quantum noise must form the highest percentage of the total. This study examined reproducibility and produced typical values for different models.

**Methods and Materials:** Variance was measured from homogeneous response function images acquired as a function of detector air kerma (DAK). A weighted 2nd order polynomial curve fit was made to variance versus DAK, giving the three noise components. Results for 309 QC tests on Fuji Innovality, Fuji Amulet(S), GEHC Essential, IMS Giotto, Hologic Selenia/Dimensions, Philips/Sectra and Siemens Inspiration systems were analyzed to give percentage of noise at clinical DAK. Typical values were calculated and, averaged across systems of the same model.
**Results:** For the Innovality (detailed data available for all), the quantum noise percentage (with 95% confidence intervals) was 94.6% (93.2%-96.0%), electronic noise was 4.4% (3.1%-5.6%) and structured noise was 1.0% (0.8%-1.2%), at the clinical DAK. Quantum noise percentage was 61.3% for the Amulet(S), 82.8% for the Essential, 84.6% for the Selenia/Dimensions, 72.4% for the Giotto, 97.3% for the Philips/Sectra and 78.1% for Inspiration. Electronic noise formed the next highest percentage (range 1.0% to 37.3%), while structured noise was low (range 0.1% to 4.1%). Coefficient of variation for the quantum percentage ranged from 1.7% to 9.7%.
**Conclusion:** Three component noise decomposition is reproducible and quickly verifies whether quantum noise is the dominant noise source. This is a useful QC test for tracking system noise performance.
*Author Disclosures:*
**H. Bosmans:** Founder; Qaelum.

**B-1843** 14:49
**Personalised estimates of mean glandular dose and image quality investigations in cone-beam computed tomography dedicated to the breast**
A. Sarno[1], G. Mettivier[1], R.M. Tucciariello[2], J.M. Boone[3], H. Bosmans[4], K. Bliznakova[5], P. Russo[1]; [1]*Naples/IT*, [2]*Pisa/IT*, [3]*Sacramento, CA/US*, [4]*Leuven/BE*, [5]*Varna/BG (sarno@na.infn.it)*

**Purpose:** To estimate the mean glandular dose (MGD) and imaging technology assessment in dedicated breast computed tomography (BCT) exams, by means of anthropomorphic digital breast phantoms produced from 3D clinical breast images.
**Methods and Materials:** We developed 200 anthropomorphic digital breast phantoms by classifying glandular, adipose and skin tissues in 3D breast images acquired at the University of California, Davis (USA), with their dedicated BCT scanner. The MGD patient-specific estimates were compared to those resulting from the adoption of the glandular/adipose homogeneous mixture and cylindrical breast geometry model. We developed a Monte Carlo code validated vs data of the AAPM TG-195. We replicated the specifications of the scanner developed at the UC Davis and input the heterogeneous breast models in the simulations. We also provided a code module for producing monoenergetic and polyenergetic breast projections of 3D digital phantoms, for investigating BCT technology.
**Results:** The average difference in MGD estimates by means of heterogeneous patient-specific and that for homogeneous breast models resulted ~7%. However, the homogeneous assumption led to a maximum mean glandular dose overestimation of 28% and a maximum dose underestimation of 16%, over the considered breast cohort.
**Conclusion:** We showed that the homogeneous breast assumption in mean glandular dose estimates in BCT leads to differences ranging between -16% and +28% when compared to patient-like heterogeneous models. We provided glandular dose distribution maps that will be a valid support for risk models. Moreover, we setup and presented a Monte Carlo software for future BCT scanner optimisations.
*Author Disclosures:*
**A. Sarno:** Grant Recipient; This work was supported by the MaXIMA project, which has received funding from the European Union's Horizon 2020 research and innovation programme under grant agreement No 692097.

**B-1844** 14:57
**The effect of overestimating glandularity on mean mammographic glandular dose**
M.E. Suleiman, P.C. Brennan, M.F. McEntee; *Sydney/AU (moe.suleiman@sydney.edu.au)*

**Purpose:** This work aimed to explore mammographic breast density (MBD) for women who attend BreastScreen NSW. Furthermore, this study explored the effect of MBD on mean glandular dose (MGD), proposing the use of MBD in the calculation of actual glandular dose (AGD).
**Methods and Materials:** Anonymised sample of mammograms (31,097) was retrieved from a central database. Patient data and examination technical parameters were exported from DICOM headers using third-party software. LIBRA software was used to estimate MBD for each mammogram. MGD was estimated using Dance et al. method, whilst AGD was calculated by replacing Dance et al.'s standard glandularities with LIBRA estimated MBDs. Linear regression analysis was used to study the correlation between MGD and AGD. Bland-Altman analysis was performed to assess their mean difference.
**Results:** MGD, AGD, and MBD medians were 1.53mGy, 1.62mGy and 8%, respectively. Median MBDs were lower than Dance's standard glandularities for all breast thickness (almost 50% for 20mm breast thickness). There was a strong positive correlation ($R^2$ = 0.987, p<0.0001) between MGD and AGD although Bland-Altman analysis revealed small statistically significant bias of 0.087mGy between MGD and AGD (p < 0.001).
**Conclusion:** Median MBD was comparable to that previously reported for Australian women (8.1%). AGD is highly correlated to MGD, although the Dance method particularly underestimates dose at smaller compressed breast thicknesses.

**B-1845** 15:05
**Accuracy testing of two partial breast imaging simulation platforms for applications in virtual clinical trials**
L. Vancoillie[1], M. Herbst[2], L. Cockmartin[1], N. Marshall[1], F. Schebesch[3], J. Vignero[1], H. Bosmans[1]; [1]*Leuven/BE*, [2]*Forchheim/DE*, [3]*Erlangen/DE (liesbeth.vancoillie@uzleuven.be)*

**Purpose:** Simulation platforms lie at the heart of virtual clinical trials and must be verified for accuracy and realism. Two platforms ('A' and 'B') developed by 2 different groups were evaluated for these aspects. Both platforms employ partial simulation, whereby lesions or objects are simulated into real images.
**Methods and Materials:** Both platforms undertake: (1) object creation (2) generation of object template including realistic resolution and scatter (3) template insertion followed by reconstruction, but there are differences in implementation. Verification measurements were made using digital mammography and tomosynthesis scans acquired on a Siemens Inspiration system. A small high contrast 0.5mm aluminium sphere and a large area contrast 10x10x0.2mm³ aluminium foil were simulated using both platforms and compared to measurements. Parameters assessed included contrast, signal different to noise ratio (SDNR), a profile through the sphere and foil in the *xy*-direction and the artefact spread function (ASF). A visual comparison was also made.
**Results:** Contrast and SDNR for the sphere showed good agreement (<5% error), while consistent deviations of ± 20 % were seen using platform B for the foil, which was also noticeable visually. There were only small differences between all profile plots and the ASF of the Al sphere, indicating good geometric modelling for both platforms.
**Conclusion:** Close correspondence was demonstrated between the simulated and the real objects, both visually and quantitatively for platform A. The deviation of the foil in platform B requires further investigation. Prior to their use in virtual clinical trials, simulation platform accuracy must be verified.
*Author Disclosures:*
**M. Herbst:** Employee; Siemens-Healthineers. **H. Bosmans:** Founder; Co-founder Qaelum NV. Research/Grant Support; The medical physics team has a research agreement with Siemens-Healthineers and GE HealthCare.

**B-1846** 15:13
**Towards clinical phase-contrast breast CT at Elettra: major SNR improvement at larger propagation distances**
L. Brombal[1], P. Delogu[2], D. Dreossi[1], B. Golosio[3], P. Oliva[4], G. Mettivier[5], L. Rigon[1], A. Taibi[6], R. Longo[1]; [1]*Trieste/IT*, [2]*Siena/IT*, [3]*Cagliari/IT*, [4]*Sassari/IT*, [5]*Naples/IT*, [6]*Ferrara/IT (luca.brombal@phd.units.it)*

**Purpose:** In-vivo phase-contrast breast CT is feasible using synchrotron radiation. Novel results towards the clinical exam based on a surgical specimen are reported, showing a major SNR improvement associated to larger propagation distances.
**Methods and Materials:** Effects of propagation distance on image quality metrics, i.e., SNR, contrast and spatial resolution, are quantitatively evaluated by scanning a large breast surgical specimen at 3 propagation distances (1.5, 2.7, 6.4 m). The sample is imaged at 30 keV with a CdTe single-photon-counting detector (60 µm pixel pitch) and the flux is adjusted to provide similar doses at all distances. Images are acquired in free-space-propagation and the Paganin's phase-retrieval algorithm is applied. Experimental results are described within a rigorous theoretical framework accounting for propagation, phase-retrieval and tomographic reconstruction processes.
**Results:** A major (4-fold) SNR increase is observed going from the shortest to the longest propagation distance while no significant differences in contrast or spatial resolution are reported. As a result, a major visibility improvement of fibroglandular or tumoural details (e.g., spicules) is demonstrated. For all the distances experimental data are in a good agreement with theoretical predictions.
**Conclusion:** Moving towards larger propagation distances offers significant advantages since it greatly improves soft tissue details' visibility (i.e., SNR) at a constant radiation dose, without altering the image appearance in terms of contrast and spatial resolution. Conversely, the same image quality can be attained by reducing the radiation dose. The proposed theoretical background is a valuable tool for propagation distance optimization towards the clinical exam.

**B-1847** 15:21
**Dose-compatible grating-based phase-contrast mammography on mastectomy specimens using a compact synchrotron source**
L. Heck[1], E. Eggl[1], S. Grandl[2], M. Dierolf[1], D. Mayr[2], K.A. Hellerhoff[2], M.F. Reiser[2], F. Pfeiffer[1], J. Herzen[1]; [1]*Garching/DE*, [2]*Munich/DE* *(lisa.heck@tum.de)*

**Purpose:** In X-ray breast imaging, the low soft-tissue contrast of conventional attenuation-based images can be improved by phase-contrast imaging. Here, the diagnostic performance of grating-based phase-contrast mammography with a compact synchrotron source was evaluated.
**Methods and Materials:** In this study, grating-based multimodal mammography on mastectomy specimens at an inverse Compton compact synchrotron source was performed. This novel X-ray source offers a quasi-monochromatic X-ray beam which is tunable in its energy and is partially coherent. Grating-based phase-contrast and dark-field images as well as conventional absorption images were acquired. Here, we examined four freshly dissected breast specimens and one mammographic accreditation phantom for a quantitative analysis.
**Results:** We found enhanced diagnostic information in monochromatic grating-based phase-contrast images while the applied dose was lower or equal to the clinically applied dose. Microcalcifications could be identified equally well at significantly reduced dose in the monochromatic images. In a quantitative analysis performed with a mammographic accreditation phantom, the monochromatic absorption-contrast images showed better or equal contrast-to-noise ratio (CNR) compared to the clinical one even at 20% reduced dose. The monochromatic grating-based dark-field images outperformed the clinical image in case of the tumor masses even at 65% reduced dose.
**Conclusion:** The demonstrated improved content of diagnostic information and increased CNR at lower or equal dose indicates that compact synchrotron sources have great potential to bring benefits to clinical imaging for mammography. This is particularly advantageous with regard to the radiation exposure, since glandular breast tissue is one of the tissues most sensitive to the applied dose.

**14:00 - 15:30** **Room K**

### Chest

### SS 1904

### Traditional and advanced pulmonary vascular imaging

*Moderators:*
M. Sánchez; Barcelona/ES
A. Nair; London/UK

**B-1848** 14:00
**Perfusion-ventilation CT via three-material differentiation in dual-layer CT: a feasibility study**
A. Sauter[1], J. Hammel[1], M. Kimm[1], D. Pfeiffer[1], F.K. Kopp[1], K. Achterhold[2], F. Pfeiffer[2], E.J. Rummeny[1], P.B. Noel[1]; [1]*Munich/DE*, [2]*Garching/DE* *(andreas.sauter@tum.de)*

**Purpose:** Evaluation of a simultaneous xenon-ventilation and gadolinium-perfusion dual-energy computed tomography (CT) examination in a pig-model using a tissue discriminating two-material decomposition, resulting in a three-material differentiation.
**Methods and Materials:** For verification, xenon and gadolinium quantification were examined in a phantom model by comparing nominal to measured concentrations. A landrace pig was examined using a dual-layer CT (DL-CT). Three scans were performed: 1) native; 2) xenon ventilation only; 3) xenon ventilation and gadolinium perfusion. An in-house-developed algorithm was used to obtain xenon- and gadolinium-density maps. Firstly, lung tissue was segmented from other tissue. Consequently, a two-material decomposition was performed for lung tissue (xenon/soft tissue) and for remaining tissue (gadolinium/soft tissue). By combining both density maps, a xenon-gadolinium density map was generated.
**Results:** Measurements of xenon and gadolinium concentrations showed accurate results. For xenon, a percentage difference between nominal and measured concentration of below 1.2% was shown. For gadolinium, a difference of below 1.3% for all concentrations >1mg/ml resulted. Using the in-house-developed algorithm, it was possible to differentiate xenon and gadolinium in a ventilation/perfusion scan of a pig, resulting in xenon and gadolinium density maps. By summation of both density maps, a three-material differentiation (xenon/gadolinium/soft tissue) can be performed and thus, xenon ventilation and gadolinium perfusion can be visualized in one CT scan.
**Conclusion:** With DL-CT, xenon and gadolinium can be quantified accurately. Via three-material differentiation, a ventilation/perfusion examination can be performed during one CT-scan, resulting in decreased radiation dose and increased accuracy compared to other previously described methods.

**B-1849** 14:08
**Fine focal spot: a technique to improve image quality of computed tomography pulmonary angiography**
K.K. Lau[1], L. Abdelganne[1], K. Buchan[1], A. Kuganesan[1], T. Lau[2]; [1]*Melbourne/AU*, [2]*Southport/AU (kenkplau@gmail.com)*

**Purpose:** Faster temporal resolution (FTR) is routinely employed in CTPA to reduce motion artefacts. CT with fine focal spot (FFS) enables fine anatomical details. The latest x-ray tube design allows the use of FFS in body, but tube rotation time (rt) needs to be reduced. The aim of this retrospective study of CTPA was to compare FFS and FTR in CTPA image quality.
**Methods and Materials:** 50 consecutive adults with FTR CTPA (0.275 second-rt and standard focal spot) and another 50 consecutive adult patients with FFS CTPA (0.33 second-rt and FFS) were included. Two radiologists blindly, randomly and independently performed qualitative review on the clarities of PAs, lungs, aortas, hearts, ribs and livers on images from 2 groups using 5 point ranking method.
**Results:** 50 FFS CTPA (19 male, mean age of 62, 13% PE incidence) and 50 FTR CTPA (23 male, mean age of 65, 6.7% PE incidence) were studied. Mann-Whitney U test showed FFS was significantly better than FTR in the image qualities of pulmonary arteries, lungs, aorta, liver and ribs.
**Conclusion:** Despite slower CT rt, FFS still has stronger positive influence on CTPA image quality than FTR. FFS may become an important CTPA scanning tool that aid the PE detection.

**B-1850** 14:16
**Do subtraction CT-derived iodine maps have the same impact on pulmonary embolism detection as dual-energy CT-derived maps?**
D. Grob[1], E.J. Smit[1], J. Prince[2], J. Kist[2], L.J. Oostveen[1], M. Prokop[1], C. Schaefer-Prokop[3], I. Sechopoulos[1], M. Brink[1]; [1]*Nijmegen/NL*, [2]*Utrecht/NL*, [3]*Amersfoort/NL (Dagmar.Grob@radboudumc.nl)*

**Purpose:** To compare the impact on pulmonary embolism (PE) detection of adding iodine maps derived from either subtraction CT (SCT) or dual-energy CT (DECT) to CT angiography (CTA).
**Methods and Materials:** In this prospective study, 274 patients with suspected PE underwent CTA+DECT, and an additional pre-contrast scan to generate the SCT-based iodine map. The SCT image sets were acquired at similar but lower dose than the DECT sets. Truth was established by expert consensus using all clinical data, including six-month follow-up. An enriched 1:1 dataset of 75 randomly selected patients with and without PE were reviewed by three radiologists and six radiology residents, who scored separately the CTA alone, the CTA+SCT and the CTA+DECT for the presence of PE. The partial areas under the receiver operating characteristic curves (pAUC) for the clinically relevant specificity region were compared using multi-reader multi-case variance.
**Results:** The pooled sensitivities were not significantly different (95% confidence intervals for CTA: 67%-89%, SCT: 70%-91%, DECT: 72%-91%). Pooled specificity was significantly higher for SCT (100%-100%) compared to the other two techniques (CTA: 89%-97%, DECT: 89%-98%, p<0.001). The pAUCs for the average reader increased equally with the addition of either iodine map (p=0.029 SCT vs. CTA, p=0.009 DECT vs. CTA, p=0.65 DECT vs. SCT). Average reading times were equivalent while the median dose-length-products were 166 mGy-cm for SCT and 177 mGy-cm for DECT (p<0.001).
**Conclusion:** Iodine maps increase PE detection accuracy over CTA alone with similar reading times. SCT has the same diagnostic performance as DECT, at similar dose, without the need for dedicated hardware.
*Author Disclosures:*
**D. Grob:** Research/Grant Support; Toshiba Medical Systems. **E.J. Smit:** Research/Grant Support; Canon Medical System. Speaker; Canon Medical System. **L.J. Oostveen:** Research/Grant Support; Canon Medical System. **M. Prokop:** Research/Grant Support; Canon Medical System. Speaker; Canon Medical System, Bracco. Other; Thirona, Veolity. **I. Sechopoulos:** Research/Grant Support; Canon Medical System, Siemens Healthineers. Speaker; Siemens Healthineers. **M. Brink:** Research/Grant Support; Canon Medical System.

**B-1851** 14:24
**Split-bolus contrast injection protocol enhances the visualisation of the thoracic vasculature during chest CT**
C. Saade; *Beirut/LB (charbel.saade@aub.edu.lb)*

**Purpose:** To investigate the visualization of mediastinal lymph nodes during thoracic CT employing a multi-phasic contrast media (CM) protocol.
**Methods and Materials:** IRB approved retrospective study consisting of 300 patients with known chest malignancy. Patients were allocated to one of two

Sunday

CM protocols:Protocol A, consisted of dual bolus(Phase 1:100mL CM followed by 100mL saline chaser) intravenously injected at 2.5mL/s; Protocol B employed 100mL of CM using a multi-phasic injection protocol (Phase 1 and 2:60mL contrast and saline, followed by phase 3 and 4:40mL contrast and saline injected at 2.5mL/s) with a fixed scan delay of 70sec for each acquisition. Attenuation profiles of the thoracic arteries and veins were calculated as well as the arterio-venous contrast ratios(AVCR). Receiver operating characteristic (ROC), visual grading characteristic (VGC) and Cohen's kappa analysis we assessed.
**Results:** Arterial opacification was up to 24%(p<0.032) higher in protocol B than A, whereas, in the veins it was significantly lower in protocol B than A; with a maximum reduction of up to 84%(p<0.0001). There was no statistical significance between the central and peripheral pulmonary arteries(>263 HU) in each protocol. Protocol B, demonstrated significant improvement in AVCR at various anatomical sites(p<0.002). Radiation dose was significantly reduced in protocol B compared to A (p<0.004). Both ROC and VGC demonstrated significantly higher Az score for protocol B compared to A (p<0.0001) with an increased inter-reader agreement from poor to excellent.
**Conclusion:** Employing a multi-phasic CM protocol significantly improves opacification of the thoracic vasculature and visualization of mediastinal lymph nodes during thoracic CT.

**B-1852** 14:32
**Lung dual-energy quantitative CT vs single-photon emission computed tomography for quantification of the perfusion**
S.A. Si-Mohamed[1], C. Moreau-Triby[1], Q. Wdowik[2], P. Douek[1], L. Boussel[1]; [1]*Bron/FR*, [2]*Lyon/FR (salim.si-mohamed@chu-lyon.fr)*

**Purpose:** Perfusion scintigraphy is the current gold standard to assess the fractional contribution of each lobe to the total lung function. We developed a quantitative CT-derived method based on iodine density maps obtained from a dual-layer CT (DLCT) acquisition and compared it with perfusion single-photon emission computed tomography-CT.
**Methods and Materials:** Fifty three patients who underwent both modalities for suspected acute and chronic pulmonary embolism were retrospectively enrolled. CT angiography was performed on a DLCT (iQon, Philips, Haifa, Israel). Lung lobes were segmented semi-automatically on conventional images, using the 'COPD' module (Intellispace portal, Haifa, Israel). The lobar masks were then transposed onto the iodine density maps using in-house software, in order to compute the absolute amount of iodine in mg per lobe and the total amount in both lungs. The relative perfusion of each lobe was obtained by dividing the amount of iodine per lobe by the total amount and compared to the gold standard using linear regression and Bland-Altman analysis.
**Results:** Linear regression between the relative pulmonary lobar perfusion showed excellent good correlation (slope=0.88, offset=2.11 and $R^2$=0.87) for 265 lobes. Linear regression for each lobe demonstrated a $R^2$ at 0.85, 0.81, 0.81, 0.70, 0.87 for the left superior, inferior and right superior, middle, inferrior lobes respectively. Bland Altman analysis demonstrated a bias at -0.10 and agreement limits [-6,01; 5,81].
**Conclusion:** Given its simplicity, we anticipate that quantitative dual-layer CT could be widely used to measure the relative lung perfusion and replace perfusion lung scintigraphy for various clinical applications.

**B-1853** 14:40
**Influence of respiratory position on pulmonary artery contrast enhancement in dual-energy computed tomography angiography**
J. Vaimel[1], M.-K. Uprus[1], N. Igumenova[2], Ä. Roose[1], A. Samarin[1]; [1]*Tallinn/EE*, [2]*Peetri/EE*

**Purpose:** Transient interruption of contrast results in suboptimal enhancement of pulmonary arteries due to increased flow of unopacified blood from the inferior vena cava as a result of patient's deep inspiration prior to the scan. The primary purpose of this study was to determine if shallow-inspiration breath-hold allows superior pulmonary artery attenuation compared to deep-inspiration breath-hold CT angiography.
**Methods and Materials:** CT pulmonary angiographies were acquired in 107 patients (58 women, 49 men; age 16-91 years, mean 67 years) in shallow-inspiration breath-hold and in 115 patients (47 women, 58 men; age 26-92 years, mean 67 years) in deep-inspiration breath-hold using 64-detector row CT scanner and dual energy protocol. Bolus tracking was used for triggering the scan. Attenuation in Hounsfield units (HU) was measured in pulmonary trunk, thoracic aorta and superior vena cava on 65keV images. Scans with pulmonary trunk attenuation 250HU and higher were considered diagnostically adequate.
**Results:** Diagnostically adequate image quality was achieved in 82.6 % of the scans (n=95) in deep-inspiration breath-hold group and 93. 4% (n=100) of the scans in shallow-inspiration breath-hold group with a mean pulmonary trunk attenuation of 384.6 HU and 406.7 HU, respectively. In patients with inadequate pulmonary trunk attenuation a pattern consistent with transient interruption of contrast were seen in 45% of the inadequate scans (n=9) in deep-inspiration group and in 25% of the inadequate scans (n=2) in shallow-inspiration group.
**Conclusion:** CT pulmonary angiography performed after shallow-inspiration breath-hold leads to higher contrast enhancement of pulmonary arteries reducing the number of diagnostically inadequate scans.

**B-1854** 14:48
**Dual-energy CT pulmonary angiography and lung perfusion imaging with ultra-low-dose contrast application**
K. Hekimoglu, K. Haberal, H. Turnaoglu, G. Kahraman; *Ankara/TR (korayhekim@yahoo.com.tr)*

**Purpose:** To prospectively evaluate the quantitative and subjective quality of low-dose contrast dual-energy CT pulmonary angiography (DE-CTPA) and lung perfusion imaging (LPI) in comparison with routine pulmonary angiography (CTPA).
**Methods and Materials:** CTPA (*n*=49) had been done with 16-slice multidetector CT (MDCT) by 60 mL contrast media (21 gr iodine). A third-generation dual-energy MDCT system was used for DE-CTPA and LPI (*n*=45) with ultra-low contrast media (7 gr iodine). Average enhancement levels, signal-to-noise ratio (SNR) and contrast-to-noise ratio (CNR) were calculated. A five-point scale was used to subjectively evaluate image quality and noise. Shapiro-Wilk test was used to evaluate the normality assumption for enhancement values. Variables are presented as median with 25% and 75% interquartile range (IQR). Comparison between groups was performed with Mann-Whitney U test and LPI added three groups with Kruskal-Wallis test. *p* values <0.05 were considered statistically significant.
**Results:** Pulmonary enhancement levels of two protocols revealed no significant difference between ultra-low-dose contrast DE-CTPA protocol and routine CTPA (*p=0.*18). The SNR and the CNR ratios also revealed no statistical difference between protocols (*p*=0.2 and *p*=0.18, respectively). The five-point score was significantly better in the DE-CTPA and LPI (p=0.007) for image quality. There was not detected any significant difference for image quality and image noise evaluation between protocols (*p*=0.3).
**Conclusion:** DE-CTPA with ultra-low-contrast media allows sufficient enhancement in pulmonary arteries combined with high-quality lung perfusion images. This allows a significant reduction in iodine load while improving diagnostic confidence.

**B-1855** 14:56
**Value of CT pulmonary angiography to predict short-term outcome in a patient with pulmonary embolism**
A.M. Osman, E. H. Abdeldayem; *Cairo/EG (Dr_osman80@yahoo.com)*

**Purpose:** To evaluate the role of CT pulmonary angiography (CTPA) in the assessment of pulmonary embolism (PE) severity and the related CT cardiac changes reflecting the clinical status of the patients and predicting the outcome.
**Methods and Materials:** This is a prospective study of 184 patients presented with suspicious acute PE. All patients underwent CTPA followed by ECHO. Pulmonary artery obstructive index (PAOI) using Qanadli Score was calculated and cardiac changes were recorded. The patients' outcome was followed up for 30 days.
**Results:** Only 150 patients completed the study; 26.7 % needed ICU admission while 13.3 % died during follow-up. There was a significant relationship between the PAOI and the risk classification, right ventricular dysfunction (RVD) diagnosed by ECHO and the patients' short outcome. We found PAOI cutoff value of 45% for mortality and 35% for ICU admission and 27.5% for RVD with 60%, 75% and 90% sensitivity and 80%, 73.3% and 68.6% specificity, respectively. CT RV/LV ratio was the most sensitive parameter to predict RV dysfunction followed by pulmonary artery diameter.
**Conclusion:** CTPA is not only used for diagnosis but also to assess the severity of PE, the effect on the right ventricular function and subsequently the need for ICU admission and prediction of the outcome.

**B-1856** 15:04
**D-dimers, Wells' scores and CTPA for pulmonary embolism: are all diagnostics used appropriately?**
C. So, P. de Souza, R. Kumar, D. Remedios; *London/UK (chunwah.so@nhs.net)*

**Purpose:** Pathways for the management of a suspected pulmonary embolism (PE) rely on pre- and post-test probability. This audit assesses adherence to established pathways, incorporating use of Wells' Score, D-dimer and CT Pulmonary Angiography (CTPA).
**Methods and Materials:** Wells' scores, D-dimer levels, CTPA requests and reports from 60 consecutive patients were retrospectively analysed. Clinical, laboratory and request data were correlated with the CTPA report to assess adherence to pathways and accuracy of supplied information.

**Results:** There was 100% concordance of the supplied D-dimer result with the laboratory value confirming the absence of gaming. In 22 cases the D-dimer was not used according to guidelines, of which 9 had a low Wells' score but no D-dimer performed. In these 9 cases, the CTPA was negative. Furthermore, 13 cases had a high Wells' score with an unnecessary D-dimer. Pulmonary embolism was identified in 17% of cases in keeping with the published pickup rates for a CTPA - all of which either had a high Wells' score, high D-dimer or both, reinforcing the value of the pathway.
**Conclusion:** The rate of non-adherence with national pathways for PE investigation is 37%, resulting in waste of laboratory and radiological resources. A clinical decision support tool which factors in risk stratification and laboratory data may reduce waste by guiding clinicians towards the most appropriate first-line investigation, radiological or otherwise.

**B-1857** 15:12
**The effect of different monochromatic image choices on the reconstruction of CT perfusion data in one-stop spectral and perfusion CT scan**
H. Wang, P. Lu, S. Yue, W. Li; *Zhengzhou/CN (1508249322@qq.com)*

**Purpose:** To assess the effect of monochromatic images with different energy levels on the reconstruction of CT perfusion data in one-stop spectral and perfusion CT scan as compared with perfusion CT scan alone.
**Methods and Materials:** Twenty patients with lung tumours underwent one-stop spectral and perfusion CT scan (including whole tumour perfusion CT and whole lung contrast-enhanced CT). In the study group, monochromatic images with energy level from 40 to 80 keV (group A1 to A5), 100 kVp-like images (corresponding 68keV, group B) generated from spectral CT were used to reconstruct CT perfusion images with primary perfusion data. Another ten patients with lung tumours underwent perfusion CT scan alone was taken as the control group (group C). CT perfusion parameters of the tumours including blood volume (BV),blood flow (BF),mean transit time (MTT) and permeability surface (PS) were compared by using One-way ANOVA and independent-t test.
**Results:** Significant differences in CT perfusion parameters except for BV were found among the study group, and the BF and MTT increased with the increase of energy level. There was no difference of all CT perfusion parameters between group B and group C. However, compared to control group, BV in group A(except for group A3),BF in group A1 to A3,MTT in group A4,A5 increased and MTT in group A1 to A2,PS in group A1,A5 decreased (P<0.05).
**Conclusion:** 100 kVp-like energy level (corresponding 68keV) was the optimal energy level to reconstruct perfusion images in the one-stop spectral and perfusion CT scan.

**B-1858** 15:20
**CT angiography for pulmonary embolism in the emergency department: diagnostic accuracy of 20ml high-concentration contrast medium**
G. Milanese[1], R. Cobelli[1], C. Manna[1], M. Silva[1], E. Rasciti[2], S. Poggesi[1], N. Sverzellati[1]; [1]*Parma/IT*, [2]*Bologna/IT (gianluca.milanese@studenti.unipr.it)*

**Purpose:** To compare the diagnostic performance of CT pulmonary angiography (CTPA) between 20 ml and 40 ml of contrast medium (CM), in patients with suspected pulmonary embolism (PE).
**Methods and Materials:** CTPAs performed by 64-row multidetector scanner with 20ml (ultra-low volume: ULV) or 40 ml (low volume: LV) of high-concentration CM (Iomeprol 400 mgI/ml; iodine delivery rate 1.2 mgI/sec) were retrieved; tube potential was optimized for angiographic scan (100 kVp). Two radiologists independently scored vascular enhancement and image noise by a 5-point Likert score. Quantitative analysis was performed by densitometric parameters, including vascular contrast enhancement (CE; densitometric threshold for diagnostic CE>250 HU), and quantitative metrics for image noise, both in central and peripheral pulmonary arteries; furthermore, the overall mean CE was calculated.
**Results:** 102 ULV and 74 LV were included. The qualitative score was overall sufficient by ULV, yet inferior than LV (p<0.001). Qualitative image noise was comparable between ULV and LV, though the inter-observer agreement was only fair for peripheral vessels. Non-diagnostic qualitative parameters was reported in 9/102 ULV, of which 7/9 were associated with major pulmonary abnormalities. Quantitative image noise was not significantly different between ULV and LV. The mean CE was lower in ULV group (p<0.001), though >250 HU in both groups.
**Conclusion:** CTPA with 20 ml allows sufficient CE of the pulmonary arteries in patients with clinical suspect of PE. Decreased image quality was mostly associated with massive PE or concomitant pleuro-pulmonary abnormalities.

**14:00 - 15:30** **Room M 1**

### Cardiac

### SS 1903b

### Discovering the right ventricle in cardiac MR

*Moderators:*
Z. Olczak; Katowice/PL
V.E. Sinitsyn; Moscow/RU

**B-1859** 14:00
**Cardiac magnetic resonance feature tracking for quantifying right ventricular deformation in type 2 diabetes mellitus patients**
B. Hu, Z.-G. Yang, Y.-K. Guo; *Chengdu/CN (brigitteyue@foxmail.com)*

**Purpose:** To determine the feasibility of deformation analysis in the right ventricle (RV) using cardiovascular magnetic resonance feature tracking (CMR-FT) in type 2 diabetes mellitus (T2DM) patients.
**Methods and Materials:** We enrolled 70 T2DM patients, including 12 with impaired right ventricular ejection fraction (RVEF ≤ 45%) and 58 with preserved RVEF (RVEF > 45%), and 22 healthy controls in this prospective study. CMR was used to determine cardiac function and RV feature-tracking parameters. Binary logistic regression was used to predict RV dysfunction. ROC analysis was used to assess the diagnostic accuracy.
**Results:** Compared with controls, RV global longitudinal peak strain (GLPS) and circumferential peak strain (GCPS) were significantly lower in T2DM patients, without or with reduced RVEF [GLPS, −10.24 (−11.41–(−9.27)) % vs. −6.84 (−8.79–(−4.68)) % vs. −7.71 (−10.15–(−4.79) %; GCPS, −4.84 (−6.05–(−3.36)) % vs. −2.45 (−4.76–4.30)) % vs. 0.59 (−3.83–3.42) %; all p < 0.01]. PS in all three directions was significantly lower at basal, mid, and apical slices in T2DM patients than in controls (all $p < 0.05$), except for radial PS at the apical slice and circumferential PS at the basal slice. GCPS was an independent predictor of RV dysfunction (odds ratio: 1.265, 95% confidence interval: 1.020-1.568; $p = 0.032$), and GLPS demonstrated greater diagnostic accuracy (area under curve: 0.813) to predict RV dysfunction at a cut-off value of less than -9.54%.
**Conclusion:** In T2DM patients, CMR-FT could quantify RV deformation and identify subclinical RV dysfunction in those with normal RVEF; further, RV strain parameters are potential predictors for RV dysfunction.

**B-1860** 14:08
**Magnetic resonance functional assessment of the right ventricle in the short-axis plane as obtained from cross-referenced atrioventricular plane localisation**
M. de Meyere, B. Dubourg, D. Delacour, S. Bejar, P.J. Michelin, J.-N. Dacher; *Rouen/FR*

**Purpose:** Axial orientation may have been favoured over short-axis orientation (SAO) for evaluating the right ventricular function. The main reason lays in the difficulty to identify the atrioventricular junction in the short-axis plane implying lower reliability of the derived volumes. Our goal was to assess the accuracy and reproducibility of SAO-derived stroke volume measurement when using cross-referenced localisation of the atrioventricular plane at the tricuspid valve.
**Methods and Materials:** 41 patients referred for various acquired and congenital cardiopathies underwent CMR including SAO bSSFP slices. Additional right ventricular vertical long-axis and four-chamber views were used for semi-automatic localisation of the tricuspid plane. Right ventricle stroke volume (RV SV) was obtained three times by two observers. Two-dimensional phase contrast MRI “through plane” was performed at the level of the pulmonary trunk to measure the pulmonary stroke volume (PSV) and used as reference to assess accuracy. Intra- and inter-observer variabilities were tested using Bland-Altman analysis.
**Results:** There was high correlation (r=0.94) and no significant difference between RV SV and PSV (83±20mL vs. 81±21mL p>0.05). The RV SV, as well as others functional parameters of the RV, showed good intra- (ICC: 0.95; COV: 6.2) and inter-observer reliabilities (ICC: 0.91; COV: 8.9).
**Conclusion:** Right ventricle stroke volume derived from SAO analysis using cross-referenced localisation of the atrioventricular plane can be routinely performed with high accuracy and reproducibility.

**B-1861** 14:16

**Cardiac magnetic resonance imaging study of right ventricular myocardial deformation in spontaneous T2DM rhesus monkey**

T. Zhu[1], C. Yushu[1], Z. Yu[1], Z. Wen[1], J. Zheng[2], G. Fabao[1]; [1]*Chengdu/CN*, [2]*St. Louis, MO/US (965967939@qq.com)*

**Purpose:** To assess right ventricular function in rhesus monkeys with spontaneous T2DM using cardiac magnetic resonance imaging.
**Methods and Materials:** Twelve male spontaneous T2DM rhesus monkeys, and age- and sex-matched nine control monkeys underwent cardiac magnetic resonance imaging scanning to evaluate biventricular function using CMR cine and CMR-tissue tracking. Glucose, insulin, triglycerides, cholesterol, lipoprotein and liver, and renal function were assessed from blood.
**Results:** There was no significant decrease in RV and LV ejection fraction in T2DM (p<0.05). However, the absolute value of radial and circumferential global peak systolic strain, systolic strain rate and three directions global peak diastolic strain rate of the RV free wall were lower in the diabetic group (p<0.05). Also, only radial and circumferential peak systolic strain and radial diastolic strain rate of LV were higher (p<0.05). Our study also demonstrated that RV deformation, especially radial and circumferential, was negatively correlated with age, weight and glycated haemoglobin level (p<0.05).
**Conclusion:** RV showed systolic and diastolic dysfunction which was consistent with that of LV in spontaneous T2DM rhesus monkeys. RV dysfunction is closely related to blood glucose level, age and weight.

**B-1862** 14:24

**Right ventricular functional assessment with cardiac MRI by comparing volume measurements between axial and short axis orientations**

D.G. Oktay, E. Peker, I. Erden; *Ankara/TR (gizemoktay@gmail.com)*

**Purpose:** Right ventricular (RV) volume and function assessment is usually performed on patients with cardiovascular diseases by using cardiac magnetic resonance imaging (CMRI). İts important to clear that which slice orientation provides better overall assessment of RV functional performance. This study is designed to evaluate right ventricular (RV) volume measurements and their reproducibility with comparing axial and short axis orientation acquisition techniques.
**Methods and Materials:** From December 2015 to December 2017, 40 patients who underwent CMR (1,5 T) and had axial and short axis acquisitions were retrospectively evaluated. Measurements of RV from data sets acquired in axial and short axis orientations were compared.
**Results:** Comparison between the axial and short axis methods, there wasn't any significant and systematic difference in the EDV, ESV and SV volumes, EF values (mean bias EDV 15.6 +/- 27,6 mL/m2 difference; ESV 8.5 +/- 15,96 ml/m2; difference; SV 7,18+/- 18,7 mL/m2 difference; EF -1,63+/- 6,5 difference). Also, there was no difference between two method in terms of intraobserver reliability in all measurements.
**Conclusion:** There is not any significant impact of the slice acquisition orientation on CMR assessment of RV volumes and the addition of the axial acquisitions extend the duration of CMRI; so its reproducibility were not clinically significant.

**B-1863** 14:32

**Balloon pulmonary angioplasty (BPA) improves right ventricular dysfunction and pulmonary haemodynamics in inoperable CTEPH**

A. Hasse[1], K. Tello[1], H. Gall[1], A. Breithecker[1], C.B. Wiedenroth[2], W. Seeger[1], E. Mayer[2], G.A. Krombach[1], F.C. Roller[1]; [1]*Giessen/DE*, [2]*Bad Nauheim/DE (alexander.hasse@mail.de)*

**Purpose:** To assess the effect of BPA on right ventricular (RV) dysfunction and pulmonary haemodynamics in patients with inoperable chronic thromboembolic pulmonary hypertension (CTEPH).
**Methods and Materials:** MRI at 1.5 Tesla and right heart catheterization were performed before and 6 months after BPA in 30 consecutive patients (mean age 63.4 ± 10.6 years; 17 female). Right ventricular function (RVEF) and feature tracking strain analysis (global longitudinal (GL), circumferential (GC) and radial (GR)) were obtained and correlated to mean pulmonary arterial pressure (mPAP) and pulmonary vascular resistance (PVR).
**Results:** RVEF (35.9% ± 10.8 to 48.4% ± 8.7), mPAP (42.1mmHg ± 8.0 to 33.1mmHg ± 8.0) and PVR (551.8 ± 192.3 to 377.7 ± 140.2) significantly decreased after BPA (p<0.0001). Moreover, RV strain parameters significantly decreased after BPA (GL -19.9 ± 5.7 to 24.0 ± 3.5, p<0.001; GC -9.4 ± 4.0 to -11.0 ± 3.5, p<0.022; GR 38.2 ± 19.1 to 50.7 ± 15.0, p=0.001) and GL strain revealed the best and significant correlations to RVEF (before BPA r = -0.75; after BPA r = -0.54), mPAP (r = 0.36; r = 0.52) and PVR (r = 0.49; r = 0.48).
**Conclusion:** BPA in patients with CTEPH leads to significant improvement of RV function and pulmonary haemodynamics. RV mechanical dysfunction is significantly improved and RV strain measures showed promising correlations to function and haemodynamics. In the course of CTEPH, strain analysis might enable new insights regarding therapy effect, therapy monitoring and prognosis.

**B-1864** 14:40

**Application of CMR derived myocardial deformation parameters to predict restrictive physiology in repaired tetralogy of Fallot patients**

X.-W. Tong[1], R. Wald[2], G.R. Karur[2], Y.-F. Cheung[1], M.-Y. Ng[1]; [1]*Hong Kong/HK*, [2]*Toronto, ON/CA (xiaowan@connect.hku.hk)*

**Purpose:** Surgically repaired tetralogy of Fallot (rTOF) patients can be subdivided into those with restrictive right ventricular (RV) physiology and those without based on main pulmonary artery (MPA) end-diastolic forward flow (EDFF). Our aim was to assess the value of cardiac magnetic resonance (CMR) feature-tracking (FT) for RV restrictive physiology pre and post pulmonary valve replacement (PVR).
**Methods and Materials:** 141 rTOF patients with PVR were prospectively recruited as part of the multicenter international CORRELATE study. All patients underwent pre-PVR MRI and 78 underwent post-PVR MRI with multiplanar steady-state free precession cine imaging and phase-contrast imaging at the MPA. CMR-FT was performed to calculate late-diastolic strain rate in longitudinal (late-LDSR), radial (late-RDSR) and circumferential (late-CDSR) planes using dedicated software. RV volumetric parameters, exercise performance with peak VO2 and its predicted value were evaluated. Mann-Whitney U-test and paired t-test were performed to compare differences between restrictive and non-restrictive groups, pre/post-PVR changes.
**Results:** Among 141 patients, 68 (48.2%) patients showed restrictive RV prior and 3 (3.8%) post PVR. EDFF-negative group showed higher late-RDSR, late-CDSR, peak VO2 but less regurgitation fraction (RF) (all p<0.05) than EDFF-positive. After PVR, predicted peak VO2 improved 8.6% in EDFF-negative (p=0.021) but not for EDFF-positive group. Both groups showed decreased RV size and increased late-RDSR (p<0.05).
**Conclusion:** EDFF-positive group showed decreased RV compliance, more RF and worse exercise performance. Prevalence of restrictive RV was common in rTOF and diminished after PVR. RV reverse remodelling after PVR with improvement in RV compliance, but only non-restrictive patients showed improved exercise.

**B-1865** 14:48

**Evaluation of ascending aorta flow pattern in patients with repaired TOF using 4D flow CMR**

S. Lee, Y.J. Kim; *Seoul/KR (sjlee37@yuhs.ac)*

**Purpose:** To evaluate characteristics of the ascending aorta flow patterns with 4D-flow MRI and to determine possible predictors of aortic dilatation late after tetralogy of Fallot (TOF) repair.
**Methods and Materials:** Total 66 subjects underwent 4D-flow MRI (44 with repaired TOF patients and 11 volunteers). Data analysis included aortic diameters, velocity, wall shear stress (WSS), flow jet angle (FJA) and flow displacement (FD) at the level of sinotubular junction (STJ) and mid-ascending aorta(AA). Differences in the parameters between patients group and volunteers were analysed. Relationship between aortic dimension and flow parameters in patients group was also analysed.
**Results:** In the repaired TOF group (M:F=25:19, mean age=25.5), aortic dimensions at sinus of valsalva (SOV), STJ and AA were significantly increased compared with the volunteer group (M:F=10:1, mean age=36.00) (P=0.002, P<0.001, P=0.013). Among the flow parameters at STJ and AA, maximal velocity ($V_{max),}$), mean velocity ($V_{mean)}$), maximal WSS ($WSS_{max)}$), mean WSS ($WSS_{mean)}$), mean WSS at axial direction ($WSS_{ax\_mean)}$), mean WSS at circular direction ($WSS_{cir\_mean)}$) were significantly decreased (P=0.002, P<0.001, P<0.001, P=0.003, P<0.001, P=0.023) and FJA was significant increased (P<0.001) in the level of STJ. And $V_{max,}$, $V_{mean}$, and $WSS_{cir\_mean}$ were significantly decreased (P=0.003, P<0.001, P=0.016) and FJA was significantly increased (P=0.003) in the level of AA. In regression test, $V_{mean}$ and WSS parameters (P=0.0019, P=0.0147 for $WSS_{max}$, P=0.0020 for $WSS_{mean}$, P=0.0300 for $WSS_{ax\_mean}$, P=0.0016 for $WSS_{cir\_mean)}$) were significantly correlated to STJ diameter. $V_{mean}$, $WSS_{mean}$ and $WSS_{cir\_mean}$ (P<0.0001, P=0.0244, P=0.0035) were significantly correlated to AA diameter.
**Conclusion:** In repaired TOF patients, WSS is decreased at ascending aorta and it was correlated with ascending aorta dilatation, especially in proximal portion.

**B-1866** 14:56

**Measurement of wall shear stress and pressure gradients using 4D-flow-MRI in patients with Fontan circulation**

A. Curta, A. Lehner, S. Ulrich, R. Dalla-Pozza, M. Fischer, N. Haas, H. Kramer; *Munich/DE (adrian.curta@med.uni-muenchen.de)*

**Purpose:** To assess feasibility of indirect measurements of wall shear stress (WSS) and pressure gradients (PG) using 4D-phase-contrast-flow(4D-flow)-MRI in the work-up of patients with a univentricular system and Fontan circulation.
**Methods and Materials:** 4D-flow-MRI was acquired in 9 patients (13,9±4,2 years old) with a Fontan circulation. Velocity encoding was set at 100 cm/s with a spatial resolution of 2.2x1.8x1.8mm and a temporal resolution of 10

Sunday frames/cardiac cycle. Vessel geometry was measured in contrast enhanced MR-angiography using 3D multiplanar reconstructions. Flow was visualized using path lines; WSS and PG were visualised using time-resolved colour-coded maps. Data was processed offline using a commercially available software.
**Results:** The PG from the Fontan tunnel (FT) to the right pulmonary artery (RPA) was lower than from the FT to the left pulmonary artery (LPA) (-0.7±1.6mmHg vs. -1.2±1.4mmHg). Average WSS was lower in the RPA than in the LPA (0.19±0.08Pa vs. 0.23±0.11Pa). There was a significant negative correlation of the angle of the RPA anastomosis to the average WSS of the RPA (P=-0.76; p=0.03) and of the angle of the sum of the angles of the LPA and RPA anastomosis to the average WSS of the LPA (P=-0.937; p=0.001) and RPA (P=-0.760; p=0.029).
**Conclusion:** Indirect measurements of WSS and PG using 4D-flow-MRI were feasible. These measurements could aid in further optimizing surgical methods in order improve flow from the FT to the pulmonary arteries to prevent complications such as protein-losing enteropathy and intrapulmonary shunts.
*Author Disclosures:*
**A. Curta:** Research/Grant Support; Circle Cardiovascular provided software free of charge for research purposes.

**B-1867** 15:04
**Pulmonary insufficiency: extending the advantage of pulmonary regurgitation volume versus pulmonary regurgitation fraction to a congenital heart disease mixed population**
M. Petrini[1], F. Secchi[2], C.B. Monti[2], M. Ali[2], P.M. Cannao[2], E. Michieletti[1], F. Sardanelli[2]; [1]*Piacenza/IT*, [2]*Milan/IT*

**Purpose:** To compare the use of pulmonary regurgitation volume (PRV) and pulmonary regurgitation fraction (PRF) in the assessment of patients with pulmonary regurgitation (PR) undergoing cardiac magnetic resonance (CMR).
**Methods and Materials:** 179 patients with PR who had undergone CMR were retrospectively evaluated. Their right ventricular diastolic (EDVI) and systolic (ESVI) volume indexes, stroke volume and ejection fraction were obtained from cine CMR sequences, while PRV and PRF were obtained by phase contrast sequences. Patients were divided into subgroups, according to their underlying pathology, and to PR severity. Correlations between PRV or PRF and RV parameters were studied through Spearman's ρ both in the main group and subgroups. Afterwards, follow-up examinations were studied when present, and correlations between PRV or PRF from the first CMR examination and volume data from the second were analysed.
**Results:** Tetralogy of Fallot was the main setting of PR (98/179). Overall, EDVI had a stronger correlation to PRV (ρ=0.592, *p*<0.001) than PRF (ρ=0.522, *p* <0.001), and ESVI had a stronger correlation to PRV (ρ=0.454, *p* <0.001) and PRF (ρ=0.406, *p* <0.001). Regarding subgroup analysis, in moderate or severe PR patients, EDVI had a significantly (*p* =0.043) stronger correlation to PRV (ρ=0.499, *p* <0.001) than PRF (ρ=0.317, *p* <0.001). Follow-up EDVI had a significant correlation to PRV (ρ=0.450, *p* =0.031), while not with PRF.
**Conclusion:** Especially when assessing moderate to severe PR, PRV may be a better indicator of right ventricular dysfunction than PRF. Moreover, PRV may be a predictor of worsening RV dilation.

**B-1868** 15:12
**Free breathing real-time cardiac MRI in children with congenital heart disease: comparison of right ventricular function with standard breath-hold imaging**
C. Treutlein, M. Zeilinger, R. Heiß, W. Wuest, O. Rompel; *Erlangen/DE (christoph.treutlein@uk-erlangen.de)*

**Purpose:** Right ventricular (RV) functional parameters in children with congenital heart disease acquired in free-breathing (FB) and single-breath-hold (SB) full cardiac cycle real-time compressed sensing (CS) cine CMR compared with standard retrospective segmented multi-breath-hold cine CMR.
**Methods and Materials:** 23 patients with biventricular congenital heart disease underwent standard segmented breath-hold cine, single-breath-hold and free-breathing CS real-time cine CMR with inline motion correction to obtain a stack of continuous axial images of the right ventricle. Functional parameters and scan time were compared. Two radiologists independently performed qualitative analysis of image quality (rated on a 5-point scale; 1 = nondiagnostic up to 5= excellent) and quantitative analysis of right ventricular volume measurements.
**Results:** All functional parameters correlated significantly (all p<0.01). Image quality score was slightly lower in SB and FB than in standard BH (4,1 ±0,8 and 4,2 ±0,8 vs. 5,5 ±0,6). Differences in functional results of the right ventricle based on Bland-Altman analysis were less than 5% (95% CI, -8.7 to 7.2). Scan time was 20s (±22) and 14s (±10) vs. 209s (±89). Overall inter-rater variability ranged from -9,0 to 8,1%.
**Conclusion:** Free-breathing and single-breath-hold real-time compressed sensing cine CMR in children with congenital heart disease could evaluate RV volume with excellent accuracy and delivers diagnostic image quality in a shorter examination time than standard segmented BH examination. It may replace multi-breath-hold standard cine CMR.

**B-1869** 15:20
**Regional right ventricular deformation in hypertrophic cardiomyopathy: assessed with cardiovascular magnetic resonance tissue tracking**
X. Li, Y. Zhigang, Y. Guo, Y. He, S. He, K. Shi, C. Xia, Z. Li; *Chengdu/CN (lixiang5861@163.com)*

**Purpose:** To evaluate the regional right ventricular (RV) systolic function in patients with hypertrophic cardiomyopathy (HCM) using cardiovascular magnetic resonance-3D tissue tracking.
**Methods and Materials:** Seventy-one HCM patients classified as late gadolinium enhancement (LGE) present or absent groups and 15 healthy controls were enrolled. The short-axis and four-chamber long-axis cine imaging as well as LGE imaging were performed. The ejection fraction of right ventricular (RVEF), global and regional (basal, mid, and apical) RV tracking strain variables were measured.
**Results:** In the HCM group, 44 patients presented with LGE and 27 without LGE. The RVEF were not comparable among all patients and controls (P=0.58), but global peak longitudinal strain (PLS) in HCM patients with LGE were significantly lower than those without LGE (-5.64±12.93% vs. -10.68±5.53%, P<0.05). Furthermore, the apical and mid regional of RV-PLS were significantly lower in HCM patients with LGE compared with those without LGE and controls (apical PLS: -6.59±14.53% vs. -10.38±7.61% vs. -12.43±6.69%, P<0.05; mid PLS: -0.13 ±23.11% versus -9.72±7.72% versus -10.28±5.36%, P<0.05). The basal RV longitudinal strain in HCM patients with LGE were significantly lower than without LGE (-6.58±20.11% vs. -10.30±6.28%, P<0.05).
**Conclusion:** In HCM, the decreased regional RV myocardial strain occurred before the RVEF reduced, which suggests that regional RV systolic function for HCM is impaired in subclinical state. Moreover, myocardial deformation of RV was more severe with the presence of LV LGE.
*Author Disclosures:*
**Y. Zhigang:** Research/Grant Support; National Natural Science Foundation of China (No. 81471721, 81471722, 81641169, 81771887, and 81771897).

**14:00 - 15:30** **Room M 4**

### GI Tract

### SS 1901b

### Gastro-oesophageal imaging: now and next

*Moderators:*
G.H. Mostbeck; Vienna/AT
T. Rohan; Brno/CZ

**B-1871** 14:00
**Significance of multidetector computed tomography in the assessment of achalasia subtypes and detection of pulmonary complications**
S. Jovanovic, A. Djuric Stefanovic, A. Simic, O. Skrobic, D. Masulovic; *Belgrade/RS (dr.sanja.jovanovic@gmail.com)*

**Purpose:** To evaluate the multidetector computed tomography (MDCT) findings in patients who have achalasia, to assess its role in differentiating three different subtypes, detecting lung involvement and extra-oesophageal thoracic complications.
**Methods and Materials:** Clinical retrospective study included 51 patients with manometrically confirmed achalasia who underwent chest X-ray and MDCT in preoperative diagnostic workup. Oesophageal wall thickness and morphology, luminal dilatation, lung changes and extra-oesophageal manifestations were analysed on MDCT. Wilcoxon, Kruskal-Wallis and Mann-Whitney test were used for assessing the differences among the achalasia subtypes.
**Results:** Fourteen (27.5%) patients had achalasia subtype 1, 21 (60.8%) had subtype 2 while 6 (11.8%) had subtype 3. Oesophageal wall thickness of the oesophageal body (EB) and distal oesophageal segment (DES) as well as nodular/lobulated appearance of DES were significantly more often found in subtype 3 (p=0.024, p<0.001, p=0.009, respectively). Oesophageal dilatation gradually decreased from subtype 1 to 3 (p=0.006). Chest X-ray revealed lung changes in 9 (17%) and MDCT in 21 (41%) patients (p=0.001), most frequently in subtype 1, with predominance of ground-glass opacities. Tracheal/carinal compression was detected in 27 (52.9%) and left atrial compression in 17 (33.3%) patients.

**Conclusion:** MDCT is a useful tool in detection of lung and extra-oesophageal thoracic complications in patients with achalasia, and a valuable additional imaging modality in differentiation of achalasia subtypes.

**B-1872** 14:08

**Value of MRI with static and dynamic sequences in the study nature extension of the tight stenosis of the oesophagus-gastric junction**

G. De Franco[1], S. Bruni[1], G. D'Alesio[2], S. Bux[1], A. Ambrosi[3], A. Vaccaro[1], V. Grimaldi[1], M. Fracella[1]; [1]*Bari/IT*, [2]*Triggiano/IT*, [3]*Valenzano/IT* *(dottguelfodefranco@yahoo.it)*

**Purpose:** Evaluation of the efficacy of MRI with static and dynamic sequences in the diagnostic procedure for the evaluation of the extension and nature of the tight stenosis of the gastric-oesophageal junction.
**Methods and Materials:** From August 2016 to February 2018, 112 patients with tight stenosis of the gastric-oesophageal junction were assessed by gastroscopy. After distension of the stomach by 700-900 cc of water and intravenous administration of hypotonic drug (1fl of n-methyl scopolamine) MRI of the oesophagus and stomach was performed with 1.5 T high-field magnet and phased-array surface coils. The protocol provided the execution of morphological sequences (true fisp / HASTE), cine -RM, DWI and VIBE with intravenous contrast medium.
**Results:** The MRI examination allowed to identify in all cases the thickening of the oesophagus-gastric junction. The identification of the parietal signal, the enhancement and the DWI signal of the wall allowed to correctly characterize: 60 fibrotic stenosis (53%), 24 acalasic stenosis (22%) and 16 neoplastic stenosis (14%). In the remaining 12 cases (10% of the total) the examination was not conclusive due to the poor collaboration/claustrophobic crisis of the patients (8 cases) or to associated comorbidities (4 cases).
**Conclusion:** The MRI of the oesophagus and the stomach has the possibility to discriminate with high accuracy the extension and nature of the tight gastric-oesophageal stenosis with very high diagnostic efficacy and allowing patients a subsequent adequate and appropriate therapeutic approach.

**B-1873** 14:16

**Accuracy of 3.0 T MRI in the preoperative T staging of patients with potentially resectable oesophageal cancer, with histopathological correlation**

Y. Lu[1], J. Qu[1], Z. Wang[1], X. Yan[2]; [1]*Zhengzhou/CN*, [2]*Shanghai/CN* *(15249675779@163.com)*

**Purpose:** To explore the value of 3.0 T MRI using multiple sequences (T2-TSE-BLADE, contrast-enhanced star-VIBE, delayed star-VIBE, and combination of the three sequences) in evaluating the preoperative T staging for potentially resectable oesophageal cancer (EC) with pathologic confirmation.
**Methods and Materials:** Patients with endoscopically biopsy-proven EC and T1/T2/T3/T4a staging by CT who were imaged on a 3.0 T scanner were prospectively enrolled. The MRI protocol included T2-TSE-BLADE, contrast-enhanced star-VIBE, and delayed star-VIBE. Readers assigned a T stage on MRI, and post-operative pathologic confirmation was considered the gold standard. Inter-reader agreement was also calculated. The diagnostic accuracy of T staging on T2-TSE-BLADE, contrast enhanced star-VIBE, delayed star-VIBE, and the combination of the three sequences were analysed and compared to post-operative pathologic T staging.
**Results:** The study included 158 patients. Inter-reader agreements of T staging were excellent for T2-TSE-BLADE (Kappa=0.818, $P<0.0001$), contrast-enhanced star-VIBE (Kappa=0.807, $P<0.0001$), delayed star-VIBE (Kappa=0.817, $P<0.0001$) and the combination of the three sequences (Kappa=0.935, $P<0.0001$). Diagnostic accuracies of delayed star-VIBE for T1/T2 staging were 95.57% and 97.47% for reader 1 and reader 2, respectively. For T3/T4 staging, diagnostic accuracy of T2-TSE-BLADE and contrast enhanced star-VIBE ranged between 88.61% and 97.47% for reader 1 and reader 2, respectively. Diagnostic accuracy of the combination of the three sequences was higher than individual sequences for both readers.
**Conclusion:** 3.0T MRI using the 3 combined sequences showed higher diagnostic accuracy in T staging of patients with potentially resectable EC compared to individual sequence.

**B-1874** 14:24

**Is the perfusion-metabolic tumour phenotype related to tumour volume and nodal stage in primary oesophageal tumours undergoing DCE-MRI and $^{18}$F-FDG PET/CT?**

S.J. Withey, K. Owczarczyk, C. Yip, M.M. Siddique, A. Green, J. Bell, G. Cook, V.J. Goh; *London/UK (samuel.withey@nhs.net)*

**Purpose:** Locoregional stage remains a relatively poor outcome discriminator in primary oesophageal cancer undergoing curative treatment. We aimed to assess the relationship between tumour perfusion, metabolism & volume with nodal stage.
**Methods and Materials:** Following ethical approval and informed consent, DCE-MRI and $^{18}$F-FDG PET/CT were performed at staging in 24 patients (18 male; mean age 64 ± 10 years; 21 adenocarcinoma, 3 squamous cell carcinoma) with primary oesophageal cancer, planned for surgery ± neoadjuvant therapy. DCE-MRI transfer constant ($K^{trans}$), $^{18}$F-FDG PET/CT maximum standardised uptake value ($SUV_{max}$) and FDG-avid mean tumour volume (MTV) were calculated. Spearman rank correlation assessed the relationship between $K^{trans}$, $SUV_{max}$ and MTV. $K^{trans}$, $SUV_{max}$ and MTV were compared in clinically and pathologically node positive patients (cN and pN respectively) using Wilcoxon rank test. Significance was at 5%.
**Results:** No correlation was observed between $K^{trans}$, $SUV_{max}$ or MTV (Spearman 0.1 and 0.01 respectively). No statistically significant difference between $K^{trans}$, $SUV_{max}$ or MTV in cN+ versus cN- or pN+ versus pN- patients.When stratifying patients by MTV (<median versus ≥median), in patients with lower metabolic tumour volume, baseline $K^{trans}$ was significantly higher in node negative patients (cN and pN, p=0.02 and p=0.04 respectively). This trend was not observed in patients with high metabolic volume cancers. In patients with lower MTV, baseline $K^{trans}$ was inversely correlated with number of pathologically involved lymph nodes (Spearman -0.65, p=0.01).
**Conclusion:** Low DCE-MRI $K^{trans}$ appears predictive of nodal involvement in lower MTV cancers.

**B-1875** 14:32

**Combination of quantitative $^{18}$F-FDG PET parameters and contrast-enhanced CT in locoregional restaging and prognostication of survival in patients with oesophageal cancer**

D. Tamandl, B. Fueger, A. Haug, A. Ba-Ssalamah; *Vienna/AT* *(dietmar.tamandl@meduniwien.ac.at)*

**Purpose:** To assess whether the combination of contrast-enhanced CT (CE-CT) and quantitative 18F-FDGPET parameters can improve locoregional restaging in oesophageal cancer (EC) after neoadjuvant therapy.
**Methods and Materials:** Eighty-eight consecutive patients with locally advanced EC, who underwent oesophagectomy after neoadjuvant chemotherapy or chemoradiotherapy were included in this retrospective study. The diagnostic accuracy of CE-CT, visual 18F-FDGPET/CT (vPET/CT) and quantitative PET parameters were assessed for local staging. Histopathology was used as the reference standard. The prognostic value on survival was assessed using Cox regression analysis.
**Results:** T,N staging: sensitivity, positive predictive value (PPV) and accuracy was 78.8%, 70.2%, 59.0% (CE-CT) and 81.1%, 81.1%, 68.2% (vPET/CT) for T staging as well as 59.5%, 75.9%, 50.0% (CE-CT) and 70.2%, 93.7%, and 67.0% (vPET/CT) for N staging, respectively. Tumour length (TL) and metabolic tumour volume (MTV) correlated with advancing T stages (p=0.002 and 0.038). CE-CT had the best sensitivity to differentiate between T3/4 and T0-2 stages (AUC 0.86, p<0.001), while MTV had the highest sensitivity to detect complete response (T0 vs. 1-4, AUC 0.77, p=0.002). The combination of CE-CT and MTV had an even superior accuracy to predict complete response (AUC 0.82, p<0.001). The imaging AJCC stage provided a better prognostication of RFS, CSS and OS than either T stage, N stage derived from CE-CT or vPET/CT, or quantitative PET parameters alone.
**Conclusion:** Combining both CE-CT and quantitative PET parameters allows for robust prediction of restaging, recurrence and survival in EC.

**B-1876** 14:40

**Correlation between dual-energy CT and perfusion CT parameters in patients with gastric carcinoma**

Y. Chai; *Zhengzhou/CN (chaiyarucyr@163.com)*

**Purpose:** To develop a dual-energy enhanced CT protocol using time attenuation curves from previously acquired perfusion CT data and to evaluate the relationship between iodine enhancement value at dual-energy CT and perfusion parameters in gastric carcinoma patients.
**Methods and Materials:** The retrospective part included the development of a dual-energy enhanced CT protocol to evaluate peak arterial and venous enhancement on the basis of time-attenuation curves from previously acquired perfusion CT data in 40 patients. The prospective part consisted of an intraindividual comparison of dual-energy CT and perfusion CT data in another 40 patients with gastric carcinoma. Iodine value and normalized iodine value in arterial and venous phase from dual-energy CT and perfusion parameters (arterial flow, AF) from perfusion CT were compared. Pearson R and linear correlation coefficients were calculated.
**Results:** The strongest intraindividual correlations in gastric carcinoma were found between iodine value in AP with AF (r= 0.73, P= 0.001). Moderate correlations were found between normalized iodine value in AP with AF (r= 0.52, P= 0.021) and between normalized iodine value in VP with AF (r = 0.57, P =0 .013). No further significant correlations were found. The volume CT dose index (10.3 mGy) and dose-length product (214.0 m Gy·cm) of dual-energy CT were lower than those of the arterial phase of perfusion CT (29.8m Gy and 654.9 m Gy·cm, respectively).

Sunday

**Conclusion:** A enhanced dual-energy CT protocol acquired from perfusion CT data sets in patients with gastric carcinoma could show good correlation between iodine value from dual-energy CT with perfusion parameters.

**B-1877** 14:48

**Clinical value of radiomics nomogram in preoperative prediction of lymph node metastasis of gastric adenocarcinoma**

R. Wang, J. Li, J. Gao; *Zhengzhou/CN (wr807115125@163.com)*

**Purpose:** To develop and validate the spectral CT-based radiomics in predicting preoperatively the lymph node metastasis (LNM) of gastric adenocarcinoma.
**Methods and Materials:** A total of 196 gastric adenocarcinoma patients confirmed by pathology and underwent preoperative spectral CT scanning were retrospectively enrolled (98 in the training set and 98 in the test set). The clinical information of these patients were recorded, and the CT values, iodine concentration (IC) values and normalized iodine concentration (nIC) values of primary lesions and lymph nodes in dual-phases were measured and calculated in the postprocessing workstation. 273 radiomics features were extracted from the dual-phases CT images in different energy level (40, 65 and 100KeV) to build a radiomics signature. Univariate and multivariable logistic regression analysis were performed to screen predictive indicators for LNM in gastric adenocarcinoma. A radiomics nomogram incorporating significant indicators was developed in the training set, and its performance was measured using the receiver operating characteristics (ROC) curves and was compared with DeLong test.
**Results:** The radiomics nomogram, which incorporated the CT-reported LN status, IC values in portal venous phases of primary lesions and two radiomics signatures (RSa40, RSp65) distinguished LNM with an area under curve (AUC) of 0.822 (95% CI, 0.739-0.906) in the training set. It yielded an AUC of 0.819 (95% CI, 0.732-0.906) and an accuracy of 0.735 (95% CI, 0.636-0.819) in the test set. There were no significant differences in AUC between two sets (p=0.9533).
**Conclusion:** The radiomics nomogram provides individualized preoperative prediction of LNM in gastric adenocarcinoma patients.
*Author Disclosures:*
**R. Wang:** Author; li Jing.

**B-1878** 14:56

**Imaging of mini/one anastomosis gastric bypass abdominal complications**

G. Scavone[1], D.C. Caltabiano[1], M.V. Raciti[2], M.C. Calcagno[1], G. Caltabiano[1], F. Gulino[1], A. Giarrizzo[1], L. Piazza[1], A. Scavone[1]; [1]*Catania/IT*, [2]*Pavia/IT (mc.calcagno@gmail.com)*

**Purpose:** Mini/One Anastomosis Gastric Bypass(MGB/OAGB) is a restrictive and malabsorptive bariatric surgical procedure; it consists of a gastric tubulization carried out along the small curvature of the stomach that is sutured to a long jejunal loop. The purpose of this retrospective study is to investigate outcome and abdominal complications evaluated by radiological techniques and to assess the role of multidetector computer tomography(MDCT) and radiographic upper gastrointestinal(UGI).
**Methods and Materials:** Since January 2005, 953 patients (female 684-71.7%, male 269-28.3%; mean 42 years old, range 18-70) underwent videolaparoscopic MGB/OAGB in our center and were evaluated with UGI series in 3th day after procedure.In clinical suspect of gastric leakage (abdominal pain, tachycardia/tachypnea, chest pain, fever and leukocytosis) and hemorrhage(hypotension, tachycardia, cold sweating, hematemesis/melena) in stable patients, MDCT with intravenous and oral contrast media administration was performed.
**Results:** MDCT detected two types of abdominal complications: early (within one month after procedure) and late (from second month to 10 years after procedure).We found 13 early complications (6 endoluminal/intra-abdominal bleedings, 4 gastric pouch leaks, 1 anastomotic dehisce, 1 anastomotic stenosis, 1 abdominal abscess) and 16 late complications (11 Weight Regain: 7 for dilatation of gastric pouch, 4 for gastro-gastric fistula, 3 excessive weight loss for malabsorption syndrome, 1 intestinal occlusion due to adherence and 1 peri-anastomotic abscess).Only 2(6.8%) complications were detected using UGI.
**Conclusion:** In presence of a reasonable clinical suspect, MDCT with oral and intravenous contrast media administration is a robust technique for diagnosis of complications, useful for surgical, interventional radiological or conservative treatment planning.

**B-1879** 15:04

**Structured CT reporting improves accuracy in diagnosing internal herniation after laparoscopic Roux-en-Y gastric bypass**

J.C. Ederveen[1], S.W. Nienhuijs[2], J. Nederend[2]; [1]*Maastricht/NL*, [2]*Eindhoven/NL (jeannette.ederveen@mumc.nl)*

**Purpose:** To confirm that structured reporting of CT scans using ten signs in clinical practice leads to a better accuracy in diagnosing internal herniation (IH), compared to non-structured reporting.
**Methods and Materials:** A cohort was used of patients who had undergone laparoscopic gastric bypass surgery between January 1, 2011 and January 1, 2018. CT scans between June 1, 2017 and October 1, 2018 were prospectively included and structured reports were made using ten signs: (1) swirl sign, (2) small-bowel obstruction, (3) clustered loops, (4) mushroom sign, (5) hurricane eye sign, (6) small bowel behind superior mesenteric artery, (7) right-sided anastomosis, (8) enlarged nodes, (9) venous congestion, and (10) mesenteric oedema. Furthermore, an overall impression of IH likelihood was given using a 5-point Likert scale. CT scans performed between January 1, 2011 and January 1, 2017, without structured reporting, were used for comparison. Accuracy was calculated using two-way contingency tables. Reoperation and three-month follow-up were used as reference.
**Results:** A total of 472 CT scans were performed in the study period, 292 in 2011-2016 without structured reporting and 180 in 2017-2018. Of these, 164 (91.1%) were reported using structured reporting. Sensitivity was 79.5% (95%-CI: 67.6-91.5%) and 86.2% (95%-CI: 73.7-98.8%), respectively (p=0.467); specificity was 87.6% (95%-CI: 83.5-91.7%) and 95.6% (95%-CI: 92.1-99.0%), respectively (p=0.011); positive predictive value was 53.0% (95%-CI: 41.0-65.1%) and 80.6% (95%-CI: 66.7-94.6%), respectively (p=0.009); negative predictive value was 96.1% (95%-CI: 93.5-98.6%) and 97.0% (95%-CI: 94.1-99.9%), respectively (p=0.644); and accuracy was 86.4% (95%-CI: 81.8-90.0%) and 93.9% (95%-CI: 88.8-96.9%), respectively (p=0.014).
**Conclusion:** Structured reporting for the diagnosis of internal herniation after bariatric surgery improves accuracy and can be implemented in clinical practice with good result.

**B-1880** 15:12

**Can routine postoperative CT scan provide reliable leakage risk stratification after laparoscopic sleeve gastrectomy (LSG)?**

D. Palumbo, G. Guazzarotti, C. Martinenghi, R. Nicoletti, A. Del Maschio, F. De Cobelli; *Milan/IT (palumbo.diego@hsr.it)*

**Purpose:** Our aim was to assess the role of routine postoperative CT scan in the early identification of factors representing potential substrates of leakage after LSG.
**Methods and Materials:** We enrolled 208 LSG patients (139F, 69M; 43±12.1 years; 43.5±6.5kg/m$^2$) who underwent contrast enhanced CT scan within 72 hours from surgery between September 2015 and September 2018.Imaging post processing included measurement of the distance between the first staple firing and pylorus (StP). We also evaluated the presence of perigastric haematoma and of any twisting of the stomach remnant.
**Results:** 9 patients suffered from gastric leak (4.3%).The mean StP was significantly lower in patients who developed gastric leak (24.2±11.9 mm vs.40.3±16.4 mm; p=.005). ROC analysis identified as best threshold for StP 29.9 mm, below which patients demonstrate a higher rate of gastric leakage (AUC:.83;Se:81.8%;Sp:75.4%).9 patients(4.3%)suffered from perigastric haematoma; they were more likely to develop gastric leakage after LSG (33.3%;p=.005).16 patients developed twist of stomach remnant (7.7%); we identified two patterns of twist: type A (10 patients,4.8%), if the twist involves the proximal third of the gastric remnant; type B (6 patients,2.9%), if it involves its middle and distal part.5 out of 6 type B patients suffered from gastric leak, while no gastric leak was found in type A group, thus suggesting that type B twisting of the gastric remnant significantly increases the probability of gastric leak after LSG (p=.004). By means of a step wise multivariate analysis we identified this CT sign as the strongest risk factor for leakage after LSG (p=.005).
**Conclusion:** Routine postoperative CT scan has a promising role in the risk stratification of patients who underwent LSG.

**14:00 - 15:30** **Room M 5**

### Neuro

### SS 1911b

### Epilepsy and epileptogenic conditions

*Moderators:*
N.N.
N. Pyatigorskaya; Paris/FR

**B-1881** 14:00

**Evaluation of visuospatial memory performance in temporal lobe epilepsy: a retrospective FMRI study**

V. Schmidbauer, O. Fösleitner, K.-H. Nenning, G. Kasprian, M. Schwarz, G. Geisl, C. Baumgartner, D. Prayer, S. Bonelli; *Vienna/AT (victor.schmidbauer@meduniwien.ac.at)*

**Purpose:** Impairment of cognitive functions is often observed in temporal lobe epilepsy (TLE). Adapted versions of the Roland's Hometown Walking (RHTW) task offer a possibility to examine visuo-spatial memory performance non-invasively by functional MRI (fMRI). The aim of this study was to test whether by means of an adapted form of the RHTW paradigm laterality of visuo-spatial memory function can be identified.
**Methods and Materials:** Between 01/2013 and 09/2017, 32 patients with the diagnosis of medically intractable TLE were examined on the same 3 Tesla MRI scanner using an adapted form of the RHTW fMRI task as part of the pre-surgical work-up. EEG revealed a right-sided seizure onset in 9/32 and a left-sided origin in 23/32. Imaging data were analyzed using spm12. Significance levels were set at $p<0.05$ family-wise error (FWE) corrected and $p<0.001$ uncorrected. Special attention was payed to mesio-temporal regions.
**Results:** Left TLE patients showed significant activations in both, right mesio-temporal structures (right posterior parahippocampal gyrus: FWE-corrected $p=0.007$/z-score=5.17) and left mesio-temporal structures (left posterior parahippocampal gyrus: FWE-corrected $p=0.026$/z-score=4.86). Right TLE patients showed significant activations only in left mesio-temporal structures (left hippocampus: uncorrected $p\leq0.001$/z-score=3.37). No significant activations were observed in right mesio-temporal structures, most likely due to the underlying disease.
**Conclusion:** Using an adapted version of the RHTW fMRI task it was possible to lateralize visuo-spatial memory function in patients with left and right TLE.

**B-1882** 14:08

**Disturbed hippocampal integration into the functional language network in patients with temporal lobe epilepsy**

K.-H. Nenning, V. Schmidbauer, O. Fösleitner, D. Prayer, C. Baumgartner, E. Pataraia, G. Langs, S. Bonelli, G. Kasprian; *Vienna/AT*

**Purpose:** Temporal lobe epilepsy (TLE) in the language dominant hemisphere may impair language function. We used functional magnetic resonance imaging (fMRI) to study the language network and its hippocampal integration in patients with left and right TLE.
**Methods and Materials:** Analysis was based on fMRI data (3T MRI, verb-generation task) of a homogeneous group of 32 patients with TLE due to unilateral hippocampal-sclerosis (HS) (17 left) and 14 healthy controls. We analyzed differences in task activation and alterations in the whole-brain language connectome. We quantified the hippocampal integration into the language network with seed-based connectivity analysis, evaluated the hippocampal involvement in the altered language connectome, and more specifically, we analyzed the functional dissociation along the anterior-posterior axis of both hippocampi.
**Results:** While no significant differences in task activation were found, wide-spread alterations of the language connectome were observed in left TLE. The diseased hippocampus, and in left TLE also the right hippocampus, showed significantly impaired connectivity. While the right hippocampus showed primarily impaired connectivity to regions associated with the task-negative default mode network, the left hippocampus showed disturbed connectivity to frontal task-positive language regions. Additionally, analysis of the functional dissociation revealed a reduced connectivity for more anterior parts of the diseased hippocampus, particularly in left TLE for hippocampal connections to inferior frontal language regions.
**Conclusion:** Our analysis revealed widespread disruptions of fronto-temporal language networks and impairment of fronto-hippocampal connectivity only in patients with left TLE. This emphasizes the critical role of the integration of the left hippocampus during language tasks.

**B-1883** 14:16

**Study of correlation between hippocampal perfusion and volume in patients with temporal lobe epilepsy for epileptic lateralisation**

Y. Zhang, H. You, Y. Lv, W. Dou, B. Hou, F. Feng; *Beijing/CN (1556222529@qq.com)*

**Purpose:** To analyse the asymmetry of hippocampal perfusion and volume quantitatively for epileptic lateralization based on the application of MRI arterial spin labelling (ASL) in cerebral blood flow (CBF) and MR automated volumetry and to assess the relationship between perfusion and volume atrophy.
**Methods and Materials:** Fifty-two patients (28 (53.8%) women; mean age 27.9±9.64 years) with temple lobe epilepsy were recruited retrospectively. Regions of interests (ROIs) were provided in hippocampal head, body and tail for average hippocampal CBF ratio measurements (CBF ratio = CBF (non-epileptogenic hippocampi)/CBF (epileptogenic hippocampi)). The hippocampal volume was automatically segmented and measured using AccuBrain®. The radios for hippocampal volume were also calculated (volume ratio = volume (non-epileptogenic hippocampi)/volume (epileptogenic hippocampi)). The logistic regression model examined the utility of perfusion and volume asymmetry in lateralizing seizure onset determined by video-EEG. The Spearman correlation was used to evaluate the relationship between perfusion and volume asymmetry.
**Results:** 45 (86.5%) and 44 (84.6%) patients showed average hippocampal CBF ratios and hippocampal volume ratios were bigger than 1, respectively. Logistic regression analysis showed that the hippocampal perfusion and volume asymmetry was the strong predictor of epileptic laterality (hippocampal perfusion asymmetry $\beta=3.455$, $P<0.001$; hippocampal volume asymmetry $\beta=3.620$, $P<0.001$). There was significant correlation between hippocampal perfusion and volume asymmetry (rs=0.33, $P=0.017$).
**Conclusion:** The hippocampal perfusion and volume asymmetry were highly correlated and both were significant predictors of epileptic lateralization in TLE patients.

**B-1884** 14:24

**DTI-MRI findings in synthetic cannabinoid users**

F.D. Gokharman, S. Aydin, S.C. Paltun, E. Fatihoglu, S. Yalcin Sahiner, P.N. Kosar, H.K. Karli Oguz; *Ankara/TR (sonaydin89@hotmail.com)*

**Purpose:** Synthetic cannabinoids (SC) are major agonists of cannabinoid receptors. There are so many different SC types based on their chemical structure, some widely known subtypes Spice, Bonzai, K2, Aroma or Kronic. These SC subtypes have become increasingly popular especially among young adults and adolescents in Europe. In the literature there is some conventional MRI findings are defined for SC abusers, such as restricted diffusion and increased T2/FLAIR signal in the corpus callosum and cerebellar peduncles. In this study, we aimed to analyse micostructural alterations in the white matter of the brains in SC users as demonstrated by diffusion tensor imaging (DTI).
**Methods and Materials:** Approval of ethical committee was obtained. Diffusion imaging data were acquired in 41 non-collinear directions to create diffusion tensor images of 22 patients (F/M: 0/22, Age range: 18-41 years, mean age: 22.7±4.8 years) who have used SCs for at least six months and more than one times in a day. We have compared the FA values with a sex and age matched, healthy control group.
**Results:** We have detected no abnormal conventional MRI findings. Patients had been using SCs for a mean of 13 months (min-max: 5-37 months), a mean of 4 times in a day (min-max:1-12). We showed that FA values acquired from left temporal lobe (inferior longitudinal fasciculus) (216,2±58,9vs263±27,4; p=0,002), and right hippocampus (224,5±61,5 vs 255±24,3; p=0,040) are lower than control group.
**Conclusion:** DTI can show micro structural white matter changes occurring in SC users, before conventional MRI sequences.

**B-1885** 14:32

**Validating the efficacy of multiple high-resolution MRI sequences with 18 FDG-PET co-registration in detecting epileptogenic lesion**

T. Krishna Moorthy[1], K. Rahmat[2], L.K.S. Lim[1], N. Ramli[1]; [1]*Petaling Jaya/MY*, [2]*Kuala Lumpur/MY (thulakm@gmail.com)*

**Purpose:** To identify the more superior magnetic resonance imaging (MRI) sequences to co-register with 18-fluorodeoxyglucose-positron emission tomography (PET) scan in order to obtain the best possible images to detect epileptogenic lesion and to guide a second look interpretation of the MRI studies.
**Methods and Materials:** Twenty-eight patients with non-lesional intractable epilepsy were included prospectively. Preliminary assessment was carried out following the standard protocol of our epilepsy unit. For each subject, the attenuation corrected PET images and four MRI sequences (FSPGR, FSPGR BRAVO, T2 FLAIR and FLAIR CUBE) were each co-registered in turn, using Statistical Parametric Mapping (SPM) 12 software implemented in MATLAB R2015b. Two neuro-radiologists were assigned to interpret, compare and detect areas of hypometabolism in the fused images. Finally, a second look MRI interpretation was done to determine the presence of MRI morphometric abnormality in the detected hypometabolic region.
**Results:** Comparison between two co-registered high resolution T1WI sequences using Wilcoxon signed-ranks test, which indicated that the FSPGR BRAVO-PET images was rated more favourable than the FSPGR-PET z=-4.5, p<0.001. Similar test was done for T2WI sequences, where FLAIR CUBE-PET images was rated more favorable than T2FLAIR-PET, z=-5.0, p<0.001. Second look guided MRI showed 53.6% change in diagnosis from non-lesional to possible-lesional study.
**Conclusion:** FSPGR BRAVO(T1WI) and FLAIR CUBE(T2WI) are the more superior T1WI and T2WI MRI sequences respectively that can be utilized in PET co-registration process in order to detect epileptogenic lesion. PET-MRI co-registration is a useful imaging variant in guiding second look MRI to detect morphometric abnormalities.

**B-1886** 14:40
**Evaluation of MRI findings in Hippocampal sclerosis with special reference to T2 relaxometry**
V. Inampudi[1], S. Nimmalapudi[2], A. Prakash[3]; [1]*Vijayawada/IN*, [2]*Kolar/IN*, [3]*Bengalore/IN (vineel.inampudi@gmail.com)*

**Purpose:** 1. To evaluate MR detectable changes of hippocampal sclerosis in patients presenting with seizure. 2. To determine the T2 relaxation time in bilateral hippocampus in control and study population.
**Methods and Materials:** We conducted a prospective analysis of 120 cases presenting with hippocampal sclerosis referred to the Department of Radiodiagnosis during November 2014 to May 2018. A Control group of 120 healthy subjects are evaluated for normal hippocampal T2 relaxation time. Patients were evaluated with Siemens Magnatom Avanto 1.5T whole body MR system with epilepsy protocol.
**Results:** A total of 120 patients with hippocampal sclerosis are included in our study, which included 64 male patients and 56 female patients. Second decade individuals are most commonly effected. Hippocampal atrophy was the most common change associated with mesial temporal sclerosis seen in 86% cases followed by FLAIR hyperintensity in 80% of cases. 100 cases had unilateral hippocampus involvement and 20 patient had bilateral hippocampus involvement. The mean T2 relaxation time in normal hippocampi was 104.15ms and a value more than three standard deviation of 110ms was set as normal cut off value. In right diseased hippocampus T2 relaxation value was 120.57 ms and left hippocampus was 119.4 ms.
**Conclusion:** MRI with specific epilepsy protocol is the investigation of choice for Mesial Temporal Sclerosis. T2 relaxometry allows quantification of hippocampal signal intensity allowing detection of even subtle changes. In bilateral disease, T2 relaxometry can indicate the side of severity which helps to determine the side to be operated.

**B-1887** 14:48
**Metabolic changes after severe TBI: $^1$H MRS study**
P. Menshchikov, A. Ivantsova, A. Manzhurtsev, M. Ublinskiy, I. Melnikov, T. Akhadov, N. Semenova; *Moscow/RU (peeterem@gmail.com)*

**Purpose:** Previous $^1$H MRS studies reported significant decrease of major neuronal marker (N-acetyl aspartate (NAA)) concentration. The possible reasons for NAA reduction are not still completely known. Disruption of the NAA synthesis from aspartate Asp may result in its decrease. Thus, the main idea of this study is the investigation of dynamic changes in NAA, Asp and glutamate Glu concentrations using $^1$H MRS.
**Methods and Materials:** We studied 2 patients groups: eight children (mean age - 14±2 years) with acute sTBI (23±4 hours after trauma) and seven patients with chronic sTBI (3 months after trauma). Control group consisted of 11 healthy children (mean age - 15±1 years) without history of any TBI. $^{Asp}$MEGA PRESS (*Menshchikov et al. 2018*) was acquired from voxels (25×25×30 mm) located in the frontal lobe (predominantly grey matter).
**Results:** NAA and Asp concentrations are reduced in both the patient groups (acute sTBI - on 65% and 61%, chronic sTBI - on 65% and 61%). Glu is significantly reduced only in acute sTBI; however, a significant decrease in the Asp/Glu ratio was found in patients with chronic sTBI.
**Conclusion:** Since stoichiometric Asp-Glu ratio is maintained by the exchange of glutamate with mitochondrial aspartate, significant reduction of [Asp]/[Glu] may indicate a disruption in the normal functioning of the malate-aspartate shuttle in chronic sTBI. Reduced Glu and Asp concentrations in acute sTBI are associated with excitotoxicity. This research was supported by RFBR 17-04-01149.

**B-1888** 14:56
**Cortical atrophy and cerebral hypoperfusion in anti-N-methyl-D-aspartate receptor encephalitis: relationship to cognitive impairment and disease severity**
Y. Li, Y. Xiang, C. Zeng, J. Wang, Y. Han, Q. Luo, Y. Zheng; *Chongqing/CN (lymzhang70@aliyun.com)*

**Purpose:** To investigate cortical morphology and cerebral perfusion changes in anti-N-methyl-D-aspartate receptor (NMDAR) encephalitis and analyze their correlations with cognitive impairment and disease severity.
**Methods and Materials:** For this prospective study between October 2012 and July 2017, 38 patients with anti-NMDAR encephalitis and 26 age- and sex-matched healthy controls underwent 3-tesla MRI with FreeSurfer image analysis to explore the cortical morphology changes and pseudo-continuous arterial spin labelling (pc-ASL) sequence to quantify cortical perfusion alterations. Meanwhile, comprehensive neuropsychological tests in participants were performed, and correlation, mediation and receiver operator characteristic (ROC) analyses were conducted to assess their relationships with cognition and disease severity (the modified Rankin scale, mRS).
**Results:** Reduced cortical volumes, thickness and perfusion ($P< .01$) were clearly indicated in patients. The bilateral hippocampus and right thalamus were more different in the subcortical areas. Correlation analysis demonstrated the significant relationship between cortical atrophy and perfusion in the left fusiform cortex (r=0.434, $P= .044$). Regional cortical atrophy and hypoperfusion were associated with cognitive deficits and clinical outcome ($P< .05$). Mediation analysis found the total cortical volume mediated the effect of epilepsy on disease severity (mRS) and in turn led to worse cognitive abilities (MoCA). ROC analysis revealed regional cortical atrophy, especially in the hippocampus, and decreased cortical perfusion discriminated significantly between the two groups ($P< .05$).
**Conclusion:** Cortical atrophy and cerebral hypoperfusion in anti-NMDAR encephalitis were discovered in our study and were closely related to cognitive deficits and disease severity. Moreover, they may have opposite predictive effects on clinical outcomes.

**B-1889** 15:04
**Predictive value of two-tensor unscented Kalman filter fractography in the reconstruction of the arcuate fasciculus in patients with gliomas involving eloquent language areas**
J. Yan, J. Cheng, J. Geng; *Zhengzhou/CN (ayyj1986124@126.com)*

**Purpose:** To preliminarily investigate the post-operative changes of arcuate fasciculus (AF) in glioma patients detected by two-tensor unscented Kalman filter tractography (UKFT) from the perspective of the usefulness as a reference for post-operative recovery of language functions.
**Methods and Materials:** Nine right-handed patients with gliomas involving eloquent language areas successfully post-operative follow-up were enrolled, and all underwent MRI protocols including 3D-T1, T2-FLAIR and DTI. UKFT was applied to reconstruct the long, anterior and posterior segment of bilateral AF. The relative ratio of AF (RRAF) was calculated according to the formula: RRAF = (volume of left $AF_{Postop}$/volume of right $AF_{Postop}$)/(volume of left $AF_{Preop}$/volume of right $AF_{Preop}$). Language assessment was conducted using the aphasia battery of Chinese. The changes in language functions were analysed according to the formula: subtraction score = (score in language $function_{Postop}$) - (score in language $function_{Preop}$).
**Results:** Post-operative RRAF of the long segment of the left AF was positively correlated with the subtraction score in AQ (r=0.777, P=0.014), comprehension (r=0.711, P=0.032) and repetition ($r_s$=0.740, P=0.023); Post-operative RRAF of the posterior segment of the left AF was positively correlated with the subtraction score in comprehension ($r_s$=0.850, P=0.007) and repetition ($r_s$=0.747, P=0.033).
**Conclusion:** UKFT can preliminarily show that increasing volume of the long segment of the left AF may be a predictor of a well restoration of post-operative language function. The restoration of post-operative long and posterior segment of the left AF may be helpful for improving language comprehending and repeating ability of patients.

**B-1890** 15:12
**Feasibility of evaluating the genetic mutation status of grade II and III gliomas by diffusion-weighted imaging**
Y. Zhang, S. Liu, H. You, Y. Wang, B. Hou, Y. Lv, F. Feng; *Beijing/CN (1556222529@qq.com)*

**Purpose:** To explore the feasibility and value of distinguishing and predicting genetic status (e.g. IDH1, MGMT, and TERT mutation status) in WHO grade II and III gliomas by diffusion-weighted MRI.
**Methods and Materials:** Sixty-eight patients with pathologically confirmed WHO grade II and III gliomas, IDH1 and MGMT, TERT mutation status (MUT/WT) were included retrospectively in this study. In all patients, normalised apparent diffusion coefficient (ADC) was estimated within T2 hyperintense lesions. Mann-Whitney U test was used to evaluate differences between genetic mutation status of grade II and III gliomas. Receiver operating characteristic (ROC) analysis was performed to assess normalised ADC in the diagnostic performance. A logistic regression model examined the utility of threshold ADC in predicting genetic mutation status.
**Results:** Normalised ADC showed significant differences between IDH1 mutation status in grade II and III gliomas (*P=0.0002*). There were no statistical differences presented in MGMT and TERT mutation status (MGMT: *P=0.03545*, TERT: *P=0.0632*). In ROC analysis, the AUC of normalised ADC for IDH1 mutation status was 0.7584 (95%CI: 0.6361-0.8806). The sensitivity and specificity were 70.37% and 85.37%, respectively, when the cutoff value of normalised ADC was set as 1.59. Logistic regression analysis showed that normalised ADC could be identified as the strong independent parameter in the differentiation of IDH1 mutation status (β= 2.629, *P*<0.001) in grade II and III gliomas.
**Conclusion:** DWI could be the potential tool for differentiating different IDH1 mutation statuses. MGMT and TERT mutation status of II and III gliomas have little influence on MR diffusion parameter.

**B-1891** 15:20
**The dorsolateral prefrontal cortex mediates the recruitment of auditory cortex in cross-modal plasticity and higher order cognitive functions in sensorineural hearing loss**
Y. Luan, G.-J. Teng; *Nanjing/CN (luan0610@hotmail.com)*

**Purpose:** The recruitment of auditory cortex in the non-auditory sensory and higher order cognitive processing was demonstrated following hearing deprivation. The dorsolateral prefrontal cortex (dlPFC), with dense anatomical connections with auditory pathway, is involved in the multi-sensory integration, auditory regulation and various cognitive functions. We aimed to verify the mediation role of the dlPFC in the cross-modal reorganization and cognitive participation of auditory cortex in the long-term sensorineural hearing loss (SNHL) by combing the functional and structural measurements.
**Methods and Materials:** 35 long-term bilateral SNHL patients and 35 well-matched controls were recruited for assessment of structural imaging, resting-state functional magnetic resonance imaging, diffusion tensor imaging and neuropsychological test.
**Results:** No difference of grey matter volume in dlPFC was found. The functional connectivity (FC) between dlPFC and auditory cortex, cuneus, fusiform, lingual gyrus and calcarine sulcus was increased in the SNHL patients. The FC in the auditory cortex was associated with the symbol digit modality test (SDMT) scores, which reflect the attention, processing speed and visual memory. Hearing-related FC with dlPFC was found particularly in visual areas. Tract-based spatial statistics (TBSS) analysis revealed decreased FA values mainly in inferior fronto-occipital fasciculus (IFOF), which showed negative correlations with dlPFC FC in auditory cortex in SNHL.
**Conclusion:** Higher functional coupling between dlPFC with auditory and visual areas, accompanied by decreased FA along the fasciculus connecting frontal cortex with temporal and occipital areas, might mediate the cross-modal plasticity via a top-down regulation and facilitate the involvement of auditory cortex into higher order cognitive processing following SNHL.

**14:00 - 15:30 Tech Gate Auditorium**

### Genitourinary

### SS 1907

### Imaging of the uterus

*Moderators:*
N.N.
K. Skrobisz; Gdansk/PL

**B-1892** 14:00
**Why guidelines are important: inter observer variability in assessing MRI signs of endometriosis between reader following ESUR guidelines vs. reader using prior domain knowledge**
S. Rajan, R. Rajan, M. Murugavel, M. Barnwal, S. Gupta, V. Venugopal, V. Mahajan; *New Delhi/IN (drsriramrajan@gmail.com)*

**Purpose:** To assess the interobserver variability in identifying the MRI signs of endometriosis as described by European society of urogenital radiology.
**Methods and Materials:** This retrospective study included 77 randomly selected cases of endometriosis diagnosed on MRI. The cases were pulled from PACS, anonymized and assigned to two radiologists with 16 years and 11 years of experience in body imaging. The radiologists identified the presence or absence of following signs in each case: Retroflexion of uterus, torus uterinus involvement, Uterosacral ligament thickening, tethering of rectum, ovaries adherent to uterus, posterior position of ovaries, cluster Of haemorrhagic cysts in ovaries, thickened round ligament, uterus and ovaries posterior to inter-ischial line, T2 Shading, restricted diffusion, hematosalpinx, vaginal vault pulled-up, bladder involvement and superficial peritoneal implants.Radiologist 1 followed the definitions of the ESUR guidelines whereas Radiologist 2 assigned the labels based on prior domain knowledge. Interobserver variability was assessed (Fleiss bounds) via the Krippendorff's alpha coefficient corrected for chance.
**Results:** The interobserver variability ranged from 'fair to good' (Fleiss bounds, 0.41-0.75) in 9 labels (retroflexion, ovaries adherent to uterus, posterior position of ovaries, haemorrhagic cluster cyst in ovaries, T2-shading, hematosalpinx, pulling up of vaginal vault, bladder involvement, superficial peritoneal implants). Five labels (torus uterinus involvement, uterosacral ligament thickening, tethering of rectum, thickened round ligament, uterus and ovaries posterior to inter-ischial line, restricted diffusion) were deemed 'poor' (<0.40)
**Conclusion:** We identified the MRI signs of endometriosis having high inter-observer variability. There is scope for improvement in the agreement if the guidelines are followed universally.

**B-1893** 14:08
**Multi-parametric magnetic resonance relaxometry as an assessment of dysmenorrhea severity in patients with adenomyosis**
C. Lin, Y. He, Y. Qi, X. Wang, H. Zhou, H. Xue, Z. Jin; *Beijing/CN (linchengyu@pumch.cn)*

**Purpose:** To determine whether dysmenorrhea described as visual analogue scale (VAS) in patients with adenomyosis can be estimated by magnetic resonance (MR) relaxometry.
**Methods and Materials:** This prospective study was approved by institutional review board and ethical committee. Written informed consents were obtained. From Nov 2017 to June 2018, 60 patients suspected of adenomyosis were continuously enrolled. Pelvic MR examinations including T1, T2 and T2* relaxometry were performed on 3T Ingenia CX (Philips Healthcare, the Netherlands) during peri-ovulatory period. No visible adenomyosis lesions were found in 22 patients, and 38 patients were finally enrolled. VAS of dysmenorrhea were collected. T1, T2, and T2* relaxation times of lesions were measured blindly by two radiologists via Intellispace Portal (version 10.1.0.64190, Philips Healthcare, the Netherlands) on slices showing maximum lesion area. Intraclass correlation coefficient (ICC) was used to evaluate interobserver reproducibility. Spearman rank correlation coefficients were calculated to demonstrate the relationship between relaxation times and VAS, and a p value <0.05 was considered statistically significant.
**Results:** ICC ranged from 0.857 to 0.988. A moderate, negative correlation was found between T2* relaxation time of lesions and VAS (r=-0.5444, p=0.0006). Weak correlations were found regarding T1 and T2 relaxation times but they are not statistically significant (r=-0.8563, p=0.6195, and r=-0.1129, p=0.4999, respectively).
**Conclusion:** T2* relaxation time of lesions in peri-ovulatory period can quantitatively assess dysmenorrhea severity in patients with adenomyosis.

**B-1894** 14:16
**Diagnostic algorithm to differentiate benign atypical leiomyomas from malignant uterine sarcomas using MR imaging**
C. Abdel Wahab[1], A.-S. Jannot[1], P.A. Bonaffini[2], M.-A. Lefrere Belda[1], A.-S. Bats[1], I. Thomassin-Naggara[1], A. Bellucci[3], C. Reinhold[4], L. Fournier[1]; [1]*Paris/FR*, [2]*Monza, QC/CA*, [3]*Issy Les Moulineaux/FR*, [4]*McGill, QC/CA (aw.cendos@gmail.com)*

**Purpose:** To build a diagnostic algorithm to differentiate malignant uterine sarcomas from benign leiomyomas with atypical presentation on MRI.
**Methods and Materials:** An IRB-approved case-control study included 156 women (51 sarcomas and 105 atypical leiomyomas) with an atypical uterine mass on MRI underwent before surgery or with MRI follow-up > 1 year. A diagnostic algorithm was developed for the prediction of malignancy. Clinical and MRI data were collected and compared with pathological findings. Two independent populations (N=43 and 59) were used for validation, one with a reader with no previous knowledge of the algorithm to evaluate its generalizability.
**Results:** Predictive imaging criteria for malignancy included enlarged lymph node or peritoneal implants (OR = 21), high signal on DWI > the endometrium (OR = ∞), ADC ≤0.905×10−3m $mm^2/s$ (OR = 178). Conversely, the presence of a portion, even partial, with low T2 signal had a VPN of 100% (p<0.0001). The accuracy of the algorithm for differentiating benign atypical leiomyomas from sarcomas was 96.8% in the training set, and 95% and 95% in the two validation populations. The algorithm, however, should not be applied to extensively haemorrhagic masses.
**Conclusion:** Beyond the previously known clinical and morphologic criteria, adding a DWI sequence with ADC on MRI better differentiates malignant sarcomas from atypical leiomyomas, to guide optimal therapeutic management. We developed an interpretation model usable in routine practice for myometrial tumours on MRI including T2 and T1 signal, DWIsignal and ADC value.

**B-1895** 14:24

**Differentiation of uterine sarcomas from benign atypical leiomyomas: diagnostic value of quantitative parameters based on diffusion-weighted imaging**

E. Kim, S. Rha, M.H. Choi, M. Choi, J.-I. Choi; *Seoul/KR (doorihyun6@gmail.com)*

**Purpose:** To investigate the diagnostic value of quantitative parameters based on diffusion-weighted imaging (DWI) for differentiation between uterine sarcoma (US) and benign atypical leiomyoma (ALM).
**Methods and Materials:** This retrospective study include 80 women (ALM=56, US=24) imaged with pelvic MRI with DWI prior to surgery. Two readers independently evaluated each lesion for qualitative features as well as quantitative parameters including the mean $ADC_{mass}$, the ADC ratio [$ADC_{mass}/ADC_{muscle}$], the relative contrast ratio (RCR) [$SI_{mass}$ on $b_{1000}/SI_{muscle}$ on $b_{1000}$], and the contrast-to-noise ratio (CNR) [($SI_{mass}$ on $b_{1000}$-$SI_{muscle}$ on $b_{1000}$)/$SD_{muscle}$ on $b_{1000}$]. The diagnostic performance of the calculated parameters in discriminating US and ALM was compared using receiver operating characteristic (ROC) analysis.
**Results:** The area under the ROC curve (AUC) for the mean $ADC_{mass}$ was most superior (0.818 [95% confidence interval (CI) (0.716-0.896)] with sensitivity (SN) and specificity (SP) of 83% and 77%), followed by the RCR (0.811 [95% CI (0.708-0.890)] with SN and SP of 79% and 78%), the CNR (0.742 [95% CI (0.632-0.833)] with SN and SP of 75% and 75%), and the ADC ratio (0.729 [95% (CI) (0.618-0.822)] with SN and SP of 50% and 91%). All four parameters showed statistically significance for discrimination between US and ALM (p≤0.006).
**Conclusion:** The clinical application of quantitative parameters based on DWI and ADC map is helpful for distinguishing US from ALM preoperatively.

**B-1896** 14:32

**Correlation between zonal oblique multislice intravoxel incoherent motion and dynamic contrast enhanced magnetic resonance imaging parameters in endometrial cancer**

C. Lin, Y. He, Y. Qi, X. Wang, H. Zhou, H. Xue, Z. Jin; *Beijing/CN (linchengyu@pumch.cn)*

**Purpose:** To investigate the correlation between parameters of Zonal Oblique Multislice (ZOOM) Intravoxel Incoherent Motion (IVIM) and Dynamic Contrast Enhanced Magnetic Resonance Imaging (DCE-MRI) in patients with endometrial cancer.
**Methods and Materials:** Institutional review board approval and informed consents were obtained. Between December 2017 and September 2018, 36 patients pathologically confirmed of endometrial cancer were included in this prospective study. Pelvic examinations were performed in a 3.0 T MR scanner (Ingenia CX, Philips Healthcare, the Netherlands), including ZOOM IVIM with 8 b values and DCE-MRI with 3 different flip angles (5° and 15° initially; and 8°sequentially afterwards). IVIM parameters (D, diffusion coefficient; Dstar, pseudo-diffusion coefficient; and f, perfusion fraction) and DCE-MRI parameters (time to peak; wash in rate; wash out rate; Ktrans, transfer constant between blood plasma and extravascular extracellular space; and Kep, transfer rate between extravascular extracellular space and blood plasma) were blindly measured by two radiologists via Intellispace Portal (version 10.1.0.64190, Philips Healthcare, the Netherlands). Spearman correlation was performed and a p value <0.05 was considered statistically significant.
**Results:** Interobserver measurement reproducibility was good to excellent. A moderate correlation was found between f and wash out rate (r=0.500, p=0.0019). Mild correlations were found between D and Ktrans (r=0.329, p=0.0498), D and Kep (r=0.411, p=0.025), f and time to peak (r=-0.358, p=0.0319), f*Dstar and wash in rate (r=0.345, p=0.0423), and f*Dstar and wash out rate (r=0.377, p=0.0255).
**Conclusion:** ZOOM IVIM perfusion-related parameters demonstrated moderate-to-mild correlation with DCE-MRI quantitative parameters in endometrial cancer.

**B-1897** 14:40

**Multi-parametric MR imaging for prediction of response to gonadotropin releasing hormone analogue in patients with adenomyosis: a preliminary study**

C. Lin, Y. He, Y. Qi, X. Wang, H. Zhou, H. Xue, Z. JIN; *Beijing/CN (linchengyu@pumch.cn)*

**Purpose:** To evaluate the feasibility of multi-parametric MR imaging in predicting the therapeutic response to gonadotropin releasing hormone analogue (GnRHa) in patients with adenomyosis.
**Methods and Materials:** 12 patients suspected of adenomyosis were continuously involved in multi-parametric pelvic MR examinations between October 2017 and May 2018. Intravoxel Incoherent Motion (IVIM) with 8 b values and Amide Proton Transfer (APT) imaging were performed on 3T Ingenia CX (Philips Healthcare, the Netherlands) during peri-ovulatory period. Visual analogue scale (VAS) of dysmenorrhea and menstrual blood volume (MBV), were collected before GnRHa injection and 3 months after. APT values and IVIM parameters (D, diffusion coefficient; Dstar, pseudo-diffusion coefficient; f, perfusion fraction) of adenomyosis lesions were blindly measured by two radiologists and compared between patients with different therapeutic responses. Receiver operating characteristic (ROC) analysis was performed.
**Results:** Among 12 patients, 6 patients achieved complete response (CR, VAS=0 and normal MBV), while 6 patients with partial response (PR, otherwise). APT values in lesions were higher in patients with PR than those with CR (2.387% vs. 1.668%, p=0.01300), and Dstar values were found lower (22.69 $mm^2s^{-1}$ vs. 84.47 $mm^2s^{-1}$, p=0.0087). AUC of APT and Dstar for differentiating PR from CR was 0.9167 (p=0.0163) and 0.9444 (p=0.0104), respectively. The feasible threshold values was estimated as 2.035% and 52.92 $mm^2s^{-1}$, with sensitivity of 83.33% and specificity of 100%, respectively.
**Conclusion:** Parameters acquired from multi-parametric MR imaging, especially APT and Dstar values showed potentials in predicting GnRHa therapeutic response in patients with adenomyosis.

**B-1898** 14:48

**Conservative treatment in patients with cervical cancer on FIGO stage IB1-IIA1 >2cm: the role of MRI in a pilot study: preliminary results**

B. Gui, L. Russo, F. Cambi, F. Petta, S. Persiani, R. De Vincenzo, R. Manfredi; *Rome/IT (lucarusso.md@gmail.com)*

**Purpose:** To evaluate the role of Magnetic Resonance (MRI) in the detection of staging and evaluation of response to “fertility-sparing” treatment in patients with early stage cervical cancer (FIGO stage IB1-IIA1 >2cm).
**Methods and Materials:** In this prospective study, 11 patients with early-stage cervical cancer (FIGO stage IB1-IIA1 >2cm) and negative pelvic lymphadenectomy, who want to preserve their fertility, have been enrolled. Patients performed colposcopy, MRI, transvaginal sonography and PET-CT at staging moment and during the follow-up. All patients enrolled underwent neoadjuvant chemotherapy (nCT), with platinum and taxanes, and instrumental evaluation. In case of clinical complete response to treatment (CR) to nCT, patients underwent conization. Follow-up MRI exam has been performed at 6 and 18 months, including conventional and diffusion-weighted sequences.
**Results:** Patients’ mean age was 30 years old (range 23-36 years old). MRI correctly identified tumor FIGO stage and classified treatment response in all 11 patients. 9 out of 11 patients had CR to nCT, 1 patient had no response to nCT, 1 patient developed disease progression after nCT. Between the 9 patients with CR to nCT, 1 patient decide to abandon the protocol to undergo radical hysterectomy, 7 patients completed study protocol without disease recurrence (1 of them completed a pregnancy) and 1 patient developed liver metastasis recurrence 6 months after conization, but she is still alive at 57 months of follow-up.
**Conclusion:** In patients with early-stage cervical cancer who undergo fertility-sparing treatment, MRI exam is very helpful to assess response to nCT and during follow-up after conization.

**B-1899** 14:56

**Quantitative MR texture analysis to distinguish leiomyosarcoma from benign leiomyoma with high signal intensity on T2-weighted imaging**

T. Yagi, A. Tasaki, T. Hirata, M. Yamazaki, N. Yoshimura, H. Aoyama; *Niigata/JP*

**Purpose:** To distinguish leiomyosarcoma (LMS) from benign leiomyoma (LM) with high signal intensity (SI) on T2-weighted imaging (T2WI) using quantitative MR texture analysis.
**Methods and Materials:** We retrospectively studied 39 patients with uterine smooth muscle tumours with high SI on T2WI, which were surgically resected and pathologically diagnosed as LM or LMS, between October 2009 and November 2012. From pathological analysis, 33 were diagnosed with LM, and six with LMS. Each tumour was manually segmented and assessed on axial or sagittal MR images on T2WI and on apparent diffusion coefficient (ADC) map using ImageJ (a software program for quantitative analysis). Quantitative MR texture parameters analysed in each lesion included T2WI skewness, T2WI kurtosis, T2WI entropy, T2WI uniformity, ADC mean, ADC variance, ADC skewness, ADC kurtosis, ADC entropy, ADC uniformity and percentile ADC values. The differences between LMs and LMSs were statistically evaluated by Mann-Whitney U test, logistic regression analysis and receiver operating characteristic curves.
**Results:** In the univariate analysis, significant differences between LMs and LMSs were observed in ADC variance, ADC entropy and ADC uniformity (P < 0.05, respectively). Compared with the LMs, the LMSs exhibited larger ADC variance and ADC entropy, but smaller ADC uniformity on the histogram. The multivariate analysis revealed that the independent differentiator was ADC entropy (P < 0.05) with moderate accuracy (area under the curve, 0.838). The best cutoff value for ADC entropy was 9.901 (sensitivity: 64%, specificity: 100%).
**Conclusion:** ADC entropy can help accurately distinguish LMSs from LMs with high SI on T2WI.

Sunday

**B-1900** 15:04
**Imaging of potential donors for uterus transplantation, with emphasis on the uterine arteries**
J. Båth, P. Dahm-Kähler, N. Kvarnström, A. Thilander Klang, M. Brännström, H. Leonhardt; *Gothenburg/SE (john.bath@vgregion.se)*

**Purpose:** To explore the optimal imaging evaluation algorithm regarding potential donors for uterus transplantation and assessing the uterine arteries.
**Methods and Materials:** In this prospective study, 10 women aged 45-62 willing to donate their uteri underwent MR imaging of the pelvis, including contrast-enhanced MR angiography (MRA), contrast-enhanced CT angiography (CTA), and digital subtraction angiography (DSA). Details of the uterine arteries were compared between the three different angiographic modalities.
**Results:** MRA was not able to adequately identify 7 out of 20 arteries. One of these arteries was questionable on CT and judged to be generally thin, but DSA finally visualized it as proximally divided in two small-calibre branches and the woman was accepted for surgery. Another MRA absent artery was identified as adequate on both CTA and DSA. There was no significant difference of average arterial lumen diameter measured by MRA, CTA and DSA. Three women were excluded from surgery because of significantly stenosed uterine arteries bilaterally. There were no method-related complications found.
**Conclusion:** Given the capacity of MRI to describe details of uterine anatomy and pathology it is valuable to start with this modality. If fully visualized from the origin into the uterus wall by MRA with a diameter of at least 1.5 mm, there is no need to move on with other angiographic methods. If not, CTA should be performed for better details of the uterine arteries. In a few cases, there may be a need for further investigation by the more invasive modality of DSA.

**B-1901** 15:12
**Diffusion tensor imaging parameters of endometrial carcinoma can predict local tumour behavior: a feasibility study**
A. Ghosh, T. Singh, V. Singla, R. Bagga, R. Srinivasan; *Chandigarh/IN (tulikardx@yahoo.com)*

**Purpose:** We evaluated diffusion tensor parameters of endometrial tumours and their correlation with myoinvasion and tumour-grade; demonstrating the feasibility of predicting myoinvasion and tumour grade, demonstrating the feasibility of predicting them, using quantitative diffusion tensor imaging.
**Methods and Materials:** This prospective study included 26 consecutive patients with endometrial carcinoma. Patients underwent DCE-MRI and DTI with 12 diffusion sensitising directions in the sagittal plane on a 1.5 tesla scanner. Fractional Anisotropy, mean, radial and axial diffusivity and eigen values 1&2 were recorded from freehand ROIs-drawn on the endometrial tumour by two radiologists separately.
**Results:** Multivariate analysis was done which showed that the mean and minimum values of mean-diffusivity, fractional anisotropy, axial-diffusivity and eigenvalue2 correlated significantly with myoinvasion. The minimum axial diffusivity of the tumour had an AUC of 0.87(0.68 to 0.97) and 0.708 (0.50 to 0.87) in predicting superficial and deep myoinvasion. Similarly FA sum obtained an AUC of 0.81 (0.61 to 0.94) in predicting type 1 versus type 2 endometrial tumours.
**Conclusion:** Diffusion Tensor Imaging parameters can be used to characterise endometrial tumours and predict local myoinvasion, thus demonstrating the utility of physiological diffusion imaging in tumour staging and characterisation.

**B-1902** 15:20
**Shear wave elastography of the endometrium: a simple tool to improve diagnostic accuracy of endometrial carcinoma from other benign endometrial diseases**
S. Mah, A. Vijayananthan, K. Rahmat, R. Muridan, Y. Abdul Aziz, C.H. Yeong; *Kuala Lumpur/MY (stephen_mah7@hotmail.com)*

**Purpose:** The incidence of endometrial cancer (EC) is increasing globally and in Malaysia, it rose from 3300/100,000 in 2003 to 4100/100,000 in 2007. We evaluated the diagnostic value of endovaginal shear wave elastography (SWE) of the endometrium in patients with abnormal uterine bleeding (AUB) in order to reduce the incidence of unnecessary invasive endometrial biopsy.
**Methods and Materials:** 43 subjects were enrolled, where shear wave elastography ultrasound were performed. There were 24 healthy controls and 19 patients who presented with AUB, where histopathology results were available. SWE average values were elaborated as $E_{max}$ and $E_{mean}$ where, “E” represents the longitudinal elasticity of a material. These values were described in kilopascal (kPa) unit. Analysis was performed for the SWE values and endometrial thickness (ET).
**Results:** The mean age of patients were 58.9 years. Highly significant correlation between the SWE values and ET was present in AUB patients ($r_s$=0.771, p<0.001). Significant difference of ET and SWE values were demonstrated between the control and AUB patients (p<0.05). Amongst the AUB patients, there was also significant difference of SWE values between the malignant and non-malignant diseases, where p=0.01. The SWE cut-off value of ≤82.5 kPa ($E_{mean}$) and ≤103.7 kPa ($E_{max}$) resulted in 100% sensitivity, 86.8% specificity and 88.4% accuracy.
**Conclusion:** Endometrial SWE serves as a clinically applicable diagnostic tool in women with AUB. With cut-off values of $E_{mean}$ 82.5 kPa and/or $E_{max}$ 103.7 kPa, EC were excluded from other non-malignant endometrial diseases. This will aid in triaging patients for invasive biopsy procedures.

### C

### Scientific and Educational Exhibits (C)

Full EPOS™ presentations are published at **epos.myESR.org** and can be cited by a Digital Object Identifier (DOI), if selected so during poster submission.

### D

### Satellite Symposia (D)

Wednesday, February 27.... 588
Thursday, February 28 ....... 591
Friday, March 1 .................. 593
Saturday, March 2.............. 596

ECR/ESR takes no responsibility for the content of the Satellite Symposia, and the opinions expressed therein do not necessarily reflect those of the ECR/ESR

### Wednesday, February 27

**10:30 - 12:00** **Studio 2019**

### jointly organised by Siemens Healthineers and Bayer HealthCare

### SY 2a

### How to overcome barriers for breast MRI screening?

*Moderator:*
L. Umutlu; Essen/DE

**Showing MRI efficacy vs mammography**
J. Barkhausen; *Lübeck/DE*

More than 30 years ago, MRI emerged as a new technique in clinical breast imaging and over the last three decades numerous clinical studies have shown excellent results for the detection and characterization of breast lesions. However, despite this tremendous gain in knowledge, breast MRI still has its share of controversies, including appropriate indications for screening and appropriate indications for preoperative breast MRI. In this lecture the results of the most recent clinical trials comparing MRI and x-ray mammography will be presented in a comprehensive manner, and the impact of these studies on daily clinical routine will be discussed. Additionally, there are possible new indications for breast MRI, bearing in mind that breast cancer subsumes different diseases with varying histologic subtypes, clinical presentations, and treatment responses. Therefore, several studies aim to investigate the associations between MR imaging features and breast cancer subtypes as well as treatment responses. The recent efforts to characterize cancer subtypes and to support individualized tumor treatment based on quantitative imaging parameters are a unique feature of multiparametric breast MRI, and offer important advantages over mammography and ultrasound.
*Learning Objectives:*
1. To gain knowledge on the results of the most recent trials comparing MRI and mammography.
2. To discuss the impact of the results on the management of breast lesions.
3. To learn about future developments and applications.

**Improving patient experience**
L. Umutlu; *Essen/DE*

**Using abbreviated protocols**
C.K. Kuhl; *Aachen/DE*

**12:15 - 13:45** **Studio 2019**

### jointly organised by Siemens Healthineers and Bayer HealthCare

### SY 2b

### Multimodality lunch symposium: from morphological to functional breast imaging

*Moderator:*
S. Zackrisson; Malmö/SE

**3D automated breast ultrasound: accuracy and diagnostic potentials**
M.J.C.M. Rutten; *'s-Hertogenbosch/NL*

Automated 3D breast ultrasound (US) is a new technique, which scans the breast automatically and almost entirely. Data sets can be stored and are available for 3D cross-correlating review. This technique may eliminate the subjectivity of conventional 2D ultrasound, and disconnects the acquisition and assessment of US data. It facilitates the possibility of double reading, review of follow-up studies and the application of computer-aided detection. Issues to be discussed are: - Implementation of automated 3D breast ultrasound in the clinical radiological setting - Technique of reading and interpretation of 3D US data sets - Standardization of assessment and reproducibility (e.g., CAD and reading software) - Diagnostic yield, sensitivity and specificity and factors influencing these, such as limitations, artefacts and pitfalls.
*Learning Objectives:*
1. To learn how to implement 3D breast US in daily practice.
2. To become familiar with the technique and assessment software.
3. To understand the accuracy and limitations of 3D breast ultrasound.
4. To become familiar with potential future developments.

**Advanced elastography in breast cancer detection**
M. Vancu; *Craiova/RO*

The 3D reconstruction of the breast has opened up a new era in breast ultrasound. This new technique has improved the quality of ultrasound images to such an extent that sonography is now a major mode of detection for breast cancer. The ACUSON S2000 ABVS overcomes the inherent limitations of the breast ultrasound investigation: a small field of view and poor reproducibility. The advantages of the ACUSON S2000 ABVS include standardization with the possibility of second-look and follow-up studies, reproducibility and operator independence. Coronal slices of the breast allow quick identification of areas with impaired architecture. The tools perform measurements more accurately, an attribute critical for follow-up studies such as in cases of chemotherapy. Cancer tissue is stiffer than normal breast tissue. The idea of using this stiffness information for diagnosis evolved into a new diagnostic imaging method for detecting tissue elasticity. Ultrasound elastography is an excellent technique for characterizing breast lesions as benign or malignant. There are two types of elastography: strain elastography, which allows for a qualitative assessment of the lesion, and shear wave elastography, which can be measured as a velocity, with a quantitative value of the stiffness. We have used elastography on over 3000 diagnostic breast ultrasound cases over several years and it has been a “virtual biopsy”. The technique has been extremely helpful in increasing confidence in breast evaluation.

**What you need to know when using contrast media in breast imaging**
E.M. Fallenberg; *Munich/DE*

Since nearly 100 years contrast agents are used in imaging. The big leap for contrast use in breast imaging was the introduction of breast MRI in the 80s. Contrast enhanced breast MRI is the most sensitive technique for detection of breast cancer today. Digital full-field mammography introduced roughly 20 years ago also paved the way for contrast enhanced mammography. In contrast to MRI, where gadolinium based contrast agents (GBCA’s) are applied, contrast enhanced mammography uses iodine containing agents. Even if contrast agents are generally judged as safe, some specialties for both of them have to be considered to prevent side effects or damage to the patient. This talk will focus on the history of contrast enhanced breast imaging and point out advantages and disadvantages of the different types of contrast agents. Recent data will address risks and concerns regarding Gd presence of GBCA’s in the brain. To conclude you will learn which patient history information and laboratory is required to securely apply different contrast agents and how to manage of the most common side effects.
*Learning Objectives:*
1. To give an overview of the advantages and disadvantages of the different available contrast agents.
2. To enable audience to prepare patients adequately for contrast administration and handle possible side-effects.

**Clinical utility of contrast enhanced dual energy mammography**
L.J. Pina Insausti; *Pamplona/ES*

Titanium Contrast Enhanced Mammography (TiCEM) is a development of digital mammography. By using dual energy (conventional low energy mammography plus high energy mammography) after the administration of a bolus of intravenous iodinated contrast medium, a morpho-functional image of the breast is acquired. This technique joins the high spatial resolution of conventional digital mammography and the functional information based on neoangiogenesis. The unique titanium filter enables consecutive TiCEM studies without tube overheating. The indications of this technique for the clinical use include: Problem -solving technique after inconclusive mammography, preoperative assessment of breast cancer, follow-up of scars after conservative treatment, follow-up of intermediate risk patients ( borderline histological lesions , such as lobular carcinoma in situ, atypical ductal or lobular hiperplasia, as well as positive family history of breast cancer). In fact, the majority of indications are those of MRI, including the contraindications for MRI (pacemakers, claustrophobic patients···). According to our clinical experience with 200 cases, TiCEM significantly increases the accuracy and sensitivity of mammography, with no loss of specificity. Unfortunately, some false negative results can be obtained in DCIS and some false positive results in papilloma and some fibroadenomas. TiCEM is contraindicated for patients allergic to iodine, for patients with renal insufficiency and for pregnant women.

*Learning Objectives:*
1. To become familiar with Titanium Contrast Enhanced Mammography (TiCEM).
2. To learn the main indications for this technique.
3. To learn the main limitations of TiCEM.

**Non-invasive phenotyping of breast cancer with novel MR biomarkers**
P.A.T. Baltzer; *Vienna/AT*

Breast imaging aims to identify breast cancer. Suspicious lesions detected by imaging nowadays undergo image-guided biopsies to secure a final diagnosis. One aim of imaging is to provide a maximum sensitivity while maintaining the highest possible specificity. False positive imaging findings undergo unnecessary biopsies and follow-up examinations and thus cause a substantial economical and medical burden. In addition, the debate on overdiagnosis is still ongoing. MRI as a method providing a multitude of image contrasts including multiple (semi-)quantitative parameters allows an accurate characterization of tissue properties. These data can not only be used to distinguish between benign and malignant findings but further to characterise therapeutically and diagnostically relevant subtypes of breast disease. The subject can be referred to as non-invasive imaging-based phenotyping of breast disease. The talk will provide an evidence-based overview on the clinical relevance and current possibilities of imaging-based phenotyping applied to the breast. In addition, future developments including PET-MRI will be discussed.

**14:00 - 15:30 Studio 2019**

### jointly organised by Siemens Healthineers and Bayer HealthCare

### SY 2c

### What should the future of breast cancer screening with digital breast tomosynthesis look like?

*Moderator:*
I. Sechopoulos; Nijmegen/NL

**Introduction**
I. Sechopoulos; *Nijmegen/NL*

The superiority of digital breast tomosynthesis (DBT) over digital mammography (DM) for cancer detection in screening has been proven in several population-based prospective screening trials. However, most of these results have been achieved with considerably longer reading times than with DM. In some cases, higher radiation doses have also been used. Is this acceptable? What are the most important features and characteristics on our wish list for the ideal breast cancer screening program with DBT in the future? In this symposium, the latest results of clinical trials will be presented, analyzed in terms of effectiveness when considering population screening, and the remaining knowledge gaps identified. We will discuss whether we should just copy the standard approach with DM for screening with DBT or whether we should go in new directions. Finally, we will review the challenges faced in the widespread implementation of DBT for population screening.

**View of future screening programmes: conclusion from the Malmö Breast Tomosynthesis Screening trial - a concept for breast screening**
S. Zackrisson; *Malmö/SE*

According to some of the important principles of screening established by WHO in 1968, a screening test should be fast, safe, efficient and acceptable for the target population. Screening with 2D mammography fulfills many of these prerequisites, but how will digital breast tomosynthesis, DBT, fit in? The organization and workflow in mammography screening is long-standing and well-established in many countries, and changes may have a great impact on these issues. This talk will focus on the facts about digital breast tomosynthesis regarding screening performance, effectiveness and possible measures to reduce reading time. When planning the Malmö Breast Tomosynthesis Screening Trial we took the principles of screening into consideration and used 1-view DBT and reduced compression. Furthermore, artificial intelligence in combination with the radiologist opens up possibilities for further future improvements.

**Comparison of two-dimensional and three-dimensional mammography performance in breast cancer screening**
D. Pavic; *Charleston, SC/US*

Aim: The study compares breast cancer screening performance using Full Field Digital Mammography images (2D) and three-dimensional Digital Breast Tomosynthesis (DBT) images, in MLO projection only (3DMLO) and as a complete DBT exam in MLO and CC projections (3D). Introduction: Radiation dose at 3D screening can be reduced by omitting separate 2D acquisition and abandoning CC projection in DBT, if 3DMLO could demonstrate comparable diagnostic performance. Material and methods: Women age 40 and older who had screening exams using Siemens a DBT unit in the period from Aug 1, 2016 to Sep 30, 2017 were included. 21 biopsy-proven cancer patients with 24 cancers were identified. Based on age and Breast Imaging Reporting and Data System (BI-RADS) density assessment (DS) they were matched with 24 biopsy-proven benign patients and 25 patients who were negative or benign on screening. Age, race and DS were collected. Lesion location, type and size, axillary lymph nodes status and pathological diagnosis were also collected for biopsy patients. Screening exams of those 70 patients, consisting of 2D and 3D mammography images, were included in the image set. Three fellowship-trained breast radiologists and two breast imaging fellows were the readers. We will compare the diagnostic performance of 3DMLO, 3D and 2D readings, separately for each reader and for all readers, by looking at recall rate, cancer detection rate, positive predictive value and area under curve (AUC).

**CAD for breast cancer screening: first experiences with a CAD integration for screen-reading of 3D mammograms and with 3D CAD for tomo-reading**
S.H. Heywang-Köbrunner; *Munich/DE*

So far, CAD has not routinely been applicable for screen-reading in organized screening programs, mostly due to excessively false positive rates (of 1 to 1/3 hit per mammogram). Tomosynthesis, which is considered the most promising method for breast cancer screening, is associated with a 10-20-fold increase in the number of images. Insufficient data exists concerning the capability of maintaining the reader’s concentration with this work load, so the need for CAD-support increases. Also, new artificial intelligence (AI) algorithms promise improved sensitivity and specificity. We present a software solution that allows integration of an AI-CAD system with the screen reading software. Mammograms are automatically assigned a degree of suspicion (1-10), based on a large number of screening mammograms. Filtering cases with high suspicion (9 or 10), either a selective third reading may be considered, or a second reader might decide to spend more time on these cases or read them first. Due to the high sensitivity for microcalcifications, less reading time can be used for cases rated 1-4. In a different study using AI-CAD for tomosynthesis an excellent sensitivity of 90-92% combined with a very low false positive rate of 9-15% was achieved in a demanding case series of 400 proven cases. These results indicate important progress compared to prior CAD systems on 2D mammograms.
*Learning Objectives:*
1. Prior limitations of CAD.
2. Possibilities for integrating a CAD system for screen reading.
3. Progress achieved by CAD-3D reading.

**The future role of synthetic mammograms: is it more than a 2D image?**
C. Van Ongeval; *Leuven/BE*

A synthetic mammography (SM) is a two-dimensional image, reconstructed from digital breast tomosynthesis (DBT) data. Simulation studies (work of Michielsen et al.) using insertion of simulated microcalcifications and mass lesions in raw data of tomosynthesis showed an impact of reconstruction algorithm on the lesion detection fraction. The algorithm of SM impacts on the presentation of line structures and high density structures like microcalcifications, resulting in increased conspicuity of lesions in the SM. In the Oslo trial (Per Skaane et al.) SM was included into the screening protocol and in this study it was concluded that improvement of the reconstruction algorithm had an impact on the lesion detection. Further work on the reconstruction algorithm of SM can improve the presentation of suspicious lesions with careful attention to the creation of false positive lesions: how far is this CAD related enhancement allowed in clinical practice? Physical-technical evaluation of the quality of SM is ongoing and will be complicated by the complex structure of the applied processing algorithms.

**Personalised breast cancer screening and the role of breast density**
M. Broeders; *Nijmegen/NL*

Breast screening programs today generally offer a mammographic examination every two years to women in a specified age range. Ongoing research is exploring the added value of personalized risk-based screening, i.e. screening strategies tailored to a woman’s individual risk of breast cancer. A vital prerequisite for personalized risk-based screening is a comprehensive breast cancer risk prediction model. The next step is to develop screening regimens for women at varying levels of risk, which may be addressed in several ways: changing starting and stopping ages, changing screening intervals, as well as offering further imaging techniques in addition to digital mammography. Using modeling techniques, the harm-benefit ratios and cost-effectiveness can then be estimated to find the optimal screening strategies. In parallel, it is essential to understand the acceptability of personalized approaches, especially for women at low risk, and the challenges in communication and implementation. Breast density is an important factor in this research area because it can contribute to risk prediction, but will also play a role in finding the optimal screening strategy. Automated measures of breast density on digital mammography are being updated rapidly, which will facilitate getting this data for all women in the target population. However, the fact that DBT may replace digital mammography in the near future raises the following questions: how does DBT perform across breast density categories? What is the relationship between automated breast density measures on DBT and breast cancer risk? What is the role of additional imaging modalities when DBT is the screening test?

*Learning Objectives:*
1. To understand the components of a personalized breast cancer screening program.
2. To explore the role of automated breast density estimates in personalized breast cancer screening with tomosynthesis.

**16:00 - 17:00 — Studio 2019**

### jointly organised by Siemens Healthineers and Bayer HealthCare

### SY 2d

### Pretherapeutic diagnostics of tumour extent

*Moderators:*
P. Clauser; Vienna/AT
E. Wenkel; Erlangen/DE

**Pretherapeutic diagnostics of tumour extent (part 1)**
E. Wenkel; *Erlangen/DE*

In pretherapeutic diagnostics to determine the extent of spread of a breast cancer (TNM staging), the interaction of the various breast imaging modalities (comprehensive mammo diagnostics) plays a particularly important role. Various clinics are working on modern staging concepts, combining current guidelines with new imaging procedures. In this symposium, two experts will each discuss pretherapeutic TNM staging diagnostics cases from their clinical routine. The audience will be actively involved in the coordination processes and is invited to participate in the discussion.

**Pretherapeutic diagnostics of tumour extent (part 2)**
P. Clauser; *Vienna/AT*

In pretherapeutic diagnostics to determine the extent of spread of a breast cancer (TNM staging), the interaction of the various breast imaging modalities (comprehensive mammo diagnostics) plays a particularly important role. Various clinics are working on modern staging concepts, combining current guidelines with new imaging procedures. In this symposium, two experts will each discuss pretherapeutic TNM staging diagnostics cases from their clinical routine. The audience will be actively involved in the coordination processes and is invited to participate in the discussion.

**12:30 - 13:30 — Room F2**

### organised by GE Healthcare

### SY 3

### Elevating radiology through precision health

*Moderator:*
M. Goyen; Hamburg/DE

**Why managing risk in oncology patients really matters: a nephrologist's perspective on precision health**
A. de Francisco; *-/ES*

**Artificial intelligence: the new way to your best CT image**
L. Macron; *Saint Denis/FR*

**How MRI with AIR™ Technology brings comfort, speed and quality**
V. Martínez de Vega; *Madrid/ES*

**12:30 - 13:30 — Room K**

### organised by Canon Medical

### SY 4

### AI, deep learning and multi-parametric assessment for advanced imaging and diagnostics

*Moderator:*
C. Oppenheim; Paris/FR

**Roadmap for artificial intelligence in radiology: recognition, reconstruction, reasoning**
B. Van Ginneken; *Nijmegen/NL*

Artificial Intelligence, in the form of deep learning, is extremely successful in automated recognition of abnormalities, in segmentation and in quantification. I will illustrate this with some recent results. Increasingly, deep neural networks are used to generate new images. They can be used for reconstruction, for denoising extremely low dose scan, for creating novel images, and for mapping images from one modality to another, for example CT to MR. The next frontier is to go beyond mappings from input data to output data and design systems that can reason based on variable inputs.

**Deep learning reconstruction: the next step in CT image quality**
M. Prokop; *Nijmegen/NL*

I will present the first clinical results of Deep Learning Reconstruction (DLR) algorithm for CT (both 320-row CT and UHR-CT), featuring a deep learning neural network that can differentiate and remove noise from signal, creating extraordinary high quality images.

**MRI and advanced algorithms like computed MRI from research to clinical practice**
L. Saba; *Cagliari/IT*

Computed MRI finally has become more practical in a clinical setting. Clinical imaging therefore may very well profit from these advanced imaging techniques. Where in the past these tools were mostly used in research it can also provide flexibility and reduction in examination time in daily practice. It also offers the possibility to offline reconstruct conventional sequences with different contrast settings and it allows to obtain quantitative values that may be used for follow up or for tissue characterization.

**Multiparametric approach for diffuse liver disease with ultrasound**
V. Vilgrain; *Clichy/FR*

Diffuse liver disease poses a major challenge to the global healthcare system. Quantitative techniques in ultrasound have recently been developed for follow-up and for therapy monitoring. Nowadays, the measurement of shear wave propagation speed is already being used to assess liver elasticity in clinical routine. In this talk we will discuss novel techniques such as the quantitative imaging of the attenuation of the longitudinal ultrasound waves (ATI), as well as the quantitative imaging of shear wave dispersion (SWD) to assess tissue parameters related to steatosis and to the viscosity of liver tissue, and present early clinical experience.

### Thursday, February 28

**12:30 - 13:30** **Room X**

### organised by Kheiron Medical Technologies

### SY 5

### Deep learning in breast cancer screening: clinical rigour, deep workflow integration and patient collaboration

*Moderator:*
C.K. Austin; Sammamish, WA/US

**Clinical rigour and the validation process for deep learning algorithms**
H. Harvey; *London/UK*

An overview of the phases of clinical studies and trials required during the algorithmic validation process, from phase I feasibility all the way through to phase IV post-deployment and post-market surveillance.

**Understanding workflow: the key to effective integration**
N. Sharma; *Leeds/UK*

Integration with, and deep vertical knowledge of, the breast screening workflow is vital to ensure effective deployment of deep learning algorithms. This talk will provide an overview of the current UK breast screening workflow, and how deep learning is poised to provide robust and effective support.

**Patient involvement in the testing and validation of deep learning systems**
L. O'Riordan; *Ipswich/UK*

Patient and Public Initiatives are key to ensuring valuable information and learning occurs from the early stages of the clinical validation and testing process. This talk will cover the patient's experiences of the breast cancer pathway, and discuss how building patient advocacy and feedback into clinical studies is a vital part of research in the development of deep learning systems.

**12:30 - 13:30** **Room N**

### organised by Siemens Healthineers (MR)

### SY 6

### Siemens Healthineers MR lunch symposium

*Moderator:*
A. Schneck; Forchheim/DE

**BioMatrix: delivering on increased consistency and higher productivity in MRI**
A. Schneck; *Forchheim/DE*

**MAGNETOM Lumina: first clinical experiences**
M.G. Mack; *Munich/DE*

**Fast MSK with CAIPI SPACE, SMS TSE and compressed sensing**
J. Fritz; *Tübingen/DE*

**Reducing variability in MRI**
L. Tanenbaum; *New York, NY/US*

**12:30 - 13:30** **Room O**

### organised by Philips (MR)

### SY 7

### Break diagnostic boundaries with speed, comfort, and confidence

*Moderator:*
A. Radder; Best/NL

**Advance neuro diagnostics and clinical outcome with Ingenia Eliton solution**
J. Savatovsky; *Paris/FR*

**Beyond helium-free operations, boost diagnostic confidence and speed with Ingenia Ambition**
C. Juli; *Uster/CH*

**12:30 - 13:30** **Studio 2019**

### organised by Hologic

### SY 8

### Advances in breast imaging: clinical use of CEDM across Europe

**Programme not available by date of publication**

**12:30 - 13:30** **Room E1**

### organised by Bracco

### SY 9

**Programme not available by date of publication**

**12:30 - 13:30** **Room Y**

### organised by Philips (IGT MOS)

### SY 10

### Redefining the potential of mobile surgical imaging

*Moderator:*
N. Bonaros; Innsbruck/AT

**Complex cases of TAVI**
N. Bonaros; *Innsbuck/AT*

**12:30 - 13:30** **Room C&T 3**

### organised by Elsevier

### SY 32

**Programme not available by date of publication**

12:30 - 13:30 Room M 2

### jointly organised by Bayer HealthCare and Siemens Healthineers (CTEPH)

### SY 12

### Driving the chronic thromboembolic pulmonary hypertension (CTEPH) patient journey through imaging

*Moderator:*
D. Gopalan; London/UK

**Welcome and introduction**
D. Gopalan; *London/UK*

**The first step to accurate diagnosis: the evolving role of DECT**
M. Rémy-Jardin; *Lille/FR*

**Balloon pulmonary angioplasty: an emerging treatment for CTEPH**
B.C. Meyer; *Hannover/DE*

**Imaging innovations and their impact on patients**
D. Gopalan; *London/UK*

**Q&A and closing**
All speakers

12:30 - 13:30 Room M 3

### organised by Bayer HealthCare (MRI 1)

### SY 13

### Primovist© (gadoxetic acid): the liver specific contrast agent

*Moderator:*
F. Caseiro Alves; Coimbra/PT

**Benefits of Primovist® in non-cirrhotic livers**
F. Caseiro Alves; *Coimbra/PT*

**Benefits of Primovist® in cirrhotic livers**
G. Brancatelli; *Palermo/IT*

**Optimisation of the workflow in liver MRI**
L. Grazioli; *Brescia/IT*

12:30 - 13:30 Room M 5

### organised by Bayer HealthCare (MRI 2)

### SY 14

### Trust the macrocyclics

*Moderator:*
M. Essig; Winnipeg, MB/CA

**Basics for high efficacy in MRI**
H. Pietsch; *Berlin/DE*

**Modern neuroimaging protocols**
M. Essig; *Winnipeg, MB/CA*

**Essentials of comprehensive MRA**
H. Kramer; *Munich/DE*

### Friday, March 1

**12:30 - 13:30** **Room X**

### organised by Infinitt

### SY 15

### Dose management systems and beyond: challenges and experiences

*Moderators:*
M. Kolodziej; Frankfurt a. Main/DE
L. Thursar; Frankfurt a. Main/DE

**Managing radiological examinations: dose management and beyond**
E. Kotter; *Freiburg/DE*

**Implementation of the radiation protection laws in Switzerland**
R. Simmler; *Glattpark/CH*

**12:30 - 13:30** **Room N**

### organised by Philips (CT)

### SY 16

### How spectral CT & AI can improve routine clinical practice

*Moderator:*
P. Coulon; Suresnes/FR

**Benefits of spectral CT in emergency department**
C. Truwit; *Minneapolis, MN/US*

**Spectral CT for routine practice CTA and cardiac CT**
P. Ball; *Belfast/UK*

**Spectral CT and AI in oncology applications**
D. Maintz; *Cologne/DE*

**12:30 - 13:30** **Room O**

### organised by Philips (US)

### SY 17

### Breakthrough innovations in ultrasound

*Moderator:*
P.S. Sidhu; London/UK

**Exploring the ultimate vascular solution with the world's first xMATRIX XL14-3 transducer**
D. Staub; *Basle/CH*

**Early clinical experience using the XL14-3 for MSK applications**
S. Konijnenberg; *Schijndel/NL*

**Early clinical experience using the XL14-3 for small parts applications**
D.A. Clevert; *Munich/DE*

**Advancing the role of ultrasound with 3D/4D CEUS in the abdomen**
D.A. Clevert; *Munich/DE*

**12:30 - 13:30** **Studio 2019**

### organised by Samsung

### SY 18

### Samsung innovations for patient safety: CEUS and paediatric DR imaging

*Moderator:*
V. Cantisani; Rome/IT

**CEUS: from guidelines to clinical practice**
V. Cantisani; *Rome/IT*

To date CEUS EFSUMB guidelines reporting on hepatic and non-hepatic applications have been published with an additional recent statement on EFSUMB CEUS use in pediatric applications and one on liver which is ongoing provided by WFUMB. Those guidelines updated the previous ones and had the purpose to inform recommended and tailored clinical practice rather than to report on research projects. Thus, they provide a kaleidoscope of current findings, formulated by a group of experts and are primarily based on surveys of the published peer-reviewed literature. In general, CEUS is most useful where an abnormality can be displayed on B-mode ultrasound (US), and the better the quality of the B-mode imaging, the better the quality of the CEUS images, although sometimes expecially in liver metastasis detection they may reveal what was not previously observed at Baseline Ultrasound. Therefore, CEUS is frequently used as an extension of conventional US (B-mode and color Doppler). Contrast studies should always be interpreted in the context of the overall clinical picture, other imaging and laboratory tests. Overall, UCAs are mainly used as vascular agents following intravenous injection and they highlight the macro- and microvascular systems. However, they can also be instilled into body cavities, both normal and pathological. According to indications and recommendations in the present lecture daily practice cases will be presented in order to enhance the benefit of CEUS application over baseline Ultrasound or even CT and MRI. Tips and tricks to tailor the examination will be provided as well.

**Balancing the risks and benefits in radiology - contrast and x-rays and paediatric blunt abdominal trauma: safety and dose reduction**
M. Sellars; *London/UK*

Contrast - enhanced ultrasound (CEUS) is a cost effective, safe alternative to computed tomography (CT) when imaging children who have sustained blunt abdominal injuries. CT still remains the gold standard for assessment of abdominal and pelvic injuries however, depending on the mechanism of injury and the severity of solid organ injury these patients are often exposed to repeated examinations involving ionising radiation and intravenous iodinated contrast agents. The use of CEUS in the follow up of severe blunt abdominal injuries in the paediatric population has evolved in the past 10 years and more recently in our institution has also proved beneficial as a first line imaging technique in children who have sustained minor blunt abdominal injuries. Although current data has shown that ultrasound contrast agents are safe for use in the adult population, few studies have evaluated safety in children. Clinicians therefore have to be prepared for the possibility of both anaphylactoid and severe anaphylactic reactions as would be the case when administering any intravenous contrast agent.

**Clinical trial to evaluate the dose reduction in paediatric radiography**
S. Oh; *Seoul/KR*

Objectives: To prove that an upgraded image post-processing engine (IPE) in S-station, exclusively installed in Samsung Digital Diagnostic X-ray system, provides achievable dose reduction at a certain lower dose than the predicate device (old IPE) used in pediatric patients. Methods: Validation study of a noise simulation tool and clinical image evaluations on clinical images and low dose images rendered by the validated noise simulation tool was done, respectively. Results: Both quantitative and qualitative analysis results strongly suggested that simulated low dose images by a noise simulation tool showed almost the same image quality as corresponding real dose images. The dose step difference were shown to have statistical significance. Conclusions: The performance of the new IPE suggests achievable dose reduction rates up to 45% relative to the predicate IPE for pediatric populations.

**12:30 - 13:30** **Room E1**

### jointly organised by Siemens Healthineers and Bayer HealthCare

### SY 19

### Clear direction in CT: advanced efficiencies for better patient care

*Moderator:*
J.E. Wildberger; Maastricht/NL

**AMACING results on prophylactic iv hydration prior to contrast administration**
J.E. Wildberger; *Maastricht/NL*

**AMACING and pre-hydration in the ESUR prevention of PC-AKI guideline**
A.J. van der Molen; *Leiden/NL*

**12:30 - 13:30** **Room Y**

### organised by Olea Medical

### SY 20

### AI, automation, visualisation: impacting radiology

**Technological trends that are changing medical imaging and the practice of radiology**
A. Davis; *New York, NY/US*

**Opportunities for AI-enabled patient care**
P. Chang; *Irvine, CA/US*

**Quick decision for a personalised healthcare**
M. Essig; *Winnipeg, MB/CA*

**12:30 - 13:30** **Room G**

### organised by Bracco

### SY 21

**Programme not available by date of publication**

**12:30 - 13:30** **Room M 1**

### organised by Bayer HealthCare (GD)

### SY 22

### The good MR exam: overcoming technical and physiological hurdles

*Moderator:*
H. Kramer; Munich/DE

**The daily challenge: how to fight artefacts**
H.H. Quick; *Essen/DE*

**The daily challenges of a good MR exam (part 1)**
F. Girard; *Pont de Roide/FR*

**The daily challenges of a good MR exam (part 2)**
T. Lamprecht; *Essen/DE*

**12:30 - 13:30** **Room M 2**

### organised by Siemens Healthineers

### SY 23

### Precision medicine in ultrasound: addressing the difficult to scan scenario with the new ACUSON sequoia

*Moderator:*
A. Nilsson; Uppsala/SE

**Precision ultrasound for an increasingly XXL population using image fusion, CEUS and improved ultrasound penetration**
A. Nilsson; *Uppsala/SE*

As an increasingly large proportion of our patient population can be classified as obese, finding, characterizing and potentially treating focal lesions is routinely becoming more difficult. Diagnostic tools, such as high quality contrast enhanced ultrasound and multi-modality fusion, in conjunction with improved ultrasound penetration, can help us personalize ultrasound to meet this challenge.

**CEUS diagnostic imaging in the paediatric population**
M. Stenzel; *Jena/DE*

An overview of current best practice of ultrasound in infants and children referencing adult patients where appropriate. Sonography is the imaging modality of choice in paediatrics because it provides a cross-sectional depiction of anatomical structures with high spatial and temporal resolution and is relatively robust handling restless and active patients. No other method can provide comparable diagnostic information. Indications for paediatric sonography include: screening, diagnosis and follow up exams. Contrast enhanced ultrasound can be used to enhance diagnostic possibilities. The two main indications for contrast enhanced ultrasound in children are the assessment of vesicoureteral reflux disease and the characterization of focal lesions, particularly in the liver.

**New horizons in breast ultrasound**
M. Daniaux; *Innsbruck/AT*

In conjunction with the superb spatial resolution of high frequency B-mode ultrasound and highly sensitive colour Doppler, advanced applications like Strain and Shear Wave Elastography provide clinicians with the tools necessary to deliver precision medicine. Strain Elastography can be used to classify a lesion according to specific recognized patterns. Shear Wave Elastography allows for the quantification of tissue stiffness by providing a velocity map which allows comparison of the shear wave velocities at multiple points. Using these three imaging modes (B-mode, Colour and Elastography) improves diagnostic confidence and may reduce the costs associated with unnecessary biopsies and improve the patient experience.

**12:30 - 13:30** **Room M 3**

### organised by Bayer Russia

### SY 24

### Game changers in brain imaging: when differential diagnosis really matters

*Moderator:*
I.E. Tyurin; Moscow/RU

**Brain cases: adults**
I. Trofimenko; *Moscow/RU*

**Brain cases: paediatric**
A.E. Tsoriev; *Yekaterinburg/RU*

**Head and neck cases**
D. Farina; *Brescia/IT*

12:30 - 13:30 **Tech Gate Auditorium**

### organised by Guerbet

### SY 30

### GBCA: how to get away from background noise

*Moderator:*
E. de Kerviler; Paris/FR

**Mechanisms of gadolinium retention in the body**
E. Lancelot; *Villepinte/FR*

**The global safety of gadolinium contrast agents**
E. De Kerviler; *Paris/FR*

**The diagnostic power of gadolinium contrast agents**
A. Radbruch; *Heidelberg/DE*

14:00 - 15:30 **Room X**

### organised by GE Healthcare

### SY 25

**Programme not available by date of publication**

14:00 - 15:30 **Room O**

### organised by Elsevier

### SY 26

### "Brain in flame"- pathology and imaging of non-infectious CNS inflammation: a masterclass from Dr. Anne Osborn

*Moderator:*
L. Farley; London/UK

**"Brain in flame"- pathology and imaging of non-infectious CNS inflammation: a masterclass from Dr. Anne Osborn**
A.G. Osborn; *Salt Lake City, UT/US*

In this special seminar we discuss the role of aberrant brain inflammation in a broad spectrum of CNS pathology, focusing on both suggestive MRI features as well as direct visualization of brain inflammatory using Translocator Protein (TSPO) PET imaging. The didactive presentation will be followed by an interactive case-based session demonstrating the use of CDSS to solve diagnostic dilemmas.

14:00 - 15:30 **Room M 1**

### organised by Carestream

### SY 27

### Advancements in volumetric extremity imaging

*Moderator:*
M. Boesen; Copenhagen/DK

**Arthro-cone beam computed tomography for the detection of osteoarthritis**
S. Boudabbous; *Geneva/CH*

Introduction: XR remain the gold standard for establishing an imaging-based diagnosis of osteoarthritis. However, the criteria of Kellgren and Lawrence classification (KLC) are delayed and lack of sensitivity and specificity. CBCT is an emerging modality in extremity's imaging allowing low dose radiation with diagnostic quality images.

Materials and Methods: 32 arthro-CBCT requested for osteoarthritis assessment, preceded by XR were performed. All Arthro-CBCT were reconstructed with Modeled Based Iterative Reconstruction and were analysed by 2 independent-blinded MSK radiologists for the overall quality (3-point Likert scale). Contrast, bone density and minimal joint space width (JSW) were evaluated. The stage of Kellgren and Lawrence was compared between ArthroCBCT and XR as well as sclerosis and erosions using 3-point Likert scale. P value was calculated using the Wilcoxon test. Finally, radiation dose was estimated and compared between the two modalities.
Results: The image quality was excellent in all cases of arthro-CBCT with excellent inter observer concordance (kappa=1). The mean of density was 1966,328,812 and 330 respectively for the contrast, the trabecular bone, the subchondral cortex and for the cartilage. An under classification of osteoarthritis was noticed with XR regarding the JSW (p= 0.02), the detection of osteophytes (<0.0001) and the KLC (P<0.0001). The dose for arthro-CBCT and XR were estimated at 4.9 and 0.05 mSv.
Conclusion: Arthro-CBCT may be a new way to assess osteoarthritis due to its high image quality and low radiation dose.

**Articular surfaces of the tibiotalar joint from different medical imaging technologies: for morphological models and custom-made prostheses**
A. Leardini; *Bologna/IT*

Total ankle replacement still suffers of failures and patient dissatisfaction, likely accounted for to the poor design and the limited number of sizes. To match better patient-specific joint anatomy, custom-made prostheses are being introduced, possibly to reduce the mismatching. Additive manufacturing, i.e. 3D printing, and biomechanical modelling and designing nowadays make this possible. For this purpose, however, reliable medical-imaging analyses are mandatory, to achieve accurate patient-specific models of the joint to be replaced, and in case of the contralateral as a reference. The aim of this study was to investigate the quality of customized morphological models of the ankle on different medical imaging technologies. A cadaver shank-foot specimen instrumented with multi-modal markers was plastered. Scans were taken in a CT standard, Dual-Energy (DE) and Cone-Beam (CB) technologies, as well as on 1.5 and 3.0 T MRI. Relevant DICOM files were used to create CAD models for the tibia and talus bones, the cartilages and the beads. Also to create bone-cartilage models, these models were registered based on the fiducial markers or 3D shape matching. Distance map analysis calculated the inter-model differences. Large distances by corresponding models were observed using surfaces derived from standard CT and 1.5 T MRI. More uniform distance maps of cartilage and bone models were obtained respectively by 3.0 T MRI and CBCT, which resulted also in best bone-cartilage models. In personalized joint replacements, these findings support the definition of subject-specific morphological models for better custom-made prostheses.

**Clinical and weight-bearing indication for CBCT of the extremities**
M. Boesen; *Copenhagen/DK*

CBCT offers the opportunity to study the bony changes in a variety of clinical and research indications of the musculoskeletal system such as 3D isotropic high resolution bone imaging of trauma to the extremities, 3D isotropic high resolution bone imaging of rheumatic diseases (Osteoarthritis, Rheumatoid Arthritis, Psoriasis Arthritis, Gout and Pseudo Gout (CPPD/HADD) with a low radiation dose. Weight-bearing CBCT can also provide important information regarding mechanical changes in knee and foot in 3D between supine and weight-bearing conditions. The lecture will summarize the potential for application of CBCT in the above mentioned conditions and show preliminary results regarding weight-bearing imaging of the patellofemoral joint and ankle.

**Extremity CT: the basics and the promise**
J. Yorkston; *Rochester, NY/US*

Recent years have seen the introduction of a number of specialized x-ray systems optimized for imaging specific anatomical regions. In particular, volumetric imaging systems designed to acquire dental, ENT and extremity images based on Cone Beam CT technology are now commercially available. This submission will review the technical aspects of these new systems with a focus on their unique capabilities. The presentation will also describe the ongoing work to improve the image quality of the Carestream Onsight system with a view to enabling various quantitative CT capabilities. Model based iterative reconstruction coupled with advanced scatter correction algorithms have resulted in a significant improvement in HU accuracy. Continuing development of semi and fully automated measurement tools hold the promise of providing the clinician with a full array of accurate 3D measurements in the foot, knee and hand in an efficient, reproducible manner that should enhance clinical decision making once fully implemented. The talk will review a number of these forthcoming capabilities. Additionally the potential for advances in patient specific instrumentation to improve surgical efficiency and patient outcomes will be discussed in the context of 3D printing of patient specific surgical implants and surgical guides. Ongoing work associated with image analysis of the high quality 3D reconstructions to detect, classify and monitor early onset OA and RA will also be highlighted.

### Saturday, March 2

**10:30 - 12:00** **C&T 2**

### organised by Elsevier

### SY 33

**Programme not available by date of publication**

**12:30 - 13:30** **Room Y**

### organised by Philips (DXR)

### SY 28

### Next generation image processing technologies for clinical excellence in digital radiography

*Moderator:*
S. Mintert; Hamburg/DE

**Philips UNIQUE 2: meeting clinical challenges by tailored image processing**
S. Specht; *Hamburg/DE*

**A paediatric radiologist's perspective**
P.-C. Krüger; *Jena/DE*

**More confident image interpretation with Philips bone suppression (by Riverain Technologies)**
D. Manke; *Hamburg/DE*

**12:30 - 13:30** **Room G**

### organised by Bracco (US)

### SY 29

**Programme not available by date of publication**

**12:30 - 13:30** **Tech Gate Auditorium**

### organised by ICIS

### SY 31

### All about CT and MRI contrast medium use for upper abdominal imaging noise

*Moderator:*
A.G. Rockall; London/UK

**Use of iodine contrast in upper abdominal imaging: stop to the confusion***
J.-P. Tasu; Poitiers/FR

**Time to clarify which gadolinium-based contrast should be used in liver MRI***
B. Taouli; New York, NY/US

**Contrast agents in interventional procedure to treat HCC and follow-up**
J.M. Lee; Seoul/KR

** The lecture will be given in French*

### E

### Authors' Index (E)

### A

**Aarts B.M., Klompenhouwer E.G., Dresen R.C., Laenen A., Beets-Tan R.G.H., Punie K., Neven P., Wildiers H., Maleux G.**
Intrahepatic mitomycin C infusion in liver-dominant metastatic breast cancer: factors influencing the outcome (SS 609), B-0559

**Aarts B.M., Klompenhouwer E.G., Dresen R.C., Deroose C., Laenen A., Beets-Tan R.G.H., Neven P., Wildiers H., Maleux G.**
Prospective monocentre pilot study of intrahepatic mitomycin C infusion after radioembolisation with Y-90 in chemo refractory liver dominant metastatic breast cancer patients (MY 8), B-0850

**Aarts S.**
Working in radiology: burnout and bore-out or engaged and passionate?: The role of technology in the job satisfaction and well-being of radiology workers (PC 13), A-0743

**Abdel Aal A.M.K., Mahmoud K., El Khudari H., Aboueldahab N., Saddekni S., Gunn A.**
Safety and outcomes of transarterial chemoembolization using small- versus medium-sized drug-eluting beads for unresectable hepatocellular carcinoma (MY 8), B-0838

**Abdel Razek N.M.**
Minimally invasive excision of suspicious breast lesions (SS 1402a), B-1156

**Abdel Wahab C., Jannot A., Bonaffini P.A., Lefrere Belda M., Bats A., Thomassin-Naggara I., Bellucci A., Reinhold C., Fournier L.**
Diagnostic algorithm to differentiate benign atypical leiomyomas from malignant uterine sarcomas using MR imaging (SS 1907), B-1894

**Abdel-Kawi M., Nasr M., Ibrahim A.**
Beyond lymph nodes: 18F FDG PET CT in detection of usual and unusual sites of extra nodal lymphoma (MY 3), B-0366

**Abdo A., Karam E., Leygnac S., Henry T., Haioun K., Khalil A., Debray M.**
Can we reduce the radiation dose with wide-volume scan mode in a 320 detector-rows CT for interstitial lung disease? (SS 1604), B-1407

**Abdolell M., Payne J.I., Brown P., Barnes P., Caines J., Tsuruda K., Talbot P., Tong O., Iles S.E.**
Defining a parsimonious breast cancer risk model to risk-stratify average-risk women for follow-up screening (SS 1002), B-0916

**Abdolell M., Payne J.I., Brown P., Barnes P., Caines J., Tsuruda K., Talbot P., Tong O., Iles S.E.**
The role of breast density in predicting interval detected breast cancer (SS 1002), B-0919

**Abela-Ridder B.**
Radiology and migrations: Importance of migration for neglected tropical diseases: WHO perspective (PC 5), A-0229

**Aberle C., Ryckx N., Treier R., Schindera S., Menz R.**
Update of the diagnostic reference levels for CT in Switzerland using dose management software (SS 1413), B-1196

**Abou Elkassem A., Abdel Aal A.M.K., Mahmoud K., Gunn A., Iirette S., Smith A.**
Liver surface nodularity score as a new predictor of overall survival in patients with HCC treated by TACE (MY 3), B-0357

**Abou Elkassem A., Abdel Aal A.M.K., Gunn A., Mahmoud K., Lirette S., Smith A.**
CT-based liver surface nodularity score for noninvasive assessment of clinically significant portal hypertension (MY 17), B-1473

**Abouelkheir R.T., Abou El-Ghar M.E., El-Diasty T.**
Dynamic contrast-enhanced MRI perspectives in the assessment of aggressiveness of urinary bladder cancer (SS 707), B-0830

**Abrar D.B., Frenken M., Klosterkemper Y., Antoch G., Schleich C.**
Rheumatoid arthritis magnetic resonance imaging score predicts therapy response (SS 610), B-0516

**Abrar D.B., Frenken M., Klosterkemper Y., Antoch G., Schleich C.**
Silent progression in patients with rheumatoid arthritis: is DAS28 remission an insufficient goal in RA? (SS 610), B-0518

**Abrar D.B., Frenken M., Sewerin P., Antoch G., Schleich C.**
Is an abbreviated version of psoriatic arthritis magnetic resonance imaging score (PsAMRIS), PsAMRIS-6, applicable for detection and monitoring of disease-related joint changes? (SS 610), B-0519

**Abuzaid M.M., Elshami W., Noorajan Z.**
Radiation dose associated with patients undergoing upper limb radiographic examination in different patient orientation: a phantom study (SS 714a), B-0644

**Abuzaid M.M., Elshami W., Sulieman A.A., Kappas C.**
Brain CT with 64- and 16-slice CT scans: comparison of image quality and radiation dose with standard protocols (SS 1814), B-1486

**Acquafredda F., Abdelhafez Y., Zhang M., Guindani M., Lee S., Sarohia D., Badawi R.D., Nardo L.**
Predictive value of MRI radiomic features in differentiating lipoma from atypical lipomatous tumour (SS 210b), B-0056

**Adams L.C.**
Native T1 mapping as an in vivo biomarker for the identification of higher grade renal cell carcinoma (SS 1407), B-1141

**Adla T., Suchanek V., Polovincak M., Miksik L., Salagovicova Z., Markova N., Biskup J., Rocek M.**
Cardiac computed tomography left ventricle myocardial strain analysis (SS 603), B-0597

**Adriaensen M.**
Imaging professionals in the EU: radiologists without borders: Chairpersons' introduction (part 1) (ESR/UEMS), A-0211

**Afadzi M., Fosså K., Andersen H., Aaløkken T.M., Martinsen A.C.T.**
Image quality evaluation of ultra-low dose chest CT using six iterative reconstructions (SS 213), B-0131

**Agarwal A.**
Sonographic mapping of brachial plexus and comparison with magnetic resonance imaging and electrophysiological studies (SS 1910b), B-1747

**Agliata G., Schicci N., Esposto Pirani P., Tagliati C., Giovagnoni A.**
Additive value of dual energy (DE) acquisition in CT follow-up of endovascular aortic repair (EVAR or TEVAR) (SS 1915), B-1719

**Agostini A., Borgheresi A., Mari A., Lorenzoni A., Ottaviani L., Maggi S., Giovagnoni A.**
Quantification of gadolinium with 3rd generation dual source dual energy CT: role of spectral separation (SS 1406), B-1118

**Ahmad Ainuddin H., Kirat Singh S.K., Rahmat K., Ramli Hamid M., Fadzli F., Ahmad Ainuddin H.**
Diffusion kurtosis imaging (DKI) in breast cancer: diagnostic accuracy of DKI-derived values in characterization of breast lesions and correlation with prognostic factors (MY 5), B-0468

**Ahmed S.A., El Barody M.M., El Malek N.A.A., El Salam Ebrahem M.A.**
Imaging surveillance in operable invasive transitional cell carcinoma of the bladder (MY 13), B-1086

**Ahmed S.A., Fouad D., Seifeldein G.S., Taleb H.A., Yehia A., El Malek N.A.A., Badary D.**
Accuracy of MDCT and laparoscopy in the prediction of peritoneal carcinomatosis index score in primary ovarian cancer (MY 3), B-0371

**Ahmed S.A., Fouad D., Taleb H.A., Badary D.**
Peritoneal carcinomatosis extent evaluation: radiologic, laparoscopic and pathologic (SS 1401b), B-1284

**Ahn H., An Y., Baek J., Suh Y., Choi H.**
Which factors affect the accuracy of MRI in predicting response and estimating residual disease after neoadjuvant chemotherapy? (SS 1902b), B-1800

**Ahn Y., Hong G., Park K., Lee C.W.**
Repeated imaging work-up in revisiting emergency patients: analysis of clinical and radiological risk factors (SS 217), B-0105

**Ailianou A.**
Imaging of eye and orbital pathologies: A. Traumatic lesions of the eye and orbit (RC 1708), A-1068

**Aissa J., Sawicki L.M., Appel E., Heusch P., Thomas C., Antoch G., Boos J.**
Dose optimisation in CT for cocaine body packing: where is the limit of extensive dose reduction? (MY 15), B-1355

**Aivazoglou L.U., Toma M.K., Arruda P.H.C., Ezzeddine O.A., Ormond A., Nico M., Silva F.**
Knee MR using a body coil is equivalent to CT in measuring the TTTG distance: removing the systematic bias (MY 9), B-0860

**Akhan O.**
Radiology and migrations: Chairperson's introduction (PC 5), A-0227

**Akhoundi N., Faghihi Langroudi T.**
Computed tomography pulmonary angiography for acute pulmonary embolism: prediction of adverse outcome and 90-day mortality in a single test (MY 4), B-0427

**Akram M.H.**
The role of radiology in major healthcare challenges faced by Pakistan: Introduction (EM 3), A-1077

**Al Kuhaimi T.S., Aljefri A., Alshaikh M., Almalki S., Bauones S.**
Do aggressive radiologic features of synovial sarcoma correlate with pathologic grading? A retrospective study (SS 210b), B-0060

**Al Raaisi A., Cormier E., Shotar E., Drir M., Clarencon F., Chiras J.**
Percutaneous stabilisation of humeral metastatic lesion (SS 1809), B-1574

**Al-Mosawe A.M.J., Al-Jiboori M.A.K.**
The value of MRI in the evaluation of peri-anal fistula (SS 201b), B-0209

**Al-Ubeidy H., Alshamari M., Widell J., Eriksson T., Lidén M.**
High-pitch, low-kVp computed tomography for ruling out pulmonary embolism with 17-mL contrast media (SS 615), B-0491

**Alagely A., Okromelidze L., Vilanilam G.K., Middlebrooks E.H., Gupta V.**
The forgotten juxta-sellar mass: ectopic pituitary adenomas (SS 1411a), B-1169

**Alagic Z., Bujila R., Srivastava S., Koskinen S.K.**
Implementation of an ultra-low-dose CT-protocol for extremities: initial experience with 106 subjects (SS 710), B-0674

**Albano D., Aliprandi A., Brioschi M., Magnani S., Messina C., Sdao S., Randelli F., Sconfienza L.M.**
The extension thickness damage (ETD) score: a pre-operative hip MR arthrography-based classification to predict type of labrum surgery (SS 1010), B-0898

**Alberich-Bayarri A.**
Everything you need to know about 3D post-processing: A. 3D post-processing in 2019 (RC 405), A-0143

**Alberich-Bayarri A.**
Artificial intelligence: presented with new lecture formats: Deep learning (DL) in medical imaging (ESOR), A-0371

**Alberich-Bayarri A.**
Investment strategies: C. Start-up in radiology ($E^3$ 1118), A-0637

**Albrecht M.H., Croissant Y., Czerny C., Vogl T.J., Zangos S.**
Robot-assisted percutaneous placement of K-Wires during minimal-invasive interventions of the spine (MY 8), B-0847

**Alcalá-Galiano A.**
MR imaging of the knee: A. Cruciate ligaments: what to know and what to do (RC 810), A-0443

**Aldoj N., Lukas S., Dewey M., Penzkofer T.**
Classification of prostate cancer on multiparametric MR imaging using 3D convolutional neural network (SS 216), B-0172

**Aldoj N., Biavati F., Rutz M., Stober S., Dewey M.**
Automatic prostate segmentation of magnetic resonance images using convolutional neural networks (MY 7), B-0802

**Alfaqih M., Ali S., Sana Ali J., Chea P., Flacke S., French R., Elentuck D.**
Hip arthroplasty and computed tomography of abdomen: a retroperitoneal indicator of post-arthroplasty hip flexor impingement (SS 1010), B-0902

**Alghamdi J.M.A., Raja S.**
Automated stroke detection and profiling on initial non-contrast CT via machine learning and textural analysis (MY 7), B-0809

**Alì M., Monti C.B., Melazzini L., Fossati B., Cavalli M., Cardani R., Meola G., Sardanelli F., Secchi F.**
Cardiac magnetic resonance in myotonic dystrophy type 1: a contribution to risk assessment (SS 1903a), B-1821

**Alkadhi H.**
Emergency radiology I: A. Acute aortic syndrome ($E^3$ 121), A-0044

**Alkadhi H.**
Cardiac imaging in structural heart disease: C. Follow-up after minimally invasive valvular repair (RC 1503), A-0879

**Alkadhi H.**
Dose reduction and image quality implications of iterative image reconstruction in CT: B. Iterative image reconstruction in clinical practice: dos and don'ts (RC 1713), A-1051

**Alkasab T.K.**
Reporting and communication today and tomorrow: challenges to implement structured reporting (RS) and deal with artificial intelligence (AI): Decision support and artificial intelligence (AI) to improve reporting in radiology (PI 1), A-0506

**Allen M.**
How to deal with the epidemics of thyroid nodules?: The surgeon intervention (MS 16), A-0988

**Almeida M.F.A., Cruz R., Begnami M.D.F.S., Barbosa P.N.V.P., Bitencourt A.G.V., Coimbra F.J.F., Chojniak R.**
Diffusion-weighted imaging of colorectal liver metastases: prospective evaluation of apparent diffusion coefficient in correlation with histologic findings (MY 3), B-0358

**Aloufi F.F.**
Prevalence of asymptomatic pacemaker and ICD lead perforation on computed tomography (MY 18), B-1670

**Alqahtani S.J.M., Meakin J.R., Palfrey R.M., Knapp K.**
The impact of different exposure factors on radiation dose of lumbar spine in five different sizes phantoms: a factorial design study (SS 714b), B-0659

**Alqahtani S.J.M., Meakin J.R., Palfrey R.M., Knapp K.M.**
Stylised phantom used in PCXMC, the validation against hybrid phantom in obesity (SS 714b), B-0662

**Alqahtani S.J.M., Meakin J.R., Palfrey R.M., Knapp K.M.**
The impact of different exposure factors on image quality of lumbar spine in five different sizes phantoms: a factorial design study (SS 1314), B-1067

### Authors' Index

**Alqattan A., McGee A., Delahunt E., Eustace S., McNulty J., Cradock A.**
Quantitative diffusion-weighted knee MR imaging and ADC mapping in healthy volunteers (SS 414), B-0409

**Alukic E., Mekis N.**
Lumbar spine imaging: can we reduce the effective and organ dose with the use of PA projection? (SS 1314), B-1068

**Alvi A., Mubarak F., Alvi A., Enam S., Shahzad M.**
Lesion and lesion habitat analysis on magnetic resonance imaging: an imaging biomarker for tumour grade and 1p19q deletion status (SS 211a), B-0090

**Amad A.**
Brain imaging in psychiatry: The epidemiology of psychiatric disorders: why is MRI needed? (MS 8b), A-0403

**Ambrogio S., Walker A., Verma P., Narracott A., Fenner J.**
Design and testing of a novel complex flow phantom for medical imaging (SS 713), B-0760

**Amelin M., Rzaev J., Moysak G., Tulupov A.**
Diffusion tensor imaging in trigeminal neuralgia caused with neurovascular conflict and in estimation of long-term results of microvascular decompression (SS 1411b), B-1266

**An H., Lee E., Perucho J.A., Chiu K.W., Chu M., Tse K.Y.**
DWI predicts surgical outcome in advanced ovarian cancer (MY 3), B-0370

**Andersen M.B., Ebbesen D., Thygesen J., Kruis M., Rasmussen F.R.**
Impact of spectral imaging in patients suspected for occult cancer: a study of 93 patients (SS 1716), B-1465

**Andreotti R.F.**
Early detection of ovarian cancer: B. ORADS: ultrasound and International Ovarian Tumor Analysis (IOTA) group models (E³ 119), A-0072

**Andres Cano I.**
Flow diverters in the treatment of intracranial aneurysms: experience at our institution (SS 309), B-0287

**Annoni A.D., Mancini M.E., Formenti A., Muscogiuri G., Guglielmo M., Mushtaq S., Pontone G., Pepi M., Andreini D.**
Follow-up of thoracic aorta diseases with concurrent evaluation of coronary arteries using wide-detector-coverage CT with low-dose protocol (SS 1815), B-1501

**Antonelli A., Manganaro L., Di Trani M.G., Capuani S., Bernardo S., Petrillo R., Catalano C.**
Reliable evaluation of ADC values in the study of normal foetal brain, using denoising correction: a prenatal MR study (SS 612), B-0607

**Antonelli A., Manganaro L., Capuani S., Grimm R., Kuehn B., de Rinaldis A., Catalano C.**
The potential of IVIM model as a marker of normal placenta microstructural changes during pregnancy: a foetal MR study (SS 1507), B-1390

**Antonuccio E.G.M., Vasquez Cuadra S.J., Pirillo S.P., Tipaldi L.**
Low tube voltage and low contrast media volume multidetector CT angiography in evaluation of abdominal aorta in EVAR follow-up: a comparison study (SS 1915), B-1724

**Aparisi Gomez M.P.**
Everything you always wanted to know about metabolic bone disease (but were afraid to ask): Could "that" be metabolic? (SF 15a), A-0881

**Apine I., Baduna M., Pitura R., Krumina G.**
ADC values of DWI and DWIBS in bowel imaging: when they are consistent and when not? (SS 1801b), B-1652

**Appel E., Thomas C., Klosterkemper Y., Aissa J., Antoch G., Boos J.**
Split-filter dual-energy CT: value of advanced monoenergetic reconstructions for the assessment of vessel stents (SS 1915), B-1726

**Appelman L., Appelman P., Bult P., Mann R.**
Targeted ultrasound as first-line examination in women with focal breast complaints (SS 702b), B-0726

**Arboleda C., Lang K., Wang Z., Köhler T., Prevrhal S., Leo C., Forte S., Kubik-Huch R., Stampanoni M.**
The feasibility of using clinically compatible grating interferometry mammography in breast cancer diagnosis (SS 1902a), B-1792

**Arendt C.T., Leithner D., Mayerhöfer M.E., Lenga L., Czerny C., Burck I., Vogl T.J., Schernthaner R.**
Double-center study on textural differences between cholesteatoma and middle ear inflammation in non-enhanced high-resolution computed tomography (SS 308), B-0299

**Arendt C.T., Beeres M., Leithner D., Tischendorf P., Langenbach M.C., Kaltenbach B., Vogl T.J., Gruber-Rouh T.**
Paediatric Hodgkin's and non-Hodgkin's lymphoma on chest MRI: do we really need intravenous contrast agent? (SS 1404), B-1204

**Argyropoulou M.I.**
Non-contrast MR angiography: ready to go?: Non-contrast MR angiography in paediatric vascular imaging (SF 8d), A-0423

**Argyropoulou M.I.**
Intensive care paediatric radiology: the very sick neonate: A. Neuroimaging in the neonatal intensive care unit (RC 512), A-0277

**Arous A., Jemel E., Krichene M., Zayani O., Mahmoud M., Drissi C., Naji S., Hammami N., Ben Hammouda M.**
Paediatric cerebellar tumours: how useful is the apparent diffusion coefficient? (SS 1411a), B-1160

**Arrigoni F., Bianchi G., Palumbo P., Mariani S., Bruno F., Zugaro L., Barile A., Masciocchi C.**
Fusion imaging of US and MRI coupled with electromagnetic virtual navigation for periradicular infiltrations of steroids in low back pain: pilot experience of a radiation-free procedure (SS 1809), B-1580

**Aryan A., Sethi K.**
Brain imaging in 43 serologically positive cases of Chikungunya virus infection with neurological complications at our institute during epidemic outbreak (SS 611), B-0537

**Asahara T., Hayashi H., Tomita E., Mihara Y., Goto S., Kanazawa Y., Kimoto N., Asakawa T., Okazaki T.**
Actual dose measurements of assistant during paediatric x-ray examination with a precisely calibrated optically stimulated luminescence dosimeter (SS 714a), B-0642

**Asbach P.**
Imaging strategies in renal tumours: C. Staging and organ-preserving strategies (RC 907), A-0574

**Ascione A., De Rubeis G., Galea N., Francone M., Catalano C.**
Predictive role of lipid panel and DLCN score in the assessment of coronary artery disease with coronary CT in patients with familial hypercholesterolemia (SS 1003), B-1019

**Aslan S., Ozdemir S.**
Radiological-pathological correlation of thyroid nodule ultrasound and cytology using the TIRADS and Bethesda classifications (SS 208), B-0122

**Asokan P., Shivalingappa S.S., Rao Kesari A.A., Ashok Kumar M., Desai I., Sampangi S.**
Determination of fractional anisotropy cut off values on diffusion tensor imaging in differentiating recurrence versus radiation necrosis in gliomas (SS 1411b), B-1269

**Aswani Y., Saifi S.**
Therapeutic implications of ultrasound of peripheral nerves in leprosy (SS 1910b), B-1749

**Atalabi O.M., Akinwumi O.**
Computed tomographic pattern of intracranial injuries among patients with *okada* (motor cycle) road traffic accidents in Southwestern Nigeria (SS 1017), B-0947

**Athanasiou A.**
Minimally-invasive local treatment of breast cancer: the time is now: Chairperson's introduction (RC 1202), A-0671

**Atri P.K., Sodhi K.S., Bhatia A., Saxena A.K., Khandelwal N.**
Model-based iterative reconstruction: is it relevant for reducing radiation dose in paediatric head CT? (SS 1412), B-1224

**Attanasio A., Corrias G., Saba L.**
OLEA/NOVA+ SyntenticMRI application on workflow in pelvis MRI studies (SS 414), B-0415

**Attye A.**
Imaging in Meniere's disease: what to believe and how to interpret: MR hydrops imaging: how to do it? (SF 15b), A-0887

**Auer T.A., Grieser C., Geisel D., Hamm B., Denecke T.**
MRI of hepatocellular adenomas: is there an additional value of using Gd-EOB for subtype differentiation? (MY 17), B-1469

**Autti T.**
Audit across Europe: directive and perspective: Implementing a clinical audit programme: the Finland experience (ESR Audit), A-0139

**Avendano D., Sutton E.J., Morris E.A., Marino M.A., Gibbs P., Martinez D.**
How should we manage breast ultrasound masses that do not enhance on MRI? (SS 702b), B-0719

**Avendano D., Jochelson M.S., Pinker-Domenig K., Marino M.A., Gluskin J., Sung J.S.**
Contrast-enhanced mammography screening women after breast-conserving therapy (SS 1902b), B-1804

**Aviram G.**
Thoracic emergencies: C. Diagnosing PE (RC 1704), A-1055

**Ayaz Ü.Y., Döğen M.E.**
Making measurements on coronal ultrasound images of infant hips during Barlow manoeuvre: a new and rapid screening method for developmental dysplasia of the hip (SS 1012), B-1053

**Aydin R.C., Bräu F., Notohamiprodjo M., Roos J.E., Leng S., Williamson E.E., Nikolaou K., Cyron C.J., Marcus R.P.**
Evaluation of blood pressure variations on CTA geometries of abdominal aortic aneurysms (SS 1915), B-1729

**Aydingoz U.**
Musculoskeletal radiology: inflammation: B. Arthropathies (E³ 221), A-0090

**Aydingoz U.**
MR imaging of the knee: C. Looking around the corners: posteromedial and posterolateral (RC 810), A-0445

**Ayres V.J., Fleury E.F., Ramalho L., Pompei L.D.**
Solitary dilated duct visualised by mammography: are we correctly classifying according to BI-RADS lexicon 5th edition? (SS 202), B-0084

**Ayuso C.**
Imaging of the complicated postoperative abdomen: A. Liver (RC 901), A-0486

**Azevedo K.**
Patient safety in medical imaging: Patient safety: beyond radiation protection (ESR/EFRS), A-0929

**Azman S.S., McDermott E., Ryan M.-L.**
Written patient information in radiology: an Irish perspective (SS 214), B-0024

### B

**Ba-Ssalamah A.**
Imaging of benign liver lesions: still difficult?: C. FNH or adenoma? (RC 101), A-0004

**Babar J.**
Challenging HRCT patterns: tips and tricks from the experts: Chairperson's introduction (SF 8b), A-0451

**Bacher K.**
Advanced clinical dosimetry in interventional radiology: The basis of clinical dosimetry in interventional radiology (EU 4), A-0748

**Bachmann Nielsen M.**
Ultrasound simulation models in training and education: where are we going?: Review of the available market of ultrasound simulators (ESR US SC/EFSUMB), A-0042

**Bachmann Nielsen M.**
US teaching to undergraduate students: why, how and from whom?: How: US teaching in the radiology department and hands-on classroom teaching using living models and simulators (ESR US SC/UES), A-0338

**Bae J., Lee D., Joo I., Jeon S., Han J.K.**
Utilisation of virtual non-contrast images derived from dual-energy CT in the evaluation of biliary stone disease: should we obtain true non-contrast images? (SS 301), B-0389

**Bae J., Lee J., Lee D., Kim H., Han J.**
Quantitative evaluation of nonalcoholic fatty liver disease using normalised local variance US in a rat model (SS 1406), B-1119

**Baeßler B.**
Dream team: C. Leading quality improvement projects (E³ 1018), A-0597

**Baeßler B.**
A new era of gadolinium-less MR: Radiomics and multiparametric MRI approaches for a better contrast in cardiac and prostate applications (ESR/ESMRMB), A-1008

**Baggiano A., Guglielmo M., Muscogiuri G., Soldi M., Del Torto A., Guaricci A.I., Pepi M., Pontone G.**
Comparison between stress CT perfusion versus FFRCT in the evaluation of suspected CAD: PERFECTION prospective study (SS 703), B-0783

**Baggiano A., Guglielmo M., Muscogiuri G., Soldi M., Del Torto A., Guaricci A.I., Pepi M., Pontone G.**
Diagnostic accuracy of combined CCTA and low-dose dynamic stress CT myocardial perfusion with a whole-heart coverage CT scanner in patients with suspected CAD (SS 1403), B-1212

**Bagheri M., Shafiei A., Farhadi F., Apolo A.B., Folio L.R., Jones E.C., Summers R.M.**
Lymph node measurement on serial imaging in clinical trials (SS 1816), B-1640

**Bagur A., Hutton C., Gyngell M.L., Robson M.D., Brady M.**
Accuracy and reproducibility of magnitude-based proton density fat fraction quantification with resolved water-fat ambiguity (SS 713), B-0763

**Bai J., Matthews R., Bangiyev L., Chimpiri A.R., Franceschi D.**
Imaging detection and characterisation of bone metastases from neuroendocrine neoplasm with simultaneously acquired Ga-68 DOTATATE PET/MRI (SS 716), B-0687

**Balaji R.**
IVIM MRI: a better tool to assess myometrial invasion in endometrial cancer (SS 216), B-0176

**Balaji R.**
Intravoxel incoherent motion imaging (IVIM) assessment of bone marrow infiltration in multiple myeloma (SS 316), B-0345

**Balaji R.**
Response assessment in multiple myeloma using intra-voxel incoherent motion (IVIM) diffusion MRI: a newer paradigm (SS 316), B-0346

**Balaji R.**
Arterial spin labelling (ASL) MRI to assess nodal metastases in head and neck cancer (SS 608), B-0636

**Balaji R., Anand V.K., Krishnamurthi G.**
MRI histogram and texture analysis as predictors of response to therapy in head and neck malignancies (SS 1816), B-1639

### Authors' Index

**Balaji R.**
Image briefly: the new aphorism for surveillance in testicular cancer (SS 1816), B-1641

**Bali M.A.**
Chronic pancreatitis: Functional evaluation of chronic pancreatitis (E³ 24B), A-0341

**Ball P.**
Spectral CT for routine practice CTA and cardiac CT (SY 16)

**Ball P.A.**
Reduced dose, reduced contrast single-acquisition TAVI imaging using novel contrast protocol and spectral low-mono-energetic imaging: a proof of concept study (SS 203), B-0197

**Ballati F., Lomoro P., Lucioni M., Di Giulio G.**
Positive predictive value for malignancy of uncertain malignant potential (B3) breast lesions diagnosed on vacuum-assisted biopsy: is surgical excision still recommended? (SS 1402a), B-1153

**Balta C., Sechopoulos I., Van Engen R., Broeders M., Veldkamp W.J.H., Reiser I.**
A new Fourier-based model observer for image quality evaluation in the central slice of simulated breast tomosynthesis images (SS 302), B-0255

**Balter S., Abrams P., Kalra S., Leidholdt E.M.**
A proposed paradigm revision for recurrent radiation safety training (SS 1413), B-1191

**Baltzer P.A.T.**
Non-invasive phenotyping of breast cancer with novel MR biomarkers (SY 2b)

**Baltzer P.A.T.**
MRI for early detection, staging and management of breast cancer: Chairperson's introduction (RC 502), A-0232

**Baltzer P.A.T.**
Hybrid and translational imaging: the bigger picture: Prostate-specific membrane antigen (PSMA) PET/MR: guiding clinical management of prostate cancer (ESR/ESHI(MT)), A-0974

**Baltzer P.A.T.**
DWI, spectroscopy and other advanced breast MR techniques: Keynote lecture (SS 1802b), K-36

**Bamberg F.**
Dead or alive: imaging of myocardial viability: C. CT (RC 1703), A-1020

**Bancroft L.W.**
Shoulder sports injuries and postoperative MRI: B. Postoperative shoulder MRI after instability surgery (TC 1328), A-0805

**Bancroft L.W.**
Shoulder sports injuries and postoperative MRI: C. Interactive case discussion (Part 2) (TC 1328), A-0807

**Bankier A.A.**
Lung nodule management in 2019: C. Management guidelines (RC 904), A-0551

**Bansal G., Jaipal A.**
Diagnostic accuracy of magnetic resonance (MRI) to predict axillary lymph node metastases in patients with breast cancer: does neo-adjuvant chemotherapy impact accuracy? (SS 602), B-0546

**Baptista M., Pinheiro C., Ribeiro Fragata I., Carvalho R., Pamplona J., Nunes A.P., Reis J.**
Direct puncture of the carotid artery for thrombectomy in acute stroke patients: a single centre experience (SS 309), B-0284

**Baptista T.**
Computational fluid dynamics in patients treated with flow-diverter devices (SS 309), B-0285

**Barabasch A., Heil P., Distelmaier M., Kuhl C.K., Bruners P.**
Safety and efficacy of IRE after major liver surgery (SS 1709), B-1437

**Barash Y., Levy T., Elazar Y., Zimlichman E., Konen E., Soffer S., Guralnik G., Amitai M.M., Klang E.**
Flagging of pathological head CT interpretations using natural language processing (NLP) (SS 1405), B-1186

**Barati M., Illing R., Kardos L., Paraskevopoulou C., Katsari K.**
Should testing for lead contamination be included in lead radiation protection apparel quality control? (SS 1413), B-1200

**Barbieri P., Cina A., Amodeo E., Manfredi R., Marrone G.**
Volumetric vs bidimensional CT assessment of sarcopenia in patients candidate for liver transplantation (SS 616a), B-0529

**Barbosa Jr. E.J.M., Kolansky A., Sui X., Jiang J.**
Lung cancer CT screening performance at a large hospital system in the US: our 5-year experience showcases the need for better systems of care (SS 204), B-0144

**Barbosa Jr. E.J.M., Osuntokun O.**
Incidental findings in thoracic CTS performed in trauma patients: an underestimated problem (SS 717), B-0736

**Barchetti G., Campa R., Pecoraro M., Barchetti F., Catalano C., Panebianco V.**
How to improve the equivocal category PIRADS score 3? Quantitative multiparametric MRI assessment of prostate cancer with histopathology correlation (SS 207), B-0156

**Barchetti G., Campa R., Pecoraro M., Ceravolo I., Catalano C., Panebianco V.**
Multiparametric MRI of the bladder: interobserver agreement and accuracy with the vesical imaging-reporting and data system (VIRADS) at a single reference centre (SS 707), B-0825

**Bardelli A.**
The role of imaging in the era of liquid biopsy: Liquid biopsy in perspective: colorectal cancer as a model system (NH 1), A-0035

**Bargellini I.**
Thoracic emergencies: D. Vascular intervention in thoracic emergencies (RC 1704), A-1056

**Barkhausen J.**
Showing MRI efficacy vs mammography (SY 2a)

**Barnacle A.**
Interventional radiology (IR) in children: what a non-interventional radiologist needs to know: A. Common IR procedures in children: state-of-the-art (RC 112), A-0061

**Barnett J.L., Pulzato I., Wilson R., Padley S., Nicholson A.G., Devaraj A.**
Using relationship between pulmonary veins and pulmonary nodules to distinguish benign intrapulmonary lymph nodes from lung cancer on CT (SS 1804), B-1605

**Barral P., Bartoli A., Piquet P., Demasi-Jacquier M., Gaudry M., Jacquier A.**
Fusion imaging to guide thoracic endovascular aortic repair (TEVAR): comparison of two methods: 2D/3D vs 3D/3D image fusion (SS 1009), B-0964

**Basilico R.**
Emergency radiology I: B. Abdominal trauma (E³ 121), A-0045

**Basilico R.**
Post-treatment emergencies in oncologic patients: B. Abdomen (E³ 926), A-0517

**Basso L., Buonomenna G., Sorrentino A., Perugin G., Rosa F., Olivero A., Testino N., Terrone C., Neumaier C.**
Contrast-enhanced computed tomography (CECT) quantitative radiological parameters to predict outcome in patients with renal cell carcinoma (RCC) (SS 1407), B-1139

**Bastarrika G.**
Cardiac: recalling the forgotten structures: Lost chamber: the right ventricle (SA 13), A-0774

**Batalov A., Zakharova N., Pogosbekyan E., Fadeeva L., Baev A., Goryaynov S., Kosyr'kova A., Potapov A., Pronin I.**
Noncontrast ASL-perfusion in pre-surgical differential diagnosis of brain gliomas (SS 211a), B-0089

**Båth J., Dahm-Kähler P., Kvarnström N., Thilander Klang A., Brännström M., Leonhardt H.**
Imaging of potential donors for uterus transplantation, with emphasis on the uterine arteries (SS 1907), B-1900

**Bauer B.**
Communicating the role of the radiologist: best practices to manage patient expectations: The patient perspective: sharing expectations and experiences (part 1) (PA), A-0825

**Bauer M.J., van Assen M., De Cecco C.N., Durden J.A., Scarabello M., Hudson H.T., Griffith L., Varga-Szemes A., Schoepf U.J.**
AI-based prediction of haematocrit values from non-contrast CT imaging data - towards fully automated CT-derived myocardial extracellular volume fraction quantification (SS 1403), B-1217

**Bauer M.J., Lavra F., Scarabello M., van Assen M., Saba L., De Cecco C.N., Schoepf U.J.**
Quantitative analysis of dynamic CTA for the detection of endoleaks after abdominal aorta aneurysm endovascular repair: a feasibility study (SS 1915), B-1721

**Bäuerle T.**
The radiological investigation of musculoskeletal tumours: C. CT and hybrid imaging (RC 1210), A-0694

**Bäuerle T.**
How to improve bone metastases and osteosarcoma assessment: Keynote lecture (SS 716), K-15

**Bayer R.R., van Assen M., Martin S.S., Savage R.H., Steinbach R., Fischer A.M., Griffith L.P., Schoepf U.J.**
CT-FFR profiles in patients without coronary artery disease: the effect of location and lumen area (SS 703), B-0781

**Bazzocchi A.**
MSK intervention: the road from low to no invasion: Preventing side effects and complications by new procedures: management by radiologists (SF 5c), A-0275

**Bazzocchi A.**
Everything you always wanted to know about metabolic bone disease (but were afraid to ask): Chairperson's introduction (SF 15a), A-0880

**Beardmore C.**
The future of radiology: what it could mean for new radiography graduates: What will radiology look like in 30 years? (BR 17), A-1045

**Beaumont H., Evans T.L., Klifa C., Hong S.R., Chadjaa M., Monostori Z., Iannessi A.**
RECIST 1.1 evaluations in a phase II clinical trial: does reader expertise represent a risk factor for measure reliability? (SS 1016), B-1023

**Beck S., Deniffel D., Riederer I., Rummeny E.J., Pfeiffer D.**
Iodine images in dual-energy CT: benchmarking quantitative iodine concentration values of the healthy liver (SS 701), B-0790

**Becker C.D.**
IT-security and GDPR: Understanding the key points of GDPR (SF 12), A-0696

**Becker C.D.**
The tumour board of the future: Maximising the added value of tumour boards (NH 16), A-0959

**Bedene A., Alukic E., Žibert J., Mekis N.**
The use of different angulation in mediolateral oblique view based on patient anatomy in mammography (SS 1914b), B-1710

**Bedlington N.**
Communicating the role of the radiologist: best practices to manage patient expectations: Chairpersons' introduction (part 1) (PA), A-0822

**Beenen L.F.M., Scheerder M.J., Bekebrede-Kauffman D., Lobe N.H.J.**
Whole-body trauma CT for critical and mass casualty incident patients: how fast can we go (SS 217), B-0099

**Beer L., Hochmair M., Haug A., Kifjak D., Schwabel B., Mayerhöfer M.E., Prosch H.**
Comparison of RECIST, iRECIST, and PERCIST for the evaluation of response to PD1/PD-L1-blockade therapy in patients with non-small cell lung cancer (SS 1016), B-1020

**Beer L., Bastati N., Pötter-Lang S., Stoyanova D., Michael E., Bican Y., Einspieler H., Ba-Ssalamah A.**
What is the effect of steatosis, iron overload and renal function on the uptake and excretion of gadoxetic acid-enhanced MRI? (SS 1001b), B-1052

**Beeres M., Scholtz J., Leithner D., Barakat A., Vogl T.J., Gruber-Rouh T., Kaltenbach B., Polkowski C., Bucher A.M.**
Evaluation of virtual grid post-processing for paediatric no-grid radiography in an unselected paediatric patient cohort (SS 1512), B-1317

**Beeres M.**
Interventional radiology (IR) in children: what a non-interventional radiologist needs to know: B. Vascular malformations: diagnosis and interventions (RC 112), A-0062

**Beets-Tan R.G.H.**
Multidisciplinary approach to cancer care: B. The colorectal team (RC 816), A-0467

**Beets-Tan R.G.H.**
Wilhelm Conrad Röntgen Honorary Lecture: Oncologic imaging: a new beginning has just begun (HL 1), A-0336

**Beets-Tan R.G.H.**
Current status and future challenges in MR-integrated radiotherapy: MR-based functional imaging (ESR/ESTRO), A-0500

**Beigelman C.**
Pleural neoplasms: B. Pleural carcinomatosis of thoracic and extra thoracic origin ($E^3$ 1322), A-0802

**Ben Daamer N., Benaissa A., Le Faucheur J.P., Faugeras F., Benadjaoud S., Kalsoum E., Tuilier T., Hosseini H., Hodel J.**
Added value of susceptibility-weighted imaging for the prediction of hemorrhagic transformation in patients with acute ischaemic stroke treated by mechanical thrombectomy (SS 311), B-0261

**Beneventi A., Casamassima N., Muollo A., Coppola A., Macchi E., Piacentino F., Ierardi A., Fontana F., Carrafiello G.**
CBCT-guided hydrogel plug percutaneous deployment before video-assisted thoracoscopic surgical resection (SS 1909), B-1808

**Bengo G.**
Computed tomography (CT) imaging in weight-bearing positions: the positives and challenges in an institutional trial (SS 614), B-0487

**Benlala I., Berger P., Girodet P.O., Dromer C., Macey J., Laurent F., Dournes G.**
Fully automated 3D quantification of the lung emphysema in COPD using 3D-UTE MRI (SS 1404), B-1202

**Bennani-Baiti B.I., Krug K.B., Giese D., Weiss K., Helbich T.H., Baltzer P.A.T.**
Gadolinium deposits in healthy women undergoing repetitive GDBCA-enhanced breast MRI in the context of high-risk breast cancer screening (SS 1802a), B-1542

**Berger C.**
Late effects in survivors of childhood cancer: Fertility issues: male and female (Part 2) (SF 17), A-1026

**Berger M.F.**
Working in radiology: burnout and bore-out or engaged and passionate?: A personal story (PC 13), A-0742

**Berlis A.**
How to organise nationwide ischaemic stroke care (and make multidisciplinary teams work): Are you experienced, trained or certified? Certification, subspecialisation, specialisation: friend or foe of the patient? (PC 16), A-0980

**Bernaerts A., Vanspauwen R., Blaivie C., van Dinther J., Zarowski A., Wuyts F., Offeciers F.E., Casselman J.W., de Foer B.**
Value of four stage vestibular hydrops grading and asymmetric perilymphatic enhancement in the diagnosis of Ménière's disease on MRI (SS 308), B-0290

**Bernardo A., Gonçalves F., Fernandes M.O.**
CT scan chest: a new approach to lung cancer (SS 1814), B-1490

**Bertalan Z., Paixao T., Ljuhar R., Ljuhar D., Dimai H., Nehrer S.**
Predicting the incidence of knee osteoarthritis using deep neural networks: data from the osteoarthritis initiative (OAI) and the multicenter osteoarthritis study (MOST) (SS 605), B-0569

**Bertani V., La Grassa M., Balestreri L., Berger N., Urbani M., Lang K., Boss A., Frauenfelder T., Marcon M.**
Outcome of microcalcifications classified as BI-RADS 3, 4a and 4b in patients with and without a history of breast cancer (SS 202), B-0085

**Bertani V., La Grassa M., Balestreri L., Berger N., Urbani M., Frauenfelder T., Boss A., Marcon M.**
Diagnostic utility of breast DCE-MRI in patients with diagnosis of atypical ductal hyperplasia after percutaneous biopsy (SS 602), B-0554

**Bertelli E., Agostini S., Verna S., Lucarini S., Savi E., Mercatelli L., Carta P., Serni S., Miele V.**
Contrast-enhanced ultrasonography (CEUS) surveillance of native kidneys in renal transplant patients: a monocentric experience (SS 607), B-0622

**Bertolini M., Trojani V., Nitrosi A., Iori M., Sassatelli R., Ortenzia O., Ghetti C.**
Implementation of channelised hotelling observer (CHO) to assess image quality of GE discovery IGS 740 angiography system (SS 1013), B-0993

**Bertolotto M.**
Imaging and management of the incidental scrotal lesions: Chairperson's introduction (SF 5d), A-0292

**Bertolotto M.**
Imaging strategies in renal tumours: A. CT, contrast-enhanced ultrasound (CEUS) and MRI: the best out of them (RC 907), A-0572

**Bertuletti M., Cosentini D., Cieno S., Di Terlizzi M., Grisanti S., Frittoli B., Berruti A., Ambrosini R., Grazioli L.**
CT response evaluation of adrenocortical carcinoma (ACC) during and after chemotherapy: which criteria? (SS 616b), B-0619

**Beyer T., Rehnitz C., Thierfelder K.M., van Rijswijk C.S.P., Martel Villagrán J., Muto M., von Falck C., Gielen J., Weber M.-A.**
European multi-centre study on technical success and long-term clinical outcome of radiofrequency ablation for the treatment of spinal osteoid osteoma and osteoblastoma (SS 1510), B-1286

**Beyer T.**
Clinical and methodological pitfalls in hybrid imaging: problems and solutions: Artefacts in hybrid imaging (ESHI(MT) 1), A-0185

**Bezzina P.**
Radiography research: a "how to" guide: How to avoid ethical issues (BR 13), A-0778

**Bhagwat K.A., Mahadevappa V.**
MR imaging of transitional lumbosacral junction: co-incidental or causative factor for the disc degeneration and herniation (SS 1910a), B-1737

**Bian Y.**
A new approach to predict grade of non-functional pancreatic neuroendocrine tumour ≤2 cm: a radiomics nomogram (SS 1805b), B-1587

**Bian Y.**
Radiomics nomogram for the preoperative prediction of lymph node metastasis in pancreatic cancer (SS 1905b), B-1834

**Bianchi G., Arrigoni F., Palumbo P., Cortellini A., Zugaro L., Cannita K., Ficorella C., Barile A., Masciocchi C.**
Retrospective multivariate analysis of the outcomes of percutaneous thermal ablation of painful bone metastasis in oncological patients (SS 1809), B-1572

**Bibbolino C.**
From morphology to function: Introduction: Radiology in Italy (EM 2), A-0579

**Bick U.**
Screening for breast cancer: C. Personalised multimodality screening with MRI (RC 902), A-0529

**Bidault F.**
Functional imaging in head and neck oncology: Chairperson's introduction ($E^3$ 126b), A-0074

**Biederer J.**
Cavitary and cystic diseases of the lung: Chairperson's introduction ($E^3$ 722), A-0373

**Bierry G.**
Imaging of chronic forefoot pain: B. Extra-articular source of pain (RC 510), A-0262

**Bijan B.**
Captain's dashboard: A. Fundamentals of imaging management and administration ($E^3$ 918), A-0556

**Bilbao J.I.**
Basic interventional radiology for non-interventionalists: let's start with biopsy!: Chairperson's introduction (SF 5b), A-0244

**Bilbao J.**
Vascular interventions in oncology (including radioembolisation): Keynote lecture (SS 609), K-12

**Bilello M., Mattay R., Mamourian A.**
Clinically-implemented, CAD-assisted decision support system to limit gadolinium injection in patients with multiple sclerosis (SS 1911a), B-1781

**Billingsley S., O'Neill C., McKean D.**
Long term efficacy of a dorsal indirect approach for CT-guided cervical nerve root injections (SS 1510), B-1288

**Binkert C.**
The old spine: challenges of imaging and treatment: C. Interventional radiology: is there still a place for vertebroplasty and kypho-/stentoplasty (RC 1610), A-0971

**Blackburn Andersen P.**
EFRS meets Denmark: Improvements in healthcare (EM 4), A-0622

**Bladowska J., Zacharzewska-Gondek A., Koltowska A., Pokryszko-Dragan A., Budrewicz S., Sasiadek M.**
Increased ADC values within normal appearing white matter as a predictor of a poor response to interferon beta therapy in patients with multiple sclerosis in the 4-year follow-up (SS 611), B-0540

**Blankenstein T.N., Grainger A., Dube B., Evans R., Robinson P.**
MRI hip findings in asymptomatic professional Rugby players, ballet dancers and age-matched controls (SS 1010), B-0900

**Blankholm A.D.**
EFRS meets Denmark: MR safety (EM 4), A-0624

**Blanks R.G., Wallis M., Alison R., Jenkins J., Kearins O., Patnick J., Given-Wilson R.M.**
Conversion to digital mammography had no impact on invasive grade 3 cancer detection in the English NHS breast cancer screening programme: analysis of 11.3 million screening tests (SS 202), B-0078

**Blanks R.G., Given-Wilson R.M., Cohen S., Jenkins J., Patnick J., Alison R., Wallis M.G.**
Can we optimise the recall rate and reduce overdiagnosis? An analysis of 11.3 million screening tests from the English NHS breast cancer screening programme (SS 202), B-0080

**Blazic I., Patil S., Konishi T., Campbell N.M., Garcia-Aguilar J., Gollub M.J.**
Clinical impact of lateral lymph nodes at pelvic MRI in patients with rectal cancer (SS 616b), B-1663

**Bloem J.L.**
Musculoskeletal: bones and soft tissues: Bone tumours (BS 1), A-0033

**Bloem J.L.**
Infection: bone and soft tissue: Infection: bone and soft tissue (E³ 25A), A-0106

**Blum A.**
Upper extremity imaging: Keynote lecture (SS 310), K-07

**Boban J.**
Altered mental state: B. Imaging in an altered mental state (RC 1311), A-0766

**Boca I., Ciurea A., Ciortea C., Berghe A.**
The value of automated breast ultrasound added to full-field digital mammography in women with dense breasts (SS 1002), B-0922

**Boccalini S., Si-Mohamed S.A., Matzuzzi M., Plaine B., Rotzinger D.C., Hanquier L., Boussel L., Revel D., Douek P.**
Characteristics of first-pass myocardial iodine distribution as assessed by dual layer CT with different injection protocols (SS 1403), B-1214

**Bodden J.H.W., Roski F., Hammel J., Mei K., Baum T., Laugerette A., Rummeny E.J., Gersing A.S., Schwaiger B.J.**
Opportunistic osteoporosis screening using hydroxyapatite-specific vertebral bone mineral density measurements derived from dual-layer CT (SS 210a), B-0047

**Boesen L., Thomsen F.B., Nørgaard N., Logager V., Bisbjerg R., Balslev I., Jakobsen H., Thomsen H.S.**
Prostate cancer risk stratification using biparametric MRI and clinical parameters improves selection of biopsy naïve men for prostate biopsies (SS 207), B-0154

**Boesen L., Logager V., Nørgaard N., Bisbjerg R., Balslev I., Thestrup K.D., Jakobsen H., Thomsen H.S., Padhani A.R.**
Assessment of diagnostic yields of significant prostate cancer by altering the prostate-specific antigen density thresholds for biopsy according to MRI findings (SS 407), B-0442

**Boesen M.**
Clinical and weight-bearing indication for CBCT of the extremities (SY 27)

**Bogaert J.**
Functional imaging of the heart: B. Ischaemic diseases (E³ 420), A-0200

**Bogveradze N., Prezzi D., Neji R., Kelly-Morland C., Verma H., Lashkhi K., Goh V.J.**
Investigating blood oxygenation level dependent (BOLD) MRI in small renal masses (SRM), and its correlation with dynamic contrast enhanced (DCE) MRI (SS 616b), B-0616

**Boland M., Rainford L.A., Byrne C., Ryan M.-L.**
An ethnographic study to investigate general practitioner's opinion of ultrasound services in primary care (SS 1414), B-1113

**Boldrini L.**
Current status and future challenges in MR-integrated radiotherapy: Clinical status of MR-integrated photon therapy (ESR/ESTRO), A-0498

**Bollen T.**
Acute pancreatitis: Atlanta Classification of acute pancreatitis (E³ 24A), A-0096

**Bollen T.L.**
Diagnosis and management of acute pancreatitis: A. The 2012 revised Atlanta Classification: what has really changed? (RC 1601), A-0925

**Bolstad K.N.**
Dose reduction and image quality implications of iterative image reconstruction in CT: Chairperson's introduction (RC 1713), A-1049

**Bonaffini P.A., Salman A., Lazaris A., Zu-Hua G., Metrakos P., Vargas H., Hoeffel C., Gallix B., Reinhold C.**
Histopathological growth patterns of colorectal cancer liver metastases: predictive value of MRI morphological features (SS 616a), B-0528

**Bonatti M., Valletta R., Lombardo F., Avesani G., Erdini F., Negri G., Zamboni G., Schifferle G., Bonatti G.**
MRI correlates of molecular classification of endometrial cancer: a preliminary study (SS 1507), B-1389

**Bonaros N.**
Complex cases of TAVI (SY 10)

**Bonneville F.**
Update on cerebrospinal fluid (CSF) diseases: C. RCVS, PRES and others (RC 911), A-0521

**Bonomo L.**
Imaging professionals in the EU: radiologists without borders: Chairpersons' introduction (part 2) (ESR/UEMS), A-0212

**Bons L.R., Sedghi Gamechi Z., Kofoed K.F., Pederson J.H., Budde R.P.J., de Bruijne M., Roos-Hesselink J.W.**
Gender-specific reference values of thoracic aortic growth (SS 203), B-0194

**Booij R., Budde R.P., Bos D., Kremer M., Dijkshoorn M., van Straten M.**
Coronary calcium scoring in CT with a calcium-aware image reconstruction technique: dose reduction by lowering x-ray tube voltage (SS 1813), B-1598

**Booz C., Yel I., Lenga L., Al Kamali A., Boettger S., Wichmann J.L., Vogl T.J., Albrecht M.H., Bodelle B.**
Artificial intelligence in bone age assessment: accuracy and clinical efficiency of a novel fully automated algorithm for bone age assessment in comparison to the Greulich Pyle method (MY 15), B-1362

**Boraschi P., Donati F., Pacciardi F., Cervelli R., Tarantini G., Urbani L., Castagna M., Caramella D., Falaschi F.**
Preoperative staging of colorectal liver metastases: is MRI of the liver needed for the surgical planning? (SS 616a), B-0526

**Boraschi P., Donati F., Tarantini G., Cervelli R., Pacciardi F., Lombardo C., Boggi U., Falaschi F., Caramella D.**
Comparison of reduced field-of-view and large field-of-view diffusion-weighted 3T MR imaging in the evaluation of solid pancreatic lesions (SS 1401a), B-1097

**Borg Grima K., Bezzina P., Rainford L.A.**
The establishment of a triage tool for patient stratifications undergoing myocardial perfusion scintigraphy stress tests in Malta (SS 1014), B-0891

**Borges A.**
Head and neck imaging: A. Cystic neck lesions (E³ 1721), A-0999

**Borggrefe J., Gebest M., Hauger M., Kabbasch C., Schlamann M., Dörner J.**
Differentiation of histologically proven intracerebral tumour entities with quantitative iodine mapping in dual-layer computed tomography (SS 1411a), B-1166

**Borralho P.**
How to deal with the epidemics of thyroid nodules?: The pathologist assessment (MS 16), A-0989

**Bortsova G., Dubost F., Van der Lugt A., Bos D., De Bruijne M.**
Automatic detection of intracranial calcifications in CT using deep learning (SS 1005b), B-0982

**Borzelli A., Paladini A., Amodio F., Cavaglià E., Niola R.**
Role of multidetector CE-CT before transcatheter endovascular embolisation in active arterial bleedings (SS 109), B-0002

**Borzelli A., Paladini A., Amodio F., Cavaglià E., Niola R.**
Tips and tricks of emergency TIPS in patients affected by severe portal hypertension (SS 109), B-0011

**Bosmans J.M.L.**
Reporting and communication today and tomorrow: challenges to implement structured reporting (RS) and deal with artificial intelligence (AI): Communication with referring physicians and patients: what is relevant? (PI 1), A-0507

**Boswijk E., Sanders K.J.C., Broeders E., de Ligt M., Vanmarkenlichtenbelt W.D., Mottaghy F., Schrauwen P., Wildberger J.E., Bucerius J.**
TSH suppressive therapy aggravates arterial inflammation: an $^{18}$F-FDG PET study in thyroid carcinoma patients (SS 315), B-0220

**Boudabbous S.**
Arthro-cone beam computed tomography for the detection of osteoarthritis (SY 27)

**Boudinaud C., Abergel A., Fontarensky M., Joubert Zakeyh J., Boyer L., Magnin B.**
Quantification of hepatic steatosis: evaluation of 4 MRI techniques vs reference method by biopsy (MY 17), B-1470

**Bouhamama A., Leporq B., Lame F., Bihane C., Sdika M., Blay J., Beuf O., Pilleul F.**
MRI-based radiomic to assess lipomatous soft tissue tumours malignancy: a pilot study (SS 1816), B-1638

**Bourgioti C., Chatoupis K., Antoniou A., Tzavara C., Panourgias E., Vergetidou M., Moulopoulos L.**
Adnexal cystic lymphangioma in patients with massive leiomyomatous uterus: a not so uncommon finding (SS 1507), B-1388

**Bourgioti C.**
Imaging in pregnancy: A. Safety issues in pregnancy: what radiologists need to know (RC 1307), A-0809

**Böven J., Boos J., Lanzman R.S., Klosterkemper Y., Kröpil P., Thomas C., Antoch G., Aissa J.**
Evaluation of a novel 3D-surface reconstruction technique in post-mortem CT after stab attacks: diagnostic accuracy and forensic impact compared to conventional CT and autopsy findings (SS 1017), B-0954

**Böven J., Boos J., Lanzman R.S., Kröpil P., Klosterkemper Y., Thomas C., Antoch G., Aissa J.**
Forensic relevance and diagnostic impact of a novel 3D-reconstruction technique (cinematic rendering) of severe injuries in postmortem computed tomography (MY 15), B-1368

**Bovenberg J.A.**
Will the General Data Protection Regulation (GDPR) hamper the secondary use of clinical imaging data for research?: Can I sell or licence imaging data to industry or spin-off companies? (ESR/BBMRI-ERIC), A-0632

**Brady A.**
Reporting and communication today and tomorrow: challenges to implement structured reporting (RS) and deal with artificial intelligence (AI): Chairpersons' introduction (part 1) (PI 1), A-0502

**Brady A.**
Clinical audit: how to deal with the legal and professional requirements: Overview on adoption of BSS throughout Europe (PI 2), A-0587

**Brady A.**
Communicating the role of the radiologist: best practices to manage patient expectations: Explaining the radiologist's role to patients (PA), A-0824

**Brady A.**
Patient safety in medical imaging: Value-based imaging and patient safety (ESR/EFRS), A-0930

**Brambilla M.**
Big data and the big picture: deep learning in optimisation of medical imaging (part B): Chairperson's introduction (EF 2), A-0324

**Brambilla M.**
Medical imaging and emerging issues in occupational radiation exposure: Selection and usage of personal protective equipment in the fluoroscopy and interventional radiology operating room (ESR/EFOMP), A-0547

**Brancatelli G.**
Benefits of Primovist® in cirrhotic livers (SY 13)

**Brancatelli G.**
Hepatocellular carcinoma: from diagnosis to treatment: Chairperson's introduction (RC 1701), A-1001

**Brandão Nascimento A.C., Araujo e Araujo A.R., Meneguette N.S., Papais Alvarenga M., Vasconcelos C.C.F., Bento C.A.M., Rafful P.P., Brandao L.A., Papais Alvarenga R.M.**
Magnetic resonance imaging findings at optic neuritis of recurrent optic-spinal phenotype, multiple sclerosis in a cohort of Brazilian patients from Rio de Janeiro (SS 611), B-0542

**Brantner P.**
The 3D printing lab from bench to bedside: Supporting the surgeon with 3D printing (SF 8f), A-0449

**Braspenningx S., Van Mieghem C., Vanhoenacker P.**
$FFR_{CT}$ vs CAD-RADS$^{TM}$ and its impact on patient management: a retrospective pilot study (MY 18), B-1679

**Brat H., Zanca F., Rizk B., Montandon S., Racine D., Meicher E., Fournier D.**
Clinical diagnostic reference levels for CT examinations: a prospective multicentre study (SS 1813), B-1600

**Bremerich J.**
Acute chest pain and cardiac imaging: C. Late gadolinium enhancement (LGE) and more: the role of MRI in and after ACS (RC 1603), A-0940

**Bremnes A., Ween B.**
Optimisation of shunt radiography (SS 1314), B-1072

**Briers E.**
Communicating the role of the radiologist: best practices to manage patient expectations: The patient perspective: sharing expectations and experiences (part 2) (PA), A-0826

**Briers E.**
Patient safety in medical imaging: Patient perspectives on patient safety in medical imaging (ESR/EFRS), A-0932

**Brillet P.-Y.**
Fibrotic lung diseases: B. Lung fibrosis in connective tissue and granulomatous diseases (RC 1204), A-0701

**Brink M.**
Thoracic emergencies: A. Chest trauma (RC 1704), A-1053

**Brito A.Q.L.C., Almeida R.P.P., Rodrigues S., Abrantes A.F., Ribeiro L., Sousa P., Azevedo K.B., Simão D.M.**
Establishment of national diagnostic reference levels for dental radiology (SS 714b), B-0658

**Brkljačić B.**
Imaging strategies in renal tumours: Chairperson's introduction (RC 907), A-0571

**Brkljačić B.**
Minimally-invasive local treatment of breast cancer: the time is now: B. Radiofrequency ablation therapy (RC 1202), A-0673

**Brkljačić B.**
US and vascular disease: a perfect match: B. Upper and lower limb: arterial district (RC 1315), A-0753

**Brkljačić B.**
Artificial intelligence: presented with new lecture formats: Introduction (ESOR), A-0368

**Broeders M.**
Personalised breast cancer screening and the role of breast density (SY 2c)

**Brombal L., Delogu P., Dreossi D., Golosio B., Oliva P., Mettivier G., Rigon L., Taibi A., Longo R.**
Towards clinical phase-contrast breast CT at Elettra: major SNR improvement at larger propagation distances (SS 1913), B-1846

**Brookes A.L.**
Forensic imaging: C. Paediatric forensic imaging (RC 814), A-0401

**Brountzos E.N.**
Multidisciplinary approach to the diabetic foot: Chairperson's introduction (MS 17), A-1062

**Brountzos E.N.**
Multidisciplinary approach to the diabetic foot: Multidisciplinary case presentation and discussion (MS 17), A-1067

**Brown G.**
Gastrointestinal radiology: B. Rectal cancer staging: key findings ($E^3$ 821), A-0391

**Brown P., Zhong J., McDermott G.M., Fronza M., Gilbert A., Appelt A., Sebag-Montefiore D., Currie S., Scarsbrook A.**
Prediction of loco-regional treatment failure after chemo-radiotherapy in anal cancer using FDG PET/CT-derived feature analysis (MY 3), B-0361

**Brugnara G., Isensee F., Wick W., Bendszus M., Maier-Hein K.H., Kickingereder P.**
Automated volumetric assessment of multiple sclerosis disease burden and activity with artificial neural networks (SS 611), B-0539

**Brumberg R.M., Emrich T., Wenzel P., Benz S., Kloeckner R., Halfmann M.C., Düber C., Kreitner K.-F.**
Feature tracking allows detection of diminishing wall motion disturbances in Takotsubo cardiomyopathy (SS 1903a), B-1824

**Bruno F., Micelli M., Panebianco L., Arrigoni F., Varrassi M., Catalucci A., Sucapane P., Ricci A., Masciocchi C.**
Treatment of essential tremor (ET) and Parkinson disease (PD) tremor with MRgFUS: preliminary results from 21 patients in a single centre (SS 1811a), B-1552

**Bubel K., Altmeyer K., Pfeifer J., Abdul-Khaliq H., Krenn T., Massmann A., Bücker A., Fries P.**
Radiation exposure of thoracic computed tomography in paediatric patients: can we always achieve a sub-mSv scan in a clinical setting? (SS 212), B-0167

**Buch K., Rapalino O., Tarbell N., Yock T., MacDonald S., Caruso P.A.**
Frequency of optic nerve involvement in paediatric intracranial germ cell tumours (SS 1412), B-1227

**Buch K., Rincon S., Krishnamoorthy K., Gomboli G., Caruso P.A.**
Deep medullary venous thrombosis in neonates correlates highly with the development of shunt-dependent hydrocephalus (SS 1412), B-1229

**Bucher A.M., Brendlin A., Scholtz J.E., Beeres M.**
Full-body 150-kV spectral-shaping low-dose CT in multiple myeloma follow-up: evaluation of image quality and radiation dose (SS 316), B-0347

**Buissink C.**
Career planning: Practising radiography around the world (BR 9), A-0532

**Bulakbasi N.**
Diffuse low-grade gliomas: new things you should know: C. Advanced imaging in low-grade gliomas (RC 1711), A-1032

**Bünting N.A., Barabasch A., Kuhl C.K.**
Intra-individual comparison of contrast enhancement rates of focal liver lesions in DCE-MRI with gadoxetic acid vs gadobutrol (SS 201a), B-0073

**Buonomenna C., Messina C., Gitto S., Biacca A., Albano D., Sconfienza L.M.**
Fat mass does not increase the precision error of trabecular bone score measurements (SS 210a), B-0052

**Burabe F.A., Luntsi G., Chigozie N.I., Nkubli F., Dlama J.Z., Ogenyi P.A.**
Sonographic estimation of amniotic fluid volume using the amniotic fluid index and the single deepest pocket in a resource-limited setting (SS 1414), B-1107

**Burck I., Goldenko M., Albrecht M.H., Lehn A., Stöver T., Harth M., Wichmann J.L., Kaltenbach B., Vogl T.J.**
Mastoid pneumatisation in children before CI: a risk factor for complications? (SS 308), B-0296

**Burck I., Schneider S., Yel I., Lehn A., Balster S., Helbig S., Stöver T., Vogl T.J., Kaltenbach B.**
Radiohistological comparison study of temporal bone specimen after CI electrode array insertion: is CBCT superior to MSCT? (MY 14), B-1247

**Burck I., Rosenzweig M., Nour Eldin N., Kvasnicka H., Stöver T., Jacobi V., Vogl T.J.**
Accessing the skull base: CT-guided submaxillary core needle biopsy for the diagnosis of skull base lesions (MY 14), B-1257

**Burger I.A.**
Whole-body imaging in metastatic urinary tract and prostate cancer: C. PET and PET/MRI in prostate cancer ($E^3$ 419), A-0197

**Burger I.A., Ferraro D.A., Müller J., Mühlematter U., Messerli M., Stolzmann P., Rupp N.J., Donati O.F., Eberli D.**
Performance of $^{68}$Ga-PSMA-11-PET/MRI guided biopsy to detect significant prostate cancer (SS 306), B-0228

**Burke A.M., Foley S.J.**
Should DR and CR projection radiography have separate LDRLs at a local hospital level? (SS 714b), B-0663

**Burulday V., Çelebi U.O., Ogden M., Akgul M.H., Dogan A., Ozveren M.F.**
Preoperative and postoperative strain elastography, and shear wave elastography findings of the sciatic nerve in patients with unilateral lumbar disc herniation (SS 1910b), B-1744

**Busse A., Neßelmann C., Hoffman A., Dohmen P.M., Weber M.-A., Meinel F.G.**
ECG-gated MR angiography at 3T for follow-up after aortic surgery involving the ascending aorta (SS 1815), B-1502

**Buvat I.**
Artificial intelligence in hybrid imaging: AI and Holomics: predicting the truth from hybrid imaging and non-imaging data (ESHI(MT) 2), A-0553

**Buytaert D., Keelson B., Van Gompel G., Casselman J., Buls N., Bacher K.**
Effective dose comparison between cone beam CT (CBCT) and MSCT for three types of head examinations (SS 1813), B-1596

**Byun S., Lee J., Min J., Shin K., You S., Cheon K., Song I., Kim K.**
Liver fibrosis staging with Cannon shear wave elastography: comparison with three other non-invasive tools using histologic criteria as a reference standard (SS 1801a), B-1538

### C

**Caballo M., Teuwen J., Mann R.M., Sechopoulos I.**
Computer-aided detection of breast masses in dedicated breast CT images using adaptive parenchyma local search and deep learning (SS 1805a), B-1523

**Caballo M., Mann R.M., Sechopoulos I.**
Automatic deep learning-based denoising filter for 4D dedicated breast CT perfusion imaging of breast cancer (SS 1805a), B-1525

**Cabuy M., Van Gompel G., Deak P.D., de Mey J., Buls N.**
Characterisation of patient skin dose in CT imaging (SS 1813), B-1597

**Calder A.D.**
Imaging in abdominal emergencies: an (evidence-based) update: B. The acute abdomen in young children (RC 412), A-0189

**Calleja M., Grech M.E., Courtier N., Couto J.G.**
Assessing dignity of patients undergoing radiotherapy for pelvic cancer (SS 714a), B-0649

**Calli C.**
State-of-the-art paediatric neuroradiology: Chairperson's introduction (RC 811), A-0428

**Calli C.**
Neurologic emergencies: Haemorrhagic stroke (SAH) (BS 9), A-0523

**Calli C.**
Neuro: Chairperson's introduction
($E^3$ 1423), A-0828

**Calliada F.**
Tips and tricks for abdominal ultrasound: Doppler imaging (US 2), A-0667

**Calvo Imirizaldu M., Ezponda Casajús A., Soriano I., Paternain Nuin A., Elorz M., Aquerreta Beola J.D.**
Comparison of image quality and radiation dose between combined ATCM and FTC technique in whole-body low-dose multidetector CT in the diagnosis of multiple myeloma
(SS 210b), B-0064

**Calvo Imirizaldu M., Ezponda Casajús A., García Baizán A., Pueyo Villoslada J.C., Rabago G., Bastarrika Alemañ G.**
Systolic high-pitch coronary CT angiography for evaluation of the coronary arteries in heart transplant recipients (SS 1003), B-1015

**Calvo Imirizaldu M., González de la Huebra Rodríguez I., Ezponda Casajús A., Etxano J., Elizalde Pérez A.M., Pina Insausti L.J.**
The accuracy of contrast-enhanced mammography: a retrospective multireader study (SS 1902a), B-1784

**Campo I., Sachs C., Bussani R., Bertolotto M., Cova M.A.**
Focal testicular lesions: multiparametric US features and association with histopathology (SS 1807), B-1659

**Campo I., Cernic S., Arban F., Pozzi Mucelli R., Sacconi F., Cova M.A.**
Pre-surgical marking under CT guidance of no-palpable, non solid/solid/partially solid pulmonary nodules: when and why to use it (SS 1909), B-1807

**Campora R., Piana Jacquot F.M., Zorza I., Agazzi G.M., Ravanelli M., Farina D.**
Staging tongue cancer with the 8$^{th}$ edition of TNM: can MR provide a surrogate of the pathologic DOI? (SS 1008), B-0970

**Camps Herrero J.**
Functional imaging of breast and female pelvis: A. Breast tumours ($E^3$ 820), A-0474

**Camps Herrero J.**
Diagnosing ductal carcinoma in situ (DCIS): B. Imaging DCIS (RC 1702), A-1028

**Cannella R., Brancatelli G., Rangaswamy B., Minervini M.I., Borhani A.A., Furlan A.**
Enhancement pattern of hepatocellular adenoma (HCA) on MR imaging performed with Gd-EOB-DTPA vs other Gd-based contrast agents (GBCAs): an intraindividual comparison
(SS 201a), B-0071

**Cannella R., Fraum T.J., Ludwig D.R., Borhani A.A., Fowler K.J., Furlan A.**
Value of targetoid appearance on T2-weighted imaging and signs of tumour vascular involvement for differentiating hepatocellular carcinoma (HCC) from other non-HCC liver malignancies (SS 1901a), B-1765

**Cannella R., Vernuccio F., Marin D., Furlan A.**
Liver imaging reporting and data system v2018: visualisation rate and diagnostic value of ancillary features favouring malignancy in observations at intermediate and high risk for HCC (SS 1901a), B-1771

**Cannizzaro E., Palumbo P., De Cataldo C., Corridore A., Torlone S., Cobianchi Bellisari F., Di Sibio A., Di Cesare E., Masciocchi C.**
Strain analysis in noncompaction morphology: usefulness in assessing an undefined cardiomyopathy (SS 603), B-0595

**Cantisani V.**
CEUS: from guidelines to clinical practice
(SY 18)

**Cao G., Duan Y., Zhu W., Liu Y.**
Brain MRI characteristics in neuromyelitis optica spectrum disorders: a multi-center study in China (MY 16), B-1429

**Caobelli F.**
Dead or alive: imaging of myocardial viability: B. Hybrid imaging (RC 1703), A-1019

**Capasso F., Coppola M., Caruso M., Stilo S., Stanzione A., Venetucci P.**
Prevention of post-partum haemorrhages in patients with morbidly adherent placenta: a monocentric experience (SS 709), B-0738

**Capocci R., Tacher V., Bosc R., Totobenazara J., Kobeiter H., Luciani A., Meyblum E., Dao T.H.**
Efficiency of mammography, tomosynthesis and breast MRI in detecting malignant extension to the nipple area complex in patients with multifocal or extensive breast DCIS
(SS 302), B-0252

**Capozzi N., Fernandez-Clotet A., Alfaro I., Ordás I., Panes J., Rimola J.**
Validation of the simplified magnetic resonance index of activity (sMaRIA) for Crohn's disease without using gadolinium (SS 1801b), B-1653

**Cappello G., Doronzio V., Romano V., Timpani A., Desi G., Cortese F., Giannini V., Mazzetti S., Regge D.**
Imaging texture features to predict neoadjuvant therapy response in locally advanced rectal cancer (SS 616b), B-0618

**Capretti I., Palumbo P., Iafrate S., Bruno F., Arrigoni F., Mariani S., Zugaro L., Barile A., Masciocchi C.**
Clinical response in hip osteoarthritis: comparison between intra-articular treatment with hybrid hyaluronic acid (HA) and combined therapy with Platelet-Rich Plasma (PRP) and HA (SS 1510), B-1295

**Caramella C.**
Imaging in oncological trials: Assessing treatment response in the era of immunotherapy (ESR/EORTC), A-0792

**Carchesio F., Iezzi R., Posa A., Veltri G., Gasbarrini A., Manfredi R.**
Operator learning curve for transradial liver cancer embolisation: implications for the initiation of a transradial access programme (SS 209), B-0109

**Carchesio F., Iezzi R., Posa A., Congedo T., Veltri G., Margaritora S., Manfredi R.**
Percutaneous radiofrequency thermal ablation (RFA) of lung neoplasms using a new 15 G internally cooled jet-tip electrode: preliminary results (SS 1909), B-1812

**Cardano G., Fighera A., Campagnola A., Zamboni G., Frulloni L., Mansueto G.**
Diagnostic accuracy of CT features and CT texture analysis in differentiating focal autoimmune pancreatitis from adenocarcinoma: a retrospective study (SS 1401a), B-1094

**Cardis E.**
CT radiation risk in children: an overview: Chairperson's introduction (EU 6), A-0933

**Cardis E.**
CT radiation risk in children: an overview: Ongoing studies and first results of the European EPI-CT study (EU 6), A-0935

**Carmona-Bozo J.C., Woitek R., Patterson A.J., Baxter G., Caraco C., Provenzano E., Fryer T.D., Graves M.J., Gilbert F.**
Is ADC a predictor of hypoxia in ER+ breast cancer? A study using $^{18}$F-FMISO PET-MRI
(SS 606), B-0501

**Carnevale A., Cossu A., Cotti E., Graziano M., Chiarello S., Giganti M.**
Combined pulmonary fibrosis and emphysema (CPFE): epidemiological and clinical features, visual scoring and quantitative CT assessment in 72 patients (SS 1604), B-1406

**Caroli A.**
Imaging biomarkers and their combinations in the era of artificial intelligence: COST action initiatives as a platform for image biomarker selection (ESR/EIBALL), A-0059

**Carolus H., Iuga A., Brosch T., Wiemker R., Klinder T., Maintz D., Höink A., Püsken M.**
Fully automatic detection and segmentation of thoracic lymph nodes in MSCT scans of the chest using deep learning (SS 1905a), B-1761

**Carrafiello G.**
From morphology to function: Emergency interventional radiology: brain and body (EM 2), A-0582

**Caruana C.J.**
Principles of imaging and radiation protection: Chairperson's introduction (E³ 1523), A-0895

**Caruana G., Taibbi A., Petta S., Cannella R., Busè G., Cutaia G., Giambelluca D., Di Marco V., Bartolotta T.V.**
Liver stiffness quantification in patients with non-alcoholic steatohepatitis: comparison of shear wave elastography and transient elastography with liver biopsy correlation (SS 1801a), B-1529

**Caruso P.A.**
Differential diagnoses you don't want to miss: B. Differential diagnoses of paediatric neck lesions (RC 1308), A-0798

**Caruso P.A., Buch K., Corre C., Eichler F.**
MRI findings in gene therapy-treated adrenoleukodystrophy (SS 1011a), B-0933

**Caruso P.A., Buch K., Bates S., Roberts D.**
MRI patterns of brain injury in neonates with pathology-proven placental abruption (SS 1412), B-1226

**Caruso P.A., Pescosolido M., Buch K., Morrow E.**
MRI findings in Christianson syndrome (SS 1412), B-1228

**Casale R.**
Preliminary results of a neural network for the automatic detection of chest X-ray incorrect exposure (SS 205), B-0020

**Casale S., Fanizza M., Paganelli C., Viselner G., Turpini E., Fiore M., Fumagalli I., Valvo F., Preda L.**
Predictive role of apparent diffusion coefficient values from diffusion weighted sequences in patients with sacral chordoma treated with carbon ions radiotherapy alone (MY 3), B-0373

**Caseiro Alves F.**
Benefits of Primovist® in non-cirrhotic livers (SY 13)

**Caseiro Alves F.**
Imaging of benign liver lesions: still difficult?: B. Liver haemangiomas and mimickers (RC 101), A-0003

**Caspers J., Turowski B., Rubbert C.**
Automated age-specific regional brain atrophy estimation: an integrated workflow (SS 1005b), B-0983

**Cassar-Pullicino V.N.**
Musculoskeletal: bones and soft tissues: Soft tissue tumours (BS 1), A-0032

**Cassar-Pullicino V.N.**
The old spine: challenges of imaging and treatment: A. Degeneration of the old spine: relevance of findings and differential diagnosis (RC 1610), A-0969

**Cassart M.**
Foetal and neonatal imaging pearls: D. Pre- and postnatal congenital cystic renal diseases (RC 912), A-0563

**Casselman J.W.**
Radiologic anatomy: head and neck: Temporal bone (BE 6), A-0319

**Casselman J.W.**
Imaging in Meniere's disease: what to believe and how to interpret: Chairperson's introduction (SF 15b), A-0885

**Casselman J.W.**
Imaging in Meniere's disease: what to believe and how to interpret: Panel discussion: Did you pick up the essentials? (SF 15b), A-0890

**Castagnoli F., Grazioli M., Puglia M., Frittoli B., Morone M., Romanini L., Donato F., Ambrosini R., Grazioli L.**
Gadoxetic acid (GA)-enhanced liver MRI and hepatic haemangiomas (HH): diagnostic performance and impact of dynamic phase on lesion characterisation (SS 1001b), B-1042

**Castañer E.**
CT and intervention in vascular pulmonary diseases: B. Evaluating the pulmonary vasculitis (E³ 822), A-0471

**Castellano E.**
Getting the balance right: radiation risk and imaging benefit in paediatric procedures: Computed tomography: are we doing enough? (EU 5), A-0873

**Catalano C.**
Imaging professionals in the EU: radiologists without borders: The benefits of harmonised training for the radiologists of tomorrow (ESR/UEMS), A-0218

**Catania D.**
Radiography audit and quality management: B. An expert in radiography quality management (RC 514), A-0209

**Catania M., Foti G., Beltramello A., Carbognin G.**
Diffusion tensor imaging with quantitative evaluation of sciatic nerve within the pelvis in patients with non-contributory lumbar spine magnetic resonance imaging in radiculopathy (SS 1910b), B-1743

**Caudal A., Lefebvre G., Guenoun D., Corcos G., Nisolle J., Vuillemin-Bodaghi V., Vande Berg B.**
Medial meniscal ossicle is associated with medial posterior root tear and ACL tear: a case-controlled multicentric SIMS study of 42 cases (SS 1810), B-1507

**Caumo F., Gennaro G., Romanucci G., Zorzi M., Fedato C., Montemezzi S.A.**
Cancer detection rate, ductal carcinomas in situ and advanced breast cancers in the 2nd round of a population-based screening with tomosynthesis and synthetic mammography (SS 302), B-0250

**Cavalieri S., Ferrari A., Villa R., Brambilla M., Stecco A., Carriero A.**
Dose optimisation in multiphasic computed tomography imaging of the liver with high-concentration contrast media (SS 701), B-0798

**Cazzato R.L.**
Imaging of chronic forefoot pain: C. Imaging-guided percutaneous treatment of forefoot pain (RC 510), A-0263

**Celeng C., Froeling M., Leiner T.**
Feasibility of T1-rho mapping as endogenous contrast agent to detect myocardial fibrosis (SS 303), B-0377

**Cerezal L.**
Elbow imaging: from detailed anatomy to pathology: C. Plica and cartilage (RC 1710), A-1048

**Cerit M., Oner A.Y., Yildiz A., Cindil E., Sendur H.N., Leventoglu S.**
Perianal fistula mapping at 3T: volumetric vs conventional MR sequences (SS 201b), B-0213

**Cewe Jönsson P.M., Thorisdottir S., Óladóttir G.L., Koskinen S.**
Imaging of penetrating extremity injuries in a Scandinavian level-1 trauma centre: a 4-year analysis of the trauma registry data from the Karolinska trauma centre, Stockholm (SS 1017), B-0955

**Chai Y.**
Application of preset adaptive statistical iterative reconstruction-V in dual-enhanced abdominal CT (SS 213), B-0139

**Chai Y.**
Diagnostic accuracy of CT for lymph node metastasis in gastric cancer: comparison of spectral parameters developed dual-energy CT and conventional CT (SS 1401b), B-1276

**Chai Y.**
Correlation between dual-energy CT and perfusion CT parameters in patients with gastric carcinoma (SS 1901b), B-1876

**Challen R., Low L., McEntee M.F.**
Dementia patient care in the medical imaging department (SS 1814), B-1496

**Chan L.C.A., Wong K.H., Tang P.Y., Tam K.Y., Cheung K.O., Chiu C.L.**
Biopsy-proven ductal carcinoma in situ radiographic features and upgrade rates
(SS 1902b), B-1798

**Chand G., Xing Y., Liu C., Wong N., Meszaros L., O'Doherty J., Ting H., Zhao J., Cook G.**
Imaging programmed cell death ligand-1 (PD-L1) expression in non-small cell lung cancer with $^{99m}$Tc-anti PDL1 sdAb SPECT
(SS 1016), B-1021

**Chang P.**
Opportunities for AI-enabled patient care
(SY 20)

**Chassagnon G.**
Pulmonary neuroendocrine proliferations and neoplasms: A. Diffuse idiopathic pulmonary neuroendocrine cell hyperplasia (DIPNECH)
(RC 504), A-0268

**Chaudhry M.B.H., Azeemuddin M., Ahmad M.N., Mubarak F., Haq T.U.**
Reliability of on-call radiology residents' interpretations in detecting intracranial aneurysms on 640 slice multidetector computed tomography angiography (MY 15), B-1367

**Chen C., Cheng J., Zhang Y.**
Enhancement of T1WI histogram in the identification with primary central nervous system lymphoma and glioblastoma
(SS 1416), B-1234

**Chen C., Cheng J., Zhang Y.**
T2WI histogram analysis of whole tumour volume for differentiating glioblastoma from primary central nervous system lymphoma
(SS 1416), B-1235

**Chen C., Ren C.P., Zhao R.C., Cheng J.L.**
Value of ADC and DCE-MRI in differentially diagnosing of angiomatous meningioma and solitary fibrous tumours/haemangiopericytoma
(MY 14), B-1264

**Chen C., Cheng J.**
Analysis the changes of white matter structure with tract-based spatial statistics according to diffusion tensor imaging in Parkinson's disease
(SS 1811a), B-1559

**Chen C., Haiwei H., Yunkun S., Xu H.**
Visualisation of the trigeminal nerve branches on enhanced 3D-SPACE-STIR sequence: initial experience (MY 14), B-1254

**Chen H., Zou T.**
Functional network-based statistics reveal abnormal resting-state functional connectivity in minimal hepatic encephalopathy
(SS 1011b), B-1041

**Chen H., Yan J., Liu K., Deng Y., Huang X., Su R., Xiaojun Z.**
Less regulated, more segregated ventral visual network in patients with major depressive disorder (MY 16), B-1430

**Chen M., Yuan H.**
Consistency of bone mineral density assessments between dual-energy CT and quantitative CT (SS 210a), B-0048

**Chen X., Xin J.**
PET/CT imaging of the novel molecular probe $^{11}$C-aminoglycerol for the diagnosis of liver fibrosis (SS 306), B-0236

**Chen X., Li H.**
DWI and high-resolution T2WI-volumetry association with lymphovascular invasion and N-stages in resectable rectal cancer
(MY 17), B-1480

**Chen X., Li L., Zhao S.**
Early detection of left ventricular involvement assessed by 3D CMR feature tracking improves risk stratification in patients with arrhythmogenic right ventricular cardiomyopathy
(SS 603), B-0593

**Chen X.J.**
Accurate pulmonary nodule detection in CT images using multi-stage 3D deep convolution neural networks (MY 7), B-0812

**Chen Y., Michalopoulou E., Gale A.G., Barrett T., Haslam P., Patel A., Ahmed H.**
Prostate imaging self-assessment and mentoring (PRISM): a prototype self-assessment scheme for radiologists (SS 407), B-0437

**Chen Y., Michalopoulou E., Gale A.G., Bernardi D.**
Improving quality assurance in DBT breast screening by visual search monitoring
(SS 1802a), B-1548

**Chen Y., Zhu Y., Guan W., Qu Y.**
MRI/US fusion transperineal prostate biopsy versus systematic biopsy for clinically significant prostate cancer detection in patients with negative ultrasound findings (SS 1007), B-0881

**Chen Y., Ma J., Wang J., Wang F., Feng S.**
A novel improved deep learning framework for liver lesion detection (MY 7), B-0810

**Chen Y., Zhu Y., Liu H., Xu K., Yu S., Su T., Zhang Z., Wang Y., Jin Z.**
Dual-layer detector CT angiography in preoperative assessment of carotid body tumours: comparison of virtual monoenergetic and polyenergetic images (SS 1808), B-1633

**Cheng S., Xue H., Jin Z.**
Locally advanced and metastatic pancreatic cancer: the role of CT quantitative imaging biomarkers for predicting outcomes of patients treated with chemotherapy (SS 1401a), B-1104

**Cheng Z., Cai X., Feng Y.**
Application of intravoxel incoherent motion imaging (IVIM) in patients with hyperuricemia
(MY 13), B-1083

**Chhabra A., Pezeshk P., Dessouky R., Morales O., Shah J.**
Dixon imaging of bone stress injury of the knee: comparison to conventional proton density-weighted MR imaging (SS 1410), B-1129

**Chianca V., Albano D., Cuocolo R., Messina C., Gitto S., Corazza A., Merli I., Brunetti A., Sconfienza L.M.**
Machine learning classification of spinal lesions: compared accuracy of texture parameters extracted by different software
(SS 1910a), B-1730

**Chilamkurthy S., Ghosh R., Tanamala S., Biviji M., Campeau N., Venugopal V.**
Automated detection of midline shift and mass effect from head CT scans using deep learning
(SS 705), B-0755

**Chin L.H.Q., Lee K.H., Ng H., Cheung M., Li Y.L., Lau H.T., Tse D.**
Development of a CT diagnostic prediction model for diagnosis of pyonephrosis
(SS 307), B-0330

**Cho C., Grosse-Siestrup C., Brinker G., Nadobny J., Niehues S., Taupitz M., Hamm B., Schlattmann P.**
Hotspot temperatures leading to burn in 3T MRI in pigs (SS 713), B-0762

**Cho E., Choi J.M., Kim J.H., Yu J., Chung J.**
Dual-arterial phases and split-bolus intravenous contrast injection: reduction of transient respiratory motion artefact on gadoxetate disodium-enhanced MRI of the liver
(SS 401), B-0397

**Cho M., An C., Aljoqiman K., Kim M.**
Non-invasive imaging diagnosis of hepatocellular carcinoma in patients with both chronic liver disease and a history of extrahepatic primary cancer (SS 1901a), B-1763

**Chodyla M., Demircioglu A., Haubold J., Bogner S., Herrmann K., Nensa F., Umutlu L.**
18F-FDG-PET/CT-based radiogenomic tumour decoding and phenotyping of non-small cell lung cancer (SS 605), B-0575

**Choi B.**
Feasibility study of synthetic diffusion-weighted MR imaging in patients with breast cancer by qualitative and quantitative assessment
(SS 1802b), B-1567

**Choi B., Lee K.Y., Kim C., Lee S., Je B., Cha J., Yong H., Kang E., Oh Y.**
Comparison of coronary lumen diameter in CT angiography: spray nitroglycerin vs sublingual nitroglycerin tablet administration
(SS 1003), B-1014

### Authors' Index

**Choi N., Kim Y., Jung N., Kang B., Lee J.**
Can 18 F-FDG PET/CT predict invasive components in DICS of breast diagnosed by needle biopsy? Comparison with variable imaging modalities including mammography, USG and MRI (MY 5), B-0469

**Chou Y.**
Using MRI or CT for preoperative evaluation of HCC makes the clinical outcome different (SS 201a), B-0075

**Choudary G., Kale P.G., Peddi S.**
Correlation of three-dimensional computerized tomographic renal volumetry with DTPA split renal function in prospective donors for renal transplantation (MY 13), B-1075

**Chriashkova J., Goncalves C., Aslam M., Perera S., Walter S., Fisher J., Podlasek A., Schmitt A.J., Grunwald I.Q.**
Can artificial intelligence improve physician sensitivity in detecting early ischaemic damage on computed tomography? (SS 1005b), B-0979

**Christie S., Ng C., Sá dos Reis C.**
Influences on radiographers' choice of immobilisation methods in paediatric radiological examinations (SS 214), B-0025

**Christou A., Koutoulidis V., Koulocheri D., Panourgias E., Zografos C., Zografos G.**
Performance of breast lesion excision system (BLES) in a complete removal of small clusters of suspicious microcalcifications proved malignant (SS 1402a), B-1157

**Chulroek T., Wangcharoenrung D., Cattapan K., Hamed K., Mitchell A.J., Harisinghani M.G., De E.J.B.**
Magnetic resonance imaging findings differentiate among periurethral bulking agent, urethral diverticulum and periurethral cyst, preventing misdiagnosis (SS 707), B-0827

**Cil B.E.**
Visceral arteries: A. Diagnosis of vascular compression syndromes (RC 115), A-0021

**Cilia F., Calvieri C., Catapano F., Coco S., Ammendola R.M., De Rubeis G., Galea N., Francone M., Catalano C.**
Evaluation of left ventricular strain assessment with feature-tracking CMR in STEMI at long term follow-up (SS 603), B-0592

**Clauser P.**
Pretherapeutic diagnostics of tumour extent (part 2) (SY 2d)

**Clauser P., Baltzer P.A.T., Kapetas P., Woitek R.A., Leone F., Pinker-Domenig K., Weber M., Bernathova M., Helbich T.H.**
Diagnostic performance of low dose contrast-enhanced dual energy mammography in comparison to contrast-enhanced MRI (SS 1802a), B-1549

**Clauser P.**
Breast imaging in pre-menopausal women: Breast cancer screening in pre-menopausal women (SF 8e), A-0426

**Clément O.**
Imaging biomarkers and their combinations in the era of artificial intelligence: Chairpersons' introduction (part 1) (ESR/EIBALL), A-0056

**Clément O.**
Professional issues for radiology departments to enable research: Chairperson's introduction (PI 3), A-0625

**Clément O.**
Contrast media guidelines: C. Ongoing evidence for acute adverse reactions (RC 1207), A-0726

**Clevert D.A.**
Advancing the role of ultrasound with 3D/4D CEUS in the abdomen (SY 17)

**Clevert D.A.**
Early clinical experience using the XL14-3 for small parts applications (SY 17)

**Clevert D.A.**
Ultrasound simulation models in training and education: where are we going?: Chairpersons' introduction (part 1) (ESR US SC/EFSUMB), A-0039

**Clevert D.A.**
How to perform ultrasound image fusion: Prostate (US 1), A-0171

**Clevert D.A.**
Tips and tricks for abdominal ultrasound: Elastography (US 2), A-0669

**Clevert D.A.**
US and vascular disease: a perfect match: A. Abdominal aorta (RC 1315), A-0752

**Clive K.**
The role of obstetric ultrasound in low resources setting: pilot survey done in Elgeyo-Marakwet and Kericho county from 2016 March to 2018 December (SS 1414), B-1111

**Cockmartin L., Lemmens K., Jacobs A., Binst J., Bosmans H.**
Multiparameter dose monitoring in mammography: the use of combined breast thickness and density determined DRLs (SS 1802a), B-1539

**Coco S., Ammendola R., Bracci A., Catapano F., Cilia F., De Rubeis G., Galea N., Francone M., Catalano C.**
Role of cardiac magnetic resonance (CMR) imaging for early detection of myocardial involvement in patients affected by Anderson Fabry disease (AFD) (SS 1903a), B-1823

**Cohen C., Gaillot K., Janot K., Cottier J., Dejobert M., Narata A.**
Does IV-tPA induce thrombus migration? A retrospective study comparing bridging therapy and thrombectomy alone (MY 8), B-0836

**Cohen C., Law-Ye B., Dormont D., Sanson M., Galanaud D., Pyatigorskaya N.**
Arterial spin labelling for the evaluation of immediate postoperative residual lesion in adult brain tumours (MY 16), B-1422

**Colarieti A., Venturini M., Perani L., Esposito A., Del Maschio A., De Cobelli F.**
US and CEUS in ovary assessment of a murine model: preliminary findings on protective role of gonadotropin releasing hormone analogue from chemotherapy-induced ovarian damage (MY 3), B-0369

**Collette L.**
Imaging in oncological trials: Trial design and imaging end-points (ESR/EORTC), A-0790

**Collocola A., Ortino M., Durante S.**
Quality controls in ultrasound: a trial to evaluate the obsolescence of ultrasound probes with a low-cost agar gel phantom (SS 1013), B-0999

**Colombi D., Chiesa S., Bodini F., Morelli N., Silva M., Sverzellati N., Franco C., Michieletti E.**
Difference between measurement of T size at computed tomography and at resected specimen: is it relevant for NSCLC stage? (SS 1016), B-1029

**Coma A.**
Imaging in abdominal emergencies: an (evidence-based) update: A. The acute abdomen in neonates (RC 412), A-0188

**Connor S.**
Differential diagnoses you don't want to miss: A. Differential diagnoses of nose and paranasal sinus lesions (RC 1308), A-0797

**Conti A., Calcagno M.C., Meo D., Modica A., Rizzo D., Mammino L., Foti P.V., Palmucci S., Basile A.**
Comparative assessment of occupational radiation exposure between transbasilic and transfemoral approach during percutaneous retrograde scleroembolisation of left varicocele (SS 1009), B-0959

**Cook G.**
Hybrid and translational imaging: the bigger picture: On the horizon: novel PET tracers with translational potential (ESR/ESHI(MT)), A-0975

**Coolen A.M.P., Korte B., Duijm L.E.M.**
Additional breast cancer detection at screening mammography through quality assurance sessions between screening radiographers and coordinating screening radiologists (SS 202), B-0081

**Copley S.J.**
Challenging HRCT patterns: tips and tricks from the experts: Mixed ground-glass opacification and reticulation (SF 8b), A-0456

**Cornelis F.**
Role of imaging in cancer of unknown primary (CUP): C. The role of the interventional radiologist (RC 516), A-0283

**Cornud F., Jemal Turki A., Barral M., Soyer P.**
Restriction of diffusion and tumor aggressiveness: a non applicable correlation for transition zone prostate cancer (SS 207), B-0155

**Corrias G., Micheletti G., Suri J., Argiolas G.M., Lucatelli P., Saba L.**
Is automatic plaque quantification reliable: an analysis of spectral multienergy CT (SS 215), B-0036

**Corrias G., Balestrieri A., Saba L.**
Is synthetic MRI of the brain feasible in a clinical setting? A comparison with standard protocol (SS 1911a), B-1775

**Corridore A., Torlone S., Micelli M., Martino M., Panebianco L., Cerrone P., Sucapane P., Splendiani A., Masciocchi C.**
3T susceptibility-weighted imaging and Parkinson's disease: new fields in neuroimaging (SS 1811a), B-1556

**Costa M., Ermacora D., Pesente S., Simonetti G., Belladonna E., Tonutti M.**
Can image registration support radiologists in breast tomosynthesis image reading? (SS 1805a), B-1521

**Costanza C., Mariscotti G., Durando M., Tagliafico A., Bergamasco L., Castellano I., Fonio P.**
Background parenchymal enhancement on breast MRI in patients undergoing neoadjuvant chemotherapy: is it a biomarker of tumour response? (SS 1402b), B-1173

**Cotten A.**
Imaging of forgotten small joints: C. Proximal tibio-fibular joint: a cause for lateral knee pain (RC 1310), A-0784

**Coudyzer W., Cockmartin L., Konings D., Busselot T., Bielen D., Vanbeckevoort D., Bosmans H.**
Patient-specific scan delay for optimised venous triggering in CT abdomen (SS 615), B-0494

**Coutinho Santos A.**
Renal transplantation: C. CT/MRI/angiography for diagnosis and intervention (RC 1607), A-0998

**Couto J.G., McClure P., McFadden S., Bezzina P., Hughes C.**
Thematic analysis of the competencies of the therapy radiographer practising in the linear accelerator (SS 714a), B-0651

**Cozzi A., Zanardo M., Monti C.B., Carbonaro L.A., Trimboli R.M., Sardanelli F.**
Technique and diagnostic performance of contrast-enhanced spectral mammography: a systematic review (MY 5), B-0465

**Cozzi D., Piccolo C.L., De Cicco M.L., Trinci M., Galluzzo M., Miele V., Ianniello S.**
Lung ultrasound in the diagnosis of pneumothorax in paediatric stable major trauma: chest x-ray is still useful? (SS 717), B-0733

**Cretese A., Como G., Girometti R., Cereser L., Giannarini G., Valotto C., Zuiani C.**
Which is the accuracy for malignancy of contrast-enhanced ultrasound of Bosniak ≥2F cystic renal lesions incidentally found on CT and MRI? (SS 1407), B-1138

**Crijns A.**
Cardiovascular effects after radiotherapy for breast cancer: the European MEDIRAD Project: Chairpersons' introduction (part 1) (EU 1), A-0014

**Crijns A.**
Cardiovascular effects after radiotherapy for breast cancer: the European MEDIRAD Project: Overview of dose-effect relationships and their use in strategies for prevention of cardiovascular effects after radiotherapy for breast cancer (EU 1), A-0016

**Crocetti L.**
Basic interventional radiology for non-interventionalists: let's start with biopsy!: How to guide your procedure (SF 5b), A-0246

**Cronin K., Ghotra S.S., Harsaker V., Gillard M., Fitzgerald J., Kieft I., Sekkelsten H., Reynolds O.**
Mapping university skills labs in radiography: students' perspectives (SS 1914a), B-1700

**Çubuk H.S., Bicakci K., Bicakci S.**
Advanced MR imaging with cognitive and vestibular stimulation in vestibular migraine (SS 1011b), B-1039

**Cui Y., Li Y., Yin Y., Li B., Dong J., Zhu J.**
Prediction of benign and malignant breast masses using digital mammograms texture features (SS 1805b), B-1520

**Cui Y., Zhuo Z., Yang X., Zhang R.**
Diffusion kurtosis imaging-derived histogram metrics for prediction of KRAS/NRAS/BRAF mutation in rectal adenocarcinoma (MY 3), B-0360

**Cunha A.J.C.D.**
Eye lens protection: new perspective (SS 1814), B-1492

**Cuocolo R., Caruso M., Di Dato F., Mollica C., Romeo V., Vallone G., Petretta M., Iorio R., Maurea S.**
Imaging follow-up in patients with biliary atresia after Kasai portoenterostomy (SS 312), B-0333

**Cuocolo R., Stanzione A., Ponsiglione A., Romeo V., Cocozza S., Brunetti A., Imbriaco M.**
Can artificial intelligence predict prostate cancer local stage on unenhanced MRI? A preliminary report (SS 1605), B-1392

**Currò F., Ricciardi M.C., Danieli R., Cavallaro M.F., Martingano P., Bertolotto M., Stacul F., Cova M.A.**
Optimisation of prostate cancer radiotherapy management with MR (MY 3), B-0368

**Curta A., Lehner A., Ulrich S., Dalla-Pozza R., Fischer M., Haas N., Kramer H.**
Measurement of wall shear stress and pressure gradients using 4D-flow-MRI in patients with Fontan circulation (SS 1903b), B-1866

**Cyran C.C.**
Imaging tumour response to immunotherapy: C. Monitoring immune response with PET (RC 116), A-0067

**Cyran C.C.**
Hybrid and translational imaging: the bigger picture: Chairpersons' introduction (part 2) (ESR/ESHI(MT)), A-0973

### D

**D'Amico N., Valbusa G., Grossi E., Fazzini D., Malasevschi A., Rigiroli F., Papa S.**
Prediction of the clinical outcome of chest ground glass area using a radiomic approach (SS 305), B-0304

**D'Onofrio M.**
Tips and tricks for abdominal ultrasound: CEUS (US 2), A-0668

**Dacher J.**
Imaging before and after aortic valve repair: Keynote lecture (SS 203), K-06

**Damen M., van Leeuwen M.S., Webb A., Klomp D.W., Arteaga de Castro C.**
MR imaging relaxation times of pancreatic tissue measured in vivo at 7.0 Tesla (SS 713), B-0766

**Damilakis J.**
Big data and the big picture: deep learning in optimisation of medical imaging (part A): Chairperson's introduction (EF 1), A-0264

**Damilakis J.**
Diagnostic reference levels (DRLs) based on clinical indication: Experience from the EUCLID (European study on clinical DRLs) Project (EU 2), A-0411

**Damilakis J.**
Medical imaging and emerging issues in occupational radiation exposure: Eye lens radiation dose and cataractogenesis (ESR/EFOMP), A-0545

**Damilakis J.**
Advanced clinical dosimetry in interventional radiology: Dose management of patients and staff in interventional radiology (EU 4), A-0751

**Damilakis J.**
Principles of imaging and radiation protection: C. Radiation protection (E³ 1523), A-0898

**Daniaux M.**
New horizons in breast ultrasound (SY 23)

**Danse E., Michoux N., Kartheuser A., Dewit O., Baldin P., Jouret-Mourin A., Jacquet L., Coche E.**
Does spectral CT help to predict the severity of ischaemic colitis? (SS 1001a), B-0944

**Danse E., Coche E.**
Abdominal emergencies: advanced imaging in daily routine?: Routine CT vs dual energy CT in acute abdominal pain: should we change our scan protocol? (SF 15c), A-0892

**Das M.**
Pulmonary neuroendocrine proliferations and neoplasms: C. Small cell lung cancer (RC 504), A-0270

**Davis A.**
Technological trends that are changing medical imaging and the practice of radiology (SY 20)

**Davis M.**
Forensic imaging: A. The chain of evidence (RC 814), A-0399

**De Cataldo C., Corridore A., Torlone S., Cannizzaro E., Palumbo P., De Matteis F., Di Luzio M., Di Cesare E., Masciocchi C.**
Retrospective evaluation of the accuracy of coronary computed tomography angiography compared to invasive coronary angiography: radiological and therapeutic approach in CAD-RADS 3/V (SS 1503), B-1309

**De Cataldo C., Torlone S., Corridore A., Palumbo P., Cannizzaro E., De Matteis F., Di Sibio A., Di Cesare E., Masciocchi C.**
Analysis of strain values in asymmetrical hypertrophic cardiomyopathy: correlations with wall thickness and extent of focal fibrosis (SS 1903a), B-1817

**De Cicco M.L., Andreoli C., Sessa B., Miele V., Ianniello S.**
Accuracy of a new system score based on lung ultrasound and clinical data in the decision of a drainage tube necessity in polytrauma patients pneumothorax (SS 717), B-0734

**De Foer B., Bernaerts A.**
Imaging in Meniere's disease: what to believe and how to interpret: MR hydrops imaging: how to interpret it? (SF 15b), A-0888

**de Francisco A.**
Why managing risk in oncology patients really matters: a nephrologist's perspective on precision health (SY 3)

**De Franco G., Bruni S., D'Alesio G., Bux S., Ambrosi A., Vaccaro A., Grimaldi V., Fracella M.**
Value of MRI with static and dynamic sequences in the study nature extension of the tight stenosis of the oesophagus-gastric junction (SS 1901b), B-1872

**De Graaf P.**
Imaging of eye and orbital pathologies: C. Benign and malignant masses of the eye and orbit (RC 1708), A-1070

**de Kerviler E.**
Oncological imaging: where are we now?: Chairperson's introduction: What are the problems of morphologic evaluation (RC 1216), A-0711

**de Kerviler E.**
The global safety of gadolinium contrast agents (SY 30)

**De Koekkoek-Doll P., Vogel W., van den Brekel M.W.M., Smit L., Beets-Tan R.G.H., Maas M.**
SUV-based lymph node selection for fine-needle aspiration guided by real-time fused ultrasound and FDG PET in head and neck cancer patients (SS 608), B-0632

**De Koekkoek-Doll P., van den Brekel M.W.M., Vogel W., Smit L., Beets-Tan R.G.H., Maas M.**
Microflow and resistance index in suspicious lymph nodes (SS 1816), B-1635

**de la Camara M.A.**
Clinical dilemmas in radiography: what graduates need to know: Professionalism in an era of social media (part 2) (BR 12), A-0690

**de Lange C., Thrane K., Thommassen K., Rydén Suther K., Ording Müller L.-S., Geier O.M., Døhlen G., Almaas R., Møller T.**
Fontan-operated adolescents have increased hepatic fibrotic markers assessed by ultrasound shear wave elastography and MR T1 mapping (SS 212), B-0168

**De Luis Pastor E., Del Alamo M., Martinez Fernandez R., Mañez Miro J., Gasca C., Rodriguez Rojas R., Pineda Pardo J., Vela L., Obeso J.**
Focused ultrasound subthalamotomy in patients with asymmetric Parkinson's disease: a pilot study (SS 1509), B-1303

**De Maeseneer M.O.**
Elbow imaging: from detailed anatomy to pathology: A. The medial and lateral epicondyle (RC 1710), A-1046

**De Magistris G., Pane F., Corvino F., Giurazza F., Coppola M., Amodio F., Silvestre M., Cavaglià E., Niola R.**
High-flow priapism: colour Doppler findings and embolisation (SS 109), B-0006

**De Martino S.R.M., Corcioni B., Gaudiano C., Ciccarese F., Vagnoni V., Schiavina R., Golfieri R.**
Is it today acceptable to perform only targeted fusion MR/US prostate biopsy in naïve patients? (SS 1007), B-0873

**De Mattia C., Ronza R., Grassi M., Pecorilla M., Colombo P.E., Siena S., Vanzulli A., Torresin A.**
An inter and intra patient radiomic study to select the most promising features aiming to precision medicine feasibility (SS 1013), B-0991

**de Meyere M., Dubourg B., Delacour D., Bejar S., Michelin P.J., Dacher J.**
Magnetic resonance functional assessment of the right ventricle in the short-axis plane as obtained from cross-referenced atrioventricular plane localisation (SS 1903b), B-1860

**de Pont L.M.H., van Steekelenburg J., van Weijnen A., Vijlbrief O.D., Blom H.M., Hammer S.**
MRI in unilateral Meniere's disease: the value of post-contrast cochlear perilymph signal intensity (SS 308), B-0298

**De Robertis Lombardi R., Grecchi A., Catania M., Beleù A., Rizzo G., Ambrosetti M.C., D'Onofrio M.**
Solid pseudopapillary neoplasms of the pancreas: clinicopathological and radiological features according to size - 20-year experience from a high-volume centre (SS 501), B-0457

**De Serio I., Jerman K., Londero V., Zuiani C.**
Architectural distorsions with microcalcifications: a mammographic malignancy predictor of B3 lesions? (SS 202), B-0086

**De Smet E., Jeurissen B., Sijbers J., Vanhevel F., Parizel P.M., Van Dyck P.**
Super-resolution reconstruction of knee MR images (SS 1410), B-1127

**De Smet E., Billiet T., Heusdens C., Parizel P.M., Van Dyck P.**
Healing of the anterior cruciate ligament following primary repair: a longitudinal DTI study (SS 1810), B-1509

**De Visschere P.**
The "prostate unit" at work: The radiologist: non-invasive explorer of the prostatic realm (MS 8a), A-0478

**Dedulle A.S.L., Rahimzadeh S., Fitousi N., Bosmans H., Jacobs J.**
Evaluating the use of a noise index to identify outliers in CT examinations (SS 213), B-0132

**Degnan A.J., Ho-Fung V.M., Nguyen J.C., Lawrence J.T.R., Kaplan S.L.**
Secondary fractures in paediatric radial head and neck fractures: common association, commonly missed (SS 1012), B-1055

**Degnan A.J., Ho-Fung V.M., Wang D., Barrera C., Ahrens-Nicklas R., Ficicioglu C.**
The value of MR spectroscopic bone marrow fat fractions in the assessment of treatment response in patients with Gaucher disease (SS 1012), B-1057

**Deike-Hofmann K., Reuter J., Paech D., Bickelhaupt S., Gnirs R., Forsting M., Heussel C., Schlemmer H.-P., Radbruch A.**
Glymphatic pathway of gadolinium-based contrast agents through the brain: overlooked and misinterpreted (MY 16), B-1421

**Dekimpe C.**
Ultrasound-guided percutaneous release of the carpal tunnel: comparison between senior vs junior operator and learning curves: a cadaveric study (SS 1510), B-1294

**Del Poggio A., Pereira C., Iadanza A., Falini A., Anzalone N.**
Diagnostic efficacy and safety of gadoteridol in neuroradiology: qualitative assessment in a large sample of MRI studies at 1,5T (SS 1911a), B-1776

**Del Rio Carrero B., Luburich Hernaiz P., Bolivar Cuevas S., Carreño García E., Vicens Zygmunt V., Molina Molina M.**
Fibrotic lung disease in MPO-ANCA positive patients (SS 1604), B-1404

**Delfino L., Damasio M., Derchi L.E.**
MRU for diagnosis of obstructive pathology of the upper urinary tract (SS 312), B-0342

**Delis H.**
Dose management in paediatric radiology: C. Optimisation in the neonate (RC 1313), A-0788

**Dell'Aversana S., Ponsiglione A., Nappi C., Stanzione A., Puglia M., D'Acierno L., Lassandro G., Cuocolo A., Imbriaco M.**
Relationship between epicardial adipose tissue and coronary vascular function in patients with normal myocardial perfusion by 82Rb PET/TC (MY 18), B-1684

**Della Corte A., Marra P., Ratti F., Giombi F., Gusmini S., Salvioni M., Venturini M., Aldrighetti L., De Cobelli F.**
Comparison between percutaneous and laparoscopic microwave ablation of hepatocellular carcinoma (SS 1709), B-1439

**Della Seta M., Collettini F., Schmelzle M., Raschzok N., Hamm B., Denecke T.**
Intra-individual comparison of the MRI contrast agents gadoxetic acid vs gadoteric acid in liver MRI of patients with HCC and underlying cirrhosis (MY 17), B-1468

**Della Vecchia G., Di Chiara A., Panzeri M., Palmisano A., Esposito A., Passoni P., Del Maschio A., De Cobelli F.**
T2-weighted signal intensity (T2SI) textural analysis in an evaluation of neoadjuvant chemoradiotherapy (CRT) response in patients with locally advanced rectal adenocarcinoma (SS 1501a), B-1345

**Della Vecchia G., Di Chiara A., Panzeri M., Palmisano A., Esposito A., Passoni P., Fiorino C., Del Maschio A., De Cobelli F.**
Rectal wall T2 relative signal intensity: a non - invasive biomarker of gastro-intestinal (GI) toxicity in patients treated with pelvic radiotherapy (RT) (MY 17), B-1485

**Dellios D.P., Pappas E.P., Seimenis I., Karaiskos P.**
Contrast-enhanced MR images employed in stereotactic radiosurgery: does susceptibility-related distortion pose a significant problem? (SS 713), B-0768

**Demaerel P.**
Inflammatory and infectious CNS pathology: A. Autoimmune encephalitis (RC 1211), A-0676

**Demircioglu A., Haubold J., Forsting M., Nassenstein K., Umutlu L., Nensa F.**
Generating dual-energy images from monoenergetic 80 kV images using a conditional generative adversarial network (SS 1005a), B-0913

**Demozzi E., Cavicchioli F., Hoogenboom M., Catania M., Foti G., Munari E., Cavalleri S., Carbognin G.**
Detection of prostate cancer: comparison between robotic-assisted "in-bore" MRI-guided biopsies and cognitive transrectal ultrasound-guided biopsies (SS 1007), B-0872

**Depetris M.A., Ibañez Sanz L., Martinez Chamorro E., Borruel Nacenta S.**
A comparative study 2007-2015 on acute appendicitis: evolution in use by diagnostic imaging techniques (SS 317), B-0268

**Depretto C., Baldari D., Cartia F., Ferranti C., Scaperrotta G.**
Affirm Prone Biopsy System 2D/3D™: tomosynthesis in the vacuum-assisted prone stereotactic biopsy (SS 302), B-0257

**Depretto C., Primolevo A., Cartia F., Ferranti C., Scaperrotta G.**
Diagnostic performance of automated breast ultrasound (ABUS) after breast-conserving surgery (SS 702b), B-0725

**Depretto C., Balestra F., Cartia F., Ferranti C., Scaperrotta G.**
Percutaneous ultrasound-guided excision with vacuum-assisted breast biopsy (VABB) system of breast lesions with imaging-histology discordance (SS 1402a), B-1155

**Derchi L.E.**
Radiology in Africa: facing challenges and opportunities: Chairpersons' introduction (part 1) (EM 1), A-0308

**Derchi L.E.**
Radiology in Africa: facing challenges and opportunities: Conclusion (part 1) (EM 1), A-0316

**Derchi L.E.**
Current status and future challenges in MR-integrated radiotherapy: Chairpersons' introduction (part 1) (ESR/ESTRO), A-0496

**Desai S.R.**
Fibrotic lung diseases: Chairperson's introduction (RC 1204), A-0699

**Desai S.R.**
Patterns of pulmonary toxicity: B. Smoking-related lung disease (E³ 1626b), A-0983

**Desbuquoit D., Dekeyzer S., Huyskens J., Nicolay S., De Smet E., Van Goethem J., Van den Hauwe L., Parizel P.M.**
Detection of intracranial haemorrhage on CT of the brain using a deep learning algorithm (SS 705), B-0753

**deSouza N.M.**
Imaging biomarkers and their combinations in the era of artificial intelligence: Chairpersons' introduction (part 2) (ESR/EIBALL), A-0057

**deSouza N.M.**
Tumour relapse in gynaecological cancer: C. Salvage treatment with image directed therapy: high-intensity focused US (HIFU) and stereotactic RT (E³ 519), A-0287

**deSouza N.M.**
Imaging in oncological trials: Chairperson's introduction (ESR/EORTC), A-0789

**Devaraj A.**
Screening with imaging: a new era?: Chairperson's introduction (SF 1), A-0027

**Devaraj A.**
Cavitary and cystic diseases of the lung: B. Langerhans cell histiocytosis (LCH) (E³ 722), A-0375

**Devaraj A.**
Lung nodule management in 2019: Chairperson's introduction (RC 904), A-0548

**Dewaguet J., Copin M., Faivre J., Deken V., Sedlmair M., Remy J., Remy-Jardin M.**
Dual-energy CT (DECT) lung tumour perfusion: can we distinguish features that can reflect prognosis? (SS 704), B-0778

**Dezman R.**
Highlighted Lectures: Getting it right: how to report an imaging study before the interventional radiology procedure (TF), A-0834

**Dhanasingh A.**
Research software in cochlear duct length estimation, Greenwood frequency mapping and electrode array length selection (SS 308), B-0297

**Di Chiara A., Palmisano A., Della Vecchia G., Esposito A., Passoni P., Fiorino C., Del Maschio A., De Cobelli F.**
Importance of tumour volume reduction rate on T2w and DWI during and after CRT for prediction of treatment response to CRT in locally advanced rectal cancer (LARC) (SS 1501a), B-1343

**Di Chiara A., Palmisano A., Esposito A., Rancoita P., Passoni P., Del Maschio A., De Cobelli F.**
Whole-tumour texture analysis of ADC maps in the prediction of pathological response of locally advanced rectal cancer (LARC) (SS 1501a), B-1344

**Di Giacomo L., Bagnacci G., Nardone V., Tini P., Gentili F., Marrelli D., Roviello F., Volterrani L., Mazzei M.A.**
Advanced gastric cancer response to neoadjuvant chemotherapy: is CT texture analysis a reliable imaging biomarker? A multi-centre study from Italian research group for gastric cancer (SS 1401b), B-1275

**Di Napoli A., Cheng S., John G., Brown M., Sokolska M., Jäger H.R.**
Arterial transit artefacts on ASL perfusion MRI in patients with carotid artery stenosis are a better predictor of recent symptoms than degree of stenosis or carotid plaque morphology (SS 711), B-0712

**Di Paola V., Russo L., Miccò M., Sbarra M., Pozzi Mucelli R., Manfredi R.**
Evaluation of periprostatic neurovascular fibres before and after radical prostatectomy by means of 1.5 T MRI diffusion tensor imaging (SS 307), B-0328

**Dick E.**
Imaging of 'foreign bodies': A. Surgical and orthopaedic devices: are they really properly positioned? (RC 417), A-0166

**Diekhoff T., Ulas S., Poddubnyy D., Schneider U., Hermann S., Biesen R., Burmester G.R., Hamm B., Hermann K.A.**
Ultra-low-dose CT detects synovitis in patients with suspected rheumatoid arthritis (SS 610), B-0514

**Dietzel M., Schulz-Wendtland R., Wenkel E., Bielowski C., Bäuerle T., Uder M., Ellmann S.**
Rethinking abbreviated breast MRI: potential to skip DCE scans by integrated semi-quantitative T2 and ADC analyses (SS 602), B-0551

**Dietzel M., Ellmann S., Clauser P., Wenkel E., Uder M., Schulz-Wendtland R., Baltzer P.A.T.**
Automated volumetric analysis of breast cancer vascularisation enables risk stratification in patients scheduled for neoadjuvant chemotherapy (SS 702a), B-0701

**Dietzel M., Ellmann S., Wenkel E., Clauser P., Schulz-Wendtland R., Uder M., Baltzer P.A.T.**
The Kaiser score, a decision rule based on BI-RADS MRI descriptors: is there an impact of histopathology on the diagnostic accuracy? (SS 702a), B-0702

**Dietzel M., Ellmann S., Wenkel E., Bielowski C., Schulz-Wendtland R., Uder M., Bäuerle T.**
Combining artificial intelligence and MRI in the management of equivocal breast lesions: the perfect couple? (SS 702a), B-0703

**Ding L., Ma L., Zhang F.**
The value of the single energy metal artefact reduction algorithm in post-surgery follow-up of patients with hip tumour prostheses (SS 1010), B-0903

**Dionisi C., Coppo M., Villa R., Brambilla M.**
Geometric efficiency: measuring methods' comparison (SS 1314), B-1071

**Djuraeva N., Ikramov A., Vakhidova N., Amirkhamzaev A., Shamirzaev K., Sultanov A.**
The primary study of low-dose 640-slice volume liver perfusion CT: a whole-organ perfusion in patients with cirrhosis of the liver before and after portosystemic shunting (SS 1501b), B-1379

**Djurdjevic T., Pereverzyev S., Lamplmayr L., Neubauer V., Kiechl-Kohlendorfer U., Wallner V., Grams A.E., Gizewski E.R.**
Prediction of neurodevelopmental outcome in preterm neonates with cerebral MR spectroscopy and DWI using neural-network-based classifiers (SS 1011a), B-0925

**Do Amaral Castro A.D., Sulla Lupinacci F.A., P.Pribeiro S., Kaiser Ururahy Nunes Fonseca E., Peixoto M.R., Larocca Skare T., Sakuma A.T., Yamauchi F., Tachibana A.**
Diagnostic value of C-reactive protein and the influence of visceral fat in patients with obesity and acute appendicitis (MY 15), B-1365

**Dodd E.**
Clinical dilemmas in radiography: what graduates need to know: Issues surrounding consent (BR 12), A-0687

**Dodd J.D.**
CT and intervention in vascular pulmonary diseases: Chairperson's introduction (E³ 822), A-0469

**Dodig D., Bartolovič N., Kovacic S., Miletic D., Rumboldt Z.**
Accuracy of virtual monochromatic images for the detection of early brain ischaemia on dual-energy CT (SS 311), B-0260

**Dogan B., Menezes G., Butler R., Neuschler E., Lavin P., Aitchison R., Tucker L.F., Otto P., Grobmyer S.**
Optoacoustic imaging is helpful in predicting breast cancer molecular subtypes (SS 1902a), B-1793

**Dogan B., Menezes G., Butler R., Neuschler E., Lavin P., Aitchison R., Tucker L.F., Otto P., Grobmyer S.**
Optoacoustic imaging features correlate with breast cancer clinicopathological prognostic factors (SS 1902a), B-1794

**Domènech-Ximenos B., Cuba Camasca V., Daunis-i-Estadella J., Pedraza Gutierrez S., Fernández-Real J.M., Puig Alcàntara J.**
Weight loss in obesity, impact on carotid intima media thickness and cardiovascular risk factors: a longitudinal study (SS 215), B-0044

**Dominguez A.**
Forensic imaging: Chairpersons' introduction (Part 1) (RC 814), A-0397

**Domínguez Paillacho I.D., Mesa Quesada J., Zurera Tendero L.J.J., Pulido Carmona C., Lombardo Galera M., Perez Montilla M.E., Espejo Herrero J.J.**
Percutaneous ablation of renal cancer: radiofrequency vs. microwave (SS 1807), B-1662

**Donato P.**
Cardiac imaging in arrhythmia and sudden cardiac death: Chairperson's introduction (E³ 526), A-0223

**Dong S., Zhu M.**
Feasibility of foetal MRI in the evaluation of foetal congenital heart disease (SS 612), B-0599

**Dong S., Ji H.**
Diffusion-weighted magnetic resonance imaging of the foetal brain in complex congenital heart disease (SS 612), B-0600

**Dong Y., Han X., Yang D., Lou W., Zhang Q., Wang W.**
Preoperative prediction of pancreatic neuroendocrine tumours' grade: the added value of contrast-enhanced ultrasound with quantitative analysis (SS 501), B-0452

**Doniselli F.M., Zanardo M., Sconfienza L.M., Sardanelli F.**
MRI principles and techniques: how and where do radiologists and radiology residents look for? (SS 713), B-0759

**Donnelly-Kehoe P., Ojeda A.A., Pascariello G., Acevedo P.A., Nallino M.B., Rodríguez Musso A.L., Gomez J.**
Open-source software multimodal neuroimage processing for presurgical assessment: benefits and comparison to proprietary software (SS 1911a), B-1780

**Donners R., Harder D.**
To biopsy or not to biopsy? A quantitative approach differentiating subfascial lipoma from atypical lipomatous tumour/well-differentiated liposarcoma on MRI (SS 210b), B-0057

**Donoso L.**
Imaging informatics: management and performance analysis: Keynote lecture (SS 1405), K-28

**Donoso L.**
Improving radiation protection in medical imaging in low- and middle-income countries: past actions and future directions: Chairpersons' introduction (part 1) (EU 3), A-0657

**Donoso L.**
Radiology and pathology: will we ever be (digitally) married?: Chairperson's introduction (SF 13a), A-0734

**Döring S.**
Why do I miss fractures in emergency?: B. Missed fractures in adults (RC 1217), A-0684

**Dorn S., Sawall S., Maier J., Polster C., Faby S., Uhrig M., Schlemmer H.-P., Kachelrieß M.**
Patient size-dependent ultralow-dose data completion scan in a whole-body photon-counting CT scanner (SS 213), B-0135

**Dorn S., Sawall S., Maier J., Kachelrieß M.**
Iodine quantification accuracy in dual-source dual-energy CT using default parameters and patient-specific calibrations (SS 313), B-0316

**Douek P.I., Rotzinger D.C., Meuli R., Dunet V., Schmidt Kobbe S.**
Impact of added CT venography performed in combination with CT pulmonary angiography on the detection of deep venous thrombosis and relevant occult CT findings (MY 15), B-1366

**Drapé J.**
Inflammatory and infectious diseases of the spine: how to differentiate from degeneration: C. Infection: imaging, indication and techniques for biopsy (RC 910), A-0543

**Dratwa C.**
Impact of a radiological consultation on the surgical management of breast cancer patients: a retrospective study of 1114 cases (SS 1802a), B-1547

**Drazinos P., Gatos I., Yarmenitis S., Theotokas I., Soultatos A., Panteleakou E., Zoumpoulis P.**
Chronic liver disease assessment with a machine learning method using parameters derived from ultrasound B-mode and shear wave elastography examination (SS 1905b), B-1837

**Dreher C., Kuder T., Windhaber S., König F., Paech D., Gnirs R., Benkert T., Schlemmer H.-P., Bickelhaupt S.**
Increasing reader confidence in cancer imaging using an advanced DWI modification: a prospective evaluation (MY 3), B-0362

**Dreher C., Kuder T., Windhaber S., König F., Paech D., Gnirs R., Benkert T., Schlemmer H.-P., Bickelhaupt S.**
Prospective evaluation of modified DWI MRI for improved image quality and tissue differentiation in oncological follow-up examinations (MY 3), B-0364

**Dreyer K.J.**
Professional issues for radiology departments to enable research: How to implement research on artificial intelligence in radiology (PI 3), A-0629

**Drinkwater K.**
Clinical audit: how to deal with the legal and professional requirements: ESR's concept and tools for clinical audit (PI 2), A-0586

**Dromain C.**
Imaging tumour response to immunotherapy: A. Imaging the immune system in cancer (RC 116), A-0065

**Dromain C.**
The tumour board of the future: How to set up and run a multidisciplinary meeting (NH 16), A-0956

**Drost F.H., Verbeek J.F.M., Alberts A.R., Roobol M.J., Schoots I.G.**
Pre- and post-MRI risk-stratification reduces prostate MRIs, biopsies and overdetection in prior negative biopsy men (SS 216), B-0181

**Du C., Liu X., Li H., Yu L., Geng D., Chen Y., Zhang J.**
Dual-targeting and execrable ultrasmall SPIONs for T1-weighted-positive MR imaging of intracranial glioblastoma by targeting lipoprotein receptor-related protein (SS 1406), B-1120

**Du J., Li X., Huang L., Huang S., Li Z.**
Combination of diffusion kurtosis imaging and conventional MRI helps to differentiate fibrotic from inflammatory bowel strictures in patients with Crohn's disease (MY 17), B-1476

**Du T., Zhu M., Qi X., Yao K., Wang L., Zhang X., Hu J., Gao J.**
Analysis of clinical, imaging and pathological characteristics in patients with pineal parenchymal tumour of intermediate differentiation (SS 1411a), B-1163

**Du Y., Ji C., Wu Y., Gu X.**
Combination of ultrasound elastography with TI-RADS in the diagnosis of small thyroid nodules (<10 mm): a new method to increase the diagnostic performance (SS 208), B-0123

**Du Plessis J.G.E.**
Peer-assisted learning to improve educational performance in radiography training (SS 1914a), B-1703

**Duba I., Khandelwal A., Weaver J., Leng S., Ferrero A., Wells M., Fletcher J.G., McCollough C., Halaweish A.F.**
Dual-energy CT dose reduction for GU CT while preserving diagnostic performance (SS 407), B-0447

**Dubost F., Adams H.H., Yilmaz P., Bortsova G., Ikram M., Niessen W.J., Vernooij M.W., De Bruijne M.**
Automated detection of enlarged perivascular spaces on brain MRI (SS 1811b), B-1690

**Duc N.M., Li C., Quang Huy H., Yang J., Keserci B.**
The effect of histogram parameters derived from quantitative T1-perfusion on predicting the high-intensity focused ultrasound outcome of uterine fibroids (SS 1509), B-1304

**Duc N.M., Keserci B., Quang Huy H., Van Phuoc L., Trong Khoan L., Duc Cuong D., Van Giang B., Dang Luu V., Duy Hue N.**
The role of magnetic resonance imaging parameters in predicting the treatment outcome of high-intensity focused ultrasound ablation of adenomyosis (SS 1509), B-1305

**Ducou Le Pointe H.**
CT radiation risk in children: an overview: A literature update (EU 6), A-0934

**Duffy S.W.**
Breast cancer screening and treatment: how much is too much?: Overdiagnosis in breast cancer screening: the epidemiologist's view (SA 16), A-0942

**Duran R.**
Hepatocellular carcinoma: from diagnosis to treatment: C. Interventional techniques (ablation, TACE, radioembolisation): when and how to treat (RC 1701), A-1004

**Durhan G., Erdemir A., Sarı S., Gültekin M., Karakaya J., Akpınar M., Yildiz F., Demirkazik F.**
Does internal mammary artery irradiation for breast cancer make significant difference in diameters of internal mammary artery? Correlation with computed tomography (SS 1902b), B-1805

**Duron L., Lecler A., Balvay D., Savatovsky J., Bergès O., Bouchouicha A., Galatoire O., Fournier L.**
Combining multiple magnetic resonance imaging sequences provides independent reproducible radiomics features (SS 1005b), B-0987

**Durum Y., Ozturk V., Ersöz N., Anık A., Karaman C.**
Is TI-RADS useful as an adult ultrasonographic malignancy risk stratification method in pediatric thyroid nodules? (SS 1012), B-1059

**Dushkova D.V., Vasilev Y.**
Structural changes of the disc of temporomandibular joint in the first stage of dysfunctions (MY 14), B-1260

**Düzgün F., Yilmaz Ovali G., Örgüc I.S.**
Spinal osseous lesions in tuberosclerosis (SS 1910b), B-1741

**Dziedataja M.**
Dose optimisation of head CT examinations in paediatrics (SS 714a), B-0647

### E

**Ebdon-Jackson S.**
Audit across Europe: directive and perspective: Clinical audit and inspection: HERCA update (ESR Audit), A-0138

**Ebel S., Hübner L., Köhler B., Kropf S., Preim B., Jung B., Grothoff M., Gutberlet M.**
Comprehensive validation of two accelerated 4D-flow MR sequences at 3T: a phantom study (SS 315), B-0221

**Ederveen J.C., Nienhuijs S.W., Nederend J.**
Structured CT reporting improves accuracy in diagnosing internal herniation after laparoscopic Roux-en-Y gastric bypass (SS 1901b), B-1879

**Edjlali M.**
Carotid disease 2.0: B. Carotid involvement in inflammatory arterial disease (RC 915), A-0509

**Edyvean S.**
Patient-specific dosimetry: B. Patient dosimetry in CT and CBCT (RC 113), A-0048

**Eggesbø H.B.**
Post-treatment imaging of the head and neck: Chairperson's introduction (RC 1208), A-0719

**Eid M.M., Othman M.H., Abdelal S., Hasan H.A., Khakifa H.E., Sayed S.**
Role of MR functional connectivity in discriminating cognitive impairment in BD (SS 1511), B-1334

**Eijgenraam S.M., Chaudhari A.S., Reijman M., Hargreaves B.A., Gold G.E., Oei E.H.G.**
Time-saving opportunities in radiology: accurate structural imaging and $T_2$ mapping of cartilage and meniscus in knee osteoarthritis using a 5-minute DESS MRI scan (SS 1410), B-1134

**Eisenblätter M.**
Quantitative imaging in oncology: A. Intra- and intertumoural heterogeneity and the impact for cancer diagnostics (E³ 1726a), A-1058

**Ekert K.**
Applying extended texture features to identify quantifiable changes in gastrointestinal stromal tumours undergoing tyrosine kinase inhibitor (TKI) therapy (SS 1401b), B-1277

**El-Diasty T.**
Renal transplantation: Chairperson's introduction (RC 1607), A-0995

**Elbadawi S.E.E.E.**
Detection and prevalence of cerebral microbleeds in middle and old age groups: a clinical-based approach (MY 16), B-1416

**Eley K.A.**
The 3D printing lab from bench to bedside: Challenges of centralised 3D printing (SF 8f), A-0450

**Elkarghali Z., Nguyen-Kim T., Spekreijse J., Trebeschi S., Haanen J.B., Blank C.U., Vogel W., Beets-Tan R.G.H.**
Using radiomics to identify invisible bone metastases on CT in melanoma patients receiving immunotherapy (SS 716), B-0685

**Ellmann S., Wenkel E., Peter S., Dietzel M., Weiland E., Janka R., Bäuerle T., Uder M.**
Combining ultrafast TWIST VIBE Dixon sequences with diffusion-weighted imaging: a clinically applicable evaluation tool with high accuracy for suspicious masses in breast MRI (MY 5), B-0474

**Ellmann S., Dietzel M., Wenkel E., Bielowski C., Schulz-Wendtland R., Uder M., Bäuerle T.**
Integration of AI into the breast MRI assessment enables objective and accurate diagnosis even by an unexperienced reader (SS 1805a), B-1518

**Elmokadem A.H., Ibrahim E.A., Gouda W.A., Abdel Razek A.**
Whole-body CT using biphasic injection protocol with adaptive statistical iterative reconstruction-V (ASiR-V) in multi-trauma patients: impact on dose reduction and image quality (SS 217), B-0100

**Elshafey R., Darwish H.S., Kamel H.A.**
Prediction of motor recovery after stroke by assessment of corticospinal tract Wallerian degeneration using diffusion tensor imaging (SS 711), B-0711

**Elugwu H.C.**
Expectant mothers' perception of prenatal sonography in southeastern population in Nigeria (MY 15), B-1358

**Elzeneini A.M., Yousef M.I., Refaat M.M.**
The role of dynamic contrast-enhanced MRI analysis of perfusion changes in hepatocellular carcinogenesis (SS 616a), B-0531

**Emiro F., De Marco P., Origgi D.**
Characterisation of iodine quantification for a fast-kVp switching dual energy CT system (SS 313), B-0318

**Emrich T., Brumberg R.M., Wenzel P., Lyschik S., Jungmann F., Rueppel S., Düber C., Kreitner K.-F.**
Long-axis T1 and T2 mapping in Takotsubo cardiomyopathy: trash or treasure? (SS 303), B-0383

**Engbersen M.P., Van 't Sant I., Van Eden W.J., Velzing J.D.R., Kok N.F., Lambregts D.M., Beets-Tan R.G.H., Aalbers A.G., Lahaye M.J.**
DW-MRI can be used to predict complete cytoreduction and survival in colorectal peritoneal carcinomatosis patients using leading predictive models (SS 216), B-0182

**Engel G., Bender Y.Y., Adams L.C., Böker S.M., Fahlenkamp U.L., Diederichs G., Wagner M., Hamm B., Makowski M.R.**
Evaluation of osseous cervical foraminal stenosis in spinal radiculopathy using susceptibility-weighted magnetic resonance imaging (SS 1910a), B-1734

**England A.**
Clinical simulation and its role in radiography education: Innovation in education: virtual education/computer-based simulator vs patient imaging (SF 9b), A-0492

**Erb-Eigner K.**
Imaging of eye and orbital pathologies: B. Infection and inflammation in the eye and orbit (RC 1708), A-1069

**Erdemir A., Öcal O., Hazirolan T.**
Could "synthetic hematocrit value" has a use on ECV calculating via cardiac MRI? A single centre study (SS 1403), B-1218

**Ereño Ealo M.J.**
State-of-the-art imaging of postoperative joints: C. Postoperative ankle and foot (E³ 1626a), A-0947

**Ertl-Wagner B.**
State-of-the-art paediatric neuroradiology: B. Imaging of developmental disorders (RC 811), A-0430

**Esser M., Kloth C., Thaiss W., Reinert C.P., Fritz J., Horger M.**
CT-response patterns and the role of CT-textural features in inoperable abdominal/retroperitoneal soft tissue sarcomas treated with trabectedin (SS 616b), B-0620

**Essig M.**
Modern neuroimaging protocols (SY 14)

**Essig M.**
Quick decision for a personalised healthcare (SY 20)

**Eulig E., Maier J., Knaup M., Koenig T., Hörndler K., Kachelrieß M.**
Deep DSA (DDSA): learning mask-free digital subtraction angiography for static and dynamic acquisition protocols using a deep convolutional neural network (SS 1005a), B-0909

**Evrimler S., Aydın H.**
Acute appendicitis: appendicocecal angle, morphological variations detected on CT imaging (SS 317), B-0269

**Ewertsen C.**
Tips and tricks for abdominal ultrasound: Fusion imaging (US 2), A-0670

**Ezponda Casajús A., Calvo Imirizaldu M., Morales M., Vivas Perez I., De La Torre M., Rodríguez-Fraile M., Sangro B., Bilbao Jaureguizar J.I.**
Same-day workup angiography and Yttrium 90 microsphere radioembolisation: feasibility and efficacy of hepatic flow redistribution (SS 209), B-0111

**Ezponda Casajús A., Calvo Imirizaldu M., García Baizán A., Paternain Nuin A., Pueyo Villoslada J.C., Gavira J.J., Bastarrika Alemañ G.**
Safety and diagnostic accuracy of regadenoson as vasodilator in stress perfusion cardiovascular magnetic resonance (SS 1403), B-1219

**Ezponda Casajús A., De Torres J., Alcaide A., Campo A., Bertó J., González J., Zulueta J., Bastarrika Alemañ G.**
Pulmonary arterial enlargement predicts long-term survival in COPD patients (SS 1804), B-1609

### F

**Fabritius M.P., Reidler P., Thierfelder K., Froelich M.F., Rotkopf L., Puhr-Westerheide D., Kunz W.G.**
Incremental value of CT perfusion for outcome prediction in acute cerebellar stroke (SS 311), B-0262

**Facal de Castro F., Mirón Mombiela R., Vucetic J., Mota Martinez J., Arana Fernandez De Moya E., Dualde Beltran D.**
Diagnostic error in emergency studies: an analysis of explanatory variables (SS 217), B-0107

**Faggioni L., Gabelloni M., Bianchi M., Neri E., Caramella D.**
Low iodine, fast kV switching dual energy thoraco-abdominal CT angiography for planning of transcatheter aortic valve implantation (TAVI): a pilot feasibility study (SS 203), B-0198

**Fahmy P.N.S., Elkalawy A., Gomaa M., Mansour S.A.**
Proton MR spectroscopy: a non-invasive tool for the detection of ovarian cancer (SS 606), B-0502

**Falaschi Z., Cavalieri S., Valente I., Mazzucca G., Carisio A., Bor S., Carriero A., Boccafoschi F., Stecco A.**
Anatomage table: a possible new tool for diagnosis and classification of Le Fort fractures (MY 14), B-1262

**Fallenberg E.M.**
New mammography: digital breast tomosynthesis and future techniques: Chairperson's introduction (RC 1302), A-0760

**Fallenberg E.M.**
Breast: C. Advanced imaging of the female breast ($E^3$ 1823), A-1086

**Fallenberg E.M.**
What you need to know when using contrast media in breast imaging (SY 2b)

**Fanciullo C., De Piano F., De Jong E., Raimondi S., Buscarino V., Rizzo S.**
Radiogenomics of non-small cell lung cancer (NSCLC): association between CT-based imaging features and EGFR and K-RAS mutations in 122 patients: an external validation (SS 704), B-0769

**Fang J., Mao J.**
Added prognostic value of pre-treatment MRI texture analysis in patients with primary nasopharyngeal carcinoma (SS 1808), B-1627

**Fang X., Wang R., Liu Y., Yuan C., Chen X., Wang Z.**
Ultra-low-dose cerebral CT perfusion for the diagnosis of acute ischemic stroke using iterative model reconstruction combined with optimised temporal resolution (SS 711), B-0715

**Fang X.**
To provide evidence for early diagnosis of pancreatic cancer: magnetic resonance spectroscopy (SS 1401a), B-1099

**Farina D.**
Head and neck cases (SY 24)

**Farina D.**
Differential diagnoses you don't want to miss: C. Differential diagnoses of soft tissue masses in adults (RC 1308), A-0799

**Faron A., Luetkens J., Schmeel F.C., Sprinkart A., Thomas D.**
Quantification of abdominal fat and skeletal muscle tissue at CT: associations between single-slice measurements and total compartment volumes (SS 1810), B-1513

**Farrugia S., van Dijk G., Mercieca S.**
Impact of body mass index on set-up variations and treatment margins for patients receiving radiotherapy to the prostate (MY 15), B-1350

**Faruch M.**
Advances in musculoskeletal techniques: whole-body MR: B. Non-oncologic applications ($E^3$ 1221), A-0647

**Fatehi M.**
Secrets of radiology communications with patients and referring physicians: Chairperson's introduction ($E^3$ 1218), A-0707

**Fathallah W.M., Habeeb M., Ghanim M.E., Bandar M.E.**
The utility of histogram analysis of whole tumour apparent diffusion coefficient map and MRI volumetry in differentiating low and high grade endometrial cancer (SS 216), B-0179

**Favia A., D'addato M., Caliolo G., Campagna C., Favia V., Carrozzo M., Spinarelli A., Scardapane A., Stabile Ianora A.**
A prospective randomised CT analysis of ankle syndesmosis injuries in Weber B and C type fractures (SS 710), B-0676

**Fedon C., Van Engen R., Binst J., Cockmartin L., Dance D., Jacobs A., Longo R., Tri Wigati K., Sechopoulos I.**
Proposal of a breast phantom for dosimetry quality control procedures in digital mammography and digital breast tomosynthesis (SS 1913), B-1838

**Fedorov A., Berezina N., Cherkashin M., Lavrenteva A.**
Performance of the competence centre for MRI and CT imaging in a network of diagnostic centres (SS 1405), B-1182

**Fehrenbach U., Fahlenkamp U.L., Kahn J., Baur A., Pavel M., Geisel D., Denecke T.**
Optimised imaging of the lower abdomen and pelvic region in hepatocyte-specific MRI: evaluation of a whole-abdomen first-pass shuttle protocol (SS 401), B-0399

**Fehrenbach U., Rodriguez Laval V., Pavel M., Steffen I., Enriquez Puga A., Jann H., Denecke T.**
Everolimus-related pneumonitis in advanced neuroendocrine tumours: correlation of radiologic findings and clinical symptoms (SS 1016), B-1024

**Feier D.S., Bastati-Huber N., Beer A., Pötter-Lang S., Fragner R.M., Ba-Ssalamah A.**
Enhancement features, blood-based bio-markers and immunohistochemical expression of hepatocyte transporters in patients with non-alcoholic fatty liver disease (NAFLD) (SS 1001b), B-1050

**Feifei G., Ge Y., Wei Y.**
Feasibility study of reducing artifacts of spine metal implants for patients at 3.0T magnetic resonance imaging (MY 9), B-0857

**Feifei G., Ge Y., Wei Y.**
Application of simultaneous multi-slice TSE in high-resolution hand and foot imaging (MY 9), B-0865

**Ferda J.**
Hybrid imaging in oncology: head to toe: Keynote lecture (SS 706), K-14

**Ferenczi Z., Papp T., Szilagyi B., Szucs P., Berenyi E., Meszar Z.**
Dendritic growth and branching of the cortical neurons can be altered by ultrasound exposition (MY 16), B-1432

**Ferrari R., Mancini Terracciano C., Voena C., Rengo M., Zerunian M., Paramatti R., Faccini R., Laghi A.**
AI automatic identification of complete- and non-responders using texture analysis of rectal cancer 3T MR images performed before, during and after neoadjuvant CRT (SS 1905b), B-1833

**Ferraro D.A., Rüschoff J.H., Müller J., Mühlematter U., Messerli M., Hüllner M., Eberli D., Rupp N.J., Burger I.A.**
PSMA expression heterogeneity of the primary tumour on IHC correlates with the detection rate of $^{68}$Ga-PSMA-11-PET in patients with biochemical recurrence (SS 306), B-0229

**Ferrer Puchol M., Gregorio A., Esteban E., Blanco F.J., Primo V., Andreo L., Villalta Santamaria M., Ramiro R., Solaz Solaz J.**
Selective intraarterial embolisation as a treatment for haemorrhoid pathology: preliminary study (SS 109), B-0003

**Ferrero G., Fiz F., Schwenck J., La Fougère C., Campi C., Sambuceti G., Fabbro E., Pickler B., Kneilling M.**
Prediction of immune-checkpoint-inhibitor therapy effectiveness by evaluation of bone marrow activity modifications: an FDG-PET/CT study (SS 716), B-0686

**Ferrero G., Fiz F., Dittman H., La Fougère C., Campi C., Sambuceti G., Sahbai S., Weissinger M., Piana M.**
Activity concentration in prostate cancer bone metastases predicts the radioisotope therapy outcome: a SPECT/CT study (SS 716), B-0689

**Feuchtner G., Langer C., Barbieri F., Beyer C., Friedrich G.J., Plank F.**
High-risk and lipid-rich, but not calcified, plaque predicts ischaemia in non-obstructive lesions (ANOCA): a CTA/CTFFR study (SS 703), B-0782

**Fighera A., Cardano G., Mazzaro A., Zamboni G., Ruzzenente A., Mansueto G.**
Is CT-texture analysis an useful instrument to predict aggressive behaviour of intrahepatic cholangiocarcinoma? (SS 616a), B-0532

**Filippiadis D.**
Interventional radiology (IR) in children: what a non-interventional radiologist needs to know: C. Osteoid osteoma: diagnosis and treatment (RC 112), A-0063

**Filippone A.**
Abdominal emergencies: Bowel obstruction (BS 5), A-0242

**Fingerle A.A., De Marco F., Willer K., Noel P.B., Sauter A., Pfeiffer D., Rummeny E.J., Herzen J., Pfeiffer F.**
A reader study in postmortem x-ray dark-field chest radiographs and correlation with conventional x-ray and CT (SS 1804), B-1612

**Fiore F., Stoia V., Somma F.**
Yttrium-90 radioembolisation in advanced-stage HCC: does portal vein thrombosis affect survival? (SS 609), B-0564

**Fiore S., Faletti R., Gentile F., Pavan L., Guarasci F., Gerboni M., Donalisio M., Gatti M., Fonio P.**
Multi-arterial phase EOB-MRI: different contrast enhancement pattern of focal nodular hyperplasia and hepatocellular carcinoma (SS 201a), B-0072

**Fischer T.**
US teaching to undergraduate students: why, how and from whom?: Why: US anatomy and pathology teaching, US in clinical practice and as a career choice (ESR US SC/UES), A-0337

**Fleury E.F., de Assis P.E.Z., Azevedo F.A., Lotufo R.**
Artificial intelligence CAD system capability to classify breast density by mammography (SS 1805a), B-1524

**Fleury E.F., de Assis P.E., Azevedo F.A., Lotufo R.**
Ability of an artificial intelligence CAD system to determine quality control of the mammographic study (SS 1805a), B-1526

**Flores Quispe S.K.J., Cavaliere A., Zulian F., Weber M., Zuliani M., Stramare R., Quaia E., Giraudo C.**
Muscle involvement in juvenile localised scleroderma: do we need a volumetric approach? (SS 610), B-0512

**Foley R.W., Ramachandran S., Akintimehin A., Williams S., Kelly P.A.**
The Ottawa subarachnoid haemorrhage rule in the decision for acute CT head: external validation in a contemporary European cohort (SS 1017), B-0950

**Foley S.J.**
Diagnostic reference levels (DRLs) based on clinical indication: Experience with a national survey based on clinical indications (EU 2), A-0412

**Foley S.J.**
Radiation protection: all you need to know: The Basic Safety Standards Directive: early experiences of implementing BSS (BR 16), A-0961

**Fontanella G., Mancinelli M., De Lucia S., Festa A., Villanacci R., Manganiello C.**
MR fistulography with percutaneous instillation of ultrasound gel and its role in the preoperative mapping: our experience (SS 201b), B-0211

**Fontanilla T.**
Abdominal emergencies: advanced imaging in daily routine?: CEUS in abdominal inflammatory and infectious conditions (-itis and abscesses) (SF 15c), A-0894

**Fonteyne V.**
The "prostate unit" at work: The radiation-oncologist: smooth irradiator of the rogue elements (MS 8a), A-0481

**Foran A., O'Shea A., Morrin M.**
Quality of same-day CT colonography following failed optical colonoscopy (SS 1001a), B-0936

**Foran A., Lee M.J.**
Are pre-MRI orbital radiographs always necessary in patients with suspected foreign bodies? (SS 1808), B-1634

**Formosa A., Portanier Mifsud C., Portelli J.L., Demicoli P.**
Investigating the impact of fragrance on patient anxiety during MRI scans (SS 414), B-0413

**Forrai G.**
The high-risk patient enigma: A. Lesions with an elevated risk for breast cancer (E³ 426a), A-0149

**Forrai G.**
Breast cancer screening and treatment: how much is too much?: Chairperson's introduction (SA 16), A-0941

**Forsting M.**
Dream team: B. Leadership in radiology (E³ 1018), A-0596

**Forstner R.**
Early detection of ovarian cancer: Chairperson's introduction (E³ 119), A-0069

**Forstner R.**
Tumour relapse in gynaecological cancer: A. Differentiating relapse from post-treatment appearances (E³ 519), A-0285

**Forton R.K., Hardy M., Sargeant A.**
The construction of care in CT (SS 214), B-0029

**Foti G., Catania M., Beltramello A., Carbognin G.**
Evaluation of glenoid labral tears: comparison between dual-energy CT arthrography and MR arthrography of the shoulder (SS 310), B-0239

**Foti G., Catania M., Beltramello A., Carbognin G.**
Identification of bone marrow oedema and osteochondral injuries of the knee: diagnostic accuracy of dual-energy CT and virtual non-calcium techniques (SS 1410), B-1128

**Fournier L.S.**
Whole-body imaging in gynaecological malignancy: Chairperson's introduction (E³ 319), A-0129

**Fournier L.S.**
Professional issues for radiology departments to enable research: How to or ganise an imaging department for clinical research (PI 3), A-0627

**Fournier L.S.**
Navigating tumour phenotype with advanced imaging analysis: Unravelling tumour heterogeneity using radiomics (SF 15d), A-0901

**Foutzitzi S., Deftereos S.P.**
Lung ultrasound, a safe & useful tool for neonatal with respiratory problems monitoring (SS 212), B-0163

**Fowler K.**
Hepatocellular carcinoma: from diagnosis to treatment: A. How to diagnose HCC: new guidelines (RC 1701), A-1002

**Fraia A., Zanatta E., Weber M., Balestro E., Stramare R., Doria A., Giraudo C., Quaia E.**
Muscle and pulmonary involvement in patients with antisynthetase syndrome: two aspects of the same disease or two different conditions? Preliminary results of an MR and HRCT study (SS 610), B-0522

**França M.M.**
US before CT in the acute abdomen?: Discussion (PS 1427), A-0838

**Franchi-Abella S.**
Paediatric oncology: The most common abdominal tumours (BS 12), A-0681

**Francone M.**
Radiologic anatomy: chest: Vasculature (BE 3), A-0124

**Francone M.**
From diagnosis to prognosis: how does cardiac imaging affect patient outcome?: A. In myocarditis (RC 403), A-0146

**Frauenfelder T.**
Everything you need to know about 3D post-processing: C. Interpretation of 3D processing results: from image to volume reading (RC 405), A-0145

**Frauenfelder T.**
Challenging HRCT patterns: tips and tricks from the experts: Perilobular consolidation (SF 8b), A-0455

**Frauenfelder T.**
Lung nodule management in 2019: A. Radiological assessment (RC 904), A-0549

**Frenken M., Abrar D., Ljimani A., Sewerin P., Klingebiel M., Antoch G., Schleich C.**
Prevention of the progressive biochemical cartilage destruction under methotrexate therapy in early rheumatoid arthritis (SS 610), B-0515

**Frenken M., Abrar D., Ljimani A., Sewerin P., Antoch G., Schleich C.**
The value of the simplified RAMRIS-5 in early-RA patients under methotrexate therapy using high-field MRI (SS 610), B-0517

**Frenken M., Abrar D., Knautz M., Latz D., Ljimani A., Antoch G., Schleich C.**
Molecular lumbar intervertebral disc alterations in patients with leg length discrepancy (SS 1910a), B-1736

**Frenzel F., Kubale R., Stroeder J., Massmann A., Jagoda P., Bücker A., Minko P.**
Prospective single-centre study to evaluate artifacts using CEUS in follow-up after EVAR: possible adverse effects on endoleak detection in comparison with CTA (SS 1915), B-1720

**Friedrich-Nel H.**
Radiographers' challenges offering imaging services in Africa: South Africa. The South African radiographer: button pusher or creative thinker? (EM 5), A-0654

**Frigerio A.**
Diagnosing ductal carcinoma in situ (DCIS): A. New radiologic-pathologic knowledge on DCIS (RC 1702), A-1027

**Frija G.**
Cardiovascular effects after radiotherapy for breast cancer: the European MEDIRAD Project: Chairpersons' introduction (part 2) (EU 1), A-0015

**Frija G., Derchi L.E., Gharbi H.A., Husseiny Salama D., Vinayak S., Niang E., Rezgui Marhoul L., Perez M.D.R., Mansouri B., Naidu K.M.**
Radiology in Africa: facing challenges and opportunities: Panel discussion: Radiology in Africa, reality and dreams (EM 1), A-0315

**Frija G.**
Diagnostic reference levels (DRLs) based on clinical indication: Chairperson's introduction (EU 2), A-0408

**Frija G.**
Diagnostic reference levels (DRLs) based on clinical indication: One shoe does not fit all: clinical indication based DRLs (EU 2), A-0410

**Frija G.**
Improving radiation protection in medical imaging in low- and middle-income countries: past actions and future directions: The International Society of Radiology's (ISR) vision (EU 3), A-0659

**Frija G.**
Patient safety in medical imaging: EuroSafe Imaging and the European Basic Safety Standards Directive: progressing patient safety (ESR/EFRS), A-0928

**Frija G.**
Artificial intelligence and radiation protection: Chairpersons' introduction (part 1) (EU 7), A-1011

**Frija G.**
Artificial intelligence and radiation protection: Artificial intelligence: a tool for quality and safety improvement in radiation protection (EU 7), A-1013

**Fritz J.**
Fast MSK with CAIPI SPACE, SMS TSE and compressed sensing (SY 6)

**Froelich M.F., Pons Lucas R., Coppenrath E., Sommer W., Treitl K.M., Saam T., Sommer N.N.**
Comparison of contrast-enhanced modified T1-weighted 3D TSE black blood and 3D MP-RAGE sequences for detection of meningeal enhancement (SS 1911a), B-1773

**Fu H., Xu R., Guo Y., Yang Z., Liu H.**
The difference of left ventricular myocardial strain in isolated LVNC and dilated cardiomyopathy with hyper-trabeculation: structure, functional and risk factors analysis (SS 212), B-0166

**Fu H., Wen L., Guo Y., Yang Z.**
CMR tissue tacking assessment of myocardial strain in non-ischaemic dilated cardiomyopathy patients with and without left ventricular hyper-trabeculations (SS 603), B-0594

**Fu J., Tang L., Li Z., Shi Q., Yan Z., Li X., Ji J.**
Diffusion kurtosis imaging evaluation of histological differentiation and Lauren classification in advanced gastric cancer (SS 616b), B-0611

**Fu J., Mengjie F., Li J., Li X., Tang L., Dong D.**
Assessment of metastatic GIST heterogeneity using texture analysis: ADC texture as a potential biomarker of 5-year survival
(SS 1401b), B-1280

**Fu R., Qiu T., Lu Q., Luo Y.**
Quantitative assessment of liver function before hepatectomy using two-dimensional shear wave elastography correlated with indocyanine green clearance test (SS 1501b), B-1370

**Fuchsjäger M.H.**
The art to transmit and to receive: how to communicate critical information to our patients: Chairpersons' introduction (Part 1)
(SF 8c), A-0414

**Fuchsjäger M.H.**
Minimally-invasive local treatment of breast cancer: the time is now: C. Cryotherapy
(RC 1202), A-0674

**Fuchsjäger M.H.**
Breast: Chairperson's introduction
(E³ 1823), A-1083

**Fujiwara M., Watanabe Y., Fujiwara T., Matsuo C., Takahashi H., Tanaka H., Ohta Y., Imai T., Tomiyama N.**
Visualisation and image quality of the chorda tympani nerve in temporal bone: comparison with conventional CT and ultra-high-resolution CT (SS 308), B-0293

**Fusaro M., Rattazzi M., Nardin C., Tiepolo M., Dorigo A., Tessarin G., Stamati G., Agostini C., Morana G.**
Acute chest pain evaluated with coronary CT: validation of new atherosclerotic markers
(SS 1003), B-1010

**Fusco R., Granata V., Pecori B., Petrillo A.**
Diffusion and perfusion MR parameters to assess preoperative short-course radiotherapy response in locally advanced rectal cancer
(SS 1501a), B-1346

**Fütterer J.J.**
Early detection of prostate cancer: C. Active surveillance: best practice (E³ 219), A-0095

### G

**Gabelloni M., D'Amore C.A., Morganti R., Faggioni L., Spinelli C., Neri E.**
Impact of ESR iGuide in the diagnostic workflow of paediatric patients with abdominal pain
(SS 205), B-0017

**Gabelloni M., Di Nasso M., D'Amore C.A., Morganti R., Faggioni L., Masi G., Neri E.**
Application of a clinical decision support system to the imaging pathway of patients with hepatocarcinoma and cholangiocarcinoma
(SS 1905b), B-1831

**Gabr A.E., Nada A., Gomaa M., Hashem R.H., Shokry A.**
The additive value of diffusion-weighted imaging in the initial workup for paediatric head and neck lesions (SS 1412), B-1232

**Gac P., Trzmielewska E., Poreba M., Mazur G., Sobieszczanska M., Poreba R.**
Ultrasonographic evaluation of changes in carotid arteries in patients with hypertension after a stroke (SS 215), B-0039

**Gagliardo C., Toia P., Schwenke M., Strehlow J., Cicero L., Cassata G., Melzer A., Preusser T., Midiri M.**
TRANS-FUSIMO: preliminary in-vivo animal results of MR-guided focused ultrasound of liver under respiratory motion (SS 1709), B-1444

**Galimzianova A., Desser T.S., Siebert S., Syed S., Kamaya A., Rubin D.**
Comparison of automated quantitative ultrasound image analysis vs expert scoring systems for prediction of malignancy in thyroid nodules (SS 1605), B-1402

**Gallagher F.A.**
Merging the best: hybrid imaging:
C. Hyperpolarised MRI (RC 806), A-0459

**Gamboa P.A.T., Ribeiro M.M., Macedo A.P.L.**
Evaluation of hippocampus volume in the elderly people by magnetic resonance imaging: comparison of two segmentation methods
(SS 414), B-0410

**Gammack P., Harper A., Alexander S., Jones J.**
An audit of the use of ultrasound scanning in investigation of paediatric inflammatory neck masses (SS 1708), B-1455

**Gangi A.**
Pain palliation in cancer patients: Chairperson's introduction (SF 9a), A-0511

**Gao A., Cheng J., Bai J., Zhang Y., Hong Y., Kong D.**
Application of histogram analysis of diffusion Kurtosis imaging to tumour grading and tissue heterogeneity (SS 1416), B-1244

**Gao A., Bai J., Zhang Y., Cheng J., Hong Y.**
Two postprocessing method of diffusion kurtosis imaging in glioma grading (SS 1911a), B-1779

**Gao B.**
The mechanism of cerebral aneurysms and treatment effect on the genesis
(SS 211b), B-0184

**Gao L., Hou Y., Ma Y., Yang L., Lu X.**
Influence of iterative model reconstruction level for measurement accuracy of the coronary stenosis caused by non-calcified plaques using low-dose CCTA: a phantom study
(SS 313), B-0315

**Gao L., Hou Y., Ma Y., Ma Q., Lu X.**
The value of multiple quantitative spectrum parameters for assessing the lymph node metastasis of lung cancer with dual-layer detector spectral CT (SS 1804), B-1604

**García Figueiras R.**
Quantitative imaging in oncology: B. Quantitative image biomarkers for targeted tumour therapies
(E³ 1726a), A-1059

**García Gorga R.**
Clinical dilemmas in radiography: what graduates need to know: Professionalism in an era of social media (part 1) (BR 12), A-0689

**García Roch C.G., García García F., Aragon Tejada F.X., Ciampi Dopazo J., Muñoz Cepeda M., Bernabéu Rodríguez M., Giovanetti González R., Roca A., Baron Rodiz P.A.**
Contrast-enhanced ultrasound (CEUS) efficiency in renal graft complication evaluation
(SS 607), B-0625

**Garcia-Castro F., Catala-March J., Brotons-Cuixat D., Llobet-Llambrich M., Sanchez-Osorio E., Alberich-Bayarri A.**
Quantification and evaluation of pre-post exercise femoral cartilage thickness and T2 changes in ultramarathon athletes
(SS 710), B-0680

**Garel C.**
Foetal and neonatal imaging pearls: B. Corpus callosum anomalies: pre- and postnatal correlation (RC 912), A-0561

**Garetier M., Grimandi R., Tissier F., Pons-Becmeur C., Ben Salem D., Borotikar B.**
Feasibility of lower limb muscle volume estimation during contraction with real-time dynamic MRI: implications to clinical diagnostics
(SS 1810), B-1511

**Gascho D., Heimer J., Thali M., Zoelch N.**
Magnetic resonance spectroscopy in muscle tissue and bone marrow to identify that a body was previously frozen and is meanwhile thawed
(SS 1810), B-1516

**Gaspar R.M.C.R., Abrantes A.F., Matos V.**
Frontiers of periosteal changes: comparison between anthropological data, clinical records and readings of X-ray medical imaging technology (SS 714a), B-0643

**Gasparotti R.**
How to implement MRI neuro advanced techniques at home: C. How to read diffusion tensor imaging (DTI) (E³ 126a), A-0026

**Gatidis S.**
Translating functional and molecular imaging in oncology: A. Functional cancer imaging with MRI
(RC 1206), A-0704

**Gatos I., Drazinos P., Yarmenitis S., Theotokas I., Kanavaki A., Manesis E., Zoumpoulis P.**
A comparative study of sound touch elastography measurement on liver's right lobe and left lobe for diagnosis of chronic liver disease using liver biopsy as gold standard (SS 1501b), B-1378

**Gatos I., Drazinos P., Yarmenitis S., Theotokas I., Soultatos A., Panteleakou E., Zoumpoulis P.**
Comparison of sound touch elastography (STE) and shear wave elastography (SWE) using liver biopsy as 'gold standard' for chronic liver disease assessment (SS 1801a), B-1530

**Gatos I., Drazinos P., Yarmenitis S., Theotokas I., Kanavaki A., Manesis E., Zoumpoulis P.**
Comparison of sound touch elastography (STE) and vibration-controlled transient elastography (VCTE) using liver biopsy as a reference for diagnosis of chronic liver disease (SS 1801a), B-1537

**Gatti M., Faletti R., Bioletto F., Palmisano A., Benedetti G., Esposito A., Fonio P.**
Two-dimensional and three-dimensional cardiac magnetic resonance feature-tracking myocardial strain analysis in acute myocarditis patients with preserved ejection fraction (SS 603), B-0598

**Gawlitza J.F.M., Michels J.D., Haubenreisser H., Henzler T., Borggrefe M., Schönberg S.O., Akin I., Trinkmann F.**
Predictors for bronchoalveolar lavage recovery rates derived from preprocedural quantified computed tomography (SS 604), B-0587

**Geão A., Pereira E.**
SPECT/CT artefacts: nuclear medicine point of view (SS 1014), B-0884

**Geão A., Pereira E.**
Dose reduction in hybrid imaging techniques in nuclear medicine (SS 1014), B-0892

**Gebauer B.**
Novelties in oncologic imaging: A. Imaging-guided liver interventions in oncology (E³ 426b), A-0191

**Gebauer B.**
Prostate biopsy and intervention: Keynote lecture (SS 1007), K-20

**Gennaro G., Bigolaro S., Hill M., Stramare R., Caumo F.**
Accuracy of mammography dosimetry in the era of the European Directive 2013/59/Euratom transposition (SS 1802a), B-1540

**Gennaro G.**
Breast imaging physics: Keynote lecture (SS 1913), K-43

**Gentili F.**
Imaging tumour response to immunotherapy: B. Monitoring immune response with CT (RC 116), A-0066

**George A.**
Early detection of ovarian cancer: A. Current guidance on screening and familial ovarian cancer (part 2) (E³ 119), A-0071

**George A.**
Early detection of prostate cancer: A. Screening for prostate cancer: where are we now? (part 1) (E³ 219), A-0092

**Gerevini S., Bozzolo E., Cao R., Della Torre E., Canti V., Cavalli G., Lanzillotta M., Dagna L., Falini A.**
Central nervous system (CNS) imaging findings in granulomatous polyangiitis (GPA), IgG4-related (IgG4-RD) and Erdheim-Chester diseases (ECD): comparison and pictorial essay (SS 1511), B-1337

**Gerevini S., Agosta F., Spinelli E., Scamarcia P., Riva N., Pagani E., Quattrini A., Falini A., Filippi M.**
MRI alterations in the brachial plexus of ALS patients (SS 1811b), B-1696

**Germano A.S.**
How to deal with the epidemics of thyroid nodules?: Chairperson's introduction (MS 16), A-0985

**Germano A.S.**
How to deal with the epidemics of thyroid nodules?: The radiologist role (MS 16), A-0987

**Germano A.S.**
How to deal with the epidemics of thyroid nodules?: Multidisciplinary case presentation and discussion: "The thyroid-team decision: when to follow and when to go beyond the guidelines" (MS 16), A-0990

**Gernhardt C., Guberina N., Burggraf M., Dietrich U., Theysohn J., Forsting M.**
Radiation exposure during radiographic and computed tomography lumbar spine imaging for adults (SS 1910a), B-1740

**Geroulakos G.**
Multidisciplinary approach to the diabetic foot: Surgical management of the diabetic foot (MS 17), A-1066

**Gersing A.S.**
The degenerative cervical spine: B. Cervical spinal stenosis and cervical spondylotic myelopathy (RC 1611), A-0949

**Gerson P.**
Radiographers' challenges offering imaging services in Africa: Chairpersons' introduction (part 2) (EM 5), A-0652

**Gerwing M., Kocman V., Helfen A., Stölting M., Greune L., Schmidt A., Heindel W., Wildgruber M., Eisenblaetter M.**
In vivo tracking of tumour-derived exosomes correlates with premetastatic cell infiltration to target-sites of metastasis (SS 1406), B-1125

**Gharbi H.A.**
Radiology in Africa: facing challenges and opportunities: Chairpersons' introduction (part 2) (EM 1), A-0309

**Gharbi H.A.**
Radiology in Africa: facing challenges and opportunities: Conclusion (part 2) (EM 1), A-0317

**Gharbi M., Aouini F., Kamoun H.**
Physicians knowledge and perceptions of radiation dose and inherent risks in computed tomography examinations (SS 1814), B-1487

**Ghosh A., Singh T., Singla V., Bagga R., Srinivasan R.**
Diffusion tensor imaging parameters of endometrial carcinoma can predict local tumour behavior: a feasibility study (SS 1907), B-1901

**Ghosh R., Chilamkurthy S., Rao P., Tanamala S., Campeau N.**
Deep learning for infarct detection and localisation from head CT scans (SS 705), B-0750

**Gianneramo C., Bruno F., Cannizzaro E., Palumbo P., Iafrate S., Mariani S., Arrigoni F., Barile A., Masciocchi C.**
Evaluation of bone loss in shoulder instability using on track/off track method: value of 3D MR arthrography in standard and ABER position (SS 310), B-0241

**Giannetto G., Pusceddu L., Doronzio V., Vani V., Romano V., Maglia C., Mazzetti S., Regge D., Russo F.**
Comparison between biparametric MRI (bp-MRI) and multiparametric MRI (mp-MRI) for the diagnosis of prostate cancer (PCa): preliminary results of a prospective randomised trial (MY 13), B-1082

**Giannotti E.**
New mammography: digital breast tomosynthesis and future techniques: C. Contrast-enhanced mammography and other developing techniques (RC 1302), A-0763

**Giannotti G., Tedesco G., Beleù A., Rizzo G., Cardobi N., De Robertis Lombardi R., D'Onofrio M.**
DWI and dynamic MRI in the study of intraductal papillary mucinous neoplasms (IPMNs): pathological comparison with tumour grade correlation (SS 501), B-0455

### Authors' Index

**Giannotti N., Kelly P., Foley S., Horgan G., Kavanagh E., O'Connell M.J., McNulty J.**
The assessment of carotid plaque morphology using magnetic resonance imaging and computed tomography as biomarkers of carotid plaque vulnerability (SS 414), B-0407

**Giasotto V., Basso L., Casaleggio A., Prono V., Terrone C., Neumaier C.E.**
Suspected lesions categorised as PIRADS 3 at multiparametric magnetic resonance (mp-MRI) for prostate cancer (PCa): should we biopsy? (SS 1007), B-0879

**Gibaud B., Cordonnier E., Pasquier G.**
Will the General Data Protection Regulation (GDPR) hamper the secondary use of clinical imaging data for research?: How to anonymise imaging data for research? (ESR/BBMRI-ERIC), A-0633

**Gibney B., Murphy M.C., Ahern D., Moughty A., Kavanagh E., Hynes D., MacMahon P.J.**
Trapezial ridge fractures: an underappreciated cause of post-traumatic wrist pain with normal initial radiographs. (SS 310), B-0246

**Giganti F., Stabile A., Giona S., Orczyk C., Moore C.M., Allen C., Kirkham A., Emberton M., Punwani S.**
Prostate cancer treated with irreversible electroporation: MRI-based volumetric analysis and oncological outcome (SS 207), B-0159

**Giganti F., Stabile A., Stavrinides V., Orczyk C., Punwani S., Allen C., Kirkham A., Emberton M., Moore C.M.**
Natural history of prostate cancer on active surveillance: stratification by MRI using the PRECISE recommendations in a UK cohort over 11 years (SS 407), B-0443

**Gilbert F.J.**
The art to transmit and to receive: how to communicate critical information to our patients: How can we improve communication with our patients? (SF 8c), A-0418

**Gilbert F.J.**
Screening for breast cancer: Chairperson's introduction (RC 902), A-0526

**Gilbert F.J.**
Decision tools and artificial intelligence in breast imaging: Keynote lecture (SS 702a), K-16

**Gilley D.**
Improving radiation protection in medical imaging in low- and middle-income countries: past actions and future directions: The International Atomic Energy Agency's (IAEA) approach (EU 3), A-0661

**Giovagnoni A.**
From morphology to function: La Radiologia Medica: the role of the journal in an international setting (EM 2), A-0584

**Giovagnoni A.**
Investment strategies: A. Budget: all these numbers ($E^3$ 1118), A-0635

**Girard F.**
The daily challenges of a good MR exam (part 1) (SY 22)

**Gitto S., Albano D., Messina C., Chianca V., Rapisarda S., Corazza A., Sconfienza L.M.**
Diagnostic performance of high-resolution ultrasound in the evaluation of intrinsic and extrinsic ligaments of the wrist in patients with previous trauma or carpal instability (MY 9), B-0868

**Gitto S., Albano D., Chianca V., Cuocolo R., Ugga L., Messina C., Sconfienza L.M.**
Machine learning classification of low-grade and high-grade chondrosarcomas based on magnetic resonance imaging-based texture analysis (MY 9), B-0870

**Gleeson F.**
Pleural neoplasms: Chairperson's introduction ($E^3$ 1322), A-0800

**Goddi A., Frattini T., Bozzetto M., Bortolotto C., Milan L., Zatelli D., Novario R., Calliada F., Remuzzi A.**
Comparison between high-frame rate vector flow and computational flow dynamics in the carotid bifurcation: evaluation of a protocol study (SS 215), B-0040

**Goh V.J.**
The role of imaging in the era of liquid biopsy: Is there still a role for imaging surveillance? And when? (NH 1), A-0037

**Goh V.J.**
Oncological imaging: where are we now?: B. Functional MRI techniques (RC 1216), A-0713

**Gohmann R.F., Krieghoff C., Seitz P., Lücke C., Hartung P., Abdel-Wahab M., Holzhey D., Thiele H., Gutberlet M.**
CT as a one stop shop for TAVI-Planning: do we still need an additional invasive coronary angiography? (SS 203), B-0196

**Gokharman F.D., Aydin S., Paltun S.C., Fatihoglu E., Yalcin Sahiner S., Kosar P.N., Karli Oguz H.K.**
DTI-MRI findings in synthetic cannabinoid users (SS 1911b), B-1884

**Goldberg S.N.**
Josef Lissner Honorary Lecture: Systemic effects of image guided tumour therapy: have we opened Pandora's box or found the Holy Grail? (HL 2), A-0602

**Golland P.**
Artificial intelligence (AI): our future cannot be predicted, but we have to be prepared: Machine learning in medical imaging going forward (SF 8a), A-0395

**Gomaa M., Moustafa A.F.I.**
Contrast-enhanced spectral mammography: is it useful in predicting invasiveness in suspicious breast micro-calcifications? (MY 5), B-0460

**Gomaa M., Kamal E., Kamal R.M., Mostafa S., Mubarak R., El-Adawy M.**
A new objective mathematical tool for assessment of response and predicting residual disease in breast cancer using CESM (SS 1402b), B-1174

**Gong Q., Deng Y., Xia C., Zhang R., Wu C., Wang Z., Zhang Y., Ma J.**
Estimation of intracranial haemorrhage volume with the deep learning method (SS 705), B-0751

**González R.L., Venugopal V., Jimenez-Pastor A., Marti-Bonmati L., Alberich-Bayarri A., Barnwal M., Mahajan V.**
Automated multiregional prostate segmentation in magnetic resonance using deeply supervised convolutional neural networks (SS 1605), B-1397

**Gonzalo Carballés M., Riaza Martin L., Coma A., Castellote Alonso A., Guillén Burrieza G., Riera L., Molino Gaete J., Piqueras Pardellans J., Vázquez E.**
The difficulties of childhood hepatocellular carcinoma MRI diagnosis (SS 312), B-0340

**Goo J.M.**
Functional imaging of the lungs: C. Lung cancer, tumour heterogeneity and tumour response ($E^3$ 520), A-0291

**Gopalan D.**
Imaging innovations and their impact on patients (SY 12)

**Gopalan D.**
Welcome and introduction (SY 12)

**Gore R.M.**
Imaging of the complicated postoperative abdomen: B. Pancreas (RC 901), A-0487

**Gorgatti T., Vit A., Sponza M., Gavrilovic V., Pellegrin A., Rosella F., Girometti R.**
Outcomes of Yttrium-90 radioembolic treatment for large monofocal hepatocarcinoma in BCLC-A stage patients: a single-centre experience (SS 201a), B-0076

**Gourtsoyianni S.**
Radiologic anatomy: abdomen: Anorectal (BE 4), A-0163

**Gourtsoyianni S.**
Role of imaging in cancer of unknown primary (CUP): Chairperson's introduction (RC 516), A-0280

**Grainger A.J.**
Wrist and foot/ankle sports injuries: B. Multimodality imaging of foot and ankle injuries in the athlete (TC 1428), A-0840

**Grainger A.J.**
Wrist and foot/ankle sports injuries: C. Interactive case discussion (Part 2) (TC 1428), A-0842

**Granata C.**
Getting the balance right: radiation risk and imaging benefit in paediatric procedures: Radiation safety in interventional procedures in children (EU 5), A-0875

**Granata C.**
CT radiation risk in children: an overview: Communicating benefits and risks to parents and carers (EU 6), A-0936

**Granzier R., Ibrahim A., Woodruff H., van Nijnatten T., de Boer M., Hulsmans F., Lambin P., Smidt M., Lobbes M.**
Radiomics based on baseline DCE-MRI is predictive of tumour pathological complete response to neoadjuvant systemic therapy in breast cancer patients (SS 1402b), B-1170

**Grassi R.**
From morphology to function: Interlude: The sirens: myth or reality? (EM 2), A-0581

**Grassi R.**
From morphology to function: Interlude: The cyclops: myth or reality? (EM 2), A-0583

**Graungaard Falkvard C.**
EFRS meets Denmark: Introduction: Across Denmark (EM 4), A-0620

**Grazioli L.**
Optimisation of the workflow in liver MRI (SY 13)

**Greco F., Girometti R., Cereser L., Como G., Zuiani C.**
Do DCE-derived quantitative parameters predict clinically significant prostate cancers? A study on patients referred to radical prostatectomy (MY 13), B-1079

**Grenier N.**
Functional imaging of the kidneys: C. Characterisation of chronic kidney diseases (CKD) (E³ 720), A-0380

**Grenier N.**
Imaging strategies in renal tumours: B. Differential diagnosis of renal masses (RC 907), A-0573

**Grenier P.A.**
Chronic obstructive pulmonary disease (COPD): A. CT phenotyping and visual assessment (E³ 922), A-0565

**Griffin E., Sheehan M., Brennan D., Looby S., Power S., O'Hare A., Brennan P., Thornton J.**
Drip, ship, retrieve and leave: a national experience in the management of patients within regional hospitals post endovascular thrombectomy for acute large vessel ischaemic stroke (SS 311), B-0265

**Griffin E., Adams N.C., Motyer R., Farrell T., O'Hare A., Looby S., Power S., Brennan P., Thornton J.**
External referrals for endovascular thrombectomy: our experience in a regional stroke centre and insights into service improvements (SS 311), B-0267

**Griffiths C., Rafferty A.R., Allen R., Noonan P.**
Impact of cardiac CT on the use of invasive diagnostic catheterisation in paediatric patients (SS 1014), B-0885

**Griffiths P.D.**
Foetal and neonatal imaging pearls: C. Foetal MR imaging of acquired brain pathology (RC 912), A-0562

**Griffiths P.D.**
Paediatric brain: Keynote lecture (SS 1011a), K-22

**Grigorieva E., Krylov V., Lukyanchikov V., Polunina N.**
4D CT-angiography as a predictor of growth of intracranial aneurysm (SS 211b), B-0186

**Grimaldi D., Monti S., Rinaldo C., Brancato V., Aiello M., Di Costanzo G., Cavaliere C., Ragozzino A., Salvatore M.**
Multiparametric MRI in prostate cancer: a radiomic study applied to different diffusion and perfusion models (SS 216), B-0174

**Grimm J.**
Oncological imaging: where are we now?: C. Assessment by molecular imaging (RC 1216), A-0714

**Grippo C., Jagmohan P., Stoeger A.M., Kapetas P., Helbich T.H., Baltzer P.A.T., Clauser P.**
External validation of the STAR (suspicion type age response of biopsy) score for risk stratification of B3 breast lesions detected at ultrasound-guided biopsy (SS 1402a), B-1150

**Grob D., Smit E.J., Prince J., Kist J., Oostveen L.J., Prokop M., Schaefer-Prokop C., Sechopoulos I., Brink M.**
Do subtraction CT-derived iodine maps have the same impact on pulmonary embolism detection as dual-energy CT-derived maps? (SS 1904), B-1850

**Grosse Hokamp N., Eck B., Holz J., Siedek F., Pinto Dos Santos D., Maintz D., Haneder S.**
Quantification of metal artefacts in CT imaging: methodological comparison of different techniques and correlation to visual perception (SS 313), B-0314

**Grosse Hokamp N., Salem J., Lennartz S., Reimer R.P., Heidenreich A., Maintz D., Haneder S.**
Kidney stone composition analysis using machine learning and spectral detector CT: an ex-vivo study (MY 13), B-1081

**Grosu S., Lorbeer R., Bamberg F., Schlett C.L., Heier M., Rospleszcz S., Peters A., Ertl-Wagner B.B., Stöcklein S.**
Association of amygdala volume and markers of cardiovascular disease (SS 211b), B-0188

**Grosu S., Rospleszcz S., Bamberg F., Schlett C.L., Peters A., Heier M., Schöppe F., Ertl-Wagner B.B., Stöcklein S.**
Extracranial determinants of white matter lesion volume: a machine learning-based top 10 list (SS 705), B-0749

**Grover S.B., Grover H., Jain P., Nag A.K., Katyan A., Antil N., Jain S.K., Mandal A.K.**
Contrast-enhanced ultrasound in breast cancer and its role in predicting immunohistochemical subtype: a novel prognostic approach (SS 702b), B-0720

**Grueneisen J., Schaarschmidt B.M., Chodyla M., Martin O., Li Y., Fendler W., Herrmann K., Forsting M., Umutlu L.**
18F-FDG PET/MRI for therapy response assessment of isolated limb perfusion in patients with soft-tissue sarcomas (SS 716), B-0690

**Gruszczynska K.**
Cardiac: recalling the forgotten structures: Friend or enemy: epicardial fat (SA 13), A-0775

**Guarasci F., Faletti R., Ruffino M.A., Fronda M., Gatti M., Varetto G., Righi D., Rispoli P., Fonio P.**
Early embolisation after carotid artery stenting: role of MR-DWI as pre-procedural predictor and discriminant between intra- and post-procedural events (SS 215), B-0038

**Guarnera A., Romano A., Moraschi M., Pierallini A., Bozzao A.**
White matter involvement in young non-demented Down's syndrome subjects: a tract-based spatial statistic analysis (SS 1411b), B-1270

**Guazzarotti G., Venturini E., Mellone R., De Gaspari A., Di Santo D., Del Maschio A., De Cobelli F.**
Parotid tumours differential diagnosis: MRI dynamic contrast-enhanced (DCE) and diffusion-weighted imaging (DWI) compared to fine needle aspiration cytology (FNAC) (SS 1008), B-0971

**Guberina N., Püllen L., Stephan T., Grueneisen J., Forsting M., Umutlu L., Reis H., Hadaschik B., Wetter A.**
Technique and first results: targeted MRI and [$^{68}$Ga]-PSMA PET/MRI ultrasound fusion-guided transperineal prostate biopsies
(SS 1007), B-0875

**Guberina N., Forsting M., Ringelstein A.**
Comparison of radiation doses imparted during multidetector CT scanners and cone beam computed tomography for the postoperative, spinal fusion assessment (SS 1813), B-1595

**Guenego A., Piotin M., Blanc R., Fahed R.**
Haemorrhagic transformation after stroke: interrater and intrarater agreement
(SS 309), B-0280

**Guenego A., Fahed R., Piotin M., Blanc R., Bonneville F., Mosimann P., Cognard C.**
Comparison of mechanical thrombectomy in single- vs. biplane angiosuites (SS 309), B-0282

**Guenego A., Zerlauth J., Meuli R., Chapot R., Mosimann P.**
Balloon-assisted coil embolisation and large stent delivery for cerebral aneurysms with a new generation of dual-lumen balloons
(SS 309), B-0288

**Guerreiro T.C.P.L., Ribeiro L., Abrantes A.F., Rodrigues S., Raposo R., Soares F., Almeida R.P.P.**
Mobile phones as a potential vehicle of nosocomial pathogens in radiology departments
(MY 15), B-1359

**Guglielmi G.**
The old spine: challenges of imaging and treatment: B. Fractures: bone fragility in the elderly, assessing osteoporosis and bone quality and differential diagnosis (RC 1610), A-0970

**Guglielmo M., Baggiano A., Muscogiuri G., Guaricci A.I., Andreini D., Alamanni F., Pepi M., Pontone G.**
Left ventricle volumes and function assessment with cardiac magnetic resonance four-dimensional flow imaging (SS 1803), B-1615

**Guglielmo M., Muscogiuri G., Baggiano A., Guaricci A.I., Andreini D., Alamanni F., Pepi M., Pontone G.**
Cardiac magnetic resonance with 4D flow imaging for mitral regurgitation severity assessment (MY 18), B-1686

**Gui B., Russo L., Cambi F., Petta F., Persiani S., De Vincenzo R., Manfredi R.**
Conservative treatment in patients with cervical cancer on FIGO stage IB1-IIA1 >2cm: the role of MRI in a pilot study: preliminary results
(SS 1907), B-1898

**Guimarães L.S.**
Dual-energy CT of the abdomen: the time is now: A. Basic principles and different approaches (E³ 1526), A-0857

**Gule-Monroe M.K., Chen M., Li J., O'Brien B.J., Chung C., Ferguson S.D., Prabhu S.S., Johnson J.M.**
Delayed FDG PET/CT in differentiating true progression from pseudo-progression in glioblastoma treated with chemoradiation
(SS 1416), B-1243

**Gulino P., Salvatore E., Bianchi A., Franzese M., Sali L., Aiello M., Cavaliere C., Salvatore M., Mascalchi M.**
Dentate and basal ganglia T1 hyperintensity after multiple gadolinium administrations and brain microstructure: an in vivo diffusion-weighted MR study in paediatric patients
(SS 1011a), B-0928

**Gumeler E., Parlak S., Yazici G., Karabulut E., Kiratli H., Karli Oguz H.K.**
Comparison of single shot echo planar imaging and turbo spin echo diffusion weighted imaging of the orbit in patients with uveal melanoma
(SS 1708), B-1445

**Gundogmus C.A., Tureli D., Asadov R., Sabet S., Andac N., Baltacioglu F.**
Endovascular treatment of intracranial carotid sidewall aneurysms by flow diversion: a single centre's experience and comparison of Fred and Pipeline stents (SS 309), B-0286

**Gunn A., Mahmoud K., El Khudari H., Salei A., Keasler E., Bready E., Winston J., Patten P., Abdel Aal A.M.K.**
Safety and outcomes of percutaneous cryoablation for large T1b renal cell carcinoma
(SS 1509), B-1301

**Gunny R.**
Late effects in survivors of childhood cancer: Neuro (SF 17), A-1024

**Guo W., Zheng S., Wu H., Liu P.C., Sun I., Hong S., Chung W., Yang W.H., Tu Y.**
BRAIMS: an incoming open source MR imaging dataset for brain instance-aware metastases segmentation (SS 705), B-0758

**Gupta K., Chandra T., Venkatesan B.**
Diagnostic performance of fifth BI-RADS edition ultrasound lexicon for prediction of malignancy in breast masses with histopathological correlation
(SS 702b), B-0717

**Gupta P., Sinha S., Dhaka N., Rajwanshi A., Vaiphei K., Kochhar R.**
Contrast-enhanced ultrasound for differentiation of pancreatic carcinoma (PC) from mass-forming chronic pancreatitis (MFCP) (SS 1401a), B-1095

**Gupta V., Verma R., Ranjan R., Belho E., Mahajan H.**
Spectrum of autoimmune encephalitis: establishing imaging patterns on FDG PET/CT for specific antibody sub-types
(SS 1511), B-1335

**Gupta V., Verma R., Ranjan R., Belho E., Mahajan H.**
Lewy body dementia and posterior cortical variant of Alzheimer's disease: distinguishing imaging patterns on FDG PET/CT and $^{99m}$Tc-TRODAT SPECT scan (SS 1811a), B-1558

**Gupta V., Maralakunte M., Grover S., Ahuja C.K.**
Role of whole brain diffusion kurtosis neuroimaging in disentangling bipolar depression from unipolar depression
(MY 16), B-1428

**Gurumoorthy M., Kaur S., Kwan Hoong N., Soo S., Rahmat K., Hongmin C.**
Accuracy comparison of breast cancer detection based on microcalcifications between full-field digital mammograms and synthetic 2D mammograms using a deep convolution neural network (MY 5), B-0475

**Gutberlet M.**
Functional imaging of the heart: Chairperson's introduction (E³ 420), A-0198

**Gutberlet M.**
Acute chest pain and cardiac imaging: A. Acute chest pain: who should see the patient first, the doctor or the CT department?
(RC 1603), A-0938

**Gutjahr R., Jost G., Pietsch H., Schmidt B., Flohr T.**
Systematic accuracy assessment of a real-time automated patient specific bolus tracking delay calculation algorithm (SS 615), B-0496

**Gutzeit A., Fröhlich J.M., Steffen F., Koh D.-M., Orasch C.**
Would it be safe to have a dog in the MRI scanner before your own examination?
(SS 217), B-0108

**Guðjónsdóttir J., Hannesdóttir G.A.**
The unjustified dose behind cropped radiographs (SS 714b), B-0656

**Gwon H.Y., Na D.G., Paik W., Yoon S.J.**
Malignancy risk of thyroid nodules with isolated macrocalcification (SS 208), B-0127

### H

**Habib Y.S., Youssef A., ElKiki H.A., ElZomor H.A., Rezk M.M.**
High-resolution MR imaging of the orbit in patients with retinoblastoma: prospective study correlated with histopathological results
(MY 15), B-1363

**Haenel A.S., Ghosn M., Karimi T., Vykoukal J., Shah D.J., Schulz D., Raizner A., Schmitz C., Alt E.**
Treatment of chronic myocardial infarction with unmodified stem cells at point of care: a feasibility study on pigs (MY 18), B-1669

**Hagberg G.**
Demystifying MRI: things you always wanted to know: C. Practical MRI: a toolkit of standard MR pulse sequences (RC 1513), A-0912

**Hahn F., Fleischmann D., Halfmann M.C., Schultheiss H., Kreitner K.-F., Wenzel P., Emrich T.**
Myocardial inflammation in DCM and mapping: an elusive connection (SS 303), B-0378

**Hahn H.K.**
Quantitative imaging in oncology: C. From quantitative imaging to radiomics and deep learning (E³ 1726a), A-1060

**Haider L., Wei-Shin E.C., Olbert E., Mangesius S., Dal-Bianco A., Leutmezer F., Prayer D., Thurnher M.**
Cranial nerve enhancement in multiple sclerosis (MS) is associated with younger age at onset and more severe disease (SS 611), B-0534

**Haller S.**
How to implement MRI neuro advanced techniques at home: B. How to read susceptibility-weighted imaging (SWI) (E³ 126a), A-0025

**Halliday K.**
Predicting fracture risk in children: Chairperson's introduction (RC 812), A-0460

**Hamdy A., Ichikawa Y., Toyomasu Y., Nagata M., Nagasawa N., Sakuma H.**
Perfusion parameters quantified with perfusion CT in pancreatic adenocarcinoma before and after neoadjuvant chemoradiotherapy: comparison between responders and non-responders (SS 1401a), B-1101

**Hamdy A., Ichikawa Y., Toyomasu Y., Nagata M., Nagasawa N., Sakuma H.**
Perfusion vs metabolism: which functional parameter should we choose in patients with pancreatic adenocarcinoma to predict response to chemoradiotherapy? (SS 1401a), B-1102

**Hamer O.**
Challenging HRCT patterns: tips and tricks from the experts: Approach to nodular pattern (SF 8b), A-0454

**Hamri H., Weiland E., Scholer M., Jolibois Z., de Bazelaire C.**
Diffusion with very high b-value in breast MRI: preliminary results (SS 1802b), B-1563

**Han D., Heuvelmans M.A., Pelgrim G., Sidorenkov G., van Ooijen P.M.A., Oudkerk M., Vliegenthart R.**
Effect of iterative reconstruction and artificial neural network denoising techniques on lung nodule volumetry in ultra-low dose computed tomography: a phantom study (SS 1004), B-1008

**Han D.Y., Ryu K.N., Park J.S., Jin W., Park S.Y., Yun S.J.**
The prevalence of Baker's cyst in relation to the arrangement pattern between medial head of gastrocnemius tendon and semimembranous tendon (SS 1410), B-1137

**Han H.**
Evaluation of usefulness of virtual calcium scoring in dual-energy cardiac CT (SS 614), B-0488

**Han P., Li W., Yang Z., Diao K., Shi R., Li Z.**
One-stop coronary CTA combined with carotid and cerebrovascular CTA using wide-detector CT: comparison with conventional protocol (SS 315), B-0218

**Han Y., Sun Y., Yan L., Wang W., Cui G.**
A radiomics strategy for amide proton transfer imaging in predicting isocitrate dehydrogenase genotype within grade II/III gliomas (SS 705), B-0757

**Hangaard S., Boesen M., Bliddal H., Wirth W.**
Do Ahlbäck scores identify subgroups with different magnitudes of cartilage thickness loss in patients with moderate to severe radiographic osteoarthritis? Data from the OAI (SS 1410), B-1135

**Hansen M.S., Becerra L., Dahl J.B., Borsook D., Mårtensson J., Christensen A.F., Nybing J.D., Havsteen I., Boesen M., Asghar M.S.**
Brain resting state connectivity in the development of secondary hyperalgesia in healthy men (SS 1011b), B-1035

**Hansen N.L., Lloyd T., Macchi A., Warren A., Barrett T., Kastner C.**
Feasibility of target dependent systematic core biopsy distribution models for transperineal MR/US fusion biopsy (SS 1007), B-0880

**Hao L.**
Histogram analysis of enhancement MRI may predict relapse of primary CNS lymphoma after surgery-combined first-line chemotherapy (SS 1411a), B-1162

**Harding L.**
Radiography audit and quality management: C. Back to basics: auditing radiographic technique (RC 514), A-0210

**Haris Phuah A., Rahmat K., Ramli N., Rozalli F.I., Fadzli F.B., Tan L., Saedon N.I., Tan M.**
Cerebral white matter structural integrity in patients with mild cognitive impairment: evaluation with diffusion tensor imaging and morphometric assessment on 3T MRI (SS 1411b), B-1274

**Harman P.A., Law C., Johnson M., Pardhan S., Grunwald I.Q., Ramavathu K.**
Resting state functional MRI connectivity comparison between Alzheimer's disease and Parkinson's disease dementia: a sub-analysis (SS 1811a), B-1555

**Hartenbach M.**
Tumour relapse in urological cancer: C. Theranostics in urological cancer (E³ 619), A-0331

**Hartmann I.**
Pleural neoplasms: A. Malignant mesothelioma (E³ 1322), A-0801

**Harvey H.**
Artificial intelligence (AI): driven by radiologists: How will the introduction of AI change the role of the radiologist? (NH 3), A-0128

**Harvey H.**
Clinical rigour and the validation process for deep learning algorithms (SY 5)

**Hashem R.H., Tomerak R.H., Tosson A.M., Abdelmoneim A.**
Ultrasound as an alternative for plain radiography in the detection of the endotracheal tube position in neonatal intensive care unit patients (SS 212), B-0162

**Hassan K.M.B., Slehria A.U., Baqir Hassan T.**
Accuracy of grey-scale ultrasound in correctly identifying acute appendicitis in comparison with surgical outcome (SS 317), B-0271

**Hasse A., Tello K., Gall H., Breithecker A., Wiedenroth C.B., Seeger W., Mayer E., Krombach G.A., Roller F.C.**
Balloon pulmonary angioplasty (BPA) improves right ventricular dysfunction and pulmonary haemodynamics in inoperable CTEPH (SS 1903b), B-1863

**Hasse F.C., Selmi-Özer B., Rupp C., Kauczor H.-U., Weber T.**
Balanced steady-state free precession MRCP is a robust alternative to respiration-navigated 3D turbo-spin-echo MRCP (SS 301), B-0385

**Haubold J., Demircioglu A., Forsting M., Umutlu L., Nensa F.**
Automated x-ray bone segmentation using a conditional adversarial network (MY 7), B-0803

**Haubold J., Demircioglu A., Gratz M., Wrede K., Glas M., Herrmann K., Forsting M., Nensa F., Umutlu L.**
Non-invasive tumour decoding and phenotyping of cerebral gliomas utilizing multiparametric $^{18}$F-FET PET-MRI and MR fingerprinting (MY 7), B-0819

**Hausegger K.**
Carotid disease 2.0: C. Carotid stent: medical history or part of the future? (RC 915), A-0510

**Hausmann D., Ignjatovic S., Kreul D., Klarhöfer M., Nickel M., Kiefer B., Kubik-Huch R.A.**
Evaluation of a compressed-sensing accelerated high-resolution VIBE sequence for an improved morphologic and functional assessment ("one-stop shop") of the uterus: initial analysis (SS 407), B-0446

**Hazell L.J., Lawrence H., Friedrich-Nel H.**
Simulation-based learning to facilitate clinical readiness in diagnostic radiography: a metasynthesis (SS 1914a), B-1707

**Hazirolan T.**
Non-contrast MR angiography: ready to go?: Non-contrast MR angiography for large vessels (SF 8d), A-0421

**He J., Zhou Q., Li X., Xu X., Ding X., Zhou X., Zhang M.**
Automatic segmentation for coronary computed tomography angiography using convolutional neural networks (SS 1405), B-1189

**He M., Xue H., JIN Z.**
Can two breath-hold 3D-MRCP replace the conventional 3D-MRCP? (SS 301), B-0386

**He M., Xue H., Jin Z.**
Comparison of reduced field-of-view (rFOV) and full FOV (fFOV) diffusion-weighted imaging (DWI) in the assessment of insulioma: image quality and WHO grading (SS 501), B-0454

**He M., Xue H., Jin Z.**
Differentiation of atypical non-functional pNET and PDAC using a CT radiomics-based nomogram (SS 616b), B-0613

**He Y., Lin C., Qi Y., Wang X., Zhou H., Xue H., Jin Z.**
Amide proton transfer MR imaging for cervical cancer: a preliminary study (MY 13), B-1074

**He Y., Lin C., Qi Y., Wang X., Zhou H., Xue H., Jin Z.**
Contrast-enhanced T1 mapping MR imaging for evaluation of myometrial invasion in endometrial cancer: initial experience (MY 13), B-1087

**Healy N., Kilburn-Toppin F., Wallis M.**
Can breast MRI aid in predicting high burden axillary disease in patients diagnosed with breast cancer? (SS 1902b), B-1801

**Healy N.A., Tanner J.A., Kilburn-Toppin F.**
CT staging in newly diagnosed breast cancer: are we adhering to current guidelines? (SS 1902b), B-1795

**Heck L., Eggl E., Grandl S., Dierolf M., Mayr D., Hellerhoff K.A., Reiser M.F., Pfeiffer F., Herzen J.**
Dose-compatible grating-based phase-contrast mammography on mastectomy specimens using a compact synchrotron source (SS 1913), B-1847

**Hegazy R.M.A., Aghahowa E.A., Yasin R., Eduarte A.**
Local diagnostic reference levels in mammography: setting a maximum/alarm dose in a diagnostic mammography unit using dose monitoring software (SS 1802a), B-1545

**Heidinger B.H., DaBreo D.C., Carroll B.J., Feldman S.A., Mohebali D., McCormick I., Matos J.D., Manning W.J., Litmanovich D.**
Presence of coronary artery calcifications on CT is associated with PE-related mortality in patients with acute PE (SS 1503), B-1306

**Heijmink S., van Muilekom E., Tillier C., Grivas N., van der Poel H.**
Radiological prediction of the chance of post-RALP urinary continence on preoperative MR prostate imaging can substantially alter patients' choice of treatment (SS 307), B-0326

**Heiß R., Mayer I., Huettel M., May M.S., Wuest W., Kopp M., Lutter C., Forst R., Hotfiel T.**
Tissue stiffness after self-myofascial release in athletes with different experience in foam rolling assessed by quantitative acoustic radiation force impulse elastography (ARFI) (SS 710), B-0684

**Heitmann S., Toomey R., Chen M., Pettka P., Molehe M., Selau Junior M., Fernandes K., Davies K., Pires Jorge J.**
Factors influencing student radiographers' assessment of chest radiograph image quality (SS 1914a), B-1704

**Hekimoglu A., Ergun O., Turan A., Turkmenoglu T.T., Hekimoglu B.**
What is the contribution of MR spectroscopy in the differential diagnosis of solitary pulmonary masses? (SS 1404), B-1206

**Hekimoglu K., Haberal K., Turnaoglu H., Kahraman G.**
Dual-energy CT pulmonary angiography and lung perfusion imaging with ultra-low-dose contrast application (SS 1904), B-1854

**Helbich T.H.**
Screening for breast cancer: B. Combined screening with mammography and ultrasound (RC 902), A-0528

**Helmberger T.**
Nonvascular interventions in the lung: Keynote lecture (SS 1909), K-42

**Helthuis J., van Doormaal T., Hillen B., Bleys R., Harteveld A., Hendrikse J., van der Toorn A., Zwanenburg J.J., van der Zwan A.**
Branching pattern of the cerebral arterial tree (SS 315), B-0215

**Hempel J., Nüßle N., Brendle C., Bender B., Bier G., Skardelly M., Richter H., Schittenhelm J., Ernemann U., Klose U.**
Diffusion kurtosis imaging parameters predicting survival in integrated molecular subtypes of diffuse glioma: an observational cohort study (SS 211a), B-0093

**Hendriks E.J., Idema S., Zwinderman A.H., Barkhof F., Vandertop W.P., Mandonnet E., Duffau H., Berger M.S., De Witt Hamer P.C.**
Preoperative resectability estimates of non-enhancing glioma by neurosurgeons and a resection probability map (SS 211a), B-0096

**Hendrikse J.**
7 Tesla MR goes clinical: Neuroimaging at 7 T: where does it make the difference? (SF 5a), A-0251

**Henner A.**
Clinical simulation and its role in radiography education: How simulation can help prepare students and have a positive impact on interprofessional working (SF 9b), A-0494

**Hermann A., Dieudonné A., Ronot M., Sanchez M., Pereira H., Chatellier G., Garin E., Lebtahi R., Vilgrain V.**
Predictive $^{99m}$Tc MAA-SPECT/CT dosimetry in patients with locally advanced inoperable hepatocellular carcinoma treated by selective internal radiotherapy with $^{90}$Y resin microspheres (MY 8), B-0848

**Hermans R.**
Post-treatment imaging of the head and neck: A. Normal findings after radiotherapy (RC 1208), A-0720

**Hermie I., Van Besien J., De Visschere P., Villeirs G.**
Predicting clinically significant prostate cancer in PI-RADS 3 lesions: a retrospective high-volume centre study (SS 207), B-0157

**Hernandez-Giron I., Schippers R., den Harder J.M., Streekstra G.J., Veldkamp W.J.H.**
Automated image quality assessment in CT using a 3D printed lung vessel phantom and structural similarity index analysis (SS 213), B-0137

**Herold C.J.**
Advanced methods for nodule evaluation: Keynote lecture (SS 1004), K-24

**Herranz M.**
Translating functional and molecular imaging in oncology: B. New PET tracers in oncology (RC 1206), A-0705

**Heussel C.P.**
Fibrotic lung diseases: C. Other causes of lung fibrosis (RC 1204), A-0702

**Heywang-Köbrunner S.H.**
CAD for breast cancer screening: first experiences with a CAD integration for screen-reading of 3D mammograms and with 3D CAD for tomo-reading (SY 2c)

**Hinzpeter R.M.M.**
Thoracic emergencies: Acute coronary syndrome (BS 2), A-0088

**Hjemly H.H.**
The future of radiology: what it could mean for new radiography graduates: Future role extension for radiographers (BR 17), A-1042

**Hodel J.**
My three top tips in neuroimaging (not only for neuroradiologists): Transient ischemic attack (SF 4), A-0158

**Hodler J.**
MSK intervention: the road from low to no invasion: Chairperson's introduction (SF 5c), A-0271

**Hoeschen C.**
Artificial intelligence and radiation protection: Chairpersons' introduction (part 2) (EU 7), A-1012

**Hoeschen C.**
Artificial intelligence and radiation protection: Artificial intelligence for scatter reduction and optimising imaging procedures (EU 7), A-1014

**Hoeschen C.**
Artificial intelligence and radiation protection: Using artificial intelligence for optimising procedures reflecting radiosusceptibility of patients (EU 7), A-1016

**Hoff S.R., Myklebust T., Hofvind S.**
Influence of annual reader volume on performance in a mammography screening program with independent double reading and consensus of digital mammograms (SS 202), B-0077

**Hoffmann A.**
Current status and future challenges in MR-integrated radiotherapy: Integration of MR and particle therapy: how far are we (ESR/ESTRO), A-0499

**Hofvind S.**
Contemporary breast imaging: C. Breast screening programmes: current evidence (RC 114), A-0009

**Hogg P.H.**
Radiation protection: all you need to know: Opportunities for optimising dose in digital radiography (BR 16), A-0963

**Holm S.**
EFRS meets Denmark: Technologically mediated patient and radiographer experience (EM 4), A-0623

**Hong G., Zou M., Yang Z.**
Visualisation of the maxillary nerve using a micro-surface coil and three-dimensional double-echo steady state with water excitation sequence (SS 1708), B-1454

**Hong J., Yoon S., Goo J., Yim J., Im J.**
Clusters of micronodules in active pulmonary tuberculosis: prevalence, patient characteristics, and natural history (SS 1604), B-1412

**Horn-Lodewyk J., Zeevaart J., Wagener J., Engelbrecht G.**
Comparative evaluation of in-house-prepared 99mTc-ethylene-dicysteine-deoxyglucose and 18F-fluorodeoxyglucose in breast cancer (SS 1014), B-0883

**Hötker A.M., Dappa E., Mazaheri Y., Ehdaie B., Zheng J., Capanu M., Hricak H., Akin O.**
The influence of background signal intensity changes on cancer detection in prostate MRI (SS 207), B-0152

**Hou P.**
Spectral imaging on a 80 mm wide-detector CT with fast kVp switching for follow-up CT angiography after EVAR planning: image quality, radiation dose and ultra-low volume contrast medium (SS 1915), B-1725

**Howard J.**
Clinical and methodological pitfalls in hybrid imaging: problems and solutions: Clinical and technical pitfalls in SPECT/CT (ESHI(MT) 1), A-0187

**Howarth N.**
Back to basics: how to interpret a chest radiograph?: A. A chest radiography reading guide (RC 1504), A-0914

**Howlett D.C.**
Audit across Europe: directive and perspective: Chairperson's introduction (ESR Audit), A-0137

**Howlett D.C.**
Clinical audit: how to deal with the legal and professional requirements: Chairperson's introduction (PI 2), A-0585

**Hrabak Paar M.**
Cardiac: recalling the forgotten structures: Wrong ways: anomalies of cardiac veins and venae cavae (SA 13), A-0776

**Hu B., Yang Z., Guo Y.**
Cardiac magnetic resonance feature tracking for quantifying right ventricular deformation in type 2 diabetes mellitus patients (SS 1903b), B-1859

**Hu F., Song B., Yang R., Wang M.**
Histogram analysis of stretched-exponential model diffusion-weighted imaging for staging of liver fibrosis: a feasibility study (SS 401), B-0405

**Hu F., Song B., Yang R., Wang M.**
Liver fibrosis and inflammation: influence on the 3D multi-echo Dixon parameters in patients with chronic liver diseases (SS 1801a), B-1535

**Hu Q.**
A computer-aided detection (CAD) system on low-dose chest CT imagesin concurrent-reader and second-reader modes: radiologist performance and reading time (MY 7), B-0815

**Hu S., Xia C., Peng W., Li Z.**
Development of a MRI radiomics model for pre-therapeutic evaluation of lymph node metastasis in rectal cancer (SS 1805b), B-1585

**Hu S., Peng W., Xia C., Li Z.**
MR imaging of rectal cancer: radiomics analysis to predict hepatic metastases (SS 1805b), B-1590

**Hu S., Peng W., Li Z.**
Comparison of automated software diameter/square detection with radiologist visual assessment for 70% stenosis on coronary CT angiography (SS 1915), B-1727

**Hu X.**
MR imaging radiomics textures for predicting the gene expression in related to recurrence risks of HCC given by GOLM1, RND1 and SETD7 (SS 1805b), B-1591

**Hu X., Hu X., Li H., Zhang L., Lu L., Bu X., Tang S., Gong Q., Huang X.**
An amplitude of low-frequency fluctuation study in first-episode drug-naive patients with major depressive disorder (SS 1511), B-1333

**Hu Y., Tao H., Chen S.**
Integrated analysis reveals disturbed metabolic pathway and identifies potential biomarker panel for the prediction of cartilage degeneration (SS 1410), B-1131

**Huang C., Mardal C.A., Tengberg L.T., Bay-Nielsen M., Bertelsen B.B.**
Fast track CT in patients that referred to emergency major abdominal surgery (SS 317), B-0275

**Huang D.Y.**
Renal transplantation: B. Multiparametric US for diagnosis and intervention (RC 1607), A-0997

**Huang J., Lyshchik A., Li J., Luo Y., Liu J., Lu Q.**
Diagnostic accuracy of CEUS LI-RADS in characterisation of small liver nodules in patients at risk for hepatocellular carcinoma (SS 1901a), B-1762

**Huang J., Yang Q., Cheng Z., Chen X., Yu T., Zhong J., Su Y., Guo H., Liang B.**
Pancreas iron overload and fat infiltration in paediatric beta-thalassemia major patients (SS 312), B-0343

**Huang L., Ran L., Xia L., Han R.**
The impact of myocardial thickness in hypertrophic cardiomyopathy using combination of CMR native T1 and T2 mapping (SS 303), B-0380

**Huang S., Yang Z., Diao K., Shi K., Gao Y., Shen M., Guo Y.**
Assessment of left ventricular deformation in restrictive cardiomyopathy with preserved ejection fraction by cardiac magnetic resonance tissue tracking (MY 18), B-1681

**Huang S., Yang Z., Shi K., Diao K., Gao Y., Guo Y., He Y., He S., Shen M.**
The diagnostic performance of novel non-contrast markers in discrimination of cardiac amyloidosis and hypertrophic cardiomyopathy on cardiovascular magnetic resonance imaging (SS 1903a), B-1820

**Huang S., Li X., Fang Z., Lin J., Zhang Z., Sun C.C., Feng S., Li Z.**
Correlation analysis between magnetisation transfer ratio with collagen area fraction in the surgical specimen of Crohn's disease (SS 1801b), B-1654

**Huang X., Hou Y., Ma Y., Gao S., Jia Z., Lu X.**
The optimal monochromatic image level of coronary CT angiography on a dual-layer detector spectral CT with half-dose contrast agent (SS 1003), B-1017

**Huber F.A., Stutz S., Vittoria De Martini I., Mannil M., Becker A.S., Winklhofer S., Burgstaller J.M., Guggenberger R.**
Texture analysis for quantitative evaluation of central lumbar spinal stenosis (SS 1910a), B-1733

**Huber F.A., Gkoumas S., Thuering T., Becce F., Guggenberger R.**
Spectral photon-counting for gout diagnosis in plain radiography: a feasibility study (SS 1013), B-0997

**Huber T., Rotkopf L., Bette S., Preibisch C., Gempt J., Pyka T., Thierfelder K., Wiestler B.**
Wavelet parameter maps of perfusion-weighted MRI correlate to tumour vascularity and cell proliferation in glioblastoma (SS 211a), B-0088

**Hunink M.G.M.**
Investment strategies: B. Value-based imaging ($E^3$ 1118), A-0636

**Hunink M.G.M.**
Working in radiology: burnout and bore-out or engaged and passionate?: Chairperson's introduction (part 1) (PC 13), A-0740

**Hur J., Park C.H., Lee S.M., Lee J.W., Hwang S.H., Seo J.S., Lee K.H., Kwon W., Im D.J.**
Hook-wire localisation vs lipiodol localisation for patients with pulmonary lesions having ground-glass opacity: the LOGIS trial (SS 704), B-0776

**Husmann L., Huellner M.W., Ledergerber B., Anagnostopoulos A., Stolzmann P., Sah B., Burger I.A., Rancic Z., Hasse B.**
Comparing the diagnostic accuracy of $^{18}$F-FDG-PET/CT, contrast-enhanced CT and combined imaging in patients with suspected vascular graft infections (SS 315), B-0224

**Husseiny Salama D.**
Radiology in Africa: facing challenges and opportunities: Structured strategies to combat radiation protection challenges in Africa. What can the ESR do? (EM 1), A-0310

### I

**Iacobellis F., Di Serafino M., Cinque T., Stavolo C., Romano L.**
Can MR be useful in the follow-up of blunt liver and spleen injuries: preliminary results (SS 217), B-0104

**Iezzi R.**
Non-contrast MR angiography: ready to go?: Chairperson's introduction (SF 8d), A-0419

**Iezzi R.**
No time to lose: aortic disease, revisited: B. Acute diagnosis and imaging in aortic dissection (RC 1515), A-0908

**Ilhan H.**
Hybrid and translational imaging: the bigger picture: Advances in theragnostics: monitoring radionuclide therapy (ESR/ESHI(MT)), A-0976

**Im S., Chung H., Kim S., Park H.**
Diagnostic value of ultrasound in paediatric veno-occlusive disease (VOD) after HSCT using the European Society for Blood and Marrow Transplantation (EBMT) criteria (SS 312), B-0336

**Inampudi V., Nimmalapudi S., Prakash A.**
Evaluation of MRI findings in Hippocampal sclerosis with special reference to T2 relaxometry (SS 1911b), B-1886

**Indo H., Yoneda T., Kurehana N., Tatewaki Y., Mutoh T., Ishiki A., Tomita N., Arai H., Taki Y.**
Quantitative MR-phase information enables classification of Alzheimer's disease stages (SS 1511), B-1329

**Ioannidis G.**
Career planning: Chairperson's introduction: What do new graduates need to look like? (BR 9), A-0530

**Iodice M., Brancato B., Carbonaro L.A., Bert A., Perassi C., Zuiani C., Sardanelli F.**
Initial experience of an online mammographic screening test (MY 5), B-0477

**Ipek A., Ünal Ö., Yildirim Erol M.**
Abnormalities concomitant with intrahepatic persistent right umbilical vein (SS 612), B-0603

**Ippolito D., Pecorelli A., Riva L., Maino C., de Vito A., Salemi I., Porta M., Talei Franzesi C., Sironi S.**
Assessing liver function: diagnostic efficacy of parenchymal enhancement and a liver volume ratio of Gd-EOB-DTPA enhanced MRI studies (SS 1501b), B-1372

**Ippolito D., Salemi I., Talei Franzesi C., Maino C., Riva L., de Vito A., Massimo C., Giandola T., Sironi S.**
Whole body low-dose CT-study combined with model based iterative reconstruction algorithm for follow-up of oncologic patients: image quality and dose deduction (SS 1716), B-1457

**Ivanac G.**
Contemporary breast imaging: Chairperson's introduction (Part 2) (RC 114), A-0006

**Ivanoski S., Vasilevska Nikodinovska V.**
Sarcopenia and osteoporosis in patients with cancer diagnosis: evaluation with CT (SS 210a), B-0050

**Ivanova D.**
Highlighted Lectures: Spinal emergencies: a look beyond trauma (TF), A-0832

**Ivashchenko O.V., Roelofs J.J., van der Most A., Veldkamp W.J.H., Hernandez Giron I.**
Influence of arm positioning on radiation dose during combined thorax and liver CT imaging: a phantom study (SS 1813), B-1592

**Izzo A., Varrassi M., Bruno F., Bianchi G., Cobianchi Bellisari F., Carducci S., Giordano A., Di Cesare E., Masciocchi C.**
Intraarticular lumbar facet joint steroid injection vs. medial branch block: which technique ensures better clinical outcome for managing lumbar back pain? A single-centre experience (SS 1809), B-1579

**Izzo A., Arrigoni F., Palumbo P., Bruno F., De Cataldo C., Mariani S., Zugaro L., Barile A., Masciocchi C.**
Is the anaesthesia a key factor for the good outcome of bone ablation procedures: a review of the last two-year experience in an MSK interventional centre (SS 1809), B-1581

### J

**Jacob S.**
Cardiovascular effects after radiotherapy for breast cancer: the European MEDIRAD Project: The MEDIRAD Early-Heart study (EU 1), A-0018

**Jacobson J.A.**
Musculoskeletal interventional procedures: B. Injectables, percutaneous tendon fenestration and tenotomy: clinical outcomes and current evidence (TC 1628), A-0992

**Jacobson J.A.**
Musculoskeletal interventional procedures: C. Interactive case discussion (Part 2) (TC 1628), A-0994

**Jäger H.R.**
How to implement MRI neuro advanced techniques at home: A. Practical approach to cerebral perfusion techniques (E³ 126a), A-0024

**Jäger H.R.**
My three top tips in neuroimaging (not only for neuroradiologists): Encephalitis (SF 4), A-0156

**Jäger H.R.**
A new era of gadolinium-less MR: Brain perfusion and angiography using ASL in comparison with gadolinium based techniques (ESR/ESMRMB), A-1010

**Jahnke P., Hamm B., Diekhoff T.**
Dose optimisation in dual-energy CT imaging of the neck: evaluation of acquisition parameters using a 3D-printed patient phantom (SS 1808), B-1630

**Jahnke P., Schwarz F.B., Ziegert M., Hamm B., Scheel M., Diekhoff T.**
Dose reduction in CT imaging of the neck - individual performance and synergetic effects of dose reduction techniques in a clinical context (SS 1808), B-1631

**Jajodia A., Aggarwal D., Rao A.S., Aggarwal R.**
Role of diffusion MRI in differentiation of residual/recurrent neck malignancies and post-treatment changes in comparison with histopathology findings (SS 1816), B-1637

**Jaly A., Ap Dafydd D., Paleri V., Hardman J.**
Theatre radiology: making the inoperable operable with real time, intraoral ultrasound guided trans oral robotic surgery (SS 1008), B-0976

**James S.L.J.**
The radiological investigation of musculoskeletal tumours: B. MRI and whole-body MRI (RC 1210), A-0693

**Jang S., Kim J., Choi S., Park S., Han J.**
Diagnostic performance of computerised 3D CT texture analysis of pancreas for the assessment of patients with diabetes (SS 501), B-0453

**Jardri R.**
Brain imaging in psychiatry: From fMRI capture of hallucinations to innovative image-guided treatments (MS 8b), A-0406

**Jargiello T.**
Thoracic emergencies: Acute aortic syndrome (BS 2), A-0086

**Jargiello T.**
Blunt polytrauma: CT protocols, CT interpretation and interventional radiology options: C. Interventional radiology in trauma: diagnosis and management (RC 817), A-0438

**Jäschke M., Thierfelder K., Klaan B., Stahnke T., Lindner T., Wree A., Weber M.-A., Langner S.**
Ultrahigh-field MR microscopy of the upper extremity of the chicken in vivo throughout the in ovo period (SS 310), B-0237

**Jaschke W.R.**
Medical imaging and emerging issues in occupational radiation exposure: Occupational exposure from interventional radiology procedures: how to measure it, how to reduce it (ESR/EFOMP), A-0546

**Jaschke W.R.**
Advanced clinical dosimetry in interventional radiology: Chairpersons' introduction (part 1) (EU 4), A-0746

**Jayaram P.R., Marshall T.**
Who gets kyphoplasties and how much height is restored: a single-centre demographic profile (SS 1510), B-1287

**Jeffery T.J., Gibson D.**
Extracted computed tomography angiography measurements as a screening tool for obstructive sleep apnoea in acute stroke patients (SS 711), B-0709

**Jeong S.Y., Lee J., Kim K.W., Jang J.K., Kwon H., Song G.W., Lee S.G.**
Estimation of the right posterior section volume in live liver donors: semi-automated CT volumetry in conjunction with portal vein segmentation (SS 701), B-0796

**Jezzard P.**
7 Tesla MR goes clinical: Why is there a benefit to higher static magnetic field in MR? (SF 5a), A-0250

**Ji J., Chen M., Hu X., Ye W., Chen C., Pang P., Lu C., Weng Q., Wang H.**
Imaging biomarkers for the differentiation of benign and malignant small solitary pulmonary nodules smaller than 1 cm based on CT texture analysis (SS 305), B-0308

**Ji J., Weng Q., Wang H., Fang S., Wu X., Chen M., Wang Z., Xu M.**
Value of TSCT features in differentiating pre- and minimally invasive from invasive adenocarcinoma presented as ground-glass nodules smaller than 3 cm (SS 704), B-0772

**Jiang H., Liu X., Chen J., Cao L., Wei Y., Song B.**
A radiomics-clinical model to diagnose hepatocellular carcinoma: prospective comparison with the EASL and LI-RADS (v2018) criteria (SS 1901a), B-1764

**Jiang J., Chen M.**
Conventional ultrasound and ultrasound elastography in non-Hodgkin lymphoma: ultrasound prognostic index compared with the international prognostic index (SS 1816), B-1642

**Jimenez-Pastor A., Alberich-Bayarri A., Garcia-Castro F., Marti-Bonmati L.**
Automatic visceral fat characterisation on CT scans through deep learning and CNN for the assessment of metabolic syndrome (SS 1905b), B-1830

**Jin P.**
Application value of dual-source CT combined with intelligent modulation and iterative reconstruction in aortic dissection imaging (SS 1815), B-1498

**Jin T.**
Application of half-dose contrast-enhanced T2-fluid-attenuated inversion recovery sequence in brain metastasis (SS 1911a), B-1774

**Jo H., Park M.**
Association between small pulmonary vascular area and mild airflow limitation in healthy smokers (SS 604), B-0582

**Johansen S.**
Neonatal chest X-ray (MY 15), B-1369

**Johnson K.J.**
Why do I miss fractures in emergency?: A. Missed fractures in children (RC 1217), A-0683

**Jokerst C., Gotway M., Panse D., Cummings K., Jensen E., Panse P.**
Understanding temporal enhancement of pulmonary nodule with dynamic contrast enhancement CT and utility of each acquisition (SS 1004), B-1007

**Joo I., Kim S., Kang T., Lee K., Lee J.**
Radiologic-pathologic correlation of non-hypervascular hypointense nodules on gadoxetic acid-enhanced MRI: a multi-centre retrospective observational study (SS 1901a), B-1767

**Joon P., Rastogi R., Agarwal A., Gupta Y., Pushkarna A.**
Comparative study of MR urethrography, sonourethrography and RGU with VCUG for diagnosis of male urethral strictures (SS 1807), B-1666

**Joseph D.Z.**
Acceptable quality dose for mammography: a guide for practitioners in developing countries (SS 1914b), B-1713

**Joseph D.Z., Nkubli F., Christian N.**
Optimization of radiation doses and image quality for mammography examination (SS 1914b), B-1714

**Jovanovic S., Djuric Stefanovic A., Simic A., Skrobic O., Masulovic D.**
Significance of multidetector computed tomography in the assessment of achalasia subtypes and detection of pulmonary complications (SS 1901b), B-1871

**Jover Sánchez J.J., Díaz Díaz J., Ferreiro Argüelles C., Núñez Valentín P., Rodríguez García E.**
Multiple sclerosis: evolution of magnetic resonance imaging diagnostic criteria from McDonald 2001 to McDonald 2017, including MAGNIMS 2016 (SS 611), B-0535

**Jreige M., Meyer M., Allenbach G., Pappon M., Nicod Lalonde M., Schaefer N., Prior J.O.**
First report from breath-holding high-resolution high-sensitivity SiPM PET/CT: are lung nodules getting more active? (SS 306), B-0235

**Juffermans J.F., Nederend I., van den Boogaard P., Roest A.A.W., Westenberg J.J., Lamb H.J.**
Age at aortic coarctation reconstruction is correlated with aortic vessel wall stiffening: an evaluation of pulse wave velocity and wall shear stress from 4D flow MRI (SS 1815), B-1506

**Juli C.**
Beyond helium-free operations, boost diagnostic confidence and speed with Ingenia Ambition (SY 7)

**Jung E.M.**
How to perform ultrasound image fusion: Liver (US 1), A-0169

**Junghanss T.**
Radiology and migrations: Clinical importance of the diseases related to migration (PC 5), A-0230

**Junker D., Steinkohl F., Sztankay B., Nagele U.**
Prostate magnetic resonance imaging with and without contrast medium: is biparametric MRI sufficient for routine examinations? (MY 13), B-1076

**Jurik A.**
Imaging of forgotten small joints: A. Sterno-clavicular joints: from trauma to inflammation (RC 1310), A-0782

**Justich C.**
The art to transmit and to receive: how to communicate critical information to our patients: How patients feel about communication (SF 8c), A-0416

### K

**Kaartinen S., Henner A., Mäkelä T.**
Patient radiation dose during coronary angiography and intervention: a multi-centre, retrospective study (SS 1014), B-0887

**Kachelrieß M.**
Big data and the big picture: deep learning in optimisation of medical imaging (part B): Deep learning in CT optimisation (EF 2), A-0326

**Kada S.**
Clinical dilemmas in radiography: what graduates need to know: Communication and compliance difficulties in imaging (BR 12), A-0688

**Käfer H., Frohn M.**
Imaging professionals in the EU: radiologists without borders: Creating a European radiology (or healthcare) workforce: the value of qualifications across borders (ESR/UEMS), A-0216

**Kahn C.E.**
Reporting and communication today and tomorrow: challenges to implement structured reporting (RS) and deal with artificial intelligence (AI): The concept of common data elements (CDE) for reporting (PI 1), A-0505

**Kahraman A., Kolu M., Kahraman B., Ozdemir Z., Karaca L., Yilmaz S.**
Postoperative complications after living donor liver transplantation: risk assessment on Gd-EOB-DTPA-enhanced 3-T MR imaging using quantitative-qualitative parameters (SS 1001b), B-1051

**Kai Y., Hui Shu Y.**
Assessment of predictors for pulmonary subsolid nodule detection in non-lung apex in ultralow dose CT reconstructed with ASiR-V (SS 1004), B-1002

**Kainberger F.**
The 3D printing lab from bench to bedside: Chairperson's introduction (SF 8f), A-0446

**Kainberger F.**
Arthritis: an imaging approach: Arthritis: an imaging approach (E³ 25D), A-0854

**Kaireit T.F., Voskrebenzev A., Gutberlet M., Freise J., Jobst B., Kauczor H.-U., Welte T., Wacker F., Vogel-Claussen J.**
Comparison of quantitative regional perfusion-weighted phase resolved functional lung (PREFUL) MRI with pulmonary perfusion DCE-MRI in COPD patients (SS 1404), B-1201

**Kaissis G., Ziegelmayer S., Lohöfer F., Weichert W., Siveke J., Rummeny E.J., Braren R.**
Quantitative magnetic resonance imaging can predict tumour subtype, therapy response and patient survival in pancreatic ductal adenocarcinoma (SS 616b), B-0615

**Kak M., Goldberg N.**
Reduced periablational inflammation for microwave ablation (MWA) compared to radiofrequency (RF) and irreversible electroporation (IRE) (SS 1709), B-1436

**Kaltenbach B., Wichmann J.L., Yel I., Albrecht M.H., Vogl T.J., Martin S.S.**
Multi-energy CT quantification of first-pass perfusion in hypervascular liver lesions: potential to differentiate hypervascular metastasis from hepatocellular carcinoma (SS 701), B-0794

**Kamali M., Clarke S.E., Costa A.F.**
Evaluation of liver MRI examinations with two dosages of gadobenate dimeglumine: a retrospective blinded intra-individual study (SS 401), B-0403

**Kamat R., Ramesh A., Nagarajan K.**
Evaluation of acoustic radiation force impulse imaging in the diagnosis of malignant cervical lymphadenopathy (SS 608), B-0631

**Kamble A.N., Kamble A.**
Novel imaging signs in detecting the 1p 19q non-co-deleted IDH wild and 1p 19q non-co-deleted IDH mutant lower grade gliomas (SS 211a), B-0092

**Kang H., Kim Y.**
Correlation of cerebrovascular reserve assessed by acetazolamide-stress SPECT with collaterals on arterial spin-labeling MRI in patients with carotid occlusive disease (MY 16), B-1414

**Kang L., Zhang X., Chen X., Li H.**
Correlation between EGFR gene mutation and CT, clinical features and pathological subtypes in ground glass lung adenocarcinoma (SS 704), B-0770

**Kantarci M.**
From diagnosis to prognosis: how does cardiac imaging affect patient outcome?: C. In coronary artery disease (RC 403), A-0148

**Kapetas P., Clauser P., Woitek R.A., Wengert G.J., Lazar M., Pinker-Domenig K., Bernathova M., Helbich T.H., Baltzer P.A.T.**
Decrease in unnecessary biopsy recommendations by the application of quantitative multiparametric breast ultrasound: a prospective study (SS 702b), B-0716

**Kaposi P.N., Youn T., Borsos P., Magyar P., Bérczi V.**
Orthopaedic metallic artefact reduction is indispensable for CT evaluation of the urinary tract after hip replacement (SS 407), B-0444

**Kaposi P.N., Budai B., Frank V., Shariati S., Folhoffer A., Abonyi M., Bérczi V.**
CT texture analysis in liver fibrosis shows a strong correlation with portal phase enhancement and can predict cirrhosis in decision tree model (SS 1805b), B-1589

**Karaca H., Soydan L., Baskak F., Sever A.**
Measurement of the depth of facial nerve at the level of stylomastoid foramen using MR imaging in Bell's palsy (SS 308), B-0295

**Karanam L.S.P., Baddam S.R., Honavar S., Palkonda V.R.**
Alternative routes of intraarterial chemoinfusion for intraocular retinoblastoma (SS 609), B-0562

**Karanam L.S.P., Baddam S.R., Alurkar A., Pamidimukkala V., Polavarapu R.**
Bilateral carotid stenting in the same sitting in high-risk patients: a single-centre study (MY 8), B-0841

**Karanam L.S.P., Baddam S.R., P. V.R., Honavar S.**
Role of intraarterial chemotherapy for retinoblastoma: our experience with review of literature (SS 1012), B-1062

**Karcaaltincaba M.**
Dual-energy CT of the abdomen: the time is now: C. Applications for abdominal organs (E³ 1526), A-0859

**Kargar S., Riederer S., Borisch E., Froemming A., Grimm R., Kawashima A., King B., Stinson E.**
Use of kZ space for sub-mm through-plane resolution in T2-weighted 2D spin-echo prostate MRI (SS 407), B-0438

**Karli Oguz K.**
Imaging of the brain: B. Acquired toxic-metabolic encephalopathies (E³ 1821), A-1076

**Kartalis N.**
Solid pancreatic neoplasms: Staging (E³ 24D), A-0853

**Kashef E.**
Blunt polytrauma: CT protocols, CT interpretation and interventional radiology options: A. CT protocols in blunt polytrauma (RC 817), A-0436

**Kasprian G.**
Diffusion tensor imaging (DTI): Keynote lecture (SS 1411b), K-30

**Kasprian G.**
Foetal and neonatal imaging pearls: A. Foetal MRI advanced technics (RC 912), A-0560

**Kassarjian A.**
Imaging of forgotten small joints: B. Symphysis pubis and its surroundings (RC 1310), A-0783

**Kastelic M., Vidmar J., Šifer K.K., Kocijančič I.**
Analysis of pleural effusion dynamics in patients with pulmonary infarction based on CTPA measurements (SS 1804), B-1608

**Kastler B.**
Pain palliation in cancer patients: Pelvic pain management with minimally invasive techniques (SF 9a), A-0514

**Kato K., Kawashima K., Suzuki T., Hamano M., Ehara S.**
Gastric variceal recurrence after balloon-occluded retrograde transvenous obliteration (SS 709), B-0742

**Katulska K.**
My three top tips in neuroimaging (not only for neuroradiologists): Multiple sclerosis (SF 4), A-0154

**Kauczor H.-U.**
Functional imaging of the lungs: B. Quantitative CT-imaging biomarkers in airways diseases (E³ 520), A-0290

**Kauczor H.-U.**
Professional issues for radiology departments to enable research: How to attract residents to research (PI 3), A-0626

**Kaufmann S., Thaiss W., Horger M., Nikolaou K., Bedke J.**
Characterisation of CT-indeterminate renal masses by contrast-enhanced ultrasound and acoustic radiation forced impulse imaging (SS 607), B-0628

**Kaul M.G., Salamon J., Jung C.S., Ittrich H., Graeser M., Adam G., Peldschus K.**
Magnetic particle imaging for pulmonary circulation time measurements (SS 615), B-0499

**Kauw F., Bennink E., De Jong H.W.A.M., Kappelle L., Horsch A.D., Velthuis B.K., Dankbaar J.W.**
Cerebrospinal fluid volume as a predictor of malignant middle cerebral artery infarction (SS 711), B-0705

**Kavanagh J., Wunder J., Ferguson P., Mohankumar R.**
Radiologic predictors of survival in soft tissue sarcoma (SS 210b), B-0066

**Kawashima J., Washizuka F., Nakano H., Shimada Y.**
The study of usefulness of blending injection method in dynamic liver CT scanning with reduced contrast medium (SS 614), B-0482

**Kazemi M., Mikalani M., Sharifian H., Moradi B.**
Diagnostic accuracy of image-guided core needle biopsy of lymphoma suspected patients (SS 608), B-0635

**Kedves A., Dr. Tóth Z., Emri M., Sipos D., Koczka V., Repa I., Kovács Á.**
Predictive value of 18F-FDG PET/CT information in the modern 3D-based radiotherapy treatment of head and neck cancer patients (SS 1014), B-0882

**Kedzierski B., Gac P., Glosna M., Poreba R., Pawlas K.**
Radiation dose and the repeatability of measurements of dimensions of the aortic valve in multidetector computed tomography performed routinely in the qualification procedure for TAVI (SS 203), B-0200

**Keeling A., Koh D.-M., O'Connor S., Boyd K., Kaiser M., Blackledge M., Messiou C.**
Lack of clinically meaningful anatomical variations in the bone marrow ADC values in diffuse myeloma allows untargeted sampling to confirm disease burden (SS 316), B-0353

**Keelson B., Buytaert D., Van Gompel G., Casselman J., Bacher K., De Brucker Y., Buls N.**
Application of the IEC3.1 standard of $CTDI_{100}$ to a supine CBCT device (SS 1808), B-1628

**Keil V.C.**
Diffuse low-grade gliomas: new things you should know: A. Molecular basis for classification, treatment and predicting outcome in low-grade gliomas (RC 1711), A-1030

**Kelly-Morland C.**
Role of imaging in cancer of unknown primary (CUP): A. MDCT: the imaging workhorse for suspected CUP (RC 516), A-0281

**Ken S., Lacroix E., Portalez D., Aziza R., Gilhodes J., Brun T.**
Multi-parametric MRI in patients with low-to-moderate risk of prostate cancer for the differentiation of adenocarcinoma and prostatitis (SS 307), B-0322

**Kenis S.F., Igashi J.B., Ibe D., Saliu A., Adams N.B., Aliyu I.**
The role cerebroplacental ratio in prediction of foetal outcome among patients with pre-eclamsia (SS 1507), B-1391

**Kenneth M., Ameda F., Byanyima R.K., Nakasujja N.**
Carotid artery plaque detected on ultrasound is associated with impaired cognitive state in the elderly: a population-based study in Wakiso district, Uganda (MY 16), B-1417

**Kertész H., Traub-Weidinger T., Rausch I., Beyer T., Cal-Gonzalez J.**
Optimising PET/CT paediatric imaging for patient dose reduction (SS 306), B-0234

**Kesari A., Swamy S.S., Sampangi S., Desai I., Ashok Kumar M., Asokan P., Kallur K.**
Denosumab response evaluation in primary osseous giant cell tumours with 18F-FDG PET/CT (SS 210b), B-0063

**Keupers M., Woussen S., Postema S., Westerlinck H., Zhang G., Cockmartin L., Bosmans H., Van Ongeval C.**
Does breast tomosynthesis increase cancer detection in a screening population with clinically elevated breast cancer risk? (SS 302), B-0251

**Khan S.U.K.**
Magnetic resonance imaging evaluation of arteritis and infarction in tuberculous meningitis (SS 1811b), B-1697

**Kharuzhyk S.**
Current status and future challenges in MR-integrated radiotherapy: Adaptive workflow: current status and challenges (ESR/ESTRO), A-0501

**Khristenko E.**
Multidisciplinary approach to cancer care: A. The soft tissues team (RC 816), A-0466

**Khurana R., Bajaj S.K.**
Evaluation of foetal modified myocardial performance index as a predictor of perinatal outcome in gestational diabetes (SS 612), B-0605

**Khurana R., Yadav A., Buxi T.B.S., Rawat K.S., Ghuman S.S.**
The forecaster of coronary atherosclerosis: black or white, fat or calcium? (MY 18), B-1668

**Kickingereder P.**
Imaging in oncological trials: Radiomics in oncology trials (ESR/EORTC), A-0793

**Kiefer L.S., Fabian J., Rospleszcz S., Storz C., Schlett C.L., Roemer F.W., Nikolaou K., Bamberg F.**
Distribution patterns of intra- and extra-myocellular fat by magnetic resonance imaging in subjects with diabetes, prediabetes and healthy controls from a population-based cohort (SS 1810), B-1514

**Kiefer L.S., Fabian J., Rospleszcz S., Storz C., Schlett C.L., Roemer F.W., Nikolaou K., Bamberg F.**
Assessment of skeletal muscle mass by magnetic resonance imaging in a population-based cohort (SS 1810), B-1515

**Kierans Z., Arrigo A., Kotze J., Van Der Velde L., Zwaagstra H., Gronning K., O'Connor M., Verzuijlen I.**
An investigation into the effectiveness of breast and gonad lead shielding in abdominal radiography (SS 1314), B-1066

**Kiessling F.M.A.**
Merging the best: hybrid imaging: B. Hybrid imaging with PET/MRI (RC 806), A-0458

**Kilburn-Toppin F.**
Breast imaging in pre-menopausal women: Physiological changes and benign breast diseases in young women (SF 8e), A-0425

**Kilburn-Toppin F.**
Breast: A. Fundamentals of mammography (E³ 1823), A-1084

**Kim D.K., An C.**
Newly appearing hepatic lesions on follow-up CT after curative-intent surgery for colorectal cancer: when and why should we order liver MRI? (SS 1401b), B-1282

**Kim D.K., Han K.**
Percutaneous cryoablation in early-stage hepatocellular carcinoma: analysis of local tumour progression factors (SS 1709), B-1443

**Kim D., Song Y.**
The comparison of hook wire vs coil localisation for video-assisted thoracoscopy surgery (SS 704), B-0775

**Kim E., Rha S., Choi M.H., Choi M., Choi J.**
Differentiation of uterine sarcomas from benign atypical leiomyomas: diagnostic value of quantitative parameters based on diffusion-weighted imaging (SS 1907), B-1895

**Kim H., Chang J., Seo D.**
Improvement of safety and image quality with a self-made airway management device for sedation and anaesthesia in paediatric heart CT (SS 714a), B-0646

**Kim H., Lee K., Ko Y., Choi J., Sim J.**
Using 2-mSv appendiceal CT in usual practice for adolescents and young adults: willingness survey of 579 radiologists, emergency physicians, and surgeons from 20 hospitals (SS 1401b), B-1285

**Kim J., Lee J.**
The effect of renal function on the intracranial gadolinium deposition in young rats (SS 312), B-0339

**Kim J., Lee C.**
Abbreviated gadoxetic acid-enhanced MRI (AGAM) including second shot arterial phase (SSAP) for hepatocellular carcinoma surveillance (SS 1001b), B-1045

**Kim J.E., Kim H., Choi D., Nickel D.**
T1 mapping for liver function evaluation in gadoxetic acid-enhanced MR imaging: comparison of look-locker inversion recovery and B1 inhomogeneity-corrected variable flip angle method (SS 401), B-0396

**Kim J., Kim M., Yoon H., Lee M., Han K., Shin H.**
Technical failure of echo-planar imaging MR elastography in paediatric liver (SS 312), B-0335

**Kim J., Kim M., Lee M., Yoon H., Shin H.**
Usefulness of mono-, bi-, and stretched-exponential model diffusion-weighted imaging for the differentiation of biliary atresia and non-biliary atresia in liver MRI (SS 312), B-0334

**Kim K.Y., Shin M.G.**
Preoperative transarterial embolisation using a gelatin sponge for benign prostatic hyperplasia: initial feasibility test (SS 709), B-0741

**Kim M., Kim S., An C., Han K., Lee S.**
Gadoxetic acid-enhanced magnetic resonance imaging for prediction of the postoperative prognosis of intrahepatic mass-forming cholangiocarcinoma (SS 1001b), B-1043

**Kim S., Kim C., Lee K.Y., Cha J.**
Accuracy of model-based iterative reconstruction for CT volumetry of part-solid nodule and pure solid nodule at various dose settings: an anthropomorphic chest phantom study (SS 1004), B-1001

**Kim S., Joo I., Kim H., Ahn S., Kang H., Jeon S., Han J.**
Response assessment of HCC after locoregional therapy on gadoxetic acid-enhanced MRI: performance of the LI-RADS TR categorisation and added value of ancillary features (SS 1901a), B-1772

**Kim T., Han H., Park J., Kim H.**
Predicting difficulties on preoperative sonographic evaluation in scheduled laparoscopic cholecystectomy in patients with acute calculous cholecystitis (SS 301), B-0392

**Kim Y., Kim M., Seo N.**
The feasibility of diffusion-weighted magnetic resonance imaging of the liver using multiplexed sensitivity encoding (SS 401), B-0402

**Kim Y., Rim J., Kim S.M., Yun B.L., Park S.Y., Ahn H.S., Kim B., Jang M.J.**
False negative on computer-aided detection application in preoperative automated breast ultrasound of breast cancer patients (SS 702a), B-0699

**Kim Y., Choi N., Kang B., Lee J., Jung N.**
Usefulness of CAD for diagnostic automated breast US in MRI detected lesions (SS 602), B-0555

**Kimoto N., Hayashi H., Asakawa T., Asahara T., Katsumata A., Kanazawa Y., Yamamoto S., Okada M.**
Effective atomic number image produced with energy-resolved photon counting detector toward the development of next-generation plain X-ray diagnosis (SS 1013), B-0994

**King A.D.**
Functional imaging in head and neck oncology: B. Incidence and prognosis of synchronous cancer or distant metastases from head and neck tumours (E³ 126b), A-0076

**King A.D.**
Post-treatment imaging of the head and neck: C. Treatment monitoring for early detection of recurrence (RC 1208), A-0722

**Kinkel K.**
Functional imaging of breast and female pelvis: Chairperson's introduction (E³ 820), A-0473

**Kirby A., Quigley A.J., Jones J., McGurk S.**
CT chest under general anaesthetic: is the degree of atelectasis improved by prone positioning? (SS 1512), B-1323

**Kirchner J., Martin O., Sawicki L.M., Umutlu L., Heusch P., Buchbender C.**
Can we replace sentinel lymph node resection in breast cancer patients by breast MRI, axillary MRI or axillary $^{18}$F-FDG PET/MRI (SS 706), B-0669

**Kirienko M., Ninatti G., Cozzi L., Gennaro N., Ricci F., Carlo Stella C., Zucali P., Chiti A., Sollini M.**
Ability of computed tomography (CT) radiomic features to differentiate lymphomas from thymic masses in the anterior mediastinum (SS 1816), B-1636

**Kirova-Nedialkova G.I.**
Highlighted Lectures: Acute aortic syndrome: intimate knowledge through intimate case (TF), A-0833

**Kitalia S., Sakwa J., Aswani E., Kanamu L.**
Evaluation of radiology request forms (MY 15), B-1356

**Kjelle E., Lysdahl K.B., Olerud H.M.**
Mobile radiography services can improve the utilisation of diagnostic imaging among nursing home residents (SS 214), B-0030

**Klein W.M., Sonnemans L., Franckenberg S., Gascho D., Prokop M., Lamers W.H., Hikspoors J.P.J.M., Thali M., Flach P.**
The histology of the right parafissural liver parenchyma: collagen is the basis for a hypoattenuating pseudolesion on CT (SS 201a), B-0068

**Klepanec A., Harsany J., Mako M., Hoferica M., Cisar J., Cabukova M., Rusina M., Krastev G.**
Endovascular treatment of patients with acute ischaemic stroke in posterior circulation: single-centre experience (SS 309), B-0281

**Klingebiel M., Schimmöller L., Ullrich T., Quentin M., Arsov C., Antoch G., Wittsack H.**
Improved diffusion weighted imaging of the prostate: RESOLVE and ZOOMit in comparison to standard EPI sequences (SS 207), B-0158

**Klosterkemper Y., Konieczny M., Hesper T., Aissa J., Antoch G., Boos J.**
Dose optimisation in spinal computed tomography for planning of scoliosis surgery (SS 1910a), B-1739

**Ko A., Park H., Lee E., Choi H., Do J., Park S., Choi B.**
Early prediction of the severity of acute pancreatitis using radiologic and clinical scoring systems with classification tree analysis (SS 501), B-0451

**Ko H., Choi J., Chae K., Park S., Jang J., Lee C.**
Relative regional air volume change as a biomarker in humidifier disinfectant-associated lung injury in children with normal-looking chest CT (SS 1512), B-1322

**Kober F.**
A new era of gadolinium-less MR: From native T1 to arterial spin labelling MRI in the heart (ESR/ESMRMB), A-1009

**Kocak B., Memis Durmaz E., Kadioglu P., Polat Korkmaz O., Comunoglu N., Tanriover N., Kocer N., Islak C., Kizilkilic O.**
Predicting response to somatostatin analogues in acromegaly: machine learning-based high-dimensional quantitative MRI texture analysis (SS 1005b), B-0986

**Kocak B., Memis Durmaz E.S., Ates E., Baykara Ulusan M., Kilickesmez O.**
Influence of segmentation margin on machine learning-based high-dimensional quantitative CT texture analysis: an experimental radiomics study on renal clear cell carcinomas (SS 1605), B-1395

**Koci M., Graber-Naidich A., Cheng X.S., Fleischmann D., Willemink M.J.**
Iodine in common contrast-enhanced CT protocols does not affect renal functionin a high-risk population with chronic kidney disease (SS 607), B-0624

**Koh D.-M.**
Imaging tumour response to immunotherapy: D. Monitoring immune response with MRI (RC 116), A-0068

**Kohler R.**
Head and neck imaging: when does it become abnormal?: B. Normative measures in the orbit (RC 908), A-0569

**Kolossvary M., Park J., Bang J., Zhang J., Lee J., Paeng J., Kubo T., Koo B., Horvat-Maurovich P.**
Radiomic analysis of coronary CT angiography images can identify invasive and radionuclide imaging markers of coronary plaque vulnerability (SS 305), B-0303

**Kondoh H., Kawai T., Mochida M., Yamaguchi T.**
Fast displaying time of electronic patient record and PACS sharing system with IHE-XDS/XDS-I and cloud technology (SS 1405), B-1187

**Konge L.**
Ultrasound simulation models in training and education: where are we going?: Gamification and virtual reality in medical simulation (ESR US SC/EFSUMB), A-0043

**König F., Rutz E., Jacobson J.A., Falkowski A.L., Magerkurth O.**
Measurement of femoural antetorsion with CT and MR: why trigonometry should be added (SS 1010), B-0893

**Konijn L.C.D., de Jong P.A., van Overhagen H., Mali W.P.**
Validation of CT imaging characteristics to differentiate intimal from medial calcifications in peripheral vessels and its application in a non-vascular cohort (SS 315), B-0222

**Konijnenberg S.**
Early clinical experience using the XL14-3 for MSK applications (SY 17)

**Koo N.**
Direct projection augmented reality-guided x-ray examination system: a preclinical study (SS 1314), B-1069

**Kool D.R.**
Post-treatment emergencies in oncologic patients: Chairperson's introduction: The role of imaging in the early detection of complications in oncologically treated patients (E³ 926), A-0515

**Korte B., Lameijer J.R.C., Duijm L.E.M.**
Two decades of biennial screening mammography: trend in the incidence of delayed breast cancer diagnosis after repeated recall for the same mammographic abnormality (SS 202), B-0082

**Korte B., Luiten J.D., Duijm L.E.M.**
Trends in frequency and outcome of high-risk breast lesions at percutaneous biopsy in women recalled at biennial screening mammography (SS 1402a), B-1149

**Kortesniemi M.**
Big data and the big picture: deep learning in optimisation of medical imaging (part B): From image quality to care outcome (EF 2), A-0327

**Kortesniemi M.**
Principles of imaging and radiation protection: A. Principles of computed tomography (E³ 1523), A-0896

**Kortesniemi M.**
Dose reduction and image quality implications of iterative image reconstruction in CT: A. Basics of iterative image reconstruction in CT (RC 1713), A-1050

**Kose S., Koç F., Hanta I.**
Correlation of USG diaphragm thickness measurements with pulmonary function and inspiratory strength tests in myotonic dystrophy type 1 (SS 1604), B-1413

**Kossov F., Olimov B., Bulychkin P., Tkachev S., Akhverdieva G., Panov V., Tyurin I.**
Macrocyclic or linear MR contrast agent: are there any differences in detection of local recurrence of prostate cancer after radical prostatectomy? (SS 607), B-0621

**Kotter E.**
Managing radiological examinations: dose management and beyond (SY 15)

**Kotter E.**
Mobile devices in radiology: B. Added value of mobile devices in education (RC 105), A-0012

**Kotter E.**
Artificial intelligence (AI): driven by radiologists: Chairperson's introduction (NH 3), A-0125

**Kotter E.**
Investment strategies: Chairperson's introduction ($E^3$ 1118), A-0634

**Kotter E.**
IT-security and GDPR: Chairperson's introduction (SF 12), A-0695

**Kraan R.B.J., Kox L., Mens M., Maas M.**
Physeal changes in youth gymnasts: a three-dimensional assessment of stress-related volume increase of the distal radial physis using magnetic resonance imaging (SS 310), B-0244

**Krainik A.**
My three top tips in neuroimaging (not only for neuroradiologists): MRI T2 brain white lesions (SF 4), A-0153

**Krainik A.**
Update on cerebrospinal fluid (CSF) diseases: D. Hypertension-associated brain changes (RC 911), A-0522

**Kramer H.**
Essentials of comprehensive MRA (SY 14)

**Kratovska A., Ponomarjova S., Ivanova P.**
Preoperative tibial artery run off score differences before endovascular or bypass surgery in patients with long popliteal artery lesions (SS 1009), B-0957

**Kraus B.**
Clinical simulation and its role in radiography education: Providing opportunities for practical ultrasound training (SF 9b), A-0495

**Kräuter C., Reiter U., Reiter C., Schmidt A., Greiser A., Masana M., Fuchsjäger M.H., Stollberger R., Reiter G.**
Alterations of the mitral valve vortex ring in ischaemic heart disease: an explorative 4D-flow study (SS 1803), B-1619

**Krestan C.R.**
Everything you always wanted to know about metabolic bone disease (but were afraid to ask): Unrevealed fractures: from missed diagnosis to opportunistic screening (SF 15a), A-0882

**Krishna Moorthy T., Rahmat K., Lim L.K.S., Ramli N.**
Validating the efficacy of multiple high-resolution MRI sequences with 18 FDG-PET co-registration in detecting epileptogenic lesion (SS 1911b), B-1885

**Kromrey M., Funayama S., Tamada D., Ichikawa S., Onishi H., Motosugi U.**
Clinical evaluation of 3D magnetic resonance cholangiopancreatography using three different methods: feasibility of compressed sensing and/or parallel imaging (SS 401), B-0404

**Kromrey M., Friedrich N., Hoffmann R.-T., Bülow R., Motosugi U., Kühn J.**
Clinical significance of pancreatic steatosis: association between pancreatic exocrine insufficiency and organ fat content (MY 17), B-1474

**Kronlage M., Knop K., Schwarz D., Godel T., Heiland S., Bendszus M., Bäumer P.**
The value of MR neurography in the differential diagnosis between amyotrophic lateral sclerosis and multifocal motor neuropathy (SS 1811a), B-1560

**Krug B., Grinstein O., Malter W., Hanstein B., Siedek F., Schmutzler R., Maintz D., Rhiem K., Hellmich M.**
Residual fibroglandular tissue (RGT) following unilateral and bilateral prophylactic mastectomies in BRCA 1/2 germline-mutation carriers (MY 5), B-0461

**Krüger P.-C.**
A paediatric radiologist's perspective (SY 28)

**Kruk-Bachonko J., Krupski W., Skoczylas T.**
Prediction of gastric and esophagogastric junction cancer response to neoadjuvant treatment by functional imaging with PCT (MY 17), B-1482

**Kshirsagar R.H., Sable N., Mahajan A., Thakur M.**
Mineralisation patterns of laryngeal cartilages: normal variations and their clinical impact (SS 1708), B-1450

**Kubicka F.M., Fleischmann-Mundt B., Kazmierczak P.M., Schneider M.J., Brooks J., Meier M., Ricke J., Kühnel F., Cyran C.C.**
MR monitoring of cytolysis and inflammatory tumour microenvironment after oncolytic adenovirus injection in a novel transgenic pancreatic adenocarcinoma mouse model (SS 606), B-0503

**Kubik-Huch R.A.**
Tumour relapse in gynaecological cancer: Chairperson's introduction ($E^3$ 519), A-0284

**Kubota K., Fujioka T., Mori M., Tsuchiya J., Tateishi U.**
Clinical value of 18F-FDG PET/CT for staging elderly and young women with preoperative breast cancer (SS 1902b), B-1796

**Kudryavtsev N.D., Malakhova M.V., Grusha Y.O., Danilov S.S., Khovrin V.V., Puzakov K.B., Galyan T.N.**
Functional multi-detector computed tomography (FMDCT) for various orbital pathologies (MY 14), B-1263

**Kuhl C.K.**
Screening with imaging: a new era?: Screening of breast cancer with abbreviated MRI (SF 1), A-0030

**Kuhl C.K.**
MRI for early detection, staging and management of breast cancer: C. Screening with abbreviated protocols (RC 502), A-0235

**Kuhl C.K.**
Multidisciplinary approach to cancer care: C. The breast team (RC 816), A-0468

**Kuhl C.K.**
Using abbreviated protocols (SY 2a)

**Kühn J., Kacprowski T., Röhnert A., Hietschold V., Bülow R., Fedders D., Hoffmann R.T., Kromrey M.**
Whole-body R2s mapping to quantify tissue in iron storage organs: associations with HFE-genotype (SS 401), B-0398

**Kühn J., Bülow R., Fedders D., Seppelt D., Hoffmann R.-T., Kromrey M.**
Assessment of whole-body fat in magnetic resonance imaging as a new approach in imaging of metabolic disorders (SS 401), B-0401

**Kuhn R., Ignacio G., Jamias J.**
Predictors for refractoriness and survival after transarterial chemoembolisation among hepatocellular cancer patients: outcomes from a novel southeast Asian cohort (SS 209), B-0118

**Kulali F., Demir M., Semiz-Oysu A., Bukte Y.**
Prediction of Bethesda category of thyroid nodule with specific sonographic findings (SS 208), B-0125

**Kulpe S., Braig E., Dierolf M., Günther B., Achterhold K., Herzen J., Rummeny E.J., Pfeiffer F., Pfeiffer D.**
K-edge subtraction imaging at the Munich compact light source (SS 1013), B-0995

**Kumar P.G., Young J., Chen H., Li Y., Qi Q., Peng X., Khoury T.**
A retrospective analysis comparing radioactive seed and wire localisation in breast surgery (SS 1402a), B-1159

**Kumar E R., Verma R., Dhamija A., Belho E., Gupta V., Mahajan H., Mahajan V.**
Diagnostic accuracy of F-18 FDG PET/CT in mediastinal lymph nodal staging of recently diagnosed carcinoma lung (SS 704), B-0777

**Kunz W.G., Rotkopf L.T., Puhr-Westerheide D., Dorn F., Tiedt S., Wollenweber F.A., Thierfelder K.M., Kemmling A., Reidler P.**
Non-contrast ASPECTS region density predicts final infarction in acute ischaemic stroke (SS 311), B-0259

**Kuru Oz D., Peker E., Kul M., Erden I., Erden A.**
MR relaxometry in chronic hepatitis b and c: the contribution of Molli T1 mapping and T2 mapping in hepatic function evaluation with Gd-EOB-DTPA-enhanced magnetic resonance (SS 1501b), B-1373

**Kurys-Denis E., Skrobas U., Brudkowska Z., Karakuła-Juchnowicz H., Krupski W., Maciejewski R.**
Regional grey matter atrophy by MR volumetry in early relapsing-remitting multiple sclerosis (RRMS) as a predictor of clinical and motor outcome (SS 611), B-0538

**Kvist O., Diaz Ruiz S., Dorniok T., Sanmartin Berglund J., Flodmark C.**
Diffusion tensor imaging of the growth plate: a preliminary study (MY 15), B-1360

**Kwon J., Kim J.**
Quantitative analysis of arterial-phase MR images in differentiating focal-type autoimmune pancreatitis from pancreatic ductal adenocarcinoma (SS 1401a), B-1096

### L

**Laghi A.**
Screening with imaging: a new era?: Screening of colorectal cancer with CT colonography (SF 1), A-0029

**Lahaye M.J.**
Tumour response assessment in abdominal imaging: B. Rectal carcinoma (RC 301), A-0108

**Lai Z., Sá dos Reis C., Sun Z.**
Dose optimisation of lumbar spine radiographs using a phantom: what are the ideal exposure parameters? (SS 1314), B-1065

**Laitinen H., Saukko E., Svedström E., Pajulo O., Niiniviita H.**
Radiation dose comparison study between conventional methods and slot-scanning system (EOS® system) on paediatric patients with scoliosis (SS 714a), B-0645

**Lam A., Aggarwal R., Liu G., Kavanagh J.**
Outcomes of long-term interval rescreening with low-dose CT for lung cancer in different risk cohorts (SS 204), B-0141

**Lamb H.J.**
Imaging professionals in the EU: radiologists without borders: Making your qualifications count at home and abroad: the European Diploma in Radiology and the CESMA (ESR/UEMS), A-0219

**Lambadiari V.**
Multidisciplinary approach to the diabetic foot: Diabetic foot: a societal problem (MS 17), A-1063

**Lambert L., Hrabak P., Novotny A., Jahoda J., Burgetova A.**
The diagnosis of internal haemorrhoids from CT colonography has a poor agreement with optical colonoscopy (SS 1001a), B-0938

**Lambin P.**
Big data and the big picture: deep learning in optimisation of medical imaging (part A): The use of radiomics in medical imaging (EF 1), A-0267

**Lambregts D.M.**
Gastrointestinal and abdominal: C. Imaging of the gastrointestinal tract (E³ 1323), A-0771

**Lameijer J.R.C., Nederend J., Voogd A.C., Tjan-Heijnen V.C., Duijm L.**
Frequency and outcome of bilateral recall at screening mammography (SS 202), B-0083

**Lampichler K., Reider L., Simbrunner B., Stadlmann A., Mandorfer M., Bucsics T., Paternostro R., Asenbaum U., Reiberger T.**
The extent of portosystemic collaterals depends on portal pressure and hepatic dysfunction rather than on PlGF-driven neoangiogenesis (SS 1501b), B-1377

**Lamprecht T.**
The daily challenges of a good MR exam (part 2) (SY 22)

**Lancelot E.**
Mechanisms of gadolinium retention in the body (SY 30)

**Lång K., Dustler M., Dahlblom V., Andersson I., Zackrisson S.**
Can artificial intelligence identify normal mammograms in screening? (SS 702a), B-0695

**Langenbach M.C., Albrecht M.H., Hammerstingl R., Gruber-Rouh T., Vogl T.J.**
MR imaging-based response evaluation of two different transarterial chemoembolisation protocols using degradable starch micorspheres in therapy of hepatocellular carcinoma (MY 8), B-0843

**Langs G.**
Artificial intelligence (AI): our future cannot be predicted, but we have to be prepared: Chairperson's introduction (SF 8a), A-0392

**Laniado M.**
US before CT in the acute abdomen?: B. Acute abdomen: CT of course! (PS 1427), A-0837

**Lanza E., Bolengo I., Poretti D., Muglia R., Ceriani R., Pedicini V.**
Bland embolisation as the sole endovascular treatment for unresectable hepatocellular carcinoma: survival analysis of 230 patients observed for seven years in a single centre (SS 609), B-0560

**Larici A.R.**
Lung cancer team: Chairperson's introduction (MS 1), A-0050

**Larici A.R.**
Lung cancer team: Multidisciplinary case presentation and discussion (MS 1), A-0055

**Larici A.R.**
Oncologic imaging: A. Lung cancer: key signs in the new TNM (E³ 621), A-0306

**Larici A.R.**
Lung nodule management in 2019: B. Computer-aided diagnosis and AI perspective (RC 904), A-0550

**Lastella G., Wiedenmann F., Monti C.B., Secchi F., Alì M., Sardanelli F.**
Ultrasound estimation of carotid intima media thickness: semiautomatic versus manual measurement (SS 215), B-0042

**Lastella G., Cannaò P.M., Ali M., Secchi F., Sardanelli F.**
Non-contrast magnetic resonance imaging in the follow-up after endovascular aortic repair (SS 1915), B-1728

**Lau K.K., Deb S., Kuganesan A.**
Efficacy of Raysum image in urolithiasis detection (SS 1407), B-1147

**Lau K.K., Abdelganne L., Buchan K., Kuganesan A., Lau T.**
Fine focal spot: a technique to improve image quality of computed tomography pulmonary angiography (SS 1904), B-1849

**Laukamp K.R., Thiele F., Pennig L., Reimer R.P., Shakirin G., Zopfs D., Timmer M., Perkuhn M., Borggrefe J.**
Fully automated segmentation of meningiomas using deep learning on multiparametric MRI: automated segmentation yields accuracies as good as manual interreader variabilities (MY 16), B-1424

**Lavens M., Wildiers H., Neven P., Nevelsteen I., Smeets A., Keupers M., Prevos R.M.A., Laenen A., Van Ongeval C.**
Longterm review of recurrence after breast cancer treatment: data from breast cancer/tumour type analysis (SS 1902b), B-1803

**Le Boulc'h M., Lusque A., Balleyguier C.S., Dratwa C., Cherel P., Kind M., Vaysse C., Le Guellec S., Boulet B.**
Imaging characterisation of breast desmoid tumours (MY 5), B-0478

**Leardini A.**
Articular surfaces of the tibiotalar joint from different medical imaging technologies: for morphological models and custom-made prostheses (SY 27)

**Lebedev S., Fournie E., Stierstorfer K., Kachelrieß M.**
Stack transition artefact removal (STAR) in cardiac CT with automatic parameter selection (SS 313), B-0312

**Leckebusch T., Wenzel P., Jungmann F., Halfmann M.C., Brumberg R.M., Düber C., Kreitner K.-F., Emrich T.**
Prognostic role of feature tracking in suspected myocardial infarction with non-obstructed coronary arteries (MINOCA): sensitive or senseless? (SS 603), B-0591

**Lecouvet F.E.**
Whole-body imaging in metastatic urinary tract and prostate cancer: A. WB-MRI: technique and reporting system Met Rads P (E³ 419), A-0195

**Lecouvet F.E.**
Advances in musculoskeletal techniques: whole-body MR: A. Oncologic application (E³ 1221), A-0646

**Lecouvet F.E.**
Imaging in oncological trials: Modern imaging-based trials: focus on oligometastatic disease and evaluation of metastatic directed therapies (ESR/EORTC), A-0791

**Lederlin M.**
Lung cancer in the era of molecular oncology and immune therapy: A. Lung adenocarcinomas with EGFR mutations (E³ 1022), A-0599

**Lee C.M., Jeon C.H., Park J.B., Kim C.W., Kwon H., Cho H.M.**
Transcatheter arterial embolisation for traumatic thoracic bleeding: 4-years' experiences in a single trauma centre (SS 1009), B-0960

**Lee C.M., Jeon C.H., Kim C.W., Kwon H., Cho H.M.**
Interventional urethral realignment to posterior urethral rupture accompanied by complex pelvic fracture (SS 1809), B-1573

**Lee C.Y., Chen Y., Chen P., Lu C., Lin W.**
Executive network integrity predicts sarcopenia in patients with Parkinson's disease (SS 1811a), B-1551

**Lee C., Osman S., Mazlan L., Mohammed F.**
Preoperative MR restaging of rectal cancer in UKMMC: correlation with surgical and histopathologic findings (SS 1501a), B-1340

**Lee D.H., Kim S., Choi A.L., Yoon C.**
Submarine sign of epidermoid cysts: a diagnostic model based on ultrasound feature (MY 9), B-0869

**Lee D., Lee J., Bae J.**
Shear wave dispersion slope obtained by shear wave elastography could help detect allograft abnormality after liver transplantation (SS 1501b), B-1375

**Lee D., Lee J.**
Gadoxetic acid-enhanced liver MR can predict tumour recurrence after curative treatment for small single hepatocellular carcinoma (MY 17), B-1466

**Lee H., Seo H.S., Lee Y.H.**
Analysing the impact of sarcopenia on brain: using correlation between paraspinal muscle and brain volume (MY 16), B-1420

**Lee H., Moon J., Hwang J., Woo J.**
Factors influencing the disagreement between the automated volumetric breast density and radiologist's visual assessment in assessment of breast density (SS 1002), B-0918

**Lee J.Y., Sabbah S., Rogalla P.**
Comparing emergency department disposition of abdominal tomogram to abdominal x-ray (SS 317), B-0277

**Lee J., Lee I., Kim J.**
Percutaneous needle biopsy for lung lesions focused on needle angle and lesion depth: 14-year results (MY 4), B-0435

**Lee J.M.**
Hepatocellular carcinoma: from diagnosis to treatment: B. Unusual appearances of HCC and lesions that simulate HCC: can we increase diagnostic confidence? (RC 1701), A-1003

**Lee J.M.**
Contrast agents in interventional procedure to treat HCC and follow-up (SY 31)

**Lee J., Kwak J., Kim E., Moon H., Park V.Y., Yoon J.**
Association of the four international guidelines for thyroid nodule management to surgical and treatment outcomes in patients with small papillary thyroid carcinoma (SS 208), B-0120

**Lee K.H.**
Imaging the acute abdomen: new insights: B. Low-dose abdominal CT for evaluating suspected appendicitis (RC 1201), A-0649

**Lee P., Mehdi A., Alsafi A., Harvey C., Lim A.K.P.**
Using contrast-enhanced ultrasound to assess diagnostically challenging and indeterminate lesions in native and transplant kidneys (SS 607), B-0627

**Lee R., Jeon C.H., Kim C.W., Kwon H., Cho H.M.**
Shattered spleen in blunt trauma: clinical results of trans-arterial embolisation, four years' experiences in a single regional trauma centre (SS 709), B-0740

**Lee S., Yoon S.**
Preoperative multi-detector CT scoring criteria for assessing pericardial, lung, and phrenic nerve invasion of thymoma (SS 1804), B-1611

**Lee S., Lee G.Y., Kim S.**
Clinical utility of fat suppressed 3-dimensional CAIPIRINHA SPACE MRI of the knee in adults (MY 9), B-0862

**Lee S.Y., Ou H., Yu C., Cheng Y.**
Safety and efficacy of small versus large drug-eluting bead transarterial chemoembolisation for hepatocellular carcinoma (SS 609), B-0561

**Lee S.**
Audit across Europe: directive and perspective: Quality improvement and change management: audit in industry (ESR Audit), A-0141

**Lee S., Kim Y.J.**
Evaluation of ascending aorta flow pattern in patients with repaired TOF using 4D flow CMR (SS 1903b), B-1865

**Legrand J., Pascart T., Norberciak L., Becce F., Budzik J.**
Dual-energy computed tomography in calcium pyrophosphate deposition: initial clinical experience (SS 210a), B-0053

**Lehmkuhl L., Grimm J., Diamantis I., Wutschke M., Lenk K., Franz M., Schulze C., Teichgräber U.K.**
Dysfunction and thrombosis of aortic valve prostheses in patients after TAVR in computed tomography (SS 203), B-0201

**Leichter I., Fialkoff B., Uziel Y., Ben-David E., Romman Z., Sosna J., Gomori J.M.**
Improved grey-white matter separation by amplitude/power coefficients derived from spectral analysis of dual-energy CT (SS 213), B-0133

**Leichter I., Ben-David E., Menat J., Peretz A., Romman Z., Gomori J.M., Sosna J.**
Accurate non-invasive assessment of carotid in-stent restenosis by dual-layer spectral CT (SS 313), B-0313

**Leimgruber A.**
Artificial intelligence in hybrid imaging: Radiomics+: prediction model using convergent data (ESHI(MT) 2), A-0552

**Leiner T.**
Non-contrast MR angiography: ready to go?: Non-contrast MR angiography techniques (SF 8d), A-0420

**Leiner T.**
Dead or alive: imaging of myocardial viability: A. MRI (RC 1703), A-1018

**Leithner D., Horvat J.V., Marino M., Bernard-Davila B., Jochelson M.S., Martinez D., Morris E.A., Thakur S., Pinker-Domenig K.**
Radiomic signatures derived from diffusion-weighted imaging for the assessment of breast cancer receptor status and molecular subtypes (SS 1402b), B-1175

**Leithner D., Horvat J.V., Marino M., Bernard-Davila B., Jochelson M.S., Martinez D., Morris E.A., Thakur S., Pinker K., Meyer-Baese A.**
Radiomic signatures with contrast-enhanced magnetic resonance imaging for the assessment of breast cancer receptor status and molecular subtypes: initial results of a multicenter study (SS 1402b), B-1176

**Leleu F., De Margerie C., de Bazelaire C., Gaudez F., Lefaucheur C., de Kerviler E.**
Contributions of the whole-body CT scan before multi-organ donation in brain dead patients (SS 1017), B-0953

**Lenga L., Booz C., Wichmann J.L., Scholtz J., Martin S.S., Vogl T.J., Albrecht M.H., Yel I.**
Single- and dual-energy CT pulmonary angiography using second- and third-generation dual-source CT systems: comparison of radiation dose and image quality (MY 4), B-0434

**Lenga L., Yel I., Booz C., Martin S.S., Scholtz J., Vogl T.J., Wichmann J.L., Albrecht M.H.**
Dual-energy CT in patients with fatty liver disease: improved assessment of hypoattenuating liver lesions using virtual monoenergetic imaging (MY 17), B-1472

**Lennartz S., Mager A., Grosse Hokamp N., Schäfer S., Maintz D., Persigehl T.**
Differentiation of benign lung nodules and metastases: a machine-learning approach using texture features from spectral detector CT-derived iodine maps and conventional images (SS 1016), B-1025

**Lennon L., Tsui S., Dick G., Hislop-Jambrich J.**
Is the technology for patient placement in CT really that useful in reducing dose and improving SNR? (SS 714b), B-0655

**Leone A.**
Spinal trauma: how to get it right: Spinal trauma: how to get it right (E³ 25C), A-0614

**Leone F., Orsi M.A., Cellina M., Presazzi A., Mariani D., Oliva G.**
False-positive recall in screening mammography: pitfalls of challenging mammogram (SS 202), B-0087

**Leonhardt H.**
Imaging of 'foreign bodies': C. The role of interventional radiology in the management of foreign bodies and following complications (RC 417), A-0168

**Lerchbaumer M.H., Marticorena Garcia S.R., Kallenbach M., Hamm B., Fischer T.**
Diagnostic performance of contrast-enhanced ultrasound (CEUS) in unclear testicular pathology (SS 1807), B-1657

**Levashkina I., Serebryakova S.V., Kitaigorodska O.V.**
Diffusion tensor imaging for vascular dementia prediction: threshold level of fractional anisotropy in liable white matter tracts (SS 1411b), B-1267

**Lewis S., Hazell L.J.**
Active learning of exposure technique in digital radiography (SS 1914a), B-1701

**Li J., Li K.**
Increased lateral meniscal slope is associated with greater risk of lateral bone contusion in noncontact ACL injury (MY 9), B-0859

**Li J., Yang H.**
Quantitative magnetic resonance imaging in patellar tendon-lateral femoral condyle friction syndrome: relationship with subtle patellofemoral instability (MY 9), B-0861

**Li J., Ma W., Wu Y., Li L.**
Development and validation of nomograms predictive of axillary nodal status to guide surgical decision-making in early-stage breast cancer (MY 5), B-0466

**Li J.**
Development and validation of a dual-energy CT-based radiomic nomogram for preoperative prediction of lymph node metastasis in patients with gastric cancer (SS 1805b), B-1584

**Li L., Ren B., Wang S., Xia C.**
Supervised one-to-one style transfer to improve the quality of CT images (SS 1005a), B-0911

**Li L., Zhao S.**
MRI characteristics and clinical outcomes of hypertrophic cardiomyopathy with infarct-like late enhancement (SS 1903a), B-1818

**Li Q., Li L., Liu K., Xia C., Xiao Y., Liu S.**
Detectability of non-solid pulmonary nodules by deep learning: results from a phantom study (SS 1004), B-1009

**Li T.**
Quantitative elastography of rectal lesions: the value of shear-wave elastography (SWE) in identifying benign and malignant rectal lesions (SS 201b), B-0206

**Li W., Zhigang Y., Han P., Li Z.**
Application of low-radiation and low-contrast dose protocol combined with high-strength ASIR-V algorithm in 'one-stop' coronary and carotid and cerebrovascular CT angiography (SS 614), B-0479

**Li X., Zhigang Y., Guo Y., He Y., He S., Shi K., Xia C., Li Z.**
Regional right ventricular deformation in hypertrophic cardiomyopathy: assessed with cardiovascular magnetic resonance tissue tracking (SS 1903b), B-1869

**Li X., Wang L.**
Small (< 4 cm) renal masses: convolutional neural network-based deep-learning approach in differentiating fat-poor renal angiomyolipoma from renal cell carcinoma at MSCT (MY 7), B-0804

**Li X., Li W.**
Deep learning with a convolutional neural network for predicting Fuhrman grade of clear cell renal cell carcinoma at MSCT (SS 1605), B-1399

**Li X., Huang S., Lu B., Huang L., Li Z., Du J., Zhong Y.**
Ability of diffusion-weighted magnetic resonance imaging to characterise bowel fibrosis depends on the degree of bowel inflammation (SS 1801b), B-1651

**Li Y., Xiang Y., Zeng C., Wang J., Han Y., Luo Q., Zheng Y.**
Cortical atrophy and cerebral hypoperfusion in anti-N-methyl-D-aspartate receptor encephalitis: relationship to cognitive impairment and disease severity (SS 1911b), B-1888

**Li Y., Yang X., Wang J., Xue H., Liang W.B., Deng Z.H., McNulty J., Lv J.C., Zhang L.**
Ante-mortem chest radiograph retrieval for forensic human identification by deep learning (SS 605), B-0573

**Li Y., Du Y., Liang X., Xue H., Jin Z.**
EGFR-targeted liposomal nanohybrid cerasomes: theranostic function and immune checkpoint inhibition in a mouse model of colorectal cancer (SS 1406), B-1121

**Li Y., Cui Y., Zhu J., Li W.**
Texture features from mammographymay predict the characterisation of breast tumours (SS 1805a), B-1519

**Li Z.**
Value of the cinematic rendering from CT data in evaluating the relationship between deep soft tissue sarcomas of the extremities and adjacent major vessels (SS 716), B-0692

**Liang M., Zhao X.**
Machine learning-based analysis of rectal cancer MRI radiomics for prediction of metachronous liver metastasis (MY 17), B-1478

**Liang S., Ma J., Xia C., Deng Y., Zhang R., Yu Z.**
Autodetection of mass and lymph node in breast mammography images with deep learning method (SS 1805a), B-1517

**Licandro R., Hofmanninger J., Weber M.-A., Menze B., Langs G.**
Whole-body MRI based lesion prediction in multiple myeloma (SS 605), B-0567

**Lieman-Sifry J., Brouha S., Yen A., Weihe E., Jacobs K., Horowitz M., Motiuk D., Hsiao A.**
Deep learning-based CAD may improve detection of pulmonary nodules while preserving a low false-positive rate (SS 1804), B-1607

**Lilleborge M., Hofvind S.**
Association between BMI and subsequent breast cancer among women with a prior false-positive without biopsy in BreastScreen Norway (SS 1002), B-0921

**Lin C., He Y., Qi Y., Wang X., Zhou H., Xue H., Jin Z.**
Multi-parametric magnetic resonance relaxometry as an assessment of dysmenorrhea severity in patients with adenomyosis (SS 1907), B-1893

**Lin C., He Y., Qi Y., Wang X., Zhou H., Xue H., Jin Z.**
Correlation between zonal oblique multislice intravoxel incoherent motion and dynamic contrast enhanced magnetic resonance imaging parameters in endometrial cancer (SS 1907), B-1896

**Lin C., He Y., Qi Y., Wang X., Zhou H., Xue H., Jin Z.**
Multi-parametric MR imaging for prediction of response to gonadotropin releasing hormone analogue in patients with adenomyosis: a preliminary study (SS 1907), B-1897

**Lin H., Lee R., Yen H., Liu C., Chen J.**
Portal vein and ligamentum teres variations in diverse gallbladder locations: is the left-sided gallbladder always a high-risk feature for pre-interventional assessment? (SS 301), B-0393

**Lin S., Lin M., Lau K.**
Efficacy of model-based iterative reconstruction in cystic fibrosis assessment on computed tomography (SS 604), B-0581

**Linck P., Kuchcinski G., Munsch F., Griffier R., Okubo G., Renou P., Dousset V., Sibon I., Tourdias T.**
Long-term neurodegeneration of substantia nigra secondary to ipsilateral infarct is quantified with iron-sensitive imaging and impacts motor outcome (MY 16), B-1415

**Linder N.P., Solty K., Hartmann A., Blüher M., Stange R., Busse H.**
Quantification of abdominal subcutaneous adipose tissue in MRI of obese patients (SS 713), B-0764

**Lindig T., Zaiss M., Herz K., Deshmane A., Bender B., Golay X., Scheffler K.**
GLINT: GlucoCEST in neoplastic tumours at 3T - first-in-man studies of GlucoCEST in glioma patients (SS 211a), B-0095

**Linn J.**
Altered mental state: A. MRI in the diagnosis of cerebral amyloid angiopathy (CAA) (RC 1311), A-0765

**Linsenmaier U.**
Imaging of 'foreign bodies': Chairperson's introduction (RC 417), A-0165

**Lisson C.S., Lisson C.G.S., Mayer-Steinacker R., von Baer A., Barth T.F.E., Baumhauer M., Beer A.J., Beer M., Schmidt S.A.**
Performance of 3D texture analysis for tissue characterisation and differential diagnosis of osteosarcoma (SS 210b), B-0061

**Litmanovich D.**
Chronic obstructive pulmonary disease (COPD): Chairperson's introduction (E³ 922), A-0564

**Littooij A.S.**
Paediatric oncology: The most common chest tumours (BS 12), A-0680

**Liu G., Wu G.**
Differentiation of clear cell and non-clear cell renal cell carcinomas by all-relevant radiomics features from multiphase CT (SS 1605), B-1394

**Liu H., Xu R., Guo Y., Yang Z., Chen L., Fu C., Li X.**
Evaluate diffuse myocardial characteristics in rabbit model based on daunorubicin-induced cardiotoxicity by CMR native T1 mapping imaging (SS 303), B-0375

**Liu H., Guo Y., Xu H., Yang Z.**
Evolution of myocardial oedema of acute and subacute myocardial infarction verified in swine model: assessed by native T1 mapping and T2 mapping (SS 303), B-0382

**Liu J., Zhang W., Cheng J.L.**
Preliminary study on changes of oxygen uptake fraction in pilots before and after hypoxia exposure (SS 1811b), B-1691

**Liu J., Zhang W., Cheng J.L.**
A resting-state functional magnetic resonance imaging study of hypoxic exposure in pilots (SS 1811b), B-1692

**Liu J., Zhang W., Cheng J.L.**
Preliminary study of hypoxic exposure effect on cerebral blood perfusion of pilots using 3D ASL (SS 1811b), B-1693

**Liu K., Tang W., Xia C., Deng Y., Zhang R., Fan L., Xiao Y., Liu S.**
Improved pulmonary nodule malignancy prediction with surrounding tissues (SS 1905a), B-1752

**Liu K., Li Z., Diao K., Peng W.**
The correlation between coronary artery stenosis and myocardial perfusion indexes: a prospective "one-stop" 256-row detector computed tomography study (MY 18), B-1677

**Liu L., Yin B., Peng W.**
Correlate of perfusion parameters on dynamic contrast-enhanced MRI with bimolecular factors and subtypes of breast cancers (SS 602), B-0552

**Liu L., Rao S., Zeng M.**
Small (<5cm) gastric sub-epithelial tumours: identification of gastrointestinal stromal tumours using CT with a practical scoring method (SS 1401b), B-1278

**Liu P., Wang Y., Jin Z., Cao J., Lin L., Yi Y.**
High concentration contrast medium injection protocol in combination with monochromatic imaging for dose reduction in spectral coronary CT angiography (SS 313), B-0320

**Liu P., Wang Y., Cao J., Lin L.**
Quantitative tissue-tracking and T1 mapping cardiac magnetic resonance (CMR) in active acromegaly (SS 414), B-0406

**Liu Q., Lu G., Cheng X., Zhang Q., Zhou C., Wang S., Liang K., Li X.**
Automatic segmentation for acute ischaemic stroke from DWI using deep convolutional neural networks (SS 1005b), B-0978

**Liu X., Yang Z., Guo Y., Ren Y., Gao Y.**
Left ventricular subclinical myocardial dysfunction in uncomplicated type 2 diabetes mellitus is associated with impaired myocardial perfusion (SS 1403), B-1222

**Liu Y., Gao P., Duan Y.**
Brain disease classification based on routine MRI using a deep learning algorithm (SS 705), B-0752

**Liu Y., Yu J., Su L.**
Comparison of the intensity and pattern of enhancement on contrast-enhanced spectral mammography between malignant and benign breast lesions (SS 1902a), B-1788

**Lo Re G., Terranova M.C., Picone D., Franchini S., Vitabile S., Midiri M.**
Semi-automatic technique for Crohn's disease diagnosis, using a supervised machine learning algorithm (SS 1905b), B-1827

**Lobbes M.B.I., Hecker J., Houben I., Pluymakers R., Jeukens C., Lalji U., Gommers S., Wildberger J.E., Nelemans P.J.**
Evaluation of single-view contrast-enhanced mammography as potential strategy for screening of patient with high breast cancer risk: a non-inferiority retrospective study
(SS 1902a), B-1787

**Lobo M.L.**
Intensive care paediatric radiology: the very sick neonate: B. Chest imaging in the ICU: tubes and catheters (RC 512), A-0278

**Loewe C.**
Thoracic emergencies: Pulmonary embolism
(BS 2), A-0087

**Loewe C.**
The art to transmit and to receive: how to communicate critical information to our patients: Chairpersons' introduction (Part 2)
(SF 8c), A-0415

**Loewe C.**
How to organise nationwide ischaemic stroke care (and make multidisciplinary teams work): Chairperson's introduction: Stroke care in Austria: small country, small challenge?
(PC 16), A-0977

**Logager V.**
Emergency radiology II: A. Urinary system trauma ($E^3$ 521), A-0202

**Logager V.**
Tumour relapse in urological cancer: Chairperson's introduction ($E^3$ 619), A-0328

**Logager V.**
Prostate MRI: the accreditation issue: B. Towards a European accreditation of prostate imaging centres ($E^3$ 1726b), A-1073

**Logudoss M., Chellathurai A.**
68 GA PSMA vs whole body diffusion weighted MR imaging in staging of high risk prostate cancer (SS 306), B-0227

**Lohmeier J., Hamm B., Makowski M.R.**
Quantitative 3D analysis of hybrid 18F-FET PET/MR-neuroimaging for differentiation between treatment response and glioma recurrence (SS 706), B-0664

**Loiseau Audirac M.P., Bader C., Castermans C., Quintana I.**
VERTIM: pilot study evaluating influence of adding nature images in imaging departments' waiting rooms, according to different aesthetic approaches, on patients' perceived anxiety
(SS 214), B-0032

**Looby S.**
The degenerative cervical spine: C. The postoperative cervical spine (RC 1611), A-0950

**Lopes R.**
Brain imaging in psychiatry: Advanced MRI and clinical applications: the structured report
(MS 8b), A-0405

**Loupa T., Brites L., Almeida C.M.D.S.**
Inoperable patients with chronic thromboembolic pulmonary hypertension: initiation a balloon pulmonary angioplasty programme
(SS 1014), B-0889

**Louw A.**
Clinical simulation and its role in radiography education: High fidelity: clinical simulation for undergraduate radiography (SF 9b), A-0493

**Løvblad K.-O.**
Stroke: interventional: Keynote lecture
(SS 311), K-08

**Lu L., Tang S., Wang X., Gong Q., Huang X.**
Altered cortical morphology of visual cortex in children with intermittent exotropia
(SS 1011b), B-1036

**Lu M.**
How preoperative sentinel lymph node contrast-enhanced ultrasound helps intraoperative sentinel lymph node biopsy in breast cancer: initial experience (MY 5), B-0459

**Lu M.**
Preliminary study of real-time three-dimensional contrast-enhanced ultrasound of sentinel lymph nodes in breast cancer (SS 702b), B-0721

**Lu Y., Qu J., Wang Z., Yan X.**
Accuracy of 3.0 T MRI in the preoperative T staging of patients with potentially resectable oesophageal cancer, with histopathological correlation (SS 1901b), B-1873

**Lu Y., Yin B.**
The diagnostic value of texture analysis in predicting WHO grades of meningiomas based on ADC maps: an attempt using decision tree and decision forest (MY 7), B-0808

**Luan Y., Teng G.**
The dorsolateral prefrontal cortex mediates the recruitment of auditory cortex in cross-modal plasticity and higher order cognitive functions in sensorineural hearing loss (SS 1911b), B-1891

**Lucertini E., Zerunian M., Caruso D., De Santis D., Moltoni G., Landolfi F., Biondi T., Iannicelli E., Laghi A.**
Magnetic resonance of rectal cancer response to therapy: comparison between 3.0 and 1.5 Tesla
(SS 1501a), B-1341

**Lucic M.A.**
Imaging professionals in the EU: radiologists without borders: It's easier than you think: the many ways to gain European CME/CPD credits
(ESR/UEMS), A-0222

**Ludwig J.M., Drescher O., Haubold J., Richly H., Forsting M., Li Y., Wetter A., Bauer S., Theysohn J.**
Low pretreatment neutrophil to lymphocyte ratio predicts better survival in uveal melanoma liver metastases undergoing hepatic chemoperfusion
(SS 209), B-0116

**Luiten J.D., Voogd A.C., Tjan-Heijnen V.C.G., Wesseling J., Luiten E.J.T., Duijm L.E.M.**
Utility of diagnostic breast excision biopsies during two decades of screening mammography
(SS 1402a), B-1158

**Luksaite R., Kliokyte R., Samuilis A., Poskus T.**
Conditional CT strategy effectiveness in diagnosing acute appendicitis (SS 317), B-0270

**Lumen N.**
The "prostate unit" at work: The urologist: knight of swords in the prostatic badlands
(MS 8a), A-0480

**Luo X., Zhang M., Ding J., Chen M.**
Pulmonary nodule size measuring difference: comparison between machine-learning based auto-measurement on CT images and gross specimens through surgery (SS 204), B-0147

**Luo Y.**
Deep convolutional neural network for histopathological classification of pancreatic neuroendocrine neoplasms (SS 1605), B-1401

**Lurie D.J.**
Demystifying MRI: things you always wanted to know: B. MR imaging basic concepts: how to turn signals into images (RC 1513), A-0911

**Lutsik N., Mendeleeva L., Solovev M., Yatsyk G., Kulikov S., Savchenko V.**
Whole-body magnetic resonance imaging (WBMRI) for the assessment of response to treatment in multiple myeloma patients after autologous haematopoietic stem cell transplantation (SS 316), B-0350

**Lv H., Wang Z., Zhao P., Wang Z.**
Long-term reactions to pulsatile tinnitus are featured by weakened short-range functional connectivity within a brain network on the right-sided temporal lobe (SS 1011b), B-1040

**Lysdahl K.B.**
Radiation protection: all you need to know: Enhancing the culture of justification
(BR 16), A-0962

### M

**M. Ali R.M.K., England A., Mercer C.E., Tootell A.K., Hogg P.H.**
Radiation risk from digital breast tomosynthesis screening: a comparison with full-field digital mammography (SS 1914b), B-1708

**Ma K.**
Explorative investigation of whole-lesion histogram MRI metrics for differentiating uterine degenerative leiomyomas and leiomyosarcomas (MY 13), B-1085

**Ma K.**
The value of ADC histogram in differentiating nasopharyngeal carcinoma from nasopharyngeal lymphoma (MY 14), B-1249

**Ma K., Cheng J., Zhang X., Song C.**
DWI-associated entire-tumour histogram analysis for the differentiation of nasopharyngeal carcinoma from nasopharyngeal lymphoma (MY 14), B-1256

**Ma Z., Cheng J.**
To study the diagnostic value of adc global gray histogram in differentiating primary central nervous system lymphoma from pleomorphic glioblastoma and single brain metastases (SS 1416), B-1238

**Maccagni D.**
Hybrid imaging: the best of both worlds: C. Optimisation/dose management in the hybrid interventional suite (RC 314), A-0112

**Mack M.G.**
MAGNETOM Lumina: first clinical experiences (SY 6)

**Mack M.G.**
Functional imaging in head and neck oncology: A. Functional imaging for locoregional tumour assessment (E³ 126b), A-0075

**Macron L.**
Artificial intelligence: the new way to your best CT image (SY 3)

**Madoff D.C.**
Pain palliation in cancer patients: Abdominal pain management with minimally invasive techniques (SF 9a), A-0513

**Maglia C., Pusceddu L., Vani V., Giannetto G., Romano V., Defeudis A., Giannini V., Mazzetti S., Regge D.**
CT texture analysis to predict response to target therapy of hepatic metastases from colorectal cancer (SS 616a), B-0527

**Magnani S., Mauri G., Di Leo G., Trimboli R.M., Poretti D., Sconfienza L.M., Sardanelli F.**
Cumulative radiation dose from contrast-enhanced CT in follow-up after EVAR (SS 1915), B-1723

**Mah S., Vijayananthan A., Rahmat K., Muridan R., Abdul Aziz Y., Yeong C.H.**
Shear wave elastography of the endometrium: a simple tool to improve diagnostic accuracy of endometrial carcinoma from other benign endometrial diseases (SS 1907), B-1902

**Mahajan H., Ganesan P.K., Vaidya A., Nanda G., Murugavel M., Venugopal V., Barnwal M., Mahajan V.**
Are radiologists bad teachers for AI algorithms: differences in the interobserver variability between consensus-defined labelling and free labelling of NIH chest x-ray14 dataset (SS 1005a), B-0910

**Mahajan V., Upadhyaya A., Venkatraman A., Venugopal V.**
Towards virtual MR imaging: predicting diffusion-weighted brain MR images from T2-weighted images using convolutional neural networks (SS 1013), B-0990

**Maher M.M.**
Diagnosis and management of acute pancreatitis: C. Intervention radiology in acute pancreatitis: why, when and how (RC 1601), A-0927

**Mahjoub S., Baur A., Pfeil J., Lee C., Rudolph M., Haas M., Cash H., Hamm B., Penzkofer T.**
Validation of the PI-RADS 2 size criterion for the prediction of significant prostate carcinomas: are adjustments to the 1.5-cm threshold warranted? (SS 207), B-0153

**Mahmood T.**
The role of radiology in major healthcare challenges faced by Pakistan: Interlude: Philanthropic cyber-knife facility in Pakistan (EM 3), A-1081

**Mahmoud K., El Khudari H., Gunn A., Keasler E., Bready E., Salei A., Winston J., Patten P., Abdel Aal A.M.K.**
Safety and efficacy of microwave ablation of stage T1 renal cell carcinoma (SS 1509), B-1296

**Mahmoud K., Gunn A., El Khudari H., Salei A., Bready E., Winston J., Keasler E., Patten P., Abdel Aal A.M.K.**
Predictors of outcomes of percutaneous cryoablation for renal cell carcinoma (SS 1509), B-1298

**Mahmoud K., Gunn A., El Khudari H., Salei A., Bready E., Winston J., Keasler E., Patten P., Abdel Aal A.M.K.**
Role of R.E.N.A.L. nephrometry and PADUA scoring systems in percutaneous ablation for renal cell carcinoma (SS 1509), B-1299

**Maier J., Dorn S., Eulig E., Sawall S., Kachelrieß M.**
Real-time patient-specific CT dose estimation for single- and dual-source CT using a deep convolutional neural network (SS 205), B-0013

**Maier S., Ernst A., Sommerwerck U., Weinreich G., Nensa F., Umutlu L., Kuehl H.**
Relevance of high-resolution computed tomography in diagnosis of infectious disease versus rejection reaction in patients after lung transplantation with acute respiratory symptoms (SS 1604), B-1411

**Maillet B.**
Imaging professionals in the EU: radiologists without borders: Putting your interests first: UEMS and ESR advocacy in the EU (part 2) (ESR/UEMS), A-0214

**Maintz D.**
Spectral CT and AI in oncology applications (SY 16)

**Majeed A.**
The role of radiology in major healthcare challenges faced by Pakistan: Interlude: Beautiful Pakistan: from sea to sky-high mountains (EM 3), A-1079

**Major V.T., Ryan S., O'Leary D.**
Exploring academic and social factors' effect on CT dose optimisation: a longitudinal study of radiographers from student to first post (SS 714b), B-0654

**Majos A.**
Functional imaging of the brain: B. Brain tumours (E³ 320), A-0135

**Malamateniou C., Mellor F., Appleyard R., McNair H., Knapp K.**
FoRRM: a pilot research mentoring scheme for radiographers in the UK: what have we achieved? An evaluation (SS 1814), B-1495

**Malamateniou C.**
Radiography research: a "how to" guide: How to produce a high-quality scientific or educational poster (BR 13), A-0781

**Malekzadeh S., Bodenmann D., Topolski A., Jouannic A., Qanadli S.D.**
A clinically driven new classification for aortic dissections (SS 1815), B-1497

**Malich A., Papageorgiou I., Wiech D.**
Whole-body MRI vs bone scan in the follow-up of oncologic patients (SS 716), B-0688

**Malich A., Kott A., Kurrat C., Feger J., Papageorgiou I.**
Bone staging of breast cancer with whole-body MRI: using low field strength without enhancer does not mitigate the diagnostic efficacy (SS 716), B-0694

**Malich A., Kurrat C., Predel D., Bang N., Papageorgiou I.**
Strain elastography ultrasound for lymph node characterisation (SS 1816), B-1643

**Malone M., Foley S.J., Rainford L.A., Toomey R.**
A comparative analysis of local diagnostic reference levels for adult projection radiography of the chest, abdomen and pelvis in Irish centres (MY 15), B-1354

**Maly Sundgren P.C.**
Cutting edge imaging and minimally invasive interventions of the spine: Imaging strategies in spine and spinal cord trauma (SF 13b), A-0756

**Mandry D.**
Functional imaging of the kidneys: A. MR measurement of split renal function (E³ 720), A-0378

**Manfrè L.**
Cutting edge imaging and minimally invasive interventions of the spine: Cutting edge minimally invasive spine interventions (SF 13b), A-0759

**Manfredi R.**
Cystic neoplasms: Other cystic pancreatic neoplasms (E³ 24C), A-0608

**Mangesius S., Hussl A., Müller C., Krismer F., Mahlknecht P., Schocke M., Gizewski E., Poewe W., Seppi K.**
Age and gender effects on brainstem MR planimetry (SS 1811b), B-1689

**Maniatis V.**
Abdominal emergencies: Perforation of the GI tract (BS 5), A-0241

**Manka R.**
Functional imaging of the heart: A. Functional techniques for clinical cardiac MR (E³ 420), A-0199

**Manke D.**
More confident image interpretation with Philips bone suppression (by Riverain Technologies) (SY 28)

**Mann R.M.**
The high-risk patient enigma: B. Value of breast MRI. Rate of underestimation and impact on treatment decision: is breast MRI increasing the number of high-risk lesions? (E³ 426a), A-0150

**Mann R.M., van Spijker G., Caballo M., Sechopoulos I.**
Dedicated breast CT may prevent unnecessary biopsies in women recalled from mammographic screening (SS 1902a), B-1790

**Mansouri B.**
Improving radiation protection in medical imaging in low- and middle-income countries: past actions and future directions: Africa's vision to improve radiation protection (EU 3), A-0663

**Mantatzis M., Thanosoulis K., Theodoridis A., Mokali J., Gianniotis C., Seimenis I.**
MRA vs DSA based models of cerebral aneurysms: how do the corresponding CFD derived flow fields compare? (SS 211b), B-0183

**Mantini C., Ricci F., Mastrodicasa D., Rotondo D., Cotroneo A.R., Gallina S.**
Aliased flow planimetry by phase-contrast CMR imaging for grading of aortic stenosis (SS 203), B-0195

**Mantini C., Caulo M., Mastrodicasa D., Marinelli D., Tartaro A., Cotroneo A.R., Di Giammarco G.**
Aortic valve bypass surgery in severe aortic valve stenosis: insights from cardiac and brain magnetic resonance imaging (SS 203), B-0203

**Mantini C., Maffei E., Toia P., Ricci F., Cotroneo A.R., Cademartiri F.**
Influence of image reconstruction parameters on cardiovascular risk reclassification by computed tomography coronary artery calcium score (SS 1503), B-1313

**Manzhurtsev A., Menshchikov P., Ublinskiy M., Melnikov I., Akhadov T., Semenova N.**
The effect of visual stimulation on the aspartate concentration in activated cortex at 3T (SS 1011b), B-1038

**Manzhurtsev A., Menshchikov P., Ublinskiy M., Semenova N., Bozhko O., Akhadov T.**
Glutamate concentration changes in response to continuous stimulation (SS 1911a), B-1778

**Mao J., Shen J.**
Evidence from in vivo MRI: ferritin heavy chain and interferon-β gene-modified mesenchymal stem cells transplanted peritumorally inhibit malignant gliomas (SS 606), B-0508

**Marcello R., Assegnati G., Di Blasi A., Cortese F., Konda D., Pofi E.**
Use of Onyx (ethylene vinyl alcohol copolymer) in haemorrhage embolisation procedures (SS 109), B-0010

**Marconi V., Lorenzon M., Girometti R., Di Mico L., Bondini F., Bracciani A., Zuiani C.**
Comparison between rapid unenhanced MRI protocol (RU-MRI) and digital breast tomosynthesis (DBT) in detecting breast cancer in a screening-like population (SS 302), B-0253

**Marcy P.-Y.**
Thyroid gland: Keynote lecture (SS 208), K-03

**Marinescu A.V., Radu R., Terecoasa E., Marinescu A.N., Tiu C., Nicula A.**
Accuracy of intracerebral haemorrhage volume calculation: comparison between manual volume segmentation, ABC/2 and sABC/2 (SS 211b), B-0191

**Marino M.A., Gucalp A., Bernard-Davila B., Keating D.M., Martinez D., Morris E., Jochelson M.S.**
Mammographic screening in male patients at high risk for breast cancer: is it worth it? (SS 1002), B-0924

**Maroldi R.**
Functional imaging in head and neck oncology: C. Is functional imaging necessary to detect distant metastases in head and neck cancers? (E³ 126b), A-0077

**Maroldi R.**
Radiologic anatomy: head and neck: Larynx (BE 6), A-0320

**Maros M.E., Siegel F., Kämpgen B., Sodmann P., Sommer W., Schönberg S.O., Henzler T., Groden C., Wenz H.**
Quality assessment of structured multi-parametric MRI reports of the prostate based on RADLEX mapping of urosurgical key information content (SS 205), B-0022

**Marra P., Della Corte A., Ratti F., Cipriani F., Gusmini S., Salvioni M., Venturini M., Aldrighetti L., De Cobelli F.**
In the ablation of colorectal cancer liver metastases with a new generation MW system nodule size >2 cm independently predicts worse local tumour progression free survival (MY 8), B-0851

**Marshall N., Bosmans H.**
Image improvement factor (Q) measured for three different antiscatter grids used in mammography imaging (SS 1913), B-1841

**Marshall N.**
Striking the balance: image quality assessment in radiological optimisation: Chairperson's introduction: The big picture, can we be objective about image quality? (RC 413), A-0181

**Martí-Bonmatí L.**
Functional imaging of the liver: Chairperson's introduction (E³ 620), A-0332

**Martí-Bonmatí L.**
Dream team: Chairperson's introduction (E³ 1018), A-0594

**Martin A.**
Career planning: The role of preceptorship in first post-radiography positions (BR 9), A-0531

**Martin C., Lassale L., Regnard N., Kuhn F.P., Larousserie F., Audard V., Biau D., Drape J.L., Feydy A.**
Diffusion-weighted imaging combined with dynamic contrast-enhanced sequences in MRI as a predictor factor of malignancy in non-fatty soft tissue tumour (SS 210b), B-0058

**Martin O., Sawicki L.M., Kirchner J., Antoch G., Herrmann K., Umutlu L., Heusch P.**
Evaluation of a fast protocol for staging patients with bronchial cancer using PET/MRI (SS 706), B-0670

**Martin O., Heusch P., Kirchner J., Ullrich T., Buchbender C., Nensa F., Herrmann K., Umutlu L., Sawicki L.M.**
Is there a connection between immunohistochemical markers and grading of lung cancer with ADC and SUV of hybrid 18F-FDG PET/MRI? (SS 706), B-0671

**Martin S.**
Musculoskeletal radiology: inflammation:
A. Inflammatory and infections in the soft tissues (E³ 221), A-0089

**Martin S.S., van Assen M., Fischer A.M., De Cecco C.N., Bauer M., Varga-Szemes A., Mastrodicasa D., Schoepf U.J.**
Value of fractional flow reserve computed from triple-rule-out CT angiography data in the emergency department (SS 703), B-0780

**Martin S.S., van Assen M., Fischer A.M., Rapaka S., Sahbaee P., Schwemmer C., Gulsun M.A., Sharma P., Schoepf U.J.**
Deep learning-based automated calcium scoring for cardiac computed tomography (MY 7), B-0820

**Martinez de Vega V.**
How MRI with AIR™ Technology brings comfort, speed and quality (SY 3)

**Martini I., Polici M., Zerunian M., Landolfi F., Panzuto F., Rinzivillo M., Annibale B., Laghi A., Iannicelli E.**
CT-texture analysis of liver metastases in PNETs vs NPNETs: correlation with histopathological findings (SS 616a), B-0525

**Martini K., Caviezel C., Schneiter D., Milanese G., Schmitt-Opitz I., Weder W., Frauenfelder T.**
Dynamic magnetic resonance imaging as an outcome predictor for lung-volume reduction surgery in patients with severe emphysema (SS 604), B-0577

**Martinoli C.**
Spine and peripheral nerve imaging: Keynote lecture (SS 1910b), K-41

**Mascherbauer J.**
Cardiac imaging in structural heart disease:
B. Defining the optimal time to treat valvular heart disease: role of MR (RC 1503), A-0878

**Masselli G.**
Emergency radiology II: B. Non-traumatic urinary tract emergencies (E³ 521), A-0203

**Masselli G.**
Imaging in pregnancy: Chairperson's introduction (RC 1307), A-0808

**Massmann A., Lepper P.M., Fries P., Wilkens H., Buecker A., Huasen B.**
Vacuum-assisted aspiration thrombectomy for treatment of acute massive pulmonary embolism (SS 1009), B-0961

**Massmann A., Frenzel F., Fries P., Kubale R., Shayesteh-Kheslat R., Buecker A.**
Prospective single-centre registry of endovascular aortic repair (EVAR) using ultra-low profile altura endograft (SS 1009), B-0963

**Mastio M., Duarte A., Raya J., Jazrawi L., Bencardino J.T.**
Can pre-operative MR imaging findings predict early failure following anterior cruciate ligament reconstruction? (SS 1810), B-1510

**Mastio M.T., Duarte Vallejo A., Raya J., Jazrawi L., Bencardino J.T.**
Rupture of the anterolateral ligament in complete acute traumatic anterior cruciate ligament tear: new insights into acute pivot shift trauma to the knee (SS 1810), B-1508

**Matos J., Mussetto I., Paparo F., Perugin Bernardi S., De Cesari M., Cevasco L., Cenderello G., Bacigalupo L., Rollandi G.A.**
Prospective comparison of transient elastography (1D-TE), shear wave elastography (2D-SWE) and magnetic resonance elastography (MRE) for assessment of liver fibrosis in HCV patients (SS 1801a), B-1528

**Matzek W.K.**
US and vascular disease: a perfect match:
C. Ultrasound as guidance for vascular interventions (RC 1315), A-0754

**Maurea S., Ginocchio M.I., Verde F., Romeo V., Mainenti P.P., Petretta M., Sarno L., Martinelli P., Brunetti A.**
Comparison of diagnostic accuracy between clinical risk factors and MRI to predict placental adhesion disorders in patients with placenta previa (SS 1507), B-1385

**Maurer M.H., de Bucourt M., Berghöfer A., Huppertz A., Rosenkrantz A.B., Streitparth F., Heverhagen J.T., Meyl T.**
Subspecialisation in radiology: effects on the diagnostic spectrum of radiologists and report turnaround time in a Swiss university hospital (SS 1405), B-1184

**Maurer M.H., Lachenmayer A., Beldi G., Candinas D., Heverhagen J.T., Kim-Fuchs C.**
Percutaneous stereotactic CT-based microwave ablation for neuroendocrine liver metastasis (SS 1709), B-1434

**Maurer M.H., Banz-Wüthrich V., Beldi G., Kim-Fuchs C., Candinas D., Tinguely P., Heverhagen J.T., Lachenmayer A.**
Stereotactic image-guided microwave ablation of hepatocellular carcinoma (SS 1709), B-1438

**Maurer M.H., Lachenmayer A., Banz-Wüthrich V., Weber S., Candinas D., Heverhagen J.T., Tinguely P.**
Stereotactic image-guided microwave ablation for malignant liver tumours: can computer-assistance broaden treatment eligibility? (SS 1709), B-1442

**May M.S., Prael I., Heiß R., Kopp M., Uder M., Wuest W.**
Late phase lung perfusion by dual energy CT: image quality and clinical findings compared to arterial perfusion (MY 4), B-0426

**Mayerhöfer M.E.**
Which whole-body exam should I choose?: Keynote lecture (SS 1716), K-33

**Mayrhofer M.T.**
Will the General Data Protection Regulation (GDPR) hamper the secondary use of clinical imaging data for research?: Can I reuse, share or give open access to existing imaging data for research? (ESR/BBMRI-ERIC), A-0631

**Mazzaro A., Gatti F.L., Bettini R., Mandruzzato N., Contro A., Mansueto G.**
Transjugular portal vein stenting: an original approach for palliative treatment of portal vein stenosis/occlusion in neoplastic patients (SS 109), B-0009

**Mazzaro A., Zamboni G., Fighera A., Campagnola A., Mansueto G.**
CT texture analysis for detection of tumour recurrence in patients resected for pancreatic adenocarcinoma (SS 1401a), B-1103

**Mazzilli A., Loria A., Castriconi R., Lusa Aguero T., Calandrino R., del Vecchio A.**
A radiation dose index monitoring (RDIM) systems comparison for CT procedures (SS 1413), B-1194

**Mc Carthy L.L., Brenner C., Sammon J., Spence L.D., O'Connor J.**
The applicability of the Greulich and Pyle atlas to a modern Irish population (SS 1012), B-1056

**McAnulla S.J., Knapp K.M., Ball S.**
Understanding diagnostic student radiographer attrition in the UK (SS 1914a), B-1698

**McDermott G.M., Hayball M., Ganeshan B., Riaz Z., Scarsbrook A.**
Automated CT texture analysis of lung cancer FDG PET-CT data using a novel software tool: preliminary results (SS 204), B-0150

**McDermott G.M., Brown P., Scarsbrook A.**
Evaluation of pre-treatment FDG PET-CT-based imaging parameters and textural features as prognostic indicators in non-small cell lung carcinoma (SS 704), B-0771

**McGinty G.**
Clinical audit: how to deal with the legal and professional requirements: Peer review as key for quality improvement: the US experience (PI 2), A-0588

**McNair H.**
Radiographers: at the heart of cancer treatment and research: Fostering excellence in radiation oncology research through a clinical research culture (EFRS/EORTC), A-0441

**McNally E.G.**
Imaging of chronic forefoot pain: A. Articular disorders (RC 510), A-0261

**McNulty J.**
Radiographers: at the heart of cancer treatment and research: An overview of the roles of radiographers in research (EFRS/EORTC), A-0439

**McNulty J.**
Clinical simulation and its role in radiography education: Clinical radiography education across Europe overview (SF 9b), A-0491

**McNulty J.**
EFRS meets Denmark: Session introduction (EM 4), A-0619

**McNulty J.**
Patient safety in medical imaging: Patient safety related education and training (ESR/EFRS), A-0931

**Meacock L.**
Musculoskeletal: A. Traumatic disorders of the musculoskeletal system ($E^3$ 1723), A-1034

**Meder J.**
Neurooncology: glioma: Keynote lecture (SS 211a), K-02

**Mehdi A.S., Islam S., Bharwani N., Sudderuddin S., Rockall A.G.**
Multiplatform reproducibility of DCE-MRI enhancement curves for adnexal masses: quality assurance study for multicentre MROC trial (SS 216), B-0177

**Meine T., Maschke S.K., Kirstein M., Renne J., Werncke T., Dewald C., Wacker F., Meyer B.C., Hinrichs J.B.**
Quantification of perfusion reduction by using 2D parametric parenchymal blood flow following partial spleen embolisation in patients with hypersplenism (SS 109), B-0001

**Meissner J., Korzowski A., Regnery S., Goerke S., Debus J., Ladd M., Schlemmer H.-P., Adeberg S., Paech D.**
Early response assessment of glioma patients to definitive chemoradiotherapy using chemical exchange saturation transfer imaging at 7 Tesla (SS 606), B-0506

**Mekkawy A.I.A., Hassan A.A., El-Sharkaway M., Kamel H.M., Thabet D.B., Nour-Eldin N.A., Naguib N.N.N., Vogl T.J.**
Transvenous pulmonary chemoembolisation (TPCE) for palliative and neoadjuvant treatment of primary lung malignancies (SS 209), B-0117

**Melazzini L., Codari M., Papini G.D.E., Beretta C., Ranucci M., Secchi F., Sardanelli F.**
Imaging requests, stroke incidence and contributing factors after open-heart surgery (SS 211b), B-0189

**Melazzini L., Codari M., Zanardo M., Bolchini M., Papini G.D., Sardanelli F.**
White matter hyperintensities volumetric burden in healthy adults: a systematic review and meta-analysis (SS 1811b), B-1687

**Meldo A.A., Utkin L.V., Moiseenko V.M., Ryabinin M.A.**
A new architecture of the computer-aided system for lung cancer diagnostics (SS 1905a), B-1760

**Melis T., Gottfriedova B., Bruna R., Willemink M.J., Koci M.**
Evaluation of the split-bolus single-pass contrast CT protocol in paediatric trauma (SS 312), B-0338

**Melnikov I., Ublinskiy M., Lukovkina O., Akhlebinina M., Akhadov T.**
Risk evaluation of malignant intracranial hypertension development in children with severe traumatic brain injury (SS 211b), B-0187

**Méndez R.J.**
Translating functional and molecular imaging in oncology: Chairperson's introduction (RC 1206), A-0703

**Meng Q.**
Application of LDCT and Lung-RADS classification in lung cancer screening of the urban population in China (MY 4), B-0432

**Meng Q., Ding J.**
Application of computer-aided diagnosis system based on 3D deep convolutional neural network in lung cancer screening (SS 1804), B-1606

**Menghini R., Zamboni G., Cybulski A., Valletta R., Marchegiani G., Mansueto G.**
Risk assessment for pancreatic fistula after pancreaticoduodenectomy with preoperative computed tomography (SS 501), B-0458

**Menshchikov P., Ivantsova A., Manzhurtsev A., Ublinskiy M., Melnikov I., Akhadov T., Semenova N.**
Metabolic changes after severe TBI: $^1$H MRS study (SS 1911b), B-1887

**Mentzel H.-J.**
Predicting fracture risk in children: B. Quantitative ultrasound (RC 812), A-0462

**Menu Y.**
Captain's dashboard: B. Being in a driver's seat and managing by objectives ($E^3$ 918), A-0557

**Meo D., Falsaperla D., Modica A., Calcagno M.C., Desiderio C., Libra F., Foti P., Palmucci S., Basile A.**
Evaluation of mismatch diameter between radial versus distal radial artery in patients suitable for vascular peripheral procedures (MY 8), B-0833

**Mereu A., Saba L.**
Effect of report length on its clarity in the stroke setting: an AI study (SS 711), B-0707

**Mereu A., Saba L.**
Update of old thyroid US reports to EU-TIRADS with AI (MY 14), B-1251

**Mershina E., Krylova N., Sinitsyn V.E., Poteshkina N., Balaeva M., Pylev A.**
Sudden cardiac death (SCD) risk stratification in hypertrophic cardiomyopathy (HCM) patients by cardiac magnetic resonance imaging (CMR) with late gadolinium enhancement (LGE) (SS 1903a), B-1819

**Mershina E.A., Moiseev S.V., Sinitsyn V.E., Pilius P.S., Moiseev A.S., Fomin V.V.**
Cardiovascular magnetic resonance (CMR) in patients with Fabry disease (SS 1903a), B-1822

**Messina C.**
Everything you always wanted to know about metabolic bone disease (but were afraid to ask): DXA: a much more powerful imaging tool than you thought (SF 15a), A-0883

**Metsälä E., Kukkes T., Rannisto K.M., Pires Jorge J., Henner A., Strøm B.**
Early detection of breast cancer education via interprofessional E-learning: the EBreast project (SS 1914b), B-1712

**Metsala E.**
Contemporary breast imaging: Chairperson's introduction (Part 1) (RC 114), A-0005

**Metsala E.**
Working in radiology: burnout and bore-out or engaged and passionate?: Chairperson's introduction (part 2) (PC 13), A-0741

**Meyer B.C.**
Balloon pulmonary angioplasty: an emerging treatment for CTEPH (SY 12)

**Meyer H., Gundermann P., Höhn A., Hamerla G., Surov A.**
Associations between whole tumour histogram analysis parameters derived from ADC maps and expression of EGFR, VEGF, Hif 1-alpha Her-2 and Histone 3 in uterine cervical cancer (SS 216), B-0175

**Meyrignac O., Losco R., Moreno R., Sewonu A., Rousseau H.**
Preliminary study for intra-abdominal liquids characterisation based on dual-energy CT (SS 701), B-0793

**Micelli M.V.M., Corridore A., Torlone S., Panebianco L., Cerrone P., Martino M., Sucapane P., Splendiani A., Masciocchi C.**
Advanced imaging technique in diagnosis of Parkinson's disease: quantitative MRI mapping of substantia nigra (SS 1811a), B-1550

**Middleton M.S., Henderson W.C., Louie A.L., Myers R., Djedjos S., Sirlin C.**
2D MRE liver stiffness values are nearly equivalent for three sequence types across four multi-centre drug-development clinical trials (SS 1801a), B-1536

**Miele V.**
From morphology to function: Italian emergency network (EM 2), A-0580

**Mikayama R., Shirasaka T., Yabuuchi H., Sakai Y., Kojima T., Kondo M., Yoshikawa H., Kato T.**
Effect of the scan mode and focal spot size on airway measurement for ultra-high-resolution computed tomography (SS 614), B-0484

**Milanese G., Cobelli R., Manna C., Silva M., Rasciti E., Poggesi S., Sverzellati N.**
CT angiography for pulmonary embolism in the emergency department: diagnostic accuracy of 20ml high-concentration contrast medium (SS 1904), B-1858

**Mildenberger P.**
Reporting and communication today and tomorrow: challenges to implement structured reporting (RS) and deal with artificial intelligence (AI): Update on developments for structured reporting: Radreport 2.0, TLAP, MRRT (PI 1), A-0504

**Miller T.T.**
Knee sports injuries and postoperative MRI: A. Sports-related injuries of the knee: what does the orthopaedic surgeon need to know? (TC 1528), A-0919

**Miller T.T.**
Knee sports injuries and postoperative MRI: C. Interactive case discussion (Part 1) (TC 1528), A-0921

**Millet I.**
Abdominal emergencies: advanced imaging in daily routine?: CT scan vs MRI in acute abdominal pain: who do we have to send to the magnet? (SF 15c), A-0893

**Millet I.**
Small bowel obstruction: improve your reading and reporting competences: A. Step 1: confirm the mechanical small bowel obstruction (SBO) (RC 1717), A-1038

**Milnerowicz M., Garcarek J., Miś M., Miś M., Kacala A.**
Temporary balloon occlusion of the internal iliac arteries as a prevention of massive haemorrhage during cesarean delivery among patients with abnormal placentation (SS 709), B-0747

**Mingliang W., Zeng M.**
Focal peliosis hepatis: imaging features of magnetic resonance imaging with diffusion-weighted imaging (SS 201a), B-0069

**Mingliang W., Zeng M.**
Solid pseudopapillary tumour of the pancreas: correlation of MR imaging appearance with biological behaviour (SS 501), B-0456

**Minotti M., De Piano F., Tofanelli L., Giugliano G., Maffini F., Mauri G., Bellomi M., Preda L., De Fiori E.**
Absence of vascularisation in thyroid nodules: new ultrasound feature for stratifying their malignancy risk (SS 1816), B-1644

**Miranda D.M.L., Mateus B., da Silva C.A., Almeida R.P., Ribeiro A.P., Brites C.T., Abrantes A.F., Ribeiro L.**
Accessibility for patients with cardiac device implantable electronic to magnetic resonance exams: a national survey (SS 414), B-0414

**Mittal K., Bansal V.**
Evaluation of sensitivity and specificity of elastography for differentiating benign from malignant cervical lymphadenopathy and comparing with B-mode and colour Doppler findings (SS 608), B-0634

**Miyazawa R., Kamo M.**
What makes a difference between life and death in patients with NOMI? Analysis of clinical data and contrast-enhanced dynamic CT findings before angiography (SS 709), B-0746

**Mlynarska-Bujny A., Bickelhaupt S., König F., Laun F., Lederer W., Daniel H., Delorme S., Schlemmer H.-P., Kuder T.**
MR mammography of suspicious lesions: influence of residual fat signal on diffusion kurtosis imaging (SS 1802b), B-1568

**Moemenam O., Njeze R., Okwudire E., Ezenwugo U.**
Comparative study of common carotid intima-media thickness in type 2 diabetics and non-diabetics in Port Harcourt, Nigeria (SS 215), B-0043

**Moerup S., Precht H., Foley S., Seegert A., Mussmann B.R.**
Impact of decentring patients for abdominal CT scan (SS 714b), B-0653

**Moghiseh M., Kumar D., Aamir R., Lowe C., Chitcholtan K., H Butler P., Sykes P., Butler A.P., Anderson N.**
Spectral CT of cancer cells with nanoparticles: in vitro and in vivo results (SS 1406), B-1117

**Mohamed R.E., Dawoud R.M., Aboelsafa A.A.**
Arterial spin-labeling and magnetic resonance spectroscopy as imaging biomarkers for the detection of epileptogenic zone in non-lesional focal impaired awareness epilepsy (MY 16), B-1423

**Mohanty H.S., Chaturvedi A.K., Jajodia A., Bind M., Rao A.S., Tripathi R., Mahawar V.**
Histopathologic and radiologic assessment of chemotherapeutic response in osteosarcoma: correlation of ADC with huvos grading (SS 716), B-0691

**Moher Alsady T., Voskrebenzev A., Greer M., Becker L., Kaireit T., Welte T., Wacker F., Gottlieb J., Vogel-Claussen J.**
MRI-derived regional flow-volume loop parameters detect early stage chronic lung allograft dysfunction (SS 604), B-0585

**Mokrane F., Chen A., Zhao B., Ammari S., Schwartz L., Houot R., Dercle L.**
Prediction of overall survival using interim 18F-FDG PET-CT and CT for response assessment of patients with Hodgkin lymphoma treated with immune checkpoint inhibitors (SS 706), B-0672

**Molinari F.**
Back to basics: how to interpret a chest radiograph?: B. Alveolar, interstitial and nodular syndromes (RC 1504), A-0915

**Molinaro S., Veglia S., Davini O., Ruggieri C.**
CTA evaluation of cervical ICA occlusion vs pseudo-occlusion in major stroke (SS 1017), B-0948

**Møller J.M., Østergaard M., Thomsen H.S., Sørensen I.J., Madsen O.R., Pedersen S.J.**
Diffusion-weighted magnetic resonance imaging in axial spondyloarthritis patients and healthy subjects: inter-MRI reliability and relation to gender, age and inflammation (SS 610), B-0520

**Monelli F., Fiocchi F., Pecchi A., Casari F., Bertucci E., Petrella E., Torricelli P.**
MRI diagnostic accuracy of placental adhesion disorders and diagnostic impact on foetal-maternal outcomes in interventional radiology-assisted delivery (SS 1507), B-1381

**Montesano M., Rengo M., Badia S., Panvini N., Picchia S., Bellini D., Carbone I., Laghi A.**
Dose team: an estimate of radiation dose reference levels for CT exams (SS 205), B-0014

**Montgomery A.**
Working in radiology: burnout and bore-out or engaged and passionate?: Dealing with daily battles: factors that lead to chronic stress and burnout (PC 13), A-0744

**Moon H., Hong G., Byeon Y., Lee C.**
Efficacy and clinical and radiological influencing factors on bone suppression by deep learning algorithm in digital chest radiograph (SS 1905a), B-1754

**Moradi Amani A., Meyer-Baese L., Tahmassebi A., Pinker-Domenig K.**
Determining the best leader nodes in Alzheimer networks (SS 1511), B-1328

**Morana G.**
State-of-the-art paediatric neuroradiology: C. Imaging in paediatric neuro-oncology (RC 811), A-0431

**Morana G.**
Pancreatic adenocarcinoma mimickers: Paraduodenal pancreatitis ($E^3$ 24E), A-1088

**Morell Hofert D., Fodor M., Braunwarth E., Haselbacher M., Schmid T., Blauth M., Gassner E., Stättner S., Primavesi F.**
Evaluation of a CT-based scoring system regarding the prognosis of blunt splenic and liver injuries: a single-centre analysis with 720 patients (SS 217), B-0102

**Morgan R.**
Visceral arteries: C. Endovascular treatment of mesenteric ischaemia (RC 115), A-0023

**Mori M., Fujioka T., Tsuchiya J., Kubota K., Tateishi U.**
Impact of 18F-FDG-PET/CT using time-of-flight in evaluating axillary lymph node metastases of breast cancer (MY 5), B-0471

**Mori T., Tanno Y., Nakai N., Yoshioka K.**
Novel rapid imaging protocol of the bilateral MCA territories for thrombectomy in acute ischaemic stroke by old-model 320-row area detector CT (SS 1017), B-0949

**Morimoto D., Hyodo T., Itou M., Fukushima H., Kamata K., Takenaka M., Kudo M., Miyagoshi K., Ishii K.**
Clinical impact of combined compressed sensing and sensitivity encoding on three-dimensional magnetic resonance cholangiopancreatography at 1.5 T (SS 414), B-0408

**Morozov S.**
Captain's dashboard: Chairperson's introduction ($E^3$ 918), A-0555

**Morozov S.**
Secrets of radiology communications with patients and referring physicians: C. Personal-branding and promotion for a radiologist ($E^3$ 1218), A-0710

**Morris E.A.**
MRI for early detection, staging and management of breast cancer: B. MR imaging biomarkers for the clinical setting (RC 502), A-0234

**Morris M.**
Reflective clinical portfolios as a tool to develop competence in radiography students (MY 15), B-1353

**Moscato F.**
The 3D printing lab from bench to bedside: Creating a 3D printing lab in radiology (SF 8f), A-0447

**Mostbeck G.H.**
GI bleeding: how can the problem be solved?: A. Acute GI bleeding (RC 501), A-0205

**Mourad C., Galant C., Libert F., Vande Berg B.**
Failure patterns in femoral head osteonecrosis: a micro-CT study with histological correlation (SS 1010), B-0894

**Mousa R.M., Fawzy F., Tantawy H., Sobhy T.**
Apparent diffusion coefficient percentage as an early predictor for evaluation of trans-catheter arterial chemoembolisation efficacy in hepatocellular carcinoma (MY 3), B-0355

**Mousseaux E.**
Cardiovascular effects after radiotherapy for breast cancer: the European MEDIRAD Project: Imaging biomarkers of cardiovascular effects of incidental cardiac radiation (EU 1), A-0019

**Muchuki C.**
Radiographers' challenges offering imaging services in Africa: Kenya. Discovering Kenyan radiographers: past, present and future (EM 5), A-0656

**Mueller-Lisse U.G., Murer S., Scheidler J., Kuhn M., Meister A., Scherr M.K., Mueller-Lisse U.L.**
MRI of the prostate at 3.0T with and without endorectal-coil: two-centre comparison in kiwi-fruit and patients (SS 407), B-0440

**Mueller-Lisse U.G., Krueger-Ziolek S., Gong B., Zimmermann H.A., Moeller K.**
Cystic-fibrosis-related lung disease: correlation of HR-CT lung morphology and regional lung function measured by electrical impedance tomography (SS 604), B-0583

**Mueller-Lisse U.G., Klingenstein A., Garip-Kuebler A., Hintschich C.**
Orbital adnexal lymphoma: diagnosis and follow-up with combined [18F]FDG-PET-CT (SS 1708), B-1451

**Muenzfeld H., Schmidt T., Posch H., Gebauer B., Hamm B., Böning G.**
Gemstone Spectral Imaging (GSI) in early CT-follow up after endovascular aortic repair (EVAR) (SS 1009), B-0962

**Muglia R., Viganò L., Gennaro N., Samà L., Colapietro F., Torzilli G., Di Tommaso L., Colombo M., Solbiati L.**
Prevalence and prognosis of intrahepatic cholangiocarcinoma (ICC) with radiological enhancement patterns that mimic hepatocellular carcinoma (HCC) (SS 616a), B-0530

**Mühlematter U.J., Schawkat K., Becker A., Müller J., Hötker A.M., Rupp N.J., Reiner C.S., Burger I.A., Donati O.F.**
Detection of extracapsular extension in high risk prostate cancer patients: multiparametric MRI vs. $^{68}$Ga-PSMA-11 PET/MRI (SS 707), B-0822

**Mukherji R., Mahajan V.**
Fuzzy PACS: linking large unorganised image and report databases for development and validation of deep learning algorithms (SS 1405), B-1183

**Mullen S.N., O'Connor M.**
Moving beyond the bookshelf: an investigation into the use of social media journal clubs in radiography practice (SS 214), B-0023

**Müller A.G., Müller F.H., Hentschel M., von Bredow C., von Bredow Y., Trenczek T.**
Detection of gut inflammation with MR and PET imaging in the insect Manduca sexta: A new screening system for effectors and inhibitors of gut inflammation (SS 606), B-0510

**Müller F.C., Bovin J.S., Gosvig K.K., Kainberger F., Rodell A., Østergaard M., Boesen M.**
Dual-energy CT for assessment of bone marrow oedema in the acute fracture: a systematic review (SS 710), B-0675

**Müller F.C., Børgesen H., Gosvig K.K., Rodell A., Booz C., Schmidt B., Krauss B., Boesen M.**
Optimising dual-energy CT parameters for virtual non-calcium imaging: a phantom study (SS 710), B-0682

**Muller H., Rae W.I.D., Friedrich-Nel H.**
Dose distribution for vascular procedures at a tertiary training hospital in South Africa (SS 1014), B-0890

**Müller L., Keber U., Mahnken A.H., Mandic R., Martini M., Seyfer P.**
USPIO contrast agents for detecting cancer: which one works best? (MY 3), B-0365

**Müller M., Reinert C.P., Nikolaou K., La Fougère C., Pfannenberg C., Gatidis S.**
Automated lung cancer detection in PET/CT using a deep learning approach (MY 7), B-0818

**Mungai F., Verrone G.B., Pietragalla M., Bonasera L., Bartolucci M., Miele V.**
Lesion-parenchyma ratio of DCE-MRI pharmacokinetic parameters for characterisation of salivary gland tumours (SS 1008), B-0975

**Muollo A., Beneventi A., Macchi E., Piacentino F., Floridi C., Fontana F., Carrafiello G., Fugazzola C.**
Fusion imaging technique in lung biopsy to demonstrate the correlation between standardised uptake value (SUV) and specimen's diagnostic value (SS 1909), B-1810

**Murchison J.T., Ritchie G., Senyszak D., van Beek E.J.R.**
Evaluation of deep learning software tool for CT-based lung nodule classification
(SS 1905a), B-1751

**Murchison J.T., Ritchie G., Senyszak D., van Beek E.J.R.**
Evaluation of deep learning software tool for CT-based lung nodule detection (SS 1905a), B-1759

**Murphy D.J.**
The heart between the lungs:
A. Cardiomyopathies (E³ 1222), A-0716

**Murugan N., Kandasamy D., Sharma R., Goyal A., Gupta A.K., Tandon N., Vara Taranikanti S., Agarwal S., Gupta N.**
Role of multiparametric 4D-MRI in detection of parathyroid lesions in primary hyperparathyroidism (SS 608), B-0640

**Musaieva K., Musaiev B., Kmetyuk Y.**
Prognostic value of restaging 18-FDG PET/CT volumetric parameters for post-index irradiation recurrence of head and neck squamous cell carcinoma (MY 14), B-1250

**Muscogiuri G., Gatti M., Dell'Aversana S., Guglielmo M., Baggiano A., Guaricci A., Pepi M., Pontone G.**
Comparison of signal intesity ratio, extent and transmurality between a novel LGE black-blood and standard LGE bright-blood sequences in patients with ischaemic heart disease
(SS 1803), B-1613

**Muscogiuri G., Gatti M., Dell'Aversana S., Pica S., Guglielmo M., Baggiano A., Guaricci A., Pontone G.**
Diagnostic accuracy of single-shot two-dimensional multisegment late gadolinium enhancement in ischaemic and non-ischaemic cardiomyopathy (SS 1803), B-1614

**Mussmann B.R.**
Radiation protection: all you need to know: Chairperson's introduction: Evolving radiographers' role in radiation protection
(BR 16), A-0960

**Mussmann B.R., Deppe S., Marschall Skov P., Foley S., Precht H.**
Organ-based tube current modulation in chest CT: effect on radiation dose and noise
(SS 614), B-0485

### N

**Nadjiri J., Kaissis G., Meurer F., Laugwitz K., Sträter A.S., Pfeiffer D., Noel P.B., Rummeny E.J., Rasper M.**
Accuracy of calcium scoring calculated from contrast-enhanced coronary computed tomography angiography using a dual-layer spectral CT (SS 1503), B-1312

**Nadrljanski M., Milosevic Z.**
Breast MRI: potential use of unenhanced morpho-functional diagnostic protocols
(SS 602), B-0549

**Nadrljanski M., Milosevic Z.**
Tumour texture parameters of IDC in neoadjuvant chemotherapy: early identification of non-responders on breast MRI
(SS 1402b), B-1172

**Nagaraj Y., Dorrius M.D., Rook M., Li Q., Guo J., Vliegenthart R., Oudkerk M., van Ooijen P.M.A.**
Multi-parametric radiomics in low-dose CT for discrimination between emphysematous and non-emphysematous lung tissue
(SS 305), B-0309

**Nair A.**
Chest: B. Inflammation and tumours of the lung (E³ 1623), A-0953

**Napoli A.**
MSK intervention: the road from low to no invasion: Avoiding side effects and complications by using MR guided high-intensity focused ultrasound (SF 5c), A-0272

**Narcisse Fidele N.**
Indications and the outcome of the mammography at Douala General Hospital
(SS 1914b), B-1711

**Nasr H., Alnajashi N., Alqarni A., Farghaly H.**
F-18 FDG PET/CT compared to Tc-99m MDP Bone scintigraphy in assessment of metastatic osseous disease in patients with breast cancer and the relation to serum CA15-3 and alkaline phosphatase (SS 1716), B-1459

**Natale L.**
Functional imaging of the heart: C. Non-ischaemic cardiopathies (E³ 420), A-0201

**Natale L.**
Cardiac imaging in structural heart disease: Chairperson's introduction (RC 1503), A-0876

**Navin P., Halaweish A.F., Schmidt B., Kim B., Wells M., Khandelwal A., Moen T., McCollough C., Fletcher J.**
Reducing radiation dose while maintaining observer performance using prior iterative reconstruction (PIR) in multiphase dual energy renal CT (SS 1407), B-1142

**Nayahangan L.**
Ultrasound simulation models in training and education: where are we going?: Needs assessment of simulations-based training in radiology (ESR US SC/EFSUMB), A-0041

**Nazarova E., Merkulov N., Mitrofanova A., Tereshenko G.**
Diffusion-weighted magnetic resonance imaging for the evaluation of Wilms tumours: radiological-pathological correlation (SS 1012), B-1061

**Nazarova E., Kupriyanov D., Tereshenko G., Smetanina N., Novichkova G.**
Magnetic resonance imaging assessment of iron status in paediatric patients with hematologic disorders (SS 1512), B-1326

**Nchimi Longang A.**
Cardiac: recalling the forgotten structures: Hide and seek: the pericardium (SA 13), A-0773

**Negru D.**
GI bleeding: how can the problem be solved?: Chairperson's introduction (RC 501), A-0204

**Fallowfield L.**
The art to transmit and to receive: how to communicate critical information to our patients: Fundamentals of physician/patient communication (SF 8c), A-0417

**Nenning K., Schmidbauer V., Fösleitner O., Prayer D., Baumgartner C., Pataraia E., Langs G., Bonelli S., Kasprian G.**
Disturbed hippocampal integration into the functional language network in patients with temporal lobe epilepsy (SS 1911b), B-1882

**Neri E.**
Big data and the big picture: deep learning in optimisation of medical imaging (part A): Imaging and dose biobanks (EF 1), A-0265

**Neri E.**
Reporting and communication today and tomorrow: challenges to implement structured reporting (RS) and deal with artificial intelligence (AI): Chairpersons' introduction (part 2)
(PI 1), A-0503

**Neri E.**
The tumour board of the future: Physical or virtual multidisciplinary meetings?
(NH 16), A-0958

**Neubauer C., Wolf J., Neubauer J.**
Influence of different examination settings on the imaging of microcalcifications in CBCT
(SS 1902a), B-1791

**Neubauer J., Reidelbach C., Kotter E., Goerke S.M.**
Comparison of patient satisfaction for wrist examinations in cone beam-computed tomography and multidetector-computed tomography (MY 9), B-0867

**Newman D.**
Radiographers' challenges offering imaging services in Africa: Chairpersons' introduction (part 1) (EM 5), A-0651

**Nguyen H., Nguyen T., Thong P.M., Ha H.S., Viet N.K., Lien L.T.T., Ngoc P.B., Hoa H.V.**
Second generation of dual source computed tomography for evaluating coronary artery lesions in Vietnamese paediatric patients with Kawasaki disease (SS 1003), B-1012

**Nguyen T.H.N., Le P.V.**
Initial results of management for acute ischaemic stroke due to large vessel occlusion by direct aspiration first-pass technique at Cho-ray hospital (SS 309), B-0278

**Niang E.H.**
Radiology in Africa: facing challenges and opportunities: How to promote radiation protection in West Africa. Needs and expected role of the ESR (EM 1), A-0312

**Nicolau C.**
Renal transplantation: A. Preoperative evaluation of donors (cadaveric or living) (RC 1607), A-0996

**Nieboer K.H.**
Blunt polytrauma: CT protocols, CT interpretation and interventional radiology options: Chairperson's introduction (RC 817), A-0435

**Niessen W.J.**
Artificial intelligence (AI): driven by radiologists: How to integrate AI technology in radiology today (NH 3), A-0127

**Niessen W.J.**
Artificial intelligence: presented with new lecture formats: Artificial intelligence (AI) and machine learning (ML) in medical imaging (ESOR), A-0370

**Niessen W.J.**
Navigating tumour phenotype with advanced imaging analysis: Role of machine learning (ML) and artificial intelligence (AI) for quantitative radiomics (SF 15d), A-0902

**Nievelstein R.A.J.**
Predicting fracture risk in children: A. Radiographs and DXA (RC 812), A-0461

**Nik-Hussin N.M.H., Robinson M., Kenyon K., Brown M., Diep P.T., Atwan M.**
On-site evaluation microscopic evaluation of unstained slides to assess adequacy of thyroid fine-needle aspiration cytology (SS 208), B-0126

**Nikitin N.A., Minin S.M., Mikheenko I., Losik D.V., Shaiakhmetova S.V., Romanov A.B.**
Hybrid $^{123}$I-mIBG SPECT/CT cardiac imaging for identification of left atrial ganglionated plexi in patients with atrial fibrillation (MY 18), B-1672

**Nikolaou K.**
Cardiac imaging in arrhythmia and sudden cardiac death: B. Preventing sudden cardiac death with CT: pure theory or new diagnostic paradigm? (E³ 526), A-0225

**Nikolic O.**
Genitourinary radiology for the general radiologist: A. Cystic pelvic masses: differential diagnosis and management (E³ 1421), A-0820

**Nilsson A.**
Precision ultrasound for an increasingly XXL population using image fusion, CEUS and improved ultrasound penetration (SY 23)

**Nissan N., Furman-Haran E., Allweis T., Menes T., Halshtok Neiman O., Faermann R., Shalmon A., Gotlieb M., Sklair M.**
Characterisation of breast parenchyma and cancer during lactation using diffusion tensor imaging (SS 1802b), B-1569

**Nocerino E.A., Nicosia L., Alì M., Monti C.B., Sardanelli F., Aliprandi A.**
Image quality of hip MR arthrography with intra-articular injection of hyaluronic acid (SS 1010), B-0895

**Nocivelli G., Ravanelli M., Ferrari M., Deganello A., Maroldi R., Farina D.**
MRI in the assessment of orbital invasion: diagnostic accuracy and impact on surgical planning (SS 1708), B-1446

**Noebauer-Huhmann I.**
Late effects in survivors of childhood cancer: MSK (SF 17), A-1023

**Nomura T., Niwa T., Hashimoto J., Imai Y.**
Visibility of bronchial artery on virtual monoenergetic image and advanced virtual monoenergetic images (SS 1804), B-1603

**Nörenberg D., Huber T., Maurus S., Jäger N., Katzmann A., Mühlberg A., Moltz J., Heinemann V., Holch J.**
Deep learning based radiomics and its usage in prediction for metastatic colorectal cancer (SS 1805b), B-1586

**Norman B., Simonovsky M., Lau F., Sall S., Golden D., Hsiao A.**
DeformationNet: unsupervised deep learning co-registration for ventricular segmentation of MR images (SS 305), B-0302

**Norton L., Maguire D., Mac a' Bháird K., Cooper G., Sheerins O., Adamson M., Kelly C., Roditi G.**
Coronary artery calcification and prediction of mortality in patients with pulmonary embolism (SS 1503), B-1307

**Notohamiprodjo M.**
Functional imaging of the kidneys: B. Renovascular diseases (RVD) (E³ 720), A-0379

**Nourzaie R., Abbas H.M. H., Monzon L., Karunanithy N., Moser S., Gkoutzios P., Ahmed I., Boolkah S., Diamantopoulos A.**
Tunnelled central venous catheters (TCVC) in infants using adult peripheral inserted central catheters (PICC) devices: ten years experience (SS 1009), B-0966

**Novello S., Reale M.L.**
Lung cancer team: Role of the oncologist: personalising the treatment (MS 1), A-0053

**Novikov M., Babkina T.**
Influence of segmentation techniques on volumetric and textural metabolic parameters of primary tumours on 18F-FDG PET-CT imaging (SS 306), B-0230

**Nowak T., Schmidt B., Euler A., Saltybaeva N., Flohr T., Alkadhi H.**
Areal bone mineral density estimation using photon-counting energy-discriminating computed tomography topograms (SS 1013), B-0996

**Ntorkou A.A., Tsili A., Goussia A., Astrakas L.G., Kaltsas A., Sofikitis N., Argyropoulou M.I.**
Apparent diffusion coefficient and magnetization transfer ratio in predicting successful sperm retrieval after mTESE (SS 1807), B-1660

**Nurili F., Acar T., Derman S., Caymaz I., Özkanli S., Bas A., Yurdaisik I., Cakiroglu H., Aras O.**
Effectiveness and safety transarterial embolisation with bleomycin-loaded PLA/PLGA microspheres for the treatment of vascular malformation using a porcine spleen model (SS 209), B-0115

**Nurili F., Bekaroglu G., İsci S., Bas A., Caymaz I., Yurdaisik I., Özkanli S., Cakiroglu H., Aras O.**
Multifunctional iron oxide nanoparticle-embedded PVP-HEC microparticles as a drug-eluding embolic material (SS 609), B-0556

**Nurili F.**
Embolisation of rabbit renal arteries to test feasibility and effects of CT-visible doxorubicin-eluting montmorillonite microparticles (SS 609), B-0565

**Nurili F., Acar T., Derman S., Bas A., Caymaz I., Özkanli S., Cakiroglu H., Yurdaisik I., Aras O.**
Catheter-directed gastric artery embolisation with octreotide acetate-loaded PLA/PLGA (poly(lactide-co-glycolide) acid) microspheres with slow sustained release properties (SS 709), B-0737

**Nurili F., Acar T., Derman S., Bas A., Caymaz I., Yurdaisik I., Cakiroglu H., Özkanli S., Aras O.**
Trans-arterial embolisation of the prostate with biodegradable flutamide-loaded PLA/PLGA microspheres for benign prostate hyperplasia: a preliminary study in normal swine model (SS 709), B-0743

**Nyhsen C.**
How to perform ultrasound image fusion: Minimise the risk of infection transmission and effective decontamination protocols in the setting of ultrasound fusion (US 1), A-0172

### O

**O'Connor J.**
Navigating tumour phenotype with advanced imaging analysis: Understanding tumour micro environment: the role of habitat imaging (SF 15d), A-0900

**O'Connor J.**
Quantitative imaging in oncology: Chairperson's introduction (E³ 1726a), A-1057

**O'Connor M.**
Radiography audit and quality management: A. Understanding PACS to support radiography quality management processes (RC 514), A-0208

**O'Connor O.J.**
Striking the balance: image quality assessment in radiological optimisation: A. Between a ROC(k) and a hard place: classical and pragmatic methods of determining clinical image quality (RC 413), A-0182

**O'Connor P.J.**
Inflammatory and infectious diseases of the spine: how to differentiate from degeneration: B. Crystals: may also affect the spine (RC 910), A-0542

**O'Leary D.**
Radiography research: a "how to" guide: Chairperson's introduction: Why is radiography research important? (BR 13), A-0777

**O'Riordan L.**
Patient involvement in the testing and validation of deep learning systems (SY 5)

**Obmann V.C., Yang K., Seyfried N., Stopchinski M., Wright K., Ghodasara S., Panda A., Chen Y., Gulani V.**
High spatiotemporal resolution free-breathing quantitative bowel perfusion imaging (SS 1801b), B-1650

**Obmann V.C., Lo W., Seyfried N., Jiang Y., Panda A., Wright K., Ropella-Panagis K., Gulani V.**
Magnetic Resonance Fingerprinting (MRF) enables simultaneous T1 and T2 mapping of the bowel wall in patients with Crohn's disease (SS 1801b), B-1655

**Obradov M.**
MSK intervention: the road from low to no invasion: The 10 most important side effects and complications in MSK interventions (SF 5c), A-0273

**Occhipinti M.**
Radiologic anatomy: chest: Mediastinal (BE 3), A-0122

**Occhipinti M.**
Back to basics: how to interpret a chest radiograph?: E. Mediastinal syndrome (RC 1504), A-0918

**Occhipinti M.**
Chest: A. Fundamentals of chest imaging (E³ 1623), A-0952

**Oechtering T.H., Panagiotopoulos A., Sieren M., Scharfschwerdt M., Fujita B., Ensminger S., Sievers H., Barkhausen J., Frydrychowicz A.**
Biological versus mechanical aortic valve prostheses: an in vitro 4D flow MRI comparison of haemodynamics (SS 203), B-0202

**Oechtering T.H., Nowak A., Sieren M., Graessner J., Kooijman-Kurfuerst H., Barkhausen J., Frydrychowicz A.**
Inter-scanner and inter-software comparison of quantitative 4D flow MRI (SS 315), B-0225

**Oei E.H.G.**
State-of-the-art imaging of postoperative joints: B. Postoperative knee (E³ 1626a), A-0946

**Oei L., Koromani F., Breda S., El Saddy S., Makurthou A., Rivadeneira F., Oei E.**
Identification of Scheuermann's disease with different radiological scoring methods for osteoporotic vertebral fractures: the Rotterdam study (MY 9), B-0856

**Offiah A.C.**
Predicting fracture risk in children: D. CT (including HRpQCT) (RC 812), A-0464

**Oganesyan A., Mershina E., Sinitsyn V.E.**
Contribution of CT lung perfusion in diagnosis of acute pulmonary embolism (SS 717), B-0730

**Oh S.**
Clinical trial to evaluate the dose reduction in paediatric radiography (SY 18)

**Ohlmeyer S., Buder T., Uder M., Wuest W.**
3D imaging of the paranasal region with a multipurpose cone beam detector system: image quality and radiation exposure (MY 14), B-1259

**Ohno Y., Fujisawa Y., Yui M., Kishida Y., Seki S., Yoshikawa T., Murakami T.**
Comparison of therapeutic outcome prediction among dynamic perfusion area-detector CT, dynamic perfusion MRI and FDG-PET/CT in small cell lung cancer patients with limited disease (MY 4), B-0417

**Ohno Y., Yui M., Kishida Y., Seki S., Yoshikawa T., Murakami T.**
Inspiratory/expiratory 3D pulmonary MRI with UTE: capability for clinical stage classification in smokers as compared with thin-section CT (MY 4), B-0422

**Ohno Y., Fujisawa Y., Sugihara N., Kishida Y., Seki S., Yoshikawa T., Koyama H., Murakami T.**
Inspiratory/expiratory xenon-enhanced CT with 3D motion analysis: utility for pulmonary functional loss assessment and clinical stage classification of COPD (SS 604), B-0579

**Ohno Y., Yaguchi A., Aoyagi K., Kishida Y., Seki S., Ueno Y., Yoshikawa T., Murakami T.**
Comparison of the capability of nodule component measurement and management on follow-up CT examinations between 3D CADv systems with and without CNN (SS 1004), B-1003

**Ohno Y., Yui M., Lee H., Koyama H., Kishida Y., Seki S., Yoshikawa T., Kassai Y., Murakami T.**
Comparison of quantitative capability among DWI with FASE and EPI sequences at 1.5T or 3T systems and FDG-PET/CT: for differentiating malignant from benign solitary pulmonary nodules (SS 1404), B-1203

**Ohno Y., Yui M., Kishida Y., Seki S., Yoshikawa T., Murakami T.**
Multi-parametric approach among CEST imaging, DWI and FDG-PET/CT for diagnosis of solitary pulmonary nodule (SS 1404), B-1209

**Ohno Y., Aoyagi K., Yui M., Kishida Y., Seki S., Yoshikawa T., Murakami T.**
Comparison of capability for TNM stage assessment among whole-body MRI and PET/MRI at different field strength and FDG-PET/CT in non-small cell lung cancer (SS 1716), B-1460

**Ohno Y., Yaguchi A., Aoyagi K., Takenaka D., Yoshikawa T., Kishida Y., Seki S., Murakami T.**
3D computer-aided CT texture analysis with machine learning: capability to play as second reader for radiological finding assessment in interstitial lung disease (SS 1905a), B-1755

**Oikarinen H., Ukkola L., Henner A.**
The reasons for practitioners' passive approach to informing patients prior to their radiological examinations (SS 1413), B-1192

**Oikonomou A.**
Cavitary and cystic diseases of the lung: C. Lymphangioleiomyomatosis (E³ 722), A-0376

**Oktay D.G., Peker E., Erden I.**
Right ventricular functional assessment with cardiac MRI by comparing volume measurements between axial and short axis orientations (SS 1903b), B-1862

**Olalla Muñoz J.R.**
Small bowel obstruction: improve your reading and reporting competences: B. Step 2: identify the SBO cause (RC 1717), A-1039

**Olasunkanmi Balogun E.**
Radiographers' challenges offering imaging services in Africa: Nigeria. Healthcare services in Nigeria: the radiographers' opportunities and challenges (EM 5), A-0653

**Oleaga Zufiría L.**
EDiR: an instrument to develop excellence in your career: Chairperson's introduction (EDiR), A-0848

**Oleaga Zufiría L.**
EDiR: an instrument to develop excellence in your career: EDiR teaser (EDiR), A-0849

**Oleaga Zufiría L.**
EDiR: an instrument to develop excellence in your career: CORE examination (Part 2) (EDiR), A-0851

**Oleaga Zufiría L.**
Imaging professionals in the EU: radiologists without borders: ETAP 2.0 (ESR/UEMS), A-0220

**Oleaga Zufiría L.**
Clinical simulation and its role in radiography education: Chairpersons' introduction (Part 1) (SF 9b), A-0489

**Oliveira M.V.L., Barros J.**
Development of 3D printed quality control tool for X-Ray beam alignment and collimation evaluation (SS 1814), B-1493

**Oluniran G., Blackwell J., Tuohy B., Colgan N.**
Phantom for independent global head SAR assessment of MRI-induced temperature change (SS 713), B-0761

**Omelchenko O., Makarchuk M., Podkovka O., Myronyak L., Rogozhyn V.**
Reward-related system fMRI activation as a monitoring tool for alcohol abuse rehabilitation (SS 1011b), B-1034

**Omoumi P.**
MR imaging of the knee: B. Meniscal tears: obvious and subtle (RC 810), A-0444

**Omura V.L.N.**
Synthesized mammography: is it as good as digital mammography to find microcalcification? (SS 302), B-0254

**Ording Müller L.-S.**
Juvenile idiopathic arthritis (JIA): C. MRI and role of contrast in the assessment of synovitis ($E^3$ 1326), A-0796

**Örnek A., Umutlu L., Kirchner J., Reichardt B., Herrmann K., Ruhlmann V.**
Inflammation focus search with 18F-FDG-PET/MRI: comparative or additive value of PET and MRI (SS 306), B-0231

**Örnek A., Umutlu L., Kirchner J., Heber D., Herrmann K., Ruhlmann V.**
Comparison evaluation of the diagnostic potential of PET/MRI and PET/CT using mIBG-Iod-124 in neuroendocrine and neuroectodermal tumours (SS 706), B-0673

**Orsatti G., Varotto A., Bonzini M., Minotti M., Morosi C., Crimì F., Giraudo C., Quaia E., Stramare R.**
Paediatric rhabdomyosarcomas: which type of radiological assessment after induction chemotherapy better predicts the survival? (SS 1012), B-1060

**Orsi M., Leone F., Cellina M., Oliva G.**
Digital breast tomosynthesis (DBT) in the assessment of false positive recalls in a single-centre breast screening programme (SS 302), B-0247

**Osborn A.G.**
"Brain in flame"- pathology and imaging of non-infectious CNS inflammation: a masterclass from Dr. Anne Osborn (SY 26)

**Osman A.M., H. Abdeldayem E.**
Value of CT pulmonary angiography to predict short-term outcome in a patient with pulmonary embolism (SS 1904), B-1855

**Owens C.**
Dose management in paediatric radiology: A. The special case of the paediatric patient: risks and justification (RC 1313), A-0786

**Owens C.**
Getting the balance right: radiation risk and imaging benefit in paediatric procedures: Chairperson's introduction (EU 5), A-0871

**Owens C.**
CT radiation risk in children: an overview: Daily practical challenges in CT for children (EU 6), A-0937

**Owens C.**
Late effects in survivors of childhood cancer: Chairperson's introduction (SF 17), A-1021

**Oyama K., Yamada A., Yoshizawa E., Ichinohe F., Komatsu D., Fujinaga Y.**
Dynamic contrast-enhanced CT diagnosis of primary liver cancers using transfer learning of pre-trained convolutional neural network: comparison with radiologists (SS 1905b), B-1836

**Oyen R.H.**
Multidisciplinary approach to cancer care: Chairperson's introduction (RC 816), A-0465

**Oyen R.H.**
Prostate cancer: Keynote lecture (SS 207), K-04

**Oz I.I., Bomers J.G., Overduin C.G., Jenniskens S.F., Sedelaar J.P.M., Paetz T., Rieder C., Schumann C., Futterer J.J.**
Focal cryoablation for recurrent prostate cancer following radiation therapy: assessment of iceball margin and signal intensity of tumour by 3D registration of MRI (SS 1007), B-0878

**Ozden C., Warncke M.L., Brunner F., Stáreková J., Koops A., Lund G.K., Kirova-Nedyalkova G.I., Adam G., Tahir E.**
Impaired left ventricular function in patients with treatment-resistant arterial hypertension evaluated by feature-tracking myocardial strain CMRI (SS 603), B-0590

**Özmen M.N.**
Renal, adrenal and urinary tract pathologies: Ureter and bladder (BS 8), A-0434

**Ozturk M., Polat A.V., Baris Y.S., Selcuk M.B.**
The diagnostic value of shear wave elastography for the differentiation of benign and malignant soft tissue tumours (SS 210b), B-0059

**Ozturk M., McDermott J.C., Laeseke P.F., Nakada S.Y., Hedican S.P., Best S., Kleedehn M.G.**
Percutaneous management of Indiana pouch stones: a single-centre retrospective study (SS 1509), B-1297

### P

**Pažanin A., Škrk D., Mekis N.**
Optimisation of collimation and its impact on patient dose in radiography of lumbal spine in anteroposterior and lateral projection (SS 1314), B-1073

**Pacifici S., Bindinelli P., Galli V., Giacobbe A., Mariotti D., Pedilarco S., Salimbeni S., Santucci G., Severi D.**
A core curriculum for the breast radiographers working in Italy (SS 1914b), B-1715

**Pacifici S., Giudice D.**
Scanxiety in follow-up breast cancer women (SS 1914b), B-1718

**Pacios Blanco R.E., Vicente Bártulos A., Manuel Fernández B., Cobo Reinoso E., Muriel García A., López-Frías López-Jurado A., Almeida N.A., Gallego Rivera J.I., Gorospe Sarasua L.**
Is it possible to establish a clinical prediction rule for computed tomography assessment in suspected bowel obstruction at the emergency department? (SS 317), B-0274

**Padhani A.R.**
Whole-body imaging in metastatic urinary tract and prostate cancer: Chairperson's introduction ($E^3$ 419), A-0194

**Padley S.P.G.**
Back to basics: how to interpret a chest radiograph?: Chairperson's introduction (RC 1504), A-0913

**Paech D., Dreher C., Regnery S., Goerke S., Unterberg A., Wick W., Bendszus M., Ladd M., Schlemmer H.-P.**
Amide proton transfer (APT) MRI is a predictor of survival and progression in high-grade glioma patients (SS 606), B-0505

**Paech D., Meissner J., Wennmann M., Radbruch A., Bendszus M., Wick W., Unterberg A., Ladd M., Schlemmer H.-P.**
Lactate-weighted imaging of brain tumours using chemical exchange saturation transfer (CEST) MRI at 7 Tesla (MY 16), B-1419

**Páez Granda D., Martínez Cutillas M.J., Díaz Pérez J., Parrilla Reverter G., Berná-Serna J., Espinosa de Rueda Ruiz M., Garcia-Villalba B., Zamarro Parra J., Felices Farias J.**
Are aneurysmal subarachnoid haemorrhage scales applicable to non-aneurysmal subarachnoid haemorrhage? (MY 16), B-1418

**Paiman E.H.M., van Eyk H.J., Bizino M.B., Dekkers I.A., de Heer P., Smit J.W.A., Jazet I.M., Lamb H.J.**
Phenotyping diabetic cardiomyopathy in South Asians and Europeans (SS 603), B-0588

**Paladini A., Beomonte Zobel D., Vallati G.E., Pizzi G., Cappelli F., Borzelli A., Amodeo E.M.**
Response to SIRT (selective internal radiation therapy) in patients affected by unresectable multifocal intra-hepatic cholangiocarcinoma: results of a preliminary study (SS 209), B-0113

**Paladini A., Vallati G.E., Pizzi G., Cappelli F., Beomonte Zobel D., Amodeo E.M., Borzelli A.**
Retrospective analysis of a cohort of patients affected by unresectable HCC treated with SIRT and liver transplant (SS 609), B-0563

**Paladini A., Vallati G.E., Pizzi G., Cappelli F., Beomonte Zobel D., Amodeo E.M., Borzelli A.**
Intermediate-stage HCC, SIRT and balloon-occlusive micro-catheter: the right way? (MY 8), B-0846

**Paladini A., Beomonte Zobel D., Falcione M., Borzelli A., Di Stasi C., Cina A., Vallati G.E., Pizzi G., Amodeo E.M.**
Minocycline hydrochloride as a soft sclerosing agent for symptomatic simple renal and hepatic cysts: our experience (SS 1709), B-1435

**Palermo M., Sambataro G., Tiralongo F., Desiderio C., Falsaperla D., Torrisi S., Basile A., Vancheri C., Palmucci S.**
To assess clinical and radiological disease severity in a population of IPF patients which differ for the presence of diffuse pulmonary ossification (DPO) on HRCT (SS 1604), B-1405

**Palmisano A., Esposito A., Benedetti G., Giannini F., Baldetti L., Del Maschio A., De Cobelli F.**
Modification of left ventricle myocardial perfusion reserve after the implantation of coronary sinus stent: a stress-rest CMR study (SS 1403), B-1221

**Palmisano A., Benedetti G., Palumbo D., Peretto G., Faletti R., Sala S., Gatti M., De Cobelli F., Esposito A.**
Cardiovascular magnetic resonance early predictors of unfavourable left ventricle remodelling in patients with acutemyocarditis (MY 18), B-1678

**Palmqvist R.**
Radiology and pathology: will we ever be (digitally) married?: What difference would there be for patients? (Part 1) (SF 13a), A-0738

**Palumbo D., Palmisano A., Faletti R., Benedetti G., Gatti M., Del Maschio A., De Cobelli F., Esposito A.**
Assessment of diagnostic performance of cardiac magnetic resonance (CMR) mapping parameters in acute myocarditis (AM) patients (SS 303), B-0381

**Palumbo D., Guazzarotti G., Martinenghi C., Nicoletti R., Del Maschio A., De Cobelli F.**
Can routine postoperative CT scan provide reliable leakage risk stratification after laparoscopic sleeve gastrectomy (LSG)? (SS 1901b), B-1880

**Palumbo P., Arrigoni F., De Cataldo C., Ruschioni M., Zugaro L., Barile A., Masciocchi C.**
Intraarticular osteoblastomas: from challenging diagnostic entity to minimally invasive treatment with magnetic resonance-guided focused ultrasound (MRgFUS) (SS 1809), B-1571

**Palumbo P., Cannizzaro E., Corridore A., De Cataldo C., Torlone S., Di Luzio M., Di Sibio A., Di Cesare E., Masciocchi C.**
Prognostic validity of stress cardiac magnetic resonance (CMR) in the intermediate-long term outcome assessment of known-CAD patients (SS 1803), B-1623

**Pambianchi G., Galea N., Carbone I., Cilia F., Catapano F., Francone M., Catalano C.**
Thoracic aortic fluid dynamics in bicuspid valve patients: correlations with valvular morphology using 4D-flow magnetic resonance imaging (SS 1815), B-1500

**Pamminger M.J., Klug G., Reinstadler S.J., Reindl M., Kremser C., Kranewitter C., Metzler B., Jaschke W.R., Mayr A.**
Pre-TAVR scanning by a comprehensive contrast-free cardiovascular magnetic resonance protocol: comparison with contrast-enhanced computed tomography (SS 615), B-0500

**Pan J., Hofmanninger J., Röhrich S., Prayer F., Holzer M., Sverzellati N., Weber M., Prosch H., Langs G.**
Unsupervised machine learning for the identification of CT imaging markers of progression in idiopathic pulmonary fibrosis (SS 305), B-0307

**Pan T., Vikram R.**
Changes on noise power spectrum by advanced CT image reconstruction techniques (SS 213), B-0134

**Pan X., Guo M.**
Clinical application of 640 slice spiral CT double low enterography in inflammatory bowel disease (SS 1801b), B-1646

**Panagiotopoulos N., Fechtrup N., Oechtering T.H., Roggenbuck U., Nassenstein K., Barkhausen J., Hunold P.**
Evaluation of the prognostic value of visually detected cardiac MRI findings in asymptomatic diabetics and healthy volunteers (SS 1803), B-1622

**Pandur A., Sipos D., Banfai B., Betlehem J., Radnai B.**
Age-adjusted D-dimer and risk-stratification in acute pulmonary embolism for reduce the unnecessary CT imaging (SS 717), B-0728

**Panebianco V.**
Tumour relapse in urological cancer: A. Prostate cancer relapse ($E^3$ 619), A-0329

**Panfili M., Calandrelli R., Pilato F., Colosimo C.**
A systematic quantitative morpho-volumetric analysis in infants with sagittal craniosynostosis and relationship with the severity of scaphocephalic deformity (SS 1412), B-1225

**Panvini N., Bellini D., Laghi A., Marin D., Patel B.N., Wang C.L., Carbone I., Mileto A.**
Diagnostic accuracy of dual-energy CT for renal mass evaluation: systematic review and meta-analysis (SS 1407), B-1148

**Panzeri M., Di Chiara A., Palmisano A., Esposito A., Passoni P., Del Maschio A., De Cobelli F.**
MRI texture analysis in predicting lymph nodal yield after neoadjuvant chemoradiotherapy in locally advanced rectal cancer (LARC) (SS 201b), B-0207

**Panzeri M., Colombo M., Papa M., Cardone G., Venturini M., Capitanio U., Montorsi F., Del Maschio A., De Cobelli F.**
Comparison of cryoablation and microwave ablation in the treatment of small renal tumours (SRT): technical success, complications and midterm clinical outcome (SS 1509), B-1302

**Panzironi G., Moffa G., Galati F., Marzocca F., Collalunga E., Pediconi F.**
Can perilesional oedema detected in MRI be considered as a biomarker for breast cancer? The role of T2-weighted sequences (SS 602), B-0553

**Paolicelli S., Virelli R., Coi L., Matino S., Telegrafo M., Stabile Ianora A., Gesualdo L., Bevilacqua V., Moschetta M.**
Calculation of total kidney volume (TKV) in autosomal dominant polycystic kidney disease (ADPKD): comparison of 3 different methods (SS 307), B-0329

**Papa M., Losa A., Suardi N., Agostini G., Maga T., Gaboardi F., Cardone G.**
Modified ABLATE-score (mABLATE): a specific nephrometric score to predict complications and relapses in percutaneous cryoablation of renal lesions (SS 1509), B-1300

**Papadaki E.**
Update on cerebrospinal fluid (CSF) diseases: B. Diagnosis and treatment of intracranial hypotension (RC 911), A-0520

**Papakonstantinou O.**
Multidisciplinary approach to the diabetic foot: Imaging evaluation of the diabetic foot (MS 17), A-1064

**Papalois V.**
Imaging professionals in the EU: radiologists without borders: Brexit means Brexit: radiologists with borders? (ESR/UEMS), A-0217

**Papanagiotou P.**
How to organise nationwide ischaemic stroke care (and make multidisciplinary teams work): What you should know being involved in a nationwide ischaemic stroke service (PC 16), A-0978

**Parimalai A.N., Chelladurai A., Logudoss M.**
Diffusion tensor imaging in intractable unilateral mesial temporal lobe epilepsy (SS 1411b), B-1271

**Parizel P.M.**
Imaging professionals in the EU: radiologists without borders: Putting your interests first: UEMS and ESR advocacy in the EU (part 1) (ESR/UEMS), A-0213

**Park E., Seo B., Han M., Kim J., Lee H., Cho K., Woo O., Song S.**
Radiogenomics of invasive breast cancer: association between ultrasound vascular imaging features and tumour genomic profiles for tumour angiogenesis (SS 1402b), B-1177

**Park J., Jung J., Yoon S.H., Hong H., Yoon J., Kim H., Goo J.M.**
CT quantification of spatial heterogeneity of boundary between fibrotic and non-fibrotic areas in idiopathic pulmonary fibrosis (SS 1604), B-1403

**Park M., Lee C., Hong G.**
Clinical significance of bedside ultrasonography and second-look ultrasonography in pediatric ileocolic intussusception (SS 317), B-0273

**Park S., Cho S., Hocheol C., Lee S.**
Distal transradial approach at the anatomical snuffbox for interventional procedures: a single-centre pilot study (SS 1009), B-0965

**Parkar A.P.**
Cavitary and cystic diseases of the lung: A. Cavitary lung lesions (E³ 722), A-0374

**Parkar A.P.**
Back to basics: how to interpret a chest radiograph?: D. Pleural syndrome (RC 1504), A-0917

**Parkar A.P.**
Chest: C. Mediastinum, pleura and chest wall (E³ 1623), A-0954

**Pasquini L., Tortora D., Rossi Espagnet M., Figà-Talamanca L., Manunza F., Occella C., Rossi A., Severino M.**
Cavernous sinus enlargement: a novel finding in Sturge-Weber syndrome (SS 1011a), B-0930

**Pasquini L., Li Q., Del Ferraro G., Peck K., Holodny A.I., Makse H.**
Functional network analysis of human language: a novel approach using task-based fMRI (SS 1011b), B-1032

**Pastorino U., Silva M., Sabia F., Sestini S., Boeri M., Sozzi G., Sverzellati N., Marchianò A.V.**
Low-dose computed tomography (LDCT) screening reduces overall and lung cancer specific mortality beyond five years (SS 204), B-0143

**Pastorino U.**
Lung cancer team: What the surgeon needs to know (MS 1), A-0054

**Patel H.T.**
Forensic imaging: Chairpersons' introduction (Part 2) (RC 814), A-0398

**Patella F., Sansone M., Franceschelli G., Fusco R., Petrillo M., Pesapane F., Zannoni S., Brambillasca P., Carrafiello G.**
Quantification of heterogeneity to classify benign parotid tumours (SS 1008), B-0974

**Patelli G., Besana F., Faietti E., Ori Belometti G., Costa C., D'Alessio A., Mauri G., Pacella C.M.**
Outcome in 73 patients with benign prostatic hyperplasia treated by an ultrasound guided transperineal laser ablation by interventional radiologists (SS 607), B-0630

**Patlas M.**
Blunt polytrauma: CT protocols, CT interpretation and interventional radiology options: B. Solid organs injuries: a tailored approach (RC 817), A-0437

**Patney A., Murugavel M., Mahajan V.**
Comparison of 90 and 180 micron resolution cone-beam CT scans in patients with artefact causing root canal filling material (SS 213), B-0140

**Patsch J.M.**
Paediatric musculoskeletal and oncologic imaging: Keynote lecture (SS 1012), K-26

**Paulo G.**
Growing radiography research: Radiography research: where should we be in 2030? (part 2) (EFRS WS), A-0861

**Paulo G.**
The future of radiology: what it could mean for new radiography graduates: Chairperson's introduction: What will radiology departments look like in the future? (BR 17), A-1041

**Pavan N.**
Imaging and management of the incidental scrotal lesions: Testicular-sparing surgery: a teamwork of radiologist and urologist (SF 5d), A-0296

**Pavic D.**
Comparison of two-dimensional and three-dimensional mammography performance in breast cancer screening (SY 2c)

**Pavlova O., Serova N.S., Davydov D., Ternovoy S.K.**
Correlation between the size of the inferior orbital wall defect and treatment tactics in orbital trauma according to the MSCT data (MY 14), B-1261

**Pecoraro M., Campa R., Barchetti G., Salvo V., Catalano C., Panebianco V.**
Is DCE useless in early detection of prostate cancer? The analysis of quantitative parameters might help radiologists to decide if findings mildly restricted on ADC map should be sampled (SS 207), B-0160

**Pecoraro M., Cipollari S., Campa R., Barchetti G., Catalano C., Panebianco V.**
Predicting clinically significant prostate cancer of the transition zone using multiparametric MRI and quantitative radiomic analysis (SS 307), B-0325

**Pecoraro M., Campa R., Barchetti G., Salvo V., Catalano C., Panebianco V.**
A quantitative approach to pinpoint prostate targets in order to reduce the number of TRUS-MRI fusion biopsy cores per patient without affecting the detection rate (SS 1007), B-0876

**Pedersen M.R.V.**
Ultrasound: continuing to make waves: A. Technology advances: from 3D to elastography (RC 1714), A-1005

**Pedersen M.R.V., Osther P.J., Nissen H., Vedsted P., Møller H., Rafaelsen S.R.**
MRI diffusion-weighted and shear wave elastography in testis (SS 1807), B-1661

**Pedersoli F., Isfort P., Zimmermann M., Schulze-Hagen M.F., Keil S., Kuhl C.K., Bruners P.**
Retrograde stent implantation of the celiac artery via the pancreaticoduodenal anastomosis (SS 109), B-0005

**Pediconi F.**
Minimally-invasive local treatment of breast cancer: the time is now: A. High-intensity focused ultrasound (HIFU) therapy (RC 1202), A-0672

**Peebles C.**
Cardiac: recalling the forgotten structures: Chairperson's introduction (SA 13), A-0772

**Peebles C.**
Dead or alive: imaging of myocardial viability: Chairperson's introduction (RC 1703), A-1017

**Peetrons P.**
Musculoskeletal interventional procedures: A. Diagnostic and therapeutic injections in the athlete: pearls and pitfalls (TC 1628), A-0991

**Peetrons P.**
Musculoskeletal interventional procedures: C. Interactive case discussion (Part 1) (TC 1628), A-0993

**Pehrson L.M.**
EFRS meets Denmark: Machine learning: a new aspect of radiography (EM 4), A-0621

**Pellegrino F., Pontrelli M., Mungari R., Fortini M., Gamberini M.R., Zatelli M.C., Ambrosio M.R., Giganti M.**
Incidence of vertebral fractures in patients with β-thalassaemia major in treatment with oral bisphosphonate (SS 210a), B-0045

**Pellegrino F., Perrucci L., Pontrelli M., Gamberini M.R., Fortini M., Gagliardi I., Zatelli M.C., Ambrosio M.R., Giganti M.**
Lumbar spine bone mineral density Z-score discrepancy by DXA and QCT in thalassaemic major patients (SS 210a), B-0046

**Pellegrino F., Tralli G., Di Stefano G., Tartari C., Tartari S., Giganti M.**
Diagnostic performance of abdominal ultrasound in right-sided acute colonic diverticulitis (SS 317), B-0272

**Peng Q., Tang W., Wu N., Huang Y., Ouyang H., Wu B.**
Reduced- and no-dose thin-section radiologic examinations: comparison of capability for nodule detection in patients having pulmonary nodules (MY 4), B-0429

**Peng W., Xu H., Li Z., Xia C., Guo Y.**
Performance of native T1 and T2 mapping cardiovascular magnetic resonance to detect myocardial oedema in patients with end stage renal disease (SS 303), B-0384

**Peng X., Yang S., Hu P., Xiao J., Pei Y., Liao W.**
Model-based accelerated T2 mapping of sacroiliac joint with 3-T MRI: a preliminary study of Grappatini (SS 710), B-0678

**Pennig L., Thiele F., Perkuhn M., Kabbasch C., Schlamann M., Borggrefe J.**
Comparison of accuracy for the prediction of infarct tissue volumes between arrival-time insensitive and arrival-time sensitive CT perfusion algorithms (SS 711), B-0710

**Perazzini C., Fonio P., Doriguzzi Breatta A., Righi D., Rossato D., Gandini G.**
Post-TIPS encephalopathy: are there any predictive factors? (SS 109), B-0004

**Pereira M.J.V., Gregório B., Cavaco A., Figueiredo J.P.**
Glioblastoma: 3D-CRT or IMRT/VMAT? (SS 714a), B-0650

**Perera M.G.R.S., Isoda H., Mizuno T., Terada M., Naito T., Tanoi C., Izumi T., Sakahara H., Naganawa S.**
Evaluation of haemodynamic and morphological biomarkers to assess the rupture risk of intracranial aneurysms using magnetic resonance fluid dynamics and computational fluid dynamics (SS 211b), B-0185

**Perez M.d.R.**
Radiology in Africa: facing challenges and opportunities: The WHO's programme in Africa: the past, the present and the future (EM 1), A-0314

**Perez M.d.R.**
Improving radiation protection in medical imaging in low- and middle-income countries: past actions and future directions: The World Health Organization's (WHO) approach (EU 3), A-0662

**Perez Riverola V., Bosch Barragan F., Prenafeta Moreno M., Cabero Moyano J., Capel Flores I., Guirao Garriga F., Barcons Vilaplana S., Rovira Gols A.**
Efficiency of biopsy criteria in thyroid nodules: the 2017 European Thyroid Imaging Reporting and Data System vs the 2014 American Thyroid Association management guidelines (SS 208), B-0121

**Perez Rodrigo S.**
Breast imaging: A. Diagnosis and management of common ductal and nipple-areolar-complex (NAC) abnormalities ($E^3$ 1521), A-0855

**Perisinakis K., Tzedakis A., Ntoufas N., Velivasaki M., Stratakis J., Damilakis J.**
The effect of scout view length on patient dose from thoracic CT examinations performed with tube current modulation (SS 1813), B-1593

**Perkuhn M., Stavrinou P., Shahzad R., Thiele F., Shakirin G., Garmpis D., Kabbasch C., Borggrefe J.**
A deep learning method for analysing post-operative MRI scans of glioblastoma patients (SS 705), B-0756

**Perkuhn M., Stavrinou P., Shahzad R., Thiele F., Shakirin G., Garmpis D., Schlamann M., Borggrefe J.**
Glioblastoma survival prediction using residual tumour volumes detected and segmented by a deep learning model in post-operative MRI (SS 1005b), B-0988

**Perl R.M., Hetzel J., Bösmüller H., Kloth C., Horger M.**
Retrospective analysis of the role of intraparenchymal blood patching and the rate of pneumothorax after percutaneous CT-guided core lung biopsy (SS 1909), B-1814

**Perucho J.A.U., Lee E.Y.P., Du R., Vardhanabhuti V., Chiu K.W.H., Wong E.M.F.**
Texture analysis of multiparametric MRI and association with tumour grading in cervical cancer (SS 216), B-0178

**Pesapane F., Grimaldi M., Rotili A., Lepanto D., Cassano E.**
Role of ultrasound in the SOUND trial: can we move away from sentinel lymph node biopsy? (SS 702b), B-0722

**Pesapane F., Cassano E., Rotili A., Penco S., Trimboli R.M., Sardanelli F.**
Screening-like single- and double-reading of DWI for breast cancer detection (SS 1802b), B-1561

**Petrini M., Secchi F., Monti C.B., Alì M., Cannao P.M., Michieletti E., Sardanelli F.**
Pulmonary insufficiency: extending the advantage of pulmonary regurgitation volume versus pulmonary regurgitation fraction to a congenital heart disease mixed population (SS 1903b), B-1867

**Petrovic D.J.**
Carotid intima-media thickness (CIMT) as a diagnostic marker of transient ischaemic attacks (TIA) (SS 215), B-0041

**Petta F., Rella R., Scrofani A.R., Bufi E., Rosignuolo M., Romani M., Belli P., Manfredi R.**
Association between background parenchymal enhancement and tumour response in breast cancer patients receiving neoadjuvant chemotherapy (SS 602), B-0547

**Pettersson M., Metsälä E.**
Radiographers' competencies for pain management in mammography
(SS 1914b), B-1716

**Pettersson O.J., Fröss-Baron K., Crona J., Sundin A.**
Towards a new model for therapy monitoring of PRRT $^{177}$Lu-DOTATATE treatment of liver metastases in PNET patients (SS 616a), B-0523

**Peynircioglu B.**
CT and intervention in vascular pulmonary diseases: C. Rendu-Osler and Behcet's diseases (E³ 822), A-0472

**Pfahler V., Gruber G.M., Kasprian G., Hilgendorff A., Förster K., Flemmer A., Ertl-Wagner B.B., Prayer D., Stöcklein S.**
Comparison of intracranial volumes of preterm-born infants and age-matched foetuses
(SS 1011a), B-0927

**Pfannenberg C.**
Clinical and methodological pitfalls in hybrid imaging: problems and solutions: Clinical pitfalls in FDG-PET/CT and PET/MR
(ESHI(MT) 1), A-0186

**Pfeil J., Lee C., Haas M., Taupitz M., Hamm B., Asbach P.**
T2-mapping with simulated T2-echo - a diagnostic alternative for 3-Tesla prostate MRI?
(SS 407), B-0441

**Pfirrmann C.W.A.**
Wrist and foot/ankle sports injuries: A. Soft tissue wrist injury in the athlete
(TC 1428), A-0839

**Pfirrmann C.W.A.**
Wrist and foot/ankle sports injuries: C. Interactive case discussion (Part 1)
(TC 1428), A-0841

**Pfirrmann C.W.A.**
State-of-the-art imaging of postoperative joints: A. Postoperative shoulder (E³ 1626a), A-0945

**Phan C.M.**
Everything you always wanted to know about metabolic bone disease (but were afraid to ask): The bone naked: texture analysis
(SF 15a), A-0884

**Phi Van V., Grgic I., Boss A., Pruschy M., Wurnig M.**
Collagen-bound and water-bound water longitudinal relaxation time by ZTE imaging as biomarker for radiation-induced bone changes in mouse model (SS 210b), B-0065

**Photopoulos G., Stowe J.G., Buissink C., O'Halloran C., Quinn M., Tschan F., Verwoolde R., Dyhre Lia A.**
Impact of a CT scan simulator on student learning (SS 1914a), B-1699

**Pialat J.**
Musculoskeletal: C. Degenerative and inflammatory disorders of the musculoskeletal system (E³ 1723), A-1036

**Pianykh O., Parke D.**
Machine learning in radiology management: predicting ED workflow overloads
(SS 1405), B-1181

**Picascia R., Berritto D., Liguori C., La Porta M., Reginelli A., Grassi R.**
Comparison of ultra-high frequency US with constrast enhanced MR Angiography and conventional US in the evaluation of carotid atheromatous plaques (SS 215), B-0034

**Picasso R., Zaottini F., Pistoia F., Miguel Perez M., Martinoli C.**
High-resolution ultrasound of the superficial sensitive nerves of the arm and the forearm: anatomy, scanning technique and clinical relevance (SS 1910b), B-1748

**Piccoli G.**
Comparison of computed tomography (CT) and contrast-enhanced ultrasound (CEUS) for the management of complex renal cysts: a single-centre experience (SS 607), B-0626

**Piccolo C.L., Cozzi D., Trinci M., Galluzzo M., De Cicco M., Miele V., Ianniello S.**
Contrast-enhanced US (CEUS) of lung abscess: how it can help (SS 717), B-0732

**Piekarek A., Podgórski P., Czarnecki M., Rydel W., Turno-Krecicka A., Misiuk-Hojło M., Zimny A.**
The assessment of diffusion tensor imaging parameters of visual pathways in patients with primary open-angle glaucoma: preliminary study
(SS 1411b), B-1265

**Pietsch H.**
Basics for high efficacy in MRI (SY 14)

**Pijnappel R.M.**
Screening for breast cancer: A. Screening with mammography and digital breast tomosynthesis alone (RC 902), A-0527

**Pijnappel R.M.**
Breast cancer screening and treatment: how much is too much?: What can the radiologist do to limit overdiagnosis? (SA 16), A-0943

**Pina Insausti L.J.**
Clinical utility of contrast enhanced dual energy mammography (SY 2b)

**Pinker-Domenig K.**
The role of imaging in the era of liquid biopsy: Combining molecular and imaging metrics in cancer (radiogenomics) (NH 1), A-0038

**Pinto dos Santos D.**
Secrets of radiology communications with patients and referring physicians: B. Marketing channels for radiology promotion
(E³ 1218), A-0709

**Pinto Ramos J.N., Silva J., Calvão-Pires P., Gil I., Marto J.P., Calado S., Branco C., Baptista J.T.C., Branco G.**
Impact of haemoglobin levels and anaemic state in the prognosis of patients at three months after mechanical thrombectomy for acute ischaemic stroke (SS 309), B-0283

**Piotrowska-Kownacka D.**
Cardiac imaging in arrhythmia and sudden cardiac death: A. The role of CMR in sudden cardiac death (E³ 526), A-0224

**Pisano A., Tipaldi M., Orgera G., Laurino F., Laghi A., Rossi M.**
Role of c-arm computed tomography-assisted adrenal vein sampling in the assessment of adrenal hypersecretion syndromes
(SS 109), B-0007

**Pittaro A., Duijts L., Wielopolski P., Vernooij M.W., Van der Lugt A., Jaddoe V.W., Kemner-van de Corput M.M.P.C., Tiddens H.A., Ciet P.**
Image quality and incidental findings of chest MRI in a large paediatric population-based study
(SS 212), B-0171

**Plakhotina N., Vázquez E., Delgado Alvarez I., Sanchez-Montanez Garcia-Carpintero A., Martinez E., Llort A., Burcet Rodríguez G., Camacho J.**
MRI in the preoperative diagnosis of medulloblastoma: correlation with molecular subgrouping (SS 1412), B-1231

**Platon A.**
Why do I miss fractures in emergency?: C. Missed musculoskeletal injuries in whole-body trauma CT (RC 1217), A-0685

**Podgorska J., Pasicz K., Zagórowicz E., Mróz A., Gołębiewski B., Kus P., Jasieniak J., Skrzyński W., Cieszanowski A.**
Perfusion fraction derived from IVIM-DWI may facilitate differentiation of moderate to severe inflammation in ulcerative colitis
(MY 17), B-1477

**Pogrebnyakov I., Trofimov I., Kukushkin A.V., Virshke E., Dolgushin B.**
The anatomic options of vessels and haemodynamic redistribution of the blood flow at selective intra-arterial chemotherapy (SIAC) in children with an intraocular retinoblastoma
(MY 8), B-0844

**Pogrebnyakov I., Dolgushin B., Trofimov I., Kukushkin A.V., Virshke E.**
The role of selective intraaterial and intravitreal chemotherapy in organ-preserving treatment of the children with an intraocular retinoblastoma
(MY 8), B-0845

**Polacin M., Gastl M., Kapos I., Gotschy A., von Spiczak J., Alkadhi H., Manka R.**
Comparison of standard 3D LGE imaging with novel short inversion time 3D LGE Imaging in patients after myocardial infarction (SS 1803), B-1616

**Polkowski C., Yel I., Langenbach M.C., Kaltenbach B., Vogl T.J., Eichler K.**
Extremity cone-beam CT of forearm and hand: evaluation of image quality, metal artefacts and radiation dose (SS 310), B-0243

**Pontana F.**
The heart between the lungs: C. Fat and calcium in the heart (E³ 1222), A-0718

**Pontone G.**
Cardiac imaging in arrhythmia and sudden cardiac death: C. Imaging of patients with arrhythmia and implantable devices (E³ 526), A-0226

**Pontone G.**
CT-FFR (fractional flow reserve) for assessment of coronary artery disease: Keynote lecture (SS 703), K-19

**Pop M.**
Comparison of Iodixanol 320, Iomeprol 350 and Iopromide 370 effectiveness in CT cardiothoracic angiography in infants (SS 212), B-0165

**Popiela T.J.**
How to organise nationwide ischaemic stroke care (and make multidisciplinary teams work): Do you need help? Train the cardiologist, neurosurgeon, interventional radiologist (PC 16), A-0981

**Popovic P.**
Basic interventional radiology for non-interventionalists: let's start with biopsy!: How to decide indications and pre-treatment evaluation (SF 5b), A-0245

**Portelli J., McNulty J., Bezzina P., Rainford L.A.**
Perceptions about the practice of seeking informed consent in paediatric imaging (SS 214), B-0027

**Portelli J.**
Radiation protection: all you need to know: The radiographers' role in benefit-risk communication (BR 16), A-0964

**Pozeg P., Meuli R., Forget J., Maeder P.**
Pediatric brain: T1-weighted signal intensity of deep brain nuclei increases with age but not with a serial administration of gadoterate meglumine (SS 1011a), B-0931

**Pozzi Mucelli R.**
Pancreatic adenocarcinoma mimickers: Autoimmune pancreatitis (E³ 24E), A-1087

**Pozzi-Mucelli R.**
How to face the tsunami of pancreatic intraductal papillary mucinous neoplasia (IPMN): Follow-up of IPMN: how many MRs do we need? (SA 5), A-0239

**Pradella M., Weikert T.J., Rapaka S., Kärgel R., Sommer G., Sauter A.W., Bremerich J., Stieltjes B., Brantner P.**
Evaluation of a novel fully automatic, artificial intelligence-based diameter measurement algorithm for the thoracic aorta (SS 305), B-0310

**Prakash A.**
Machine learning can combine B mode ultrasound and strain elastography features to better characterise BIRADS 3 and 4 lesions (SS 1805a), B-1522

**Pranjkovic T., Peric D., Milotic V., Kos V.**
Low diagnostic accuracy of abdominal ultrasound in acute kidney injury of intrinsic and prerenal aetiology (SS 317), B-0276

**Prassopoulos P.K.**
Radiologic anatomy: abdomen: Peritoneum and mesentery (BE 4), A-0164

**Prayer F., Röhrich S., Hofmanninger J., Willenpart A., Langs G., Prosch H.**
Evaluation of intra-scanner test-retest variability of machine learning features in low-dose computed tomography of interstitial lung disease (MY 7), B-0811

**Precht H.**
Forensic imaging: B. Post-mortem cardiac angiography (RC 814), A-0400

**Precht H.**
Growing radiography research: Radiography research: where should we be in 2030? (part 3) (EFRS WS), A-0862

**Prokop M.**
Deep learning reconstruction: the next step in CT image quality (SY 4)

**Prokop M.**
Screening with imaging: a new era?: Screening of lung cancer with low dose CT (SF 1), A-0028

**Prokop M.**
Writing reports: a survival guide: A. Lung nodule: managing uncertainty (E³ 1321), A-0732

**Prokop M.**
The tumour board of the future: Chairperson's introduction (NH 16), A-0955

**Prosch H.**
Pulmonary neuroendocrine proliferations and neoplasms: B. Carcinoid tumours (RC 504), A-0269

**Prosch H.**
Post-treatment emergencies in oncologic patients: A. Chest (E³ 926), A-0516

**Prosch H.**
Lung cancer in the era of molecular oncology and immune therapy: Chairperson's introduction (E³ 1022), A-0598

**Pruvo J.-P.**
Brain imaging in psychiatry: Chairperson's introduction (MS 8b), A-0402

**Pruvo J.-P.**
Brain imaging in psychiatry: Strengths of structural and functional MRI in psychiatry (MS 8b), A-0404

**Pruvo J.-P.**
Brain imaging in psychiatry: Multidisciplinary case presentation and discussion (MS 8b), A-0407

**Pruvo J.-P.**
How to organise nationwide ischaemic stroke care (and make multidisciplinary teams work): Do you like to travel? Move the patient or the doctor? (PC 16), A-0979

**Puglielli E., Manente G., Assetta M., Pulsone P., Di Mizio V.**
Evaluation of pial collaterals on multiphase CT angiography: impact on the prediction of infarct size and outcome in acute ischaemic stroke (SS 711), B-0708

**Puglielli E., Di Bartolomeo M., Assetta M., Navarra F., Di Matteo M., Galliano M., Manente G.**
Value of spot sign (SS) on CT angiography (CTA) such indicator of early haematoma expansion in the spontaneous intracerebral haemorrhage (ICH)- some therapeutic considerations (SS 1017), B-0951

**Pumberger M., Fuchs M., Engelhard N., Hermann K.A., Putzier M., Makowski M.R., Hamm B., Diekhoff T.**
Dual-energy computed tomography detects disk injuries in patients with vertebral fractures: a prospective diagnostic accuracy study (SS 1910a), B-1738

**Punwani S.**
Early detection of prostate cancer: B. Pre-biopsy detection and new techniques for detection in prostate cancer (E³ 219), A-0094

**Purcell Y.**
Artificial intelligence: presented with new lecture formats: My experience: one-year fellowship (ESOR), A-0372

**Pusceddu L., Giannetto G., Romano V., Vani V., Cortese F., Desi G., Mazzetti S., Regge D., Russo F.**
Pitfalls in reporting fast biparametric magnetic resonance imaging of the prostate: what kind of cancers do we miss? A preliminary retrospective analysis (MY 13), B-1093

**Puylaert J.B.C.M.**
Imaging of 'foreign bodies': B. Did I swallow that? US and CT of sharp foreign bodies penetrating stomach and bowel (RC 417), A-0167

**Puylaert J.B.C.M.**
US before CT in the acute abdomen?: A. Acute abdomen: US first! (PS 1427), A-0836

**Pyra K.K.**
Post-treatment emergencies in oncologic patients: C. How can interventional radiologists help in the management of oncological treatment complications? (E³ 926), A-0518

### Q

**Qi W., Shanshan R., Miao Z., Ye S., Lei C., Nan H.**
A feasible method to set coherent image quality between different CT scanners with a general phantom for low dose lung cancer CT screening (SS 1004), B-1006

**Qi Y., Wang Z.**
The most accurate measurement of TT-TG distance is through the 2/5 point of femoral trochlear notch (MY 9), B-0863

**Qin S., Li X., Qi Z., Mei Y., Quan X.**
Assessment of tumour hypoxia response to sorafenib in rabbit $VX_2$ liver tumour xenografts by tissue-oxygen-level-dependent MR imaging (SS 606), B-0504

**Qiu B., Guo J., Kraeima J., Borra R., Witjes M.J.H., van Ooijen P.M.A.**
Automatic segmentation of the mandible from CT for 3D virtual surgical planning using a convolutional neural network (SS 605), B-0574

**Qiu H.S., Chu J.P., Zhao J., Hu M.S., Wu C.**
Revealing the microstructure abnormalities of spinocerebellar ataxia by fMRI and DKI: from ROI based analysis to a VBA and TBSS study (SS 1411b), B-1273

**Qu J., Ma L., Lu Y.**
Volumetric DCE-MRI radiomics nomogram can predict tumour response in patients with oesophageal cancer treated by neoadjuvant chemotherapy (SS 616b), B-0610

**Quarantelli M., Lanzillo R., Comerci M., Cassiano M., Costabile T., Prinster A., Megna R., Brescia Morra V., Brunetti A.**
Voxel-based analysis of relaxation rates in multiple sclerosis: correlates of cognitive impairment and physical disability (SS 611), B-0543

**Quick H.H.**
The daily challenge: how to fight artefacts (SY 22)

### R

**Rabelo B.C.S., Tinoco Alvim de Souza F., Ramos Botelho Antunes P., Carvalho de Siqueira E., Alvares de Campos M., Vianna Cancado L., Vianna Cançado A., Barbosa Alvares M.**
Thyroid nodules: diagnostic performance of ATA and TIRADS on risk stratification for malignancy (SS 208), B-0129

**Raciti M.V., Franconeri A., Buizza G., Fontana G., Viselner G., Iannalfi A., D'Ippolito E., Farina L.M., Preda L.**
IVIM-DWI MRI in meningiomas: a tool to detect early microstructural changes during and after proton-therapy (MY 3), B-0372

**Radbruch A.**
The diagnostic power of gadolinium contrast agents (SY 30)

**Radovic N., Ivanac G., Biondic Spoljar I., Divjak E., Dumic Cule I., Brkljacic B.**
Effect of breast-conserving surgery with radiotherapy on the apparent diffusion coefficient of fibroglandular tissue at MRI (MY 5), B-0463

**Radzina M., Lioznovs A., Jukna A.**
Carotid plaque instability and neovascularisation assessment using contrast-enhanced ultrasound and superb micro-vascular imaging (SS 215), B-0037

**Radzina M.**
Collateral blood supply role in acute stroke outcomes (SS 311), B-0264

**Rafailidis V., Chryssogonidis I., Grisan E., Xerras C., Cheimariotis G., Tegos T., Rafailidis D., Sidhu P., Charitanti-Kouridou A.**
Imaging carotid artery-vulnerable plaque with ultrasound and contrast-enhanced ultrasound: correlation of cerebrovascular symptoms with quantitative and multi-parametric indexes (MY 18), B-1685

**Ragab Y., Emad Y., Anbar A., Khalil M., Rasker J.**
Cam vs pincer femoroacetabular impingement: which type is associated with more hip structural damage? An exploratory cross-sectional study using MRI (SS 1010), B-0899

**Raimondi E., Picchia S., Kouvelakis K., Khan K., Cunningham D., Koh D.-M., Bali M.**
Early prediction of response of CT textural analysis in unresectable liver metastases of colorectal cancer treated with Cetuximab chemotherapy (SS 616a), B-0524

**Rainford L.A.**
Career planning: The importance of professional registration and CPD (BR 9), A-0533

**Rainford L.A.**
Growing radiography research: Radiography research: where should we be in 2030? (part 1) (EFRS WS), A-0860

**Raissaki M.**
Imaging in abdominal emergencies: an (evidence-based) update: C. Polytrauma: differences between adult and paediatric protocols (RC 412), A-0190

**Raissaki M.**
Paediatric radiology for the general radiologist: A. Pitfalls in paediatric chest and abdomen (E³ 1621), A-0923

**Rajabzadeh F., Jangjoo A., Jamialahmadi T., Goshayeshi L., Nemati M., Ghafarzadegan K.**
Comparison of liver stiffness evaluated by shear wave elastography in morbidly obese patients prior to bariatric surgery with liver biopsy (SS 1501b), B-1380

**Rajamohan N., Sharma R., Devasenathipathy K., Goyal A., Bhalla A.S., Parshad R., Jain D.**
Imaging of thymic lesions in patients with myasthenia gravis: is dual energy CT superior to MRI? (SS 1404), B-1207

**Rajan S., Mahajan H., Barnwal M., Mahajan V., Venugopal V.**
Time to replace T2-STIR with diffusion-weighted imaging for visualisation of nerve disorders? (SS 1910b), B-1750

**Rajan S., Rajan R., Murugavel M., Barnwal M., Gupta S., Venugopal V., Mahajan V.**
Why guidelines are important: inter observer variability in assessing MRI signs of endometriosis between reader following ESUR guidelines vs. reader using prior domain knowledge (SS 1907), B-1892

**Rajapakse C.**
Predicting fracture risk in children: C. Bone quantification with MRI (RC 812), A-0463

**Rajasekaran M., Rathinasamy R., Viyannan M., Bala Lakshmoji D.**
Spleen ARFI: a noninvasive tool for detection of oesophageal varices in cirrhotics (SS 1501b), B-1376

**Ramaesh R., Browning G., Wakelin S., Ward E., Gibbs F., Patel D.**
The utility of F18-choline PET/CT for the localisation of parathyroid adenomata in patients with primary hyperparathyroidism (PHP) with negative or equivocal conventional imaging (SS 608), B-0639

**Ramanauskas N., Dementaviciene J., Barušauskas D., Bialopetravicius J., Armaitis J., Rimeika G.**
Deep learning based chest x-ray whole-image search and retrieval (SS 1405), B-1190

**Ramanauskas N., Dementaviciene J., Armaitis J., Barušauskas D., Bialopetravicius J., Rimeika G., Stankeviciene J.**
Towards a deep learning model for exhaustive chest x-ray pathology classification (SS 1905a), B-1757

**Ramlaul A.**
Informed consent in diagnostic imaging: an evaluation of current practice (SS 214), B-0026

**Ramos A.**
My three top tips in neuroimaging (not only for neuroradiologists): Hydrocephalus (SF 4), A-0160

**Rampal P., Sachdev N., Taneja A.**
MRI evaluation of ankle joint in juvenile idiopathic arthritis (SS 610), B-0513

**Rana A.I.**
The role of radiology in major healthcare challenges faced by Pakistan: Role of radiology in developing a living donor liver transplant programme (EM 3), A-1080

**Rana P., Sodhi K.S., Bhatia A., Saxena A.K., Suri D., Singh S.**
Diagnostic utility of 3 Tesla thorax magnetic resonance imaging in HIV-positive children (SS 212), B-0169

**Rance B.**
Imaging biomarkers and their combinations in the era of artificial intelligence: Building and discovering biomarkers with AI (ESR/EIBALL), A-0058

**Rangan K.B.K., Gambhir S.**
Role of 18 F FDG PET-CT in documenting the disease burden in brain, spinal and lymph node tuberculosis (SS 306), B-0233

**Ranschaert E.R.**
Mobile devices in radiology: A. Security and confidentiality aspects of mobile computing (RC 105), A-0011

**Ranschaert E.R.**
IT-security and GDPR: Security aspects when using mobile devices and/or social media (SF 12), A-0698

**Rasouly N., Kastler B., Hélénon O., Habib Geryes B., Comte A.**
In vitro and in vivo quantification of urinary stone volume using attenuation threshold-based optimized ultra-low-dose CT method (SS 1807), B-1664

**Ratib O.**
Mobile devices in radiology: Chairperson's introduction (RC 105), A-0010

**Ratib O.**
Artificial intelligence in hybrid imaging: Sharing is caring: on the need for open research data (ESHI(MT) 2), A-0554

**Ratib O.**
IT-security and GDPR: Issues related to patient consent to allow access to the data (SF 12), A-0697

**Ratib O.**
Hybrid and translational imaging: the bigger picture: Chairpersons' introduction (part 1) (ESR/ESHI(MT)), A-0972

**Raudner M.W., Wielandner A., Uyanik-Ünal K., Stelzmüller M., Zuckermann A., Loewe C., Beitzke D.**
Quantitative cardiac magnetic resonance in the detection of early cardiac transplant rejection - MRI compared with endomyocardial biopsy (SS 303), B-0374

**Ravaglia V., Venturi G., Scrittori N., Farnedi S.**
A method to optimize DSA clinical protocols (SS 1413), B-1198

**Razansky D.**
Translating functional and molecular imaging in oncology: C. Optical imaging in cancer (RC 1206), A-0706

**Reekers J.A.**
Visceral arteries: Chairperson's introduction (RC 115), A-0020

**Reekers J.A.**
Cone-beam, 4D and more: new diagnostic tools for vascular diseases: A. The role of intra-procedural perfusion assessment in peripheral arterial disease ($E^3$ 1226), A-0664

**Regge D.**
The role of imaging in the era of liquid biopsy: Chairperson's introduction (NH 1), A-0034

**Regge D.**
The tumour board of the future: Preparation for tumour boards: how to increase efficiency of the radiologist? (NH 16), A-0957

**Regine R., Palmieri F., Zanfardino F., Fatigati G., Martino A., Cornacchia M., Cozzolino P.**
Transperineal thermoablation in patients with benign prostatic hyperplasia: our experience (MY 13), B-1080

**Rehani M.M.**
Improving radiation protection in medical imaging in low- and middle-income countries: past actions and future directions: Chairpersons' introduction (part 2) (EU 3), A-0658

**Rehani M.M.**
Improving radiation protection in medical imaging in low- and middle-income countries: past actions and future directions: Patient doses in large part of the LMI countries and way forward: The International Organization for Medical Physics' (IOMP) vision (EU 3), A-0660

**Rehnitz C., Wuennemann F.**
Ability of T2 mapping at 3T MRI for biochemical assessment of normal and damaged glenoid cartilage of the shoulder: an arthroscopy controlled prospective study (SS 310), B-0242

**Rehwald R., Moriconi S., Smith L., Sudre C.H., Cardoso M.J., Gillard J.H., Jäger H.R.**
Prevalence of incidental intracranial vascular findings in a tri-ethnic population-based study (SS 1811b), B-1688

**Reichardt B., Örnek A., Nicolas V., Maier C.**
MRI-CT fusion imaging before high-selective lumbar sympathicolysis (MY 8), B-0839

**Reichardt B., Örnek A., Nicolas V., Maier C.**
Lumbar sympathetic trunk in MRI and CT: its visibility and distance to two anatomical landmarks for pre-interventional planning (MY 8), B-0840

**Reichardt B., Örnek A., Nicolas V.**
Cinematic MRI of the wrist: initial study for instability of the wrist in correlation to fluoroscopy and x-ray findings (MY 9), B-0866

**Reidler P., Puhr-Westerheide D., Fabritius M.P., Rotkopf L., Apel D., Tiedt S., Wollenweber F.A., Thierfelder K., Kunz W.G.**
Clinical decision support based on automated non-contrast CT density measurements in patients with acute ischaemic stroke (SS 711), B-0713

**Reijnierse M.**
Musculoskeletal interventions: Keynote lecture (SS 1510), K-31

**Reimer R.P., Reimer P., Mahnken A.H.**
MRI-based texture analysis has the potential to predict response to transarterial radioembolization at baseline (MY 3), B-0354

**Reimer R.P., Lichtenstein T.E., Flatten D., Maintz D., Borggrefe J., Grosse Hokamp N.**
Virtual monoenergetic images from spectral detector CT (SDCT) enable radiation dose reduction in unenhanced cranial CT (SS 1911a), B-1783

**Reiter C., Kräuter C., Kolesnik E., Reiter G., Greiser A., Schmidt A., Scherr D., Fuchsjäger M.H., Reiter U.**
4D- vs 2D-flow imaging in the evaluation of transmitral velocity profiles: comparison with echocardiography (SS 1803), B-1620

**Reiter G., Kovacs G., Reiter C., Kräuter C., Greiser A., Olschewski H., Fuchsjäger M.H., Reiter U.**
4D-flow-based monitoring of pulmonary pressure in patients with pulmonary hypertension: a feasibility study (SS 1013), B-0989

**Reiter U., Kovacs G., Reiter C., Kräuter C., Greiser A., Olschewski H., Fuchsjäger M.H., Reiter G.**
Magnetic resonance 4D-flow derived mean pulmonary arterial pressure at 3T: a validation study (SS 713), B-0767

**Rella R., Scrofani A.R., Petta F., Bufi E., Conti M., Giuliani M., Belli P., Manfredi R.**
Association between background parenchymal enhancement in breast MR imaging and outcome of breast cancer patients treated with neoadjuvant chemotherapy (SS 602), B-0548

**Rémy-Jardin M.**
The first step to accurate diagnosis: the evolving role of DECT (SY 12)

**Rémy-Jardin M.**
Chest emergencies: Keynote lecture (SS 717), K-17

**Rémy-Jardin M.**
Functional imaging of the lungs: A. Perfusion methods for vascular and airway diseases (CT/MRI) (E³ 520), A-0289

**Ren B., Li L., Wang S., Xia C.**
Semi-supervised multi-to-one style transfer to improve the quality of CT images (SS 205), B-0018

**Resnik A., Žibert J., Mekis N.**
Pelvis imaging: achieveing dose reduction with different patient position (SS 1314), B-1070

**Revel M.-P.**
Oncologic imaging: B. Incidental findings in oncologic patients (E³ 621), A-0307

**Revel M.-P.**
Challenging HRCT patterns: tips and tricks from the experts: The mosaic attenuation pattern (SF 8b), A-0452

**Revel M.-P.**
Thoracic emergencies: B. Haemoptysis (RC 1704), A-1054

**Rezgui Marhoul L.**
Radiology in Africa: facing challenges and opportunities: Most important challenges for imaging in North Africa (EM 1), A-0313

**Riaz S.**
Ultrasound: continuing to make waves: B. Early pregnancy guidelines (RC 1714), A-1006

**Ribeiro L.**
Radiography research: a "how to" guide: How to write a good scientific abstract (BR 13), A-0780

**Ribeiro L., Abrantes A.F., Azevedo K.B., Lesyuk O., Rodrigues S.I., Almeida R.P., Viriato A.C., Reis M.V.**
Patient perceptions of radiographer communication skills in CT examinations (SS 614), B-0489

**Ribeiro L., Abrantes A.F., Rodrigues S., Arrais J.P., Almeida R.P., Azevedo K.B., Lesyuk O.**
Expectations of radiography students regarding the new undergraduate course in medical imaging and radiotherapy (SS 714a), B-0652

**Ribeiro M.M.**
Contemporary breast imaging: B. Breast imaging in young females: the role of MRI (RC 114), A-0008

**Ricardi U.**
Current status and future challenges in MR-integrated radiotherapy: Chairpersons' introduction (part 2) (ESR/ESTRO), A-0497

**Riccabona M.**
Intensive care paediatric radiology: the very sick neonate: C. The role of imaging in emergencies in the critically ill neonate: abdominal US applications and beyond (RC 512), A-0279

**Ricci P.**
Imaging of benign liver lesions: still difficult?: Chairperson's introduction (RC 101), A-0001

**Ricci P.**
Imaging professionals in the EU: radiologists without borders: Patient safety and job security for life: CME/CPD in Europe (ESR/UEMS), A-0221

**Richenberg J.**
Prostate MRI: the accreditation issue: Chairperson's introduction (E³ 1726b), A-1071

**Ricoeur A., Caudrelier J., Buy X., Garnon J., Koch G., Cazzato R., Gangi A.**
Image-guided percutaneous techniques in the treatment of aggressive vertebral haemangiomas: clinical outcomes and therapeutic strategy (MY 8), B-0834

**Riffel P., Jawhar M., Gawlik K., Michaely H.J., Hofmann W., Schönberg S.O., Reiter A.**
Distinct bone marrow pattern in MR imaging of advanced systemic mastocytosis is associated with poor prognosis (SS 316), B-0352

**Riga M.G., Orsatti G., Varotto A., Crimì F., Weber M., Stramare R., Zucchetta P., Quaia E., Giraudo C.**
Should we still look for a correlation between ADC and SUV in lymphoma: a volumetric [18F] FDG-PET/MR study of paediatric Hodgkin's lymphoma (MY 15), B-1364

**Riklund K.**
Imaging professionals in the EU: radiologists without borders: To be or not to be at the table: why advocacy matters for radiologists (ESR/UEMS), A-0215

**Riklund K.**
Radiology and pathology: will we ever be (digitally) married?: What difference would there be for patients? (Part 2) (SF 13a), A-0739

**Rimola J.**
Gastrointestinal radiology: A. Inflammatory bowel disease (E³ 821), A-0390

**Ringl H.**
Dual-energy CT of the abdomen: the time is now: B. Applications for genitourinary system (E³ 1526), A-0858

**Risi V., Zanardo M., Trimboli R.M., Monti C.B., Di Leo G., Carbonaro L.A., Sardanelli F.**
Improving technical quality in mammography (MY 15), B-1352

**Rivière L., Rossi L., Lam Giang T., Laas E., Bataillon G., Cherel P., Brisse H., Reyal F., Malhaire C.**
Can MRI including DCE and DWI improve characterisation of endometrial carcinosarcoma? (MY 13), B-1090

**Rizzo S.**
Novelties in oncologic imaging: B. Radiomics: the role of imaging (E³ 426b), A-0192

**Robinson C., Deshpande A., Rutty G., Morgan B.**
Post-mortem computed tomography angiography (PMCTA): optimising a targeted PMCTA protocol (SS 614), B-0483

**Robinson E., Mak M., Jackson D., Kent B., Santis G., Nair A.**
HRCT does not offer added discriminatory value for eosinophilic lung diseases in patients presenting to a severe asthma clinic (SS 1604), B-1410

**Robinson L.**
Communicating the role of the radiologist: best practices to manage patient expectations: Best practices: tools to optimise communication with patients (PA), A-0827

**Robinson S.**
Hybrid imaging: the best of both worlds: B. Clinical impact of hybrid interventional imaging (RC 314), A-0111

**Rocher L.**
Imaging and management of the incidental scrotal lesions: Incidental testicular lesions: the role of MRI and ESUR recommendations (SF 5d), A-0295

**Rocher L.**
Late effects in survivors of childhood cancer: Fertility issues: male and female (Part 1) (SF 17), A-1025

**Rockall A.G., Lee P., Lu H., Arshad M.A., Avesani G., Curry E., Thornton A., Kanavati F., Fotopoulou C., Aboagye E.O.**
A mathematical-descriptor of tumour mesoscopic structure from CT images annotates prognostic and molecular phenotypes of epithelial ovarian cancer (SS 1507), B-1387

**Rockall A.G.**
Early detection of ovarian cancer: A. Current guidance on screening and familial ovarian cancer (part 1) (E³ 119), A-0070

**Rockall A.G.**
Functional imaging of breast and female pelvis: C. Ovarian tumours (E³ 820), A-0476

**Roditi G.**
Non-contrast MR angiography: ready to go?: Peripheral non-contrast MR angiography (SF 8d), A-0422

**Rodriguez Ruiz A., Kallenberg M., Gubern-Merida A., Karssemeijer N., Mann R.M.**
Artificial intelligence detecting breast cancer on mammography: does breast density play a role? (SS 702a), B-0697

**Rodriguez Ruiz A., Fedon C., Michielsen K., Sechopoulos I.**
Scatter radiation correction in digital breast tomosynthesis with a deep learning convolutional neural network (SS 1805a), B-1527

**Rogalla P., Hoppel B.E., Masakazu M., Zhou J., Farrell C., Kandel S.**
Image quality comparison between iterative reconstruction methods and AI-based reconstruction for CT chest (SS 305), B-0306

**Rogalla P., Hoppel B.E., Noro K., Yamazaki Y., Utsunomiya K., Shimomura Y., Sugiyama S.**
Automated CT abdominal imaging protocol selection using natural language processing and machine learning (SS 1905b), B-1832

**Rohan T., Andrasina T., Litavcova A., Juza T., Zavadil J., Richter S., Valek V.**
Is PET MRI valuable in inflammation or fever of unknown aetiology? (SS 306), B-0232

**Röhrich S., Hofmanninger J., Pan J., Prayer F., Prosch H., Langs G.**
Scanner parameter induced variability of radiomics features in routine chest CT data (SS 1005a), B-0905

**Roman M.S., Awadallah M.Y., Ahmed A.F., Fawzi F.S., Shawali H.A.S., Elsayed R.F.**
Introducing 3D modelling of MRI in preoperative mapping of perianal fistula: how it could help the surgeons? (SS 201b), B-0212

**Romano S.**
Small bowel obstruction: improve your reading and reporting competences: Chairperson's introduction (RC 1717), A-1037

**Romanowski C.A.J.**
Update on cerebrospinal fluid (CSF) diseases: A. Imaging strategies for hydrocephalus (RC 911), A-0519

**Romanucci G., Caumo F., Zorzi M., Bricolo P., Cugola L., Brunelli S., Mariotto R., Montemezzi S.A., Fornasa F.**
Stratifying tomosynthesis screening results by age groups and by first or subsequent screening (SS 302), B-0248

**Romanucci G., Montemezzi S.A., Brunelli S., Bricolo P., Cugola L., Caumo F.**
Stratifying tomosynthesis screening results by breast density (SS 302), B-0249

**Romei C., Turturici L., Tavanti L., Marletta M., Wielopolski P., Colagrande S., Caramella D., Falaschi F., Ciet P.**
Differentiation of inflammatory andfibrotic ground-glass opacity (GGO) with MRI: a pilot study (SS 1404), B-1205

**Romeo V., Cavaliere C., Imbriaco M., Stanzione A., Cuocolo R., Pignata A., Picariello V., Nicolai E., Salvatore M.**
Clinical impact of breast and whole body simultaneous PET/MRI for staging of breast cancer lesions >2 cm (SS 706), B-0668

**Romeo V., Cuocolo R., Ugga L., Cocozza S., Verde F., Dell'Aversana S., Elefante A., Staibano S., Brunetti A.**
Machine learning analysis in the prediction of tumour grade and nodal involvement in oropharyngeal and oral cavity squamocellular carcinoma using CT-derived texture features (SS 1808), B-1626

**Rong P., Gu Q., Feng Z.**
Radiomics machine learning classifiers for predicting Ki-67 proliferation index in non-small cell lung cancer (MY 4), B-0419

**Ronot M.**
Imaging the acute abdomen: new insights: A. Acute mesenteric ischaemia (RC 1201), A-0648

**Ronot M.**
Liver and biliary MRI: protocols and technical frontiers: Keynote lecture (SS 401), K-11

**Rosella F., Gorgatti T., Vit A., Gavrilovic V., Pellegrin A., Sponza M.**
Efficacy of drug-eluting stents ELUVIA in treatment of high grade of femoropopliteal lesions (SS 1009), B-0967

**Rosendahl K.**
US teaching to undergraduate students: why, how and from whom?: From whom: the importance of radiologists delivering US teaching and staying experts in this field (ESR US SC/UES), A-0339

**Ross J.S.**
Cutting edge imaging and minimally invasive interventions of the spine: Spinal CSF leaks: the precise "roadmap" for clinicians (SF 13b), A-0758

**Rossi A.**
Neuro: A. Congenital and white matter disorders of the brain (E³ 1423), A-0829

**Rossi A.**
Paediatric brain imaging: A. Head and neck emergencies in children (E³ 1921), A-1101

**Rossi D., Alterio D., Volpe S., Pounou F.A.K., Giannitto C., Ansarin M., Bellomi M., Jereczek-Fossa B.A., Preda L.**
Impact of radiologic imaging revision in the multidisciplinary management of head and neck cancer patient (SS 1008), B-0973

**Rossi G.**
Lung cancer team: Role of the pathologist: making the most of the sample (MS 1), A-0052

**Rovira-Cañellas A.**
My three top tips in neuroimaging (not only for neuroradiologists): Acute disseminated encephalomyelitis (ADEM) (SF 4), A-0155

**Rovira-Cañellas A.**
Head and neck imaging: B. Non-traumatic head and neck emergencies (E³ 1721), A-1000

**Royle L., Anjari M., Sasikumar S., SinhaRay R., Mak S., Subesinghe M., Murphy D., Breen R., Nair A.**
Applying British Thoracic Society 2015 compared to Fleischner Society 2017 recommendations for lung nodule management leads to different discharge and PET-CT referral rates (SS 204), B-0142

**Rubbert C., Meineke A., Sawicki L.M., Klosterkemper Y., Antoch G., Boos J.**
Potential of a machine learning model for dose optimisation in chest CT quality assurance (SS 1404), B-1210

**Rübenthaler J.**
How to perform ultrasound image fusion: Kidney (US 1), A-0170

**Rubin D.L., Akdogan M.U., Altindag C., Alkim E.**
ePAD: A platform to enable machine learning and AI application development in medical imaging (SS 1005a), B-0906

**Ruby L., Sanabria S.J., Mutschler T., Martini K., Frauenfelder T., Klingmüller V., Rominger M.**
Musculoskeletal elastography: exploration of confounders in a challenging field (SS 1810), B-1512

**Rud E., Flatabø T., Baco E., Sandbække G.**
CT urography for examining the upper urinary tract in case of haematuria: time to reconsider? (SS 307), B-0332

**Rud E., Ottoson F., Flatabo T., Baco E.**
Whole-body MRI for detecting bone metastases in treatment naïve high-risk prostate cancer: cracking nuts with a sledgehammer? (SS 707), B-0821

**Rud E., Langberg C.W., Baco E., Sandbække G.**
Testicular cancer follow-up: less is more (SS 1807), B-1656

**Ruggeri V., Mariscotti G., Durando M., Costanza C., Bergamasco L., Castellano I., Fonio P.**
MRI assessment of axillary lymph nodes after neoadjuvant chemotherapy based on breast cancer subtype (SS 1902b), B-1799

**Ruggieri C., Veglia S., Ruffino A., Ciccone G., Evangelista A., Davini O.**
Role of the cardiosynchronised computed tomography angiography in the dissection of descending thoracic aorta (SS 1815), B-1499

**Russe M.F., Kocher N., Kellner E., Reisert M., Bamberg F., Kotter E.**
Optimising the dataset for deep learning with x-ray images in musculoskeletal radiology (SS 605), B-0568

**Rutten M.J.C.M.**
3D automated breast ultrasound: accuracy and diagnostic potentials (SY 2b)

**Ryan J.W., MacMahon P.J., Bolster F.**
Mass casualty incidents: are you ready? An evidenced-based radiology MCI protocol template (SS 217), B-0098

**Ryan M.-L.**
Audit across Europe: directive and perspective: Implementing a clinical audit programme: the radiographic perspective (ESR Audit), A-0140

### S

**Sá dos Reis C., Gremion I., Schmidt K., Richli Meystre N.**
What criteria should be considered by radiographers while assessing the image quality of breast implant mammograms? (SS 1914b), B-1709

**Saade C.**
Patient habitus and renal volume have no effect on a reduced patient-specific contrast media administration during renal CTA: impact on contrast media, radiation dose and image quality (SS 614), B-0481

**Saade C., El-Merhi F.**
Patient lung volume is significantly related to contrast media volume during CT pulmonary angiography when employing a patient-specific contrast protocol (SS 717), B-0727

**Saade C., Khalifeh S., Karout L., Naffaa L.**
Multidetector liver CT: improved image quality, decreased radiation and contrast media dose with peristaltic contrast media injection system (SS 701), B-0799

**Saade C., Haydar A.**
A reduced contrast media injection protocol significantly improves the visualisation of liver vasculature at reduced radiation dose during hepatic CTA (SS 701), B-0800

**Saade C.**
An augmented patient-specific approach to administration of contrast agent for CT renal angiography (MY 15), B-1351

**Saade C.**
Split-bolus contrast injection protocol enhances the visualisation of the thoracic vasculature during chest CT (SS 1904), B-1851

**Saake M., Schmidle A., Kopp M., Hanspach J., Hepp T., Nagel A., Doerfler A., Uder M., Baeuerle T.**
MRI T1w brain signal intensity and relaxation times in individuals with prior exposure to gadobutrol (SS 1911a), B-1777

**Saba L.**
MRI and advanced algorithms like computed MRI from research to clinical practice (SY 4)

**Saba L.**
Carotid disease 2.0: A. Carotid plaque imaging: tool or fool? (RC 915), A-0508

**Sabatino V., Russo U., d'Amuri F.V., Bevilacqua A., Pagnini F., Milanese G., Nizzoli R., Pedrazzi G., De Filippo M.**
Pneumothorax and pulmonary haemorrhage after CT-guided lung biopsy: incidence, clinical significance and correlation (MY 8), B-0842

**Sachs C., Giudici F., Bortul M., Zanconati F., Gasparini C., Assante M., Tonutti M., Cova M.A.**
Relevance of arbitration in the management of discrepant screening mammography: Trieste's Breast Unit experience in 2013-2014 biennium (MY 5), B-0464

**Sadate A., Occean B., Larbi A., Fabbro-Peray P., Beregi J.**
Systematic review and meta-analysis of randomized trials on the impact of lung cancer screening by low-dose computed tomography in populations highly exposed to tobacco (MY 4), B-0431

**Sadotti G., Notaro D., Zini C., Bellini M.**
"SpineJack®" percutaneous placement in magerl A2 and A3 traumatic vertebral compression fractures of the thoracolumbar spine (SS 1809), B-1578

**Saez F.**
EDiR: an instrument to develop excellence in your career: CORE examination (Part 1) (EDiR), A-0850

**Saez F.**
Paediatric radiology for the general radiologist: B. Paediatric MSK imaging: normal variants or real injuries? ($E^3$ 1621), A-0924

**Saez F.**
Musculoskeletal: Chairperson's introduction ($E^3$ 1723), A-1033

**Sahin C., Oc Y., Hasanefendioglu Bayrak A.**
Percutaneous CT guided RFA treatment of osteoid osteoma in children and adults: it has high success rate in children too (SS 1809), B-1576

**Said N.H., Adel L., Chalabi N.A., El Sheikh R., Aboelmagd H.**
Automated breast ultrasound versus breast tomosynthesis in further evaluation of recalled dense breasts after screening mammograms (SS 1002), B-0923

**Saidléar C.**
Dose management in paediatric radiology: Chairperson's introduction (RC 1313), A-0785

**Sajid S., Nepal P., Alam S.I., Warfa M., Sajid S.**
Evaluation of MRI findings for association between patella baja and quadriceps fat pad oedema (SS 1410), B-1130

**Sala E.**
Whole-body imaging in gynaecological malignancy: C. Advanced imaging techniques in metastatic gynaecological cancer ($E^3$ 319), A-0132

**Sala E.**
Functional imaging of breast and female pelvis: B. Tumours of the uterus ($E^3$ 820), A-0475

**Sala E.**
Navigating tumour phenotype with advanced imaging analysis: Chairperson's introduction (SF 15d), A-0899

**Sala E.**
Quantitative imaging in oncology: D. Imaging heterogeneity and genomic variability in ovarian cancer (E³ 1726a), A-1061

**Salah F., Tabbara A., Al Arab N., Sibahi A., Asmar K., Tamim H., Makki M., Hourani R.**
Can MR and CT imaging features differentiate benign from malignant meningiomas? (SS 1411a), B-1168

**Salamon J., Well L., Geier K.I., Späth L., Kaul M., Herrmann J., Mautner V., Adam G., Derlin T.**
Characterisation of peripheral nerve sheath tumours in patients with neurofibromatosis type 1 using diffusion-weighted magnetic resonance imaging (SS 1416), B-1240

**Salem A., Grueneisen J., Schaarschmidt B.M., Chodyla M., Martin O., Rischpler C., Forsting M., Herrmann K., Umutlu L.**
Comparison of $^{68}$Ga-DOTATOC PET-MRI and MRI-DWI for whole body staging of patients with neuroendocrine tumours (SS 1716), B-1456

**Salgado R.**
Cardiac imaging: B. Imaging cardiac valves (E³ 921), A-0484

**Salgado R.**
Cardiac imaging in structural heart disease: A. CT-guided planning of minimally invasive procedures (RC 1503), A-0877

**Saltybaeava N., Marcon M., Berger N., Kolditz D., Steiding C., Boss A., Alkadhi H.**
First clinical application of spiral breast CT with photon-counting detector: patient-specific radiation dose assessment (SS 1802a), B-1541

**Samara E.**
Patient-specific dosimetry: Chairperson's introduction (RC 113), A-0046

**Samei E.**
Striking the balance: image quality assessment in radiological optimisation: B. Bridging the gap between physical and clinical image quality (RC 413), A-0183

**Sammut S.J., Cook N., Mahmood N., Balasubramaniam R., Thomas B., James A.**
Magnetic resonance enterography: is routine administration of gadolinium-based IV contrast really needed for the assessment of Crohn's disease? (SS 1801b), B-1647

**Samuelsson C., Kälvesten J., Wilczek M., Brismar T.B.**
Metacarpal bone diameter increases constantly, but escalated resorption of the inner surface at menopause explains decreased bone mineral density - a study in 1492 adult women (SS 210a), B-0049

**Sánchez P.S.**
The value of core-biopsy of nodules less than 10 mm (SS 1909), B-1811

**Sánchez Casanueva R.M.**
Advanced clinical dosimetry in interventional radiology: Real time patient dose monitoring (EU 4), A-0750

**Sánchez-Montañez A.**
Paediatric brain imaging: B. Acute neurological child beyond trauma (E³ 1921), A-1102

**Sanderink W., Strobbe L.J., Bult P., Sechopoulos I., Karssemeijer N., Mann R.M.**
Dependence of MRI-based size estimates of small invasive breast cancers on patient and tumour characteristics (SS 602), B-0545

**Sanderink W., Teuwen J., Sechopoulos I., Karssemeijer N., Appelman L., Weiland E., Mann R.M.**
Evaluation of breast lesions with simultaneous multi-slice DWI in comparison to routine read-out segmented DWI (SS 1802b), B-1564

**Sandiramourty S., Nguyen Troung Thanh H., Greffier J., Larbi A., Frandon J., Beregi J.P., Gris J., Pereira F.**
Radial diffusivity role in classification of patients with antiphospholipid syndrome and "normal" radiological exams (SS 1811b), B-1695

**Sanei Taheri M., ArabAhmadi M., Haghighatkhah H., Ranjbar M., Amiri M.**
The relationship between cognitive function and magnetic resonance spectroscopy findings in Parkinson disease patients (MY 16), B-1433

**Santiago I.**
Imaging of benign liver lesions: still difficult?: A. Hepatic cysts: always simple? (RC 101), A-0002

**Santiago I.**
Gastrointestinal and abdominal: A. Hepatobiliary system (E³ 1323), A-0769

**Santinha J., Vourtsis A., Uysal A., Moreira J., Papanikolaou N.**
Automatic detection of breast lesions on ABUS using a novel transfer learning approach: comparison between three different networks (SS 702a), B-0700

**Santonocito E., Trombatore C., Travali M., Santonocito S.M., Di Cataldo A., Foti P.V., Basile A., Palmucci S.**
Abdominal calcium score in the evaluation with computed tomography of the risk of leakage of the colon anastomoses (SS 1001a), B-0943

**Santos J., Carmo S., Cardoso A.R., Almeida C.M.D.S.**
The impact of imaging receptor technology on workflow, patient dose values and image quality of bedside chest radiography (SS 1314), B-1063

**Santos J., Teles P.M., Sousa M.C., Paulo G., Venancio J., Caseiro Alves F., Figueira A., Rosario P., Vaz P.**
Portuguese diagnostic reference levels national survey (SS 1814), B-1494

**Santos J.**
Clinical dilemmas in radiography: what graduates need to know: Chairperson's introduction: What are the current issues in radiography? (BR 12), A-0686

**Santos R.**
Ultrasound: continuing to make waves: C. Role of ultrasound in biomechanics and sports science (RC 1714), A-1007

**Santos R.A.M., Ferraz H.I.**
Muscular changes with ageing: ultrasound evaluation (SS 1414), B-1110

**Santos R.A., Couceiro F.**
Relation between fascia and pain by ultrasound (SS 1414), B-1108

**Santos R.A., Fernandes R.**
Obstetric ultrasound - knowledge and perception of pregnant women in Portugal (MY 15), B-1357

**Sapinho I.**
How to deal with the epidemics of thyroid nodules?: The endocrinologist perspective (MS 16), A-0986

**Sappey-Marinier D.**
Principles of imaging and radiation protection: B. Principles of magnetic resonance imaging (E³ 1523), A-0897

**Sarangi P.K., Parida S., Mohanty B.K., Mangaraj S., Mohanty J., Swain B.M.**
Diagnostic utility of mean peak systolic velocity of superior thyroid artery (STA PSV) in the differential diagnosis of thyrotoxicosis (MY 14), B-1253

**Sardanelli F.**
MRI for early detection, staging and management of breast cancer: A. Preoperative staging with MRI: results of preoperative MRI (MIPA) trial (RC 502), A-0233

**Sarkodie B.D.**
Use of MRI for age verification of U-17 footballers: the Ghana study (MY 9), B-0871

**Sarno A., Mettivier G., Tucciariello R.M., Boone J.M., Bosmans H., Bliznakova K., Russo P.**
Personalised estimates of mean glandular dose and image quality investigations in cone-beam computed tomography dedicated to the breast (SS 1913), B-1843

**Sauer M., Tennstedt P., Berliner C., Huland H., Adam G., Beyersdorff D.**
Correlation of erectile dysfunction after radical prostatectomy with findings of preoperative multiparametric prostate MRI (MY 13), B-1089

**Saukko E., Niiniviita H., Henner A., Nieminen M.T.**
Comparison of radiation dose and image quality between an image intensifier and flat panel detector system in ERCP (SS 1014), B-0886

**Sauter A., Hammel J., Kimm M., Pfeiffer D., Kopp F.K., Achterhold K., Pfeiffer F., Rummeny E.J., Noel P.B.**
Perfusion-ventilation CT via three-material differentiation in dual-layer CT: a feasibility study (SS 1904), B-1848

**Sauter A., Kopp F.K., Dangelmaier J., Deniffel D., Meurer F., Bippus R., Rummeny E.J., Noel P.B.**
Sparse-sampling computed tomography (SpSCT) for the detection of pulmonary embolism (SS 213), B-0138

**Savatovsky J.**
Advance neuro diagnostics and clinical outcome with Ingenia Eliton solution (SY 7)

**Savintseva Z., Skvortcova T., Zakhs D.V.**
Correlation between [$^{11}$C]methionine PET uptake and apparent diffusion coefficient in patients with gliomas (SS 1411a), B-1164

**Sawall S., Dorn S., Maier J., Faby S., Uhrig M., Schlemmer H.-P., Kachelrieß M.**
Does iodine CNR improve when switching from today's energy integrating CT to tomorrow's photon-counting CT? (SS 313), B-0319

**Sawall S., Dorn S., Maier J., Faby S., Uhrig M., Schlemmer H.-P., Kachelrieß M.**
Optimal iodine CNR in a whole body photon-counting CT scanner (SS 313), B-0321

**Sawicki L.M., Buddensieck C., Boos J., Kirchner J., Ullrich T., Rabenalt R., Albers P., Antoch G., Hautzel H.**
Prospective comparison of whole-body MRI and $^{68}$Ga-PSMA PET/CT for the detection of biochemical recurrence of prostate cancer (SS 1716), B-1462

**Scaglione M.**
Abdominal emergencies: advanced imaging in daily routine?: Chairperson's introduction (SF 15c), A-0891

**Scaglione M.**
Acute abdomen: Keynote lecture (SS 317), K-09

**Scarsbrook A.**
Merging the best: hybrid imaging: A. Hybrid imaging with SPECT/CT (RC 806), A-0457

**Scavone G., Raciti M.V., Calcagno M.C., Caltabiano D.C., Galvano G., Scavone A.**
Mare Nostrum and tuberculosis: the role of chest x-ray and clinical-laboratory data among Mediterranean migrants on landing (MY 4), B-0436

**Scavone G., Caltabiano D.C., Raciti M.V., Calcagno M.C., Caltabiano G., Gulino F., Giarrizzo A., Piazza L., Scavone A.**
Imaging of mini/one anastomosis gastric bypass abdominal complications (SS 1901b), B-1878

**Schaar J., Dijkstra H., Wielema M., Dorrius M., Sijens P.**
Automatic cluster analysis of breast lesions assessed with diffusion-weighted imaging (SS 702a), B-0704

**Schaarschmidt B.M., Martin O., Kirchner J., Grueneisen J., Heusch P., Forsting M., Antoch G., Herrmann K., Umutlu L.**
Does PET/MR improve lesion detection in oncology: a prospective unicentre study in 1003 examinations (SS 1716), B-1461

**Schachtner B., Ingrisch M., Gresser E., Schneider M.J., Schreier A., Solyanik O., Magistro G., Nörenberg D.**
Feature rejection for radiomic analyses based on variations of tumour segmentation masks (SS 1605), B-1400

**Schaefer-Prokop C.M.**
Radiologic anatomy: chest: Lungs (BE 3), A-0123

**Schaefer-Prokop C.M.**
Functional imaging of the lungs: Chairperson's introduction (E³ 520), A-0288

**Schaefer-Prokop C.M.**
Patterns of pulmonary toxicity: A. Drug-induced lung disease (E³ 1626b), A-0982

**Schäfer J.P.**
No time to lose: aortic disease, revisited: C. Endovascular treatment in aortic dissection (RC 1515), A-0909

**Schäfer J.**
Getting the balance right: radiation risk and imaging benefit in paediatric procedures: Dose reduction strategies in paediatric PET/CT and PET/MR examinations (EU 5), A-0874

**Schelb P., Kohl S., Radtke J., Hohenfellner M., Schlemmer H.-P., Maier-Hein K., Bonekamp D.**
Deep learning for prostate cancer segmentation and detection: diffusion MRI U-net compared to clinical PI-RADS assessment (MY 7), B-0813

**Schelb P., Kohl S., Radtke J., Wiesenfarth M., Hohenfellner M., Schlemmer H.-P., Maier-Hein K., Bonekamp D.**
Benefit of diagnostic support of PI-RADS assessment by quantitative ADC measurements and radiomics in MR diagnosis of prostate cancer (MY 13), B-1077

**Schernthaner R.**
Cone-beam, 4D and more: new diagnostic tools for vascular diseases: B. CT 4D imaging after (T)EVAR (E³ 1226), A-0665

**Schiaffino S., Tagliafico A., Gristina L., Massone E., De Giorgis S., Garlaschi A., Calabrese M.**
The value of automated breast ultrasound (ABUS) coronal view (SS 702b), B-0724

**Schiaffino S., Calabrese M., Melani E.F., Massone E., Villa A., Di Leo G., Zanardo M., Sardanelli F.**
Should atypical ductal hyperplasia be surgically excised after percutaneous needle biopsy: systematic review and meta-analysis (SS 1402a), B-1151

**Schillebeeckx J.**
Clinical audit: how to deal with the legal and professional requirements: Can newer IT-developments including artificial intelligence (AI) help to improve quality in radiology? (PI 2), A-0589

**Schima W.**
How to face the tsunami of pancreatic intraductal papillary mucinous neoplasia (IPMN): IPMN: when to watch and when to resect (SA 5), A-0237

**Schima W.**
Gastrointestinal and abdominal: B. Pancreas and spleen (E³ 1323), A-0770

**Schimmöller L., Ziayee F., Laqua N., Ullrich T., Klingebiel M., Quentin M., Antoch G., Arsov C.**
Impact of dynamic contrast-enhanced (DCE) MRI on prostate cancer detection: is it worth the effort? (SS 207), B-0161

**Schimmöller L., Ziayee F., Ullrich T., Laqua N., Antoch G., Albers P., Arsov C.**
Value of dynamic contrast enhanced (DCE) MR imaging in PI-RADS 4 patients (SS 307), B-0327

**Schimmöller L., Ullrich T., Mones F., Quentin M., Rabenalt R., Albers P., Antoch G., Arsov C.**
MP-MRI can exclude prostate cancer progression in patients under active surveillance (SS 707), B-0828

**Schindera S.T.**
Diagnostic reference levels (DRLs) based on clinical indication: Update of the national DRLs for CT in Switzerland (EU 2), A-0413

**Schlemmer H.-P.**
The role of imaging in the era of liquid biopsy: New imaging tools for cancer detection and characterisation (NH 1), A-0036

**Schlemmer H.-P.**
Novelties in oncologic imaging: C. Is PET/MRI better than PET/CT plus MRI? (E³ 426b), A-0193

**Schlötelburg W., Kriedemann M., Wirth C., Bley T.A., Veldhoen S.**
Comparison of suprapubic vs. transurethral bladder access in voiding cystourethrography (SS 312), B-0337

**Schmaranzer F., Helfenstein R., Zeng G., Lerch T., Siebenrock K., Tannast M., Kim Y., Zheng G.**
Automatic MRI-based 3D models of hip cartilage using a 3D U-net-like fully convolutional network for improved morphologic and biochemical analyses (SS 605), B-0571

**Schmaranzer F., Lerch T., Cullmann J.L., Schmaranzer E., Heverhagen J.T., Siebenrock K., Tannast M.**
Traction MR arthrography of the hip for characterisation of femoral head necrosis and resulting femoral cartilage damage in patients eligible for joint-preserving surgery (SS 1010), B-0896

**Schmaranzer F., Lerch T., Siebenrock K., Tannast M., Schmaranzer E.**
Postoperative, traction MR arthrography in patients with persisting pain after arthroscopic FAI correction reveals high prevalence of osseous deformities and intra-articular lesions (SS 1010), B-0897

**Schmaranzer F., Lerch T., Cullmann Bastian J.L., Siebenrock K., Tannast M., Heverhagen J.T., Ith M., Jung B.**
3D T1 mapping of hip cartilage: a comparison of a new inversion-recovery-based method with conventional dual-flip angle acquisition (SS 1010), B-0901

**Schmidbauer V., Geisl G., Goeral K., Klebermaß-Schrehof K., Berger A., Prayer D., Kasprian G.**
Synthetic MRI detects delayed myelination in preterm neonates (SS 1011a), B-0926

**Schmidbauer V., Fösleitner O., Nenning K., Kasprian G., Schwarz M., Geisl G., Baumgartner C., Prayer D., Bonelli S.**
Evaluation of visuospatial memory performance in temporal lobe epilepsy: a retrospective FMRI study (SS 1911b), B-1881

**Schmidt N., Nesic I., Moor M., Cyriac J., Zumofen D., Stieltjes B., Blackham K.**
Completeness of trauma head computed tomography reports: possibilities for improvement using machine learning (SS 211b), B-0193

**Schmidt N., Zumofen D., Jadczak A., Stieltjes B., Blackham K.**
Is there a clinical need for structured reporting or even more? Results from a clinical questionnaire in the context of head trauma CT (SS 1017), B-0946

**Schmidt Kobbe S.**
Imaging the acute abdomen: new insights: C. Acute colonic diverticulitis (RC 1201), A-0650

**Schneck A.**
BioMatrix: delivering on increased consistency and higher productivity in MRI (SY 6)

**Schoennagel B.P., Yamamura J., Kording F., Fischer R., Adam G., Kooijman H., Ruprecht C., Fehrs K., Tavares de Sousa M.**
Foetal dynamic phase-contrast MR angiography using ultrasound gating and comparison with Doppler ultrasound measurements (SS 612), B-0601

**Schöllnast H.**
Oncological imaging: where are we now?: A. CT perfusion techniques (RC 1216), A-0712

**Scholtz J., Baliyan V., Hedgire S., Mercaldo N.D., Takx R.A., Hoffmann MD MPH U., Vogl T.J., Ghoshhajra B.**
Randomised trial comparing transdermal with lingual and sublingual nitroglycerin administration for coronary vasodilation in coronary CT-angiography (SS 1003), B-1013

**Scholtz J., Günther D., Vogl T.J.**
Additional diagnostic value of dynamic MRI sequences in patients with temporomandibular joint dysfunction compared to static imaging (SS 1708), B-1448

**Schönberg S.O.**
Artificial intelligence (AI): our future cannot be predicted, but we have to be prepared: Which impact does AI have on medicine? (SF 8a), A-0396

**Schöppe F., Rospleszcz S., Bamberg F., Schlett C.L., Rathmann W., Ladwig K., Peters A., Ertl-Wagner B.B., Stöcklein S.**
Machine learning-based exploration of extracranial determinants of grey matter volume in the KORA-MRI study (SS 1911a), B-1782

**Schulz A., Joelsen-Hatlehol E.S., Brudvik K.W., Hanekamp B., Aasand K.K., Viktil E., Johansen C.K., Dormagen J.**
Preoperative detection of colorectal liver metastases: DWI alone or combined with MDCT is no substitute for Gd-EOB-DTPA-enhanced MRI (SS 1001b), B-1047

**Schulz-Menger J.**
7 Tesla MR goes clinical: Cardiovascular and abdomen MR at 7 T - still a challenge? (SF 5a), A-0253

**Schuppert C., von Krüchten R., Hirsch J.G., Hoinkiss D.C., Selder S., von Stackelberg O., Kauczor H.-U., Bamberg F., Schlett C.L.**
Protocol repetition in whole-body MR imaging within a large population-based cohort study: behavior of radiologic technologists and potential of automated image quality assessment (SS 205), B-0019

**Schurink N.W., Berbee M., van Griethuysen J.J.M., van Elmpt W., Maas M., Lahaye M.J., Bakers F.C.H., Beets-Tan R.G.H., Lambregts D.M.**
Multiparametric combined FDG-PET/CT and MR imaging to predict response to chemoradiotherapy in rectal cancer: whole tumour versus sub-volume analysis (MY 17), B-1484

**Schwaiger B.J., Gersing A.S., Cervantes B., Knebel C., Dangelmaier J., Pfeiffer D., Rummeny E.J., Karampinos D., Woertler K.**
Diffusion tensor imaging and tractography acquired with magnetic resonance imaging for preoperative assessment of benign peripheral nerve sheath tumours (SS 1910b), B-1746

**Schwartz F.R., Ramirez Giraldo J., Gutjahr R., Boll D., Marin D., Hurwitz L.**
Real-time patient-specific scan initiation for CT of the abdominal aorta: impact on image quality (SS 615), B-0493

**Schwartz F.R., Ramirez Giraldo J.C., Gutjahr R., Marin D., Boll D., Hurwitz L.**
Real-time patient specific scan delay in CTA of pulmonary embolism: impact on image quality (SS 717), B-0729

**Schwartz F.R., Shaw B., Lerebours R., Vernuccio F., Gonzalez F., Hurwitz-Koweek L., Ravindra K., Marin D.**
Kidney volumes in living donors: does size really matter? (SS 1407), B-1140

**Schwartz F.R., Ramirez Giraldo J.C., Gutjahr R., Boll D., Marin D., Hurwitz L.**
Real-time patient-specific scan initiation for CT of the thoracic aorta: impact on image quality (SS 1815), B-1503

**Schwarz F.B., Ziegert M., Hamm B., Scheel M., Jahnke P.**
Tissue equivalency in radiopaque 3D printing of patient phantoms over a wide range of radiation energies - applications in imaging and dosimetry (SS 1413), B-1199

**Schwyzer M., Ferraro D., Mühlematter U., Curioni-Fontecedro A., Hüllner M., Von Schulthess G.K., Kaufmann P.A., Burger I.A., Messerli M.**
Automated detection of lung cancer at ultralow dose PET/CT by deep neural networks: initial results (MY 7), B-0817

**Sconfienza L.M.**
The radiological investigation of musculoskeletal tumours: A. Radiographs and ultrasound (RC 1210), A-0692

**Screaton N.J.**
CT and intervention in vascular pulmonary diseases: A. CT imaging of pulmonary hypertension (E³ 822), A-0470

**Sechopoulos I.**
Introduction (SY 2c)

**Sechopoulos I.**
Patient-specific dosimetry: A. Breast imaging dosimetry (RC 113), A-0047

**Secil M.**
Imaging and management of the incidental scrotal lesions: Incidental extra-testicular lesions: can we characterise them? (SF 5d), A-0294

**Secinaro A.**
Late effects in survivors of childhood cancer: Cardiothoracic complications (SF 17), A-1022

**Sedghi Gamechi Z., Arias-Lorza A.M., Holst Pedersen J., Kofoed K.F., Bos D., De Bruijne M.**
3D quantitative analysis of the aorta and pulmonary artery on non-contrast CT (SS 615), B-0497

**Sedlaczek O.L.**
Imaging tumour response to immunotherapy: Chairperson's introduction (RC 116), A-0064

**Sedlaczek O.L.**
Lung cancer in the era of molecular oncology and immune therapy: B. PD-L1 positive lung tumours (E³ 1022), A-0600

**Sefidbakht S., Bijan B., Keshavarz P., Amini M., Jalli R., Zarei F., Abbasi H.R., Iranpour P.**
Teaching temporal bone anatomy CT scan as a psychomotor skill: a quasi-experimental study (SS 308), B-0291

**Sefidbakht S., Bijan B., Bagheri S., Etemadi Z., Esmaeilian S.**
Role of fetal MRI in diagnosis of genitourinary anomalies: is there an added value? (SS 612), B-0602

**Seidensticker M.**
Basic interventional radiology for non-interventionalists: let's start with biopsy!: How to follow-up on your patients (SF 5b), A-0248

**Seilern und Aspang J., Kristen K., Platzgummer H., Hartenbach F., Wiedermann J.**
Reliability of ultrasonography measurement of the anterior talofibular ligament (ATFL) length in healthy subjects, based on examinee experience and patient positioning (SS 710), B-0677

**Seitz P., Gottschling S., Abdel-Wahab M., Thiele H., Borger M., Holzhey D., Gohmann R.F., Krieghoff C., Gutberlet M.**
Evaluation of coronary arteries with prospectively-ECG-triggered-High-Pitch-CT in patients undergoing TAVI-planning (MY 18), B-1683

**Seker F., Pfaff J., Schönenberger S., Herweh C., Nagel S., Ringleb P.A., Bendszus M., Möhlenbruch M.A.**
Clinical outcome after thrombectomy in stroke patients with premorbid modified Rankin scale scores of 3 and 4: a cohort study with 136 patients (SS 311), B-0263

**Seliverstova E., Seliverstov Y., Konovalov R., Krotenkova M., Illarioshkin S.**
DMN-neuronal activity pattern in patients with PD in early stages (SS 1811a), B-1554

**Sellars M.**
Balancing the risks and benefits in radiology - contrast and x-rays and paediatric blunt abdominal trauma: safety and dose reduction (SY 18)

**Seo H.S.**
Perivertebral muscle as index of sarcopenia: comparison with previous muscle imaging and functional indexes (MY 9), B-0852

**Seo J.B.**
Chronic obstructive pulmonary disease (COPD): B. Quantitative imaging biomarkers (E³ 922), A-0566

**Seppelt D., Ittermann T., Haubold A., Fedders D., Hoffmann R.-T., Kolb C., Kühn J.**
Feasibility of volume index to assess liver volume: comparison of ultrasound and computed tomography (SS 701), B-0797

**Seraydarmansour O., Taher Rakei Z.**
MR quantification of the fatty fraction from IDEAL IQ sequence in the assessment of muscle atrophy in rotator cuff tears (SS 310), B-0238

**Sertorio F., Signa S., De Cesari M., Damasio M.B., Magnano G.M.**
Role of whole-body magnetic resonance imaging in identifying potential diagnostic clues in children with fever of unknown origin (SS 312), B-0341

**Sertorio F., De Cesari M., Mazzoni M., Pistorio A., Urru A., Magnaguagno F., Malattia C., Magnano G.M.**
Use of magnetic resonance imaging to predict progression of juvenile idiopathic arthritis in paediatric patients in clinical remission (SS 1012), B-1054

**Seuri R.**
Getting the balance right: radiation risk and imaging benefit in paediatric procedures: Guidelines for the use of conventional radiographs in children and adolescents (EU 5), A-0872

**Seuss H., Janka R., Dankerl P., Cavallaro A., Uder M., Hammon M.**
Evaluation of a semi-automated segmentation tool for volumetric analysis of the renal cortex and medulla in non-contrast T1-weighted MR images (MY 13), B-1088

**Severoni S.**
Radiology and migrations: Impact of migration on health: WHO perspective (PC 5), A-0228

**Shafaat O., Faeghi F., Bagheri A., Rabbani M., Jabbari K.**
Evaluating the diagnostic value of different imaging modalities in cerebral venous thrombosis (SS 1811b), B-1694

**Shanahan M., Geso M., Potter A., Lennie G.**
Scoliosis dose comparison using current clinical protocols (SS 714b), B-0657

**Sharma N., Schofield S., Fletcher M., Hammond R.L., N. Larson A., Bakken A.S., Handberg E., Hopland N., Iles S.E.**
Mammography positioning errors: a multi-centre study (SS 1802a), B-1543

**Sharma N.**
Understanding workflow: the key to effective integration (SY 5)

**Sharma S., Bansal R., Jain S., Sethi P.**
Comparative evaluation of strain ratio on sonographic elastography and T2* values on 3Tesla MRI in differentiating malignant from benign axillary lymph nodes in breast cancer (SS 1902b), B-1797

**Shelmerdine S.C.**
Juvenile idiopathic arthritis (JIA): A. Conventional radiography: still a helpful method? (E³ 1326), A-0794

**Shelmerdine S.C., Arthurs O.J., Wade A., Jones R., Norman W., Taylor A., Sebire N.J., Chitty L.S.**
What does perinatal autopsy add after antenatal ultrasound and post-mortem MRI investigations (SS 612), B-0606

**Shelmerdine S.C., Hutchinson J.C., Sebire N.J., Arthurs O.J.**
Post-mortem ultrasound-guided biopsy for perinatal death investigation: a pilot study (SS 1512), B-1319

**Shen M., Yang Z., Diao K., Jiang L., Huang S., Hu B., Guo Y.**
Left ventricular global strain analysis in arrhythmogenic right ventricular cardiomyopathy with or without syncope: a cardiac magnetic resonance imaging study (MY 18), B-1674

**Shen M., Yang Z., Diao K., Zhao Q., Jiang L., Huang S., Guo Y.**
Left ventricular function impairment and deformation assessment of post-transcatheter aortic valve implantation (TAVI): a systematic review and meta-analysis (MY 18), B-1680

**Shen M., Yang Z., Diao K., Jiang L., Hu B., Huang S., Guo Y.**
Functional assessment and prognostic value of cardiac magnetic resonance in arrhythmogenic right ventricular cardiomyopathy (SS 1903a), B-1825

**Shen Q., Heikkinen N., Kärkkäinen O., Kononen M., Gröhn H., Liu Y., Zhang Z., Tolmunen T., Vanninen R.**
Excessive alcohol consumption in adolescent men compromises frontal white matter integrity via disruption of myelin (and axons): a combined DTI-metabolic study (SS 1411b), B-1272

**Shi K., Yang Z., Guo Y., Huang S., Li X.**
Noninvasive assessment of myocardial oxygenation after acute myocardial infarction with breath-hold-induced blood oxygen level-dependent imaging (SS 1803), B-1618

**Shi R., Yang Z., Guo Y., Zhao Q., Gao Y., Han P.**
Comparison of the incidence of in-stent restenosis and the newly developed plaques after stenting between diabetic and non-diabetic patients: evaluation with DSCT angiography (MY 18), B-1676

**Shi Z., Chen G., Zhou C., Lu G., Zhang L.**
Development and validation of prediction model based on hemodynamic derived from computed tomography angiography for rupture risk of small unruptured intracranial aneurysms (SS 315), B-0217

**Shi Z., Pan C., Zhou C., Fan D., Li X., Lu G., Zhang L.**
Development and validation of a deep neural network-based computer-assisted detection system for cerebral aneurysms in CT angiography (SS 705), B-0754

**Shin S., Cho S., Kim W., Kim H., Kim G., Ryeom H., Lee S.M., Shin K.M.**
Diagnostic performance of MRI- vs MDCT-categorised T3cd/T4 for identifying high-risk stage II or stage III colon cancers: a pilot study (SS 1001a), B-0942

**Shokry S., Moustafa A.F.I., Hawanna M.A.M., Hussein M.M., Shokry A.**
Diffusion-weighted MR imaging diagnostic merits in post-therapeutic assessment of musculoskeletal soft tissue sarcoma (SS 716), B-0693

**Shorikov M., Sergeeva O.N., Frantsev D., Lapteva M., Virshke E., Dolgushin B.**
Overrepresentation of uncommon biliary confluence types in hilar cholagiocarcinoma patients (SS 301), B-0395

**Shu S., Xianhong X., Ganghua T.**
Cell death PET imaging of rat liver cirrhosis with $^{68}$Ga-NOTA-PEG$_3$-Duramycin (SS 1406), B-1124

**Si-Mohamed S.A., Dupuis N., Rotzinger D.C., Boccalini S., Yagil Y., Coulon P., Shapira N., Douek P., Boussel L.**
Virtual non contrast imaging of aortic intramural hematoma for replacing the non contrast CT imaging (SS 717), B-0731

**Si-Mohamed S.A., Tatard-Leitman V., Coulon P., Douek P., Noel P.B., Boussel L.**
Spectral photon-counting CT multi-phase liver imaging with dual contrast agent (SS 1406), B-1116

**Si-Mohamed S.A., Moreau-Triby C., Wdowik Q., Douek P., Boussel L.**
Lung dual-energy quantitative CT vs single-photon emission computed tomography for quantification of the perfusion (SS 1904), B-1852

**Siddique U.**
The role of radiology in major healthcare challenges faced by Pakistan: Many faces of tuberculosis (EM 3), A-1082

**Siddiqui I.A., Foley K.G., Palaniappan B., Gibson A., Pearce T., Davies G.J.**
One-year results from the South Wales Rapid Diagnostic Clinic for patients with serious but non-specific symptoms of cancer (SS 1016), B-1027

**Sidhu P.S.**
Ultrasound simulation models in training and education: where are we going?: Chairpersons' introduction (part 2) (ESR US SC/EFSUMB), A-0040

**Sidhu P.S.**
Imaging and management of the incidental scrotal lesions: Incidental testicular lesions: can we characterise them at multiparametric US? (SF 5d), A-0293

**Sidhu P.S.**
Functional imaging of the liver: B. Diagnostic and staging of diffuse liver diseases using multiparametric US (E³ 620), A-0334

**Sidhu P.S.**
Luigi Oliva Honorary Lecture: Contrast enhanced ultrasound in paediatrics: ready for clinical practice? (HL 3), A-0847

**Siegel E.L.**
Artificial intelligence (AI): driven by radiologists: The impact of AI technologies in patient care: advantages and limitations (NH 3), A-0126

**Sieren M., Brenne F., Hering A., Schenk M., Oechtering T.H., Barkhausen J., Frydrychowicz A.**
Rapid study assessment in follow-up whole-body computed tomography in patients with multiple myeloma using a dedicated bone subtraction software (SS 316), B-0349

**Sigl B., Jockwitz C., Mathys C., Eickhoff S., Hartmann C.J., Rubbert C., Turowski B., Caspers S., Caspers J.**
Functional connectivity alterations of dorsal premotor cortex subregions in Parkinson's disease (SS 1811a), B-1553

**Silva M.**
Lung cancer team: Imaging and staging (MS 1), A-0051

**Silva M.**
Lung cancer in the era of molecular oncology and immune therapy: C. ALK-rearranged and other mutations of lung adenocarcinomas (E³ 1022), A-0601

**Silva V.M.F., Ramos I., Moreira J., Marques M.**
Comparison of MRI acoustic noise levels in different types of sequences (SS 414), B-0412

**Simelius-Nieminen S., Malmivirta H., Seiko-Vänttinen V., Saukko E.**
Infection control: developing aseptic methods and creating a guideline for interventional radiology unit (SS 1014), B-0888

**Simeonov G.**
Diagnostic reference levels (DRLs) based on clinical indication: European Commission perspective (EU 2), A-0409

**Simmler R.**
Implementation of the radiation protection laws in Switzerland (SY 15)

**Simon J., Szilveszter B., Ahres A., Kolossvary M., Jablonkai B., Merkely B., Andrássy P., Maurovich- Horvat P.**
Coronary CT based on-site FFR might be a useful tool to identify haemodynamically relevant non-culprit lesions in patients who suffered myocardial infarction (SS 703), B-0786

**Singh A., George U.B., Pandian J.D.**
Pattern of leptomeningeal collateral circulation on perfusion computed tomography in patients with acute ischaemic stroke (SS 711), B-0714

**Singh D.K., Grover S.B., Kumar N., Nayak B.K., Tomar S., Suman S., Katyan A., Rajani H., Misra R.N.**
Recovering from non-specific low back pain despair: ultrasound-guided intervention in iliolumbar syndrome (SS 1510), B-1291

**Singh D.K., Kumar N., Tomar S., Nayak B.K., Suman S., Katyan A., Mittal M.K., Grover S.B., Misra R.N.**
Ultrasound-guided distension hydrotherapy with graded physiotherapy in primary adhesive capsulitis: a pilot study (SS 1510), B-1293

**Singh M., Singh T., Singla V., Jain V.**
Role of shear wave elastography of placenta in the prediction of preeclampsia in high-risk pregnancies (SS 1507), B-1382

**Sinitsyn V.E.**
Abdominal emergencies: Vascular emergencies (BS 5), A-0243

**Sipos D., Pandur A., Kedves A., Varga V., Csima M., Repa I., Kovács Á.**
Burnout level of personnel working in radiology departments in Hungary (SS 214), B-0031

**Sjöberg J.**
Striking the balance: image quality assessment in radiological optimisation: C. Sharing the message: image quality optimisation for multiple scanners and clinics (RC 413), A-0184

**Skaane P.**
New mammography: digital breast tomosynthesis and future techniques: B. Clinical validation of digital breast tomosynthesis and results in the last 10 years: where do we stand? (RC 1302), A-0762

**Skehan S.**
Cystic neoplasms: Intraductal papillary neoplasms (E³ 24C), A-0607

**Skripnik A., Fokin V., Mironchuk R., Trufanov G., Moiseeva O., Irtyuga O., Uspenskiy V., Malev E., Murtazalieva P.**
ECG-gated CTA of thorax aorta with extended data postprocessing in ascending aorta aneurism diagnosis (SS 1815), B-1505

**Smet M., Breysem L., Wang M., Bosmans H., Marshall N., Rodríguez Pérez S.**
Impact of detector pixel size on clinical image quality of digital neonatal chest X-ray images (SS 1512), B-1318

**Smirnov A., Sharaev M., Melnikova-Pitskhelauri T., Pogosbekyan E., Fadeeva L., Pitskhelauri D., Pronin I.**
Preoperative brain mapping: task-based and resting-state fMRI matching (SS 1011b), B-1037

**Smirnova A.V., Lukina O.V., Kuzmin A., Tkachev A., Plakhotina N., Cherkashin M., Anishkin M.**
Changes in high-grade gliomas after different kinds of radiation therapy depending on IDH status during chemotherapy in combination with bevacizumab (SS 211a), B-0091

**Smit E.J., Murayama K., Galen T., Pegge S., Kluijtmans L., Ikeda Y., Katada K., Meijer F.J., Prokop M.**
Improved diagnostic performance of CT perfusion imaging in acute stroke using advanced noise suppression and Bayesian perfusion algorithms (SS 711), B-0706

**Smithuis R.**
Small bowel obstruction: improve your reading and reporting competences: C. Step 3: look for bowel ischaemia (RC 1717), A-1040

**Smits M.**
Radiographers: at the heart of cancer treatment and research: Establishing the scientific and clinical value of advanced imaging (EFRS/EORTC), A-0440

**Smits M.**
Diffuse low-grade gliomas: new things you should know: B. Imaging patterns suggestive of different (molecular) subtypes of low-grade gliomas (RC 1711), A-1031

**Snaith B.**
Growing radiography research: The clinical research radiographer: essential for our profession or facilitating the research of others? (EFRS WS), A-0863

**Snoeckx A., Cant J., Franck C., Carpentier K., Luyckx E., Nicolay S., Spinhoven M.J., Van Hoyweghen A., Parizel P.M.**
Lesion measurement on a combined "all-in-one" computed tomography window on chest scans: effect on intra- and interobserver variability (SS 204), B-0145

**Snoeckx A.**
Pleural neoplasms: C. Other pleural and juxta pleural malignancies (E³ 1322), A-0803

**So C., de Souza P., Kumar R., Remedios D.**
D-dimers, Wells' scores and CTPA for pulmonary embolism: are all diagnostics used appropriately? (SS 1904), B-1856

**Sodano C., Clauser P., Kapetas P., Helbich T.H., Baltzer P.A.T.**
Breast 1H-MR-spectroscopy to distinguish benign from malignant breast lesions: an updated systematic review and meta-analysis with clinical implications (SS 1802b), B-1570

**Soesbe T.C., Lewis M.A., Xi Y., Browning T., Ananthakrishnan L., Lenkinski R.E., Leyendecker J.R.**
Differentiating and segmenting isoattenuating gallstones from bile with dual-layer spectral CT: an ex vivo phantom study (SS 301), B-0391

**Soesbe T.C., Lewis M.A., Ananthakrishnan L., Leyendecker J.R., Xi Y., Yokoo T., Lenkinski R.E.**
Measuring liver fat fraction with dual-layer spectral CT material attenuation decomposition (MAD) plots: an iodine-independent method for imaging hepatic steatosis with CT (SS 701), B-0792

**Sohaib A.**
Early detection of prostate cancer: A. Screening for prostate cancer: where are we now? (part 2) (E³ 219), A-0093

**Sokurenko M., Trofimova T., Khalikov A.**
Assessment of the reproducibility of foetal proton magnetic resonance spectroscopy at different sequence settings (SS 612), B-0608

**Sokurenko M., Trofimova T., Khalikov A.**
Assessment of the dynamics of foetal brain metabolites with proton magnetic resonance spectroscopy (SS 612), B-0609

**Solopova A.E., Sdvizhkov A.M., Makatsaria A.D.**
Ovarian cancer staging and resectability assessment: prospective comparison between diffusion-weighted MRI and MSCT (SS 216), B-0173

**Solopova A.E., Gurov S.N.**
Ovarian cancer neoadjuvant treatment response evaluation: value of diffusion-weighted MRI (SS 216), B-0180

**Son J., Hwang S., Park M.S., Park S., Kim M.J., Choi J., Han K.**
Imaging features of hepatocellular carcinoma: qualitative and quantitative comparisons between hepatobiliary and extracellular contrast-enhanced MRI (SS 1901a), B-1766

**Song C., Cheng J., Zhang Y.**
Application of RESOLVE-DWI ADC maps based on whole-tumour histogram analysis in differentiating common parotid gland tumours (MY 14), B-1252

**Song J., Zhang F., Ji J., Chen M., Weng Q., Yang X., Xu M., Lu C., Zhao Z.**
Orthotopic hepatocellular carcinoma: molecular imaging-monitored intratumoural hyperthermia-enhanced direct oncolytic virotherapy (SS 1406), B-1126

**Song T., Li Z.**
Virtual monochromatic images in low-tube current dual-energy spectral imaging combined with adaptive statistical iterative reconstruction V in head CT angiography (SS 315), B-0223

**Song Y., Park H., Yu M., Kim Y., Jung S., Jeon H.**
Diagnostic efficacy of acute necrotising pancreatitis on early CT scan and correlation with laboratory exams (SS 501), B-0449

**Sorantin E.**
Everything you need to know about 3D post-processing: Chairperson's introduction
(RC 405), A-0142

**Soyer P.**
Imaging in pregnancy: C. Imaging the placenta
(RC 1307), A-0811

**Spadarella G., Bonomo L., Monti C.B., Sardanelli F., Secchi F.**
Psoas muscle cross-sectional areas and aortic valve calcification in patients enrolled for transcatheter aortic valve implantation (TAVI)
(SS 203), B-0199

**Specht S.**
Philips UNIQUE 2: meeting clinical challenges by tailored image processing (SY 28)

**Spiliopoulos S.C.**
Multidisciplinary approach to the diabetic foot: Interventional radiological treatment (IR)
(MS 17), A-1065

**Spink C., Avanesov M., Schmidt T., Grass M., Schoen G., Adam G., Ittrich H., Bannas P.**
Radiation dose reduction during angiographic adrenal vein sampling using a new imaging technology (SS 209), B-0119

**Spoor D.S.**
Cardiovascular effects after radiotherapy for breast cancer: the European MEDIRAD Project: Optimisation of multivariable prediction models for major cardiac events after radiotherapy for breast cancer (EU 1), A-0017

**Sporea I., Bende F.B., Popescu A.S., Sirli R., Danila M.**
Are there different cut-off values for staging liver fibrosis using 2D-SWE implemented on different systems from the same manufacturer?
(SS 1801a), B-1533

**Spurny M., Jiang Y., Schübel R., Nonnenmacher T., Schlett C.L., von Stackelberg O., Kauczor H.-U., Kuhn T., Nattenmüller J.**
Changes in bone marrow fat upon dietary induced weight loss (SS 210a), B-0051

**Srivastava D.N., Monga A., Madhusudhan K., Malla S.**
Balloon vesselplasty vs percutaneous vertebroplasty in chronic compression fractures of the dorsolumbar spine (SS 1809), B-1577

**Staal F., Houwers J.B., Heeres B., de Boer M., Lambregts D.M., Maas M., Beets-Tan R.G.H.**
Findings of sinusoidal obstruction syndrome on gadoxetic-acid-enhanced MRI in patients with chemotherapy for colorectal liver metastases are poorly reproducible between radiologists
(SS 1001b), B-1049

**Stacul F.**
Contrast media guidelines: B. Iodine-based contrast media in myeloma patients
(RC 1207), A-0725

**Stacul F.**
Contrast agents for imaging: not only iodine: Keynote lecture (SS 607), K-13

**Stafrace S.**
Intensive care paediatric radiology: the very sick neonate: Chairperson's introduction
(RC 512), A-0276

**Stajgis M.**
My three top tips in neuroimaging (not only for neuroradiologists): Chairperson's introduction
(SF 4), A-0152

**Stajgis M.**
Renal, adrenal and urinary tract pathologies: Adrenal pathologies (BS 8), A-0433

**Stanzione A., Ponsiglione A., Cocozza S., Cuocolo R., Picchi S.G., Romeo V., Stilo S., Brunetti A., Imbriaco M.**
Prostate cancer local staging: a comparison of short dual pulse and biparametric MRI vs multiparametric MRI (SS 707), B-0823

**Stáreková J., Scherz B., Avanesov M., Weinrich J.M., Müllerleile K., Radunski U., Adam G., Lund G.K., Tahir E.**
Diastolic dysfunction in competitive triathletes following a strenuous endurance sports event
(MY 18), B-1682

**Starmans M.P.A., van der Voort S.R., Vos M., Incekara F., Visser J.J., Smits M., Thomeer M.G., Niessen W.J., Klein S.**
Fully automatic construction of optimal radiomics workflows (SS 1005a), B-0908

**Starmans M.P.A., Blazevic A., van der Voort S.R., Brabander T., Hofland J., Niessen W.J., de Herder W.W., Klein S.**
Prediction of surgery requirement in mesenteric fibrosis on CT using a radiomics approach
(SS 1401b), B-1279

**Starmans M.P.A., Miclea R.L., van der Voort S.R., Niessen W.J., Klein S., Thomeer M.G.**
Classification of malignant and benign liver tumours using a radiomics approach
(SS 201a), B-0067

**Staub D.**
Exploring the ultimate vascular solution with the world's first xMATRIX XL14-3 transducer
(SY 17)

**Steinbach L.**
Shoulder sports injuries and postoperative MRI: A. Shoulder injuries in the throwing athlete
(TC 1328), A-0804

**Steinbach L.**
Shoulder sports injuries and postoperative MRI: C. Interactive case discussion (Part 1)
(TC 1328), A-0806

**Steinfelder E.**
Will the General Data Protection Regulation (GDPR) hamper the secondary use of clinical imaging data for research?: Why is secondary use of existing imaging data important for research progress? (ESR/BBMRI-ERIC), A-0630

**Stenzel M.**
CEUS diagnostic imaging in the paediatric population (SY 23)

**Stern E.J.**
Thoracic imaging: new approaches and techniques: Keynote lecture (SS 1804), K-38

**Steveson C., Schuzer J., Acharya T., Bronson K., Rollison S., Bandettini W., Shanbhag S., Chen M.Y.**
Deep learning reconstruction for calcium scoring reduces radiation dose while maintaining accuracy of Agatston scores (SS 305), B-0300

**Steveson C., Schuzer J.L., Acharya T., Bronson K., Rollison S., Bandettini W., Shanbhag S., Chen M.Y.**
The effect of deep learning reconstruction on calcium scoring (SS 1414), B-1114

**Stieltjes B.**
Artificial intelligence (AI): our future cannot be predicted, but we have to be prepared: AI for lesion detection and characterisation
(SF 8a), A-0394

**Stoecklein S., Stöcklein V., Schöppe F., Thon N., Kreth F., Ertl-Wagner B.B., Tonn J., Liu H.**
Altered whole brain connectivity as a potential marker of disease burden in glioma patients
(SS 211a), B-0094

**Stoker J.**
US before CT in the acute abdomen?: Chairperson's introduction (PS 1427), A-0835

**Storz C., Zitzelsberger T., Rospleszcz S., Nikolaou K., von Knobelsdorff F., Peters A., Schulz-Menger J., Bamberg F., Schlett C.L.**
Independent association of extracellular volume with myocardial strain assessed by CMR in subjects without history of cardiovascular disease from the general western population
(SS 603), B-0589

**Storz C., Rothenbacher T., Rospleszcz S., Linseisen J., Messmann H., De Cecco C.N., Peters A., Schlett C.L., Bamberg F.**
Whole-body MRI: prevalence and extent of asymptomatic diverticular disease and the association of dietary habits, cardiometabolic and constitutional risk factors
(SS 1001a), B-0945

### Authors' Index

**Stoupis C.**
Gastrointestinal and abdominal: Chairperson's introduction (E³ 1323), A-0768

**Stowe J.G.**
The future of radiology: what it could mean for new radiography graduates: Evolving imaging technologies (BR 17), A-1043

**Stowe J.G., O'Halloran C.**
Integrating CT simulation into radiography undergraduate training (SS 1914a), B-1702

**Stranieri G., Gallo G., De Pascale A., Gned D., Bini M., Basile D., Checcucci E., Porpiglia F., Veltri A.**
Local staging in prostate cancer (PCa): the role of capsular bulging and the length of capsular contact (LCC) to predict the risk of extracapsular extention (ECE) at final pathology (SS 707), B-0824

**Stranieri G., Priola A., Basile D., Priola S., Veltri A.**
Incidental adrenal masses (IAM) at basal CT or enhanced CT: differentiating non-functioning adenomas from tumour lesion using histogram and texture analysis (TA) (SS 1407), B-1144

**Strickland N.H.**
Mobile devices in radiology: C. Recommendations for reporting on mobile devices (RC 105), A-0013

**Stroszczynski C.**
GI bleeding: how can the problem be solved?: B. Occult and overt GI bleeding: the role of radiology (RC 501), A-0206

**Strudwick R.M., Newton-Hughes A., Gibson S., Harris J., Gradwell M., Hyde E., Harvey-Lloyd J., O'Regan T.J., Hendry J.**
Value-based practice (VBP) training for radiographers (SS 214), B-0033

**Su T., Jin L.**
Leaving-tail microcoil localisation for small pulmonary nodules prior to thoracoscopy (SS 1909), B-1813

**Su T., Chen Y., Chen X., Zhang T., Shi X., Xue H., Zhang Z., Jin Z.**
Preliminary diffusion kurtosis imaging study of head and neck squamous cell carcinomas associated with histologic grades (MY 14), B-1246

**Suarez Vega V.M., Caballeros Lam F.M., Domínguez Echávarri P., Garcia de Eulate R., Gallardo Madueno G., Alonso-Burgos A., Sobrido C., Alvarez de Sierra B., Perez N.**
Comparison between high-resolution 3D-REAL-IR and 3D-FLAIR sequences in the assessment of endolymphatic hydrops in 3 Tesla (SS 308), B-0294

**Sudarkina A., Dergilev A., Gorbunov N., Kozlov V.**
Quantitative analysis of diffusion weighted magnetic resonance imaging in lung nodules characterisation (SS 1004), B-1005

**Sudarski S., Henzler T., Schäfer D., Viergutz T., Terboven T., Schönberg S.O., Haubenreisser H.**
Incidental findings in whole-body CT: a retrospective analysis in over 1000 resuscitation room patients (SS 217), B-0106

**Sudarski S., Schäfer D., Henzler T., Terboven T., Viergutz T., Schönberg S.O., Haubenreisser H.**
Iatrogenic injuries and misplaced foreign material found in whole-body CT: a retrospective analysis in over 1000 resuscitation room patients (SS 717), B-0735

**Suganchand Rikabchand S.K.**
Role of acoustic radiation force impulse (ARFI) in differentiating benign and malignant thyroid nodules (SS 208), B-0128

**Suganchand Rikabchand S.K., Mohammed Kashif R.**
Role of shearwave elastography in assessment of cervical lymph nodes (SS 608), B-0637

**Suganchand Rikabchand S.**
Investigating the appropriateness of radiological investigation of lumbar spine and determining relationship between appropriateness and imaging findings in a tertiary hospital (MY 9), B-0855

**Sugiyama M., Takehara Y., Alley M., Unno N., Katahashi K., Wakayama T., Nozaki A., Naganawa S., Sakahara H.**
Flow volume of the abdominal visceral arteries increase after EVAR treatment (SS 1915), B-1722

**Sukupova L.**
Radiation burden of patients with hepatocellular carcinoma from multiple TACE and CT procedures (SS 1813), B-1602

**Suleiman M.E., Brennan P.C., McEntee M.F.**
The effect of overestimating glandularity on mean mammographic glandular dose (SS 1913), B-1844

**Sullivan Y., Fraser L., Woodhouse G.**
A retrospective review of justification of computed tomography examinations in Northern Ireland (SS 1413), B-1193

**Sum R., Lau K.K.**
Gelfoam slurry tract embolisation after computed tomography-guided percutaneous lung biopsy: does it prevent major pneumothorax? (SS 1909), B-1815

**Sun W., Ota H., Chibana S., Sato H., Sugimura K., Takase K.**
The role of 4D flow MRI in evaluating the outcome of balloon pulmonary angioplasty (MY 4), B-0421

**Sundell V., Hippeläinen E., Peltonen J.I.**
Automatic analysis and result presentation workflow for diagnostic X-ray quality assurance (SS 1013), B-0998

**Sung P., Yoon S.**
CT bronchovascular bundle thickening sign as a predictor of survival in patients with peripheral small cell lung cancer 3 cm or smaller (SS 704), B-0773

**Svanteson M., Afadzi M., Martinsen A.C., Kløw N.E., Lysvik E.K., Bjørklund K., Enden T.R.**
Assessment of coronary in-stent stenosis using high-resolution scan mode and dual-energy CT: a phantom study (SS 614), B-0480

**Sverzellati N.**
Challenging HRCT patterns: tips and tricks from the experts: Inter-and intralobular septal thickening (SF 8b), A-0453

**Sverzellati N.**
Fibrotic lung diseases: A. Idiopathic pulmonary fibrosis (RC 1204), A-0700

**Sverzellati N.**
Writing reports: a survival guide: B. Interstitial lung disease: searching for clarity (E³ 1321), A-0733

**Swarup M., Upreti L.**
Usefulness of CT perfusion generated time-density curves and perfusion parameters in the diagnosis and differentiation of parotid neoplasms (SS 1008), B-0977

**Swinnen J., Keupers M., Prevos R.M., Postema S., Lavens M., Thywissen T., De Beul P., Soens J., Van Ongeval C.**
Survey: imaging surveillance among Belgian breast clinics for primary locoregional breast cancer in non-high-risk women (MY 5), B-0473

**Syysmäki H., Wood P., Laaksonen-Heikkilä R.**
Radiographer as RIS/PACS administrator: promoting professional networking and continuing professional education by the society of radiographers in Finland (SS 1414), B-1115

**Szatmari P., Pytlewski P., Papanikolaou N., Illing R.**
Operational performance optimisation by means of an imaging analytics platform: a case study (SS 1405), B-1185

### T

**Tack D.**
Errare humanum est: A. Errors in chest radiograph ($E^3$ 721), A-0356

**Tack D.**
Back to basics: how to interpret a chest radiograph?: C. Lobar atelectasis (RC 1504), A-0916

**Taghavi M., Trebeschi S., Meek D., Lambregts D.M., Verhoef C., Houwers J.B., van der Heide U.A., Beets-Tan R.G.H., Maas M.**
Machine learning-based analysis of CT radiomics model for the prediction of colorectal metachronous liver metastases (SS 1805b), B-1582

**Tagliafico A.**
Elbow imaging: from detailed anatomy to pathology: B. Biceps and triceps (RC 1710), A-1047

**Tagliafico A., Rossi F., Valdora F., Grandis M., Benedetti L., Bignotti B., Schenone A., Martinoli C.**
Pilot study on radiomics of peripheral nerves: feasibility of quantitative imaging phenotyping on magnetic resonance imaging (SS 1910b), B-1745

**Taha N., Zidan D., Geneidi E.**
Should multiparametric MRI with semi-quantitative perfusion replace triphasic CT in follow-up post TACE (SS 401), B-0400

**Taina M.**
Neurologic emergencies: Ischaemic stroke: diagnosis (BS 9), A-0524

**Takahashi S., Ueno Y., Sofue K., Negi N., Kagawa K., Murakami T., Tanaka U.**
Reassessment the ability of renal CE-CT for avoiding pathological upstaging of clinical T1 to pathological T3 renal cancer in the era of robotic partial nephrectomy (SS 1407), B-1145

**Takahashi S., Itoh T., Ueno Y., Negi N., Kagawa K., Sofue K., Tanaka U., Kusaka A., Murakami T.**
Low energy imaging for depicting renal venous tumour thrombus on multi-phasic contrast-enhanced dual-energy CT with $3^{rd}$ generation dual-source CT scanner (SS 1407), B-1146

**Takashima H., Yoshimoto M., Imamura R., Akatsuka Y., Nakanishi M., Yoneyama M., Hatakenaka M., Yamashita T.**
Advantages of T2* relaxation time with ultra-short TE on intervertebral disc degeneration (SS 1910a), B-1735

**Takenaka D., Fujii K., Sugihara N., Seki S., Yoshikawa T., Ohno Y.**
Ultra-high-resolution CT vs state-of-the-art area-detector CT: can higher spatial resolution improve nodule detection capability at same radiation dose in chest nodule phantom study? (MY 4), B-0424

**Takenaka D., Higashida A., Kono Y., Hashimoto T., Maeda H., Sakamoto S.**
The utility of the ultra-high-resolution CT to evaluate the detailed bronchial tree for COPD (MY 4), B-0425

**Talarczyk S., Uhlenbrock D.F., Haage P., Stückle C.A.**
Morphological changes of disc herniation following CT-guided periarticular infiltration (SS 1510), B-1289

**Talei Franzesi C., Riva L., Ippolito D., Cangiotti C., de Vito A., Maino C., Porta M., Sironi S.**
Improving CAD-RADS score with new iterative model reconstruction algorithm in low dose CCTA: inter-observer agreement and comparison with hybrid iterative reconstruction algorithm (SS 1503), B-1310

**Tali E.T.**
Inflammatory and infectious CNS pathology: Chairperson's introduction (RC 1211), A-0675

**Tam M.**
The 3D printing lab from bench to bedside: Cardiovascular applications of 3D printing (SF 8f), A-0448

**Tamandl D., Fueger B., Haug A., Ba-Ssalamah A.**
Combination of quantitative $^{18}$F-FDG PET parameters and contrast-enhanced CT in locoregional restaging and prognostication of survival in patients with oesophageal cancer (SS 1901b), B-1875

**Tamburrano C., Busacca M.**
Coll-Ha versus bone marrow stimulation for chondral and osteochondral lesion: a 2-year randomized controlled trial (MY 9), B-0864

**Tanamala S., Chilamkurthy S., Biviji M., Ghosh R., Rao P.**
Prospective evaluation of a deep learning algorithm deployed in an urban imaging centre to notify clinicians of head CT scans with critical abnormalities (SS 705), B-0748

**Tanamala S., Chilamkurthy S., Biviji M., Rao P., Ghosh R.**
Automated detection and localisation of pneumocephalus in head CT scan (SS 1005b), B-0981

**Tanenbaum L.**
Reducing variability in MRI (SY 6)

**Tang C., Lu M., Schoepf U.J., Tesche C., Bauer M., Nance J.W., Griffith L., Zhou C., Zhang L.**
Coronary computed tomography angiography-derived fractional flow reserve in anomalous origin of the right coronary artery from the left coronary sinus (SS 703), B-0784

**Tang L., Chang W., Chen M.**
A preliminary study of predicting molecular subtypes of breast cancer by the radiomics features of contrast-enhanced ultrasound (MY 7), B-0807

**Tang L., Chen Y., Du Z., Zhong Z., Chen Q., Luo J., Yang L.**
Factors that influence performance of the contrast-enhanced ultrasound prediction model in 1023 breast lesions: a multi-center study (MY 5), B-0462

**Tang W.**
Contrast-enhanced digital mammography, magnetic resonance imaging and mammography alone: a comparison of diagnostic performance in symptomatic women (SS 1902a), B-1785

**Tanturri de Horatio L.**
Juvenile idiopathic arthritis (JIA): B. Ultrasound for detecting and grading of inflammation in JIA ($E^3$ 1326), A-0795

**Tanyildizi Y., Kronfeld A., Neu M., Wingerter A., Russo A., Khawaja K., Tropine A., Brockmann M.A., Faber J.**
ASL perfusion MRI reveals reduced cerebral blood flow in the hippocampal region in patients with former childhood medulloblastoma (SS 1416), B-1241

**Tao B., Xiao Y., Zhang W., Yao L., Lui S.**
Altered cortical thickness related to single nucleotide polymorphisms (SNPs) in the major histocompatibility complex (MHC) in antipsychotic-naive schizophrenia (SS 1511), B-1330

**Taouli B.**
Functional imaging of the liver: C. Characterisation of liver tumours and prediction of tumour response ($E^3$ 620), A-0335

**Taouli B.**
Time to clarify which gadolinium-based contrast should be used in liver MRI (SY 31)

**Tasu J.-P.**
Use of iodine contrast in upper abdominal imaging: stop to the confusion (SY 31)

**Tavakoli Taba S., Baran P., Nesterets Y., Pacile S., Wienbeck S., Dullin C., Arfelli F., Lewis S., Tromba G., Gureyev T., Brennan P.**
Propagation-based computed tomography and cone-beam computed tomography of the breast: a comparison of radiological image quality (SS 1902a), B-1789

**Taylor A.M.**
Cardiac imaging: A. Grown-ups with congenital heart disease (E³ 921), A-0483

**Taylor S.A.**
Radiologic anatomy: abdomen: Small bowel (BE 4), A-0162

**Teerasamit W.S.R.V.**
Predicting progression to hypervascular HCC in hypovascular hypointense nodules on gadoxetic acid-enhanced MR images in patients with chronic liver disease (SS 1901a), B-1769

**Teh J.**
Acute trauma: patterns in the peripheral skeleton: Acute trauma: patterns in the peripheral skeleton (E³ 25E), A-1089

**Teixeira S.R., Arruda E.R., Saade S.M., Jackson O., Pollock A.N., Feygin T.**
Prenatal MRI assessment of swallowing and pharyngeal structures in foetuses with facial clefts (SS 612), B-0604

**Temel E., Cindil E., Ozhan Oktar S., Demir N.B., Sendur H.N., Haznedaroğlu S., Satis H., Bilici Salman R.**
Evaluation of quantitative shear wave sonoelastography of major salivary glands in Sjögren's syndrome (SS 1708), B-1453

**Ter-Karapetyan A., Wielpütz M.O., Kauczor H.-U., Triphan S.**
Quantitative perfusion MRI of the lung in COPD: the problem of short-term repeatability (MY 4), B-0420

**Tessarin G., Fusaro M., Bortolanza C., Morana G., Quaia E.**
HTA of patient management with and without triple rule out CT protocol (SS 1003), B-1011

**Thaha R., Jogi S., Rajan S., Singh A., Mahajan V., Mehndiratta A., Venugopal V.K., Mahajan H.**
Retrospective comparative study to assess the pitfalls of CartiGram and the complementary role of FSPD in the evaluation of cartilage lesions of the knee joint (SS 1410), B-1132

**Thaha R., Jogi S., Rajan S., Venugopal V., Bhattacharjee R., Mehndiratta A., Singh A., Barnwal M., Mahajan V., Nanda G.**
Evaluating variability of T2 values of the cartilage, menisci and muscles around knee joint on CartiGram sequence at 1.5 T and 3.0 T MR (SS 1410), B-1133

**Tham J., Harkness E.F., Stocking K., Brentnall A.R., Cuzick J., Evans D.G., Astley S.M.**
Reproducibility and measurement error in automated breast density assessment (SS 1002), B-0917

**Theilig D., Steffen I., Denecke T., Hamm B., Geisel D.**
Predicting liver failure after extended liver resection following portal vein embolisation with gadoxetic acid-enhanced MRI (SS 1501b), B-1374

**Thoduka S., Mercogliano S., Czernohorsky V., Spahr N., Schierz J., Schenk A., Abolmaali N.**
Post-SIRT $^{90}$Y-PET/CT showing variable deposition of $^{90}$Y in various liver tumour types (SS 609), B-0558

**Thoeny H.C.**
Functional imaging of the kidneys: Chairperson's introduction (E³ 720), A-0377

**Thoeny H.C.**
Renal, adrenal and urinary tract pathologies: Renal pathologies (BS 8), A-0432

**Thoeny H.C.**
Prostate MRI: the accreditation issue: C. Towards a certified radiologist (E³ 1726b), A-1074

**Thomassin-Naggara I.**
Early detection of ovarian cancer: C. ORADS: MRI (E³ 119), A-0073

**Thomassin-Naggara I.**
Breast imaging in pre-menopausal women: Chairperson's introduction (SF 8e), A-0424

**Thompson J.**
Radiography research: a "how to" guide: How to critically appraise a research article (BR 13), A-0779

**Thomsen H.S.**
Contrast media guidelines: Chairperson's introduction (RC 1207), A-0723

**Thorisdottir S., Oladottir G., Nummela M., Koskinen S.**
Diagnostic performance of CT and the use of GI contrast material for the detection of hollow viscus injury after penetrating abdominal trauma (SS 217), B-0101

**Thrall J.H.**
Dream team: A. How to find the best people and make them a part of a long-time success (E³ 1018), A-0595

**Thurnher M.M.**
My three top tips in neuroimaging (not only for neuroradiologists): Meningitis (SF 4), A-0157

**Thurnher M.M.**
Inflammatory and infectious CNS pathology: C. Inflammatory and infectious myelitis (RC 1211), A-0678

**Thurnher M.M.**
Cutting edge imaging and minimally invasive interventions of the spine: Advanced imaging of spinal cord lesions: do we need DWI, perfusion and spectroscopy? (SF 13b), A-0757

**Thurnher M.M.**
Neuro: C. Tumours of the brain and spine (E³ 1423), A-0831

**Thurnher M.M.**
Imaging of the brain: A. Stroke mimics (E³ 1821), A-1075

**Tian S., Yuan H.**
Application of 3.0T magnetic resonance diffusion tensor imaging in diagnosis of discogenic low back pain (SS 1910b), B-1742

**Tiberia F., Rengo M., Steri L., Bellini D., Trionfera G., Carbone I., Laghi A.**
Effectiveness of bowel preparation without diet restriction for optimal quality CT colonography: a validation study (SS 1001a), B-0940

**Tilliridou V., Murchison J., van Beek E.J.R., Newby D.E., Williams M.C.**
Implications of incidental coronary artery calcification on thoracic computed tomography (SS 1503), B-1316

**Tipaldi M., Pignatelli M., Orgera G., Laurino F., Laghi A., Rossi M.**
Long-term follow-up of giant visceral artery aneurysms endovascular treatment: a single centre experience (SS 109), B-0008

**Tipaldi M., Krokidis M., Orgera G., Rebonato A., Ambrogi C., Rossi M.**
Trans arterial embolization of non-variceal upper gastrointestinal bleeding: is the use of ethylene-vinyl alcohol Copolymer as safe as Coils? (MY 8), B-0849

**Tipaldi M., Orgera G., Laurino F., Ronconi E., Krokidis M., Zolovkins A., Laghi A., Rossi M.**
CT-guided lung biopsy: a simplified classification and scoring system to assess appropriateness of indication according to nodules' 18-FDG uptake, dimension and localisation (SS 1909), B-1809

**Tiralongo F., Palermo M., Falsaperla D., Stefano A., Torrisi S.E., Foti P., Basile A., Vancheri C., Palmucci S.**
Correlation between pulmonary function tests, histogram-based analysis indexes and visual HRCT scores in a population of IPF patients (MY 4), B-0418

**Titova A.M., Fokin V.A., Trufanov G.E., Ivanova M.O.**
DECT in the diagnosis of the efficiency of chelation therapy in patients with iron overload (SS 701), B-0791

**Tolan D.J.M.**
Imaging of the complicated postoperative abdomen: C. Bowel (RC 901), A-0488

**Tomehy M., Wazan M., Abdul Jabar A., Abbass E., Mishah N.**
Effect of adding charcoal capsule to abdominal ultrasound preparation on image quality (SS 1414), B-1105

**Tomita E., Hayashi H., Asahara T., Shitakubo Y., Sakuragawa K., Ikushima H., Kanazawa Y., Okazaki T., Hashizume T.**
Evaluation of dose to risk organ during brachytherapy for cervical cancer using a novel passive rectum dosimeter (SS 714a), B-0648

**Tomita H., Yamashiro T., Tsubakimoto M., Iida G., Murayama S.**
Texture analysis for differentiating between nasopharyngeal cancer and nasopharyngeal malignant lymphoma on unenhanced CT (SS 1808), B-1629

**Tomppert A., May M.S., Wiesmüller M., Heiß R., Nagel A., Uder M., Wuest W.**
Perfusion MRI with high spatial and temporal resolution in the head and neck region using golden angle radial sampling (MY 14), B-1245

**Tonelli P., Boeri C., Cirone D., Bianchi S., Miele V., Nori Cucchiari J.**
Reliability of contrast-enhanced spectral mammography (CESM) in the pre-surgical evaluation of breast cancer: CESM vs histological maximum diameter (MY 5), B-0470

**Tong X., Wald R., Karur G.R., Cheung Y., Ng M.**
Application of CMR derived myocardial deformation parameters to predict restrictive physiology in repaired tetralogy of Fallot patients (SS 1903b), B-1864

**Topcuoglu E.D., Topcuoglu O.M., Semiz Oysu A., Bukte Y.**
Gadoterate meglumine, a macrocyclic agent causes gadolinium retention in the brain of children: a case-control study (SS 1011a), B-0929

**Topcuoglu O.M., Selcuk N., Sarikaya B.**
Transarterial radioembolisation with Yttrium90 glass microspheres without temporary or permanent occlusion of the cystic artery appears to be safe in terms of adverse effects (SS 609), B-0557

**Torkzad M.R., Riddell A., Koh D.-M.**
Assessment of abbreviated MRI of liver for initial follow-up of patients with liver metastases from colorectal cancer (SS 1001b), B-1046

**Torlone S., Corridore A., Martino M., Micelli M., Panebianco L., Totaro R., Splendiani A., Masciocchi C.**
Deep grey matter and multiple sclerosis: is there a correlation between MR imaging and disability? (SS 611), B-0544

**Toth D., Kittinger J., Lampichler K., Loewe C., Schernthaner R.**
Curved surface reformations - diagnostic accuracy and time-efficiency of a new peripheral CTA reformation technique (SS 315), B-0219

**Toufique Y., Bouhali O., Doherty J.O., Goracy J., Delakis I.**
Monte Carlo simulation of dual-energy CT systems: initial validation of radiation dose measurements (SS 1813), B-1601

**Tourdias T.**
Functional imaging of the brain: A. Stroke (E³ 320), A-0134

**Tran T.T., Kristiansen C.H., Haidl F., Roy S., Lindstrom J.C., Ashraf H., Lauritzen P.**
Determining optimal scan delay in indirect CT venography: time to peak attenuation correlates with cardiac output and heart rate (SS 615), B-0492

**Trattnig S.**
7 Tesla MR goes clinical: Chairperson's introduction (SF 5a), A-0249

**Trattnig S.**
7 Tesla MR goes clinical: MSK imaging at 7 T: what are the additional clinical benefits (SF 5a), A-0252

**Travali M., Foti P., Ognibene N.M.G., Palmucci S., Puzzo L., Inserra G., Zanoli L., Parisi S., Basile A.**
Can diffusion-weighted imaging (DWI) in MR enterography discriminate inflammatory strictures from fibrotic strictures in Crohn's disease? (SS 1801b), B-1648

**Traykova N.I.**
Communicating the role of the radiologist: best practices to manage patient expectations: Chairpersons' introduction (part 2) (PA), A-0823

**Traykova N.I.**
Head and neck cancer: Keynote lecture (SS 1008), K-23

**Treutlein C., Zeilinger M., Heiß R., Wuest W., Rompel O.**
Free breathing real-time cardiac MRI in children with congenital heart disease: comparison of right ventricular function with standard breath-hold imaging (SS 1903b), B-1868

**Trianni A.**
Patient-specific dosimetry: C. Patient dose in fluoroscopy and interventional (RC 113), A-0049

**Trianni A.**
Radiation protection technology and management: Keynote lecture (SS 1413), K-29

**Triantopoulou C.**
Acute pancreatitis: Role of imaging (E³ 24A), A-0097

**Tridente D.M., Heidinger B.H., DaBreo D.C., Bankier A.A., Litmanovich D.**
CT measurements of expiratory central airway collapse in COPD patients with suspected tracheobronchomalacia: comparison between transverse and MPR images (SS 604), B-0580

**Trimboli R.M.**
Breast imaging: B. Techniques, artefacts and pitfalls in breast MRI (E³ 1521), A-0856

**Tripathi P., Guo W., Yang C., Rai B., Zeng M., Yu H.**
Clinical feasibility assessment of T3 sub-stage in rectal cancer using MRI (SS 1501a), B-1339

**Tripathi P., Hu D., Zeng M., Guo W., Rao S., Rai B.**
MRI-detected extramural vascular invasion is a strong risk factor in predicting distant metastasis in rectal cancer (MY 17), B-1479

**Trisoglio A., Soligo E., Berardo S., Sukhovei L., Bor S., Buoni G., Stecco A., Carriero A.**
Evaluation of STIR, T1 DIXON "in and out of phase" and DWI sequences, to propose a standardised protocol of the MRWB in patients affected by myeloma (MY 3), B-0367

**Trofimenko I.**
Brain cases: adults (SY 24)

**Trojanowska A.**
Radiologic anatomy: head and neck: Neck spaces (BE 6), A-0318

**Trojanowska A.**
Post-treatment imaging of the head and neck: B. Normal findings after surgery (RC 1208), A-0721

**Truwit C.**
Benefits of spectral CT in emergency department (SY 16)

**Tsapaki V.**
Dose management in paediatric radiology: B. Optimisation and technology in paediatric projection radiography, interventional and CT scanning (RC 1313), A-0787

**Tschauner S., Marterer R., Nagy E., Apfaltrer G., Singer G., Riccabona M., Sorantin E.**
Comparison of extremity cone beam-computed tomography and multidetector-computed tomography in injured children (MY 15), B-1361

**Tsetis D.K.**
GI bleeding: how can the problem be solved?: C. When is the interventional radiologist needed? (RC 501), A-0207

**Tsoi C., Law E.K.C., Lee R.K.L., Ng A.W.H., Griffith J.F.**
Prospective randomised controlled trial comparing a battery-powered and a manual drill in bone biopsy (SS 1510), B-1292

**Tsoriev A.E.**
Brain cases: paediatric (SY 24)

**Tsoumakidou G.**
Pain palliation in cancer patients: Pain from bone metastases (SF 9a), A-0512

**Tsuchiya K., Watanabe W., Abe A., Uchida K., Handa J., Goto S.**
Consecutive acquisition of MRDSA and perfusion MRI in cases of brain metastasis through the addition of a supplementary dose of a Gd-based contrast agent at 3T (SS 1411a), B-1167

**Tsuchiya M., Masui T., Katayama M., Terauchi K., Sasaki M., Kawamura K., Yamada T., Sakahara H.**
Texture analysis and machine learning for differentiation between phyllodes tumours of the breast from fibroadenomas in non-contrast MRI (SS 1402b), B-1180

**Tsuda M.**
Adaptive statistical iterative reconstruction for computed tomography of the spine (SS 1814), B-1489

**Tunariu N.**
Whole-body imaging in metastatic urinary tract and prostate cancer: B. WB-MRI and response assessment (E³ 419), A-0196

**Tuncyurek O., Garces-Descovich A., Duran E.E., Cataldo T.E., Poylin V.Y., Mortele K.J.**
Structured vs narrative reporting of pelvic MRI in perianal fistulising disease: impact on clarity, completeness, and surgical planning (SS 201b), B-0210

**Turkay R., Inci E.**
Cognitive-MR-US fusion prostate biopsy: which one is worthier to perform? (SS 1007), B-0877

### U

**Uberoi R.**
Cone-beam, 4D and more: new diagnostic tools for vascular diseases: C. How cone-beam CT can change your practice in interventional radiology (E³ 1226), A-0666

**Ud Din N.**
The role of radiology in major healthcare challenges faced by Pakistan: Imaging of oral cancer (EM 3), A-1078

**Uematsu T.**
Non-enhanced breast MRI (DWIBS mammography) for screening women with dense breasts: a feasibility study (SS 602), B-0550

**Uller W., Wohlgemuth W.A., Goessmann H., Müller-Wille R., Kirchner G.**
Partial splenic artery embolisation using ethylene vinyl alcohol copolymer in patients with portosplenomesenteric thromboses and portal hypertension (SS 709), B-0739

**Ulus S., Kovan O., Arslan A., Elpen P., Aribal M.E.**
A new technique in mammography: self-compression-survey of patient contentedness (SS 1802a), B-1546

**Umar M.S., Joseph D., Auwal A., Charbel S., Ogenyi P.A., Njokwu G., Fati M.A., Moi A.S., Shem S.L.**
Real-time sonography as a teaching aid of anatomy in undergraduate radiography institutions in Northern Nigeria (SS 1414), B-1109

**Umutlu L.**
Improving patient experience (SY 2a)

**Umutlu L.**
Whole-body imaging in gynaecological malignancy: B. PET/CT and PET/MRI in cervix and endometrial cancer: current status (E³ 319), A-0131

**Umutlu L.**
Role of imaging in cancer of unknown primary (CUP): B. Role of PET/CT in suspected CUP (RC 516), A-0282

### V

**Vaimel J., Uprus M., Igumenova N., Roose Ä., Samarin A.**
Influence of respiratory position on pulmonary artery contrast enhancement in dual-energy computed tomography angiography (SS 1904), B-1853

**Valdora F., De Luca C., Bignotti B., Rossi F., Matos J., Calabrese M., Tagliafico A.**
Radiomics analysis of breast cancer on digital breast tomosynthesis (SS 1402b), B-1178

**Valletta R., Faccioli N., Tagliamonte M., Bonatti M., Mansueto G.**
Role of CT colonography in differentiating sigmoid cancer versus chronic diverticular disease (SS 1001a), B-0937

**van Assen M., De Cecco C.N., Eid M., Bauer M.J., Scarabello M., von Knebel Doeberitz P., Oudkerk M., Vliegenthart R., Schoepf U.J.**
The prognostic value of CT myocardial perfusion and CT-FFR for MACE in patients with coronary artery disease (SS 703), B-0787

**van Assen M., De Cecco C.N., Martin S.S., Fischer A., Steinbach R., Oudkerk M., Vliegenthart R., Schoepf U.J.**
Relationship between coronary CT angiography-derived fractional flow reserve and dynamic CT myocardial perfusion imaging in patients with coronary artery disease (SS 703), B-0789

**van Assen M., De Cecco C.N., Sahbaee P., Griffith P., Bauer M.J., Varga-Szemes A., Oudkerk M., Vliegenthart R., Schoepf U.J.**
Feasibility of extracellular volume quantification using dual-energy CT (SS 1403), B-1215

**van Beers B.E.**
Functional imaging of the liver: A. Diagnostic and staging of diffuse liver diseases using functional MRI (E³ 620), A-0333

**Van Buchem M.A.**
Functional imaging of the brain: C. Cerebral small vessel diseases (E³ 320), A-0136

**Van Buchem M.A.**
Altered mental state: Chairperson's introduction (RC 1311), A-0764

**van den Hauwe L.**
My three top tips in neuroimaging (not only for neuroradiologists): Microbleeds (SF 4), A-0161

**van der Molen A.J.**
AMACING and pre-hydration in the ESUR prevention of PC-AKI guideline (SY 19)

**van der Molen A.J.**
Contrast media guidelines: A. Post-contrast acute kidney injury (PC-AKI) (RC 1207), A-0724

**Van der Voet J., Schiphof D., Vroegindeweij D., Bierma - Zeinstra S., Runhaar J., Oei E.**
Association between baseline meniscal extrusion on MRI and long-term incident knee osteoarthritis in two different cohorts (SS 1410), B-1136

**van der Werf N.R., Vonder M., van Hamersvelt R.W., Greuter M., Si-Mohamed S.A., Douek P., Boussel L., Leiner T., Willemink M.J.**
Detectability of small coronary calcifications: a comparison between dual-layer and spectral photon-counting CT (SS 313), B-0311

**van der Werf N.R., Vonder M., van Hamersvelt R.W., Willemink M.J., Greuter M., Leiner T.**
Effect of dose and iterative reconstruction on coronary calcium scores at different heart rates for dual-layer CT (SS 313), B-0317

**van der Zijden T.**
Neurologic emergencies: Ischaemic stroke: treatment (BS 9), A-0525

**van Diest P.J.**
Radiology and pathology: will we ever be (digitally) married?: Are digital pathology and AI the definitive factors for the merging? (SF 13a), A-0735

**van Diest P.J.**
Radiology and pathology: will we ever be (digitally) married?: How would the integration work? (Part 1) (SF 13a), A-0736

**Van Ginneken B.**
Roadmap for artificial intelligence in radiology: recognition, reconstruction, reasoning (SY 4)

**Van Ginneken B.**
Big data and the big picture: deep learning in optimisation of medical imaging (part B): Computer analysis in chest imaging: from rule-based to machine learning to deep learning (EF 2), A-0325

**Van Ginneken B.**
Artificial intelligence (AI): our future cannot be predicted, but we have to be prepared: Basics of machine learning and deep learning (SF 8a), A-0393

**Van Goethem J.**
Cutting edge imaging and minimally invasive interventions of the spine: Chairperson's introduction (SF 13b), A-0755

**Van Goethem J.**
The degenerative cervical spine: A. Degenerative uncovertebral and facet disease (RC 1611), A-0948

**Van Gompel G., Mahmoudi S., Brussaard C.C., De Mey J., Buls N.**
Optimising CT acquisition parameters for the quantification of lung emphysema (SS 213), B-0136

**van Hamersvelt R.W., Voskuil M., de Jong P.A., Willemink M.J., Leiner T.**
A patient specific lumped parametric model based approach to fractional flow reserve derived from coronary computed tomography angiography (SS 703), B-0788

**Van Hecke W., Ribbens A., Billiet T., Wang T., Ly L., Beadnall H.N., Barnett M.H.**
Radiological reading: man vs machine (SS 1005b), B-0984

**van Kroonenburgh A.M.J.L., van Kuijk S.M.J., Jacobi-Postma A.A.**
The intrinsic iodine content of the thyroid is correlated to the HU value of the thyroid on true non-contrast scans (SS 1708), B-1447

**van Kroonenburgh A.M.J.L., van Kuijk S.M.J., Jacobi-Postma A.A.**
Virtual non-contrast reconstructions derived from DECT cannot replace true native scans in head and neck imaging (SS 1808), B-1625

**Van Leemput K.**
Big data and the big picture: deep learning in optimisation of medical imaging (part A): Statistical methods for analysis of multidimensional imaging data (EF 1), A-0266

**van Ommen F., Kauw F., Bennink E., Dankbaar J.W., De Jong H.W.A.M.**
Effect of prolonged acquisition intervals on CT brain perfusion analyses (SS 211b), B-0192

**Van Ongeval C.**
The future role of synthetic mammograms: is it more than a 2D image? (SY 2c)

**Van Ongeval C.**
Breast imaging in pre-menopausal women: Pregnancy-associated breast cancer (PABC) (SF 8e), A-0427

**Van Ongeval C.**
Breast: B. Breast cancer diagnosis and interventions (E³ 1823), A-1085

**van Ooijen P.M.A.**
Everything you need to know about 3D post-processing: B. Making better use of your 3D package: tips and tricks (RC 405), A-0144

**van Ooijen P.M.A.**
Captain's dashboard: C. Challenging the status quo and making new things happen (E³ 918), A-0558

**van Ooijen P.M.A.**
Professional issues for radiology departments to enable research: How to deal with legal requirements in research activities (PI 3), A-0628

**van Os M.**
Radiographers: at the heart of cancer treatment and research: Radiation therapy quality assurance (RTQA) in clinical trials: the role of the radiographer/radiation therapy technologist (EFRS/EORTC), A-0442

**van Wijk Y., Jochems A., Primakov S., Lobbes M.B., Lambin P., Woodruff H.**
Radiomics and deep learning can aid classification of contrast-enhanced spectral mammograms (SS 1402b), B-1179

**Vancoillie L., Cockmartin L., Marshall N., Bosmans H.**
Comparative phantom tests of digital mammograms, breast tomosynthesis and synthetic mammograms (SS 1913), B-1840

**Vancoillie L., Herbst M., Cockmartin L., Marshall N., Schebesch F., Vignero J., Bosmans H.**
Accuracy testing of two partial breast imaging simulation platforms for applications in virtual clinical trials (SS 1913), B-1845

**Vancu M.**
Advanced elastography in breast cancer detection (SY 2b)

**Vandecaveye V.**
Whole-body imaging in gynaecological malignancy: A. WB MRI for staging and treatment planning in ovarian cancer (E³ 319), A-0130

**Vanhavere F.**
Advanced clinical dosimetry in interventional radiology: Real time staff dose monitoring (EU 4), A-0749

**Vanhoenacker F.M.H.M.**
The radiological investigation of musculoskeletal tumours: Chairperson's introduction (RC 1210), A-0691

**Vano E., Fernandez J.M., Sánchez-Casanueva R.M., Ten J.**
Using an automatic patient dose registry to help in the optimisation of interventional radiology practice (SS 1413), B-1195

**Vaño E.**
Advanced clinical dosimetry in interventional radiology: Chairpersons' introduction (part 2) (EU 4), A-0747

**Varga-Szemes A., Bauer M., van Assen M., De Cecco C.N., Suranyi P., Griffith L., Schoepf U.J.**
Machine learning-based prediction of haematocrit values from native MRI myocardial T1-maps to avoid blood sampling for extracellular volume fraction analysis (SS 1005a), B-0914

**Vargas H.A.**
Tumour relapse in gynaecological cancer: B. Planning exenterative surgery (E³ 519), A-0286

**Vargas H.A.**
Tumour relapse in urological cancer: B. Non prostate urological cancer relapse (E³ 619), A-0330

**Vassallo E.**
Head and neck imaging: when does it become abnormal?: C. Anatomical variations in the face and neck (RC 908), A-0570

**Vassallo L., Giannini V., Defeudis A., Mazzetti S., Cappello G., Regge D.**
CT texture analysis to predict response to target therapy of hepatic metastases from colorectal cancer (SS 1905b), B-1828

**Vasyliv I., Rohovyk N., Marushchak O., Rohovyk M.**
Predictive model of lung severity in children with cystic fibrosis using Brasfield and Chrispin-Norman scoring systems (SS 604), B-0584

**Vavasseur A., Muscari F., Otal P., Rousseau H., Dercle L., Mokrane F.**
Blended learning using radiology short video based lecture series improves medical school student performance and satisfaction (SS 1405), B-1188

**Vázquez E.**
Foetal and neonatal imaging pearls: Chairperson's introduction (RC 912), A-0559

**Vázquez E.**
Paediatric oncology: The most common brain tumours (BS 12), A-0679

**Veillon F.**
Head and neck imaging: when does it become abnormal?: A. Normative measures in the temporal bone (RC 908), A-0568

**Velasco González A., Münnich N., Rusche T., Buerke B., Görlich D., Sauerland C., Heindel W.**
Circle of Willis anatomy does not influence the results of mechanical thrombectomy with balloon guide catheter (SS 311), B-0266

**Veldhuis W.B.**
Radiology and pathology: will we ever be (digitally) married?: How would the integration work? (Part 2) (SF 13a), A-0737

**Velthuis B.K.**
From diagnosis to prognosis: how does cardiac imaging affect patient outcome?: B. In non-ischaemic cardiomyopathy (RC 403), A-0147

**Veltri G., Iezzi R., Posa A., Carchesio F., Gasbarrini A., Manfredi R.**
Transarterial chemoembolisation with degradable starch microspheres (DSM-TACE) as second-line treatment in HCC patients dismissing or ineligible for sorafenib (SS 209), B-0110

**Veltri G., Iezzi R., Posa A., Carchesio F., Gasbarrini A., Manfredi R.**
Locoregional treatments for unresectable early stage HCC in patients with high-risk for intraprocedural bleeding: is single-step combined therapy safe and feasible? (SS 209), B-0114

**Venkatesh S.K.**
Tumour response assessment in abdominal imaging: A. Colorectal liver metastases (RC 301), A-0107

**Venturini E., Losio C., Rodighiero M., Tacchini S., Panzeri M., Ravelli S., Schiani E., Panizza P.**
Digital breast tomosynthesis with photon-counting technology: preliminary data from a clinical comparison study (SS 302), B-0256

**Venugopal V., Mallya Y., Jagannatha V., Mahajan V., Mahajan H.**
Combined traditional image processing and deep learning approach for automated detection of tears of the anterior cruciate ligament: is it the game changer for AI in musculoskeletal MRI? (SS 605), B-0572

**Venugopal V., Vaidhya K., Mahajan V., Mahajan H., Vaidya S., Devalla A.R.**
Opening the "Black Box": radiological insights into a deep neural network for lung nodule characterisation (SS 1005a), B-0912

**Venugopal V., Tadepalli M., Reddy B., Modi A., Gupta S., Warier P., Rao P., Mahajan H., Mahajan V.**
Automated classification of chest x-rays as normal/abnormal using a high sensitivity deep learning algorithm (SS 1905a), B-1753

**Verbeke S.**
The "prostate unit" at work: The pathologist: guardian of the truth (MS 8a), A-0479

**Verhagen M.V., Watson T.A., Neriman D., Punwani S., Taylor S., Daw S., Shankar A., Menezes L., Humphries P.D.**
PET-MRI is non-inferior to PET-CT in paediatric and adolescent Hodgkin's lymphoma for staging and early response assessment (SS 1512), B-1324

**Vernooij M.**
Altered mental state: C. MRI in the diagnosis of Alzheimer's disease (RC 1311), A-0767

**Vernooij M.**
Neuro: B. Neurovascular disorders and trauma of the brain (E³ 1423), A-0830

**Vernuccio F., Rosenberg M., Meyer M., Nelson R.C., Marin D.**
Negative biopsy of focal hepatic lesions: decision tree model for patient management (SS 201a), B-0070

**Vernuccio F., Godfrey D., Meyer M., Williamson H.V., Niedzwiecki D., Ronald J., Palta M., Marin D.**
Imaging response assessment and outcomes in hepatocellular carcinoma after stereotactic body radiotherapy: iRECIST as a potential substitute for traditional criteria (SS 616a), B-0533

**Vernuccio F., Cannella R., Meyer M., Choudhoury K.R., Bashir M.R., Furlan A., Marin D.**
Diagnostic performance of hepatobiliary phase hypointensity and major imaging features for the diagnosis of HCC in lesions between 10 and 19 mm, and arterial phase hyperenhancement (SS 1901a), B-1768

**Verschakelen J.A.**
Secrets of radiology communications with patients and referring physicians: A. Communication is all that really matters (E³ 1218), A-0708

**Verschakelen J.A.**
Patterns of pulmonary toxicity: C. Inhalation: lung injury beyond smoking (E³ 1626b), A-0984

**Verstraete K.**
Musculoskeletal: bones and soft tissues: Bone marrow diseases (BS 1), A-0031

**Verstraete K.**
Bone, joint and soft tissue infection: A. Osteomyelitis (RC 410), A-0178

**Vértes M., Juhász I.Z., Nguyen D.T., Csobay-Novák C., Nemes B., Hüttl K., Dósa E.**
Long-term results of the aortoiliac kissing stents and risk factors of in-stent restenosis (SS 1009), B-0958

**Vicentin I., Ronza R., Pecorilla M., De Mattia C., Sgrazzutti C., Colombo P.E., Torresin A., Siena S., Vanzulli A.**
Polmonary metastases from pancreatic cancer: morphologic and quantitative differences compared to lung metastases from colorectal cancer (SS 1016), B-1028

**Vicini S., Rengo M., Tiberia F., Bellini D., Trionfera G., Carbone I., Laghi A., Steri L.**
Polyp detection rate as a quality measure in CT colonography: analysis of the performance of a CT colonography service using a reduced bowel preparation without dietary restriction (SS 1001a), B-0935

**Vignale D., Palmisano A., Del Maschio A., De Cobelli F., Esposito A.**
Cardiac CT in the quantification of myocardial extracellular volume fraction (ECV) with a semiautomatic method: comparison with cardiac MR (SS 1403), B-1216

**Vijayakumar D.H., Kulkarni M.M.**
Utility of 3T MRI whole-body DWI in onco-imaging for the detection of metastasis: comparison with PET/CT (SS 1716), B-1464

**Vikestad K.G.**
The future of radiology: what it could mean for new radiography graduates: Changing the delivery of radiology, responding to the needs of our patients (BR 17), A-1044

**Vilar J.**
Errare humanum est: B. Errors in CT of the chest (E³ 721), A-0357

**Vilar J.**
Chest: Chairperson's introduction (E³ 1623), A-0951

**Vilares Morgado P.M.**
Basic interventional radiology for non-interventionalists: let's start with biopsy!: How to do your procedure (SF 5b), A-0247

**Vilela P.**
My three top tips in neuroimaging (not only for neuroradiologists): Venous sinus thrombosis (SF 4), A-0159

**Vilgrain V.**
Multiparametric approach for diffuse liver disease with ultrasound (SY 4)

**Vilgrain V.**
Artificial intelligence: presented with new lecture formats: ESOR in action 2019 (ESOR), A-0369

**Villeirs G.M.**
Early detection of prostate cancer: Chairperson's introduction (E³ 219), A-0091

**Villeirs G.M.**
The "prostate unit" at work: Chairperson's introduction: My PSA is elevated...what now? (MS 8a), A-0477

**Villeirs G.M.**
The "prostate unit" at work: Multidisciplinary case presentation and discussion (MS 8a), A-0482

**Vinayak S.**
Radiology in Africa: facing challenges and opportunities: Is imaging underused in Africa? East Africa as an example. Solutions: what can the ESR do? (EM 1), A-0311

**Vinnicombe S.J.**
The high-risk patient enigma: C. Can surgery be avoided? (E³ 426a), A-0151

**Vliegenthart R.**
Acute chest pain and cardiac imaging: B. Myocardial perfusion in acute coronary syndrome (ACS): necessity or luxury? (RC 1603), A-0939

**Vo D.T., Phan C.C., Nguyen L.T.T., Le H.M.D., Nguyen T.T.**
Anal fistula: the role of MR imaging in preoperative evaluation (SS 201b), B-0214

**Vock P.**
Medical imaging and emerging issues in occupational radiation exposure: Is fear of radiation-induced occupational cancer irrational? (ESR/EFOMP), A-0544

**Voicu I., Napolitano A., Vinci M., Diomedi-Camassei F., Carducci C., Carai A., Mastronuzzi A., Toma P., Colafati G.**
Diffusion kurtosis imaging (DKI) utility in differentiating low- and high-grade gliomas in paediatric patients: further experience (SS 1512), B-1327

**Vollenbrock S.E., van Dieren J.M., Voncken F.E.M., van Turenhout S.T., Peppelenbosch-Kodach L.L., Hartemink K.J., Aleman B.M.P., Beets-Tan R.G.H., Bartels-Rutten A.**
Added value of MRI to endoscopic and endosonographic response assessment after neoadjuvant chemoradiotherapy in oesophageal cancer: a pilot study (MY 17), B-1483

**Volpe A., Romeo V., Ginocchio M.I., Maurea S., Mainenti P.P., Petretta M., Sarno L., Martinelli P., Brunetti A.**
Imaging detection of placenta adhesion disorder in patients with placenta previa: comparison between US and MR findings (SS 1507), B-1383

**von Falck C., Hinrichs J.B., Wacker F.**
Presence of intravascular contrast material after intraarticular injection for CT arthrography (SS 710), B-0681

**von Knebel Doeberitz P., Pannell T., van Assen M., Albrecht M.H., De Cecco C.N., Duguay T., Bayer R.R., Tesche C., Schoepf U.J.**
Coronary CT angiography derived plaque quantification with artificial intelligence CT fractional flow reserve for the identification of lesion-specific ischaemia (SS 305), B-0301

**von Krüchten R., Lorbeer R., Schuppert C., Schulz H., Kauczor H.-U., Peters A., Karrasch S., Bamberg F., Schlett C.**
Lung volumes and its association with subclinical impairment in whole-body MR imaging (SS 1404), B-1208

**Voormolen M., van der Zijden T., D'Archambeau O., Van den Bergh F., Mondelaers A., Loos C., Vanacker P., Baar I., Yperzeele L.**
Double aspiration thrombectomy in endovascular recanalisation of acute ischaemic stroke patients (SS 309), B-0279

**Vourtsis A.**
Breast density and risk stratification: Keynote lecture (SS 1002), K-21

**Vullierme M.**
How to face the tsunami of pancreatic intraductal papillary mucinous neoplasia (IPMN): Diagnosis of IPMN: the mimickers and how to sort them out (SA 5), A-0238

### W

**Wagner W., Wuennemann F., Biederer J., Konietzke P., Stiller W., Wielpütz M.O., Kauczor H.-U., Tromba G., Dullin C.**
Synchrotron phase-contrast imaging of the lung: a proof-of-concept study on porcine lungs in a human-scale chest phantom (SS 1804), B-1610

**Wald C., Aldoj N., Feger S., Hartmann N., Lukas S., Zargaran Tavakoli S., Dewey M.**
Automated coronary artery segmentation using a U-net deep learning architecture on cardiac CT (SS 1003), B-1016

**Wall A., Kucharczyk S.R., Hennessy W., O'Connor M., McNulty J.**
The opinions of American radiography educators on lead shielding use in conventional radiography (SS 1914a), B-1706

**Wallis M.G.**
Breast cancer screening and treatment: how much is too much?: Are there breast cancers we do not have to treat? (SA 16), A-0944

**Wallis M.G.**
Diagnosing ductal carcinoma in situ (DCIS): C. Diagnosing DCIS with biopsy (RC 1702), A-1029

**Wallis M.G., Clements K., Hilton B., Litherland J., Maxwell A.J., Sharma N., Thompson A.**
The mammographic features of 13,213 women with screen-detected non-invasive breast disease (SS 202), B-0079

**Wallström J., Geterud K., Kohestani K., Godtman R., Socratous A., Hellström M., Hugosson J.**
Evaluation of dynamic contrast enhancement in PIRADSv2: a report from the Gothenburg 2 screening study for prostate cancer (SS 307), B-0324

**Walsh C.**
Dose reduction and image quality implications of iterative image reconstruction in CT: C. Image quality assessment of iterative reconstruction: pitfalls and future directions (RC 1713), A-1052

**Wang F., Wang Q.**
Correlation between single-kidney glomerular filtration rate (GFR) with ultra-low-dose CT perfusion and 99mTc-DTPA renal scan in healthy and tumour-bearing kidney (SS 307), B-0331

**Wang F., Liu J., Wang Y., Zhou Y., Liu C., Liang D., Xie L.**
Epithelial ovarian cancer: tumour staging and detection of lymph node metastasis using ADC histogram parameters (SS 1507), B-1386

**Wang H., Lu Y.**
Diagnostic value of MR imaging with metal artefact reduction sequences in local recurrence of malignant bone tumour after joint replacement (SS 210b), B-0062

**Wang H., Lai J., Li J., Gu R., Liu F., Hu Y., Jiang X., Mei J., Su F.**
Does establishing preoperative nomogram including ultrasonographic findings help predict the likelihood of malignancy in patients with microcalcifications? (SS 702b), B-0718

**Wang H., Zhang F., Fu W., Guan J., Guo Y.**
Radiomic analysis of multiparametric MRI for preoperative evaluation of pathological grade in bladder cancer (SS 707), B-0831

**Wang H., Zou T., Xia C., Borrego P., Sousa P., Zhang M.**
On the generalisability of deep learning across populations and abnormalities: a case study of the Stanford MURA and CheXNet algorithms (SS 1005a), B-0907

**Wang H., Yue S., Lu P., Li W.**
The effect of different monochromatic image choices on the accuracy of CT perfusion values for lung tumour in one-stopspectral and perfusion CT scan (SS 704), B-0779

**Wang H., Lu P., Yue S., Li W.**
The effect of different monochromatic image choices on the reconstruction of CT perfusion data in one-stop spectral and perfusion CT scan (SS 1904), B-1857

**Wang L., Li R., Yan F., Qiang J., Han Q., Chen X.**
Volumetric iodine density using dual-layer spectral CT for liver fibrosis staging and histopathological correlation (SS 1801a), B-1531

**Wang M., Feng S., Li Z.P.**
Prediction of type 2 diabetes mellitus using noninvasive MRI quantitation of visceral abdominal adiposity tissue volume (SS 414), B-0411

**Wang M., Wang Y., Wang Y., Yu M., Wang M., Jin Z.**
Application of artificial intelligence-based image optimisation algorithm in aorta CTA with low tube voltage and reduced contrast medium volume (SS 205), B-0012

**Wang R., Li J., Gao J.**
Clinical value of radiomics nomogram in preoperative prediction of lymph node metastasis of gastric adenocarcinoma (SS 1901b), B-1877

**Wang S., Li N., Yu C.**
The application of hepatic artery-targeting guidewire technique combined with indirect portography to transjugular intrahepatic portosystemic shunt (SS 709), B-0745

**Wang S., Fan G.**
Characterising resting-state networks in bilateral severe-to-profound sensorineural hearing loss infants within an early sensitive period (SS 1412), B-1230

**Wang S., Wang Z., Zhang S., Fu Y., Hu T., Peng W.**
Assistant effects of a computer-aided diagnosis system based on 3D convolutional neural network on LDCT reading (MY 7), B-0801

**Wang S., Gong J., Yu H., Peng W.**
CT-based radiomics predict immune-iherapy response in patients with non-small-cell lung cancer (SS 1905a), B-1758

**Wang W., Cheng J.**
A whole-tumour histogram analysis of apparent diffusion coefficient maps obtained using 3.0T MRI for distinguishing uterine endometrial carcinoma from endometrial polyps (MY 7), B-0814

**Wang W., Cheng J.**
A whole-tumour histogram analysis of apparent diffusion coefficient maps for distinguishing lateral ventricle central neurocytoma from ependymoma (SS 1416), B-1239

**Wang W., Dong Y., Yao Z., Zhang Q., Yang D., Yu J.**
A sparse representation radiomics method based on multi-modality ultrasound images for hepatocellular carcinoma diagnosis and PD1 prediction (SS 1905b), B-1835

**Wang W., Ding Y., Yang L., Zeng M., Rao S., Tian J.**
Radiomics of gadoxetic acid-enhanced MRI: a predictive biomarker for CK19-positive hepatocellular carcinoma (MY 17), B-1467

**Wang Y., Yong C., Wielopolski P., van der Wiel-Kooij E.C., Tiddens H.A., Ciet P.**
Assessment of lung volume and central airways dimension using MRI in children: a comparison with plethysmography, CT and PFT (SS 212), B-0170

**Wang Y., Yao J., Raymond C., Ellingson B.M.**
Association between tumour acidity and hypervascularity within human gliomas using pH-weighted amine CEST-EPI and DSC-perfusion MRI at (SS 1411a), B-1165

**Wang Z.**
Ultrasound-guided vacuum-assisted resection: could it be enough for the diagnosis and treatment of intraductal papilloma? (MY 5), B-0467

**Watanabe A.T., May R.C., Sen L.C., Kapoor M., Leung J.**
Artificial intelligence software to improve mammography workflow (SS 702a), B-0696

**Watanabe A.T., Lim V., Weise E., Vu H.**
Improved cancer detection using artificial intelligence: a retrospective evaluation of missed cancers on mammography (SS 702a), B-0698

**Waterton J.C.**
Imaging biomarkers and their combinations in the era of artificial intelligence: Role of AI in the introduction of imaging biomarkers: accelerator or obstacle? (ESR/EIBALL), A-0060

**Wathén T., Paalimäki-Paakki K., Henner A.**
Women's experience of pain in screening mammography (SS 1914b), B-1717

**Wawrzyniak P., Bobek-Billewicz B., Hebda A., Awramienko A., Mazgaj P.**
Tissue sodium concentration (TSC) in normal brain: preliminary study (SS 1511), B-1338

**Webb A.**
Demystifying MRI: things you always wanted to know: A. Basic MR: the building blocks of pulse sequences (RC 1513), A-0910

**Webb A.**
MRI: safety, phantoms and quantification: Keynote lecture (SS 713), K-18

**Weber L., Kristiansson I., Hansson M., Siemund R., Geijer H., Geijer M.**
Exposure levels from radiology procedures used in myeloma diagnostics (SS 1413), B-1197

**Weber M.-A.**
Musculoskeletal: B. Bone tumours ($E^3$ 1723), A-1035

**Weber T.**
Radiology and migrations: Radiology of the diseases related to migration (PC 5), A-0231

**Wegner F., Friedrich T., von Gladiss A., Buzug T.M., Barkhausen J., Haegele J.**
Magnetic particle imaging: artefact-free imaging of the in-stent lumen of coronary stents (SS 615), B-0498

**Wei Y., Song B., Jiang H., Chen J.**
Can intravoxel incoherent motion diffusion-weighted imaging be use for preoperative assessment of microvascular invasion in hepatocellular carcinoma (MY 3), B-0356

**Weidekamm C.**
Knee sports injuries and postoperative MRI: B. ACL reconstruction and cartilage repair (TC 1528), A-0920

**Weidekamm C.**
Knee sports injuries and postoperative MRI: C. Interactive case discussion (Part 2) (TC 1528), A-0922

**Weikert T.J., Nesic I., Cyriac J., Moor M., Bremerich J., Sauter A.W., Sommer G., Stieltjes B.**
Generation of a curated dataset from unstructured reports using natural language processing, illustrated on CT reports regarding pulmonary embolism (SS 1005a), B-0904

**Weikert T.J., Pradella M., Durlak F., Sperl J.I., Stieltjes B., Cyriac J., Bremerich J., Sommer G., Sauter A.W.**
Assessing the precision of an AI-powered algorithm for automatic detection and 3D segmentation of primary tumours in NSCLC (SS 1016), B-1030

**Weikert T.J., Winkel D.J., Bremerich J., Stieltjes B., Sauter A.W., Sommer G.**
AI-powered detection of pulmonary embolism in CT pulmonary angiograms: a validation study of the diagnostic performance of prototype algorithms (SS 1404), B-1211

**Weinrich J.M., Maas K.J., Stáreková J., Intert L., Sehner S., Regier M., Adam G., Quitzke A.**
Feasibility of sub-milliSievert CT of the skeletal pelvis: a human cadaver study (SS 710), B-0679

**Weishaupt D.**
Focal liver lesions: characterisation: Keynote lecture (SS 201a), K-01

**Weiss C.M., Di Gaetano E., Plataroti S., Cattarin E., Bragagnolo D., Morana G.**
Second-line vacuum-assisted excision biopsy with 8G needle for the management of B3 microcalcifications: preliminary results (SS 1402a), B-1154

**Wen B., Cheng J., Zhu J.**
The value of high-resolution diffusion-weighted imaging in differentiating lingual squamous cell carcinoma and lingual lymphoma (SS 1008), B-0969

**Wen B.**
A preliminary study of CT spectral imaging on patients with aortic dissection using revolution CT (SS 1815), B-1504

**Wen D., Xia C.**
Development of a multiparametric radiomics combined T2WI and DWI to staging rectal cancer (SS 1805b), B-1588

**Wen Q., Hou Y., Ma Y., Ma Q., Lu X.**
Application of dual-layer detector spectral CT in differentiating benign and malignant lung nodules (SS 204), B-0149

**Wen Q., Hou Y., Yang L., Ma Y., Lu X.**
Accuracy and variability of coronary CT angiography with iterative model reconstruction for plaque burden assessment compared with IVUS: a pulsating cardiac phantom study (SS 1503), B-1308

**Wenkel E.**
Pretherapeutic diagnostics of tumour extent (part 1) (SY 2d)

**Wennmann M., Kintzelé L., Piraud M., Hofmanninger J., Menze B., Kauczor H.-U., Hillengass J., Langs G., Weber M.-A.**
Quantifying the change of total tumour volume in whole-body MRI over time improves risk stratification of smouldering multiple myeloma patients (SS 316), B-0344

**Wennmann M., Kintzele L., Hielscher T., Langs G., Kauczor H.-U., Merz M., Hillengass J., Menze B., Weber M.-A.**
Spatial heterogeneity in growth dynamics and local volumetric biomarkers for risk stratification and dissemination behaviour in smouldering multiple myeloma (SS 316), B-0348

**Werner S., Gast R., Grimmer R., Horger M.**
Comparison of computer-aided semi-automated volumetry with manual volumetry of part-solid pulmonary nodules (SS 204), B-0146

**Westerland O.A., Amlani A., Siddique M., Streetly M., El-Najjar I., Cook G., Goh V.J.**
Comparison of 18F-FDG PET/CT and whole-body MRI in assessing newly diagnosed myeloma (SS 316), B-0351

**Weston M.**
Imaging in pregnancy: B. Imaging acute abdomen in pregnancy (RC 1307), A-0810

**Weston M.**
Genitourinary radiology for the general radiologist: B. Gynaecological emergencies (E³ 1421), A-0821

**Weston W.**
Are the referrals being made for the investigation of pulmonary nodules appropriate according to the BTS guidelines? (SS 1004), B-1000

**Westphalen A.C., Fazel F., Nguyen H., Cabarrus M., Hanley-Knutson, K., Shinohara K., Carroll P.R.**
PSAD and ADC values improve the diagnostic accuracy of PI-RADS v2 (SS 707), B-0829

**Wetscherek M.T.A., Rutschke W., Frank C., Grothoff M., Lurz P., Thiele H., Gutberlet M., Lücke C.**
T1 and T2 mapping assessment of chronic myocarditis vs dilated cardiomyopathy, hypertensive heart disease and healthy controls (SS 303), B-0379

**Wetzl M., Weinmann D., Kopp M., Roth J., Heiß R., Wuest W., Rompel O., Uder M., May M.S.**
Evaluation of ultra low dose CT examinations of the paediatric chest using spectral shaping and an iterative reconstruction algorithm (SS 1512), B-1321

**Whitlow C.T.**
Artificial intelligence and radiation protection: Artificial intelligence for intelligent reconstruction methods for radiation protection measures (EU 7), A-1015

**Widmann G., Degenhart G., Al-Ekrish A., Puelacher W., Feuchtner G., Jaschke W.R., Jacobs R., Pauwels R.**
Dose management in computed tomography using the as-low-as-diagnostically acceptable image quality approach (SS 1813), B-1599

**Wielandner A., Gremmel F., Laggner R., Senn D., Zuckermann A., Loewe C., Beitzke D.**
Cardiac magnetic resonance for response prediction to Levosimendan treatment in patients with acute decompensated heart failure prior to cardiac surgery (SS 1803), B-1617

**Wielema M., Dorrius M.D., De Bock G.H., Pijnappel R.M., Oudkerk M., Sijens P.E.**
Diagnostic performance of region of interest methods in diffusion-weighted imaging of breast lesions: a systematic review and meta-analysis (SS 1802b), B-1565

**Wielpütz M.O.**
Chronic obstructive pulmonary disease (COPD): C. Is there a role for MRI? (E³ 922), A-0567

**Wienbeck S., Fischer U., Lotz J., Uhlig J.**
Post-contrast cone-beam breast-CT without the prior pre-contrast scan: can we reduce radiation exposure while maintaining diagnostic accuracy? (SS 1802a), B-1544

**Wiesmüller M., Wuest W., Heiß R., May M.S., Uder M., Laun F.**
Comparison of lesion detection and image quality between non-EPI diffusion weighted imaging and multi-shot EPI diffusion weighted imaging of cholesteatoma (SS 308), B-0289

**Wigati K., Bosmans H., Soejoko D.S., Cockmartin L., Jacobs A., Binst J., Lemmens K., Marshall N.**
Device-specific reference values for modulation transfer function (MTF) quality control measurements of digital mammography detectors (SS 1913), B-1839

**Wigati K., Bosmans H., Soejoko D.S., Lemmens K., Binst J., Jacobs A., Cockmartin L., Marshall N.**
Analysis of noise decomposition results in mammography quality control: is quantum noise the dominant noise source? (SS 1913), B-1842

**Wildberger J.E.**
The heart between the lungs: Chairperson's introduction (E³ 1222), A-0715

**Wildberger J.E.**
AMACING results on prophylactic iv hydration prior to contrast administration (SY 19)

**Wilkinson L.S., Warren L.M., Burnside E., Young K., Patel C., Massat N.J., Myles J., Duffy S.**
Mammographic parenchymal pattern: correlation with age, breast density and prediction of cancer detection and nodal status in a UK screening population (SS 1002), B-0920

**Williams M.**
Cardiac CT in search of coronary artery disease: Keynote lecture (SS 1003), K-25

**Williams M.**
The heart between the lungs: B. Coronary artery disease (E³ 1222), A-0717

**Wilson D.J.**
Bone, joint and soft tissue infection: C. Pyomyositis and other soft tissue infections (RC 410), A-0180

**Wilson D.J.**
MSK intervention: the road from low to no invasion: Radiation exposure: from reduction to avoidance (SF 5c), A-0274

**Wilson D.J., Allen G.M., Bullock S.A., Denton J.**
Lumbar nerve root blocks using MR: the effectiveness and safety of ultrasound/MRI fusion image guidance (SS 1510), B-1290

**Winkel D.J., Chabin G., Cyriac J., Stieltjes B., Weikert T.J.**
Reproducibility of fully automated liver volumetric analyses with artificial intelligence: towards laboratory radiology (MY 7), B-0816

**Winkel D.J., Heye T., Benz M.R., Stieltjes B., Boll D., Hofmann V.**
Prostate cancer lesion characterisation: rapid acquisition using compressed sensing in combination with semi-automatic lesion segmentation for dual-parameter decision support (MY 13), B-1092

**Winkelmann M.T.O., Bongers M.N.**
Differentiation of adrenal adenomas from adrenal metastases using single-phased dual energy CT: a comparison to MRI (SS 1407), B-1143

**Wirth S.**
Why do I miss fractures in emergency?: Chairperson's introduction (RC 1217), A-0682

**Wisselink H.J., Pelgrim G., Rook M., Oudkerk M., Vliegenthart R.**
Dose reduction in CT emphysema densitometry enabled by iterative reconstruction and post-scan noise reduction: a phantom study (SS 604), B-0586

**Withey S.J., Owczarczyk K., Yip C., Siddique M., Green A., Bell J., Cook G., Goh V.J.**
Does perfusion-metabolic tumour phenotype predict for neoadjuvant therapy response in primary oesophageal tumours undergoing DCE-MRI and $^{18}$F-FDG PET/CT? (SS 616b), B-0617

**Withey S.J., Owczarczyk K., Yip C., Siddique M.M., Green A., Bell J., Cook G., Goh V.J.**
Is the perfusion-metabolic tumour phenotype related to tumour volume and nodal stage in primary oesophageal tumours undergoing DCE-MRI and $^{18}$F-FDG PET/CT? (SS 1901b), B-1874

**Witkowska-Wrobel A., Aristovich K., Faulkner M., Avery J., Holder D.**
Imaging the seizure onset zone and fast electrical circuit activity in epilepsy with electrical impedance tomography (MY 16), B-1425

**Witowski J., Złahoda-Huzior A., Stanuch M., Grochowska A., Pędziwiatr M.**
In-house automatic liver segmentation from CT based on a deep convolutional neural network with data augmentation (SS 1805b), B-1583

**Wnuk E., Maj E., Jabłońska-Pawlak A., Rowinski O.**
Validation of exophthalmos MRI measurements in patients with Graves orbitopathy, compared to opthalmometry results (SS 1708), B-1452

**Woerner T., Raczeck P., Buecker A., Schneider G.**
Non-breath hold dynamic contrast enhanced abdominal and thoracic MRI in paediatric oncologic patients using a GRASP-sequence at 3T (SS 1012), B-1058

**Woerner T., Buecker A., Schneider G.**
Whole body MR imaging and MR-guided biopsy in initial diagnosis and follow-up of paediatric malignancies: comparison with other imaging modalities (SS 1716), B-1463

**Woitek R.A., McLean M.A., Grist J., Gill A., Abraham J., Gilbert F., Caldas C., Brindle K., Gallagher F.A.**
Response of breast cancer to neoadjuvant treatment assessed using hyperpolarised $^{13}$C MRI (SS 1902b), B-1802

**Wolf F.**
No time to lose: aortic disease, revisited: A. Diagnosis and treatment of abdominal aortic aneurysms (RC 1515), A-0907

**Wolf N.**
State-of-the-art paediatric neuroradiology: A. Imaging myelin maturation disorders (RC 811), A-0429

**Wolff L., Berkhemer O., van Es A., van Walsum T., Van Zwam W., Dippel D.W., Majoie C.B., Van der Lugt A.**
Validation of automated ASPECTS software for detection of early ischaemic brain changes on non-contrast CT scans (SS 311), B-0258

**Wong Y., Wang L., Wu C.**
Characteristics and predictive factors of delayed events in patients of blunt splenic trauma designated for non-operative management (SS 217), B-0103

**Wörtler K.**
Bone tumours: Bone tumours (E³ 25B), A-0351

**Woussen S., Westerlinck H., Keupers M., Prevos R.M., Thywissen T., Van Ongeval C.**
Impact of the use of a breast cancer risk assessment tool on the intensive screening of women clinically referred to as intermediate to high risk (SS 1002), B-0915

**Wu C., Lee S., Seo J., Lee S., Lee H., Hwang H., Cho Y., Kim N.**
Comparison of consistency of nodule detection in repeated chest PA X-ray images between radiologists and deep learning model (SS 605), B-0576

**Wu H., Zhang G.**
Axial spondyloarthritis: dual-energy virtual noncalcium CT for detection of bone marrow oedema in the sacroiliac joints (SS 610), B-0521

**Wu J., Huang H., He M., Fan Q., Ma M., Lin L.**
Non head-injured paediatrics in the emergency department: clinical predictors of abnormal head CT findings (SS 1017), B-0952

**Wu R., Shi R., An D., Wu C., Chen B., Wu L., Xu J.**
Intravoxel incoherent motion diffusion-weighted imaging for the assessment of myocardial fibrosis in hypertrophic cardiomyopathy (SS 1903a), B-1816

**Wu Y., Lin W., Lu C.**
Extended memory network degeneration in Parkinson's disease patients with lean muscle loss (MY 16), B-1431

**Wuennemann F., Rehnitz C.**
Value of 3D-multi-echo-data-image-combination (MEDIC) for evaluation of SLAP lesions at 3T-MRI of the shoulder with arthroscopic correlation (SS 310), B-0240

### X

**Xia C., Alsurayhi A., Vonder M., Pelgrim G., Rook M., Oudkerk M., Vliegenthart R.**
Reliability of coronary calcium scoring on low-dose chest CT: comparison with ECG-triggered cardiac CT based on third-generation dual-source CT (SS 1503), B-1311

**Xianying Z., Yinchen W.**
MRI quantitative parameters of small-bowel perfusion for early diagnosing and assessing activity of Crohn's disease: a preliminary study (SS 1801b), B-1649

**Xiao Y.**
Locoregional heparinisation for patients with acute deep venous thrombosis of lower extremity: safety and efficacy evaluation (SS 209), B-0112

**Xiao Y., Zeng J., Fu G., Tang B., Yang C., Lui S.**
Heterogeneity of brain structure alterations in patients with never-treated first episode schizophrenia (SS 1511), B-1332

**Xiaochun Z., Shuxia W.**
Preoperative prediction of regional lymph node metastasis in thoracic oesophageal carcinoma with 18F-FDG PET/CT and contrast-enhanced CT texture analysis: a nomogram model (MY 3), B-0363

**Xie C., Ather S., Mansour R., Gleeson F., Chowdhury R.**
Dual-energy CT in the diagnosis of occult acute scaphoid injury: a direct comparison with MRI (SS 310), B-0245

**Xie Q., Zhou X., Lin L.**
Association between coronary artery calcium score and cardiovascular magnetic resonance imaging parameters in patients with end-stage renal disease (SS 1503), B-1315

**Xie S., Li J., Wang H., Shen W.**
Liver function evaluation with Gd-EOB-DTPA-enhanced MRI: can visual scoring replace signal measurement? (SS 1501b), B-1371

**Xie S., Li Q., Qi H., Cheng Y., Shen W.**
Differentiating mild and substantial fibrosis from normal subjects: comparison of diffusion-kurtosis imaging and conventional diffusion-weighted imaging (SS 1801a), B-1534

**Xing L.**
To determine the image quality of non-linear blending (NLB) technique in aortic angiography acquired by novel injection protocol compared with linear blending (LB) technique (SS 615), B-0495

**Xu C.**
The value of RESOLVE on 3.0 T MRI for predicting the early efficacy of concurrent radiochemotherapy of advanced stage nasopharyngeal carcinoma patients (SS 1008), B-0972

**Xu E., Luo L., Li K., Zeng Q., Tan L., Long Y., Huang Q., Zheng R.**
Value of ultrasound fusion imaging for the thermal ablation of medium-sized hepatocellular carcinoma (SS 1709), B-1440

**Xu E., Long Y., Yan R., Li K., Luo L., Zeng Q., Tan L., Zhang M., Zheng R.**
Radiofrequency ablation of liver cancers adjacent to the gallbladder without additional invasive measures under contrast-enhanced ultrasound monitoring: a preliminary study (SS 1709), B-1441

**Xu H., Guo Y., Xie L., Xu R., Yang M., Li H., Fu H., Fu C.**
Myocardial characterisation using CT extracellular volume in swine acute myocardial infarction model: comparison with CMR T1 mapping and histology (SS 303), B-0376

**Xu H.M., Liu Y., Yuan H.**
Arterial spin labelling MRI in evaluating perioperative cerebral perfusion of carotid endarterectomy with different post labelling delays: comparison with CT perfusion imaging (SS 211b), B-0190

**Xu K., Zhang Y.**
Histogram analysis of CE-MRI for differential diagnosis of pleomorphic adenomas and malignant tumour of parotid gland (SS 1008), B-0968

**Xu Q., Zhou C., Ye C., Tao G., Zhao W., Ren D., Li X., Zhao L., Lu G.**
Computed tomography-based radiomics analysis to assess treatment response after perioperative neoadjuvant therapy (MY 7), B-0805

**Xu S., Anderson V., Li M., Peretti J., Garcia C., Choyke P.L., Pinto P., Turkbeyi B., Wood B.J.**
Focal prostate cancer ablation "out of the rectum and out of MRI": new tools for fusion-guidanceand "super-activesurveillance" (MY 8), B-0837

**Xu X., Teng G.**
Altered resting-state functional connectivity of the anterior cingulate cortex in anxious rats post-acoustic trauma (SS 1011b), B-1033

**Xu X., Sui X., Song W., Song L., Huang Y., Jin Z.**
Diagnostic value of quantitative dual-source CT dual-energy iodine maps combined with morphological CT features in assessing histological subtypes of lung cancer (MY 4), B-0423

**Xu X., Sui X., Song L., Huang Y., Jin Z., Song W.**
Assessment of EGFR mutation status in patients with non-small-cell lung cancer using different iodine quantification methods in dual-energy CT: areal vs volumetric analyses (MY 4), B-0430

**Xu X., Sui X., Song L., Huang Y., Jin Z., Song W.**
Feasibility of low-dose CT with spectral shaping and third-generation iterative reconstruction in evaluating interstitial lung diseases associated with connective tissue disease (SS 1604), B-1409

**Xuesong D.**
Bladder perivascular epithelioid cell tumour: dynamic CT and MRI analyses of 8 cases with 2-year follow-up (SS 1807), B-1665

### Y

**Yadav P., Kulkarni V., Patil Y., Harit S.**
Diffusion-weighted imaging with background suppression in detection of breast malignancies: a non-invasive, non-contrast and non-radiation technique (SS 1802b), B-1562

**Yadu N., Saxena S.**
Role of dynamic contrast-enhanced MRI in early detection of gallbladder carcinoma (SS 301), B-0387

**Yagami K., Hirota M., Kato H., Okada F., Suzuki S.**
Radiation dose reduction using copper filters (SS 714b), B-0660

**Yagi T., Tasaki A., Hirata T., Yamazaki M., Yoshimura N., Aoyama H.**
Quantitative MR texture analysis to distinguish leiomyosarcoma from benign leiomyoma with high signal intensity on T2-weighted imaging (SS 1907), B-1899

**Yamashiro T., Tsubakimoto M., Xu Y., Morita Y., Tomita H., Murayama S.**
Ultra-high-resolution CT for temporal bone imaging: a comparison to cone-beam CT (SS 308), B-0292

**Yan J., Cheng J., Liu F.**
Whole tumour histogram analysis of apparent diffusion coefficient for evaluation of histological grade and BRAF mutation status in pleomorphic xanthoastrocytomas (SS 1416), B-1236

**Yan J., Cheng J., Liu F.**
Prediction of the BRAF mutation status of pleomorphic xanthoastrocytomas using pre-operative multi-parametric MRI (SS 1416), B-1237

**Yan J., Zhang Z., Cheng J.**
Machine learning-based radiomics to predict molecular subgroups of medulloblastoma (SS 1416), B-1242

**Yan J., Cheng J., Geng J.**
Predictive value of two-tensor unscented Kalman filter fractography in the reconstruction of the arcuate fasciculus in patients with gliomas involving eloquent language areas (SS 1911b), B-1889

**Yan L., Chuan X., Xia C., Wang S., Chen K.**
Deep learning for automatic detection of fractures on chest CT scans after blunt trauma (SS 605), B-0566

**Yang B., Zhang W., Tao B., Liu J., Lui S.**
Grey matter network changes with ageing in a large group of never-treated patients with schizophrenia (MY 16), B-1427

**Yang D.D., Riley B., Tavare A.N., Hare S., Parthipun A.**
Comparing volume doubling time and $SUV_{max}$ for benign and malignant lung nodules (SS 1004), B-1004

**Yang H., Hong Y., Gong Yong J., Lee Y.K., Kim S.K., You J.H., Park E.**
Retrospective study of foot and ankle dual-energy CT in gouty arthritis patients without first metatarsophalangeal joint uric acid crystal deposition (SS 210a), B-0054

**Yang H., Hong Y., Gong Yong J., Lee Y., Kim S., You J., Park E.**
Importance of minimal HU setting in the foot and ankle DECT: frequency and the common site of submillimeter artefact noted in patients in gout free patients (SS 710), B-0683

**Yang J., He B.**
Effect of dual-energy CT single-energy imaging on image quality of mesenteric vessels and segmental thickening intestinal wall (SS 1401b), B-1283

**Yang I., Wu B.**
Correlation between T2WI texture features of primary tumour and nodal metastasis in rectal cancer (SS 201b), B-0204

**Yang I., Wu B.**
The role of intravoxel incoherent motion and diffusion kurtosis imaging in evaluating pathological complete response to neoadjuvant chemoradiotherapy in rectal cancer (SS 616b), B-0612

**Yang L., Jiang T.**
Value of CT image texture analysis in the differential diagnosis of benign and malignant parotid tumours (SS 1808), B-1624

**Yang L., Li H., Quan C., Zhou L., Geng D., Li Y.**
Quantitative brain lesion distributions in MOG-Ab-positive and AQP4-Ab-positive patients: overlaps and differences (SS 611), B-0541

**Yang Y., Liu H.**
Computerised texture analysis predicts histological invasiveness within lung adenocarcinoma manifesting as pure ground-glass nodules (SS 704), B-0774

**Yang Y., Han Q., Chen X., Yan F.**
Assessment of pancreatic ductal adenocarcinoma lesion using virtual monoenergetic images from dual-layer spectral detector CT (SS 1401a), B-1098

**Yang Y., Han Q., Chen X., Dong H.**
Evaluation of vascular and local lymph node invasion, resectability of pancreatic ductal adenocarcinoma with spectral detector CT (SS 1401a), B-1100

**Yao K.B.**
Radiographers' challenges offering imaging services in Africa: Côte d'Ivoire. Professional practice of radiology and imaging in Africa: radiographers for more commitment and responsibility in patients' safety (EM 5), A-0655

**Yap A., Rahmat K., Muridan R., Seow P., Fong C., Ong L., Tan L., Ramli N.**
Evaluation of white matter maturation in children with developmental delay using diffusion tensor imaging (DTI) (SS 1412), B-1233

**Yar O., Onur M.R., Idilman I., Akpinar E., Akata D.**
Investigation the causes of overscanning in z-axis in thorax, abdominopelvic and thoraco-abdominopelvic CT examinations (SS 1813), B-1594

**Yates A., Cromwell P., Hughes N.M., Skehan S., Evoy D., McDermott E., Prichard R.**
Correlation between preoperative 4D CT parathyroid volumes and perioperative PTH levels (SS 608), B-0633

**Yazbek S., Atat C., Abdel Hay J., Moussa R.**
Functional MRI study of language organisation in left-handed and right-handed trilingual subjects (SS 1011b), B-1031

**Ye Z., Xiong X., Chen J., Cao L., Duan T., Zhang Z., Song B.**
The value of MRI-PDFF in detecting and grading hepatic steatosis in patients with nonalcoholic fatty liver disease: a meta-analysis (MY 17), B-1471

**Yee A.Y.M., Norlisah R., Chng L.S.**
Role of ultrasound elastography in differentiating tuberculosis and malignant lymph nodes (SS 608), B-0641

**Yel I., Lenga L., Wichmann J.L., Albrecht M.H., Vogl T.J., Martin S.S.**
Value of static first-pass dual-energy CT perfusion analysis in patients with acute pancreatitis (SS 501), B-0448

**Yeoh H., Lee J.Y., Lee Y., Park D.W., Kim T., Ahn G.Y., Bae S., Kim Y.S., Kim H.Y.**
Association of cerebral microbleeds with vasculitis in patients with systemic lupus erythematosus (SS 1511), B-1336

**Yeshua T., Rebibo S., Sfez K., Safran O., Liebergall M., Leichter I.**
A novel machine learning approach for assessing osteoporotic fracture risk by texture analysis of femoural neck radiographs (SS 1013), B-0992

**Yévenes Aravena S., Cárdenas Loguercio G., Casas Muñoz J., Araya Campos F., Garrido Inostroza C., Manríquez Galán V., Guzmán Rojas R., Naser Nassar M., Castro Caperán D.**
Correspondence analysis between different reference lines used in magnetic resonance defecography of pelvic organs prolapse or the measurement of prolapse of pelvic organs with POPQ (SS 607), B-0629

**Yi X., Zhang Y., Liu L.**
CT based machine learning radiomics for differentiating tumour grade in clear cell renal cell carcinoma (SS 1605), B-1393

**Yin C., Joshua A., Crumbaker M., Emmett L.**
Modulation of PSMA expression by androgen deprivation therapy (ADT): serial PSMA PET in men with hormone sensitive and castrate resistant prostate cancer commencing androgen blockade (SS 306), B-0226

**Yin P., Hong N.**
A triple-classification radiomics model for the differentiation of primary chordoma, giant cell tumour, and metastatic tumour of sacrum based on magnetic resonance imaging (SS 1910a), B-1731

**Yin P., Hong N.**
Comparison of radiomics nomograms for the differentiation of sacral chordoma and sacral giant cell tumour based on 3D computed tomography and multiparametric magnetic resonance imaging (SS 1910a), B-1732

**Ying M.T.C., Cheng S.C.H., Ahuja A.T.**
Diagnostic performance of computer-aided quantification of intranodal vascularity in differentiating common neck lymph node diseases (SS 608), B-0638

**Yoen H., Kim S.H., Yoon J.H., Hur B.Y., Kim J.H., Park H.E., Oh H., Han J.K.**
Prognostic value of tumour regression grade on MR in rectal cancer: a large-scale, single-centre experience (SS 1501a), B-1342

**Yoo J., Lee J., Kang H., Ahn S.**
Reproducibility of shear wave dispersion imaging for evaluation of non-alcoholic fatty liver disease (SS 1801a), B-1532

**Yoon J., Lee E., Lee S.M., Chang W., Lee J.**
Double low-dose spectral liver CT in patients at high risk of HCC (SS 701), B-0795

**Yoon S., Park Y., Choi Y., Ahn S., Chang J., Kim S., Lee S.**
Radiomics MRI phenotyping with machine learning to predict the grade of lower grade gliomas: a study focused on non-enhancing tumours (SS 211a), B-0097

**Yorkston J.**
Extremity CT: the basics and the promise (SY 27)

**You C., Gu Y.J., Chen S., Shen X.X., Peng W.J.**
Radiologic and clinicopathologic associations with upgrade to malignancy in high-risk breast lesions (SS 1402a), B-1152

**Yousry T.A.**
Functional imaging of the brain: Chairperson's introduction (E³ 320), A-0133

**Yu Q., Quan X., Lu X.**
Quantitative assessment of the relationship between spatial distribution of emphysema and pulmonary function in COPD patients by an automatic analysis software (SS 604), B-0578

**Yu S., Chu P., Smith-Bindman R.**
Clinical indications for computer tomography (CT) diagnostic reference levels (DRLs) (SS 205), B-0015

**Yu S., Chu P., Smith-Bindman R.**
Pediatric Computer Tomography (CT) Diagnostic Reference Levels (DRLs) by age and size (SS 1512), B-1320

**Yuan D.H.**
Multislice spiral CT images combined with CEA, lymphocyte and neutrophil ratio predict recurrence and metastasis postoperative of rectal cancer (SS 201b), B-0208

**Yuan J., Yang L., Wang M., Feng Z., Zhang S.**
Terahertz time-domain spectroscopy of collagen I and tendon (MY 9), B-0854

**Yuanyuan S.**
The value of whole tumour volume-based ADC histogram analysis of differential diagnosis in paediatric posterior fossa tumours (SS 1411a), B-1161

**Yule H.**
Contemporary breast imaging: A. Tomosynthesis (RC 114), A-0007

**Yun S.G., An Y.Y.**
Predictive factors on ultrasound for the early tumour recurrence of breast cancer after breast-conserving treatment (SS 702b), B-0723

**Yunqian H., Jiang J., Chen M.**
Contrast-enhanced perfusion patterns in predicting stroke in vulnerable plaque: a cohort study of carotid stenosis in Chinese patients (SS 215), B-0035

**Yusuf M.**
Patient-centric initiative to reduce radiation dose to paediatric patients in computer tomography for improved patient safety in a tertiary care facility in Pakistan (SS 205), B-0021

### Z

**Zackrisson S.**
View of future screening programmes: conclusion from the Malmö Breast Tomosynthesis Screening trial - a concept for breast screening (SY 2c)

**Zackrisson S.**
New mammography: digital breast tomosynthesis and future techniques: A. Should we abandon 2D mammography? (RC 1302), A-0761

**Zagrodzka M., Lichtarski M., Lewicki A., Niezgoda S., Stachura E., Brzezinski J.**
Accuracy of mpMRI/US fusion prostate biopsy in diagnosis of clinically significant carcinomas with histopathological verification (SS 1007), B-0874

**Zalaudek M., Marik W., Juras V., Riedl M., Hager B., Bieri O., Deligianni X., Weber M., Trattnig S.**
7T T2*-relaxometry detects early tendon degeneration in patients with diabetes mellitus type 1 (DM1) and shows correlations with BMI and age (SS 210a), B-0055

**Zamboni G.**
Chronic pancreatitis: How to diagnose and classify (E³ 24B), A-0340

**Zamboni G.**
Diagnosis and management of acute pancreatitis: B. Role of imaging in diagnosis of acute pancreatitis: new trends (RC 1601), A-0926

**Zanardo M., Capaldo A., Codari M., Catania D., Cornacchione P., Durante S.**
Radiographers and scientific research: the Italian experience (SS 214), B-0028

**Zanardo M., Doniselli F.M., Monti C.B., Durante S., Sconfienza L.M., Sardanelli F.**
MRI principles and technical issues: where do European radiographers study from? (SS 414), B-0416

**Zanardo M., Doniselli F.M., Di Leo G., Esseridou A., Tritella S., Mattiuz C., Menicagli L., Sardanelli F.**
Using the lean body weight (LBW) instead of total body weight (TBW) to dose the iodinated contrast medium (ICM) for abdominal CT: a randomised controlled trial (RCT) (SS 615), B-0490

**Zanelli E., Cereser L., GIannarini G., Ficarra V., Zuiani C., Girometti R.**
Multiparametric MRI outperforms three popular clinical models in predicting pT3 prostate cancer in patients undergoing radical prostatectomy (MY 13), B-1078

**Zanetti M.**
Bone, joint and soft tissue infection: B. Septic arthritis (RC 410), A-0179

**Zarb F.**
Clinical simulation and its role in radiography education: Chairpersons' introduction (Part 2) (SF 9b), A-0490

**Zarb F.**
Growing radiography research: Research is nothing without effective dissemination (EFRS WS), A-0865

**Zargaran S., Wozniak F., Dewey M.**
Automatic noise and vessel density measurement for CT angiography quality assurance (SS 205), B-0016

**Zarowski A.**
Imaging in Meniere's disease: what to believe and how to interpret: Meniere's disease: the clinician's perspective (SF 15b), A-0886

**Zarowski A.**
Imaging in Meniere's disease: what to believe and how to interpret: MR hydrops imaging: what are the benefits for the surgeon and the patient? (SF 15b), A-0889

**Zawaideh J.P., Smith J., Bruining A., Moyle P.L., Sala E., Addley H.C., Freeman S.**
MRI criteria and sequence evaluation for the diagnosis of placental adhesive disorder among radiologists with different reporting experience (SS 1507), B-1384

**Zawaideh J.P., Trambaiolo Antonelli C., Olivero A., Bertolotto M., Derchi L.E.**
The added value of scrotal palpation during testicular ultrasound (US): a technical note (SS 1807), B-1658

**Zech C.J.**
Imaging of the complicated postoperative abdomen: Chairperson's introduction (RC 901), A-0485

**Zeilinger M.**
Hybrid imaging: the best of both worlds: A. PET/CT (RC 314), A-0110

**Zelefsky M.J., Zakian K., Wibmer A.G., Tyagi N., Kollmeier M., Hunt M., Sala E., Mejias Vargas H.**
Prostate cancer and benign tissue response to hypofractionated SBRT monitored by multi-parameteric MRI (SS 307), B-0323

**Zeng J., Zhang W., Xiao Y., Liu J., Tang B., Fu G., Lui S.**
Drug effects on brain structure in acute and chronic schizophrenia (SS 1511), B-1331

**Zeng Y., Deng K., Yang H., Tan Y., Liu J., Geng D., Zhang J.**
Noise-optimised virtual monoenergetic imaging of dual-energy CT: effect on metal artefact reduction in patients with lumbar internal fixation (MY 9), B-0858

**Zerunian M., De Santis D., Caruso D., Biondi T., Funicelli G., Rosati E., Zucchelli A., De Dominicis C., Laghi A.**
Non-small-cell lung cancer treated with pembrolizumab: correlation between texture analysis and iRECIST (SS 1016), B-1022

**Zhang H., Liu X., Zhang P., Zhang G.**
To categorise ovarian epithelial cancers and predict clinical outcome using multiparametric radiomics (SS 1605), B-1396

**Zhang H., Xu L.**
The differential diagnosis of benign and malignant solid pulmonary nodules by CDT-VIBE early dynamic contrast-enhancement of MRI (SS 204), B-0148

**Zhang H., Li W., Tong T.**
Comparison of intravoxel incoherent motion DWI, diffusion kurtosis imaging, and conventional DWI in predicting the chemotherapeutic response of colorectal liver metastases (MY 3), B-0359

**Zhang H., Wang Z., Liu J.**
The effects of weight-bearing and non-weight-bearing anteroposterior DR of feet in hallux valgus deformity (SS 1314), B-1064

**Zhang J.**
The value of low-dose dynamic CT myocardial perfusion imaging for accurate evaluation of microvascular obstruction in patients with acute myocardial infarction (SS 1403), B-1213

**Zhang J., Li Z., Xia C.**
T2* value analysis of achilles tendons disease with ultra-short echotime (UTE) MRI (MY 9), B-0853

**Zhang L., Guo Y., Yang Z., Li X., Fu C., Xu H., Yang M., Xu R., Xie L.**
Quantitative T2 mapping for detecting myocardial oedema and its association with myocardial injury biomarkers in acute myocardial infarction swine models (MY 18), B-1675

**Zhang S., Wang W., Su X., Sun H., Yue Q., Gong Q.**
Meta-analytic investigations of grey matter alterations in patients with Tourette syndrome (MY 16), B-1426

**Zhang X., Cheng J., Ma K.**
Utility of histogram analysis of ADC maps in differential diagnosis of Pleomorphic adenoma and Acinar cell carcinoma originated from parotid gland (MY 14), B-1248

**Zhang Y., Yang Z., Guo Y., Diao K., Shen M., Li X., Xu K., Yu H., Xia C.**
Assessment of myocardial deformation in a rabbit model of diabetes mellitus by cardiac magnetic resonance tissue tracking (SS 603), B-0596

**Zhang Y., Yang Z., Guo Y., Xu H., Peng W., Xia C., Li Z.**
Assessment of right ventricular deformation in end stage renal disease patients by cardiac magnetic resonance tissue tracking (MY 18), B-1673

**Zhang Y., You H., Lv Y., Dou W., Hou B., Feng F.**
Study of correlation between hippocampal perfusion and volume in patients with temporal lobe epilepsy for epileptic lateralisation (SS 1911b), B-1883

**Zhang Y., Liu S., You H., Wang Y., Hou B., Lv Y., Feng F.**
Feasibility of evaluating the genetic mutation status of grade II and III gliomas by diffusion-weighted imaging (SS 1911b), B-1890

**Zhang Z., Jiang H., Cao L., Ye Z., Chen J., Song B.**
A predictive model for early recurrence of hepatocellular carcinoma after tumour resection based on whole lesion MR imaging radiomics features (SS 201a), B-0074

**Zhang Z., Chen J., Jiang H., Cao L., Ye Z., Song B., Duan T.**
Peritumoural radiomics features from gadoxetic acid-enhanced MR imaging predicting early recurrence of hepatocellular carcinoma (SS 1001b), B-1044

**Zhao C.**
Three-dimensional shear wave elastography for differentiating benign from malignant thyroid nodules (MY 14), B-1255

**Zhao L., Zhao X., Ma X.**
Prediction for early recurrence of intrahepatic mass-forming cholangiocarcinoma: qualitative and quantitative MRI combined with prognostic immunohistochemical marker (SS 301), B-0394

**Zhao M.**
A GPC3-specific aptamer-mediated MR probe for hepatocellular carcinoma (SS 606), B-0507

**Zhao Q., Xu R., Yang Z., Guo Y.**
Magnetic resonance first-pass perfusion imaging for assessment of myocardial microvascular dysfunction in pediatric patients with acute leukemia (SS 1903a), B-1826

**Zhao X., Huang M., Zhu J., Cheng J.**
Diagnosis of biliary stone disease: compressed-sensing-accelerated 3D-MRCP versus conventional MRCP (SS 301), B-0390

**Zheng L., Wang Q., Wang Z.**
High-resolution MR image of intracranial blood vessels wall using 3D VISTA sequence (SS 315), B-0216

**Zheng L., Zhang L., Zhang G.**
Pilot study of three-dimensional MRI in the evaluation of eye shape of high myopia and classification of posterior staphyloma (SS 1708), B-1449

**Zheng S., Guo J., Rook M., Cui X., Veldhuis R.N.J., Oudkerk M., van Ooijen P.M.A.**
Automatic pulmonary nodule detection in low-dose CT scans using convolutional neural networks based on maximum intensity projection (SS 1905a), B-1756

**Zholdybay Z., Akhmetova G., Akhmetova G., Panina A., Ainakulova A.**
Lung cancer screening with low dose computed tomography: the republic of Kazakhstan experience (SS 1016), B-1026

**Zhong J., Arunsingh M., Brown P., Dyker K., Prestwich R., Currie S., Vaidyanathan S., Scarsbrook A.**
Prediction of patient outcome in head and neck squamous carcinoma following chemo(radiation): comparison of 4 different FDG PET-CT response assessment interpretative criteria (SS 706), B-0666

**Zhong J., Brown P., Nelstrop H.L., Prestwich R., McDermott G.M., Currie S., Vaidyanathan S., Scarsbrook A.**
Performance of FDG PET-based textural features for prediction of loco-regional recurrence in advanced larynx and hypopharynx squamous cell carcinoma (SS 706), B-0667

**Zhou F., Wang Y., Schoepf U.J., Tesche C., Xu L., Hou Y., Yan J., Zhang J.Y., Zhang L.J.**
Diagnostic performance of fractional flow reserve derived from coronary computed tomography angiography in myocardial bridging: a comparison with invasive fractional flow reserve (SS 703), B-0785

**Zhou J., Dan H., Cheng G.**
Feasibility study of whole breast texture analysis for predicting tumour response to neoadjuvant chemotherapy (SS 1402b), B-1171

**Zhou W., Chai M., Wong W., Yap P., Lai Y., Lee T., Leung S., Lee J., Ng M.**
Prognostic value of myocardial perfusion reserve index in patients with coronary microvascular disease: a multi-centre study (SS 1403), B-1220

**Zhou X., Xiao B., Hu H., Tang J.**
The development of a novel manganese dioxide-based nanoparticles and real-time monitoring of its anticancer effect by MRI (MY 5), B-0472

**Zhu B., Jiang T., Peng P., Li M.**
Can dual-energy CT replace perfusion CT for the diagnosis of the solid pulmonary nodules? (SS 204), B-0151

**Zhu L., Denecke T., Asbach P., Hamm B., Xue H., Jin Z.**
Relapse of type 1 autoimmune pancreatitis after steroid therapy: imaging findings and risk factors (SS 501), B-0450

**Zhu L., Sun Z., Jin Z., Qian T., Nickel D., Makowski M., Asbach P., Hamm B., Xue H.**
T1 mapping of autoimmune pancreatitis as a quantitative outcome biomarker (MY 17), B-1475

**Zhu T., Yushu C., Yu Z., Wen Z., Zheng J., Fabao G.**
Cardiac magnetic resonance imaging study of right ventricular myocardial deformation in spontaneous T2DM rhesus monkey (SS 1903b), B-1861

**Zhu Y., Chen Y., Guan W.**
The experimental study of prostate cancer imaging using PSMA targeting ultrasound/MRI dual modality nanobubbles (SS 606), B-0511

**Zhu Z., Zhao X., Zhou C.**
Wide-detector axial scan on revolution CT for combining application of ASIR-V and ASIR on unenhanced abdominal CT in vivo: the image quality and radiation dose evaluation (SS 407), B-0445

**Zhu Z., Zhao X., Zhou C.**
Perfusion MRI prediction of progrossion-free survival in patients with hepatocellular carcinoma treat with TACE (MY 8), B-0832

**Zhuravlev K.N., Sinitsyn V.E., Shpektor A.V.**
Coronary calcium screening with non-gated standard and low-dose chest CT in comparison with ECG-gated calcium scoring CT (SS 1503), B-1314

**Zimny A.**
Inflammatory and infectious CNS pathology: B. Infectious encephalitis (RC 1211), A-0677

**Zini C., Notaro D., Sadotti G., Bellini M.**
Percutaneous intervertebral disc coagulation therapy (PDCT) by plasma light: a new method for the treatment of lumbar and cervical disc herniation (SS 1809), B-1575

**Zins M.**
Visceral arteries: B. Acute and chronic mesenteric ischaemia (RC 115), A-0022

**Zins M.**
Tumour response assessment in abdominal imaging: C. Pancreatic adenocarcinoma (RC 301), A-0109

**Zins M.**
How to face the tsunami of pancreatic intraductal papillary mucinous neoplasia (IPMN): Chairperson's introduction (SA 5), A-0236

**Zins M.**
How to face the tsunami of pancreatic intraductal papillary mucinous neoplasia (IPMN): Panel discussion: Management of IPMN: what is the Achilles heel of our current concept? (SA 5), A-0240

**Zins M.**
Solid pancreatic neoplasms: Diagnosis ($E^3$ 24D), A-0852

**Zolda P.**
Growing radiography research: Turning your research idea into reality: opportunities for European funding (EFRS WS), A-0864

**Zorzenon I., Ukmar M., Gennari A., Zdjelar A., Bottaro L., Sartori A., Dinoto A., Cova M.A.**
Evaluation of signal intensity in dentate nucleus and globus pallidus after repeated administrations of gadolinium in multiple sclerosis patients (SS 611), B-0536

**Zubler V.**
Inflammatory and infectious diseases of the spine: how to differentiate from degeneration: A. Spondyloarthritis: a diagnostic chameleon (RC 910), A-0541

**Zugni F., Funicelli L., Bertani E., Bellomi M.**
Lymphadenectomy of small intestine neuroendocrine neoplasias (si-NEN): testing the most recent preoperative classification with contrast-enhanced computed tomography (CT) (SS 1401b), B-1281

**Zur G., Lesman-Segev O., Schlesinger I., Eran A., Kahn I.**
Structural integrity impact of focused ultrasound thalamotomy and correlation to behavioural outcomes for tremor disorders (SS 1811a), B-1557

**Zwierko B., Dura M., Zuchowski P., Jeka S.**
Applying the low-dose protocol in high resolution computed tomography examination in patients with rheumatoid arthritis (SS 1604), B-1408

### F

### List of Authors & Co-Authors (F)

### List of Authors & Co-Authors

### A

Aalbers A.G.: B-0182
Aaløkken T.M.: B-0131
Aamir R.: B-1117
Aarts B.M.: B-0559, B-0850
Aarts S.: A-0743
Aasand K.K.: B-1047
Abbas H.M. H.: B-0966
Abbasi H.R.: B-0291
Abbass E.: B-1105
Abdel Aal A.M.K.: B-0357, B-0838, B-1296, B-1298, B-1299, B-1301, B-1473
Abdel Hay J.: B-1031
Abdel Razek A.: B-0100
Abdel Razek N.M.: B-1156
Abdel Wahab C.: B-1894
Abdelal S.: B-1334
Abdelganne L.: B-1849
Abdelhafez Y.: B-0056
Abdel-Kawi M.: B-0366
Abdelmoneim A.: B-0162
Abdel-Wahab M.: B-0196, B-1683
Abdo A.: B-1407
Abdolell M.: B-0916, B-0919
Abdul Aziz Y.F.: B-1902
Abdul Jabar A.: B-1105
Abdul-Khaliq H.: B-0167
Abe A.: B-1167
Abela-Ridder B.: A-0229
Abergel A.: B-1470
Aberle C.: B-1196
Aboagye E.O.: B-1387
Aboelmagd H.: B-0923
Aboelsafa A.A.: B-1423
Abolmaali N.: B-0558
Abonyi M.: B-1589
Abou El-Ghar M.E.: B-0830
Abou Elkassem A.: B-0357, B-1473
Aboueldahab N.: B-0838
Abouelkheir R.T.: B-0830
Abraham J.: B-1802
Abrams P.: B-1191
Abrantes A.F.: B-0414, B-0489, B-0643, B-0652, B-0658, B-1359
Abrar D.B.: B-0515, B-0516, B-0517, B-0518, B-0519, B-1736
Abuzaid M.M.: B-0644, B-1486
Acar T.: B-0115, B-0737, B-0743
Acevedo P.A.: B-1780
Acharya T.: B-0300, B-1114
Achterhold K.: B-0995, B-1848
Acquafredda F.: B-0056
Adam G.: B-0119, B-0499, B-0590, B-0601, B-0679, B-1089, B-1240, B-1682
Adams H.H.: B-1690
Adams L.C.: B-1141, B-1734
Adams N.B.: B-1391
Adams N.C.: B-0267
Adamson M.: B-1307
Addley H.C.: B-1384
Adeberg S.: B-0506
Adel L.: B-0923
Adla T.: B-0597
Adriaensen M.: A-0211
Afadzi M.: B-0131, B-0480
Agarwal A.: B-1666, B-1747
Agarwal S.: B-0640
Agazzi G.M.: B-0970
Aggarwal D.: B-1637
Aggarwal R.: B-0141, B-1637
Aghahowa E.A.: B-1545
Agliata G.: B-1719
Agosta F.: B-1696
Agostini A.: B-1118
Agostini C.: B-1010
Agostini G.A.: B-1300
Agostini S.: B-0622
Ahern D.: B-0246
Ahmad Ainuddin H.: B-0468
Ahmad M.N.: B-1367
Ahmed A.F.: B-0212
Ahmed H.U.: B-0437
Ahmed I.: B-0966
Ahmed S.A.: B-0371, B-1086, B-1284
Ahn G.Y.: B-1336
Ahn H.S.: B-0699, B-1800
Ahn S.J.: B-1532, B-1772
Ahn S.S.: B-0097
Ahn Y.: B-0105
Ahrens-Nicklas R.: B-1057
Ahres A.: B-0786
Ahuja A.T.: B-0638
Ahuja C.K.: B-1428
Aiello M.: B-0174, B-0928
Ailianou A.: A-1068
Ainakulova A.: B-1026
Aissa J.: B-0954, B-1355, B-1368, B-1726, B-1739
Aitchison R.: B-1793, B-1794
Aivazoglou L.U.: B-0860
Akata D.: A-0355, B-1594
Akatsuka Y.: B-1735
Akdogan M.U.: B-0906
Akgul M.H.: B-1744
Akhadov T.: B-0187, B-1038, B-1778, B-1887
Akhan O.: A-0227
Akhlebinina M.: B-0187
Akhmetova G.: B-1026
Akhoundi N.: B-0427
Akhverdieva G.: B-0621
Akin I.: B-0587
Akin O.: B-0152
Akintimehin A.: B-0950
Akinwumi O.: B-0947
Akpinar E.: B-1594
Akpınar M.: B-1805
Akram M.H.: A-1077
Al Arab N.: B-1168
Al Kamali A.: B-1362
Al Kuhaimi T.S.: B-0060
Al Raaisi A.: B-1574
Alagely A.: B-1169
Alagic Z.: B-0674
Alam S.I.: B-1130
Alamanni F.: B-1615, B-1686
Albano D.: B-0052, B-0868, B-0870, B-0898, B-1730
Alberich-Bayarri A.: A-0143, A-0371, A-0637, B-0680, B-1397, B-1830
Albers P.: B-0327, B-0828, B-1462
Alberts A.R.: B-0181
Albrecht M.H.: B-0296, B-0301, B-0434, B-0448, B-0794, B-0843, B-0847, B-1362, B-1472
Alcaide A.B.: B-1609
Alcalá-Galiano A.: A-0443
Aldoj N.: B-0172, B-0802, B-1016
Aldrighetti L.: B-0851, B-1439
Al-Ekrish A.: B-1599
Aleman B.M.P.: B-1483
Alexander S.: B-1455
Alfaqih M.: B-0902
Alfaro I.: B-1653
Alghamdi J.M.A.: B-0809
Alì M.: B-0042, B-0895, B-1728, B-1821, B-1867
Ali S.: B-0902
Aliprandi A.: B-0895, B-0898
Alison R.: B-0078, B-0080
Aliyu I.: B-1391
Aljefri A.: B-0060
Al-Jiboori M.A.K.: B-0209
Aljoqiman K.S.: B-1763
Alkadhi H.: A-0044, A-0879, A-1051, B-0996, B-1541, B-1616
Alkasab T.K.: A-0506
Alkim E.: B-0906
Allen C.: B-0159, B-0443
Allen G.M.: B-1290
Allen M.: A-0988
Allen R.A.: B-0885
Allenbach G.: B-0235
Alley M.: B-1722
Allweis T.: B-1569
Almaas R.: B-0168
Almalki S.: B-0060
Almeida C.M.D.S.: B-0889, B-1063
Almeida M.F.A.: B-0358
Almeida N.A.: B-0274
Almeida R.P.P.: B-0414, B-0489, B-0652, B-0658, B-1359
Al-Mosawe A.M.J.: B-0209
Alnajashi N.: B-1459
Alonso-Burgos A.: B-0294
Aloufi F.F.: B-1670
Alqahtani S.J.M.: B-0659, B-0662, B-1067
Alqarni A.: B-1459
Alqattan A.: B-0409
Alsafi A.: B-0627
Alshaikh M.: B-0060
Alshamari M.: B-0491
Alsurayhi A.: B-1311
Alt E.: B-1669
Alterio D.: B-0973
Altindag C.: B-0906
Altmeyer K.: B-0167
Al-Ubeidy H.: B-0491
Alukic E.: B-1068, B-1710
Alurkar A.: B-0841
Alvares de Campos M.: B-0129
Alvarez de Sierra B.: B-0294
Alvi A.: B-0090
Amad A.: A-0403
Ambrogi C.: B-0849
Ambrogio S.: B-0760
Ambrosetti M.C.: B-0457
Ambrosi A.: B-1872
Ambrosini R.: B-0619, B-1042
Ambrosio M.R.: B-0045, B-0046
Ameda F.: B-1417
Amelin M.: B-1266
Amini M.: B-0291
Amiri M.: B-1433
Amirkhamzaev A.: B-1379
Amitai M.M.: B-1186
Amlani A.: B-0351
Ammari S.: B-0672
Ammendola R.M.: B-0592, B-1823
Amodeo E.M.: B-0113, B-0529, B-0563, B-0846, B-1435
Amodio F.: B-0002, B-0006, B-0011
An C.: B-1043, B-1282
An C.S.: B-1763
An D.-A.: B-1816
An H.: B-0370
An Y.Y.: B-0723, B-1800
Anagnostopoulos A.: B-0224
Anand V.K.: B-1639
Ananthakrishnan L.: B-0391, B-0792
Anbar A.: B-0899
Andac N.: B-0286
Andersen H.K.: B-0131
Andersen M.B.: B-1465
Anderson N.: B-1117
Anderson V.: B-0837
Andersson I.: B-0695
Andrasina T.: B-0232
Andrássy P.: B-0786
Andreini D.: B-1501, B-1615, B-1686
Andreo L.: B-0003
Andreoli C.: B-0734
Andreotti R.F.: A-0072
Andres Cano I.: B-0287
Anık A.: B-1059
Anishkin M.: B-0091
Anjari M.: B-0142
Annibale B.: B-0525
Annoni A.D.: B-1501
Ansarin M.: B-0973
Antil N.: B-0720

### List of Authors & Co-Authors

Antoch G.: *B-0158, B-0161, B-0327, B-0515, B-0516, B-0517, B-0518, B-0519, B-0670, B-0828, B-0954, B-1210, B-1355, B-1368, B-1461, B-1462, B-1726, B-1736, B-1739*
Antonelli A.: *B-0607, B-1390*
Antoniou A.: *B-1388*
Antonuccio E.G.M.: *B-1724*
Anzalone N.: *B-1776*
Aouini F.: *B-1487*
Aoyagi K.: *B-1003, B-1460, B-1755*
Aoyama H.: *B-1899*
Ap Dafydd D.: *B-0976*
Aparisi Gomez M.P.: *A-0881*
Apel D.: *B-0713*
Apfaltrer G.: *B-1361*
Apine I.: *B-1652*
Apolo A.B.: *B-1640*
Appel E.: *B-1355, B-1726*
Appelman L.: *B-0726, B-1564*
Appelman P.: *B-0726*
Appelt A.: *B-0361*
Appleyard R.: *B-1495*
Aquerreta Beola J.D.: *B-0064*
ArabAhmadi M.: *B-1433*
Aragon Tejada F.X.: *B-0625*
Arai H.: *B-1329*
Arana Fernandez De Moya E.: *B-0107*
Aras O.: *B-0115, B-0556, B-0737, B-0743*
Araujo e Araujo A.C.R.: *B-0542*
Araya Campos F.: *B-0629*
Arban F.: *B-1807*
Arbelaez P.: *B-0570*
Arboleda C.: *B-1792*
Arendt C.T.: *B-0299, B-1204*
Arfelli F.: *B-1789*
Argiolas G.M.: *B-0036*
Argyropoulou M.I.: *A-0277, A-0423, B-1660*
Arias-Lorza A.M.: *B-0497*
Aribal M.E.: *B-1546*
Aristovich K.: *B-1425*
Armaitis J.: *B-1190, B-1757*
Arous A.: *B-1160*
Arrais J.P.: *B-0652*
Arrigo A.: *B-1066*
Arrigoni F.: *B-0241, B-1295, B-1552, B-1571, B-1572, B-1580, B-1581*
Arruda E.R.: *B-0604*
Arruda P.H.C.: *B-0860*
Arshad M.A.: *B-1387*
Arslan A.: *B-1546*
Arsov C.: *B-0158, B-0161, B-0327, B-0828*
Arteaga de Castro C.: *B-0766*
Arthurs O.J.: *B-0606, B-1319*
Arunsingh M.: *B-0666*
Aryan A.: *B-0537*
Asadov R.: *B-0286*
Asahara T.: *B-0642, B-0648, B-0994*
Asakawa T.: *B-0642, B-0994*
Asbach P.: *A-0574, B-0441, B-0450, B-1475*
Ascione A.: *B-1019*
Asenbaum U.: *B-1377*
Asghar M.S.: *B-1035*
Ashok Kumar M.: *B-0063, B-1269*
Ashraf H.: *B-0492*
Aslam M.: *B-0979*
Aslan S.: *B-0122*
Asmar K.: *B-1168*
Asokan P.: *B-0063, B-1269*
Assante M.: *B-0464*
Assegnati G.: *B-0010*
Assetta M.: *B-0708, B-0951*
Astley S.M.: *B-0917*
Astrakas L.G.: *B-1660*
Aswani E.: *B-1356*
Aswani Y.: *B-1749*
Atalabi O.M.: *B-0947*
Atat C.: *B-1031*
Ates E.: *B-1395*
Athanasiou A.: *A-0671*
Ather S.: *B-0245*
Atri P.K.: *B-1224*
Attanasio A.: *B-0415*
Attye A.: *A-0887*
Atwan M.: *B-0126*
Audard V.: *B-0058*
Auer T.A.: *B-1469*
Autti T.: *A-0139*
Auwal A.: *B-1109*
Avanesov M.: *B-0119, B-1682*
Avendano D.: *B-0719, B-1804*
Avery J.: *B-1425*
Avesani G.: *B-1387, B-1389*
Aviram G.: *A-1055*
Awadallah M.Y.: *B-0212*
Awramienko A.: *B-1338*
Ayaz Ü.Y.: *B-1053*
Aydın H.: *B-0269*
Aydin R.C.: *B-1729*
Aydin S.: *B-1884*
Aydingoz U.: *A-0090, A-0445*
Ayres V.J.: *B-0084*
Ayuso C.: *A-0486*
Azeemuddin M.: *B-1367*
Azevedo F.A.: *B-1524, B-1526*
Azevedo K.: *A-0929*
Azevedo K.B.: *B-0489, B-0652, B-0658*
Aziza R.: *B-0322*
Azman S.S.: *B-0024*

### B

Baar I.: *B-0279*
Babar J.: *A-0451*
Babkina T.: *B-0230*
Bacher K.: *A-0748, B-1596, B-1628*
Bachmann Nielsen M.: *A-0042, A-0338*
Bacigalupo L.: *B-1528*
Baco E.: *B-0332, B-0821, B-1656*
Badary D.: *B-0371, B-1284*
Badawi R.D.: *B-0056*
Baddam S.R.: *B-0562, B-0841, B-1062*
Bader C.: *B-0032*
Badia S.: *B-0014*
Baduna M.: *B-1652*
Bae J.: *B-0389, B-1119, B-1375*
Bae S.-C.: *B-1336*
Baek J.H.: *B-1800*
Baeßler B.: *A-0597, A-1008*
Baeuerle T.: *B-1777*
Baev A.: *B-0089*
Bagga R.: *B-1901*
Baggiano A.: *B-0783, B-1212, B-1613, B-1614, B-1615, B-1686*
Bagheri A.: *B-1694*
Bagheri M.: *B-1640*
Bagheri S.: *B-0602*
Bagnacci G.: *B-1275*
Bagur A.: *B-0763*
Bai J.: *B-0687, B-1244, B-1779*
Bajaj S.K.: *B-0605*
Bakers F.C.H.: *B-1484*
Bakken A.S.: *B-1543*
Bala Lakshmoji D.: *B-1376*
Balaeva M.: *B-1819*
Balaji R.: *B-0176, B-0345, B-0346, B-0636, B-1639, B-1641*
Balasubramaniam R.: *B-1647*
Baldari D.: *B-0257*
Baldetti L.: *B-1221*
Baldin P.: *B-0944*
Balestra F.: *B-1155*
Balestreri L.: *B-0085, B-0554*
Balestrieri A.: *B-1775*
Balestro E.: *B-0522*
Bali M.A.: *A-0341, B-0524*
Baliyan V.: *B-1013*
Ball P.: *SY 16*
Ball P.A.: *B-0197*
Ball S.: *B-1698*
Ballati F.: *B-1153*
Balleyguier C.S.: *B-0478*
Balslev I.: *B-0154, B-0442*
Balster S.: *B-1247*
Balta C.: *B-0255*
Baltacioglu F.: *B-0286*
Balter S.: *B-1191*
Baltzer P.A.T.: *A-0232, A-0974, B-0701, B-0702, B-0716, B-1150, B-1542, B-1549, B-1570, K-36, SY 2b*
Balvay D.: *B-0987*
Bamberg F.: *A-1020, B-0019, B-0188, B-0568, B-0589, B-0749, B-0945, B-1208, B-1514, B-1515, B-1782*
Bancroft L.W.: *A-0805, A-0807*
Bandar M.E.: *B-0179*
Bandettini W.P.: *B-0300, B-1114*
Banfai B.: *B-0728*
Bang J.-I.: *B-0303*
Bang N.: *B-1643*
Bangiyev L.: *B-0687*
Bankier A.A.: *A-0551, B-0580*
Bannas P.: *B-0119*
Bansal G.: *B-0546*
Bansal R.: *B-1797*
Bansal V.: *B-0634*
Banz-Wüthrich V.: *B-1438, B-1442*
Baptista J.T.C.: *B-0283*
Baptista M.: *B-0284*
Baptista T.: *B-0285*
Baqir Hassan T.: *B-0271*
Barabasch A.: *B-0073, B-1437*
Barakat A.: *B-1317*
Baran P.: *B-1789*
Barash Y.: *B-1186*
Barati M.: *B-1200*
Barbieri F.: *B-0782*
Barbieri P.: *B-0529*
Barbosa Alvares M.C.: *B-0129*
Barbosa Jr. E.J.M.: *B-0144, B-0736*
Barbosa P.N.V.P.: *B-0358*
Barchetti F.: *B-0156*
Barchetti G.: *B-0156, B-0160, B-0325, B-0825, B-0876*
Barcons Vilaplana S.: *B-0121*
Bardelli A.: *A-0035*
Bargellini I.: *A-1056*
Barile A.: *B-0241, B-1295, B-1571, B-1572, B-1580, B-1581*
Baris Y.S.: *B-0059*
Barkhausen J.: *B-0202, B-0225, B-0349, B-0498, B-1622, SY 2a*
Barkhof F.: *B-0096*
Barnacle A.: *A-0061*
Barnes P.: *B-0916, B-0919*
Barnett J.L.: *B-1605*
Barnett M.H.: *B-0984*
Barnwal M.: *B-0910, B-1133, B-1397, B-1750, B-1892*
Baron Rodiz P.A.: *B-0625*
Barral M.: *B-0155*
Barral P.-A.: *B-0964*
Barrera C.: *B-1057*
Barrett T.: *B-0437, B-0880*
Barros J.: *B-1493*
Bartels-Rutten A.: *B-1483*
Barth T.F.E.: *B-0061*
Bartoli A.: *B-0964*
Bartolotta T.V.: *B-1529*
Bartolovič N.: *B-0260*
Bartolucci M.: *B-0975*
Barušauskas D.: *B-1190, B-1757*
Bas A.: *B-0115, B-0556, B-0737, B-0743*
Bashir M.R.: *B-1768*
Basile A.: *B-0418, B-0833, B-0943, B-0959, B-1405, B-1648*
Basile D.: *B-0824, B-1144*
Basilico R.: *A-0045, A-0517*
Baskak F.: *B-0295*
Ba-Ssalamah A.: *A-0004, B-1050, B-1052, B-1875*
Basso L.: *B-0879, B-1139*
Bastarrika Alemañ G.: *B-1015, B-1219, B-1609*
Bastarrika G.: *A-0774*

### List of Authors & Co-Authors

Bastati N.: *B-1052*
Bastati-Huber N.: *B-1050*
Bataillon G.: *B-1090*
Batalov A.: *B-0089*
Bates S.: *B-1226*
Båth J.: *B-1900*
Bats A.-S.: *B-1894*
Bauer B.: *A-0825*
Bauer M.J.: *B-0780, B-0784, B-0787, B-0914, B-1215, B-1217, B-1721*
Bauer S.: *B-0116*
Bäuerle T.: *A-0694, B-0474, B-0551, B-0703, B-1518, K-15*
Baum T.: *B-0047*
Bäumer P.: *B-1560*
Baumgartner C.: *B-1881, B-1882*
Baumhauer M.: *B-0061*
Bauones S.: *B-0060*
Baur A.: *B-0153, B-0399*
Baxter G.: *B-0501*
Bayer R.R.: *B-0301, B-0781*
Baykara Ulusan M.: *B-1395*
Bay-Nielsen M.: *B-0275*
Bazzocchi A.: *A-0275, A-0880*
Beadnall H.N.: *B-0984*
Beardmore C.: *A-1045*
Beaumont H.: *B-1023*
Becce F.: *B-0053, B-0997*
Becerra L.: *B-1035*
Beck S.: *B-0790*
Becker A.: *B-0822*
Becker A.S.: *B-1733*
Becker C.D.: *A-0696, A-0959*
Becker L.: *B-0585*
Bedene A.: *B-1710*
Bedke J.: *B-0628*
Bedlington N.: *A-0822*
Beenen L.F.M.: *B-0099*
Beer A.: *B-1050*
Beer A.J.: *B-0061*
Beer L.: *B-1020, B-1052*
Beer M.: *B-0061*
Beeres M.: *A-0062, B-0347, B-1204, B-1317*
Beets-Tan R.G.H.: *A-0336, A-0467, A-0500, B-0182, B-0559, B-0632, B-0685, B-0850, B-1049, B-1483, B-1484, B-1582, B-1635*
Begnami M.D.F.S.: *B-0358*
Beigelman C.: *A-0802*
Beitzke D.: *B-0374, B-1617*
Bejar S.: *B-1860*
Bekaroglu G.: *B-0556*
Bekebrede-Kauffman D.: *B-0099*
Beldi G.: *B-1434, B-1438*
Beleù A.: *B-0455, B-0457*
Belho E.: *B-0777, B-1335, B-1558*
Bell J.: *B-0617, B-1874*
Belladonna E.: *B-1521*
Belli P.: *B-0547, B-0548*
Bellini D.: *B-0014, B-0935, B-0940, B-1148*
Bellini M.: *B-1575, B-1578*
Bellomi M.: *B-0973, B-1281, B-1644*
Bellucci A.: *B-1894*
Beltramello A.: *B-0239, B-1128, B-1743*
Ben Daamer N.: *B-0261*
Ben Hammouda M.: *B-1160*
Ben Salem D.: *B-1511*
Benadjaoud S.: *B-0261*
Benaissa A.: *B-0261*
Bencardino J.T.: *B-1508, B-1510*
Ben-David E.: *B-0133, B-0313*
Bende F.B.: *B-1533*
Bender B.: *B-0093, B-0095*
Bender Y.Y.: *B-1734*
Bendszus M.: *B-0263, B-0505, B-0539, B-1419, B-1560*
Benedetti G.: *B-0381, B-0598, B-1221, B-1678*
Benedetti L.: *B-1745*
Beneventi A.: *B-1808, B-1810*
Bengo G.J.: *B-0487*
Benkert T.: *B-0362, B-0364*
Benlala I.: *B-1202*
Bennani-Baiti B.I.: *B-1542*
Bennink E.: *B-0192, B-0705*
Bento C.A.M.: *B-0542*
Benz M.R.: *B-1092*
Benz S.: *B-1824*
Beomonte Zobel D.: *B-0113, B-0563, B-0846, B-1435*
Berardo S.: *B-0367*
Berbee M.: *B-1484*
Bérczi V.: *B-0444, B-1589*
Beregi J.P.: *B-0431, B-1695*
Berenyi E.: *B-1432*
Beretta C.: *B-0189*
Berezina N.: *B-1182*
Bergamasco L.: *B-1173, B-1799*
Berger A.: *B-0926*
Berger C.: *A-1026*
Berger M.F.: *A-0742*
Berger M.S.: *B-0096*
Berger N.: *B-0085, B-0554, B-1541*
Berger P.: *B-1202*
Bergès O.: *B-0987*
Berghe A.-S.: *B-0922*
Berghöfer A.: *B-1184*
Berkhemer O.: *B-0258*
Berliner C.: *B-1089*
Berlis A.: *A-0980*
Bernabéu Rodríguez M.: *B-0625*
Bernaerts A.: *A-0888, B-0290*
Bernard-Davila B.: *B-0924, B-1175, B-1176*
Bernardi D.: *B-1548*
Bernardo A.: *B-1490*
Bernardo S.: *B-0607*
Berná-Serna J.D.D.: *B-1418*
Bernathova M.: *B-0716, B-1549*
Berritto D.: *B-0034*
Berruti A.: *B-0619*
Bert A.: *B-0477*
Bertalan Z.: *B-0569*
Bertani E.: *B-1281*
Bertani V.: *B-0085, B-0554*
Bertelli E.: *B-0622*
Bertelsen B.B.: *B-0275*
Bertó J.: *B-1609*
Bertolini M.: *B-0993*
Bertolotto M.: *A-0292, A-0572, B-0368, B-1658, B-1659*
Bertucci E.: *B-1381*
Bertuletti M.: *B-0619*
Besana F.: *B-0630*
Best S.: *B-1297*
Betlehem J.: *B-0728*
Bette S.: *B-0088*
Bettini R.: *B-0009*
Beuf O.: *B-1638*
Bevilacqua A.: *B-0842*
Bevilacqua V.: *B-0329*
Beyer C.: *B-0782*
Beyer T.: *A-0185, B-0234, B-1286*
Beyersdorff D.: *B-1089*
Bezzina P.: *A-0778, B-0027, B-0651, B-0891*
Bhagwat K.A.: *B-1737*
Bhalla A.S.: *B-1207*
Bharwani N.: *B-0177*
Bhatia A.: *B-0169, B-1224*
Bhattacharjee R.: *B-1133*
Biacca A.: *B-0052*
Bialopetravicius J.: *B-1190, B-1757*
Bian Y.: *B-1587, B-1834*
Bianchi A.: *B-0928*
Bianchi G.: *B-1572, B-1579, B-1580*
Bianchi M.: *B-0198*
Bianchi S.: *B-0470*
Biau D.: *B-0058*
Biavati F.: *B-0802*
Bibbolino C.: *A-0579*
Bicakci K.: *B-1039*
Bicakci S.: *B-1039*
Bican Y.: *B-1052*
Bick U.: *A-0529*
Bickelhaupt S.: *B-0362, B-0364, B-1421, B-1568*
Bidault F.: *A-0074*
Biederer J.: *A-0373, B-1610*
Bielen D.: *B-0494*
Bielowski C.: *B-0551, B-0703, B-1518*
Bier G.: *B-0093*
Bieri O.: *B-0055*
Bierma - Zeinstra S.: *B-1136*
Bierry G.: *A-0262*
Biesen R.: *B-0514*
Bignotti B.: *B-1178, B-1745*
Bigolaro S.: *B-1540*
Bihane C.: *B-1638*
Bijan B.: *A-0556, B-0291, B-0602*
Bilbao J.I.: *A-0244, K-12*
Bilbao Jaureguizar J.I.: *B-0111*
Bilello M.: *B-1781*
Bilici Salman R.: *B-1453*
Billiet T.: *B-0984, B-1509*
Billingsley S.: *B-1288*
Bind M.: *B-0691*
Bindinelli P.: *B-1715*
Bini M.: *B-0824*
Binkert C.: *A-0971*
Binst J.: *B-1539, B-1838, B-1839, B-1842*
Bioletto F.: *B-0598*
Biondi T.: *B-1022, B-1341*
Biondic Spoljar I.: *B-0463*
Bippus R.: *B-0138*
Bisbjerg R.: *B-0154, B-0442*
Biskup J.: *B-0597*
Bitencourt A.G.V.: *B-0358*
Biviji M.: *B-0748, B-0755, B-0981*
Bizino M.B.: *B-0588*
Bjørklund K.: *B-0480*
Blackburn Andersen P.: *A-0622*
Blackham K.: *B-0193, B-0946*
Blackledge M.: *B-0353*
Blackwell J.: *B-0761*
Bladowska J.: *B-0540*
Blaivie C.: *B-0290*
Blanc R.: *B-0280, B-0282*
Blanco F.J.: *B-0003*
Blank C.U.: *B-0685*
Blankenstein T.N.: *B-0900*
Blankholm A.D.: *A-0624*
Blanks R.G.: *B-0078, B-0080*
Blauth M.: *B-0102*
Blay J.-Y.: *B-1638*
Blazevic A.: *B-1279*
Blazic I.: *B-1663*
Bley T.A.: *B-0337*
Bleys R.: *B-0215*
Bliddal H.: *B-1135*
Bliznakova K.: *B-1843*
Bloem J.L.: *A-0033, A-0106*
Blom H.M.: *B-0298*
Blüher M.: *B-0764*
Blum A.: *K-07*
Boban J.: *A-0766*
Bobek-Billewicz B.: *B-1338*
Boca I.: *B-0922*
Boccafoschi F.: *B-1262*
Boccalini S.: *B-0731, B-1214*
Bodden J.H.W.: *B-0047*
Bodelle B.: *B-1362*
Bodenmann D.: *B-1497*
Bodini F.C.: *B-1029*
Boeri C.: *B-0470*
Boeri M.: *B-0143*
Boesen L.: *B-0154, B-0442*
Boesen M.: *B-0675, B-0682, B-1035, B-1135, SY 27*
Boettger S.: *B-1362*
Bogaert J.: *A-0200*
Boggi U.: *B-1097*
Bogner S.: *B-0575*
Bogveradze N.: *B-0616*
Böker S.M.: *B-1734*
Boland M.: *B-1113*
Bolchini M.: *B-1687*
Boldrini L.: *A-0498*

Bolengo I.: *B-0560*
Bolivar Cuevas S.: *B-1404*
Boll D.: *B-0493, B-0729, B-1092, B-1503*
Bollen T.: *A-0096*
Bollen T.L.: *A-0925*
Bolstad K.N.: *A-1049*
Bolster F.: *B-0098*
Bomers J.G.: *B-0878*
Bonaffini P.A.: *B-0528*
Bonaffini P.A.A.: *B-1894*
Bonaros N.: *SY 10*
Bonasera L.: *B-0975*
Bonatti G.: *B-1389*
Bonatti M.: *B-0937, B-1389*
Bondini F.: *B-0253*
Bonekamp D.: *B-0813, B-1077*
Bonelli S.: *B-1881, B-1882*
Bongers M.N.: *B-1143*
Böning G.: *B-0962*
Bonneville F.: *A-0521, B-0282*
Bonomo L.: *A-0212, B-0199*
Bons L.R.: *B-0194*
Bonzini M.: *B-1060*
Booij R.: *B-1598*
Boolkah S.: *B-0966*
Boone J.M.: *B-1843*
Boos J.: *B-0954, B-1210, B-1355, B-1368, B-1462, B-1726, B-1739*
Booz C.: *B-0434, B-0682, B-1362, B-1472*
Bor S.: *B-0367, B-1262*
Boraschi P.: *B-0526, B-1097*
Borg Grima K.: *B-0891*
Borger M.: *B-1683*
Borges A.: *A-0999*
Børgesen H.: *B-0682*
Borggrefe J.: *B-0710, B-0756, B-0988, B-1166, B-1424, B-1783*
Borggrefe M.: *B-0587*
Borgheresi A.: *B-1118*
Borhani A.A.: *B-0071, B-1765*
Borisch E.: *B-0438*
Borotikar B.: *B-1511*
Borra R.: *B-0574*
Borralho P.: *A-0989*
Borrego P.: *B-0907*
Borruel Nacenta S.: *B-0268*
Borsook D.: *B-1035*
Borsos P.: *B-0444*
Bortolanza C.: *B-1011*
Bortolotto C.: *B-0040*
Bortsova G.: *B-0982, B-1690*
Bortul M.: *B-0464*
Borzelli A.: *B-0002, B-0011, B-0113, B-0563, B-0846, B-1435*
Bos D.: *B-0497, B-0982, B-1598*
Bosc R.: *B-0252*
Bosch Barragan F.: *B-0121*
Bosmans H.: *B-0132, B-0251, B-0494, B-1318, B-1539, B-1839, B-1840, B-1841, B-1842, B-1843, B-1845*
Bosmans J.M.L.: *A-0507*
Bösmüller H.: *B-1814*
Boss A.: *B-0065, B-0085, B-0554, B-1541*
Boswijk E.: *B-0220*
Bottaro L.: *B-0536*
Bouchouicha A.: *B-0987*
Boudabbous S.: *SY 27*
Boudinaud C.: *B-1470*
Bouhali O.: *B-1601*
Bouhamama A.: *B-1638*
Boulet B.: *B-0478*
Bourgioti C.: *A-0809, B-1388*
Boussel L.: *B-0311, B-0731, B-1116, B-1214, B-1852*
Böven J.: *B-0954, B-1368*
Bovenberg J.A.: *A-0632*
Bovin J.S.: *B-0675*
Boyd K.: *B-0353*
Boyer L.: *B-1470*
Bozhko O.: *B-1778*
Bozzao A.: *B-1270*
Bozzetto M.: *B-0040*
Bozzolo E.: *B-1337*
Brabander T.: *B-1279*
Bracci A.: *B-1823*
Bracciani A.: *B-0253*
Brady A.: *A-0079, A-0502, A-0587, A-0824, A-0930*
Brady M.: *B-0763*
Bragagnolo D.S.: *B-1154*
Braig E.: *B-0995*
Brambilla M.: *A-0324, A-0547, B-0798, B-1071*
Brambillasca P.: *B-0974*
Brancatelli G.: *A-1001, B-0071, SY 13*
Brancato B.: *B-0477*
Brancato V.: *B-0174*
Branco C.: *B-0283*
Branco G.: *B-0283*
Brandao L.A.: *B-0542*
Brandão Nascimento A.C.: *B-0542*
Brännström M.: *B-1900*
Brantner P.: *A-0449, B-0310*
Braren R.: *B-0615*
Braspenningx S.: *B-1679*
Brat H.: *B-1600*
Bräu F.: *B-1729*
Braunwarth E.: *B-0102*
Bready E.: *B-1296, B-1298, B-1299, B-1301*
Breda S.: *B-0856*
Breen R.: *B-0142*
Breithecker A.: *B-1863*
Bremerich J.: *A-0940, B-0310, B-0904, B-1030, B-1211*
Bremnes A.: *B-1072*
Brendle C.: *B-0093*
Brendlin A.: *B-0347*
Brennan D.: *B-0265*
Brennan P.: *B-0265, B-0267, B-1789*
Brennan P.C.: *B-1844*
Brenne F.: *B-0349*
Brenner C.: *B-1056*
Brentnall A.R.: *B-0917*
Brescia Morra V.: *B-0543*
Breysem L.: *B-1318*
Bricolo P.: *B-0248, B-0249*
Briers E.: *A-0826, A-0932*
Brillet P.-Y.: *A-0701*
Brindle K.: *B-1802*
Brink M.: *A-1053, B-1850*
Brinker G.: *B-0762*
Brioschi M.: *B-0898*
Brismar T.B.: *B-0049*
Brisse H.: *B-1090*
Brites C.T.: *B-0414*
Brites L.: *B-0889*
Brito A.Q.L.C.: *B-0658*
Brkljačić B.: *A-0368, A-0571, A-0673, A-0753, B-0463*
Brockmann M.A.: *B-1241*
Broeders E.: *B-0220*
Broeders M.: *B-0255, SY 2c*
Brombal L.: *B-1846*
Bronson K.: *B-0300, B-1114*
Brookes A.L.: *A-0401*
Brooks J.: *B-0503*
Brosch T.: *B-1761*
Brotons-Cuixat D.: *B-0680*
Brouha S.: *B-1607*
Brountzos E.N.: *A-1062, A-1067*
Brown G.: *A-0391*
Brown M.: *B-0126, B-0712*
Brown P.: *B-0361, B-0666, B-0667, B-0771, B-0916, B-0919*
Browning G.: *B-0639*
Browning T.: *B-0391*
Brudkowska Z.: *B-0538*
Brudvik K.W.: *B-1047*
Brugnara G.: *B-0539*
Bruining A.: *B-1384*
Brumberg R.M.: *B-0383, B-0591, B-1824*
Brun T.: *B-0322*
Bruna R.: *B-0338*
Brunelli S.: *B-0248, B-0249*
Bruners P.: *B-0005, B-1437*
Brunetti A.: *B-0543, B-0823, B-1383, B-1385, B-1392, B-1626, B-1730*
Bruni S.: *B-1872*
Brunner F.: *B-0590*
Bruno F.: *B-0241, B-1295, B-1552, B-1579, B-1580, B-1581*
Brussaard C.C.: *B-0136*
Brzezinski J.: *B-0874*
Bu X.: *B-1333*
Bubel K.: *B-0167*
Bucerius J.: *B-0220*
Buch K.: *B-0933, B-1226, B-1227, B-1228, B-1229*
Buchan K.: *B-1849*
Buchbender C.: *B-0669, B-0671*
Bucher A.M.: *B-0347, B-1317*
Bücker A.: *B-0167, B-1720*
Bucsics T.: *B-1377*
Budai B.: *B-1589*
Budde R.P.: *B-1598*
Budde R.P.J.: *B-0194*
Buddensieck C.: *B-1462*
Buder T.: *B-1259*
Budrewicz S.: *B-0540*
Budzik J.-F.: *B-0053*
Buecker A.: *B-0961, B-0963, B-1058, B-1463*
Buerke B.: *B-0266*
Bufi E.: *B-0547, B-0548*
Buissink C.: *A-0532, B-1699*
Buizza G.: *B-0372*
Bujila R.: *B-0674*
Bukte Y.: *B-0125, B-0929*
Bulakbasi N.: *A-1032*
Bullock S.A.: *B-1290*
Bülow R.: *B-0398, B-0401, B-1474*
Buls N.: *B-0136, B-1596, B-1597, B-1628*
Bult P.: *B-0545, B-0726*
Bulychkin P.: *B-0621*
Bünting N.A.: *B-0073*
Buoni G.: *B-0367*
Buonomenna C.: *B-0052*
Buonomenna G.: *B-1139*
Burabe F.A.: *B-1107*
Burcet Rodríguez G.: *B-1231*
Burck I.: *B-0296, B-0299, B-1247, B-1257*
Burger I.A.: *A-0197, B-0224, B-0228, B-0229, B-0817, B-0822*
Burgetova A.: *B-0938*
Burggraf M.: *B-1740*
Burgstaller J.M.: *B-1733*
Burke A.M.: *B-0663*
Burmester G.R.: *B-0514*
Burnside E.: *B-0920*
Burulday V.: *B-1744*
Busacca M.: *B-0864*
Buscarino V.: *B-0769*
Busè G.: *B-1529*
Bussani R.: *B-1659*
Busse A.: *B-1502*
Busse H.: *B-0764*
Busselot T.: *B-0494*
Butler A.P.: *B-1117*
Butler R.: *B-1793, B-1794*
Buvat I.: *A-0553*
Bux S.: *B-1872*
Buxi T.B.S.: *B-1668*
Buy X.: *B-0834*
Buytaert D.: *B-1596, B-1628*
Buzug T.M.: *B-0498*
Byanyima R.K.: *B-1417*
Byeon Y.: *B-1754*
Byrne C.: *B-1113*
Byun S.H.: *B-1538*

### C

Caballeros Lam F.M.: *B-0294*
Caballo M.: *B-1523, B-1525, B-1790*
Cabarrus M.: *B-0829*
Cabero Moyano J.: *B-0121*
Cabukova M.: *B-0281*
Cabuy M.: *B-1597*
Cademartiri F.: *B-1313*
Cai X.: *B-1083*
Caines J.: *B-0916, B-0919*
Cakiroglu H.: *B-0115, B-0556, B-0737, B-0743*
Calabrese M.: *B-0724, B-1151, B-1178*
Calado S.: *B-0283*
Calandrelli R.: *B-1225*
Calandrino R.: *B-1194*
Calcagno M.C.: *B-0436, B-0833, B-0959, B-1878*
Caldas C.: *B-1802*
Calder A.D.: *A-0189*
Cal-Gonzalez J.: *B-0234*
Caliolo G.: *B-0676*
Calleja M.: *B-0649*
Calli C.: *A-0428, A-0523, A-0828*
Calliada F.: *A-0667, B-0040*
Caltabiano D.C.: *B-0436, B-1878*
Caltabiano G.: *B-1878*
Calvão-Pires P.: *B-0283*
Calvieri C.: *B-0592*
Calvo Imirizaldu M.: *B-0064, B-0111, B-1015, B-1219, B-1784*
Camacho J.: *B-1231*
Cambi F.: *B-1898*
Campa R.: *B-0156, B-0160, B-0325, B-0825, B-0876*
Campagna C.: *B-0676*
Campagnola A.: *B-1094, B-1103*
Campbell N.M.: *B-1663*
Campeau N.: *B-0750, B-0755*
Campi C.: *B-0686, B-0689*
Campo A.: *B-1609*
Campo I.: *B-1659, B-1807*
Campora R.A.: *B-0970*
Camps Herrero J.: *A-0474, A-1028*
Candinas D.: *B-1434, B-1438, B-1442*
Cangiotti C.: *B-1310*
Cannao P.M.: *B-1728, B-1867*
Cannella R.: *B-0071, B-1529, B-1765, B-1768, B-1771*
Cannita K.: *B-1572*
Cannizzaro E.: *B-0241, B-0595, B-1309, B-1623, B-1817*
Cant J.: *B-0145*
Canti V.: *B-1337*
Cantisani V.: *SY 18*
Cao G.: *B-1429*
Cao J.: *B-0320, B-0406*
Cao L.: *B-0074, B-1044, B-1471, B-1764*
Cao R.: *B-1337*
Caobelli F.: *A-1019*
Capaldo A.: *B-0028*
Capanu M.: *B-0152*
Capasso F.: *B-0738*
Capel Flores I.: *B-0121*
Capitanio U.: *B-1302*
Capocci R.: *B-0252*
Capozzi N.: *B-1653*
Cappelli F.: *B-0113, B-0563, B-0846*
Cappello G.: *B-0618, B-1828*
Capretti I.: *B-1295*
Capuani S.: *B-0607, B-1390*
Caraco C.: *B-0501*
Carai A.: *B-1327*
Caramella C.: *A-0792*
Caramella D.: *B-0198, B-0526, B-1097, B-1205*
Carbognin G.: *B-0239, B-0872, B-1128, B-1743*
Carbonaro L.A.: *B-0465, B-0477, B-1352*
Carbone I.: *B-0014, B-0935, B-0940, B-1148, B-1500*
Carchesio F.: *B-0109, B-0110, B-0114, B-1812*
Cardani R.: *B-1821*
Cardano G.: *B-0532, B-1094*
Cárdenas Loguercio G.E.: *B-0629*
Cardis E.: *A-0933, A-0935*
Cardobi N.: *B-0455*
Cardone G.: *B-1300, B-1302*
Cardoso A.R.: *B-1063*
Cardoso M.J.: *B-1688*
Carducci C.: *B-1327*
Carducci S.: *B-1579*
Carisio A.: *B-1262*
Carlo Stella C.: *B-1636*
Carmo S.: *B-1063*
Carmona-Bozo J.C.: *B-0501*
Carnevale A.: *B-1406*
Caroli A.: *A-0059*
Carolus H.: *B-1761*
Carpentier K.: *B-0145*
Carrafiello G.: *A-0582, B-0974, B-1808, B-1810*
Carreño García E.: *B-1404*
Carriero A.: *B-0367, B-0798, B-1262*
Carroll B.J.: *B-1306*
Carroll P.R.: *B-0829*
Carrozzo M.: *B-0676*
Carta P.: *B-0622*
Cartia F.: *B-0257, B-0725, B-1155*
Caruana C.J.: *A-0895*
Caruana G.: *B-1529*
Caruso D.: *B-1022, B-1341*
Caruso M.: *B-0333, B-0738*
Caruso P.A.: *A-0798, B-0933, B-1226, B-1227, B-1228, B-1229*
Carvalho de Siqueira E.: *B-0129*
Carvalho R.: *B-0284*
Casale R.: *B-0020*
Casale S.: *B-0373*
Casaleggio A.: *B-0879*
Casamassima N.: *B-1808*
Casari F.: *B-1381*
Casas Muñoz J.S.: *B-0629*
Caseiro Alves F.: *A-0003, B-1494, SY 13*
Cash H.: *B-0153*
Caspers J.: *B-0983, B-1553*
Caspers S.: *B-1553*
Cassano E.: *B-0722, B-1561*
Cassar-Pullicino V.N.: *A-0032, A-0969*
Cassart M.: *A-0563*
Cassata G.: *B-1444*
Casselman J.W.: *A-0319, A-0885, A-0890, B-0290, B-1596, B-1628*
Cassiano M.T.: *B-0543*
Castagna M.: *B-0526*
Castagnoli F.: *B-1042*
Castañer E.: *A-0471*
Castellano E.: *A-0873*
Castellano I.: *B-1173, B-1799*
Castellote Alonso A.: *B-0340*
Castermans C.: *B-0032*
Castriconi R.: *B-1194*
Castro Caperán D.: *B-0629*
Catala-March J.: *B-0680*
Catalano C.: *A-0218, B-0156, B-0160, B-0325, B-0592, B-0607, B-0825, B-0876, B-1019, B-1390, B-1500, B-1823*
Cataldo T.E.: *B-0210*
Catalucci A.: *B-1552*
Catania D.: *A-0209, B-0028*
Catania M.: *B-0239, B-0457, B-0872, B-1128, B-1743*
Catapano F.: *B-0592, B-1500, B-1823*
Cattapan K.: *B-0827*
Cattarin E.: *B-1154*
Caudal A.: *B-1507*
Caudrelier J.: *B-0834*
Caulo M.: *B-0203*
Caumo F.: *B-0248, B-0249, B-0250, B-1540*
Cavaco A.: *B-0650*
Cavaglià E.: *B-0002, B-0006, B-0011*
Cavaliere A.: *B-0512*
Cavaliere C.: *B-0174, B-0668, B-0928*
Cavalieri S.: *B-0798, B-1262*
Cavallaro A.: *B-1088*
Cavallaro M.F.: *B-0368*
Cavalleri S.: *B-0872*
Cavalli G.: *B-1337*
Cavalli M.: *B-1821*
Cavicchioli F.M.: *B-0872*
Caviezel C.: *B-0577*
Caymaz I.: *B-0115, B-0556, B-0737, B-0743*
Cazzato R.L.: *A-0263, B-0834*
Çelebi U.O.: *B-1744*
Celeng C.: *B-0377*
Cellina M.: *B-0087, B-0247*
Cenderello G.: *B-1528*
Ceravolo I.: *B-0825*
Cereser L.: *B-1078, B-1079, B-1138*
Cerezal L.: *A-1048*
Ceriani R.: *B-0560*
Cerit M.: *B-0213*
Cernic S.: *B-1807*
Cerrone P.: *B-1550, B-1556*
Cervantes B.: *B-1746*
Cervelli R.: *B-0526, B-1097*
Cevasco L.: *B-1528*
Cewe Jönsson P.M.: *B-0955*
Cha J.: *B-1001, B-1014*
Chabin G.: *B-0816*
Chadjaa M.: *B-1023*
Chae K.J.: *B-1322*
Chai M.Y.C.: *B-1220*
Chai Y.: *B-0139, B-1276, B-1876*
Chalabi N.A.: *B-0923*
Challen R.: *B-1496*
Chan L.C.A.: *B-1798*
Chand G.: *B-1021*
Chandra T.: *B-0717*
Chang J.H.: *B-0097*
Chang J.I.: *B-0646*
Chang P.: *SY 20*
Chang Sen L.Q.: *B-0696*
Chang W.: *B-0795, B-0807*
Chapot R.: *B-0288*
Charbel S.: *B-1109*
Charitanti-Kouridou A.: *B-1685*
Chassagnon G.: *A-0268*
Chatellier G.: *B-0848*
Chatoupis K.: *B-1388*
Chaturvedi A.K.: *B-0691*
Chaudhari A.S.: *B-1134*
Chaudhry M.B.H.: *B-1367*
Chea P.: *B-0902*
Checcucci E.: *B-0824*
Cheimariotis G.-A.: *B-1685*
Chelladurai A.: *B-1271*
Chellathurai A.: *B-0227*
Chen A.: *B-0672*
Chen B.: *B-1816*
Chen C.: *B-0308, B-1234, B-1235, B-1254, B-1264, B-1559*
Chen G.: *B-0217*
Chen H.: *B-1159, B-1430*
Chen H.-J.: *B-1041*
Chen J.: *B-0074, B-0356, B-1044, B-1471, B-1764*
Chen J.-L.: *B-0393*
Chen K.: *B-0566*
Chen L.: *B-0375*
Chen M.: *B-0035, B-0048, B-0147, B-0308, B-0772, B-0807, B-1126, B-1243, B-1642, B-1704*
Chen M.Y.: *B-0300, B-1114*
Chen P.-C.: *B-1551*
Chen Q.: *B-0462*
Chen S.: *B-1131, B-1152*
Chen X.: *B-0236, B-0343, B-0593, B-0715, B-0770, B-1098, B-1100, B-1246, B-1480, B-1531*
Chen X.J.: *B-0812*
Chen Y.: *B-0437, B-0462, B-0511, B-0810, B-0881, B-1120, B-1246, B-1548, B-1633, B-1650*
Chen Y.-S.: *B-1551*
Cheng G.: *B-1171*

Cheng J.: *B-0390, B-0814, B-0969, B-1234, B-1235, B-1236, B-1237, B-1238, B-1239, B-1242, B-1244, B-1248, B-1252, B-1256, B-1559, B-1779, B-1889*
Cheng J.L.: *B-1691, B-1692, B-1693*
Cheng J.-L.: *B-1264*
Cheng S.C.H.: *B-0638*
Cheng S.F.: *B-0712*
Cheng S.-H.: *B-1104*
Cheng X.: *B-0978*
Cheng X.S.: *B-0624*
Cheng Y.: *B-1534*
Cheng Y.-F.: *B-0561*
Cheng Z.: *B-0343, B-1083*
Cheon K.S.: *B-1538*
Cherel P.: *B-0478, B-1090*
Cherkashin M.: *B-0091, B-1182*
Cheung K.O.: *B-1798*
Cheung M.: *B-0330*
Cheung Y.-F.: *B-1864*
Chhabra A.: *B-1129*
Chianca V.: *B-0868, B-0870, B-1730*
Chiarello S.: *B-1406*
Chibana S.: *B-0421*
Chiesa S.: *B-1029*
Chigozie N.I.: *B-1107*
Chilamkurthy S.: *B-0748, B-0750, B-0755, B-0981*
Chimpiri A.R.: *B-0687*
Chin L.H.Q.: *B-0330*
Chiras J.: *B-1574*
Chitcholtan K.: *B-1117*
Chiti A.: *B-1636*
Chitty L.S.: *B-0606*
Chiu C.L.: *B-1798*
Chiu K.W.H.: *B-0178*
Chiu K.W.-H.: *B-0370*
Chng L.S.: *B-0641*
Cho C.-H.: *B-0762*
Cho E.-S.: *B-0397*
Cho H.M.: *B-0740, B-0960, B-1573*
Cho K.R.: *B-1177*
Cho M.J.: *B-1763*
Cho S.B.: *B-0965*
Cho S.H.: *B-0942*
Cho Y.: *B-0576*
Chodyla M.: *B-0575, B-0690, B-1456*
Choi A.L.: *B-0869*
Choi B.: *B-1014*
Choi B.H.: *B-1567*
Choi B.I.: *B-0451*
Choi D.S.: *B-0396*
Choi H.J.: *B-1800*
Choi H.W.: *B-0451*
Choi J.: *B-1322*
Choi J.-I.: *B-1895*
Choi J.M.: *B-0397*
Choi J.Y.: *B-1285*
Choi J.-Y.: *B-1766*
Choi M.H.: *B-1895*
Choi N.: *B-0469, B-0555*
Choi S.-Y.: *B-0453*
Choi Y.S.: *B-0097*
Chojniak R.: *B-0358*
Chou Y.-C.: *B-0075*
Choudary G.V.: *B-1075*
Choudhoury K.R.: *B-1768*
Chowdhury R.: *B-0245*
Choyke P.L.: *B-0837*
Chriashkova J.: *B-0979*
Christensen A.F.: *B-1035*
Christian N.: *B-1714*
Christie S.: *B-0025*
Christou A.: *B-1157*
Chryssogonidis I.: *B-1685*
Chu J.P.: *B-1273*
Chu M.: *B-0370*
Chu P.: *B-0015, B-1320*
Chuan X.: *B-0566*
Chulroek T.: *B-0827*
Chung C.: *B-1243*
Chung H.: *B-0336*
Chung J.-J.: *B-0397*
Chung W.-Y.: *B-0758*
Ciampi Dopazo J.J.: *B-0625*
Ciccarese F.: *B-0873*
Ciccone G.: *B-1499*
Cicero L.: *B-1444*
Cieno S.: *B-0619*
Cieszanowski A.: *B-1477*
Ciet P.: *B-0170, B-0171, B-1205*
Cil B.E.: *A-0021*
Cilia F.: *B-0592, B-1500, B-1823*
Cina A.: *B-0529, B-1435*
Cindil E.: *B-0213, B-1453*
Cinque T.: *B-0104*
Ciortea C.: *B-0922*
Cipollari S.: *B-0325*
Cipriani F.: *B-0851*
Cirone D.: *B-0470*
Cisar J.: *B-0281*
Ciurea A.I.: *B-0922*
Clarencon F.: *B-1574*
Clarke S.E.: *B-0403*
Clauser P.: *A-0426, B-0701, B-0702, B-0716, B-1150, B-1549, B-1570, SY 2d*
Clément O.: *A-0056, A-0625, A-0726*
Clements K.: *B-0079*
Clevert D.A.: *A-0039, A-0171, A-0669, A-0752, SY 17*
Clive K.: *B-1111*
Cobelli R.: *B-1858*
Cobianchi Bellisari F.: *B-0595, B-1579*
Cobo Reinoso E.: *B-0274*
Coche E.: *A-0892, B-0944*
Cockmartin L.: *B-0251, B-0494, B-1539, B-1838, B-1839, B-1840, B-1842, B-1845*
Coco S.: *B-0592, B-1823*
Cocozza S.: *B-0823, B-1392, B-1626*
Codari M.: *B-0028, B-0189, B-1687*
Cognard C.: *B-0282*
Cohen C.: *B-0836, B-1422*
Cohen S.: *B-0080*
Coi L.: *B-0329*
Coimbra F.J.F.: *B-0358*
Colafati G.S.: *B-1327*
Colagrande S.: *B-1205*
Colapietro F.: *B-0530*
Colarieti A.: *B-0369*
Colgan N.: *B-0761*
Collalunga E.: *B-0553*
Collette L.: *A-0790*
Collettini F.: *B-1468*
Collocola A.: *B-0999*
Colombi D.: *B-1029*
Colombo M.: *B-0530, B-1302*
Colombo P.E.: *B-0991, B-1028*
Colosimo C.: *B-1225*
Coma A.: *A-0188, B-0340*
Comerci M.: *B-0543*
Como G.: *B-1079, B-1138*
Comte A.: *B-1664*
Comunoglu N.: *B-0986*
Congedo T.: *B-1812*
Connor S.: *A-0797*
Conti A.: *B-0959*
Conti M.: *B-0548*
Contro A.: *B-0009*
Cook G.: *A-0975, B-0351, B-0617, B-1021, B-1874*
Cook N.: *B-1647*
Coolen A.M.P.: *B-0081*
Cooper G.: *B-1307*
Copin M.-C.: *B-0778*
Copley S.J.: *A-0456*
Coppenrath E.: *B-1773*
Coppo M.: *B-1071*
Coppola A.: *B-1808*
Coppola M.: *B-0006, B-0738*
Corazza A.: *B-0868, B-1730*
Corcioni B.: *B-0873*
Corcos G.: *B-1507*
Cordonnier E.: *A-0633*
Cormier E.: *B-1574*
Cornacchia M.: *B-1080*
Cornacchione P.: *B-0028*
Cornelis F.: *A-0283*
Cornud F.: *B-0155*
Corre C.: *B-0933*
Corrias G.: *B-0036, B-0415, B-1775*
Corridore A.: *B-0544, B-0595, B-1309, B-1550, B-1556, B-1623, B-1817*
Cortellini A.: *B-1572*
Cortese F.: *B-0010, B-0618, B-1093*
Corvino F.: *B-0006*
Cosentini D.: *B-0619*
Cossu A.: *B-1406*
Costa A.F.: *B-0403*
Costa C.: *B-0630*
Costa M.: *B-1521*
Costabile T.: *B-0543*
Costanza C.: *B-1173, B-1799*
Cotroneo A.R.: *B-0195*
Cotroneo A.R.R.: *B-0203, B-1313*
Cotten A.: *A-0784*
Cotti E.: *B-1406*
Cottier J.P.: *B-0836*
Couceiro F.: *B-1108*
Coudyzer W.: *B-0494*
Coulon P.: *B-0731, B-1116*
Courtier N.: *B-0649*
Coutinho Santos A.: *A-0998*
Couto J.G.: *B-0649, B-0651*
Cova M.A.A.: *B-0368, B-0464, B-0536, B-1659, B-1807*
Cozzi A.: *B-0465*
Cozzi D.: *B-0732, B-0733*
Cozzi L.: *B-1636*
Cozzolino P.: *B-1080*
Cradock A.: *B-0409*
Cretese A.: *B-1138*
Crijns A.: *A-0014, A-0016*
Crimì F.: *B-1060, B-1364*
Crocetti L.: *A-0246*
Croissant Y.: *B-0847*
Cromwell P.: *B-0633*
Crona J.: *B-0523*
Cronin K.: *B-1700*
Crumbaker M.: *B-0226*
Cruz R.: *B-0358*
Csima M.: *B-0031*
Csobay-Novák C.: *B-0958*
Cuba Camasca V.: *B-0044*
Çubuk H.S.: *B-1039*
Cugola L.: *B-0248, B-0249*
Cui G.-B.: *B-0757*
Cui X.: *B-1756*
Cui Y.: *B-0360, B-1519*
Cullmann Bastian J.L.: *B-0901*
Cullmann J.L.: *B-0896*
Cummings K.: *B-1007*
Cunha A.J.C.D.: *B-1492*
Cunningham D.: *B-0524*
Cuocolo A.: *B-1684*
Cuocolo R.: *B-0333, B-0668, B-0823, B-0870, B-1392, B-1626, B-1730*
Curioni-Fontecedro A.: *B-0817*
Currie S.: *B-0361, B-0666, B-0667*
Currò F.: *B-0368*
Curry E.: *B-1387*
Curta A.: *B-1866*
Cutaia G.: *B-1529*
Cuzick J.: *B-0917*
Cybulski A.J.: *B-0458*
Cyran C.C.: *A-0067, A-0973, B-0503*
Cyriac J.: *B-0193, B-0816, B-0904, B-1030*
Cyron C.J.: *B-1729*
Czarnecki M.: *B-1265*
Czernohorsky V.: *B-0558*
Czerny C.: *B-0299, B-0847*

### D

da Silva C.A.: *B-0414*
DaBreo D.C.: *B-0580, B-1306*
Dacher J.-N.: *B-1860, K-06*
D'Acierno L.: *B-1684*
D'addato M.: *B-0676*
Dagna L.: *B-1337*
Dahl J.B.: *B-1035*
Dahlblom V.: *B-0695*
Dahm-Kähler P.: *B-1900*
Dal-Bianco A.: *B-0534*
D'Alesio G.: *B-1872*
D'Alessio A.: *B-0630*
Dalla-Pozza R.: *B-1866*
Damasio M.B.: *B-0341, B-0342*
Damen M.: *B-0766*
D'Amico N.C.: *B-0304*
Damilakis J.: *A-0264, A-0411, A-0545, A-0751, A-0898, B-1593*
D'Amore C.A.: *B-0017, B-1831*
d'Amuri F.V.: *B-0842*
Dan H.: *B-1171*
Dance D.: *B-1838*
Dang Luu V.: *B-1305*
Dangelmaier J.: *B-0138, B-1746*
Daniaux M.: *SY 23*
Daniel H.: *B-1568*
Danieli R.: *B-0368*
Danila M.: *B-1533*
Danilov S.S.: *B-1263*
Dankbaar J.W.: *B-0192, B-0705*
Dankerl P.: *B-1088*
Danse E.: *A-0892, B-0944*
Dao T.H.: *B-0252*
Dappa E.: *B-0152*
D'Archambeau O.: *B-0279*
Darwish H.S.: *B-0711*
Das M.: *A-0270*
Daunis-i-Estadella J.: *B-0044*
Davies G.J.: *B-1027*
Davies K.: *B-1704*
Davini O.: *B-0948, B-1499*
Davis A.: *SY 20*
Davis M.: *A-0399*
Davydov D.: *B-1261*
Daw S.: *B-1324*
Dawoud R.M.: *B-1423*
de Assis P.E.: *B-1526*
de Assis P.E.Z.: *B-1524*
de Bazelaire C.: *B-0953, B-1563*
De Beul P.: *B-0473*
De Bock G.H.: *B-1565*
de Boer M.: *B-1049, B-1170*
De Brucker Y.: *B-1628*
de Bruijne M.: *B-0194, B-0497, B-0982, B-1690*
de Bucourt M.: *B-1184*
De Cataldo C.: *B-0595, B-1309, B-1571, B-1581, B-1623, B-1817*
De Cecco C.N.: *B-0301, B-0780, B-0787, B-0789, B-0914, B-0945, B-1215, B-1217, B-1721*
De Cesari M.: *B-0341, B-1054, B-1528*
De Cicco M.L.: *B-0732, B-0733, B-0734*
De Cobelli F.: *B-0207, B-0369, B-0381, B-0851, B-0971, B-1216, B-1221, B-1302, B-1343, B-1344, B-1345, B-1439, B-1485, B-1678, B-1880*
De Dominicis C.: *B-1022*
De E.J.B.: *B-0827*
De Filippo M.: *B-0842*
De Fiori E.: *B-1644*
De Foer B.: *A-0888, B-0290*
de Francisco A.: *SY 3*
De Franco G.: *B-1872*
De Gaspari A.: *B-0971*
De Giorgis S.: *B-0724*
De Graaf P.: *A-1070*
de Heer P.: *B-0588*
de Herder W.W.: *B-1279*
De Jong E.: *B-0769*
De Jong H.W.A.M.: *B-0192, B-0705*
de Jong P.A.: *B-0222, B-0788*
de Kerviler E.: *A-0711, B-0953, SY 30*
De Koekkoek-Doll P.: *B-0632, B-1635*
de la Camara M.A.: *A-0690*
De La Torre M.: *B-0111*
de Lange C.: *B-0168*
de Ligt M.: *B-0220*
De Luca C.: *B-1178*
De Lucia S.: *B-0211*
De Luis Pastor E.: *B-1303*
De Maeseneer M.O.: *A-1046*
De Magistris G.: *B-0006*
De Marco F.: *B-1612*
De Marco P.: *B-0318*
De Margerie C.: *B-0953*
De Martino S.R.M.: *B-0873*
De Matteis F.: *B-1309, B-1817*
De Mattia C.: *B-0991, B-1028*
De Mey J.: *B-0136, B-1597*
de Meyere M.: *B-1860*
De Pascale A.: *B-0824*
De Piano F.: *B-0769, B-1644*
de Pont L.M.H.: *B-0298*
de Rinaldis A.: *B-1390*
De Robertis Lombardi R.: *B-0455, B-0457*
De Rubeis G.: *B-0592, B-1019, B-1823*
De Santis D.: *B-1022, B-1341*
De Serio I.: *B-0086*
De Smet E.: *B-0753, B-1127, B-1509*
de Souza P.: *B-1856*
De Torres J.P.: *B-1609*
De Vincenzo R.: *B-1898*
De Visschere P.: *A-0478, B-0157*
de Vito A.: *B-1310, B-1372, B-1457*
De Witt Hamer P.C.: *B-0096*
Deak P.D.: *B-1597*
Deb S.: *B-1147*
Debray M.-P.: *B-1407*
Debus J.: *B-0506*
Deckert C.: *B-0623*
Dedulle A.S.L.: *B-0132*
Defeudis A.: *B-0527, B-1828*
Deftereos S.P.: *B-0163*
Deganello A.: *B-1446*
Degenhart G.: *B-1599*
Degnan A.J.: *B-1055, B-1057*
Deike-Hofmann K.: *B-1421*
Dejobert M.: *B-0836*
Deken V.: *B-0778*
Dekeyzer S.: *B-0753*
Dekimpe C.: *B-1294*
Dekkers I.A.: *B-0588*
Del Alamo M.: *B-1303*
Del Ferraro G.: *B-1032*
Del Maschio A.: *B-0207, B-0369, B-0381, B-0971, B-1216, B-1221, B-1302, B-1343, B-1344, B-1345, B-1485, B-1880*
Del Poggio A.: *B-1776*
Del Rio Carrero B.: *B-1404*
Del Torto A.: *B-0783, B-1212*
del Vecchio A.: *B-1194*
Delacour D.: *B-1860*
Delahunt E.: *B-0409*
Delakis I.: *B-1601*
Delfino L.: *B-0342*
Delgado Alvarez I.: *B-1231*
Deligianni X.: *B-0055*
Delis H.: *A-0788*
Della Corte A.: *B-0851, B-1439*
Della Seta M.: *B-1468*
Della Torre E.: *B-1337*
Della Vecchia G.: *B-1343, B-1345, B-1485*
Dell'Aversana S.: *B-1613, B-1614, B-1626, B-1684*
Dellios D.P.: *B-0768*
Delogu P.: *B-1846*
Delorme S.: *B-1568*
Demaerel P.: *A-0676*
Demasi-Jacquier M.: *B-0964*
Dementaviciene J.: *B-1190, B-1757*
Demicoli P.: *B-0413*
Demir M.: *B-0125*
Demir N.B.: *B-1453*
Demircioglu A.: *B-0575, B-0803, B-0819, B-0913*
Demirkazik F.: *B-1805*
Demozzi E.: *B-0872*
den Harder J.M.: *B-0137*
Denecke T.: *B-0399, B-0450, B-1024, B-1374, B-1468, B-1469*
Deng K.: *B-0858*
Deng Y.: *B-0751, B-1430, B-1517, B-1752*
Deng Z.H.: *B-0573*
Deniffel D.: *B-0138, B-0790*
Denton J.: *B-1290*
Depetris M.A.: *B-0268*
Deppe S.: *B-0485*
Depretto C.: *B-0257, B-0725, B-1155*
Derchi L.E.: *A-0308, A-0315, A-0316, A-0355, A-0496, B-0342, B-1658*
Dercle L.: *B-0672, B-1188*
Dergilev A.: *B-1005*
Derlin T.: *B-1240*
Derman S.: *B-0115, B-0737, B-0743*
Deroose C.: *B-0850*
Desai I.: *B-0063, B-1269*
Desai S.R.: *A-0699, A-0983*
Desbuquoit D.: *B-0753*
Deshmane A.: *B-0095*
Deshpande A.: *B-0483*
Desi G.: *B-0618*
Desi G.L.: *B-1093*
Desiderio C.: *B-0833, B-1405*
deSouza N.M.: *A-0057, A-0287, A-0789*
Desser T.S.: *B-1402*
Dessouky R.: *B-1129*
Devalla A.R.: *B-0912*
Devaraj A.: *A-0027, A-0375, A-0548, B-1605*
Devasenathipathy K.: *B-1207*
Dewaguet J.: *B-0778*
Dewald C.: *B-0001*
Dewey M.: *B-0016, B-0172, B-0802, B-1016*
Dewit O.: *B-0944*
Dezman R.: *A-0834*
Dhaka N.: *B-1095*
Dhamija A.: *B-0777*
Dhanasingh A.: *B-0297*
Di Bartolomeo M.: *B-0951*
Di Blasi A.: *B-0010*
Di Cataldo A.: *B-0943*
Di Cesare E.: *B-0595, B-1309, B-1579, B-1623, B-1817*
Di Chiara A.: *B-0207, B-1343, B-1344, B-1345, B-1485*
Di Costanzo G.: *B-0174*
Di Dato F.: *B-0333*
Di Gaetano E.: *B-1154*
Di Giacomo L.: *B-1275*
Di Giammarco G.: *B-0203*
Di Giulio G.: *B-1153*
Di Leo G.: *B-0490, B-1151, B-1352, B-1723*
Di Luzio M.: *B-1309, B-1623*
Di Marco V.: *B-1529*
Di Matteo M.: *B-0951*
Di Mico L.: *B-0253*
Di Mizio V.: *B-0708*
Di Napoli A.: *B-0712*
Di Nasso M.: *B-1831*
Di Paola V.: *B-0328*
Di Santo D.: *B-0971*
Di Serafino M.: *B-0104*
Di Sibio A.: *B-0595, B-1623, B-1817*
Di Stasi C.: *B-1435*
Di Stefano G.: *B-0272*
Di Terlizzi M.: *B-0619*
Di Tommaso L.: *B-0530*
Di Trani M.G.: *B-0607*
Diamantis I.: *B-0201*
Diamantopoulos A.: *B-0966*
Diao K.: *B-0218, B-0596, B-1674, B-1677, B-1680, B-1681, B-1820, B-1825*
Díaz Díaz J.: *B-0535*

Díaz Pérez J.: *B-1418*
Diaz Ruiz S.: *B-1360*
Dick E.: *A-0166*
Dick G.: *B-0655*
Diederichs G.: *B-1734*
Diekhoff T.: *B-0514, B-1630, B-1631, B-1738*
Diep P.-T.: *B-0126*
Dierolf M.: *B-0995, B-1847*
Dietrich U.: *B-1740*
Dietzel M.: *B-0474, B-0551, B-0701, B-0702, B-0703, B-1518*
Dieudonné A.: *B-0848*
Dijkshoorn M.L.: *B-1598*
Dijkstra H.: *B-0704*
Dimai H.-P.: *B-0569*
Ding J.: *B-0147, B-1606*
Ding L.: *B-0903*
Ding X.: *B-1189*
Ding Y.: *B-1467*
Dinoto A.: *B-0536*
Diomedi-Camassei F.: *B-1327*
Dionisi C.: *B-1071*
Dippel D.W.: *B-0258*
D'Ippolito E.: *B-0372*
Distelmaier M.: *B-1437*
Dittman H.: *B-0689*
Divjak E.: *B-0463*
Djedjos S.: *B-1536*
Djuraeva N.: *B-1379*
Djurdjevic T.: *B-0925*
Djuric Stefanovic A.: *B-1871*
Dlama J.Z.: *B-1107*
Do Amaral Castro A.D.A.: *B-1365*
Do J.H.: *B-0451*
Dodd E.: *A-0687*
Dodd J.D.: *A-0469*
Dodig D.: *B-0260*
Doerfler A.: *B-1777*
Dogan A.: *B-1744*
Dogan B.: *B-1793, B-1794*
Döğen M.E.: *B-1053*
Doherty J.O.: *B-1601*
Døhlen G.: *B-0168*
Dohmen P.M.: *B-1502*
Dolgushin B.: *B-0395, B-0844, B-0845*
Domènech-Ximenos B.: *B-0044*
Dominguez A.: *A-0397*
Domínguez Echávarri P.: *B-0294*
Domínguez Paillacho I.D.: *B-1662*
Donalisio M.: *B-0072*
Donati F.: *B-0526, B-1097*
Donati O.F.: *B-0228, B-0822*
Donato F.: *B-1042*
Donato P.: *A-0223*
Dong D.: *B-1280*
Dong H.: *B-1100*
Dong S.-Z.: *B-0599, B-0600*
Dong Y.: *B-0452, B-1835*
Doniselli F.M.: *B-0416, B-0490, B-0759*
Donnelly-Kehoe P.: *B-1780*
Donners R.: *B-0057*
D'Onofrio M.: *A-0668, B-0455, B-0457*
Donoso L.: *A-0657, A-0734, K-28*
Doria A.: *B-0522*
Dorigo A.: *B-1010*
Doriguzzi Breatta A.: *B-0004*
Döring S.: *A-0684*
Dormagen J.B.: *B-1047*
Dormont D.: *B-1422*
Dorn F.: *B-0259*
Dorn S.: *B-0013, B-0135, B-0316, B-0319, B-0321*
Dörner J.: *B-1166*
Dorniok T.: *B-1360*
Doronzio V.M.: *B-0618, B-1082*
Dorrius M.: *B-0704*
Dorrius M.D.: *B-0309, B-1565*
Dósa E.: *B-0958*
Dou W.: *B-1883*
Douek P.: *B-0311, B-0731, B-1116, B-1214, B-1852*
Douek P.I.: *B-1366*
Dournes G.: *B-1202*
Dousset V.: *B-1415*
Dr. Tóth Z.: *B-0882*
Drape J.L.: *B-0058*
Drapé J.-L.: *A-0543*
Dratwa C.: *B-0478, B-1547*
Drazinos P.: *B-1378, B-1530, B-1537, B-1837*
Dreher C.: *B-0362, B-0364, B-0505*
Dreossi D.: *B-1846*
Drescher O.: *B-0116*
Dresen R.C.: *B-0559, B-0850*
Dreyer K.J.: *A-0629*
Drinkwater K.: *A-0586*
Drir M.: *B-1574*
Drissi C.: *B-1160*
Dromain C.: *A-0065, A-0956*
Dromer C.: *B-1202*
Drost F.-J.H.: *B-0181*
Du C.: *B-1120*
Du J.: *B-1476, B-1651*
Du Plessis J.G.E.: *B-1703*
Du R.: *B-0178*
Du T.: *B-1163*
Du Y.: *B-0123, B-1121*
Du Z.: *B-0462*
Dualde Beltran D.: *B-0107*
Duan T.: *B-1044, B-1471*
Duan Y.: *B-0752, B-1429*
Duarte A.: *B-1510*
Duarte Vallejo A.: *B-1508*
Duba I.: *B-0447*
Dube B.: *B-0900*
Düber C.: *B-0383, B-0591, B-1824*
Dubost F.: *B-0982, B-1690*
Dubourg B.: *B-1860*
Duc Cuong D.: *B-1305*
Duc N.M.: *B-1304, B-1305*
Ducou Le Pointe H.: *A-0934*
Duffau H.: *B-0096*
Duffy S.: *B-0920*
Duffy S.W.: *A-0942*
Duguay T.M.: *B-0301*
Duijm L.: *B-0083*
Duijm L.E.M.: *B-0081, B-0082, B-1149, B-1158*
Duijts L.: *B-0171*
Dullin C.: *B-1610, B-1789*
Dumic Cule I.: *B-0463*
Dunet V.: *B-1366*
Dupuis N.: *B-0731*
Dura M.: *B-1408*
Duran E.E.: *B-0210*
Duran R.: *A-1004*
Durando M.: *B-1173, B-1799*
Durante S.: *B-0028, B-0416, B-0999*
Durden J.A.: *B-1217*
Durhan G.: *B-1805*
Durlak F.: *B-1030*
Duron L.: *B-0987*
Durum Y.: *B-1059*
Dushkova D.V.: *B-1260*
Dustler M.: *B-0695*
Duy Hue N.: *B-1305*
Düzgün F.: *B-1741*
Dyhre Lia A.: *B-1699*
Dyker K.: *B-0666*
Dziedataja M.: *B-0647*

### E

Ebbesen D.: *B-1465*
Ebdon-Jackson S.: *A-0138*
Ebel S.: *B-0221*
Eberli D.: *B-0228, B-0229*
Eck B.: *B-0314*
Ederveen J.C.: *B-1879*
Edjlali M.: *A-0509*
Eduarte A.: *B-1545*
Edyvean S.: *A-0048*
Eggesbø H.B.: *A-0719*
Eggl E.: *B-1847*
Ehara S.: *B-0742*
Ehdaie B.: *B-0152*
Eichler F.: *B-0933*
Eichler K.: *B-0243*
Eickhoff S.: *B-1553*
Eid M.: *B-0787*
Eid M.M.: *B-1334*
Eijgenraam S.M.: *B-1134*
Einspieler H.: *B-1052*
Eisenblaetter M.: *B-1125*
Eisenblätter M.: *A-1058*
Ekert K.: *B-1277*
El Barody M.M.: *B-1086*
El Khudari H.: *B-0838, B-1296, B-1298, B-1299, B-1301*
El Malek N.A.A.: *B-0371, B-1086*
El Saddy S.: *B-0856*
El Salam Ebrahem M.A.: *B-1086*
El Sheikh R.: *B-0923*
El-Adawy M.: *B-1174*
Elazar Y.: *B-1186*
Elbadawi S.E.E.E.: *B-1416*
El-Diasty T.: *A-0995, B-0830*
Elefante A.: *B-1626*
Elentuck D.: *B-0902*
Eley K.A.: *A-0450*
Elizalde Pérez A.M.: *B-1784*
Elkalawy A.: *B-0502*
Elkarghali Z.: *B-0685*
ElKiki H.A.: *B-1363*
Ellingson B.M.: *B-1165*
Ellmann S.: *B-0474, B-0551, B-0701, B-0702, B-0703, B-1518*
El-Merhi F.: *B-0727*
Elmokadem A.H.: *B-0100*
El-Najjar I.: *B-0351*
Elorz M.: *B-0064*
Elpen P.: *B-1546*
Elsayed R.F.: *B-0212*
Elshafey R.: *B-0711*
Elshami W.: *B-0644, B-1486*
El-Sharkaway M.: *B-0117*
Elugwu H.C.: *B-1358*
Elzeneini A.M.: *B-0531*
ElZomor H.E.A.: *B-1363*
Emad Y.: *B-0899*
Emberton M.: *B-0159, B-0443*
Emiro F.: *B-0318*
Emmett L.: *B-0226*
Emri M.: *B-0882*
Emrich T.: *B-0378, B-0383, B-0591, B-1824*
Enam S.A.: *B-0090*
Enden T.R.: *B-0480*
Engbersen M.P.: *B-0182*
Engel G.: *B-1734*
Engelbrecht G.: *B-0883*
Engelhard N.: *B-1738*
England A.: *A-0492, B-1708*
Enriquez Puga A.: *B-1024*
Ensminger S.: *B-0202*
Eran A.: *B-1557*
Erb-Eigner K.: *A-1069*
Erdemir A.: *B-1218, B-1805*
Erden A.: *B-1373*
Erden I.: *B-1373, B-1862*
Erdini F.: *B-1389*
Ereño Ealo M.J.: *A-0947*
Ergun O.: *B-1206*
Eriksson T.: *B-0491*
Ermacora D.: *B-1521*
Ernemann U.: *B-0093*
Ernst A.: *B-1411*
Ersöz N.: *B-1059*
Ertl-Wagner B.: *A-0430*
Ertl-Wagner B.B.: *B-0094, B-0188, B-0749, B-0927, B-1782*
Esmaeilian S.: *B-0602*
Espejo Herrero J.J.: *B-1662*
Espinosa de Rueda Ruiz M.: *B-1418*

Esposito A.: B-0207, B-0369, B-0381, B-0598, B-1216, B-1221, B-1343, B-1344, B-1345, B-1485, B-1678
Esposto Pirani P.: B-1719
Esser M.: B-0620
Esseridou A.: B-0490
Essig M.: SY 14, SY 20
Esteban E.: B-0003
Estrampes C.: A-0355
Etemadi Z.: B-0602
Etxano J.: B-1784
Euler A.: B-0996
Eulig E.: B-0013, B-0909
Eustace S.: B-0409
Evangelista A.: B-1499
Evans D.G.: B-0917
Evans R.: B-0900
Evans T.L.: B-1023
Evoy D.: B-0633
Evrimler S.: B-0269
Ewertsen C.: A-0670
Ezenwugo U.: B-0043
Ezponda Casajús A.: B-0064, B-0111, B-1015, B-1219, B-1609, B-1784
Ezzeddine O.A.: B-0860

### F

Fabao G.: B-1861
Fabbro E.: B-0686
Fabbro-Peray P.: B-0431
Faber J.: B-1241
Fabian J.: B-1514, B-1515
Fabritius M.P.: B-0262, B-0713
Faby S.: B-0135, B-0319, B-0321
Facal de Castro F.: B-0107
Faccini R.: B-1833
Faccioli N.: B-0937
Fadeeva L.: B-0089, B-1037
Fadzli F.: B-0468
Fadzli F.B.: B-1274
Faeghi F.: B-1694
Faermann R.: B-1569
Faggioni L.: B-0017, B-0198, B-1831
Faghihi Langroudi T.: B-0427
Fahed R.: B-0280, B-0282
Fahlenkamp U.L.: B-0399, B-1734
Fahmy P.N.S.: B-0502
Faietti E.: B-0630
Faivre J.-B.: B-0778
Falaschi F.: B-0526, B-1097, B-1205
Falaschi Z.: B-1262
Falcione M.: B-1435
Faletti R.: B-0038, B-0072, B-0381, B-0598, B-1678
Falini A.: B-1337, B-1696, B-1776
Falkowski A.L.: B-0893
Fallenberg E.M.: A-0760, A-1086, SY 2b
Fallowfield L.: A-0417
Falsaperla D.: B-0418, B-0833, B-1405
Fan D.: B-0754
Fan G.: B-1230
Fan L.: B-1752
Fan Q.: B-0952
Fanciullo C.: B-0769
Fang J.: B-1627
Fang S.: B-0772
Fang X.: B-0715, B-1099
Fang Z.: B-1654
Fanizza M.: B-0373
Farghaly H.: B-1459
Farhadi F.: B-1640
Farina D.: A-0799, B-0970, B-1446, SY 24
Farina L.M.: B-0372
Farnedi S.: B-1198
Faron A.: B-1513
Farrell C.: B-0306
Farrell T.: B-0267
Farrugia S.: B-1350
Faruch M.: A-0647
Fatehi M.: A-0707
Fathallah W.M.: B-0179
Fati M.A.: B-1109
Fatigati G.: B-1080
Fatihoglu E.: B-1884
Faugeras F.: B-0261
Faulkner M.: B-1425
Favia A.: B-0676
Favia V.: B-0676
Fawzi F.S.: B-0212
Fawzy F.: B-0355
Fazel F.: B-0829
Fazzini D.: B-0304
Fechtrup N.: B-1622
Fedato C.: B-0250
Fedders D.: B-0398, B-0401, B-0797
Fedon C.: B-1527, B-1838
Fedorov A.: B-1182
Feger J.: B-0694
Feger S.: B-1016
Fehrenbach U.: B-0399, B-1024
Fehrs K.: B-0601
Feier D.S.: B-1050
Feifei G.: B-0857, B-0865
Feldman S.A.: B-1306
Felices Farias J.M.: B-1418
Fendler W.: B-0690
Feng F.: B-1883, B-1890
Feng S.: B-0810, B-1654
Feng S.-T.: B-0411
Feng Y.: B-1083
Feng Z.: B-0419, B-0854
Fenner J.: B-0760
Ferda J.: K-14
Ferenczi Z.: B-1432
Ferguson P.: B-0066
Ferguson S.D.: B-1243
Fernandes K.: B-1704
Fernandes M.O.: B-1490
Fernandes R.: B-1357
Fernandez J.M.: B-1195
Fernandez-Clotet A.: B-1653
Fernández-Real J.M.: B-0044
Ferranti C.: B-0257, B-0725, B-1155
Ferrari A.: B-0798
Ferrari M.: B-1446
Ferrari R.: B-1833
Ferraro D.: B-0817
Ferraro D.A.: B-0228, B-0229
Ferraz H.I.: B-1110
Ferreiro Argüelles C.: B-0535
Ferrer Puchol M.D.: B-0003
Ferrero A.: B-0447
Ferrero G.: B-0686, B-0689
Festa A.: B-0211
Feuchtner G.: B-0782, B-1599
Feydy A.: B-0058
Feygin T.: B-0604
Fialkoff B.: B-0133
Ficarra V.: B-1078
Ficicioglu C.: B-1057
Ficorella C.: B-1572
Figà-Talamanca L.: B-0930
Fighera A.: B-0532, B-1094, B-1103
Figueira A.R.: B-1494
Figueiredo J.P.: B-0650
Filippi M.: B-1696
Filippiadis D.: A-0063
Filippone A.: A-0242
Fingerle A.A.: B-1612
Fiocchi F.: B-1381
Fiore F.: B-0564
Fiore M.R.: B-0373
Fiore S.: B-0072
Fiorino C.: B-1343, B-1485
Fischer A.: B-0789
Fischer A.M.: B-0780, B-0781, B-0820
Fischer M.: B-1866
Fischer R.: B-0601
Fischer T.: A-0337, B-1657
Fischer U.: B-1544
Fisher J.: B-0979
Fitousi N.: B-0132
Fitzgerald J.: B-1700
Fiz F.: B-0686, B-0689
Flach P.: B-0068
Flacke S.: B-0902
Flatabø T.: B-0332, B-0821
Flatten D.: B-1783
Fleischmann D.: B-0378, B-0624
Fleischmann-Mundt B.: B-0503
Flemmer A.: B-0927
Fletcher J.G.: B-0447, B-1142
Fletcher M.: B-1543
Fleury E.F.C.: B-0084, B-1524, B-1526
Flodmark C.-E.: B-1360
Flohr T.: B-0496, B-0996
Flores Quispe S.K.J.: B-0512
Floridi C.: B-1810
Fodor M.: B-0102
Fokin V.: B-1505
Fokin V.A.: B-0791
Foley K.G.: B-1027
Foley R.W.: B-0950
Foley S.: B-0407, B-0485, B-0653
Foley S.J.: A-0412, A-0961, B-0663, B-1354
Folhoffer A.: B-1589
Folio L.R.: B-1640
Fomin V.V.: B-1822
Fong C.Y.: B-1233
Fonio P.: B-0004, B-0038, B-0072, B-0598, B-1173, B-1799
Fontana F.: B-1808, B-1810
Fontana G.: B-0372
Fontanella G.: B-0211
Fontanilla T.: A-0894
Fontarensky M.: B-1470
Fonteyne V.: A-0481
Foran A.: B-0936, B-1634
Forget J.: B-0931
Formenti A.: B-1501
Formosa A.: B-0413
Fornasa F.: B-0248
Forrai G.: A-0149, A-0941
Forst R.: B-0684
Förster K.: B-0927
Forsting M.: A-0596, B-0116, B-0690, B-0803, B-0819, B-0875, B-0913, B-1421, B-1456, B-1461, B-1595, B-1740
Forstner R.: A-0069, A-0285
Forte S.: B-1792
Fortini M.: B-0045, B-0046
Forton R.K.: B-0029
Fösleitner O.: B-1881, B-1882
Fosså K.: B-0131
Fossati B.: B-1821
Foti G.: B-0239, B-0872, B-1128, B-1743
Foti P.V.: B-0418, B-0833, B-0943, B-0959, B-1648
Fotopoulou C.: B-1387
Fouad D.: B-0371, B-1284
Fournie E.: B-0312
Fournier D.: B-1600
Fournier L.: B-0987, B-1894
Fournier L.S.: A-0129, A-0627, A-0901
Foutzitzi S.: B-0163
Fowler K.: A-1002
Fowler K.J.: B-1765
Fracella M.R.: B-1872
Fragner R.M.: B-1050
Fraia A.S.: B-0522
França M.M.: A-0838
Franceschelli G.: B-0974
Franceschi D.: B-0687
Franchi-Abella S.: A-0681
Franchini S.: B-1827
Franck C.: B-0145
Franckenberg S.: B-0068
Franco C.: B-1029
Francone M.: A-0124, A-0146, B-0592, B-1019, B-1500, B-1823
Franconeri A.: B-0372
Frandon J.: B-1695

Frank C.: *B-0379*
Frank V.: *B-1589*
Frantsev D.: *B-0395*
Franz M.: *B-0201*
Franzese M.: *B-0928*
Fraser L.: *B-1193*
Frattini T.: *B-0040*
Frauenfelder T.: *A-0145, A-0455, A-0549, B-0085, B-0554, B-0577, B-1512*
Fraum T.J.: *B-1765*
Freeman S.: *B-1384*
Freise J.: *B-1201*
French R.: *B-0902*
Frenken M.: *B-0515, B-0516, B-0517, B-0518, B-0519, B-1736*
Frenzel F.: *B-0963, B-1720*
Friedrich G.J.: *B-0782*
Friedrich N.: *B-1474*
Friedrich T.: *B-0498*
Friedrich-Nel H.: *A-0654, B-0890, B-1707*
Fries P.: *B-0167, B-0961, B-0963*
Frigerio A.: *A-1027*
Frija G.: *A-0015, A-0315, A-0408, A-0410, A-0659, A-0928, A-1011, A-1013*
Frittoli B.: *B-0619, B-1042*
Fritz J.: *B-0620, SY 6*
Froelich M.F.: *B-0262, B-1773*
Froeling M.: *B-0377*
Froemming A.: *B-0438*
Fröhlich J.M.: *B-0108*
Frohn M.: *A-0216*
Fronda M.: *B-0038*
Fronza M.: *B-0361*
Fröss-Baron K.: *B-0523*
Frulloni L.: *B-1094*
Frydrychowicz A.: *B-0202, B-0225, B-0349*
Fryer T.D.: *B-0501*
Fu C.: *B-0375, B-0376, B-1675*
Fu G.: *B-1331, B-1332*
Fu H.: *B-0166, B-0376, B-0594*
Fu J.: *B-0611, B-1280*
Fu R.: *B-1370*
Fu W.: *B-0831*
Fu Y.: *B-0801*
Fuchs M.: *B-1738*
Fuchsjäger M.H.: *A-0355, A-0414, A-0674, A-1083, B-0767, B-0767, B-0989, B-1619, B-1620*
Fueger B.: *B-1875*
Fugazzola C.: *B-1810*
Fujii K.: *B-0424*
Fujinaga Y.: *B-1836*
Fujioka T.: *B-0471, B-1796*
Fujisawa Y.: *B-0417, B-0579*
Fujita B.: *B-0202*
Fujiwara M.: *B-0293*
Fujiwara T.: *B-0293*
Fukushima H.: *B-0408*
Fumagalli I.C.: *B-0373*
Funayama S.: *B-0404*
Funicelli G.: *B-1022*
Funicelli L.: *B-1281*
Furlan A.: *B-0071, B-1765, B-1768, B-1771*
Furman-Haran E.: *B-1569*
Fusaro M.: *B-1010, B-1011*
Fusco R.: *B-0974, B-1346*
Fütterer J.J.: *A-0095, B-0878*

### G

Gabelloni M.: *B-0017, B-0198, B-1831*
Gaboardi F.: *B-1300*
Gabr A.E.: *B-1232*
Gac P.: *B-0039, B-0200*
Gagliardi I.: *B-0046*
Gagliardo C.: *B-1444*
Gaillot K.: *B-0836*
Galanaud D.: *B-1422*
Galant C.: *B-0894*
Galati F.: *B-0553*
Galatoire O.: *B-0987*
Gale A.G.: *B-0437, B-1548*
Galea N.: *B-0592, B-1019, B-1500, B-1823*
Galen T.: *B-0706*
Galimzianova A.: *B-1402*
Gall H.: *B-1863*
Gallagher F.A.: *A-0459, B-1802*
Gallardo Madueno G.: *B-0294*
Gallego Rivera J.I.: *B-0274*
Galli V.: *B-1715*
Galliano M.: *B-0951*
Gallina S.: *B-0195*
Gallix B.: *B-0528*
Gallo G.: *B-0824*
Galluzzo M.: *B-0732, B-0733*
Galvano G.: *B-0436*
Galyan T.N.: *B-1263*
Gamberini M.R.: *B-0045, B-0046*
Gambhir S.: *B-0233*
Gamboa P.A.T.: *B-0410*
Gammack P.: *B-1455*
Gandini G.: *B-0004*
Ganesan P.K.: *B-0910*
Ganeshan B.: *B-0150*
Ganghua T.: *B-1124*
Gangi A.: *A-0511, B-0834*
Gao A.: *B-1244, B-1779*
Gao B.-L.: *B-0184*
Gao J.: *B-1163, B-1877*
Gao L.: *B-0315, B-1604*
Gao P.: *B-0752*
Gao S.: *B-1017*
Gao Y.: *B-1222, B-1676, B-1681, B-1820*
Garcarek J.: *B-0747*
Garces-Descovich A.: *B-0210*
García Baizán A.: *B-1015, B-1219*
Garcia C.: *B-0837*
Garcia de Eulate R.: *B-0294*
García Figueiras R.: *A-1059*
García García F.: *B-0625*
García Gorga R.: *A-0689*
García Roch C.G.R.: *B-0625*
Garcia-Aguilar J.: *B-1663*
Garcia-Castro F.: *B-0680, B-1830*
Garcia-Villalba B.: *B-1418*
Garel C.: *A-0561*
Garetier M.: *B-1511*
Garin E.: *B-0848*
Garip-Kuebler A.: *B-1451*
Garlaschi A.: *B-0724*
Garmpis D.: *B-0756, B-0988*
Garnon J.: *B-0834*
Garrido Inostroza C.: *B-0629*
Gasbarrini A.: *B-0109, B-0110, B-0114*
Gasca C.: *B-1303*
Gascho D.: *B-0068, B-1516*
Gaspar R.C.M.C.R.: *B-0643*
Gasparini C.: *B-0464*
Gasparotti R.: *A-0026*
Gassner E.-M.: *B-0102*
Gast R.: *B-0146*
Gastl M.: *B-1616*
Gatidis S.: *A-0704, B-0818*
Gatos I.: *B-1378, B-1530, B-1537, B-1837*
Gatti F.L.: *B-0009*
Gatti M.: *B-0038, B-0072, B-0381, B-0598, B-1613, B-1614, B-1678*
Gaudez F.: *B-0953*
Gaudiano C.: *B-0873*
Gaudry M.: *B-0964*
Gavira J.J.: *B-1219*
Gavrilovic V.: *B-0076, B-0967*
Gawlik K.: *B-0352*
Gawlitza J.F.M.: *B-0587*
Ge Y.: *B-0857, B-0865*
Geão A.: *B-0884, B-0892*
Gebauer B.: *A-0191, B-0962, K-20*
Gebest M.: *B-1166*
Geier K.I.: *B-1240*
Geier O.M.: *B-0168*
Geijer H.: *B-1197*
Geijer M.: *B-1197*
Geisel D.: *B-0399, B-1374, B-1469*
Geisl G.: *B-0926, B-1881*
Gempt J.: *B-0088*
Geneidi E.: *B-0400*
Geng D.: *B-0541, B-0858, B-1120*
Geng J.: *B-1889*
Gennari A.: *B-0536*
Gennaro G.: *B-0250, B-1540, K-43*
Gennaro N.: *B-0530, B-1636*
Gentile F.: *B-0072*
Gentili F.: *A-0066, B-1275*
George A.: *A-0071, A-0092*
George U.B.: *B-0714*
Gerboni M.: *B-0072*
Gerevini S.: *B-1337, B-1696*
Germano A.S.: *A-0985, A-0987, A-0990*
Gernhardt C.: *B-1740*
Geroulakos G.: *A-1066*
Gersing A.S.: *A-0949, B-0047, B-1746*
Gerson P.: *A-0652*
Gerwing M.: *B-1125*
Geso M.: *B-0657*
Gesualdo L.: *B-0329*
Geterud K.: *B-0324*
Ghafarzadegan K.: *B-1380*
Ghanim M.E.: *B-0179*
Gharbi H.A.: *A-0309, A-0315, A-0317*
Gharbi M.: *B-1487*
Ghetti C.: *B-0993*
Ghodasara S.: *B-1650*
Ghosh A.: *B-1901*
Ghosh R.: *B-0748, B-0750, B-0755, B-0981*
Ghoshhajra B.: *B-1013*
Ghosn M.: *B-1669*
Ghotra S.S.: *B-1700*
Ghuman S.S.: *B-1668*
Giacobbe A.: *B-1715*
Giambelluca D.: *B-1529*
Giandola T.: *B-1457*
Glannarini G.: *B-1078, B-1138*
Gianneramo C.: *B-0241*
Giannetto G.: *B-0527, B-1082, B-1093*
Giannini F.: *B-1221*
Giannini V.: *B-0527, B-0618, B-1828*
Gianniotis C.: *B-0183*
Giannitto C.: *B-0973*
Giannotti E.: *A-0763*
Giannotti G.: *B-0455*
Giannotti N.: *B-0407*
Giarrizzo A.: *B-1878*
Giasotto V.: *B-0879*
Gibaud B.: *A-0633*
Gibbs F.: *B-0639*
Gibbs P.: *B-0719*
Gibney B.: *B-0246*
Gibson A.: *B-1027*
Gibson D.: *B-0709*
Gibson S.: *B-0033*
Gielen J.: *B-1286*
Giese D.: *B-1542*
Giganti F.: *B-0159, B-0443*
Giganti M.: *B-0045, B-0046, B-0272, B-1406*
Gil I.: *B-0283*
Gilbert A.: *B-0361*
Gilbert F.: *B-0501*
Gilbert F.J.: *A-0418, A-0526, B-1802, K-16*
Gilhodes J.: *B-0322*
Gill A.: *B-1802*
Gillard J.H.: *B-1688*
Gillard M.: *B-1700*
Gilley D.: *A-0661*
Ginocchio M.I.: *B-1383, B-1385*
Giombi F.: *B-1439*
Giona S.: *B-0159*
Giordano A.V.: *B-1579*
Giovagnoni A.: *A-0584, A-0635, B-1118, B-1719*
Giovanetti González R.: *B-0625*
Girard F.: *SY 22*
Giraudo C.: *B-0512, B-0522, B-1060, B-1364*
Girodet P.O.: *B-1202*

### List of Authors & Co-Authors

Girometti R.: *B-0076, B-0253, B-1078, B-1079, B-1138*
Gitto S.: *B-0052, B-0868, B-0870, B-1730*
Giudice D.: *B-1718*
Giudici F.: *B-0464*
Giugliano G.: *B-1644*
Giuliani M.: *B-0548*
Giurazza F.: *B-0006*
Given-Wilson R.M.: *B-0078, B-0080*
Gizewski E.R.: *B-1689*
Gizewski E.R.R.: *B-0925*
Gkoumas S.: *B-0997*
Gkoutzios P.: *B-0966*
Glas M.: *B-0819*
Gleeson F.: *A-0800, B-0245*
Glosna M.: *B-0200*
Gluskin J.: *B-1804*
Gned D.: *B-0824*
Gnirs R.: *B-0362, B-0364, B-1421*
Goddi A.: *B-0040*
Godel T.: *B-1560*
Godfrey D.: *B-0533*
Godtman R.: *B-0324*
Goeral K.: *B-0926*
Goerke S.: *B-0505, B-0506*
Goerke S.M.: *B-0867*
Goessmann H.: *B-0739*
Goh V.J.: *A-0037, A-0713, B-0351, B-0616, B-0617, B-1874*
Gohmann R.F.: *B-0196, B-1683*
Gokharman F.D.: *B-1884*
Golay X.: *B-0095*
Gold G.E.: *B-1134*
Goldberg N.: *B-1436*
Goldberg S.N.: *A-0602*
Golden D.: *B-0302*
Goldenko M.: *B-0296*
Gołębiewski B.: *B-1477*
Golfieri R.: *B-0873*
Golland P.: *A-0395*
Gollub M.J.: *B-1663*
Golosio B.: *B-1846*
Gomaa M.: *B-0460, B-0502, B-1174, B-1232*
Gomboli G.: *B-1229*
Gomez J.C.: *B-1780*
Gommers S.: *B-1787*
Gomori J.M.: *B-0133, B-0313*
Goncalves C.: *B-0979*
Gonçalves F.: *B-1490*
Gong B.: *B-0583*
Gong J.: *B-1758*
Gong Q.: *B-0751, B-1036, B-1333, B-1426*
Gong Yong J.: *B-0054, B-0683*
González de la Huebra Rodríguez I.: *B-1784*
Gonzalez F.: *B-1140*
González J.: *B-1609*
González R.L.: *B-1397*
Gonzalo Carballés M.: *B-0340*
Goo J.M.: *A-0291, B-1403, B-1412*
Gopalan D.: *SY 12*
Goracy J.: *B-1601*
Gorbunov N.: *B-1005*
Gore R.M.: *A-0487*
Gorgatti T.: *B-0076, B-0967*
Görlich D.: *B-0266*
Gorospe Sarasua L.: *B-0274*
Goryaynov S.: *B-0089*
Goshayeshi L.: *B-1380*
Gosvig K.K.: *B-0675, B-0682*
Gotlieb M.: *B-1569*
Goto S.: *B-0642, B-1167*
Gotschy A.: *B-1616*
Gottfriedova B.: *B-0338*
Gottlieb J.: *B-0585*
Gottschling S.: *B-1683*
Gotway M.: *B-1007*
Gouda W.A.: *B-0100*
Gourtsoyianni S.: *A-0163, A-0280*
Goussia A.: *B-1660*
Goyal A.: *B-0640, B-1207*
Graber-Naidich A.: *B-0624*
Gradwell M.: *B-0033*
Graeser M.: *B-0499*
Graessner J.: *B-0225*
Grainger A.: *B-0900*
Grainger A.J.: *A-0840, A-0842*
Grams A.E.: *B-0925*
Granata C.: *A-0875, A-0936*
Granata V.: *B-1346*
Grandis M.: *B-1745*
Grandl S.: *B-1847*
Granzier R.: *B-1170*
Grass M.: *B-0119*
Grassi M.: *B-0991*
Grassi R.: *A-0581, A-0583, B-0034*
Gratz M.: *B-0819*
Graungaard Falkvard C.: *A-0620*
Graves M.J.: *B-0501*
Graziano M.: *B-1406*
Grazioli L.: *B-0619, B-1042, SY 13*
Grazioli M.: *B-1042*
Grecchi A.: *B-0457*
Grech M.E.: *B-0649*
Greco F.: *B-1079*
Green A.: *B-0617, B-1874*
Greer M.: *B-0585*
Greffier J.: *B-1695*
Gregorio A.: *B-0003*
Gregório B.: *B-0650*
Greiser A.: *B-0767, B-0989, B-1619, B-1620*
Gremion I.: *B-1709*
Gremmel F.: *B-1617*
Grenier N.: *A-0380, A-0573*
Grenier P.A.: *A-0565*
Gresser E.: *B-1400*
Greune L.: *B-1125*
Greuter M.: *B-0311, B-0317*
Grgic I.: *B-0065*
Grieser C.: *B-1469*
Griffier R.: *B-1415*
Griffin E.: *B-0265, B-0267*
Griffith J.F.: *B-1292*
Griffith L.P.: *B-0781, B-0784, B-0914, B-1217*
Griffith P.: *B-1215*
Griffiths C.: *B-0885*
Griffiths P.D.: *A-0562, K-22*
Grigorieva E.: *B-0186*
Grimaldi D.: *B-0174*
Grimaldi M.C.: *B-0722*
Grimaldi V.: *B-1872*
Grimandi R.: *B-1511*
Grimm J.: *A-0714, B-0201*
Grimm R.: *B-0438, B-1390*
Grimmer R.: *B-0146*
Grinstein O.: *B-0461*
Grippo C.: *B-1150*
Gris J.-C.: *B-1695*
Grisan E.: *B-1685*
Grisanti S.: *B-0619*
Grist J.: *B-1802*
Gristina L.: *B-0724*
Grivas N.: *B-0326*
Grob D.: *B-1850*
Grobmyer S.: *B-1793, B-1794*
Grochowska A.: *B-1583*
Groden C.: *B-0022*
Gröhn H.: *B-1272*
Gronning K.: *B-1066*
Grosse Hokamp N.: *B-0314, B-1025, B-1081, B-1783*
Grosse-Siestrup C.: *B-0762*
Grossi E.: *B-0304*
Grosu S.: *B-0188, B-0749*
Grothoff M.: *B-0221, B-0379*
Grover H.: *B-0720*
Grover S.: *B-1428*
Grover S.B.: *B-0720, B-1291, B-1293*
Gruber G.M.: *B-0927*
Gruber-Rouh T.: *B-0843, B-1204, B-1317*
Grueneisen J.: *B-0690, B-0875, B-1456, B-1461*
Grunwald I.Q.: *B-0979, B-1555*
Grusha Y.O.: *B-1263*
Gruszczynska K.: *A-0775*
Gu Q.: *B-0419*
Gu R.: *B-0718*
Gu X.: *B-0123*
Gu Y.J.: *B-1152*
Guan J.: *B-0831*
Guan W.: *B-0511, B-0881*
Guarasci F.: *B-0038, B-0072*
Guaricci A.I.: *B-0783, B-1212, B-1613, B-1614, B-1615, B-1686*
Guarnera A.: *B-1270*
Guazzarotti G.: *B-0971, B-1880*
Guberina N.: *B-0875, B-1595, B-1740*
Gubern-Merida A.: *B-0697*
Gucalp A.: *B-0924*
Guðjónsdóttir J.: *B-0656*
Guenego A.: *B-0280, B-0282, B-0288*
Guenoun D.: *B-1507*
Guerreiro T.C.P.L.: *B-1359*
Guggenberger R.: *B-0997, B-1733*
Guglielmi G.: *A-0970*
Guglielmo M.: *B-0783, B-1212, B-1501, B-1613, B-1614, B-1615, B-1686*
Gui B.: *B-1898*
Guillén Burrieza G.: *B-0340*
Guimarães L.S.: *A-0857*
Guindani M.: *B-0056*
Guirao Garriga F.J.: *B-0121*
Gulani V.: *B-1650, B-1655*
Gule-Monroe M.K.: *B-1243*
Gulino F.: *B-1878*
Gulino P.: *B-0928*
Gulsun M.A.: *B-0820*
Gültekin M.: *B-1805*
Gumeler E.: *B-1445*
Gundermann P.: *B-0175*
Gundogmus C.A.: *B-0286*
Gunn A.: *B-0357, B-0838, B-1296, B-1298, B-1299, B-1301, B-1473*
Gunny R.: *A-1024*
Günther B.: *B-0995*
Günther D.: *B-1448*
Guo H.: *B-0343*
Guo J.: *B-0309, B-0574, B-1756*
Guo M.: *B-1646*
Guo W.: *B-1339, B-1479*
Guo W.-Y.: *B-0758*
Guo Y.: *B-0166, B-0375, B-0376, B-0384, B-0831, B-1674, B-1680, B-1825, B-1869*
Guo Y.-K.: *B-0382, B-0594, B-0596, B-1222, B-1618, B-1673, B-1675, B-1676, B-1681, B-1820, B-1826, B-1859*
Gupta A.K.: *B-0640*
Gupta K.: *B-0717*
Gupta N.: *B-0640*
Gupta P.: *B-1095*
Gupta S.: *B-1753, B-1892*
Gupta V.: *B-0777, B-1169, B-1335, B-1428, B-1558*
Gupta Y.: *B-1666*
Guralnik G.: *B-1186*
Gureyev T.: *B-1789*
Gurov S.N.: *B-0180*
Gurumoorthy M.: *B-0475*
Gusmini S.: *B-0851, B-1439*
Gutberlet M.: *A-0198, A-0938, B-0196, B-0221, B-0379, B-1201, B-1683*
Gutjahr R.: *B-0493, B-0496, B-0729, B-1503*
Gutzeit A.: *B-0108*
Guzmán Rojas R.: *B-0629*
Gwon H.Y.: *B-0127*
Gyngell M.L.: *B-0763*

### H

H Butler P.: *B-1117*
H. Abdeldayem E.: *B-1855*
Ha H.S.: *B-1012*
Haage P.: *B-1289*
Haanen J.B.: *B-0685*
Haas M.: *B-0153, B-0441*

### List of Authors & Co-Authors

Haas N.: *B-1866*
Habeeb M.: *B-0179*
Haberal K.M.: *B-1854*
Habib Geryes B.: *B-1664*
Habib Y.S.: *B-1363*
Hadaschik B.A.: *B-0875*
Haegele J.: *B-0498*
Haenel A.S.: *B-1669*
Hagberg G.: *A-0912*
Hager B.: *B-0055*
Haghighatkhah H.: *B-1433*
Hahn F.: *B-0378*
Hahn H.K.: *A-1060*
Haider L.: *B-0534*
Haidl F.: *B-0492*
Haioun K.: *B-1407*
Haiwei H.: *B-1254*
Hakanen J.: *A-0745*
Halaweish A.F.: *B-0447, B-1142*
Halfmann M.C.: *B-0378, B-0591, B-1824*
Haller S.: *A-0025*
Halliday K.: *A-0460*
Halshtok Neiman O.: *B-1569*
Hamano M.: *B-0742*
Hamdy A.: *B-1101, B-1102*
Hamed K.: *B-0827*
Hamer O.: *A-0454*
Hamerla G.: *B-0175*
Hamm B.: *B-0153, B-0441, B-0450, B-0514, B-0664, B-0762, B-0962, B-1199, B-1374, B-1468, B-1469, B-1475, B-1630, B-1631, B-1657, B-1734, B-1738*
Hammami N.: *B-1160*
Hammel J.: *B-0047, B-1848*
Hammer S.: *B-0298*
Hammerstingl R.: *B-0843*
Hammon M.: *B-1088*
Hammond R.L.: *B-1543*
Hamri H.: *B-1563*
Han D.: *B-1008*
Han D.Y.: *B-1137*
Han H.: *B-0488*
Han H.Y.: *B-0392*
Han J.K.: *B-0453, B-1119, B-1772*
Han J.K.K.: *B-0389, B-1342*
Han K.: *B-0335, B-1043, B-1443, B-1766*
Han M.-R.: *B-1177*
Han P.: *B-0218, B-0479*
Han P.-L.: *B-1676*
Han Q.: *B-1098, B-1100, B-1531*
Han X.: *B-0452*
Han Y.: *B-0757, B-1888*
Handa J.: *B-1167*
Handberg E.: *B-1543*
Haneder S.: *B-0314, B-1081*
Hanekamp B.: *B-1047*
Hangaard S.: *B-1135*
Hanley-Knutson, K.: *B-0829*
Hannesdóttir G.A.: *B-0656*
Hanquier L.: *B-1214*
Hansen M.S.: *B-1035*
Hansen N.L.: *B-0880*
Hanspach J.: *B-1777*
Hansson M.: *B-1197*
Hanstein B.: *B-0461*
Hanta I.: *B-1413*
Hao L.: *B-1162*
Haq T.U.: *B-1367*
Harder D.: *B-0057*
Harding L.: *A-0210*
Hardman J.: *B-0976*
Hardy M.: *B-0029*
Hare S.: *B-1004*
Hargreaves B.A.: *B-1134*
Haris Phuah A.: *B-1274*
Harisinghani M.G.: *B-0827*
Harit S.: *B-1562*
Harkness E.F.: *B-0917*
Harman P.A.: *B-1555*
Harper A.: *B-1455*
Harris J.: *B-0033*
Harsaker V.: *B-1700*
Harsany J.: *B-0281*
Hartemink K.J.: *B-1483*
Hartenbach F.: *B-0677*
Hartenbach M.: *A-0331*
Harteveld A.: *B-0215*
Harth M.: *B-0296*
Hartmann A.: *B-0764*
Hartmann C.J.: *B-1553*
Hartmann I.: *A-0801*
Hartmann N.-A.: *B-1016*
Hartung P.: *B-0196*
Harvey C.: *B-0627*
Harvey H.: *A-0128, SY 5*
Harvey-Lloyd J.: *B-0033*
Hasan H.A.: *B-1334*
Hasanefendioglu Bayrak A.: *B-1576*
Haselbacher M.: *B-0102*
Hashem R.H.: *B-0162, B-1232*
Hashimoto J.: *B-1603*
Hashimoto T.: *B-0425*
Hashizume T.: *B-0648*
Haslam P.: *B-0437*
Hassan A.A.-Q.: *B-0117*
Hassan K.M.B.: *B-0271*
Hasse A.: *B-1863*
Hasse B.: *B-0224*
Hasse F.C.: *B-0385*
Hatakenaka M.: *B-1735*
Haubenreisser H.: *B-0106, B-0587, B-0735*
Haubold A.: *B-0797*
Haubold J.: *B-0116, B-0575, B-0803, B-0819, B-0913*
Haug A.: *B-1020, B-1875*
Hauger M.: *B-1166*
Hausegger K.A.: *A-0510*
Hausmann D.: *B-0446*
Hautzel H.: *B-1462*
Havsteen I.: *B-1035*
Hawanna M.A.M.: *B-0693*
Hayashi H.: *B-0642, B-0648, B-0994*
Hayball M.: *B-0150*
Haydar A.: *B-0800*
Hazell L.J.: *B-1701, B-1707*
Hazirolan T.: *A-0421, B-1218*
Haznedaroğlu S.: *B-1453*
He B.: *B-1283*
He J.: *B-1189*
He M.: *B-0386, B-0454, B-0613, B-0952*
He S.: *B-1820, B-1869*
He Y.: *B-1074, B-1087, B-1820, B-1869, B-1893, B-1896, B-1897*
Healy N.: *B-1801*
Healy N.A.: *B-1795*
Hebda A.: *B-1338*
Heber D.: *B-0673*
Heck L.: *B-1847*
Hecker J.: *B-1787*
Hedgire S.: *B-1013*
Hedican S.P.: *B-1297*
Heeres B.: *B-1049*
Hegazy R.M.A.: *B-1545*
Heidenreich A.: *B-1081*
Heidinger B.H.: *B-0580, B-1306*
Heier M.: *B-0188, B-0749*
Heijmink S.: *B-0326*
Heikkinen N.: *B-1272*
Heil P.: *B-1437*
Heiland S.: *B-1560*
Heimer J.: *B-1516*
Heindel W.: *B-0266, B-1125*
Heinemann V.: *B-1586*
Heiß R.: *B-0289, B-0426, B-0684, B-1245, B-1321, B-1868*
Heitmann S.O.: *B-1704*
Hekimoglu A.: *B-1206*
Hekimoglu B.: *B-1206*
Hekimoglu K.: *B-1854*
Helbich T.H.: *A-0528, B-0716, B-1150, B-1542, B-1549, B-1570*
Helbig S.: *B-1247*
Hélénon O.: *B-1664*
Helfen A.: *B-1125*
Helfenstein R.: *B-0571*
Hellerhoff K.A.: *B-1847*
Hellmich M.: *B-0461*
Hellström M.: *B-0324*
Helmberger T.K.: *K-42*
Helthuis J.: *B-0215*
Hempel J.-M.: *B-0093*
Henderson W.C.: *B-1536*
Hendriks E.J.: *B-0096*
Hendrikse J.: *A-0251, B-0215*
Hendry J.: *B-0033*
Henner A.: *A-0494, B-0886, B-0887, B-1192, B-1712, B-1717*
Hennessy W.: *B-1706*
Henry T.: *B-1407*
Hentschel M.: *B-0510*
Henzler T.: *B-0022, B-0106, B-0587, B-0735*
Hepp T.: *B-1777*
Herbst M.: *B-1845*
Hering A.: *B-0349*
Hermann A.-L.: *B-0848*
Hermann K.-G.A.: *B-0514, B-1738*
Hermann S.: *B-0514*
Hermans R.: *A-0720*
Hermie I.: *B-0157*
Hernandez Giron I.: *B-1592*
Hernandez-Giron I.: *B-0137*
Herold C.J.: *K-24*
Herranz M.: *A-0705*
Herrmann J.: *B-1240*
Herrmann K.: *B-0231, B-0575, B-0670, B-0671, B-0673, B-0690, B-0819, B-1456, B-1461*
Herweh C.: *B-0263*
Herz K.: *B-0095*
Herzen J.: *B-0995, B-1612, B-1847*
Hesper T.: *B-1739*
Hetzel J.: *B-1814*
Heusch P.: *B-0669, B-0670, B-0671, B-1355, B-1461*
Heusdens C.H.: *B-1509*
Heussel C.P.: *A-0702, B-1421*
Heuvelmans M.A.: *B-1008*
Heverhagen J.T.: *B-0896, B-0901, B-1184, B-1434, B-1438, B-1442*
Heye T.: *B-1092*
Heywang-Köbrunner S.H.: *SY 2c*
Hielscher T.: *B-0348*
Hietschold V.: *B-0398*
Higashida A.: *B-0425*
Hikspoors J.P.J.M.: *B-0068*
Hilgendorff A.: *B-0927*
Hill M.: *B-1540*
Hillen B.: *B-0215*
Hillengass J.: *B-0344, B-0348*
Hilton B.: *B-0079*
Hinrichs J.B.: *B-0001, B-0681*
Hintschich C.: *B-1451*
Hinzpeter R.M.M.: *A-0088*
Hippeläinen E.: *B-0998*
Hirata T.: *B-1899*
Hirota M.: *B-0660*
Hirsch J.G.: *B-0019*
Hislop-Jambrich J.: *B-0655*
Hjemly H.H.: *A-1042*
Hoa H.V.: *B-1012*
Hocheol C.: *B-0965*
Hochmair M.: *B-1020*
Hodel J.: *A-0158, B-0261*
Hodler J.: *A-0271*
Hoeffel C.: *B-0528*
Hoeschen C.: *A-1012, A-1014, A-1016*
Hoferica M.: *B-0281*
Hoff S.R.: *B-0077*
Hoffman A.: *B-1502*
Hoffmann A.: *A-0499*
Hoffmann MD MPH U.: *B-1013*
Hoffmann R.-T.: *B-0398, B-0401, B-0797, B-1474*
Hofland J.: *B-1279*

Hofmann V.: *B-1092*
Hofmann W.-K.: *B-0352*
Hofmanninger J.: *B-0307, B-0344, B-0567, B-0811, B-0905*
Ho-Fung V.M.: *B-1055, B-1057*
Hofvind S.: *A-0009, B-0077, B-0921*
Hogg P.H.: *A-0963, B-1708*
Hohenfellner M.: *B-0813, B-1077*
Höhn A.K.: *B-0175*
Höink A.: *B-1761*
Hoinkiss D.C.: *B-0019*
Holch J.: *B-1586*
Holder D.: *B-1425*
Holm S.: *A-0623*
Holodny A.I.: *B-1032*
Holst Pedersen J.: *B-0497*
Holz J.: *B-0314*
Holzer M.: *B-0307*
Holzhey D.: *B-0196, B-1683*
Honavar S.: *B-0562, B-1062*
Hong G.: *B-1454*
Hong G.-S.: *B-0105, B-0273, B-1754*
Hong H.: *B-1403*
Hong J.H.: *B-1412*
Hong N.: *B-1731, B-1732*
Hong S.: *B-0758*
Hong S.R.: *B-1023*
Hong Y.: *B-1244, B-1779*
Hong Y.-S.: *B-0054, B-0683*
Hongmin C.: *B-0475*
Hoogenboom M.: *B-0872*
Hopland N.-M.: *B-1543*
Hoppel B.E.: *B-0306, B-1832*
Horgan G.: *B-0407*
Horger M.: *B-0146, B-0620, B-0623, B-0628, B-1814*
Hörndler K.: *B-0909*
Horn-Lodewyk J.: *B-0883*
Horowitz M.: *B-1607*
Horsch A.D.: *B-0705*
Horvat J.V.: *B-1175, B-1176*
Horvat-Maurovich P.: *B-0303*
Hosseini H.: *B-0261*
Hotfiel T.: *B-0684*
Hötker A.M.: *B-0152, B-0822*
Hou B.: *B-1883, B-1890*
Hou P.: *B-1725*
Hou Y.: *B-0149, B-0315, B-0785, B-1017, B-1308, B-1604*
Houben I.: *B-1787*
Houot R.: *B-0672*
Hourani R.: *B-1168*
Houwers J.B.: *B-1049, B-1582*
Howard J.: *A-0187*
Howarth N.: *A-0914*
Howlett D.C.: *A-0137, A-0585*
Hrabak P.: *B-0938*
Hrabak Paar M.: *A-0776*
Hricak H.: *B-0152*
Hsiao A.: *B-0302, B-1607*
Hu B.: *B-1674, B-1825, B-1859*
Hu D.: *B-1479*
Hu F.: *B-0405, B-1535*
Hu H.: *B-0472*
Hu J.: *B-1163*
Hu M.S.: *B-1273*
Hu P.: *B-0678*
Hu Q.: *B-0815*
Hu S.: *B-1585, B-1590, B-1727*
Hu T.: *B-0801*
Hu X.: *B-0308, B-1333, B-1591*
Hu Y.: *B-0718, B-1131*
Huang C.: *B-0275*
Huang D.Y.: *A-0997*
Huang H.: *B-0952*
Huang J.: *B-0343, B-1762*
Huang L.: *B-0380, B-1476, B-1651*
Huang M.: *B-0390*
Huang Q.: *B-1440*
Huang S.: *B-1476, B-1618, B-1651, B-1654, B-1674, B-1680, B-1681, B-1820, B-1825*
Huang X.: *B-1017, B-1036, B-1333, B-1430*
Huang Y.: *B-0423, B-0429, B-0430, B-1409*
Huasen B.: *B-0961*
Huber F.A.: *B-0997, B-1733*
Huber T.: *B-0088, B-1586*
Hübner L.: *B-0221*
Hudson H.T.: *B-1217*
Huellner M.W.: *B-0224*
Huettel M.: *B-0684*
Hughes C.: *B-0651*
Hughes N.M.: *B-0633*
Hugosson J.: *B-0324*
Hui Shu Y.: *B-1002*
Huland H.: *B-1089*
Hüllner M.: *B-0229, B-0817*
Hulsmans F.J.: *B-1170*
Humphries P.D.: *B-1324*
Hunink M.G.M.: *A-0636, A-0740*
Hunold P.: *B-1622*
Hunt M.: *B-0323*
Huppertz A.: *B-1184*
Hur B.Y.: *B-1342*
Hur J.: *B-0776*
Hurwitz L.: *B-0493, B-0729, B-1503*
Hurwitz-Koweek L.: *B-1140*
Husmann L.: *B-0224*
Hussein M.M.: *B-0693*
Husseiny Salama D.: *A-0310, A-0315*
Hussl A.: *B-1689*
Hutchinson J.C.: *B-1319*
Hüttl K.: *B-0958*
Hutton C.: *B-0763*
Huyskens J.: *B-0753*
Hwang H.J.: *B-0576*
Hwang J.Y.: *B-0918*
Hwang S.: *B-1766*
Hwang S.H.: *B-0776*
Hyde E.: *B-0033*
Hynes D.: *B-0246*
Hyodo T.: *B-0408*

### I

Iacobellis F.: *B-0104*
Iadanza A.: *B-1776*
Iafrate S.: *B-0241, B-1295*
Iannalfi A.: *B-0372*
Iannessi A.: *B-1023*
Iannicelli E.: *B-0525, B-1341*
Ianniello S.: *B-0732, B-0733, B-0734*
Ibañez Sanz L.: *B-0268*
Ibe D.: *B-1391*
Ibrahim A.: *B-0366, B-1170*
Ibrahim E.A.: *B-0100*
Ichikawa S.: *B-0404*
Ichikawa Y.: *B-1101, B-1102*
Ichinohe F.: *B-1836*
Idema S.: *B-0096*
Idilman I.: *B-1594*
Ierardi A.M.: *B-1808*
Iezzi R.: *A-0419, A-0908, B-0109, B-0110, B-0114, B-1812*
Igashi J.B.: *B-1391*
Ignacio G.M.: *B-0118*
Ignjatovic S.: *B-0446*
Igumenova N.: *B-1853*
Iida G.: *B-1629*
Ikeda Y.: *B-0706*
Ikram M.A.: *B-1690*
Ikramov A.: *B-1379*
Ikushima H.: *B-0648*
Iles S.E.: *B-0916, B-0919, B-1543*
Ilhan H.: *A-0976*
Illarioshkin S.: *B-1554*
Illing R.: *B-1185, B-1200*
Im D.J.: *B-0776*
Im J.-G.: *B-1412*
Im S.A.: *B-0336*
Imai T.: *B-0293*
Imai Y.: *B-1603*
Imamura R.: *B-1735*
Imbriaco M.: *B-0668, B-0823, B-1392, B-1684*
Inampudi V.: *B-1886*
Incekara F.: *B-0908*
Inci E.: *B-0877*
Indo H.: *B-1329*
Ingrisch M.: *B-1400*
Inserra G.: *B-1648*
Intert L.: *B-0679*
Ioannidis G.: *A-0530*
Iodice M.: *B-0477*
Iori M.: *B-0993*
Iorio R.: *B-0333*
Ipek A.: *B-0603*
Ippolito D.: *B-1310, B-1372, B-1457*
Iranpour P.: *B-0291*
Irtyuga O.: *B-1505*
İsci S.: *B-0556*
Isensee F.: *B-0539*
Isfort P.: *B-0005*
Ishii K.: *B-0408*
Ishiki A.: *B-1329*
Islak C.: *B-0986*
Islam S.: *B-0177*
Isoda H.: *B-0185*
Ith M.: *B-0901*
Itoh T.: *B-1146*
Itou M.: *B-0408*
Ittermann T.: *B-0797*
Ittrich H.: *B-0119, B-0499*
Iuga A.-I.: *B-1761*
Ivanac G.: *A-0006, B-0463*
Ivanoski S.: *B-0050*
Ivanova D.: *A-0832*
Ivanova M.O.: *B-0791*
Ivanova P.: *B-0957*
Ivantsova A.: *B-1887*
Ivashchenko O.V.: *B-1592*
Izumi T.: *B-0185*
Izzo A.: *B-1579, B-1581*

### J

Jabbari K.: *B-1694*
Jablonkai B.: *B-0786*
Jabłońska-Pawlak A.: *B-1452*
Jackson D.: *B-1410*
Jackson O.: *B-0604*
Jacob S.: *A-0018*
Jacobi V.: *B-1257*
Jacobi-Postma A.A.: *B-1447, B-1625*
Jacobs A.: *B-1539, B-1838, B-1839, B-1842*
Jacobs J.: *B-0132*
Jacobs K.: *B-1607*
Jacobs R.: *B-1599*
Jacobson J.A.: *A-0992, A-0994, B-0893*
Jacquet L.: *B-0944*
Jacquier A.: *B-0964*
Jadczak A.: *B-0946*
Jaddoe V.W.: *B-0171*
Jagannatha V.: *B-0572*
Jäger H.R.: *A-0024, A-0156, A-1010, B-0712, B-1688*
Jäger N.: *B-1586*
Jagmohan P.: *B-1150*
Jagoda P.: *B-1720*
Jahnke P.: *B-1199, B-1630, B-1631*
Jahoda J.: *B-0938*
Jain D.: *B-1207*
Jain P.: *B-0720*
Jain S.: *B-1797*
Jain S.K.: *B-0720*
Jain V.: *B-1382*
Jaipal A.: *B-0546*
Jajodia A.: *B-0691, B-1637*
Jakobsen H.: *B-0154, B-0442*
Jalli R.: *B-0291*
Jaly A.: *B-0976*
James A.: *B-1647*
James S.L.J.: *A-0693*
Jamialahmadi T.: *B-1380*
Jamias J.: *B-0118*

Jang J.J.: *B-1322*
Jang J.K.: *B-0796*
Jang M.J.: *B-0699*
Jang S.: *B-0453*
Jangjoo A.: *B-1380*
Janka R.: *B-0474, B-1088*
Jann H.: *B-1024*
Jannot A.-S.: *B-1894*
Janot K.: *B-0836*
Jardri R.: *A-0406*
Jargiello T.: *A-0086, A-0438*
Jäschke M.: *B-0237*
Jaschke W.R.: *A-0546, A-0746, B-0500, B-1599*
Jasieniak J.: *B-1477*
Jawhar M.: *B-0352*
Jayaram P.R.: *B-1287*
Jazet I.M.: *B-0588*
Jazrawi L.: *B-1508, B-1510*
Je B.-K.: *B-1014*
Jeffery T.J.: *B-0709*
Jeka S.: *B-1408*
Jemal Turki A.: *B-0155*
Jemel E.: *B-1160*
Jenkins J.: *B-0078, B-0080*
Jenniskens S.F.: *B-0878*
Jensen E.: *B-1007*
Jeon C.H.: *B-0740, B-0960, B-1573*
Jeon H.J.: *B-0449*
Jeon S.K.: *B-0389, B-1772*
Jeong S.Y.: *B-0796*
Jereczek-Fossa B.A.: *B-0973*
Jerman K.: *B-0086*
Jeukens C.: *B-1787*
Jeurissen B.: *B-1127*
Jezzard P.: *A-0250*
Ji C.: *B-0123*
Ji H.: *B-0600*
Ji J.: *B-0308, B-0611, B-0772, B-1126*
Jia Z.: *B-1017*
Jiang H.: *B-0074, B-0356, B-1044, B-1764*
Jiang J.: *B-0035, B-0144, B-1642*
Jiang L.: *B-1674, B-1680, B-1825*
Jiang T.: *B-0151, B-1624*
Jiang X.: *B-0718*
Jiang Y.: *B-0051, B-1655*
Jimenez-Pastor A.: *B-1397, B-1830*
Jin L.: *B-1813*
Jin P.: *B-1498*
Jin T.: *B-1774*
Jin W.: *B-1137*
Jin Z.: *B-0012, B-0320, B-0386, B-0423, B-0430, B-0454, B-0613, B-1074, B-1087, B-1121, B-1246, B-1409, B-1633, B-1893, B-1896, B-1897*
Jin Z.-Y.: *B-0450, B-1104, B-1475*
Jo H.H.: *B-0582*
Jobst B.: *B-1201*
Jochelson M.S.: *B-0924, B-1175, B-1176, B-1804*
Jochems A.: *B-1179*
Jockwitz C.: *B-1553*
Joelsen-Hatlehol E.S.: *B-1047*
Jogi S.: *B-1132, B-1133*
Johansen C.K.: *B-1047*
Johansen S.: *B-1369*
John G.: *B-0712*
Johnson J.M.: *B-1243*
Johnson K.J.: *A-0683*
Johnson M.: *B-1555*
Jokerst C.: *B-1007*
Jolibois Z.: *B-1563*
Jones E.C.: *B-1640*
Jones J.: *B-1323, B-1455*
Jones R.: *B-0606*
Joo I.: *B-0389, B-1767, B-1772*
Joon P.: *B-1666*
Joseph D.Z.: *B-1109, B-1713, B-1714*
Joshua A.: *B-0226*
Jost G.: *B-0496*
Jouannic A.-M.: *B-1497*
Joubert Zakeyh J.: *B-1470*
Jouret-Mourin A.: *B-0944*
Jovanovic S.: *B-1871*
Jover Sánchez J.J.: *B-0535*
Jreige M.: *B-0235*
Juffermans J.F.: *B-1506*
Juhász I.Z.: *B-0958*
Jukna A.: *B-0037*
Juli C.: *SY 7*
Jung B.: *B-0221, B-0901*
Jung C.S.: *B-0499*
Jung E.M.: *A-0169*
Jung J.: *B-1403*
Jung N.Y.: *B-0469, B-0555*
Jung S.I.: *B-0449*
Junghanss T.: *A-0230*
Jungmann F.: *B-0383, B-0591*
Junker D.: *B-1076*
Juras V.: *B-0055*
Jurik A.-G.: *A-0782*
Justich C.: *A-0416*
Juza T.: *B-0232*

### K

Kaartinen S.: *B-0887*
Kabbasch C.: *B-0710, B-0756, B-1166*
Kacala A.: *B-0747*
Kachelrieß M.: *A-0326, B-0013, B-0135, B-0312, B-0316, B-0319, B-0321, B-0909*
Kacprowski T.: *B-0398*
Kada S.: *A-0688*
Kadioglu P.: *B-0986*
Käfer H.: *A-0216*
Kagawa K.: *B-1145, B-1146*
Kahn C.E.: *A-0505*
Kahn I.: *B-1557*
Kahn J.: *B-0399*
Kahraman A.S.: *B-1051*
Kahraman B.: *B-1051*
Kahraman G.: *B-1854*
Kai Y.: *B-1002*
Kainberger F.: *A-0446, A-0854, B-0675*
Kaireit T.F.: *B-0585, B-1201*
Kaiser M.: *B-0353*
Kaiser Ururahy Nunes Fonseca E.: *B-1365*
Kaissis G.: *B-0615, B-1312*
Kak M.: *B-1436*
Kale P.G.: *B-1075*
Kallenbach M.: *B-1657*
Kallenberg M.: *B-0697*
Kallur K.: *B-0063*
Kalra S.: *B-1191*
Kalsoum E.: *B-0261*
Kaltenbach B.: *B-0243, B-0296, B-0794, B-1204, B-1247, B-1317*
Kaltsas A.: *B-1660*
Kälvesten J.: *B-0049*
Kamal E.: *B-1174*
Kamal R.M.: *B-1174*
Kamali M.: *B-0403*
Kamat R.: *B-0631*
Kamata K.: *B-0408*
Kamaya A.: *B-1402*
Kamble A.: *B-0092*
Kamble A.N.: *B-0092*
Kamel H.A.: *B-0711*
Kamel H.M.: *B-0117*
Kamo M.: *B-0746*
Kamoun H.: *B-1487*
Kämpgen B.: *B-0022*
Kanamu L.: *B-1356*
Kanavaki A.: *B-1378, B-1537*
Kanavati F.: *B-1387*
Kanazawa Y.: *B-0642, B-0648, B-0994*
Kandasamy D.: *B-0640*
Kandel S.: *B-0306*
Kang B.J.: *B-0469, B-0555*
Kang E.-Y.: *B-1014*
Kang H.: *B-1414*
Kang H.-J.: *B-1532, B-1772*
Kang L.: *B-0770*
Kang T.W.: *B-1767*
Kantarci M.: *A-0148*
Kapetas P.: *B-0716, B-1150, B-1549, B-1570*
Kaplan S.L.: *B-1055*
Kapoor M.: *B-0696*
Kapos I.: *B-1616*
Kaposi P.N.: *B-0444, B-1589*
Kappas C.: *B-1486*
Kappelle L.J.: *B-0705*
Karabulut E.: *B-1445*
Karaca H.: *B-0295*
Karaca L.: *B-1051*
Karaiskos P.: *B-0768*
Karakaya J.: *B-1805*
Karakuła-Juchnowicz H.: *B-0538*
Karam E.: *B-1407*
Karaman C.Z.: *B-1059*
Karampinos D.C.: *B-1746*
Karanam L.S.P.: *B-0562, B-0841, B-1062*
Karcaaltincaba M.: *A-0859*
Kardos L.: *B-1200*
Kargar S.: *B-0438*
Kärgel R.: *B-0310*
Karimi T.: *B-1669*
Kärkkäinen O.: *B-1272*
Karli Oguz H.K.K.: *B-1445, B-1884*
Karli Oguz K.: *A-1076*
Karout L.: *B-0799*
Karrasch S.: *B-1208*
Karssemeijer N.: *B-0545, B-0697, B-1564*
Kartalis N.: *A-0853*
Kartheuser A.: *B-0944*
Karunanithy N.: *B-0966*
Karur G.R.: *B-1864*
Kashef E.: *A-0436*
Kasprian G.: *A-0560, B-0926, B-0927, B-1881, B-1882, K-30*
Kassai Y.: *B-1203*
Kassarjian A.: *A-0783*
Kastelic M.: *B-1608*
Kastler B.: *A-0514, B-1664*
Kastner C.: *B-0880*
Katada K.: *B-0706*
Katahashi K.: *B-1722*
Katayama M.: *B-1180*
Kato H.: *B-0660*
Kato K.: *B-0742*
Kato T.: *B-0484*
Katsari K.: *B-1200*
Katsumata A.: *B-0994*
Katulska K.: *A-0154*
Katyan A.: *B-0720, B-1291, B-1293*
Katzmann A.: *B-1586*
Kauczor H.-U.: *A-0290, A-0626, B-0019, B-0051, B-0344, B-0348, B-0385, B-0420, B-1201, B-1208, B-1610*
Kaufmann P.A.: *B-0817*
Kaufmann S.: *B-0628*
Kaul M.: *B-1240*
Kaul M.G.: *B-0499*
Kaur S.: *B-0475*
Kauw F.: *B-0192, B-0705*
Kavanagh E.: *B-0246, B-0407*
Kavanagh J.: *B-0066, B-0141*
Kawai T.: *B-1187*
Kawamura K.: *B-1180*
Kawashima A.: *B-0438*
Kawashima J.: *B-0482*
Kawashima K.: *B-0742*
Kazemi M.A.: *B-0635*
Kazmierczak P.M.: *B-0503*
Kearins O.: *B-0078*
Keasler E.: *B-1296, B-1298, B-1299, B-1301*
Keating D.M.: *B-0924*
Keber U.: *B-0365*
Kedves A.: *B-0031, B-0882*
Kedzierski B.: *B-0200*
Keeling A.: *B-0353*
Keelson B.: *B-1596, B-1628*
Keil S.: *B-0005*
Keil V.C.: *A-1030*

Kellner E.: *B-0568*
Kelly C.: *B-1307*
Kelly P.: *B-0407*
Kelly P.A.: *B-0950*
Kelly-Morland C.: *A-0281, B-0616*
Kemmling A.: *B-0259*
Kemner-van de Corput M.M.P.C.: *B-0171*
Ken S.: *B-0322*
Kenis S.F.: *B-1391*
Kenneth M.: *B-1417*
Kent B.: *B-1410*
Kenyon K.: *B-0126*
Kertész H.: *B-0234*
Kesari A.: *B-0063*
Keserci B.: *B-1304, B-1305*
Keshavarz P.: *B-0291*
Keupers M.: *B-0251, B-0473, B-0915, B-1803*
Khakifa H.E.: *B-1334*
Khalifeh S.: *B-0799*
Khalikov A.: *B-0608, B-0609*
Khalil A.: *B-1407*
Khalil M.R.: *B-0899*
Khan K.: *B-0524*
Khan S.U.K.: *B-1697*
Khandelwal A.: *B-0447, B-1142*
Khandelwal N.: *B-1224*
Kharuzhyk S.: *A-0501*
Khawaja K.: *B-1241*
Khoury T.: *B-1159*
Khovrin V.V.: *B-1263*
Khristenko E.: *A-0466*
Khurana R.: *B-0605, B-1668*
Kickingereder P.: *A-0793, B-0539*
Kiechl-Kohlendorfer U.: *B-0925*
Kiefer B.: *B-0446*
Kiefer L.S.: *B-1514, B-1515*
Kieft I.: *B-1700*
Kierans Z.: *B-1066*
Kiessling F.M.A.: *A-0458*
Kifjak D.: *B-1020*
Kilburn-Toppin F.: *A-0425, A-1084, B-1795, B-1801*
Kilickesmez O.: *B-1395*
Kim B.: *B-0699, B-1142*
Kim C.: *B-1001, B-1014*
Kim C.W.: *B-0740, B-0960, B-1573*
Kim D.: *B-0775*
Kim D.K.: *B-1282, B-1443*
Kim E.H.: *B-1895*
Kim E.-K.: *B-0120*
Kim G.-C.: *B-0942*
Kim H.: *B-0646, B-1119, B-1403*
Kim H.-C.: *B-1772*
Kim H.J.: *B-0392, B-0942, B-1285*
Kim H.O.: *B-0396*
Kim H.Y.: *B-1336*
Kim J.: *B-0334, B-0435*
Kim J.E.: *B-0396*
Kim J.H.: *B-0397, B-0453, B-1096, B-1342*
Kim J.-K.: *B-0335*
Kim J.R.: *B-0339*
Kim J.S.: *B-1177*
Kim J.W.: *B-1045*
Kim K.H.: *B-1538*
Kim K.W.: *B-0796*
Kim K.Y.: *B-0741*
Kim M.-J.: *B-0334, B-0335, B-0402, B-1043, B-1763, B-1766*
Kim N.: *B-0576*
Kim S.: *B-0336, B-0862, B-0869, B-1043, B-1772*
Kim S.H.: *B-0097, B-1342*
Kim S.K.: *B-0054, B-0683*
Kim S.-K.: *B-1001*
Kim S.M.: *B-0699*
Kim S.Y.: *B-1767*
Kim T.: *B-1336*
Kim T.-K.: *B-0392*
Kim W.H.: *B-0942*
Kim Y.: *B-0469, B-0555, B-0699, B-1414*
Kim Y.J.: *B-0449, B-1865*
Kim Y.-J.: *B-0571*
Kim Y.S.: *B-1336*
Kim Y.Y.: *B-0402*
Kim-Fuchs C.: *B-1434, B-1438*
Kimm M.: *B-1848*
Kimoto N.: *B-0642, B-0994*
Kind M.: *B-0478*
King A.D.: *A-0076, A-0722*
King B.: *B-0438*
Kinkel K.: *A-0473*
Kintzelé L.: *B-0344, B-0348*
Kirat Singh S.K.: *B-0468*
Kiratli H.: *B-1445*
Kirby A.: *B-1323*
Kirchner G.: *B-0739*
Kirchner J.: *B-0231, B-0669, B-0670, B-0671, B-0673, B-1461, B-1462*
Kirienko M.: *B-1636*
Kirkham A.: *B-0159, B-0443*
Kirova-Nedialkova G.I.: *A-0833*
Kirova-Nedyalkova G.I.: *B-0590*
Kirstein M.: *B-0001*
Kishida Y.: *B-0417, B-0422, B-0579, B-1003, B-1203, B-1209, B-1460, B-1755*
Kist J.: *B-1850*
Kitaigorodska O.V.: *B-1267*
Kitalia S.: *B-1356*
Kittinger J.: *B-0219*
Kizilkilic O.: *B-0986*
Kjelle E.: *B-0030*
Klaan B.: *B-0237*
Klang E.: *B-1186*
Klarhöfer M.: *B-0446*
Klebermaß-Schrehof K.: *B-0926*
Kleedehn M.G.: *B-1297*
Klein S.: *B-0067, B-0908, B-1279*
Klein W.M.: *B-0068*
Klepanec A.: *B-0281*
Klifa C.: *B-1023*
Klinder T.: *B-1761*
Klingebiel M.: *B-0158, B-0161, B-0515*
Klingenstein A.: *B-1451*
Klingmüller V.: *B-1512*
Kliokyte R.: *B-0270*
Kloeckner R.: *B-1824*
Klomp D.W.: *B-0766*
Klompenhouwer E.G.: *B-0559, B-0850*
Klose U.: *B-0093*
Klosterkemper Y.: *B-0516, B-0518, B-0954, B-1210, B-1368, B-1726, B-1739*
Kloth C.: *B-0620, B-1814*
Kløw N.E.: *B-0480*
Klug G.: *B-0500*
Kluijtmans L.: *B-0706*
Kmetyuk Y.: *B-1250*
Knapp K.: *B-0659, B-1495*
Knapp K.M.: *B-0662, B-1067, B-1698*
Knaup M.: *B-0909*
Knautz M.: *B-1736*
Knebel C.: *B-1746*
Kneilling M.: *B-0686*
Knop K.C.: *B-1560*
Ko A.: *B-0451*
Ko H.: *B-1322*
Ko Y.: *B-1285*
Kobeiter H.: *B-0252*
Kober F.: *A-1009*
Koç F.: *B-1413*
Kocak B.: *B-0986, B-1395*
Kocer N.: *B-0986*
Koch G.: *B-0834*
Kocher N.: *B-0568*
Kochhar R.: *B-1095*
Koci M.: *B-0338, B-0624*
Kocijančič I.: *B-1608*
Kocman V.: *B-1125*
Koczka V.: *B-0882*
Koenig T.: *B-0909*
Kofoed K.F.: *B-0194, B-0497*
Koh D.-M.: *A-0068, B-0108, B-0353, B-0524, B-1046*
Kohestani K.: *B-0324*
Kohl S.: *B-0813, B-1077*
Köhler B.: *B-0221*
Kohler R.: *A-0569*
Köhler T.: *B-1792*
Kojima T.: *B-0484*
Kok N.F.: *B-0182*
Kolansky A.: *B-0144*
Kolb C.: *B-0797*
Kolditz D.: *B-1541*
Kolesnik E.: *B-1620*
Kollmeier M.: *B-0323*
Kolossvary M.: *B-0303, B-0786*
Koltowska A.: *B-0540*
Kolu M.: *B-1051*
Komatsu D.: *B-1836*
Konda D.: *B-0010*
Kondo M.: *B-0484*
Kondoh H.: *B-1187*
Konen E.: *B-1186*
Kong D.: *B-1244*
Konge L.: *A-0043*
Konieczny M.: *B-1739*
Konietzke P.: *B-1610*
König F.: *B-0362, B-0364, B-0893, B-1568*
Konijn L.C.D.: *B-0222*
Konijnenberg S.E.: *SY 17*
Konings D.: *B-0494*
Konishi T.: *B-1663*
Kono Y.: *B-0425*
Kononen M.: *B-1272*
Konovalov R.: *B-1554*
Koo B.-K.: *B-0303*
Koo N.-H.: *B-1069*
Kooijman H.: *B-0601*
Kooijman-Kurfuerst H.: *B-0225*
Kool D.R.: *A-0515*
Koops A.: *B-0590*
Kopp F.K.: *B-0138, B-1848*
Kopp M.: *B-0426, B-0684, B-1321, B-1777*
Kording F.: *B-0601*
Koromani F.: *B-0856*
Korte B.: *B-0081, B-0082, B-1149*
Kortesniemi M.: *A-0327, A-0896, A-1050*
Korzowski A.: *B-0506*
Kos V.: *B-0276*
Kosar P.N.: *B-1884*
Kose S.: *B-1413*
Koskinen S.K.: *B-0101, B-0674, B-0955*
Kossov F.: *B-0621*
Kosyr'kova A.: *B-0089*
Kott A.: *B-0694*
Kotter E.: *A-0012, A-0125, A-0634, A-0695, B-0568, B-0867, SY 15*
Kotze J.: *B-1066*
Koulocheri D.: *B-1157*
Koutoulidis V.: *B-1157*
Kouvelakis K.: *B-0524*
Kovacic S.: *B-0260*
Kovács Á.: *B-0031, B-0882*
Kovacs G.: *B-0767, B-0989*
Kovan O.: *B-1546*
Kox L.: *B-0244*
Koyama H.: *B-0579, B-1203*
Kozlov V.: *B-1005*
Kraan R.B.J.: *B-0244*
Kraeima J.: *B-0574*
Krainik A.: *A-0153, A-0522*
Kramer H.: *B-1866, SY 14*
Kranewitter C.: *B-0500*
Krastev G.: *B-0281*
Kratovska A.: *B-0957*
Kraus B.: *A-0495*
Krauss B.: *B-0682*
Kräuter C.: *B-0767, B-0989, B-1619, B-1620*
Kreitner K.-F.: *B-0378, B-0383, B-0591, B-1824*
Kremer M.: *B-1598*
Kremser C.: *B-0500*
Krenn T.: *B-0167*
Krestan C.R.: *A-0882*
Kreth F.-W.: *B-0094*

Kreul D.: *B-0446*
Krichene M.A.: *B-1160*
Kriedemann M.: *B-0337*
Krieghoff C.: *B-0196, B-1683*
Krishna Moorthy T.: *B-1885*
Krishnamoorthy K.: *B-1229*
Krishnamurthi G.: *B-1639*
Krismer F.: *B-1689*
Kristen K.-H.: *B-0677*
Kristiansen C.H.: *B-0492*
Kristiansson I.: *B-1197*
Krokidis M.: *B-0849, B-1809*
Krombach G.A.: *B-1863*
Kromrey M.-L.: *B-0398, B-0401, B-0404, B-1474*
Kronfeld A.: *B-1241*
Kronlage M.: *B-1560*
Kropf S.: *B-0221*
Kröpil P.: *B-0954, B-1368*
Krotenkova M.: *B-1554*
Krueger-Ziolek S.: *B-0583*
Krug B.: *B-0461*
Krug K.B.: *B-1542*
Krüger P.-C.: *SY 28*
Kruis M.: *B-1465*
Kruk-Bachonko J.: *B-1482*
Krumina G.: *B-1652*
Krupski W.: *B-0538, B-1482*
Krylov V.: *B-0186*
Krylova N.: *B-1819*
Kshirsagar R.H.: *B-1450*
Kubale R.: *B-0963, B-1720*
Kubicka F.M.: *B-0503*
Kubik-Huch R.A.: *A-0284, B-0446, B-1792*
Kubo T.: *B-0303*
Kubota K.: *B-0471, B-1796*
Kucharczyk S.R.: *B-1706*
Kuchcinski G.: *B-1415*
Kuder T.: *B-1568*
Kuder T.A.: *B-0362, B-0364*
Kudo M.: *B-0408*
Kudryavtsev N.D.: *B-1263*
Kuehl H.: *B-1411*
Kuehn B.: *B-1390*
Kuganesan A.: *B-1147, B-1849*
Kuhl C.K.: *A-0030, A-0235, A-0468, B-0005, B-0073, B-1437, SY 2a*
Kuhn F.P.: *B-0058*
Kühn J.-P.: *B-0398, B-0401, B-0797, B-1474*
Kuhn M.: *B-0440*
Kuhn R.: *B-0118*
Kuhn T.: *B-0051*
Kühnel F.: *B-0503*
Kukkes T.: *B-1712*
Kukushkin A.V.: *B-0844, B-0845*
Kul M.: *B-1373*
Kulali F.: *B-0125*
Kulikov S.: *B-0350*
Kulkarni M.M.: *B-1464*
Kulkarni V.: *B-1562*
Kulpe S.: *B-0995*
Kumar D.: *B-1117*
Kumar E R.: *B-0777*
Kumar N.: *B-1291, B-1293*
Kumar P.G.: *B-1159*
Kumar R.: *B-1856*
Kunz W.G.: *B-0259, B-0262, B-0713*
Kupriyanov D.: *B-1326*
Kurehana N.: *B-1329*
Kurrat C.: *B-0694, B-1643*
Kuru Oz D.: *B-1373*
Kurys-Denis E.: *B-0538*
Kus P.: *B-1477*
Kusaka A.: *B-1146*
Kuzmin A.: *B-0091*
Kvarnström N.: *B-1900*
Kvasnicka H.: *B-1257*
Kvist O.: *B-1360*
Kwak J.Y.: *B-0120*
Kwan Hoong N.: *B-0475*
Kwon H.: *B-0740, B-0960, B-1573*
Kwon H.-J.: *B-0796*
Kwon J.H.: *B-1096*
Kwon W.: *B-0776*

### L

La Fougère C.: *B-0686, B-0689, B-0818*
La Grassa M.: *B-0085, B-0554*
La Porta M.: *B-0034*
Laaksonen-Heikkilä R.: *B-1115*
Laas E.: *B-1090*
Lachenmayer A.: *B-1434, B-1438, B-1442*
Lacroix E.: *B-0322*
Ladd M.: *B-0505, B-0506, B-1419*
Ladwig K.-H.: *B-1782*
Laenen A.: *B-0559, B-0850, B-1803*
Laeseke P.F.: *B-1297*
Laggner R.: *B-1617*
Laghi A.: *A-0029, B-0007, B-0008, B-0014, B-0525, B-0935, B-0940, B-1022, B-1148, B-1341, B-1809, B-1833*
Lahaye M.J.: *A-0108, B-0182, B-1484*
Lai J.: *B-0718*
Lai Y.T.: *B-1220*
Lai Z.H.: *B-1065*
Laitinen H.: *B-0645*
Lalji U.: *B-1787*
Lam A.: *B-0141*
Lam Giang T.: *B-1090*
Lamb H.J.: *A-0219, B-0588, B-1506*
Lambadiari V.: *A-1063*
Lambert L.: *B-0938*
Lambin P.: *A-0267, B-1170, B-1179*
Lambregts D.M.: *A-0771, B-0182, B-1049, B-1484, B-1582*
Lame F.: *B-1638*
Lameijer J.R.C.: *B-0082, B-0083*
Lamers W.H.: *B-0068*
Lampichler K.: *B-0219, B-1377*
Lamplmayr L.: *B-0925*
Lamprecht T.: *SY 22*
Lancelot E.: *SY 30*
Landolfi F.: *B-0525, B-1341*
Lang K.: *B-0085, B-0695, B-1792*
Langberg C.W.: *B-1656*
Langenbach M.C.: *B-0243, B-0843, B-1204*
Langer C.: *B-0782*
Langner S.: *B-0237*
Langs G.: *A-0392, B-0307, B-0344, B-0348, B-0567, B-0811, B-0905, B-1882*
Laniado M.: *A-0837*
Lanza E.: *B-0560*
Lanzillo R.: *B-0543*
Lanzillotta M.: *B-1337*
Lanzman R.S.: *B-0954, B-1368*
Lapteva M.: *B-0395*
Laqua N.: *B-0161, B-0327*
Larbi A.: *B-0431, B-1695*
Larici A.R.: *A-0050, A-0055, A-0306, A-0550*
Larocca Skare T.: *B-1365*
Larousserie F.: *B-0058*
Lashkhi K.: *B-0616*
Lassale L.: *B-0058*
Lassandro G.: *B-1684*
Lastella G.: *B-0042, B-1728*
Latz D.: *B-1736*
Lau F.: *B-0302*
Lau H.T.: *B-0330*
Lau K.: *B-0581*
Lau K.K.: *B-1815*
Lau K.K.-P.: *B-1147, B-1849*
Lau T.: *B-1849*
Laugerette A.: *B-0047*
Laugwitz K.-L.: *B-1312*
Laukamp K.R.: *B-1424*
Laun F.: *B-0289, B-1568*
Laurent F.: *B-1202*
Laurino F.: *B-0007, B-0008, B-1809*
Lauritzen P.: *B-0492*
Lavens M.: *B-0473, B-1803*
Lavin P.: *B-1793, B-1794*
Lavra F.: *B-1721*
Lavrenteva A.: *B-1182*
Law C.: *B-1555*
Law E.K.C.: *B-1292*
Lawrence H.: *B-1707*
Lawrence J.T.R.: *B-1055*
Law-Ye B.: *B-1422*
Lazar M.: *B-0716*
Lazaris A.: *B-0528*
Le Boulc'h M.: *B-0478*
Le Faucheur J.P.: *B-0261*
Le Guellec S.: *B-0478*
Le H.M.D.: *B-0214*
Le P.V.: *B-0278*
Leardini A.: *SY 27*
Lebedev S.: *B-0312*
Lebtahi R.: *B-0848*
Leckebusch T.: *B-0591*
Lecler A.: *B-0987*
Lecouvet F.E.: *A-0195, A-0646, A-0791*
Lederer W.: *B-1568*
Ledergerber B.: *B-0224*
Lederlin M.: *A-0599*
Lee C.H.: *B-0153, B-0441, B-1045, B-1322*
Lee C.M.: *B-0960, B-1573*
Lee C.W.: *B-0105, B-0273, B-1754*
Lee C.Y.: *B-1340, B-1551*
Lee D.H.: *B-0389, B-0869, B-1119, B-1375, B-1466*
Lee E.: *B-0370*
Lee E.S.: *B-0451, B-0795*
Lee E.Y.P.: *B-0178*
Lee G.Y.: *B-0862*
Lee H.: *B-1420*
Lee H.J.: *B-0576*
Lee H.J.L.: *B-0918*
Lee H.Y.: *B-1177, B-1203*
Lee I.J.: *B-0435*
Lee J.: *B-0796*
Lee J.C.Y.: *B-1220*
Lee J.E.: *B-1538*
Lee J.H.: *B-0120, B-0435*
Lee J.M.: *A-1003, B-0303, B-0469, B-0555, B-0795, B-1466, B-1532, B-1767, SY 31*
Lee J.S.: *B-0339*
Lee J.W.: *B-0776*
Lee J.Y.: *B-0277, B-1119, B-1336, B-1375*
Lee K.B.: *B-1767*
Lee K.H.: *A-0649, B-0330, B-0776, B-1285*
Lee K.Y.: *B-1001*
Lee K.Y.Y.: *B-1014*
Lee M.J.: *B-1634*
Lee M.-J.: *B-0334, B-0335*
Lee P.: *B-0627, B-1387*
Lee R.: *B-0740*
Lee R.-C.: *B-0393*
Lee R.K.L.: *B-1292*
Lee S.: *A-0141, B-0056, B-0862, B-0965, B-1043, B-1865*
Lee S.G.: *B-0796*
Lee S.H.: *B-1014, B-1611*
Lee S.-K.: *B-0097*
Lee S.M.: *B-0576, B-0776, B-0795, B-0942*
Lee S.Y.: *B-0561*
Lee T.F.: *B-1220*
Lee Y.H.: *B-1420*
Lee Y.-J.: *B-1336*
Lee Y.K.: *B-0054, B-0683*
Lefaucheur C.: *B-0953*
Lefebvre G.: *B-1507*
Lefrere Belda M.-A.: *B-1894*
Legrand J.: *B-0053*
Lehmkuhl L.: *B-0201*
Lehn A.: *B-0296, B-1247*
Lehner A.: *B-1866*
Lei C.: *B-1006*
Leichter I.: *B-0133, B-0313, B-0992*
Leidholdt E.M.: *B-1191*
Leimgruber A.: *A-0552*
Leiner T.: *A-0420, A-1018, B-0311, B-0317, B-0377, B-0788*

Leithner D.: *B-0299, B-1175, B-1176, B-1204, B-1317*
Leleu F.: *B-0953*
Lemmens K.: *B-1539, B-1839, B-1842*
Leng S.: *B-0447, B-1729*
Lenga L.: *B-0299, B-0434, B-0448, B-1362, B-1472*
Lenk K.: *B-0201*
Lenkinski R.E.: *B-0391, B-0792*
Lennartz S.: *B-1025, B-1081*
Lennie G.: *B-0657*
Lennon L.: *B-0655*
Leo C.: *B-1792*
Leone A.: *A-0614*
Leone F.: *B-0087, B-0247, B-1549*
Leonhardt H.: *A-0168, B-1900*
Lepanto D.: *B-0722*
Leporq B.: *B-1638*
Lepper P.M.: *B-0961*
Lerch T.: *B-0571, B-0896, B-0897, B-0901*
Lerchbaumer M.H.: *B-1657*
Lerebours R.: *B-1140*
Lesman-Segev O.: *B-1557*
Lesyuk O.: *B-0489, B-0652*
Leung J.: *B-0696*
Leung S.T.B.: *B-1220*
Leutmezer F.: *B-0534*
Levashkina I.: *B-1267*
Leventoglu S.: *B-0213*
Levy T.: *B-1186*
Lewicki A.: *B-0874*
Lewis M.A.: *B-0391, B-0792*
Lewis S.: *B-1701, B-1789*
Leyendecker J.R.: *B-0391, B-0792*
Leygnac S.: *B-1407*
Li C.: *B-1304*
Li H.: *B-0376, B-0541, B-0770, B-1120, B-1333, B-1480*
Li J.: *B-0466, B-0718, B-0859, B-0861, B-1243, B-1280, B-1371, B-1584, B-1877*
Li J.-W.: *B-1762*
Li K.: *B-0859, B-1440, B-1441*
Li L.: *B-0018, B-0466, B-0593, B-0911, B-1009, B-1818*
Li M.: *B-0151, B-0837*
Li N.: *B-0745*
Li Q.: *B-0309, B-1009, B-1032, B-1534*
Li R.: *B-1531*
Li T.: *B-0206*
Li W.: *B-0218, B-0359, B-0479, B-0779, B-1399, B-1519, B-1857*
Li X.: *B-0375, B-0504, B-0596, B-0611, B-0754, B-0804, B-0805, B-0978, B-1189, B-1280, B-1399, B-1476, B-1618, B-1651, B-1654, B-1869*
Li X.-S.: *B-1675*
Li Y.: *B-0116, B-0541, B-0573, B-0690, B-1121, B-1159, B-1349, B-1519, B-1888*
Li Y.L.: *B-0330*
Li Z.: *B-0218, B-0223, B-0384, B-0479, B-0611, B-0692, B-0853, B-1476, B-1585, B-1590, B-1651, B-1673, B-1677, B-1727, B-1869*
Li Z.P.: *B-0411, B-1654*
Liang B.: *B-0343*
Liang D.: *B-1386*
Liang K.: *B-0978*
Liang M.: *B-1478*
Liang S.: *B-1517*
Liang W.B.: *B-0573*
Liang X.: *B-1121*
Liao W.: *B-0678*
Libert F.: *B-0894*
Libra F.: *B-0833*
Licandro R.: *B-0567*
Lichtarski M.: *B-0874*
Lichtenstein T.E.: *B-1783*
Lidén M.: *B-0491*
Liebergall M.: *B-0992*
Lieman-Sifry J.: *B-1607*
Lien L.T.T.: *B-1012*
Liguori C.: *B-0034*
Lilleborge M.: *B-0921*
Lim A.K.P.: *B-0627*
Lim L.K.S.: *B-1885*
Lim V.: *B-0698*
Lin C.: *B-1074, B-1087, B-1893, B-1896, B-1897*
Lin H.-Y.: *B-0393*
Lin J.J.: *B-1654*
Lin L.: *B-0320, B-0406, B-0952, B-1315*
Lin M.: *B-0581*
Lin S.: *B-0581*
Lin W.-C.: *B-1431, B-1551*
Linck P.-A.: *B-1415*
Linder N.P.: *B-0764*
Lindig T.: *B-0095*
Lindner T.: *B-0237*
Lindstrom J.C.: *B-0492*
Linn J.: *A-0765*
Linseisen J.: *B-0945*
Linsenmaier U.: *A-0165*
Lioznovs A.: *B-0037*
lirette S.: *B-0357, B-1473*
Lisson C.G.S.: *B-0061*
Lisson C.S.: *B-0061*
Litavcova A.: *B-0232*
Litherland J.: *B-0079*
Litmanovich D.: *A-0564, B-0580, B-1306*
Littooij A.S.: *A-0680*
Liu C.: *B-1021, B-1386*
Liu C.-A.: *B-0393*
Liu F.: *B-0718, B-1236, B-1237*
Liu G.: *B-0141, B-1394*
Liu H.: *B-0094, B-0166, B-0375, B-0382, B-0774, B-1633*
Liu J.: *B-0858, B-1064, B-1331, B-1386, B-1427, B-1691, B-1692, B-1693*
Liu J.-B.: *B-1762*
Liu K.: *B-1009, B-1430, B-1677, B-1752*
Liu L.: *B-0552, B-1278, B-1393*
Liu P.: *B-0320, B-0406*
Liu P.C.: *B-0758*
Liu Q.: *B-0978*
Liu S.: *B-1009, B-1752, B-1890*
Liu X.: *B-1120, B-1222, B-1396, B-1764*
Liu Y.: *B-0190, B-0715, B-0752, B-1272, B-1429, B-1788*
Ljimani A.: *B-0515, B-0517, B-1736*
Ljuhar D.: *B-0569*
Ljuhar R.: *B-0569*
Llobet-Llambrich M.: *B-0680*
Llort A.: *B-1231*
Lloyd T.: *B-0880*
Lo Re G.: *B-1827*
Lo W.-C.: *B-1655*
Lobbes M.: *B-1170*
Lobbes M.B.: *B-1179*
Lobbes M.B.I.: *B-1787*
Lobe N.H.J.: *B-0099*
Lobo M.L.: *A-0278*
Loewe C.: *A-0087, A-0415, A-0977, B-0219, B-0374, B-1617*
Logager V.: *A-0202, A-0328, A-1073, B-0154, B-0442*
Logudoss M.: *B-0227, B-1271*
Lohmeier J.: *B-0664*
Lohöfer F.: *B-0615*
Loiseau Audirac M.P.: *B-0032*
Lombardo C.: *B-1097*
Lombardo F.: *B-1389*
Lombardo Galera M.S.: *B-1662*
Lomoro P.: *B-1153*
Londero V.: *B-0086*
Long Y.: *B-1440, B-1441*
Longo R.: *B-1838, B-1846*
Looby S.: *A-0950, B-0265, B-0267*
Loos C.: *B-0279*
Lopes R.: *A-0405*
López-Frías López-Jurado A.: *B-0274*
Lorbeer R.: *B-0188, B-1208*
Lorenzon M.: *B-0253*
Lorenzoni A.: *B-1118*
Loria A.: *B-1194*
Losa A.: *B-1300*
Losco R.: *B-0793*
Losik D.V.: *B-1672*
Losio C.: *B-0256*
Lotufo R.: *B-1524, B-1526*
Lotz J.: *B-1544*
Lou W.: *B-0452*
Louie A.L.: *B-1536*
Loupa T.: *B-0889*
Louw A.: *A-0493*
Løvblad K.-O.: *K-08*
Low L.-F.: *B-1496*
Lowe C.: *B-1117*
Lu B.: *B-1651*
Lu C.: *B-0308, B-1126*
Lu C.-H.: *B-1431, B-1551*
Lu G.: *B-0217, B-0754, B-0805, B-0978*
Lu H.: *B-1387*
Lu L.: *B-1036, B-1333*
Lu M.: *B-0459, B-0721*
Lu M.J.: *B-0784*
Lu P.: *B-0779, B-1857*
Lu Q.: *B-1370, B-1762*
Lu X.: *B-0149, B-0315, B-0578, B-1017, B-1308, B-1604*
Lu Y.: *B-0062, B-0610, B-0808, B-1873*
Luan Y.: *B-1891*
Luburich Hernaiz P.: *B-1404*
Lucarini S.: *B-0622*
Lucatelli P.: *B-0036*
Lucertini E.: *B-1341*
Luciani A.: *B-0252*
Lucic M.A.: *A-0222*
Lucioni M.: *B-1153*
Lücke C.: *B-0196, B-0379*
Ludwig D.R.: *B-1765*
Ludwig J.M.: *B-0116*
Luetkens J.A.: *B-1513*
Lui S.: *B-1330, B-1331, B-1332, B-1427*
Luiten E.J.T.: *B-1158*
Luiten J.D.: *B-1149, B-1158*
Lukas S.: *B-0172, B-1016*
Lukina O.V.: *B-0091*
Lukovkina O.: *B-0187*
Luksaite R.: *B-0270*
Lukyanchikov V.: *B-0186*
Lumen N.: *A-0480*
Lund G.K.: *B-0590, B-1682*
Luntsi G.: *B-1107*
Luo J.: *B-0462*
Luo L.: *B-1440, B-1441*
Luo Q.: *B-1888*
Luo X.: *B-0147*
Luo Y.: *B-1370, B-1401, B-1762*
Lurie D.J.: *A-0911*
Lurz P.: *B-0379*
Lusa Aguero T.: *B-1194*
Lusque A.: *B-0478*
Lutsik N.: *B-0350*
Lutter C.: *B-0684*
Luyckx E.: *B-0145*
Lv H.: *B-1040*
Lv J.C.: *B-0573*
Lv Y.: *B-1883, B-1890*
Ly L.: *B-0984*
Lyschik S.: *B-0383*
Lysdahl K.B.: *A-0962, B-0030*
Lyshchik A.: *B-1762*
Lysvik E.K.: *B-0480*

### M

M. Ali R.M.K.: *B-1708*
Ma J.: *B-0751, B-0810, B-1517*
Ma K.: *B-1085, B-1248, B-1249, B-1256*
Ma L.: *B-0610, B-0903*
Ma M.: *B-0952*
Ma Q.: *B-0149, B-1604*
Ma W.: *B-0466*
Ma X.: *B-0394*
Ma Y.: *B-0149, B-0315, B-1017, B-1308, B-1604*

Ma Z.: *B-1238*
Maas K.J.: *B-0679*
Maas M.: *B-0244, B-0632, B-1049, B-1484, B-1582, B-1635*
Mac a' Bháird K.: *B-1307*
Maccagni D.: *A-0112*
Macchi A.: *B-0880*
Macchi E.: *B-1808, B-1810*
MacDonald S.: *B-1227*
Macedo A.P.L.: *B-0410*
Macey J.: *B-1202*
Maciejewski R.: *B-0538*
Mack M.G.: *A-0075, SY 6*
MacMahon P.J.: *B-0098, B-0246*
Macron L.: *SY 3*
Madhusudhan K.: *B-1577*
Madoff D.C.: *A-0513*
Madsen O.R.: *B-0520*
Maeda H.: *B-0425*
Maeder P.: *B-0931*
Maffei E.: *B-1313*
Maffini F.: *B-1644*
Maga T.: *B-1300*
Mager A.: *B-1025*
Magerkurth O.: *B-0893*
Maggi S.: *B-1118*
Magistro G.: *B-1400*
Maglia C.: *B-0527, B-1082*
Magnaguagno F.: *B-1054*
Magnani S.: *B-0898, B-1723*
Magnano G.M.: *B-0341*
Magnano G.M.M.: *B-1054*
Magnin B.: *B-1470*
Maguire D.: *B-1307*
Magyar P.: *B-0444*
Mah S.Y.: *B-1902*
Mahadevappa V.: *B-1737*
Mahajan A.: *B-1450*
Mahajan H.: *B-0572, B-0777, B-0910, B-0912, B-1132, B-1335, B-1558, B-1750, B-1753*
Mahajan V.: *B-0140, B-0572, B-0777, B-0910, B-0912, B-0990, B-1132, B-1133, B-1183, B-1397, B-1750, B-1753, B-1892*
Mahawar V.: *B-0691*
Maher M.M.: *A-0927*
Mahjoub S.: *B-0153*
Mahlknecht P.: *B-1689*
Mahmood N.: *B-1647*
Mahmood T.: *A-1081*
Mahmoud K.: *B-0357, B-0838, B-1296, B-1298, B-1299, B-1301, B-1473*
Mahmoud M.: *B-1160*
Mahmoudi S.: *B-0136*
Mahnken A.H.: *B-0354, B-0365*
Maier C.: *B-0839, B-0840*
Maier J.: *B-0013, B-0135, B-0316, B-0319, B-0321, B-0909*
Maier S.: *B-1411*
Maier-Hein K.: *B-0813, B-1077*
Maier-Hein K.H.: *B-0539*
Maillet B.: *A-0214*
Mainenti P.P.: *B-1383, B-1385*
Maino C.: *B-1310, B-1372, B-1457*
Maintz D.: *B-0314, B-0461, B-1025, B-1081, B-1761, B-1783, SY 16*
Maj E.: *B-1452*
Majeed A.: *A-1079*
Majoie C.B.: *B-0258*
Major V.T.: *B-0654*
Majos A.: *A-0135*
Mak M.: *B-1410*
Mak S.M.: *B-0142*
Makarchuk M.: *B-1034*
Makatsaria A.D.: *B-0173*
Mäkelä T.: *B-0887*
Makki M.: *B-1168*
Mako M.: *B-0281*
Makowski M.: *B-1475*
Makowski M.R.: *B-0664, B-1734, B-1738*
Makse H.: *B-1032*
Makurthou A.: *B-0856*
Malakhova M.V.: *B-1263*
Malamateniou C.: *A-0781, B-1495*
Malasevschi A.: *B-0304*
Malattia C.: *B-1054*
Malekzadeh S.: *B-1497*
Maleux G.: *B-0559, B-0850*
Malev E.: *B-1505*
Malhaire C.: *B-1090*
Mali W.P.: *B-0222*
Malich A.: *B-0688, B-0694, B-1643*
Malla S.: *B-1577*
Mallya Y.: *B-0572*
Malmivirta H.: *B-0888*
Malone M.: *B-1354*
Malter W.: *B-0461*
Maly Sundgren P.C.: *A-0756*
Mammino L.: *B-0959*
Mamourian A.: *B-1781*
Mancinelli M.: *B-0211*
Mancini M.E.: *B-1501*
Mancini Terracciano C.: *B-1833*
Mandal A.K.: *B-0720*
Mandic R.: *B-0365*
Mandonnet E.: *B-0096*
Mandorfer M.: *B-1377*
Mandruzzato N.: *B-0009*
Mandry D.: *A-0378*
Manente G.: *B-0708, B-0951*
Manesis E.: *B-1378, B-1537*
Mañez Miro J.U.: *B-1303*
Manfrè L.: *A-0759*
Manfredi R.: *A-0608, B-0109, B-0110, B-0114, B-0328, B-0529, B-0547, B-0548, B-1812, B-1898*
Manganaro L.: *B-0607, B-1390*
Manganiello C.A.T.: *B-0211*
Mangaraj S.: *B-1253*
Mangesius S.: *B-0534, B-1689*
Maniatis V.: *A-0241*
Manka R.: *A-0199, B-1616*
Manke D.: *SY 28*
Mann R.: *B-0726*
Mann R.M.: *A-0150, B-0545, B-0697, B-1523, B-1525, B-1564, B-1790*
Manna C.: *B-1858*
Mannil M.: *B-1733*
Manning W.J.: *B-1306*
Manríquez Galán V.: *B-0629*
Mansour R.: *B-0245*
Mansour S.A.: *B-0502*
Mansouri B.: *A-0315, A-0663*
Mansueto G.: *B-0009, B-0458, B-0532, B-0937, B-1094, B-1103*
Mantatzis M.: *B-0183*
Mantini C.: *B-0195, B-0203, B-1313*
Manuel Fernández B.: *B-0274*
Manunza F.: *B-0930*
Manzhurtsev A.: *B-1038, B-1778, B-1887*
Mao J.: *B-0508, B-1627*
Maralakunte M.: *B-1428*
Marcello R.: *B-0010*
Marchegiani G.: *B-0458*
Marchianò A.V.: *B-0143*
Marcon M.: *B-0085, B-0554, B-1541*
Marconi V.: *B-0253*
Marcus R.P.: *B-1729*
Marcy P.-Y.: *K-03*
Mardal C.A.: *B-0275*
Margaritora S.: *B-1812*
Mari A.: *B-1118*
Mariani D.: *B-0087*
Mariani S.: *B-0241, B-1295, B-1580, B-1581*
Marik W.: *B-0055*
Marin D.: *B-0070, B-0493, B-0533, B-0729, B-1140, B-1148, B-1503, B-1768, B-1771*
Marinelli D.: *B-0203*
Marinescu A.N.: *B-0191*
Marinescu A.V.V.: *B-0191*
Marino M.A.: *B-0719, B-1175, B-1176, B-1804*
Marino M.A.A.: *B-0924*
Mariotti D.: *B-1715*
Mariotto R.: *B-0248*
Mariscotti G.: *B-1173, B-1799*
Markova N.: *B-0597*
Marletta M.: *B-1205*
Maroldi R.: *A-0077, A-0320, B-1446*
Maros M.E.: *B-0022*
Marques M.: *B-0412*
Marra P.: *B-0851, B-1439*
Marrelli D.: *B-1275*
Marrone G.: *B-0529*
Marschall Skov P.: *B-0485*
Marshall N.: *A-0181, B-1318, B-1839, B-1840, B-1841, B-1842, B-1845*
Marshall T.: *B-1287*
Martel Villagrán J.: *B-1286*
Mårtensson J.: *B-1035*
Marterer R.: *B-1361*
Martí-Bonmatí L.: *A-0332, A-0594, B-1397, B-1830*
Marticorena Garcia S.R.: *B-1657*
Martin A.: *A-0531*
Martin C.: *B-0058*
Martin O.: *B-0669, B-0670, B-0671, B-0690, B-1456, B-1461*
Martin S.: *A-0089*
Martin S.S.: *B-0434, B-0448, B-0780, B-0781, B-0789, B-0794, B-0820, B-1472*
Martinelli P.: *B-1383, B-1385*
Martinenghi C.: *B-1880*
Martinez Chamorro E.: *B-0268*
Martínez Cutillas M.J.: *B-1418*
Martinez D.: *B-0719, B-0924, B-1175, B-1176*
Martinez de Vega V.: *SY 3*
Martinez E.: *B-1231*
Martinez Fernandez R.: *B-1303*
Martingano P.: *B-0368*
Martini I.: *B-0525*
Martini K.: *B-0577, B-1512*
Martini M.-L.: *B-0365*
Martino A.: *B-1080*
Martino M.: *B-0544, B-1550, B-1556*
Martinoli C.: *B-1745, B-1748, K-41*
Martinsen A.C.: *B-0480*
Martinsen A.C.T.: *B-0131*
Marto J.P.: *B-0283*
Marushchak O.: *B-0584*
Marzocca F.: *B-0553*
Masakazu M.: *B-0306*
Masana M.: *B-1619*
Mascalchi M.: *B-0928*
Mascherbauer J.: *A-0878*
Maschke S.K.: *B-0001*
Masciocchi C.: *B-0241, B-0544, B-0595, B-1295, B-1309, B-1550, B-1552, B-1556, B-1571, B-1572, B-1579, B-1580, B-1581, B-1623, B-1817*
Masi G.: *B-1831*
Massat N.J.: *B-0920*
Masselli G.: *A-0203, A-0808*
Massimo C.: *B-1457*
Massmann A.: *B-0167, B-0961, B-0963, B-1720*
Massone E.: *B-0724, B-1151*
Mastio M.: *B-1510*
Mastio M.T.: *B-1508*
Mastrodicasa D.: *B-0195, B-0203, B-0780*
Mastronuzzi A.: *B-1327*
Masui T.: *B-1180*
Masulovic D.: *B-1871*
Mateus B.M.M.: *B-0414*
Mathys C.: *B-1553*
Matino S.: *B-0329*
Matos J.: *B-1178, B-1528*
Matos J.D.: *B-1306*
Matos V.: *B-0643*
Matsuo C.: *B-0293*
Mattay R.: *B-1781*
Matthews R.: *B-0687*
Mattiuz C.: *B-0490*
Matzek W.K.: *A-0754*
Matzuzzi M.: *B-1214*
Maurea S.: *B-0333, B-1383, B-1385*

Maurer M.H.: *B-1184, B-1434, B-1438, B-1442*
Mauri G.: *B-0630, B-1644, B-1723*
Maurovich- Horvat P.: *B-0786*
Maurus S.: *B-1586*
Mautner V.-F.: *B-1240*
Maxwell A.J.: *B-0079*
May M.S.: *B-0289, B-0426, B-0684, B-1245, B-1321*
Mayer E.: *B-1863*
Mayer I.: *B-0684*
Mayerhöfer M.E.: *B-0299, B-1020, K-33*
Mayer-Steinacker R.: *B-0061*
Mayo R.C.: *B-0696*
Mayr A.: *B-0500*
Mayr D.: *B-1847*
Mayrhofer M.T.: *A-0631*
Mazaheri Y.: *B-0152*
Mazgaj P.: *B-1338*
Mazlan L.: *B-1340*
Mazur G.: *B-0039*
Mazzaro A.: *B-0009, B-0532, B-1103*
Mazzei M.A.: *B-1275*
Mazzetti S.: *B-0527, B-0618, B-1082, B-1093, B-1828*
Mazzilli A.: *B-1194*
Mazzoni M.: *B-1054*
Mazzucca G.: *B-1262*
Mc Carthy L.L.: *B-1056*
McAnulla S.J.: *B-1698*
McClure P.: *B-0651*
McCollough C.: *B-0447, B-1142*
McCormick I.: *B-1306*
McDermott E.: *B-0024, B-0633*
McDermott G.M.: *B-0150, B-0361, B-0667, B-0771*
McDermott J.C.: *B-1297*
McEntee M.F.: *B-1496, B-1844*
McFadden S.: *B-0651*
McGee A.: *B-0409*
McGinty G.: *A-0588*
McGurk S.: *B-1323*
McKean D.: *B-1288*
McLean M.A.: *B-1802*
McNair H.: *A-0441, B-1495*
McNally E.G.: *A-0261*
McNulty J.: *A-0439, A-0491, A-0619, A-0931, B-0027, B-0407, B-0409, B-0573, B-1706*
Meacock L.: *A-1034*
Meakin J.R.: *B-0659, B-0662, B-1067*
Meder J.-F.: *K-02*
Meek D.: *B-1582*
Megna R.: *B-0543*
Mehdi A.S.: *B-0177, B-0627*
Mehndiratta A.: *B-1132, B-1133*
Mei J.: *B-0718*
Mei K.: *B-0047*
Mei Y.: *B-0504*
Meicher E.: *B-1600*
Meier M.: *B-0503*
Meijer F.J.: *B-0706*
Meine T.: *B-0001*
Meineke A.: *B-1210*
Meinel F.G.: *B-1502*
Meissner J.-E.: *B-0506, B-1419*
Meister A.: *B-0440*
Mejias Vargas H.: *B-0323*
Mekis N.: *B-1068, B-1070, B-1073, B-1710*
Mekkawy A.I.A.: *B-0117*
Melani E.F.: *B-1151*
Melazzini L.: *B-0189, B-1687, B-1821*
Meldo A.A.: *B-1760*
Melis T.: *B-0338*
Mellone R.: *B-0971*
Mellor F.: *B-1495*
Melnikov I.: *B-0187, B-1038, B-1887*
Melnikova-Pitskhelauri T.: *B-1037*
Melzer A.: *B-1444*
Memis Durmaz E.S.: *B-0986, B-1395*
Menat J.: *B-0313*
Mendeleeva L.: *B-0350*
Méndez R.J.: *A-0703*
Meneguette N.S.: *B-0542*
Menes T.: *B-1569*
Menezes G.: *B-1793, B-1794*
Menezes L.: *B-1324*
Meng Q.: *B-0432, B-1606*
Menghini R.: *B-0458*
Mengjie F.: *B-1280*
Menicagli L.: *B-0490*
Mens M.: *B-0244*
Menshchikov P.: *B-1038, B-1778, B-1887*
Mentzel H.-J.: *A-0462*
Menu Y.: *A-0557*
Menz R.: *B-1196*
Menze B.: *B-0344, B-0348, B-0567*
Meo D.: *B-0833, B-0959*
Meola G.: *B-1821*
Mercaldo N.D.: *B-1013*
Mercatelli L.: *B-0622*
Mercer C.E.: *B-1708*
Mercieca S.: *B-1350*
Mercogliano S.: *B-0558*
Mereu A.: *B-0707, B-1251*
Merkely B.: *B-0786*
Merkulov N.: *B-1061*
Merli I.: *B-1730*
Mershina E.: *B-0730, B-1819*
Mershina E.A.: *B-1822*
Merz M.: *B-0348*
Mesa Quesada J.: *B-1662*
Messerli M.: *B-0228, B-0229, B-0817*
Messina C.: *A-0883, B-0052, B-0868, B-0870, B-0898, B-1730*
Messiou C.: *B-0353*
Messmann H.: *B-0945*
Meszar Z.: *B-1432*
Meszaros L.: *B-1021*
Metrakos P.: *B-0528*
Metsala E.: *A-0005, A-0741, B-1712, B-1716*
Mettivier G.: *B-1843, B-1846*
Metzler B.: *B-0500*
Meuli R.: *B-0288, B-0931, B-1366*
Meurer F.: *B-0138, B-1312*
Meyblum E.: *B-0252*
Meyer B.C.: *B-0001, SY 12*
Meyer H.-J.: *B-0175*
Meyer M.: *B-0070, B-0235, B-0533, B-1768*
Meyer-Baese A.: *B-1176*
Meyer-Baese L.: *B-1328*
Meyl T.P.: *B-1184*
Meyrignac O.: *B-0793*
Miao Z.: *B-1006*
Miccò M.: *B-0328*
Micelli M.V.M.: *B-0544, B-1550, B-1552, B-1556*
Michael E.: *B-1052*
Michaely H.J.: *B-0352*
Michalopoulou E.: *B-0437, B-1548*
Micheletti G.: *B-0036*
Michelin P.J.: *B-1860*
Michels J.D.: *B-0587*
Michieletti E.: *B-1029, B-1867*
Michielsen K.: *B-1527*
Michoux N.: *B-0944*
Miclea R.L.: *B-0067*
Middlebrooks E.H.: *B-1169*
Middleton M.S.: *B-1536*
Midiri M.: *B-1444, B-1827*
Miele V.: *A-0580, B-0470, B-0622, B-0732, B-0733, B-0734, B-0975*
Miguel Perez M.: *B-1748*
Mihara Y.: *B-0642*
Mikalani M.: *B-0635*
Mikayama R.: *B-0484*
Mikheenko I.: *B-1672*
Miksik L.: *B-0597*
Milan L.: *B-0040*
Milanese G.: *B-0577, B-0842, B-1858*
Mildenberger P.: *A-0504*
Miletic D.: *B-0260*
Mileto A.: *B-1148*
Miller T.T.: *A-0919, A-0921*
Millet I.: *A-0893, A-1038*
Milnerowicz M.: *B-0747*
Milosevic Z.: *B-0549, B-1172*
Milotic V.: *B-0276*
Min J.H.: *B-1538*
Minervini M.I.: *B-0071*
Mingliang W.: *B-0069, B-0456*
Minin S.M.: *B-1672*
Minko P.: *B-1720*
Minotti M.: *B-1060, B-1644*
Miranda D.M.L.: *B-0414*
Mirón Mombiela R.: *B-0107*
Mironchuk R.: *B-1505*
Miś M.: *B-0747*
Mishah N.: *B-1105*
Misiuk-Hojło M.: *B-1265*
Misra R.N.: *B-1291, B-1293*
Mitchell A.J.: *B-0827*
Mitrofanova A.: *B-1061*
Mittal K.: *B-0634*
Mittal M.K.: *B-1293*
Miyagoshi K.: *B-0408*
Miyazawa R.: *B-0746*
Mizuno T.: *B-0185*
Mlynarska-Bujny A.: *B-1568*
Mochida M.: *B-1187*
Modi A.: *B-1753*
Modica A.: *B-0833, B-0959*
Moeller K.: *B-0583*
Moemenam O.: *B-0043*
Moen T.: *B-1142*
Moerup S.: *B-0653*
Moffa G.: *B-0553*
Moghiseh M.: *B-1117*
Mohamed R.E.-E.-D.: *B-1423*
Mohammed F.: *B-1340*
Mohammed Kashif R.: *B-0637*
Mohankumar R.: *B-0066*
Mohanty B.K.: *B-1253*
Mohanty H.S.: *B-0691*
Mohanty J.: *B-1253*
Mohebali D.: *B-1306*
Moher Alsady T.: *B-0585*
Möhlenbruch M.A.: *B-0263*
Moi A.S.: *B-1109*
Moiseenko V.M.: *B-1760*
Moiseev A.S.: *B-1822*
Moiseev S.V.: *B-1822*
Moiseeva O.: *B-1505*
Mokali J.: *B-0183*
Mokrane F.Z.: *B-1188*
Mokrane F.-Z.: *B-0672*
Molehe M.M.: *B-1704*
Molina Molina M.: *B-1404*
Molinari F.: *A-0915*
Molinaro S.: *B-0948*
Molino Gaete J.A.: *B-0340*
Møller H.: *B-1661*
Møller J.M.: *B-0520*
Møller T.: *B-0168*
Mollica C.: *B-0333*
Moltoni G.: *B-1341*
Moltz J.: *B-1586*
Mondelaers A.: *B-0279*
Monelli F.: *B-1381*
Mones F.: *B-0828*
Monga A.: *B-1577*
Monostori Z.: *B-1023*
Montandon S.: *B-1600*
Montemezzi S.A.: *B-0248, B-0249, B-0250*
Montesano M.: *B-0014*
Montgomery A.: *A-0744*
Monti C.B.: *B-0042, B-0199, B-0416, B-0465, B-0895, B-1352, B-1821, B-1867*
Monti S.: *B-0174*
Montorsi F.: *B-1302*
Monzon L.: *B-0966*
Moon H.H.: *B-1754*
Moon H.J.: *B-0120*
Moon J.H.: *B-0918*
Moor M.: *B-0193, B-0904*
Moore C.M.: *B-0159, B-0443*

Moradi Amani A.: *B-1328*
Moradi B.: *B-0635*
Morales M.I.: *B-0111*
Morales O.: *B-1129*
Morana G.: *A-0431, A-1088, B-1010, B-1011, B-1154*
Moraschi M.: *B-1270*
Moreau-Triby C.: *B-1852*
Moreira J.: *B-0412*
Moreira J.M.: *B-0700*
Morell Hofert D.: *B-0102*
Morelli N.: *B-1029*
Moreno R.: *B-0793*
Morgan B.: *B-0483*
Morgan R.: *A-0023*
Morganti R.: *B-0017, B-1831*
Mori M.: *B-0471, B-1796*
Mori T.: *B-0949*
Moriconi S.: *B-1688*
Morimoto D.: *B-0408*
Morita Y.: *B-0292*
Morone M.: *B-1042*
Morosi C.: *B-1060*
Morozov S.: *A-0555, A-0710*
Morrin M.: *B-0936*
Morris E.: *B-0924*
Morris E.A.: *A-0234, B-0719, B-1175, B-1176*
Morris M.: *B-1353*
Morrow E.: *B-1228*
Mortele K.J.: *B-0210*
Moscato F.: *A-0447*
Moschetta M.: *B-0329*
Moser S.: *B-0966*
Mosimann P.: *B-0282, B-0288*
Mostafa S.: *B-1174*
Mostbeck G.H.: *A-0205*
Mota Martinez J.: *B-0107*
Motiuk D.: *B-1607*
Motosugi U.: *B-0404, B-1474*
Mottaghy F.: *B-0220*
Motyer R.: *B-0267*
Moughty A.: *B-0246*
Moulopoulos L.A.: *B-1388*
Mourad C.: *B-0894*
Mousa R.M.: *B-0355*
Moussa R.: *B-1031*
Mousseaux E.: *A-0019*
Moustafa A.F.I.: *B-0460, B-0693*
Moyle P.L.: *B-1384*
Moysak G.: *B-1266*
Mróz A.: *B-1477*
Mubarak F.: *B-0090, B-1367*
Mubarak R.: *B-1174*
Muchuki C.: *A-0656*
Mueller-Lisse U.G.: *B-0440, B-0583, B-1451*
Mueller-Lisse U.L.: *B-0440*
Muenzfeld H.: *B-0962*
Muglia R.: *B-0530, B-0560*
Mühlberg A.: *B-1586*
Mühlematter U.: *B-0228, B-0229, B-0817*
Mühlematter U.J.: *B-0822*
Mukherji R.: *B-1183*
Mullen S.N.: *B-0023*
Müller A.G.: *B-0510*
Müller C.: *B-1689*
Müller F.C.: *B-0675, B-0682*
Müller F.H.: *B-0510*
Muller H.: *B-0890*
Müller J.: *B-0228, B-0229, B-0822*
Müller L.: *B-0365*
Müller M.: *B-0818*
Müllerleile K.: *B-1682*
Müller-Wille R.: *B-0739*
Munari E.: *B-0872*
Mungai F.: *B-0975*
Mungari R.: *B-0045*
Münnich N.: *B-0266*
Muñoz Cepeda M.A.: *B-0625*
Munsch F.: *B-1415*
Muollo A.: *B-1808, B-1810*
Murakami T.: *B-0417, B-0422, B-0579, B-1003, B-1145, B-1146, B-1203, B-1209, B-1460, B-1755*
Murayama K.: *B-0706*
Murayama S.: *B-0292, B-1629*
Murchison J.T.: *B-1316, B-1751, B-1759*
Murer S.: *B-0440*
Muridan R.: *B-1233, B-1902*
Muriel García A.: *B-0274*
Murphy D.: *B-0142*
Murphy D.J.: *A-0716*
Murphy M.C.: *B-0246*
Murtazalieva P.: *B-1505*
Murugan N.: *B-0640*
Murugavel M.: *B-0140, B-0910, B-1892*
Musaiev B.: *B-1250*
Musaieva K.: *B-1250*
Muscari F.: *B-1188*
Muscogiuri G.: *B-0783, B-1212, B-1501, B-1613, B-1614, B-1615, B-1686*
Mushtaq S.: *B-1501*
Mussetto I.: *B-1528*
Mussmann B.R.: *A-0960, B-0485, B-0653*
Muto M.: *B-1286*
Mutoh T.: *B-1329*
Mutschler T.: *B-1512*
Myers R.: *B-1536*
Myklebust T.Å.: *B-0077*
Myles J.: *B-0920*
Myronyak L.: *B-1034*

### N

N. Larson A.: *B-1543*
Na D.G.: *B-0127*
Nada A.: *B-1232*
Nadjiri J.: *B-1312*
Nadobny J.: *B-0762*
Nadrljanski M.: *B-0549, B-1172*
Naffaa L.: *B-0799*
Nag A.K.: *B-0720*
Naganawa S.: *B-0185, B-1722*
Nagaraj Y.: *B-0309*
Nagarajan K.: *B-0631*
Nagasawa N.: *B-1101, B-1102*
Nagata M.: *B-1101, B-1102*
Nagel A.: *B-1245, B-1777*
Nagel S.: *B-0263*
Nagele U.: *B-1076*
Naguib N.N.N.: *B-0117*
Nagy E.: *B-1361*
Naidu K.M.: *A-0315*
Nair A.: *A-0953, B-0142, B-1410*
Naito T.: *B-0185*
Naji S.: *B-1160*
Nakada S.Y.: *B-1297*
Nakai N.: *B-0949*
Nakanishi M.: *B-1735*
Nakano H.: *B-0482*
Nakasujja N.: *B-1417*
Nallino M.B.B.: *B-1780*
Nan H.: *B-1006*
Nance J.W.: *B-0784*
Nanda G.: *B-0910, B-1133*
Napoli A.: *A-0272*
Napolitano A.: *B-1327*
Nappi C.: *B-1684*
Narata A.-P.: *B-0836*
Narcisse Fidele N.N.: *B-1711*
Nardin C.: *B-1010*
Nardo L.: *B-0056*
Nardone V.: *B-1275*
Narracott A.: *B-0760*
Naser Nassar M.: *B-0629*
Nasr H.: *B-1459*
Nasr M.: *B-0366*
Nassenstein K.: *B-0913, B-1622*
Natale L.: *A-0201, A-0876*
Nattenmüller J.: *B-0051*
Navarra F.: *B-0951*
Navin P.: *B-1142*
Nayahangan L.: *A-0041*
Nayak B.K.: *B-1291, B-1293*
Nazarova E.: *B-1061, B-1326*
Nchimi Longang A.: *A-0773*
Nederend I.: *B-1506*
Nederend J.: *B-0083, B-1879*
Negi N.: *B-1145, B-1146*
Negri G.: *B-1389*
Negru D.: *A-0204*
Nehrer S.: *B-0569*
Neji R.: *B-0616*
Nelemans P.J.: *B-1787*
Nelson R.C.: *B-0070*
Nelstrop H.L.: *B-0667*
Nemati M.: *B-1380*
Nemes B.: *B-0958*
Nenning K.-H.: *B-1881, B-1882*
Nensa F.: *B-0575, B-0671, B-0803, B-0819, B-0913, B-1411*
Nepal P.: *B-1130*
Neri E.: *A-0265, A-0503, A-0958, B-0017, B-0198, B-1831*
Neriman D.: *B-1324*
Nesic I.: *B-0193, B-0904*
Neßelmann C.: *B-1502*
Nesterets Y.: *B-1789*
Neu M.-A.: *B-1241*
Neubauer C.: *B-1791*
Neubauer J.: *B-0867, B-1791*
Neubauer V.: *B-0925*
Neumaier C.E.: *B-0879, B-1139*
Neuschler E.: *B-1793, B-1794*
Nevelsteen I.: *B-1803*
Neven P.: *B-0559, B-0850, B-1803*
Newby D.E.: *B-1316*
Newman D.: *A-0651*
Newton-Hughes A.: *B-0033*
Ng A.W.H.: *B-1292*
Ng C.: *B-0025*
Ng H.T.: *B-0330*
Ng M.-Y.: *B-1220, B-1864*
Ngoc P.B.: *B-1012*
Nguyen D.T.: *B-0958*
Nguyen H.: *B-0829, B-1012*
Nguyen J.C.: *B-1055*
Nguyen L.T.T.: *B-0214*
Nguyen T.: *B-1012*
Nguyen T.H.N.: *B-0278*
Nguyen T.T.: *B-0214*
Nguyen Troung Thanh H.: *B-1695*
Nguyen-Kim T.D.L.: *B-0685*
Niang E.H.: *A-0312, A-0315*
Nicholson A.G.: *B-1605*
Nickel D.: *B-0396, B-1475*
Nickel M.: *B-0446*
Nico M.: *B-0860*
Nicod Lalonde M.: *B-0235*
Nicolai E.: *B-0668*
Nicolas V.: *B-0839, B-0840, B-0866*
Nicolau C.: *A-0996*
Nicolay S.: *B-0145, B-0753*
Nicoletti R.: *B-1880*
Nicosia L.: *B-0895*
Nicula A.: *B-0191*
Nieboer K.H.: *A-0435*
Niedzwiecki D.: *B-0533*
Niehues S.M.: *B-0762*
Nieminen M.T.: *B-0886*
Nienhuijs S.W.: *B-1879*
Niessen W.J.: *A-0127, A-0370, A-0902, B-0067, B-0908, B-1279, B-1690*
Nievelstein R.A.J.: *A-0461*
Niezgoda S.: *B-0874*
Niiniviita H.: *B-0645, B-0886*
Nik-Hussin N.M.H.: *B-0126*
Nikitin N.A.: *B-1672*
Nikolaou K.: *A-0225, B-0589, B-0628, B-0818, B-1514, B-1515, B-1729*
Nikolic O.: *A-0820*
Nilsson A.: *SY 23*
Nimmalapudi S.: *B-1886*

Ninatti G.: *B-1636*
Niola R.: *B-0002, B-0006, B-0011*
Nisolle J.-F.: *B-1507*
Nissan N.: *B-1569*
Nissen H.: *B-1661*
Nitrosi A.: *B-0993*
Niwa T.: *B-1603*
Nizzoli R.: *B-0842*
Njeze R.: *B-0043*
Njokwu G.: *B-1109*
Nkubli F.: *B-1107, B-1714*
Nocerino E.A.: *B-0895*
Nocivelli G.: *B-1446*
Noebauer-Huhmann I.-M.: *A-1023*
Noel P.B.: *B-0138, B-1116, B-1312, B-1612, B-1848*
Nomura T.: *B-1603*
Nonnenmacher T.: *B-0051*
Noonan P.: *B-0885*
Noorajan Z.: *B-0644*
Norberciak L.: *B-0053*
Nörenberg D.: *B-1400, B-1586*
Nørgaard N.: *B-0154, B-0442*
Nori Cucchiari J.: *B-0470*
Norlisah R.: *B-0641*
Norman B.: *B-0302*
Norman W.: *B-0606*
Noro K.: *B-1832*
Norton L.: *B-1307*
Notaro D.: *B-1575, B-1578*
Notohamiprodjo M.: *A-0379, B-1729*
Nour Eldin N.-E.: *B-1257*
Nour-Eldin N.-E.A.: *B-0117*
Nourzaie R.: *B-0966*
Novario R.: *B-0040*
Novello S.: *A-0053*
Novichkova G.: *B-1326*
Novikov M.: *B-0230*
Novotny A.: *B-0938*
Nowak A.: *B-0225*
Nowak T.: *B-0996*
Nozaki A.: *B-1722*
Ntorkou A.A.: *B-1660*
Ntoufas N.: *B-1593*
Nummela M.: *B-0101*
Nunes A.P.: *B-0284*
Núñez Valentín P.: *B-0535*
Nurili F.: *B-0115, B-0556, B-0565, B-0737, B-0743*
Nüßle N.-C.: *B-0093*
Nybing J.D.: *B-1035*
Nyhsen C.: *A-0172*

### O

O'Brien B.J.: *B-1243*
Obeso J.A.: *B-1303*
Obmann V.C.: *B-1650, B-1655*
Obradov M.: *A-0273*
Oc Y.: *B-1576*
Öcal O.: *B-1218*
Occean B.: *B-0431*
Occella C.: *B-0930*
Occhipinti M.: *A-0122, A-0918, A-0952*
O'Connell M.J.: *B-0407*
O'Connor J.: *A-0900, A-1057, B-1056*
O'Connor M.: *A-0208, B-0023, B-1066, B-1706*
O'Connor O.J.: *A-0182*
O'Connor P.J.: *A-0542*
O'Connor S.: *B-0353*
O'Doherty J.: *B-1021*
Oechtering T.H.: *B-0202, B-0225, B-0349, B-1622*
Oei E.: *B-0856, B-1136*
Oei E.H.G.: *A-0946, B-1134*
Oei L.: *B-0856*
Offeciers F.E.: *B-0290*
Offiah A.C.: *A-0464*
Oganesyan A.: *B-0730*
Ogden M.: *B-1744*
Ogenyi P.A.: *B-1107, B-1109*
Ognibene N.M.G.: *B-1648*
Oh H.J.: *B-1342*
Oh Y.-W.: *B-1014*
Oh S.: *SY 18*
O'Halloran C.: *B-1699, B-1702*
O'Hare A.: *B-0265, B-0267*
Ohlmeyer S.: *B-1259*
Ohno Y.: *B-0417, B-0422, B-0424, B-0579, B-1003, B-1203, B-1209, B-1460, B-1755*
Ohta Y.: *B-0293*
Oikarinen H.: *B-1192*
Oikonomou A.: *A-0376*
Ojeda A.A.: *B-1780*
Okada F.: *B-0660*
Okada M.: *B-0994*
Okazaki T.: *B-0642, B-0648*
Okromelidze L.: *B-1169*
Oktay D.G.: *B-1862*
Okubo G.: *B-1415*
Okwudire E.: *B-0043*
Oladottir G.L.: *B-0101, B-0955*
Olalla Muñoz J.R.: *A-1039*
Olasunkanmi Balogun E.: *A-0653*
Olbert E.: *B-0534*
Oleaga Zufiría L.: *A-0220, A-0489, A-0848, A-0849, A-0851*
O'Leary D.: *A-0777, B-0654*
Olerud H.M.: *B-0030*
Olimov B.: *B-0621*
Oliva G.: *B-0087, B-0247*
Oliva P.: *B-1846*
Oliveira M.V.L.: *B-1493*
Olivero A.: *B-1139, B-1658*
Olschewski H.: *B-0767, B-0989*
Oluniran G.: *B-0761*
Omelchenko O.: *B-1034*
Omoumi P.: *A-0444*
Omura V.L.N.: *B-0254*
O'Neill C.: *B-1288*
Oner A.Y.: *B-0213*
Ong L.C.: *B-1233*
Onishi H.: *B-0404*
Onur M.R.: *B-1594*
Oostveen L.J.: *B-1850*
Oppenheim C.: *SY 4*
Orasch C.: *B-0108*
Orczyk C.: *B-0159, B-0443*
Ordás I.: *B-1653*
Ording Müller L.-S.: *A-0796, B-0168*
O'Regan T.J.: *B-0033*
Orgera G.: *B-0007, B-0008, B-0849, B-1809*
Örgüc I.S.S.: *B-1741*
Ori Belometti G.: *B-0630*
Origgi D.: *B-0318*
O'Riordan L.: *SY 5*
Ormond A.: *B-0860*
Örnek A.: *B-0231, B-0673, B-0839, B-0840, B-0866*
Orsatti G.: *B-1060, B-1364*
Orsi M.A.: *B-0087, B-0247*
Ortenzia O.: *B-0993*
Ortino M.: *B-0999*
Osborn A.G.: *SY 26*
O'Shea A.: *B-0936*
Osman A.M.: *B-1855*
Osman S.S.: *B-1340*
Østergaard M.: *B-0520, B-0675*
Osther P.J.: *B-1661*
Osuntokun O.: *B-0736*
Ota H.: *B-0421*
Otal P.: *B-1188*
Othman M.H.: *B-1334*
Ottaviani L.: *B-1118*
Otto P.: *B-1793, B-1794*
Ottoson F.: *B-0821*
Ou H.-Y.: *B-0561*
Oudkerk M.: *B-0309, B-0586, B-0787, B-0789, B-1008, B-1215, B-1311, B-1565, B-1756*
Ouyang H.: *B-0429*
Overduin C.G.: *B-0878*
Owczarczyk K.: *B-0617, B-1874*
Owens C.: *A-0786, A-0871, A-0937, A-1021*
Oyama K.: *B-1836*
Oyen R.H.: *A-0465, K-04*
Oz I.I.: *B-0878*
Ozdemir S.: *B-0122*
Ozdemir Z.M.: *B-1051*
Ozden C.: *B-0590*
Ozhan Oktar S.: *B-1453*
Özkanli S.: *B-0115, B-0556, B-0737, B-0743*
Özmen M.N.: *A-0434*
Ozturk M.: *B-0059, B-1297*
Ozturk V.S.: *B-1059*
Ozveren M.F.: *B-1744*

### P

P. V.R.: *B-1062*
P.Pribeiro S.: *B-1365*
Paalimäki-Paakki K.: *B-1717*
Pacciardi F.: *B-0526, B-1097*
Pacella C.M.: *B-0630*
Pacifici S.: *B-1715, B-1718*
Pacile S.: *B-1789*
Pacios Blanco R.E.: *B-0274*
Padhani A.R.: *A-0194*
Padhani A.R.R.: *B-0442*
Padley S.: *B-1605*
Padley S.P.G.: *A-0913*
Paech D.: *B-0362, B-0364, B-0505, B-0506, B-1419, B-1421*
Paeng J.C.: *B-0303*
Paetz T.: *B-0878*
Páez Granda D.: *B-1418*
Paganelli C.: *B-0373*
Pagani E.: *B-1696*
Pagnini F.: *B-0842*
Paik W.: *B-0127*
Paiman E.H.M.: *B-0588*
Paixao T.: *B-0569*
Pajulo O.: *B-0645*
Paladini A.: *B-0002, B-0011, B-0113, B-0563, B-0846, B-1435*
Palaniappan B.: *B-1027*
Paleri V.: *B-0976*
Palermo M.: *B-0418, B-1405*
Palfrey R.M.: *B-0659, B-0662, B-1067*
Palkonda V.R.: *B-0562*
Palmer C.: *A-0355*
Palmieri F.: *B-1080*
Palmisano A.: *B-0207, B-0381, B-0598, B-1216, B-1221, B-1343, B-1344, B-1345, B-1485, B-1678*
Palmqvist R.: *A-0738*
Palmucci S.: *B-0418, B-0833, B-0943, B-0959, B-1405, B-1648*
Palta M.: *B-0533*
Paltun S.C.: *B-1884*
Palumbo D.: *B-0381, B-1678, B-1880*
Palumbo P.: *B-0241, B-0595, B-1295, B-1309, B-1571, B-1572, B-1580, B-1581, B-1623, B-1817*
Pambianchi G.: *B-1500*
Pamidimukkala V.: *B-0841*
Pamminger M.J.: *B-0500*
Pamplona J.: *B-0284*
Pan C.: *B-0754*
Pan J.: *B-0307, B-0905*
Pan T.: *B-0134*
Pan X.: *B-1646*
Panagiotopoulos A.: *B-0202*
Panagiotopoulos N.: *B-1622*
Panda A.: *B-1650, B-1655*
Pandian J.D.: *B-0714*
Pandur A.: *B-0031, B-0728*
Pane F.: *B-0006*
Panebianco L.: *B-0544, B-1550, B-1552, B-1556*
Panebianco V.: *A-0329, B-0156, B-0160, B-0325, B-0825, B-0876*
Panes J.: *B-1653*
Panfili M.: *B-1225*
Pang P.: *B-0308*

Panina A.: *B-1026*
Panizza P.: *B-0256*
Pannell T.: *B-0301*
Panourgias E.: *B-1157, B-1388*
Panov V.: *B-0621*
Panse D.: *B-1007*
Panse P.: *B-1007*
Panteleakou E.: *B-1530, B-1837*
Panvini N.: *B-0014, B-1148*
Panzeri M.M.: *B-0207, B-0256, B-1302, B-1345, B-1485*
Panzironi G.: *B-0553*
Panzuto F.: *B-0525*
Paolicelli S.: *B-0329*
Papa M.: *B-1300, B-1302*
Papa S.: *B-0304*
Papadaki E.: *A-0520*
Papageorgiou I.: *B-0688, B-0694, B-1643*
Papais Alvarenga M.: *B-0542*
Papais Alvarenga R.M.: *B-0542*
Papakonstantinou O.: *A-1064*
Papalois V.: *A-0217*
Papanagiotou P.: *A-0978*
Papanikolaou N.: *B-0700, B-1185*
Paparo F.: *B-1528*
Papini G.D.: *B-1687*
Papini G.D.E.: *B-0189*
Papp T.: *B-1432*
Pappas E.P.: *B-0768*
Pappon M.: *B-0235*
Paramatti R.: *B-1833*
Paraskevopoulou C.: *B-1200*
Pardhan S.: *B-1555*
Parida S.: *B-1253*
Parimalai A.N.: *B-1271*
Parisi S.: *B-1648*
Parizel P.M.: *A-0213, B-0145, B-0753, B-1127, B-1509*
Park C.H.: *B-0776*
Park D.W.: *B-1336*
Park E.H.: *B-0054, B-0683*
Park E.K.: *B-1177*
Park H.: *B-0336*
Park H.E.: *B-1342*
Park H.J.: *B-0451*
Park H.S.: *B-0449*
Park J.: *B-0303, B-1403*
Park J.B.: *B-0960*
Park J.S.: *B-0392, B-1137*
Park K.: *B-0105*
Park M.J.: *B-0582*
Park M.-S.: *B-1766*
Park M.Y.: *B-0273*
Park S.: *B-1322, B-1766*
Park S.B.: *B-0451*
Park S.-E.: *B-0965*
Park S.J.: *B-0453*
Park S.Y.: *B-0699, B-1137*
Park V.Y.: *B-0120*
Park Y.W.: *B-0097*
Parkar A.P.: *A-0374, A-0917, A-0954*
Parke D.: *B-1181*
Parlak S.: *B-1445*
Parrilla Reverter G.: *B-1418*
Parshad R.: *B-1207*
Parthipun A.: *B-1004*
Pascariello G.: *B-1780*
Pascart T.: *B-0053*
Pasicz K.: *B-1477*
Pasquier G.: *A-0633*
Pasquini L.: *B-0930, B-1032*
Passoni P.: *B-0207, B-1343, B-1344, B-1345, B-1485*
Pastorino U.: *A-0054, B-0143*
Pataraia E.: *B-1882*
Patel A.: *B-0437*
Patel B.N.: *B-1148*
Patel C.: *B-0920*
Patel D.: *B-0639*
Patel H.T.: *A-0398*
Patella F.: *B-0974*
Patelli G.: *B-0630*
Paternain Nuin A.: *B-0064, B-1219*
Paternostro R.: *B-1377*
Patil S.: *B-1663*
Patil Y.: *B-1562*
Patlas M.: *A-0437*
Patney A.: *B-0140*
Patnick J.: *B-0078, B-0080*
Patsch J.M.: *K-26*
Patten P.: *B-1296, B-1298, B-1299, B-1301*
Patterson A.J.: *B-0501*
Paulo G.: *A-0861, A-1041, B-1494*
Pauwels R.: *B-1599*
Pavan L.J.: *B-0072*
Pavan N.: *A-0296*
Pavel M.: *B-0399, B-1024*
Pavic D.: *SY 2c*
Pavlova O.: *B-1261*
Pawlas K.: *B-0200*
Payne J.I.: *B-0916, B-0919*
Pažanin A.: *B-1073*
Pearce T.: *B-1027*
Pecchi A.: *B-1381*
Peck K.: *B-1032*
Pecoraro M.: *B-0156, B-0160, B-0325, B-0825, B-0876*
Pecorelli A.: *B-1372*
Pecori B.: *B-1346*
Pecorilla M.: *B-0991, B-1028*
Peddi S.: *B-1075*
Pedersen M.R.V.: *A-1005, B-1661*
Pedersen S.J.: *B-0520*
Pedersoli F.: *B-0005*
Pederson J.H.: *B-0194*
Pedicini V.: *B-0560*
Pediconi F.: *A-0672, B-0553*
Pedilarco S.: *B-1715*
Pedraza Gutierrez S.: *B-0044*
Pedrazzi G.: *B-0842*
Pędziwiatr M.: *B-1583*
Peebles C.: *A-0772, A-1017*
Peetrons P.: *A-0991, A-0993*
Pegge S.: *B-0706*
Pehrson L.M.: *A-0621*
Pei Y.: *B-0678*
Peixoto M.R.: *B-1365*
Peker E.: *B-1373, B-1862*
Peldschus K.: *B-0499*
Pelgrim G.J.: *B-0586, B-1008, B-1311*
Pellegrin A.: *B-0076, B-0967*
Pellegrino F.: *B-0045, B-0046, B-0272*
Peltonen J.I.: *B-0998*
Penco S.: *B-1561*
Peng P.: *B-0151*
Peng Q.: *B-0429*
Peng W.: *B-0384, B-0552, B-0801, B-1585, B-1590, B-1673, B-1677, B-1727, B-1758*
Peng W.J.: *B-1152*
Peng X.: *B-0678, B-1159*
Pennig L.: *B-0710, B-1424*
Penzkofer T.: *B-0153, B-0172*
Pepi M.: *B-0783, B-1212, B-1501, B-1613, B-1615, B-1686*
Peppelenbosch-Kodach L.L.: *B-1483*
Perani L.: *B-0369*
Perassi C.: *B-0477*
Perazzini C.: *B-0004*
Pereira C.: *B-1776*
Pereira E.: *B-0884, B-0892*
Pereira F.: *B-1695*
Pereira H.: *B-0848*
Pereira M.J.V.: *B-0650*
Perera M.G.R.S.: *B-0185*
Perera S.: *B-0979*
Peretti J.: *B-0837*
Peretto G.: *B-1678*
Peretz A.: *B-0313*
Pereverzyev S.: *B-0925*
Perez M.D.R.: *A-0314, A-0315, A-0662*
Perez Montilla M.E.: *B-1662*
Perez N.: *B-0294*
Perez Riverola V.: *B-0121*
Perez Rodrigo S.: *A-0855*
Peric D.: *B-0276*
Perisinakis K.: *B-1593*
Perkuhn M.: *B-0710, B-0756, B-0988, B-1424*
Perl R.M.: *B-1814*
Perrucci L.: *B-0046*
Persiani S.: *B-1898*
Persigehl T.: *B-1025*
Perucho J.A.: *B-0370*
Perucho J.A.U.: *B-0178*
Perugin Bernardi S.: *B-1528*
Perugin G.: *B-1139*
Pesapane F.: *B-0722, B-0974, B-1561*
Pescosolido M.: *B-1228*
Pesente S.: *B-1521*
Peter S.: *B-0474*
Peters A.: *B-0188, B-0589, B-0749, B-0945, B-1208, B-1782*
Petrella E.: *B-1381*
Petretta M.: *B-0333, B-1383, B-1385*
Petrillo A.: *B-1346*
Petrillo M.: *B-0974*
Petrillo R.: *B-0607*
Petrini M.: *B-1867*
Petrovic D.J.: *B-0041*
Petta F.: *B-0547, B-0548, B-1898*
Petta S.: *B-1529*
Pettersson M.: *B-1716*
Pettersson O.J.: *B-0523*
Pettka P.: *B-1704*
Peynircioglu B.: *A-0472*
Pezeshk P.: *B-1129*
Pfaff J.: *B-0263*
Pfahler V.: *B-0927*
Pfannenberg C.: *A-0186, B-0818*
Pfeifer J.: *B-0167*
Pfeiffer D.: *B-0790, B-0995, B-1312, B-1612, B-1746, B-1848*
Pfeiffer F.: *B-0995, B-1612, B-1847, B-1848*
Pfeil J.: *B-0153, B-0441*
Pfirrmann C.W.A.: *A-0839, A-0841, A-0945*
Phan C.C.: *B-0214*
Phan C.M.: *A-0884*
Phi Van V.: *B-0065*
Photopoulos G.: *B-1699*
Piacentino F.: *B-1808, B-1810*
Pialat J.-B.: *A-1036*
Piana Jacquot F.M.: *B-0970*
Piana M.: *B-0689*
Pianykh O.: *B-1181*
Piazza L.: *B-1878*
Pica S.: *B-1614*
Picariello V.: *B-0668*
Picascia R.: *B-0034*
Picasso R.: *B-1748*
Picchi S.G.: *B-0823*
Picchia S.: *B-0014, B-0524*
Piccoli G.: *B-0626*
Piccolo C.L.: *B-0732, B-0733*
Pickler B.: *B-0686*
Picone D.: *B-1827*
Piekarek A.: *B-1265*
Pierallini A.: *B-1270*
Pietragalla M.: *B-0975*
Pietsch H.: *B-0496, SY 14*
Pignata A.: *B-0668*
Pignatelli M.: *B-0008*
Pijnappel R.M.: *A-0527, A-0943, B-1565*
Pilato F.: *B-1225*
Pilius P.S.: *B-1822*
Pilleul F.: *B-1638*
Pina Insausti L.J.: *B-1784, SY 2b*
Pineda Pardo J.A.: *B-1303*
Pinheiro C.: *B-0284*
Pinker K.: *B-1176*
Pinker-Domenig K.: *A-0038, B-0716, B-1175, B-1328, B-1549, B-1804*
Pinto dos Santos D.: *A-0709, B-0314*
Pinto P.: *B-0837*
Pinto Ramos J.N.: *B-0283*

Piotin M.: *B-0280, B-0282*
Piotrowska-Kownacka D.: *A-0224*
Piqueras Pardellans J.: *B-0340*
Piquet P.: *B-0964*
Piraud M.: *B-0344*
Pires Jorge J.: *B-1704, B-1712*
Pirillo S.P.: *B-1724*
Pisano A.: *B-0007*
Pistoia F.: *B-1748*
Pistorio A.: *B-1054*
Pitskhelauri D.: *B-1037*
Pittaro A.: *B-0171*
Pitura R.: *B-1652*
Pizzi G.: *B-0113, B-0563, B-0846, B-1435*
Plaine B.: *B-1214*
Plakhotina N.: *B-0091, B-1231*
Plank F.: *B-0782*
Plataroti S.: *B-1154*
Platon A.: *A-0685*
Platzgummer H.: *B-0677*
Pluymakers R.: *B-1787*
Poddubnyy D.: *B-0514*
Podgorska J.: *B-1477*
Podgórski P.: *B-1265*
Podkovka O.: *B-1034*
Podlasek A.: *B-0979*
Poewe W.: *B-1689*
Pofi E.: *B-0010*
Poggesi S.: *B-1858*
Pogosbekyan E.: *B-0089, B-1037*
Pogrebnyakov I.: *B-0844, B-0845*
Pokryszko-Dragan A.: *B-0540*
Polacin M.: *B-1616*
Polat A.V.: *B-0059*
Polat Korkmaz O.: *B-0986*
Polavarapu R.: *B-0841*
Polici M.: *B-0525*
Polkowski C.: *B-0243, B-1317*
Pollock A.N.: *B-0604*
Polovincak M.: *B-0597*
Polster C.: *B-0135*
Polunina N.: *B-0186*
Pompei L.D.M.: *B-0084*
Ponomarjova S.: *B-0957*
Pons Lucas R.: *B-1773*
Pons-Becmeur C.: *B-1511*
Ponsiglione A.: *B-0823, B-1392, B-1684*
Pontana F.: *A-0718*
Pontone G.: *A-0226, B-0783, B-1212, B-1501, B-1613, B-1614, B-1615, B-1686, K-19*
Pontrelli M.: *B-0045, B-0046*
Pop M.: *B-0165*
Popescu A.S.: *B-1533*
Popiela T.J.: *A-0981*
Popovic P.: *A-0245*
Poreba M.: *B-0039*
Poreba R.: *B-0039, B-0200*
Poretti D.: *B-0560, B-1723*
Porpiglia F.: *B-0824*
Porta M.: *B-1310, B-1372*
Portalez D.: *B-0322*
Portanier Mifsud C.: *B-0413*
Portelli J.: *A-0964, B-0027*
Portelli J.L.: *B-0413*
Posa A.: *B-0109, B-0110, B-0114, B-1812*
Posch H.: *B-0962*
Poskus T.: *B-0270*
Postema S.: *B-0251, B-0473*
Potapov A.: *B-0089*
Poteshkina N.: *B-1819*
Potter A.: *B-0657*
Pötter-Lang S.: *B-1050, B-1052*
Pounou F.A.K.: *B-0973*
Power S.: *B-0265, B-0267*
Poylin V.Y.: *B-0210*
Pozeg P.: *B-0931*
Pozzi Mucelli R.: *A-1087, B-0328, B-1807*
Pozzi-Mucelli R.: *A-0239*
Prabhu S.S.: *B-1243*
Pradella M.: *B-0310, B-1030*
Prael I.: *B-0426*
Prakash A.: *B-1522, B-1886*
Pranjkovic T.: *B-0276*
Prassopoulos P.K.: *A-0164*
Prayer D.: *B-0534, B-0926, B-0927, B-1881, B-1882*
Prayer F.: *B-0307, B-0811, B-0905*
Precht H.: *A-0400, A-0862, B-0485, B-0653*
Preda L.: *B-0372, B-0373, B-0973, B-1644*
Predel D.: *B-1643*
Preibisch C.: *B-0088*
Preim B.: *B-0221*
Prenafeta Moreno M.: *B-0121*
Presazzi A.: *B-0087*
Prestwich R.: *B-0666, B-0667*
Preusser T.: *B-1444*
Prevos R.M.A.: *B-0473, B-1803*
Prevos R.M.-A.: *B-0915*
Prevrhal S.: *B-1792*
Prezzi D.: *B-0616*
Prichard R.: *B-0633*
Primakov S.: *B-1179*
Primavesi F.: *B-0102*
Primo V.: *B-0003*
Primolevo A.: *B-0725*
Prince J.: *B-1850*
Prinster A.: *B-0543*
Priola A.M.: *B-1144*
Priola S.: *B-1144*
Prior J.O.: *B-0235*
Prokop M.: *A-0028, A-0732, A-0955, B-0706, B-1850, SY 4*
Pronin I.: *B-0089, B-1037*
Prono V.: *B-0879*
Prosch H.: *A-0269, A-0516, A-0598, B-0307, B-0811, B-0905, B-1020*
Provenzano E.: *B-0501*
Pruschy M.: *B-0065*
Pruvo J.-P.: *A-0402, A-0404, A-0407, A-0979*
Puelacher W.: *B-1599*
Pueyo Villoslada J.C.: *B-1015, B-1219*
Puglia M.: *B-1042, B-1684*
Puglielli E.: *B-0708, B-0951*
Puhr-Westerheide D.: *B-0259, B-0262, B-0713*
Puig Alcàntara J.: *B-0044*
Pulido Carmona C.: *B-1662*
Püllen L.: *B-0875*
Pulsone P.: *B-0708*
Pulzato I.: *B-1605*
Pumberger M.: *B-1738*
Punie K.: *B-0559*
Punwani S.: *A-0094, B-0159, B-0443, B-1324*
Purcell Y.: *A-0372*
Pusceddu L.: *B-0527, B-1082, B-1093*
Pushkarna A.: *B-1666*
Püsken M.: *B-1761*
Putzier M.: *B-1738*
Puylaert J.B.C.M.: *A-0167, A-0836*
Puzakov K.B.: *B-1263*
Puzzo L.: *B-1648*
Pyatigorskaya N.: *B-1422*
Pyka T.: *B-0088*
Pylev A.: *B-1819*
Pyra K.K.: *A-0518*
Pytlewski P.: *B-1185*

### Q

Qanadli S.D.D.: *B-1497*
Qi H.: *B-1534*
Qi Q.: *B-1159*
Qi W.: *B-1006*
Qi X.: *B-1163*
Qi Y.: *B-0863, B-1074, B-1087, B-1893, B-1896, B-1897*
Qi Z.: *B-0504*
Qian T.-Y.: *B-1475*
Qiang J.: *B-1531*
Qin S.: *B-0504*
Qiu B.: *B-0574*
Qiu H.S.: *B-1273*
Qiu T.: *B-1370*
Qu J.: *B-0610, B-1873*
Qu Y.: *B-0881*
Quaia E.: *B-0512, B-0522, B-1011, B-1060, B-1364*
Quan C.: *B-0541*
Quan X.: *B-0504, B-0578*
Quang Huy H.: *B-1304, B-1305*
Quarantelli M.: *B-0543*
Quattrini A.: *B-1696*
Quentin M.: *B-0158, B-0161, B-0828*
Quick H.H.: *SY 22*
Quigley A.J.: *B-1323*
Quinn M.: *B-1699*
Quintana I.: *B-0032*
Quitzke A.: *B-0679*

### R

Rabago G.: *B-1015*
Rabbani M.: *B-1694*
Rabelo B.C.S.: *B-0129*
Rabenalt R.: *B-0828, B-1462*
Racine D.: *B-1600*
Raciti M.V.: *B-0436, B-1878*
Raciti M.V.V.: *B-0372*
Raczeck P.: *B-1058*
Radbruch A.: *B-1419, B-1421, SY 30*
Radnai B.: *B-0728*
Radovic N.: *B-0463*
Radtke J.-P.: *B-0813, B-1077*
Radu R.: *B-0191*
Radunski U.: *B-1682*
Radzina M.: *B-0037, B-0264*
Rae W.I.D.: *B-0890*
Rafaelsen S.R.: *B-1661*
Rafailidis D.: *B-1685*
Rafailidis V.: *B-1685*
Rafferty A.R.: *B-0885*
Rafful P.P.: *B-0542*
Ragab Y.: *B-0899*
Ragozzino A.: *B-0174*
Rahimzadeh S.: *B-0132*
Rahmat K.: *B-0468, B-0475, B-1233, B-1274, B-1885, B-1902*
Rai B.: *B-1339, B-1479*
Raimondi E.: *B-0524*
Raimondi S.: *B-0769*
Rainford L.A.: *A-0533, A-0860, B-0027, B-0891, B-1113, B-1354*
Raissaki M.: *A-0190, A-0923*
Raizner A.: *B-1669*
Raja S.: *B-0809*
Rajabzadeh F.: *B-1380*
Rajamohan N.: *B-1207*
Rajan R.S.: *B-1892*
Rajan S.: *B-1132, B-1133, B-1750, B-1892*
Rajani H.: *B-1291*
Rajapakse C.: *A-0463*
Rajasekaran M.: *B-1376*
Rajwanshi A.: *B-1095*
Ramachandran S.: *B-0950*
Ramaesh R.: *B-0639*
Ramalho L.: *B-0084*
Ramanauskas N.: *B-1190, B-1757*
Ramavathu K.: *B-1555*
Ramesh A.: *B-0631*
Ramirez Giraldo J.C.: *B-0493, B-0729, B-1503*
Ramiro R.: *B-0003*
Ramlaul A.: *B-0026*
Ramli Hamid M.T.: *B-0468*
Ramli N.: *B-1233, B-1274, B-1885*
Ramos A.: *A-0160*
Ramos Botelho Antunes P.: *B-0129*
Ramos I.M.: *B-0412*
Rampal P.: *B-0513*
Ran L.: *B-0380*
Rana A.I.: *A-1080*
Rana P.: *B-0169*
Rance B.: *A-0058*
Rancic Z.: *B-0224*
Rancoita P.M.: *B-1344*

Randelli F.: *B-0898*
Rangan K.B.K.: *B-0233*
Rangaswamy B.: *B-0071*
Ranjan R.: *B-1335, B-1558*
Ranjbar M.: *B-1433*
Rannisto K.M.J.: *B-1712*
Ranschaert E.R.: *A-0011, A-0698*
Ranucci M.: *B-0189*
Rao A.S.: *B-0691, B-1637*
Rao Kesari A.A.: *B-1269*
Rao P.: *B-0748, B-0750, B-0981, B-1753*
Rao S.: *B-1278, B-1479*
Rao S.-X.: *B-1467*
Rapaka S.: *B-0310, B-0820*
Rapalino O.: *B-1227*
Rapisarda S.: *B-0868*
Raposo R.: *B-1359*
Raschzok N.: *B-1468*
Rasciti E.: *B-1858*
Rasker J.: *B-0899*
Rasmussen F.R.: *B-1465*
Rasouly N.: *B-1664*
Rasper M.: *B-1312*
Rastogi R.: *B-1666*
Rathinasamy R.: *B-1376*
Rathmann W.: *B-1782*
Ratib O.: *A-0010, A-0554, A-0697, A-0972*
Rattazzi M.: *B-1010*
Ratti F.: *B-0851, B-1439*
Raudner M.W.: *B-0374*
Rausch I.: *B-0234*
Ravaglia V.: *B-1198*
Ravanelli M.: *B-0970, B-1446*
Ravelli S.: *B-0256*
Ravindra K.: *B-1140*
Rawat K.S.: *B-1668*
Raya J.: *B-1508, B-1510*
Raymond C.: *B-1165*
Razansky D.: *A-0706*
Reale M.L.: *A-0053*
Rebibo S.: *B-0992*
Rebonato A.: *B-0849*
Reddy B.: *B-1753*
Reekers J.A.: *A-0020, A-0664*
Refaat M.M.: *B-0531*
Regge D.: *A-0034, A-0957, B-0527, B-0618, B-1082, B-1093, B-1828*
Regier M.: *B-0679*
Regine R.: *B-1080*
Reginelli A.: *B-0034*
Regnard N.-E.: *B-0058*
Regnery S.: *B-0505, B-0506*
Rehani M.M.: *A-0658, A-0660*
Rehnitz C.: *B-0240, B-0242, B-1286*
Rehwald R.: *B-1688*
Reiberger T.: *B-1377*
Reichardt B.: *B-0231, B-0839, B-0840, B-0866*
Reidelbach C.: *B-0867*
Reider L.: *B-1377*
Reidler P.: *B-0259, B-0262, B-0713*
Reijman M.: *B-1134*
Reijnierse M.: *K-31*
Reimer P.: *B-0354*
Reimer R.P.: *B-0354, B-1081, B-1424, B-1783*
Reindl M.: *B-0500*
Reiner C.S.: *B-0822*
Reinert C.P.: *B-0620, B-0818*
Reinhold C.: *B-0528, B-1894*
Reinstadler S.J.: *B-0500*
Reis H.: *B-0875*
Reis J.: *B-0284*
Reis M.V.: *B-0489*
Reiser I.: *B-0255*
Reiser M.F.: *B-1847*
Reisert M.: *B-0568*
Reiter A.: *B-0352*
Reiter C.: *B-0767, B-0989, B-1619, B-1620*
Reiter G.: *B-0767, B-0989, B-1619, B-1620*
Reiter U.: *B-0767, B-0989, B-1619, B-1620*
Rella R.: *B-0547, B-0548*
Remedios D.: *B-1856*
Remuzzi A.: *B-0040*
Remy J.: *B-0778*
Rémy-Jardin M.: *A-0289, B-0778, K-17, SY 12*
Ren B.: *B-0018, B-0911*
Ren C.-P.: *B-1264*
Ren D.: *B-0805*
Ren Y.: *B-1222*
Rengo M.: *B-0014, B-0935, B-0940, B-1833*
Renne J.: *B-0001*
Renou P.: *B-1415*
Repa I.: *B-0031, B-0882*
Resnik A.: *B-1070*
Reuter J.: *B-1421*
Revel D.: *B-1214*
Revel M.-P.: *A-0307, A-0452, A-1054*
Reyal F.: *B-1090*
Reynolds O.: *B-1700*
Rezgui Marhoul L.: *A-0313, A-0315*
Rezk M.M.: *B-1363*
Rha S.E.: *B-1895*
Rhiem K.: *B-0461*
Riaz S.: *A-1006*
Riaz Z.: *B-0150*
Riaza Martin L.: *B-0340*
Ribbens A.: *B-0984*
Ribeiro A.P.: *B-0414*
Ribeiro Fragata I.M.: *B-0284*
Ribeiro L.: *B-0414*
Ribeiro L.: *A-0780, B-0489, B-0652, B-0658, B-1359*
Ribeiro M.M.: *A-0008, B-0410*
Ricardi U.: *A-0497*
Riccabona M.: *A-0279, B-1361*
Ricci A.: *B-1552*
Ricci F.: *B-0195, B-1313, B-1636*
Ricci P.: *A-0001, A-0221*
Ricciardi M.C.: *B-0368*
Richenberg J.: *A-1071*
Richli Meystre N.: *B-1709*
Richly H.: *B-0116*
Richter H.: *B-0093*
Richter S.: *B-0232*
Ricke J.: *B-0503*
Ricoeur A.: *B-0834*
Riddell A.: *B-1046*
Rieder C.: *B-0878*
Riederer I.: *B-0790*
Riederer S.: *B-0438*
Riedl M.: *B-0055*
Riera L.: *B-0340*
Riffel P.: *B-0352*
Riga M.G.: *B-1364*
Righi D.: *B-0004, B-0038*
Rigiroli F.: *B-0304*
Rigon L.: *B-1846*
Riklund K.: *A-0215, A-0739*
Riley B.: *B-1004*
Rim J.: *B-0699*
Rimeika G.: *B-1190, B-1757*
Rimola J.: *A-0390, B-1653*
Rinaldo C.: *B-0174*
Rincon S.: *B-1229*
Ringelstein A.: *B-1595*
Ringl H.: *A-0858*
Ringleb P.A.: *B-0263*
Rinzivillo M.: *B-0525*
Rischpler C.: *B-1456*
Risi V.: *B-1352*
Rispoli P.: *B-0038*
Ritchie G.: *B-1751, B-1759*
Riva L.: *B-1310, B-1372, B-1457*
Riva N.: *B-1696*
Rivadeneira F.: *B-0856*
Rivière L.: *B-1090*
Rizk B.: *B-1600*
Rizzo D.: *B-0959*
Rizzo G.: *B-0455, B-0457*
Rizzo S.: *A-0192, B-0769*
Roberts D.: *B-1226*
Robinson C.: *B-0483*
Robinson E.: *B-1410*
Robinson L.: *A-0827*
Robinson M.: *B-0126*
Robinson P.: *B-0900*
Robinson S.: *A-0111*
Robson M.D.: *B-0763*
Roca A.: *B-0625*
Rocek M.: *B-0597*
Rocher L.: *A-0295, A-1025*
Rockall A.G.: *A-0070, A-0476, B-0177, B-1387*
Rodell A.: *B-0675, B-0682*
Rodighiero M.G.: *B-0256*
Roditi G.: *A-0422, B-1307*
Rodrigues S.: *B-0652, B-0658, B-1359*
Rodrigues S.I.: *B-0489*
Rodríguez García E.: *B-0535*
Rodriguez Laval V.: *B-1024*
Rodríguez Musso A.L.: *B-1780*
Rodríguez Pérez S.: *B-1318*
Rodriguez Rojas R.: *B-1303*
Rodriguez Ruiz A.: *B-0697, B-1527*
Rodríguez-Fraile M.: *B-0111*
Roelofs J.J.: *B-1592*
Roemer F.W.: *B-1514, B-1515*
Roest A.A.W.: *B-1506*
Rogalla P.: *B-0277, B-0306, B-1832*
Roggenbuck U.: *B-1622*
Rogozhyn V.: *B-1034*
Rohan T.: *B-0232*
Röhnert A.: *B-0398*
Rohovyk M.: *B-0584*
Rohovyk N.: *B-0584*
Röhrich S.: *B-0307, B-0811, B-0905*
Rollandi G.A.A.: *B-1528*
Roller F.C.: *B-1863*
Rollison S.: *B-0300, B-1114*
Roman M.S.: *B-0212*
Romani M.: *B-0547*
Romanini L.: *B-1042*
Romano A.: *B-1270*
Romano L.: *B-0104*
Romano S.: *A-1037*
Romano V.: *B-0527, B-0618, B-1082, B-1093*
Romanov A.B.: *B-1672*
Romanowski C.A.J.: *A-0519*
Romanucci G.: *B-0248, B-0249, B-0250*
Romei C.: *B-1205*
Romeo V.: *B-0333, B-0668, B-0823, B-1383, B-1385, B-1392, B-1626*
Rominger M.: *B-1512*
Romman Z.: *B-0133, B-0313*
Rompel O.: *B-1321, B-1868*
Ronald J.: *B-0533*
Ronconi E.: *B-1809*
Rong P.: *B-0419*
Ronot M.: *A-0648, B-0848, K-11*
Ronza R.: *B-0991, B-1028*
Roobol M.J.: *B-0181*
Rook M.: *B-0309, B-0586, B-1311, B-1756*
Roos J.E.: *B-1729*
Roose Ä.: *B-1853*
Roos-Hesselink J.W.: *B-0194*
Ropella-Panagis K.: *B-1655*
Rosa F.: *B-1139*
Rosario P.: *B-1494*
Rosati E.: *B-1022*
Rosella F.: *B-0076, B-0967*
Rosenberg M.: *B-0070*
Rosendahl K.: *A-0339*
Rosenkrantz A.B.: *B-1184*
Rosenzweig M.: *B-1257*
Rosignuolo M.: *B-0547*
Roski F.: *B-0047*
Rospleszcz S.: *B-0188, B-0589, B-0749, B-0945, B-1514, B-1515, B-1782*
Ross J.S.: *A-0758*
Rossato D.: *B-0004*
Rossi A.: *A-0829, A-1101, B-0930*
Rossi D.: *B-0973*
Rossi Espagnet M.C.: *B-0930*
Rossi F.: *B-1178, B-1745*
Rossi G.: *A-0052*

Rossi L.: *B-1090*
Rossi M.: *B-0007, B-0008, B-0849, B-1809*
Roth J.: *B-1321*
Rothenbacher T.: *B-0945*
Rotili A.: *B-0722, B-1561*
Rotkopf L.: *B-0088, B-0262, B-0713*
Rotkopf L.T.: *B-0259*
Rotondo D.: *B-0195*
Rotzinger D.C.: *B-0731, B-1214, B-1366*
Rousseau H.: *B-0793, B-1188*
Roviello F.: *B-1275*
Rovira Gols A.: *B-0121*
Rovira-Cañellas A.: *A-0155, A-1000*
Rowinski O.: *B-1452*
Roy S.: *B-0492*
Royle L.: *B-0142*
Rozalli F.I.: *B-1274*
Rubbert C.: *B-0983, B-1210, B-1553*
Rübenthaler J.: *A-0170*
Rubin D.: *B-1402*
Rubin D.L.: *B-0906*
Ruby L.: *B-1512*
Rud E.: *B-0332, B-0821, B-1656*
Rudolph M.: *B-0153*
Rueppel S.: *B-0383*
Ruffino A.: *B-1499*
Ruffino M.A.: *B-0038*
Ruggeri V.: *B-1799*
Ruggieri C.: *B-0948, B-1499*
Ruhlmann V.: *B-0231, B-0673*
Rumboldt Z.: *B-0260*
Rummeny E.J.: *B-0047, B-0138, B-0615, B-0790, B-0995, B-1312, B-1612, B-1746, B-1848*
Runhaar J.: *B-1136*
Rupp C.: *B-0385*
Rupp N.J.: *B-0228, B-0229, B-0822*
Ruprecht C.: *B-0601*
Rusche T.: *B-0266*
Ruschioni M.: *B-1571*
Rüschoff J.H.: *B-0229*
Rusina M.: *B-0281*
Russe M.F.: *B-0568*
Russo A.: *B-1241*
Russo F.: *B-1082, B-1093*
Russo L.: *B-0328, B-1898*
Russo P.: *B-1843*
Russo U.: *B-0842*
Rutschke W.: *B-0379*
Rutten M.J.C.M.: *SY 2b*
Rutty G.: *B-0483*
Rutz E.: *B-0893*
Rutz M.: *B-0802*
Ruzzenente A.: *B-0532*
Ryabinin M.A.: *B-1760*
Ryan J.W.: *B-0098*
Ryan M.-L.: *A-0140, B-0024, B-1113*
Ryan S.: *B-0654*
Ryckx N.: *B-1196*
Rydel W.: *B-1265*
Rydén Suther K.: *B-0168*
Ryeom H.-K.: *B-0942*
Ryu K.N.: *B-1137*
Rzaev J.: *B-1266*

### S

Sá dos Reis C.: *B-0025, B-1065, B-1709*
Saade C.: *B-0481, B-0727, B-0799, B-0800, B-1351, B-1851*
Saade S.M.: *B-0604*
Saake M.: *B-1777*
Saam T.: *B-1773*
Saba L.: *A-0508, B-0036, B-0415, B-0707, B-1251, B-1721, B-1775, SY 4*
Sabatino V.: *B-0842*
Sabbah S.: *B-0277*
Sabet S.: *B-0286*
Sabia F.: *B-0143*
Sable N.: *B-1450*
Sacconi F.: *B-1807*
Sachdev N.: *B-0513*
Sachs C.: *B-0464, B-1659*
Sadate A.: *B-0431*
Saddekni S.: *B-0838*
Sadotti G.: *B-1575, B-1578*
Saedon N.I.: *B-1274*
Saez F.: *A-0850, A-0924, A-1033*
Safran O.: *B-0992*
Sah B.-R.: *B-0224*
Sahbaee P.: *B-0820, B-1215*
Sahbai S.: *B-0689*
Sahin C.: *B-1576*
Said N.H.: *B-0923*
Saidléar C.: *A-0785*
Saifi S.: *B-1749*
Sajid S.: *B-1130*
Sakahara H.: *B-0185, B-1180, B-1722*
Sakai Y.: *B-0484*
Sakamoto S.: *B-0425*
Sakuma A.T.: *B-1365*
Sakuma H.: *B-1101, B-1102*
Sakuragawa K.: *B-0648*
Sakwa J.: *B-1356*
Sala E.: *A-0132, A-0475, A-0899, A-1061, B-0323, B-1384*
Sala S.: *B-1678*
Salagovicova Z.: *B-0597*
Salah F.: *B-1168*
Salamon J.: *B-0499, B-1240*
Salei A.: *B-1296, B-1298, B-1299, B-1301*
Salem A.: *B-1456*
Salem J.: *B-1081*
Salemi I.: *B-1372, B-1457*
Salgado R.: *A-0484, A-0877*
Sali L.: *B-0928*
Salimbeni S.: *B-1715*
Saliu A.: *B-1391*
Sall S.: *B-0302*
Salman A.: *B-0528*
Saltybaeava N.: *B-1541*
Saltybaeva N.: *B-0996*
Salvatore E.: *B-0928*
Salvatore M.: *B-0174, B-0668, B-0928*
Salvioni M.: *B-0851, B-1439*
Salvo V.: *B-0160, B-0876*
Samà L.: *B-0530*
Samara E.: *A-0046*
Samarin A.: *B-1853*
Sambataro G.: *B-1405*
Sambuceti G.: *B-0686, B-0689*
Samei E.: *A-0183*
Sammon J.: *B-1056*
Sammut S.J.: *B-1647*
Sampangi S.: *B-0063, B-1269*
Samuelsson C.: *B-0049*
Samuilis A.: *B-0270*
Sana Ali J.: *B-0902*
Sanabria S.J.: *B-1512*
Sánchez Casanueva R.M.: *A-0750*
Sanchez M.: *B-0848*
Sánchez P.S.: *B-1811*
Sánchez-Casanueva R.M.: *B-1195*
Sánchez-Montañez A.: *A-1102*
Sanchez-Montanez Garcia-Carpintero A.: *B-1231*
Sanchez-Osorio E.: *B-0680*
Sandbækk G.: *B-0332, B-1656*
Sanderink W.: *B-0545, B-1564*
Sanders K.J.C.: *B-0220*
Sandiramourty S.: *B-1695*
Sanei Taheri M.: *B-1433*
Sangro B.: *B-0111*
Sanmartin Berglund J.: *B-1360*
Sanson M.: *B-1422*
Sansone M.: *B-0974*
Santiago I.: *A-0002, A-0769*
Santinha J.: *B-0700*
Santis G.: *B-1410*
Santonocito E.F.: *B-0943*
Santonocito S.M.: *B-0943*
Santos J.: *A-0686, B-1063, B-1494*
Santos R.: *A-1007*
Santos R.A.M.: *B-1108, B-1110, B-1357*
Santucci G.: *B-1715*
Sapinho I.: *A-0986*
Sappey-Marinier D.: *A-0897*
Sarangi P.K.: *B-1253*
Sardanelli F.: *A-0233, B-0042, B-0189, B-0199, B-0416, B-0465, B-0477, B-0490, B-0759, B-0895, B-1151, B-1352, B-1561, B-1687, B-1723, B-1728, B-1821, B-1867*
Sargeant A.: *B-0029*
Sarı S.: *B-1805*
Sarikaya B.: *B-0557*
Sarkodie B.D.: *B-0871*
Sarno A.: *B-1843*
Sarno L.: *B-1383, B-1385*
Sarohia D.: *B-0056*
Sartori A.: *B-0536*
Sasaki M.: *B-1180*
Sasiadek M.: *B-0540*
Sasikumar S.: *B-0142*
Sassatelli R.: *B-0993*
Satis H.: *B-1453*
Sato H.: *B-0421*
Sauer M.: *B-1089*
Sauerland C.: *B-0266*
Saukko E.: *B-0645, B-0886, B-0888*
Sauter A.: *B-0138, B-1612, B-1848*
Sauter A.W.: *B-0310, B-0904, B-1030, B-1211*
Savage R.H.: *B-0781*
Savatovsky J.: *B-0987, SY 7*
Savchenko V.: *B-0350*
Savi E.: *B-0622*
Savintseva Z.: *B-1164*
Sawall S.: *B-0013, B-0135, B-0316, B-0319, B-0321*
Sawicki L.M.: *B-0669, B-0670, B-0671, B-1210, B-1355, B-1462*
Saxena A.K.: *B-0169, B-1224*
Saxena S.: *B-0387*
Sayed S.: *B-1334*
Sbarra M.: *B-0328*
Scaglione M.: *A-0891, K-09*
Scamarcia P.G.: *B-1696*
Scaperrotta G.: *B-0257, B-0725, B-1155*
Scarabello M.: *B-0787, B-1217, B-1721*
Scardapane A.: *B-0676*
Scarsbrook A.: *A-0457, B-0150, B-0361, B-0666, B-0667, B-0771*
Scavone A.: *B-0436, B-1878*
Scavone G.: *B-0436, B-1878*
Schaar J.: *B-0704*
Schaarschmidt B.M.: *B-0690, B-1456, B-1461*
Schachtner B.: *B-1400*
Schaefer N.: *B-0235*
Schaefer-Prokop C.M.: *A-0123, A-0288, A-0982, B-1850*
Schäfer D.: *B-0106, B-0735*
Schäfer J.: *A-0874*
Schäfer J.P.: *A-0909*
Schäfer S.: *B-1025*
Scharfschwerdt M.: *B-0202*
Schawkat K.: *B-0822*
Schebesch F.: *B-1845*
Scheel M.: *B-1199, B-1631*
Scheerder M.J.: *B-0099*
Scheffler K.: *B-0095*
Scheidler J.: *B-0440*
Schelb P.: *B-0813, B-1077*
Schenk A.: *B-0558*
Schenk M.: *B-0349*
Schenone A.: *B-1745*
Schernthaner R.: *A-0665, B-0219, B-0299*
Scherr D.: *B-1620*
Scherr M.K.: *B-0440*
Scherz B.: *B-1682*
Schiaffino S.: *B-0724, B-1151*
Schiani E.: *B-0256*
Schiavina R.: *B-0873*
Schicci N.: *B-1719*
Schierz J.-H.: *B-0558*

Schifferle G.: *B-1389*
Schillebeeckx J.: *A-0589*
Schima W.: *A-0237, A-0770*
Schimmöller L.: *B-0158, B-0161, B-0327, B-0828*
Schindera S.: *B-1196*
Schindera S.T.: *A-0413*
Schiphof D.: *B-1136*
Schippers R.: *B-0137*
Schittenhelm J.: *B-0093*
Schlamann M.: *B-0710, B-0988, B-1166*
Schlattmann P.: *B-0762*
Schleich C.: *B-0515, B-0516, B-0517, B-0518, B-0519, B-1736*
Schlemmer H.-P.: *A-0036, A-0193, B-0135, B-0319, B-0321, B-0362, B-0364, B-0505, B-0506, B-0813, B-1077, B-1419, B-1421, B-1568*
Schlesinger I.: *B-1557*
Schlett C.L.: *B-0019, B-0051, B-0188, B-0589, B-0749, B-0945, B-1208, B-1514, B-1515, B-1782*
Schlötelburg W.: *B-0337*
Schmaranzer E.: *B-0896, B-0897*
Schmaranzer F.: *B-0571, B-0896, B-0897, B-0901*
Schmeel F.C.: *B-1513*
Schmelzle M.: *B-1468*
Schmid T.: *B-0102*
Schmidbauer V.: *B-0926, B-1881, B-1882*
Schmidle A.: *B-1777*
Schmidt A.: *B-1125, B-1619, B-1620*
Schmidt B.: *B-0496, B-0682, B-0996, B-1142*
Schmidt K.: *B-1709*
Schmidt Kobbe S.: *A-0650, B-1366*
Schmidt N.: *B-0193, B-0946*
Schmidt S.A.: *B-0061*
Schmidt T.: *B-0119, B-0962*
Schmitt A.J.: *B-0979*
Schmitt-Opitz I.: *B-0577*
Schmitz C.: *B-1669*
Schmutzler R.: *B-0461*
Schneck A.: *SY 6*
Schneider G.: *B-1058, B-1463*
Schneider M.J.: *B-0503, B-1400*
Schneider S.: *B-1247*
Schneider U.: *B-0514*
Schneiter D.: *B-0577*
Schocke M.: *B-1689*
Schoen G.: *B-0119*
Schoennagel B.P.: *B-0601*
Schoepf U.J.: *B-0301, B-0780, B-0781, B-0784, B-0785, B-0787, B-0789, B-0820, B-0914, B-1215, B-1217, B-1721*
Schofield S.: *B-1543*
Scholer M.: *B-1563*
Schöllnast H.: *A-0712*
Scholtz J.E.: *B-0347*
Scholtz J.-E.: *B-0434, B-1013, B-1317, B-1448, B-1472*
Schönberg S.O.: *A-0396, B-0022, B-0106, B-0352, B-0587, B-0735*
Schönenberger S.: *B-0263*
Schoots I.G.: *B-0181*
Schöppe F.: *B-0094, B-0749, B-1782*
Schrauwen P.: *B-0220*
Schreier A.: *B-1400*
Schübel R.: *B-0051*
Schultheiss H.-P.: *B-0378*
Schulz A.: *B-1047*
Schulz D.: *B-1669*
Schulz H.: *B-1208*
Schulze C.: *B-0201*
Schulze-Hagen M.F.: *B-0005*
Schulz-Menger J.: *A-0253, B-0589*
Schulz-Wendtland R.: *B-0551, B-0701, B-0702, B-0703, B-1518*
Schumann C.: *B-0878*
Schuppert C.: *B-0019, B-1208*
Schurink N.W.: *B-1484*
Schuzer J.L.: *B-0300, B-1114*
Schwabel B.K.: *B-1020*
Schwaiger B.J.: *B-0047, B-1746*
Schwartz F.R.: *B-0493, B-0729, B-1140, B-1503*
Schwartz L.: *B-0672*
Schwarz D.: *B-1560*
Schwarz F.B.: *B-1199, B-1631*
Schwarz M.: *B-1881*
Schwemmer C.: *B-0820*
Schwenck J.: *B-0686*
Schwenke M.: *B-1444*
Schwyzer M.: *B-0817*
Sconfienza L.M.: *A-0692, B-0052, B-0416, B-0759, B-0868, B-0870, B-0898, B-1723, B-1730*
Screaton N.J.: *A-0470*
Scrittori N.: *B-1198*
Scrofani A.R.: *B-0547, B-0548*
Sdao S.: *B-0898*
Sdika M.: *B-1638*
Sdvizhkov A.M.: *B-0173*
Sebag-Montefiore D.: *B-0361*
Sebire N.J.: *B-0606, B-1319*
Secchi F.: *B-0042, B-0189, B-0199, B-1728, B-1821, B-1867*
Sechopoulos I.: *A-0047, B-0255, B-0545, B-1523, B-1525, B-1527, B-1564, B-1790, B-1838, B-1850, SY 2c*
Secil M.: *A-0294*
Secinaro A.: *A-1022*
Sedelaar J.P.M.: *B-0878*
Sedghi Gamechi Z.: *B-0194, B-0497*
Sedlaczek O.L.: *A-0064, A-0600*
Sedlmair M.: *B-0778*
Seeger W.: *B-1863*
Seegert A.: *B-0653*
Sefidbakht S.: *B-0291, B-0602*
Sehner S.: *B-0679*
Seidensticker M.: *A-0248*
Seifeldein G.S.: *B-0371*
Seiko-Vänttinen V.: *B-0888*
Seilern und Aspang J.: *B-0677*
Seimenis I.: *B-0183, B-0768*
Seitz P.: *B-0196, B-1683*
Seker F.: *B-0263*
Seki S.: *B-0417, B-0422, B-0424, B-0579, B-1003, B-1203, B-1209, B-1460, B-1755*
Sekkelsten H.: *B-1700*
Selau Junior M.: *B-1704*
Selcuk M.B.: *B-0059*
Selcuk N.A.: *B-0557*
Selder S.: *B-0019*
Seliverstov Y.: *B-1554*
Seliverstova E.: *B-1554*
Sellars M.: *SY 18*
Selmi-Özer B.: *B-0385*
Semenova N.: *B-1038, B-1778, B-1887*
Semiz Oysu A.: *B-0929*
Semiz-Oysu A.: *B-0125*
Sendur H.N.: *B-0213, B-1453*
Senn D.: *B-1617*
Senyszak D.: *B-1751, B-1759*
Seo B.K.: *B-1177*
Seo D.-K.: *B-0646*
Seo H.S.: *B-0852, B-1420*
Seo J.B.: *A-0566, B-0576*
Seo J.S.: *B-0776*
Seo N.: *B-0402*
Seow P.C.: *B-1233*
Seppelt D.: *B-0401, B-0797*
Seppi K.: *B-1689*
Seraydarmansour O.: *B-0238*
Serebryakova S.V.: *B-1267*
Sergeeva O.N.: *B-0395*
Serni S.: *B-0622*
Serova N.S.: *B-1261*
Sertorio F.: *B-0341, B-1054*
Sessa B.: *B-0734*
Sestini S.: *B-0143*
Sethi K.: *B-0537*
Sethi P.: *B-1797*
Seuri R.: *A-0872*
Seuss H.: *B-1088*
Sever A.: *B-0295*
Severi D.: *B-1715*
Severino M.: *B-0930*
Severoni S.: *A-0228*
Sewerin P.: *B-0515, B-0517, B-0519*
Sewonu A.: *B-0793*
Seyfer P.: *B-0365*
Seyfried N.: *B-1650, B-1655*
Sfez K.: *B-0992*
Sgrazzutti C.: *B-1028*
Shafaat O.: *B-1694*
Shafiei A.: *B-1640*
Shah D.J.: *B-1669*
Shah J.: *B-1129*
Shahzad M.: *B-0090*
Shahzad R.: *B-0756, B-0988*
Shaiakhmetova S.V.: *B-1672*
Shakirin G.: *B-0756, B-0988, B-1424*
Shalmon A.: *B-1569*
Shamirzaev K.: *B-1379*
Shanahan M.: *B-0657*
Shanbhag S.: *B-0300, B-1114*
Shankar A.: *B-1324*
Shanshan R.: *B-1006*
Shapira N.: *B-0731*
Sharaev M.: *B-1037*
Shariati S.: *B-1589*
Sharifian H.: *B-0635*
Sharma N.: *B-0079, B-1543, SY 5*
Sharma P.: *B-0820*
Sharma R.: *B-0640, B-1207*
Sharma S.: *B-1797*
Shaw B.: *B-1140*
Shawali H.A.S.: *B-0212*
Shayesteh-Kheslat R.: *B-0963*
Sheehan M.: *B-0265*
Sheerins O.: *B-1307*
Shelmerdine S.C.: *A-0794, B-0606, B-1319*
Shem S.L.: *B-1109*
Shen J.: *B-0508*
Shen M.-T.: *B-0596, B-1674, B-1680, B-1681, B-1820, B-1825*
Shen Q.: *B-1272*
Shen W.: *B-1371, B-1534*
Shen X.X.: *B-1152*
Shi K.: *B-1618, B-1681, B-1820, B-1869*
Shi Q.: *B-0611*
Shi R.: *B-0218, B-1676*
Shi R.-Y.: *B-1816*
Shi X.: *B-1246*
Shi Z.: *B-0217, B-0754*
Shimada Y.: *B-0482*
Shimomura Y.: *B-1832*
Shin H.J.: *B-0334*
Shin H.-J.: *B-0335*
Shin K.M.: *B-0942*
Shin K.S.: *B-1538*
Shin M.G.: *B-0741*
Shin S.: *B-0942*
Shinohara K.: *B-0829*
Shirasaka T.: *B-0484*
Shitakubo Y.: *B-0648*
Shivalingappa S.S.: *B-1269*
Shokry A.: *B-0693, B-1232*
Shokry S.: *B-0693*
Shorikov M.: *B-0395*
Shotar E.: *B-1574*
Shpektor A.V.: *B-1314*
Shu S.: *B-1124*
Shuxia W.: *B-0363*
Sibahi A.: *B-1168*
Sibon I.: *B-1415*
Siddique M.M.: *B-0351, B-0617, B-1874*
Siddique U.: *A-1082*
Siddiqui I.A.: *B-1027*
Sidhu P.S.: *A-0040, A-0293, A-0334, A-0847, B-1685*
Sidorenkov G.: *B-1008*
Siebenrock K.: *B-0571, B-0896, B-0897, B-0901*
Siebert S.: *B-1402*

Siedek F.: *B-0314, B-0461*
Siegel E.L.: *A-0126*
Siegel F.: *B-0022*
Siemund R.: *B-1197*
Siena S.: *B-0991, B-1028*
Sieren M.: *B-0202, B-0225, B-0349*
Sievers H.-H.: *B-0202*
Šifer K.K.: *B-1608*
Sigl B.: *B-1553*
Signa S.: *B-0341*
Sijbers J.: *B-1127*
Sijens P.: *B-0704*
Sijens P.E.: *B-1565*
Silva F.: *B-0860*
Silva J.: *B-0283*
Silva M.: *A-0051, A-0601, B-0143, B-1029, B-1858*
Silva V.M.F.: *B-0412*
Silvestre M.: *B-0006*
Sim J.Y.: *B-1285*
Simão D.M.: *B-0658*
Simbrunner B.: *B-1377*
Simelius-Nieminen S.: *B-0888*
Simeonov G.: *A-0409*
Simic A.: *B-1871*
Simmler R.: *SY 15*
Si-Mohamed S.A.: *B-0311, B-0731, B-1116, B-1214, B-1852*
Simon J.: *B-0786*
Simonetti G.: *B-1521*
Simonovsky M.: *B-0302*
Singer G.: *B-1361*
Singh A.: *B-0714, B-1132, B-1133*
Singh D.K.: *B-1291, B-1293*
Singh M.: *B-1382*
Singh S.: *B-0169*
Singh T.: *B-1382, B-1901*
Singla V.: *B-1382, B-1901*
Sinha S.: *B-1095*
SinhaRay R.: *B-0142*
Sinitsyn V.E.: *A-0243, B-0730, B-1314, B-1819, B-1822*
Sipos D.: *B-0031, B-0728, B-0882*
Sirli R.: *B-1533*
Sirlin C.B.: *B-1536*
Sironi S.: *B-1310, B-1372, B-1457*
Siveke J.: *B-0615*
Sjöberg J.: *A-0184*
Skaane P.: *A-0762*
Skardelly M.: *B-0093*
Skehan S.: *A-0607, B-0633*
Sklair M.: *B-1569*
Skoczylas T.: *B-1482*
Skripnik A.: *B-1505*
Škrk D.: *B-1073*
Skrobas U.: *B-0538*
Skrobic O.: *B-1871*
Skrzyński W.: *B-1477*
Skvortcova T.: *B-1164*
Slehria A.U.: *B-0271*
Smeets A.: *B-1803*
Smet M.H.: *B-1318*
Smetanina N.: *B-1326*
Smidt M.: *B-1170*
Smirnov A.: *B-1037*
Smirnova A.V.: *B-0091*
Smit E.J.: *B-0706, B-1850*
Smit J.W.A.: *B-0588*
Smit L.: *B-0632, B-1635*
Smith A.: *B-0357, B-1473*
Smith J.: *B-1384*
Smith L.: *B-1688*
Smith-Bindman R.: *B-0015, B-1320*
Smithuis R.: *A-1040*
Smits M.: *A-0440, A-1031, B-0908*
Snaith B.: *A-0863*
Snoeckx A.: *A-0803, B-0145*
So C.W.: *B-1856*
Soares F.: *B-1359*
Sobhy T.: *B-0355*
Sobieszczanska M.: *B-0039*
Sobrido C.: *B-0294*
Socratous A.: *B-0324*
Sodano C.: *B-1570*
Sodhi K.S.: *B-0169, B-1224*
Sodmann P.: *B-0022*
Soejoko D.S.: *B-1839, B-1842*
Soens J.: *B-0473*
Soesbe T.C.: *B-0391, B-0792*
Soffer S.: *B-1186*
Sofikitis N.: *B-1660*
Sofue K.: *B-1145, B-1146*
Sohaib A.: *A-0093*
Sokolska M.: *B-0712*
Sokurenko M.: *B-0608, B-0609*
Solaz Solaz J.: *B-0003*
Solbiati L.: *B-0530*
Soldi M.: *B-0783, B-1212*
Soligo E.: *B-0367*
Sollini M.: *B-1636*
Solopova A.E.: *B-0173, B-0180*
Solovev M.: *B-0350*
Solty K.: *B-0764*
Solyanik O.: *B-1400*
Somma F.: *B-0564*
Sommer G.: *B-0310, B-0904, B-1030, B-1211*
Sommer N.N.: *B-1773*
Sommer W.: *B-0022, B-1773*
Sommerwerck U.: *B-1411*
Son J.: *B-1766*
Song B.: *B-0074, B-0356, B-0405, B-1044, B-1471, B-1535, B-1764*
Song C.: *B-1252, B-1256*
Song G.W.: *B-0796*
Song I.S.: *B-1538*
Song J.: *B-1126*
Song L.: *B-0423, B-0430, B-1409*
Song S.E.: *B-1177*
Song T.: *B-0223*
Song W.: *B-0423, B-0430, B-1409*
Song Y.G.: *B-0775*
Song Y.S.: *B-0449*
Sonnemans L.: *B-0068*
Soo S.W.: *B-0475*
Sorantin E.: *A-0142, B-1361*
Sørensen I.J.: *B-0520*
Soriano I.: *B-0064*
Sorrentino A.: *B-1139*
Sosna J.: *B-0133, B-0313*
Soultatos A.: *B-1530, B-1837*
Sousa M.C.: *B-1494*
Sousa P.: *B-0658, B-0907*
Soydan L.: *B-0295*
Soyer P.: *A-0811, B-0155*
Sozzi G.: *B-0143*
Spadarella G.: *B-0199*
Spahr N.: *B-0558*
Späth L.: *B-1240*
Specht S.: *SY 28*
Spekreijse J.: *B-0685*
Spence L.D.: *B-1056*
Sperl J.I.: *B-1030*
Spiliopoulos S.C.: *A-1065*
Spinarelli A.: *B-0676*
Spinelli C.: *B-0017*
Spinelli E.G.: *B-1696*
Spinhoven M.J.: *B-0145*
Spink C.: *B-0119*
Splendiani A.: *B-0544, B-1550, B-1556*
Sponza M.: *B-0076, B-0967*
Spoor D.S.: *A-0017*
Sporea I.: *B-1533*
Sprinkart A.M.: *B-1513*
Spurny M.: *B-0051*
Srinivasan R.: *B-1901*
Srivastava D.N.: *B-1577*
Srivastava S.: *B-0674*
Staal F.: *B-1049*
Stabile A.: *B-0159, B-0443*
Stabile Ianora A.A.: *B-0329, B-0676*
Stachura E.: *B-0874*
Stacul F.: *A-0725, B-0368, K-13*
Stadlmann A.: *B-1377*
Stafrace S.: *A-0276*
Stahnke T.: *B-0237*
Staibano S.: *B-1626*
Stajgis M.: *A-0152, A-0433*
Stamati G.: *B-1010*
Stampanoni M.: *B-1792*
Stange R.: *B-0764*
Stankeviciene J.: *B-1757*
Stanuch M.: *B-1583*
Stanzione A.: *B-0668, B-0738, B-0823, B-1392, B-1684*
Stáreková J.: *B-0590, B-0679, B-1682*
Starmans M.P.A.: *B-0067, B-0908, B-1279*
Stättner S.: *B-0102*
Staub D.: *SY 17*
Stavolo C.: *B-0104*
Stavrinides V.: *B-0443*
Stavrinou P.: *B-0756, B-0988*
Stecco A.: *B-0367, B-0798, B-1262*
Stefano A.: *B-0418*
Steffen F.: *B-0108*
Steffen I.: *B-1024, B-1374*
Steiding C.: *B-1541*
Steinbach L.: *A-0804, A-0806*
Steinbach R.: *B-0781, B-0789*
Steinfelder E.: *A-0630*
Steinkohl F.: *B-1076*
Stelzmüller M.-E.: *B-0374*
Stenzel M.: *SY 23*
Stephan T.: *B-0875*
Steri L.: *B-0935, B-0940*
Stern E.J.: *K-38*
Steveson C.: *B-0300, B-1114*
Stieltjes B.: *A-0394, B-0193, B-0310, B-0816, B-0904, B-0946, B-1030, B-1092, B-1211*
Stierstorfer K.: *B-0312*
Stiller W.: *B-1610*
Stilo S.: *B-0738, B-0823*
Stinson E.: *B-0438*
Stober S.: *B-0802*
Stocking K.: *B-0917*
Stöcklein S.: *B-0188, B-0749, B-0927, B-1782*
Stöcklein V.: *B-0094*
Stoecklein S.: *B-0094*
Stoeger A.M.: *B-1150*
Stoia V.: *B-0564*
Stoker J.: *A-0835*
Stollberger R.: *B-1619*
Stölting M.: *B-1125*
Stolzmann P.: *B-0224, B-0228*
Stopchinski M.: *B-1650*
Storz C.: *B-0589, B-0945, B-1514, B-1515*
Stoupis C.: *A-0768*
Stöver T.: *B-0296, B-1247, B-1257*
Stowe J.G.: *A-1043, B-1699, B-1702*
Stoyanova D.P.: *B-1052*
Stramare R.: *B-0512, B-0522, B-1060, B-1364, B-1540*
Stranieri G.: *B-0824, B-1144*
Stratakis J.: *B-1593*
Sträter A.S.: *B-1312*
Streekstra G.J.: *B-0137*
Streetly M.: *B-0351*
Strehlow J.: *B-1444*
Streitparth F.: *B-1184*
Strickland N.H.: *A-0013*
Strobbe L.J.: *B-0545*
Stroeder J.: *B-1720*
Strøm B.: *B-1712*
Stroszczynski C.: *A-0206*
Strudwick R.M.: *B-0033*
Stückle C.A.: *B-1289*
Stutz S.: *B-1733*
Su F.: *B-0718*
Su L.: *B-1788*
Su R.: *B-1430*
Su T.: *B-1246, B-1633, B-1813*
Su X.: *B-1426*
Su Y.: *B-0343*
Suardi N.: *B-1300*

Suarez Vega V.M.: *B-0294*
Subesinghe M.: *B-0142*
Sucapane P.: *B-1550, B-1552, B-1556*
Suchanek V.: *B-0597*
Sudarkina A.: *B-1005*
Sudarski S.: *B-0106, B-0735*
Sudderuddin S.: *B-0177*
Sudre C.H.: *B-1688*
Suganchand Rikabchand S.K.: *B-0128, B-0637, B-0855*
Sugihara N.: *B-0424, B-0579*
Sugimura K.: *B-0421*
Sugiyama M.: *B-1722*
Sugiyama S.: *B-1832*
Suh Y.J.: *B-1800*
Sui X.: *B-0144, B-0423, B-0430, B-1409*
Sukhovei L.: *B-0367*
Sukupova L.: *B-1602*
Suleiman M.E.: *B-1844*
Sulieman A.A.: *B-1486*
Sulla Lupinacci F.A.: *B-1365*
Sullivan Y.: *B-1193*
Sultanov A.: *B-1379*
Sum R.: *B-1815*
Suman S.: *B-1291, B-1293*
Summers R.M.: *B-1640*
Sun C.C.: *B-1654*
Sun H.: *B-1426*
Sun I.-J.: *B-0758*
Sun W.: *B-0421*
Sun Y.-Z.: *B-0757*
Sun Z.: *B-1065*
Sun Z.-Y.: *B-1475*
Sundell V.-M.: *B-0998*
Sundin A.: *B-0523*
Sung J.S.: *B-1804*
Sung P.: *B-0773*
Suranyi P.: *B-0914*
Suri D.: *B-0169*
Suri J.: *B-0036*
Surov A.: *B-0175*
Sutton E.J.: *B-0719*
Suzuki S.: *B-0660*
Suzuki T.: *B-0742*
Svanteson M.: *B-0480*
Svedström E.: *B-0645*
Sverzellati N.: *A-0453, A-0700, A-0733, B-0143, B-0307, B-1029, B-1858*
Swain B.M.: *B-1253*
Swamy S.S.: *B-0063*
Swarup M.S.: *B-0977*
Swinnen J.: *B-0473*
Syed S.: *B-1402*
Sykes P.: *B-1117*
Syysmäki H.: *B-1115*
Szatmari P.: *B-1185*
Szilagyi B.: *B-1432*
Szilveszter B.: *B-0786*
Sztankay B.: *B-1076*
Szucs P.: *B-1432*

### T

Tabbara A.: *B-1168*
Tacchini S.: *B-0256*
Tacher V.: *B-0252*
Tachibana A.: *B-1365*
Tack D.: *A-0356, A-0916*
Tadepalli M.: *B-1753*
Taghavi M.: *B-1582*
Tagliafico A.: *A-1047, B-0724, B-1173, B-1178, B-1745*
Tagliamonte M.: *B-0937*
Tagliati C.: *B-1719*
Taha N.: *B-0400*
Taher Rakei Z.: *B-0238*
Tahir E.: *B-0590, B-1682*
Tahmassebi A.: *B-1328*
Taibbi A.: *B-1529*
Taibi A.: *B-1846*
Taina M.: *A-0524*
Takahashi H.: *B-0293*
Takahashi S.: *B-1145, B-1146*
Takase K.: *B-0421*
Takashima H.: *B-1735*
Takehara Y.: *B-1722*
Takenaka D.: *B-0424, B-0425, B-1755*
Takenaka M.: *B-0408*
Taki Y.: *B-1329*
Takx R.A.: *B-1013*
Talarczyk S.: *B-1289*
Talbot P.: *B-0916, B-0919*
Taleb H.A.: *B-0371, B-1284*
Talei Franzesi C.: *B-1310, B-1372, B-1457*
Tali E.T.: *A-0675*
Tam K.Y.: *B-1798*
Tam M.: *A-0448*
Tamada D.: *B-0404*
Tamandl D.: *B-1875*
Tamburrano C.: *B-0864*
Tamim H.: *B-1168*
Tan L.: *B-1440, B-1441*
Tan L.K.: *B-1233, B-1274*
Tan M.P.: *B-1274*
Tan Y.: *B-0858*
Tanaka H.: *B-0293*
Tanaka U.: *B-1145, B-1146*
Tanamala S.: *B-0748, B-0750, B-0755, B-0981*
Tandon N.: *B-0640*
Taneja A.: *B-0513*
Tanenbaum L.: *SY 6*
Tang B.: *B-1331, B-1332*
Tang C.X.: *B-0784*
Tang J.: *B-0472*
Tang L.: *B-0462, B-0611, B-0807, B-1280*
Tang P.Y.: *B-1798*
Tang S.: *B-1036, B-1333*
Tang W.: *B-0429, B-1752, B-1785*
Tannast M.: *B-0571, B-0896, B-0897, B-0901*
Tanner J.A.: *B-1795*
Tanno Y.: *B-0949*
Tanoi C.: *B-0185*
Tanriover N.: *B-0986*
Tantawy H.: *B-0355*
Tanturri de Horatio L.: *A-0795*
Tanyildizi Y.: *B-1241*
Tao B.: *B-1330, B-1427*
Tao G.: *B-0805*
Tao H.: *B-1131*
Taouli B.: *A-0335, SY 31*
Tarantini G.: *B-0526, B-1097*
Tarbell N.: *B-1227*
Tartari C.: *B-0272*
Tartari S.: *B-0272*
Tartaro A.: *B-0203*
Tasaki A.: *B-1899*
Tasu J.-P.: *SY 31*
Tatard-Leitman V.: *B-1116*
Tateishi U.: *B-0471, B-1796*
Tatewaki Y.: *B-1329*
Taupitz M.: *B-0441, B-0762*
Tavakoli Taba S.: *B-1789*
Tavanti L.: *B-1205*
Tavare A.N.: *B-1004*
Tavares de Sousa M.: *B-0601*
Taylor A.M.: *A-0483, B-0606*
Taylor S.: *B-1324*
Taylor S.A.: *A-0162*
Tedesco G.: *B-0455*
Teerasamit W.S.H.R.T.V.S.: *B-1769*
Tegos T.: *B-1685*
Teh J.: *A-1089*
Teichgräber U.K.: *B-0201*
Teixeira S.R.: *B-0604*
Telegrafo M.: *B-0329*
Teles P.M.: *B-1494*
Tello K.: *B-1863*
Temel E.: *B-1453*
Ten J.I.: *B-1195*
Teng G.-J.: *B-1033, B-1891*
Tengberg L.T.: *B-0275*
Tennstedt P.: *B-1089*
Terada M.: *B-0185*
Terauchi K.: *B-1180*
Terboven T.: *B-0106, B-0735*
Terecoasa E.: *B-0191*
Tereshenko G.: *B-1061, B-1326*
Ter-Karapetyan A.: *B-0420*
Ternovoy S.K.: *B-1261*
Terranova M.C.: *B-1827*
Terrone C.: *B-0879, B-1139*
Tesche C.: *B-0301, B-0784, B-0785*
Tessarin G.: *B-1010, B-1011*
Testino N.: *B-1139*
Teuwen J.: *B-1523, B-1564*
Thabet D.B.: *B-0117*
Thaha R.: *B-1132, B-1133*
Thaiss W.: *B-0620, B-0628*
Thakur M.: *B-1450*
Thakur S.: *B-1175, B-1176*
Thali M.: *B-0068, B-1516*
Tham J.-L.: *B-0917*
Thanosoulis K.: *B-0183*
Theilig D.: *B-1374*
Theodoridis A.: *B-0183*
Theotokas I.: *B-1378, B-1530, B-1537, B-1837*
Thestrup K.-C.D.: *B-0442*
Theysohn J.: *B-0116, B-1740*
Thiele F.: *B-0710, B-0756, B-0988, B-1424*
Thiele H.: *B-0196, B-0379, B-1683*
Thierfelder K.: *B-0088, B-0237, B-0262, B-0713*
Thierfelder K.M.: *B-0259, B-1286*
Thilander Klang A.: *B-1900*
Thoduka S.: *B-0558*
Thoeny H.C.: *A-0377, A-0432, A-1074*
Thomas B.: *B-1647*
Thomas C.: *B-0954, B-1355, B-1368, B-1726*
Thomas D.: *B-1513*
Thomassin-Naggara I.: *A-0073, A-0424, B-1894*
Thomeer M.G.: *B-0067, B-0908*
Thommassen K.: *B-0168*
Thompson A.: *B-0079*
Thompson J.: *A-0779*
Thomsen F.B.: *B-0154*
Thomsen H.S.: *A-0723, B-0154, B-0442, B-0520*
Thon N.: *B-0094*
Thong P.M.: *B-1012*
Thorisdottir S.: *B-0101, B-0955*
Thornton A.: *B-1387*
Thornton J.: *B-0265, B-0267*
Thrall J.H.: *A-0595*
Thrane K.J.: *B-0168*
Thuering T.: *B-0997*
Thurnher M.M.: *A-0157, A-0678, A-0757, A-0831, A-1075, B-0534*
Thygesen J.: *B-1465*
Thywissen T.: *B-0473, B-0915*
Tian J.: *B-1467*
Tian S.: *B-1742*
Tiberia F.: *B-0935, B-0940*
Tiddens H.A.: *B-0170, B-0171*
Tiedt S.: *B-0259, B-0713*
Tiepolo M.: *B-1010*
Tillier C.: *B-0326*
Tilliridou V.: *B-1316*
Timmer M.: *B-1424*
Timpani A.: *B-0618*
Ting H.H.: *B-1021*
Tinguely P.: *B-1438, B-1442*
Tini P.: *B-1275*
Tinoco Alvim de Souza F.: *B-0129*
Tipaldi L.: *B-1724*
Tipaldi M.A.: *B-0007, B-0008, B-0849, B-1809*
Tiralongo F.: *B-0418, B-1405*
Tischendorf P.: *B-1204*
Tissier F.: *B-1511*
Titova A.M.: *B-0791*
Tiu C.: *B-0191*
Tjan-Heijnen V.C.: *B-0083*
Tjan-Heijnen V.C.G.: *B-1158*
Tkachev A.: *B-0091*
Tkachev S.: *B-0621*
Tofanelli L.: *B-1644*

Toia P.: *B-1313, B-1444*
Tolan D.J.M.: *A-0488*
Tolmunen T.: *B-1272*
Toma M.K.: *B-0860*
Toma P.: *B-1327*
Tomar S.: *B-1291, B-1293*
Tomehy M.: *B-1105*
Tomerak R.H.: *B-0162*
Tomita E.: *B-0642, B-0648*
Tomita H.: *B-0292, B-1629*
Tomita N.: *B-1329*
Tomiyama N.: *B-0293*
Tomppert A.: *B-1245*
Tonelli P.: *B-0470*
Tong O.: *B-0916, B-0919*
Tong T.: *B-0359*
Tong X.-W.: *B-1864*
Tonn J.-C.: *B-0094*
Tonutti M.: *B-0464, B-1521*
Toomey R.: *B-1354, B-1704*
Tootell A.K.: *B-1708*
Topcuoglu E.D.: *B-0929*
Topcuoglu O.M.: *B-0557, B-0929*
Topolski A.: *B-1497*
Torkzad M.R.: *B-1046*
Torlone S.: *B-0544, B-0595, B-1309, B-1550, B-1556, B-1623, B-1817*
Torres F.: *B-0570*
Torresin A.: *B-0991, B-1028*
Torricelli P.: *B-1381*
Torrisi S.E.: *B-0418, B-1405*
Tortora D.: *B-0930*
Torzilli G.: *B-0530*
Tosson A.M.: *B-0162*
Totaro R.: *B-0544*
Toth D.: *B-0219*
Totobenazara J.-L.: *B-0252*
Toufique Y.: *B-1601*
Tourdias T.: *A-0134, B-1415*
Toyomasu Y.: *B-1101, B-1102*
Tralli G.: *B-0272*
Trambaiolo Antonelli C.: *B-1658*
Tran T.T.: *B-0492*
Trattnig S.: *A-0249, A-0252, B-0055*
Traub-Weidinger T.: *B-0234*
Travali M.: *B-0943, B-1648*
Traykova N.I.: *A-0823, K-23*
Trebeschi S.: *B-0685, B-1582*
Treier R.: *B-1196*
Treitl K.M.: *B-1773*
Trenczek T.E.: *B-0510*
Treutlein C.: *B-1868*
Tri Wigati K.: *B-1838*
Triana G.: *B-0570*
Trianni A.: *A-0049, K-29*
Triantopoulou C.: *A-0097*
Tridente D.M.: *B-0580*
Trimboli R.M.: *A-0856, B-0465, B-1352, B-1561, B-1723*
Trinci M.: *B-0732, B-0733*
Trinkmann F.: *B-0587*
Trionfera G.: *B-0935, B-0940*
Tripathi P.: *B-1339, B-1479*
Tripathi R.: *B-0691*
Triphan S.: *B-0420*
Trisoglio A.: *B-0367*
Tritella S.: *B-0490*
Trofimenko I.: *SY 24*
Trofimov I.: *B-0844, B-0845*
Trofimova T.: *B-0608, B-0609*
Trojani V.: *B-0993*
Trojanowska A.: *A-0318, A-0721*
Tromba G.: *B-1610, B-1789*
Trombatore C.: *B-0943*
Trong Khoan L.: *B-1305*
Tropine A.: *B-1241*
Trufanov G.E.: *B-0791, B-1505*
Truwit C.: *SY 16*
Trzmielewska E.: *B-0039*
Tsapaki V.: *A-0787*
Tschan F.: *B-1699*
Tschauner S.: *B-1361*
Tse D.: *B-0330*
Tse K.Y.: *B-0370*
Tsetis D.K.: *A-0207*
Tsili A.: *B-1660*
Tsoi C.: *B-1292*
Tsoriev A.E.: *SY 24*
Tsoumakidou G.: *A-0512*
Tsubakimoto M.: *B-0292, B-1629*
Tsuchiya J.: *B-0471, B-1796*
Tsuchiya K.: *B-1167*
Tsuchiya M.: *B-1180*
Tsuda M.: *B-1489*
Tsui S.: *B-0655*
Tsuruda K.: *B-0916, B-0919*
Tu Y.-C.: *B-0758*
Tucciariello R.M.: *B-1843*
Tucker L.F.: *B-1793, B-1794*
Tuilier T.: *B-0261*
Tulupov A.: *B-1266*
Tunariu N.: *A-0196*
Tuncyurek O.: *B-0210*
Tuohy B.: *B-0761*
Turan A.: *B-1206*
Tureli D.: *B-0286*
Turkay R.: *B-0877*
Turkbeyi B.: *B-0837*
Turkmenoglu T.T.: *B-1206*
Turnaoglu H.: *B-1854*
Turno-Krecicka A.: *B-1265*
Turowski B.: *B-0983, B-1553*
Turpini E.: *B-0373*
Turturici L.: *B-1205*
Tyagi N.: *B-0323*
Tyurin I.E.: *B-0621, SY 24*
Tzavara C.: *B-1388*
Tzedakis A.: *B-1593*

### U

Uberoi R.: *A-0666*
Ublinskiy M.: *B-0187, B-1038, B-1778, B-1887*
Uchida K.: *B-1167*
Ud Din N.: *A-1078*
Uder M.: *B-0289, B-0426, B-0474, B-0551, B-0701, B-0702, B-0703, B-1088, B-1245, B-1259, B-1321, B-1518, B-1777*
Uematsu T.: *B-0550*
Ueno Y.: *B-1003, B-1145, B-1146*
Ugga L.: *B-0870, B-1626*
Uhlenbrock D.F.P.: *B-1289*
Uhlig J.: *B-1544*
Uhrig M.: *B-0135, B-0319, B-0321*
Ukkola L.: *B-1192*
Ukmar M.: *B-0536*
Ulas S.-T.: *B-0514*
Uller W.: *B-0739*
Ullrich T.: *B-0158, B-0161, B-0327, B-0671, B-0828, B-1462*
Ulrich S.: *B-1866*
Ulus S.: *B-1546*
Umar M.S.: *B-1109*
Umutlu L.: *A-0131, A-0282, B-0231, B-0575, B-0669, B-0670, B-0671, B-0673, B-0690, B-0803, B-0819, B-0875, B-0913, B-1411, B-1456, B-1461, SY 2a*
Ünal Ö.: *B-0603*
Unno N.: *B-1722*
Unterberg A.: *B-0505, B-1419*
Upadhyaya A.: *B-0990*
Upreti L.: *B-0977*
Uprus M.-K.: *B-1853*
Urbani L.: *B-0526*
Urbani M.: *B-0085, B-0554*
Urru A.: *B-1054*
Uspenskiy V.: *B-1505*
Utkin L.V.: *B-1760*
Utsunomiya K.: *B-1832*
Uyanik-Ünal K.: *B-0374*
Uysal A.: *B-0700*
Uziel Y.: *B-0133*

### V

Vaccaro A.: *B-1872*
Vagnoni V.: *B-0873*
Vaidhya K.: *B-0912*
Vaidya A.: *B-0910*
Vaidya S.: *B-0912*
Vaidyanathan S.: *B-0666, B-0667*
Vaimel J.: *B-1853*
Vaiphei K.: *B-1095*
Vakhidova N.: *B-1379*
Valbusa G.: *B-0304*
Valdora F.: *B-1178, B-1745*
Válek V.: *B-0232*
Valente I.: *B-1262*
Vallati G.E.: *B-0113, B-0563, B-0846, B-1435*
Valletta R.: *B-0458, B-0937, B-1389*
Vallone G.: *B-0333*
Valotto C.: *B-1138*
Valvo F.: *B-0373*
van Assen M.: *B-0301, B-0780, B-0781, B-0787, B-0789, B-0820, B-0914, B-1215, B-1217, B-1721*
van Beek E.J.R.: *B-1316, B-1751, B-1759*
van Beers B.E.: *A-0333*
Van Besien J.: *B-0157*
Van Buchem M.A.: *A-0136, A-0764*
Van den Bergh F.: *B-0279*
van den Boogaard P.: *B-1506*
van den Brekel M.W.M.: *B-0632, B-1635*
van den Hauwe L.: *A-0161, B-0753*
van der Heide U.A.: *B-1582*
Van der Lugt A.: *B-0171, B-0258, B-0982*
van der Molen A.J.: *A-0724, SY 19*
van der Most A.: *B-1592*
van der Poel H.: *B-0326*
van der Toorn A.: *B-0215*
Van Der Velde L.: *B-1066*
Van der Voet J.: *B-1136*
van der Voort S.R.: *B-0067, B-0908, B-1279*
van der Werf N.R.: *B-0311, B-0317*
van der Wiel-Kooij E.C.: *B-0170*
van der Zijden T.: *A-0525, B-0279*
van der Zwan A.: *B-0215*
van Dieren J.M.: *B-1483*
van Diest P.J.: *A-0735, A-0736*
van Dijk G.: *B-1350*
van Dinther J.: *B-0290*
van Doormaal T.: *B-0215*
Van Dyck P.: *B-1127, B-1509*
Van Eden W.J.: *B-0182*
van Elmpt W.: *B-1484*
Van Engen R.: *B-0255, B-1838*
van Es A.: *B-0258*
van Eyk H.J.: *B-0588*
Van Giang B.: *B-1305*
Van Ginneken B.: *A-0325, A-0393, SY 4*
Van Goethem J.: *A-0755, A-0948, B-0753*
Van Gompel G.: *B-0136, B-1596, B-1597, B-1628*
van Griethuysen J.J.M.: *B-1484*
van Hamersvelt R.W.: *B-0311, B-0317, B-0788*
Van Hecke W.: *B-0984*
Van Hoyweghen A.: *B-0145*
van Kroonenburgh A.M.J.L.: *B-1447, B-1625*
van Kuijk S.M.J.: *B-1447, B-1625*
Van Leemput K.: *A-0266*
van Leeuwen M.S.: *B-0766*
Van Mieghem C.: *B-1679*
van Muilekom E.: *B-0326*
van Nijnatten T.: *B-1170*
van Ommen F.: *B-0192*
Van Ongeval C.: *A-0427, A-1085, B-0251, B-0473, B-0915, B-1803, SY 2c*
van Ooijen P.M.A.: *A-0144, A-0558, A-0628, B-0309, B-0574, B-1008, B-1756*
van Os M.: *A-0442*
van Overhagen H.: *B-0222*
Van Phuoc L.: *B-1305*
van Rijswijk C.S.P.: *B-1286*
van Spijker G.: *B-1790*

van Steekelenburg J.: *B-0298*
van Straten M.: *B-1598*
Van 't Sant I.: *B-0182*
van Turenhout S.T.: *B-1483*
van Walsum T.: *B-0258*
van Weijnen A.: *B-0298*
van Wijk Y.: *B-1179*
Van Zwam W.: *B-0258*
Vanacker P.: *B-0279*
Vanbeckevoort D.: *B-0494*
Vancheri C.: *B-0418, B-1405*
Vancoillie L.: *B-1840, B-1845*
Vancu M.: *SY 2b*
Vande Berg B.: *B-0894, B-1507*
Vandecaveye V.: *A-0130*
Vandertop W.P.: *B-0096*
Vanhavere F.: *A-0749*
Vanhevel F.: *B-1127*
Vanhoenacker F.M.H.M.: *A-0691*
Vanhoenacker P.: *B-1679*
Vani V.: *B-0527, B-1082, B-1093*
Vanmarkenlichtenbelt W.D.: *B-0220*
Vanninen R.: *B-1272*
Vaño E.: *A-0747, B-1195*
Vanspauwen R.: *B-0290*
Vanzulli A.: *B-0991, B-1028*
Vara Taranikanti S.: *B-0640*
Vardhanabhuti V.: *B-0178*
Varetto G.: *B-0038*
Varga V.: *B-0031*
Vargas H.A.: *A-0286, A-0330, B-0528*
Varga-Szemes A.: *B-0780, B-0914, B-1215, B-1217*
Varotto A.: *B-1060, B-1364*
Varrassi M.: *B-1552, B-1579*
Vasconcelos C.C.F.: *B-0542*
Vasilev Y.: *B-1260*
Vasilevska Nikodinovska V.: *B-0050*
Vasquez Cuadra S.J.: *B-1724*
Vassallo E.: *A-0570*
Vassallo L.: *B-1828*
Vasyliv I.: *B-0584*
Vavasseur A.: *B-1188*
Vaysse C.: *B-0478*
Vaz P.: *B-1494*
Vázquez E.: *A-0559, A-0679, B-0340, B-1231*
Vedsted P.: *B-1661*
Veglia S.: *B-0948, B-1499*
Veillon F.: *A-0568*
Vela L.: *B-1303*
Velasco González A.: *B-0266*
Veldhoen S.: *B-0337*
Veldhuis R.N.J.: *B-1756*
Veldhuis W.B.: *A-0737*
Veldkamp W.J.H.: *B-0137, B-0255, B-1592*
Velivasaki M.: *B-1593*
Velthuis B.K.: *A-0147, B-0705*
Veltri A.: *B-0824, B-1144*
Veltri G.: *B-0109, B-0110, B-0114, B-1812*
Velzing J.D.R.: *B-0182*
Venancio J.: *B-1494*
Venetucci P.: *B-0738*
Venkatesan B.: *B-0717*
Venkatesh S.K.: *A-0107*
Venkatraman A.: *B-0990*
Venturi G.: *B-1198*
Venturini E.: *B-0256, B-0971*
Venturini M.: *B-0369, B-0851, B-1302, B-1439*
Venugopal V.: *B-0755, B-0910, B-0990, B-1397, B-1750, B-1753*
Venugopal V.K.: *B-0572, B-0912, B-1132, B-1133, B-1892*
Verbeek J.F.M.: *B-0181*
Verbeke S.: *A-0479*
Verde F.: *B-1385, B-1626*
Vergetidou M.: *B-1388*
Verhagen M.V.: *B-1324*
Verhoef C.: *B-1582*
Verma H.: *B-0616*
Verma P.: *B-0760*
Verma R.: *B-0777, B-1335, B-1558*
Verna S.: *B-0622*
Vernooij M.: *A-0767, A-0830*
Vernooij M.W.: *B-0171, B-1690*
Vernuccio F.: *B-0070, B-0533, B-1140, B-1768, B-1771*
Verrone G.B.: *B-0975*
Verschakelen J.A.: *A-0708, A-0984*
Verstraete K.: *A-0031, A-0178*
Vértes M.: *B-0958*
Verwoolde R.: *B-1699*
Verzuijlen I.: *B-1066*
Vianna Cançado A.: *B-0129*
Vianna Cancado L.: *B-0129*
Vicens Zygmunt V.: *B-1404*
Vicente Bártulos A.: *B-0274*
Vicentin I.: *B-1028*
Vicini S.: *B-0935*
Vidmar J.: *B-1608*
Viergutz T.: *B-0106, B-0735*
Viet N.K.: *B-1012*
Viganò L.: *B-0530*
Vignale D.: *B-1216*
Vignero J.: *B-1845*
Vijayakumar D.H.: *B-1464*
Vijayananthan A.: *B-1902*
Vijlbrief O.D.: *B-0298*
Vikestad K.G.: *A-1044*
Vikram R.: *B-0134*
Viktil E.: *B-1047*
Vilanilam G.K.: *B-1169*
Vilar J.: *A-0357, A-0951*
Vilares Morgado P.M.: *A-0247*
Vilela P.: *A-0159*
Vilgrain V.: *A-0369, B-0848, SY 4*
Villa A.: *B-1151*
Villa R.: *B-0798, B-1071*
Villalta Santamaria M.: *B-0003*
Villanacci R.: *B-0211*
Villeirs G.: *B-0157*
Villeirs G.M.: *A-0091, A-0477, A-0482*
Vinayak S.: *A-0311, A-0315*
Vinci M.: *B-1327*
Vinnicombe S.J.: *A-0151*
Virelli R.: *B-0329*
Viriato A.C.: *B-0489*
Virshke E.: *B-0395, B-0844, B-0845*
Viselner G.: *B-0372, B-0373*
Visser J.J.: *B-0908*
Vit A.: *B-0076, B-0967*
Vitabile S.: *B-1827*
Vittoria De Martini I.: *B-1733*
Vivas Perez I.: *B-0111*
Viyannan M.: *B-1376*
Vliegenthart R.: *A-0939, B-0309, B-0586, B-0787, B-0789, B-1008, B-1215, B-1311*
Vo D.T.: *B-0214*
Vock P.: *A-0544*
Voena C.: *B-1833*
Vogel W.: *B-0632, B-0685, B-1635*
Vogel-Claussen J.: *B-0585, B-1201*
Vogl T.J.: *B-0117, B-0243, B-0296, B-0299, B-0434, B-0448, B-0794, B-0843, B-0847, B-1013, B-1204, B-1247, B-1257, B-1317, B-1362, B-1448, B-1472*
Voicu I.P.: *B-1327*
Vollenbrock S.E.: *B-1483*
Volpe A.: *B-1383*
Volpe S.: *B-0973*
Volterrani L.: *B-1275*
von Baer A.: *B-0061*
von Bredow C.-R.: *B-0510*
von Bredow Y.M.: *B-0510*
von Falck C.: *B-0681, B-1286*
von Gladiss A.: *B-0498*
von Knebel Doeberitz P.: *B-0301, B-0787*
von Knobelsdorff F.: *B-0589*
von Krüchten R.: *B-0019, B-1208*
Von Schulthess G.K.: *B-0817*
von Spiczak J.: *B-1616*
von Stackelberg O.: *B-0019, B-0051*
Voncken F.E.M.: *B-1483*
Vonder M.: *B-0311, B-0317, B-1311*
Voogd A.C.: *B-0083, B-1158*
Voormolen M.: *B-0279*
Vos M.: *B-0908*
Voskrebenzev A.: *B-0585, B-1201*
Voskuil M.: *B-0788*
Vourtsis A.: *B-0700, K-21*
Vroegindeweij D.: *B-1136*
Vu H.: *B-0698*
Vucetic J.: *B-0107*
Vuillemin-Bodaghi V.: *B-1507*
Vullierme M.-P.: *A-0238*
Vykoukal J.: *B-1669*

### W

Wacker F.: *B-0001, B-0585, B-0681, B-1201*
Wade A.: *B-0606*
Wagener J.: *B-0883*
Wagner M.: *B-1734*
Wagner W.: *B-1610*
Wakayama T.: *B-1722*
Wakelin S.: *B-0639*
Wald C.: *B-1016*
Wald R.M.: *B-1864*
Walker A.: *B-0760*
Wall A.: *B-1706*
Wallis M.G.: *A-0944, A-1029, B-0078, B-0079, B-0080, B-1801*
Wallner V.: *B-0925*
Wallström J.: *B-0324*
Walsh C.: *A-1052*
Walter S.: *B-0979*
Wang C.L.: *B-1148*
Wang D.-J.: *B-1057*
Wang F.: *B-0331, B-0810, B-1386*
Wang H.: *B-0062, B-0308, B-0718, B-0772, B-0779, B-0831, B-0907, B-1371, B-1857*
Wang J.: *B-0573, B-0810, B-1888*
Wang L.: *B-0804, B-1163, B-1531*
Wang L.-J.: *B-0103*
Wang M.: *B-0012, B-0405, B-0411, B-0854, B-1318, B-1535*
Wang Q.: *B-0216, B-0331*
Wang R.: *B-0715, B-1877*
Wang S.: *B-0018, B-0566, B-0745, B-0801, B-0911, B-0978, B-1230, B-1758*
Wang T.: *B-0984*
Wang W.: *B-0757, B-0814, B-1239, B-1426, B-1467, B-1835*
Wang W.-P.: *B-0452*
Wang X.: *B-1036, B-1074, B-1087, B-1893, B-1896, B-1897*
Wang Y.: *B-0012, B-0170, B-0320, B-0406, B-1165, B-1386, B-1633, B-1890*
Wang Y.N.: *B-0012, B-0785*
Wang Z.: *B-0216, B-0715, B-0751, B-0772, B-0801, B-0863, B-1040, B-1064, B-1792, B-1873*
Wang Z.L.: *B-0467*
Wangcharoenrung D.: *B-0827*
Ward E.: *B-0639*
Warfa M.: *B-1130*
Warier P.: *B-1753*
Warncke M.L.: *B-0590*
Warren A.: *B-0880*
Warren L.M.: *B-0920*
Washizuka F.: *B-0482*
Watanabe A.T.: *B-0696, B-0698*
Watanabe W.: *B-1167*
Watanabe Y.: *B-0293*
Waterton J.C.: *A-0060*
Wathén T.: *B-1717*
Watson T.A.: *B-1324*
Wawrzyniak P.: *B-1338*
Wazan M.: *B-1105*
Wdowik Q.: *B-1852*
Weaver J.: *B-0447*
Webb A.: *A-0910, B-0766, K-18*
Weber L.: *B-1197*

Weber M.: *B-0055, B-0307, B-0512, B-0522, B-1364, B-1549*
Weber M.-A.: *A-1035, B-0237, B-0344, B-0348, B-0567, B-1286, B-1502*
Weber S.: *B-1442*
Weber T.: *A-0231, B-0385*
Weder W.: *B-0577*
Ween B.: *B-1072*
Wegner F.: *B-0498*
Wei Y.: *B-0356, B-0857, B-0865, B-1764*
Weichert W.: *B-0615*
Weidekamm C.: *A-0920, A-0922*
Weihe E.: *B-1607*
Weikert T.J.: *B-0310, B-0816, B-0904, B-1030, B-1211*
Weiland E.: *B-0474, B-1563, B-1564*
Weinmann D.: *B-1321*
Weinreich G.: *B-1411*
Weinrich J.M.: *B-0679, B-1682*
Weise E.: *B-0698*
Weishaupt D.: *K-01*
Wei-Shin E.C.: *B-0534*
Weiss C.M.: *B-1154*
Weiss K.: *B-1542*
Weissinger M.: *B-0689*
Well L.: *B-1240*
Wells M.: *B-0447, B-1142*
Welte T.: *B-0585, B-1201*
Wen B.: *B-0969, B-1504*
Wen D.: *B-1588*
Wen L.: *B-0594*
Wen Q.: *B-0149, B-1308*
Wen Z.: *B-1861*
Weng Q.: *B-0308, B-0772, B-1126*
Wengert G.J.: *B-0716*
Wenkel E.: *B-0474, B-0551, B-0701, B-0702, B-0703, B-1518, SY 2d*
Wennmann M.: *B-0344, B-0348, B-1419*
Wenz H.: *B-0022*
Wenzel P.: *B-0378, B-0383, B-0591, B-1824*
Werncke T.: *B-0001*
Werner S.: *B-0146, B-0623*
Wesseling J.: *B-1158*
Westenberg J.J.: *B-1506*
Westerland O.A.: *B-0351*
Westerlinck H.: *B-0251, B-0915*
Weston M.: *A-0810, A-0821*
Weston W.: *B-1000*
Westphalen A.C.: *B-0829*
Wetscherek M.T.A.: *B-0379*
Wetter A.: *B-0116, B-0875*
Wetzl M.: *B-1321*
Whitlow C.T.: *A-1015*
Wibmer A.G.: *B-0323*
Wichmann J.L.: *B-0296, B-0434, B-0448, B-0794, B-1362, B-1472*
Wick W.: *B-0505, B-0539, B-1419*
Widell J.: *B-0491*
Widmann G.: *B-1599*
Wiech D.: *B-0688*
Wiedenmann F.: *B-0042*
Wiedenroth C.B.: *B-1863*
Wiedermann J.: *B-0677*
Wielandner A.: *B-0374, B-1617*
Wielema M.: *B-0704, B-1565*
Wielopolski P.: *B-1205*
Wielopolski P.A.: *B-0170, B-0171*
Wielpütz M.O.: *A-0567, B-0420, B-1610*
Wiemker R.: *B-1761*
Wienbeck S.: *B-1544, B-1789*
Wiesenfarth M.: *B-1077*
Wiesmüller M.: *B-0289, B-1245*
Wiestler B.: *B-0088*
Wigati K.T.: *B-1839, B-1842*
Wilczek M.: *B-0049*
Wildberger J.E.: *A-0715, B-0220, B-1787, SY 19*
Wildgruber M.: *B-1125*
Wildiers H.: *B-0559, B-0850, B-1803*
Wilkens H.: *B-0961*
Wilkinson L.S.: *B-0920*
Willemink M.J.: *B-0311, B-0317, B-0338, B-0624, B-0788*
Willenpart A.: *B-0811*
Willer K.: *B-1612*
Williams M.: *A-0717, K-25*
Williams M.C.: *B-1316*
Williams S.: *B-0950*
Williamson E.E.: *B-1729*
Williamson H.V.: *B-0533*
Wilson D.J.: *A-0180, A-0274, B-1290*
Wilson R.: *B-1605*
Windhaber S.: *B-0362, B-0364*
Wingerter A.: *B-1241*
Winkel D.J.: *B-0816, B-1092, B-1211*
Winkelmann M.T.O.: *B-1143*
Winklhofer S.: *B-1733*
Winston J.: *B-1296, B-1298, B-1299, B-1301*
Wirth C.: *B-0337*
Wirth S.: *A-0682*
Wirth W.: *B-1135*
Wisselink H.J.: *B-0586*
Withey S.J.: *B-0617, B-1874*
Witjes M.J.H.: *B-0574*
Witkowska-Wrobel A.: *B-1425*
Witowski J.: *B-1583*
Wittsack H.-J.: *B-0158*
Wnuk E.: *B-1452*
Woerner T.: *B-1058, B-1463*
Woertler K.: *B-1746*
Wohlgemuth W.A.: *B-0739*
Woitek R.: *B-0501*
Woitek R.A.: *B-0716, B-1549, B-1802*
Wolf F.: *A-0907*
Wolf J.: *B-1791*
Wolf N.: *A-0429*
Wolff L.: *B-0258*
Wollenweber F.A.: *B-0259, B-0713*
Wong E.M.F.: *B-0178*
Wong K.H.: *B-1798*
Wong N.: *B-1021*
Wong W.W.: *B-1220*
Wong Y.-C.: *B-0103*
Woo J.-Y.: *B-0918*
Woo O.H.: *B-1177*
Wood B.J.: *B-0837*
Wood P.: *B-1115*
Woodhouse G.: *B-1193*
Woodruff H.: *B-1170, B-1179*
Wörtler K.: *A-0351*
Woussen S.: *B-0251, B-0915*
Wozniak F.: *B-0016*
Wrede K.: *B-0819*
Wree A.: *B-0237*
Wright K.: *B-1650, B-1655*
Wu B.: *B-0204, B-0429, B-0612*
Wu C.: *B-0751, B-1273*
Wu C.-H.: *B-0103*
Wu C.J.: *B-0576*
Wu C.-W.: *B-1816*
Wu G.: *B-1394*
Wu H.: *B-0521*
Wu H.-M.: *B-0758*
Wu J.: *B-0952*
Wu L.: *B-1816*
Wu N.: *B-0429*
Wu R.: *B-1816*
Wu X.: *B-0772*
Wu Y.: *B-0123, B-0466*
Wu Y.-N.: *B-1431*
Wuennemann F.: *B-0240, B-0242, B-1610*
Wuest W.: *B-0289, B-0426, B-0684, B-1245, B-1259, B-1321, B-1868*
Wunder J.: *B-0066*
Wurnig M.: *B-0065*
Wutschke M.: *B-0201*
Wuyts F.: *B-0290*

### X

Xerras C.: *B-1685*
Xi Y.: *B-0391, B-0792*
Xia C.: *B-0018, B-0384, B-0566, B-0596, B-0751, B-0853, B-0907, B-0911, B-1009, B-1311, B-1517, B-1585, B-1588, B-1590, B-1673, B-1752, B-1869*
Xia L.: *B-0380*
Xiang Y.: *B-1888*
Xianhong X.: *B-1124*
Xianying Z.: *B-1649*
Xiao B.: *B-0472*
Xiao J.: *B-0678*
Xiao Y.: *B-1009, B-1330, B-1331, B-1332, B-1752*
Xiao Y.-D.: *B-0112*
Xiaochun Z.: *B-0363*
Xiaojun Z.: *B-1430*
Xie C.: *B-0245*
Xie L.: *B-1386, B-1675*
Xie L.J.: *B-0376*
Xie Q.: *B-1315*
Xie S.: *B-1371, B-1534*
Xin J.: *B-0236*
Xing L.: *B-0495*
Xing Y.: *B-1021*
Xiong X.: *B-1471*
Xu C.: *B-0972*
Xu E.: *B-1440, B-1441*
Xu H.: *B-0376, B-0382, B-0384, B-1254, B-1673, B-1675*
Xu H.M.: *B-0190*
Xu J.: *B-1816*
Xu K.: *B-0596, B-0968, B-1633*
Xu L.: *B-0148, B-0785*
Xu M.: *B-0772, B-1126*
Xu Q.: *B-0805*
Xu R.: *B-0166, B-0375, B-0376, B-1675, B-1826*
Xu S.: *B-0837*
Xu X.: *B-0423, B-0430, B-1189, B-1409*
Xu X.-M.: *B-1033*
Xu Y.: *B-0292*
Xue H.: *B-0386, B-0454, B-0573, B-0613, B-1074, B-1087, B-1121, B-1246, B-1893, B-1896, B-1897*
Xue H.-D.: *B-0450, B-1104, B-1475*
Xuesong D.: *B-1665*

### Y

Yabuuchi H.: *B-0484*
Yadav A.: *B-1668*
Yadav P.: *B-1562*
Yadu N.: *B-0387*
Yagami K.: *B-0660*
Yagi T.: *B-1899*
Yagil Y.: *B-0731*
Yaguchi A.: *B-1003, B-1755*
Yalcin Sahiner S.: *B-1884*
Yamada A.: *B-1836*
Yamada T.: *B-1180*
Yamaguchi T.: *B-1187*
Yamamoto S.: *B-0994*
Yamamura J.: *B-0601*
Yamashiro T.: *B-0292, B-1629*
Yamashita T.: *B-1735*
Yamauchi F.: *B-1365*
Yamazaki M.: *B-1899*
Yamazaki Y.: *B-1832*
Yan F.: *B-1098, B-1531*
Yan J.: *B-0785, B-1236, B-1237, B-1242, B-1430, B-1889*
Yan L.: *B-0566*
Yan L.-F.: *B-0757*
Yan R.: *B-1441*
Yan X.: *B-1873*
Yan Z.: *B-0611*
Yang B.: *B-1427*
Yang C.: *B-1332, B-1339*
Yang D.: *B-0452, B-1835*

Yang D.D.: *B-1004*
Yang H.: *B-0054, B-0683, B-0858*
Yang H.-T.: *B-0861*
Yang J.: *B-1283, B-1304*
Yang K.: *B-1650*
Yang L.: *B-0204, B-0315, B-0462, B-0541, B-0612, B-0854, B-1308, B-1467, B-1624*
Yang M.: *B-0376*
Yang M.-X.: *B-1675*
Yang Q.: *B-0343*
Yang R.: *B-0405, B-1535*
Yang S.: *B-0678*
Yang W.H.: *B-0758*
Yang X.: *B-0360, B-0573, B-1126*
Yang Y.: *B-0774, B-1098, B-1100*
Yang Z.: *B-0166, B-0375, B-0594, B-1454, B-1674, B-1680, B-1825*
Yang Z.-G.: *B-0218, B-0382, B-0596, B-1222, B-1618, B-1673, B-1675, B-1676, B-1681, B-1820, B-1826, B-1859*
Yao J.: *B-1165*
Yao K.: *B-1163*
Yao K.B.: *A-0655*
Yao L.: *B-1330*
Yao Z.: *B-1835*
Yap A.: *B-1233*
Yap P.M.: *B-1220*
Yar O.: *B-1594*
Yarmenitis S.: *B-1378, B-1530, B-1537, B-1837*
Yasin R.: *B-1545*
Yates A.: *B-0633*
Yatsyk G.: *B-0350*
Yazbek S.: *B-1031*
Yazici G.: *B-1445*
Ye C.: *B-0805*
Ye S.: *B-1006*
Ye W.: *B-0308*
Ye Z.: *B-0074, B-1044, B-1349, B-1471*
Yee A.Y.M.: *B-0641*
Yehia A.: *B-0371*
Yel I.: *B-0243, B-0434, B-0448, B-0794, B-1247, B-1362, B-1472*
Yen A.: *B-1607*
Yen H.-H.: *B-0393*
Yeoh H.: *B-1336*
Yeong C.H.: *B-1902*
Yeshua T.: *B-0992*
Yévenes Aravena S.: *B-0629*
Yi X.: *B-1393*
Yi Y.: *B-0320*
Yildirim Erol M.: *B-0603*
Yildiz A.: *B-0213*
Yildiz F.: *B-1805*
Yilmaz Ovali G.: *B-1741*
Yilmaz P.: *B-1690*
Yilmaz S.: *B-1051*
Yim J.-J.: *B-1412*
Yin B.: *B-0552, B-0808*
Yin C.: *B-0226*
Yin P.: *B-1731, B-1732*
Yinchen W.: *B-1649*
Ying M.T.C.: *B-0638*
Yip C.: *B-0617, B-1874*
Yock T.: *B-1227*
Yoen H.: *B-1342*
Yokoo T.: *B-0792*
Yoneda T.: *B-1329*
Yoneyama M.: *B-1735*
Yong C.: *B-0170*
Yong H.: *B-1014*
Yoo J.: *B-1532*
Yoon C.S.: *B-0869*
Yoon H.: *B-0334, B-0335*
Yoon J.H.: *B-0120, B-0795, B-1342*
Yoon J.-H.: *B-1403*
Yoon S.: *B-0773*
Yoon S.E.: *B-0097*
Yoon S.H.: *B-1403, B-1412, B-1611*
Yoon S.J.: *B-0127*
Yorkston J.: *SY 27*
Yoshikawa H.: *B-0484*
Yoshikawa T.: *B-0417, B-0422, B-0424, B-0579, B-1003, B-1203, B-1209, B-1460, B-1755*
Yoshimoto M.: *B-1735*
Yoshimura N.: *B-1899*
Yoshioka K.: *B-0949*
Yoshizawa E.: *B-1836*
You C.: *B-1152*
You H.: *B-1883, B-1890*
You J.H.: *B-0054, B-0683*
You S.K.: *B-1538*
Youn T.: *B-0444*
Young J.: *B-1159*
Young K.: *B-0920*
Yousef M.I.: *B-0531*
Yousry T.A.: *A-0133*
Youssef A.: *B-1363*
Yperzeele L.: *B-0279*
Yu C.: *B-0745*
Yu C.-Y.: *B-0561*
Yu H.: *B-0596, B-1339, B-1758*
Yu J.: *B-1788, B-1835*
Yu J.-S.: *B-0397*
Yu L.: *B-1120*
Yu M.: *B-0012*
Yu M.H.: *B-0449*
Yu Q.: *B-0578*
Yu S.: *B-0015, B-1320, B-1633*
Yu T.: *B-0343*
Yu Z.: *B-1517, B-1861*
Yuan C.: *B-0715*
Yuan D.H.: *B-0208*
Yuan H.: *B-0048, B-0190, B-1742*
Yuan J.: *B-0854*
Yuanyuan S.: *B-1161*
Yue Q.: *B-1426*
Yue S.W.: *B-0779, B-1857*
Yui M.: *B-0417, B-0422, B-1203, B-1209, B-1460*
Yule H.: *A-0007*
Yun B.L.: *B-0699*
Yun S.G.: *B-0723*
Yun S.J.: *B-1137*
Yunkun S.: *B-1254*
Yunqian H.: *B-0035*
Yurdaisik I.: *B-0115, B-0556, B-0737, B-0743*
Yushu C.: *B-1861*
Yusuf M.: *B-0021*

### Z

Zacharzewska-Gondek A.: *B-0540*
Zackrisson S.: *A-0761, B-0695, SY 2c*
Zagórowicz E.: *B-1477*
Zagrodzka M.: *B-0874*
Zaiss M.: *B-0095*
Zakharova N.: *B-0089*
Zakhs D.V.: *B-1164*
Zakian K.: *B-0323*
Zalaudek M.: *B-0055*
Zamarro Parra J.: *B-1418*
Zamboni G.: *A-0340, A-0926, B-0458, B-0532, B-1094, B-1103, B-1389*
Zanardo M.: *B-0028, B-0416, B-0465, B-0490, B-0759, B-1151, B-1352, B-1687*
Zanatta E.: *B-0522*
Zanca F.: *B-1600*
Zanconati F.: *B-0464*
Zanelli E.: *B-1078*
Zanetti M.: *A-0179*
Zanfardino F.: *B-1080*
Zangos S.: *B-0847*
Zannoni S.: *B-0974*
Zanoli L.: *B-1648*
Zaottini F.: *B-1748*
Zarb F.: *A-0490, A-0865*
Zarei F.: *B-0291*
Zargaran S.: *B-0016*
Zargaran Tavakoli S.: *B-1016*
Zarowski A.: *A-0886, A-0889, B-0290*
Zatelli D.: *B-0040*
Zatelli M.C.: *B-0045, B-0046*
Zavadil J.: *B-0232*
Zawaideh J.P.: *B-1384, B-1658*
Zayani O.: *B-1160*
Zdjelar A.: *B-0536*
Zech C.J.: *A-0485*
Zeevaart J.R.: *B-0883*
Zeilinger M.: *A-0110, B-1868*
Zelefsky M.J.: *B-0323*
Zeng C.: *B-1888*
Zeng G.: *B-0571*
Zeng J.: *B-1331, B-1332*
Zeng M.: *B-0069, B-0456, B-1278, B-1339, B-1467, B-1479*
Zeng Q.: *B-1440, B-1441*
Zeng Y.: *B-0858*
Zerlauth J.-B.: *B-0288*
Zerunian M.: *B-0525, B-1022, B-1341, B-1833*
Zhang F.: *B-0831, B-1126*
Zhang F.-L.: *B-0903*
Zhang G.: *B-0251, B-0521, B-1396, B-1449*
Zhang H.: *B-0148, B-0359, B-1064, B-1396*
Zhang J.: *B-0303, B-0858, B-1120, B-1213*
Zhang J.-G.: *B-0853*
Zhang J.Y.: *B-0785*
Zhang L.: *B-0217, B-0573, B-0754, B-1333, B-1449, B-1675*
Zhang L.J.: *B-0784, B-0785*
Zhang M.: *B-0056, B-0147, B-0907, B-1189, B-1441*
Zhang P.: *B-1396*
Zhang Q.: *B-0452, B-0978, B-1835*
Zhang R.: *B-0360, B-0751, B-1517, B-1752*
Zhang S.: *B-0801, B-0854, B-1426*
Zhang T.: *B-1246*
Zhang W.: *B-1330, B-1331, B-1427*
Zhang W.-S.: *B-1691, B-1692, B-1693*
Zhang X.: *B-0770, B-1163, B-1248, B-1256*
Zhang Y.: *B-0596, B-0751, B-0968, B-1234, B-1235, B-1244, B-1252, B-1393, B-1673, B-1779, B-1883, B-1890*
Zhang Z.: *B-0074, B-1044, B-1242, B-1246, B-1272, B-1471, B-1633, B-1654*
Zhao B.: *B-0672*
Zhao C.-K.: *B-1255*
Zhao J.: *B-1021, B-1273*
Zhao L.: *B-0394, B-0805*
Zhao M.: *B-0507*
Zhao P.: *B-1040*
Zhao Q.: *B-1676, B-1680, B-1826*
Zhao R.-C.: *B-1264*
Zhao S.: *B-0593, B-1818*
Zhao W.: *B-0805*
Zhao X.: *B-0390, B-0394, B-0832, B-1478*
Zhao X.-M.: *B-0445*
Zhao Z.: *B-1126*
Zheng G.: *B-0571*
Zheng J.: *B-0152, B-1861*
Zheng L.: *B-0216, B-1449*
Zheng R.: *B-1440, B-1441*
Zheng S.: *B-1756*
Zheng S.-Q.: *B-0758*
Zheng Y.: *B-1888*
Zhigang Y.: *B-0479, B-1869*
Zholdybay Z.: *B-1026*
Zhong J.: *B-0343, B-0361, B-0666, B-0667*
Zhong Y.-K.: *B-1651*
Zhong Z.: *B-0462*
Zhou C.: *B-0217, B-0445, B-0754, B-0805, B-0832, B-0978*
Zhou C.S.: *B-0784*
Zhou F.: *B-0785*
Zhou H.: *B-1074, B-1087, B-1893, B-1896, B-1897*
Zhou J.: *B-0306*
Zhou J.-Y.: *B-1171*
Zhou L.: *B-0541*
Zhou Q.: *B-1189*
Zhou W.: *B-1220*
Zhou X.: *B-0472, B-1189, B-1315*
Zhou Y.: *B-1386*
Zhu B.: *B-0151*

Zhu J.: *B-0390, B-0969, B-1519*
Zhu L.: *B-0450, B-1475*
Zhu M.: *B-0599, B-1163*
Zhu T.: *B-1861*
Zhu W.: *B-1429*
Zhu Y.: *B-0511, B-0881, B-1633*
Zhu Z.: *B-0445, B-0832*
Zhuo Z.: *B-0360*
Zhuravlev K.N.: *B-1314*
Ziayee F.: *B-0161, B-0327*
Žibert J.: *B-1070, B-1710*
Zidan D.: *B-0400*
Ziegelmayer S.: *B-0615*
Ziegert M.: *B-1199, B-1631*
Zimlichman E.: *B-1186*
Zimmermann H.A.: *B-0583*
Zimmermann M.: *B-0005*
Zimny A.: *A-0677, B-1265*
Zini C.: *B-1575, B-1578*
Zins M.: *A-0022, A-0109, A-0236, A-0240, A-0852*
Zitzelsberger T.: *B-0589*
Złahoda-Huzior A.: *B-1583*
Zoelch N.: *B-1516*
Zografos C.: *B-1157*
Zografos G.: *B-1157*
Zolda P.: *A-0864*
Zolovkins A.: *B-1809*
Zopfs D.: *B-1424*
Zorza I.: *B-0970*
Zorzenon I.: *B-0536*
Zorzi M.: *B-0248, B-0250*
Zou M.: *B-1454*
Zou T.: *B-0907*
Zou T.-X.: *B-1041*
Zoumpoulis P.: *B-1378, B-1530, B-1537, B-1837*
Zubler V.: *A-0541*
Zucali P.: *B-1636*
Zucchelli A.: *B-1022*
Zucchetta P.: *B-1364*
Zuchowski P.: *B-1408*
Zuckermann A.: *B-0374, B-1617*
Zugaro L.: *B-1295, B-1571, B-1572, B-1580, B-1581*
Zugni F.: *B-1281*
Zu-Hua G.: *B-0528*
Zuiani C.: *B-0086, B-0253, B-0477, B-1078, B-1079, B-1138*
Zulian F.: *B-0512*
Zuliani M.: *B-0512*
Zulueta J.: *B-1609*
Zumofen D.: *B-0193, B-0946*
Zur G.: *B-1557*
Zurera Tendero L.J.J.: *B-1662*
Zwaagstra H.: *B-1066*
Zwanenburg J.J.: *B-0215*
Zwierko B.: *B-1408*
Zwinderman A.H.: *B-0096*

### G

### List of Moderators (G)

### List of Moderators

### A

Adam E.J.: *RC 514*
Adriaensen M.: *MY 9*
Akinci D'Antonoli T.: *SS 1910a*
Akram M.H.: *EM 3*
Albazaz R.: *SS 201a*
Albrecht T.: *SS 1709*
Alcalá-Galiano A.: *SS 1510*
Alexopoulou E.: *RC 112*
Amosova E.: *SS 501*
Andrasina T.: *SS 1709*
Annoni A.D.: *SS 1915*
Antoch G.: *RC 806*
Arkink E.: *RC 1308*
Asbach P.: *MY 13*
Aström G.K.O.: *SS 210a*
Austin C.: *SY 5*

### B

Baeßler B.: *SS 605*
Bali M.A.: *SS 201a*
Balleyguier C.S.: *BS 8*
Bancroft L.W.: *TC 1328, TC 1428, TC 1528, TC 1628*
Barter S.: *SS 1402a*
Ba-Ssalamah A.: *$E^3$ 24E, SS 1901a*
Basta Nikolic M.: *SS 1507*
Beardmore C.: *EFRS/EORTC*
Becker M.: *BE 6*
Beigelman C.: *SS 1604*
Belfield J.: *SS 1007*
Bell J.K.: *PI 3*
Bellin M.-F.: *SS 1407*
Benegas M.: *SS 1604*
Benndorf M.: *SS 1605*
Bérczi V.: *RC 1315, MY 8*
Bernathova M.: *SS 1902a*
Bick U.: *SS 1805a*
Biddle D.: *SS 614*
Binkert C.A.: *$E^3$ 1226*
Björkman-Burtscher I.M.: *SS 414*
Blachar A.: *SS 701*
Blanco Barrio A.: *SS 317*
Blanco Sequeiros R.: *SS 605*
Blazic I.: *SS 1401b*
Bloem J.L.: *RC 910*
Blomqvist L.K.: *MY 17*
Bluekens A.M.J.: *SS 202*
Boban J.: *SS 1511*
Bodner G.: *SS 1910b*
Boesen M.: *SS 610, SY 27*
Bogveradze N.: *SS 1716*
Bolstad K.N.: *SS 1813*
Bonaros N.: *SY 10*
Bonomo L.: *SS 714b*
Booth T.C.: *SS 705*
Boric I.: *SS 310*
Bortolotto C.C.: *SS 1405*
Bowden L.: *SS 1413*
Bozzetti F.: *SS 311*
Brader P.: *$E^3$ 426b*
Brady A.: *ESR/EFRS*
Brambilla M.: *EF 2, ESR/EFOMP*
Brink M.: *SS 717*

### C

Cantisani V.: *SY 18*
Caroff J.: *SS 309*
Caruso D.: *SS 616b*
Caseiro Alves F.: *SY 13*
Cassar-Pullicino V.N.: *$E^3$ 25A, $E^3$ 25B, $E^3$ 25C, $E^3$ 25D, $E^3$ 25E*
Cavedon C.: *SS 1013*
Celeng C.: *SS 203*
Chassagnon G.: *SS 1804*
Chelu R.G.: *SS 1803*
Chidambaranathan N.: *RC 1611*
Choudhary S.: *SS 710*
Christodoulou D.: *SS 1901a*
Cicchetti G.: *SS 1016*
Cieszanowski A.: *SS 616b*
Clauser P.: *SS 602, SY 2d*
Cocozza S.: *SS 1811a*
Cokkinos D.D.: *SS 317*
Cook G.: *SS 1716*
Coolen J.: *SS 1404*
Coulon P.: *SY 16*
Courcoutsakis N.A.: *SS 301*
Croisille P.: *RC 1603*
Curic J.: *SS 1509*
Curvo-Semedo L.: *SS 1001a*
Cvetko D.: *SS 709*
Cyran C.C.: *ESHI$^{MT}$ 1, ESHI$^{MT}$ 2*

### D

Damilakis J.: *EF 1*
D'Anastasi M.: *MY 3*
De Kerviler E.: *SY 30*
de Lange C.E.: *RC 412*
De Visschere P.: *SS 307*
Delorme S.: *US 2*
Demaerel P.: *MY 16*
Demirkazik F.B.: *SS 204*
Denecke T.: *SS 1801a*
Derchi L.E.: *EM 1, EM 2, EM 3*
Deutschmann H.A.: *SS 315*
Dewey M.: *CT 2, CT 10, MY 7*
Dežman R.: *SS 1509*
Diaz O.: *SS 1805a*
Diogo M.C.: *SS 1011a*
Djilas D.: *SS 1402b*
Dodd J.D.: *SS 604*
Donato P.: *SS 1503*
Dondelinger R.F.: *SS 1009*
Dormagen J.B.: *SS 717*
Dromain C.: *SS 1401b*
Drudi F.M.: *SS 1807*
Due-Tønnessen P.: *SS 711*
Duran R.: *SS 209*

### E

Edyvean S.: *SS 213*
Egger K.: *SS 705*
El-Diasty T.: *SS 307*
Ergen F.B.: *SS 1010*
Erkan B.: *SS 1017*
Ertürk S.M.: *RC 301*
Esen G.: *SS 1002*
Essig M.: *SY 14*
Etxano J.: *SS 302*

### F

Fahlenkamp U.: *SS 710*
Fallenberg E.M.: *MY 5*
Farchione A.: *SS 1905a*
Farley L.: *SY 26*
Farrugia Wismayer E.: *SS 714a*
Fatehi M.: *SS 205*
Feydy A.: *SS 1910a*
França M.M.: *SS 1801a*
Franchi P.: *SS 204*
Francone M.: *MY 18*
Frija G.: *EU 2*
Fütterer J.J.: *SS 407*

### G

Gagarina N.: *SS 203*
Gallagher F.A.: *ESHI$^{M}$ 1*
Garbajs M.: *SS 1501b*
García Santos J.M.: *SS 1914a*
Gatidis S.: *SS 1406*
Gennaro G.: *SS 1913*
Gerson P.: *EM 5*
Gharbi H.A.: *EM 1*
Ghaye B.: *RC 1704*
Gheonea I.-A.: *SS 1402b*
Giannotti E.: *SS 702b*
Gilligan P.: *EF 1*
Goh V.J.: *CT 6*
Golay X.: *SS 1411a*
Gopalan D.: *SY 12*
Govindaraj J.: *SS 1808*
Goyen M.: *SY 3*
Grainger A.J.: *TC 1328, TC 1428, TC 1528, TC 1628*
Grassi R.: *EM 2*
Grazioli L.: *SS 616a*
Grover S.B.: *RC 1714*
Grunwald I.Q.: *SS 311*
Guggenberger R.: *SS 1410*

### H

Haage P.: *RC 915*
Hahn H.K.: *ESHI$^{MT}$ 2*
Haider L.: *SS 1811b*
Heiss P.: *SS 709*
Heiß R.: *SS 603*
Hendriks E.J.: *SS 1411a*
Herlihy T.: *RC 1714*
Hirvonen J.: *SS 1005b*

### I

Iacobellis F.: *SS 201b*
Isaac A.: *SS 210b*
Ivanac G.: *SS 602*

### J

Jackson V.P.: *CT 2*
Jalaguier A.: *SS 1402a*
Jaschke W.R.: *ESR/EFOMP*

### K

Kallifatidis A.: *RC 403*
Kalmar P.I.: *SS 1815*
Kantarci M.: *SS 1003*
Karantanas A.H.: *BS 1*
Karpenko A.K.: *SS 716*
Kartalis N.: *SS 1401a*
Katulska K.: *SS 1017*
Kaya T.: *SS 1510*
Kazmierczak P.M.: *SS 706*
Keavey E.: *SS 1913*
Kenigsberg K.: *SS 1411b*
Kiessling F.M.A.: *SS 1406*
Kilburn-Toppin F.: *$E^3$ 426a*
King A.D.: *SS 1008*
Kintzelé L.: *SS 716*
Klauß M.: *SS 1401a*
Koç A.M.: *SS 611*
Kolodziej M.: *SY 15*
Kortesniemi M.: *EF 2*
Koutalonis M.: *SS 1802a*
Kozak O.W.: *SS 1807*
Kralik I.: *SS 1802a*
Kramer H.: *SY 22*
Krokidis M.: *RC 1515*
Krupiński M.: *SS 1003*

### L

Laghi A.: *MY 3*
Lança L.J.O.C.: *SS 714b*
Lassalle L.: *SS 310*
Lauenstein T.C.: *$E^3$ 24C, MY 17*
Lehotská V.: *SS 202*
Lell M.: *MY 14*
Lemmerling M.M.: *RC 1708*
Lenghel L.M.: *SS 608*

### List of Moderators

Llorens R.: *SS 612*
Logager V.: *SS 214*
Luciani A.: *SS 616a*
Lücke C.: *SS 1503*
Lupescu I.G.: *$E^3$ 24D*
Lurie D.J.: *SS 713*

### M

Maguire S.: *SS 313*
Mahmood U.: *CT 6, CT 10*
Maier A.: *SS 201b*
Malamateniou C.: *SS 414*
Malone D.E.: *RC 1201*
Maly Sundgren P.C.: *$E^3$ 126a*
Manfredi R.: *$E^3$ 24A*
Marincek B.: *BS 5*
Markiet K.: *MY 13, SS 308*
Marolt Music M.: *SS 702b*
Marticorena Garcia S.R.: *SS 301*
Martin S.S.: *SS 1005a*
Martinoli C.: *MY 9*
Masselli G.: *SS 1507*
Matos C.: *$E^3$ 24B*
McGinty G.: *PI 2*
McNulty J.: *EM 4, EFRS WS, ESR/EFRS*
Mentzel H.-J.: *SS 312*
Merhemic Z.: *RC 911*
Miletić D.: *SS 1501a*
Minoiu C.A.: *SS 703*
Mintert S.: *SY 28*
Mitreska N.: *SS 1012*
Mizzi D.: *SS 1914b*
Molinari F.: *SS 704*
Montet X.: *SS 1016*
Morozov S.: *SS 1405*
Mostbeck G.H.: *SS 1901b*
Moyle P.L.: *SS 302*
Mück F.G.: *SS 217*
Müller-Eschner M.: *SS 1915*
Mussmann B.R.: *SS 1314*
Mytsyk Y.: *SS 607*

### N

Nair A.: *SS 1904*
Neri E.: *SS 1005a*
Nevalainen M.: *SS 1810*
Newman D.: *EM 5*
Niessen W.J.: *SS 1805b*
Nightingale J.M.: *EFRS WS, SS 1814*
Nikolaou K.: *SS 1403*
Nilsson A.: *SY 23*
Njagulj V.: *RC 410, SS 1010*
Nyhsen C.: *ESR US SC/UES*

### O

Oei E.: *SS 210a*
Olczak Z.: *SS 1903b*
Oppenheim C.: *SY 4*
Ording Müller L.-S.: *BS 12*
O'Reilly G.: *SS 1813*
Orsi M.A.: *SS 1902b*
Otero-García M.: *SS 216*
Ozgen Mocan B.: *SS 308*

### P

Palkó A.: *BE 4*
Papadaki E.: *SS 1416*
Parkar A.P.: *BS 2*
Pavlica P.: *SS 707*
Pedersen R.: *RC 1610*
Penzkofer T.: *SS 707*
Pershina E.: *SS 1903a*
Pesapane F.: *SS 1902a*
Pinto dos Santos D.: *SS 1905b*
Piqueras J.: *SS 1512*
Prayer D.: *SS 612*
Prayer F.: *SS 312*
Precht H.: *EM 4*
Prokop M.: *SS 1905b*
Pronin I.N.: *RC 1711*
Puchner S.: *SS 609*
Puech P.: *SS 207*
Pulzato I.: *SS 1004*
Pusceddu C.: *SS 1909*
Pyatigorskaya N.: *SS 1911b*

### R

Radder A.: *SY 7*
Radovic N.: *SS 1802b*
Raissaki M.: *SS 614*
Ramanathan S.: *SS 1407*
Ratib O.: *SF 12*
Rausch I.: *SS 606*
Reiner C.S.: *SS 401*
Reiser M.F.: *$E^3$ 1626a*
Regier M.: *SS 1004*
Reponen J.: *SS 1414*
Revelli M.: *SS 610*
Riera L.: *SS 212*
Riklund K.: *RC 314*
Rockall A.G.: *SY 31*
Roding T.: *RC 314*
Rohan T.: *SS 1901b*
Ronot M.: *SS 401*
Rosendahl K.: *$E^3$ 1326*
Rossi A.: *SS 1403*
Russe M.F.: *SS 1805b*

### S

Saat R.: *MY 14*
Salgado R.: *SS 603*
Samara E.: *SS 213*
Sánchez M.: *SS 1904*
Sánchez Montañez A.: *SS 1412*
Santos J.: *MY 15*
Santos R.A.M.: *SS 1914a*
Sappey-Marinier D.: *ESR/ESMRMB*
Sartor H.: *SS 1002*
Scapin E.: *SS 208*
Scarsbrook A.: *SS 306*
Schaarschmidt B.M.: *SS 216*
Schimmöller L.: *SS 1007*
Schlett C.L.: *SS 305*
Schneck A.: *SY 6*
Schwarz-Nemec U.: *SS 1012*
Sechopoulos I.: *SY 2c*
Seimenis I.: *RC 1513*
Seliverstova E.: *SS 1011b*
Sella T.: *SS 702a*
Seuri R.: *SS 714a*
Severino M.: *MY 16*
Sidhu P.S.: *US 1, SY 17*
Silva M.: *RC 504*
Simeonov G.: *EU 2*
Singhal A.: *SS 208*
Sinitsyn V.E.: *SS 1903b*
Sjöberg J.: *SS 1013*
Skrobisz K.: *SS 1907*
Smits M.: EFRS/EORTC, ESR/EORTC
Snaith B.: *SS 214*
Sofia C.: *SS 701*
Sosna J.: *$E^3$ 1526*
Springer E.: *SS 1810*
Stajgis M.: *SS 217*
Steinfelder E.: *ESR/BBMRI-ERIC*
Stojanov D.: *SS 1911a*
Strobl F.F.: *SS 1009*
Sudoł-Szopińska I.: *RC 1710*
Suomi V.: *SS 205*
Sutter R.: *RC 1310*
Svare A.: *TF*

### T

Taibbi A.: *SS 501*
Takx R.A.P.: *SS 703*
Tarnoki D.L.: *SS 215*
Teneva T.: *TF*
Thieme N.: *RC 908*
Thompson J.: *SS 1014*
Thurner P.: *SS 711*
Thursar L.: *SY 15*
Toledano-Massiah S.: *MY 4*
Torregrosa Andrés A.: *SS 1001b*
Tresoldi S.: *SS 615*
Trianni A.: *SS 1413*
Triantopoulou C.: *RC 1601*
Trimboli R.M.: *MY 5*
Triulzi F.M.: *SS 1412*
Tsitskari M.: *SS 109*
Tüdös Z.: *SS 1909*
Tyurin I.E.: *$E^3$ 1626b, SY 24*
Tzalonikou M.: *RC 810*

### U

Umutlu L.: *SY 2a*

### V

Válek V.: *ESR US SC/UES*
van der Hoorn A.: *SS 1008*
van der Lugt A.: *ESR/BBMRI-ERIC*
Van Ongeval C.: *RC 1702*
van Osch M.J.P.: *ESR/ESMRMB*
Vasco Aragão M.F.: *SS 1811a*
Vasilevska-Nikodinovska V.: *SS 1410*
Velonakis G.: *SS 1809*
Verbist B.: *SS 1816*
Vernuccio F.: *SS 1001b*
Vidjak V.: *SS 609*
Vilar J.: *BE 3*
Vilgrain V.: *ESOR*

### W

Walecki J.: *BS 9*
Wenkel E.: *SY 2d*
Wielpütz M.O.: *MY 4*
Wildberger J.E.: *SY 19*
Woitek R.: *SS 306*
Wolf F.: *MY 18*
Wörtler K.: *RC 510*

### Y

Yakimov A.: *SS 1011b*
Yalynska T.: *SS 212*
Yousry T.A.: *SS 611*

### Z

Zackrisson S.: *SY 2b*
Zaitoun M.M.A.: *SS 211b*
Zanirato Rambaldi G.: *SS 1816*
Zarb F.: *RC 514*
Zelenak K.: *SS 309*
Zins M.: *MY 7*
